# Supplementary material for: DESS deconstructed: Is EDTA solely responsible for protection of high molecular weight DNA in this common tissue preservative?
Source: PLoS One. 2020 Aug 20;15(8):e0237356. doi: 10.1371/journal.pone.0237356 (PMC7440624; doi:10.1371/journal.pone.0237356)
Supplement: S1 Table — The barcode region of the mitochondrial COI gene was sequenced from two specimens of each taxon used in this study to confirm species identifications. The values listed are percent identities to the best match found in the Barcode of Life Datasystem (BOLD). Specimen IDs for both the Ocean Genome Legacy online catalog and best matches found in BOLD are presented. (PDF) [file pone.0237356.s001.pdf]

**OGL specimen ID**	**Record ID of best match in BOLD**	**Species ID of best match in BOLD**	**Sequence identity (%)**
S29192	ECMOL342-11	*Mytilus edulis*	99.21
S29193	ECMOL335-11	*Mytilus edulis*	99.17
S29199	GBCMD20877-19	*Faxonius virilis*	100
S29200	GBCMD20877-19	*Faxonius virilis*	100
S29206	NBPOL001-08	*Alitta virens*	100
S29207	NBPOL001-08	*Alitta virens*	100
