# Supplementary material for: DESS deconstructed: Is EDTA solely responsible for protection of high molecular weight DNA in this common tissue preservative?
Source: PLoS One. 2020 Aug 20;15(8):e0237356. doi: 10.1371/journal.pone.0237356 (PMC7440624; doi:10.1371/journal.pone.0237356)
Supplement: S2 Table — Values are presented for tissues of Mytilus edulis, Faxonius virilis and Alitta virens extracted immediately after dissection (fresh) or stored for one day (1 d), three months (3 m) or six months (6 m) in preservative treatments containing DMSO (D), EDTA (E) and/or saturated NaCl (SS) or 95% ethanol (EtOH). N/a indicates samples for which data were not collected. (PDF) [file pone.0237356.s002.pdf]

Sample ID	Taxa	Replicate	Treatment	Time Interval	A260/A280 Ratio	Yield (µg)	Total Normalized Yield (µg DNA/mg tissue)	nY (µg DNA/ mg tissue)	%R	TapeStation Filename
1	Mytilus edulis	1	DESS	1 d	1.97	6.79	0.7670	0.3795	49.53	Mussel and Seaworm T1 Data.gDNA
2	Mytilus edulis	2	DESS	1 d	2.09	20.1	1.6681	0.7743	46.44	Mussel and Seaworm T1 Data.gDNA
3	Mytilus edulis	3	DESS	1 d	2.05	10.1	0.8363	0.4256	50.9	Mussel and Seaworm T1 Data.gDNA
4	Mytilus edulis	4	DESS	1 d	2.12	9.72	0.8858	0.4347	49.09	Mussel and Seaworm T1 Data.gDNA
5	Mytilus edulis	5	DESS	1 d	2.08	9.11	0.7781	0.3459	44.44	Mussel and Seaworm T1 Data.gDNA
6	Mytilus edulis	1	DE	1 d	2.10	7.67	0.7166	0.2737	38.16	Mussel and Seaworm T1 Data.gDNA
7	Mytilus edulis	2	DE	1 d	2.10	4.6	0.3874	0.1701	43.93	Mussel and Seaworm T1 Data.gDNA
8	Mytilus edulis	3	DE	1 d	2.13	6.03	0.6120	0.2263	36.96	Mussel and Seaworm T1 Data.gDNA
9	Mytilus edulis	4	DE	1 d	1.95	8.41	0.8437	0.3421	40.58	Mussel and Seaworm T1 Data.gDNA
10	Mytilus edulis	5	DE	1 d	1.99	15.9	1.6550	0.6037	36.56	Mussel and Seaworm T1 Data.gDNA
11	Mytilus edulis	1	DSS	1 d	2.03	30.2	4.1210	0.1014	2.47	Mussel and Seaworm T1 Data.gDNA
12	Mytilus edulis	2	DSS	1 d	1.92	16.4	0.8879	0.3968	44.83	Mussel and Seaworm T1 Data.gDNA
13	Mytilus edulis	3	DSS	1 d	1.94	16.1	1.3521	0.0312	2.32	Mussel and Seaworm T1 Data.gDNA
14	Mytilus edulis	4	DSS	1 d	1.98	14.4	1.3461	0.1767	13.14	Mussel and Seaworm T1 Data.gDNA
15	Mytilus edulis	5	DSS	1 d	1.95	14.2	1.6528	0.0324	1.97	Mussel and Seaworm T1 Data.gDNA
16	Mytilus edulis	1	ESS	1 d	2.08	8.42	0.6268	0.2732	43.61	Mussel and Seaworm T1 Data.gDNA
17	Mytilus edulis	2	ESS	1 d	1.95	3.24	0.3262	0.1399	43.06	Mussel and Seaworm T1 Data.gDNA
18	Mytilus edulis	3	ESS	1 d	2.08	7.71	0.6279	0.2785	44.31	Mussel and Seaworm T1 Data.gDNA
19	Mytilus edulis	4	ESS	1 d	1.73	2.45	0.1799	0.0837	46.59	Mussel and Seaworm T1 Data.gDNA
20	Mytilus edulis	5	ESS	1 d	2.20	17.9	1.6574	0.7491	45.28	Mussel and Seaworm T1 Data.gDNA
21	Mytilus edulis	1	D	1 d	2.00	20.3	0.8495	0.0151	1.79	Mussel and Seaworm T1 Data.gDNA
22	Mytilus edulis	2	D	1 d	1.97	12.8	0.4758	0.0054	1.13	Mussel and Seaworm T1 Data.gDNA
23	Mytilus edulis	3	D	1 d	2.00	34.6	1.5212	0.2748	18.05	Mussel and Seaworm T1 Data.gDNA
24	Mytilus edulis	4	D	1 d	2.01	11.8	0.5751	0.0093	1.61	Mussel and Seaworm T1 Data.gDNA
25	Mytilus edulis	5	D	1 d	1.95	8.38	0.5688	0.0193	3.39	Mussel and Seaworm T1 Data.gDNA
26	Mytilus edulis	1	E	1 d	2.01	9.56	0.6368	0.2431	38.19	Mussel and Seaworm T1 Data.gDNA
27	Mytilus edulis	2	E	1 d	2.08	5.07	0.3306	0.1259	37.95	Mussel and Seaworm T1 Data.gDNA
28	Mytilus edulis	3	E	1 d	2.02	6.95	0.6463	0.2344	36.25	Mussel and Seaworm T1 Data.gDNA
29	Mytilus edulis	4	E	1 d	1.64	6.82	0.5056	0.1868	36.94	Mussel and Seaworm T1 Data.gDNA
30	Mytilus edulis	5	E	1 d	2.01	5.33	0.3449	0.1430	41.44	Mussel and Seaworm T1 Data.gDNA
31	Mytilus edulis	1	SS	1 d	2.02	25.7	1.4701	0.2042	13.87	Mussel and Seaworm T1 Data.gDNA
32	Mytilus edulis	2	SS	1 d	1.99	6.59	0.4212	0.1636	38.89	Mussel and Seaworm T1 Data.gDNA
33	Mytilus edulis	3	SS	1 d	1.94	17	1.4510	0.3815	26.29	Mussel and Seaworm T1 Data.gDNA
34	Mytilus edulis	4	SS	1 d	1.97	25.1	1.2370	0.2114	17.06	Mussel and Seaworm T1 Data.gDNA
35	Mytilus edulis	5	SS	1 d	2.03	16.2	1.1434	0.1983	17.38	Mussel and Seaworm T1 Data.gDNA
36	Mytilus edulis	1	EtOH	1 d	1.99	57.1	1.2501	0.2780	22.32	Mussel and Seaworm T1 Data.gDNA
37	Mytilus edulis	2	EtOH	1 d	2.07	53.7	1.1878	0.3119	26.25	Mussel and Seaworm T1 Data.gDNA
38	Mytilus edulis	3	EtOH	1 d	1.99	28.5	0.8115	0.2155	26.53	Mussel and Seaworm T1 Data.gDNA
39	Mytilus edulis	4	EtOH	1 d	2.00	25.4	0.8248	0.2549	30.93	Mussel and Seaworm T1 Data.gDNA
40	Mytilus edulis	5	EtOH	1 d	1.97	26.4	0.8789	0.1169	13.3	Mussel and Seaworm T1 Data.gDNA
41	Mytilus edulis	1	Fresh	1 d	1.89	27.6	0.8903	0.2071	23.22	Mussel and Seaworm T1 Data.gDNA
42	Mytilus edulis	2	Fresh	1 d	2.06	26	1.1017	0.3555	32.25	Mussel and Seaworm T1 Data.gDNA
43	Mytilus edulis	3	Fresh	1 d	2.01	26.6	0.9236	0.3361	36.43	Mussel and Seaworm T1 Data.gDNA
44	Mytilus edulis	4	Fresh	1 d	2.03	15.2	0.5588	0.1996	35.73	Mussel and Seaworm T1 Data.gDNA
45	Mytilus edulis	5	Fresh	1 d	1.95	72.3	2.1327	0.3982	18.61	Mussel and Seaworm T1 Data.gDNA
136	Mytilus edulis	1	DESS	3 m	1.99	20.1	1.1258	0.6217	54.96	2018-12-03-01.gDNA
137	Mytilus edulis	2	DESS	3 m	2.01	18.6	1.2696	0.5843	45.98	2018-12-03-01.gDNA
138	Mytilus edulis	3	DESS	3 m	1.98	19.5	1.2556	0.6136	48.96	2018-12-03-01.gDNA
139	Mytilus edulis	4	DESS	3 m	1.99	21.3	1.1273	0.6510	57.88	2018-12-03-01.gDNA
140	Mytilus edulis	5	DESS	3 m	1.93	26.3	1.9180	0.9189	47.77	2018-12-03-01.gDNA
141	Mytilus edulis	1	DE	3 m	1.94	17	0.8926	0.2641	29.6	2018-12-03-01.gDNA
142	Mytilus edulis	2	DE	3 m	1.88	28.7	1.6439	0.8878	53.95	2018-12-03-01.gDNA
143	Mytilus edulis	3	DE	3 m	1.89	30.5	1.6884	0.8913	52.92	2018-12-03-01.gDNA
144	Mytilus edulis	4	DE	3 m	1.99	39.3	1.7532	0.6959	39.84	2018-12-03-01.gDNA
145	Mytilus edulis	5	DE	3 m	2.00	3.47	0.1740	0.0233	13.34	2018-12-03-01.gDNA
146	Mytilus edulis	1	DSS	3 m	2.14	0.561	0.0282	0.0011	3.96	2019-07-29-01.gDNA

Sample ID	Taxa	Replicate	Treatment	Time Interval	A260/A280 Ratio	Yield (µg)	Total Normalized Yield (µg DNA/mg tissue)	nY (µg DNA/ mg tissue)	%R	TapeStation Filename
147	Mytilus edulis	2	DSS	3 m	2.19	0.502	0.0305	0.0005	1.68	2019-07-29-01.gDNA
148	Mytilus edulis	3	DSS	3 m	2.16	0.551	0.0477	0.0010	2.02	2019-07-29-01.gDNA
149	Mytilus edulis	4	DSS	3 m	2.13	0.66	0.0492	0.0011	2.22	2019-07-29-01.gDNA
150	Mytilus edulis	5	DSS	3 m	2.16	0.447	0.0298	0.0017	5.8	2019-07-29-01.gDNA
151	Mytilus edulis	1	ESS	3 m	2.00	3.79	0.3211	0.1788	55.71	2018-12-03-01.gDNA
152	Mytilus edulis	2	ESS	3 m	1.95	12.3	0.9733	0.6188	63.63	2018-12-03-01.gDNA
153	Mytilus edulis	3	ESS	3 m	1.97	17.4	1.0131	0.5409	53.56	2018-12-03-01.gDNA
154	Mytilus edulis	4	ESS	3 m	2.01	34	2.0856	0.7238	34.81	2018-12-03-01.gDNA
155	Mytilus edulis	5	ESS	3 m	1.98	8.79	0.5783	0.3671	63.5	2018-12-03-01.gDNA
156	Mytilus edulis	1	D	3 m	2.09	2.52	0.1255	0.0016	1.27	2020-06-10-01.gDNA
157	Mytilus edulis	2	D	3 m	2.13	1.04	0.0393	0.0006	1.64	2020-06-10-01.gDNA
158	Mytilus edulis	3	D	3 m	2.08	2.27	0.1371	0.0014	1.01	2020-06-10-01.gDNA
159	Mytilus edulis	4	D	3 m	2.12	1.42	0.0648	0.0012	1.78	2020-06-10-01.gDNA
160	Mytilus edulis	5	D	3 m	2.09	1.75	0.0812	0.0010	1.26	2020-06-10-01.gDNA
161	Mytilus edulis	1	E	3 m	1.84	44.5	1.8122	0.5457	30.16	2018-12-03-01.gDNA
162	Mytilus edulis	2	E	3 m	1.89	10.5	0.4283	0.2394	55.73	2018-12-03-01.gDNA
163	Mytilus edulis	3	E	3 m	1.85	13	0.6153	0.3517	57.21	2018-12-03-01.gDNA
164	Mytilus edulis	4	E	3 m	1.96	9.04	0.3941	0.2075	52.7	2018-12-03-01.gDNA
165	Mytilus edulis	5	E	3 m	1.87	20.3	0.9199	0.4894	53.17	2018-12-03-01.gDNA
166	Mytilus edulis	1	SS	3 m	2.05	0.598	0.0329	0.0001	0.42	2019-07-29-01.gDNA
167	Mytilus edulis	2	SS	3 m	2.02	1.31	0.0608	0.0001	0.14	2019-07-29-01.gDNA
168	Mytilus edulis	3	SS	3 m	2.14	0.662	0.0292	0.0001	0.33	2019-07-29-01.gDNA
169	Mytilus edulis	4	SS	3 m	2.00	0.183	0.0094	0.0001	0.91	2019-07-29-01.gDNA
170	Mytilus edulis	5	SS	3 m	2.02	0.452	0.0162	0.0002	0.99	2019-07-29-01.gDNA
171	Mytilus edulis	1	EtOH	3 m	2.00	42.2	1.0489	0.0390	3.73	2018-12-03-01.gDNA
172	Mytilus edulis	2	EtOH	3 m	2.03	45.9	1.0375	0.0359	3.47	2018-12-03-01.gDNA
173	Mytilus edulis	3	EtOH	3 m	2.01	63.7	1.7405	0.4399	25.29	2018-12-03-01.gDNA
174	Mytilus edulis	4	EtOH	3 m	2.00	44.8	1.0043	0.1175	11.69	2018-12-03-01.gDNA
175	Mytilus edulis	5	EtOH	3 m	2.01	57.3	1.3900	0.3105	22.41	2018-12-03-01.gDNA
176	Mytilus edulis	1	Fresh	3 m	2.02	35.9	1.0257	0.4086	39.91	2018-12-03-01.gDNA
177	Mytilus edulis	2	Fresh	3 m	1.98	30.9	0.9537	0.3488	36.49	2018-12-03-01.gDNA
178	Mytilus edulis	3	Fresh	3 m	1.99	24	0.8000	0.2577	32.25	2018-12-03-01.gDNA
179	Mytilus edulis	4	Fresh	3 m	2.05	24.1	0.7902	0.2662	33.76	2018-12-03-01.gDNA
180	Mytilus edulis	5	Fresh	3 m	2.03	21.6	0.6353	0.1191	18.73	2018-12-03-01.gDNA
181	Mytilus edulis	1	DESS	6 m	2.08	11.4	0.8600	0.4051	47.28	Lounsberry_T5_Samples_AllSpecies_AllReplicates_3.19.19.gDNA
182	Mytilus edulis	2	DESS	6 m	1.99	29.2	2.4778	0.9419	37.98	Lounsberry_T5_Samples_AllSpecies_AllReplicates_3.19.19.gDNA
183	Mytilus edulis	3	DESS	6 m	2.04	24	2.1977	0.9615	43.67	Lounsberry_T5_Samples_AllSpecies_AllReplicates_3.19.19.gDNA
184	Mytilus edulis	4	DESS	6 m	2.05	17.6	1.1895	0.6035	50.74	Lounsberry_T5_Samples_AllSpecies_AllReplicates_3.19.19.gDNA
185	Mytilus edulis	5	DESS	6 m	2.03	14.6	0.6910	0.3872	55.98	Lounsberry_T5_Samples_AllSpecies_AllReplicates_3.19.19.gDNA
186	Mytilus edulis	1	DE	6 m	1.99	36.2	1.6026	0.9430	58.75	T5_Initials&Errors_4-3-2019_p2.gDNA
187	Mytilus edulis	2	DE	6 m	1.89	27.4	1.0822	0.5964	55.2	Lounsberry_T5_Samples_AllSpecies_AllReplicates_3.19.19.gDNA
188	Mytilus edulis	3	DE	6 m	1.80	26.2	1.3502	0.6545	48.4	Lounsberry_T5_Samples_AllSpecies_AllReplicates_3.19.19.gDNA
189	Mytilus edulis	4	DE	6 m	1.95	11.6	0.5907	0.3575	60.52	Lounsberry_T5_Samples_AllSpecies_AllReplicates_3.19.19.gDNA
190	Mytilus edulis	5	DE	6 m	1.93	31.8	1.7399	0.6019	34.7	Lounsberry_T5_Samples_AllSpecies_AllReplicates_3.19.19.gDNA
191	Mytilus edulis	1	DSS	6 m	2.03	1.34	0.0827	0.0032	3.84	Lounsberry_T5_Samples_AllSpecies_AllReplicates_3.19.19.gDNA
192	Mytilus edulis	2	DSS	6 m	1.89	0.31	0.0138	0.0004	3.11	2020-06-10-01.gDNA
193	Mytilus edulis	3	DSS	6 m	2.13	0.361	0.0221	0.0013	5.89	2020-06-10-01.gDNA
194	Mytilus edulis	4	DSS	6 m	1.75	0.276	0.0139	0.0004	3.08	2020-06-10-01.gDNA
195	Mytilus edulis	5	DSS	6 m	1.98	1.24	0.0742	0.0018	2.39	Lounsberry_T5_Samples_AllSpecies_AllReplicates_3.19.19.gDNA
196	Mytilus edulis	1	ESS	6 m	1.99	20.9	1.7829	0.8701	48.82	Lounsberry_T5_Samples_AllSpecies_AllReplicates_3.19.19.gDNA
197	Mytilus edulis	2	ESS	6 m	2.01	28.6	1.8554	0.4548	24.52	Lounsberry_T5_Samples_AllSpecies_AllReplicates_3.19.19.gDNA
198	Mytilus edulis	3	ESS	6 m	1.98	25.3	1.7943	0.8581	47.97	Lounsberry_T5_Samples_AllSpecies_AllReplicates_3.19.19.gDNA
199	Mytilus edulis	4	ESS	6 m	2.04	13	0.9456	0.5579	58.85	Lounsberry_T5_Samples_AllSpecies_AllReplicates_3.19.19.gDNA
200	Mytilus edulis	5	ESS	6 m	1.98	8.03	0.8065	0.3606	44.72	Lounsberry_T5_Samples_AllSpecies_AllReplicates_3.19.19.gDNA
201	Mytilus edulis	1	D	6 m	2.26	0.998	0.0572	0.0009	1.51	Lounsberry_T5_Samples_AllSpecies_AllReplicates_3.19.19.gDNA
202	Mytilus edulis	2	D	6 m	2.10	3.64	0.2052	0.0215	10.45	Lounsberry_T5_Samples_AllSpecies_AllReplicates_3.19.19.gDNA

Sample ID	Taxa	Replicate	Treatment	Time Interval	A260/A280 Ratio	Yield (µg)	Total Normalized Yield (µg DNA/mg tissue)	nY (µg DNA/ mg tissue)	%R	TapeStation Filename
203	Mytilus edulis	3	D	6 m	2.64	0.393	0.0158	0.0005	3.05	2020-06-10-01.gDNA
204	Mytilus edulis	4	D	6 m	2.14	0.386	0.0186	0.0004	2.32	2020-06-10-01.gDNA
205	Mytilus edulis	5	D	6 m	2.54	0.396	0.0214	0.0007	3.37	2020-06-10-01.gDNA
206	Mytilus edulis	1	E	6 m	1.91	55.3	2.9006	0.7868	27.08	Lounsberry_T5_Samples_AllSpecies_AllReplicates_3.19.19.gDNA
207	Mytilus edulis	2	E	6 m	2.16	7.05	0.3977	0.2319	58.29	Lounsberry_T5_Samples_AllSpecies_AllReplicates_3.19.19.gDNA
208	Mytilus edulis	3	E	6 m	1.94	24.3	1.0569	0.2731	25.9	Lounsberry_T5_Samples_AllSpecies_AllReplicates_3.19.19.gDNA
209	Mytilus edulis	4	E	6 m	2.01	33.7	1.5374	0.7162	46.53	Lounsberry_T5_Samples_AllSpecies_AllReplicates_3.19.19.gDNA
210	Mytilus edulis	5	E	6 m	1.94	24	1.0145	0.5706	56.02	Lounsberry_T5_Samples_AllSpecies_AllReplicates_3.19.19.gDNA
211	Mytilus edulis	1	SS	6 m	2.82	0.172	0.0091	0.0002	1.97	2020-06-10-01.gDNA
212	Mytilus edulis	2	SS	6 m	2.36	0.184	0.0068	0.0002	2.83	2020-06-10-01.gDNA
213	Mytilus edulis	3	SS	6 m	4.62	0.143	0.0074	0.0002	2.69	2020-06-10-01.gDNA
214	Mytilus edulis	4	SS	6 m	-6.59	0.15	0.0069	0.0002	3.51	2020-06-10-01.gDNA
215	Mytilus edulis	5	SS	6 m	2.86	0.243	0.0100	0.0004	3.5	2020-06-10-01.gDNA
216	Mytilus edulis	1	EtOH	6 m	2.00	37.9	1.2670	0.1300	10.27	Lounsberry_T5_Samples_AllSpecies_AllReplicates_3.19.19.gDNA
217	Mytilus edulis	2	EtOH	6 m	2.00	30.7	1.2010	0.2343	19.53	Lounsberry_T5_Samples_AllSpecies_AllReplicates_3.19.19.gDNA
218	Mytilus edulis	3	EtOH	6 m	1.89	45.8	1.6867	0.2081	12.33	Lounsberry_T5_Samples_AllSpecies_AllReplicates_3.19.19.gDNA
219	Mytilus edulis	4	EtOH	6 m	2.05	23.6	0.8044	0.0487	6.07	Lounsberry_T5_Samples_AllSpecies_AllReplicates_3.19.19.gDNA
220	Mytilus edulis	5	EtOH	6 m	2.04	6.88	0.2233	0.0118	5.27	Lounsberry_T5_Samples_AllSpecies_AllReplicates_3.19.19.gDNA
221	Mytilus edulis	1	Fresh	6 m	2.04	81.1	2.9068	0.7240	24.95	T5_Initials&Errors_4-3-2019.gDNA
222	Mytilus edulis	2	Fresh	6 m	2.04	412	12.7950	1.7019	13.28	T5_Initials&Errors_4-3-2019.gDNA
223	Mytilus edulis	3	Fresh	6 m	1.96	36.7	1.2483	0.4184	33.58	2019-07-29-01.gDNA
224	Mytilus edulis	4	Fresh	6 m	2.05	149	4.7003	1.1230	23.91	T5_Initials&Errors_4-3-2019.gDNA
225	Mytilus edulis	5	Fresh	6 m	2.04	230	6.7449	1.7683	26.23	T5_Initials&Errors_4-3-2019.gDNA
226	Mytilus edulis	6	DESS	1 d	2.10	1.67	0.1351	0.0640	47.27	2018-09-27-01.gDNA
227	Mytilus edulis	7	DESS	1 d	2.04	3.43	0.3520	0.1478	41.86	2018-09-27-01.gDNA
228	Mytilus edulis	6	DE	1 d	2.05	6.51	0.5253	0.1727	32.91	2018-09-27-01.gDNA
229	Mytilus edulis	7	DE	1 d	2.03	4.25	0.4382	0.1969	44.9	2018-09-27-01.gDNA
230	Mytilus edulis	6	DSS	1 d	1.97	14.8	1.2111	0.1890	15.56	2018-09-27-01.gDNA
231	Mytilus edulis	7	DSS	1 d	1.93	13.4	1.8508	0.0493	2.66	2018-09-27-01.gDNA
232	Mytilus edulis	6	ESS	1 d	2.06	1.51	0.1043	0.0475	45.48	2018-09-27-01.gDNA
233	Mytilus edulis	7	ESS	1 d	2.05	4.12	0.2934	0.1431	48.75	2018-09-27-01.gDNA
234	Mytilus edulis	6	D	1 d	2.02	9.96	0.6610	0.0115	1.73	2018-09-27-01.gDNA
235	Mytilus edulis	7	D	1 d	1.99	4.61	0.1868	0.0073	3.87	2018-09-27-01.gDNA
236	Mytilus edulis	6	E	1 d	2.05	3.49	0.2106	0.0779	36.9	2018-09-27-01.gDNA
237	Mytilus edulis	7	E	1 d	2.08	4.46	0.2786	0.1343	48.08	2018-09-27-01.gDNA
238	Mytilus edulis	6	SS	1 d	1.98	21.1	1.8107	0.5938	32.82	2018-09-27-01.gDNA
239	Mytilus edulis	7	SS	1 d	1.92	27	1.9168	0.3969	20.68	2018-09-27-01.gDNA
240	Mytilus edulis	6	EtOH	1 d	2.02	61.6	1.2525	0.3192	25.48	2018-09-27-01.gDNA
241	Mytilus edulis	7	EtOH	1 d	1.99	34	0.8066	0.1509	18.69	2018-09-27-01.gDNA
242	Mytilus edulis	6	Fresh	1 d	2.04	39	1.5984	0.4918	30.73	2018-09-27-01.gDNA
243	Mytilus edulis	7	Fresh	1 d	1.99	12.8	0.4571	0.1579	34.56	2018-09-27-01.gDNA
280	Mytilus edulis	6	DESS	3 m	2.03	7.76	0.6121	0.3510	57.39	2018-12-03-01.gDNA
281	Mytilus edulis	7	DESS	3 m	2.04	7.99	0.5000	0.3079	61.59	2018-12-03-01.gDNA
282	Mytilus edulis	6	DE	3 m	1.92	24.7	1.1966	0.5959	49.87	2018-12-03-01.gDNA
283	Mytilus edulis	7	DE	3 m	1.91	38	1.3138	0.5497	41.85	2018-12-03-01.gDNA
284	Mytilus edulis	6	DSS	3 m	2.14	0.715	0.0435	0.0020	4.49	2019-07-29-01.gDNA
285	Mytilus edulis	7	DSS	3 m	2.16	0.593	0.0344	0.0007	2.05	2019-07-29-01.gDNA
286	Mytilus edulis	6	ESS	3 m	1.99	10.2	0.5340	0.2995	55.78	2018-12-03-01.gDNA
287	Mytilus edulis	7	ESS	3 m	1.97	11.6	0.5756	0.3577	61.97	2018-12-03-01.gDNA
288	Mytilus edulis	6	D	3 m	2.13	1.55	0.0615	0.0011	1.77	2020-06-10-01.gDNA
289	Mytilus edulis	7	D	3 m	2.08	2.21	0.1096	0.0009	0.86	2020-06-10-01.gDNA
290	Mytilus edulis	6	E	3 m	1.85	6.16	0.3789	0.2196	57.9	2018-12-03-01.gDNA
291	Mytilus edulis	7	E	3 m	1.87	28.7	1.0829	0.4981	46.19	2018-12-03-01.gDNA
292	Mytilus edulis	6	SS	3 m	2.13	0.613	0.7787	0.0061	0.78	2019-07-29-01.gDNA
293	Mytilus edulis	7	SS	3 m	2.16	0.728	0.0304	0.0005	1.71	2019-07-29-01.gDNA
294	Mytilus edulis	6	EtOH	3 m	2.03	50.6	1.3264	0.2857	21.64	2018-12-03-01.gDNA

Sample ID	Taxa	Replicate	Treatment	Time Interval	A260/A280 Ratio	Yield (µg)	Total Normalized Yield (µg DNA/mg tissue)	nY (µg DNA/ mg tissue)	%R	TapeStation Filename
295	Mytilus edulis	7	EtOH	3 m	2.02	40.6	0.8602	0.1661	19.33	2018-12-03-01.gDNA
296	Mytilus edulis	6	Fresh	3 m	2.03	15.1	0.6113	0.2105	34.45	2018-12-03-01.gDNA
297	Mytilus edulis	7	Fresh	3 m	2.02	18.3	0.6100	0.2500	40.96	2018-12-03-01.gDNA
298	Mytilus edulis	6	DESS	6 m	1.99	40.9	2.5242	0.7344	29.18	Lounsberry_T5_Samples_AllSpecies_AllReplicates_3.19.19.gDNA
299	Mytilus edulis	7	DESS	6 m	2.01	25.6	1.8876	0.7521	40.03	Lounsberry_T5_Samples_AllSpecies_AllReplicates_3.19.19.gDNA
300	Mytilus edulis	6	DE	6 m	2.06	12.7	0.4233	0.1377	32.4	Lounsberry_T5_Samples_AllSpecies_AllReplicates_3.19.19.gDNA
301	Mytilus edulis	7	DE	6 m	1.95	28.3	1.2334	0.6102	49.55	Lounsberry_T5_Samples_AllSpecies_AllReplicates_3.19.19.gDNA
302	Mytilus edulis	6	DSS	6 m	2.17	0.578	0.0226	0.0011	4.69	2020-06-10-01.gDNA
303	Mytilus edulis	7	DSS	6 m	2.09	0.219	0.0149	0.0004	2.71	2020-06-10-01.gDNA
304	Mytilus edulis	6	ESS	6 m	1.98	43.6	2.5921	0.8323	32.11	Lounsberry_T5_Samples_AllSpecies_AllReplicates_3.19.19.gDNA
305	Mytilus edulis	7	ESS	6 m	1.98	52.6	3.0740	0.8065	26.13	Lounsberry_T5_Samples_AllSpecies_AllReplicates_3.19.19.gDNA
306	Mytilus edulis	6	D	6 m	2.11	2.73	0.0896	0.0062	6.93	2020-06-10-01.gDNA
307	Mytilus edulis	7	D	6 m	2.12	0.945	0.0327	0.0008	2.43	2020-06-10-01.gDNA
308	Mytilus edulis	6	E	6 m	1.90	28	1.1763	0.6344	53.69	Lounsberry_T5_Samples_AllSpecies_AllReplicates_3.19.19.gDNA
309	Mytilus edulis	7	E	6 m	1.90	63.9	2.3948	0.4610	19.29	Lounsberry_T5_Samples_AllSpecies_AllReplicates_3.19.19.gDNA
310	Mytilus edulis	6	SS	6 m	1.66	0.269	0.0115	0.0004	3.82	2020-06-10-01.gDNA
311	Mytilus edulis	7	SS	6 m	1.86	0.242	0.0088	0.0003	3.92	2020-06-10-01.gDNA
312	Mytilus edulis	6	EtOH	6 m	2.02	78.5	3.3300	1.1496	34.57	Lounsberry_T5_Samples_AllSpecies_AllReplicates_3.19.19.gDNA
313	Mytilus edulis	7	EtOH	6 m	2.03	40.7	1.3783	0.5351	38.86	Lounsberry_T5_Samples_AllSpecies_AllReplicates_3.19.19.gDNA
314	Mytilus edulis	6	Fresh	6 m	2.06	133	4.7500	0.9714	20.49	T5_Initials&Errors_4-3-2019.gDNA
315	Mytilus edulis	7	Fresh	6 m	2.04	27.8	0.9754	0.3825	39.18	T5_Initials&Errors_4-3-2019.gDNA
316	Faxonius virilis	6	DESS	1 d	2.16	0.766	0.0326	0.0085	25.99	2018-09-26-01.gDNA
317	Faxonius virilis	7	DESS	1 d	2.15	1.39	0.0595	0.0138	23.07	2018-09-26-01.gDNA
318	Faxonius virilis	6	DE	1 d	2.07	1.02	0.0420	0.0189	44.94	2018-09-26-01.gDNA
319	Faxonius virilis	7	DE	1 d	2.12	1.1	0.0431	0.0205	47.34	2018-09-26-01.gDNA
320	Faxonius virilis	6	DSS	1 d	2.15	2.08	0.0536	0.0051	9.45	2018-09-26-01.gDNA
321	Faxonius virilis	7	DSS	1 d	2.17	0.725	0.0263	0.0016	6.05	2018-09-26-01.gDNA
322	Faxonius virilis	6	ESS	1 d	2.20	0.804	0.0230	0.0070	30.57	2018-09-26-01.gDNA
323	Faxonius virilis	7	ESS	1 d	2.19	0.694	0.0195	0.0037	18.85	2018-09-26-01.gDNA
324	Faxonius virilis	6	D	1 d	2.11	2.6	0.0908	0.0145	15.97	2018-09-26-01.gDNA
325	Faxonius virilis	7	D	1 d	2.08	1.75	0.0772	0.0237	30.55	2018-09-26-01.gDNA
326	Faxonius virilis	6	E	1 d	2.21	0.71	0.0323	0.0079	24.49	2018-09-26-01.gDNA
327	Faxonius virilis	7	E	1 d	2.09	0.413	0.0139	0.0028	20.35	2018-09-26-01.gDNA
328	Faxonius virilis	6	SS	1 d	1.95	1.41	0.0536	0.0137	25.54	2018-09-26-01.gDNA
329	Faxonius virilis	7	SS	1 d	2.01	2.75	0.0815	0.0043	5.23	2018-09-26-01.gDNA
330	Faxonius virilis	6	EtOH	1 d	2.11	2.99	0.0573	0.0171	29.76	2018-09-26-01.gDNA
331	Faxonius virilis	7	EtOH	1 d	2.02	1.56	0.0251	0.0078	30.96	2018-09-26-01.gDNA
332	Faxonius virilis	6	Fresh	1 d	2.19	0.596	0.0177	0.0081	45.78	2018-09-26-01.gDNA
333	Faxonius virilis	7	Fresh	1 d	2.17	0.53	0.0185	0.0066	35.93	2018-09-26-01.gDNA
370	Faxonius virilis	6	DESS	3 m	2.29	1.42	0.0725	0.0108	14.97	2018-12-06-01.gDNA
371	Faxonius virilis	7	DESS	3 m	2.27	0.509	0.0207	0.0037	18.03	2018-12-06-01.gDNA
372	Faxonius virilis	6	DE	3 m	2.13	0.705	0.0280	0.0090	32.2	2018-12-06-01.gDNA
373	Faxonius virilis	7	DE	3 m	2.37	0.763	0.0352	0.0148	41.96	2018-12-06-01.gDNA
374	Faxonius virilis	6	DSS	3 m	2.23	0.481	0.0143	0.0007	4.76	2019-07-29-02.gDNA
375	Faxonius virilis	7	DSS	3 m	2.33	0.856	0.0424	0.0018	4.28	2019-07-29-02.gDNA
376	Faxonius virilis	6	ESS	3 m	2.28	0.506	0.0209	0.0027	12.96	2018-12-06-01.gDNA
377	Faxonius virilis	7	ESS	3 m	2.24	0.554	0.0212	0.0049	22.98	2018-12-06-01.gDNA
378	Faxonius virilis	6	D	3 m	3.99	0.53	0.0187	0.0002	1.15	2019-07-29-02.gDNA
379	Faxonius virilis	7	D	3 m	2.14	1.03	0.0361	0.0006	1.77	2019-07-29-02.gDNA
380	Faxonius virilis	6	E	3 m	2.55	1.23	0.0454	0.0231	50.8	2018-12-06-01.gDNA
381	Faxonius virilis	7	E	3 m	1.75	0.841	0.0326	0.0152	46.73	2018-12-06-01.gDNA
382	Faxonius virilis	6	SS	3 m	-11.76	0.243	0.0084	0.0003	3.76	2019-07-29-02.gDNA
383	Faxonius virilis	7	SS	3 m	1.97	1.72	0.0520	0.0008	1.57	2019-07-29-03.gDNA
384	Faxonius virilis	6	EtOH	3 m	2.10	3.54	0.0654	0.0156	23.89	2018-12-06-01.gDNA
385	Faxonius virilis	7	EtOH	3 m	2.11	2.95	0.0664	0.0277	41.58	2018-12-06-01.gDNA
386	Faxonius virilis	6	Fresh	3 m	1.97	2.85	0.0819	0.0359	43.74	2018-12-06-01.gDNA

Sample ID	Taxa	Replicate	Treatment	Time Interval	A260/A280 Ratio	Yield (µg)	Total Normalized Yield (µg DNA/mg tissue)	nY (µg DNA/ mg tissue)	%R	TapeStation Filename
387	Faxonius virilis	7	Fresh	3 m	2.19	1.03	0.0337	0.0137	40.65	2018-12-06-01.gDNA
388	Faxonius virilis	6	DESS	6 m	2.24	4.83	0.1633	0.0818	50.16	Lounsberry_T5_Samples_AllSpecies_AllReplicates_3.19.19.gDNA
389	Faxonius virilis	7	DESS	6 m	2.27	8.64	0.3033	0.1664	54.81	T5_Initials&Errors_4-3-2019_p2.gDNA
390	Faxonius virilis	6	DE	6 m	2.07	6.87	0.1919	0.0405	21.11	Lounsberry_T5_Samples_AllSpecies_AllReplicates_3.19.19.gDNA
391	Faxonius virilis	7	DE	6 m	2.13	12.3	0.3110	0.1788	57.25	Lounsberry_T5_Samples_AllSpecies_AllReplicates_3.19.19.gDNA
392	Faxonius virilis	6	DSS	6 m	2.20	1.88	0.0230	0.0010	4.4	Lounsberry_T5_Samples_AllSpecies_AllReplicates_3.19.19.gDNA
393	Faxonius virilis	7	DSS	6 m	2.19	0.317	0.0106	0.0006	5.48	Lounsberry_T5_Samples_AllSpecies_AllReplicates_3.19.19.gDNA
394	Faxonius virilis	6	ESS	6 m	2.26	4.47	0.1396	0.0556	39.72	Lounsberry_T5_Samples_AllSpecies_AllReplicates_3.19.19.gDNA
395	Faxonius virilis	7	ESS	6 m	2.15	3.38	0.0949	0.0497	52.24	Lounsberry_T5_Samples_AllSpecies_AllReplicates_3.19.19.gDNA
396	Faxonius virilis	6	D	6 m	2.00	0.316	0.0040	0.0003	7.42	Lounsberry_T5_Samples_AllSpecies_AllReplicates_3.19.19.gDNA
397	Faxonius virilis	7	D	6 m	-4.26	0.215	0.0063	0.0001	1.7	2020-06-10-01.gDNA
398	Faxonius virilis	6	E	6 m	1.98	6.92	0.0928	0.0307	33.14	Lounsberry_T5_Samples_AllSpecies_AllReplicates_3.19.19.gDNA
399	Faxonius virilis	7	E	6 m	2.18	9.73	0.1696	0.0891	52.57	Lounsberry_T5_Samples_AllSpecies_AllReplicates_3.19.19.gDNA
400	Faxonius virilis	6	SS	6 m	0.67	0.234	0.0054	0.0002	4.31	2020-06-10-01.gDNA
401	Faxonius virilis	7	SS	6 m	0.94	0.185	0.0067	0.0003	5.07	2020-06-10-01.gDNA
402	Faxonius virilis	6	EtOH	6 m	2.08	26.4	0.8938	0.3961	44.3	Lounsberry_T5_Samples_AllSpecies_AllReplicates_3.19.19.gDNA
403	Faxonius virilis	7	EtOH	6 m	2.09	29.7	1.0505	0.4421	41.99	Lounsberry_T5_Samples_AllSpecies_AllReplicates_3.19.19.gDNA
404	Faxonius virilis	6	Fresh	6 m	2.00	9.88	0.3304	0.1552	46.95	T5_Initials&Errors_4-3-2019.gDNA
405	Faxonius virilis	7	Fresh	6 m	2.09	4.59	0.1417	0.0247	17.44	T5_Initials&Errors_4-3-2019_p2.gDNA
451	Faxonius virilis	1	DESS	1 d	2.20	0.749	0.0330	0.0103	31.1	2018-09-26-01.gDNA
452	Faxonius virilis	2	DESS	1 d	2.16	0.514	0.0181	0.0036	19.85	2018-09-26-01.gDNA
453	Faxonius virilis	3	DESS	1 d	2.12	0.718	0.0228	0.0067	29.47	2018-09-26-01.gDNA
454	Faxonius virilis	4	DESS	1 d	2.18	0.657	0.0219	0.0063	28.65	2018-09-26-01.gDNA
455	Faxonius virilis	5	DESS	1 d	2.17	0.748	0.0186	0.0051	27.45	2018-09-26-01.gDNA
456	Faxonius virilis	1	DE	1 d	2.27	1.04	0.0454	0.0168	37.01	2018-09-26-01.gDNA
457	Faxonius virilis	2	DE	1 d	2.07	1.28	0.0435	0.0157	36.01	2018-09-26-01.gDNA
458	Faxonius virilis	3	DE	1 d	2.29	0.688	0.0168	0.0063	37.65	2018-09-26-01.gDNA
459	Faxonius virilis	4	DE	1 d	2.15	0.877	0.0255	0.0100	39.49	2018-09-26-01.gDNA
460	Faxonius virilis	5	DE	1 d	2.20	0.782	0.0280	0.0116	41.44	2018-09-26-01.gDNA
461	Faxonius virilis	1	DSS	1 d	2.09	5.05	0.2222	0.1201	54.08	2018-09-26-01.gDNA
462	Faxonius virilis	2	DSS	1 d	2.00	38.5	1.4551	0.2695	18.52	2018-09-26-01.gDNA
463	Faxonius virilis	3	DSS	1 d	2.06	7.46	0.2192	0.0673	30.65	2018-09-26-01.gDNA
464	Faxonius virilis	4	DSS	1 d	2.13	4.18	0.1644	0.0775	47.09	2018-09-26-01.gDNA
465	Faxonius virilis	5	DSS	1 d	2.01	3.04	0.0987	0.0126	12.74	2018-09-26-01.gDNA
466	Faxonius virilis	1	ESS	1 d	2.17	0.724	0.0253	0.0058	23.12	2018-09-26-01.gDNA
467	Faxonius virilis	2	ESS	1 d	2.12	2.5	0.1319	0.0565	42.77	2018-09-26-01.gDNA
468	Faxonius virilis	3	ESS	1 d	2.15	0.64	0.0295	0.0096	32.44	2018-09-26-01.gDNA
469	Faxonius virilis	4	ESS	1 d	2.02	0.491	0.0174	0.0058	33.58	2018-09-26-01.gDNA
470	Faxonius virilis	5	ESS	1 d	2.32	0.376	0.0155	0.0031	19.86	2018-09-26-01.gDNA
471	Faxonius virilis	1	D	1 d	1.96	7.2	0.2826	0.0356	12.61	2018-09-26-01.gDNA
472	Faxonius virilis	2	D	1 d	1.98	2.19	0.1267	0.0201	15.85	2018-09-26-01.gDNA
473	Faxonius virilis	3	D	1 d	1.94	8.12	0.3577	0.0555	15.57	2018-09-26-01.gDNA
474	Faxonius virilis	4	D	1 d	2.08	0.808	0.0267	0.0025	9.3	2018-09-26-01.gDNA
475	Faxonius virilis	5	D	1 d	2.04	1.27	0.0637	0.0148	23.12	2018-09-26-01.gDNA
476	Faxonius virilis	1	E	1 d	2.25	0.979	0.0300	0.0147	49.11	2018-09-26-01.gDNA
477	Faxonius virilis	2	E	1 d	2.20	0.588	0.0199	0.0062	31.5	2018-09-26-01.gDNA
478	Faxonius virilis	3	E	1 d	2.37	0.578	0.0226	0.0097	42.82	2018-09-26-01.gDNA
479	Faxonius virilis	4	E	1 d	2.29	1.07	0.0431	0.0207	48.1	2018-09-26-01.gDNA
480	Faxonius virilis	5	E	1 d	2.15	0.596	0.0196	0.0083	42.33	2018-09-26-01.gDNA
481	Faxonius virilis	1	SS	1 d	2.04	4.34	0.2216	0.1159	52.3	2018-09-26-01.gDNA
482	Faxonius virilis	2	SS	1 d	1.92	2.82	0.0885	0.0051	5.74	2018-09-26-01.gDNA
483	Faxonius virilis	3	SS	1 d	1.96	4.21	0.1254	0.0825	65.74	2018-09-26-01.gDNA
484	Faxonius virilis	4	SS	1 d	2.12	1.93	0.0753	0.0358	47.65	2018-09-26-01.gDNA
485	Faxonius virilis	5	SS	1 d	1.99	4.5	0.1714	0.0506	29.6	2018-09-26-01.gDNA
486	Faxonius virilis	1	EtOH	1 d	2.10	2.73	0.0832	0.0399	47.89	2018-09-26-01.gDNA
487	Faxonius virilis	2	EtOH	1 d	2.08	4.91	0.0883	0.0236	26.74	2018-09-26-01.gDNA

Sample ID	Taxa	Replicate	Treatment	Time Interval	A260/A280 Ratio	Yield (µg)	Total Normalized Yield (µg DNA/mg tissue)	nY (µg DNA/ mg tissue)	%R	TapeStation Filename
488	Faxonius virilis	3	EtOH	1 d	2.14	0.944	0.0176	0.0061	34.86	2018-09-26-01.gDNA
489	Faxonius virilis	4	EtOH	1 d	2.22	0.96	0.0183	0.0082	44.83	2018-09-26-01.gDNA
490	Faxonius virilis	5	EtOH	1 d	2.21	1.72	0.0349	0.0142	40.86	2018-09-26-01.gDNA
491	Faxonius virilis	1	Fresh	1 d	2.06	3.16	0.1141	0.0437	38.14	2018-09-26-01.gDNA
492	Faxonius virilis	2	Fresh	1 d	2.08	0.656	0.0207	0.0074	35.66	2018-09-26-01.gDNA
493	Faxonius virilis	3	Fresh	1 d	2.14	0.656	0.0239	0.0069	28.58	2018-09-26-01.gDNA
494	Faxonius virilis	4	Fresh	1 d	2.13	0.966	0.0345	0.0139	40.36	2018-09-26-01.gDNA
495	Faxonius virilis	5	Fresh	1 d	2.11	1.19	0.0406	0.0222	54.54	2018-09-26-01.gDNA
586	Faxonius virilis	1	DESS	3 m	2.16	1.17	0.0409	0.0204	49.87	2018-12-06-01.gDNA
587	Faxonius virilis	2	DESS	3 m	2.19	0.739	0.0322	0.0099	30.77	2018-12-06-01.gDNA
588	Faxonius virilis	3	DESS	3 m	2.14	0.603	0.0247	0.0025	10.05	2018-12-06-01.gDNA
589	Faxonius virilis	4	DESS	3 m	2.13	0.49	0.0221	0.0035	15.73	2018-12-06-01.gDNA
590	Faxonius virilis	5	DESS	3 m	2.19	1.04	0.0351	0.0065	18.54	2018-12-06-01.gDNA
591	Faxonius virilis	1	DE	3 m	2.15	0.613	0.0172	0.0015	8.86	2018-12-06-01.gDNA
592	Faxonius virilis	2	DE	3 m	2.17	0.782	0.0287	0.0094	32.78	2018-12-06-01.gDNA
593	Faxonius virilis	3	DE	3 m	2.02	4.41	0.1965	0.1154	58.78	2018-12-06-01.gDNA
594	Faxonius virilis	4	DE	3 m	2.05	0.62	0.0265	0.0087	32.9	2018-12-06-01.gDNA
595	Faxonius virilis	5	DE	3 m	1.95	3.58	0.1462	0.0429	29.37	2018-12-06-01.gDNA
596	Faxonius virilis	1	DSS	3 m	2.24	0.302	0.0091	0.0001	1.36	2019-07-29-02.gDNA
597	Faxonius virilis	2	DSS	3 m	2.14	0.702	0.0202	0.0002	1.15	2019-07-29-02.gDNA
598	Faxonius virilis	3	DSS	3 m	1.32	0.253	0.0109	0.0008	7.36	2019-07-29-02.gDNA
599	Faxonius virilis	4	DSS	3 m	2.26	0.221	0.0099	0.0004	4.19	2019-07-29-02.gDNA
600	Faxonius virilis	5	DSS	3 m	2.92	0.115	0.0039	0.0001	3.86	2019-07-29-02.gDNA
601	Faxonius virilis	1	ESS	3 m	2.05	1.51	0.0658	0.0369	55.99	2018-12-06-01.gDNA
602	Faxonius virilis	2	ESS	3 m	2.15	0.904	0.0397	0.0185	46.48	2018-12-06-01.gDNA
603	Faxonius virilis	3	ESS	3 m	2.19	1.17	0.0637	0.0054	8.57	2018-12-18-01.gDNA
604	Faxonius virilis	4	ESS	3 m	2.04	3.1	0.1536	0.0912	59.29	2018-12-06-01.gDNA
605	Faxonius virilis	5	ESS	3 m	2.17	0.784	0.0254	0.0074	29.02	2018-12-06-01.gDNA
606	Faxonius virilis	1	D	3 m	1.71	0.208	0.0075	0.0002	2.14	2019-07-29-02.gDNA
607	Faxonius virilis	2	D	3 m	2.06	0.332	0.0128	0.0001	1.01	2019-07-29-02.gDNA
608	Faxonius virilis	3	D	3 m	2.09	0.38	0.0210	0.0004	1.97	2019-07-29-02.gDNA
609	Faxonius virilis	4	D	3 m	2.12	0.594	0.0258	0.0007	2.57	2019-07-29-02.gDNA
610	Faxonius virilis	5	D	3 m	2.29	0.266	0.0139	0.0001	0.99	2019-07-29-02.gDNA
611	Faxonius virilis	1	E	3 m	2.05	0.527	0.0208	0.0096	45.89	2018-12-06-01.gDNA
612	Faxonius virilis	2	E	3 m	1.99	0.676	0.0258	0.0139	53.85	2018-12-06-01.gDNA
613	Faxonius virilis	3	E	3 m	1.97	1.2	0.0637	0.0379	59.17	2018-12-06-01.gDNA
614	Faxonius virilis	4	E	3 m	2.00	1.47	0.0789	0.0442	55.91	2018-12-06-01.gDNA
615	Faxonius virilis	5	E	3 m	2.02	4.2	0.1415	0.0708	49.85	2018-12-06-01.gDNA
616	Faxonius virilis	1	SS	3 m	2.91	0.194	0.0064	0.0002	2.71	2019-07-29-03.gDNA
617	Faxonius virilis	2	SS	3 m	2.15	0.207	0.0058	0.0002	3.92	2019-07-29-03.gDNA
618	Faxonius virilis	3	SS	3 m	2.48	0.959	0.0350	0.0016	4.68	2019-07-29-03.gDNA
619	Faxonius virilis	4	SS	3 m	1.97	0.967	0.0270	0.0003	0.97	2019-07-29-03.gDNA
620	Faxonius virilis	5	SS	3 m	3.21	0.342	0.0108	0.0004	3.25	2019-07-29-03.gDNA
621	Faxonius virilis	1	EtOH	3 m	2.13	2.28	0.0082	0.0025	30.24	2018-12-06-01.gDNA
622	Faxonius virilis	2	EtOH	3 m	2.11	2.2	0.0462	0.0214	46.32	2018-12-06-01.gDNA
623	Faxonius virilis	3	EtOH	3 m	2.09	2.9	0.0550	0.0260	47.24	2018-12-06-01.gDNA
624	Faxonius virilis	4	EtOH	3 m	1.99	5.54	0.1621	0.0819	50.54	2018-12-06-01.gDNA
625	Faxonius virilis	5	EtOH	3 m	2.08	5.05	0.0860	0.0267	31.12	2018-12-06-01.gDNA
626	Faxonius virilis	1	Fresh	3 m	2.16	0.898	0.0283	0.0100	35.26	2018-12-06-01.gDNA
627	Faxonius virilis	2	Fresh	3 m	2.13	1.75	0.0651	0.0249	38.43	2018-12-06-01.gDNA
628	Faxonius virilis	3	Fresh	3 m	1.88	3.24	0.1200	0.0137	11.44	2018-12-06-01.gDNA
629	Faxonius virilis	4	Fresh	3 m	2.01	3.46	0.1134	0.0521	45.8	2018-12-06-01.gDNA
630	Faxonius virilis	5	Fresh	3 m	2.19	0.982	0.0294	0.0094	32.03	2018-12-06-01.gDNA
631	Faxonius virilis	1	DESS	6 m	2.13	3.37	0.1107	0.0532	48.18	Lounsberry_T5_Samples_AllSpecies_AllReplicates_3.19.19.gDNA
632	Faxonius virilis	2	DESS	6 m	2.14	4.72	0.2757	0.1484	53.85	Lounsberry_T5_Samples_AllSpecies_AllReplicates_3.19.19.gDNA
633	Faxonius virilis	3	DESS	6 m	2.16	2.16	0.0748	0.0357	47.97	Lounsberry_T5_Samples_AllSpecies_AllReplicates_3.19.19.gDNA

Sample ID	Taxa	Replicate	Treatment	Time Interval	A260/A280 Ratio	Yield (µg)	Total Normalized Yield (µg DNA/mg tissue)	nY (µg DNA/ mg tissue)	%R	TapeStation Filename
634	Faxonius virilis	4	DESS	6 m	1.89	2.01	0.0802	0.0407	51.03	Lounsberry_T5_Samples_AllSpecies_AllReplicates_3.19.19.gDNA
635	Faxonius virilis	5	DESS	6 m	2.21	2.21	0.0841	0.0419	49.87	Lounsberry_T5_Samples_AllSpecies_AllReplicates_3.19.19.gDNA
636	Faxonius virilis	1	DE	6 m	2.10	2.46	0.0673	0.0192	28.54	Lounsberry_T5_Samples_AllSpecies_AllReplicates_3.19.19.gDNA
637	Faxonius virilis	2	DE	6 m	1.67	3.08	0.1481	0.0798	53.8	Lounsberry_T5_Samples_AllSpecies_AllReplicates_3.19.19.gDNA
638	Faxonius virilis	3	DE	6 m	2.15	8.53	0.2264	0.1295	57.22	Lounsberry_T5_Samples_AllSpecies_AllReplicates_3.19.19.gDNA
639	Faxonius virilis	4	DE	6 m	1.91	8.09	0.2670	0.1567	58.68	Lounsberry_T5_Samples_AllSpecies_AllReplicates_3.19.19.gDNA
640	Faxonius virilis	5	DE	6 m	1.98	5	0.2088	0.1144	54.83	Lounsberry_T5_Samples_AllSpecies_AllReplicates_3.19.19.gDNA
641	Faxonius virilis	1	DSS	6 m	2.18	0.177	0.0051	0.0004	6.93	2020-06-10-01.gDNA
642	Faxonius virilis	2	DSS	6 m	2.08	0.227	0.0097	0.0003	2.59	2020-06-10-01.gDNA
643	Faxonius virilis	3	DSS	6 m	1.57	0.255	0.0094	0.0002	2.02	2020-06-10-01.gDNA
644	Faxonius virilis	4	DSS	6 m	2.11	0.356	0.0089	0.0005	5.17	2020-06-10-01.gDNA
645	Faxonius virilis	5	DSS	6 m	2.12	0.43	0.0121	0.0004	3.69	2020-06-10-01.gDNA
646	Faxonius virilis	1	ESS	6 m	2.08	4.91	0.1536	0.0804	52.34	Lounsberry_T5_Samples_AllSpecies_AllReplicates_3.19.19.gDNA
647	Faxonius virilis	2	ESS	6 m	1.86	7.74	0.2702	0.0960	35.58	Lounsberry_T5_Samples_AllSpecies_AllReplicates_3.19.19.gDNA
648	Faxonius virilis	3	ESS	6 m	2.17	2.83	0.0754	0.0402	53.44	Lounsberry_T5_Samples_AllSpecies_AllReplicates_3.19.19.gDNA
649	Faxonius virilis	4	ESS	6 m	2.18	0.865	0.0328	0.0077	23.31	Lounsberry_T5_Samples_AllSpecies_AllReplicates_3.19.19.gDNA
650	Faxonius virilis	5	ESS	6 m	2.12	2.98	0.1163	0.0573	49.5	Lounsberry_T5_Samples_AllSpecies_AllReplicates_3.19.19.gDNA
651	Faxonius virilis	1	D	6 m	5.94	0.225	0.0063	0.0002	2.74	2020-06-10-01.gDNA
652	Faxonius virilis	2	D	6 m	3.08	0.192	0.0095	0.0003	2.77	2020-06-10-01.gDNA
653	Faxonius virilis	3	D	6 m	1.76	0.268	0.0098	0.0002	2.09	2020-06-10-01.gDNA
654	Faxonius virilis	4	D	6 m	2.08	0.312	0.0128	0.0004	2.87	2020-06-10-01.gDNA
655	Faxonius virilis	5	D	6 m	1.67	0.233	0.0088	0.0002	2.32	2020-06-10-01.gDNA
656	Faxonius virilis	1	E	6 m	1.97	12.7	0.3234	0.1780	55.3	Lounsberry_T5_Samples_AllSpecies_AllReplicates_3.19.19.gDNA
657	Faxonius virilis	2	E	6 m	1.78	6.55	0.4081	0.2523	61.8	Lounsberry_T5_Samples_AllSpecies_AllReplicates_3.19.19.gDNA
658	Faxonius virilis	3	E	6 m	2.05	14.1	0.4117	0.2438	59.19	Lounsberry_T5_Samples_AllSpecies_AllReplicates_3.19.19.gDNA
659	Faxonius virilis	4	E	6 m	2.35	19.3	0.7000	0.3989	57.04	Lounsberry_T5_Samples_AllSpecies_AllReplicates_3.19.19.gDNA
660	Faxonius virilis	5	E	6 m	1.99	8.72	0.2655	0.1632	61.4	Lounsberry_T5_Samples_AllSpecies_AllReplicates_3.19.19.gDNA
661	Faxonius virilis	1	SS	6 m	18.98	0.192	0.0068	0.0002	3.42	2020-06-10-01.gDNA
662	Faxonius virilis	2	SS	6 m	-5.17	0.164	0.0057	0.0003	5.12	2020-06-10-01.gDNA
663	Faxonius virilis	3	SS	6 m	2.30	0.148	0.0007	0.0000	3.93	2020-06-10-01.gDNA
664	Faxonius virilis	4	SS	6 m	1.45	0.176	0.0052	0.0002	3.06	2020-06-10-01.gDNA
665	Faxonius virilis	5	SS	6 m	0.78	0.281	0.0079	0.0004	4.77	2020-06-10-01.gDNA
666	Faxonius virilis	1	EtOH	6 m	2.07	50.2	1.4378	0.5757	40.14	Lounsberry_T5_Samples_AllSpecies_AllReplicates_3.19.19.gDNA
667	Faxonius virilis	2	EtOH	6 m	2.00	8.63	0.2112	0.1021	48.33	Lounsberry_T5_Samples_AllSpecies_AllReplicates_3.19.19.gDNA
668	Faxonius virilis	3	EtOH	6 m	2.07	25.2	0.7751	0.2824	36.37	Lounsberry_T5_Samples_AllSpecies_AllReplicates_3.19.19.gDNA
669	Faxonius virilis	4	EtOH	6 m	2.14	8.33	0.1989	0.1067	53.7	Lounsberry_T5_Samples_AllSpecies_AllReplicates_3.19.19.gDNA
670	Faxonius virilis	5	EtOH	6 m	2.18	5.97	0.1390	0.0626	45.06	Lounsberry_T5_Samples_AllSpecies_AllReplicates_3.19.19.gDNA
671	Faxonius virilis	1	Fresh	6 m	2.01	26.4	0.8381	0.3746	44.7	T5_Initials&Errors_4-3-2019.gDNA
672	Faxonius virilis	2	Fresh	6 m	2.06	3.71	0.1253	0.0622	49.51	T5_Initials&Errors_4-3-2019.gDNA
673	Faxonius virilis	3	Fresh	6 m	2.02	11	0.3806	0.1529	40.01	T5_Initials&Errors_4-3-2019.gDNA
674	Faxonius virilis	4	Fresh	6 m	2.20	0.224	0.0072	0.0016	22.27	T5_Initials&Errors_4-3-2019.gDNA
675	Faxonius virilis	5	Fresh	6 m	2.01	3.33	0.0920	0.0475	51.65	T5_Initials&Errors_4-3-2019.gDNA
676	Alitta virens	1	DESS	1 d	2.13	0.691	0.0261	0.0088	33.56	Mussel and Seaworm T1 Data.gDNA
677	Alitta virens	2	DESS	1 d	2.12	1.8	0.0867	0.0354	40.77	Mussel and Seaworm T1 Data.gDNA
678	Alitta virens	3	DESS	1 d	1.93	0.901	0.0423	0.0129	30.41	Mussel and Seaworm T1 Data.gDNA
679	Alitta virens	4	DESS	1 d	1.85	1.31	0.0430	0.0187	43.75	Mussel and Seaworm T1 Data.gDNA
680	Alitta virens	5	DESS	1 d	2.10	3.15	1.2631	0.7178	56.91	Mussel and Seaworm T1 Data.gDNA
681	Alitta virens	1	DE	1 d	2.14	3.05	0.1406	0.0687	48.98	Mussel and Seaworm T1 Data.gDNA
682	Alitta virens	2	DE	1 d	2.15	2.06	0.0670	0.0255	38.01	Mussel and Seaworm T1 Data.gDNA
683	Alitta virens	3	DE	1 d	2.16	1.18	0.0617	0.0241	39.19	Mussel and Seaworm T1 Data.gDNA
684	Alitta virens	4	DE	1 d	1.83	2.26	0.0734	0.0317	43.08	Mussel and Seaworm T1 Data.gDNA
685	Alitta virens	5	DE	1 d	2.11	3.42	0.1603	0.0759	47.29	Mussel and Seaworm T1 Data.gDNA
686	Alitta virens	1	DSS	1 d	1.87	1.14	0.0347	0.0025	7.12	Mussel and Seaworm T1 Data.gDNA
687	Alitta virens	2	DSS	1 d	2.11	1.85	0.0573	0.0040	6.97	Mussel and Seaworm T1 Data.gDNA
688	Alitta virens	3	DSS	1 d	2.03	0.859	0.0345	0.0026	7.46	Mussel and Seaworm T1 Data.gDNA
689	Alitta virens	4	DSS	1 d	1.95	1.07	0.0341	0.0021	6.31	Mussel and Seaworm T1 Data.gDNA

Sample ID	Taxa	Replicate	Treatment	Time Interval	A260/A280 Ratio	Yield (µg)	Total Normalized Yield (µg DNA/mg tissue)	nY (µg DNA/ mg tissue)	%R	TapeStation Filename
690	Alitta virens	5	DSS	1 d	2.05	1.1	0.0565	0.0048	8.52	Mussel and Seaworm T1 Data.gDNA
691	Alitta virens	1	ESS	1 d	2.06	2.57	0.0950	0.0532	55.88	Mussel and Seaworm T1 Data.gDNA
692	Alitta virens	2	ESS	1 d	2.07	3.68	0.1235	0.0715	57.97	Mussel and Seaworm T1 Data.gDNA
693	Alitta virens	3	ESS	1 d	1.94	2.07	0.0847	0.0404	47.72	Mussel and Seaworm T1 Data.gDNA
694	Alitta virens	4	ESS	1 d	1.95	2.04	0.0792	0.0442	55.9	Mussel and Seaworm T1 Data.gDNA
695	Alitta virens	5	ESS	1 d	2.05	5.44	0.2375	0.1454	61.18	Mussel and Seaworm T1 Data.gDNA
696	Alitta virens	1	D	1 d	2.19	0.812	0.0353	0.0018	5.12	Mussel and Seaworm T1 Data.gDNA
697	Alitta virens	2	D	1 d	2.14	2.54	0.1395	0.0048	3.44	Mussel and Seaworm T1 Data.gDNA
698	Alitta virens	3	D	1 d	2.18	1.16	0.0545	0.0052	9.51	Mussel and Seaworm T1 Data.gDNA
699	Alitta virens	4	D	1 d	2.08	0.97	0.0423	0.0008	1.9	Mussel and Seaworm T1 Data.gDNA
700	Alitta virens	5	D	1 d	2.16	1.35	0.0555	0.0017	3.01	Mussel and Seaworm T1 Data.gDNA
701	Alitta virens	1	E	1 d	2.01	6.16	0.2730	0.1130	41.31	Mussel and Seaworm T1 Data.gDNA
702	Alitta virens	2	E	1 d	2.13	5.38	0.1680	0.0884	52.67	Mussel and Seaworm T1 Data.gDNA
703	Alitta virens	3	E	1 d	2.10	5	0.2263	0.0950	42.07	Mussel and Seaworm T1 Data.gDNA
704	Alitta virens	4	E	1 d	2.12	2.56	0.0990	0.0445	44.9	Mussel and Seaworm T1 Data.gDNA
705	Alitta virens	5	E	1 d	2.10	5.22	0.2203	0.0992	45.04	Mussel and Seaworm T1 Data.gDNA
706	Alitta virens	1	SS	1 d	1.88	1.92	0.0561	0.0092	16.46	Mussel and Seaworm T1 Data.gDNA
707	Alitta virens	2	SS	1 d	2.09	2.12	0.0774	0.0172	22.21	Mussel and Seaworm T1 Data.gDNA
708	Alitta virens	3	SS	1 d	2.04	1.87	0.0716	0.0256	35.79	Mussel and Seaworm T1 Data.gDNA
709	Alitta virens	4	SS	1 d	2.01	0.936	0.0334	0.0039	11.69	Mussel and Seaworm T1 Data.gDNA
710	Alitta virens	5	SS	1 d	2.10	1.96	0.0557	0.0111	19.79	Mussel and Seaworm T1 Data.gDNA
711	Alitta virens	1	EtOH	1 d	2.02	3.77	0.0992	0.0242	24.38	Mussel and Seaworm T1 Data.gDNA
712	Alitta virens	2	EtOH	1 d	1.95	9.21	0.2346	0.0780	33.25	Mussel and Seaworm T1 Data.gDNA
713	Alitta virens	3	EtOH	1 d	2.07	7.33	0.1973	0.0589	29.86	Mussel and Seaworm T1 Data.gDNA
714	Alitta virens	4	EtOH	1 d	2.02	2.36	0.0687	0.0189	27.52	Mussel and Seaworm T1 Data.gDNA
715	Alitta virens	5	EtOH	1 d	2.00	5.12	0.1369	0.0345	25.31	Mussel and Seaworm T1 Data.gDNA
716	Alitta virens	1	Fresh	1 d	2.06	7.68	0.2037	0.0459	22.57	Mussel and Seaworm T1 Data.gDNA
717	Alitta virens	2	Fresh	1 d	2.03	11.6	0.4203	0.1000	23.75	Mussel and Seaworm T1 Data.gDNA
718	Alitta virens	3	Fresh	1 d	2.11	5.7	0.1691	0.0902	53.37	Mussel and Seaworm T1 Data.gDNA
719	Alitta virens	4	Fresh	1 d	1.98	10.6	0.2953	0.1604	54.56	Mussel and Seaworm T1 Data.gDNA
720	Alitta virens	5	Fresh	1 d	2.07	8.16	0.2702	0.1404	51.95	Mussel and Seaworm T1 Data.gDNA
811	Alitta virens	1	DESS	3 m	1.81	6.45	0.2246	0.0359	15.9	2019-07-29-03.gDNA
812	Alitta virens	2	DESS	3 m	1.78	3.93	0.1362	0.0065	4.76	2019-07-29-03.gDNA
813	Alitta virens	3	DESS	3 m	1.74	1.26	0.0438	0.0017	3.96	2019-07-29-03.gDNA
814	Alitta virens	4	DESS	3 m	1.83	7	0.2500	0.0235	9.4	2019-07-29-03.gDNA
815	Alitta virens	5	DESS	3 m	1.80	2.63	0.1229	0.0110	8.96	2019-07-29-03.gDNA
816	Alitta virens	1	DE	3 m	1.87	0.13	0.0051	0.0001	2.17	2019-07-29-04.gDNA
817	Alitta virens	2	DE	3 m	1.61	0.199	0.0094	0.0003	3.07	2019-07-29-04.gDNA
818	Alitta virens	3	DE	3 m	2.00	1.26	0.0506	0.0003	0.54	2019-07-29-04.gDNA
819	Alitta virens	4	DE	3 m	1.84	0.169	0.0063	0.0002	3.34	2019-07-29-04.gDNA
820	Alitta virens	5	DE	3 m	1.98	0.318	0.0136	0.0003	2.09	2019-07-29-04.gDNA
821	Alitta virens	1	DSS	3 m	1.62	0.123	0.0047	0.0001	2.58	2020-06-10-01.gDNA
822	Alitta virens	2	DSS	3 m	1.25	0.125	0.0053	0.0001	2.09	2020-06-10-01.gDNA
823	Alitta virens	3	DSS	3 m	1.19	0.115	0.0044	0.0001	1.93	2020-06-10-01.gDNA
824	Alitta virens	4	DSS	3 m	1.15	0.153	0.0054	0.0001	2.25	2020-06-10-01.gDNA
825	Alitta virens	5	DSS	3 m	1.65	0.144	0.0055	0.0002	3.45	2020-06-10-01.gDNA
826	Alitta virens	1	ESS	3 m	1.79	6.25	0.1919	0.0203	10.61	2019-07-29-04.gDNA
827	Alitta virens	2	ESS	3 m	1.87	3.94	0.1616	0.0053	3.3	2019-07-29-04.gDNA
828	Alitta virens	3	ESS	3 m	1.74	2.95	0.1039	0.0134	12.94	2019-07-29-04.gDNA
829	Alitta virens	4	ESS	3 m	1.72	4.05	0.1221	0.0013	1.09	2019-07-29-04.gDNA
830	Alitta virens	5	ESS	3 m	1.81	3.7	0.1463	0.0207	14.13	2019-07-29-05.gDNA
831	Alitta virens	1	D	3 m	2.21	0.137	0.0052	0.0001	2.46	2020-06-10-01.gDNA
832	Alitta virens	2	D	3 m	2.15	0.16	0.0073	0.0002	2.59	2020-06-10-01.gDNA
833	Alitta virens	3	D	3 m	2.62	0.148	0.0048	0.0001	1.75	2020-06-10-01.gDNA
834	Alitta virens	4	D	3 m	1.44	0.143	0.0063	0.0001	2.3	2020-06-10-01.gDNA
835	Alitta virens	5	D	3 m	1.43	0.125	0.0051	0.0002	3.57	2020-06-10-01.gDNA

Sample ID	Taxa	Replicate	Treatment	Time Interval	A260/A280 Ratio	Yield (µg)	Total Normalized Yield (µg DNA/mg tissue)	nY (µg DNA/ mg tissue)	%R	TapeStation Filename
836	Alitta virens	1	E	3 m	1.84	2.04	0.0785	0.0001	0.16	2019-07-29-05.gDNA
837	Alitta virens	2	E	3 m	1.77	2.49	0.1130	0.0005	0.43	2019-07-29-05.gDNA
838	Alitta virens	3	E	3 m	1.48	0.186	0.0060	0.0001	2.16	2019-07-29-05.gDNA
839	Alitta virens	4	E	3 m	1.86	4.81	0.1918	0.0004	0.21	2019-07-29-05.gDNA
840	Alitta virens	5	E	3 m	1.67	0.203	0.0078	0.0001	1.85	2019-07-29-05.gDNA
841	Alitta virens	1	SS	3 m	1.42	0.121	0.0043	0.0002	5.48	2019-07-29-06.gDNA
842	Alitta virens	2	SS	3 m	1.53	0.113	0.0031	0.0002	6.79	2019-07-29-06.gDNA
843	Alitta virens	3	SS	3 m	2.13	0.155	0.0049	0.0002	3.79	2019-07-29-06.gDNA
844	Alitta virens	4	SS	3 m	1.75	0.216	0.0072	0.0005	6.53	2019-07-29-06.gDNA
845	Alitta virens	5	SS	3 m	1.33	0.122	0.0049	0.0002	3.64	2019-07-29-06.gDNA
846	Alitta virens	1	EtOH	3 m	1.97	7.15	0.1738	0.0258	14.79	2019-07-29-06.gDNA
847	Alitta virens	2	EtOH	3 m	2.00	5.76	0.1152	0.0156	13.56	2019-07-29-06.gDNA
848	Alitta virens	3	EtOH	3 m	2.03	6.78	0.1614	0.0162	10.02	2019-07-29-06.gDNA
849	Alitta virens	4	EtOH	3 m	2.05	10.7	0.2271	0.0100	4.39	2019-07-29-06.gDNA
850	Alitta virens	5	EtOH	3 m	2.02	6.92	0.1312	0.0341	26.04	2019-07-30-02.gDNA
851	Alitta virens	1	Fresh	3 m	2.03	5.53	0.1784	0.0684	38.33	2019-07-30-03.gDNA
852	Alitta virens	2	Fresh	3 m	2.02	4.05	0.1506	0.0606	40.34	2019-07-30-03.gDNA
853	Alitta virens	3	Fresh	3 m	2.04	14.4	0.4768	0.1361	28.55	2019-07-30-03.gDNA
854	Alitta virens	4	Fresh	3 m	2.01	3.9	0.1413	0.0576	40.7	2019-07-30-02.gDNA
855	Alitta virens	5	Fresh	3 m	2.08	6.07	0.1921	0.0826	42.96	2019-07-30-02.gDNA
856	Alitta virens	1	DESS	6 m	1.61	0.346	0.0185	0.0010	5.44	2019-07-30-01.gDNA
857	Alitta virens	2	DESS	6 m	n/a	n/a	n/a	n/a	n/a	n/a
858	Alitta virens	3	DESS	6 m	2.56	0.594	0.0256	0.0012	4.58	2019-07-30-01.gDNA
859	Alitta virens	4	DESS	6 m	1.83	0.258	0.0093	0.0002	1.93	2019-07-30-01.gDNA
860	Alitta virens	5	DESS	6 m	2.26	0.755	0.0288	0.0039	13.57	2019-07-30-01.gDNA
861	Alitta virens	1	DE	6 m	n/a	n/a	n/a	n/a	n/a	n/a
862	Alitta virens	2	DE	6 m	2.21	0.245	0.0070	0.0002	2.63	2020-06-10-01.gDNA
863	Alitta virens	3	DE	6 m	1.75	0.245	0.0086	0.0006	6.9	2020-06-10-01.gDNA
864	Alitta virens	4	DE	6 m	1.46	0.174	0.0092	0.0005	4.96	2020-06-10-01.gDNA
865	Alitta virens	5	DE	6 m	1.47	0.186	0.0072	0.0001	1.2	2020-06-10-01.gDNA
866	Alitta virens	1	DSS	6 m	2.88	0.164	0.0084	0.0002	2.57	2020-06-10-01.gDNA
867	Alitta virens	2	DSS	6 m	2.39	0.0888	0.0038	0.0001	3	2020-06-10-01.gDNA
868	Alitta virens	3	DSS	6 m	1.08	0.156	0.0075	0.0002	3.1	2020-06-10-01.gDNA
869	Alitta virens	4	DSS	6 m	2.69	0.145	0.0048	0.0001	3.12	2020-06-10-01.gDNA
870	Alitta virens	5	DSS	6 m	4.82	0.132	0.0060	0.0001	1.93	2020-06-10-01.gDNA
871	Alitta virens	1	ESS	6 m	1.86	0.837	0.0454	0.0080	17.67	2019-07-30-01.gDNA
872	Alitta virens	2	ESS	6 m	1.32	0.239	0.0099	0.0002	1.68	2020-06-10-02.gDNA
873	Alitta virens	3	ESS	6 m	1.97	0.639	0.0261	0.0061	23.25	2019-07-30-01.gDNA
874	Alitta virens	4	ESS	6 m	1.75	2.11	0.0577	0.0065	11.22	2019-07-30-01.gDNA
875	Alitta virens	5	ESS	6 m	1.6	0.365	0.0112	0.0034	30.2	2019-07-30-01.gDNA
876	Alitta virens	1	D	6 m	2.9	0.0846	0.0036	0.0001	1.88	2020-06-10-01.gDNA
877	Alitta virens	2	D	6 m	2.28	0.119	0.0045	0.0004	8.1	2020-06-10-01.gDNA
878	Alitta virens	3	D	6 m	1.97	0.15	0.0069	0.0002	2.68	2020-06-10-01.gDNA
879	Alitta virens	4	D	6 m	1.64	0.187	0.0091	0.0009	10.21	2020-06-10-01.gDNA
880	Alitta virens	5	D	6 m	1.78	0.117	0.0054	0.0001	1.66	2020-06-10-01.gDNA
881	Alitta virens	1	E	6 m	1.23	0.213	0.0025	0.0000	1.79	2020-06-10-01.gDNA
882	Alitta virens	2	E	6 m	3.51	0.147	0.0049	0.0001	2.89	2020-06-10-01.gDNA
883	Alitta virens	3	E	6 m	1.48	0.145	0.0061	0.0002	2.94	2020-06-10-01.gDNA
884	Alitta virens	4	E	6 m	1.38	0.123	0.0028	0.0001	2.45	2020-06-10-01.gDNA
885	Alitta virens	5	E	6 m	1.8	0.799	0.0408	0.0013	3.07	Lounsberry_T5_Samples_AllSpecies_AllReplicates_3.19.19.gDNA
886	Alitta virens	1	SS	6 m	1.52	0.177	0.0073	0.0009	12.23	2020-06-10-02.gDNA
887	Alitta virens	2	SS	6 m	1.79	0.146	0.0037	0.0001	2.02	2020-06-10-02.gDNA
888	Alitta virens	3	SS	6 m	2.09	0.156	0.0043	0.0001	2.2	2020-06-10-02.gDNA
889	Alitta virens	4	SS	6 m	1.39	0.155	0.0034	0.0001	3.48	2020-06-10-02.gDNA
890	Alitta virens	5	SS	6 m	2.47	0.0969	0.0029	0.0000	0.72	2020-06-10-02.gDNA
891	Alitta virens	1	EtOH	6 m	2.06	26.6	0.7488	0.0996	13.32	T5_Worm_EtOH&Initials_4-26-2019.gDNA

Sample ID	Taxa	Replicate	Treatment	Time Interval	A260/A280 Ratio	Yield (µg)	Total Normalized Yield (µg DNA/mg tissue)	nY (µg DNA/ mg tissue)	%R	TapeStation Filename
892	Alitta virens	2	EtOH	6 m	2.03	17.9	0.4974	0.0278	5.61	T5_Worm_EtOH&Initials_4-26-2019.gDNA
893	Alitta virens	3	EtOH	6 m	1.93	30.1	0.8963	0.2495	27.83	T5_Worm_EtOH&Initials_4-26-2019.gDNA
894	Alitta virens	4	EtOH	6 m	2	9.58	0.3034	0.0051	1.68	2019-07-30-01.gDNA
895	Alitta virens	5	EtOH	6 m	2.12	34.6	0.8873	0.1067	12.01	T5_Worm_EtOH&Initials_4-26-2019.gDNA
896	Alitta virens	1	Fresh	6 m	2.05	20.4	0.6415	0.3302	51.26	T5_Worm_EtOH&Initials_4-26-2019.gDNA
897	Alitta virens	2	Fresh	6 m	2.04	19.1	0.5335	0.1704	31.88	T5_Worm_EtOH&Initials_4-26-2019.gDNA
898	Alitta virens	3	Fresh	6 m	1.94	21.7	0.6439	0.2338	36.29	T5_Worm_EtOH&Initials_4-26-2019.gDNA
899	Alitta virens	4	Fresh	6 m	1.99	26.1	0.7861	0.2916	37.02	T5_Worm_EtOH&Initials_4-26-2019.gDNA
900	Alitta virens	5	Fresh	6 m	2.14	23.1	0.7726	0.3645	47.39	T5_Worm_EtOH&Initials_4-26-2019.gDNA
901	Alitta virens	6	DESS	1 d	2.02	4.89	0.1503	0.0685	45.53	2018-09-27-01.gDNA
902	Alitta virens	7	DESS	1 d	2.16	1.23	0.0409	0.0171	41.93	2018-09-27-01.gDNA
903	Alitta virens	8	DESS	1 d	2.09	1.43	0.0405	0.0187	46.17	2018-09-27-01.gDNA
904	Alitta virens	9	DESS	1 d	2.08	1.75	0.0444	0.0178	40.04	2018-09-27-01.gDNA
905	Alitta virens	10	DESS	1 d	2.01	1.72	0.0460	0.0156	33.9	2018-09-27-01.gDNA
906	Alitta virens	6	DE	1 d	2.09	0.952	0.0315	0.0046	14.61	2018-09-27-01.gDNA
907	Alitta virens	7	DE	1 d	2.16	1.48	0.0584	0.0221	37.83	2018-09-27-01.gDNA
908	Alitta virens	8	DE	1 d	2.18	0.609	0.0230	0.0017	7.53	2018-09-27-01.gDNA
909	Alitta virens	9	DE	1 d	2.1	2.89	0.1063	0.0508	47.85	2018-09-27-01.gDNA
910	Alitta virens	10	DE	1 d	2.13	2.34	0.0897	0.0391	43.42	2018-09-27-01.gDNA
911	Alitta virens	6	DSS	1 d	2.04	3.26	0.1126	0.0063	5.63	2018-09-27-01.gDNA
912	Alitta virens	7	DSS	1 d	2.14	1.19	0.0427	0.0032	7.5	2018-09-27-01.gDNA
913	Alitta virens	8	DSS	1 d	2.09	0.875	0.3109	0.0229	7.36	2018-09-27-01.gDNA
914	Alitta virens	9	DSS	1 d	2.13	2.57	0.0730	0.0073	9.93	2018-09-27-01.gDNA
915	Alitta virens	10	DSS	1 d	1.92	1.19	0.0445	0.0032	7.06	2018-09-27-01.gDNA
916	Alitta virens	6	ESS	1 d	2.04	1.97	0.0673	0.0311	46.07	2018-09-27-01.gDNA
917	Alitta virens	7	ESS	1 d	2.07	1.79	0.0553	0.0286	51.63	2018-09-27-01.gDNA
918	Alitta virens	8	ESS	1 d	2.06	1.31	0.0376	0.0179	47.47	2018-09-27-01.gDNA
919	Alitta virens	9	ESS	1 d	2	3.63	0.0972	0.0431	44.42	2018-09-27-01.gDNA
920	Alitta virens	10	ESS	1 d	1.87	3.92	0.1076	0.0510	47.44	2018-09-27-01.gDNA
921	Alitta virens	6	D	1 d	2.11	0.462	0.0246	0.0007	3.02	2018-09-27-01.gDNA
922	Alitta virens	7	D	1 d	2.12	0.613	0.0337	0.0008	2.26	2018-09-27-01.gDNA
923	Alitta virens	8	D	1 d	2.08	1.12	0.0587	0.0012	1.97	2018-09-27-01.gDNA
924	Alitta virens	9	D	1 d	2.14	1.19	0.0504	0.0017	3.41	2018-09-27-01.gDNA
925	Alitta virens	10	D	1 d	2.14	0.634	0.0315	0.0010	3.23	2018-09-27-01.gDNA
926	Alitta virens	6	E	1 d	1.97	8.36	0.3537	0.1857	52.47	2018-09-27-01.gDNA
927	Alitta virens	7	E	1 d	2.04	5.55	0.2266	0.1029	45.42	2018-09-27-01.gDNA
928	Alitta virens	8	E	1 d	2.03	4.92	0.2178	0.1115	51.13	2018-09-27-01.gDNA
929	Alitta virens	9	E	1 d	2.02	9.09	0.4114	0.1855	45.07	2018-09-27-01.gDNA
930	Alitta virens	10	E	1 d	2.02	7.6	0.2421	0.1348	55.7	2018-09-27-01.gDNA
931	Alitta virens	6	SS	1 d	1.8	2.12	0.0581	0.0134	23.05	2018-09-27-01.gDNA
932	Alitta virens	7	SS	1 d	2.03	0.944	0.0313	0.0058	18.65	2018-09-27-01.gDNA
933	Alitta virens	8	SS	1 d	1.98	2.24	0.0661	0.0167	25.35	2018-09-27-01.gDNA
934	Alitta virens	9	SS	1 d	2.1	1.67	0.0433	0.0085	19.63	2018-09-27-01.gDNA
935	Alitta virens	10	SS	1 d	2	2.72	0.1005	0.0292	29.06	2018-09-27-01.gDNA
936	Alitta virens	6	EtOH	1 d	1.98	6.49	0.1543	0.0449	29.13	2018-09-27-01.gDNA
937	Alitta virens	7	EtOH	1 d	2.07	6	0.1208	0.0395	32.71	2018-09-27-01.gDNA
938	Alitta virens	8	EtOH	1 d	2.06	8.11	0.1150	0.0352	30.58	2018-09-27-01.gDNA
939	Alitta virens	9	EtOH	1 d	2.06	8.17	0.1679	0.0442	26.32	2018-09-27-01.gDNA
940	Alitta virens	10	EtOH	1 d	2	6.23	0.1356	0.0440	32.45	2018-09-27-01.gDNA
941	Alitta virens	6	Fresh	1 d	2	11.9	0.4457	0.1678	37.78	2018-09-27-01.gDNA
942	Alitta virens	7	Fresh	1 d	2.06	5.93	0.2103	0.1142	54.35	2018-09-27-01.gDNA
943	Alitta virens	8	Fresh	1 d	1.91	22	0.8560	0.3249	37.87	2018-09-27-01.gDNA
944	Alitta virens	9	Fresh	1 d	2.03	16.3	0.5362	0.2109	39.32	2018-09-27-01.gDNA
945	Alitta virens	10	Fresh	1 d	1.88	9.14	0.2704	0.1142	42.24	2018-09-27-01.gDNA
1036	Alitta virens	6	DESS	3 m	1.95	2.18	0.0769	0.0066	8.55	2019-07-29-03.gDNA
1037	Alitta virens	7	DESS	3 m	1.97	5.85	0.2244	0.1500	66.76	2019-07-29-03.gDNA

Sample ID	Taxa	Replicate	Treatment	Time Interval	A260/A280 Ratio	Yield (µg)	Total Normalized Yield (µg DNA/mg tissue)	nY (µg DNA/ mg tissue)	%R	TapeStation Filename
1038	Alitta virens	8	DESS	3 m	1.88	3.2	0.1244	0.0486	38.97	2019-07-29-03.gDNA
1039	Alitta virens	9	DESS	3 m	1.88	5.97	0.1684	0.0063	3.75	2019-07-29-03.gDNA
1040	Alitta virens	10	DESS	3 m	1.84	3.5	0.1521	0.0192	12.67	2019-07-29-04.gDNA
1041	Alitta virens	6	DE	3 m	1.26	0.132	0.0038	0.0001	2.22	2019-07-29-04.gDNA
1042	Alitta virens	7	DE	3 m	1.87	2.33	0.0696	0.0004	0.54	2019-07-29-04.gDNA
1043	Alitta virens	8	DE	3 m	1.81	1.56	0.0813	0.0007	0.83	2019-07-29-04.gDNA
1044	Alitta virens	9	DE	3 m	2.08	0.944	0.0322	0.0001	0.45	2019-07-29-04.gDNA
1045	Alitta virens	10	DE	3 m	1.78	0.283	0.0118	0.0003	2.28	2019-07-29-04.gDNA
1046	Alitta virens	6	DSS	3 m	2.35	0.105	0.0044	0.0001	2.66	2020-06-10-01.gDNA
1047	Alitta virens	7	DSS	3 m	1.75	0.121	0.0053	0.0001	2.31	2020-06-10-01.gDNA
1048	Alitta virens	8	DSS	3 m	1.35	0.137	0.0059	0.0002	3.26	2020-06-10-01.gDNA
1049	Alitta virens	9	DSS	3 m	0.88	0.151	0.0063	0.0002	3.53	2020-06-10-01.gDNA
1050	Alitta virens	10	DSS	3 m	0.9	0.118	0.0042	0.0001	2.25	2020-06-10-01.gDNA
1051	Alitta virens	6	ESS	3 m	1.9	1.88	0.0600	0.0013	2.08	2019-07-29-05.gDNA
1052	Alitta virens	7	ESS	3 m	1.86	1.9	0.0766	0.0175	22.8	2019-07-29-05.gDNA
1053	Alitta virens	8	ESS	3 m	1.88	1.84	0.0822	0.0087	10.5	2019-07-29-05.gDNA
1054	Alitta virens	9	ESS	3 m	1.79	3.03	0.1102	0.0157	14.31	2019-07-29-05.gDNA
1055	Alitta virens	10	ESS	3 m	1.85	4.54	0.1248	0.0110	8.82	2019-07-29-05.gDNA
1056	Alitta virens	6	D	3 m	1.44	0.222	0.0104	0.0003	2.48	2020-06-10-01.gDNA
1057	Alitta virens	7	D	3 m	2.13	0.184	0.0081	0.0002	2.32	2020-06-10-01.gDNA
1058	Alitta virens	8	D	3 m	1.37	0.132	0.0059	0.0002	3.06	2020-06-10-01.gDNA
1059	Alitta virens	9	D	3 m	1.58	0.0987	0.0041	0.0000	0.95	2020-06-10-01.gDNA
1060	Alitta virens	10	D	3 m	1.39	0.162	0.0079	0.0003	3.66	2020-06-10-01.gDNA
1061	Alitta virens	6	E	3 m	2.35	0.187	0.0058	0.0001	1.74	2019-07-29-05.gDNA
1062	Alitta virens	7	E	3 m	1.73	1.62	0.0520	0.0004	0.78	2019-07-29-05.gDNA
1063	Alitta virens	8	E	3 m	1.36	0.186	0.0061	0.0002	2.82	2019-07-29-05.gDNA
1064	Alitta virens	9	E	3 m	1.27	0.262	0.0057	0.0000	0.44	2019-07-29-05.gDNA
1065	Alitta virens	10	E	3 m	2.01	0.443	0.0124	0.0002	1.58	2019-07-29-06.gDNA
1066	Alitta virens	6	SS	3 m	1.6	0.106	0.0042	0.0002	5.17	2019-07-29-06.gDNA
1067	Alitta virens	7	SS	3 m	1.36	0.133	0.0047	0.0002	4.35	2019-07-29-06.gDNA
1068	Alitta virens	8	SS	3 m	1.56	0.153	0.0067	0.0002	3.48	2019-07-29-06.gDNA
1069	Alitta virens	9	SS	3 m	1.58	0.157	0.0055	0.0003	4.92	2019-07-29-06.gDNA
1070	Alitta virens	10	SS	3 m	0.94	0.127	0.0040	0.0003	7.79	2019-07-29-06.gDNA
1071	Alitta virens	6	EtOH	3 m	2.07	7.03	0.0994	0.0250	25.18	2019-07-30-02.gDNA
1072	Alitta virens	7	EtOH	3 m	2.07	3.96	0.0565	0.0177	31.23	2019-07-30-02.gDNA
1073	Alitta virens	8	EtOH	3 m	2.06	1.37	0.0253	0.0023	8.99	2019-07-30-02.gDNA
1074	Alitta virens	9	EtOH	3 m	2.09	5.06	0.0712	0.0047	6.54	2019-07-30-02.gDNA
1075	Alitta virens	10	EtOH	3 m	2.05	9.02	0.1414	0.0447	31.6	2019-07-30-03.gDNA
1076	Alitta virens	6	Fresh	3 m	2.06	5.12	0.1455	0.0344	23.68	2019-07-30-02.gDNA
1077	Alitta virens	7	Fresh	3 m	2.1	2.28	0.0789	0.0429	54.44	2019-07-30-02.gDNA
1078	Alitta virens	8	Fresh	3 m	2.06	4.32	0.1143	0.0495	43.4	2019-07-30-02.gDNA
1079	Alitta virens	9	Fresh	3 m	2.04	7.2	0.2006	0.0866	43.21	2019-07-30-03.gDNA
1080	Alitta virens	10	Fresh	3 m	2.07	5.79	0.1548	0.0759	49.02	
1081	Alitta virens	6	DESS	6 m	1.84	4.46	0.2318	0.1039	44.96	2019-07-30-01.gDNA
1082	Alitta virens	7	DESS	6 m	1.69	2.58	0.0842	0.0086	10.22	2019-07-30-01.gDNA
1083	Alitta virens	8	DESS	6 m	1.61	1.22	0.0554	0.0091	16.44	2019-07-30-01.gDNA
1084	Alitta virens	9	DESS	6 m	1.84	1.49	0.0598	0.0067	11.24	2019-07-30-01.gDNA
1085	Alitta virens	10	DESS	6 m	1.81	6.69	0.2858	0.0247	8.64	2019-07-30-01.gDNA
1086	Alitta virens	6	DE	6 m	1.74	0.171	0.0047	0.0006	13.27	2020-06-10-01.gDNA
1087	Alitta virens	7	DE	6 m	1.7	0.459	0.0209	0.0018	8.51	2020-06-10-01.gDNA
1088	Alitta virens	8	DE	6 m	2.6	0.281	0.0103	0.0004	4.16	2020-06-10-01.gDNA
1089	Alitta virens	9	DE	6 m	1.43	0.739	0.0364	0.0012	3.2	2020-06-10-01.gDNA
1090	Alitta virens	10	DE	6 m	1.51	0.375	0.0164	0.0002	1.41	2020-06-10-01.gDNA
1091	Alitta virens	6	DSS	6 m	1.26	0.115	0.0043	0.0001	3.17	2020-06-10-01.gDNA
1092	Alitta virens	7	DSS	6 m	1.26	0.149	0.0073	0.0002	2.4	2020-06-10-01.gDNA
1093	Alitta virens	8	DSS	6 m	1.58	0.151	0.0074	0.0004	5.21	2020-06-10-01.gDNA

Sample ID	Taxa	Replicate	Treatment	Time Interval	A260/A280 Ratio	Yield (µg)	Total Normalized Yield (µg DNA/mg tissue)	nY (µg DNA/ mg tissue)	%R	TapeStation Filename
1094	Alitta virens	9	DSS	6 m	1.3	0.157	0.0071	0.0003	3.62	2020-06-10-01.gDNA
1095	Alitta virens	10	DSS	6 m	2.12	0.128	0.0050	0.0001	2.35	2020-06-10-01.gDNA
1096	Alitta virens	6	ESS	6 m	1.39	0.196	0.0028	0.0002	6.74	2019-07-30-02.gDNA
1097	Alitta virens	7	ESS	6 m	1.97	0.926	0.0339	0.0074	21.86	2019-07-30-02.gDNA
1098	Alitta virens	8	ESS	6 m	1.81	3.72	0.1598	0.0455	28.41	2019-07-30-02.gDNA
1099	Alitta virens	9	ESS	6 m	1.67	0.866	0.0446	0.0146	32.63	2019-07-30-02.gDNA
1100	Alitta virens	10	ESS	6 m	1.4	0.589	0.0185	0.0008	4.45	2019-07-30-02.gDNA
1101	Alitta virens	6	D	6 m	1.51	0.217	0.0131	0.0014	11	2020-06-10-01.gDNA
1102	Alitta virens	7	D	6 m	1.76	0.202	0.0091	0.0008	9	2020-06-10-01.gDNA
1103	Alitta virens	8	D	6 m	1.86	0.175	0.0074	0.0005	6.97	2020-06-10-01.gDNA
1104	Alitta virens	9	D	6 m	1.71	0.132	0.0083	0.0002	2.23	2020-06-10-01.gDNA
1105	Alitta virens	10	D	6 m	1.85	0.145	0.0079	0.0003	3.32	2020-06-10-01.gDNA
1106	Alitta virens	6	E	6 m	1.32	0.181	0.0067	0.0002	2.64	2020-06-10-02.gDNA
1107	Alitta virens	7	E	6 m	1.27	0.154	0.0073	0.0003	3.55	2020-06-10-02.gDNA
1108	Alitta virens	8	E	6 m	1.86	2.63	0.1443	0.0375	25.98	2020-06-10-02.gDNA
1109	Alitta virens	9	E	6 m	1.56	0.438	0.0258	0.0006	2.43	2020-06-10-02.gDNA
1110	Alitta virens	10	E	6 m	1.06	0.245	0.0123	0.0005	4.18	2020-06-10-02.gDNA
1111	Alitta virens	6	SS	6 m	1.12	0.172	0.0068	0.0003	3.8	2020-06-10-02.gDNA
1112	Alitta virens	7	SS	6 m	1.87	0.154	0.0062	0.0002	3.16	2020-06-10-02.gDNA
1113	Alitta virens	8	SS	6 m	1.83	0.138	0.0058	0.0001	2.35	2020-06-10-02.gDNA
1114	Alitta virens	9	SS	6 m	3.64	0.119	0.0055	0.0001	2.74	2020-06-10-02.gDNA
1115	Alitta virens	10	SS	6 m	1.23	0.222	0.0090	0.0006	6.96	2020-06-10-03.gDNA
1116	Alitta virens	6	EtOH	6 m	2.05	30.6	0.9508	0.1600	16.85	T5_Worm_EtOH&Initials_4-26-2019.gDNA
1117	Alitta virens	7	EtOH	6 m	2.04	3.87	0.1098	0.0110	9.96	T5_Worm_EtOH&Initials_4-26-2019.gDNA
1118	Alitta virens	8	EtOH	6 m	1.69	2.59	0.0828	0.0099	11.86	T5_Worm_EtOH&Initials_4-26-2019.gDNA
1119	Alitta virens	9	EtOH	6 m	1.96	2.08	0.0674	0.0077	11.34	T5_Worm_EtOH&Initials_4-26-2019.gDNA
1120	Alitta virens	10	EtOH	6 m	1.98	14.3	0.4951	0.0415	8.42	T5_Worm_EtOH&Initials_4-26-2019.gDNA
1121	Alitta virens	6	Fresh	6 m	1.96	17.3	0.5966	0.2703	45.37	T5_Worm_EtOH&Initials_4-26-2019.gDNA
1122	Alitta virens	7	Fresh	6 m	1.94	17.5	0.6206	0.1628	26.28	T5_Worm_EtOH&Initials_4-26-2019.gDNA
1123	Alitta virens	8	Fresh	6 m	2.01	17.9	0.5375	0.2099	38.99	T5_Worm_EtOH&Initials_4-26-2019.gDNA
1124	Alitta virens	9	Fresh	6 m	2.05	10.8	0.3612	0.1893	52.58	T5_Worm_EtOH&Initials_4-26-2019.gDNA
1125	Alitta virens	10	Fresh	6 m	2.01	13.5	0.3814	0.1780	46.61	T5_Worm_EtOH&Initials_4-26-2019.gDNA
