# Supplementary material for: DESS deconstructed: Is EDTA solely responsible for protection of high molecular weight DNA in this common tissue preservative?
Source: PLoS One. 2020 Aug 20;15(8):e0237356. doi: 10.1371/journal.pone.0237356 (PMC7440624; doi:10.1371/journal.pone.0237356)
Supplement: S3 Table — Average %R (avg) and standard deviation (SD) for tissues of Mytilus edulis, Faxonius virilis and Alitta virens extracted immediately after dissection (fresh) or stored for one day (1 d), three months (3 m) or six months (6 m) in preservative treatments containing DMSO (D), EDTA (E) and/or saturated NaCl (SS) or 95% ethanol (EtOH). (PDF) [file pone.0237356.s003.pdf]

		1 day											
		Yield (µg DNA)			Total Normalized Yield (µg DNA/mg tissue)			nY (µg DNA/mg tissue)			%R		
		mean	sd	*N*	mean	sd	*N*	mean	sd	*N*	mean	sd	*N*
Mytilus edulis	DESS	8.7029	5.9717	7	0.7747	0.4834	7	0.3674	0.2289	7	47.08	3.14	7
	DE	7.6243	3.9459	7	0.7397	0.4333	7	0.2837	0.1538	7	39.14	4.27	7
	DSS	17.0714	5.8846	7	1.7745	1.0795	7	0.1395	0.1309	7	11.85	15.62	7
	ESS	6.4786	5.6672	7	0.5451	0.5307	7	0.2450	0.2389	7	45.30	1.93	7
	D	14.6357	10.0298	7	0.6912	0.4172	7	0.0489	0.0997	7	4.51	6.05	7
	E	5.9543	2.0091	7	0.4219	0.1746	7	0.1636	0.0605	7	39.39	4.19	7
	SS	19.8129	7.2080	7	1.3500	0.4960	7	0.3071	0.1570	7	23.86	9.22	7
	EtOH	40.9571	15.8462	7	1.0017	0.2159	7	0.2353	0.0782	7	23.36	5.84	7
	Fresh	31.3571	20.0233	7	1.0947	0.5910	7	0.3066	0.1221	7	30.22	6.78	7
Faxonius virilis	DESS	0.7917	0.2778	7	0.0295	0.0146	7	0.0077	0.0034	7	26.51	3.91	7
	DE	0.9696	0.2016	7	0.0349	0.0113	7	0.0143	0.0051	7	40.55	4.26	7
	DSS	8.7193	13.3091	7	0.3199	0.5064	7	0.0791	0.0949	7	25.51	18.96	7
	ESS	0.8899	0.7247	7	0.0374	0.0419	7	0.0131	0.0192	7	28.74	8.62	7
	D	3.4197	2.9664	7	0.1465	0.1243	7	0.0238	0.0172	7	17.57	7.09	7
	E	0.7049	0.2364	7	0.0259	0.0099	7	0.0101	0.0059	7	36.96	11.52	7
	SS	3.1371	1.2346	7	0.1168	0.0603	7	0.0440	0.0424	7	33.11	23.24	7
	EtOH	2.2591	1.4130	7	0.0464	0.0301	7	0.0167	0.0119	7	36.56	8.08	7
	Fresh	1.1077	0.9347	7	0.0386	0.0344	7	0.0155	0.0136	7	39.86	8.31	7
Alitta virens	DESS	1.8872	1.2492	10	0.1783	0.3829	10	0.0931	0.2201	10	41.30	7.65	10
	DE	2.0241	0.9468	10	0.0812	0.0441	10	0.0344	0.0247	10	36.78	14.22	10
	DSS	1.5104	0.8050	10	0.0801	0.0846	10	0.0059	0.0062	10	7.39	1.17	10
	ESS	2.8420	1.2807	10	0.0985	0.0549	10	0.0526	0.0358	10	51.57	5.78	10
	D	1.0851	0.5876	10	0.0526	0.0327	10	0.0020	0.0016	10	3.69	2.25	10
	E	5.9840	1.9118	10	0.2438	0.0879	10	0.1161	0.0433	10	47.58	4.97	10
	SS	1.8500	0.5539	10	0.0593	0.0210	10	0.0141	0.0083	10	22.17	6.76	10
	EtOH	6.2790	2.1015	10	0.1430	0.0482	10	0.0422	0.0169	10	29.15	3.18	10
	Fresh	10.9010	5.0185	10	0.3677	0.2084	10	0.1469	0.0776	10	41.78	11.98	10

		3 months											
		Yield (µg DNA)			Total Normalized Yield (µg DNA/mg tissue)			nY (µg DNA/mg tissue)			%R		
		mean	sd	*N*	mean	sd	*N*	mean	sd	*N*	mean	sd	*N*
Mytilus edulis	DESS	17.3643	6.9406	7	1.1155	0.4690	7	0.5783	0.2038	7	53.50	5.94	7
	DE	25.9529	12.5080	7	1.2375	0.5615	7	0.5583	0.3196	7	40.20	14.61	7
	DSS	0.5756	0.0910	7	0.0376	0.0089	7	0.0012	0.0005	7	3.17	1.58	7
	ESS	14.0114	9.7129	7	0.8687	0.5909	7	0.4410	0.1927	7	55.57	10.03	7
	D	1.8229	0.5308	7	0.0884	0.0364	7	0.0011	0.0003	7	1.37	0.37	7
	E	18.8857	13.6520	7	0.8045	0.5217	7	0.3645	0.1459	7	50.44	9.76	7
	SS	0.6494	0.3423	7	0.1368	0.2835	7	0.0010	0.0022	7	0.75	0.53	7
	EtOH	49.3000	8.4751	7	1.2011	0.3020	7	0.1992	0.1516	7	15.37	9.07	7
	Fresh	24.2714	7.1435	7	0.7752	0.1679	7	0.2658	0.0932	7	33.79	7.37	7
Faxonius virilis	DESS	0.8530	0.3611	7	0.0355	0.0179	7	0.0082	0.0063	7	22.57	13.61	7
	DE	1.6390	1.6285	7	0.0683	0.0721	7	0.0288	0.0404	7	33.84	14.93	7
	DSS	0.4186	0.2732	7	0.0158	0.0127	7	0.0006	0.0006	7	3.85	2.12	7
	ESS	1.2183	0.9004	7	0.0558	0.0471	7	0.0238	0.0320	7	33.61	20.47	7
	D	0.4771	0.2798	7	0.0194	0.0095	7	0.0003	0.0002	7	1.66	0.62	7
	E	1.4491	1.2580	7	0.0584	0.0422	7	0.0307	0.0219	7	51.74	4.84	7
	SS	0.6617	0.5780	7	0.0208	0.0178	7	0.0005	0.0005	7	2.98	1.33	7
	EtOH	3.4943	1.3170	7	0.0699	0.0472	7	0.0288	0.0250	7	38.70	10.23	7
	Fresh	2.0300	1.1284	7	0.0674	0.0392	7	0.0228	0.0160	7	35.34	11.55	7
Alitta virens	DESS	4.1970	1.9863	10	0.1524	0.0663	10	0.0309	0.0444	10	17.37	20.18	10
	DE	0.7325	0.7644	10	0.0284	0.0288	10	0.0003	0.0002	10	1.75	1.08	10
	DSS	0.1292	0.0162	10	0.0051	0.0007	10	0.0001	0.0000	10	2.63	0.58	10
	ESS	3.4080	1.3968	10	0.1180	0.0406	10	0.0115	0.0073	10	10.06	6.64	10
	D	0.1512	0.0339	10	0.0065	0.0019	10	0.0002	0.0001	10	2.51	0.81	10
	E	1.2427	1.5308	10	0.0479	0.0631	10	0.0002	0.0002	10	1.22	0.93	10
	SS	0.1403	0.0321	10	0.0049	0.0012	10	0.0003	0.0001	10	5.19	1.46	10
	EtOH	6.3750	2.5859	10	0.1203	0.0601	10	0.0196	0.0131	10	17.23	10.34	10
	Fresh	5.8660	3.2954	10	0.1833	0.1092	10	0.0695	0.0289	10	40.46	8.94	10

		6 months											
		Yield (µg DNA)			Total Normalized Yield (µg DNA/mg tissue)			nY (µg DNA/mg tissue)			%R		
		mean	sd	*N*	mean	sd	*N*	mean	sd	*N*	mean	sd	*N*
Mytilus edulis	DESS	23.3286	10.0011	7	1.6897	0.7693	7	0.6837	0.2320	7	43.55	8.85	7
	DE	24.8857	9.3142	7	1.1461	0.4908	7	0.5573	0.2518	7	48.50	11.14	7
	DSS	0.6177	0.4738	7	0.0349	0.0301	7	0.0012	0.0010	7	3.67	1.24	7
	ESS	27.4329	15.9590	7	1.8358	0.8126	7	0.6772	0.2140	7	40.45	12.99	7
	D	1.3554	1.3033	7	0.0629	0.0681	7	0.0044	0.0078	7	4.29	3.22	7
	E	33.7500	19.5948	7	1.4969	0.8675	7	0.5249	0.2132	7	40.97	16.38	7
	SS	0.2004	0.0503	7	0.0087	0.0017	7	0.0003	0.0001	7	3.18	0.71	7
	EtOH	37.7257	22.1245	7	1.4130	0.9664	7	0.3311	0.3996	7	18.13	13.59	7
	Fresh	152.8000	134.0373	7	4.8744	4.0518	7	1.0128	0.5618	7	25.95	8.45	7
Faxonius virilis	DESS	3.9914	2.3694	7	0.1560	0.0963	7	0.0811	0.0545	7	50.84	2.63	7
	DE	6.6186	3.4301	7	0.2029	0.0795	7	0.1027	0.0590	7	47.35	15.62	7
	DSS	0.5203	0.6055	7	0.0112	0.0056	7	0.0005	0.0003	7	4.33	1.71	7
	ESS	3.8821	2.1433	7	0.1261	0.0753	7	0.0553	0.0283	7	43.73	11.35	7
	D	0.2516	0.0483	7	0.0082	0.0029	7	0.0002	0.0001	7	3.13	1.94	7
	E	11.1457	4.5552	7	0.3387	0.1980	7	0.1937	0.1203	7	54.35	9.91	7
	SS	0.1971	0.0457	7	0.0055	0.0023	7	0.0002	0.0001	7	4.24	0.81	7
	EtOH	22.0614	15.8466	7	0.6723	0.5016	7	0.2811	0.1984	7	44.27	5.64	7
	Fresh	8.4477	8.7750	7	0.2736	0.2819	7	0.1170	0.1283	7	38.93	13.61	7
Alitta virens	DESS	2.0437	2.1947	9	0.0888	0.1001	9	0.0177	0.0332	9	13.00	12.82	9
	DE	0.3194	0.1844	9	0.0134	0.0100	9	0.0006	0.0005	9	5.14	3.89	9
	DSS	0.1386	0.0231	10	0.0062	0.0016	10	0.0002	0.0001	10	3.05	0.91	10
	ESS	1.0487	1.0845	10	0.0410	0.0454	10	0.0093	0.0135	10	17.81	11.24	10
	D	0.1529	0.0419	10	0.0075	0.0027	10	0.0005	0.0004	10	5.71	3.72	10
	E	0.5075	0.7740	10	0.0254	0.0435	10	0.0041	0.0117	10	5.19	7.33	10
	SS	0.1536	0.0338	10	0.0055	0.0019	10	0.0003	0.0003	10	3.97	3.33	10
	EtOH	17.2220	12.5778	10	0.5039	0.3534	10	0.0719	0.0819	10	11.89	7.00	10
	Fresh	18.7400	4.4677	10	0.5875	0.1415	10	0.2401	0.0710	10	41.37	8.64	10
