# Supplementary figures and images for: DESS deconstructed: Is EDTA solely responsible for protection of high molecular weight DNA in this common tissue preservative?
Source: PLoS One. 2020 Aug 20;15(8):e0237356. doi: 10.1371/journal.pone.0237356 (PMC7440624; doi:10.1371/journal.pone.0237356)

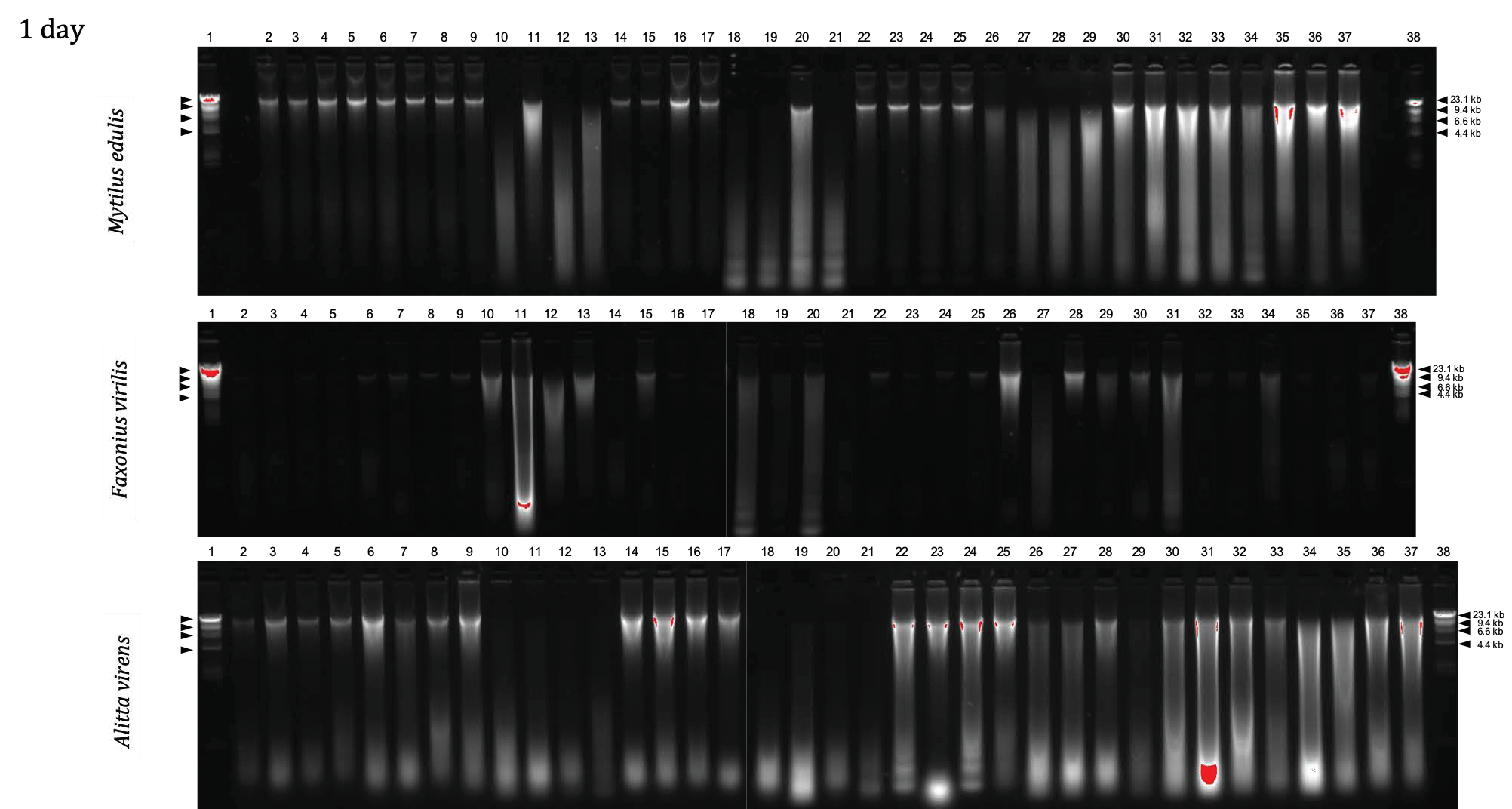

Supplement: S1 Fig — Tissues of three taxa, Mytilus edulis, Faxonius virilis and Alitta virens, were stored for six months at room temperature in DESS (lanes 2–5), six DESS-variant solutions (DE, lanes 6–9; DSS, lanes 10–13; ESS, lanes 14–17; D, lanes 18–21; E, lanes 22–25; SS, lanes 26–29) and 95% ethanol (lanes 30–33). DNA extracts from fresh tissues are displayed in lanes 34–37. Lanes 1 and 38 contain 0.16 μg of λ DNA-HindIII Digest DNA Ladder (New England BioLabs; Ipswich, MA). D, DMSO; E, EDTA; SS, saturated NaCl; EtOH, 95% ethanol; Fresh, untreated tissue extracted immediately after dissection. (TIF) [file pone.0237356.s004.tif]

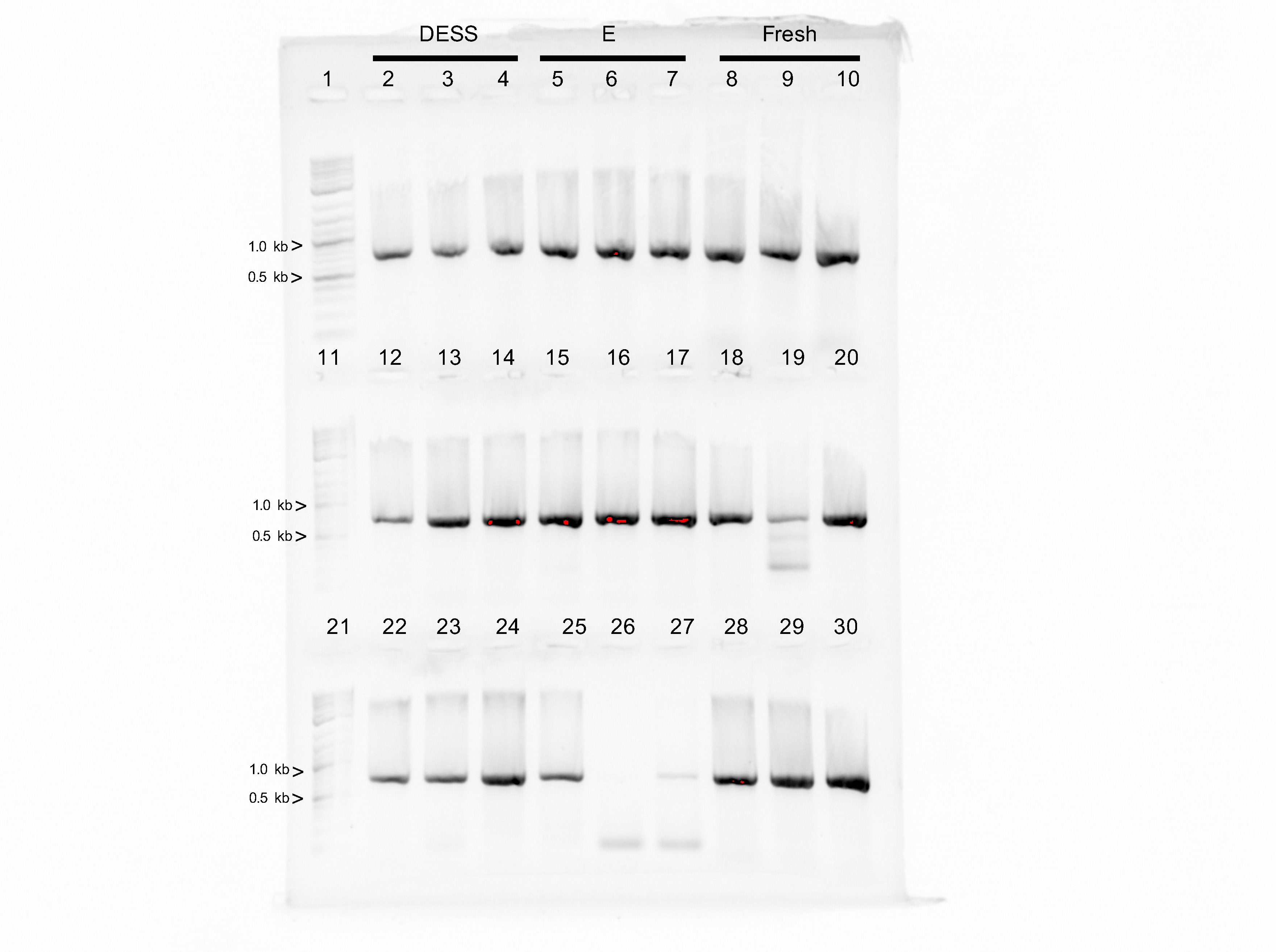

Supplement: S2 Fig — Select DNA extracts from all three taxa were PCR amplified after storage for six months. Mytilus edulis tissues stored in DESS (lanes 2–4), E (lanes 5–7) or fresh (8–10); Foxonius virilis tissues stored in DESS (12–14), E (lanes 15–17) or fresh (18–20); Alitta virens tissues stored in DESS (lanes 22–24), E (25–27) or fresh (28–30). Lanes 1, 11 and 21 contain 0.05 μg of Quick-Load Purple 1 kb Plus DNA Ladder (New England BioLabs; Ipswich, MA). (TIFF) [file pone.0237356.s005.tiff]
