# Supplementary material for: DESS deconstructed: Is EDTA solely responsible for protection of high molecular weight DNA in this common tissue preservative?
Source: PLoS One. 2020 Aug 20;15(8):e0237356. doi: 10.1371/journal.pone.0237356 (PMC7440624; doi:10.1371/journal.pone.0237356)
Supplement: S3 Fig — Tissues of Mytilus edulis, Faxonius virilis and Alitta virens were stored for 1 day, three months and six months at room temperature in DESS (lanes 2–5), six DESS-variant solutions (DE, lanes 6–9; DSS, lanes 10–13; ESS, lanes 14–17; D, lanes 18–21; E, lanes 22–25; SS, lanes 26–29) and 95% EtOH (lanes 30–33). DNA extracts from fresh tissues are displayed in lanes 34–37. Lanes 1 and 38 contain 0.16 μg of λ DNA-HindIII Digest DNA Ladder (New England BioLabs; Ipswich, MA). D, DMSO; E, EDTA; SS, saturated NaCl; EtOH, 95% ethanol; Fresh, untreated tissue extracted immediately after dissection. (PDF) [file pone.0237356.s006.pdf]

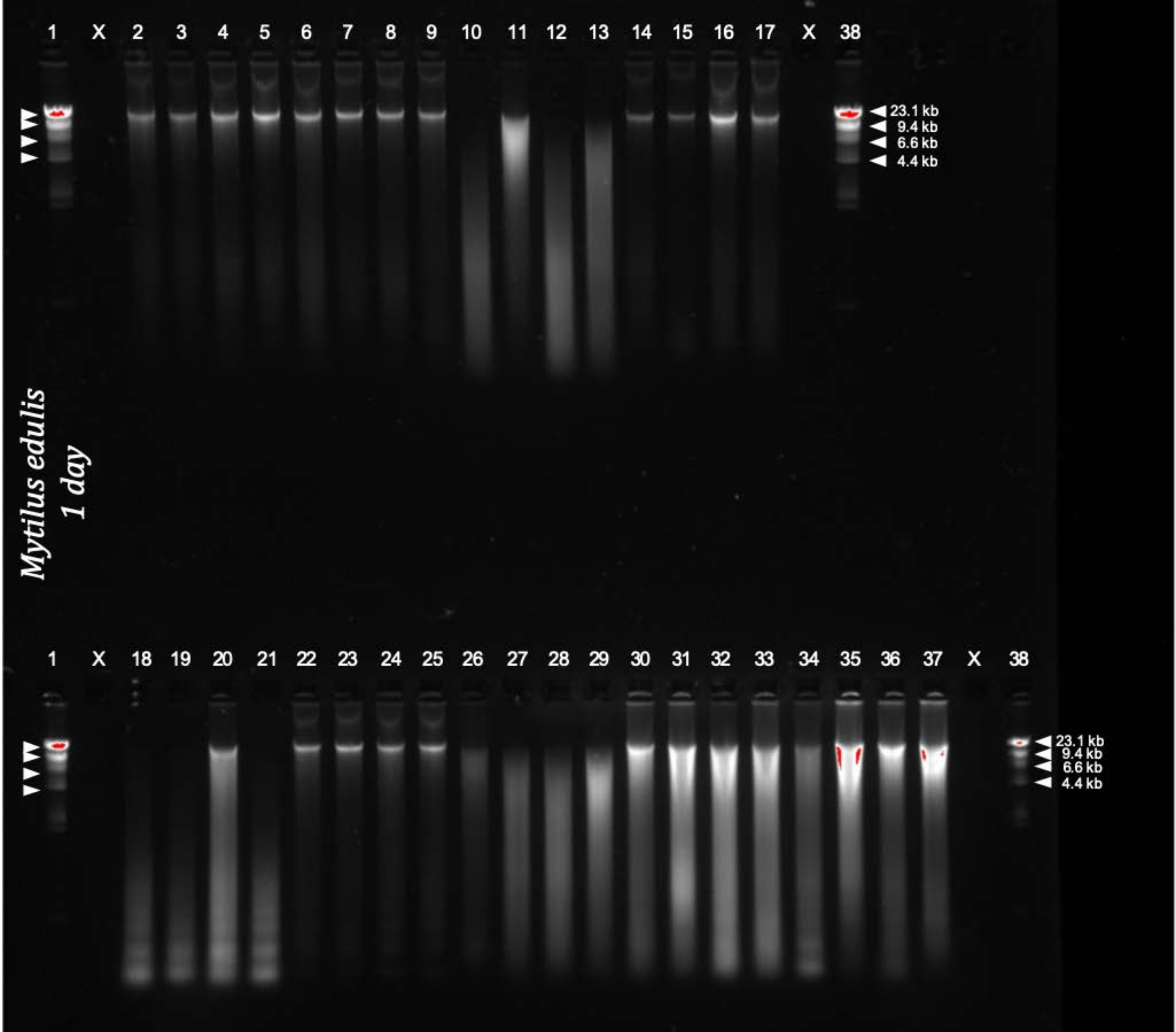
1 X 2 3 4 5 6 7 8 9 10 11 12 13 14 15 16 17 X 38
23.1 kb
9.4 kb
6.6 kb
4.4 kb
Mytilus edulis
1 day
1 X 18 19 20 21 22 23 24 25 26 27 28 29 30 31 32 33 34 35 36 37 X 38
23.1 kb
9.4 kb
6.6 kb
4.4 kb

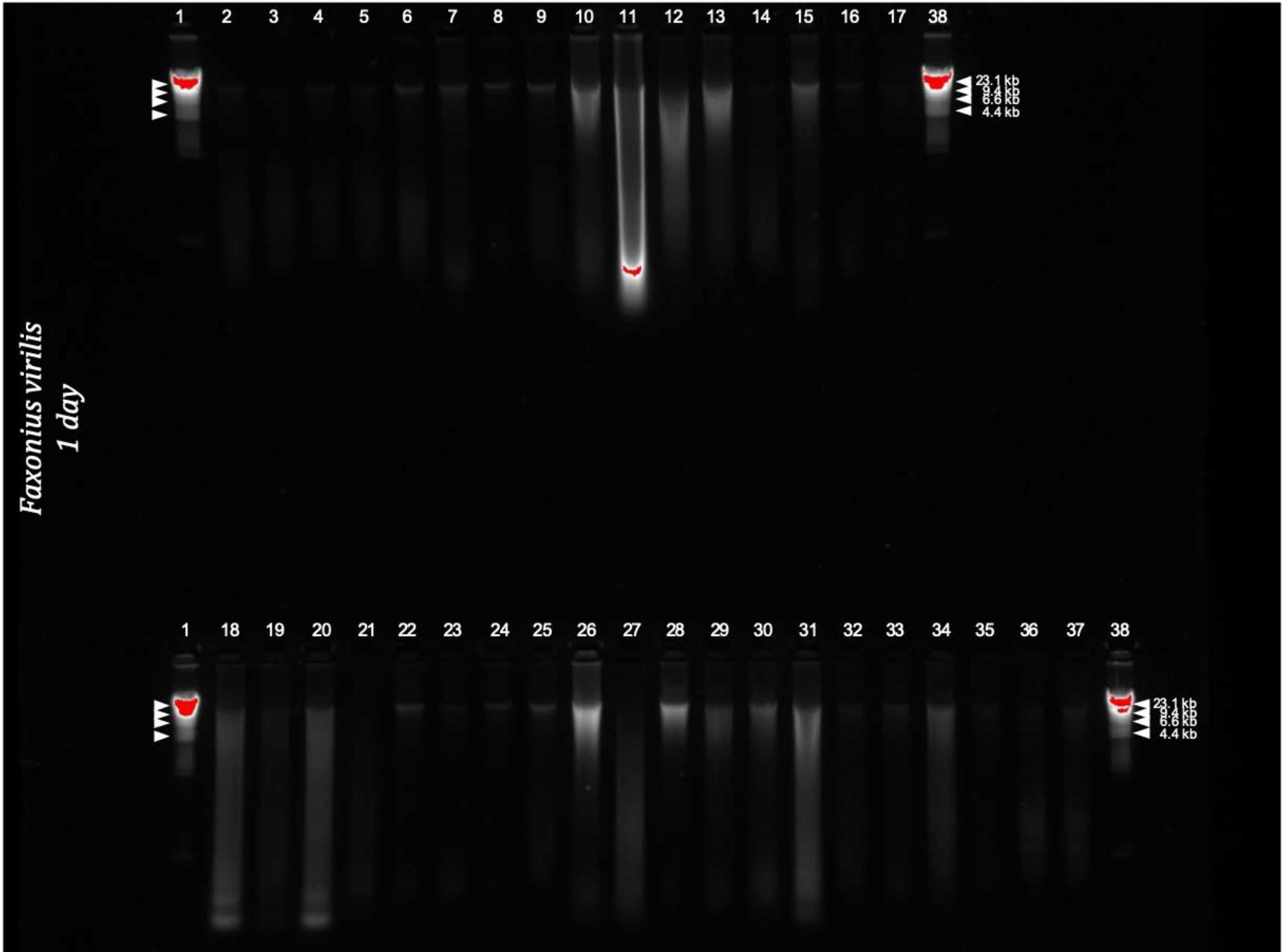
1 2 3 4 5 6 7 8 9 10 11 12 13 14 15 16 17 38
23.1 kb
9.4 kb
6.6 kb
4.4 kb
Faxonius virilis
1 day
1 18 19 20 21 22 23 24 25 26 27 28 29 30 31 32 33 34 35 36 37 38
23.1 kb
9.4 kb
6.6 kb
4.4 kb

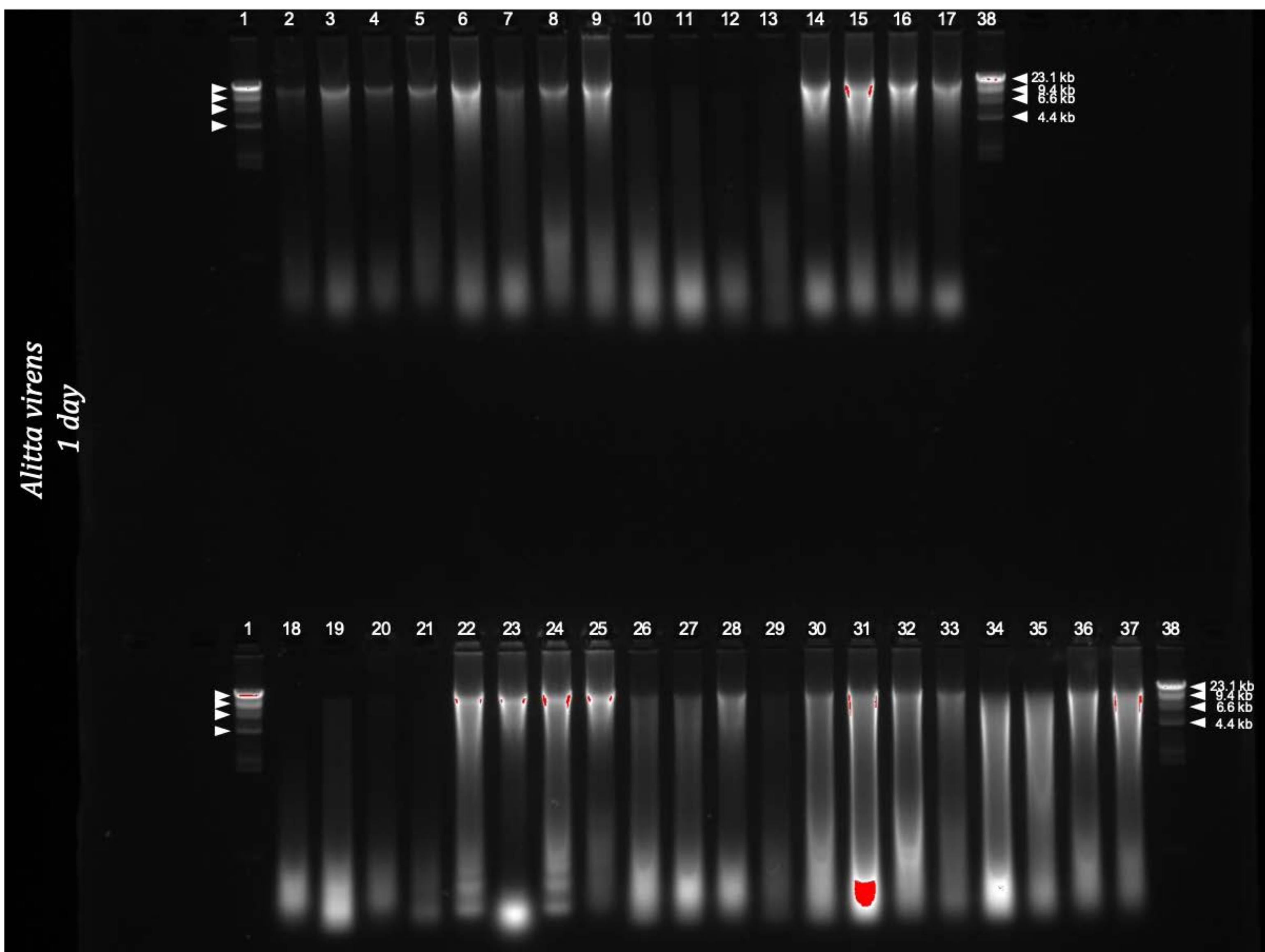

1 2 3 4 5 6 7 8 9 10 11 12 13 14 15 16 17 38
23.1 kb
9.4 kb
6.6 kb
4.4 kb
Alitta virens
1 day
1 18 19 20 21 22 23 24 25 26 27 28 29 30 31 32 33 34 35 36 37 38
23.1 kb
9.4 kb
6.6 kb
4.4 kb

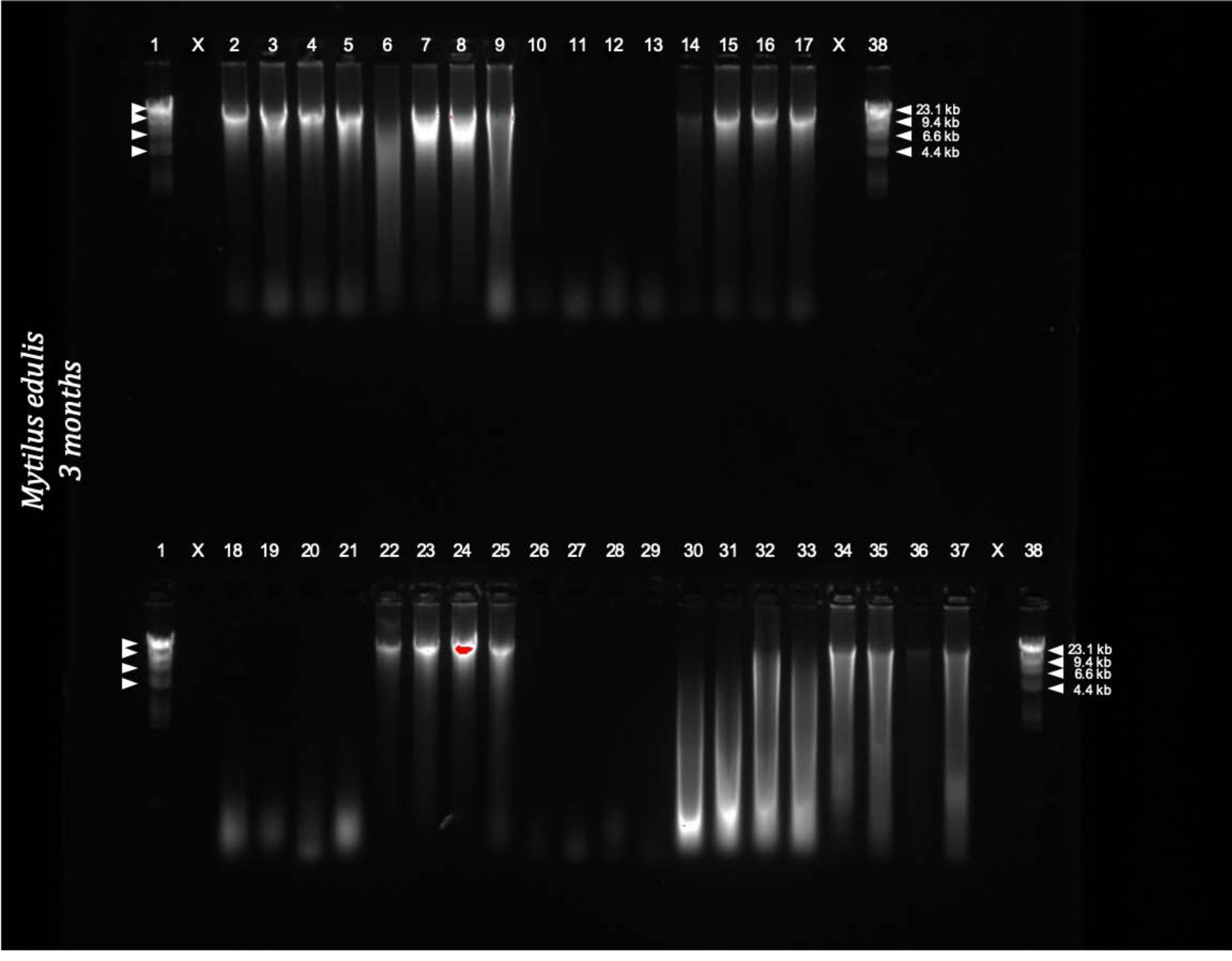
Mytilus edulis
3 months
1 X 2 3 4 5 6 7 8 9 10 11 12 13 14 15 16 17 X 38
23.1 kb
9.4 kb
6.6 kb
4.4 kb
1 X 18 19 20 21 22 23 24 25 26 27 28 29 30 31 32 33 34 35 36 37 X 38
23.1 kb
9.4 kb
6.6 kb
4.4 kb

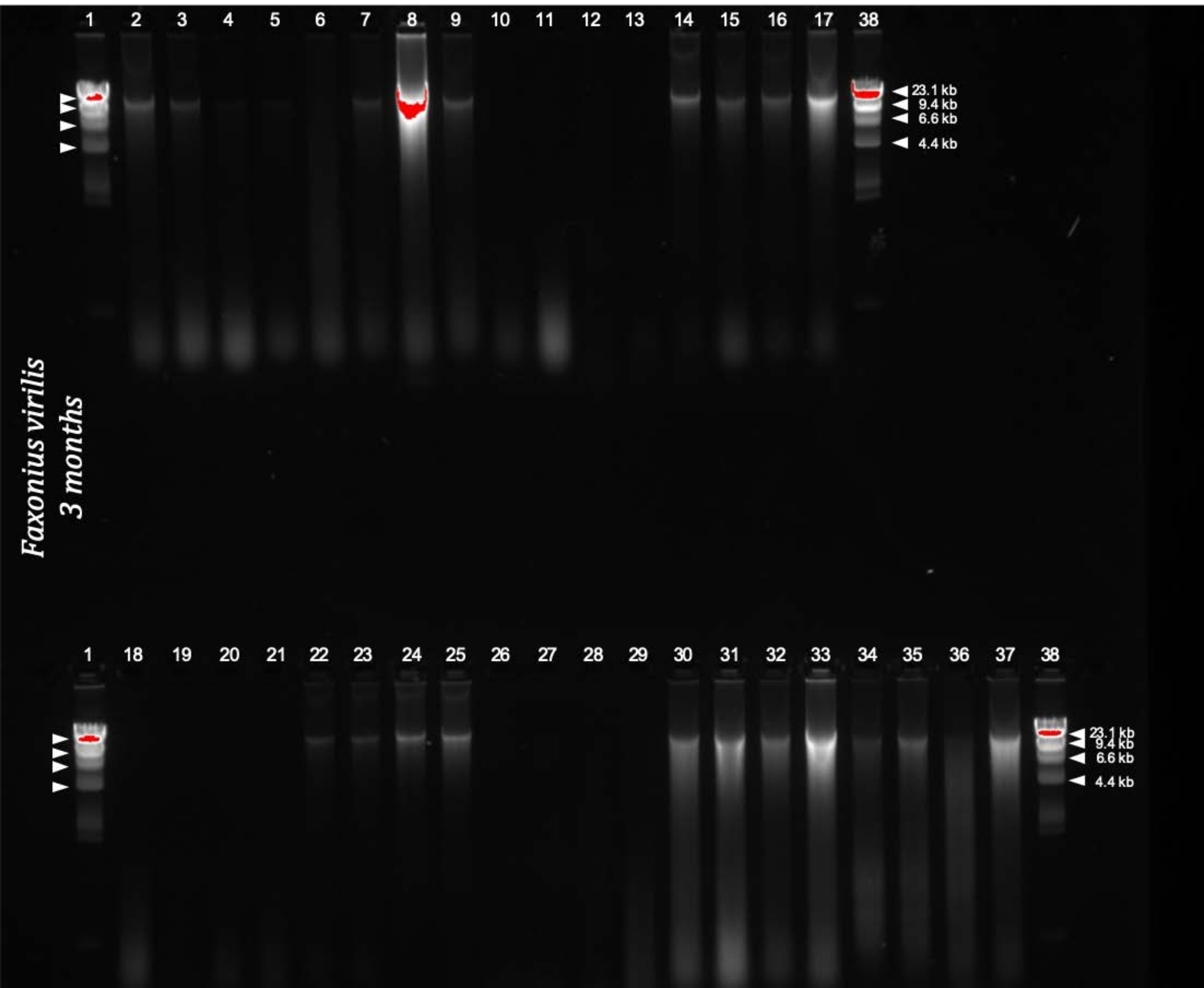

Faxonius virilis
3 months
1 2 3 4 5 6 7 8 9 10 11 12 13 14 15 16 17 38
23.1 kb
9.4 kb
6.6 kb
4.4 kb
1 18 19 20 21 22 23 24 25 26 27 28 29 30 31 32 33 34 35 36 37 38
23.1 kb
9.4 kb
6.6 kb
4.4 kb

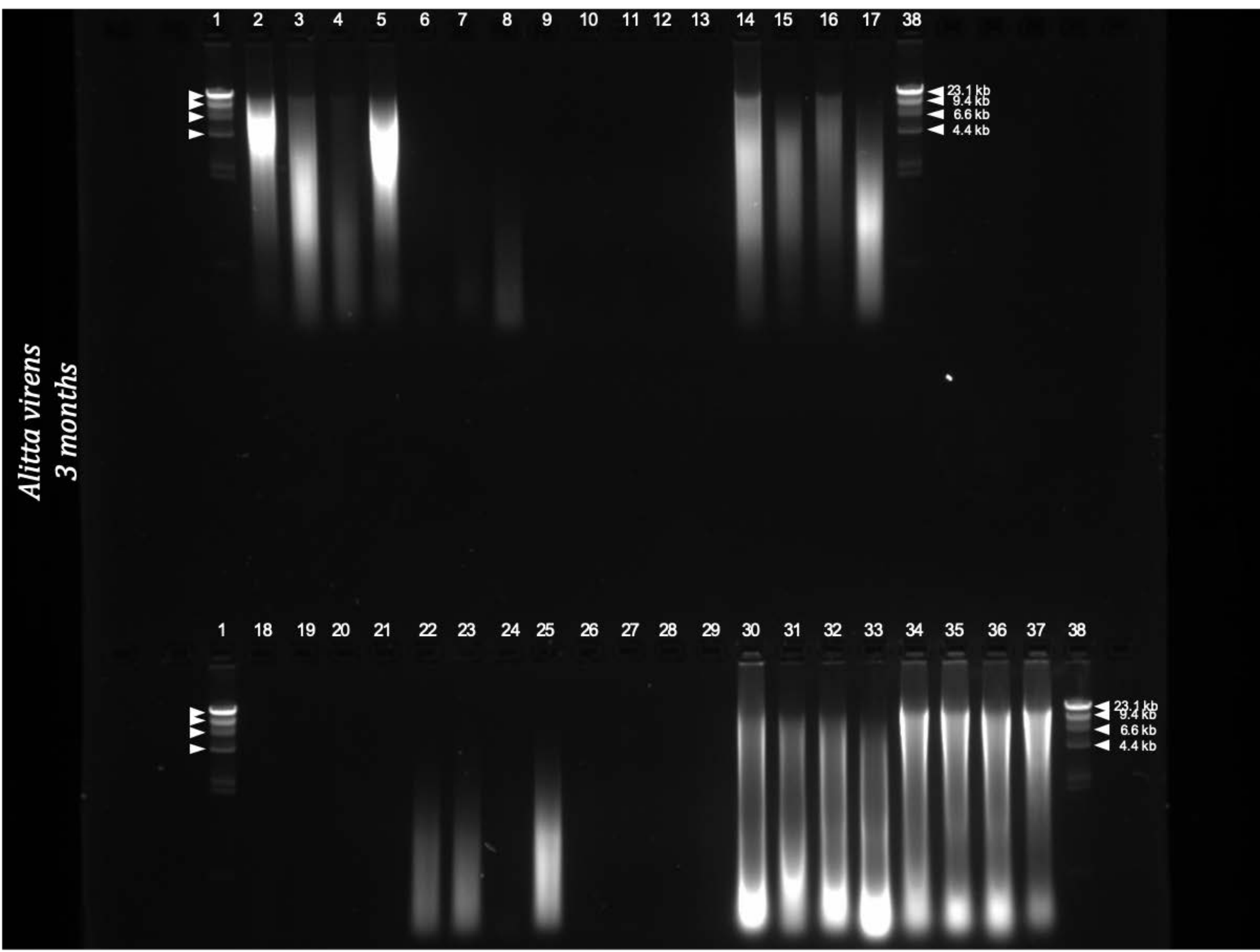
Alitta virens
3 months
1 2 3 4 5 6 7 8 9 10 11 12 13 14 15 16 17 38
23.1 kb
9.4 kb
6.6 kb
4.4 kb
1 18 19 20 21 22 23 24 25 26 27 28 29 30 31 32 33 34 35 36 37 38
23.1 kb
9.4 kb
6.6 kb
4.4 kb

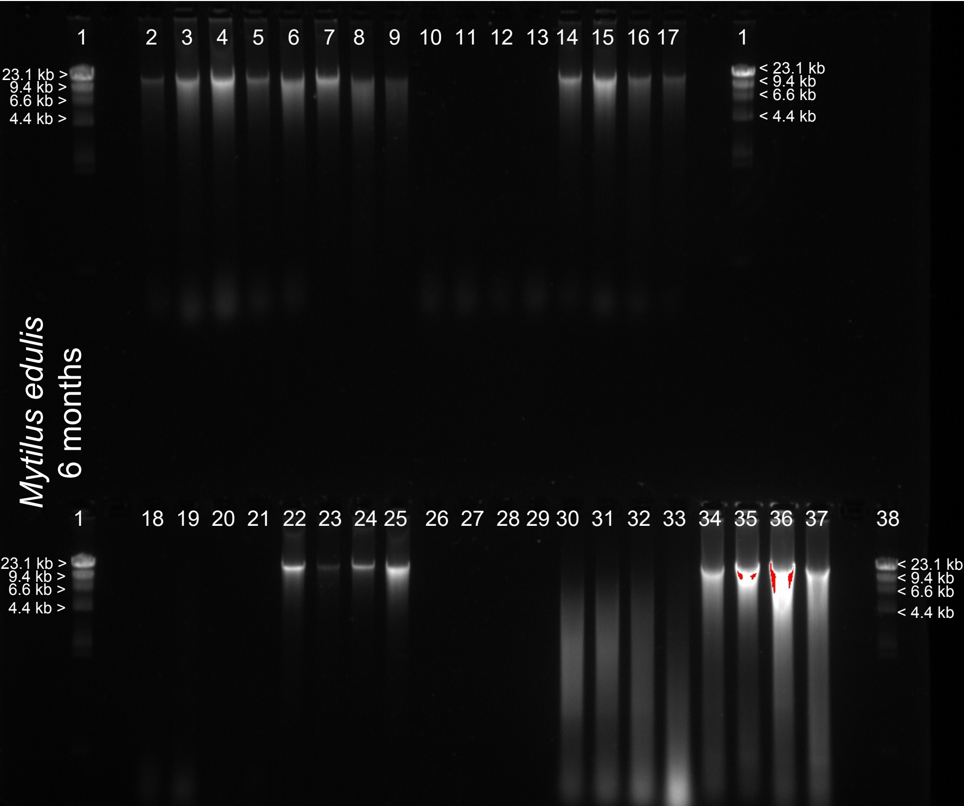

1
2 3 4 5 6 7 8 9 10 11 12 13 14 15 16 17
1
23.1 kb >
9.4 kb >
6.6 kb >
4.4 kb >
< 23.1 kb
< 9.4 kb
< 6.6 kb
< 4.4 kb
Mytilus edulis
6 months
1
18 19 20 21 22 23 24 25 26 27 28 29 30 31 32 33 34 35 36 37
38
23.1 kb >
9.4 kb >
6.6 kb >
4.4 kb >
< 23.1 kb
< 9.4 kb
< 6.6 kb
< 4.4 kb

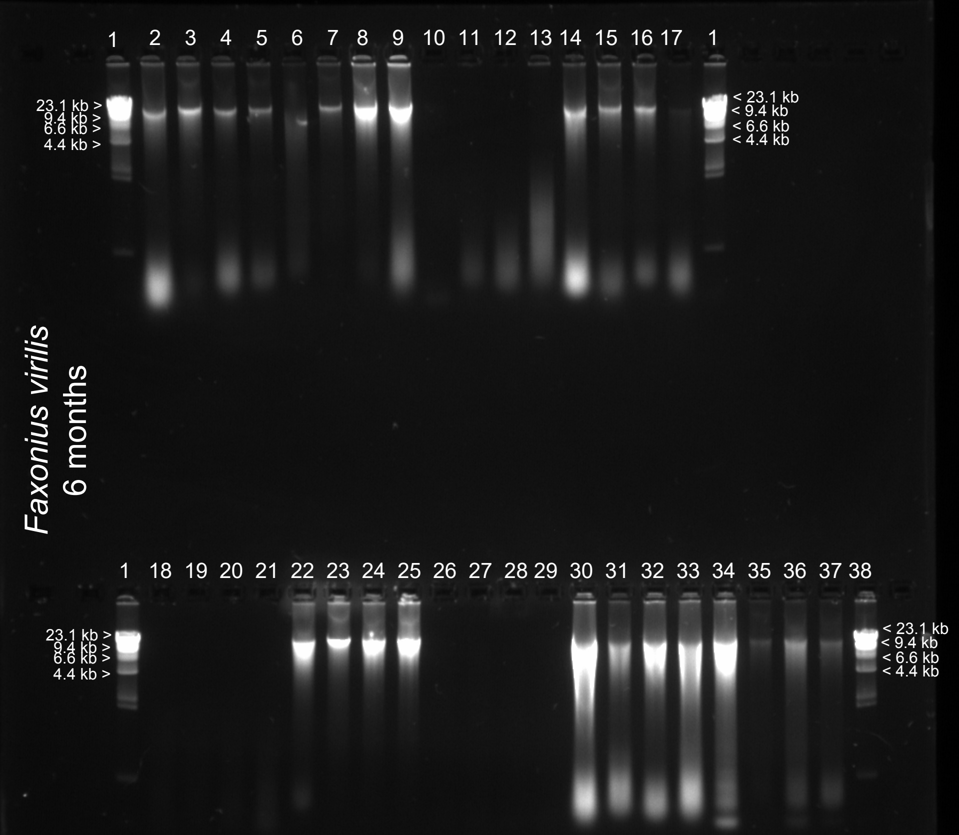

1 2 3 4 5 6 7 8 9 10 11 12 13 14 15 16 17 1
23.1 kb >
9.4 kb >
6.6 kb >
4.4 kb >
< 23.1 kb
< 9.4 kb
< 6.6 kb
< 4.4 kb
Faxonius virilis
6 months
1 18 19 20 21 22 23 24 25 26 27 28 29 30 31 32 33 34 35 36 37 38
23.1 kb >
9.4 kb >
6.6 kb >
4.4 kb >
< 23.1 kb
< 9.4 kb
< 6.6 kb
< 4.4 kb

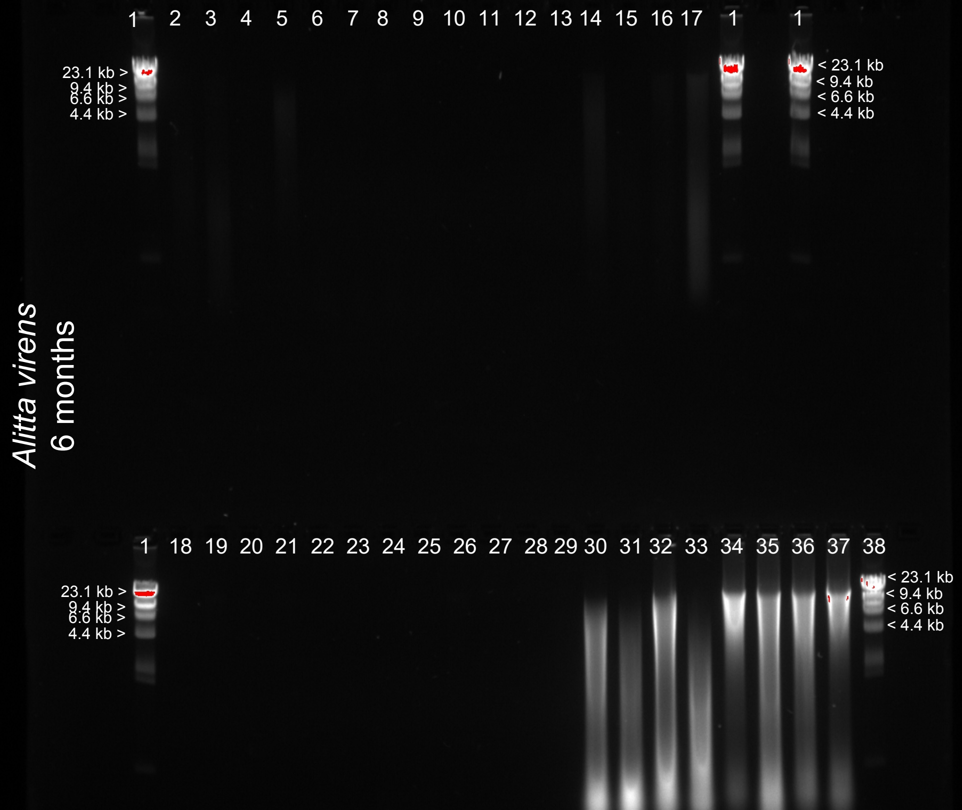
Alitta virens
6 months
1 2 3 4 5 6 7 8 9 10 11 12 13 14 15 16 17 1 1
23.1 kb >
9.4 kb >
6.6 kb >
4.4 kb >
< 23.1 kb
< 9.4 kb
< 6.6 kb
< 4.4 kb
1 18 19 20 21 22 23 24 25 26 27 28 29 30 31 32 33 34 35 36 37 38
23.1 kb >
9.4 kb >
6.6 kb >
4.4 kb >
< 23.1 kb
< 9.4 kb
< 6.6 kb
< 4.4 kb
