# Supplementary material for: DESS deconstructed: Is EDTA solely responsible for protection of high molecular weight DNA in this common tissue preservative?
Source: PLoS One. 2020 Aug 20;15(8):e0237356. doi: 10.1371/journal.pone.0237356 (PMC7440624; doi:10.1371/journal.pone.0237356)
Supplement: S4 Fig — TapeStation analysis was carried out on DNAs extracted from tissues of Mytilus edulis, Faxonius virilis and Alitta virens that were extracted immediately from fresh tissue or stored for 1 day, 3 months or 6 months at room temperature in DESS, six DESS-variant solutions or 95% ethanol. We provide the gel, sample information and electropherogram including region analysis for each sample analyzed. Values can be cross referenced with S2 Table. (PDF) [file pone.0237356.s007.pdf]

Filename: 2018-09-26-01.gDNA

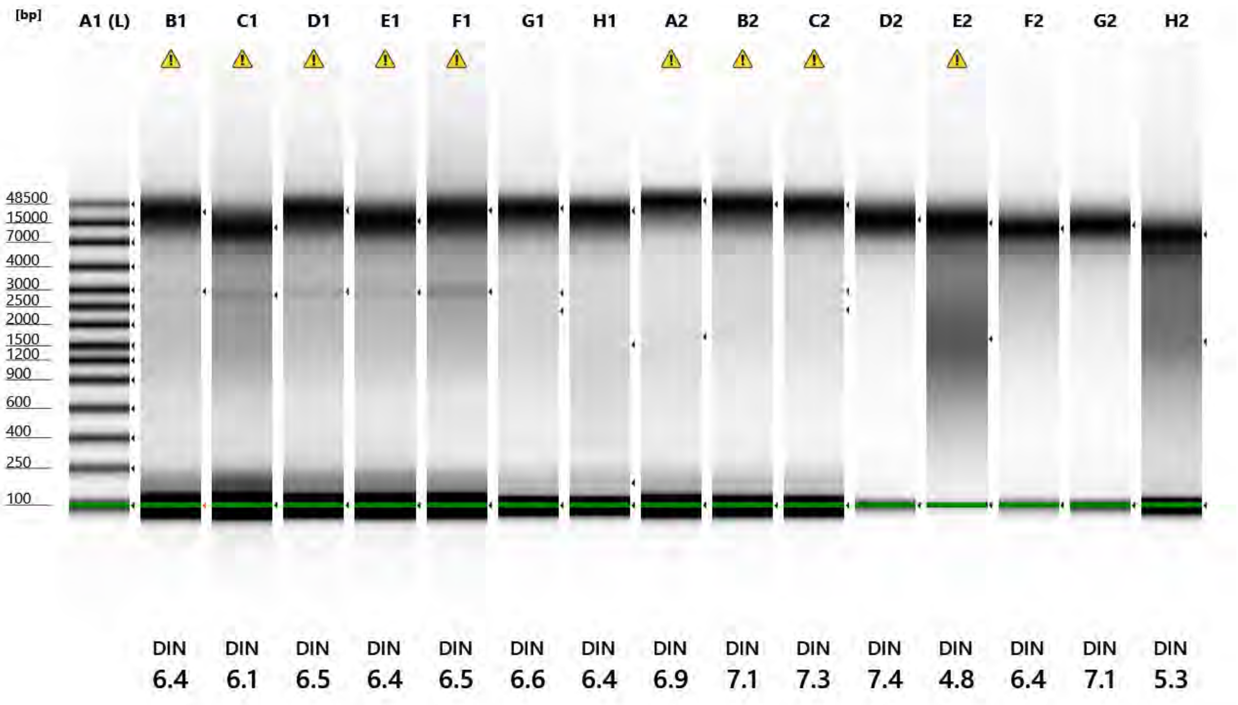

Default image (Contrast 50%), Image is Scaled to Sample

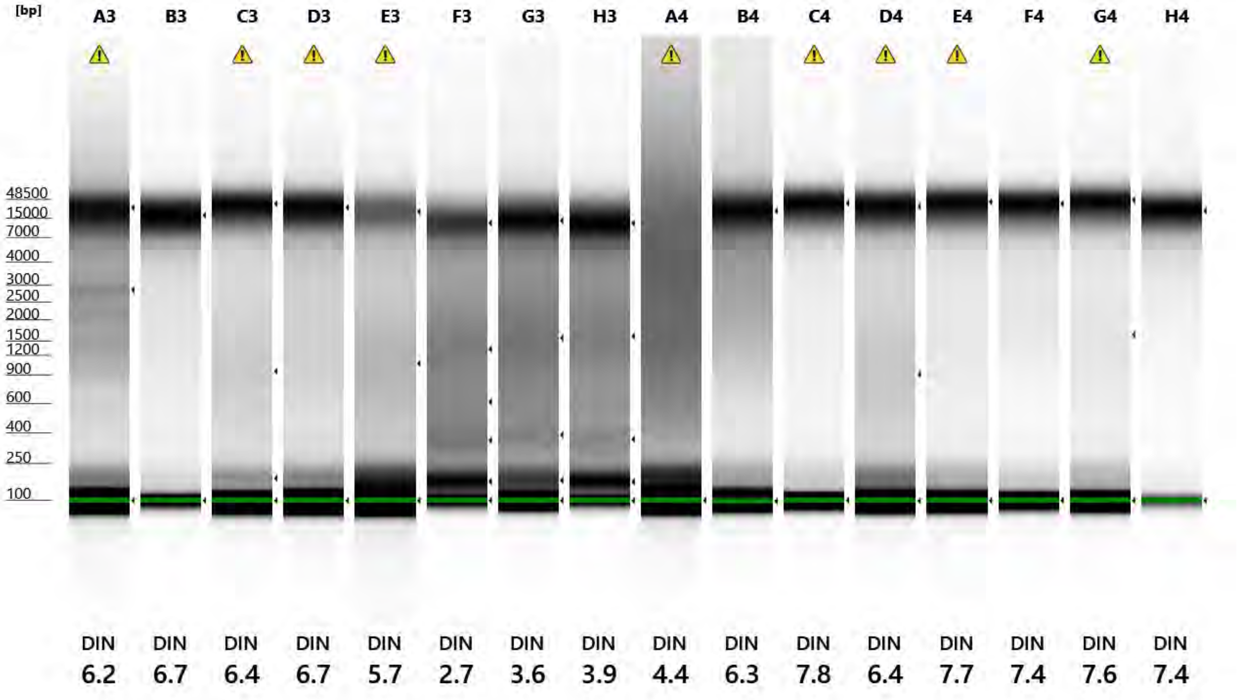

Default image (Contrast 50%), Image is Scaled to Sample

 © Agilent Technologies, Inc. 2017

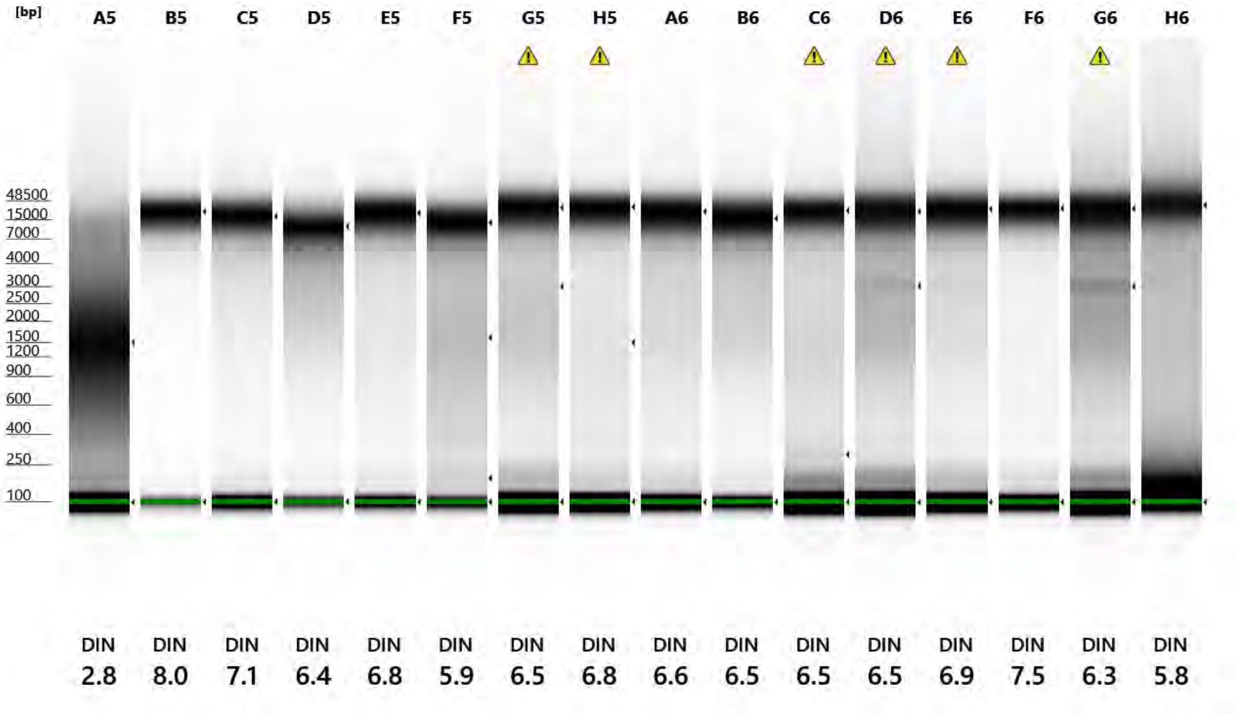

Default image (Contrast 50%), Image is Scaled to Sample

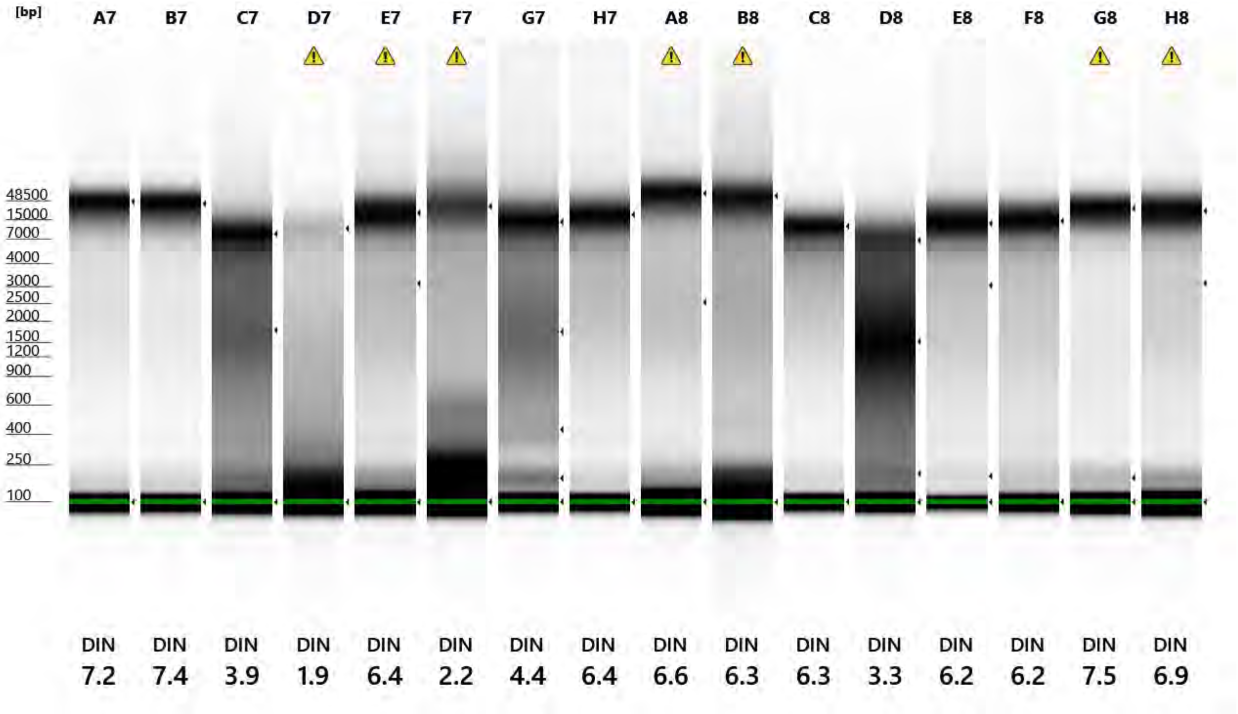

Default image (Contrast 50%), Image is Scaled to Sample

Sample Info

Well	DIN	Conc. [ng/µl]	Sample Description	Alert	Observations
A1	-	85.9	Ladder		Ladder
B1	6.4	7.49	C451	⚠	Sample concentration outside recommended range
C1	6.1	5.14	C452	⚠	Sample concentration outside recommended range
D1	6.5	7.18	C453	⚠	Sample concentration outside recommended range
E1	6.4	6.57	C454	⚠	Sample concentration outside recommended range
F1	6.5	7.48	C455	⚠	Sample concentration outside recommended range
G1	6.6	10.4	C456		
H1	6.4	12.8	C457		
A2	6.9	6.88	C458	⚠	Sample concentration outside recommended range
B2	7.1	8.77	C459	⚠	Sample concentration outside recommended range
C2	7.3	7.82	C460	⚠	Sample concentration outside recommended range
D2	7.4	50.5	C461		
E2	4.8	385	C462	⚠	Sample concentration outside functional range for DIN
F2	6.4	74.6	C463		
G2	7.1	41.8	C464		
H2	5.3	30.4	C465		
A3	6.2	7.24	C466	⚠	Sample concentration outside recommended range
B3	6.7	25.0	C467		
C3	6.4	6.40	C468	⚠	Sample concentration outside recommended range
D3	6.7	4.91	C469	⚠	Sample concentration outside functional range for DIN
E3	5.7	3.76	C470	⚠	Sample concentration outside functional range for DIN
F3	2.7	72.0	C471		
G3	3.6	21.9	C472		
H3	3.9	81.2	C473		
A4	4.4	8.08	C474	⚠	Sample concentration outside recommended range
B4	6.3	12.7	C475		
C4	7.8	9.79	C476	⚠	Sample concentration outside recommended range
D4	6.4	5.88	C477	⚠	Sample concentration outside recommended range
E4	7.7	5.78	C478	⚠	Sample concentration outside recommended range
F4	7.4	10.7	C479		
G4	7.6	5.96	C480	⚠	Sample concentration outside recommended range
H4	7.4	43.4	C481		
A5	2.8	28.2	C482		
B5	8.0	42.1	C483		
C5	7.1	19.3	C484		
D5	6.4	45.0	C485		
E5	6.8	27.3	C486		
F5	5.9	49.1	C487		
G5	6.5	9.44	C488	⚠	Sample concentration outside recommended range
H5	6.8	9.60	C489	⚠	Sample concentration outside recommended range
A6	6.6	17.2	C490		
B6	6.5	31.6	C491		
C6	6.5	6.56	C492	⚠	Sample concentration outside recommended range
D6	6.5	6.56	C493	⚠	Sample concentration outside recommended range
E6	6.9	9.66	C494	⚠	Sample concentration outside recommended range
F6	7.5	11.9	C495		
G6	6.3	7.66	C316	⚠	Sample concentration outside recommended range
H6	5.8	13.9	C317		
A7	7.2	10.2	C318		
B7	7.4	11.0	C319		
C7	3.9	20.8	C320		
D7	1.9	7.25	C321	⚠	Sample concentration outside recommended range
E7	6.4	8.04	C322	⚠	Sample concentration outside recommended range
F7	2.2	6.94	C323	⚠	Sample concentration outside recommended range
G7	4.4	26.0	C324		
H7	6.4	17.5	C325		
A8	6.6	7.10	C326	⚠	Sample concentration outside recommended range
B8	6.3	4.13	C327	⚠	Sample concentration outside functional range for DIN
C8	6.3	14.1	C328		
D8	3.3	27.5	C329		
E8	6.2	29.9	C330		
F8	6.2	15.6	C331		
G8	7.5	5.96	C332	⚠	Sample concentration outside recommended range
H8	6.9	5.30	C333	⚠	Sample concentration outside recommended range

 © Agilent Technologies, Inc. 2017

A1: Ladder

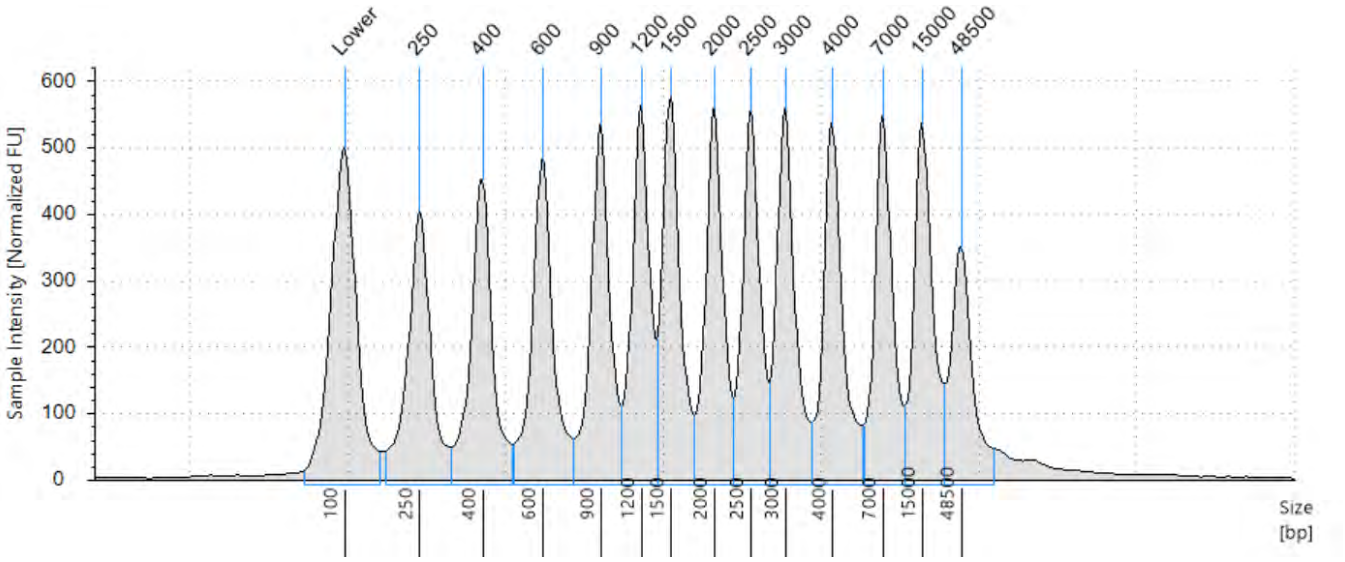

Sample Table

Well	DIN	Conc. [ng/µl]	Sample Description	Alert	Observations
A1	-	85.9	Ladder		Ladder

Peak Table

Size [bp]	Calibrated Conc. [ng/µl]	Assigned Conc. [ng/µl]	% Integrated Area	From [bp]	To [bp]	Peak Comment	Observations
100	8.50	8.50	-	61	153		Lower Marker
250	6.10	-	7.17	166	315		
400	6.40	-	7.51	315	488		
600	6.71	-	7.88	492	742		
900	6.76	-	7.94	742	1034		
1200	6.82	-	8.01	1034	1349		
1500	6.97	-	8.18	1349	1756		
2000	6.71	-	7.88	1756	2255		
2500	6.64	-	7.80	2255	2769		
3000	6.84	-	8.03	2769	3523		
4000	6.85	-	8.04	3523	5544		
7000	6.64	-	7.79	5631	10820		
15000	6.75	-	7.93	10820	23200		
48500	4.97	-	5.83	23200	>60000		

 © Agilent Technologies, Inc. 2017

B1: C451

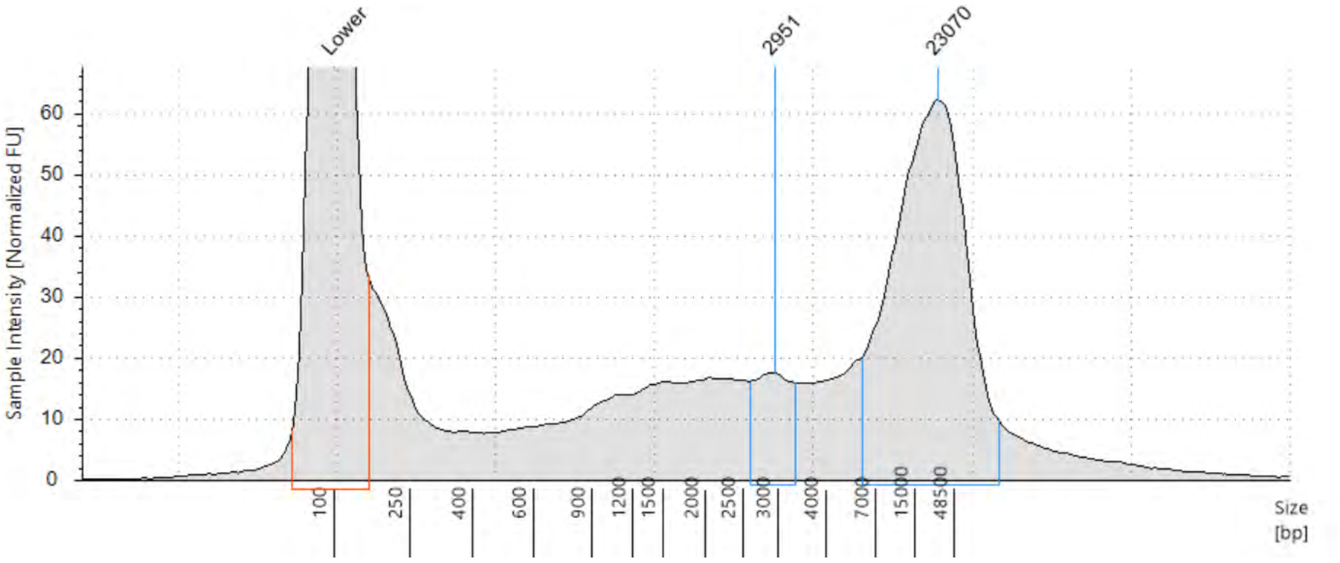

Sample Table

Well	DIN	Conc. [ng/µl]	Sample Description	Alert	Observations
B1	6.4	7.49	C451	⚠	Sample concentration outside recommended range

Peak Table

Size [bp]	Calibrated Conc. [ng/µl]	Assigned Conc. [ng/µl]	% Integrated Area	From [bp]	To [bp]	Peak Comment	Observations
100	8.50	8.50	-	61	152		Lower Marker
2951	0.447	-	12.36	2594	3335		
23070	3.14	-	86.87	5965	>60000		
-	-	-	-	-	-		Sample Well

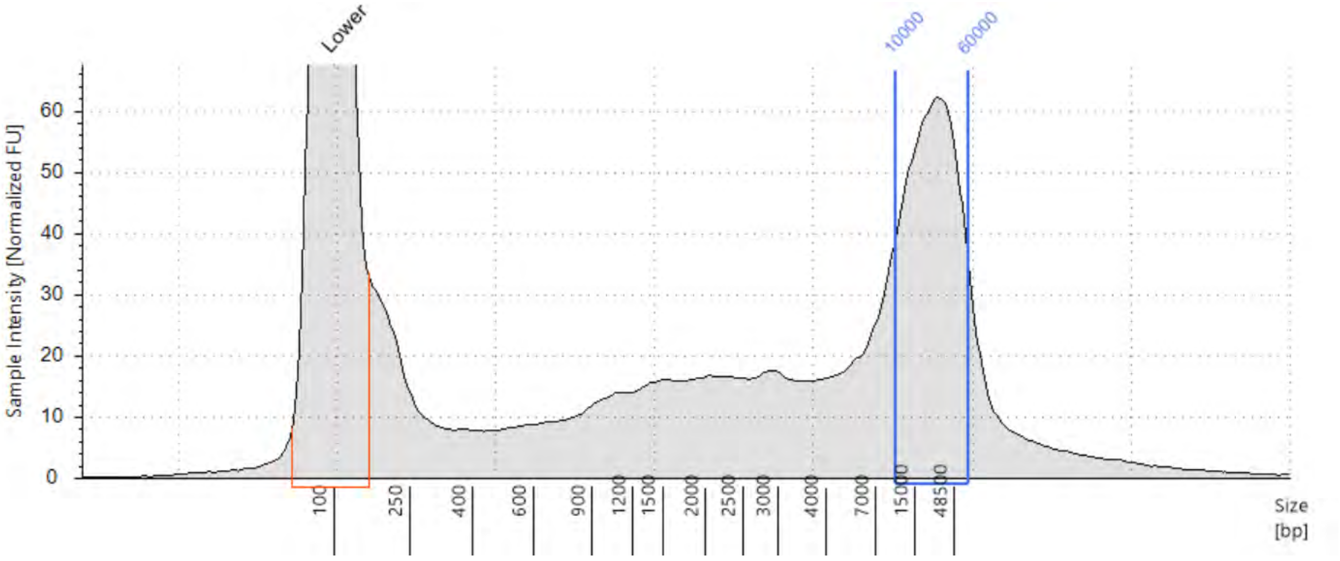

Region Table

From [bp]	To [bp]	Average Size [bp]	Conc. [ng/µl]	Region Molarity [nmol/l]	% of Total	Region Comment	Color
10000	60000	26374	2.33	0.180	31.10		■

 © Agilent Technologies, Inc. 2017

C1: C452

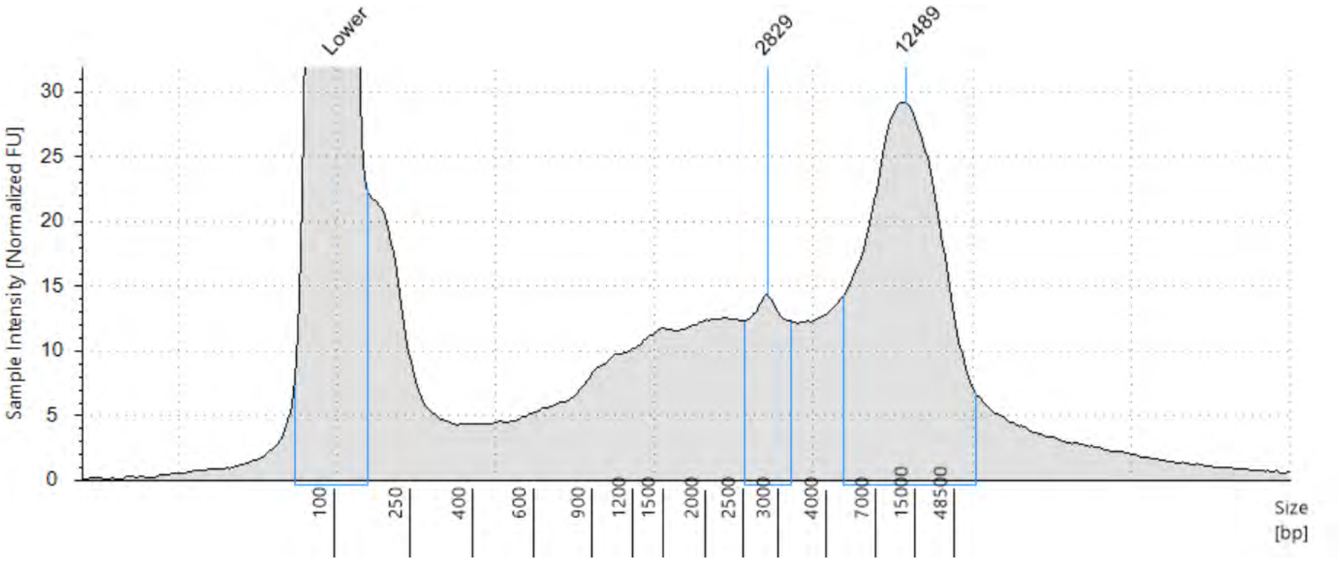

Sample Table

Well	DIN	Conc. [ng/µl]	Sample Description	Alert	Observations
C1	6.1	5.14	C452	⚠	Sample concentration outside recommended range

Peak Table

Size [bp]	Calibrated Conc. [ng/µl]	Assigned Conc. [ng/µl]	% Integrated Area	From [bp]	To [bp]	Peak Comment	Observations
100	8.50	8.50	-	61	147		Lower Marker
2829	0.393	-	18.41	2519	3233		
12489	1.72	-	80.63	4863	>60000		
-	-	-	-	-	-		Sample Well

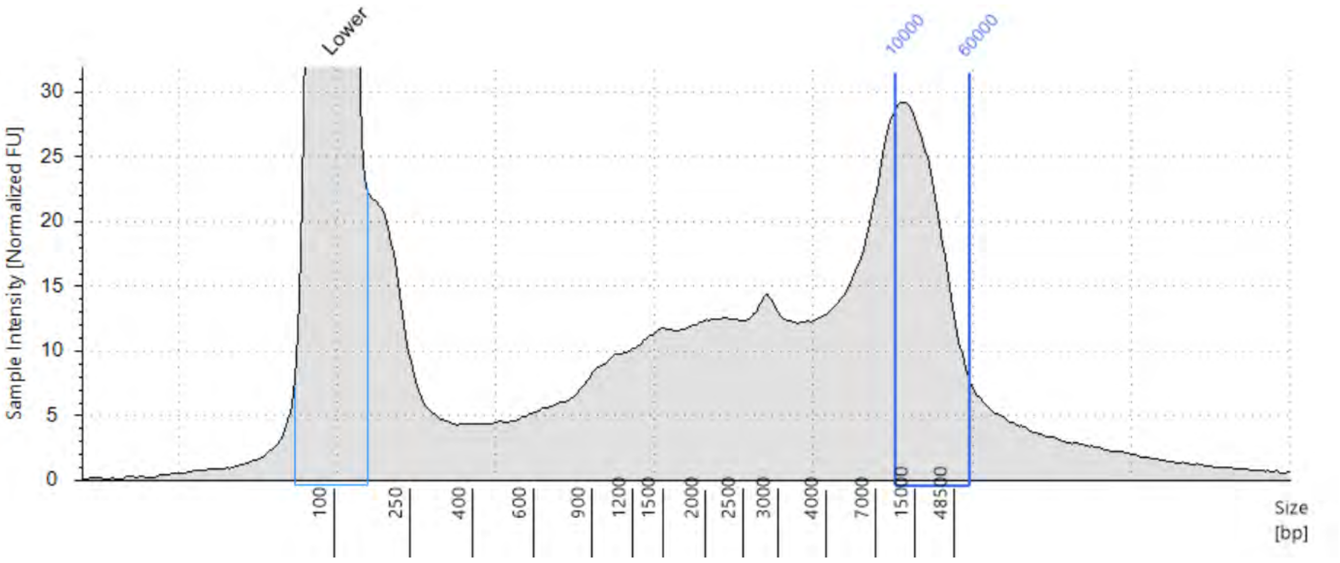

Region Table

From [bp]	To [bp]	Average Size [bp]	Conc. [ng/µl]	Region Molarity [nmol/l]	% of Total	Region Comment	Color
10000	60000	21569	1.02	0.0940	19.85		■

D1: C453

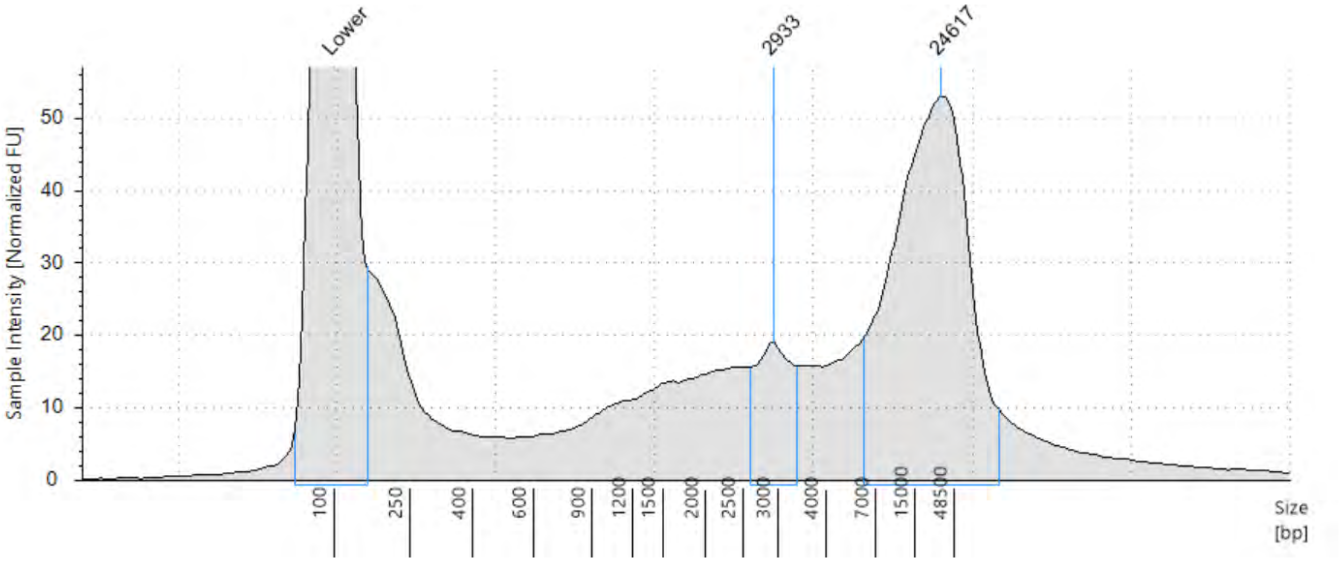

Sample Table

Well	DIN	Conc. [ng/µl]	Sample Description	Alert	Observations
D1	6.5	7.18	C453	⚠	Sample concentration outside recommended range

Peak Table

Size [bp]	Calibrated Conc. [ng/µl]	Assigned Conc. [ng/µl]	% Integrated Area	From [bp]	To [bp]	Peak Comment	Observations
100	8.50	8.50	-	61	147		Lower Marker
2933	0.488	-	14.36	2593	3343		
24617	2.90	-	85.19	6037	>60000		
-	-	-	-	-	-		Sample Well

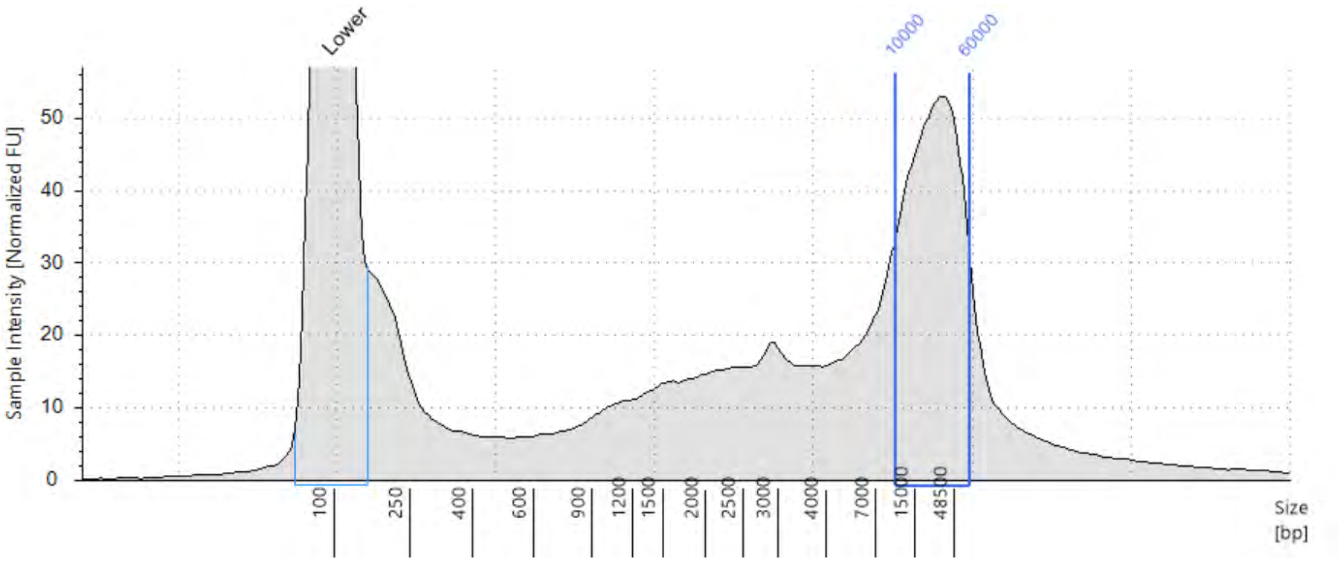

Region Table

From [bp]	To [bp]	Average Size [bp]	Conc. [ng/µl]	Region Molarity [nmol/l]	% of Total	Region Comment	Color
10000	60000	26664	2.12	0.163	29.47		■

 © Agilent Technologies, Inc. 2017

E1: C454

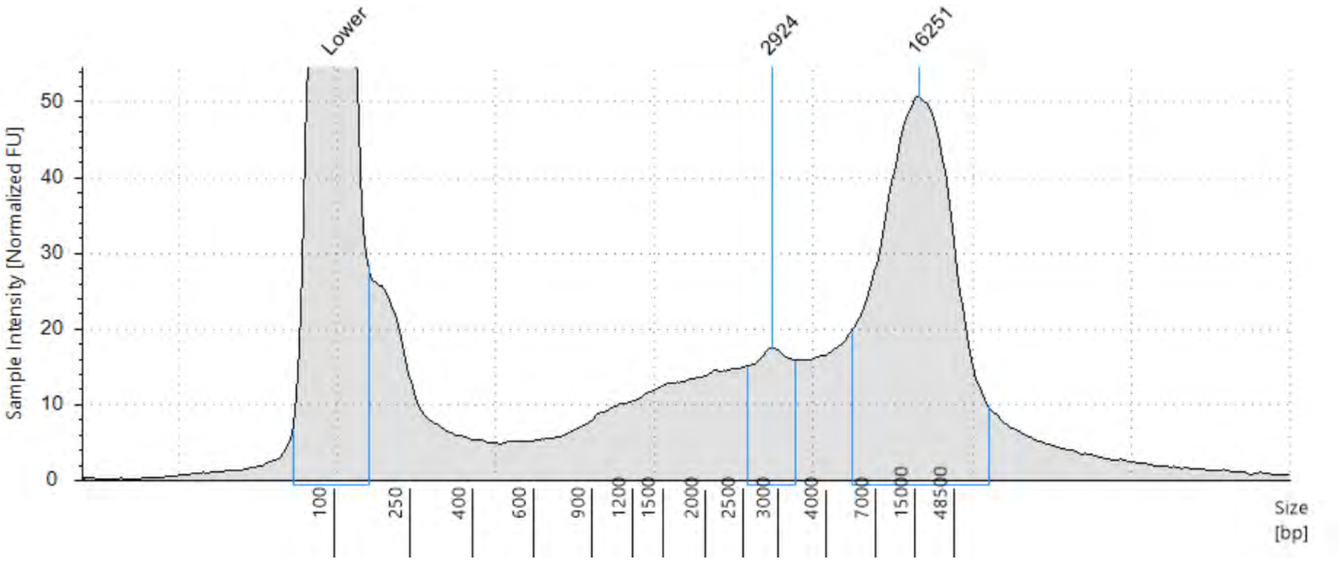

Sample Table

Well	DIN	Conc. [ng/µl]	Sample Description	Alert	Observations
E1	6.4	6.57	C454	⚠	Sample concentration outside recommended range

Peak Table

Size [bp]	Calibrated Conc. [ng/µl]	Assigned Conc. [ng/µl]	% Integrated Area	From [bp]	To [bp]	Peak Comment	Observations
100	8.50	8.50	-	61	152		Lower Marker
2924	0.466	-	14.49	2569	3327		
16251	2.73	-	84.84	5345	>60000		
-	-	-	-	-	-		Sample Well

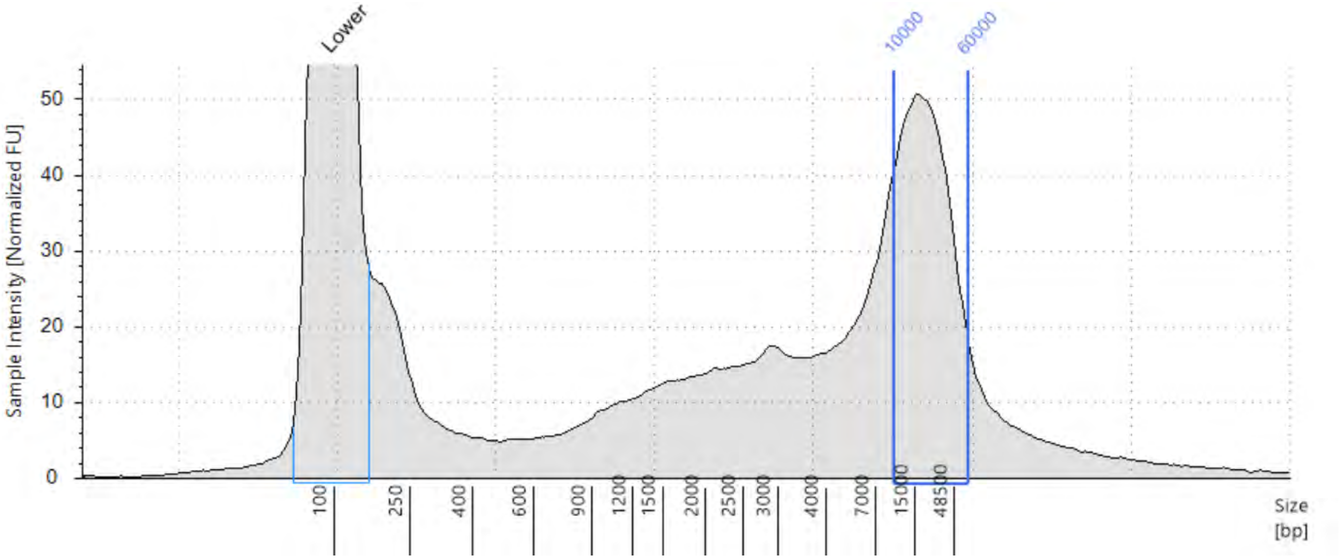

Region Table

From [bp]	To [bp]	Average Size [bp]	Conc. [ng/µl]	Region Molarity [nmol/l]	% of Total	Region Comment	Color
10000	60000	23446	1.88	0.160	28.65		■

 © Agilent Technologies, Inc. 2017

F1: C455

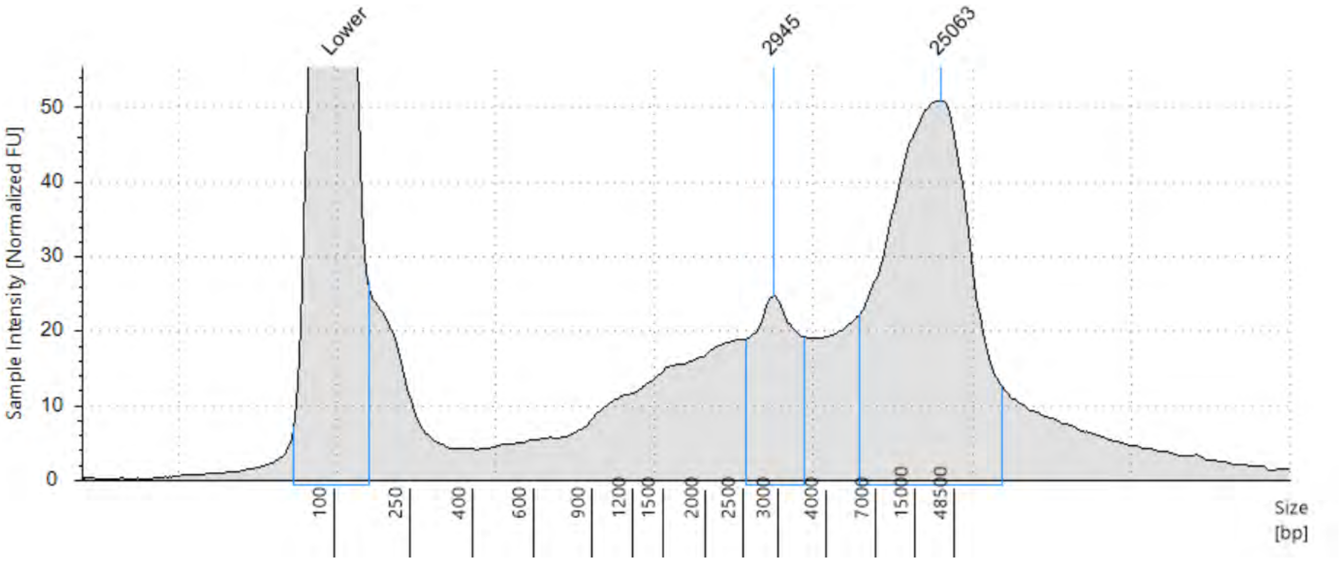

Sample Table

Well	DIN	Conc. [ng/µl]	Sample Description	Alert	Observations
F1	6.5	7.48	C455	⚠	Sample concentration outside recommended range

Peak Table

Size [bp]	Calibrated Conc. [ng/µl]	Assigned Conc. [ng/µl]	% Integrated Area	From [bp]	To [bp]	Peak Comment	Observations
100	8.50	8.50	-	62	152		Lower Marker
2945	0.743	-	19.55	2550	3527		
25063	2.99	-	78.73	5860	>60000		
-	-	-	-	-	-		Sample Well

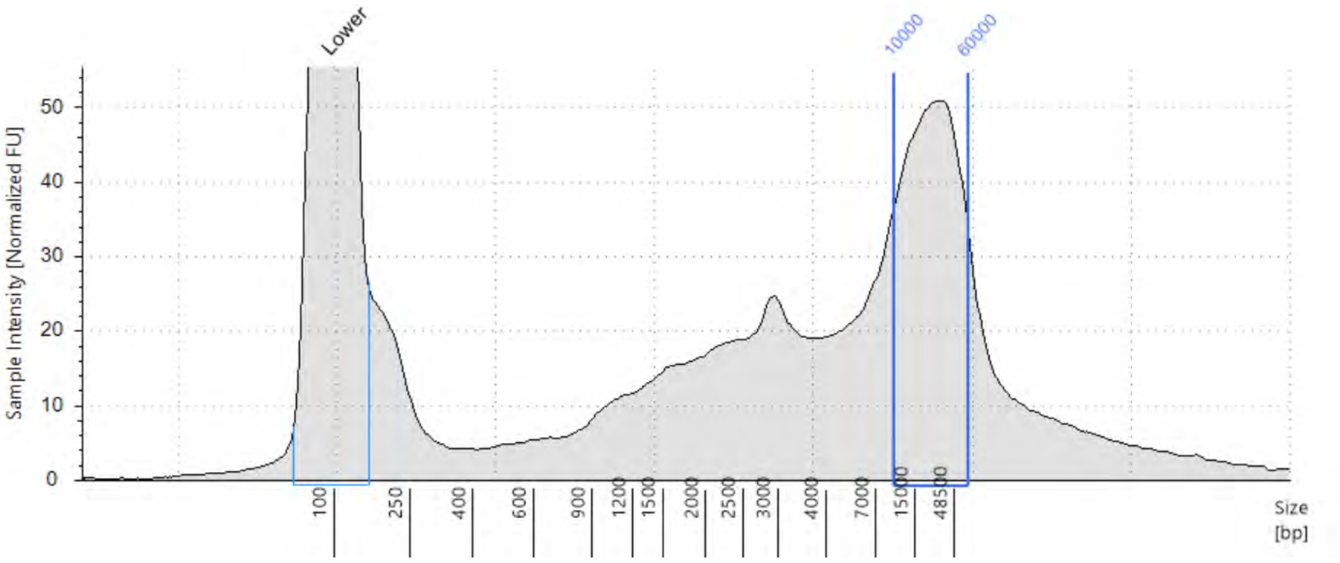

Region Table

From [bp]	To [bp]	Average Size [bp]	Conc. [ng/µl]	Region Molarity [nmol/l]	% of Total	Region Comment	Color
10000	60000	26479	2.05	0.161	27.45		■

© Agilent Technologies, Inc. 2017

G1: C456

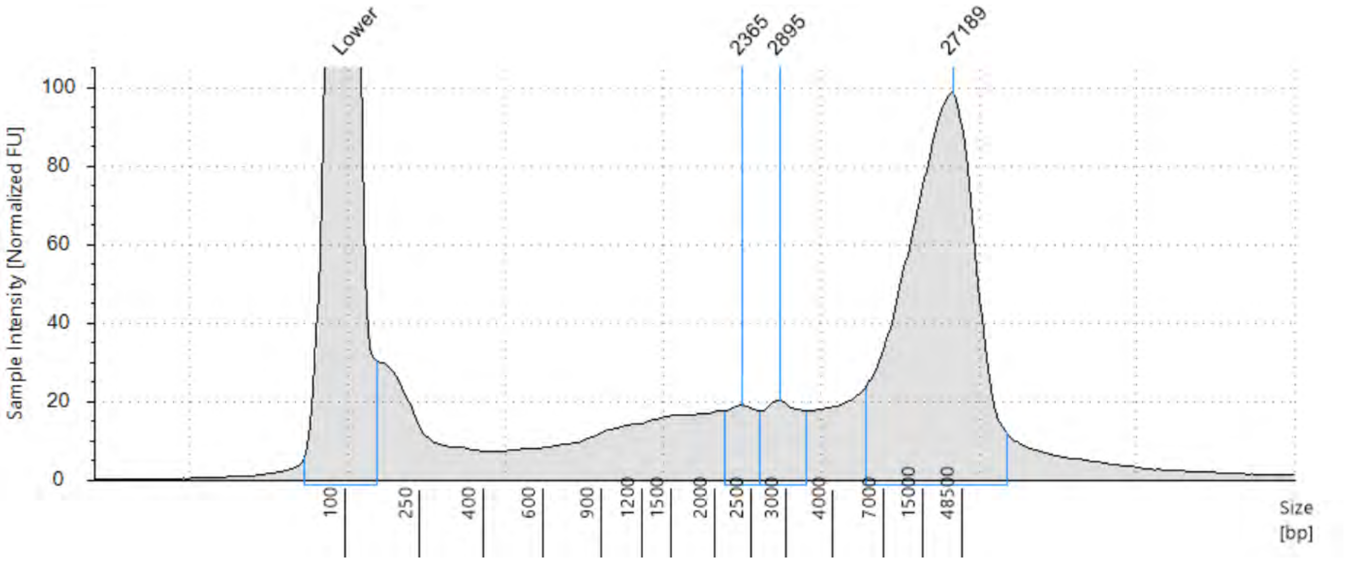

Sample Table

Well	DIN	Conc. [ng/µl]	Sample Description	Alert	Observations
G1	6.6	10.4	C456		

Peak Table

Size [bp]	Calibrated Conc. [ng/µl]	Assigned Conc. [ng/µl]	% Integrated Area	From [bp]	To [bp]	Peak Comment	Observations
100	8.50	8.50	-	62	147		Lower Marker
2365	0.430	-	6.87	2119	2618		
2895	0.555	-	8.86	2618	3373		
27189	5.20	-	82.95	5734	>60000		
-	-	-	-	-	-		Sample Well

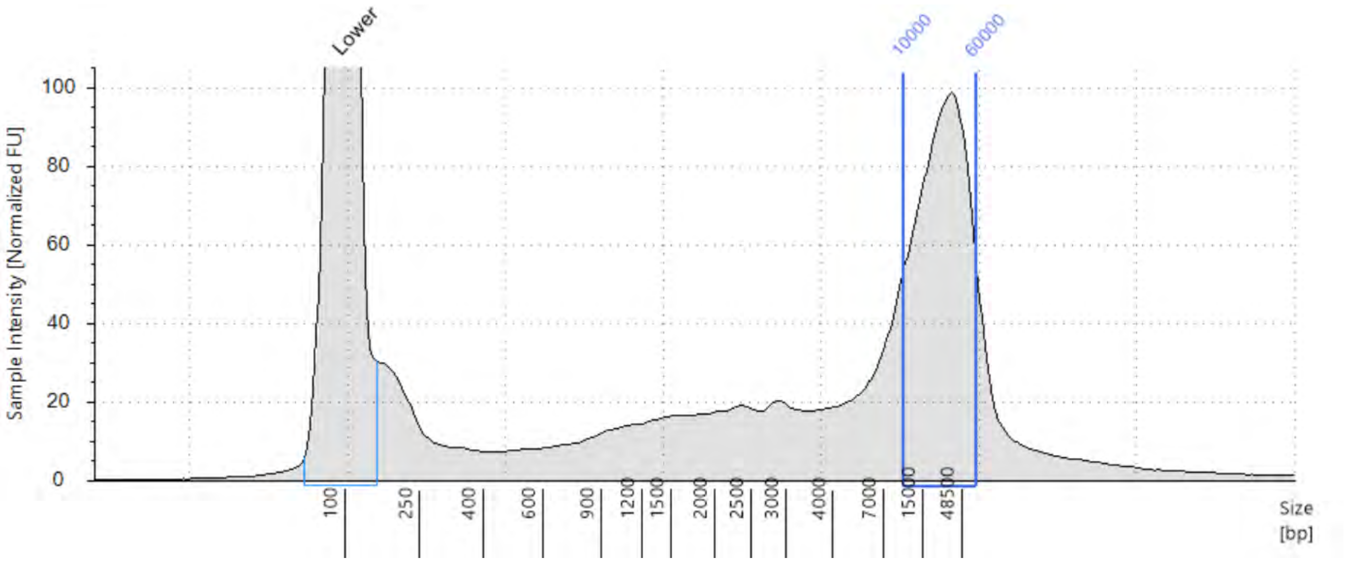

Region Table

From [bp]	To [bp]	Average Size [bp]	Conc. [ng/µl]	Region Molarity [nmol/l]	% of Total	Region Comment	Color
10000	60000	27321	3.85	0.285	37.01		

H1: C457

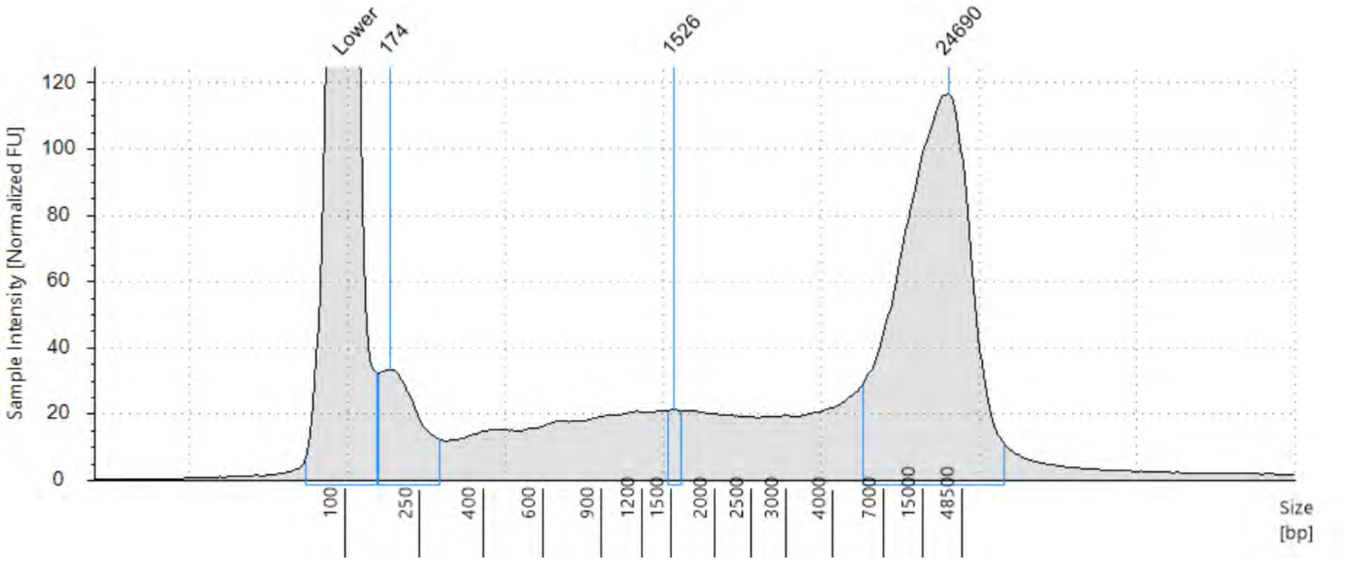

Sample Table

Well	DIN	Conc. [ng/µl]	Sample Description	Alert	Observations
H1	6.4	12.8	C457		

Peak Table

Size [bp]	Calibrated Conc. [ng/µl]	Assigned Conc. [ng/µl]	% Integrated Area	From [bp]	To [bp]	Peak Comment	Observations
100	8.50	8.50	-	62	147		Lower Marker
174	0.978	-	12.24	149	290		
1526	0.201	-	2.52	1451	1597		
24690	6.18	-	77.34	5546	>60000		
-	-	-	-	-	-		Sample Well

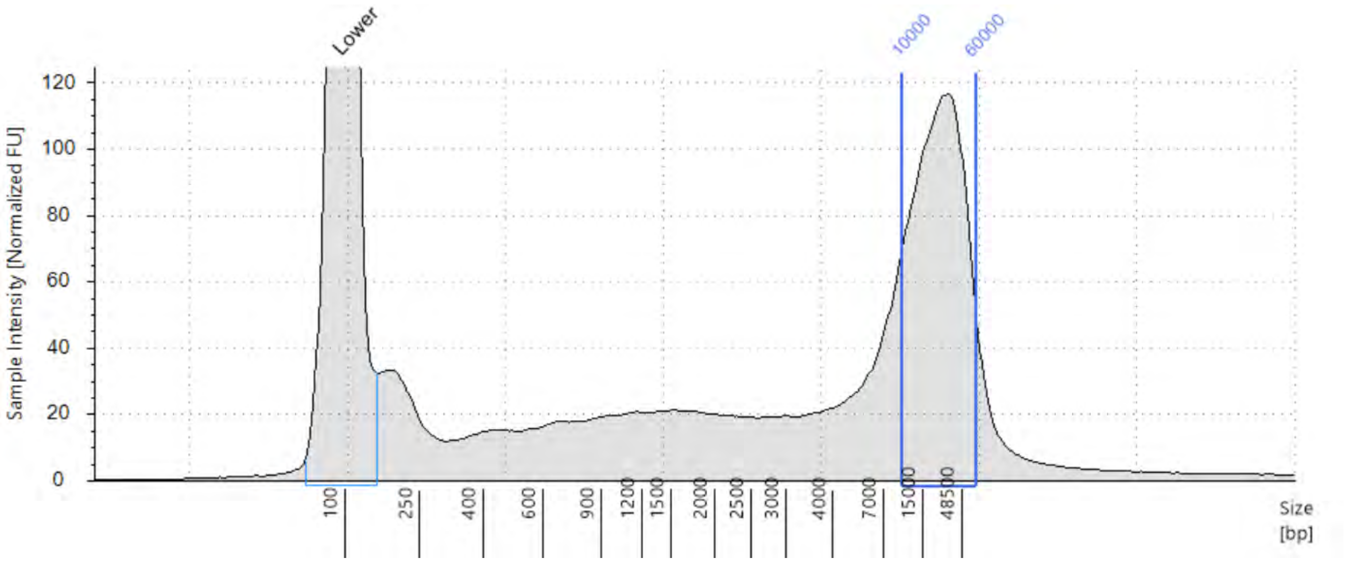

Region Table

From [bp]	To [bp]	Average Size [bp]	Conc. [ng/µl]	Region Molarity [nmol/l]	% of Total	Region Comment	Color
10000	60000	25984	4.61	0.356	36.01		

© Agilent Technologies, Inc. 2017

A2: C458

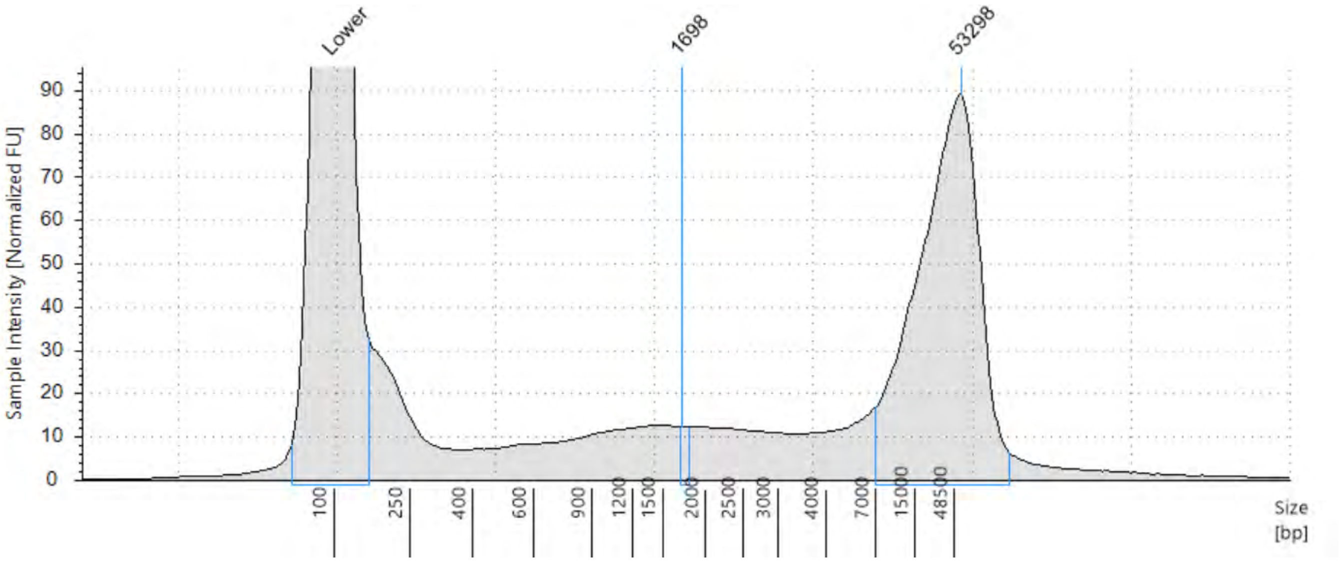

Sample Table

Well	DIN	Conc. [ng/µl]	Sample Description	Alert	Observations
A2	6.9	6.88	C458	⚠	Sample concentration outside recommended range

Peak Table

Size [bp]	Calibrated Conc. [ng/µl]	Assigned Conc. [ng/µl]	% Integrated Area	From [bp]	To [bp]	Peak Comment	Observations
100	8.50	8.50	-	59	152		Lower Marker
1698	0.0644	-	1.77	1682	1778		
53298	3.54	-	97.19	6937	>60000		
-	-	-	-	-	-		Sample Well

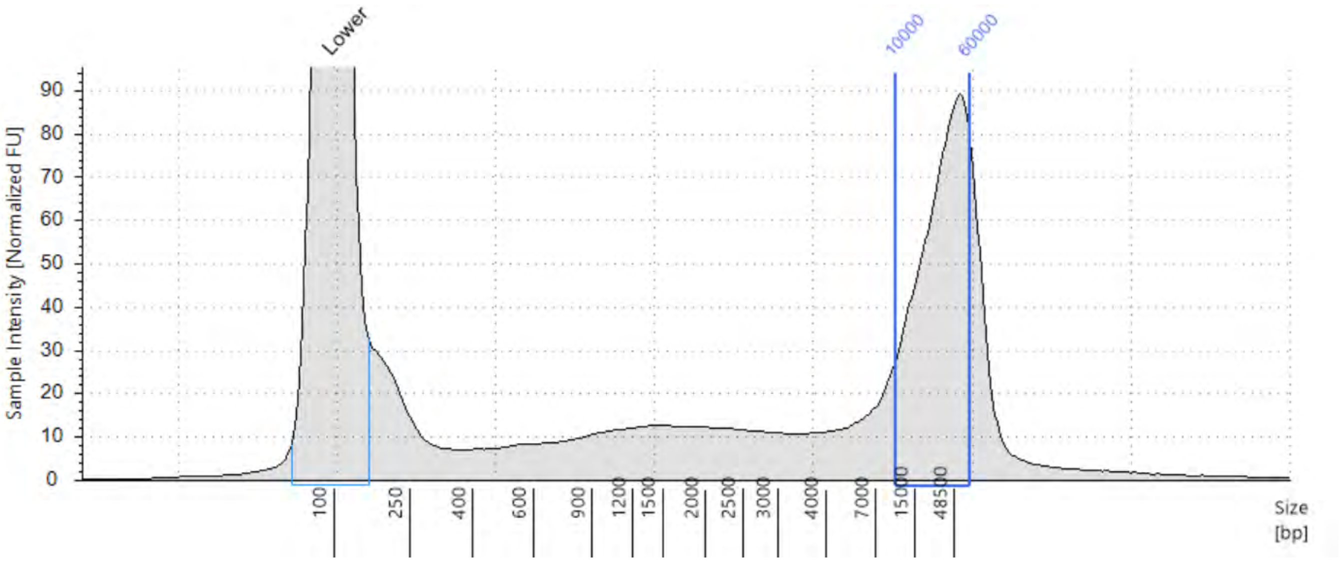

Region Table

From [bp]	To [bp]	Average Size [bp]	Conc. [ng/µl]	Region Molarity [nmol/l]	% of Total	Region Comment	Color
10000	60000	31238	2.59	0.172	37.65		■

B2: C459

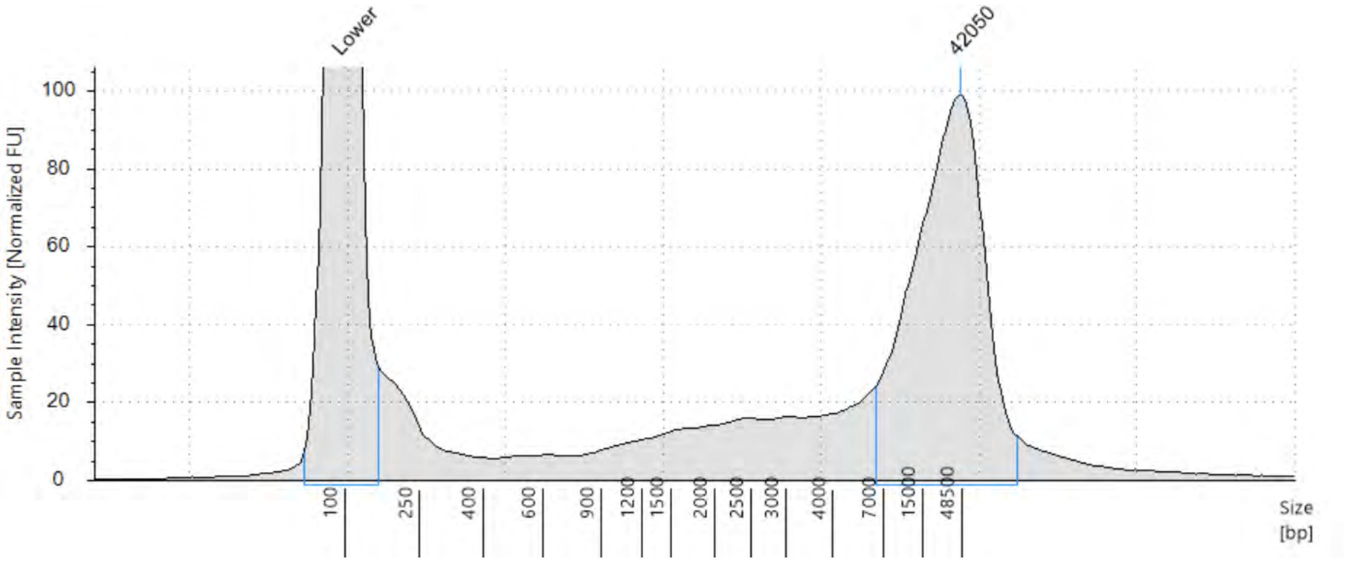

Sample Table

Well	DIN	Conc. [ng/µl]	Sample Description	Alert	Observations
B2	7.1	8.77	C459	⚠	Sample concentration outside recommended range

Peak Table

Size [bp]	Calibrated Conc. [ng/µl]	Assigned Conc. [ng/µl]	% Integrated Area	From [bp]	To [bp]	Peak Comment	Observations
100	8.50	8.50	-	61	152		Lower Marker
42050	4.83	-	98.92	6451	>60000		
-	-	-	-	-	-		Sample Well

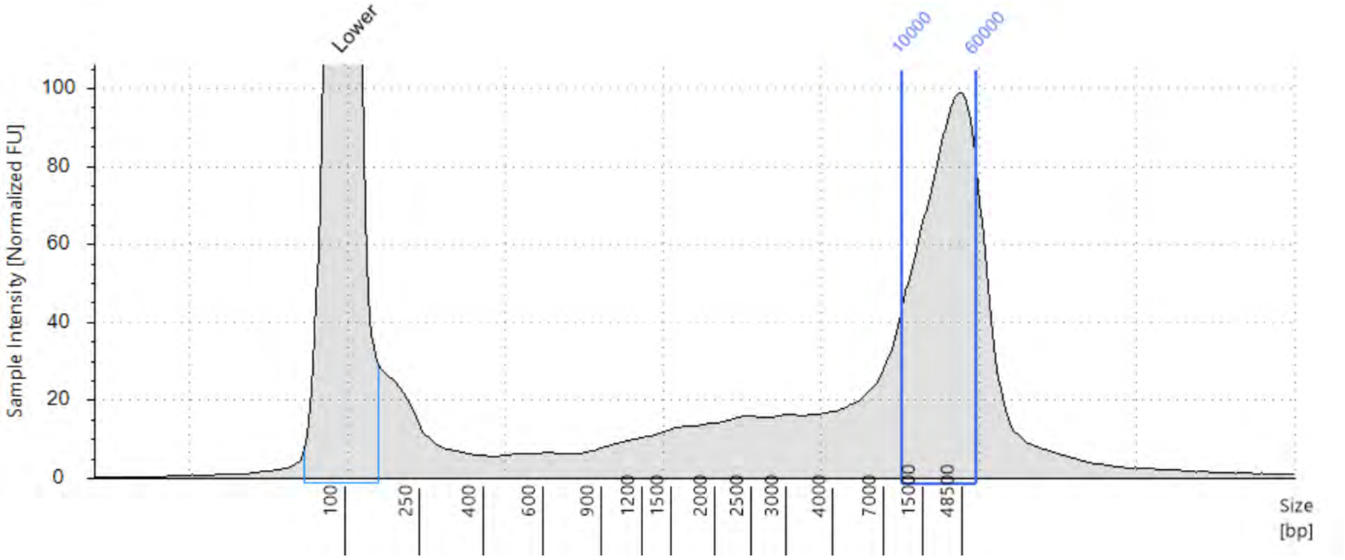

Region Table

From [bp]	To [bp]	Average Size [bp]	Conc. [ng/µl]	Region Molarity [nmol/l]	% of Total	Region Comment	Color
10000	60000	29397	3.46	0.244	39.49		

C2: C460

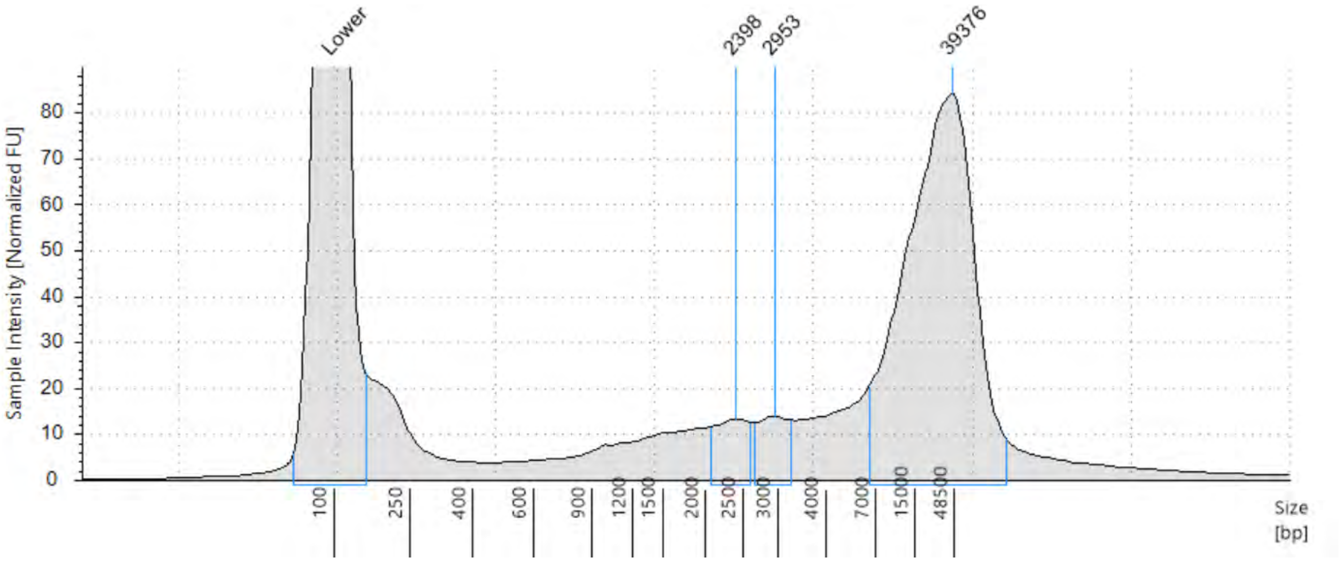

Sample Table

Well	DIN	Conc. [ng/µl]	Sample Description	Alert	Observations
C2	7.3	7.82	C460	⚠	Sample concentration outside recommended range

Peak Table

Size [bp]	Calibrated Conc. [ng/µl]	Assigned Conc. [ng/µl]	% Integrated Area	From [bp]	To [bp]	Peak Comment	Observations
100	8.50	8.50	-	61	147		Lower Marker
2398	0.324	-	6.40	2057	2612		
2953	0.308	-	6.09	2669	3257		
39376	4.34	-	85.69	6493	>60000		
-	-	-	-	-	-		Sample Well

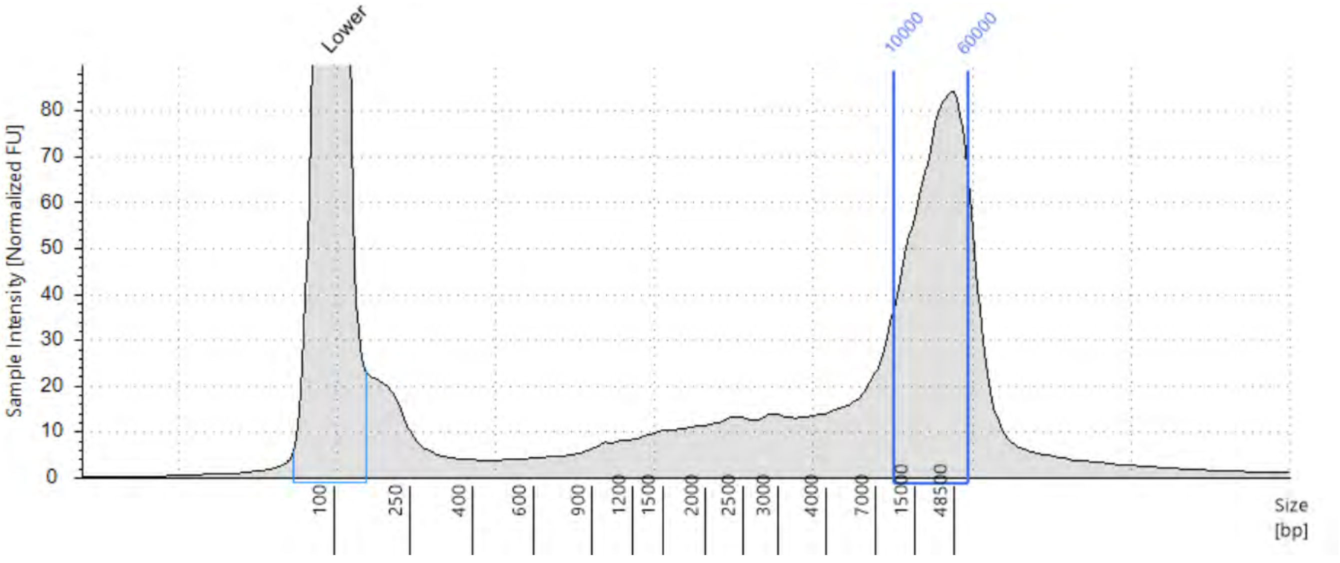

Region Table

From [bp]	To [bp]	Average Size [bp]	Conc. [ng/µl]	Region Molarity [nmol/l]	% of Total	Region Comment	Color
10000	60000	29404	3.24	0.227	41.44		■

© Agilent Technologies, Inc. 2017

D2: C461

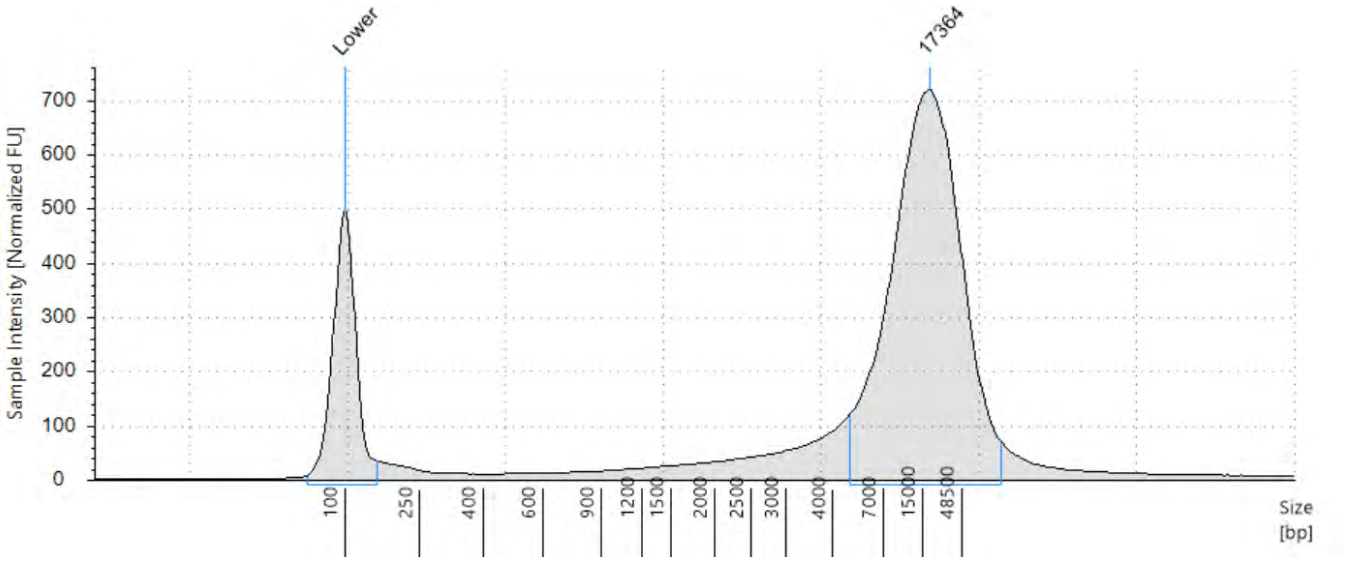

Sample Table

Well	DIN	Conc. [ng/µl]	Sample Description	Alert	Observations
D2	7.4	50.5	C461		

Peak Table

Size [bp]	Calibrated Conc. [ng/µl]	Assigned Conc. [ng/µl]	% Integrated Area	From [bp]	To [bp]	Peak Comment	Observations
100	8.50	8.50	-	63	147		Lower Marker
17364	38.2	-	95.22	4831	>60000		
-	-	-	-	-	-		Sample Well

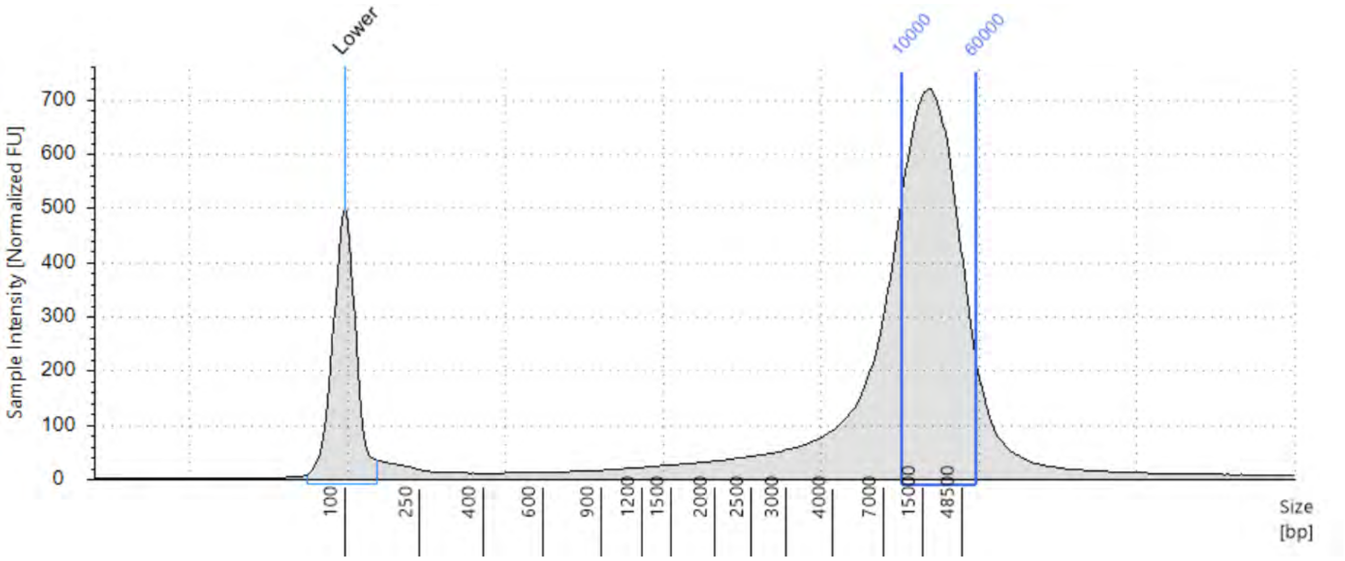

Region Table

From [bp]	To [bp]	Average Size [bp]	Conc. [ng/µl]	Region Molarity [nmol/l]	% of Total	Region Comment	Color
10000	60000	23236	27.3	2.30	54.08		

 © Agilent Technologies, Inc. 2017

E2: C462

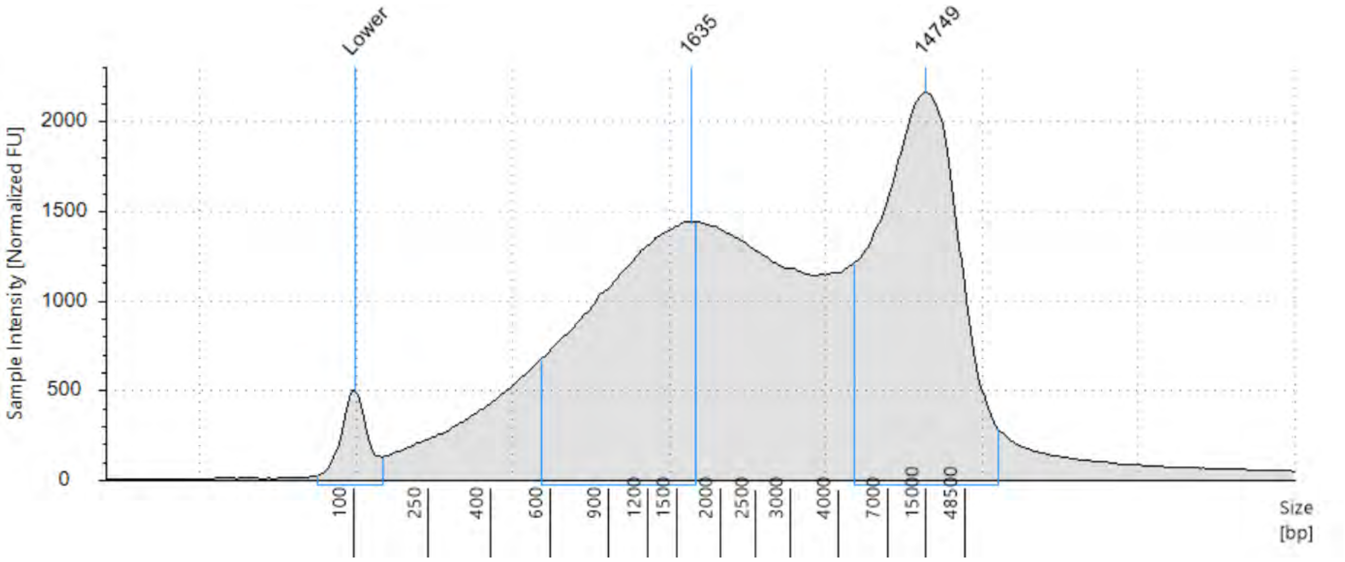

Sample Table

Well	DIN	Conc. [ng/µl]	Sample Description	Alert	Observations
E2	4.8	385	C462	⚠	Sample concentration outside functional range for DIN

Peak Table

Size [bp]	Calibrated Conc. [ng/µl]	Assigned Conc. [ng/µl]	% Integrated Area	From [bp]	To [bp]	Peak Comment	Observations
100	8.50	8.50	-	64	140		Lower Marker
1635	99.6	-	44.67	559	1681		
14749	121	-	54.35	4743	>60000		
-	-	-	-	-	-		Sample Well

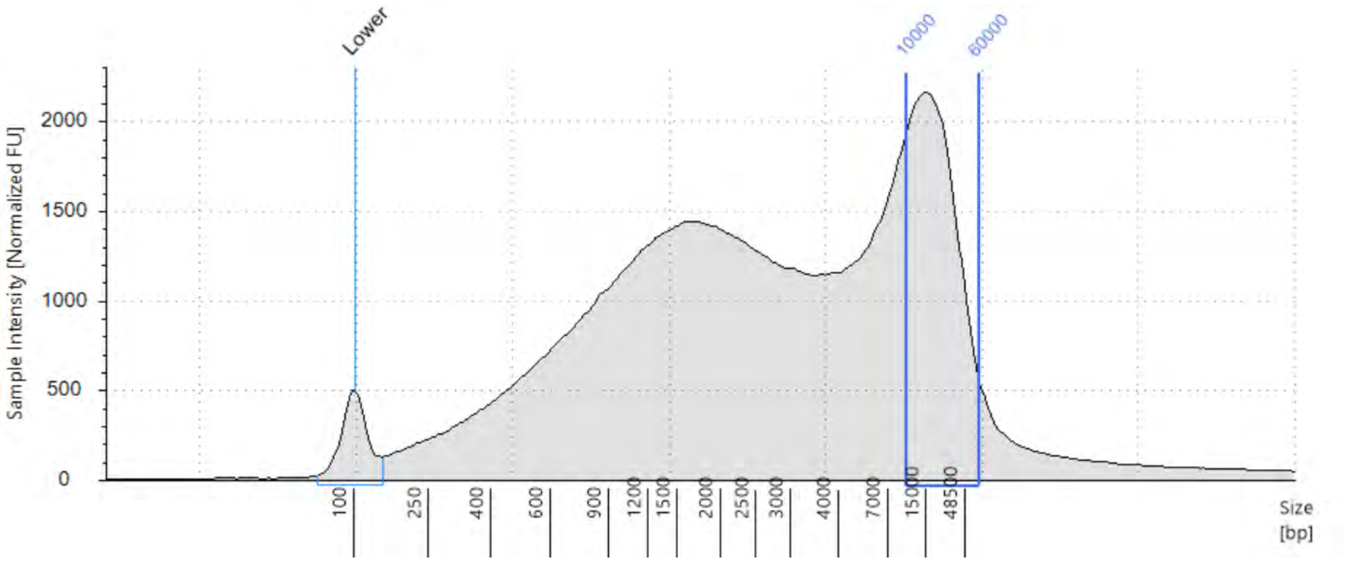

Region Table

From [bp]	To [bp]	Average Size [bp]	Conc. [ng/µl]	Region Molarity [nmol/l]	% of Total	Region Comment	Color
10000	60000	22025	71.3	6.31	18.52		■

 © Agilent Technologies, Inc. 2017

F2: C463

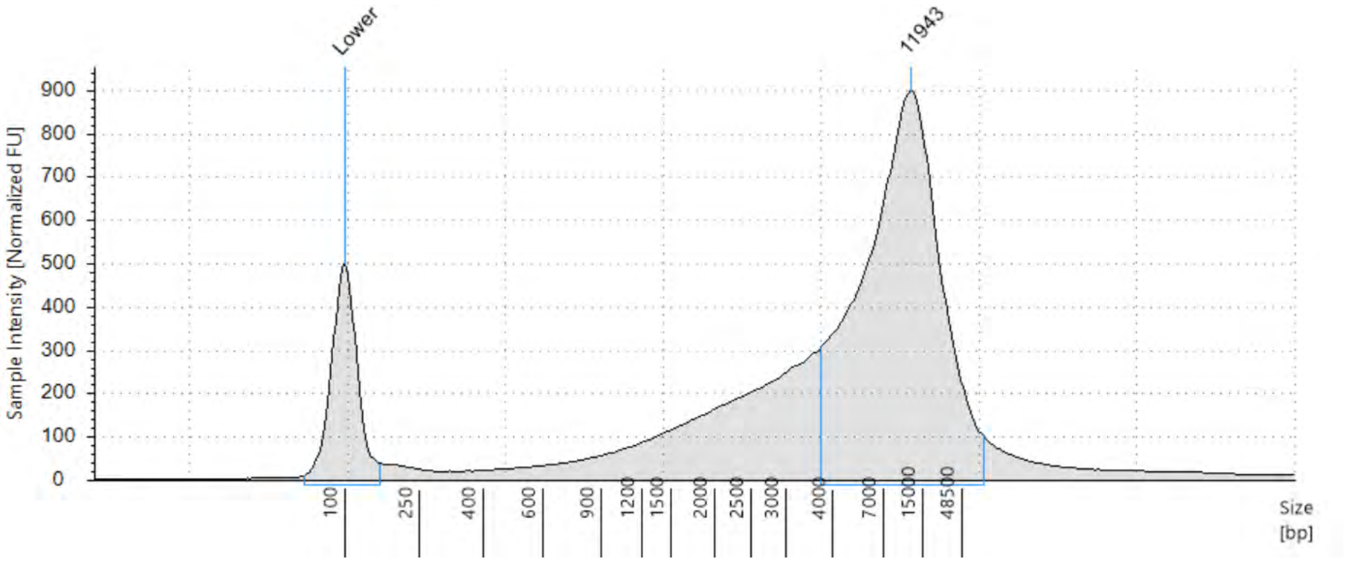

Sample Table

Well	DIN	Conc. [ng/µl]	Sample Description	Alert	Observations
F2	6.4	74.6	C463		

Peak Table

Size [bp]	Calibrated Conc. [ng/µl]	Assigned Conc. [ng/µl]	% Integrated Area	From [bp]	To [bp]	Peak Comment	Observations
100	8.50	8.50	-	61	153		Lower Marker
11943	46.3	-	98.74	3708	>60000		
-	-	-	-	-	-		Sample Well

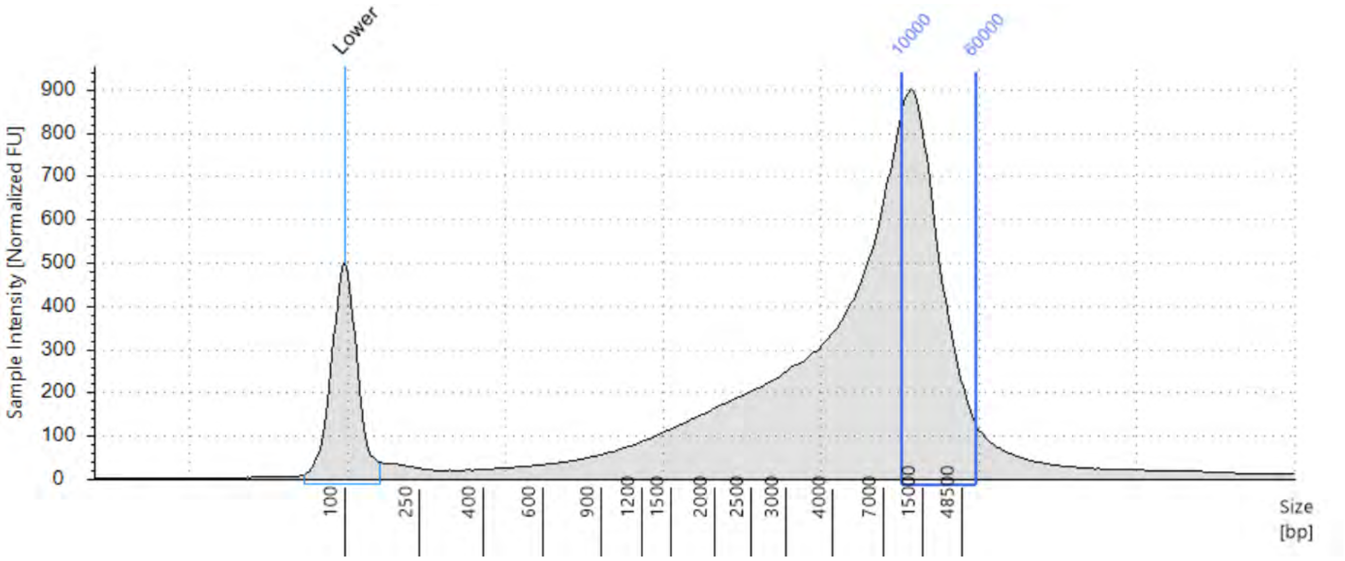

Region Table

From [bp]	To [bp]	Average Size [bp]	Conc. [ng/µl]	Region Molarity [nmol/l]	% of Total	Region Comment	Color
10000	60000	19661	22.9	2.21	30.65		

G2: C464

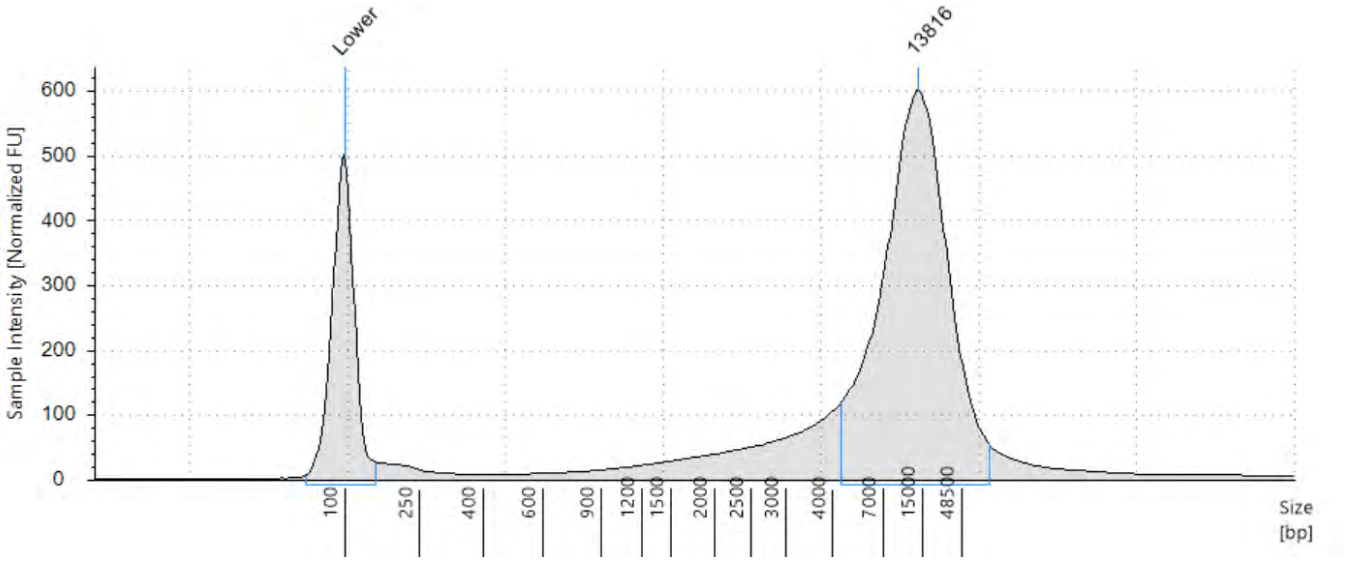

Sample Table

Well	DIN	Conc. [ng/µl]	Sample Description	Alert	Observations
G2	7.1	41.8	C464		

Peak Table

Size [bp]	Calibrated Conc. [ng/µl]	Assigned Conc. [ng/µl]	% Integrated Area	From [bp]	To [bp]	Peak Comment	Observations
100	8.50	8.50	-	62	146		Lower Marker
13816	30.3	-	94.91	4355	>60000		
-	-	-	-	-	-		Sample Well

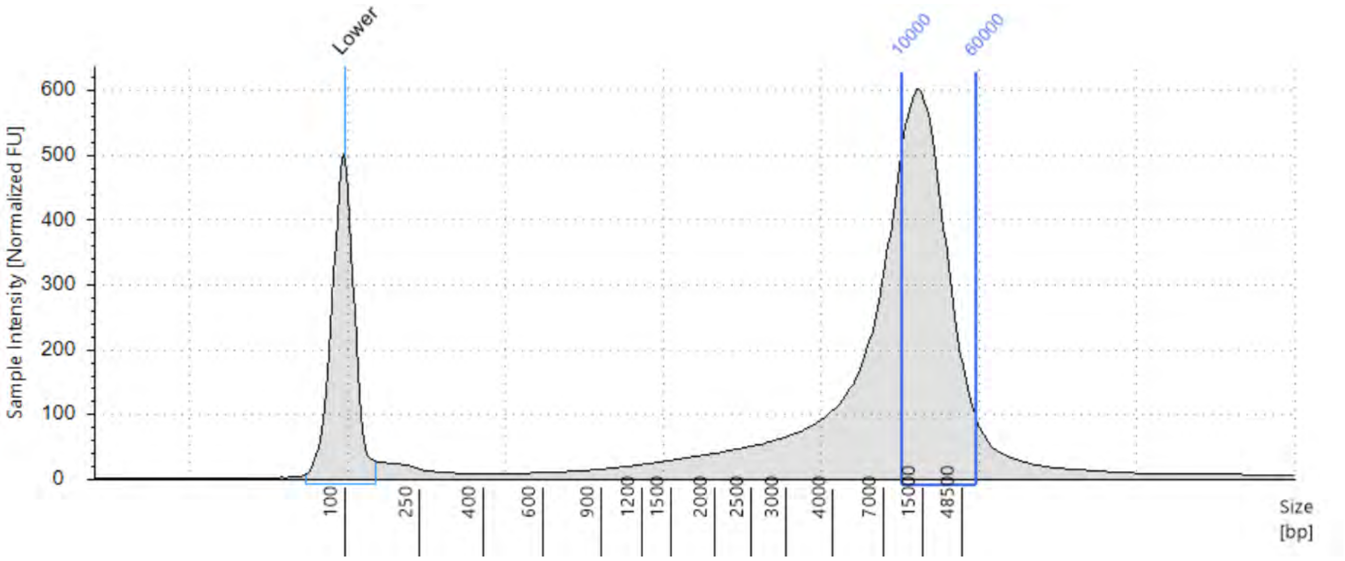

Region Table

From [bp]	To [bp]	Average Size [bp]	Conc. [ng/µl]	Region Molarity [nmol/l]	% of Total	Region Comment	Color
10000	60000	20249	19.7	1.84	47.09		

H2: C465

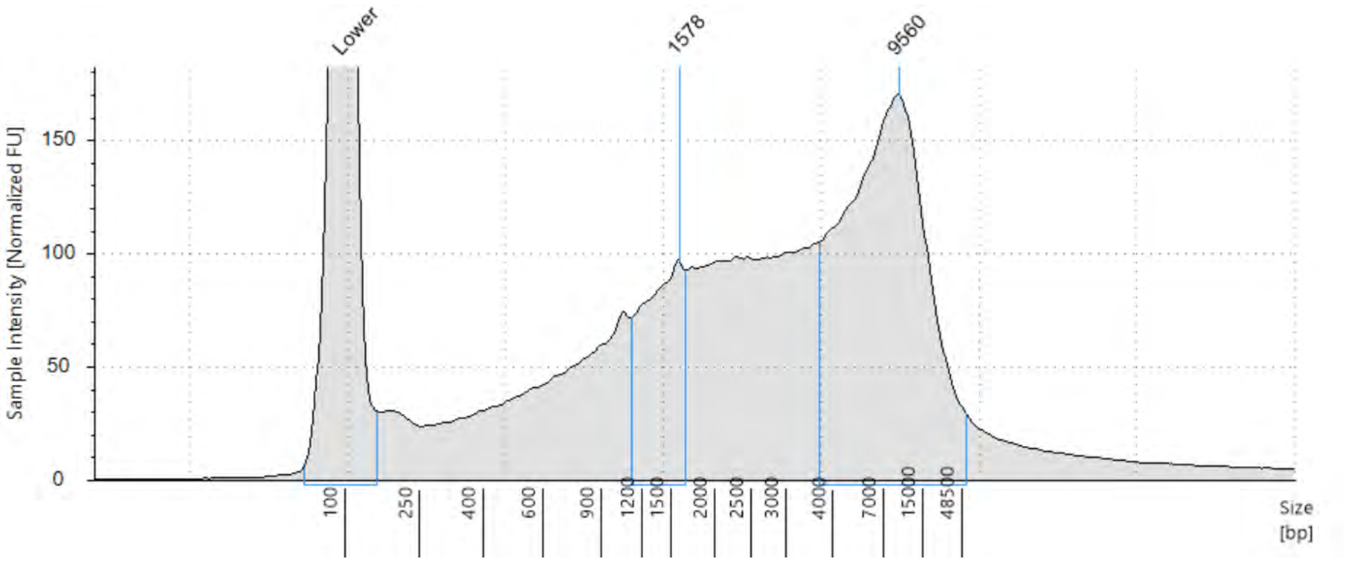

Sample Table

Well	DIN	Conc. [ng/µl]	Sample Description	Alert	Observations
H2	5.3	30.4	C465		

Peak Table

Size [bp]	Calibrated Conc. [ng/µl]	Assigned Conc. [ng/µl]	% Integrated Area	From [bp]	To [bp]	Peak Comment	Observations
100	8.50	8.50	-	61	147		Lower Marker
1578	2.98	-	20.64	1110	1651		
9560	10.8	-	74.79	3662	52732		
-	-	-	-	-	-		Sample Well

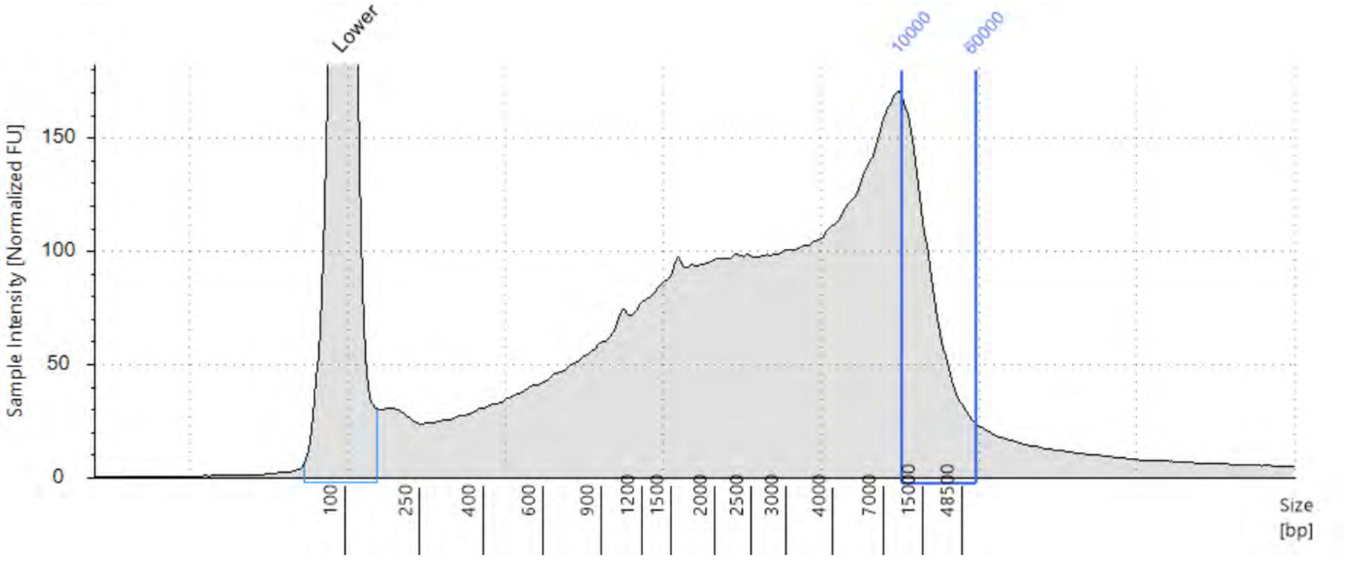

Region Table

From [bp]	To [bp]	Average Size [bp]	Conc. [ng/µl]	Region Molarity [nmol/l]	% of Total	Region Comment	Color
10000	60000	19314	3.87	0.393	12.74		

 © Agilent Technologies, Inc. 2017

A3: C466

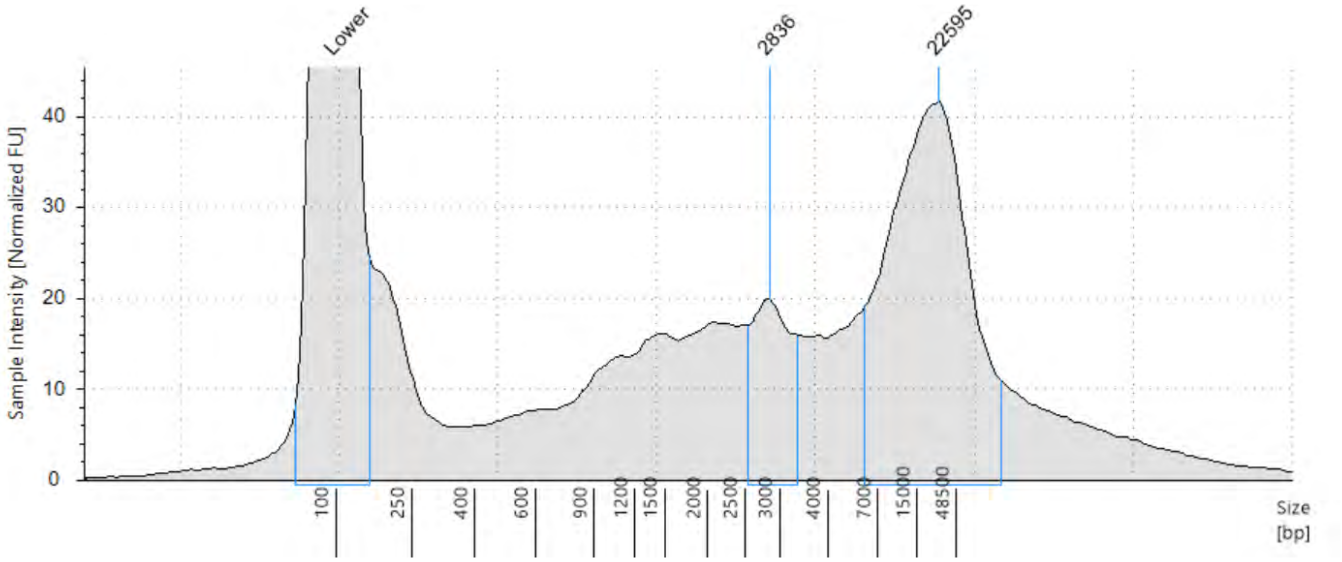

Sample Table

Well	DIN	Conc. [ng/µl]	Sample Description	Alert	Observations
A3	6.2	7.24	C466	⚠	Sample concentration outside recommended range

Peak Table

Size [bp]	Calibrated Conc. [ng/µl]	Assigned Conc. [ng/µl]	% Integrated Area	From [bp]	To [bp]	Peak Comment	Observations
100	8.50	8.50	-	61	150		Lower Marker
2836	0.555	-	18.57	2543	3328		
22595	2.41	-	80.65	6007	>60000		
-	-	-	-	-	-		Sample Well

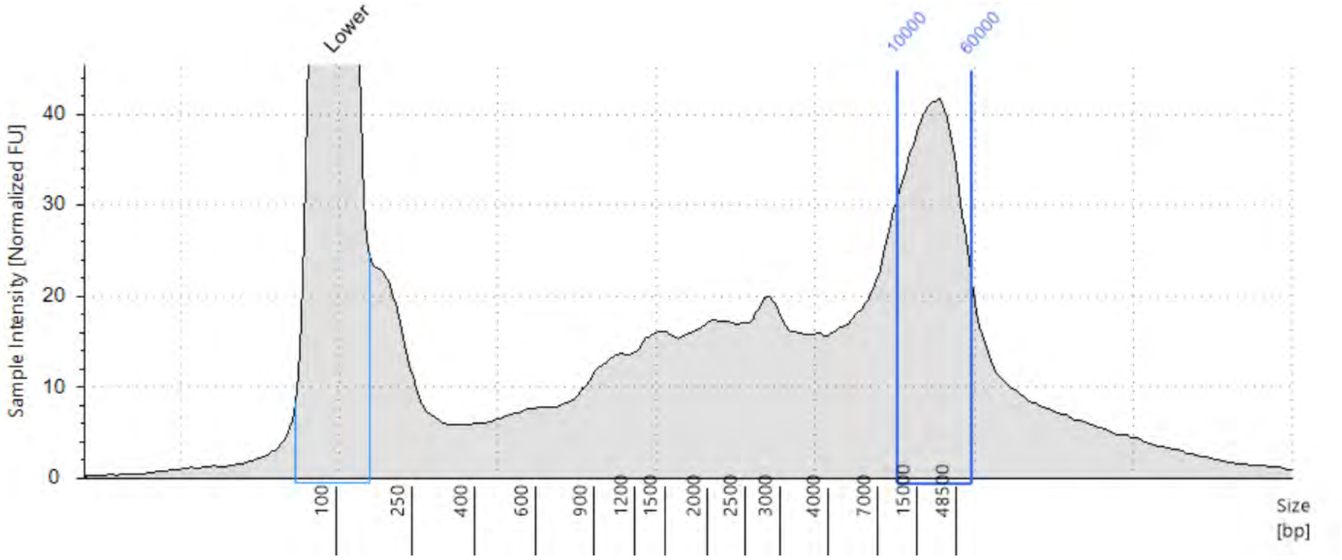

Region Table

From [bp]	To [bp]	Average Size [bp]	Conc. [ng/µl]	Region Molarity [nmol/l]	% of Total	Region Comment	Color
10000	60000	25331	1.67	0.134	23.12		■

 © Agilent Technologies, Inc. 2017

B3: C467

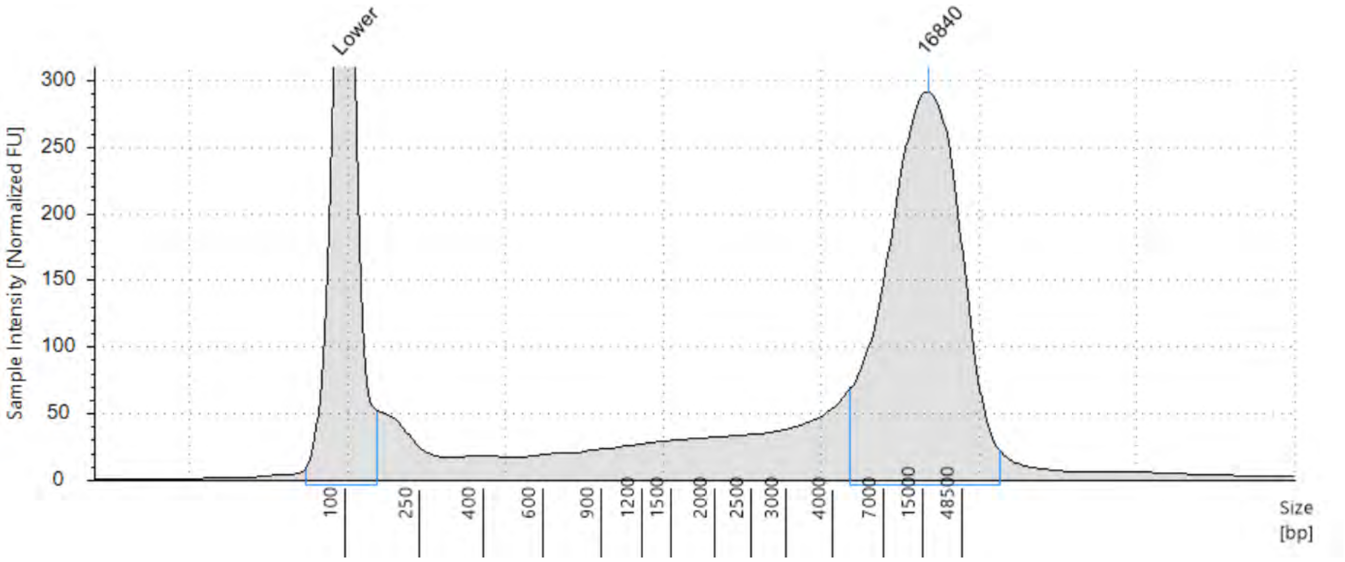

Sample Table

Well	DIN	Conc. [ng/µl]	Sample Description	Alert	Observations
B3	6.7	25.0	C467		

Peak Table

Size [bp]	Calibrated Conc. [ng/µl]	Assigned Conc. [ng/µl]	% Integrated Area	From [bp]	To [bp]	Peak Comment	Observations
100	8.50	8.50	-	62	149		Lower Marker
16840	15.4	-	94.78	4798	>60000		
-	-	-	-	-	-		Sample Well

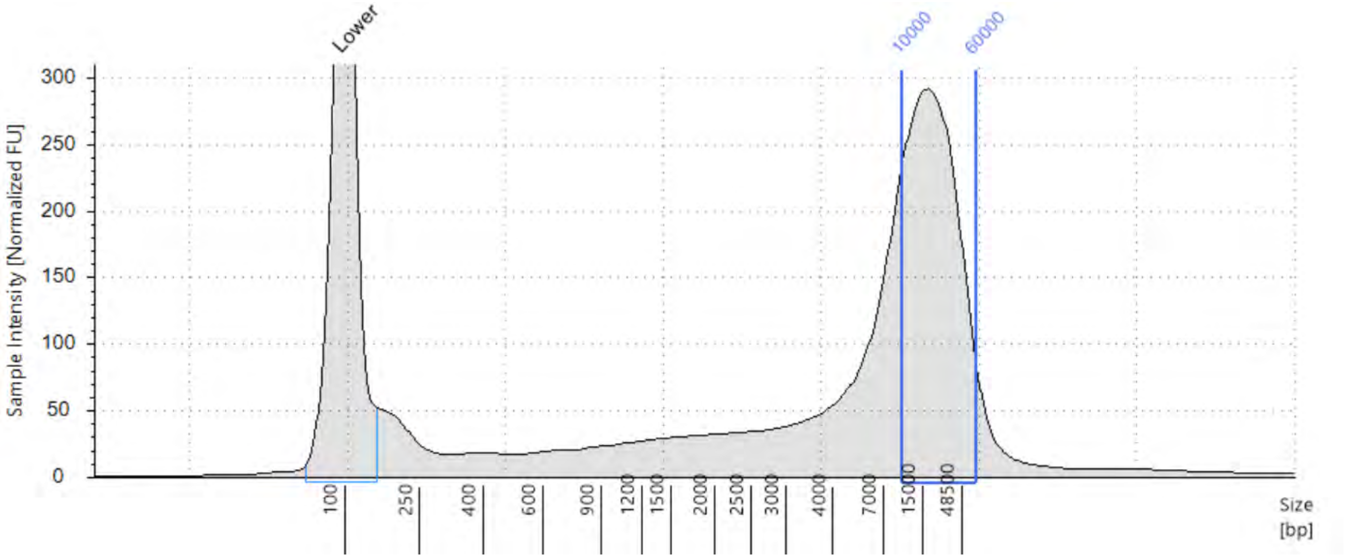

Region Table

From [bp]	To [bp]	Average Size [bp]	Conc. [ng/µl]	Region Molarity [nmol/l]	% of Total	Region Comment	Color
10000	60000	23294	10.7	0.900	42.77		

© Agilent Technologies, Inc. 2017

C3: C468

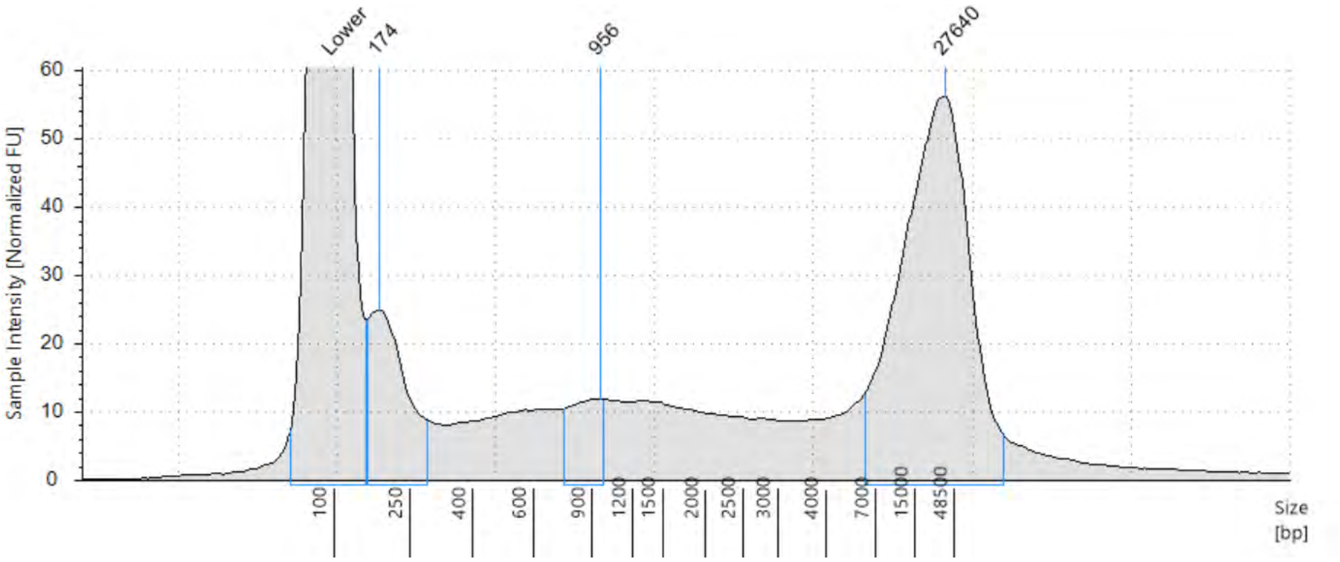

Sample Table

Well	DIN	Conc. [ng/µl]	Sample Description	Alert	Observations
C3	6.4	6.40	C468	⚠	Sample concentration outside recommended range

Peak Table

Size [bp]	Calibrated Conc. [ng/µl]	Assigned Conc. [ng/µl]	% Integrated Area	From [bp]	To [bp]	Peak Comment	Observations
100	8.50	8.50	-	59	147		Lower Marker
174	0.661	-	17.46	150	284		
956	0.272	-	7.17	744	975		
27640	2.73	-	72.20	6200	>60000		
-	-	-	-	-	-		Sample Well

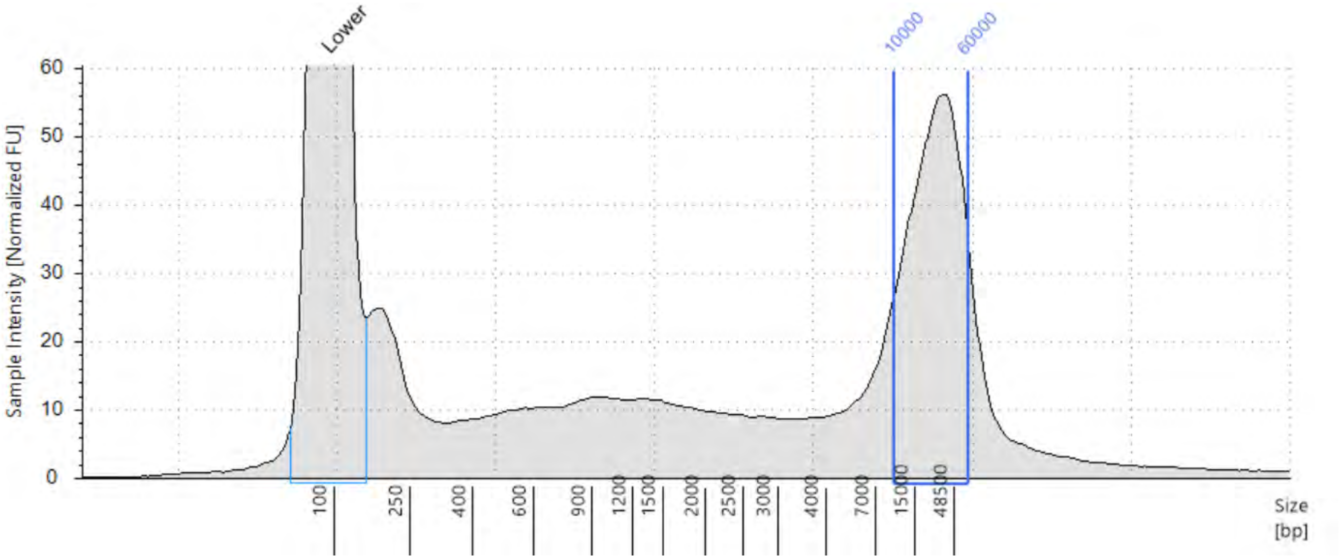

Region Table

From [bp]	To [bp]	Average Size [bp]	Conc. [ng/µl]	Region Molarity [nmol/l]	% of Total	Region Comment	Color
10000	60000	27979	2.08	0.151	32.44		■

 © Agilent Technologies, Inc. 2017

D3: C469

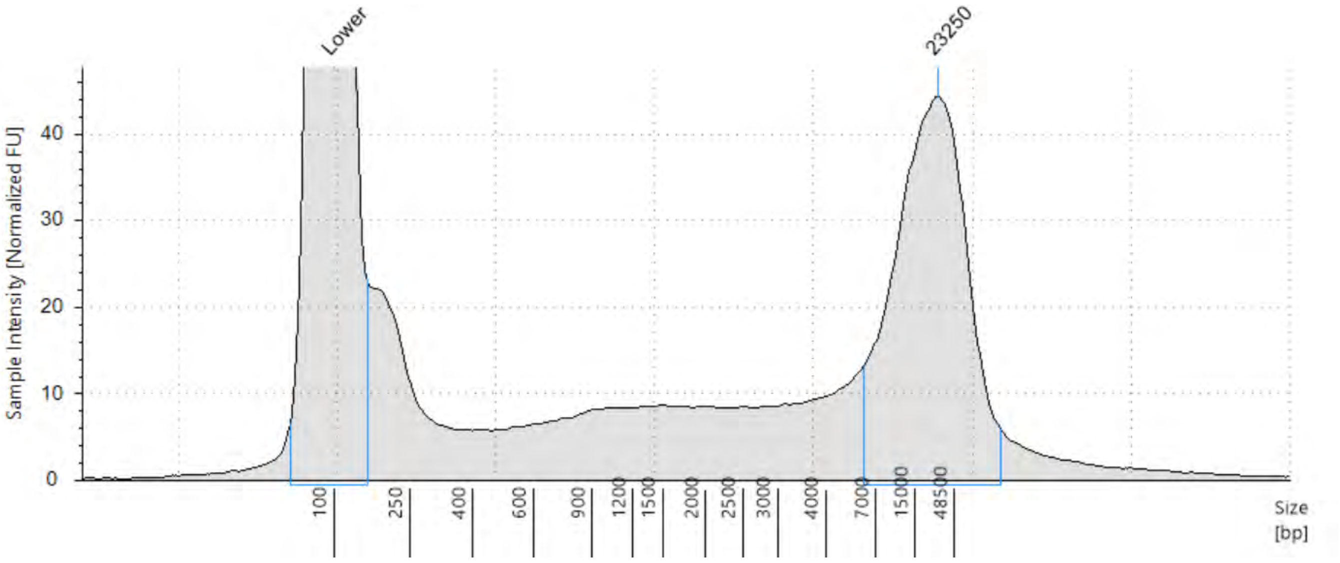

Sample Table

Well	DIN	Conc. [ng/µl]	Sample Description	Alert	Observations
D3	6.7	4.91	C469	⚠	Sample concentration outside functional range for DIN

Peak Table

Size [bp]	Calibrated Conc. [ng/µl]	Assigned Conc. [ng/µl]	% Integrated Area	From [bp]	To [bp]	Peak Comment	Observations
100	8.50	8.50	-	59	150		Lower Marker
23250	2.19	-	98.39	6116	>60000		
-	-	-	-	-	-		Sample Well

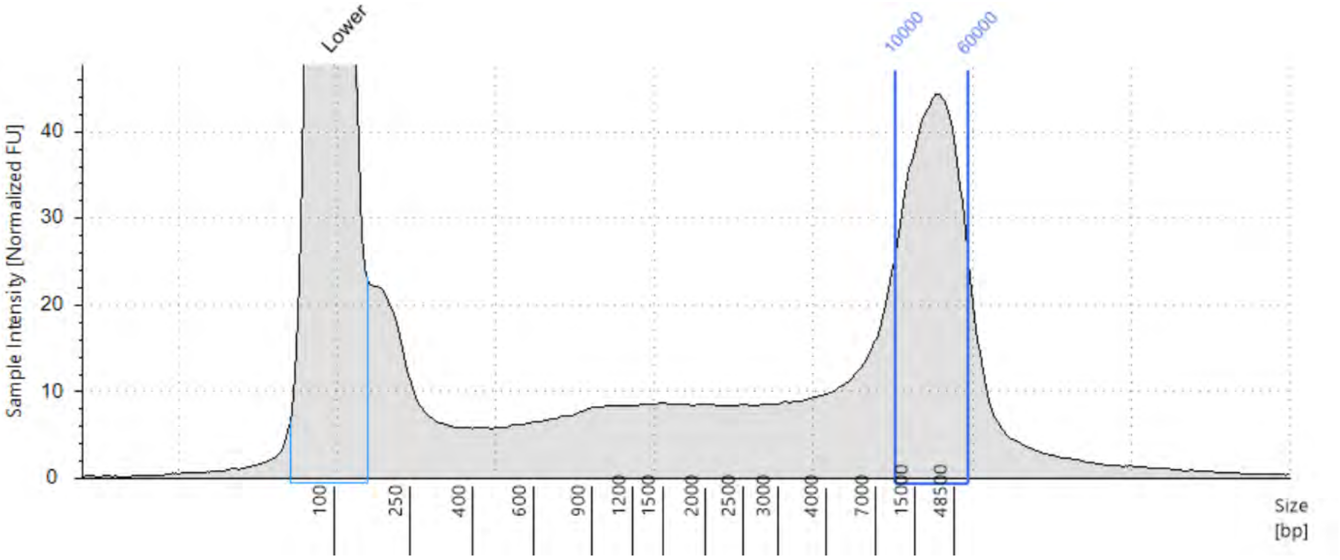

Region Table

From [bp]	To [bp]	Average Size [bp]	Conc. [ng/µl]	Region Molarity [nmol/l]	% of Total	Region Comment	Color
10000	60000	26372	1.65	0.127	33.58		■

E3: C470

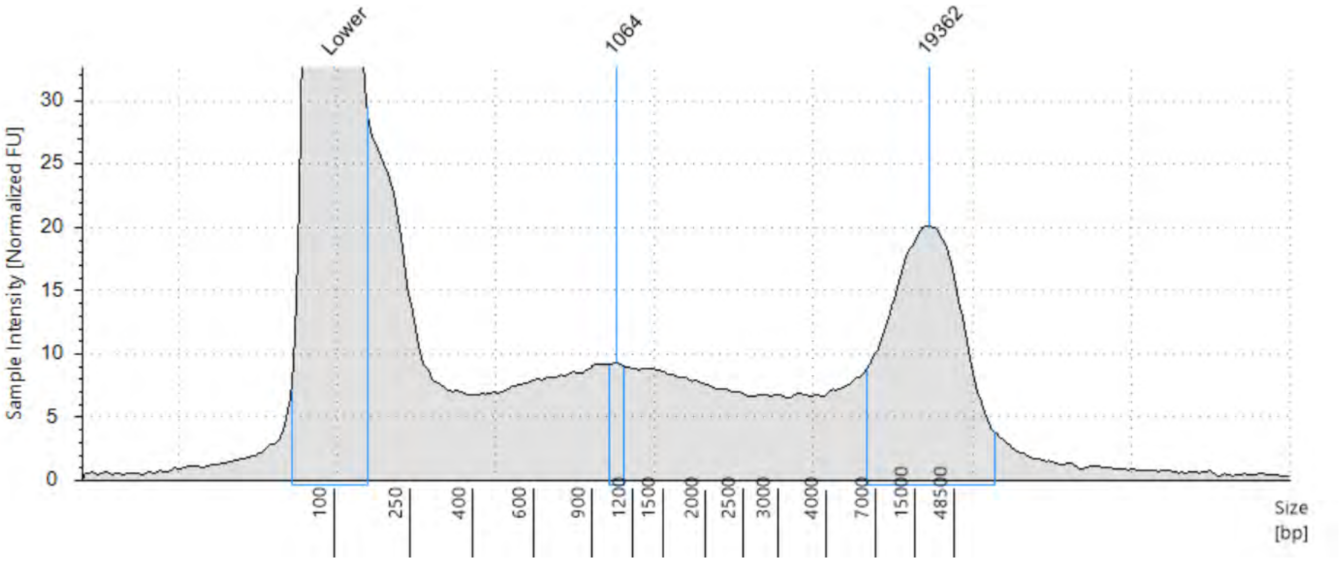

Sample Table

Well	DIN	Conc. [ng/µl]	Sample Description	Alert	Observations
E3	5.7	3.76	C470	⚠	Sample concentration outside functional range for DIN

Peak Table

Size [bp]	Calibrated Conc. [ng/µl]	Assigned Conc. [ng/µl]	% Integrated Area	From [bp]	To [bp]	Peak Comment	Observations
100	8.50	8.50	-	60	149		Lower Marker
1064	0.0787	-	7.19	1013	1117		
19362	1.02	-	92.81	6285	>60000		

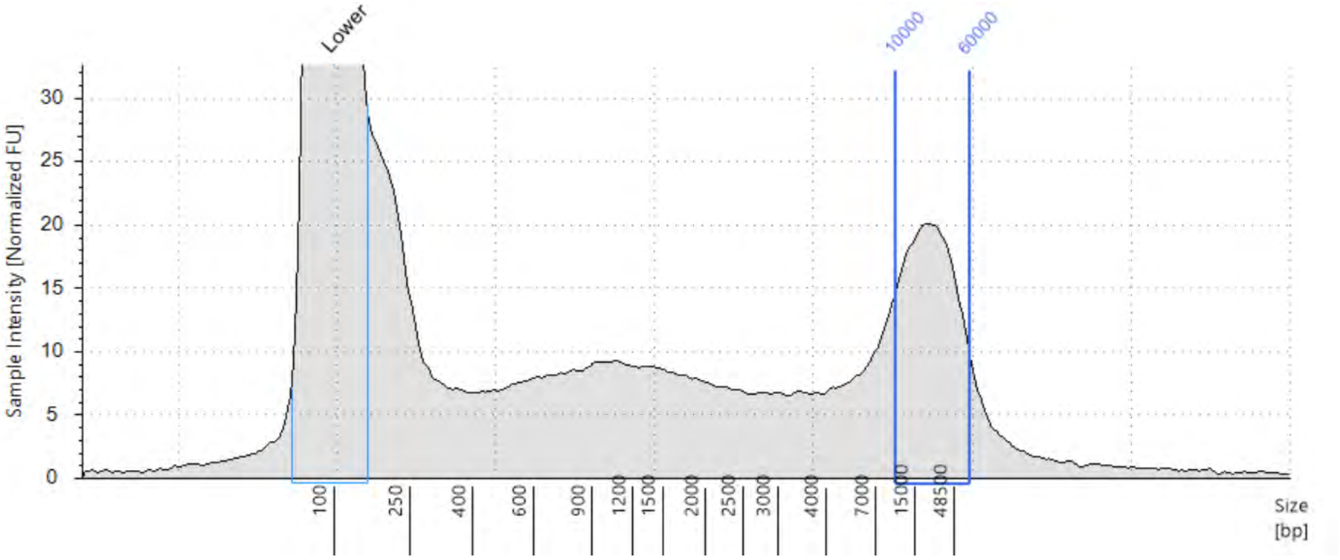

Region Table

From [bp]	To [bp]	Average Size [bp]	Conc. [ng/µl]	Region Molarity [nmol/l]	% of Total	Region Comment	Color
10000	60000	25137	0.747	0.0609	19.86		■

F3: C471

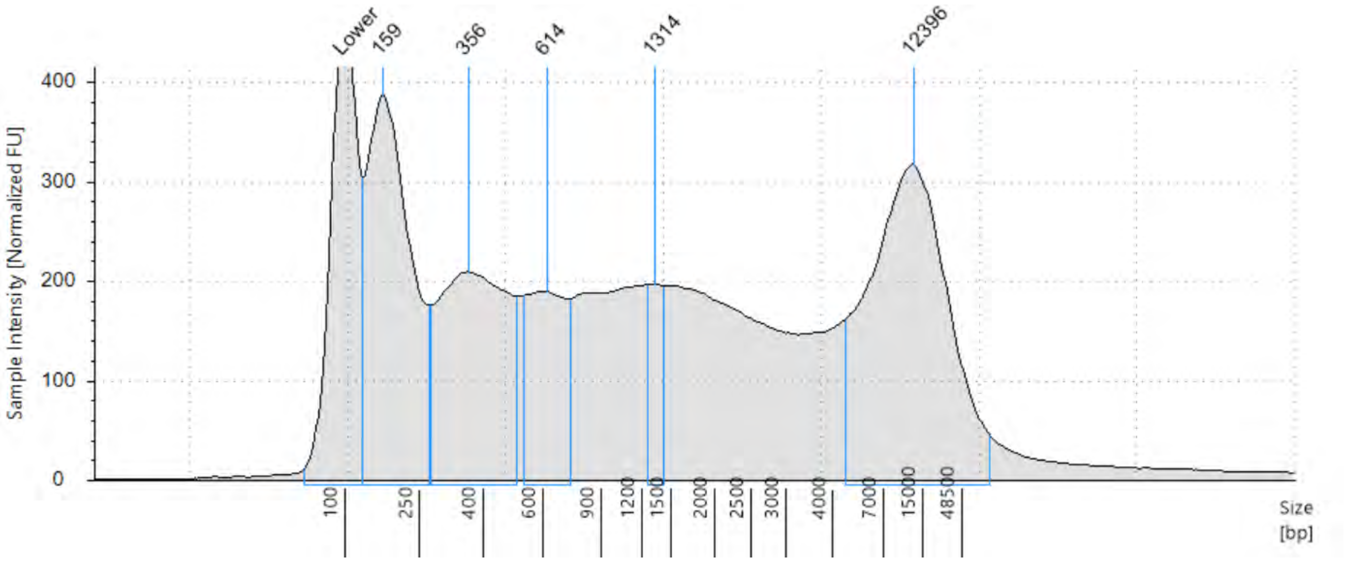

Sample Table

Well	DIN	Conc. [ng/µl]	Sample Description	Alert	Observations
F3	2.7	72.0	C471		

Peak Table

Size [bp]	Calibrated Conc. [ng/µl]	Assigned Conc. [ng/µl]	% Integrated Area	From [bp]	To [bp]	Peak Comment	Observations
100	8.50	8.50	-	61	124		Lower Marker
159	11.2	-	24.74	124	268		
356	9.49	-	21.06	270	501		
614	4.94	-	10.97	525	721		
1314	1.94	-	4.30	1248	1413		
12396	16.4	-	36.42	4543	>60000		
-	-	-	-	-	-		Sample Well

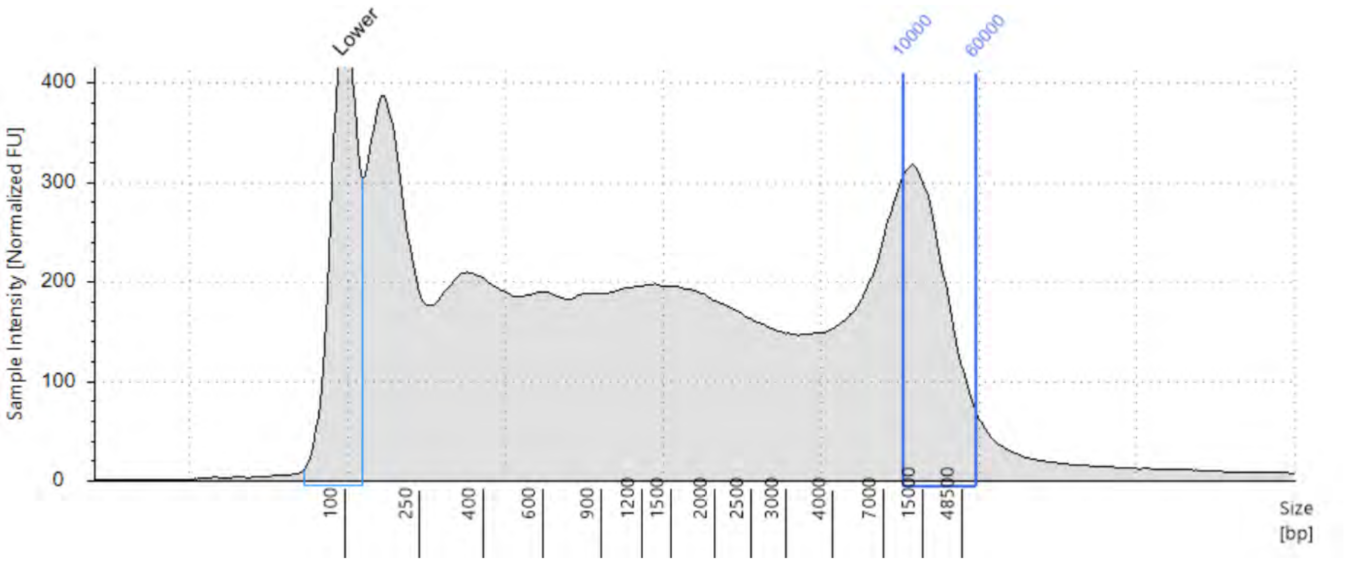

Region Table

From [bp]	To [bp]	Average Size [bp]	Conc. [ng/µl]	Region Molarity [nmol/l]	% of Total	Region Comment	Color
10000	60000	20837	9.08	0.849	12.61		

G3: C472

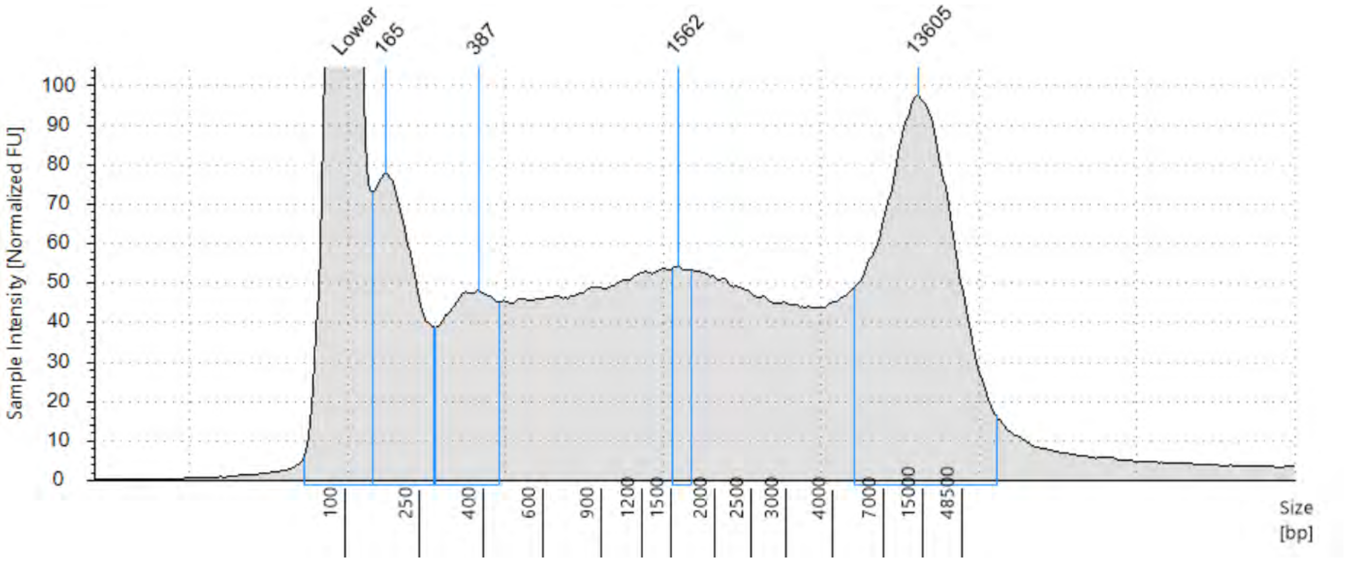

Sample Table

Well	DIN	Conc. [ng/µl]	Sample Description	Alert	Observations
G3	3.6	21.9	C472		

Peak Table

Size [bp]	Calibrated Conc. [ng/µl]	Assigned Conc. [ng/µl]	% Integrated Area	From [bp]	To [bp]	Peak Comment	Observations
100	8.50	8.50	-	61	140		Lower Marker
165	2.44	-	21.50	140	278		
387	1.81	-	15.95	281	447		
1562	0.692	-	6.09	1505	1712		
13605	5.65	-	49.71	5044	>60000		
-	-	-	-	-	-		Sample Well

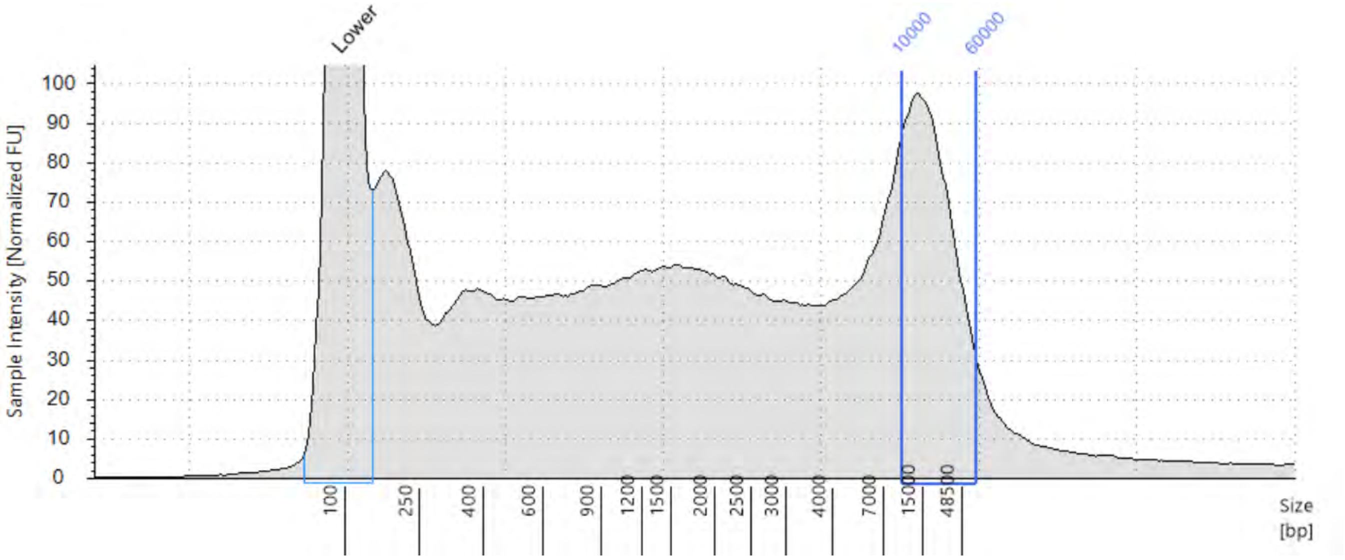

Region Table

From [bp]	To [bp]	Average Size [bp]	Conc. [ng/µl]	Region Molarity [nmol/l]	% of Total	Region Comment	Color
10000	60000	22456	3.47	0.307	15.85		

H3: C473

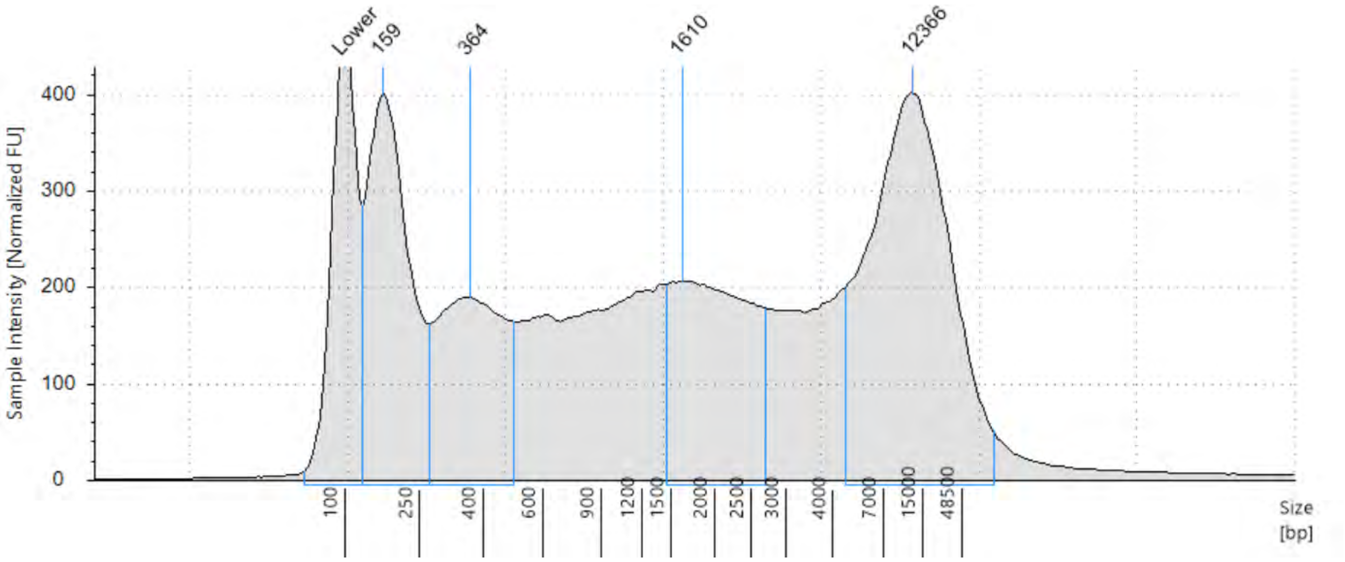

Sample Table

Well	DIN	Conc. [ng/µl]	Sample Description	Alert	Observations
H3	3.9	81.2	C473		

Peak Table

Size [bp]	Calibrated Conc. [ng/µl]	Assigned Conc. [ng/µl]	% Integrated Area	From [bp]	To [bp]	Peak Comment	Observations
100	8.50	8.50	-	62	124		Lower Marker
159	11.9	-	21.32	124	268		
364	9.03	-	16.14	268	494		
1610	11.6	-	20.68	1450	2694		
12366	22.7	-	40.63	4599	>60000		
-	-	-	-	-	-		Sample Well

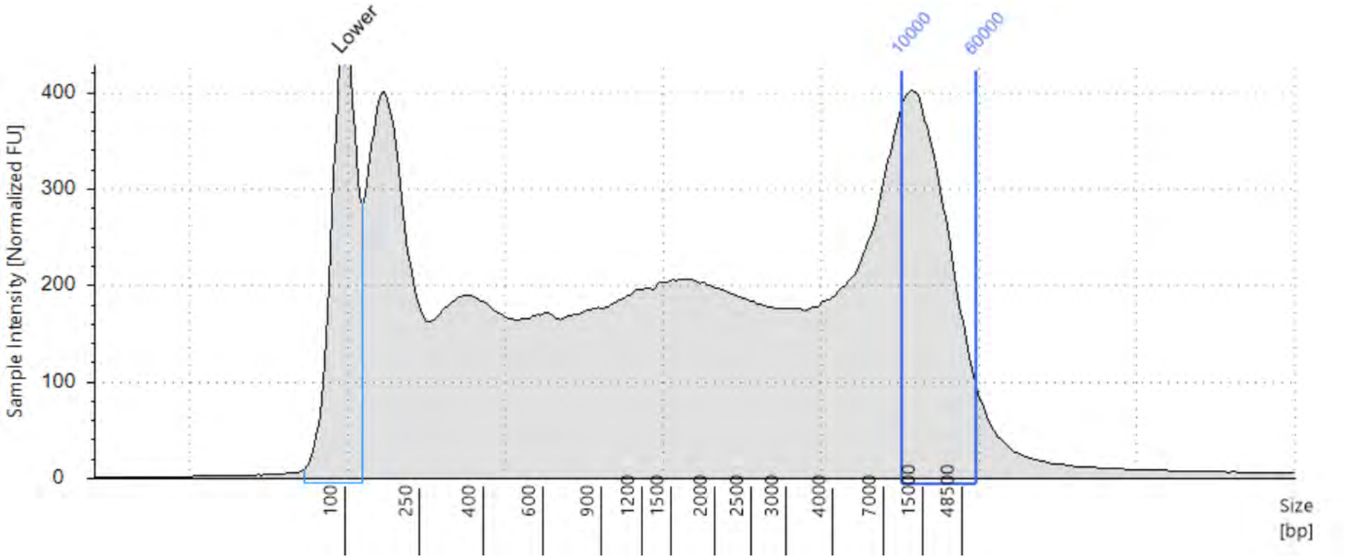

Region Table

From [bp]	To [bp]	Average Size [bp]	Conc. [ng/µl]	Region Molarity [nmol/l]	% of Total	Region Comment	Color
10000	60000	21687	12.6	1.14	15.57		

 © Agilent Technologies, Inc. 2017

A4: C474

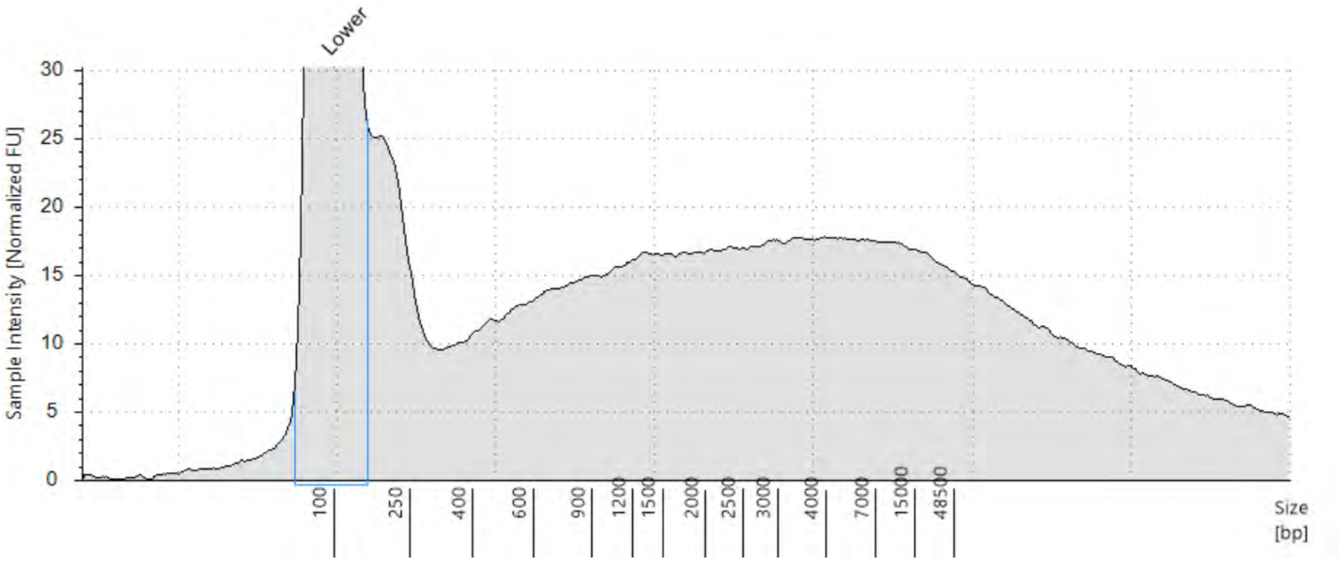

Sample Table

Well	DIN	Conc. [ng/µl]	Sample Description	Alert	Observations
A4	4.4	8.08	C474	⚠	Sample concentration outside recommended range

Peak Table

Size [bp]	Calibrated Conc. [ng/µl]	Assigned Conc. [ng/µl]	% Integrated Area	From [bp]	To [bp]	Peak Comment	Observations
100	8.50	8.50	-	62	149		Lower Marker
-	-	-	-	-	-		Sample Well

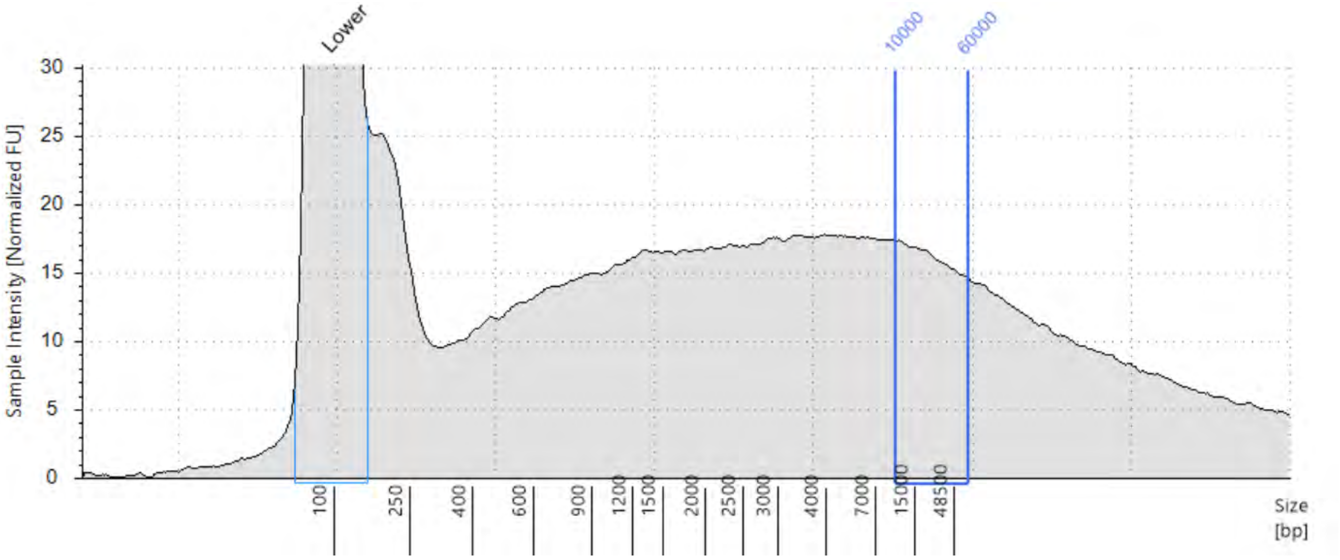

Region Table

From [bp]	To [bp]	Average Size [bp]	Conc. [ng/µl]	Region Molarity [nmol/l]	% of Total	Region Comment	Color
10000	60000	26328	0.751	0.0619	9.30		■

© Agilent Technologies, Inc. 2017

B4: C475

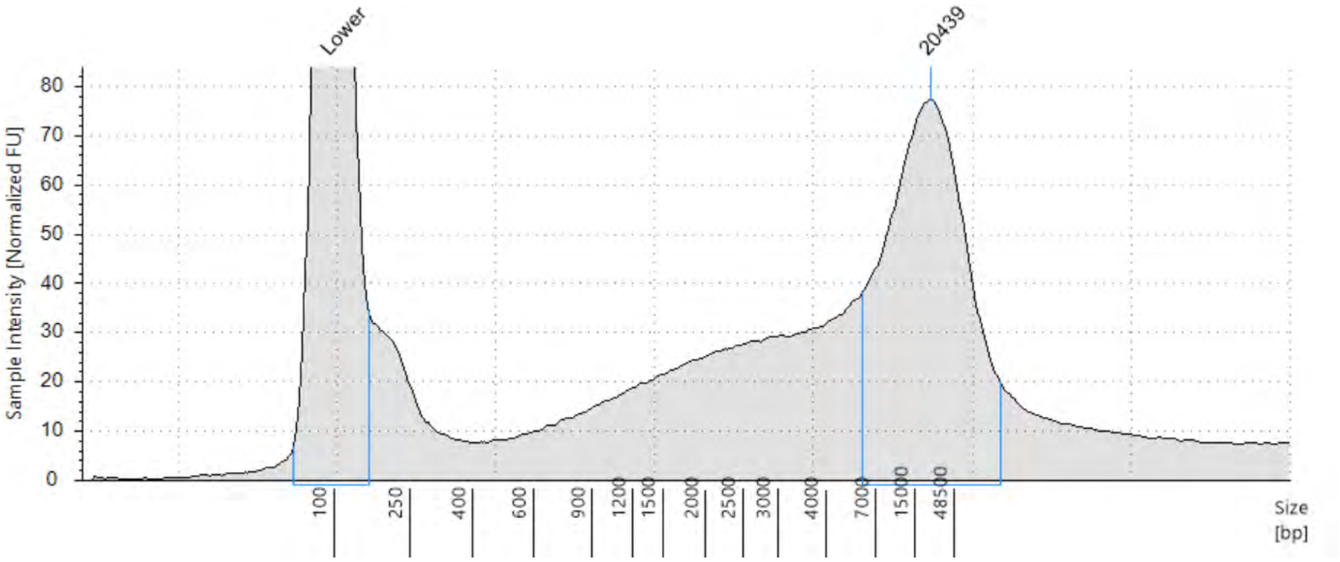

Sample Table

Well	DIN	Conc. [ng/µl]	Sample Description	Alert	Observations
B4	6.3	12.7	C475		

Peak Table

Size [bp]	Calibrated Conc. [ng/µl]	Assigned Conc. [ng/µl]	% Integrated Area	From [bp]	To [bp]	Peak Comment	Observations
100	8.50	8.50	-	62	155		Lower Marker
20439	4.29	-	62.08	6019	>60000		
-	-	-	-	-	-		Sample Well

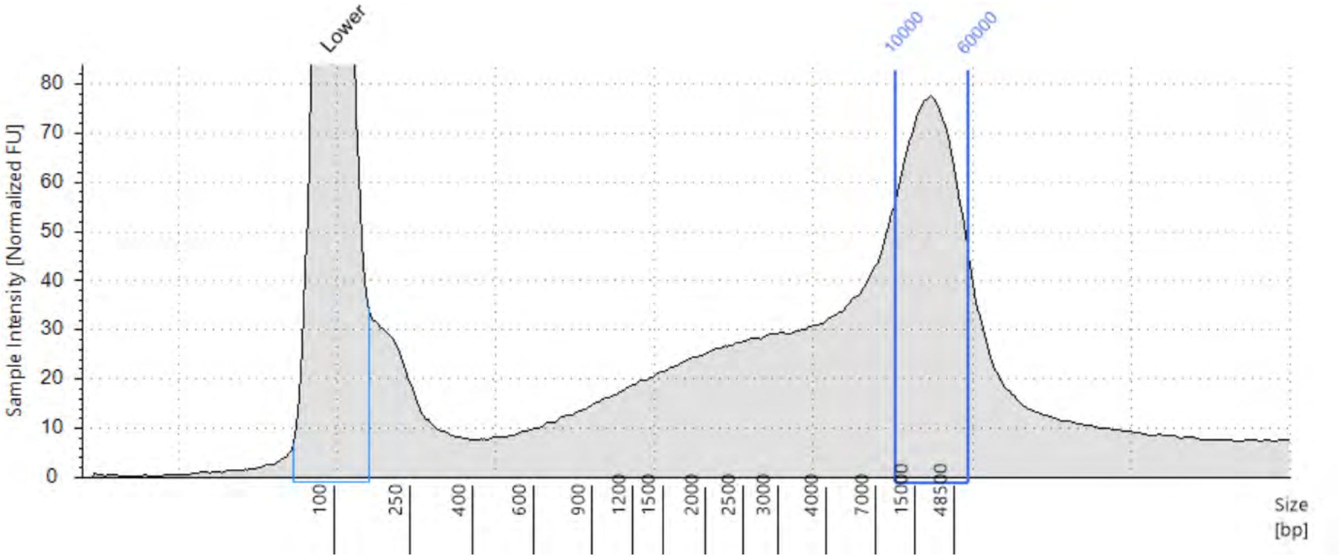

Region Table

From [bp]	To [bp]	Average Size [bp]	Conc. [ng/µl]	Region Molarity [nmol/l]	% of Total	Region Comment	Color
10000	60000	25715	2.94	0.234	23.12		

C4: C476

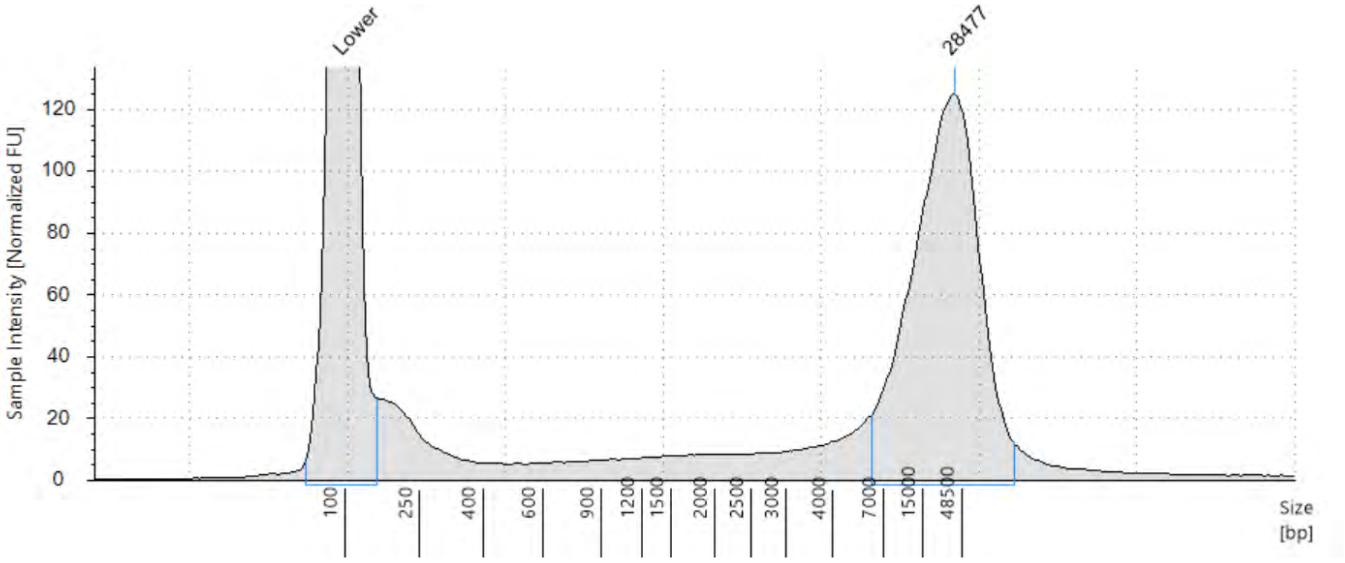

Sample Table

Well	DIN	Conc. [ng/µl]	Sample Description	Alert	Observations
C4	7.8	9.79	C476	⚠	Sample concentration outside recommended range

Peak Table

Size [bp]	Calibrated Conc. [ng/µl]	Assigned Conc. [ng/µl]	% Integrated Area	From [bp]	To [bp]	Peak Comment	Observations
100	8.50	8.50	-	62	146		Lower Marker
28477	6.38	-	98.33	6091	>60000		
-	-	-	-	-	-		Sample Well

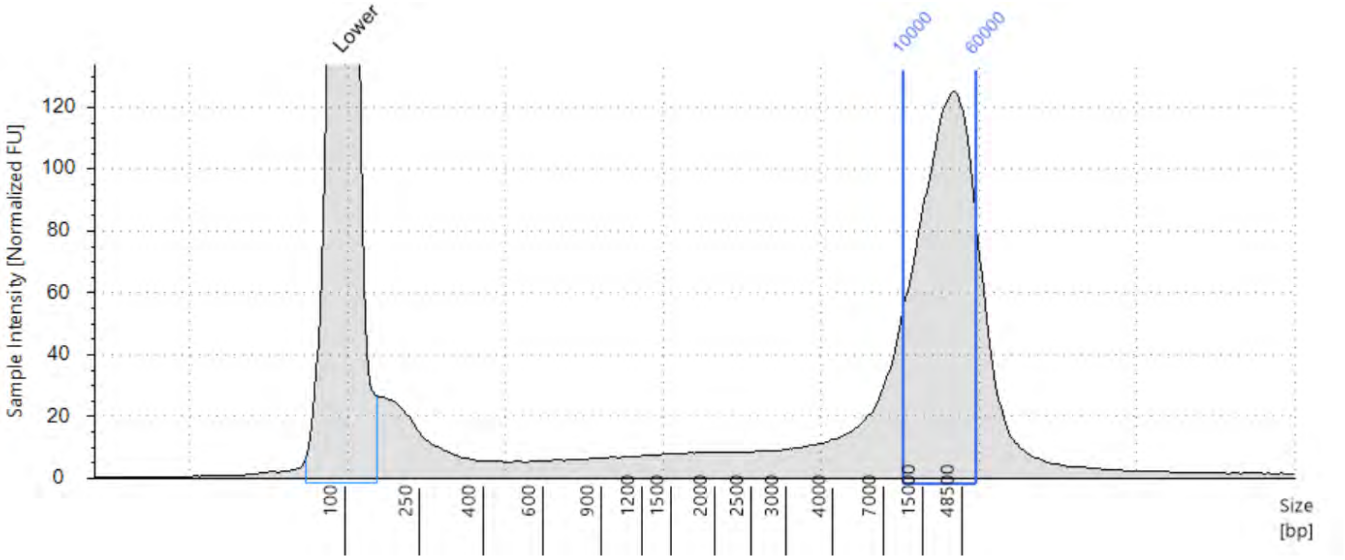

Region Table

From [bp]	To [bp]	Average Size [bp]	Conc. [ng/µl]	Region Molarity [nmol/l]	% of Total	Region Comment	Color
10000	60000	28295	4.81	0.345	49.11		■

D4: C477

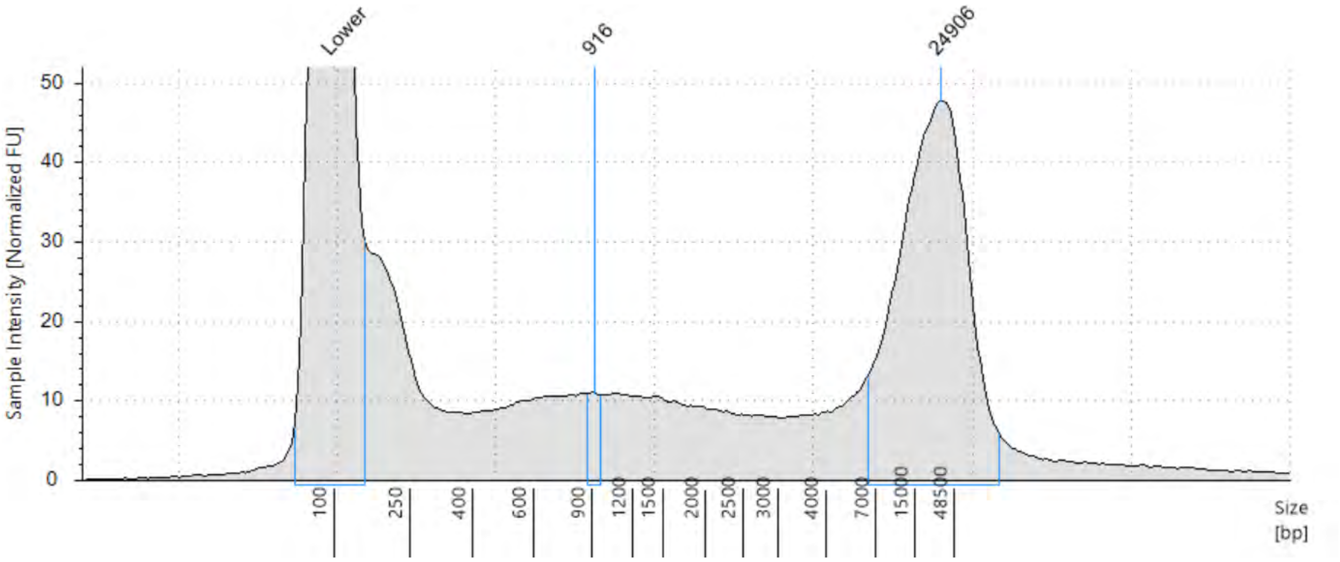

Sample Table

Well	DIN	Conc. [ng/µl]	Sample Description	Alert	Observations
D4	6.4	5.88	C477	⚠	Sample concentration outside recommended range

Peak Table

Size [bp]	Calibrated Conc. [ng/µl]	Assigned Conc. [ng/µl]	% Integrated Area	From [bp]	To [bp]	Peak Comment	Observations
100	8.50	8.50	-	62	146		Lower Marker
916	0.0884	-	3.48	874	952		
24906	2.40	-	94.61	6402	>60000		
-	-	-	-	-	-		Sample Well

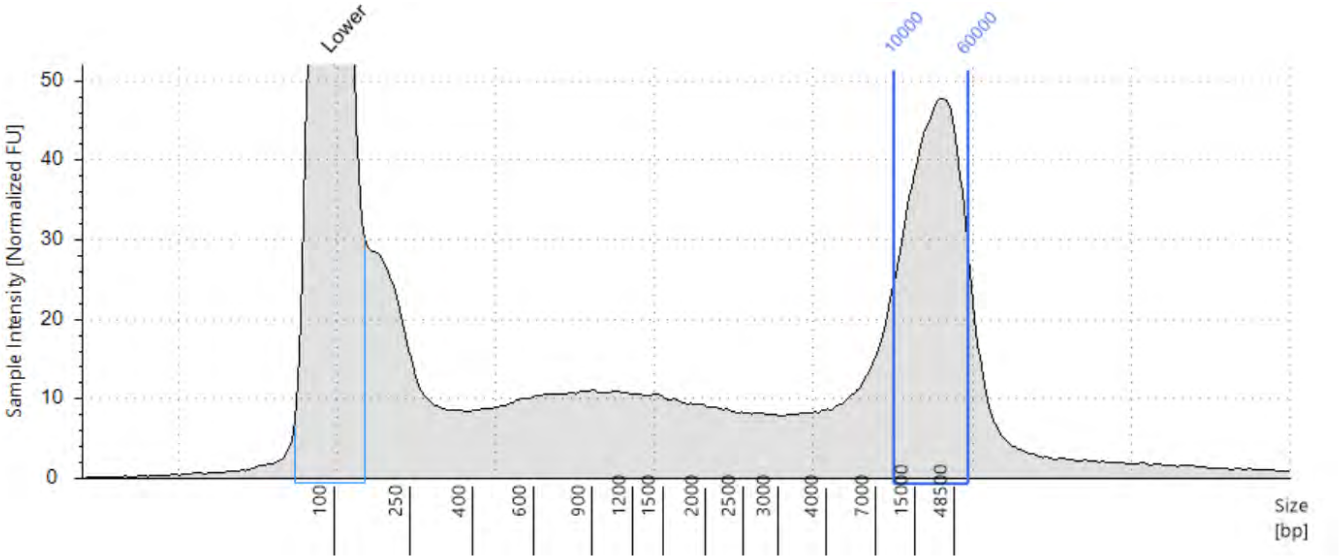

Region Table

From [bp]	To [bp]	Average Size [bp]	Conc. [ng/µl]	Region Molarity [nmol/l]	% of Total	Region Comment	Color
10000	60000	27115	1.85	0.140	31.50		■

 © Agilent Technologies, Inc. 2017

E4: C478

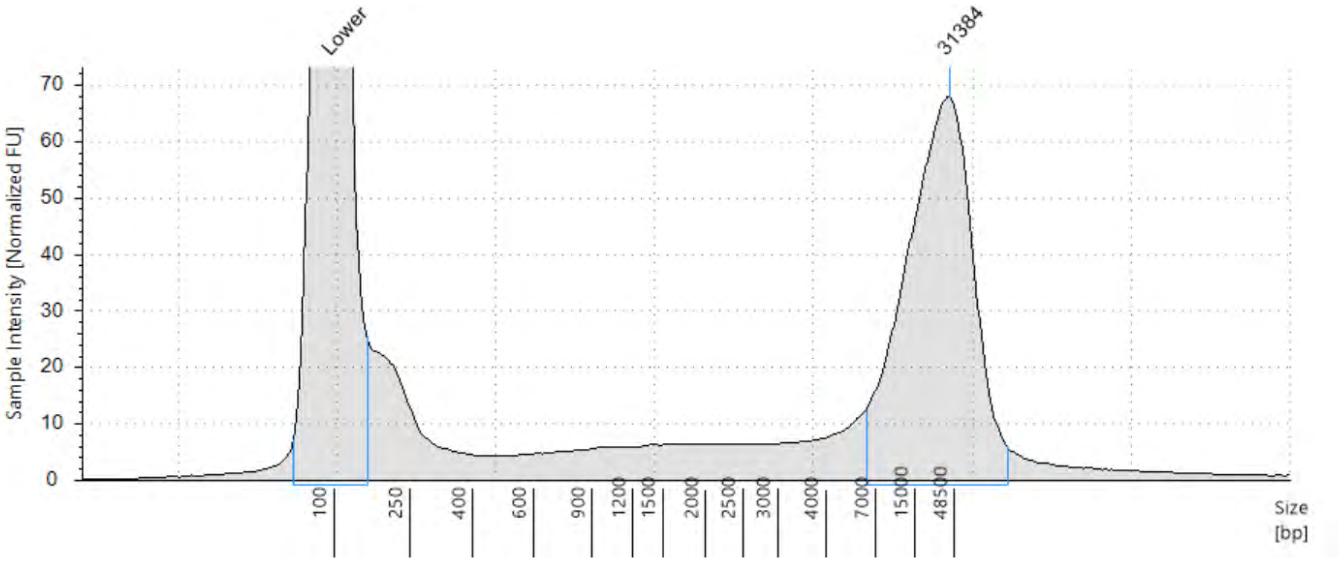

Sample Table

Well	DIN	Conc. [ng/µl]	Sample Description	Alert	Observations
E4	7.7	5.78	C478	⚠	Sample concentration outside recommended range

Peak Table

Size [bp]	Calibrated Conc. [ng/µl]	Assigned Conc. [ng/µl]	% Integrated Area	From [bp]	To [bp]	Peak Comment	Observations
100	8.50	8.50	-	61	150		Lower Marker
31384	3.28	-	96.31	6256	>60000		
-	-	-	-	-	-		Sample Well

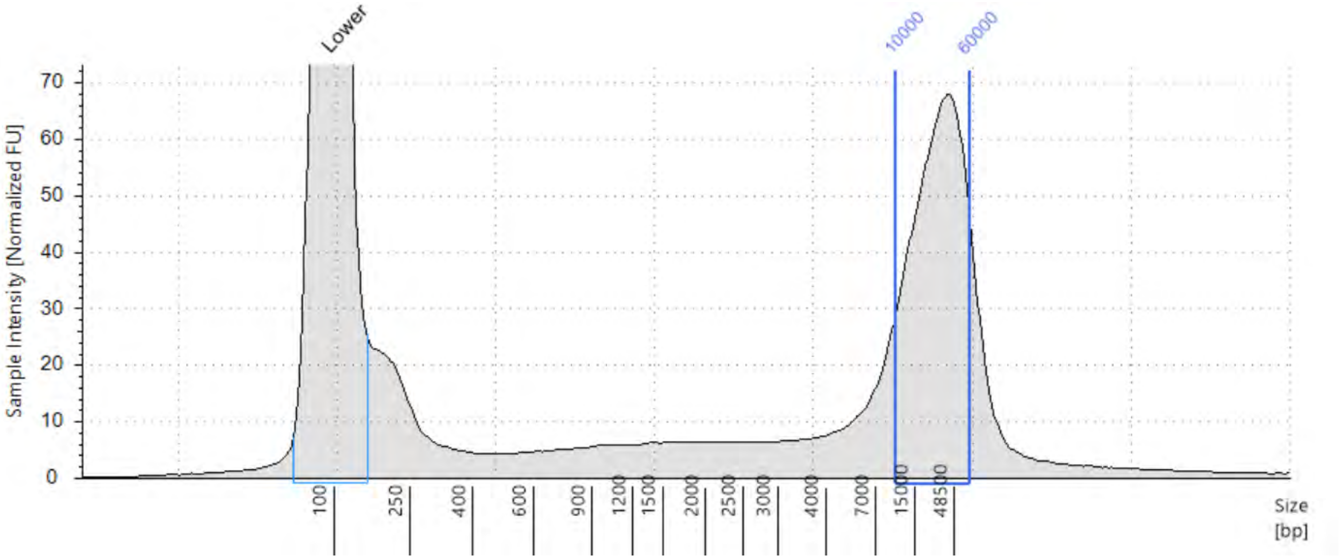

Region Table

From [bp]	To [bp]	Average Size [bp]	Conc. [ng/µl]	Region Molarity [nmol/l]	% of Total	Region Comment	Color
10000	60000	28656	2.48	0.176	42.82		■

© Agilent Technologies, Inc. 2017

F4: C479

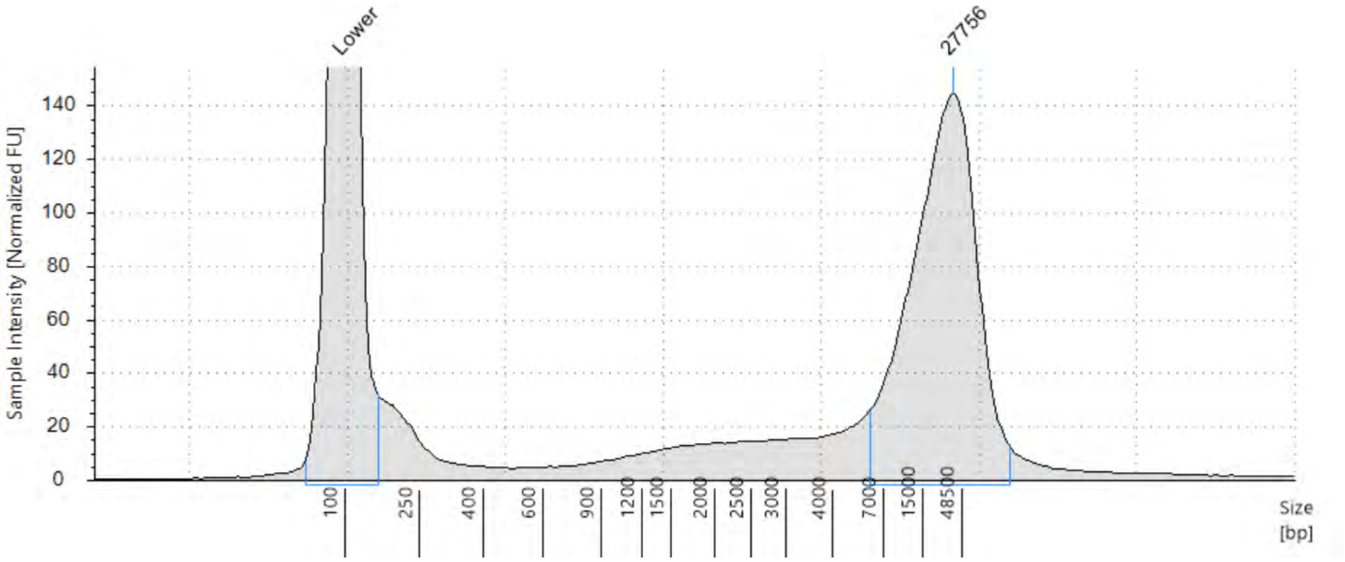

Sample Table

Well	DIN	Conc. [ng/µl]	Sample Description	Alert	Observations
F4	7.4	10.7	C479		

Peak Table

Size [bp]	Calibrated Conc. [ng/µl]	Assigned Conc. [ng/µl]	% Integrated Area	From [bp]	To [bp]	Peak Comment	Observations
100	8.50	8.50	-	62	149		Lower Marker
27756	6.68	-	97.23	6071	>60000		
-	-	-	-	-	-		Sample Well

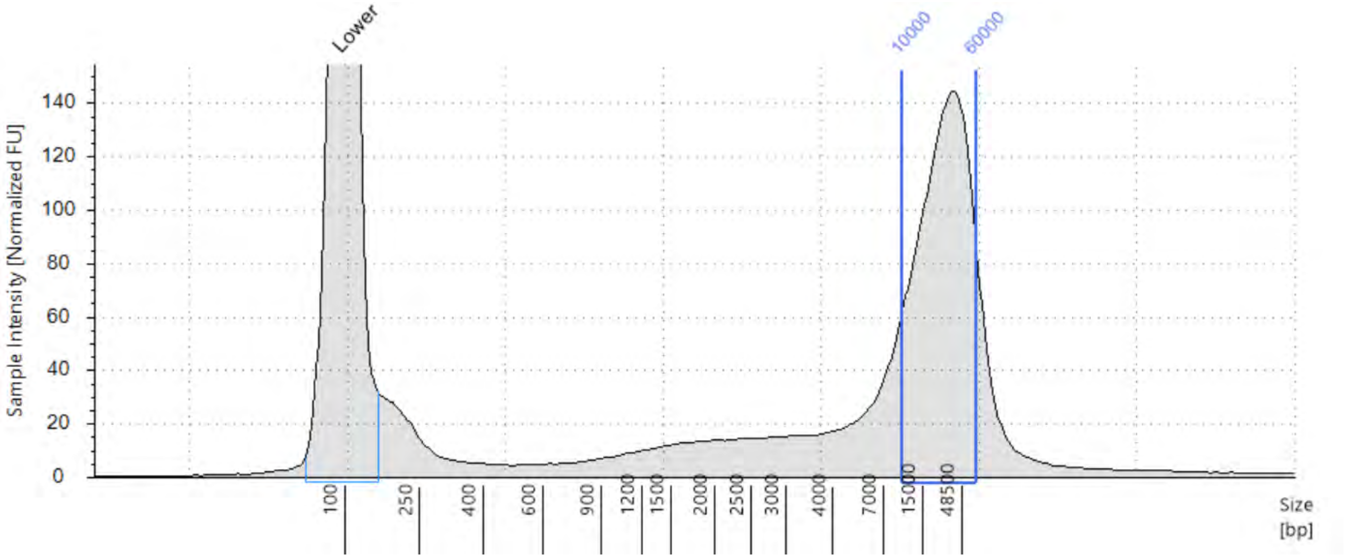

Region Table

From [bp]	To [bp]	Average Size [bp]	Conc. [ng/µl]	Region Molarity [nmol/l]	% of Total	Region Comment	Color
10000	60000	28583	5.15	0.366	48.10		

G4: C480

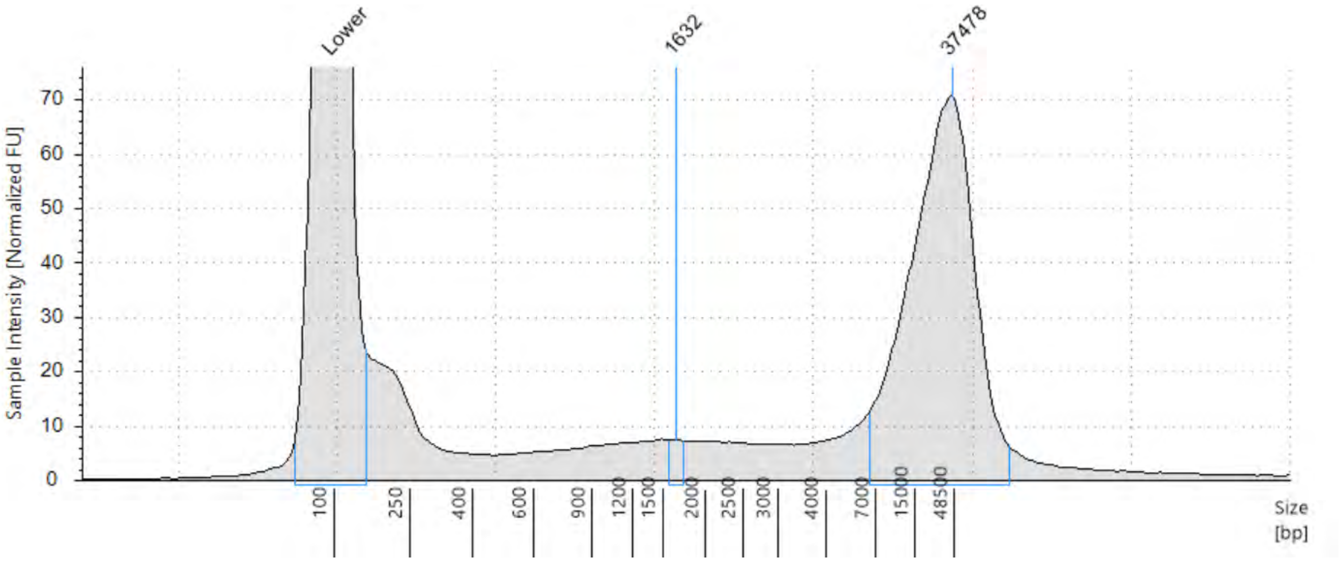

Sample Table

Well	DIN	Conc. [ng/µl]	Sample Description	Alert	Observations
G4	7.6	5.96	C480	⚠	Sample concentration outside recommended range

Peak Table

Size [bp]	Calibrated Conc. [ng/µl]	Assigned Conc. [ng/µl]	% Integrated Area	From [bp]	To [bp]	Peak Comment	Observations
100	8.50	8.50	-	62	147		Lower Marker
1632	0.0665	-	1.91	1560	1708		
37478	3.35	-	95.91	6510	>60000		
-	-	-	-	-	-		Sample Well

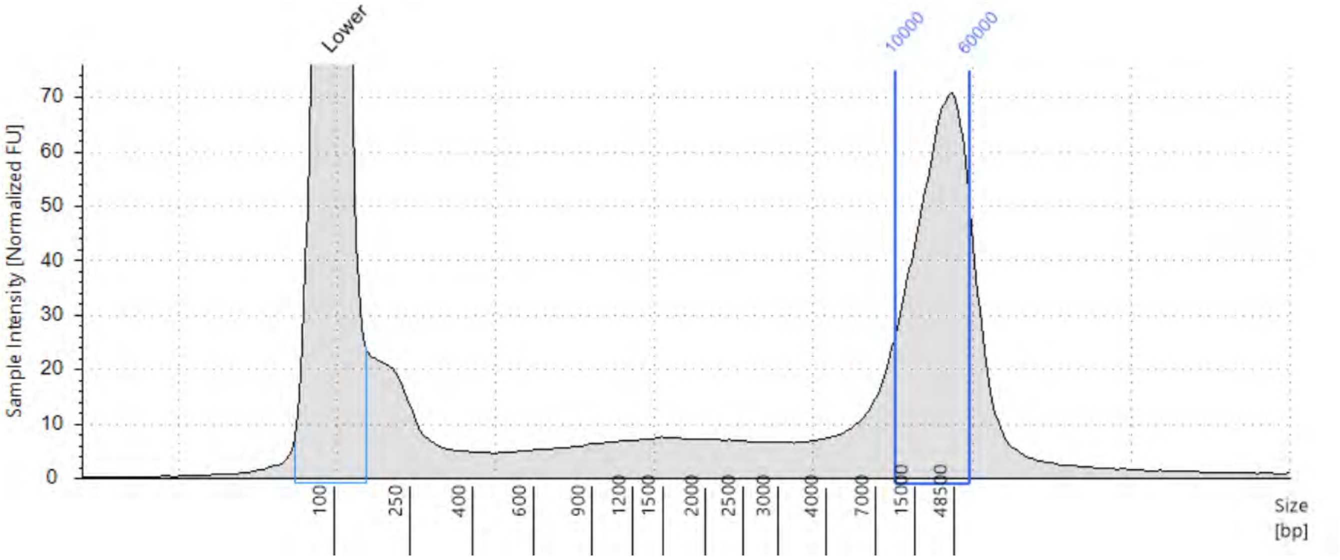

Region Table

From [bp]	To [bp]	Average Size [bp]	Conc. [ng/µl]	Region Molarity [nmol/l]	% of Total	Region Comment	Color
10000	60000	29448	2.52	0.175	42.33		■

 © Agilent Technologies, Inc. 2017

H4: C481

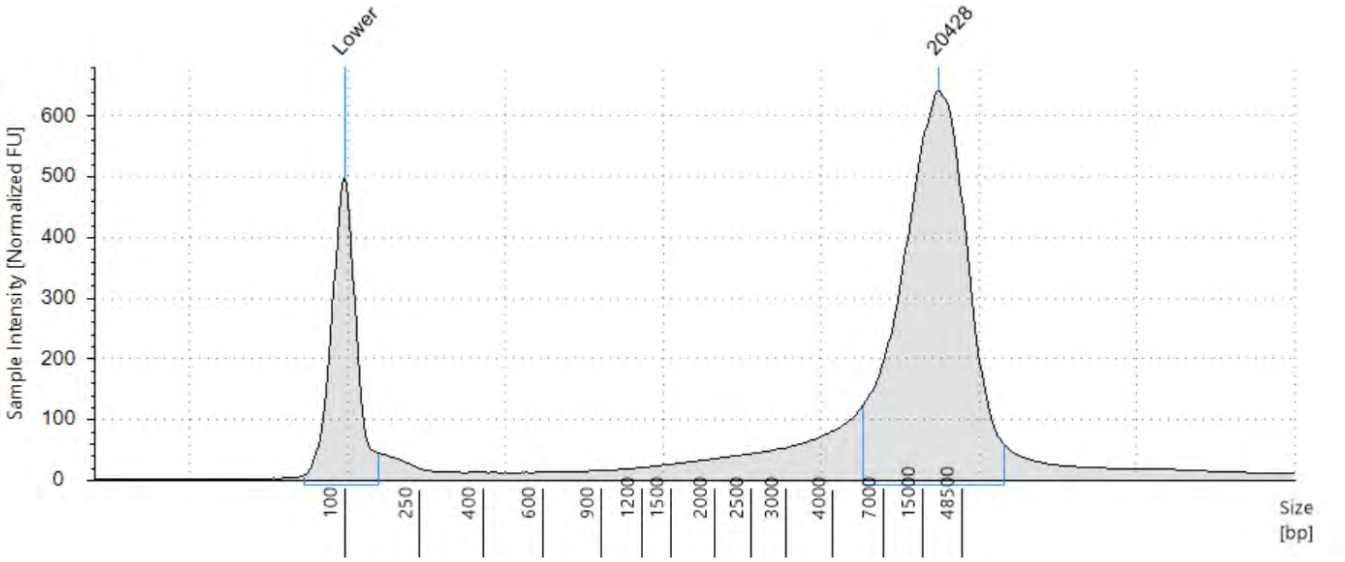

Sample Table

Well	DIN	Conc. [ng/µl]	Sample Description	Alert	Observations
H4	7.4	43.4	C481		

Peak Table

Size [bp]	Calibrated Conc. [ng/µl]	Assigned Conc. [ng/µl]	% Integrated Area	From [bp]	To [bp]	Peak Comment	Observations
100	8.50	8.50	-	61	149		Lower Marker
20428	29.5	-	87.51	5561	>60000		
-	-	-	-	-	-		Sample Well

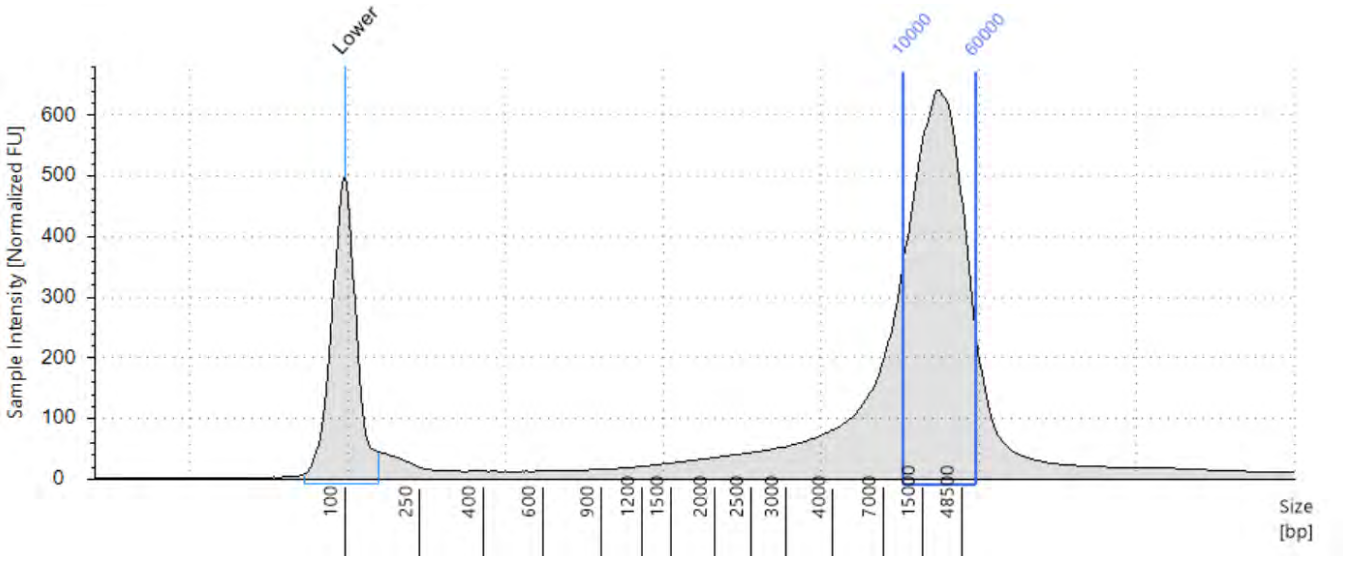

Region Table

From [bp]	To [bp]	Average Size [bp]	Conc. [ng/µl]	Region Molarity [nmol/l]	% of Total	Region Comment	Color
10000	60000	25058	22.7	1.78	52.30		

A5: C482

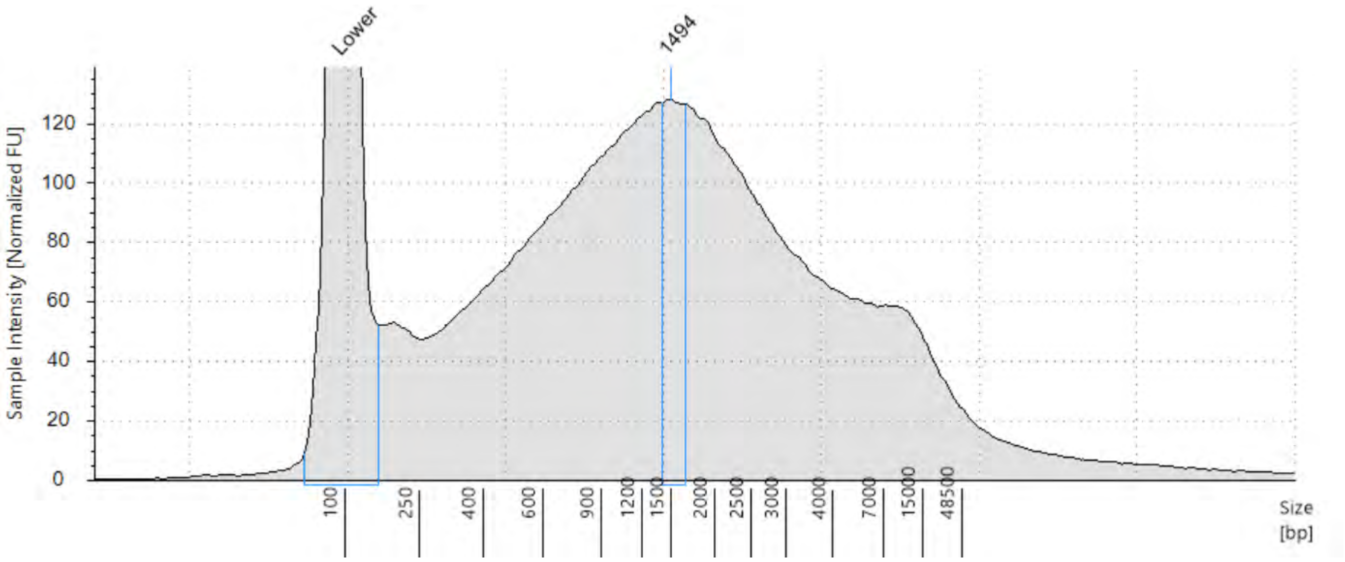

Sample Table

Well	DIN	Conc. [ng/µl]	Sample Description	Alert	Observations
A5	2.8	28.2	C482		

Peak Table

Size [bp]	Calibrated Conc. [ng/µl]	Assigned Conc. [ng/µl]	% Integrated Area	From [bp]	To [bp]	Peak Comment	Observations
100	8.50	8.50	-	61	150		Lower Marker
1494	1.84	-	97.10	1403	1654		
-	-	-	-	-	-		Sample Well

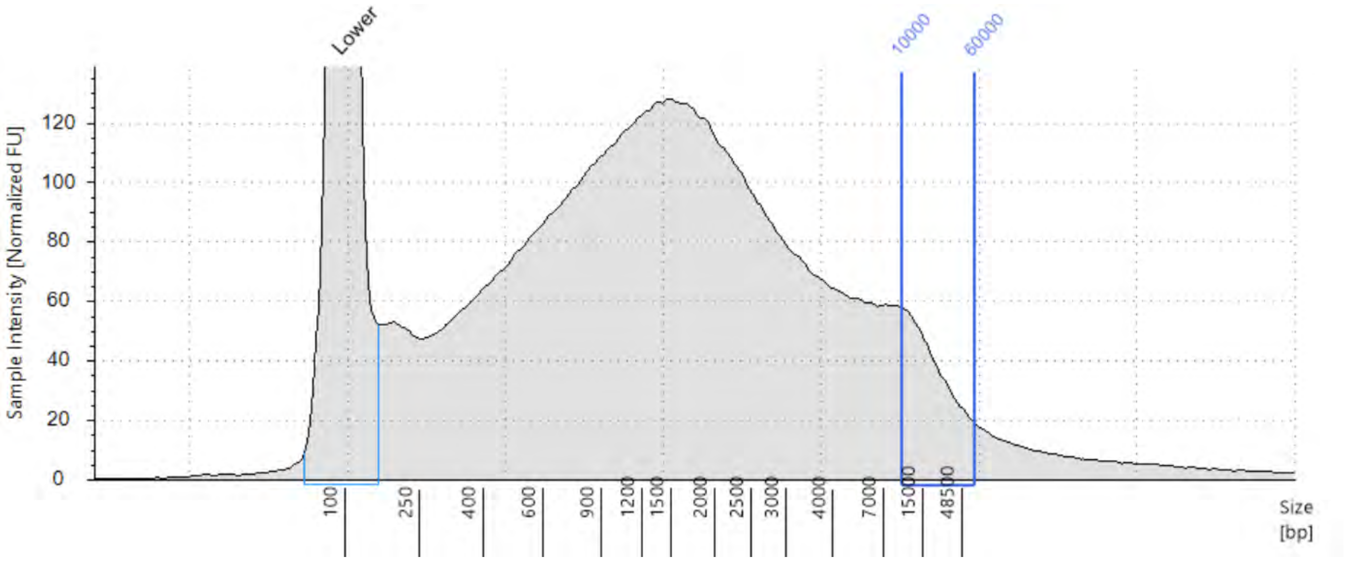

Region Table

From [bp]	To [bp]	Average Size [bp]	Conc. [ng/µl]	Region Molarity [nmol/l]	% of Total	Region Comment	Color
10000	60000	22553	1.62	0.148	5.74		

 © Agilent Technologies, Inc. 2017

B5: C483

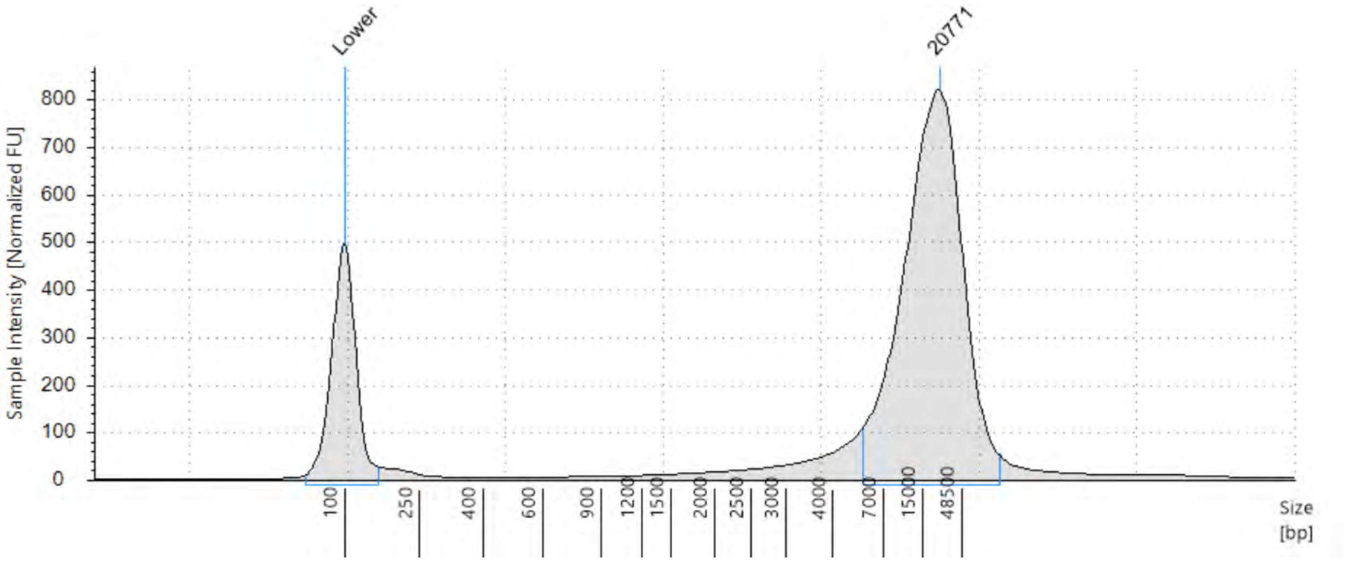

Sample Table

Well	DIN	Conc. [ng/µl]	Sample Description	Alert	Observations
B5	8.0	42.1	C483		

Peak Table

Size [bp]	Calibrated Conc. [ng/µl]	Assigned Conc. [ng/µl]	% Integrated Area	From [bp]	To [bp]	Peak Comment	Observations
100	8.50	8.50	-	62	151		Lower Marker
20771	34.3	-	96.19	5555	>60000		
-	-	-	-	-	-		Sample Well

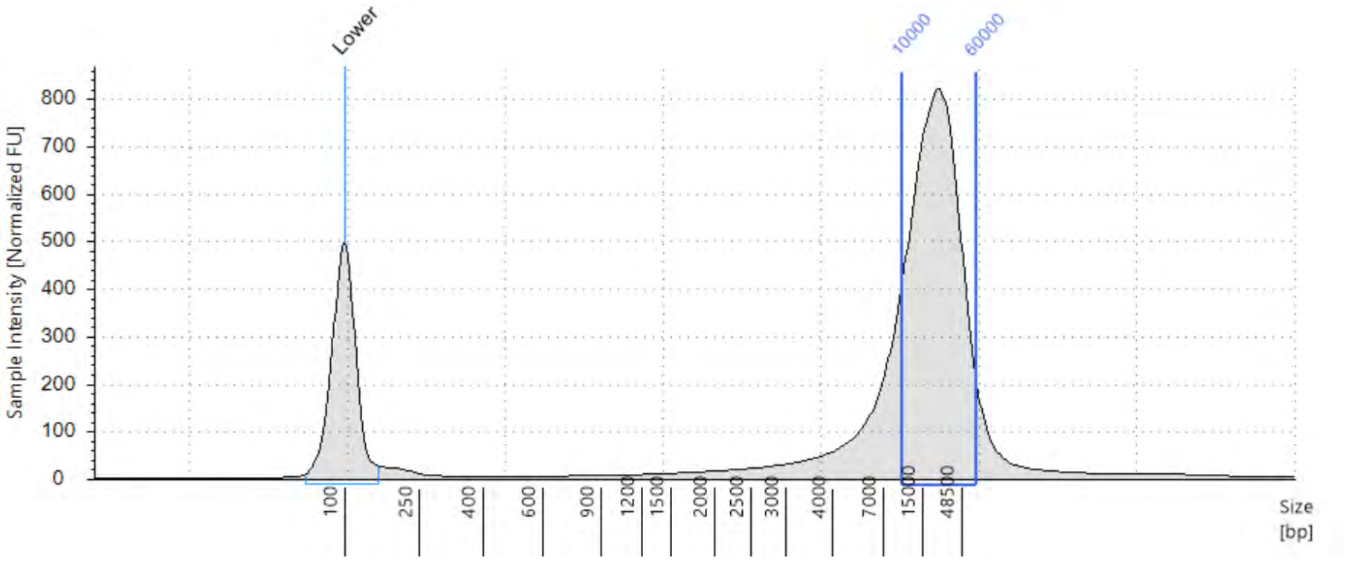

Region Table

From [bp]	To [bp]	Average Size [bp]	Conc. [ng/µl]	Region Molarity [nmol/l]	% of Total	Region Comment	Color
10000	60000	23997	27.7	2.23	65.74		

 © Agilent Technologies, Inc. 2017

C5: C484

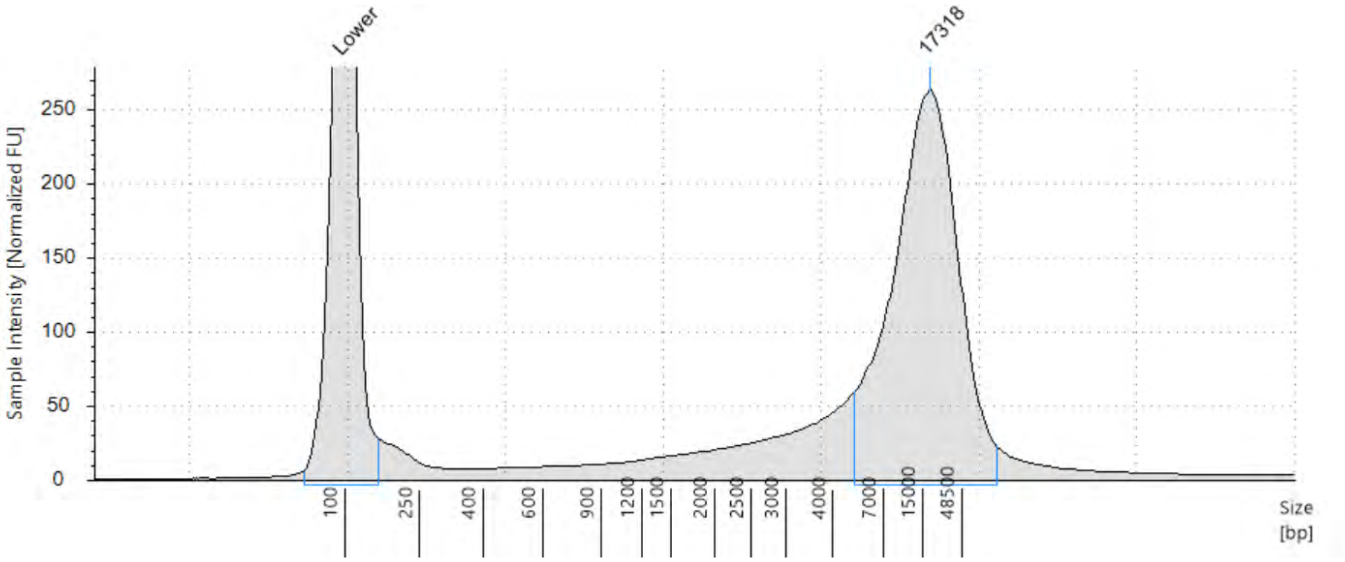

Sample Table

Well	DIN	Conc. [ng/µl]	Sample Description	Alert	Observations
C5	7.1	19.3	C484		

Peak Table

Size [bp]	Calibrated Conc. [ng/µl]	Assigned Conc. [ng/µl]	%Integrated Area	From [bp]	To [bp]	Peak Comment	Observations
100	8.50	8.50	-	61	151		Lower Marker
17318	12.5	-	93.18	5039	>60000		
-	-	-	-	-	-		Sample Well

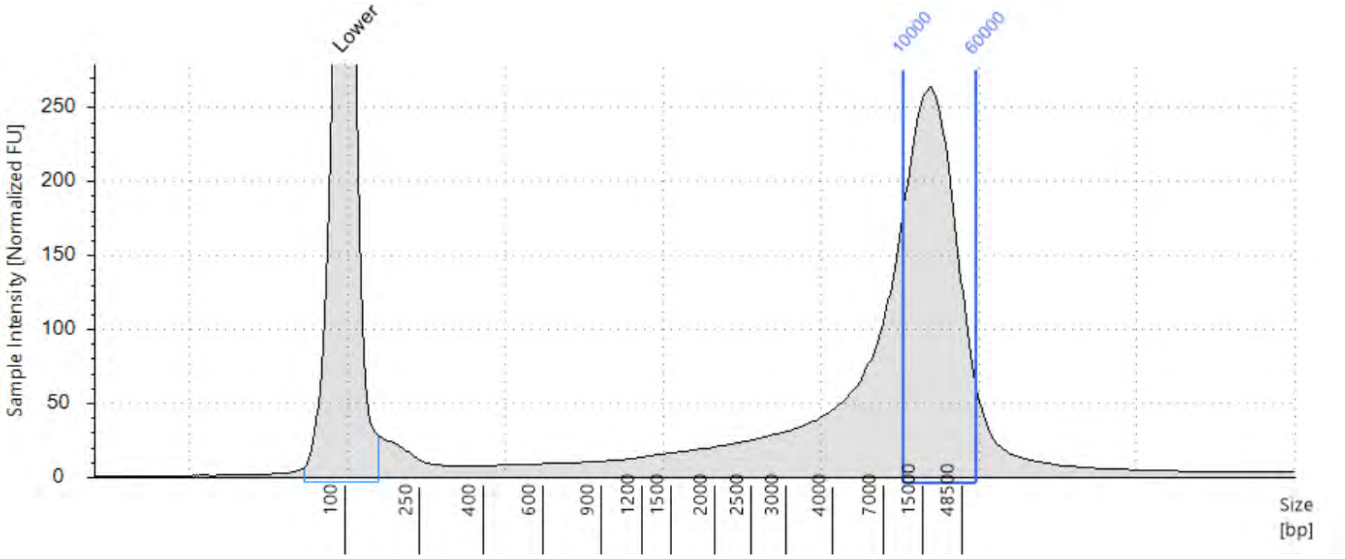

Region Table

From [bp]	To [bp]	Average Size [bp]	Conc. [ng/µl]	Region Molarity [nmol/l]	% of Total	Region Comment	Color
10000	60000	22476	9.19	0.789	47.65		

© Agilent Technologies, Inc. 2017

D5: C485

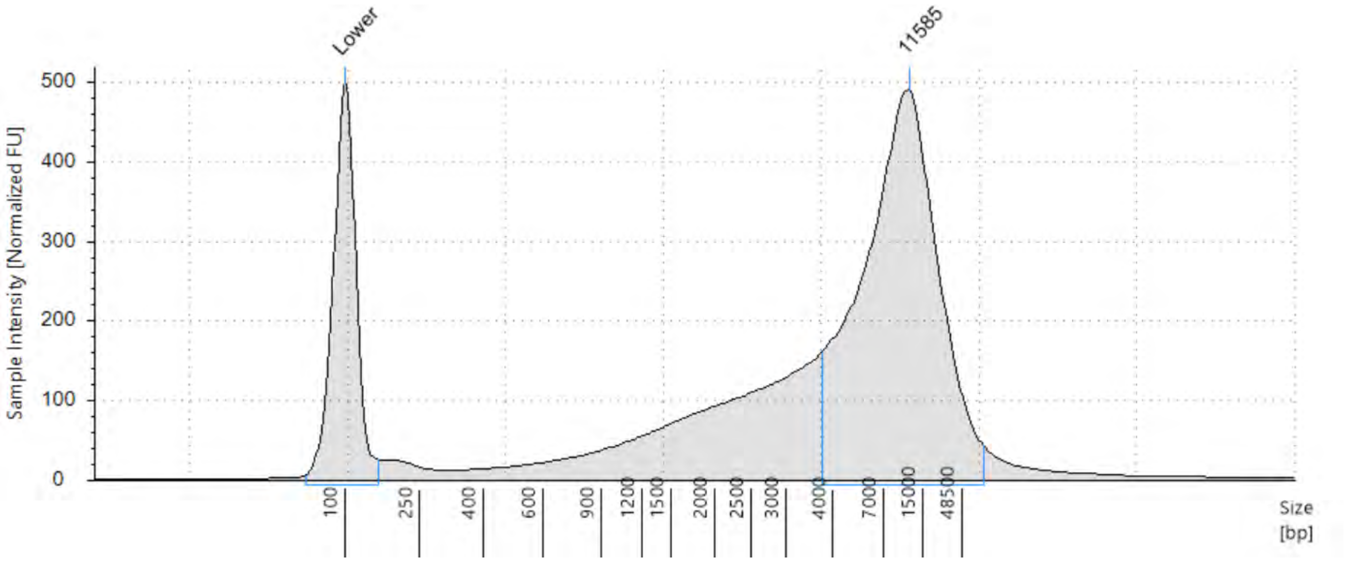

Sample Table

Well	DIN	Conc. [ng/µl]	Sample Description	Alert	Observations
D5	6.4	45.0	C485		

Peak Table

Size [bp]	Calibrated Conc. [ng/µl]	Assigned Conc. [ng/µl]	% Integrated Area	From [bp]	To [bp]	Peak Comment	Observations
100	8.50	8.50	-	62	149		Lower Marker
11585	27.8	-	97.74	3737	>60000		
-	-	-	-	-	-		Sample Well

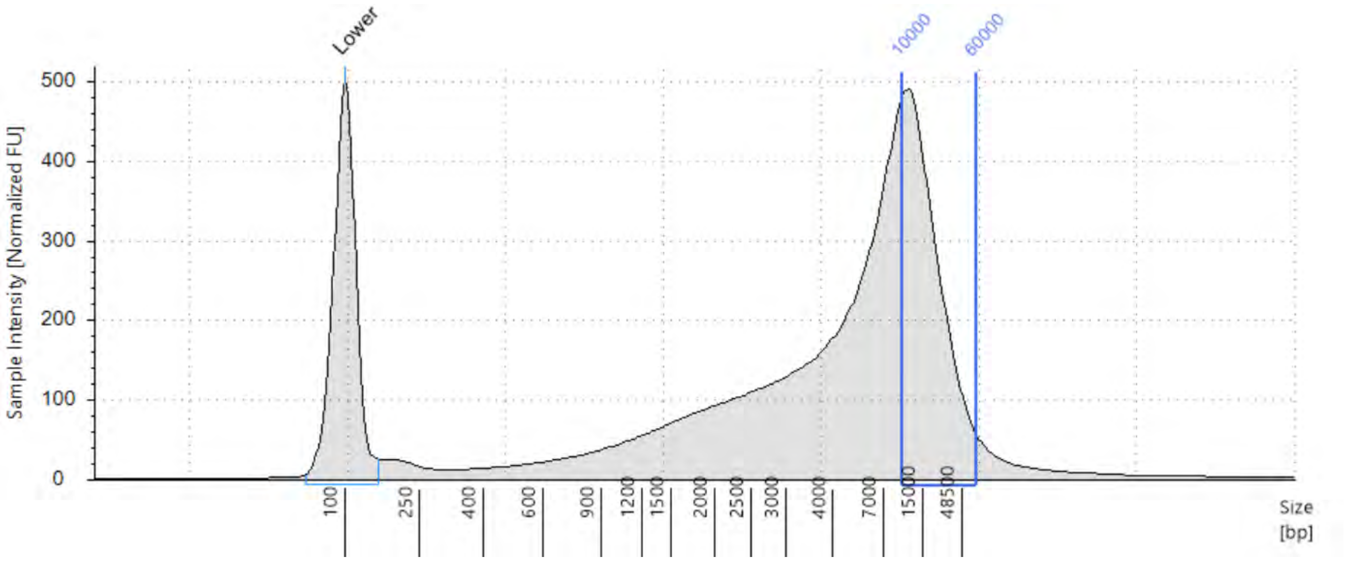

Region Table

From [bp]	To [bp]	Average Size [bp]	Conc. [ng/µl]	Region Molarity [nmol/l]	% of Total	Region Comment	Color
10000	60000	19328	13.3	1.31	29.60		

E5: C486

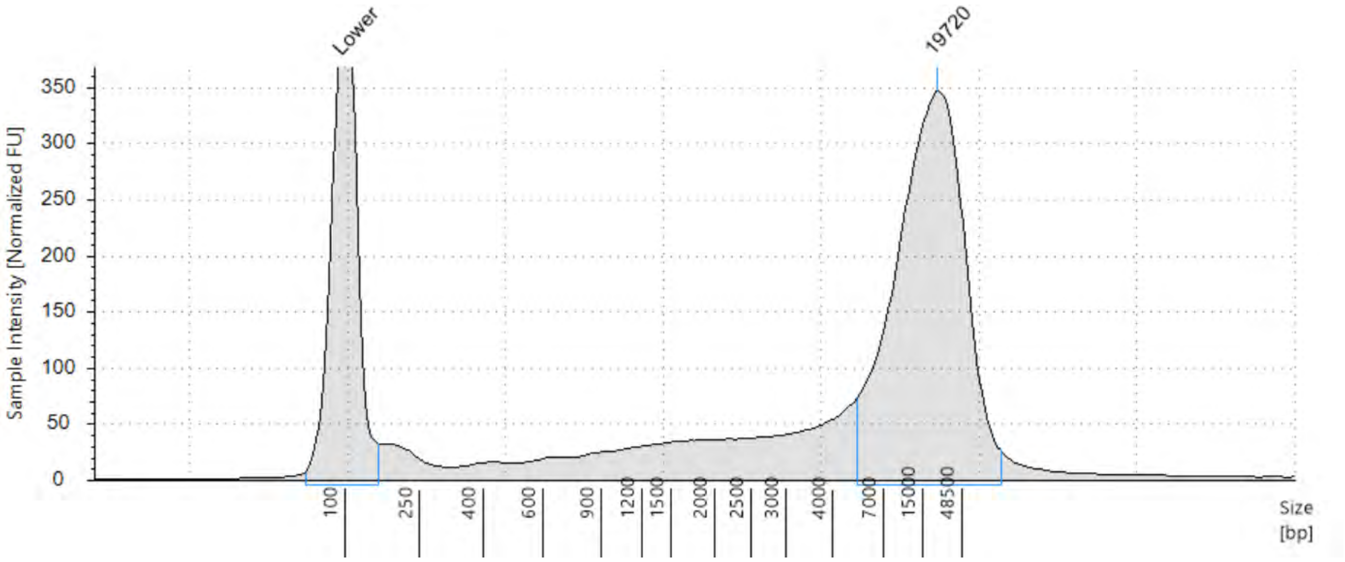

Sample Table

Well	DIN	Conc. [ng/µl]	Sample Description	Alert	Observations
E5	6.8	27.3	C486		

Peak Table

Size [bp]	Calibrated Conc. [ng/µl]	Assigned Conc. [ng/µl]	% Integrated Area	From [bp]	To [bp]	Peak Comment	Observations
100	8.50	8.50	-	62	149		Lower Marker
19720	17.5	-	97.04	5241	>60000		
-	-	-	-	-	-		Sample Well

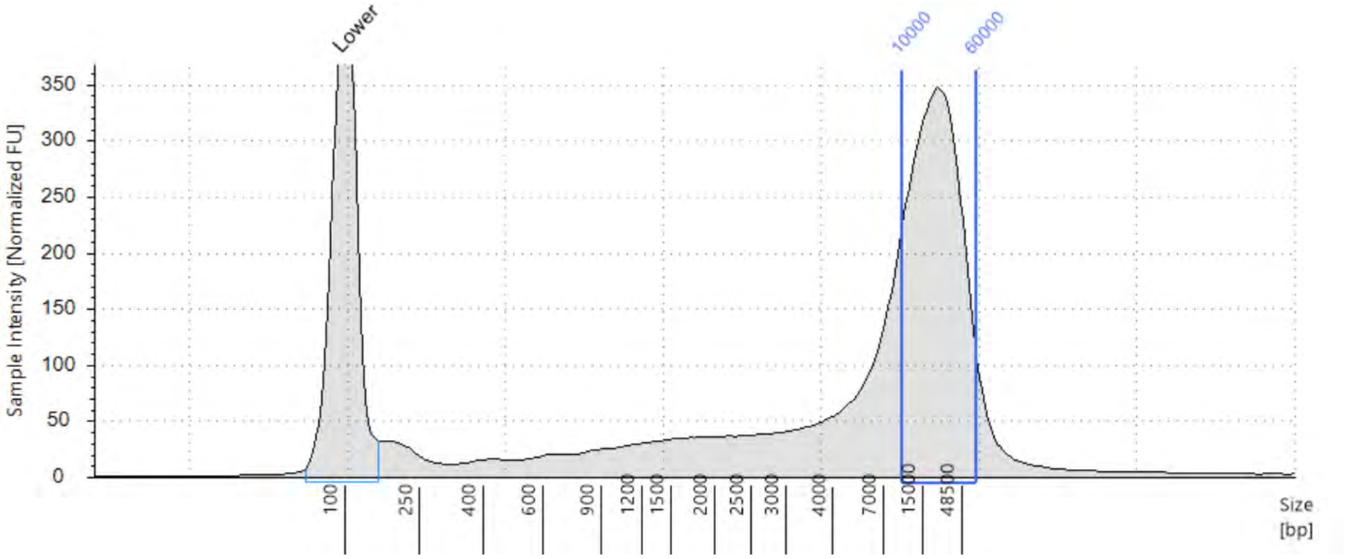

Region Table

From [bp]	To [bp]	Average Size [bp]	Conc. [ng/µl]	Region Molarity [nmol/l]	% of Total	Region Comment	Color
10000	60000	24469	13.1	1.05	47.89		

F5: C487

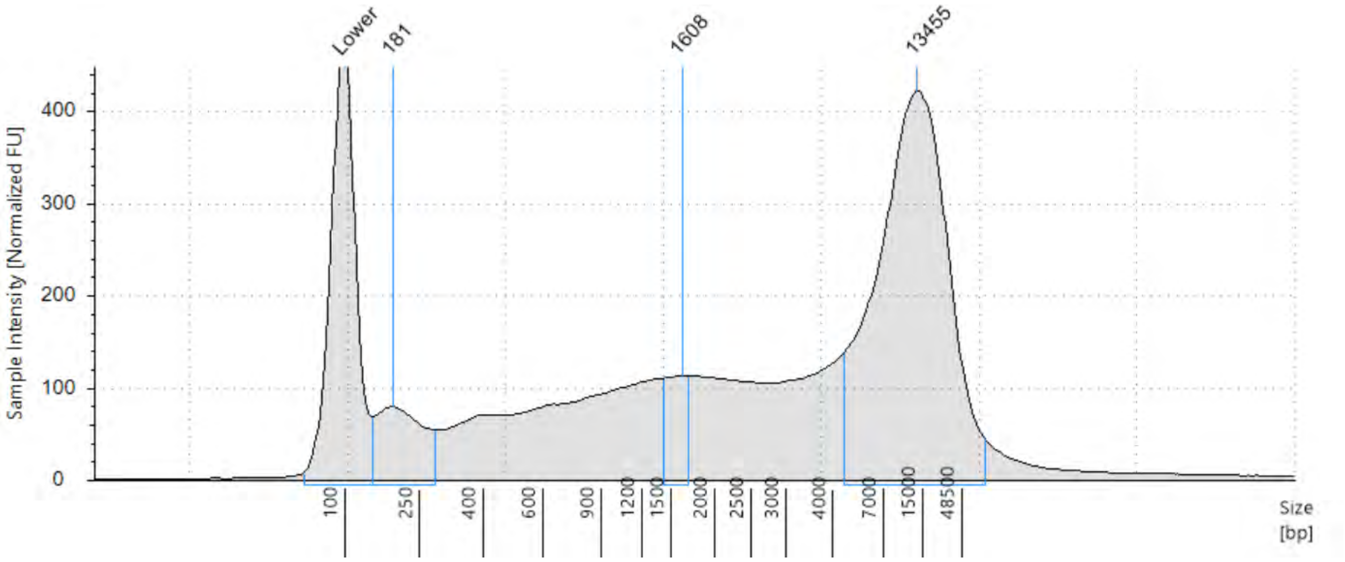

Sample Table

Well	DIN	Conc. [ng/µl]	Sample Description	Alert	Observations
F5	5.9	49.1	C487		

Peak Table

Size [bp]	Calibrated Conc. [ng/µl]	Assigned Conc. [ng/µl]	% Integrated Area	From [bp]	To [bp]	Peak Comment	Observations
100	8.50	8.50	-	61	141		Lower Marker
181	2.61	-	9.92	141	281		
1608	1.74	-	6.61	1420	1683		
13455	21.2	-	80.84	4489	>60000		
-	-	-	-	-	-		Sample Well

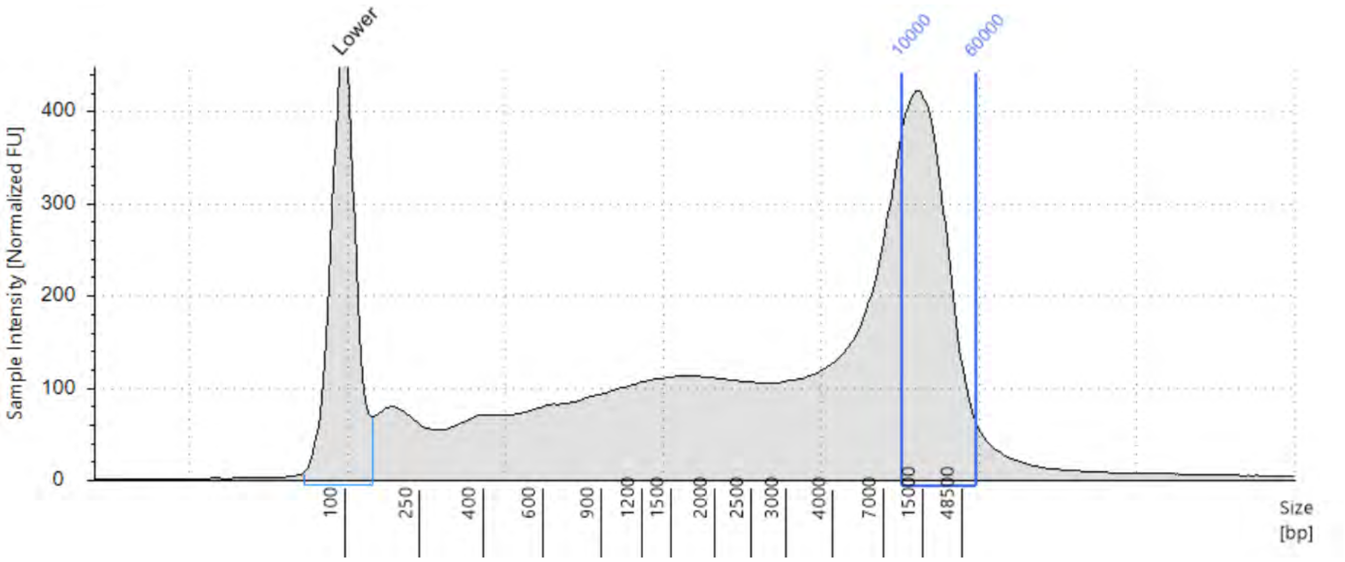

Region Table

From [bp]	To [bp]	Average Size [bp]	Conc. [ng/µl]	Region Molarity [nmol/l]	% of Total	Region Comment	Color
10000	60000	20143	13.1	1.23	26.74		

G5: C488

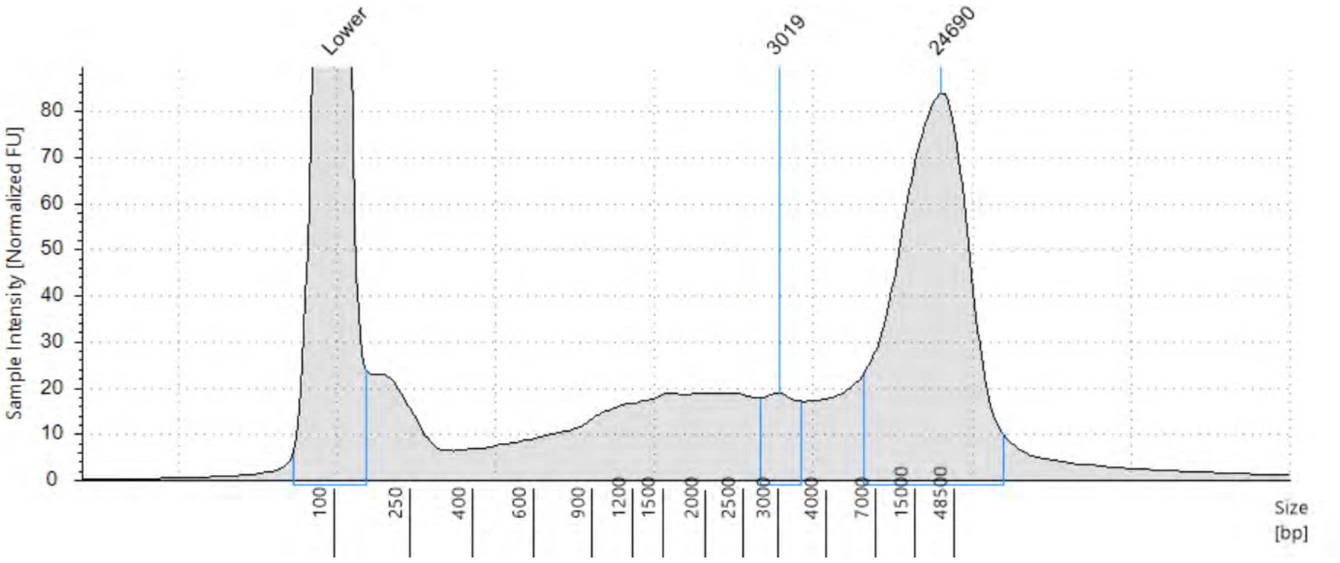

Sample Table

Well	DIN	Conc. [ng/µl]	Sample Description	Alert	Observations
G5	6.5	9.44	C488	⚠	Sample concentration outside recommended range

Peak Table

Size [bp]	Calibrated Conc. [ng/µl]	Assigned Conc. [ng/µl]	% Integrated Area	From [bp]	To [bp]	Peak Comment	Observations
100	8.50	8.50	-	61	147		Lower Marker
3019	0.461	-	9.15	2747	3451		
24690	4.41	-	87.62	6081	>60000		
-	-	-	-	-	-		Sample Well

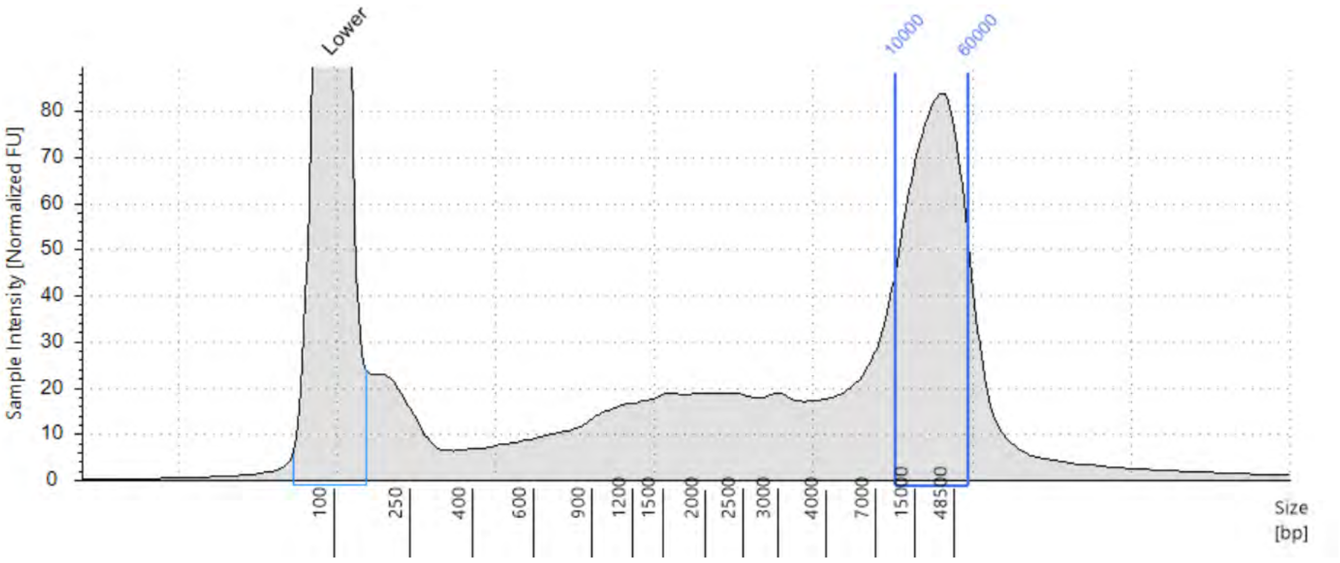

Region Table

From [bp]	To [bp]	Average Size [bp]	Conc. [ng/µl]	Region Molarity [nmol/l]	% of Total	Region Comment	Color
10000	60000	27001	3.29	0.247	34.86		■

H5: C489

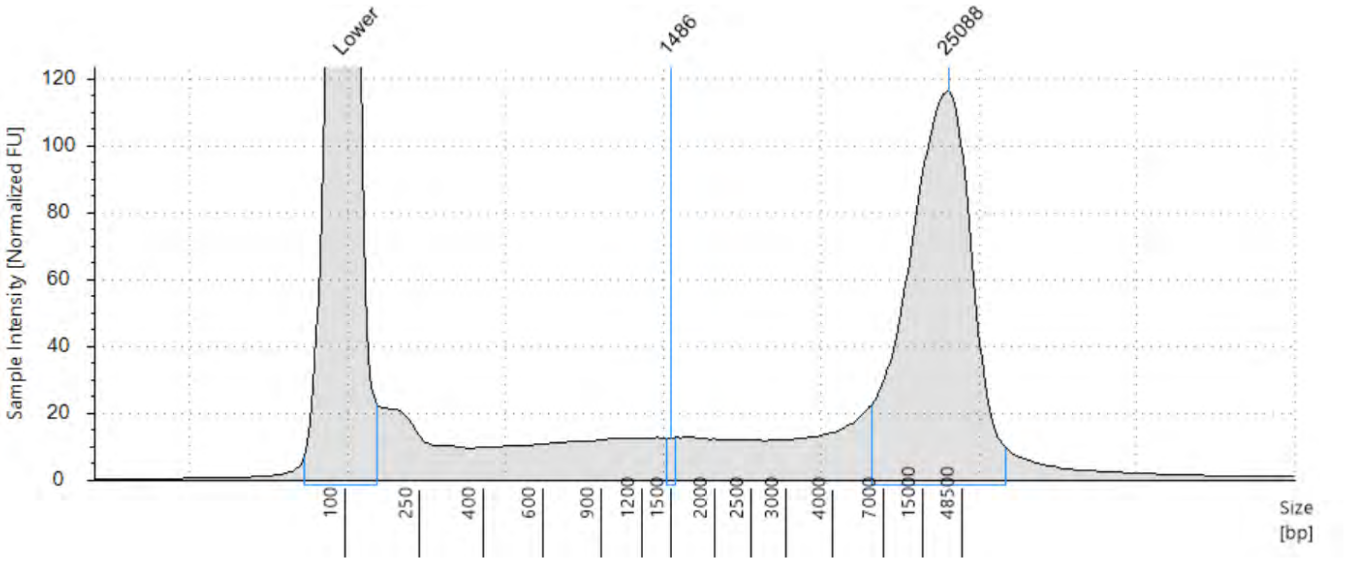

Sample Table

Well	DIN	Conc. [ng/µl]	Sample Description	Alert	Observations
H5	6.8	9.60	C489	⚠	Sample concentration outside recommended range

Peak Table

Size [bp]	Calibrated Conc. [ng/µl]	Assigned Conc. [ng/µl]	% Integrated Area	From [bp]	To [bp]	Peak Comment	Observations
100	8.50	8.50	-	61	147		Lower Marker
1486	0.0734	-	1.31	1440	1529		
25088	5.35	-	95.77	6057	>60000		
-	-	-	-	-	-		Sample Well

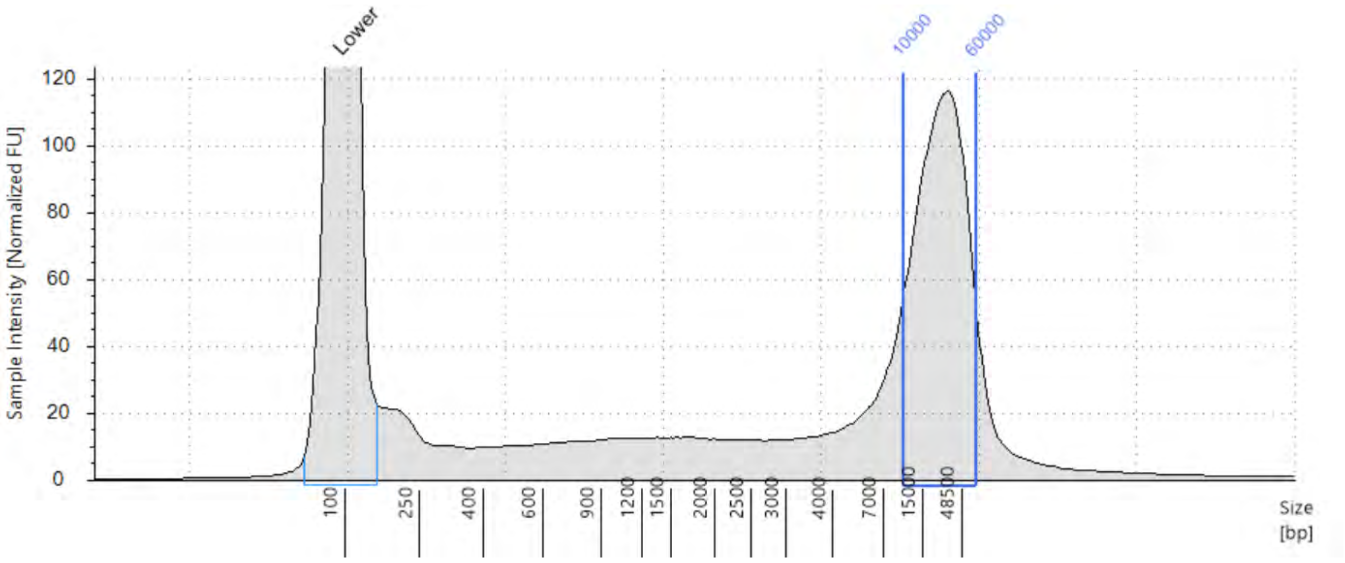

Region Table

From [bp]	To [bp]	Average Size [bp]	Conc. [ng/µl]	Region Molarity [nmol/l]	% of Total	Region Comment	Color
10000	60000	26605	4.30	0.323	44.83		■

A6: C490

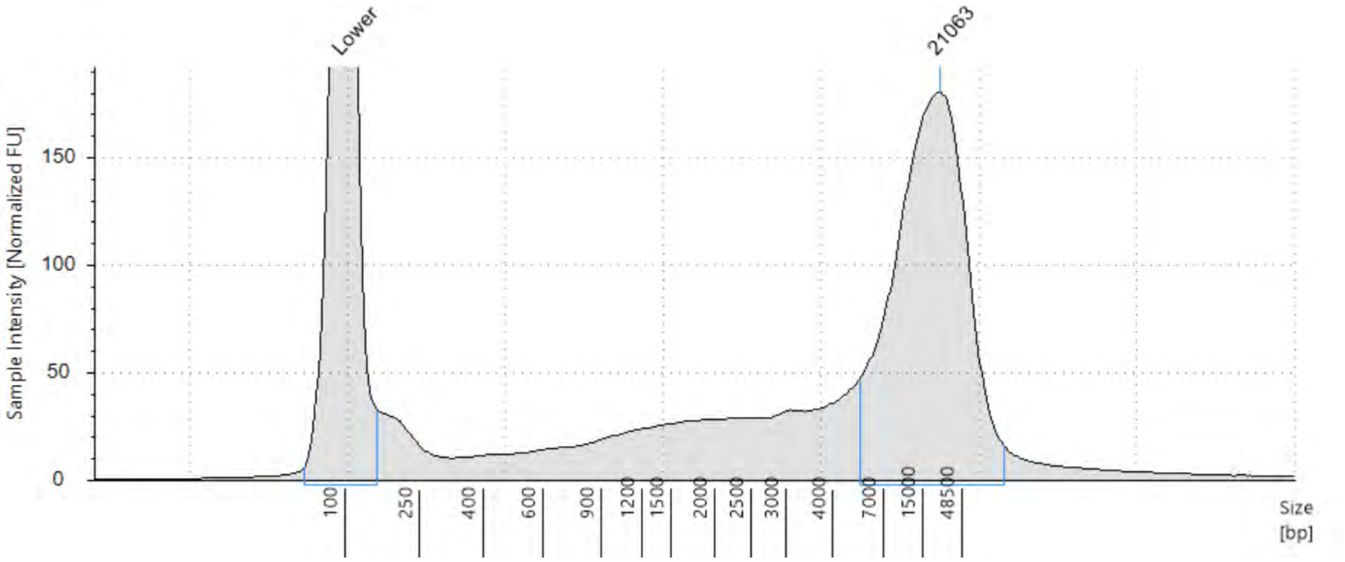

Sample Table

Well	DIN	Conc. [ng/µl]	Sample Description	Alert	Observations
A6	6.6	17.2	C490		

Peak Table

Size [bp]	Calibrated Conc. [ng/µl]	Assigned Conc. [ng/µl]	% Integrated Area	From [bp]	To [bp]	Peak Comment	Observations
100	8.50	8.50	-	62	149		Lower Marker
21063	9.67	-	97.41	5354	>60000		
-	-	-	-	-	-		Sample Well

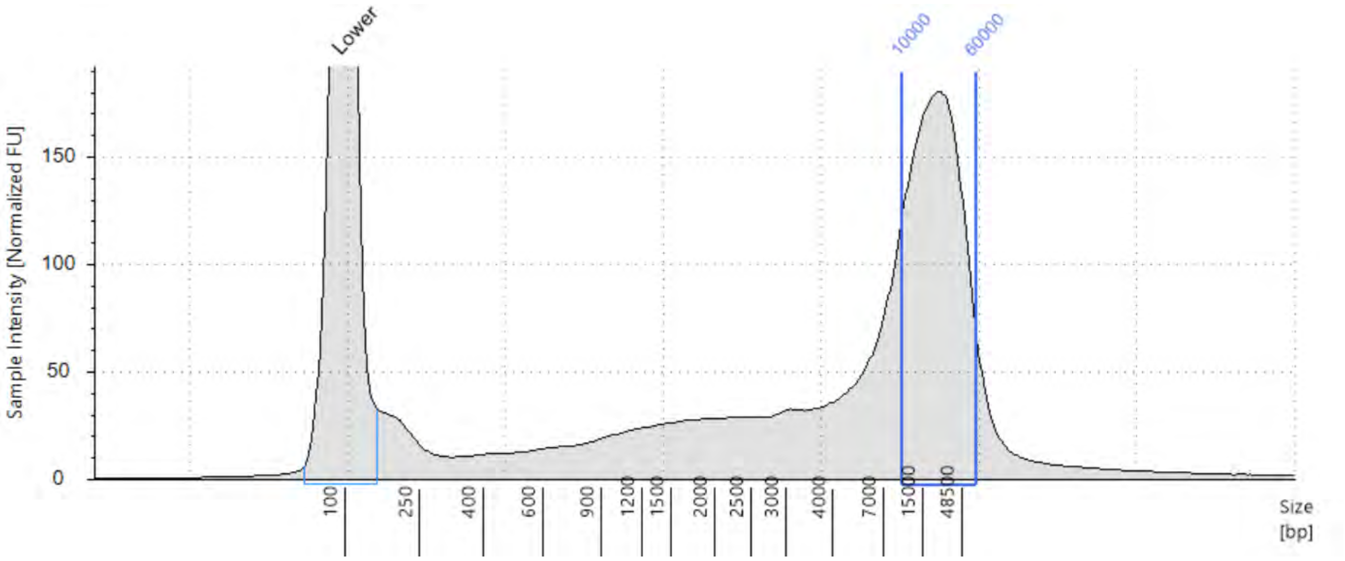

Region Table

From [bp]	To [bp]	Average Size [bp]	Conc. [ng/µl]	Region Molarity [nmol/l]	% of Total	Region Comment	Color
10000	60000	24834	7.02	0.559	40.86		

B6: C491

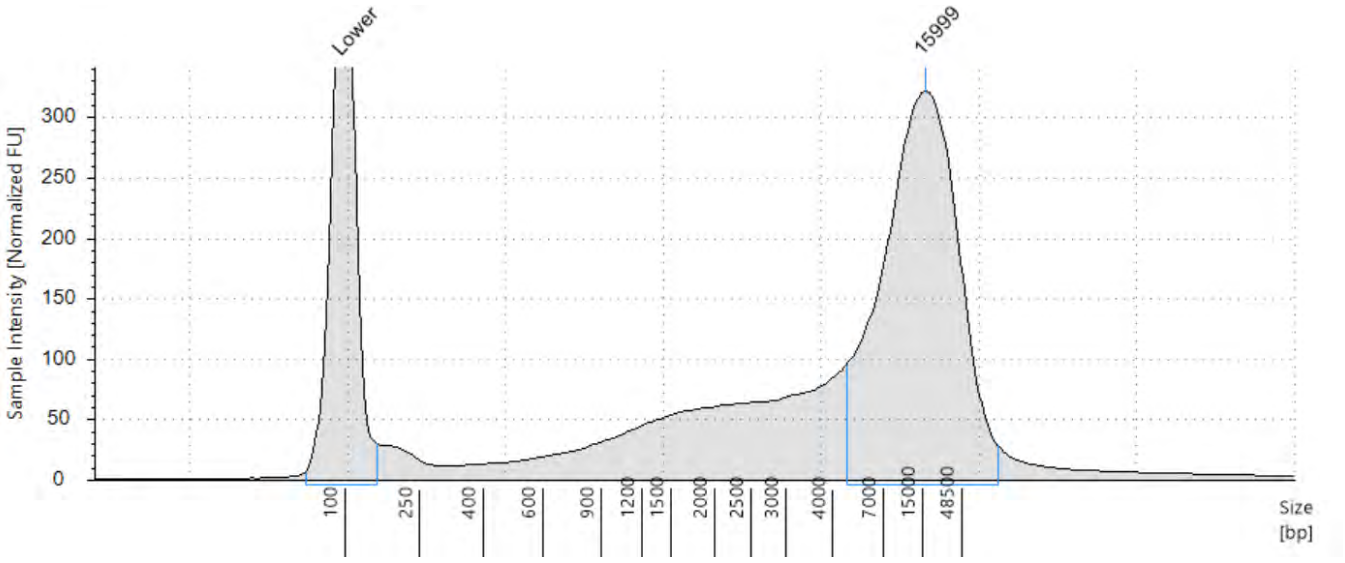

Sample Table

Well	DIN	Conc. [ng/µl]	Sample Description	Alert	Observations
B6	6.5	31.6	C491		

Peak Table

Size [bp]	Calibrated Conc. [ng/µl]	Assigned Conc. [ng/µl]	% Integrated Area	From [bp]	To [bp]	Peak Comment	Observations
100	8.50	8.50	-	62	149		Lower Marker
15999	18.2	-	96.30	4669	>60000		
-	-	-	-	-	-		Sample Well

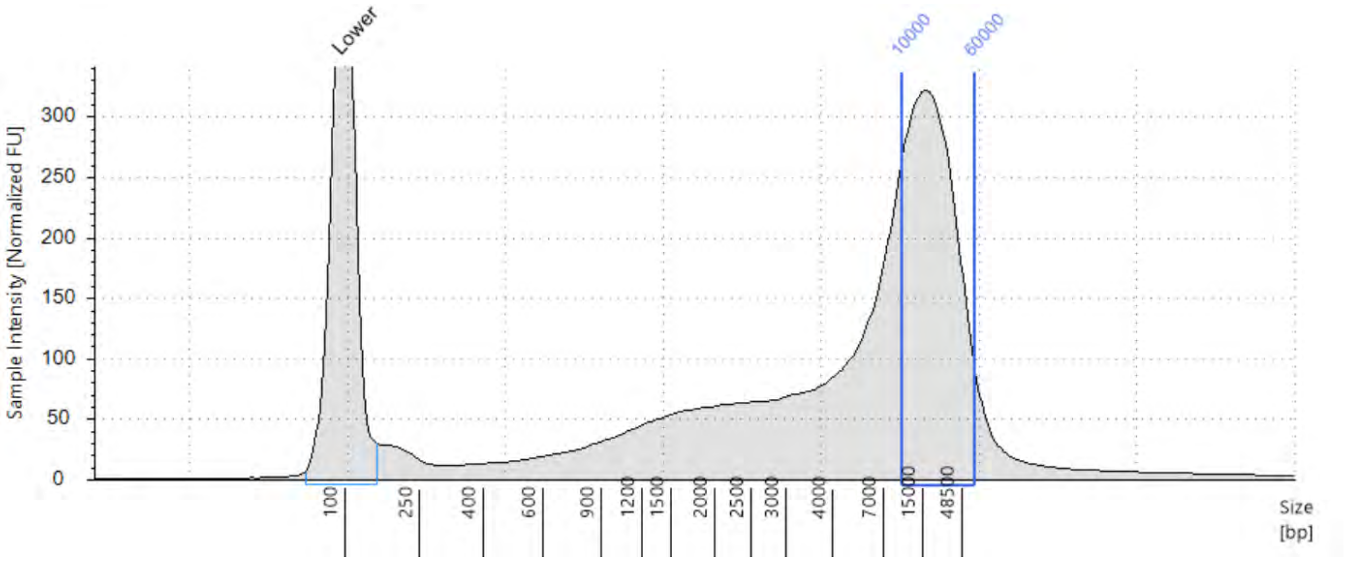

Region Table

From [bp]	To [bp]	Average Size [bp]	Conc. [ng/µl]	Region Molarity [nmol/l]	% of Total	Region Comment	Color
10000	60000	22894	12.1	1.03	38.14		

 © Agilent Technologies, Inc. 2017

C6: C492

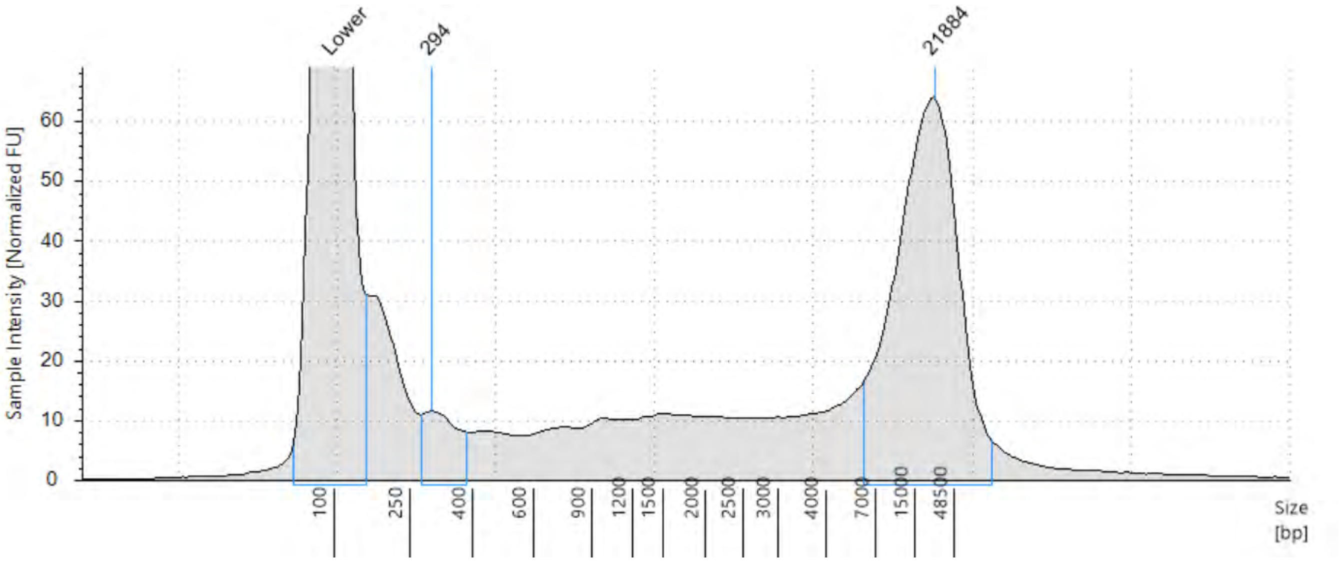

Sample Table

Well	DIN	Conc. [ng/µl]	Sample Description	Alert	Observations
C6	6.5	6.56	C492	⚠	Sample concentration outside recommended range

Peak Table

Size [bp]	Calibrated Conc. [ng/µl]	Assigned Conc. [ng/µl]	% Integrated Area	From [bp]	To [bp]	Peak Comment	Observations
100	8.50	8.50	-	61	147		Lower Marker
294	0.284	-	8.66	271	380		
21884	2.94	-	89.68	6071	>60000		
-	-	-	-	-	-		Sample Well

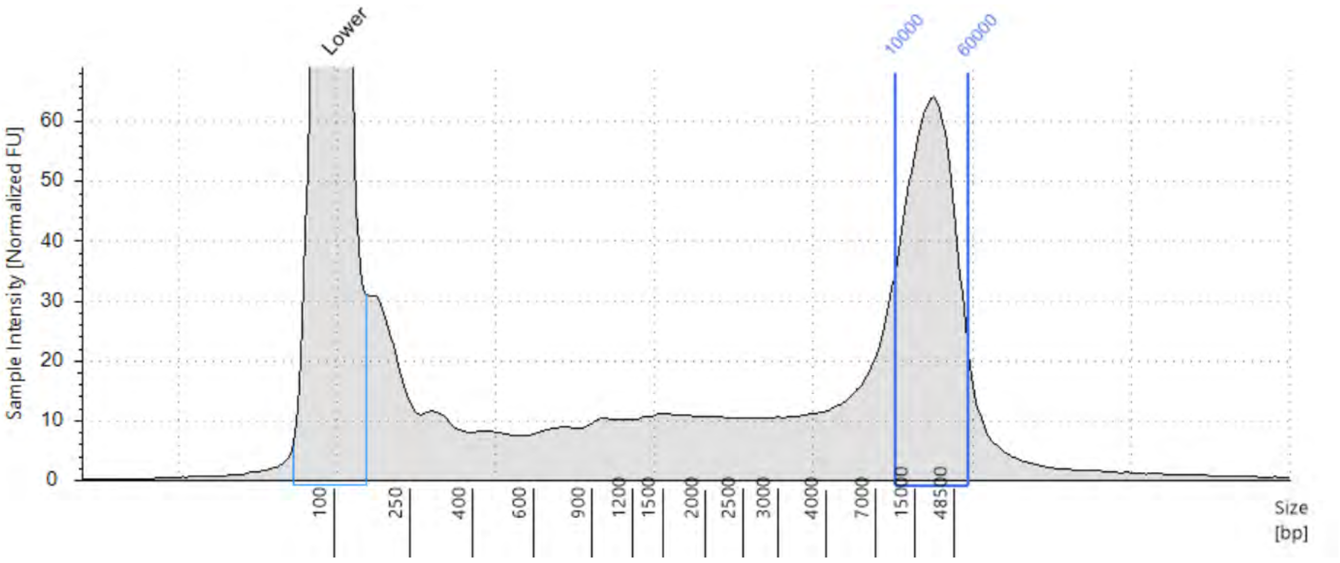

Region Table

From [bp]	To [bp]	Average Size [bp]	Conc. [ng/µl]	Region Molarity [nmol/l]	% of Total	Region Comment	Color
10000	60000	24838	2.34	0.186	35.66		■

D6: C493

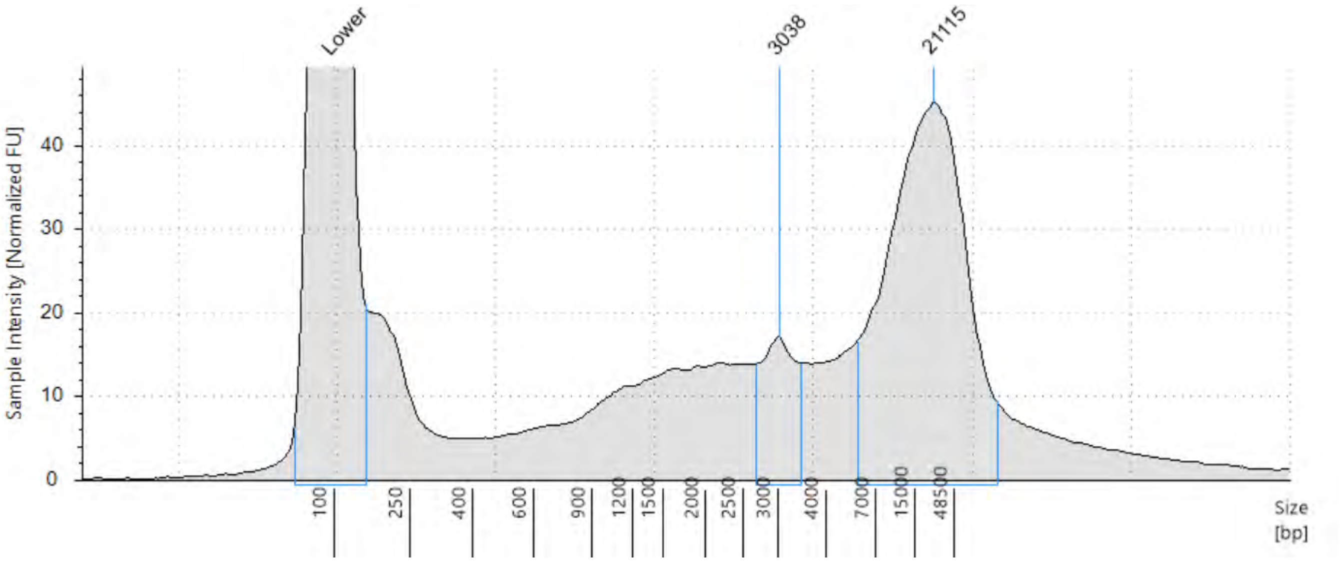

Sample Table

Well	DIN	Conc. [ng/µl]	Sample Description	Alert	Observations
D6	6.5	6.56	C493	⚠	Sample concentration outside recommended range

Peak Table

Size [bp]	Calibrated Conc. [ng/µl]	Assigned Conc. [ng/µl]	% Integrated Area	From [bp]	To [bp]	Peak Comment	Observations
100	8.50	8.50	-	62	146		Lower Marker
3038	0.441	-	13.93	2688	3466		
21115	2.65	-	83.80	5719	>60000		
-	-	-	-	-	-		Sample Well

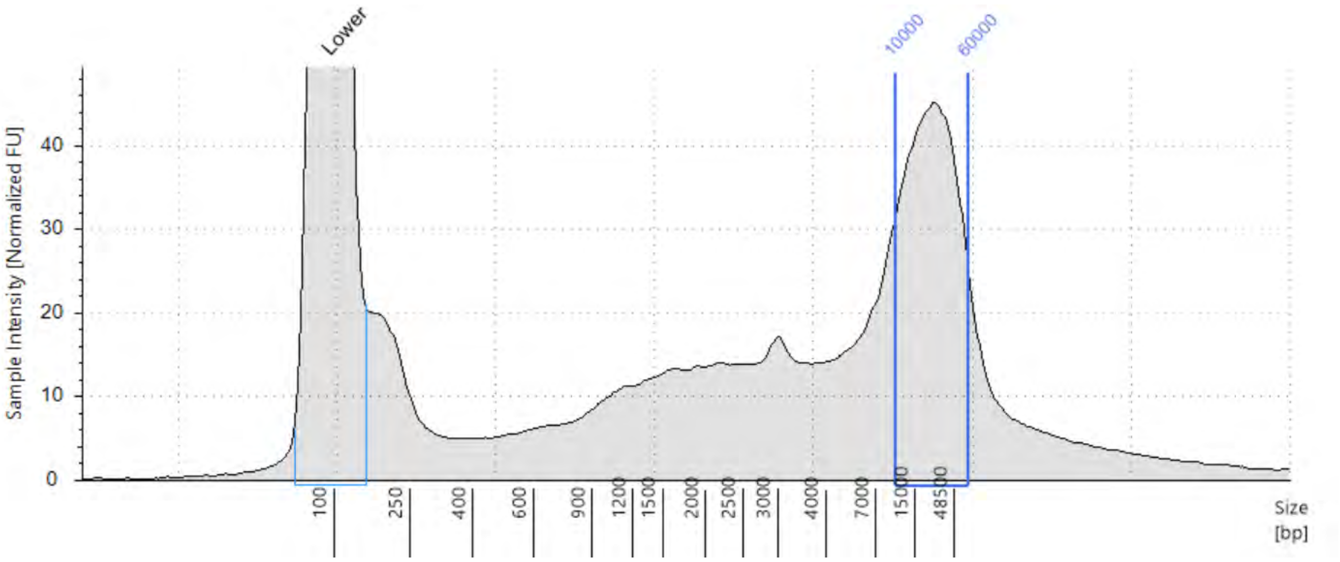

Region Table

From [bp]	To [bp]	Average Size [bp]	Conc. [ng/µl]	Region Molarity [nmol/l]	% of Total	Region Comment	Color
10000	60000	25701	1.88	0.149	28.58		■

E6: C494

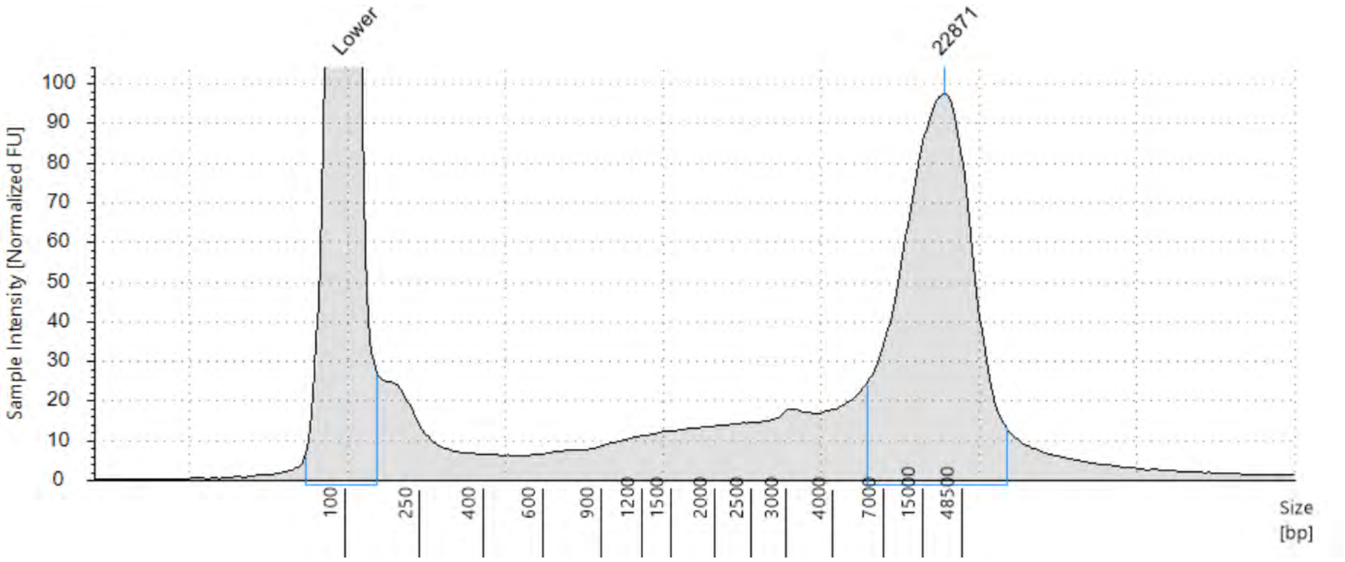

Sample Table

Well	DIN	Conc. [ng/µl]	Sample Description	Alert	Observations
E6	6.9	9.66	C494	⚠	Sample concentration outside recommended range

Peak Table

Size [bp]	Calibrated Conc. [ng/µl]	Assigned Conc. [ng/µl]	% Integrated Area	From [bp]	To [bp]	Peak Comment	Observations
100	8.50	8.50	-	62	149		Lower Marker
22871	5.22	-	98.74	5806	>60000		
-	-	-	-	-	-		Sample Well

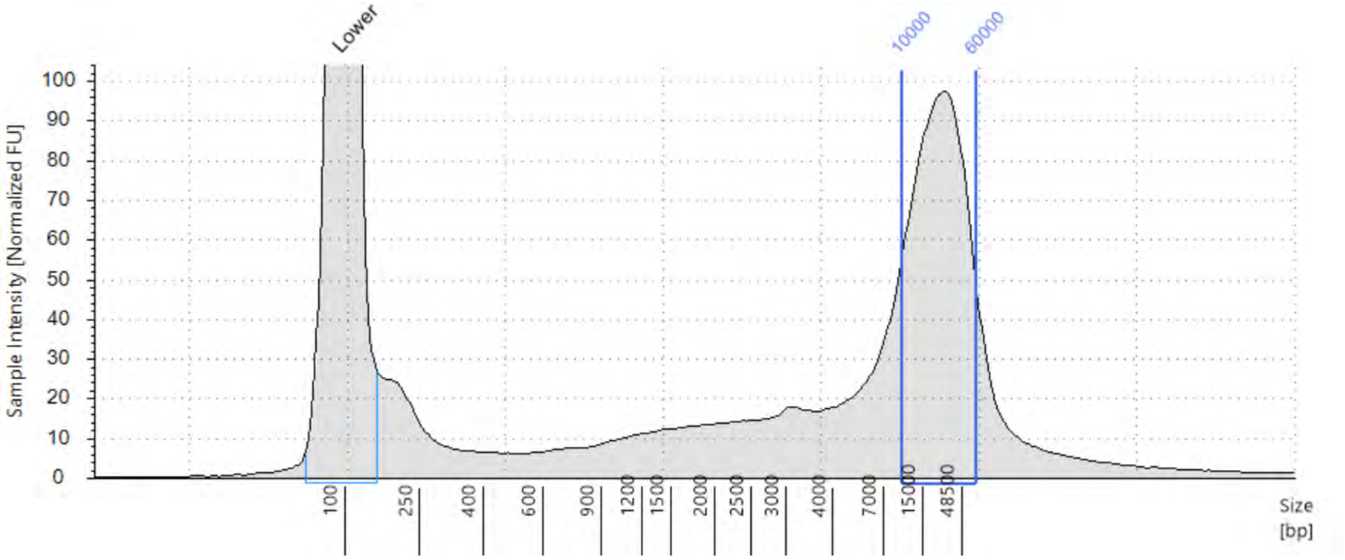

Region Table

From [bp]	To [bp]	Average Size [bp]	Conc. [ng/µl]	Region Molarity [nmol/l]	% of Total	Region Comment	Color
10000	60000	25994	3.90	0.301	40.36		

F6: C495

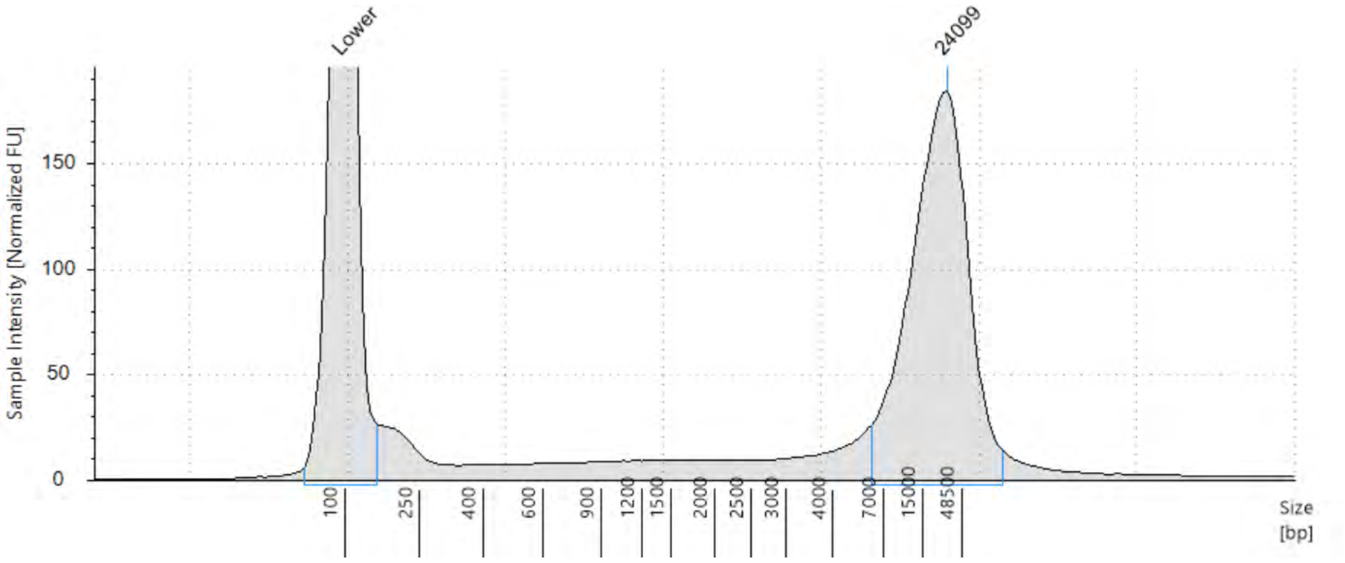

Sample Table

Well	DIN	Conc. [ng/µl]	Sample Description	Alert	Observations
F6	7.5	11.9	C495		

Peak Table

Size [bp]	Calibrated Conc. [ng/µl]	Assigned Conc. [ng/µl]	% Integrated Area	From [bp]	To [bp]	Peak Comment	Observations
100	8.50	8.50	-	62	149		Lower Marker
24099	7.90	-	95.26	6140	>60000		
-	-	-	-	-	-		Sample Well

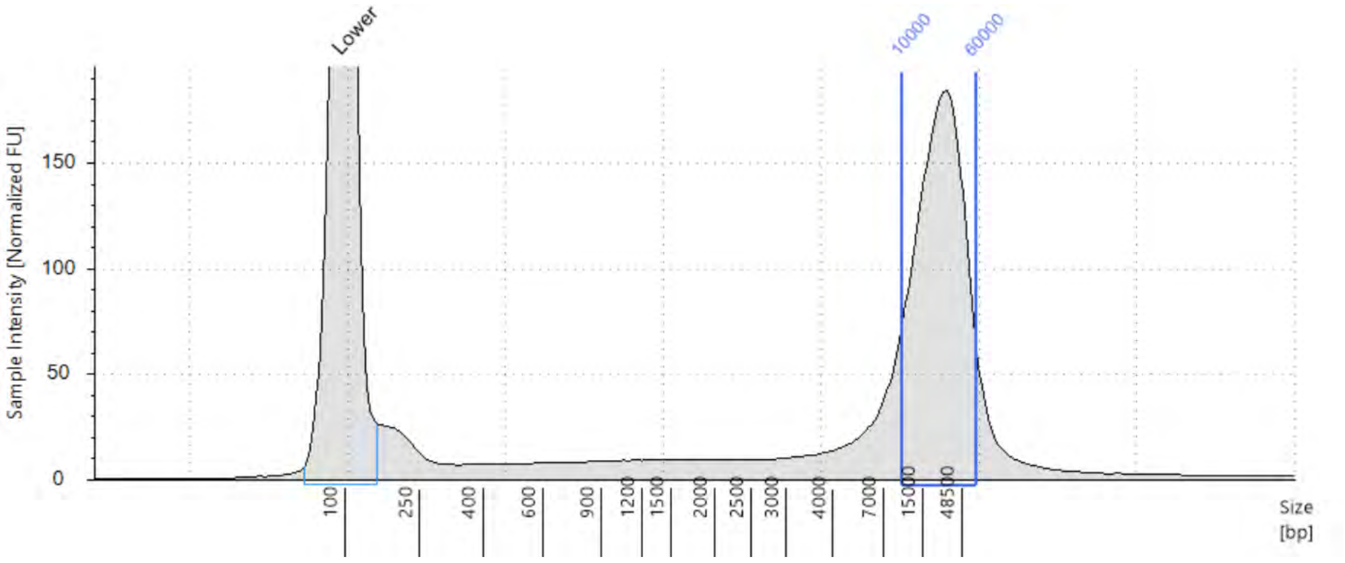

Region Table

From [bp]	To [bp]	Average Size [bp]	Conc. [ng/µl]	Region Molarity [nmol/l]	% of Total	Region Comment	Color
10000	60000	26185	6.49	0.484	54.54		

© Agilent Technologies, Inc. 2017

G6: C316

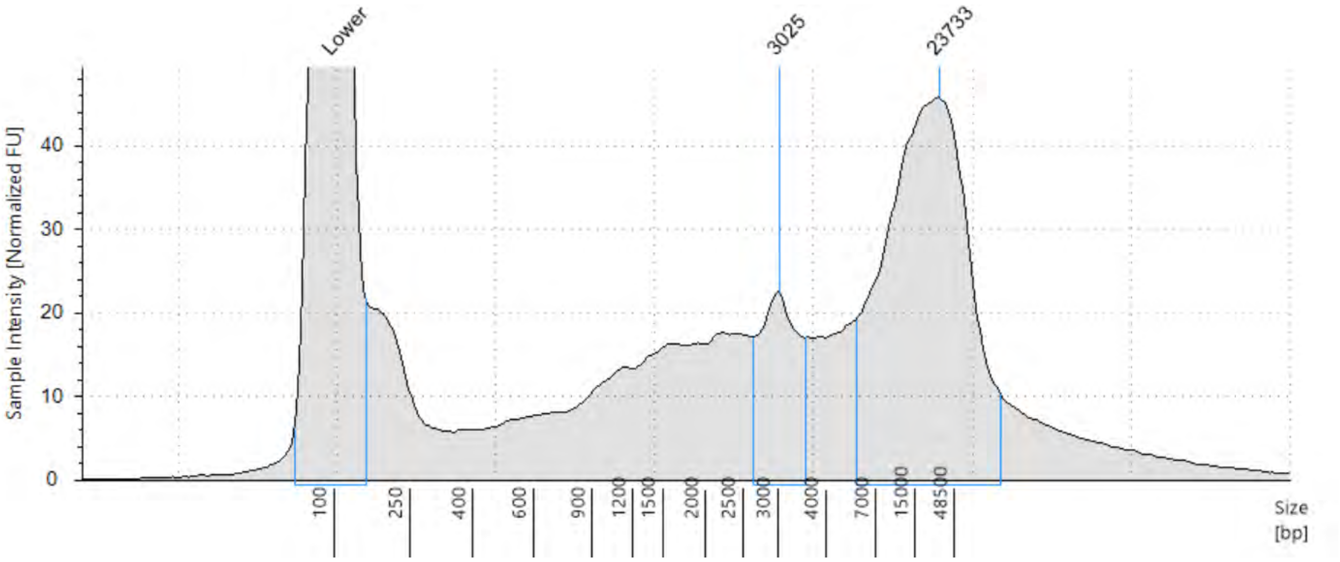

Sample Table

Well	DIN	Conc. [ng/µl]	Sample Description	Alert	Observations
G6	6.3	7.66	C316	⚠	Sample concentration outside recommended range

Peak Table

Size [bp]	Calibrated Conc. [ng/µl]	Assigned Conc. [ng/µl]	% Integrated Area	From [bp]	To [bp]	Peak Comment	Observations
100	8.50	8.50	-	62	147		Lower Marker
3025	0.657	-	18.19	2637	3542		
23733	2.92	-	80.77	5625	>60000		
-	-	-	-	-	-		Sample Well

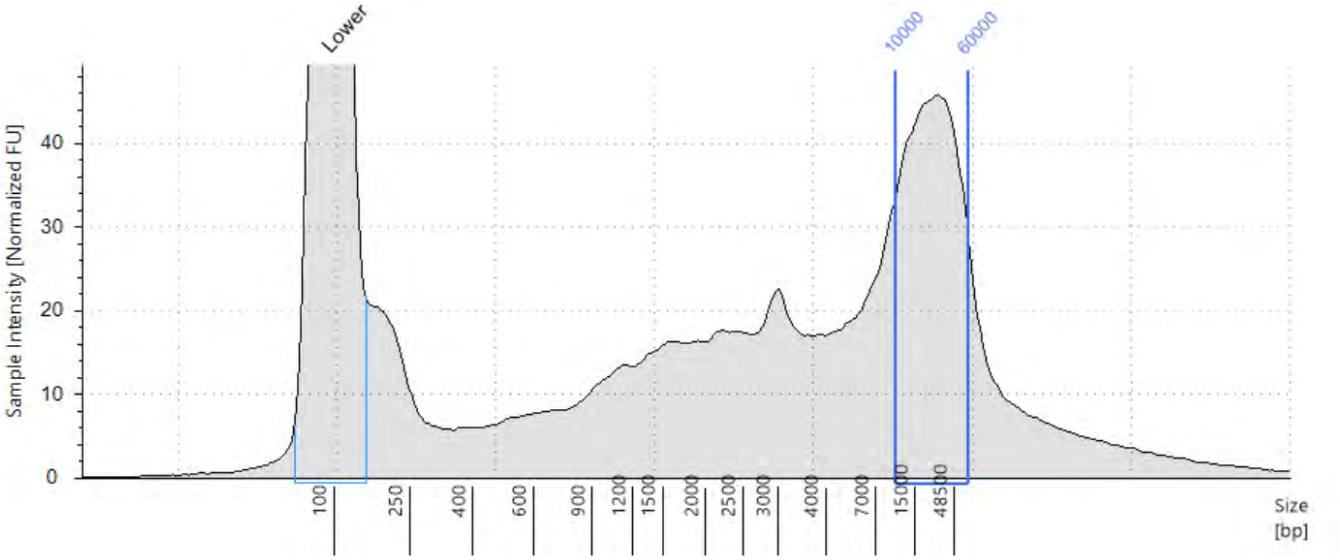

Region Table

From [bp]	To [bp]	Average Size [bp]	Conc. [ng/µl]	Region Molarity [nmol/l]	% of Total	Region Comment	Color
10000	60000	26310	1.99	0.156	25.99		■

 © Agilent Technologies, Inc. 2017

H6: C317

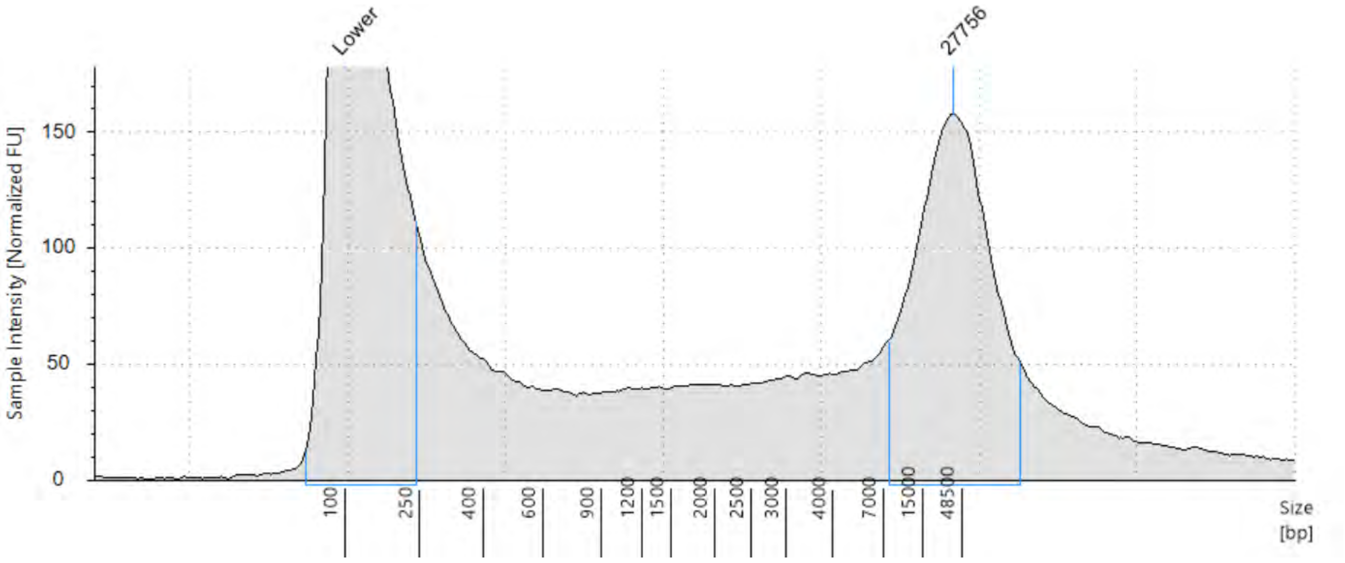

Sample Table

Well	DIN	Conc. [ng/µl]	Sample Description	Alert	Observations
H6	5.8	13.9	C317		

Peak Table

Size [bp]	Calibrated Conc. [ng/µl]	Assigned Conc. [ng/µl]	% Integrated Area	From [bp]	To [bp]	Peak Comment	Observations
100	8.50	8.50	-	62	238		Lower Marker
27756	4.71	-	97.50	7721	>60000		
-	-	-	-	-	-		Sample Well

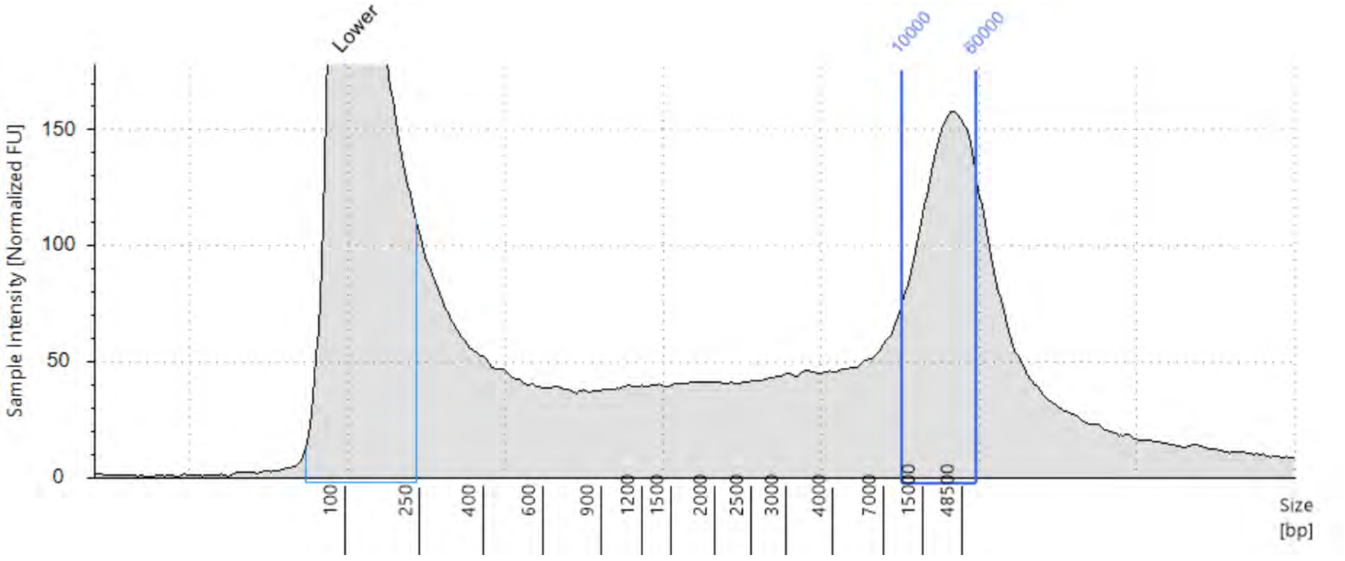

Region Table

From [bp]	To [bp]	Average Size [bp]	Conc. [ng/µl]	Region Molarity [nmol/l]	% of Total	Region Comment	Color
10000	60000	29087	3.21	0.228	23.07		

 © Agilent Technologies, Inc. 2017

A7: C318

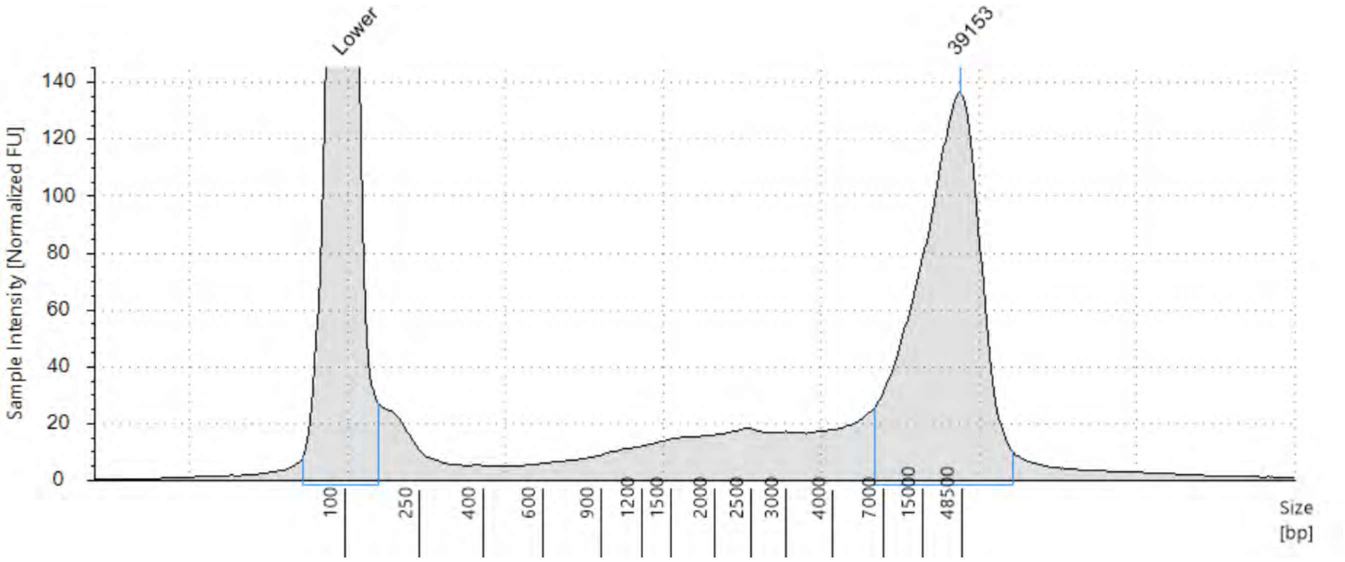

Sample Table

Well	DIN	Conc. [ng/µl]	Sample Description	Alert	Observations
A7	7.2	10.2	C318		

Peak Table

Size [bp]	Calibrated Conc. [ng/µl]	Assigned Conc. [ng/µl]	% Integrated Area	From [bp]	To [bp]	Peak Comment	Observations
100	8.50	8.50	-	60	150		Lower Marker
39153	6.04	-	98.23	6350	>60000		
-	-	-	-	-	-		Sample Well

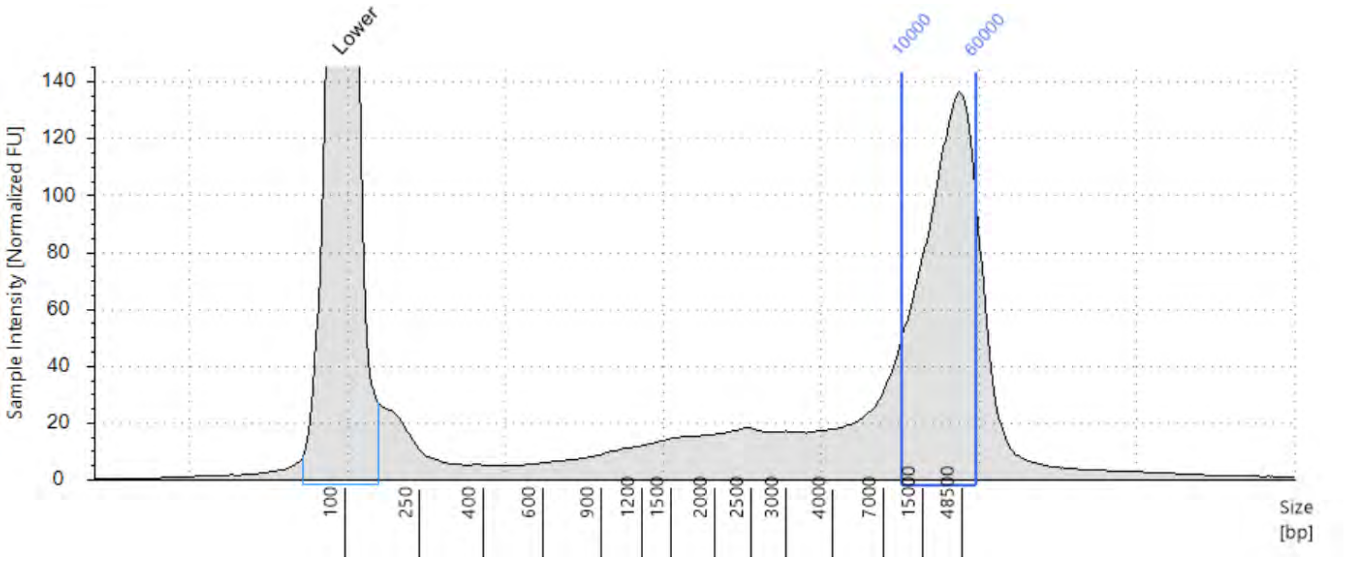

Region Table

From [bp]	To [bp]	Average Size [bp]	Conc. [ng/µl]	Region Molarity [nmol/l]	% of Total	Region Comment	Color
10000	60000	30280	4.60	0.312	44.94		

© Agilent Technologies, Inc. 2017

B7: C319

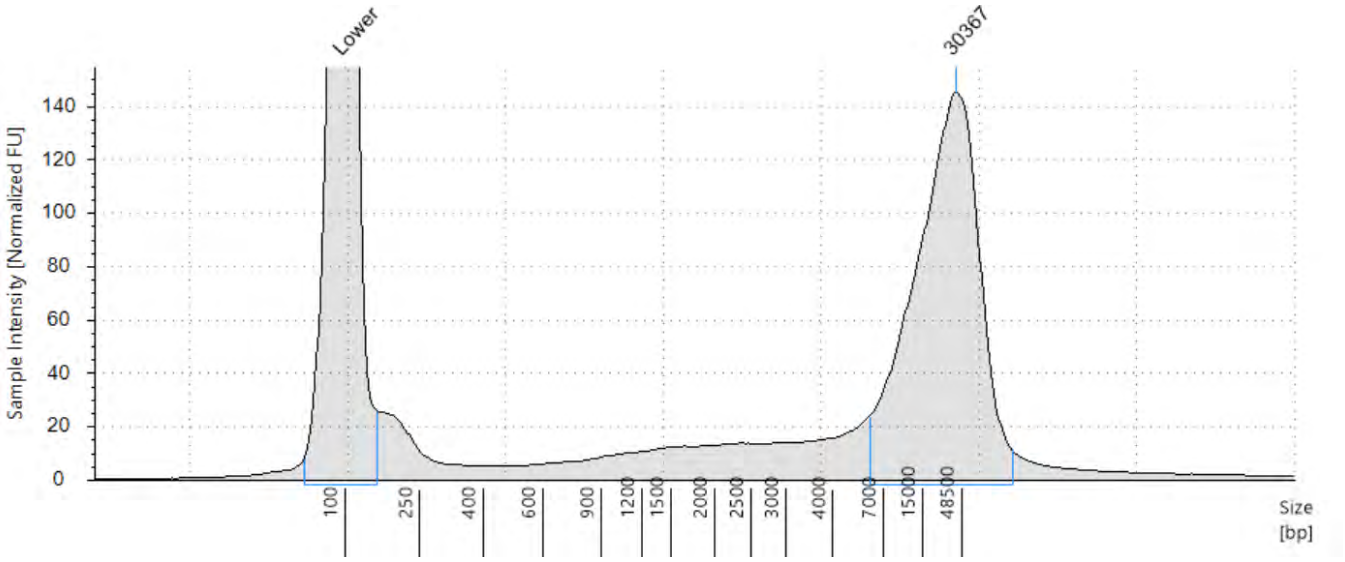

Sample Table

Well	DIN	Conc. [ng/µl]	Sample Description	Alert	Observations
B7	7.4	11.0	C319		

Peak Table

Size [bp]	Calibrated Conc. [ng/µl]	Assigned Conc. [ng/µl]	% Integrated Area	From [bp]	To [bp]	Peak Comment	Observations
100	8.50	8.50	-	60	147		Lower Marker
30367	6.90	-	95.84	5998	>60000		
-	-	-	-	-	-		Sample Well

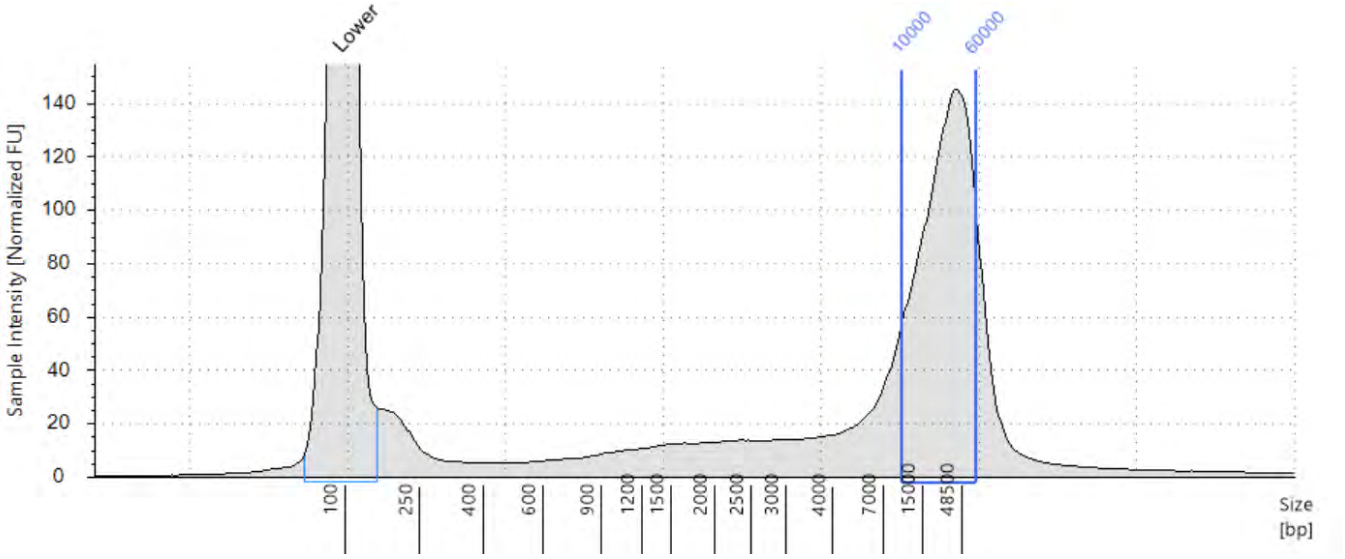

Region Table

From [bp]	To [bp]	Average Size [bp]	Conc. [ng/µl]	Region Molarity [nmol/l]	% of Total	Region Comment	Color
10000	60000	29663	5.23	0.360	47.34		

C7: C320

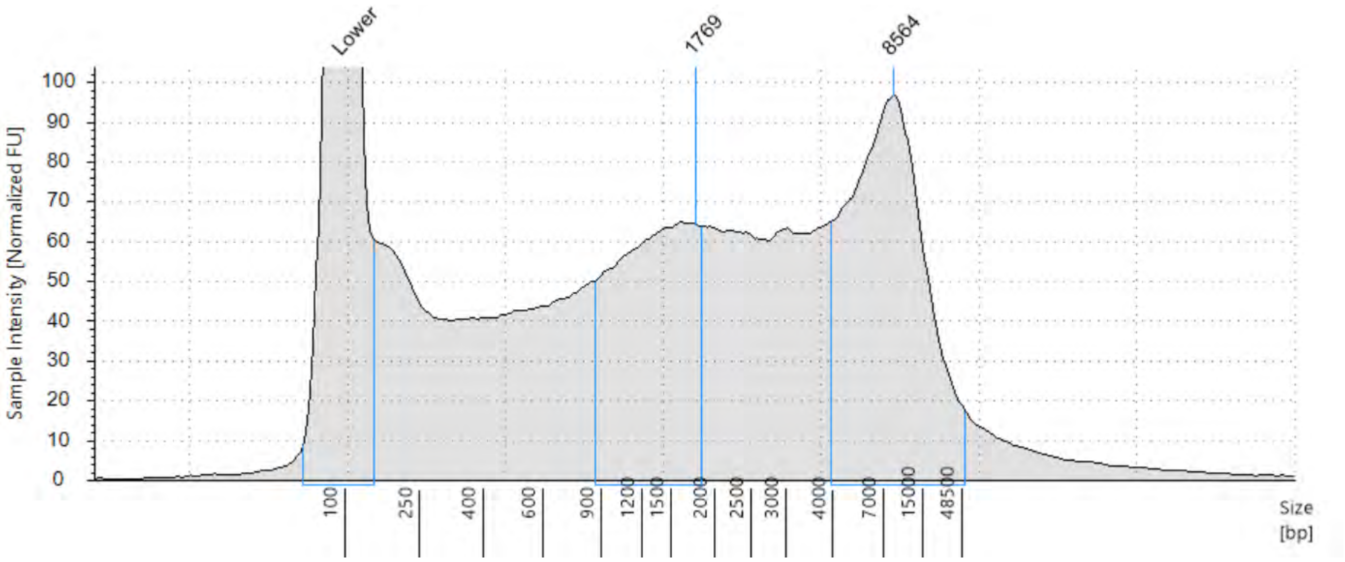

Sample Table

Well	DIN	Conc. [ng/µl]	Sample Description	Alert	Observations
C7	3.9	20.8	C320		

Peak Table

Size [bp]	Calibrated Conc. [ng/µl]	Assigned Conc. [ng/µl]	% Integrated Area	From [bp]	To [bp]	Peak Comment	Observations
100	8.50	8.50	-	61	144		Lower Marker
1769	3.88	-	41.87	858	1835		
8564	5.33	-	57.61	3938	51550		
-	-	-	-	-	-		Sample Well

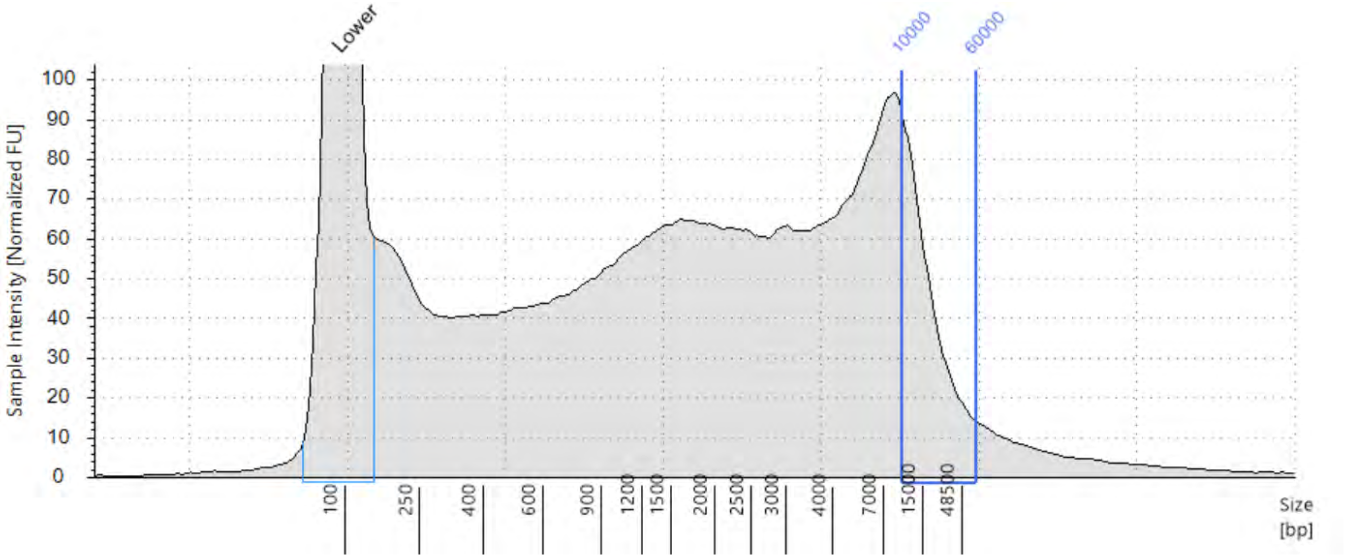

Region Table

From [bp]	To [bp]	Average Size [bp]	Conc. [ng/µl]	Region Molarity [nmol/l]	% of Total	Region Comment	Color
10000	60000	19619	1.96	0.199	9.45		

 © Agilent Technologies, Inc. 2017

D7: C321

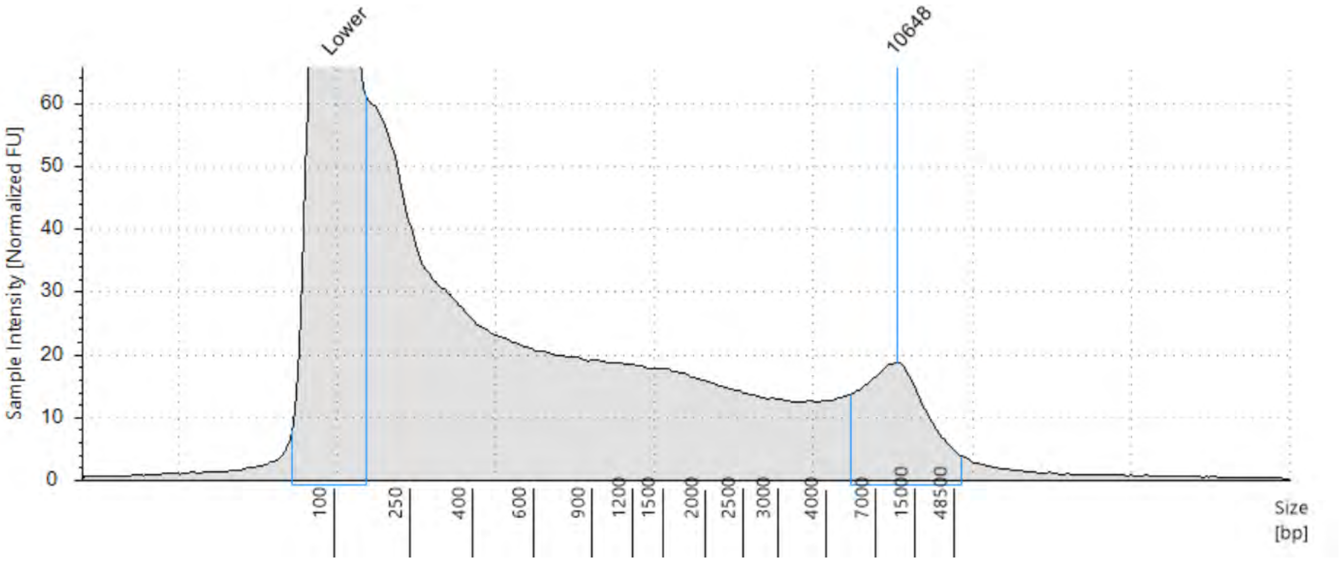

Sample Table

Well	DIN	Conc. [ng/µl]	Sample Description	Alert	Observations
D7	1.9	7.25	C321	⚠	Sample concentration outside recommended range

Peak Table

Size [bp]	Calibrated Conc. [ng/µl]	Assigned Conc. [ng/µl]	% Integrated Area	From [bp]	To [bp]	Peak Comment	Observations
100	8.50	8.50	-	60	147		Lower Marker
10648	0.833	-	100.00	5268	54487		

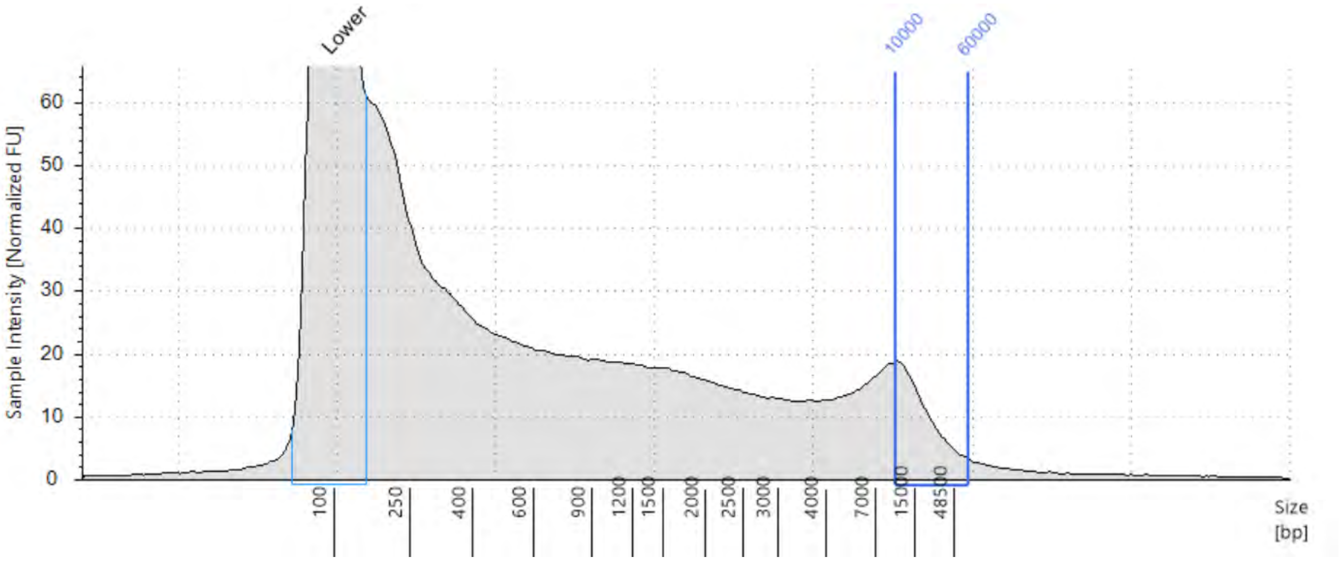

Region Table

From [bp]	To [bp]	Average Size [bp]	Conc. [ng/µl]	Region Molarity [nmol/l]	% of Total	Region Comment	Color
10000	60000	19865	0.438	0.0449	6.05		■

E7: C322

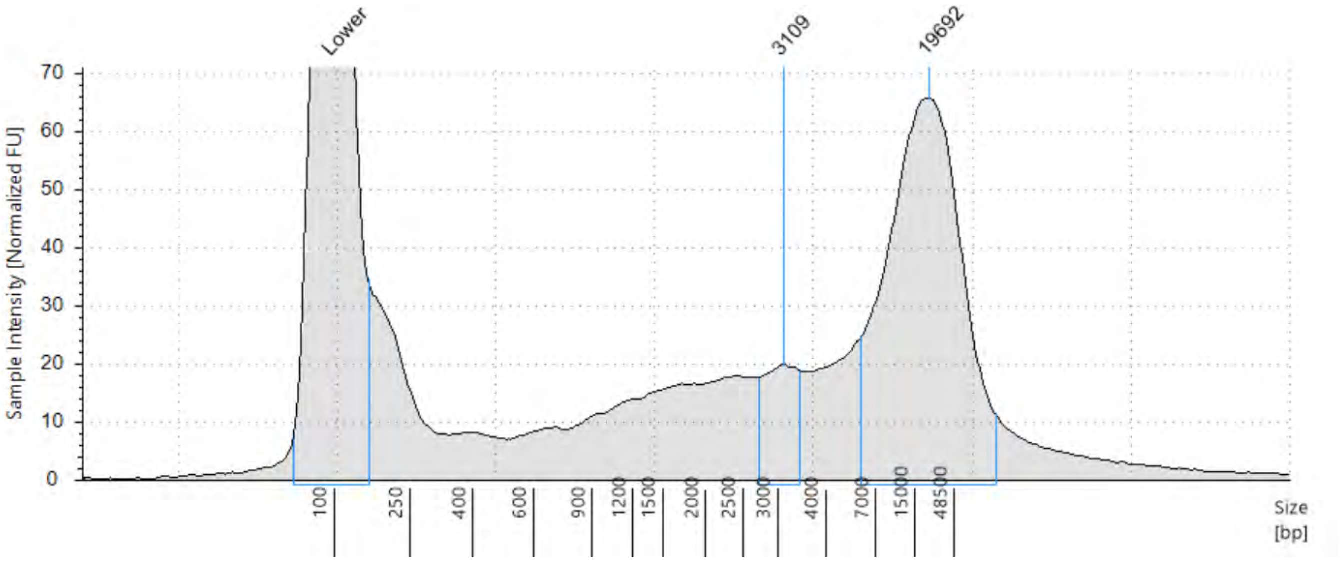

Sample Table

Well	DIN	Conc. [ng/µl]	Sample Description	Alert	Observations
E7	6.4	8.04	C322	⚠	Sample concentration outside recommended range

Peak Table

Size [bp]	Calibrated Conc. [ng/µl]	Assigned Conc. [ng/µl]	% Integrated Area	From [bp]	To [bp]	Peak Comment	Observations
100	8.50	8.50	-	61	151		Lower Marker
3109	0.450	-	11.72	2720	3405		
19692	3.38	-	87.88	5903	>60000		
-	-	-	-	-	-		Sample Well

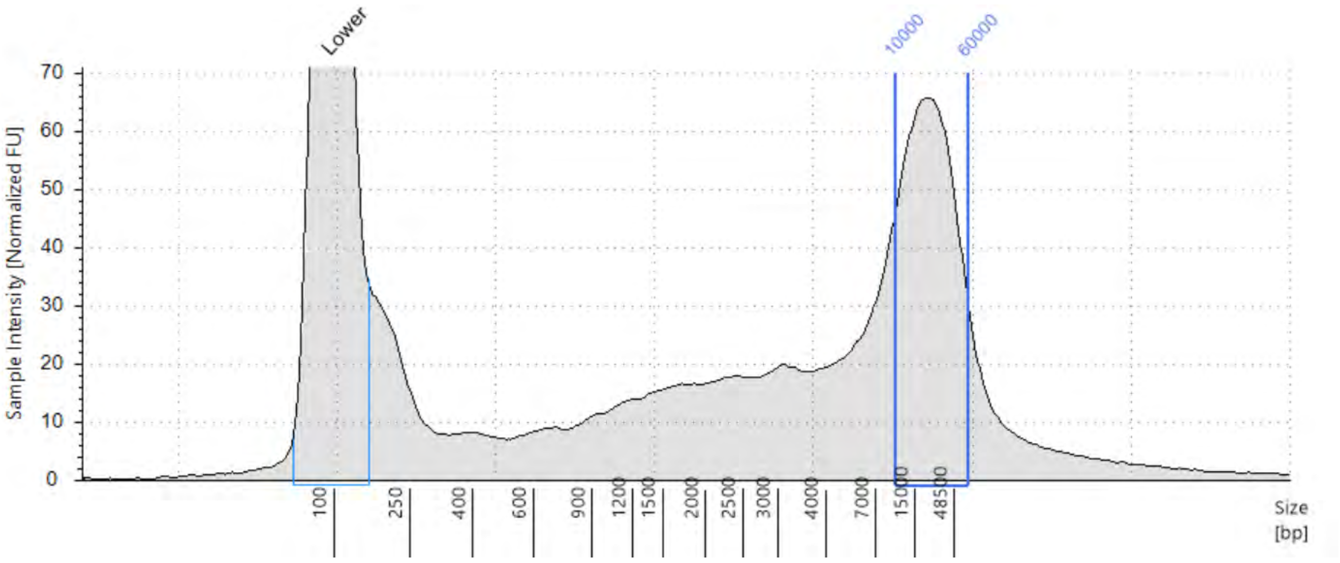

Region Table

From [bp]	To [bp]	Average Size [bp]	Conc. [ng/µl]	Region Molarity [nmol/l]	% of Total	Region Comment	Color
10000	60000	24884	2.46	0.199	30.57		■

F7: C323

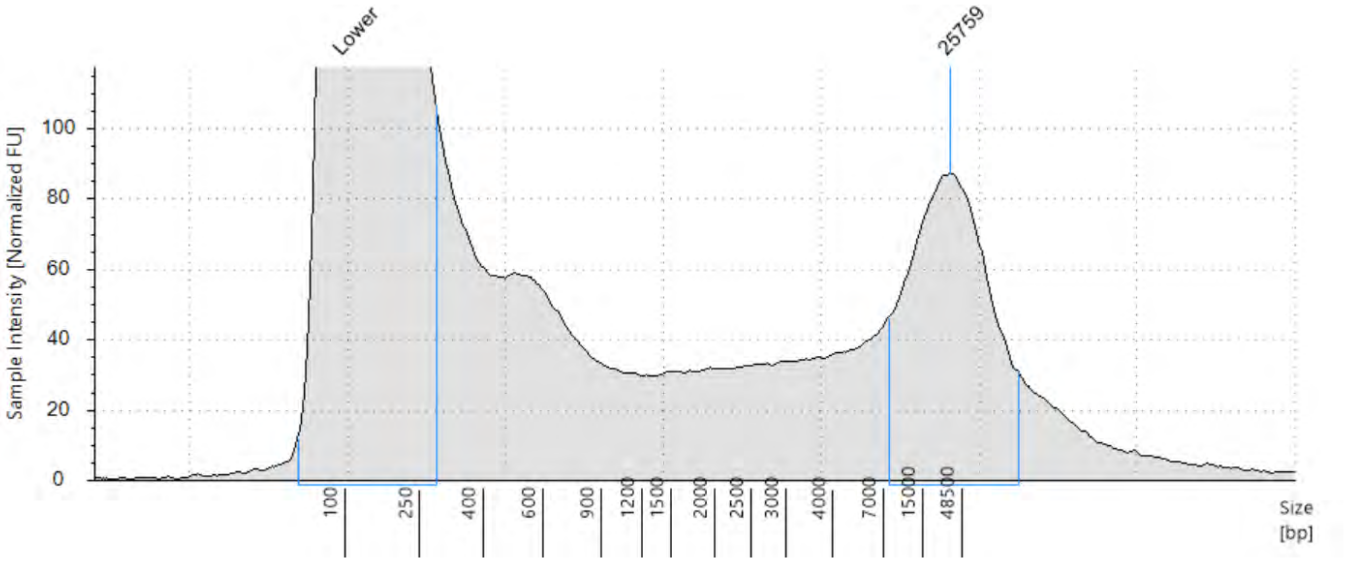

Sample Table

Well	DIN	Conc. [ng/µl]	Sample Description	Alert	Observations
F7	2.2	6.94	C323	⚠	Sample concentration outside recommended range

Peak Table

Size [bp]	Calibrated Conc. [ng/µl]	Assigned Conc. [ng/µl]	% Integrated Area	From [bp]	To [bp]	Peak Comment	Observations
100	8.50	8.50	-	56	282		Lower Marker
25759	1.91	-	98.90	7795	>60000		
-	-	-	-	-	-		Sample Well

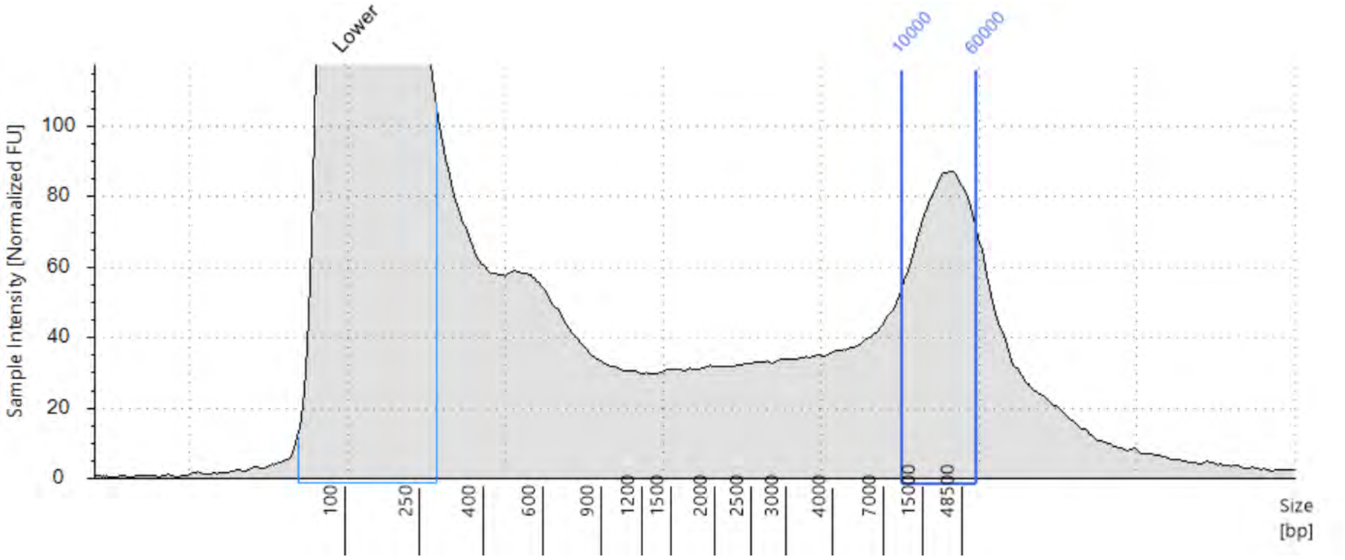

Region Table

From [bp]	To [bp]	Average Size [bp]	Conc. [ng/µl]	Region Molarity [nmol/l]	% of Total	Region Comment	Color
10000	60000	28003	1.31	0.0964	18.85		■

 © Agilent Technologies, Inc. 2017

G7: C324

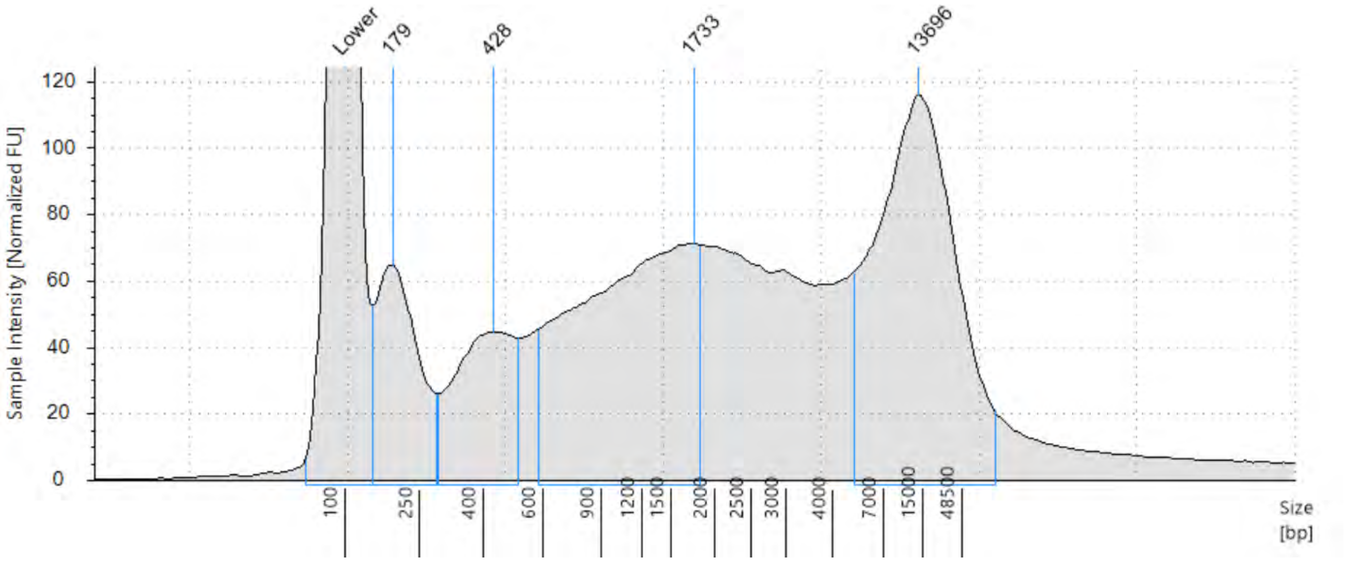

Sample Table

Well	DIN	Conc. [ng/µl]	Sample Description	Alert	Observations
G7	4.4	26.0	C324		

Peak Table

Size [bp]	Calibrated Conc. [ng/µl]	Assigned Conc. [ng/µl]	% Integrated Area	From [bp]	To [bp]	Peak Comment	Observations
100	8.50	8.50	-	62	140		Lower Marker
179	2.03	-	11.23	140	283		
428	1.97	-	10.88	286	505		
1733	6.20	-	34.26	581	1813		
13696	6.90	-	38.14	5010	>60000		
-	-	-	-	-	-		Sample Well

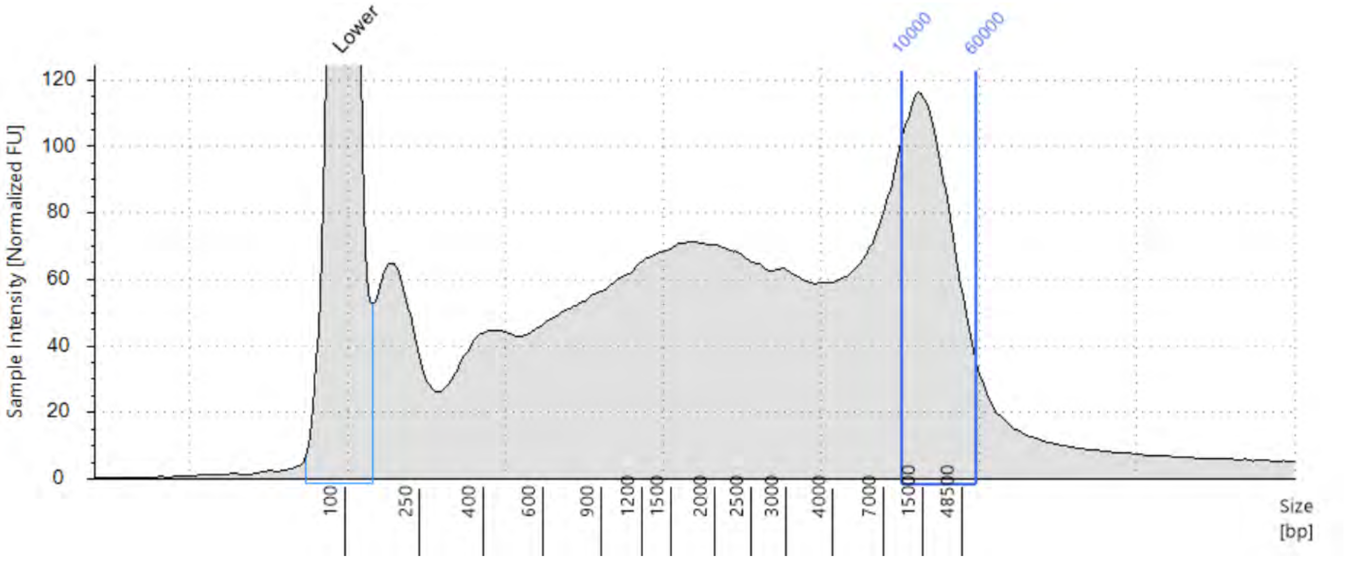

Region Table

From [bp]	To [bp]	Average Size [bp]	Conc. [ng/µl]	Region Molarity [nmol/l]	% of Total	Region Comment	Color
10000	60000	22428	4.15	0.365	15.97		

H7: C325

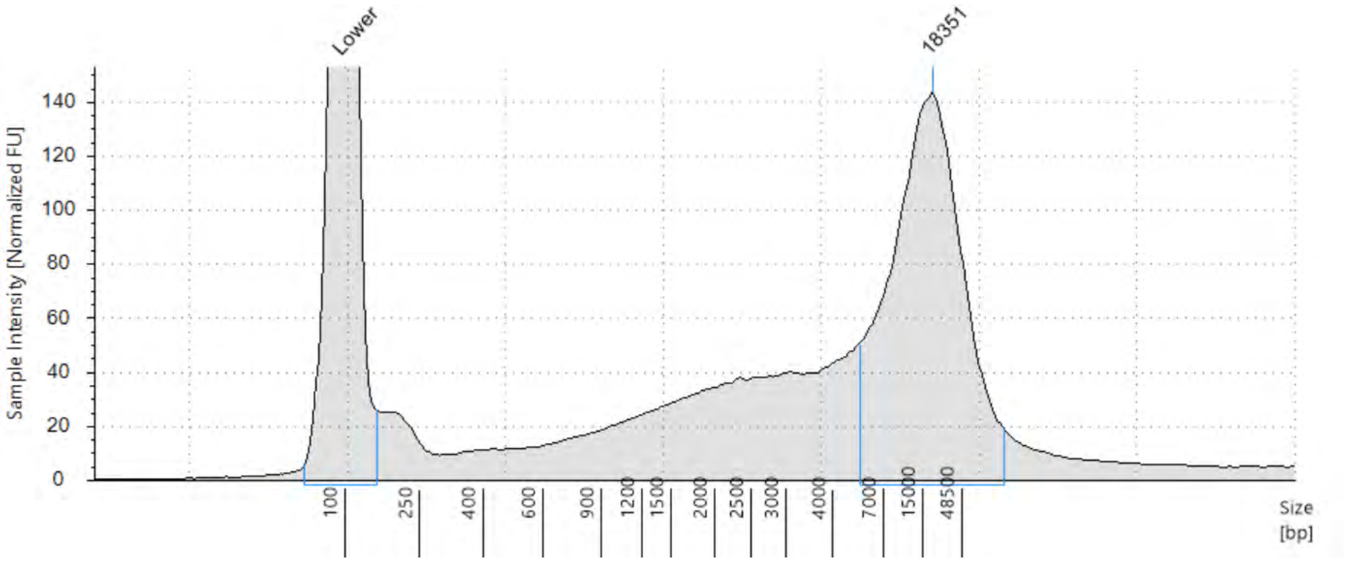

Sample Table

Well	DIN	Conc. [ng/µl]	Sample Description	Alert	Observations
H7	6.4	17.5	C325		

Peak Table

Size [bp]	Calibrated Conc. [ng/µl]	Assigned Conc. [ng/µl]	% Integrated Area	From [bp]	To [bp]	Peak Comment	Observations
100	8.50	8.50	-	62	147		Lower Marker
18351	7.77	-	85.24	5393	>60000		
-	-	-	-	-	-		Sample Well

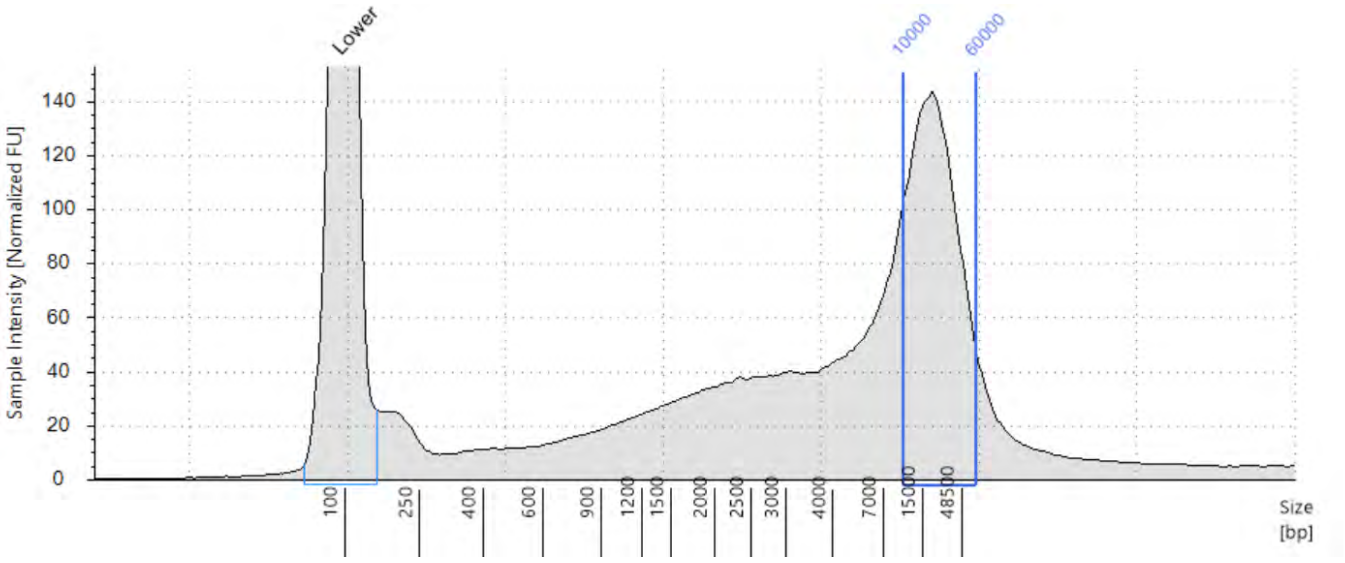

Region Table

From [bp]	To [bp]	Average Size [bp]	Conc. [ng/µl]	Region Molarity [nmol/l]	% of Total	Region Comment	Color
10000	60000	23451	5.36	0.449	30.55		

 © Agilent Technologies, Inc. 2017

A8: C326

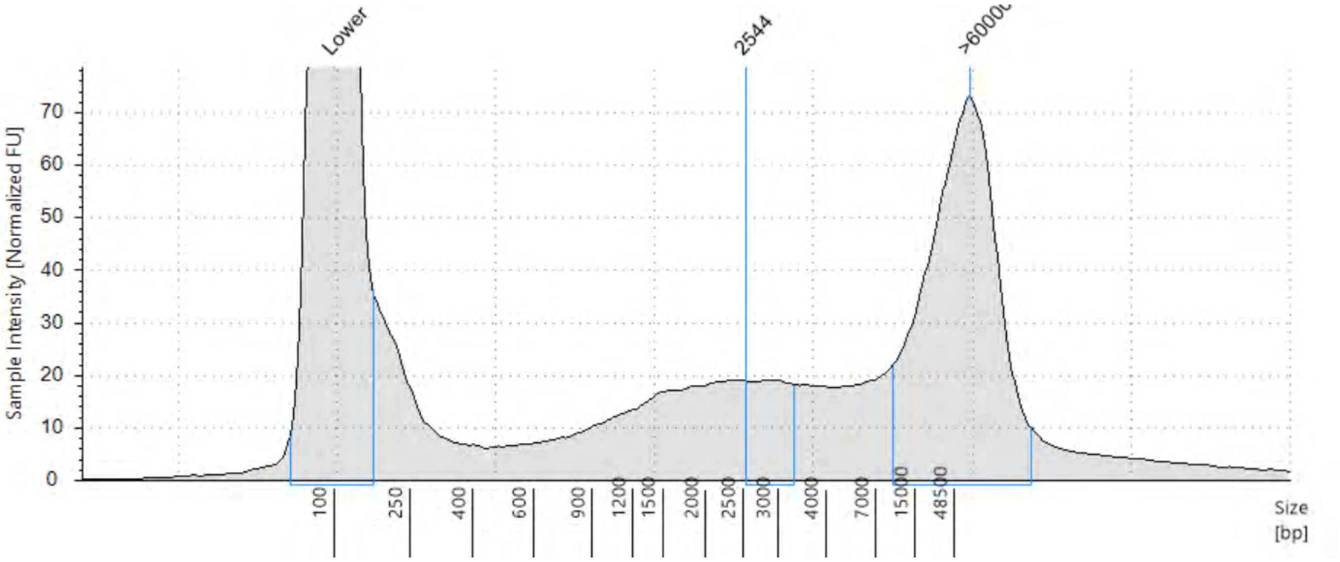

Sample Table

Well	DIN	Conc. [ng/µl]	Sample Description	Alert	Observations
A8	6.6	7.10	C326	⚠	Sample concentration outside recommended range

Peak Table

Size [bp]	Calibrated Conc. [ng/µl]	Assigned Conc. [ng/µl]	% Integrated Area	From [bp]	To [bp]	Peak Comment	Observations
100	8.50	8.50	-	58	160		Lower Marker
2544	0.443	-	12.73	2544	3292		
>60000	2.98	-	85.71	9732	>60000		
-	-	-	-	-	-		Sample Well

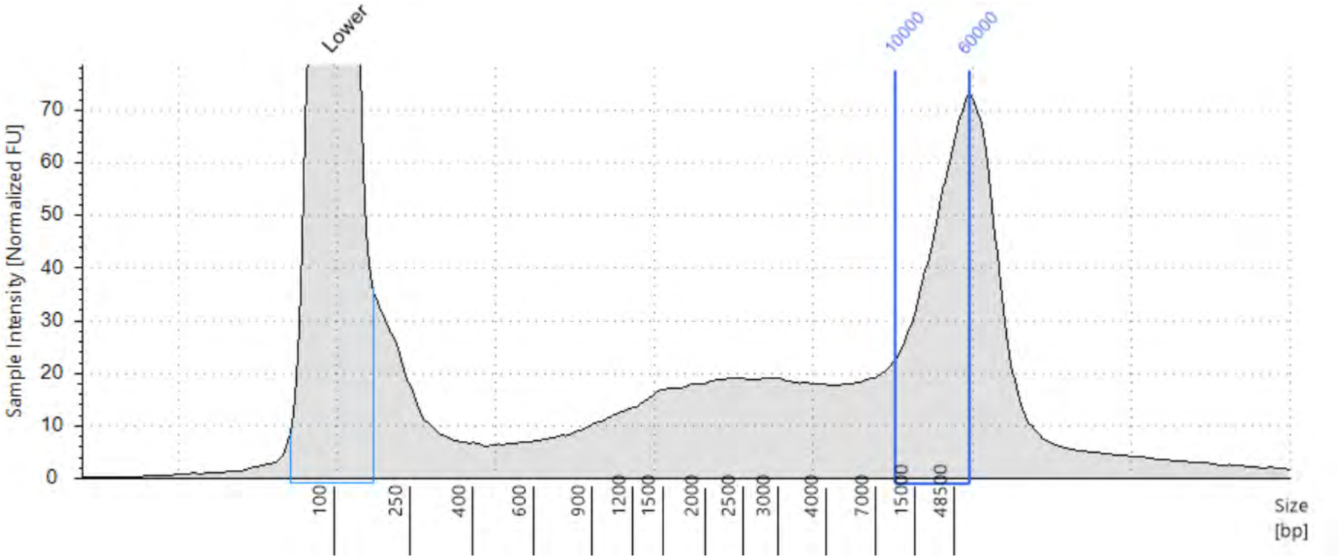

Region Table

From [bp]	To [bp]	Average Size [bp]	Conc. [ng/µl]	Region Molarity [nmol/l]	% of Total	Region Comment	Color
10000	60000	32579	1.74	0.113	24.49		■

© Agilent Technologies, Inc. 2017

B8: C327

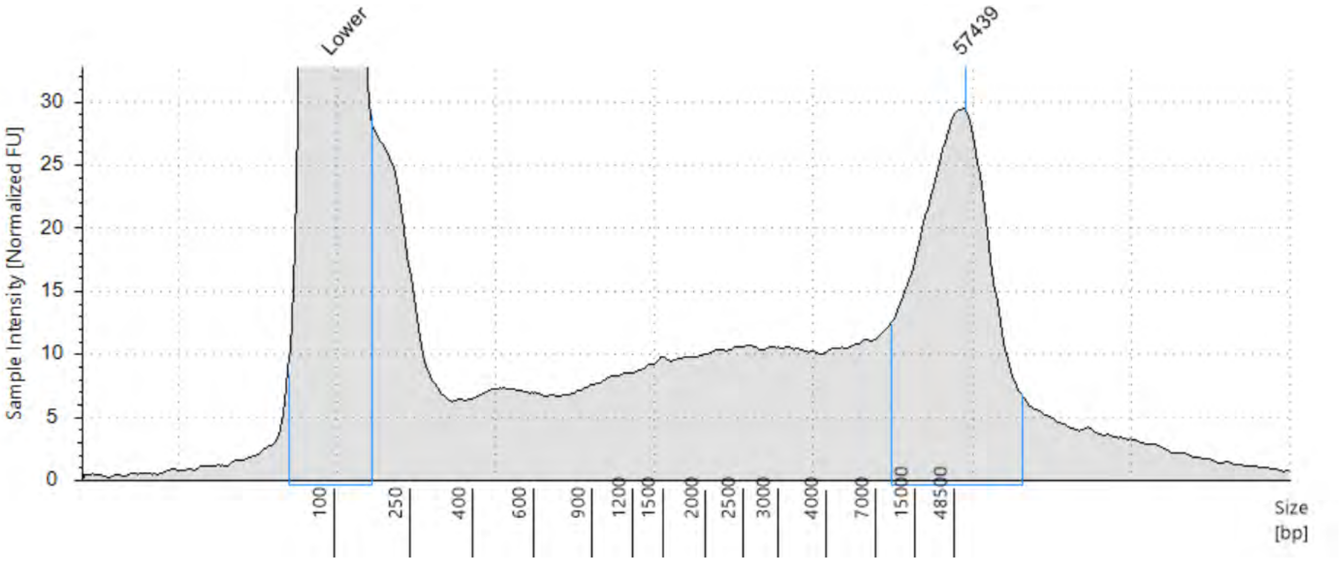

Sample Table

Well	DIN	Conc. [ng/µl]	Sample Description	Alert	Observations
B8	6.3	4.13	C327	⚠	Sample concentration outside functional range for DIN

Peak Table

Size [bp]	Calibrated Conc. [ng/µl]	Assigned Conc. [ng/µl]	% Integrated Area	From [bp]	To [bp]	Peak Comment	Observations
100	8.50	8.50	-	58	157		Lower Marker
57439	1.27	-	100.00	9503	>60000		

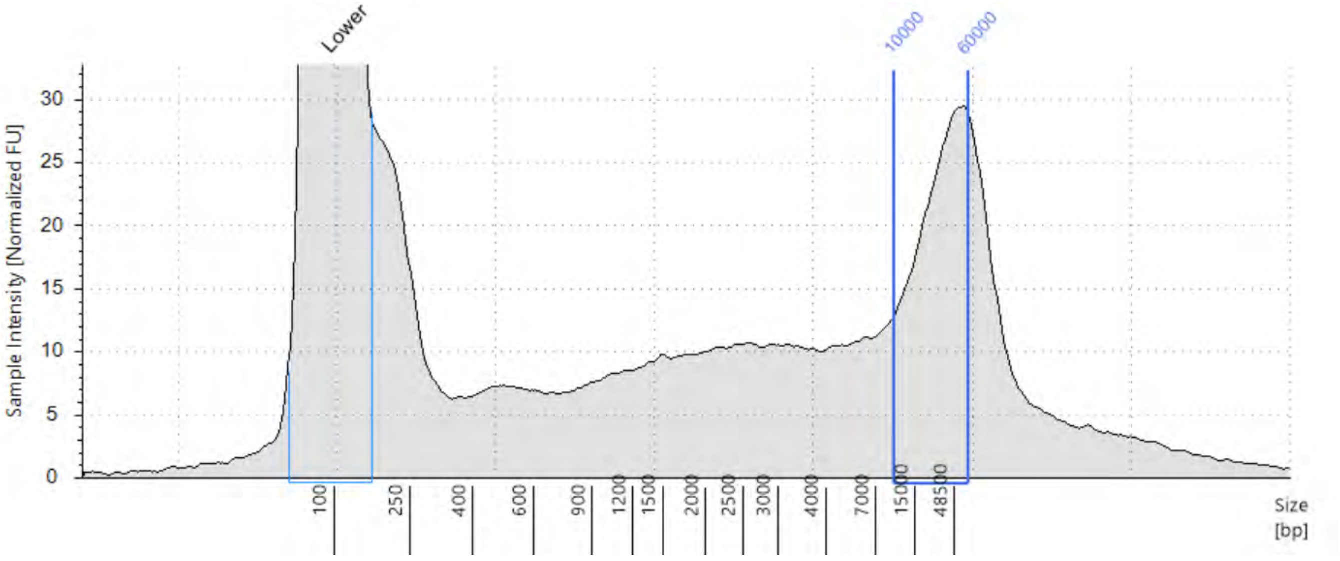

Region Table

From [bp]	To [bp]	Average Size [bp]	Conc. [ng/µl]	Region Molarity [nmol/l]	% of Total	Region Comment	Color
10000	60000	30611	0.841	0.0592	20.35		■

C8: C328

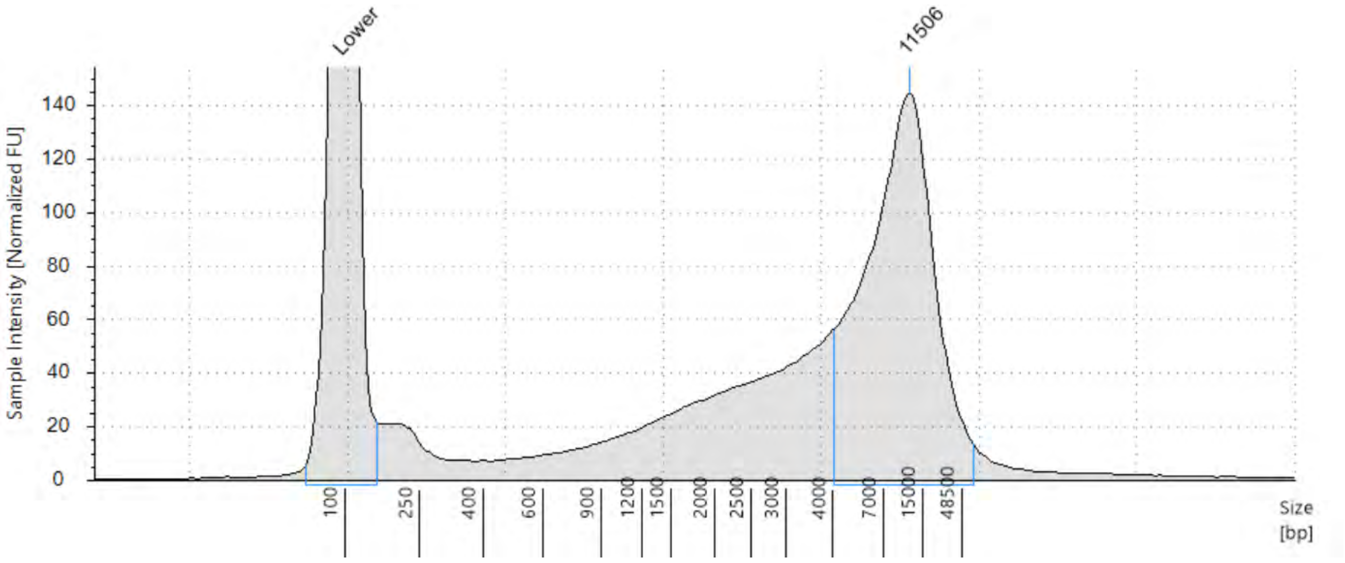

Sample Table

Well	DIN	Conc. [ng/µl]	Sample Description	Alert	Observations
C8	6.3	14.1	C328		

Peak Table

Size [bp]	Calibrated Conc. [ng/µl]	Assigned Conc. [ng/µl]	% Integrated Area	From [bp]	To [bp]	Peak Comment	Observations
100	8.50	8.50	-	62	146		Lower Marker
11506	7.47	-	100.00	3994	57987		

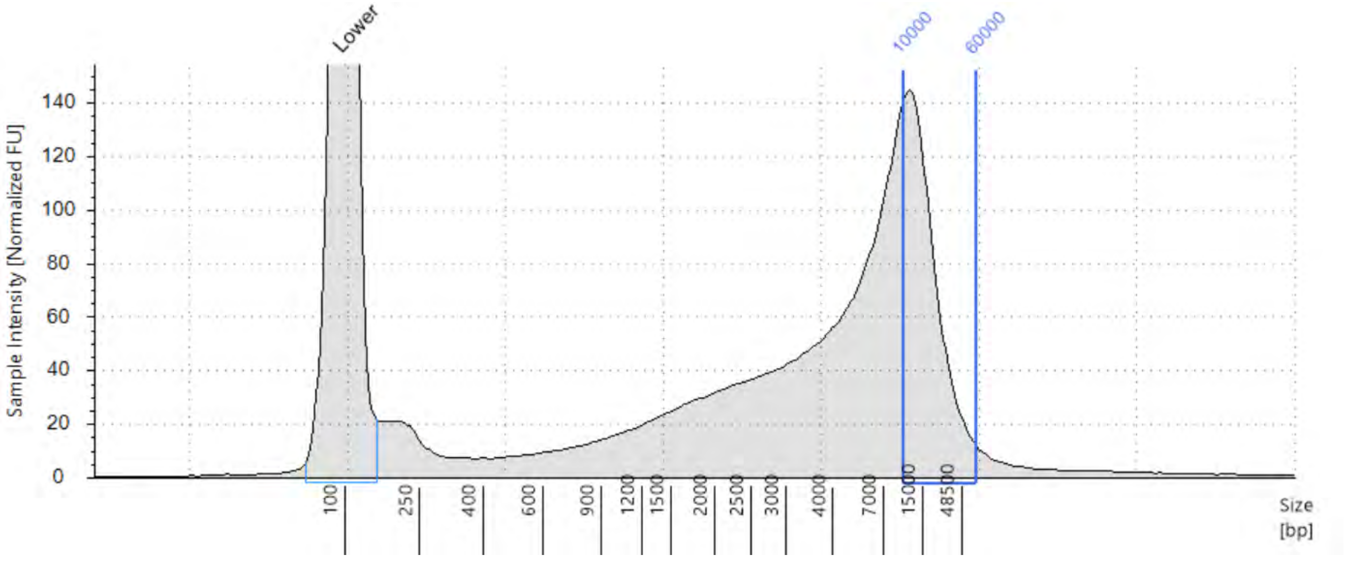

Region Table

From [bp]	To [bp]	Average Size [bp]	Conc. [ng/µl]	Region Molarity [nmol/l]	% of Total	Region Comment	Color
10000	60000	17954	3.60	0.372	25.54		

D8: C329

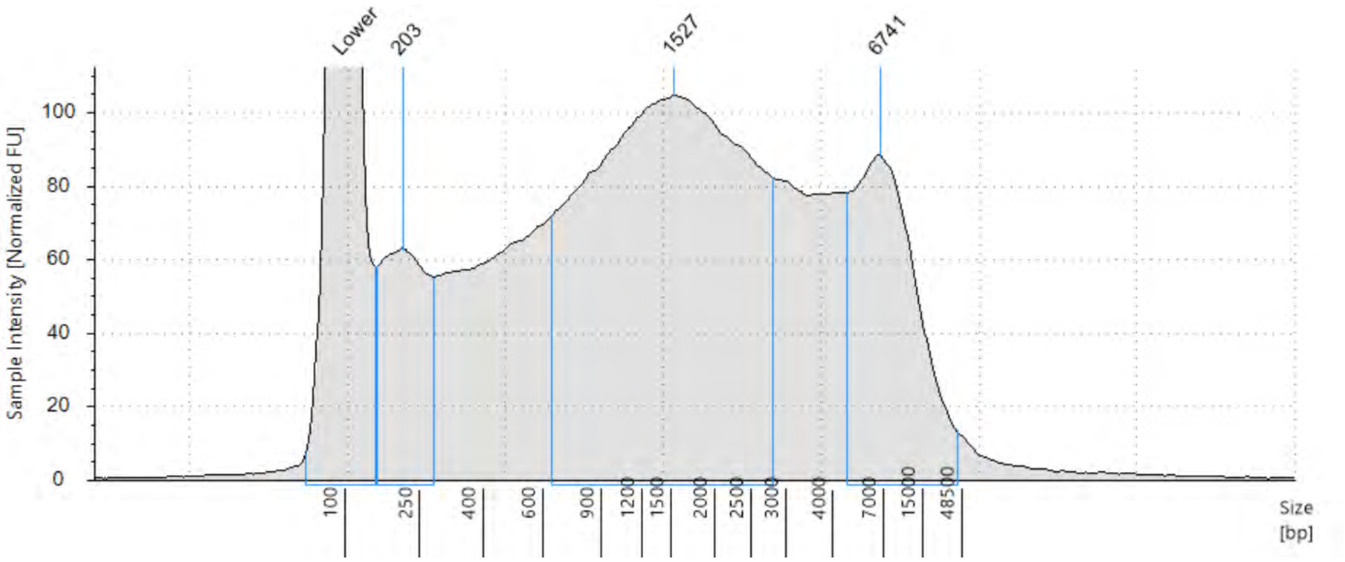

Sample Table

Well	DIN	Conc. [ng/µl]	Sample Description	Alert	Observations
D8	3.3	27.5	C329		

Peak Table

Size [bp]	Calibrated Conc. [ng/µl]	Assigned Conc. [ng/µl]	% Integrated Area	From [bp]	To [bp]	Peak Comment	Observations
100	8.50	8.50	-	62	145		Lower Marker
203	2.16	-	11.30	147	276		
1527	12.7	-	66.73	634	2794		
6741	4.19	-	21.97	4657	33759		

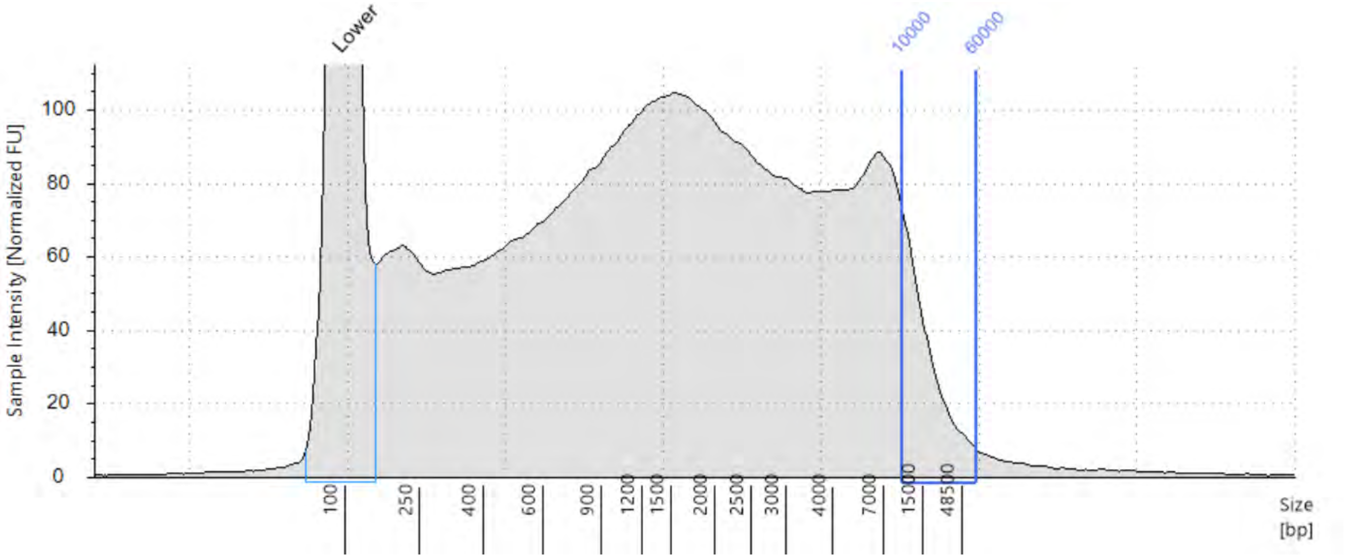

Region Table

From [bp]	To [bp]	Average Size [bp]	Conc. [ng/µl]	Region Molarity [nmol/l]	% of Total	Region Comment	Color
10000	60000	18803	1.44	0.151	5.23		

 © Agilent Technologies, Inc. 2017

E8: C330

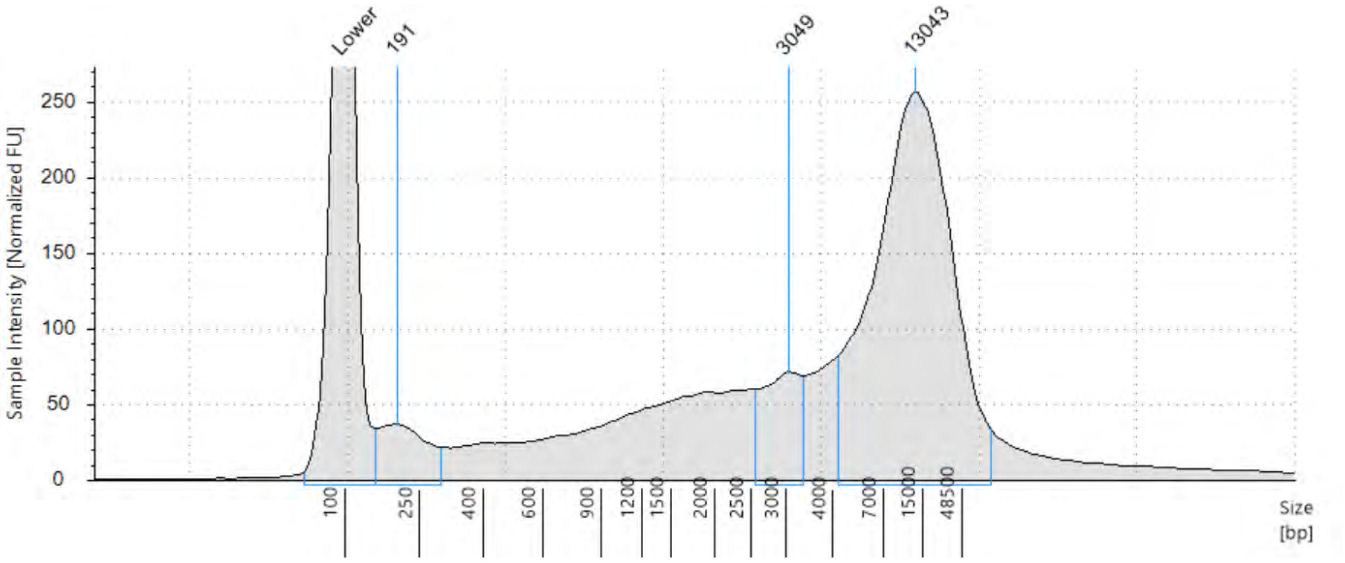

Sample Table

Well	DIN	Conc. [ng/µl]	Sample Description	Alert	Observations
E8	6.2	29.9	C330		

Peak Table

Size [bp]	Calibrated Conc. [ng/µl]	Assigned Conc. [ng/µl]	% Integrated Area	From [bp]	To [bp]	Peak Comment	Observations
100	8.50	8.50	-	62	146		Lower Marker
191	1.30	-	6.89	146	294		
3049	2.07	-	10.98	2563	3339		
13043	15.0	-	79.52	4262	>60000		
-	-	-	-	-	-		Sample Well

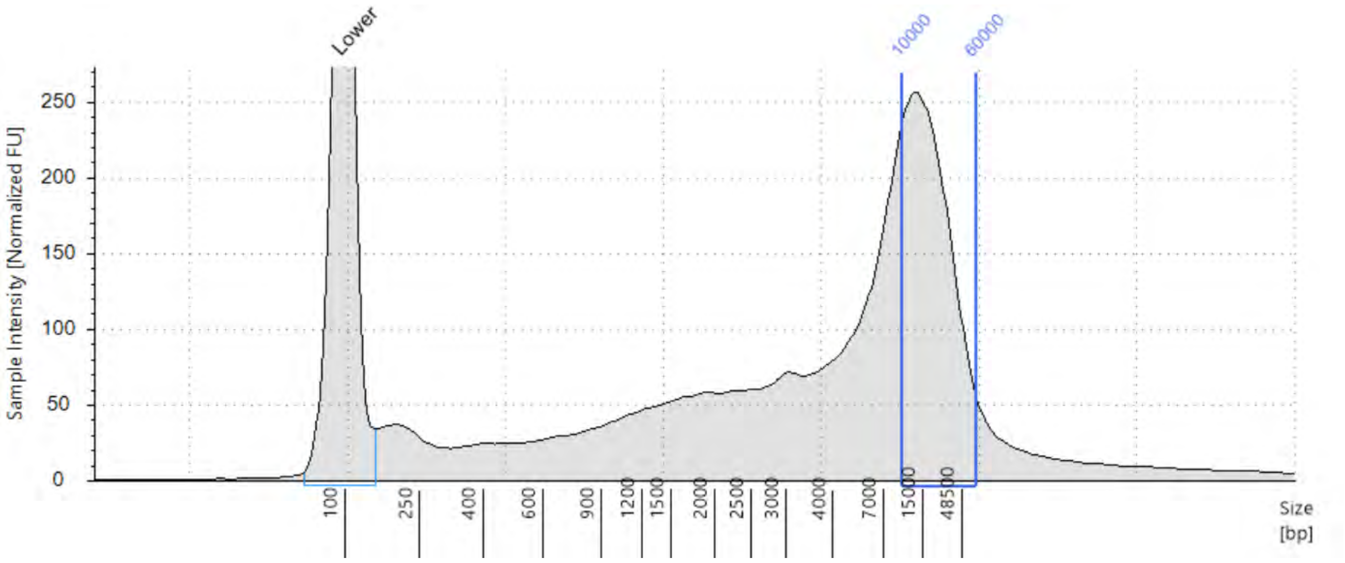

Region Table

From [bp]	To [bp]	Average Size [bp]	Conc. [ng/µl]	Region Molarity [nmol/l]	% of Total	Region Comment	Color
10000	60000	21568	8.90	0.797	29.76		

© Agilent Technologies, Inc. 2017

F8: C331

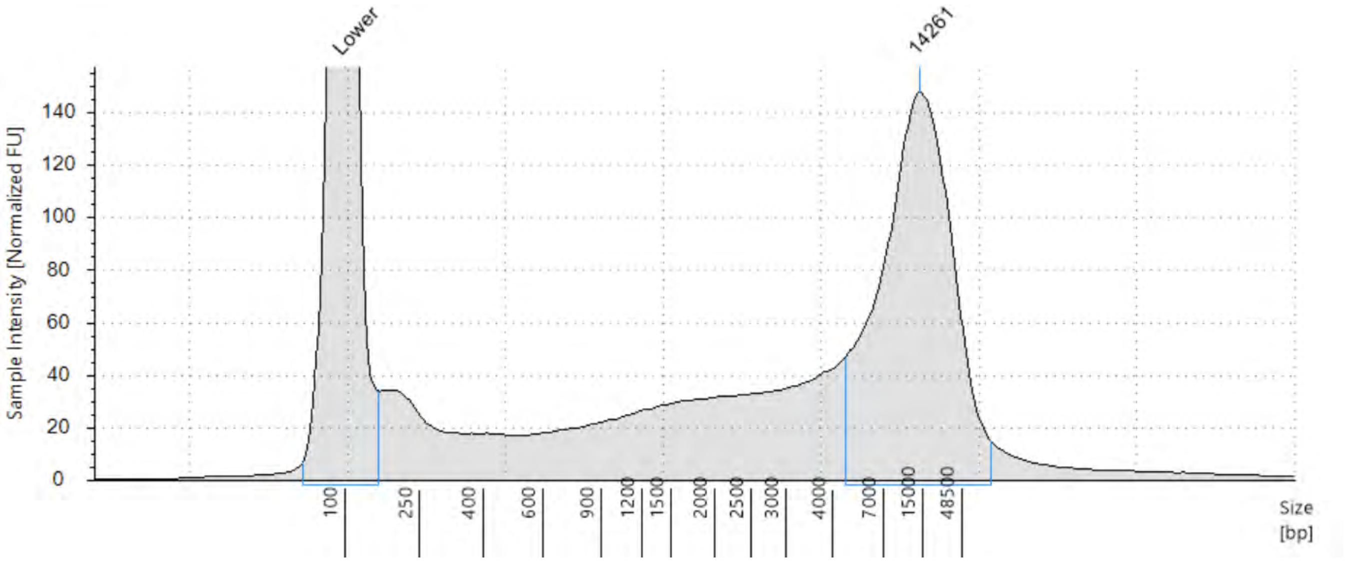

Sample Table

Well	DIN	Conc. [ng/µl]	Sample Description	Alert	Observations
F8	6.2	15.6	C331		

Peak Table

Size [bp]	Calibrated Conc. [ng/µl]	Assigned Conc. [ng/µl]	% Integrated Area	From [bp]	To [bp]	Peak Comment	Observations
100	8.50	8.50	-	60	148		Lower Marker
14261	7.55	-	99.23	4573	>60000		
-	-	-	-	-	-		Sample Well

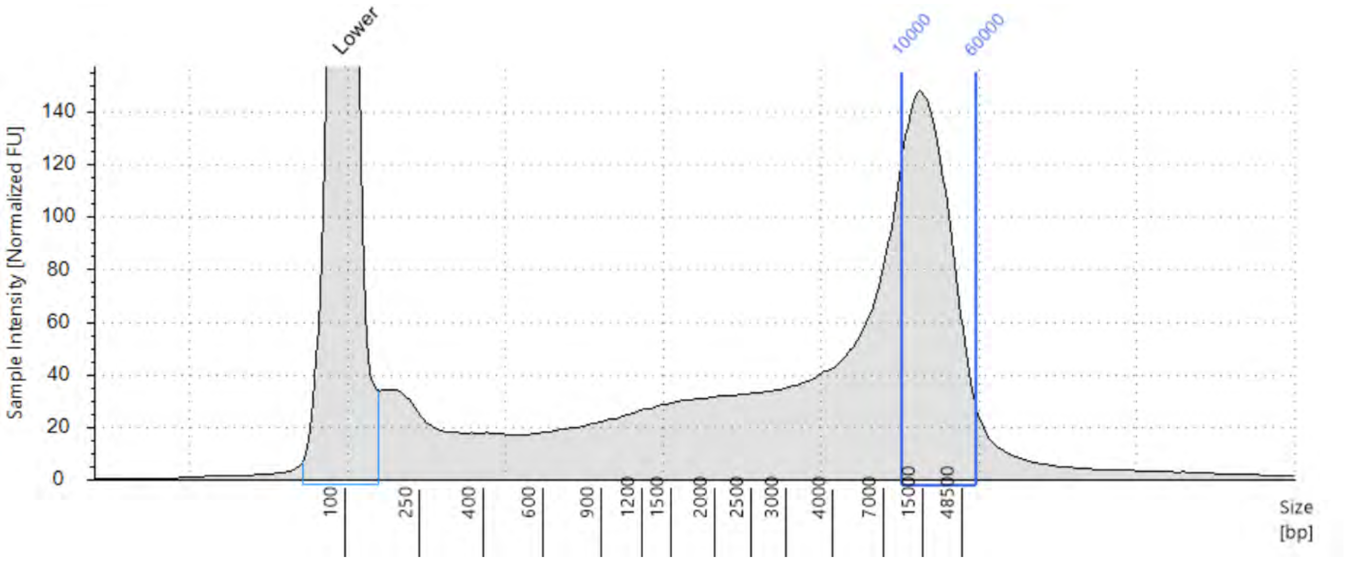

Region Table

From [bp]	To [bp]	Average Size [bp]	Conc. [ng/µl]	Region Molarity [nmol/l]	% of Total	Region Comment	Color
10000	60000	21556	4.84	0.432	30.96		

G8: C332

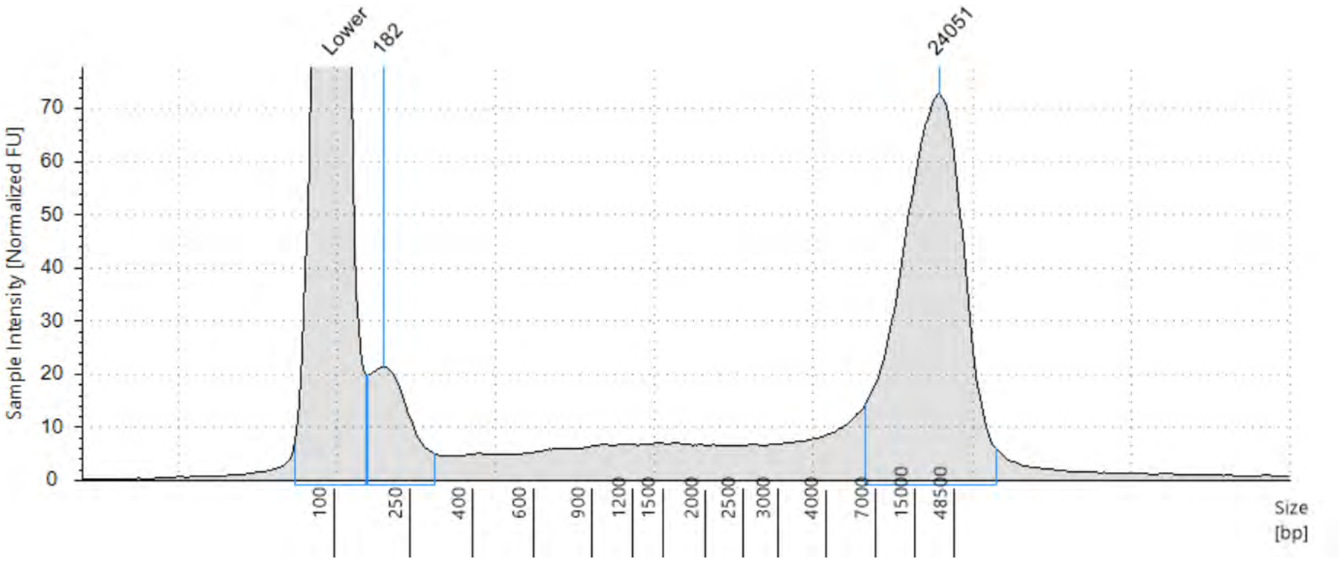

Sample Table

Well	DIN	Conc. [ng/µl]	Sample Description	Alert	Observations
G8	7.5	5.96	C332	⚠	Sample concentration outside recommended range

Peak Table

Size [bp]	Calibrated Conc. [ng/µl]	Assigned Conc. [ng/µl]	% Integrated Area	From [bp]	To [bp]	Peak Comment	Observations
100	8.50	8.50	-	62	147		Lower Marker
182	0.625	-	15.34	149	298		
24051	3.39	-	83.15	6204	>60000		
-	-	-	-	-	-		Sample Well

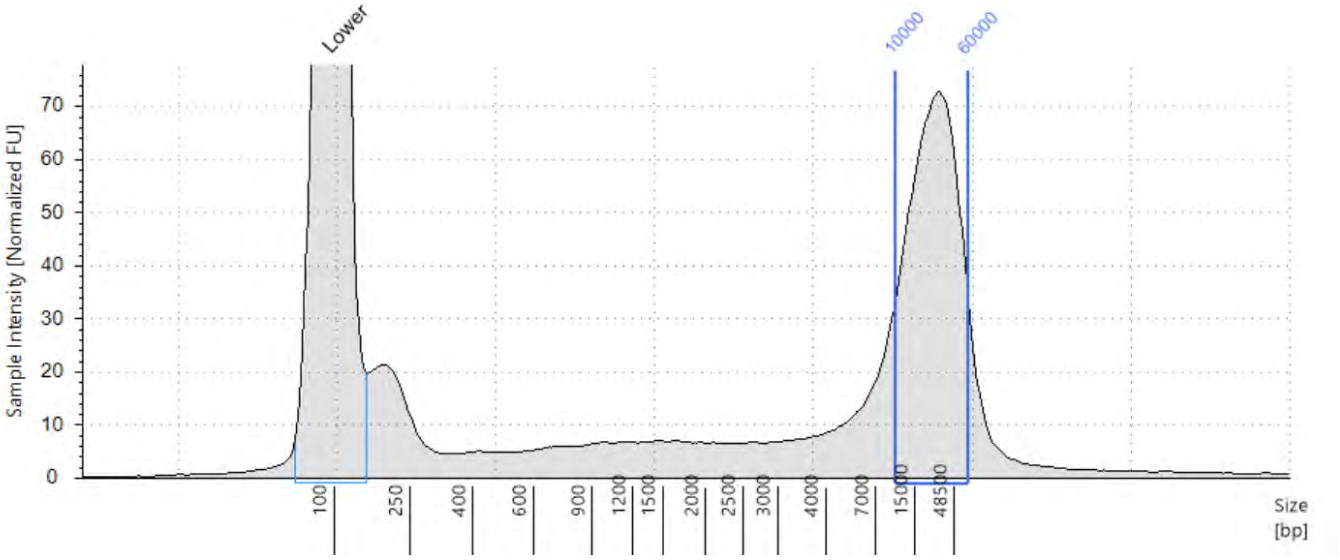

Region Table

From [bp]	To [bp]	Average Size [bp]	Conc. [ng/µl]	Region Molarity [nmol/l]	% of Total	Region Comment	Color
10000	60000	26456	2.73	0.205	45.78		■

H8: C333

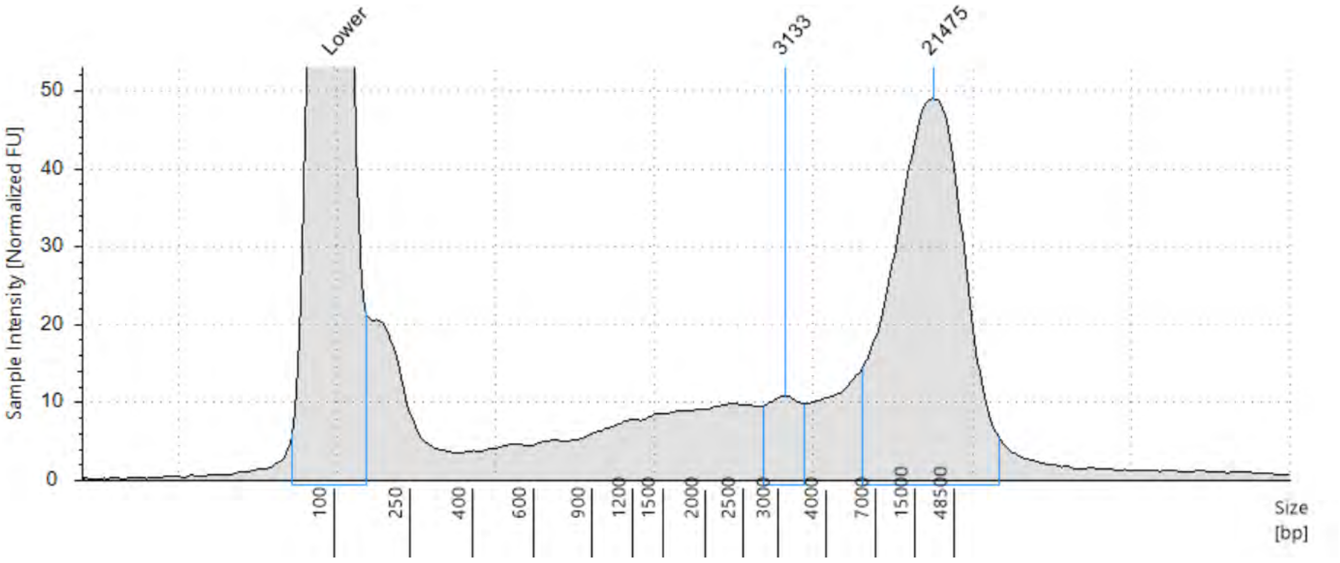

Sample Table

Well	DIN	Conc. [ng/µl]	Sample Description	Alert	Observations
H8	6.9	5.30	C333	⚠	Sample concentration outside recommended range

Peak Table

Size [bp]	Calibrated Conc. [ng/µl]	Assigned Conc. [ng/µl]	% Integrated Area	From [bp]	To [bp]	Peak Comment	Observations
100	8.50	8.50	-	60	147		Lower Marker
3133	0.266	-	9.45	2774	3523		
21475	2.52	-	89.53	6052	>60000		
-	-	-	-	-	-		Sample Well

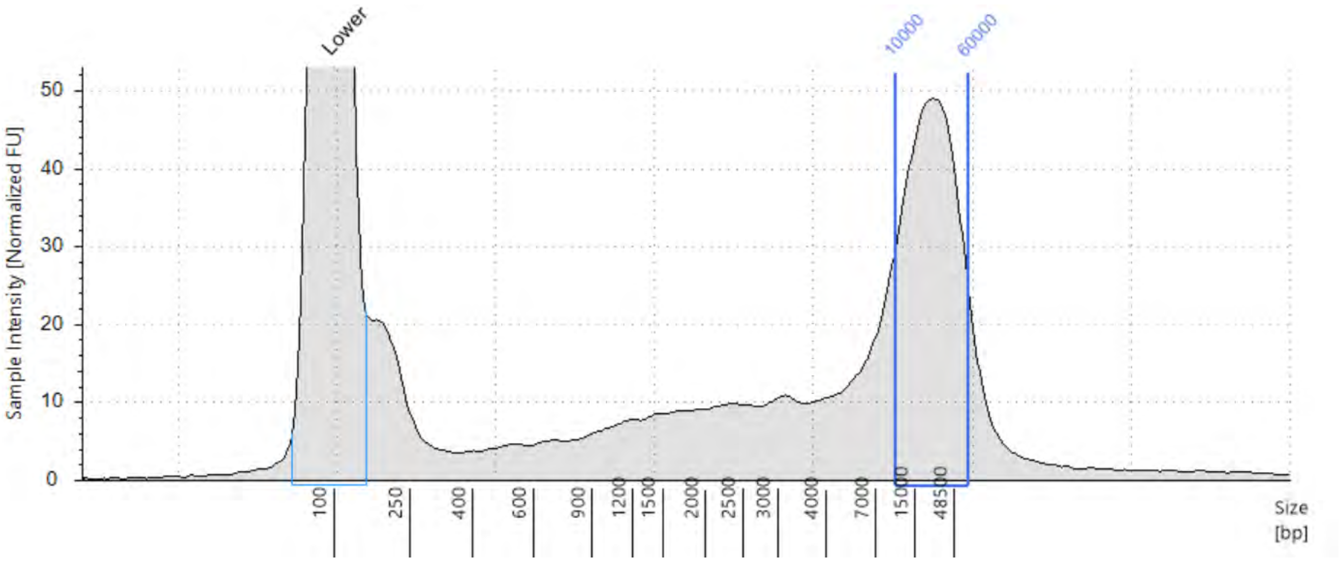

Region Table

From [bp]	To [bp]	Average Size [bp]	Conc. [ng/µl]	Region Molarity [nmol/l]	% of Total	Region Comment	Color
10000	60000	25685	1.90	0.149	35.93		■

Filename: 2018-09-27-01.gDNA

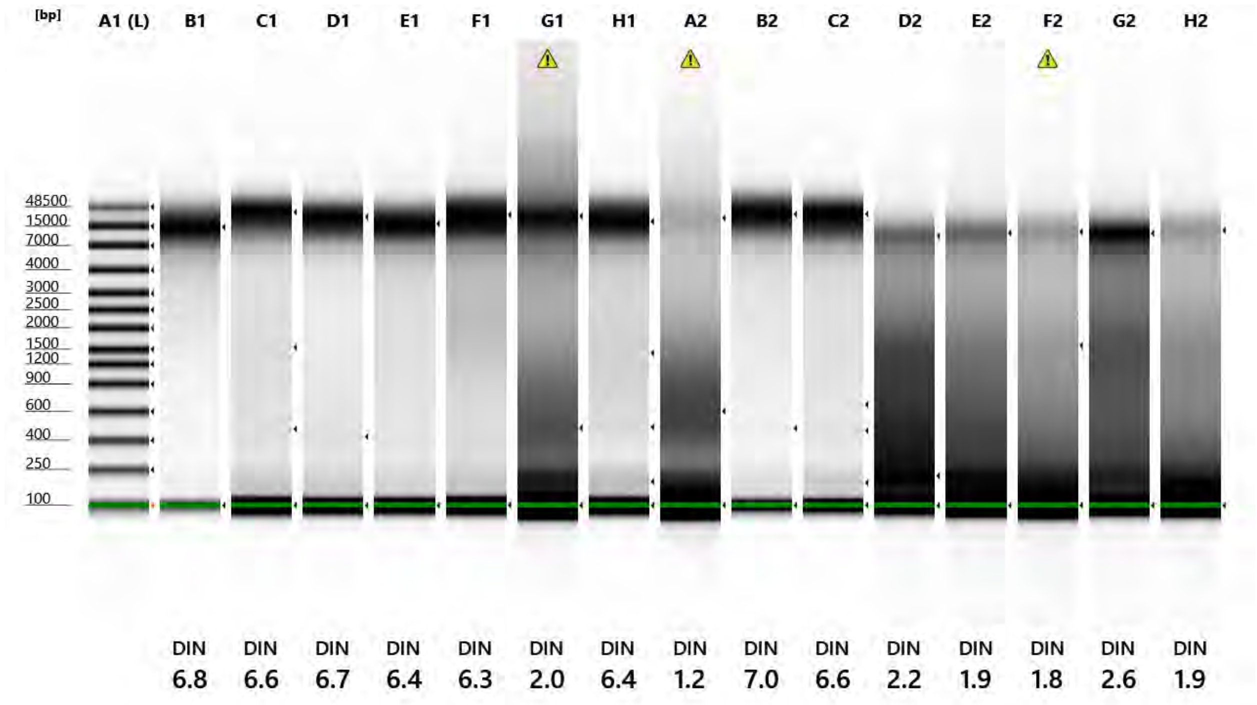

Default image (Contrast 50%), Image is Scaled to Sample

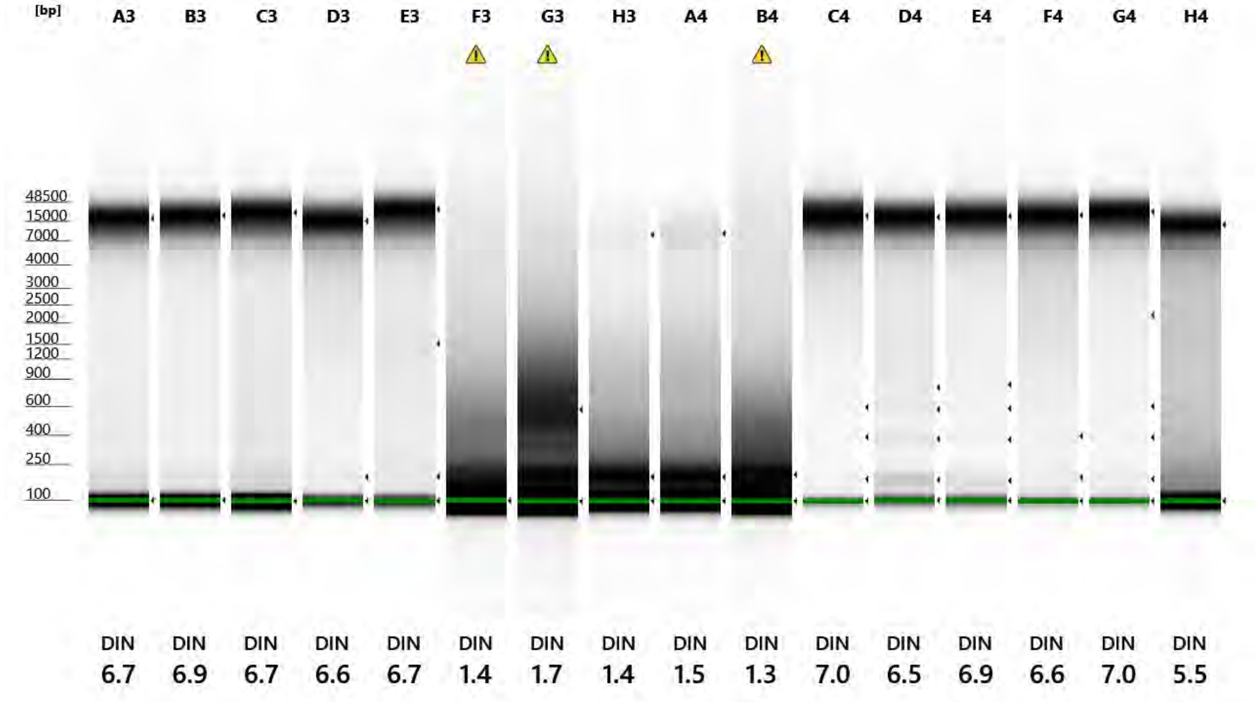

Default image (Contrast 50%), Image is Scaled to Sample

 © Agilent Technologies, Inc. 2017

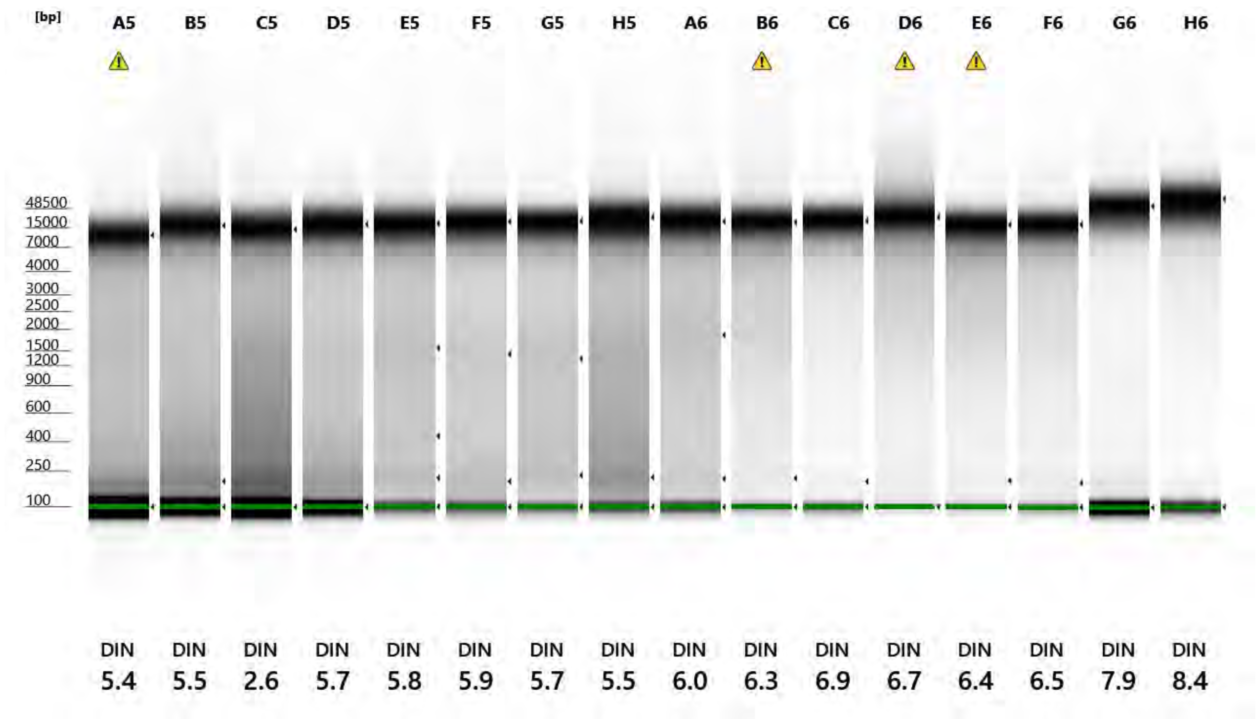

Default image (Contrast 50%), Image is Scaled to Sample

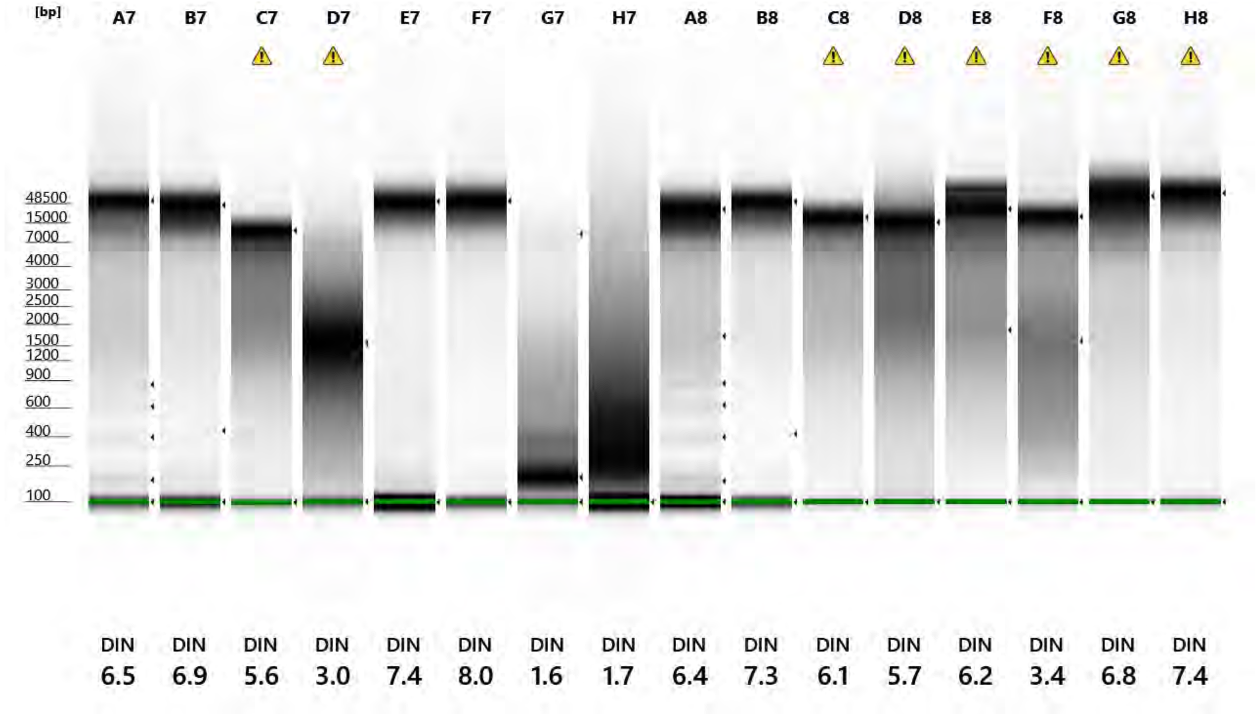

Default image (Contrast 50%), Image is Scaled to Sample

 © Agilent Technologies, Inc. 2017

Sample Info

Well	DIN	Conc. [ng/µl]	Sample Description	Alert	Observations
A1	-	97.5	Ladder		Ladder
B1	6.8	48.9	W901		
C1	6.6	12.3	W902		
D1	6.7	14.3	W903		
E1	6.4	17.5	W904		
F1	6.3	17.2	W905		
G1	2.0	9.52	W906	⚠	Sample concentration outside recommended range
H1	6.4	14.8	W907		
A2	1.2	6.09	W908	⚠	Sample concentration outside recommended range
B2	7.0	28.9	W909		
C2	6.6	23.4	W910		
D2	2.2	32.6	W911		
E2	1.9	11.9	W912		
F2	1.8	8.75	W913	⚠	Sample concentration outside recommended range
G2	2.6	25.7	W914		
H2	1.9	11.9	W915		
A3	6.7	19.7	W916		
B3	6.9	17.9	W917		
C3	6.7	13.1	W918		
D3	6.6	36.3	W919		
E3	6.7	39.2	W920		
F3	1.4	4.62	W921	⚠	Sample concentration outside functional range for DIN
G3	1.7	6.13	W922	⚠	Sample concentration outside recommended range
H3	1.4	11.2	W923		
A4	1.5	11.9	W924		
B4	1.3	6.34	W925	⚠	Sample concentration outside recommended range
C4	7.0	83.6	W926		
D4	6.5	55.5	W927		
E4	6.9	49.2	W928		
F4	6.6	90.9	W929		
G4	7.0	76.0	W930		
H4	5.5	21.2	W931		
A5	5.4	9.44	W932	⚠	Sample concentration outside recommended range
B5	5.5	22.4	W933		
C5	2.6	16.7	W934		
D5	5.7	27.2	W935		
E5	5.8	64.9	W936		
F5	5.9	60.0	W937		
G5	5.7	81.1	W938		
H5	5.5	81.7	W939		
A6	6.0	62.3	W940		
B6	6.3	119	W941	⚠	Sample concentration outside recommended range
C6	6.9	59.3	W942		
D6	6.7	220	W943	⚠	Sample concentration outside recommended range
E6	6.4	163	W944	⚠	Sample concentration outside recommended range
F6	6.5	91.4	W945		
G6	7.9	16.7	M226		
H6	8.4	34.3	M227		
A7	6.5	65.1	M228		
B7	6.9	42.5	M229		
C7	5.6	148	M230	⚠	Sample concentration outside recommended range
D7	3.0	134	M231	⚠	Sample concentration outside recommended range
E7	7.4	15.1	M232		
F7	8.0	41.2	M233		
G7	1.6	99.6	M234		
H7	1.7	46.1	M235		
A8	6.4	34.9	M236		
B8	7.3	44.6	M237		
C8	6.1	211	M238	⚠	Sample concentration outside recommended range
D8	5.7	270	M239	⚠	Sample concentration outside recommended range
E8	6.2	616	M240	⚠	Sample concentration outside functional range for DIN
F8	3.4	340	M241	⚠	Sample concentration outside functional range for DIN
G8	6.8	390	M242	⚠	Sample concentration outside functional range for DIN
H8	7.4	128	M243	⚠	Sample concentration outside recommended range

 © Agilent Technologies, Inc. 2017

A1: Ladder

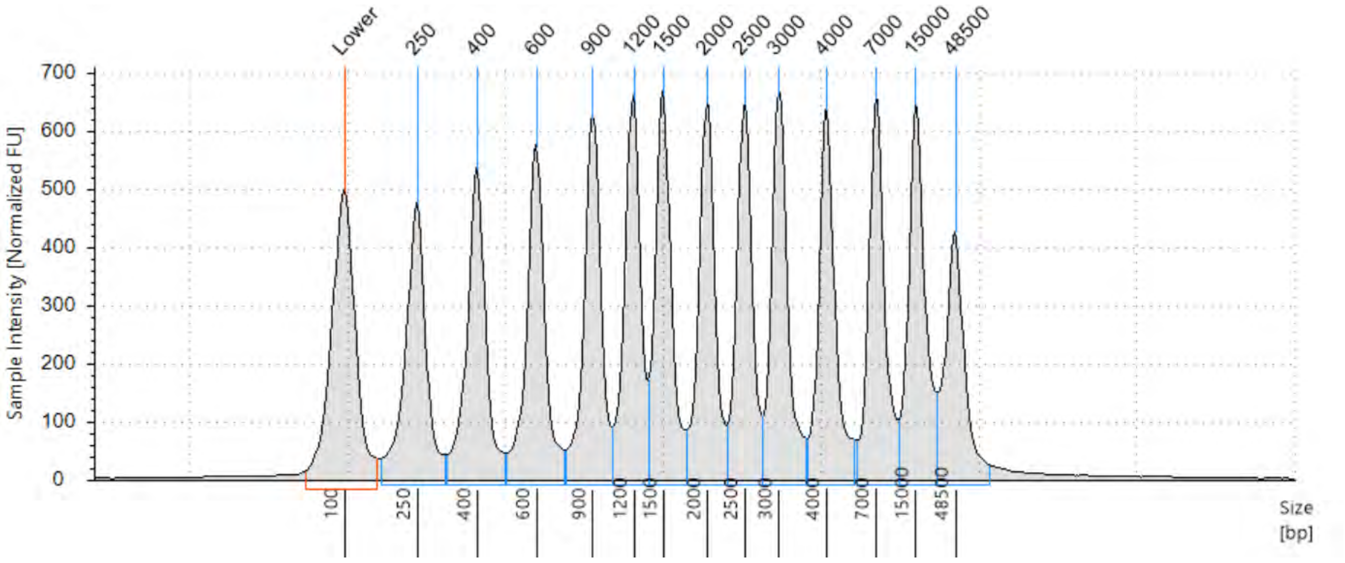

Sample Table

Well	DIN	Conc. [ng/µl]	Sample Description	Alert	Observations
A1	-	97.5	Ladder		Ladder

Peak Table

Size [bp]	Calibrated Conc. [ng/µl]	Assigned Conc. [ng/µl]	% Integrated Area	From [bp]	To [bp]	Peak Comment	Observations
100	8.50	8.50	-	61	149		Lower Marker
250	6.99	-	7.21	159	310		
400	7.26	-	7.49	313	483		
600	7.48	-	7.72	488	731		
900	7.62	-	7.85	738	1029		
1200	7.78	-	8.02	1029	1342		
1500	7.89	-	8.13	1342	1748		
2000	7.57	-	7.80	1748	2245		
2500	7.40	-	7.63	2245	2749		
3000	7.90	-	8.14	2749	3536		
4000	7.56	-	7.79	3565	5459		
7000	7.70	-	7.94	5631	10943		
15000	7.95	-	8.20	10943	22849		
48500	5.89	-	6.07	22849	>60000		

 © Agilent Technologies, Inc. 2017

B1: W901

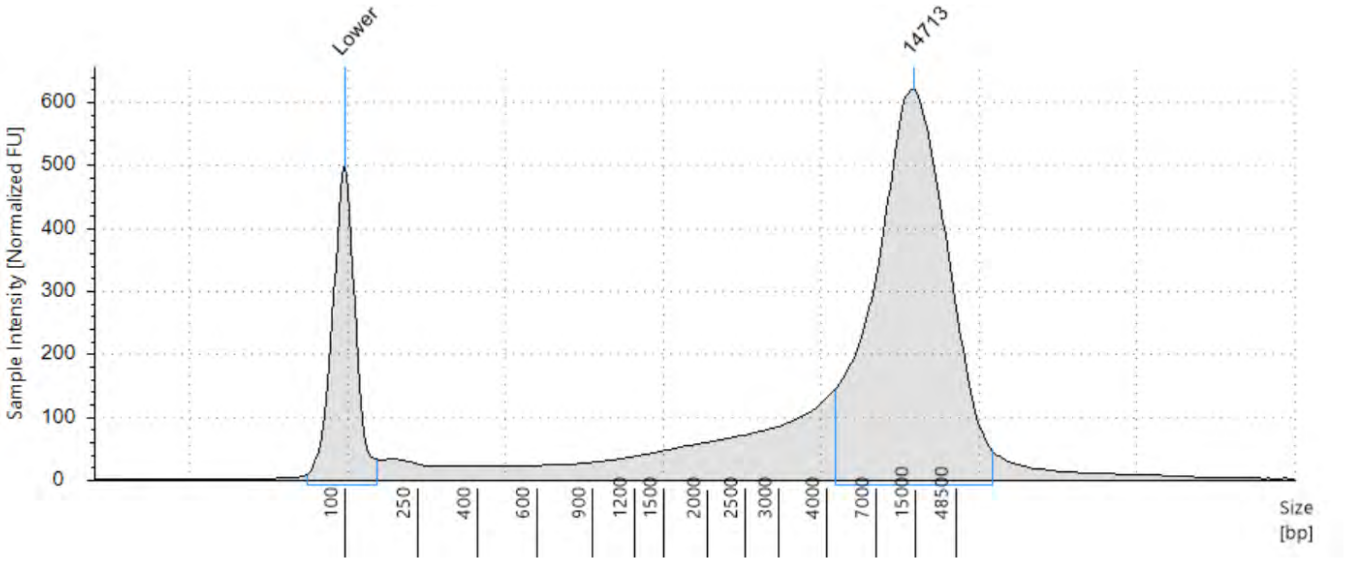

Sample Table

Well	DIN	Conc. [ng/µl]	Sample Description	Alert	Observations
B1	6.8	48.9	W901		

Peak Table

Size [bp]	Calibrated Conc. [ng/µl]	Assigned Conc. [ng/µl]	% Integrated Area	From [bp]	To [bp]	Peak Comment	Observations
100	8.50	8.50	-	62	151		Lower Marker
14713	34.0	-	99.77	4435	>60000		
-	-	-	-	-	-		Sample Well

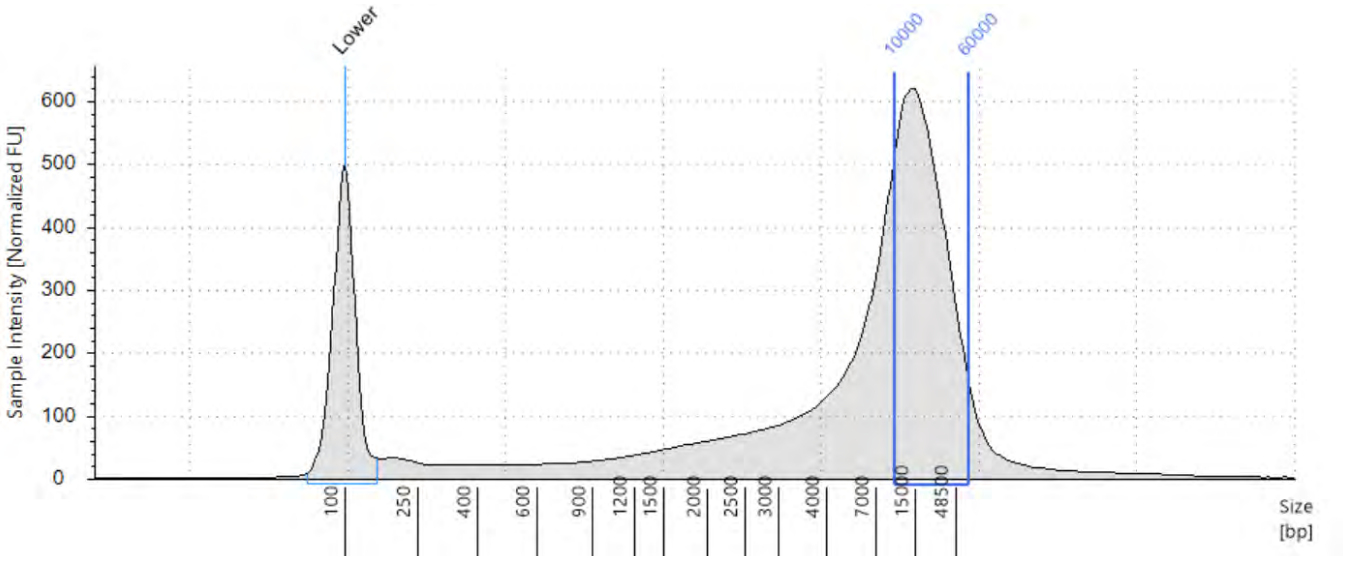

Region Table

From [bp]	To [bp]	Average Size [bp]	Conc. [ng/µl]	Region Molarity [nmol/l]	% of Total	Region Comment	Color
10000	60000	21966	22.3	1.96	45.53		

 © Agilent Technologies, Inc. 2017

C1: W902

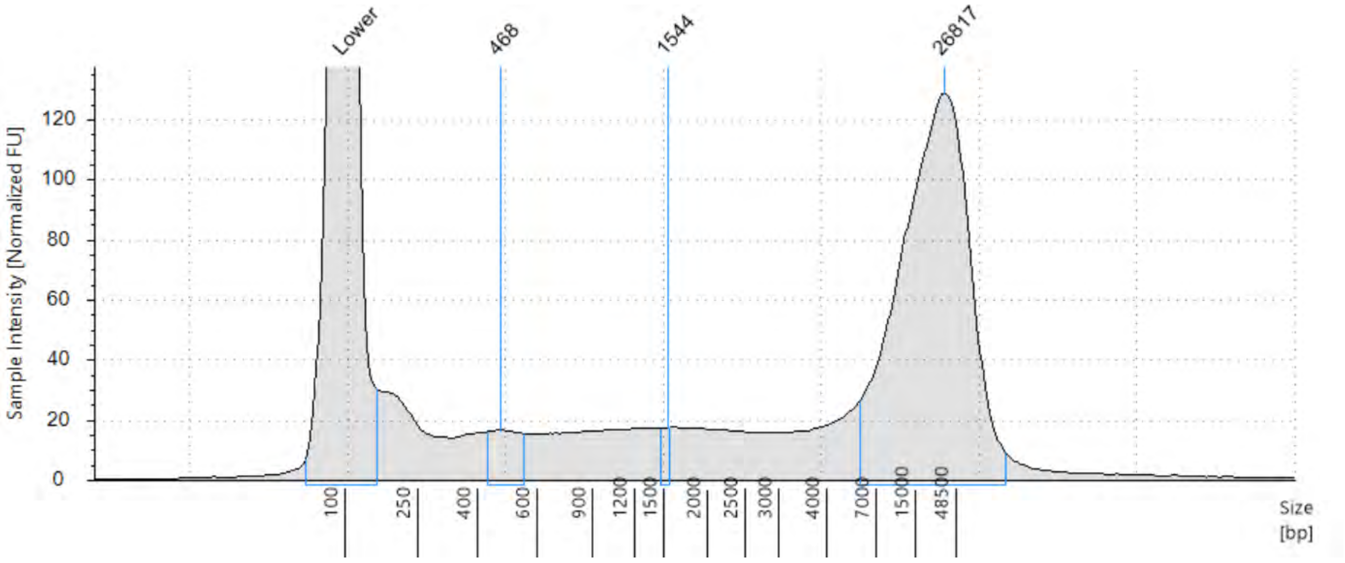

Sample Table

Well	DIN	Conc. [ng/µl]	Sample Description	Alert	Observations
C1	6.6	12.3	W902		

Peak Table

Size [bp]	Calibrated Conc. [ng/µl]	Assigned Conc. [ng/µl]	% Integrated Area	From [bp]	To [bp]	Peak Comment	Observations
100	8.50	8.50	-	61	149		Lower Marker
468	0.383	-	5.28	426	549		
1544	0.104	-	1.43	1473	1558		
26817	6.76	-	93.11	5878	>60000		
-	-	-	-	-	-		Sample Well

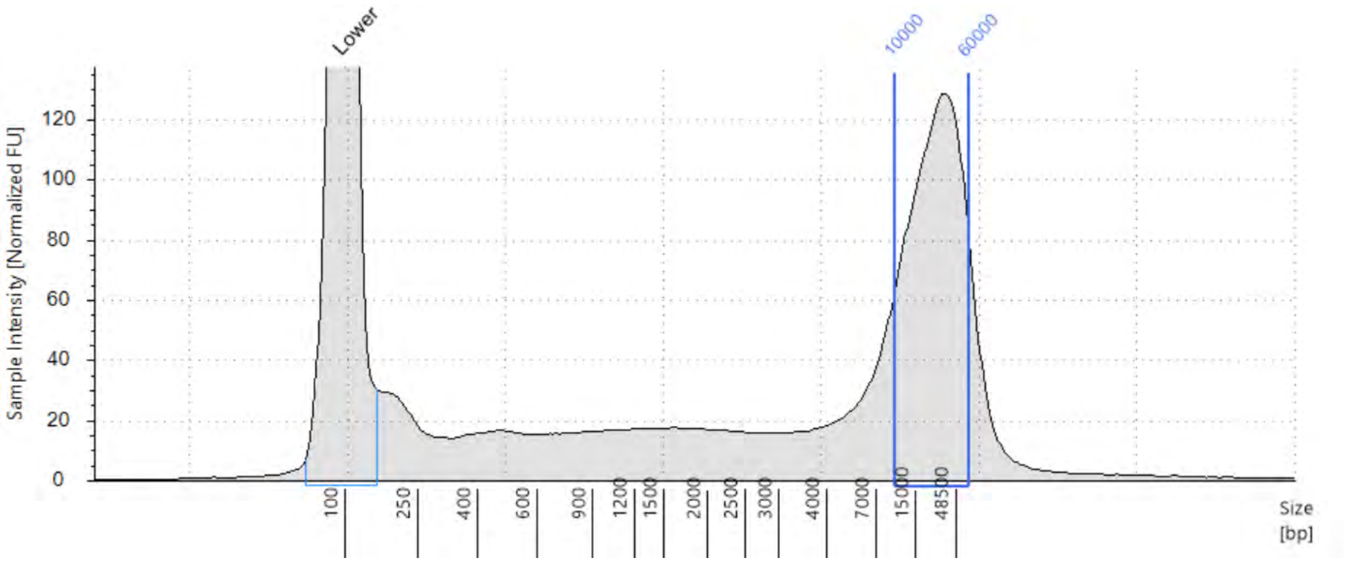

Region Table

From [bp]	To [bp]	Average Size [bp]	Conc. [ng/µl]	Region Molarity [nmol/l]	% of Total	Region Comment	Color
10000	60000	27750	5.14	0.378	41.93		

D1: W903

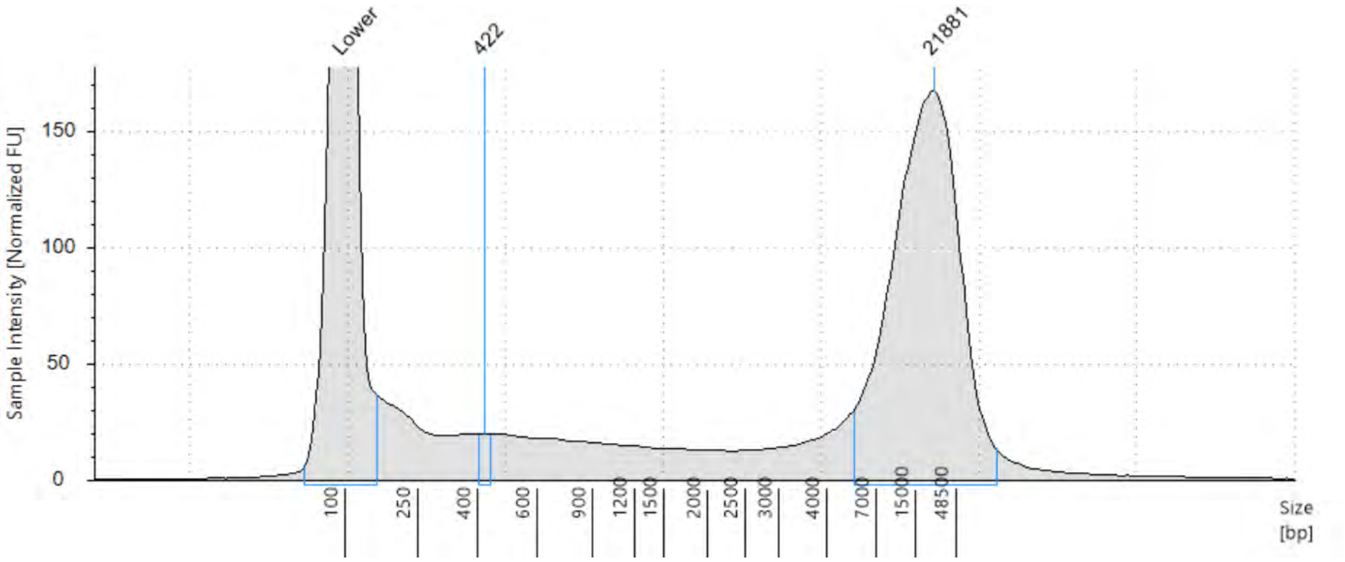

Sample Table

Well	DIN	Conc. [ng/µl]	Sample Description	Alert	Observations
D1	6.7	14.3	W903		

Peak Table

Size [bp]	Calibrated Conc. [ng/µl]	Assigned Conc. [ng/µl]	% Integrated Area	From [bp]	To [bp]	Peak Comment	Observations
100	8.50	8.50	-	60	149		Lower Marker
422	0.173	-	1.97	402	438		
21881	8.58	-	97.93	5462	>60000		
-	-	-	-	-	-		Sample Well

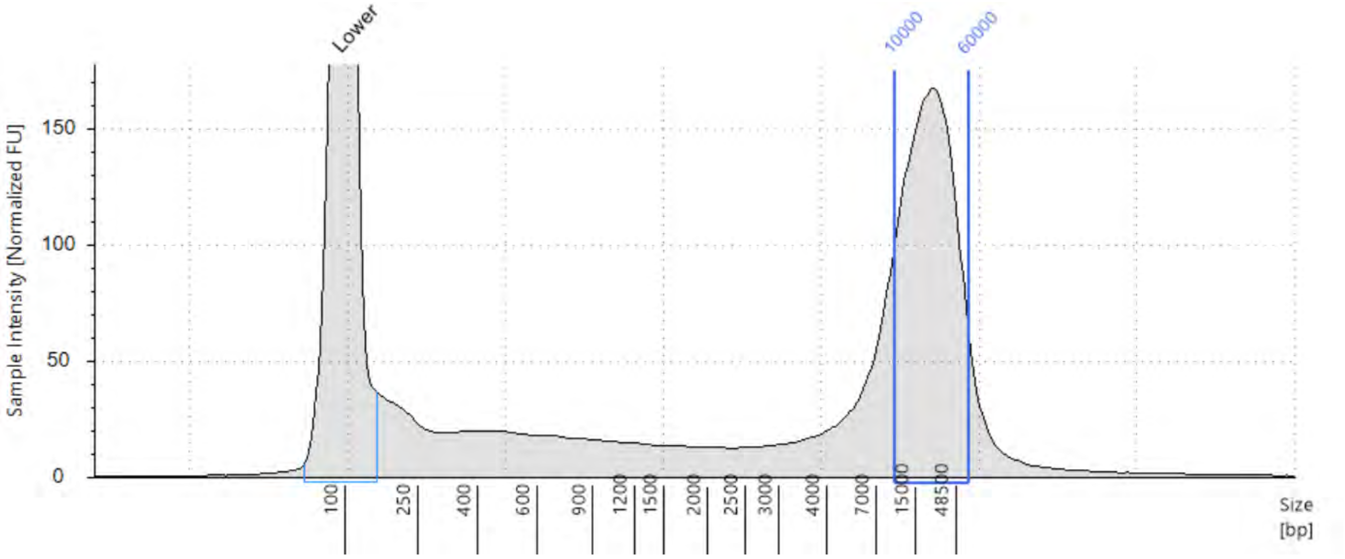

Region Table

From [bp]	To [bp]	Average Size [bp]	Conc. [ng/µl]	Region Molarity [nmol/l]	% of Total	Region Comment	Color
10000	60000	24927	6.62	0.524	46.17		■

E1: W904

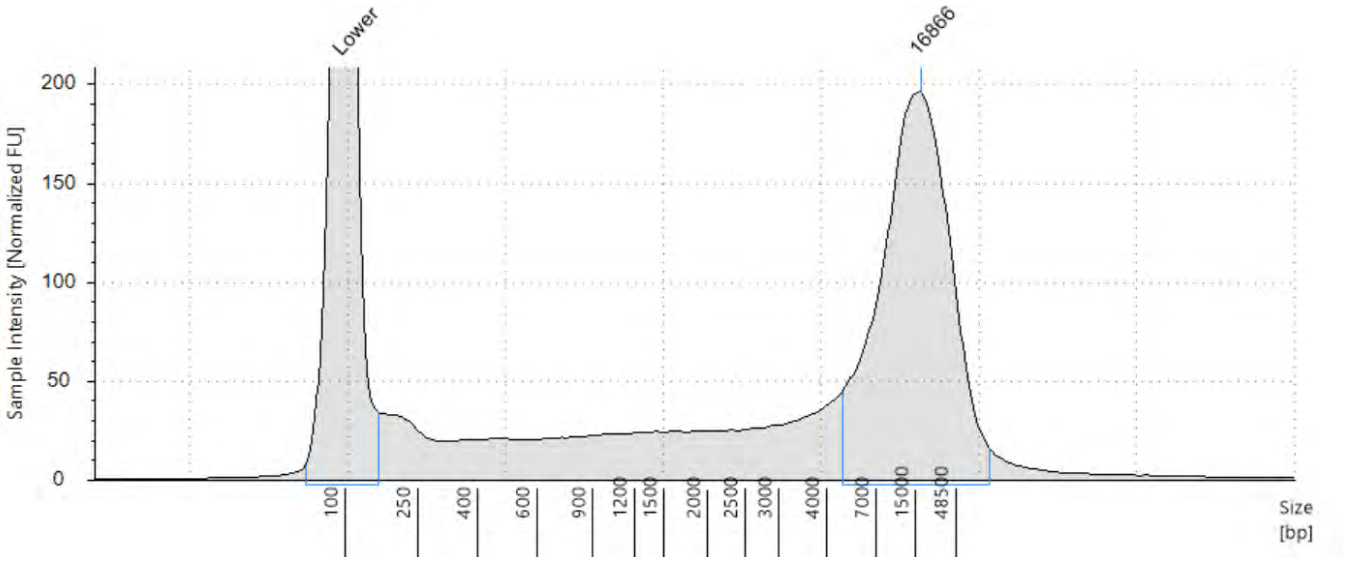

Sample Table

Well	DIN	Conc. [ng/µl]	Sample Description	Alert	Observations
E1	6.4	17.5	W904		

Peak Table

Size [bp]	Calibrated Conc. [ng/µl]	Assigned Conc. [ng/µl]	% Integrated Area	From [bp]	To [bp]	Peak Comment	Observations
100	8.50	8.50	-	61	151		Lower Marker
16866	9.99	-	100.00	4775	>60000		

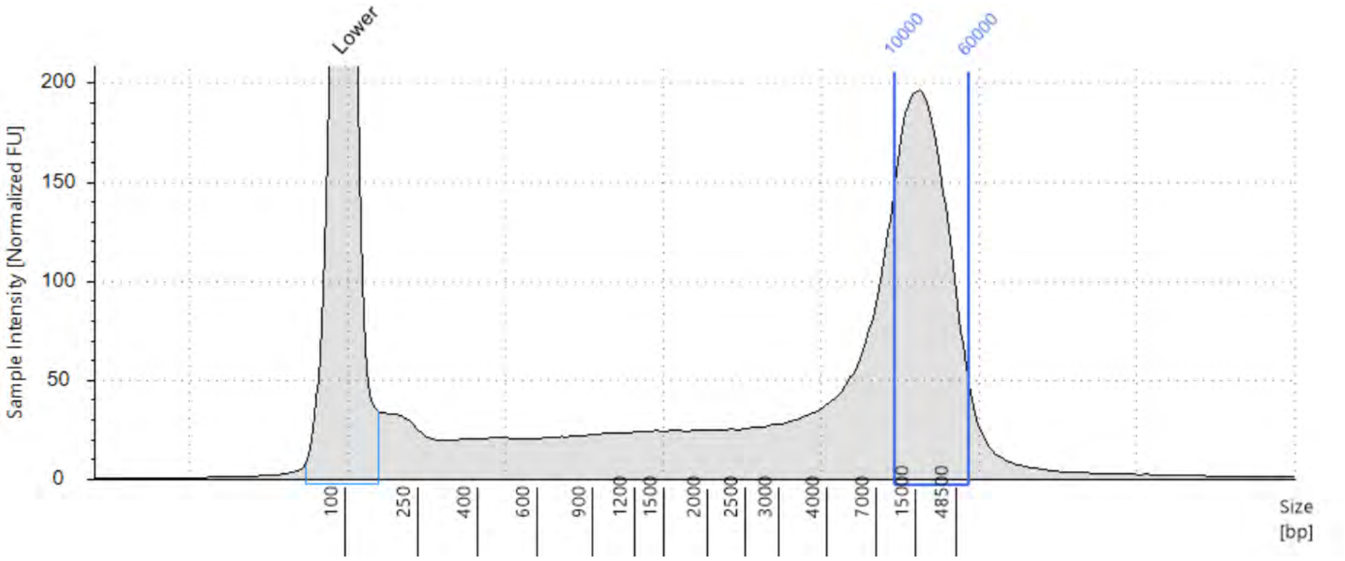

Region Table

From [bp]	To [bp]	Average Size [bp]	Conc. [ng/µl]	Region Molarity [nmol/l]	% of Total	Region Comment	Color
10000	60000	22543	7.02	0.602	40.04		

F1: W905

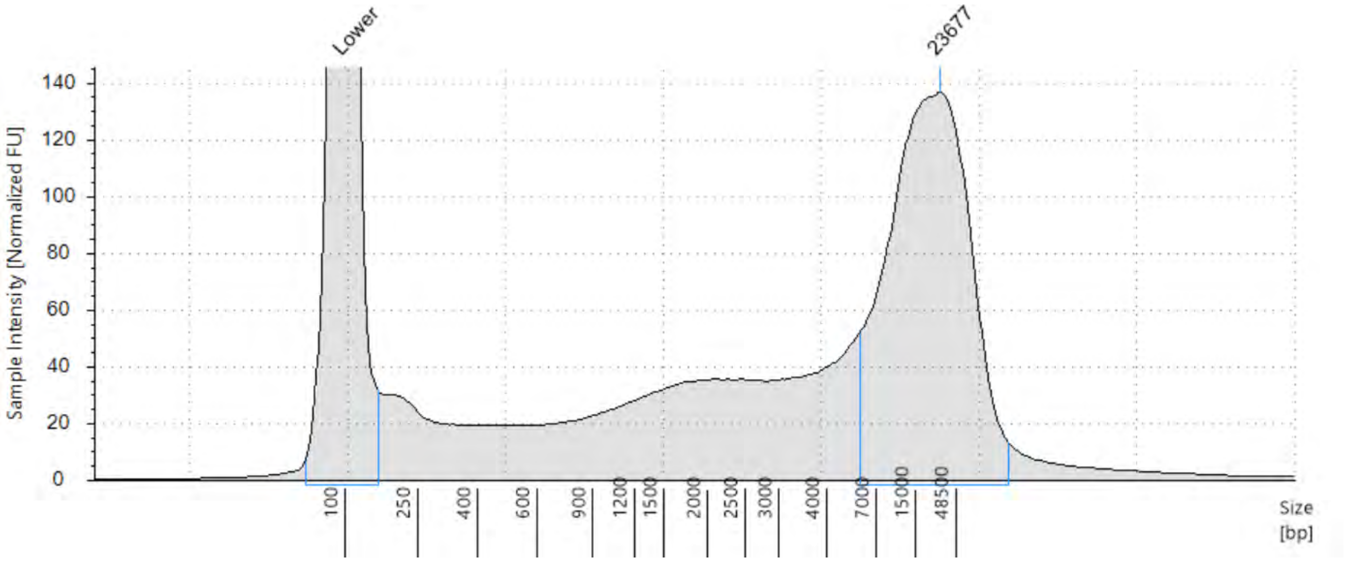

Sample Table

Well	DIN	Conc. [ng/µl]	Sample Description	Alert	Observations
F1	6.3	17.2	W905		

Peak Table

Size [bp]	Calibrated Conc. [ng/µl]	Assigned Conc. [ng/µl]	% Integrated Area	From [bp]	To [bp]	Peak Comment	Observations
100	8.50	8.50	-	60	152		Lower Marker
23677	8.30	-	99.78	5798	>60000		
-	-	-	-	-	-		Sample Well

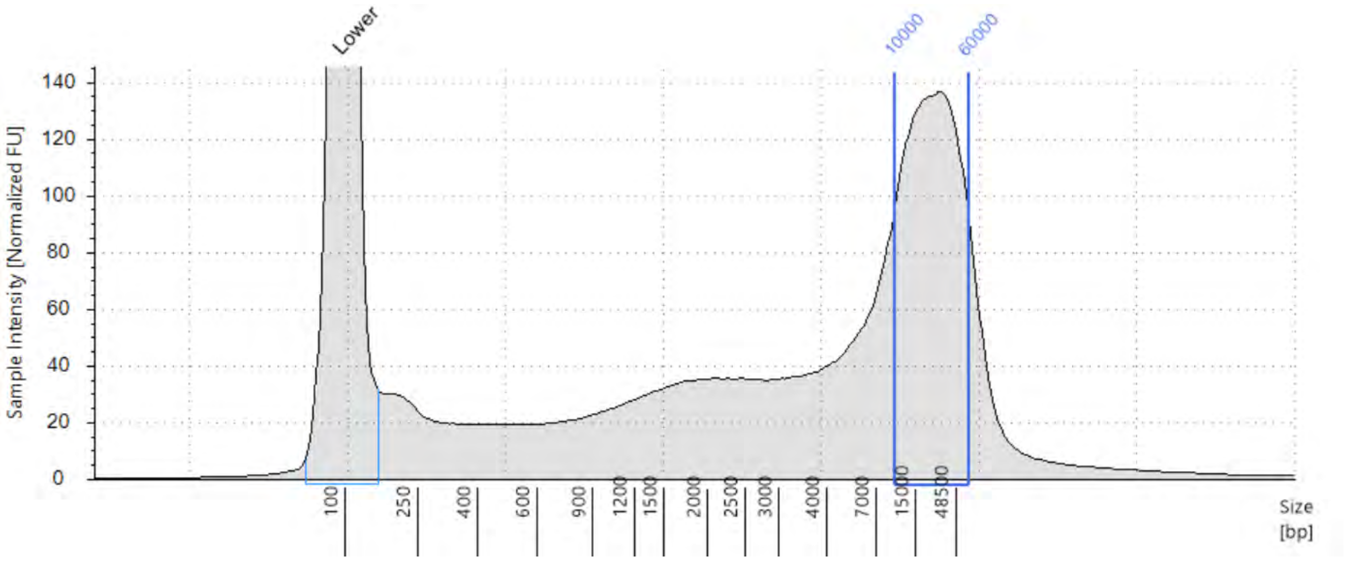

Region Table

From [bp]	To [bp]	Average Size [bp]	Conc. [ng/µl]	Region Molarity [nmol/l]	% of Total	Region Comment	Color
10000	60000	26378	5.83	0.452	33.90		

© Agilent Technologies, Inc. 2017

G1: W906

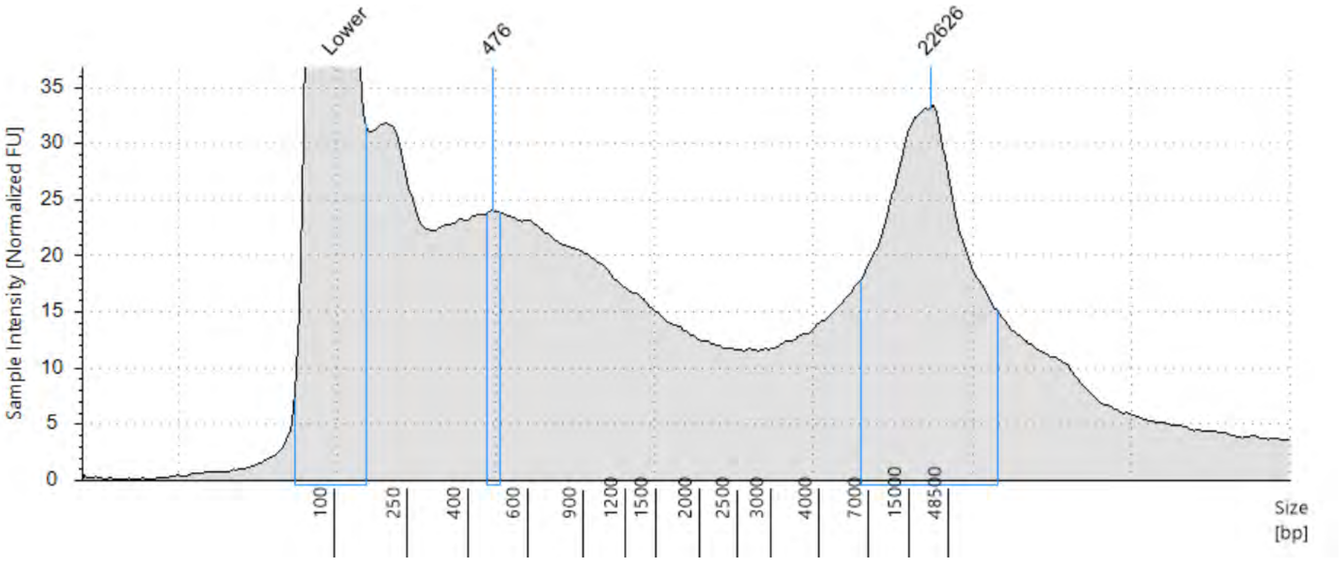

Sample Table

Well	DIN	Conc. [ng/µl]	Sample Description	Alert	Observations
G1	2.0	9.52	W906	⚠	Sample concentration outside recommended range

Peak Table

Size [bp]	Calibrated Conc. [ng/µl]	Assigned Conc. [ng/µl]	% Integrated Area	From [bp]	To [bp]	Peak Comment	Observations
100	8.50	8.50	-	61	149		Lower Marker
476	0.224	-	6.83	454	498		
22626	2.10	-	64.04	6368	>60000		
-	-	-	-	-	-		Sample Well

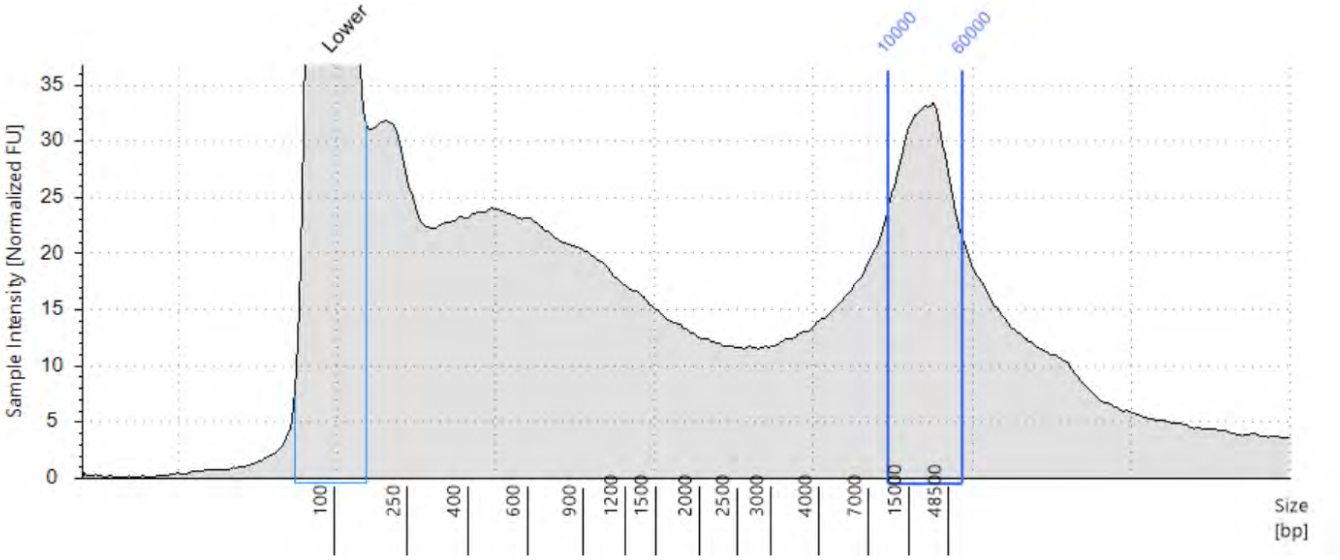

Region Table

From [bp]	To [bp]	Average Size [bp]	Conc. [ng/µl]	Region Molarity [nmol/l]	% of Total	Region Comment	Color
10000	60000	25858	1.39	0.112	14.61		■

H1: W907

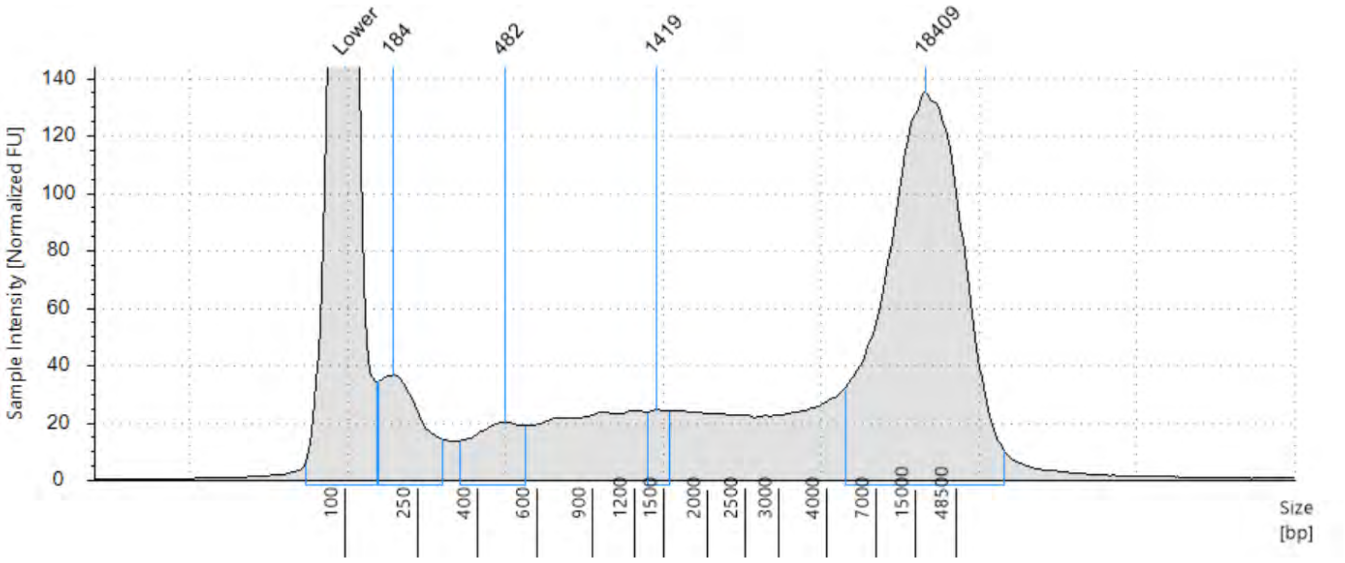

Sample Table

Well	DIN	Conc. [ng/µl]	Sample Description	Alert	Observations
H1	6.4	14.8	W907		

Peak Table

Size [bp]	Calibrated Conc. [ng/µl]	Assigned Conc. [ng/µl]	% Integrated Area	From [bp]	To [bp]	Peak Comment	Observations
100	8.50	8.50	-	60	149		Lower Marker
184	1.14	-	11.19	152	305		
482	0.739	-	7.26	346	556		
1419	0.356	-	3.49	1334	1563		
18409	7.92	-	77.72	4926	>60000		
-	-	-	-	-	-		Sample Well

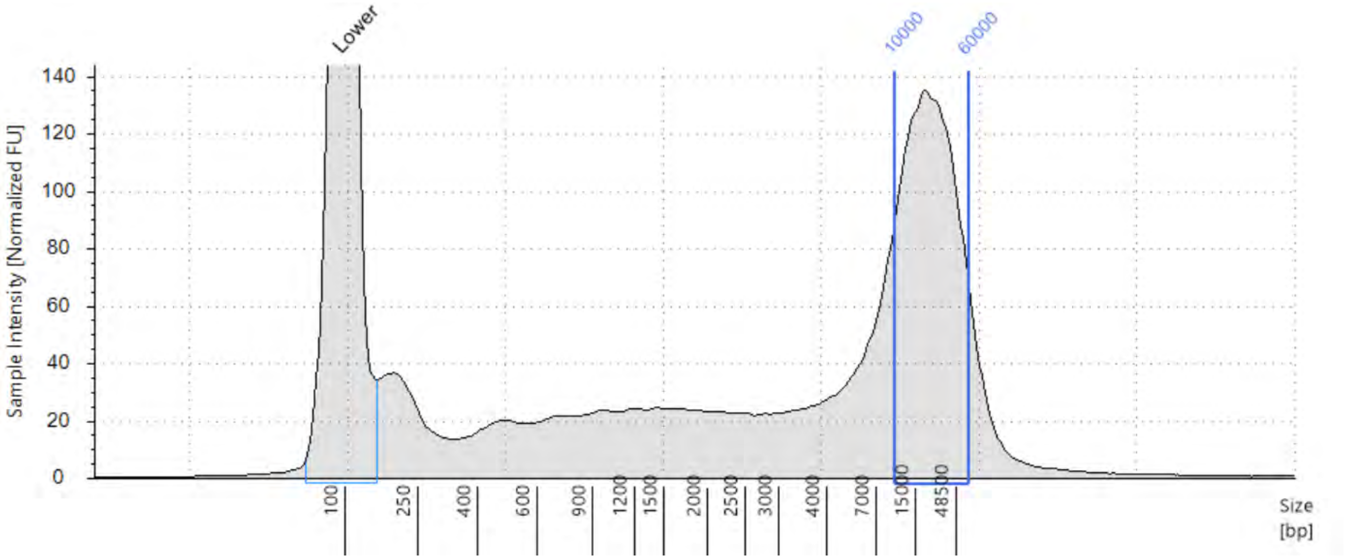

Region Table

From [bp]	To [bp]	Average Size [bp]	Conc. [ng/µl]	Region Molarity [nmol/l]	% of Total	Region Comment	Color
10000	60000	24938	5.61	0.451	37.83		

 © Agilent Technologies, Inc. 2017

A2: W908

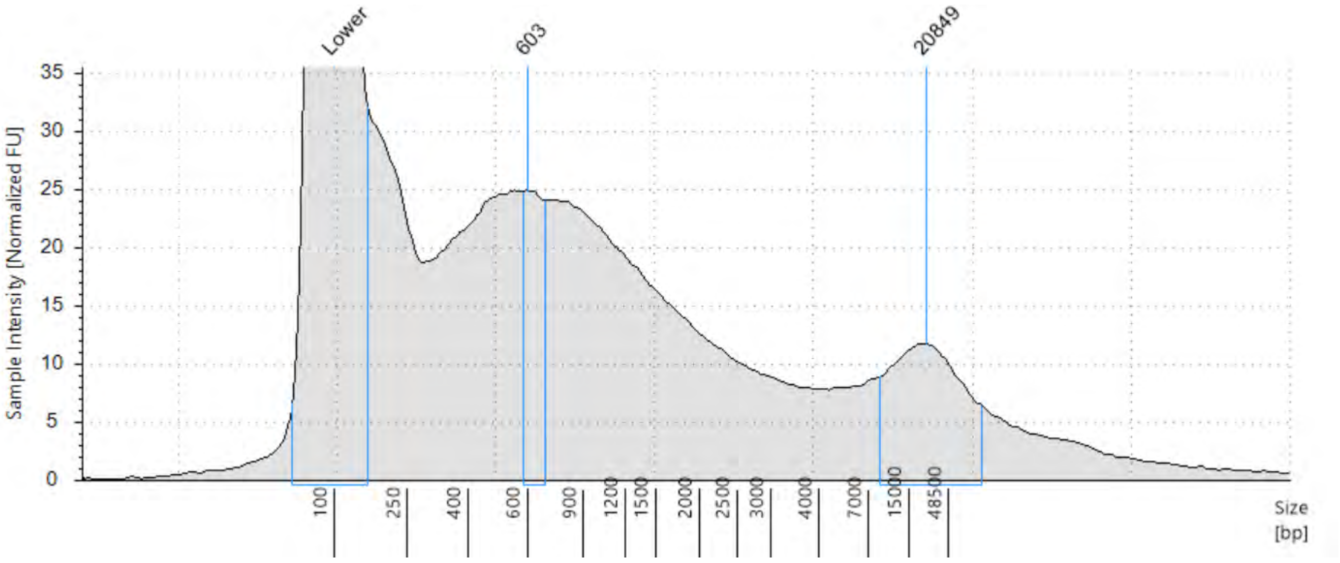

Sample Table

Well	DIN	Conc. [ng/µl]	Sample Description	Alert	Observations
A2	1.2	6.09	W908	⚠	Sample concentration outside recommended range

Peak Table

Size [bp]	Calibrated Conc. [ng/µl]	Assigned Conc. [ng/µl]	% Integrated Area	From [bp]	To [bp]	Peak Comment	Observations
100	8.50	8.50	-	59	153		Lower Marker
603	0.327	-	33.63	586	680		
20849	0.580	-	59.71	8624	>60000		
-	-	-	-	-	-		Sample Well

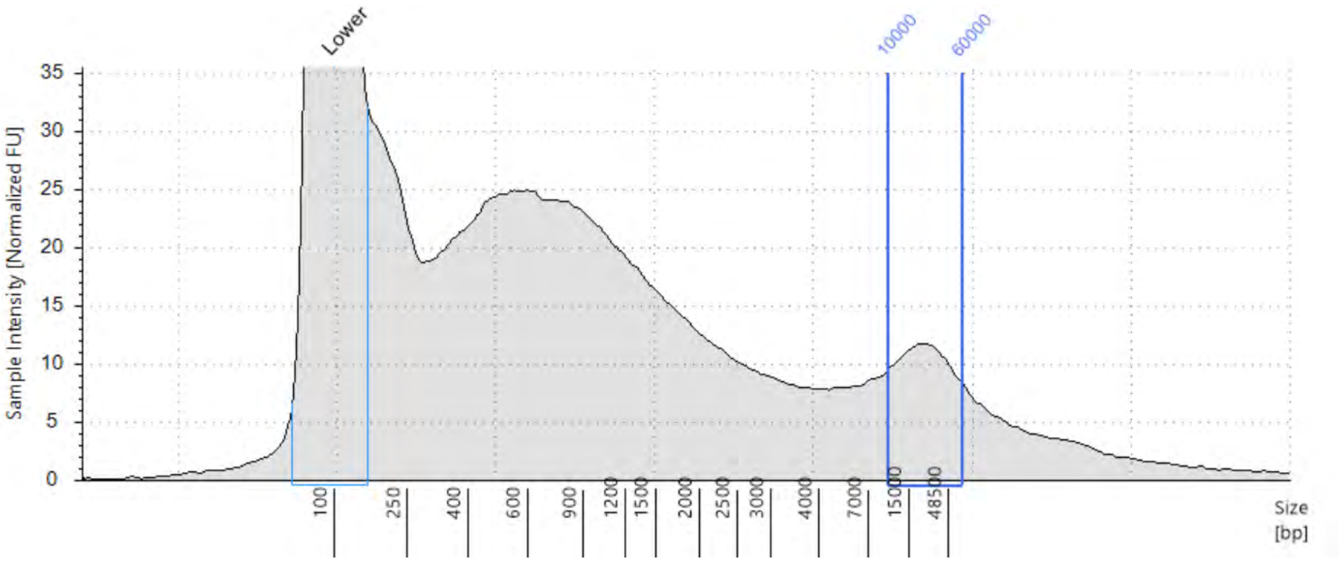

Region Table

From [bp]	To [bp]	Average Size [bp]	Conc. [ng/µl]	Region Molarity [nmol/l]	% of Total	Region Comment	Color
10000	60000	25808	0.458	0.0381	7.53		■

B2: W909

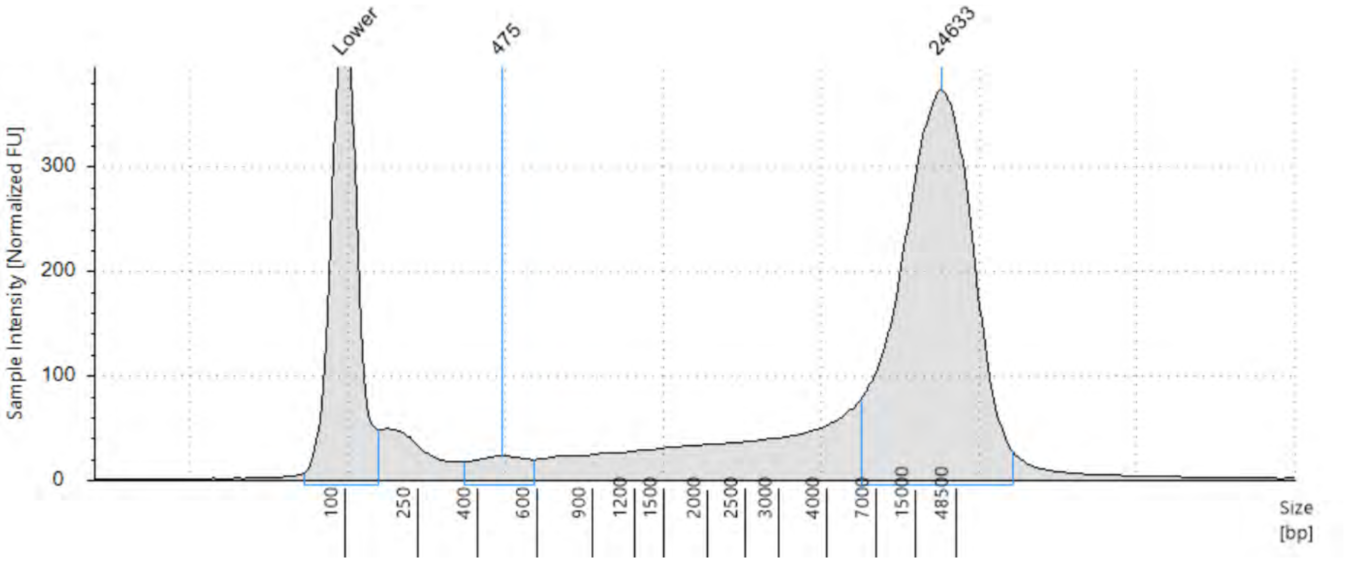

Sample Table

Well	DIN	Conc. [ng/µl]	Sample Description	Alert	Observations
B2	7.0	28.9	W909		

Peak Table

Size [bp]	Calibrated Conc. [ng/µl]	Assigned Conc. [ng/µl]	% Integrated Area	From [bp]	To [bp]	Peak Comment	Observations
100	8.50	8.50	-	60	152		Lower Marker
475	0.843	-	4.23	359	592		
24633	19.1	-	95.77	5912	>60000		

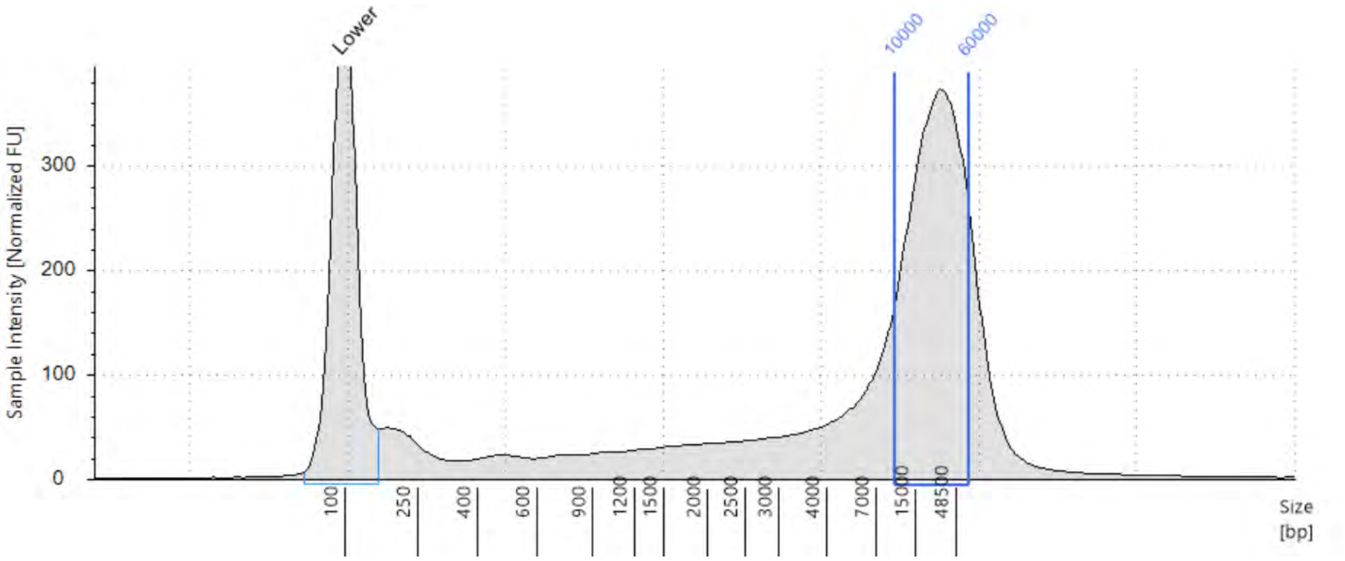

Region Table

From [bp]	To [bp]	Average Size [bp]	Conc. [ng/µl]	Region Molarity [nmol/l]	% of Total	Region Comment	Color
10000	60000	27737	13.8	1.00	47.85		■

 © Agilent Technologies, Inc. 2017

C2: W910

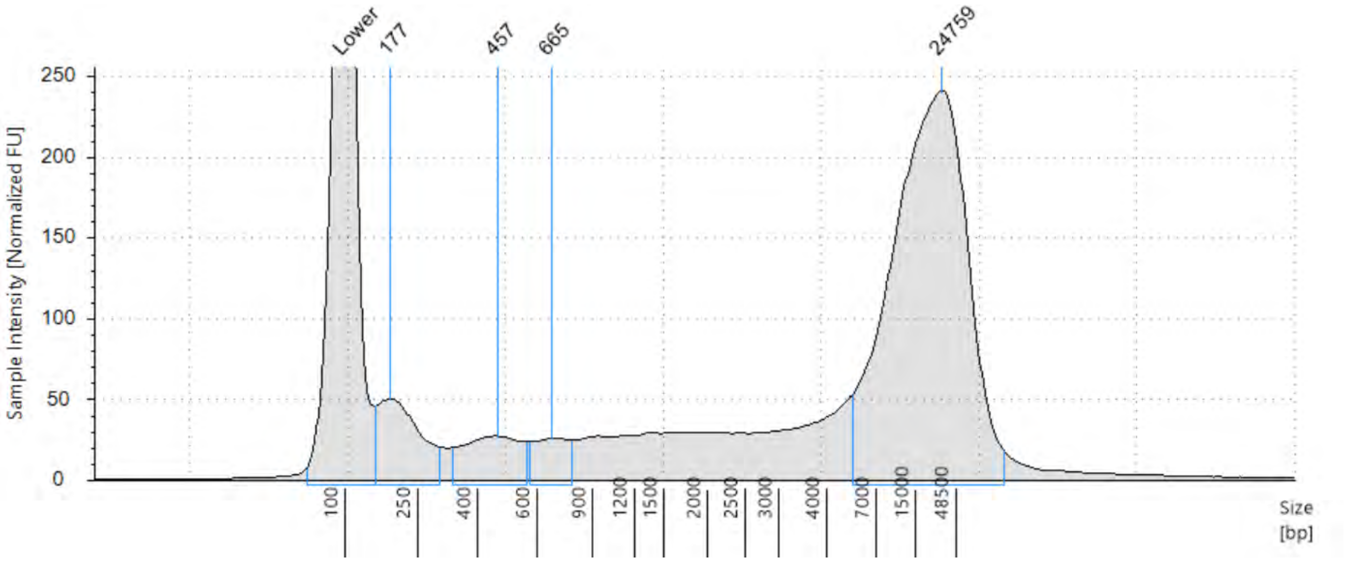

Sample Table

Well	DIN	Conc. [ng/µl]	Sample Description	Alert	Observations
C2	6.6	23.4	W910		

Peak Table

Size [bp]	Calibrated Conc. [ng/µl]	Assigned Conc. [ng/µl]	% Integrated Area	From [bp]	To [bp]	Peak Comment	Observations
100	8.50	8.50	-	62	146		Lower Marker
177	1.59	-	9.14	146	297		
457	1.15	-	6.62	327	558		
665	0.662	-	3.81	574	771		
24759	13.9	-	79.91	5359	>60000		
-	-	-	-	-	-		Sample Well

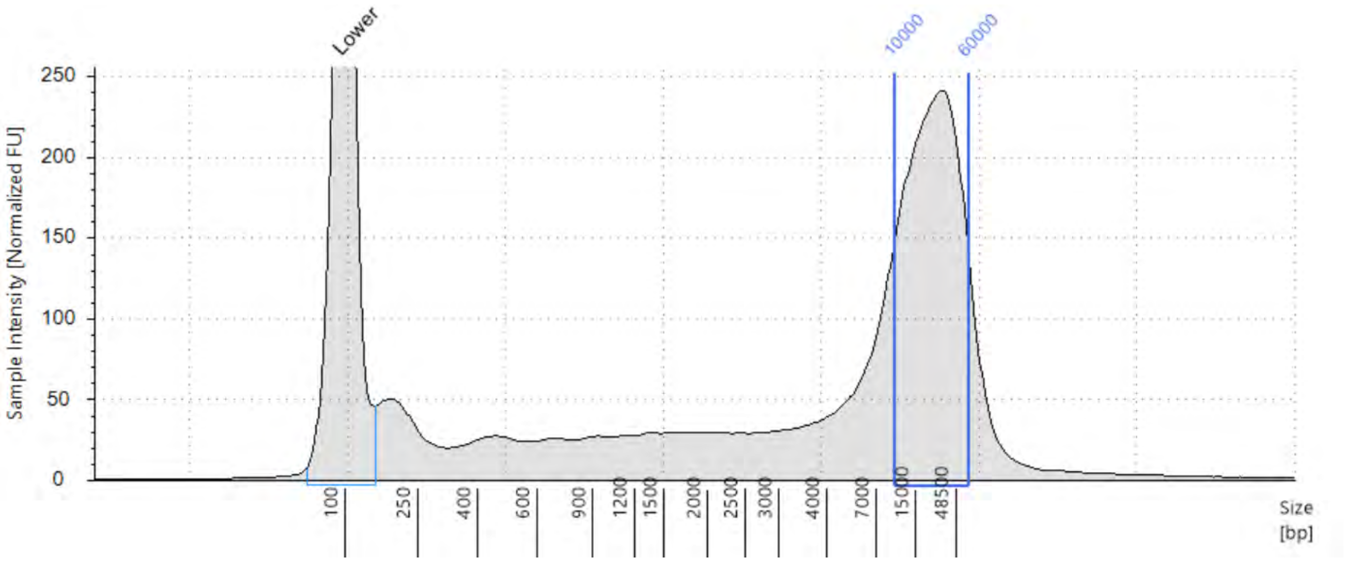

Region Table

From [bp]	To [bp]	Average Size [bp]	Conc. [ng/µl]	Region Molarity [nmol/l]	% of Total	Region Comment	Color
10000	60000	26273	10.2	0.782	43.42		

D2: W911

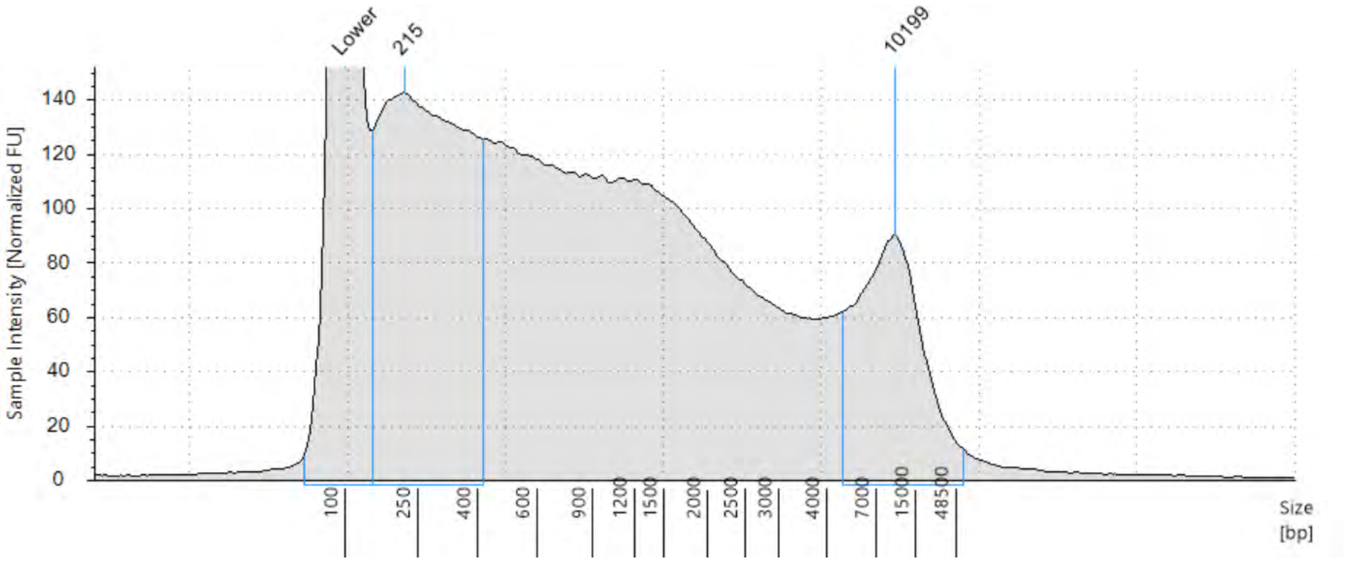

Sample Table

Well	DIN	Conc. [ng/µl]	Sample Description	Alert	Observations
D2	2.2	32.6	W911		

Peak Table

Size [bp]	Calibrated Conc. [ng/µl]	Assigned Conc. [ng/µl]	% Integrated Area	From [bp]	To [bp]	Peak Comment	Observations
100	8.50	8.50	-	60	141		Lower Marker
215	8.76	-	68.60	141	417		
10199	4.01	-	31.40	4835	54834		

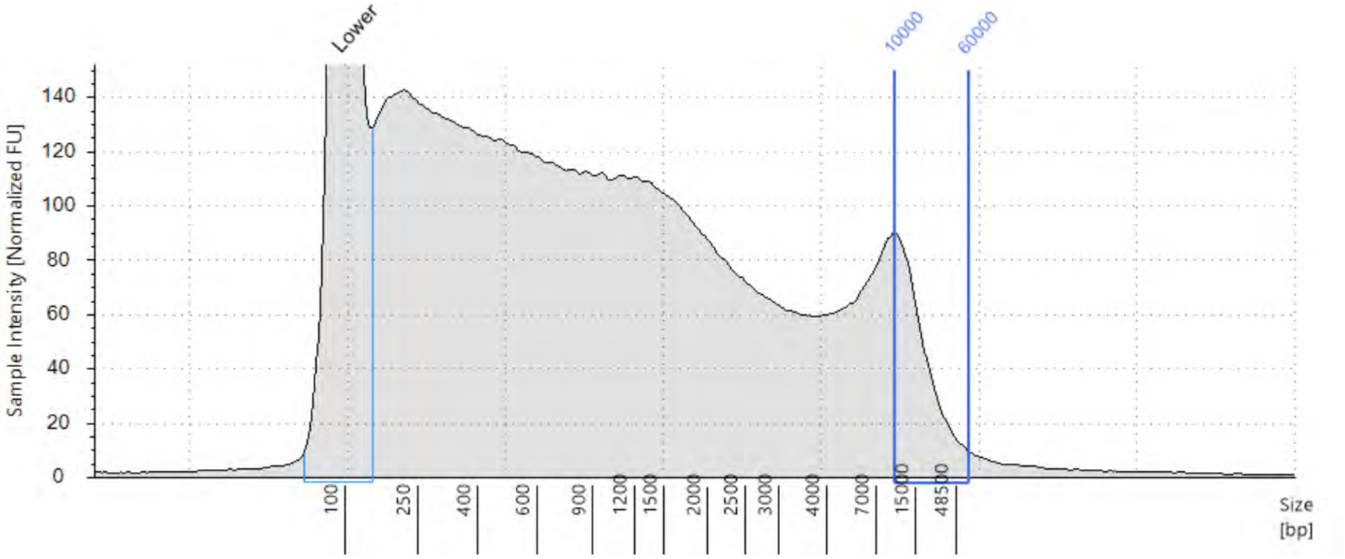

Region Table

From [bp]	To [bp]	Average Size [bp]	Conc. [ng/µl]	Region Molarity [nmol/l]	% of Total	Region Comment	Color
10000	60000	18620	1.83	0.192	5.63		

E2: W912

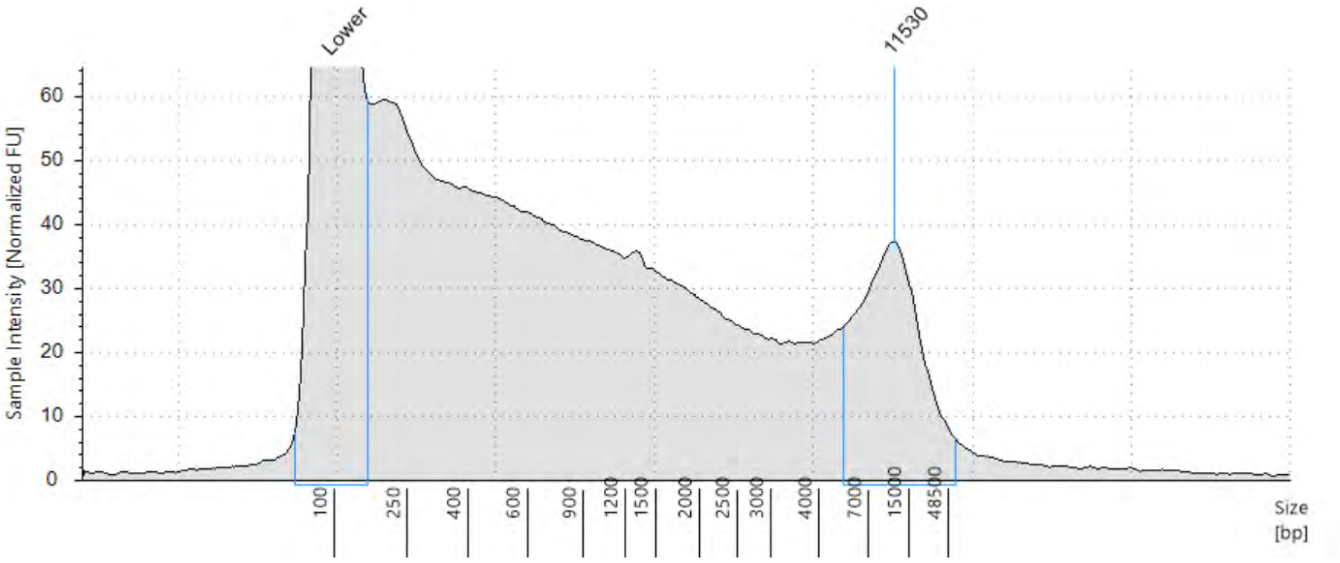

Sample Table

Well	DIN	Conc. [ng/µl]	Sample Description	Alert	Observations
E2	1.9	11.9	W912		

Peak Table

Size [bp]	Calibrated Conc. [ng/µl]	Assigned Conc. [ng/µl]	% Integrated Area	From [bp]	To [bp]	Peak Comment	Observations
100	8.50	8.50	-	61	151		Lower Marker
11530	1.61	-	100.00	5239	53766		

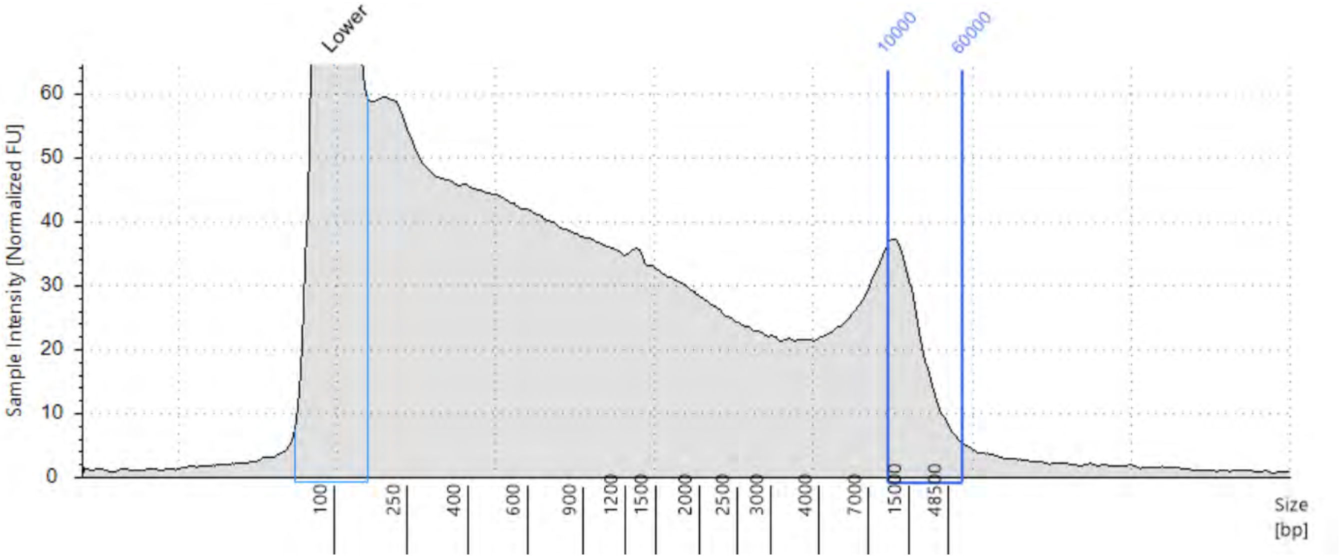

Region Table

From [bp]	To [bp]	Average Size [bp]	Conc. [ng/µl]	Region Molarity [nmol/l]	% of Total	Region Comment	Color
10000	60000	19272	0.891	0.0928	7.50		

F2: W913

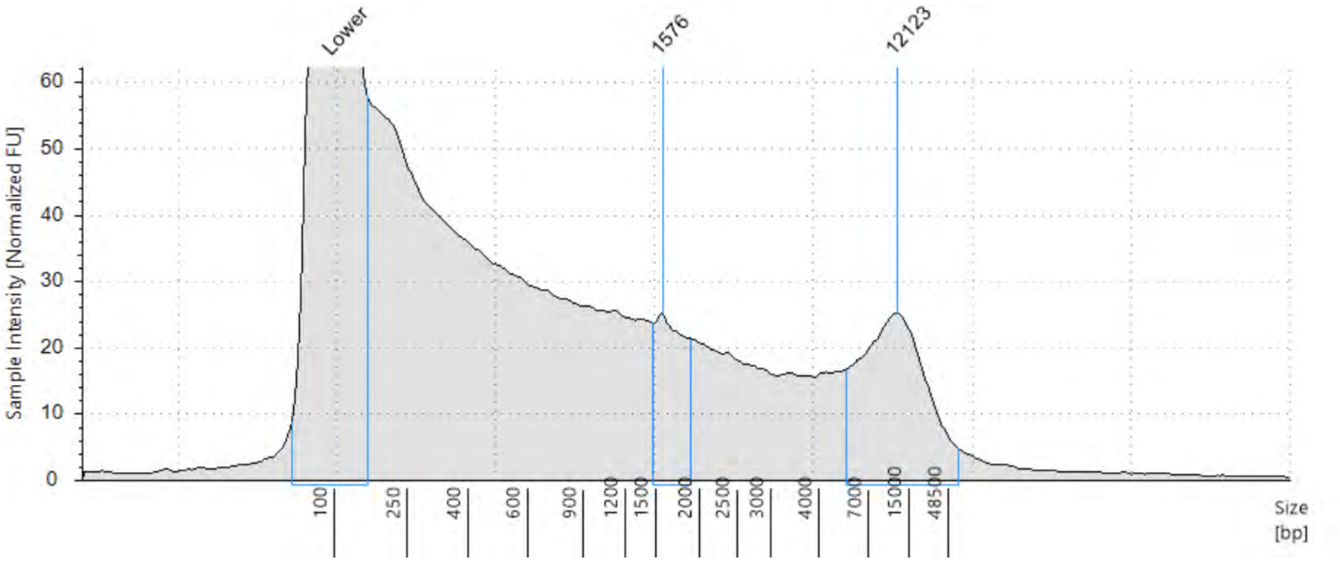

Sample Table

Well	DIN	Conc. [ng/µl]	Sample Description	Alert	Observations
F2	1.8	8.75	W913	⚠	Sample concentration outside recommended range

Peak Table

Size [bp]	Calibrated Conc. [ng/µl]	Assigned Conc. [ng/µl]	% Integrated Area	From [bp]	To [bp]	Peak Comment	Observations
100	8.50	8.50	-	58	152		Lower Marker
1576	0.493	-	31.38	1477	1888		
12123	1.08	-	68.62	5443	56577		

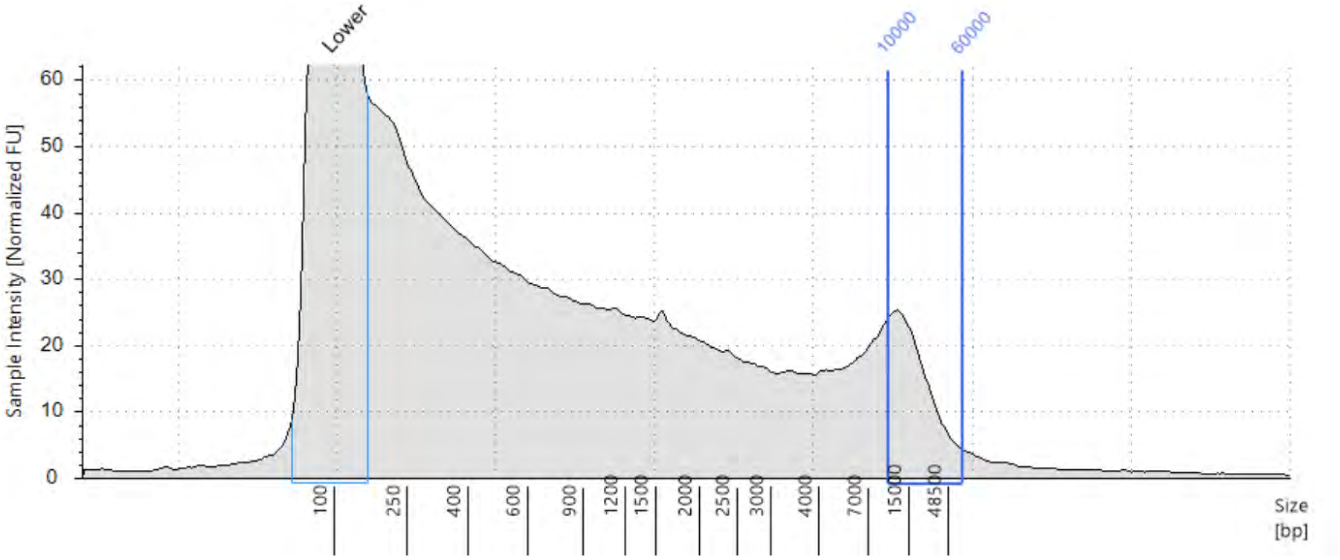

Region Table

From [bp]	To [bp]	Average Size [bp]	Conc. [ng/µl]	Region Molarity [nmol/l]	% of Total	Region Comment	Color
10000	60000	19759	0.644	0.0649	7.36		■

G2: W914

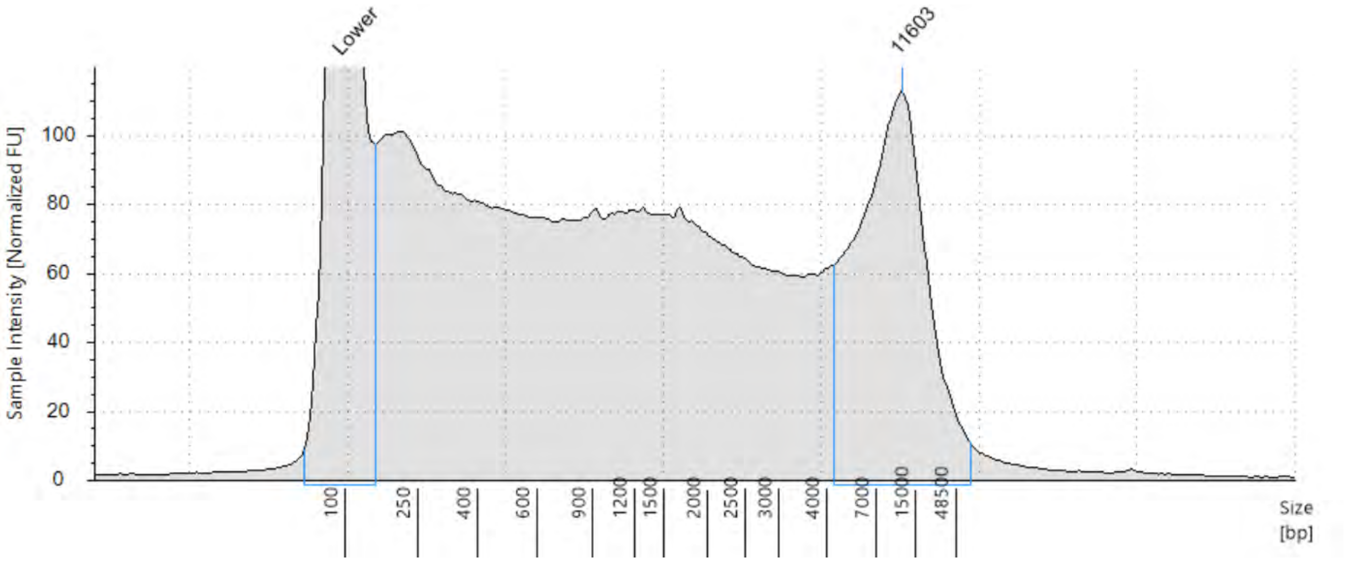

Sample Table

Well	DIN	Conc. [ng/µl]	Sample Description	Alert	Observations
G2	2.6	25.7	W914		

Peak Table

Size [bp]	Calibrated Conc. [ng/µl]	Assigned Conc. [ng/µl]	% Integrated Area	From [bp]	To [bp]	Peak Comment	Observations
100	8.50	8.50	-	60	147		Lower Marker
11603	5.34	-	100.00	4388	>60000		

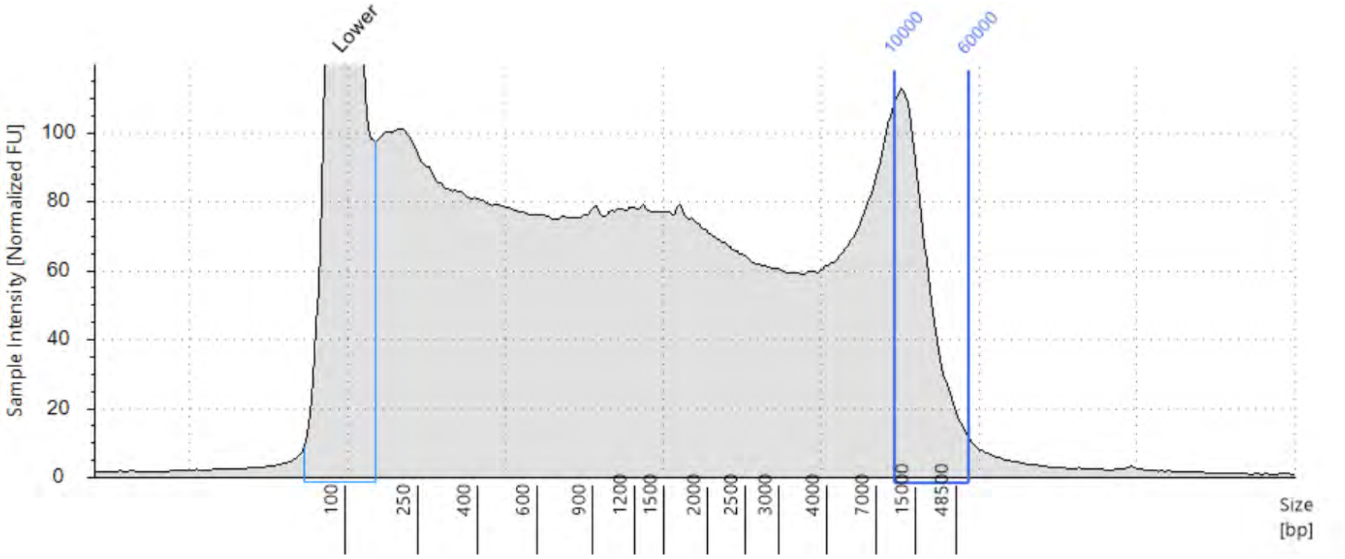

Region Table

From [bp]	To [bp]	Average Size [bp]	Conc. [ng/µl]	Region Molarity [nmol/l]	% of Total	Region Comment	Color
10000	60000	18584	2.56	0.263	9.93		

© Agilent Technologies, Inc. 2017

H2: W915

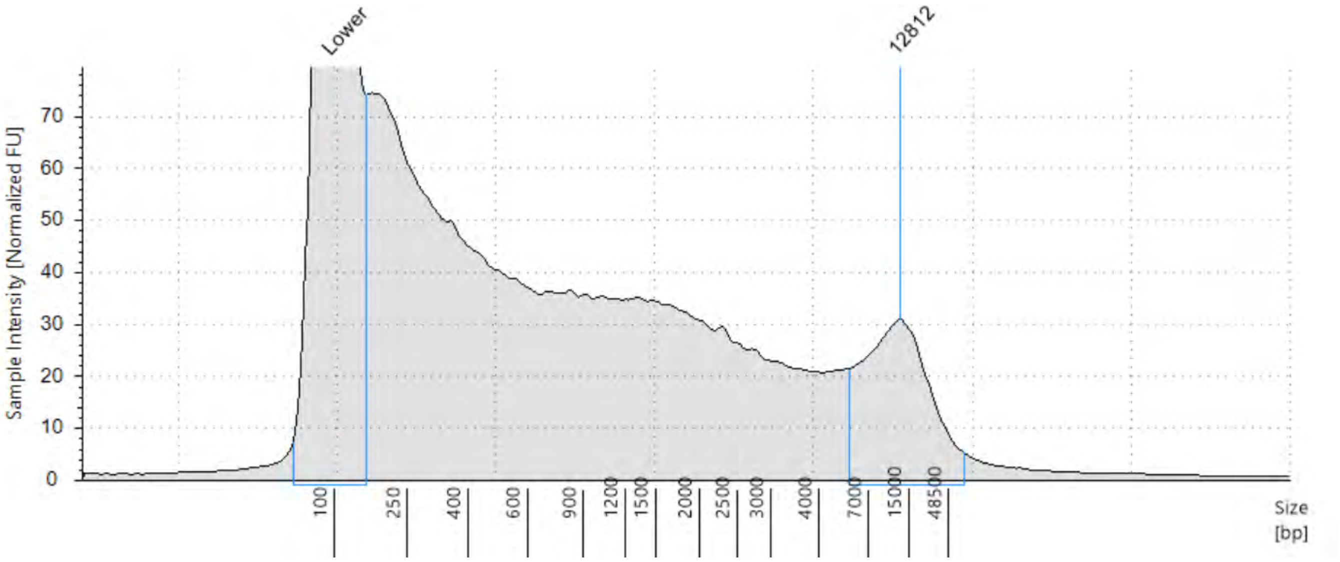

Sample Table

Well	DIN	Conc. [ng/µl]	Sample Description	Alert	Observations
H2	1.9	11.9	W915		

Peak Table

Size [bp]	Calibrated Conc. [ng/µl]	Assigned Conc. [ng/µl]	% Integrated Area	From [bp]	To [bp]	Peak Comment	Observations
100	8.50	8.50	-	60	149		Lower Marker
12812	1.37	-	100.00	5631	>60000		

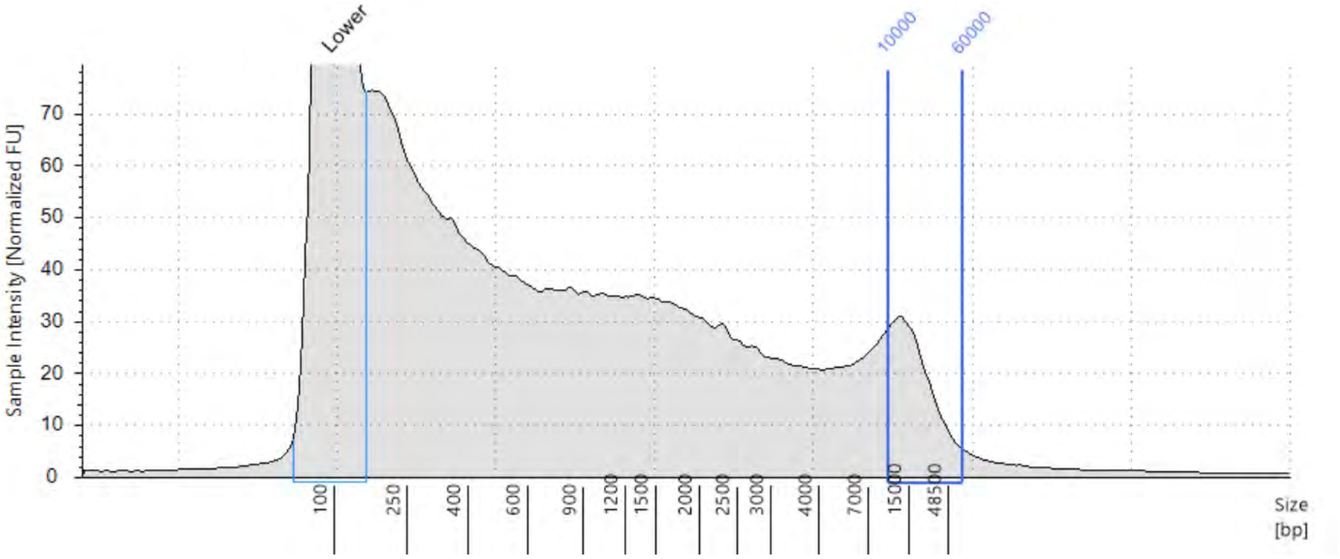

Region Table

From [bp]	To [bp]	Average Size [bp]	Conc. [ng/µl]	Region Molarity [nmol/l]	% of Total	Region Comment	Color
10000	60000	20029	0.844	0.0833	7.06		

A3: W916

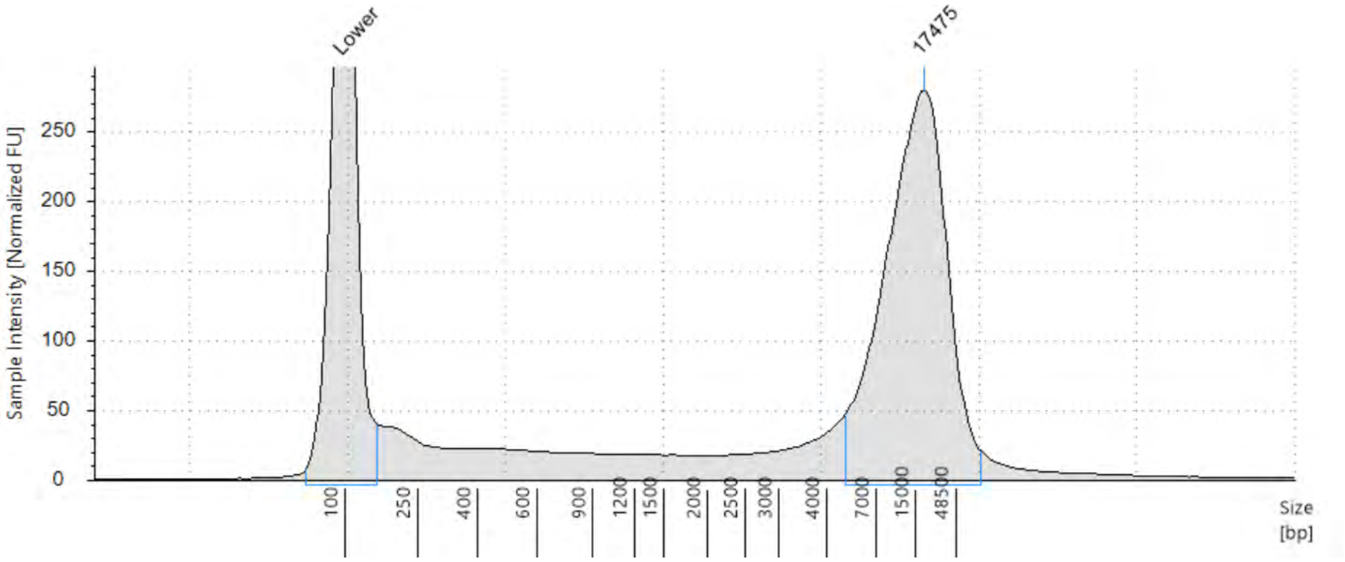

Sample Table

Well	DIN	Conc. [ng/µl]	Sample Description	Alert	Observations
A3	6.7	19.7	W916		

Peak Table

Size [bp]	Calibrated Conc. [ng/µl]	Assigned Conc. [ng/µl]	% Integrated Area	From [bp]	To [bp]	Peak Comment	Observations
100	8.50	8.50	-	60	150		Lower Marker
17475	12.5	-	98.48	4911	>60000		
-	-	-	-	-	-		Sample Well

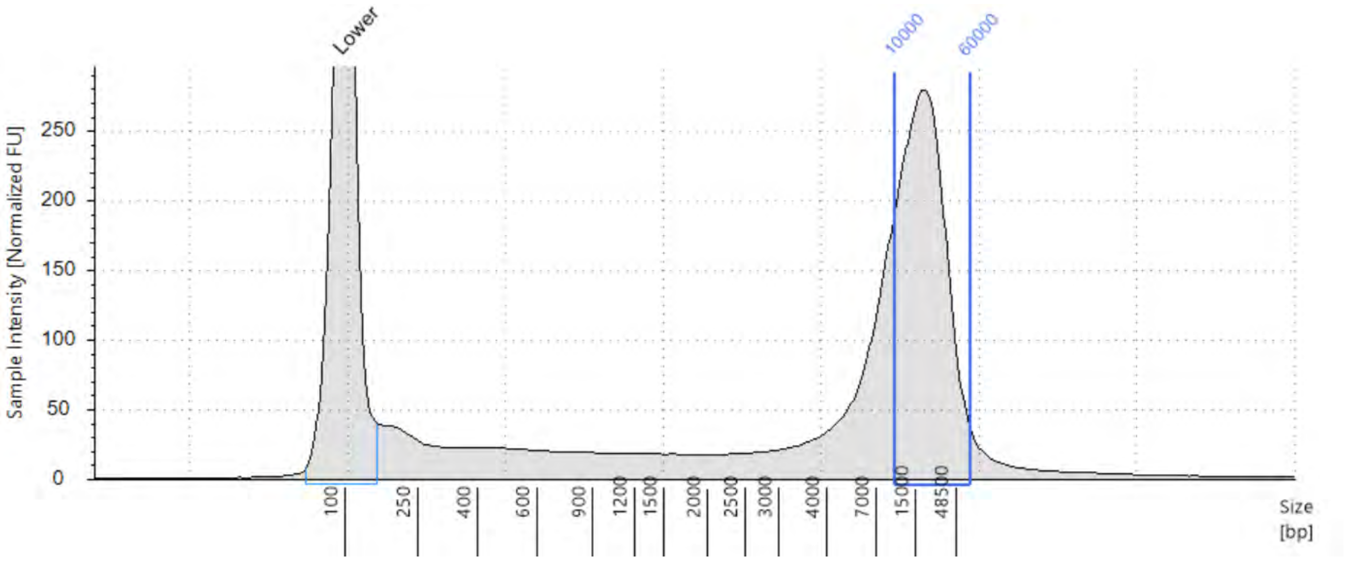

Region Table

From [bp]	To [bp]	Average Size [bp]	Conc. [ng/µl]	Region Molarity [nmol/l]	% of Total	Region Comment	Color
10000	60000	21067	9.09	0.802	46.07		

B3: W917

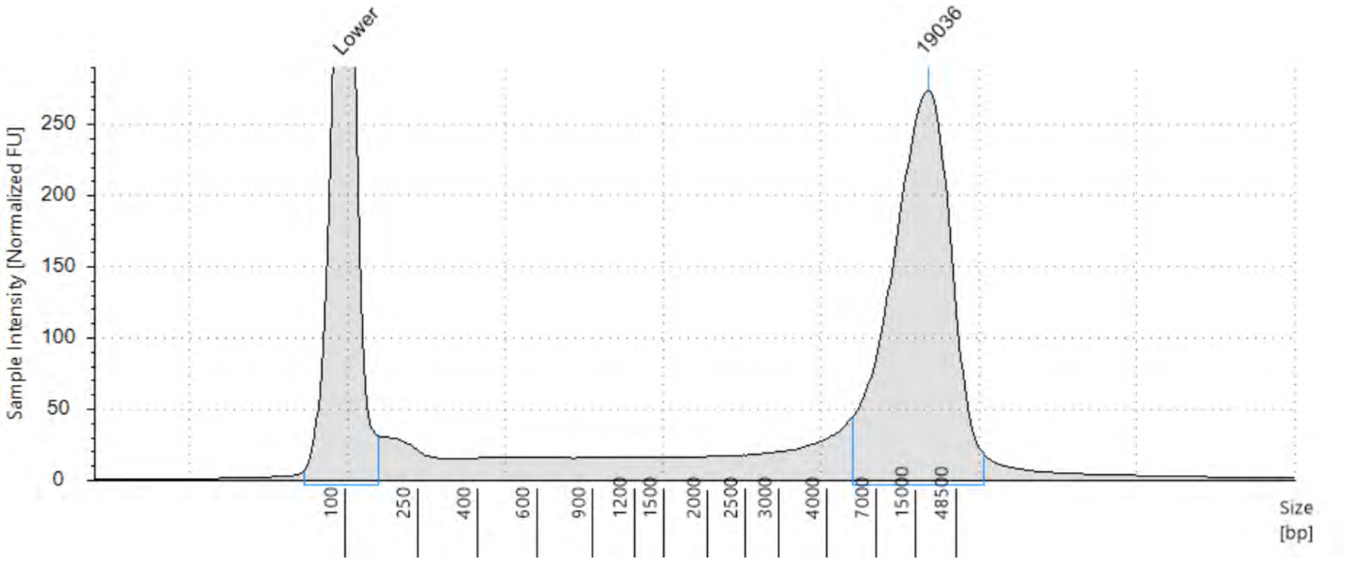

Sample Table

Well	DIN	Conc. [ng/µl]	Sample Description	Alert	Observations
B3	6.9	17.9	W917		

Peak Table

Size [bp]	Calibrated Conc. [ng/µl]	Assigned Conc. [ng/µl]	% Integrated Area	From [bp]	To [bp]	Peak Comment	Observations
100	8.50	8.50	-	60	151		Lower Marker
19036	11.7	-	99.00	5390	>60000		
-	-	-	-	-	-		Sample Well

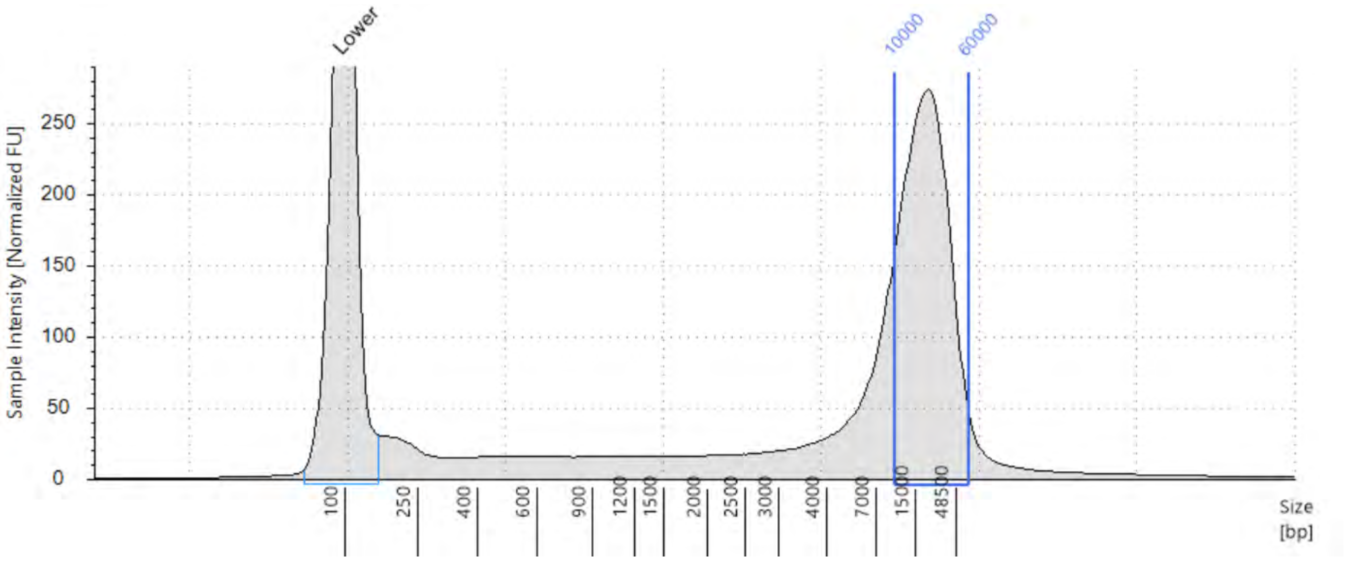

Region Table

From [bp]	To [bp]	Average Size [bp]	Conc. [ng/µl]	Region Molarity [nmol/l]	% of Total	Region Comment	Color
10000	60000	22289	9.26	0.783	51.63		

C3: W918

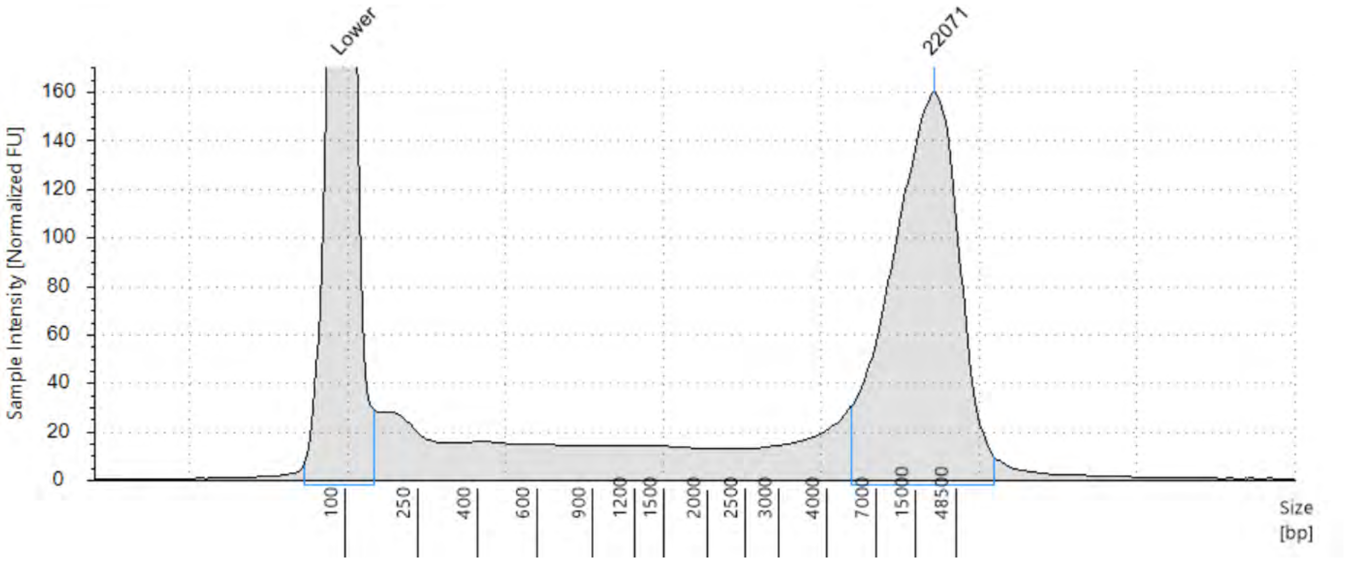

Sample Table

Well	DIN	Conc. [ng/µl]	Sample Description	Alert	Observations
C3	6.7	13.1	W918		

Peak Table

Size [bp]	Calibrated Conc. [ng/µl]	Assigned Conc. [ng/µl]	% Integrated Area	From [bp]	To [bp]	Peak Comment	Observations
100	8.50	8.50	-	61	148		Lower Marker
22071	8.09	-	99.67	5387	>60000		
-	-	-	-	-	-		Sample Well

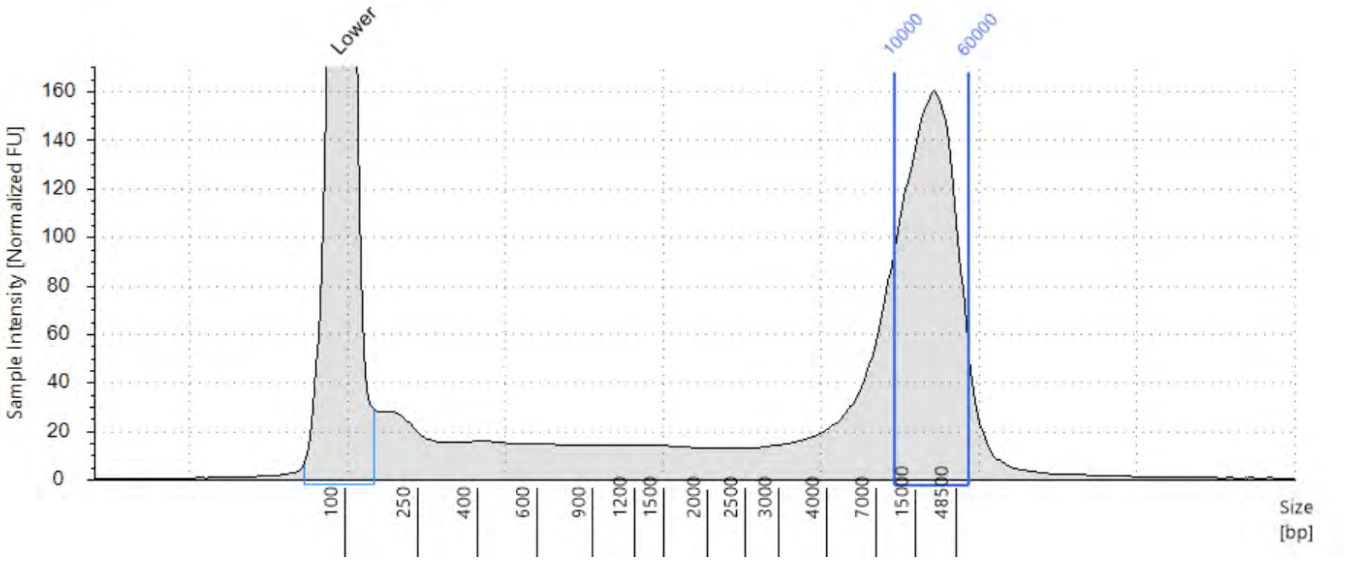

Region Table

From [bp]	To [bp]	Average Size [bp]	Conc. [ng/µl]	Region Molarity [nmol/l]	% of Total	Region Comment	Color
10000	60000	24923	6.23	0.493	47.47		

 © Agilent Technologies, Inc. 2017

D3: W919

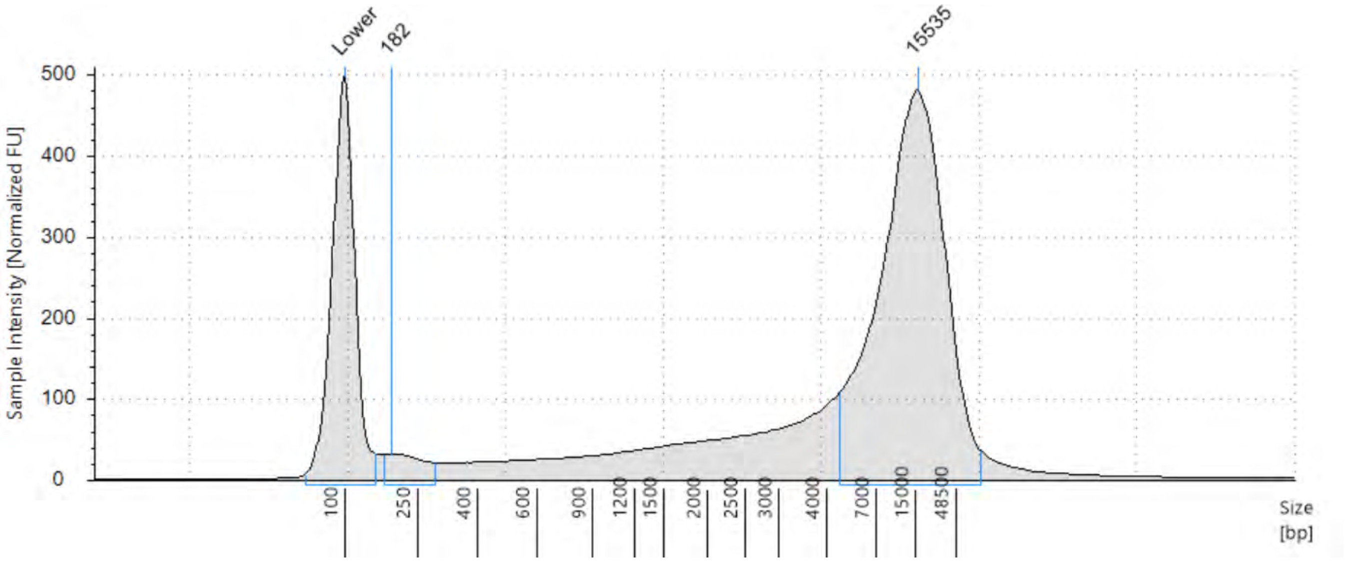

Sample Table

Well	DIN	Conc. [ng/µl]	Sample Description	Alert	Observations
D3	6.6	36.3	W919		

Peak Table

Size [bp]	Calibrated Conc. [ng/µl]	Assigned Conc. [ng/µl]	% Integrated Area	From [bp]	To [bp]	Peak Comment	Observations
100	8.50	8.50	-	61	148		Lower Marker
182	0.902	-	3.67	164	289		
15535	23.4	-	95.22	4660	>60000		
-	-	-	-	-	-		Sample Well

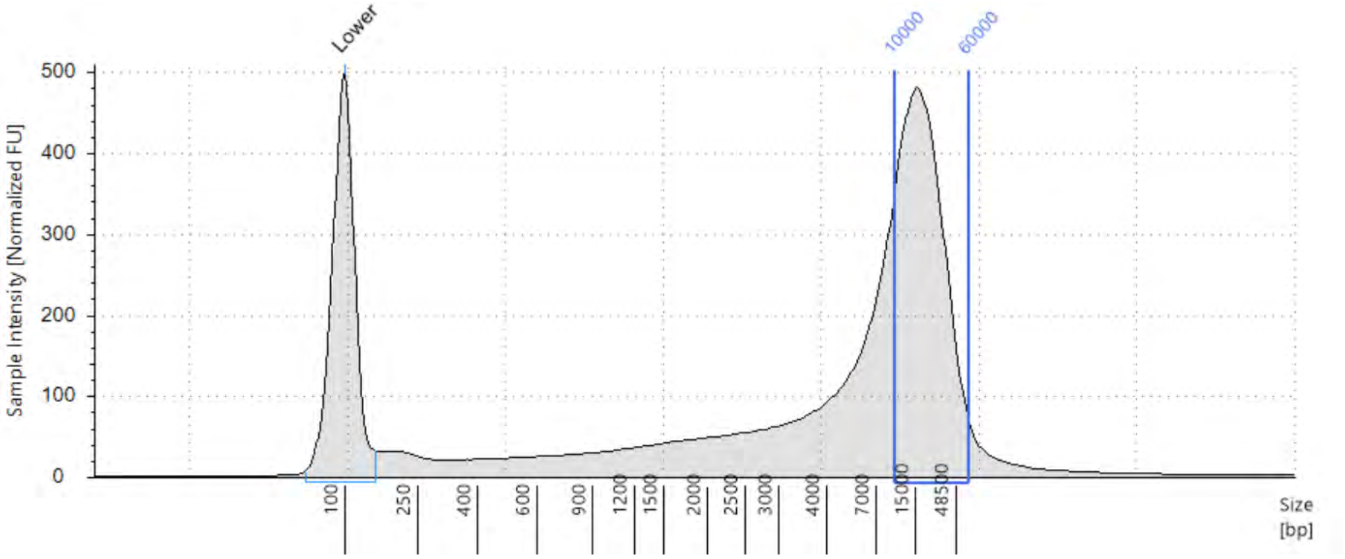

Region Table

From [bp]	To [bp]	Average Size [bp]	Conc. [ng/µl]	Region Molarity [nmol/l]	% of Total	Region Comment	Color
10000	60000	20860	16.1	1.45	44.42		

© Agilent Technologies, Inc. 2017

E3: W920

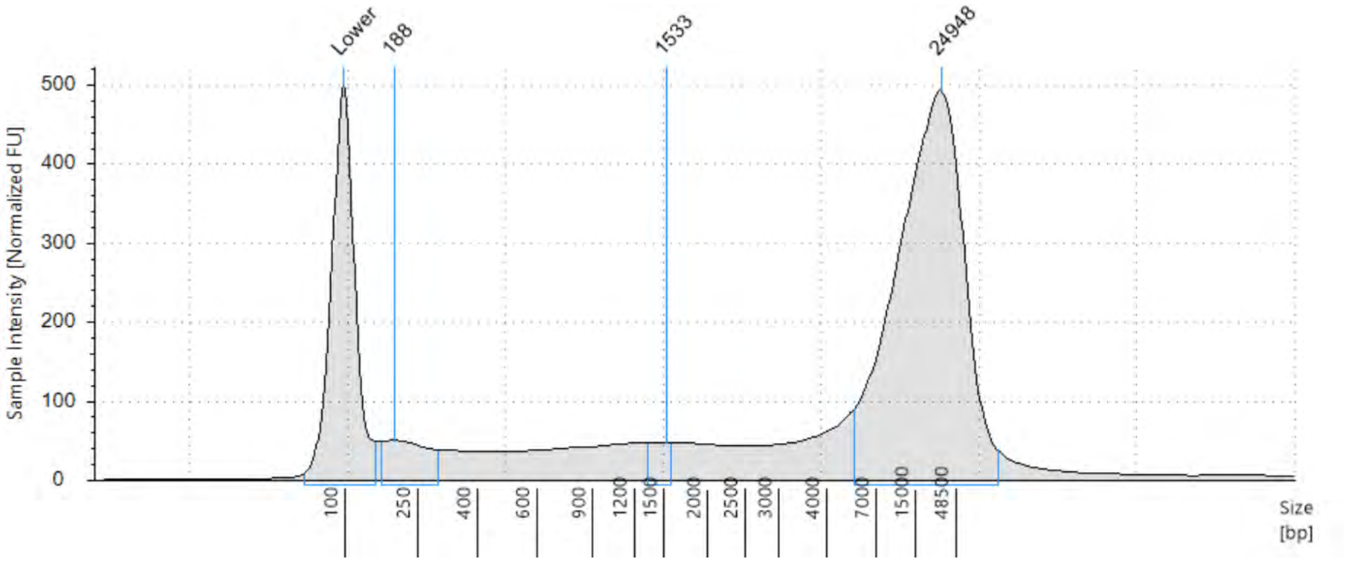

Sample Table

Well	DIN	Conc. [ng/µl]	Sample Description	Alert	Observations
E3	6.7	39.2	W920		

Peak Table

Size [bp]	Calibrated Conc. [ng/µl]	Assigned Conc. [ng/µl]	% Integrated Area	From [bp]	To [bp]	Peak Comment	Observations
100	8.50	8.50	-	61	148		Lower Marker
188	1.58	-	5.87	161	294		
1533	0.707	-	2.62	1336	1574		
24948	24.1	-	89.35	5537	>60000		
-	-	-	-	-	-		Sample Well

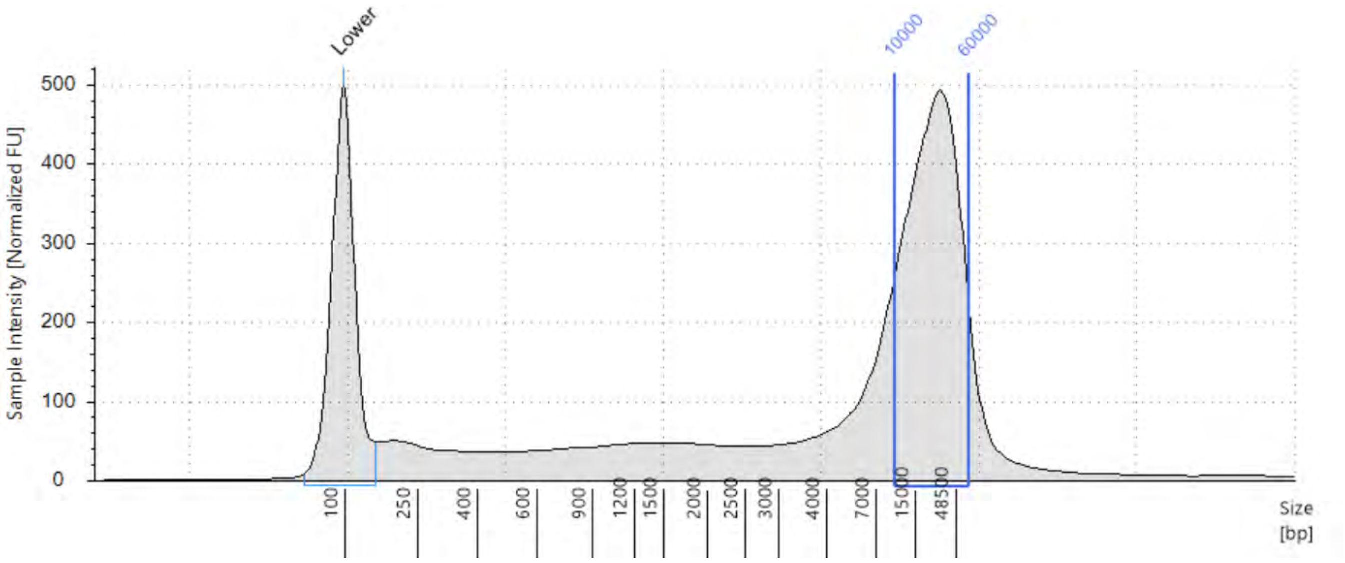

Region Table

From [bp]	To [bp]	Average Size [bp]	Conc. [ng/µl]	Region Molarity [nmol/l]	% of Total	Region Comment	Color
10000	60000	26183	18.6	1.42	47.44		

 © Agilent Technologies, Inc. 2017

F3: W921

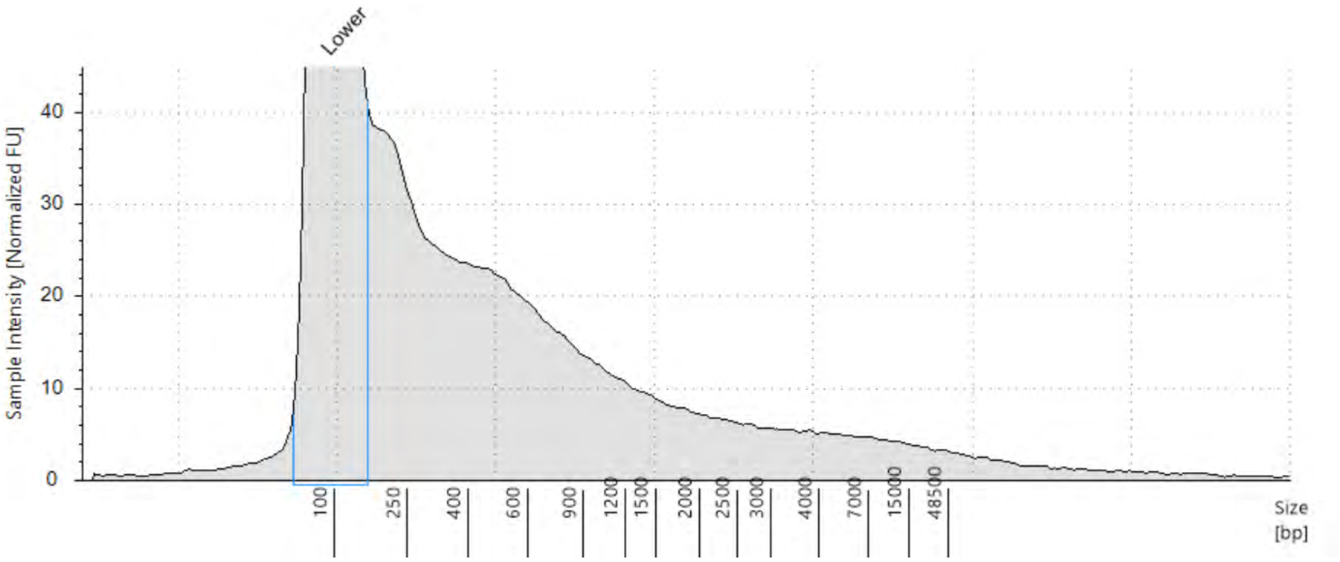

Sample Table

Well	DIN	Conc. [ng/µl]	Sample Description	Alert	Observations
F3	1.4	4.62	W921	⚠	Sample concentration outside functional range for DIN

Peak Table

Size [bp]	Calibrated Conc. [ng/µl]	Assigned Conc. [ng/µl]	% Integrated Area	From [bp]	To [bp]	Peak Comment	Observations
100	8.50	8.50	-	60	153		Lower Marker

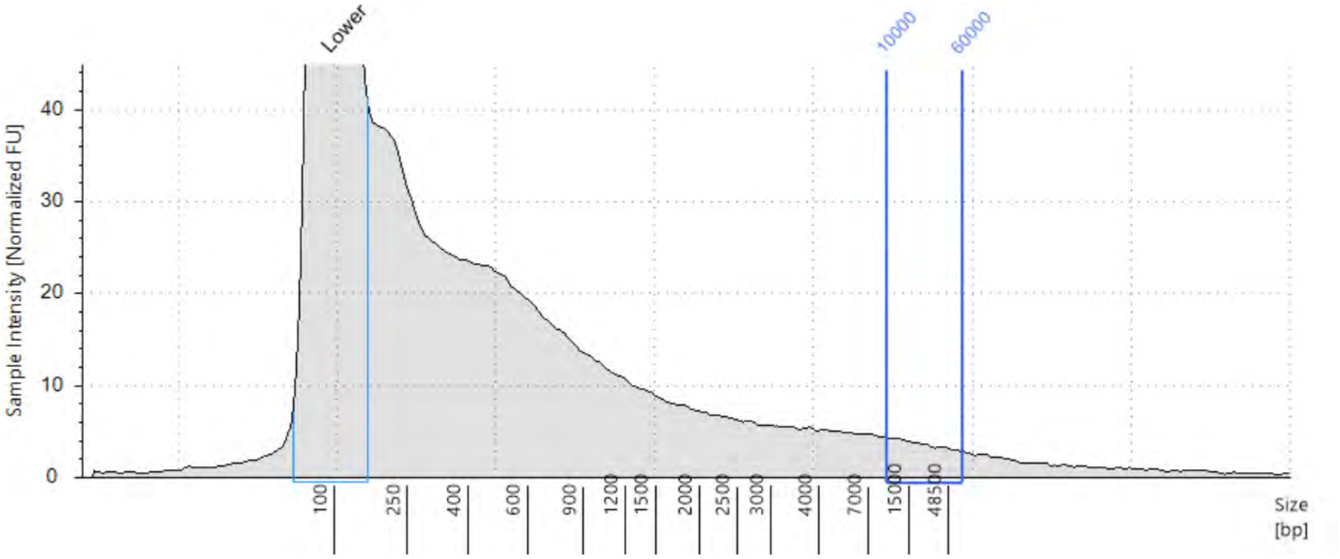

Region Table

From [bp]	To [bp]	Average Size [bp]	Conc. [ng/µl]	Region Molarity [nmol/l]	% of Total	Region Comment	Color
10000	60000	25257	0.139	0.0131	3.02		■

© Agilent Technologies, Inc. 2017

G3: W922

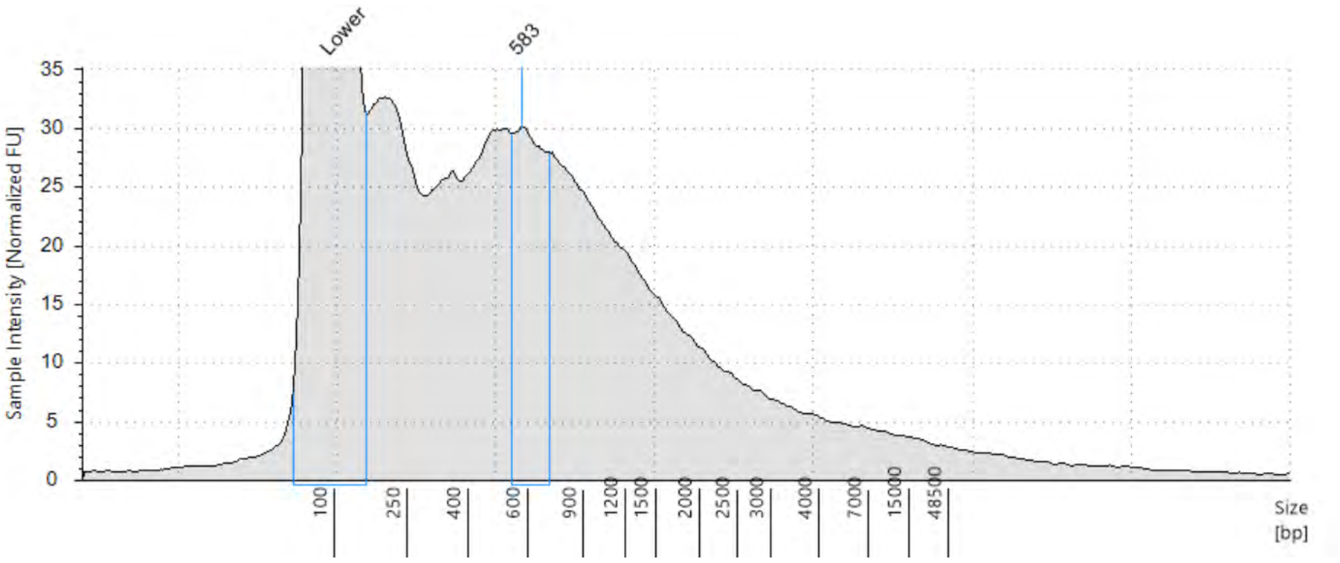

Sample Table

Well	DIN	Conc. [ng/µl]	Sample Description	Alert	Observations
G3	1.7	6.13	W922	⚠	Sample concentration outside recommended range

Peak Table

Size [bp]	Calibrated Conc. [ng/µl]	Assigned Conc. [ng/µl]	% Integrated Area	From [bp]	To [bp]	Peak Comment	Observations
100	8.50	8.50	-	61	151		Lower Marker
583	0.684	-	100.00	546	708		

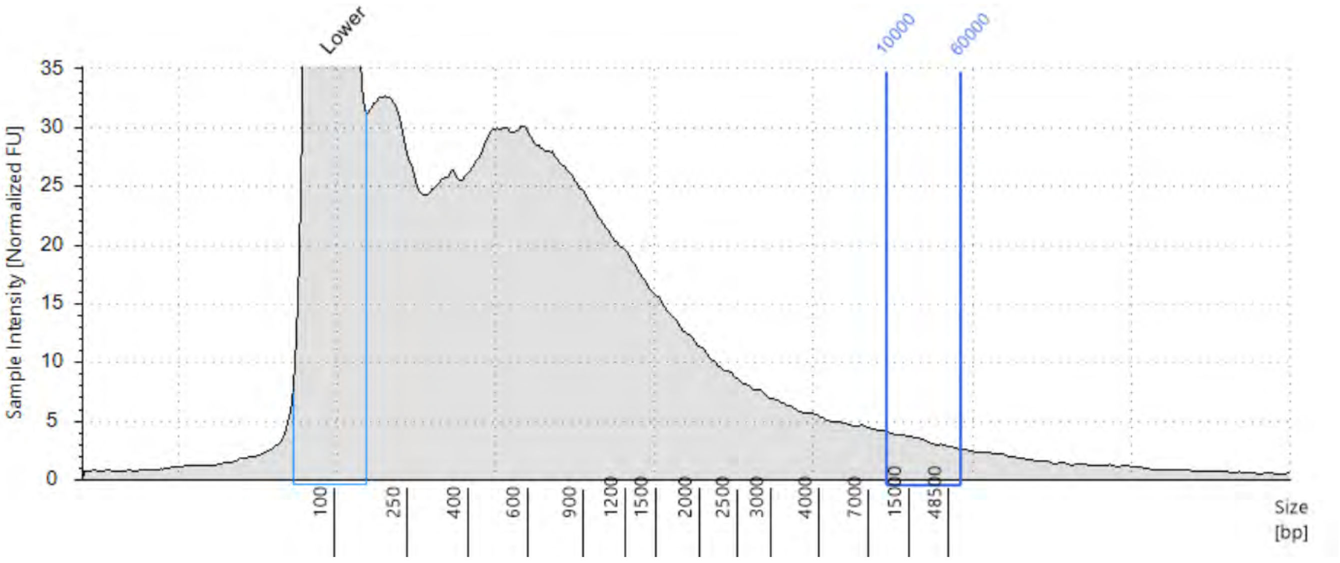

Region Table

From [bp]	To [bp]	Average Size [bp]	Conc. [ng/µl]	Region Molarity [nmol/l]	% of Total	Region Comment	Color
10000	60000	25001	0.139	0.0130	2.26		■

H3: W923

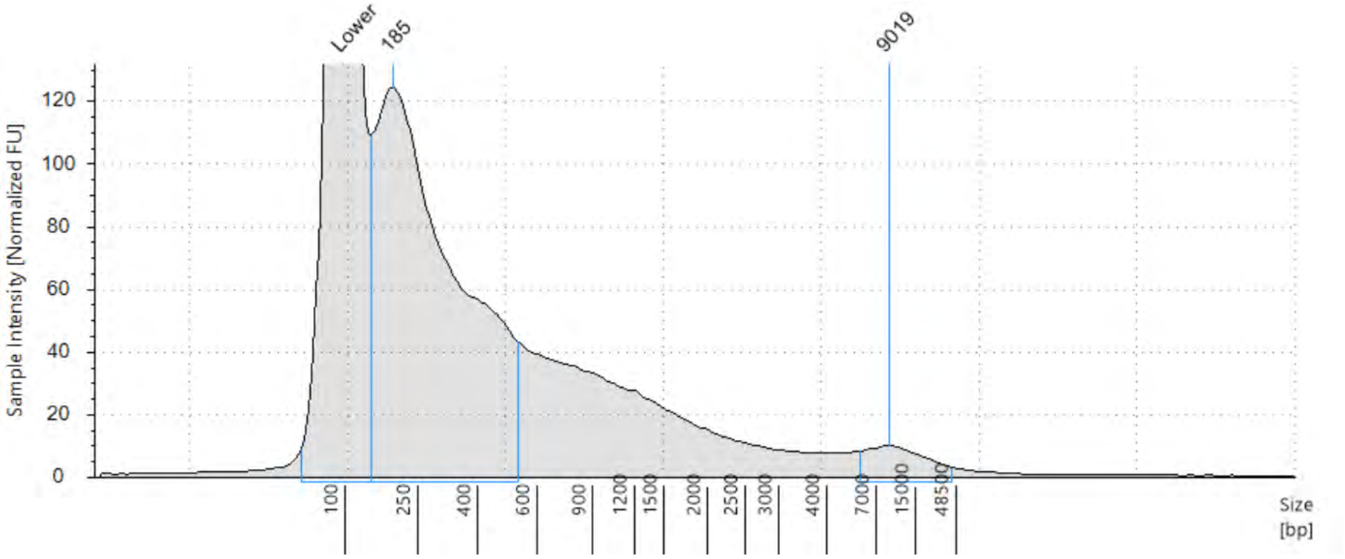

Sample Table

Well	DIN	Conc. [ng/µl]	Sample Description	Alert	Observations
H3	1.4	11.2	W923		

Peak Table

Size [bp]	Calibrated Conc. [ng/µl]	Assigned Conc. [ng/µl]	% Integrated Area	From [bp]	To [bp]	Peak Comment	Observations
100	8.50	8.50	-	59	141		Lower Marker
185	6.85	-	94.83	141	534		
9019	0.373	-	5.17	5888	37696		

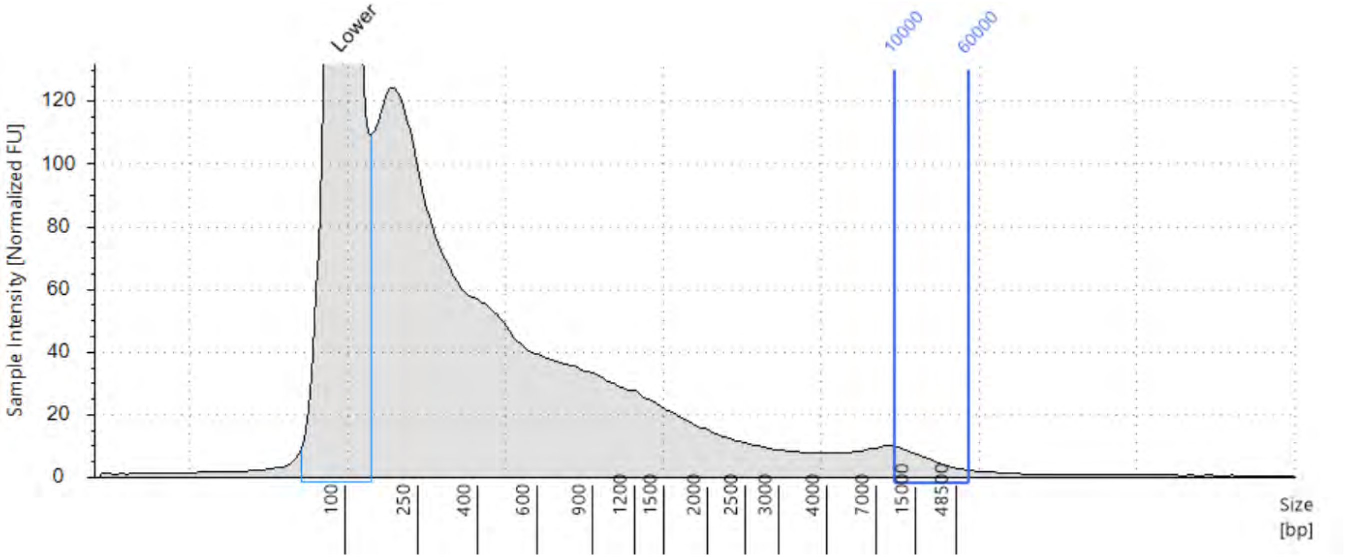

Region Table

From [bp]	To [bp]	Average Size [bp]	Conc. [ng/µl]	Region Molarity [nmol/l]	% of Total	Region Comment	Color
10000	60000	20514	0.222	0.0235	1.97		

 © Agilent Technologies, Inc. 2017

A4: W924

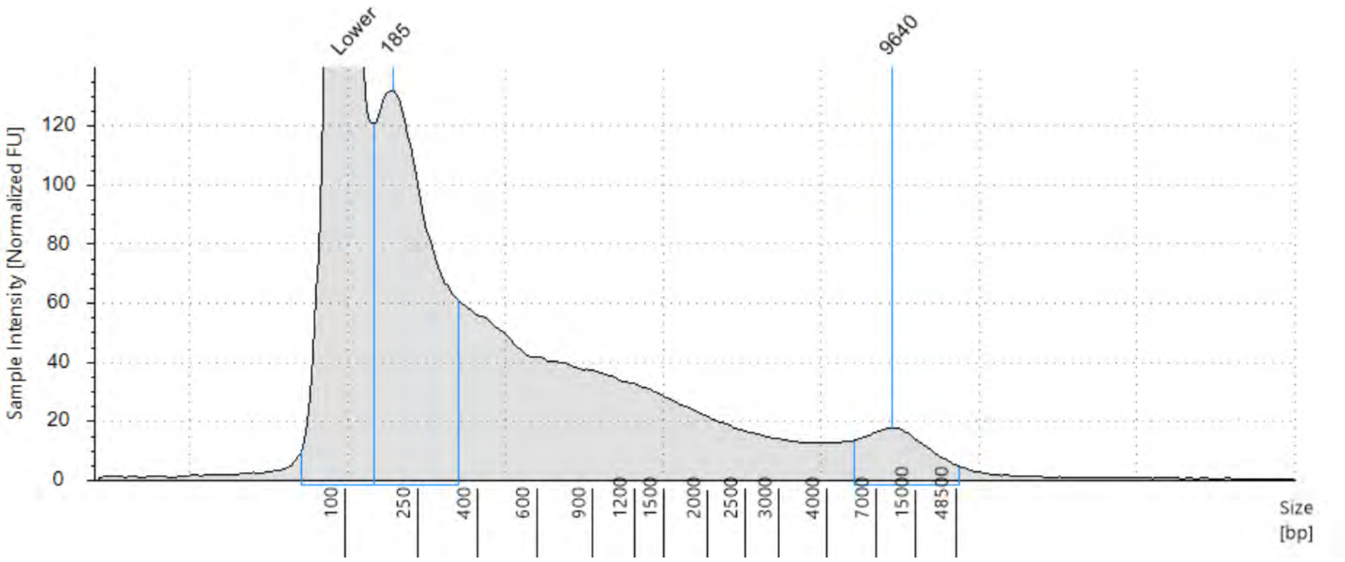

Sample Table

Well	DIN	Conc. [ng/µl]	Sample Description	Alert	Observations
A4	1.5	11.9	W924		

Peak Table

Size [bp]	Calibrated Conc. [ng/µl]	Assigned Conc. [ng/µl]	% Integrated Area	From [bp]	To [bp]	Peak Comment	Observations
100	8.50	8.50	-	59	146		Lower Marker
185	4.76	-	86.77	146	348		
9640	0.725	-	13.23	5502	52639		

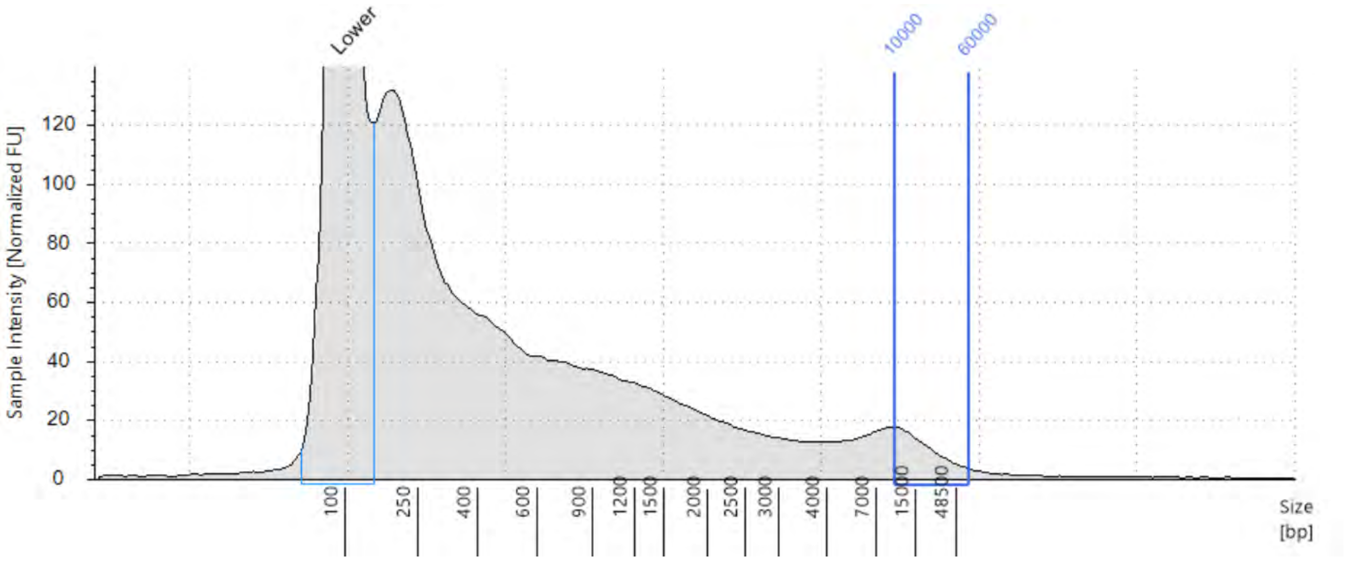

Region Table

From [bp]	To [bp]	Average Size [bp]	Conc. [ng/µl]	Region Molarity [nmol/l]	% of Total	Region Comment	Color
10000	60000	20654	0.407	0.0408	3.41		

© Agilent Technologies, Inc. 2017

B4: W925

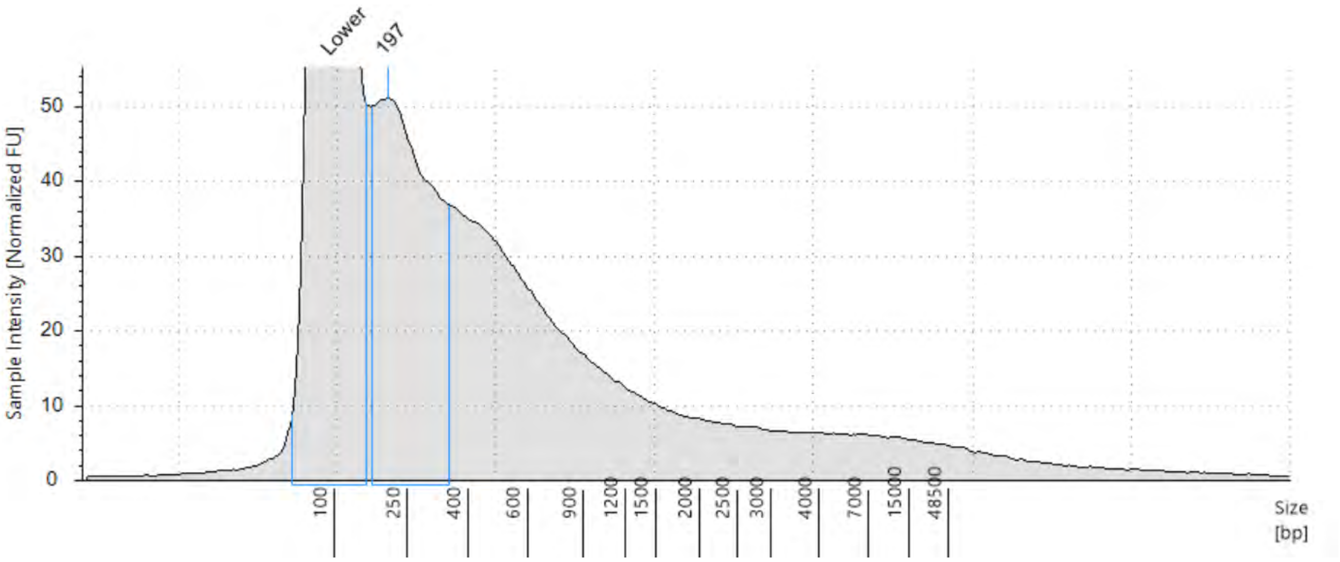

Sample Table

Well	DIN	Conc. [ng/µl]	Sample Description	Alert	Observations
B4	1.3	6.34	W925	⚠	Sample concentration outside recommended range

Peak Table

Size [bp]	Calibrated Conc. [ng/µl]	Assigned Conc. [ng/µl]	% Integrated Area	From [bp]	To [bp]	Peak Comment	Observations
100	8.50	8.50	-	60	150		Lower Marker
197	2.04	-	100.00	164	347		

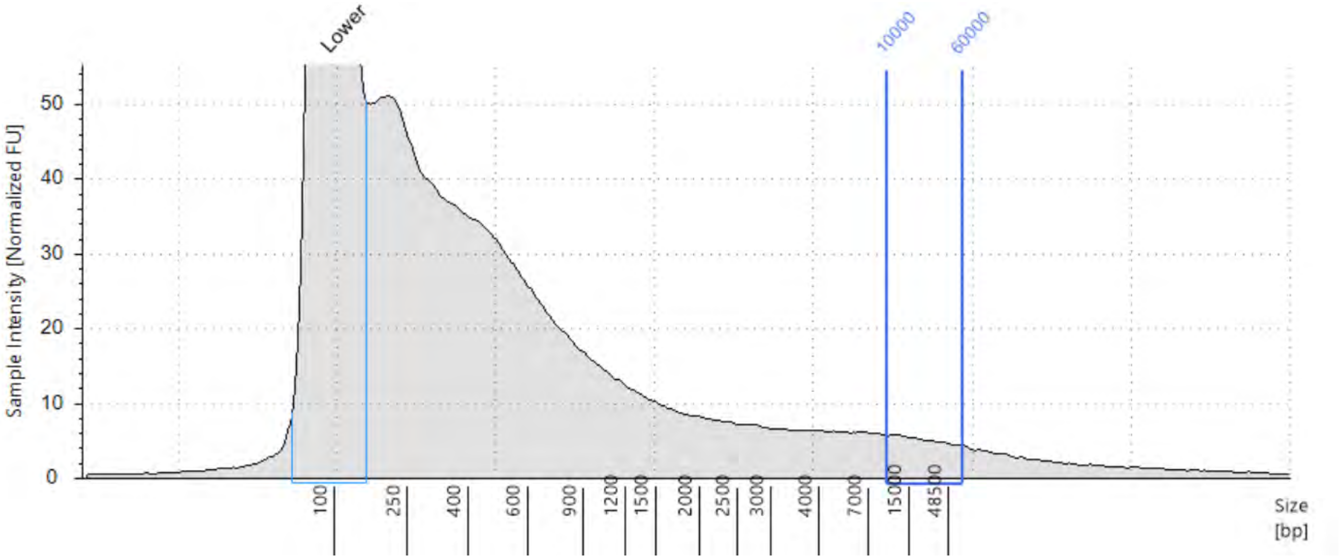

Region Table

From [bp]	To [bp]	Average Size [bp]	Conc. [ng/µl]	Region Molarity [nmol/l]	% of Total	Region Comment	Color
10000	60000	25785	0.205	0.0185	3.23		■

C4: W926

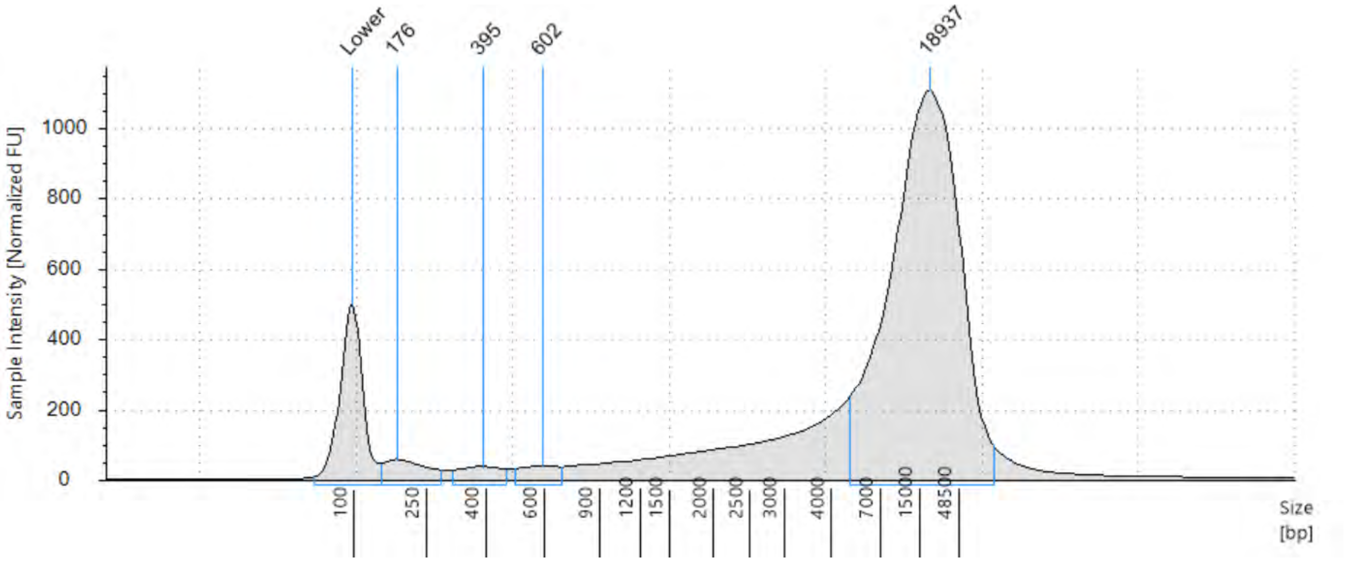

Sample Table

Well	DIN	Conc. [ng/µl]	Sample Description	Alert	Observations
C4	7.0	83.6	W926		

Peak Table

Size [bp]	Calibrated Conc. [ng/µl]	Assigned Conc. [ng/µl]	% Integrated Area	From [bp]	To [bp]	Peak Comment	Observations
100	8.50	8.50	-	61	143		Lower Marker
176	1.72	-	2.69	143	285		
395	1.13	-	1.76	313	468		
602	1.09	-	1.70	495	690		
18937	59.1	-	92.13	5018	>60000		
-	-	-	-	-	-		Sample Well

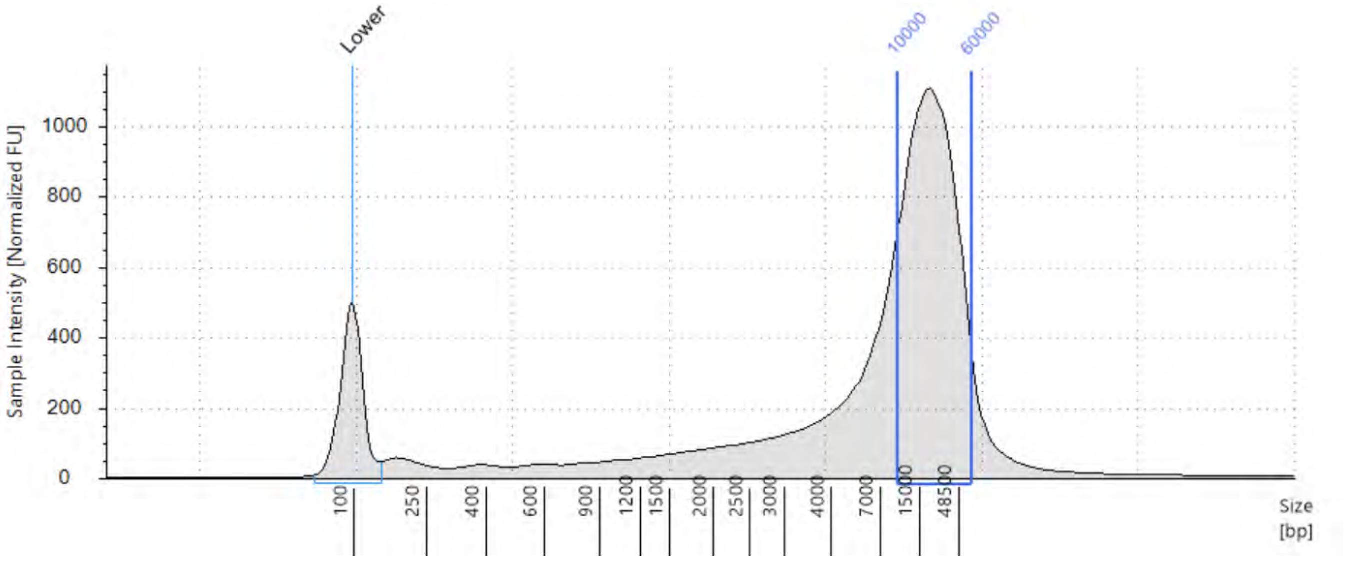

Region Table

From [bp]	To [bp]	Average Size [bp]	Conc. [ng/µl]	Region Molarity [nmol/l]	% of Total	Region Comment	Color
10000	60000	24231	43.9	3.55	52.47		

 © Agilent Technologies, Inc. 2017

D4: W927

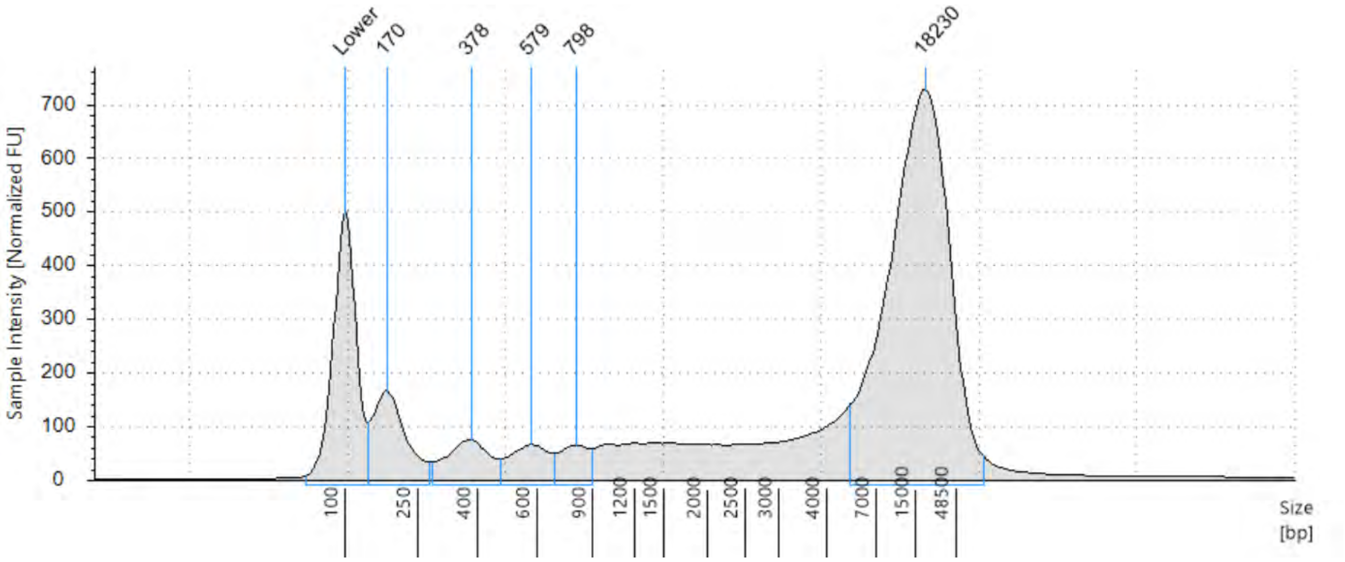

Sample Table

Well	DIN	Conc. [ng/µl]	Sample Description	Alert	Observations
D4	6.5	55.5	W927		

Peak Table

Size [bp]	Calibrated Conc. [ng/µl]	Assigned Conc. [ng/µl]	% Integrated Area	From [bp]	To [bp]	Peak Comment	Observations
100	8.50	8.50	-	61	134		Lower Marker
170	3.93	-	9.15	134	273		
378	2.29	-	5.33	279	467		
579	1.84	-	4.27	467	682		
798	1.41	-	3.29	682	897		
18230	32.8	-	76.22	5222	>60000		
-	-	-	-	-	-		Sample Well

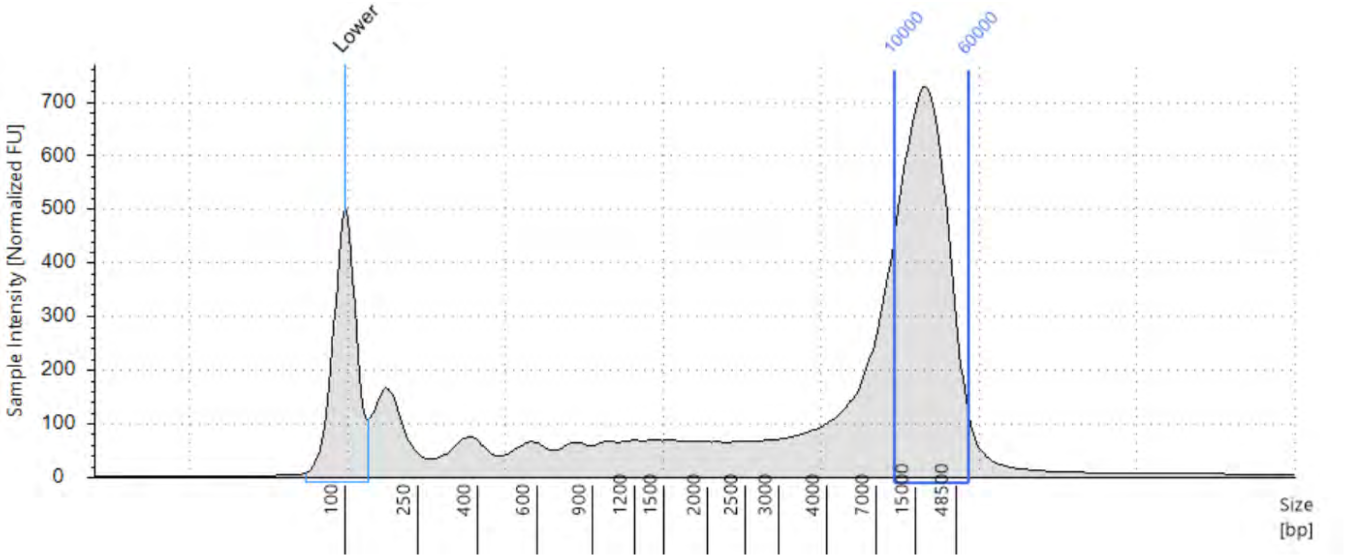

Region Table

From [bp]	To [bp]	Average Size [bp]	Conc. [ng/µl]	Region Molarity [nmol/l]	% of Total	Region Comment	Color
10000	60000	21879	25.2	2.17	45.42		

E4: W928

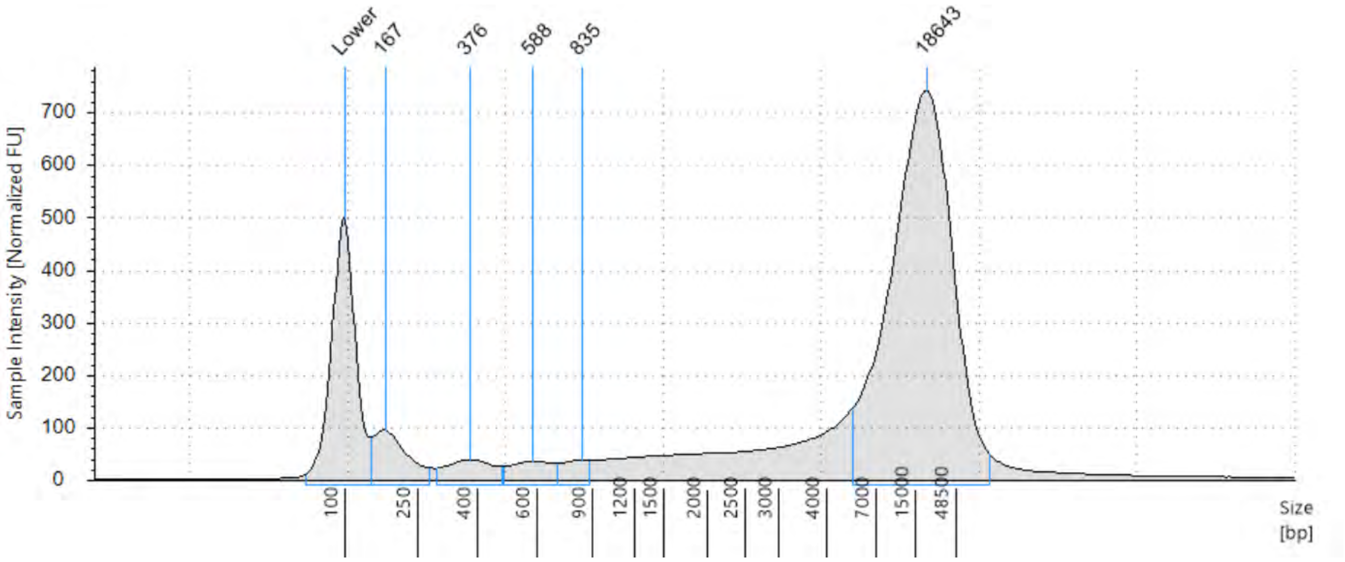

Sample Table

Well	DIN	Conc. [ng/µl]	Sample Description	Alert	Observations
E4	6.9	49.2	W928		

Peak Table

Size [bp]	Calibrated Conc. [ng/µl]	Assigned Conc. [ng/µl]	% Integrated Area	From [bp]	To [bp]	Peak Comment	Observations
100	8.50	8.50	-	61	141		Lower Marker
167	2.20	-	5.71	141	277		
376	1.21	-	3.15	292	474		
588	1.04	-	2.69	478	700		
835	0.672	-	1.75	700	877		
18643	32.7	-	84.99	5354	>60000		
-	-	-	-	-	-		Sample Well

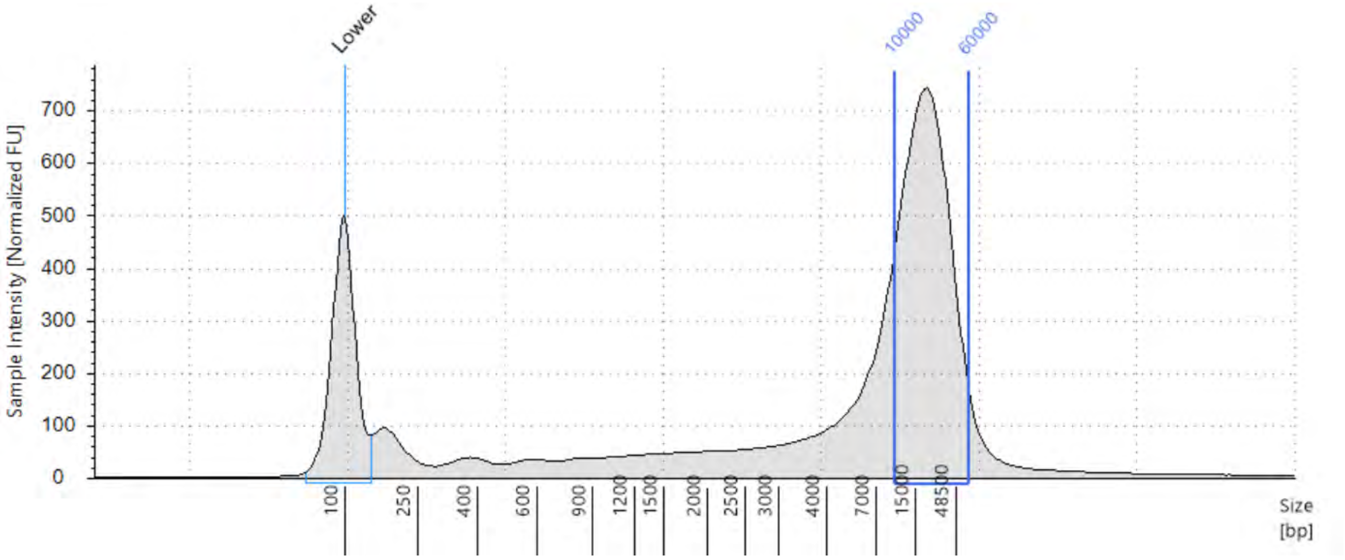

Region Table

From [bp]	To [bp]	Average Size [bp]	Conc. [ng/µl]	Region Molarity [nmol/l]	% of Total	Region Comment	Color
10000	60000	22837	25.2	2.10	51.13		

 © Agilent Technologies, Inc. 2017

F4: W929

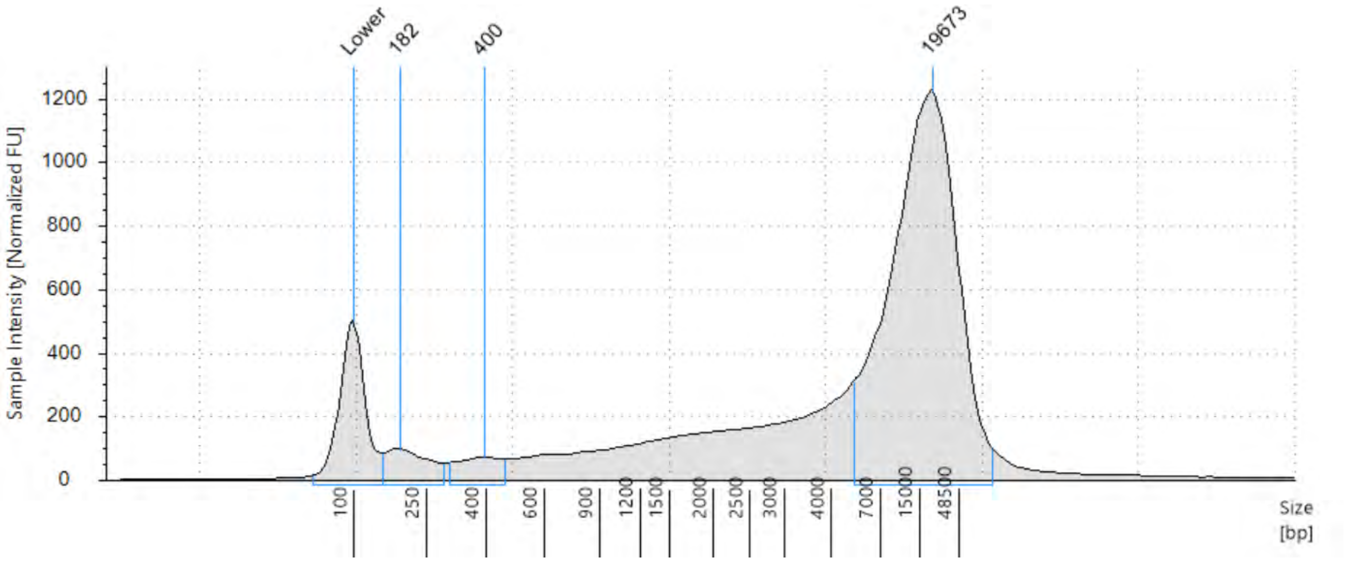

Sample Table

Well	DIN	Conc. [ng/µl]	Sample Description	Alert	Observations
F4	6.6	90.9	W929		

Peak Table

Size [bp]	Calibrated Conc. [ng/µl]	Assigned Conc. [ng/µl]	% Integrated Area	From [bp]	To [bp]	Peak Comment	Observations
100	8.50	8.50	-	60	146		Lower Marker
182	2.75	-	4.49	146	291		
400	2.02	-	3.30	304	460		
19673	55.2	-	90.21	5207	>60000		
-	-	-	-	-	-		Sample Well

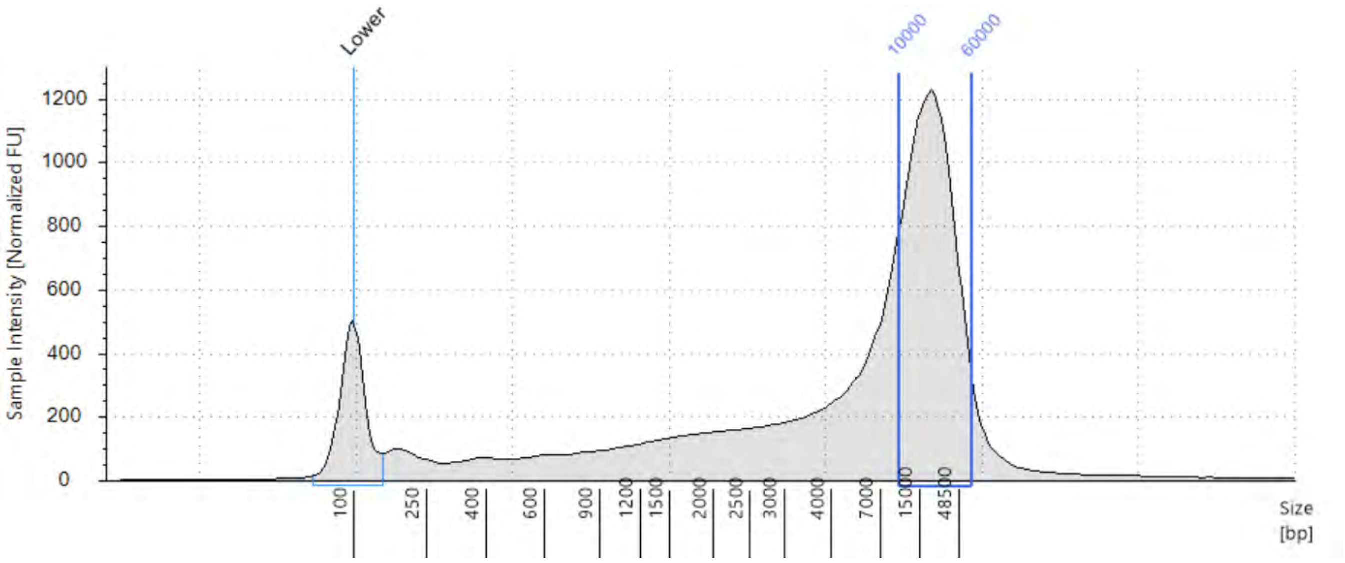

Region Table

From [bp]	To [bp]	Average Size [bp]	Conc. [ng/µl]	Region Molarity [nmol/l]	% of Total	Region Comment	Color
10000	60000	23203	41.0	3.41	45.07		

© Agilent Technologies, Inc. 2017

G4: W930

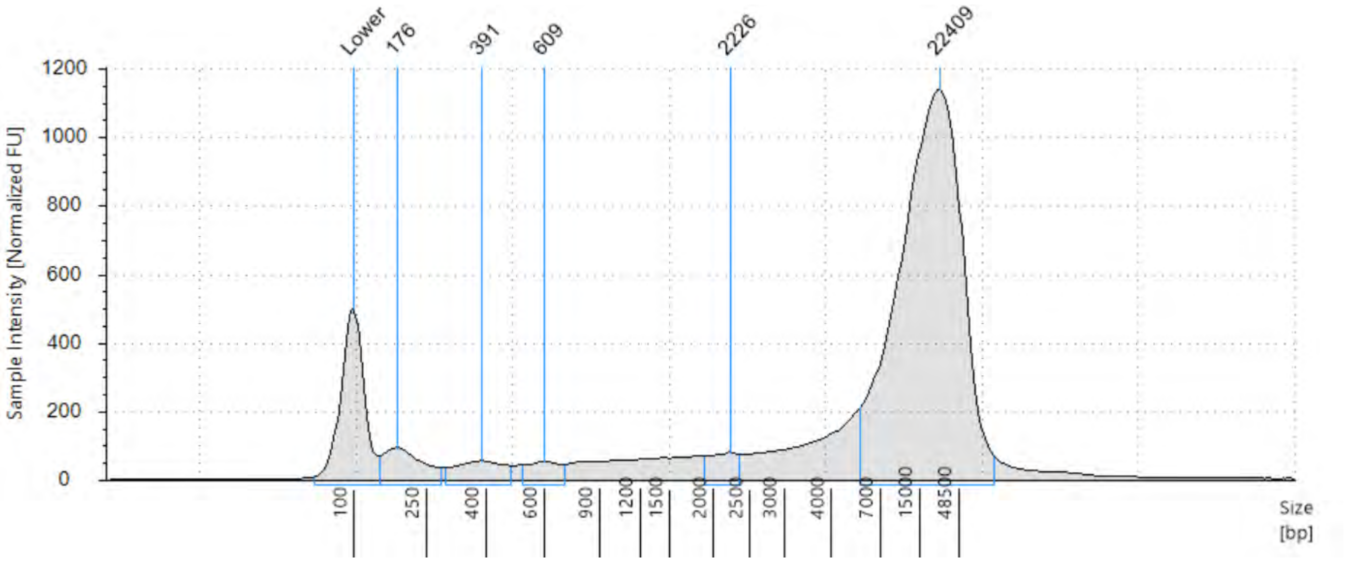

Sample Table

Well	DIN	Conc. [ng/µl]	Sample Description	Alert	Observations
G4	7.0	76.0	W930		

Peak Table

Size [bp]	Calibrated Conc. [ng/µl]	Assigned Conc. [ng/µl]	% Integrated Area	From [bp]	To [bp]	Peak Comment	Observations
100	8.50	8.50	-	61	141		Lower Marker
176	2.53	-	4.14	141	282		
391	1.87	-	3.06	294	482		
609	1.28	-	2.09	519	705		
2226	1.65	-	2.69	1898	2357		
22409	52.9	-	86.24	5623	>60000		
-	-	-	-	-	-		Sample Well

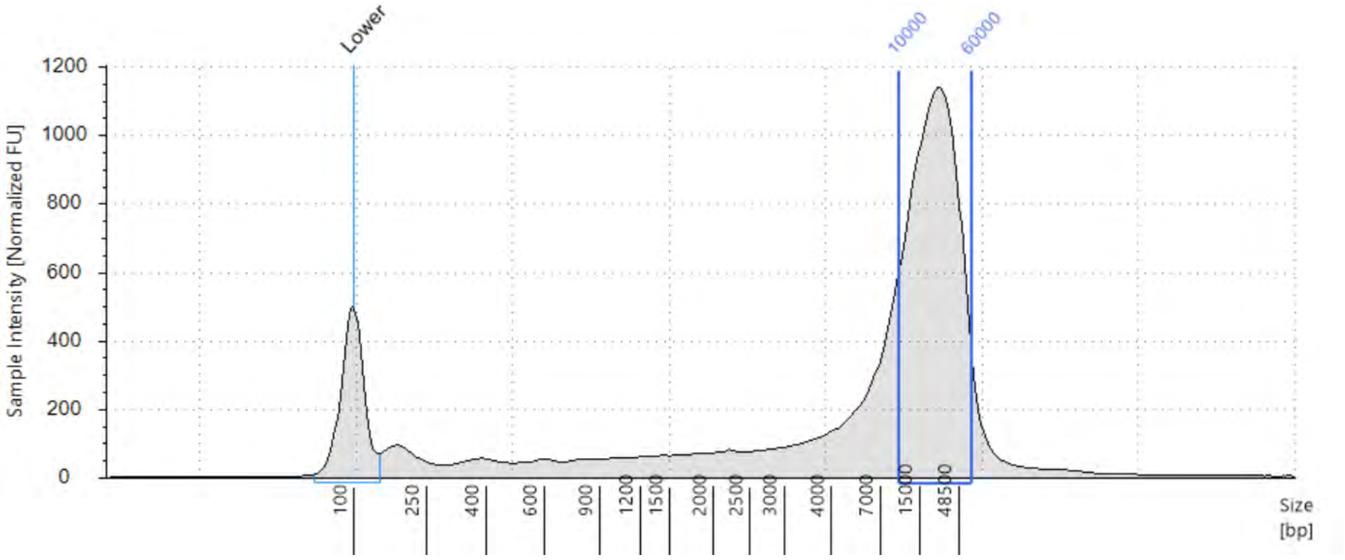

Region Table

From [bp]	To [bp]	Average Size [bp]	Conc. [ng/µl]	Region Molarity [nmol/l]	% of Total	Region Comment	Color
10000	60000	24954	42.3	3.32	55.70		

 © Agilent Technologies, Inc. 2017

H4: W931

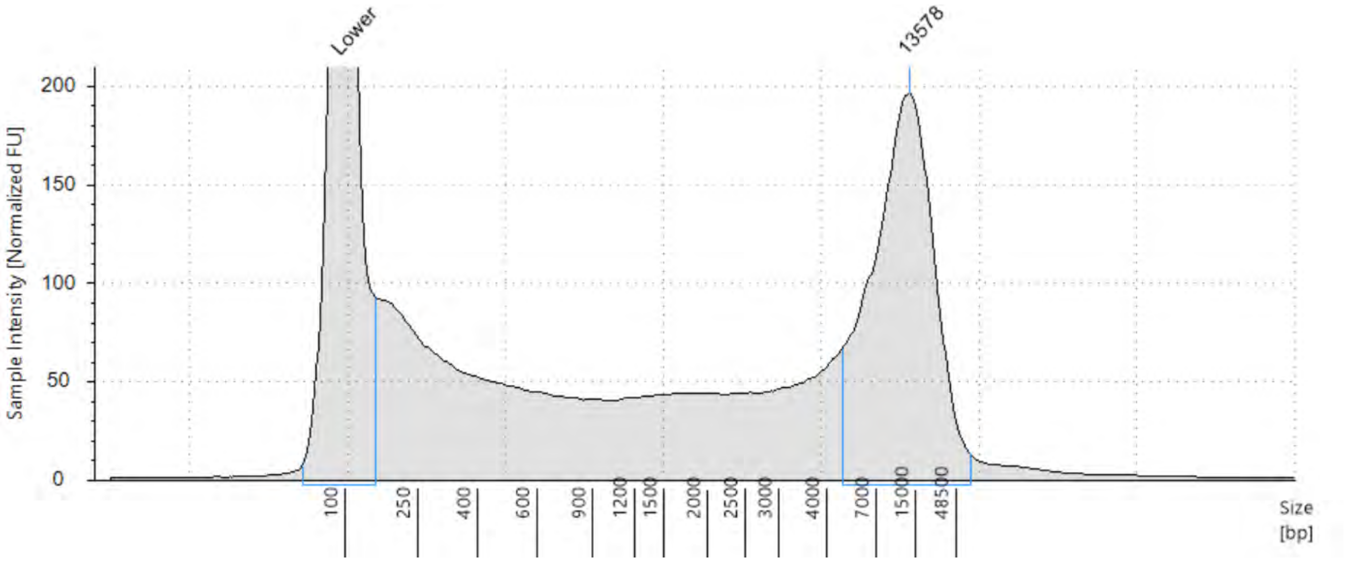

Sample Table

Well	DIN	Conc. [ng/µl]	Sample Description	Alert	Observations
H4	5.5	21.2	W931		

Peak Table

Size [bp]	Calibrated Conc. [ng/µl]	Assigned Conc. [ng/µl]	% Integrated Area	From [bp]	To [bp]	Peak Comment	Observations
100	8.50	8.50	-	59	150		Lower Marker
13578	7.93	-	99.26	4822	>60000		
-	-	-	-	-	-		Sample Well

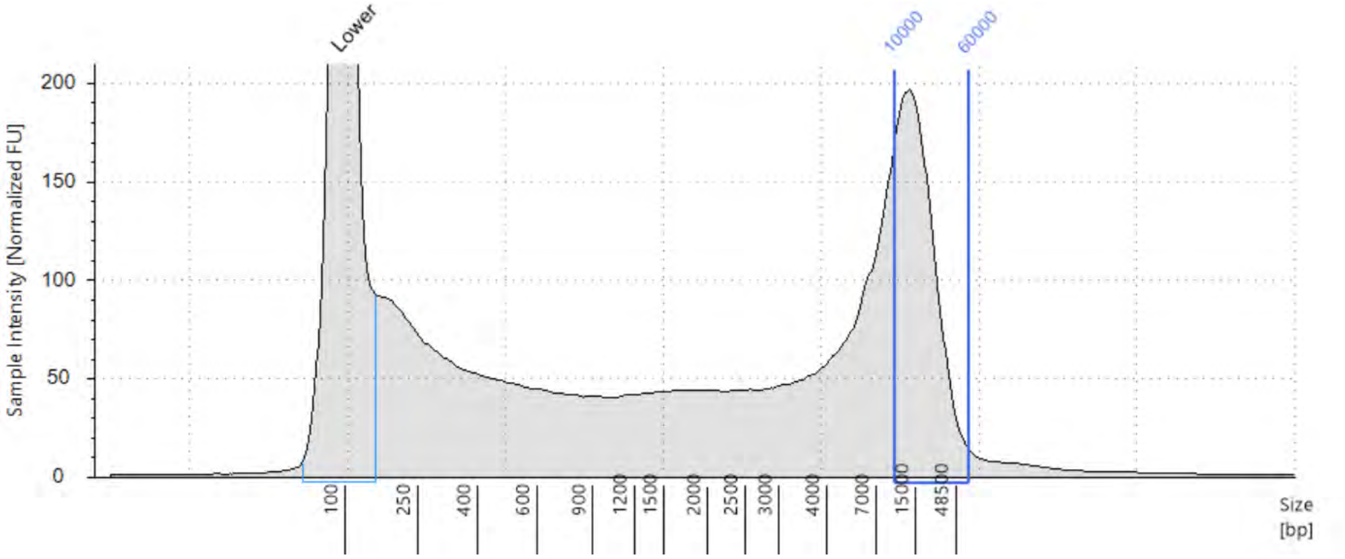

Region Table

From [bp]	To [bp]	Average Size [bp]	Conc. [ng/µl]	Region Molarity [nmol/l]	% of Total	Region Comment	Color
10000	60000	18572	4.89	0.481	23.05		

A5: W932

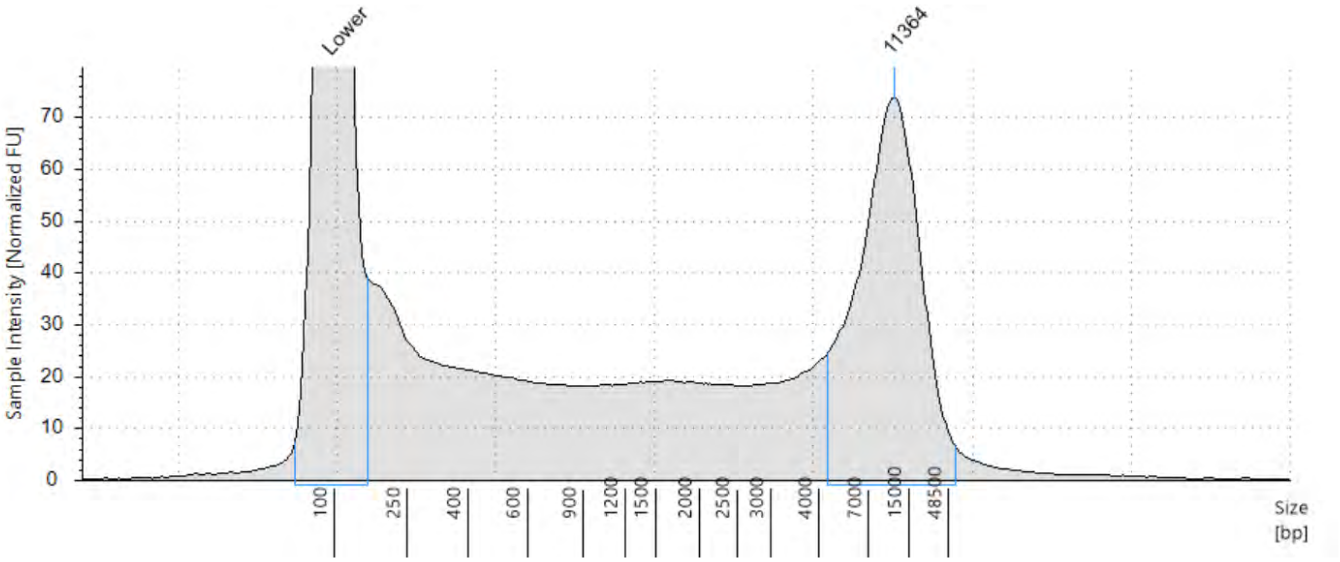

Sample Table

Well	DIN	Conc. [ng/µl]	Sample Description	Alert	Observations
A5	5.4	9.44	W932	⚠	Sample concentration outside recommended range

Peak Table

Size [bp]	Calibrated Conc. [ng/µl]	Assigned Conc. [ng/µl]	%Integrated Area	From [bp]	To [bp]	Peak Comment	Observations
100	8.50	8.50	-	61	151		Lower Marker
11364	3.34	-	100.00	4382	54331		

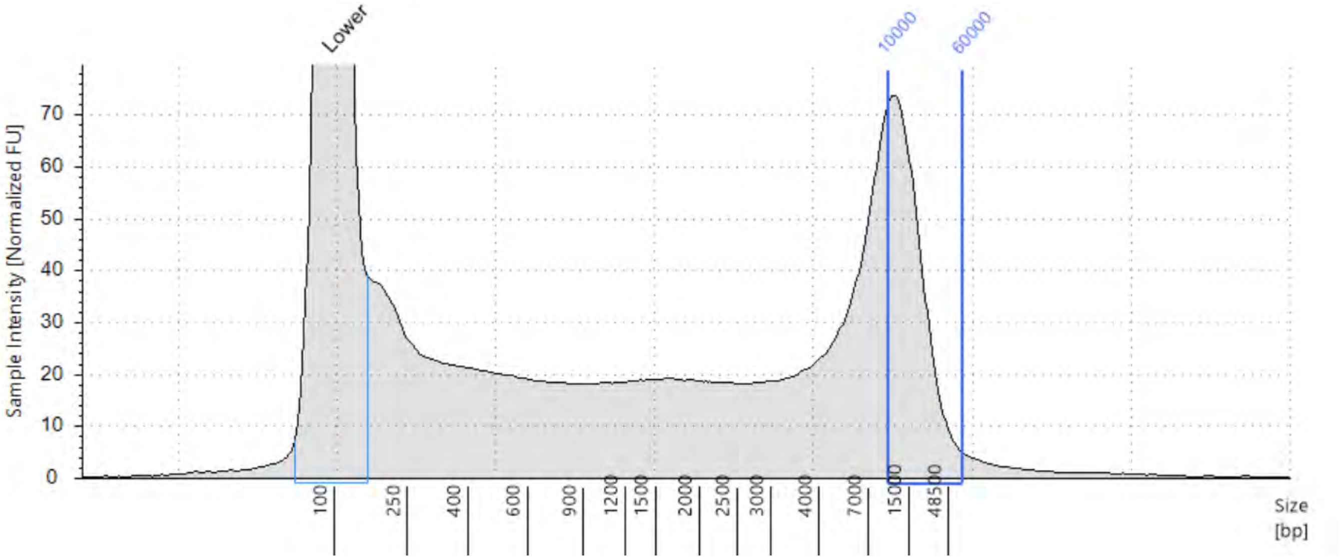

Region Table

From [bp]	To [bp]	Average Size [bp]	Conc. [ng/µl]	Region Molarity [nmol/l]	% of Total	Region Comment	Color
10000	60000	17669	1.76	0.181	18.65		■

 © Agilent Technologies, Inc. 2017

B5: W933

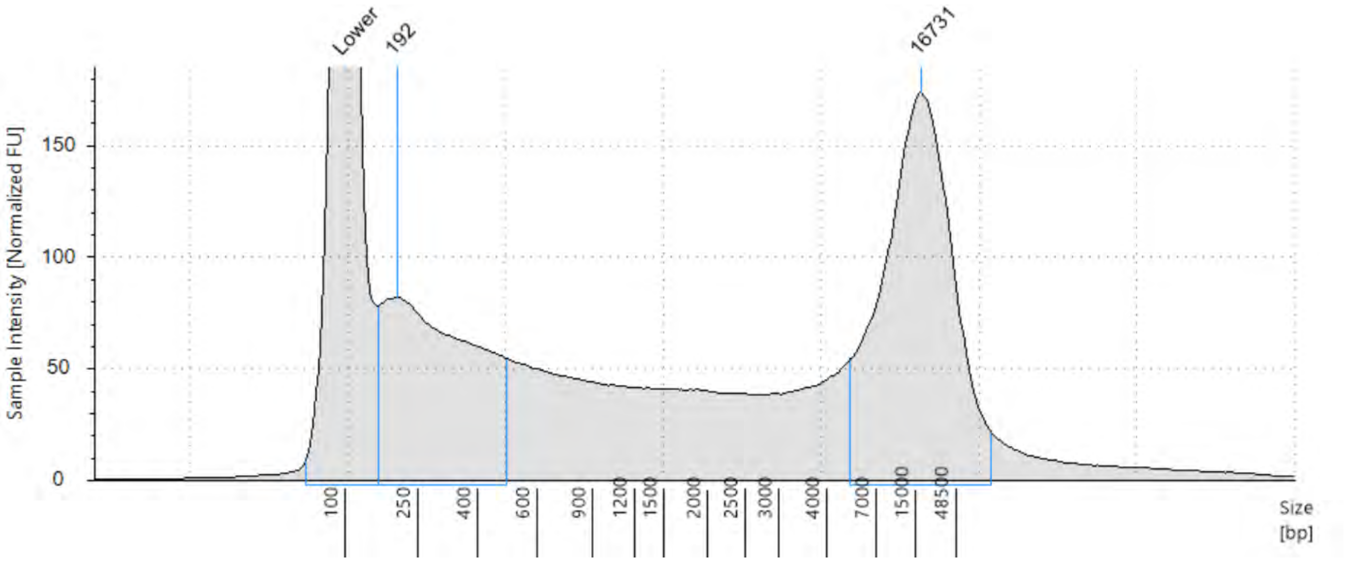

Sample Table

Well	DIN	Conc. [ng/µl]	Sample Description	Alert	Observations
B5	5.5	22.4	W933		

Peak Table

Size [bp]	Calibrated Conc. [ng/µl]	Assigned Conc. [ng/µl]	% Integrated Area	From [bp]	To [bp]	Peak Comment	Observations
100	8.50	8.50	-	61	151		Lower Marker
192	5.03	-	38.05	151	485		
16731	8.10	-	61.20	5160	>60000		
-	-	-	-	-	-		Sample Well

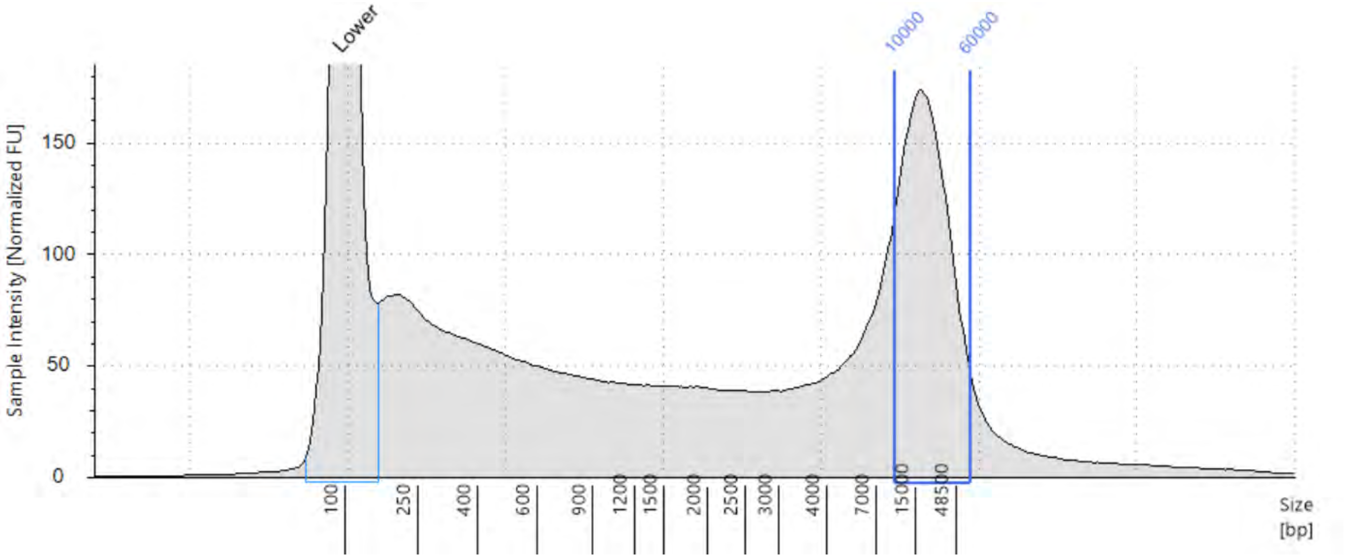

Region Table

From [bp]	To [bp]	Average Size [bp]	Conc. [ng/µl]	Region Molarity [nmol/l]	% of Total	Region Comment	Color
10000	60000	22714	5.67	0.485	25.35		

C5: W934

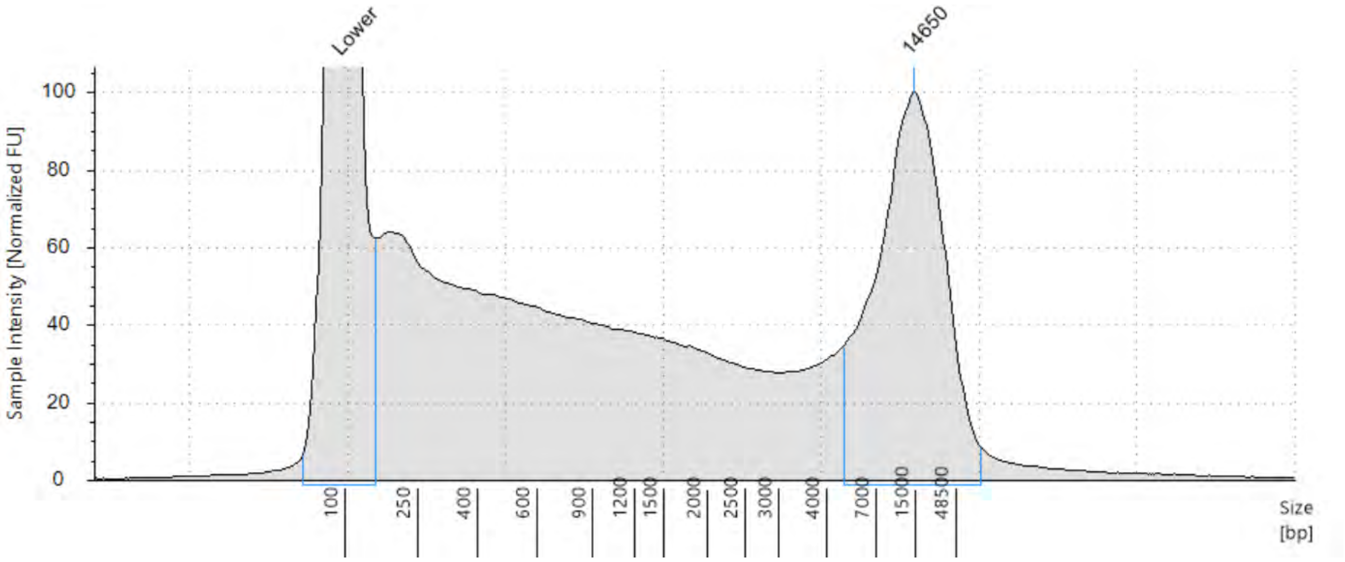

Sample Table

Well	DIN	Conc. [ng/µl]	Sample Description	Alert	Observations
C5	2.6	16.7	W934		

Peak Table

Size [bp]	Calibrated Conc. [ng/µl]	Assigned Conc. [ng/µl]	% Integrated Area	From [bp]	To [bp]	Peak Comment	Observations
100	8.50	8.50	-	59	147		Lower Marker
14650	4.88	-	100.00	4865	>60000		

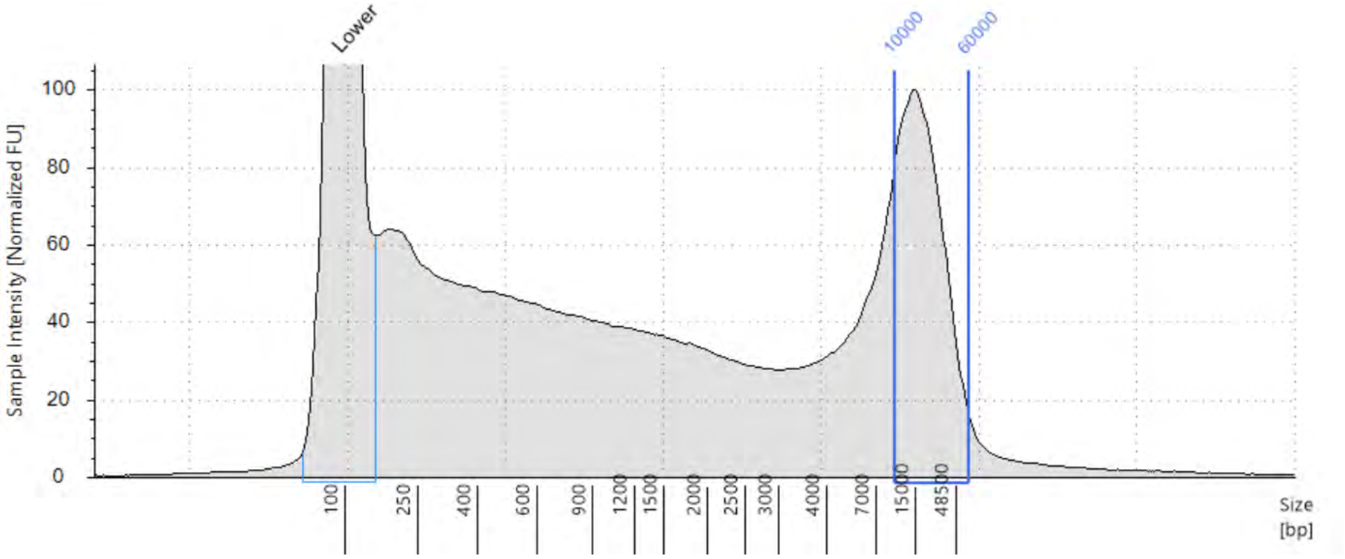

Region Table

From [bp]	To [bp]	Average Size [bp]	Conc. [ng/µl]	Region Molarity [nmol/l]	% of Total	Region Comment	Color
10000	60000	21063	3.28	0.298	19.63		

D5: W935

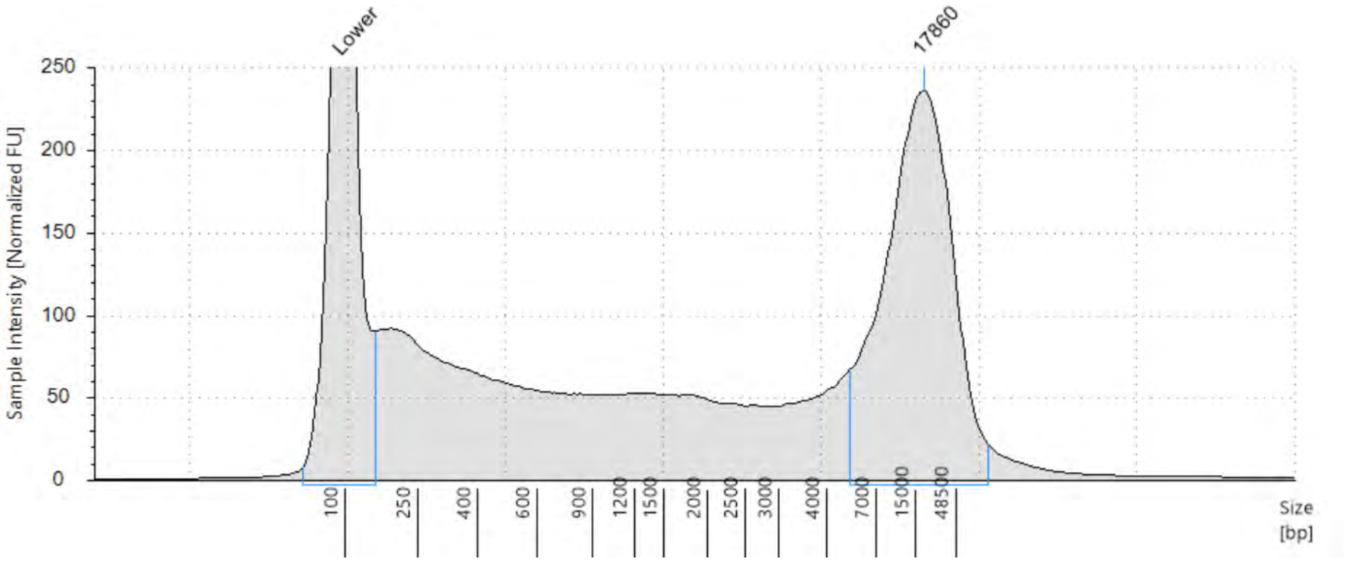

Sample Table

Well	DIN	Conc. [ng/µl]	Sample Description	Alert	Observations
D5	5.7	27.2	W935		

Peak Table

Size [bp]	Calibrated Conc. [ng/µl]	Assigned Conc. [ng/µl]	% Integrated Area	From [bp]	To [bp]	Peak Comment	Observations
100	8.50	8.50	-	59	147		Lower Marker
17860	10.9	-	99.31	5213	>60000		
-	-	-	-	-	-		Sample Well

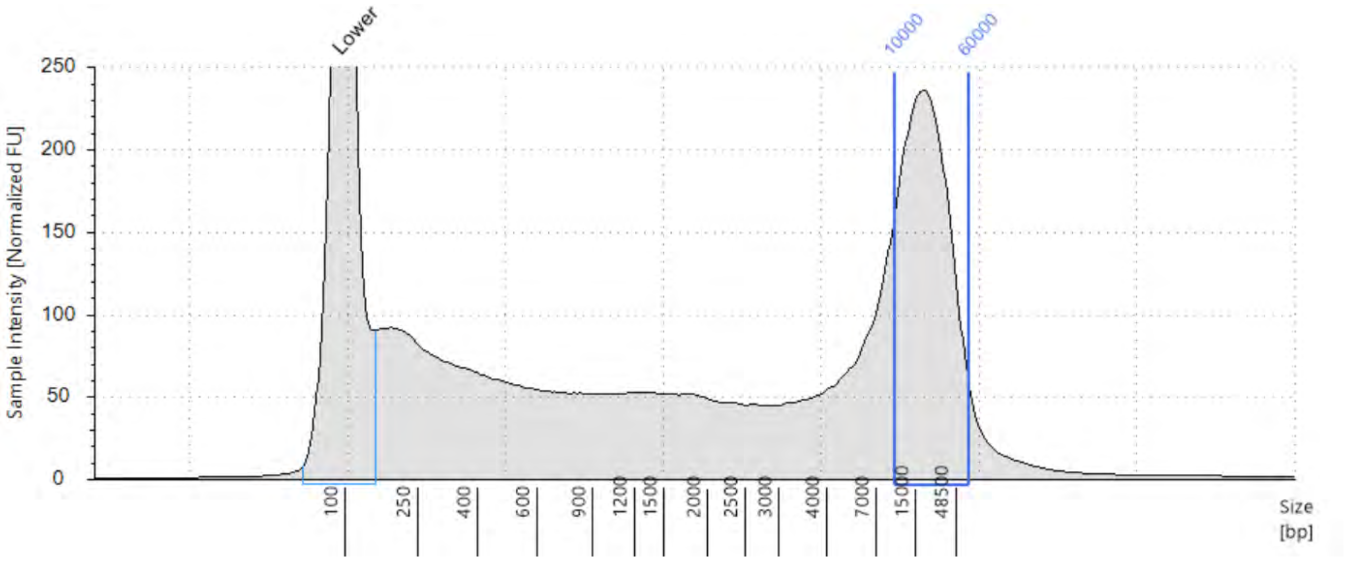

Region Table

From [bp]	To [bp]	Average Size [bp]	Conc. [ng/µl]	Region Molarity [nmol/l]	% of Total	Region Comment	Color
10000	60000	22829	7.90	0.666	29.06		

E5: W936

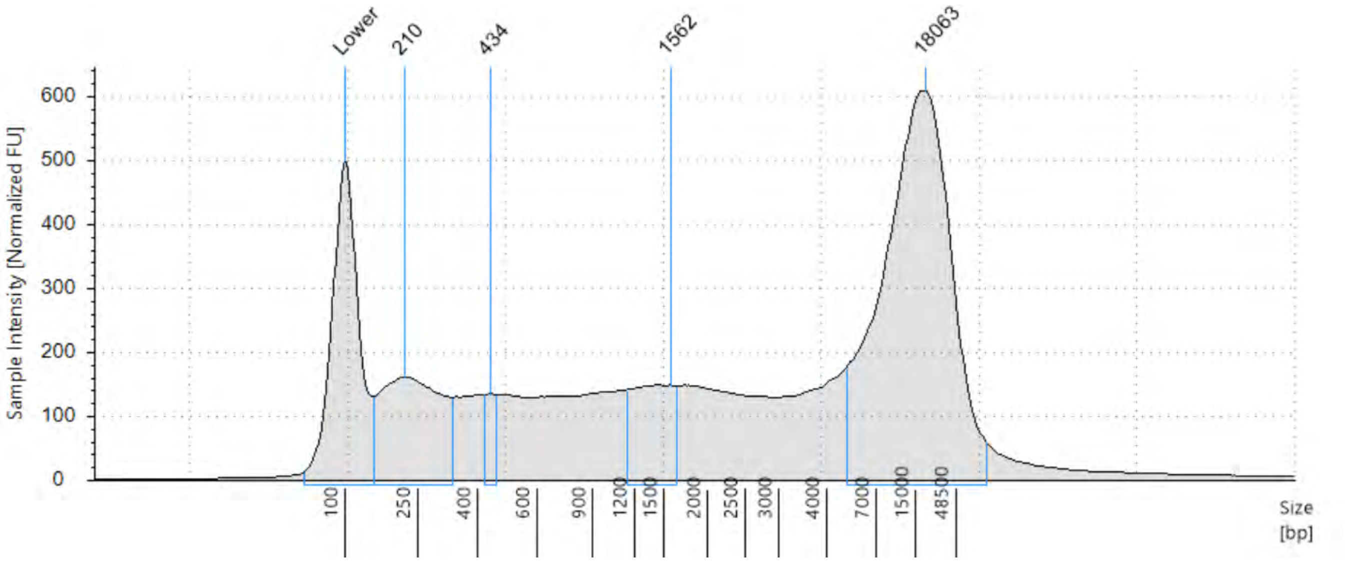

Sample Table

Well	DIN	Conc. [ng/µl]	Sample Description	Alert	Observations
E5	5.8	64.9	W936		

Peak Table

Size [bp]	Calibrated Conc. [ng/µl]	Assigned Conc. [ng/µl]	% Integrated Area	From [bp]	To [bp]	Peak Comment	Observations
100	8.50	8.50	-	60	144		Lower Marker
210	6.28	-	16.23	144	326		
434	0.997	-	2.58	418	455		
1562	4.12	-	10.66	1139	1633		
18063	26.7	-	69.01	4988	>60000		
-	-	-	-	-	-		Sample Well

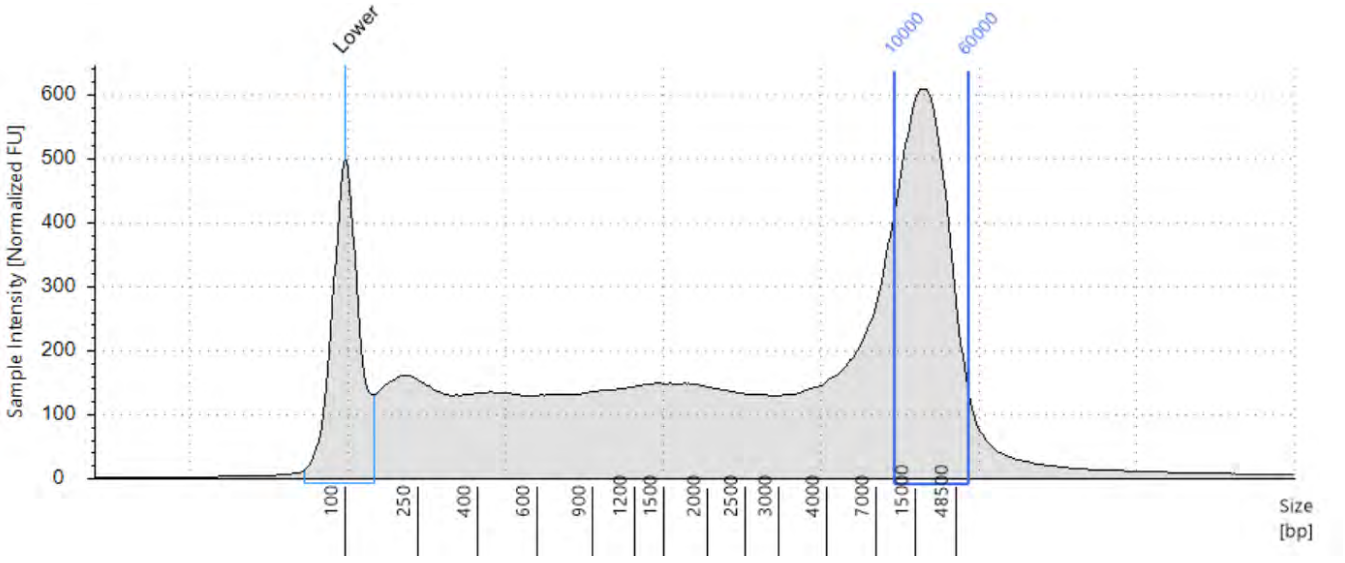

Region Table

From [bp]	To [bp]	Average Size [bp]	Conc. [ng/µl]	Region Molarity [nmol/l]	% of Total	Region Comment	Color
10000	60000	22246	18.9	1.62	29.13		

 © Agilent Technologies, Inc. 2017

F5: W937

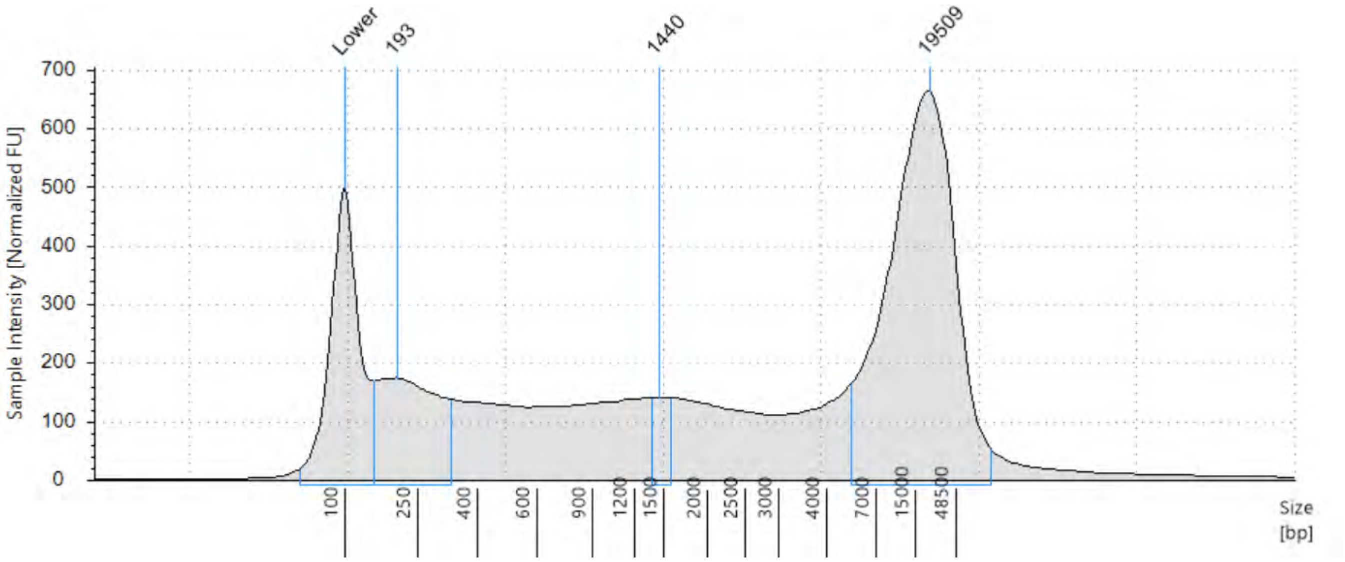

Sample Table

Well	DIN	Conc. [ng/µl]	Sample Description	Alert	Observations
F5	5.9	60.0	W937		

Peak Table

Size [bp]	Calibrated Conc. [ng/µl]	Assigned Conc. [ng/µl]	% Integrated Area	From [bp]	To [bp]	Peak Comment	Observations
100	8.50	8.50	-	57	144		Lower Marker
193	6.40	-	18.65	144	327		
1440	1.45	-	4.22	1369	1569		
19509	26.3	-	76.56	5292	>60000		
-	-	-	-	-	-		Sample Well

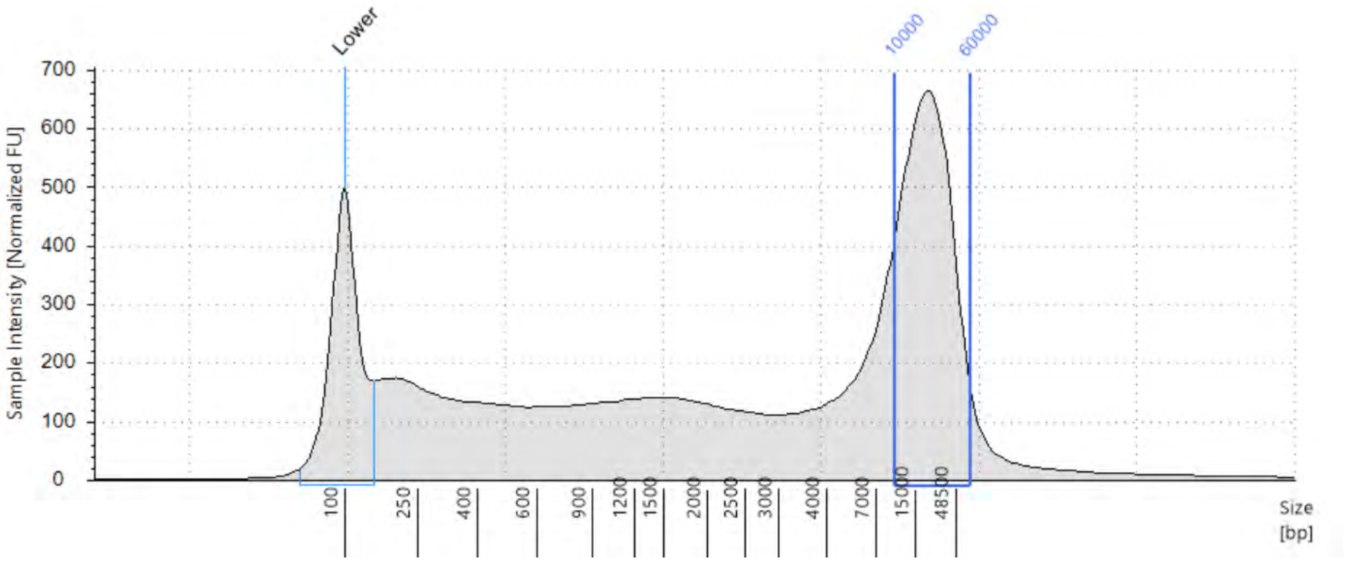

Region Table

From [bp]	To [bp]	Average Size [bp]	Conc. [ng/µl]	Region Molarity [nmol/l]	% of Total	Region Comment	Color
10000	60000	23228	19.6	1.63	32.71		

G5: W938

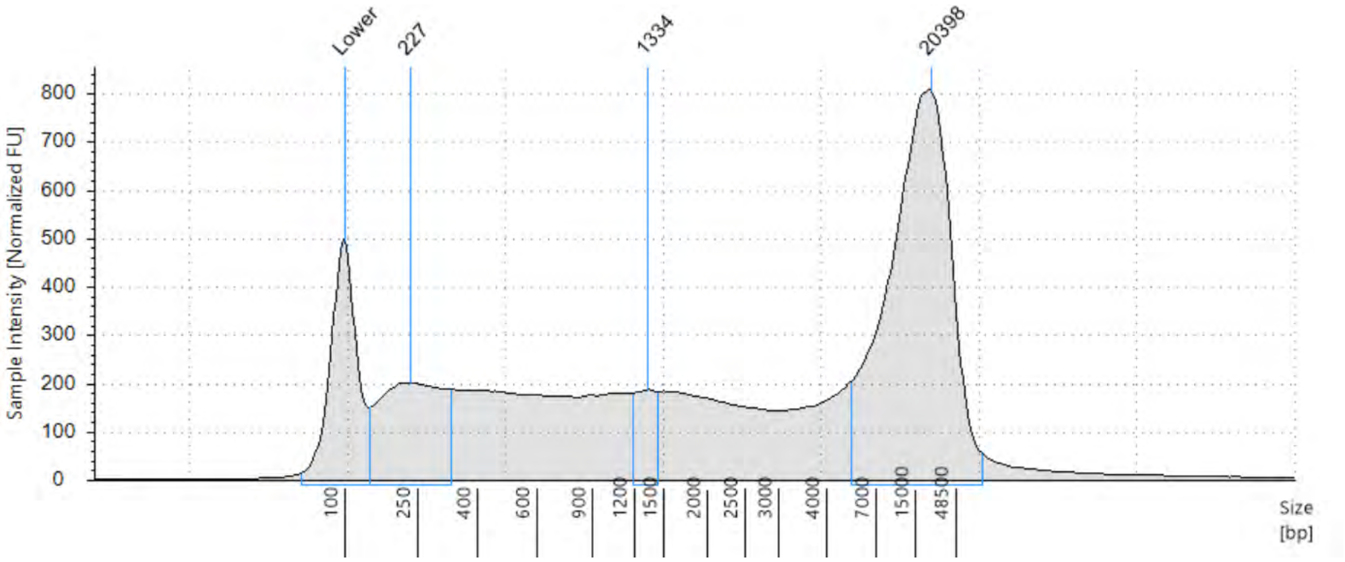

Sample Table

Well	DIN	Conc. [ng/µl]	Sample Description	Alert	Observations
G5	5.7	81.1	W938		

Peak Table

Size [bp]	Calibrated Conc. [ng/µl]	Assigned Conc. [ng/µl]	% Integrated Area	From [bp]	To [bp]	Peak Comment	Observations
100	8.50	8.50	-	58	137		Lower Marker
227	8.62	-	19.46	137	326		
1334	2.67	-	6.03	1192	1433		
20398	32.8	-	74.17	5296	>60000		
-	-	-	-	-	-		Sample Well

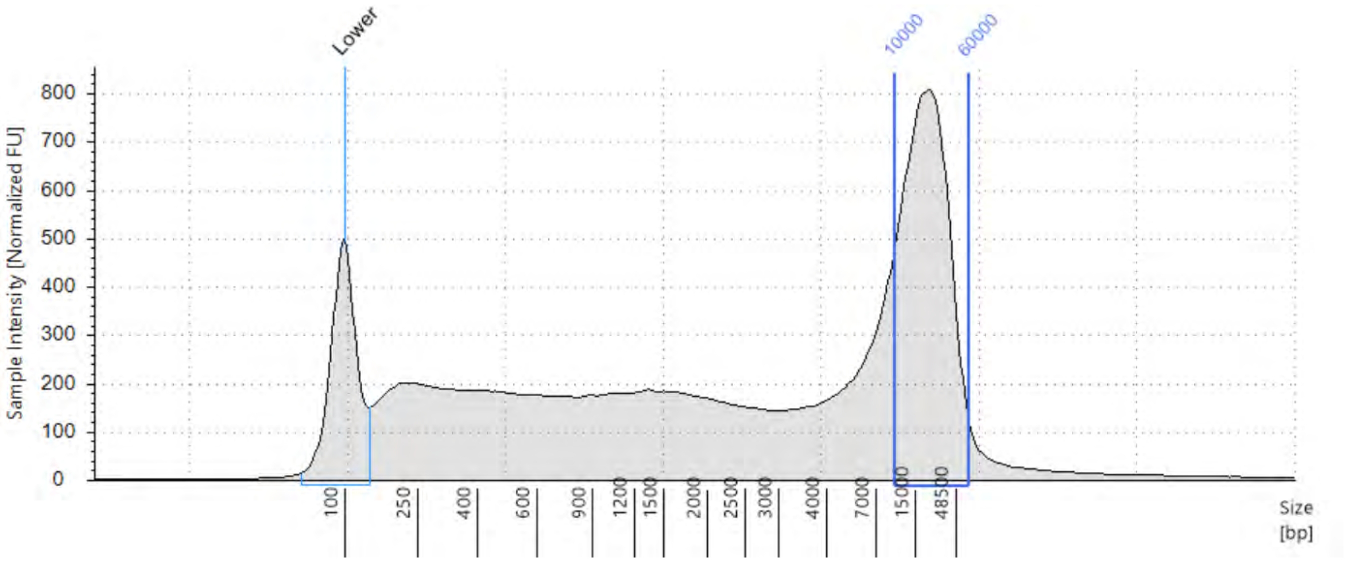

Region Table

From [bp]	To [bp]	Average Size [bp]	Conc. [ng/µl]	Region Molarity [nmol/l]	% of Total	Region Comment	Color
10000	60000	22367	24.8	2.08	30.58		

© Agilent Technologies, Inc. 2017

H5: W939

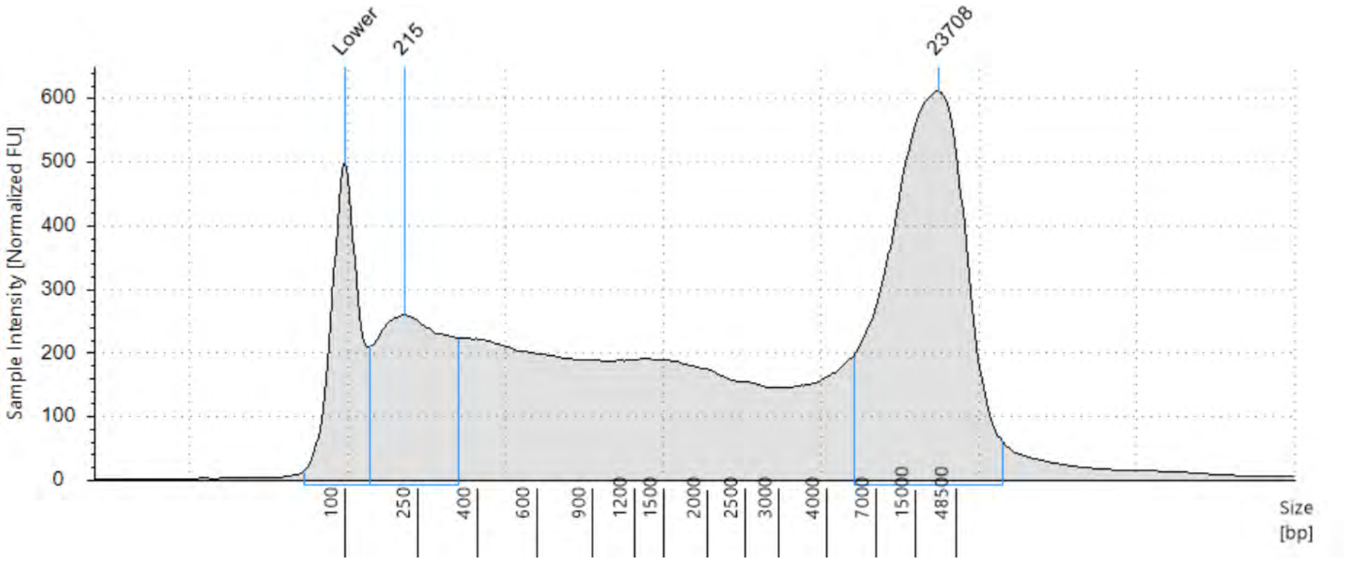

Sample Table

Well	DIN	Conc. [ng/µl]	Sample Description	Alert	Observations
H5	5.5	81.7	W939		

Peak Table

Size [bp]	Calibrated Conc. [ng/µl]	Assigned Conc. [ng/µl]	% Integrated Area	From [bp]	To [bp]	Peak Comment	Observations
100	8.50	8.50	-	59	137		Lower Marker
215	11.5	-	27.64	137	346		
23708	30.1	-	72.06	5462	>60000		
-	-	-	-	-	-		Sample Well

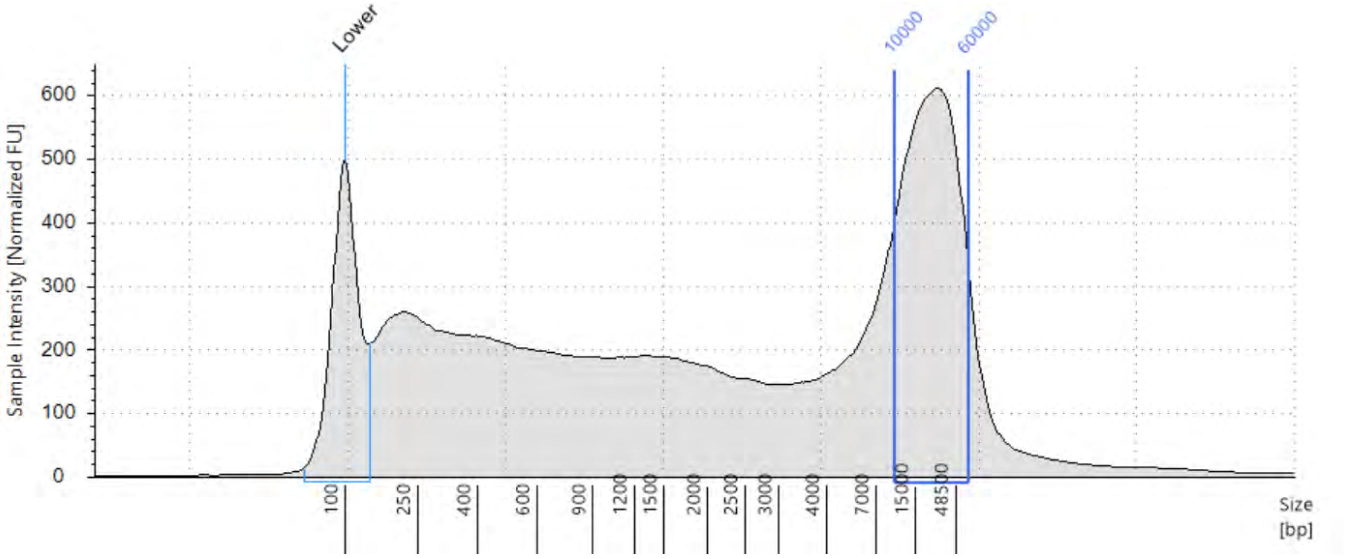

Region Table

From [bp]	To [bp]	Average Size [bp]	Conc. [ng/µl]	Region Molarity [nmol/l]	% of Total	Region Comment	Color
10000	60000	26057	21.5	1.67	26.32		

A6: W940

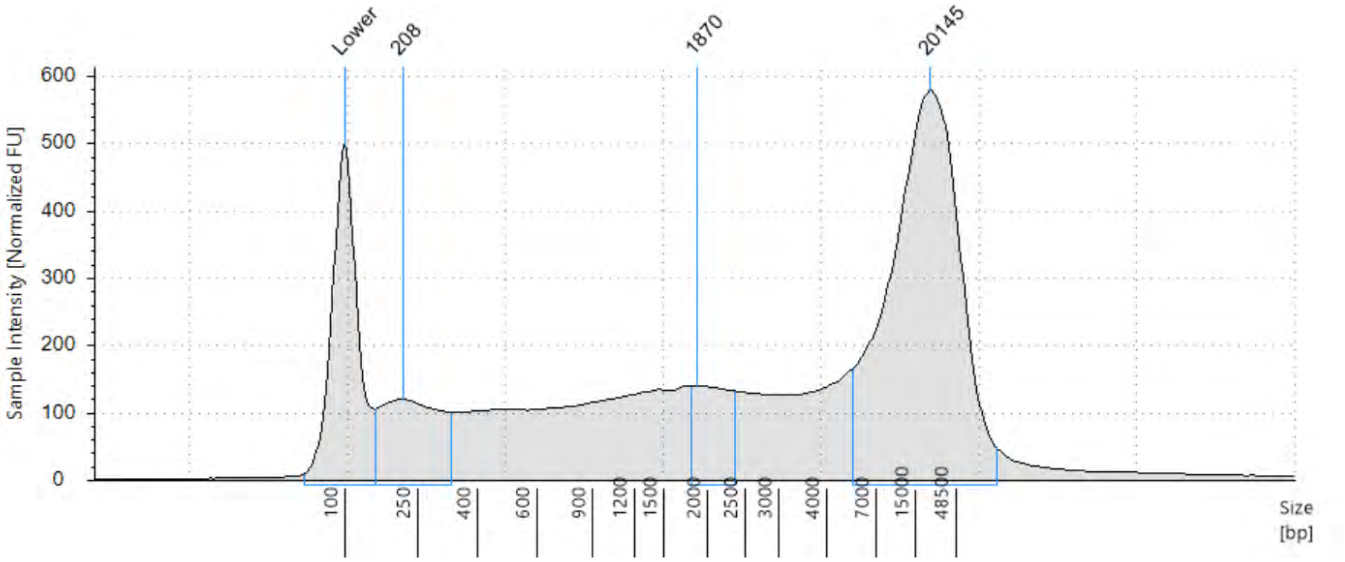

Sample Table

Well	DIN	Conc. [ng/µl]	Sample Description	Alert	Observations
A6	6.0	62.3	W940		

Peak Table

Size [bp]	Calibrated Conc. [ng/µl]	Assigned Conc. [ng/µl]	% Integrated Area	From [bp]	To [bp]	Peak Comment	Observations
100	8.50	8.50	-	60	147		Lower Marker
208	4.87	-	13.57	147	326		
1870	3.44	-	9.60	1804	2356		
20145	27.1	-	75.68	5430	>60000		
-	-	-	-	-	-		Sample Well

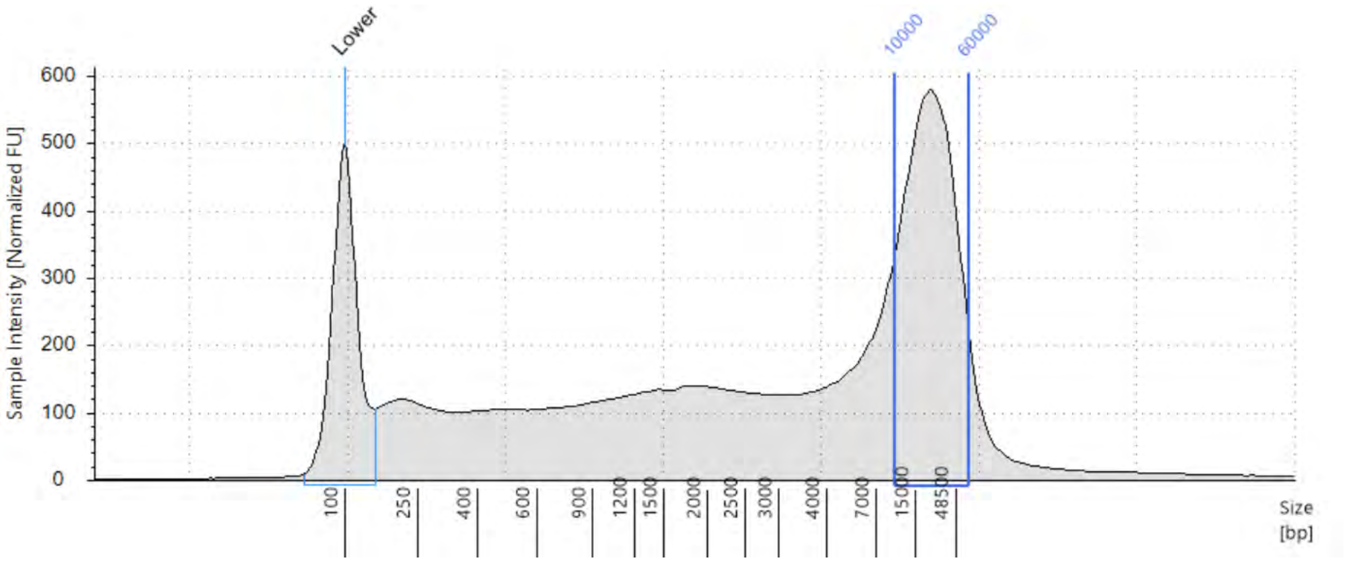

Region Table

From [bp]	To [bp]	Average Size [bp]	Conc. [ng/µl]	Region Molarity [nmol/l]	% of Total	Region Comment	Color
10000	60000	25034	20.2	1.59	32.45		

B6: W941

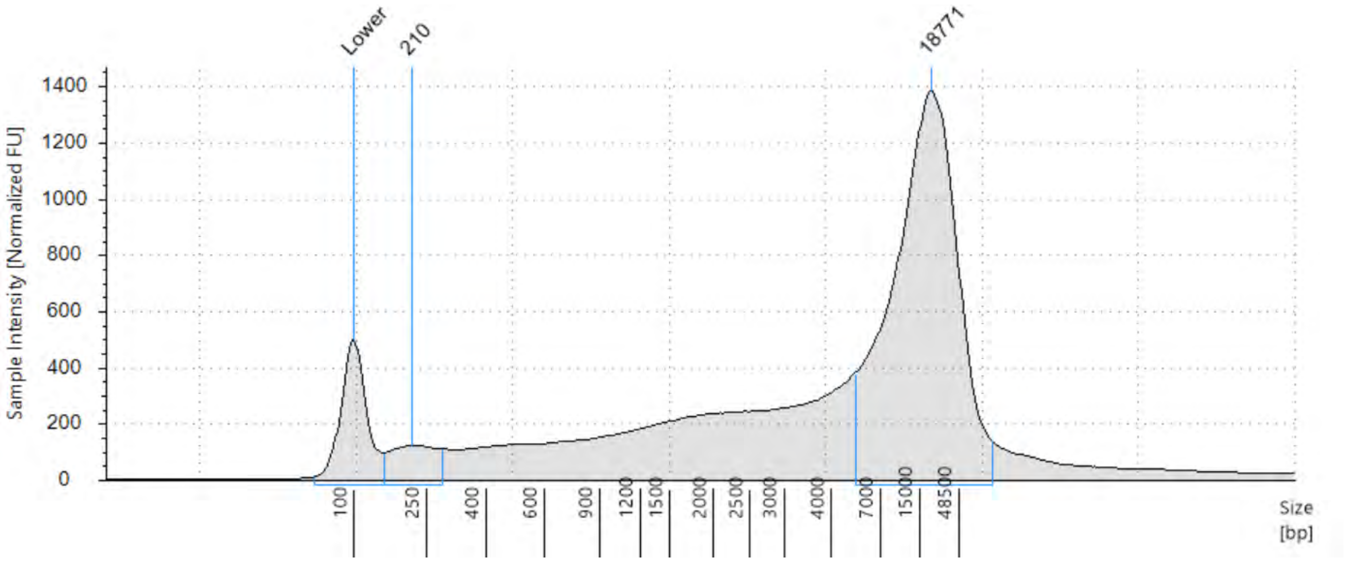

Sample Table

Well	DIN	Conc. [ng/µl]	Sample Description	Alert	Observations
B6	6.3	119	W941	⚠	Sample concentration outside recommended range

Peak Table

Size [bp]	Calibrated Conc. [ng/µl]	Assigned Conc. [ng/µl]	% Integrated Area	From [bp]	To [bp]	Peak Comment	Observations
100	8.50	8.50	-	61	146		Lower Marker
210	3.82	-	5.89	146	284		
18771	60.8	-	93.85	5258	>60000		
-	-	-	-	-	-		Sample Well

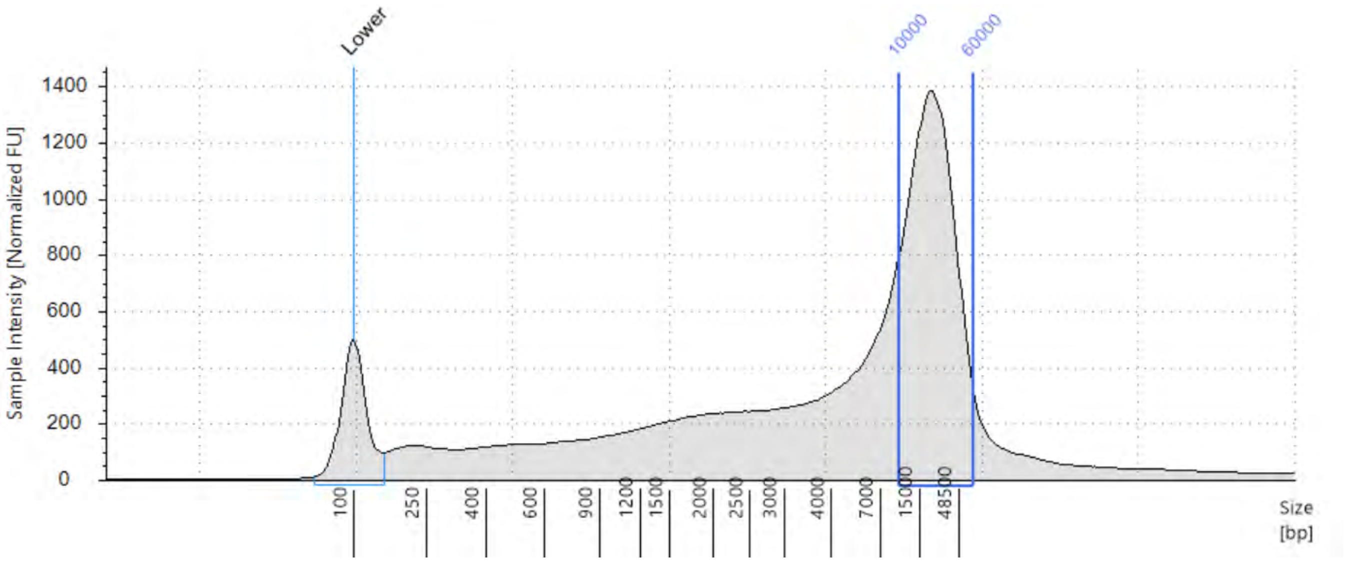

Region Table

From [bp]	To [bp]	Average Size [bp]	Conc. [ng/µl]	Region Molarity [nmol/l]	% of Total	Region Comment	Color
10000	60000	23560	44.8	3.65	37.78		■

 © Agilent Technologies, Inc. 2017

C6: W942

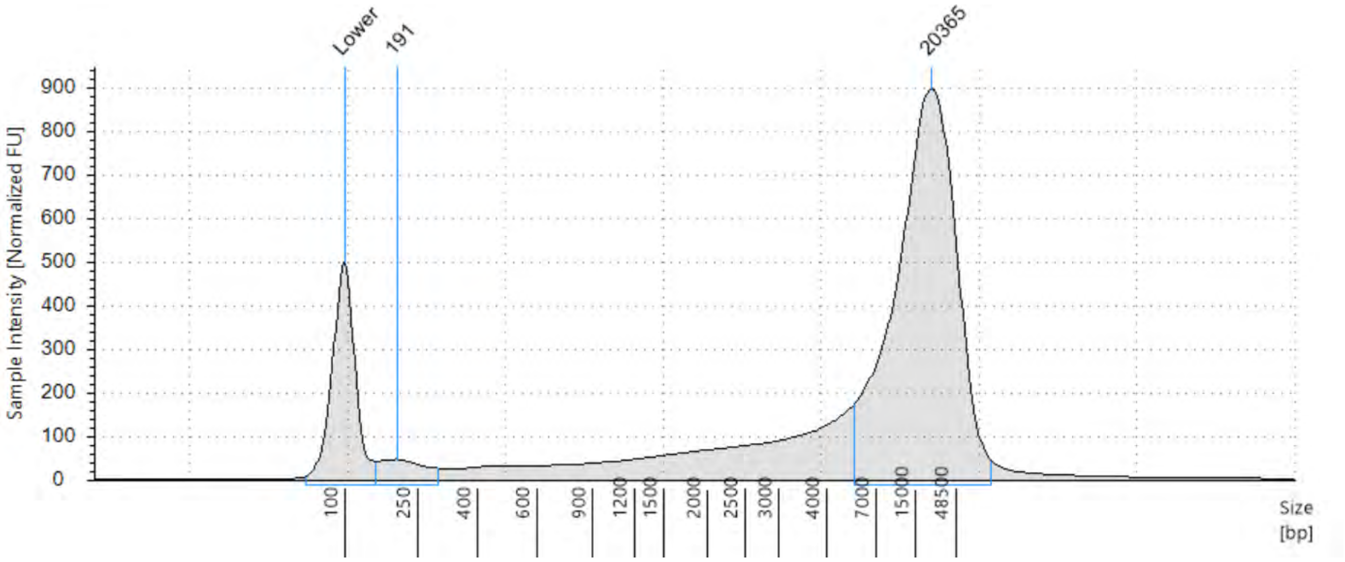

Sample Table

Well	DIN	Conc. [ng/µl]	Sample Description	Alert	Observations
C6	6.9	59.3	W942		

Peak Table

Size [bp]	Calibrated Conc. [ng/µl]	Assigned Conc. [ng/µl]	% Integrated Area	From [bp]	To [bp]	Peak Comment	Observations
100	8.50	8.50	-	60	147		Lower Marker
191	1.52	-	3.57	147	293		
20365	40.8	-	96.05	5449	>60000		
-	-	-	-	-	-		Sample Well

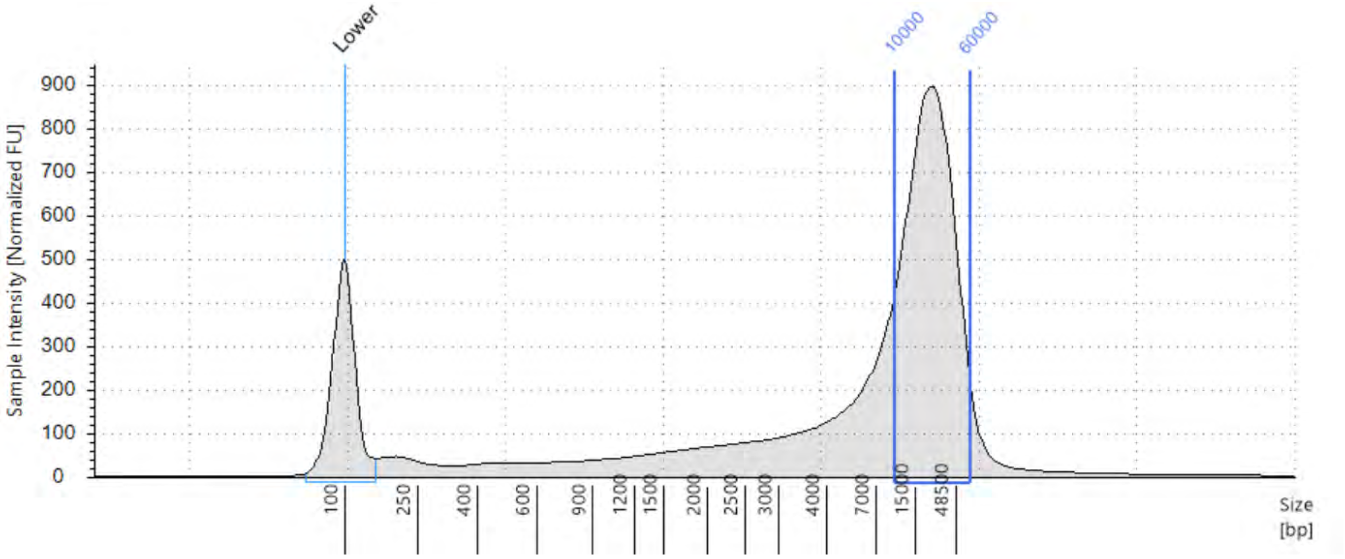

Region Table

From [bp]	To [bp]	Average Size [bp]	Conc. [ng/µl]	Region Molarity [nmol/l]	% of Total	Region Comment	Color
10000	60000	24265	32.2	2.55	54.35		■

© Agilent Technologies, Inc. 2017

D6: W943

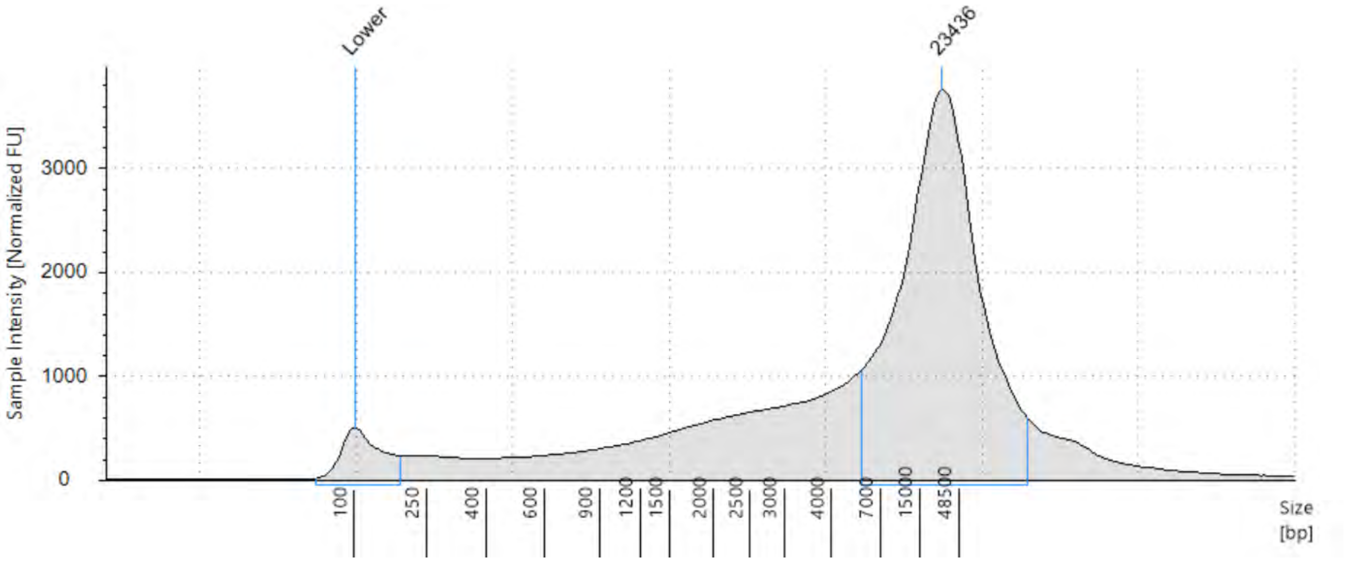

Sample Table

Well	DIN	Conc. [ng/µl]	Sample Description	Alert	Observations
D6	6.7	220	W943	⚠	Sample concentration outside recommended range

Peak Table

Size [bp]	Calibrated Conc. [ng/µl]	Assigned Conc. [ng/µl]	% Integrated Area	From [bp]	To [bp]	Peak Comment	Observations
100	8.50	8.50	-	62	180		Lower Marker
23436	127	-	99.18	5703	>60000		
-	-	-	-	-	-		Sample Well

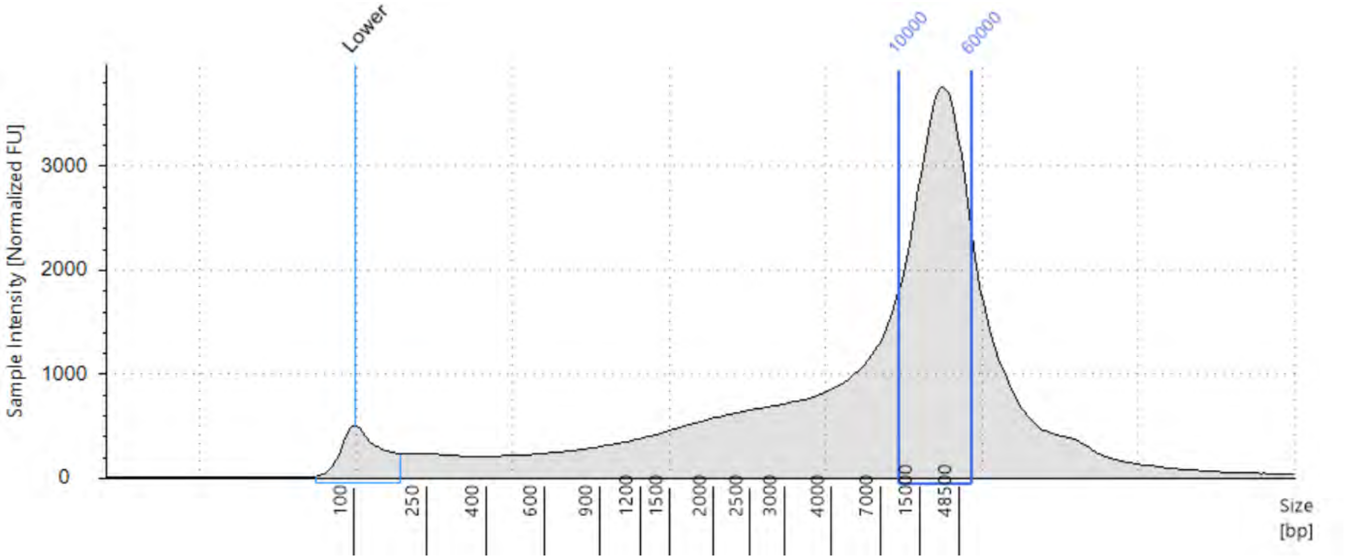

Region Table

From [bp]	To [bp]	Average Size [bp]	Conc. [ng/µl]	Region Molarity [nmol/l]	% of Total	Region Comment	Color
10000	60000	27179	83.5	6.15	37.87		■

E6: W944

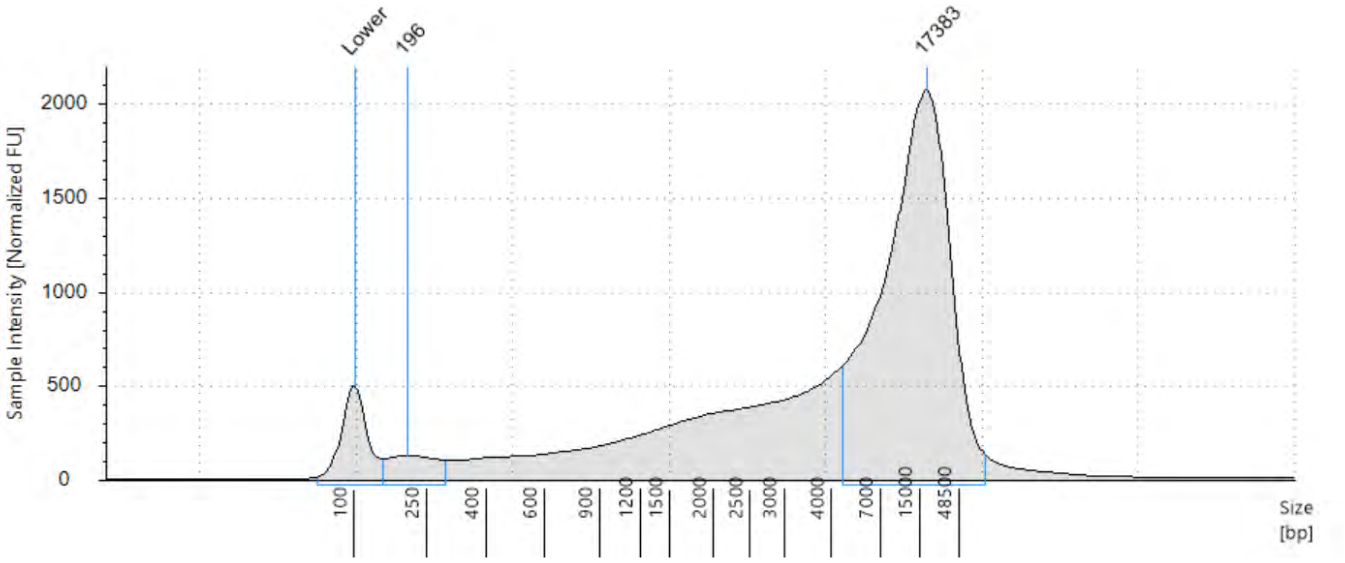

Sample Table

Well	DIN	Conc. [ng/µl]	Sample Description	Alert	Observations
E6	6.4	163	W944	⚠	Sample concentration outside recommended range

Peak Table

Size [bp]	Calibrated Conc. [ng/µl]	Assigned Conc. [ng/µl]	% Integrated Area	From [bp]	To [bp]	Peak Comment	Observations
100	8.50	8.50	-	62	144		Lower Marker
196	4.21	-	4.27	144	290		
17383	93.9	-	95.35	4587	>60000		
-	-	-	-	-	-		Sample Well

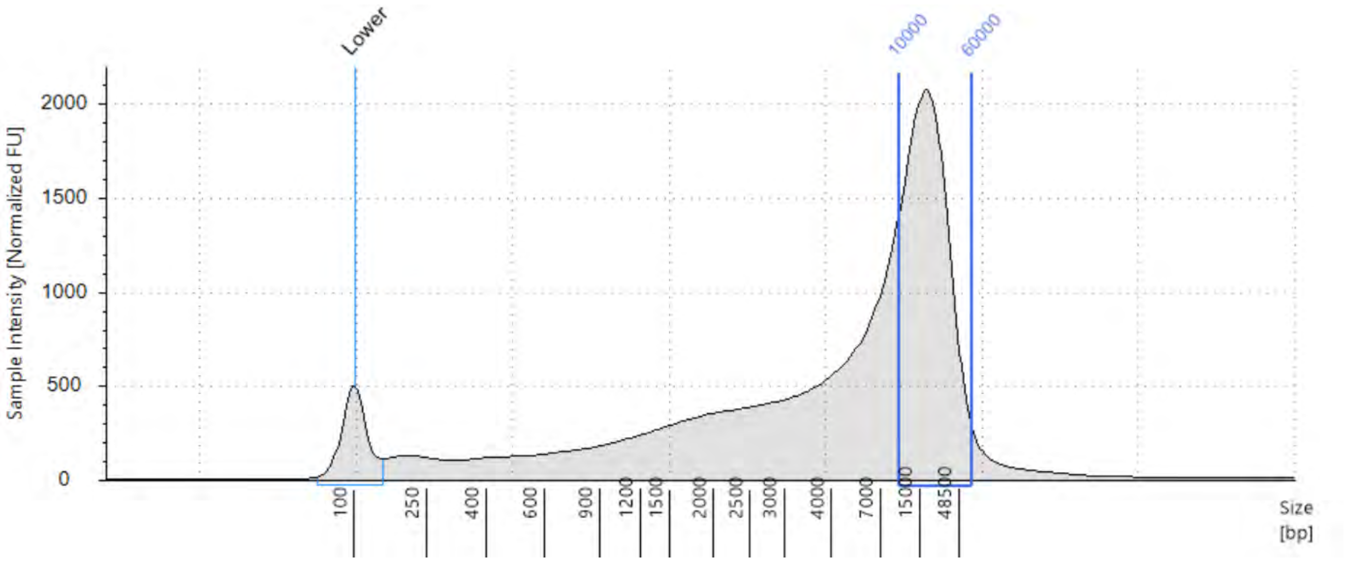

Region Table

From [bp]	To [bp]	Average Size [bp]	Conc. [ng/µl]	Region Molarity [nmol/l]	% of Total	Region Comment	Color
10000	60000	21102	64.1	5.68	39.32		■

 © Agilent Technologies, Inc. 2017

F6: W945

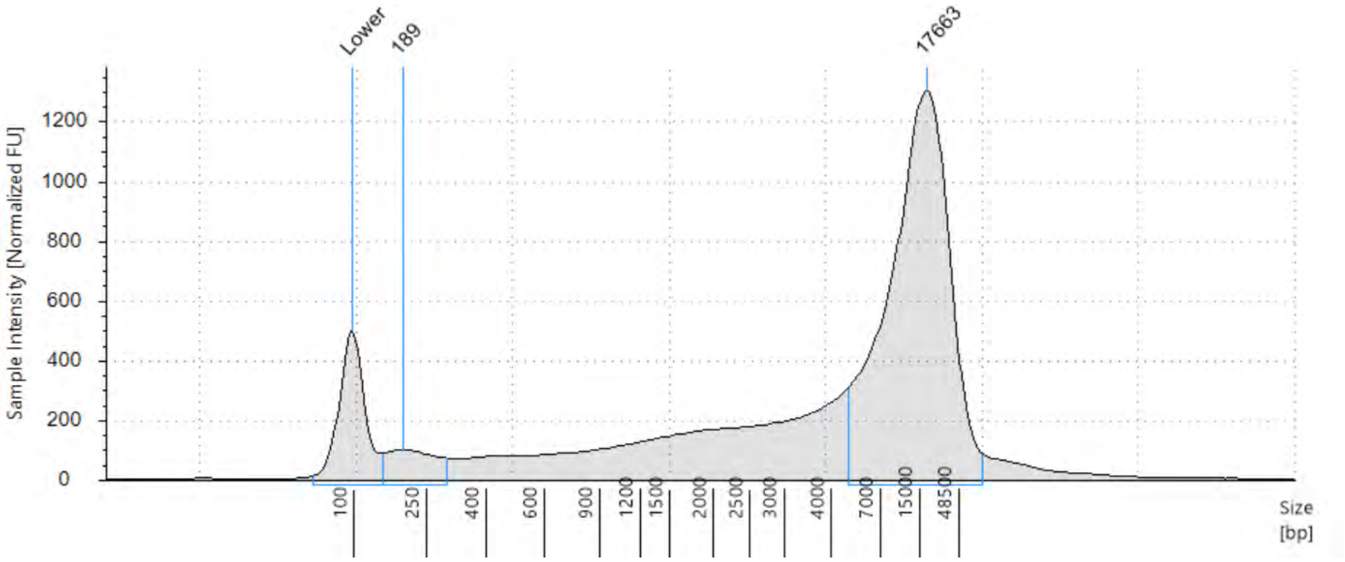

Sample Table

Well	DIN	Conc. [ng/µl]	Sample Description	Alert	Observations
F6	6.5	91.4	W945		

Peak Table

Size [bp]	Calibrated Conc. [ng/µl]	Assigned Conc. [ng/µl]	% Integrated Area	From [bp]	To [bp]	Peak Comment	Observations
100	8.50	8.50	-	60	146		Lower Marker
189	3.33	-	5.89	146	298		
17663	53.1	-	93.79	4941	>60000		
-	-	-	-	-	-		Sample Well

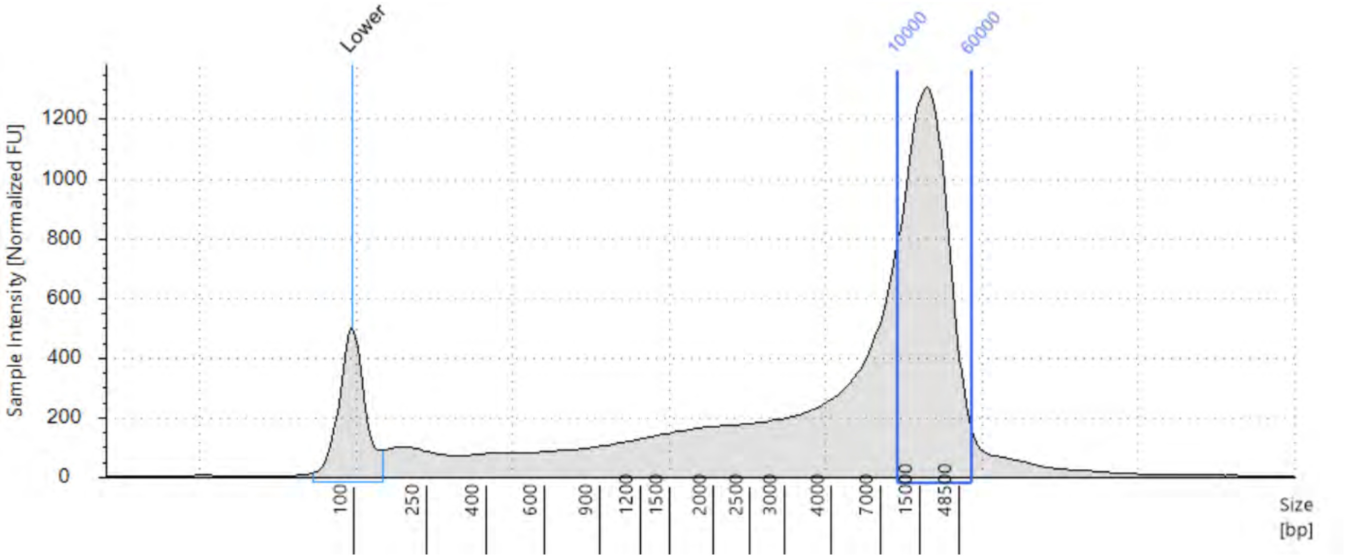

Region Table

From [bp]	To [bp]	Average Size [bp]	Conc. [ng/µl]	Region Molarity [nmol/l]	% of Total	Region Comment	Color
10000	60000	21178	38.6	3.37	42.24		

 © Agilent Technologies, Inc. 2017

G6: M226

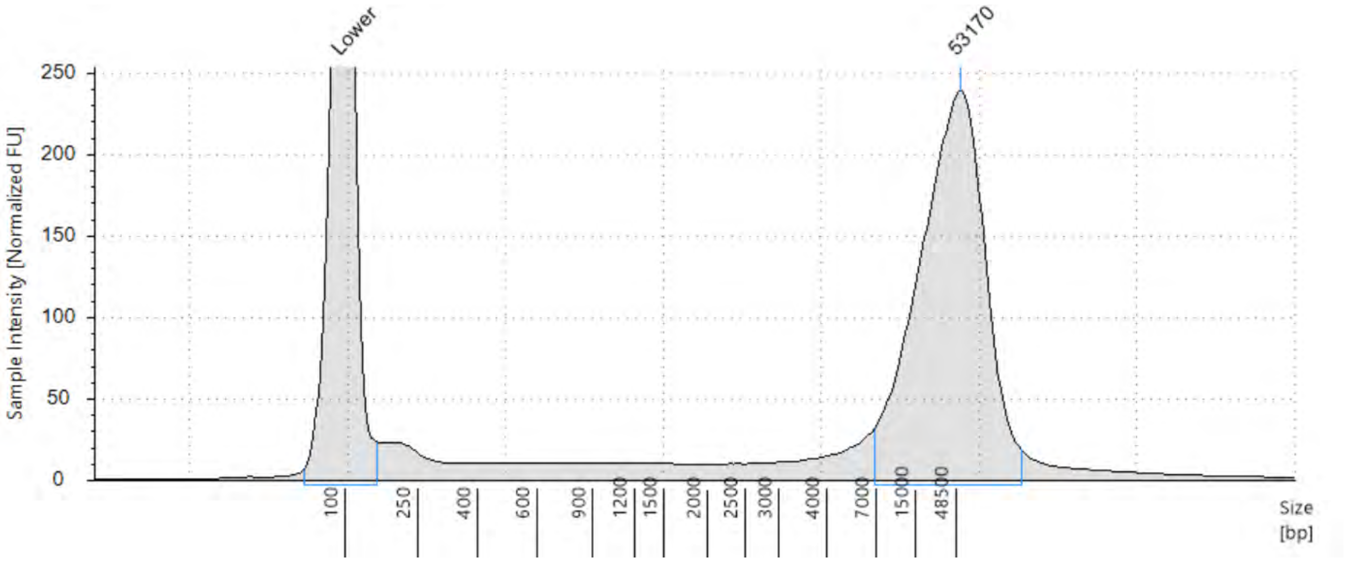

Sample Table

Well	DIN	Conc. [ng/µl]	Sample Description	Alert	Observations
G6	7.9	16.7	M226		

Peak Table

Size [bp]	Calibrated Conc. [ng/µl]	Assigned Conc. [ng/µl]	% Integrated Area	From [bp]	To [bp]	Peak Comment	Observations
100	8.50	8.50	-	61	151		Lower Marker
53170	11.9	-	99.09	6941	>60000		
-	-	-	-	-	-		Sample Well

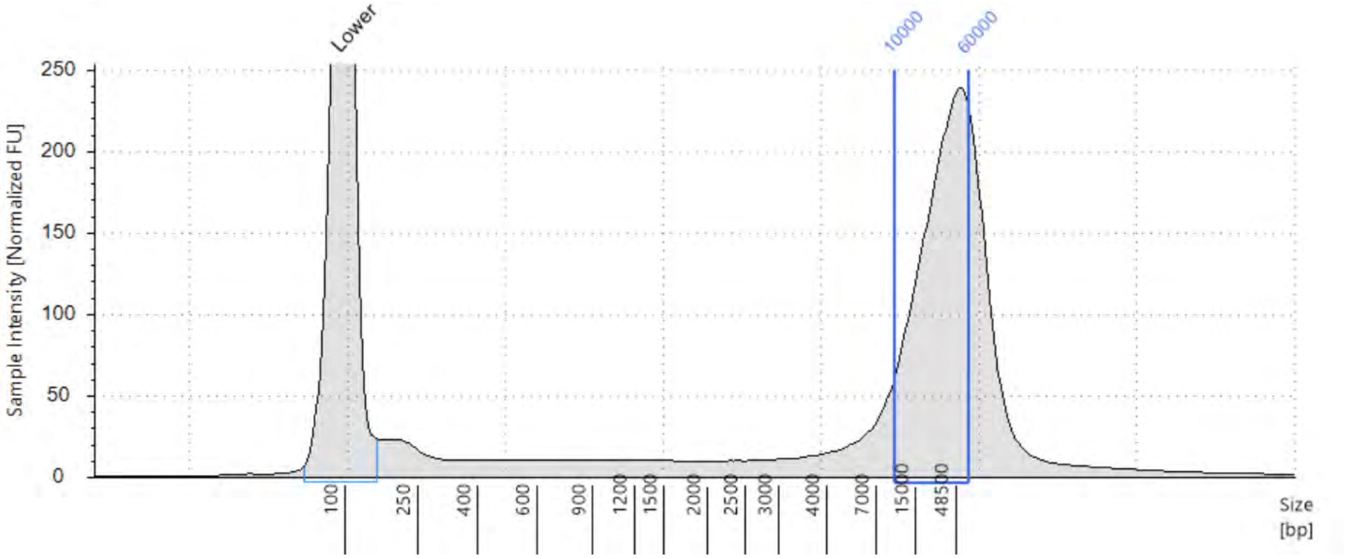

Region Table

From [bp]	To [bp]	Average Size [bp]	Conc. [ng/µl]	Region Molarity [nmol/l]	% of Total	Region Comment	Color
10000	60000	31524	7.91	0.510	47.27		

H6: M227

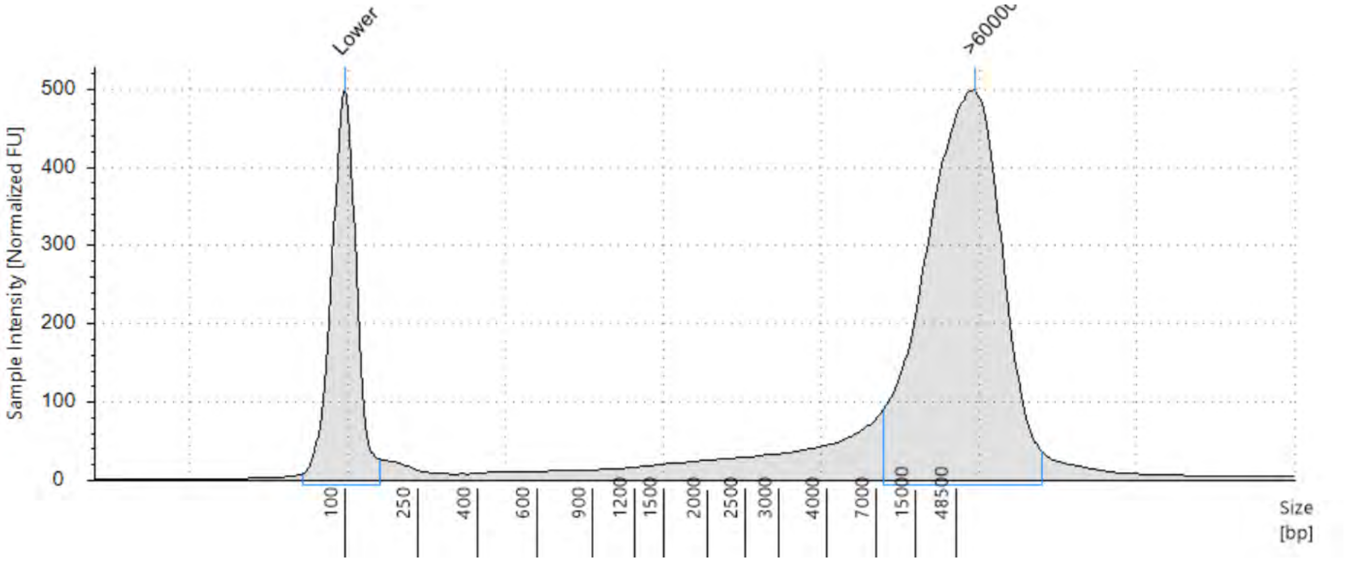

Sample Table

Well	DIN	Conc. [ng/µl]	Sample Description	Alert	Observations
H6	8.4	34.3	M227		

Peak Table

Size [bp]	Calibrated Conc. [ng/µl]	Assigned Conc. [ng/µl]	% Integrated Area	From [bp]	To [bp]	Peak Comment	Observations
100	8.50	8.50	-	59	155		Lower Marker
>60000	26.5	-	99.58	8100	>60000		
-	-	-	-	-	-		Sample Well

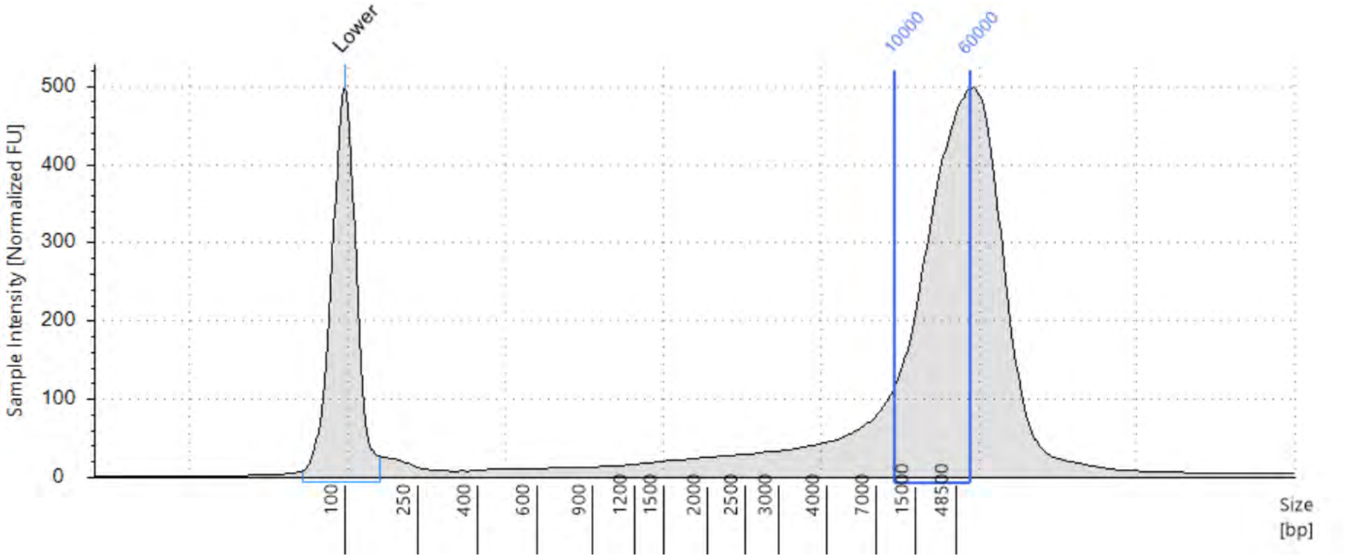

Region Table

From [bp]	To [bp]	Average Size [bp]	Conc. [ng/µl]	Region Molarity [nmol/l]	% of Total	Region Comment	Color
10000	60000	32036	14.4	0.914	41.86		

A7: M228

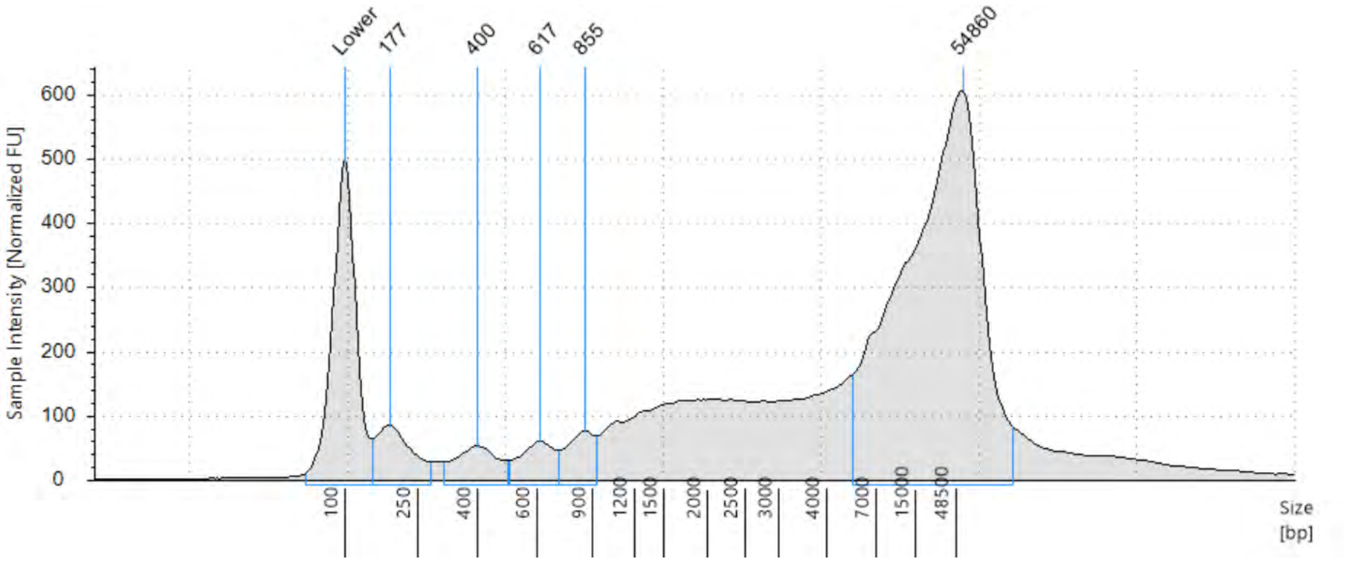

Sample Table

Well	DIN	Conc. [ng/µl]	Sample Description	Alert	Observations
A7	6.5	65.1	M228		

Peak Table

Size [bp]	Calibrated Conc. [ng/µl]	Assigned Conc. [ng/µl]	% Integrated Area	From [bp]	To [bp]	Peak Comment	Observations
100	8.50	8.50	-	61	141		Lower Marker
177	2.15	-	5.23	141	278		
400	1.52	-	3.71	306	492		
617	1.45	-	3.54	496	709		
855	1.48	-	3.61	709	924		
54860	34.2	-	83.22	5424	>60000		
-	-	-	-	-	-		Sample Well

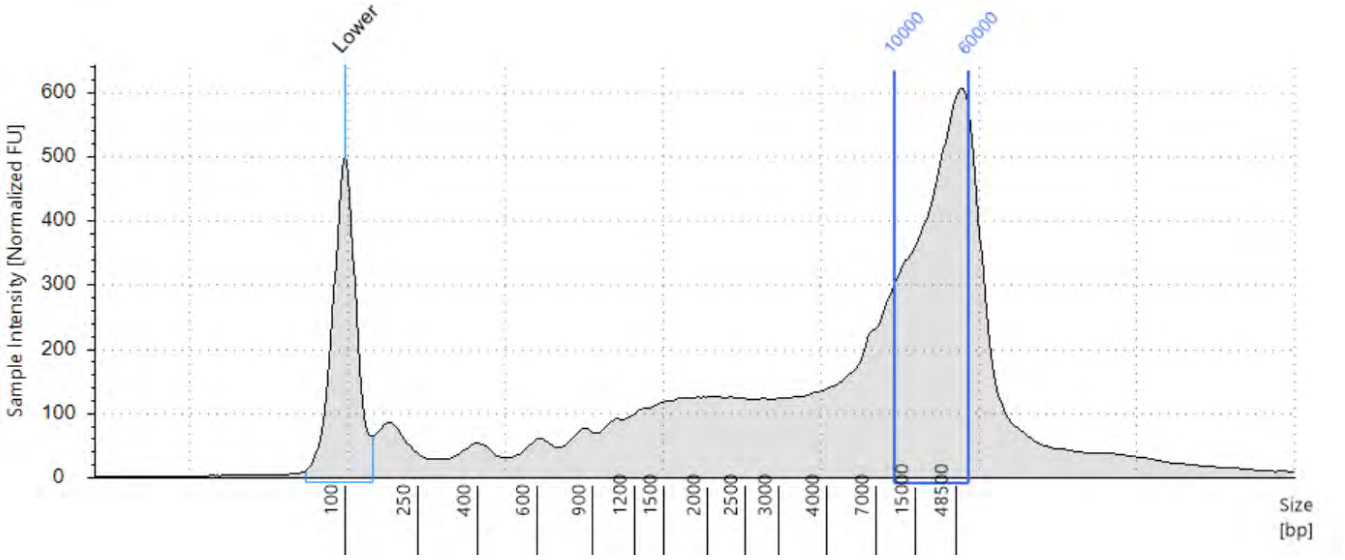

Region Table

From [bp]	To [bp]	Average Size [bp]	Conc. [ng/µl]	Region Molarity [nmol/l]	% of Total	Region Comment	Color
10000	60000	30452	21.4	1.48	32.91		

© Agilent Technologies, Inc. 2017

B7: M229

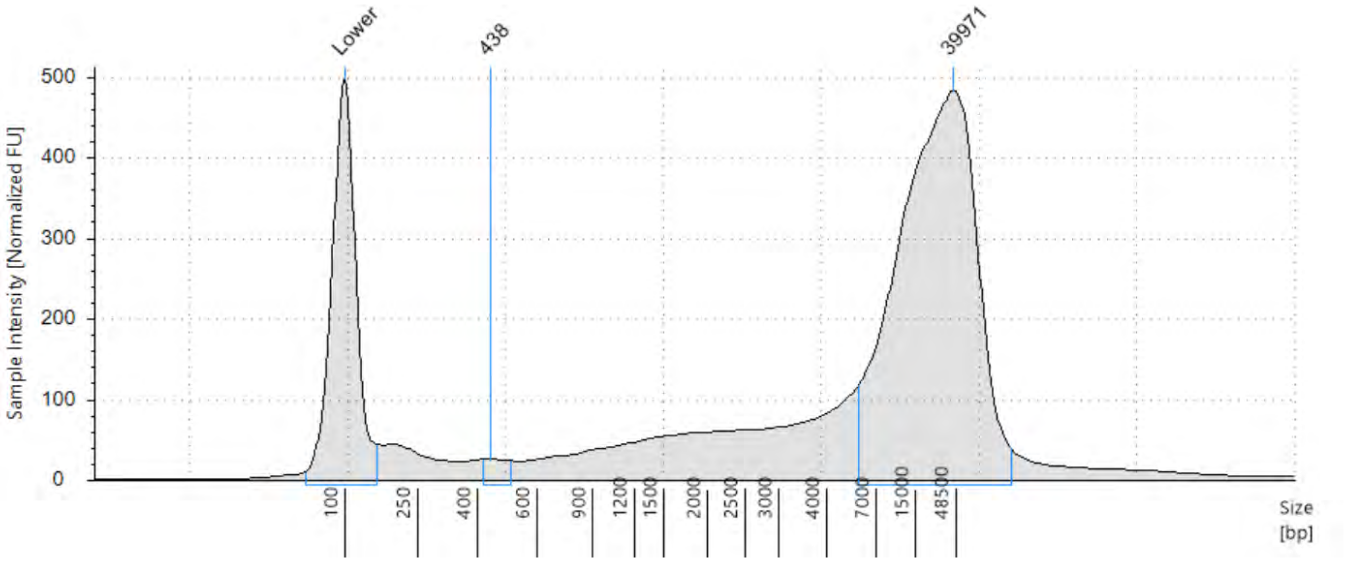

Sample Table

Well	DIN	Conc. [ng/µl]	Sample Description	Alert	Observations
B7	6.9	42.5	M229		

Peak Table

Size [bp]	Calibrated Conc. [ng/µl]	Assigned Conc. [ng/µl]	% Integrated Area	From [bp]	To [bp]	Peak Comment	Observations
100	8.50	8.50	-	61	151		Lower Marker
438	0.405	-	1.46	418	504		
39971	27.2	-	98.18	5762	>60000		
-	-	-	-	-	-		Sample Well

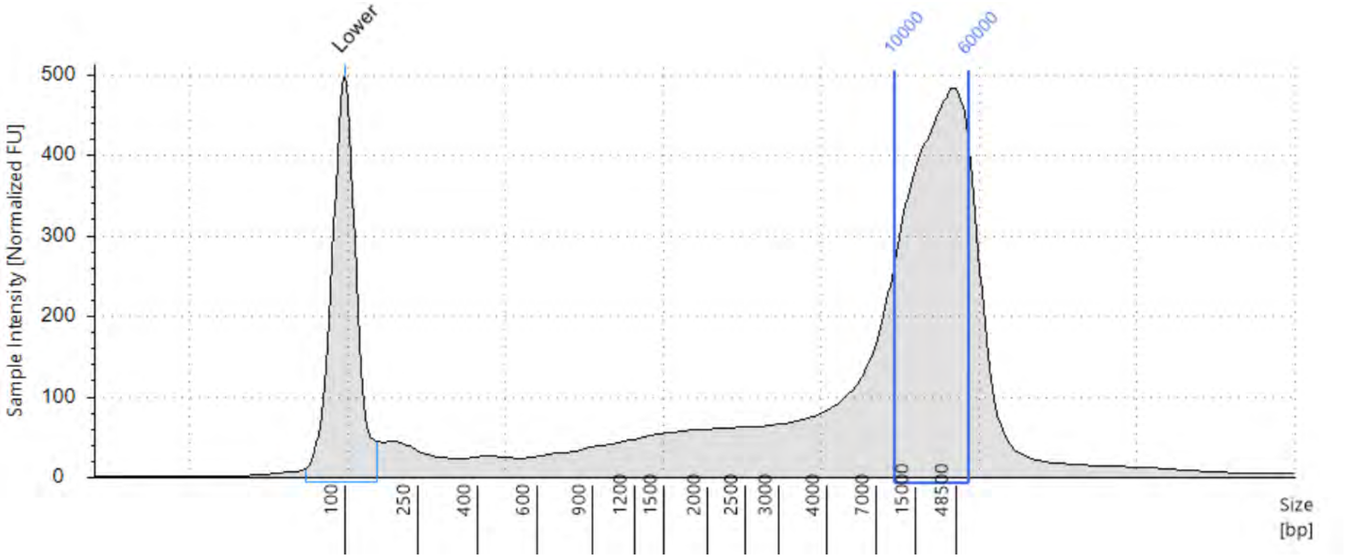

Region Table

From [bp]	To [bp]	Average Size [bp]	Conc. [ng/µl]	Region Molarity [nmol/l]	% of Total	Region Comment	Color
10000	60000	28564	19.1	1.38	44.90		

C7: M230

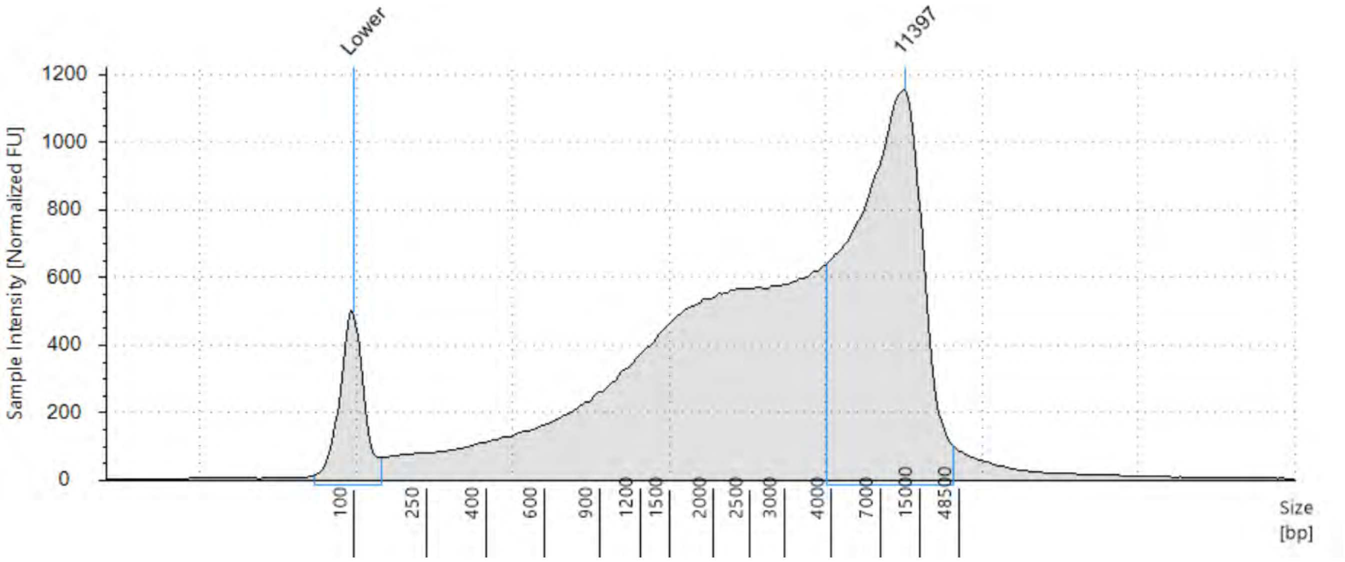

Sample Table

Well	DIN	Conc. [ng/µl]	Sample Description	Alert	Observations
C7	5.6	148	M230	⚠	Sample concentration outside recommended range

Peak Table

Size [bp]	Calibrated Conc. [ng/µl]	Assigned Conc. [ng/µl]	% Integrated Area	From [bp]	To [bp]	Peak Comment	Observations
100	8.50	8.50	-	61	143		Lower Marker
11397	58.6	-	100.00	3908	31780		

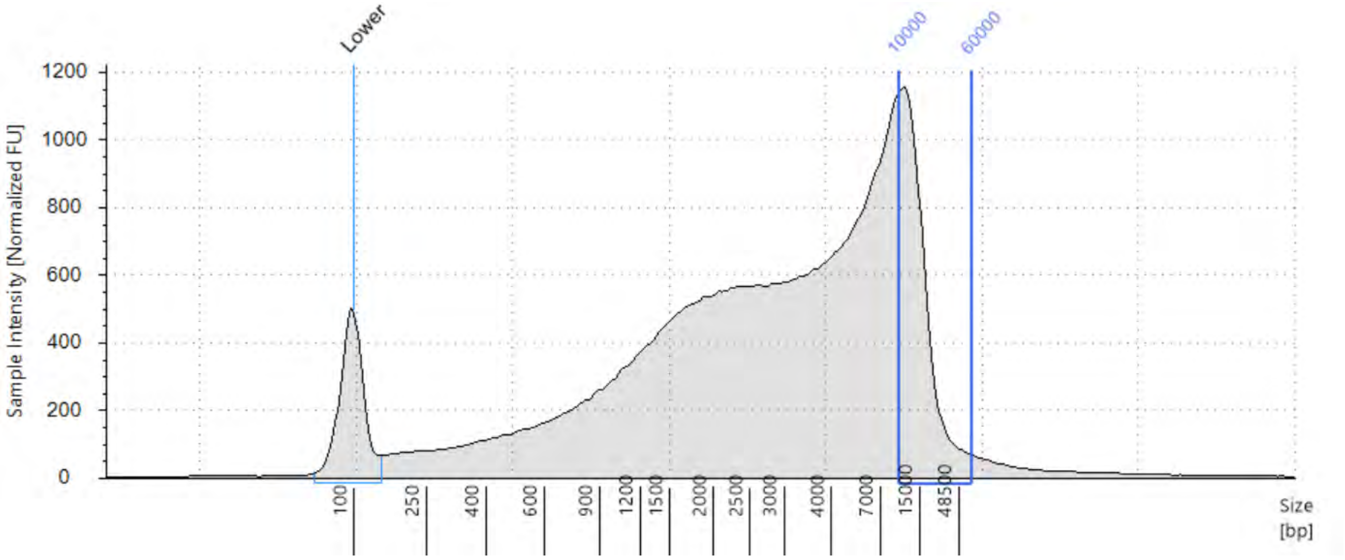

Region Table

From [bp]	To [bp]	Average Size [bp]	Conc. [ng/µl]	Region Molarity [nmol/l]	% of Total	Region Comment	Color
10000	60000	16185	23.1	2.56	15.56		■

D7: M231

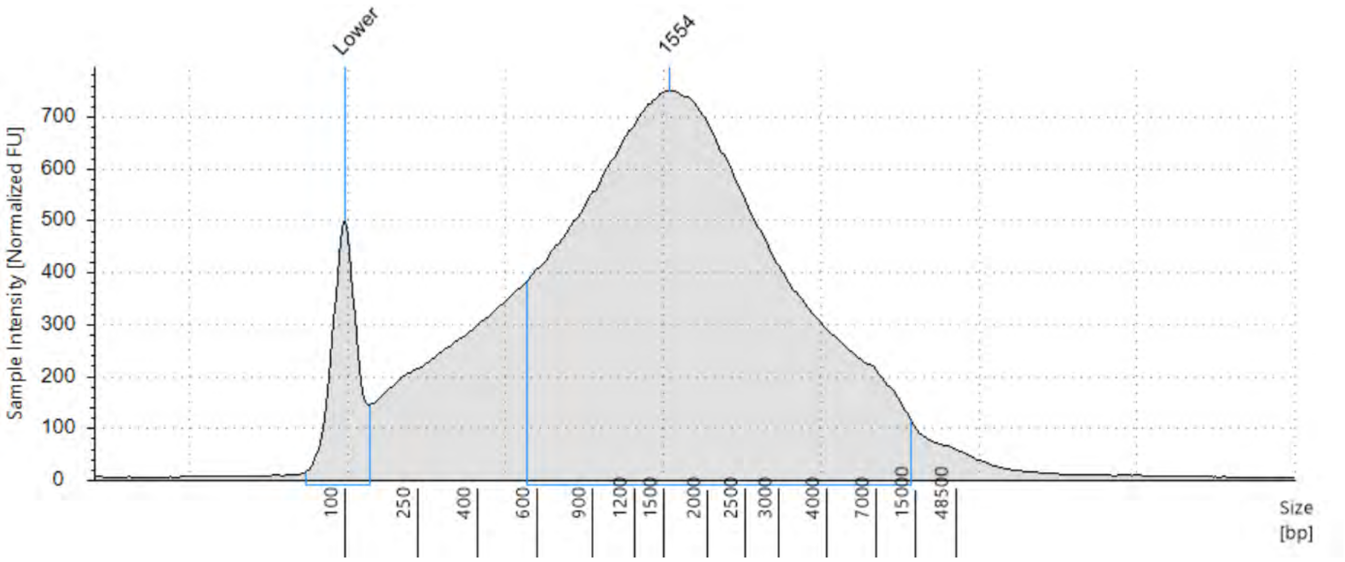

Sample Table

Well	DIN	Conc. [ng/µl]	Sample Description	Alert	Observations
D7	3.0	134	M231	⚠	Sample concentration outside recommended range

Peak Table

Size [bp]	Calibrated Conc. [ng/µl]	Assigned Conc. [ng/µl]	% Integrated Area	From [bp]	To [bp]	Peak Comment	Observations
100	8.50	8.50	-	61	136		Lower Marker
1554	108	-	100.00	561	13638		

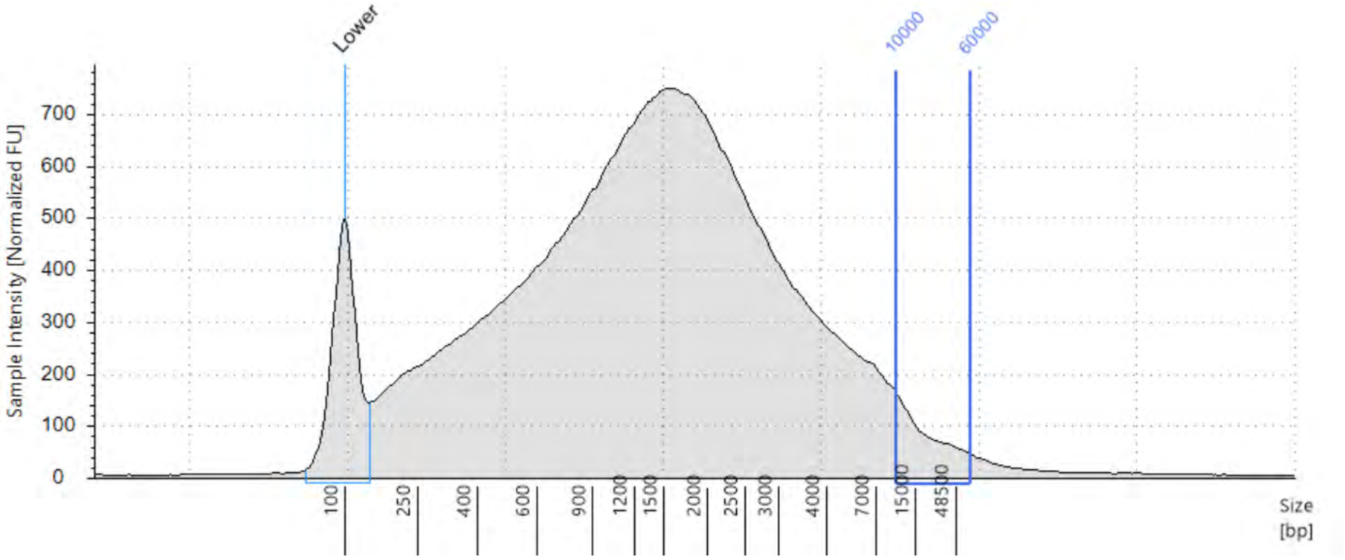

Region Table

From [bp]	To [bp]	Average Size [bp]	Conc. [ng/µl]	Region Molarity [nmol/l]	% of Total	Region Comment	Color
10000	60000	22374	3.57	0.349	2.66		■

E7: M232

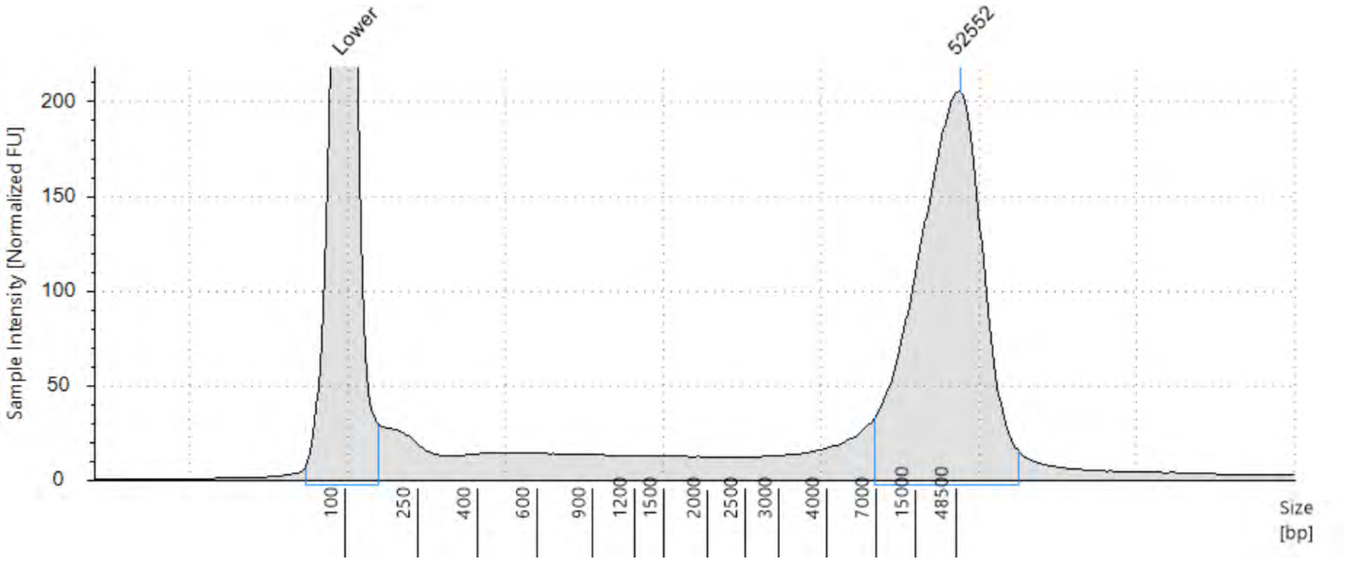

Sample Table

Well	DIN	Conc. [ng/µl]	Sample Description	Alert	Observations
E7	7.4	15.1	M232		

Peak Table

Size [bp]	Calibrated Conc. [ng/µl]	Assigned Conc. [ng/µl]	% Integrated Area	From [bp]	To [bp]	Peak Comment	Observations
100	8.50	8.50	-	61	154		Lower Marker
52552	9.78	-	97.74	6954	>60000		
-	-	-	-	-	-		Sample Well

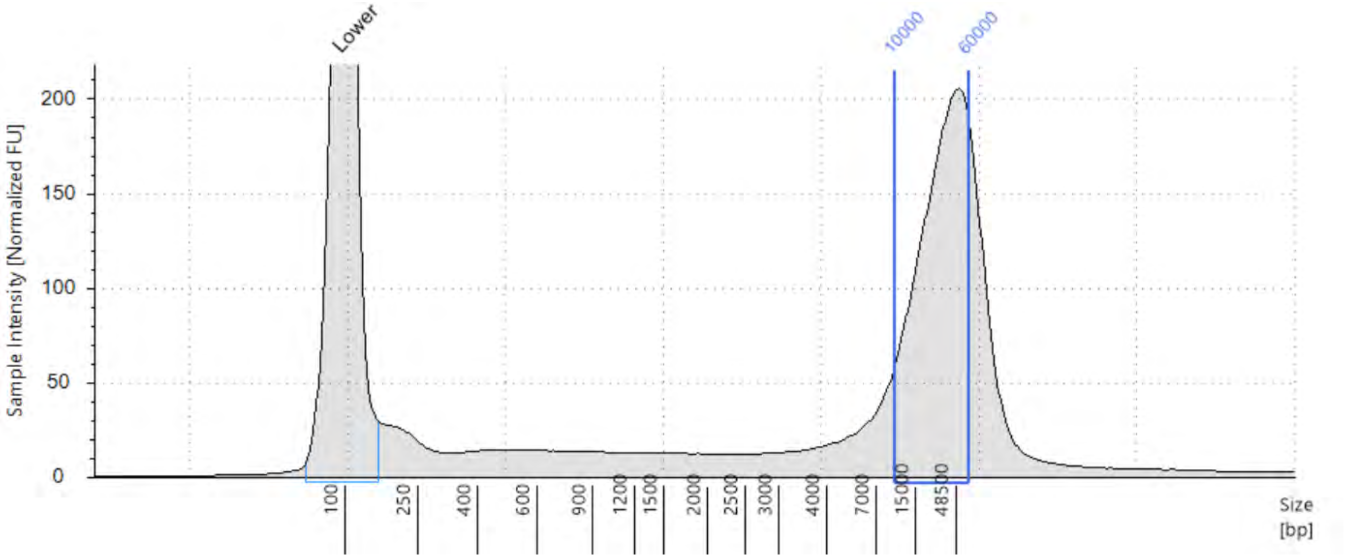

Region Table

From [bp]	To [bp]	Average Size [bp]	Conc. [ng/µl]	Region Molarity [nmol/l]	% of Total	Region Comment	Color
10000	60000	31315	6.88	0.450	45.48		■

F7: M233

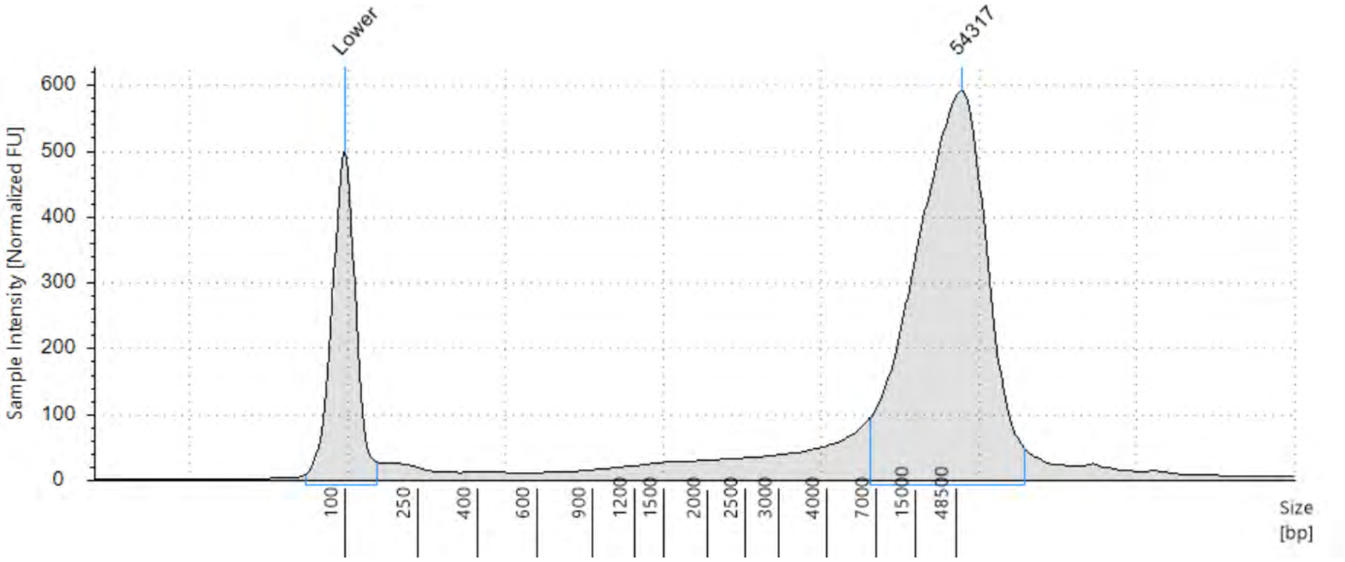

Sample Table

Well	DIN	Conc. [ng/µl]	Sample Description	Alert	Observations
F7	8.0	41.2	M233		

Peak Table

Size [bp]	Calibrated Conc. [ng/µl]	Assigned Conc. [ng/µl]	%Integrated Area	From [bp]	To [bp]	Peak Comment	Observations
100	8.50	8.50	-	61	151		Lower Marker
54317	30.8	-	99.13	6627	>60000		
-	-	-	-	-	-		Sample Well

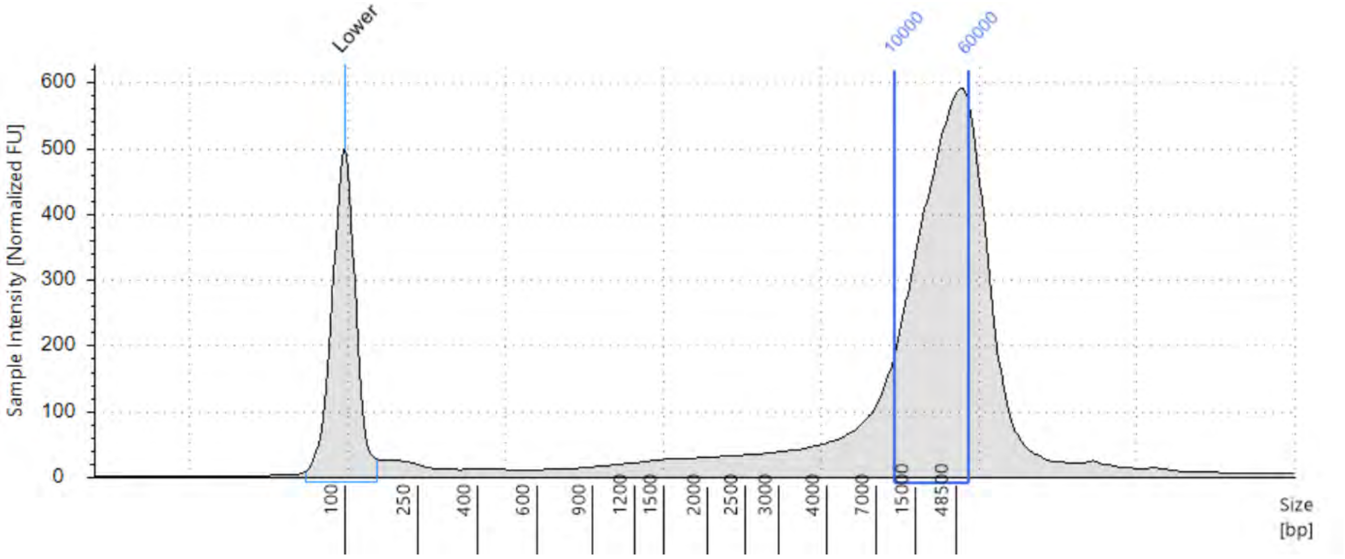

Region Table

From [bp]	To [bp]	Average Size [bp]	Conc. [ng/µl]	Region Molarity [nmol/l]	% of Total	Region Comment	Color
10000	60000	30668	20.1	1.34	48.75		

G7: M234

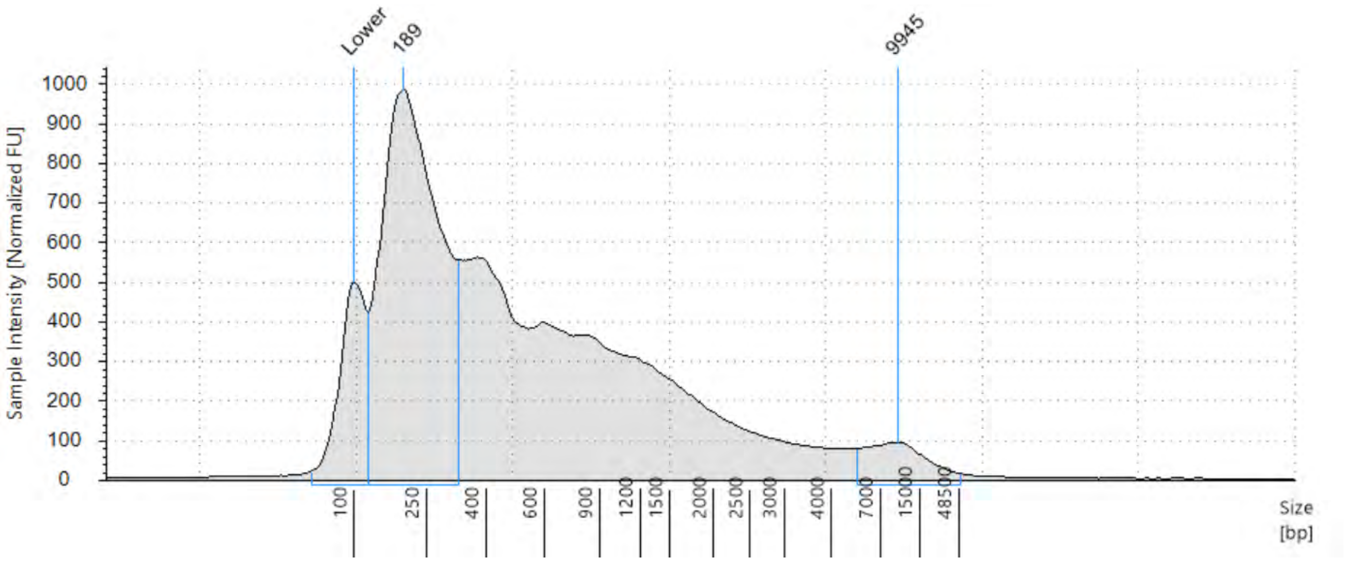

Sample Table

Well	DIN	Conc. [ng/µl]	Sample Description	Alert	Observations
G7	1.6	99.6	M234		

Peak Table

Size [bp]	Calibrated Conc. [ng/µl]	Assigned Conc. [ng/µl]	% Integrated Area	From [bp]	To [bp]	Peak Comment	Observations
100	8.50	8.50	-	58	121		Lower Marker
189	37.4	-	91.26	121	325		
9945	3.58	-	8.74	5421	49727		

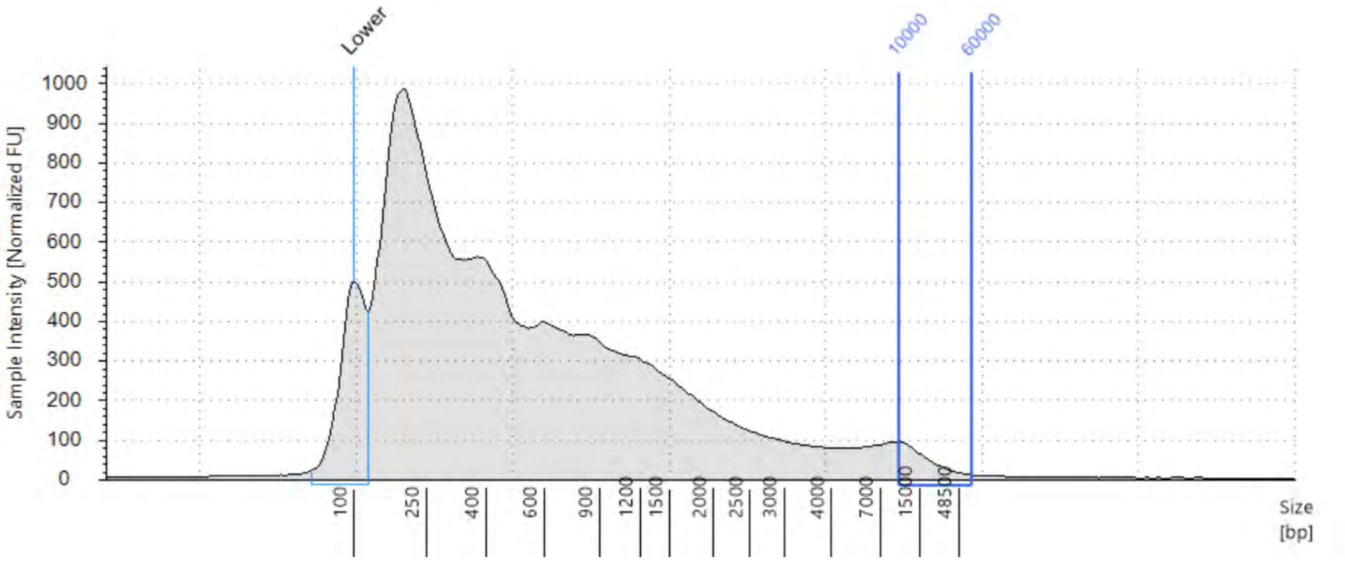

Region Table

From [bp]	To [bp]	Average Size [bp]	Conc. [ng/µl]	Region Molarity [nmol/l]	% of Total	Region Comment	Color
10000	60000	18915	1.73	0.190	1.73		

H7: M235

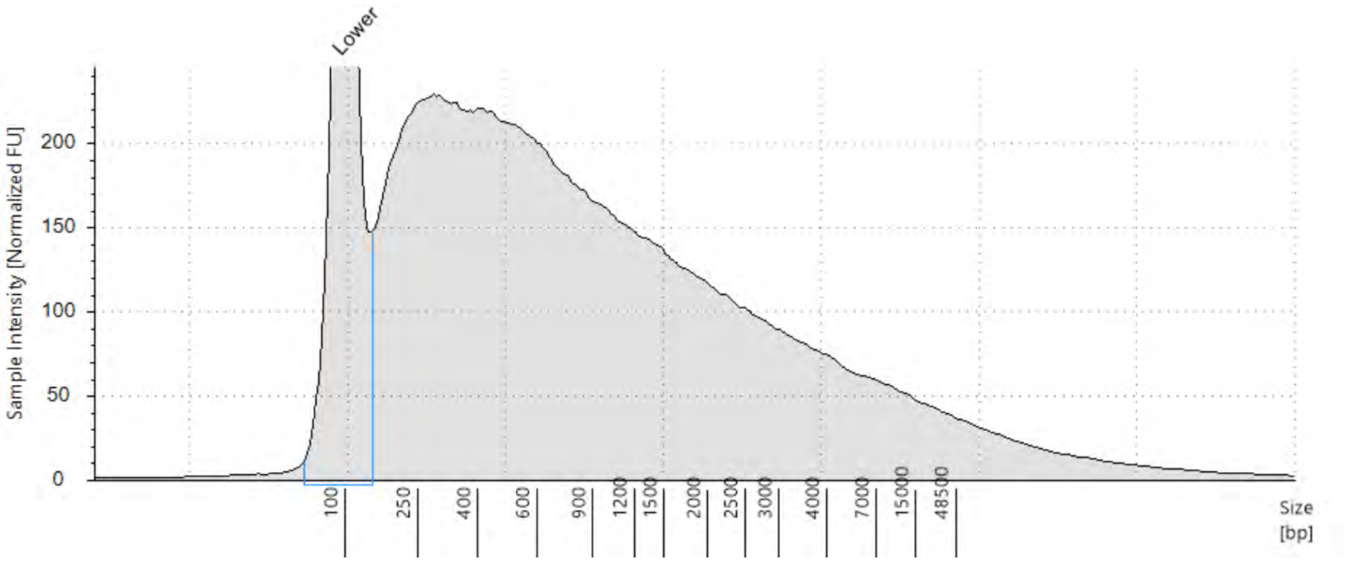

Sample Table

Well	DIN	Conc. [ng/µl]	Sample Description	Alert	Observations
H7	1.7	46.1	M235		

Peak Table

Size [bp]	Calibrated Conc. [ng/µl]	Assigned Conc. [ng/µl]	% Integrated Area	From [bp]	To [bp]	Peak Comment	Observations
100	8.50	8.50	-	60	141		Lower Marker

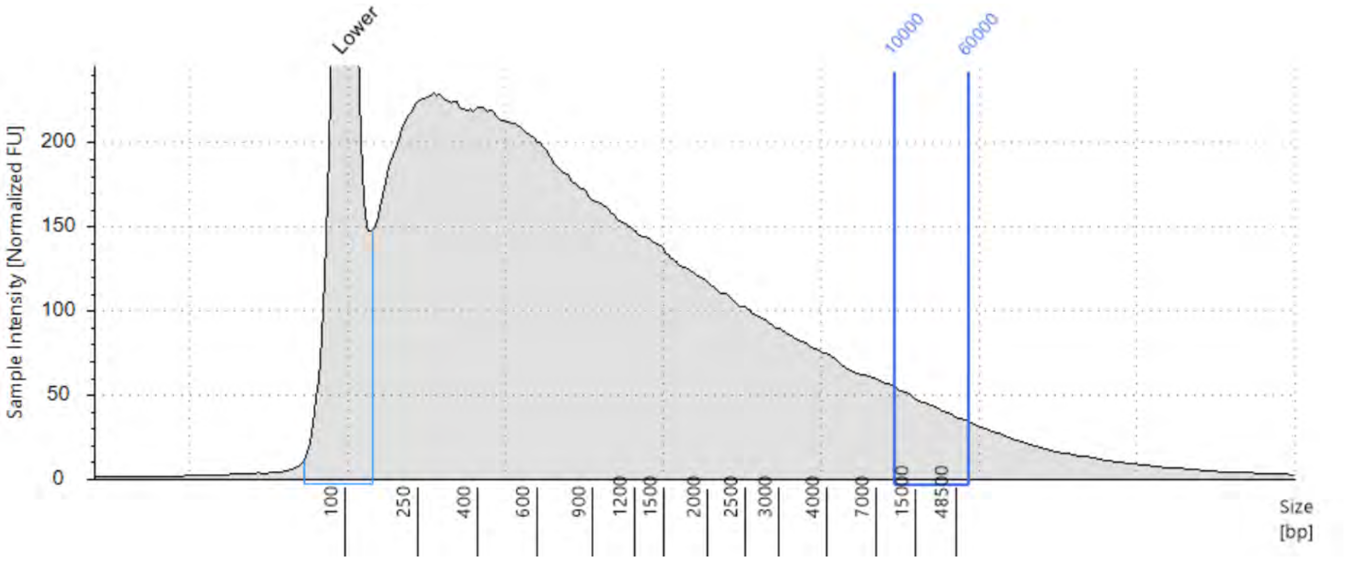

Region Table

From [bp]	To [bp]	Average Size [bp]	Conc. [ng/µl]	Region Molarity [nmol/l]	% of Total	Region Comment	Color
10000	60000	24963	1.79	0.155	3.87		

A8: M236

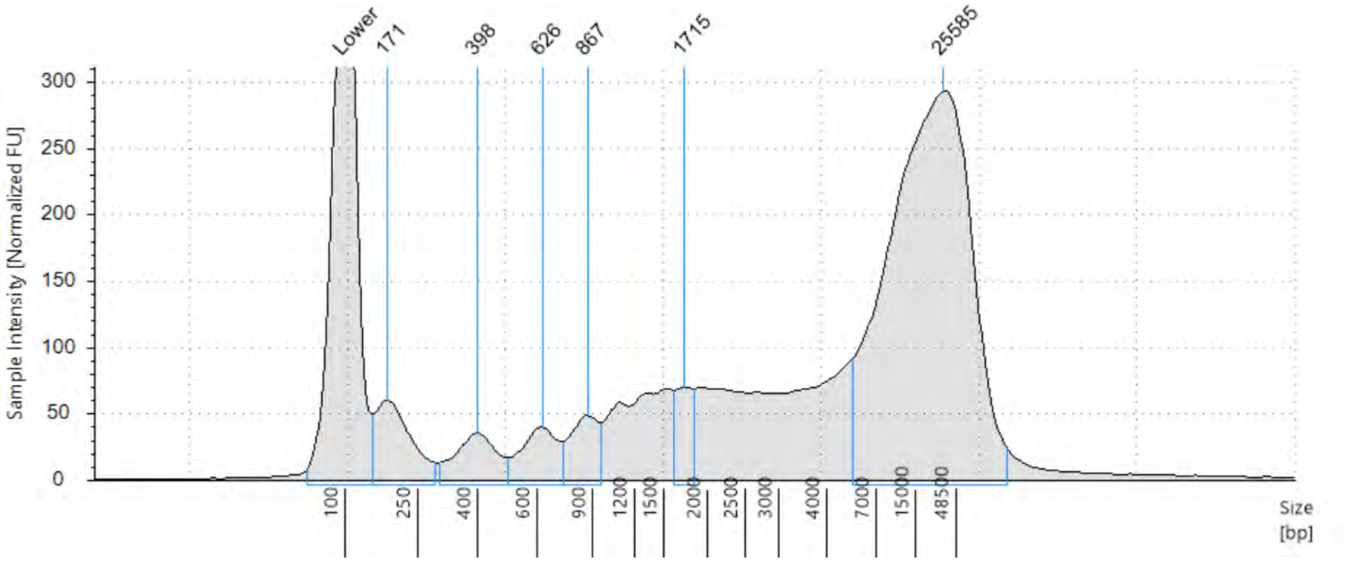

Sample Table

Well	DIN	Conc. [ng/µl]	Sample Description	Alert	Observations
A8	6.4	34.9	M236		

Peak Table

Size [bp]	Calibrated Conc. [ng/µl]	Assigned Conc. [ng/µl]	% Integrated Area	From [bp]	To [bp]	Peak Comment	Observations
100	8.50	8.50	-	62	141		Lower Marker
171	1.60	-	6.53	141	286		
398	1.03	-	4.19	296	490		
626	1.06	-	4.33	490	726		
867	1.04	-	4.24	726	955		
1715	0.977	-	3.97	1597	1826		
25585	18.8	-	76.49	5323	>60000		
-	-	-	-	-	-		Sample Well

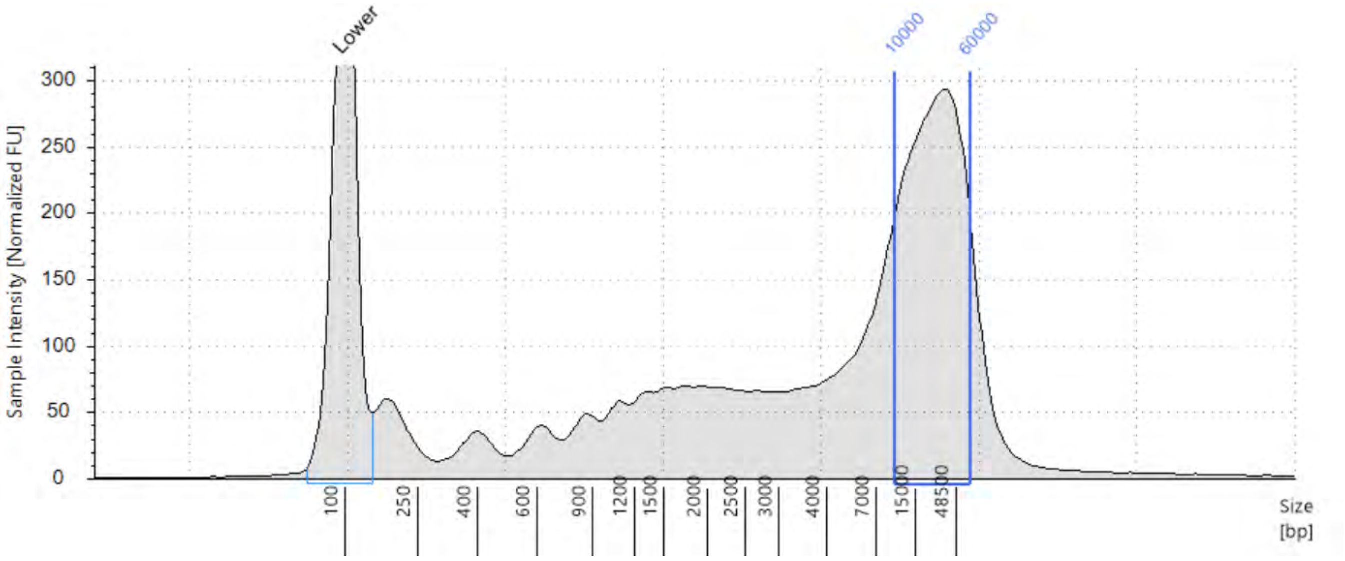

Region Table

From [bp]	To [bp]	Average Size [bp]	Conc. [ng/µl]	Region Molarity [nmol/l]	% of Total	Region Comment	Color
10000	60000	26846	12.9	0.982	36.90		

B8: M237

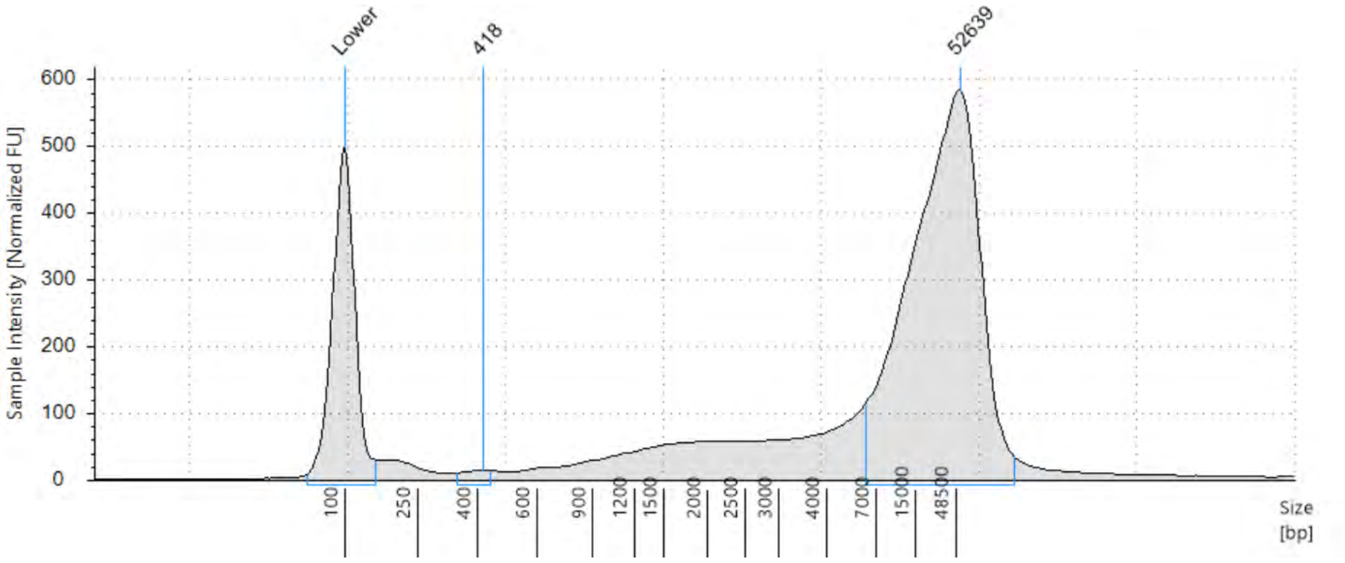

Sample Table

Well	DIN	Conc. [ng/µl]	Sample Description	Alert	Observations
B8	7.3	44.6	M237		

Peak Table

Size [bp]	Calibrated Conc. [ng/µl]	Assigned Conc. [ng/µl]	% Integrated Area	From [bp]	To [bp]	Peak Comment	Observations
100	8.50	8.50	-	62	148		Lower Marker
418	0.254	-	0.82	341	438		
52639	30.5	-	98.47	6222	>60000		
-	-	-	-	-	-		Sample Well

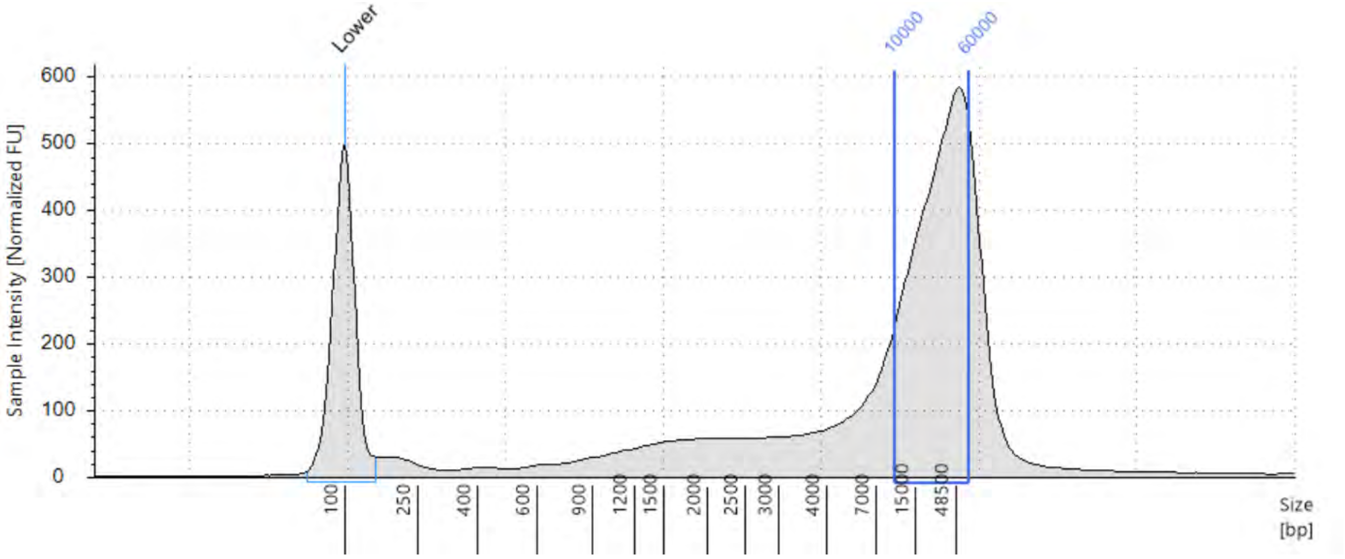

Region Table

From [bp]	To [bp]	Average Size [bp]	Conc. [ng/µl]	Region Molarity [nmol/l]	% of Total	Region Comment	Color
10000	60000	30469	21.5	1.46	48.08		■

C8: M238

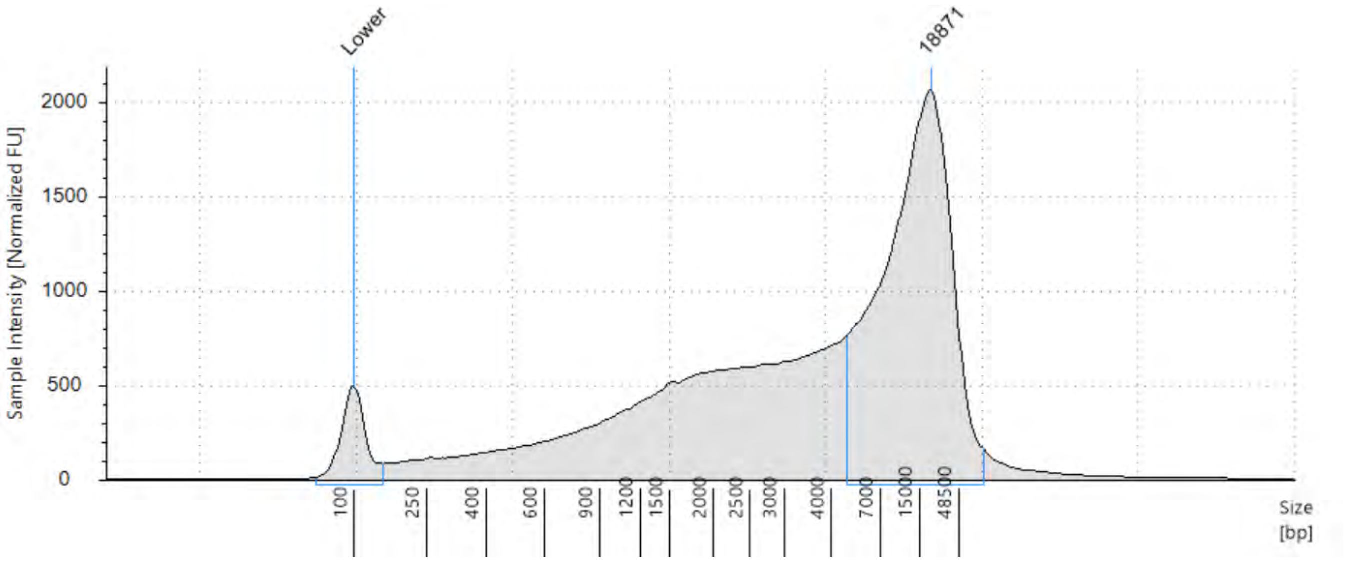

Sample Table

Well	DIN	Conc. [ng/µl]	Sample Description	Alert	Observations
C8	6.1	211	M238	⚠	Sample concentration outside recommended range

Peak Table

Size [bp]	Calibrated Conc. [ng/µl]	Assigned Conc. [ng/µl]	% Integrated Area	From [bp]	To [bp]	Peak Comment	Observations
100	8.50	8.50	-	62	146		Lower Marker
18871	102	-	99.53	4836	>60000		
-	-	-	-	-	-		Sample Well

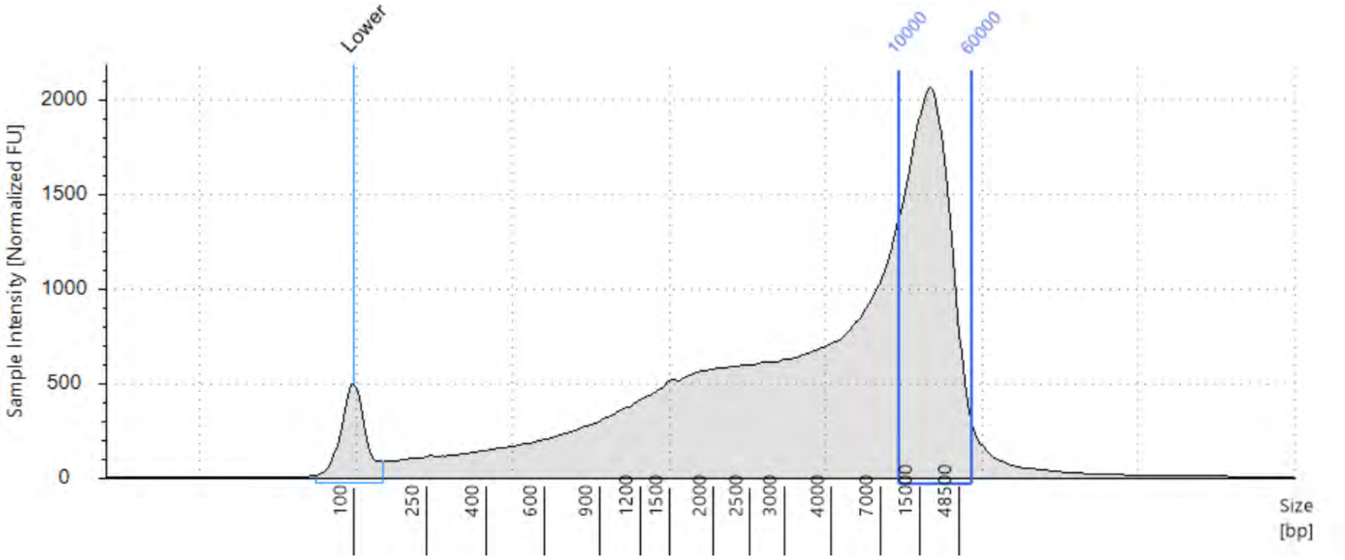

Region Table

From [bp]	To [bp]	Average Size [bp]	Conc. [ng/µl]	Region Molarity [nmol/l]	% of Total	Region Comment	Color
10000	60000	21736	69.2	5.99	32.82		■

 © Agilent Technologies, Inc. 2017

D8: M239

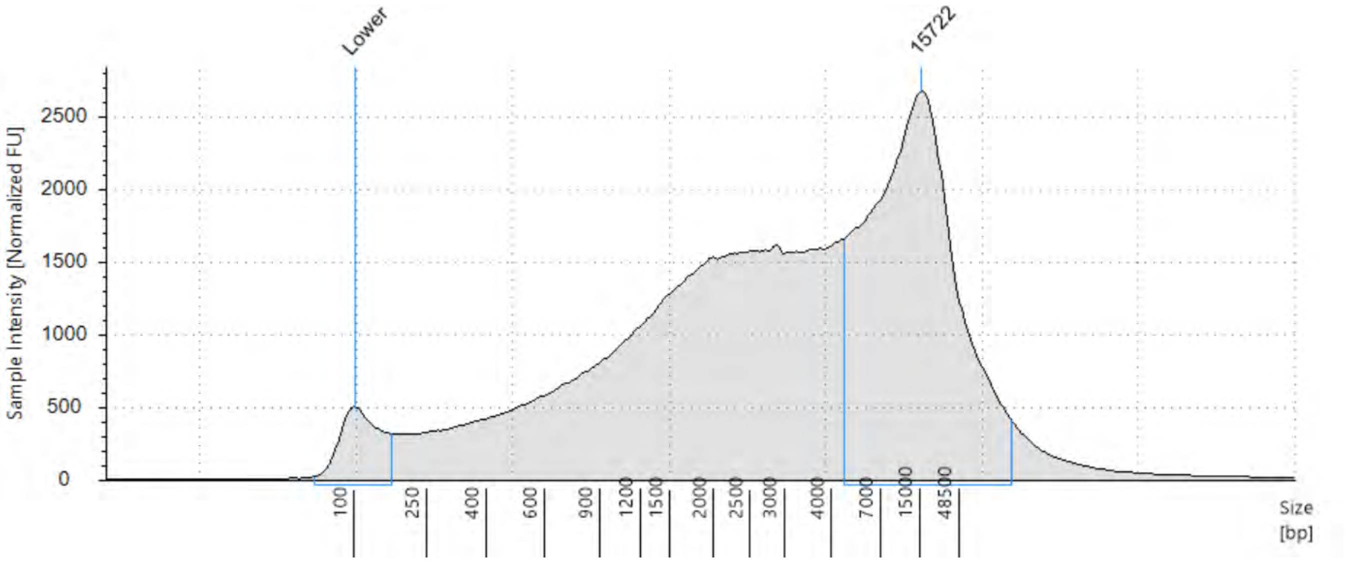

Sample Table

Well	DIN	Conc. [ng/µl]	Sample Description	Alert	Observations
D8	5.7	270	M239	⚠	Sample concentration outside recommended range

Peak Table

Size [bp]	Calibrated Conc. [ng/µl]	Assigned Conc. [ng/µl]	% Integrated Area	From [bp]	To [bp]	Peak Comment	Observations
100	8.50	8.50	-	60	159		Lower Marker
15722	103	-	99.73	4617	>60000		
-	-	-	-	-	-		Sample Well

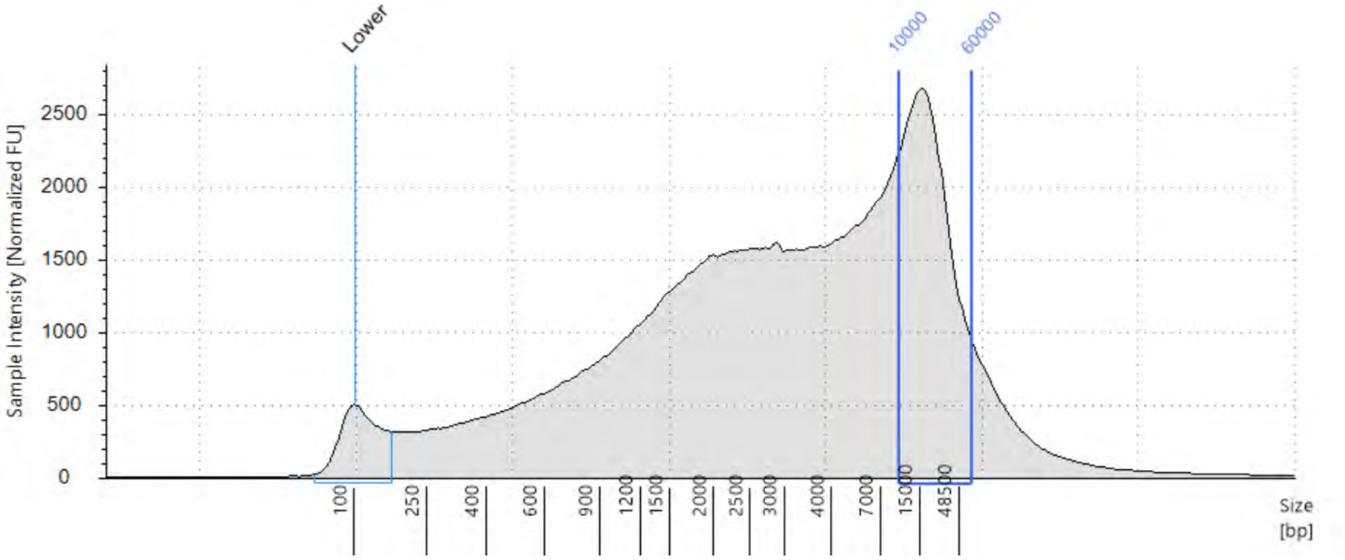

Region Table

From [bp]	To [bp]	Average Size [bp]	Conc. [ng/µl]	Region Molarity [nmol/l]	% of Total	Region Comment	Color
10000	60000	22556	55.9	4.87	20.68		■

E8: M240

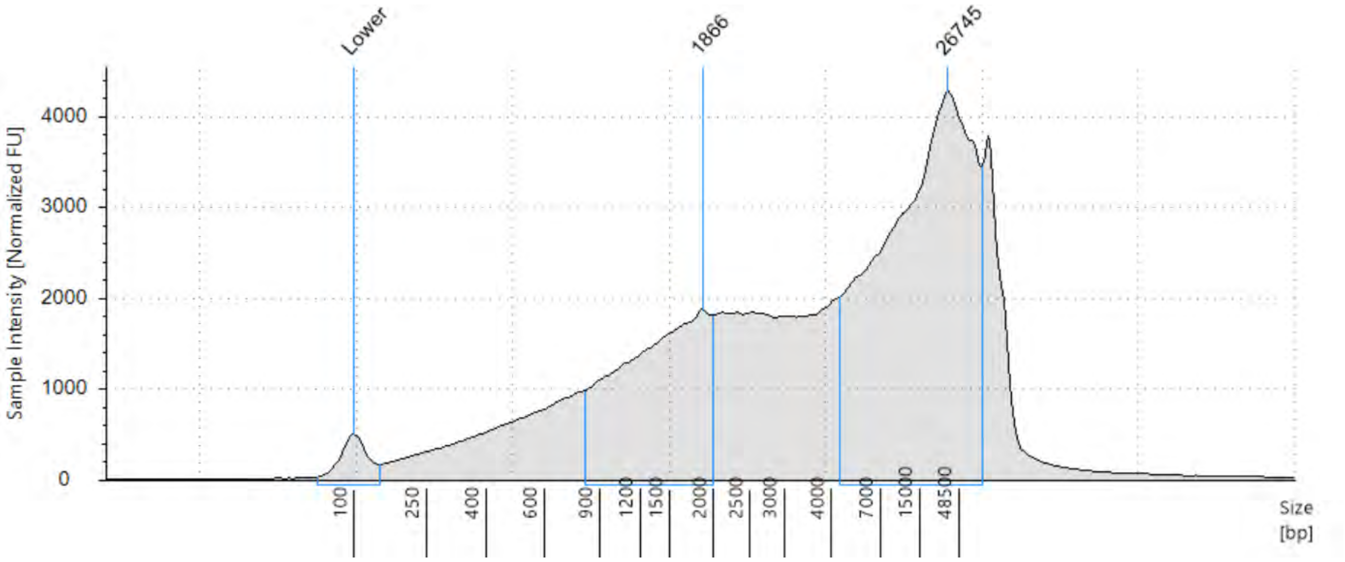

Sample Table

Well	DIN	Conc. [ng/µl]	Sample Description	Alert	Observations
E8	6.2	616	M240	⚠	Sample concentration outside functional range for DIN

Peak Table

Size [bp]	Calibrated Conc. [ng/µl]	Assigned Conc. [ng/µl]	% Integrated Area	From [bp]	To [bp]	Peak Comment	Observations
100	8.50	8.50	-	63	139		Lower Marker
1866	108	-	29.43	809	2005		
26745	259	-	70.57	4418	>60000		

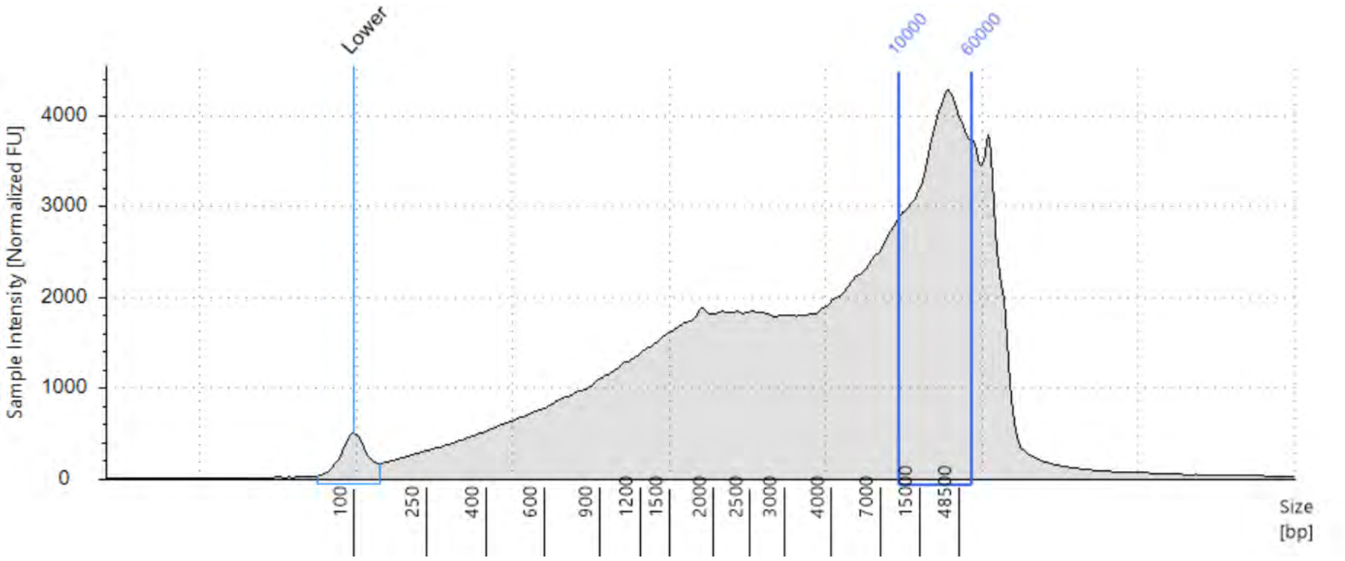

Region Table

From [bp]	To [bp]	Average Size [bp]	Conc. [ng/µl]	Region Molarity [nmol/l]	% of Total	Region Comment	Color
10000	60000	28250	157	11.5	25.48		■

F8: M241

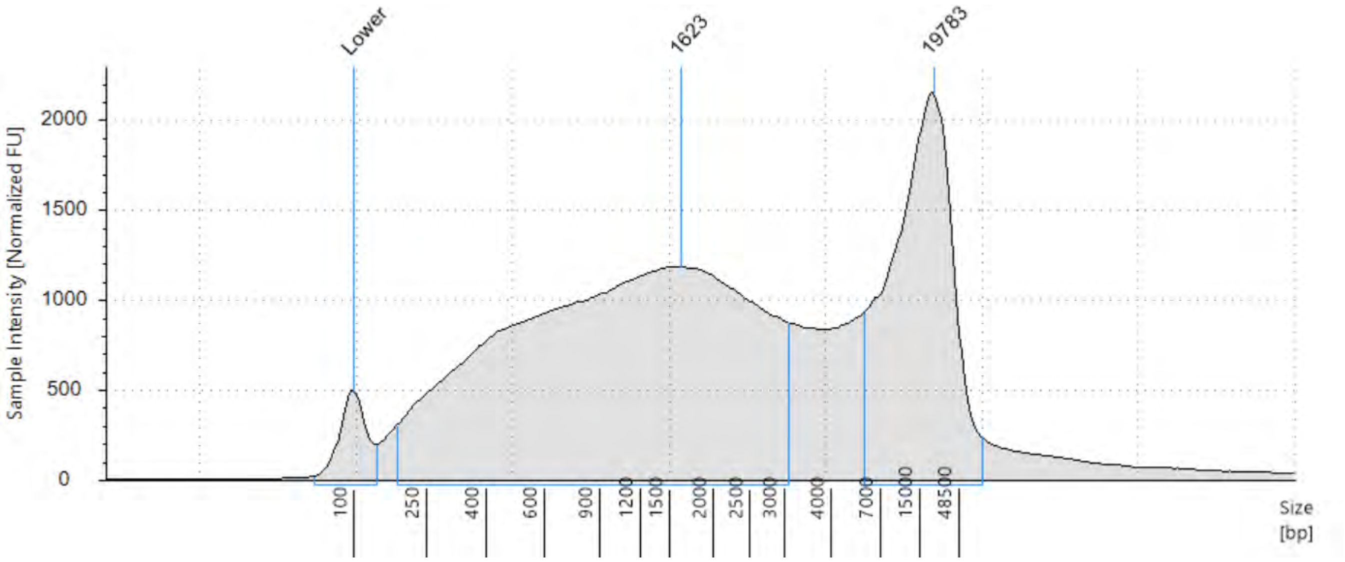

Sample Table

Well	DIN	Conc. [ng/µl]	Sample Description	Alert	Observations
F8	3.4	340	M241	⚠	Sample concentration outside functional range for DIN

Peak Table

Size [bp]	Calibrated Conc. [ng/µl]	Assigned Conc. [ng/µl]	% Integrated Area	From [bp]	To [bp]	Peak Comment	Observations
100	8.50	8.50	-	61	134		Lower Marker
1623	201	-	69.79	174	3091		
19783	86.4	-	30.01	5862	>60000		
-	-	-	-	-	-		Sample Well

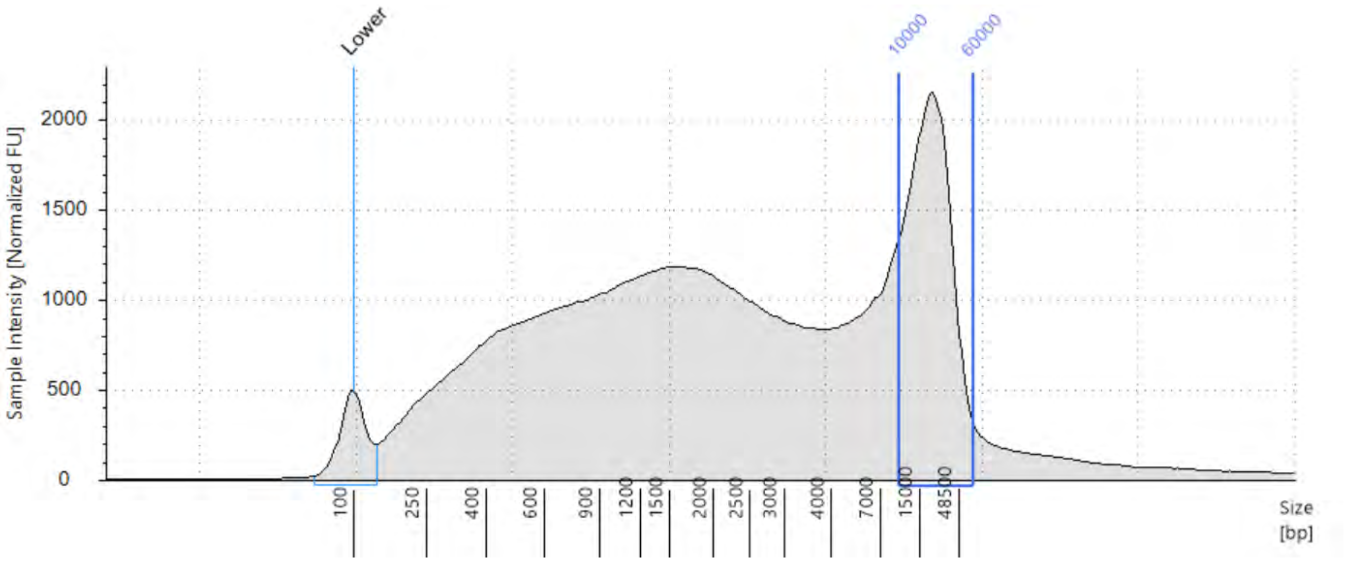

Region Table

From [bp]	To [bp]	Average Size [bp]	Conc. [ng/µl]	Region Molarity [nmol/l]	% of Total	Region Comment	Color
10000	60000	22074	63.6	5.42	18.69		■

G8: M242

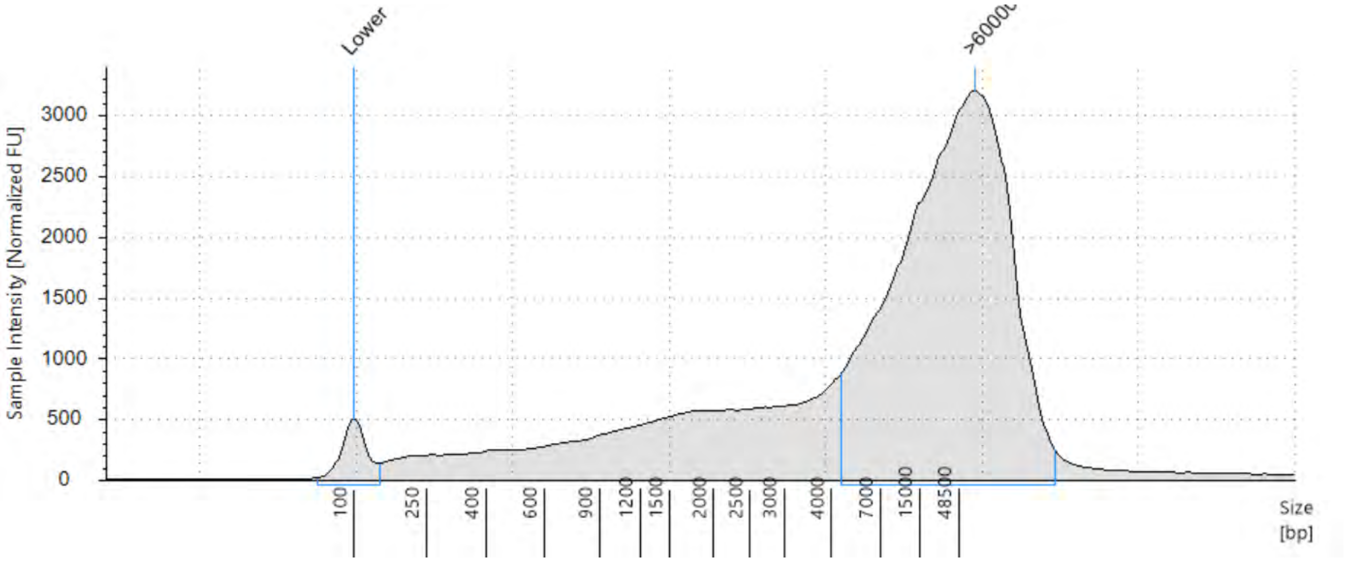

Sample Table

Well	DIN	Conc. [ng/µl]	Sample Description	Alert	Observations
G8	6.8	390	M242	⚠	Sample concentration outside functional range for DIN

Peak Table

Size [bp]	Calibrated Conc. [ng/µl]	Assigned Conc. [ng/µl]	% Integrated Area	From [bp]	To [bp]	Peak Comment	Observations
100	8.50	8.50	-	63	139		Lower Marker
>60000	262	-	97.47	4460	>60000		
-	-	-	-	-	-		Sample Well

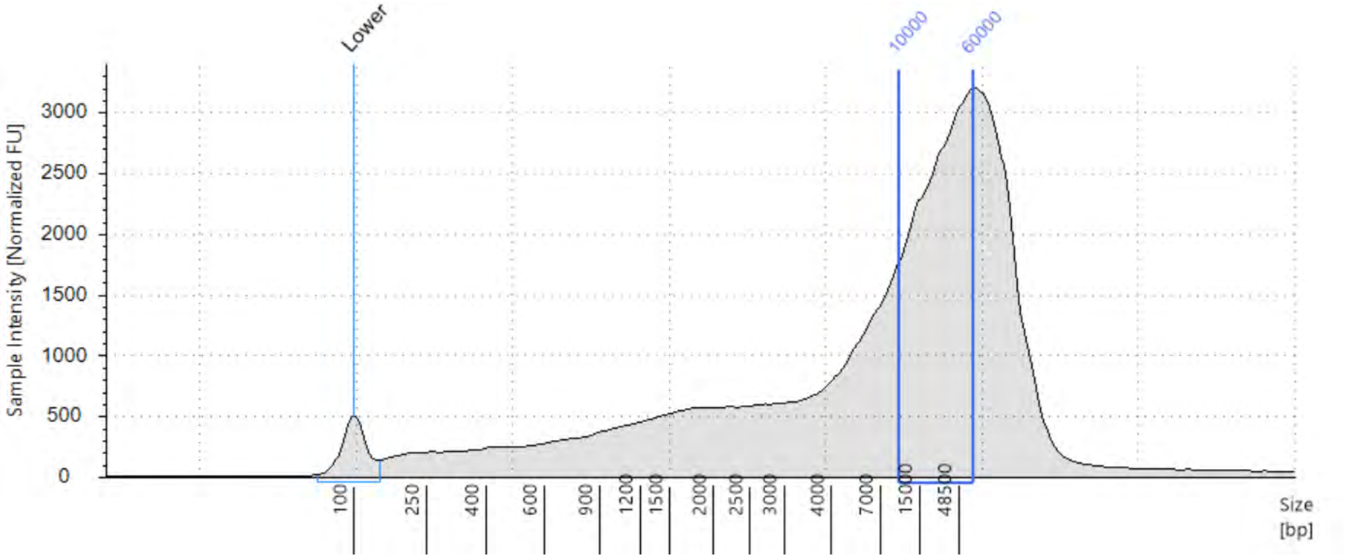

Region Table

From [bp]	To [bp]	Average Size [bp]	Conc. [ng/µl]	Region Molarity [nmol/l]	% of Total	Region Comment	Color
10000	60000	28998	120	8.66	30.73		■

H8: M243

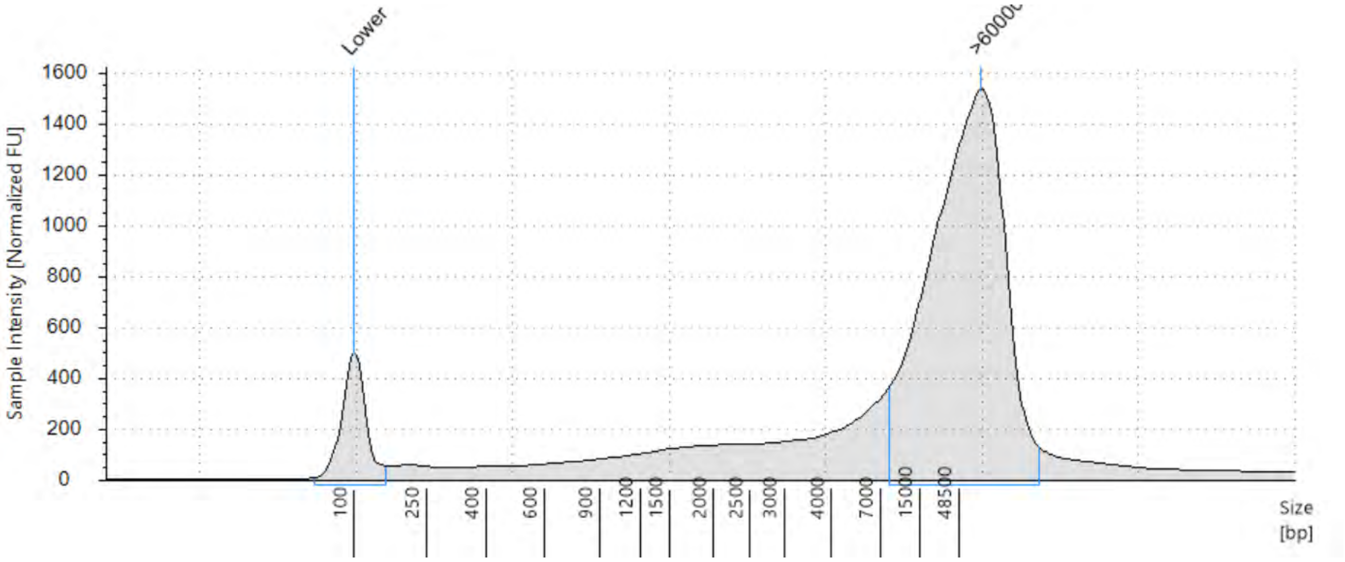

Sample Table

Well	DIN	Conc. [ng/µl]	Sample Description	Alert	Observations
H8	7.4	128	M243	⚠	Sample concentration outside recommended range

Peak Table

Size [bp]	Calibrated Conc. [ng/µl]	Assigned Conc. [ng/µl]	% Integrated Area	From [bp]	To [bp]	Peak Comment	Observations
100	8.50	8.50	-	60	150		Lower Marker
>60000	82.0	-	95.06	8360	>60000		
-	-	-	-	-	-		Sample Well

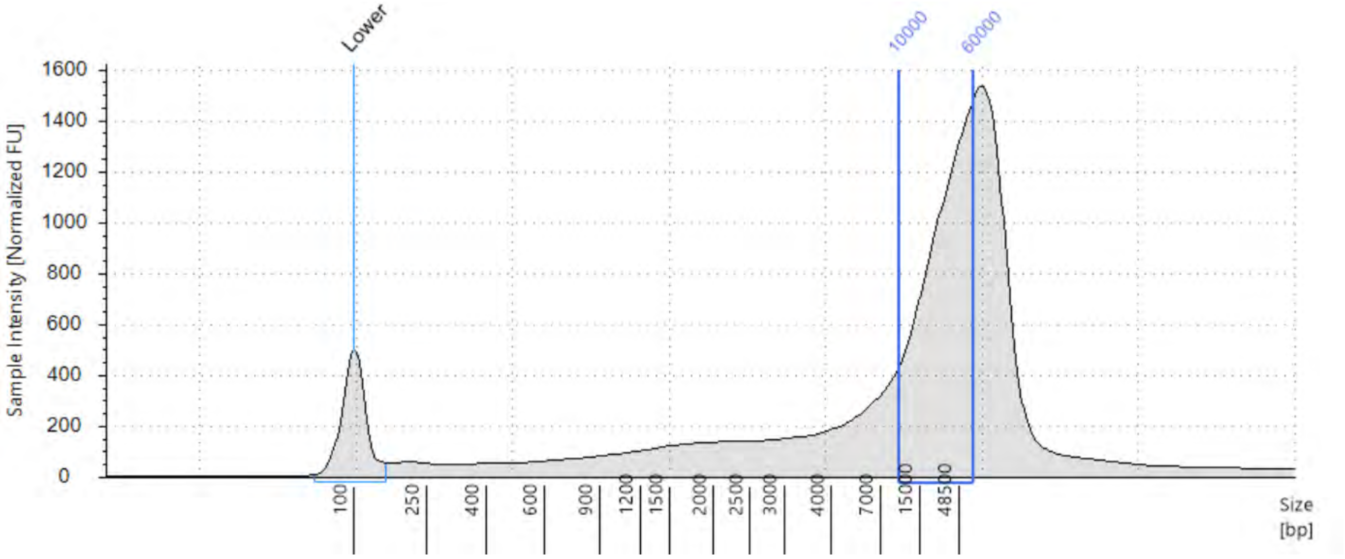

Region Table

From [bp]	To [bp]	Average Size [bp]	Conc. [ng/µl]	Region Molarity [nmol/l]	% of Total	Region Comment	Color
10000	60000	31819	44.2	2.88	34.56		■

Filename: 2018-12-03-01.gDNA

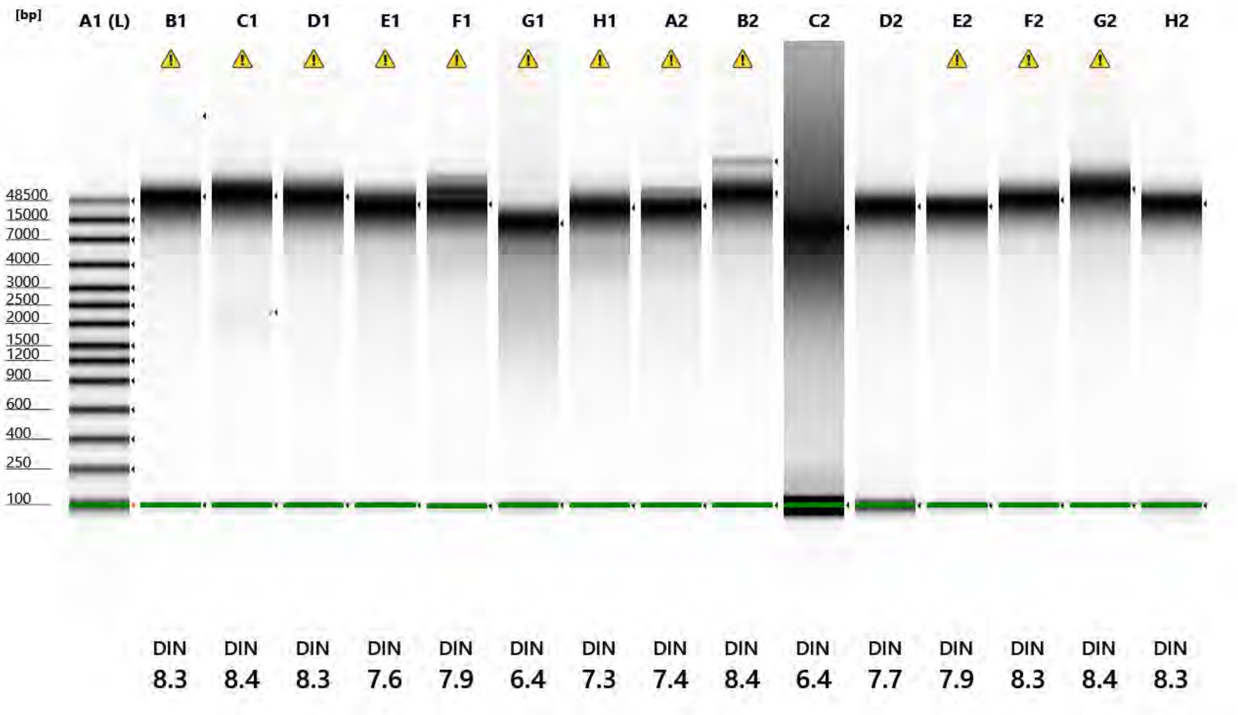

Default image (Contrast 50%), Image is Scaled to Sample

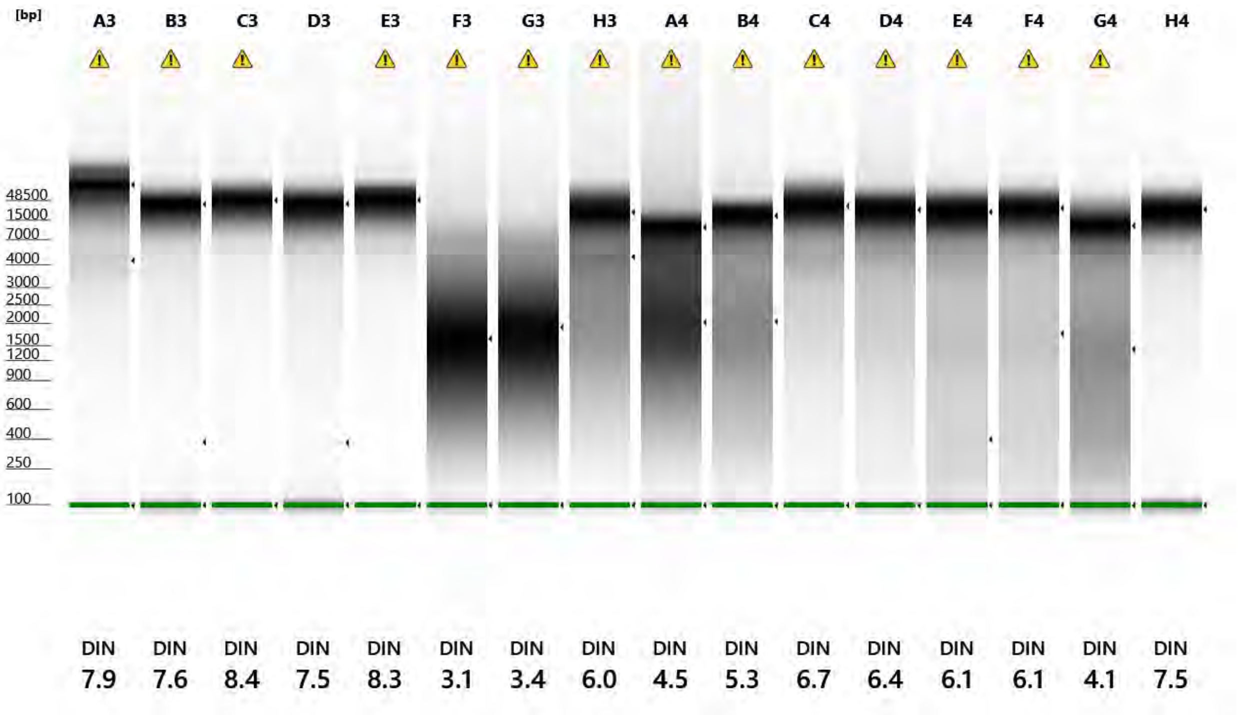

Default image (Contrast 50%), Image is Scaled to Sample

 © Agilent Technologies, Inc. 2017

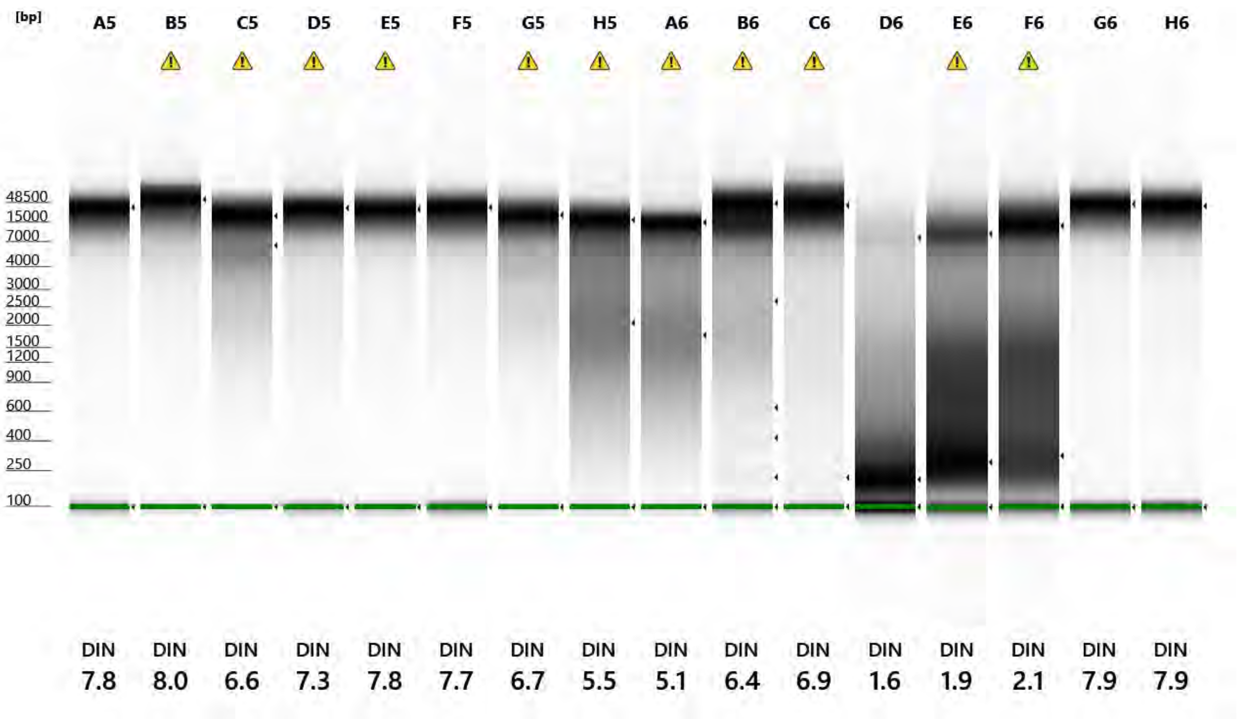

Default image (Contrast 50%), Image is Scaled to Sample

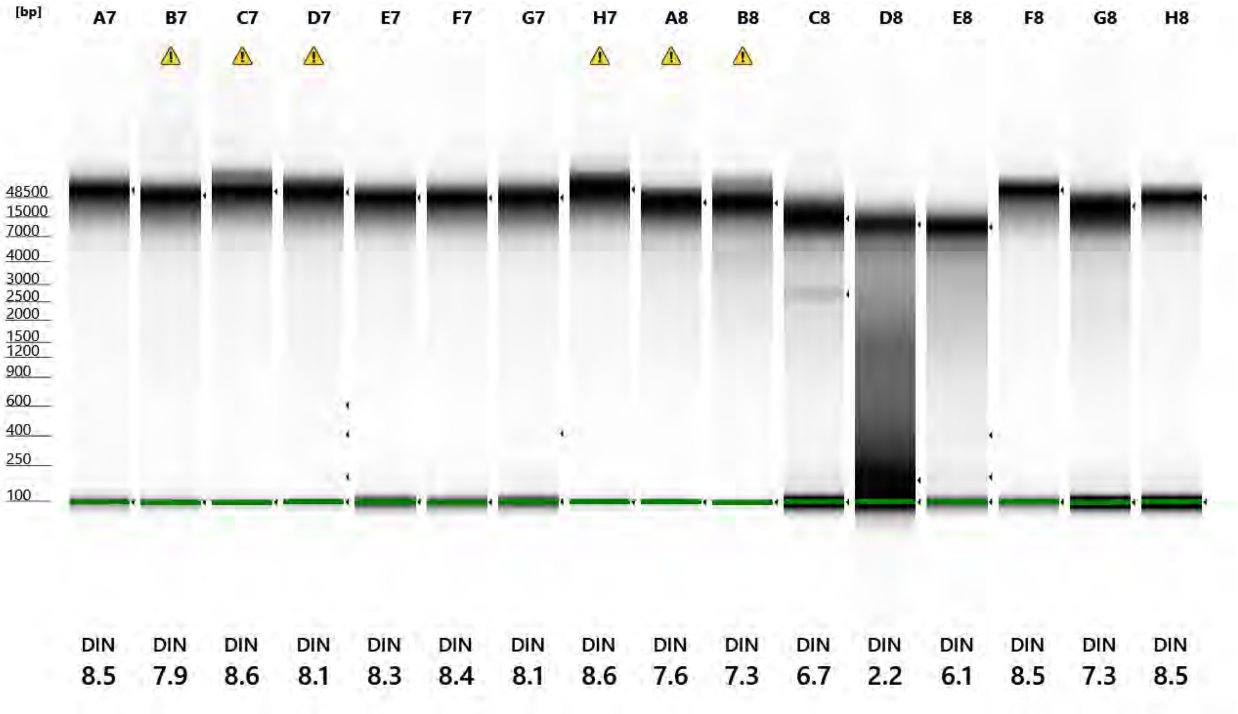

Default image (Contrast 50%), Image is Scaled to Sample

Sample Info

Well	DIN	Conc. [ng/µl]	Sample Description	Alert	Observations
A1	-	90.1	Ladder		Ladder
B1	8.3	201	M136	⚠	Sample concentration outside recommended range
C1	8.4	186	M137	⚠	Sample concentration outside recommended range
D1	8.3	195	M138	⚠	Sample concentration outside recommended range
E1	7.6	213	M139	⚠	Sample concentration outside recommended range
F1	7.9	263	M140	⚠	Sample concentration outside recommended range
G1	6.4	170	M141	⚠	Sample concentration outside recommended range
H1	7.3	287	M142	⚠	Sample concentration outside recommended range
A2	7.4	305	M143	⚠	Sample concentration outside functional range for DIN
B2	8.4	393	M144	⚠	Sample concentration outside functional range for DIN
C2	6.4	34.7	M145		
D2	7.7	37.9	M151		
E2	7.9	123	M152	⚠	Sample concentration outside recommended range
F2	8.3	174	M153	⚠	Sample concentration outside recommended range
G2	8.4	340	M154	⚠	Sample concentration outside functional range for DIN
H2	8.3	87.9	M155		
A3	7.9	445	M161	⚠	Sample concentration outside functional range for DIN
B3	7.6	105	M162	⚠	Sample concentration outside recommended range
C3	8.4	130	M163	⚠	Sample concentration outside recommended range
D3	7.5	90.4	M164		
E3	8.3	203	M165	⚠	Sample concentration outside recommended range
F3	3.1	422	M171	⚠	Sample concentration outside functional range for DIN
G3	3.4	459	M172	⚠	Sample concentration outside functional range for DIN
H3	6.0	637	M173	⚠	Sample concentration outside functional range for DIN
A4	4.5	448	M174	⚠	Sample concentration outside functional range for DIN
B4	5.3	573	M175	⚠	Sample concentration outside functional range for DIN
C4	6.7	359	M176	⚠	Sample concentration outside functional range for DIN
D4	6.4	309	M177	⚠	Sample concentration outside functional range for DIN
E4	6.1	240	M178	⚠	Sample concentration outside recommended range
F4	6.1	241	M179	⚠	Sample concentration outside recommended range
G4	4.1	216	M180	⚠	Sample concentration outside recommended range
H4	7.5	77.6	M280		
A5	7.8	79.9	M281		
B5	8.0	247	M282	⚠	Sample concentration outside recommended range
C5	6.6	380	M283	⚠	Sample concentration outside functional range for DIN
D5	7.3	102	M286	⚠	Sample concentration outside recommended range
E5	7.8	116	M287	⚠	Sample concentration outside recommended range
F5	7.7	61.6	M290		
G5	6.7	287	M291	⚠	Sample concentration outside recommended range
H5	5.5	506	M294	⚠	Sample concentration outside functional range for DIN
A6	5.1	406	M295	⚠	Sample concentration outside functional range for DIN
B6	6.4	151	M296	⚠	Sample concentration outside recommended range
C6	6.9	183	M297	⚠	Sample concentration outside recommended range
D6	1.6	57.5	Sarah_361		
E6	1.9	147	Sarah_362	⚠	Sample concentration outside recommended range
F6	2.1	180	Sarah_363	⚠	Sample concentration outside recommended range
G6	7.9	49.5	Sarah_364		
H6	7.9	46.6	Sarah_365		
A7	8.5	66.3	Sarah_366		
B7	7.9	103	Sarah_367	⚠	Sample concentration outside recommended range
C7	8.6	182	Sarah_368	⚠	Sample concentration outside recommended range
D7	8.1	157	Sarah_369	⚠	Sample concentration outside recommended range
E7	8.3	35.7	Sarah_373		
F7	8.4	43.1	Sarah_374		
G7	8.1	39.5	Sarah_375		
H7	8.6	189	Sarah_379	⚠	Sample concentration outside recommended range
A8	7.6	219	Sarah_380	⚠	Sample concentration outside recommended range
B8	7.3	419	Sarah_381	⚠	Sample concentration outside functional range for DIN
C8	6.7	27.0	Sarah_I46		
D8	2.2	54.2	Sarah_I47		
E8	6.1	60.6	Sarah_I48		
F8	8.5	47.8	Sarah_8_rerun		
G8	7.3	23.6	Sarah_65_rerun		
H8	8.5	14.2	Sarah_67_rerun		

© Agilent Technologies, Inc. 2017

A1: Ladder

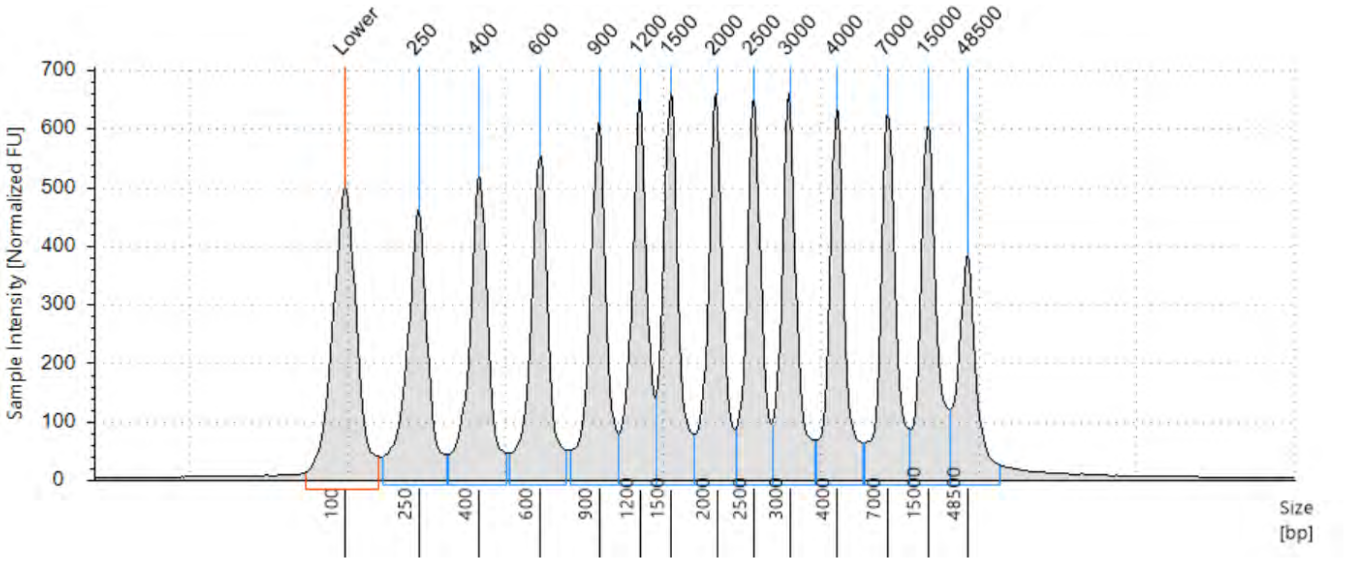

Sample Table

Well	DIN	Conc. [ng/µl]	Sample Description	Alert	Observations
A1	-	90.1	Ladder		Ladder

Peak Table

Size [bp]	Calibrated Conc. [ng/µl]	Assigned Conc. [ng/µl]	% Integrated Area	From [bp]	To [bp]	Peak Comment	Observations
100	8.50	8.50	-	61	151		Lower Marker
250	6.79	-	7.61	159	310		
400	6.95	-	7.79	313	481		
600	6.99	-	7.85	490	718		
900	7.18	-	8.05	738	1029		
1200	7.21	-	8.09	1029	1342		
1500	7.27	-	8.15	1342	1740		
2000	7.16	-	8.03	1740	2245		
2500	6.87	-	7.71	2245	2739		
3000	7.08	-	7.95	2739	3494		
4000	6.84	-	7.67	3523	5332		
7000	6.85	-	7.68	5413	10659		
15000	6.97	-	7.82	10659	22849		
48500	4.91	-	5.51	22849	>60000		
-	-	-	-	-	-		Sample Well

 © Agilent Technologies, Inc. 2017

B1: M136

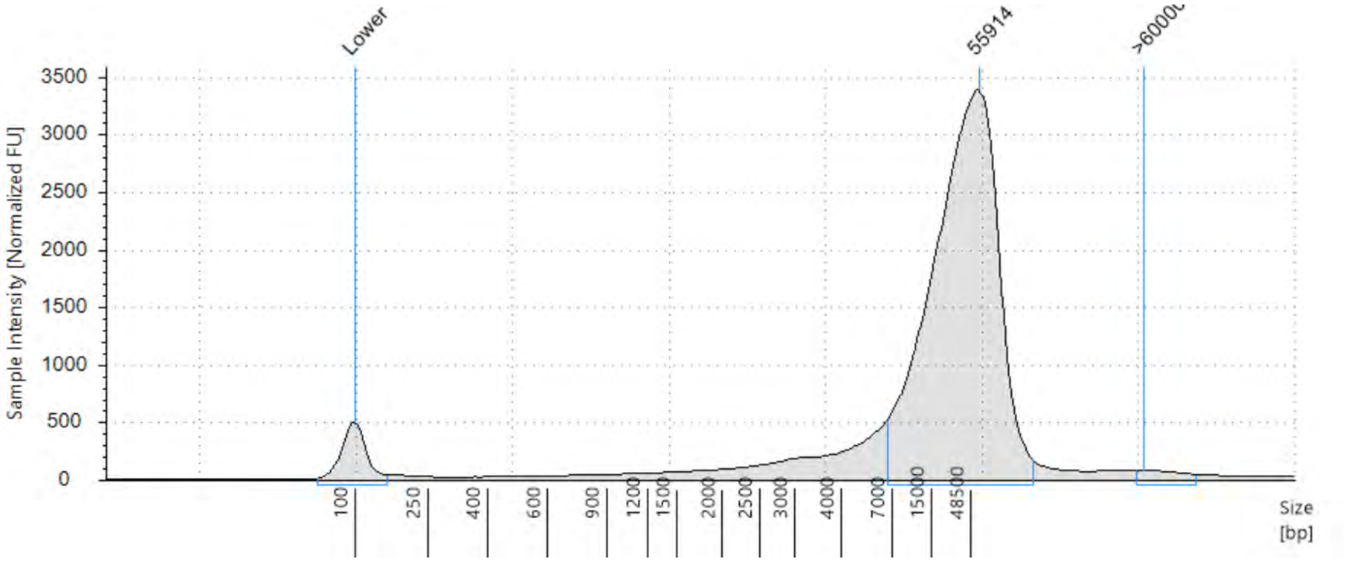

Sample Table

Well	DIN	Conc. [ng/µl]	Sample Description	Alert	Observations
B1	8.3	201	M136	⚠	Sample concentration outside recommended range

Peak Table

Size [bp]	Calibrated Conc. [ng/µl]	Assigned Conc. [ng/µl]	% Integrated Area	From [bp]	To [bp]	Peak Comment	Observations
100	8.50	8.50	-	62	150		Lower Marker
55914	160	-	94.48	6743	>60000		
>60000	2.37	-	1.40	>60000	>60000		
-	-	-	-	-	-		Sample Well

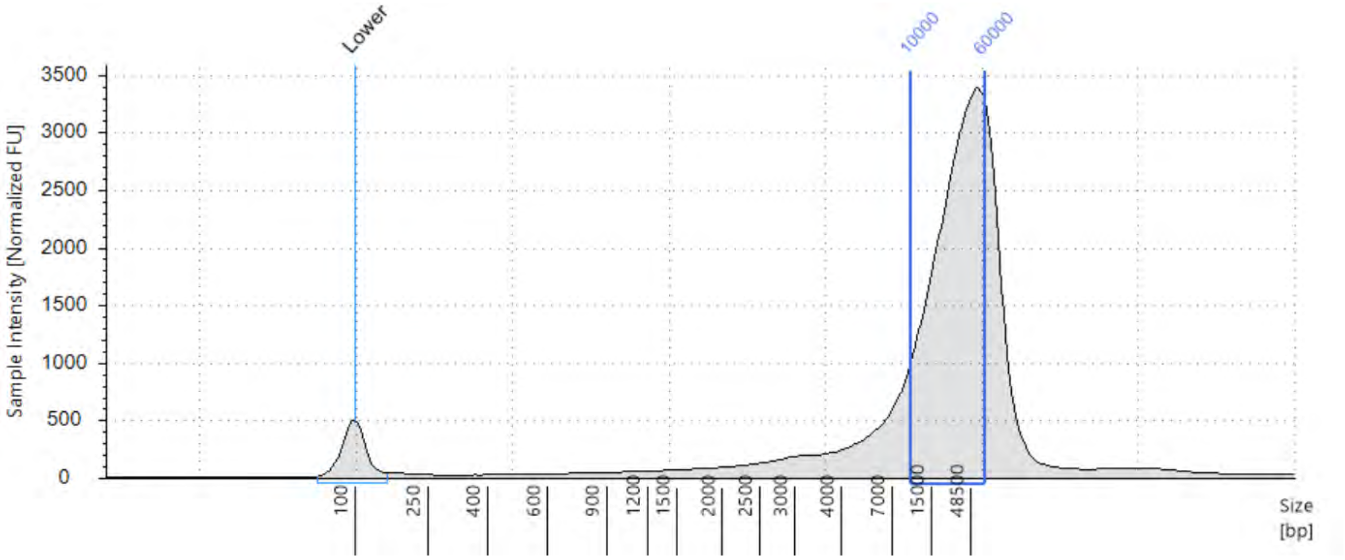

Region Table

From [bp]	To [bp]	Average Size [bp]	Conc. [ng/µl]	Region Molarity [nmol/l]	% of Total	Region Comment	Color
10000	60000	31433	111	7.20	54.96		■

C1: M137

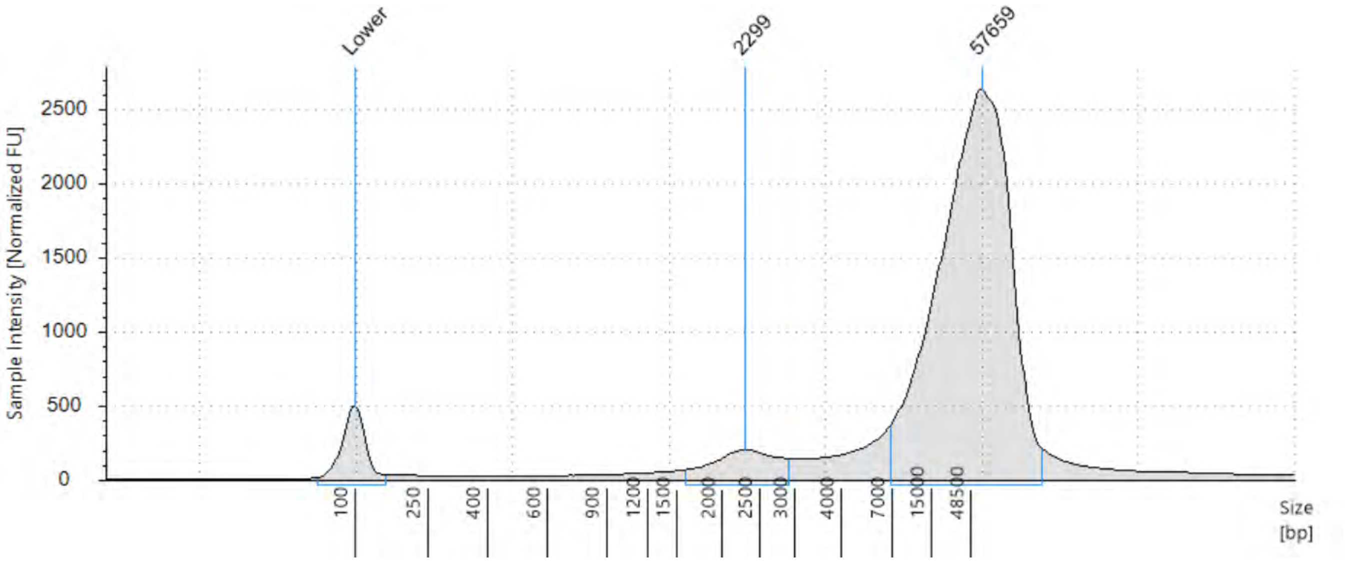

Sample Table

Well	DIN	Conc. [ng/µl]	Sample Description	Alert	Observations
C1	8.4	186	M137	⚠	Sample concentration outside recommended range

Peak Table

Size [bp]	Calibrated Conc. [ng/µl]	Assigned Conc. [ng/µl]	% Integrated Area	From [bp]	To [bp]	Peak Comment	Observations
100	8.50	8.50	-	62	148		Lower Marker
2299	9.42	-	5.78	1585	2907		
57659	145	-	88.93	6964	>60000		
-	-	-	-	-	-		Sample Well

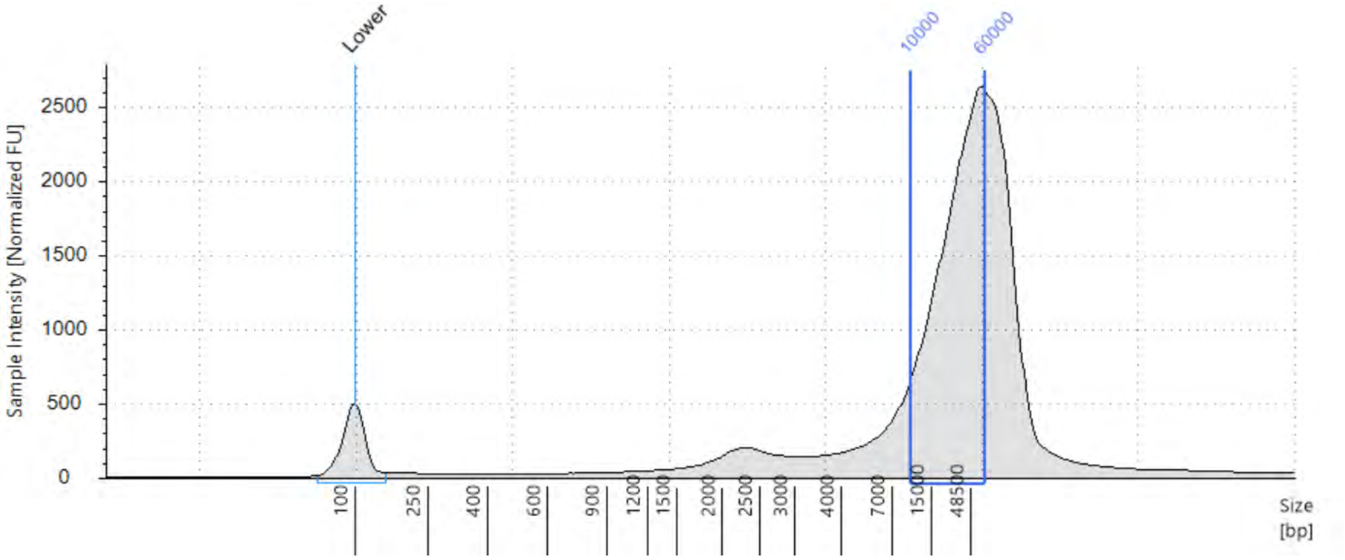

Region Table

From [bp]	To [bp]	Average Size [bp]	Conc. [ng/µl]	Region Molarity [nmol/l]	% of Total	Region Comment	Color
10000	60000	32796	85.6	5.38	45.98		■

D1: M138

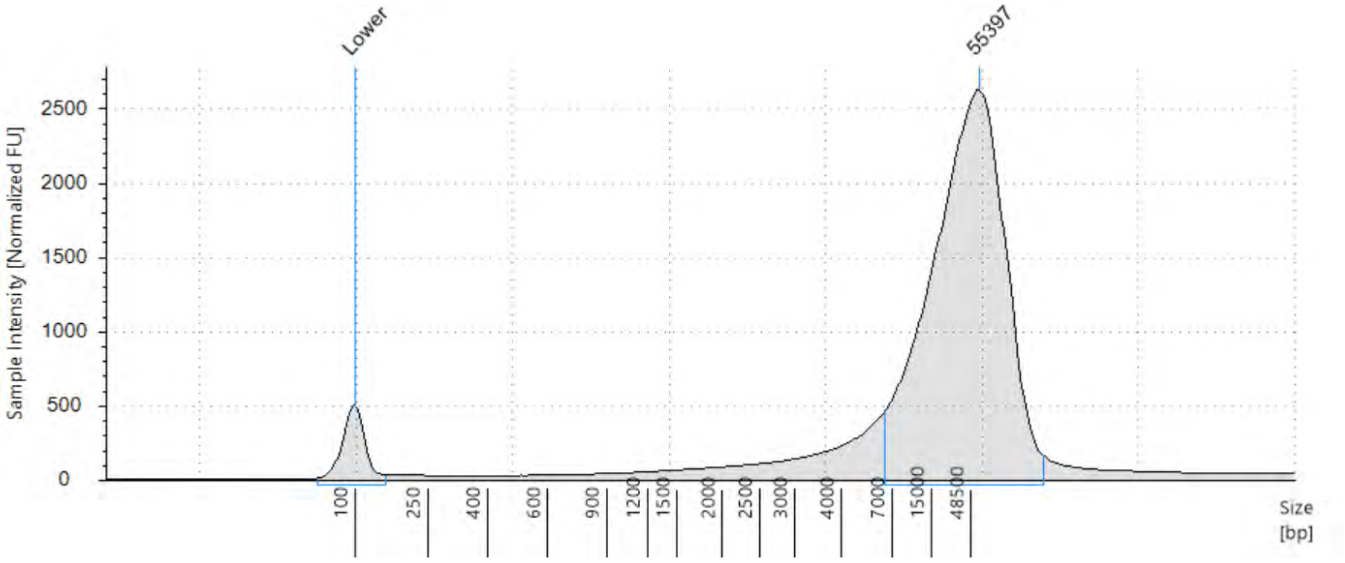

Sample Table

Well	DIN	Conc. [ng/µl]	Sample Description	Alert	Observations
D1	8.3	195	M138	⚠	Sample concentration outside recommended range

Peak Table

Size [bp]	Calibrated Conc. [ng/µl]	Assigned Conc. [ng/µl]	% Integrated Area	From [bp]	To [bp]	Peak Comment	Observations
100	8.50	8.50	-	63	148		Lower Marker
55397	151	-	91.78	6532	>60000		
-	-	-	-	-	-		Sample Well

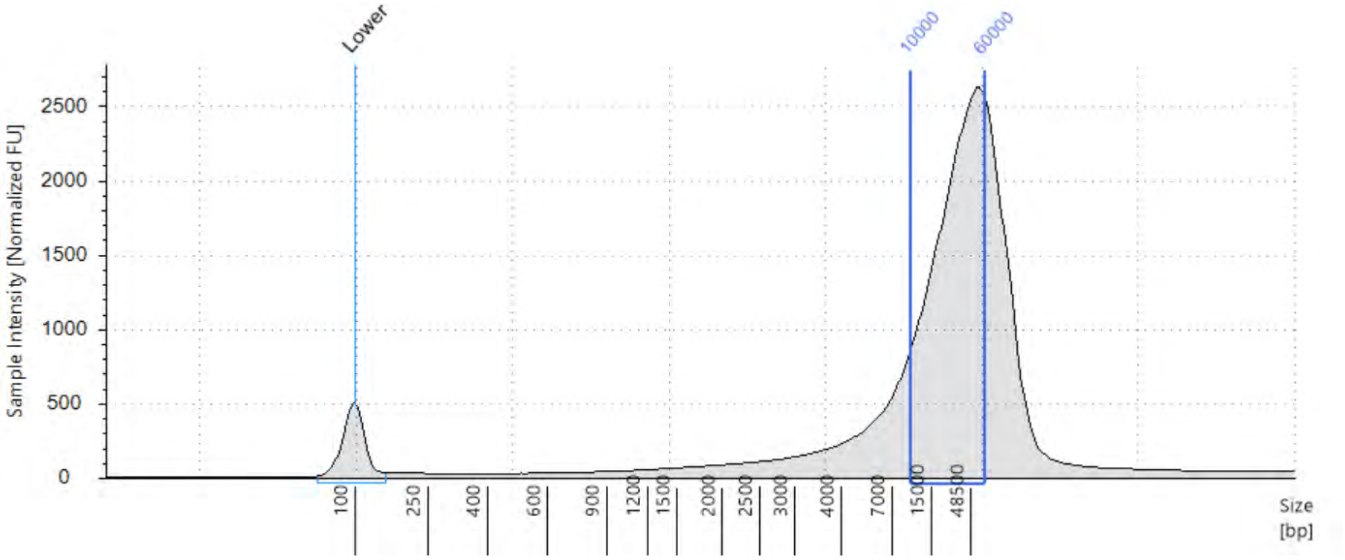

Region Table

From [bp]	To [bp]	Average Size [bp]	Conc. [ng/µl]	Region Molarity [nmol/l]	% of Total	Region Comment	Color
10000	60000	31740	95.3	6.22	48.96		■

E1: M139

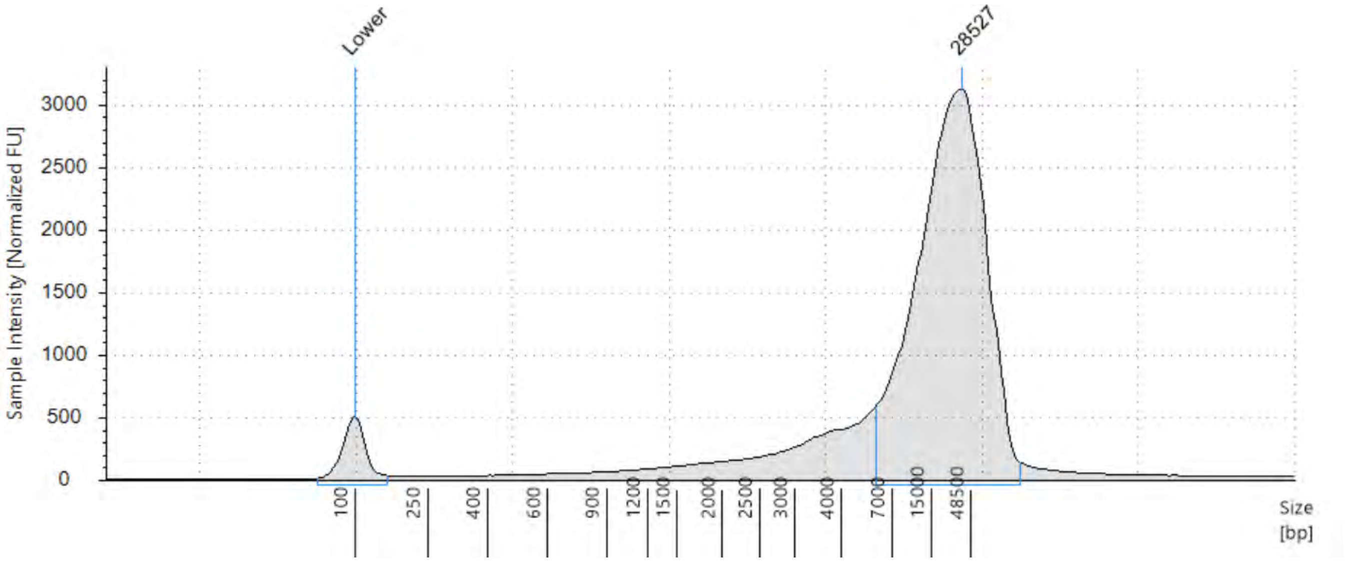

Sample Table

Well	DIN	Conc. [ng/µl]	Sample Description	Alert	Observations
E1	7.6	213	M139	⚠	Sample concentration outside recommended range

Peak Table

Size [bp]	Calibrated Conc. [ng/µl]	Assigned Conc. [ng/µl]	% Integrated Area	From [bp]	To [bp]	Peak Comment	Observations
100	8.50	8.50	-	62	150		Lower Marker
28527	162	-	96.18	5888	>60000		
-	-	-	-	-	-		Sample Well

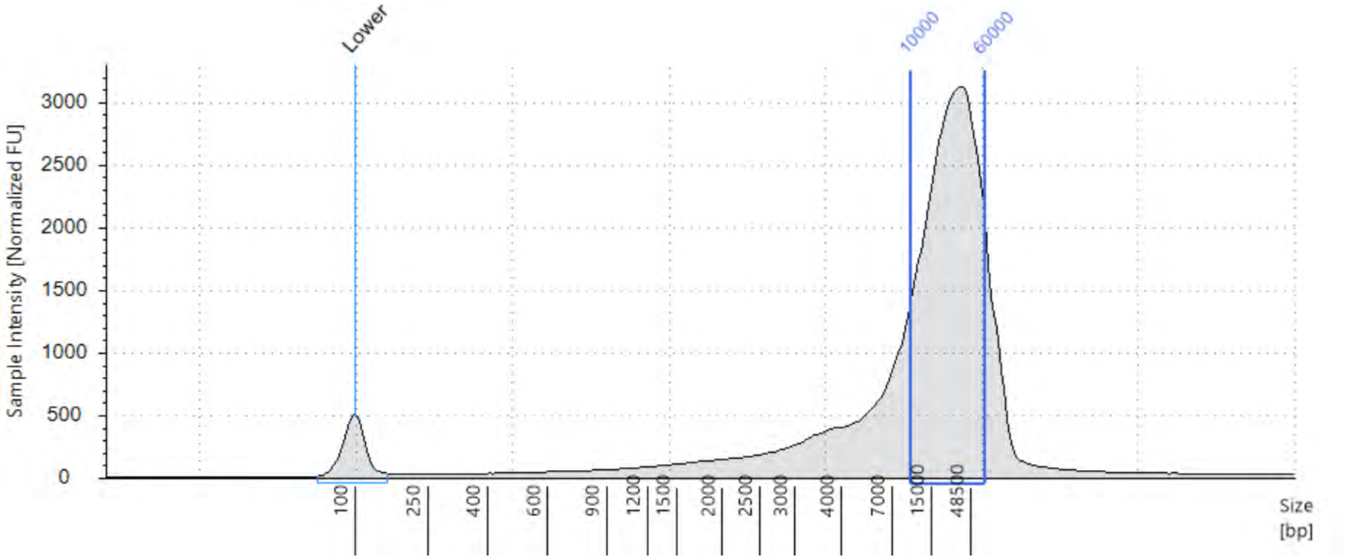

Region Table

From [bp]	To [bp]	Average Size [bp]	Conc. [ng/µl]	Region Molarity [nmol/l]	% of Total	Region Comment	Color
10000	60000	28296	123	8.83	57.88		■

F1: M140

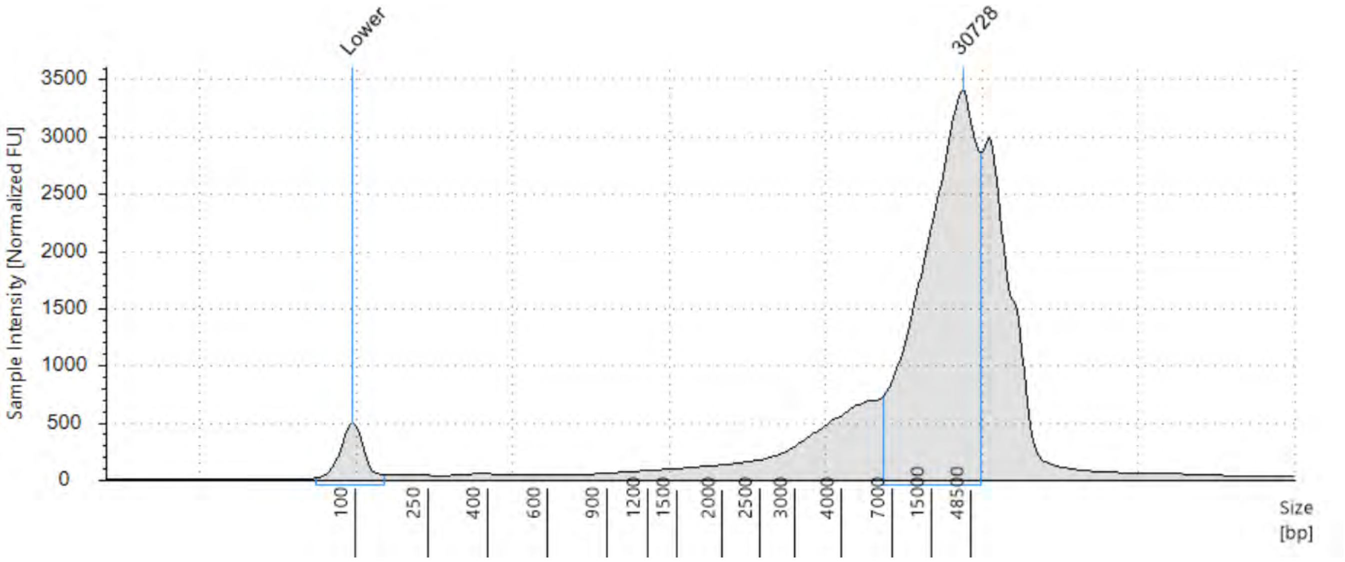

Sample Table

Well	DIN	Conc. [ng/µl]	Sample Description	Alert	Observations
F1	7.9	263	M140	⚠	Sample concentration outside recommended range

Peak Table

Size [bp]	Calibrated Conc. [ng/µl]	Assigned Conc. [ng/µl]	% Integrated Area	From [bp]	To [bp]	Peak Comment	Observations
100	8.50	8.50	-	62	148		Lower Marker
30728	137	-	93.89	6476	58153		
-	-	-	-	-	-		Sample Well

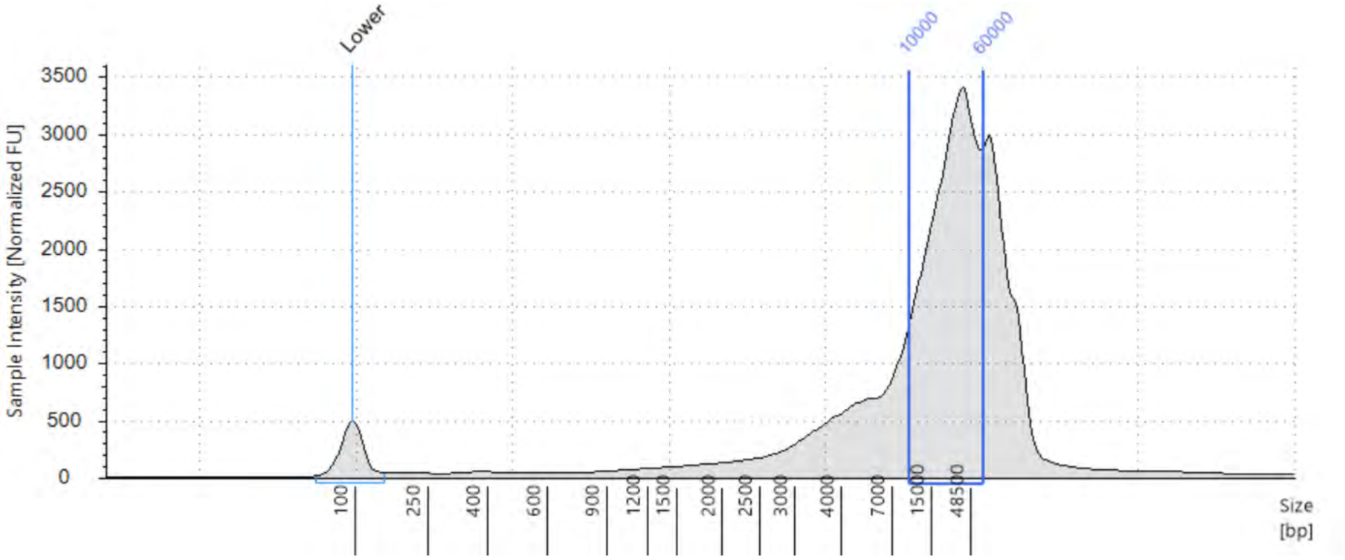

Region Table

From [bp]	To [bp]	Average Size [bp]	Conc. [ng/µl]	Region Molarity [nmol/l]	% of Total	Region Comment	Color
10000	60000	29264	126	8.74	47.77		■

 © Agilent Technologies, Inc. 2017

G1: M141

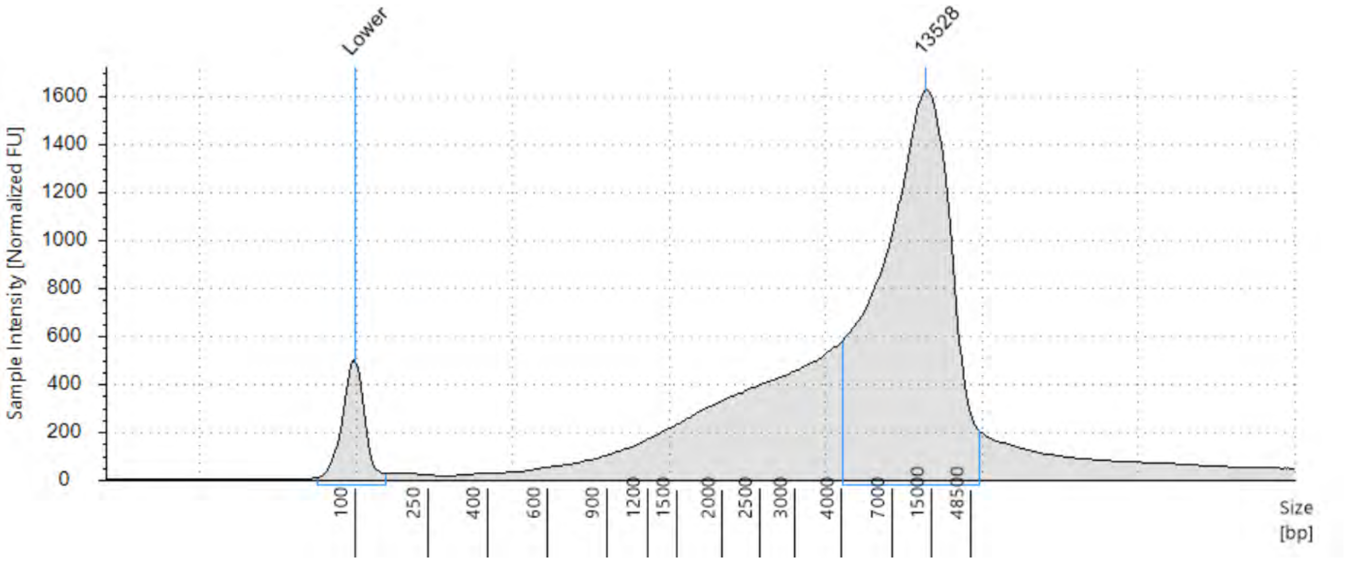

Sample Table

Well	DIN	Conc. [ng/µl]	Sample Description	Alert	Observations
G1	6.4	170	M141	⚠	Sample concentration outside recommended range

Peak Table

Size [bp]	Calibrated Conc. [ng/µl]	Assigned Conc. [ng/µl]	% Integrated Area	From [bp]	To [bp]	Peak Comment	Observations
100	8.50	8.50	-	62	148		Lower Marker
13528	89.2	-	89.48	4066	55914		
-	-	-	-	-	-		Sample Well

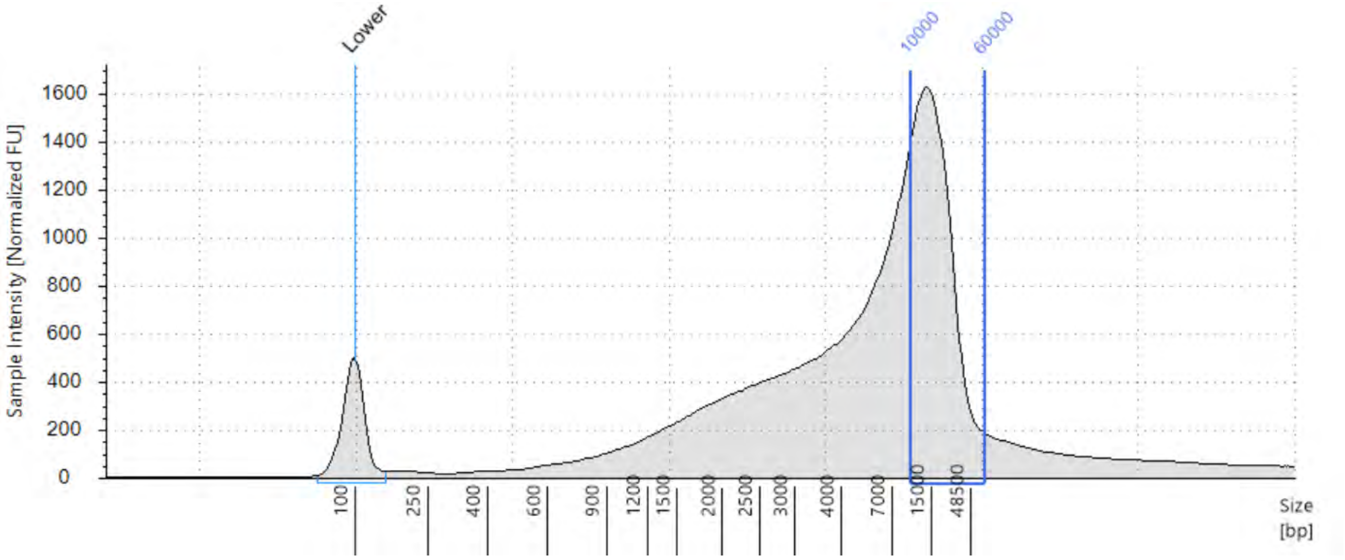

Region Table

From [bp]	To [bp]	Average Size [bp]	Conc. [ng/µl]	Region Molarity [nmol/l]	% of Total	Region Comment	Color
10000	60000	18834	50.3	4.87	29.60		■

H1: M142

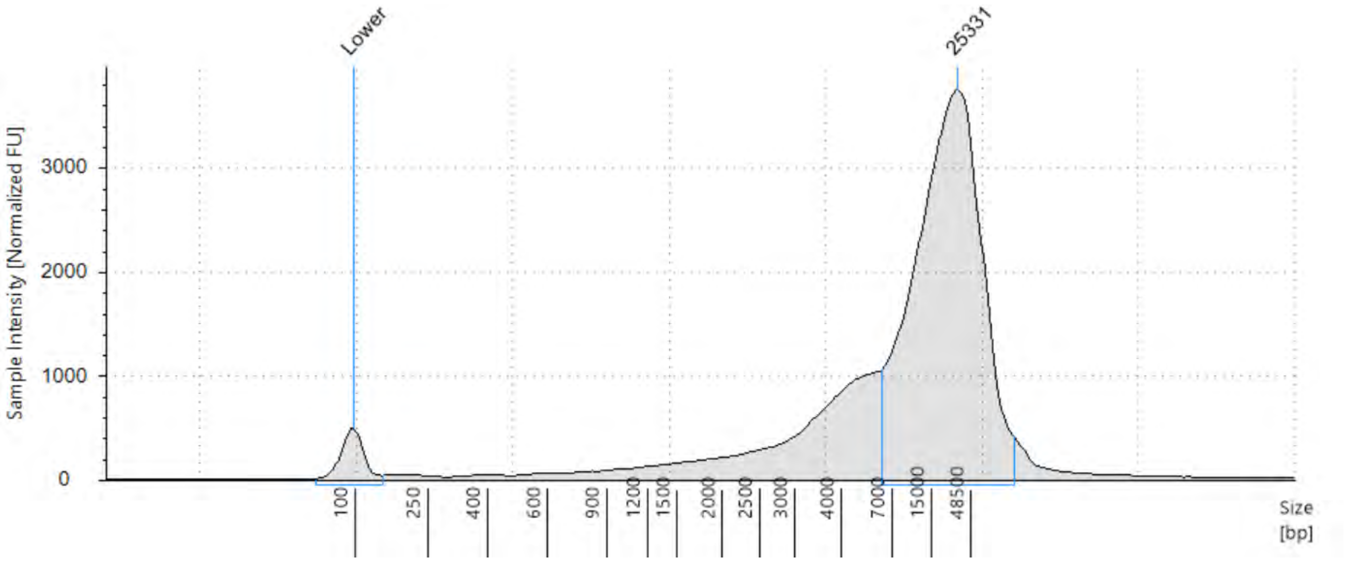

Sample Table

Well	DIN	Conc. [ng/µl]	Sample Description	Alert	Observations
H1	7.3	287	M142	⚠	Sample concentration outside recommended range

Peak Table

Size [bp]	Calibrated Conc. [ng/µl]	Assigned Conc. [ng/µl]	% Integrated Area	From [bp]	To [bp]	Peak Comment	Observations
100	8.50	8.50	-	62	145		Lower Marker
25331	199	-	97.43	6301	>60000		
-	-	-	-	-	-		Sample Well

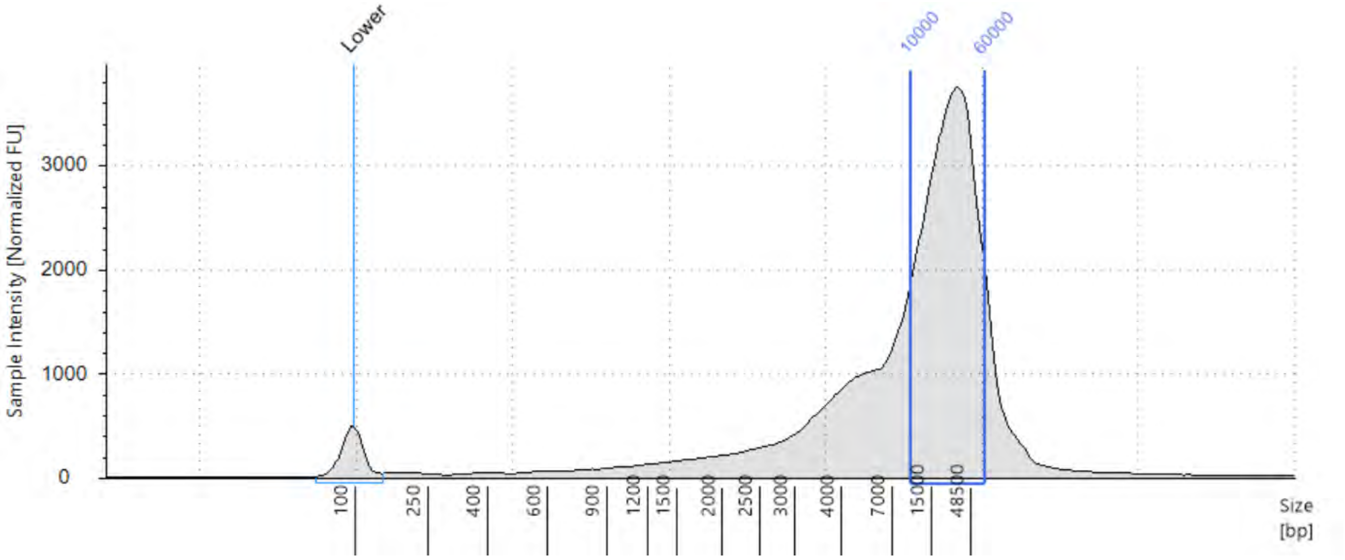

Region Table

From [bp]	To [bp]	Average Size [bp]	Conc. [ng/µl]	Region Molarity [nmol/l]	% of Total	Region Comment	Color
10000	60000	26753	155	11.6	53.95		■

A2: M143

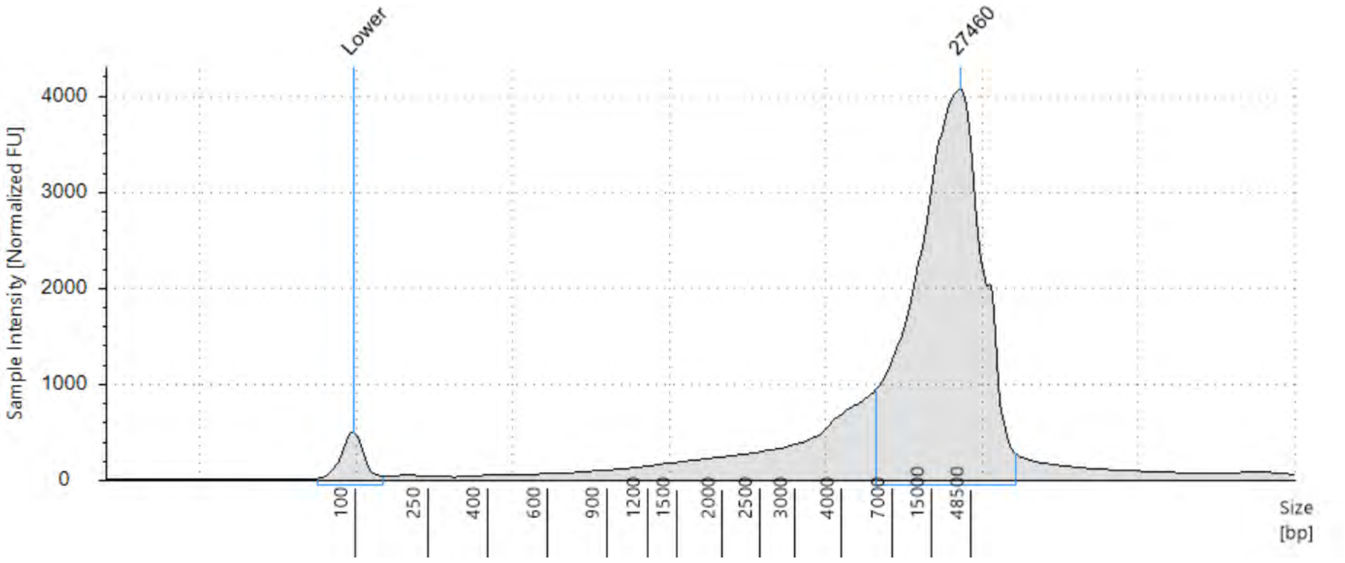

Sample Table

Well	DIN	Conc. [ng/µl]	Sample Description	Alert	Observations
A2	7.4	305	M143	⚠	Sample concentration outside functional range for DIN

Peak Table

Size [bp]	Calibrated Conc. [ng/µl]	Assigned Conc. [ng/µl]	% Integrated Area	From [bp]	To [bp]	Peak Comment	Observations
100	8.50	8.50	-	63	145		Lower Marker
27460	213	-	94.51	5916	>60000		
-	-	-	-	-	-		Sample Well

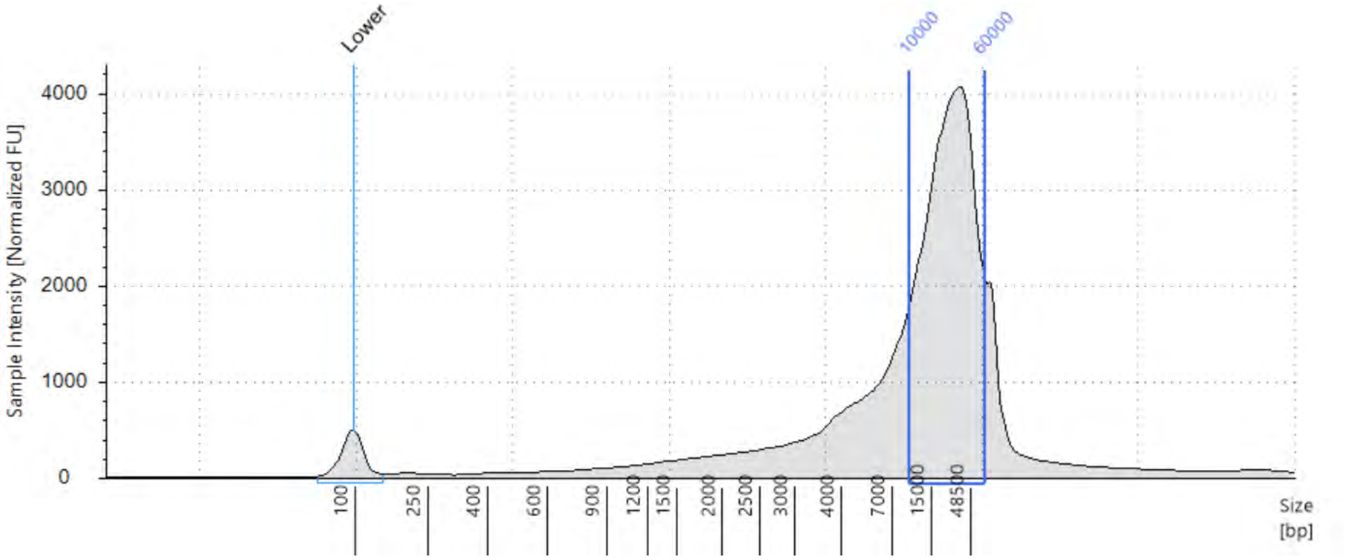

Region Table

From [bp]	To [bp]	Average Size [bp]	Conc. [ng/µl]	Region Molarity [nmol/l]	% of Total	Region Comment	Color
10000	60000	27276	161	11.8	52.92		■

B2: M144

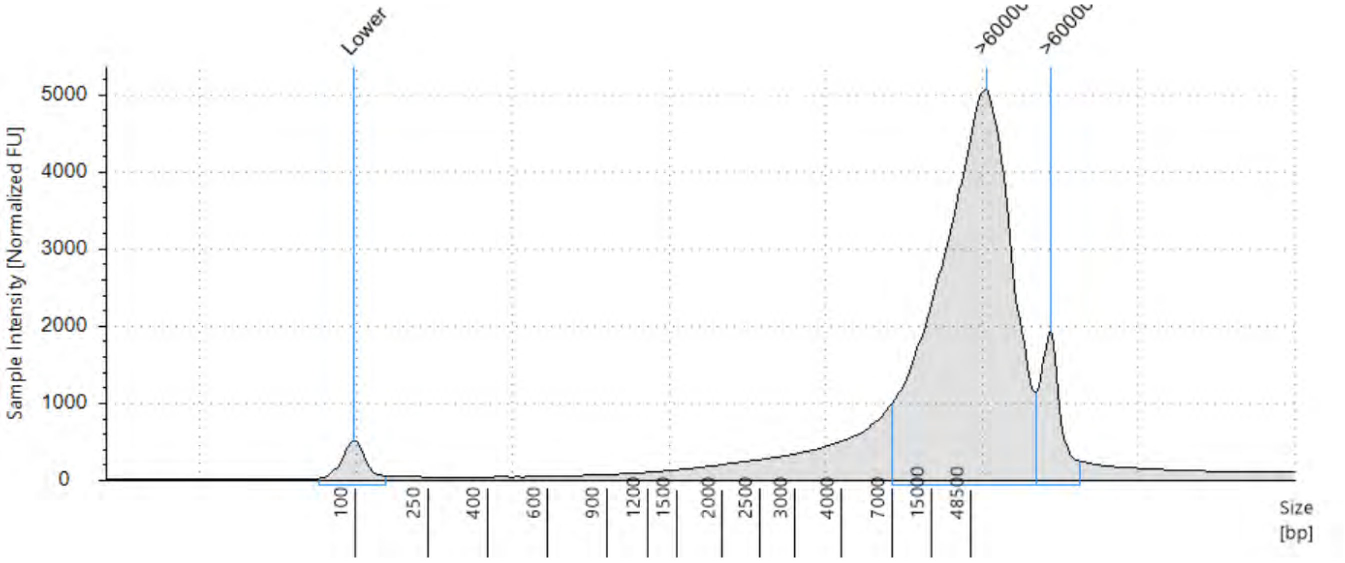

Sample Table

Well	DIN	Conc. [ng/µl]	Sample Description	Alert	Observations
B2	8.4	393	M144	⚠	Sample concentration outside functional range for DIN

Peak Table

Size [bp]	Calibrated Conc. [ng/µl]	Assigned Conc. [ng/µl]	% Integrated Area	From [bp]	To [bp]	Peak Comment	Observations
100	8.50	8.50	-	64	150		Lower Marker
>60000	275	-	80.83	7152	>60000		
>60000	29.3	-	8.62	>60000	>60000		
-	-	-	-	-	-		Sample Well

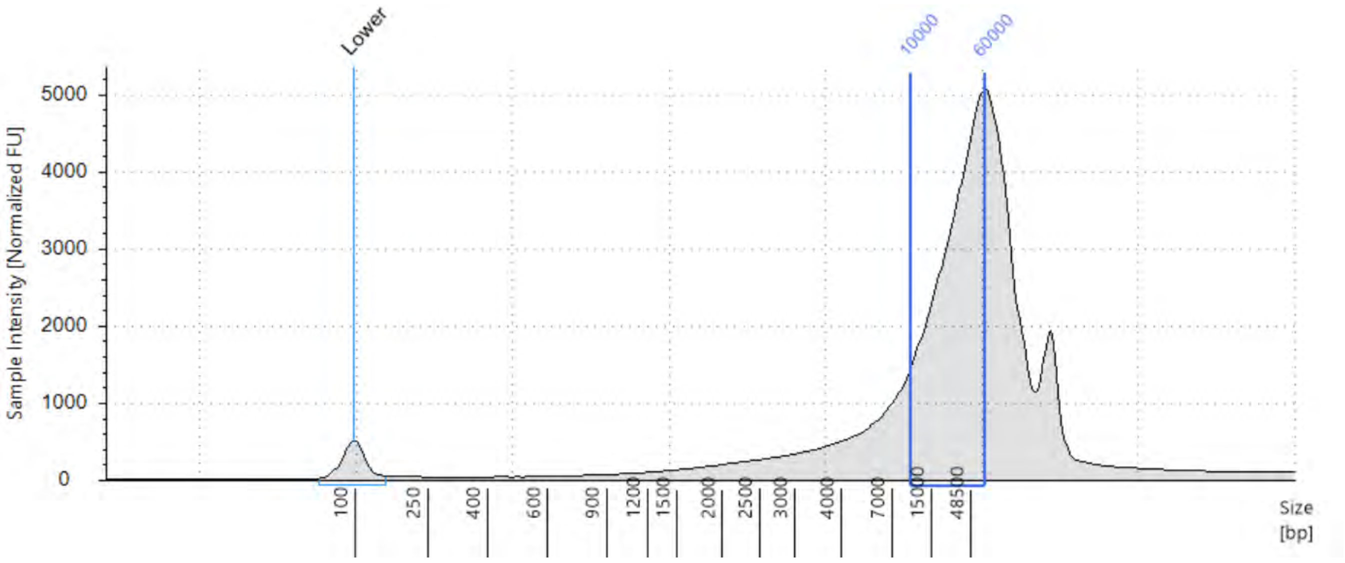

Region Table

From [bp]	To [bp]	Average Size [bp]	Conc. [ng/µl]	Region Molarity [nmol/l]	% of Total	Region Comment	Color
10000	60000	32520	156	10.0	39.84		■

C2: M145

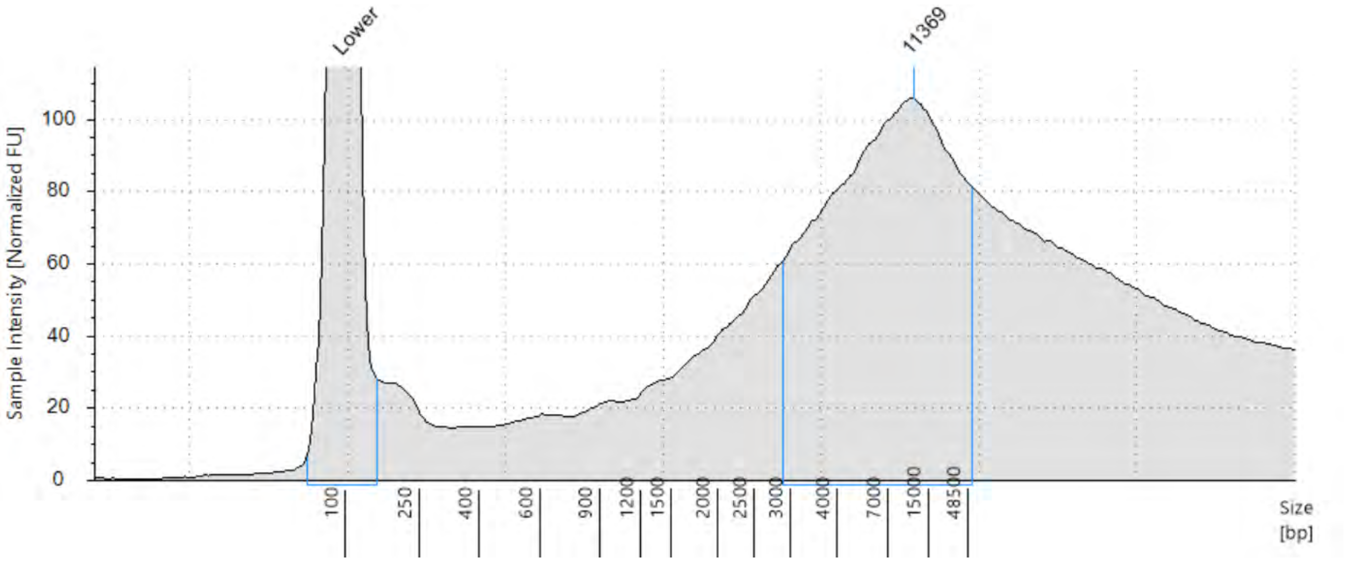

Sample Table

Well	DIN	Conc. [ng/µl]	Sample Description	Alert	Observations
C2	6.4	34.7	M145		

Peak Table

Size [bp]	Calibrated Conc. [ng/µl]	Assigned Conc. [ng/µl]	% Integrated Area	From [bp]	To [bp]	Peak Comment	Observations
100	8.50	8.50	-	62	147		Lower Marker
11369	10.8	-	50.07	2890	52491		
-	-	-	-	-	-		Sample Well

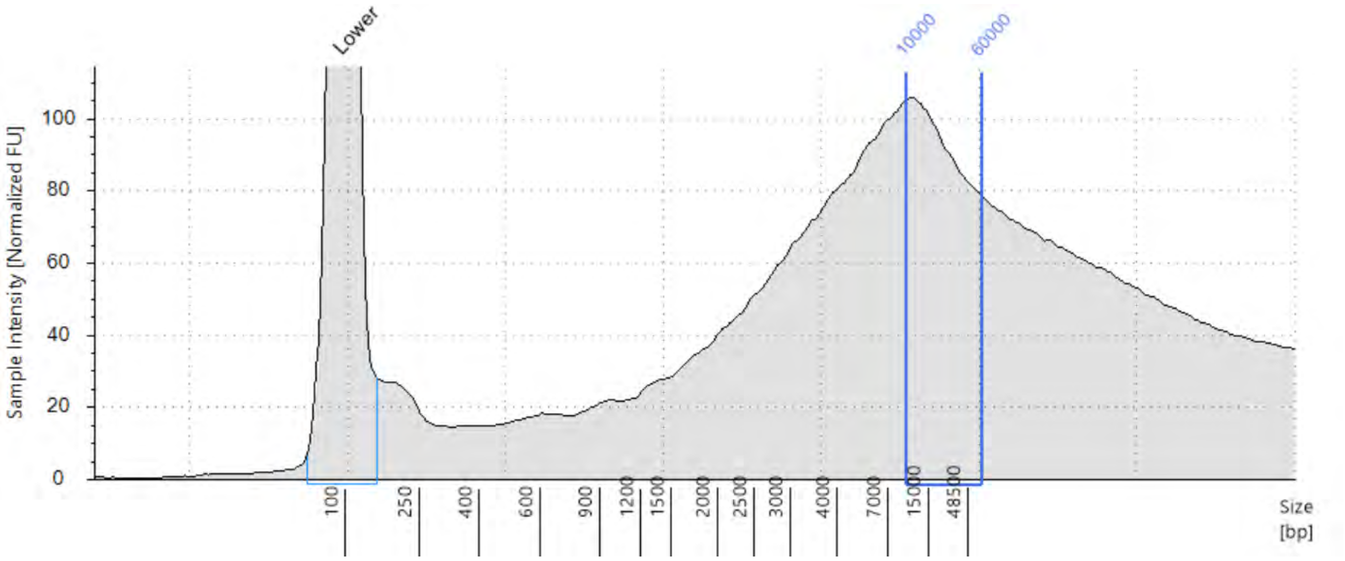

Region Table

From [bp]	To [bp]	Average Size [bp]	Conc. [ng/µl]	Region Molarity [nmol/l]	% of Total	Region Comment	Color
10000	60000	25289	4.64	0.390	13.34		

D2: M151

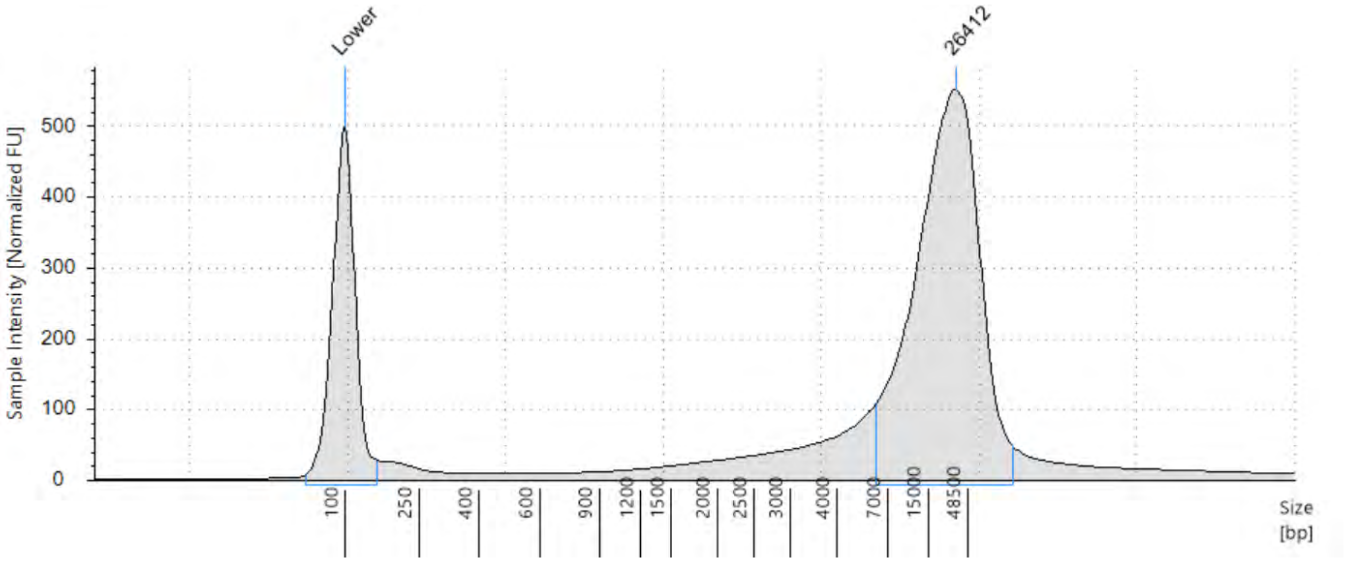

Sample Table

Well	DIN	Conc. [ng/µl]	Sample Description	Alert	Observations
D2	7.7	37.9	M151		

Peak Table

Size [bp]	Calibrated Conc. [ng/µl]	Assigned Conc. [ng/µl]	% Integrated Area	From [bp]	To [bp]	Peak Comment	Observations
100	8.50	8.50	-	62	151		Lower Marker
26412	26.5	-	90.80	6179	>60000		
>60000	0.250	-	0.85	>60000	>60000		
-	-	-	-	-	-		Sample Well

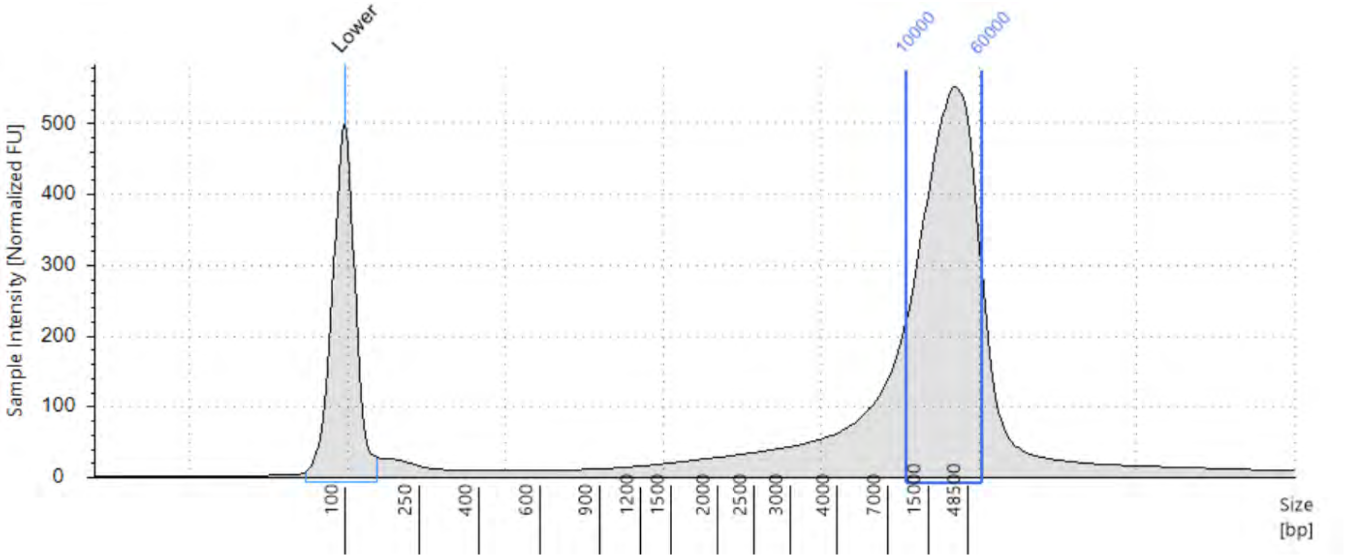

Region Table

From [bp]	To [bp]	Average Size [bp]	Conc. [ng/µl]	Region Molarity [nmol/l]	% of Total	Region Comment	Color
10000	60000	28095	21.1	1.51	55.71		

E2: M152

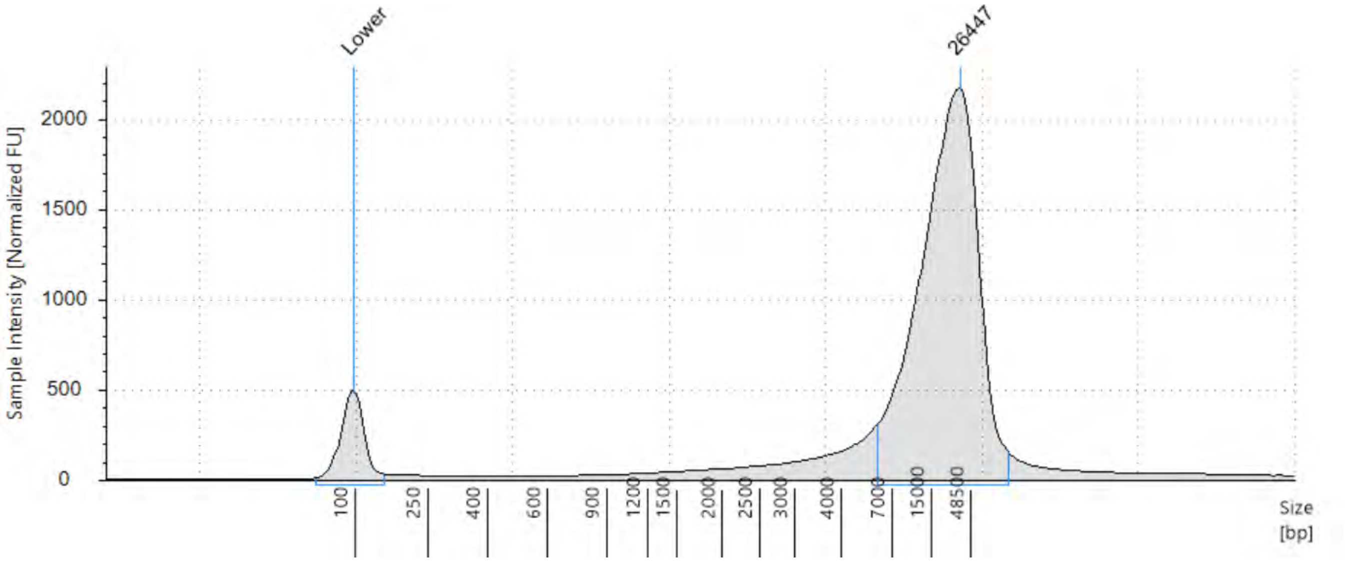

Sample Table

Well	DIN	Conc. [ng/µl]	Sample Description	Alert	Observations
E2	7.9	123	M152	⚠	Sample concentration outside recommended range

Peak Table

Size [bp]	Calibrated Conc. [ng/µl]	Assigned Conc. [ng/µl]	% Integrated Area	From [bp]	To [bp]	Peak Comment	Observations
100	8.50	8.50	-	62	148		Lower Marker
26447	93.3	-	92.81	6024	>60000		
-	-	-	-	-	-		Sample Well

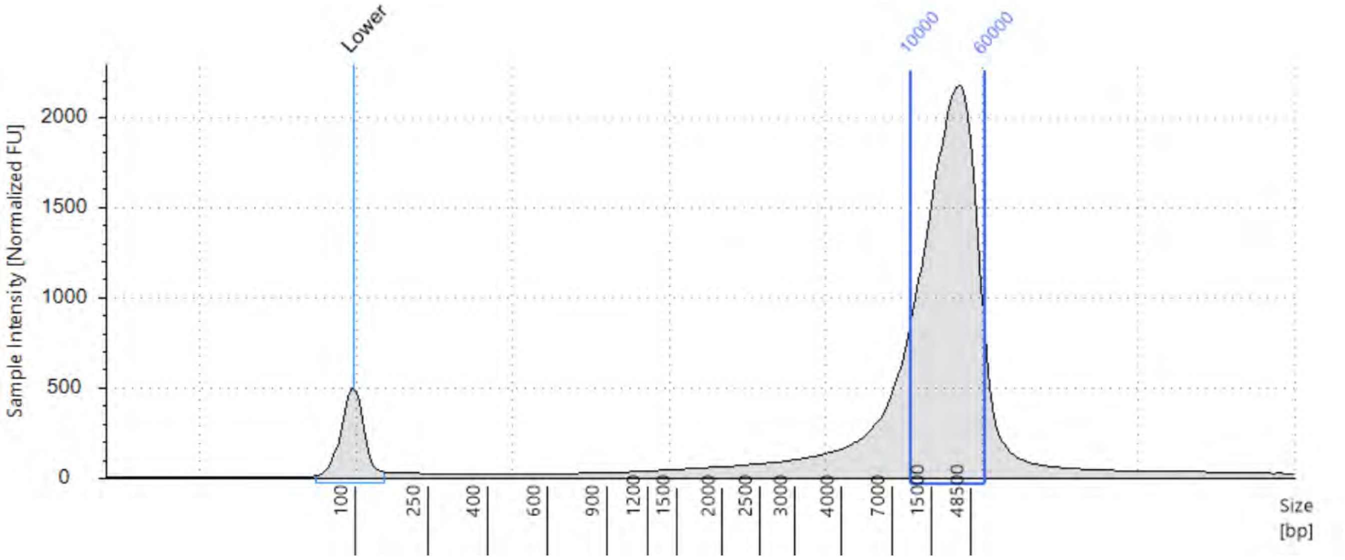

Region Table

From [bp]	To [bp]	Average Size [bp]	Conc. [ng/µl]	Region Molarity [nmol/l]	% of Total	Region Comment	Color
10000	60000	27170	78.2	5.70	63.63		■

© Agilent Technologies, Inc. 2017

F2: M153

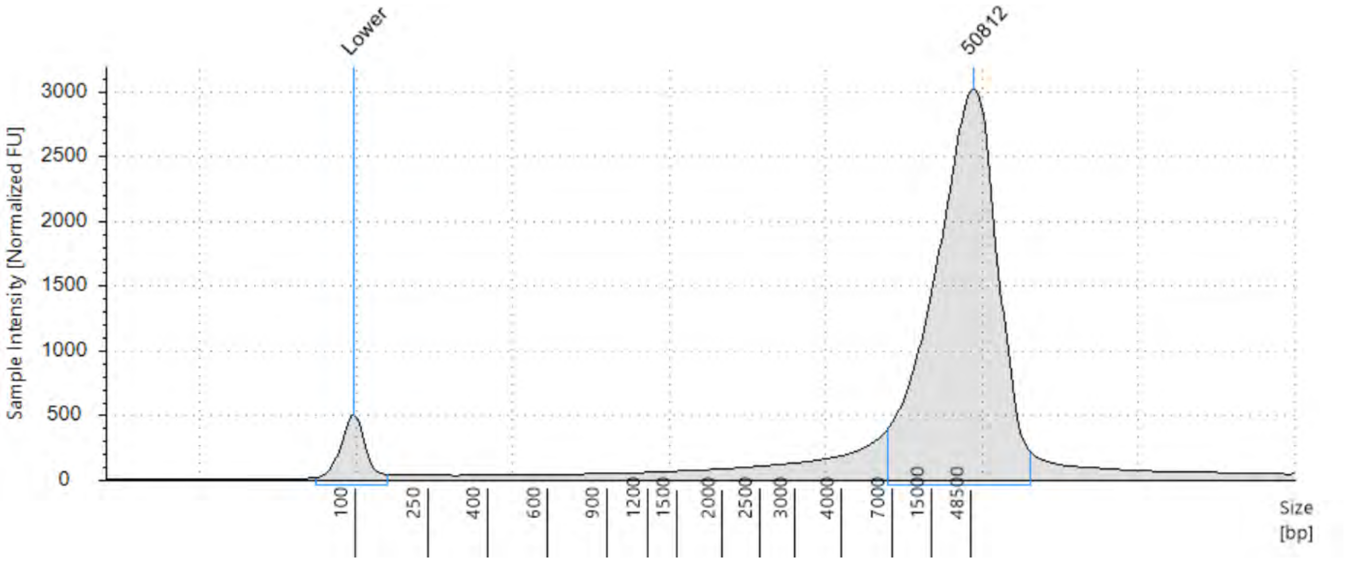

Sample Table

Well	DIN	Conc. [ng/µl]	Sample Description	Alert	Observations
F2	8.3	174	M153	⚠	Sample concentration outside recommended range

Peak Table

Size [bp]	Calibrated Conc. [ng/µl]	Assigned Conc. [ng/µl]	% Integrated Area	From [bp]	To [bp]	Peak Comment	Observations
100	8.50	8.50	-	62	152		Lower Marker
50812	133	-	94.86	6777	>60000		
-	-	-	-	-	-		Sample Well

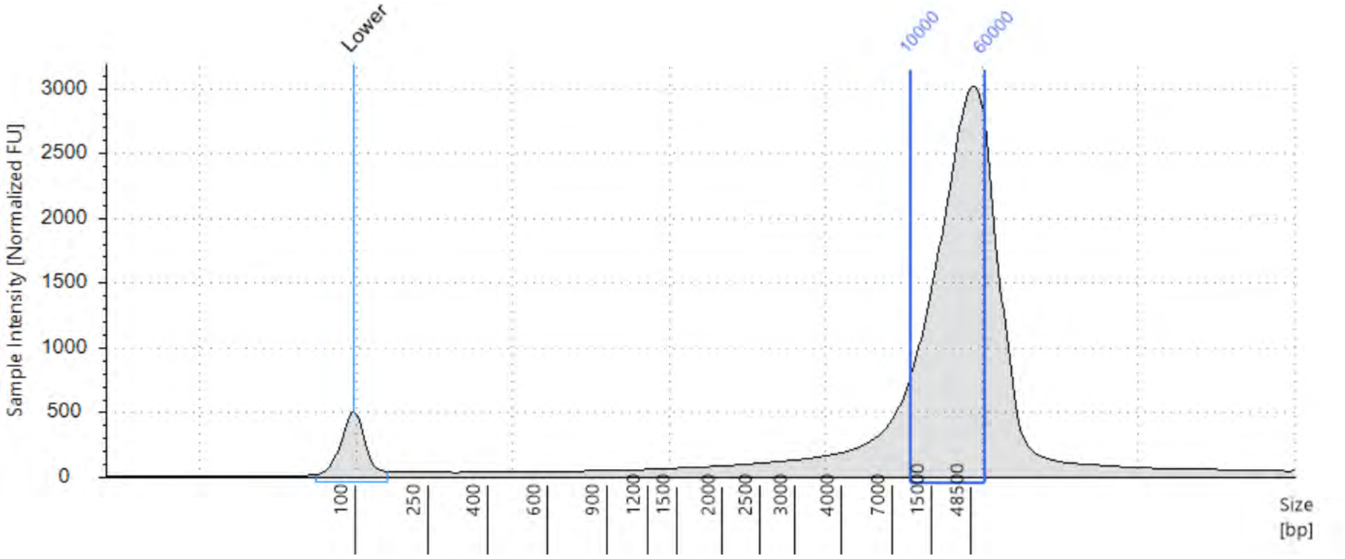

Region Table

From [bp]	To [bp]	Average Size [bp]	Conc. [ng/µl]	Region Molarity [nmol/l]	% of Total	Region Comment	Color
10000	60000	31626	92.9	5.97	53.56		■

G2: M154

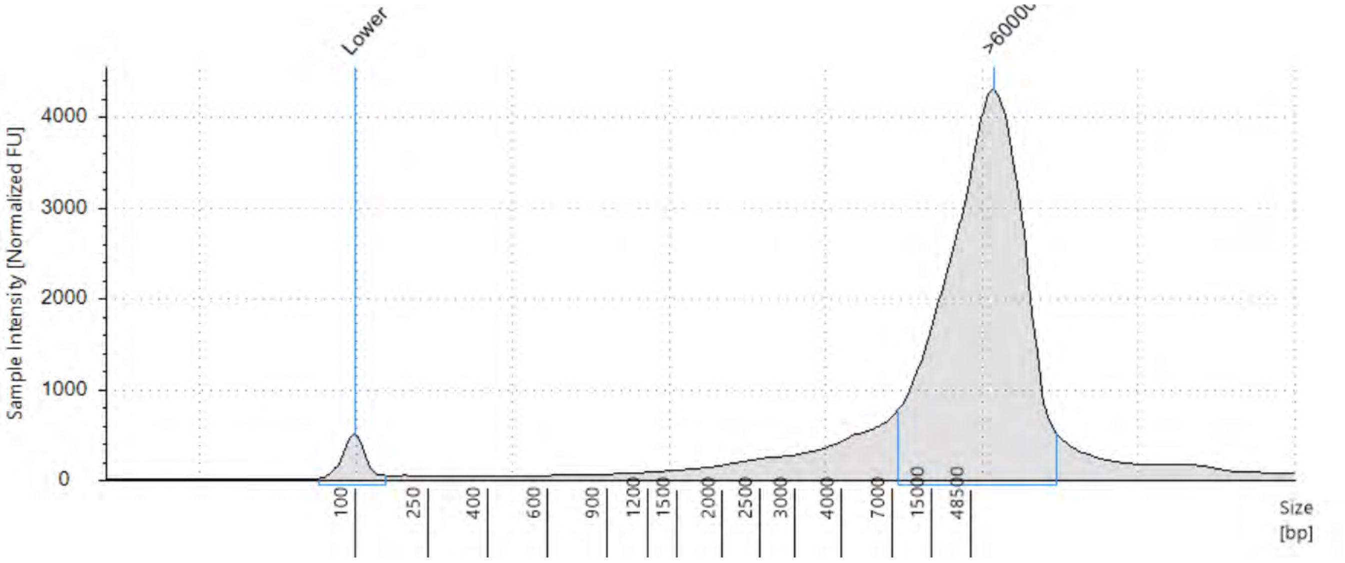

Sample Table

Well	DIN	Conc. [ng/µl]	Sample Description	Alert	Observations
G2	8.4	340	M154	⚠	Sample concentration outside functional range for DIN

Peak Table

Size [bp]	Calibrated Conc. [ng/µl]	Assigned Conc. [ng/µl]	% Integrated Area	From [bp]	To [bp]	Peak Comment	Observations
100	8.50	8.50	-	63	148		Lower Marker
>60000	253	-	94.69	8017	>60000		
-	-	-	-	-	-		Sample Well

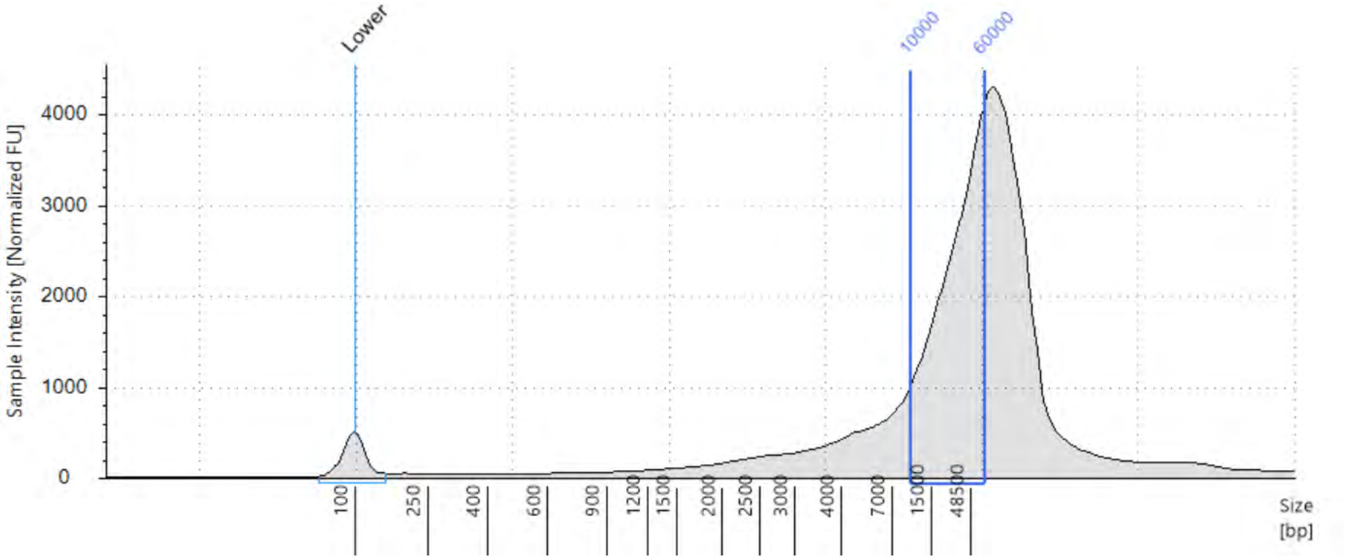

Region Table

From [bp]	To [bp]	Average Size [bp]	Conc. [ng/µl]	Region Molarity [nmol/l]	% of Total	Region Comment	Color
10000	60000	32628	118	7.56	34.81		■

 © Agilent Technologies, Inc. 2017

H2: M155

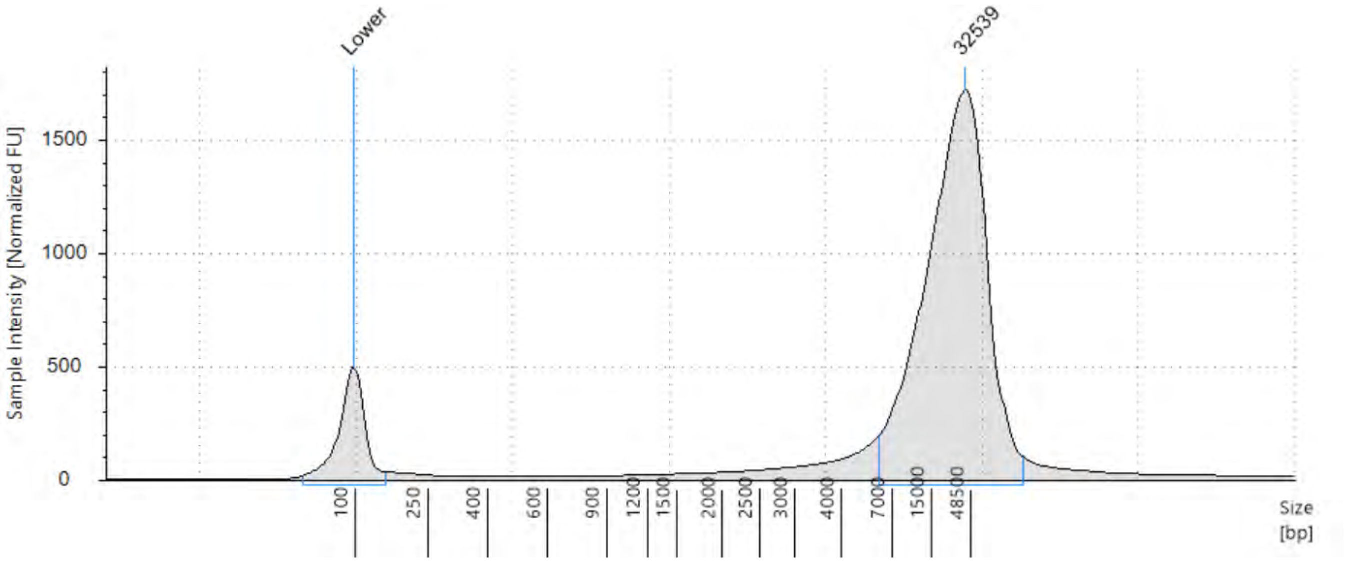

Sample Table

Well	DIN	Conc. [ng/µl]	Sample Description	Alert	Observations
H2	8.3	87.9	M155		

Peak Table

Size [bp]	Calibrated Conc. [ng/µl]	Assigned Conc. [ng/µl]	% Integrated Area	From [bp]	To [bp]	Peak Comment	Observations
100	8.50	8.50	-	53	149		Lower Marker
32539	71.2	-	95.92	6149	>60000		
-	-	-	-	-	-		Sample Well

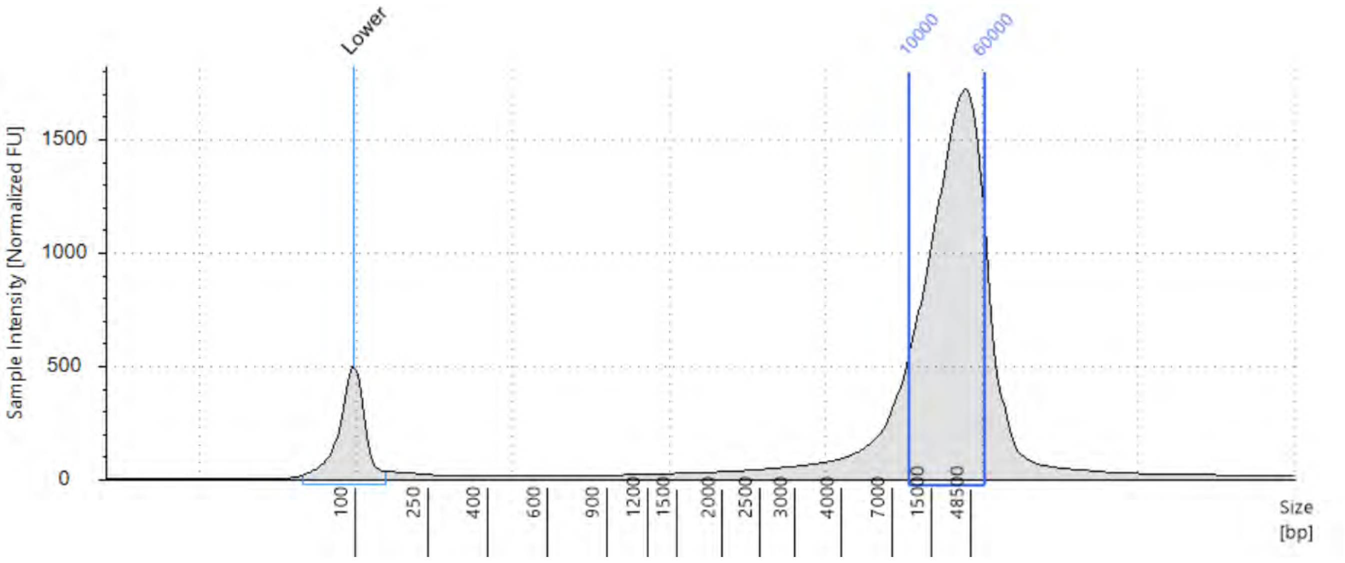

Region Table

From [bp]	To [bp]	Average Size [bp]	Conc. [ng/µl]	Region Molarity [nmol/l]	% of Total	Region Comment	Color
10000	60000	29831	55.8	3.77	63.50		

A3: M161

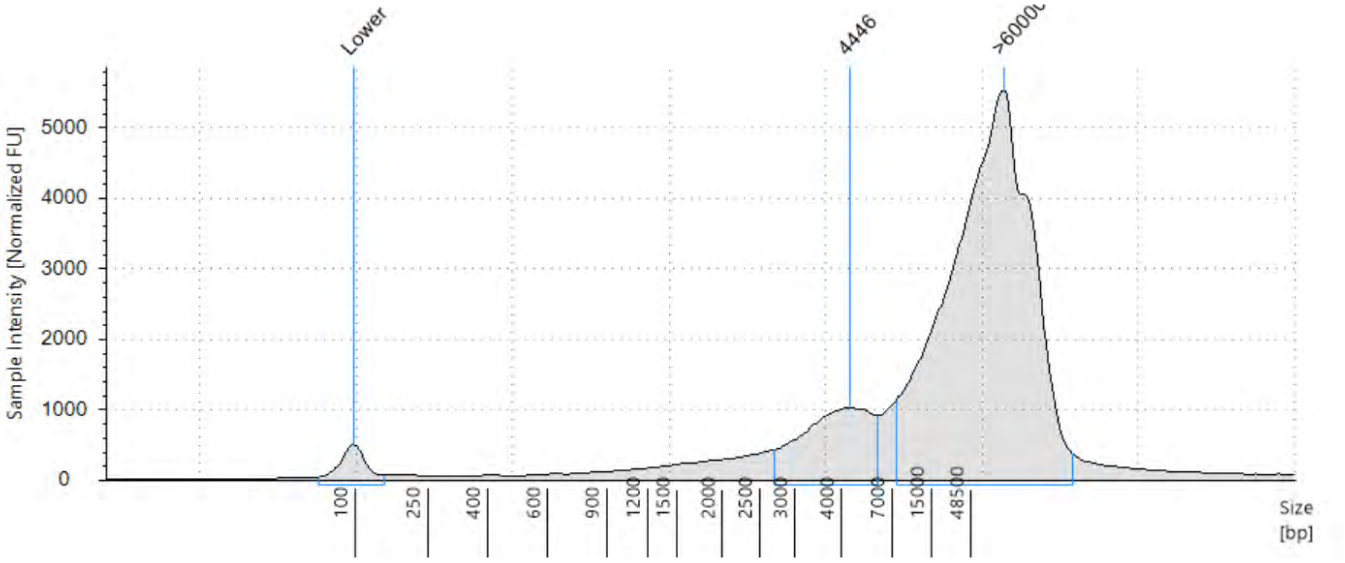

Sample Table

Well	DIN	Conc. [ng/µl]	Sample Description	Alert	Observations
A3	7.9	445	M161	⚠	Sample concentration outside functional range for DIN

Peak Table

Size [bp]	Calibrated Conc. [ng/µl]	Assigned Conc. [ng/µl]	% Integrated Area	From [bp]	To [bp]	Peak Comment	Observations
100	8.50	8.50	-	64	146		Lower Marker
4446	51.2	-	13.05	2706	6007		
>60000	322	-	82.04	7737	>60000		
-	-	-	-	-	-		Sample Well

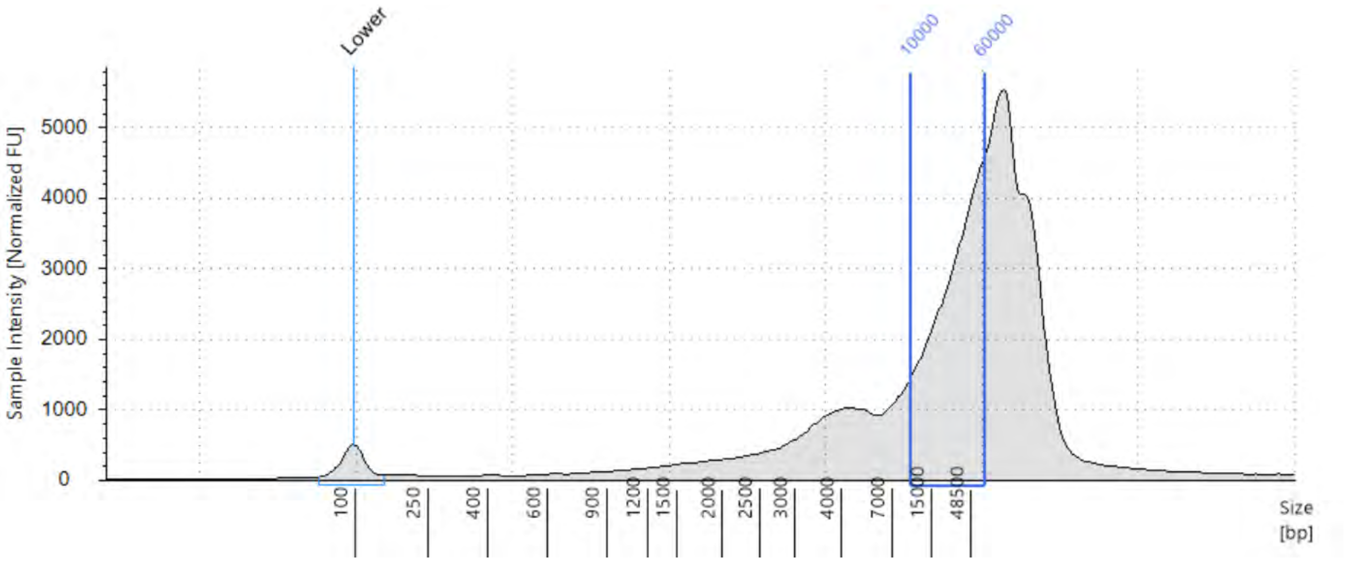

Region Table

From [bp]	To [bp]	Average Size [bp]	Conc. [ng/µl]	Region Molarity [nmol/l]	% of Total	Region Comment	Color
10000	60000	31883	134	8.82	30.16		

 © Agilent Technologies, Inc. 2017

B3: M162

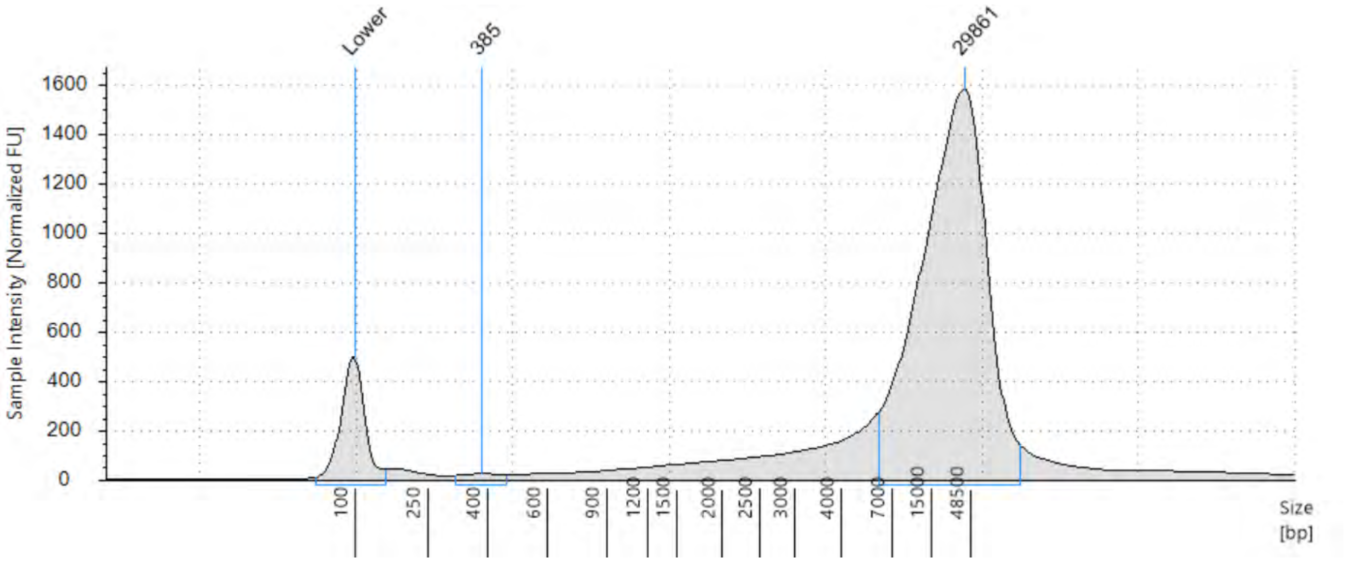

Sample Table

Well	DIN	Conc. [ng/µl]	Sample Description	Alert	Observations
B3	7.6	105	M162	⚠	Sample concentration outside recommended range

Peak Table

Size [bp]	Calibrated Conc. [ng/µl]	Assigned Conc. [ng/µl]	% Integrated Area	From [bp]	To [bp]	Peak Comment	Observations
100	8.50	8.50	-	61	148		Lower Marker
385	0.618	-	0.78	312	455		
29861	75.6	-	95.94	6038	>60000		
-	-	-	-	-	-		Sample Well

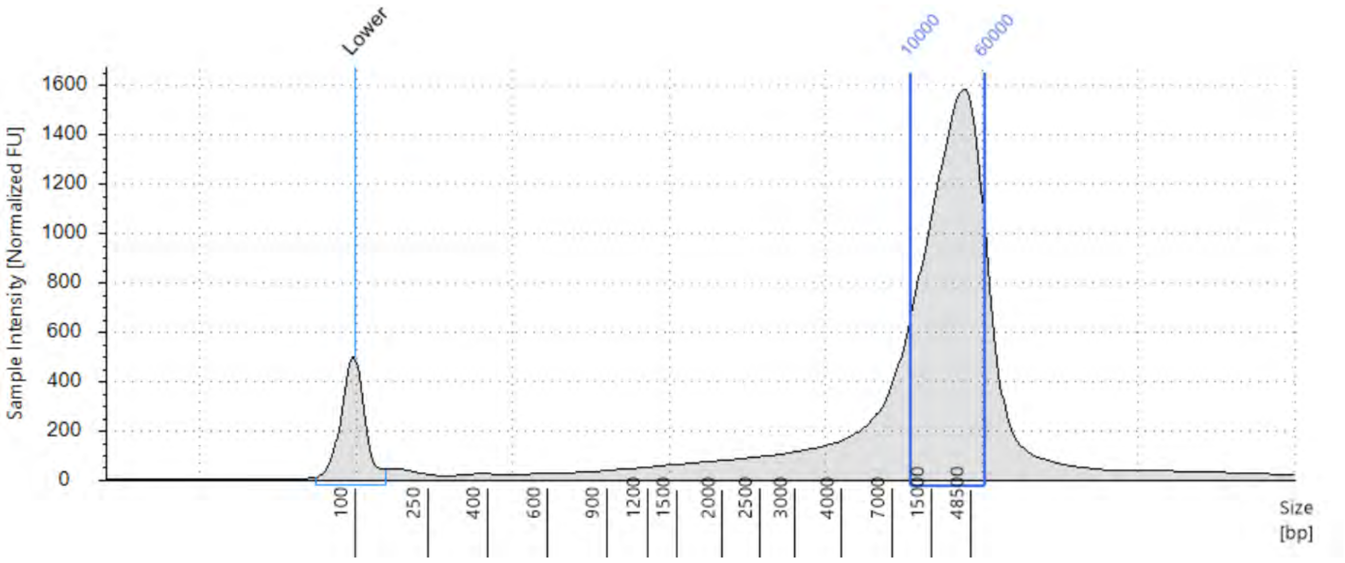

Region Table

From [bp]	To [bp]	Average Size [bp]	Conc. [ng/µl]	Region Molarity [nmol/l]	% of Total	Region Comment	Color
10000	60000	28689	58.7	4.15	55.73		■

 © Agilent Technologies, Inc. 2017

C3: M163

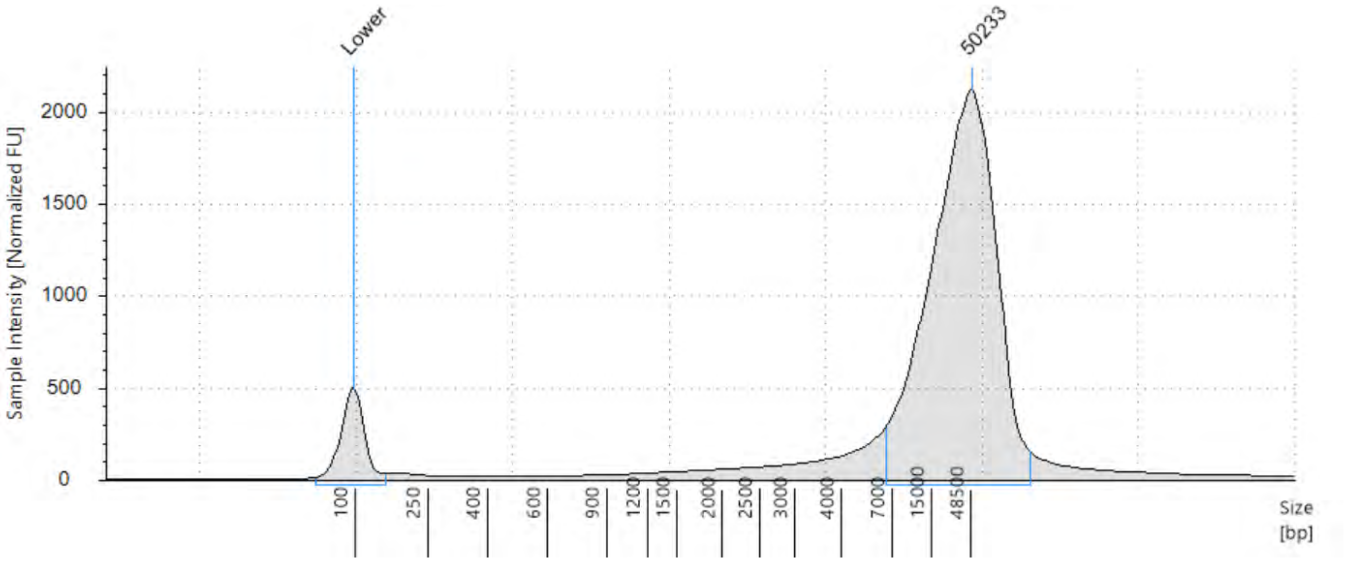

Sample Table

Well	DIN	Conc. [ng/µl]	Sample Description	Alert	Observations
C3	8.4	130	M163	⚠	Sample concentration outside recommended range

Peak Table

Size [bp]	Calibrated Conc. [ng/µl]	Assigned Conc. [ng/µl]	% Integrated Area	From [bp]	To [bp]	Peak Comment	Observations
100	8.50	8.50	-	62	149		Lower Marker
50233	104	-	95.91	6574	>60000		
-	-	-	-	-	-		Sample Well

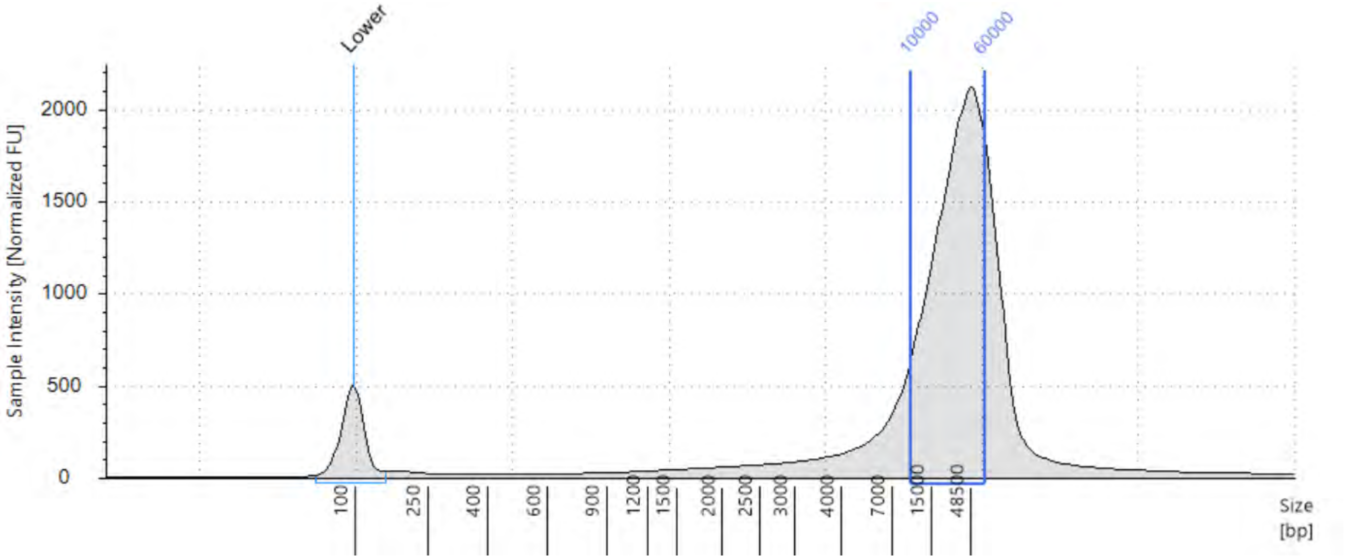

Region Table

From [bp]	To [bp]	Average Size [bp]	Conc. [ng/µl]	Region Molarity [nmol/l]	% of Total	Region Comment	Color
10000	60000	30904	74.3	4.92	57.21		■

D3: M164

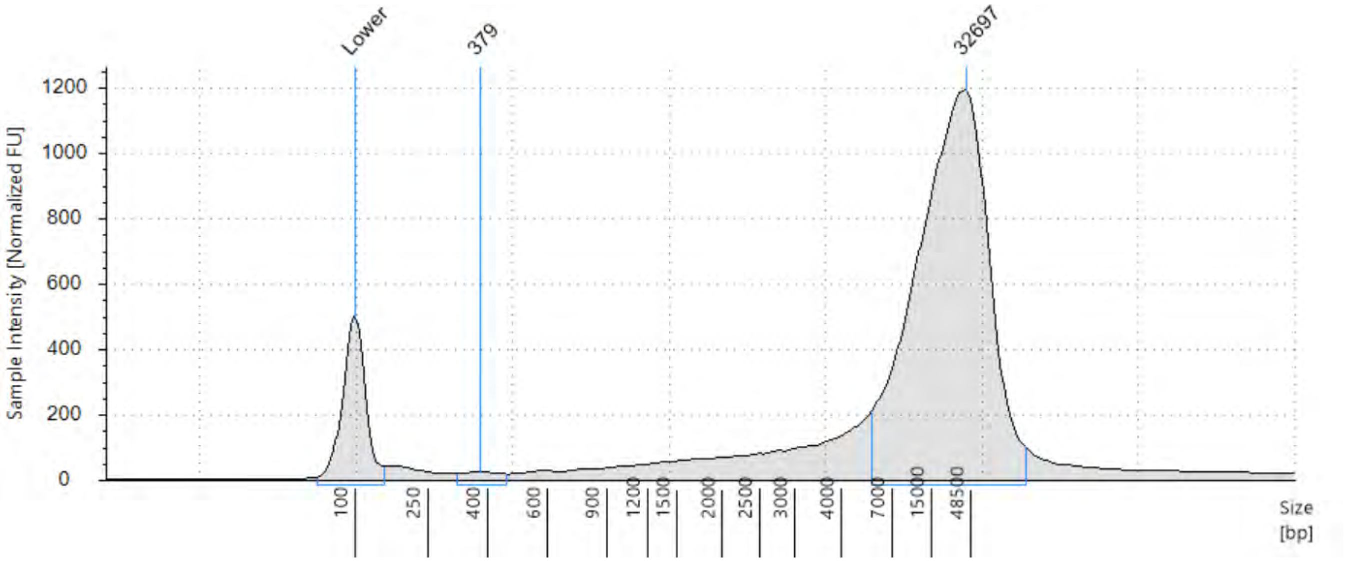

Sample Table

Well	DIN	Conc. [ng/µl]	Sample Description	Alert	Observations
D3	7.5	90.4	M164		

Peak Table

Size [bp]	Calibrated Conc. [ng/µl]	Assigned Conc. [ng/µl]	% Integrated Area	From [bp]	To [bp]	Peak Comment	Observations
100	8.50	8.50	-	62	146		Lower Marker
379	0.602	-	0.88	317	458		
32697	65.2	-	95.64	5613	>60000		
-	-	-	-	-	-		Sample Well

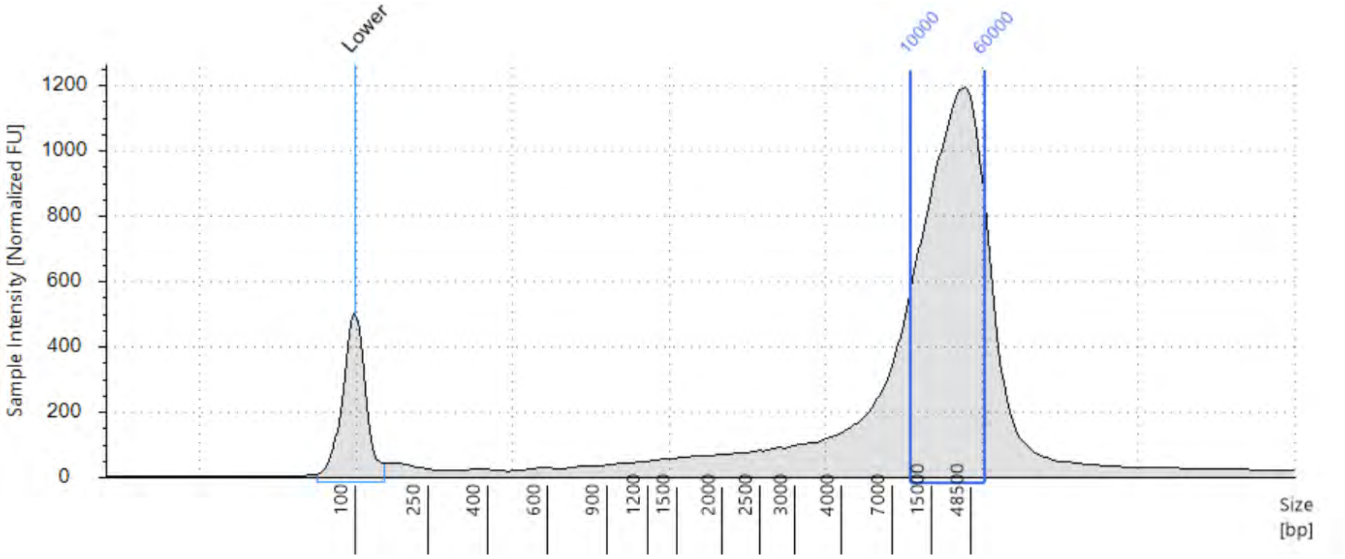

Region Table

From [bp]	To [bp]	Average Size [bp]	Conc. [ng/µl]	Region Molarity [nmol/l]	% of Total	Region Comment	Color
10000	60000	28402	47.6	3.42	52.70		

 © Agilent Technologies, Inc. 2017

E3: M165

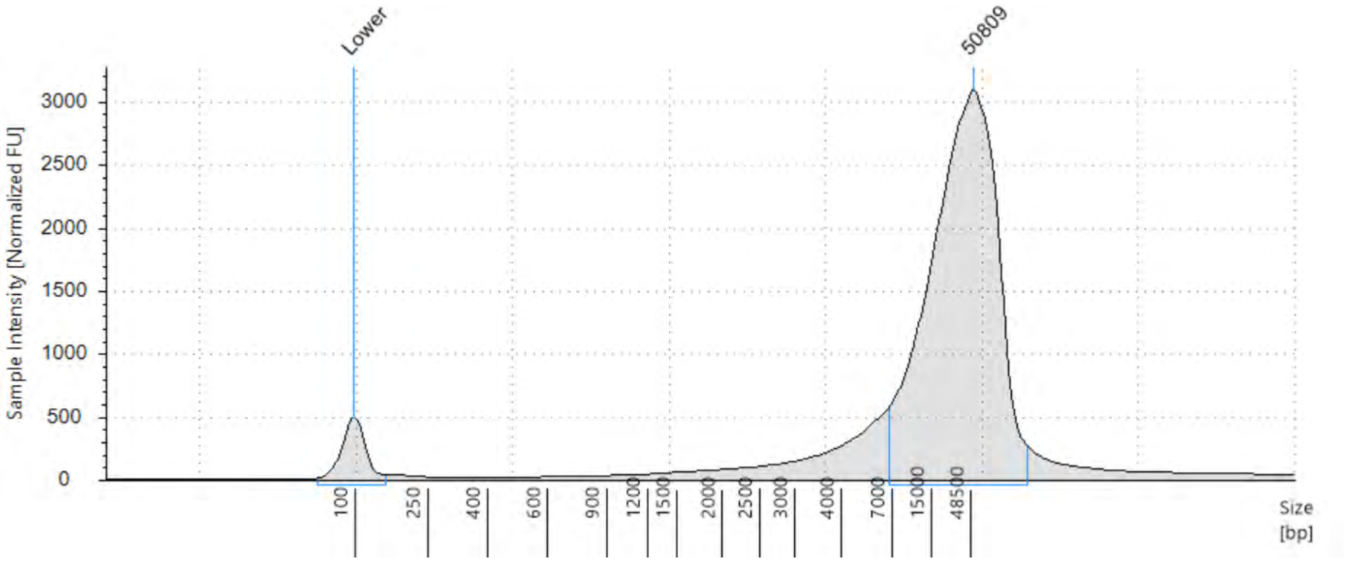

Sample Table

Well	DIN	Conc. [ng/µl]	Sample Description	Alert	Observations
E3	8.3	203	M165	⚠	Sample concentration outside recommended range

Peak Table

Size [bp]	Calibrated Conc. [ng/µl]	Assigned Conc. [ng/µl]	% Integrated Area	From [bp]	To [bp]	Peak Comment	Observations
100	8.50	8.50	-	63	149		Lower Marker
50809	156	-	95.40	6805	>60000		
-	-	-	-	-	-		Sample Well

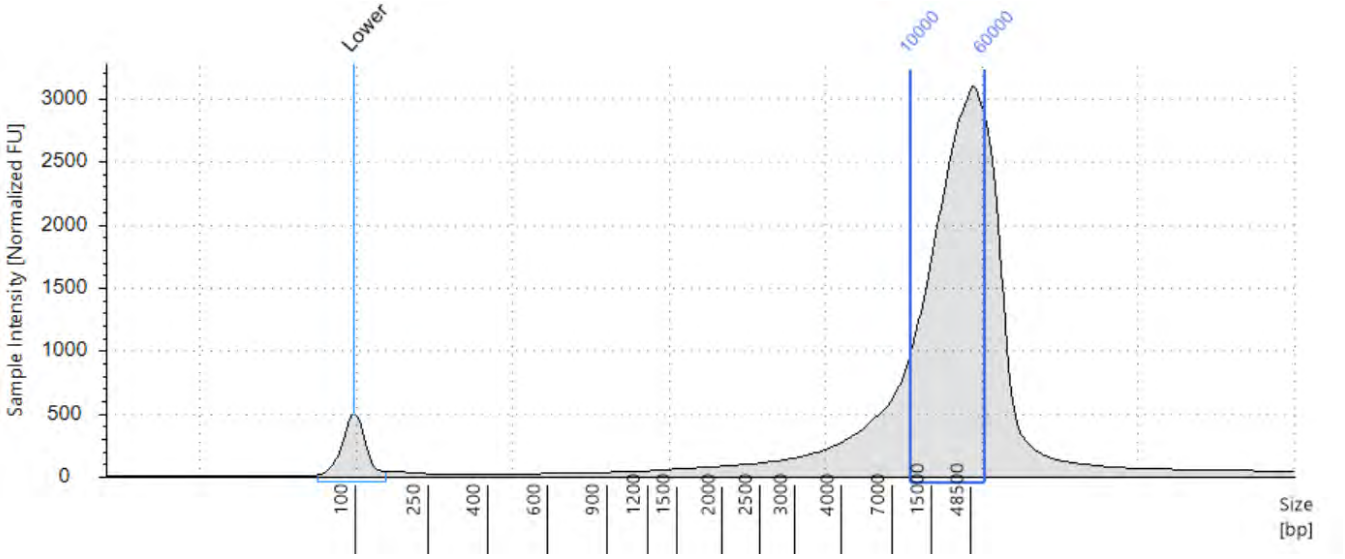

Region Table

From [bp]	To [bp]	Average Size [bp]	Conc. [ng/µl]	Region Molarity [nmol/l]	% of Total	Region Comment	Color
10000	60000	30668	108	7.16	53.17		■

F3: M171

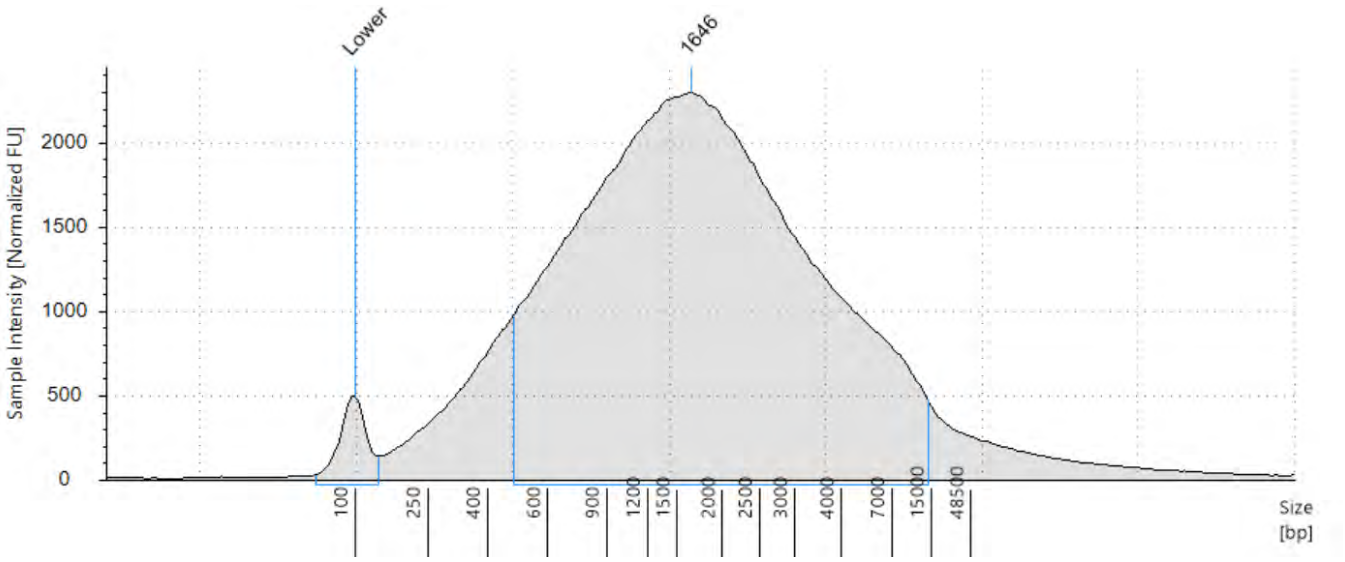

Sample Table

Well	DIN	Conc. [ng/µl]	Sample Description	Alert	Observations
F3	3.1	422	M171	⚠	Sample concentration outside functional range for DIN

Peak Table

Size [bp]	Calibrated Conc. [ng/µl]	Assigned Conc. [ng/µl]	% Integrated Area	From [bp]	To [bp]	Peak Comment	Observations
100	8.50	8.50	-	61	136		Lower Marker
1646	364	-	100.00	476	14076		

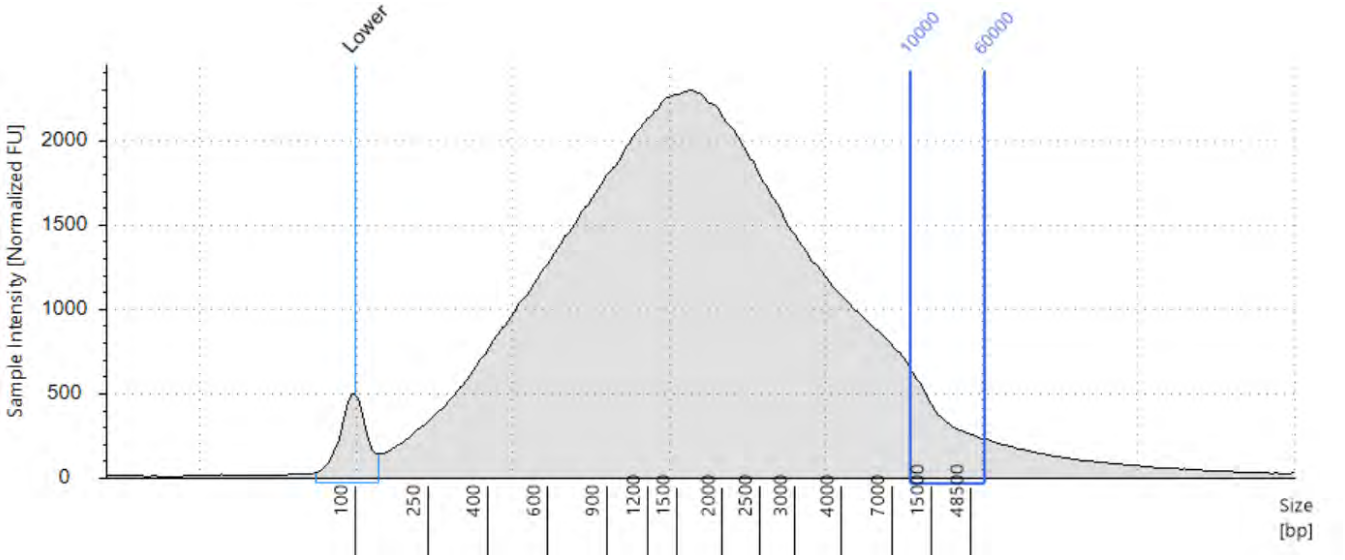

Region Table

From [bp]	To [bp]	Average Size [bp]	Conc. [ng/µl]	Region Molarity [nmol/l]	% of Total	Region Comment	Color
10000	60000	22911	15.7	1.48	3.73		■

G3: M172

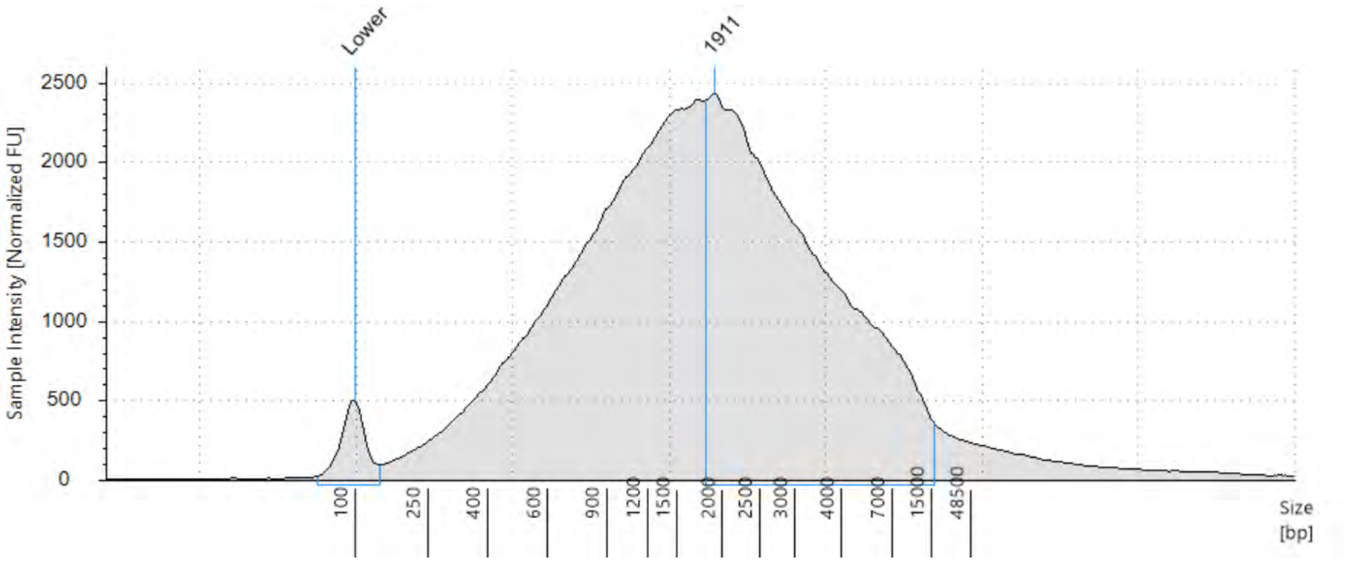

Sample Table

Well	DIN	Conc. [ng/µl]	Sample Description	Alert	Observations
G3	3.4	459	M172	⚠	Sample concentration outside functional range for DIN

Peak Table

Size [bp]	Calibrated Conc. [ng/µl]	Assigned Conc. [ng/µl]	% Integrated Area	From [bp]	To [bp]	Peak Comment	Observations
100	8.50	8.50	-	62	138		Lower Marker
1911	207	-	100.00	1799	16161		

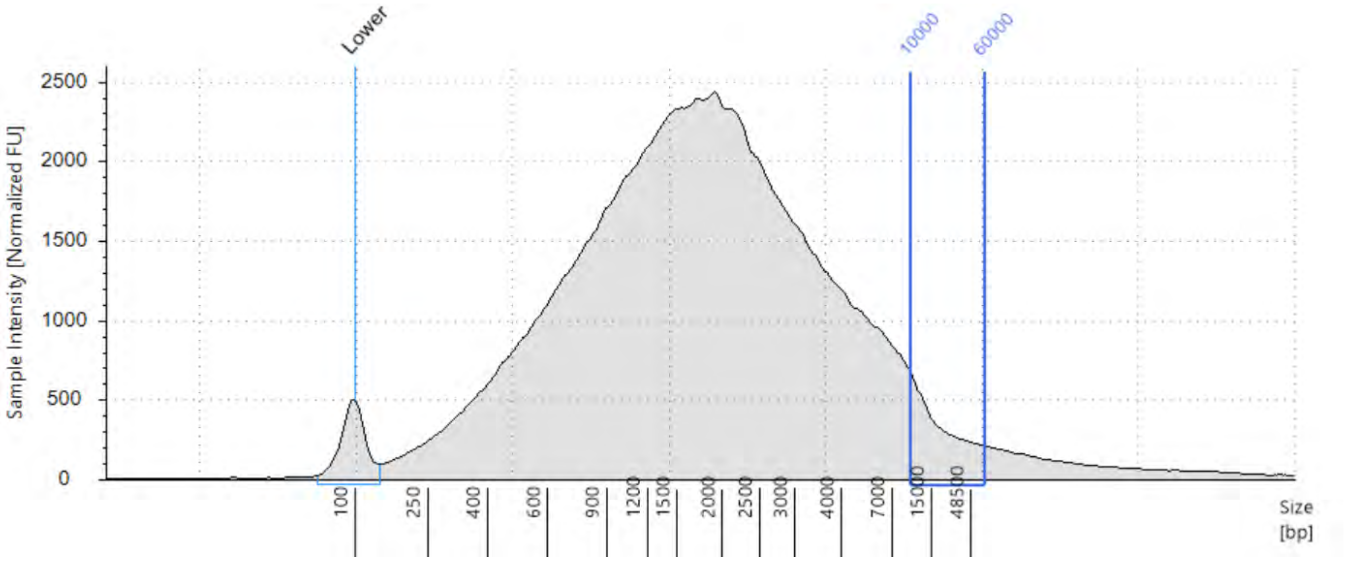

Region Table

From [bp]	To [bp]	Average Size [bp]	Conc. [ng/µl]	Region Molarity [nmol/l]	% of Total	Region Comment	Color
10000	60000	22559	15.9	1.52	3.47		

© Agilent Technologies, Inc. 2017

H3: M173

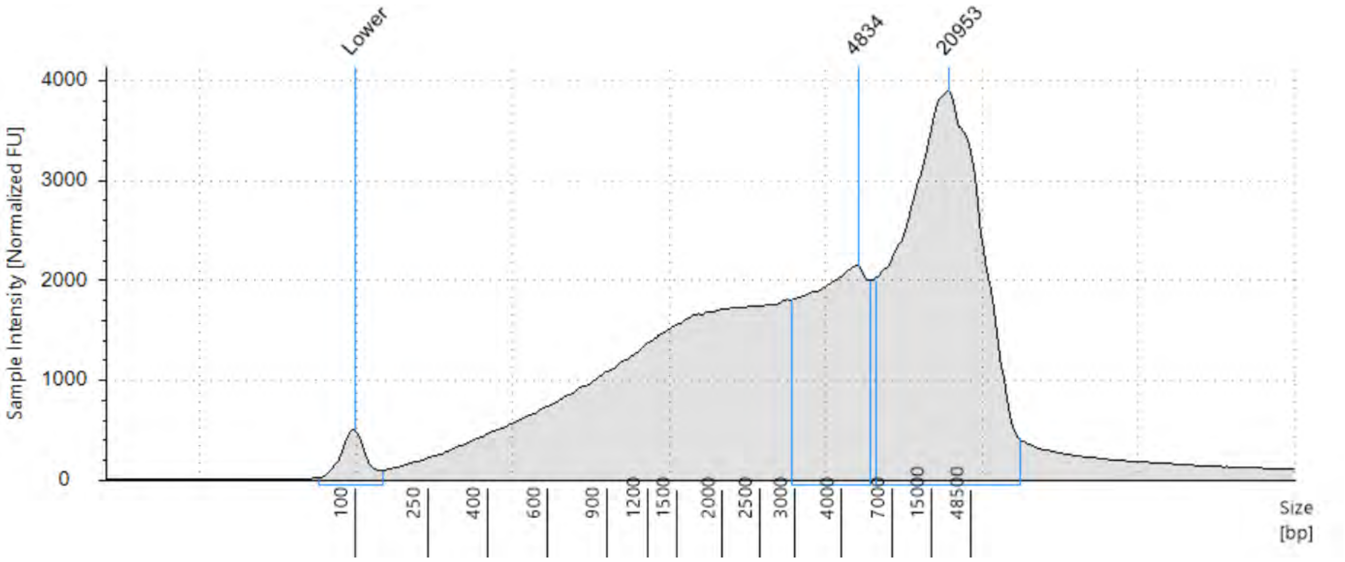

Sample Table

Well	DIN	Conc. [ng/µl]	Sample Description	Alert	Observations
H3	6.0	637	M173	⚠	Sample concentration outside functional range for DIN

Peak Table

Size [bp]	Calibrated Conc. [ng/µl]	Assigned Conc. [ng/µl]	% Integrated Area	From [bp]	To [bp]	Peak Comment	Observations
100	8.50	8.50	-	63	143		Lower Marker
4834	99.6	-	28.76	2960	5530		
20953	237	-	68.51	5870	>60000		
-	-	-	-	-	-		Sample Well

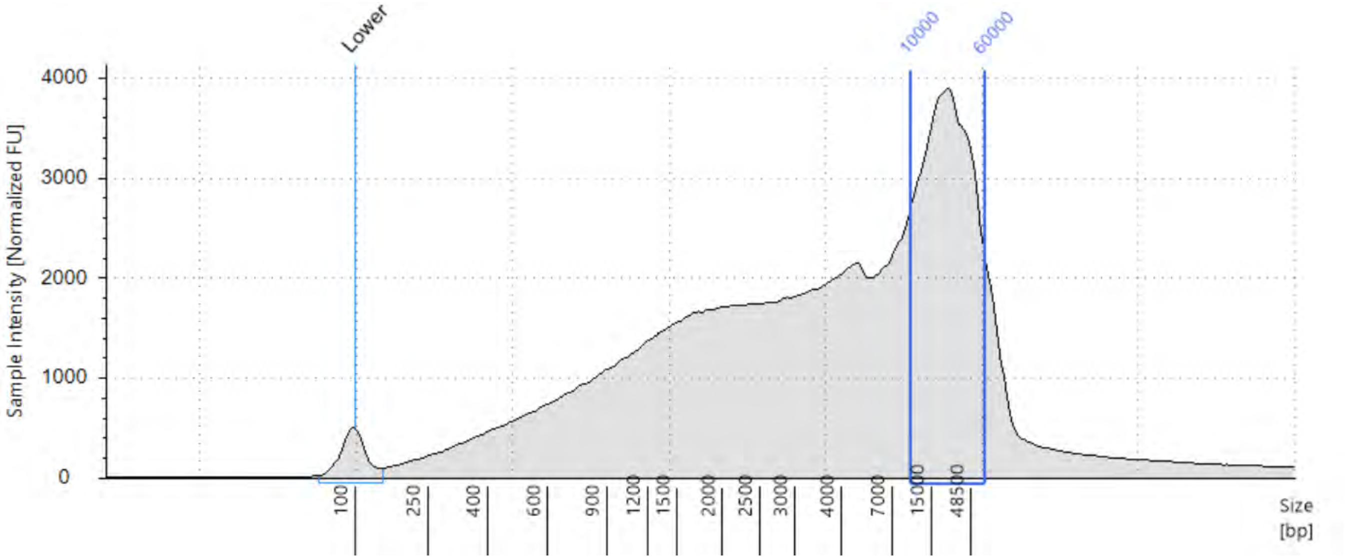

Region Table

From [bp]	To [bp]	Average Size [bp]	Conc. [ng/µl]	Region Molarity [nmol/l]	% of Total	Region Comment	Color
10000	60000	26025	161	12.6	25.29		■

 © Agilent Technologies, Inc. 2017

A4: M174

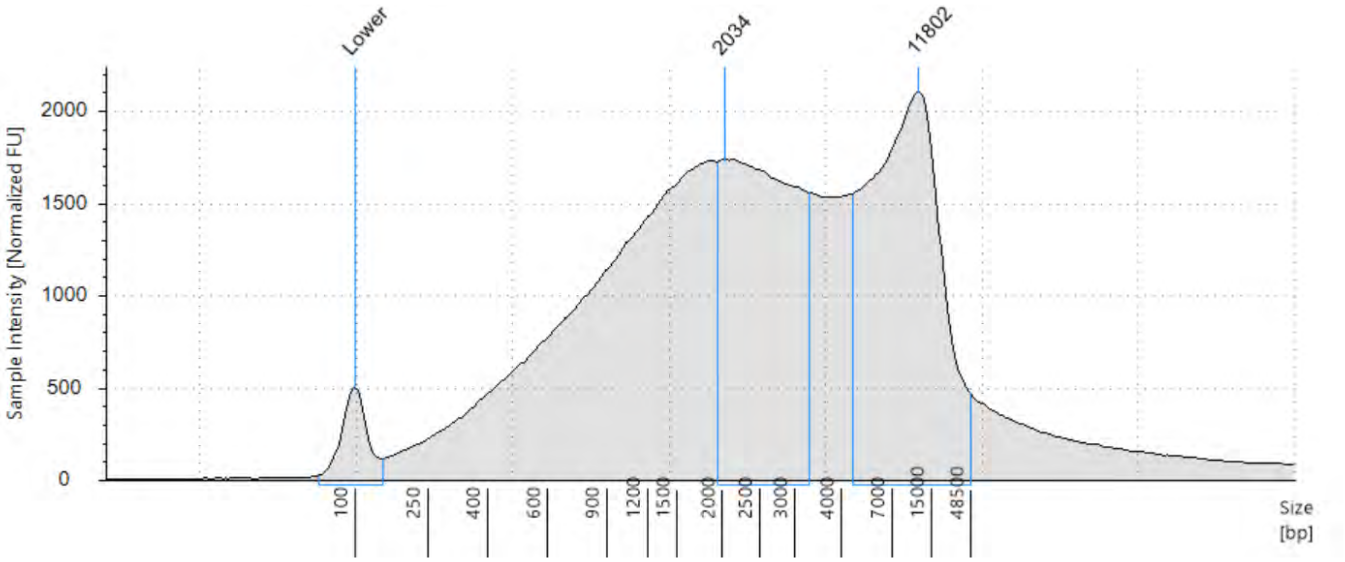

Sample Table

Well	DIN	Conc. [ng/µl]	Sample Description	Alert	Observations
A4	4.5	448	M174	⚠	Sample concentration outside functional range for DIN

Peak Table

Size [bp]	Calibrated Conc. [ng/µl]	Assigned Conc. [ng/µl]	% Integrated Area	From [bp]	To [bp]	Peak Comment	Observations
100	8.50	8.50	-	63	143		Lower Marker
2034	91.7	-	45.62	1950	3283		
11802	108	-	53.68	4579	48537		
-	-	-	-	-	-		Sample Well

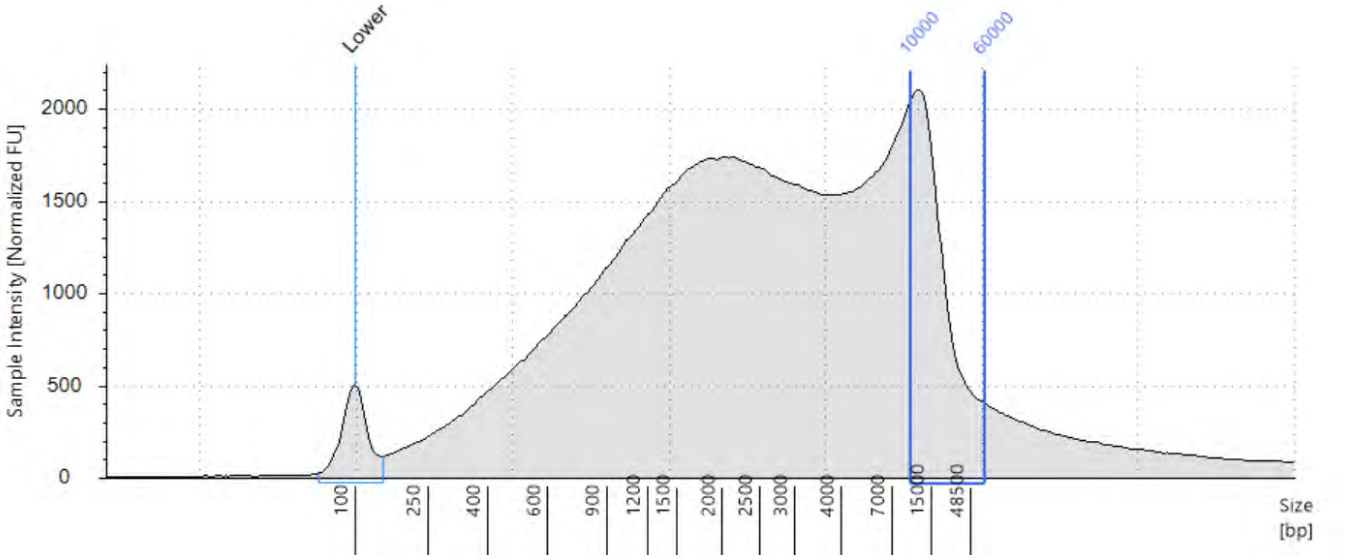

Region Table

From [bp]	To [bp]	Average Size [bp]	Conc. [ng/µl]	Region Molarity [nmol/l]	% of Total	Region Comment	Color
10000	60000	19605	52.4	5.22	11.69		■

B4: M175

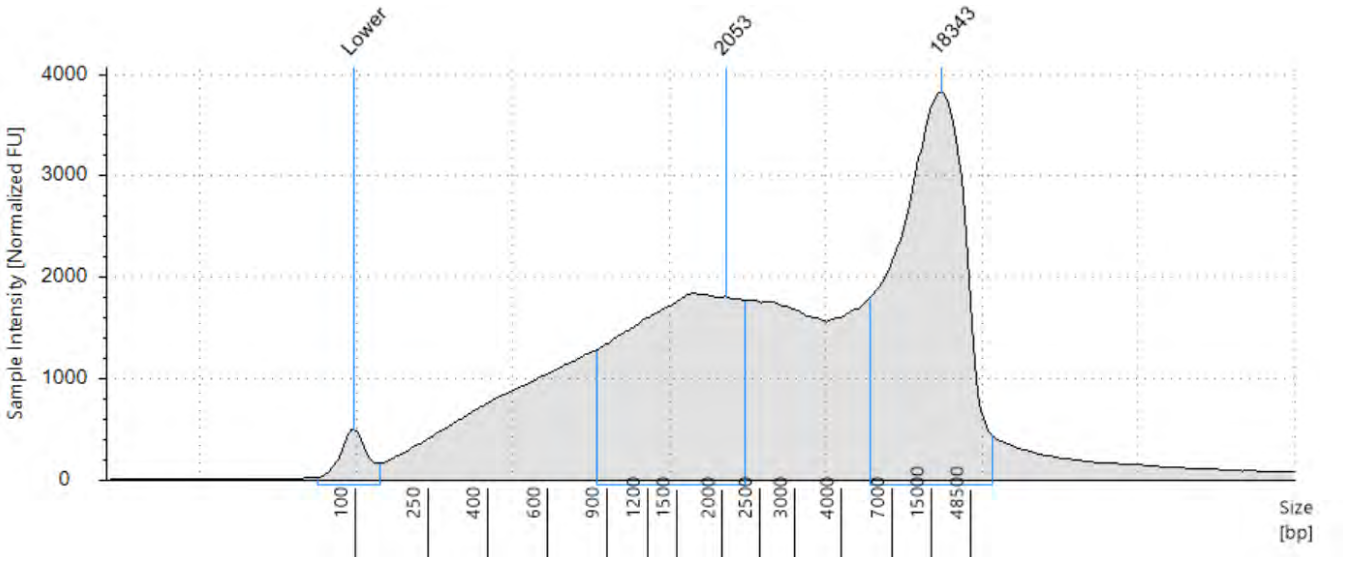

Sample Table

Well	DIN	Conc. [ng/µl]	Sample Description	Alert	Observations
B4	5.3	573	M175	⚠	Sample concentration outside functional range for DIN

Peak Table

Size [bp]	Calibrated Conc. [ng/µl]	Assigned Conc. [ng/µl]	% Integrated Area	From [bp]	To [bp]	Peak Comment	Observations
100	8.50	8.50	-	64	140		Lower Marker
2053	145	-	43.97	850	2298		
18343	182	-	55.18	5509	>60000		
-	-	-	-	-	-		Sample Well

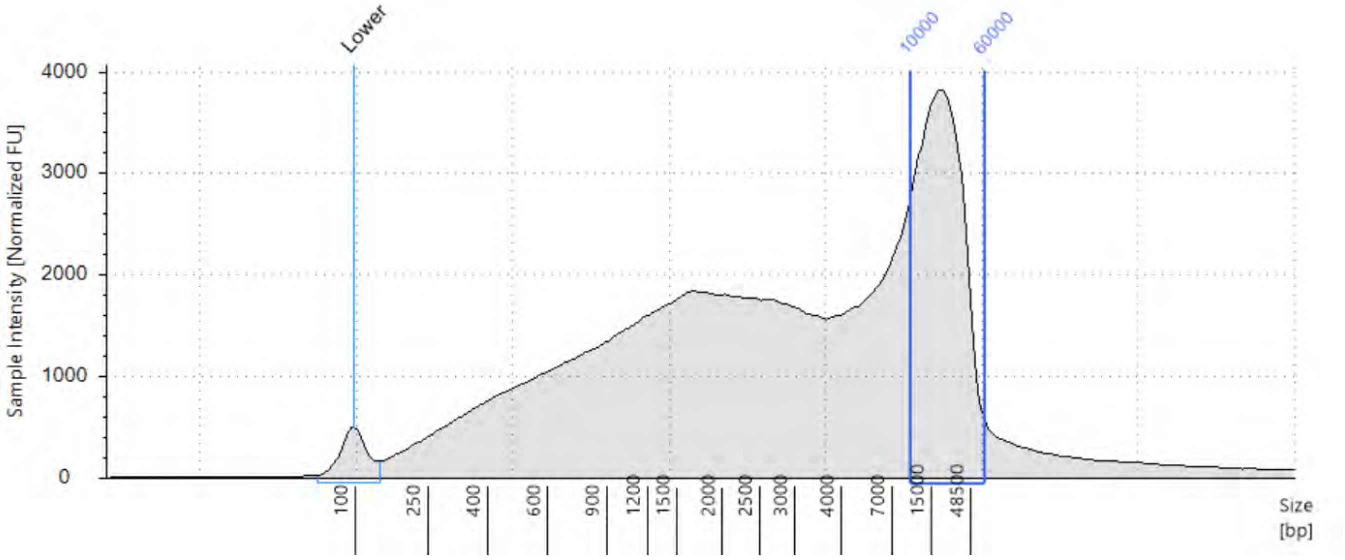

Region Table

From [bp]	To [bp]	Average Size [bp]	Conc. [ng/µl]	Region Molarity [nmol/l]	% of Total	Region Comment	Color
10000	60000	21934	128	11.1	22.41		■

 © Agilent Technologies, Inc. 2017

C4: M176

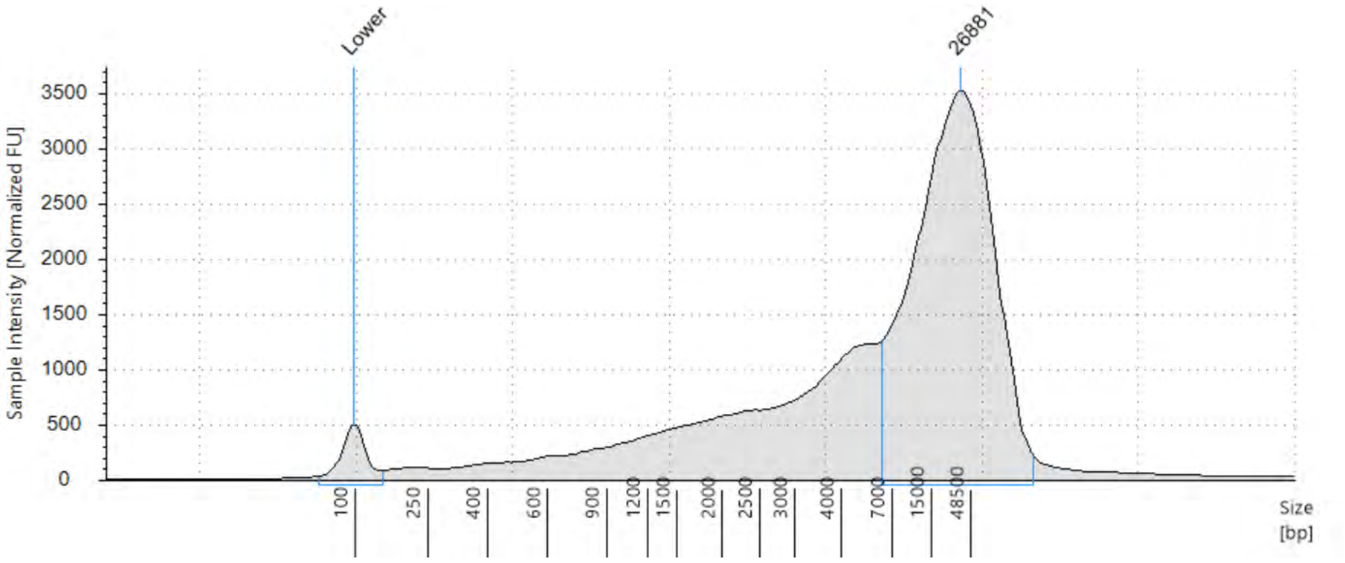

Sample Table

Well	DIN	Conc. [ng/µl]	Sample Description	Alert	Observations
C4	6.7	359	M176	⚠	Sample concentration outside functional range for DIN

Peak Table

Size [bp]	Calibrated Conc. [ng/µl]	Assigned Conc. [ng/µl]	% Integrated Area	From [bp]	To [bp]	Peak Comment	Observations
100	8.50	8.50	-	64	143		Lower Marker
26881	210	-	95.63	6246	>60000		
-	-	-	-	-	-		Sample Well

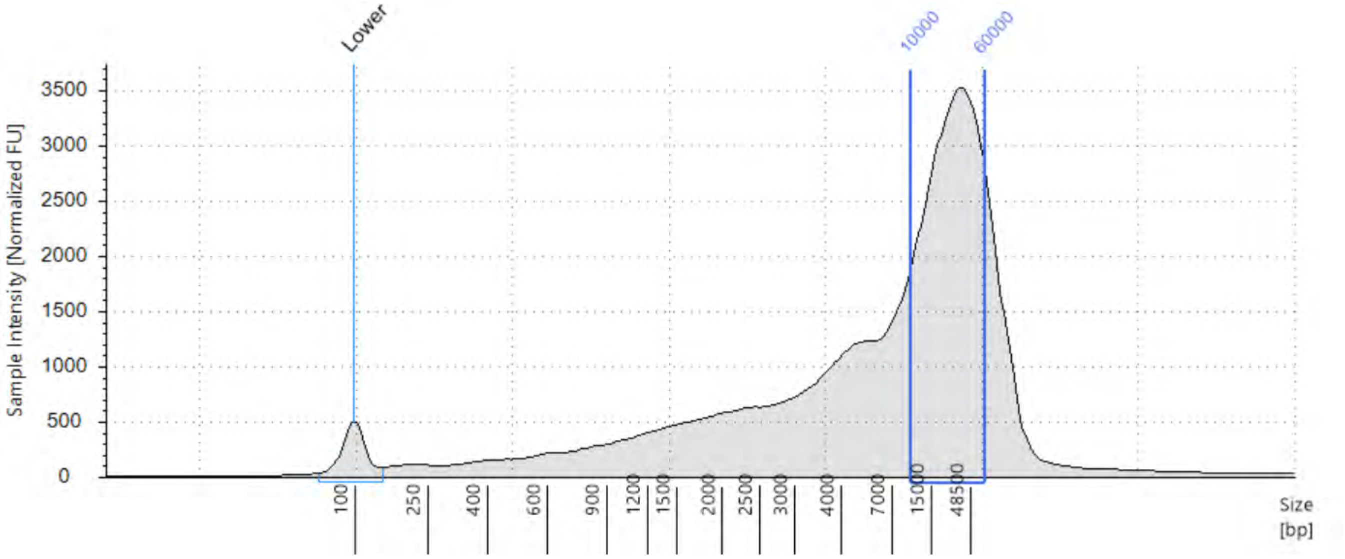

Region Table

From [bp]	To [bp]	Average Size [bp]	Conc. [ng/µl]	Region Molarity [nmol/l]	% of Total	Region Comment	Color
10000	60000	28190	143	10.4	39.91		■

D4: M177

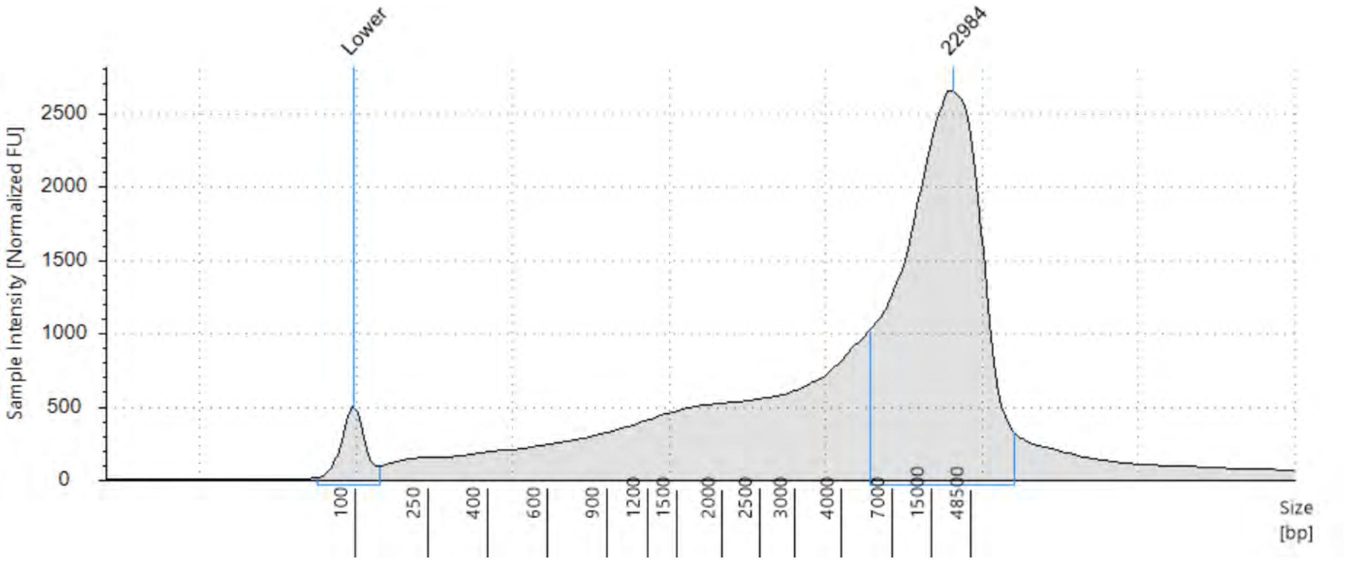

Sample Table

Well	DIN	Conc. [ng/µl]	Sample Description	Alert	Observations
D4	6.4	309	M177	⚠	Sample concentration outside functional range for DIN

Peak Table

Size [bp]	Calibrated Conc. [ng/µl]	Assigned Conc. [ng/µl]	% Integrated Area	From [bp]	To [bp]	Peak Comment	Observations
100	8.50	8.50	-	63	138		Lower Marker
22984	157	-	94.27	5509	>60000		
-	-	-	-	-	-		Sample Well

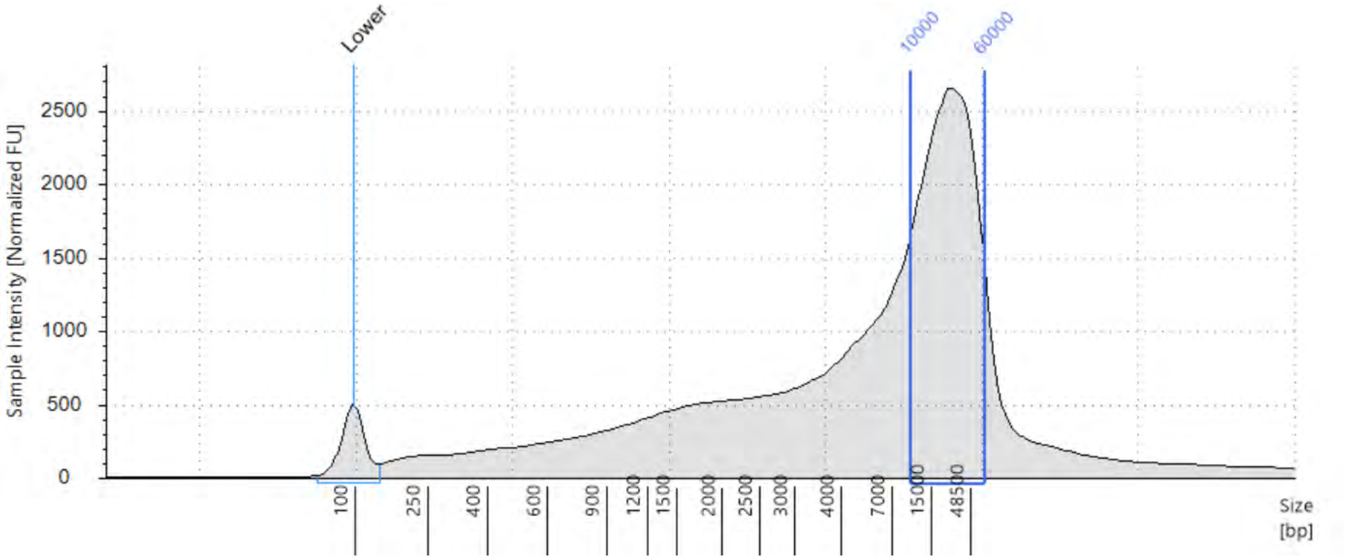

Region Table

From [bp]	To [bp]	Average Size [bp]	Conc. [ng/µl]	Region Molarity [nmol/l]	% of Total	Region Comment	Color
10000	60000	26553	113	8.62	36.49		■

E4: M178

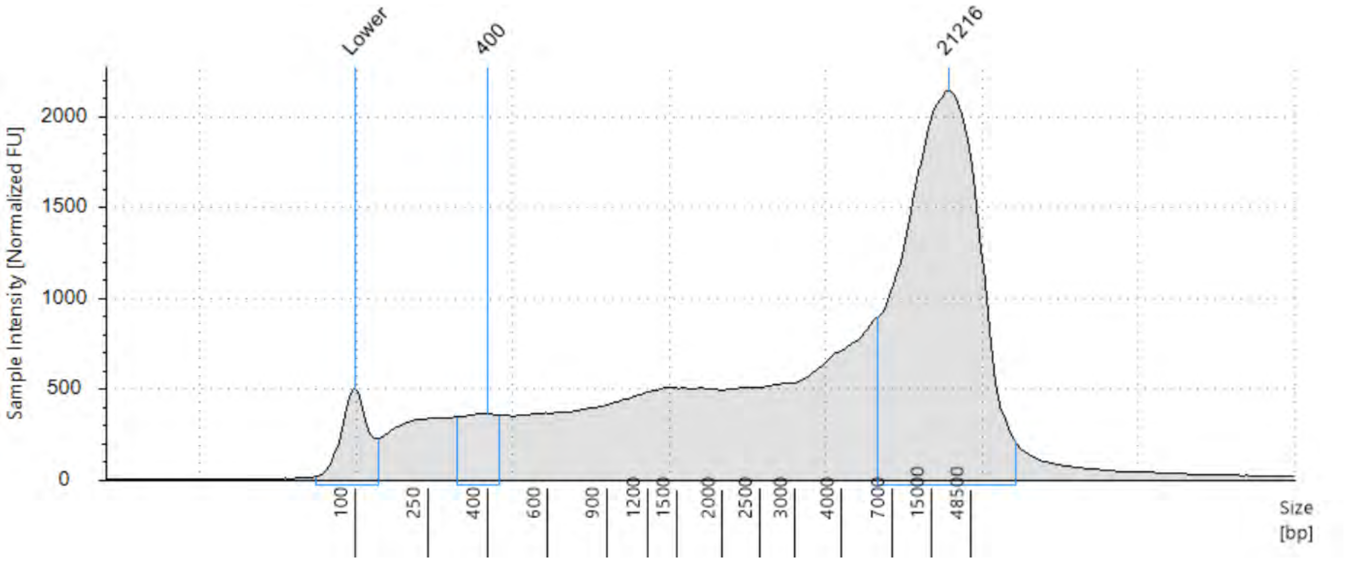

Sample Table

Well	DIN	Conc. [ng/µl]	Sample Description	Alert	Observations
E4	6.1	240	M178	⚠	Sample concentration outside recommended range

Peak Table

Size [bp]	Calibrated Conc. [ng/µl]	Assigned Conc. [ng/µl]	% Integrated Area	From [bp]	To [bp]	Peak Comment	Observations
100	8.50	8.50	-	61	134		Lower Marker
400	8.41	-	7.14	317	434		
21216	106	-	89.77	5997	>60000		
-	-	-	-	-	-		Sample Well

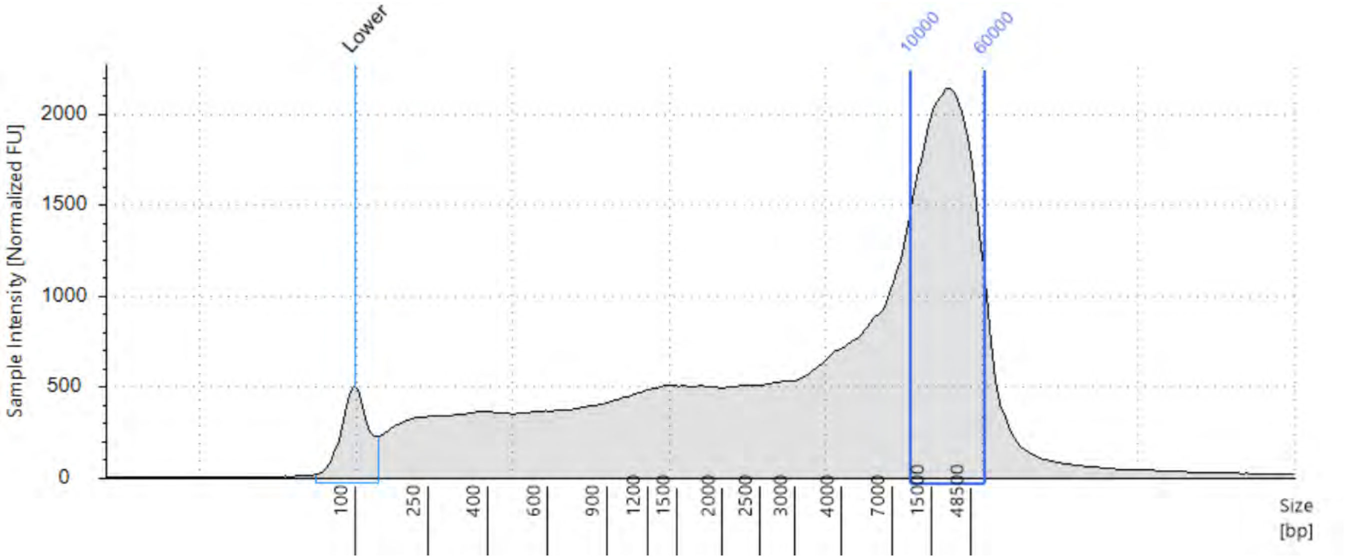

Region Table

From [bp]	To [bp]	Average Size [bp]	Conc. [ng/µl]	Region Molarity [nmol/l]	% of Total	Region Comment	Color
10000	60000	25911	77.3	6.02	32.25		■

F4: M179

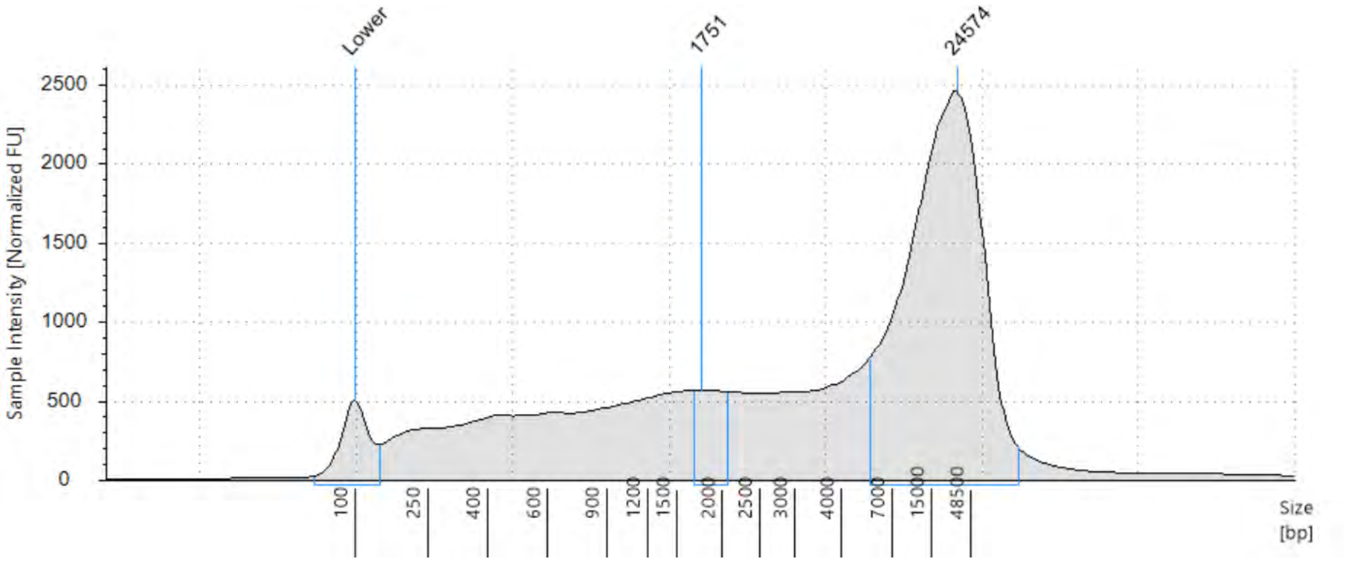

Sample Table

Well	DIN	Conc. [ng/µl]	Sample Description	Alert	Observations
F4	6.1	241	M179	⚠	Sample concentration outside recommended range

Peak Table

Size [bp]	Calibrated Conc. [ng/µl]	Assigned Conc. [ng/µl]	% Integrated Area	From [bp]	To [bp]	Peak Comment	Observations
100	8.50	8.50	-	60	138		Lower Marker
1751	10.2	-	7.91	1674	2071		
24574	113	-	88.02	5495	>60000		
-	-	-	-	-	-		Sample Well

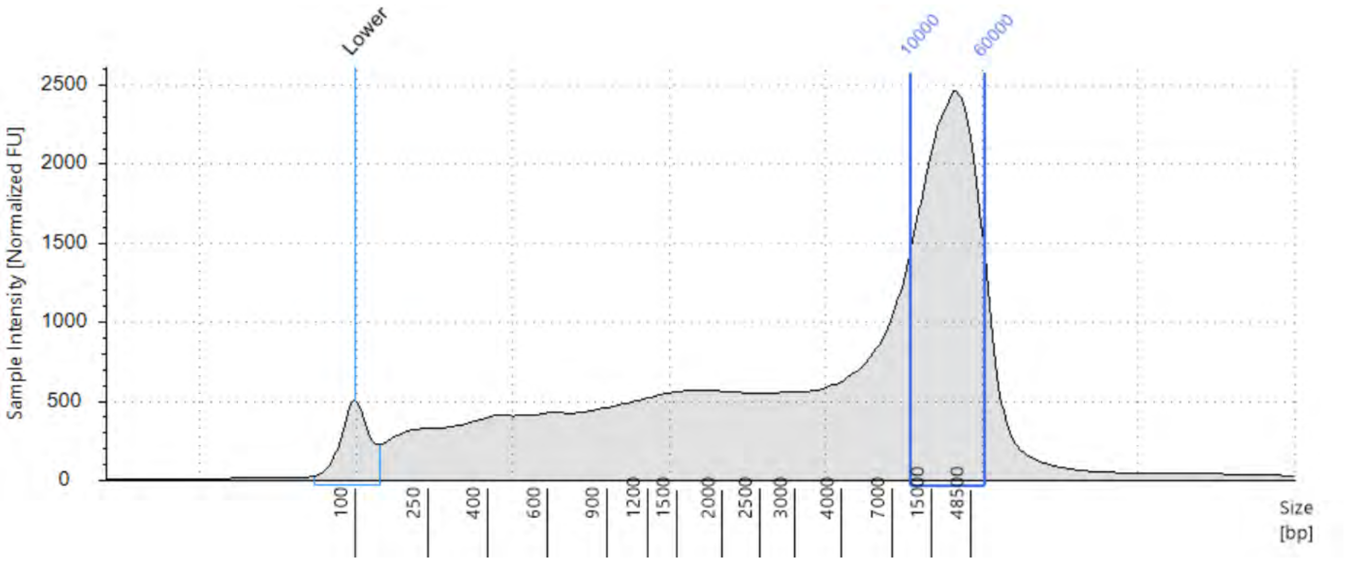

Region Table

From [bp]	To [bp]	Average Size [bp]	Conc. [ng/µl]	Region Molarity [nmol/l]	% of Total	Region Comment	Color
10000	60000	26522	81.2	6.19	33.76		■

G4: M180

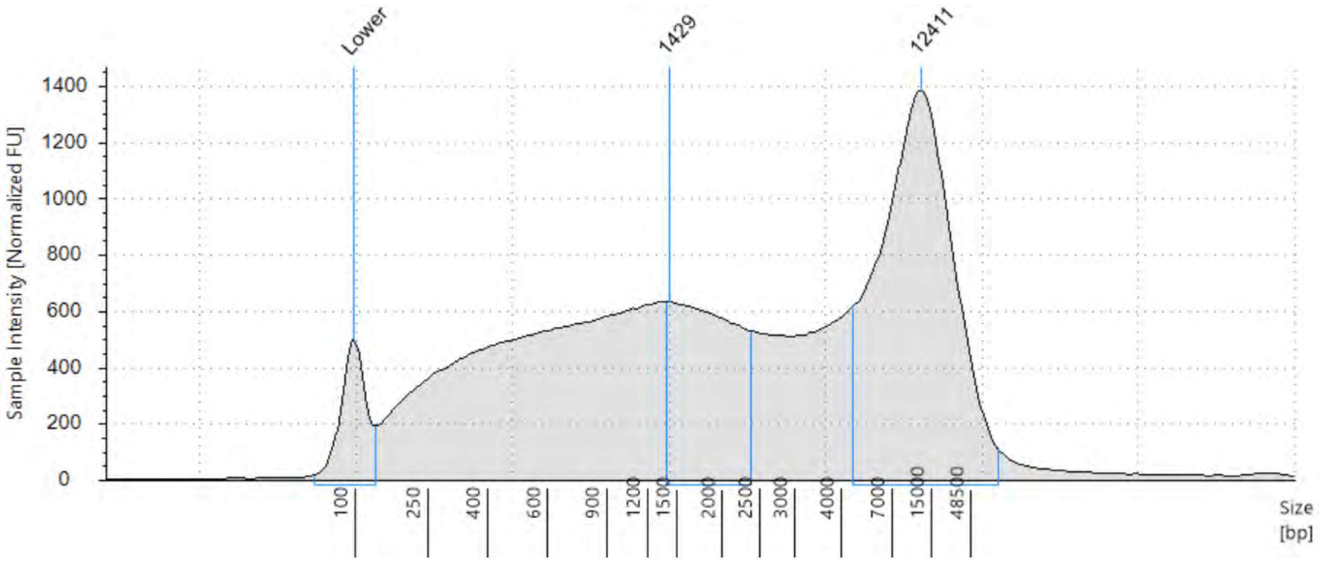

Sample Table

Well	DIN	Conc. [ng/µl]	Sample Description	Alert	Observations
G4	4.1	216	M180	⚠	Sample concentration outside recommended range

Peak Table

Size [bp]	Calibrated Conc. [ng/µl]	Assigned Conc. [ng/µl]	% Integrated Area	From [bp]	To [bp]	Peak Comment	Observations
100	8.50	8.50	-	61	132		Lower Marker
1429	30.0	-	29.54	1386	2394		
12411	70.7	-	69.60	4595	>60000		
-	-	-	-	-	-		Sample Well

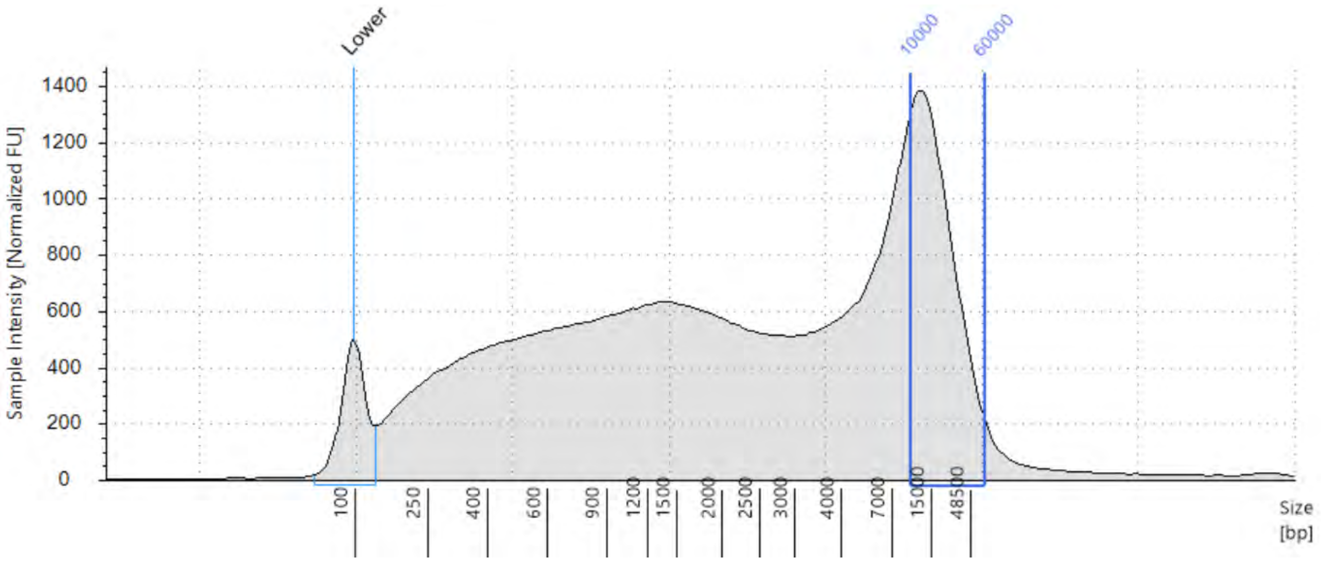

Region Table

From [bp]	To [bp]	Average Size [bp]	Conc. [ng/µl]	Region Molarity [nmol/l]	% of Total	Region Comment	Color
10000	60000	20294	40.5	3.83	18.73		■

 © Agilent Technologies, Inc. 2017

H4: M280

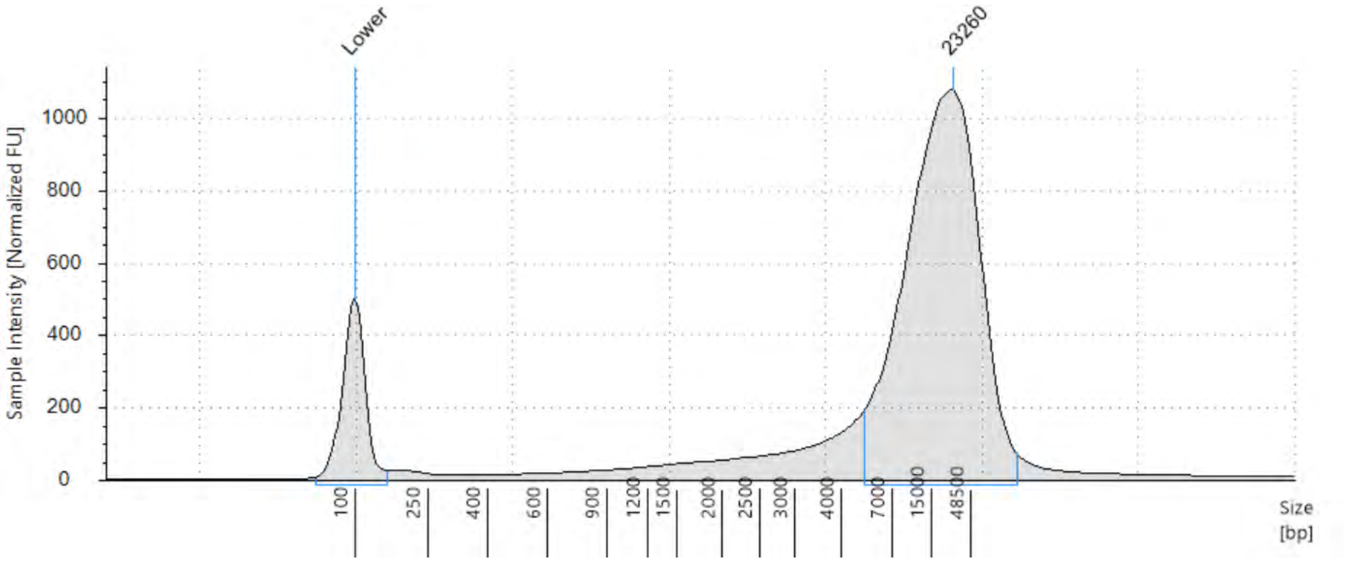

Sample Table

Well	DIN	Conc. [ng/µl]	Sample Description	Alert	Observations
H4	7.5	77.6	M280		

Peak Table

Size [bp]	Calibrated Conc. [ng/µl]	Assigned Conc. [ng/µl]	% Integrated Area	From [bp]	To [bp]	Peak Comment	Observations
100	8.50	8.50	-	61	151		Lower Marker
23260	60.3	-	96.96	5209	>60000		
-	-	-	-	-	-		Sample Well

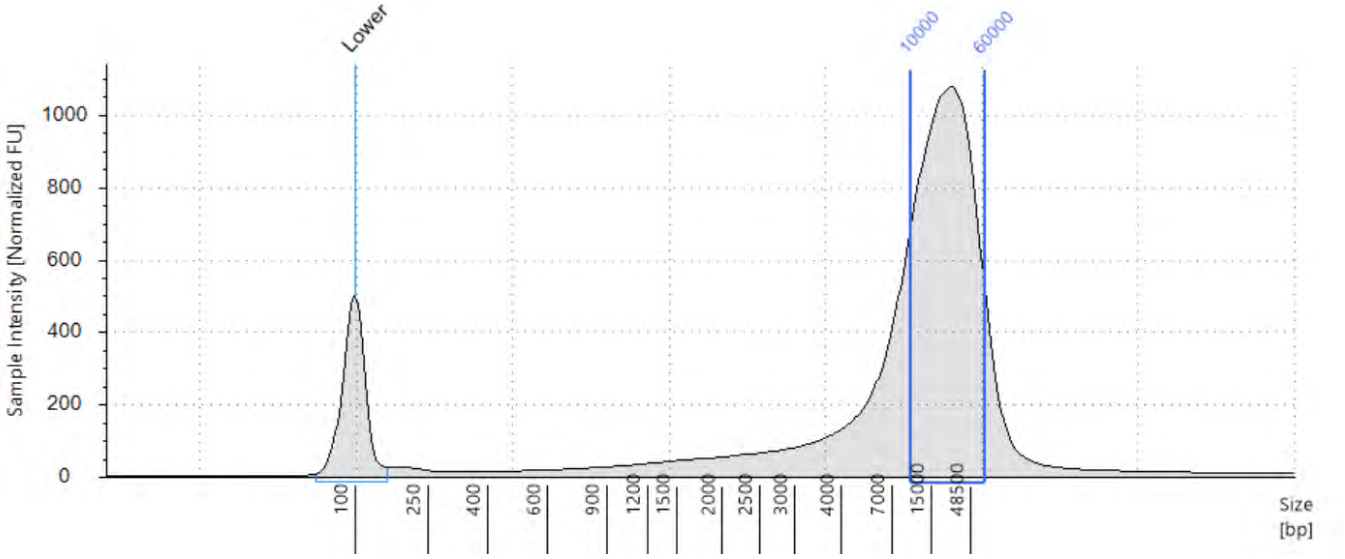

Region Table

From [bp]	To [bp]	Average Size [bp]	Conc. [ng/µl]	Region Molarity [nmol/l]	% of Total	Region Comment	Color
10000	60000	25732	44.5	3.47	57.39		

A5: M281

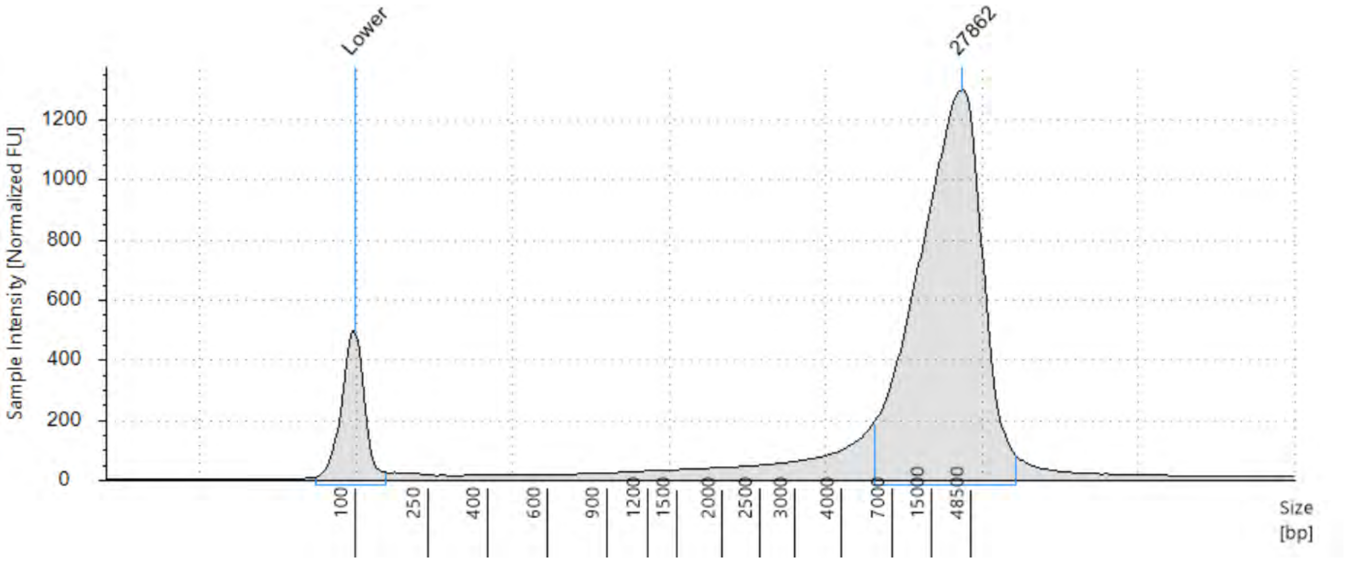

Sample Table

Well	DIN	Conc. [ng/µl]	Sample Description	Alert	Observations
A5	7.8	79.9	M281		

Peak Table

Size [bp]	Calibrated Conc. [ng/µl]	Assigned Conc. [ng/µl]	% Integrated Area	From [bp]	To [bp]	Peak Comment	Observations
100	8.50	8.50	-	61	148		Lower Marker
27862	62.3	-	97.29	5783	>60000		
-	-	-	-	-	-		Sample Well

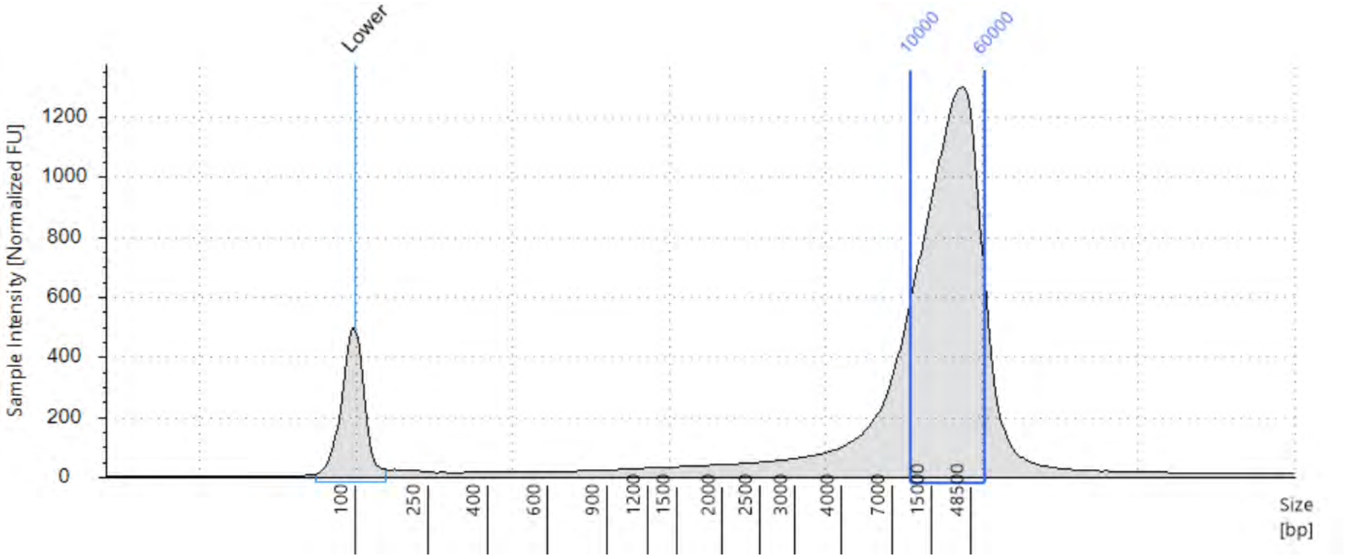

Region Table

From [bp]	To [bp]	Average Size [bp]	Conc. [ng/µl]	Region Molarity [nmol/l]	% of Total	Region Comment	Color
10000	60000	27687	49.2	3.56	61.59		

© Agilent Technologies, Inc. 2017

B5: M282

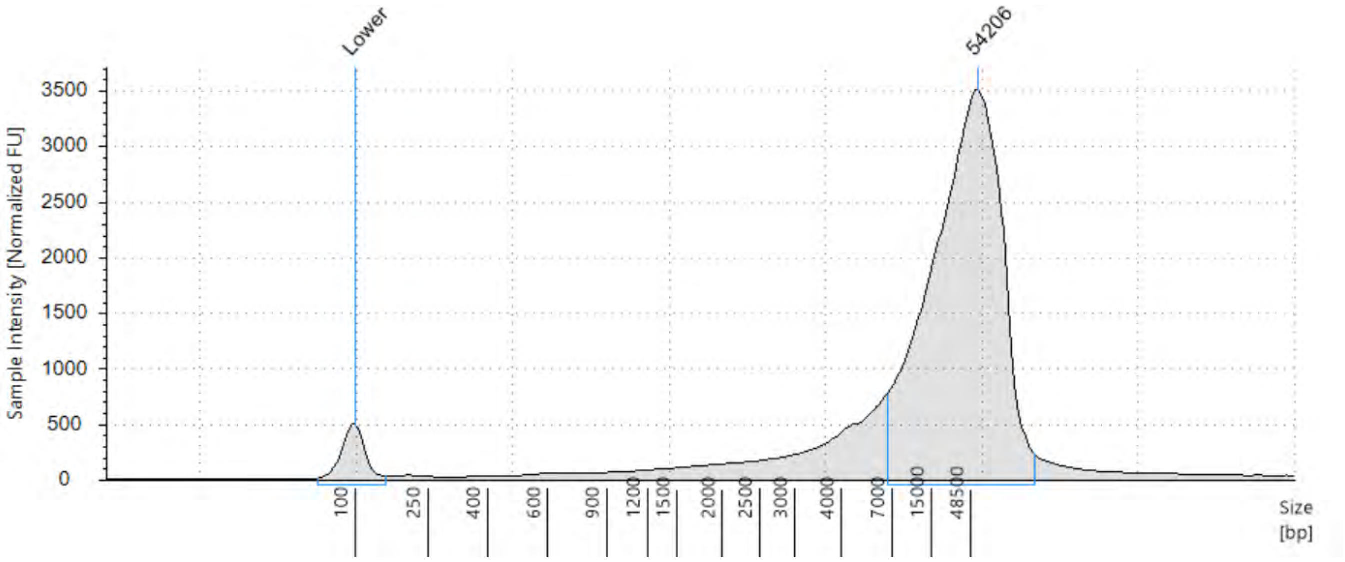

Sample Table

Well	DIN	Conc. [ng/µl]	Sample Description	Alert	Observations
B5	8.0	247	M282	⚠	Sample concentration outside recommended range

Peak Table

Size [bp]	Calibrated Conc. [ng/µl]	Assigned Conc. [ng/µl]	% Integrated Area	From [bp]	To [bp]	Peak Comment	Observations
100	8.50	8.50	-	62	148		Lower Marker
54206	186	-	95.52	6700	>60000		
-	-	-	-	-	-		Sample Well

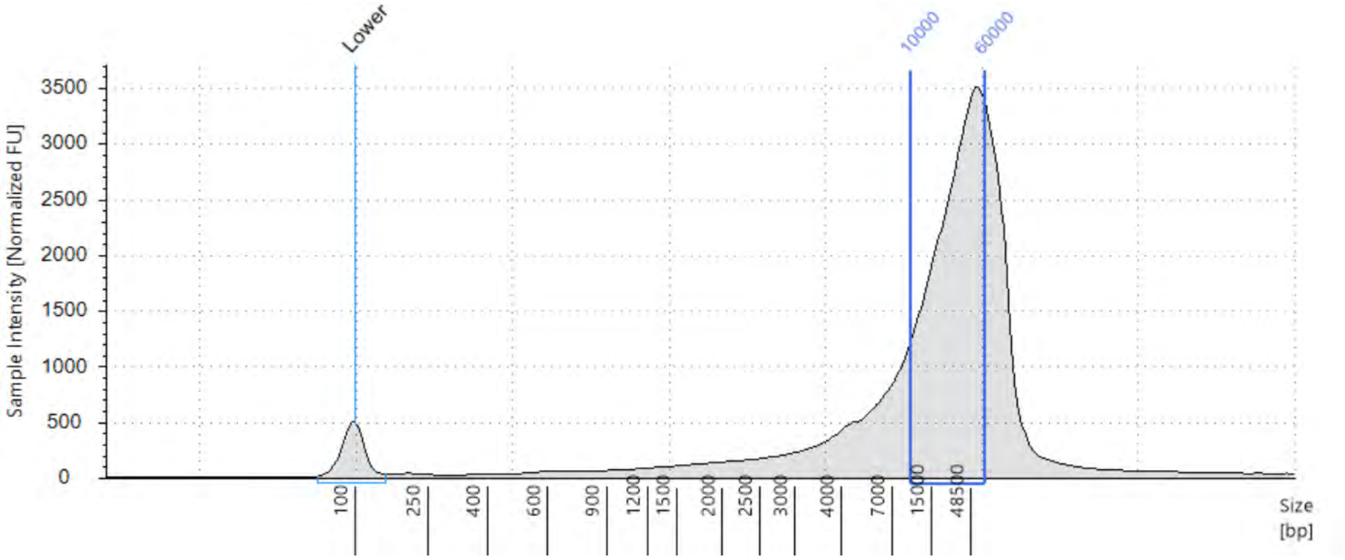

Region Table

From [bp]	To [bp]	Average Size [bp]	Conc. [ng/µl]	Region Molarity [nmol/l]	% of Total	Region Comment	Color
10000	60000	31419	123	8.17	49.87		■

 © Agilent Technologies, Inc. 2017

C5: M283

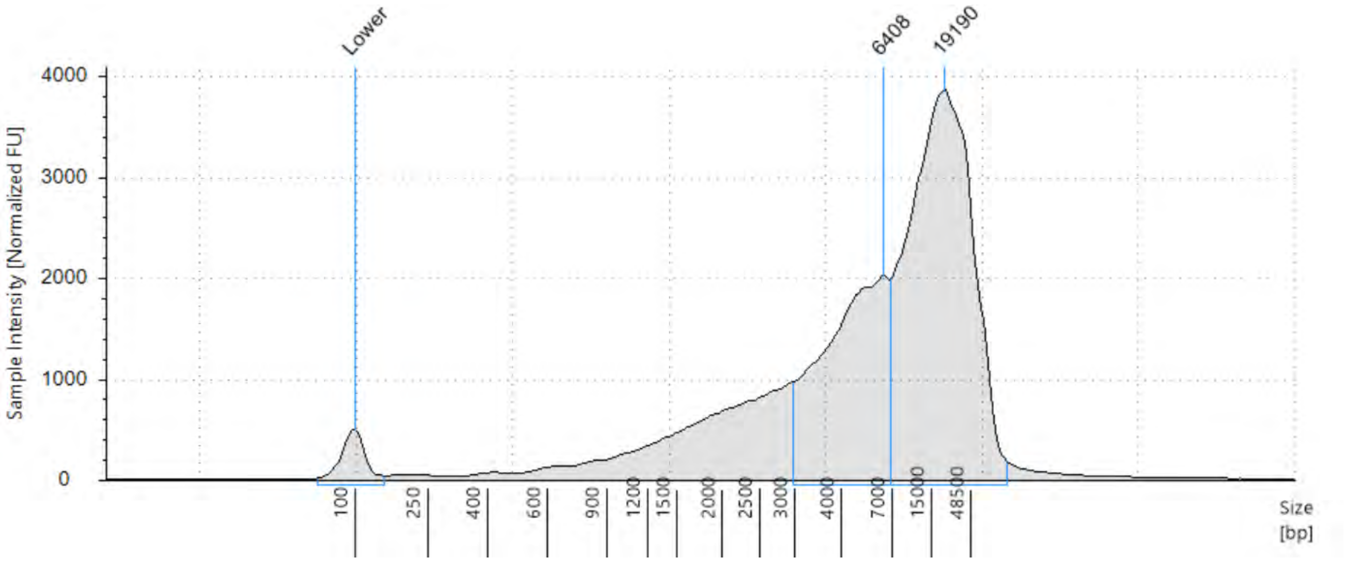

Sample Table

Well	DIN	Conc. [ng/µl]	Sample Description	Alert	Observations
C5	6.6	380	M283	⚠	Sample concentration outside functional range for DIN

Peak Table

Size [bp]	Calibrated Conc. [ng/µl]	Assigned Conc. [ng/µl]	% Integrated Area	From [bp]	To [bp]	Peak Comment	Observations
100	8.50	8.50	-	62	145		Lower Marker
6408	101	-	33.89	2965	6902		
19190	196	-	65.60	6902	>60000		
-	-	-	-	-	-		Sample Well

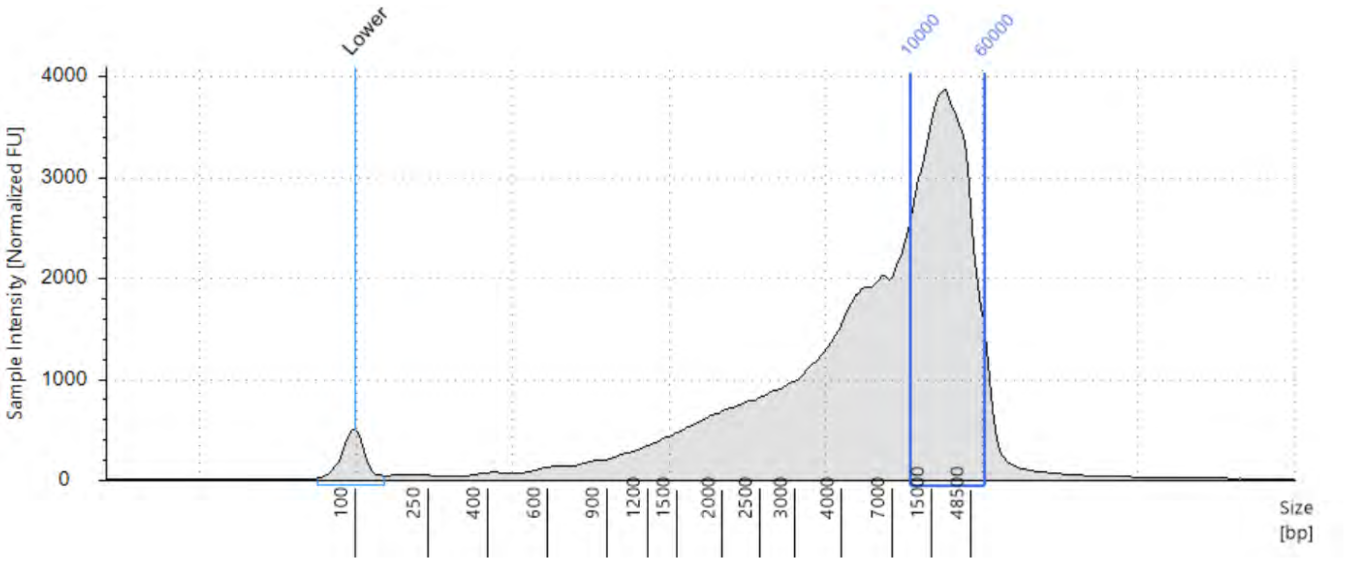

Region Table

From [bp]	To [bp]	Average Size [bp]	Conc. [ng/µl]	Region Molarity [nmol/l]	% of Total	Region Comment	Color
10000	60000	24430	159	12.9	41.85		■

D5: M286

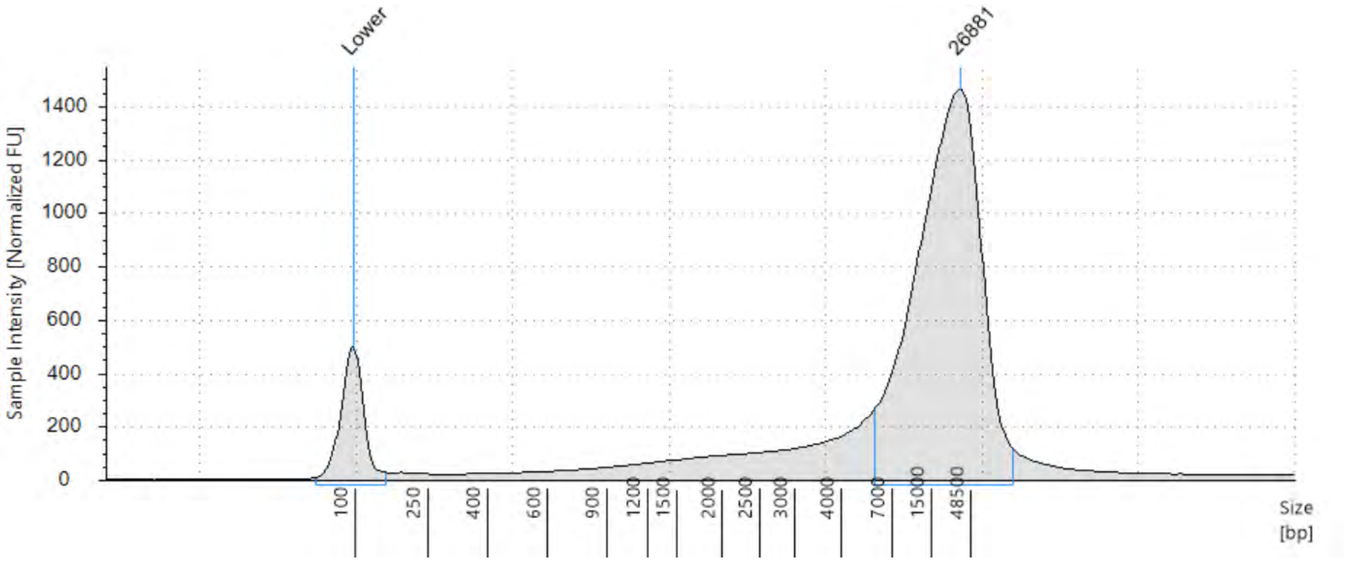

Sample Table

Well	DIN	Conc. [ng/µl]	Sample Description	Alert	Observations
D5	7.3	102	M286	⚠	Sample concentration outside recommended range

Peak Table

Size [bp]	Calibrated Conc. [ng/µl]	Assigned Conc. [ng/µl]	% Integrated Area	From [bp]	To [bp]	Peak Comment	Observations
100	8.50	8.50	-	61	148		Lower Marker
26881	72.7	-	95.19	5797	>60000		
-	-	-	-	-	-		Sample Well

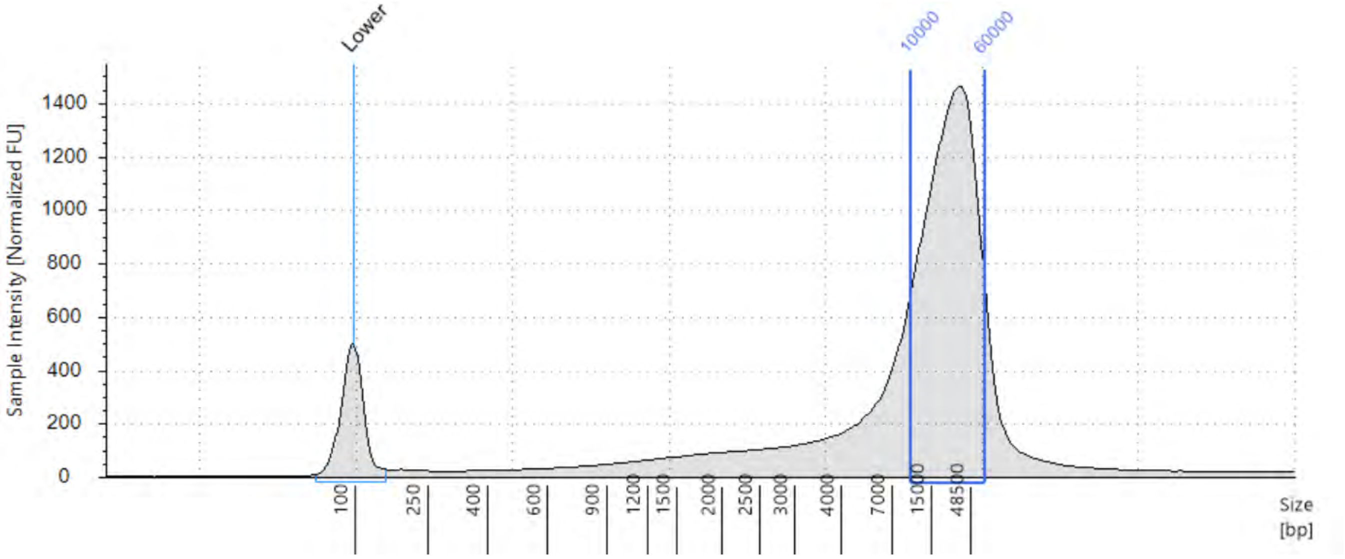

Region Table

From [bp]	To [bp]	Average Size [bp]	Conc. [ng/µl]	Region Molarity [nmol/l]	% of Total	Region Comment	Color
10000	60000	27144	57.2	4.21	55.78		■

E5: M287

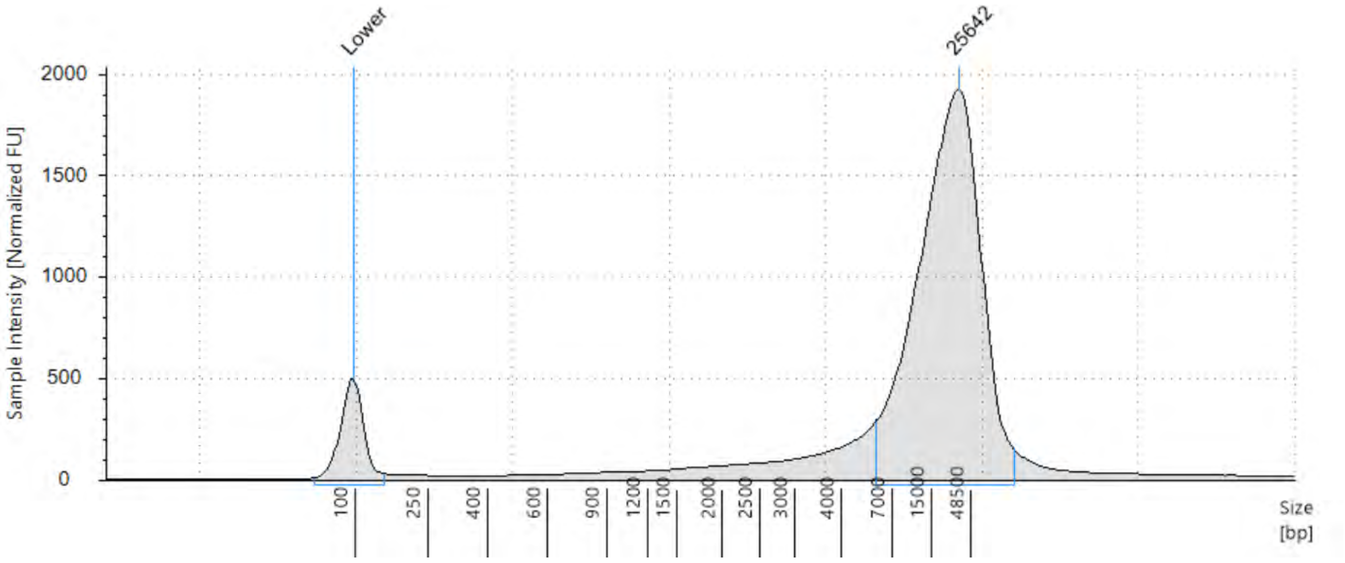

Sample Table

Well	DIN	Conc. [ng/µl]	Sample Description	Alert	Observations
E5	7.8	116	M287	⚠	Sample concentration outside recommended range

Peak Table

Size [bp]	Calibrated Conc. [ng/µl]	Assigned Conc. [ng/µl]	% Integrated Area	From [bp]	To [bp]	Peak Comment	Observations
100	8.50	8.50	-	61	148		Lower Marker
25642	89.6	-	96.69	5935	>60000		
-	-	-	-	-	-		Sample Well

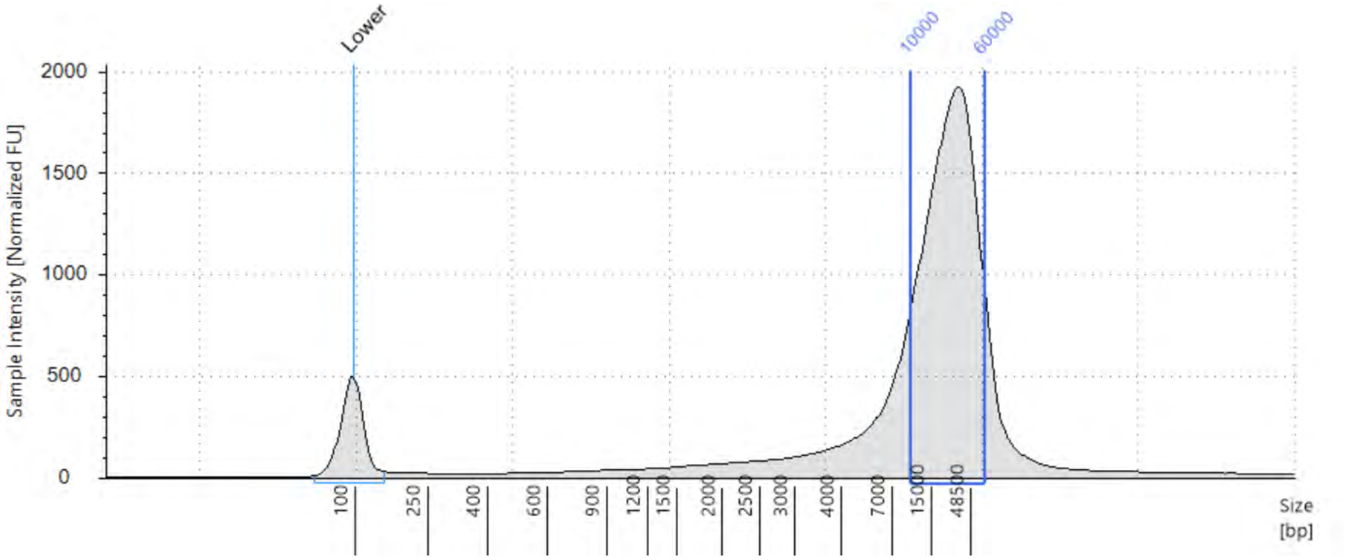

Region Table

From [bp]	To [bp]	Average Size [bp]	Conc. [ng/µl]	Region Molarity [nmol/l]	% of Total	Region Comment	Color
10000	60000	27290	72.1	5.29	61.97		■

© Agilent Technologies, Inc. 2017

F5: M290

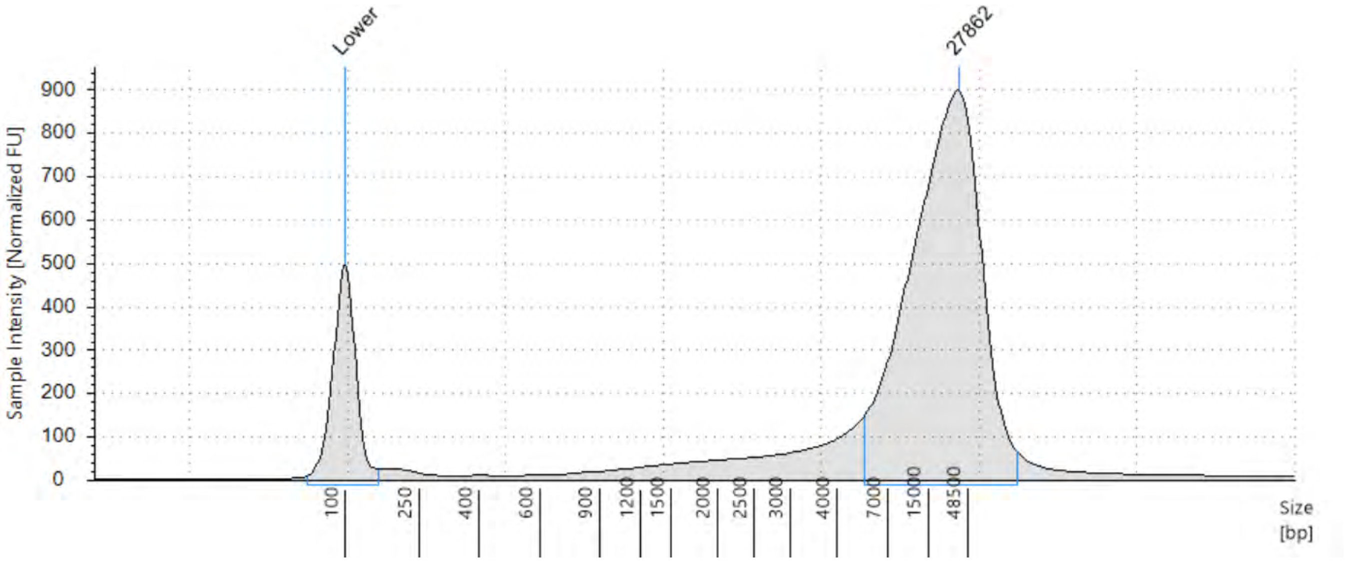

Sample Table

Well	DIN	Conc. [ng/µl]	Sample Description	Alert	Observations
F5	7.7	61.6	M290		

Peak Table

Size [bp]	Calibrated Conc. [ng/µl]	Assigned Conc. [ng/µl]	% Integrated Area	From [bp]	To [bp]	Peak Comment	Observations
100	8.50	8.50	-	62	151		Lower Marker
27862	47.7	-	99.07	5448	>60000		
-	-	-	-	-	-		Sample Well

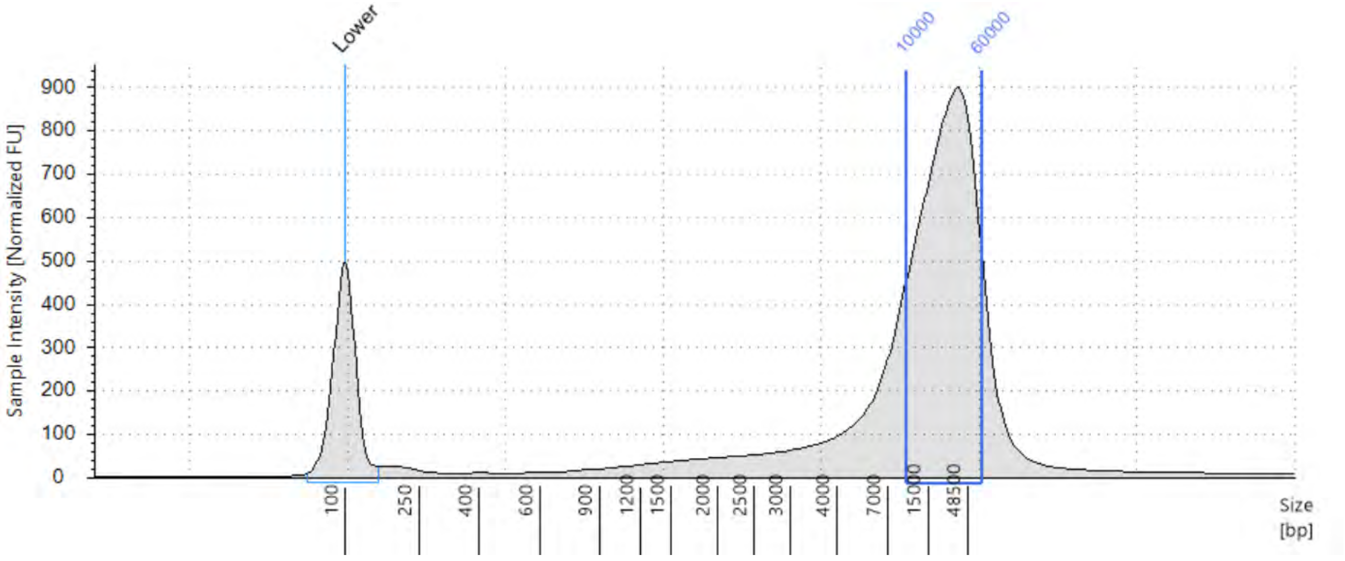

Region Table

From [bp]	To [bp]	Average Size [bp]	Conc. [ng/µl]	Region Molarity [nmol/l]	% of Total	Region Comment	Color
10000	60000	27587	35.7	2.61	57.90		

G5: M291

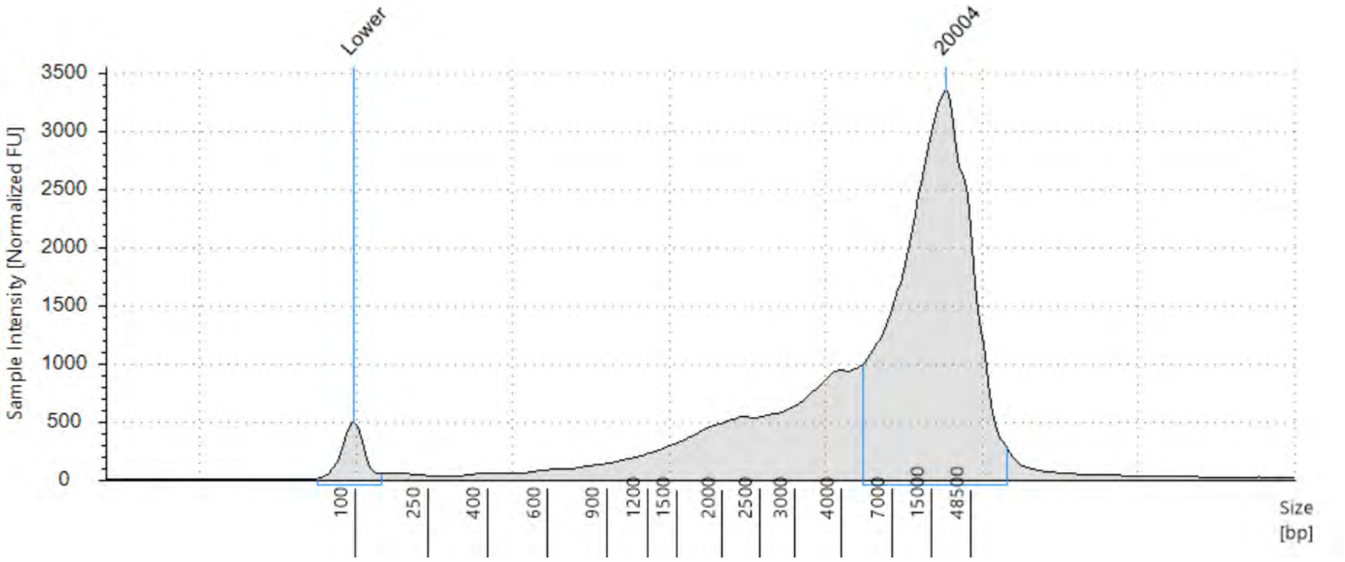

Sample Table

Well	DIN	Conc. [ng/µl]	Sample Description	Alert	Observations
G5	6.7	287	M291	⚠	Sample concentration outside recommended range

Peak Table

Size [bp]	Calibrated Conc. [ng/µl]	Assigned Conc. [ng/µl]	% Integrated Area	From [bp]	To [bp]	Peak Comment	Observations
100	8.50	8.50	-	63	143		Lower Marker
20004	184	-	98.31	5123	>60000		
-	-	-	-	-	-		Sample Well

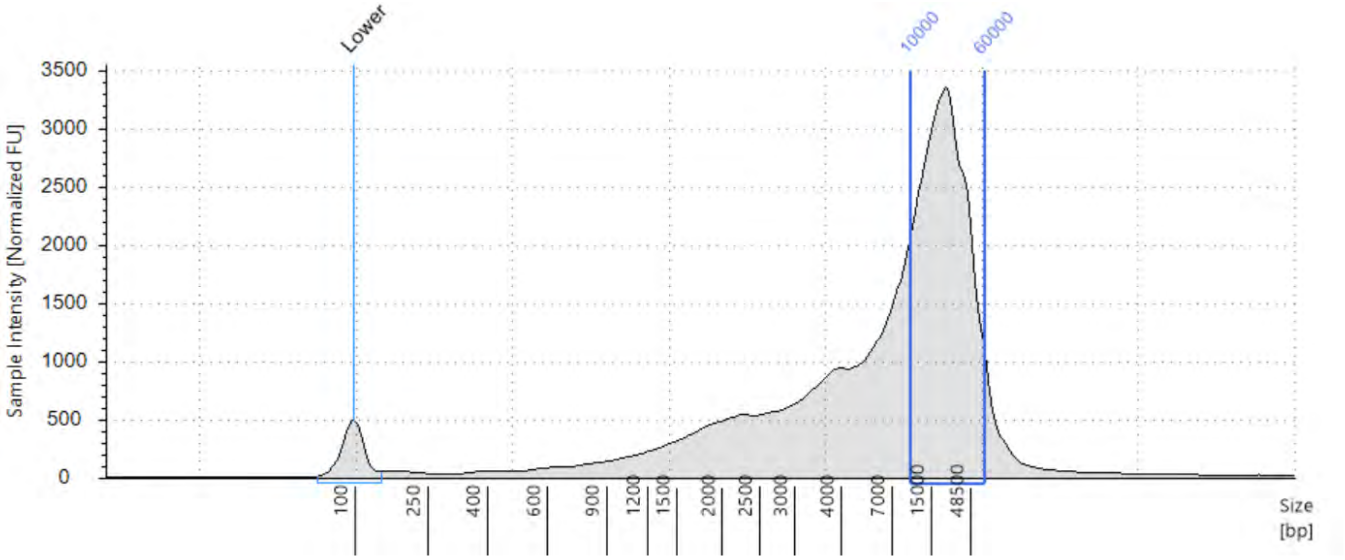

Region Table

From [bp]	To [bp]	Average Size [bp]	Conc. [ng/µl]	Region Molarity [nmol/l]	% of Total	Region Comment	Color
10000	60000	23981	132	10.8	46.19		■

 © Agilent Technologies, Inc. 2017

H5: M294

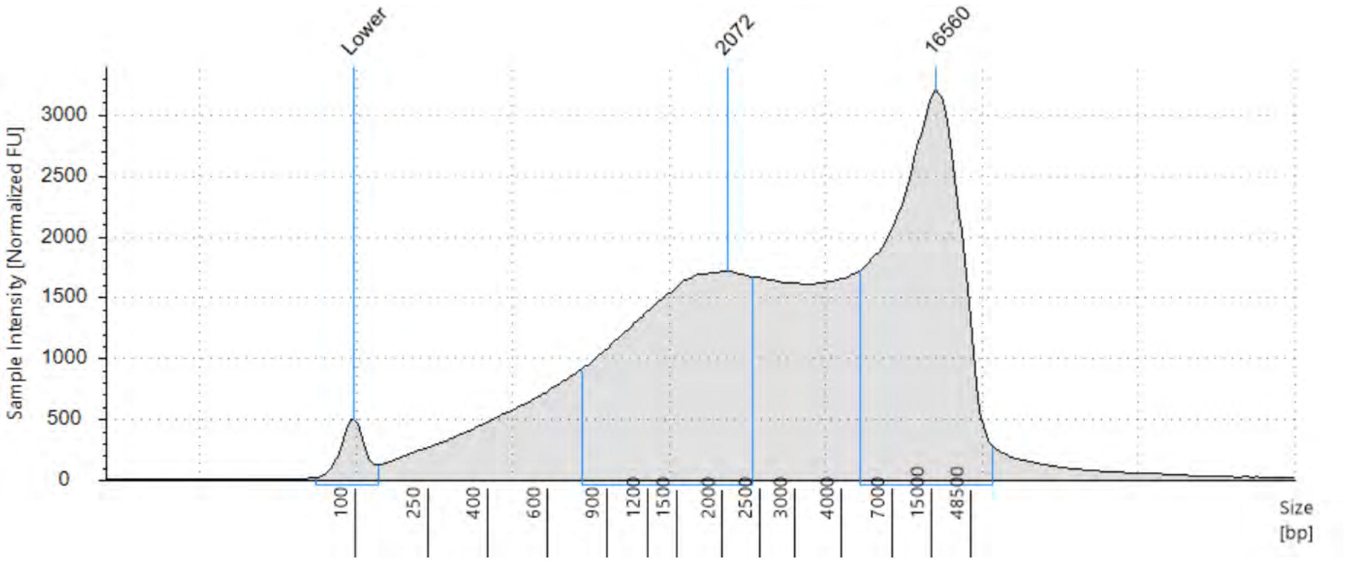

Sample Table

Well	DIN	Conc. [ng/µl]	Sample Description	Alert	Observations
H5	5.5	506	M294	⚠	Sample concentration outside functional range for DIN

Peak Table

Size [bp]	Calibrated Conc. [ng/µl]	Assigned Conc. [ng/µl]	% Integrated Area	From [bp]	To [bp]	Peak Comment	Observations
100	8.50	8.50	-	62	136		Lower Marker
2072	154	-	47.03	762	2401		
16560	174	-	52.97	4967	>60000		

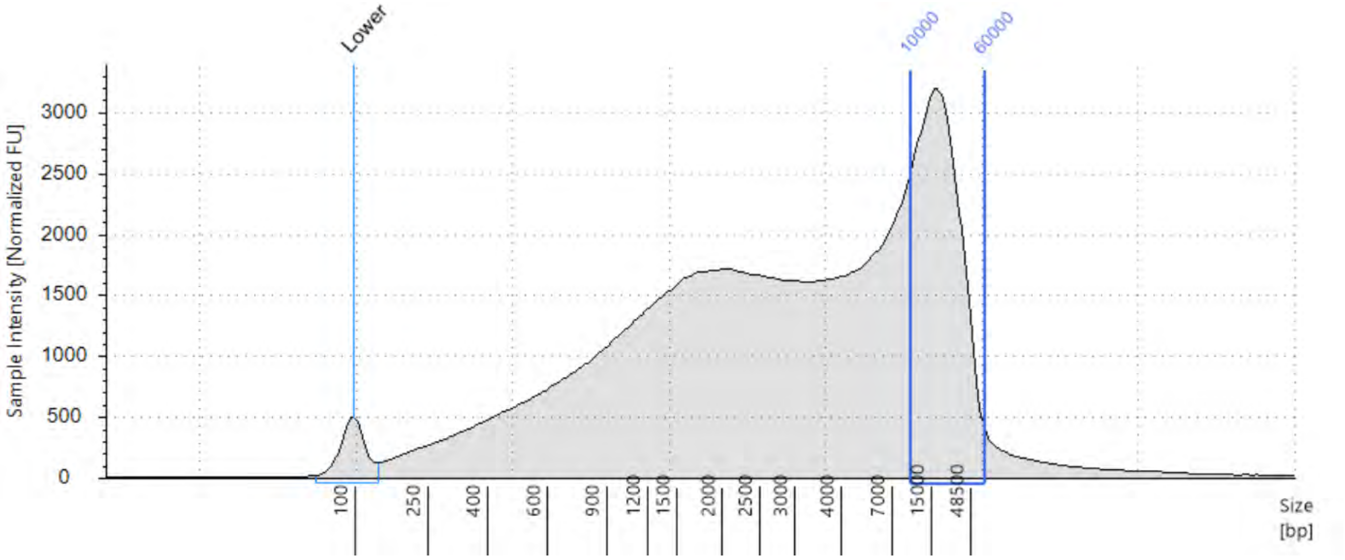

Region Table

From [bp]	To [bp]	Average Size [bp]	Conc. [ng/µl]	Region Molarity [nmol/l]	% of Total	Region Comment	Color
10000	60000	21136	109	9.77	21.64		

A6: M295

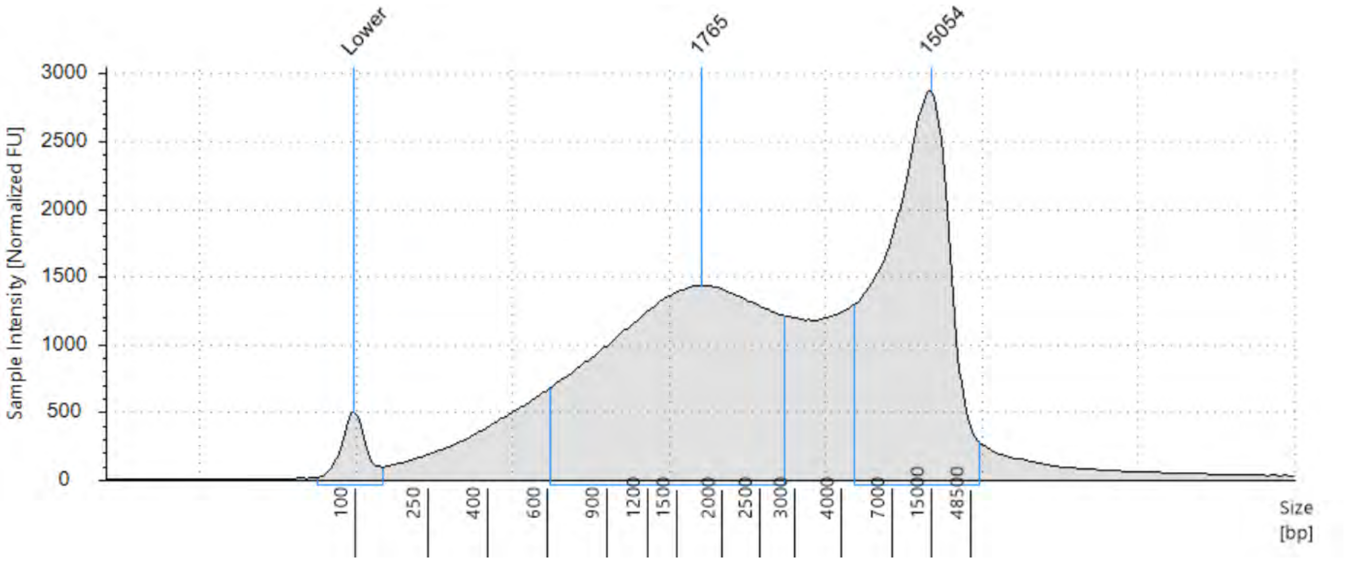

Sample Table

Well	DIN	Conc. [ng/µl]	Sample Description	Alert	Observations
A6	5.1	406	M295	⚠	Sample concentration outside functional range for DIN

Peak Table

Size [bp]	Calibrated Conc. [ng/µl]	Assigned Conc. [ng/µl]	% Integrated Area	From [bp]	To [bp]	Peak Comment	Observations
100	8.50	8.50	-	63	145		Lower Marker
1765	171	-	55.75	617	2844		
15054	136	-	44.17	4675	55890		
-	-	-	-	-	-		Sample Well

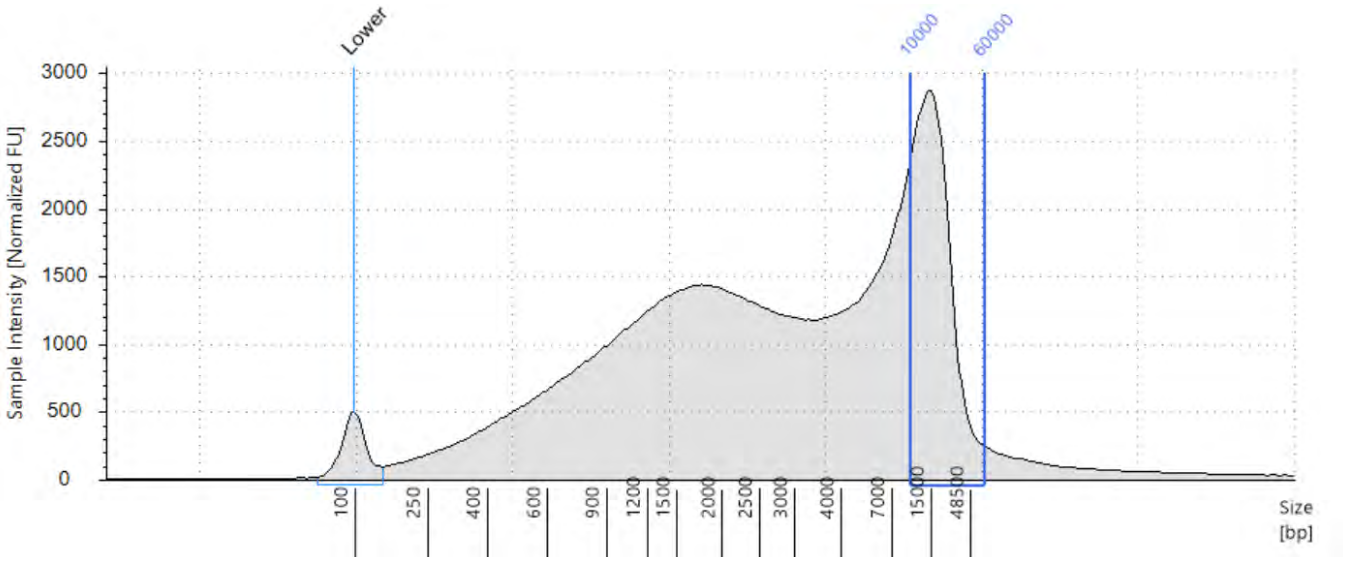

Region Table

From [bp]	To [bp]	Average Size [bp]	Conc. [ng/µl]	Region Molarity [nmol/l]	% of Total	Region Comment	Color
10000	60000	18345	78.4	7.69	19.33		■

B6: M296

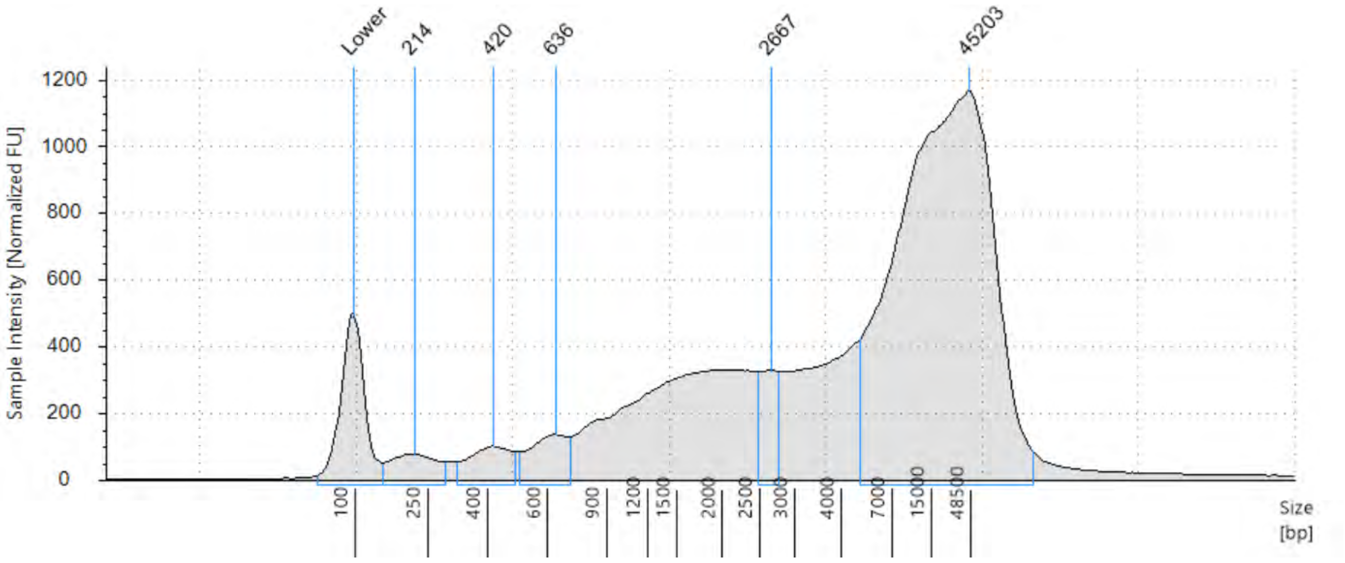

Sample Table

Well	DIN	Conc. [ng/µl]	Sample Description	Alert	Observations
B6	6.4	151	M296	⚠	Sample concentration outside recommended range

Peak Table

Size [bp]	Calibrated Conc. [ng/µl]	Assigned Conc. [ng/µl]	% Integrated Area	From [bp]	To [bp]	Peak Comment	Observations
100	8.50	8.50	-	62	145		Lower Marker
214	2.47	-	2.52	145	291		
420	2.96	-	3.02	317	485		
636	3.69	-	3.76	499	704		
2667	4.41	-	4.49	2489	2760		
45203	83.8	-	85.43	4987	>60000		
-	-	-	-	-	-		Sample Well

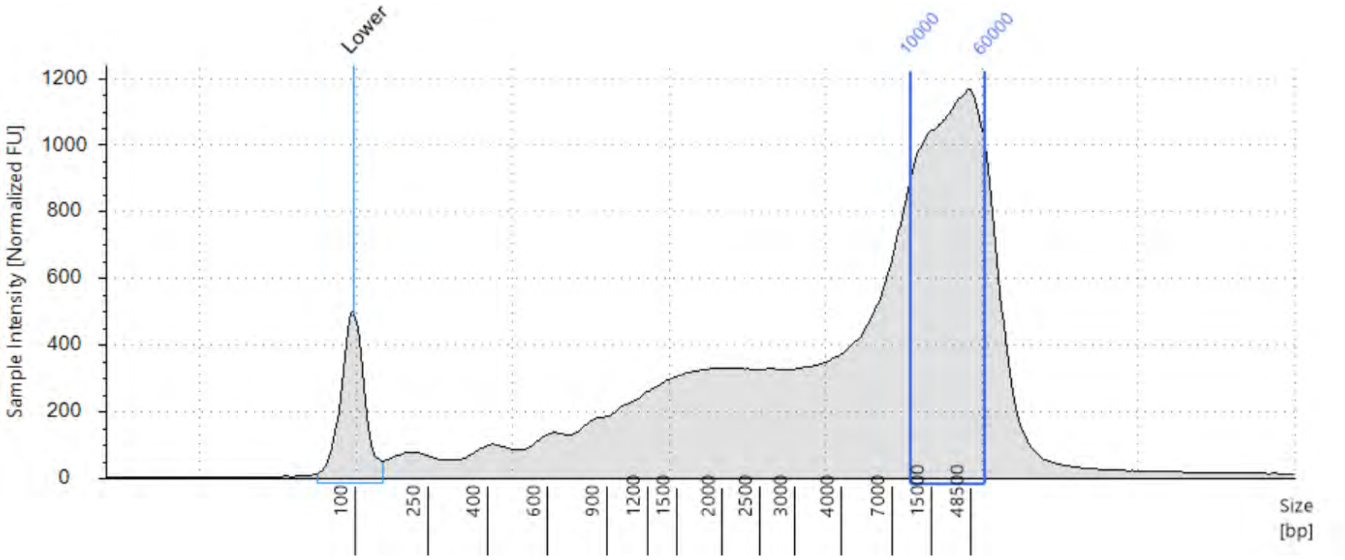

Region Table

From [bp]	To [bp]	Average Size [bp]	Conc. [ng/µl]	Region Molarity [nmol/l]	% of Total	Region Comment	Color
10000	60000	27314	52.0	3.97	34.45		■

 © Agilent Technologies, Inc. 2017

C6: M297

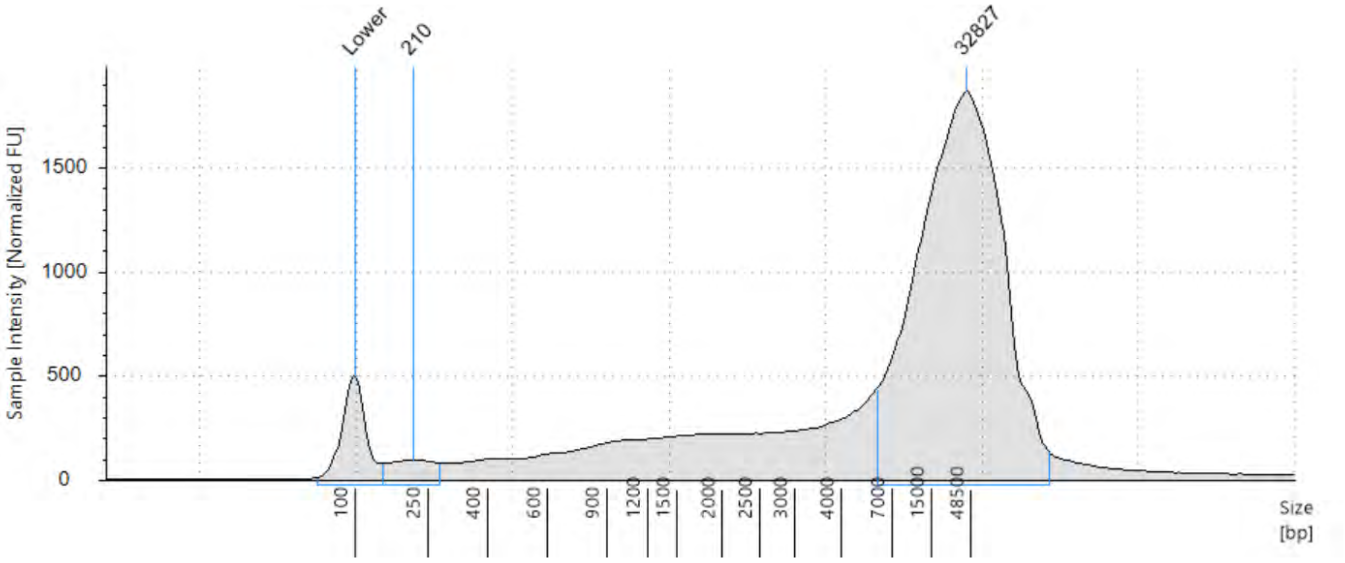

Sample Table

Well	DIN	Conc. [ng/µl]	Sample Description	Alert	Observations
C6	6.9	183	M297	⚠	Sample concentration outside recommended range

Peak Table

Size [bp]	Calibrated Conc. [ng/µl]	Assigned Conc. [ng/µl]	% Integrated Area	From [bp]	To [bp]	Peak Comment	Observations
100	8.50	8.50	-	62	143		Lower Marker
210	3.08	-	2.42	143	277		
32827	119	-	93.47	5939	>60000		
-	-	-	-	-	-		Sample Well

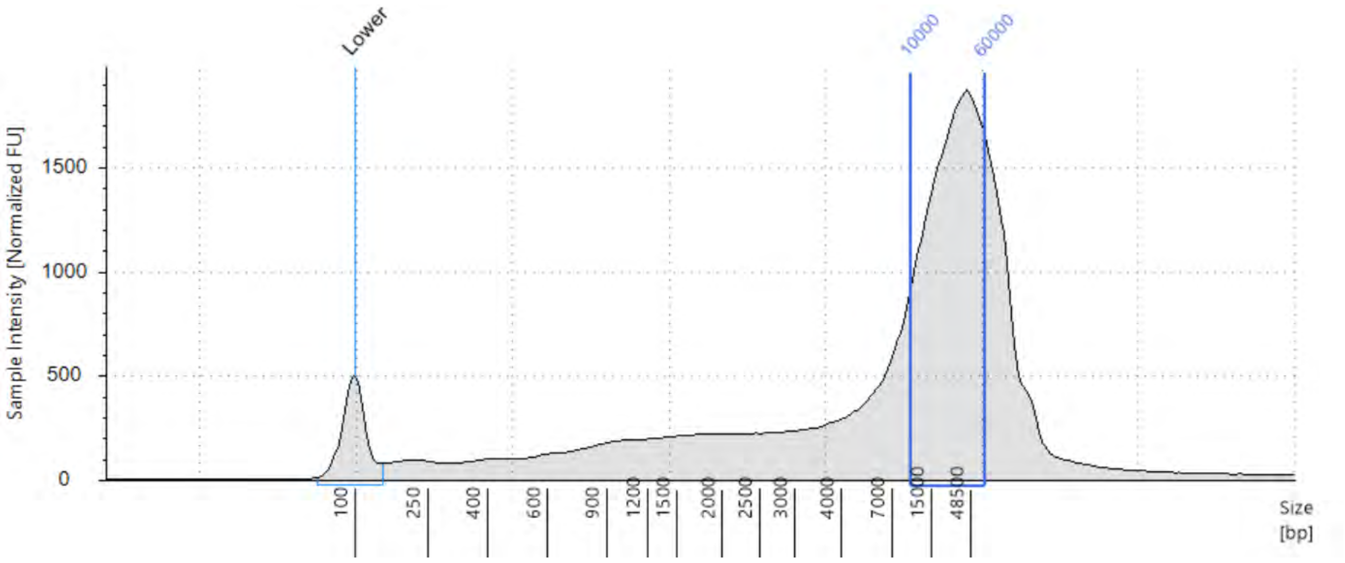

Region Table

From [bp]	To [bp]	Average Size [bp]	Conc. [ng/µl]	Region Molarity [nmol/l]	% of Total	Region Comment	Color
10000	60000	28687	75.0	5.40	40.96		■

Filename: 2018-12-06-01.gDNA

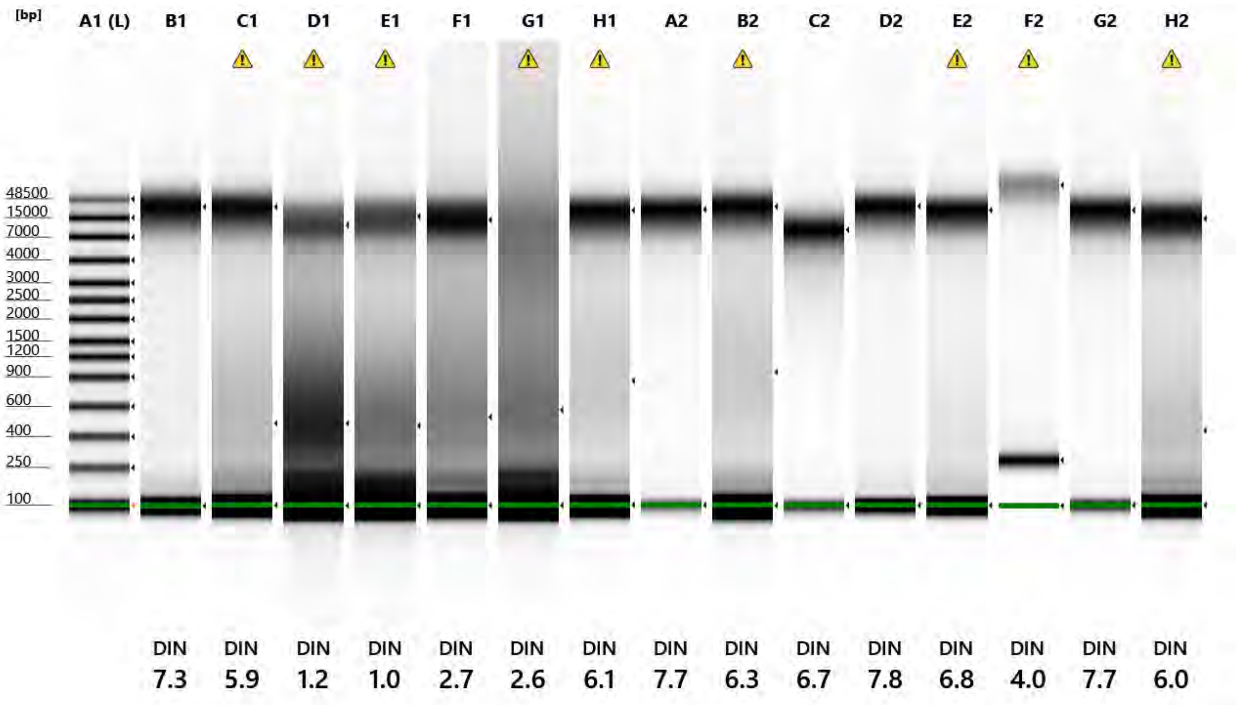

Default image (Contrast 50%), Image is Scaled to Sample

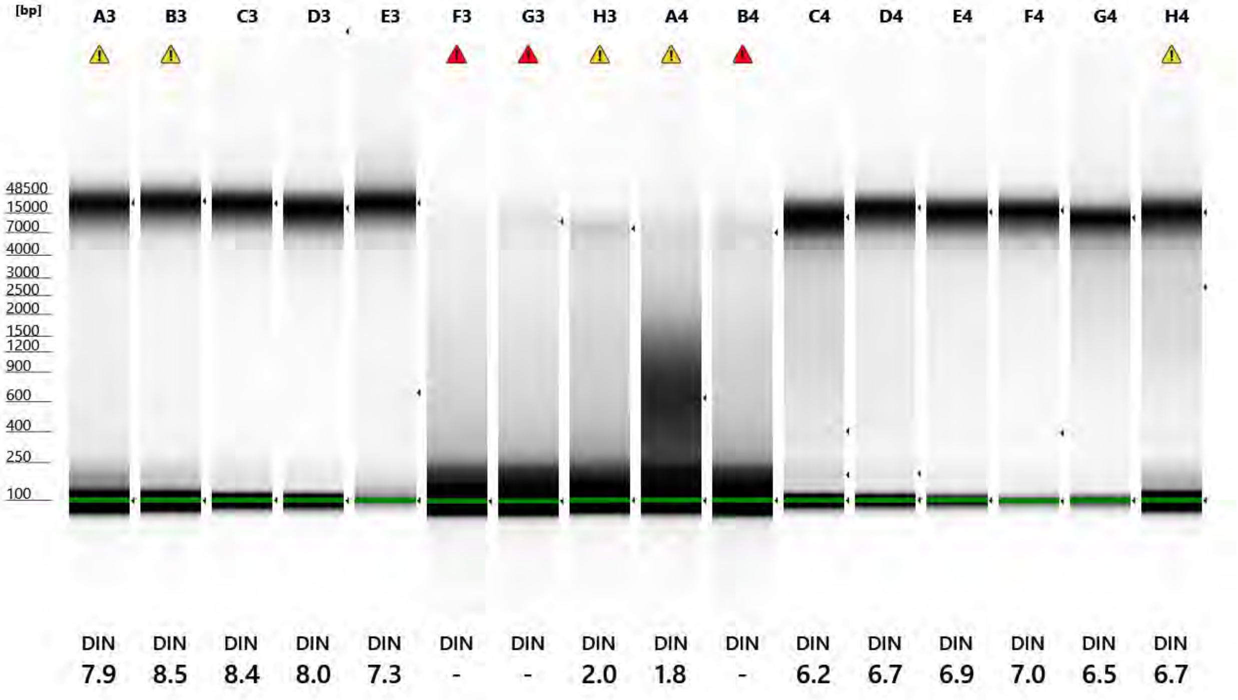

Default image (Contrast 50%), Image is Scaled to Sample

 © Agilent Technologies, Inc. 2017

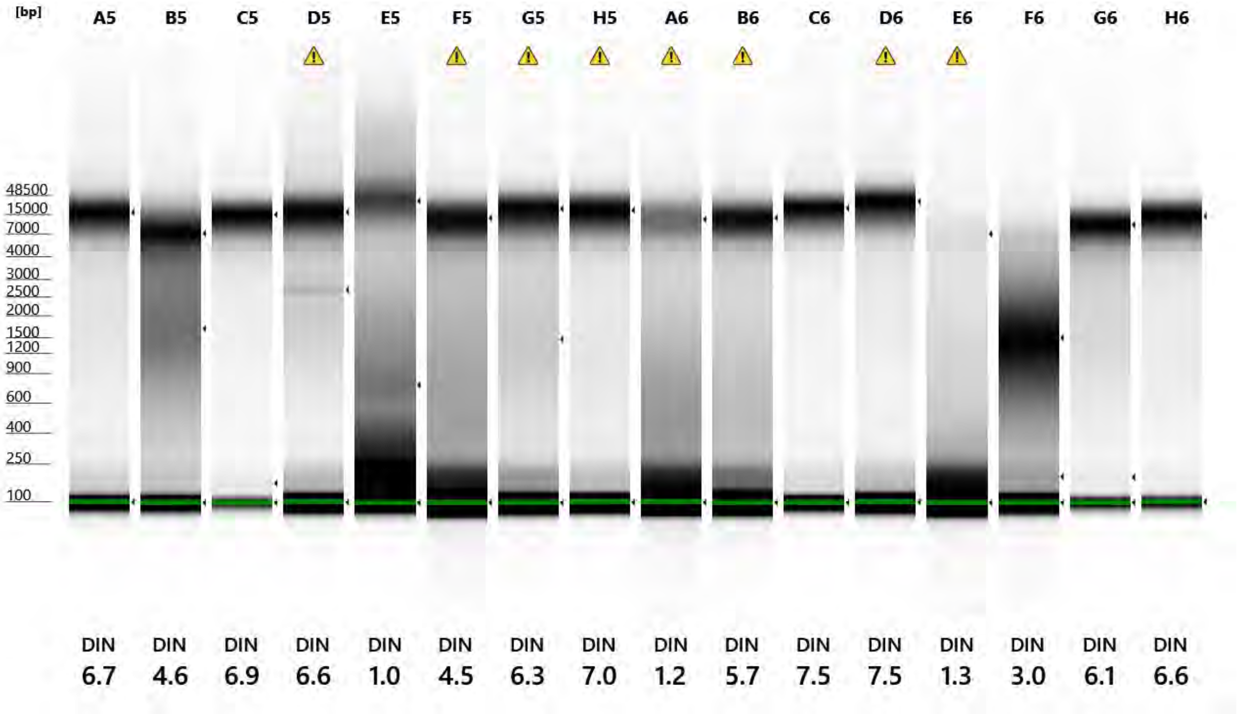

Default image (Contrast 50%), Image is Scaled to Sample

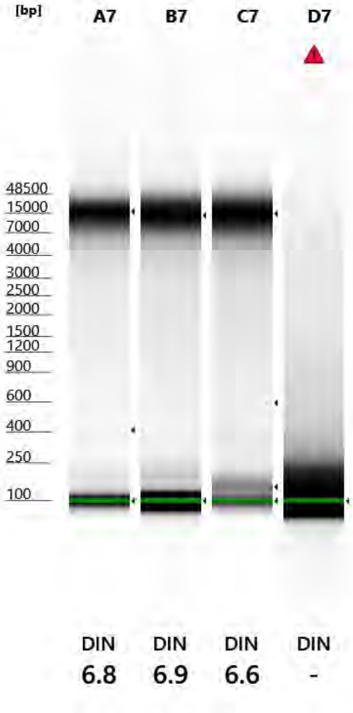

Default image (Contrast 50%), Image is Scaled to Sample

 © Agilent Technologies, Inc. 2017

Sample Info

Well	DIN	Conc. [ng/µl]	Sample Description	Alert	Observations
A1	-	55.8	Ladder		Ladder
B1	7.3	11.7	C586		
C1	5.9	7.39	C587	⚠	Sample concentration outside recommended range
D1	1.2	6.03	C588	⚠	Sample concentration outside recommended range
E1	1.0	4.90	C589	⚠	Sample concentration outside functional range for DIN
F1	2.7	10.4	C590		
G1	2.6	6.13	C591	⚠	Sample concentration outside recommended range
H1	6.1	7.82	C592	⚠	Sample concentration outside recommended range
A2	7.7	44.1	C593		
B2	6.3	6.20	C594	⚠	Sample concentration outside recommended range
C2	6.7	35.8	C595		
D2	7.8	15.1	C601		
E2	6.8	9.04	C602	⚠	Sample concentration outside recommended range
F2	4.0	2650	C603	⚠	Sample concentration outside functional range for DIN
G2	7.7	31.0	C604		
H2	6.0	7.84	C605	⚠	Sample concentration outside recommended range
A3	7.9	5.27	C611	⚠	Sample concentration outside recommended range
B3	8.5	6.76	C612	⚠	Sample concentration outside recommended range
C3	8.4	12.0	C613		
D3	8.0	14.7	C614		
E3	7.3	42.0	C615		
F3	-	2.37	C616	▲	Sample concentration outside functional range for DIN
G3	-	2.43	C617	▲	Sample concentration outside functional range for DIN
H3	2.0	4.29	C618	⚠	Sample concentration outside functional range for DIN
A4	1.8	9.50	C619	⚠	Sample concentration outside recommended range
B4	-	2.77	C620	▲	Sample concentration outside functional range for DIN
C4	6.2	22.8	C621		
D4	6.7	22.0	C622		
E4	6.9	29.0	C623		
F4	7.0	55.4	C624		
G4	6.5	50.5	C625		
H4	6.7	8.98	C626	⚠	Sample concentration outside recommended range
A5	6.7	17.5	C627		
B5	4.6	32.4	C628		
C5	6.9	34.6	C629		
D5	6.6	9.82	C630	⚠	Sample concentration outside recommended range
E5	1.0	14.2	C370		
F5	4.5	5.09	C371	⚠	Sample concentration outside recommended range
G5	6.3	7.05	C372	⚠	Sample concentration outside recommended range
H5	7.0	7.63	C373	⚠	Sample concentration outside recommended range
A6	1.2	5.06	C376	⚠	Sample concentration outside recommended range
B6	5.7	5.54	C377	⚠	Sample concentration outside recommended range
C6	7.5	12.3	C380		
D6	7.5	8.41	C381	⚠	Sample concentration outside recommended range
E6	1.3	3.02	C382	⚠	Sample concentration outside functional range for DIN
F6	3.0	18.7	C383		
G6	6.1	35.4	C384		
H6	6.6	29.5	C385		
A7	6.8	28.5	C386		
B7	6.9	10.3	C387		
C7	6.6	51.0	Sarah_49_rerun		
D7	-	1.60	buffer	▲	Sample concentration outside functional range for DIN

© Agilent Technologies, Inc. 2017

A1: Ladder

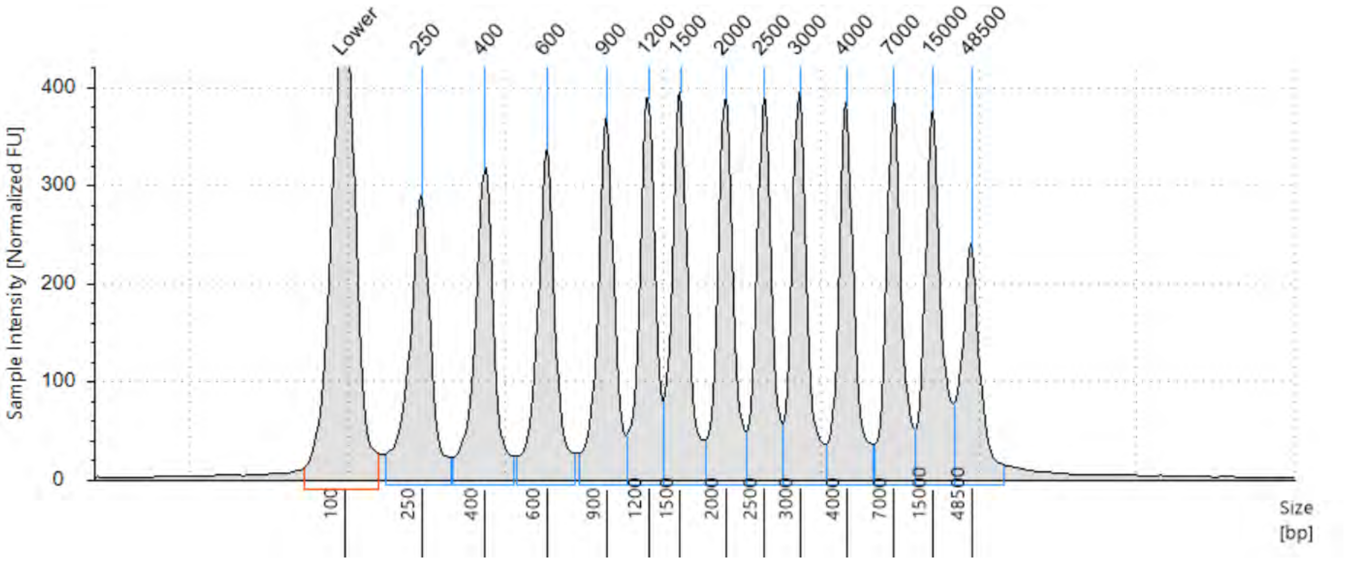

Sample Table

Well	DIN	Conc. [ng/µl]	Sample Description	Alert	Observations
A1	-	55.8	Ladder		Ladder

Peak Table

Size [bp]	Calibrated Conc. [ng/µl]	Assigned Conc. [ng/µl]	% Integrated Area	From [bp]	To [bp]	Peak Comment	Observations
100	8.50	8.50	-	62	148		Lower Marker
250	4.30	-	7.78	163	310		
400	4.28	-	7.74	313	483		
600	4.31	-	7.80	492	721		
900	4.38	-	7.93	742	1029		
1200	4.38	-	7.93	1029	1335		
1500	4.53	-	8.20	1335	1755		
2000	4.39	-	7.94	1755	2254		
2500	4.29	-	7.75	2254	2758		
3000	4.38	-	7.92	2758	3523		
4000	4.26	-	7.71	3523	5469		
7000	4.25	-	7.69	5559	10659		
15000	4.39	-	7.94	10659	22254		
48500	3.14	-	5.68	22254	>60000		

B1: C586

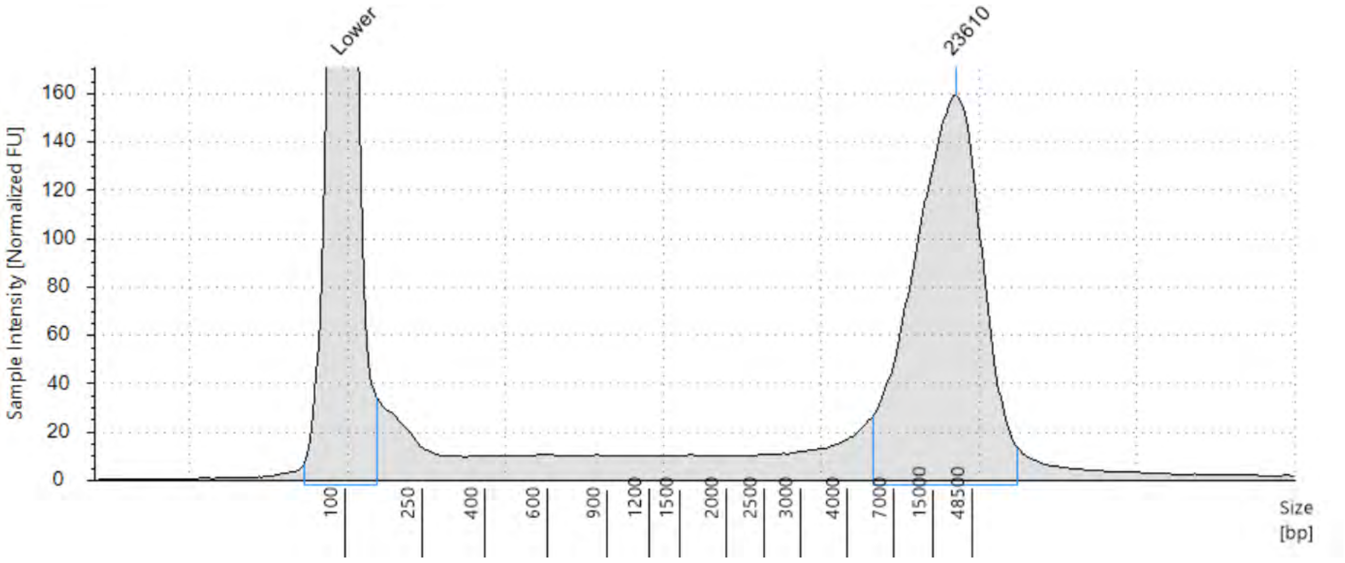

Sample Table

Well	DIN	Conc. [ng/µl]	Sample Description	Alert	Observations
B1	7.3	11.7	C586		

Peak Table

Size [bp]	Calibrated Conc. [ng/µl]	Assigned Conc. [ng/µl]	% Integrated Area	From [bp]	To [bp]	Peak Comment	Observations
100	8.50	8.50	-	63	149		Lower Marker
23610	7.69	-	96.08	5607	>60000		
-	-	-	-	-	-		Sample Well

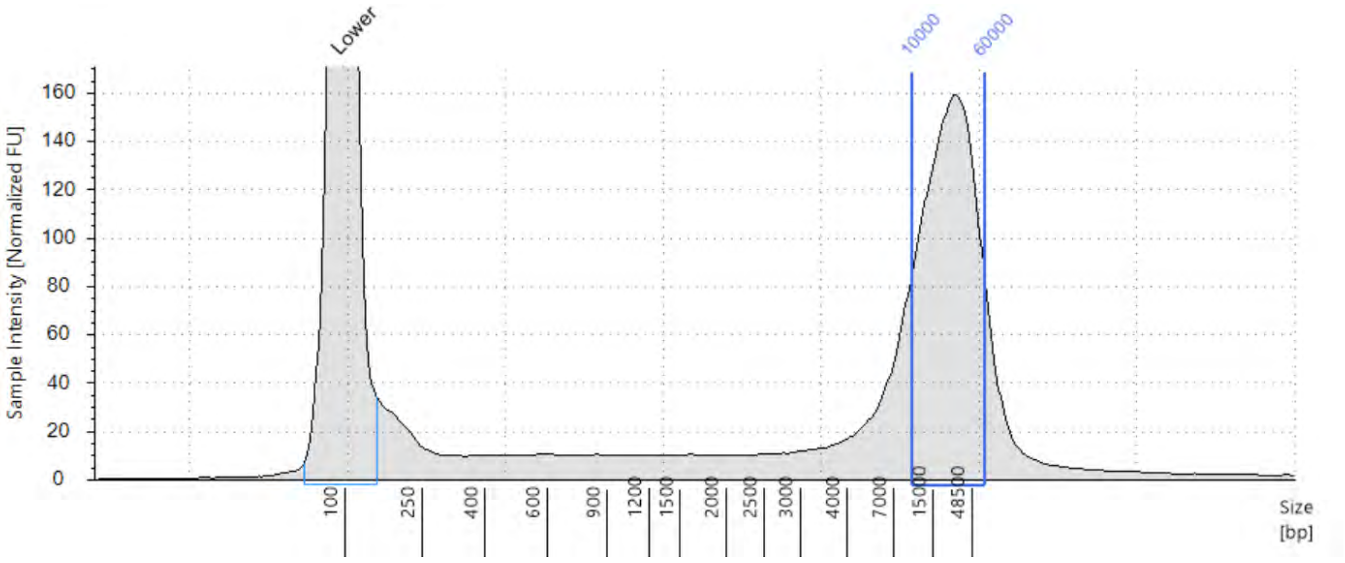

Region Table

From [bp]	To [bp]	Average Size [bp]	Conc. [ng/µl]	Region Molarity [nmol/l]	% of Total	Region Comment	Color
10000	60000	26192	5.83	0.448	49.87		

© Agilent Technologies, Inc. 2017

C1: C587

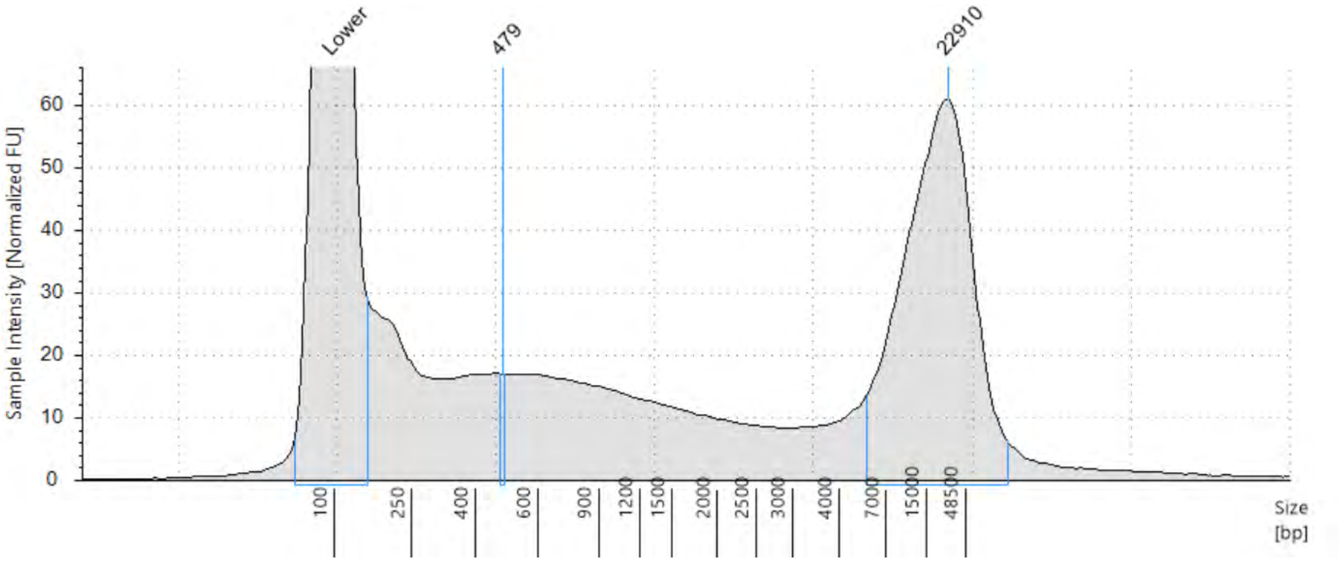

Sample Table

Well	DIN	Conc. [ng/µl]	Sample Description	Alert	Observations
C1	5.9	7.39	C587	⚠	Sample concentration outside recommended range

Peak Table

Size [bp]	Calibrated Conc. [ng/µl]	Assigned Conc. [ng/µl]	% Integrated Area	From [bp]	To [bp]	Peak Comment	Observations
100	8.50	8.50	-	62	148		Lower Marker
479	0.0567	-	1.76	470	483		
22910	3.05	-	94.75	5524	>60000		
-	-	-	-	-	-		Sample Well

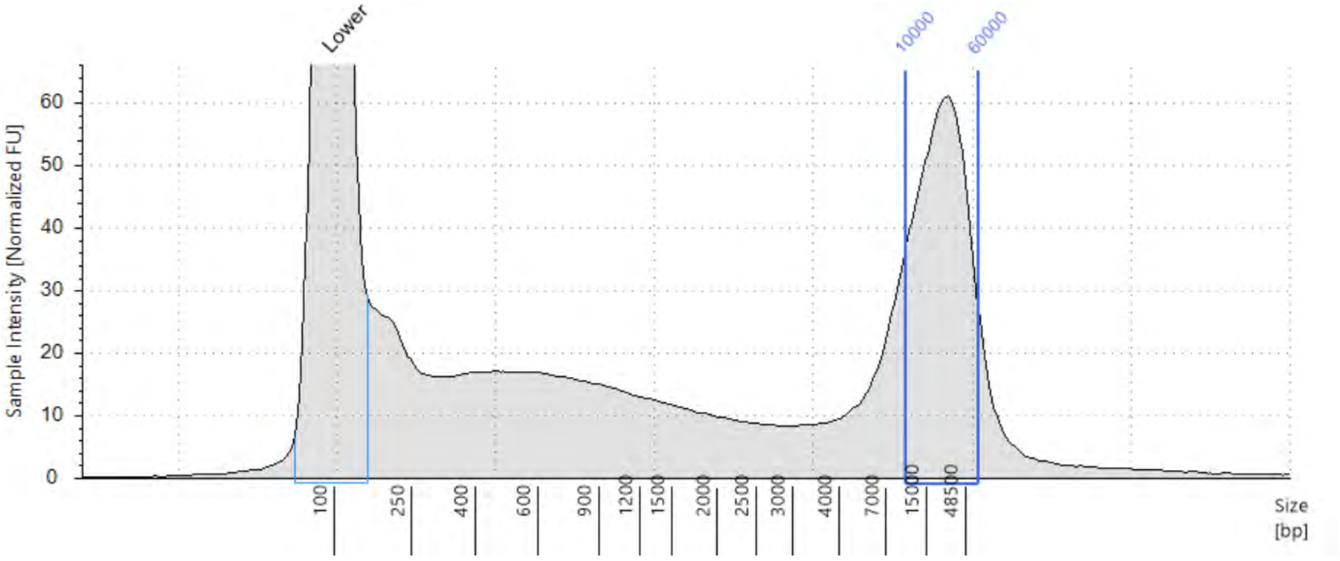

Region Table

From [bp]	To [bp]	Average Size [bp]	Conc. [ng/µl]	Region Molarity [nmol/l]	% of Total	Region Comment	Color
10000	60000	25329	2.27	0.180	30.77		■

© Agilent Technologies, Inc. 2017

D1: C588

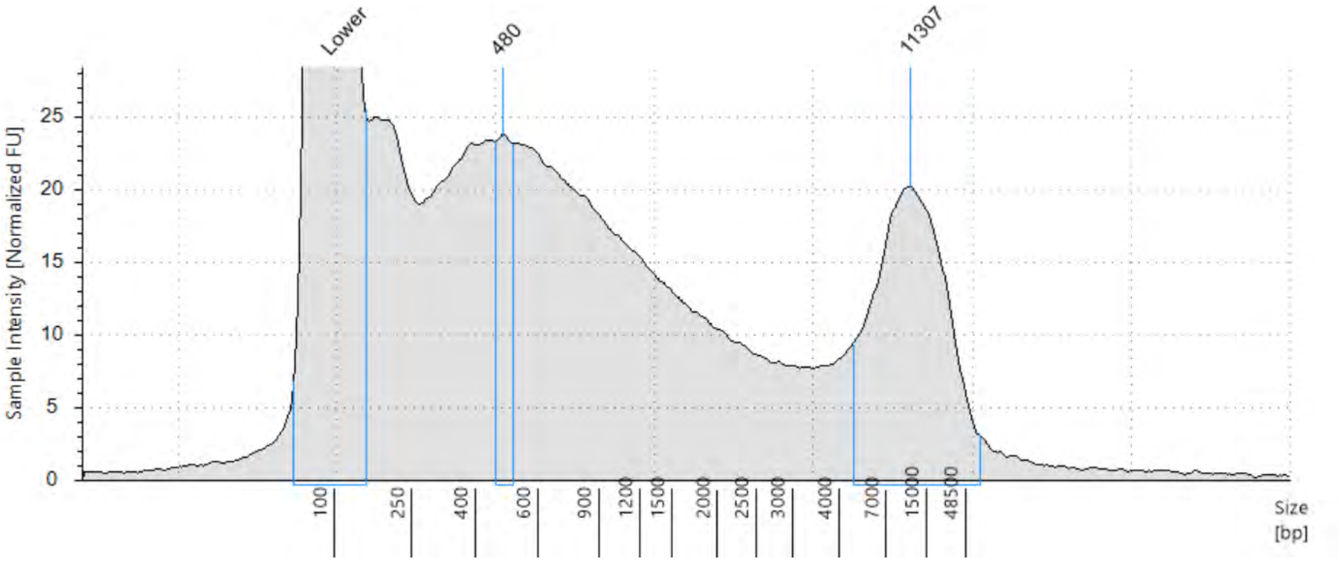

Sample Table

Well	DIN	Conc. [ng/µl]	Sample Description	Alert	Observations
D1	1.2	6.03	C588	⚠	Sample concentration outside recommended range

Peak Table

Size [bp]	Calibrated Conc. [ng/µl]	Assigned Conc. [ng/µl]	% Integrated Area	From [bp]	To [bp]	Peak Comment	Observations
100	8.50	8.50	-	62	145		Lower Marker
480	0.280	-	20.86	459	515		
11307	1.06	-	79.14	4756	>60000		

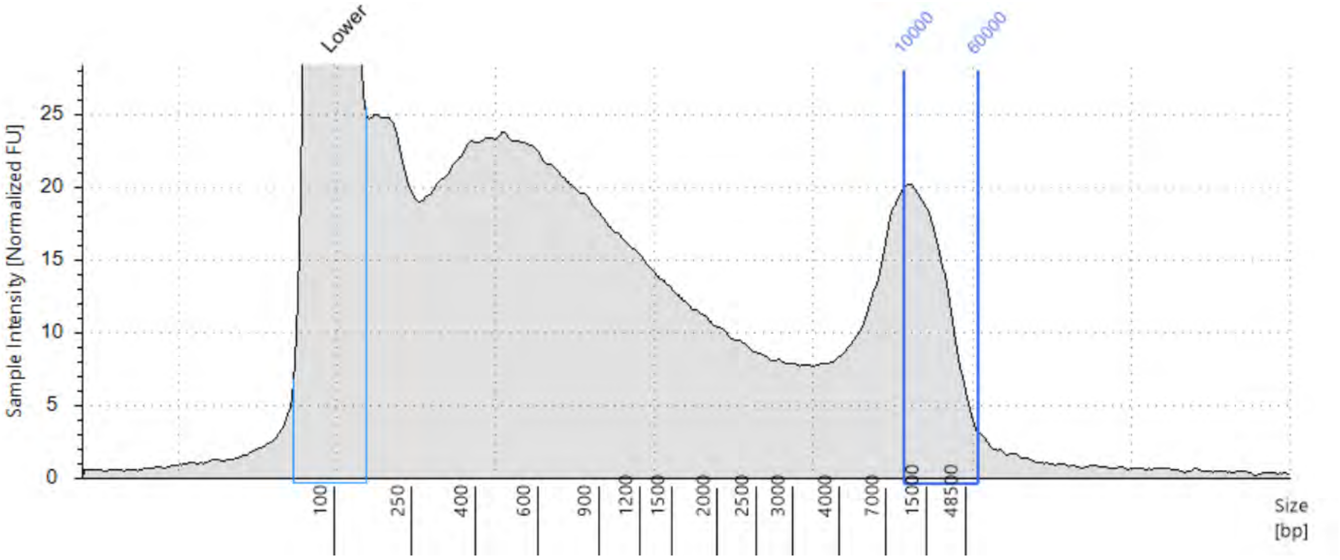

Region Table

From [bp]	To [bp]	Average Size [bp]	Conc. [ng/µl]	Region Molarity [nmol/l]	% of Total	Region Comment	Color
10000	60000	20128	0.606	0.0596	10.05		■

 © Agilent Technologies, Inc. 2017

E1: C589

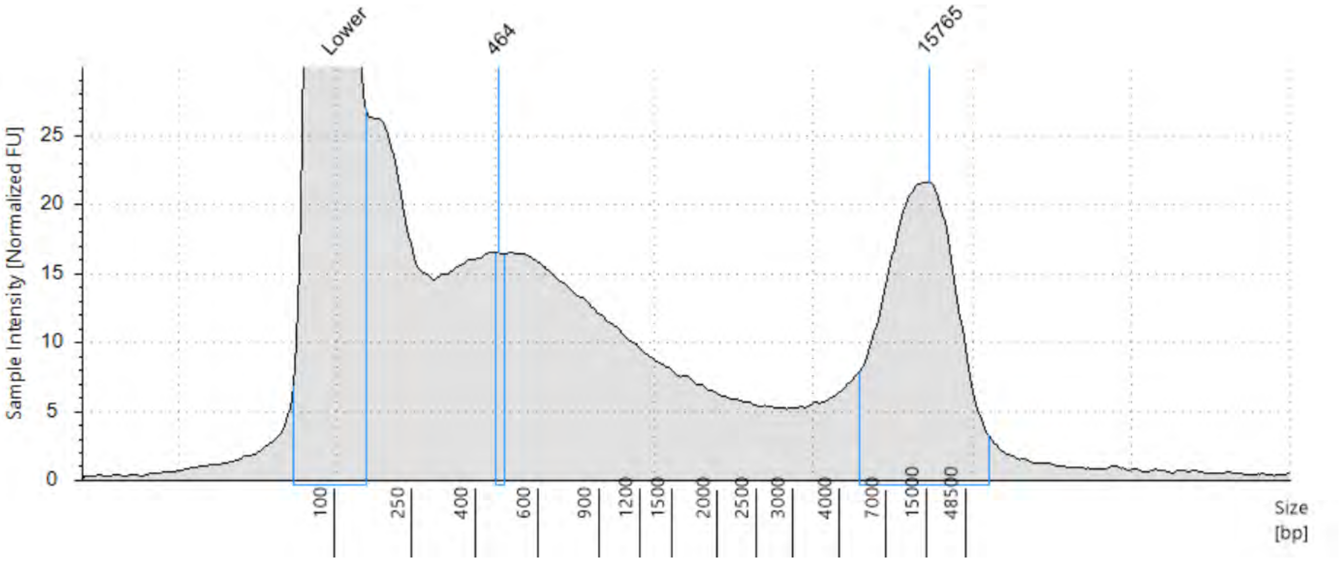

Sample Table

Well	DIN	Conc. [ng/µl]	Sample Description	Alert	Observations
E1	1.0	4.90	C589	⚠	Sample concentration outside functional range for DIN

Peak Table

Size [bp]	Calibrated Conc. [ng/µl]	Assigned Conc. [ng/µl]	% Integrated Area	From [bp]	To [bp]	Peak Comment	Observations
100	8.50	8.50	-	62	146		Lower Marker
464	0.114	-	8.78	456	486		
15765	1.16	-	89.37	5092	>60000		
-	-	-	-	-	-		Sample Well

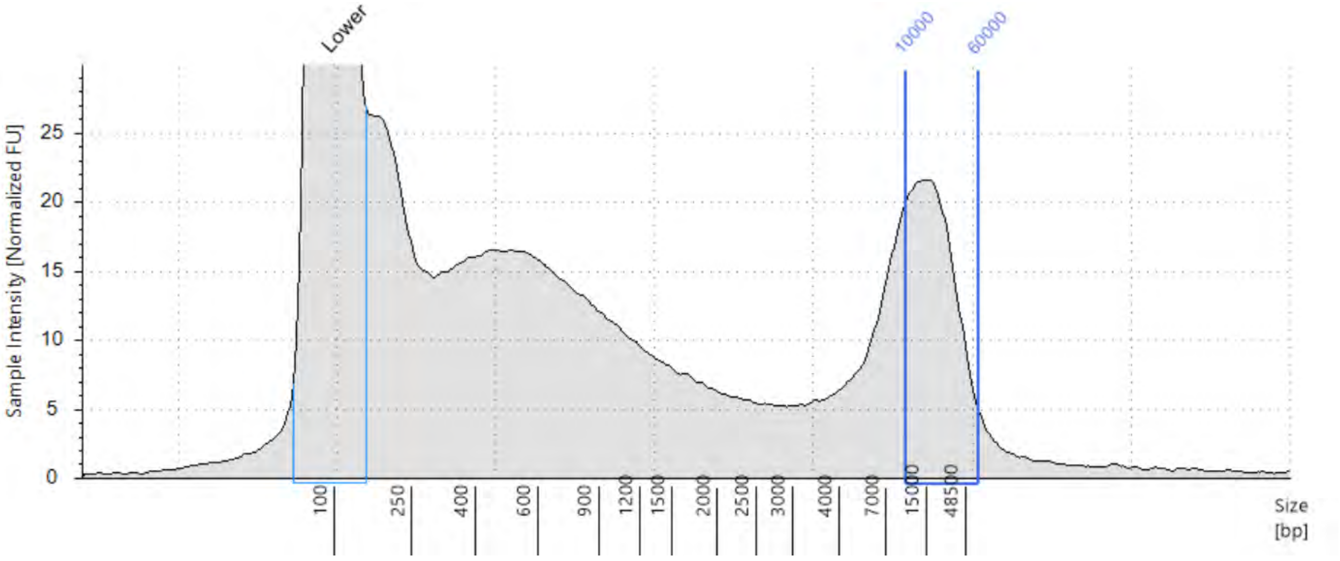

Region Table

From [bp]	To [bp]	Average Size [bp]	Conc. [ng/µl]	Region Molarity [nmol/l]	% of Total	Region Comment	Color
10000	60000	21399	0.771	0.0710	15.73		■

 © Agilent Technologies, Inc. 2017

F1: C590

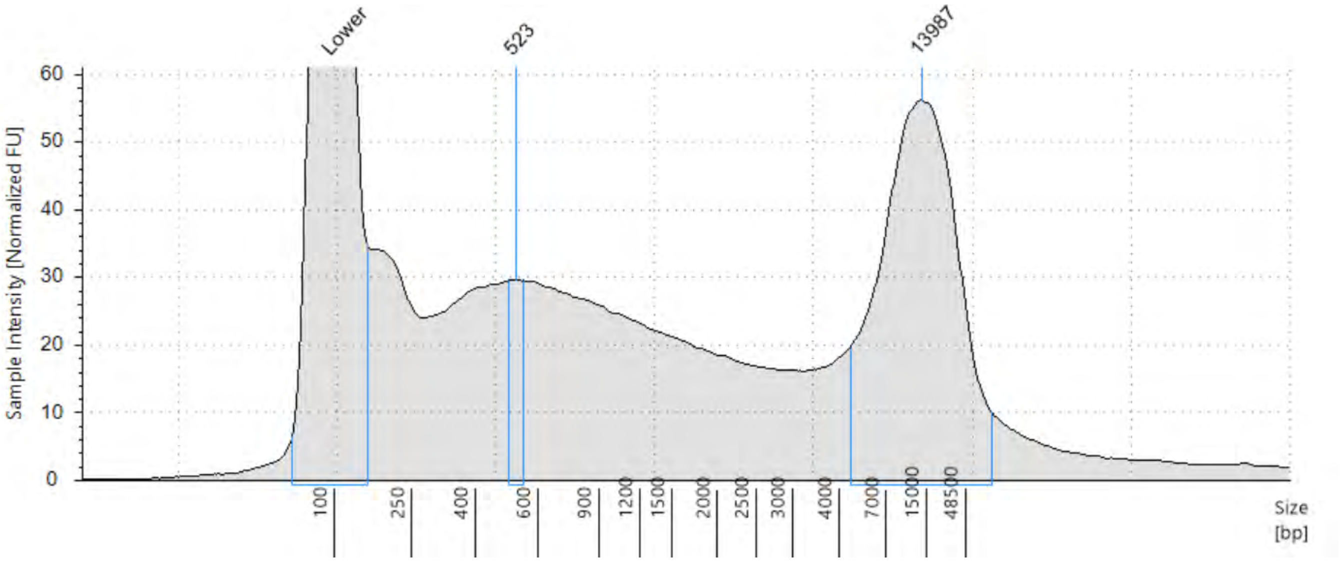

Sample Table

Well	DIN	Conc. [ng/µl]	Sample Description	Alert	Observations
F1	2.7	10.4	C590		

Peak Table

Size [bp]	Calibrated Conc. [ng/µl]	Assigned Conc. [ng/µl]	% Integrated Area	From [bp]	To [bp]	Peak Comment	Observations
100	8.50	8.50	-	61	148		Lower Marker
523	0.266	-	7.85	500	547		
13987	3.04	-	89.76	4643	>60000		
-	-	-	-	-	-		Sample Well

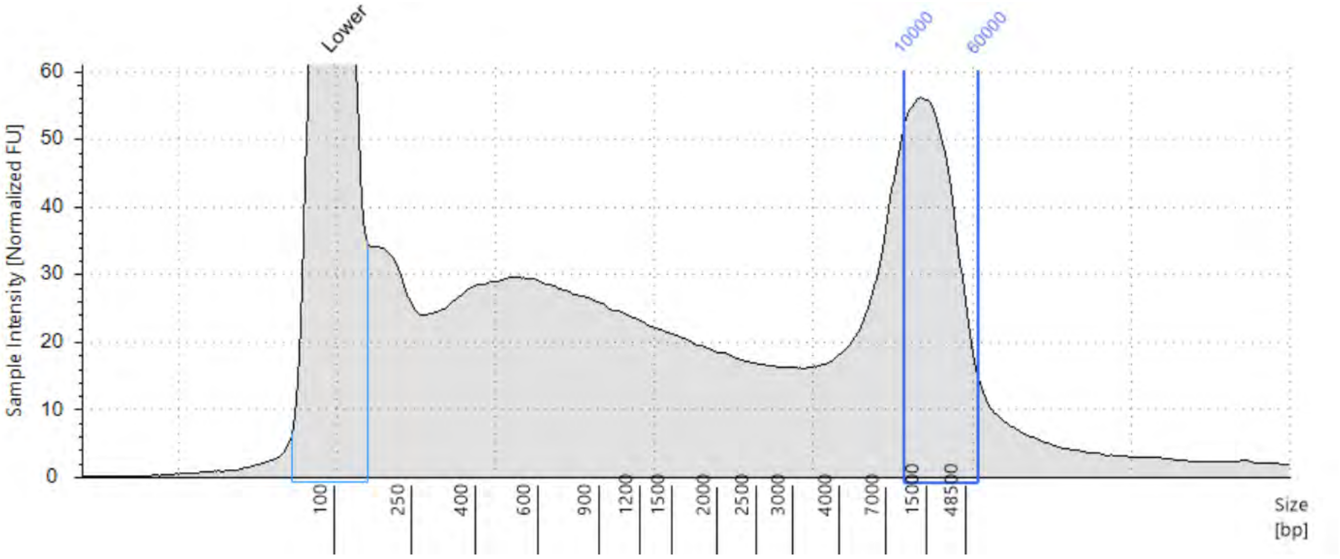

Region Table

From [bp]	To [bp]	Average Size [bp]	Conc. [ng/µl]	Region Molarity [nmol/l]	% of Total	Region Comment	Color
10000	60000	22049	1.93	0.172	18.54		

 © Agilent Technologies, Inc. 2017

G1: C591

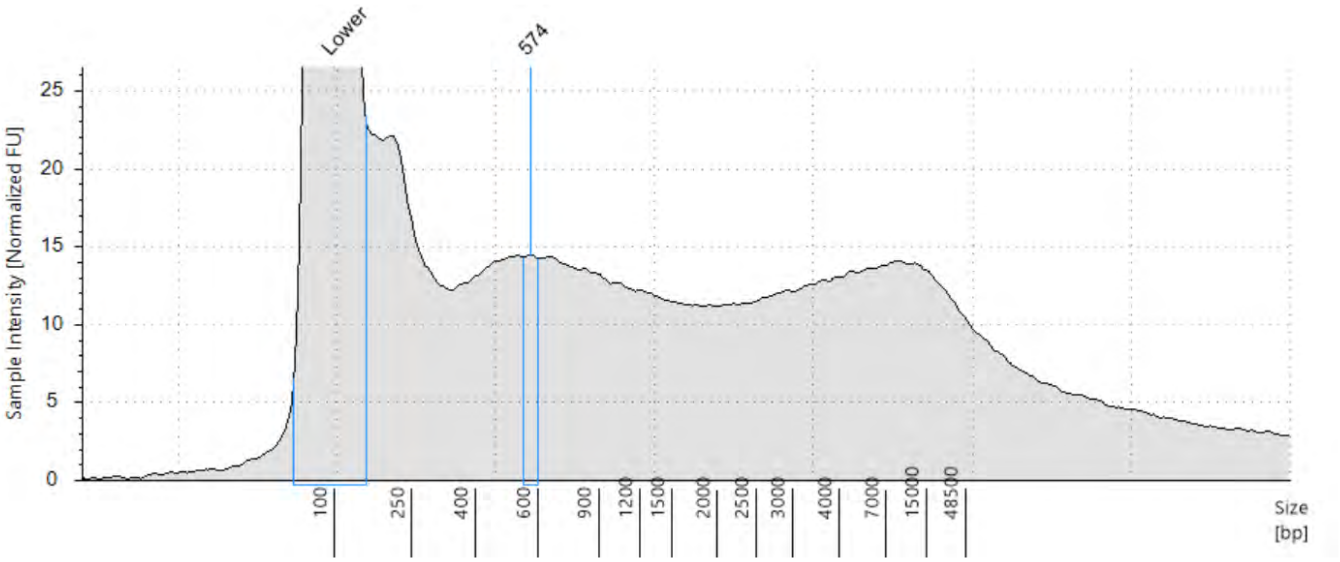

Sample Table

Well	DIN	Conc. [ng/µl]	Sample Description	Alert	Observations
G1	2.6	6.13	C591	⚠	Sample concentration outside recommended range

Peak Table

Size [bp]	Calibrated Conc. [ng/µl]	Assigned Conc. [ng/µl]	% Integrated Area	From [bp]	To [bp]	Peak Comment	Observations
100	8.50	8.50	-	62	146		Lower Marker
574	0.133	-	14.92	548	600		
-	-	-	-	-	-		Sample Well

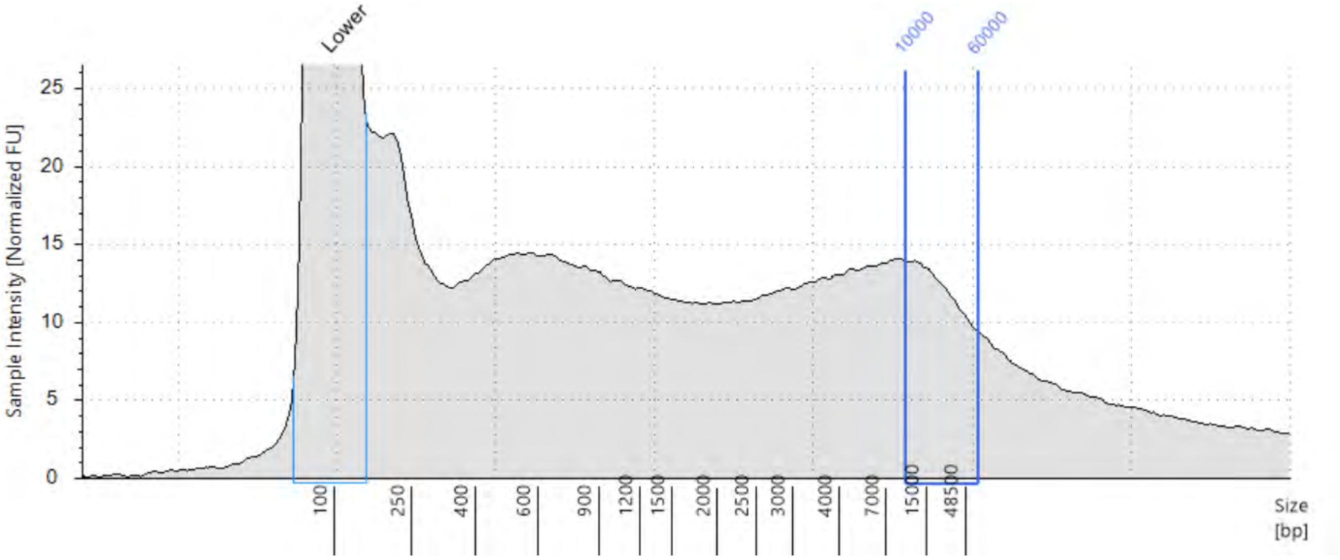

Region Table

From [bp]	To [bp]	Average Size [bp]	Conc. [ng/µl]	Region Molarity [nmol/l]	% of Total	Region Comment	Color
10000	60000	24784	0.543	0.0480	8.86		■

© Agilent Technologies, Inc. 2017

H1: C592

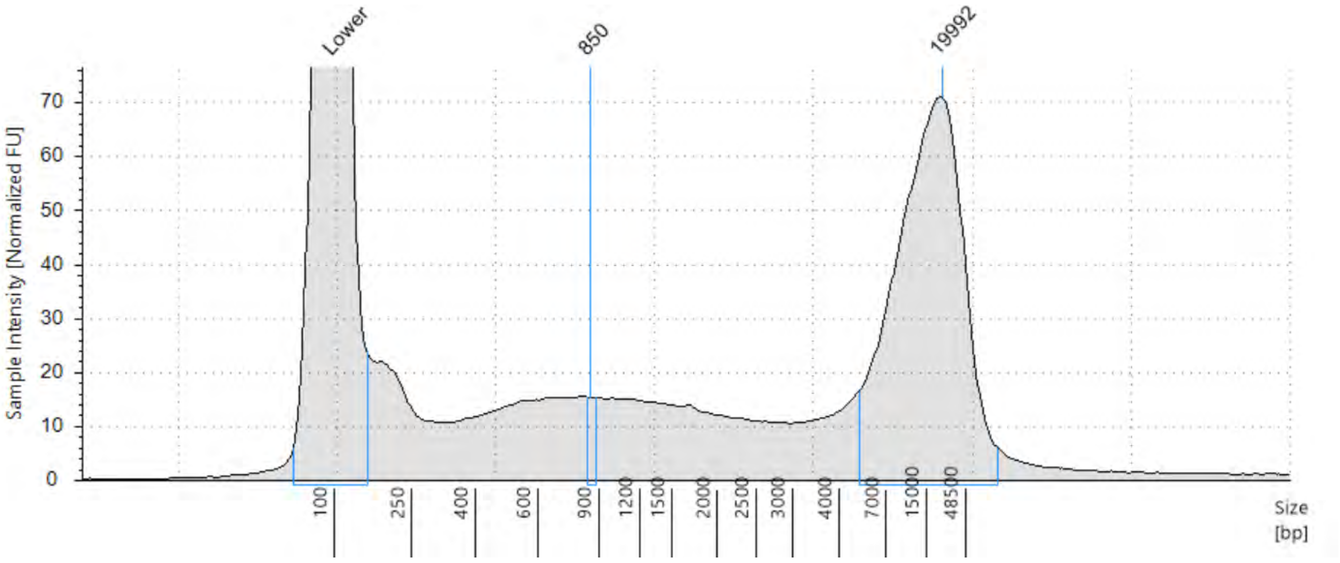

Sample Table

Well	DIN	Conc. [ng/µl]	Sample Description	Alert	Observations
H1	6.1	7.82	C592	⚠	Sample concentration outside recommended range

Peak Table

Size [bp]	Calibrated Conc. [ng/µl]	Assigned Conc. [ng/µl]	% Integrated Area	From [bp]	To [bp]	Peak Comment	Observations
100	8.50	8.50	-	61	146		Lower Marker
850	0.0908	-	2.42	835	882		
19992	3.50	-	93.24	5092	>60000		
-	-	-	-	-	-		Sample Well

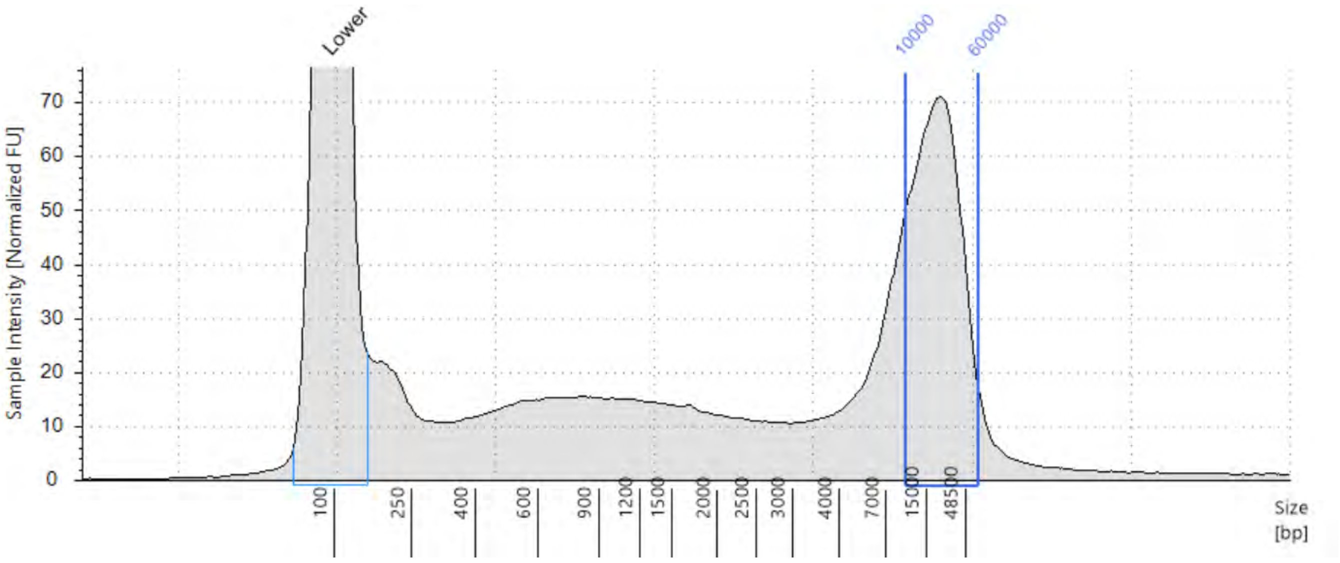

Region Table

From [bp]	To [bp]	Average Size [bp]	Conc. [ng/µl]	Region Molarity [nmol/l]	% of Total	Region Comment	Color
10000	60000	22792	2.56	0.219	32.78		■

A2: C593

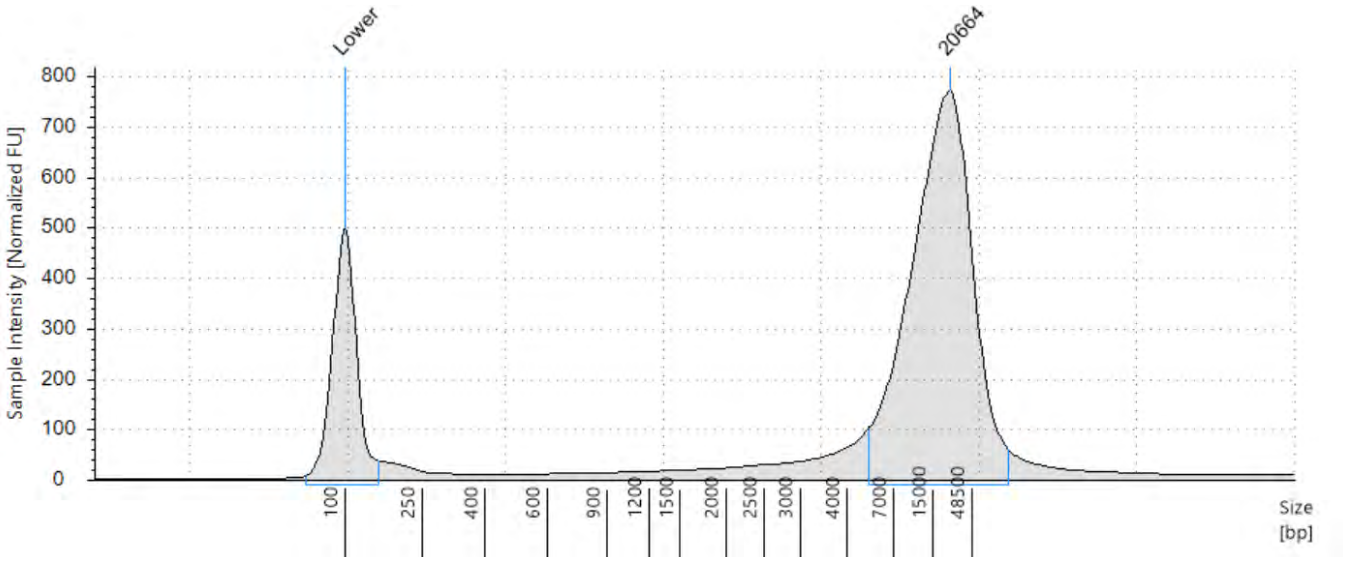

Sample Table

Well	DIN	Conc. [ng/µl]	Sample Description	Alert	Observations
A2	7.7	44.1	C593		

Peak Table

Size [bp]	Calibrated Conc. [ng/µl]	Assigned Conc. [ng/µl]	% Integrated Area	From [bp]	To [bp]	Peak Comment	Observations
100	8.50	8.50	-	63	147		Lower Marker
20664	33.6	-	95.00	5203	>60000		
-	-	-	-	-	-		Sample Well

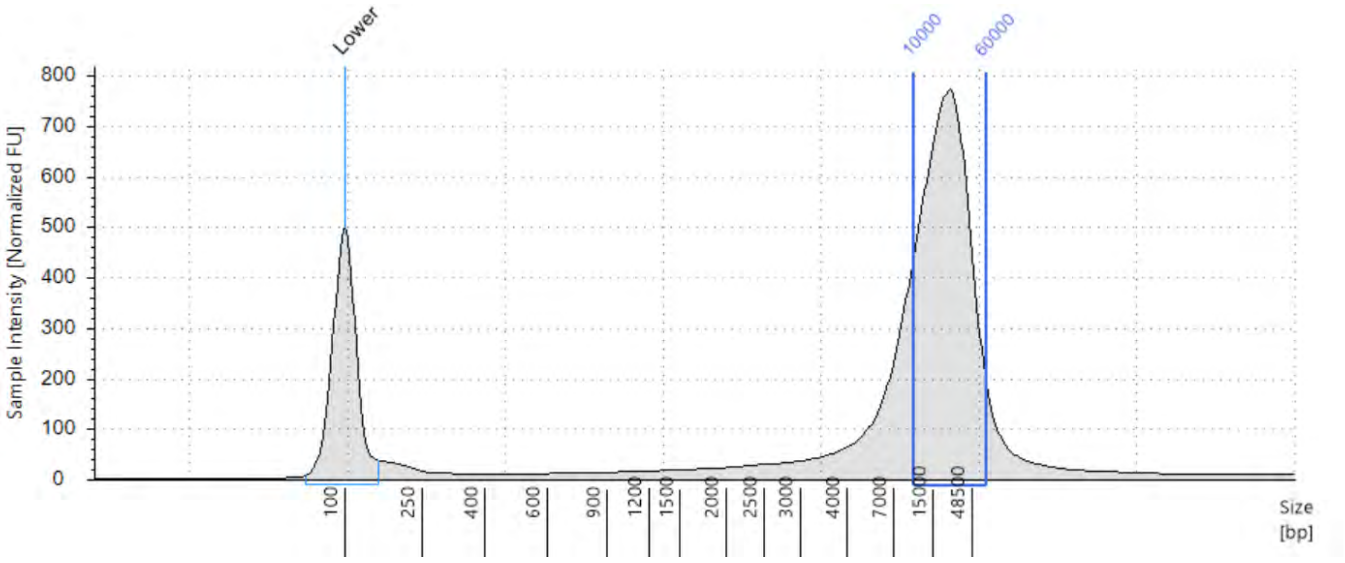

Region Table

From [bp]	To [bp]	Average Size [bp]	Conc. [ng/µl]	Region Molarity [nmol/l]	% of Total	Region Comment	Color
10000	60000	23493	25.9	2.12	58.78		

 © Agilent Technologies, Inc. 2017

B2: C594

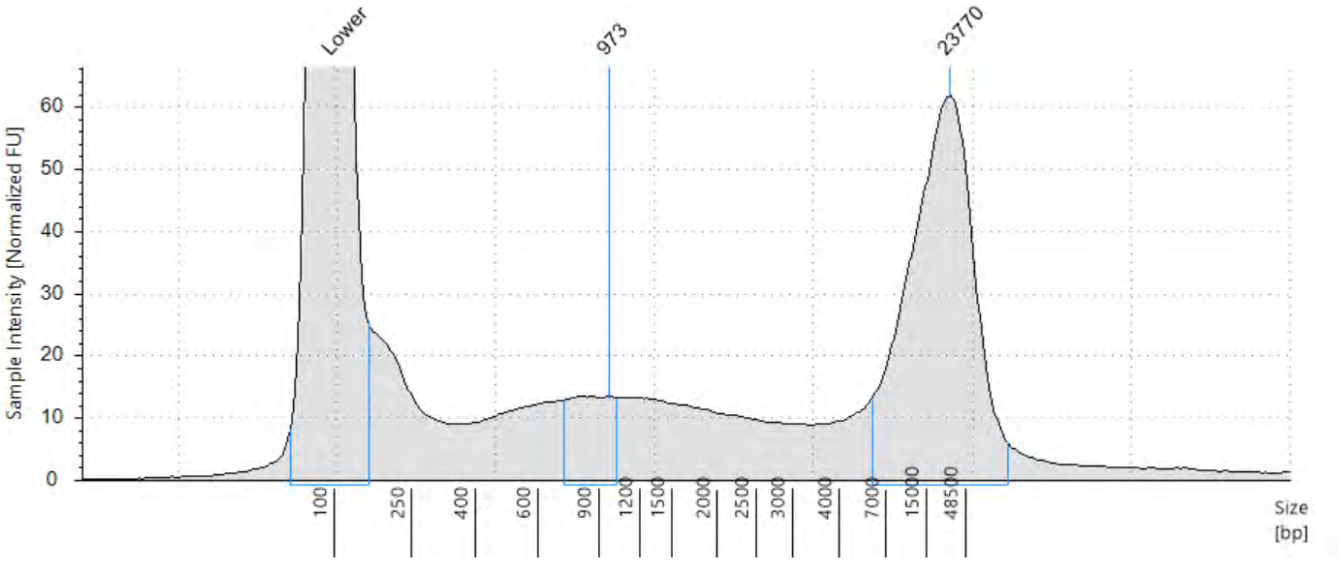

Sample Table

Well	DIN	Conc. [ng/µl]	Sample Description	Alert	Observations
B2	6.3	6.20	C594	⚠	Sample concentration outside recommended range

Peak Table

Size [bp]	Calibrated Conc. [ng/µl]	Assigned Conc. [ng/µl]	% Integrated Area	From [bp]	To [bp]	Peak Comment	Observations
100	8.50	8.50	-	60	151		Lower Marker
973	0.382	-	12.20	721	1021		
23770	2.62	-	83.66	5951	>60000		
-	-	-	-	-	-		Sample Well

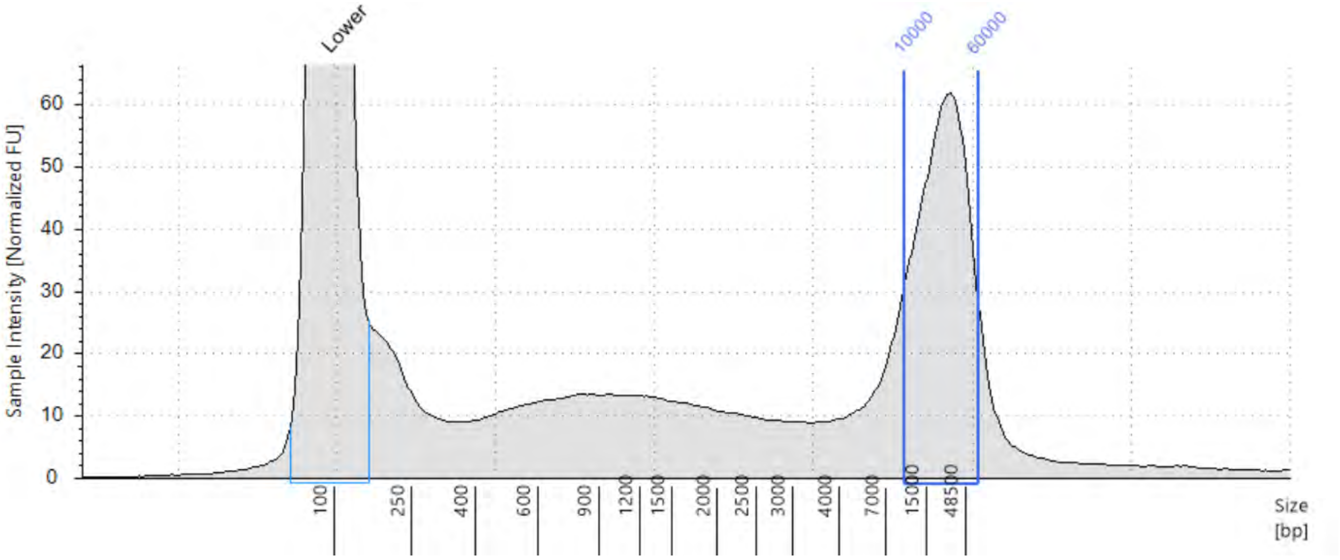

Region Table

From [bp]	To [bp]	Average Size [bp]	Conc. [ng/µl]	Region Molarity [nmol/l]	% of Total	Region Comment	Color
10000	60000	26550	2.04	0.155	32.90		■

 © Agilent Technologies, Inc. 2017

C2: C595

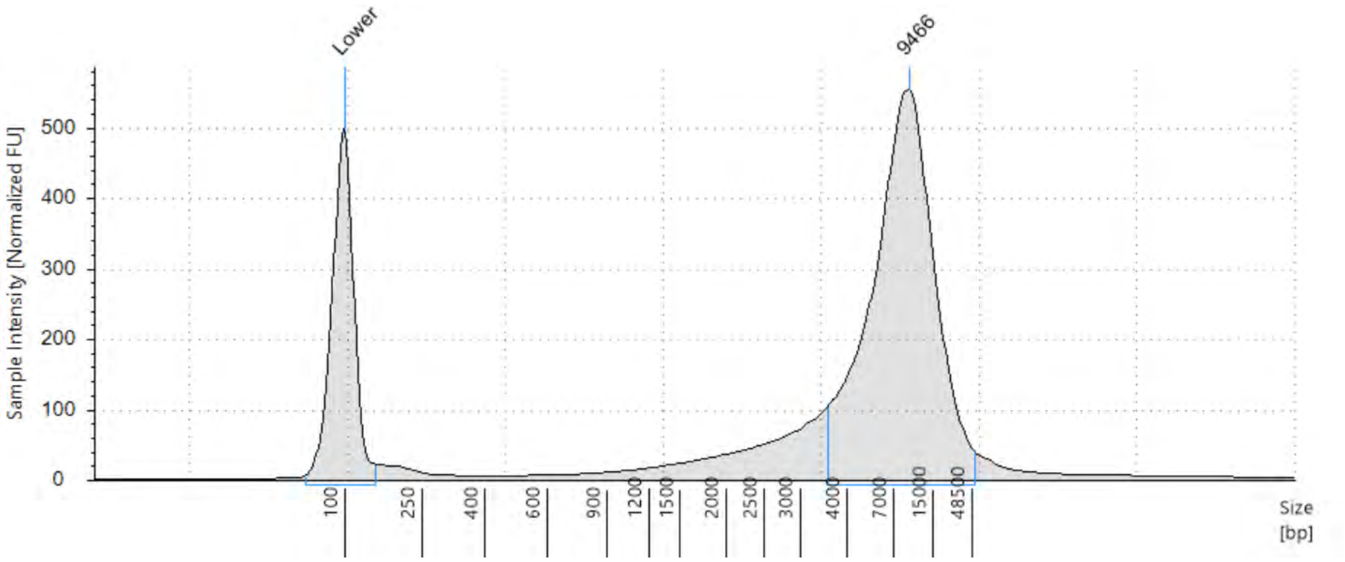

Sample Table

Well	DIN	Conc. [ng/µl]	Sample Description	Alert	Observations
C2	6.7	35.8	C595		

Peak Table

Size [bp]	Calibrated Conc. [ng/µl]	Assigned Conc. [ng/µl]	% Integrated Area	From [bp]	To [bp]	Peak Comment	Observations
100	8.50	8.50	-	63	145		Lower Marker
9466	26.9	-	96.16	3587	52031		
-	-	-	-	-	-		Sample Well

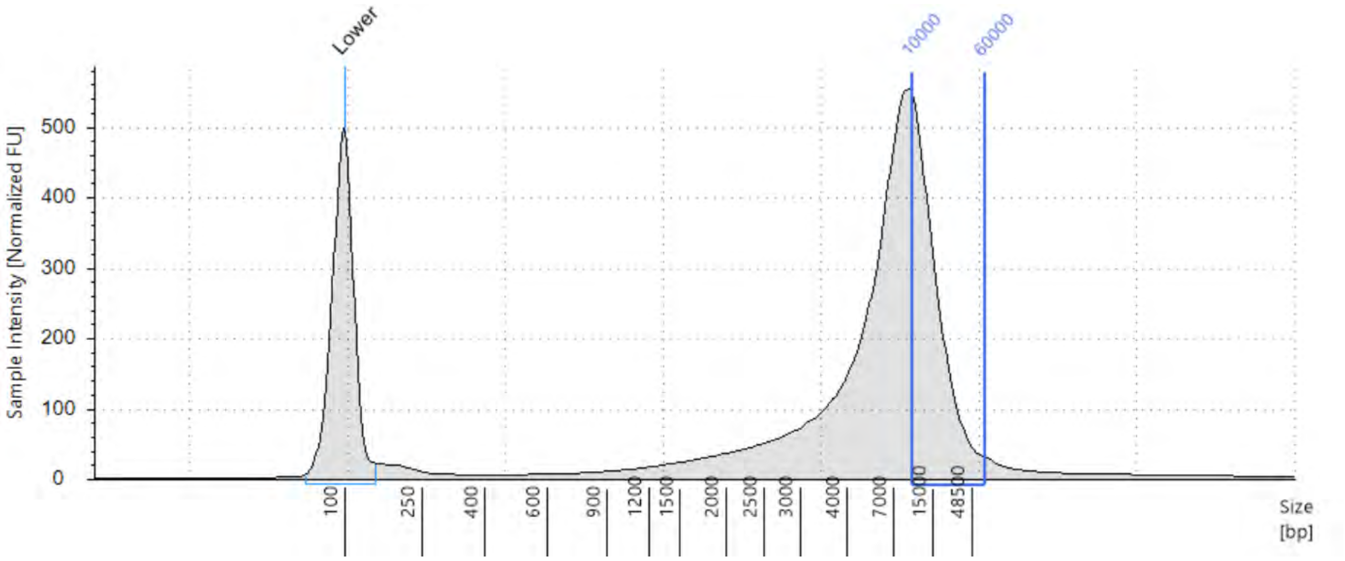

Region Table

From [bp]	To [bp]	Average Size [bp]	Conc. [ng/µl]	Region Molarity [nmol/l]	% of Total	Region Comment	Color
10000	60000	16897	10.5	1.13	29.37		

 © Agilent Technologies, Inc. 2017

D2: C601

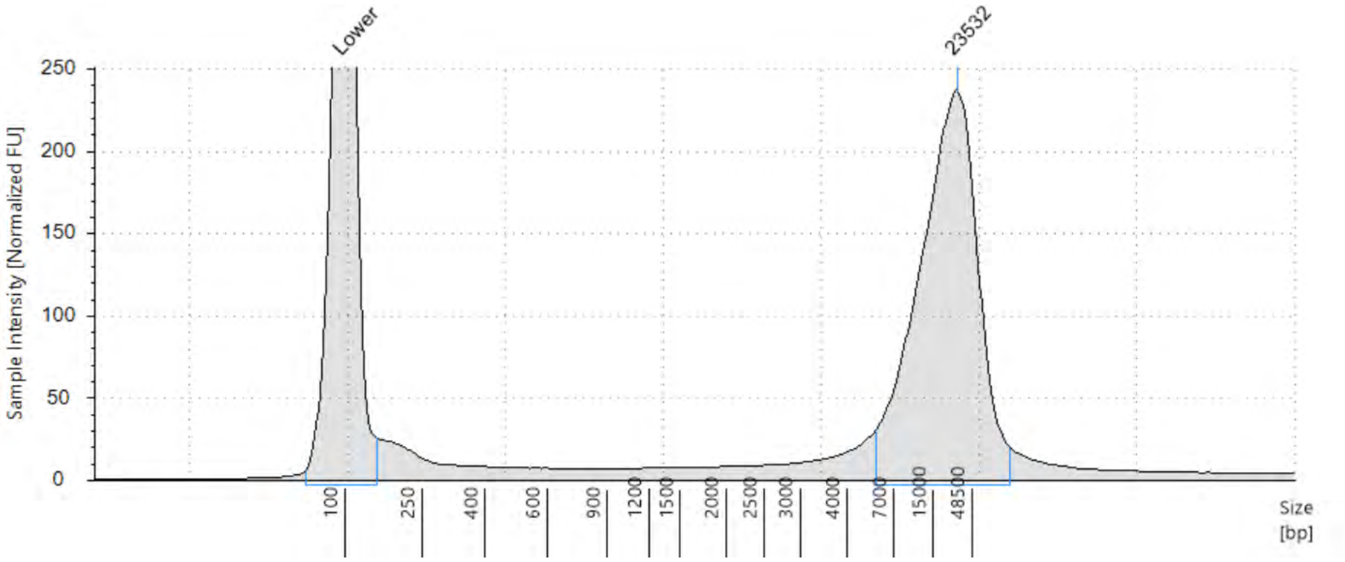

Sample Table

Well	DIN	Conc. [ng/µl]	Sample Description	Alert	Observations
D2	7.8	15.1	C601		

Peak Table

Size [bp]	Calibrated Conc. [ng/µl]	Assigned Conc. [ng/µl]	%Integrated Area	From [bp]	To [bp]	Peak Comment	Observations
100	8.50	8.50	-	62	145		Lower Marker
23532	10.5	-	93.81	5615	>60000		
-	-	-	-	-	-		Sample Well

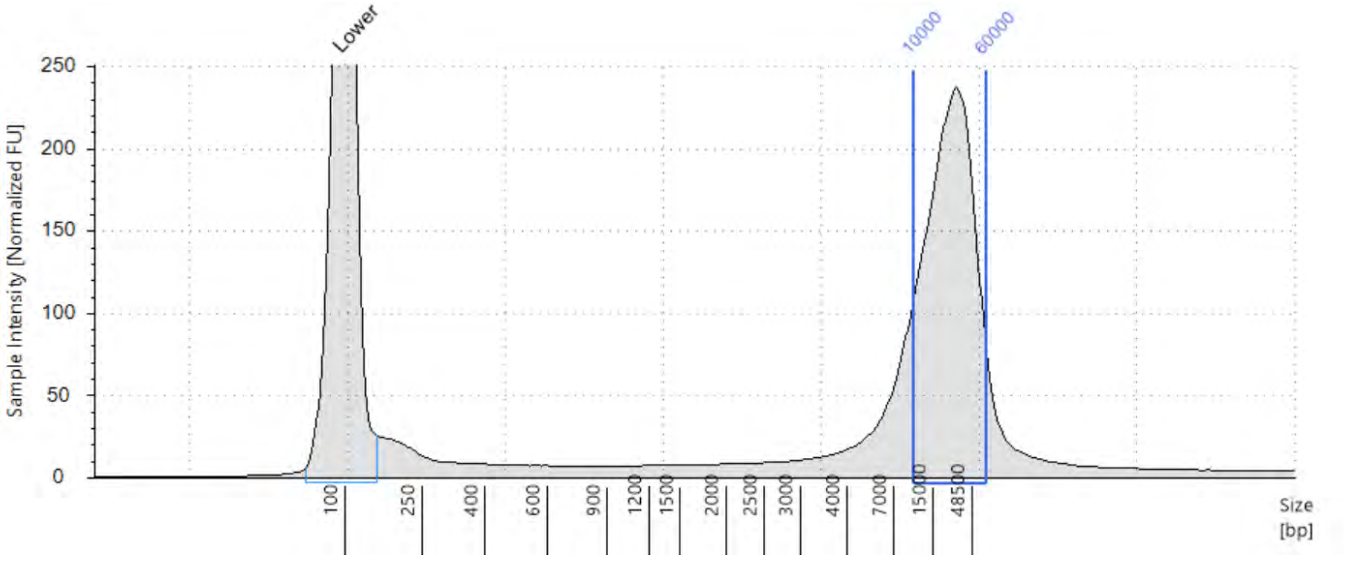

Region Table

From [bp]	To [bp]	Average Size [bp]	Conc. [ng/µl]	Region Molarity [nmol/l]	% of Total	Region Comment	Color
10000	60000	25609	8.46	0.647	55.99		

E2: C602

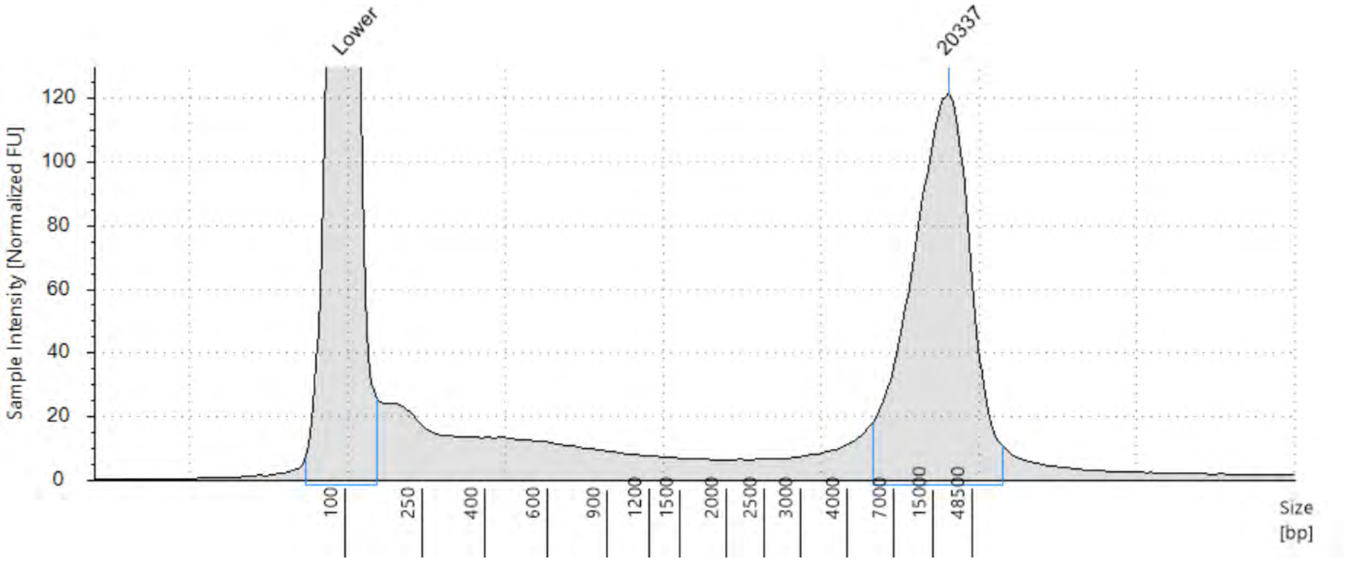

Sample Table

Well	DIN	Conc. [ng/µl]	Sample Description	Alert	Observations
E2	6.8	9.04	C602	⚠	Sample concentration outside recommended range

Peak Table

Size [bp]	Calibrated Conc. [ng/µl]	Assigned Conc. [ng/µl]	% Integrated Area	From [bp]	To [bp]	Peak Comment	Observations
100	8.50	8.50	-	62	148		Lower Marker
20337	5.23	-	94.03	5459	>60000		
-	-	-	-	-	-		Sample Well

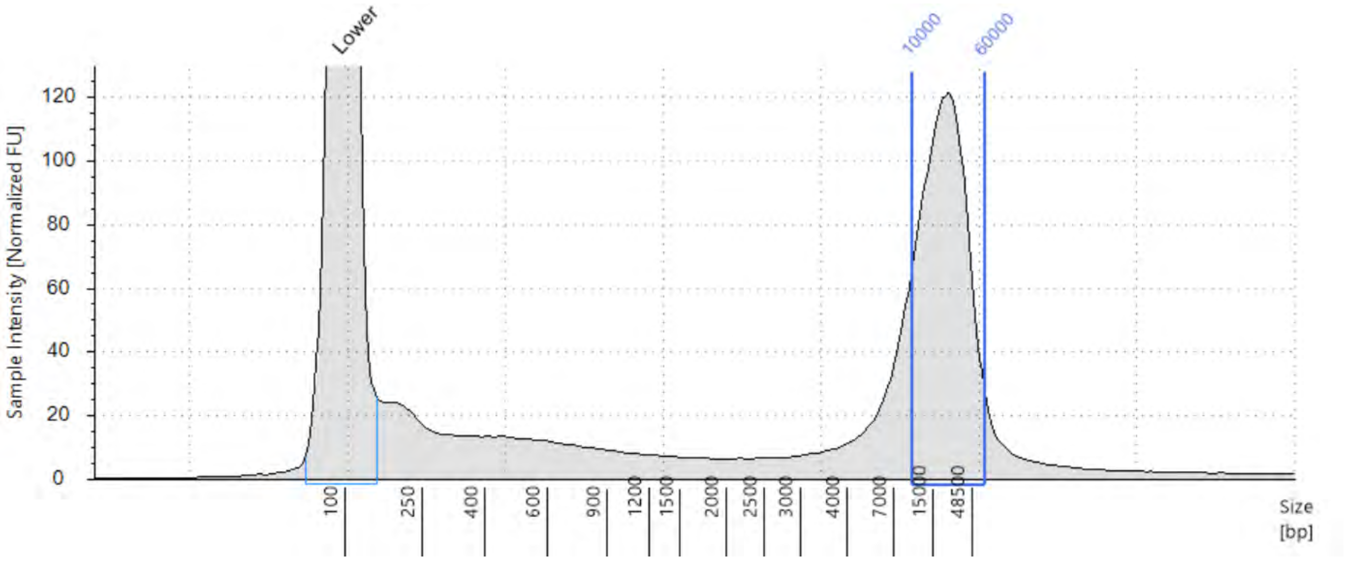

Region Table

From [bp]	To [bp]	Average Size [bp]	Conc. [ng/µl]	Region Molarity [nmol/l]	% of Total	Region Comment	Color
10000	60000	22948	4.20	0.352	46.48		■

G2: C604

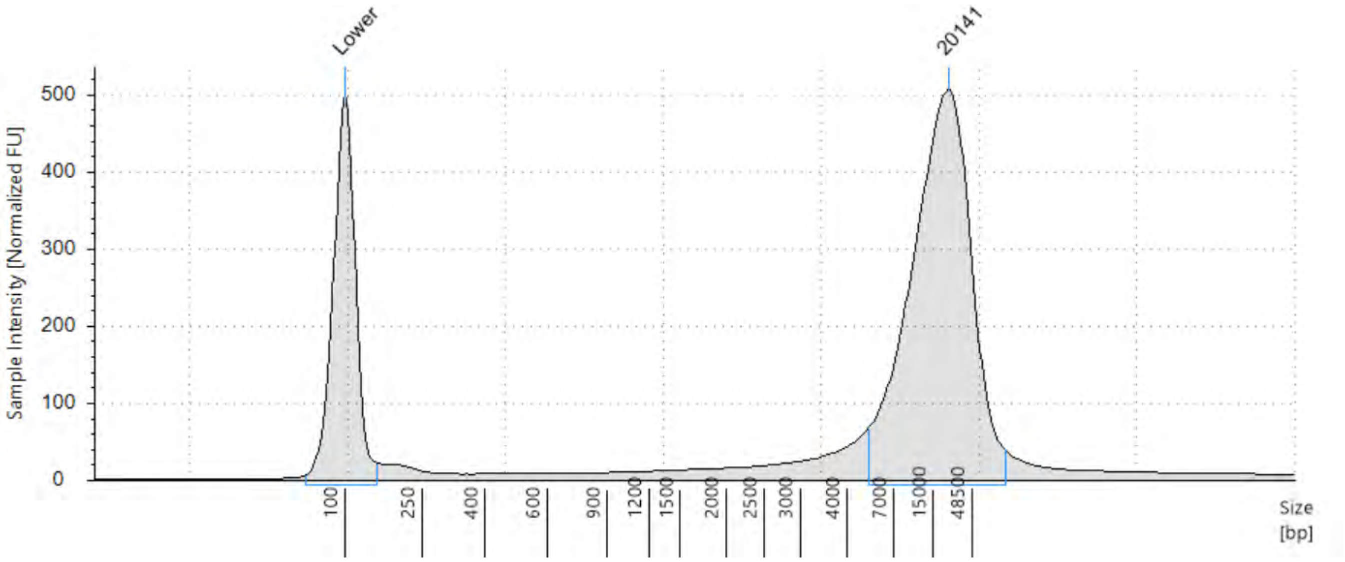

Sample Table

Well	DIN	Conc. [ng/µl]	Sample Description	Alert	Observations
G2	7.7	31.0	C604		

Peak Table

Size [bp]	Calibrated Conc. [ng/µl]	Assigned Conc. [ng/µl]	% Integrated Area	From [bp]	To [bp]	Peak Comment	Observations
100	8.50	8.50	-	63	147		Lower Marker
20141	23.3	-	93.62	5216	>60000		
-	-	-	-	-	-		Sample Well

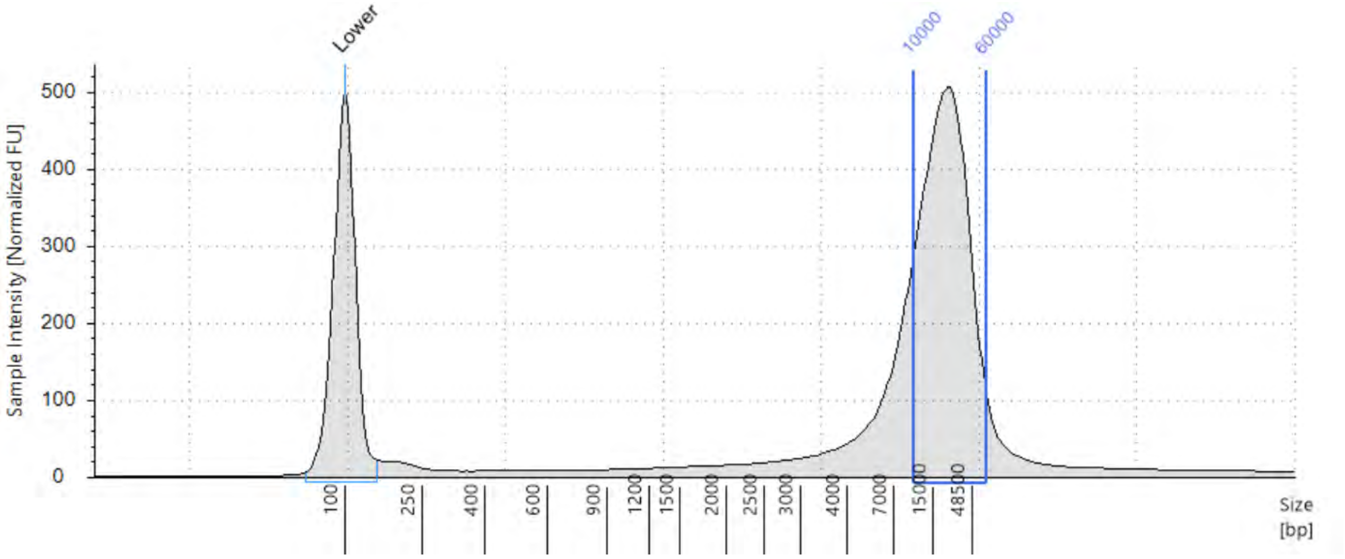

Region Table

From [bp]	To [bp]	Average Size [bp]	Conc. [ng/µl]	Region Molarity [nmol/l]	% of Total	Region Comment	Color
10000	60000	22981	18.4	1.53	59.29		

© Agilent Technologies, Inc. 2017

H2: C605

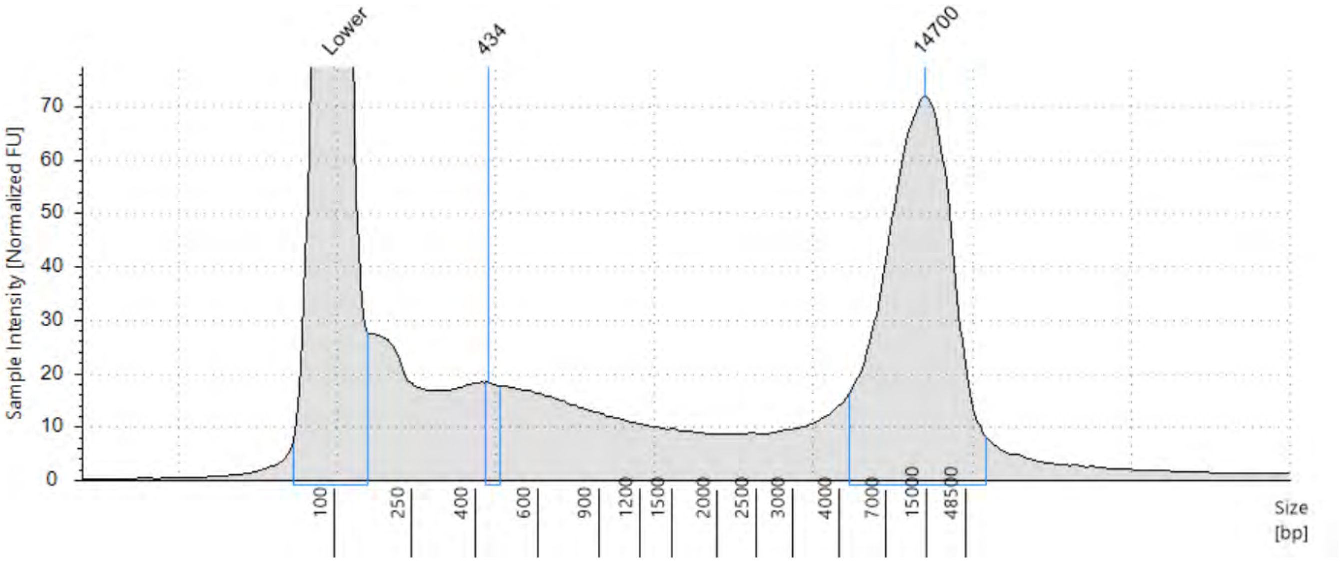

Sample Table

Well	DIN	Conc. [ng/µl]	Sample Description	Alert	Observations
H2	6.0	7.84	C605	⚠	Sample concentration outside recommended range

Peak Table

Size [bp]	Calibrated Conc. [ng/µl]	Assigned Conc. [ng/µl]	% Integrated Area	From [bp]	To [bp]	Peak Comment	Observations
100	8.50	8.50	-	61	149		Lower Marker
434	0.164	-	4.25	426	467		
14700	3.46	-	89.48	4540	>60000		
-	-	-	-	-	-		Sample Well

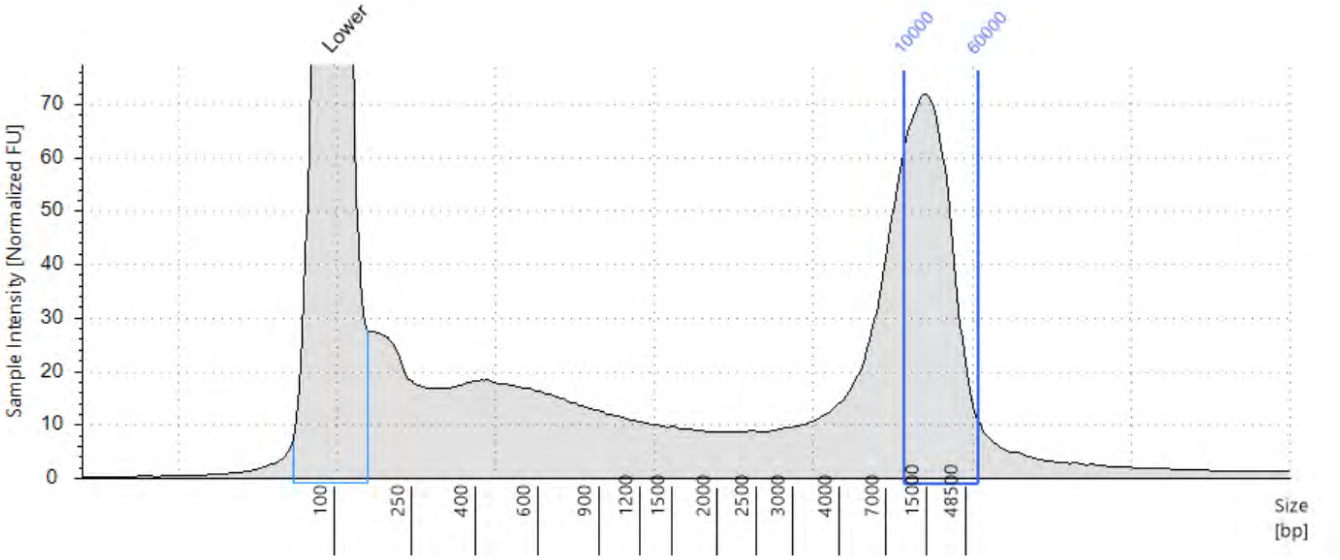

Region Table

From [bp]	To [bp]	Average Size [bp]	Conc. [ng/µl]	Region Molarity [nmol/l]	% of Total	Region Comment	Color
10000	60000	20141	2.27	0.212	29.02		■

A3: C611

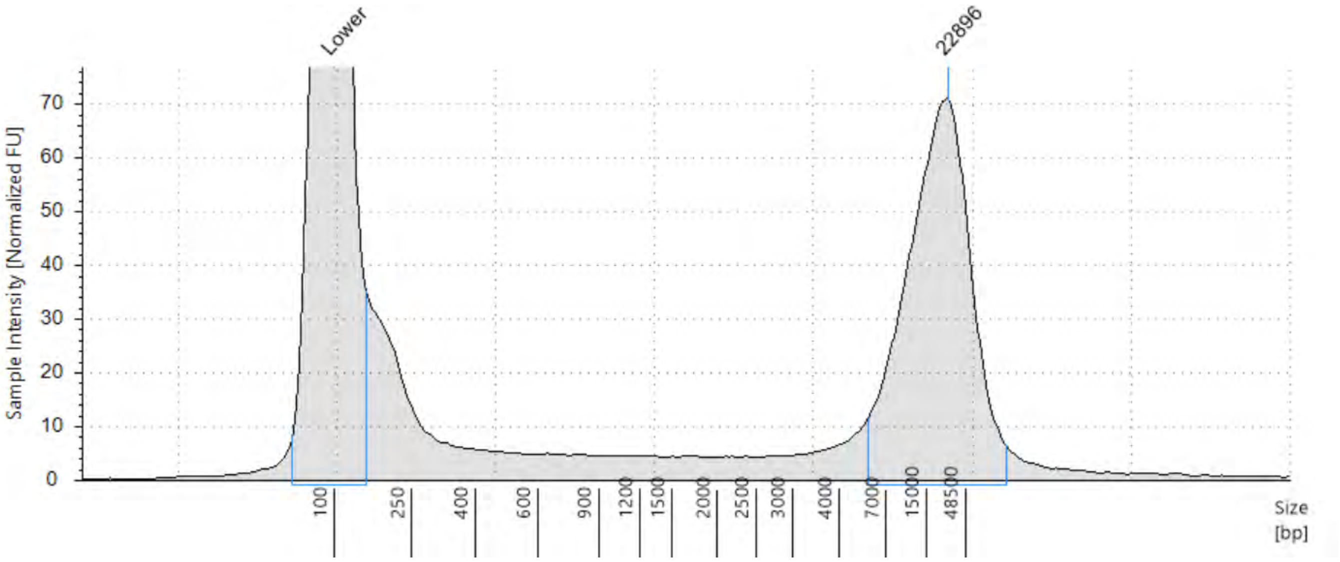

Sample Table

Well	DIN	Conc. [ng/µl]	Sample Description	Alert	Observations
A3	7.9	5.27	C611	⚠	Sample concentration outside recommended range

Peak Table

Size [bp]	Calibrated Conc. [ng/µl]	Assigned Conc. [ng/µl]	% Integrated Area	From [bp]	To [bp]	Peak Comment	Observations
100	8.50	8.50	-	61	148		Lower Marker
22896	3.10	-	93.59	5694	>60000		
-	-	-	-	-	-		Sample Well

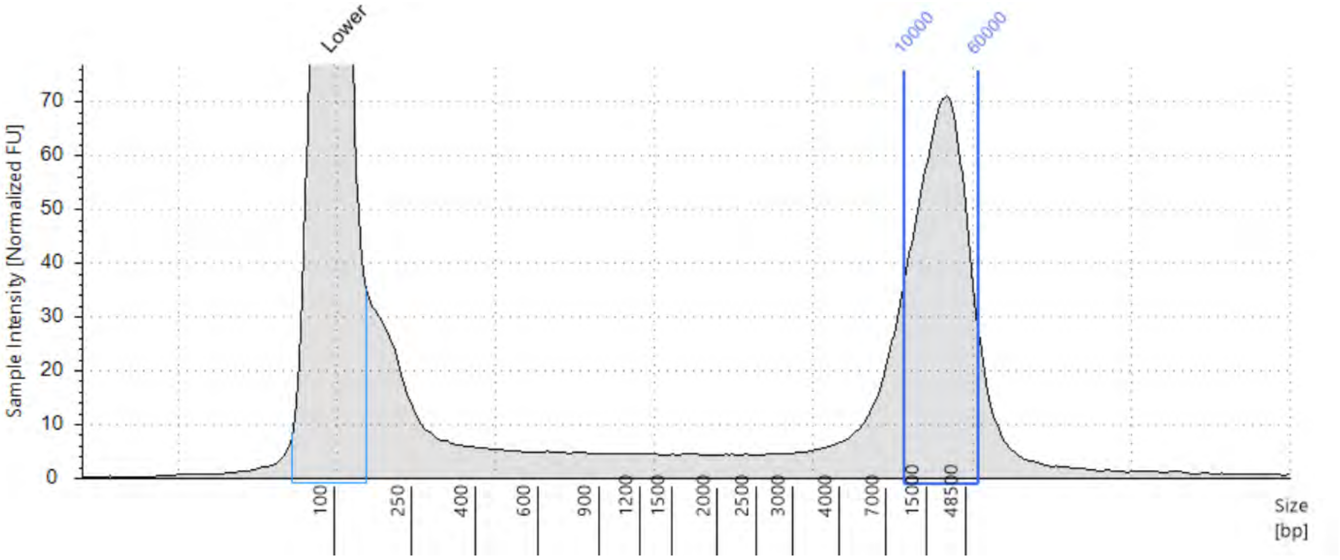

Region Table

From [bp]	To [bp]	Average Size [bp]	Conc. [ng/µl]	Region Molarity [nmol/l]	% of Total	Region Comment	Color
10000	60000	25211	2.42	0.190	45.89		■

 © Agilent Technologies, Inc. 2017

B3: C612

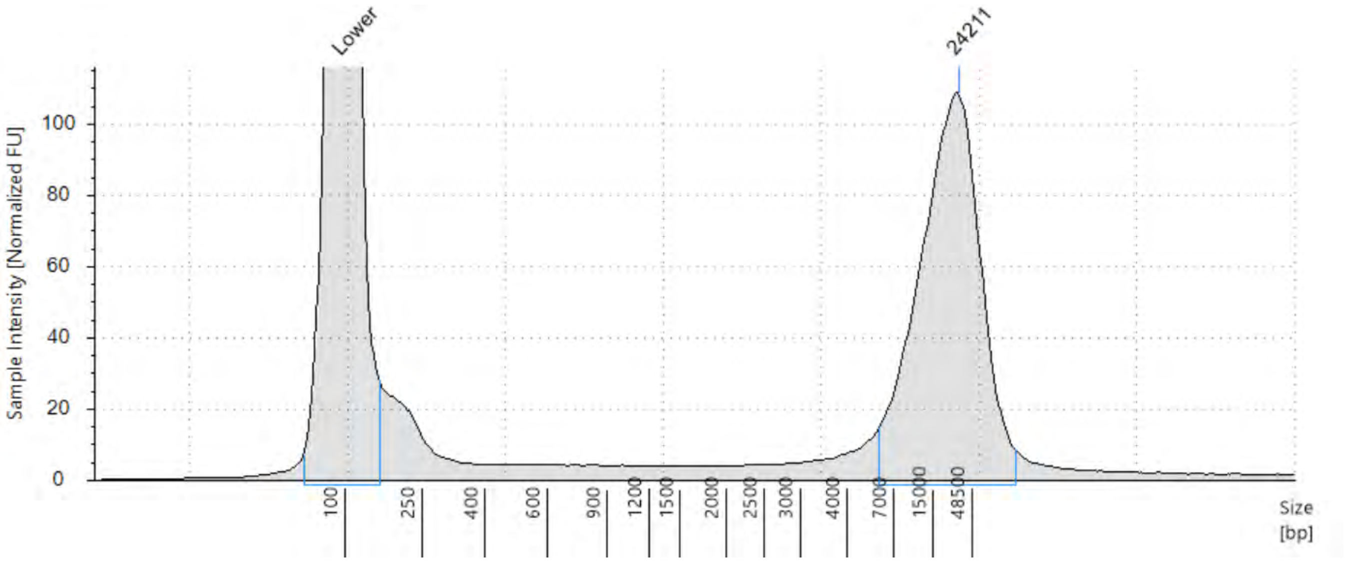

Sample Table

Well	DIN	Conc. [ng/µl]	Sample Description	Alert	Observations
B3	8.5	6.76	C612	⚠	Sample concentration outside recommended range

Peak Table

Size [bp]	Calibrated Conc. [ng/µl]	Assigned Conc. [ng/µl]	% Integrated Area	From [bp]	To [bp]	Peak Comment	Observations
100	8.50	8.50	-	62	152		Lower Marker
24211	4.58	-	92.52	5925	>60000		
-	-	-	-	-	-		Sample Well

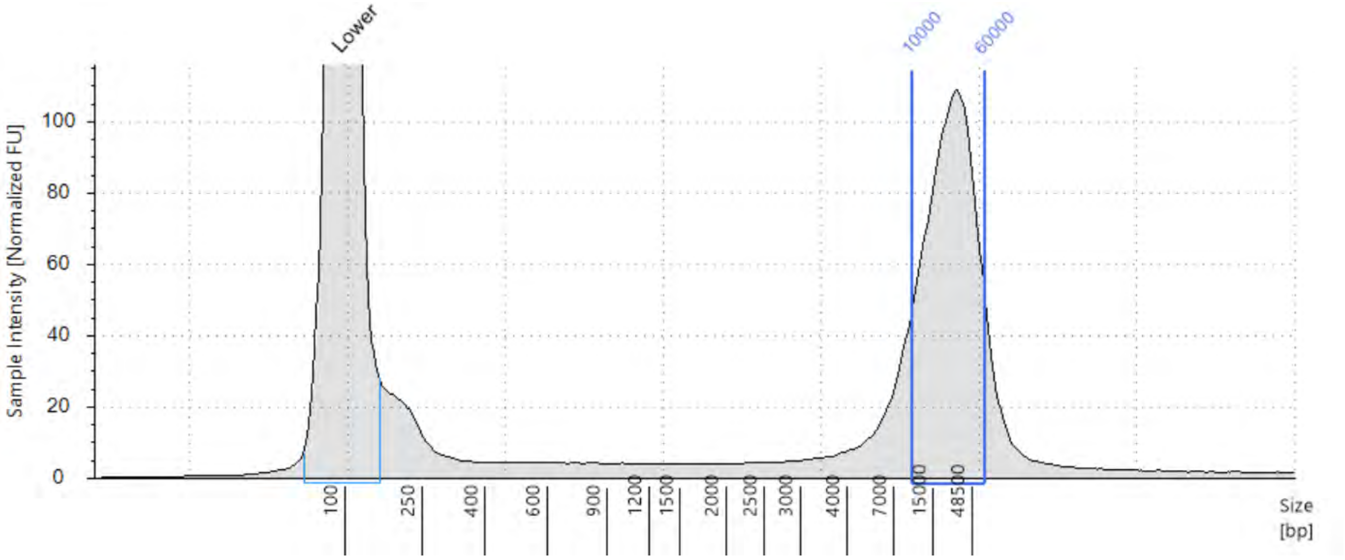

Region Table

From [bp]	To [bp]	Average Size [bp]	Conc. [ng/µl]	Region Molarity [nmol/l]	% of Total	Region Comment	Color
10000	60000	26219	3.64	0.276	53.85		■

C3: C613

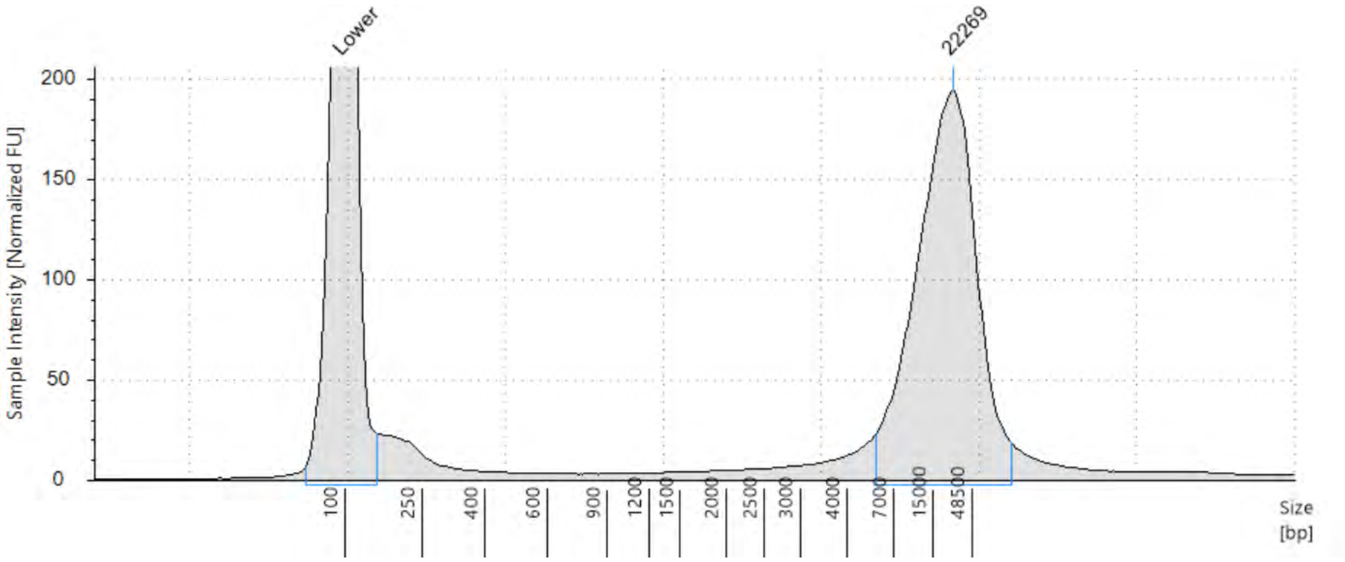

Sample Table

Well	DIN	Conc. [ng/µl]	Sample Description	Alert	Observations
C3	8.4	12.0	C613		

Peak Table

Size [bp]	Calibrated Conc. [ng/µl]	Assigned Conc. [ng/µl]	% Integrated Area	From [bp]	To [bp]	Peak Comment	Observations
100	8.50	8.50	-	62	146		Lower Marker
22269	8.85	-	93.21	5666	>60000		
-	-	-	-	-	-		Sample Well

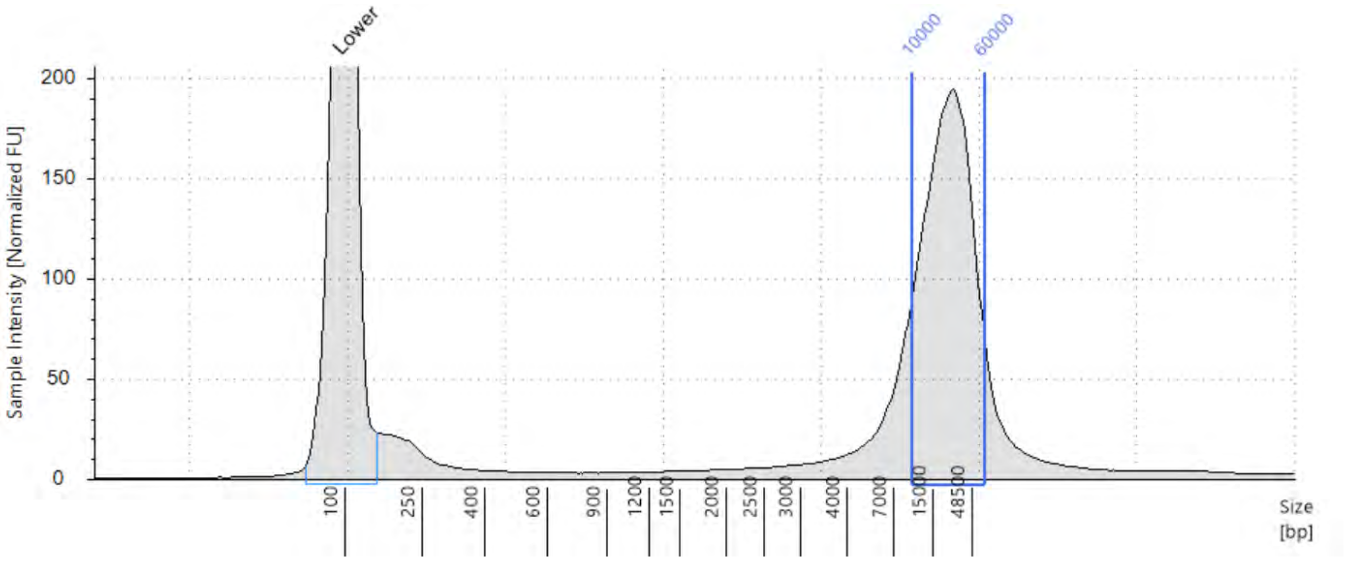

Region Table

From [bp]	To [bp]	Average Size [bp]	Conc. [ng/µl]	Region Molarity [nmol/l]	% of Total	Region Comment	Color
10000	60000	24963	7.13	0.559	59.17		

D3: C614

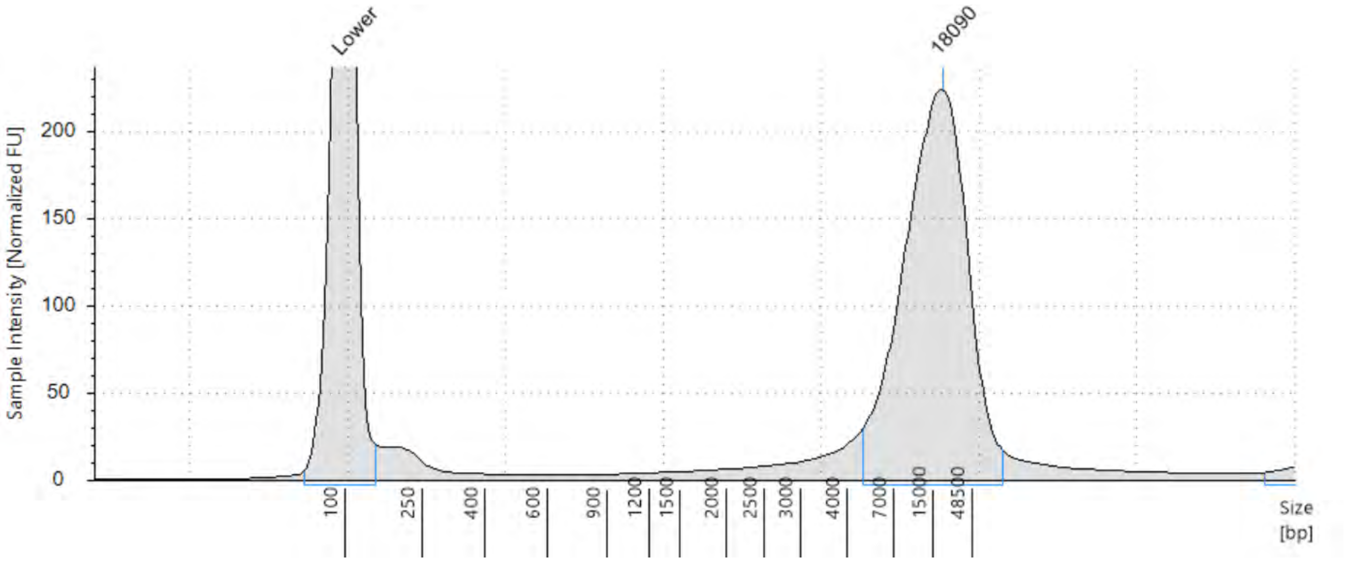

Sample Table

Well	DIN	Conc. [ng/µl]	Sample Description	Alert	Observations
D3	8.0	14.7	C614		

Peak Table

Size [bp]	Calibrated Conc. [ng/µl]	Assigned Conc. [ng/µl]	% Integrated Area	From [bp]	To [bp]	Peak Comment	Observations
100	8.50	8.50	-	62	145		Lower Marker
18090	11.0	-	90.43	4914	>60000		
>60000	0.170	-	1.40	>60000	>60000		
-	-	-	-	-	-		Sample Well

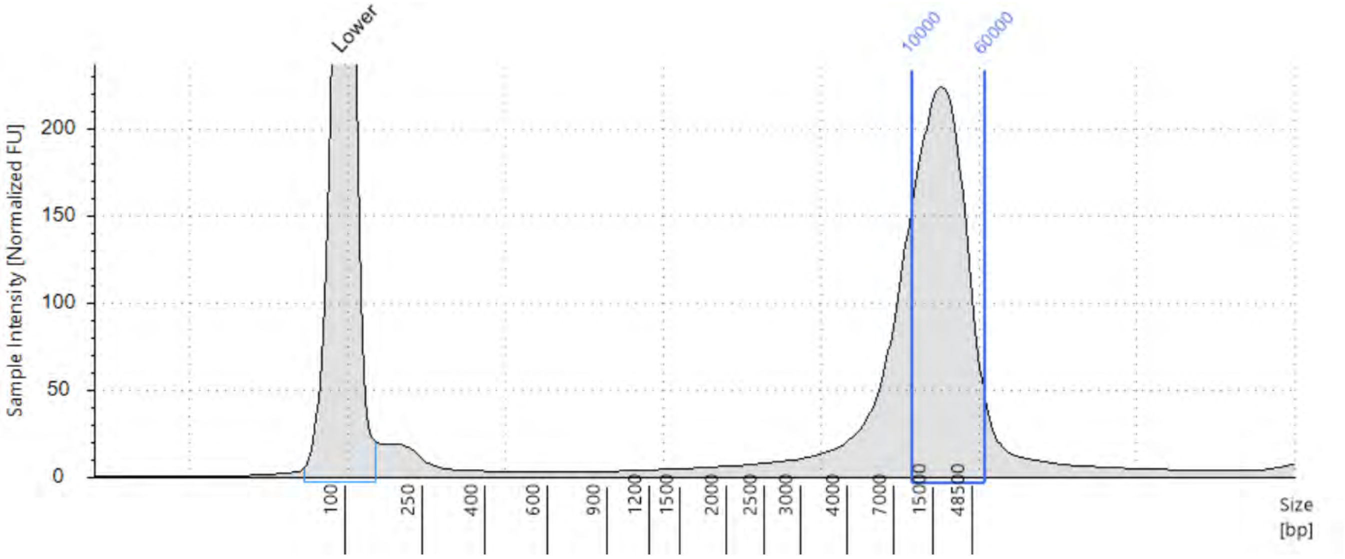

Region Table

From [bp]	To [bp]	Average Size [bp]	Conc. [ng/µl]	Region Molarity [nmol/l]	% of Total	Region Comment	Color
10000	60000	22079	8.24	0.712	55.91		

© Agilent Technologies, Inc. 2017

E3: C615

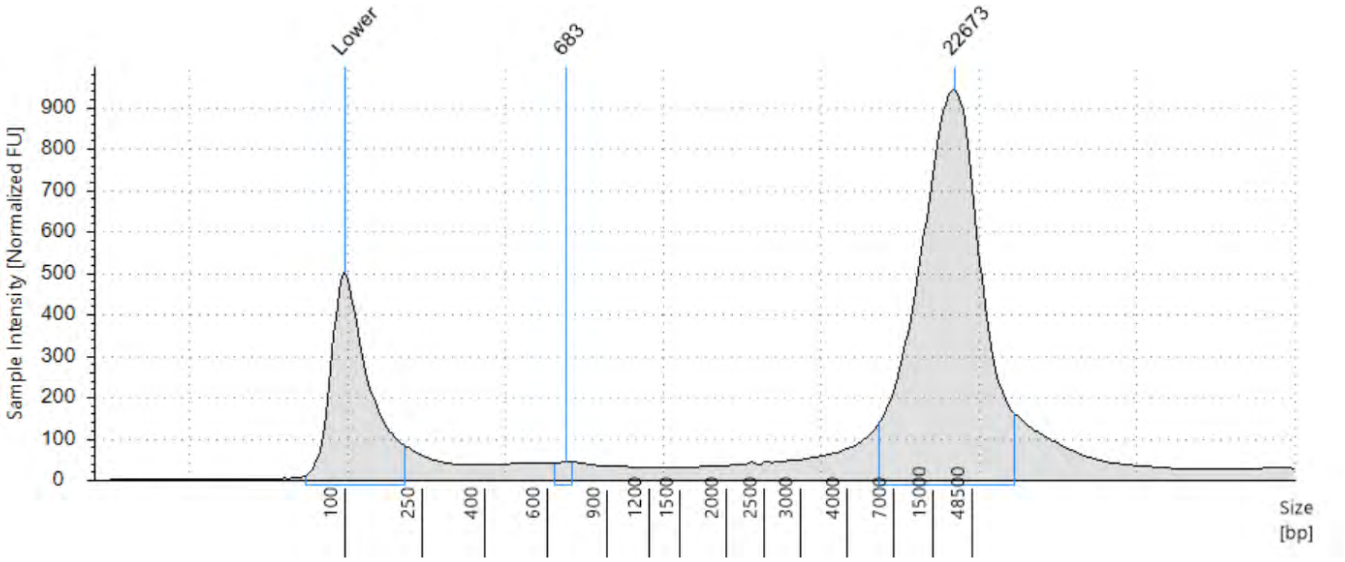

Sample Table

Well	DIN	Conc. [ng/µl]	Sample Description	Alert	Observations
E3	7.3	42.0	C615		

Peak Table

Size [bp]	Calibrated Conc. [ng/µl]	Assigned Conc. [ng/µl]	% Integrated Area	From [bp]	To [bp]	Peak Comment	Observations
100	8.50	8.50	-	63	206		Lower Marker
683	0.299	-	1.01	635	714		
22673	26.8	-	90.10	5911	>60000		
-	-	-	-	-	-		Sample Well

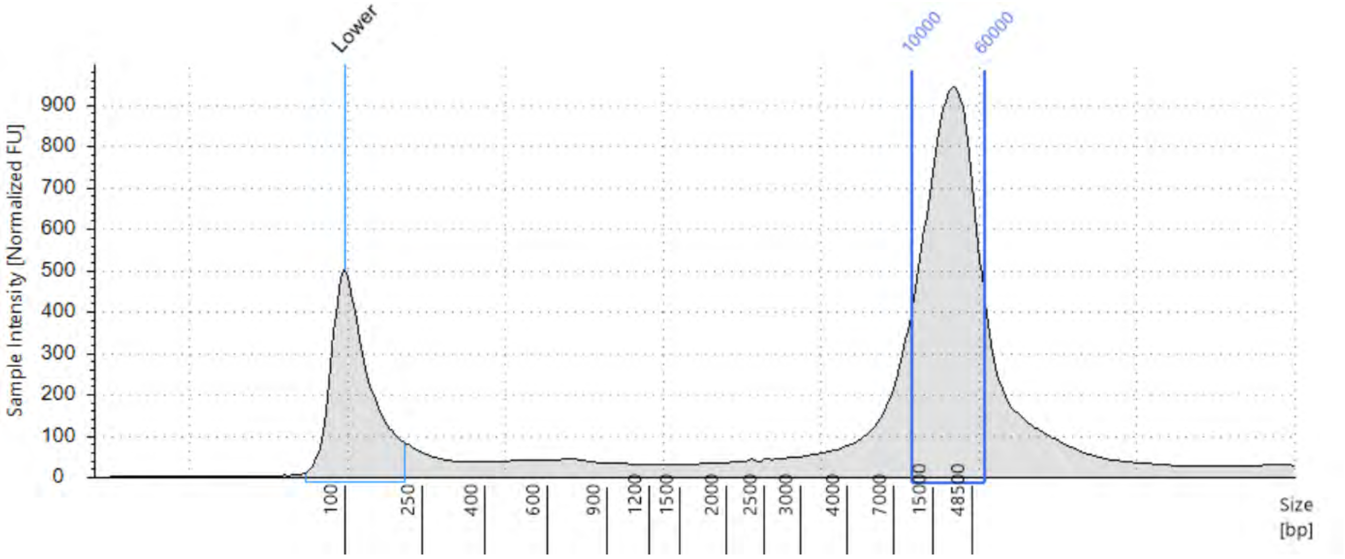

Region Table

From [bp]	To [bp]	Average Size [bp]	Conc. [ng/µl]	Region Molarity [nmol/l]	% of Total	Region Comment	Color
10000	60000	25952	21.0	1.59	49.85		

C4: C621

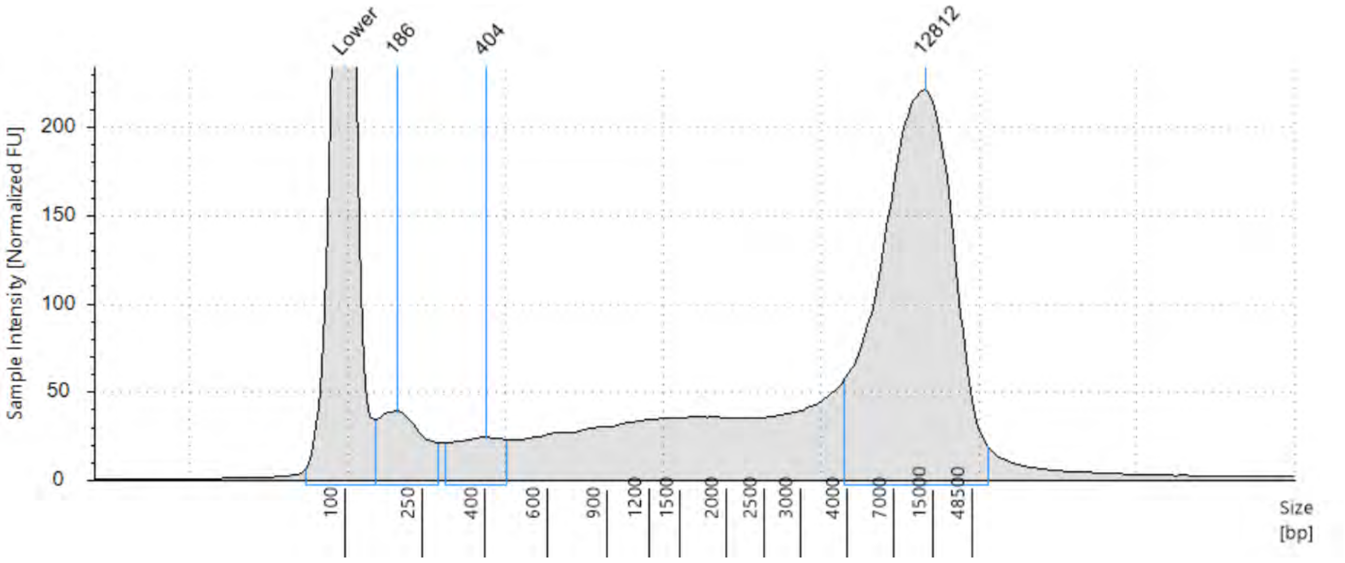

Sample Table

Well	DIN	Conc. [ng/µl]	Sample Description	Alert	Observations
C4	6.2	22.8	C621		

Peak Table

Size [bp]	Calibrated Conc. [ng/µl]	Assigned Conc. [ng/µl]	% Integrated Area	From [bp]	To [bp]	Peak Comment	Observations
100	8.50	8.50	-	63	143		Lower Marker
186	1.33	-	8.85	143	283		
404	0.915	-	6.09	297	462		
12812	12.5	-	82.95	3933	>60000		
-	-	-	-	-	-		Sample Well

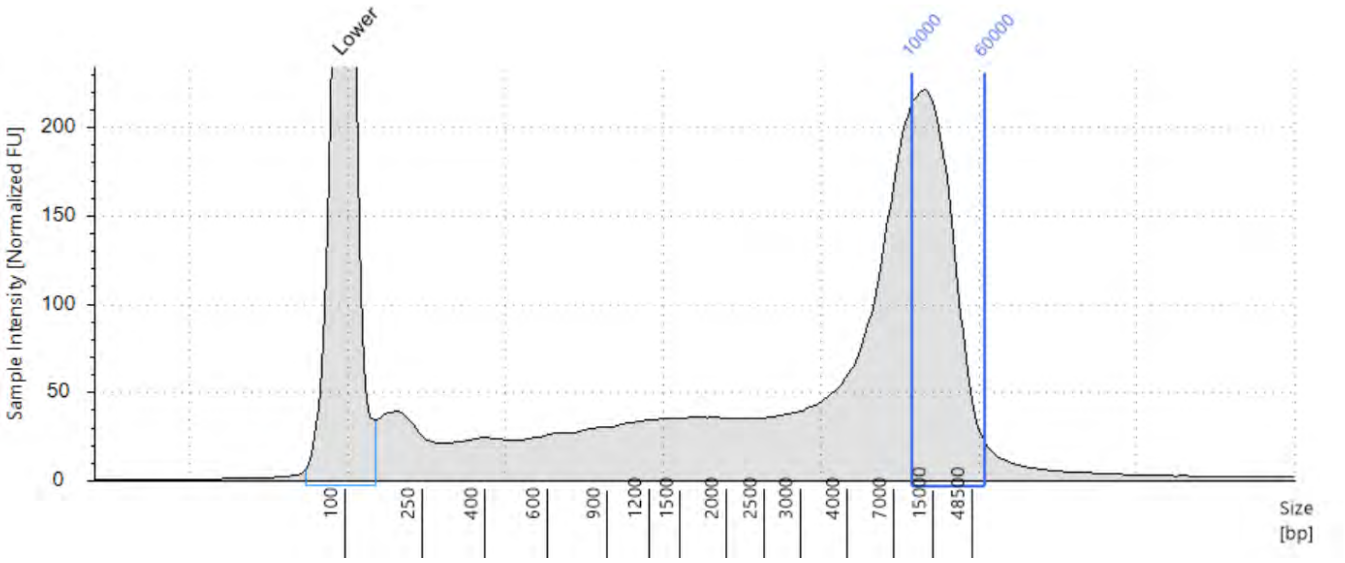

Region Table

From [bp]	To [bp]	Average Size [bp]	Conc. [ng/µl]	Region Molarity [nmol/l]	% of Total	Region Comment	Color
10000	60000	18770	6.91	0.676	30.24		■

 © Agilent Technologies, Inc. 2017

D4: C622

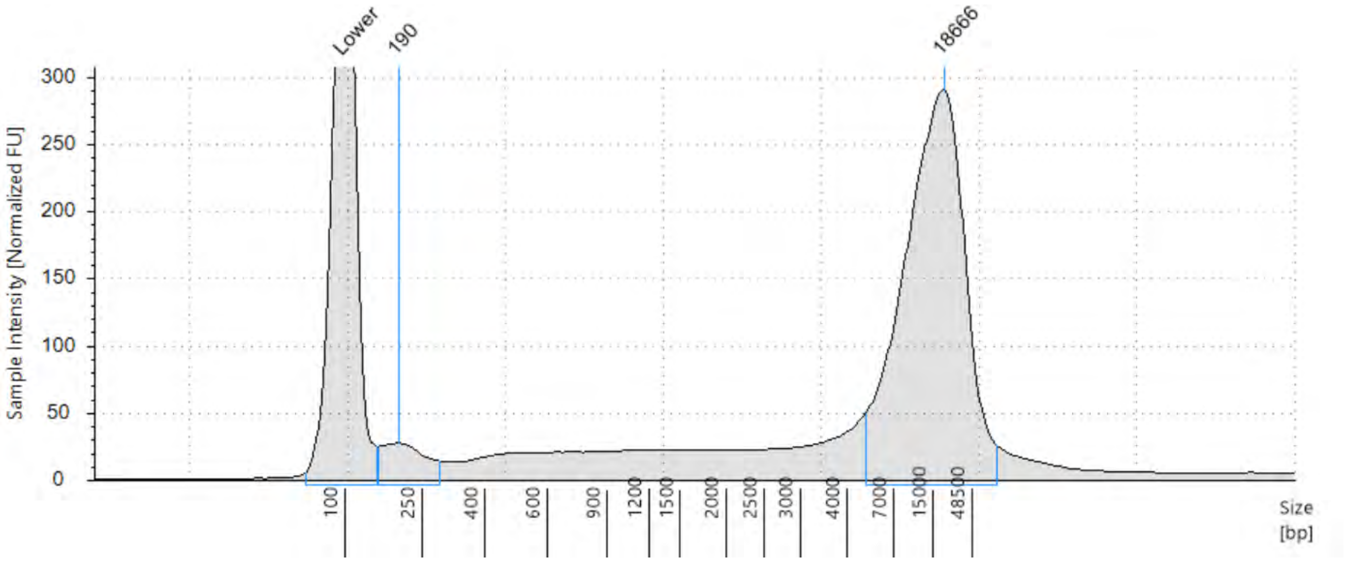

Sample Table

Well	DIN	Conc. [ng/µl]	Sample Description	Alert	Observations
D4	6.7	22.0	C622		

Peak Table

Size [bp]	Calibrated Conc. [ng/µl]	Assigned Conc. [ng/µl]	% Integrated Area	From [bp]	To [bp]	Peak Comment	Observations
100	8.50	8.50	-	63	145		Lower Marker
190	0.885	-	5.87	147	285		
18666	13.6	-	90.03	5063	>60000		
-	-	-	-	-	-		Sample Well

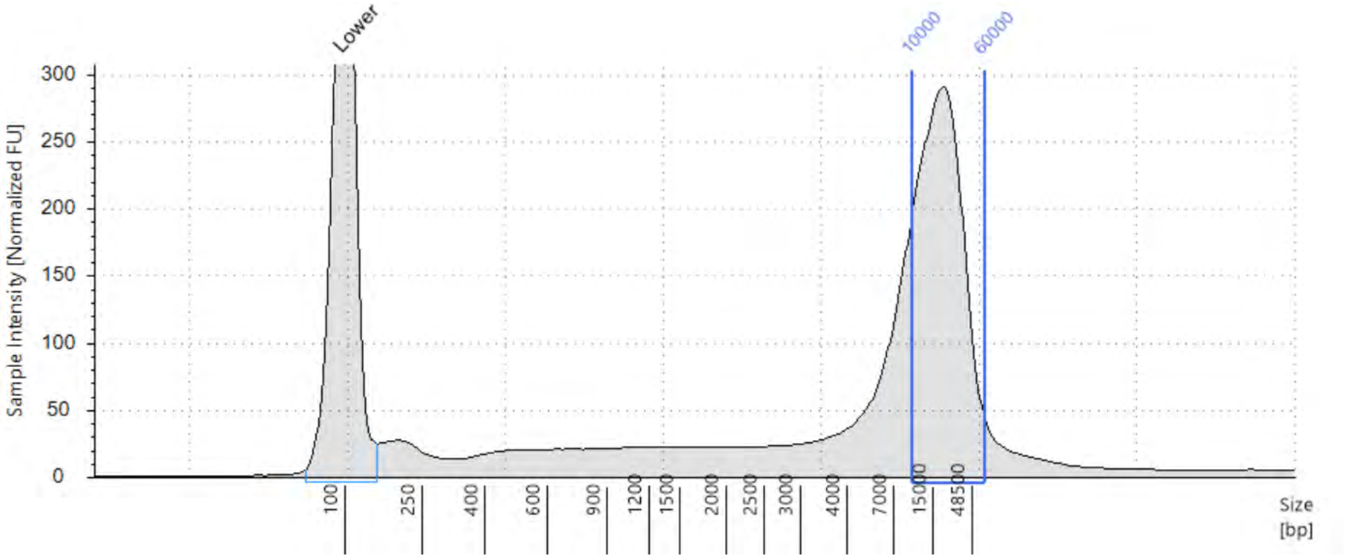

Region Table

From [bp]	To [bp]	Average Size [bp]	Conc. [ng/µl]	Region Molarity [nmol/l]	% of Total	Region Comment	Color
10000	60000	21110	10.2	0.907	46.32		

E4: C623

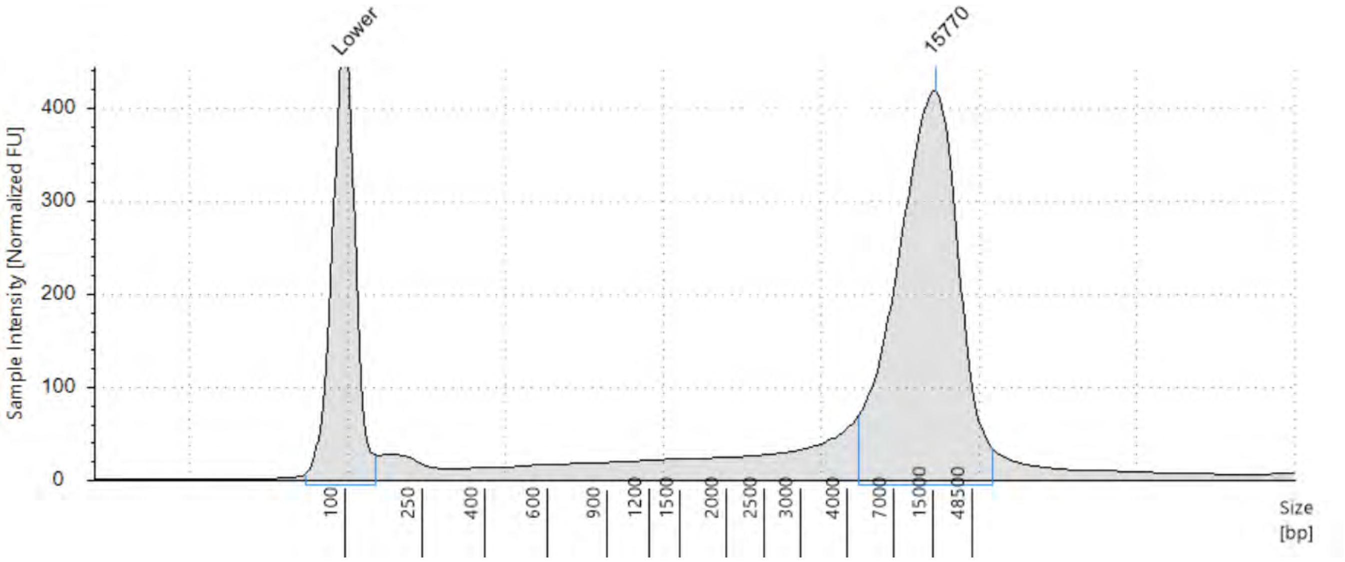

Sample Table

Well	DIN	Conc. [ng/µl]	Sample Description	Alert	Observations
E4	6.9	29.0	C623		

Peak Table

Size [bp]	Calibrated Conc. [ng/µl]	Assigned Conc. [ng/µl]	% Integrated Area	From [bp]	To [bp]	Peak Comment	Observations
100	8.50	8.50	-	63	145		Lower Marker
15770	19.8	-	94.01	4626	>60000		
-	-	-	-	-	-		Sample Well

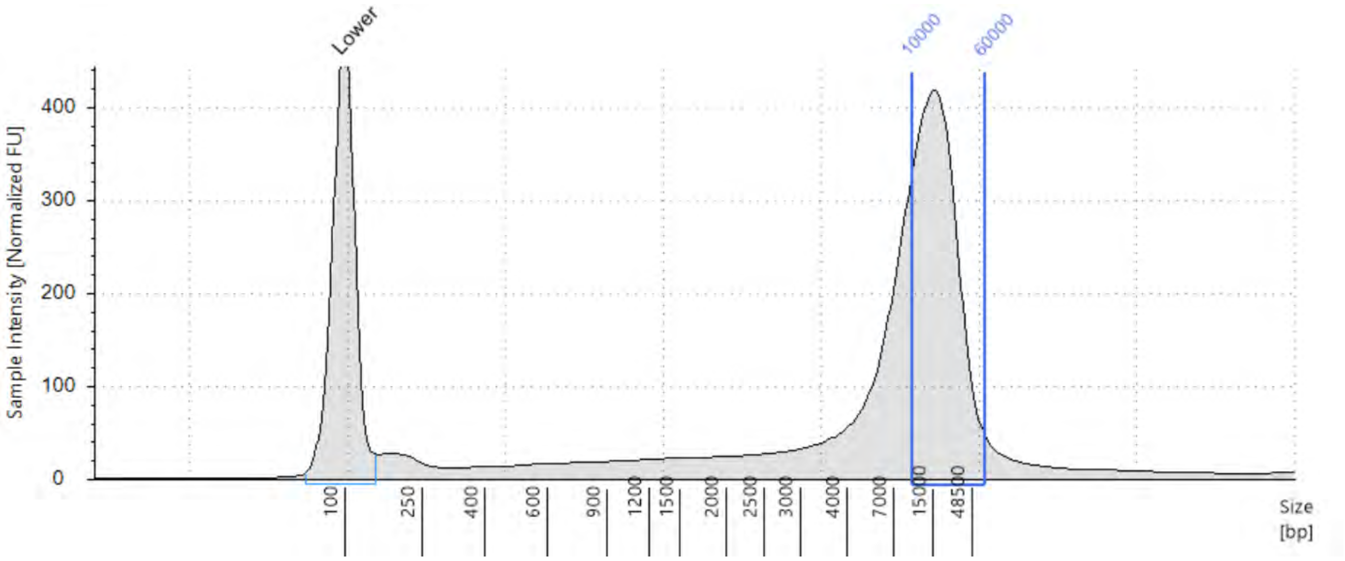

Region Table

From [bp]	To [bp]	Average Size [bp]	Conc. [ng/µl]	Region Molarity [nmol/l]	% of Total	Region Comment	Color
10000	60000	19650	13.7	1.28	47.24		

 © Agilent Technologies, Inc. 2017

F4: C624

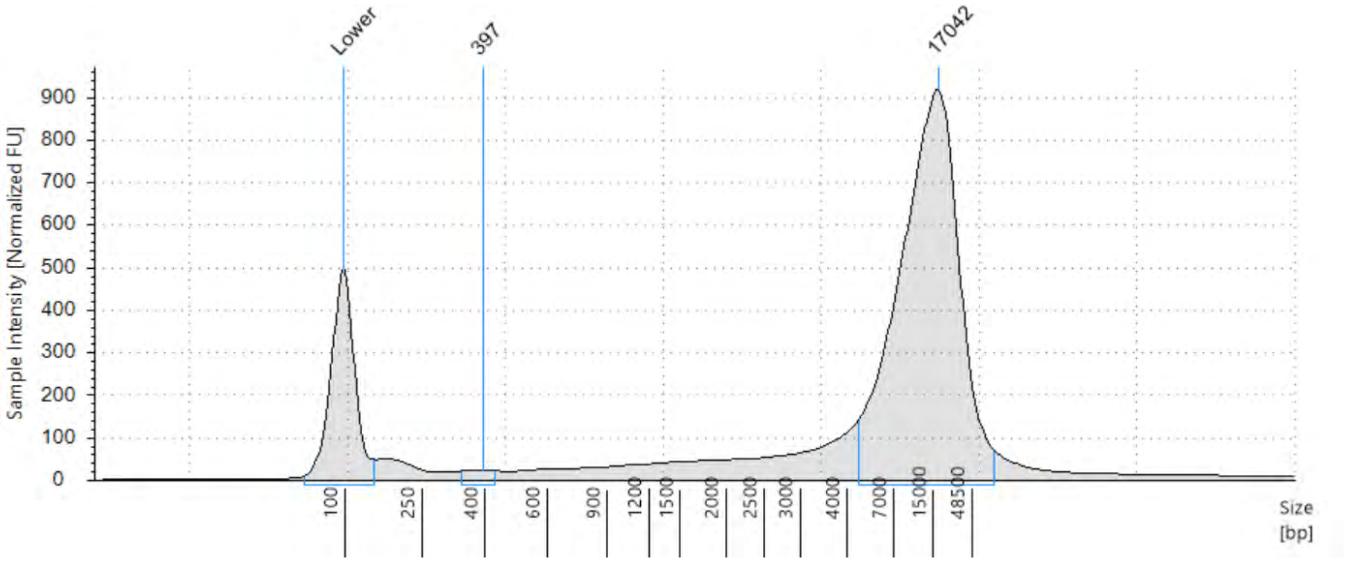

Sample Table

Well	DIN	Conc. [ng/µl]	Sample Description	Alert	Observations
F4	7.0	55.4	C624		

Peak Table

Size [bp]	Calibrated Conc. [ng/µl]	Assigned Conc. [ng/µl]	% Integrated Area	From [bp]	To [bp]	Peak Comment	Observations
100	8.50	8.50	-	63	145		Lower Marker
397	0.421	-	0.98	338	431		
17042	39.5	-	91.57	4701	>60000		
-	-	-	-	-	-		Sample Well

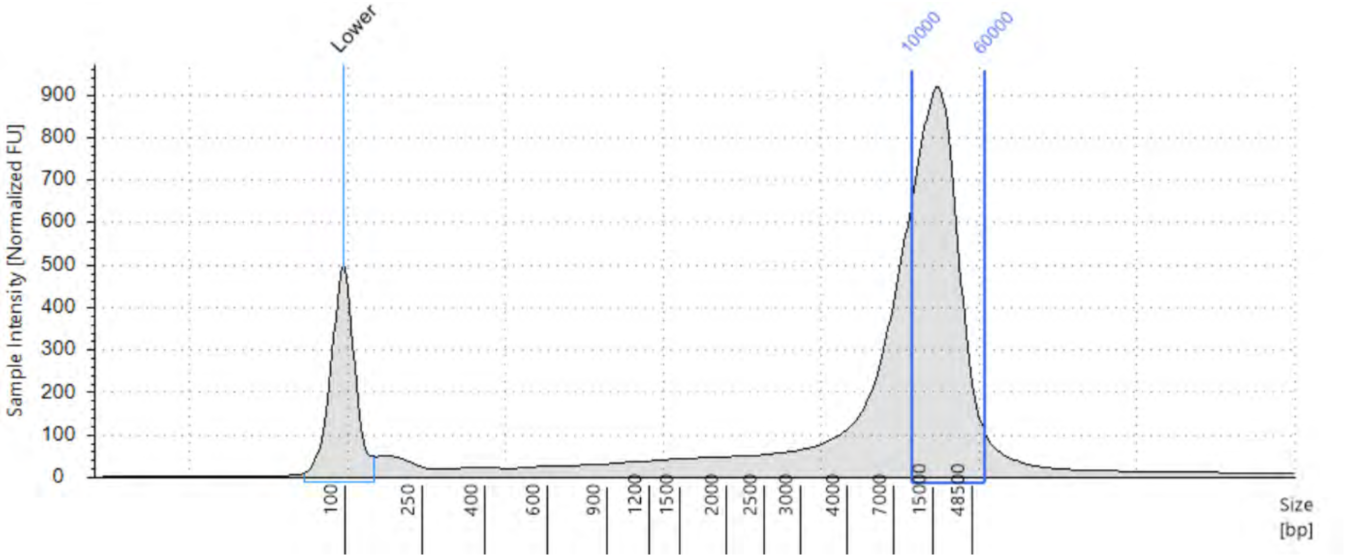

Region Table

From [bp]	To [bp]	Average Size [bp]	Conc. [ng/µl]	Region Molarity [nmol/l]	% of Total	Region Comment	Color
10000	60000	19989	28.0	2.58	50.54		■

 © Agilent Technologies, Inc. 2017

G4: C625

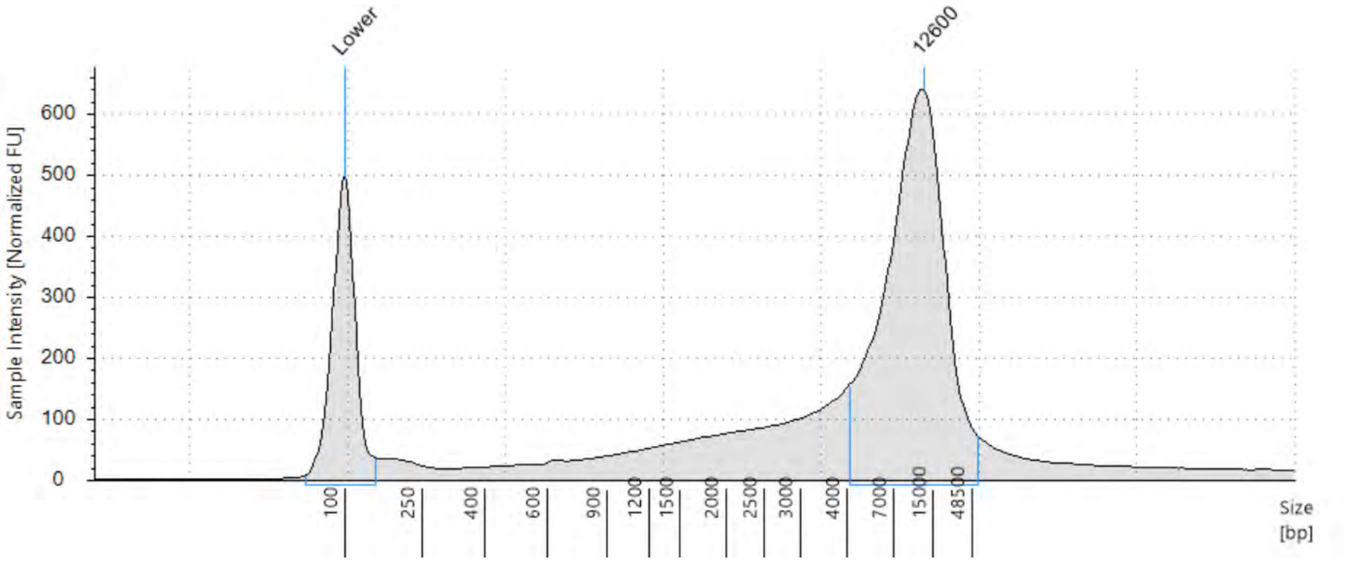

Sample Table

Well	DIN	Conc. [ng/µl]	Sample Description	Alert	Observations
G4	6.5	50.5	C625		

Peak Table

Size [bp]	Calibrated Conc. [ng/µl]	Assigned Conc. [ng/µl]	% Integrated Area	From [bp]	To [bp]	Peak Comment	Observations
100	8.50	8.50	-	63	145		Lower Marker
12600	28.1	-	90.36	4131	54398		
-	-	-	-	-	-		Sample Well

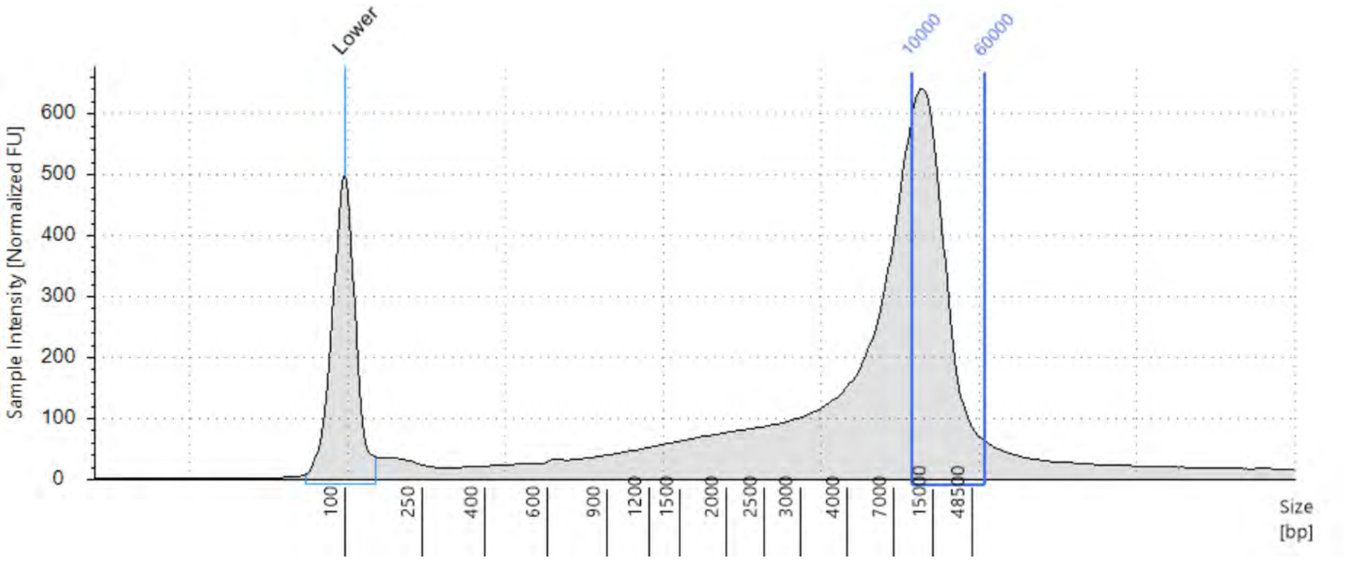

Region Table

From [bp]	To [bp]	Average Size [bp]	Conc. [ng/µl]	Region Molarity [nmol/l]	% of Total	Region Comment	Color
10000	60000	17784	15.7	1.61	31.12		

H4: C626

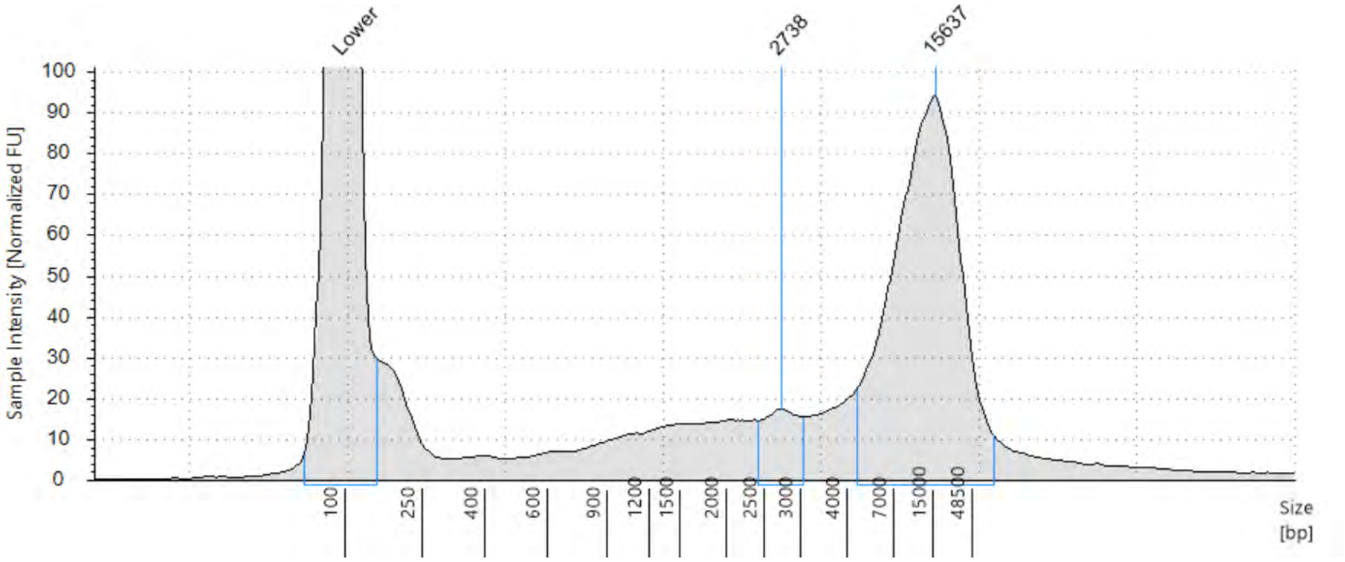

Sample Table

Well	DIN	Conc. [ng/µl]	Sample Description	Alert	Observations
H4	6.7	8.98	C626	⚠	Sample concentration outside recommended range

Peak Table

Size [bp]	Calibrated Conc. [ng/µl]	Assigned Conc. [ng/µl]	% Integrated Area	From [bp]	To [bp]	Peak Comment	Observations
100	8.50	8.50	-	61	146		Lower Marker
2738	0.461	-	8.39	2422	3074		
15637	4.75	-	86.42	4546	>60000		
-	-	-	-	-	-		Sample Well

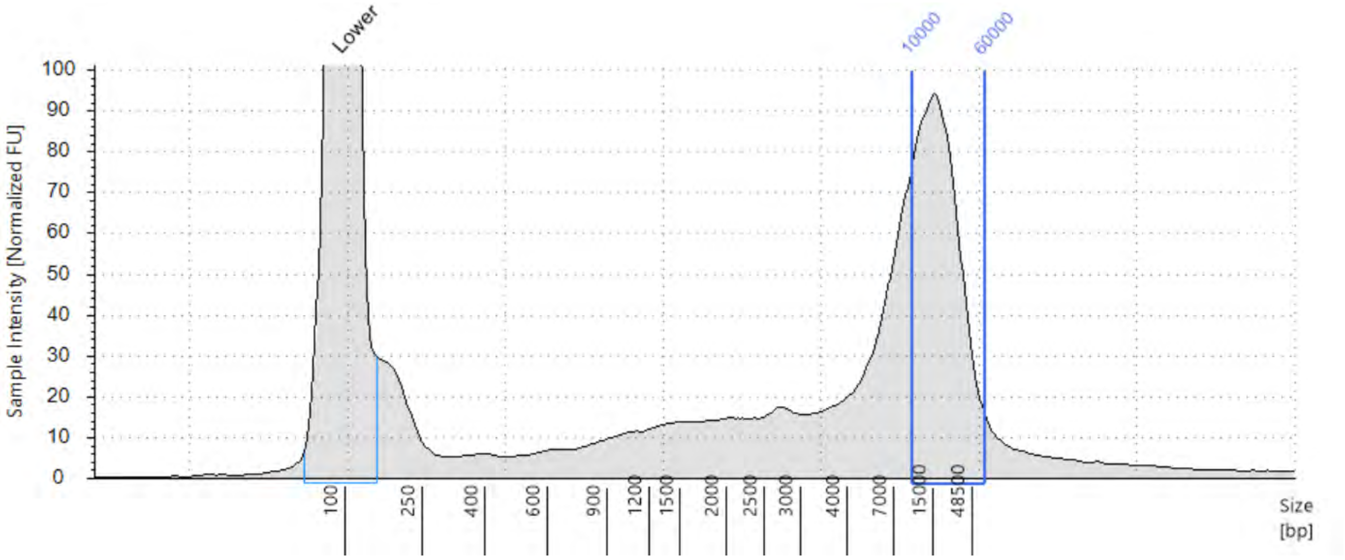

Region Table

From [bp]	To [bp]	Average Size [bp]	Conc. [ng/µl]	Region Molarity [nmol/l]	% of Total	Region Comment	Color
10000	60000	20495	3.17	0.294	35.26		■

 © Agilent Technologies, Inc. 2017

A5: C627

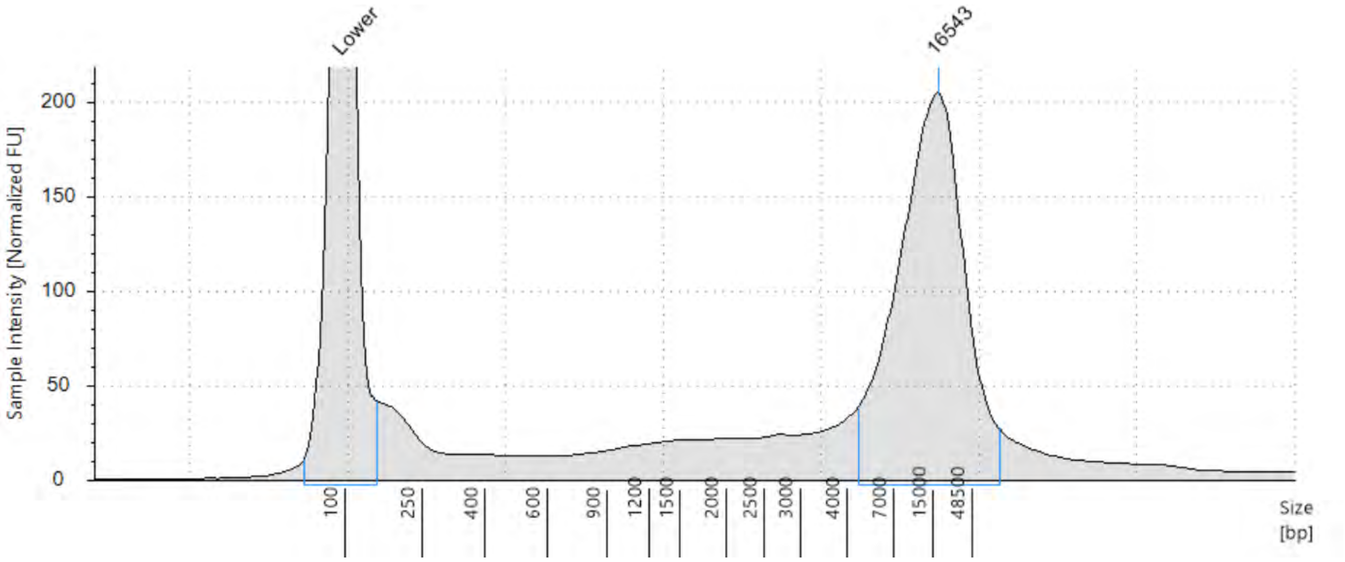

Sample Table

Well	DIN	Conc. [ng/µl]	Sample Description	Alert	Observations
A5	6.7	17.5	C627		

Peak Table

Size [bp]	Calibrated Conc. [ng/µl]	Assigned Conc. [ng/µl]	% Integrated Area	From [bp]	To [bp]	Peak Comment	Observations
100	8.50	8.50	-	62	145		Lower Marker
16543	9.69	-	95.25	4586	>60000		
-	-	-	-	-	-		Sample Well

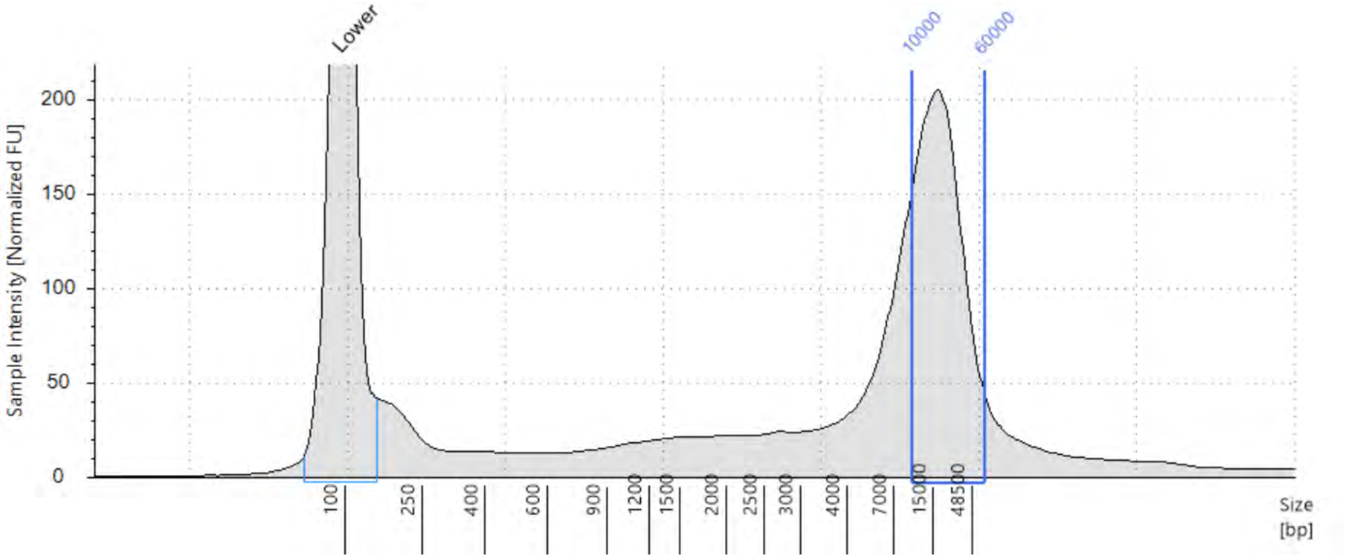

Region Table

From [bp]	To [bp]	Average Size [bp]	Conc. [ng/µl]	Region Molarity [nmol/l]	% of Total	Region Comment	Color
10000	60000	21273	6.71	0.602	38.43		

B5: C628

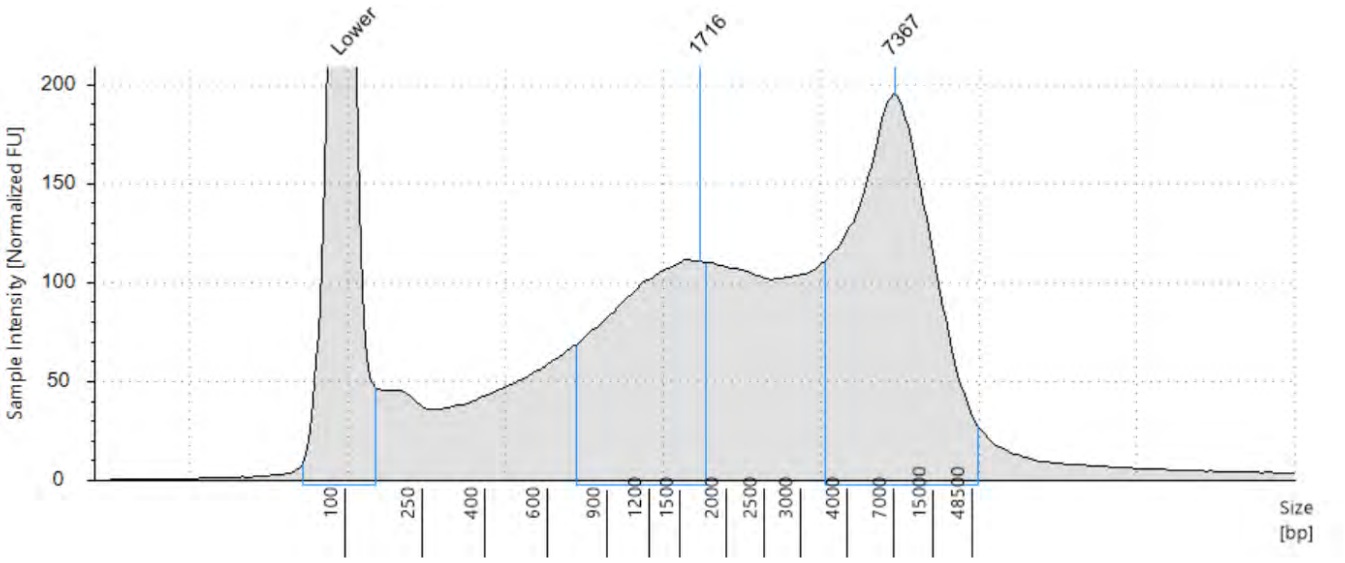

Sample Table

Well	DIN	Conc. [ng/µl]	Sample Description	Alert	Observations
B5	4.6	32.4	C628		

Peak Table

Size [bp]	Calibrated Conc. [ng/µl]	Assigned Conc. [ng/µl]	% Integrated Area	From [bp]	To [bp]	Peak Comment	Observations
100	8.50	8.50	-	62	147		Lower Marker
1716	7.27	-	38.36	735	1776		
7367	11.4	-	60.19	3519	54348		
-	-	-	-	-	-		Sample Well

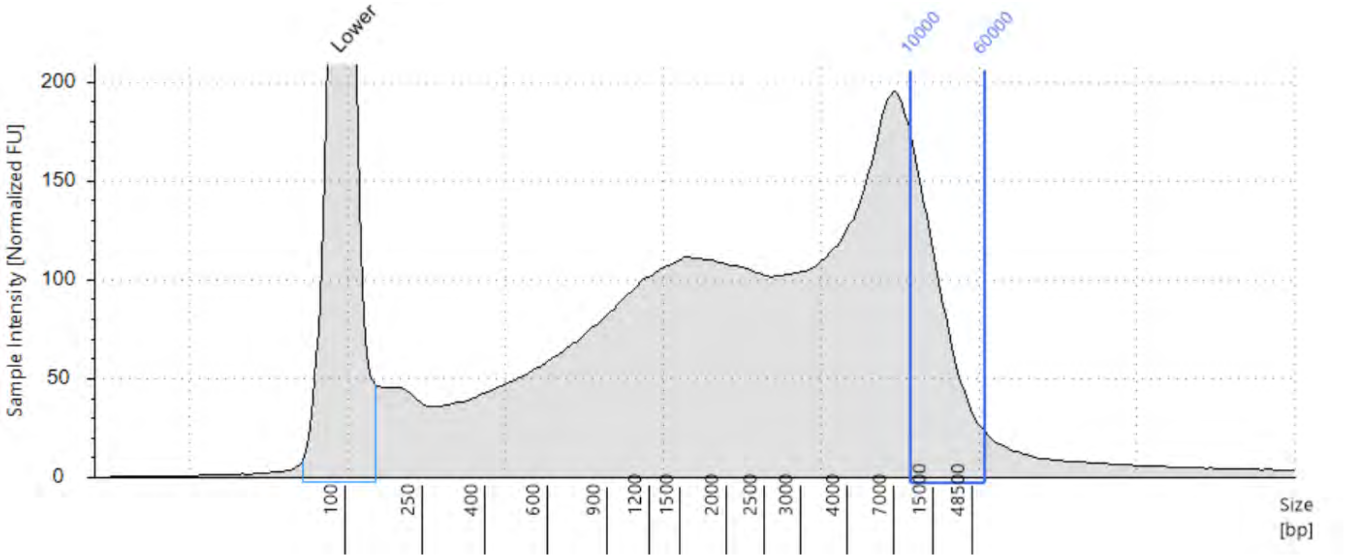

Region Table

From [bp]	To [bp]	Average Size [bp]	Conc. [ng/µl]	Region Molarity [nmol/l]	% of Total	Region Comment	Color
10000	60000	19087	3.71	0.378	11.44		

C5: C629

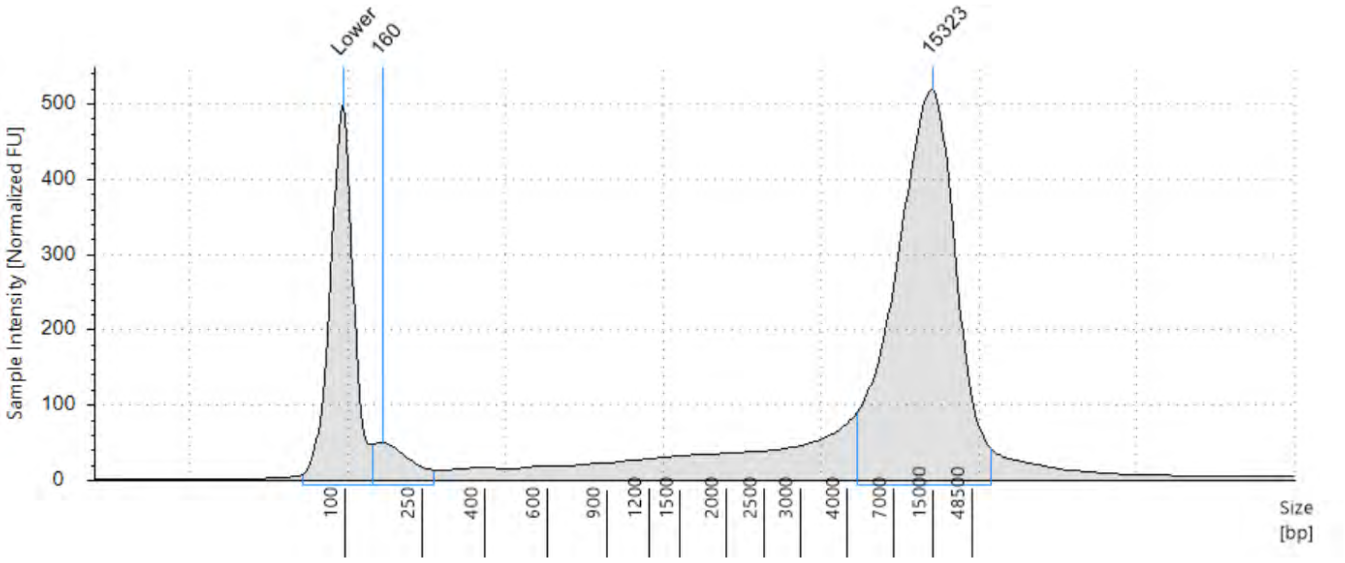

Sample Table

Well	DIN	Conc. [ng/µl]	Sample Description	Alert	Observations
C5	6.9	34.6	C629		

Peak Table

Size [bp]	Calibrated Conc. [ng/µl]	Assigned Conc. [ng/µl]	% Integrated Area	From [bp]	To [bp]	Peak Comment	Observations
100	8.50	8.50	-	62	140		Lower Marker
160	1.29	-	5.07	140	275		
15323	23.2	-	91.36	4599	>60000		
-	-	-	-	-	-		Sample Well

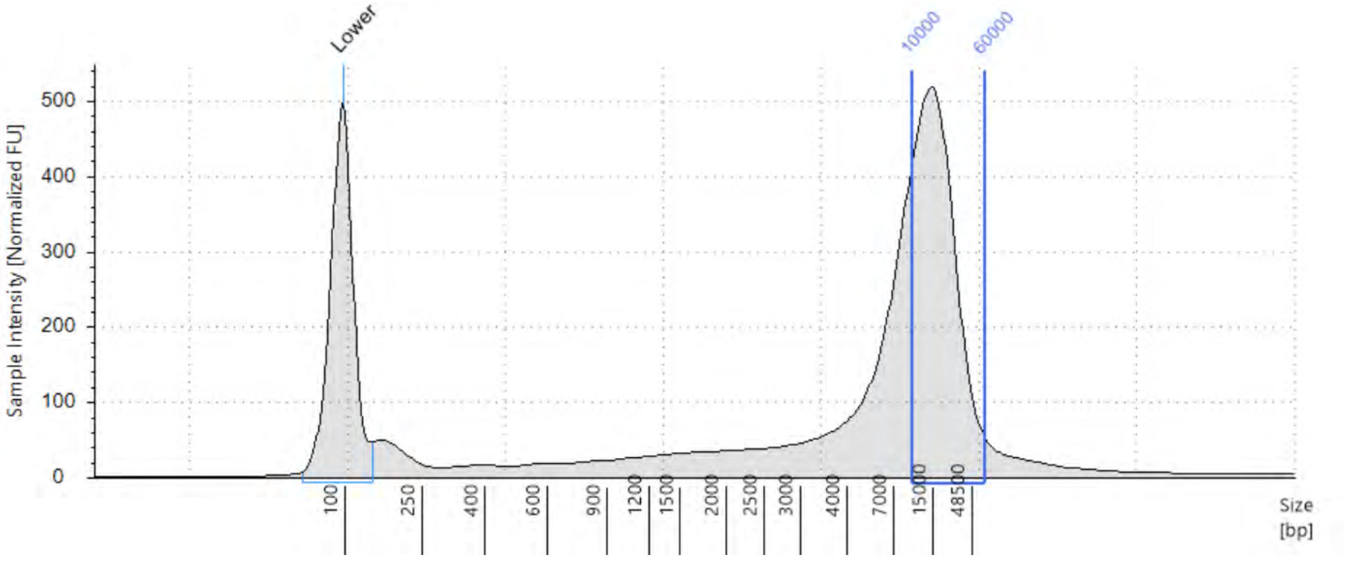

Region Table

From [bp]	To [bp]	Average Size [bp]	Conc. [ng/µl]	Region Molarity [nmol/l]	% of Total	Region Comment	Color
10000	60000	19262	15.9	1.51	45.80		■

© Agilent Technologies, Inc. 2017

D5: C630

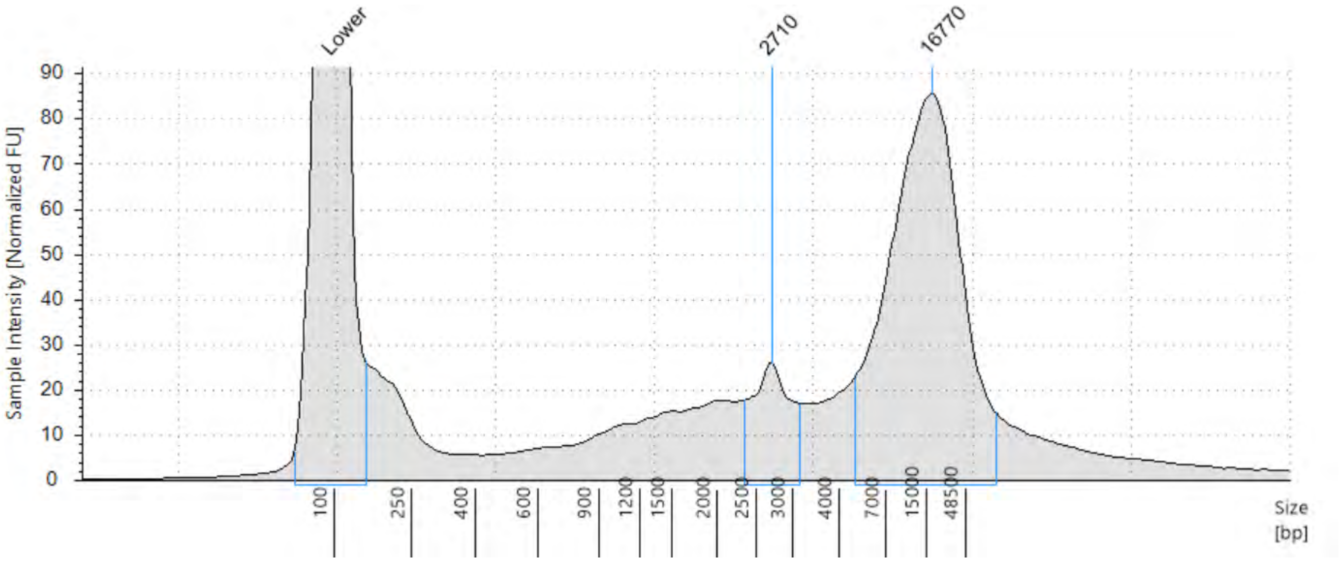

Sample Table

Well	DIN	Conc. [ng/µl]	Sample Description	Alert	Observations
D5	6.6	9.82	C630	⚠	Sample concentration outside recommended range

Peak Table

Size [bp]	Calibrated Conc. [ng/µl]	Assigned Conc. [ng/µl]	% Integrated Area	From [bp]	To [bp]	Peak Comment	Observations
100	8.50	8.50	-	63	145		Lower Marker
2710	0.720	-	12.74	2341	3138		
16770	4.65	-	82.42	4862	>60000		
-	-	-	-	-	-		Sample Well

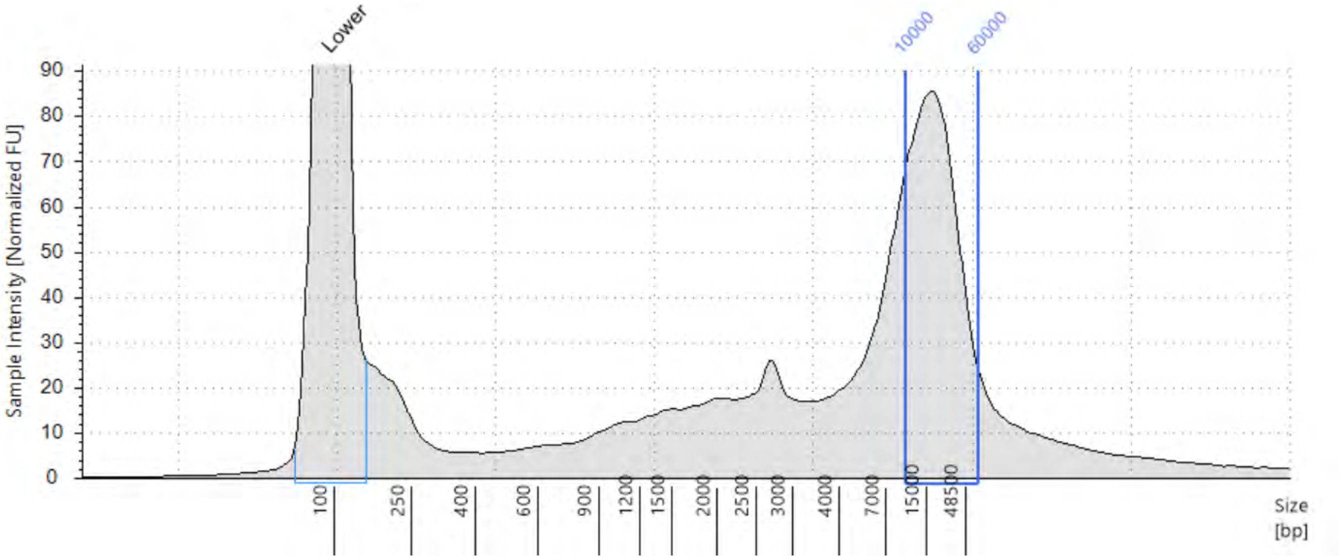

Region Table

From [bp]	To [bp]	Average Size [bp]	Conc. [ng/µl]	Region Molarity [nmol/l]	% of Total	Region Comment	Color
10000	60000	22312	3.15	0.274	32.03		■

E5: C370

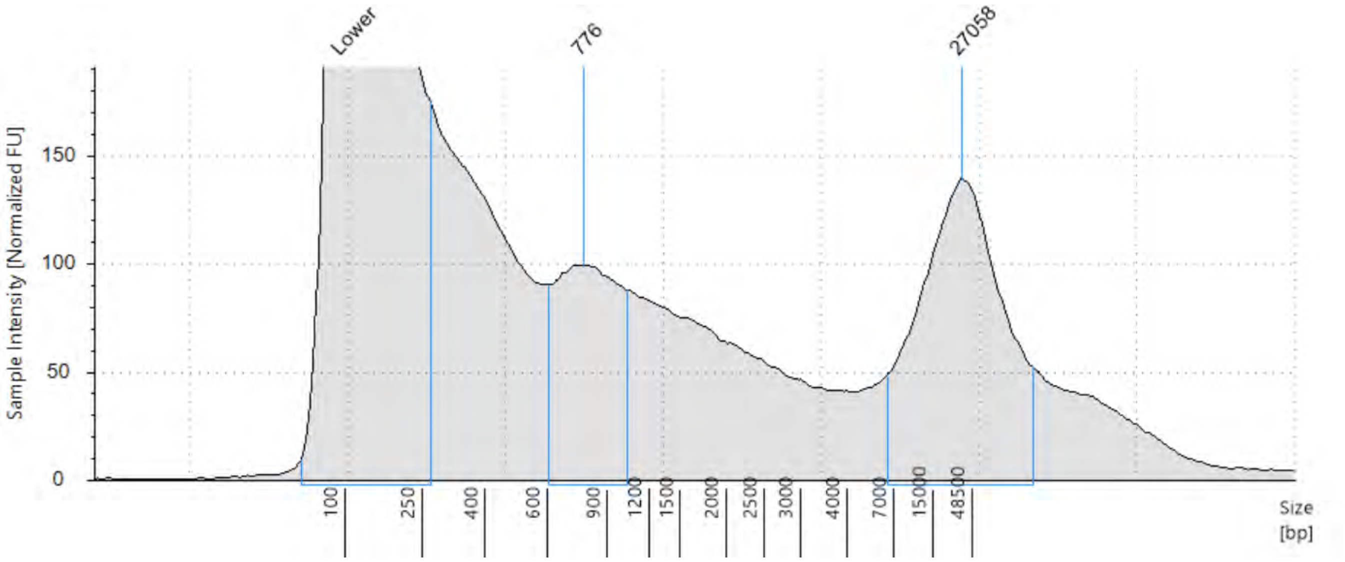

Sample Table

Well	DIN	Conc. [ng/µl]	Sample Description	Alert	Observations
E5	1.0	14.2	C370		

Peak Table

Size [bp]	Calibrated Conc. [ng/µl]	Assigned Conc. [ng/µl]	% Integrated Area	From [bp]	To [bp]	Peak Comment	Observations
100	8.50	8.50	-	61	269		Lower Marker
776	1.88	-	34.47	612	1044		
27058	3.36	-	61.64	6577	>60000		
-	-	-	-	-	-		Sample Well

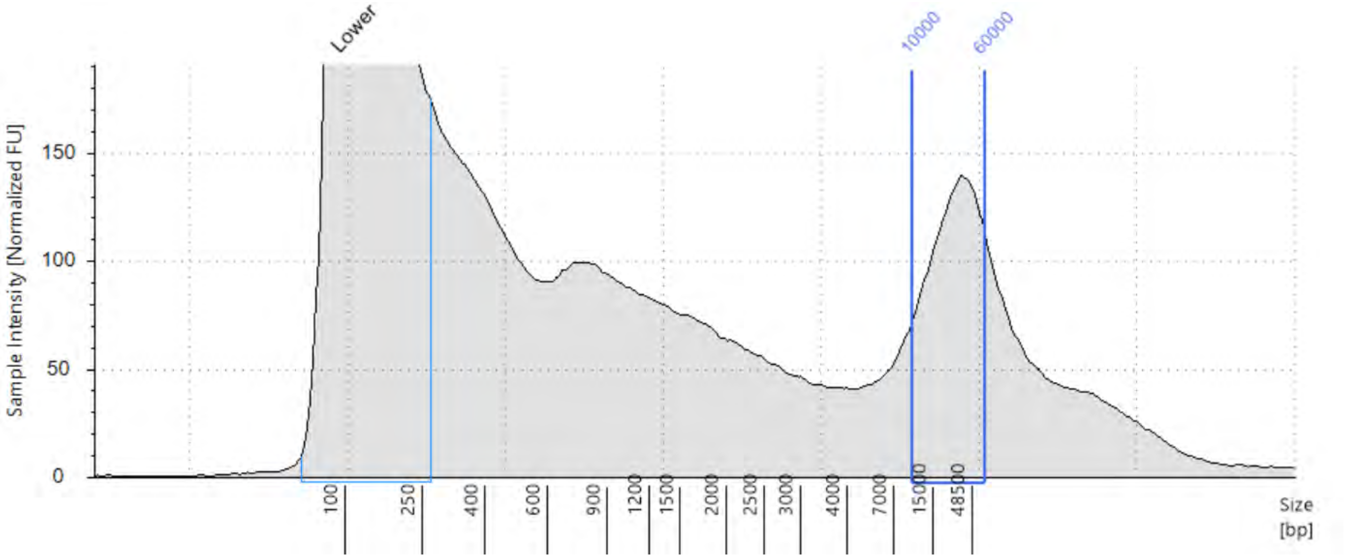

Region Table

From [bp]	To [bp]	Average Size [bp]	Conc. [ng/µl]	Region Molarity [nmol/l]	% of Total	Region Comment	Color
10000	60000	28441	2.12	0.154	14.97		

 © Agilent Technologies, Inc. 2017

F5: C371

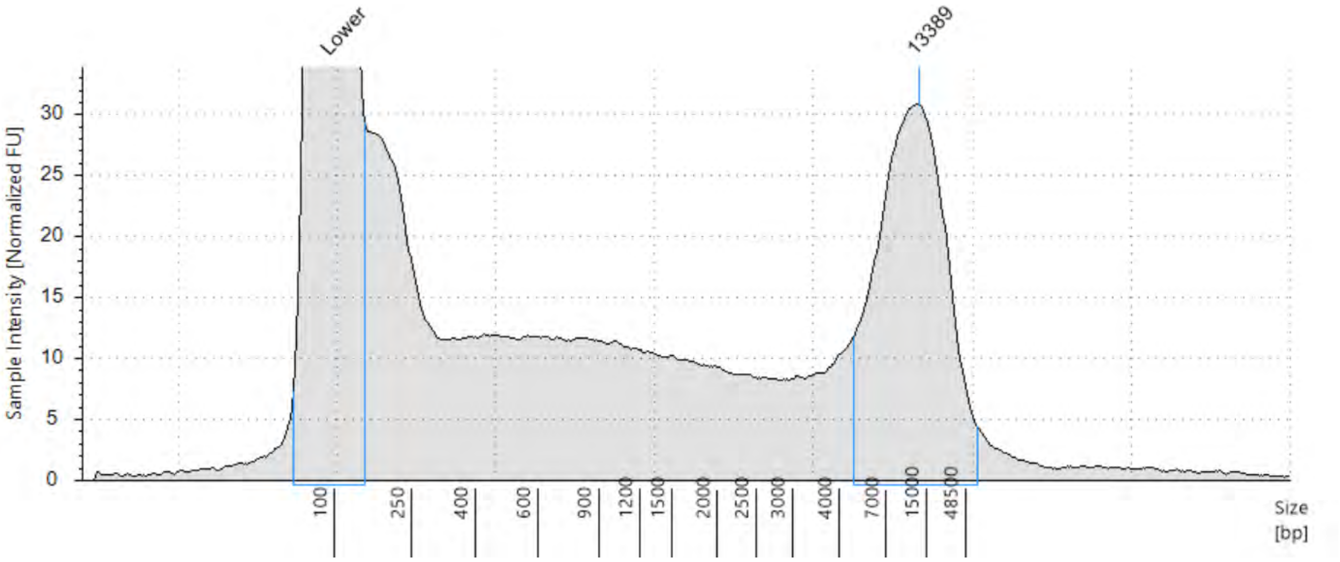

Sample Table

Well	DIN	Conc. [ng/µl]	Sample Description	Alert	Observations
F5	4.5	5.09	C371	⚠	Sample concentration outside recommended range

Peak Table

Size [bp]	Calibrated Conc. [ng/µl]	Assigned Conc. [ng/µl]	% Integrated Area	From [bp]	To [bp]	Peak Comment	Observations
100	8.50	8.50	-	63	147		Lower Marker
13389	1.52	-	100.00	4850	>60000		

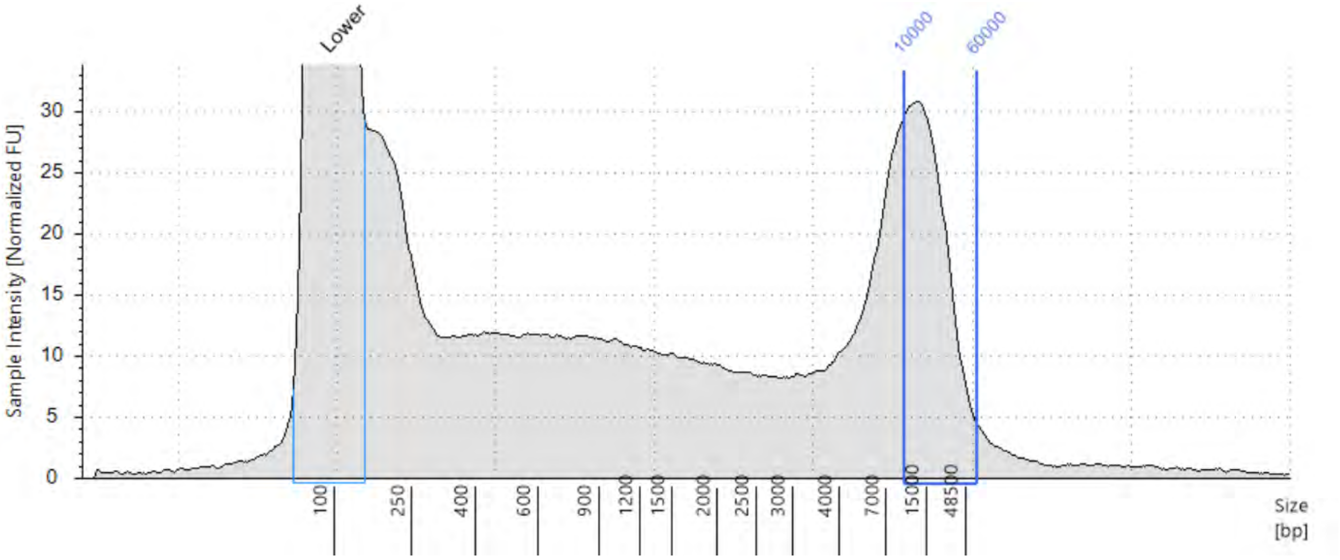

Region Table

From [bp]	To [bp]	Average Size [bp]	Conc. [ng/µl]	Region Molarity [nmol/l]	% of Total	Region Comment	Color
10000	60000	19511	0.918	0.0903	18.03		■

G5: C372

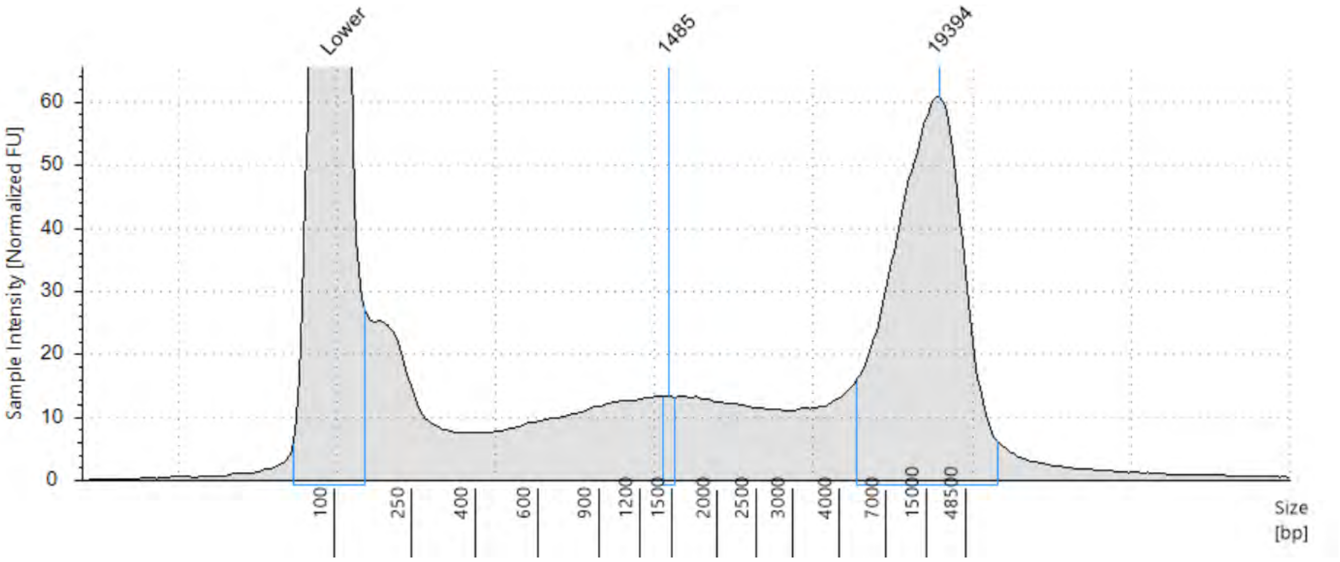

Sample Table

Well	DIN	Conc. [ng/µl]	Sample Description	Alert	Observations
G5	6.3	7.05	C372	⚠	Sample concentration outside recommended range

Peak Table

Size [bp]	Calibrated Conc. [ng/µl]	Assigned Conc. [ng/µl]	% Integrated Area	From [bp]	To [bp]	Peak Comment	Observations
100	8.50	8.50	-	63	145		Lower Marker
1485	0.111	-	3.24	1416	1539		
19394	3.20	-	93.29	5016	>60000		
-	-	-	-	-	-		Sample Well

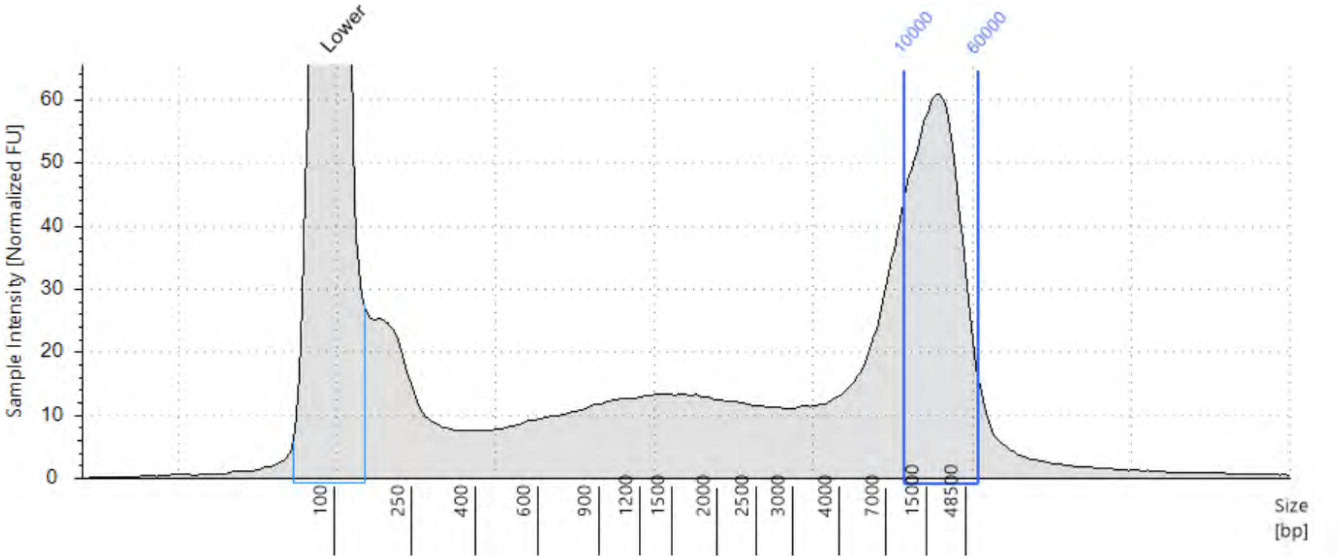

Region Table

From [bp]	To [bp]	Average Size [bp]	Conc. [ng/µl]	Region Molarity [nmol/l]	% of Total	Region Comment	Color
10000	60000	23080	2.27	0.193	32.20		■

H5: C373

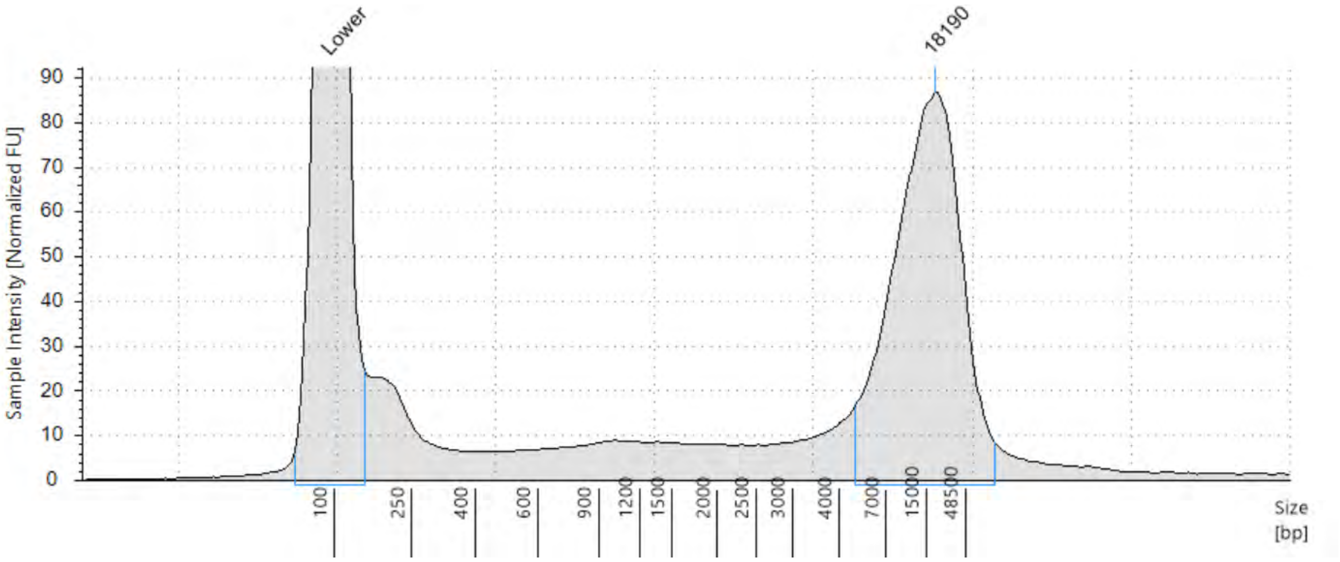

Sample Table

Well	DIN	Conc. [ng/µl]	Sample Description	Alert	Observations
H5	7.0	7.63	C373	⚠	Sample concentration outside recommended range

Peak Table

Size [bp]	Calibrated Conc. [ng/µl]	Assigned Conc. [ng/µl]	% Integrated Area	From [bp]	To [bp]	Peak Comment	Observations
100	8.50	8.50	-	63	145		Lower Marker
18190	4.39	-	96.80	4902	>60000		
-	-	-	-	-	-		Sample Well

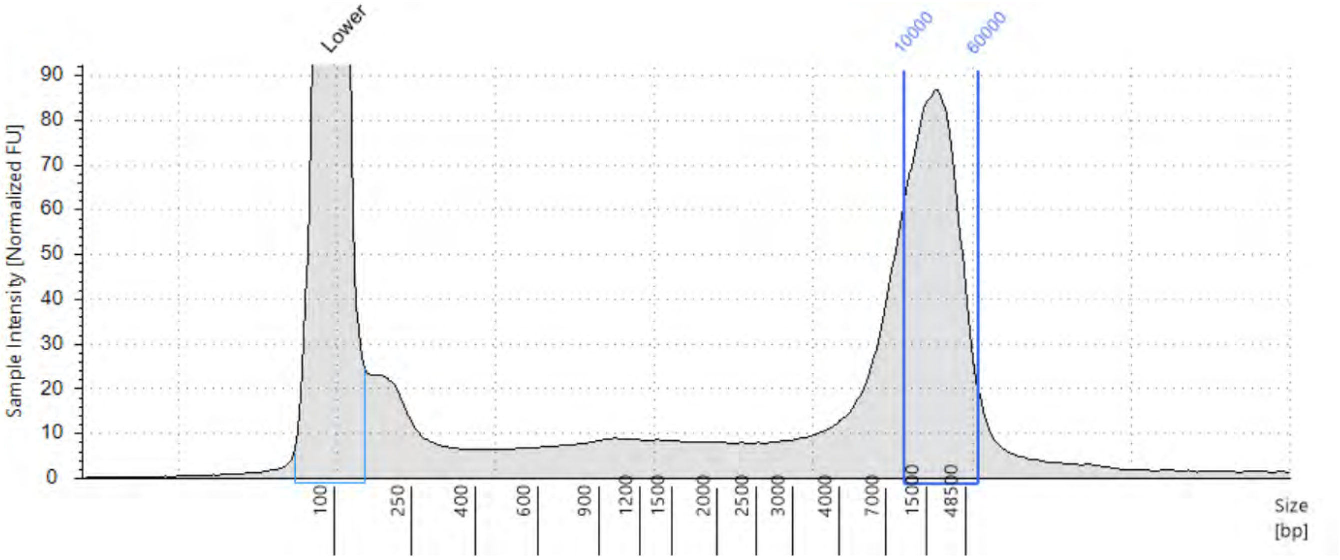

Region Table

From [bp]	To [bp]	Average Size [bp]	Conc. [ng/µl]	Region Molarity [nmol/l]	% of Total	Region Comment	Color
10000	60000	22362	3.20	0.277	41.96		■

A6: C376

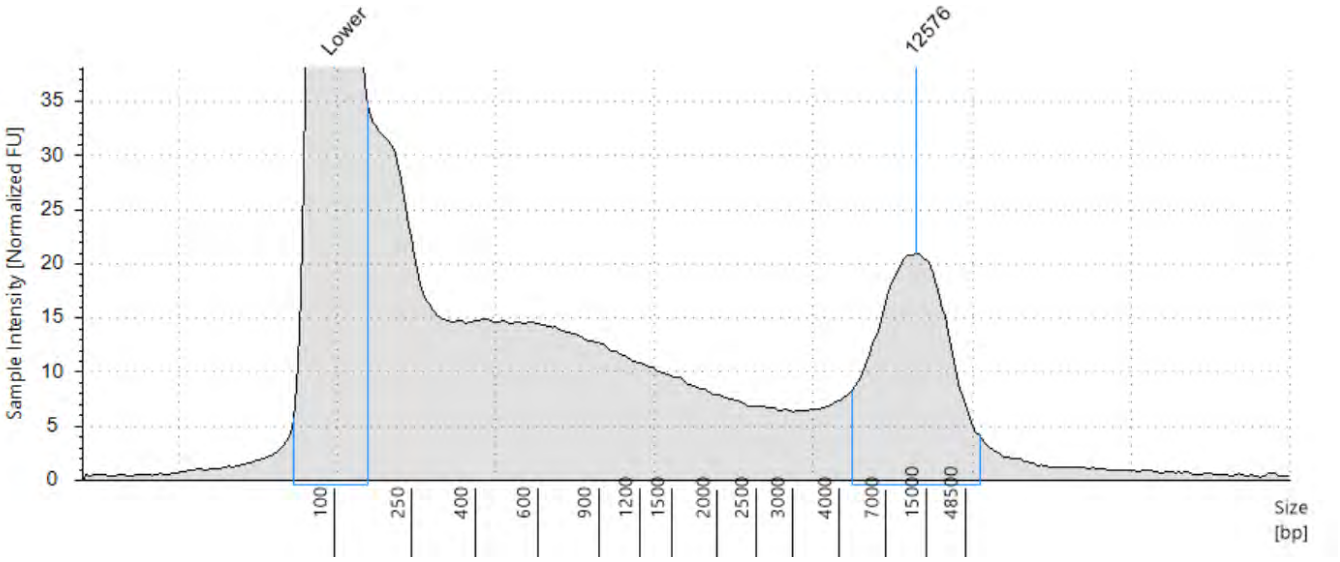

Sample Table

Well	DIN	Conc. [ng/µl]	Sample Description	Alert	Observations
A6	1.2	5.06	C376	⚠	Sample concentration outside recommended range

Peak Table

Size [bp]	Calibrated Conc. [ng/µl]	Assigned Conc. [ng/µl]	% Integrated Area	From [bp]	To [bp]	Peak Comment	Observations
100	8.50	8.50	-	62	150		Lower Marker
12576	1.12	-	100.00	4693	>60000		

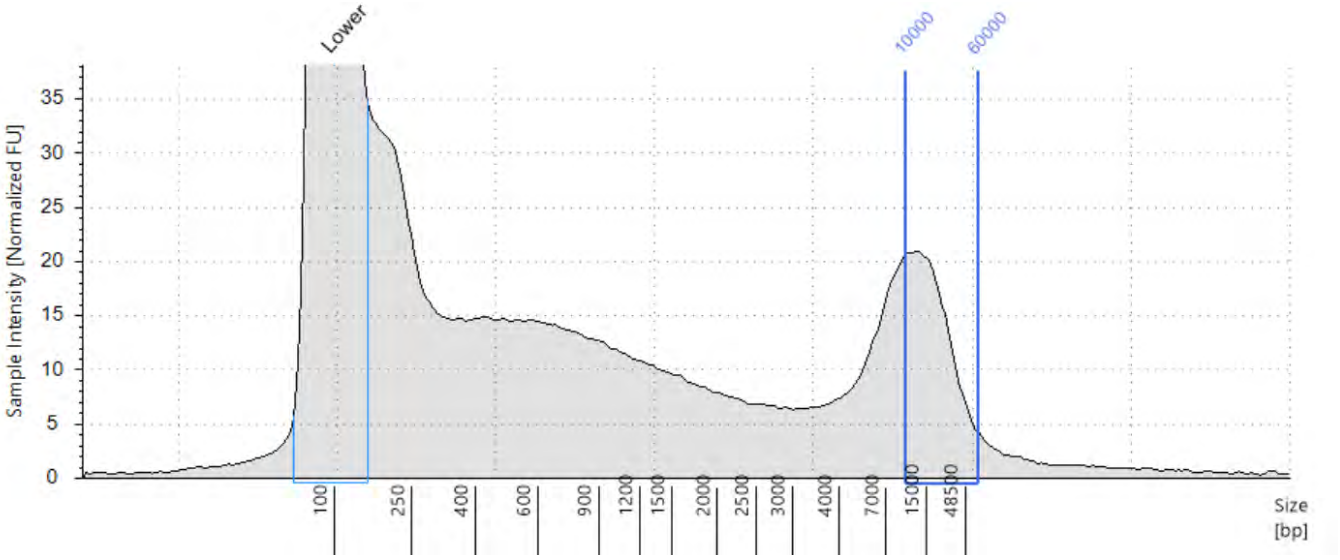

Region Table

From [bp]	To [bp]	Average Size [bp]	Conc. [ng/µl]	Region Molarity [nmol/l]	% of Total	Region Comment	Color
10000	60000	20487	0.656	0.0629	12.96		■

 © Agilent Technologies, Inc. 2017

B6: C377

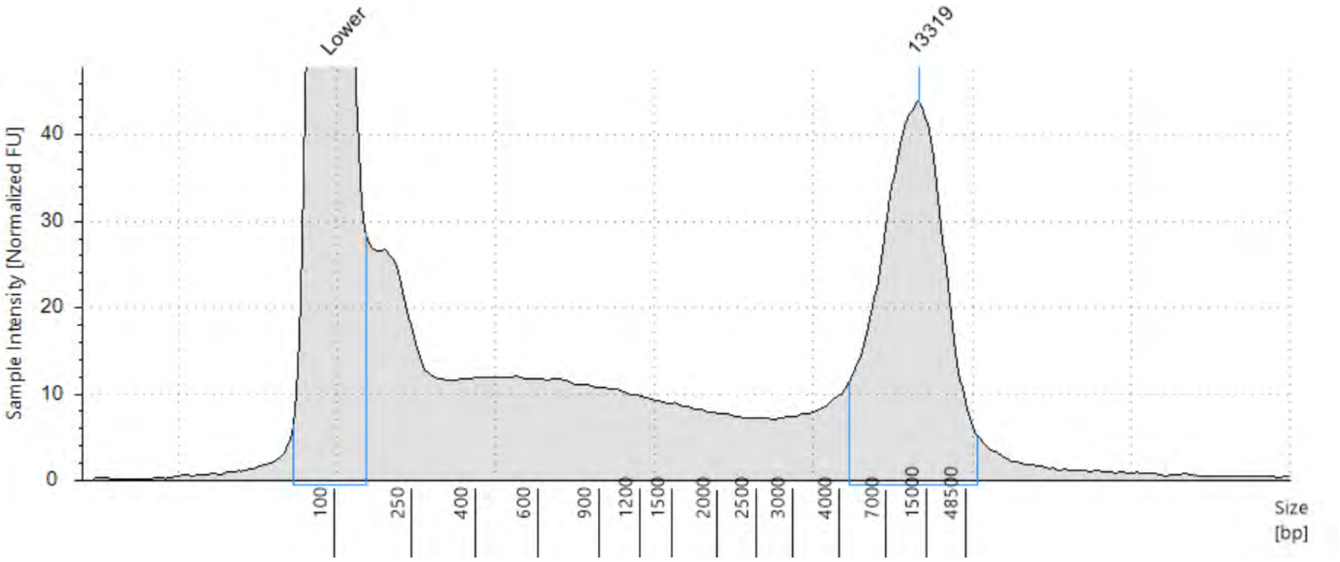

Sample Table

Well	DIN	Conc. [ng/µl]	Sample Description	Alert	Observations
B6	5.7	5.54	C377	⚠	Sample concentration outside recommended range

Peak Table

Size [bp]	Calibrated Conc. [ng/µl]	Assigned Conc. [ng/µl]	% Integrated Area	From [bp]	To [bp]	Peak Comment	Observations
100	8.50	8.50	-	63	147		Lower Marker
13319	2.07	-	97.83	4582	>60000		
-	-	-	-	-	-		Sample Well

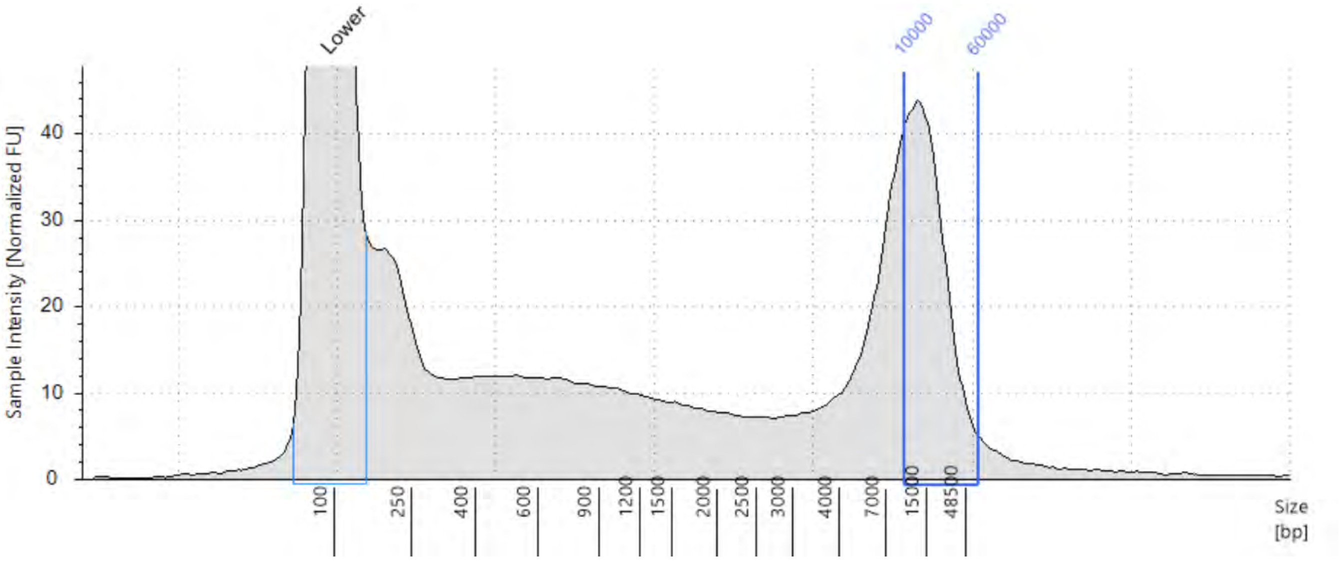

Region Table

From [bp]	To [bp]	Average Size [bp]	Conc. [ng/µl]	Region Molarity [nmol/l]	% of Total	Region Comment	Color
10000	60000	18806	1.27	0.127	22.98		■

 © Agilent Technologies, Inc. 2017

C6: C380

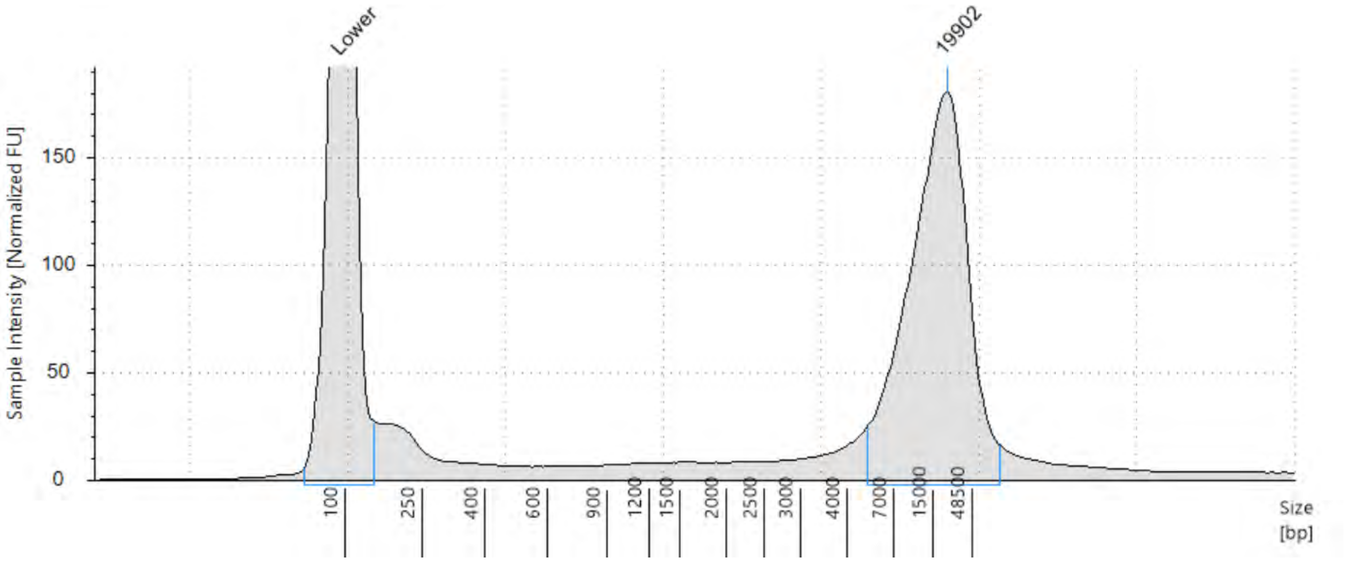

Sample Table

Well	DIN	Conc. [ng/µl]	Sample Description	Alert	Observations
C6	7.5	12.3	C380		

Peak Table

Size [bp]	Calibrated Conc. [ng/µl]	Assigned Conc. [ng/µl]	% Integrated Area	From [bp]	To [bp]	Peak Comment	Observations
100	8.50	8.50	-	63	145		Lower Marker
19902	8.02	-	93.81	5181	>60000		
-	-	-	-	-	-		Sample Well

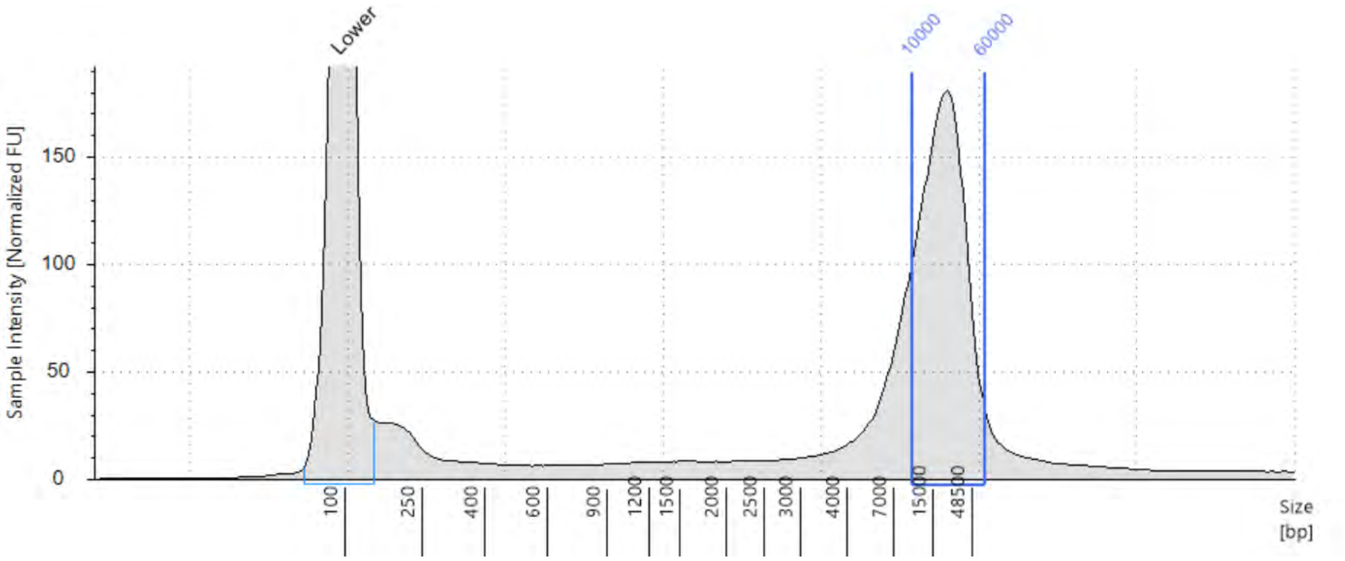

Region Table

From [bp]	To [bp]	Average Size [bp]	Conc. [ng/µl]	Region Molarity [nmol/l]	% of Total	Region Comment	Color
10000	60000	22527	6.26	0.526	50.80		

D6: C381

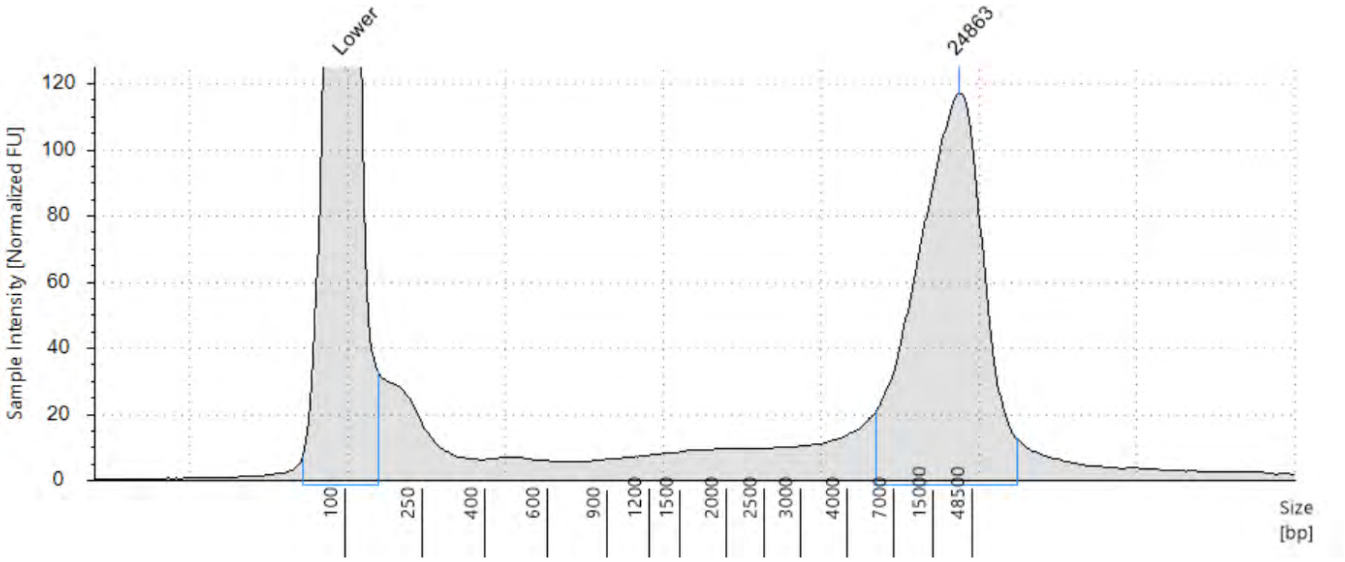

Sample Table

Well	DIN	Conc. [ng/µl]	Sample Description	Alert	Observations
D6	7.5	8.41	C381	⚠	Sample concentration outside recommended range

Peak Table

Size [bp]	Calibrated Conc. [ng/µl]	Assigned Conc. [ng/µl]	% Integrated Area	From [bp]	To [bp]	Peak Comment	Observations
100	8.50	8.50	-	60	148		Lower Marker
24863	5.14	-	97.35	5680	>60000		
-	-	-	-	-	-		Sample Well

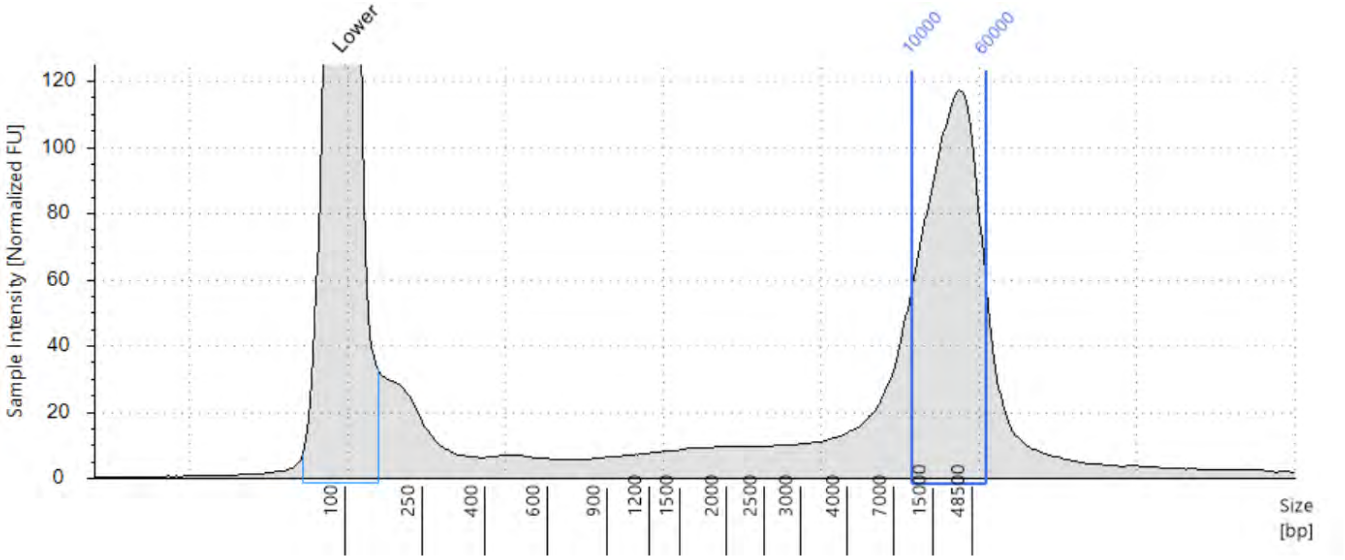

Region Table

From [bp]	To [bp]	Average Size [bp]	Conc. [ng/µl]	Region Molarity [nmol/l]	% of Total	Region Comment	Color
10000	60000	26618	3.93	0.298	46.73		■

G6: C384

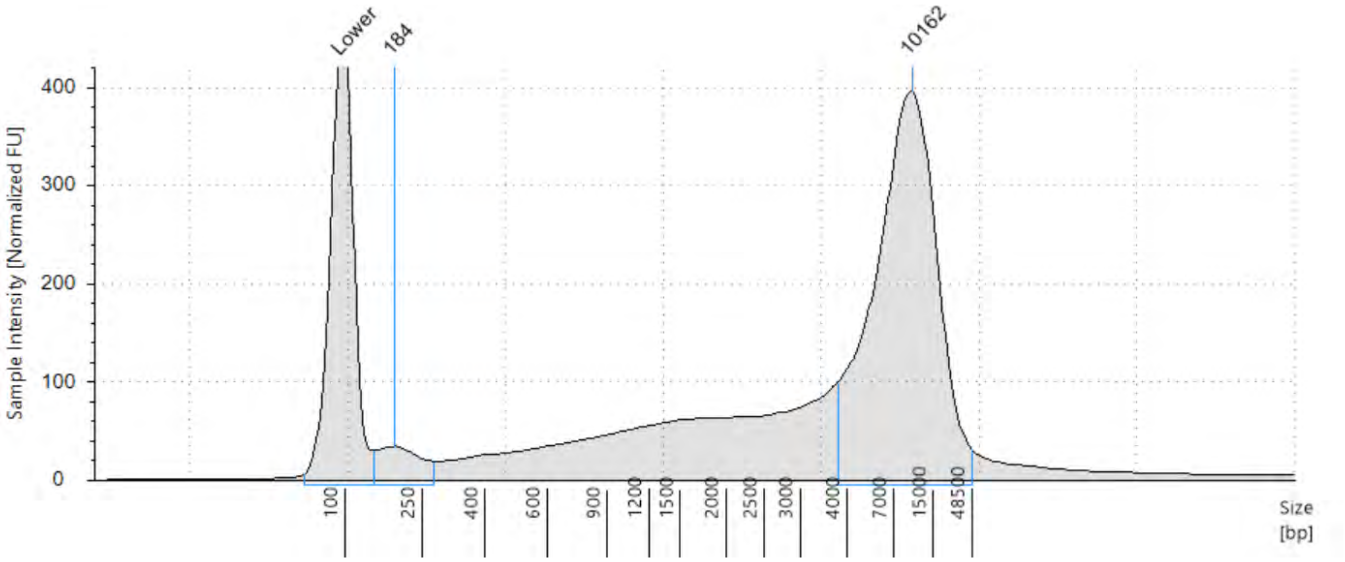

Sample Table

Well	DIN	Conc. [ng/µl]	Sample Description	Alert	Observations
G6	6.1	35.4	C384		

Peak Table

Size [bp]	Calibrated Conc. [ng/µl]	Assigned Conc. [ng/µl]	% Integrated Area	From [bp]	To [bp]	Peak Comment	Observations
100	8.50	8.50	-	63	142		Lower Marker
184	1.07	-	5.09	145	277		
10162	19.2	-	91.36	3804	49059		
-	-	-	-	-	-		Sample Well

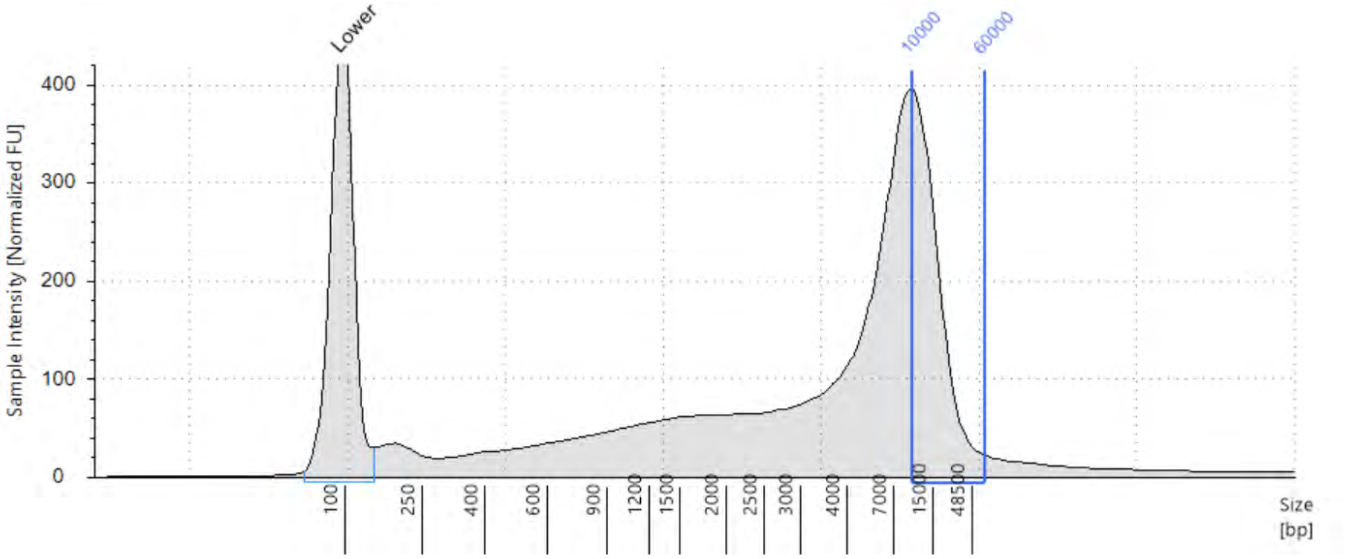

Region Table

From [bp]	To [bp]	Average Size [bp]	Conc. [ng/µl]	Region Molarity [nmol/l]	% of Total	Region Comment	Color
10000	60000	16638	8.46	0.918	23.89		

H6: C385

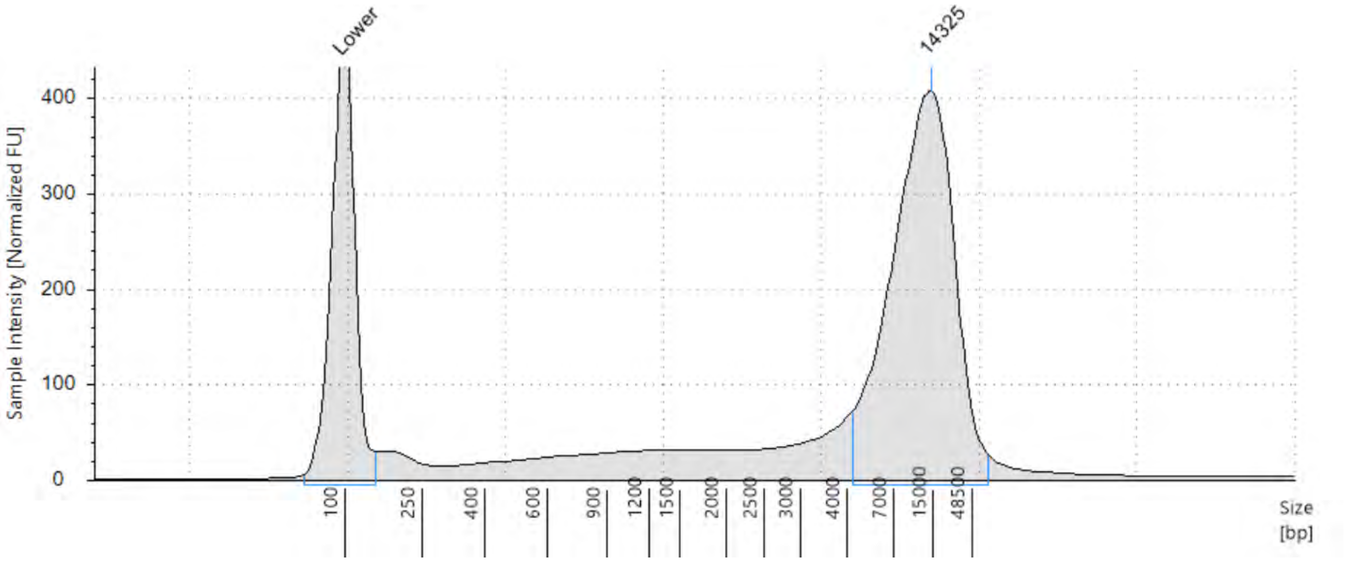

Sample Table

Well	DIN	Conc. [ng/µl]	Sample Description	Alert	Observations
H6	6.6	29.5	C385		

Peak Table

Size [bp]	Calibrated Conc. [ng/µl]	Assigned Conc. [ng/µl]	% Integrated Area	From [bp]	To [bp]	Peak Comment	Observations
100	8.50	8.50	-	61	143		Lower Marker
14325	19.6	-	95.90	4255	>60000		
-	-	-	-	-	-		Sample Well

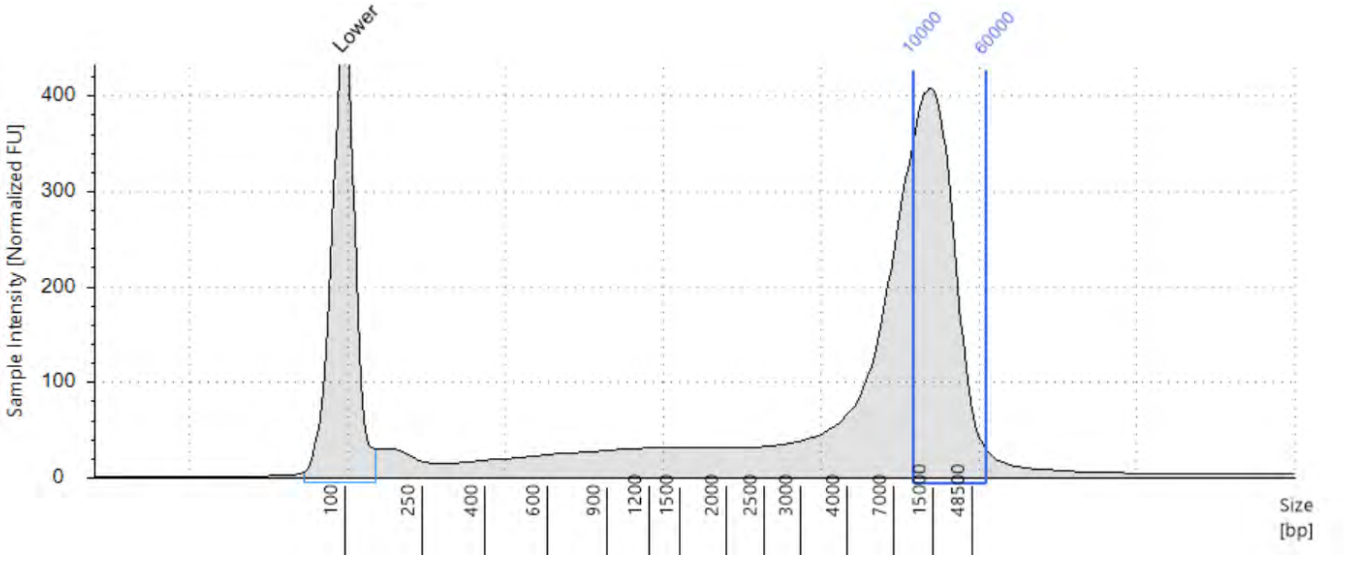

Region Table

From [bp]	To [bp]	Average Size [bp]	Conc. [ng/µl]	Region Molarity [nmol/l]	% of Total	Region Comment	Color
10000	60000	18493	12.3	1.19	41.58		■

A7: C386

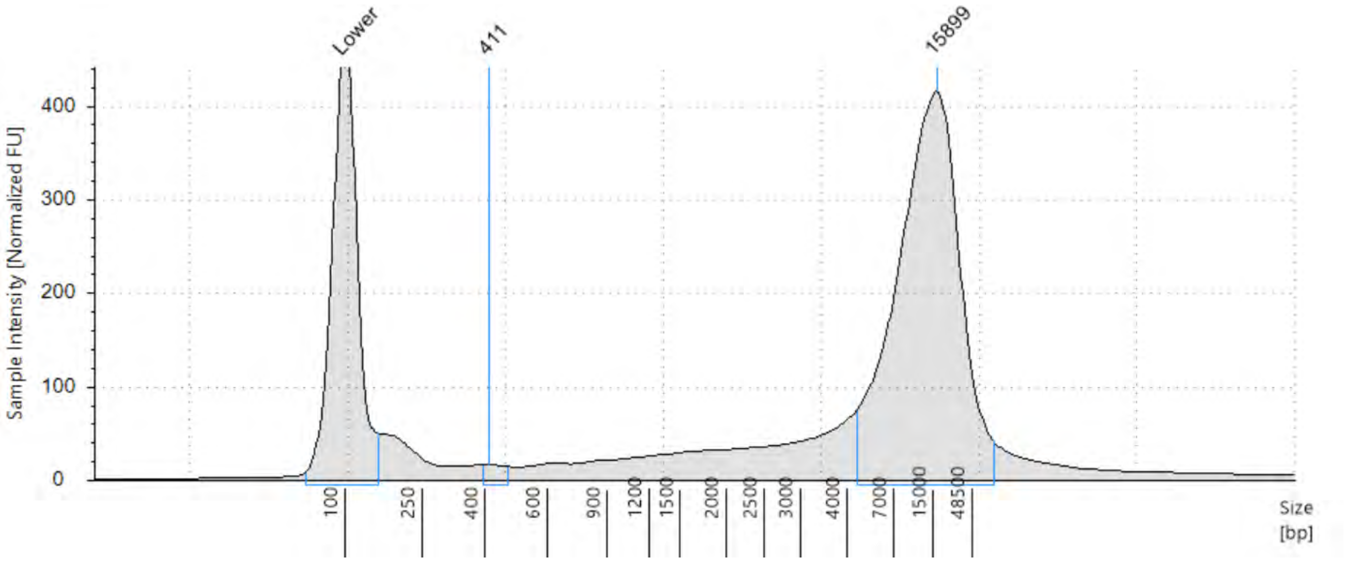

Sample Table

Well	DIN	Conc. [ng/µl]	Sample Description	Alert	Observations
A7	6.8	28.5	C386		

Peak Table

Size [bp]	Calibrated Conc. [ng/µl]	Assigned Conc. [ng/µl]	% Integrated Area	From [bp]	To [bp]	Peak Comment	Observations
100	8.50	8.50	-	62	148		Lower Marker
411	0.219	-	1.12	392	462		
15899	18.3	-	93.93	4542	>60000		
-	-	-	-	-	-		Sample Well

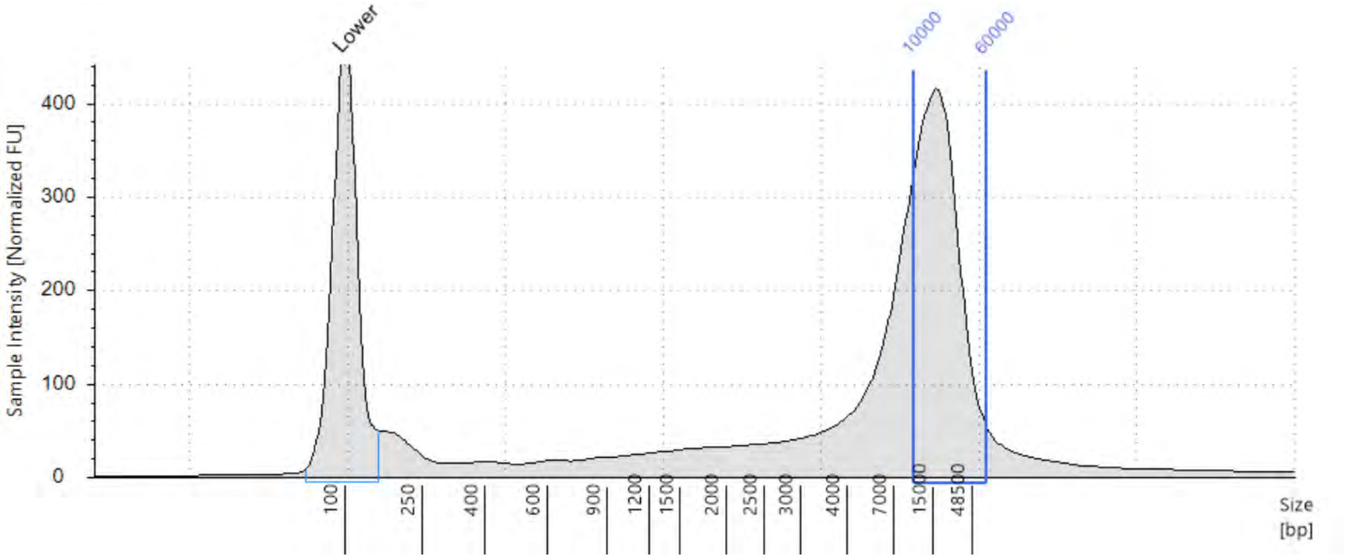

Region Table

From [bp]	To [bp]	Average Size [bp]	Conc. [ng/µl]	Region Molarity [nmol/l]	% of Total	Region Comment	Color
10000	60000	19973	12.5	1.16	43.74		

B7: C387

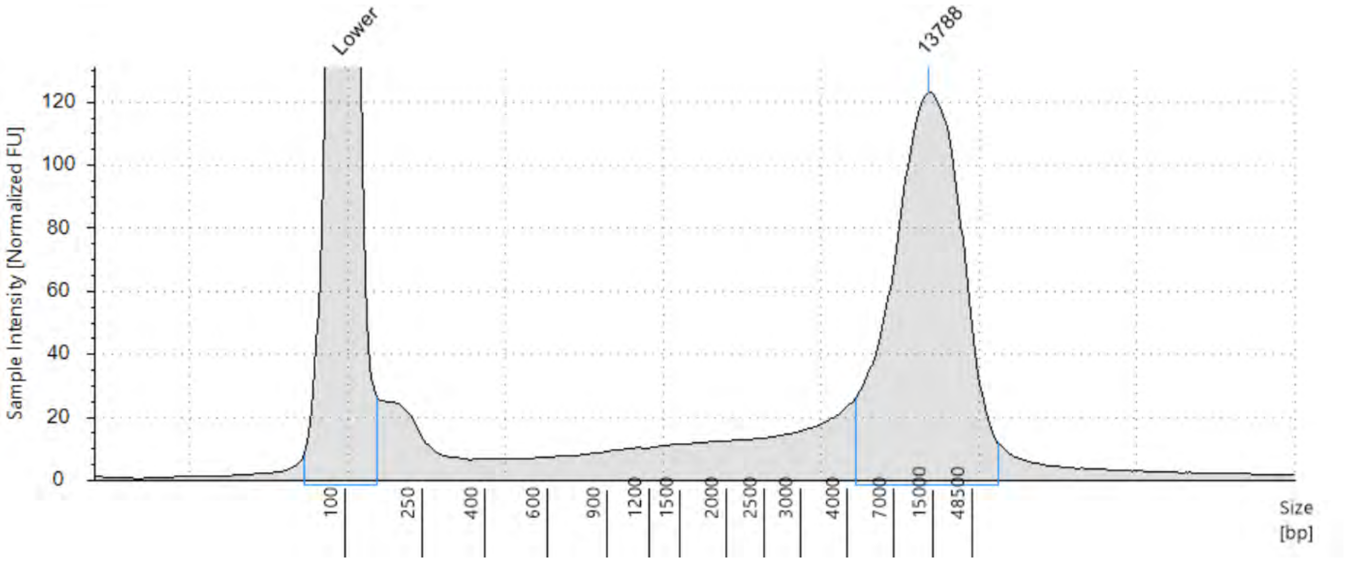

Sample Table

Well	DIN	Conc. [ng/µl]	Sample Description	Alert	Observations
B7	6.9	10.3	C387		

Peak Table

Size [bp]	Calibrated Conc. [ng/µl]	Assigned Conc. [ng/µl]	% Integrated Area	From [bp]	To [bp]	Peak Comment	Observations
100	8.50	8.50	-	62	148		Lower Marker
13788	6.24	-	96.08	4439	>60000		
-	-	-	-	-	-		Sample Well

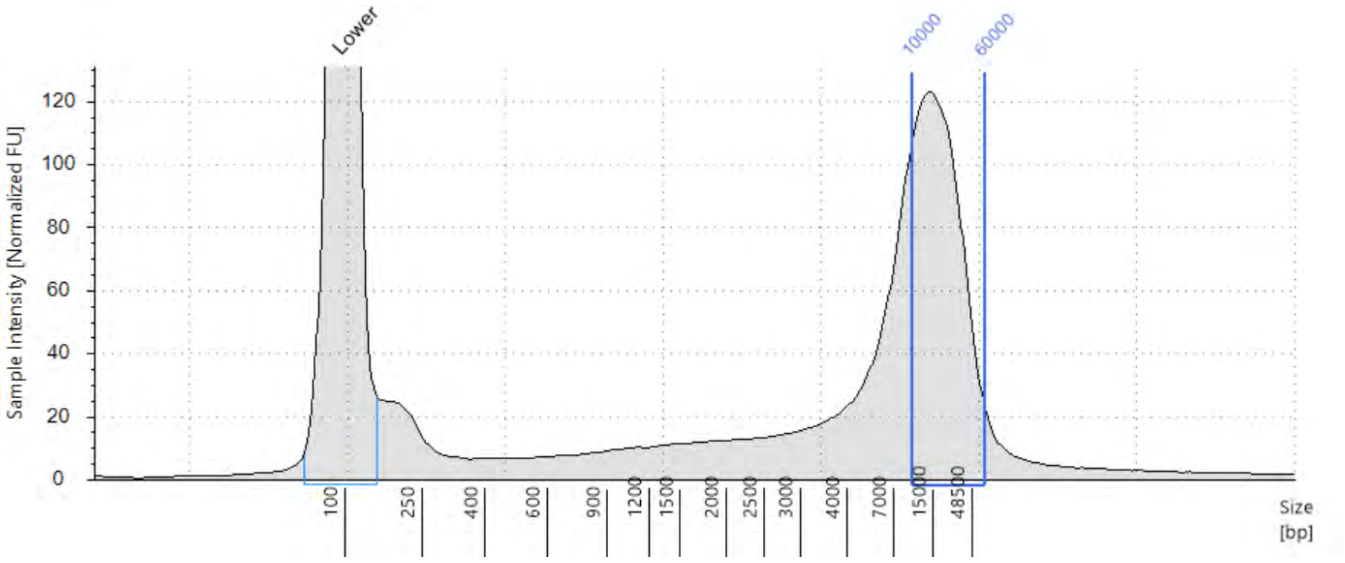

Region Table

From [bp]	To [bp]	Average Size [bp]	Conc. [ng/µl]	Region Molarity [nmol/l]	% of Total	Region Comment	Color
10000	60000	20999	4.18	0.381	40.65		

 © Agilent Technologies, Inc. 2017

Filename: 2018-12-18-01.gDNA

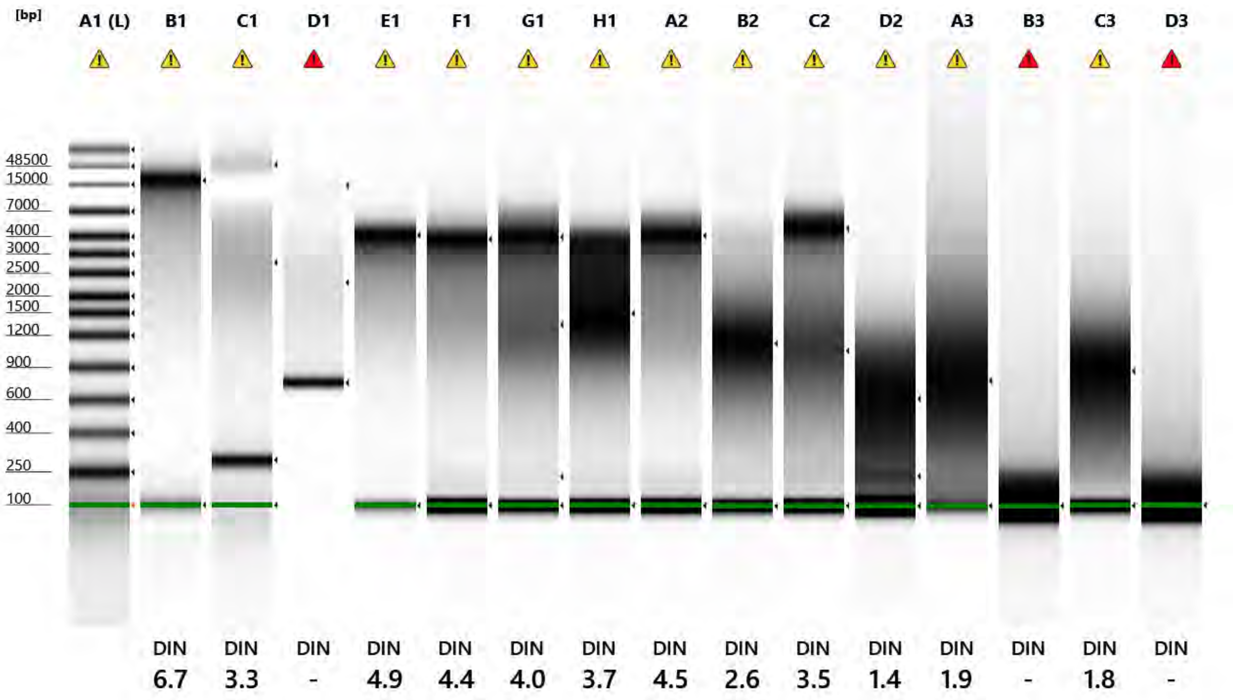

Default image (Contrast 50%), Image is Scaled to Sample

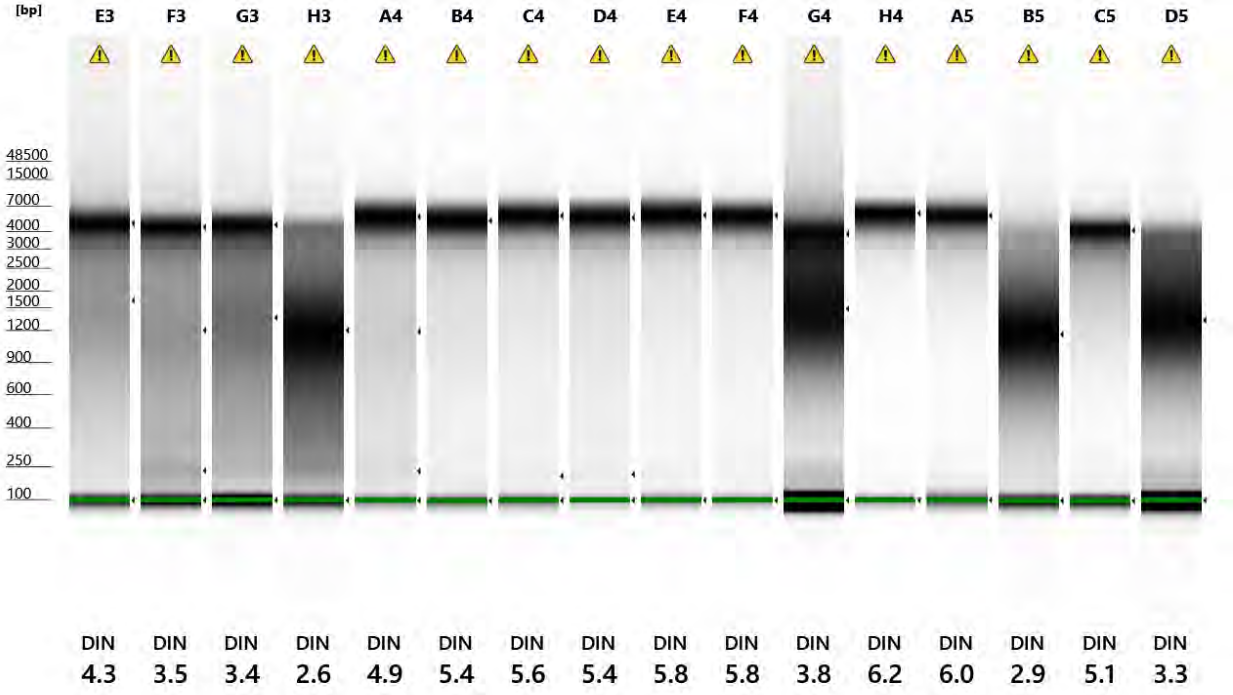

Default image (Contrast 50%), Image is Scaled to Sample

© Agilent Technologies, Inc. 2017

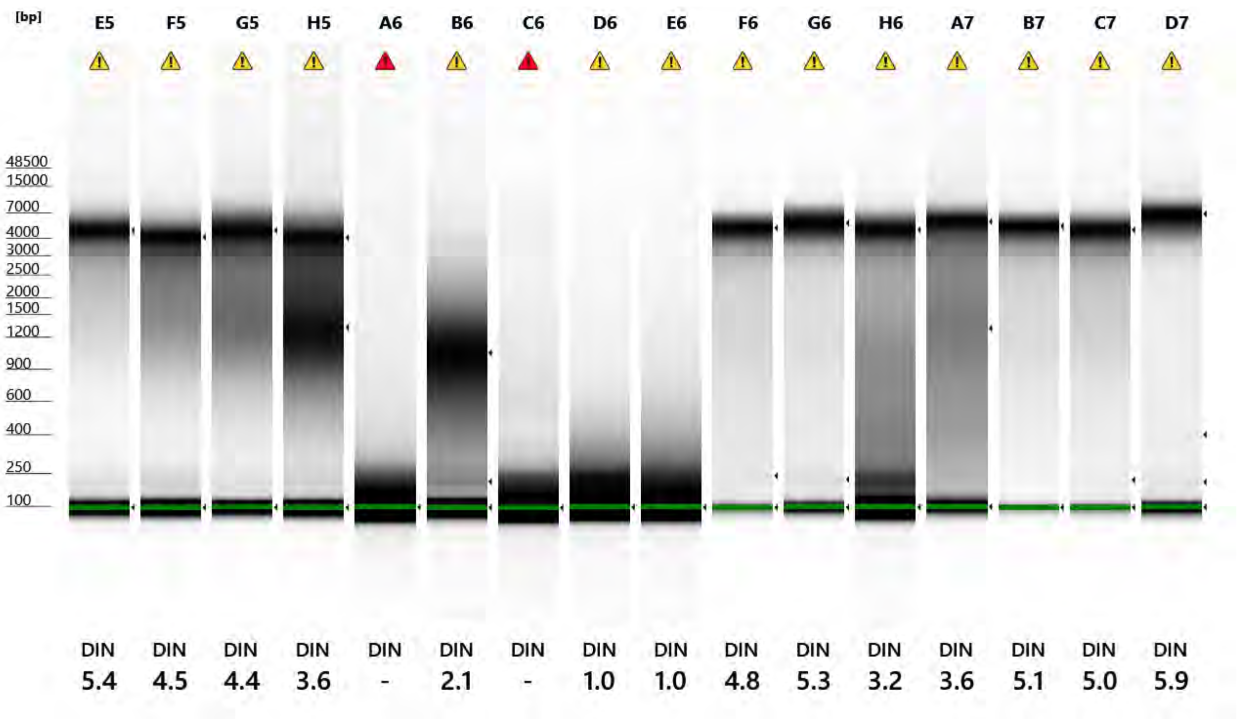

Default image (Contrast 50%), Image is Scaled to Sample

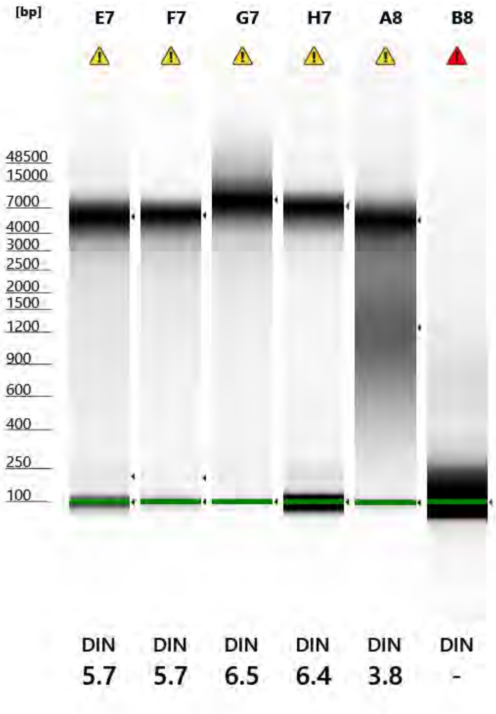

Default image (Contrast 50%), Image is Scaled to Sample

 © Agilent Technologies, Inc. 2017

Sample Info

Well	DIN	Conc. [ng/µl]	Sample Description	Alert	Observations
A1	-	67.9	Ladder	⚠	Issue with ladder peak detection (too many peaks detected); Ladder
B1	6.7	56.5	W811	⚠	Issue with ladder peak detection (too many peaks detected)
C1	3.3	57.1	W812	⚠	Issue with ladder peak detection (too many peaks detected)
D1	-		W813	▲	Marker(s) not detected; Issue with ladder peak detection (too many peaks detected)
E1	4.9	69.1	W814	⚠	Issue with ladder peak detection (too many peaks detected)
F1	4.4	24.7	W815	⚠	Issue with ladder peak detection (too many peaks detected)
G1	4.0	54.2	W826	⚠	Issue with ladder peak detection (too many peaks detected)
H1	3.7	46.4	W827	⚠	Issue with ladder peak detection (too many peaks detected)
A2	4.5	26.4	W828	⚠	Issue with ladder peak detection (too many peaks detected)
B2	2.6	48.1	W829	⚠	Issue with ladder peak detection (too many peaks detected)
C2	3.5	43.9	W830	⚠	Issue with ladder peak detection (too many peaks detected)
D2	1.4	18.2	W836	⚠	Issue with ladder peak detection (too many peaks detected)
A3	1.9	98.0	W837	⚠	Issue with ladder peak detection (too many peaks detected)
B3	-	1.89	W838	▲	Sample concentration outside functional range for DIN; Issue with ladder peak detection (too many peaks detected)
C3	1.8	51.9	W839	⚠	Issue with ladder peak detection (too many peaks detected)
D3	-	2.25	W840	▲	Sample concentration outside functional range for DIN; Issue with ladder peak detection (too many peaks detected)
E3	4.3	78.0	W846	⚠	Issue with ladder peak detection (too many peaks detected)
F3	3.5	64.1	W847	⚠	Issue with ladder peak detection (too many peaks detected)
G3	3.4	59.0	W848	⚠	Issue with ladder peak detection (too many peaks detected)
H3	2.6	104	W849	⚠	Sample concentration outside recommended range; Issue with ladder peak detection (too many peaks detected)
A4	4.9	89.6	W850	⚠	Issue with ladder peak detection (too many peaks detected)
B4	5.4	60.2	W851	⚠	Issue with ladder peak detection (too many peaks detected)
C4	5.6	57.8	W852	⚠	Issue with ladder peak detection (too many peaks detected)
D4	5.4	109	W853	⚠	Sample concentration outside recommended range; Issue with ladder peak detection (too many peaks detected)
E4	5.8	61.1	W854	⚠	Issue with ladder peak detection (too many peaks detected)
F4	5.8	72.7	W855	⚠	Issue with ladder peak detection (too many peaks detected)
G4	3.8	20.4	W1036	⚠	Issue with ladder peak detection (too many peaks detected)
H4	6.2	59.5	W1037	⚠	Issue with ladder peak detection (too many peaks detected)
A5	6.0	35.6	W1038	⚠	Issue with ladder peak detection (too many peaks detected)
B5	2.9	79.6	W1039	⚠	Issue with ladder peak detection (too many peaks detected)
C5	5.1	33.8	W1040	⚠	Issue with ladder peak detection (too many peaks detected)
D5	3.3	21.5	W1051	⚠	Issue with ladder peak detection (too many peaks detected)
E5	5.4	19.5	W1052	⚠	Issue with ladder peak detection (too many peaks detected)
F5	4.5	19.0	W1053	⚠	Issue with ladder peak detection (too many peaks detected)
G5	4.4	36.2	W1054	⚠	Issue with ladder peak detection (too many peaks detected)
H5	3.6	34.7	W1055	⚠	Issue with ladder peak detection (too many peaks detected)
A6	-	1.84	W1061	▲	Sample concentration outside functional range for DIN; Issue with ladder peak detection (too many peaks detected)
B6	2.1	19.8	W1062	⚠	Issue with ladder peak detection (too many peaks detected)
C6	-	1.60	W1063	▲	Sample concentration outside functional range for DIN; Issue with ladder peak detection (too many peaks detected)
D6	1.0	3.10	W1064	⚠	Sample concentration outside functional range for DIN; Issue with ladder peak detection (too many peaks detected)
E6	1.0	3.67	W1065	⚠	Sample concentration outside functional range for DIN; Issue with ladder peak detection (too many peaks detected)
F6	4.8	71.3	W1071	⚠	Issue with ladder peak detection (too many peaks detected)
G6	5.3	31.4	W1072	⚠	Issue with ladder peak detection (too many peaks detected)
H6	3.2	11.0	W1073	⚠	Issue with ladder peak detection (too many peaks detected)
A7	3.6	50.6	W1074	⚠	Issue with ladder peak detection (too many peaks detected)
B7	5.1	107	W1075	⚠	Sample concentration outside recommended range; Issue with ladder peak detection (too many peaks detected)
C7	5.0	86.3	W1076	⚠	Issue with ladder peak detection (too many peaks detected)
D7	5.9	25.0	W1077	⚠	Issue with ladder peak detection (too many peaks detected)
E7	5.7	47.7	W1078	⚠	Issue with ladder peak detection (too many peaks detected)
F7	5.7	103	W1079	⚠	Sample concentration outside recommended range; Issue with ladder peak detection (too many peaks detected)
G7	6.5	312	W1080	⚠	Sample concentration outside functional range for DIN; Issue with ladder peak detection (too many peaks detected)
H7	6.4	11.7	C603	⚠	Issue with ladder peak detection (too many peaks detected)
A8	3.8	402	C462	⚠	Sample concentration outside functional range for DIN; Issue with ladder peak detection (too many peaks detected)

B8	-	1.23	buffer		Sample concentration outside functional range for DIN; Issue with ladder peak detection (too many peaks detected)

© Agilent Technologies, Inc. 2017

A1: Ladder

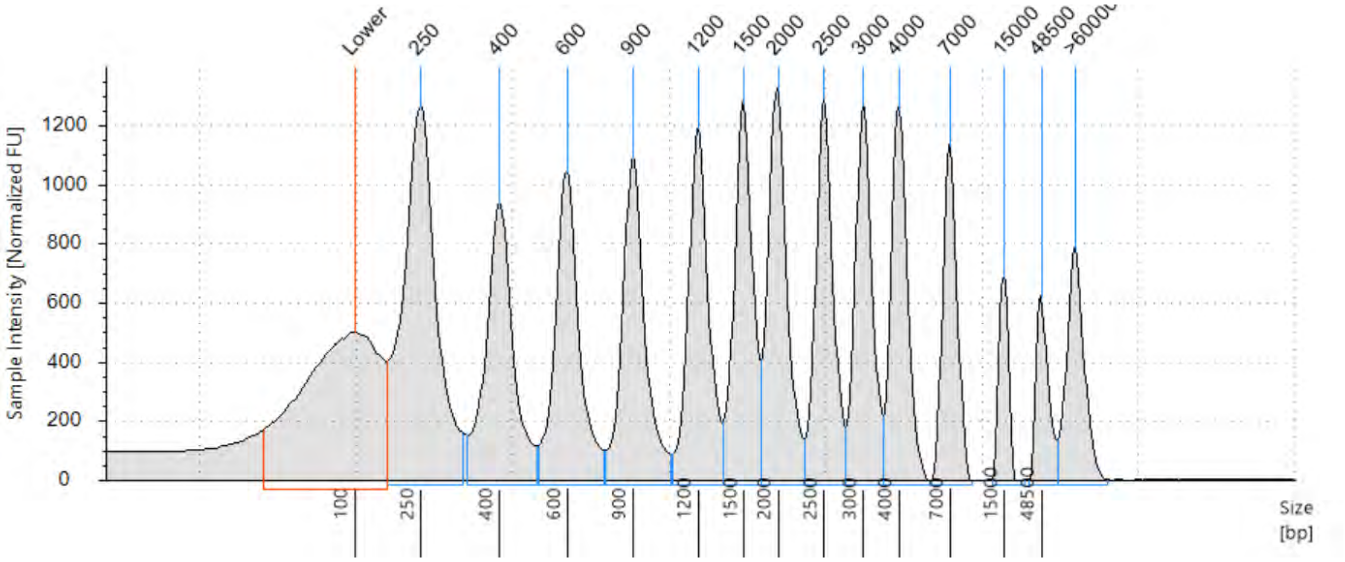

Sample Table

Well	DIN	Conc. [ng/µl]	Sample Description	Alert	Observations
A1	-	67.9	Ladder	⚠	Issue with ladder peak detection (too many peaks detected); Ladder

Peak Table

Size [bp]	Calibrated Conc. [ng/µl]	Assigned Conc. [ng/µl]	% Integrated Area	From [bp]	To [bp]	Peak Comment	Observations
100	8.50	8.50	-	29	157		Lower Marker
250	9.13	-	14.08	157	321		
400	5.45	-	8.40	329	498		
600	5.36	-	8.27	502	754		
900	5.30	-	8.17	760	1064		
1200	5.26	-	8.11	1071	1359		
1500	5.22	-	8.05	1359	1742		
2000	5.54	-	8.54	1742	2281		
2500	4.89	-	7.54	2281	2765		
3000	4.74	-	7.31	2765	3542		
4000	4.51	-	6.95	3542	5696		
7000	3.50	-	5.39	5696	9495		
15000	1.40	-	2.16	12636	19974		
48500	1.62	-	2.49	21262	>60000		
>60000	2.95	-	4.54	>60000	>60000		

© Agilent Technologies, Inc. 2017

H7: C603

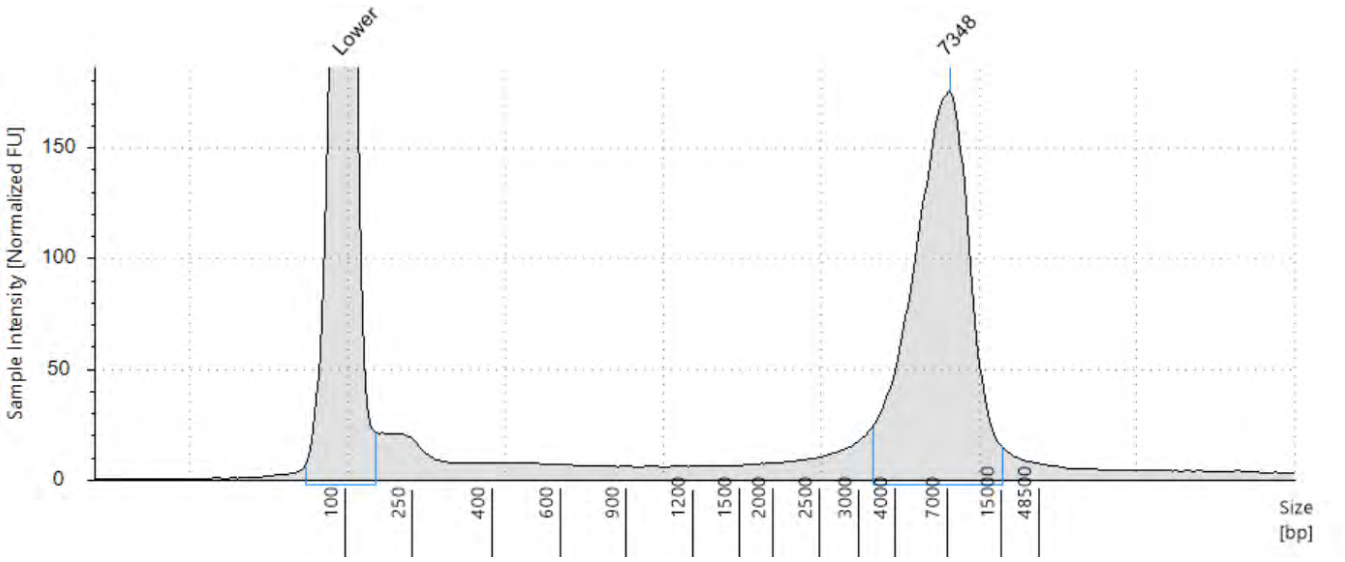

Sample Table

Well	DIN	Conc. [ng/µl]	Sample Description	Alert	Observations
H7	6.4	11.7	C603	⚠	Issue with ladder peak detection (too many peaks detected)

Peak Table

Size [bp]	Calibrated Conc. [ng/µl]	Assigned Conc. [ng/µl]	% Integrated Area	From [bp]	To [bp]	Peak Comment	Observations
100	8.50	8.50	-	58	154		Lower Marker
7348	7.68	-	92.47	3341	15121		
-	-	-	-	-	-		Sample Well

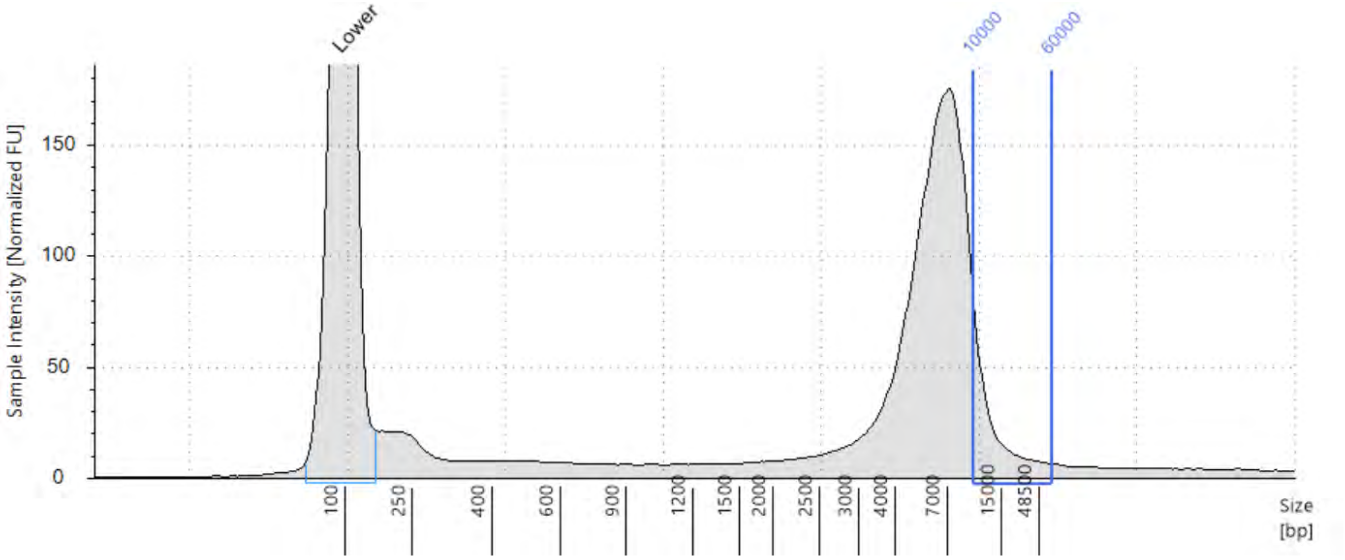

Region Table

From [bp]	To [bp]	Average Size [bp]	Conc. [ng/µl]	Region Molarity [nmol/l]	% of Total	Region Comment	Color
10000	60000	16638	1.00	0.120	8.57		■

Filename: 2019-07-29-01.gDNA

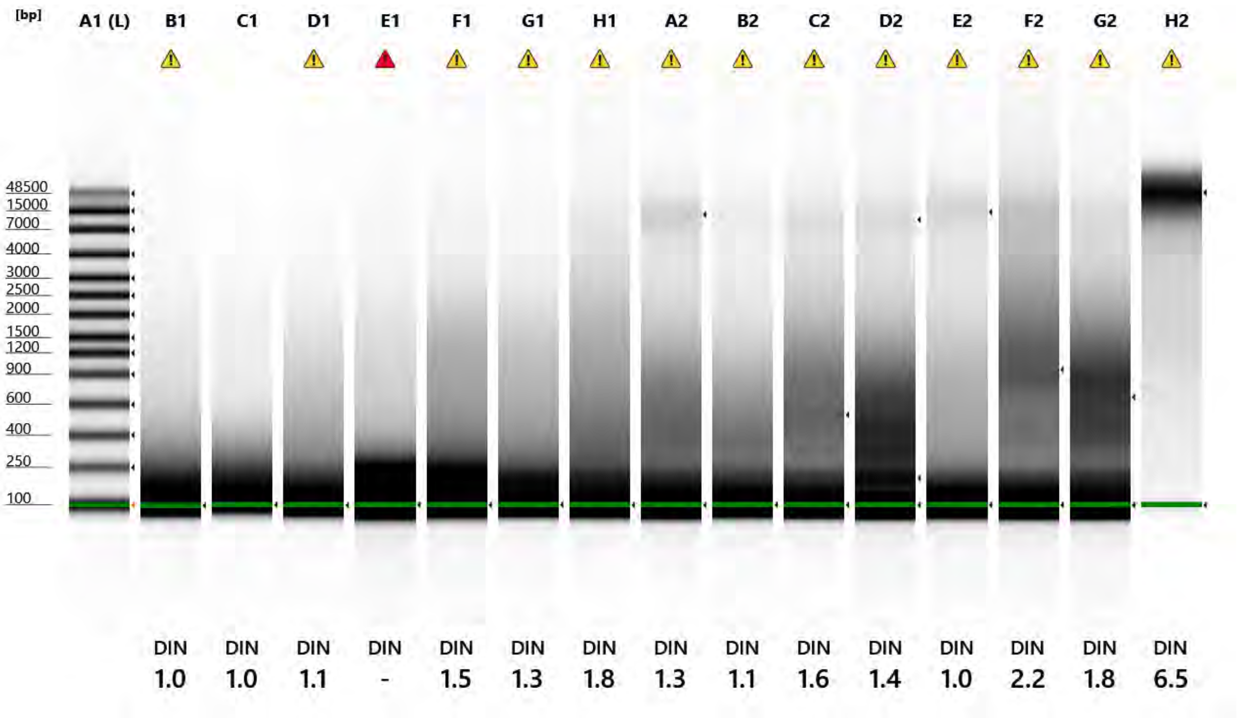

Default image (Contrast 50%), Image is Scaled to Sample

Sample Info

Well	DIN	Conc. [ng/µl]	Sample Description	Alert	Observations
A1	-	69.6	Ladder		Ladder
B1	1.0	5.98	M-T4-S-166	⚠	Sample concentration outside recommended range
C1	1.0	13.1	M-T4-S-167		
D1	1.1	6.62	M-T4-S-168	⚠	Sample concentration outside recommended range
E1	-	1.83	M-T4-S-169	▲	Sample concentration outside functional range for DIN
F1	1.5	4.52	M-T4-S-170	⚠	Sample concentration outside functional range for DIN
G1	1.3	6.13	M-T4-S-292	⚠	Sample concentration outside recommended range
H1	1.8	7.28	M-T4-S-293	⚠	Sample concentration outside recommended range
A2	1.3	5.61	M-T4-DS-146	⚠	Sample concentration outside recommended range
B2	1.1	5.02	M-T4-DS-147	⚠	Sample concentration outside recommended range
C2	1.6	5.51	M-T4-DS-148	⚠	Sample concentration outside recommended range
D2	1.4	6.60	M-T4-DS-149	⚠	Sample concentration outside recommended range
E2	1.0	4.47	M-T4-DS-150	⚠	Sample concentration outside functional range for DIN
F2	2.2	7.15	M-T4-DS-284	⚠	Sample concentration outside recommended range
G2	1.8	5.93	M-T4-DS-285	⚠	Sample concentration outside recommended range
H2	6.5	367	M-T5-Initial-223	⚠	Sample concentration outside functional range for DIN

 © Agilent Technologies, Inc. 2017

A1: Ladder

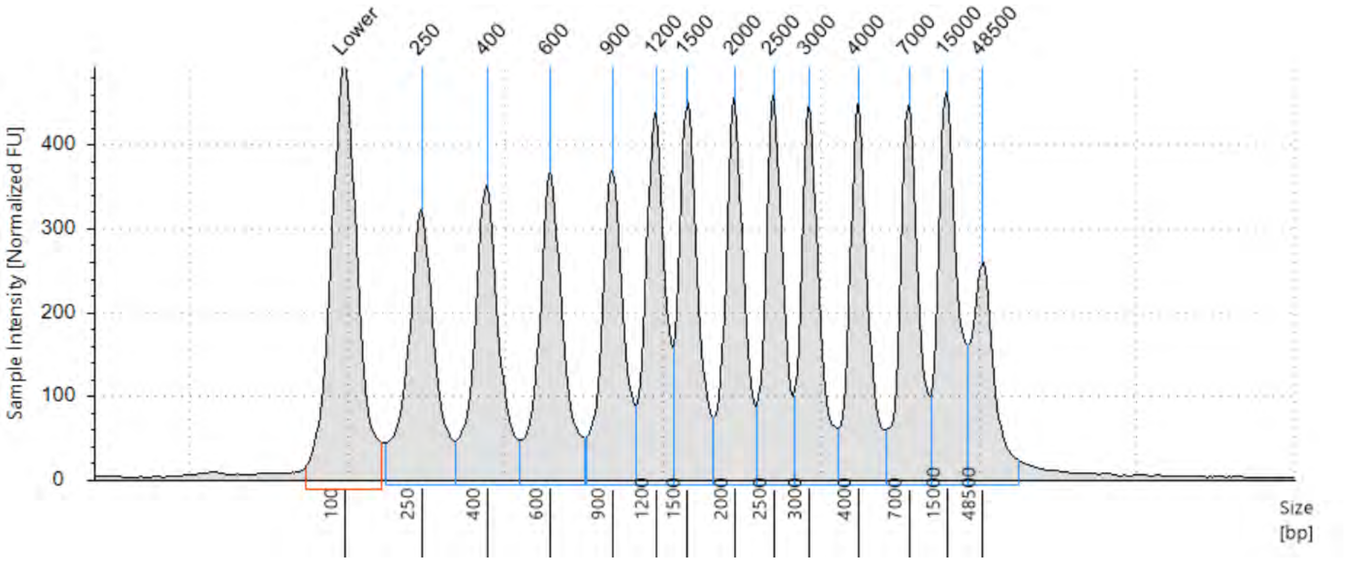

Sample Table

Well	DIN	Conc. [ng/µl]	Sample Description	Alert	Observations
A1	-	69.6	Ladder		Ladder

Peak Table

Size [bp]	Calibrated Conc. [ng/µl]	Assigned Conc. [ng/µl]	% Integrated Area	From [bp]	To [bp]	Peak Comment	Observations
100	8.50	8.50	-	62	153		Lower Marker
250	5.52	-	7.94	163	319		
400	5.57	-	8.02	319	494		
600	5.71	-	8.21	494	752		
900	5.17	-	7.44	758	1049		
1200	5.58	-	8.02	1049	1361		
1500	5.64	-	8.11	1361	1754		
2000	5.58	-	8.02	1754	2272		
2500	5.26	-	7.56	2272	2777		
3000	5.31	-	7.63	2777	3548		
4000	5.36	-	7.71	3548	5413		
7000	5.40	-	7.76	5413	10820		
15000	5.72	-	8.22	10820	22580		
48500	3.73	-	5.36	22580	>60000		

B1: M-T4-S-166

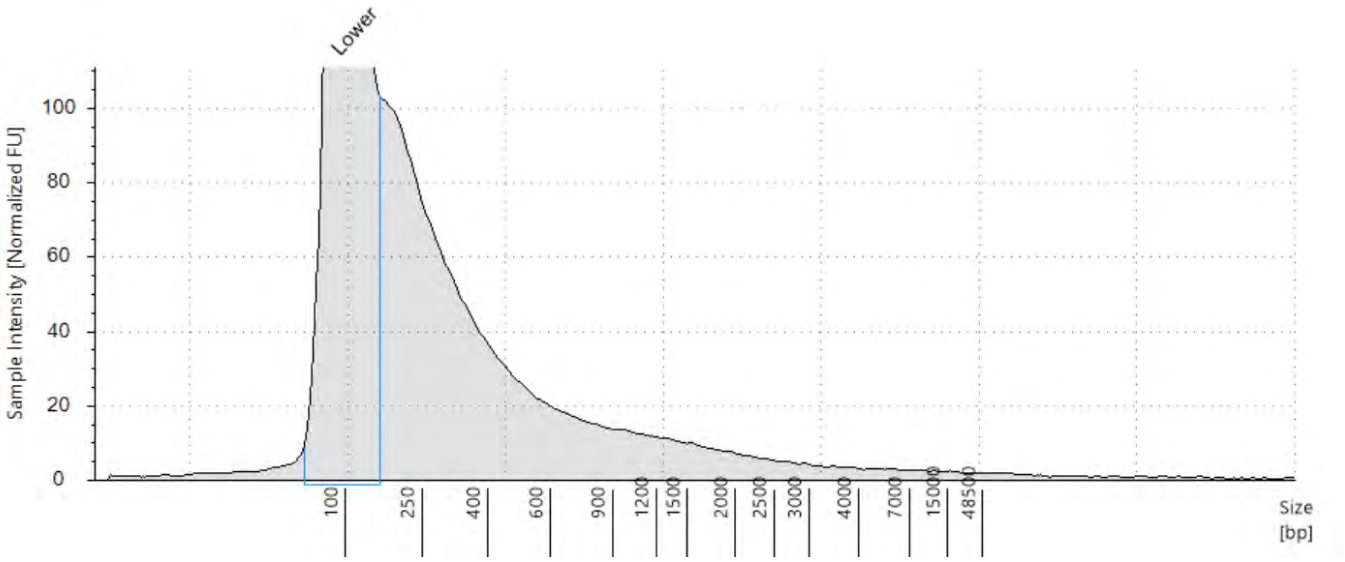

Sample Table

Well	DIN	Conc. [ng/µl]	Sample Description	Alert	Observations
B1	1.0	5.98	M-T4-S-166	⚠	Sample concentration outside recommended range

Peak Table

Size [bp]	Calibrated Conc. [ng/µl]	Assigned Conc. [ng/µl]	% Integrated Area	From [bp]	To [bp]	Peak Comment	Observations
100	8.50	8.50	-	62	154		Lower Marker

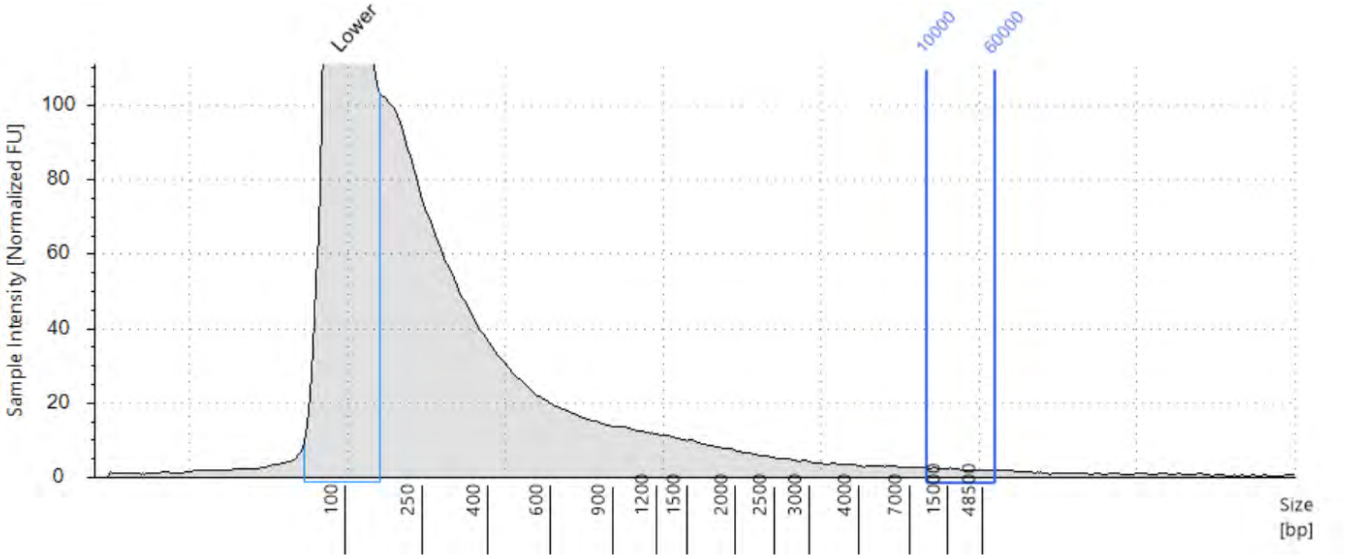

Region Table

From [bp]	To [bp]	Average Size [bp]	Conc. [ng/µl]	Region Molarity [nmol/l]	% of Total	Region Comment	Color
10000	60000	24619	0.0250	0.00606	0.42		■

C1: M-T4-S-167

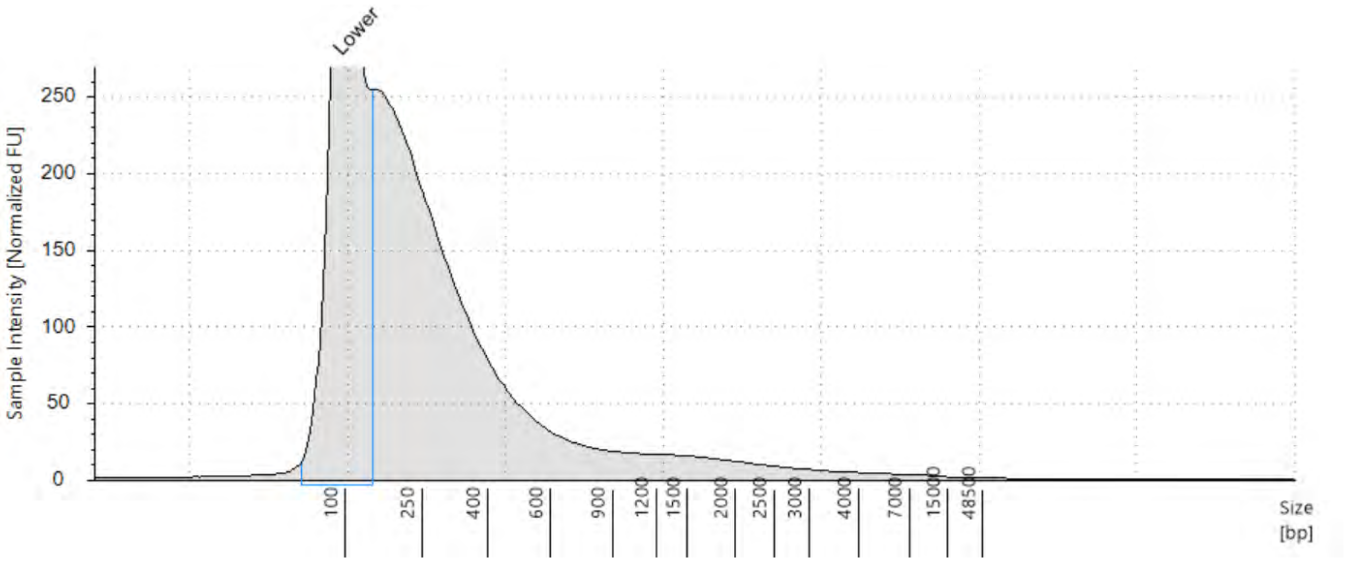

Sample Table

Well	DIN	Conc. [ng/µl]	Sample Description	Alert	Observations
C1	1.0	13.1	M-T4-S-167		

Peak Table

Size [bp]	Calibrated Conc. [ng/µl]	Assigned Conc. [ng/µl]	% Integrated Area	From [bp]	To [bp]	Peak Comment	Observations
100	8.50	8.50	-	59	138		Lower Marker

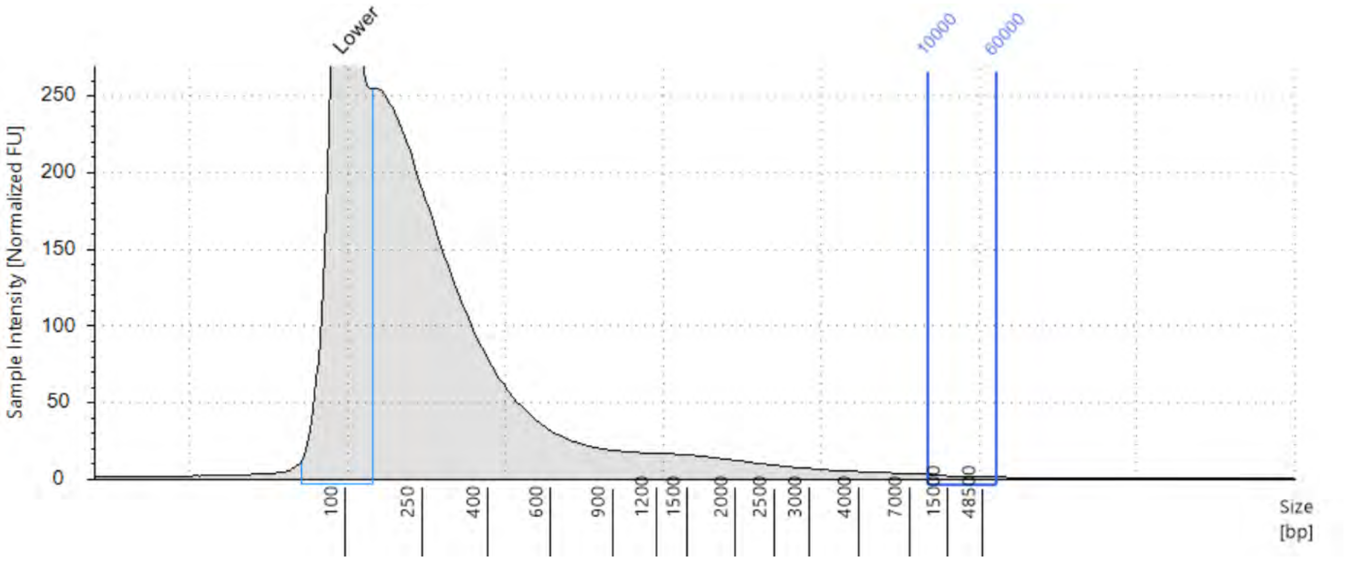

Region Table

From [bp]	To [bp]	Average Size [bp]	Conc. [ng/µl]	Region Molarity [nmol/l]	% of Total	Region Comment	Color
10000	60000	23561	0.0183	0.00491	0.14		

 © Agilent Technologies, Inc. 2017

D1: M-T4-S-168

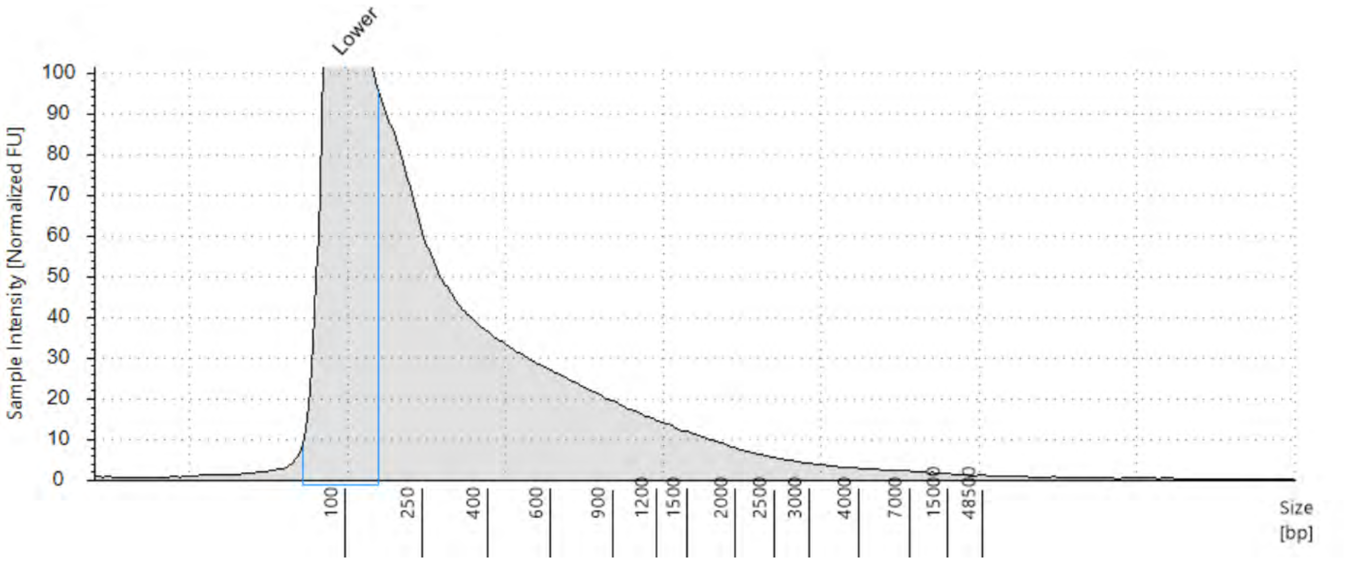

Sample Table

Well	DIN	Conc. [ng/µl]	Sample Description	Alert	Observations
D1	1.1	6.62	M-T4-S-168	⚠	Sample concentration outside recommended range

Peak Table

Size [bp]	Calibrated Conc. [ng/µl]	Assigned Conc. [ng/µl]	% Integrated Area	From [bp]	To [bp]	Peak Comment	Observations
100	8.50	8.50	-	60	150		Lower Marker

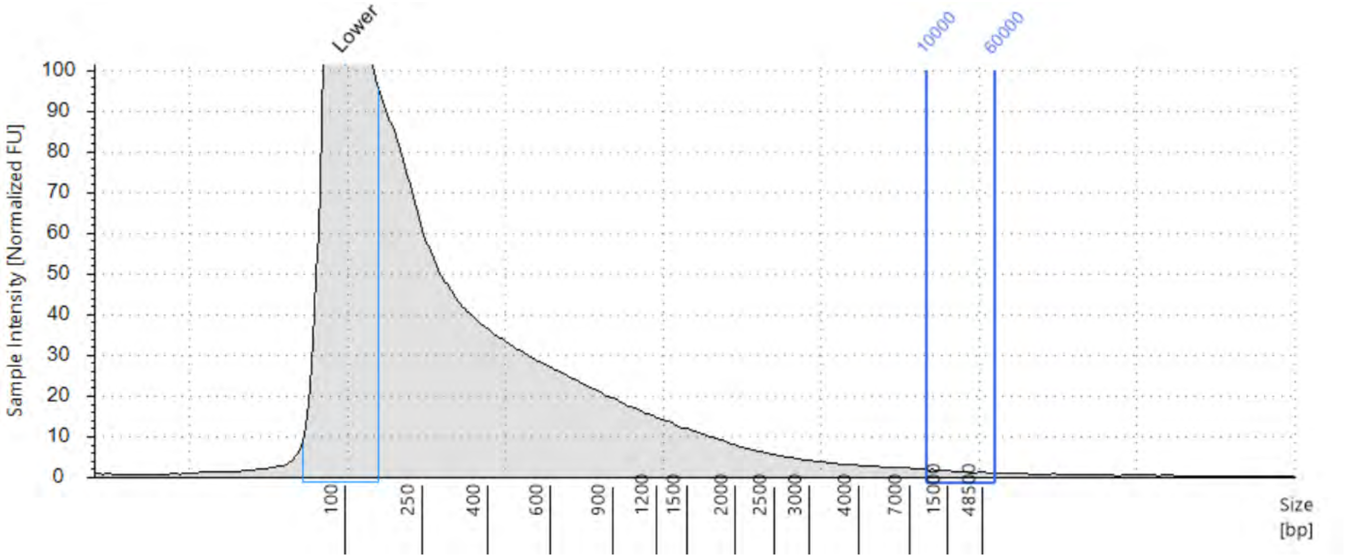

Region Table

From [bp]	To [bp]	Average Size [bp]	Conc. [ng/µl]	Region Molarity [nmol/l]	% of Total	Region Comment	Color
10000	60000	24285	0.0219	0.00426	0.33		■

 © Agilent Technologies, Inc. 2017

E1: M-T4-S-169

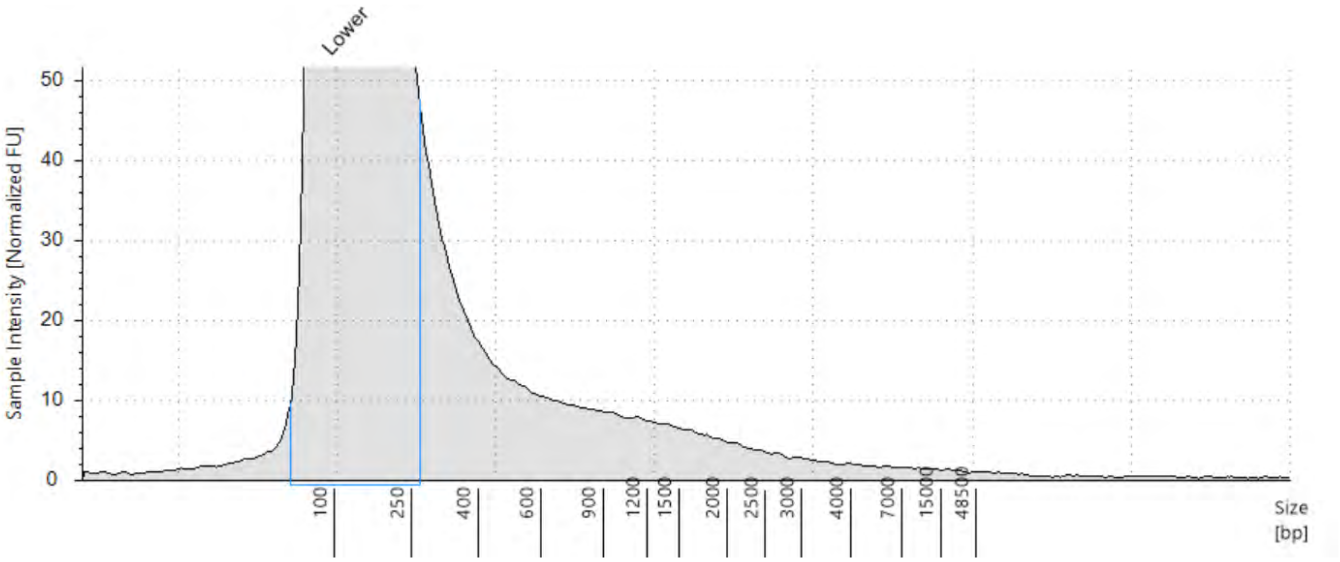

Sample Table

Well	DIN	Conc. [ng/µl]	Sample Description	Alert	Observations
E1	-	1.83	M-T4-S-169	▲	Sample concentration outside functional range for DIN

Peak Table

Size [bp]	Calibrated Conc. [ng/µl]	Assigned Conc. [ng/µl]	% Integrated Area	From [bp]	To [bp]	Peak Comment	Observations
100	8.50	8.50	-	59	265		Lower Marker

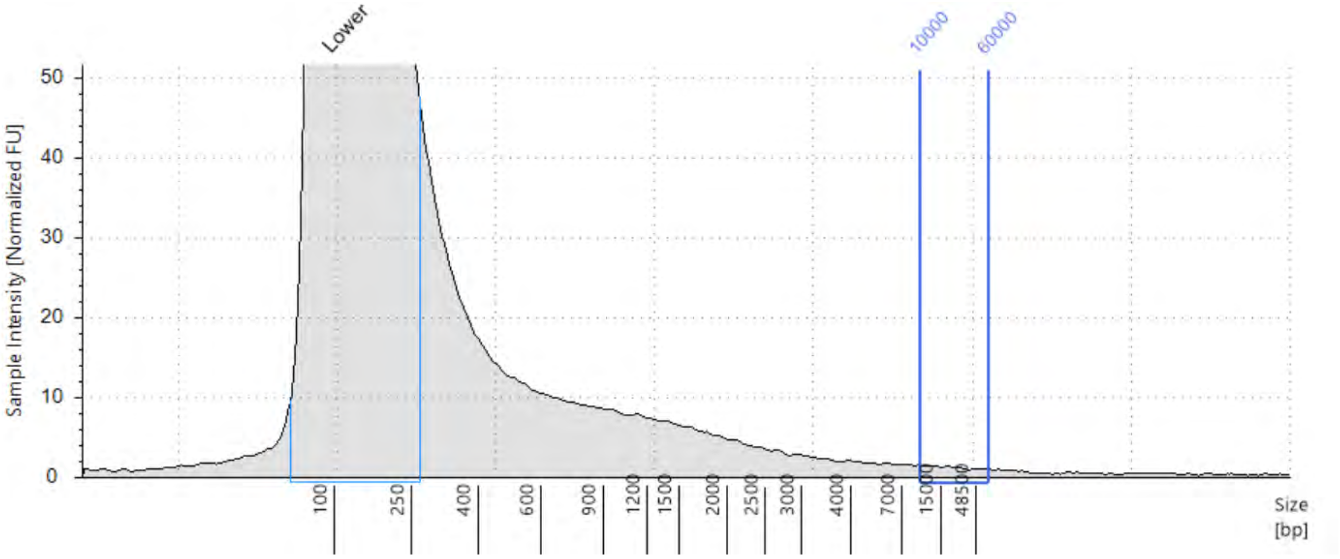

Region Table

From [bp]	To [bp]	Average Size [bp]	Conc. [ng/µl]	Region Molarity [nmol/l]	% of Total	Region Comment	Color
10000	60000	24874	0.0167	0.00281	0.91		■

 © Agilent Technologies, Inc. 2017

F1: M-T4-S-170

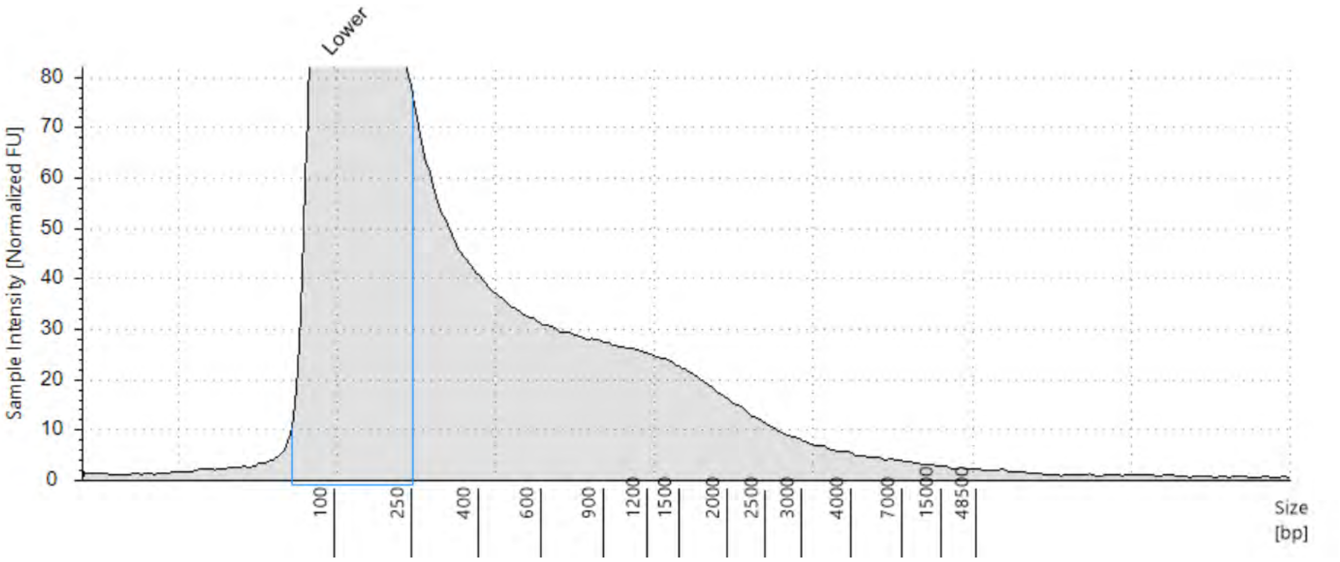

Sample Table

Well	DIN	Conc. [ng/µl]	Sample Description	Alert	Observations
F1	1.5	4.52	M-T4-S-170	⚠	Sample concentration outside functional range for DIN

Peak Table

Size [bp]	Calibrated Conc. [ng/µl]	Assigned Conc. [ng/µl]	% Integrated Area	From [bp]	To [bp]	Peak Comment	Observations
100	8.50	8.50	-	60	253		Lower Marker

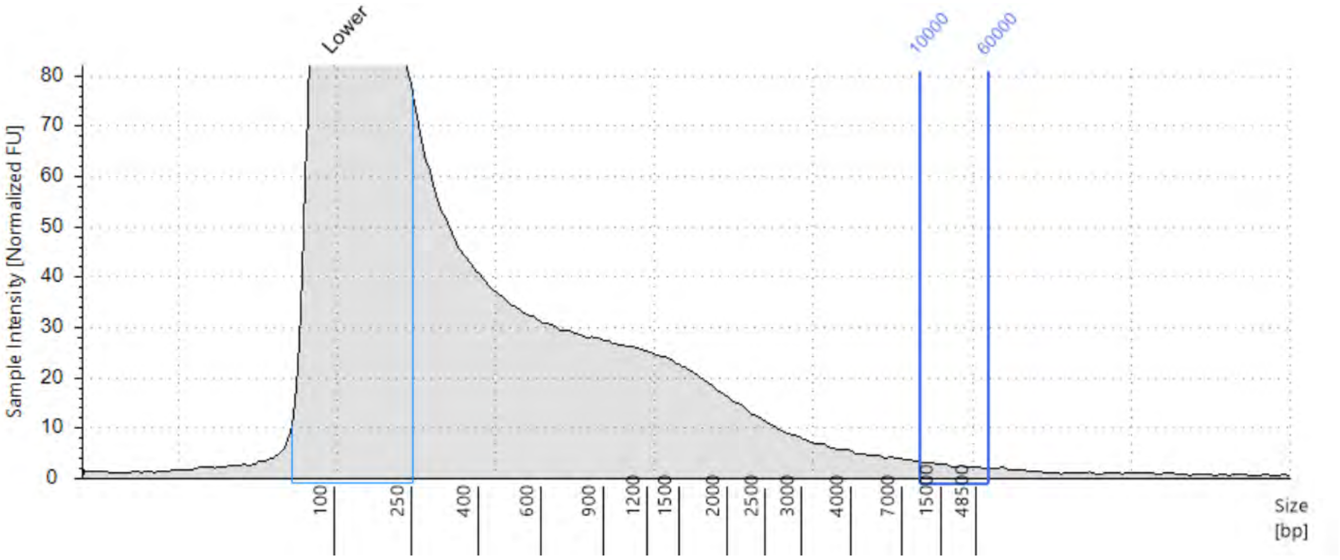

Region Table

From [bp]	To [bp]	Average Size [bp]	Conc. [ng/µl]	Region Molarity [nmol/l]	% of Total	Region Comment	Color
10000	60000	24263	0.0447	0.00552	0.99		■

 © Agilent Technologies, Inc. 2017

G1: M-T4-S-292

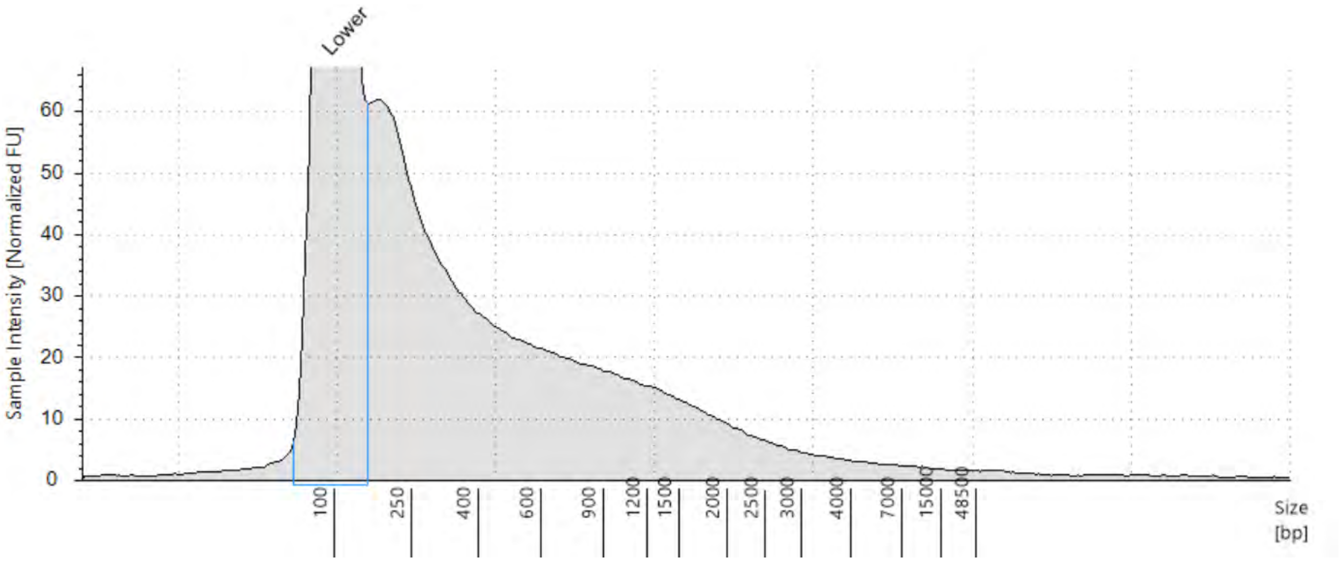

Sample Table

Well	DIN	Conc. [ng/µl]	Sample Description	Alert	Observations
G1	1.3	6.13	M-T4-S-292	⚠	Sample concentration outside recommended range

Peak Table

Size [bp]	Calibrated Conc. [ng/µl]	Assigned Conc. [ng/µl]	% Integrated Area	From [bp]	To [bp]	Peak Comment	Observations
100	8.50	8.50	-	61	148		Lower Marker

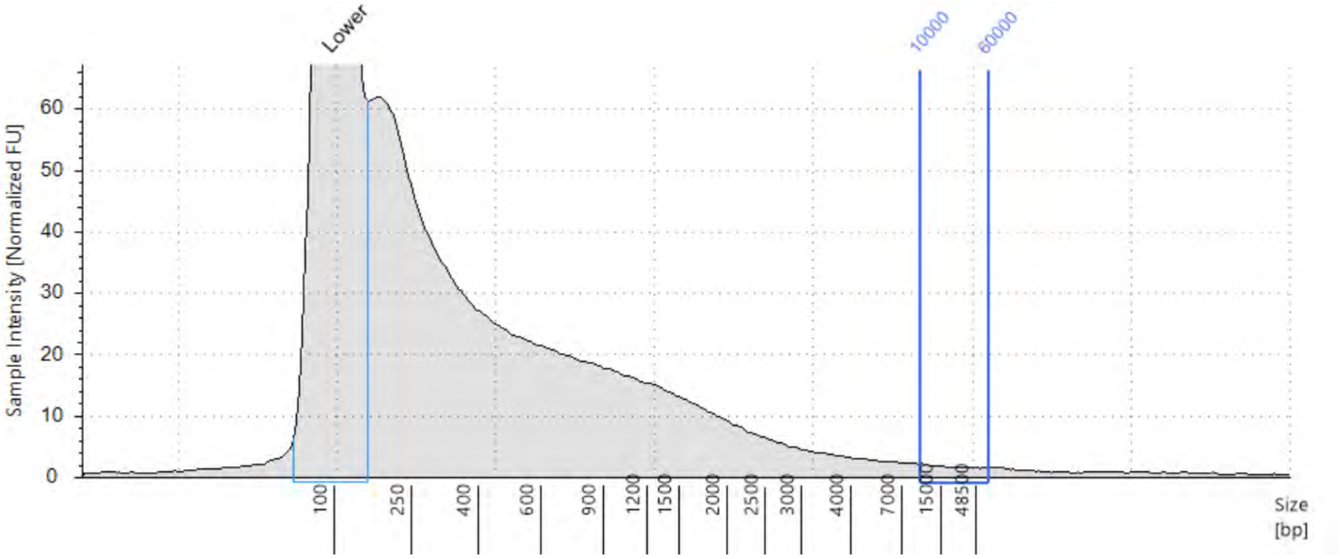

Region Table

From [bp]	To [bp]	Average Size [bp]	Conc. [ng/µl]	Region Molarity [nmol/l]	% of Total	Region Comment	Color
10000	60000	25590	0.0479	0.00595	0.78		■

H1: M-T4-S-293

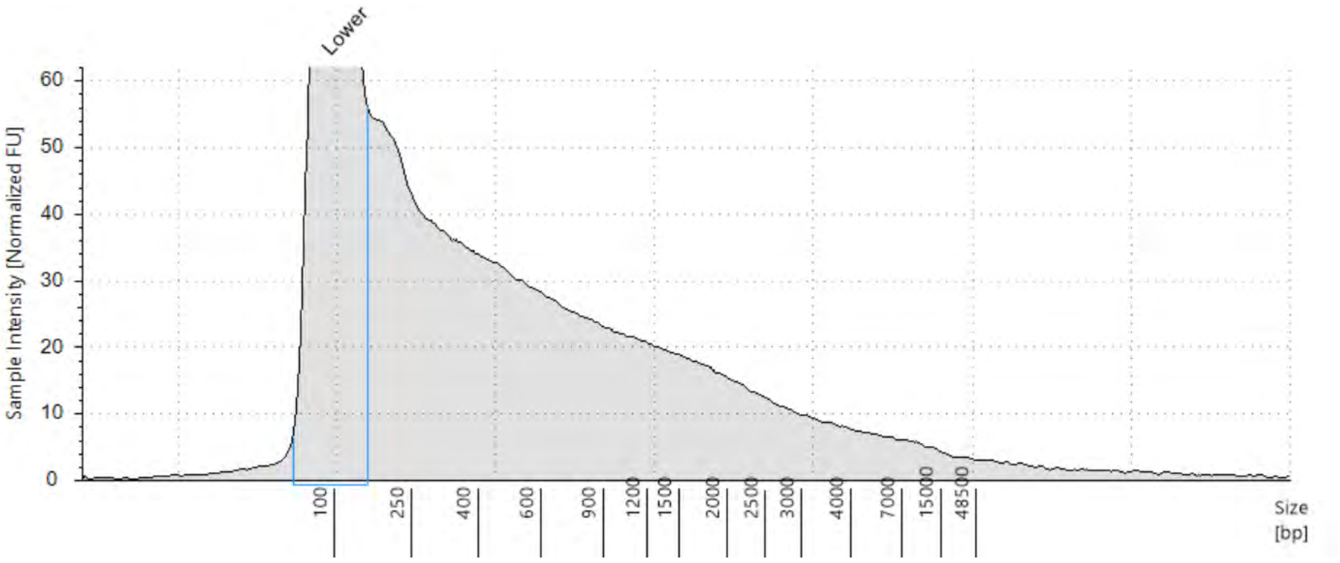

Sample Table

Well	DIN	Conc. [ng/µl]	Sample Description	Alert	Observations
H1	1.8	7.28	M-T4-S-293	⚠	Sample concentration outside recommended range

Peak Table

Size [bp]	Calibrated Conc. [ng/µl]	Assigned Conc. [ng/µl]	% Integrated Area	From [bp]	To [bp]	Peak Comment	Observations
100	8.50	8.50	-	61	149		Lower Marker

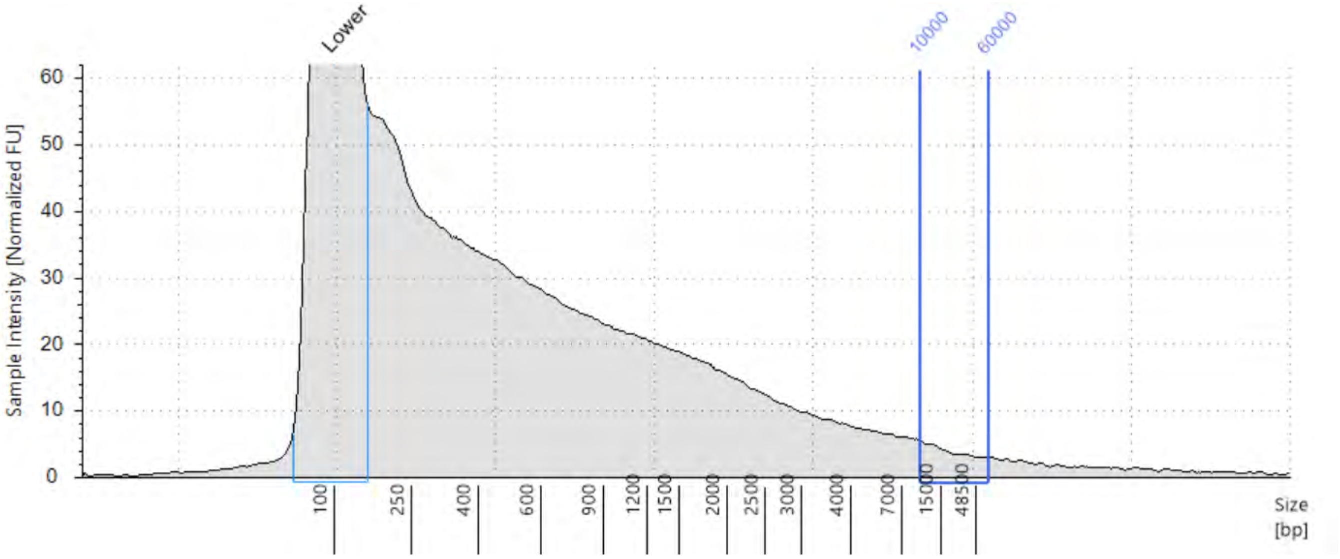

Region Table

From [bp]	To [bp]	Average Size [bp]	Conc. [ng/µl]	Region Molarity [nmol/l]	% of Total	Region Comment	Color
10000	60000	23991	0.125	0.0140	1.71		■

A2: M-T4-DS-146

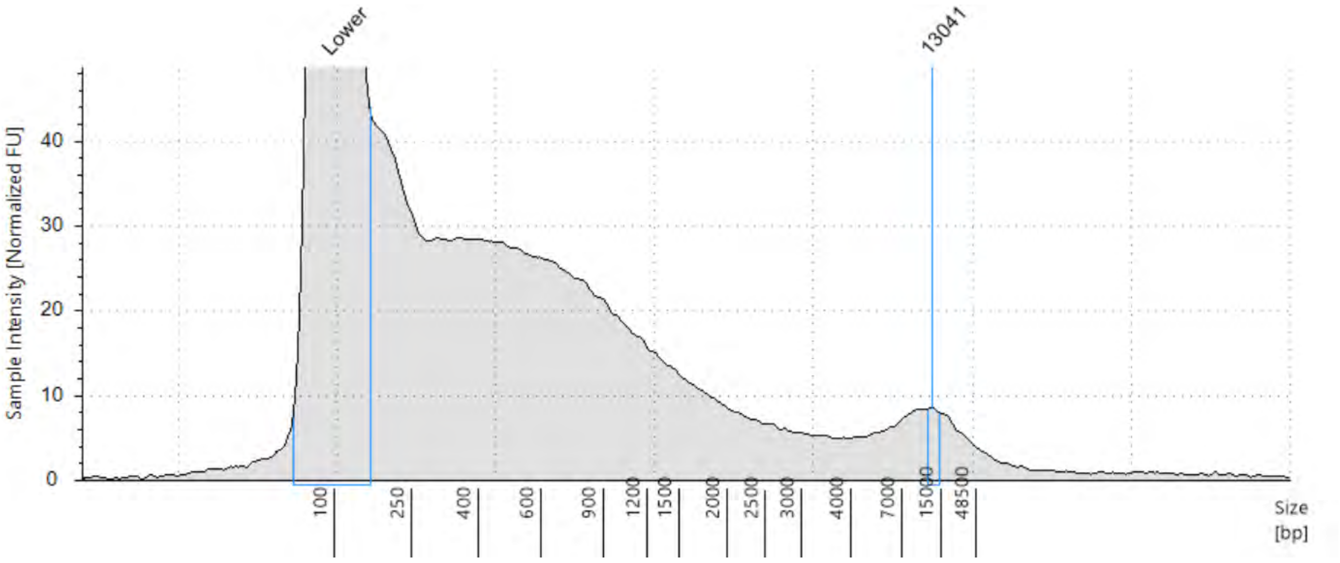

Sample Table

Well	DIN	Conc. [ng/µl]	Sample Description	Alert	Observations
A2	1.3	5.61	M-T4-DS-146	⚠	Sample concentration outside recommended range

Peak Table

Size [bp]	Calibrated Conc. [ng/µl]	Assigned Conc. [ng/µl]	% Integrated Area	From [bp]	To [bp]	Peak Comment	Observations
100	8.50	8.50	-	61	153		Lower Marker
13041	0.0612	-	100.00	11687	14956		

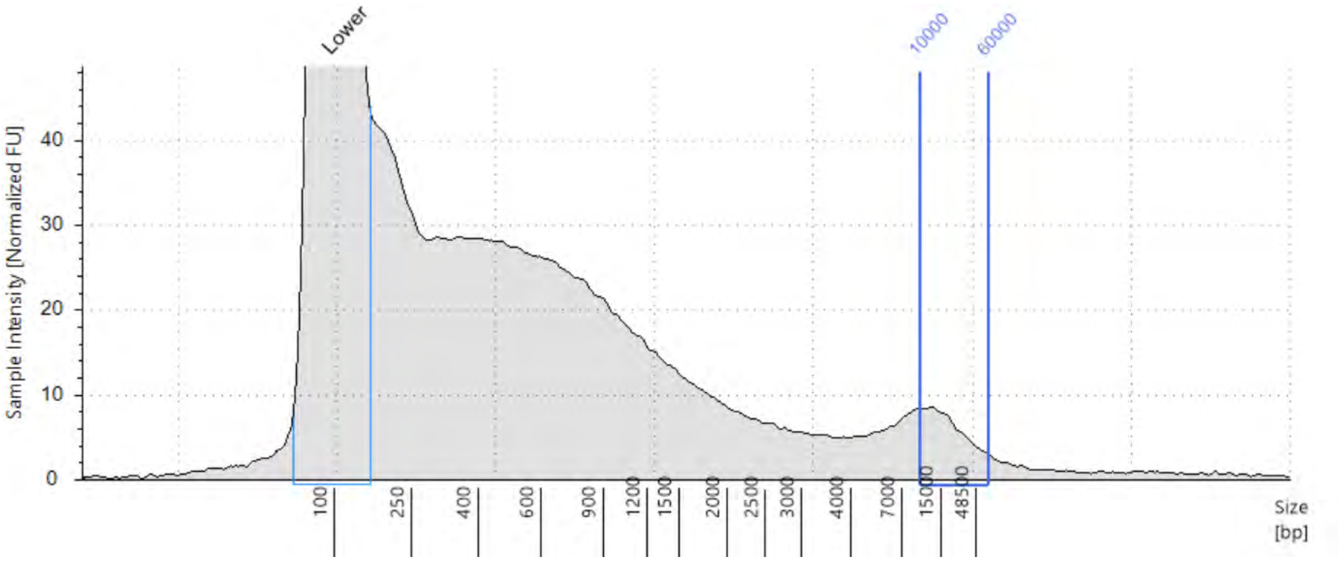

Region Table

From [bp]	To [bp]	Average Size [bp]	Conc. [ng/µl]	Region Molarity [nmol/l]	% of Total	Region Comment	Color
10000	60000	21874	0.222	0.0219	3.96		■

B2: M-T4-DS-147

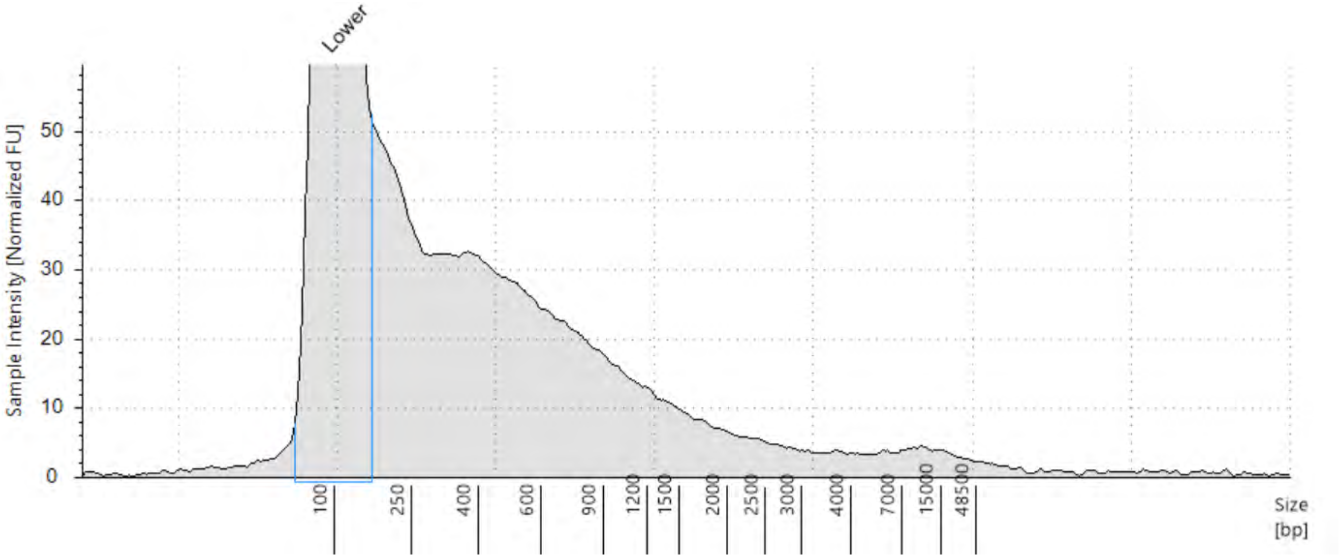

Sample Table

Well	DIN	Conc. [ng/µl]	Sample Description	Alert	Observations
B2	1.1	5.02	M-T4-DS-147	⚠	Sample concentration outside recommended range

Peak Table

Size [bp]	Calibrated Conc. [ng/µl]	Assigned Conc. [ng/µl]	% Integrated Area	From [bp]	To [bp]	Peak Comment	Observations
100	8.50	8.50	-	62	155		Lower Marker

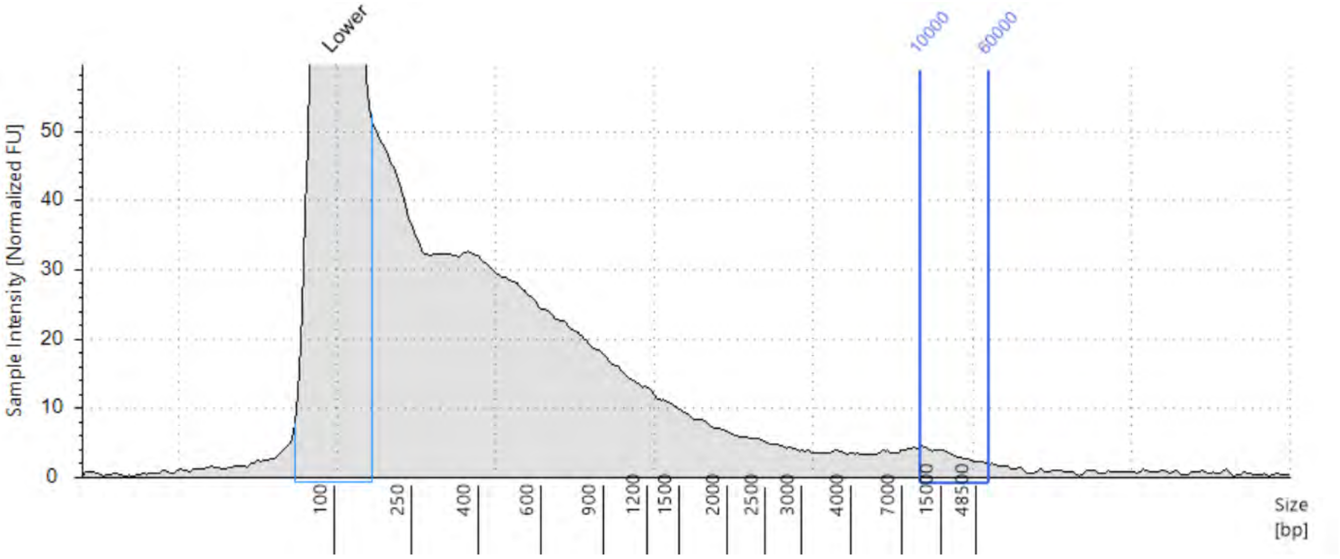

Region Table

From [bp]	To [bp]	Average Size [bp]	Conc. [ng/µl]	Region Molarity [nmol/l]	% of Total	Region Comment	Color
10000	60000	23079	0.0841	0.0111	1.68		■

 © Agilent Technologies, Inc. 2017

C2: M-T4-DS-148

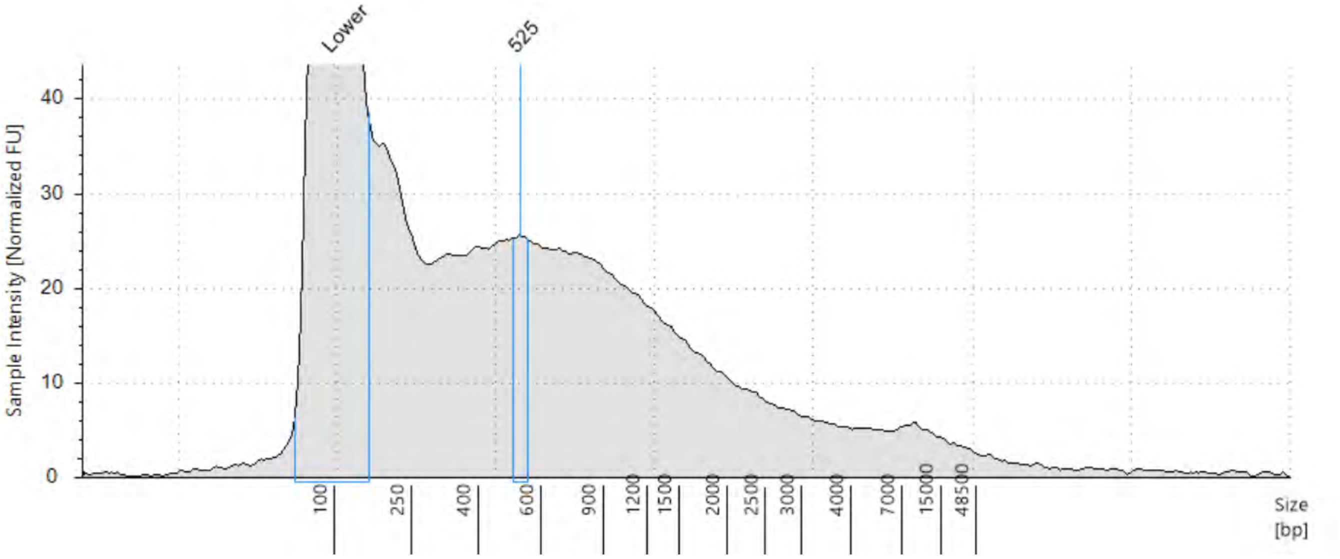

Sample Table

Well	DIN	Conc. [ng/µl]	Sample Description	Alert	Observations
C2	1.6	5.51	M-T4-DS-148	⚠	Sample concentration outside recommended range

Peak Table

Size [bp]	Calibrated Conc. [ng/µl]	Assigned Conc. [ng/µl]	% Integrated Area	From [bp]	To [bp]	Peak Comment	Observations
100	8.50	8.50	-	63	150		Lower Marker
525	0.224	-	100.00	503	549		

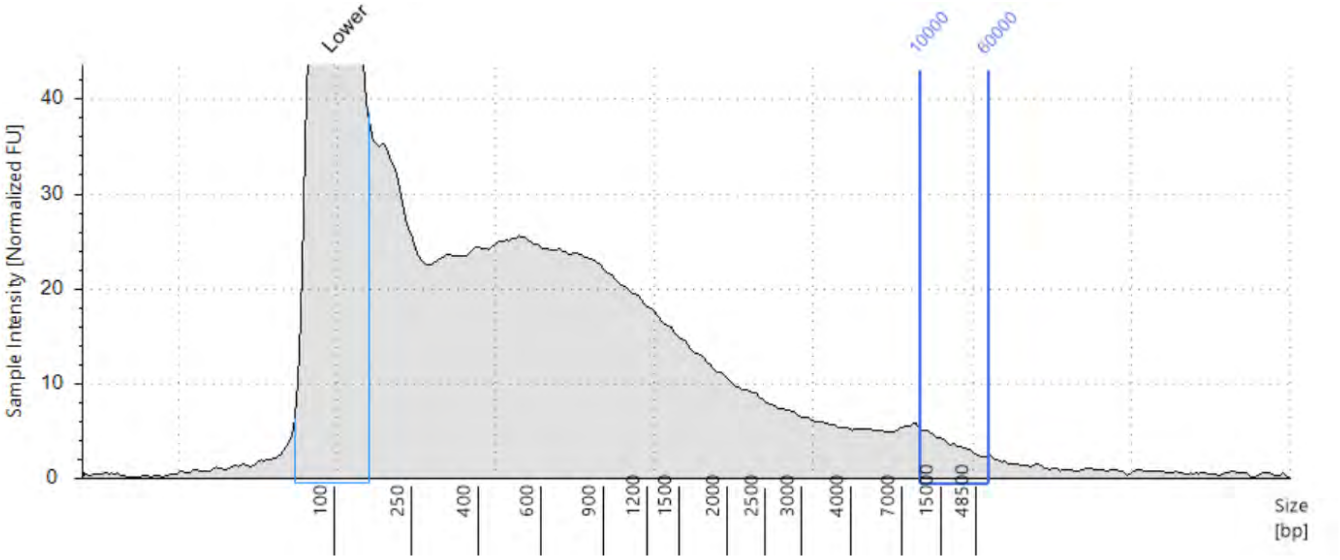

Region Table

From [bp]	To [bp]	Average Size [bp]	Conc. [ng/µl]	Region Molarity [nmol/l]	% of Total	Region Comment	Color
10000	60000	23009	0.112	0.0138	2.02		■

D2: M-T4-DS-149

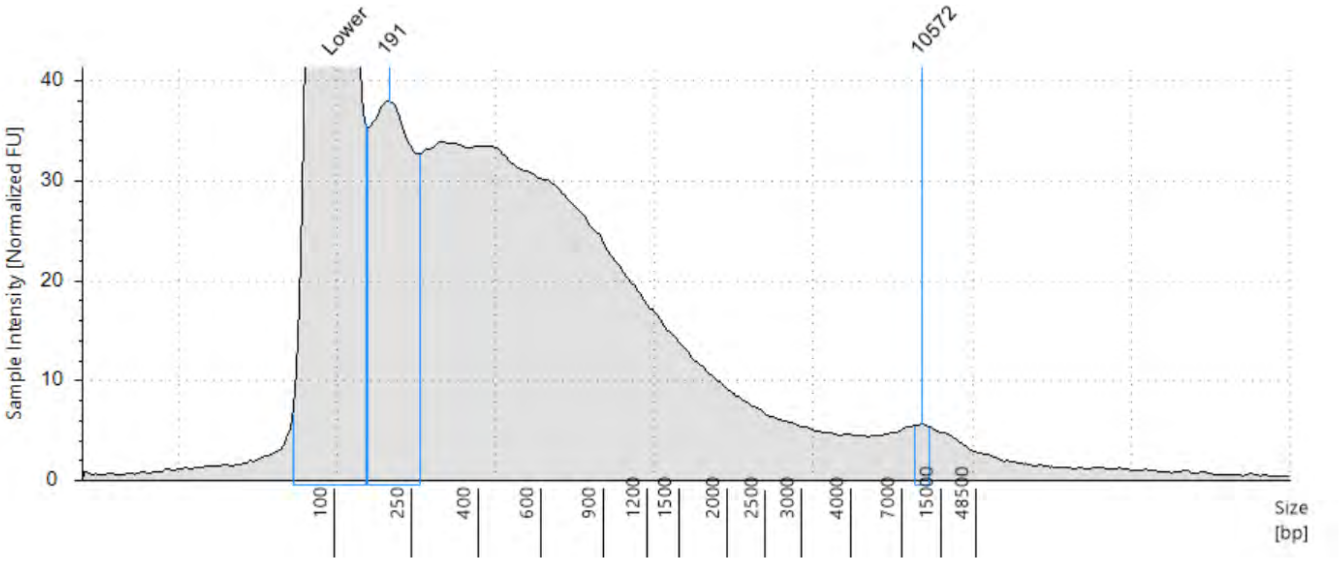

Sample Table

Well	DIN	Conc. [ng/µl]	Sample Description	Alert	Observations
D2	1.4	6.60	M-T4-DS-149	⚠	Sample concentration outside recommended range

Peak Table

Size [bp]	Calibrated Conc. [ng/µl]	Assigned Conc. [ng/µl]	% Integrated Area	From [bp]	To [bp]	Peak Comment	Observations
100	8.50	8.50	-	61	146		Lower Marker
191	1.15	-	96.19	149	264		
10572	0.0455	-	3.81	9211	12133		

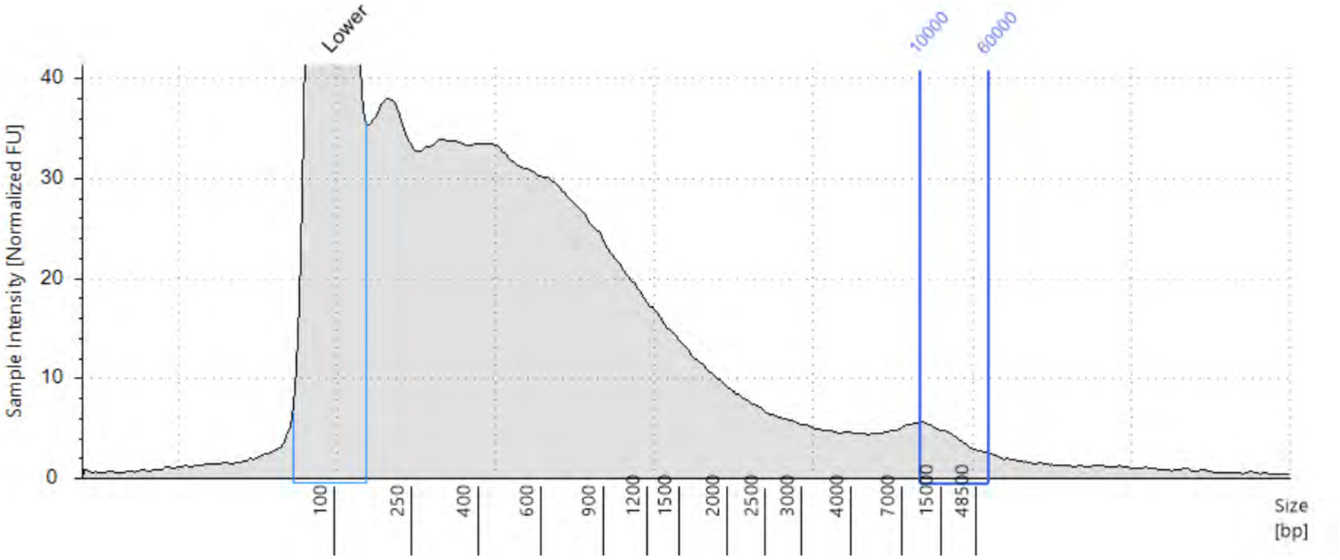

Region Table

From [bp]	To [bp]	Average Size [bp]	Conc. [ng/µl]	Region Molarity [nmol/l]	% of Total	Region Comment	Color
10000	60000	22823	0.146	0.0160	2.22		

E2: M-T4-DS-150

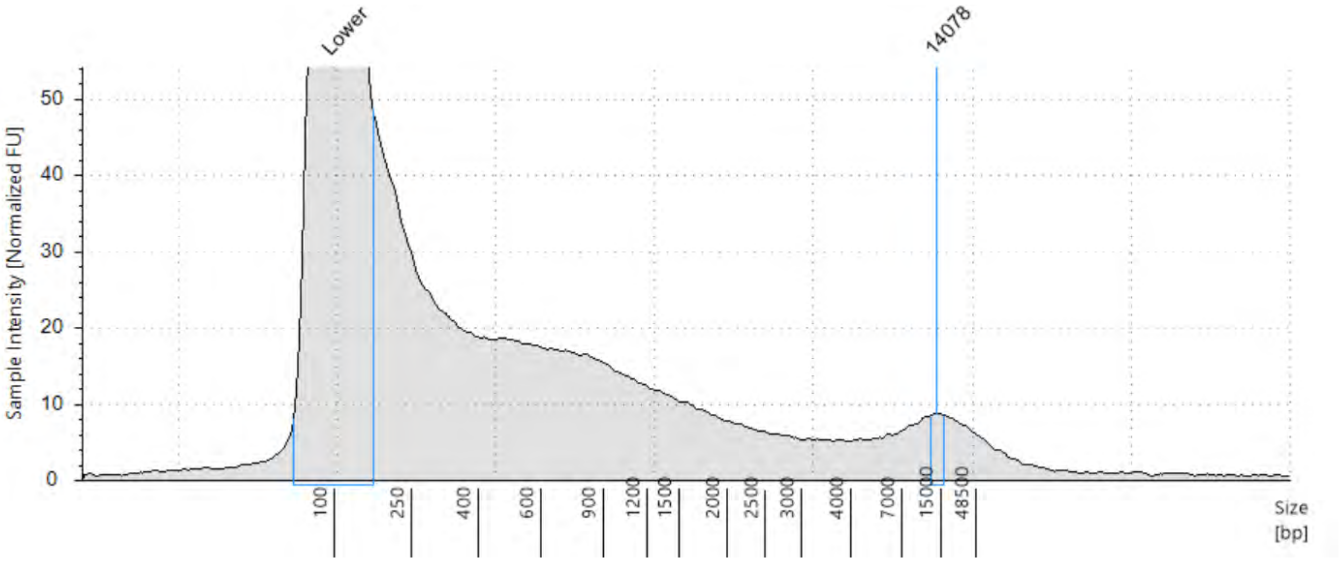

Sample Table

Well	DIN	Conc. [ng/µl]	Sample Description	Alert	Observations
E2	1.0	4.47	M-T4-DS-150	⚠	Sample concentration outside functional range for DIN

Peak Table

Size [bp]	Calibrated Conc. [ng/µl]	Assigned Conc. [ng/µl]	% Integrated Area	From [bp]	To [bp]	Peak Comment	Observations
100	8.50	8.50	-	62	159		Lower Marker
14078	0.0674	-	100.00	12257	16170		

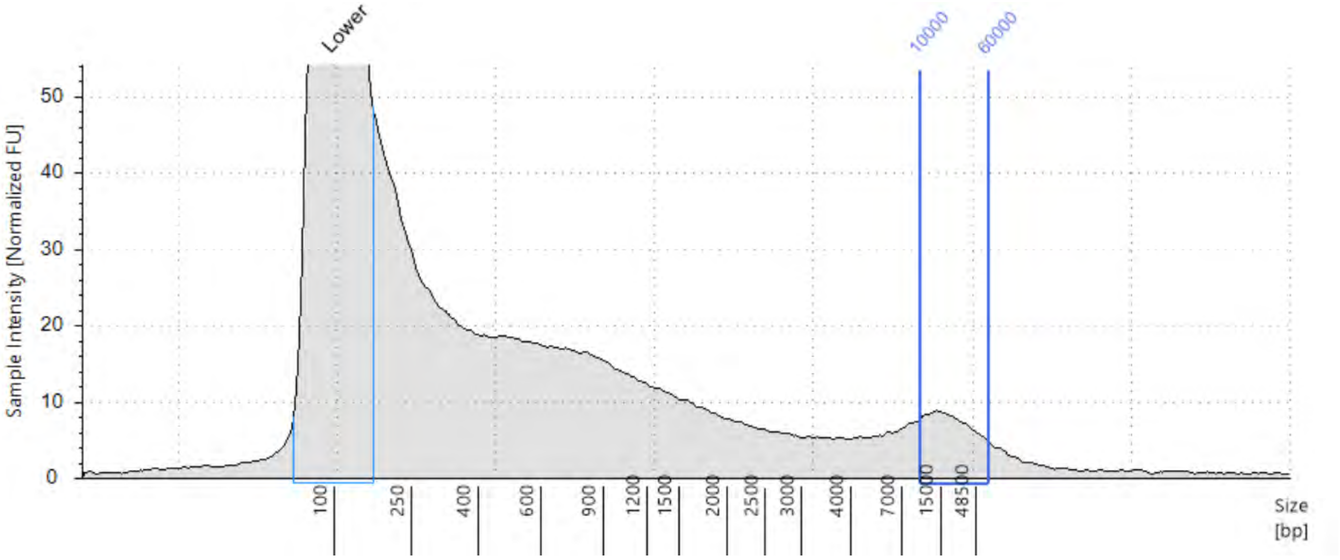

Region Table

From [bp]	To [bp]	Average Size [bp]	Conc. [ng/µl]	Region Molarity [nmol/l]	% of Total	Region Comment	Color
10000	60000	23921	0.259	0.0234	5.80		■

© Agilent Technologies, Inc. 2017

F2: M-T4-DS-284

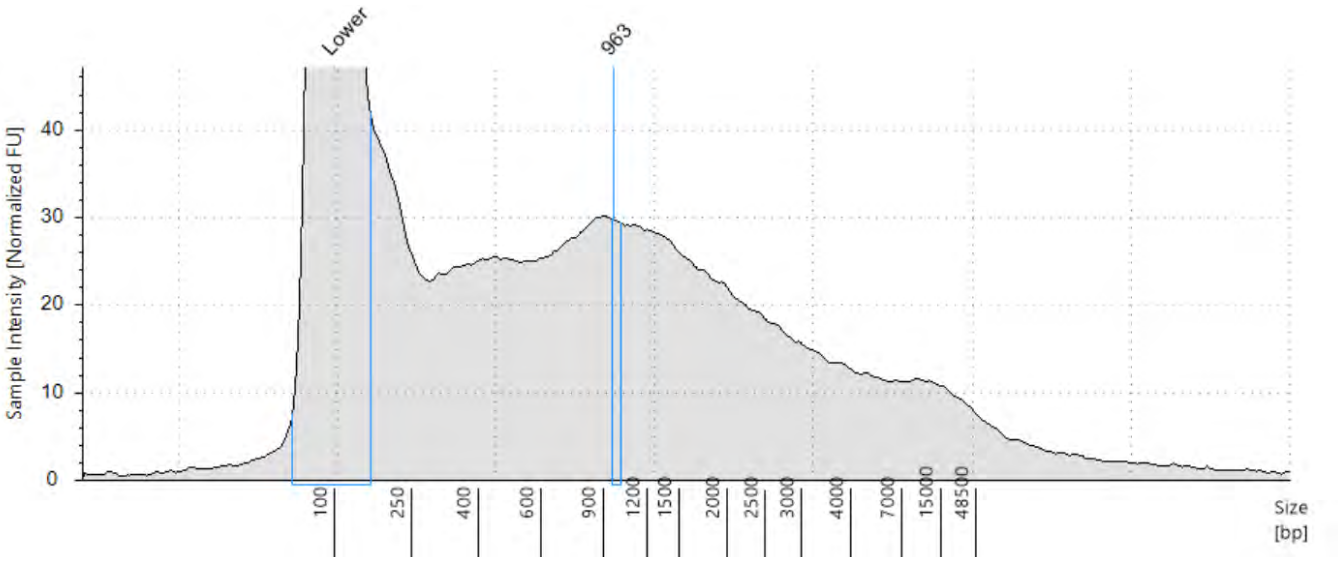

Sample Table

Well	DIN	Conc. [ng/µl]	Sample Description	Alert	Observations
F2	2.2	7.15	M-T4-DS-284	⚠	Sample concentration outside recommended range

Peak Table

Size [bp]	Calibrated Conc. [ng/µl]	Assigned Conc. [ng/µl]	% Integrated Area	From [bp]	To [bp]	Peak Comment	Observations
100	8.50	8.50	-	61	154		Lower Marker
963	0.157	-	100.00	954	1008		

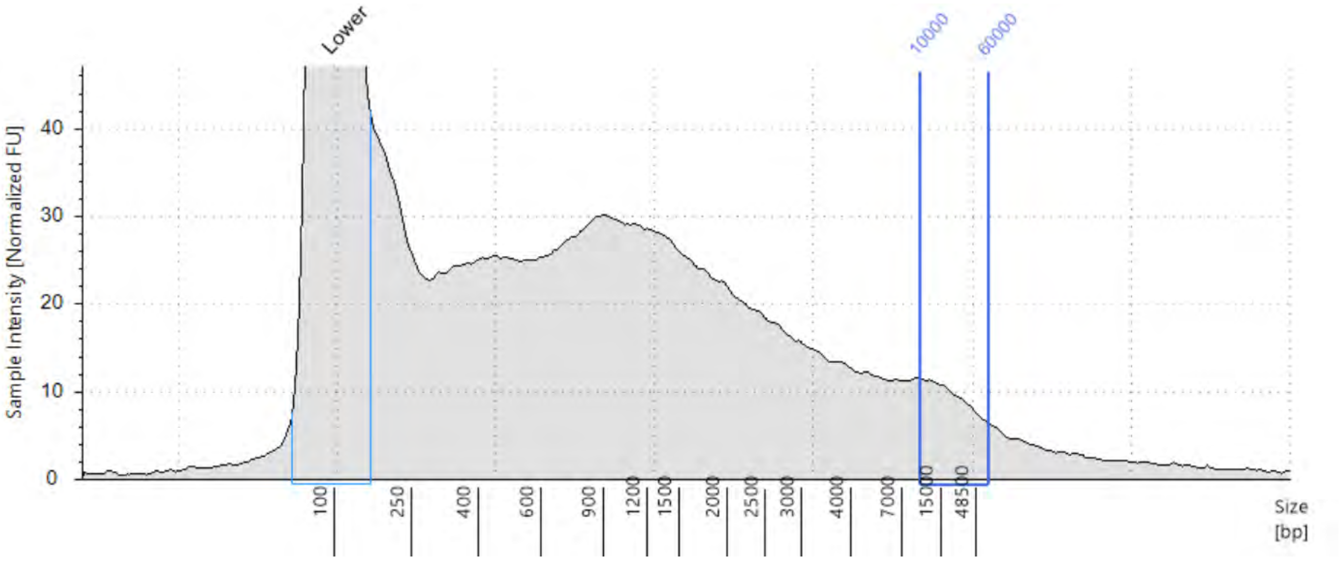

Region Table

From [bp]	To [bp]	Average Size [bp]	Conc. [ng/µl]	Region Molarity [nmol/l]	% of Total	Region Comment	Color
10000	60000	23813	0.321	0.0319	4.49		

G2: M-T4-DS-285

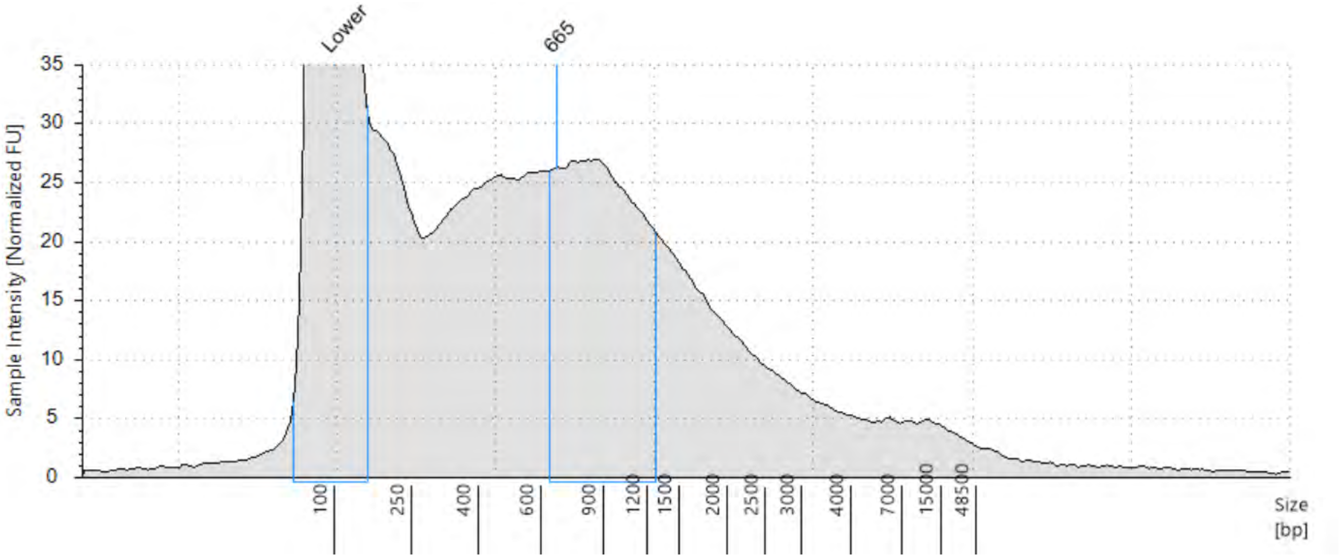

Sample Table

Well	DIN	Conc. [ng/µl]	Sample Description	Alert	Observations
G2	1.8	5.93	M-T4-DS-285	⚠	Sample concentration outside recommended range

Peak Table

Size [bp]	Calibrated Conc. [ng/µl]	Assigned Conc. [ng/µl]	% Integrated Area	From [bp]	To [bp]	Peak Comment	Observations
100	8.50	8.50	-	62	149		Lower Marker
665	1.63	-	100.00	635	1285		

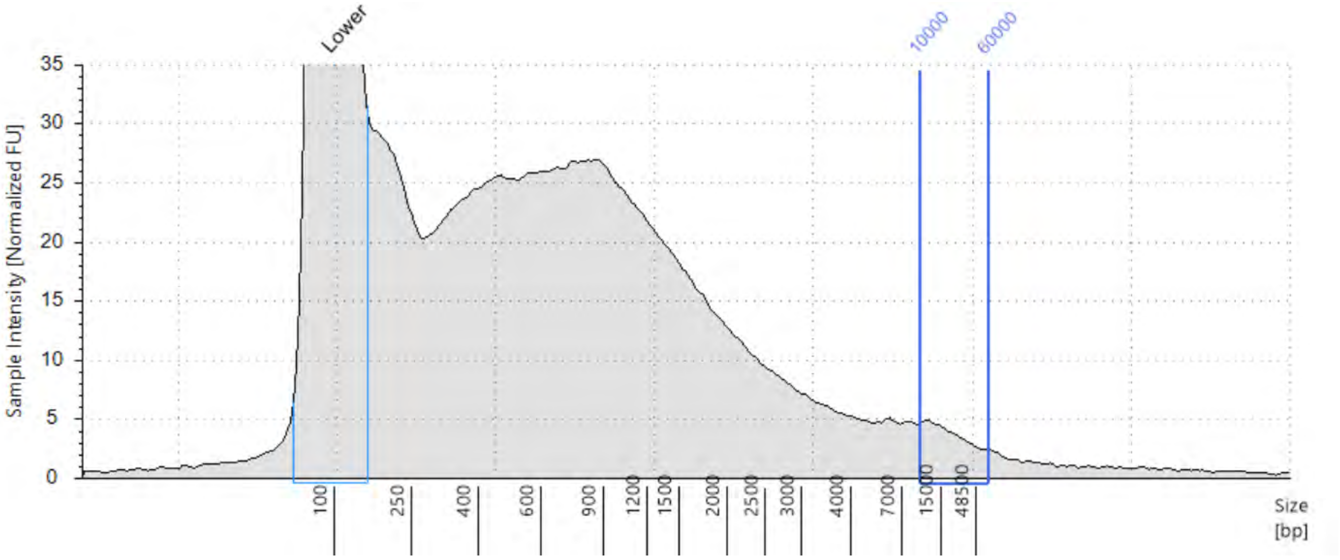

Region Table

From [bp]	To [bp]	Average Size [bp]	Conc. [ng/µl]	Region Molarity [nmol/l]	% of Total	Region Comment	Color
10000	60000	23013	0.121	0.0138	2.05		■

H2: M-T5-Initial-223

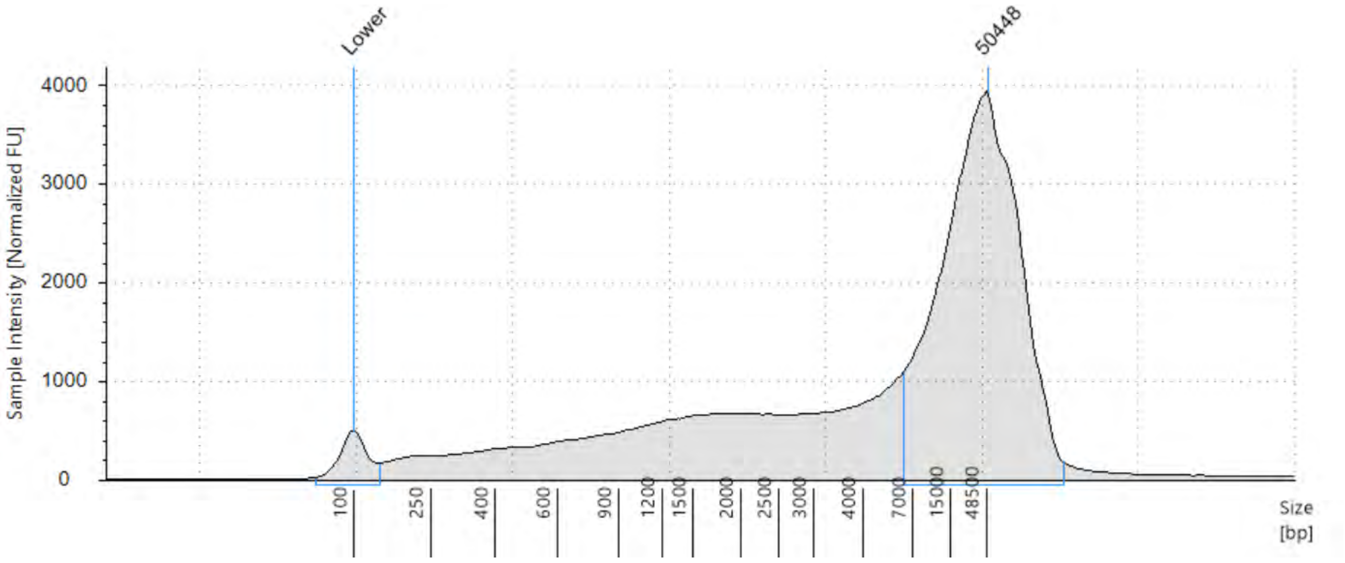

Sample Table

Well	DIN	Conc. [ng/µl]	Sample Description	Alert	Observations
H2	6.5	367	M-T5-Initial-223	⚠	Sample concentration outside functional range for DIN

Peak Table

Size [bp]	Calibrated Conc. [ng/µl]	Assigned Conc. [ng/µl]	% Integrated Area	From [bp]	To [bp]	Peak Comment	Observations
100	8.50	8.50	-	63	137		Lower Marker
50448	210	-	98.48	6339	>60000		
-	-	-	-	-	-		Sample Well

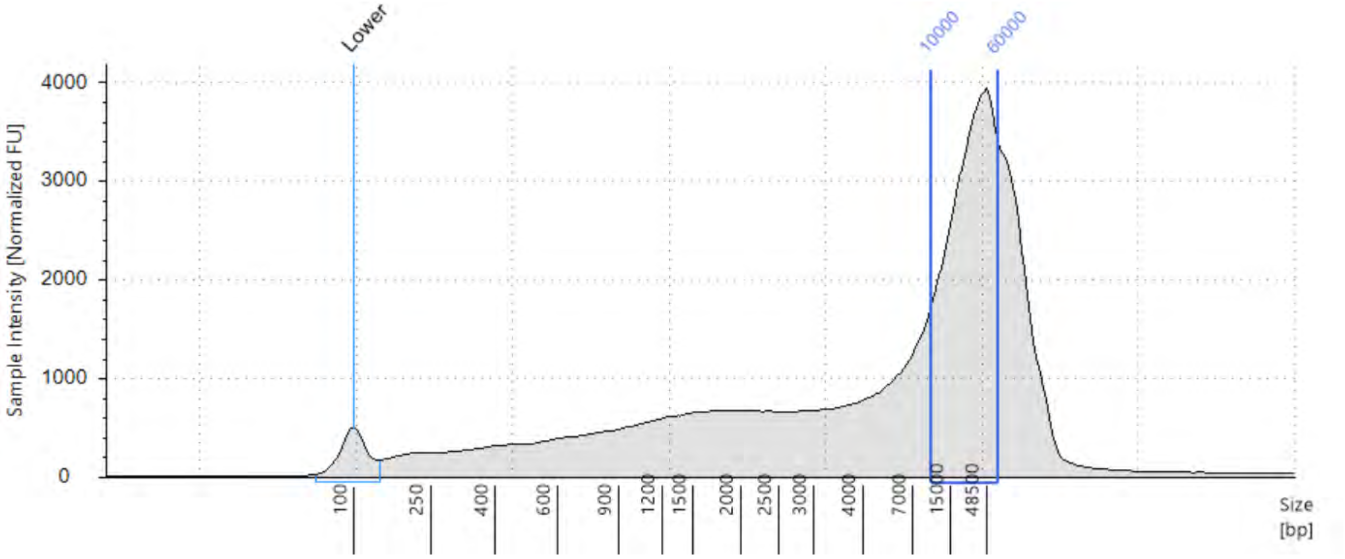

Region Table

From [bp]	To [bp]	Average Size [bp]	Conc. [ng/µl]	Region Molarity [nmol/l]	% of Total	Region Comment	Color
10000	60000	29269	123	8.77	33.58		■

© Agilent Technologies, Inc. 2017

Filename: 2019-07-29-02.gDNA

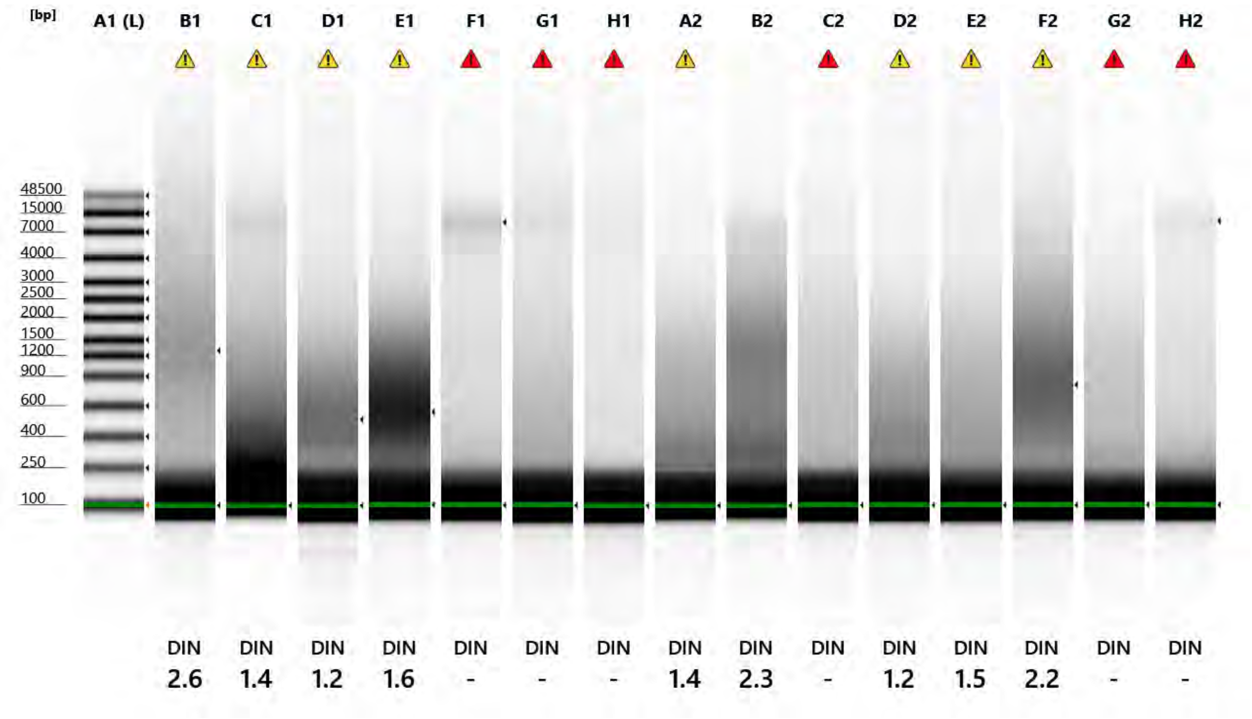

Default image (Contrast 50%), Image is Scaled to Sample

Sample Info

Well	DIN	Conc. [ng/µl]	Sample Description	Alert	Observations
A1	-	74.3	Ladder		Ladder
B1	2.6	4.81	C-T4-DS-374	⚠	Sample concentration outside functional range for DIN
C1	1.4	8.56	C-T4-DS-375	⚠	Sample concentration outside recommended range
D1	1.2	3.02	C-T4-DS-596	⚠	Sample concentration outside functional range for DIN
E1	1.6	7.02	C-T4-DS-597	⚠	Sample concentration outside recommended range
F1	-	2.53	C-T4-DS-598	▲	Sample concentration outside functional range for DIN
G1	-	2.21	C-T4-DS-599	▲	Sample concentration outside functional range for DIN
H1	-	1.15	C-T4-DS-600	▲	Sample concentration outside functional range for DIN
A2	1.4	5.30	C-T4-D-378	⚠	Sample concentration outside recommended range
B2	2.3	10.3	C-T4-D-379		
C2	-	2.08	C-T4-D-606	▲	Sample concentration outside functional range for DIN
D2	1.2	3.32	C-T4-D-607	⚠	Sample concentration outside functional range for DIN
E2	1.5	3.80	C-T4-D-608	⚠	Sample concentration outside functional range for DIN
F2	2.2	5.94	C-T4-D-609	⚠	Sample concentration outside recommended range
G2	-	2.66	C-T4-D-610	▲	Sample concentration outside functional range for DIN
H2	-	2.43	C-T4-S-382	▲	Sample concentration outside functional range for DIN

© Agilent Technologies, Inc. 2017

A1: Ladder

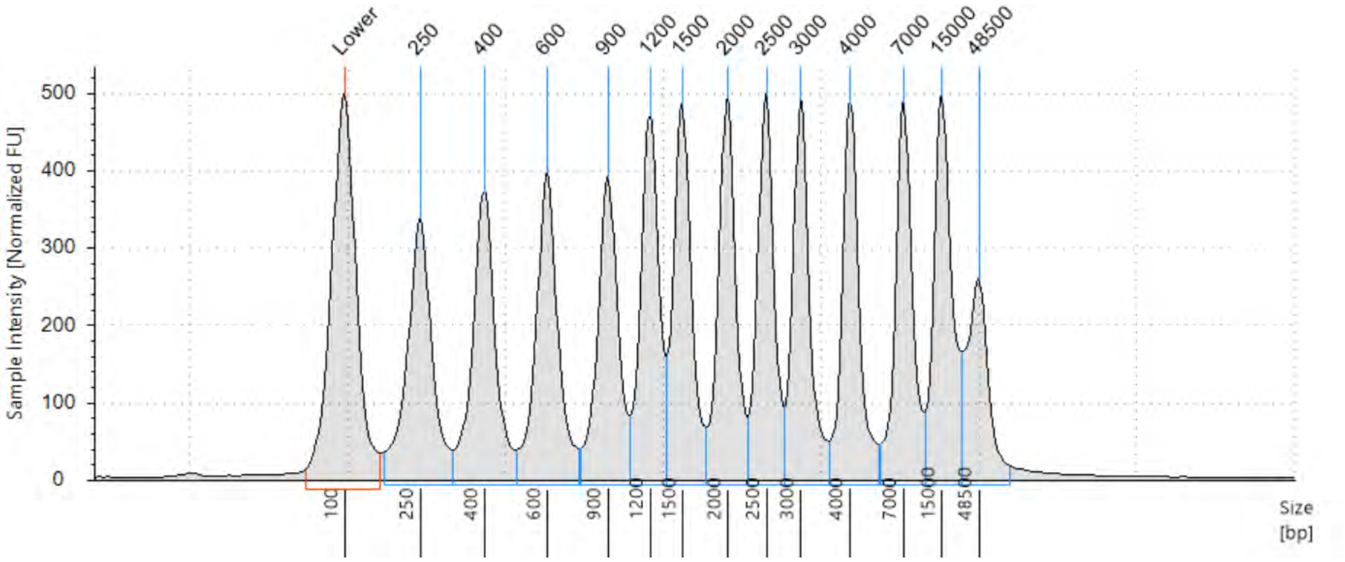

Sample Table

Well	DIN	Conc. [ng/µl]	Sample Description	Alert	Observations
A1	-	74.3	Ladder		Ladder

Peak Table

Size [bp]	Calibrated Conc. [ng/µl]	Assigned Conc. [ng/µl]	% Integrated Area	From [bp]	To [bp]	Peak Comment	Observations
100	8.50	8.50	-	62	152		Lower Marker
250	5.72	-	7.71	162	318		
400	5.79	-	7.80	318	490		
600	5.99	-	8.07	490	742		
900	5.52	-	7.44	749	1044		
1200	6.01	-	8.11	1044	1342		
1500	6.16	-	8.30	1342	1740		
2000	5.96	-	8.03	1740	2236		
2500	5.79	-	7.80	2236	2749		
3000	5.79	-	7.81	2749	3548		
4000	5.70	-	7.68	3548	5407		
7000	5.73	-	7.73	5485	10820		
15000	6.21	-	8.37	10820	22573		
48500	3.81	-	5.14	22573	>60000		

 © Agilent Technologies, Inc. 2017

B1: C-T4-DS-374

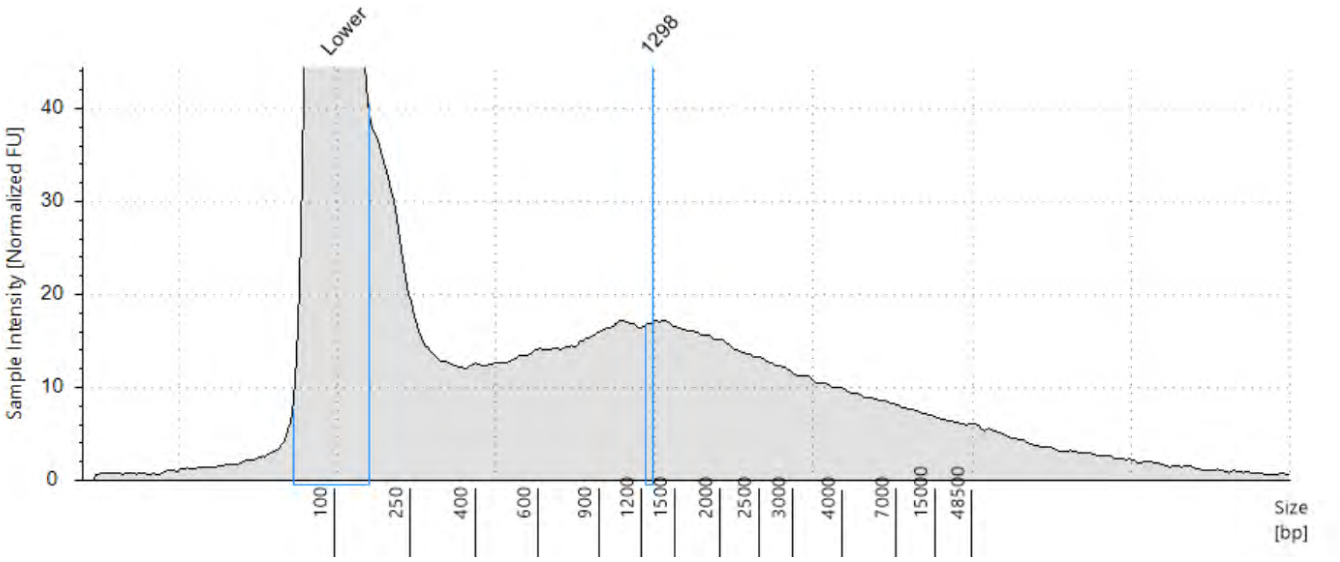

Sample Table

Well	DIN	Conc. [ng/µl]	Sample Description	Alert	Observations
B1	2.6	4.81	C-T4-DS-374	⚠	Sample concentration outside functional range for DIN

Peak Table

Size [bp]	Calibrated Conc. [ng/µl]	Assigned Conc. [ng/µl]	% Integrated Area	From [bp]	To [bp]	Peak Comment	Observations
100	8.50	8.50	-	61	153		Lower Marker
1298	0.0743	-	100.00	1240	1298		

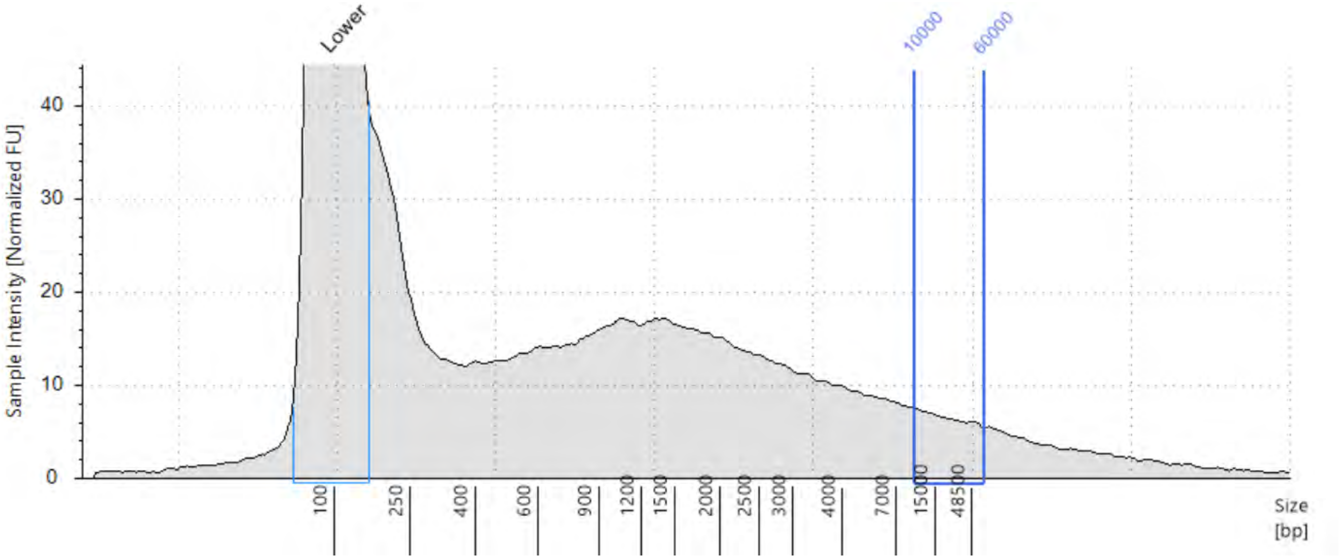

Region Table

From [bp]	To [bp]	Average Size [bp]	Conc. [ng/µl]	Region Molarity [nmol/l]	% of Total	Region Comment	Color
10000	60000	25567	0.229	0.0214	4.76		■

 © Agilent Technologies, Inc. 2017

C1: C-T4-DS-375

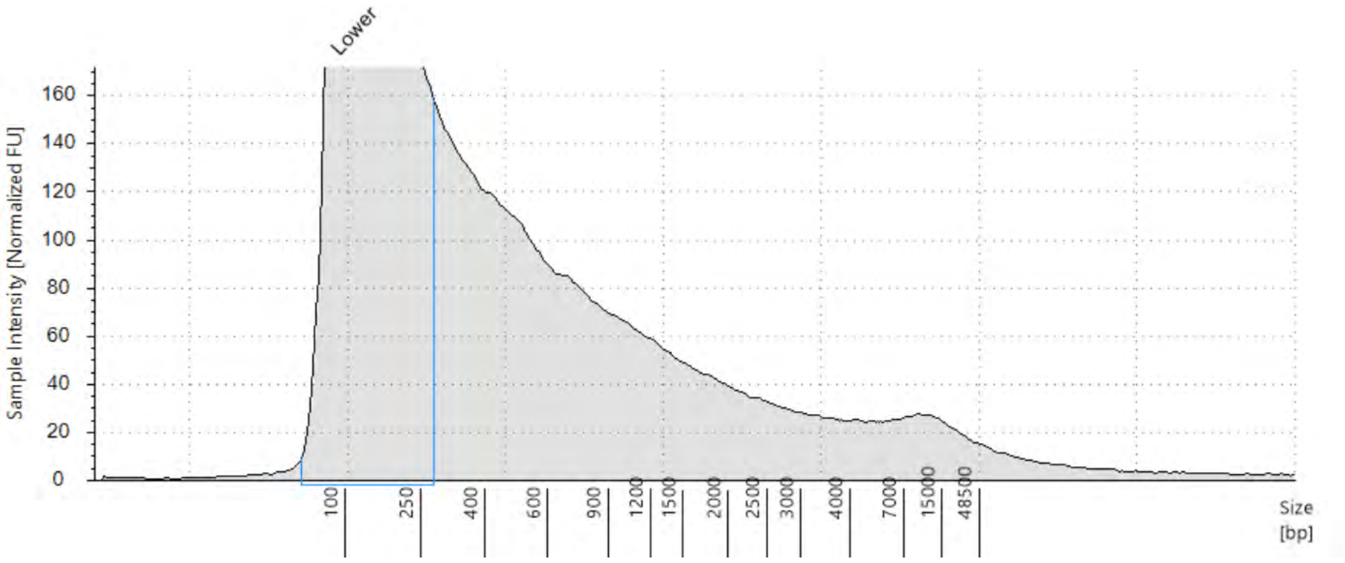

Sample Table

Well	DIN	Conc. [ng/µl]	Sample Description	Alert	Observations
C1	1.4	8.56	C-T4-DS-375	⚠	Sample concentration outside recommended range

Peak Table

Size [bp]	Calibrated Conc. [ng/µl]	Assigned Conc. [ng/µl]	% Integrated Area	From [bp]	To [bp]	Peak Comment	Observations
100	8.50	8.50	-	60	280		Lower Marker
-	-	-	-	-	-		Sample Well

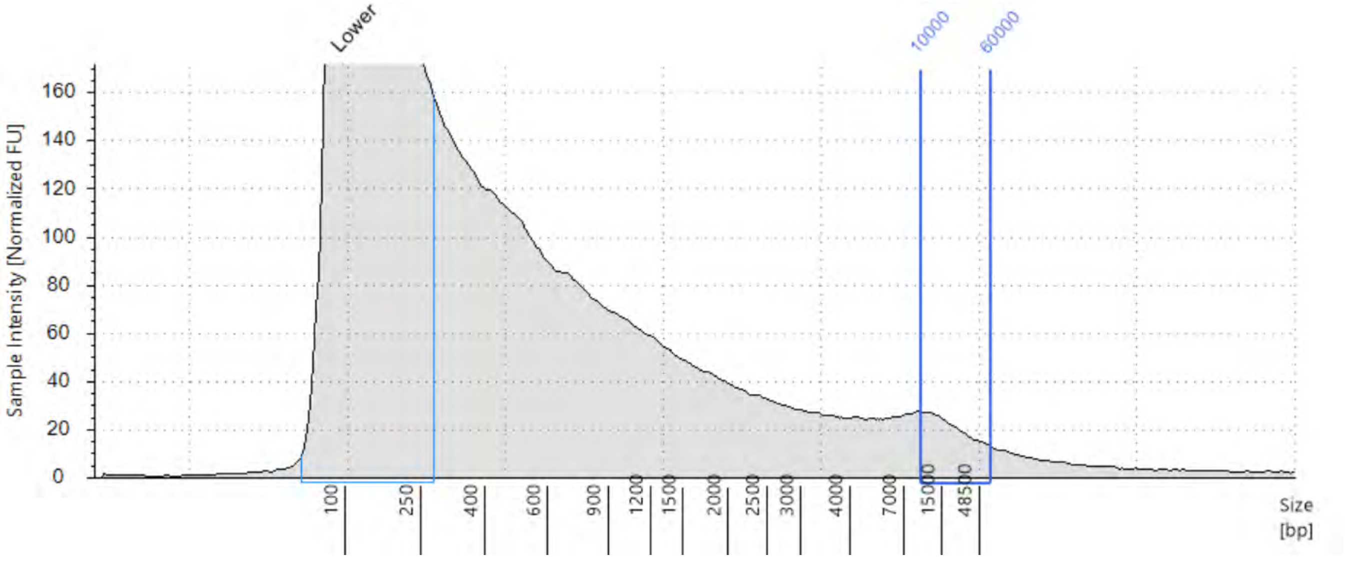

Region Table

From [bp]	To [bp]	Average Size [bp]	Conc. [ng/µl]	Region Molarity [nmol/l]	% of Total	Region Comment	Color
10000	60000	23287	0.367	0.0329	4.28		■

 © Agilent Technologies, Inc. 2017

D1: C-T4-DS-596

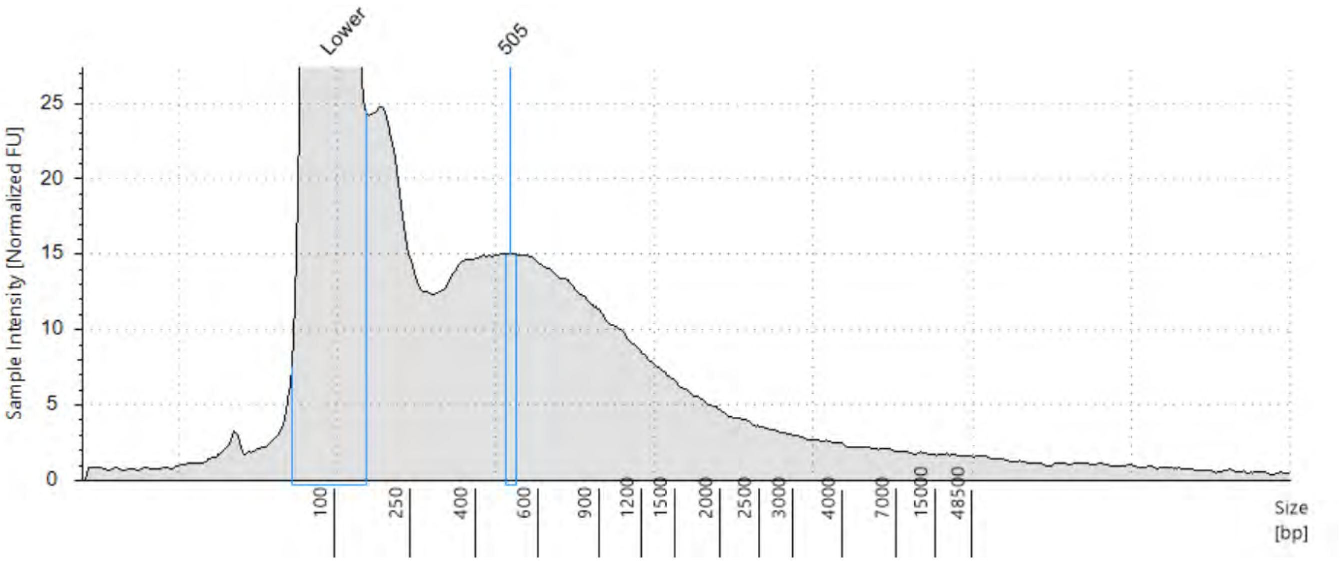

Sample Table

Well	DIN	Conc. [ng/µl]	Sample Description	Alert	Observations
D1	1.2	3.02	C-T4-DS-596	⚠	Sample concentration outside functional range for DIN

Peak Table

Size [bp]	Calibrated Conc. [ng/µl]	Assigned Conc. [ng/µl]	% Integrated Area	From [bp]	To [bp]	Peak Comment	Observations
100	8.50	8.50	-	61	148		Lower Marker
505	0.108	-	100.00	492	528		

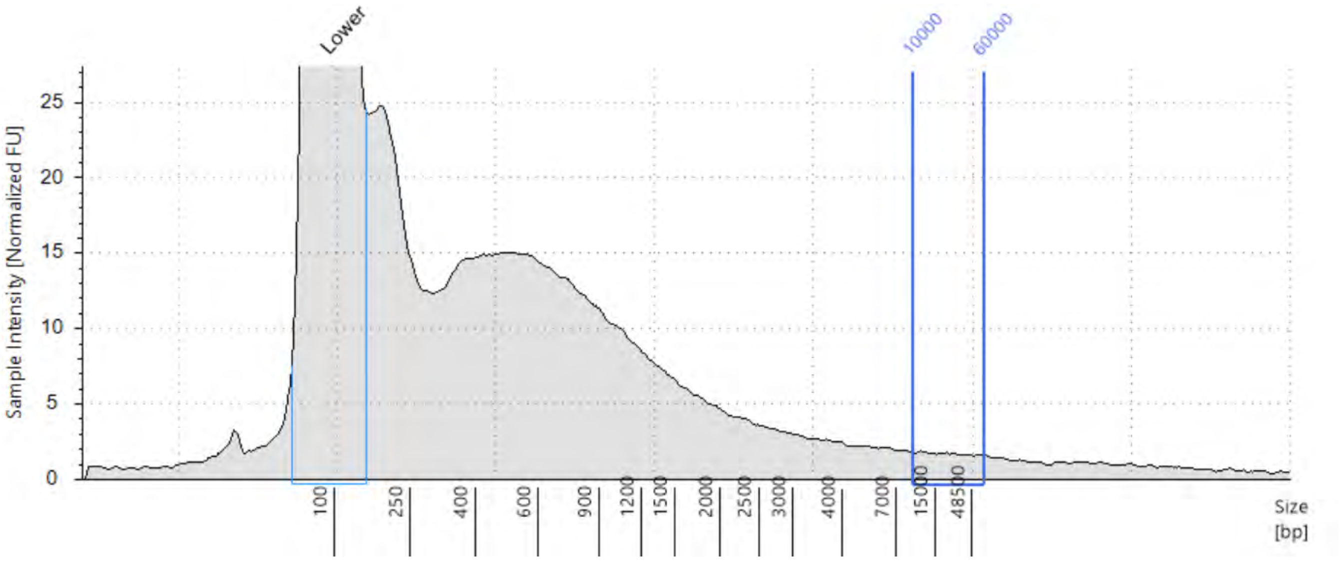

Region Table

From [bp]	To [bp]	Average Size [bp]	Conc. [ng/µl]	Region Molarity [nmol/l]	% of Total	Region Comment	Color
10000	60000	25982	0.0410	0.00576	1.36		■

E1: C-T4-DS-597

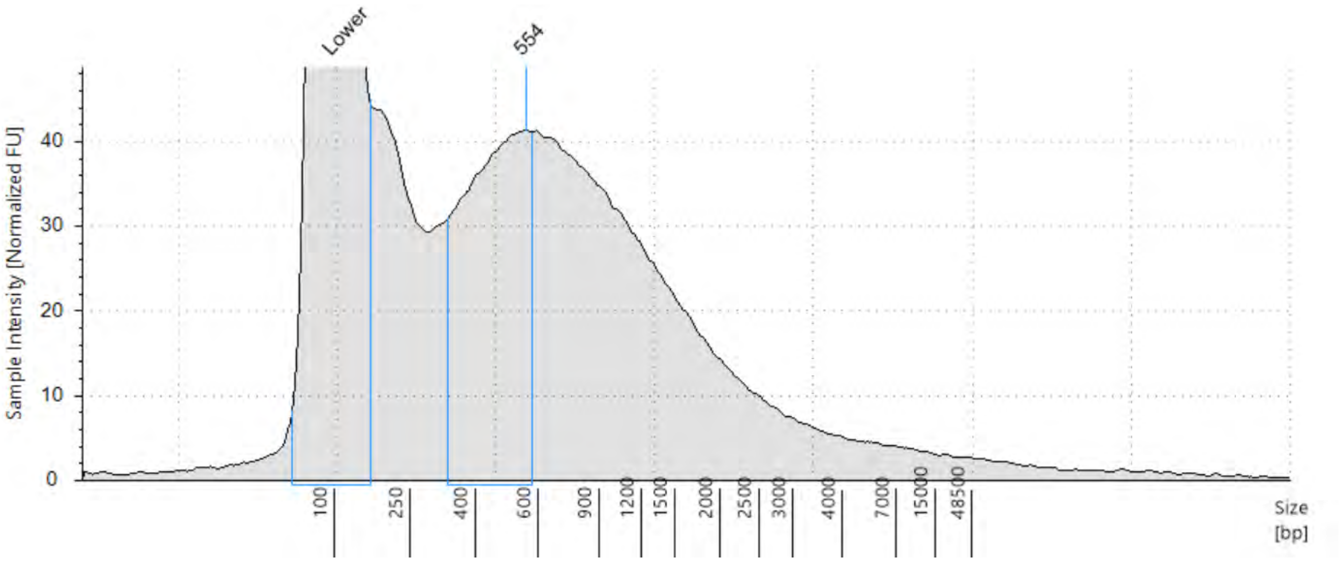

Sample Table

Well	DIN	Conc. [ng/µl]	Sample Description	Alert	Observations
E1	1.6	7.02	C-T4-DS-597	⚠	Sample concentration outside recommended range

Peak Table

Size [bp]	Calibrated Conc. [ng/µl]	Assigned Conc. [ng/µl]	% Integrated Area	From [bp]	To [bp]	Peak Comment	Observations
100	8.50	8.50	-	60	154		Lower Marker
554	1.70	-	100.00	329	574		

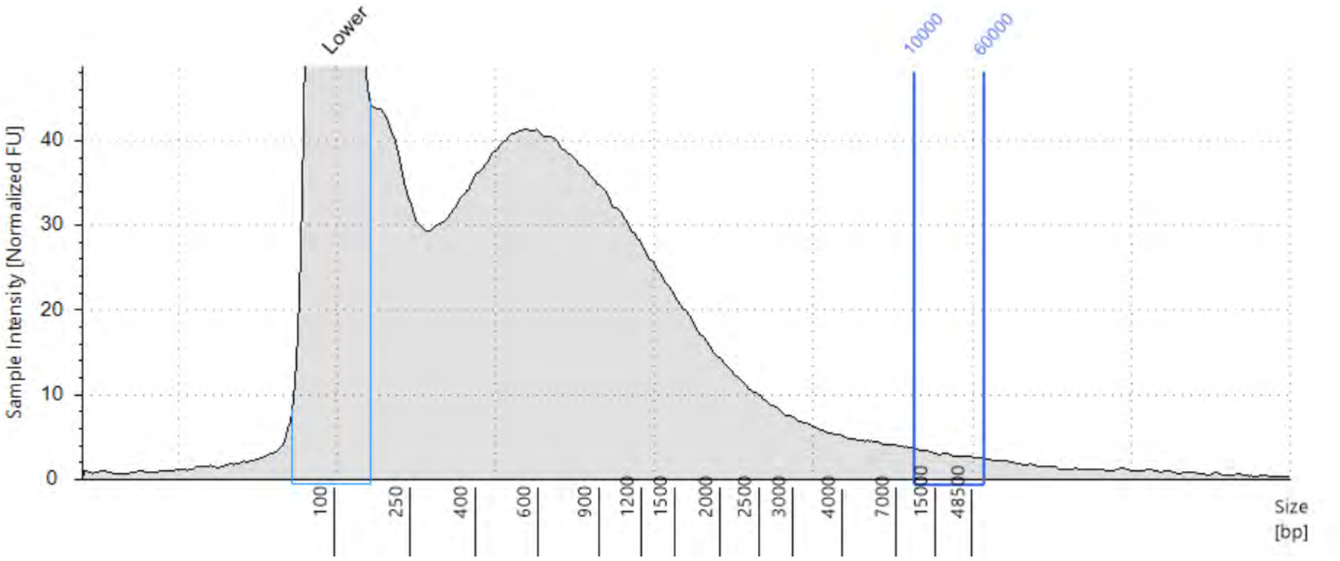

Region Table

From [bp]	To [bp]	Average Size [bp]	Conc. [ng/µl]	Region Molarity [nmol/l]	% of Total	Region Comment	Color
10000	60000	25145	0.0810	0.00929	1.15		■

© Agilent Technologies, Inc. 2017

F1: C-T4-DS-598

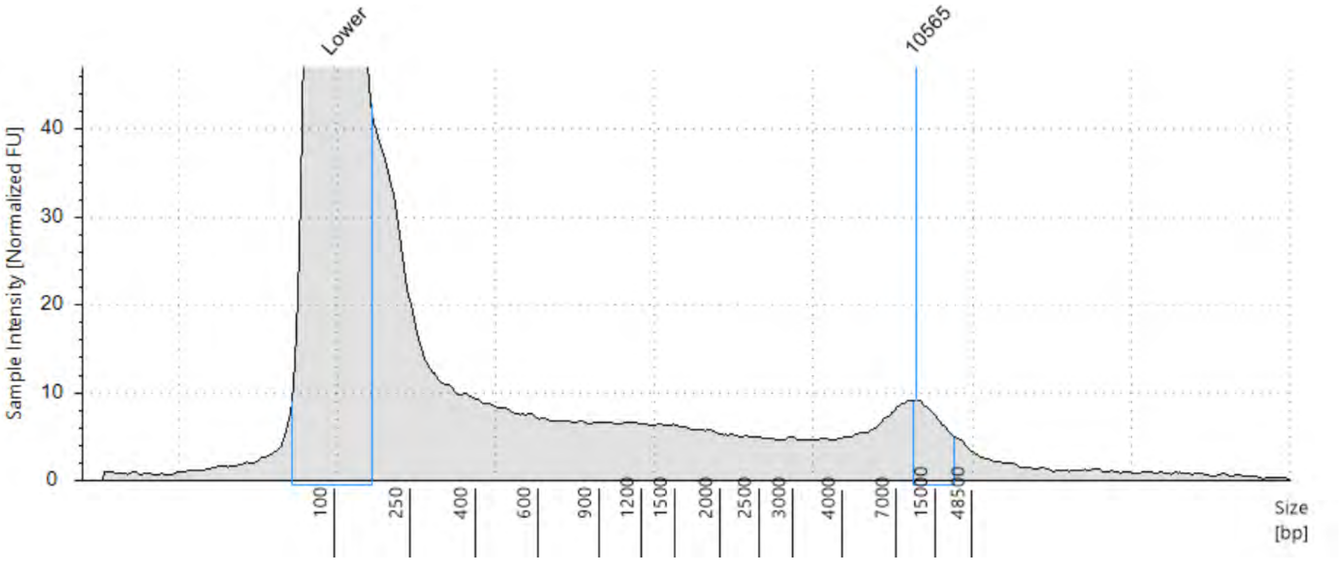

Sample Table

Well	DIN	Conc. [ng/µl]	Sample Description	Alert	Observations
F1	-	2.53	C-T4-DS-598	▲	Sample concentration outside functional range for DIN

Peak Table

Size [bp]	Calibrated Conc. [ng/µl]	Assigned Conc. [ng/µl]	% Integrated Area	From [bp]	To [bp]	Peak Comment	Observations
100	8.50	8.50	-	60	158		Lower Marker
10565	0.148	-	100.00	9751	22332		

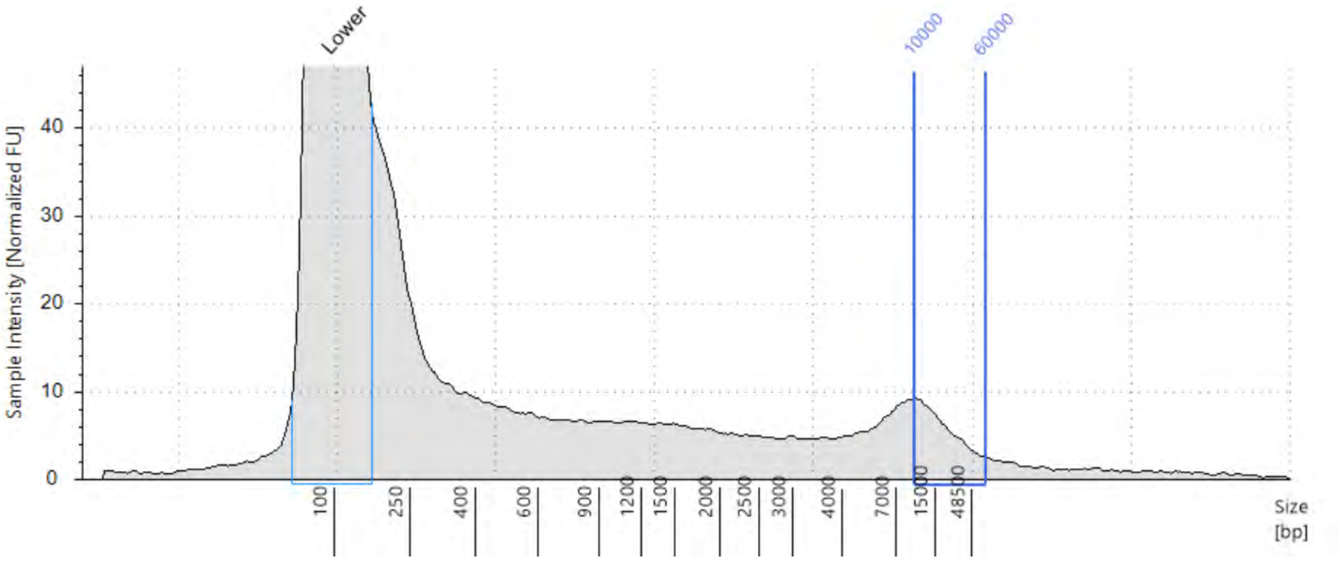

Region Table

From [bp]	To [bp]	Average Size [bp]	Conc. [ng/µl]	Region Molarity [nmol/l]	% of Total	Region Comment	Color
10000	60000	21311	0.186	0.0203	7.36		■

G1: C-T4-DS-599

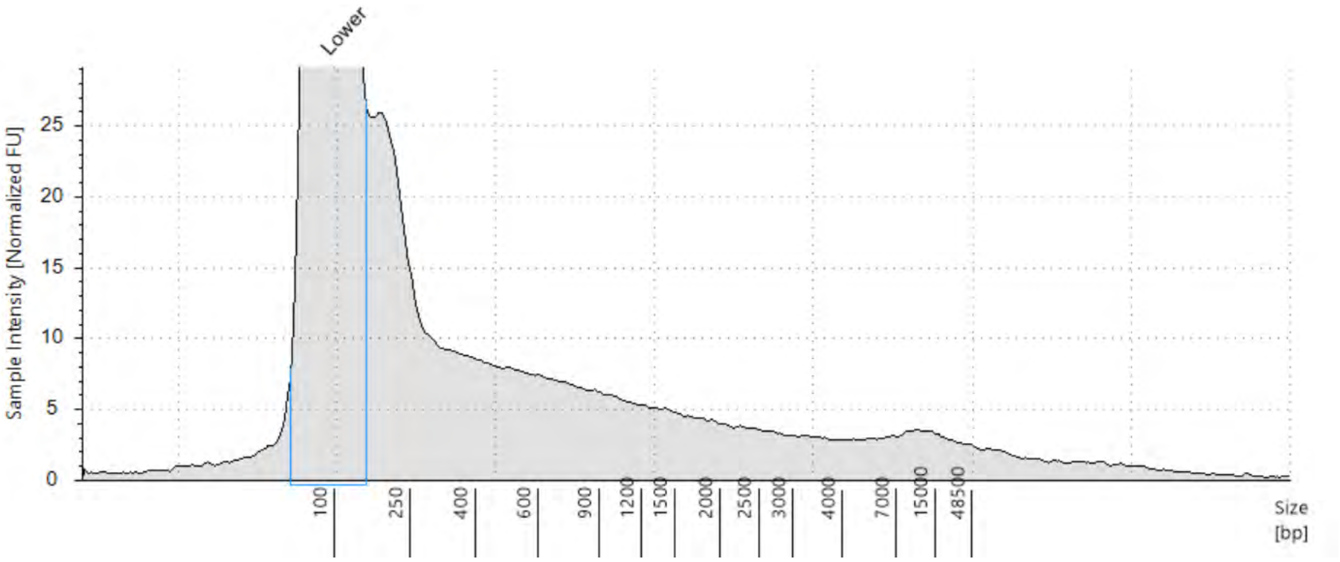

Sample Table

Well	DIN	Conc. [ng/µl]	Sample Description	Alert	Observations
G1	-	2.21	C-T4-DS-599	▲	Sample concentration outside functional range for DIN

Peak Table

Size [bp]	Calibrated Conc. [ng/µl]	Assigned Conc. [ng/µl]	% Integrated Area	From [bp]	To [bp]	Peak Comment	Observations
100	8.50	8.50	-	60	149		Lower Marker

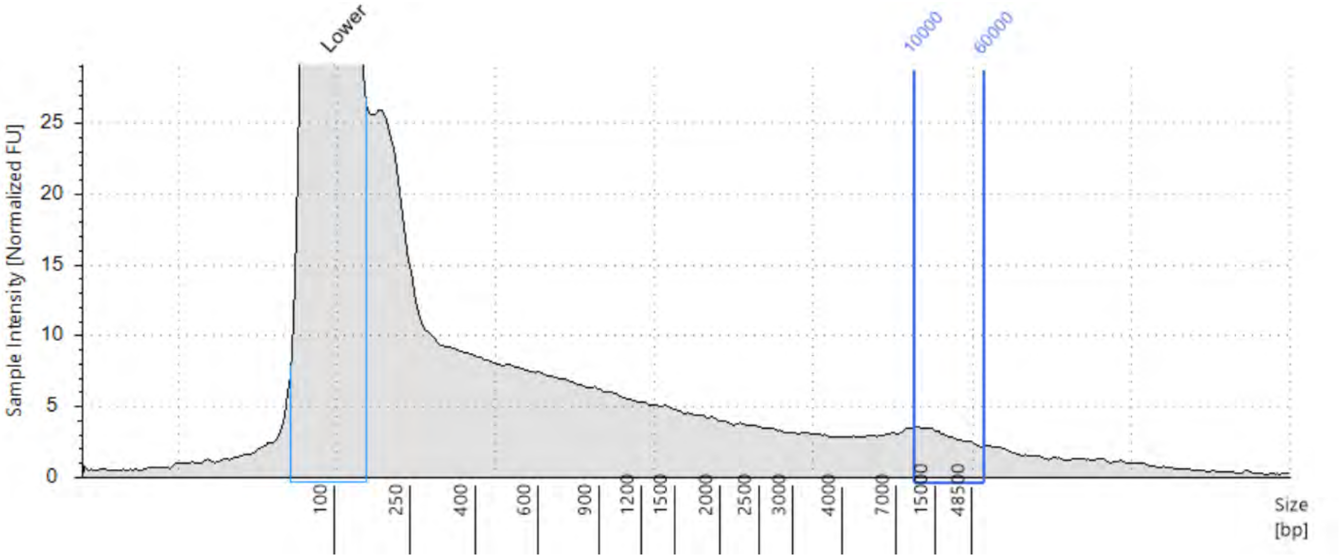

Region Table

From [bp]	To [bp]	Average Size [bp]	Conc. [ng/µl]	Region Molarity [nmol/l]	% of Total	Region Comment	Color
10000	60000	24258	0.0927	0.00995	4.19		■

H1: C-T4-DS-600

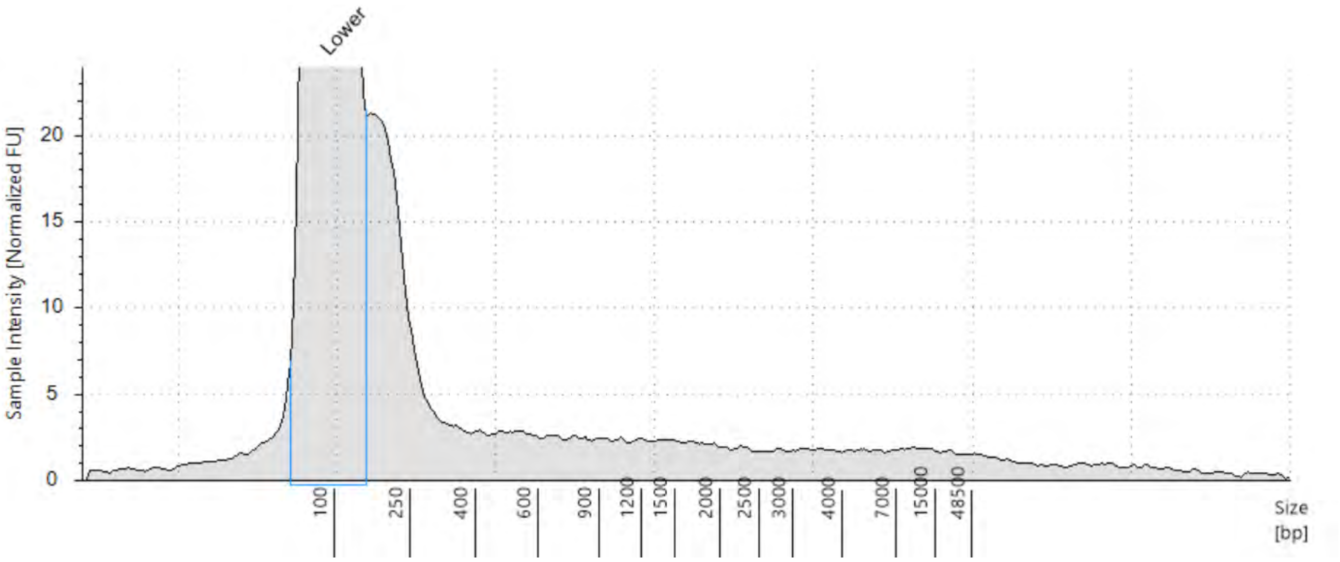

Sample Table

Well	DIN	Conc. [ng/µl]	Sample Description	Alert	Observations
H1	-	1.15	C-T4-DS-600	▲	Sample concentration outside functional range for DIN

Peak Table

Size [bp]	Calibrated Conc. [ng/µl]	Assigned Conc. [ng/µl]	% Integrated Area	From [bp]	To [bp]	Peak Comment	Observations
100	8.50	8.50	-	60	148		Lower Marker

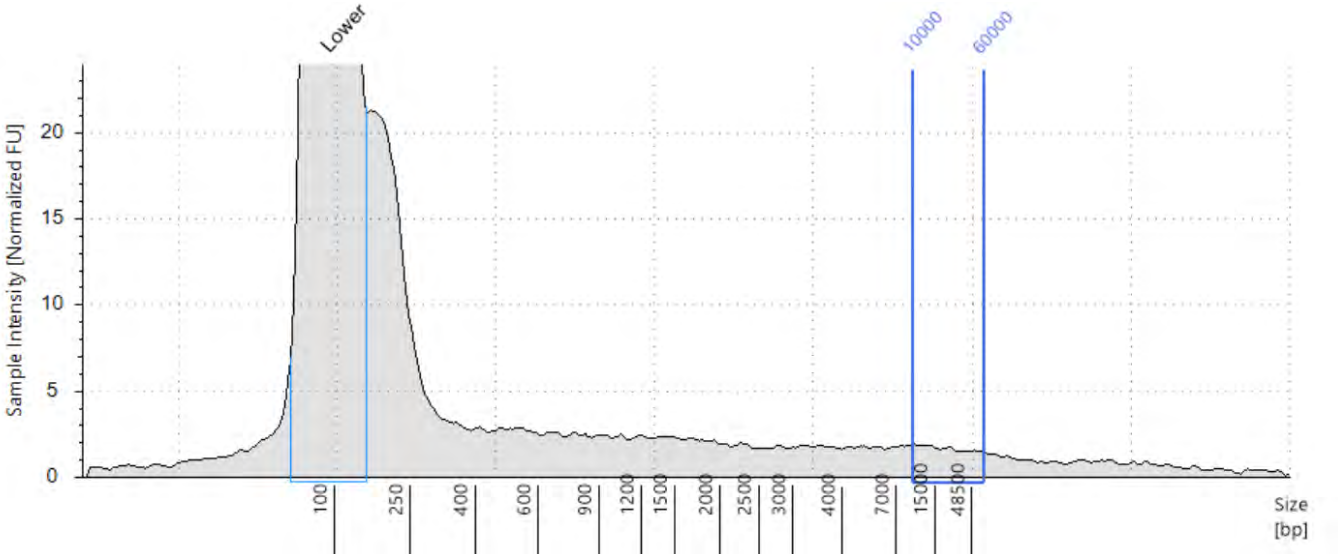

Region Table

From [bp]	To [bp]	Average Size [bp]	Conc. [ng/µl]	Region Molarity [nmol/l]	% of Total	Region Comment	Color
10000	60000	25516	0.0445	0.00563	3.86		■

© Agilent Technologies, Inc. 2017

A2: C-T4-D-378

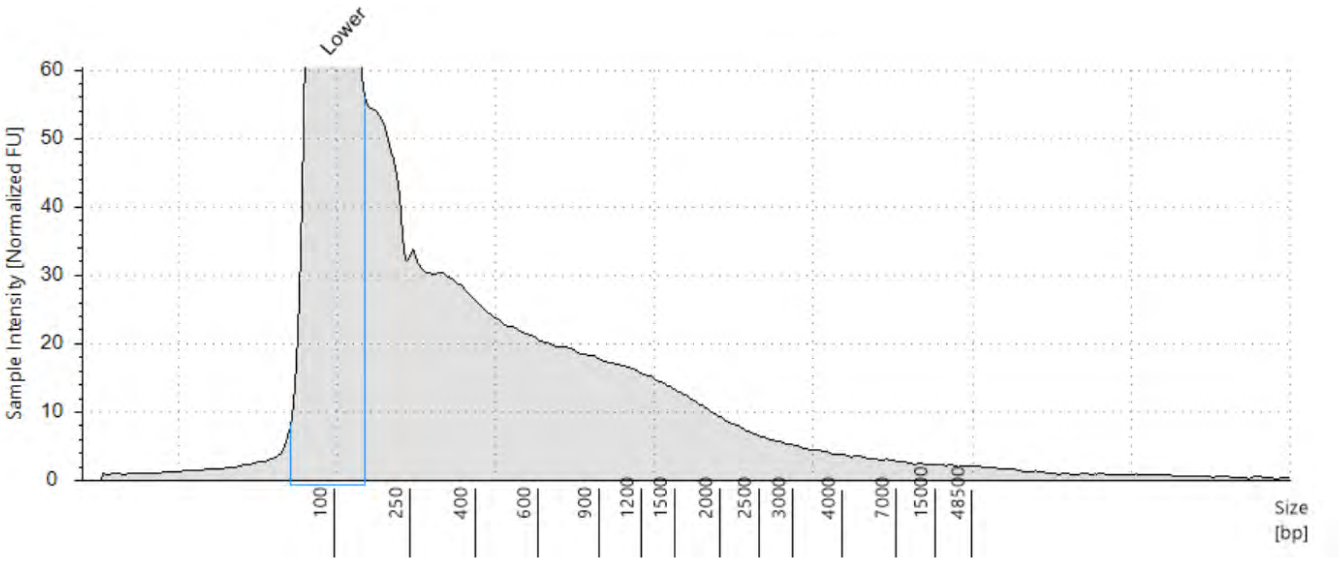

Sample Table

Well	DIN	Conc. [ng/µl]	Sample Description	Alert	Observations
A2	1.4	5.30	C-T4-D-378	⚠	Sample concentration outside recommended range

Peak Table

Size [bp]	Calibrated Conc. [ng/µl]	Assigned Conc. [ng/µl]	% Integrated Area	From [bp]	To [bp]	Peak Comment	Observations
100	8.50	8.50	-	60	148		Lower Marker

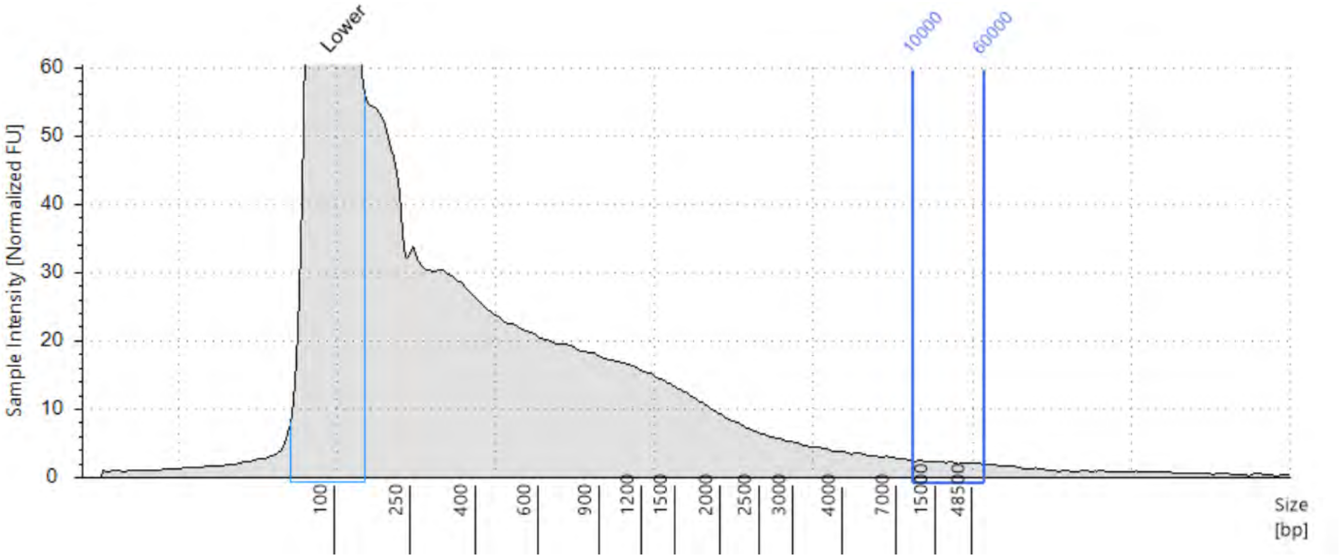

Region Table

From [bp]	To [bp]	Average Size [bp]	Conc. [ng/µl]	Region Molarity [nmol/l]	% of Total	Region Comment	Color
10000	60000	25853	0.0609	0.00714	1.15		■

B2: C-T4-D-379

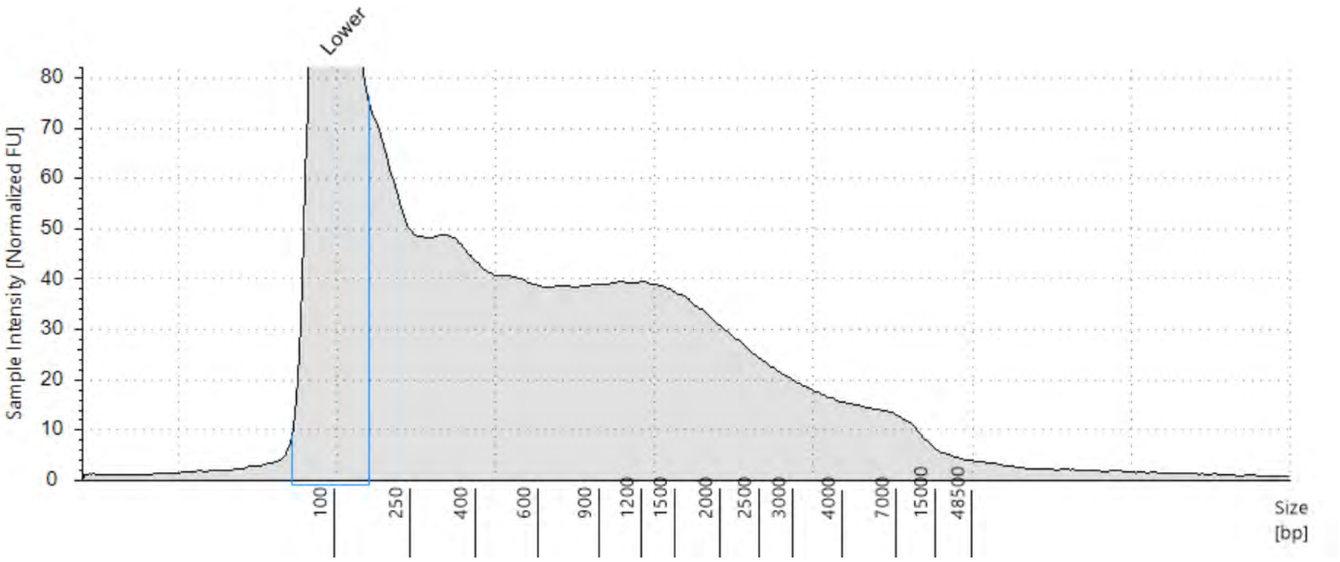

Sample Table

Well	DIN	Conc. [ng/µl]	Sample Description	Alert	Observations
B2	2.3	10.3	C-T4-D-379		

Peak Table

Size [bp]	Calibrated Conc. [ng/µl]	Assigned Conc. [ng/µl]	%Integrated Area	From [bp]	To [bp]	Peak Comment	Observations
100	8.50	8.50	-	61	153		Lower Marker

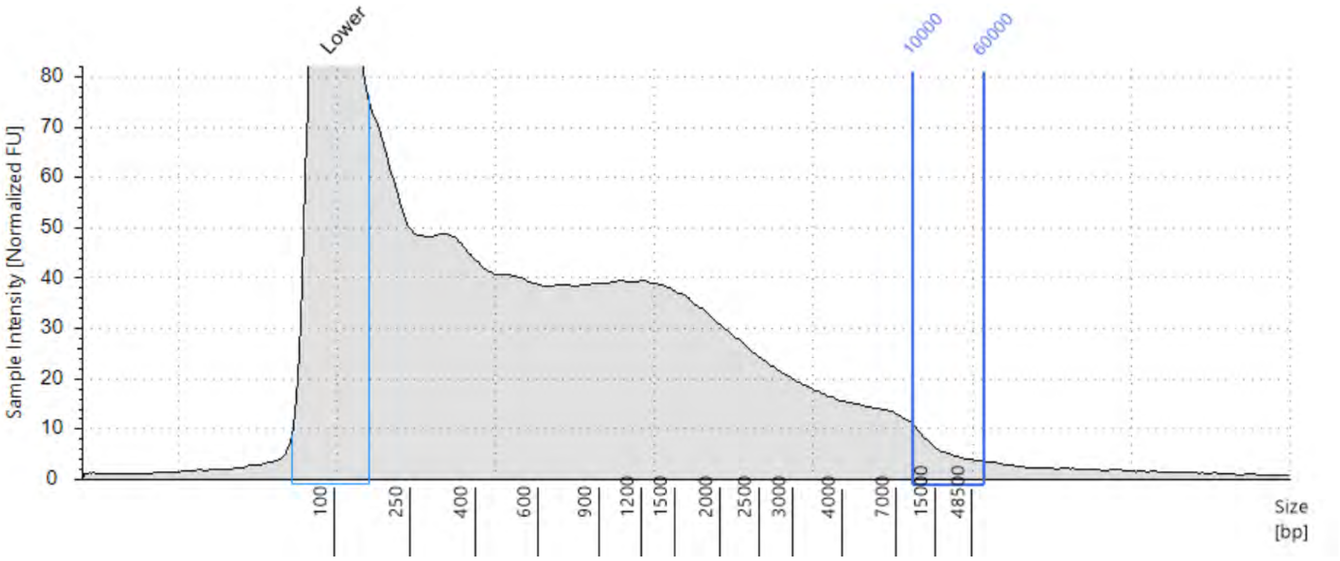

Region Table

From [bp]	To [bp]	Average Size [bp]	Conc. [ng/µl]	Region Molarity [nmol/l]	% of Total	Region Comment	Color
10000	60000	22420	0.181	0.0200	1.77		

© Agilent Technologies, Inc. 2017

C2: C-T4-D-606

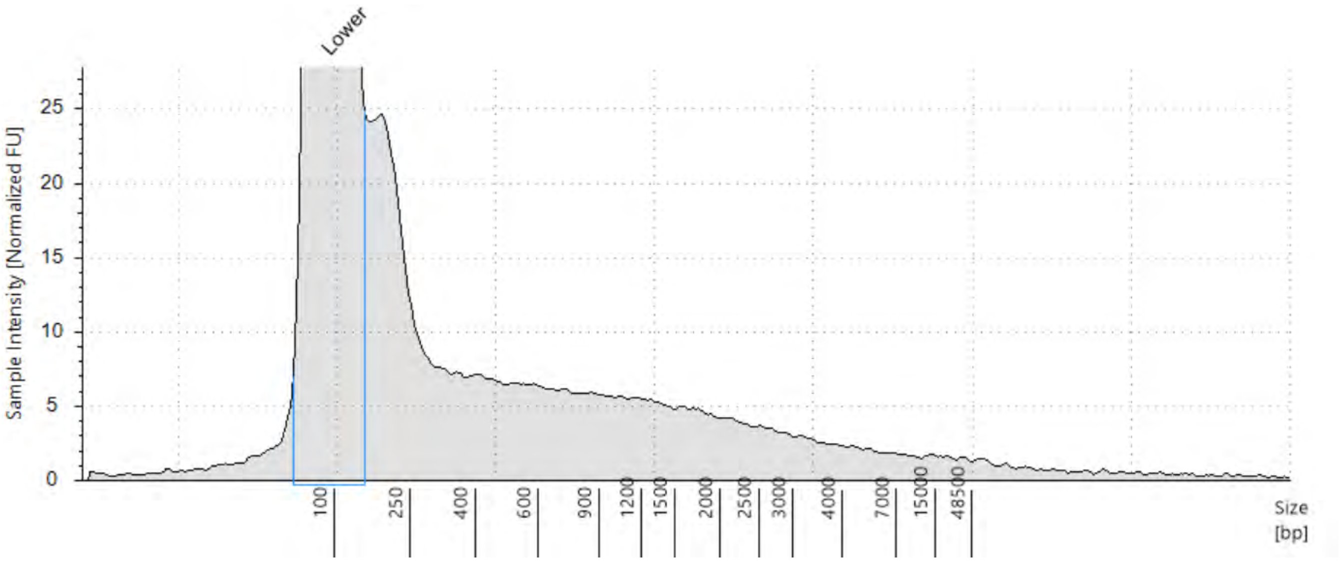

Sample Table

Well	DIN	Conc. [ng/µl]	Sample Description	Alert	Observations
C2	-	2.08	C-T4-D-606	▲	Sample concentration outside functional range for DIN

Peak Table

Size [bp]	Calibrated Conc. [ng/µl]	Assigned Conc. [ng/µl]	% Integrated Area	From [bp]	To [bp]	Peak Comment	Observations
100	8.50	8.50	-	61	146		Lower Marker

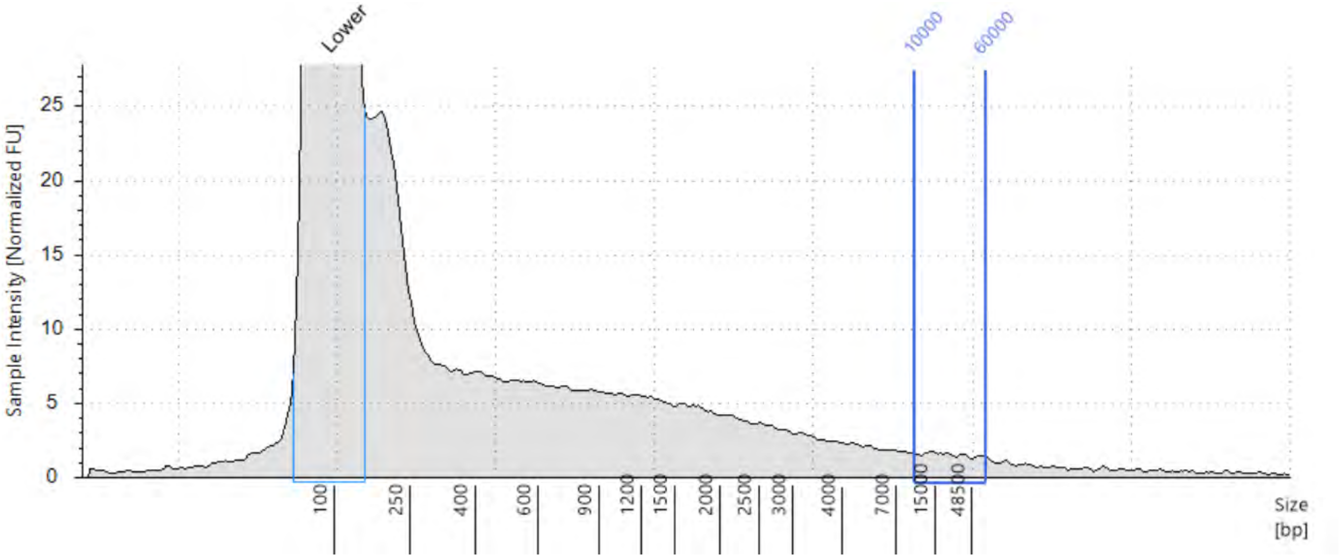

Region Table

From [bp]	To [bp]	Average Size [bp]	Conc. [ng/µl]	Region Molarity [nmol/l]	% of Total	Region Comment	Color
10000	60000	25991	0.0445	0.00547	2.14		■

 © Agilent Technologies, Inc. 2017

D2: C-T4-D-607

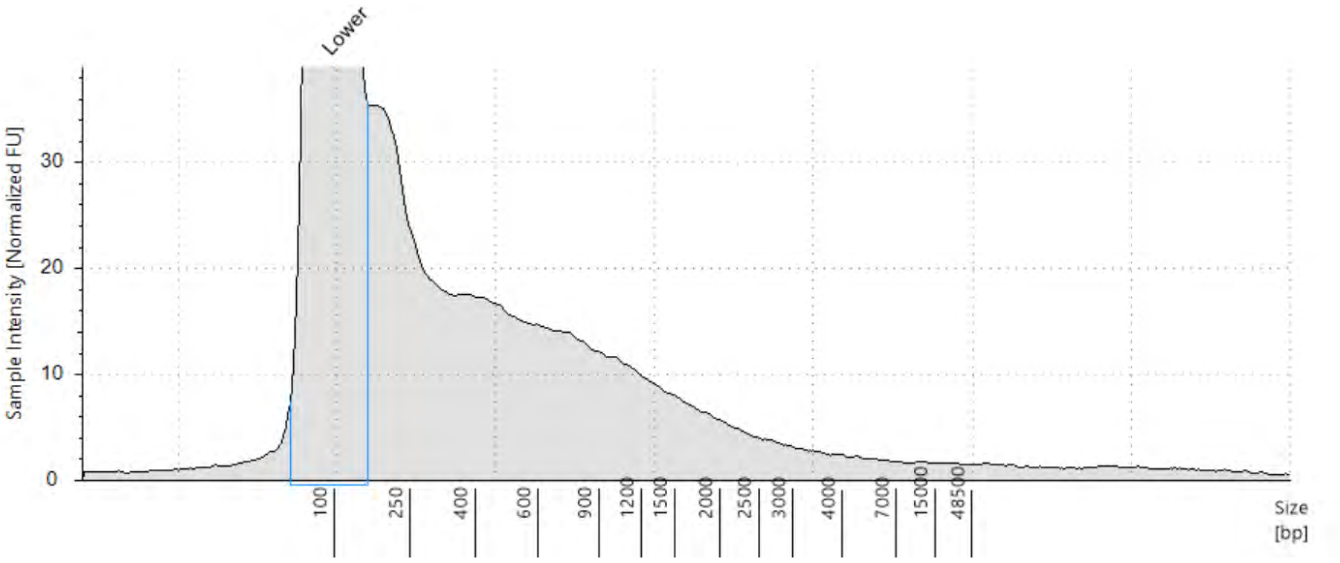

Sample Table

Well	DIN	Conc. [ng/µl]	Sample Description	Alert	Observations
D2	1.2	3.32	C-T4-D-607	⚠	Sample concentration outside functional range for DIN

Peak Table

Size [bp]	Calibrated Conc. [ng/µl]	Assigned Conc. [ng/µl]	% Integrated Area	From [bp]	To [bp]	Peak Comment	Observations
100	8.50	8.50	-	59	149		Lower Marker

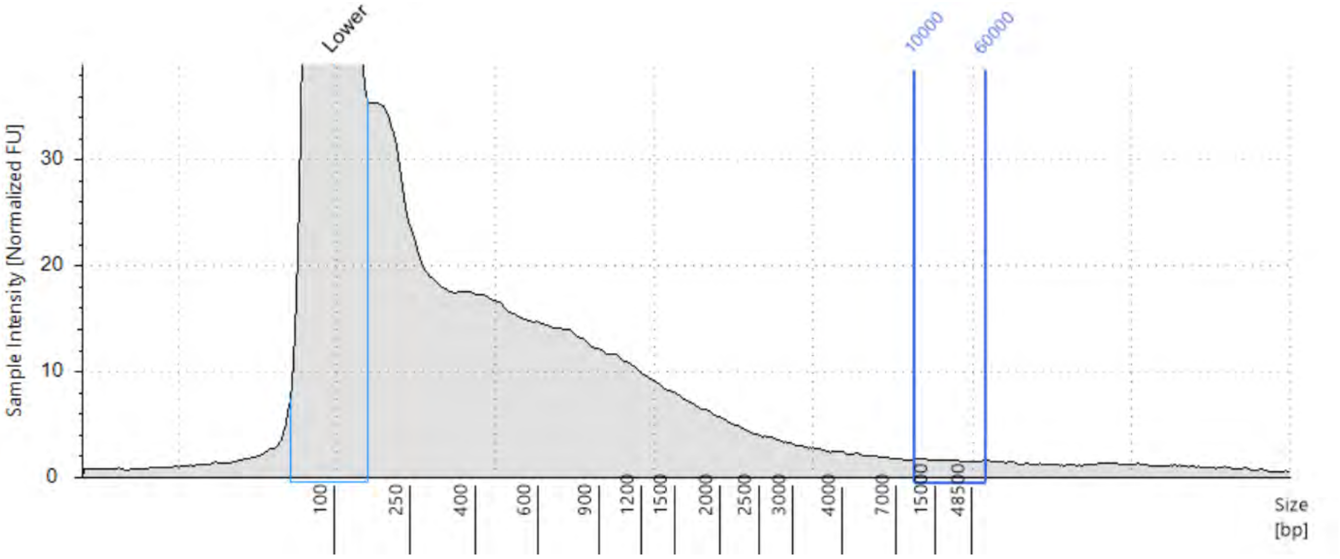

Region Table

From [bp]	To [bp]	Average Size [bp]	Conc. [ng/µl]	Region Molarity [nmol/l]	% of Total	Region Comment	Color
10000	60000	26106	0.0337	0.00509	1.01		■

© Agilent Technologies, Inc. 2017

E2: C-T4-D-608

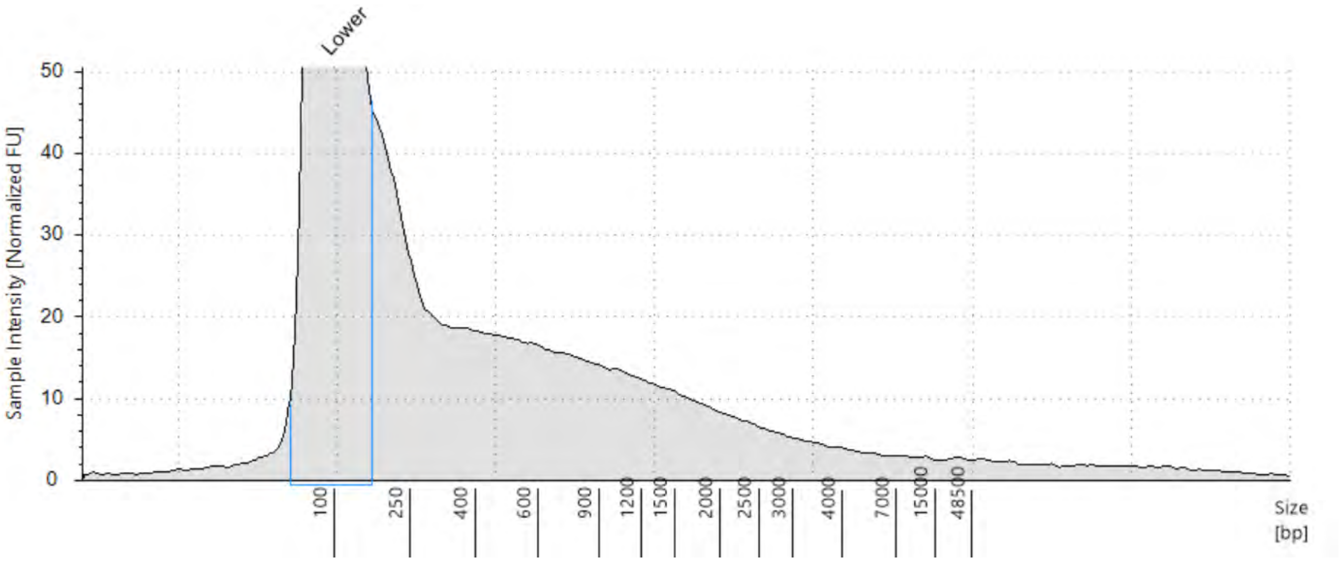

Sample Table

Well	DIN	Conc. [ng/µl]	Sample Description	Alert	Observations
E2	1.5	3.80	C-T4-D-608	⚠	Sample concentration outside functional range for DIN

Peak Table

Size [bp]	Calibrated Conc. [ng/µl]	Assigned Conc. [ng/µl]	% Integrated Area	From [bp]	To [bp]	Peak Comment	Observations
100	8.50	8.50	-	59	157		Lower Marker

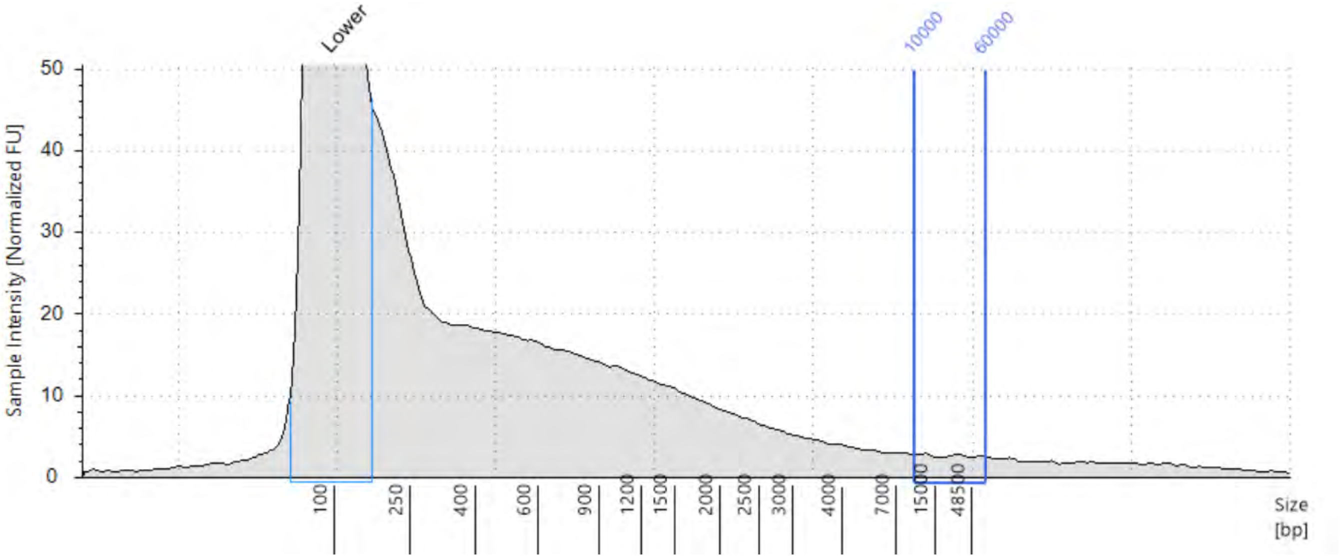

Region Table

From [bp]	To [bp]	Average Size [bp]	Conc. [ng/µl]	Region Molarity [nmol/l]	% of Total	Region Comment	Color
10000	60000	26702	0.0749	0.00747	1.97		■

© Agilent Technologies, Inc. 2017

F2: C-T4-D-609

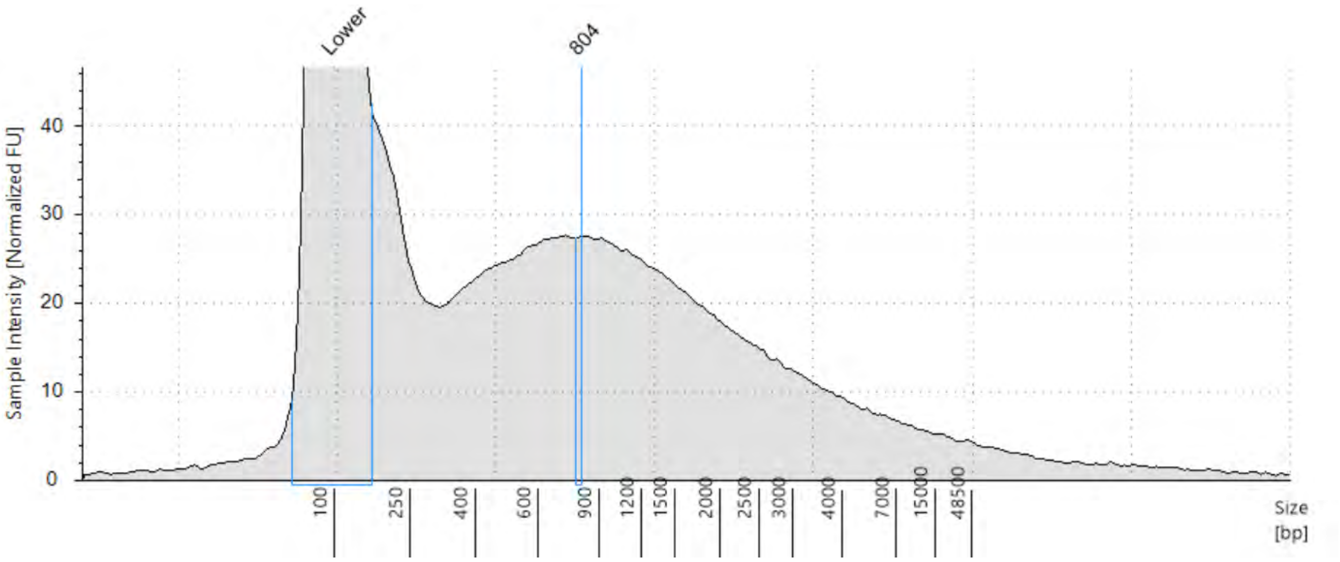

Sample Table

Well	DIN	Conc. [ng/µl]	Sample Description	Alert	Observations
F2	2.2	5.94	C-T4-D-609	⚠	Sample concentration outside recommended range

Peak Table

Size [bp]	Calibrated Conc. [ng/µl]	Assigned Conc. [ng/µl]	% Integrated Area	From [bp]	To [bp]	Peak Comment	Observations
100	8.50	8.50	-	60	158		Lower Marker
804	0.100	-	100.00	774	804		

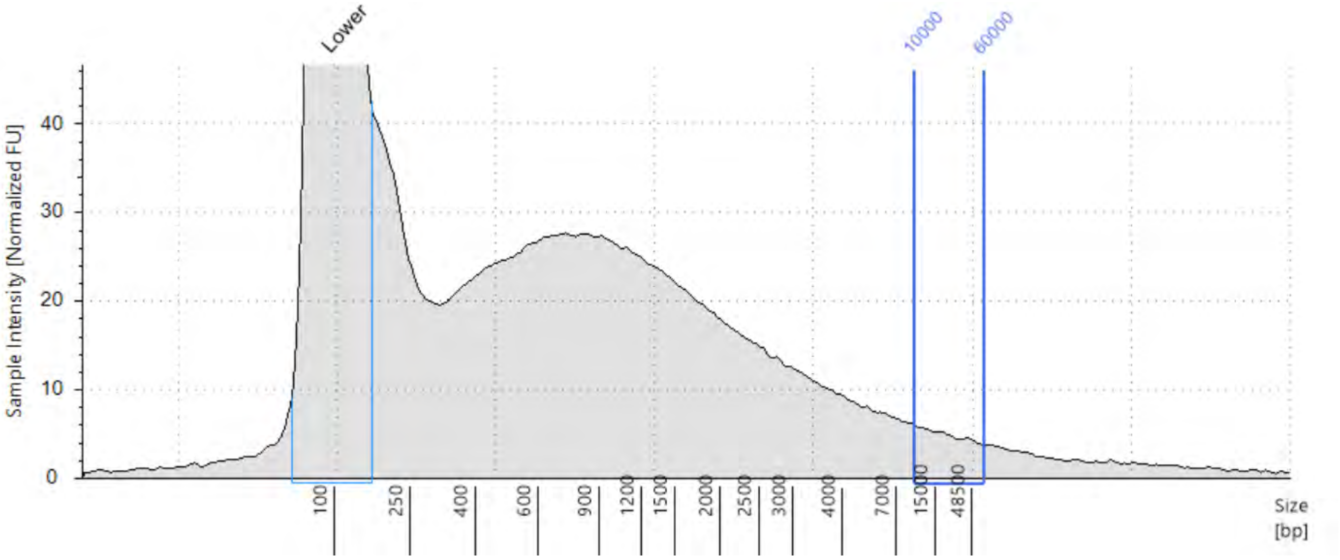

Region Table

From [bp]	To [bp]	Average Size [bp]	Conc. [ng/µl]	Region Molarity [nmol/l]	% of Total	Region Comment	Color
10000	60000	25071	0.153	0.0153	2.57		■

G2: C-T4-D-610

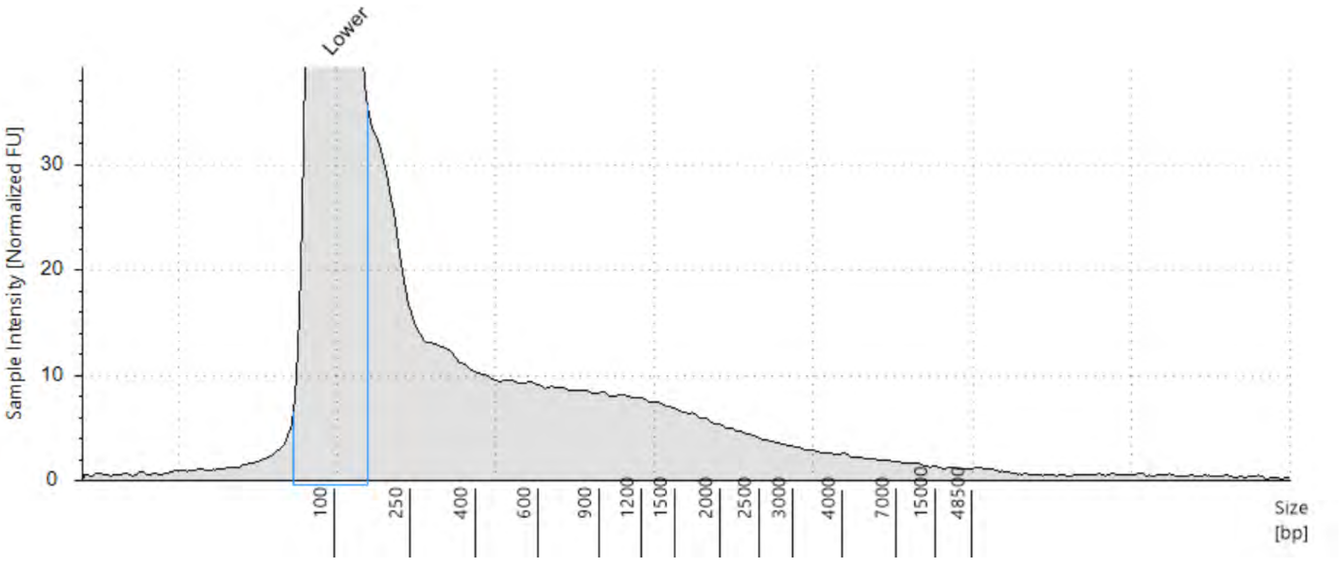

Sample Table

Well	DIN	Conc. [ng/µl]	Sample Description	Alert	Observations
G2	-	2.66	C-T4-D-610	▲	Sample concentration outside functional range for DIN

Peak Table

Size [bp]	Calibrated Conc. [ng/µl]	Assigned Conc. [ng/µl]	% Integrated Area	From [bp]	To [bp]	Peak Comment	Observations
100	8.50	8.50	-	61	149		Lower Marker

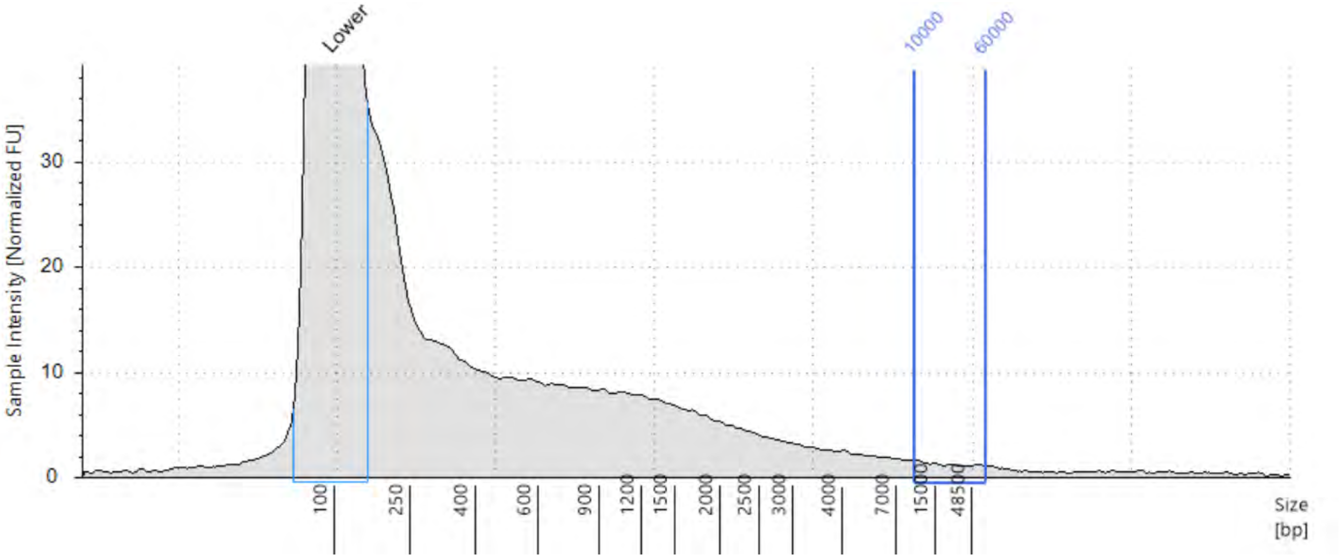

Region Table

From [bp]	To [bp]	Average Size [bp]	Conc. [ng/µl]	Region Molarity [nmol/l]	% of Total	Region Comment	Color
10000	60000	25440	0.0263	0.00437	0.99		■

H2: C-T4-S-382

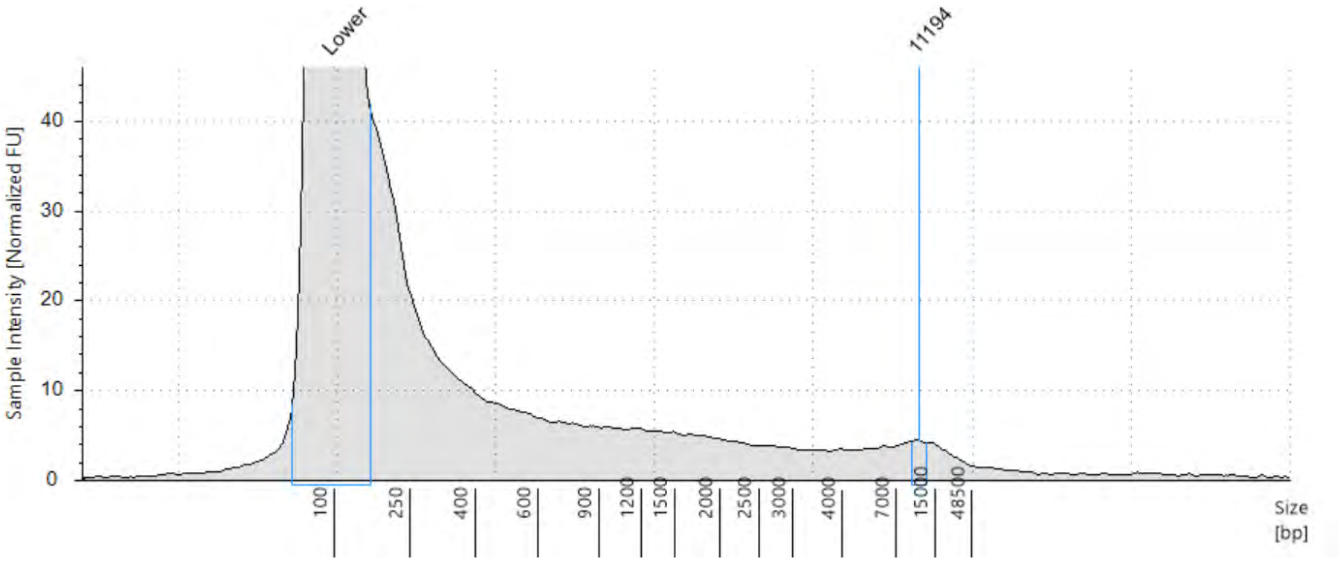

Sample Table

Well	DIN	Conc. [ng/µl]	Sample Description	Alert	Observations
H2	-	2.43	C-T4-S-382	▲	Sample concentration outside functional range for DIN

Peak Table

Size [bp]	Calibrated Conc. [ng/µl]	Assigned Conc. [ng/µl]	% Integrated Area	From [bp]	To [bp]	Peak Comment	Observations
100	8.50	8.50	-	60	153		Lower Marker
11194	0.0319	-	100.00	9737	12869		

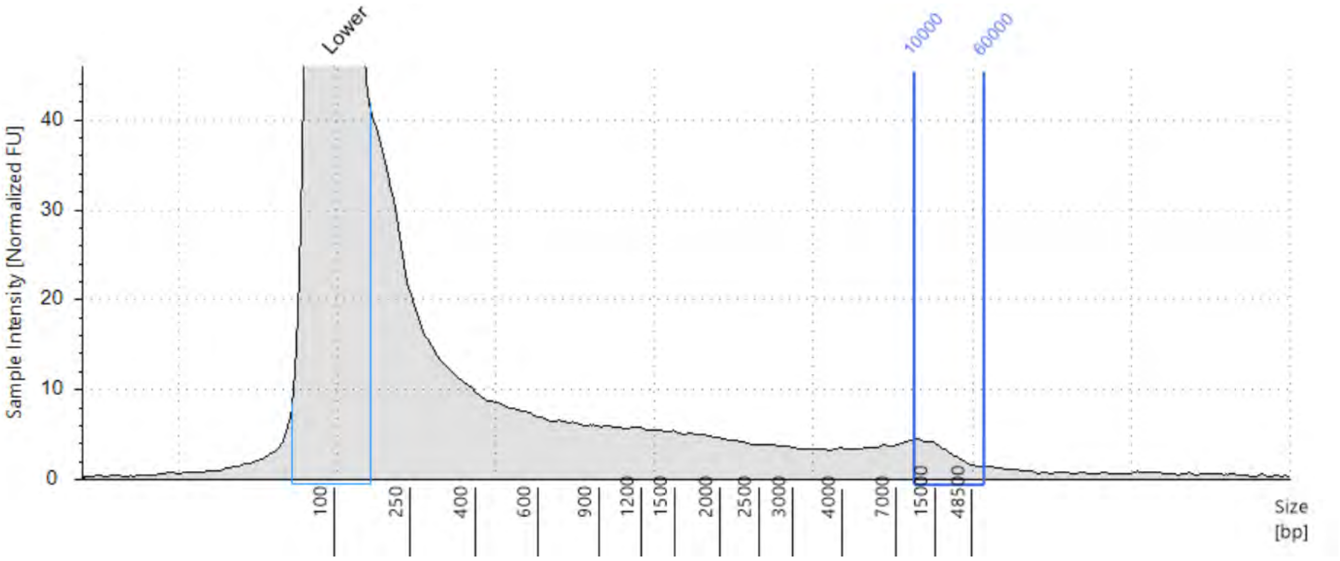

Region Table

From [bp]	To [bp]	Average Size [bp]	Conc. [ng/µl]	Region Molarity [nmol/l]	% of Total	Region Comment	Color
10000	60000	21044	0.0912	0.0110	3.76		■

© Agilent Technologies, Inc. 2017

Filename: 2019-07-29-03.gDNA

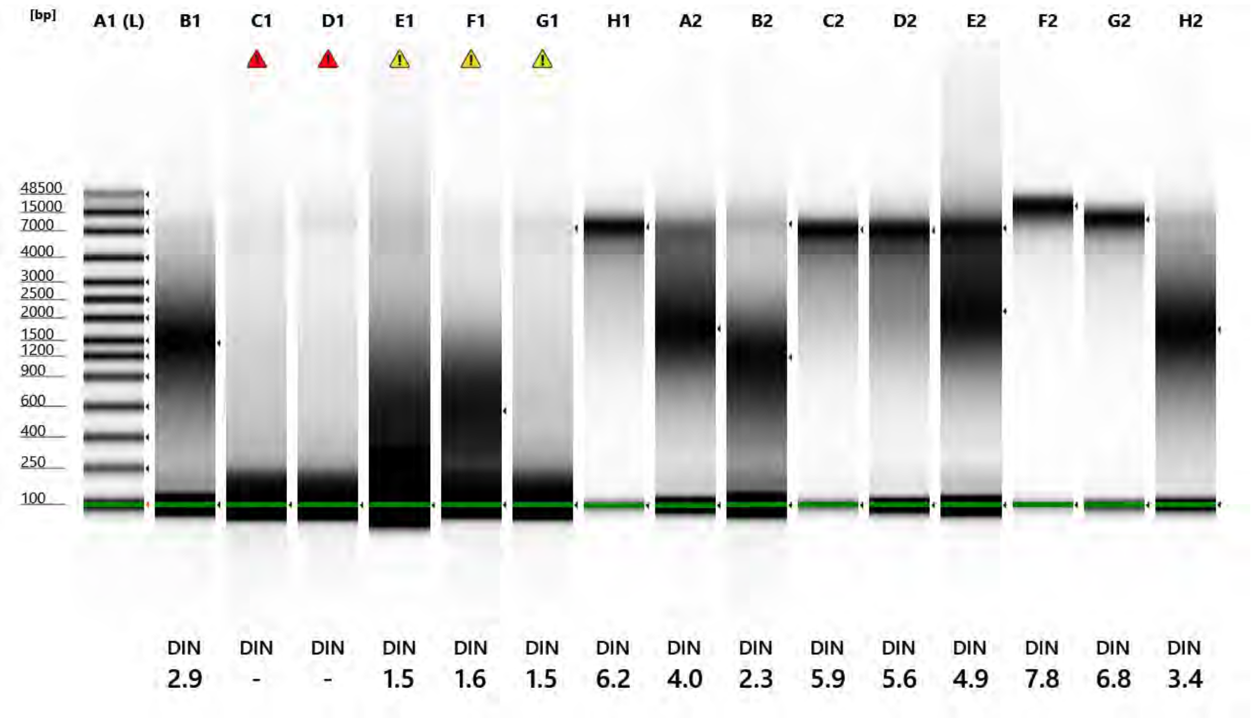

Default image (Contrast 50%), Image is Scaled to Sample

Sample Info

Well	DIN	Conc. [ng/µl]	Sample Description	Alert	Observations
A1	-	65.3	Ladder		Ladder
B1	2.9	17.2	C-T4-S-383		
C1	-	1.94	C-T4-S-616	▲	Sample concentration outside functional range for DIN
D1	-	2.07	C-T4-S-617	▲	Sample concentration outside functional range for DIN
E1	1.5	9.59	C-T4-S-618	⚠	Sample concentration outside recommended range
F1	1.6	9.67	C-T4-S-619	⚠	Sample concentration outside recommended range
G1	1.5	3.42	C-T4-S-620	⚠	Sample concentration outside functional range for DIN
H1	6.2	64.5	W-T4-DES-811		
A2	4.0	39.3	W-T4-DES-812		
B2	2.3	12.6	W-T4-DES-813		
C2	5.9	70.0	W-T4-DES-814		
D2	5.6	26.3	W-T4-DES-815		
E2	4.9	21.8	W-T4-DES-1036		
F2	7.8	58.5	W-T4-DES-1037		
G2	6.8	32.0	W-T4-DES-1038		
H2	3.4	59.7	W-T4-DES-1039		

 © Agilent Technologies, Inc. 2017

A1: Ladder

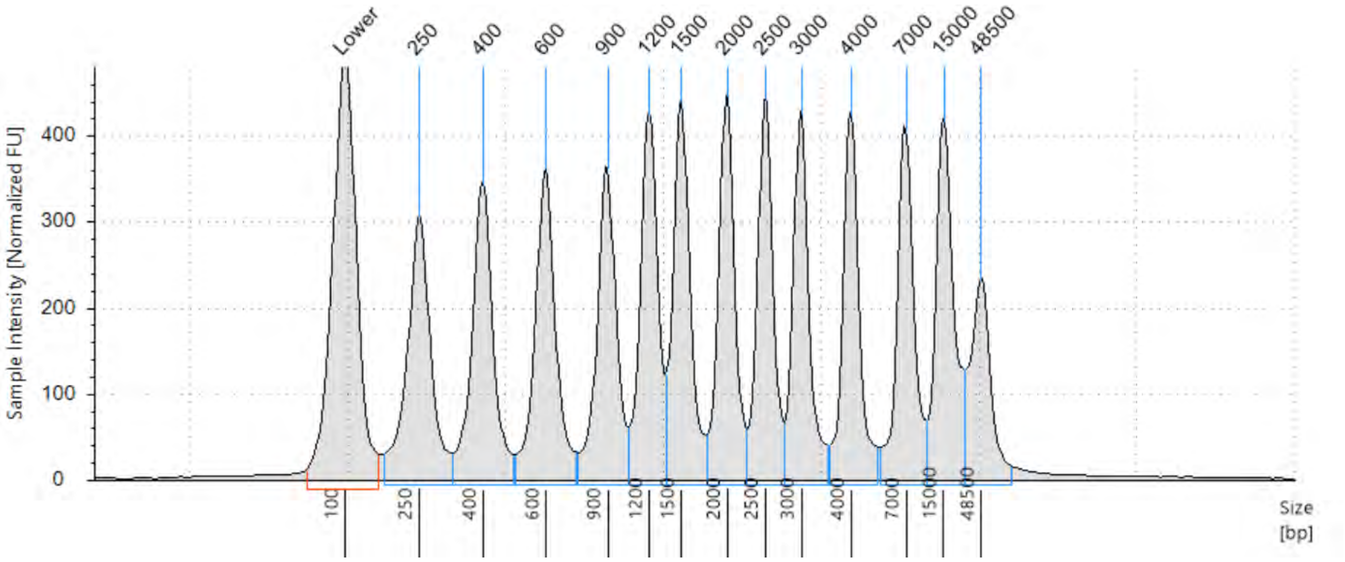

Sample Table

Well	DIN	Conc. [ng/µl]	Sample Description	Alert	Observations
A1	-	65.3	Ladder		Ladder

Peak Table

Size [bp]	Calibrated Conc. [ng/µl]	Assigned Conc. [ng/µl]	% Integrated Area	From [bp]	To [bp]	Peak Comment	Observations
100	8.50	8.50	-	62	151		Lower Marker
250	5.10	-	7.83	162	319		
400	5.16	-	7.91	319	488		
600	5.29	-	8.11	492	735		
900	5.00	-	7.67	742	1039		
1200	5.39	-	8.27	1039	1348		
1500	5.47	-	8.39	1348	1755		
2000	5.24	-	8.04	1755	2245		
2500	5.07	-	7.78	2245	2758		
3000	4.92	-	7.54	2758	3492		
4000	4.91	-	7.54	3520	5254		
7000	4.92	-	7.55	5407	10690		
15000	5.31	-	8.14	10690	22917		
48500	3.40	-	5.22	22917	>60000		

B1: C-T4-S-383

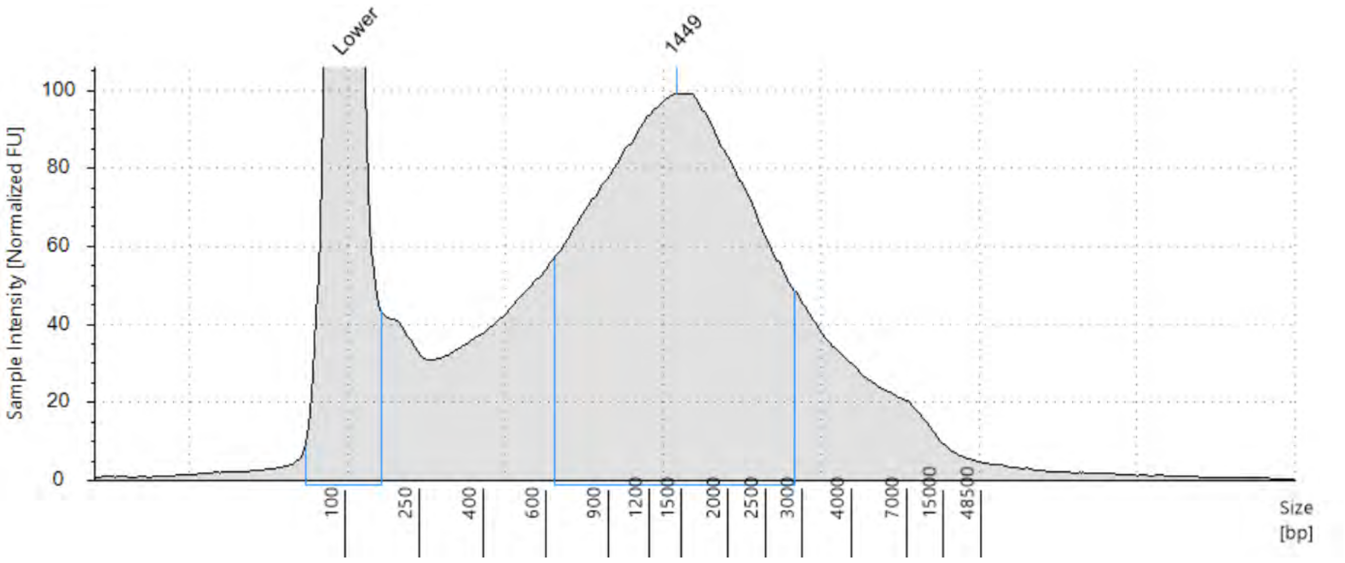

Sample Table

Well	DIN	Conc. [ng/µl]	Sample Description	Alert	Observations
B1	2.9	17.2	C-T4-S-383		

Peak Table

Size [bp]	Calibrated Conc. [ng/µl]	Assigned Conc. [ng/µl]	% Integrated Area	From [bp]	To [bp]	Peak Comment	Observations
100	8.50	8.50	-	61	156		Lower Marker
1449	10.8	-	100.00	634	2914		

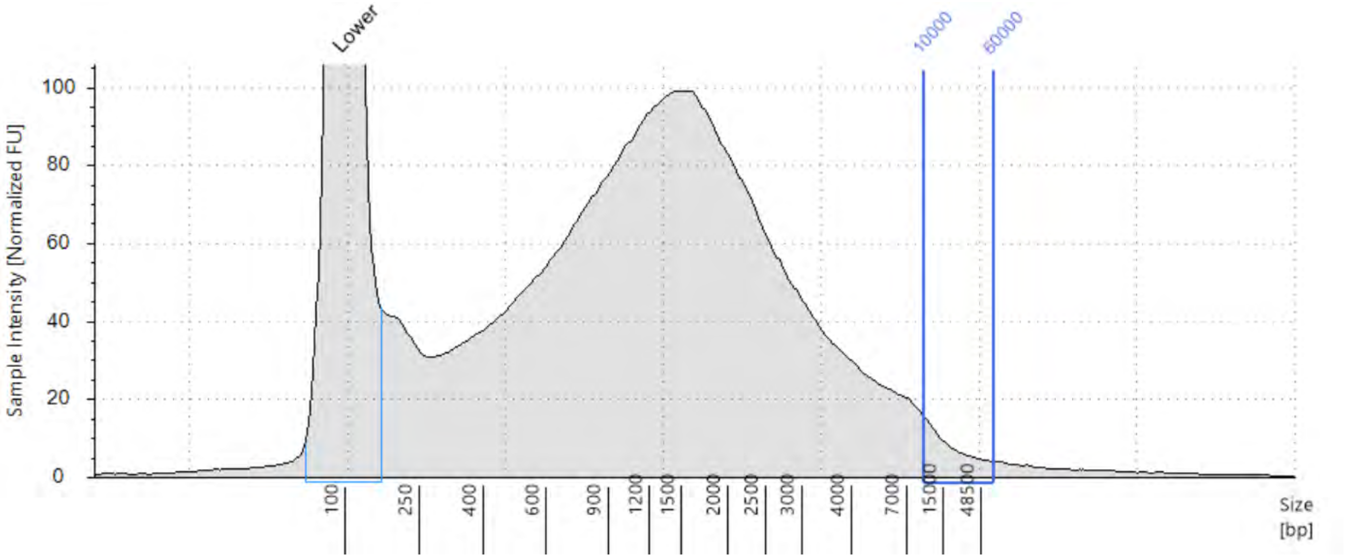

Region Table

From [bp]	To [bp]	Average Size [bp]	Conc. [ng/µl]	Region Molarity [nmol/l]	% of Total	Region Comment	Color
10000	60000	21714	0.270	0.0298	1.57		

 © Agilent Technologies, Inc. 2017

C1: C-T4-S-616

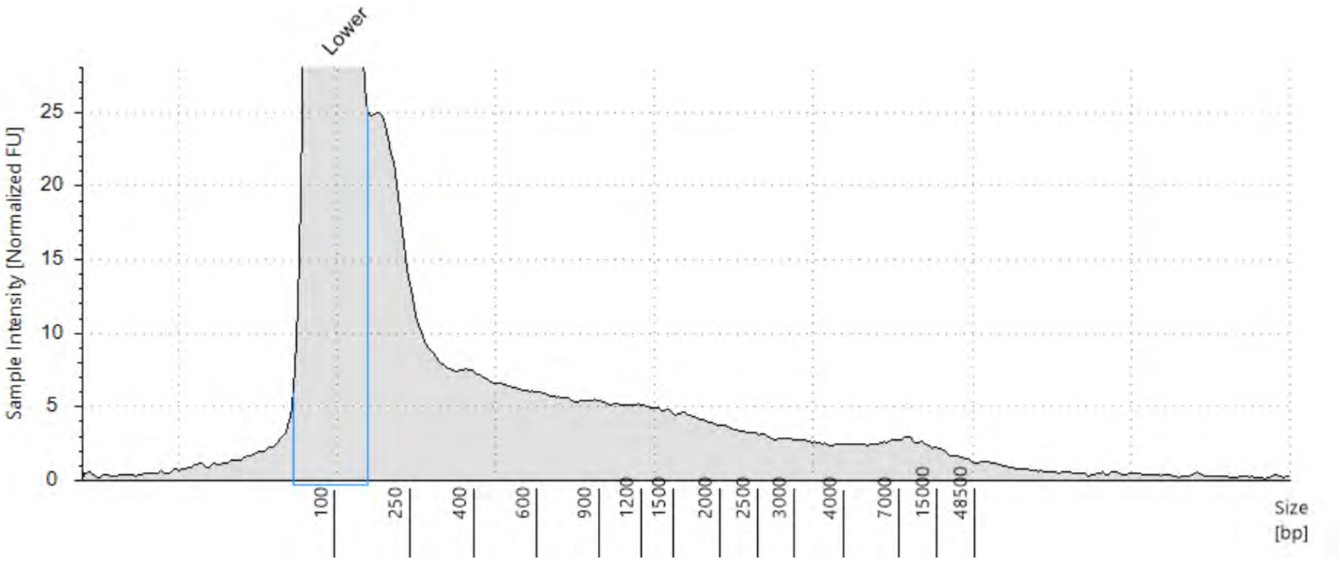

Sample Table

Well	DIN	Conc. [ng/µl]	Sample Description	Alert	Observations
C1	-	1.94	C-T4-S-616	▲	Sample concentration outside functional range for DIN

Peak Table

Size [bp]	Calibrated Conc. [ng/µl]	Assigned Conc. [ng/µl]	% Integrated Area	From [bp]	To [bp]	Peak Comment	Observations
100	8.50	8.50	-	60	150		Lower Marker

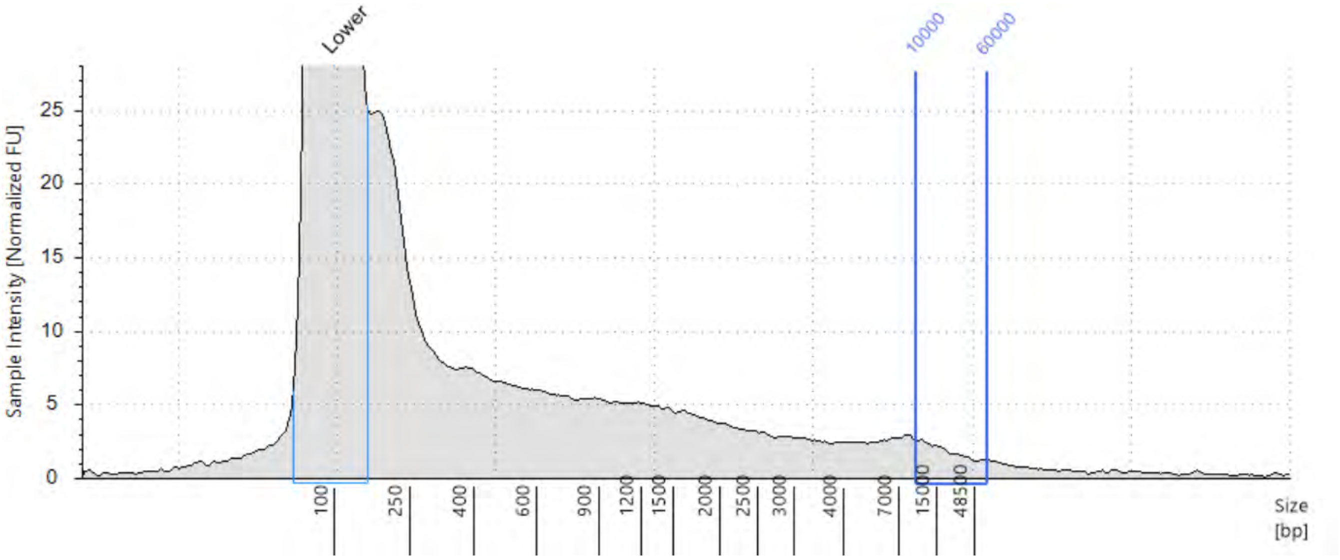

Region Table

From [bp]	To [bp]	Average Size [bp]	Conc. [ng/µl]	Region Molarity [nmol/l]	% of Total	Region Comment	Color
10000	60000	23567	0.0526	0.00694	2.71		■

 © Agilent Technologies, Inc. 2017

D1: C-T4-S-617

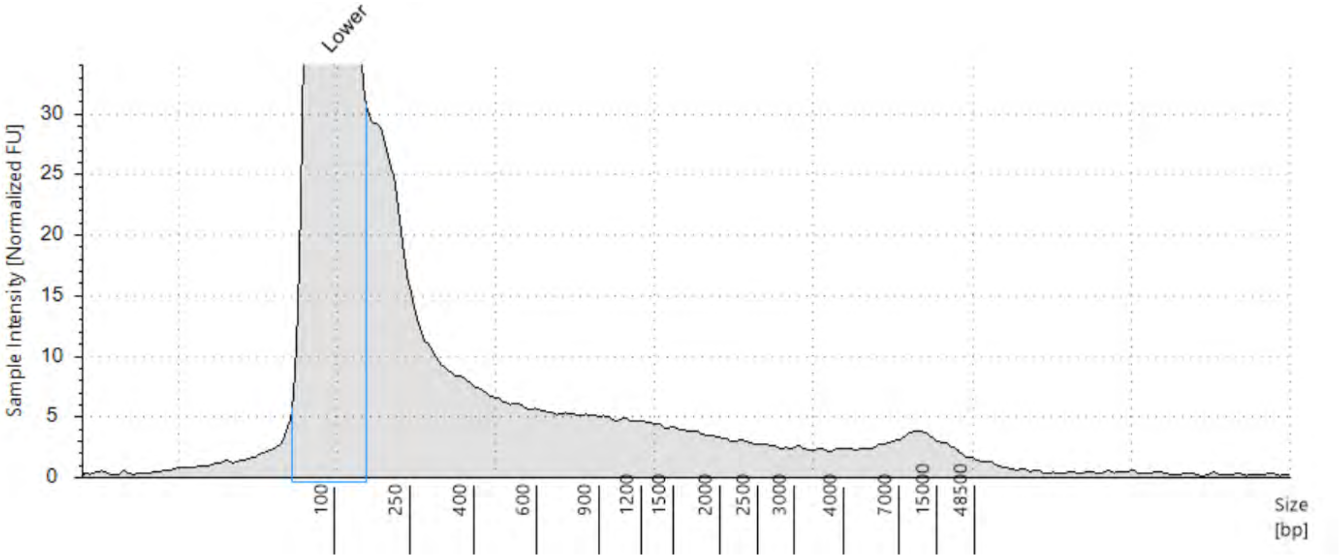

Sample Table

Well	DIN	Conc. [ng/µl]	Sample Description	Alert	Observations
D1	-	2.07	C-T4-S-617	▲	Sample concentration outside functional range for DIN

Peak Table

Size [bp]	Calibrated Conc. [ng/µl]	Assigned Conc. [ng/µl]	% Integrated Area	From [bp]	To [bp]	Peak Comment	Observations
100	8.50	8.50	-	60	149		Lower Marker

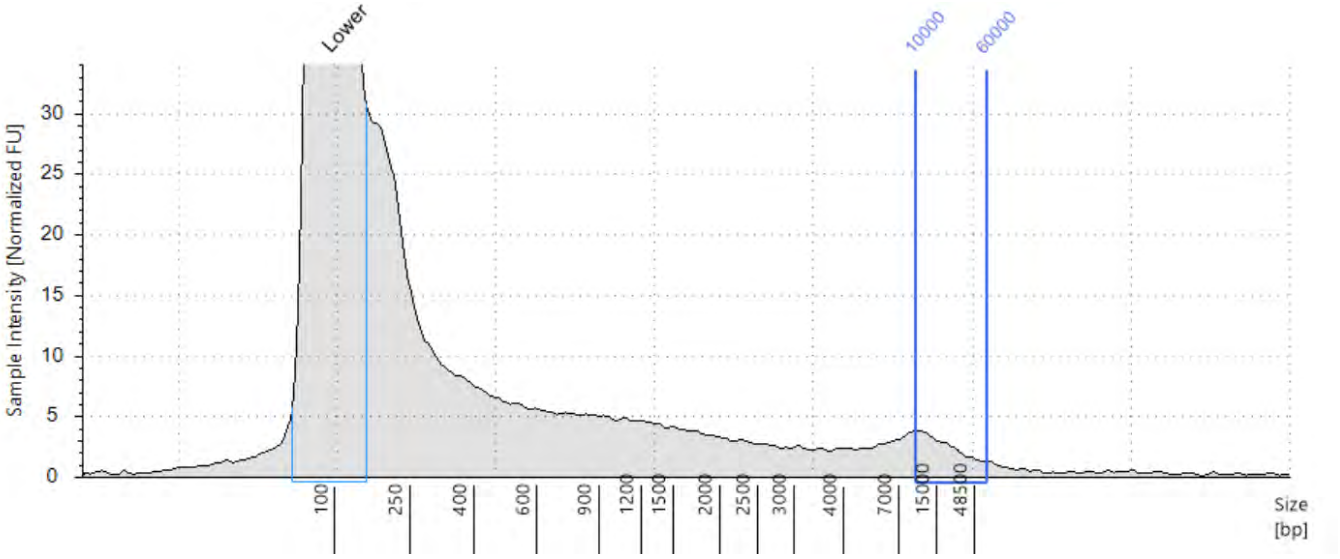

Region Table

From [bp]	To [bp]	Average Size [bp]	Conc. [ng/µl]	Region Molarity [nmol/l]	% of Total	Region Comment	Color
10000	60000	22171	0.0810	0.00994	3.92		■

E1: C-T4-S-618

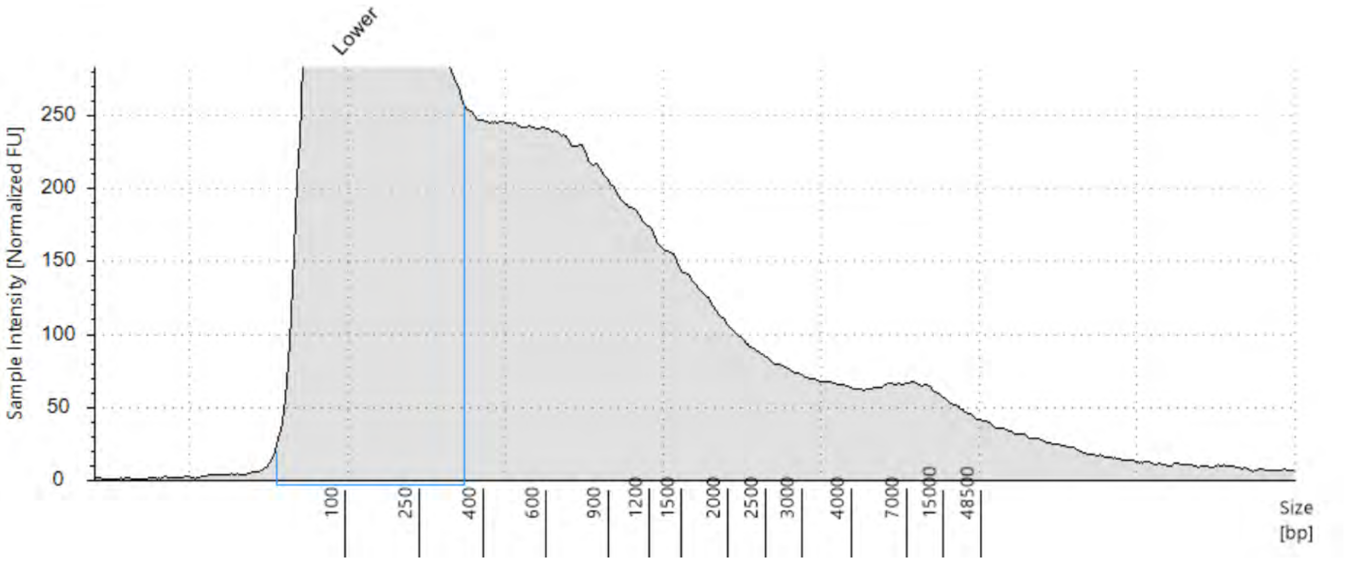

Sample Table

Well	DIN	Conc. [ng/µl]	Sample Description	Alert	Observations
E1	1.5	9.59	C-T4-S-618	⚠	Sample concentration outside recommended range

Peak Table

Size [bp]	Calibrated Conc. [ng/µl]	Assigned Conc. [ng/µl]	% Integrated Area	From [bp]	To [bp]	Peak Comment	Observations
100	8.50	8.50	-	43	349		Lower Marker

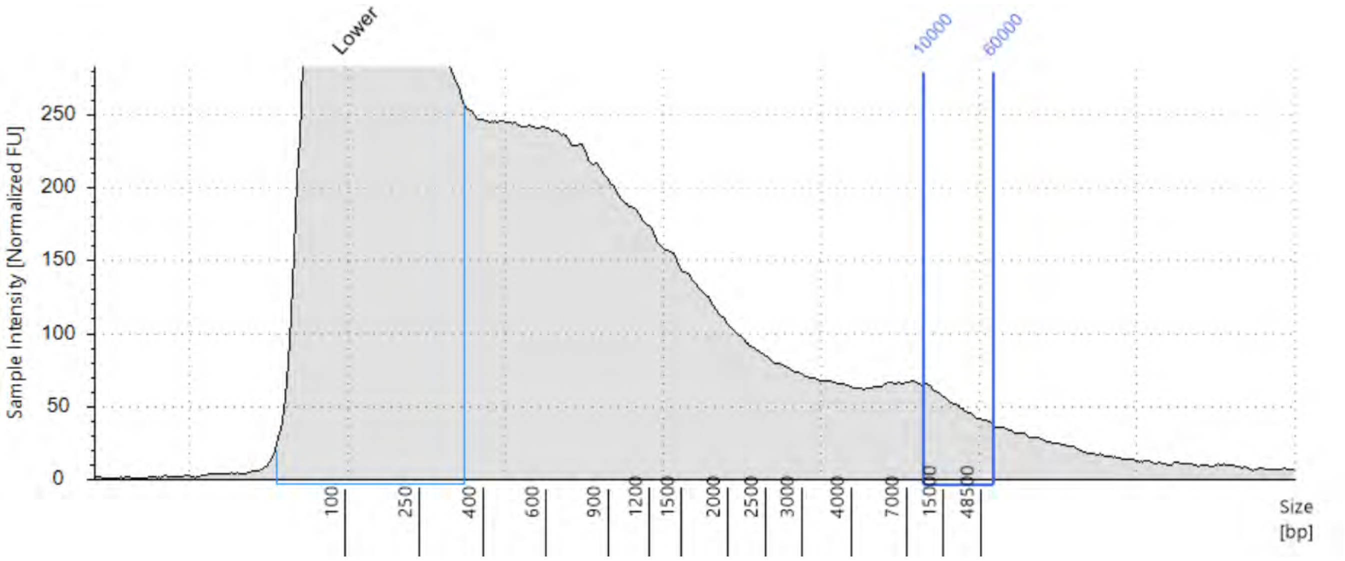

Region Table

From [bp]	To [bp]	Average Size [bp]	Conc. [ng/µl]	Region Molarity [nmol/l]	% of Total	Region Comment	Color
10000	60000	24625	0.449	0.0381	4.68		■

© Agilent Technologies, Inc. 2017

F1: C-T4-S-619

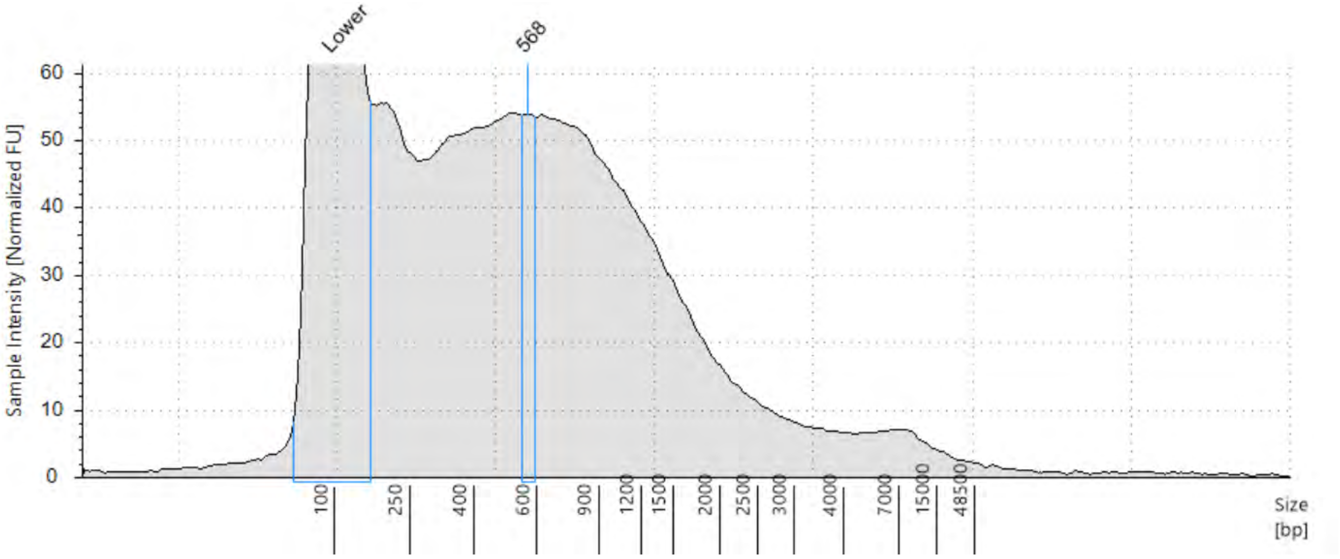

Sample Table

Well	DIN	Conc. [ng/µl]	Sample Description	Alert	Observations
F1	1.6	9.67	C-T4-S-619	⚠	Sample concentration outside recommended range

Peak Table

Size [bp]	Calibrated Conc. [ng/µl]	Assigned Conc. [ng/µl]	% Integrated Area	From [bp]	To [bp]	Peak Comment	Observations
100	8.50	8.50	-	60	155		Lower Marker
568	0.444	-	100.00	544	594		

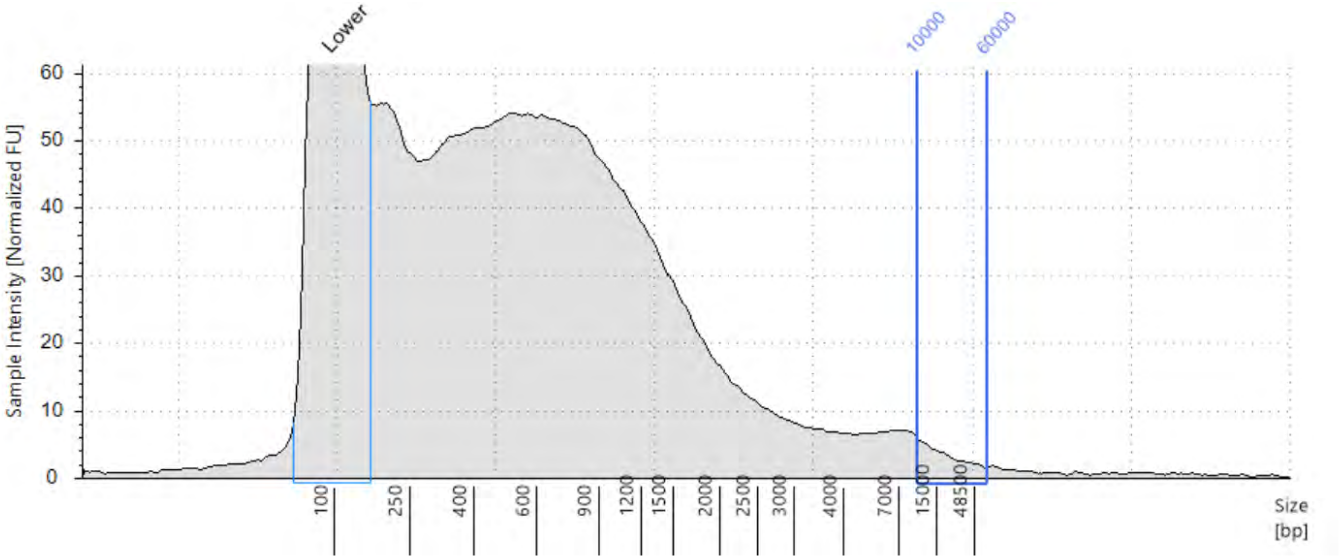

Region Table

From [bp]	To [bp]	Average Size [bp]	Conc. [ng/µl]	Region Molarity [nmol/l]	% of Total	Region Comment	Color
10000	60000	21684	0.0941	0.0123	0.97		■

G1: C-T4-S-620

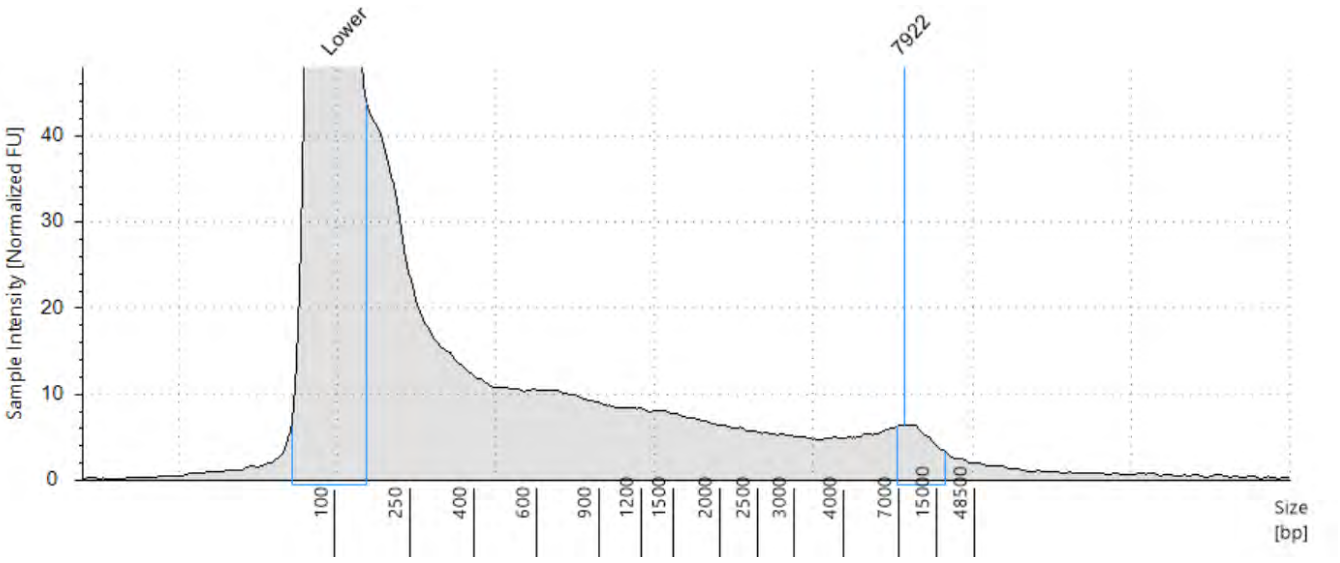

Sample Table

Well	DIN	Conc. [ng/µl]	Sample Description	Alert	Observations
G1	1.5	3.42	C-T4-S-620	⚠	Sample concentration outside functional range for DIN

Peak Table

Size [bp]	Calibrated Conc. [ng/µl]	Assigned Conc. [ng/µl]	% Integrated Area	From [bp]	To [bp]	Peak Comment	Observations
100	8.50	8.50	-	59	150		Lower Marker
7922	0.137	-	100.00	6949	18153		

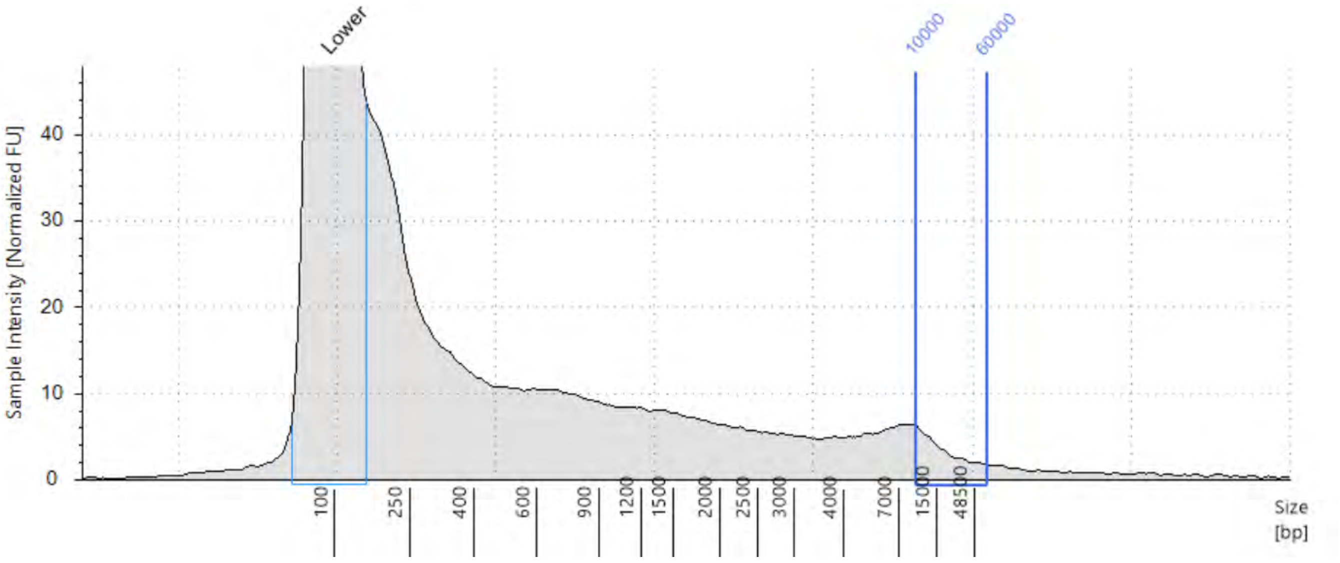

Region Table

From [bp]	To [bp]	Average Size [bp]	Conc. [ng/µl]	Region Molarity [nmol/l]	% of Total	Region Comment	Color
10000	60000	21931	0.111	0.0130	3.25		■

H1: W-T4-DES-811

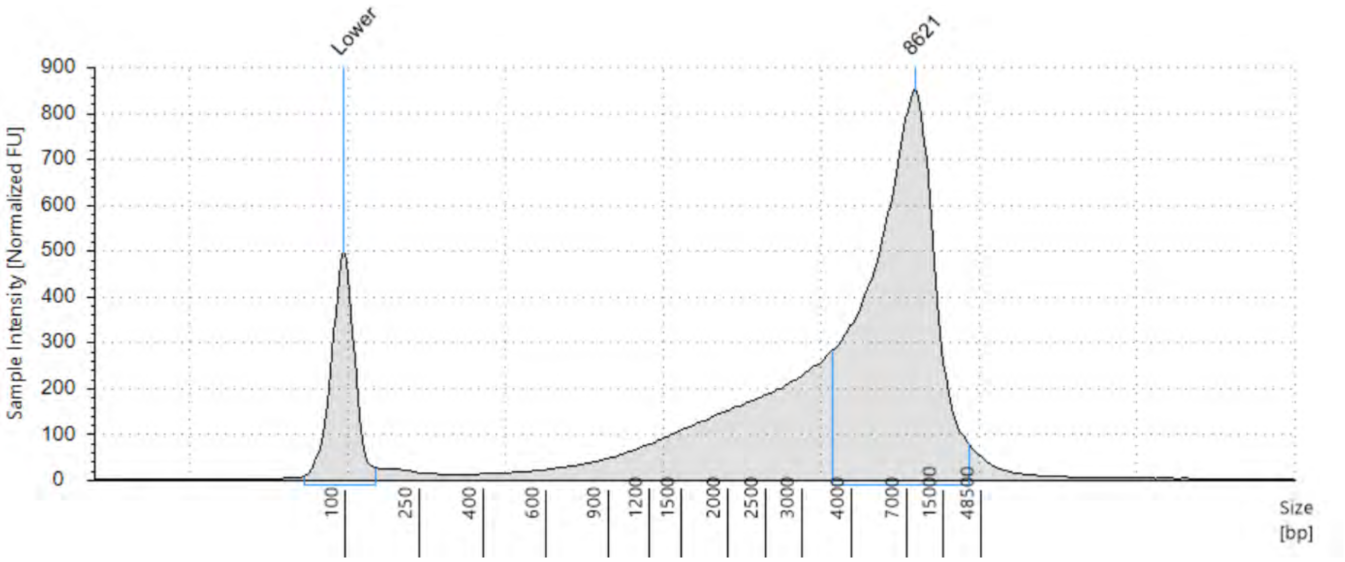

Sample Table

Well	DIN	Conc. [ng/µl]	Sample Description	Alert	Observations
H1	6.2	64.5	W-T4-DES-811		

Peak Table

Size [bp]	Calibrated Conc. [ng/µl]	Assigned Conc. [ng/µl]	% Integrated Area	From [bp]	To [bp]	Peak Comment	Observations
100	8.50	8.50	-	62	149		Lower Marker
8621	39.9	-	100.00	3619	25836		

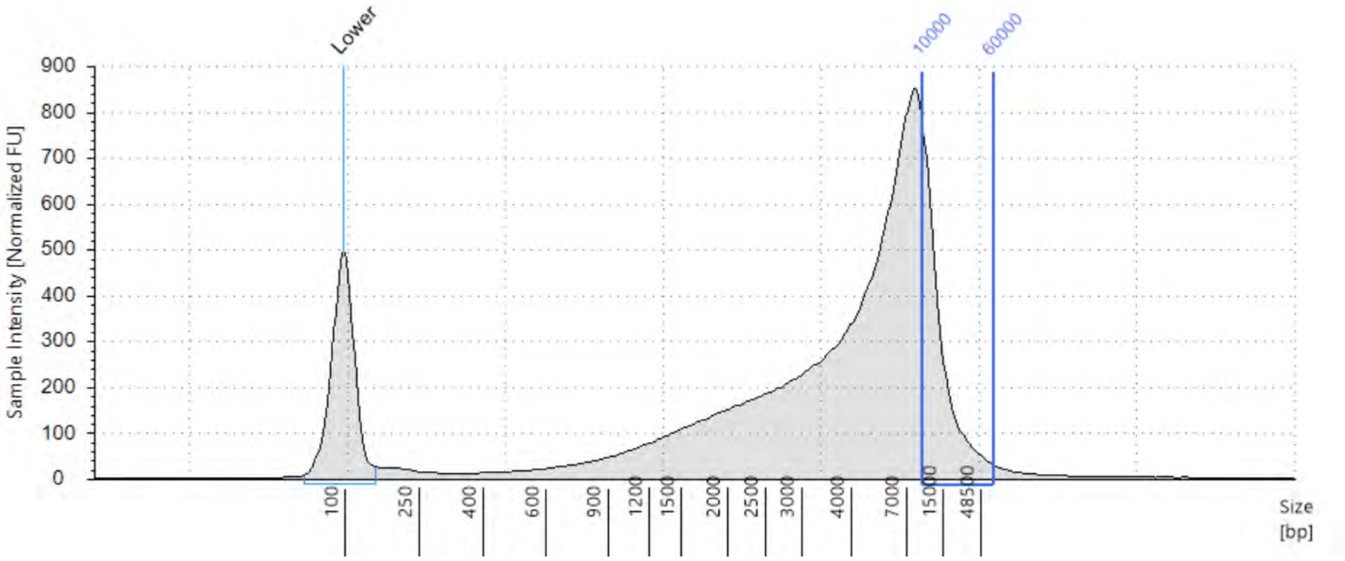

Region Table

From [bp]	To [bp]	Average Size [bp]	Conc. [ng/µl]	Region Molarity [nmol/l]	% of Total	Region Comment	Color
10000	60000	16382	10.3	1.16	15.90		■

 © Agilent Technologies, Inc. 2017

A2: W-T4-DES-812

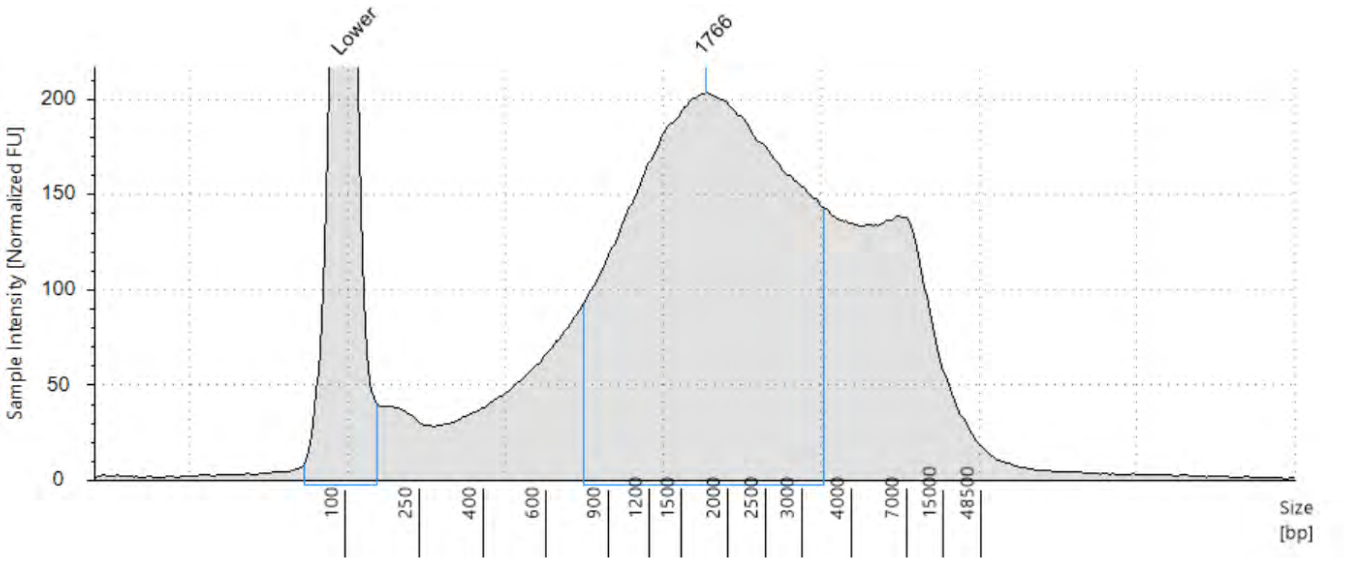

Sample Table

Well	DIN	Conc. [ng/µl]	Sample Description	Alert	Observations
A2	4.0	39.3	W-T4-DES-812		

Peak Table

Size [bp]	Calibrated Conc. [ng/µl]	Assigned Conc. [ng/µl]	% Integrated Area	From [bp]	To [bp]	Peak Comment	Observations
100	8.50	8.50	-	62	152		Lower Marker
1766	23.7	-	100.00	776	3429		

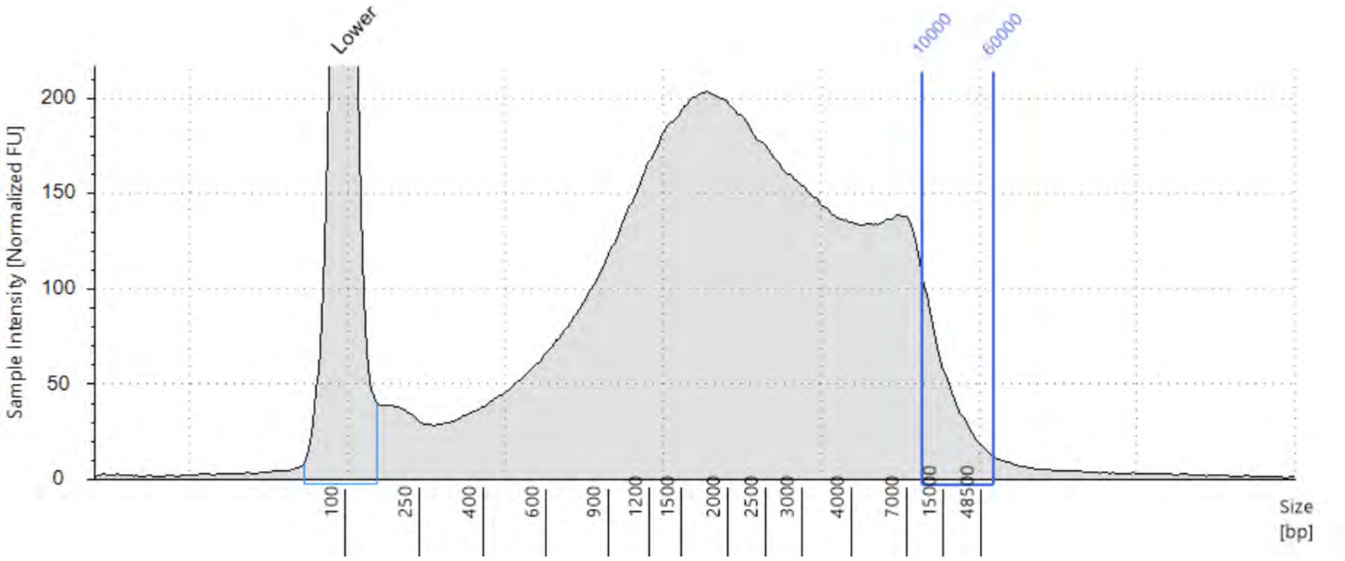

Region Table

From [bp]	To [bp]	Average Size [bp]	Conc. [ng/µl]	Region Molarity [nmol/l]	% of Total	Region Comment	Color
10000	60000	19223	1.87	0.195	4.76		

 © Agilent Technologies, Inc. 2017

B2: W-T4-DES-813

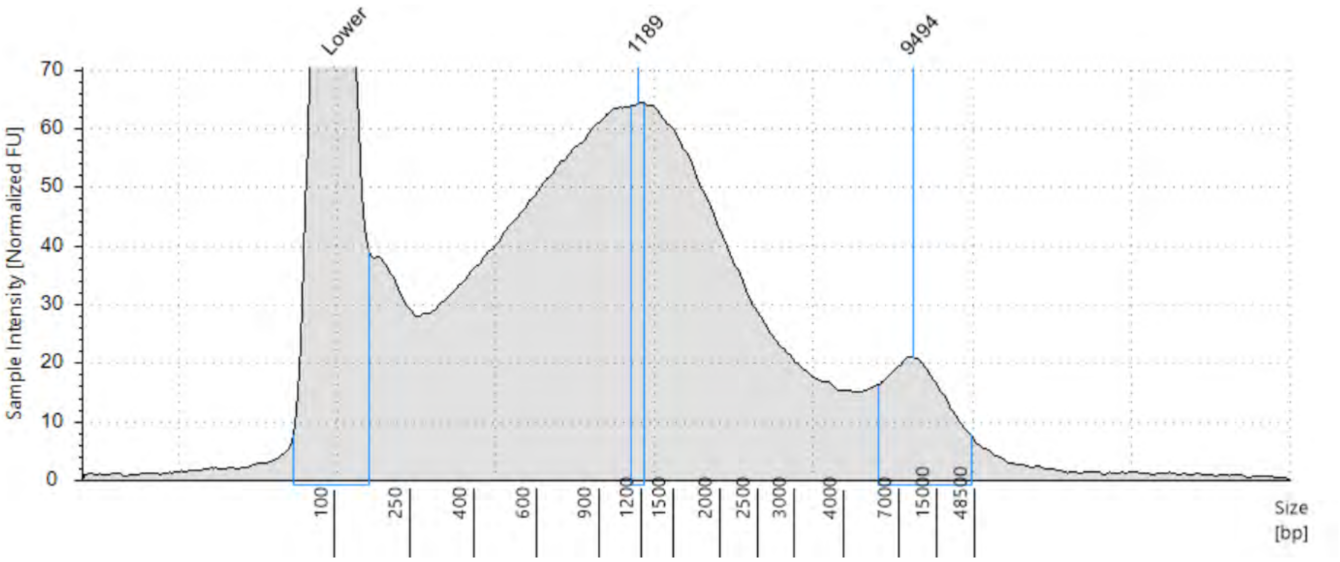

Sample Table

Well	DIN	Conc. [ng/µl]	Sample Description	Alert	Observations
B2	2.3	12.6	W-T4-DES-813		

Peak Table

Size [bp]	Calibrated Conc. [ng/µl]	Assigned Conc. [ng/µl]	%Integrated Area	From [bp]	To [bp]	Peak Comment	Observations
100	8.50	8.50	-	61	154		Lower Marker
1189	0.501	-	37.59	1135	1234		
9494	0.832	-	62.41	5783	37719		

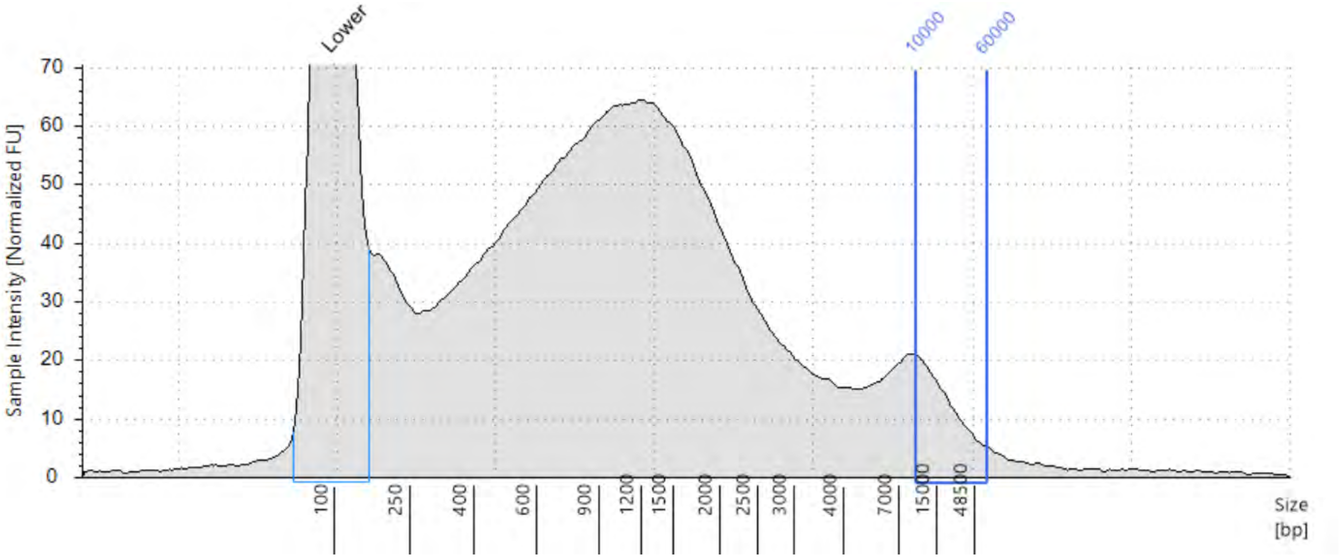

Region Table

From [bp]	To [bp]	Average Size [bp]	Conc. [ng/µl]	Region Molarity [nmol/l]	% of Total	Region Comment	Color
10000	60000	21163	0.498	0.0508	3.96		■

C2: W-T4-DES-814

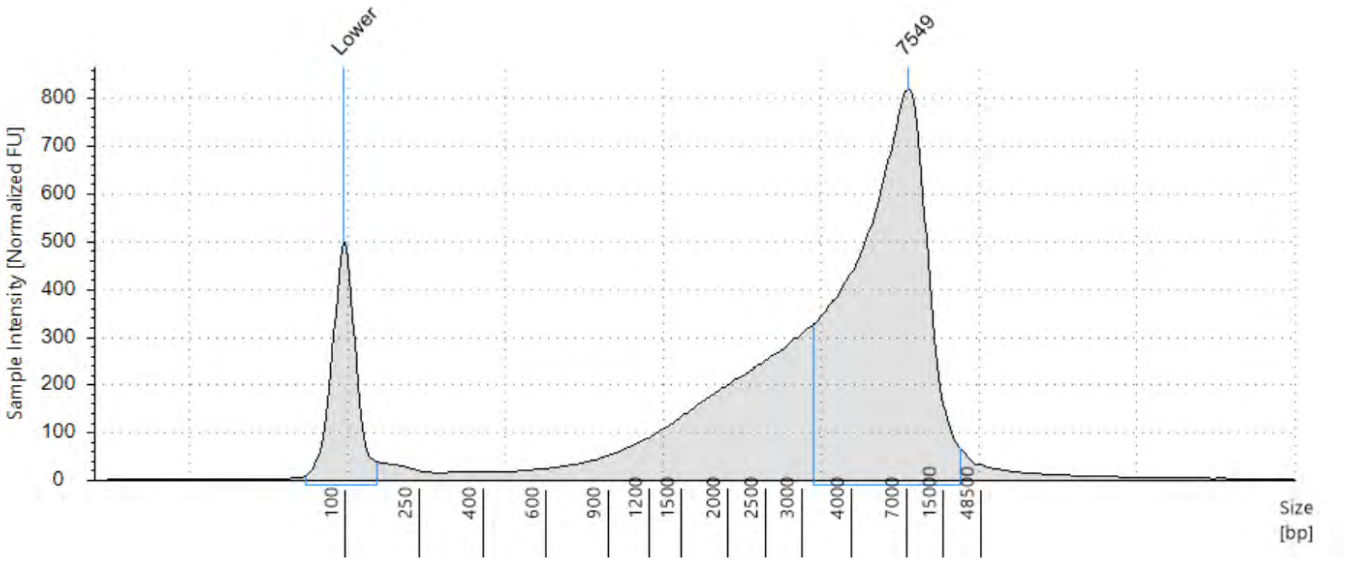

Sample Table

Well	DIN	Conc. [ng/µl]	Sample Description	Alert	Observations
C2	5.9	70.0	W-T4-DES-814		

Peak Table

Size [bp]	Calibrated Conc. [ng/µl]	Assigned Conc. [ng/µl]	% Integrated Area	From [bp]	To [bp]	Peak Comment	Observations
100	8.50	8.50	-	62	151		Lower Marker
7549	43.4	-	100.00	3227	21536		

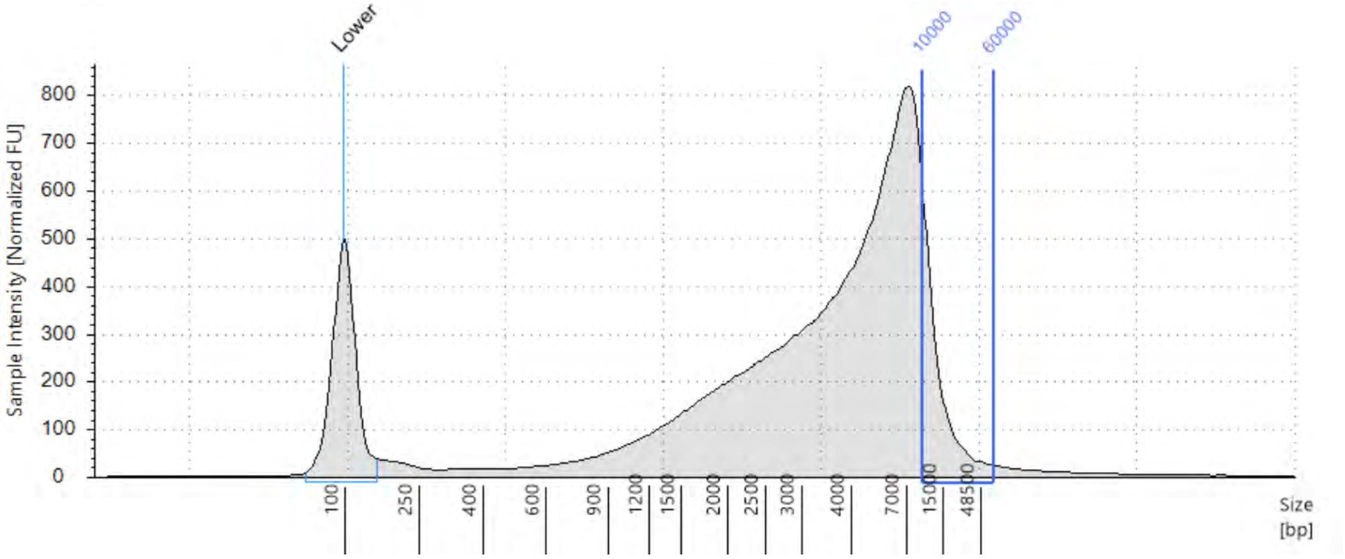

Region Table

From [bp]	To [bp]	Average Size [bp]	Conc. [ng/µl]	Region Molarity [nmol/l]	% of Total	Region Comment	Color
10000	60000	16159	6.58	0.766	9.40		■

D2: W-T4-DES-815

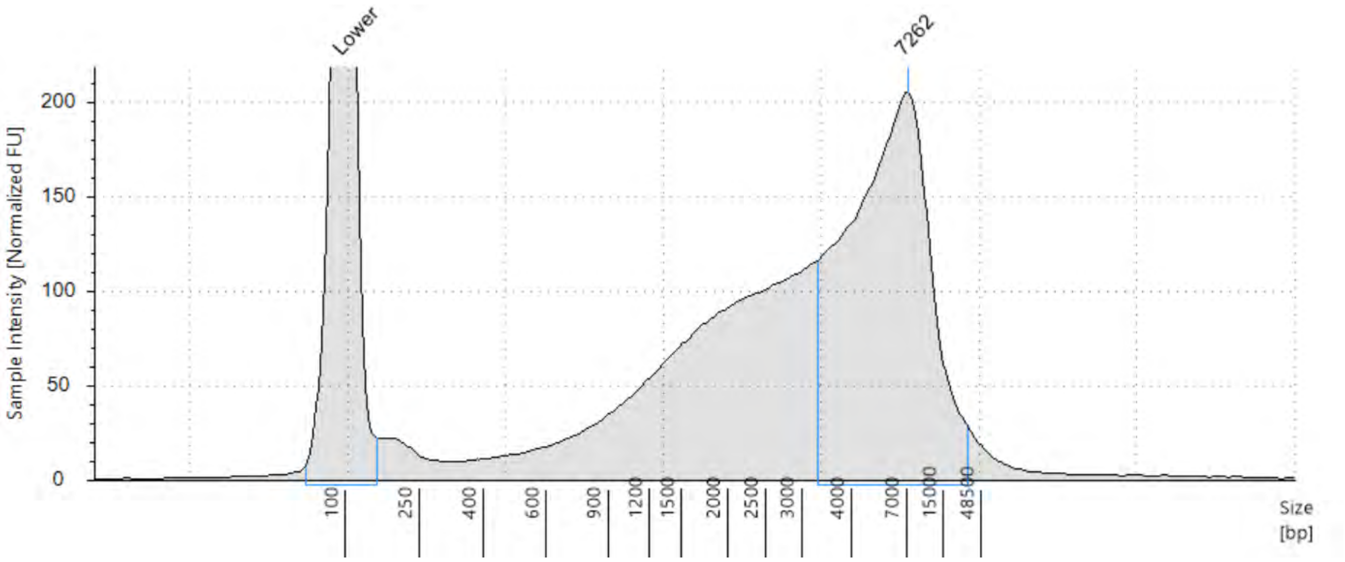

Sample Table

Well	DIN	Conc. [ng/µl]	Sample Description	Alert	Observations
D2	5.6	26.3	W-T4-DES-815		

Peak Table

Size [bp]	Calibrated Conc. [ng/µl]	Assigned Conc. [ng/µl]	% Integrated Area	From [bp]	To [bp]	Peak Comment	Observations
100	8.50	8.50	-	61	148		Lower Marker
7262	12.9	-	100.00	3295	25033		

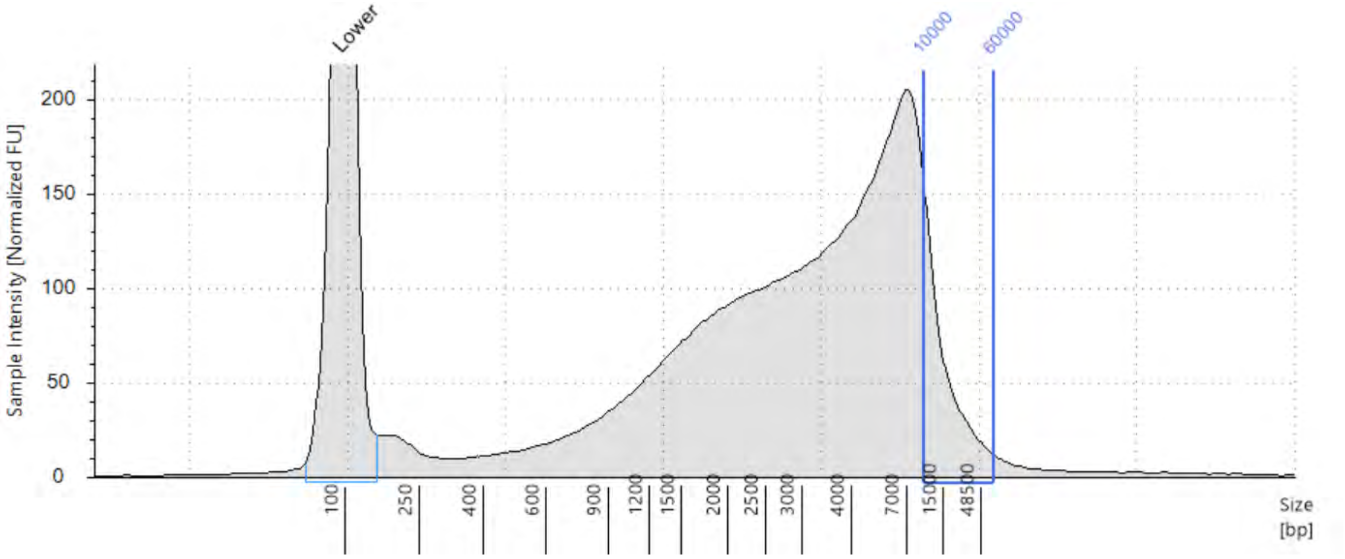

Region Table

From [bp]	To [bp]	Average Size [bp]	Conc. [ng/µl]	Region Molarity [nmol/l]	% of Total	Region Comment	Color
10000	60000	18331	2.36	0.255	8.96		

E2: W-T4-DES-1036

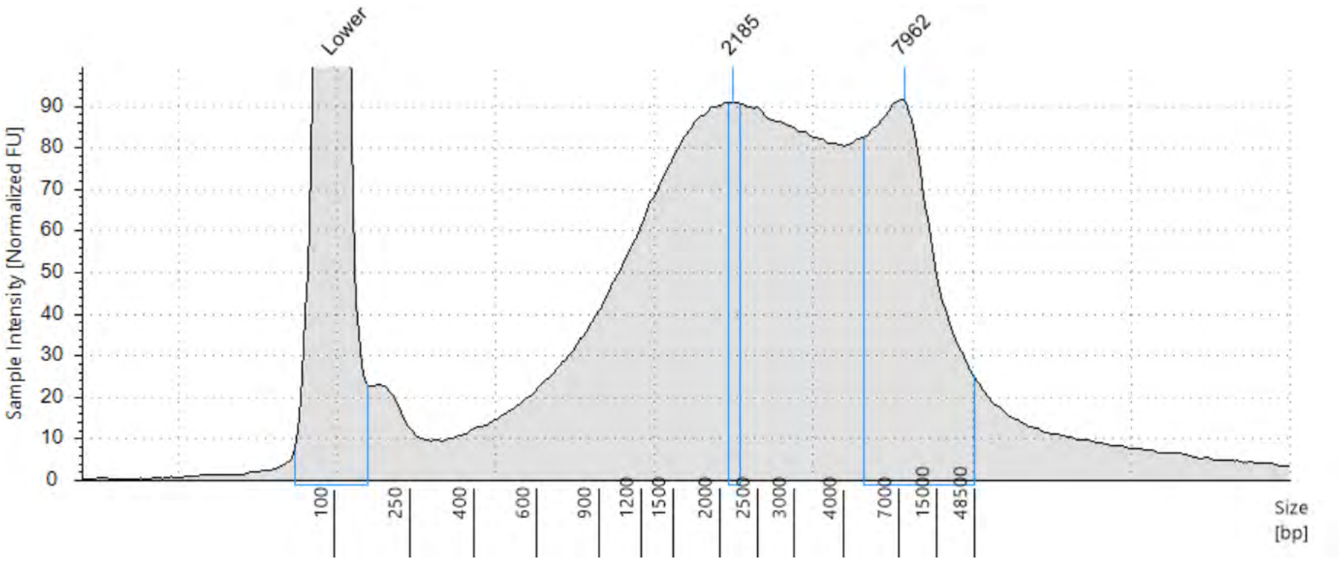

Sample Table

Well	DIN	Conc. [ng/µl]	Sample Description	Alert	Observations
E2	4.9	21.8	W-T4-DES-1036		

Peak Table

Size [bp]	Calibrated Conc. [ng/µl]	Assigned Conc. [ng/µl]	% Integrated Area	From [bp]	To [bp]	Peak Comment	Observations
100	8.50	8.50	-	61	150		Lower Marker
2185	0.782	-	14.52	2115	2275		
7962	4.60	-	85.48	4912	48856		

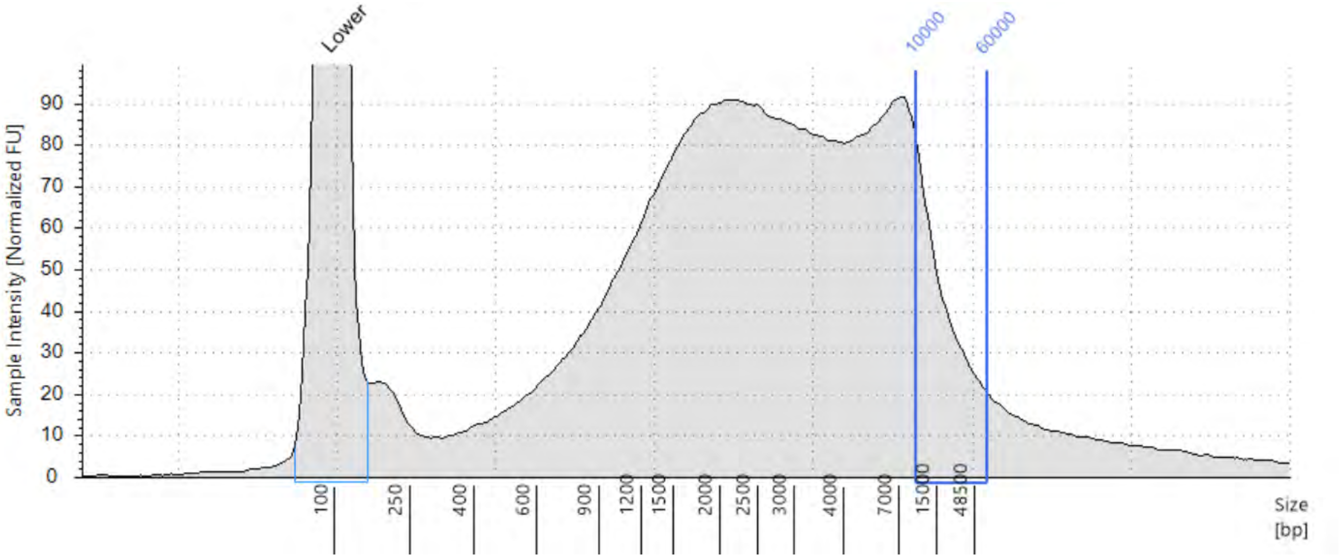

Region Table

From [bp]	To [bp]	Average Size [bp]	Conc. [ng/µl]	Region Molarity [nmol/l]	% of Total	Region Comment	Color
10000	60000	21947	1.86	0.178	8.55		

F2: W-T4-DES-1037

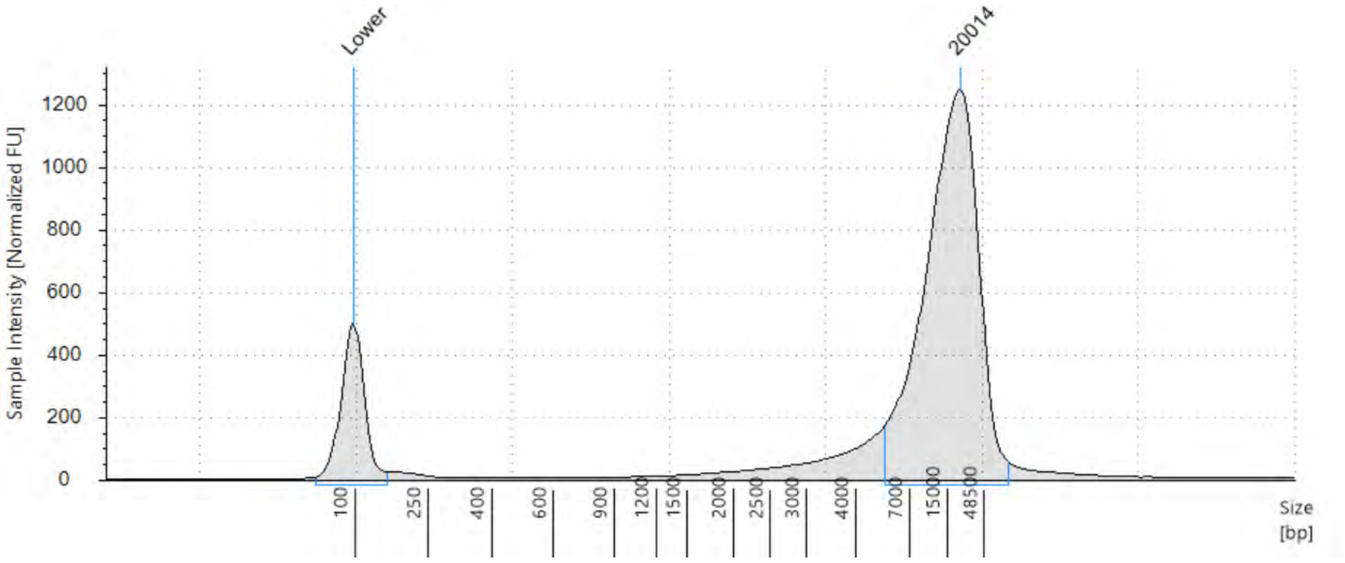

Sample Table

Well	DIN	Conc. [ng/µl]	Sample Description	Alert	Observations
F2	7.8	58.5	W-T4-DES-1037		

Peak Table

Size [bp]	Calibrated Conc. [ng/µl]	Assigned Conc. [ng/µl]	% Integrated Area	From [bp]	To [bp]	Peak Comment	Observations
100	8.50	8.50	-	61	150		Lower Marker
20014	48.7	-	99.70	5450	>60000		
-	-	-	-	-	-		Sample Well

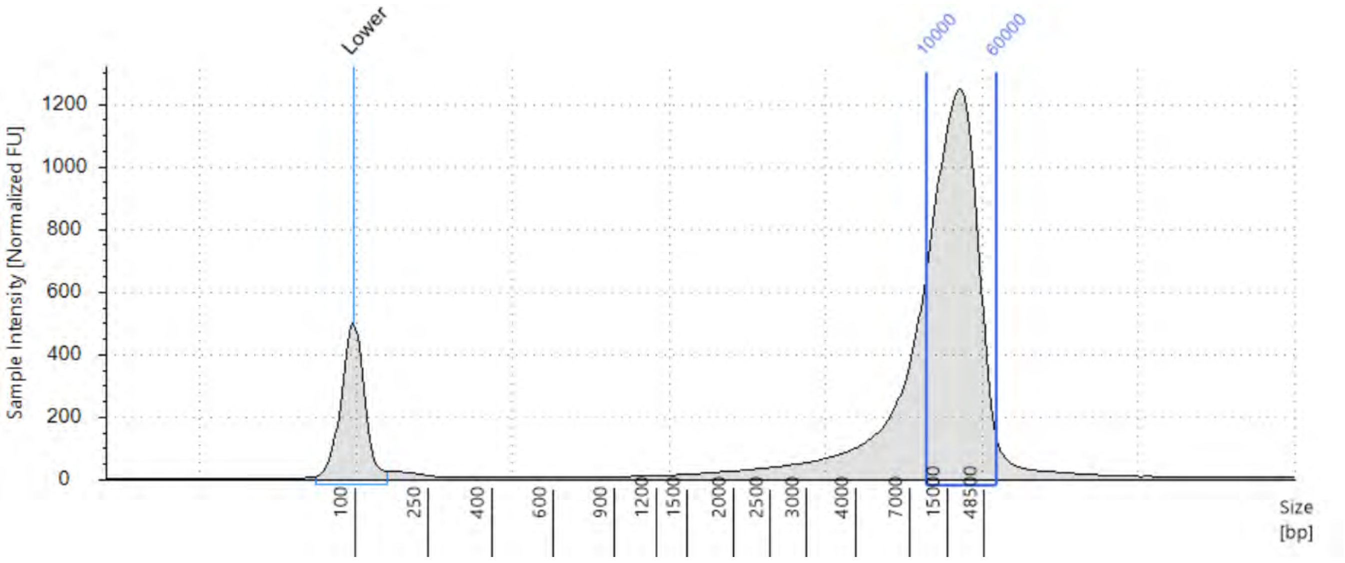

Region Table

From [bp]	To [bp]	Average Size [bp]	Conc. [ng/µl]	Region Molarity [nmol/l]	% of Total	Region Comment	Color
10000	60000	21664	39.1	3.34	66.76		

© Agilent Technologies, Inc. 2017

G2: W-T4-DES-1038

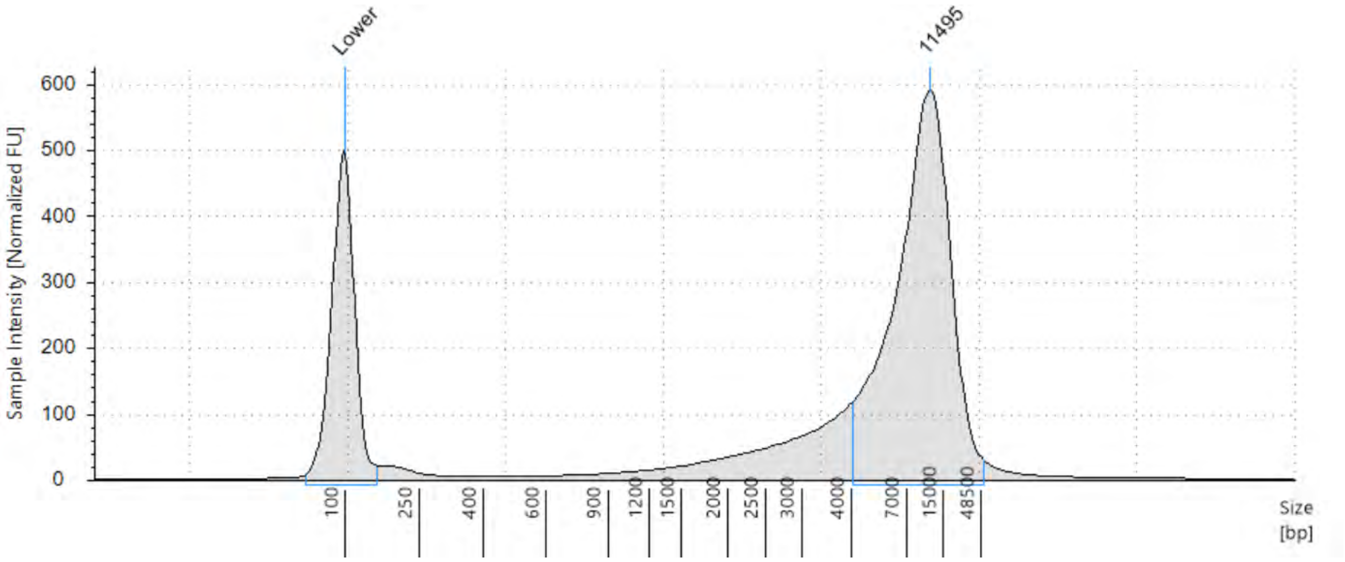

Sample Table

Well	DIN	Conc. [ng/µl]	Sample Description	Alert	Observations
G2	6.8	32.0	W-T4-DES-1038		

Peak Table

Size [bp]	Calibrated Conc. [ng/µl]	Assigned Conc. [ng/µl]	%Integrated Area	From [bp]	To [bp]	Peak Comment	Observations
100	8.50	8.50	-	61	150		Lower Marker
11495	24.1	-	100.00	4097	50737		

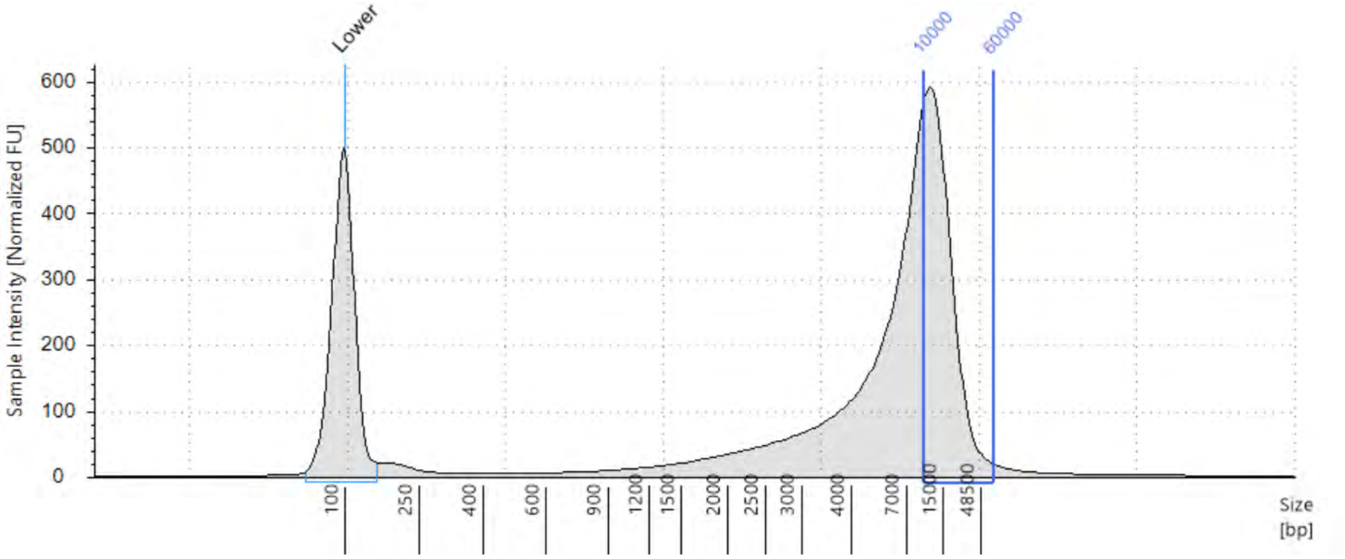

Region Table

From [bp]	To [bp]	Average Size [bp]	Conc. [ng/µl]	Region Molarity [nmol/l]	% of Total	Region Comment	Color
10000	60000	16018	12.5	1.36	38.97		

© Agilent Technologies, Inc. 2017

H2: W-T4-DES-1039

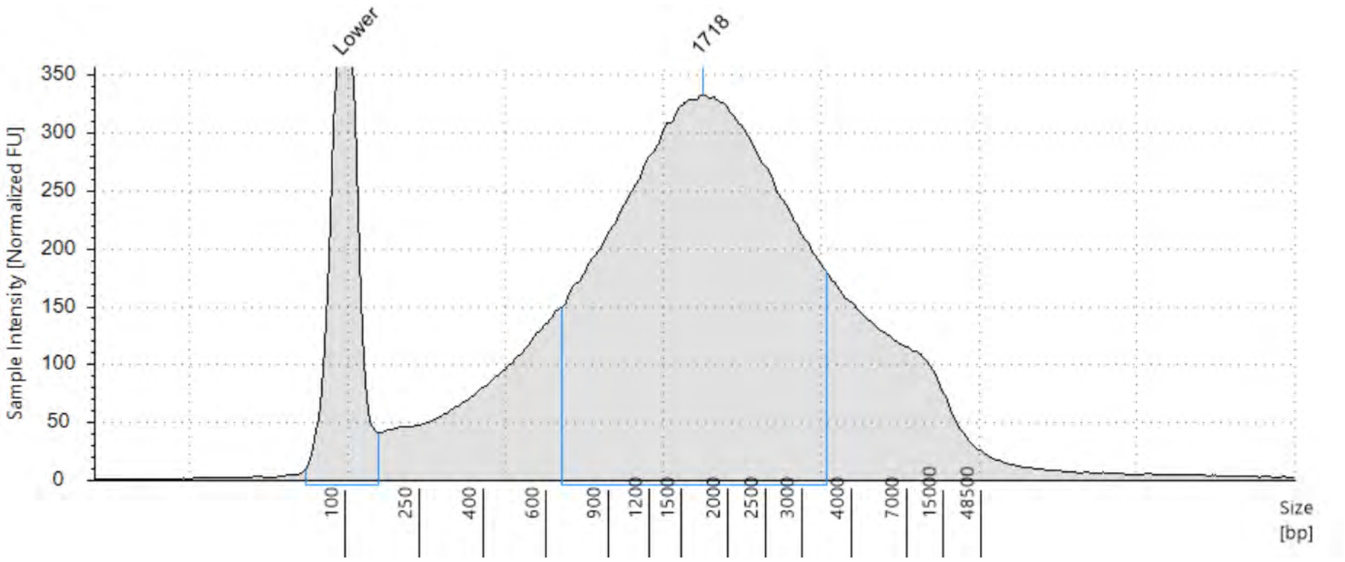

Sample Table

Well	DIN	Conc. [ng/µl]	Sample Description	Alert	Observations
H2	3.4	59.7	W-T4-DES-1039		

Peak Table

Size [bp]	Calibrated Conc. [ng/µl]	Assigned Conc. [ng/µl]	%Integrated Area	From [bp]	To [bp]	Peak Comment	Observations
100	8.50	8.50	-	61	151		Lower Marker
1718	40.9	-	100.00	665	3466		

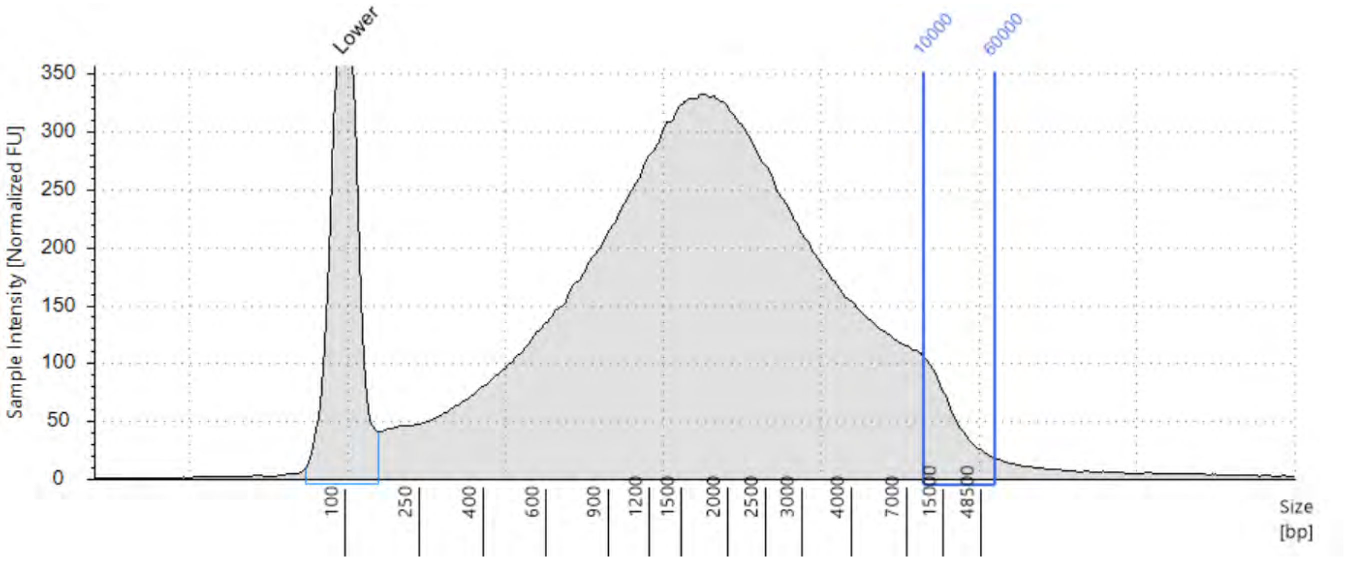

Region Table

From [bp]	To [bp]	Average Size [bp]	Conc. [ng/µl]	Region Molarity [nmol/l]	% of Total	Region Comment	Color
10000	60000	19706	2.24	0.236	3.75		

Filename: 2019-07-29-04.gDNA

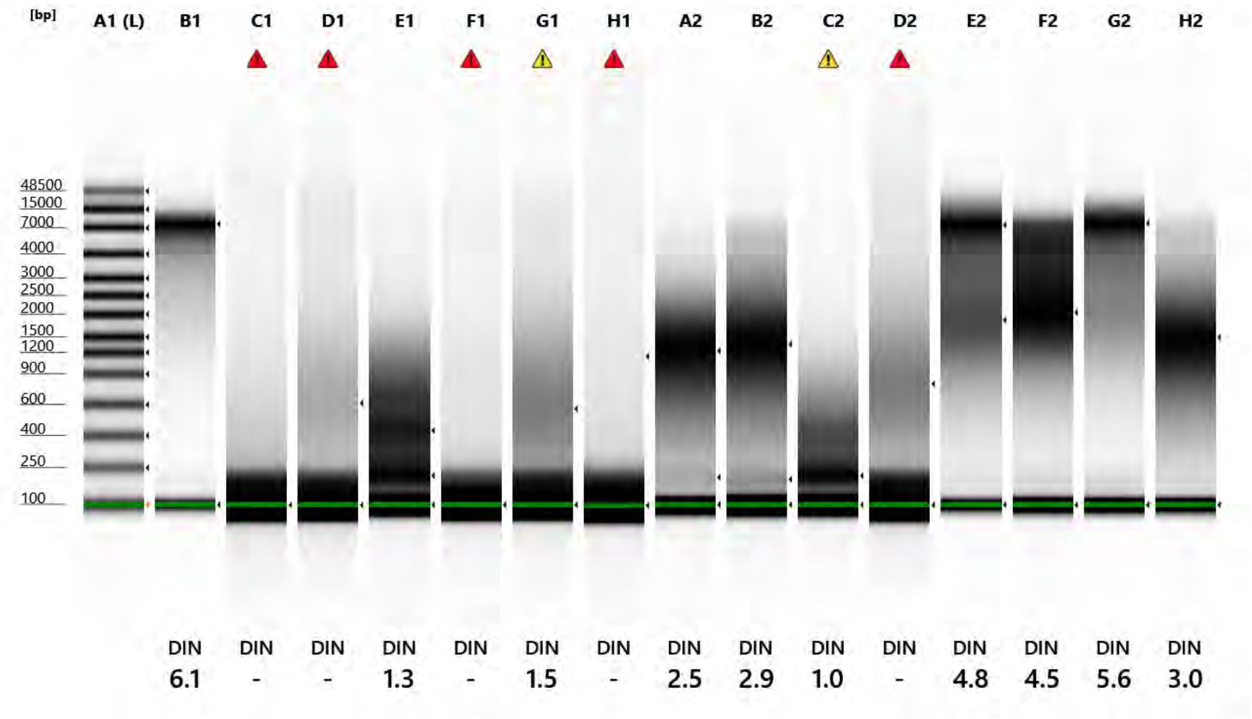

Default image (Contrast 50%), Image is Scaled to Sample

Sample Info

Well	DIN	Conc. [ng/µl]	Sample Description	Alert	Observations
A1	-	87.8	Ladder		Ladder
B1	6.1	35.0	W-T4-DES-1040		
C1	-	1.30	W-T4-DE-816	▲	Sample concentration outside functional range for DIN
D1	-	1.99	W-T4-DE-817	▲	Sample concentration outside functional range for DIN
E1	1.3	12.6	W-T4-DE-818		
F1	-	1.69	W-T4-DE-819	▲	Sample concentration outside functional range for DIN
G1	1.5	3.18	W-T4-DE-820	⚠	Sample concentration outside functional range for DIN
H1	-	1.32	W-T4-DE-1041	▲	Sample concentration outside functional range for DIN
A2	2.5	23.3	W-T4-DE-1042		
B2	2.9	15.6	W-T4-DE-1043		
C2	1.0	9.44	W-T4-DE-1044	⚠	Sample concentration outside recommended range
D2	-	2.83	W-T4-DE-1045	▲	Sample concentration outside functional range for DIN
E2	4.8	62.5	W-T4-ES-826		
F2	4.5	39.4	W-T4-ES-827		
G2	5.6	29.5	W-T4-ES-828		
H2	3.0	40.5	W-T4-ES-829		

 © Agilent Technologies, Inc. 2017

A1: Ladder

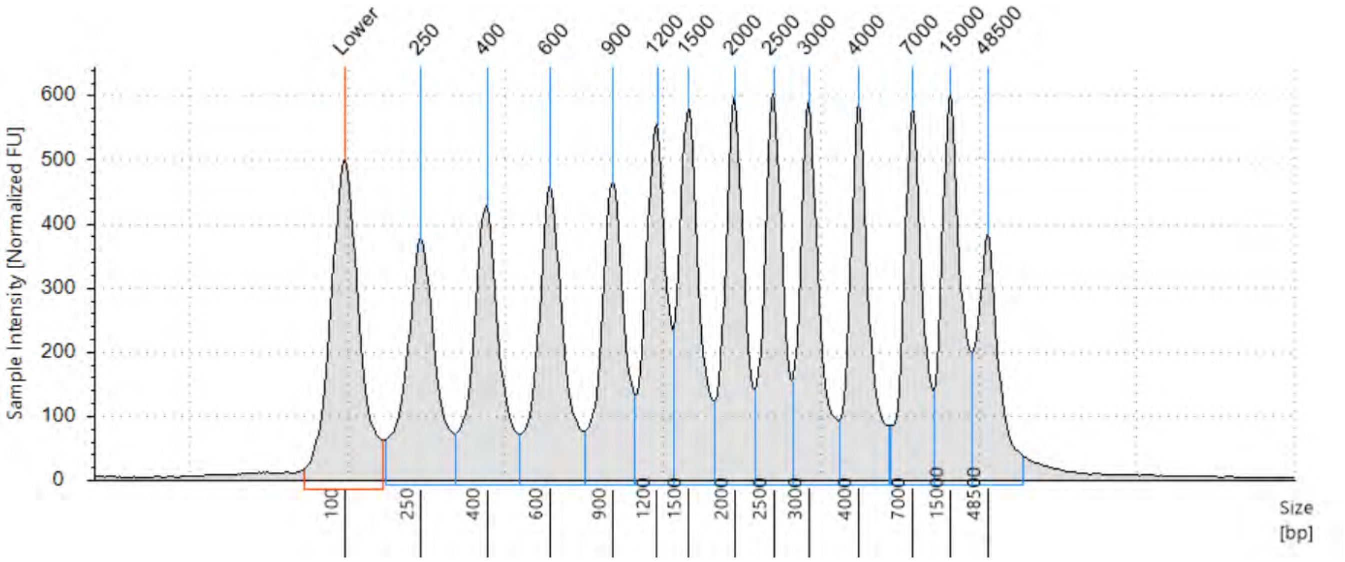

Sample Table

Well	DIN	Conc. [ng/µl]	Sample Description	Alert	Observations
A1	-	87.8	Ladder		Ladder

Peak Table

Size [bp]	Calibrated Conc. [ng/µl]	Assigned Conc. [ng/µl]	% Integrated Area	From [bp]	To [bp]	Peak Comment	Observations
100	8.50	8.50	-	61	157		Lower Marker
250	6.59	-	7.52	162	319		
400	6.82	-	7.79	319	494		
600	7.08	-	8.09	494	748		
900	6.44	-	7.35	755	1039		
1200	7.02	-	8.02	1039	1348		
1500	7.31	-	8.35	1348	1762		
2000	7.03	-	8.03	1762	2254		
2500	6.80	-	7.76	2254	2758		
3000	7.02	-	8.02	2758	3577		
4000	6.82	-	7.79	3577	5485		
7000	6.60	-	7.54	5564	10820		
15000	7.08	-	8.09	10820	23208		
48500	4.95	-	5.66	23208	>60000		

 © Agilent Technologies, Inc. 2017

B1: W-T4-DES-1040

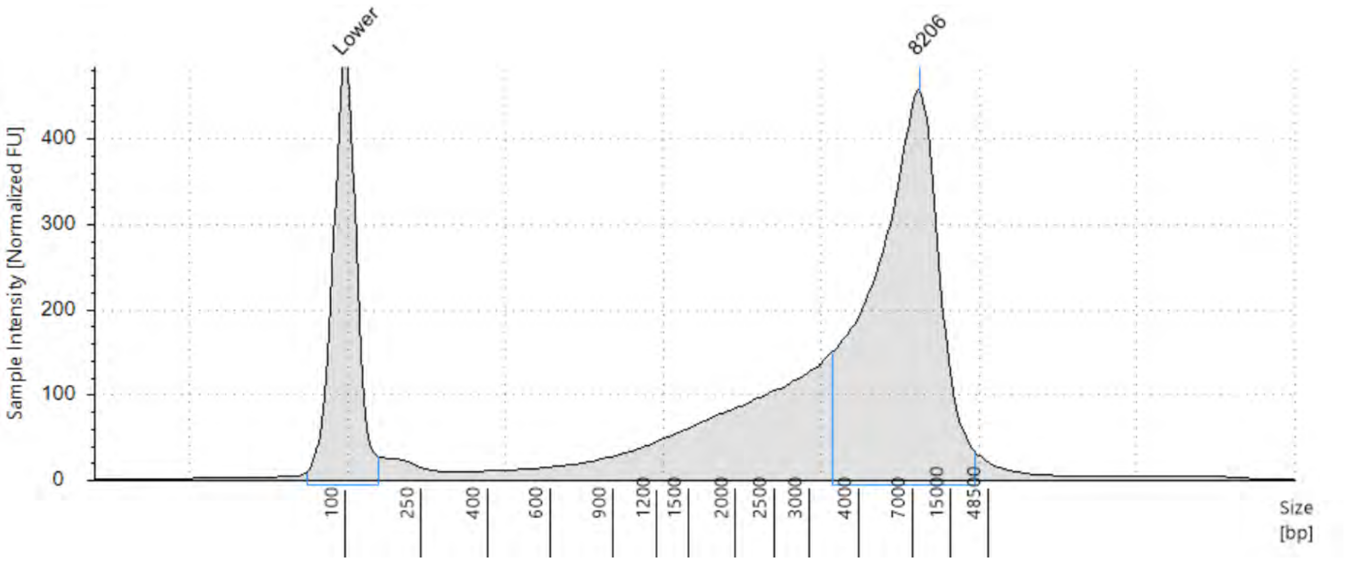

Sample Table

Well	DIN	Conc. [ng/µl]	Sample Description	Alert	Observations
B1	6.1	35.0	W-T4-DES-1040		

Peak Table

Size [bp]	Calibrated Conc. [ng/µl]	Assigned Conc. [ng/µl]	% Integrated Area	From [bp]	To [bp]	Peak Comment	Observations
100	8.50	8.50	-	63	149		Lower Marker
8206	21.6	-	100.00	3424	24596		

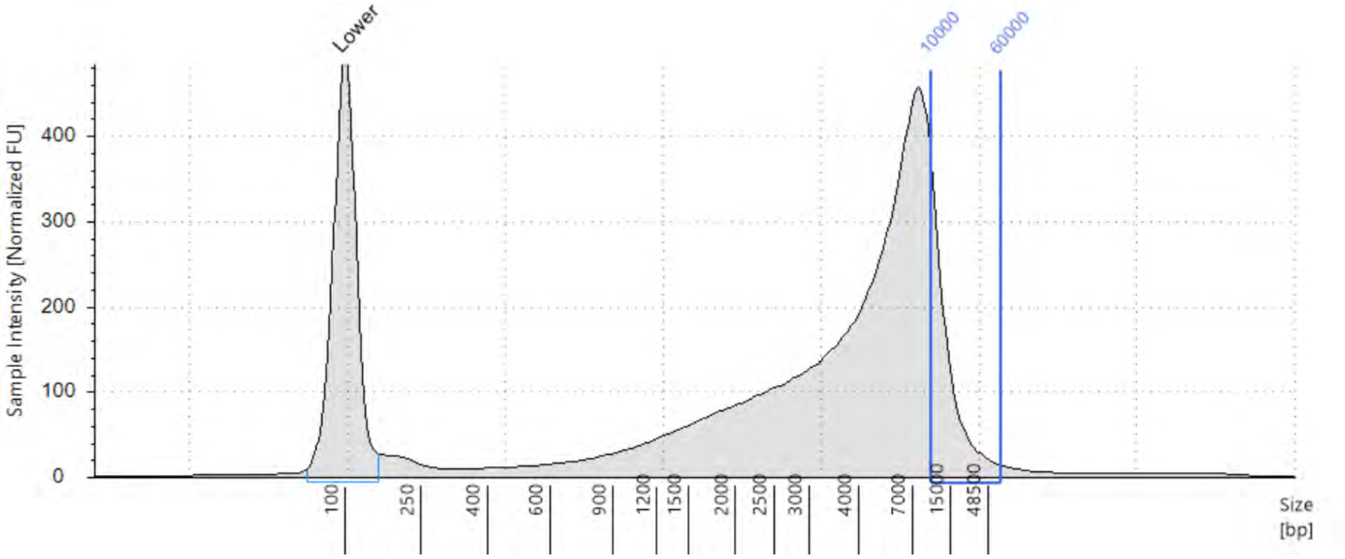

Region Table

From [bp]	To [bp]	Average Size [bp]	Conc. [ng/µl]	Region Molarity [nmol/l]	% of Total	Region Comment	Color
10000	60000	16165	4.43	0.510	12.67		

© Agilent Technologies, Inc. 2017

C1: W-T4-DE-816

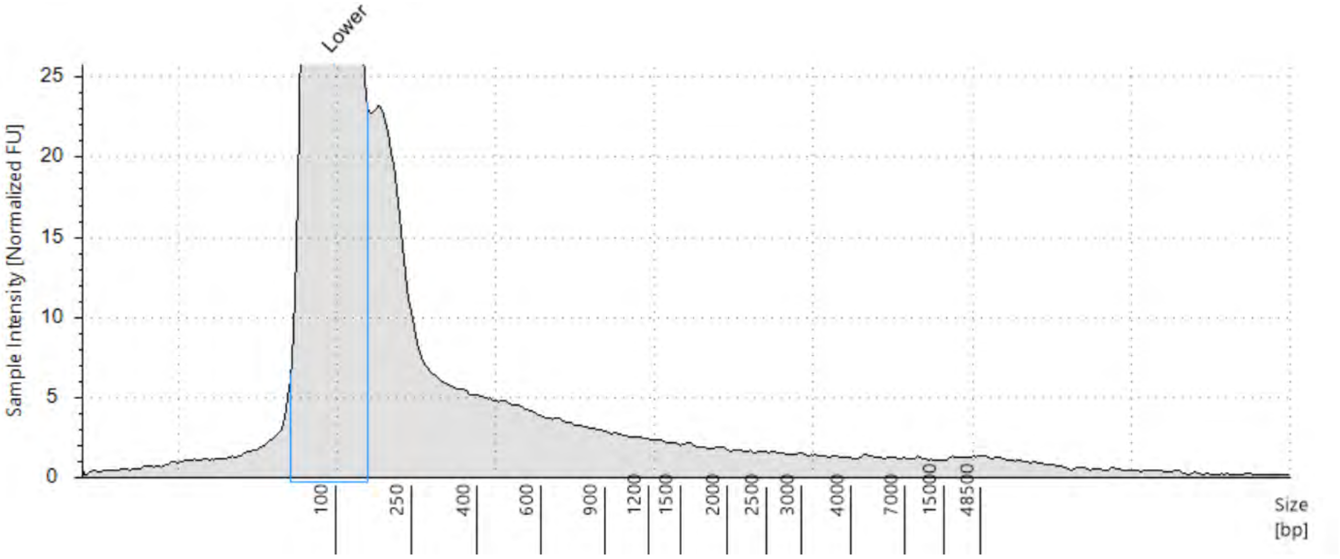

Sample Table

Well	DIN	Conc. [ng/µl]	Sample Description	Alert	Observations
C1	-	1.30	W-T4-DE-816	▲	Sample concentration outside functional range for DIN

Peak Table

Size [bp]	Calibrated Conc. [ng/µl]	Assigned Conc. [ng/µl]	% Integrated Area	From [bp]	To [bp]	Peak Comment	Observations
100	8.50	8.50	-	59	150		Lower Marker

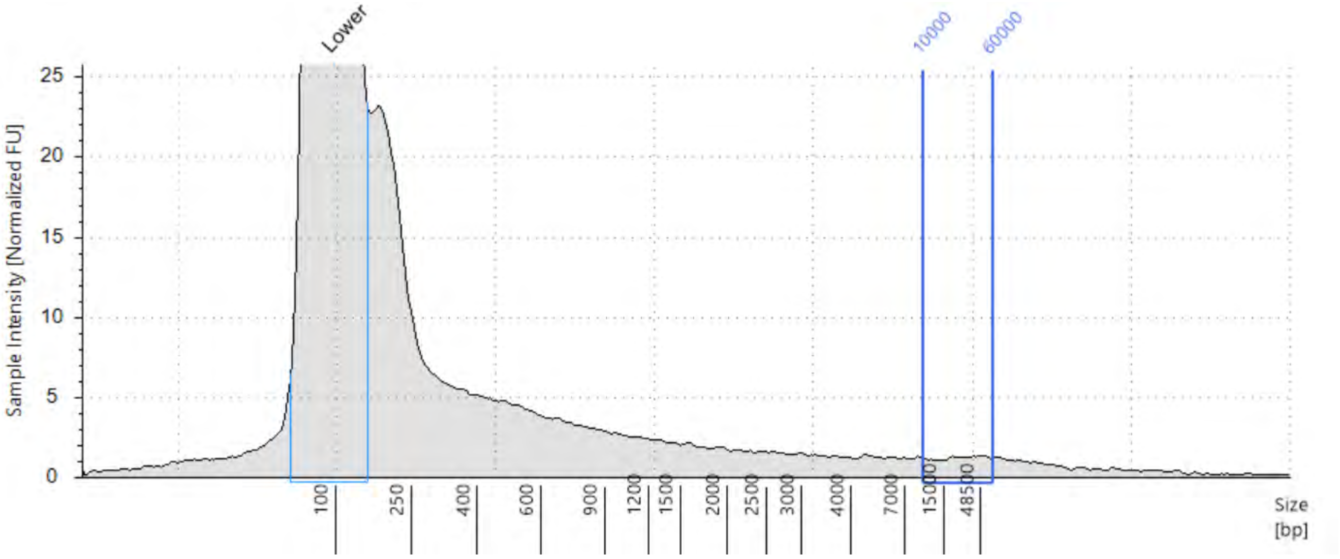

Region Table

From [bp]	To [bp]	Average Size [bp]	Conc. [ng/µl]	Region Molarity [nmol/l]	% of Total	Region Comment	Color
10000	60000	27251	0.0282	0.00387	2.17		■

D1: W-T4-DE-817

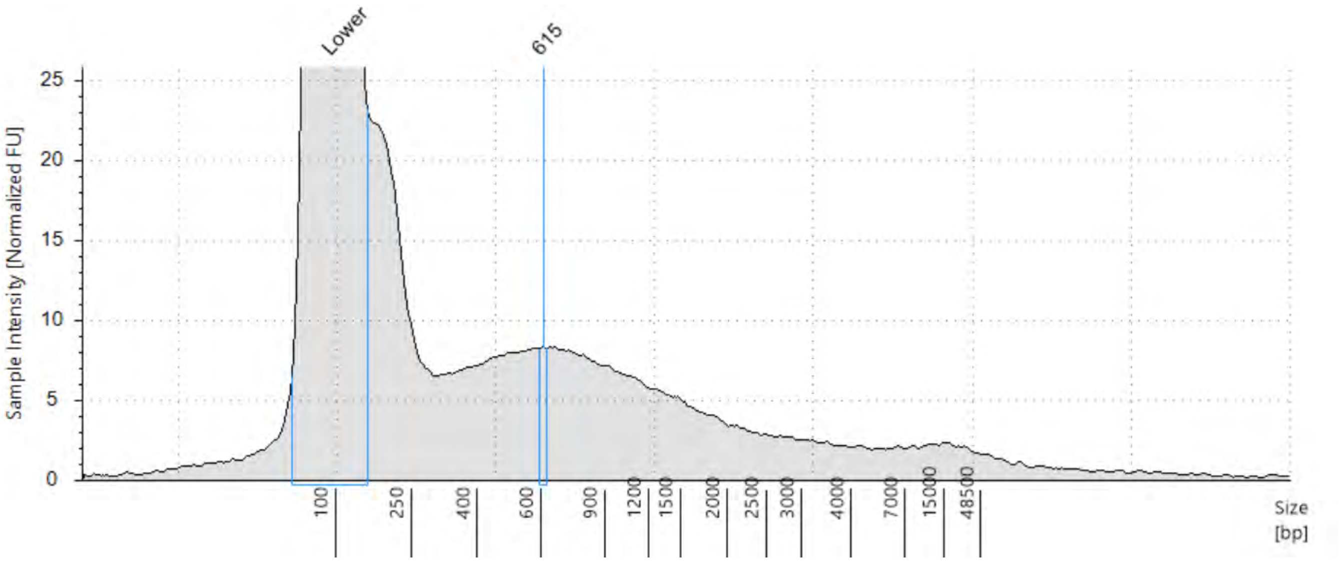

Sample Table

Well	DIN	Conc. [ng/µl]	Sample Description	Alert	Observations
D1	-	1.99	W-T4-DE-817	▲	Sample concentration outside functional range for DIN

Peak Table

Size [bp]	Calibrated Conc. [ng/µl]	Assigned Conc. [ng/µl]	% Integrated Area	From [bp]	To [bp]	Peak Comment	Observations
100	8.50	8.50	-	60	150		Lower Marker
615	0.0395	-	100.00	599	626		

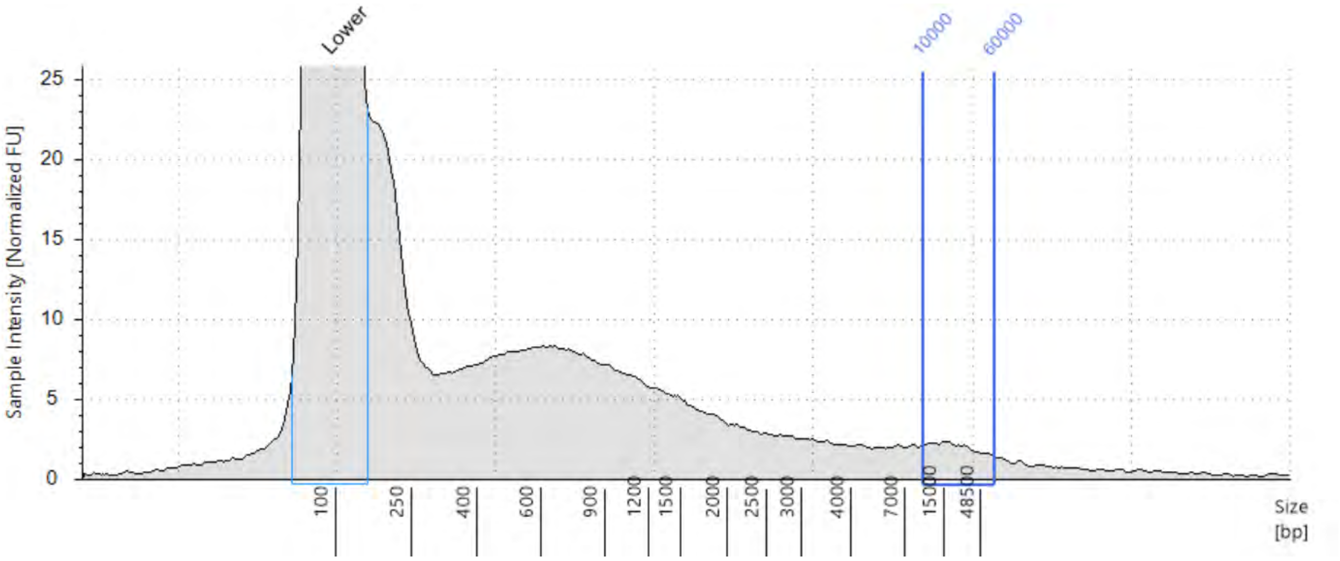

Region Table

From [bp]	To [bp]	Average Size [bp]	Conc. [ng/µl]	Region Molarity [nmol/l]	% of Total	Region Comment	Color
10000	60000	24877	0.0609	0.00699	3.07		■

 © Agilent Technologies, Inc. 2017

E1: W-T4-DE-818

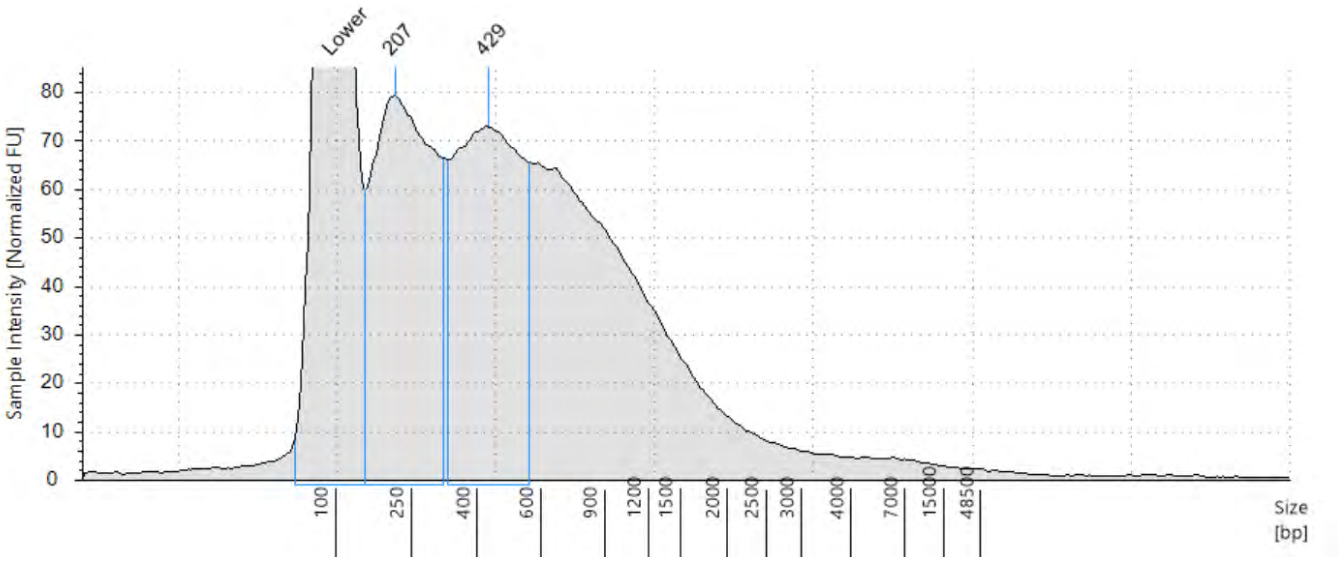

Sample Table

Well	DIN	Conc. [ng/µl]	Sample Description	Alert	Observations
E1	1.3	12.6	W-T4-DE-818		

Peak Table

Size [bp]	Calibrated Conc. [ng/µl]	Assigned Conc. [ng/µl]	% Integrated Area	From [bp]	To [bp]	Peak Comment	Observations
100	8.50	8.50	-	62	144		Lower Marker
207	3.41	-	50.22	144	317		
429	3.38	-	49.78	327	558		

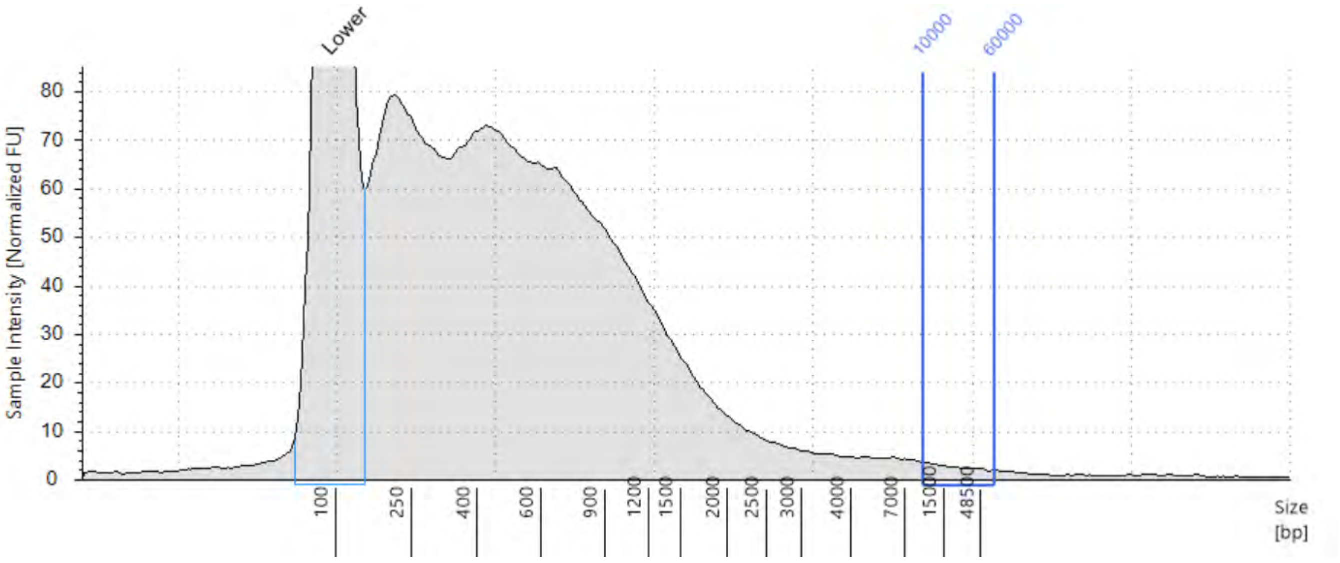

Region Table

From [bp]	To [bp]	Average Size [bp]	Conc. [ng/µl]	Region Molarity [nmol/l]	% of Total	Region Comment	Color
10000	60000	24180	0.0675	0.00947	0.54		

 © Agilent Technologies, Inc. 2017

F1: W-T4-DE-819

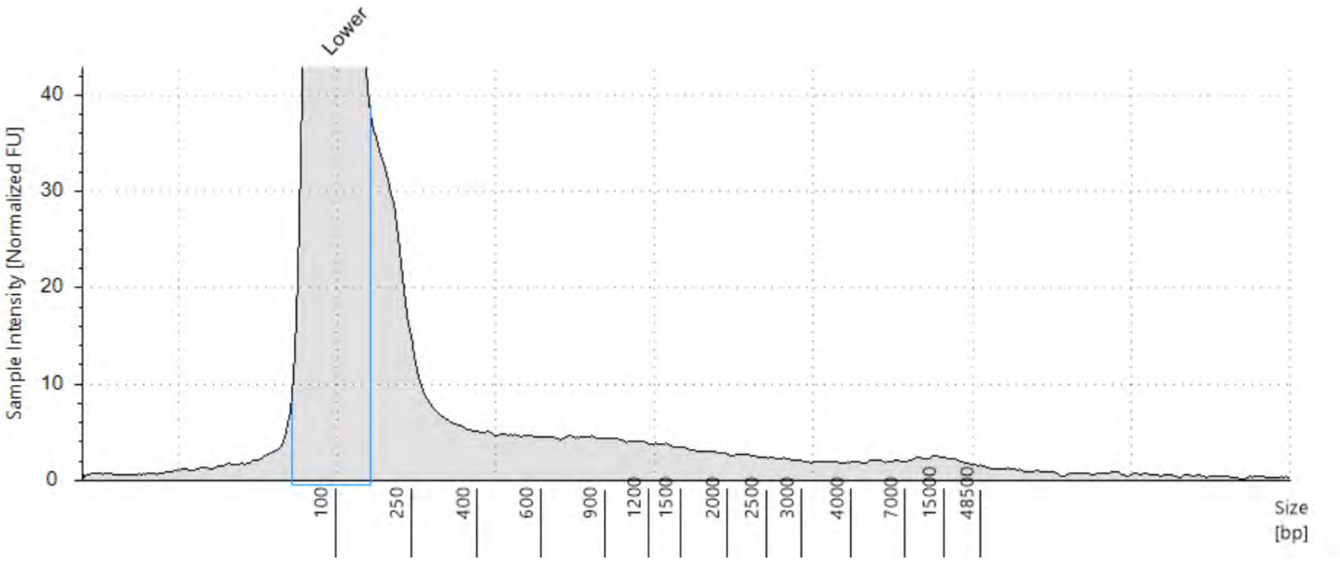

Sample Table

Well	DIN	Conc. [ng/µl]	Sample Description	Alert	Observations
F1	-	1.69	W-T4-DE-819	▲	Sample concentration outside functional range for DIN

Peak Table

Size [bp]	Calibrated Conc. [ng/µl]	Assigned Conc. [ng/µl]	% Integrated Area	From [bp]	To [bp]	Peak Comment	Observations
100	8.50	8.50	-	60	154		Lower Marker

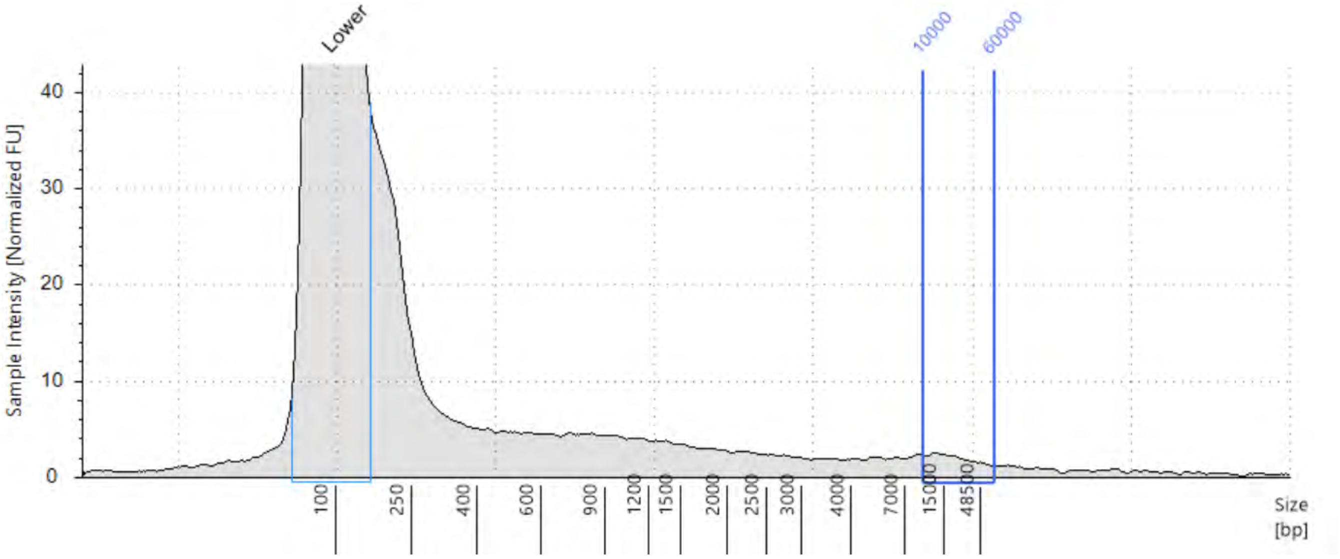

Region Table

From [bp]	To [bp]	Average Size [bp]	Conc. [ng/µl]	Region Molarity [nmol/l]	% of Total	Region Comment	Color
10000	60000	23490	0.0565	0.00646	3.34		■

© Agilent Technologies, Inc. 2017

G1: W-T4-DE-820

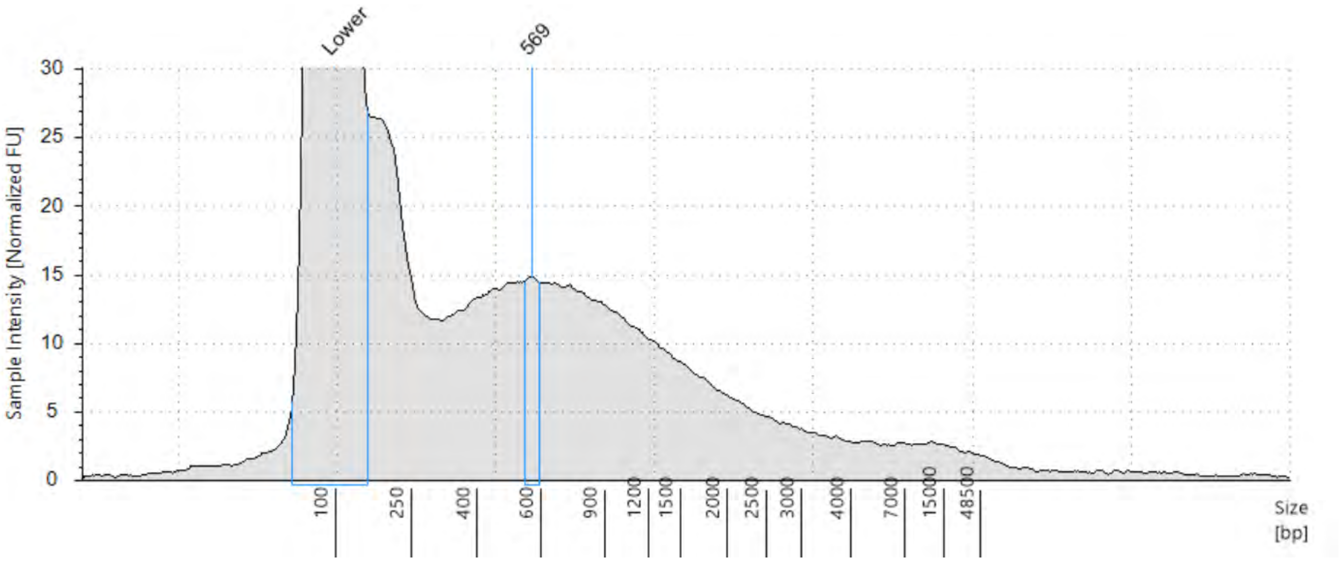

Sample Table

Well	DIN	Conc. [ng/µl]	Sample Description	Alert	Observations
G1	1.5	3.18	W-T4-DE-820	⚠	Sample concentration outside functional range for DIN

Peak Table

Size [bp]	Calibrated Conc. [ng/µl]	Assigned Conc. [ng/µl]	% Integrated Area	From [bp]	To [bp]	Peak Comment	Observations
100	8.50	8.50	-	60	150		Lower Marker
569	0.130	-	100.00	544	595		

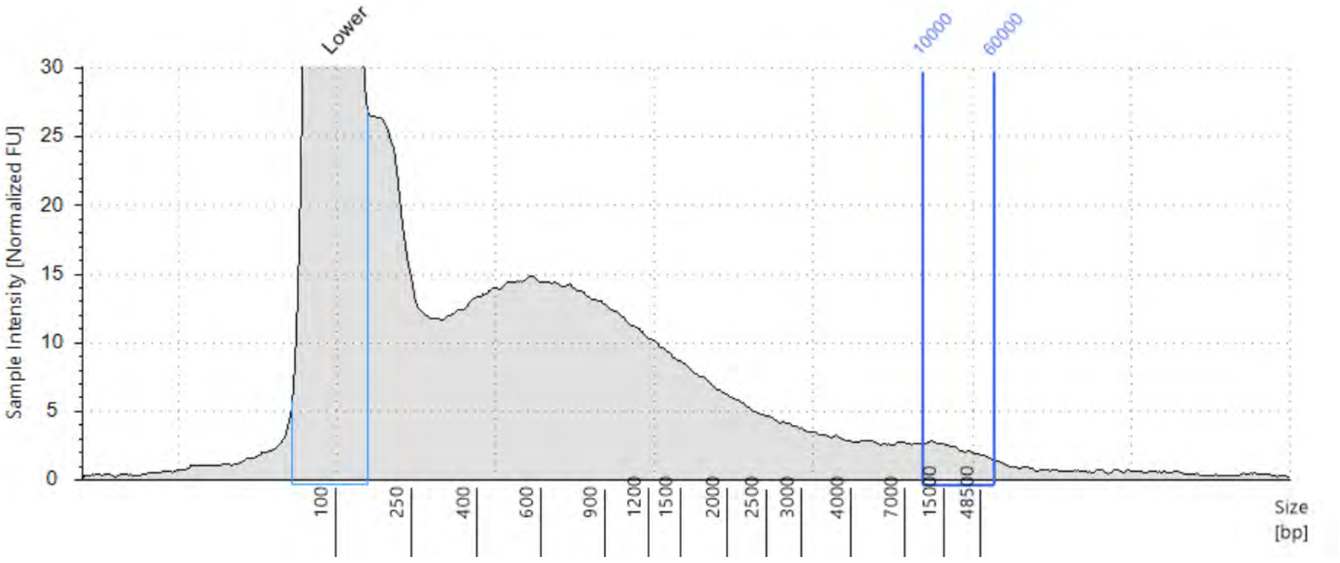

Region Table

From [bp]	To [bp]	Average Size [bp]	Conc. [ng/µl]	Region Molarity [nmol/l]	% of Total	Region Comment	Color
10000	60000	24302	0.0666	0.00790	2.09		■

H1: W-T4-DE-1041

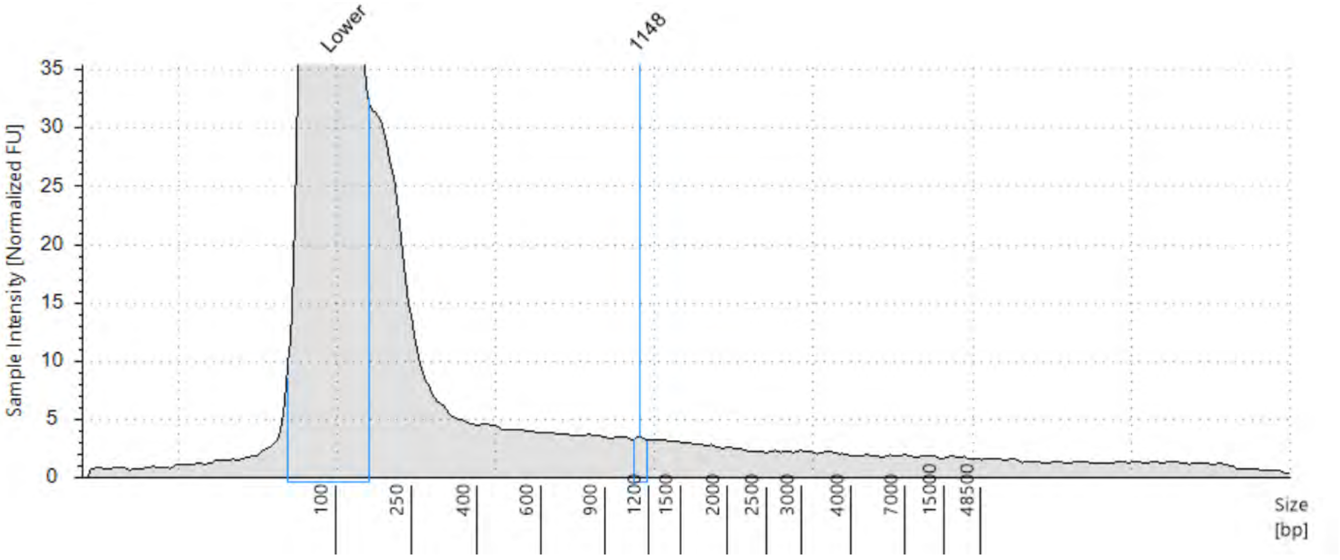

Sample Table

Well	DIN	Conc. [ng/µl]	Sample Description	Alert	Observations
H1	-	1.32	W-T4-DE-1041	▲	Sample concentration outside functional range for DIN

Peak Table

Size [bp]	Calibrated Conc. [ng/µl]	Assigned Conc. [ng/µl]	% Integrated Area	From [bp]	To [bp]	Peak Comment	Observations
100	8.50	8.50	-	58	154		Lower Marker
1148	0.0197	-	100.00	1098	1200		

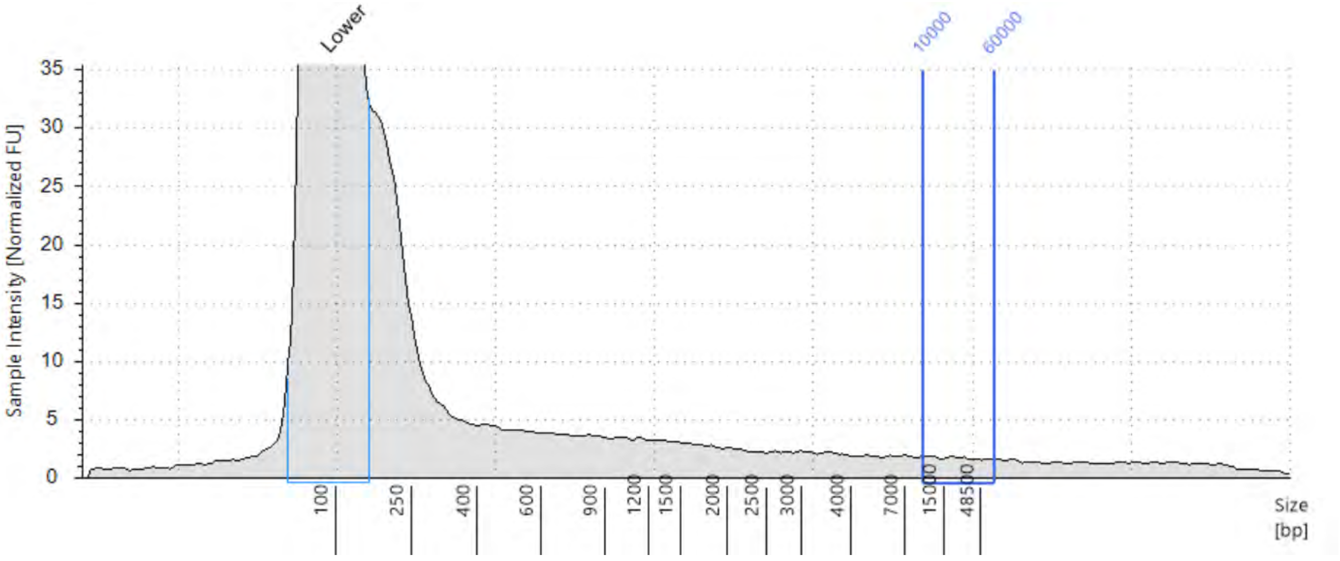

Region Table

From [bp]	To [bp]	Average Size [bp]	Conc. [ng/µl]	Region Molarity [nmol/l]	% of Total	Region Comment	Color
10000	60000	25783	0.0292	0.00507	2.22		■

© Agilent Technologies, Inc. 2017

A2: W-T4-DE-1042

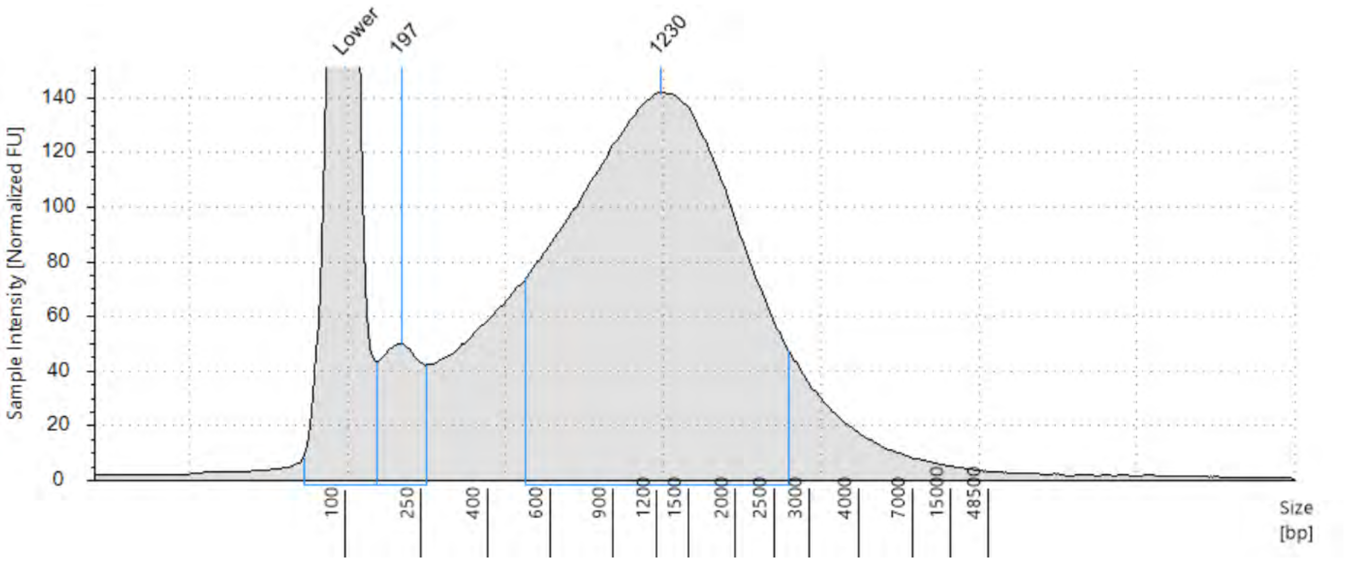

Sample Table

Well	DIN	Conc. [ng/µl]	Sample Description	Alert	Observations
A2	2.5	23.3	W-T4-DE-1042		

Peak Table

Size [bp]	Calibrated Conc. [ng/µl]	Assigned Conc. [ng/µl]	% Integrated Area	From [bp]	To [bp]	Peak Comment	Observations
100	8.50	8.50	-	60	148		Lower Marker
197	1.37	-	7.54	148	260		
1230	16.9	-	92.46	512	2700		

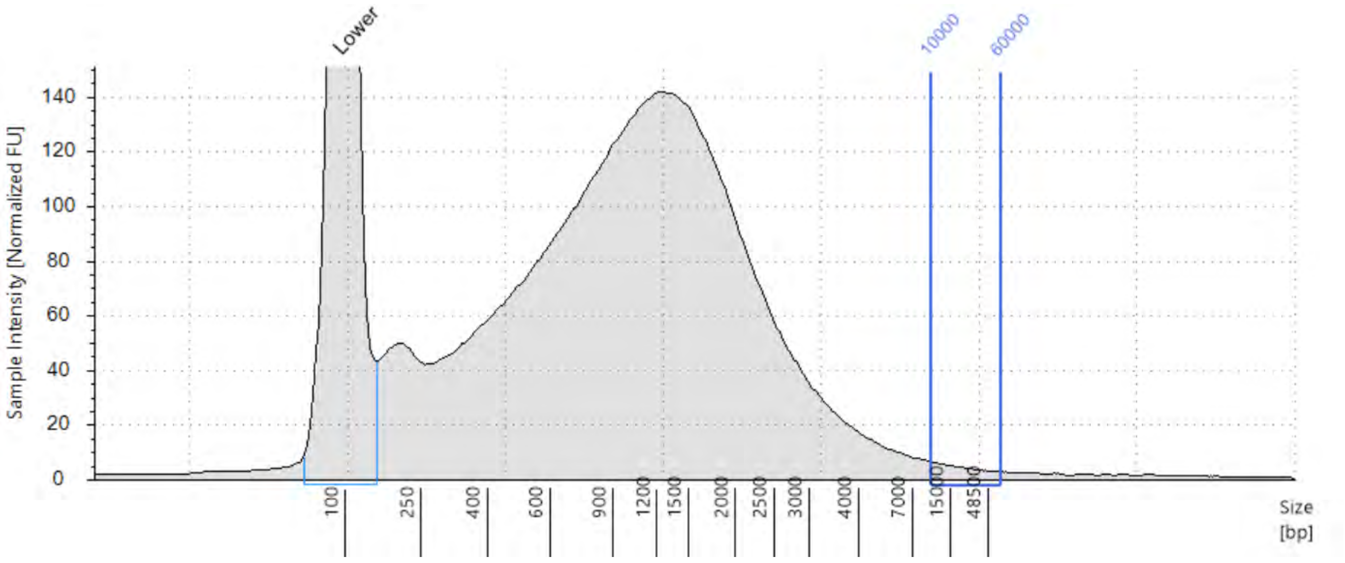

Region Table

From [bp]	To [bp]	Average Size [bp]	Conc. [ng/µl]	Region Molarity [nmol/l]	% of Total	Region Comment	Color
10000	60000	23055	0.126	0.0163	0.54		

 © Agilent Technologies, Inc. 2017

B2: W-T4-DE-1043

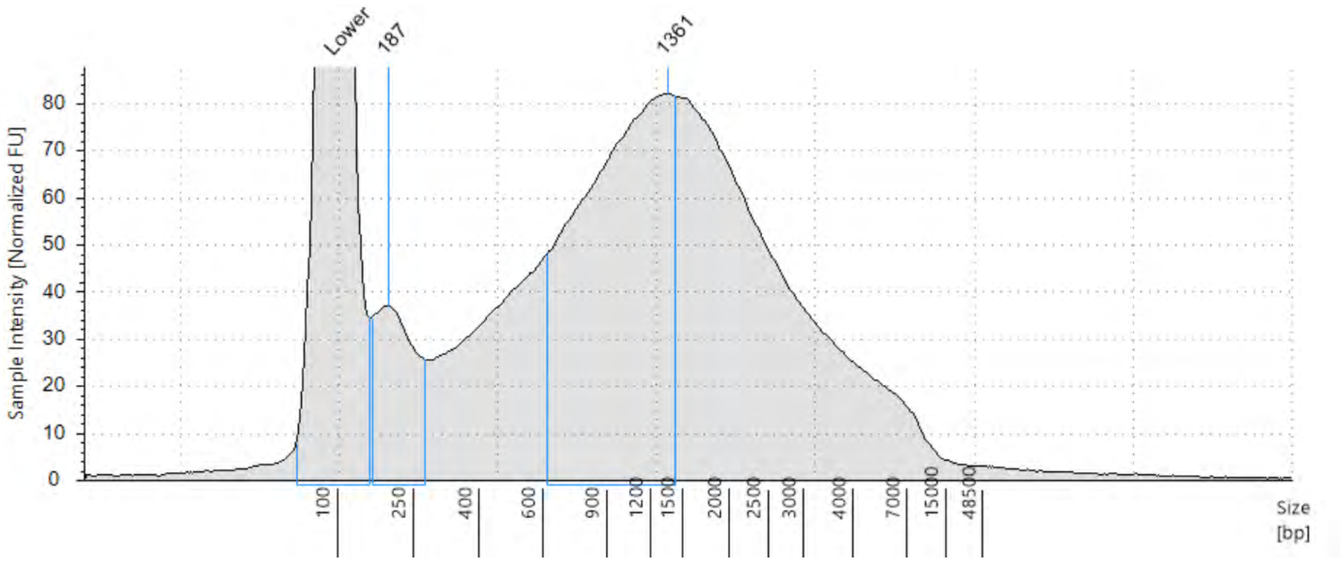

Sample Table

Well	DIN	Conc. [ng/µl]	Sample Description	Alert	Observations
B2	2.9	15.6	W-T4-DE-1043		

Peak Table

Size [bp]	Calibrated Conc. [ng/µl]	Assigned Conc. [ng/µl]	% Integrated Area	From [bp]	To [bp]	Peak Comment	Observations
100	8.50	8.50	-	61	148		Lower Marker
187	1.06	-	16.67	153	273		
1361	5.29	-	83.33	619	1430		

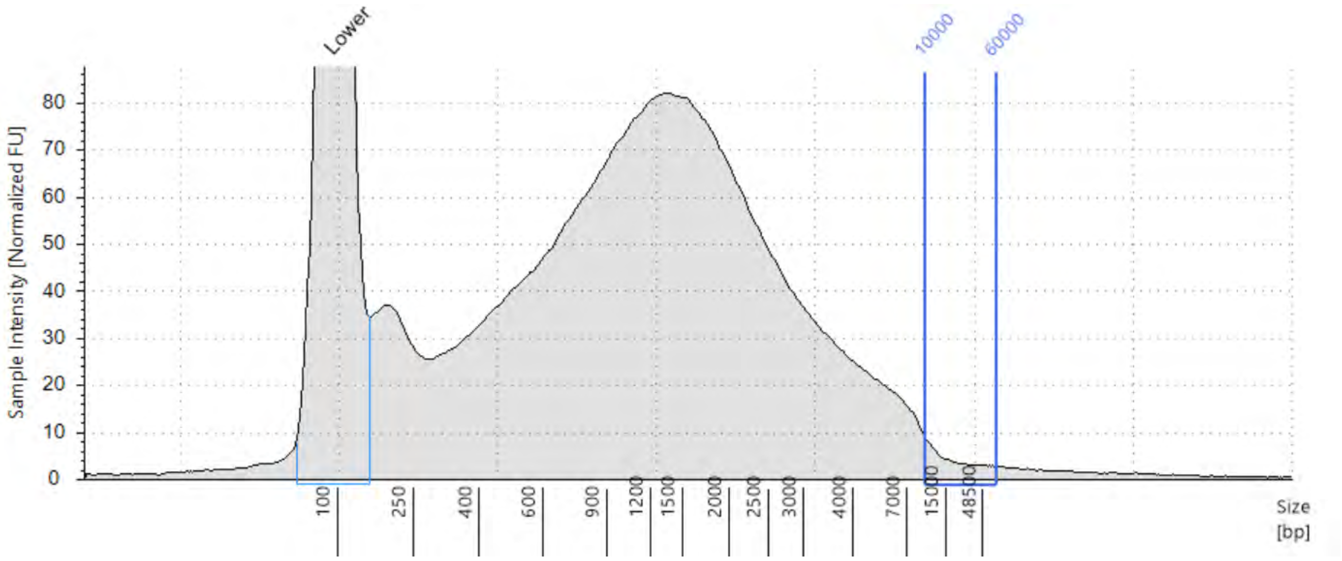

Region Table

From [bp]	To [bp]	Average Size [bp]	Conc. [ng/µl]	Region Molarity [nmol/l]	% of Total	Region Comment	Color
10000	60000	23155	0.130	0.0163	0.83		

C2: W-T4-DE-1044

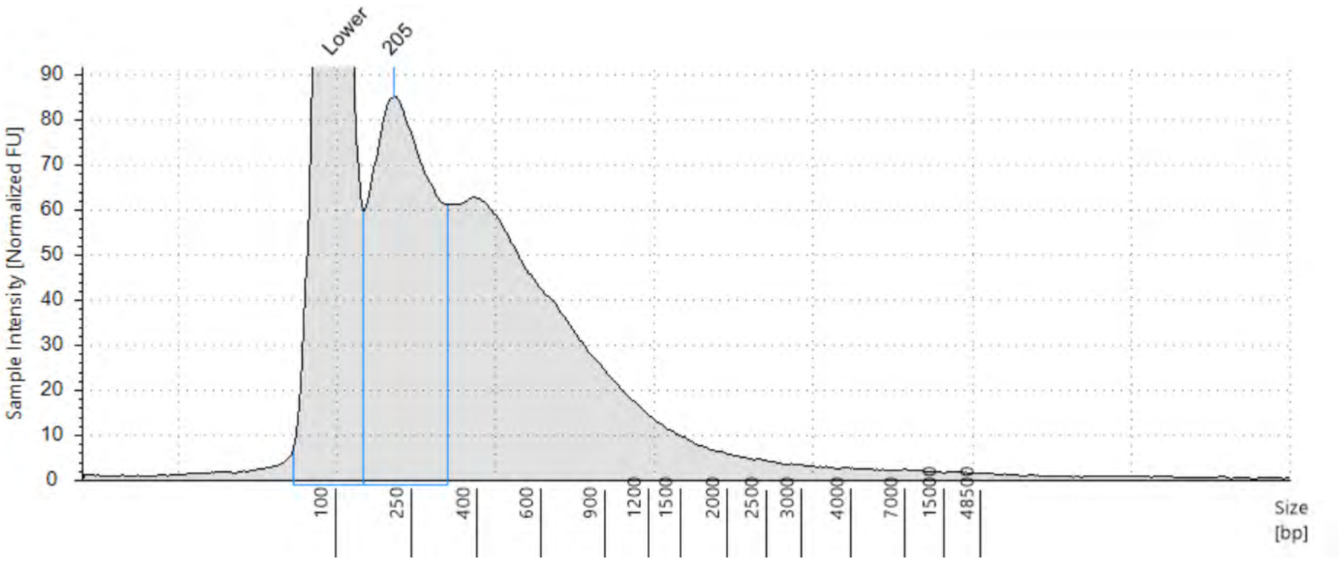

Sample Table

Well	DIN	Conc. [ng/µl]	Sample Description	Alert	Observations
C2	1.0	9.44	W-T4-DE-1044	⚠	Sample concentration outside recommended range

Peak Table

Size [bp]	Calibrated Conc. [ng/µl]	Assigned Conc. [ng/µl]	% Integrated Area	From [bp]	To [bp]	Peak Comment	Observations
100	8.50	8.50	-	61	142		Lower Marker
205	3.68	-	100.00	142	324		

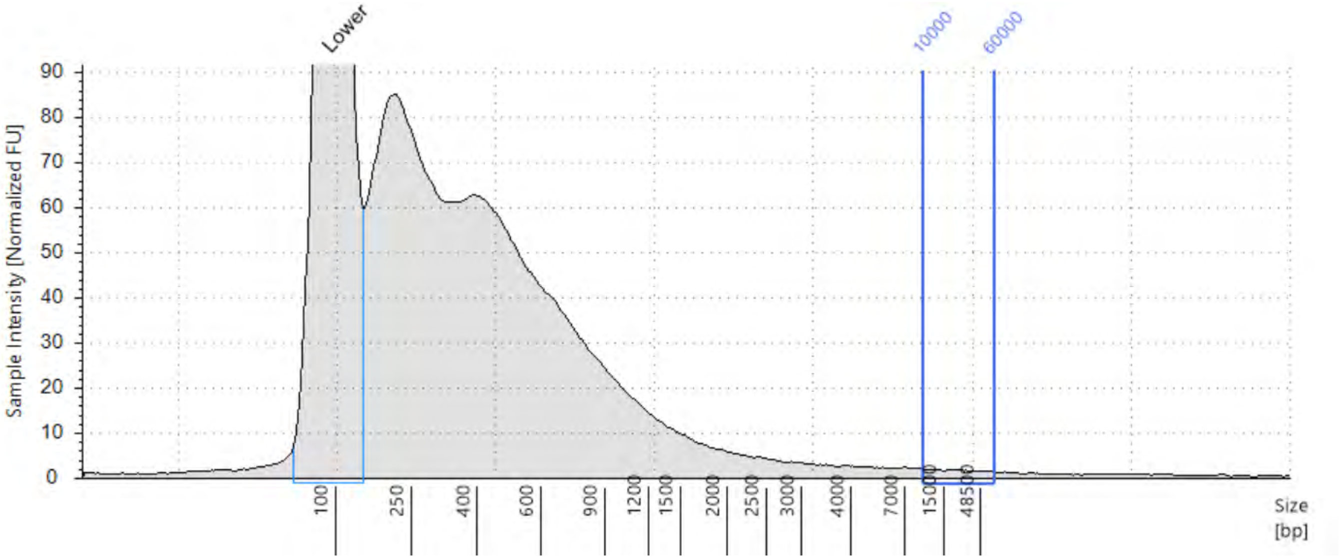

Region Table

From [bp]	To [bp]	Average Size [bp]	Conc. [ng/µl]	Region Molarity [nmol/l]	% of Total	Region Comment	Color
10000	60000	25711	0.0422	0.00568	0.45		■

 © Agilent Technologies, Inc. 2017

D2: W-T4-DE-1045

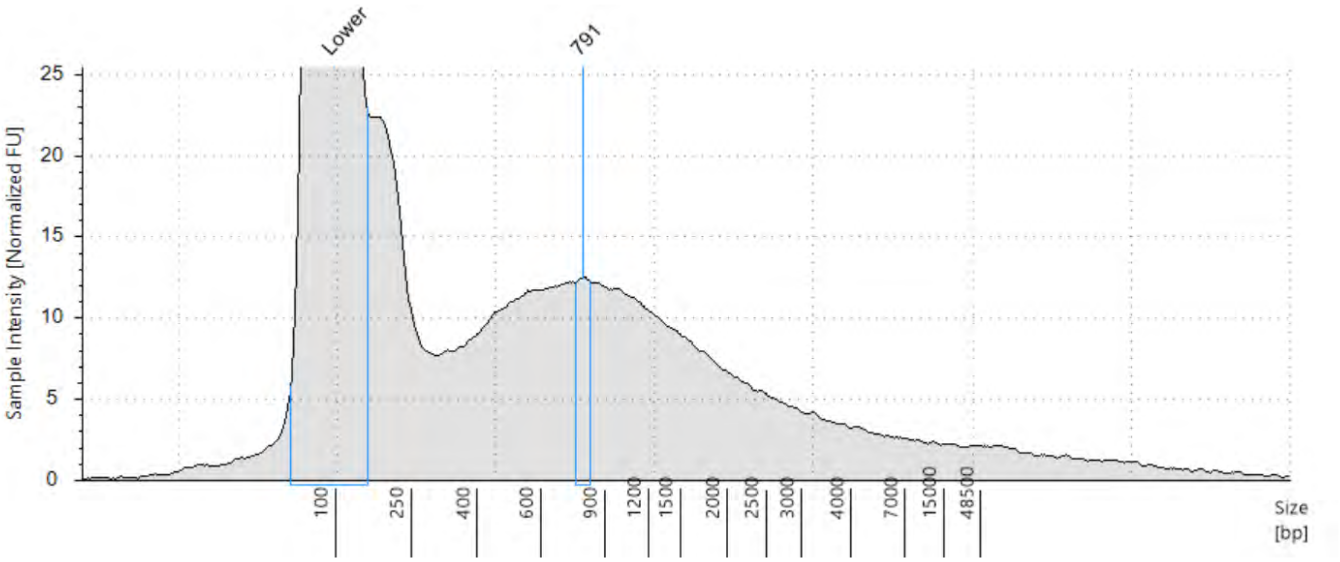

Sample Table

Well	DIN	Conc. [ng/µl]	Sample Description	Alert	Observations
D2	-	2.83	W-T4-DE-1045	▲	Sample concentration outside functional range for DIN

Peak Table

Size [bp]	Calibrated Conc. [ng/µl]	Assigned Conc. [ng/µl]	% Integrated Area	From [bp]	To [bp]	Peak Comment	Observations
100	8.50	8.50	-	59	149		Lower Marker
791	0.111	-	100.00	755	828		

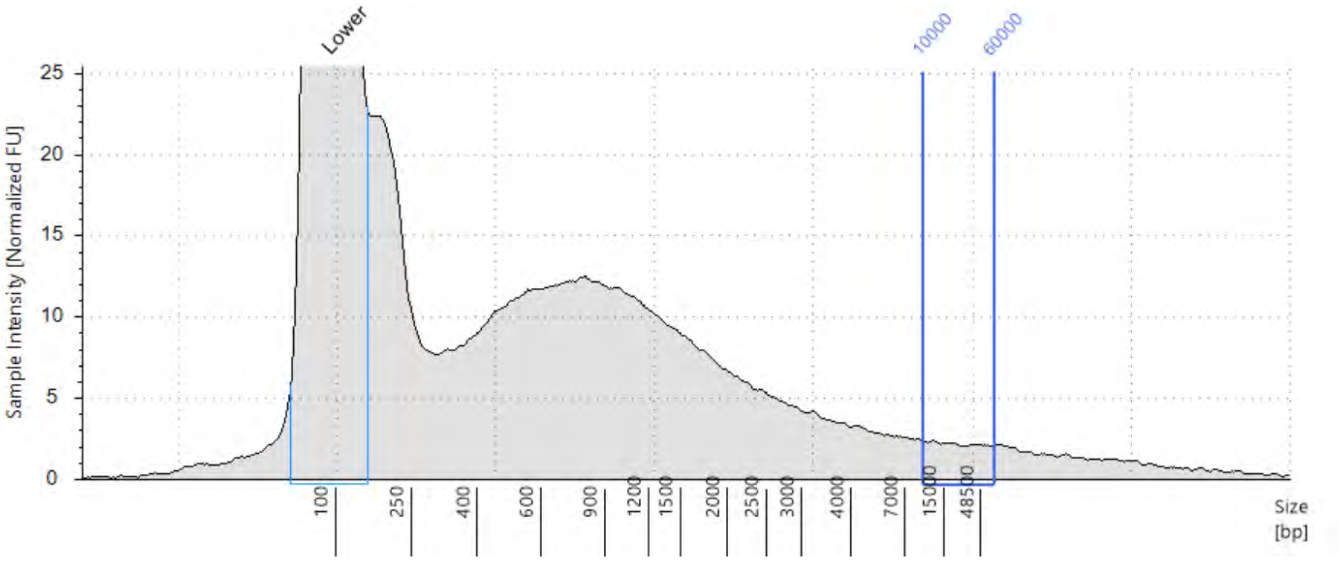

Region Table

From [bp]	To [bp]	Average Size [bp]	Conc. [ng/µl]	Region Molarity [nmol/l]	% of Total	Region Comment	Color
10000	60000	25979	0.0647	0.00747	2.28		■

E2: W-T4-ES-826

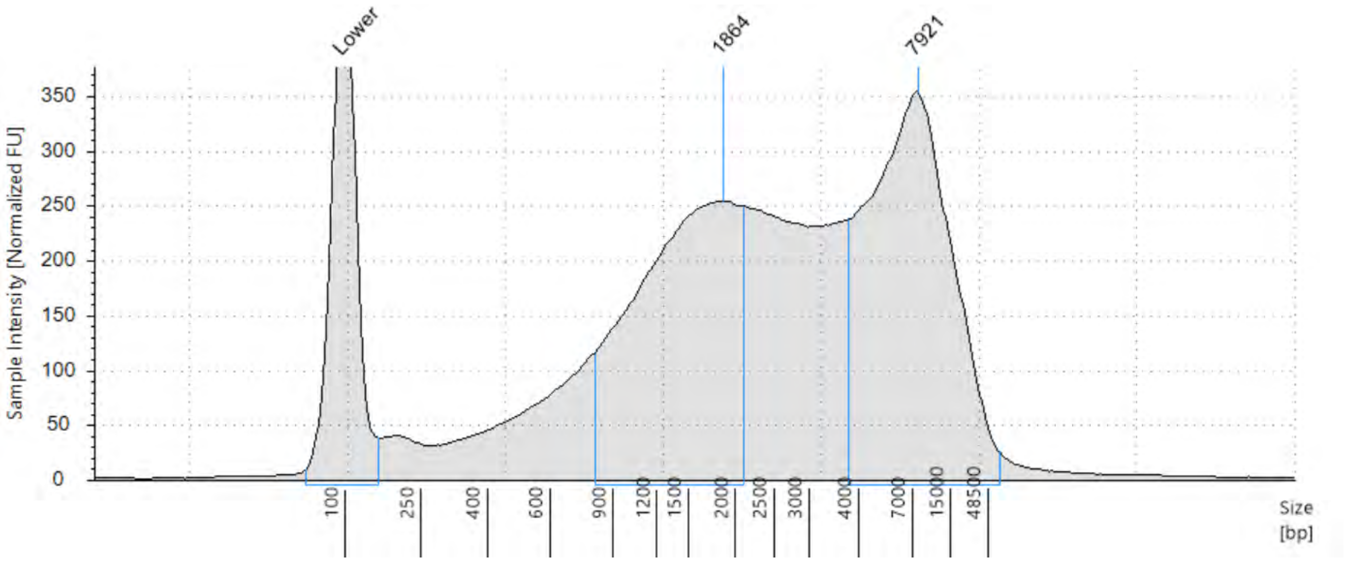

Sample Table

Well	DIN	Conc. [ng/µl]	Sample Description	Alert	Observations
E2	4.8	62.5	W-T4-ES-826		

Peak Table

Size [bp]	Calibrated Conc. [ng/µl]	Assigned Conc. [ng/µl]	% Integrated Area	From [bp]	To [bp]	Peak Comment	Observations
100	8.50	8.50	-	62	149		Lower Marker
1864	18.7	-	46.78	801	2109		
7921	21.3	-	53.22	3770	59017		

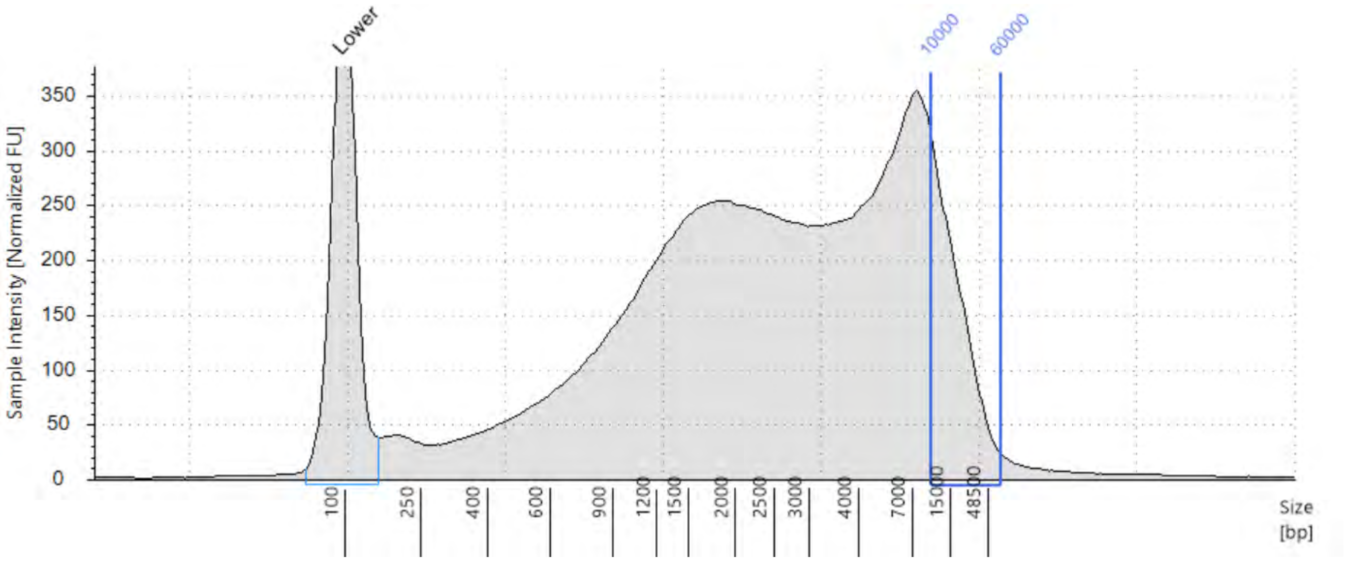

Region Table

From [bp]	To [bp]	Average Size [bp]	Conc. [ng/µl]	Region Molarity [nmol/l]	% of Total	Region Comment	Color
10000	60000	18319	6.62	0.680	10.61		

 © Agilent Technologies, Inc. 2017

F2: W-T4-ES-827

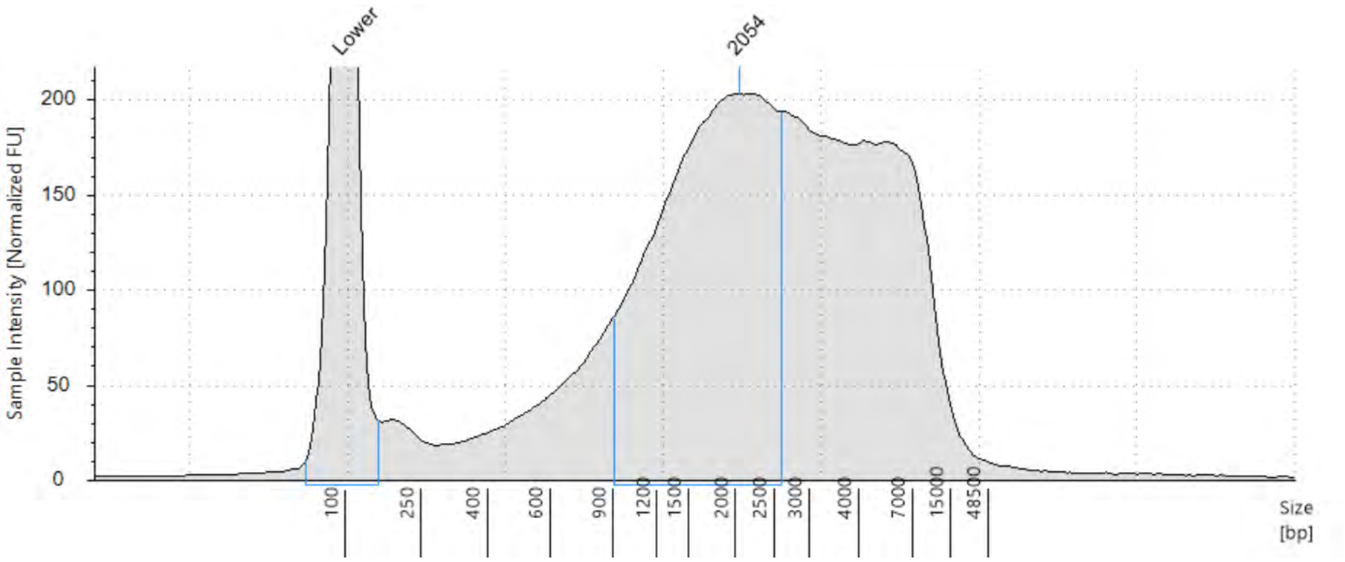

Sample Table

Well	DIN	Conc. [ng/µl]	Sample Description	Alert	Observations
F2	4.5	39.4	W-T4-ES-827		

Peak Table

Size [bp]	Calibrated Conc. [ng/µl]	Assigned Conc. [ng/µl]	% Integrated Area	From [bp]	To [bp]	Peak Comment	Observations
100	8.50	8.50	-	62	150		Lower Marker
2054	17.0	-	100.00	905	2606		

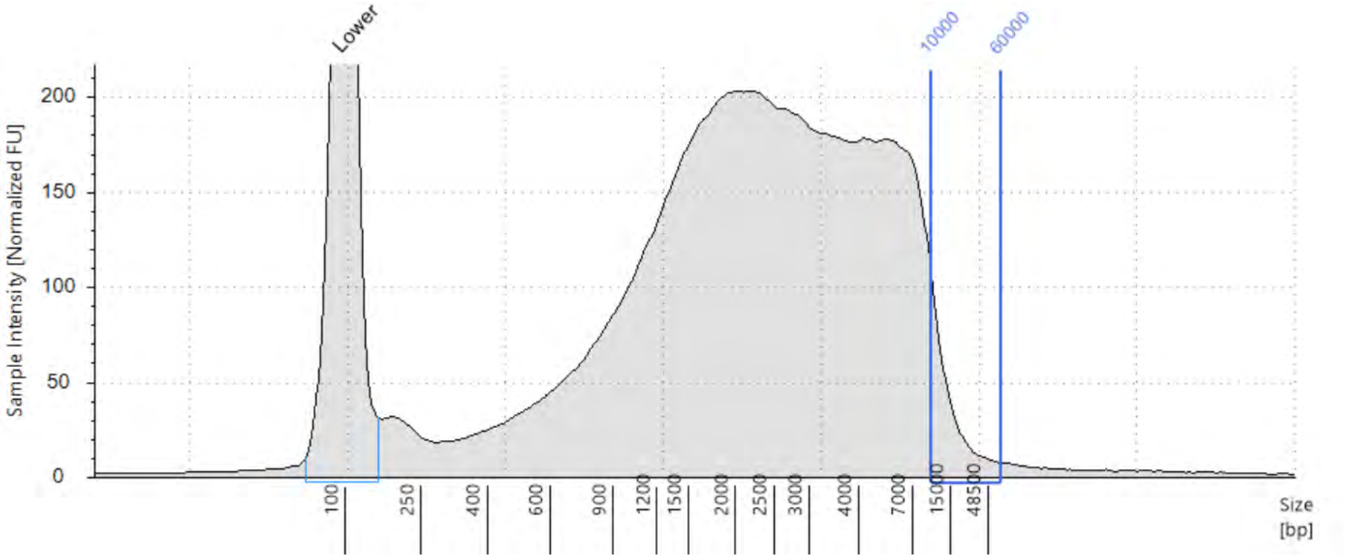

Region Table

From [bp]	To [bp]	Average Size [bp]	Conc. [ng/µl]	Region Molarity [nmol/l]	% of Total	Region Comment	Color
10000	60000	17509	1.30	0.150	3.30		

G2: W-T4-ES-828

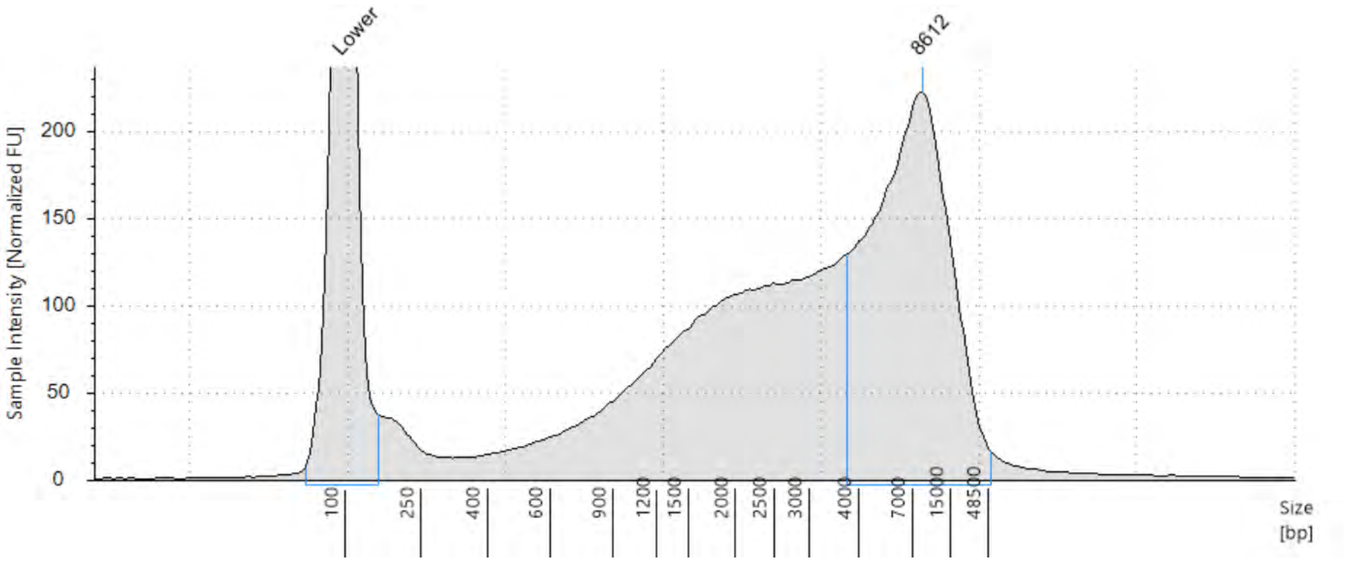

Sample Table

Well	DIN	Conc. [ng/µl]	Sample Description	Alert	Observations
G2	5.6	29.5	W-T4-ES-828		

Peak Table

Size [bp]	Calibrated Conc. [ng/µl]	Assigned Conc. [ng/µl]	% Integrated Area	From [bp]	To [bp]	Peak Comment	Observations
100	8.50	8.50	-	62	149		Lower Marker
8612	12.5	-	100.00	3746	51539		

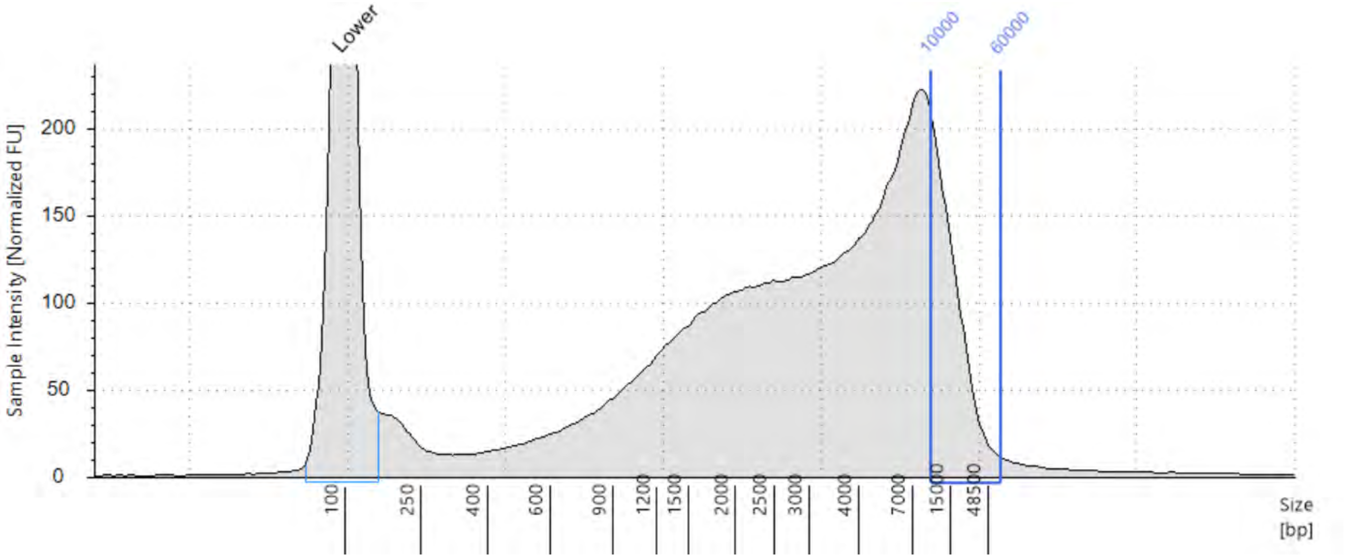

Region Table

From [bp]	To [bp]	Average Size [bp]	Conc. [ng/µl]	Region Molarity [nmol/l]	% of Total	Region Comment	Color
10000	60000	17018	3.81	0.409	12.94		

 © Agilent Technologies, Inc. 2017

H2: W-T4-ES-829

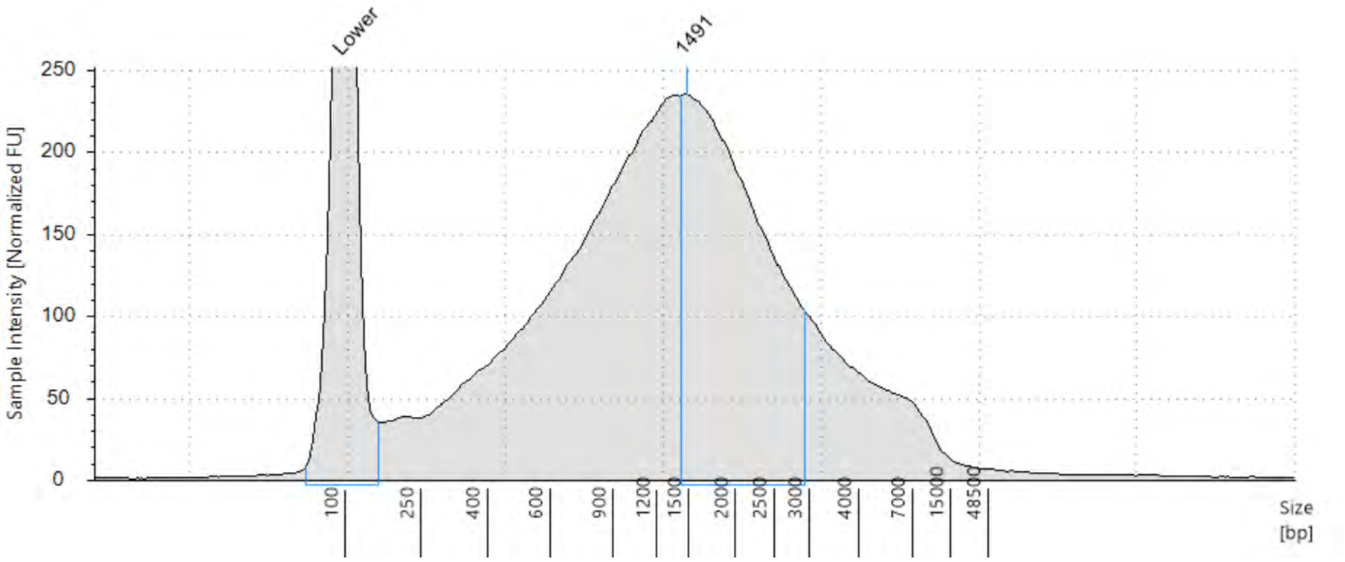

Sample Table

Well	DIN	Conc. [ng/µl]	Sample Description	Alert	Observations
H2	3.0	40.5	W-T4-ES-829		

Peak Table

Size [bp]	Calibrated Conc. [ng/µl]	Assigned Conc. [ng/µl]	%Integrated Area	From [bp]	To [bp]	Peak Comment	Observations
100	8.50	8.50	-	62	149		Lower Marker
1491	13.9	-	100.00	1421	2933		

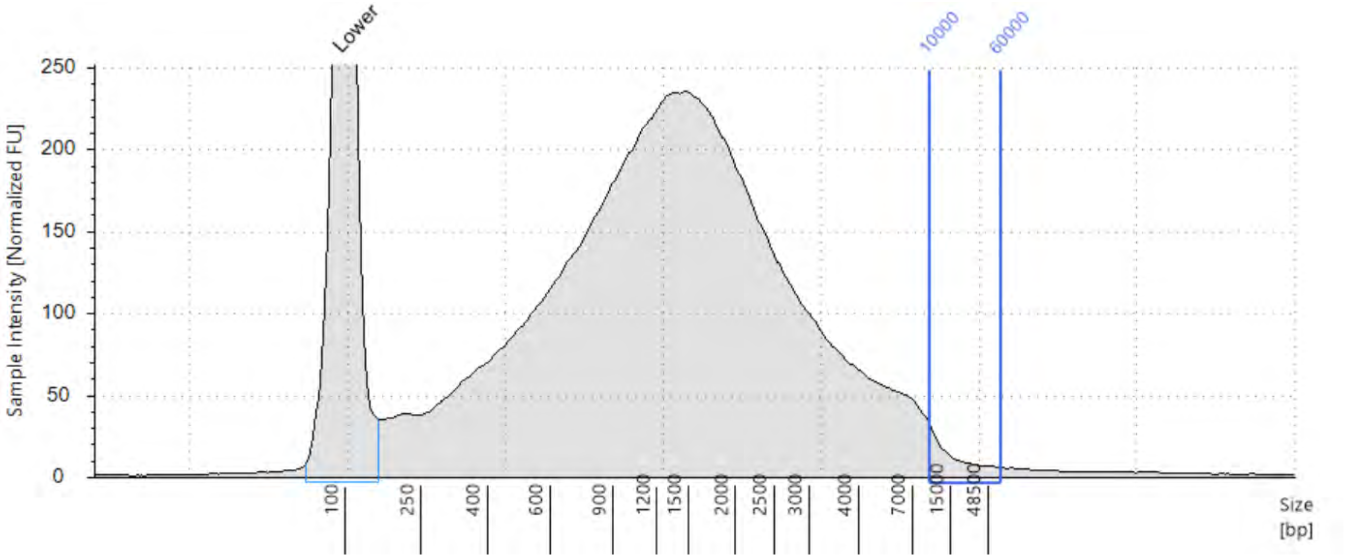

Region Table

From [bp]	To [bp]	Average Size [bp]	Conc. [ng/µl]	Region Molarity [nmol/l]	% of Total	Region Comment	Color
10000	60000	20691	0.440	0.0545	1.09		

Filename: 2019-07-29-05.gDNA

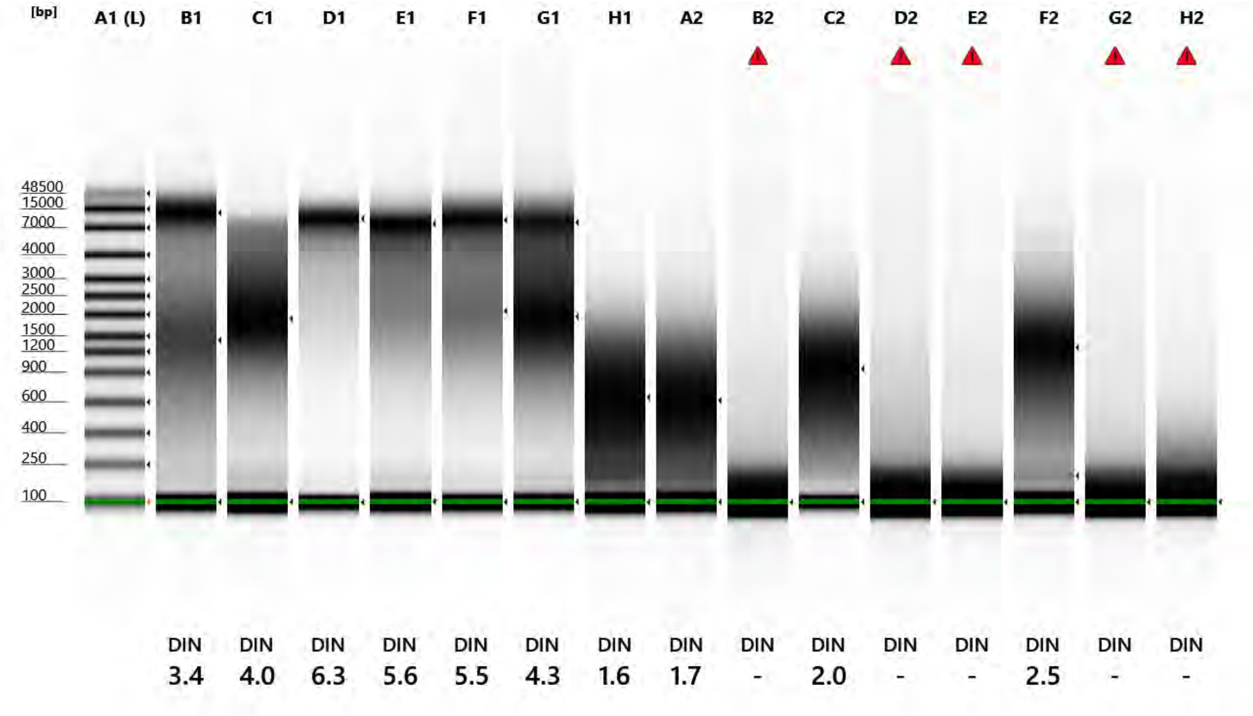

Default image (Contrast 50%), Image is Scaled to Sample

Sample Info

Well	DIN	Conc. [ng/µl]	Sample Description	Alert	Observations
A1	-	91.5	Ladder		Ladder; Sizing Changed
B1	3.4	37.0	W-T4-ES-830		DIN Edited (Ladder sizing changed)
C1	4.0	18.8	W-T4-ES-1051		DIN Edited (Ladder sizing changed)
D1	6.3	19.0	W-T4-ES-1052		DIN Edited (Ladder sizing changed)
E1	5.6	18.4	W-T4-ES-1053		DIN Edited (Ladder sizing changed)
F1	5.5	30.3	W-T4-ES-1054		DIN Edited (Ladder sizing changed)
G1	4.3	45.4	W-T4-ES-1055		DIN Edited (Ladder sizing changed)
H1	1.6	20.4	W-T4-E-836		DIN Edited (Ladder sizing changed)
A2	1.7	24.9	W-T4-E-837		DIN Edited (Ladder sizing changed)
B2	-	1.86	W-T4-E-838	▲	Sample concentration outside functional range for DIN; DIN Edited (Ladder sizing changed)
C2	2.0	48.1	W-T4-E-839		DIN Edited (Ladder sizing changed)
D2	-	2.03	W-T4-E-840	▲	Sample concentration outside functional range for DIN; DIN Edited (Ladder sizing changed)
E2	-	1.87	W-T4-E-1061	▲	Sample concentration outside functional range for DIN; DIN Edited (Ladder sizing changed)
F2	2.5	16.2	W-T4-E-1062		DIN Edited (Ladder sizing changed)
G2	-	1.86	W-T4-E-1063	▲	Sample concentration outside functional range for DIN; DIN Edited (Ladder sizing changed)
H2	-	2.62	W-T4-E-1064	▲	Sample concentration outside functional range for DIN; DIN Edited (Ladder sizing changed)

 © Agilent Technologies, Inc. 2017

A1: Ladder

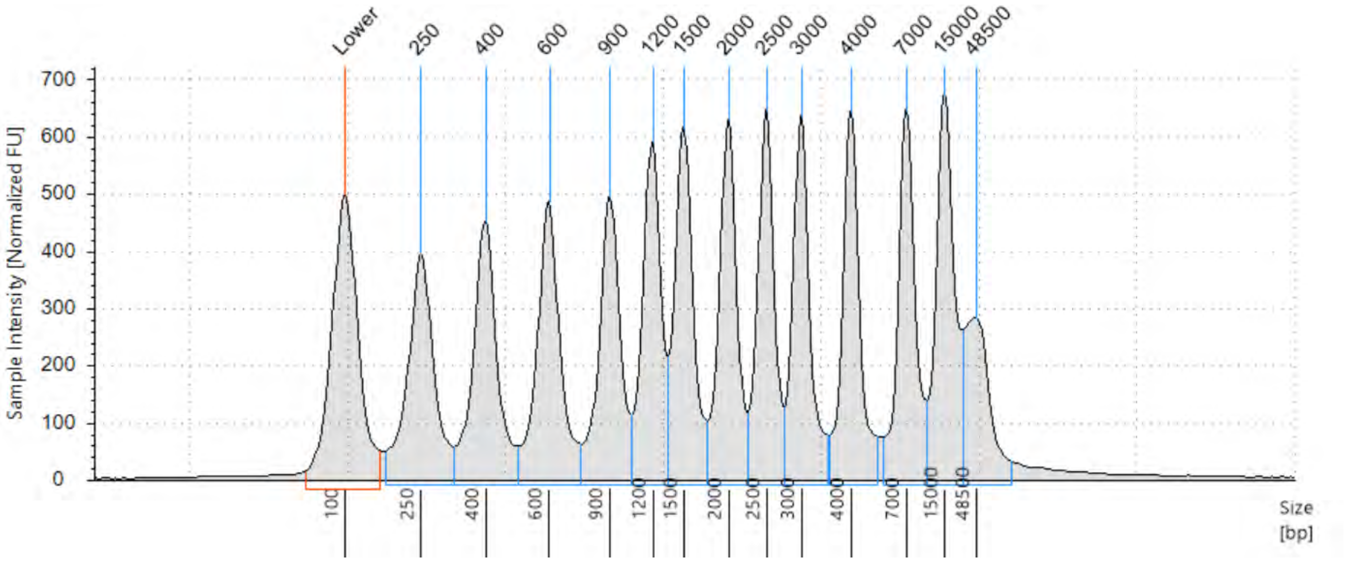

Sample Table

Well	DIN	Conc. [ng/µl]	Sample Description	Alert	Observations
A1	-	91.5	Ladder		Ladder; Sizing Changed

Peak Table

Size [bp]	Calibrated Conc. [ng/µl]	Assigned Conc. [ng/µl]	% Integrated Area	From [bp]	To [bp]	Peak Comment	Observations
100	8.50	8.50	-	62	152		Lower Marker
250	6.69	-	7.35	162	318		
400	6.95	-	7.63	318	490		
600	7.26	-	7.97	490	742		
900	6.83	-	7.50	742	1044		
1200	7.18	-	7.88	1044	1335		
1500	7.64	-	8.38	1335	1748		
2000	7.29	-	8.00	1748	2236		
2500	7.07	-	7.76	2236	2729		
3000	7.21	-	7.92	2729	3492		
4000	7.12	-	7.82	3520	5218		
7000	7.21	-	7.91	5518	10530		
15000	7.97	-	8.75	10530	21381		
48500	4.67	-	5.13	21381	>60000		

 © Agilent Technologies, Inc. 2017

B1: W-T4-ES-830

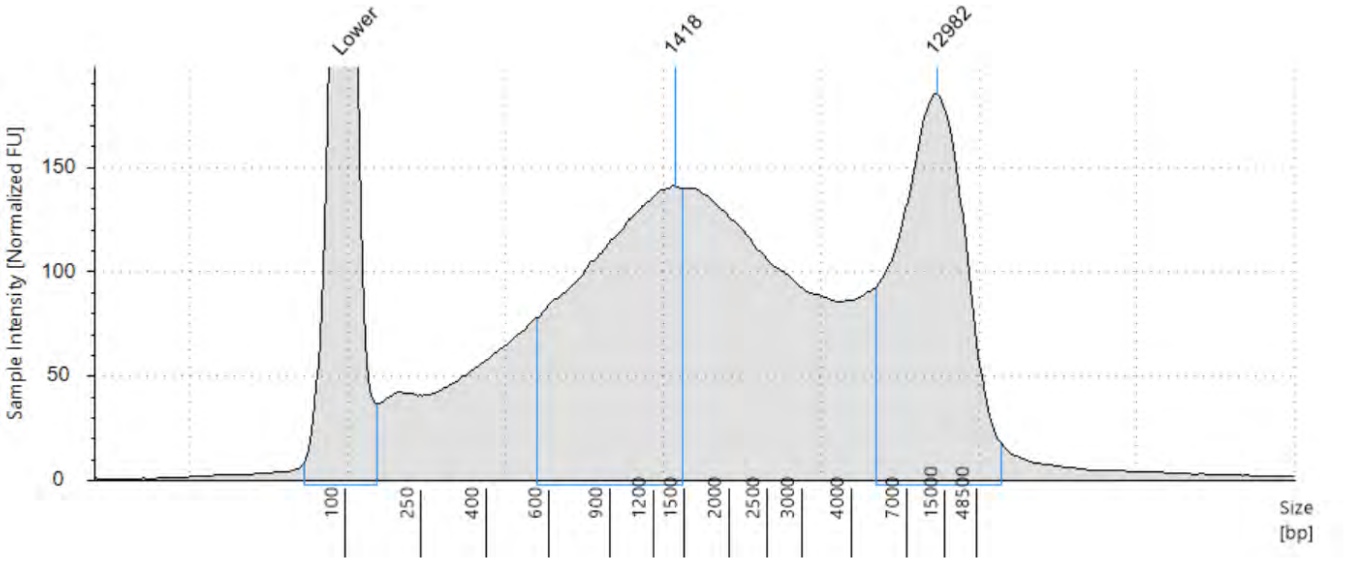

Sample Table

Well	DIN	Conc. [ng/µl]	Sample Description	Alert	Observations
B1	3.4	37.0	W-T4-ES-830		DIN Edited (Ladder sizing changed)

Peak Table

Size [bp]	Calibrated Conc. [ng/µl]	Assigned Conc. [ng/µl]	% Integrated Area	From [bp]	To [bp]	Peak Comment	Observations
100	8.50	8.50	-	61	147		Lower Marker
1418	10.1	-	52.65	558	1489		
12982	9.05	-	47.35	5153	>60000		

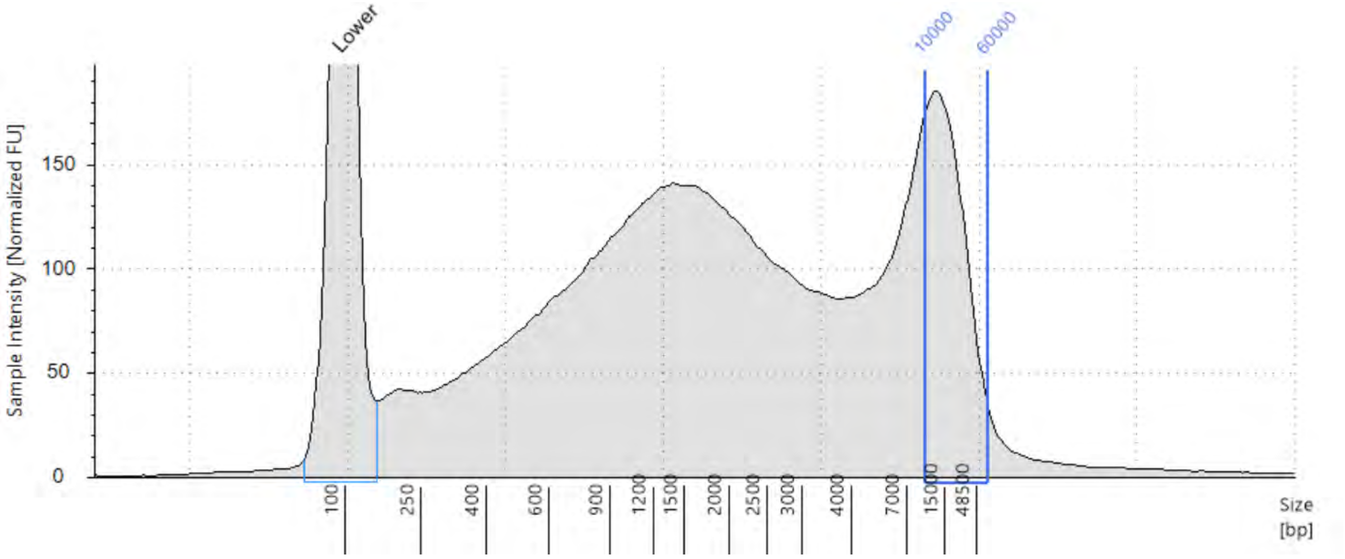

Region Table

From [bp]	To [bp]	Average Size [bp]	Conc. [ng/µl]	Region Molarity [nmol/l]	% of Total	Region Comment	Color
10000	60000	19753	5.23	0.507	14.13		

C1: W-T4-ES-1051

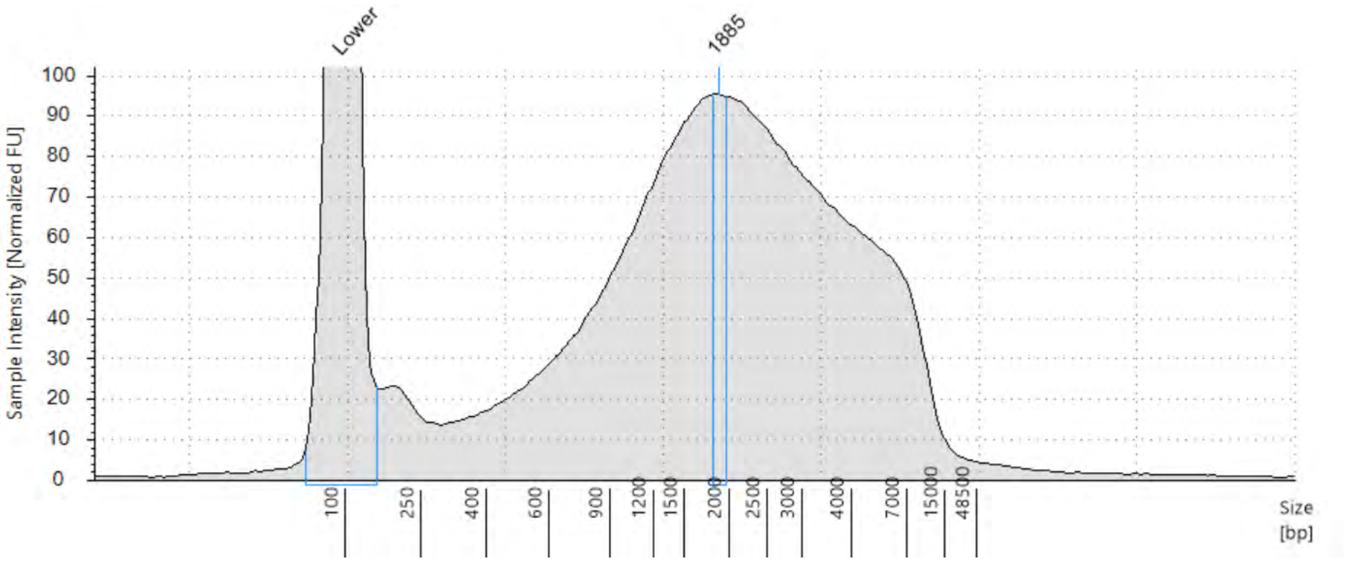

Sample Table

Well	DIN	Conc. [ng/µl]	Sample Description	Alert	Observations
C1	4.0	18.8	W-T4-ES-1051		DIN Edited (Ladder sizing changed)

Peak Table

Size [bp]	Calibrated Conc. [ng/µl]	Assigned Conc. [ng/µl]	% Integrated Area	From [bp]	To [bp]	Peak Comment	Observations
100	8.50	8.50	-	62	146		Lower Marker
1885	0.914	-	100.00	1803	1970		

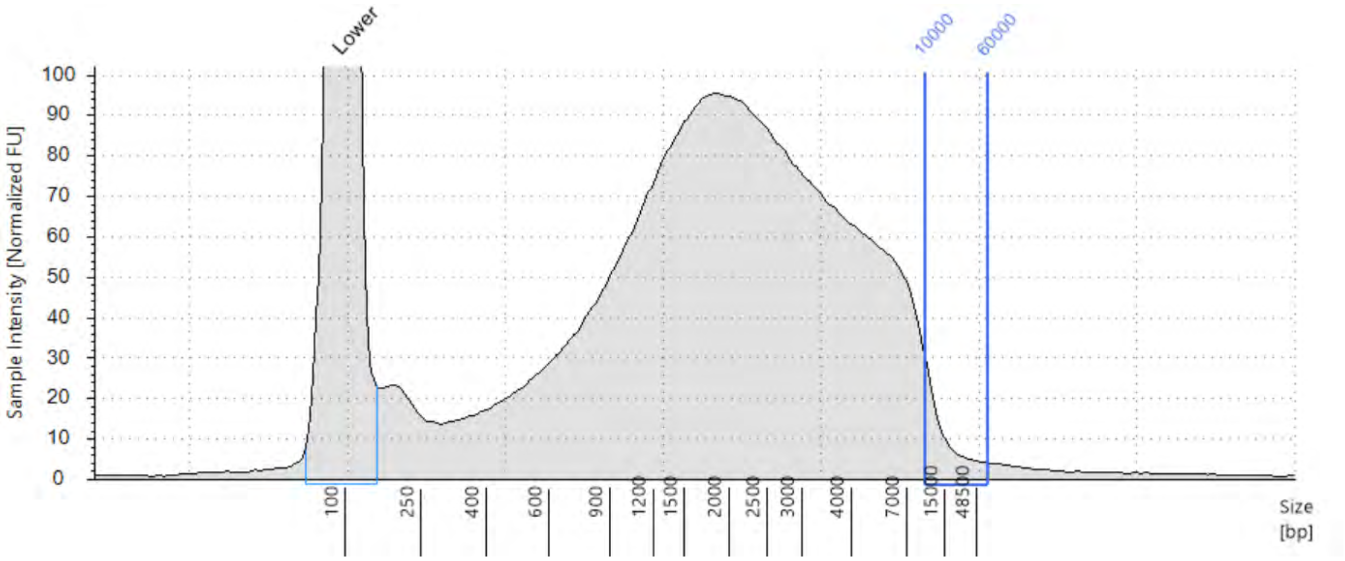

Region Table

From [bp]	To [bp]	Average Size [bp]	Conc. [ng/µl]	Region Molarity [nmol/l]	% of Total	Region Comment	Color
10000	60000	18702	0.392	0.0459	2.08		

© Agilent Technologies, Inc. 2017

D1: W-T4-ES-1052

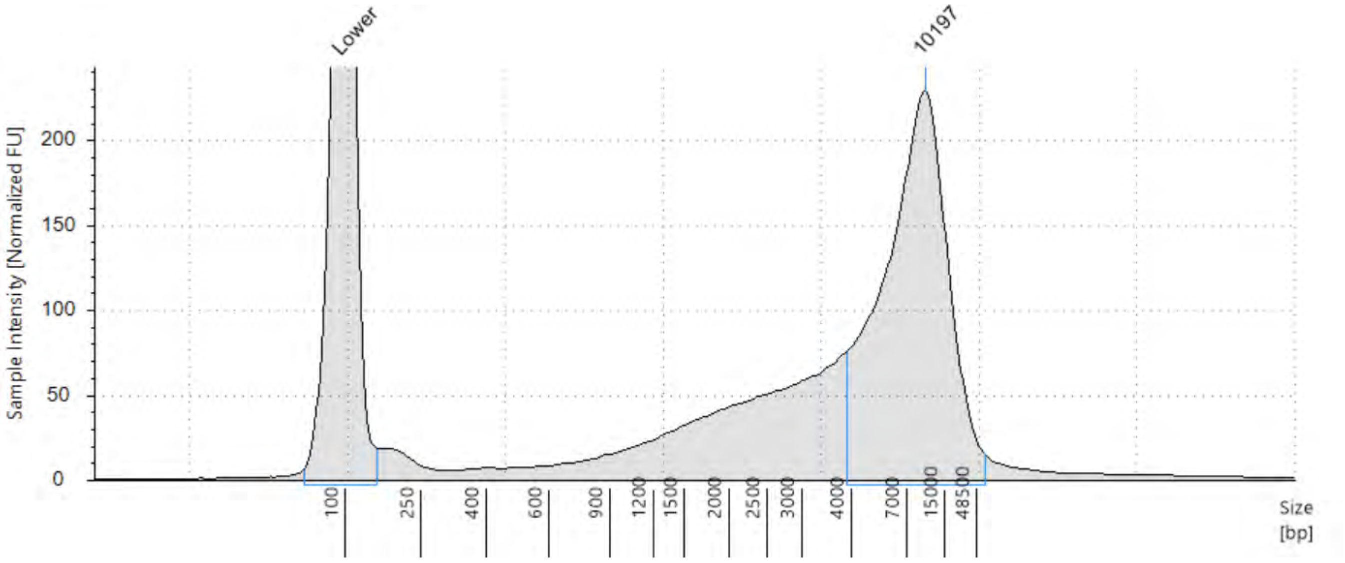

Sample Table

Well	DIN	Conc. [ng/µl]	Sample Description	Alert	Observations
D1	6.3	19.0	W-T4-ES-1052		DIN Edited (Ladder sizing changed)

Peak Table

Size [bp]	Calibrated Conc. [ng/µl]	Assigned Conc. [ng/µl]	% Integrated Area	From [bp]	To [bp]	Peak Comment	Observations
100	8.50	8.50	-	62	149		Lower Marker
10197	11.0	-	100.00	3917	57803		

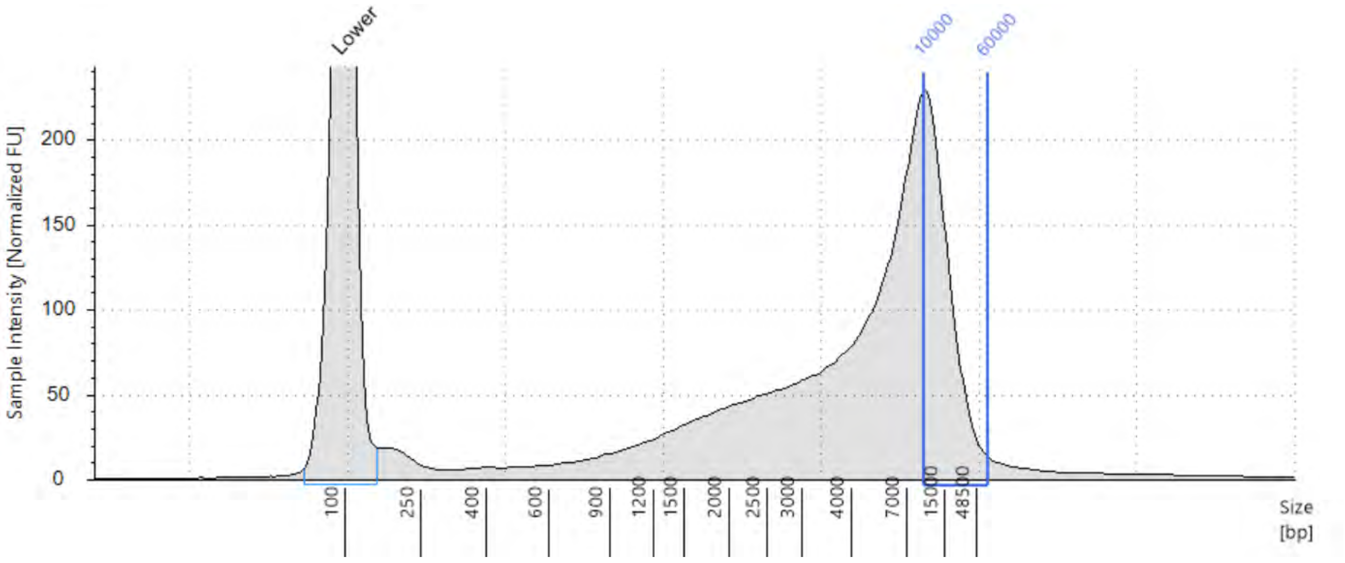

Region Table

From [bp]	To [bp]	Average Size [bp]	Conc. [ng/µl]	Region Molarity [nmol/l]	% of Total	Region Comment	Color
10000	60000	16544	4.34	0.474	22.80		■

 © Agilent Technologies, Inc. 2017

E1: W-T4-ES-1053

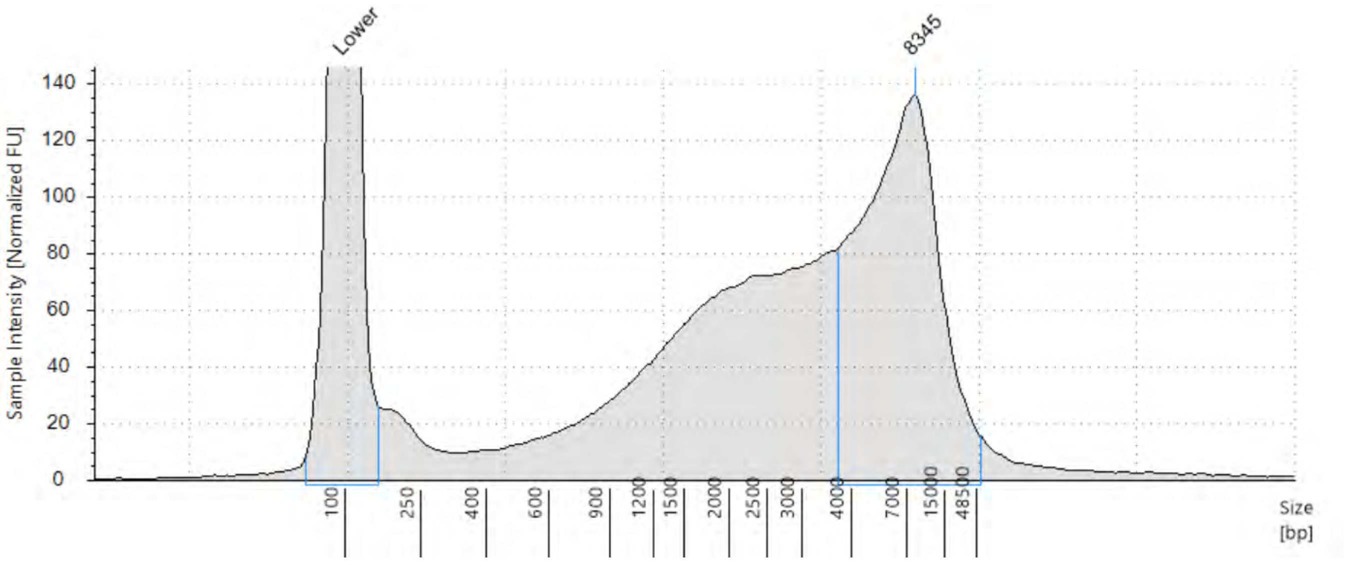

Sample Table

Well	DIN	Conc. [ng/µl]	Sample Description	Alert	Observations
E1	5.6	18.4	W-T4-ES-1053		DIN Edited (Ladder sizing changed)

Peak Table

Size [bp]	Calibrated Conc. [ng/µl]	Assigned Conc. [ng/µl]	% Integrated Area	From [bp]	To [bp]	Peak Comment	Observations
100	8.50	8.50	-	62	149		Lower Marker
8345	7.55	-	100.00	3704	52153		

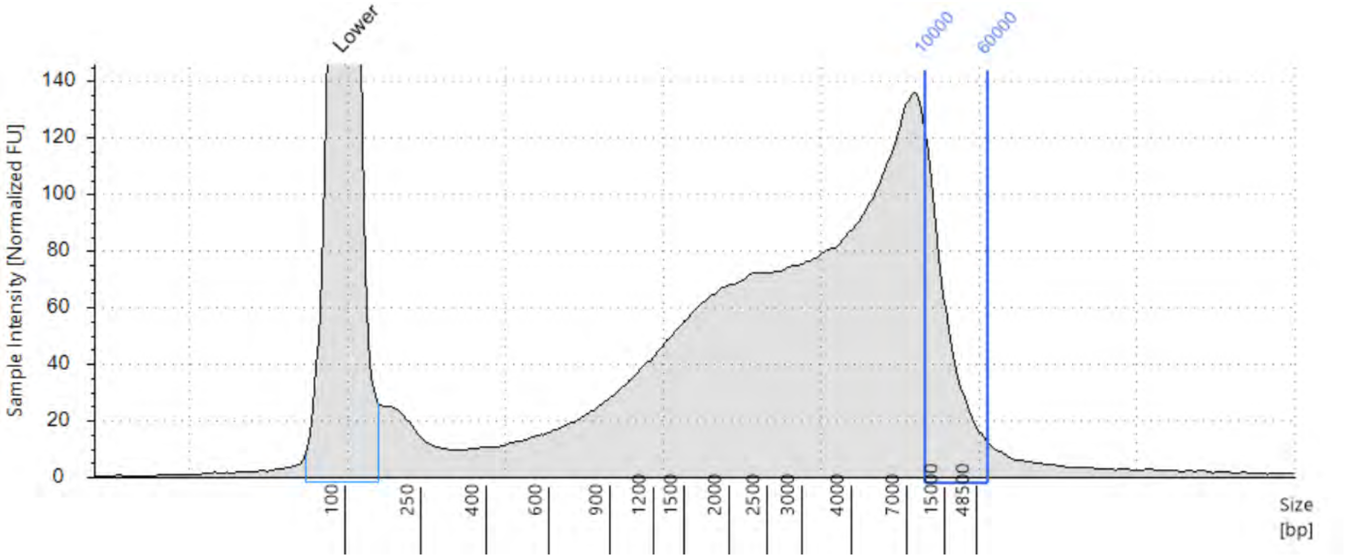

Region Table

From [bp]	To [bp]	Average Size [bp]	Conc. [ng/µl]	Region Molarity [nmol/l]	% of Total	Region Comment	Color
10000	60000	17677	1.94	0.212	10.50		■

 © Agilent Technologies, Inc. 2017

F1: W-T4-ES-1054

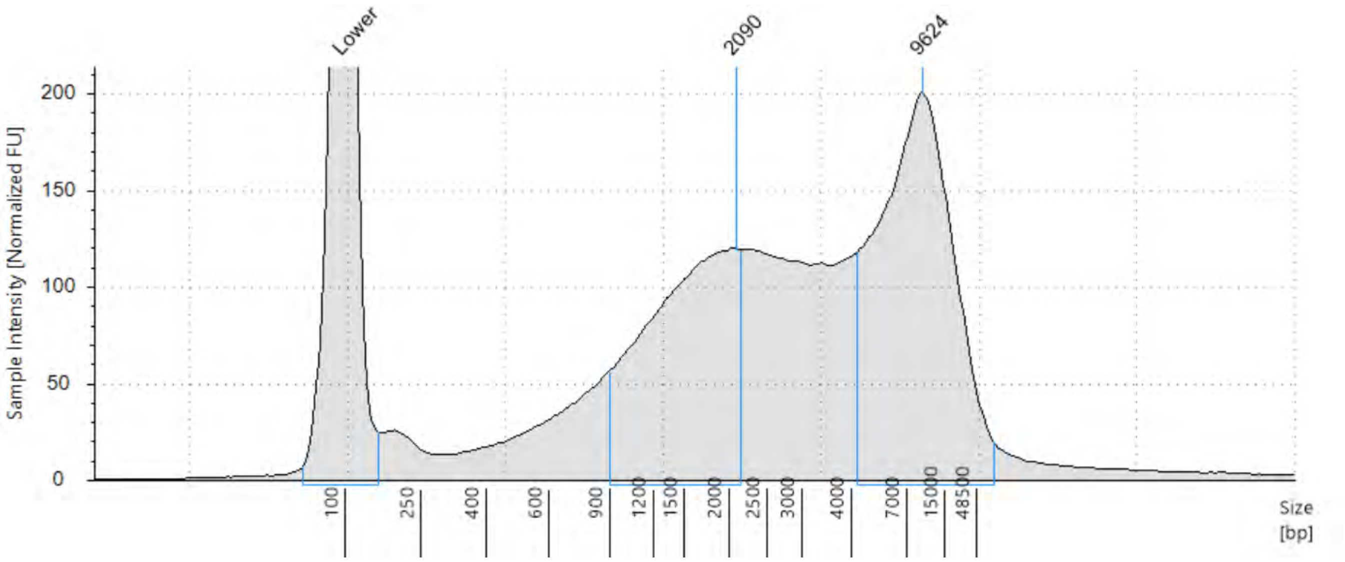

Sample Table

Well	DIN	Conc. [ng/µl]	Sample Description	Alert	Observations
F1	5.5	30.3	W-T4-ES-1054		DIN Edited (Ladder sizing changed)

Peak Table

Size [bp]	Calibrated Conc. [ng/µl]	Assigned Conc. [ng/µl]	% Integrated Area	From [bp]	To [bp]	Peak Comment	Observations
100	8.50	8.50	-	60	150		Lower Marker
2090	7.59	-	41.50	897	2158		
9624	10.7	-	58.50	4247	>60000		

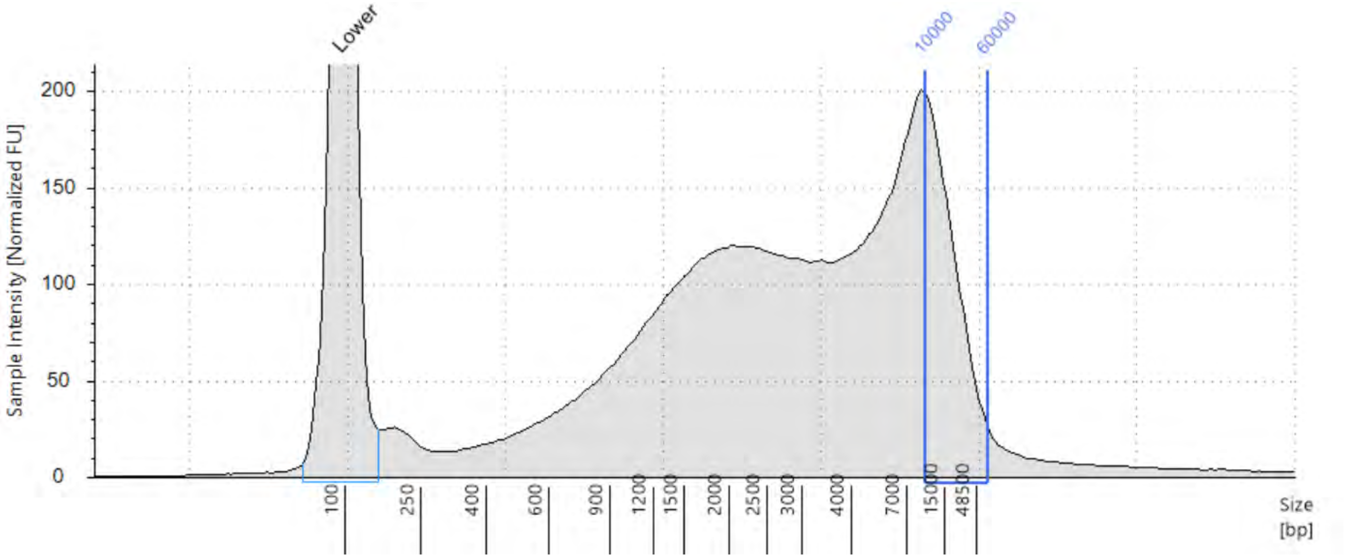

Region Table

From [bp]	To [bp]	Average Size [bp]	Conc. [ng/µl]	Region Molarity [nmol/l]	% of Total	Region Comment	Color
10000	60000	18593	4.33	0.443	14.31		

 © Agilent Technologies, Inc. 2017

G1: W-T4-ES-1055

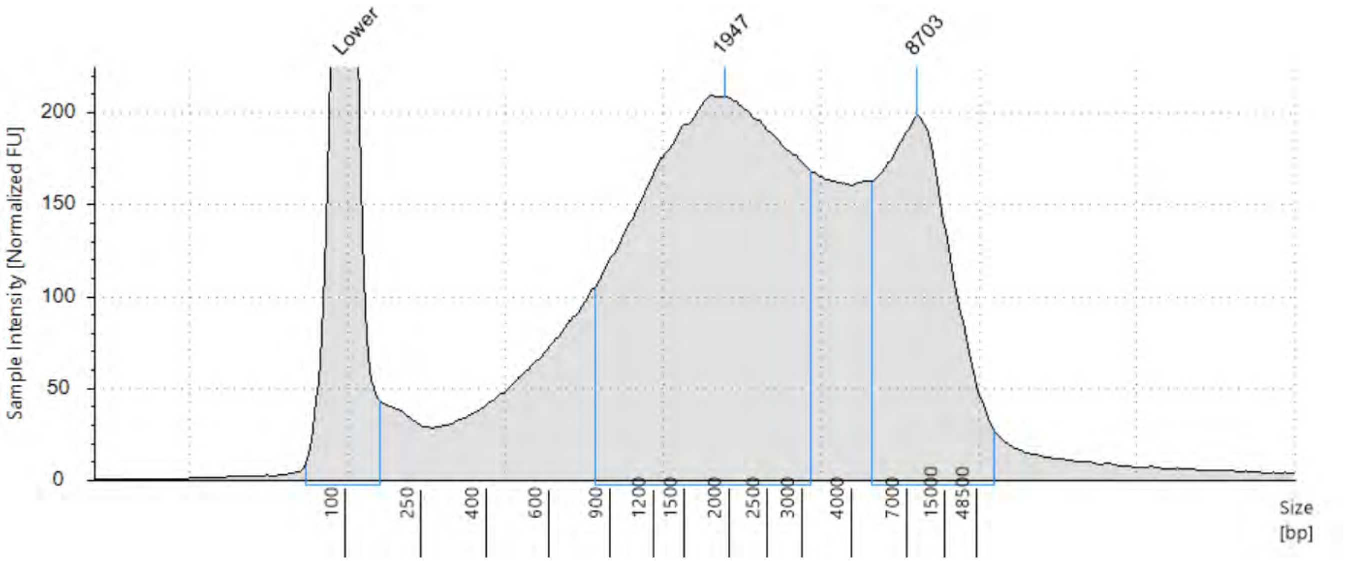

Sample Table

Well	DIN	Conc. [ng/µl]	Sample Description	Alert	Observations
G1	4.3	45.4	W-T4-ES-1055		DIN Edited (Ladder sizing changed)

Peak Table

Size [bp]	Calibrated Conc. [ng/µl]	Assigned Conc. [ng/µl]	% Integrated Area	From [bp]	To [bp]	Peak Comment	Observations
100	8.50	8.50	-	62	152		Lower Marker
1947	22.4	-	69.54	813	3173		
8703	9.81	-	30.46	4934	>60000		

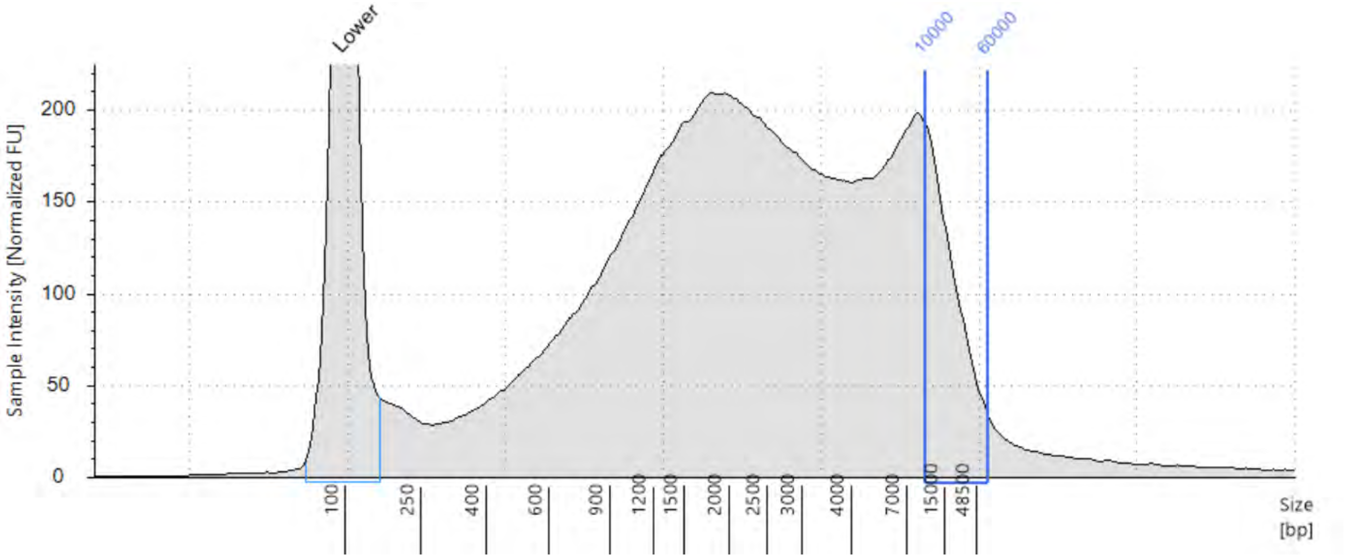

Region Table

From [bp]	To [bp]	Average Size [bp]	Conc. [ng/µl]	Region Molarity [nmol/l]	% of Total	Region Comment	Color
10000	60000	19379	4.00	0.402	8.82		

© Agilent Technologies, Inc. 2017

H1: W-T4-E-836

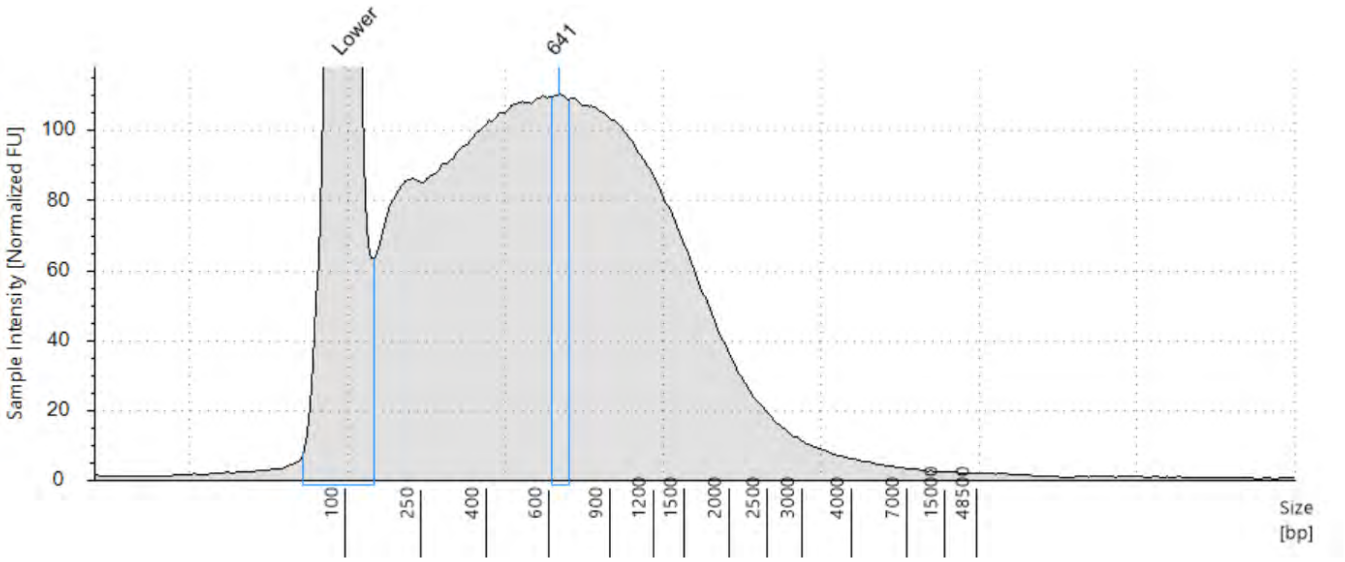

Sample Table

Well	DIN	Conc. [ng/µl]	Sample Description	Alert	Observations
H1	1.6	20.4	W-T4-E-836		DIN Edited (Ladder sizing changed)

Peak Table

Size [bp]	Calibrated Conc. [ng/µl]	Assigned Conc. [ng/µl]	% Integrated Area	From [bp]	To [bp]	Peak Comment	Observations
100	8.50	8.50	-	61	142		Lower Marker
641	1.18	-	100.00	612	684		

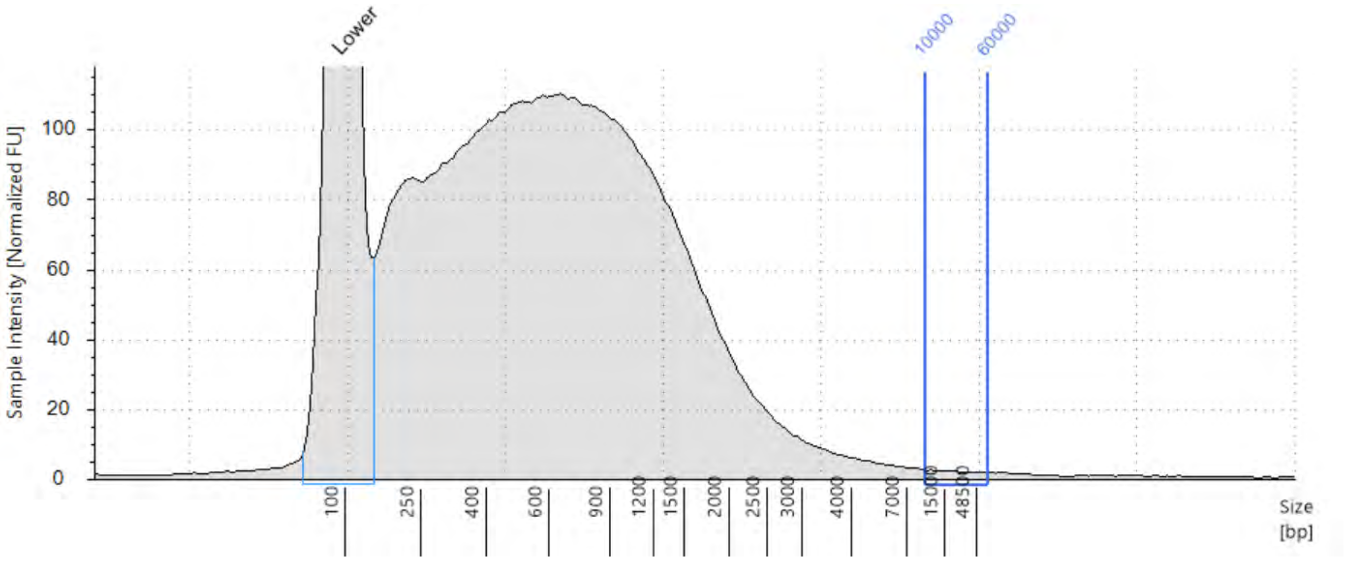

Region Table

From [bp]	To [bp]	Average Size [bp]	Conc. [ng/µl]	Region Molarity [nmol/l]	% of Total	Region Comment	Color
10000	60000	24520	0.0318	0.00730	0.16		

© Agilent Technologies, Inc. 2017

A2: W-T4-E-837

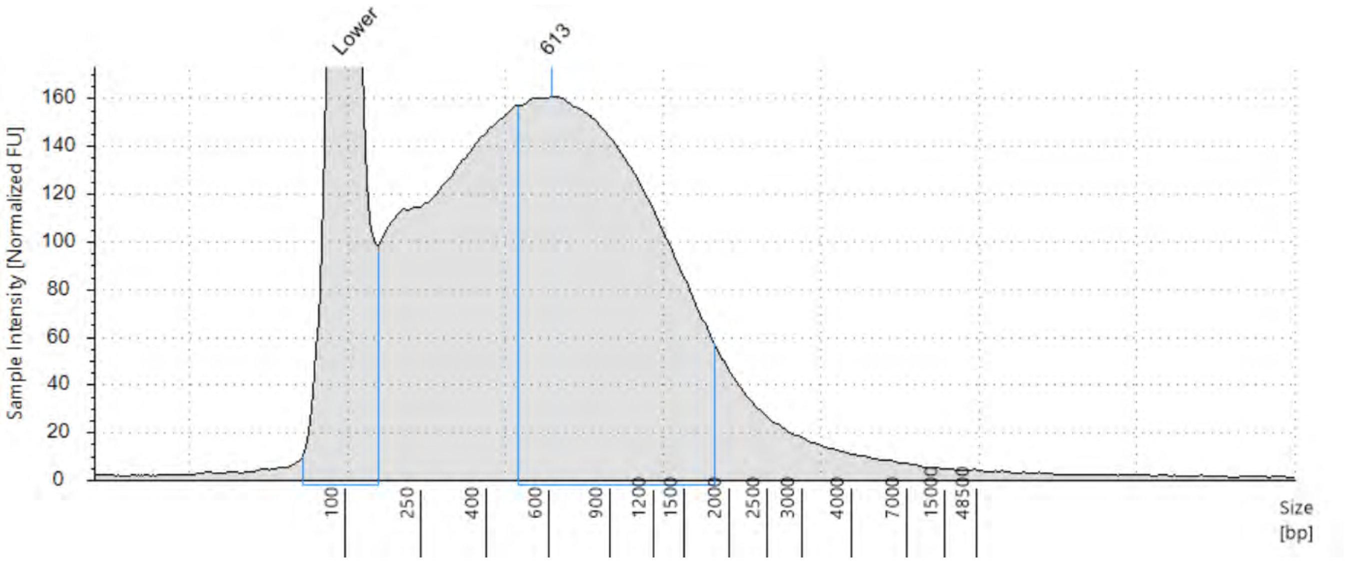

Sample Table

Well	DIN	Conc. [ng/µl]	Sample Description	Alert	Observations
A2	1.7	24.9	W-T4-E-837		DIN Edited (Ladder sizing changed)

Peak Table

Size [bp]	Calibrated Conc. [ng/µl]	Assigned Conc. [ng/µl]	% Integrated Area	From [bp]	To [bp]	Peak Comment	Observations
100	8.50	8.50	-	60	150		Lower Marker
613	13.3	-	100.00	490	1834		

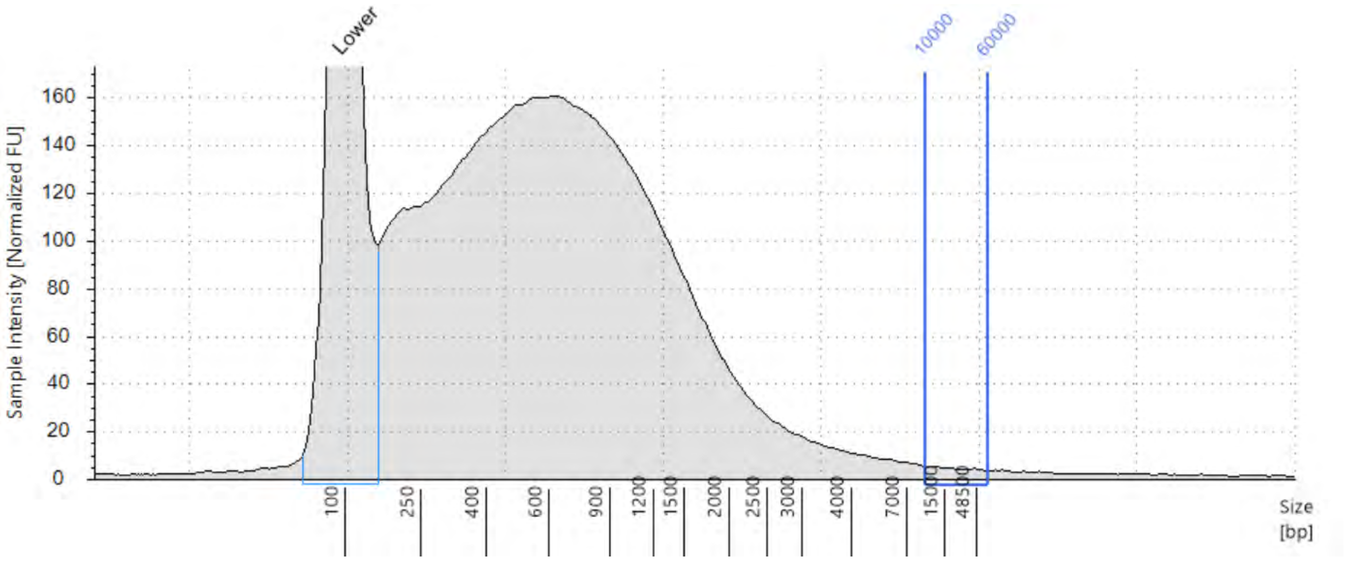

Region Table

From [bp]	To [bp]	Average Size [bp]	Conc. [ng/µl]	Region Molarity [nmol/l]	% of Total	Region Comment	Color
10000	60000	24110	0.106	0.0127	0.43		

 © Agilent Technologies, Inc. 2017

B2: W-T4-E-838

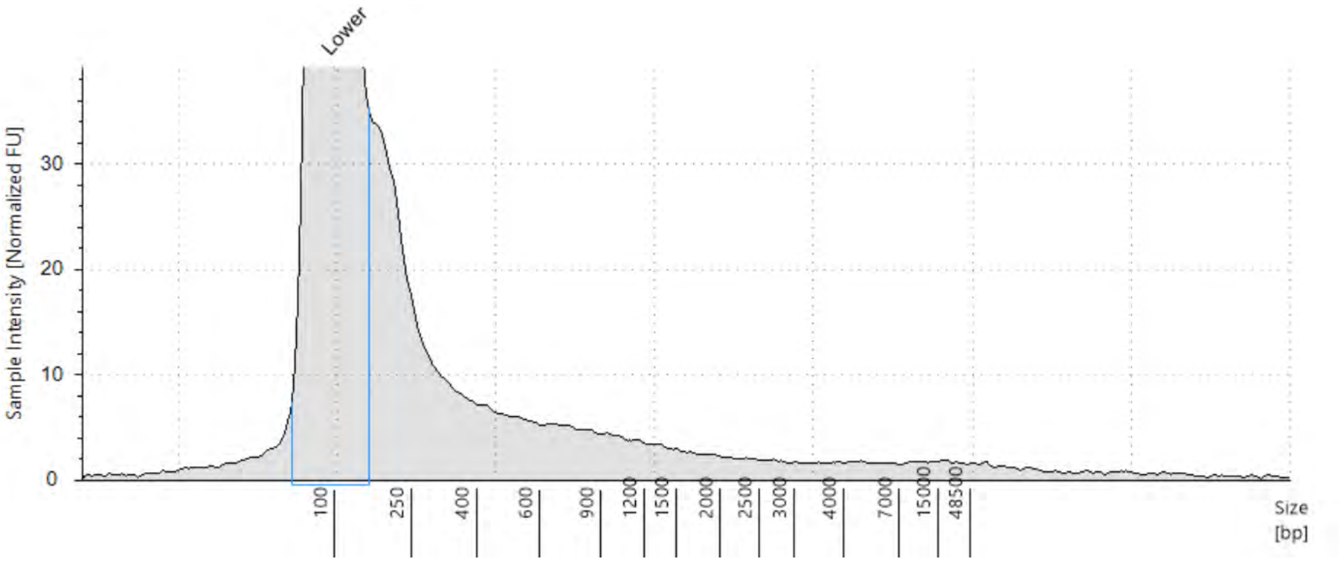

Sample Table

Well	DIN	Conc. [ng/µl]	Sample Description	Alert	Observations
B2	-	1.86	W-T4-E-838	▲	Sample concentration outside functional range for DIN; DIN Edited (Ladder sizing changed)

Peak Table

Size [bp]	Calibrated Conc. [ng/µl]	Assigned Conc. [ng/µl]	% Integrated Area	From [bp]	To [bp]	Peak Comment	Observations
100	8.50	8.50	-	60	152		Lower Marker

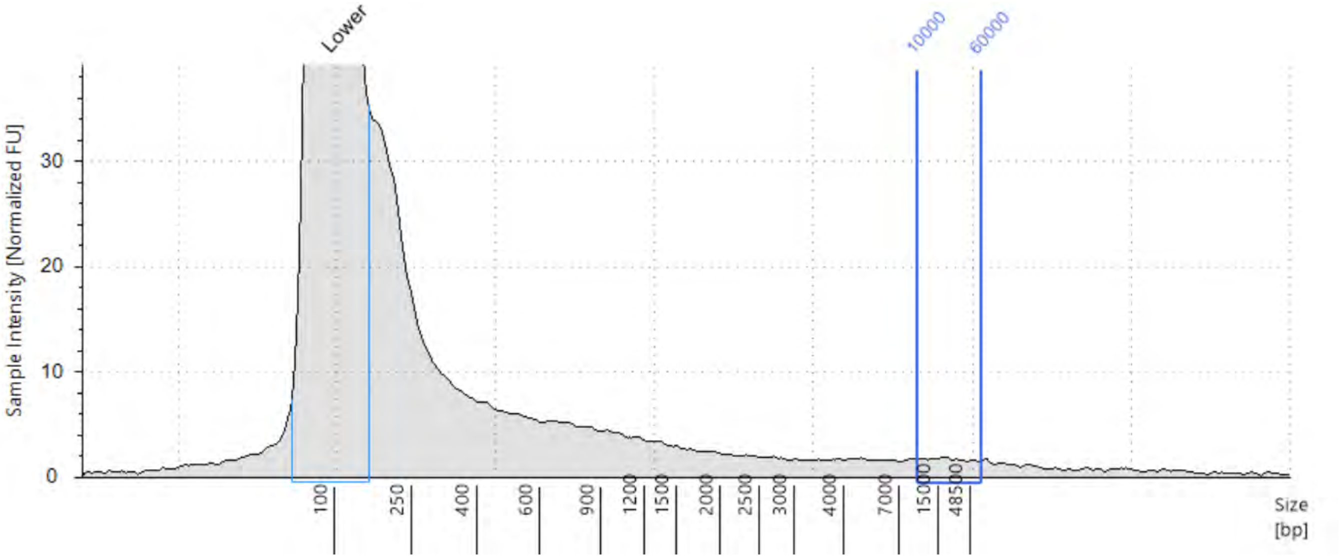

Region Table

From [bp]	To [bp]	Average Size [bp]	Conc. [ng/µl]	Region Molarity [nmol/l]	% of Total	Region Comment	Color
10000	60000	25010	0.0402	0.00529	2.16		■

 © Agilent Technologies, Inc. 2017

C2: W-T4-E-839

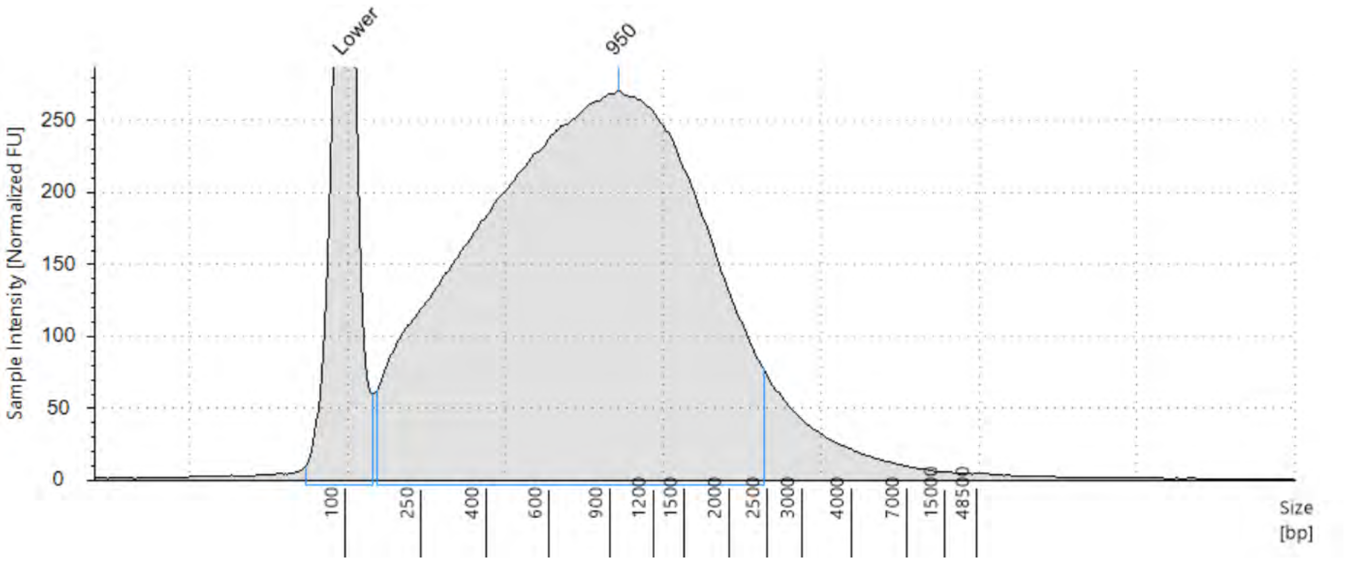

Sample Table

Well	DIN	Conc. [ng/µl]	Sample Description	Alert	Observations
C2	2.0	48.1	W-T4-E-839		DIN Edited (Ladder sizing changed)

Peak Table

Size [bp]	Calibrated Conc. [ng/µl]	Assigned Conc. [ng/µl]	% Integrated Area	From [bp]	To [bp]	Peak Comment	Observations
100	8.50	8.50	-	62	140		Lower Marker
950	45.2	-	100.00	147	2448		

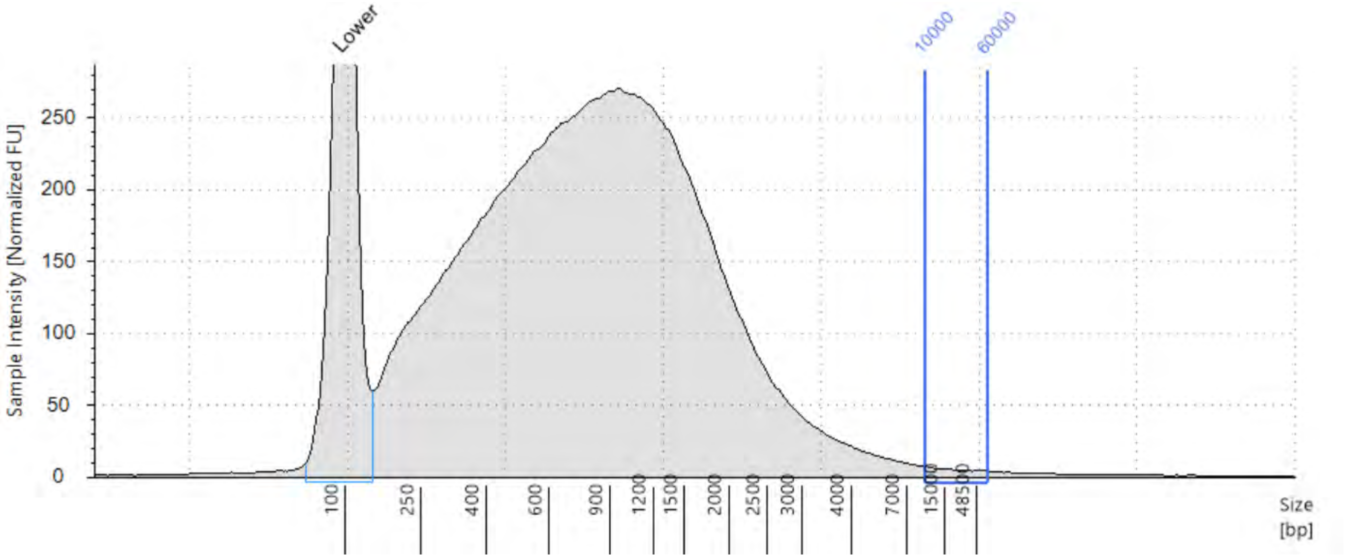

Region Table

From [bp]	To [bp]	Average Size [bp]	Conc. [ng/µl]	Region Molarity [nmol/l]	% of Total	Region Comment	Color
10000	60000	23500	0.103	0.0182	0.21		

D2: W-T4-E-840

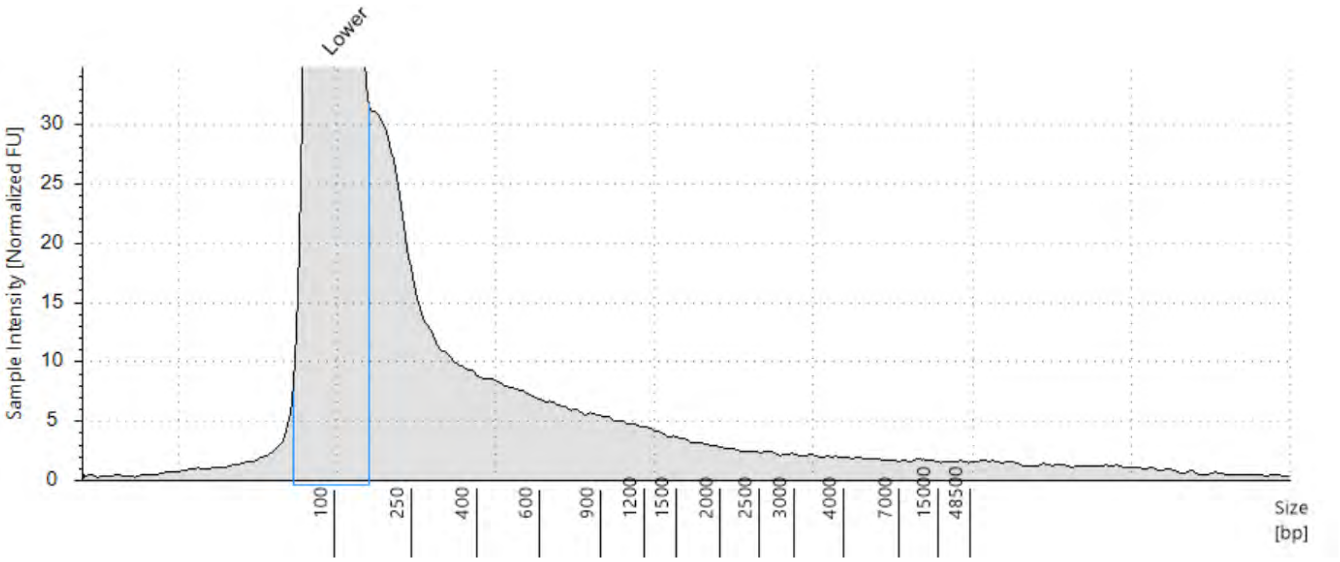

Sample Table

Well	DIN	Conc. [ng/µl]	Sample Description	Alert	Observations
D2	-	2.03	W-T4-E-840	▲	Sample concentration outside functional range for DIN; DIN Edited (Ladder sizing changed)

Peak Table

Size [bp]	Calibrated Conc. [ng/µl]	Assigned Conc. [ng/µl]	% Integrated Area	From [bp]	To [bp]	Peak Comment	Observations
100	8.50	8.50	-	60	152		Lower Marker

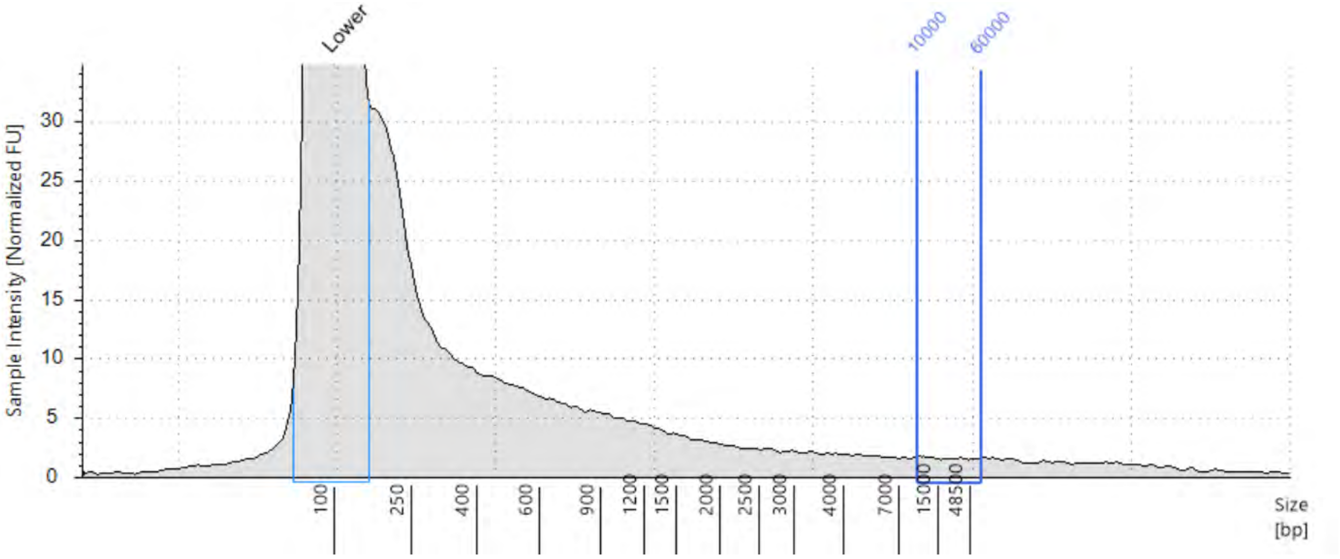

Region Table

From [bp]	To [bp]	Average Size [bp]	Conc. [ng/µl]	Region Molarity [nmol/l]	% of Total	Region Comment	Color
10000	60000	25434	0.0376	0.00491	1.85		■

E2: W-T4-E-1061

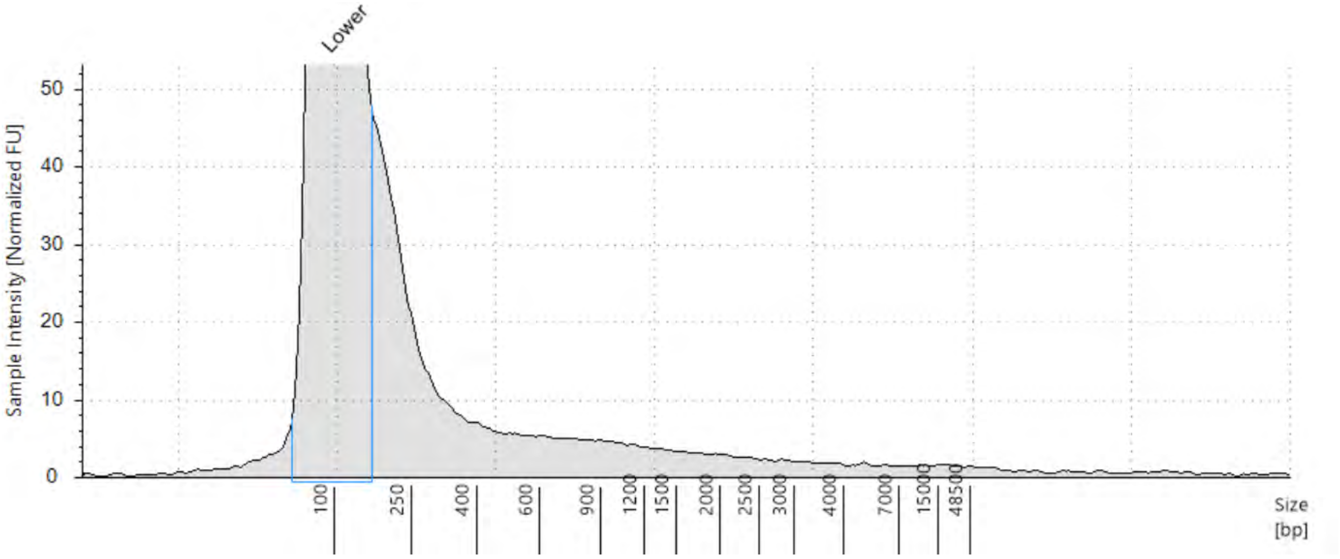

Sample Table

Well	DIN	Conc. [ng/µl]	Sample Description	Alert	Observations
E2	-	1.87	W-T4-E-1061	▲	Sample concentration outside functional range for DIN; DIN Edited (Ladder sizing changed)

Peak Table

Size [bp]	Calibrated Conc. [ng/µl]	Assigned Conc. [ng/µl]	% Integrated Area	From [bp]	To [bp]	Peak Comment	Observations
100	8.50	8.50	-	60	158		Lower Marker

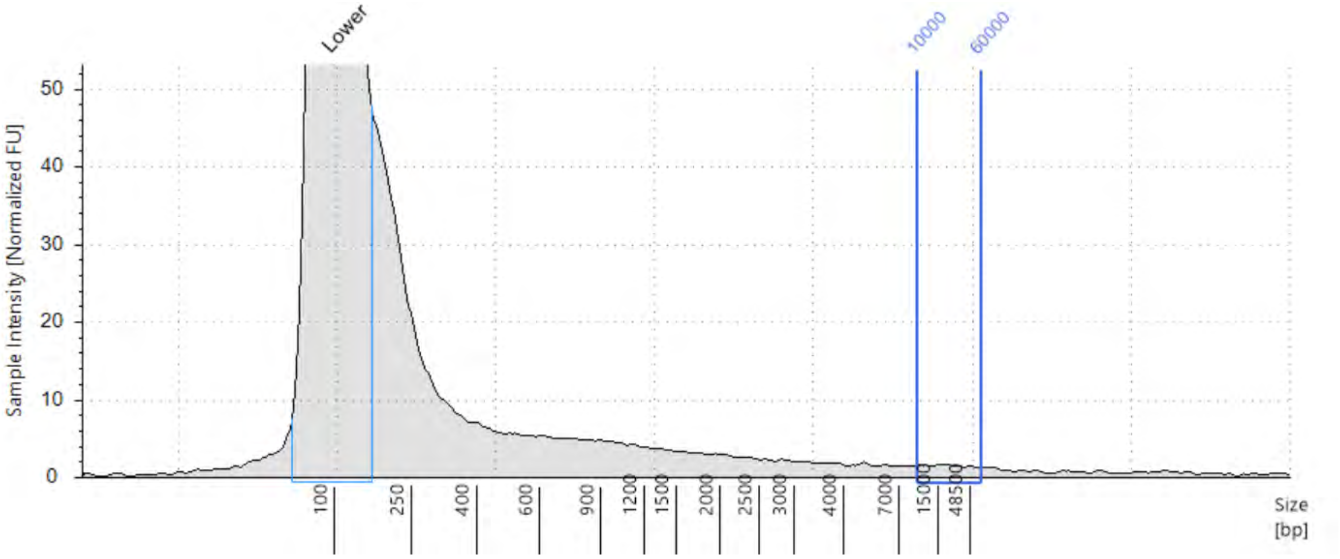

Region Table

From [bp]	To [bp]	Average Size [bp]	Conc. [ng/µl]	Region Molarity [nmol/l]	% of Total	Region Comment	Color
10000	60000	24904	0.0324	0.00403	1.74		■

F2: W-T4-E-1062

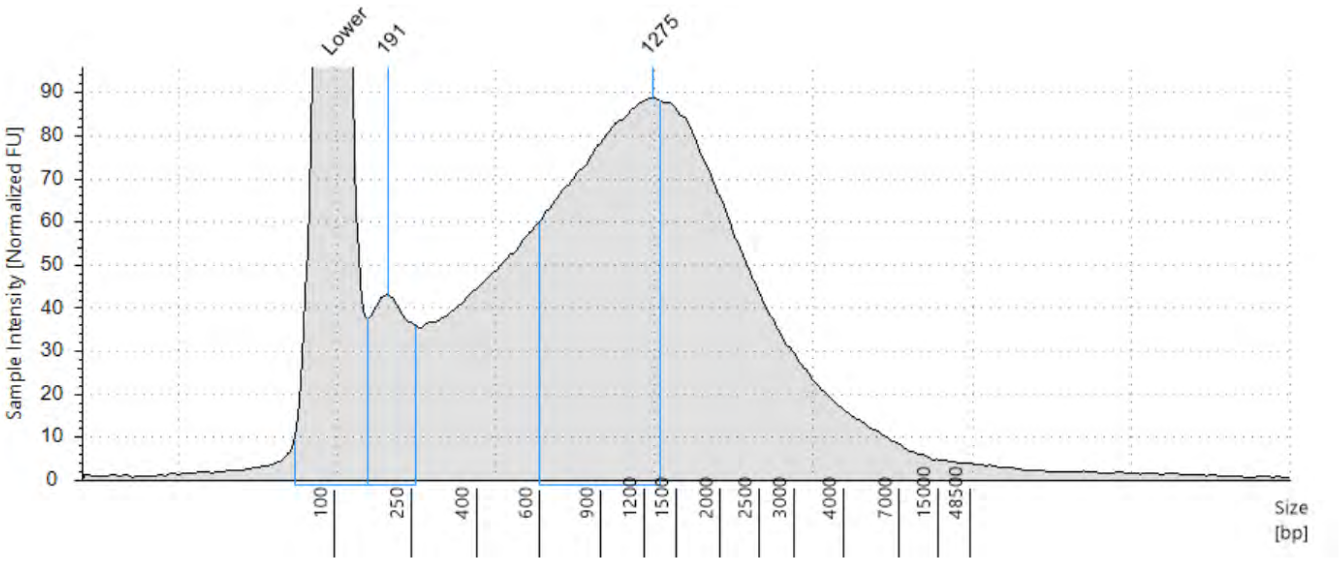

Sample Table

Well	DIN	Conc. [ng/µl]	Sample Description	Alert	Observations
F2	2.5	16.2	W-T4-E-1062		DIN Edited (Ladder sizing changed)

Peak Table

Size [bp]	Calibrated Conc. [ng/µl]	Assigned Conc. [ng/µl]	% Integrated Area	From [bp]	To [bp]	Peak Comment	Observations
100	8.50	8.50	-	62	149		Lower Marker
191	1.16	-	17.26	149	259		
1275	5.54	-	82.74	599	1338		

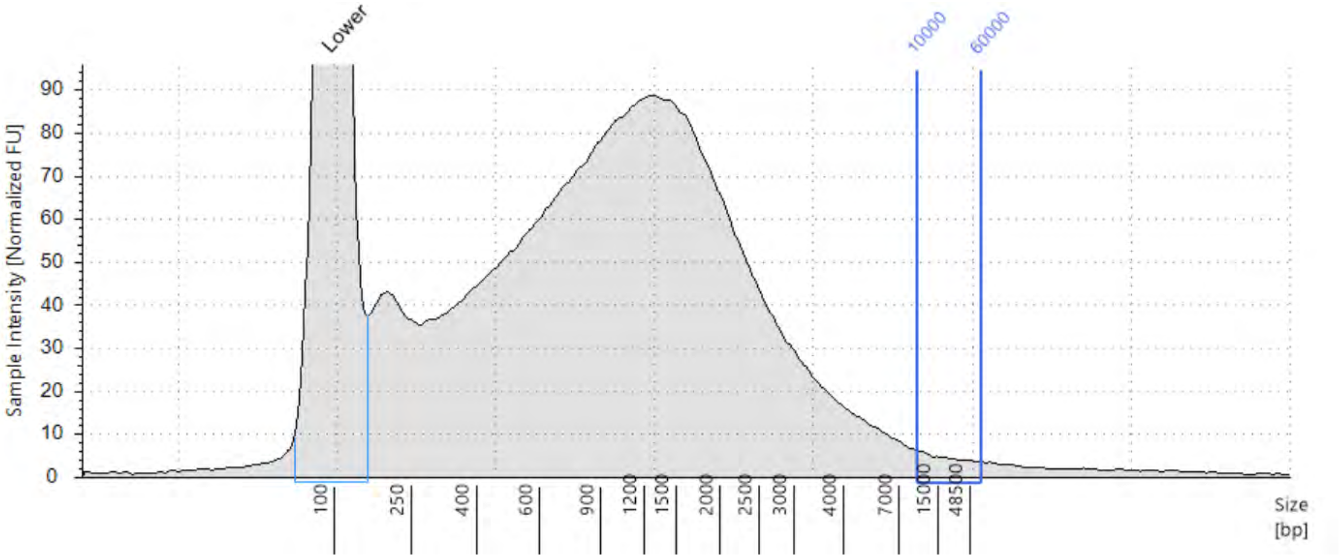

Region Table

From [bp]	To [bp]	Average Size [bp]	Conc. [ng/µl]	Region Molarity [nmol/l]	% of Total	Region Comment	Color
10000	60000	23726	0.126	0.0152	0.78		

G2: W-T4-E-1063

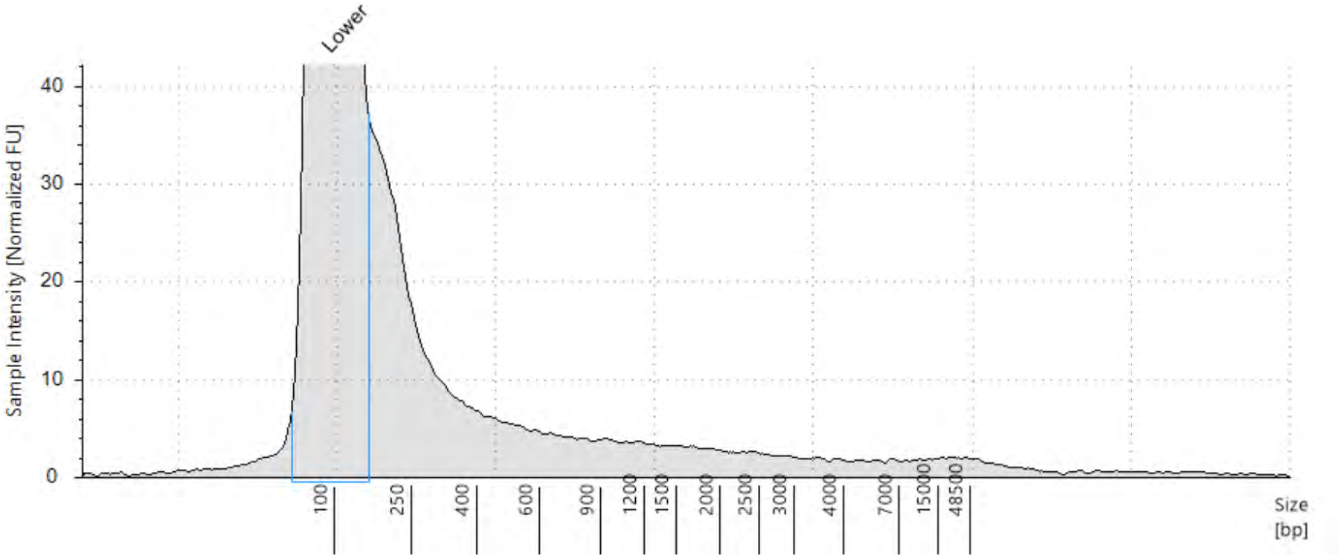

Sample Table

Well	DIN	Conc. [ng/µl]	Sample Description	Alert	Observations
G2	-	1.86	W-T4-E-1063	▲	Sample concentration outside functional range for DIN; DIN Edited (Ladder sizing changed)

Peak Table

Size [bp]	Calibrated Conc. [ng/µl]	Assigned Conc. [ng/µl]	% Integrated Area	From [bp]	To [bp]	Peak Comment	Observations
100	8.50	8.50	-	60	151		Lower Marker

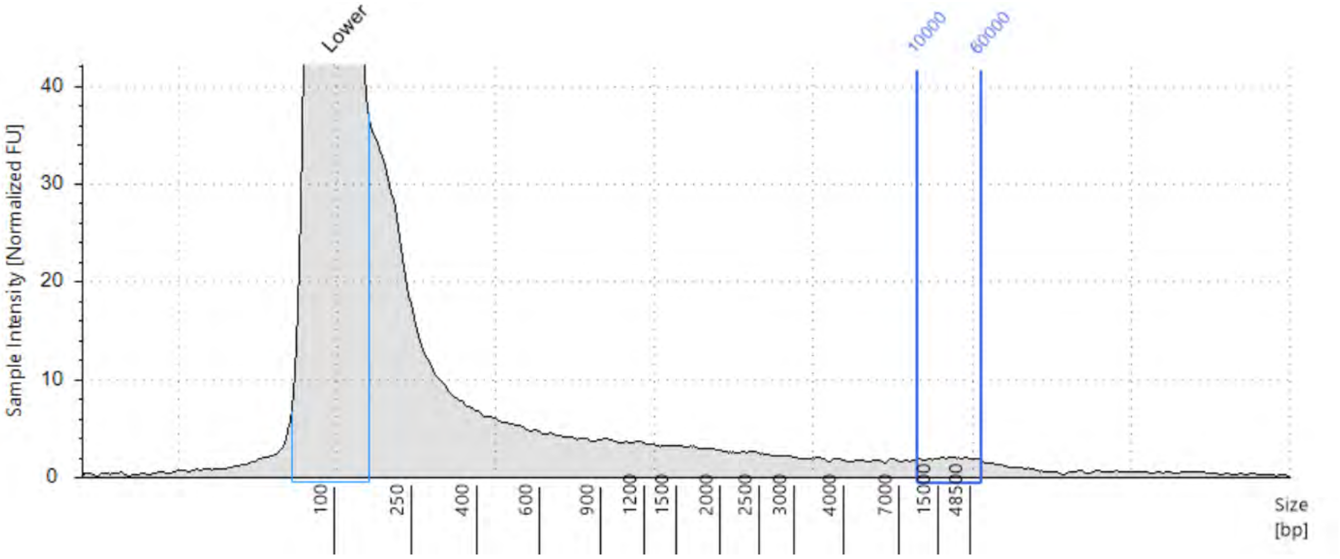

Region Table

From [bp]	To [bp]	Average Size [bp]	Conc. [ng/µl]	Region Molarity [nmol/l]	% of Total	Region Comment	Color
10000	60000	25609	0.0524	0.00560	2.82		■

H2: W-T4-E-1064

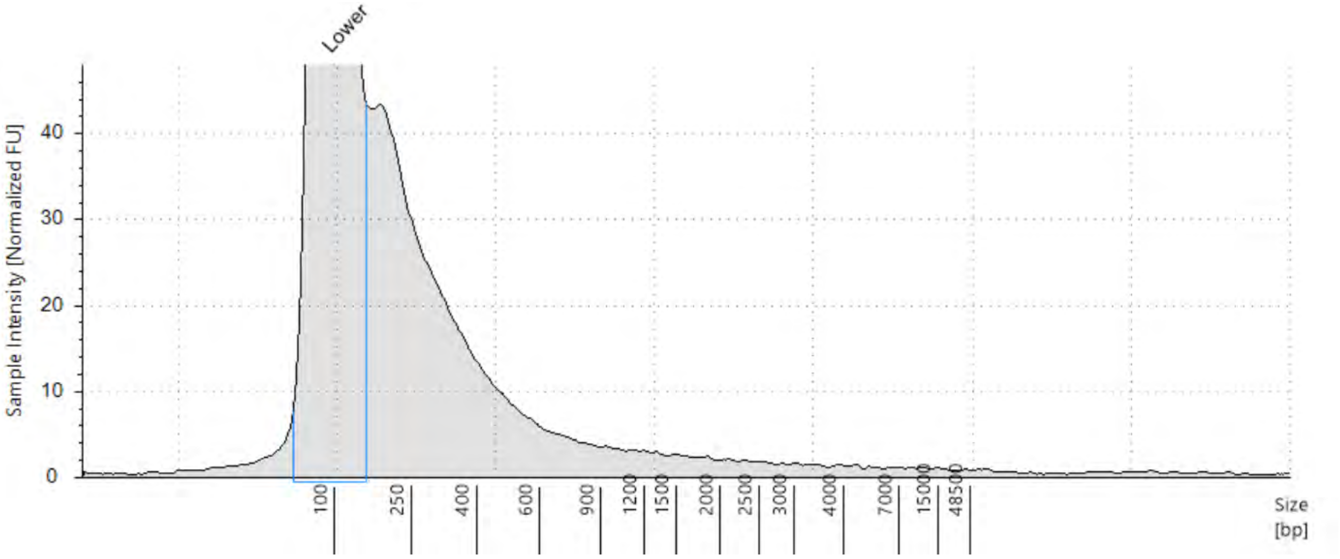

Sample Table

Well	DIN	Conc. [ng/µl]	Sample Description	Alert	Observations
H2	-	2.62	W-T4-E-1064	▲	Sample concentration outside functional range for DIN; DIN Edited (Ladder sizing changed)

Peak Table

Size [bp]	Calibrated Conc. [ng/µl]	Assigned Conc. [ng/µl]	% Integrated Area	From [bp]	To [bp]	Peak Comment	Observations
100	8.50	8.50	-	61	146		Lower Marker

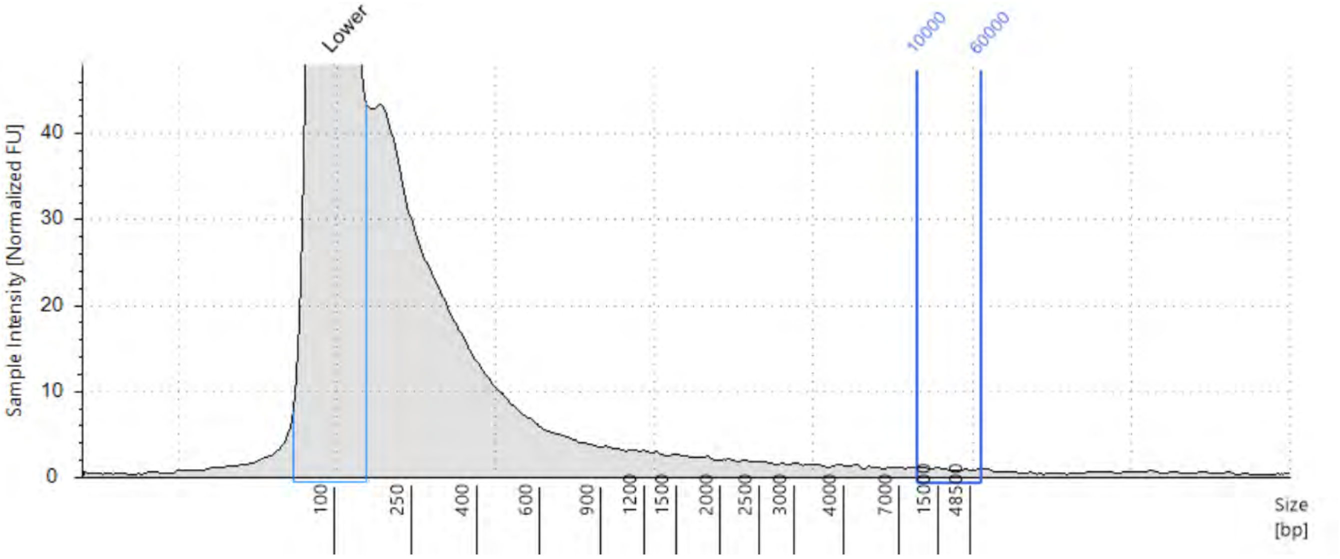

Region Table

From [bp]	To [bp]	Average Size [bp]	Conc. [ng/µl]	Region Molarity [nmol/l]	% of Total	Region Comment	Color
10000	60000	24491	0.0115	0.00301	0.44		■

Filename: 2019-07-29-06.gDNA

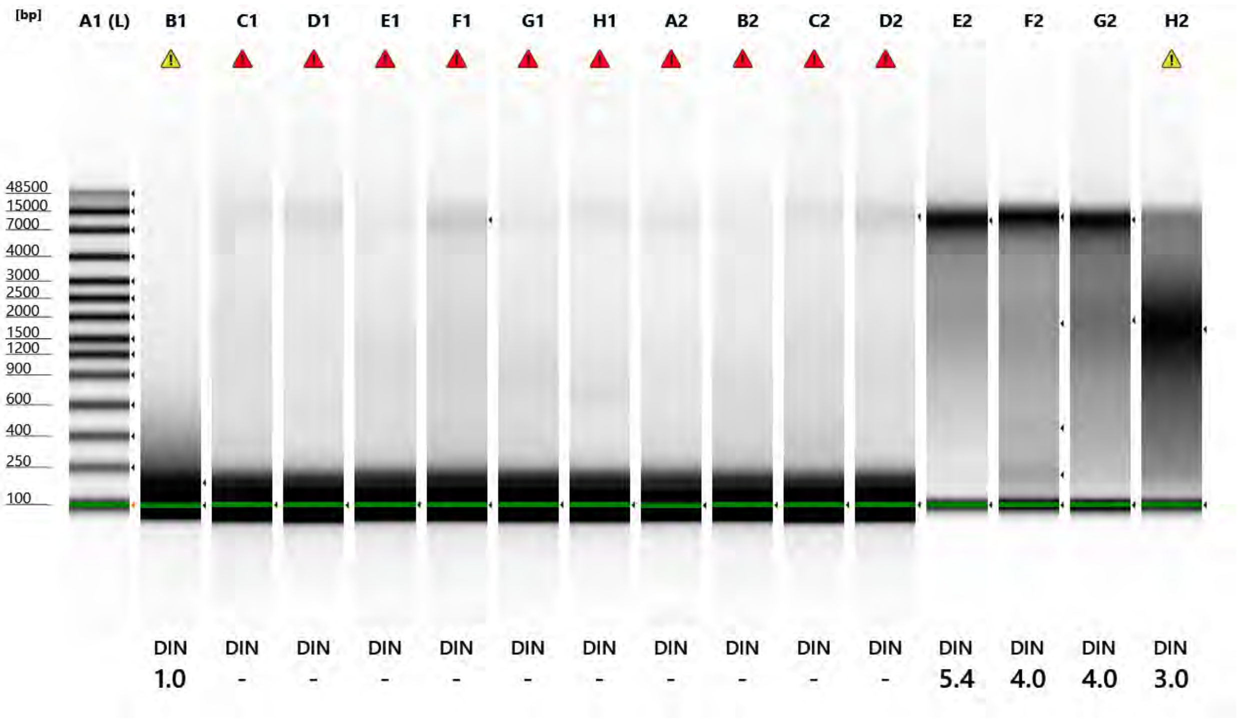

Default image (Contrast 50%), Image is Scaled to Sample

Sample Info

Well	DIN	Conc. [ng/µl]	Sample Description	Alert	Observations
A1	-	80.8	Ladder		Ladder
B1	1.0	4.43	W-T4-E-1065	⚠	Sample concentration outside functional range for DIN
C1	-	1.21	W-T4-S-841	▲	Sample concentration outside functional range for DIN
D1	-	1.13	W-T4-S-842	▲	Sample concentration outside functional range for DIN
E1	-	1.55	W-T4-S-843	▲	Sample concentration outside functional range for DIN
F1	-	2.16	W-T4-S-844	▲	Sample concentration outside functional range for DIN
G1	-	1.22	W-T4-S-845	▲	Sample concentration outside functional range for DIN
H1	-	1.06	W-T4-S-1066	▲	Sample concentration outside functional range for DIN
A2	-	1.33	W-T4-S-1067	▲	Sample concentration outside functional range for DIN
B2	-	1.53	W-T4-S-1068	▲	Sample concentration outside functional range for DIN
C2	-	1.57	W-T4-S-1069	▲	Sample concentration outside functional range for DIN
D2	-	1.27	W-T4-S-1070	▲	Sample concentration outside functional range for DIN
E2	5.4	71.5	W-T4-EtOH-846		
F2	4.0	57.6	W-T4-EtOH-847		
G2	4.0	67.8	W-T4-EtOH-848		
H2	3.0	107	W-T4-EtOH-849	⚠	Sample concentration outside recommended range

© Agilent Technologies, Inc. 2017

A1: Ladder

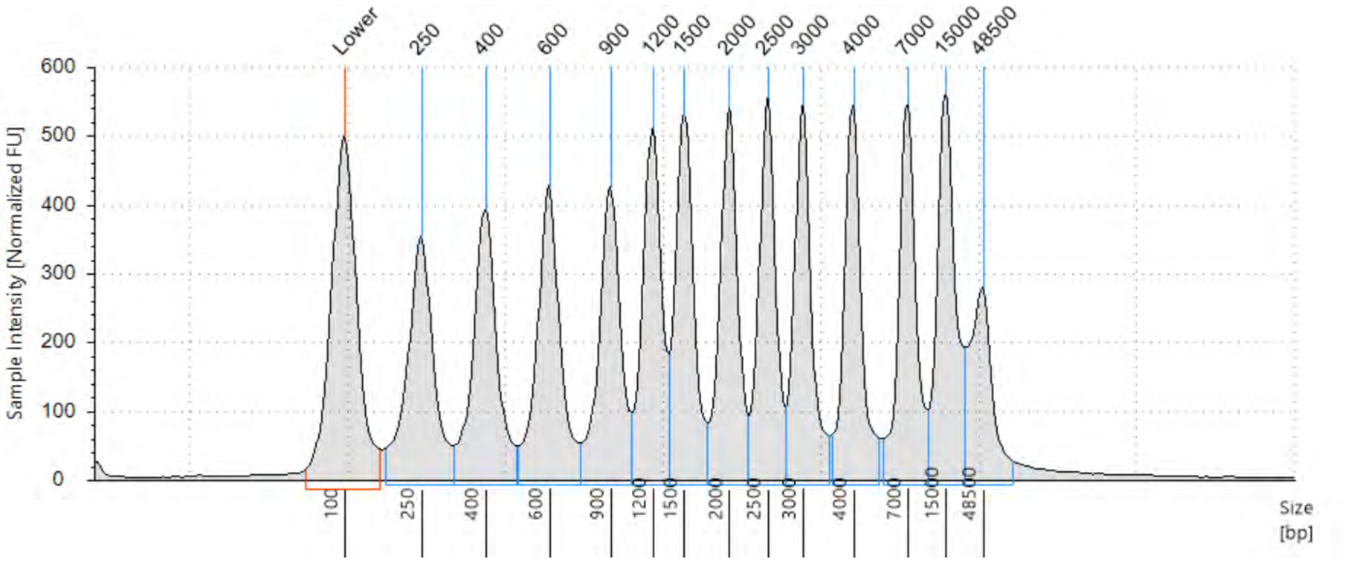

Sample Table

Well	DIN	Conc. [ng/µl]	Sample Description	Alert	Observations
A1	-	80.8	Ladder		Ladder

Peak Table

Size [bp]	Calibrated Conc. [ng/µl]	Assigned Conc. [ng/µl]	% Integrated Area	From [bp]	To [bp]	Peak Comment	Observations
100	8.50	8.50	-	62	151		Lower Marker
250	6.04	-	7.55	163	318		
400	6.20	-	7.75	318	488		
600	6.46	-	8.07	492	738		
900	6.01	-	7.52	738	1034		
1200	6.59	-	8.24	1034	1348		
1500	6.57	-	8.21	1348	1740		
2000	6.43	-	8.03	1740	2236		
2500	6.26	-	7.82	2236	2739		
3000	6.26	-	7.83	2739	3478		
4000	6.15	-	7.68	3532	5218		
7000	6.19	-	7.73	5442	10530		
15000	6.70	-	8.37	10530	21962		
48500	4.16	-	5.20	21962	>60000		

© Agilent Technologies, Inc. 2017

B1: W-T4-E-1065

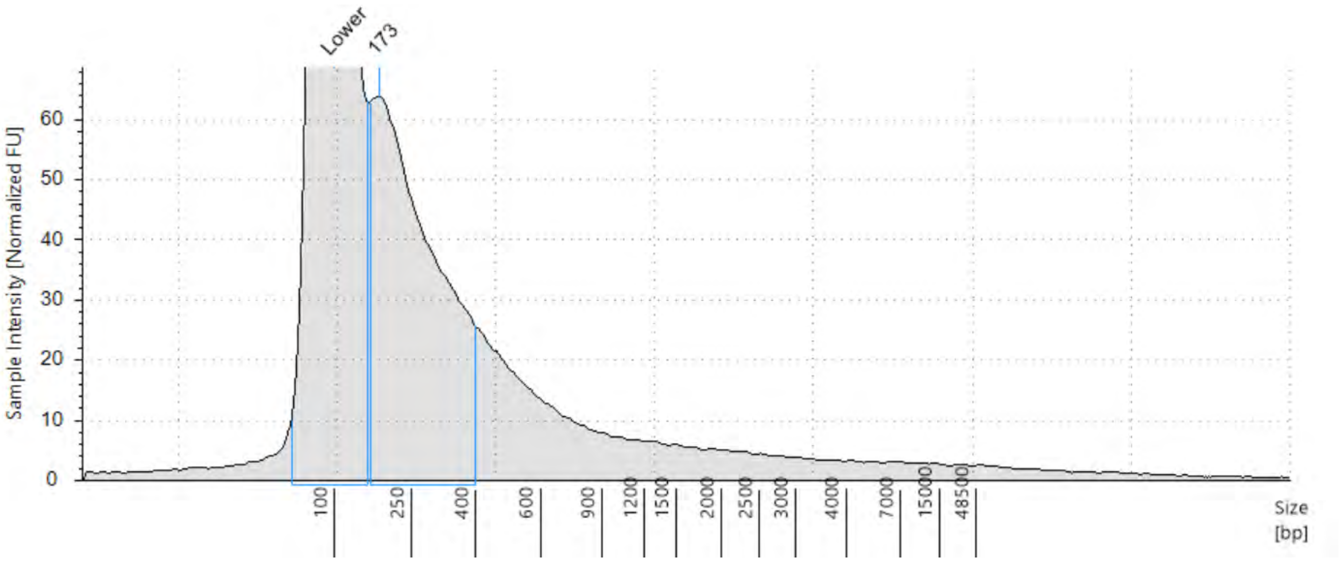

Sample Table

Well	DIN	Conc. [ng/µl]	Sample Description	Alert	Observations
B1	1.0	4.43	W-T4-E-1065	⚠	Sample concentration outside functional range for DIN

Peak Table

Size [bp]	Calibrated Conc. [ng/µl]	Assigned Conc. [ng/µl]	% Integrated Area	From [bp]	To [bp]	Peak Comment	Observations
100	8.50	8.50	-	61	150		Lower Marker
173	2.50	-	100.00	155	403		

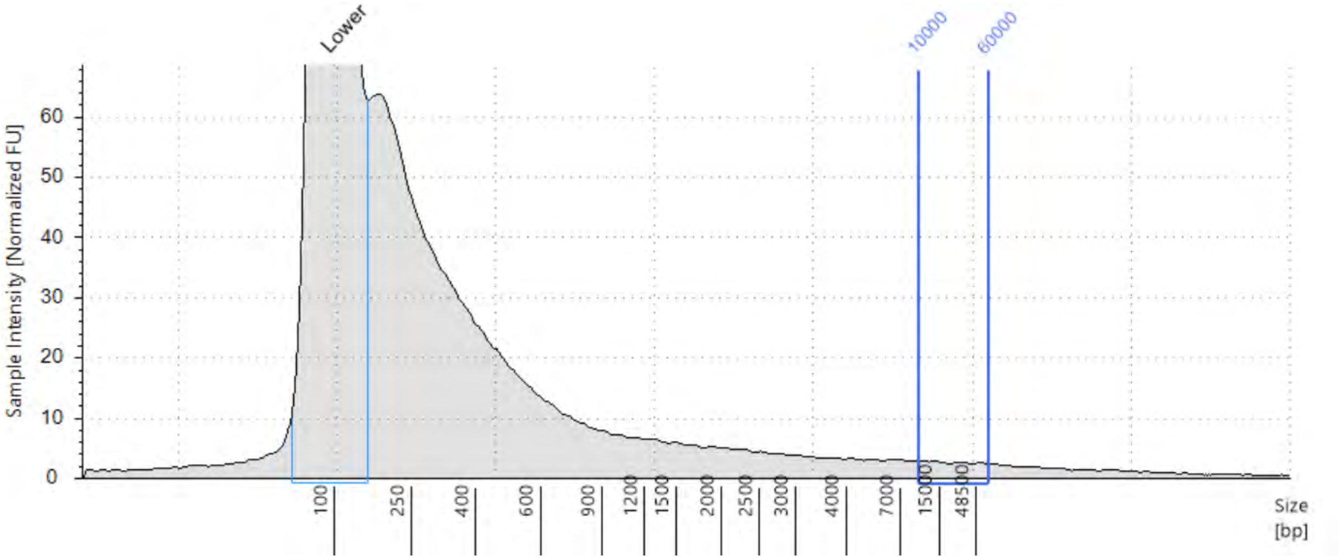

Region Table

From [bp]	To [bp]	Average Size [bp]	Conc. [ng/µl]	Region Molarity [nmol/l]	% of Total	Region Comment	Color
10000	60000	26032	0.0698	0.00789	1.58		■

© Agilent Technologies, Inc. 2017

C1: W-T4-S-841

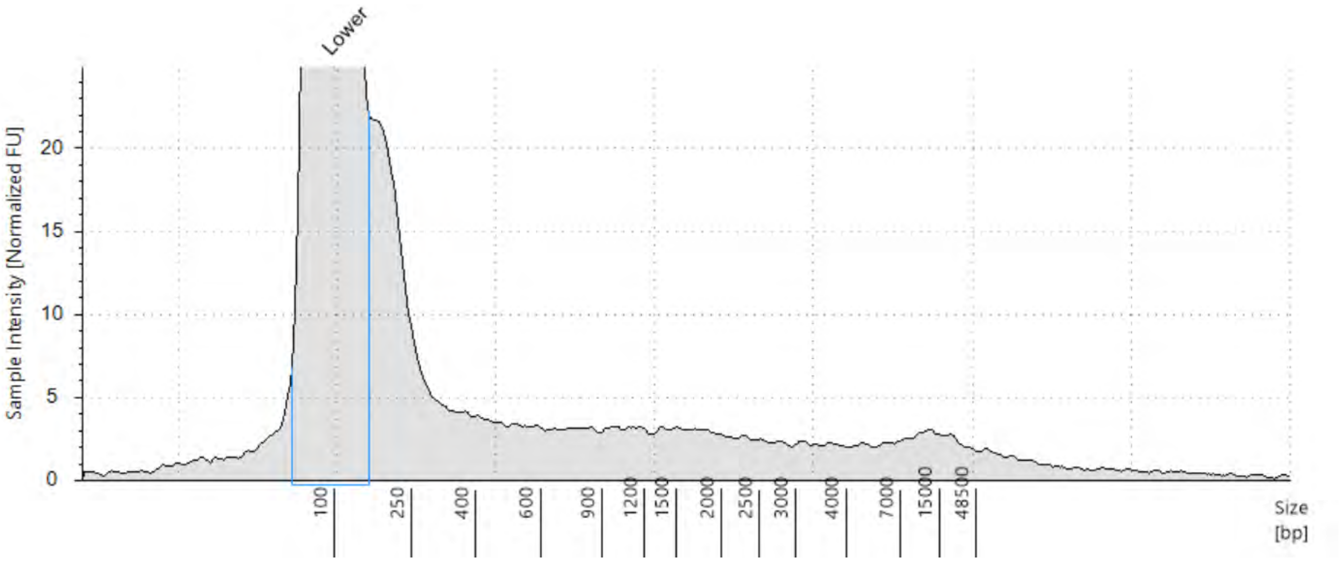

Sample Table

Well	DIN	Conc. [ng/µl]	Sample Description	Alert	Observations
C1	-	1.21	W-T4-S-841	▲	Sample concentration outside functional range for DIN

Peak Table

Size [bp]	Calibrated Conc. [ng/µl]	Assigned Conc. [ng/µl]	% Integrated Area	From [bp]	To [bp]	Peak Comment	Observations
100	8.50	8.50	-	60	151		Lower Marker

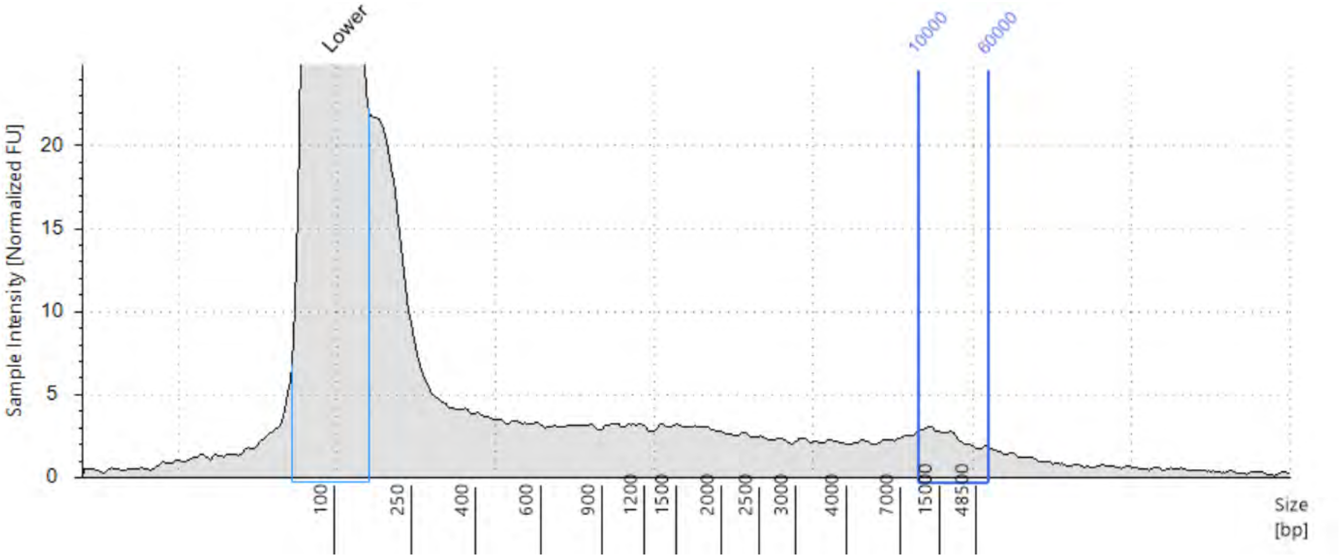

Region Table

From [bp]	To [bp]	Average Size [bp]	Conc. [ng/µl]	Region Molarity [nmol/l]	% of Total	Region Comment	Color
10000	60000	24301	0.0662	0.00829	5.48		■

 © Agilent Technologies, Inc. 2017

D1: W-T4-S-842

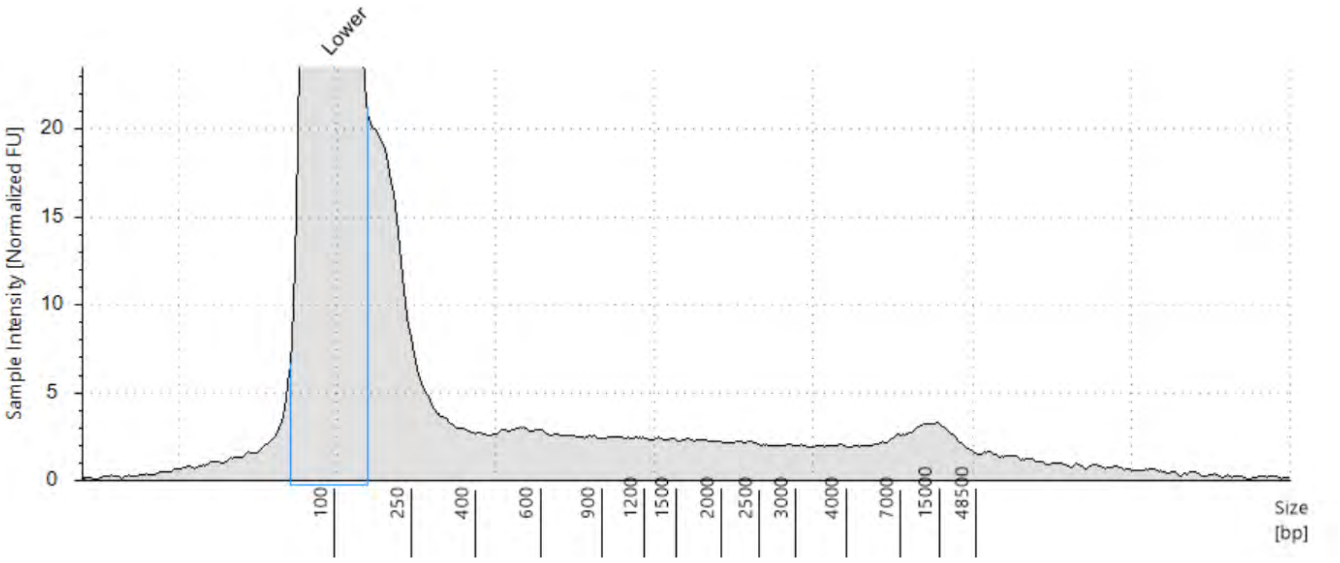

Sample Table

Well	DIN	Conc. [ng/µl]	Sample Description	Alert	Observations
D1	-	1.13	W-T4-S-842	▲	Sample concentration outside functional range for DIN

Peak Table

Size [bp]	Calibrated Conc. [ng/µl]	Assigned Conc. [ng/µl]	% Integrated Area	From [bp]	To [bp]	Peak Comment	Observations
100	8.50	8.50	-	60	149		Lower Marker

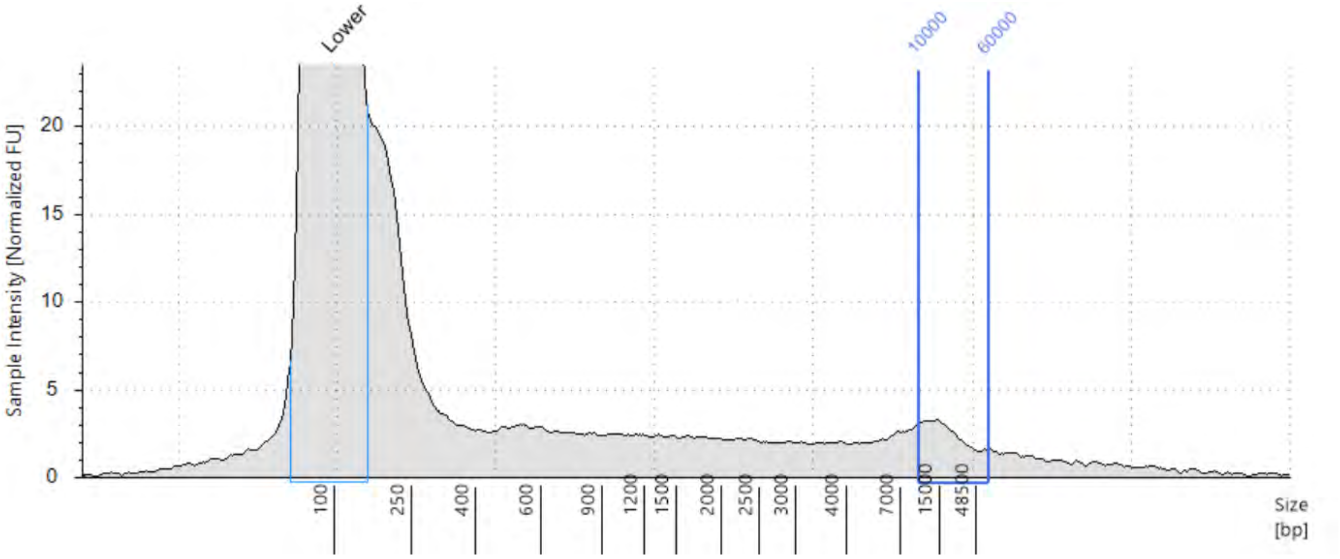

Region Table

From [bp]	To [bp]	Average Size [bp]	Conc. [ng/µl]	Region Molarity [nmol/l]	% of Total	Region Comment	Color
10000	60000	22539	0.0769	0.00907	6.79		■

E1: W-T4-S-843

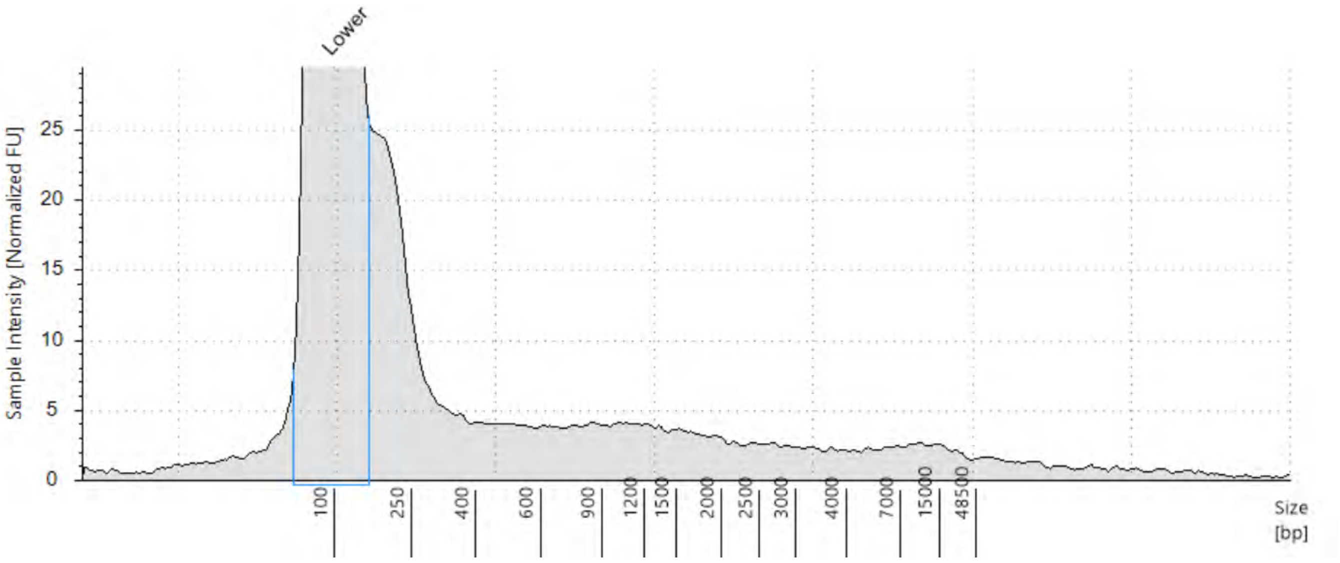

Sample Table

Well	DIN	Conc. [ng/µl]	Sample Description	Alert	Observations
E1	-	1.55	W-T4-S-843	▲	Sample concentration outside functional range for DIN

Peak Table

Size [bp]	Calibrated Conc. [ng/µl]	Assigned Conc. [ng/µl]	% Integrated Area	From [bp]	To [bp]	Peak Comment	Observations
100	8.50	8.50	-	62	150		Lower Marker

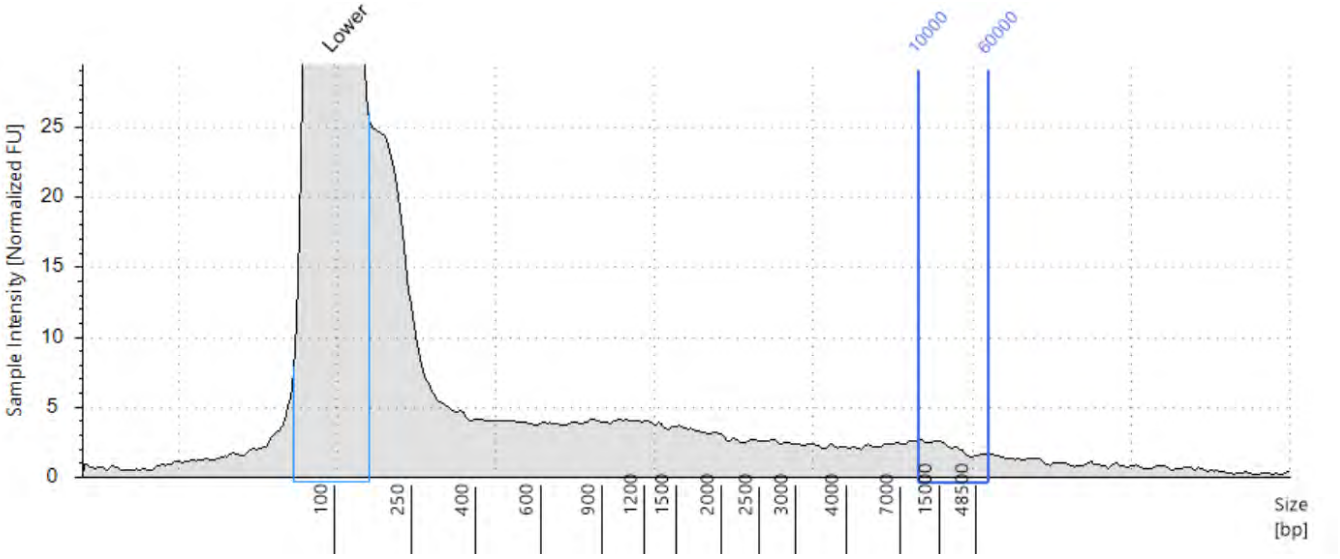

Region Table

From [bp]	To [bp]	Average Size [bp]	Conc. [ng/µl]	Region Molarity [nmol/l]	% of Total	Region Comment	Color
10000	60000	23857	0.0590	0.00721	3.79		■

 © Agilent Technologies, Inc. 2017

F1: W-T4-S-844

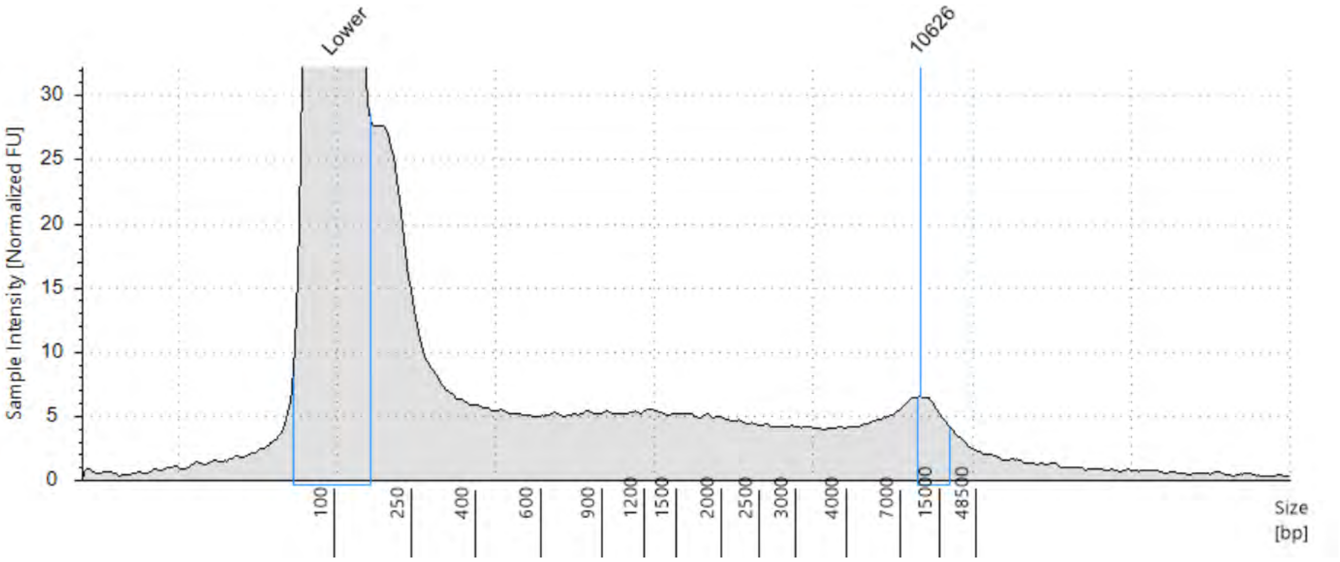

Sample Table

Well	DIN	Conc. [ng/µl]	Sample Description	Alert	Observations
F1	-	2.16	W-T4-S-844	▲	Sample concentration outside functional range for DIN

Peak Table

Size [bp]	Calibrated Conc. [ng/µl]	Assigned Conc. [ng/µl]	% Integrated Area	From [bp]	To [bp]	Peak Comment	Observations
100	8.50	8.50	-	61	153		Lower Marker
10626	0.0987	-	100.00	9789	18879		

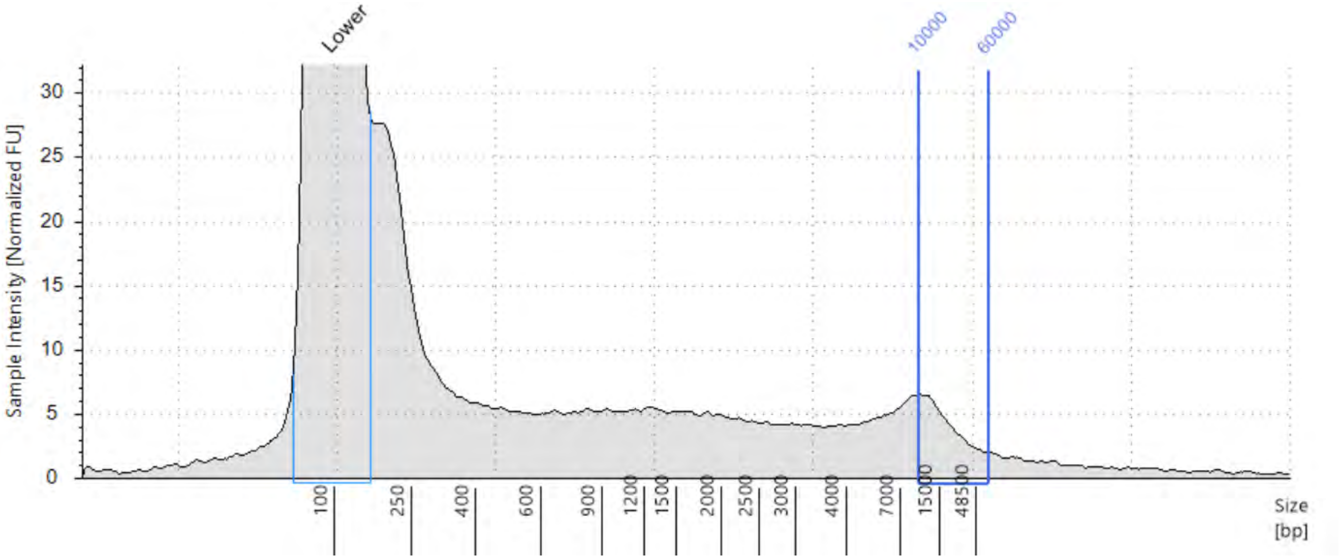

Region Table

From [bp]	To [bp]	Average Size [bp]	Conc. [ng/µl]	Region Molarity [nmol/l]	% of Total	Region Comment	Color
10000	60000	21219	0.141	0.0154	6.53		■

 © Agilent Technologies, Inc. 2017

G1: W-T4-S-845

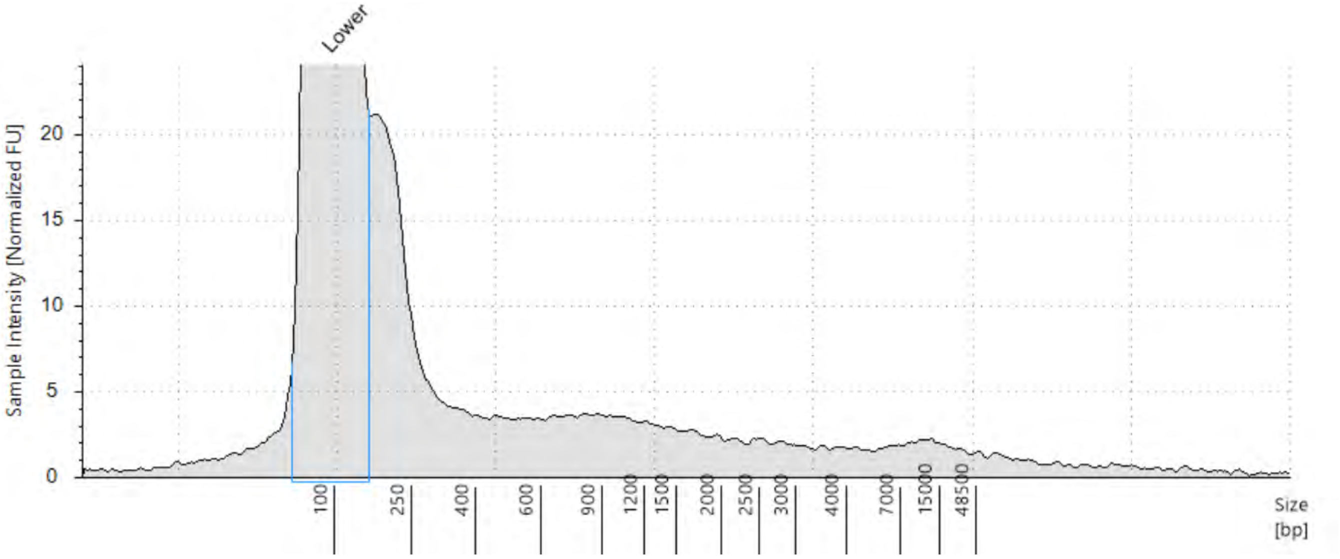

Sample Table

Well	DIN	Conc. [ng/µl]	Sample Description	Alert	Observations
G1	-	1.22	W-T4-S-845	▲	Sample concentration outside functional range for DIN

Peak Table

Size [bp]	Calibrated Conc. [ng/µl]	Assigned Conc. [ng/µl]	% Integrated Area	From [bp]	To [bp]	Peak Comment	Observations
100	8.50	8.50	-	60	151		Lower Marker

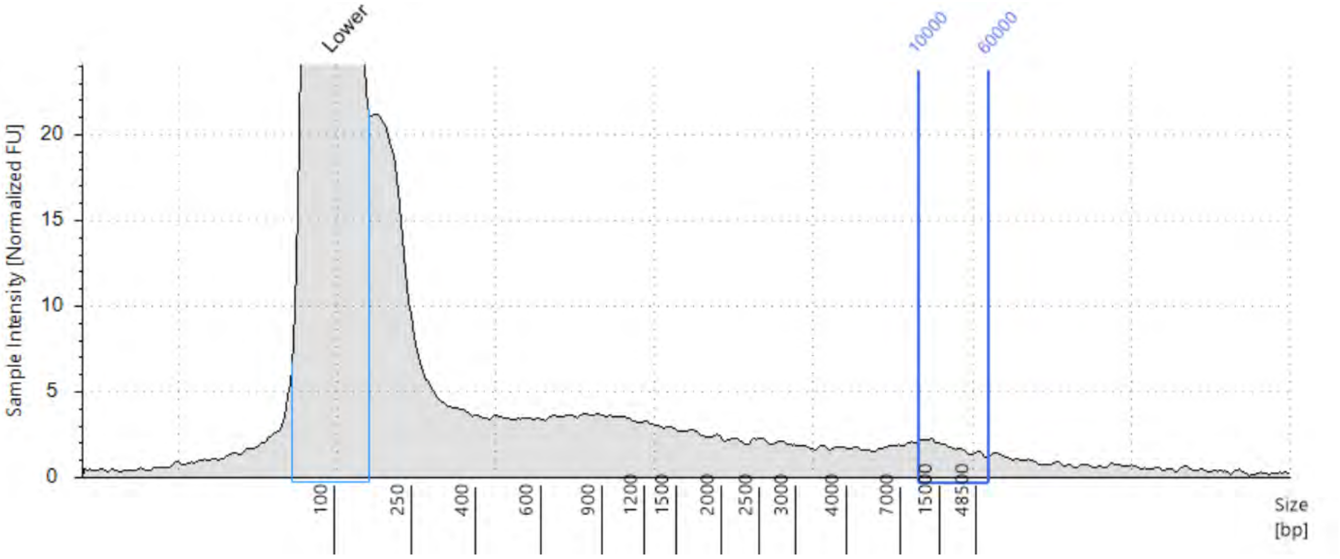

Region Table

From [bp]	To [bp]	Average Size [bp]	Conc. [ng/µl]	Region Molarity [nmol/l]	% of Total	Region Comment	Color
10000	60000	24278	0.0444	0.00605	3.64		■

H1: W-T4-S-1066

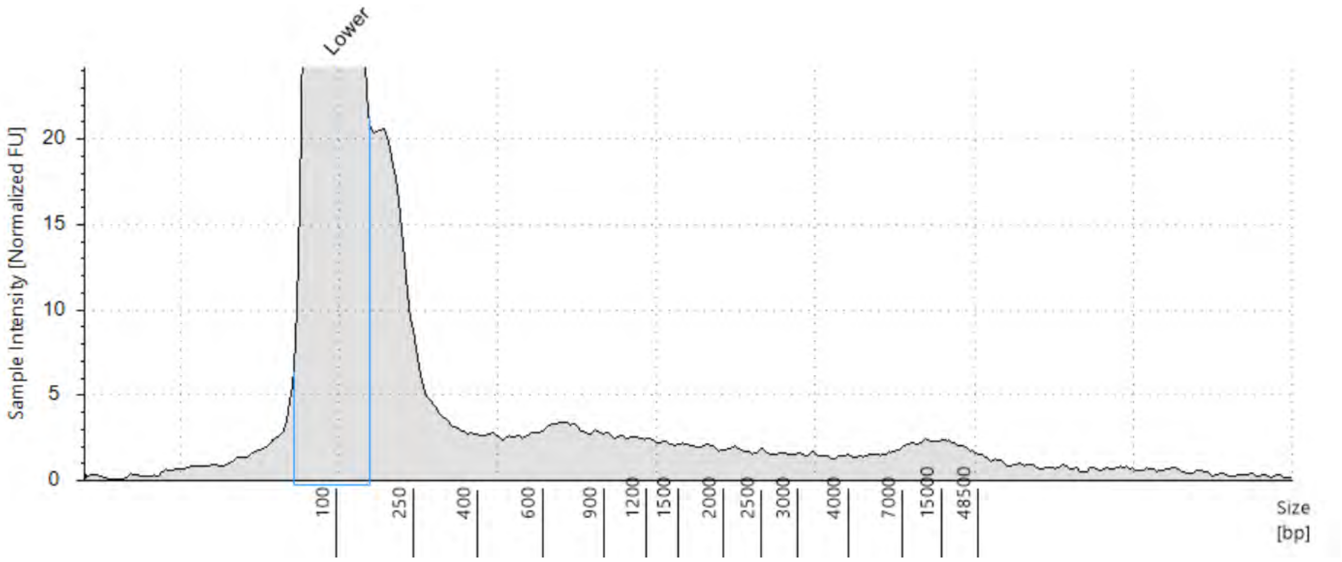

Sample Table

Well	DIN	Conc. [ng/µl]	Sample Description	Alert	Observations
H1	-	1.06	W-T4-S-1066	▲	Sample concentration outside functional range for DIN

Peak Table

Size [bp]	Calibrated Conc. [ng/µl]	Assigned Conc. [ng/µl]	% Integrated Area	From [bp]	To [bp]	Peak Comment	Observations
100	8.50	8.50	-	60	148		Lower Marker

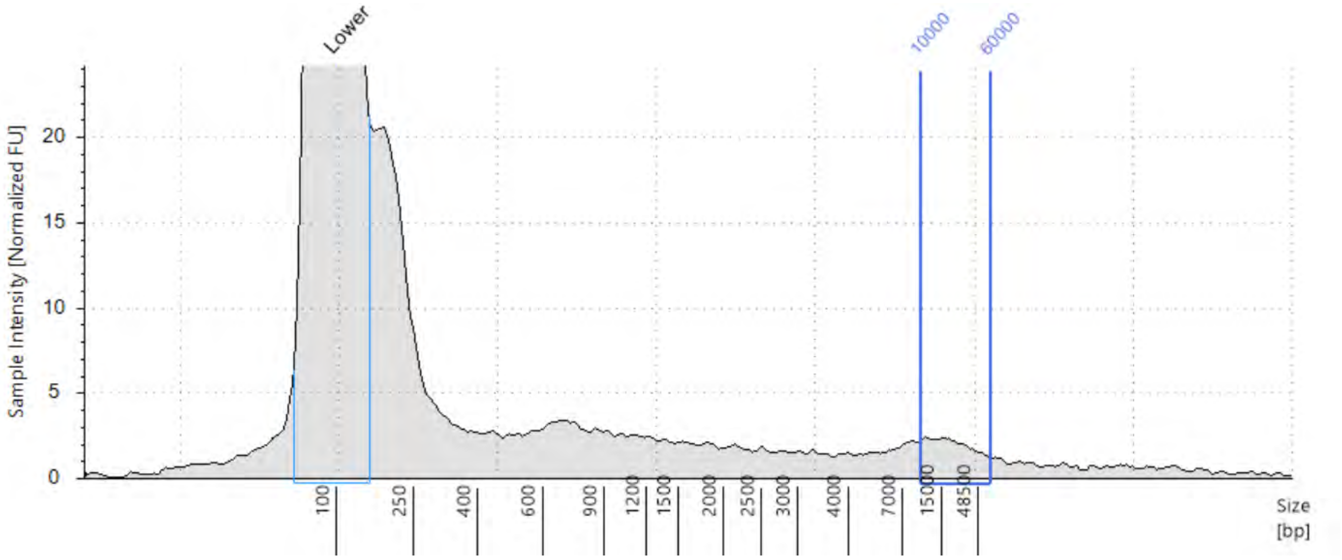

Region Table

From [bp]	To [bp]	Average Size [bp]	Conc. [ng/µl]	Region Molarity [nmol/l]	% of Total	Region Comment	Color
10000	60000	23950	0.0548	0.00700	5.17		■

 © Agilent Technologies, Inc. 2017

A2: W-T4-S-1067

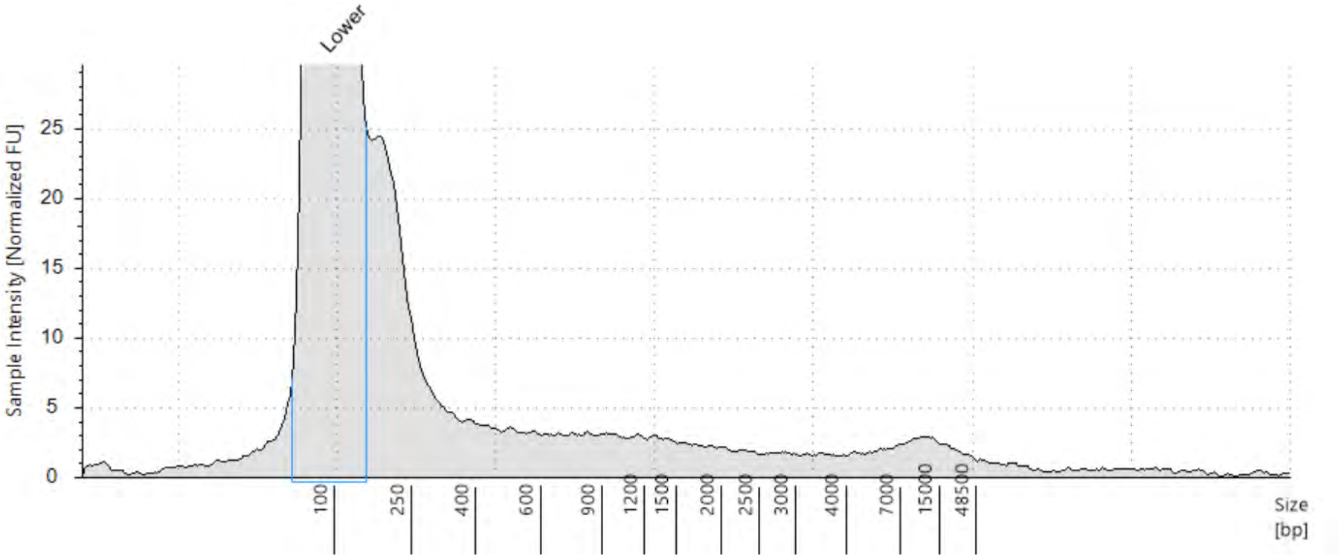

Sample Table

Well	DIN	Conc. [ng/µl]	Sample Description	Alert	Observations
A2	-	1.33	W-T4-S-1067	▲	Sample concentration outside functional range for DIN

Peak Table

Size [bp]	Calibrated Conc. [ng/µl]	Assigned Conc. [ng/µl]	% Integrated Area	From [bp]	To [bp]	Peak Comment	Observations
100	8.50	8.50	-	62	150		Lower Marker

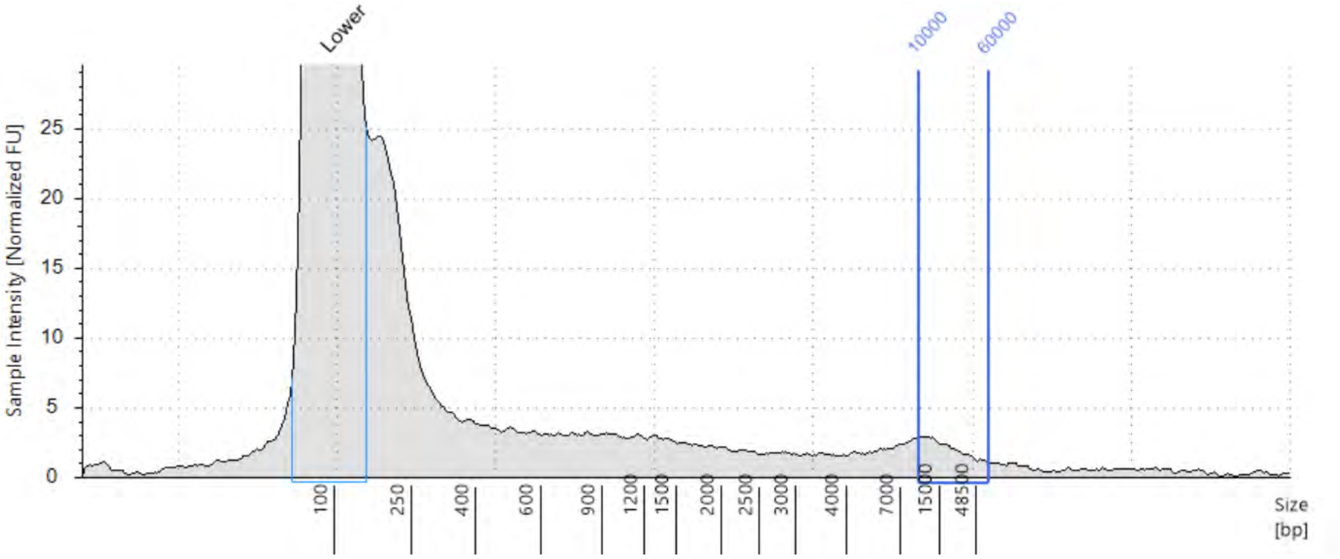

Region Table

From [bp]	To [bp]	Average Size [bp]	Conc. [ng/µl]	Region Molarity [nmol/l]	% of Total	Region Comment	Color
10000	60000	22673	0.0578	0.00767	4.35		■

 © Agilent Technologies, Inc. 2017

B2: W-T4-S-1068

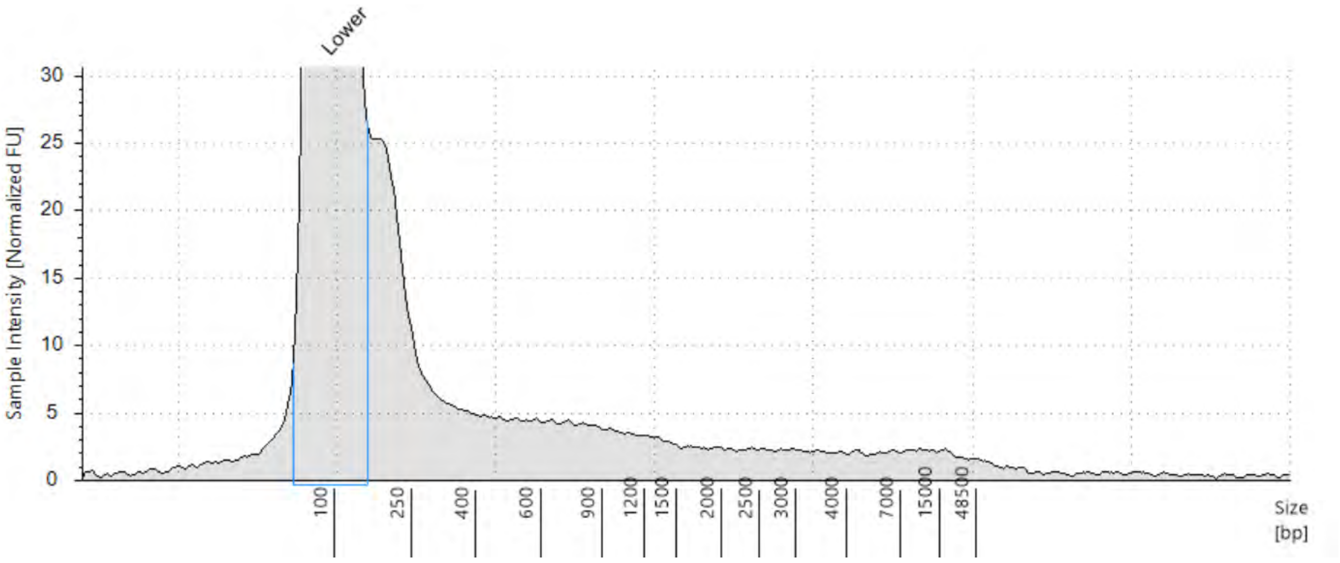

Sample Table

Well	DIN	Conc. [ng/µl]	Sample Description	Alert	Observations
B2	-	1.53	W-T4-S-1068	▲	Sample concentration outside functional range for DIN

Peak Table

Size [bp]	Calibrated Conc. [ng/µl]	Assigned Conc. [ng/µl]	% Integrated Area	From [bp]	To [bp]	Peak Comment	Observations
100	8.50	8.50	-	62	150		Lower Marker

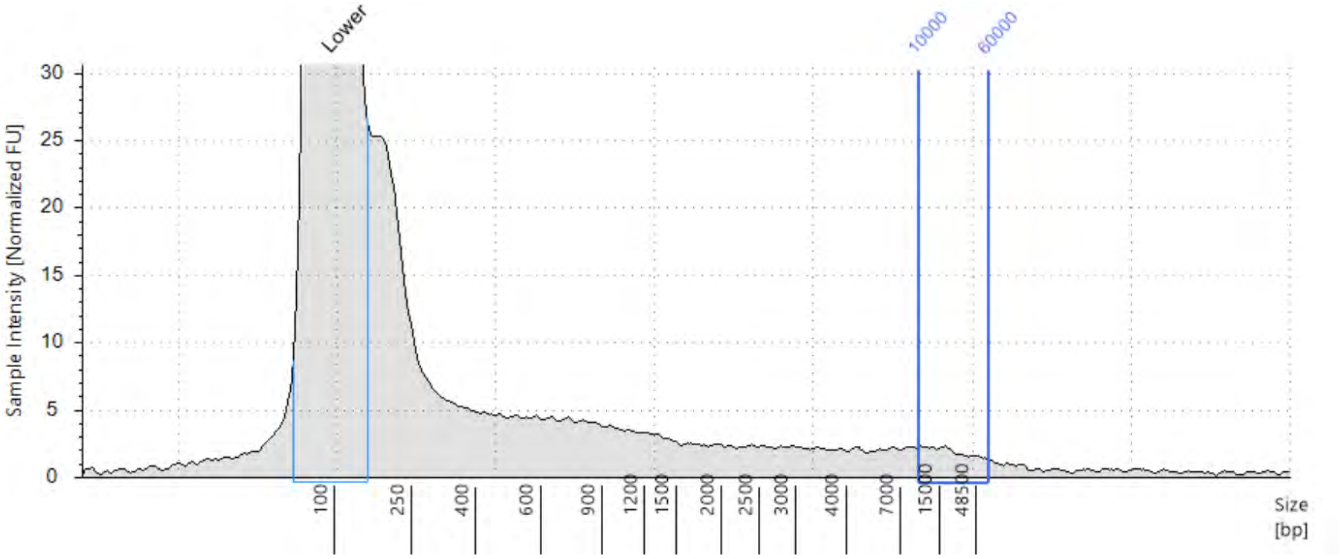

Region Table

From [bp]	To [bp]	Average Size [bp]	Conc. [ng/µl]	Region Molarity [nmol/l]	% of Total	Region Comment	Color
10000	60000	24781	0.0533	0.00651	3.48		■

C2: W-T4-S-1069

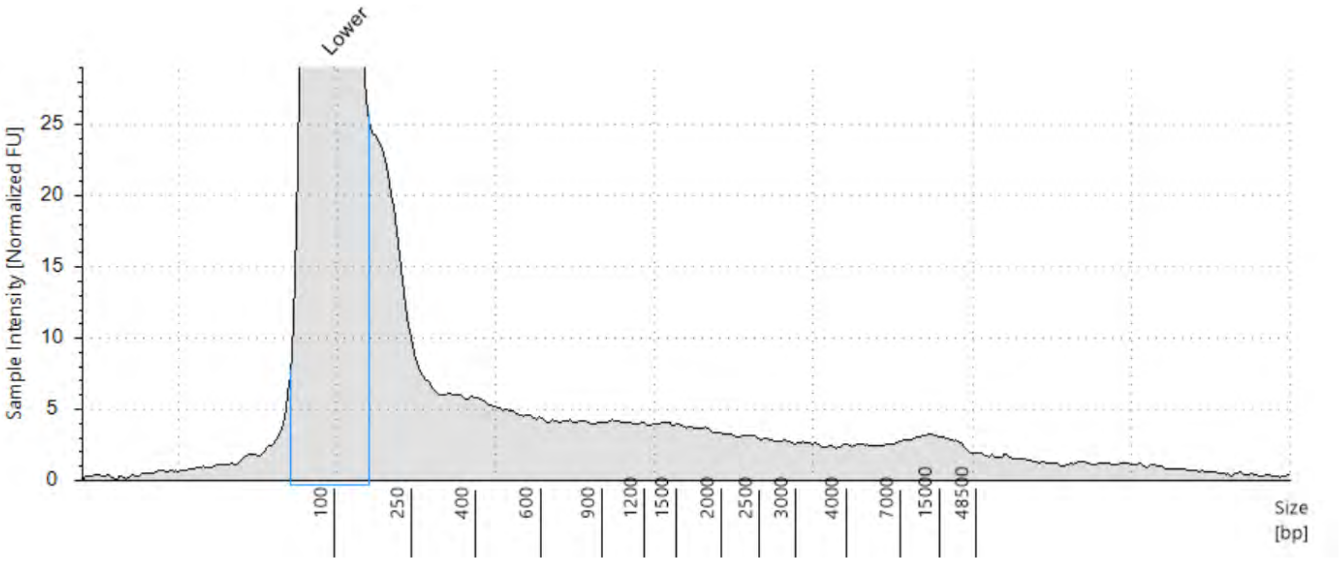

Sample Table

Well	DIN	Conc. [ng/µl]	Sample Description	Alert	Observations
C2	-	1.57	W-T4-S-1069	▲	Sample concentration outside functional range for DIN

Peak Table

Size [bp]	Calibrated Conc. [ng/µl]	Assigned Conc. [ng/µl]	% Integrated Area	From [bp]	To [bp]	Peak Comment	Observations
100	8.50	8.50	-	59	150		Lower Marker

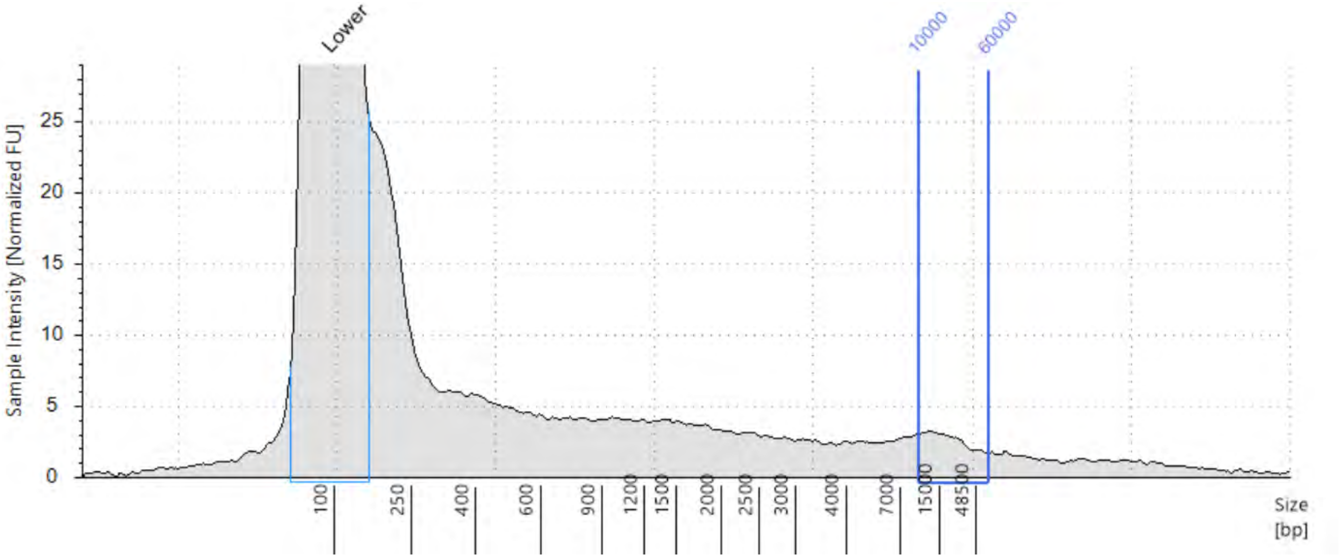

Region Table

From [bp]	To [bp]	Average Size [bp]	Conc. [ng/µl]	Region Molarity [nmol/l]	% of Total	Region Comment	Color
10000	60000	23451	0.0772	0.00876	4.92		■

© Agilent Technologies, Inc. 2017

D2: W-T4-S-1070

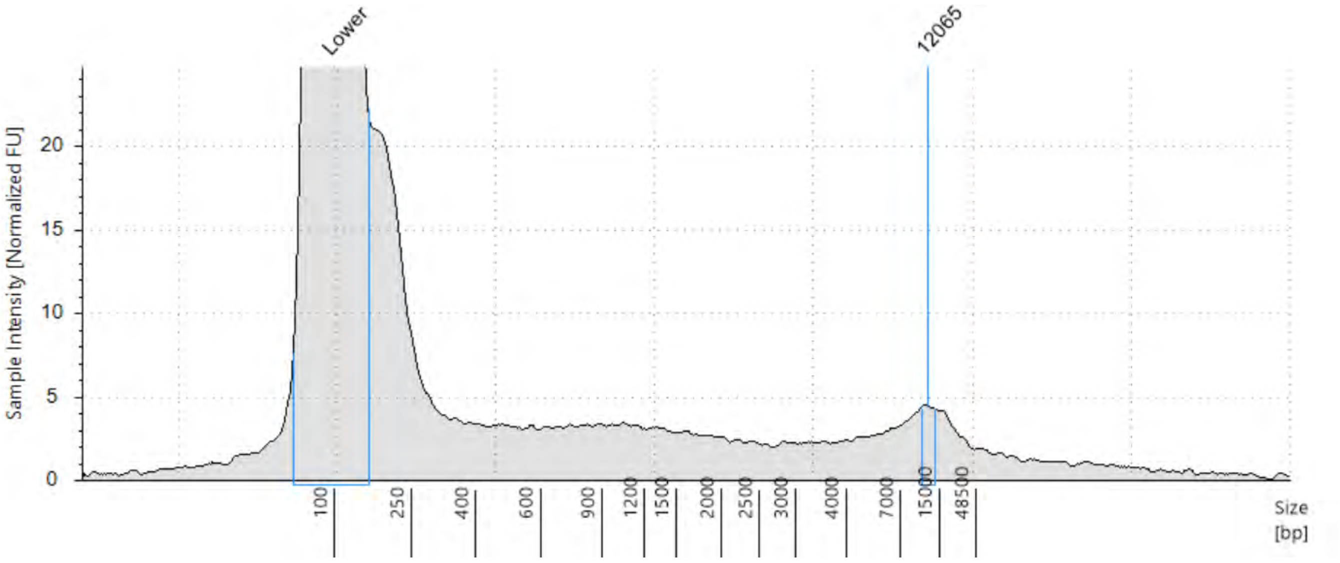

Sample Table

Well	DIN	Conc. [ng/µl]	Sample Description	Alert	Observations
D2	-	1.27	W-T4-S-1070	▲	Sample concentration outside functional range for DIN

Peak Table

Size [bp]	Calibrated Conc. [ng/µl]	Assigned Conc. [ng/µl]	% Integrated Area	From [bp]	To [bp]	Peak Comment	Observations
100	8.50	8.50	-	61	151		Lower Marker
12065	0.0324	-	100.00	10530	13824		

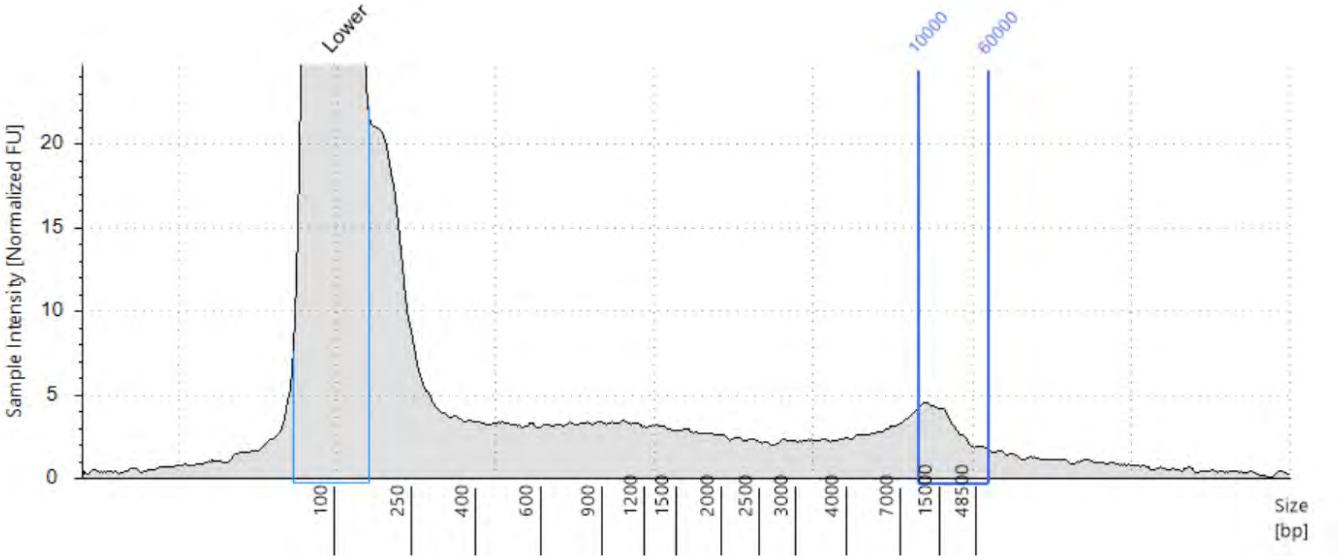

Region Table

From [bp]	To [bp]	Average Size [bp]	Conc. [ng/µl]	Region Molarity [nmol/l]	% of Total	Region Comment	Color
10000	60000	22300	0.0990	0.0115	7.79		■

 © Agilent Technologies, Inc. 2017

E2: W-T4-EtOH-846

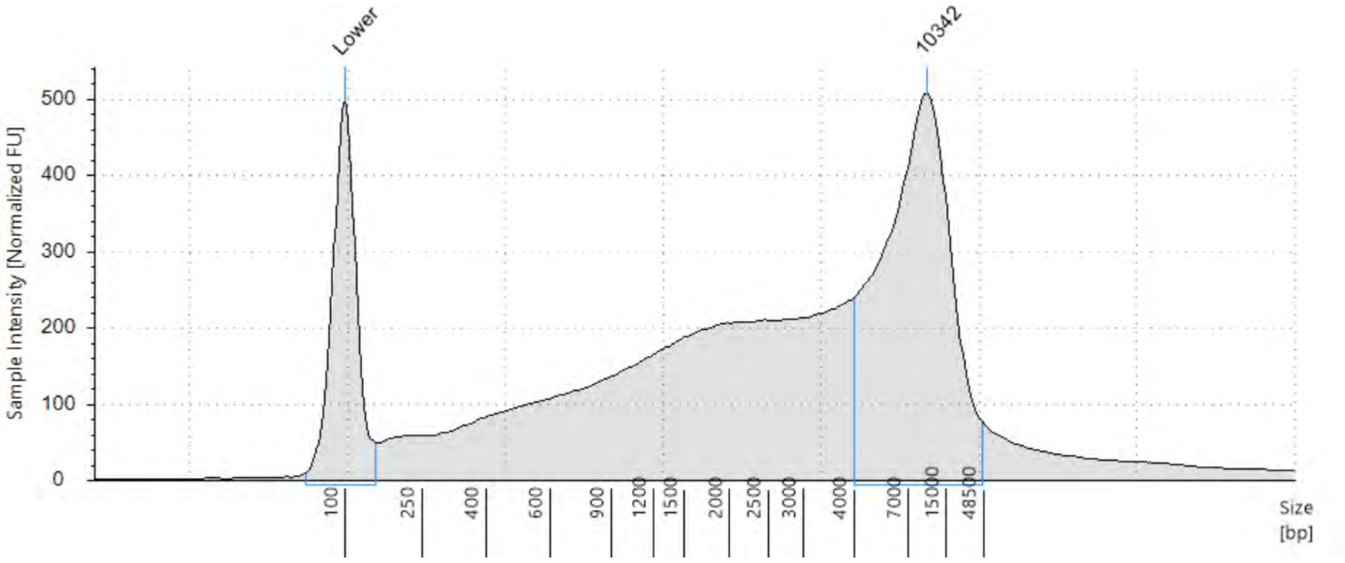

Sample Table

Well	DIN	Conc. [ng/µl]	Sample Description	Alert	Observations
E2	5.4	71.5	W-T4-EtOH-846		

Peak Table

Size [bp]	Calibrated Conc. [ng/µl]	Assigned Conc. [ng/µl]	% Integrated Area	From [bp]	To [bp]	Peak Comment	Observations
100	8.50	8.50	-	63	145		Lower Marker
10342	25.0	-	97.82	4052	47606		
-	-	-	-	-	-		Sample Well

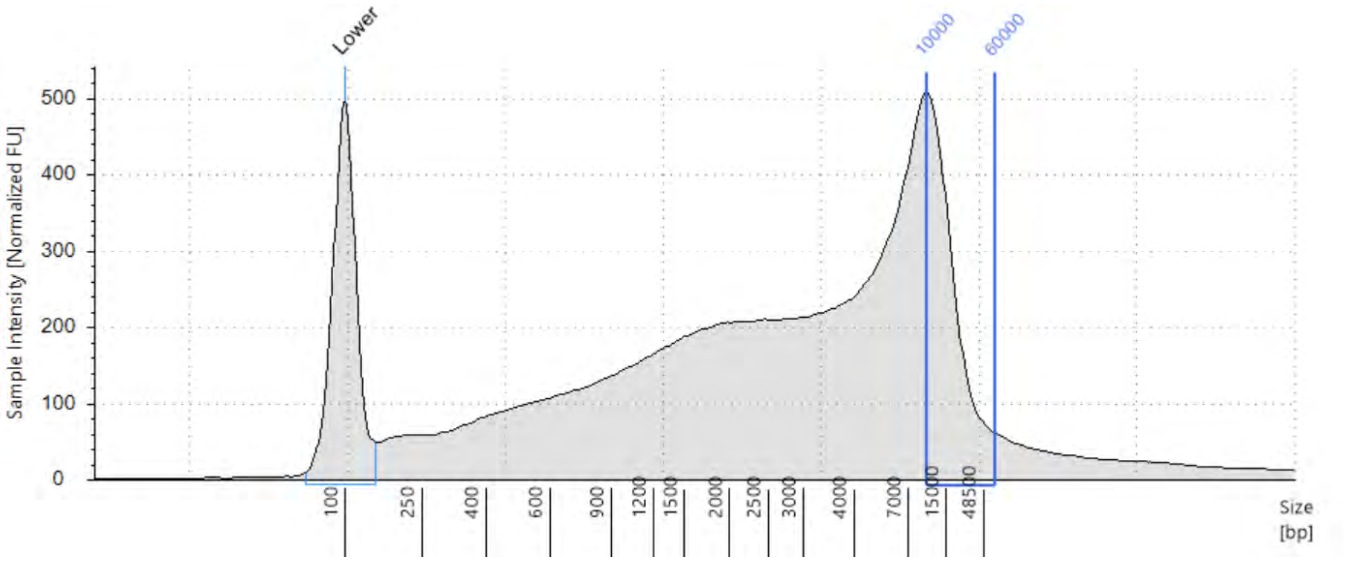

Region Table

From [bp]	To [bp]	Average Size [bp]	Conc. [ng/µl]	Region Molarity [nmol/l]	% of Total	Region Comment	Color
10000	60000	18065	10.6	1.11	14.79		

F2: W-T4-EtOH-847

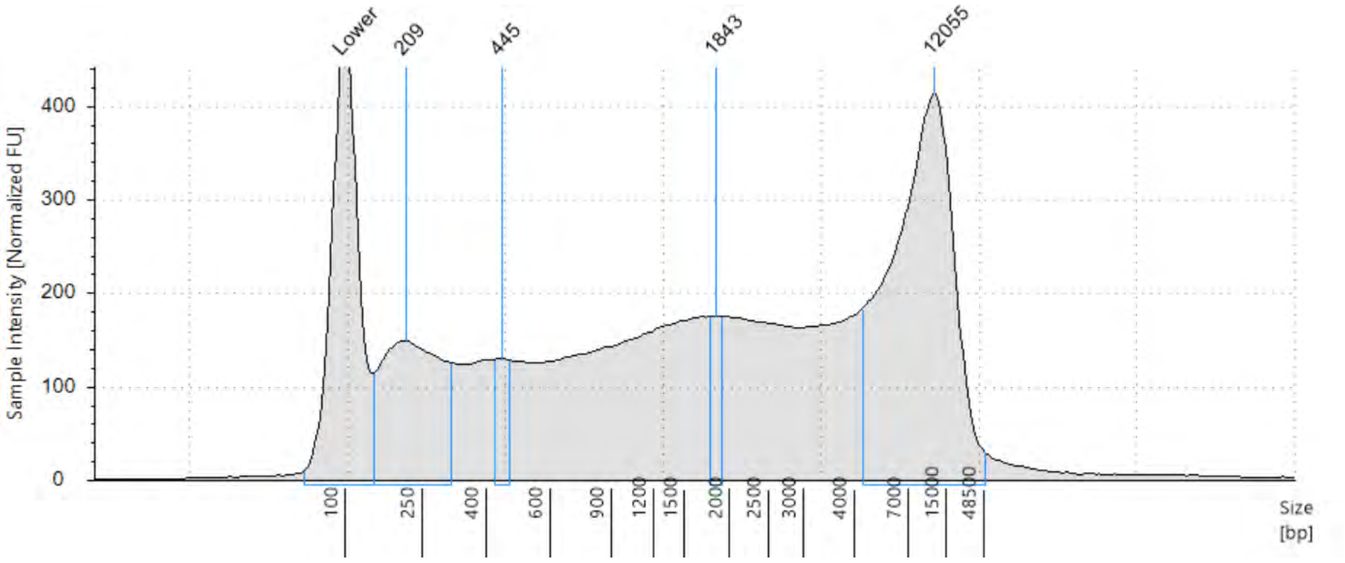

Sample Table

Well	DIN	Conc. [ng/µl]	Sample Description	Alert	Observations
F2	4.0	57.6	W-T4-EtOH-847		

Peak Table

Size [bp]	Calibrated Conc. [ng/µl]	Assigned Conc. [ng/µl]	% Integrated Area	From [bp]	To [bp]	Peak Comment	Observations
100	8.50	8.50	-	62	142		Lower Marker
209	5.84	-	23.47	142	310		
445	1.09	-	4.37	426	465		
1843	1.22	-	4.88	1779	1910		
12055	16.7	-	67.27	4404	50650		

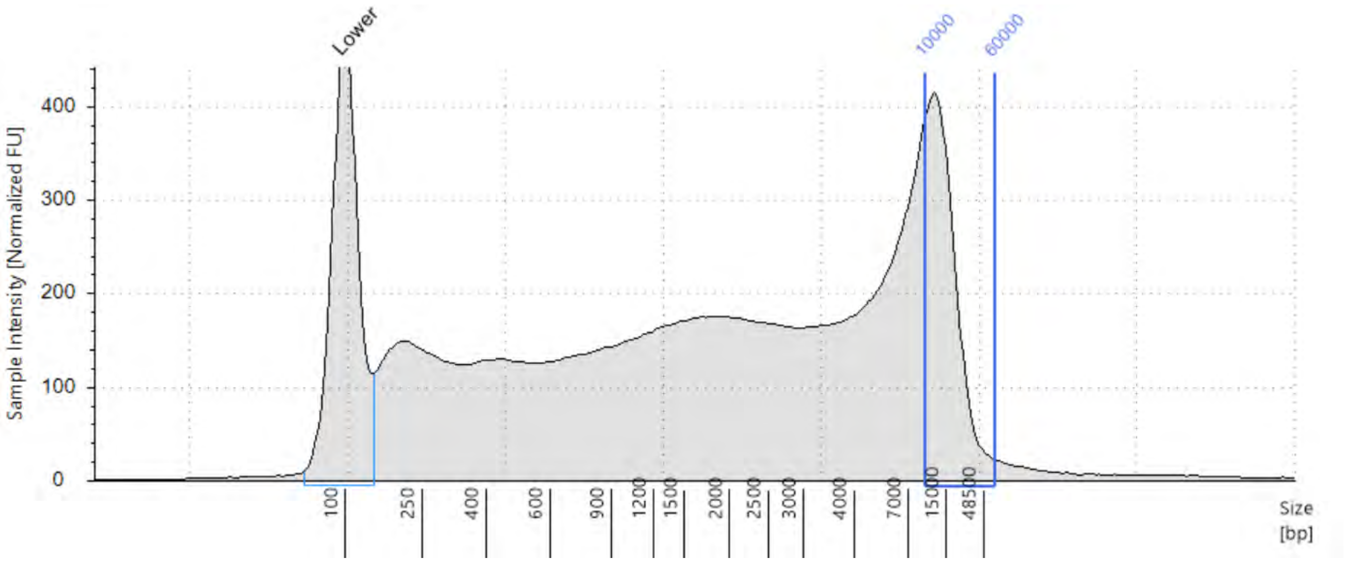

Region Table

From [bp]	To [bp]	Average Size [bp]	Conc. [ng/µl]	Region Molarity [nmol/l]	% of Total	Region Comment	Color
10000	60000	16609	7.81	0.841	13.56		

G2: W-T4-EtOH-848

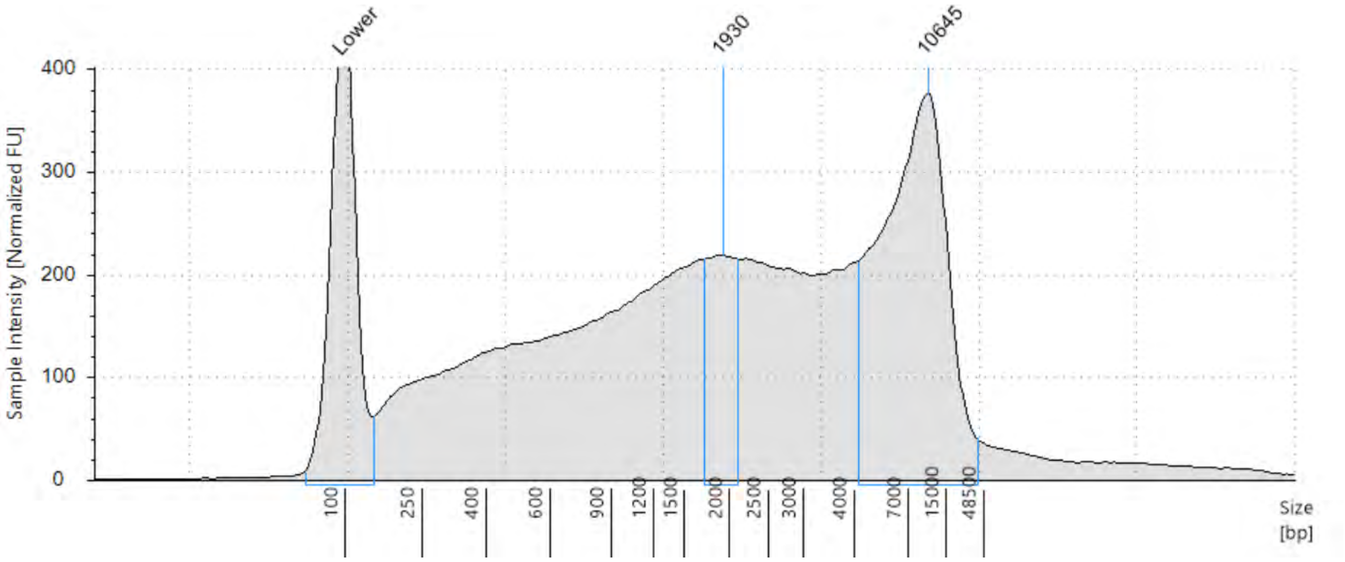

Sample Table

Well	DIN	Conc. [ng/µl]	Sample Description	Alert	Observations
G2	4.0	67.8	W-T4-EtOH-848		

Peak Table

Size [bp]	Calibrated Conc. [ng/µl]	Assigned Conc. [ng/µl]	% Integrated Area	From [bp]	To [bp]	Peak Comment	Observations
100	8.50	8.50	-	62	141		Lower Marker
1930	4.52	-	20.37	1708	2097		
10645	17.7	-	79.63	4244	31455		

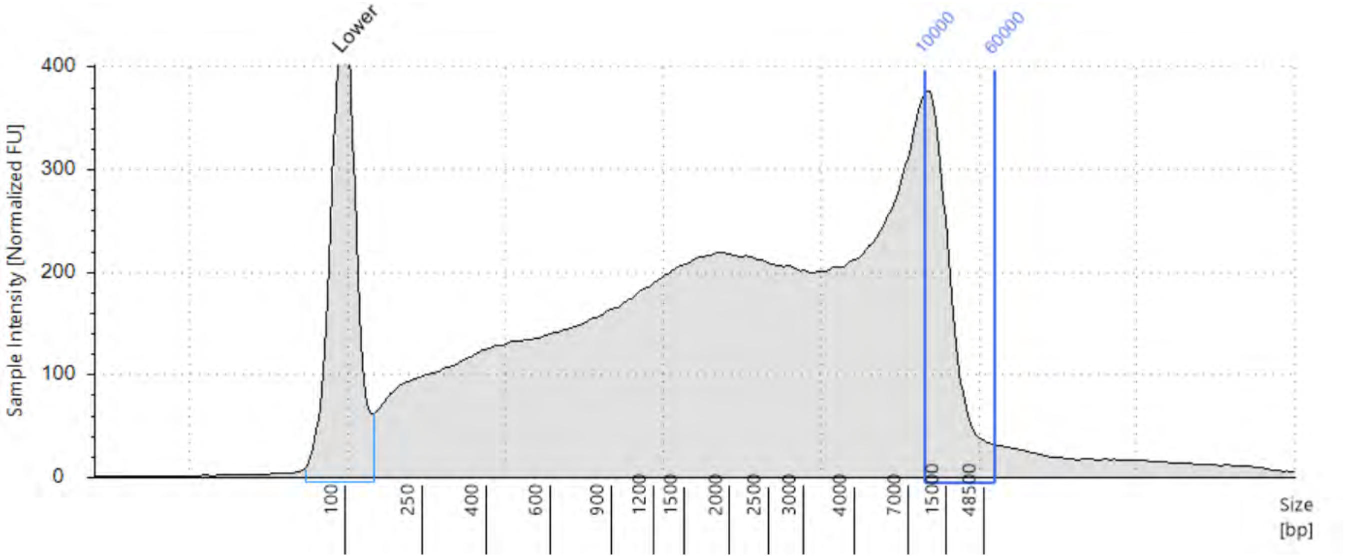

Region Table

From [bp]	To [bp]	Average Size [bp]	Conc. [ng/µl]	Region Molarity [nmol/l]	% of Total	Region Comment	Color
10000	60000	16796	6.80	0.755	10.02		

H2: W-T4-EtOH-849

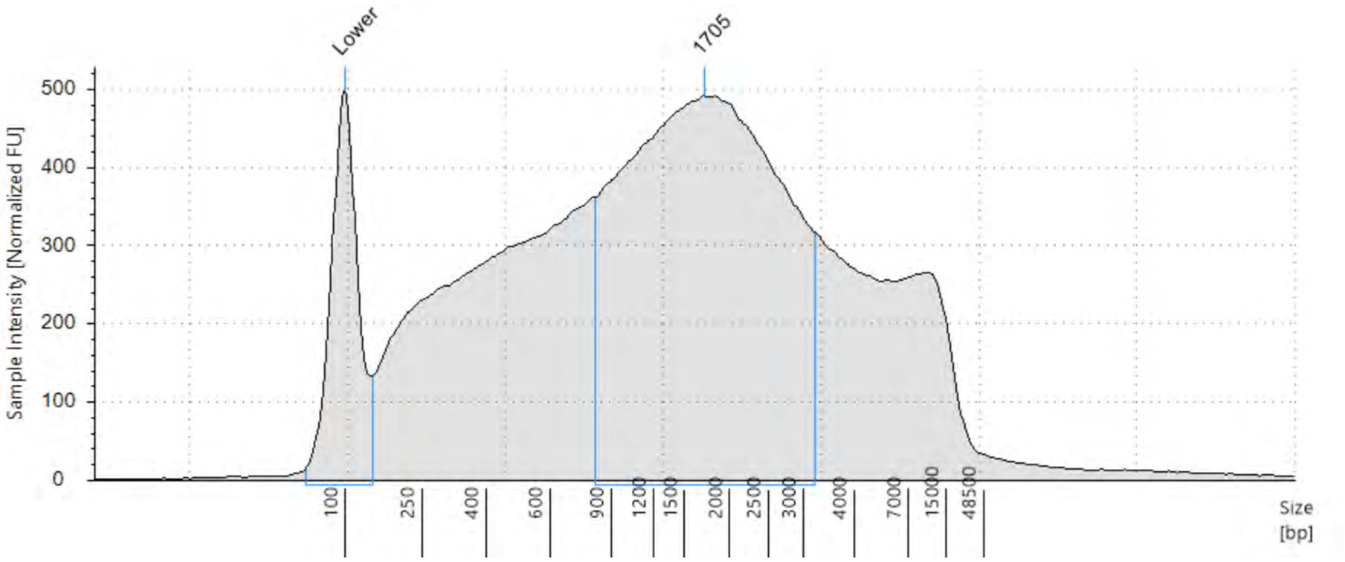

Sample Table

Well	DIN	Conc. [ng/µl]	Sample Description	Alert	Observations
H2	3.0	107	W-T4-EtOH-849	⚠	Sample concentration outside recommended range

Peak Table

Size [bp]	Calibrated Conc. [ng/µl]	Assigned Conc. [ng/µl]	% Integrated Area	From [bp]	To [bp]	Peak Comment	Observations
100	8.50	8.50	-	62	139		Lower Marker
1705	52.0	-	100.00	814	3208		

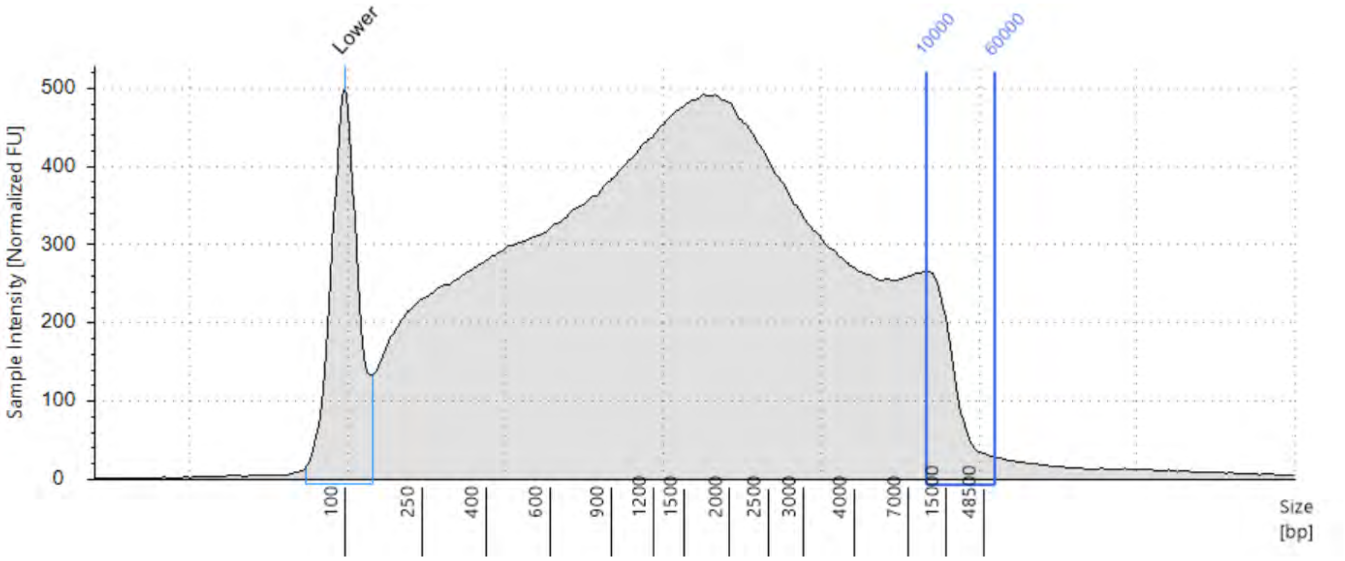

Region Table

From [bp]	To [bp]	Average Size [bp]	Conc. [ng/µl]	Region Molarity [nmol/l]	% of Total	Region Comment	Color
10000	60000	17427	4.69	0.526	4.39		■

Filename: 2019-07-30-01.gDNA

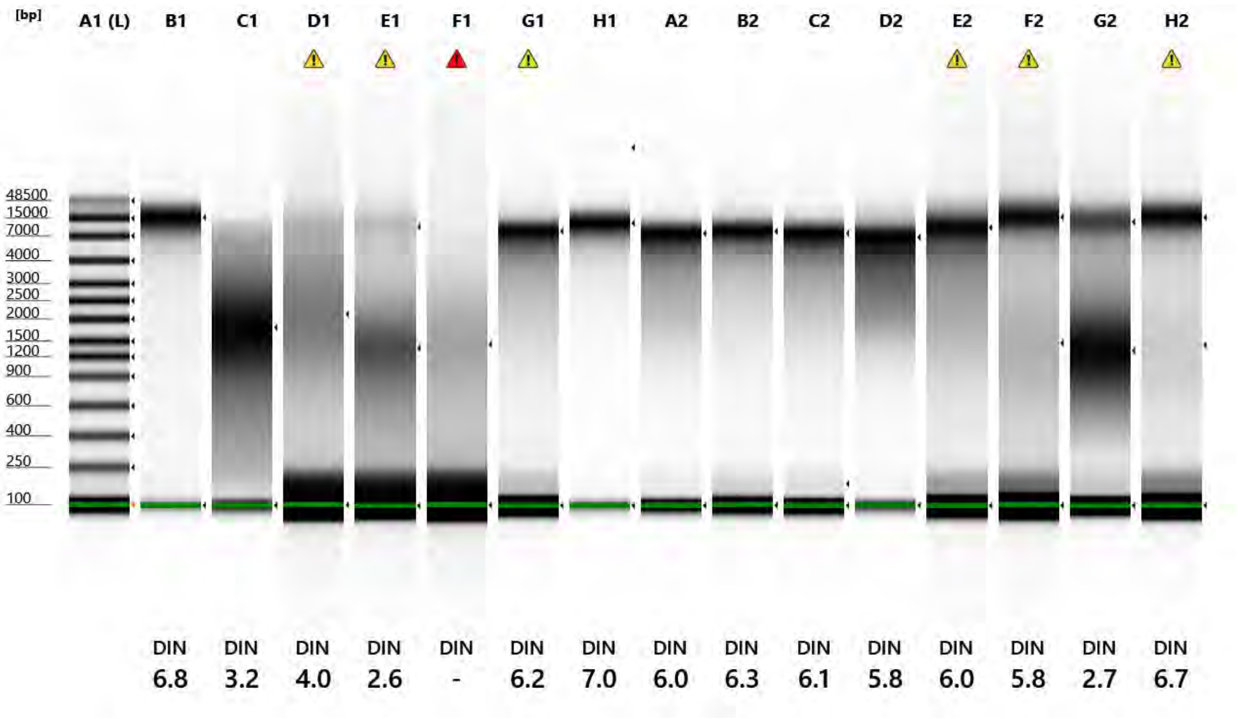

Default image (Contrast 50%), Image is Scaled to Sample

Sample Info

Well	DIN	Conc. [ng/µl]	Sample Description	Alert	Observations
A1	-	33.0	Ladder		Ladder
B1	6.8	57.9	W-T4-Initial-1080		
C1	3.2	95.8	W-T5-EtOH-894		
D1	4.0	3.46	W-T5-DES-856	⚠	Sample concentration outside functional range for DIN
E1	2.6	5.94	W-T5-DES-858	⚠	Sample concentration outside recommended range
F1	-	2.58	W-T5-DES-859	▲	Sample concentration outside functional range for DIN
G1	6.2	7.55	W-T5-DES-860	⚠	Sample concentration outside recommended range
H1	7.0	44.6	W-T5-DES-1081		
A2	6.0	25.8	W-T5-DES-1082		
B2	6.3	12.2	W-T5-DES-1083		
C2	6.1	14.9	W-T5-DES-1084		
D2	5.8	66.9	W-T5-DES-1085		
E2	6.0	8.37	W-T5-ES-871	⚠	Sample concentration outside recommended range
F2	5.8	6.39	W-T5-ES-873	⚠	Sample concentration outside recommended range
G2	2.7	21.1	W-T5-ES-874		
H2	6.7	3.65	W-T5-ES-875	⚠	Sample concentration outside functional range for DIN

A1: Ladder

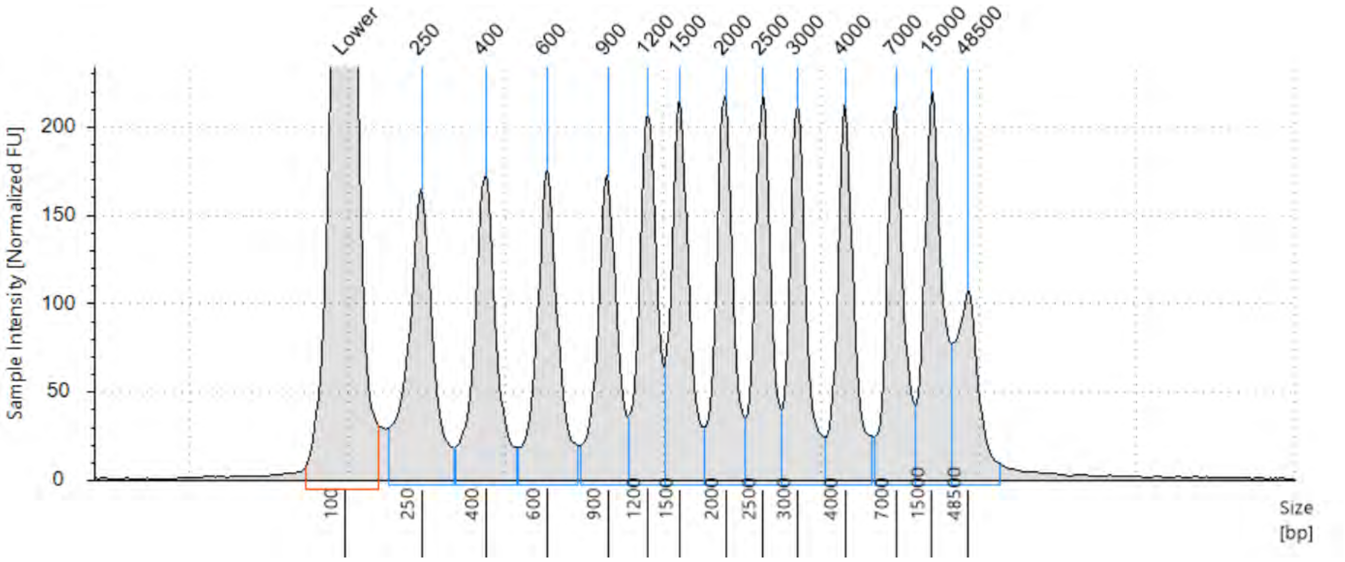

Sample Table

Well	DIN	Conc. [ng/µl]	Sample Description	Alert	Observations
A1	-	33.0	Ladder		Ladder

Peak Table

Size [bp]	Calibrated Conc. [ng/µl]	Assigned Conc. [ng/µl]	% Integrated Area	From [bp]	To [bp]	Peak Comment	Observations
100	8.50	8.50	-	63	149		Lower Marker
250	2.84	-	8.63	167	316		
400	2.65	-	8.03	319	490		
600	2.63	-	7.97	494	738		
900	2.41	-	7.30	752	1049		
1200	2.62	-	7.96	1049	1354		
1500	2.69	-	8.16	1354	1747		
2000	2.61	-	7.92	1747	2254		
2500	2.51	-	7.61	2254	2758		
3000	2.50	-	7.58	2758	3536		
4000	2.49	-	7.55	3536	5370		
7000	2.51	-	7.62	5531	10690		
15000	2.79	-	8.46	10690	22917		
48500	1.72	-	5.22	22917	>60000		

C1: W-T5-EtOH-894

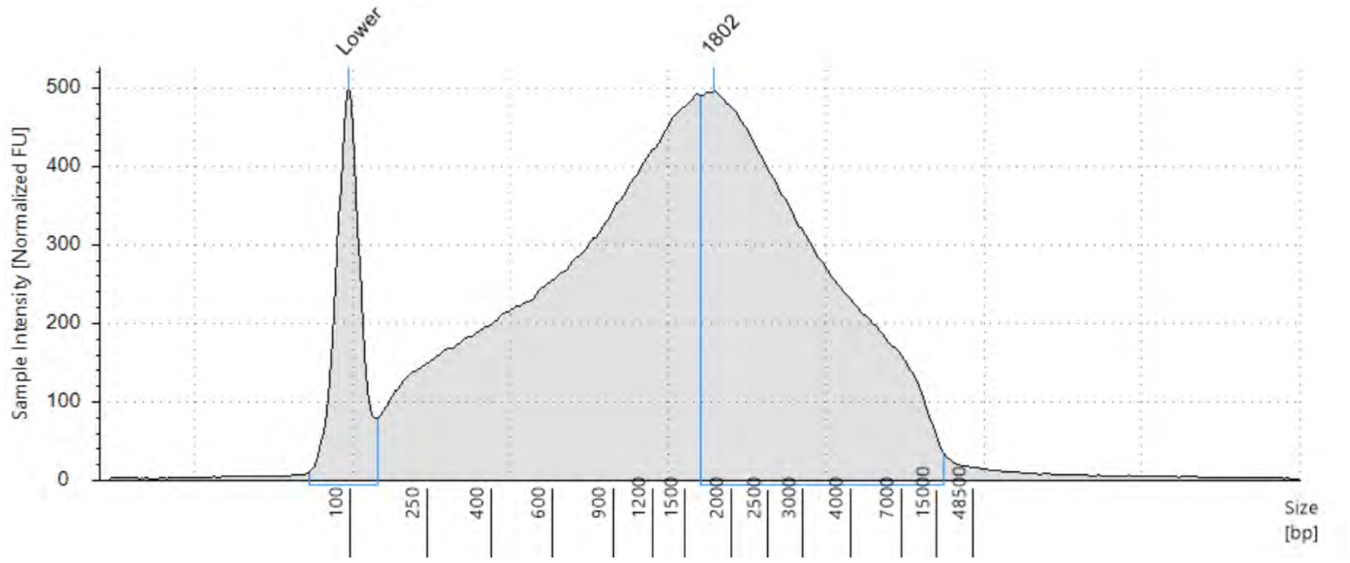

Sample Table

Well	DIN	Conc. [ng/µl]	Sample Description	Alert	Observations
C1	3.2	95.8	W-T5-EtOH-894		

Peak Table

Size [bp]	Calibrated Conc. [ng/µl]	Assigned Conc. [ng/µl]	% Integrated Area	From [bp]	To [bp]	Peak Comment	Observations
100	8.50	8.50	-	63	140		Lower Marker
1802	43.2	-	100.00	1670	17531		

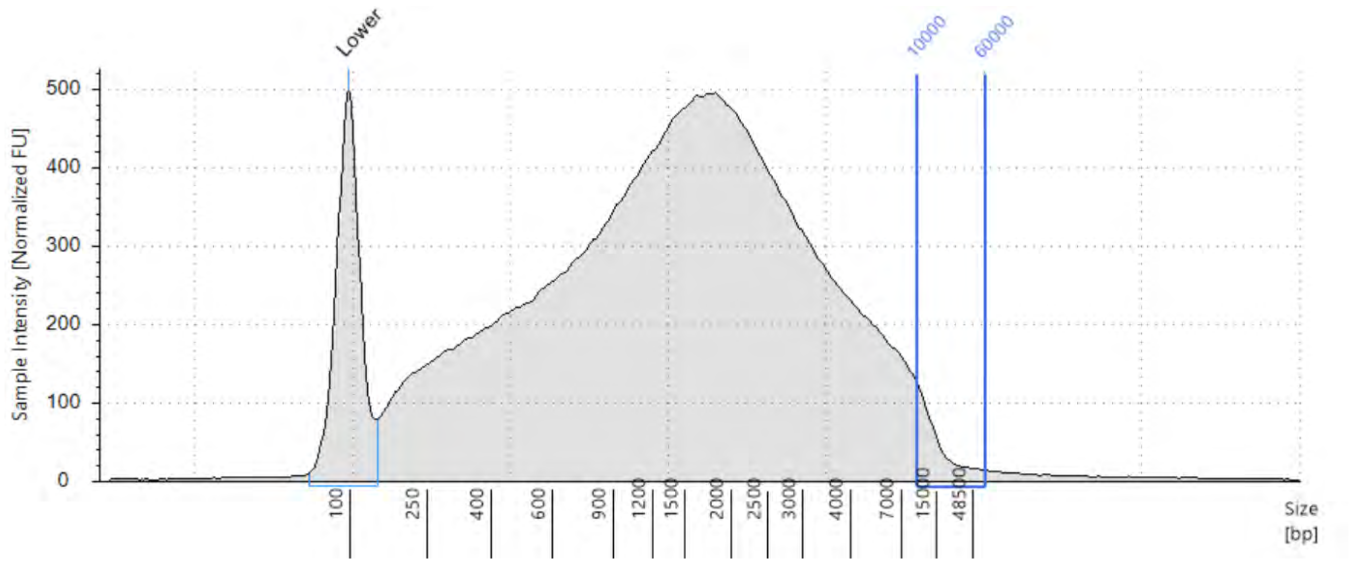

Region Table

From [bp]	To [bp]	Average Size [bp]	Conc. [ng/µl]	Region Molarity [nmol/l]	% of Total	Region Comment	Color
10000	60000	18574	1.61	0.188	1.68		

 © Agilent Technologies, Inc. 2017

D1: W-T5-DES-856

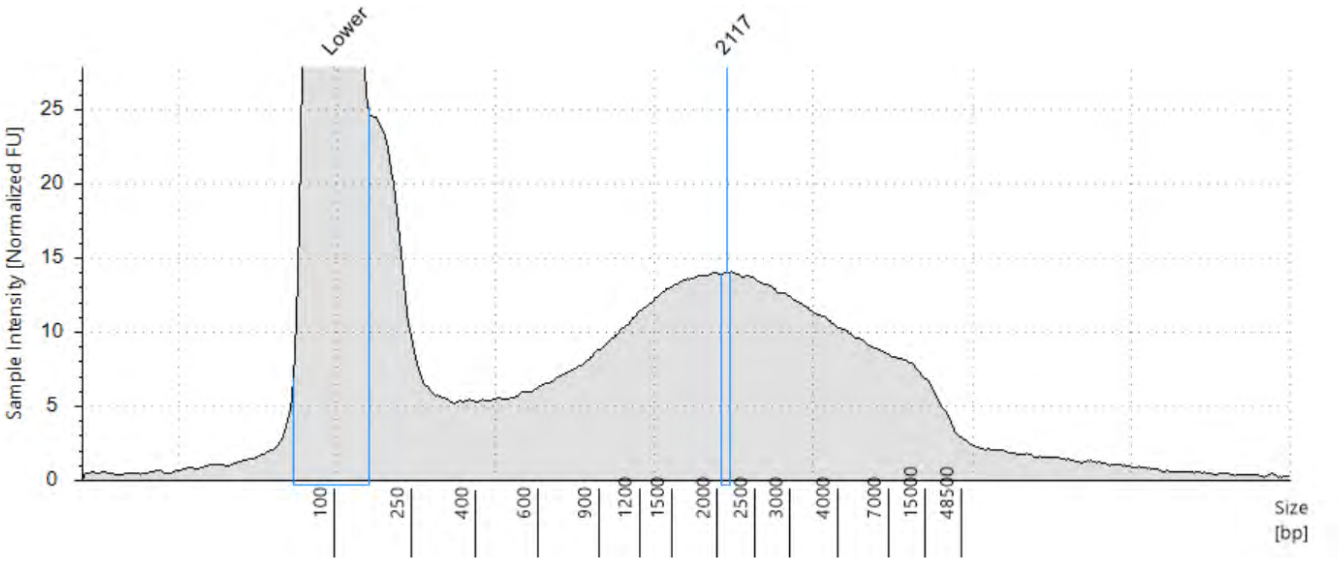

Sample Table

Well	DIN	Conc. [ng/µl]	Sample Description	Alert	Observations
D1	4.0	3.46	W-T5-DES-856	⚠	Sample concentration outside functional range for DIN

Peak Table

Size [bp]	Calibrated Conc. [ng/µl]	Assigned Conc. [ng/µl]	% Integrated Area	From [bp]	To [bp]	Peak Comment	Observations
100	8.50	8.50	-	61	150		Lower Marker
2117	0.0758	-	100.00	2050	2151		

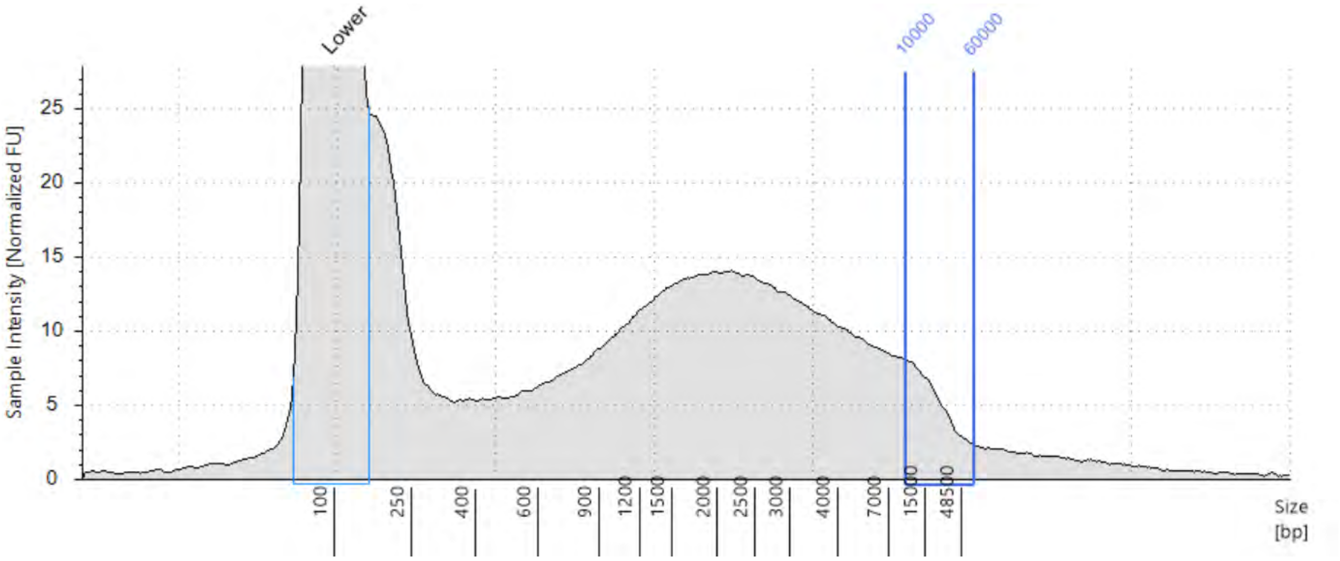

Region Table

From [bp]	To [bp]	Average Size [bp]	Conc. [ng/µl]	Region Molarity [nmol/l]	% of Total	Region Comment	Color
10000	60000	21813	0.188	0.0190	5.44		■

E1: W-T5-DES-858

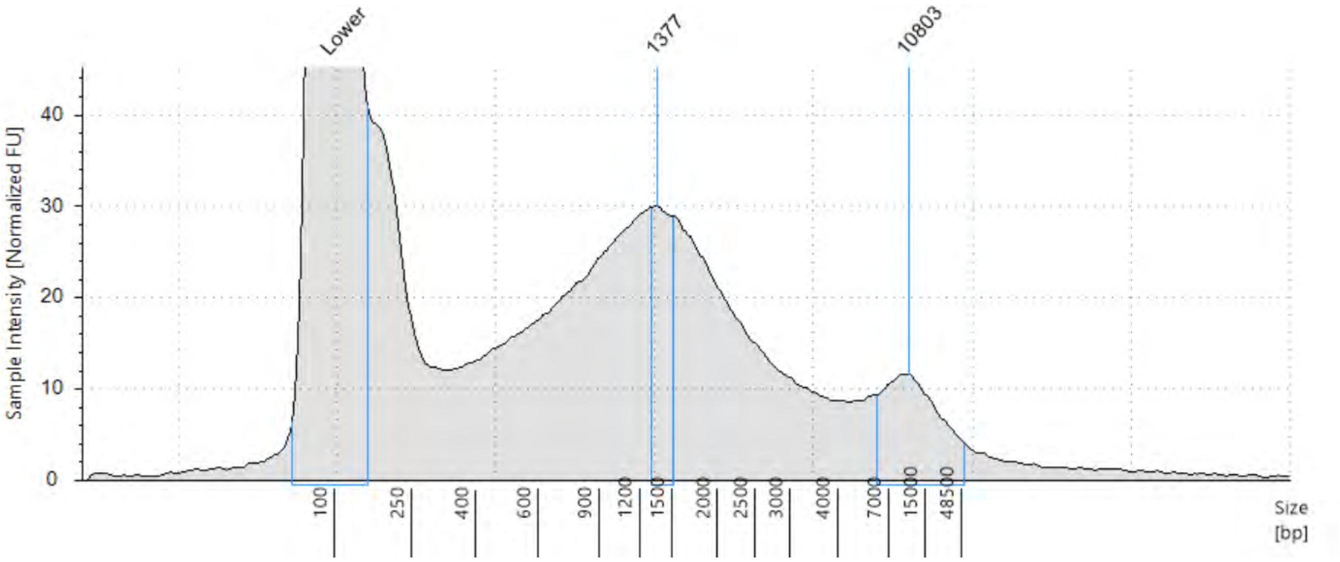

Sample Table

Well	DIN	Conc. [ng/µl]	Sample Description	Alert	Observations
E1	2.6	5.94	W-T5-DES-858	⚠	Sample concentration outside recommended range

Peak Table

Size [bp]	Calibrated Conc. [ng/µl]	Assigned Conc. [ng/µl]	% Integrated Area	From [bp]	To [bp]	Peak Comment	Observations
100	8.50	8.50	-	62	152		Lower Marker
1377	0.388	-	48.08	1314	1523		
10803	0.419	-	51.92	6249	51608		

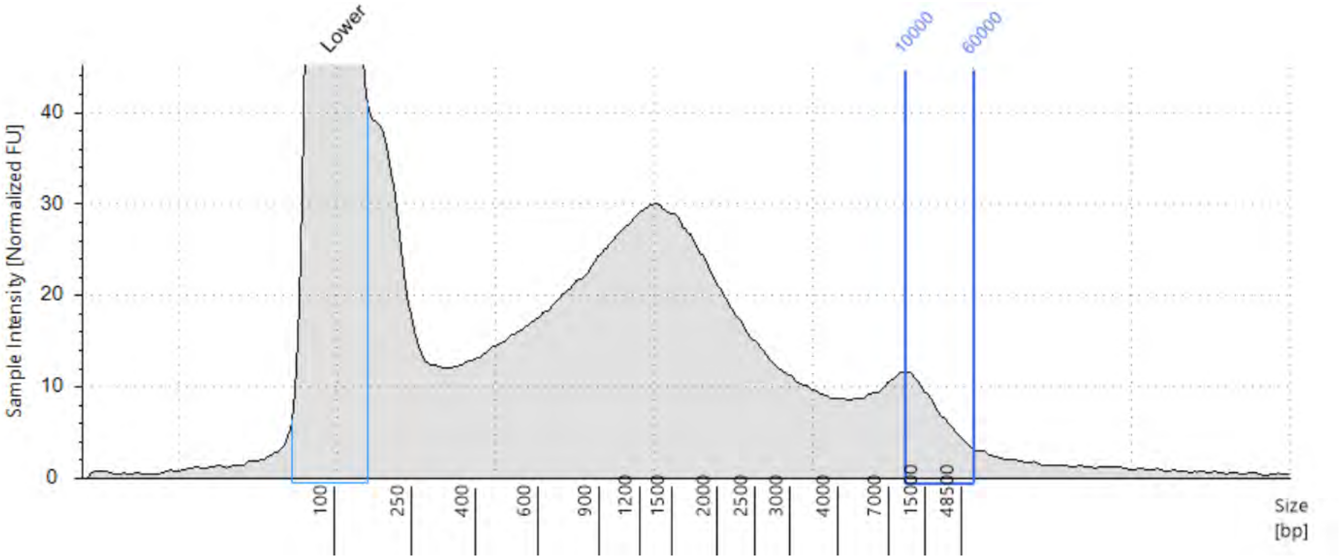

Region Table

From [bp]	To [bp]	Average Size [bp]	Conc. [ng/µl]	Region Molarity [nmol/l]	% of Total	Region Comment	Color
10000	60000	21780	0.272	0.0273	4.58		■

F1: W-T5-DES-859

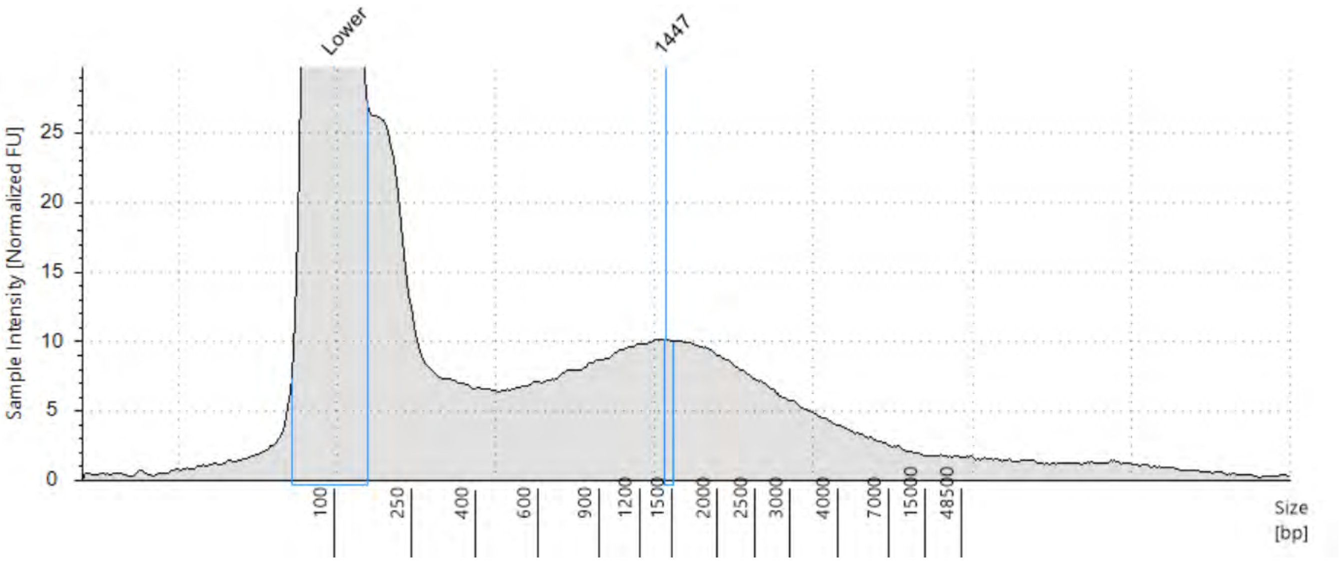

Sample Table

Well	DIN	Conc. [ng/µl]	Sample Description	Alert	Observations
F1	-	2.58	W-T5-DES-859	▲	Sample concentration outside functional range for DIN

Peak Table

Size [bp]	Calibrated Conc. [ng/µl]	Assigned Conc. [ng/µl]	% Integrated Area	From [bp]	To [bp]	Peak Comment	Observations
100	8.50	8.50	-	60	150		Lower Marker
1447	0.0522	-	100.00	1433	1515		

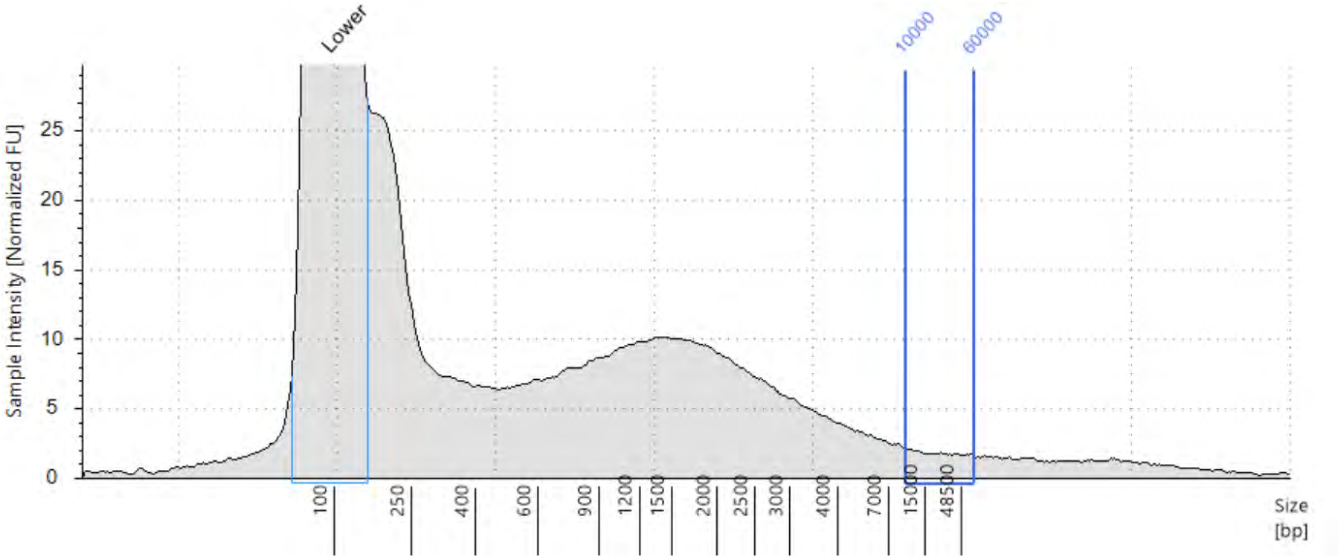

Region Table

From [bp]	To [bp]	Average Size [bp]	Conc. [ng/µl]	Region Molarity [nmol/l]	% of Total	Region Comment	Color
10000	60000	26325	0.0498	0.00580	1.93		■

 © Agilent Technologies, Inc. 2017

G1: W-T5-DES-860

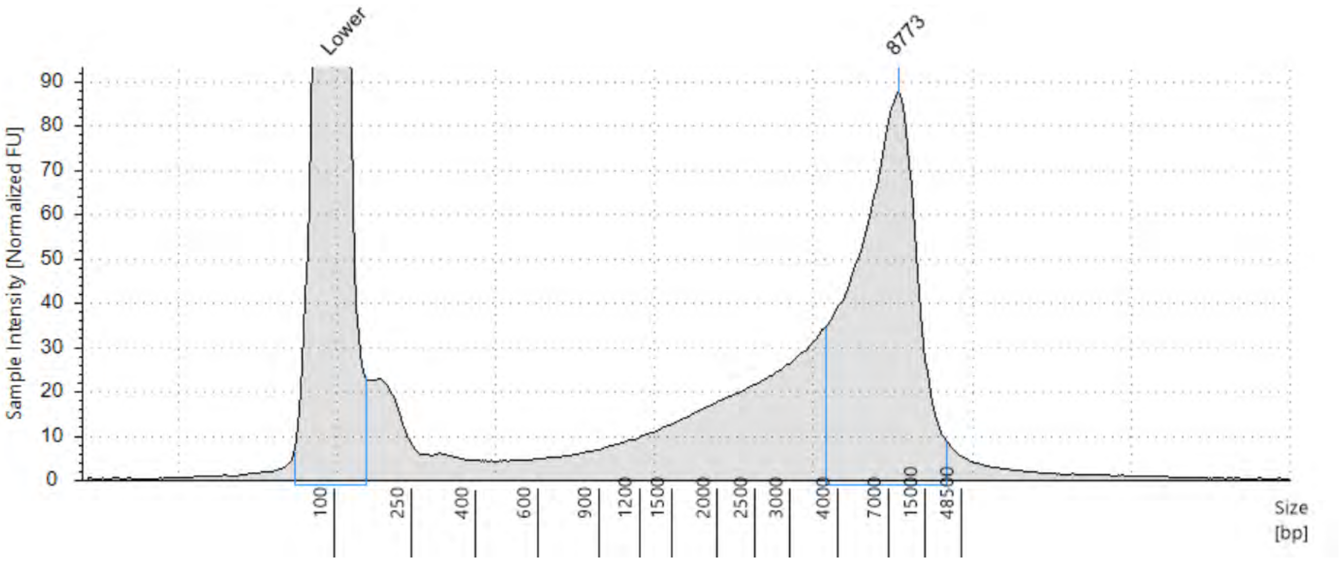

Sample Table

Well	DIN	Conc. [ng/µl]	Sample Description	Alert	Observations
G1	6.2	7.55	W-T5-DES-860	⚠	Sample concentration outside recommended range

Peak Table

Size [bp]	Calibrated Conc. [ng/µl]	Assigned Conc. [ng/µl]	% Integrated Area	From [bp]	To [bp]	Peak Comment	Observations
100	8.50	8.50	-	63	147		Lower Marker
8773	3.87	-	100.00	3745	24284		

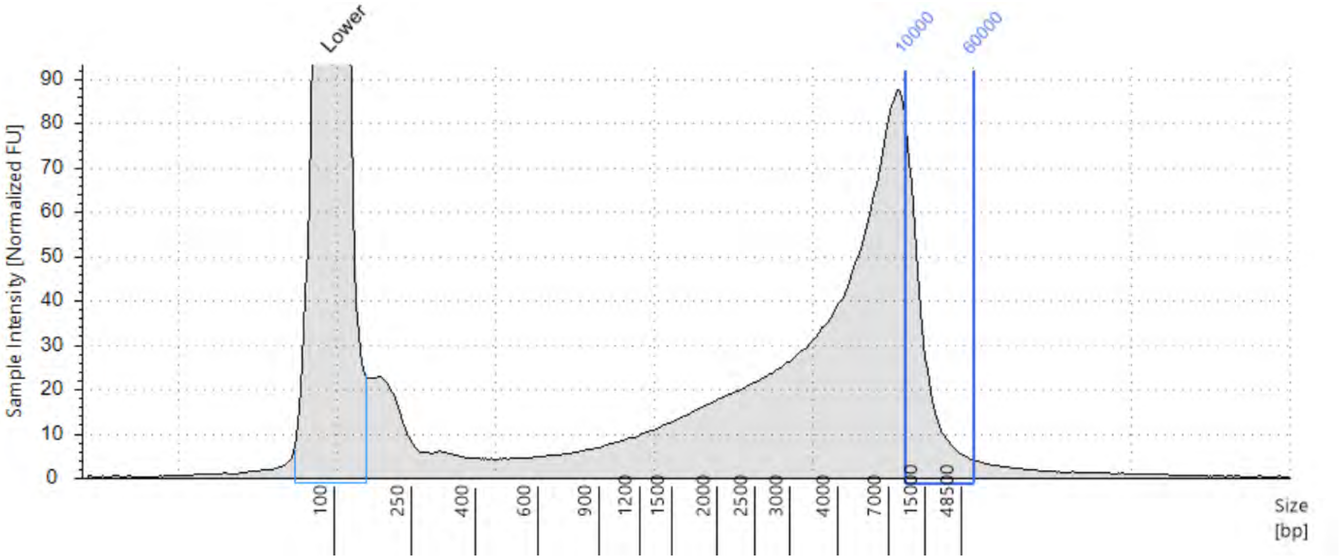

Region Table

From [bp]	To [bp]	Average Size [bp]	Conc. [ng/µl]	Region Molarity [nmol/l]	% of Total	Region Comment	Color
10000	60000	16499	1.02	0.119	13.57		■

 © Agilent Technologies, Inc. 2017

H1: W-T5-DES-1081

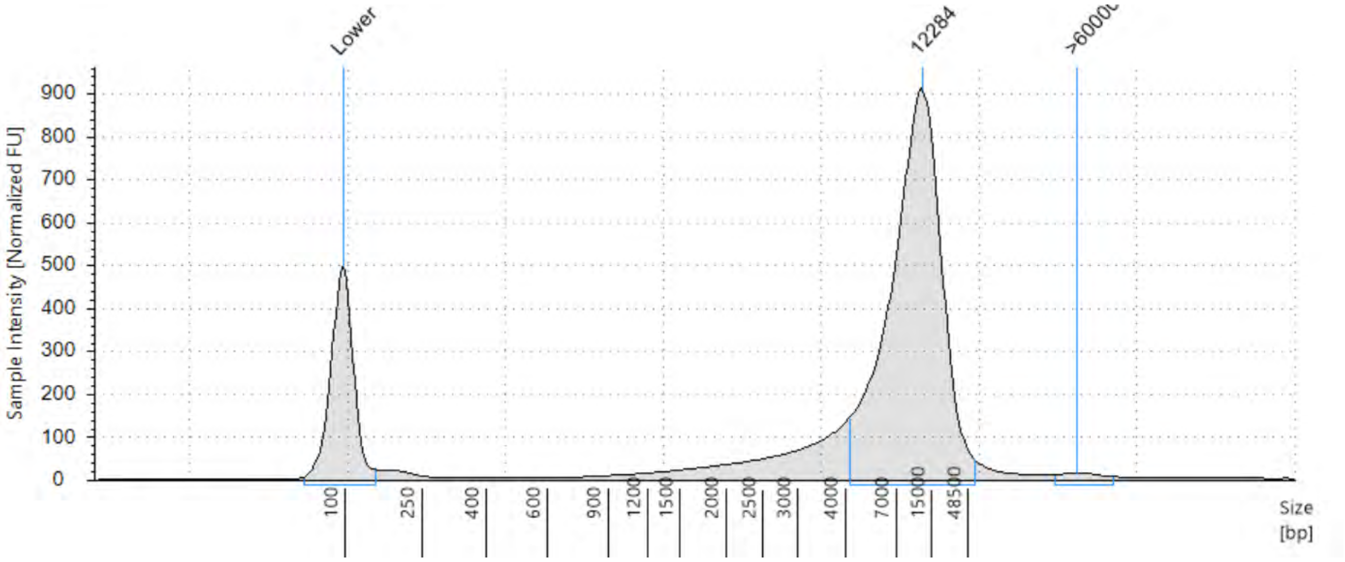

Sample Table

Well	DIN	Conc. [ng/µl]	Sample Description	Alert	Observations
H1	7.0	44.6	W-T5-DES-1081		

Peak Table

Size [bp]	Calibrated Conc. [ng/µl]	Assigned Conc. [ng/µl]	% Integrated Area	From [bp]	To [bp]	Peak Comment	Observations
100	8.50	8.50	-	63	147		Lower Marker
12284	35.0	-	97.55	4231	54714		
>60000	0.423	-	1.18	>60000	>60000		
-	-	-	-	-	-		Sample Well

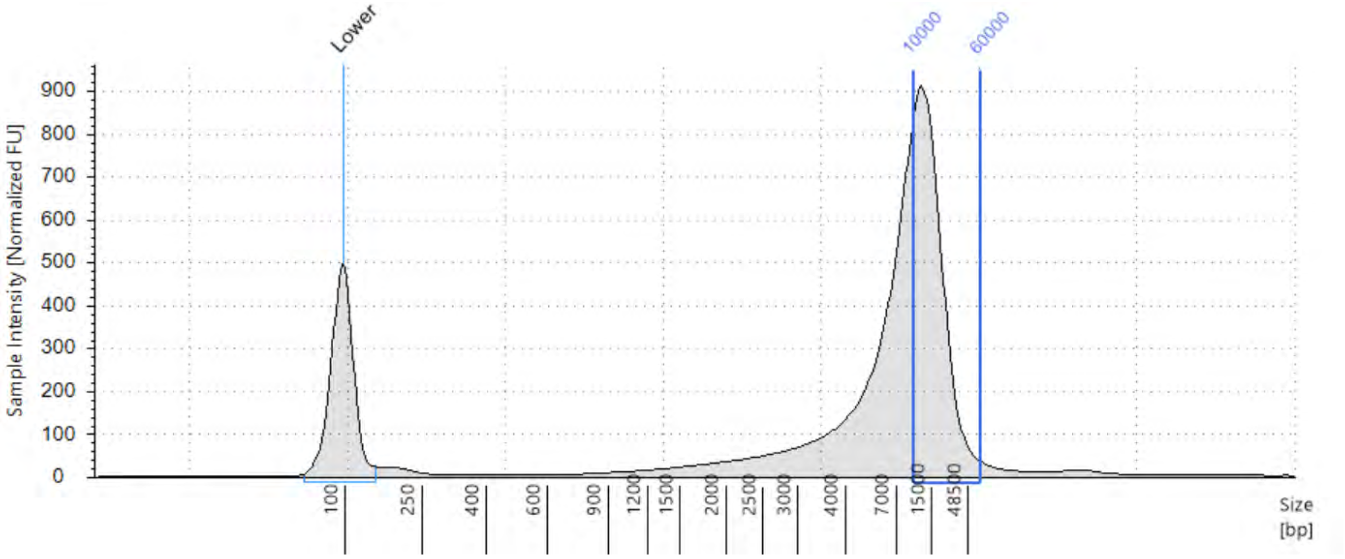

Region Table

From [bp]	To [bp]	Average Size [bp]	Conc. [ng/µl]	Region Molarity [nmol/l]	% of Total	Region Comment	Color
10000	60000	16628	20.0	2.12	44.96		

 © Agilent Technologies, Inc. 2017

A2: W-T5-DES-1082

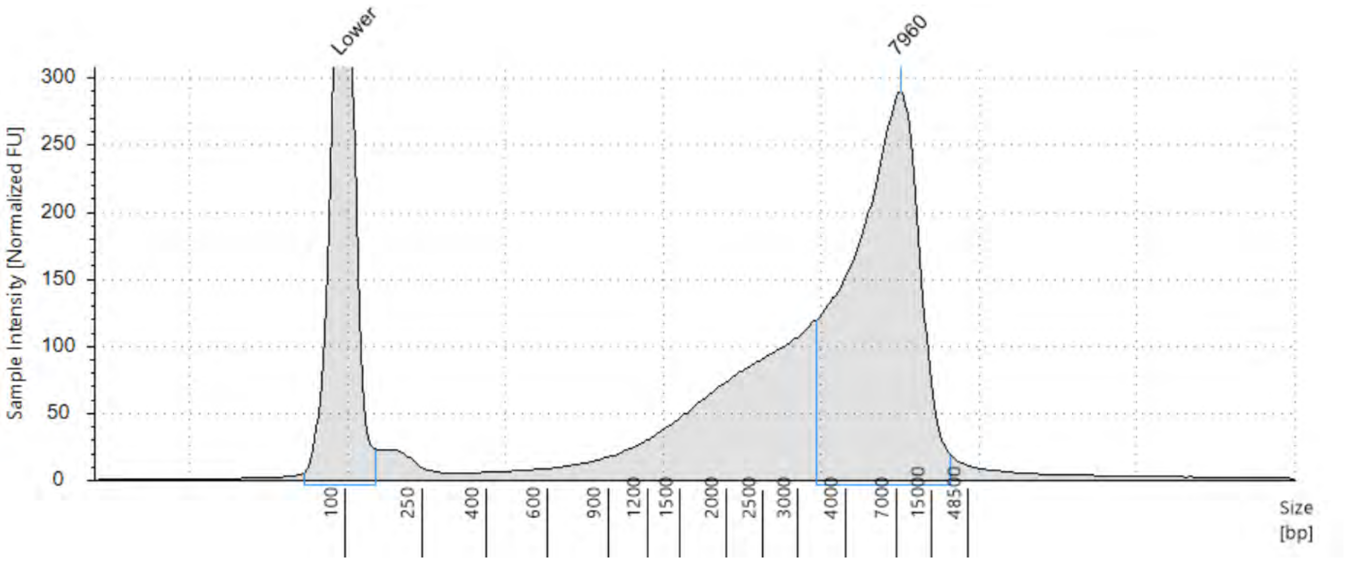

Sample Table

Well	DIN	Conc. [ng/µl]	Sample Description	Alert	Observations
A2	6.0	25.8	W-T5-DES-1082		

Peak Table

Size [bp]	Calibrated Conc. [ng/µl]	Assigned Conc. [ng/µl]	% Integrated Area	From [bp]	To [bp]	Peak Comment	Observations
100	8.50	8.50	-	63	147		Lower Marker
7960	15.1	-	100.00	3383	22602		

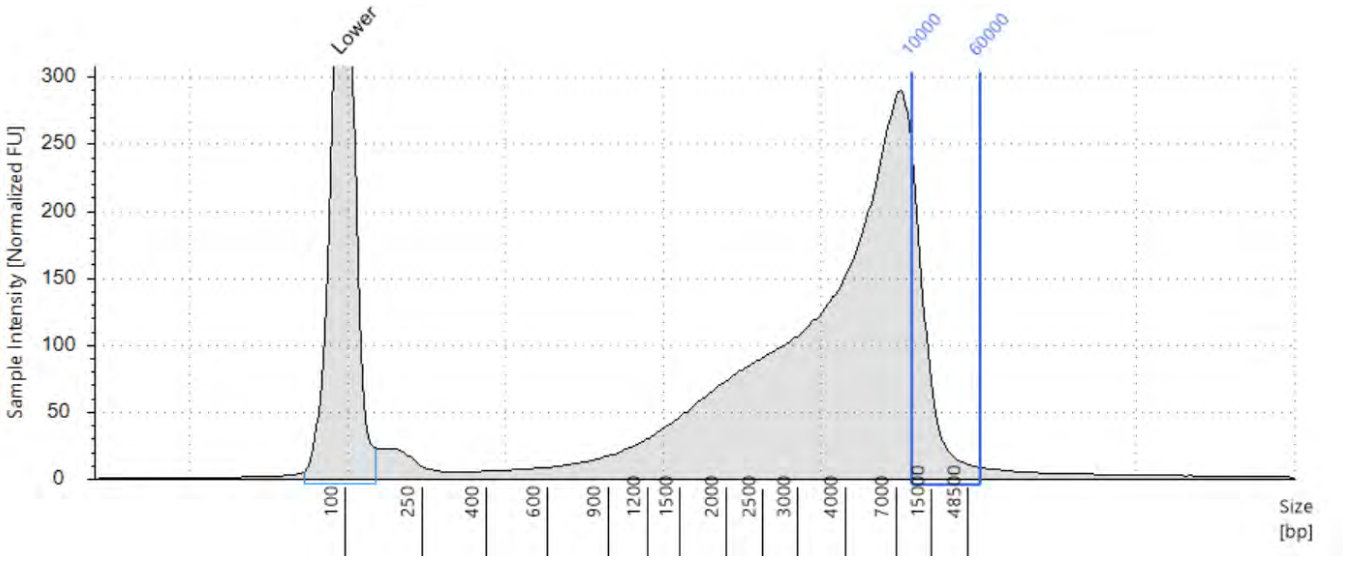

Region Table

From [bp]	To [bp]	Average Size [bp]	Conc. [ng/µl]	Region Molarity [nmol/l]	% of Total	Region Comment	Color
10000	60000	15742	2.64	0.310	10.22		

 © Agilent Technologies, Inc. 2017

B2: W-T5-DES-1083

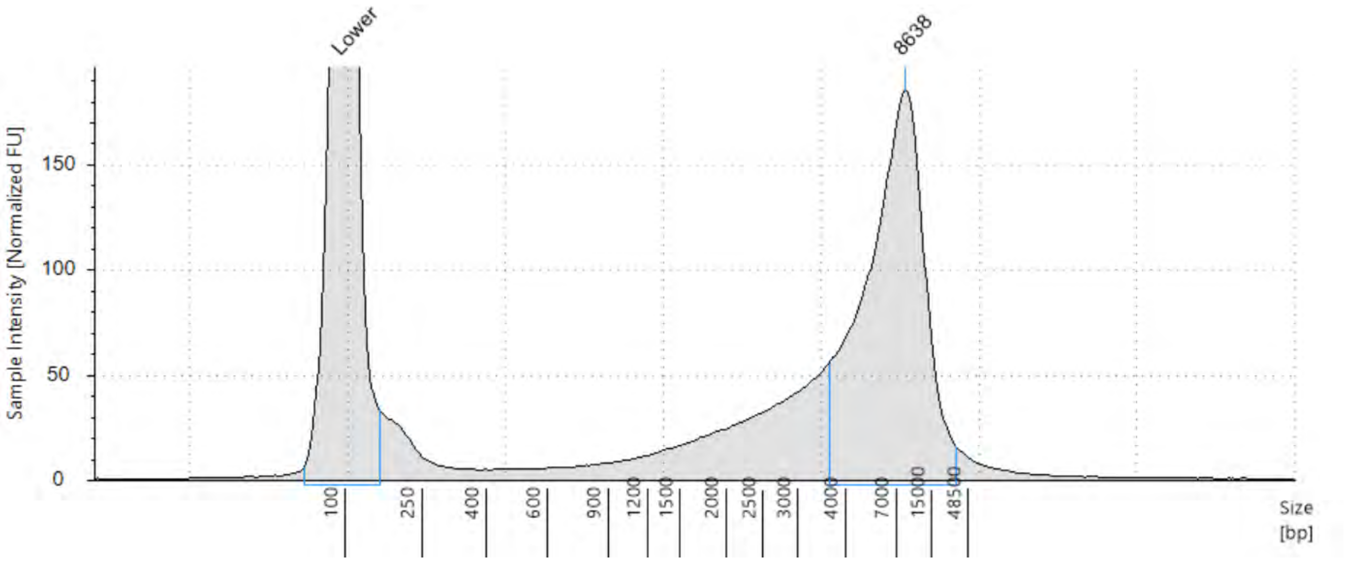

Sample Table

Well	DIN	Conc. [ng/µl]	Sample Description	Alert	Observations
B2	6.3	12.2	W-T5-DES-1083		

Peak Table

Size [bp]	Calibrated Conc. [ng/µl]	Assigned Conc. [ng/µl]	% Integrated Area	From [bp]	To [bp]	Peak Comment	Observations
100	8.50	8.50	-	62	152		Lower Marker
8638	7.49	-	100.00	3636	25414		

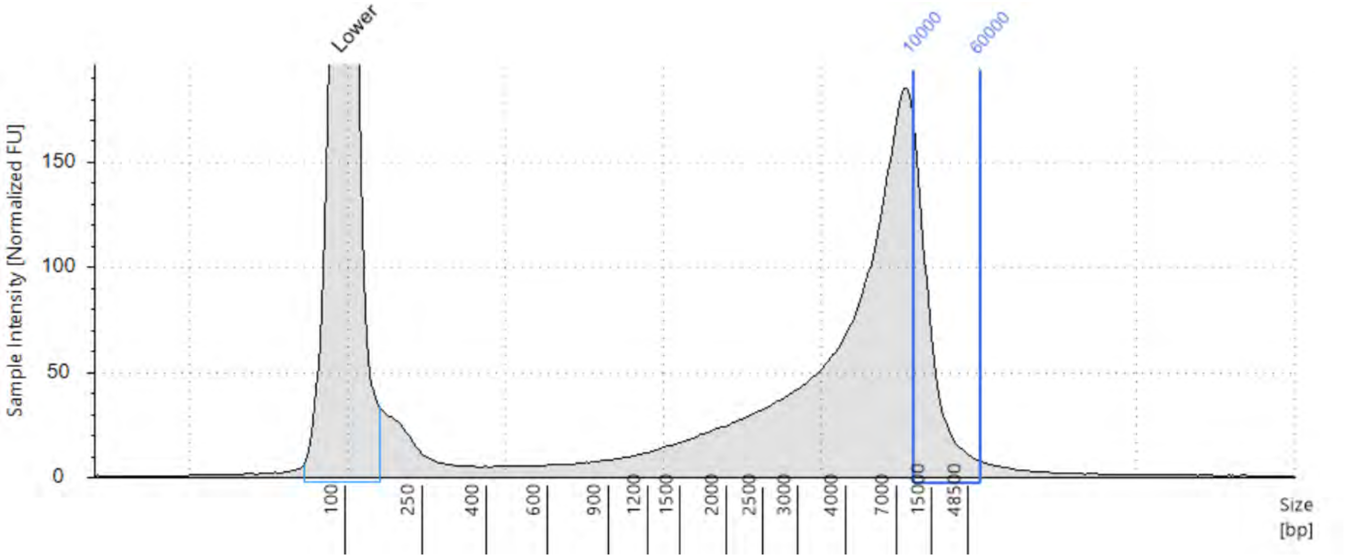

Region Table

From [bp]	To [bp]	Average Size [bp]	Conc. [ng/µl]	Region Molarity [nmol/l]	% of Total	Region Comment	Color
10000	60000	16374	2.00	0.227	16.44		■

C2: W-T5-DES-1084

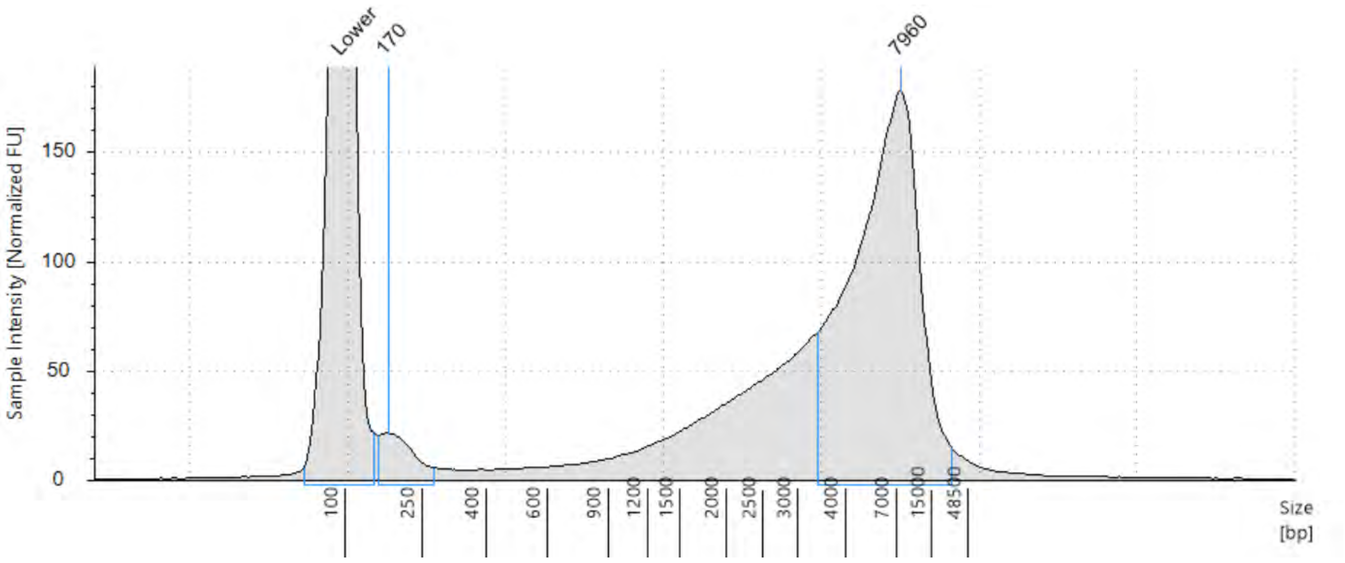

Sample Table

Well	DIN	Conc. [ng/µl]	Sample Description	Alert	Observations
C2	6.1	14.9	W-T5-DES-1084		

Peak Table

Size [bp]	Calibrated Conc. [ng/µl]	Assigned Conc. [ng/µl]	%Integrated Area	From [bp]	To [bp]	Peak Comment	Observations
100	8.50	8.50	-	62	145		Lower Marker
170	0.525	-	5.52	152	276		
7960	8.98	-	94.48	3411	23256		

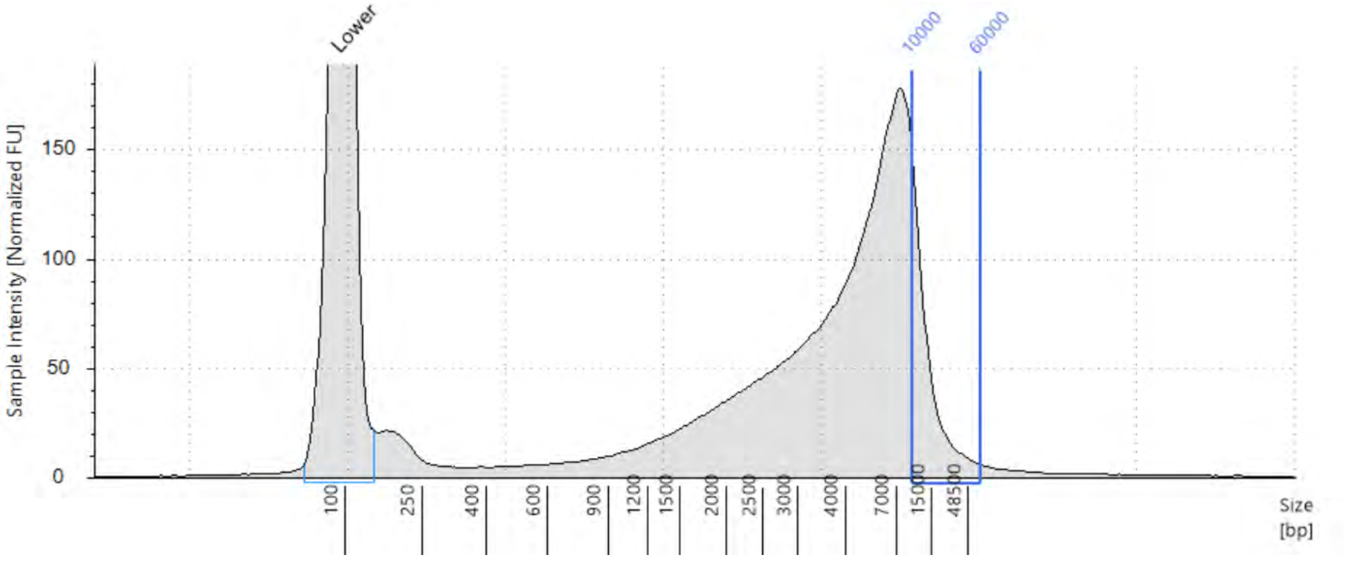

Region Table

From [bp]	To [bp]	Average Size [bp]	Conc. [ng/µl]	Region Molarity [nmol/l]	% of Total	Region Comment	Color
10000	60000	16497	1.67	0.192	11.24		

D2: W-T5-DES-1085

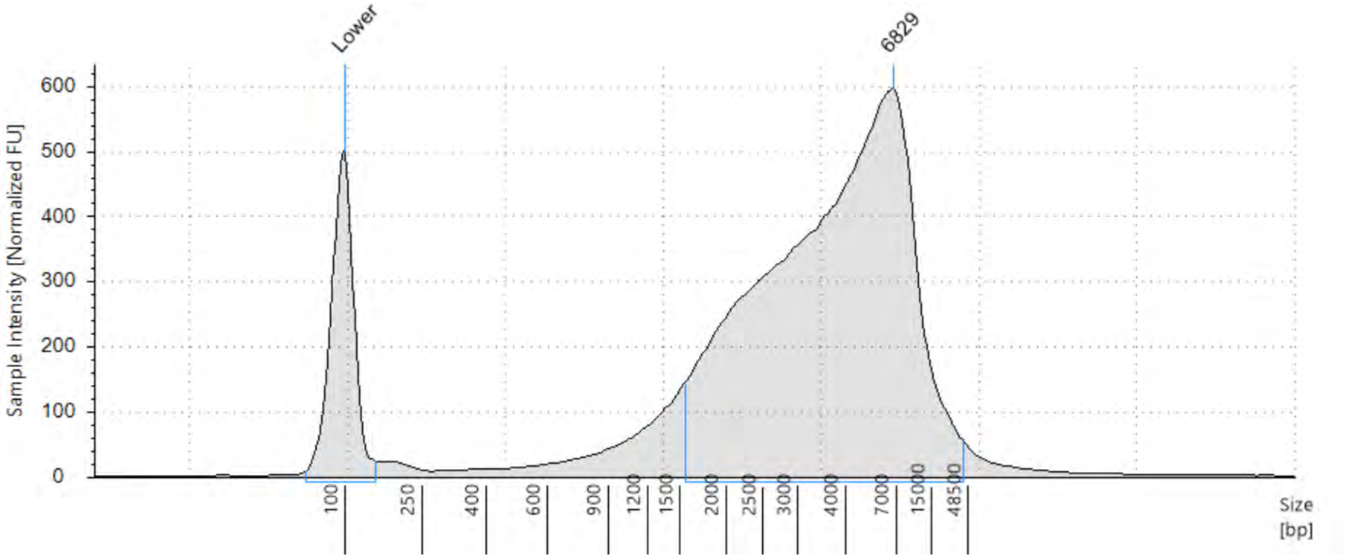

Sample Table

Well	DIN	Conc. [ng/µl]	Sample Description	Alert	Observations
D2	5.8	66.9	W-T5-DES-1085		

Peak Table

Size [bp]	Calibrated Conc. [ng/µl]	Assigned Conc. [ng/µl]	%Integrated Area	From [bp]	To [bp]	Peak Comment	Observations
100	8.50	8.50	-	63	145		Lower Marker
6829	59.8	-	100.00	1549	31927		

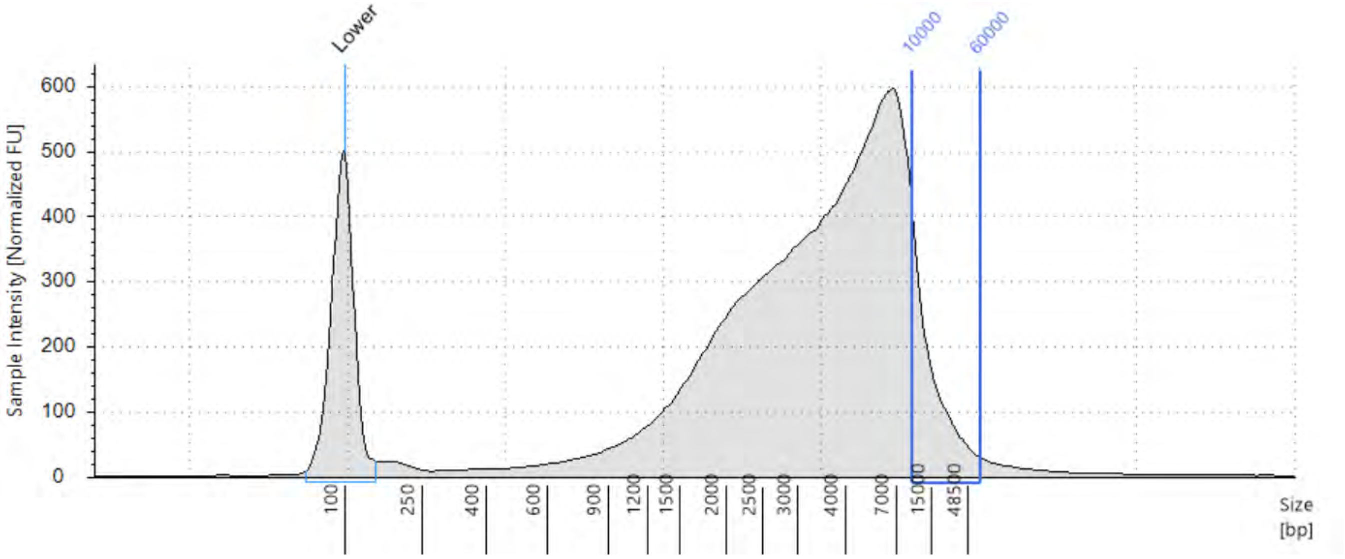

Region Table

From [bp]	To [bp]	Average Size [bp]	Conc. [ng/µl]	Region Molarity [nmol/l]	% of Total	Region Comment	Color
10000	60000	18458	5.79	0.616	8.64		

© Agilent Technologies, Inc. 2017

E2: W-T5-ES-871

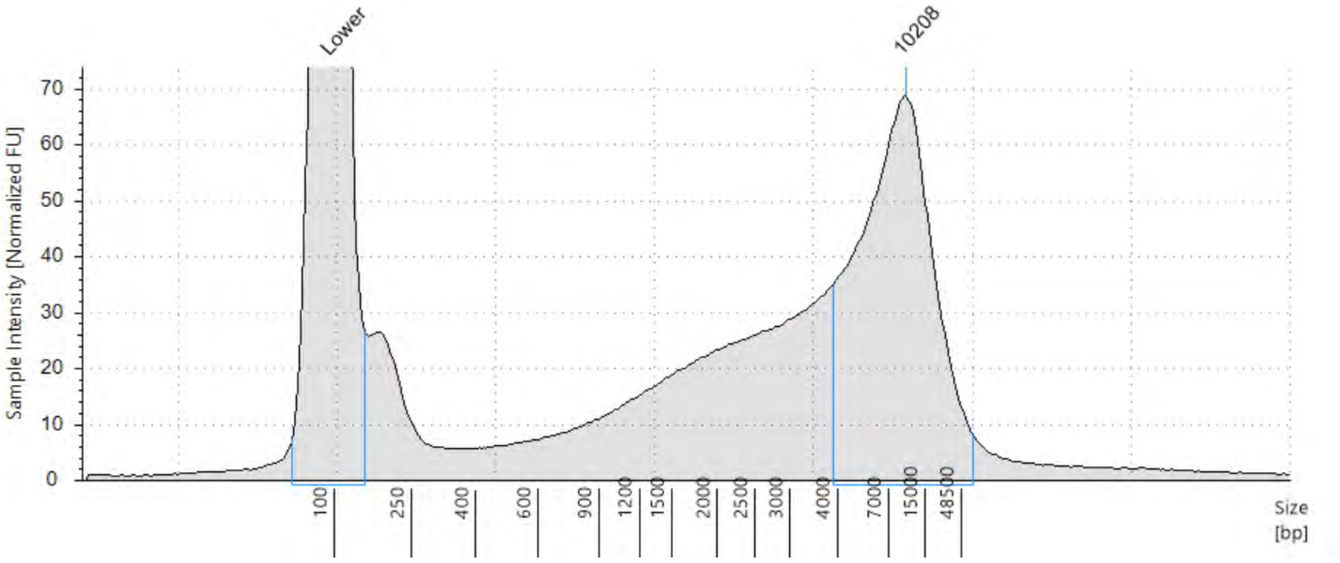

Sample Table

Well	DIN	Conc. [ng/µl]	Sample Description	Alert	Observations
E2	6.0	8.37	W-T5-ES-871	⚠	Sample concentration outside recommended range

Peak Table

Size [bp]	Calibrated Conc. [ng/µl]	Assigned Conc. [ng/µl]	% Integrated Area	From [bp]	To [bp]	Peak Comment	Observations
100	8.50	8.50	-	62	147		Lower Marker
10208	3.60	-	100.00	3941	>60000		

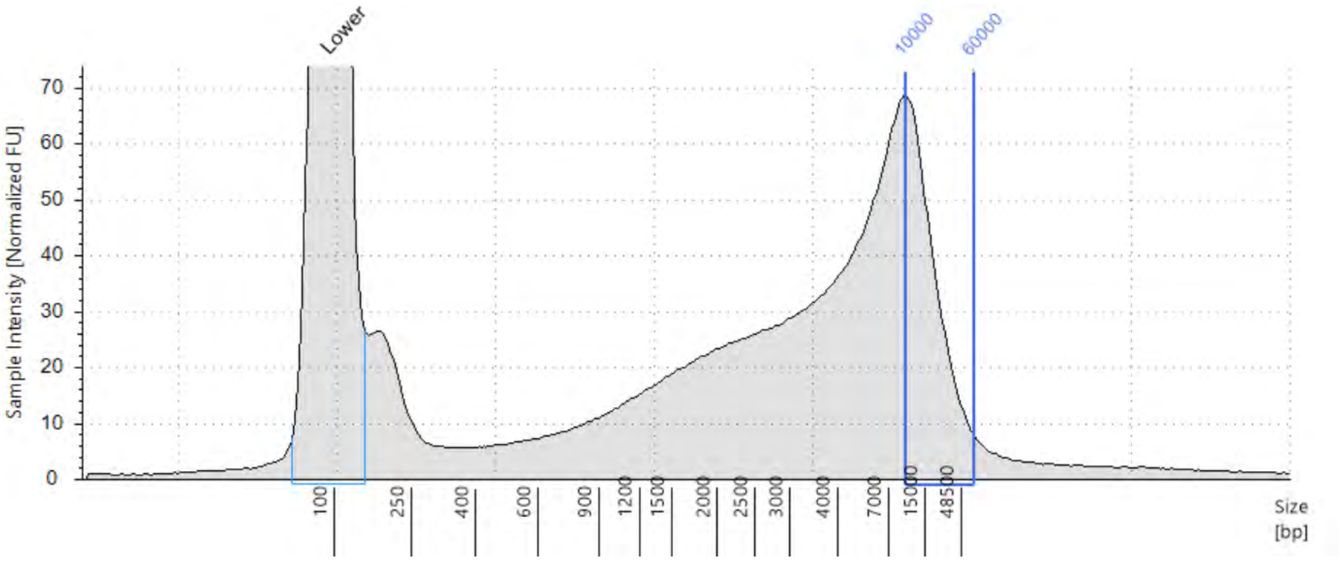

Region Table

From [bp]	To [bp]	Average Size [bp]	Conc. [ng/µl]	Region Molarity [nmol/l]	% of Total	Region Comment	Color
10000	60000	19145	1.48	0.149	17.67		■

 © Agilent Technologies, Inc. 2017

F2: W-T5-ES-873

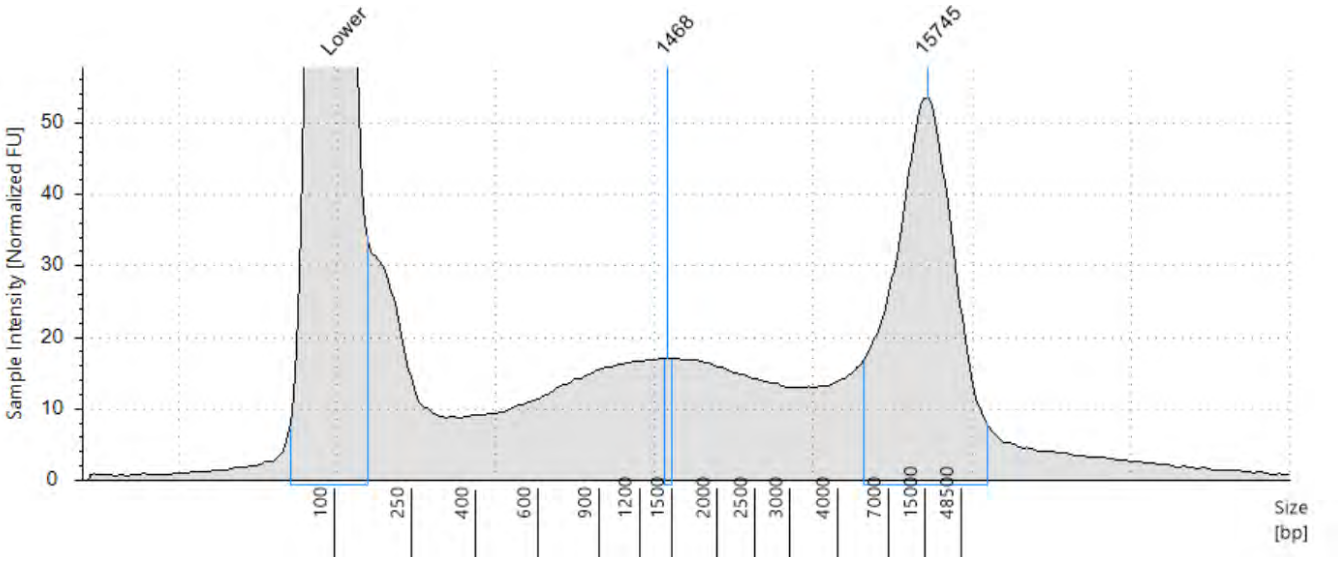

Sample Table

Well	DIN	Conc. [ng/µl]	Sample Description	Alert	Observations
F2	5.8	6.39	W-T5-ES-873	⚠	Sample concentration outside recommended range

Peak Table

Size [bp]	Calibrated Conc. [ng/µl]	Assigned Conc. [ng/µl]	% Integrated Area	From [bp]	To [bp]	Peak Comment	Observations
100	8.50	8.50	-	59	150		Lower Marker
1468	0.0878	-	3.95	1427	1509		
15745	2.12	-	95.53	5387	>60000		
-	-	-	-	-	-		Sample Well

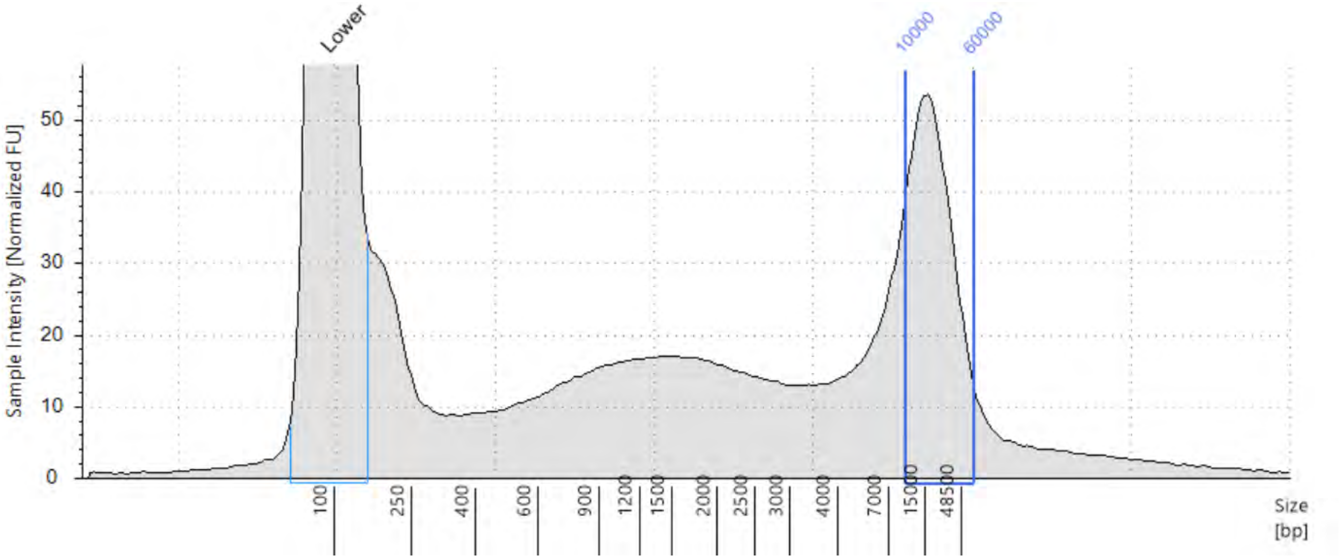

Region Table

From [bp]	To [bp]	Average Size [bp]	Conc. [ng/µl]	Region Molarity [nmol/l]	% of Total	Region Comment	Color
10000	60000	22271	1.49	0.130	23.25		■

G2: W-T5-ES-874

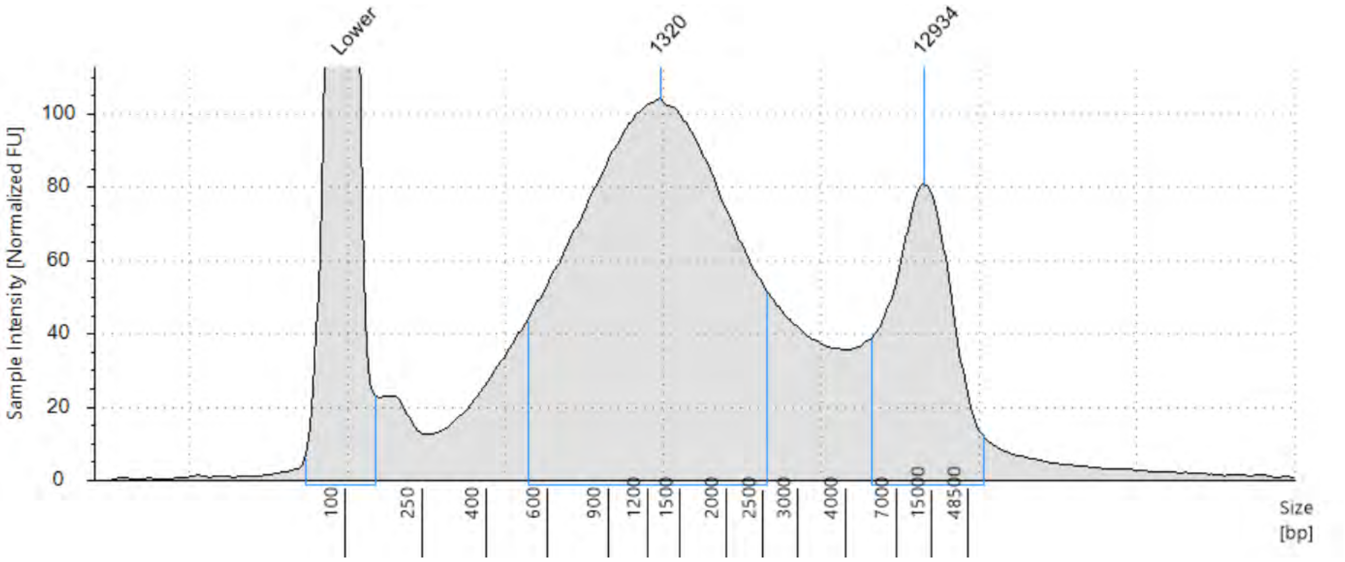

Sample Table

Well	DIN	Conc. [ng/µl]	Sample Description	Alert	Observations
G2	2.7	21.1	W-T5-ES-874		

Peak Table

Size [bp]	Calibrated Conc. [ng/µl]	Assigned Conc. [ng/µl]	% Integrated Area	From [bp]	To [bp]	Peak Comment	Observations
100	8.50	8.50	-	63	147		Lower Marker
1320	12.3	-	76.62	530	2564		
12934	3.74	-	23.38	5374	>60000		

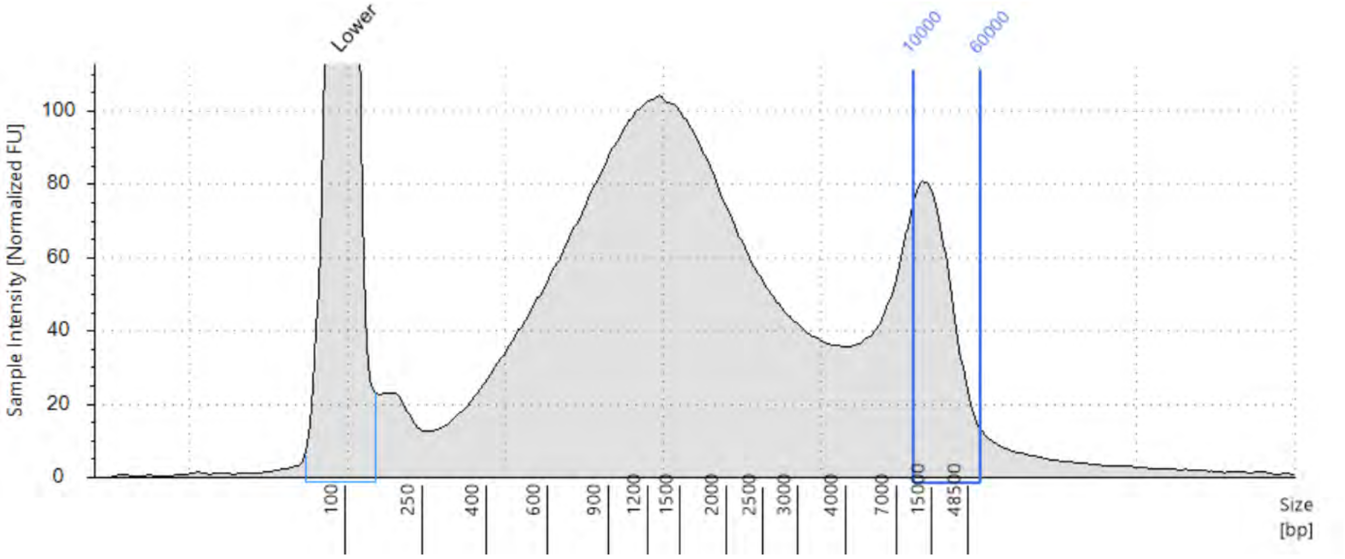

Region Table

From [bp]	To [bp]	Average Size [bp]	Conc. [ng/µl]	Region Molarity [nmol/l]	% of Total	Region Comment	Color
10000	60000	19959	2.37	0.229	11.22		

H2: W-T5-ES-875

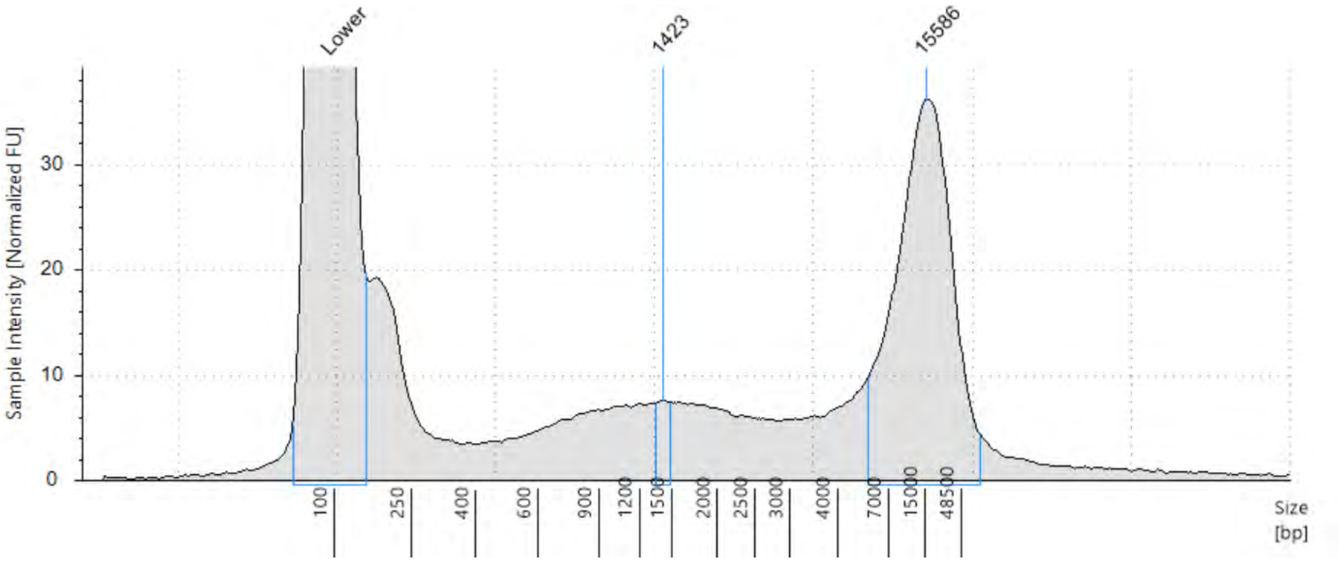

Sample Table

Well	DIN	Conc. [ng/µl]	Sample Description	Alert	Observations
H2	6.7	3.65	W-T5-ES-875	⚠	Sample concentration outside functional range for DIN

Peak Table

Size [bp]	Calibrated Conc. [ng/µl]	Assigned Conc. [ng/µl]	% Integrated Area	From [bp]	To [bp]	Peak Comment	Observations
100	8.50	8.50	-	62	147		Lower Marker
1423	0.0670	-	4.31	1358	1491		
15586	1.49	-	95.69	5623	>60000		

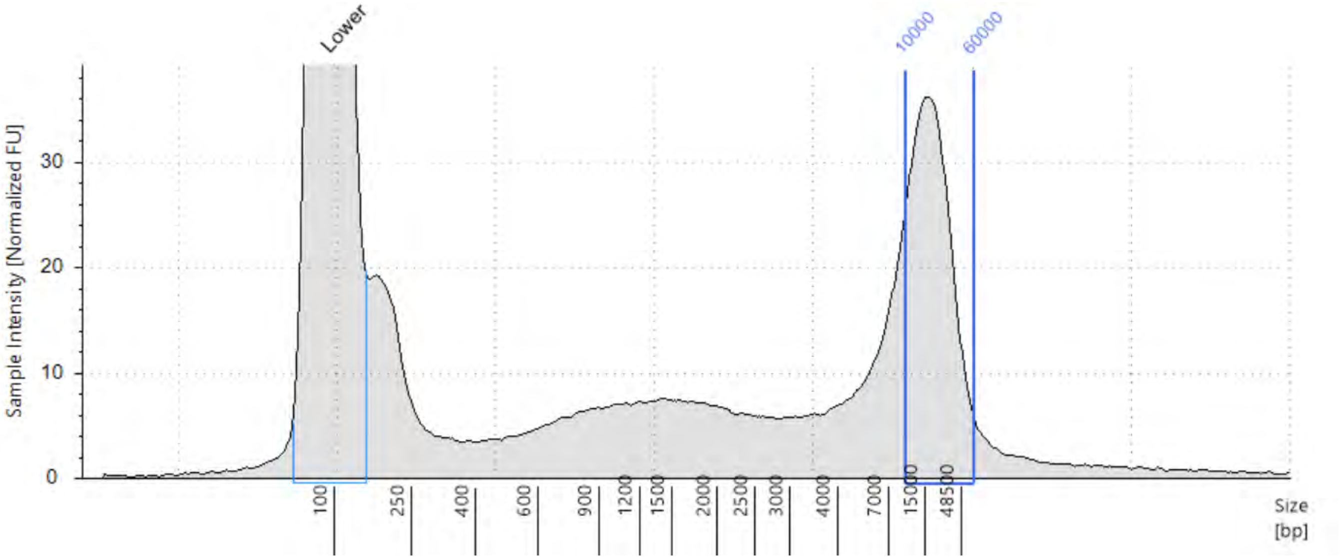

Region Table

From [bp]	To [bp]	Average Size [bp]	Conc. [ng/µl]	Region Molarity [nmol/l]	% of Total	Region Comment	Color
10000	60000	21078	1.10	0.0996	30.20		■

Filename: 2019-07-30-02.gDNA

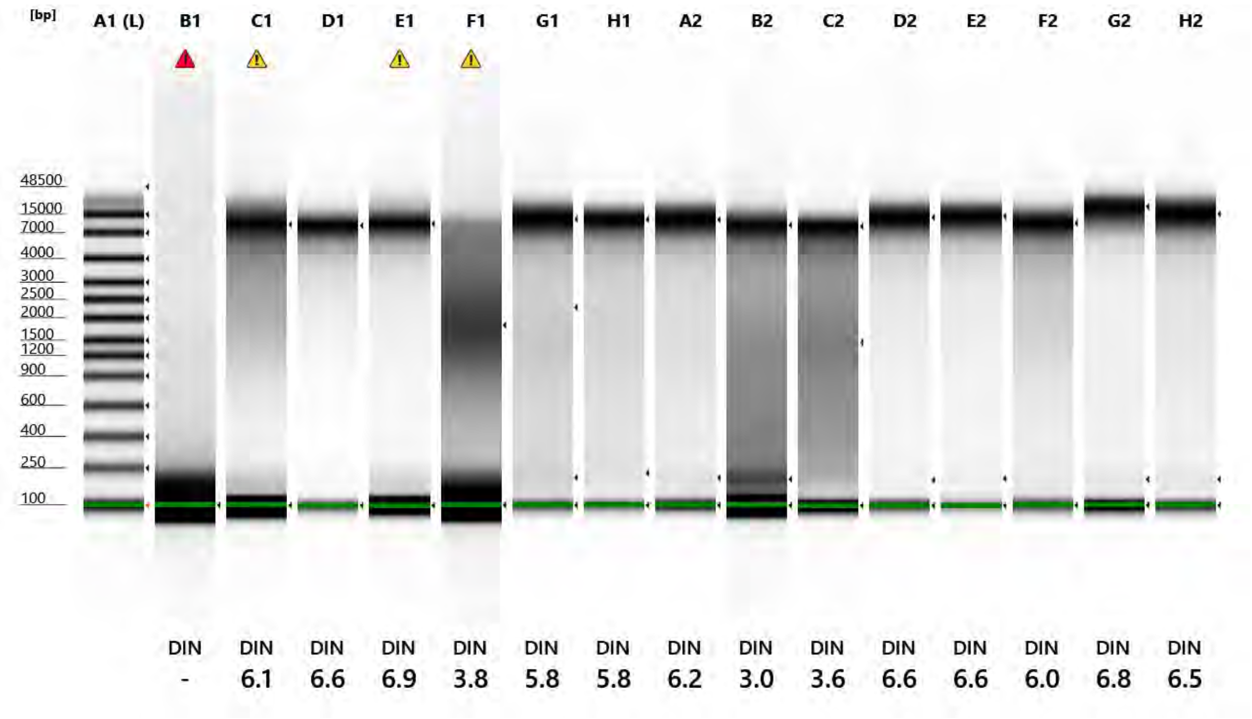

Default image (Contrast 50%), Image is Scaled to Sample

Sample Info

Well	DIN	Conc. [ng/µl]	Sample Description	Alert	Observations
A1	-	70.1	Ladder		Ladder; Sizing Changed
B1	-	1.96	W-T5-ES-1096		Sample concentration outside functional range for DIN; DIN Edited (Ladder sizing changed)
C1	6.1	9.26	W-T5-ES-1097		Sample concentration outside recommended range; DIN Edited (Ladder sizing changed)
D1	6.6	37.2	W-T5-ES-1098		DIN Edited (Ladder sizing changed)
E1	6.9	8.66	W-T5-ES-1099		Sample concentration outside recommended range; DIN Edited (Ladder sizing changed)
F1	3.8	5.89	W-T5-ES-1100		Sample concentration outside recommended range; DIN Edited (Ladder sizing changed)
G1	5.8	69.2	W-T4-EtOH-850		DIN Edited (Ladder sizing changed)
H1	5.8	70.3	W-T4-EtOH-1071		DIN Edited (Ladder sizing changed)
A2	6.2	39.6	W-T4-EtOH-1072		DIN Edited (Ladder sizing changed)
B2	3.0	13.7	W-T4-EtOH-1073		DIN Edited (Ladder sizing changed)
C2	3.6	50.6	W-T4-EtOH-1074		DIN Edited (Ladder sizing changed)
D2	6.6	39.0	W-T4-Initial-854		DIN Edited (Ladder sizing changed)
E2	6.6	60.7	W-T4-Initial-855		DIN Edited (Ladder sizing changed)
F2	6.0	51.2	W-T4-Initial-1076		DIN Edited (Ladder sizing changed)
G2	6.8	22.8	W-T4-Initial-1077		DIN Edited (Ladder sizing changed)
H2	6.5	43.2	W-T4-Initial-1078		DIN Edited (Ladder sizing changed)

 © Agilent Technologies, Inc. 2017

A1: Ladder

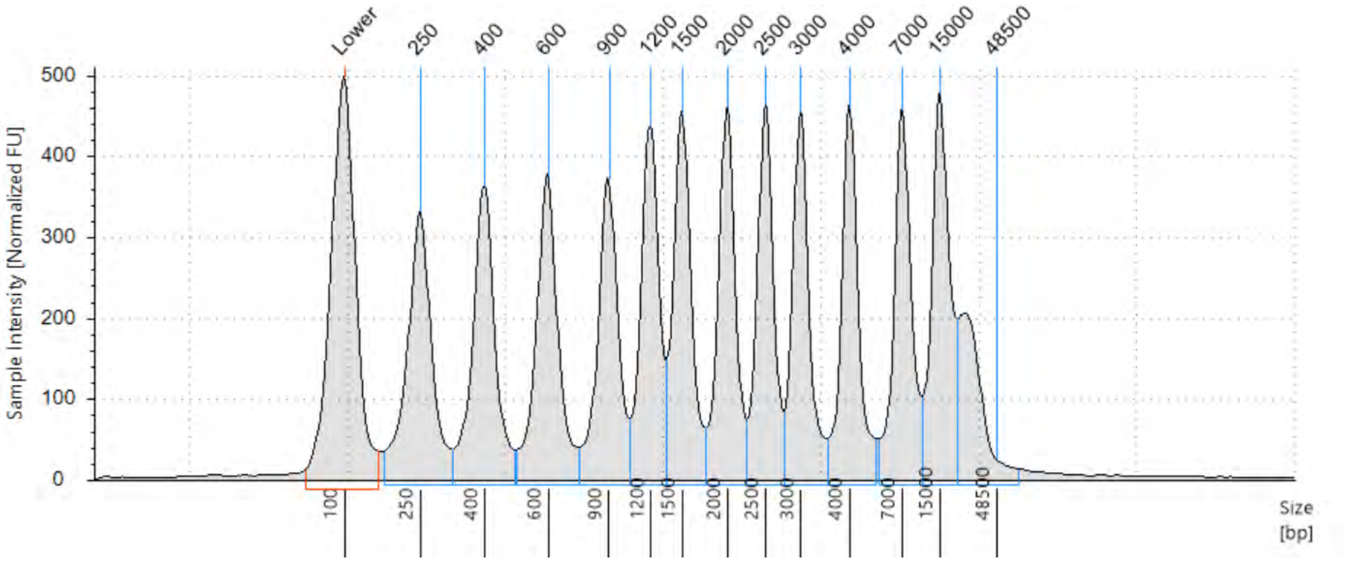

Sample Table

Well	DIN	Conc. [ng/µl]	Sample Description	Alert	Observations
A1	-	70.1	Ladder		Ladder; Sizing Changed

Peak Table

Size [bp]	Calibrated Conc. [ng/µl]	Assigned Conc. [ng/µl]	% Integrated Area	From [bp]	To [bp]	Peak Comment	Observations
100	8.50	8.50	-	62	151		Lower Marker
250	5.57	-	7.94	163	316		
400	5.62	-	8.01	316	488		
600	5.68	-	8.09	492	738		
900	5.29	-	7.54	738	1044		
1200	5.61	-	8.01	1044	1348		
1500	5.74	-	8.18	1348	1747		
2000	5.53	-	7.89	1747	2236		
2500	5.43	-	7.74	2236	2758		
3000	5.33	-	7.60	2758	3520		
4000	5.39	-	7.69	3520	5330		
7000	5.50	-	7.84	5485	10530		
15000	6.06	-	8.65	10530	21368		
48500	3.38	-	4.82	21368	>60000		

© Agilent Technologies, Inc. 2017

B1: W-T5-ES-1096

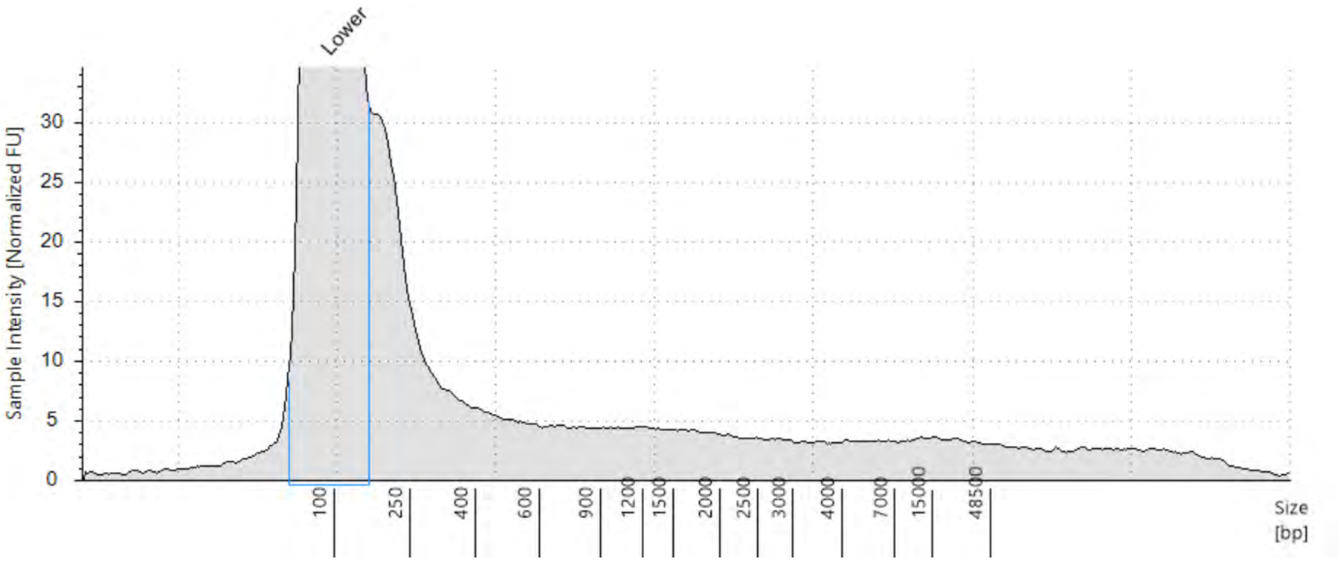

Sample Table

Well	DIN	Conc. [ng/µl]	Sample Description	Alert	Observations
B1	-	1.96	W-T5-ES-1096	▲	Sample concentration outside functional range for DIN; DIN Edited (Ladder sizing changed)

Peak Table

Size [bp]	Calibrated Conc. [ng/µl]	Assigned Conc. [ng/µl]	% Integrated Area	From [bp]	To [bp]	Peak Comment	Observations
100	8.50	8.50	-	58	153		Lower Marker

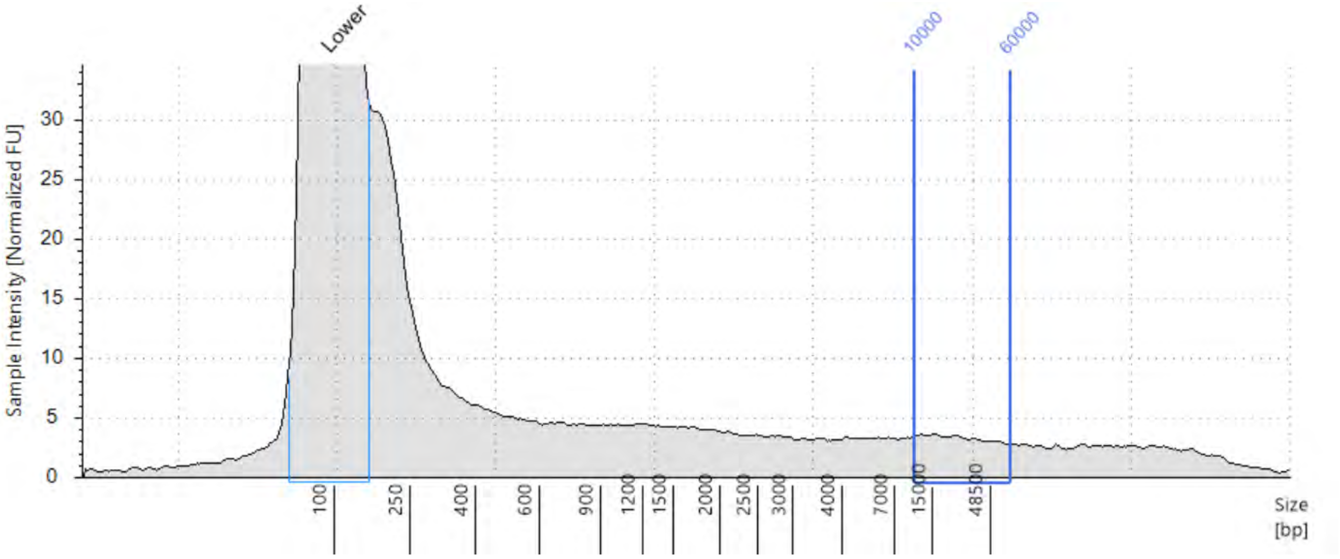

Region Table

From [bp]	To [bp]	Average Size [bp]	Conc. [ng/µl]	Region Molarity [nmol/l]	% of Total	Region Comment	Color
10000	60000	29883	0.132	0.0115	6.74		■

C1: W-T5-ES-1097

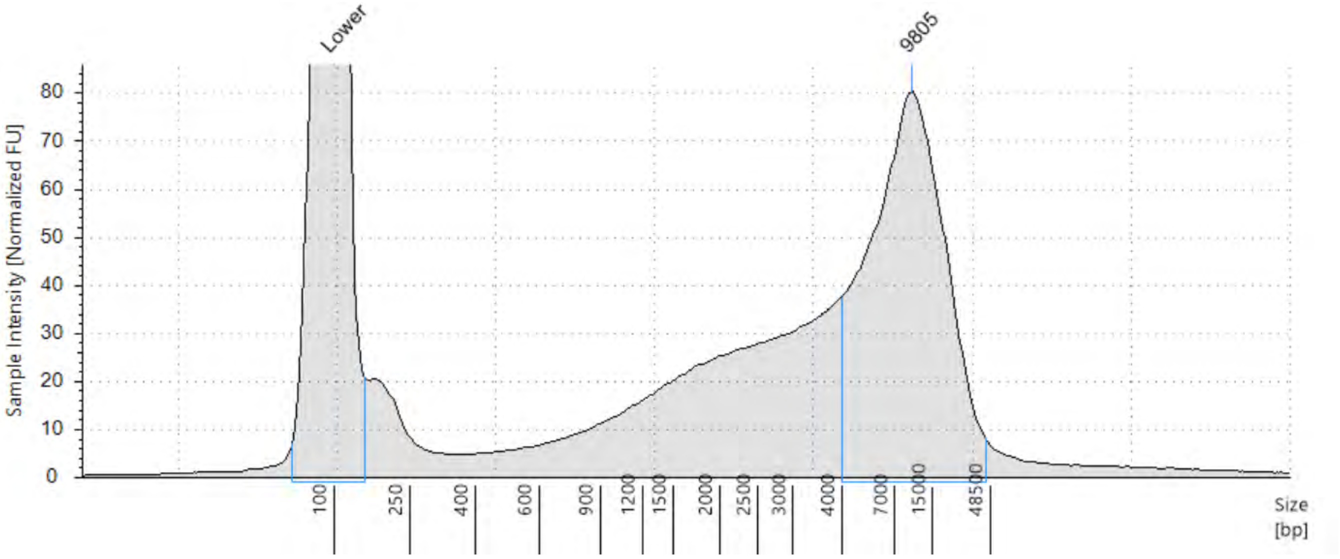

Sample Table

Well	DIN	Conc. [ng/µl]	Sample Description	Alert	Observations
C1	6.1	9.26	W-T5-ES-1097	⚠	Sample concentration outside recommended range; DIN Edited (Ladder sizing changed)

Peak Table

Size [bp]	Calibrated Conc. [ng/µl]	Assigned Conc. [ng/µl]	% Integrated Area	From [bp]	To [bp]	Peak Comment	Observations
100	8.50	8.50	-	61	146		Lower Marker
9805	4.36	-	99.57	4012	43884		
-	-	-	-	-	-		Sample Well

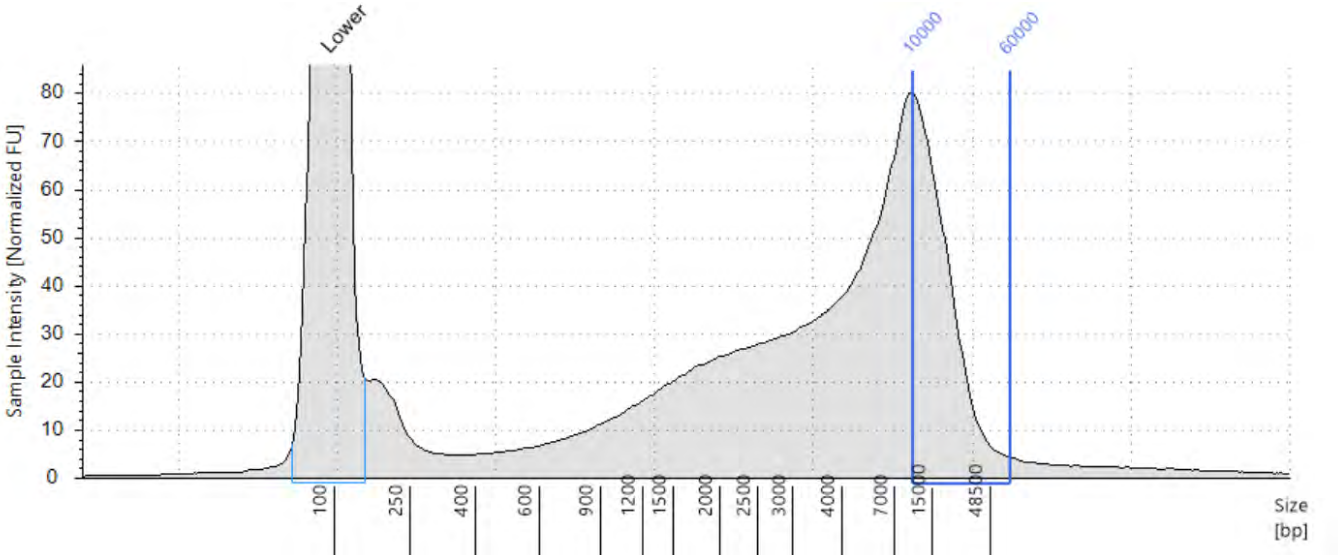

Region Table

From [bp]	To [bp]	Average Size [bp]	Conc. [ng/µl]	Region Molarity [nmol/l]	% of Total	Region Comment	Color
10000	60000	19319	2.02	0.200	21.86		■

D1: W-T5-ES-1098

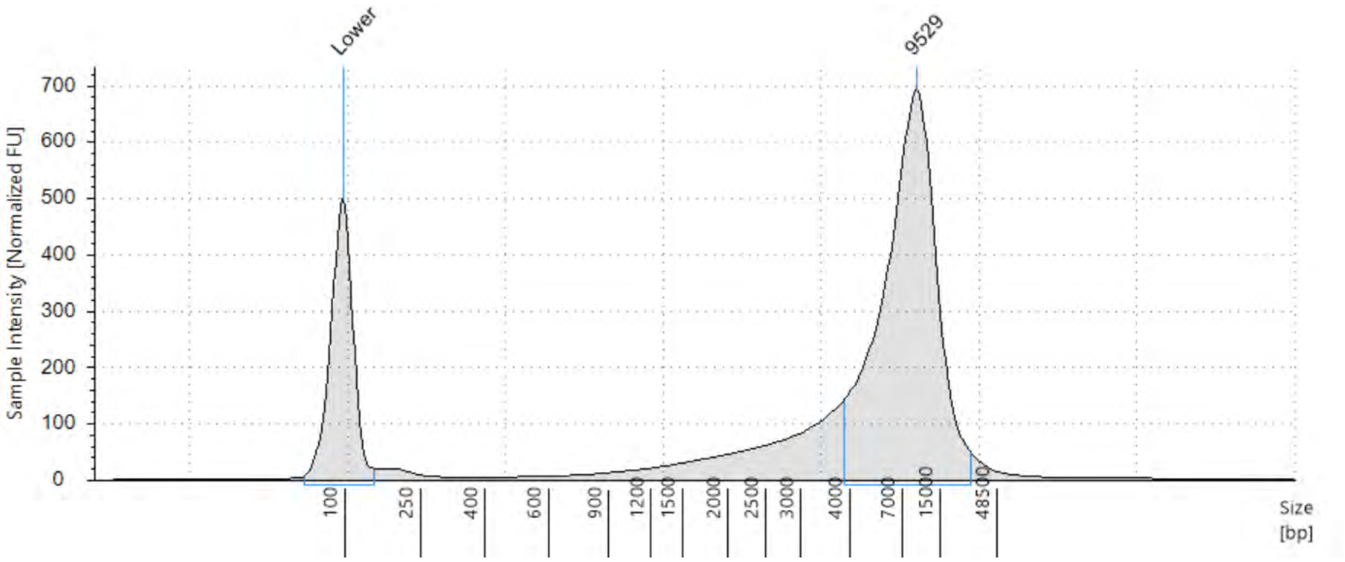

Sample Table

Well	DIN	Conc. [ng/µl]	Sample Description	Alert	Observations
D1	6.6	37.2	W-T5-ES-1098		DIN Edited (Ladder sizing changed)

Peak Table

Size [bp]	Calibrated Conc. [ng/µl]	Assigned Conc. [ng/µl]	% Integrated Area	From [bp]	To [bp]	Peak Comment	Observations
100	8.50	8.50	-	61	145		Lower Marker
9529	28.0	-	100.00	3868	28047		

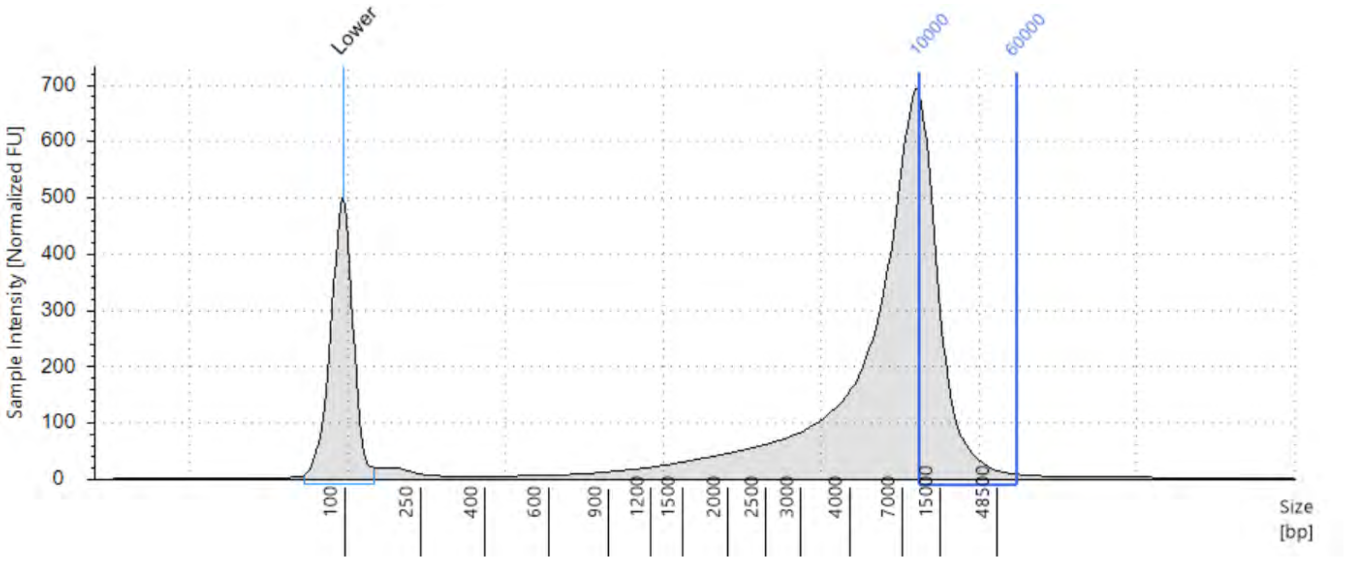

Region Table

From [bp]	To [bp]	Average Size [bp]	Conc. [ng/µl]	Region Molarity [nmol/l]	% of Total	Region Comment	Color
10000	60000	15769	10.6	1.21	28.41		■

E1: W-T5-ES-1099

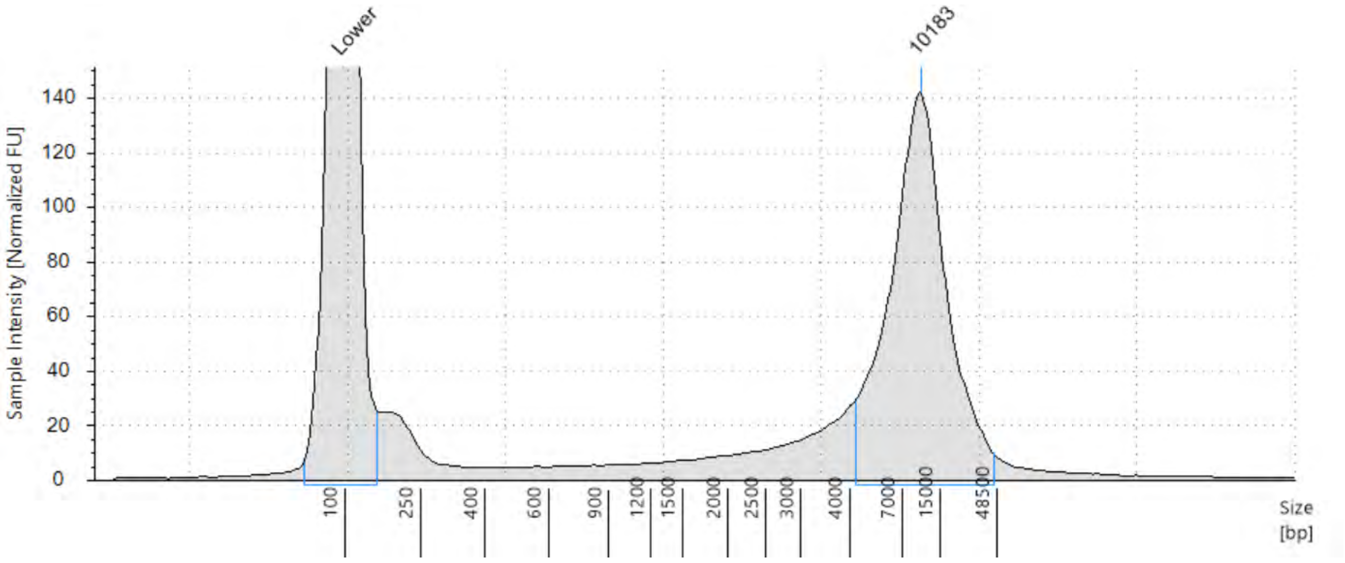

Sample Table

Well	DIN	Conc. [ng/µl]	Sample Description	Alert	Observations
E1	6.9	8.66	W-T5-ES-1099	⚠	Sample concentration outside recommended range; DIN Edited (Ladder sizing changed)

Peak Table

Size [bp]	Calibrated Conc. [ng/µl]	Assigned Conc. [ng/µl]	% Integrated Area	From [bp]	To [bp]	Peak Comment	Observations
100	8.50	8.50	-	61	150		Lower Marker
10183	5.69	-	100.00	4245	44991		

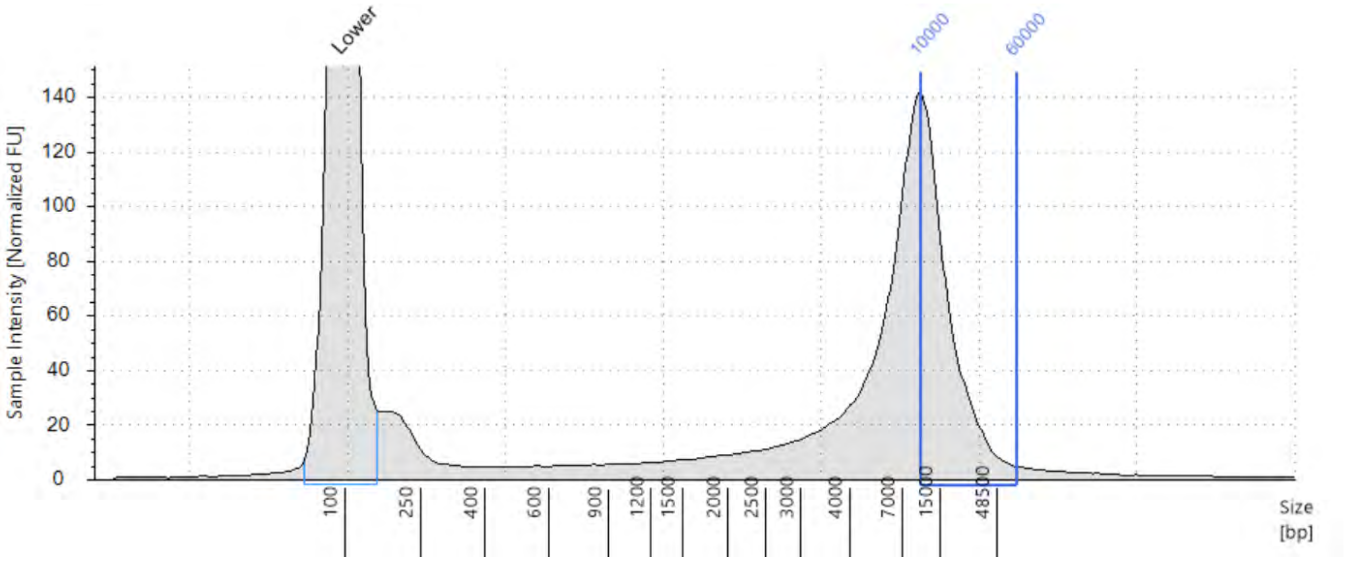

Region Table

From [bp]	To [bp]	Average Size [bp]	Conc. [ng/µl]	Region Molarity [nmol/l]	% of Total	Region Comment	Color
10000	60000	18442	2.83	0.289	32.63		■

F1: W-T5-ES-1100

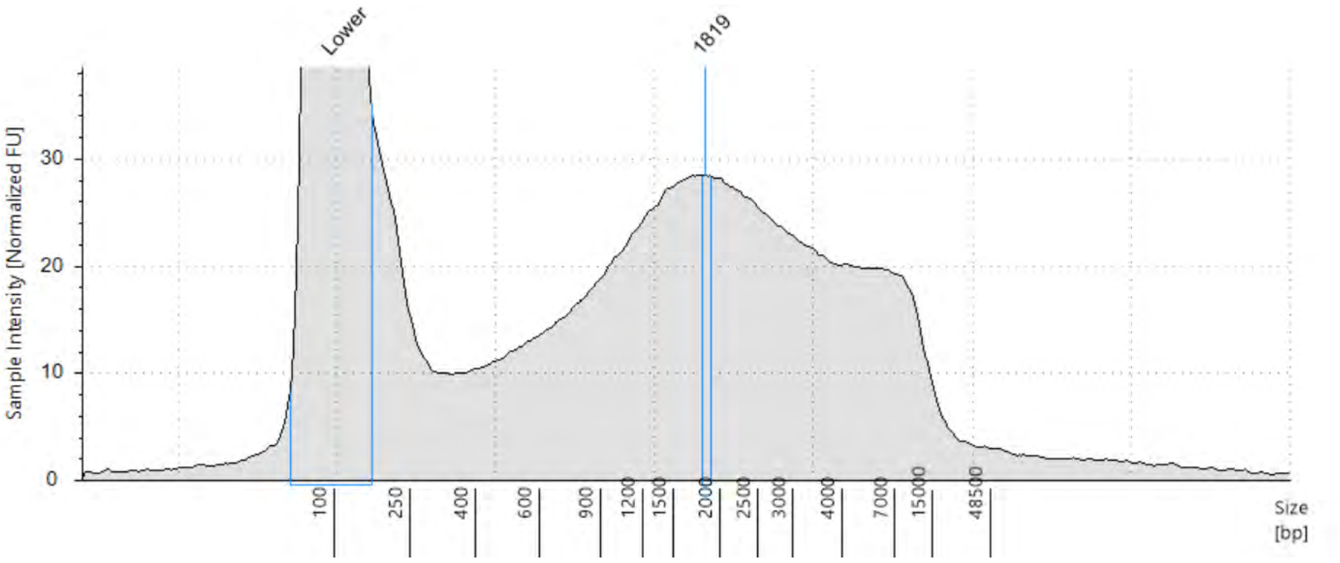

Sample Table

Well	DIN	Conc. [ng/µl]	Sample Description	Alert	Observations
F1	3.8	5.89	W-T5-ES-1100	⚠	Sample concentration outside recommended range; DIN Edited (Ladder sizing changed)

Peak Table

Size [bp]	Calibrated Conc. [ng/µl]	Assigned Conc. [ng/µl]	% Integrated Area	From [bp]	To [bp]	Peak Comment	Observations
100	8.50	8.50	-	59	159		Lower Marker
1819	0.161	-	100.00	1788	1898		

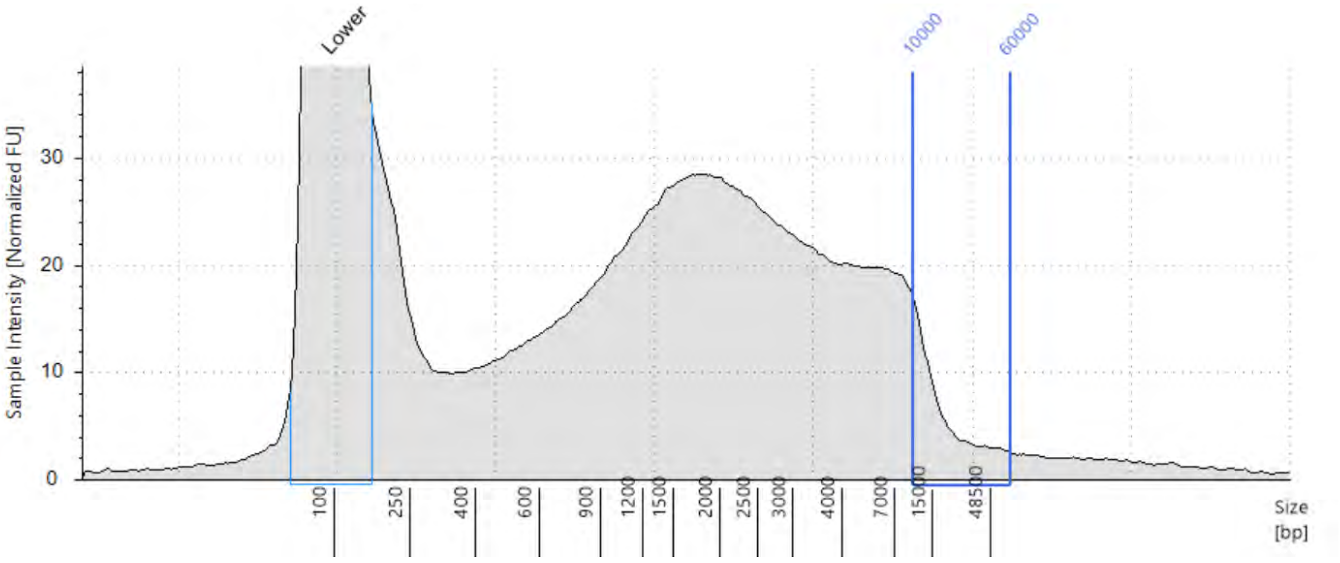

Region Table

From [bp]	To [bp]	Average Size [bp]	Conc. [ng/µl]	Region Molarity [nmol/l]	% of Total	Region Comment	Color
10000	60000	22978	0.262	0.0275	4.45		■

 © Agilent Technologies, Inc. 2017

G1: W-T4-EtOH-850

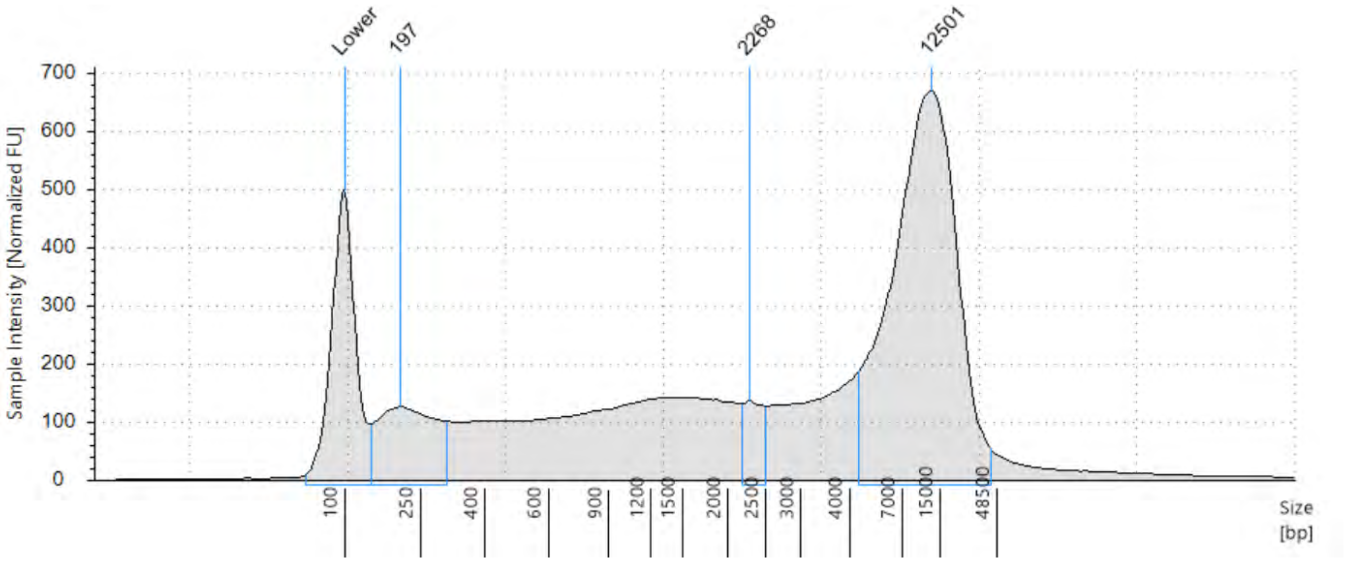

Sample Table

Well	DIN	Conc. [ng/µl]	Sample Description	Alert	Observations
G1	5.8	69.2	W-T4-EtOH-850		DIN Edited (Ladder sizing changed)

Peak Table

Size [bp]	Calibrated Conc. [ng/µl]	Assigned Conc. [ng/µl]	% Integrated Area	From [bp]	To [bp]	Peak Comment	Observations
100	8.50	8.50	-	63	138		Lower Marker
197	5.18	-	13.45	138	302		
2268	2.02	-	5.26	2163	2493		
12501	31.0	-	80.50	4415	41605		
-	-	-	-	-	-		Sample Well

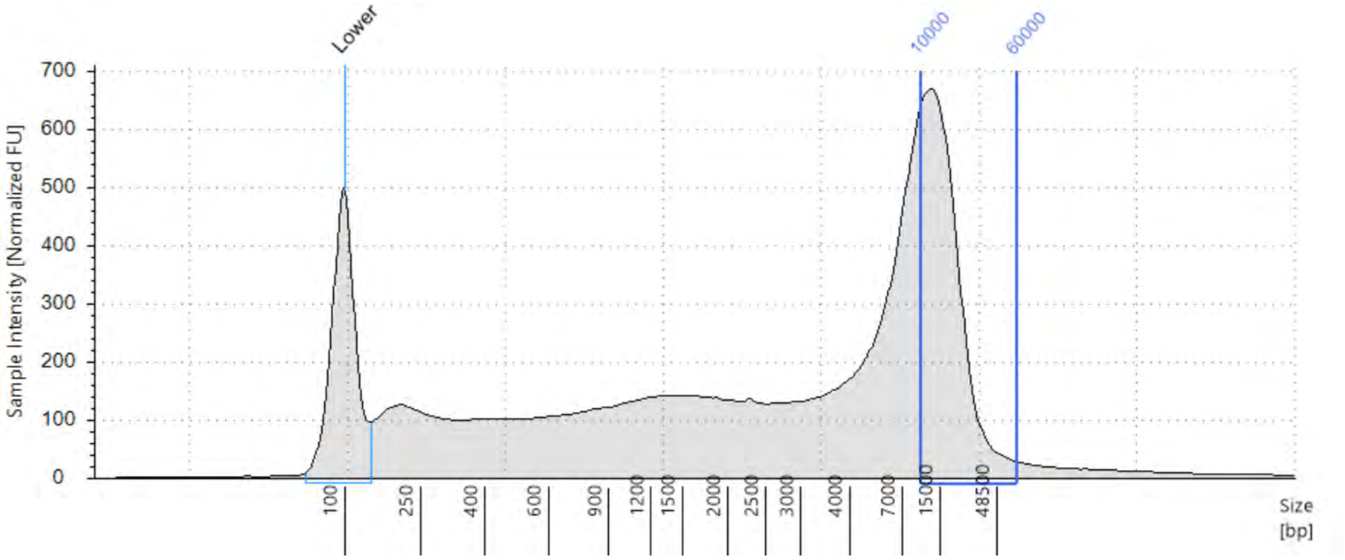

Region Table

From [bp]	To [bp]	Average Size [bp]	Conc. [ng/µl]	Region Molarity [nmol/l]	% of Total	Region Comment	Color
10000	60000	18419	18.0	1.79	26.04		

H1: W-T4-EtOH-1071

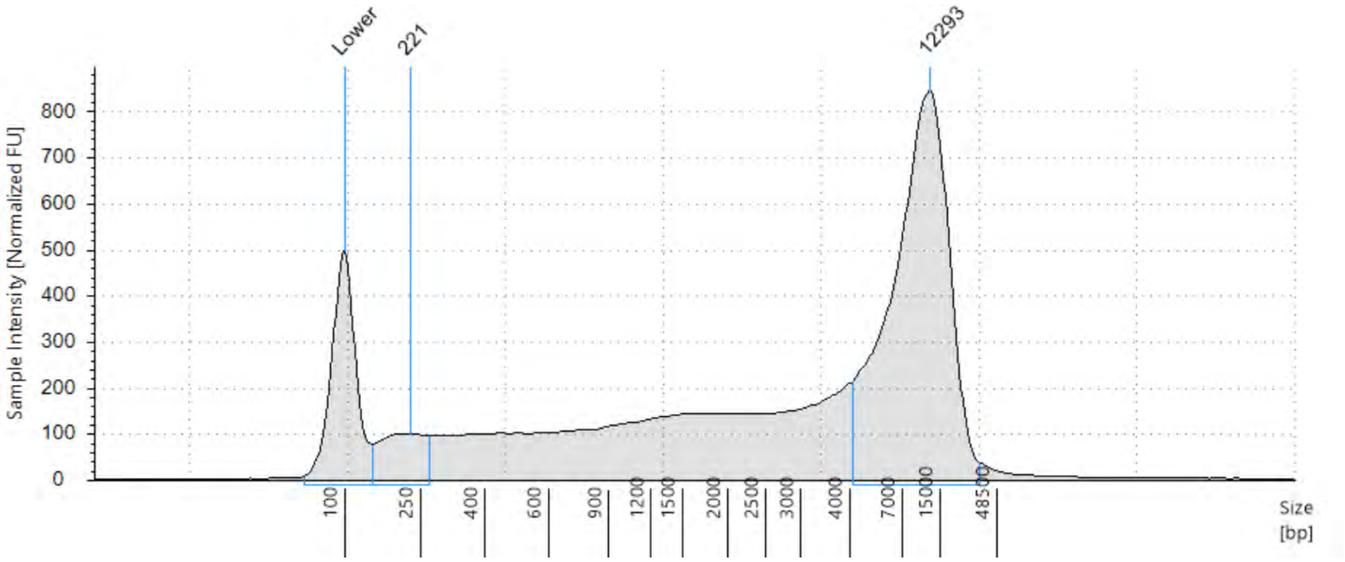

Sample Table

Well	DIN	Conc. [ng/µl]	Sample Description	Alert	Observations
H1	5.8	70.3	W-T4-EtOH-1071		DIN Edited (Ladder sizing changed)

Peak Table

Size [bp]	Calibrated Conc. [ng/µl]	Assigned Conc. [ng/µl]	% Integrated Area	From [bp]	To [bp]	Peak Comment	Observations
100	8.50	8.50	-	62	142		Lower Marker
221	3.21	-	8.57	142	267		
12293	34.2	-	91.43	4120	33165		

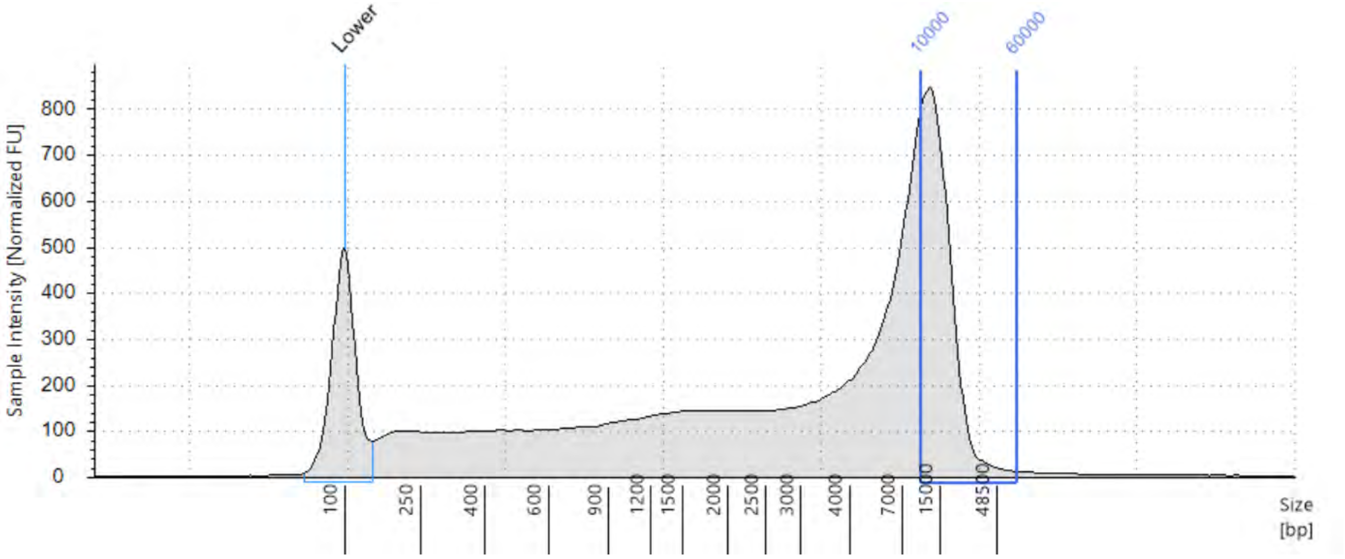

Region Table

From [bp]	To [bp]	Average Size [bp]	Conc. [ng/µl]	Region Molarity [nmol/l]	% of Total	Region Comment	Color
10000	60000	16243	17.7	1.91	25.18		

A2: W-T4-EtOH-1072

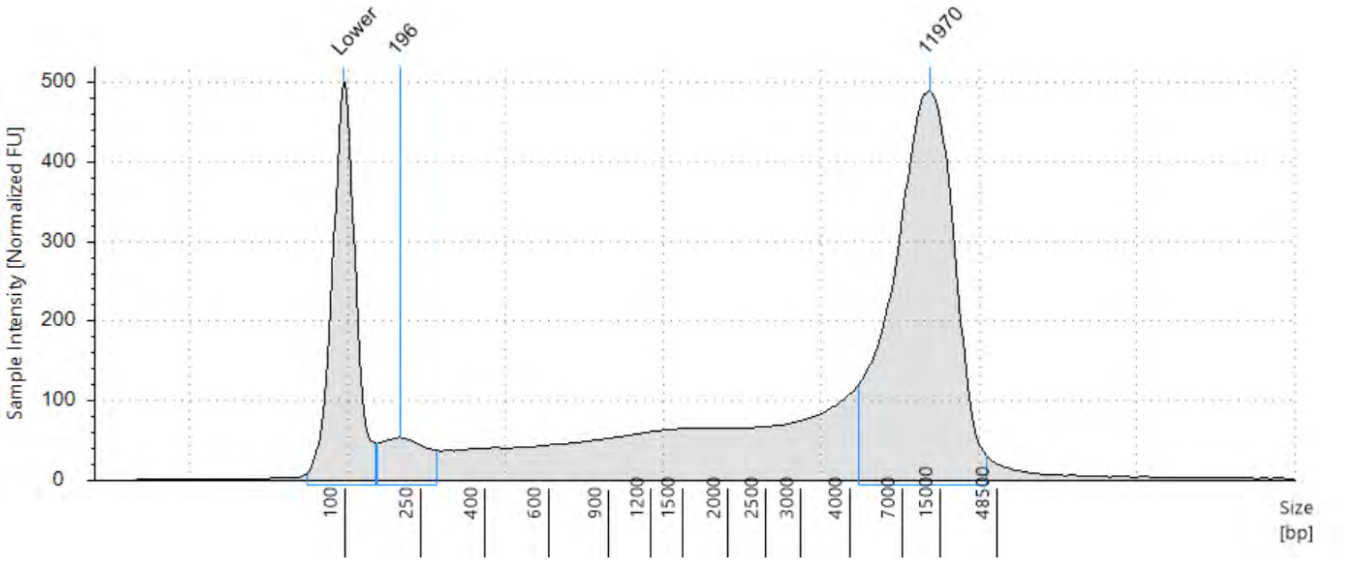

Sample Table

Well	DIN	Conc. [ng/µl]	Sample Description	Alert	Observations
A2	6.2	39.6	W-T4-EtOH-1072		DIN Edited (Ladder sizing changed)

Peak Table

Size [bp]	Calibrated Conc. [ng/µl]	Assigned Conc. [ng/µl]	% Integrated Area	From [bp]	To [bp]	Peak Comment	Observations
100	8.50	8.50	-	64	147		Lower Marker
196	1.73	-	7.26	149	282		
11970	22.0	-	92.47	4419	37857		
-	-	-	-	-	-		Sample Well

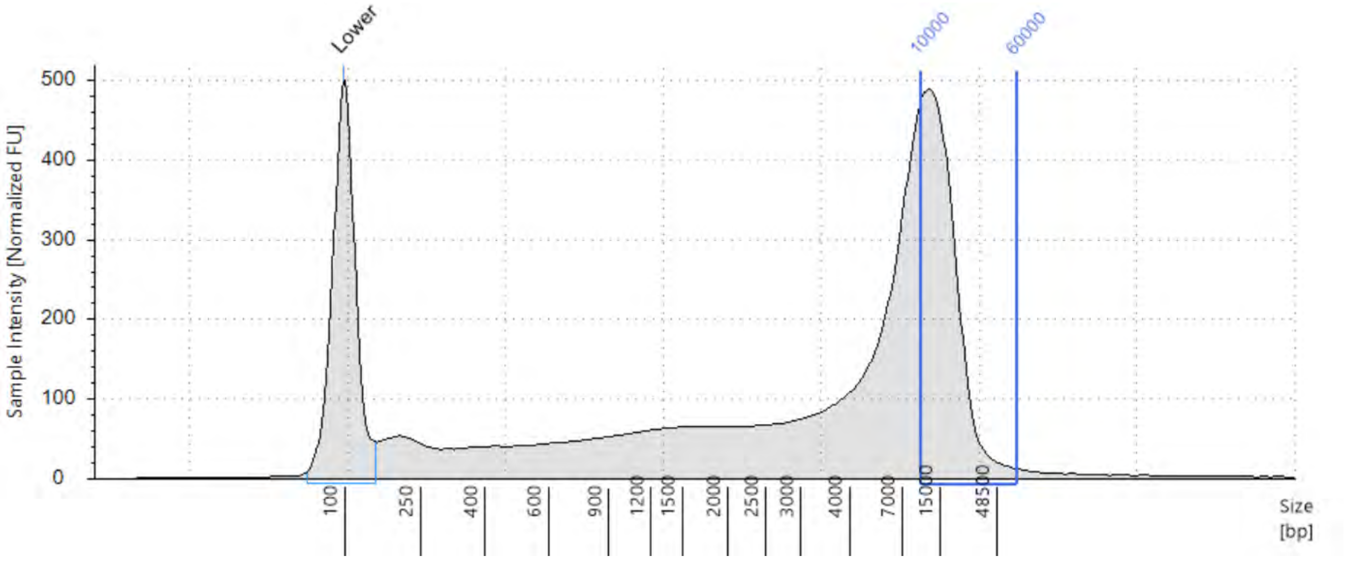

Region Table

From [bp]	To [bp]	Average Size [bp]	Conc. [ng/µl]	Region Molarity [nmol/l]	% of Total	Region Comment	Color
10000	60000	17561	12.4	1.26	31.23		

 © Agilent Technologies, Inc. 2017

B2: W-T4-EtOH-1073

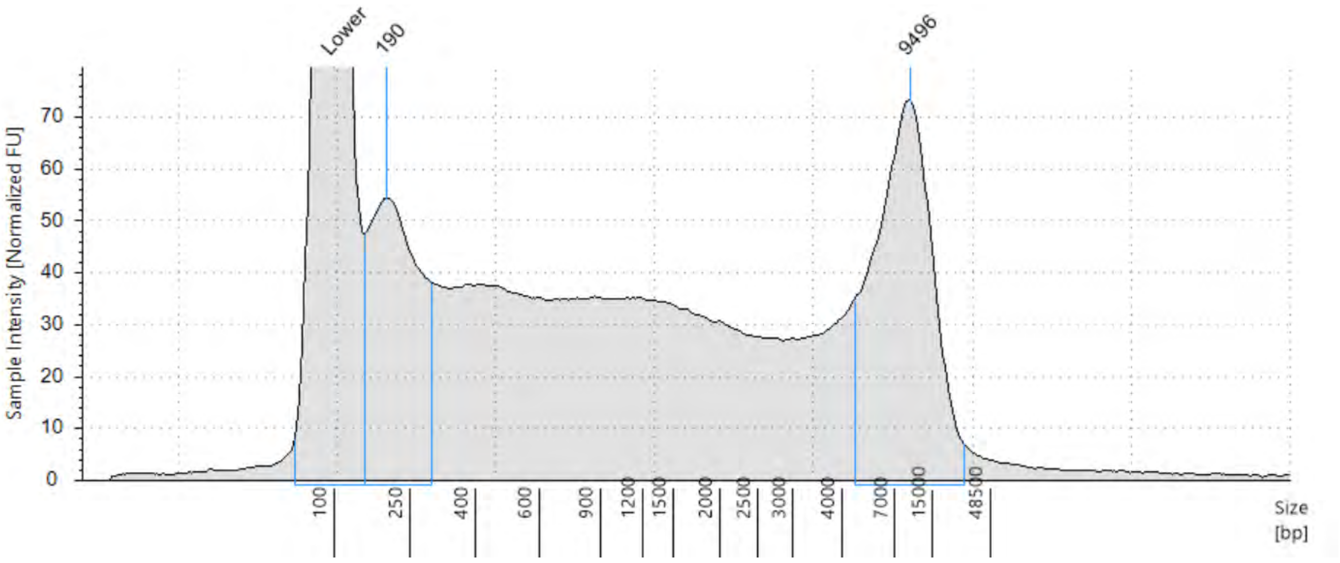

Sample Table

Well	DIN	Conc. [ng/µl]	Sample Description	Alert	Observations
B2	3.0	13.7	W-T4-EtOH-1073		DIN Edited (Ladder sizing changed)

Peak Table

Size [bp]	Calibrated Conc. [ng/µl]	Assigned Conc. [ng/µl]	% Integrated Area	From [bp]	To [bp]	Peak Comment	Observations
100	8.50	8.50	-	63	145		Lower Marker
190	1.99	-	39.44	145	291		
9496	3.05	-	60.56	4549	27663		

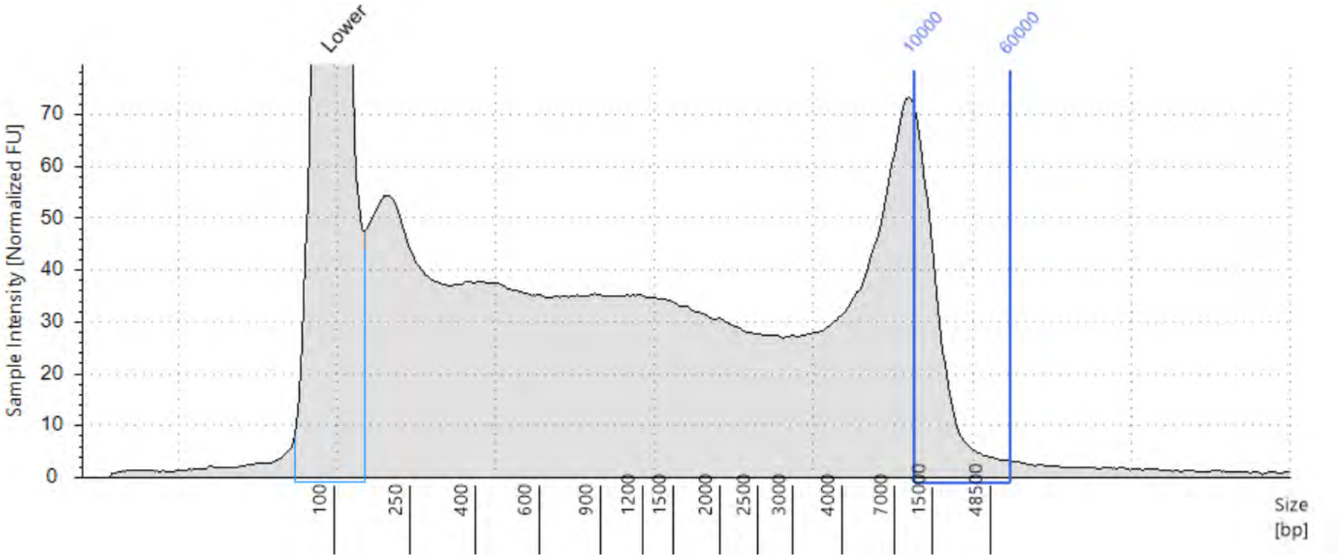

Region Table

From [bp]	To [bp]	Average Size [bp]	Conc. [ng/µl]	Region Molarity [nmol/l]	% of Total	Region Comment	Color
10000	60000	17396	1.23	0.139	8.99		■

 © Agilent Technologies, Inc. 2017

C2: W-T4-EtOH-1074

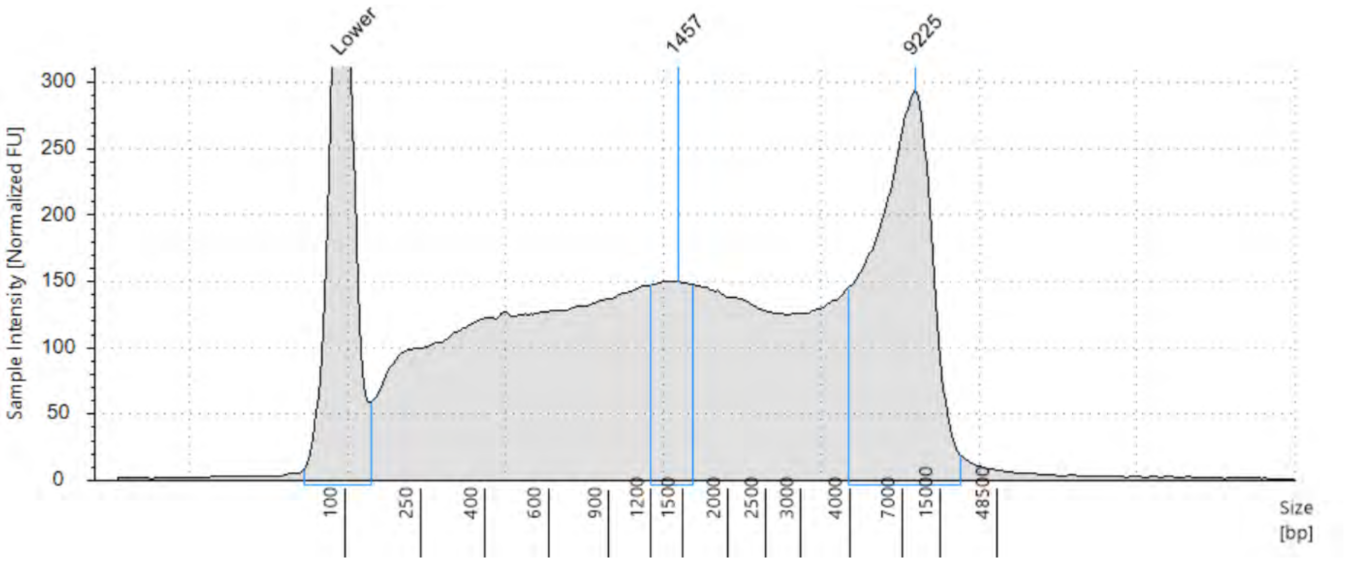

Sample Table

Well	DIN	Conc. [ng/µl]	Sample Description	Alert	Observations
C2	3.6	50.6	W-T4-EtOH-1074		DIN Edited (Ladder sizing changed)

Peak Table

Size [bp]	Calibrated Conc. [ng/µl]	Assigned Conc. [ng/µl]	%Integrated Area	From [bp]	To [bp]	Peak Comment	Observations
100	8.50	8.50	-	63	140		Lower Marker
1457	3.99	-	23.72	1204	1603		
9225	12.8	-	76.28	3967	22933		

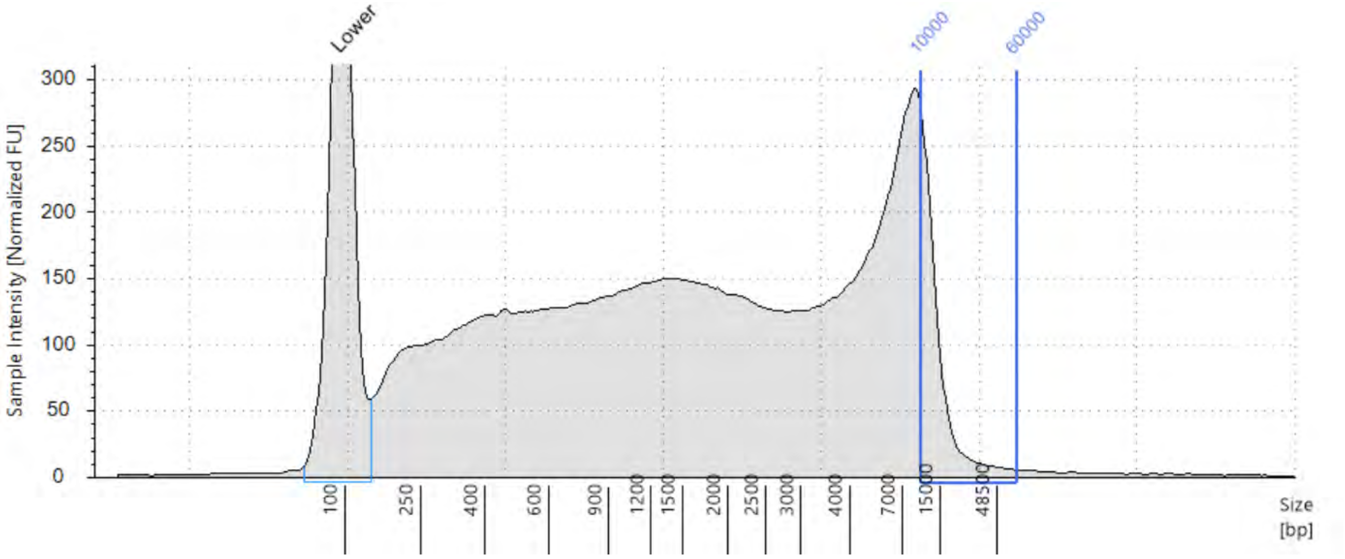

Region Table

From [bp]	To [bp]	Average Size [bp]	Conc. [ng/µl]	Region Molarity [nmol/l]	% of Total	Region Comment	Color
10000	60000	15903	3.31	0.398	6.54		

 © Agilent Technologies, Inc. 2017

D2: W-T4-Initial-854

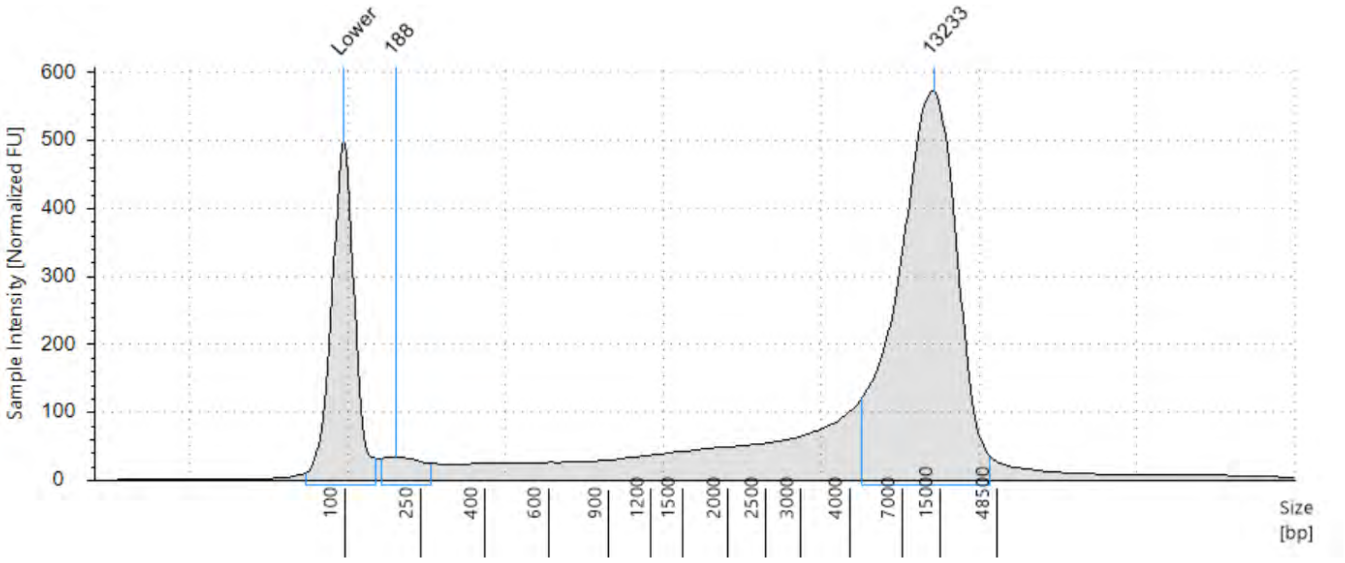

Sample Table

Well	DIN	Conc. [ng/µl]	Sample Description	Alert	Observations
D2	6.6	39.0	W-T4-Initial-854		DIN Edited (Ladder sizing changed)

Peak Table

Size [bp]	Calibrated Conc. [ng/µl]	Assigned Conc. [ng/µl]	% Integrated Area	From [bp]	To [bp]	Peak Comment	Observations
100	8.50	8.50	-	64	147		Lower Marker
188	0.926	-	3.46	157	270		
13233	25.5	-	95.34	4565	41521		
-	-	-	-	-	-		Sample Well

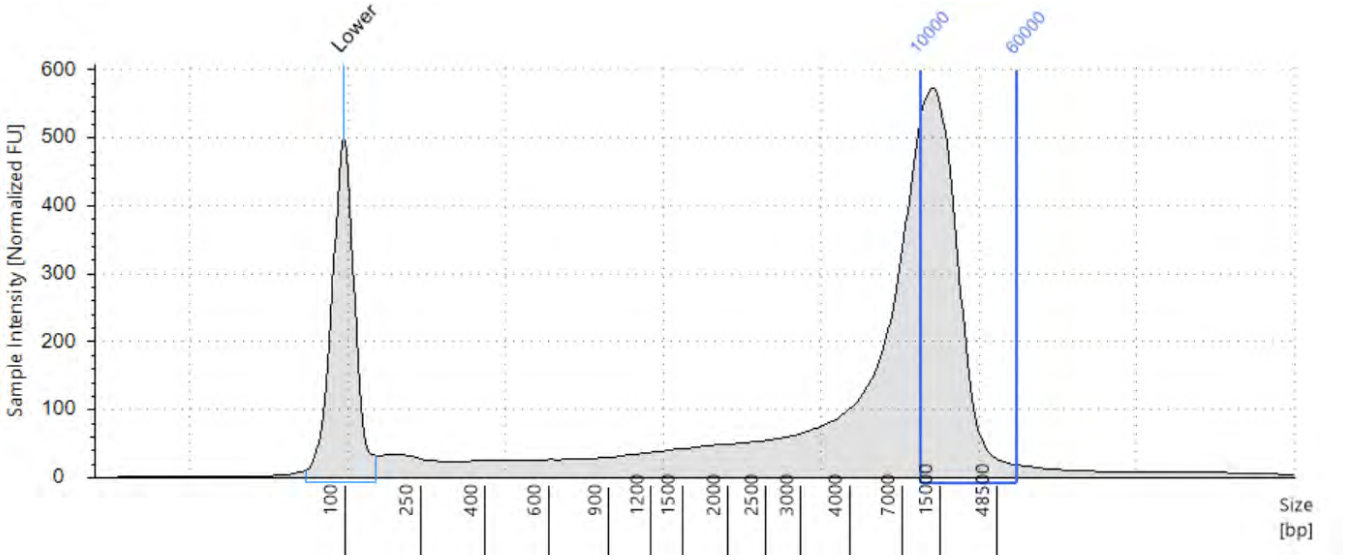

Region Table

From [bp]	To [bp]	Average Size [bp]	Conc. [ng/µl]	Region Molarity [nmol/l]	% of Total	Region Comment	Color
10000	60000	17972	15.9	1.59	40.70		

 © Agilent Technologies, Inc. 2017

E2: W-T4-Initial-855

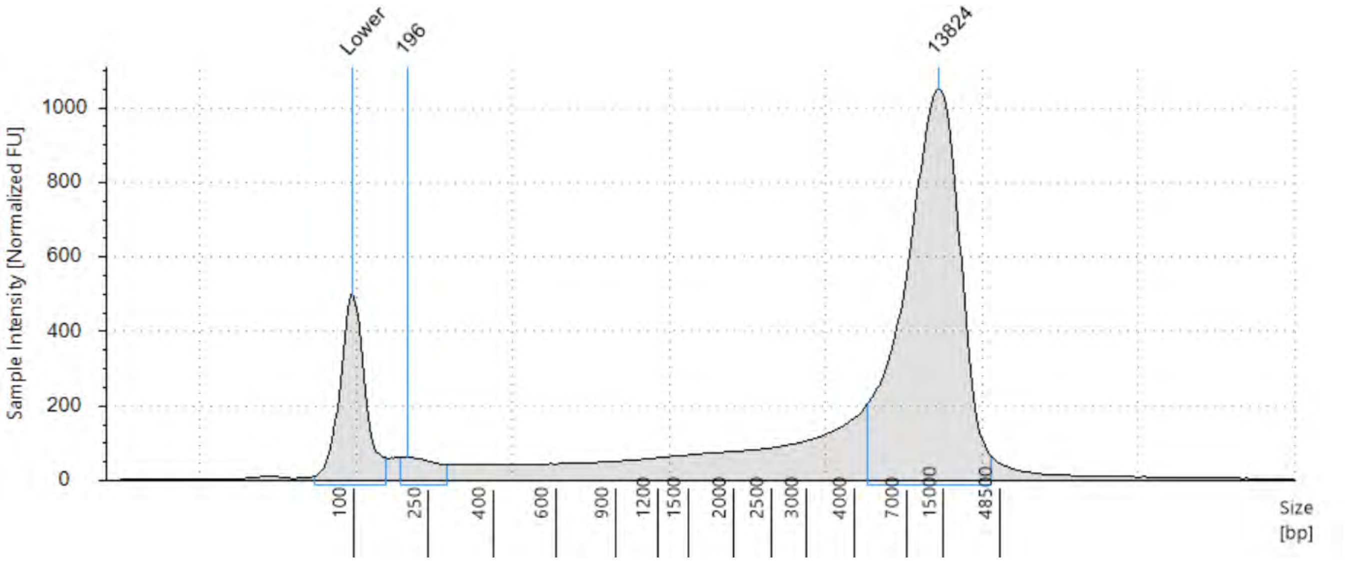

Sample Table

Well	DIN	Conc. [ng/µl]	Sample Description	Alert	Observations
E2	6.6	60.7	W-T4-Initial-855		DIN Edited (Ladder sizing changed)

Peak Table

Size [bp]	Calibrated Conc. [ng/µl]	Assigned Conc. [ng/µl]	% Integrated Area	From [bp]	To [bp]	Peak Comment	Observations
100	8.50	8.50	-	63	151		Lower Marker
196	1.40	-	3.36	180	287		
13824	40.3	-	96.44	4684	40553		
-	-	-	-	-	-		Sample Well

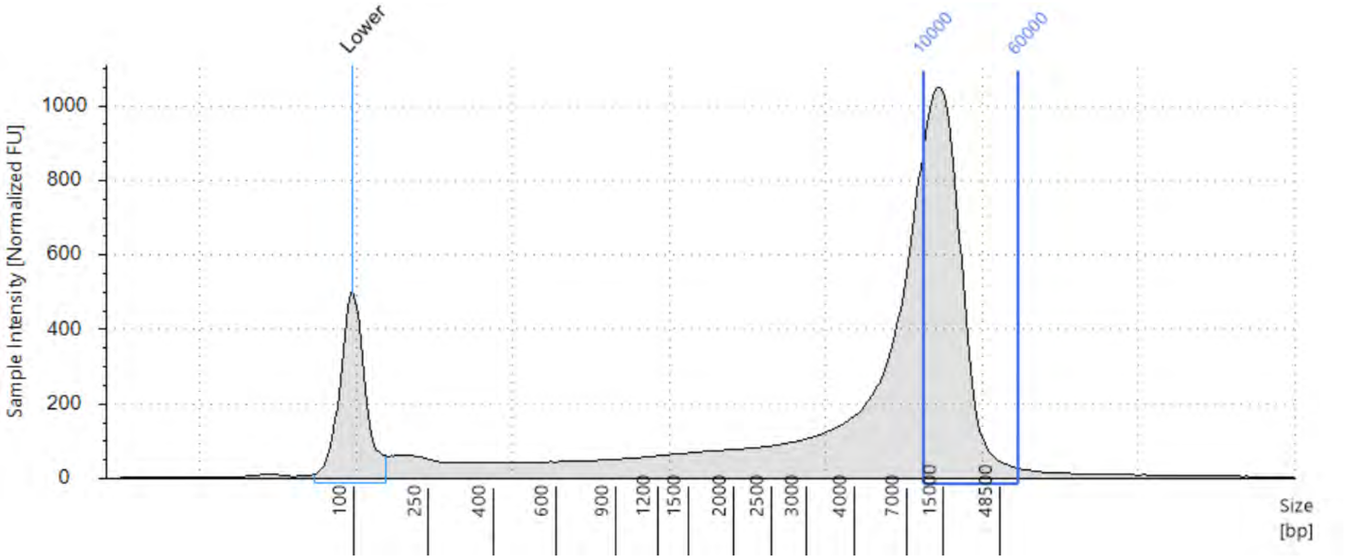

Region Table

From [bp]	To [bp]	Average Size [bp]	Conc. [ng/µl]	Region Molarity [nmol/l]	% of Total	Region Comment	Color
10000	60000	17999	26.1	2.57	42.96		

 © Agilent Technologies, Inc. 2017

F2: W-T4-Initial-1076

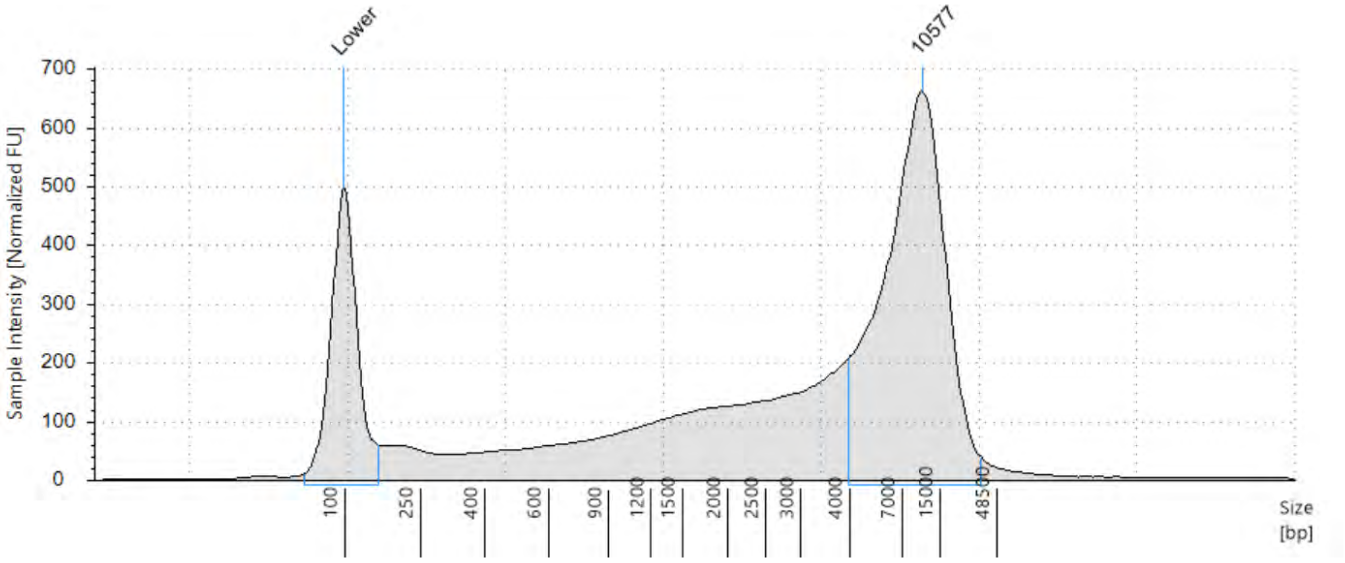

Sample Table

Well	DIN	Conc. [ng/µl]	Sample Description	Alert	Observations
F2	6.0	51.2	W-T4-Initial-1076		DIN Edited (Ladder sizing changed)

Peak Table

Size [bp]	Calibrated Conc. [ng/µl]	Assigned Conc. [ng/µl]	% Integrated Area	From [bp]	To [bp]	Peak Comment	Observations
100	8.50	8.50	-	62	151		Lower Marker
10577	26.7	-	100.00	3988	34598		

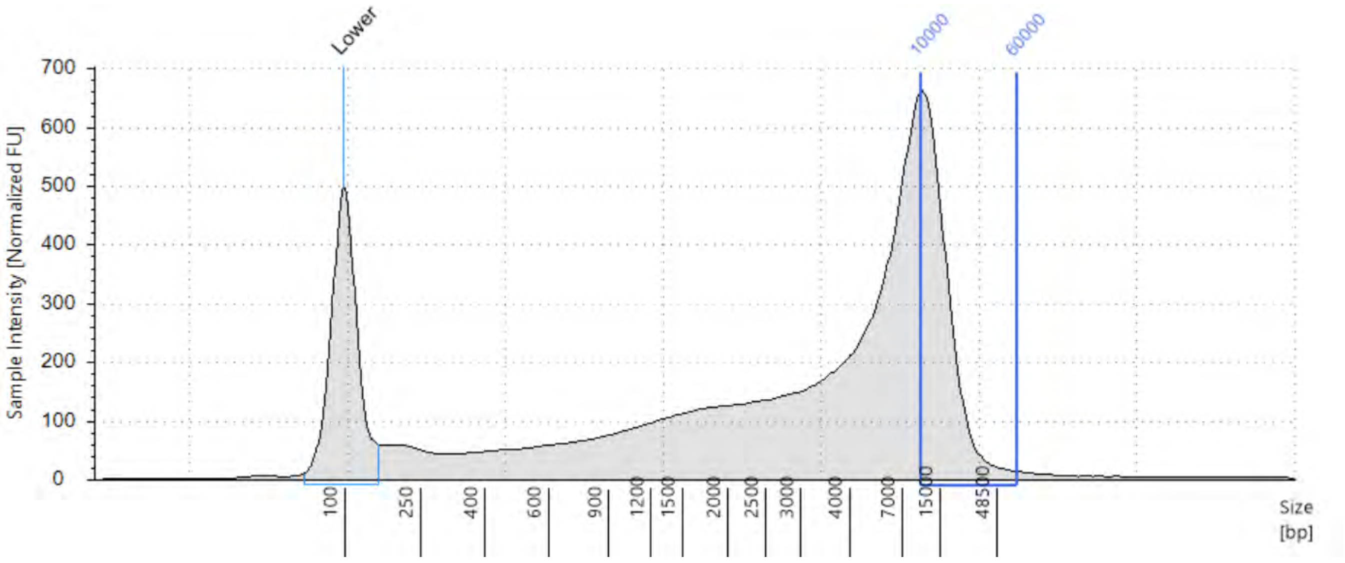

Region Table

From [bp]	To [bp]	Average Size [bp]	Conc. [ng/µl]	Region Molarity [nmol/l]	% of Total	Region Comment	Color
10000	60000	16554	12.1	1.31	23.68		

 © Agilent Technologies, Inc. 2017

G2: W-T4-Initial-1077

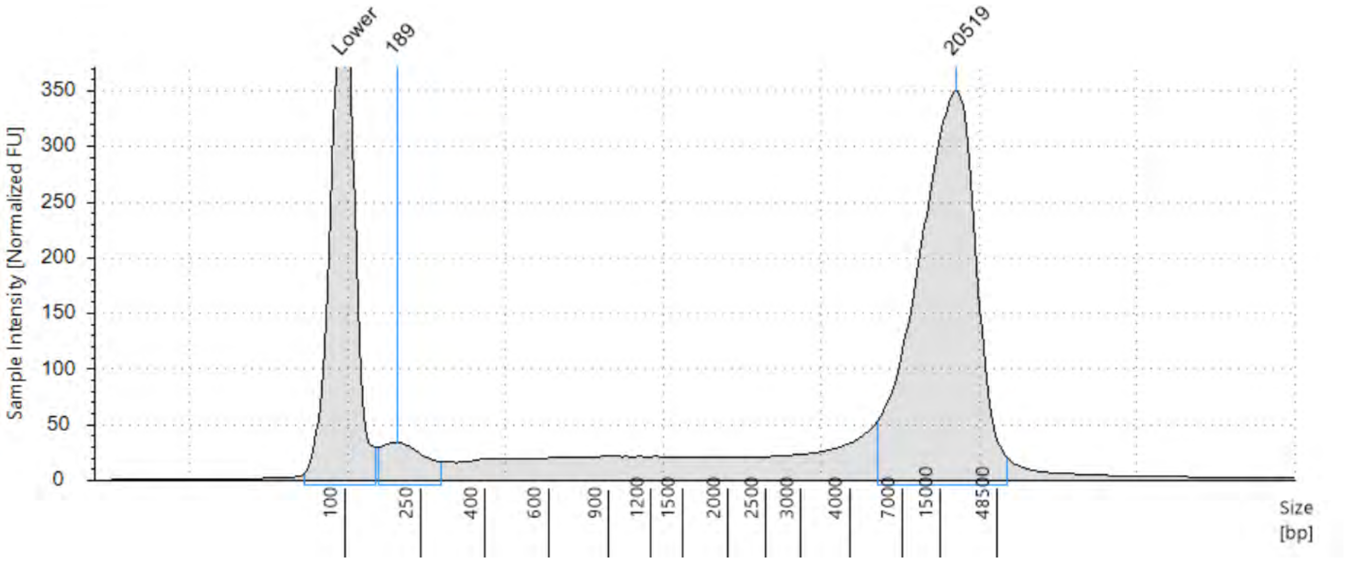

Sample Table

Well	DIN	Conc. [ng/µl]	Sample Description	Alert	Observations
G2	6.8	22.8	W-T4-Initial-1077		DIN Edited (Ladder sizing changed)

Peak Table

Size [bp]	Calibrated Conc. [ng/µl]	Assigned Conc. [ng/µl]	% Integrated Area	From [bp]	To [bp]	Peak Comment	Observations
100	8.50	8.50	-	61	146		Lower Marker
189	1.07	-	6.48	150	292		
20519	15.3	-	92.93	5374	54458		
-	-	-	-	-	-		Sample Well

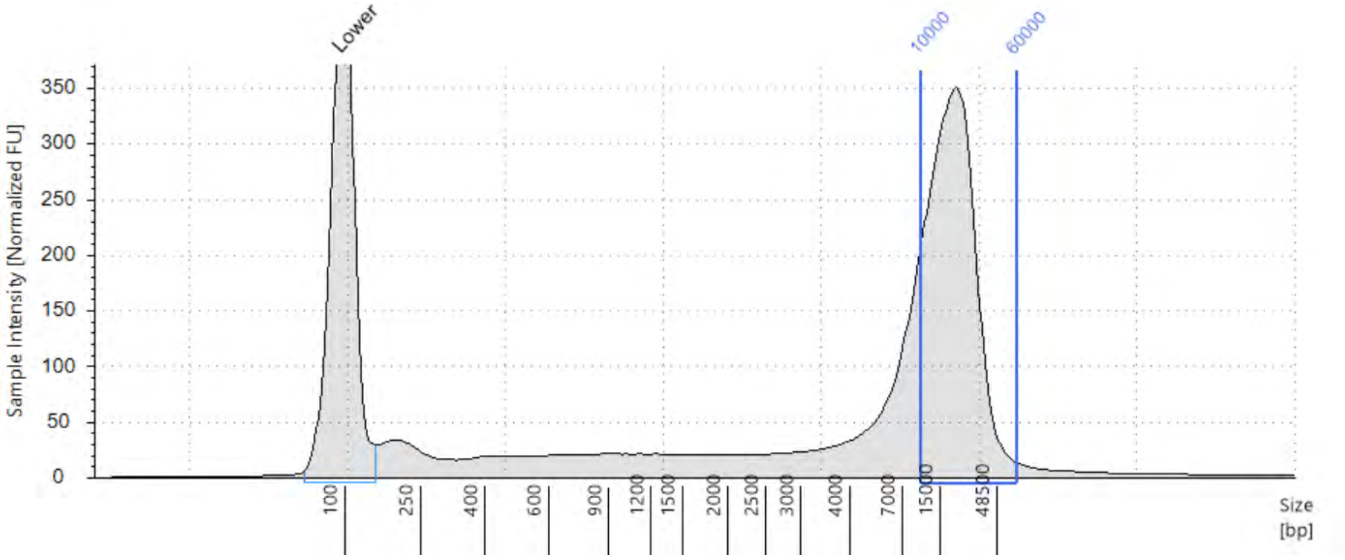

Region Table

From [bp]	To [bp]	Average Size [bp]	Conc. [ng/µl]	Region Molarity [nmol/l]	% of Total	Region Comment	Color
10000	60000	21731	12.4	1.04	54.44		

H2: W-T4-Initial-1078

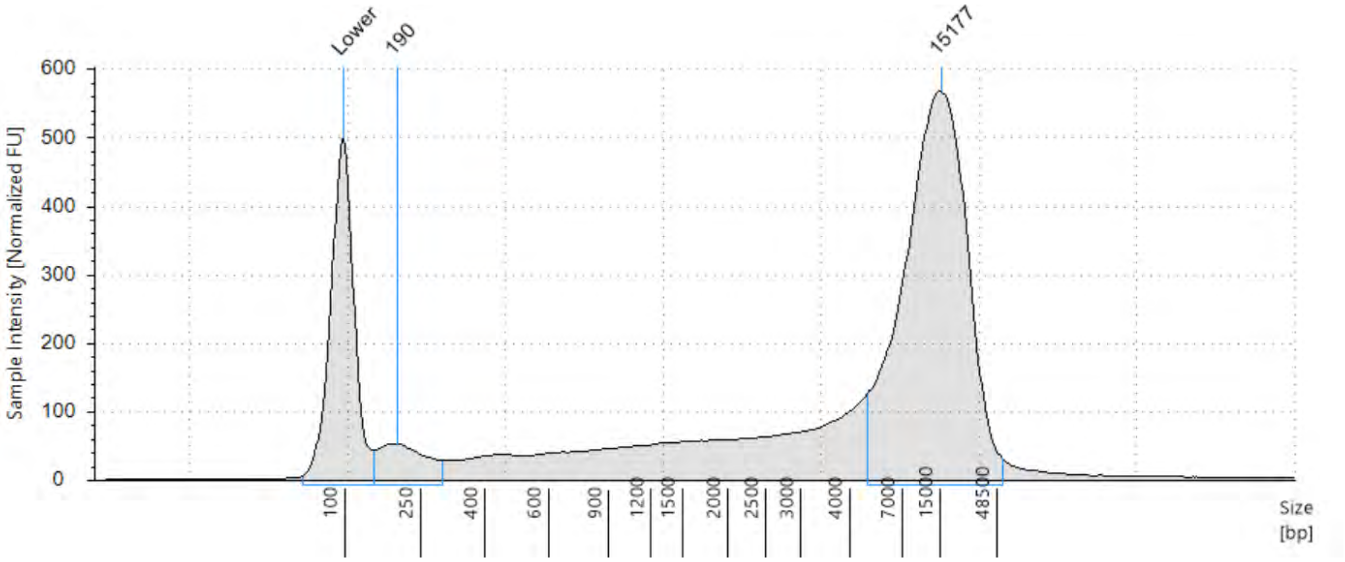

Sample Table

Well	DIN	Conc. [ng/µl]	Sample Description	Alert	Observations
H2	6.5	43.2	W-T4-Initial-1078		DIN Edited (Ladder sizing changed)

Peak Table

Size [bp]	Calibrated Conc. [ng/µl]	Assigned Conc. [ng/µl]	%Integrated Area	From [bp]	To [bp]	Peak Comment	Observations
100	8.50	8.50	-	61	143		Lower Marker
190	1.78	-	6.17	143	293		
15177	26.7	-	92.61	4838	51354		
-	-	-	-	-	-		Sample Well

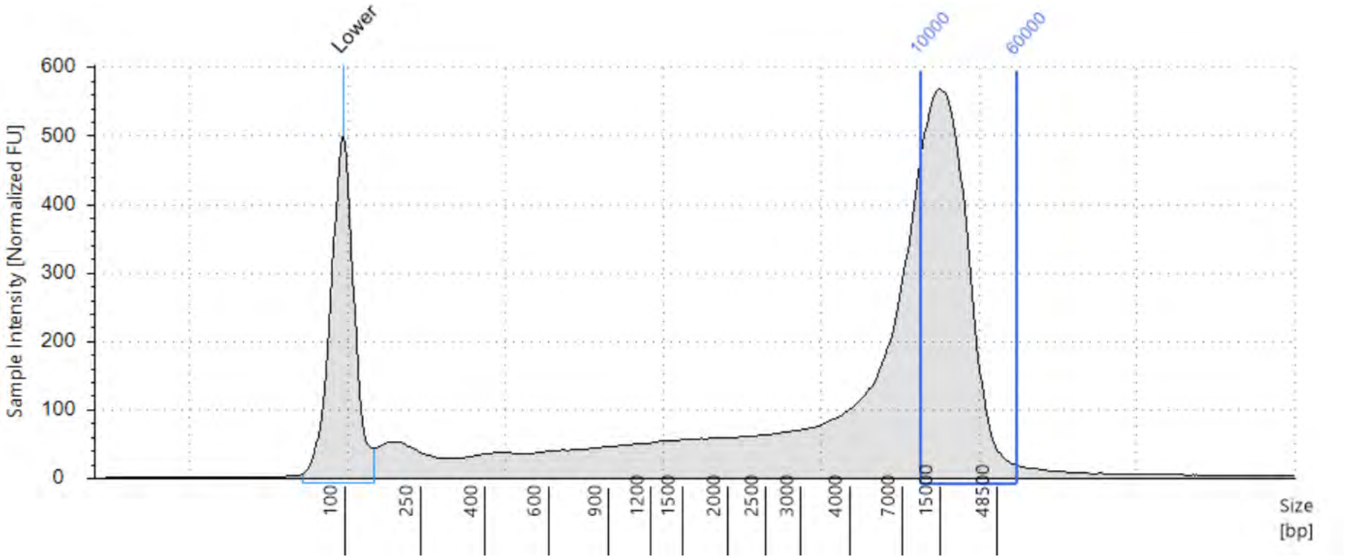

Region Table

From [bp]	To [bp]	Average Size [bp]	Conc. [ng/µl]	Region Molarity [nmol/l]	% of Total	Region Comment	Color
10000	60000	19809	18.7	1.72	43.40		

Filename: 2019-07-30-03.gDNA

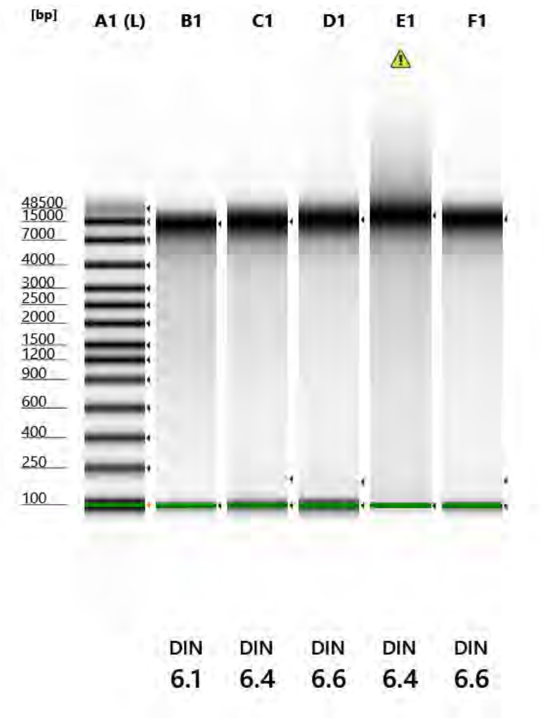

Default image (Contrast 50%), Image is Scaled to Sample

Sample Info

Well	DIN	Conc. [ng/µl]	Sample Description	Alert	Observations
A1	-	61.1	Ladder		Ladder
B1	6.1	90.2	W-T4-EtOH-1075		
C1	6.4	55.3	W-T4-Initial-851		
D1	6.6	40.5	W-T4-Initial-852		
E1	6.4	144	W-T4-Initial-853	⚠	Sample concentration outside recommended range
F1	6.6	72.0	W-T4-Initial-1079		

 © Agilent Technologies, Inc. 2017

A1: Ladder

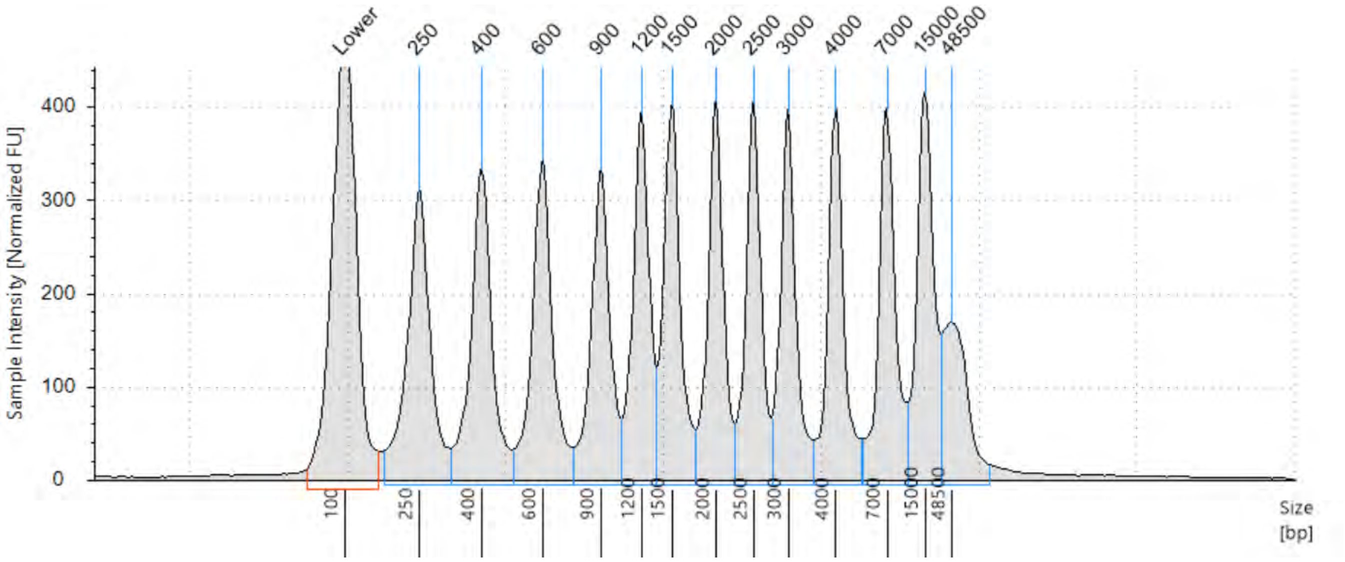

Sample Table

Well	DIN	Conc. [ng/µl]	Sample Description	Alert	Observations
A1	-	61.1	Ladder		Ladder

Peak Table

Size [bp]	Calibrated Conc. [ng/µl]	Assigned Conc. [ng/µl]	% Integrated Area	From [bp]	To [bp]	Peak Comment	Observations
100	8.50	8.50	-	63	149		Lower Marker
250	5.15	-	8.46	162	316		
400	5.04	-	8.28	316	492		
600	5.01	-	8.23	492	738		
900	4.58	-	7.52	738	1039		
1200	4.84	-	7.95	1039	1342		
1500	4.99	-	8.20	1342	1755		
2000	4.68	-	7.68	1755	2236		
2500	4.54	-	7.46	2236	2749		
3000	4.49	-	7.37	2749	3507		
4000	4.58	-	7.52	3507	5292		
7000	4.80	-	7.88	5370	10530		
15000	5.11	-	8.40	10530	20825		
48500	3.07	-	5.05	20825	>60000		

 © Agilent Technologies, Inc. 2017

B1: W-T4-EtOH-1075

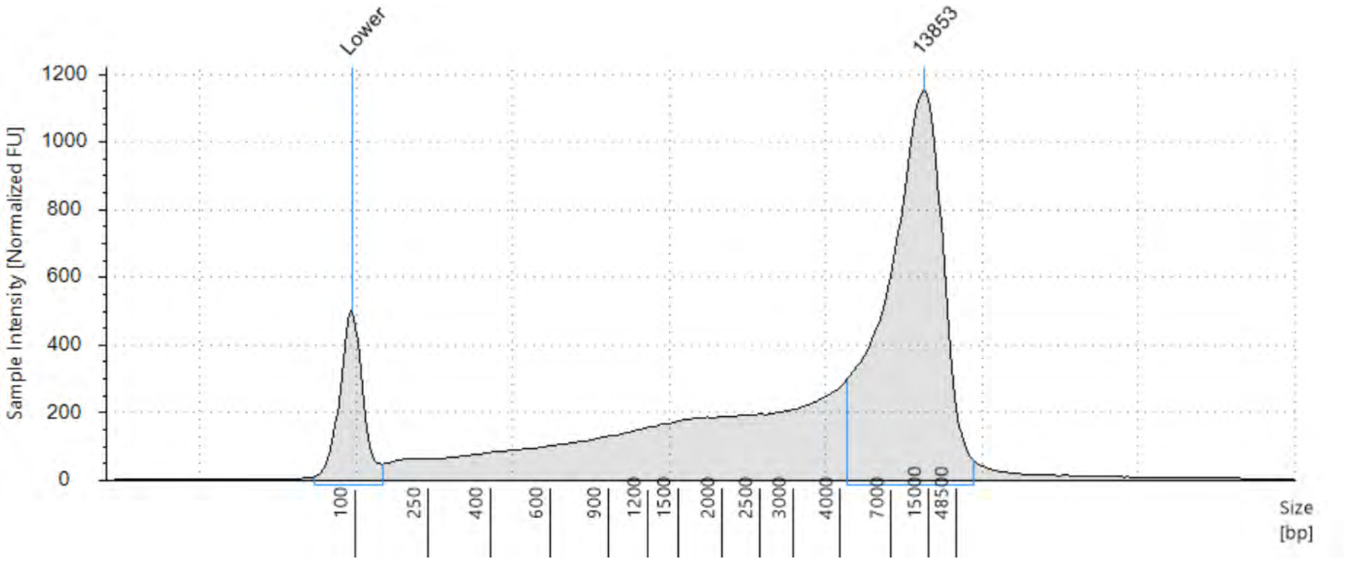

Sample Table

Well	DIN	Conc. [ng/µl]	Sample Description	Alert	Observations
B1	6.1	90.2	W-T4-EtOH-1075		

Peak Table

Size [bp]	Calibrated Conc. [ng/µl]	Assigned Conc. [ng/µl]	% Integrated Area	From [bp]	To [bp]	Peak Comment	Observations
100	8.50	8.50	-	62	144		Lower Marker
13853	48.2	-	100.00	4382	>60000		

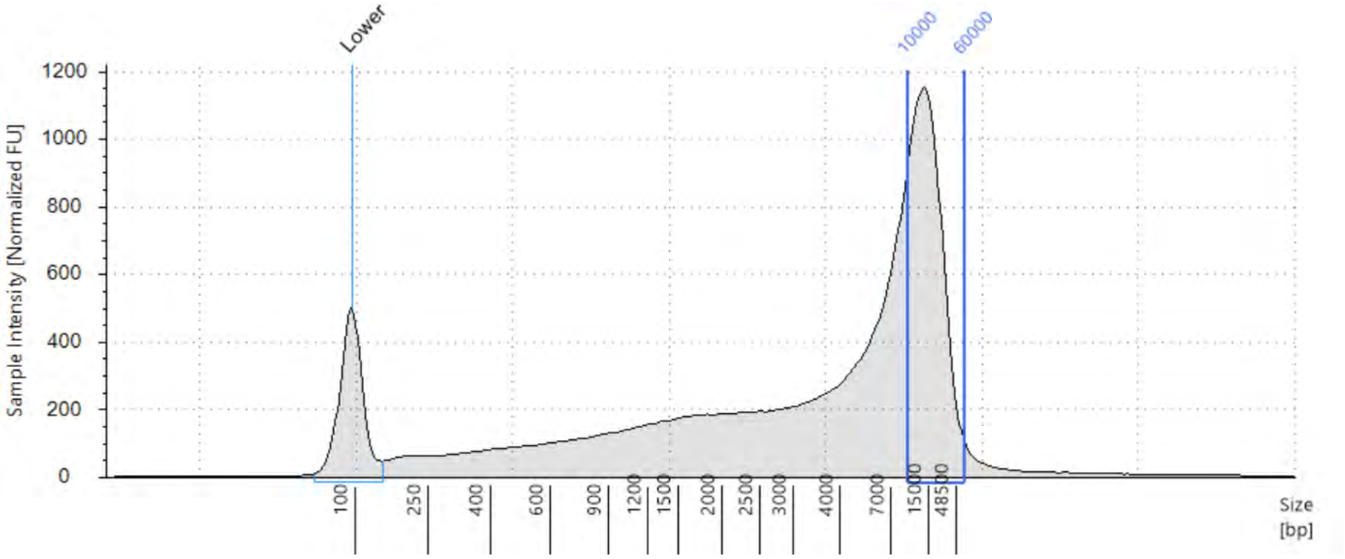

Region Table

From [bp]	To [bp]	Average Size [bp]	Conc. [ng/µl]	Region Molarity [nmol/l]	% of Total	Region Comment	Color
10000	60000	17500	28.5	2.93	31.60		

© Agilent Technologies, Inc. 2017

C1: W-T4-Initial-851

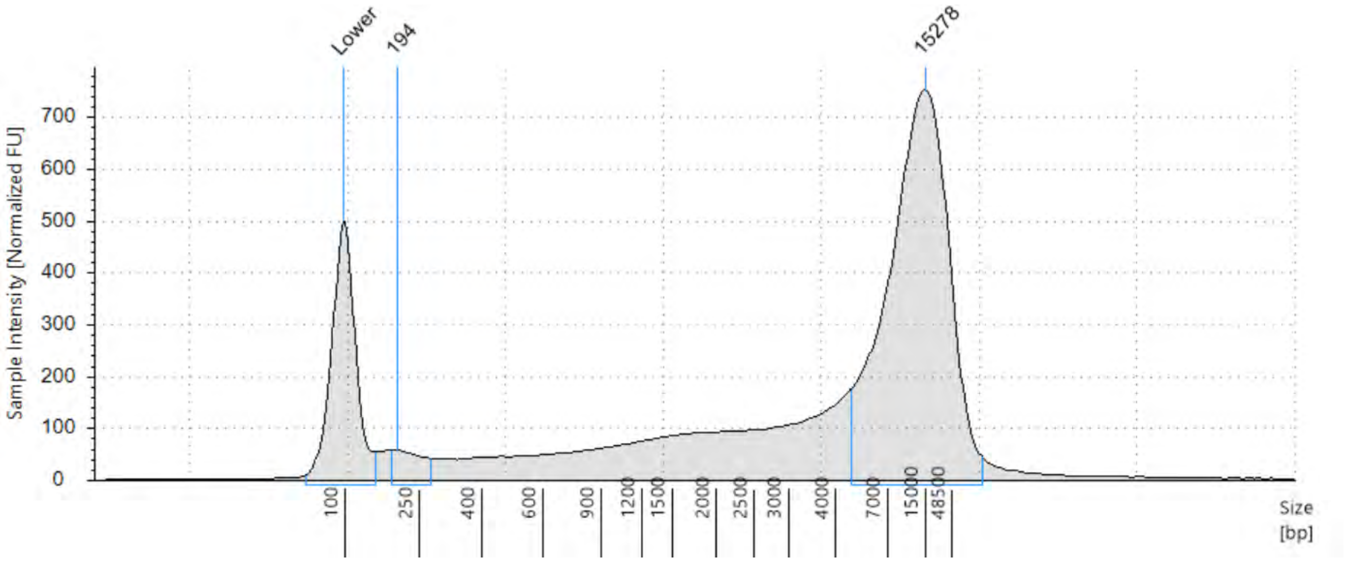

Sample Table

Well	DIN	Conc. [ng/µl]	Sample Description	Alert	Observations
C1	6.4	55.3	W-T4-Initial-851		

Peak Table

Size [bp]	Calibrated Conc. [ng/µl]	Assigned Conc. [ng/µl]	% Integrated Area	From [bp]	To [bp]	Peak Comment	Observations
100	8.50	8.50	-	63	146		Lower Marker
194	1.22	-	3.56	179	273		
15278	32.9	-	95.83	4771	>60000		
-	-	-	-	-	-		Sample Well

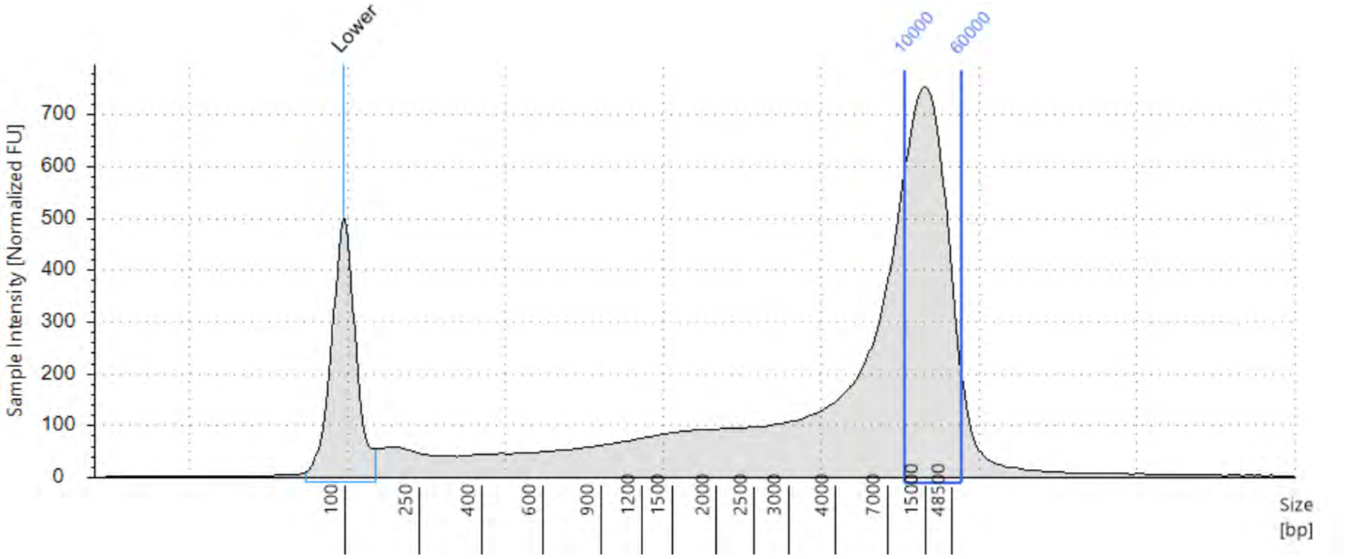

Region Table

From [bp]	To [bp]	Average Size [bp]	Conc. [ng/µl]	Region Molarity [nmol/l]	% of Total	Region Comment	Color
10000	60000	20698	21.2	1.97	38.33		

© Agilent Technologies, Inc. 2017

D1: W-T4-Initial-852

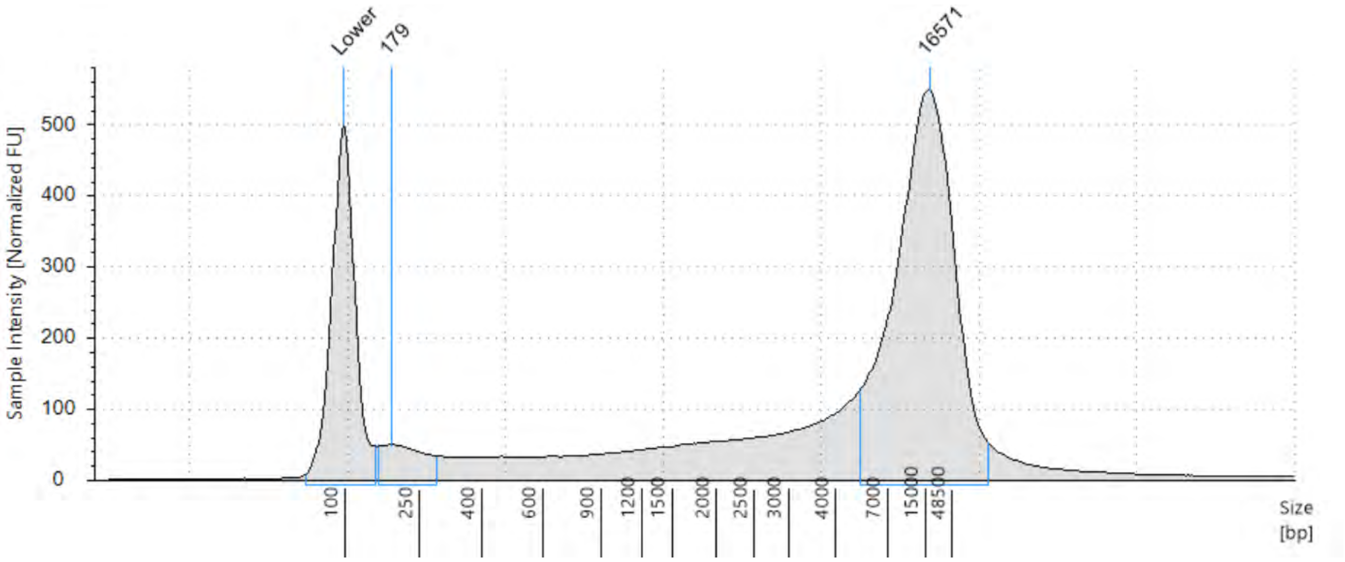

Sample Table

Well	DIN	Conc. [ng/µl]	Sample Description	Alert	Observations
D1	6.6	40.5	W-T4-Initial-852		

Peak Table

Size [bp]	Calibrated Conc. [ng/µl]	Assigned Conc. [ng/µl]	% Integrated Area	From [bp]	To [bp]	Peak Comment	Observations
100	8.50	8.50	-	62	146		Lower Marker
179	1.57	-	6.04	151	287		
16571	24.1	-	92.34	5210	>60000		
-	-	-	-	-	-		Sample Well

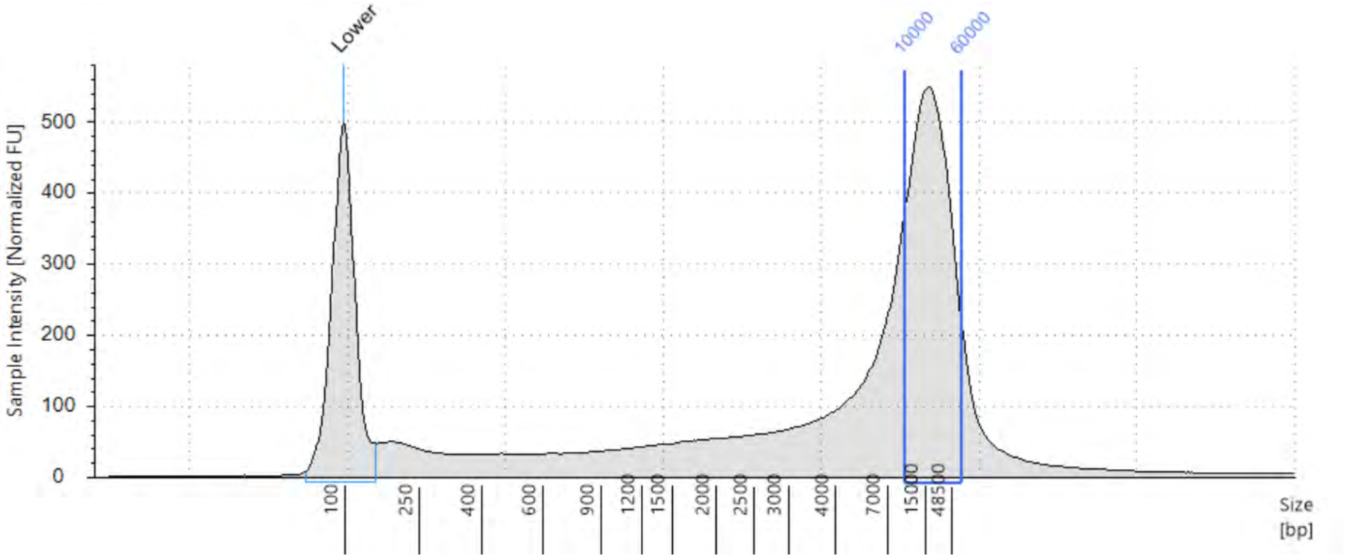

Region Table

From [bp]	To [bp]	Average Size [bp]	Conc. [ng/µl]	Region Molarity [nmol/l]	% of Total	Region Comment	Color
10000	60000	22250	16.3	1.45	40.34		

© Agilent Technologies, Inc. 2017

E1: W-T4-Initial-853

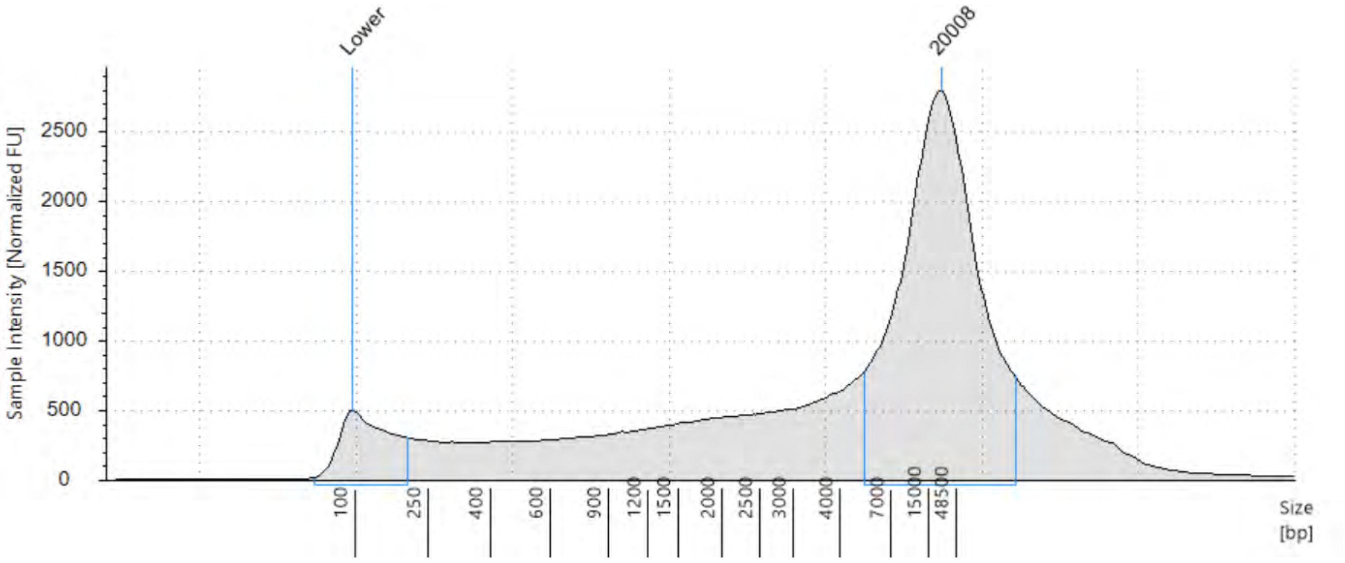

Sample Table

Well	DIN	Conc. [ng/µl]	Sample Description	Alert	Observations
E1	6.4	144	W-T4-Initial-853	⚠	Sample concentration outside recommended range

Peak Table

Size [bp]	Calibrated Conc. [ng/µl]	Assigned Conc. [ng/µl]	% Integrated Area	From [bp]	To [bp]	Peak Comment	Observations
100	8.50	8.50	-	62	198		Lower Marker
20008	73.8	-	98.33	5273	>60000		
-	-	-	-	-	-		Sample Well

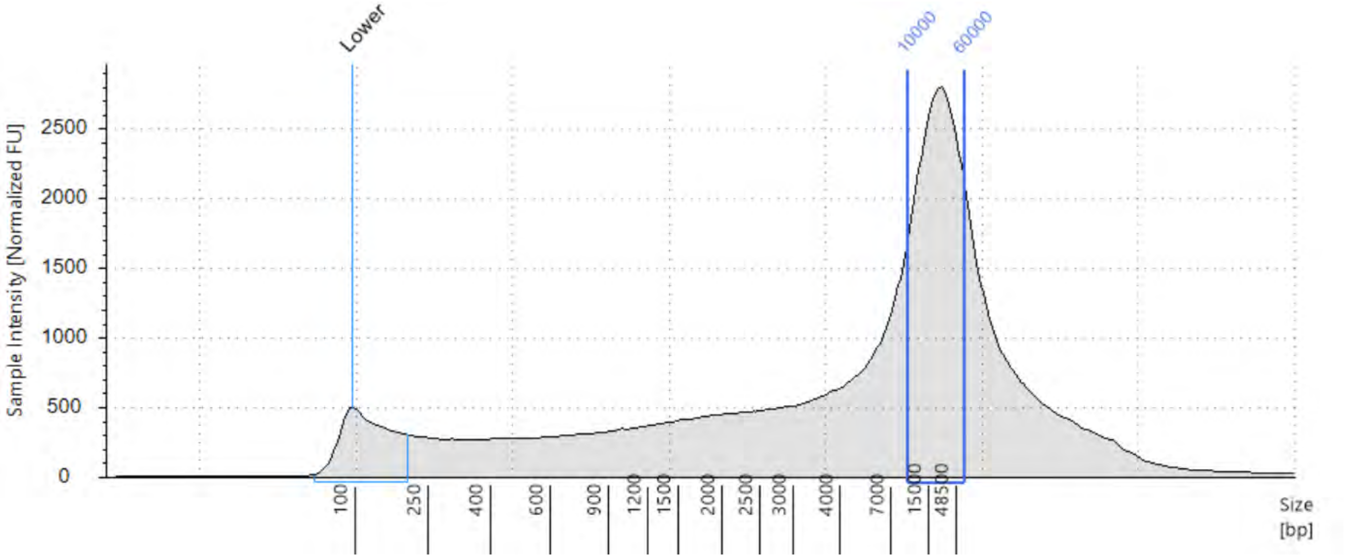

Region Table

From [bp]	To [bp]	Average Size [bp]	Conc. [ng/µl]	Region Molarity [nmol/l]	% of Total	Region Comment	Color
10000	60000	25050	41.1	3.38	28.55		■

F1: W-T4-Initial-1079

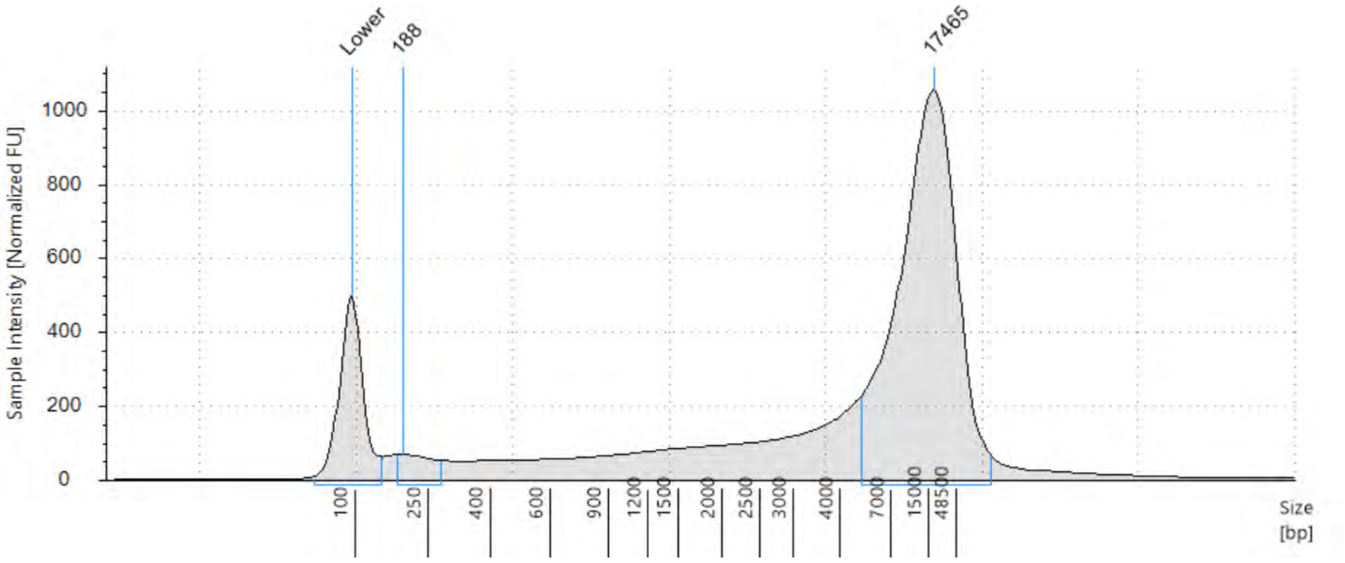

Sample Table

Well	DIN	Conc. [ng/µl]	Sample Description	Alert	Observations
F1	6.6	72.0	W-T4-Initial-1079		

Peak Table

Size [bp]	Calibrated Conc. [ng/µl]	Assigned Conc. [ng/µl]	% Integrated Area	From [bp]	To [bp]	Peak Comment	Observations
100	8.50	8.50	-	62	144		Lower Marker
188	1.70	-	3.60	173	279		
17465	45.2	-	95.74	5120	>60000		
-	-	-	-	-	-		Sample Well

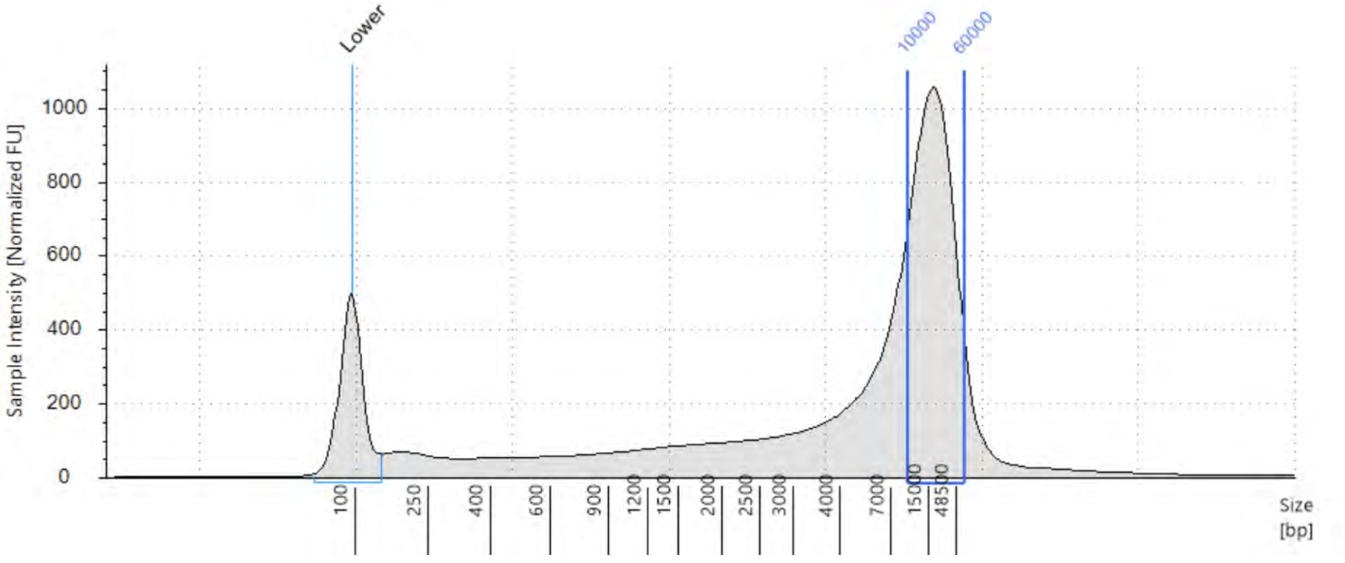

Region Table

From [bp]	To [bp]	Average Size [bp]	Conc. [ng/µl]	Region Molarity [nmol/l]	% of Total	Region Comment	Color
10000	60000	22188	31.1	2.76	43.21		

© Agilent Technologies, Inc. 2017

Filename: 2020-06-10-01.gDNA

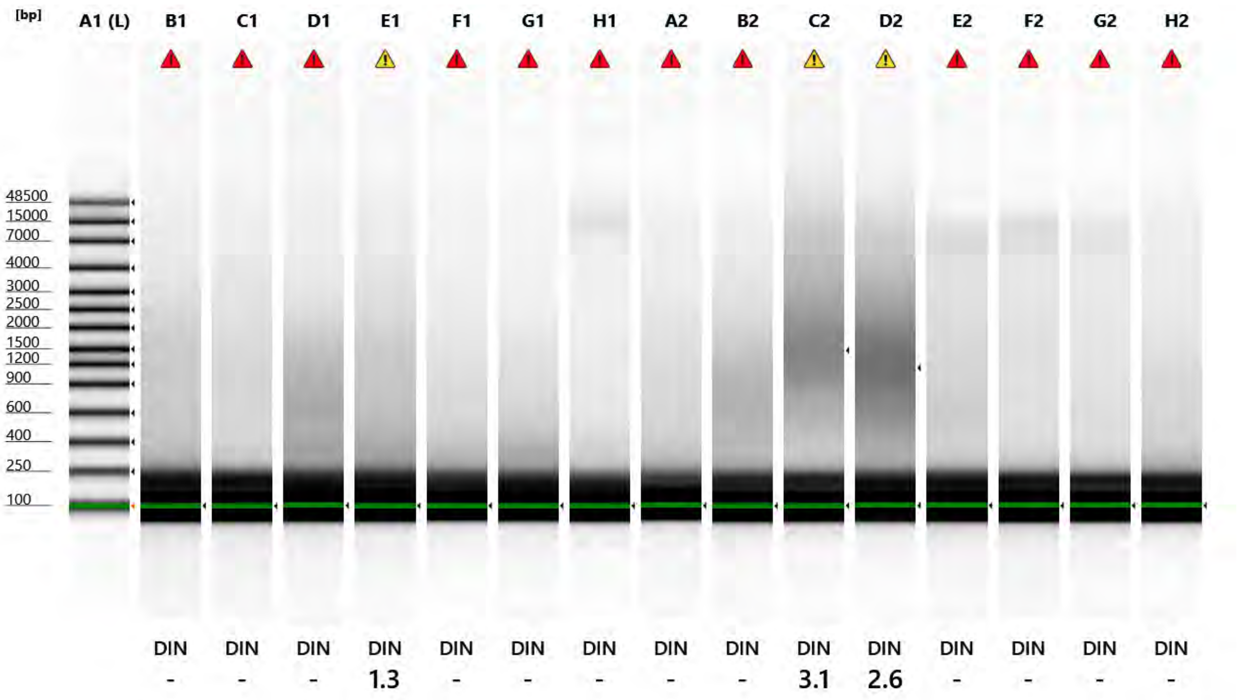

Default image (Contrast 50%), Image is Scaled to Sample

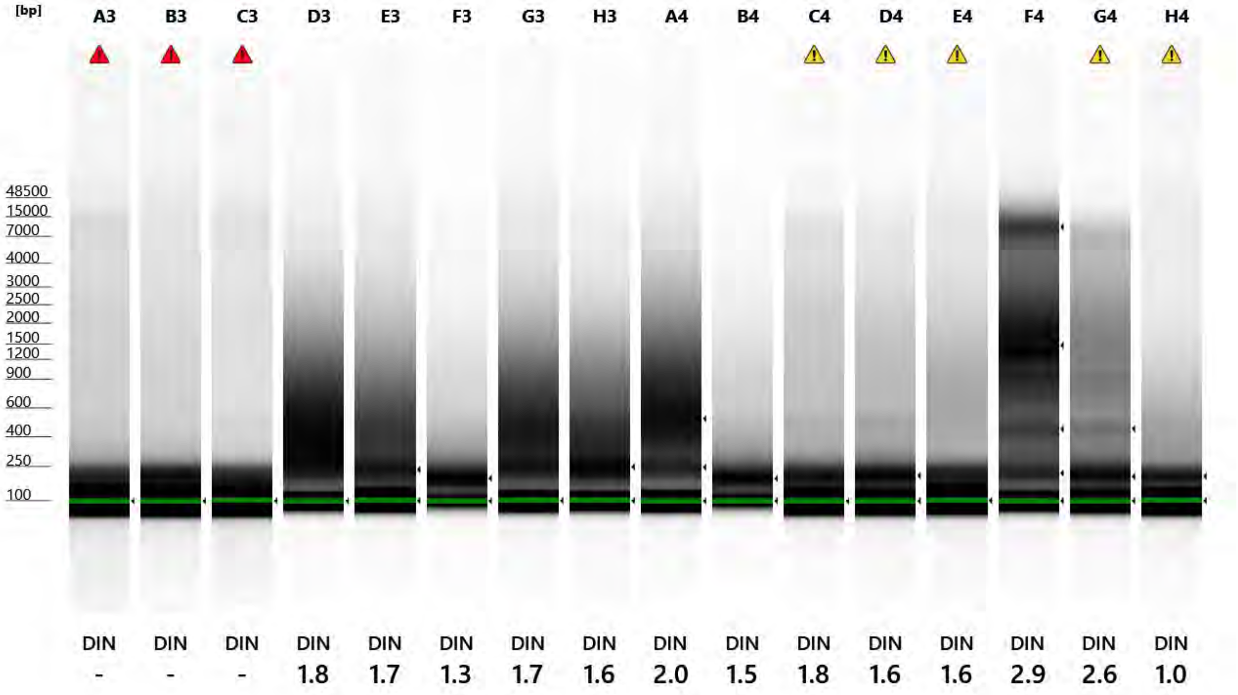

Default image (Contrast 50%), Image is Scaled to Sample

© Agilent Technologies, Inc. 2017

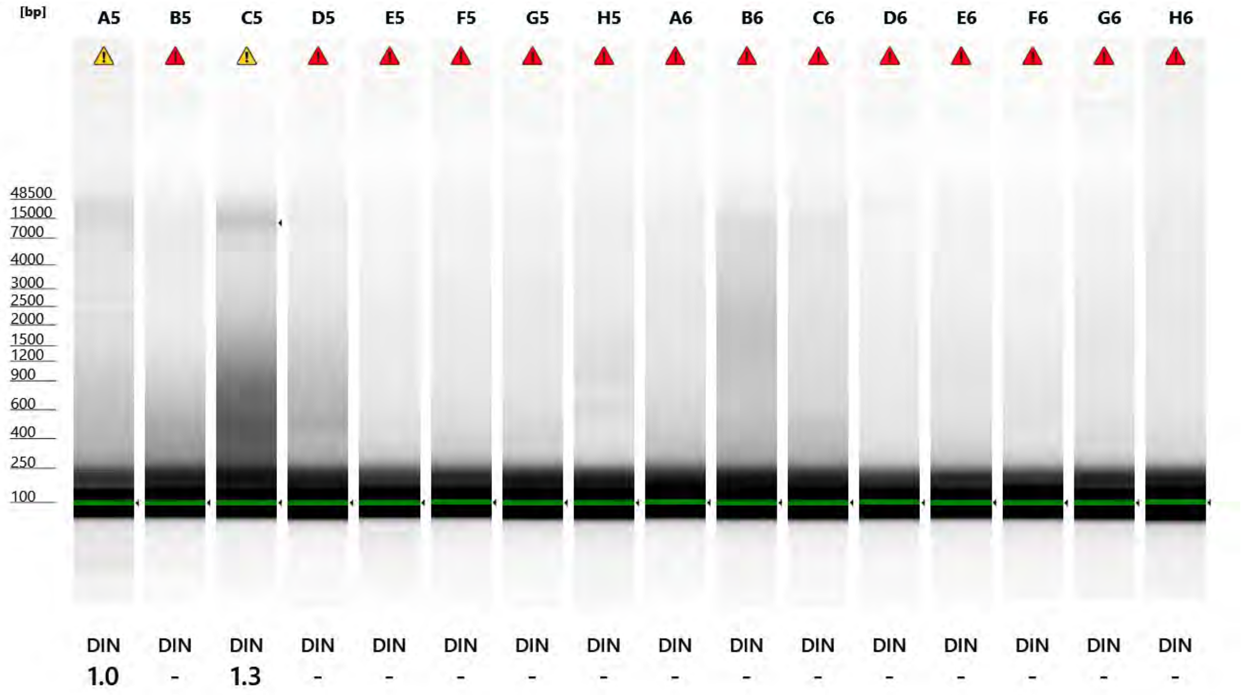

Default image (Contrast 50%), Image is Scaled to Sample

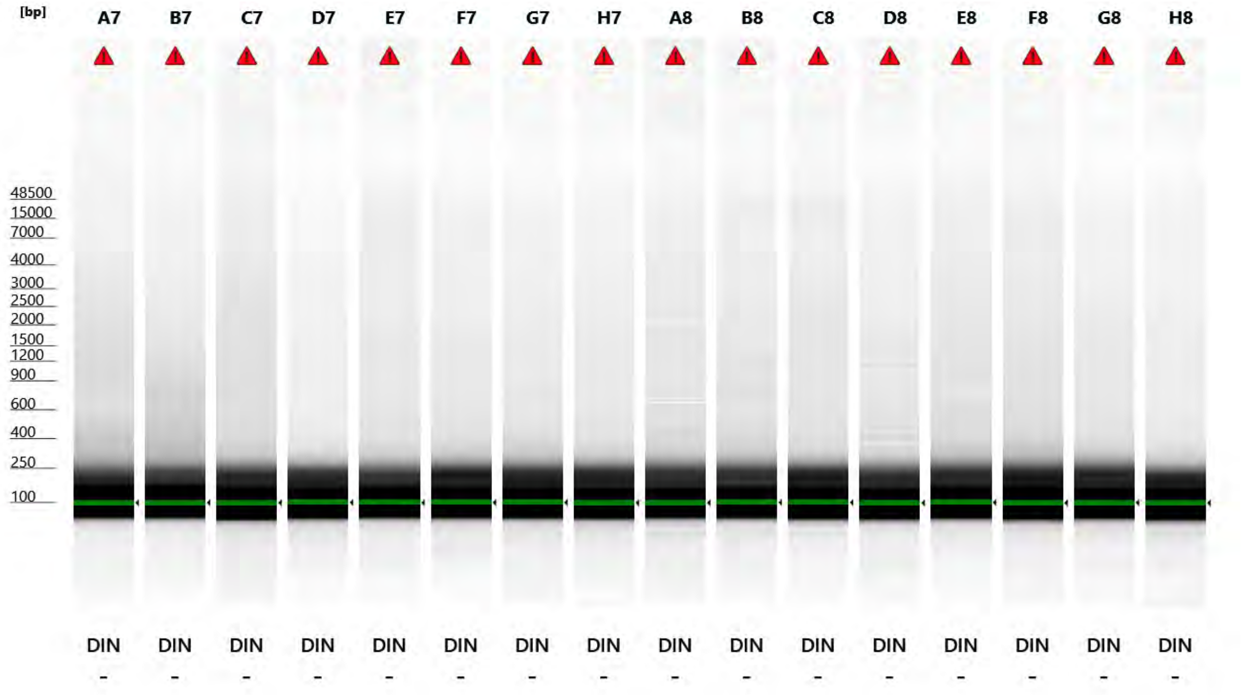

Default image (Contrast 50%), Image is Scaled to Sample

 © Agilent Technologies, Inc. 2017

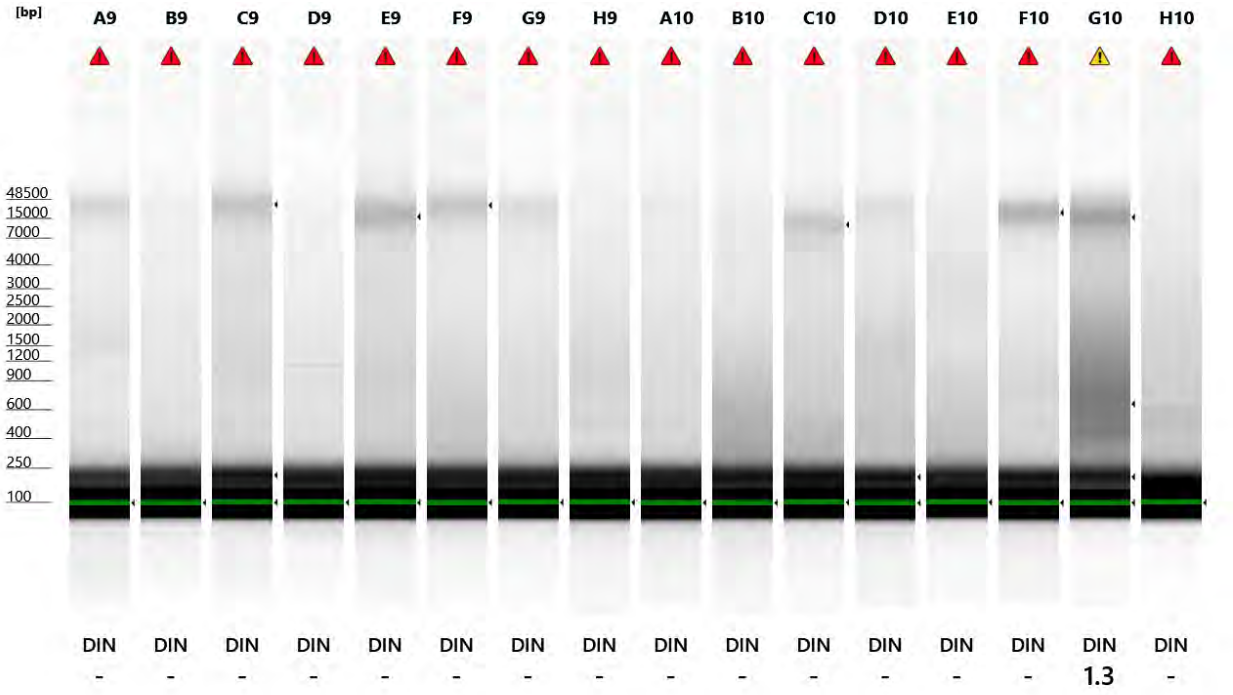

Default image (Contrast 50%), Image is Scaled to Sample

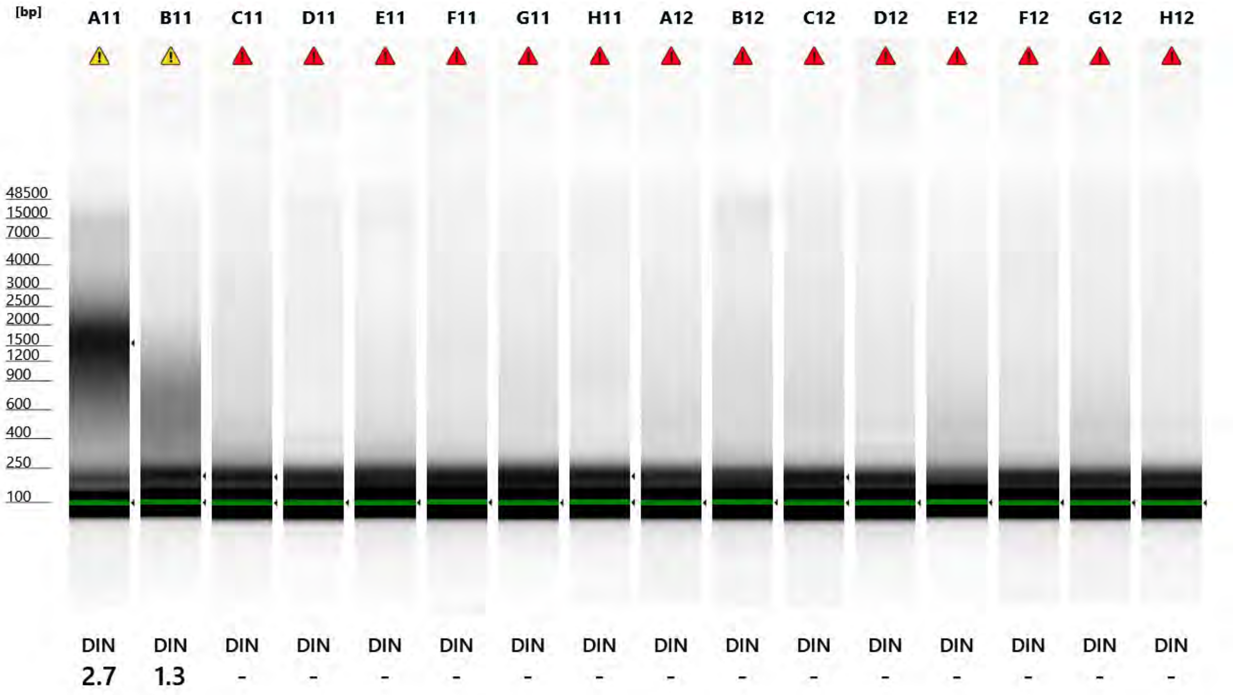

Default image (Contrast 50%), Image is Scaled to Sample

© Agilent Technologies, Inc. 2017

Sample Info

Well	DIN	Conc. [ng/µl]	Sample Description	Alert	Observations
A1	-	79.9	Ladder		Ladder
B1	-	2.25	C-651-T5-D		Sample concentration outside functional range for DIN
C1	-	1.92	C-652-T5-D		Sample concentration outside functional range for DIN
D1	-	2.68	C-653-T5-D		Sample concentration outside functional range for DIN
E1	1.3	3.12	C-654-T5-D		Sample concentration outside functional range for DIN
F1	-	2.33	C-655-T5-D		Sample concentration outside functional range for DIN
G1	-	2.15	C-397-T5-D		Sample concentration outside functional range for DIN
H1	-	1.77	C-641-T5-DSS		Sample concentration outside functional range for DIN
A2	-	2.27	C-642-T5-DSS		Sample concentration outside functional range for DIN
B2	-	2.55	C-643-T5-DSS		Sample concentration outside functional range for DIN
C2	3.1	3.56	C-644-T5-DSS		Sample concentration outside functional range for DIN
D2	2.6	4.30	C-645-T5-DSS		Sample concentration outside functional range for DIN
E2	-	1.92	C-661-T5-SS		Sample concentration outside functional range for DIN
F2	-	1.64	C-662-T5-SS		Sample concentration outside functional range for DIN
G2	-	1.48	C-663-T5-SS		Sample concentration outside functional range for DIN
H2	-	1.76	C-664-T5-SS		Sample concentration outside functional range for DIN
A3	-	2.81	C-665-T5-SS		Sample concentration outside functional range for DIN
B3	-	2.34	C-400-T5-SS		Sample concentration outside functional range for DIN
C3	-	1.85	C-401-T5-SS		Sample concentration outside functional range for DIN
D3	1.8	25.2	M-156-T4-D		
E3	1.7	10.4	M-157-T4-D		
F3	1.3	22.7	M-158-T4-D		
G3	1.7	14.2	M-159-T4-D		
H3	1.6	17.5	M-160-T4-D		
A4	2.0	15.5	M-288-T4-D		
B4	1.5	22.1	M-289-T4-D		
C4	1.8	3.93	M-203-T5-D		Sample concentration outside functional range for DIN
D4	1.6	3.86	M-204-T5-D		Sample concentration outside functional range for DIN
E4	1.6	3.96	M-205-T5-D		Sample concentration outside functional range for DIN
F4	2.9	27.3	M-306-T5-D		
G4	2.6	9.45	M-307-T5-D		Sample concentration outside recommended range
H4	1.0	3.10	M-192-T5-DSS		Sample concentration outside functional range for DIN
A5	1.0	3.61	M-193-T5-DSS		Sample concentration outside functional range for DIN
B5	-	2.76	M-194-T5-DSS		Sample concentration outside functional range for DIN
C5	1.3	5.78	M-302-T5-DSS		Sample concentration outside recommended range
D5	-	2.19	M-303-T5-DSS		Sample concentration outside functional range for DIN
E5	-	1.72	M-211-T5-SS		Sample concentration outside functional range for DIN
F5	-	1.84	M-212-T5-SS		Sample concentration outside functional range for DIN
G5	-	1.43	M-213-T5-SS		Sample concentration outside functional range for DIN
H5	-	1.50	M-214-T5-SS		Sample concentration outside functional range for DIN
A6	-	2.43	M-215-T5-SS		Sample concentration outside functional range for DIN
B6	-	2.69	M-310-T5-SS		Sample concentration outside functional range for DIN
C6	-	2.42	M-311-T5-SS		Sample concentration outside functional range for DIN
D6	-	1.37	S-831-T4-D		Sample concentration outside functional range for DIN
E6	-	1.60	S-832-T4-D		Sample concentration outside functional range for DIN
F6	-	1.48	S-833-T4-D		Sample concentration outside functional range for DIN
G6	-	1.43	S-834-T4-D		Sample concentration outside functional range for DIN
H6	-	1.25	S-835-T4-D		Sample concentration outside functional range for DIN
A7	-	2.22	S-1056-T4-D		Sample concentration outside functional range for DIN
B7	-	1.84	S-1057-T4-D		Sample concentration outside functional range for DIN
C7	-	1.32	S-1058-T4-D		Sample concentration outside functional range for DIN
D7	-	0.987	S-1059-T4-D		Sample concentration outside functional range for DIN
E7	-	1.62	S-1060-T4-D		Sample concentration outside functional range for DIN
F7	-	1.23	S-821-T4-DSS		Sample concentration outside functional range for DIN
G7	-	1.25	S-822-T4-DSS		Sample concentration outside functional range for DIN
H7	-	1.15	S-823-T4-DSS		Sample concentration outside functional range for DIN
A8	-	1.53	S-824-T4-DSS		Sample concentration outside functional range for DIN
B8	-	1.44	S-825-T4-DSS		Sample concentration outside functional range for DIN
C8	-	1.05	S-1046-T4-DSS		Sample concentration outside functional range for DIN
D8	-	1.21	S-1047-T4-DSS		Sample concentration outside functional range for DIN
E8	-	1.37	S-1048-T4-DSS		Sample concentration outside functional range for DIN
F8	-	1.51	S-1049-T4-DSS		Sample concentration outside functional range for DIN
G8	-	1.18	S-1050-T4-DSS		Sample concentration outside functional range for DIN

 © Agilent Technologies, Inc. 2017

H8	-	0.846	S-876-T5-D		Sample concentration outside functional range for DIN
A9	-	1.19	S-877-T5-D		Sample concentration outside functional range for DIN
B9	-	1.50	S-878-T5-D		Sample concentration outside functional range for DIN
C9	-	1.87	S-879-T5-D		Sample concentration outside functional range for DIN
D9	-	1.17	S-880-T5-D		Sample concentration outside functional range for DIN
E9	-	2.17	S-1101-T5-D		Sample concentration outside functional range for DIN
F9	-	2.02	S-1102-T5-D		Sample concentration outside functional range for DIN
G9	-	1.75	S-1103-T5-D		Sample concentration outside functional range for DIN
H9	-	1.32	S-1104-T5-D		Sample concentration outside functional range for DIN
A10	-	1.45	S-1105-T5-D		Sample concentration outside functional range for DIN
B10	-	2.45	S-862-T5-DE		Sample concentration outside functional range for DIN
C10	-	2.45	S-863-T5-DE		Sample concentration outside functional range for DIN
D10	-	1.74	S-864-T5-DE		Sample concentration outside functional range for DIN
E10	-	1.86	S-865-T5-DE		Sample concentration outside functional range for DIN
F10	-	1.71	S-1086-T5-DE		Sample concentration outside functional range for DIN
G10	1.3	4.59	S-1087-T5-DE		Sample concentration outside functional range for DIN
H10	-	2.81	S-1088-T5-DE		Sample concentration outside functional range for DIN
A11	2.7	7.39	S-1089-T5-DE		Sample concentration outside recommended range
B11	1.3	3.75	S-1090-T5-DE		Sample concentration outside functional range for DIN
C11	-	1.64	S-866-T5-DSS		Sample concentration outside functional range for DIN
D11	-	0.888	S-867-T5-DSS		Sample concentration outside functional range for DIN
E11	-	1.56	S-868-T5-DSS		Sample concentration outside functional range for DIN
F11	-	1.45	S-869-T5-DSS		Sample concentration outside functional range for DIN
G11	-	1.32	S-870-T5-DSS		Sample concentration outside functional range for DIN
H11	-	1.15	S-1091-T5-DSS		Sample concentration outside functional range for DIN
A12	-	1.49	S-1092-T5-DSS		Sample concentration outside functional range for DIN
B12	-	1.51	S-1093-T5-DSS		Sample concentration outside functional range for DIN
C12	-	1.57	S-1094-T5-DSS		Sample concentration outside functional range for DIN
D12	-	1.28	S-1095-T5-DSS		Sample concentration outside functional range for DIN
E12	-	2.13	S-881-T5-E		Sample concentration outside functional range for DIN
F12	-	1.47	S-882-T5-E		Sample concentration outside functional range for DIN
G12	-	1.45	S-883-T5-E		Sample concentration outside functional range for DIN
H12	-	1.23	S-884-T5-E		Sample concentration outside functional range for DIN

 © Agilent Technologies, Inc. 2017

A1: Ladder

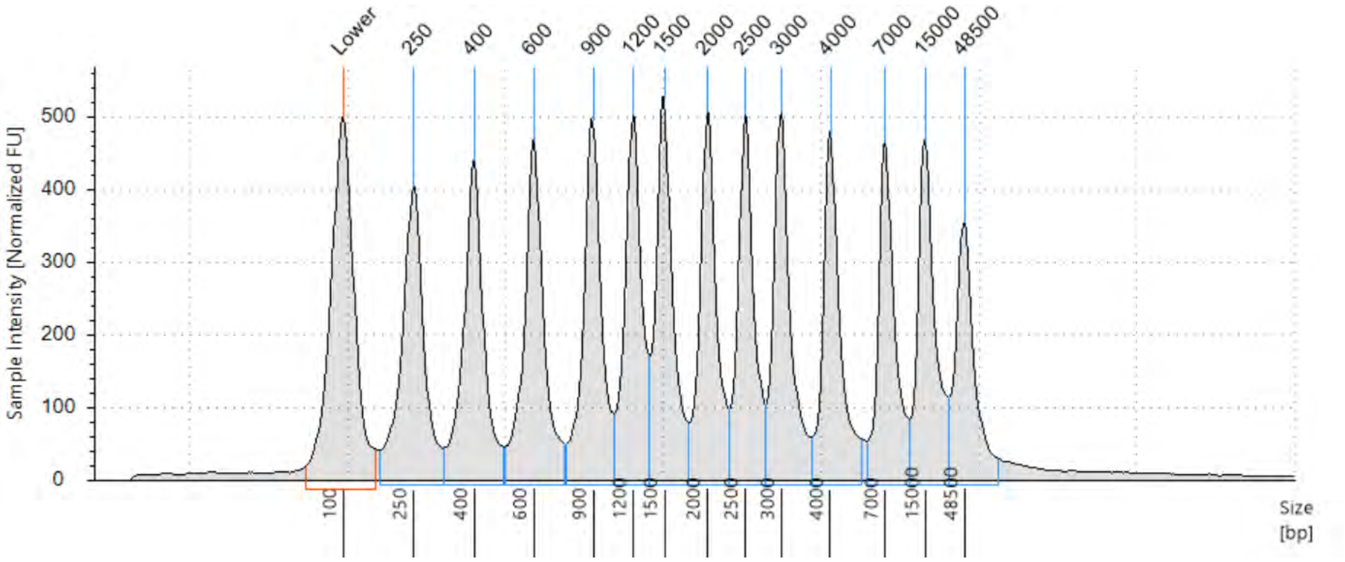

Sample Table

Well	DIN	Conc. [ng/µl]	Sample Description	Alert	Observations
A1	-	79.9	Ladder		Ladder

Peak Table

Size [bp]	Calibrated Conc. [ng/µl]	Assigned Conc. [ng/µl]	% Integrated Area	From [bp]	To [bp]	Peak Comment	Observations
100	8.50	8.50	-	61	153		Lower Marker
250	6.41	-	8.17	161	315		
400	6.45	-	8.23	315	490		
600	6.59	-	8.41	494	742		
900	6.44	-	8.21	749	1049		
1200	6.13	-	7.82	1049	1348		
1500	6.52	-	8.31	1348	1763		
2000	6.08	-	7.75	1763	2272		
2500	5.84	-	7.44	2272	2766		
3000	6.19	-	7.90	2766	3577		
4000	5.83	-	7.43	3577	5475		
7000	5.46	-	6.96	5862	11058		
15000	5.73	-	7.31	11058	23121		
48500	4.76	-	6.06	23121	>60000		

B1: C-651-T5-D

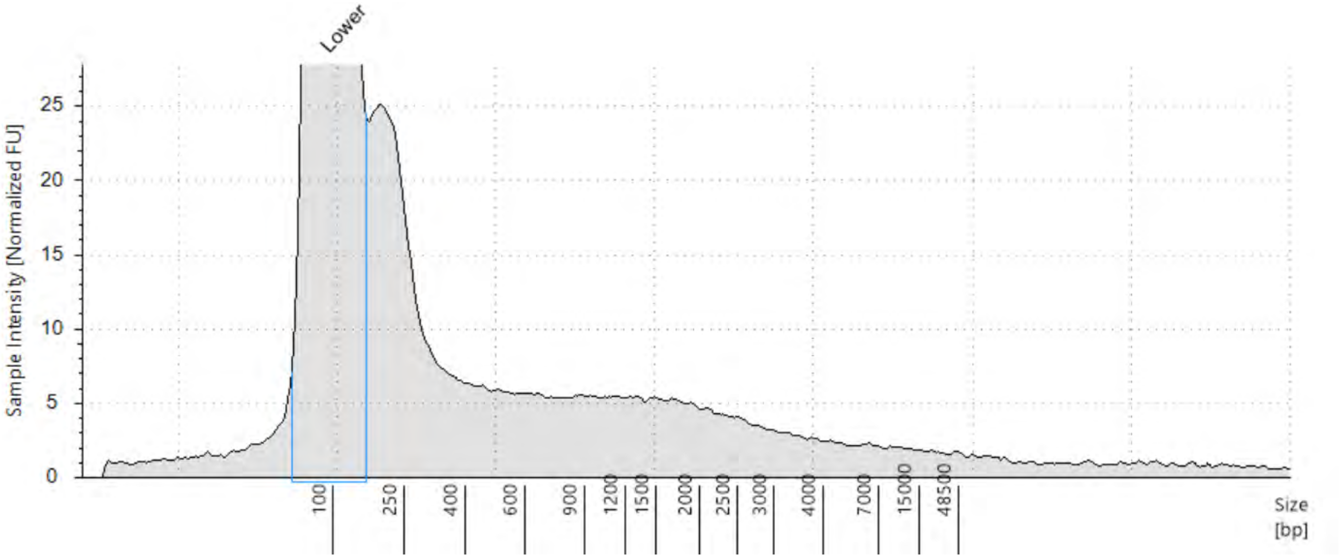

Sample Table

Well	DIN	Conc. [ng/µl]	Sample Description	Alert	Observations
B1	-	2.25	C-651-T5-D	▲	Sample concentration outside functional range for DIN

Peak Table

Size [bp]	Calibrated Conc. [ng/µl]	Assigned Conc. [ng/µl]	% Integrated Area	From [bp]	To [bp]	Peak Comment	Observations
100	8.50	8.50	-	58	154		Lower Marker

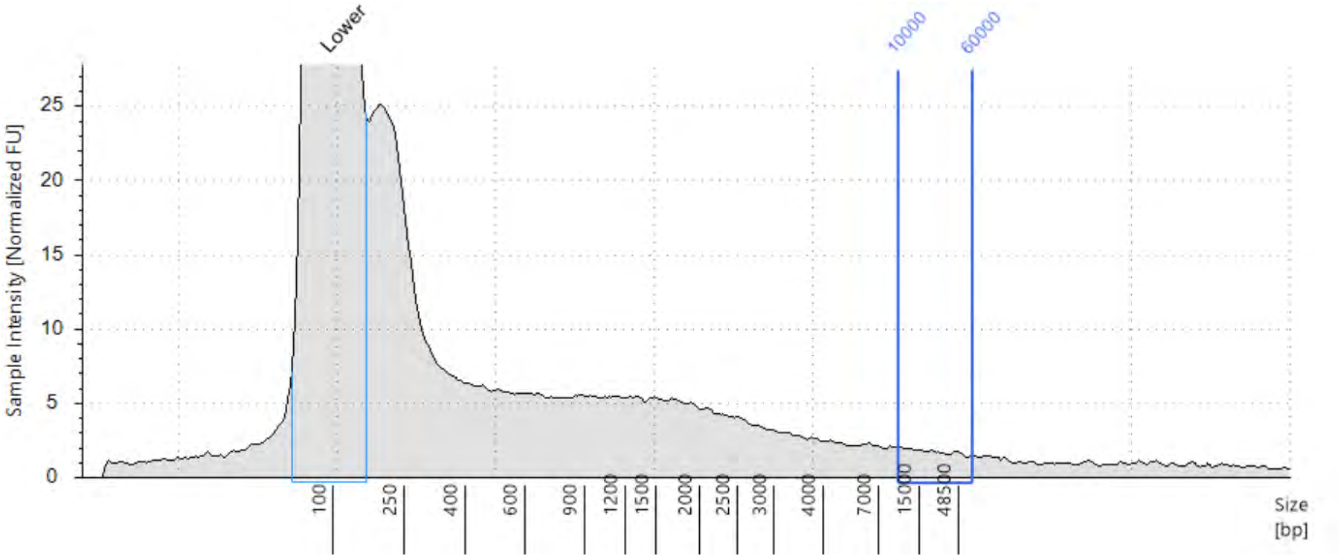

Region Table

From [bp]	To [bp]	Average Size [bp]	Conc. [ng/µl]	Region Molarity [nmol/l]	% of Total	Region Comment	Color
10000	60000	25143	0.0616	0.00664	2.74		■

C1: C-652-T5-D

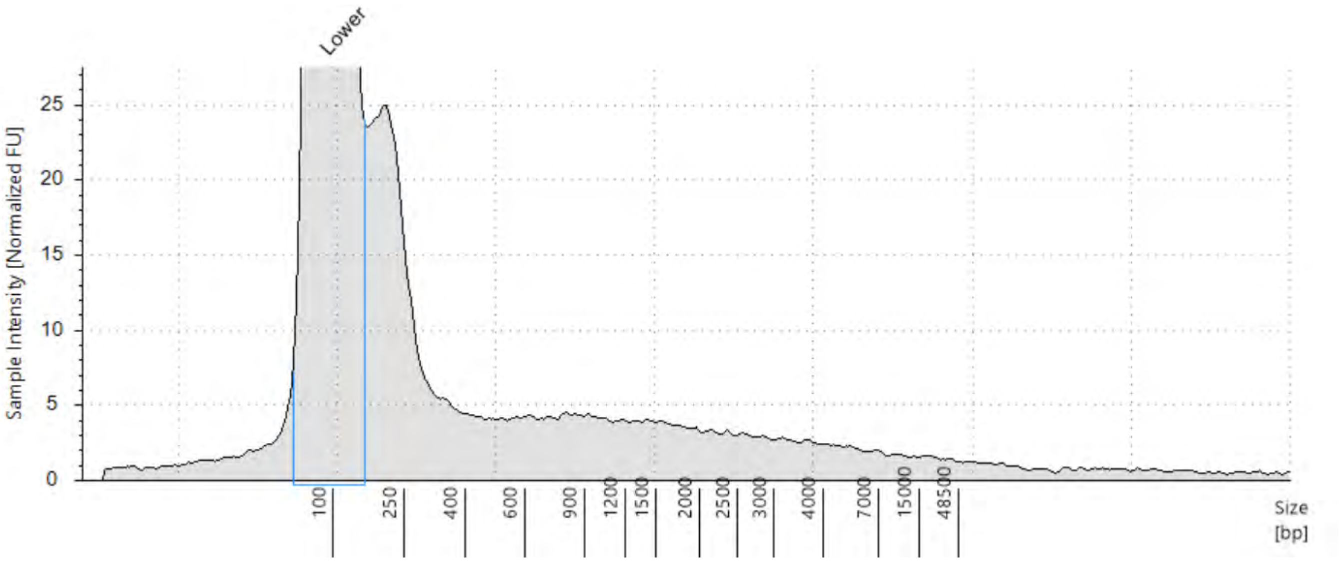

Sample Table

Well	DIN	Conc. [ng/µl]	Sample Description	Alert	Observations
C1	-	1.92	C-652-T5-D	▲	Sample concentration outside functional range for DIN

Peak Table

Size [bp]	Calibrated Conc. [ng/µl]	Assigned Conc. [ng/µl]	% Integrated Area	From [bp]	To [bp]	Peak Comment	Observations
100	8.50	8.50	-	60	151		Lower Marker

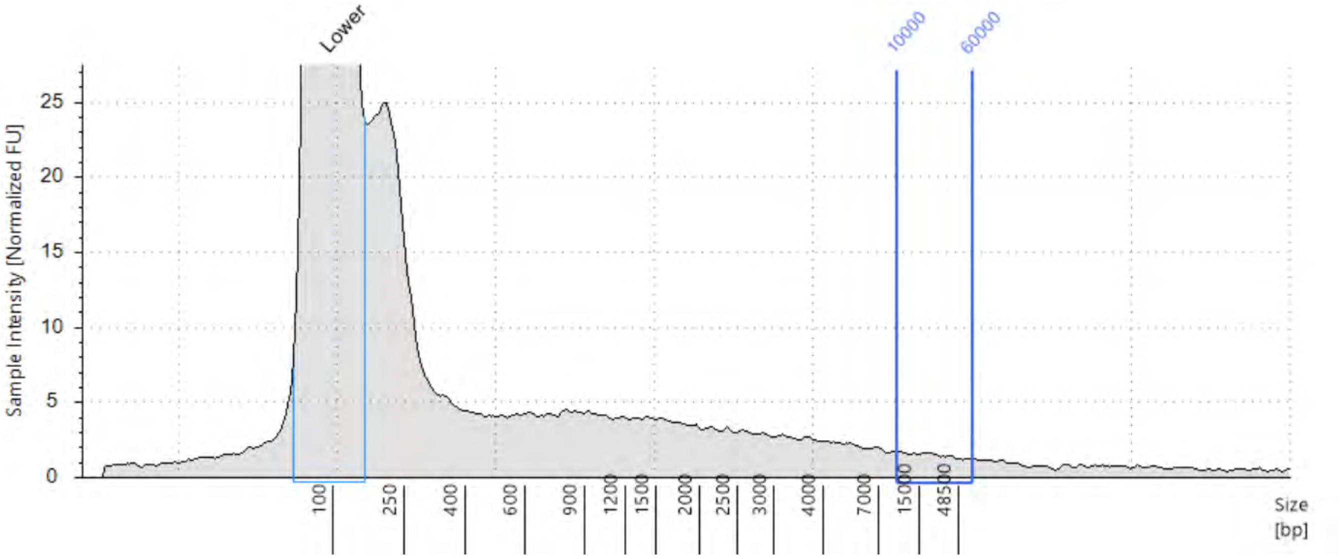

Region Table

From [bp]	To [bp]	Average Size [bp]	Conc. [ng/µl]	Region Molarity [nmol/l]	% of Total	Region Comment	Color
10000	60000	25302	0.0533	0.00572	2.77		■

D1: C-653-T5-D

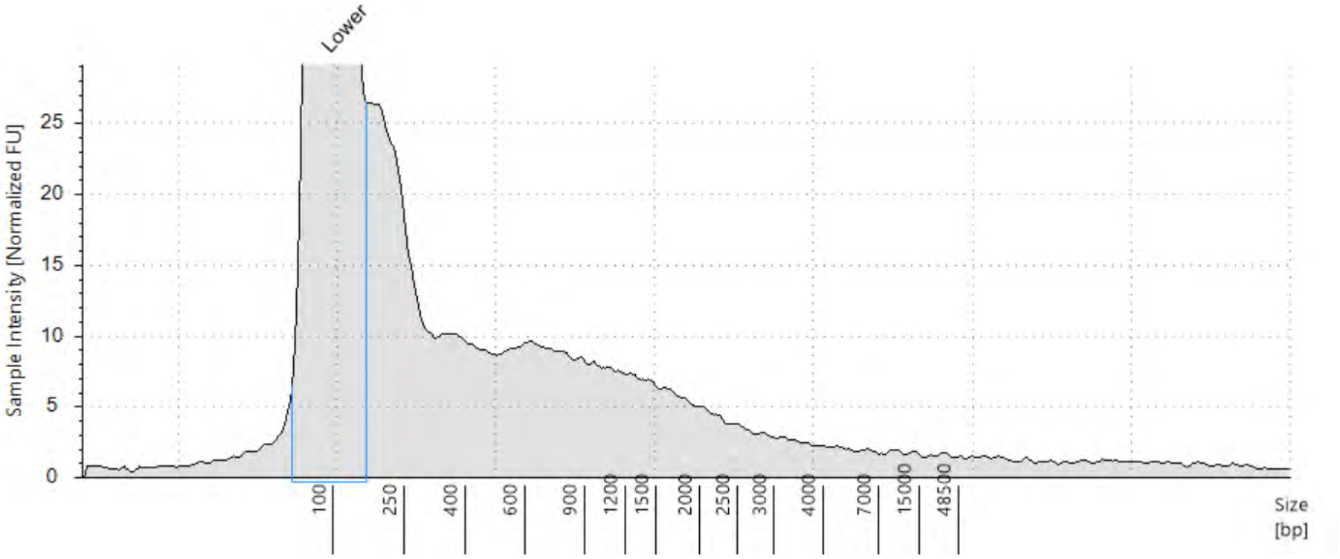

Sample Table

Well	DIN	Conc. [ng/µl]	Sample Description	Alert	Observations
D1	-	2.68	C-653-T5-D	▲	Sample concentration outside functional range for DIN

Peak Table

Size [bp]	Calibrated Conc. [ng/µl]	Assigned Conc. [ng/µl]	% Integrated Area	From [bp]	To [bp]	Peak Comment	Observations
100	8.50	8.50	-	58	152		Lower Marker

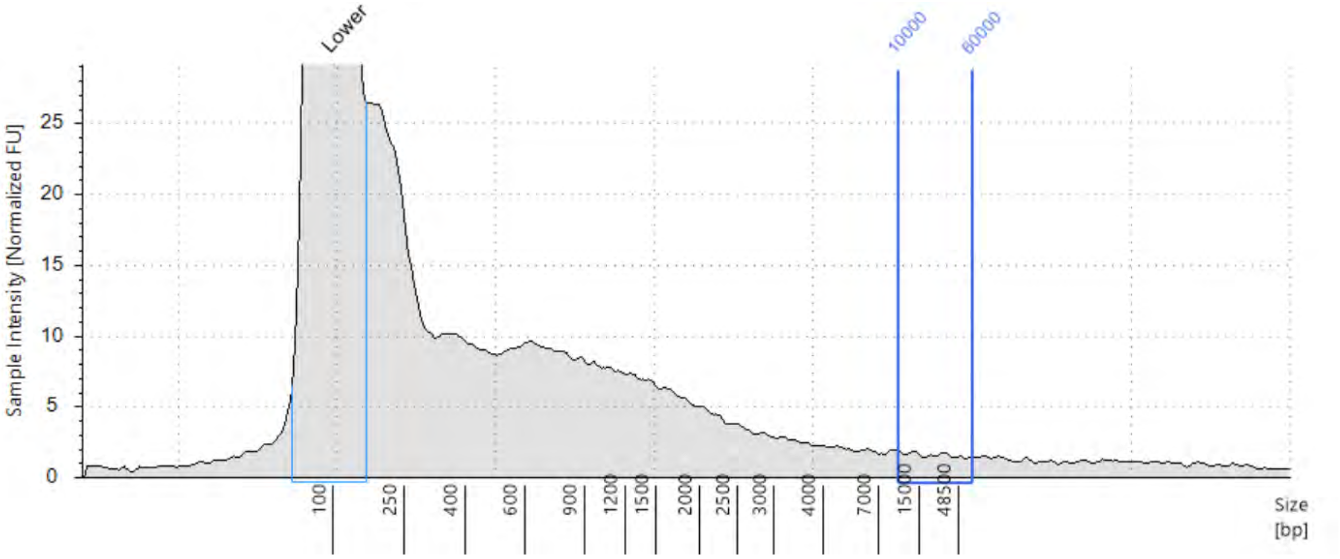

Region Table

From [bp]	To [bp]	Average Size [bp]	Conc. [ng/µl]	Region Molarity [nmol/l]	% of Total	Region Comment	Color
10000	60000	25757	0.0561	0.00600	2.09		■

 © Agilent Technologies, Inc. 2017

E1: C-654-T5-D

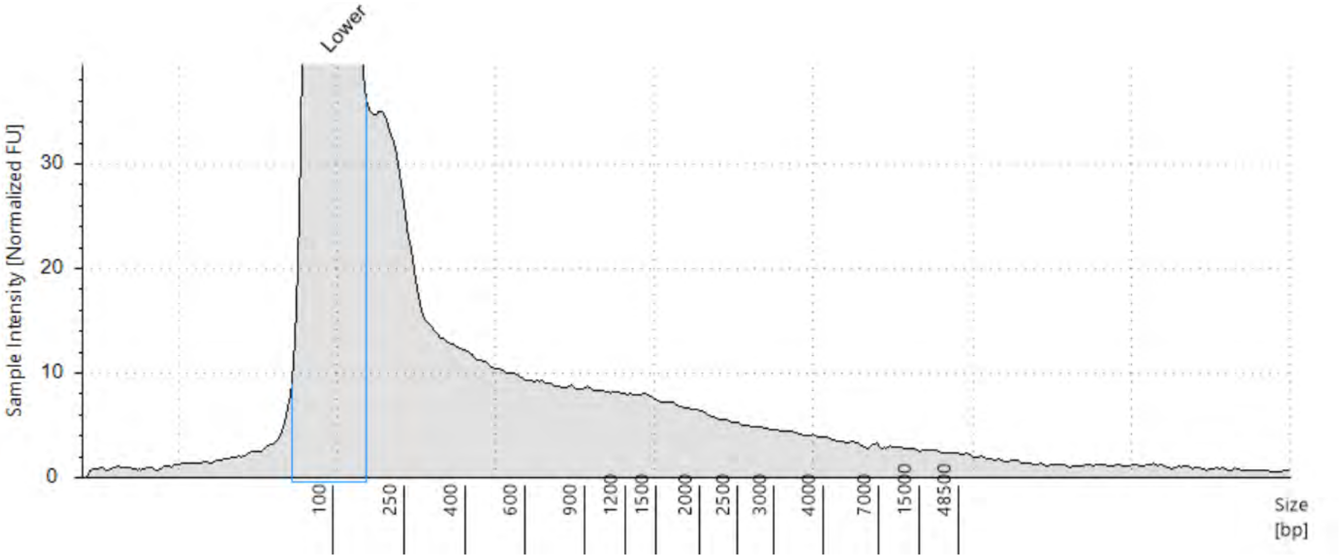

Sample Table

Well	DIN	Conc. [ng/µl]	Sample Description	Alert	Observations
E1	1.3	3.12	C-654-T5-D	⚠	Sample concentration outside functional range for DIN

Peak Table

Size [bp]	Calibrated Conc. [ng/µl]	Assigned Conc. [ng/µl]	% Integrated Area	From [bp]	To [bp]	Peak Comment	Observations
100	8.50	8.50	-	58	154		Lower Marker

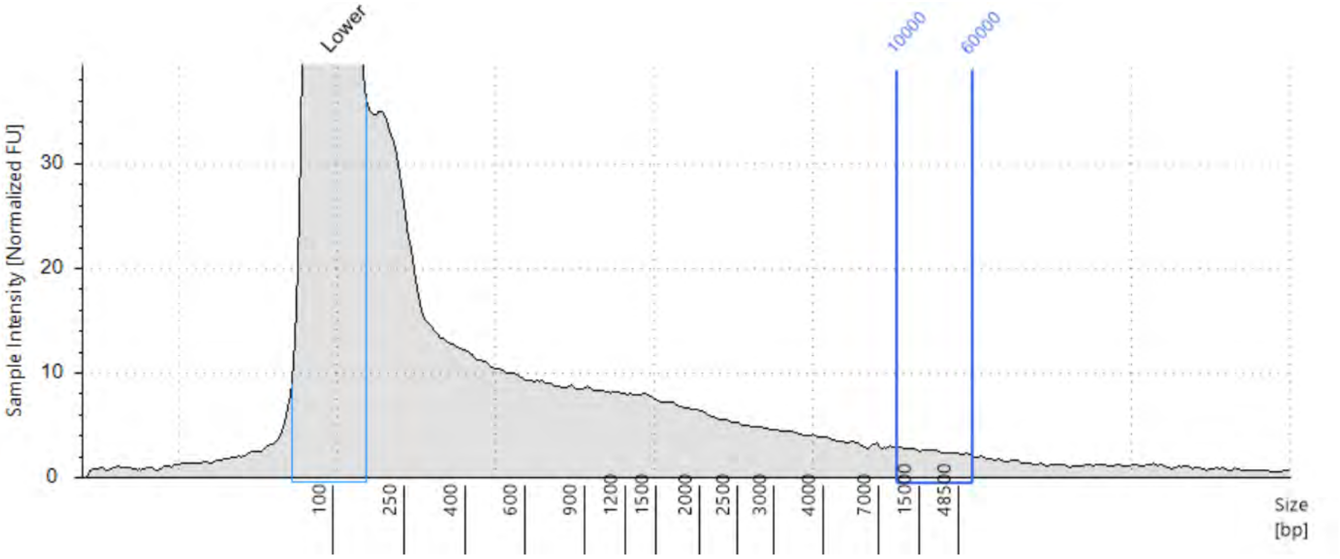

Region Table

From [bp]	To [bp]	Average Size [bp]	Conc. [ng/µl]	Region Molarity [nmol/l]	% of Total	Region Comment	Color
10000	60000	25636	0.0897	0.00889	2.87		■

F1: C-655-T5-D

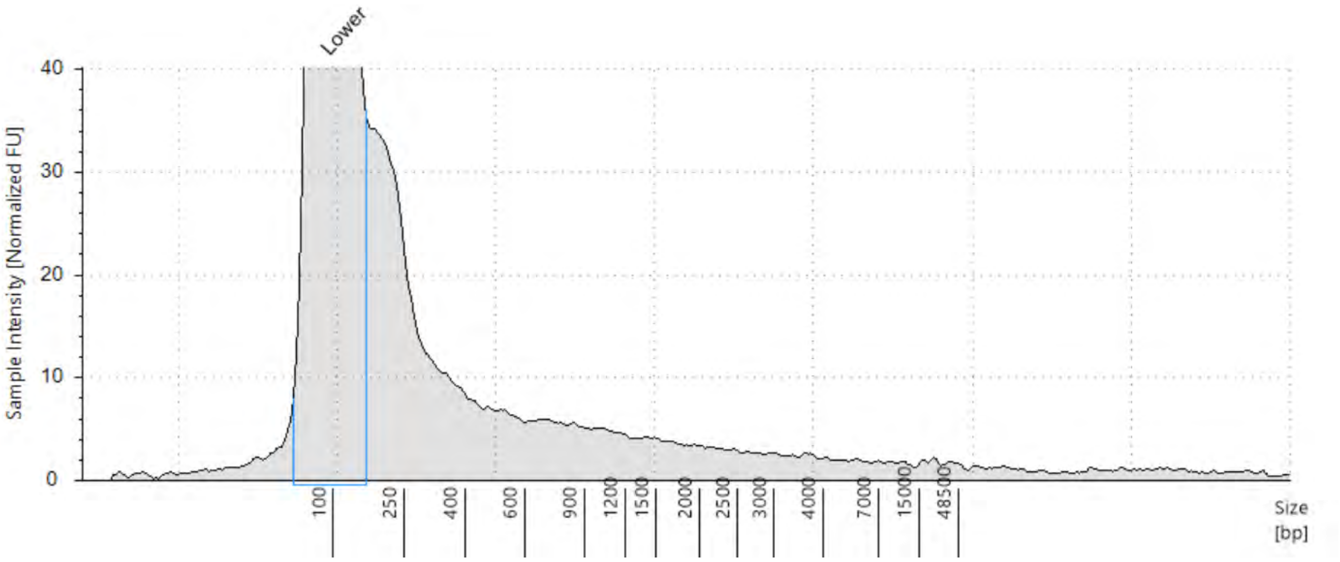

Sample Table

Well	DIN	Conc. [ng/µl]	Sample Description	Alert	Observations
F1	-	2.33	C-655-T5-D	▲	Sample concentration outside functional range for DIN

Peak Table

Size [bp]	Calibrated Conc. [ng/µl]	Assigned Conc. [ng/µl]	% Integrated Area	From [bp]	To [bp]	Peak Comment	Observations
100	8.50	8.50	-	60	153		Lower Marker

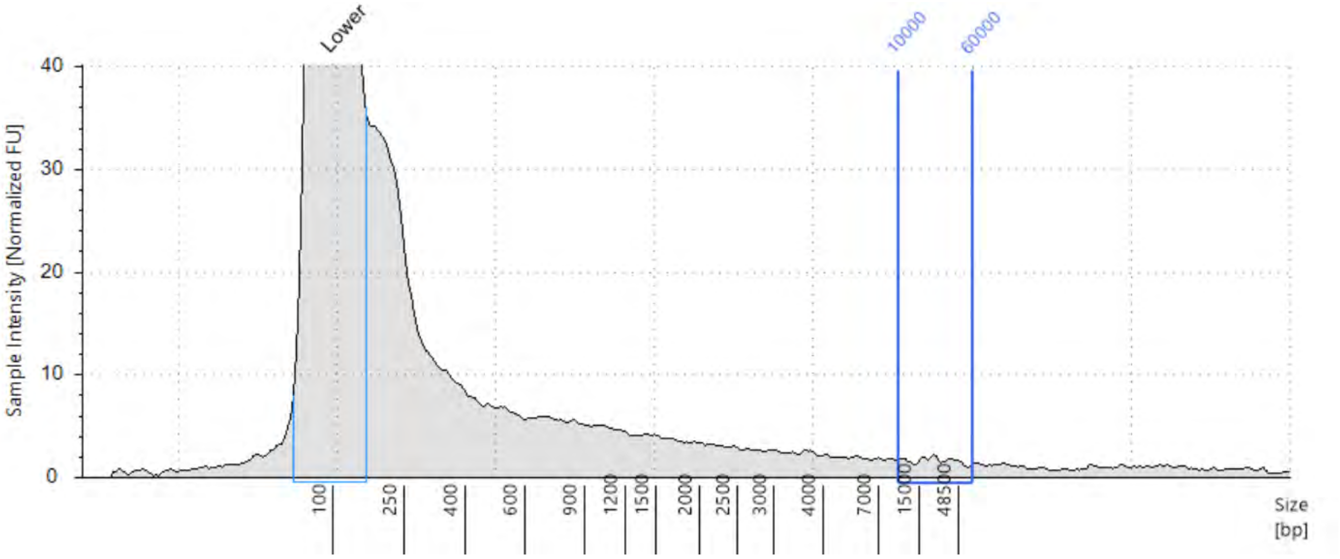

Region Table

From [bp]	To [bp]	Average Size [bp]	Conc. [ng/µl]	Region Molarity [nmol/l]	% of Total	Region Comment	Color
10000	60000	25034	0.0540	0.00605	2.32		■

G1: C-397-T5-D

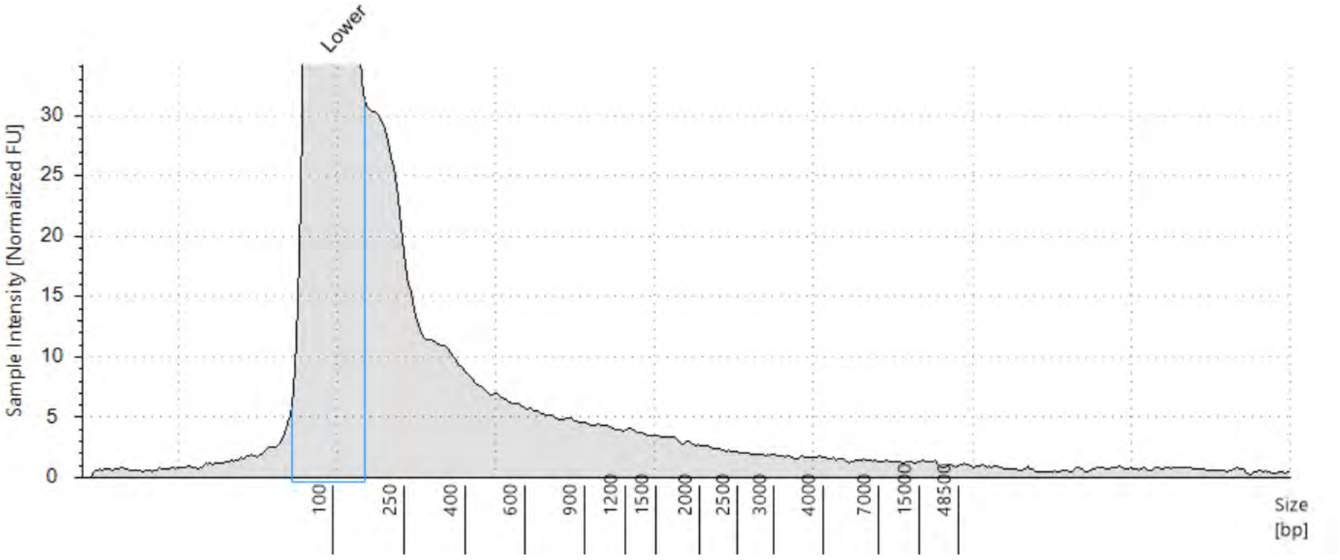

Sample Table

Well	DIN	Conc. [ng/µl]	Sample Description	Alert	Observations
G1	-	2.15	C-397-T5-D	▲	Sample concentration outside functional range for DIN

Peak Table

Size [bp]	Calibrated Conc. [ng/µl]	Assigned Conc. [ng/µl]	% Integrated Area	From [bp]	To [bp]	Peak Comment	Observations
100	8.50	8.50	-	58	151		Lower Marker

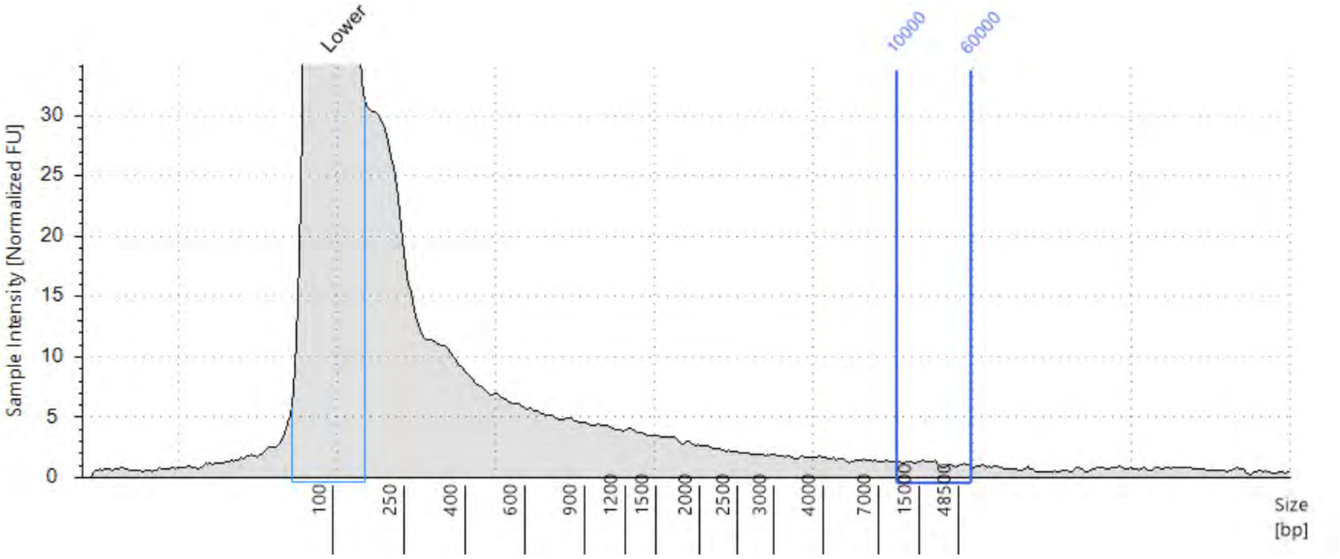

Region Table

From [bp]	To [bp]	Average Size [bp]	Conc. [ng/µl]	Region Molarity [nmol/l]	% of Total	Region Comment	Color
10000	60000	25051	0.0365	0.00443	1.70		■

H1: C-641-T5-DSS

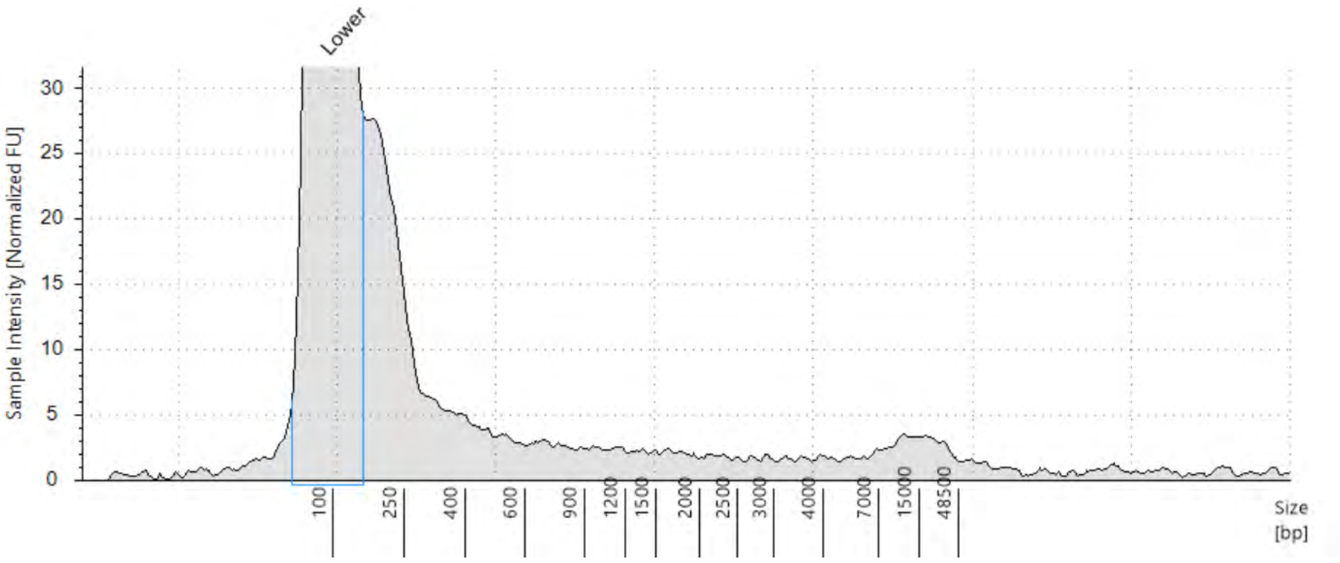

Sample Table

Well	DIN	Conc. [ng/µl]	Sample Description	Alert	Observations
H1	-	1.77	C-641-T5-DSS	▲	Sample concentration outside functional range for DIN

Peak Table

Size [bp]	Calibrated Conc. [ng/µl]	Assigned Conc. [ng/µl]	% Integrated Area	From [bp]	To [bp]	Peak Comment	Observations
100	8.50	8.50	-	59	148		Lower Marker

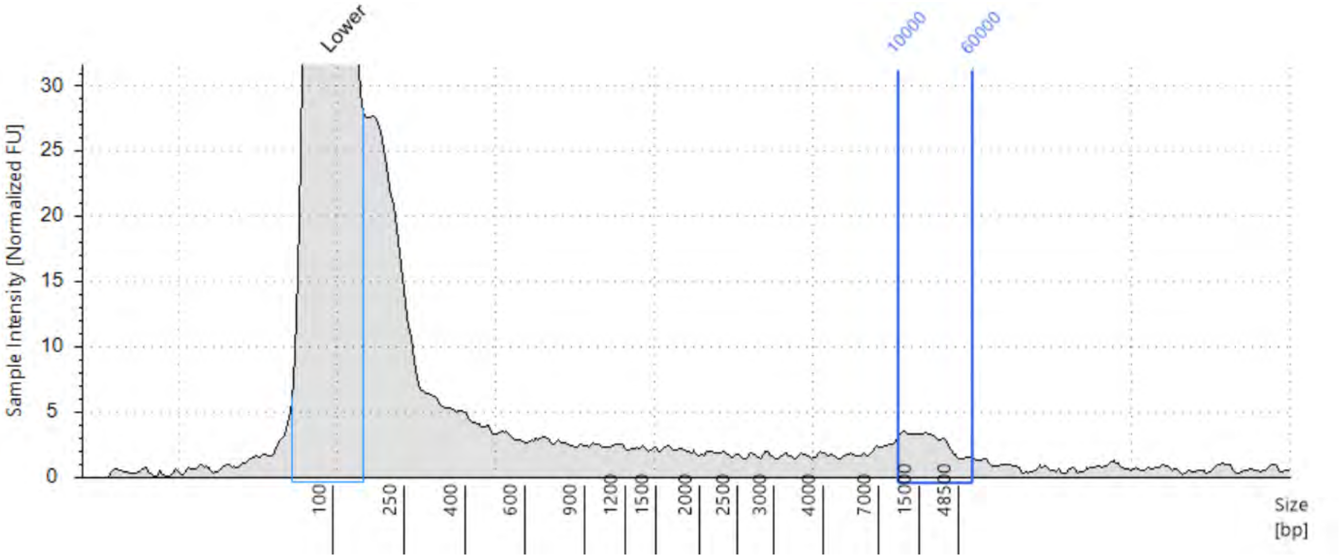

Region Table

From [bp]	To [bp]	Average Size [bp]	Conc. [ng/µl]	Region Molarity [nmol/l]	% of Total	Region Comment	Color
10000	60000	22386	0.123	0.0121	6.93		■

 © Agilent Technologies, Inc. 2017

A2: C-642-T5-DSS

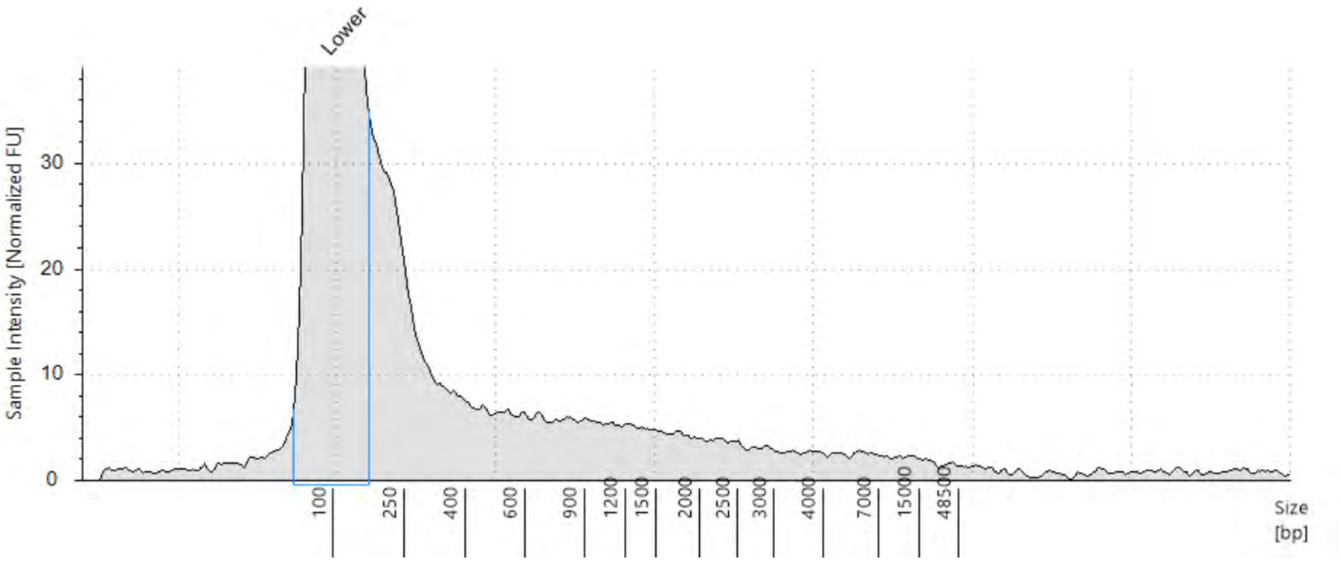

Sample Table

Well	DIN	Conc. [ng/µl]	Sample Description	Alert	Observations
A2	-	2.27	C-642-T5-DSS	▲	Sample concentration outside functional range for DIN

Peak Table

Size [bp]	Calibrated Conc. [ng/µl]	Assigned Conc. [ng/µl]	% Integrated Area	From [bp]	To [bp]	Peak Comment	Observations
100	8.50	8.50	-	60	159		Lower Marker

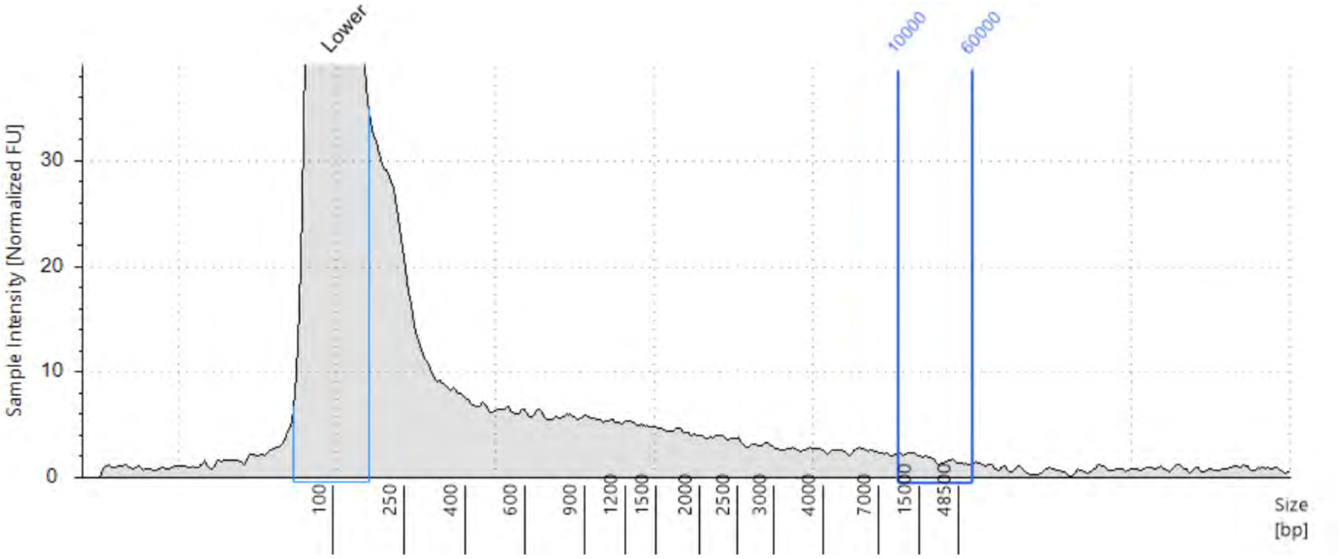

Region Table

From [bp]	To [bp]	Average Size [bp]	Conc. [ng/µl]	Region Molarity [nmol/l]	% of Total	Region Comment	Color
10000	60000	24138	0.0589	0.00661	2.59		■

B2: C-643-T5-DSS

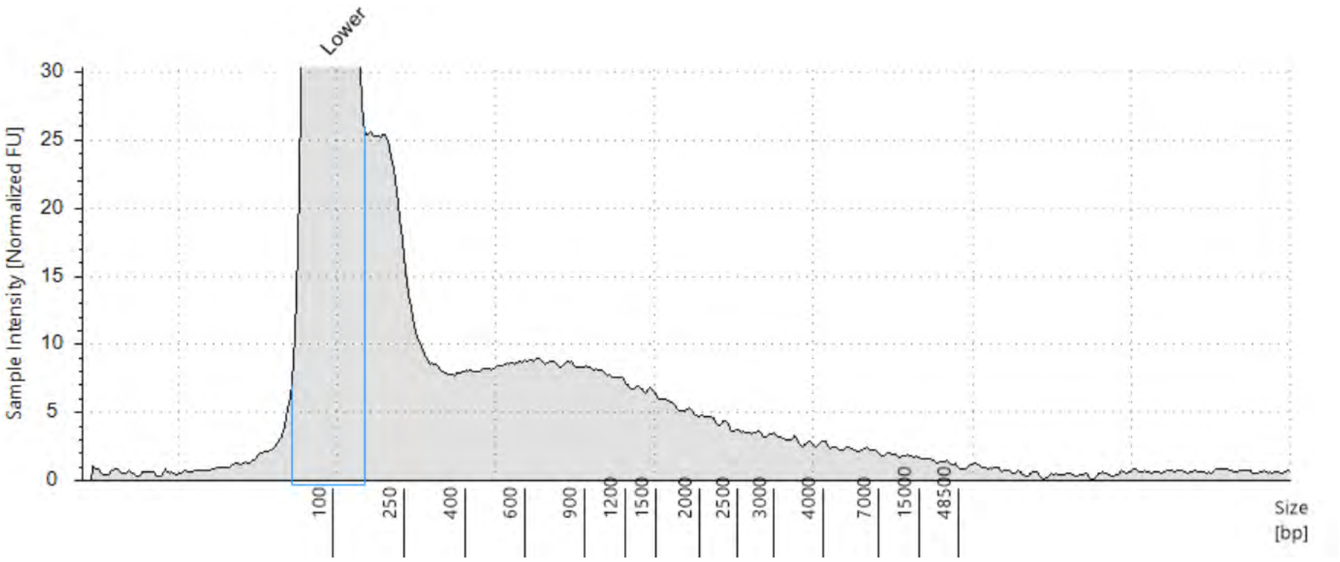

Sample Table

Well	DIN	Conc. [ng/µl]	Sample Description	Alert	Observations
B2	-	2.55	C-643-T5-DSS	▲	Sample concentration outside functional range for DIN

Peak Table

Size [bp]	Calibrated Conc. [ng/µl]	Assigned Conc. [ng/µl]	% Integrated Area	From [bp]	To [bp]	Peak Comment	Observations
100	8.50	8.50	-	58	151		Lower Marker

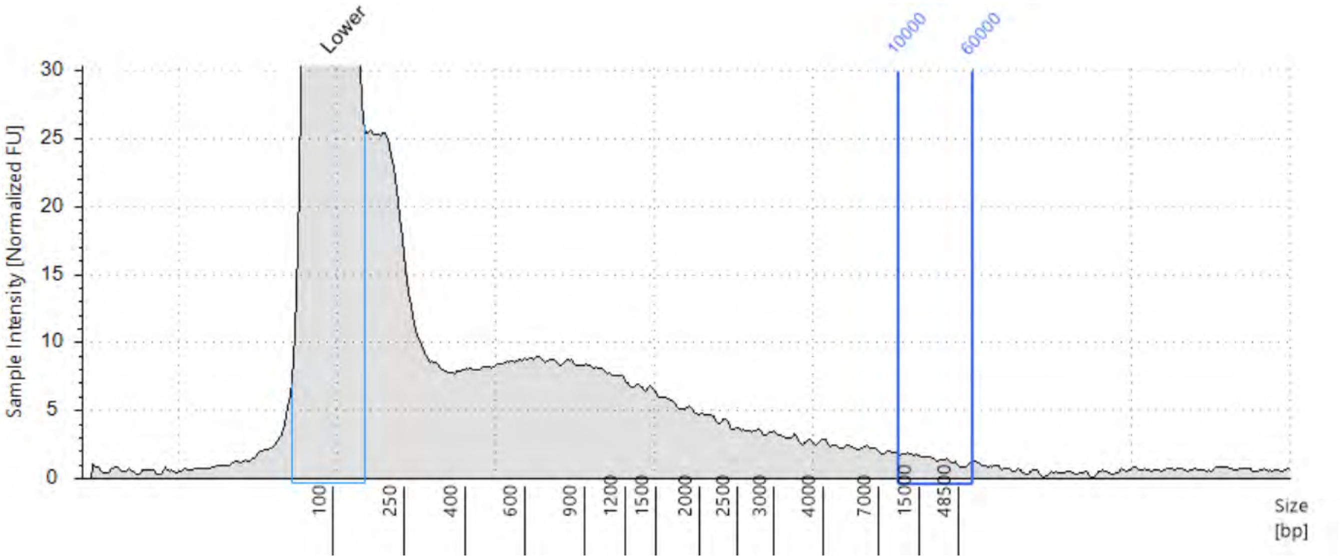

Region Table

From [bp]	To [bp]	Average Size [bp]	Conc. [ng/µl]	Region Molarity [nmol/l]	% of Total	Region Comment	Color
10000	60000	23278	0.0515	0.00585	2.02		■

C2: C-644-T5-DSS

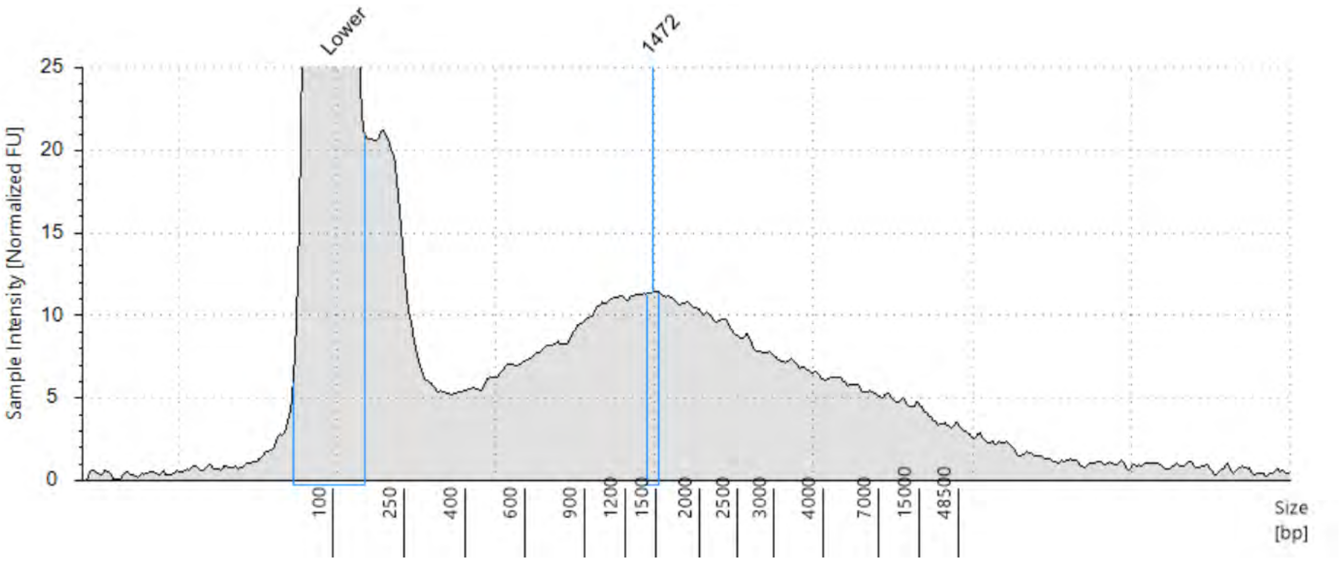

Sample Table

Well	DIN	Conc. [ng/µl]	Sample Description	Alert	Observations
C2	3.1	3.56	C-644-T5-DSS	⚠	Sample concentration outside functional range for DIN

Peak Table

Size [bp]	Calibrated Conc. [ng/µl]	Assigned Conc. [ng/µl]	% Integrated Area	From [bp]	To [bp]	Peak Comment	Observations
100	8.50	8.50	-	59	151		Lower Marker
1472	0.104	-	63.83	1401	1529		
-	-	-	-	-	-		Sample Well

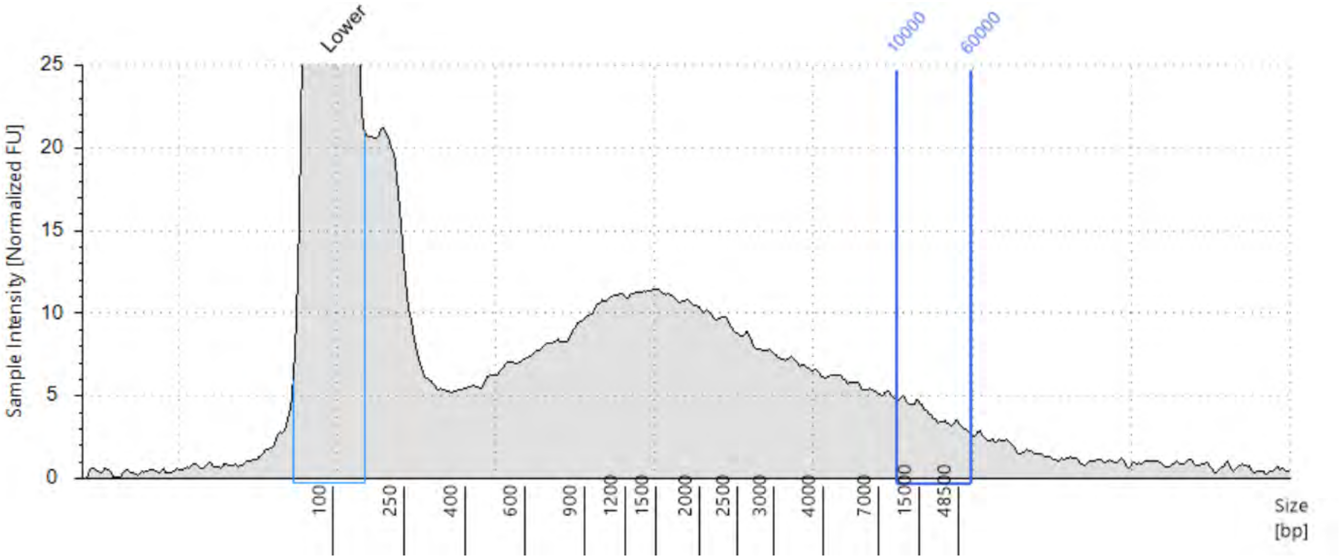

Region Table

From [bp]	To [bp]	Average Size [bp]	Conc. [ng/µl]	Region Molarity [nmol/l]	% of Total	Region Comment	Color
10000	60000	24107	0.184	0.0169	5.17		■

D2: C-645-T5-DSS

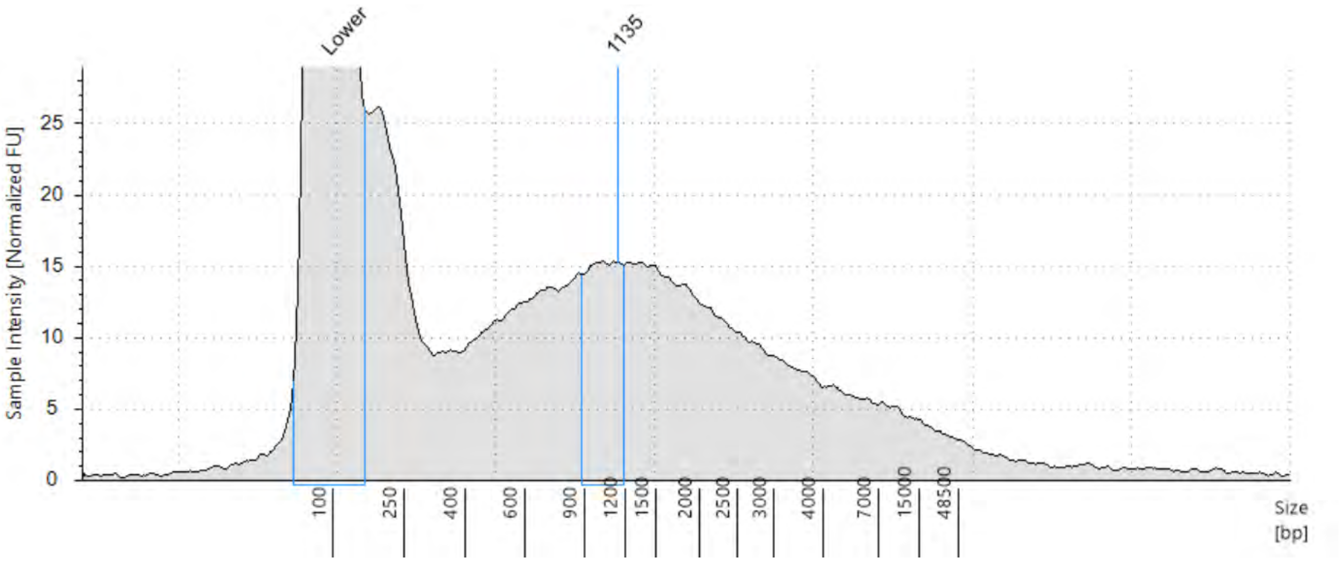

Sample Table

Well	DIN	Conc. [ng/µl]	Sample Description	Alert	Observations
D2	2.6	4.30	C-645-T5-DSS	⚠	Sample concentration outside functional range for DIN

Peak Table

Size [bp]	Calibrated Conc. [ng/µl]	Assigned Conc. [ng/µl]	% Integrated Area	From [bp]	To [bp]	Peak Comment	Observations
100	8.50	8.50	-	59	149		Lower Marker
1135	0.425	-	90.76	880	1192		
-	-	-	-	-	-		Sample Well

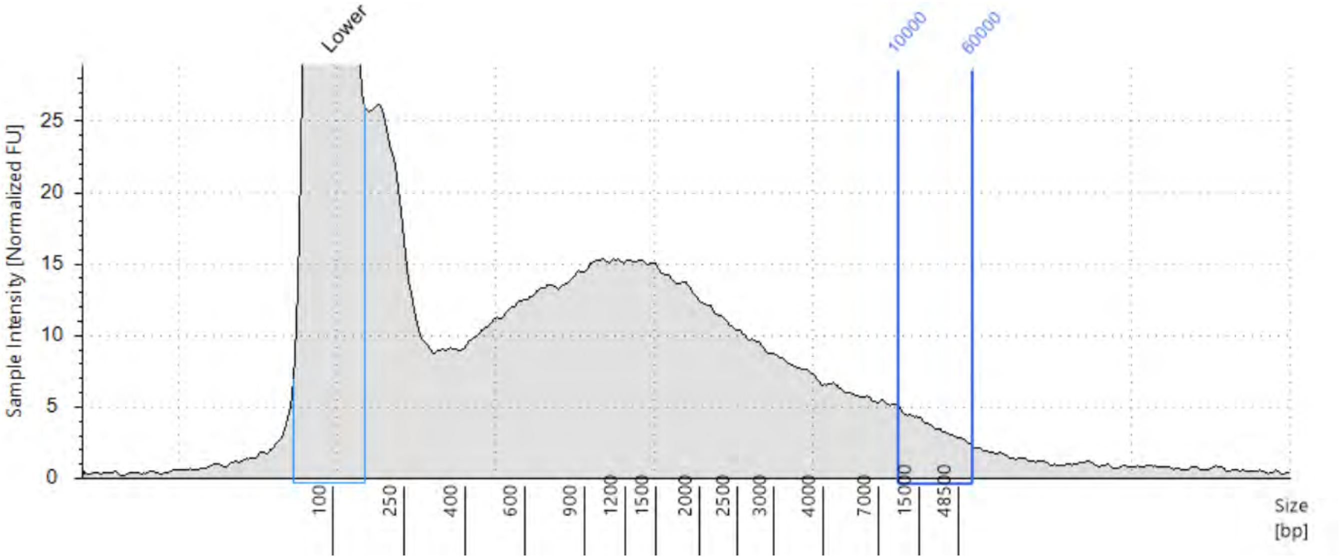

Region Table

From [bp]	To [bp]	Average Size [bp]	Conc. [ng/µl]	Region Molarity [nmol/l]	% of Total	Region Comment	Color
10000	60000	23800	0.159	0.0154	3.69		

E2: C-661-T5-SS

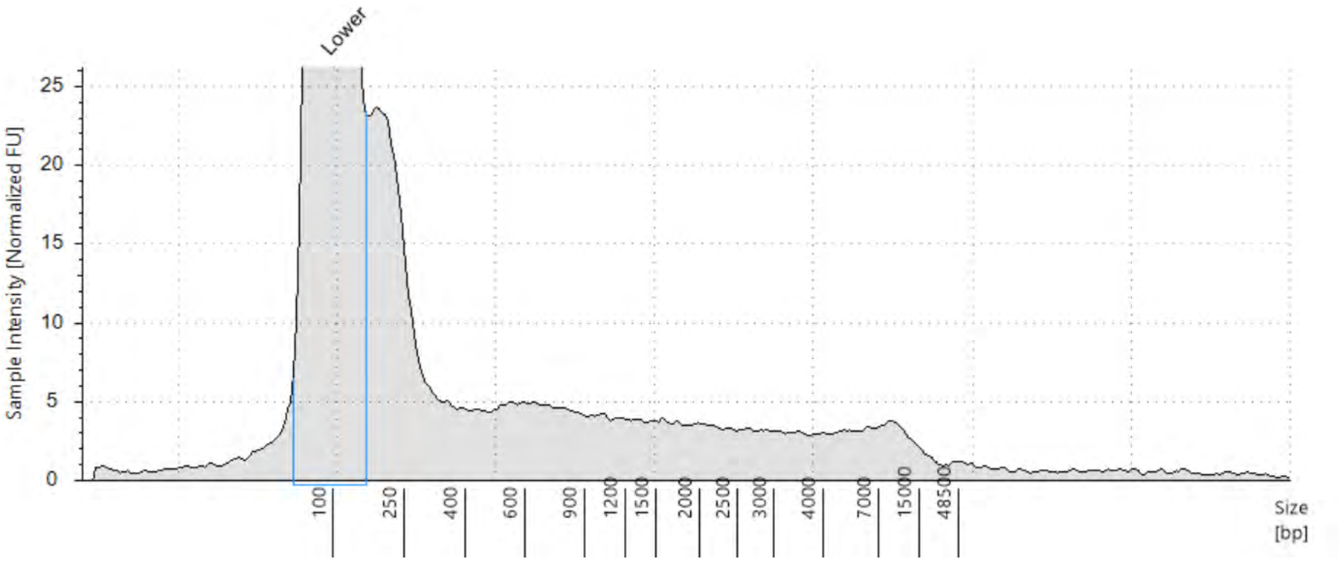

Sample Table

Well	DIN	Conc. [ng/µl]	Sample Description	Alert	Observations
E2	-	1.92	C-661-T5-SS	▲	Sample concentration outside functional range for DIN

Peak Table

Size [bp]	Calibrated Conc. [ng/µl]	Assigned Conc. [ng/µl]	% Integrated Area	From [bp]	To [bp]	Peak Comment	Observations
100	8.50	8.50	-	59	152		Lower Marker

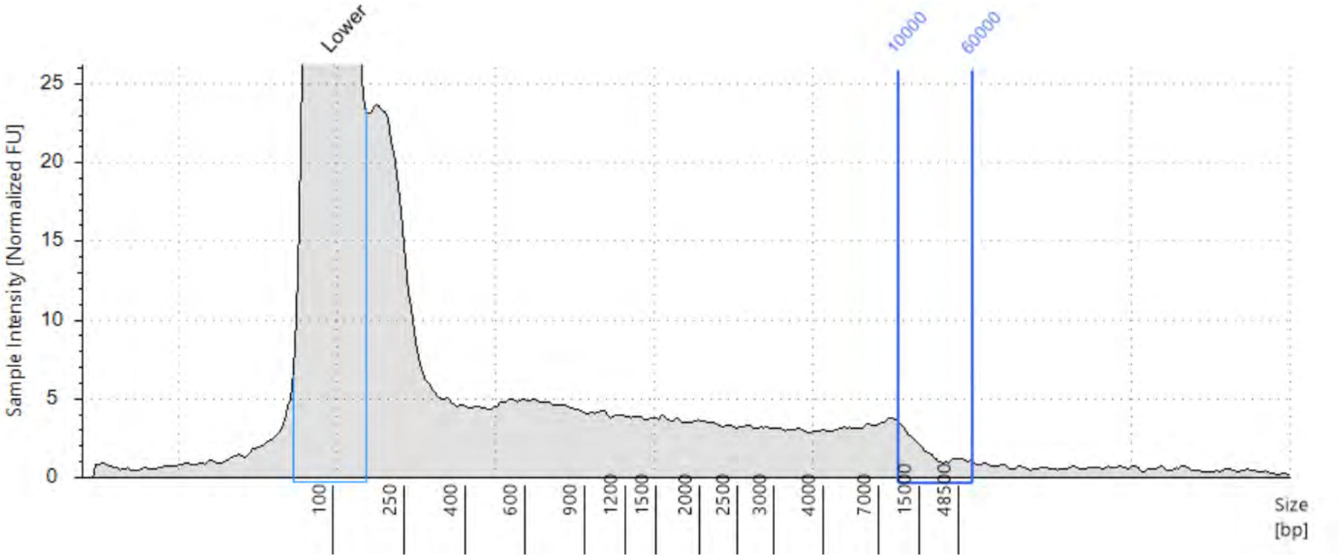

Region Table

From [bp]	To [bp]	Average Size [bp]	Conc. [ng/µl]	Region Molarity [nmol/l]	% of Total	Region Comment	Color
10000	60000	21969	0.0657	0.00773	3.42		■

F2: C-662-T5-SS

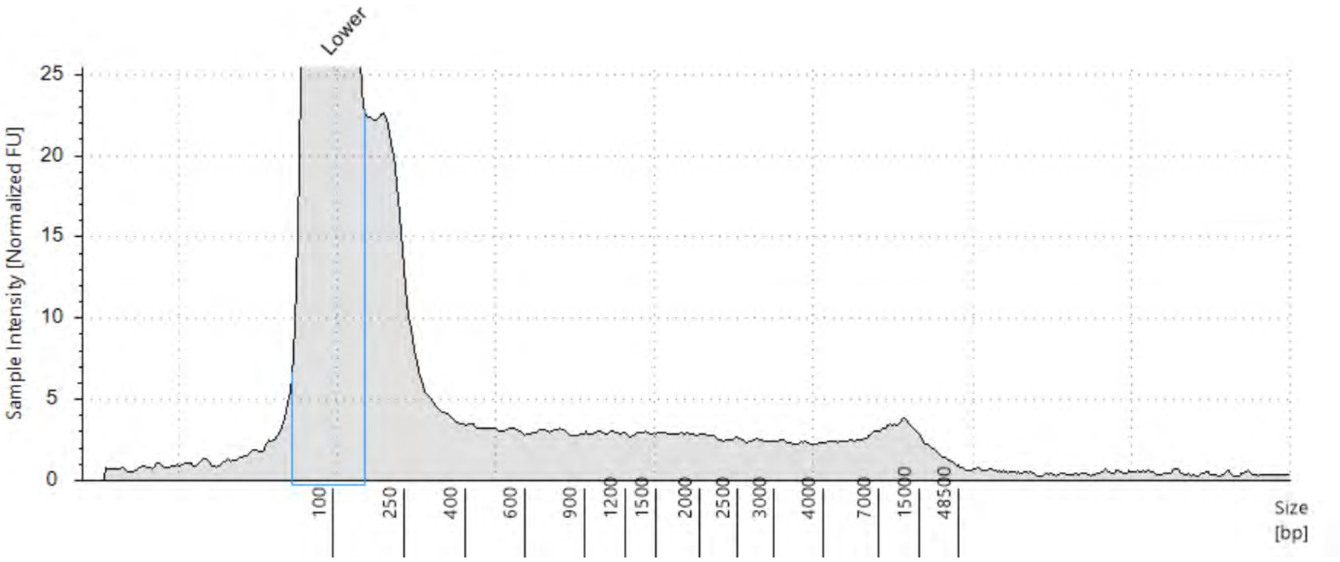

Sample Table

Well	DIN	Conc. [ng/µl]	Sample Description	Alert	Observations
F2	-	1.64	C-662-T5-SS	▲	Sample concentration outside functional range for DIN

Peak Table

Size [bp]	Calibrated Conc. [ng/µl]	Assigned Conc. [ng/µl]	% Integrated Area	From [bp]	To [bp]	Peak Comment	Observations
100	8.50	8.50	-	59	151		Lower Marker

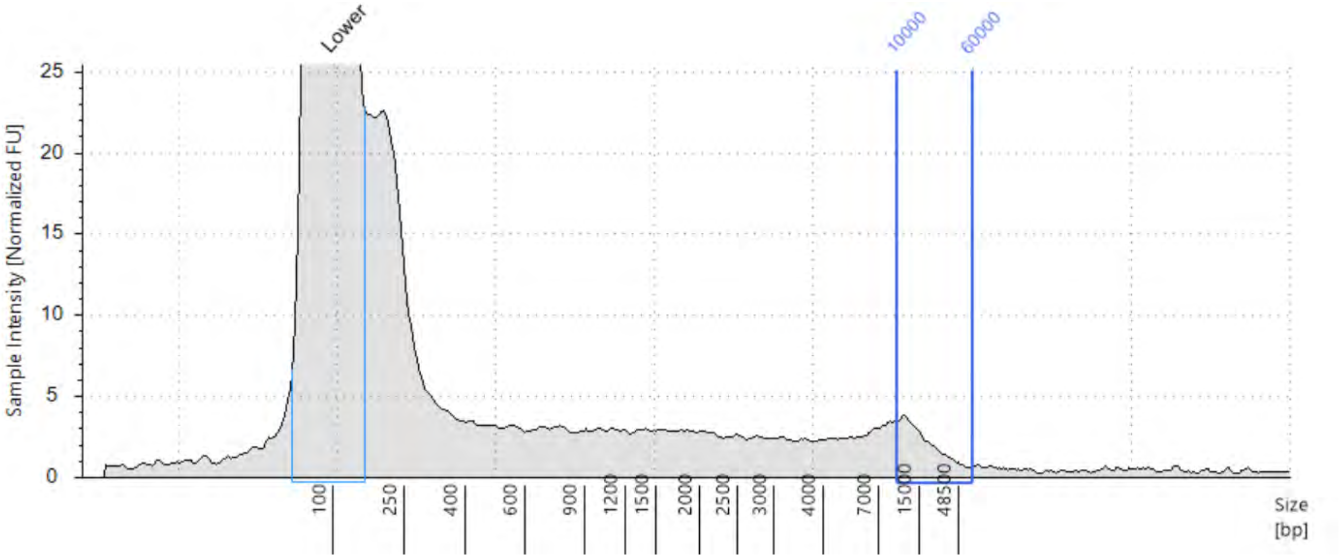

Region Table

From [bp]	To [bp]	Average Size [bp]	Conc. [ng/µl]	Region Molarity [nmol/l]	% of Total	Region Comment	Color
10000	60000	19318	0.0842	0.00990	5.12		■

 © Agilent Technologies, Inc. 2017

G2: C-663-T5-SS

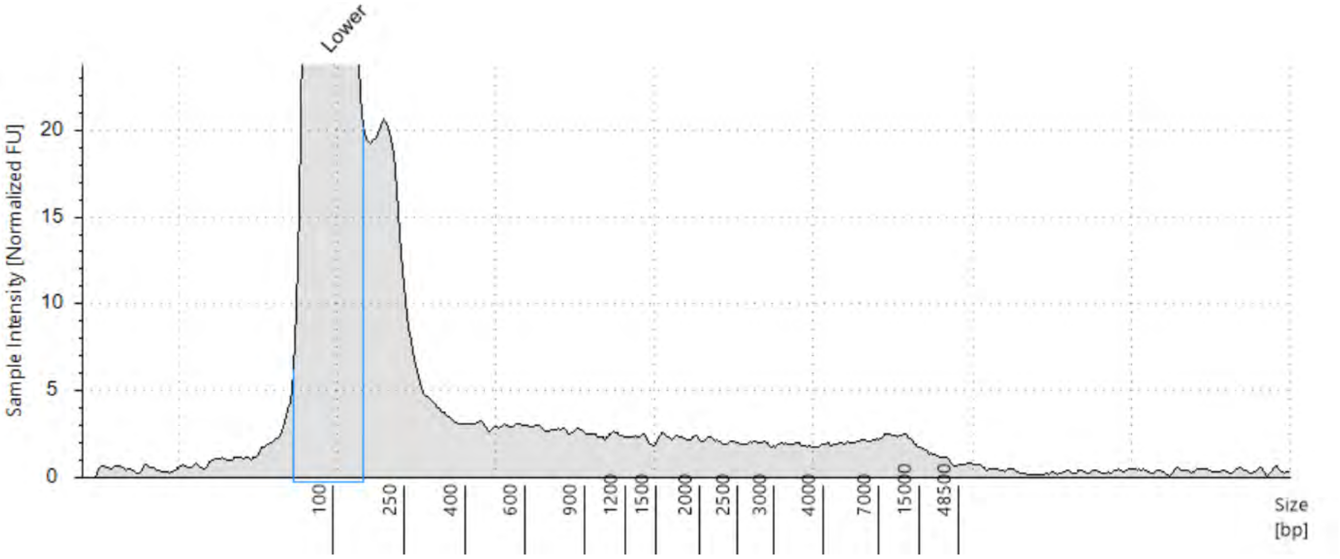

Sample Table

Well	DIN	Conc. [ng/µl]	Sample Description	Alert	Observations
G2	-	1.48	C-663-T5-SS	▲	Sample concentration outside functional range for DIN

Peak Table

Size [bp]	Calibrated Conc. [ng/µl]	Assigned Conc. [ng/µl]	% Integrated Area	From [bp]	To [bp]	Peak Comment	Observations
100	8.50	8.50	-	60	148		Lower Marker

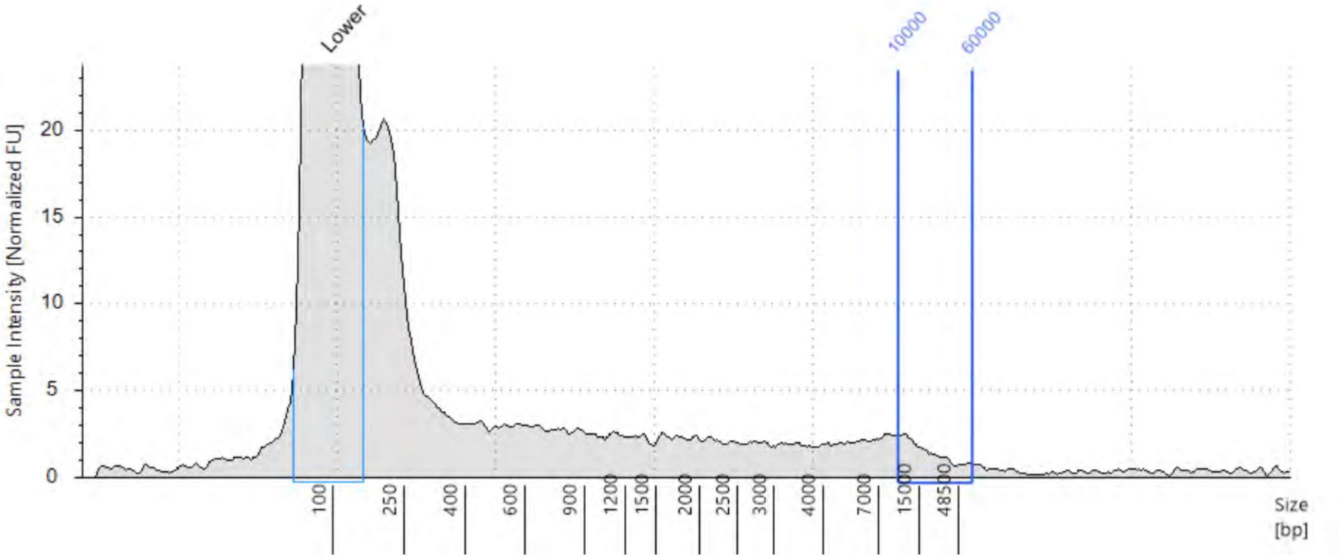

Region Table

From [bp]	To [bp]	Average Size [bp]	Conc. [ng/µl]	Region Molarity [nmol/l]	% of Total	Region Comment	Color
10000	60000	21377	0.0580	0.00665	3.93		■

H2: C-664-T5-SS

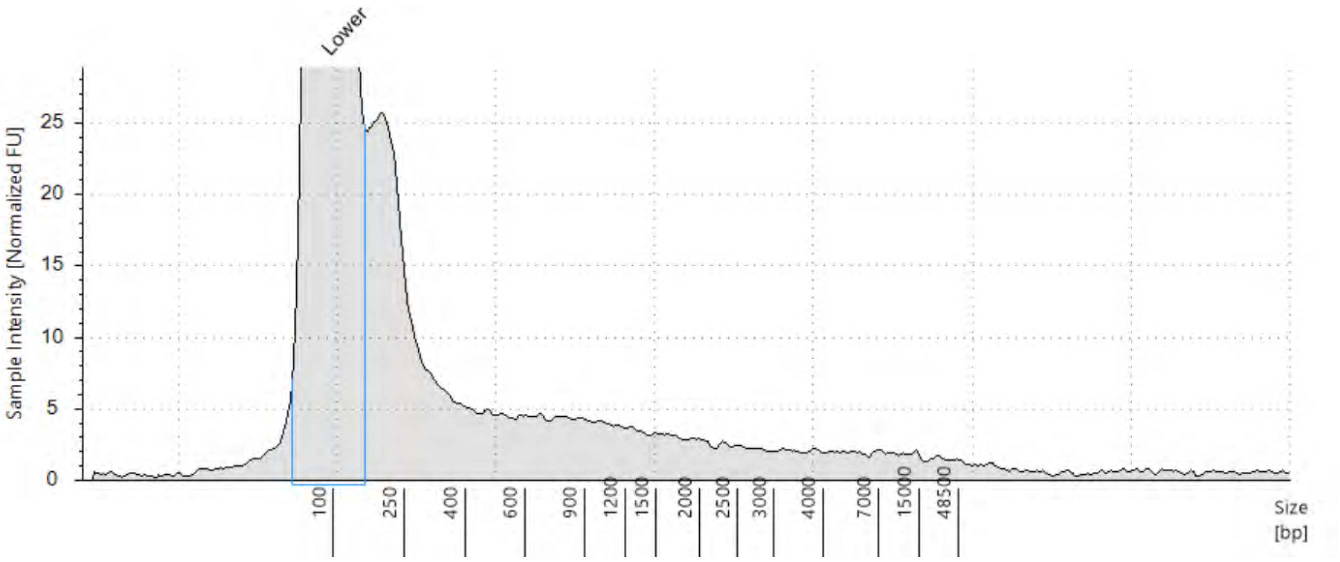

Sample Table

Well	DIN	Conc. [ng/µl]	Sample Description	Alert	Observations
H2	-	1.76	C-664-T5-SS	▲	Sample concentration outside functional range for DIN

Peak Table

Size [bp]	Calibrated Conc. [ng/µl]	Assigned Conc. [ng/µl]	% Integrated Area	From [bp]	To [bp]	Peak Comment	Observations
100	8.50	8.50	-	58	148		Lower Marker

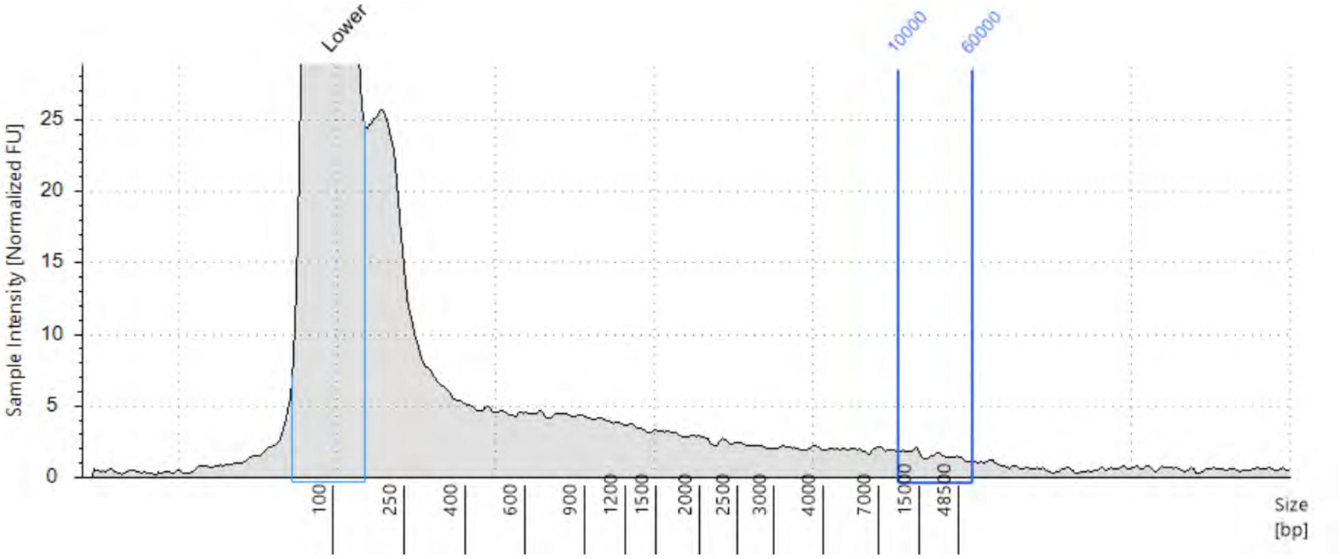

Region Table

From [bp]	To [bp]	Average Size [bp]	Conc. [ng/µl]	Region Molarity [nmol/l]	% of Total	Region Comment	Color
10000	60000	24205	0.0538	0.00614	3.06		■

 © Agilent Technologies, Inc. 2017

A3: C-665-T5-SS

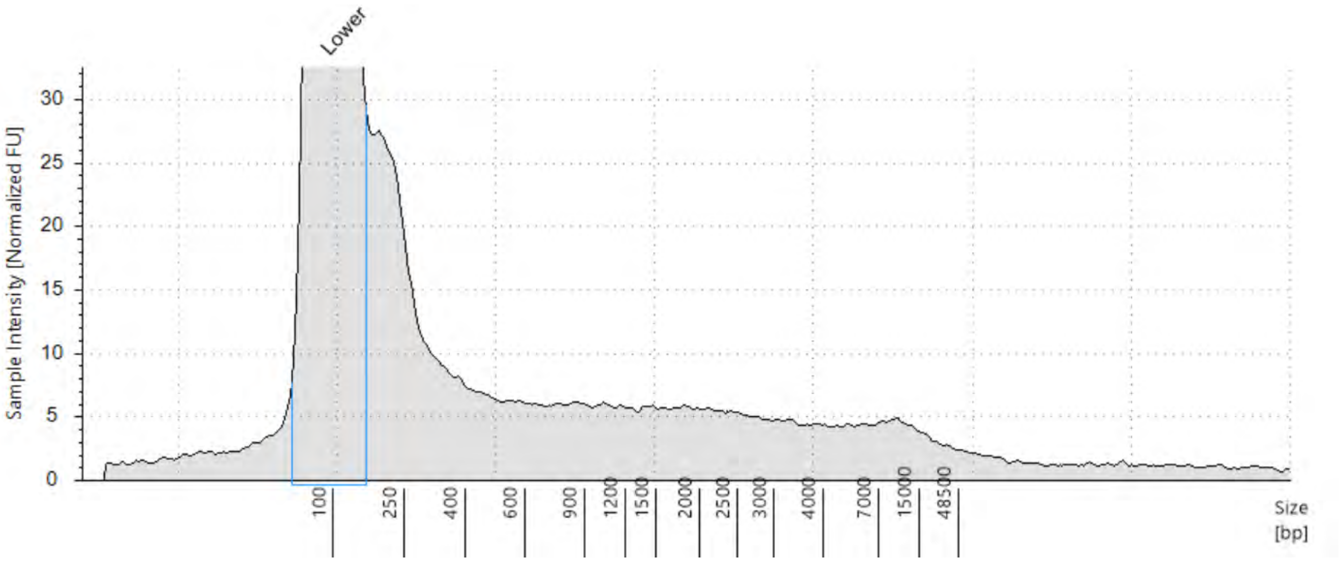

Sample Table

Well	DIN	Conc. [ng/µl]	Sample Description	Alert	Observations
A3	-	2.81	C-665-T5-SS	▲	Sample concentration outside functional range for DIN

Peak Table

Size [bp]	Calibrated Conc. [ng/µl]	Assigned Conc. [ng/µl]	% Integrated Area	From [bp]	To [bp]	Peak Comment	Observations
100	8.50	8.50	-	58	154		Lower Marker

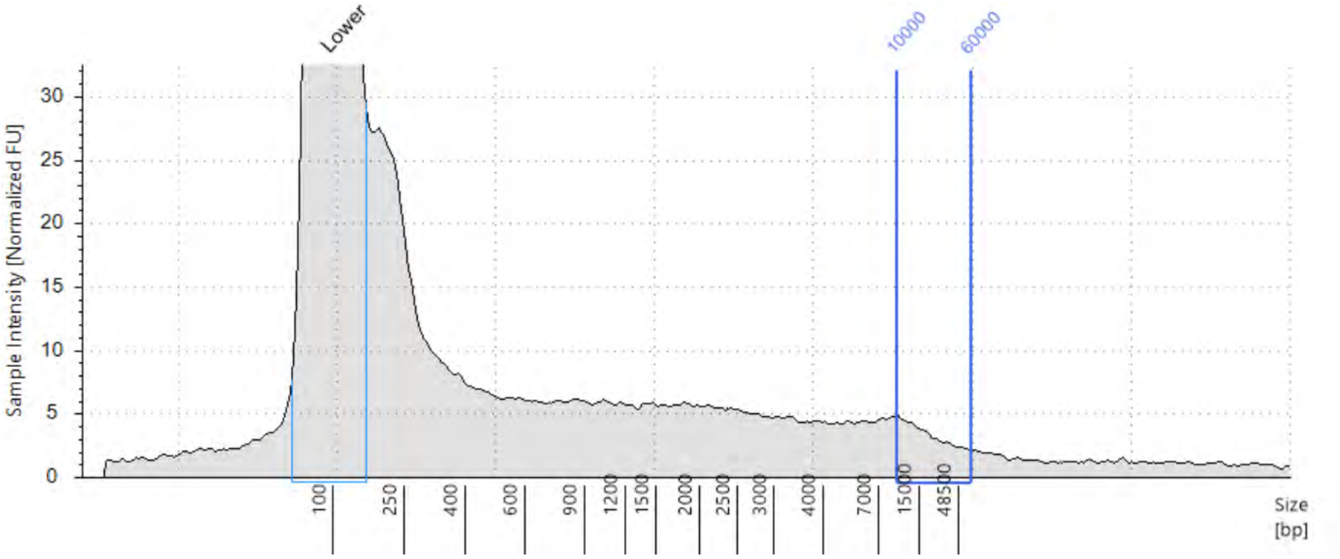

Region Table

From [bp]	To [bp]	Average Size [bp]	Conc. [ng/µl]	Region Molarity [nmol/l]	% of Total	Region Comment	Color
10000	60000	22982	0.134	0.0133	4.77		■

© Agilent Technologies, Inc. 2017

B3: C-400-T5-SS

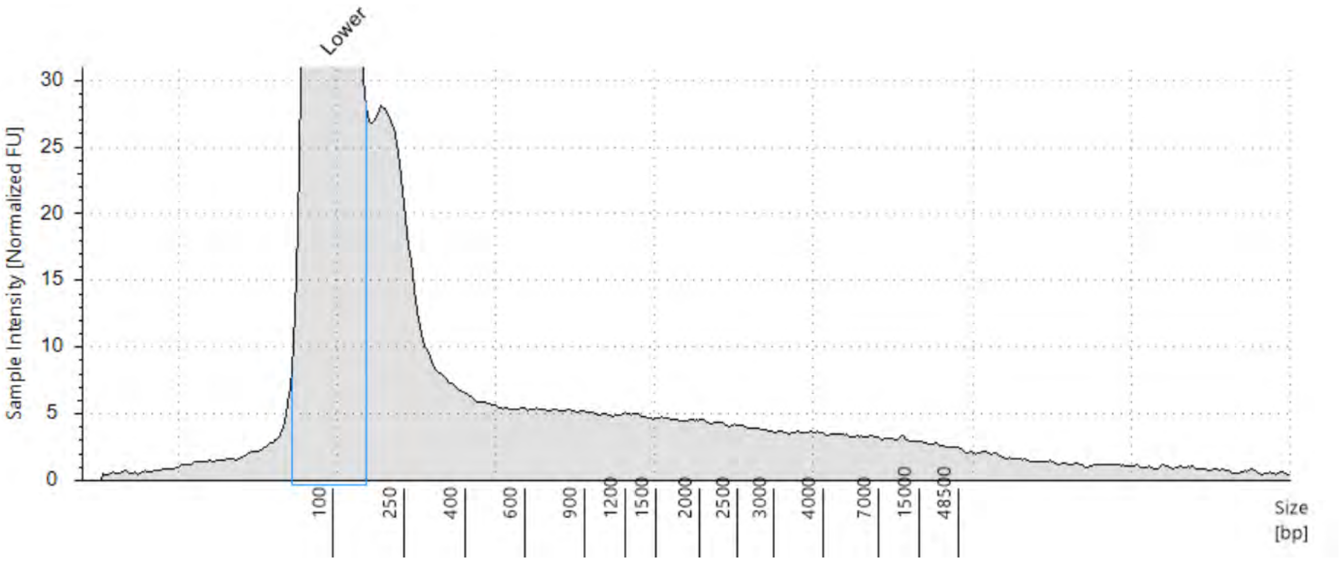

Sample Table

Well	DIN	Conc. [ng/µl]	Sample Description	Alert	Observations
B3	-	2.34	C-400-T5-SS	▲	Sample concentration outside functional range for DIN

Peak Table

Size [bp]	Calibrated Conc. [ng/µl]	Assigned Conc. [ng/µl]	% Integrated Area	From [bp]	To [bp]	Peak Comment	Observations
100	8.50	8.50	-	58	154		Lower Marker

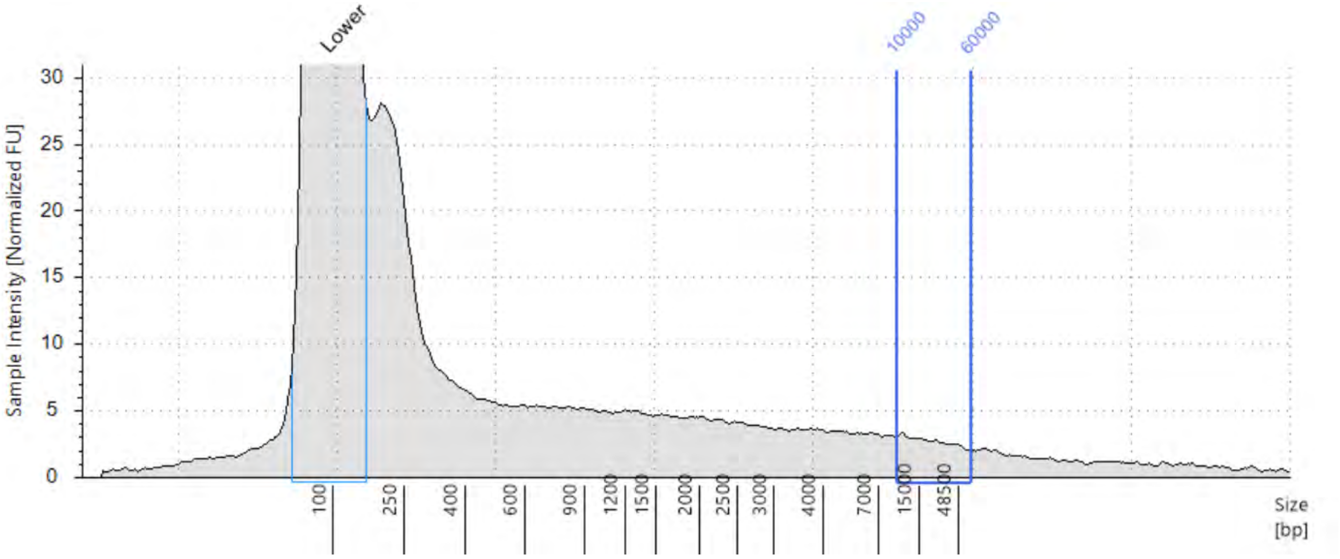

Region Table

From [bp]	To [bp]	Average Size [bp]	Conc. [ng/µl]	Region Molarity [nmol/l]	% of Total	Region Comment	Color
10000	60000	24896	0.101	0.00998	4.31		■

© Agilent Technologies, Inc. 2017

C3: C-401-T5-SS

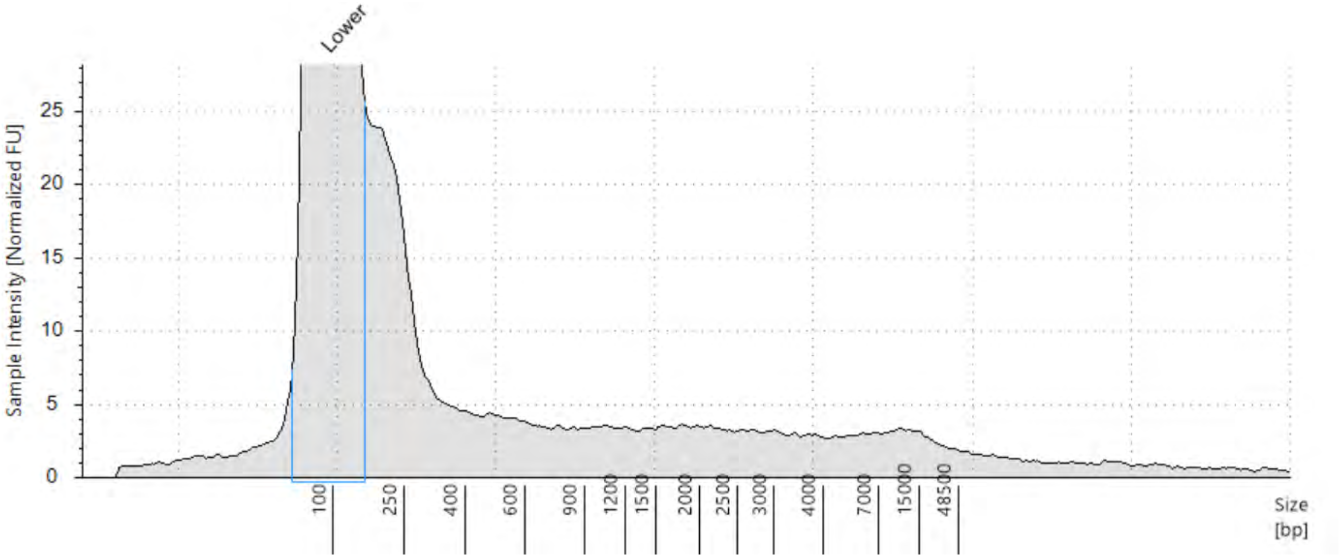

Sample Table

Well	DIN	Conc. [ng/µl]	Sample Description	Alert	Observations
C3	-	1.85	C-401-T5-SS	▲	Sample concentration outside functional range for DIN

Peak Table

Size [bp]	Calibrated Conc. [ng/µl]	Assigned Conc. [ng/µl]	% Integrated Area	From [bp]	To [bp]	Peak Comment	Observations
100	8.50	8.50	-	59	151		Lower Marker

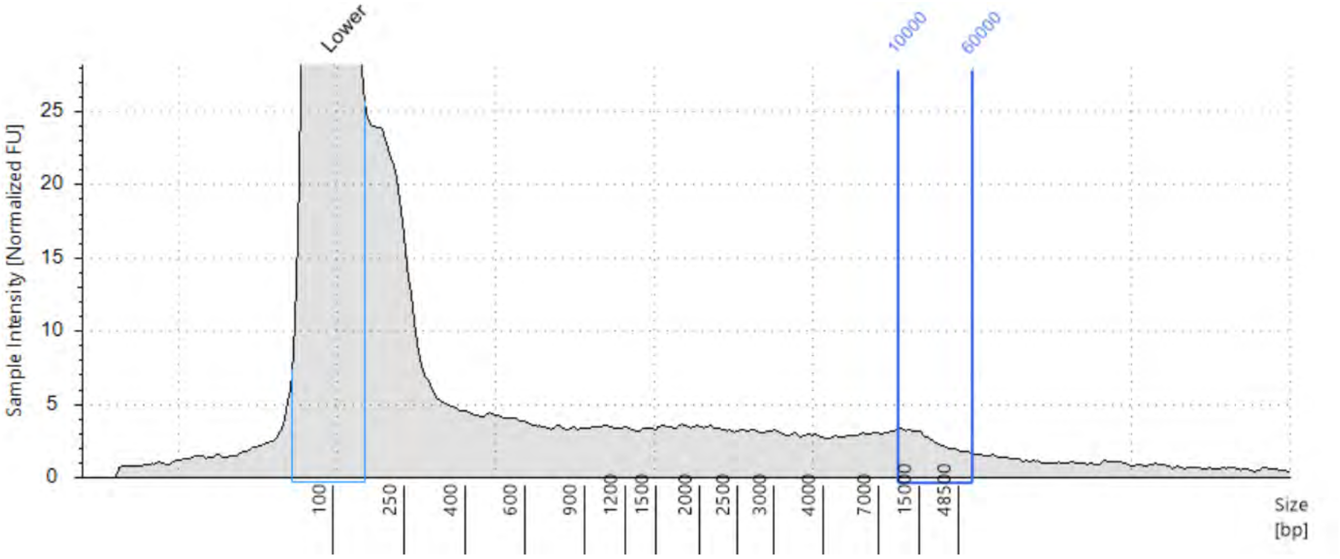

Region Table

From [bp]	To [bp]	Average Size [bp]	Conc. [ng/µl]	Region Molarity [nmol/l]	% of Total	Region Comment	Color
10000	60000	23567	0.0940	0.00993	5.07		■

© Agilent Technologies, Inc. 2017

D3: M-156-T4-D

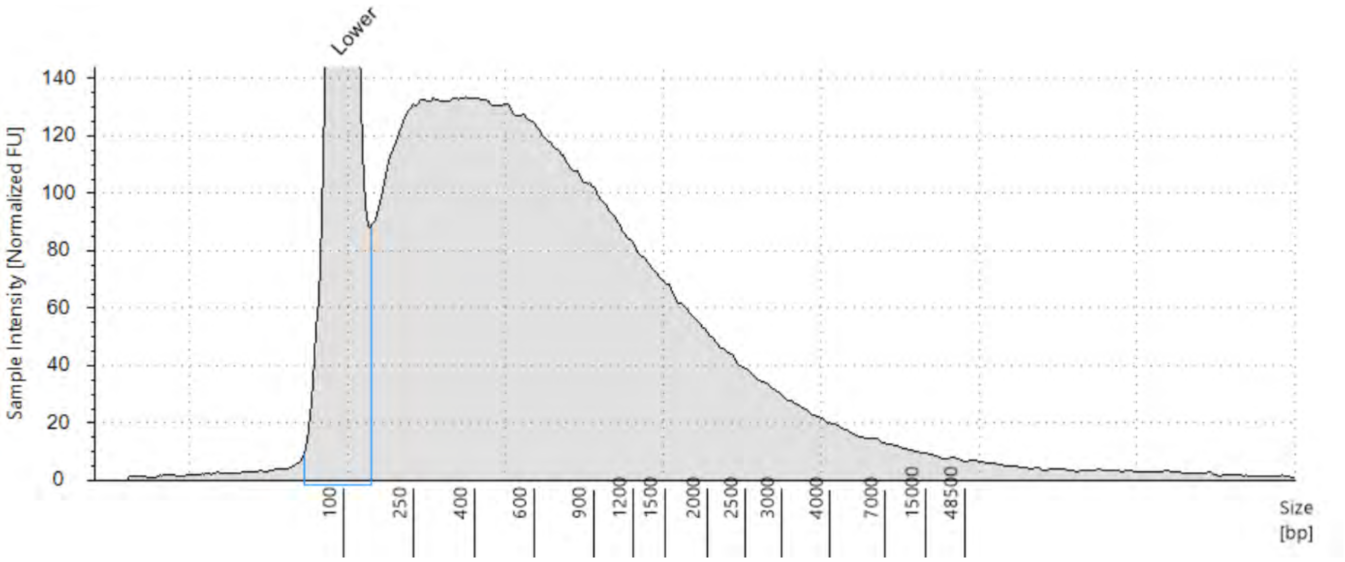

Sample Table

Well	DIN	Conc. [ng/µl]	Sample Description	Alert	Observations
D3	1.8	25.2	M-156-T4-D		

Peak Table

Size [bp]	Calibrated Conc. [ng/µl]	Assigned Conc. [ng/µl]	% Integrated Area	From [bp]	To [bp]	Peak Comment	Observations
100	8.50	8.50	-	60	143		Lower Marker

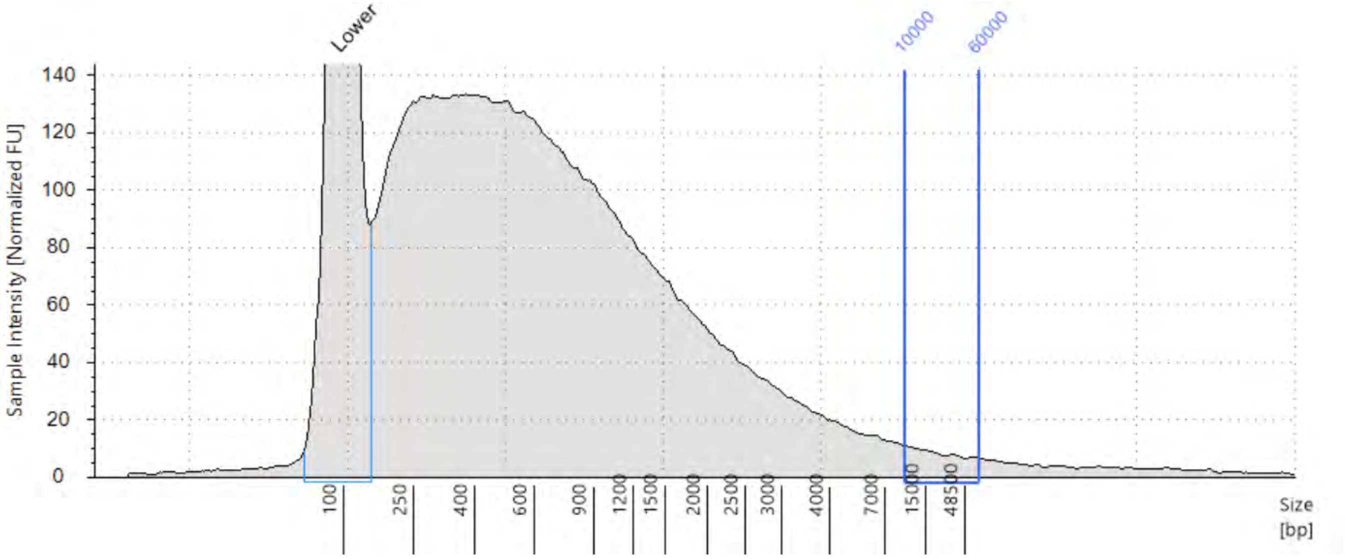

Region Table

From [bp]	To [bp]	Average Size [bp]	Conc. [ng/µl]	Region Molarity [nmol/l]	% of Total	Region Comment	Color
10000	60000	24369	0.320	0.0316	1.27		

 © Agilent Technologies, Inc. 2017

E3: M-157-T4-D

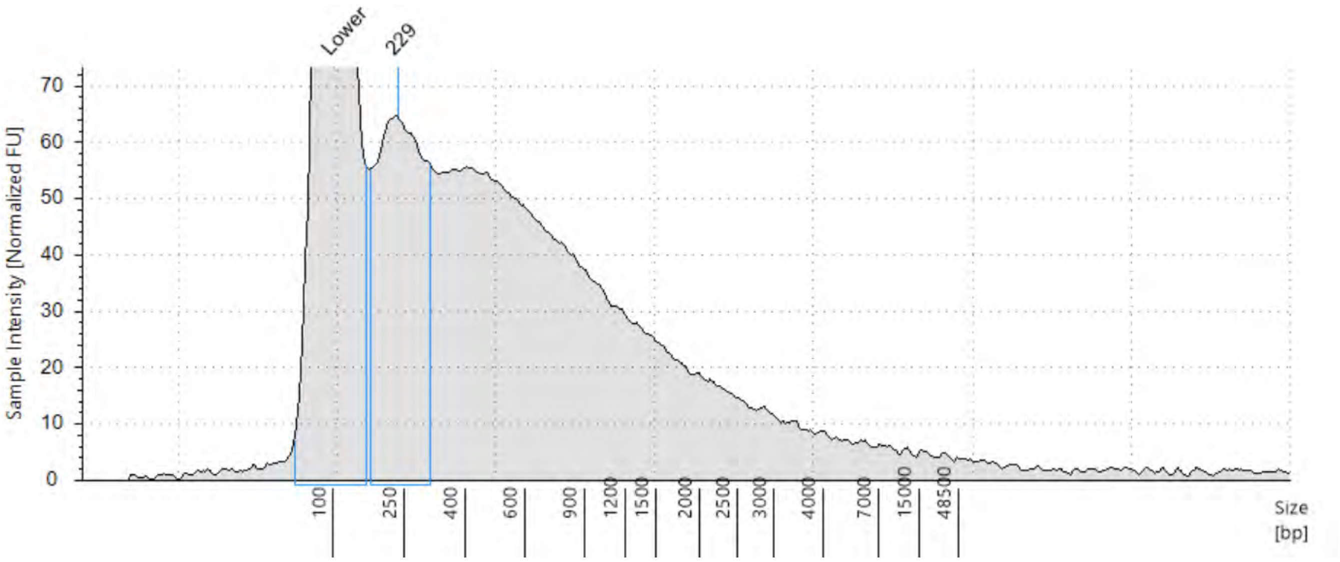

Sample Table

Well	DIN	Conc. [ng/µl]	Sample Description	Alert	Observations
E3	1.7	10.4	M-157-T4-D		

Peak Table

Size [bp]	Calibrated Conc. [ng/µl]	Assigned Conc. [ng/µl]	% Integrated Area	From [bp]	To [bp]	Peak Comment	Observations
100	8.50	8.50	-	60	153		Lower Marker
229	2.21	-	100.00	161	305		

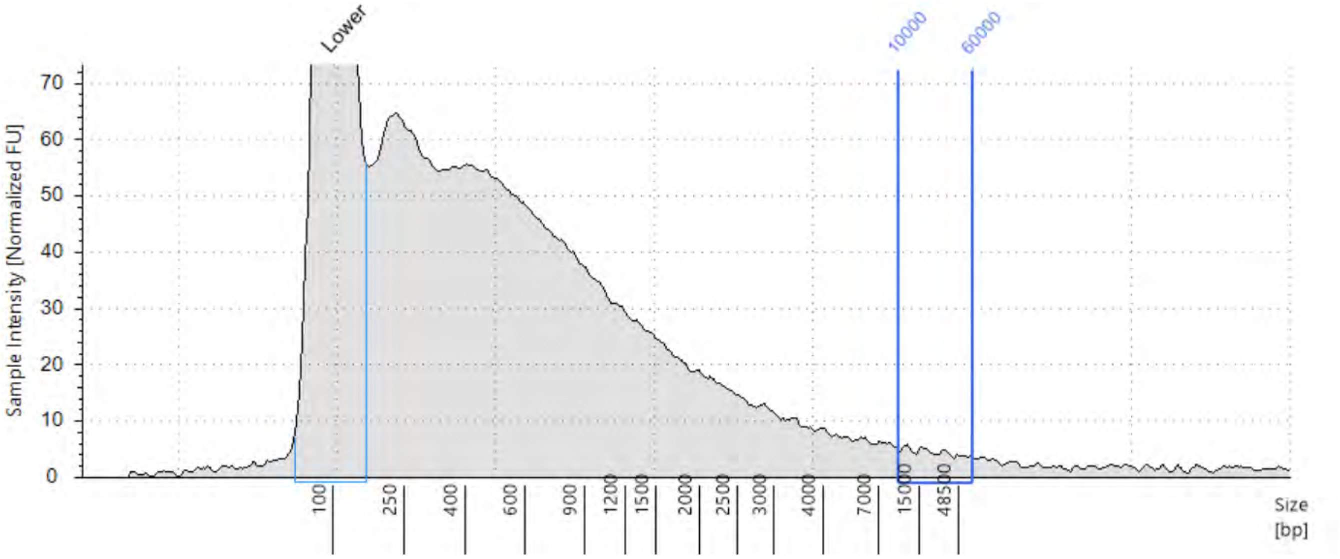

Region Table

From [bp]	To [bp]	Average Size [bp]	Conc. [ng/µl]	Region Molarity [nmol/l]	% of Total	Region Comment	Color
10000	60000	24936	0.171	0.0163	1.64		

F3: M-158-T4-D

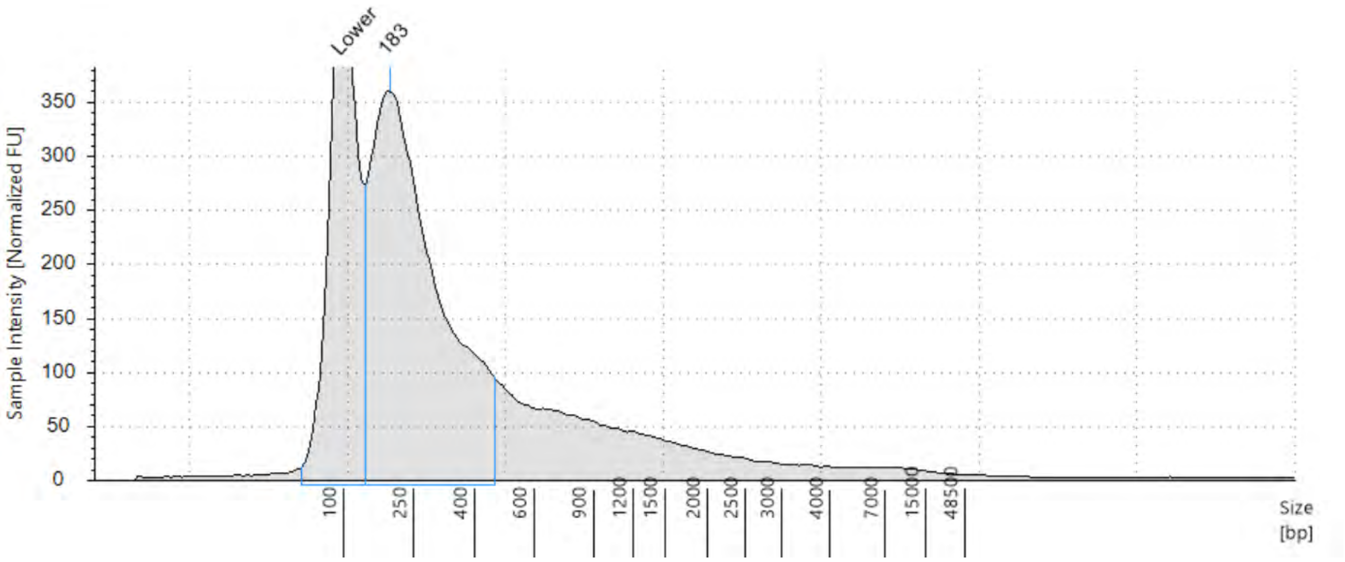

Sample Table

Well	DIN	Conc. [ng/µl]	Sample Description	Alert	Observations
F3	1.3	22.7	M-158-T4-D		

Peak Table

Size [bp]	Calibrated Conc. [ng/µl]	Assigned Conc. [ng/µl]	%Integrated Area	From [bp]	To [bp]	Peak Comment	Observations
100	8.50	8.50	-	58	133		Lower Marker
183	14.9	-	100.00	133	458		

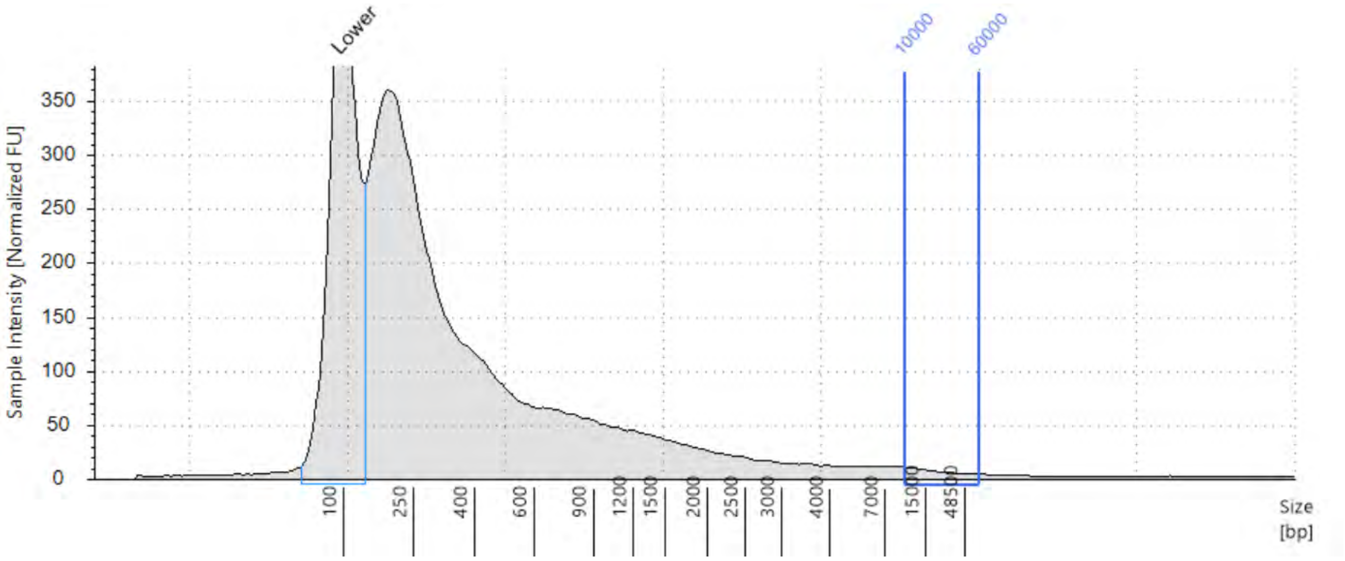

Region Table

From [bp]	To [bp]	Average Size [bp]	Conc. [ng/µl]	Region Molarity [nmol/l]	% of Total	Region Comment	Color
10000	60000	22587	0.229	0.0239	1.01		■

G3: M-159-T4-D

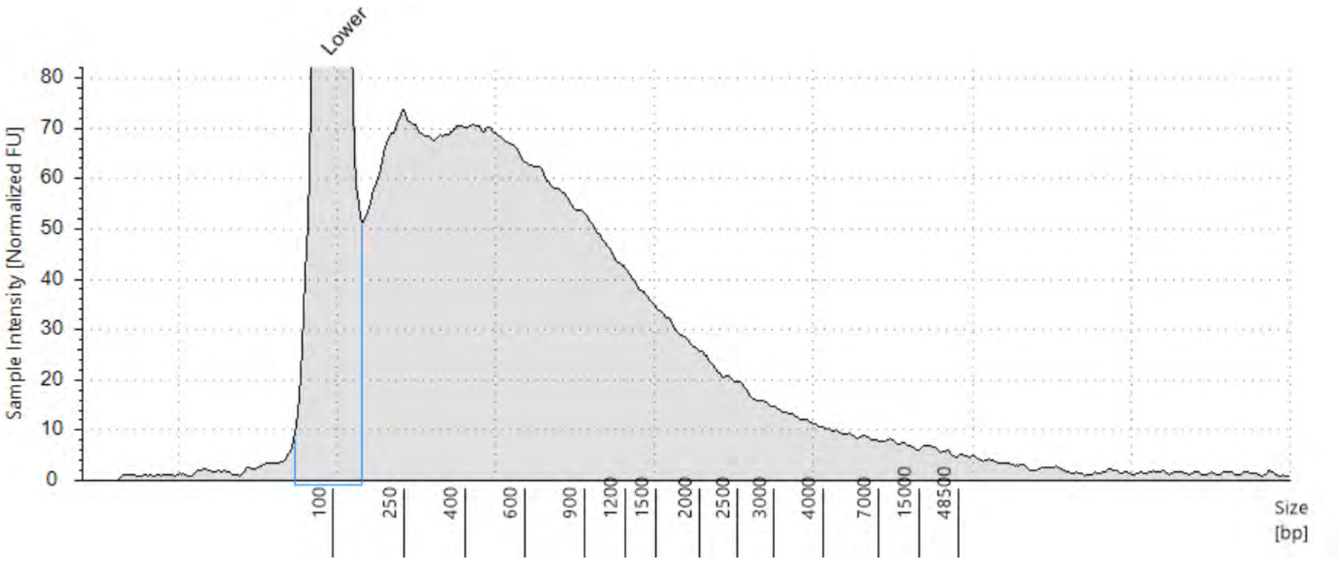

Sample Table

Well	DIN	Conc. [ng/µl]	Sample Description	Alert	Observations
G3	1.7	14.2	M-159-T4-D		

Peak Table

Size [bp]	Calibrated Conc. [ng/µl]	Assigned Conc. [ng/µl]	% Integrated Area	From [bp]	To [bp]	Peak Comment	Observations
100	8.50	8.50	-	60	145		Lower Marker

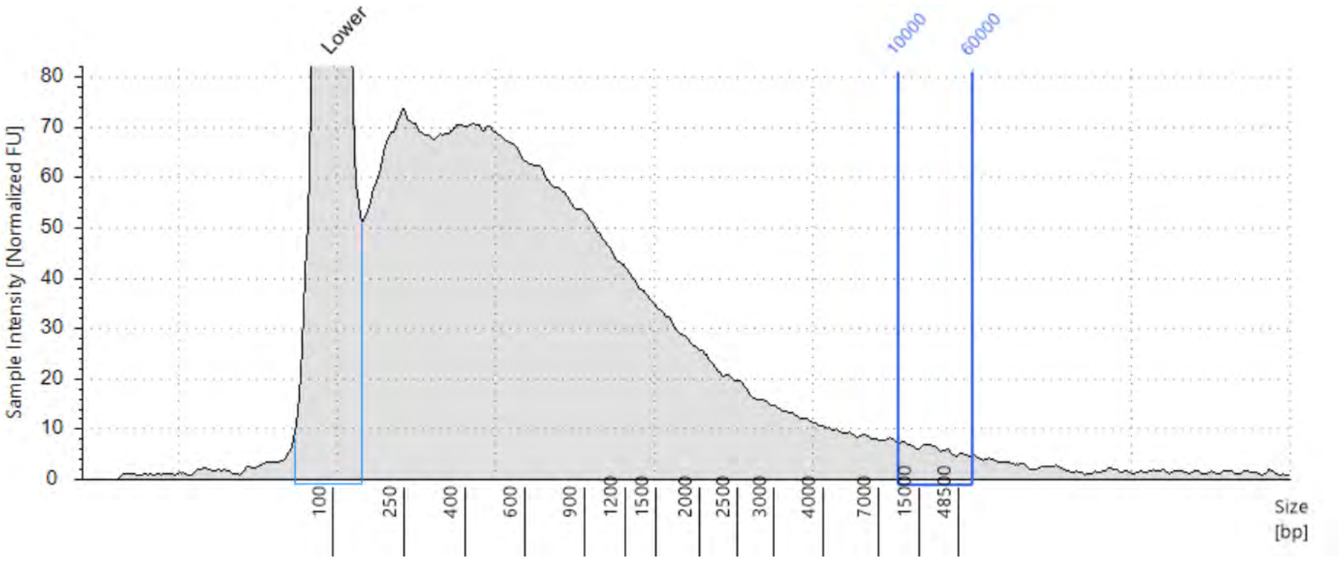

Region Table

From [bp]	To [bp]	Average Size [bp]	Conc. [ng/µl]	Region Molarity [nmol/l]	% of Total	Region Comment	Color
10000	60000	24460	0.254	0.0240	1.78		■

 © Agilent Technologies, Inc. 2017

H3: M-160-T4-D

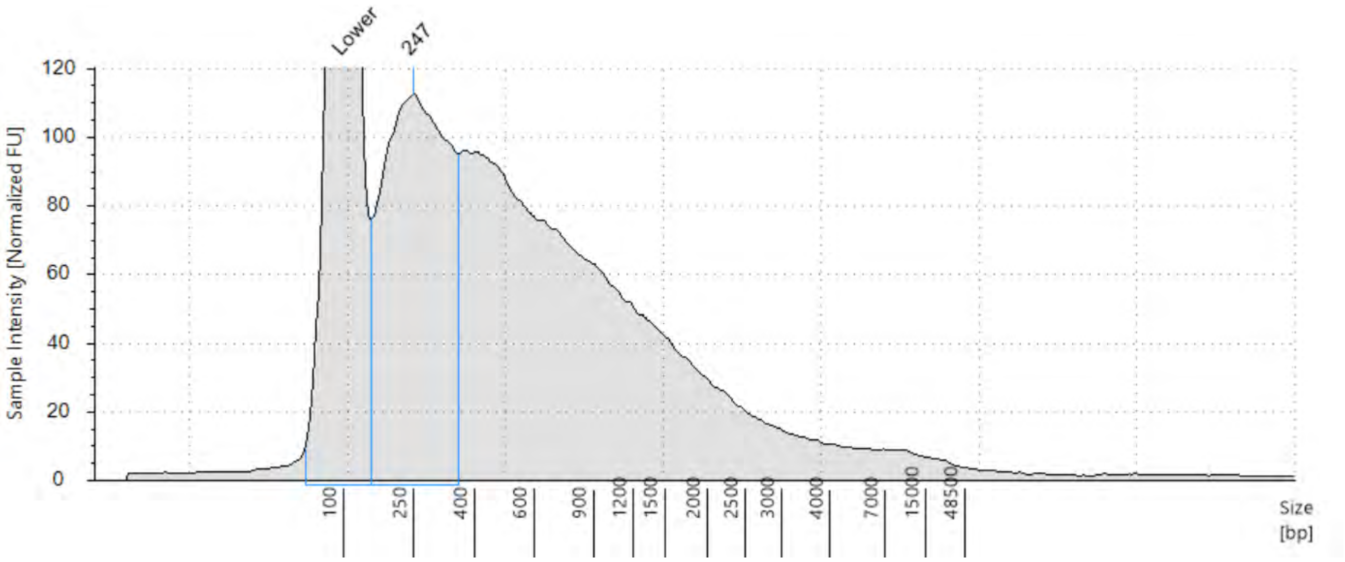

Sample Table

Well	DIN	Conc. [ng/µl]	Sample Description	Alert	Observations
H3	1.6	17.5	M-160-T4-D		

Peak Table

Size [bp]	Calibrated Conc. [ng/µl]	Assigned Conc. [ng/µl]	% Integrated Area	From [bp]	To [bp]	Peak Comment	Observations
100	8.50	8.50	-	60	143		Lower Marker
247	5.47	-	100.00	143	352		

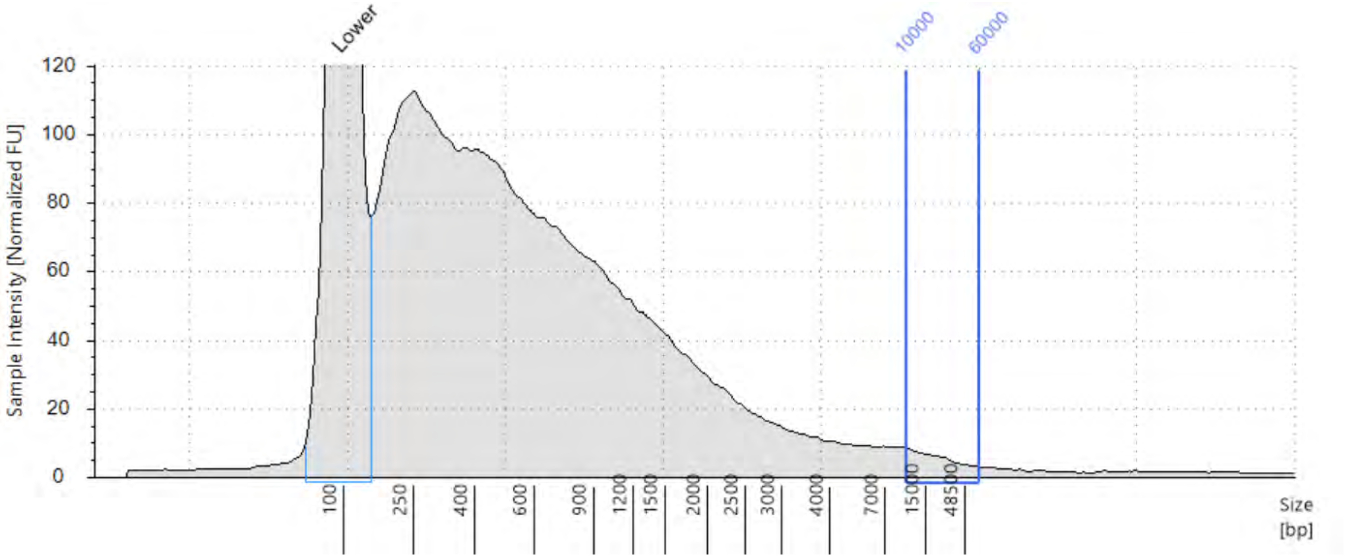

Region Table

From [bp]	To [bp]	Average Size [bp]	Conc. [ng/µl]	Region Molarity [nmol/l]	% of Total	Region Comment	Color
10000	60000	22186	0.220	0.0235	1.26		

A4: M-288-T4-D

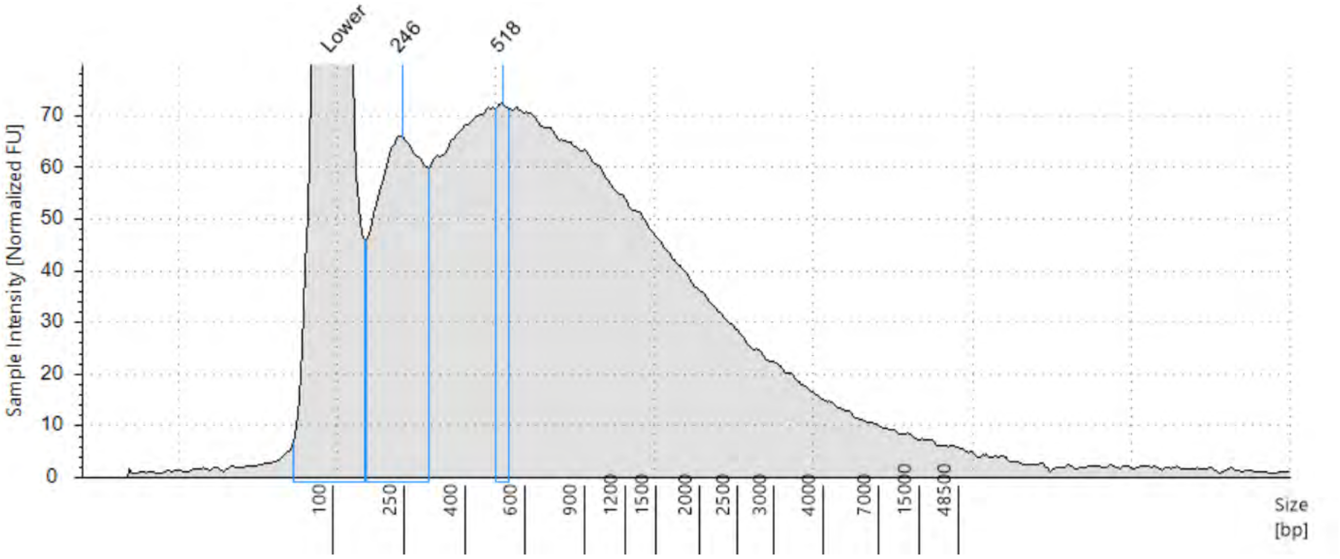

Sample Table

Well	DIN	Conc. [ng/µl]	Sample Description	Alert	Observations
A4	2.0	15.5	M-288-T4-D		

Peak Table

Size [bp]	Calibrated Conc. [ng/µl]	Assigned Conc. [ng/µl]	% Integrated Area	From [bp]	To [bp]	Peak Comment	Observations
100	8.50	8.50	-	60	150		Lower Marker
246	2.36	-	77.89	153	302		
518	0.670	-	22.11	494	542		

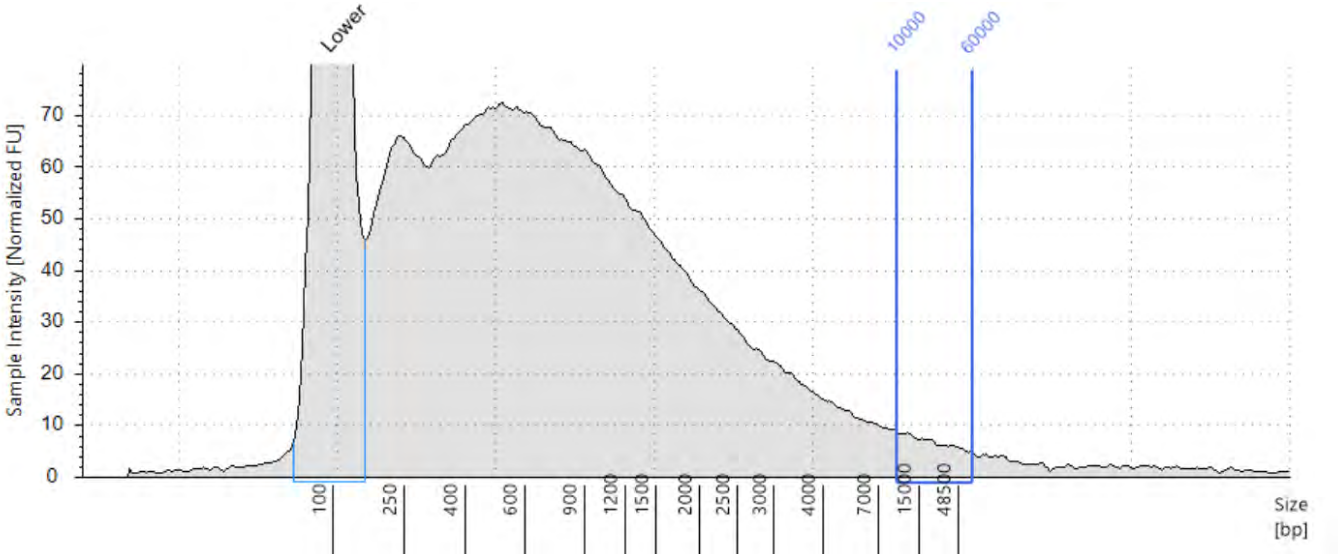

Region Table

From [bp]	To [bp]	Average Size [bp]	Conc. [ng/µl]	Region Molarity [nmol/l]	% of Total	Region Comment	Color
10000	60000	24296	0.274	0.0265	1.77		

B4: M-289-T4-D

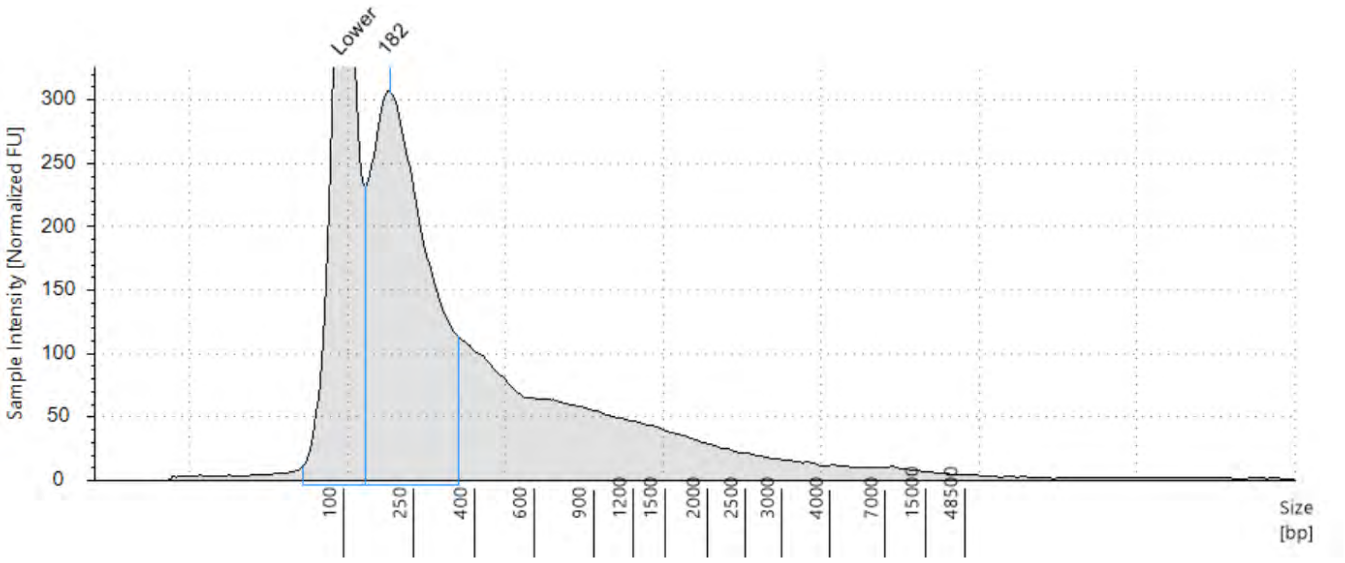

Sample Table

Well	DIN	Conc. [ng/µl]	Sample Description	Alert	Observations
B4	1.5	22.1	M-289-T4-D		

Peak Table

Size [bp]	Calibrated Conc. [ng/µl]	Assigned Conc. [ng/µl]	% Integrated Area	From [bp]	To [bp]	Peak Comment	Observations
100	8.50	8.50	-	59	133		Lower Marker
182	11.7	-	100.00	133	353		

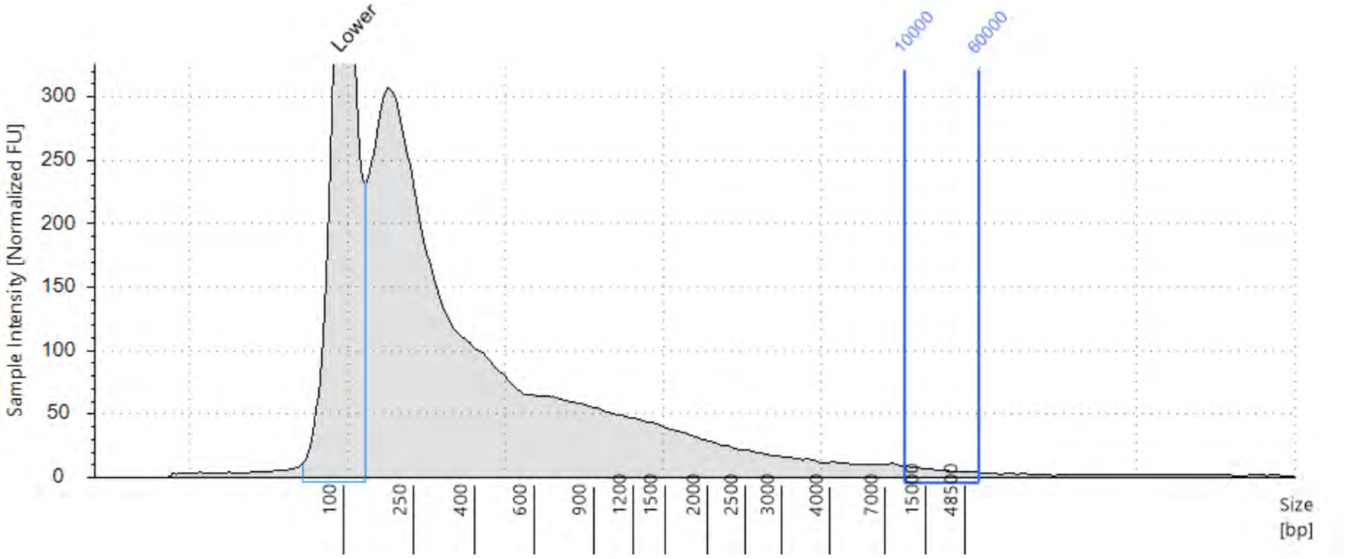

Region Table

From [bp]	To [bp]	Average Size [bp]	Conc. [ng/µl]	Region Molarity [nmol/l]	% of Total	Region Comment	Color
10000	60000	23218	0.190	0.0201	0.86		

C4: M-203-T5-D

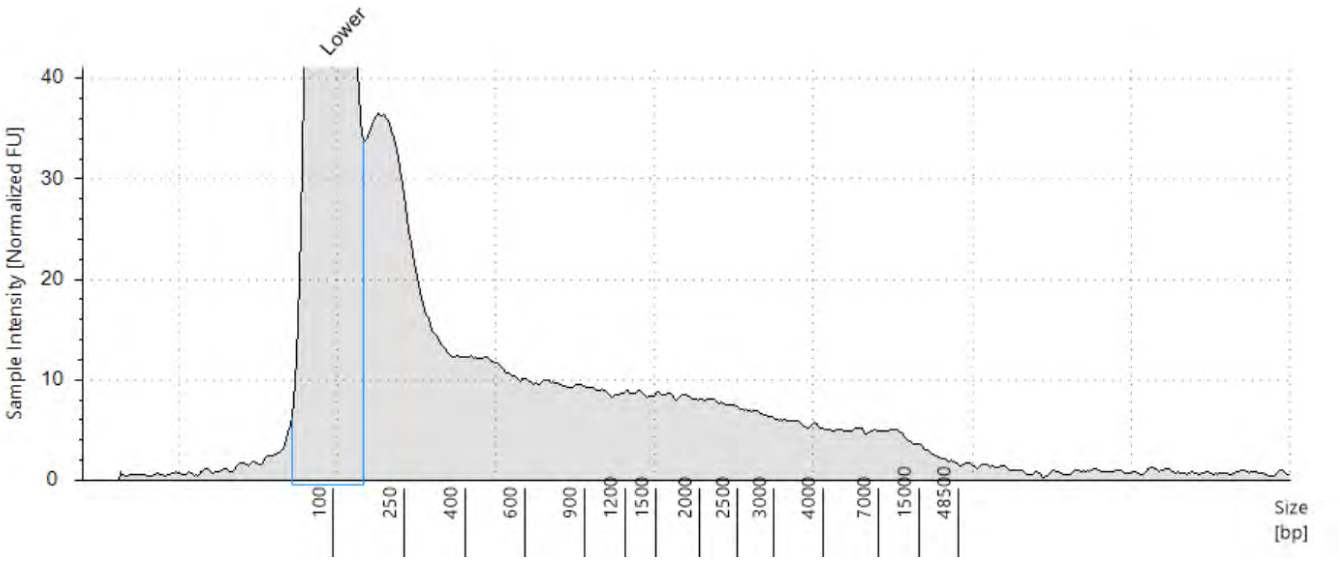

Sample Table

Well	DIN	Conc. [ng/µl]	Sample Description	Alert	Observations
C4	1.8	3.93	M-203-T5-D	⚠	Sample concentration outside functional range for DIN

Peak Table

Size [bp]	Calibrated Conc. [ng/µl]	Assigned Conc. [ng/µl]	% Integrated Area	From [bp]	To [bp]	Peak Comment	Observations
100	8.50	8.50	-	59	150		Lower Marker

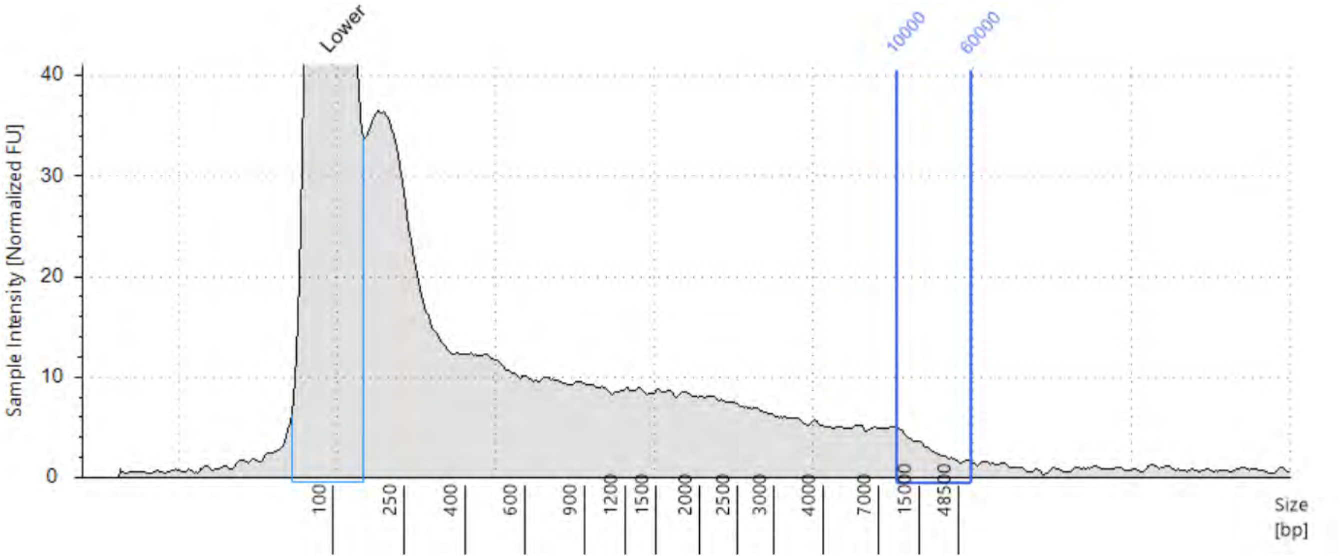

Region Table

From [bp]	To [bp]	Average Size [bp]	Conc. [ng/µl]	Region Molarity [nmol/l]	% of Total	Region Comment	Color
10000	60000	21727	0.120	0.0126	3.05		■

D4: M-204-T5-D

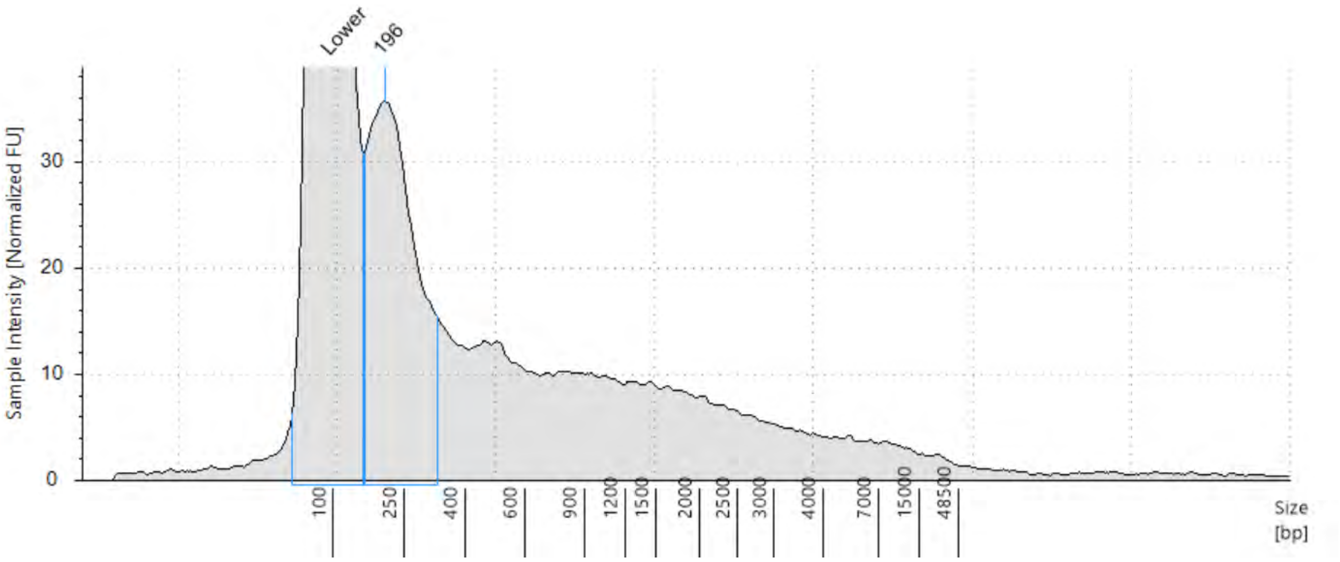

Sample Table

Well	DIN	Conc. [ng/µl]	Sample Description	Alert	Observations
D4	1.6	3.86	M-204-T5-D	⚠	Sample concentration outside functional range for DIN

Peak Table

Size [bp]	Calibrated Conc. [ng/µl]	Assigned Conc. [ng/µl]	% Integrated Area	From [bp]	To [bp]	Peak Comment	Observations
100	8.50	8.50	-	59	148		Lower Marker
196	1.30	-	100.00	150	323		

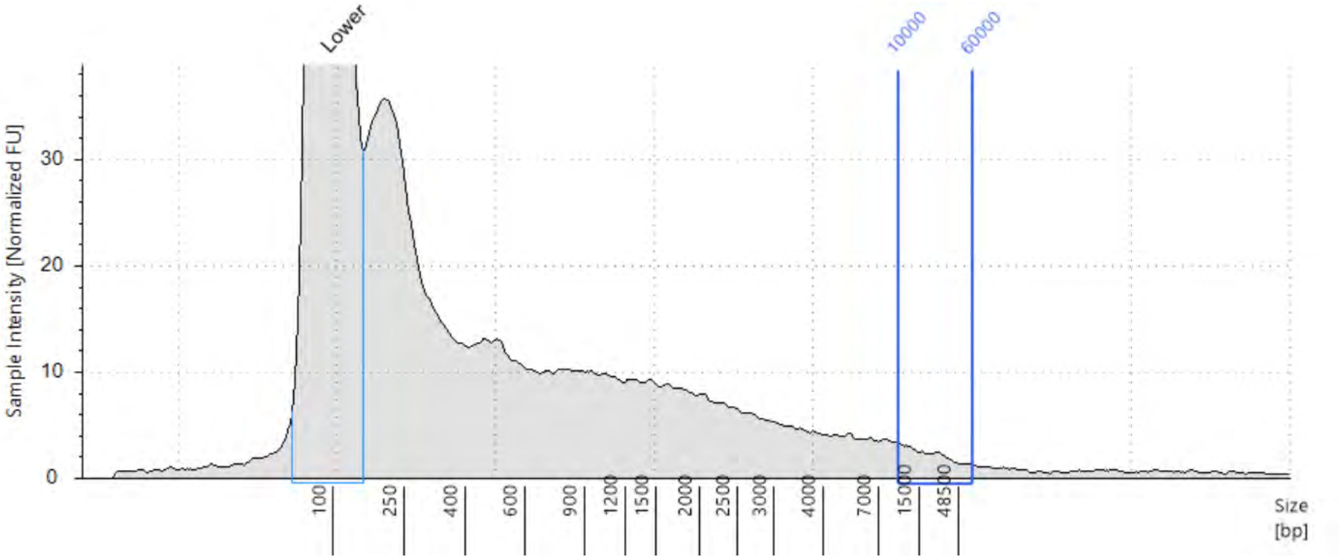

Region Table

From [bp]	To [bp]	Average Size [bp]	Conc. [ng/µl]	Region Molarity [nmol/l]	% of Total	Region Comment	Color
10000	60000	22520	0.0897	0.00966	2.32		■

 © Agilent Technologies, Inc. 2017

E4: M-205-T5-D

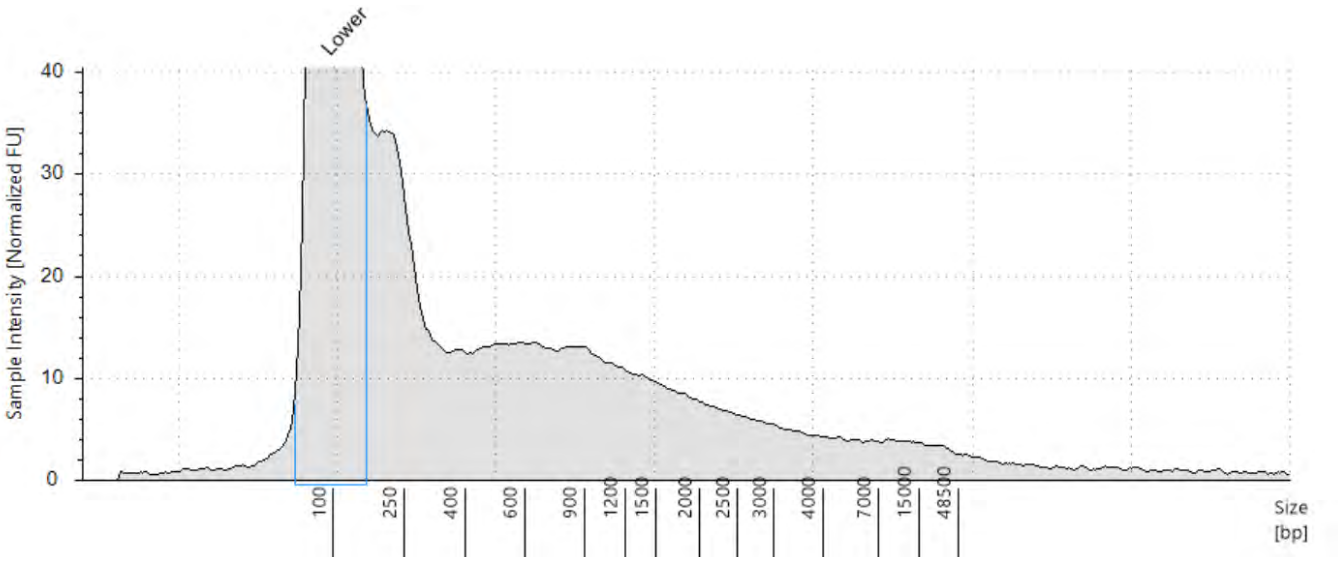

Sample Table

Well	DIN	Conc. [ng/µl]	Sample Description	Alert	Observations
E4	1.6	3.96	M-205-T5-D	⚠	Sample concentration outside functional range for DIN

Peak Table

Size [bp]	Calibrated Conc. [ng/µl]	Assigned Conc. [ng/µl]	% Integrated Area	From [bp]	To [bp]	Peak Comment	Observations
100	8.50	8.50	-	60	153		Lower Marker

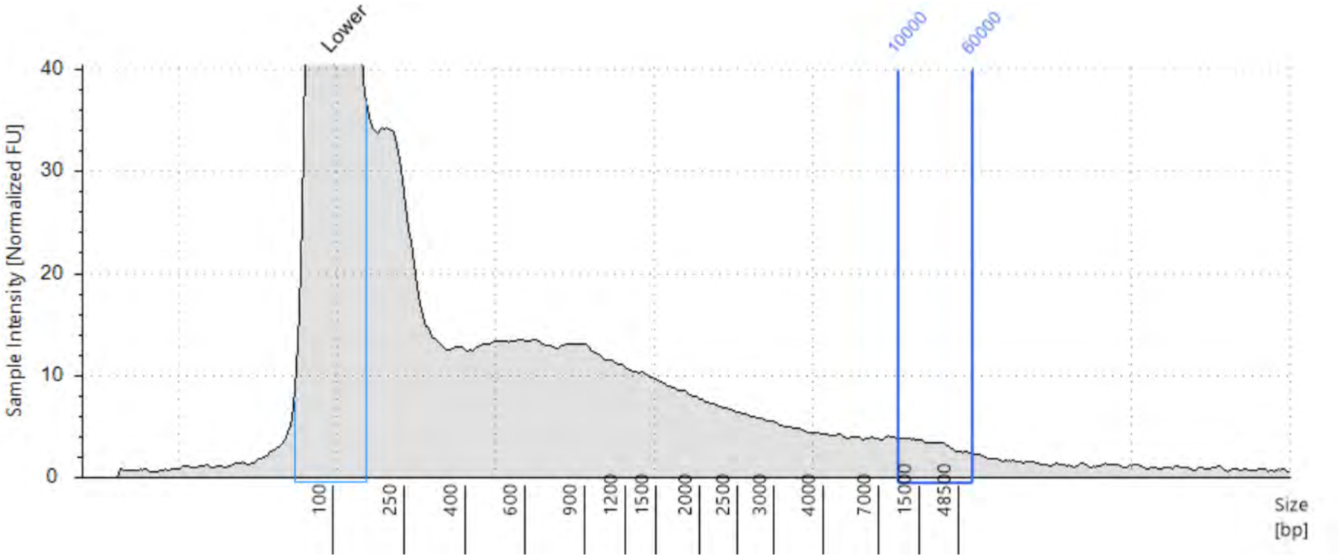

Region Table

From [bp]	To [bp]	Average Size [bp]	Conc. [ng/µl]	Region Molarity [nmol/l]	% of Total	Region Comment	Color
10000	60000	24170	0.133	0.0129	3.37		■

 © Agilent Technologies, Inc. 2017

F4: M-306-T5-D

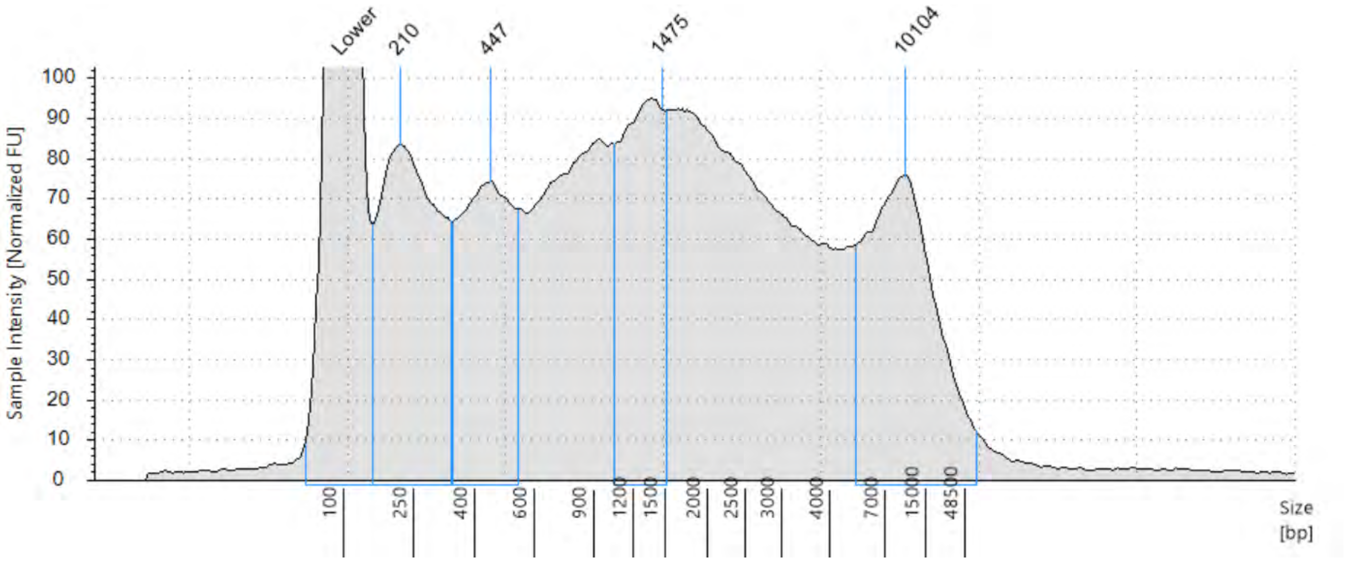

Sample Table

Well	DIN	Conc. [ng/µl]	Sample Description	Alert	Observations
F4	2.9	27.3	M-306-T5-D		

Peak Table

Size [bp]	Calibrated Conc. [ng/µl]	Assigned Conc. [ng/µl]	% Integrated Area	From [bp]	To [bp]	Peak Comment	Observations
100	8.50	8.50	-	60	145		Lower Marker
210	3.66	-	27.03	145	332		
447	2.88	-	21.30	339	538		
1475	3.05	-	22.56	1041	1517		
10104	3.94	-	29.11	5143	58326		

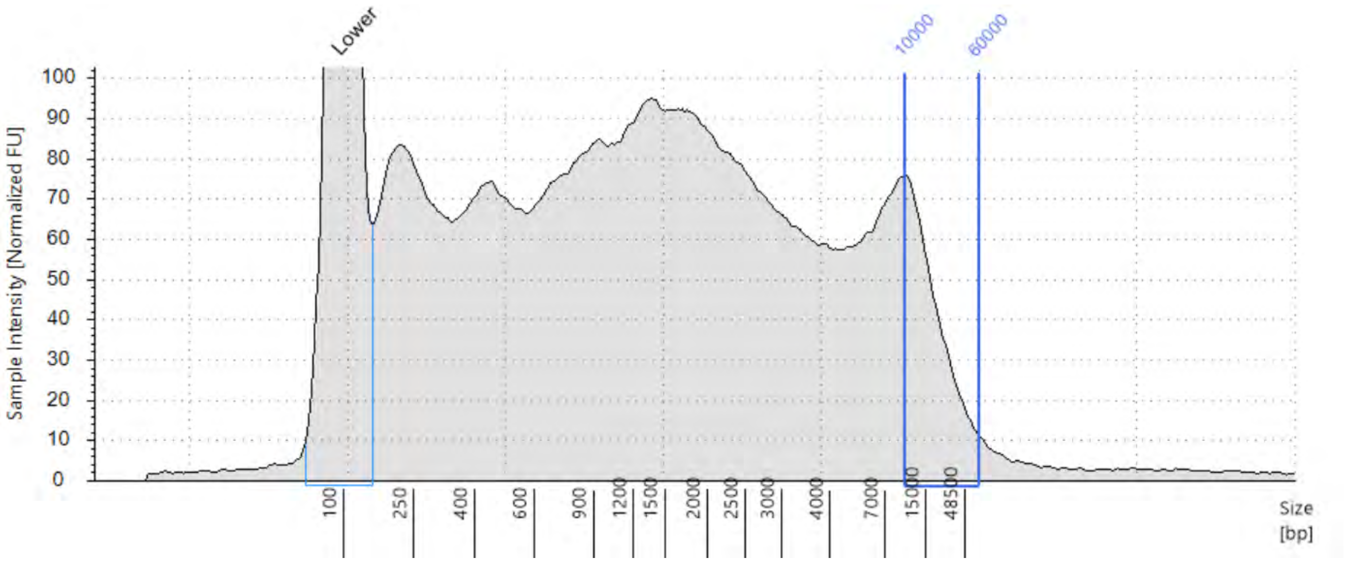

Region Table

From [bp]	To [bp]	Average Size [bp]	Conc. [ng/µl]	Region Molarity [nmol/l]	% of Total	Region Comment	Color
10000	60000	19513	1.89	0.190	6.93		

G4: M-307-T5-D

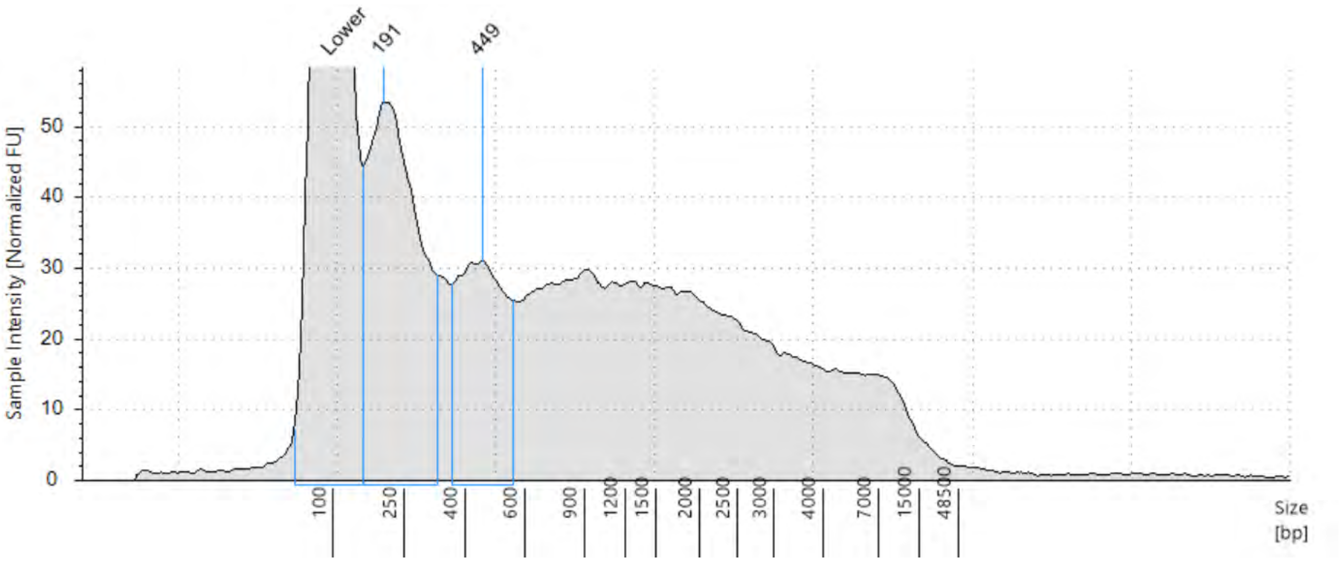

Sample Table

Well	DIN	Conc. [ng/µl]	Sample Description	Alert	Observations
G4	2.6	9.45	M-307-T5-D	⚠	Sample concentration outside recommended range

Peak Table

Size [bp]	Calibrated Conc. [ng/µl]	Assigned Conc. [ng/µl]	% Integrated Area	From [bp]	To [bp]	Peak Comment	Observations
100	8.50	8.50	-	60	147		Lower Marker
191	2.11	-	64.75	147	323		
449	1.15	-	35.25	363	554		

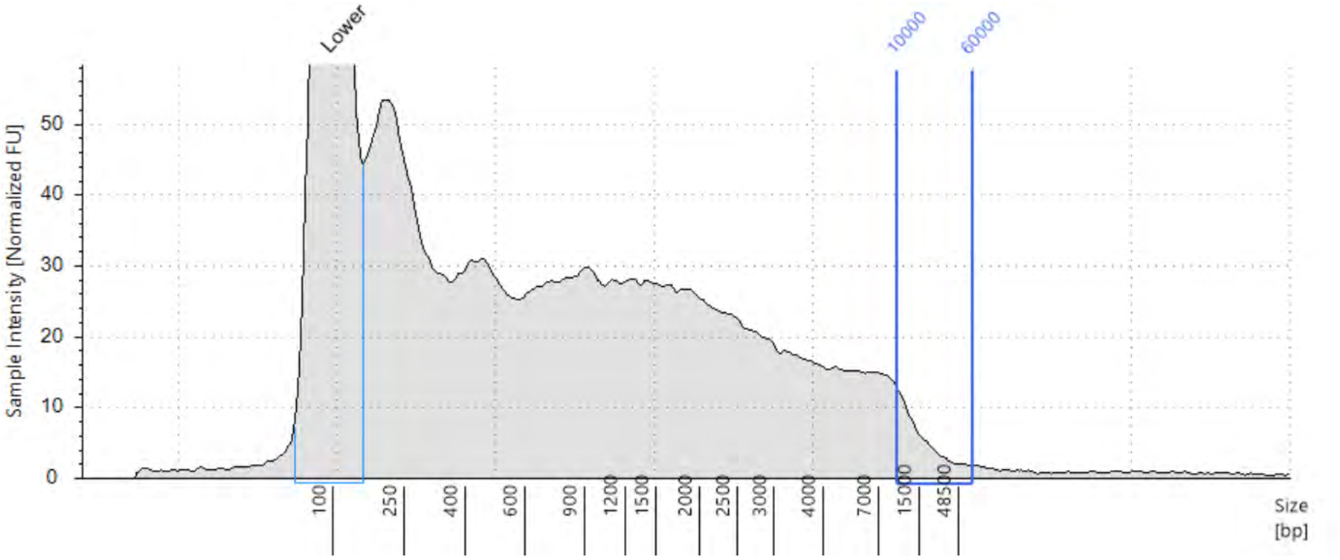

Region Table

From [bp]	To [bp]	Average Size [bp]	Conc. [ng/µl]	Region Molarity [nmol/l]	% of Total	Region Comment	Color
10000	60000	18754	0.230	0.0259	2.43		■

 © Agilent Technologies, Inc. 2017

H4: M-192-T5-DSS

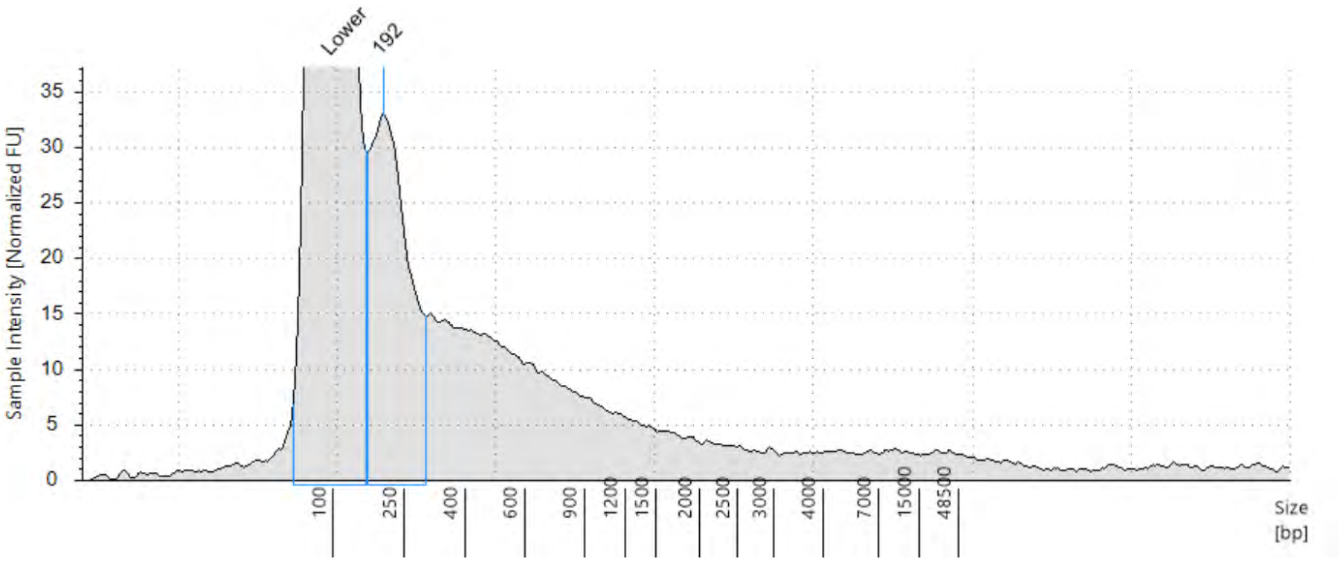

Sample Table

Well	DIN	Conc. [ng/µl]	Sample Description	Alert	Observations
H4	1.0	3.10	M-192-T5-DSS	⚠	Sample concentration outside functional range for DIN

Peak Table

Size [bp]	Calibrated Conc. [ng/µl]	Assigned Conc. [ng/µl]	% Integrated Area	From [bp]	To [bp]	Peak Comment	Observations
100	8.50	8.50	-	59	152		Lower Marker
192	0.939	-	100.00	155	295		

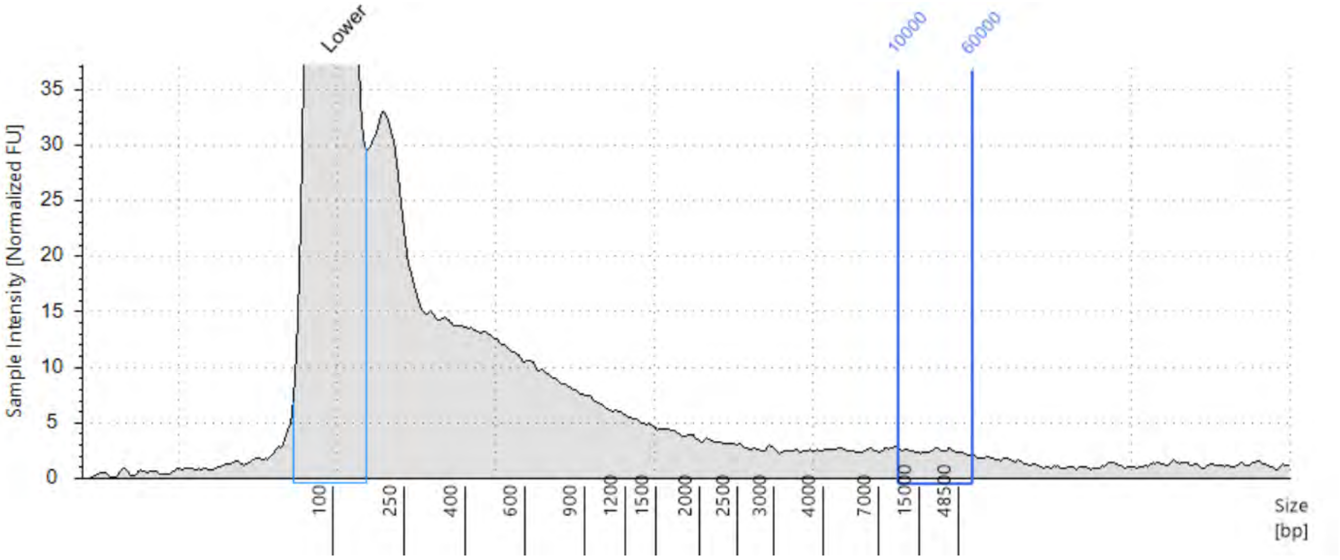

Region Table

From [bp]	To [bp]	Average Size [bp]	Conc. [ng/µl]	Region Molarity [nmol/l]	% of Total	Region Comment	Color
10000	60000	25999	0.0961	0.00915	3.11		■

A5: M-193-T5-DSS

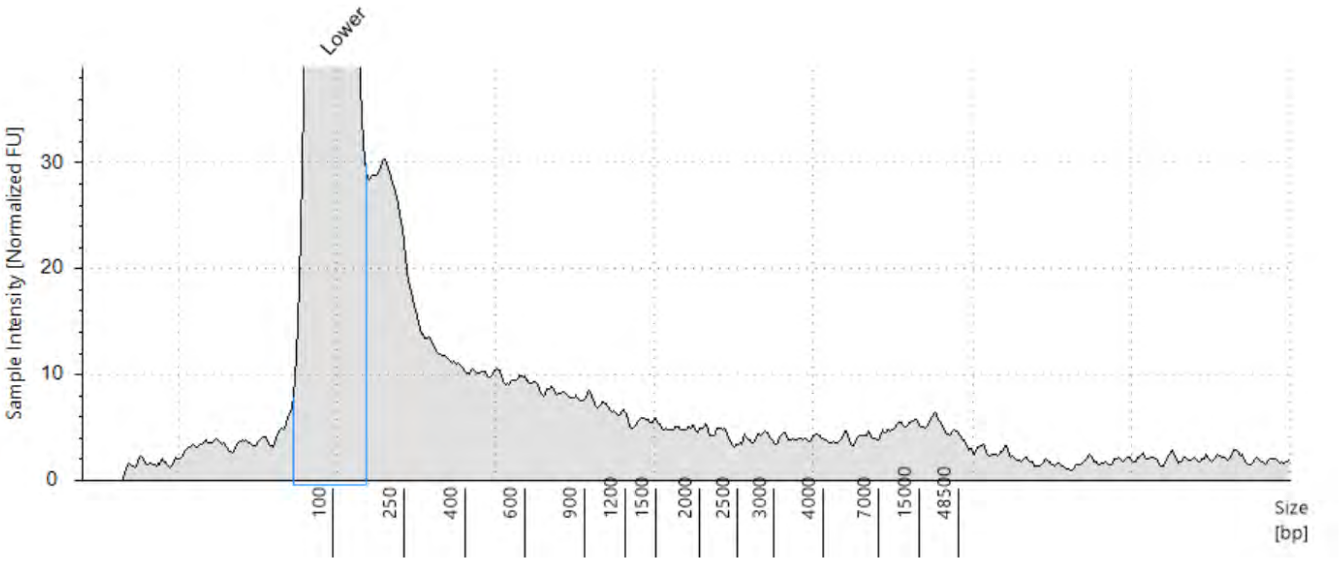

Sample Table

Well	DIN	Conc. [ng/µl]	Sample Description	Alert	Observations
A5	1.0	3.61	M-193-T5-DSS	⚠	Sample concentration outside functional range for DIN

Peak Table

Size [bp]	Calibrated Conc. [ng/µl]	Assigned Conc. [ng/µl]	% Integrated Area	From [bp]	To [bp]	Peak Comment	Observations
100	8.50	8.50	-	60	153		Lower Marker

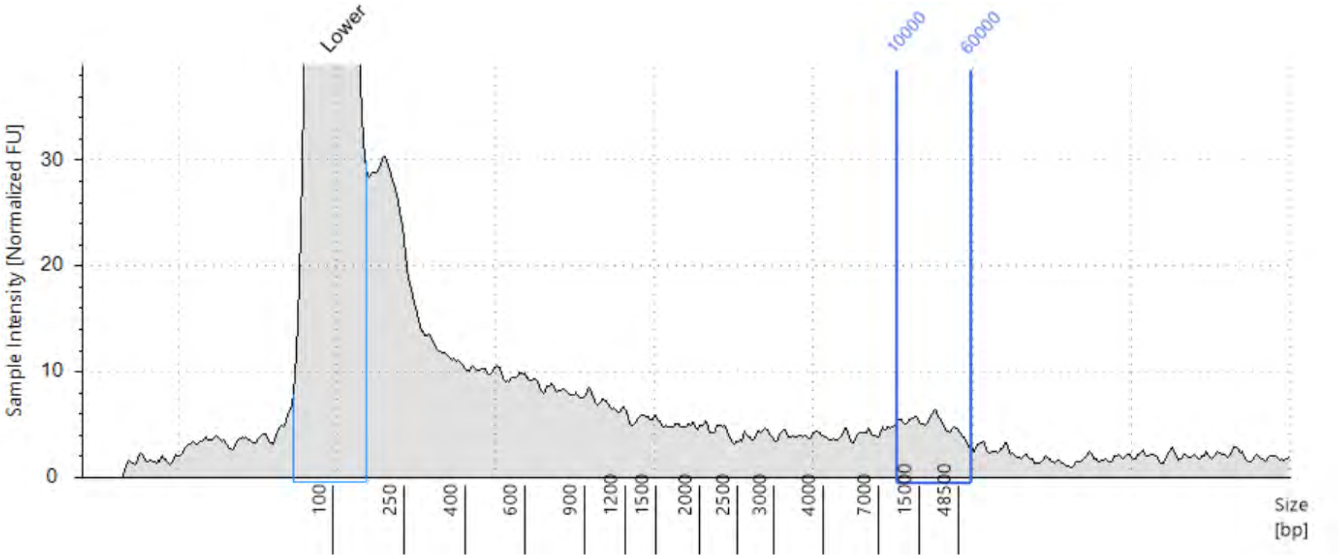

Region Table

From [bp]	To [bp]	Average Size [bp]	Conc. [ng/µl]	Region Molarity [nmol/l]	% of Total	Region Comment	Color
10000	60000	24342	0.213	0.0193	5.89		■

B5: M-194-T5-DSS

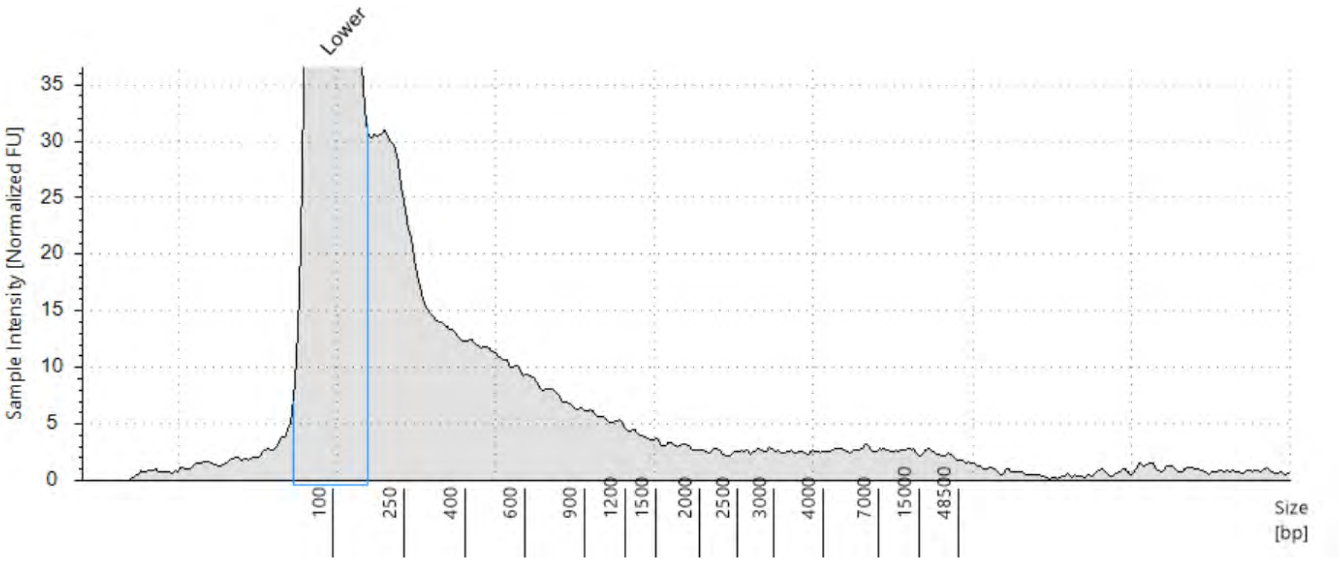

Sample Table

Well	DIN	Conc. [ng/µl]	Sample Description	Alert	Observations
B5	-	2.76	M-194-T5-DSS	▲	Sample concentration outside functional range for DIN

Peak Table

Size [bp]	Calibrated Conc. [ng/µl]	Assigned Conc. [ng/µl]	% Integrated Area	From [bp]	To [bp]	Peak Comment	Observations
100	8.50	8.50	-	60	156		Lower Marker

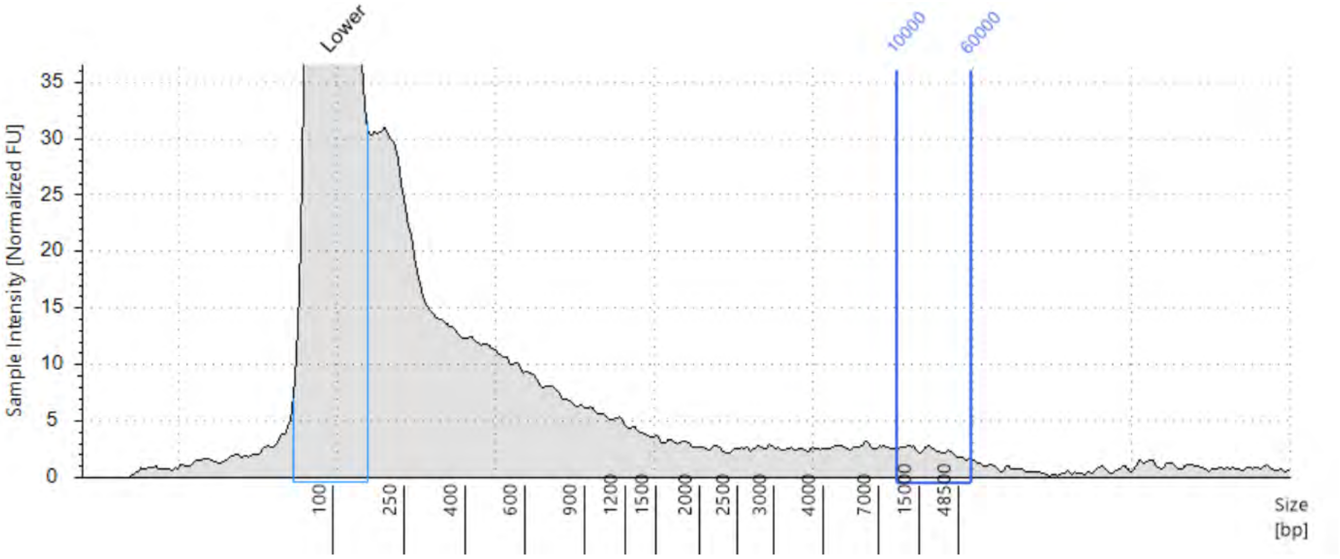

Region Table

From [bp]	To [bp]	Average Size [bp]	Conc. [ng/µl]	Region Molarity [nmol/l]	% of Total	Region Comment	Color
10000	60000	24072	0.0850	0.00893	3.08		■

C5: M-302-T5-DSS

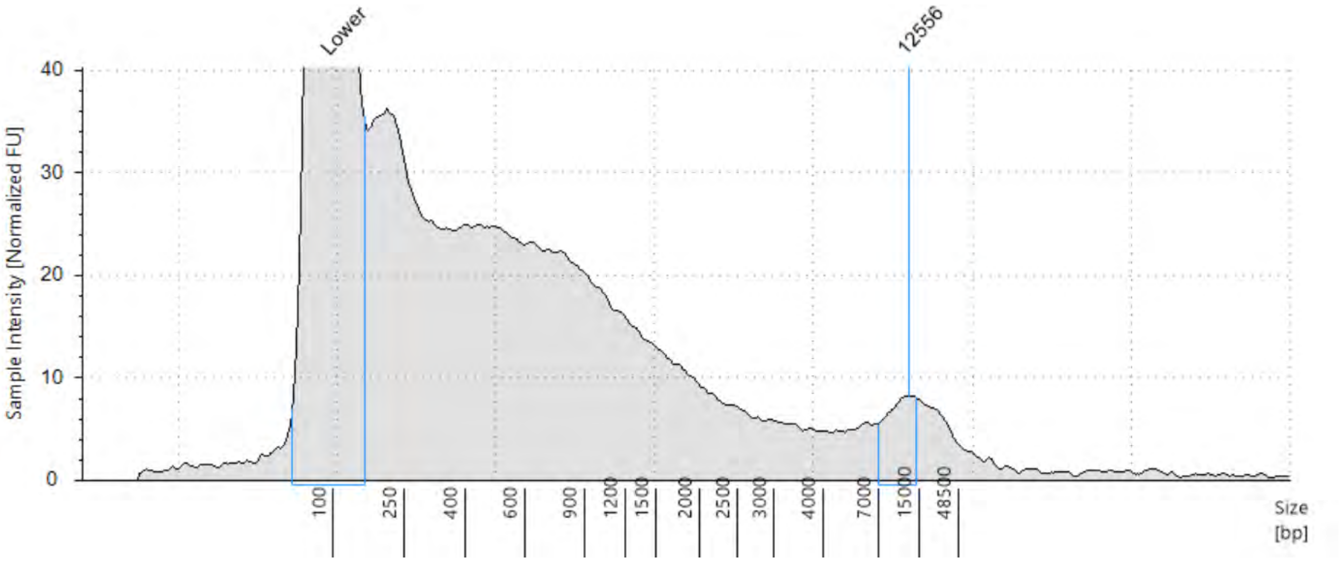

Sample Table

Well	DIN	Conc. [ng/µl]	Sample Description	Alert	Observations
C5	1.3	5.78	M-302-T5-DSS	⚠	Sample concentration outside recommended range

Peak Table

Size [bp]	Calibrated Conc. [ng/µl]	Assigned Conc. [ng/µl]	% Integrated Area	From [bp]	To [bp]	Peak Comment	Observations
100	8.50	8.50	-	59	150		Lower Marker
12556	0.169	-	100.00	7000	14257		

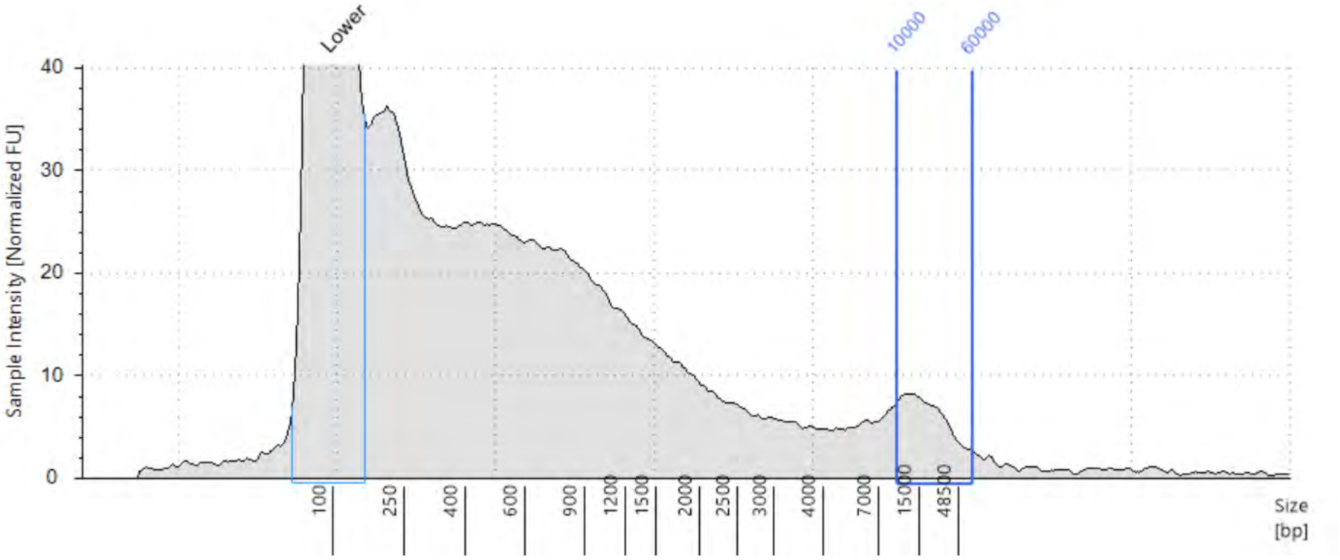

Region Table

From [bp]	To [bp]	Average Size [bp]	Conc. [ng/µl]	Region Molarity [nmol/l]	% of Total	Region Comment	Color
10000	60000	21832	0.271	0.0261	4.69		■

D5: M-303-T5-DSS

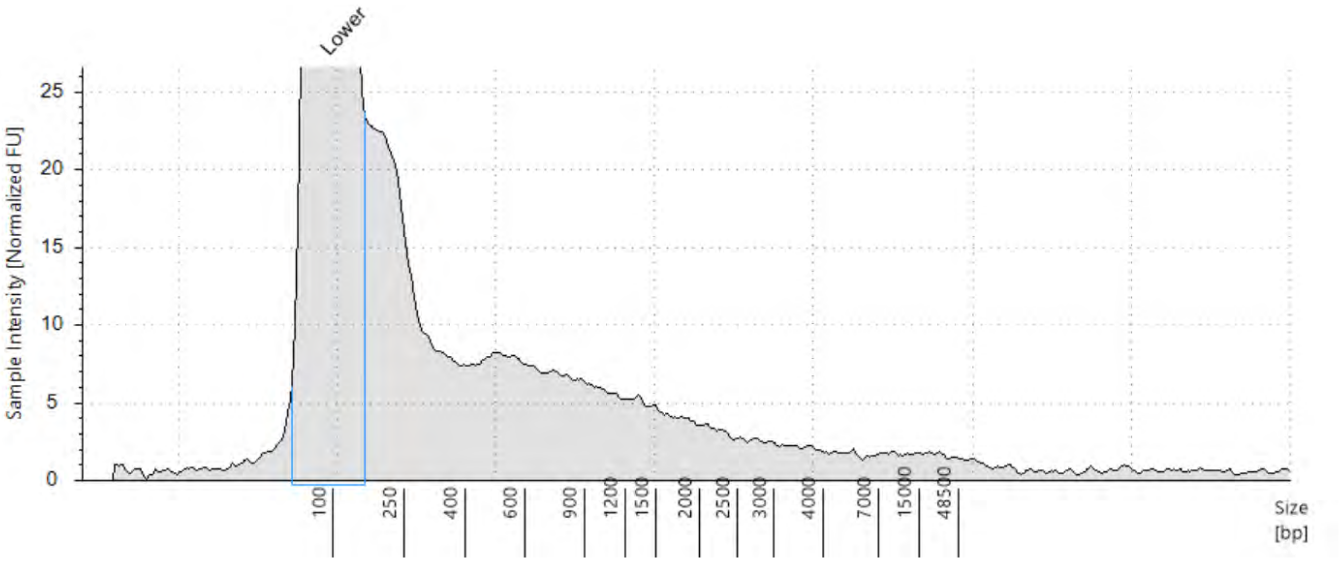

Sample Table

Well	DIN	Conc. [ng/µl]	Sample Description	Alert	Observations
D5	-	2.19	M-303-T5-DSS	▲	Sample concentration outside functional range for DIN

Peak Table

Size [bp]	Calibrated Conc. [ng/µl]	Assigned Conc. [ng/µl]	% Integrated Area	From [bp]	To [bp]	Peak Comment	Observations
100	8.50	8.50	-	58	151		Lower Marker

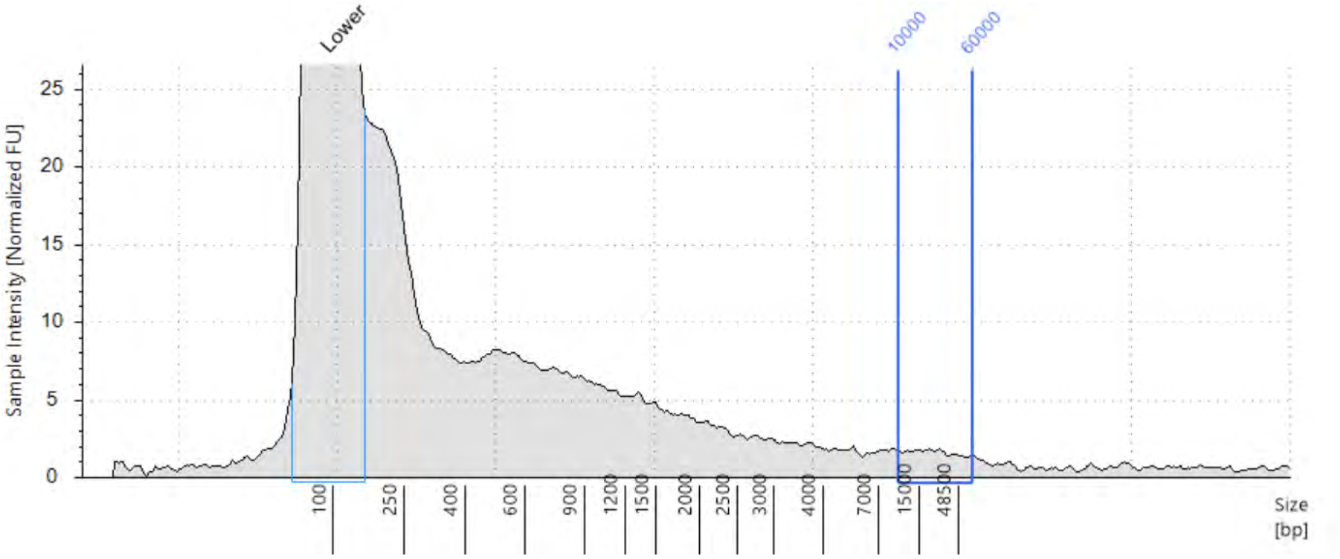

Region Table

From [bp]	To [bp]	Average Size [bp]	Conc. [ng/µl]	Region Molarity [nmol/l]	% of Total	Region Comment	Color
10000	60000	25131	0.0594	0.00622	2.71		■

 © Agilent Technologies, Inc. 2017

E5: M-211-T5-SS

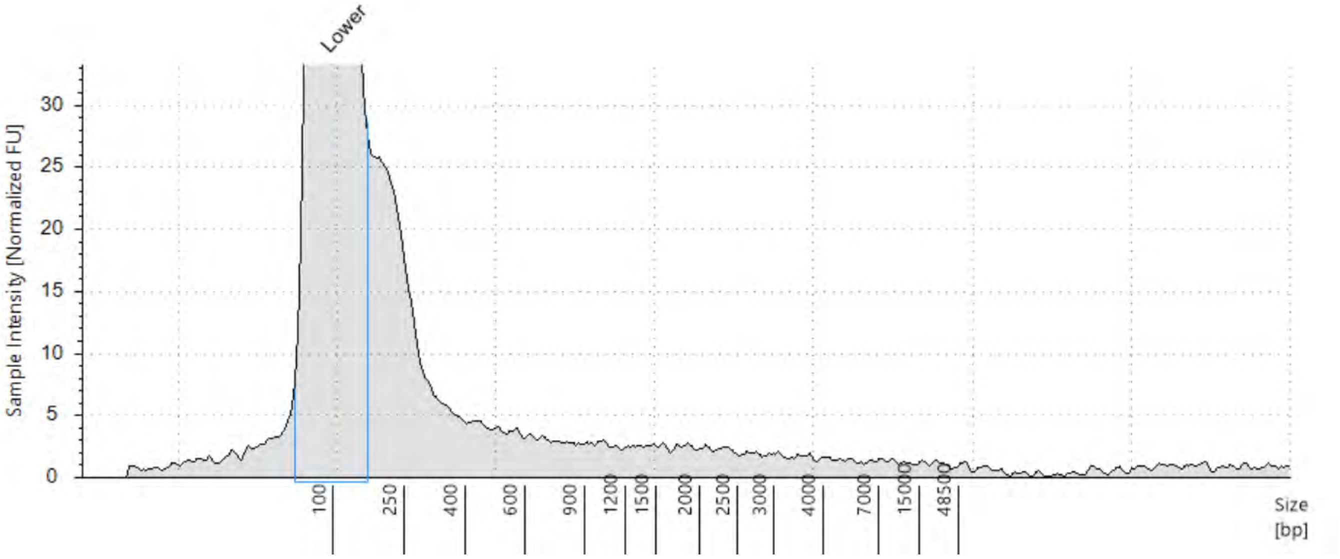

Sample Table

Well	DIN	Conc. [ng/µl]	Sample Description	Alert	Observations
E5	-	1.72	M-211-T5-SS	▲	Sample concentration outside functional range for DIN

Peak Table

Size [bp]	Calibrated Conc. [ng/µl]	Assigned Conc. [ng/µl]	% Integrated Area	From [bp]	To [bp]	Peak Comment	Observations
100	8.50	8.50	-	60	155		Lower Marker

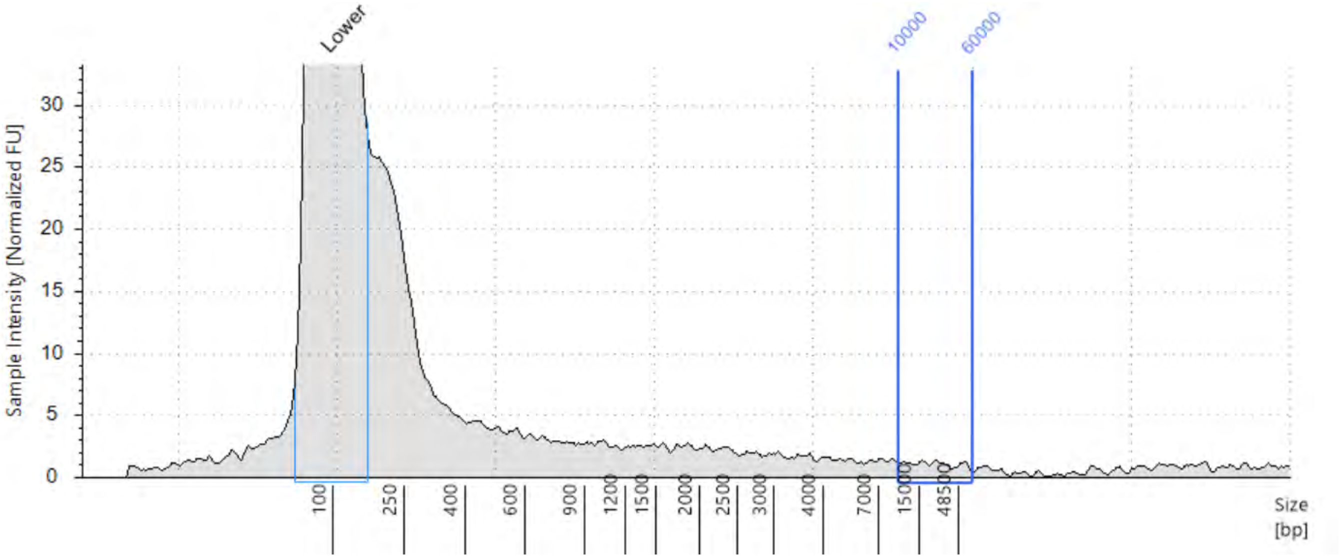

Region Table

From [bp]	To [bp]	Average Size [bp]	Conc. [ng/µl]	Region Molarity [nmol/l]	% of Total	Region Comment	Color
10000	60000	25523	0.0339	0.00410	1.97		■

F5: M-212-T5-SS

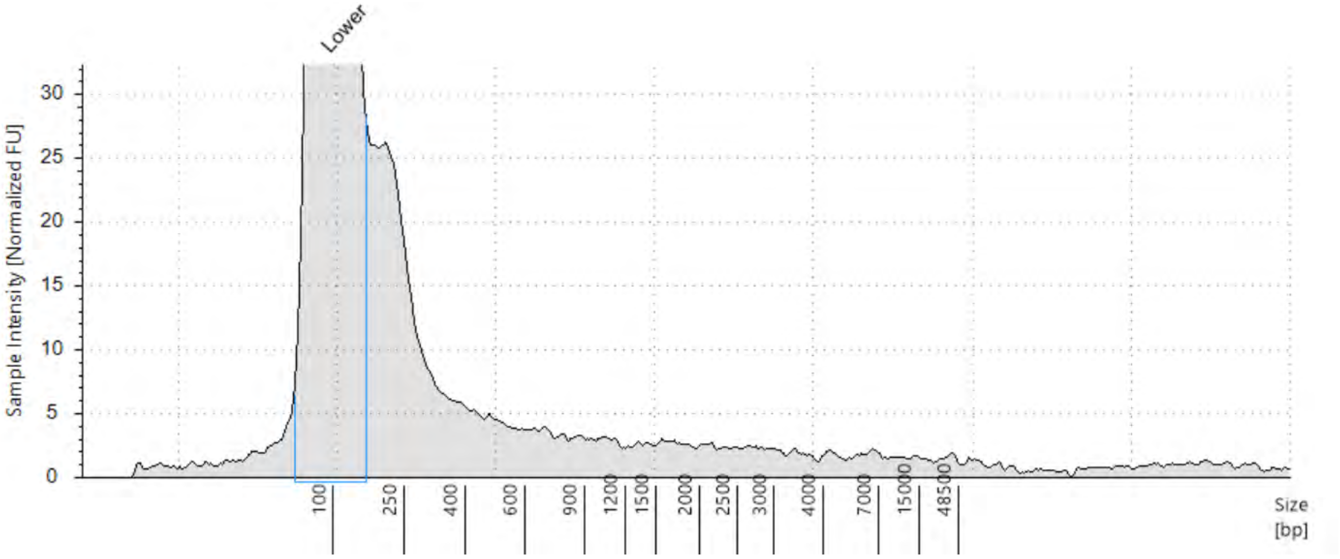

Sample Table

Well	DIN	Conc. [ng/µl]	Sample Description	Alert	Observations
F5	-	1.84	M-212-T5-SS	▲	Sample concentration outside functional range for DIN

Peak Table

Size [bp]	Calibrated Conc. [ng/µl]	Assigned Conc. [ng/µl]	% Integrated Area	From [bp]	To [bp]	Peak Comment	Observations
100	8.50	8.50	-	60	153		Lower Marker

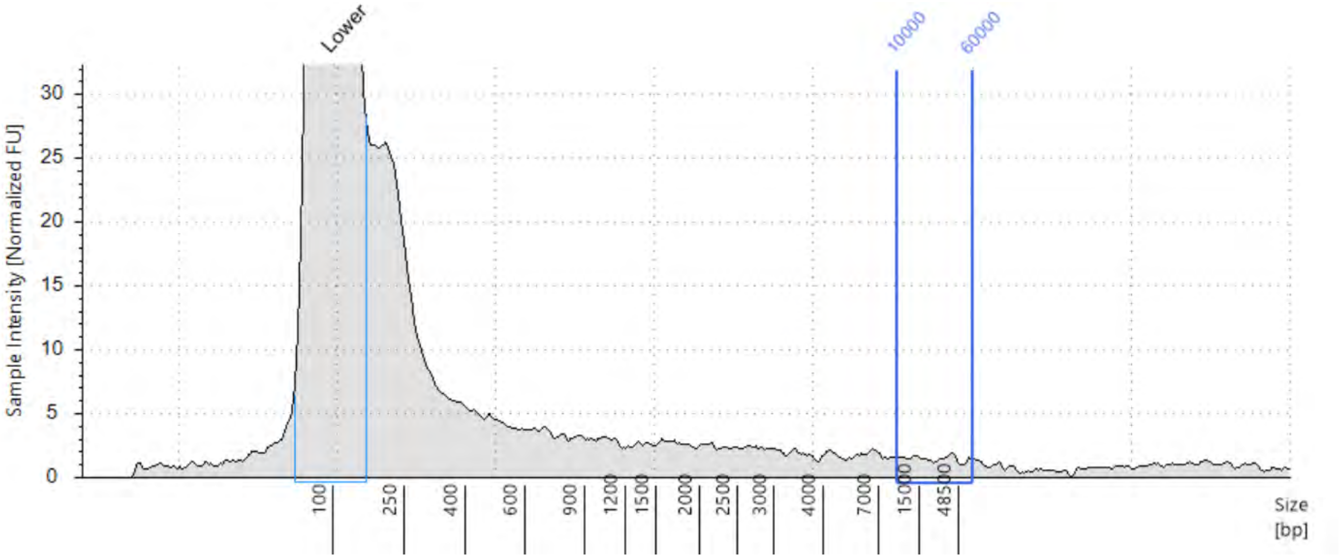

Region Table

From [bp]	To [bp]	Average Size [bp]	Conc. [ng/µl]	Region Molarity [nmol/l]	% of Total	Region Comment	Color
10000	60000	25686	0.0520	0.00560	2.83		■

 © Agilent Technologies, Inc. 2017

G5: M-213-T5-SS

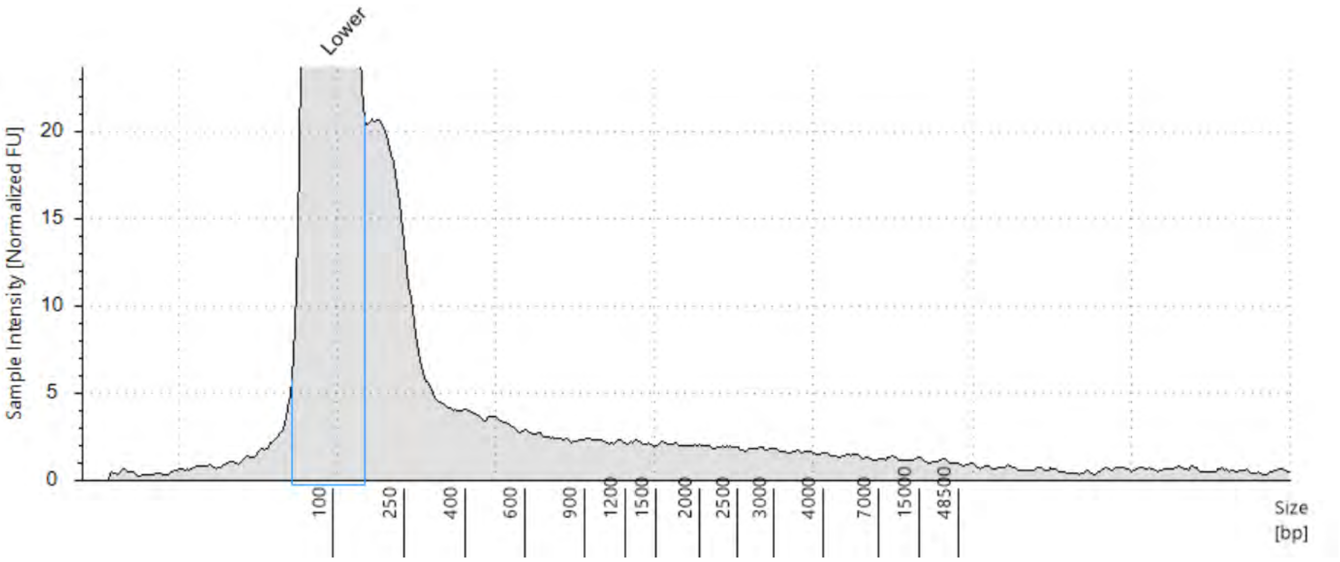

Sample Table

Well	DIN	Conc. [ng/µl]	Sample Description	Alert	Observations
G5	-	1.43	M-213-T5-SS	▲	Sample concentration outside functional range for DIN

Peak Table

Size [bp]	Calibrated Conc. [ng/µl]	Assigned Conc. [ng/µl]	% Integrated Area	From [bp]	To [bp]	Peak Comment	Observations
100	8.50	8.50	-	58	151		Lower Marker

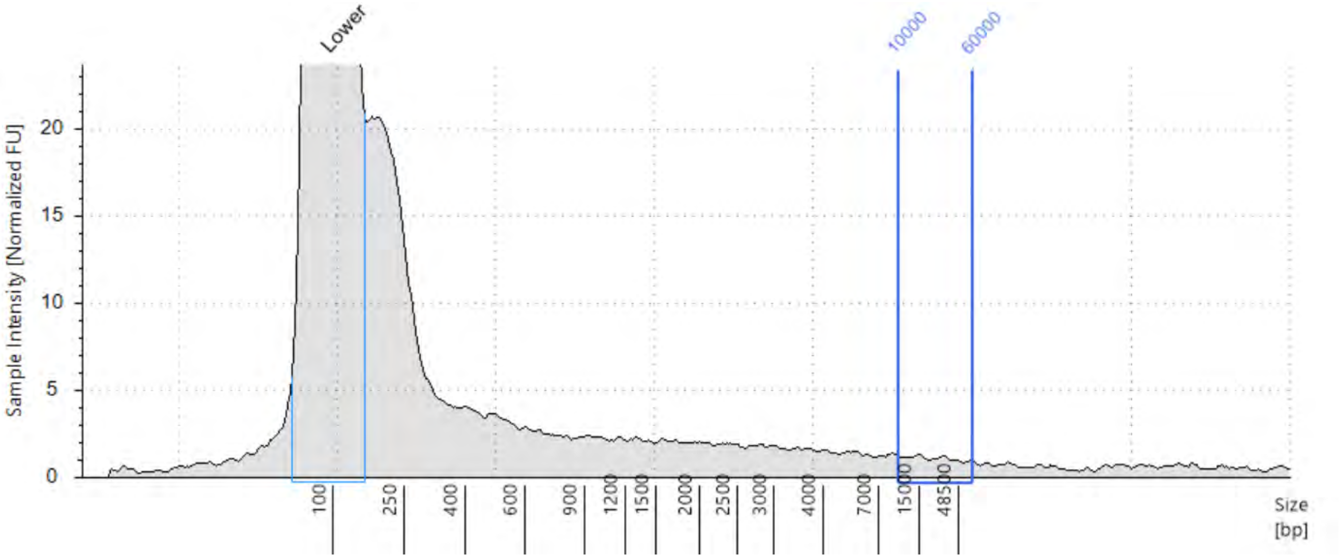

Region Table

From [bp]	To [bp]	Average Size [bp]	Conc. [ng/µl]	Region Molarity [nmol/l]	% of Total	Region Comment	Color
10000	60000	25429	0.0386	0.00425	2.69		■

 © Agilent Technologies, Inc. 2017

H5: M-214-T5-SS

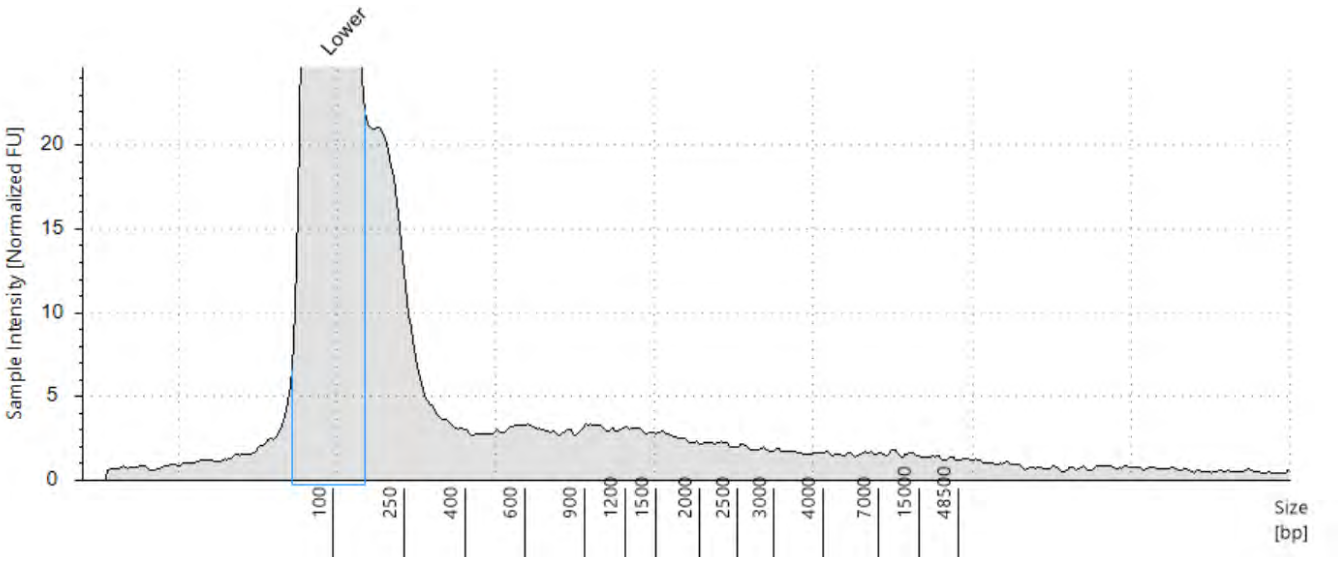

Sample Table

Well	DIN	Conc. [ng/µl]	Sample Description	Alert	Observations
H5	-	1.50	M-214-T5-SS	▲	Sample concentration outside functional range for DIN

Peak Table

Size [bp]	Calibrated Conc. [ng/µl]	Assigned Conc. [ng/µl]	% Integrated Area	From [bp]	To [bp]	Peak Comment	Observations
100	8.50	8.50	-	58	151		Lower Marker

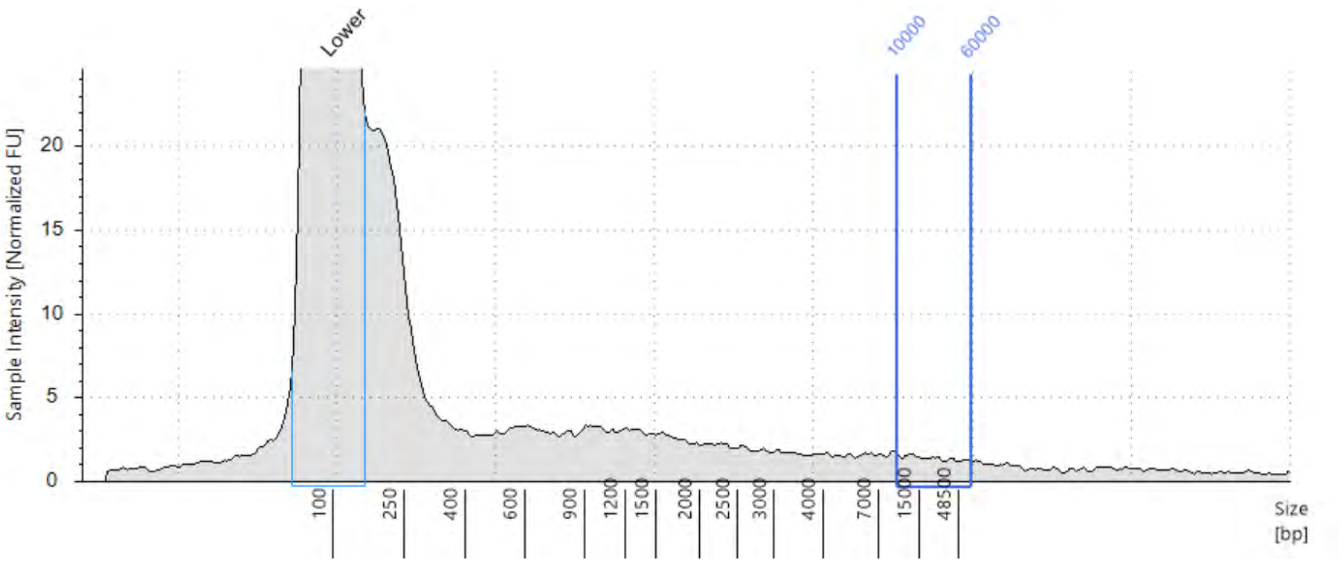

Region Table

From [bp]	To [bp]	Average Size [bp]	Conc. [ng/µl]	Region Molarity [nmol/l]	% of Total	Region Comment	Color
10000	60000	25393	0.0527	0.00545	3.51		■

 © Agilent Technologies, Inc. 2017

A6: M-215-T5-SS

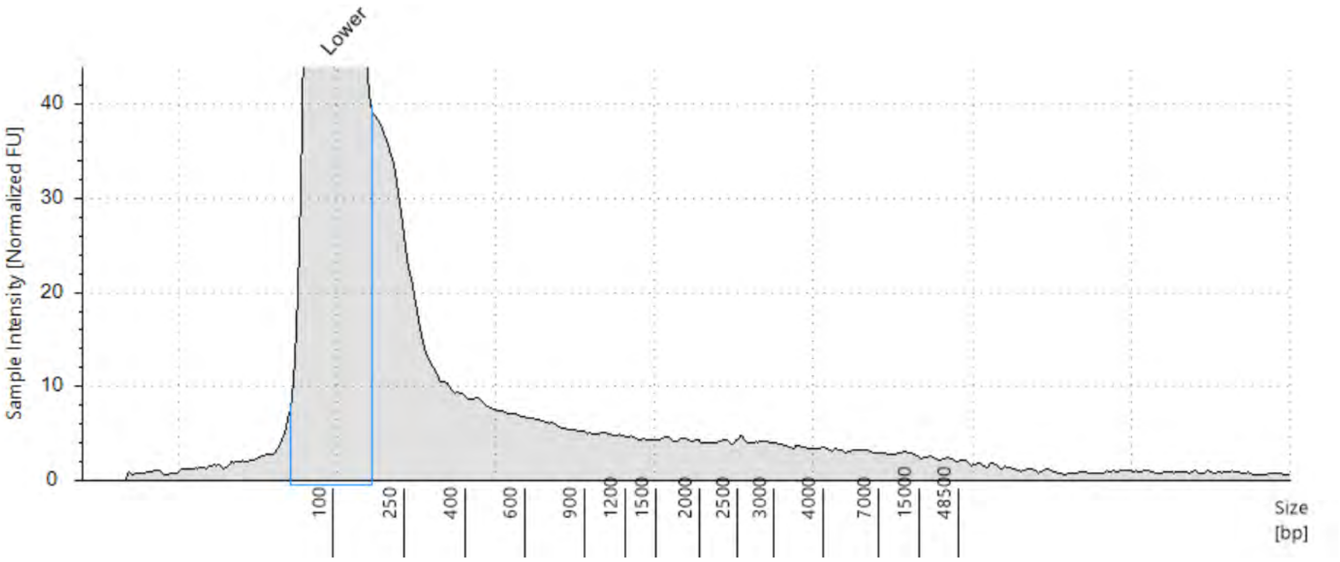

Sample Table

Well	DIN	Conc. [ng/µl]	Sample Description	Alert	Observations
A6	-	2.43	M-215-T5-SS	▲	Sample concentration outside functional range for DIN

Peak Table

Size [bp]	Calibrated Conc. [ng/µl]	Assigned Conc. [ng/µl]	% Integrated Area	From [bp]	To [bp]	Peak Comment	Observations
100	8.50	8.50	-	58	164		Lower Marker

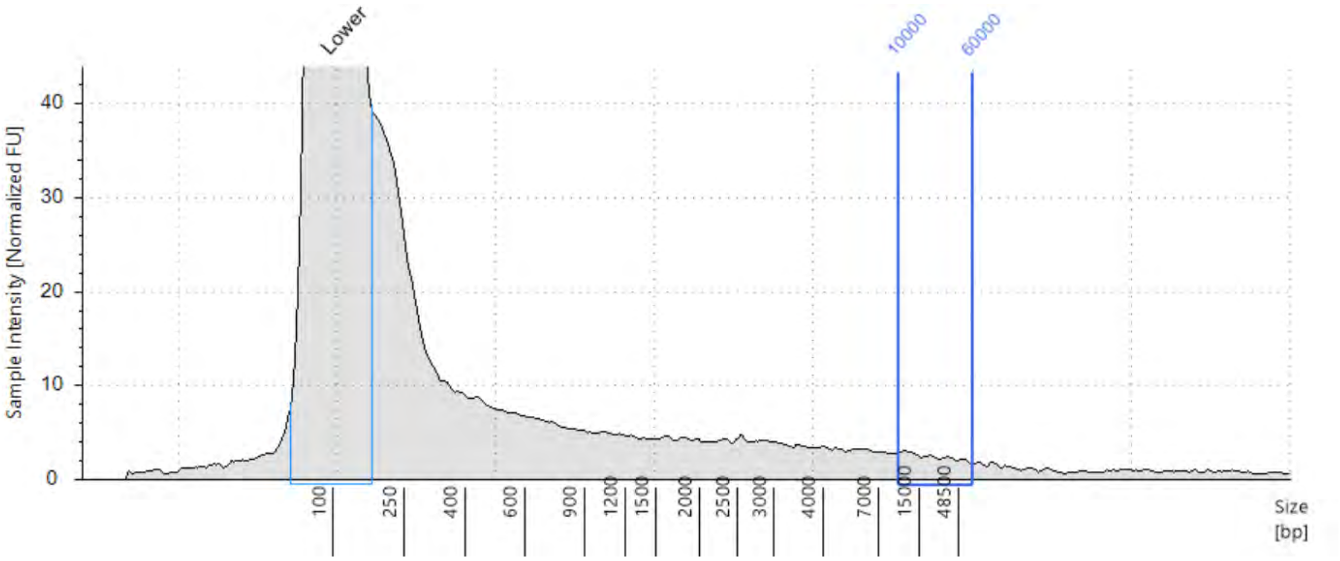

Region Table

From [bp]	To [bp]	Average Size [bp]	Conc. [ng/µl]	Region Molarity [nmol/l]	% of Total	Region Comment	Color
10000	60000	24564	0.0850	0.00820	3.50		■

B6: M-310-T5-SS

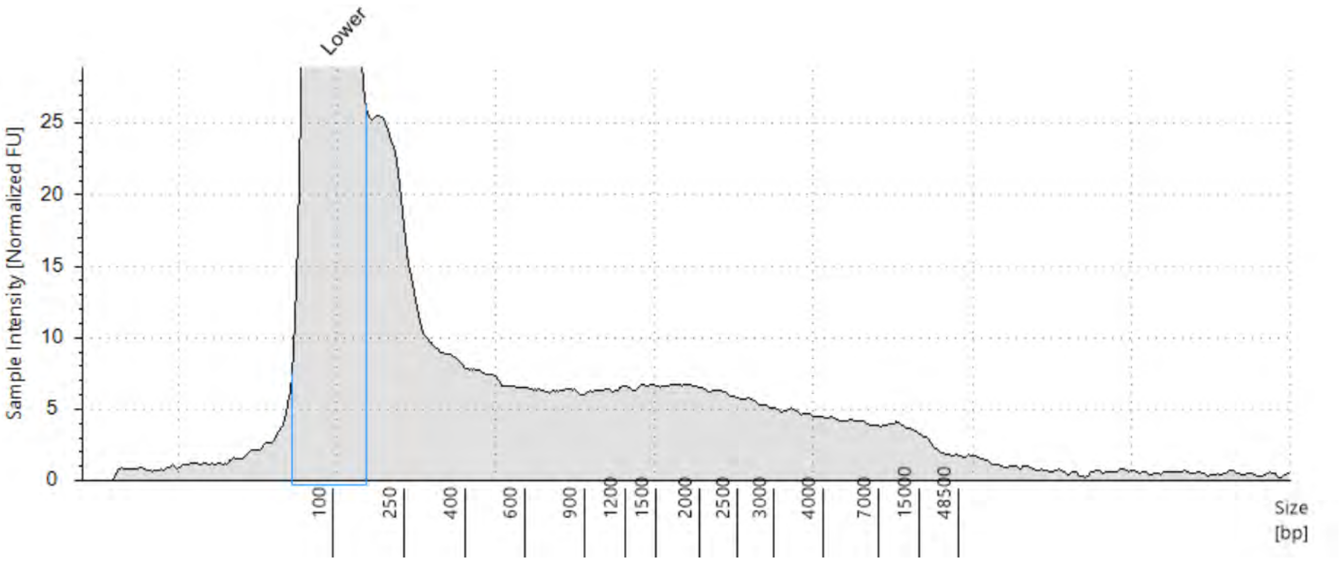

Sample Table

Well	DIN	Conc. [ng/µl]	Sample Description	Alert	Observations
B6	-	2.69	M-310-T5-SS	▲	Sample concentration outside functional range for DIN

Peak Table

Size [bp]	Calibrated Conc. [ng/µl]	Assigned Conc. [ng/µl]	% Integrated Area	From [bp]	To [bp]	Peak Comment	Observations
100	8.50	8.50	-	59	153		Lower Marker

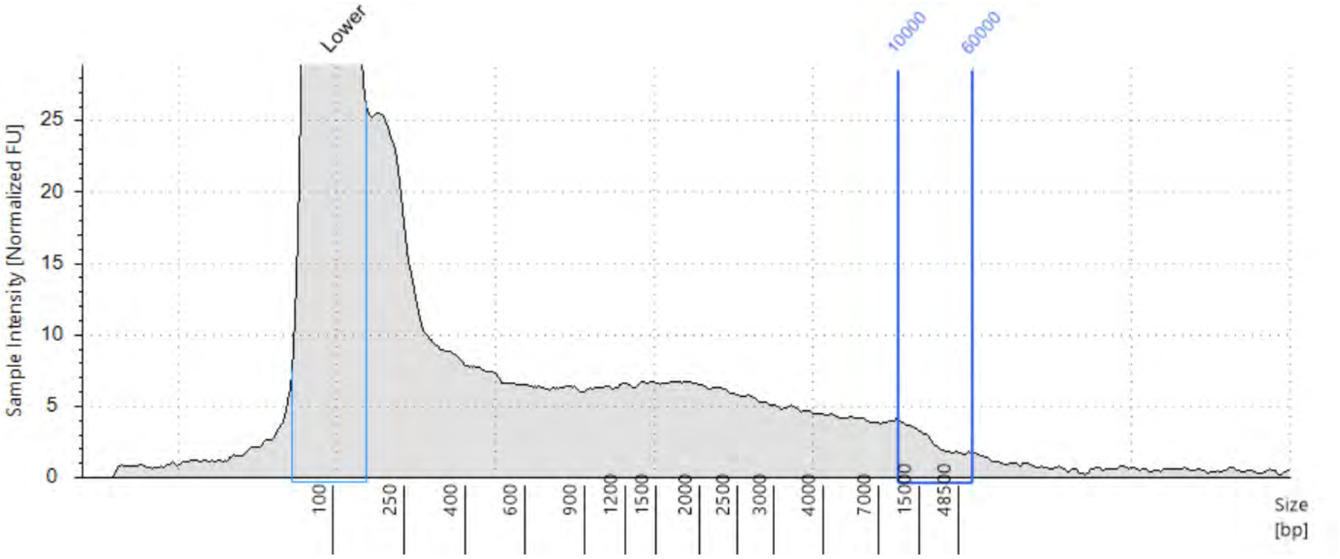

Region Table

From [bp]	To [bp]	Average Size [bp]	Conc. [ng/µl]	Region Molarity [nmol/l]	% of Total	Region Comment	Color
10000	60000	22406	0.103	0.0110	3.82		■

C6: M-311-T5-SS

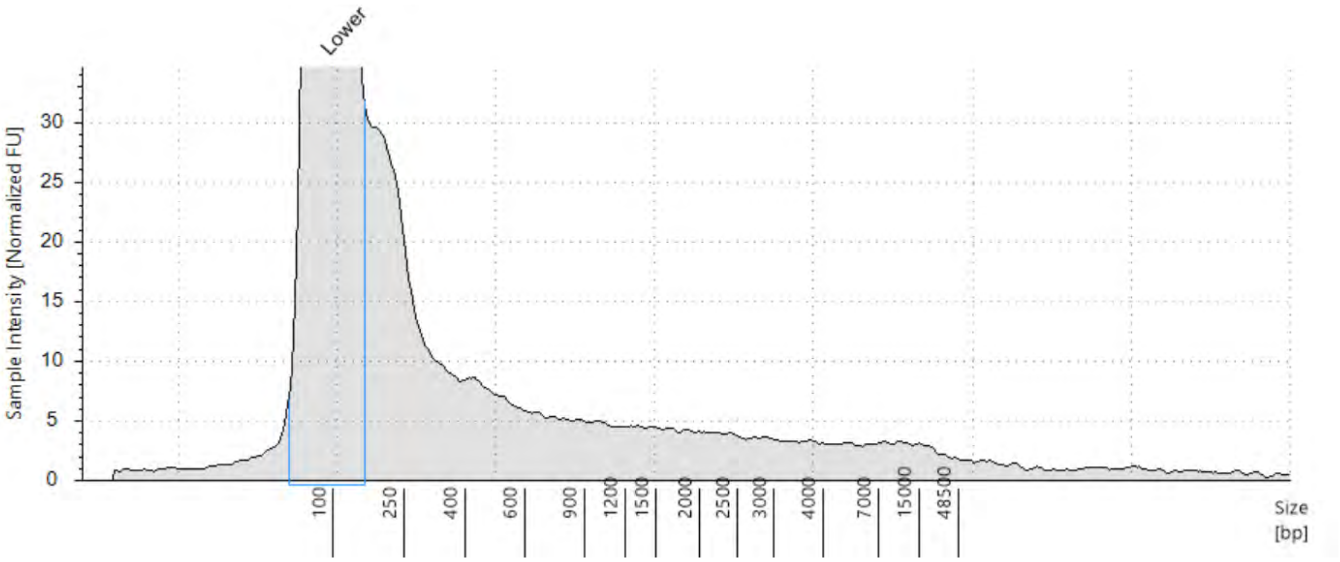

Sample Table

Well	DIN	Conc. [ng/µl]	Sample Description	Alert	Observations
C6	-	2.42	M-311-T5-SS	▲	Sample concentration outside functional range for DIN

Peak Table

Size [bp]	Calibrated Conc. [ng/µl]	Assigned Conc. [ng/µl]	% Integrated Area	From [bp]	To [bp]	Peak Comment	Observations
100	8.50	8.50	-	57	151		Lower Marker

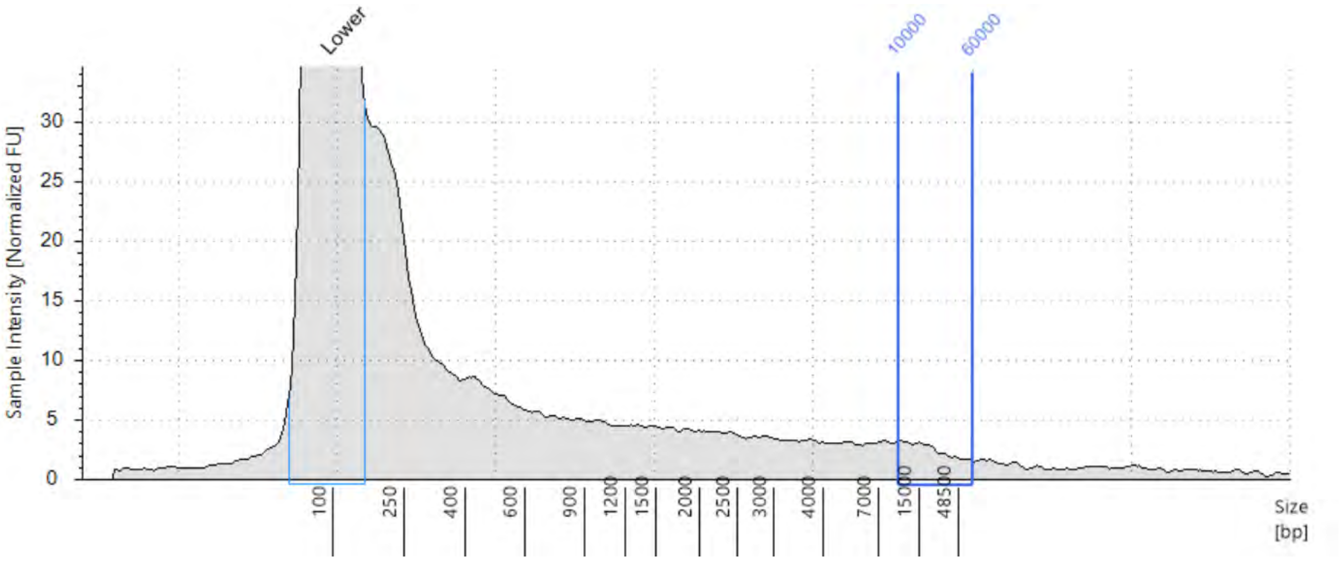

Region Table

From [bp]	To [bp]	Average Size [bp]	Conc. [ng/µl]	Region Molarity [nmol/l]	% of Total	Region Comment	Color
10000	60000	23166	0.0948	0.00965	3.92		■

 © Agilent Technologies, Inc. 2017

D6: S-831-T4-D

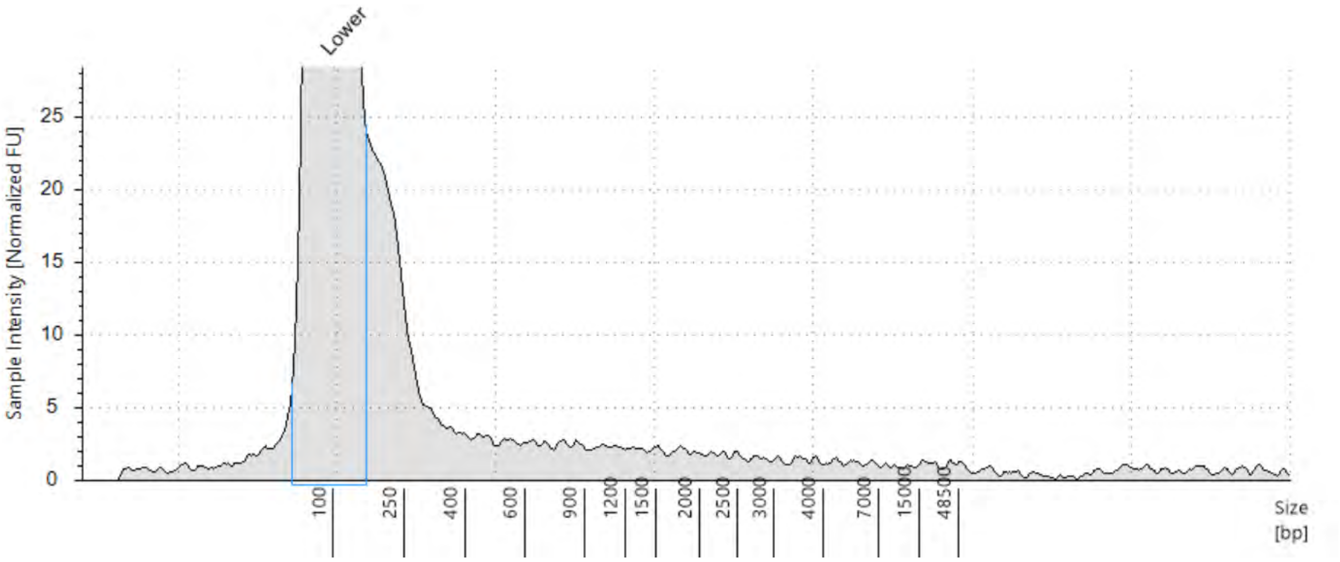

Sample Table

Well	DIN	Conc. [ng/µl]	Sample Description	Alert	Observations
D6	-	1.37	S-831-T4-D	▲	Sample concentration outside functional range for DIN

Peak Table

Size [bp]	Calibrated Conc. [ng/µl]	Assigned Conc. [ng/µl]	% Integrated Area	From [bp]	To [bp]	Peak Comment	Observations
100	8.50	8.50	-	59	153		Lower Marker

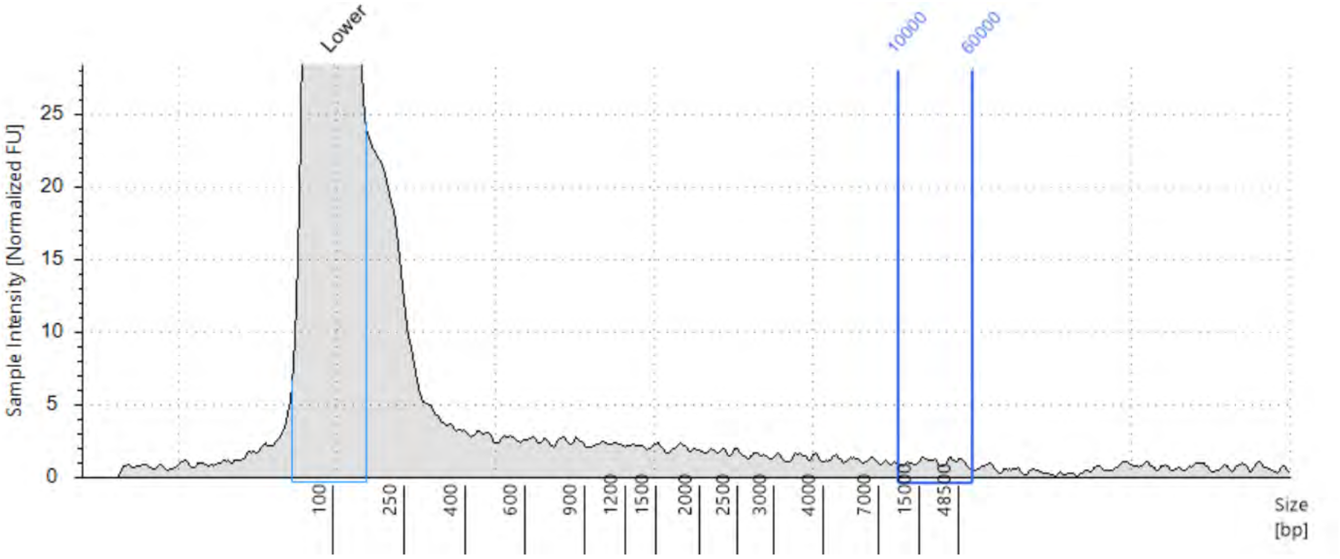

Region Table

From [bp]	To [bp]	Average Size [bp]	Conc. [ng/µl]	Region Molarity [nmol/l]	% of Total	Region Comment	Color
10000	60000	25908	0.0336	0.00388	2.46		■

 © Agilent Technologies, Inc. 2017

E6: S-832-T4-D

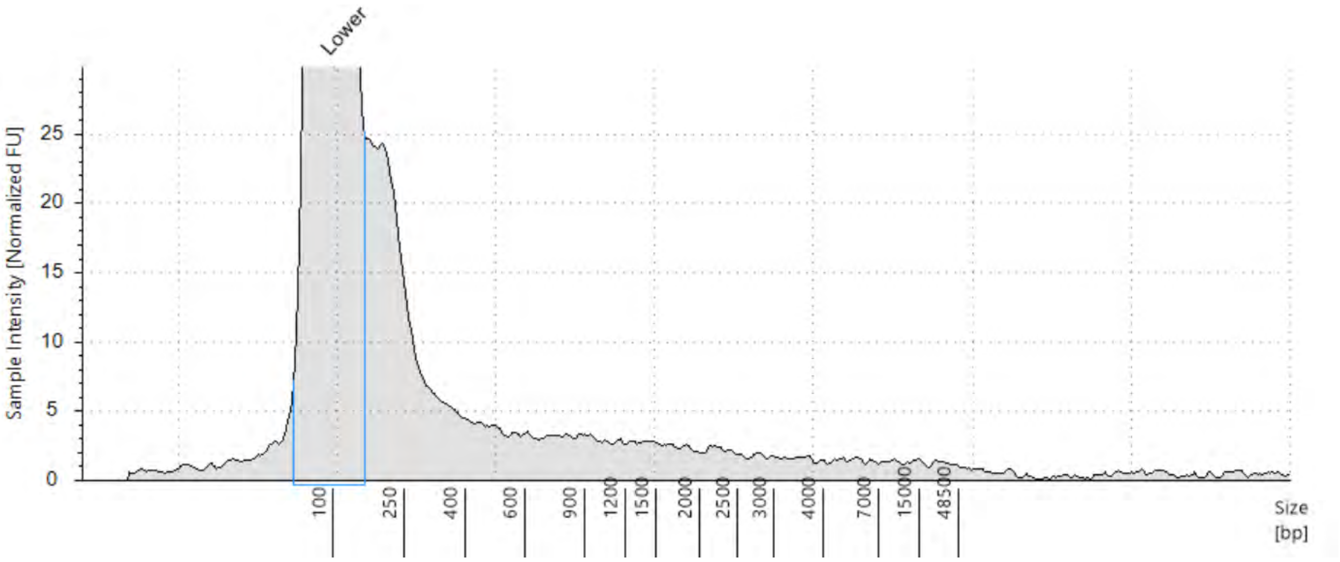

Sample Table

Well	DIN	Conc. [ng/µl]	Sample Description	Alert	Observations
E6	-	1.60	S-832-T4-D	▲	Sample concentration outside functional range for DIN

Peak Table

Size [bp]	Calibrated Conc. [ng/µl]	Assigned Conc. [ng/µl]	% Integrated Area	From [bp]	To [bp]	Peak Comment	Observations
100	8.50	8.50	-	60	150		Lower Marker

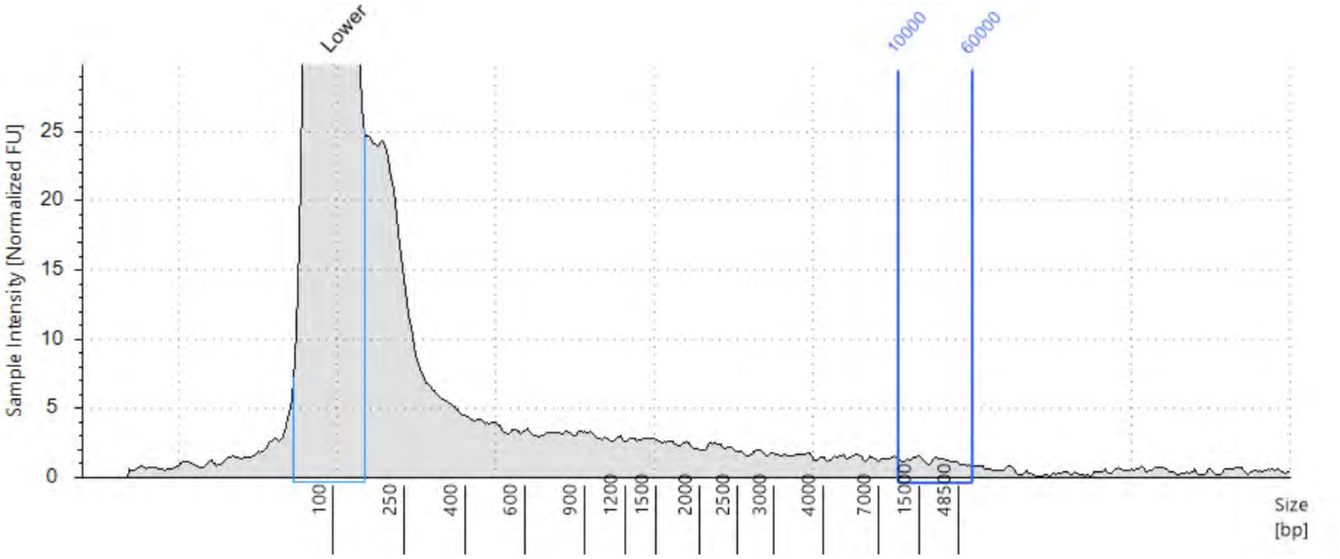

Region Table

From [bp]	To [bp]	Average Size [bp]	Conc. [ng/µl]	Region Molarity [nmol/l]	% of Total	Region Comment	Color
10000	60000	24726	0.0415	0.00478	2.59		■

 © Agilent Technologies, Inc. 2017

F6: S-833-T4-D

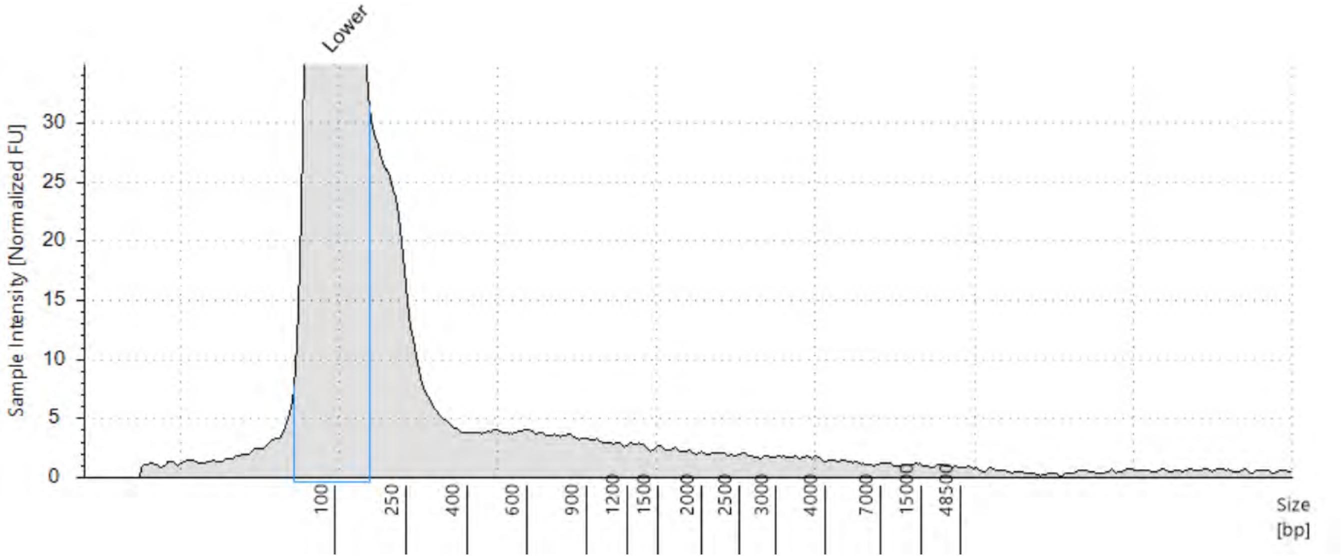

Sample Table

Well	DIN	Conc. [ng/µl]	Sample Description	Alert	Observations
F6	-	1.48	S-833-T4-D	▲	Sample concentration outside functional range for DIN

Peak Table

Size [bp]	Calibrated Conc. [ng/µl]	Assigned Conc. [ng/µl]	% Integrated Area	From [bp]	To [bp]	Peak Comment	Observations
100	8.50	8.50	-	59	158		Lower Marker

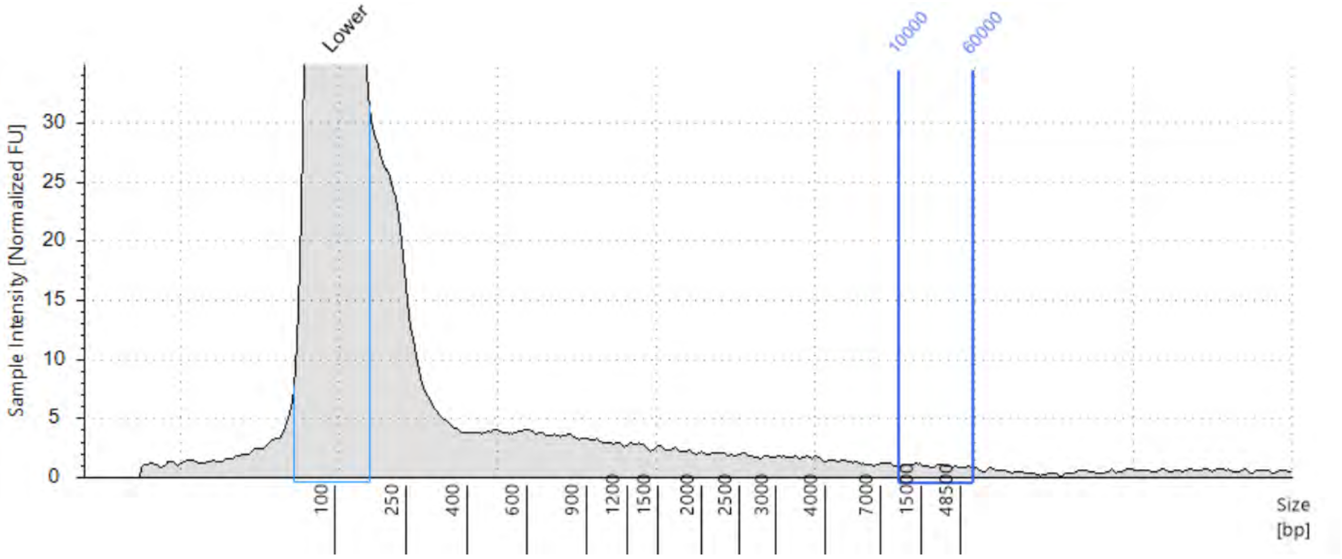

Region Table

From [bp]	To [bp]	Average Size [bp]	Conc. [ng/µl]	Region Molarity [nmol/l]	% of Total	Region Comment	Color
10000	60000	25458	0.0259	0.00345	1.75		■

G6: S-834-T4-D

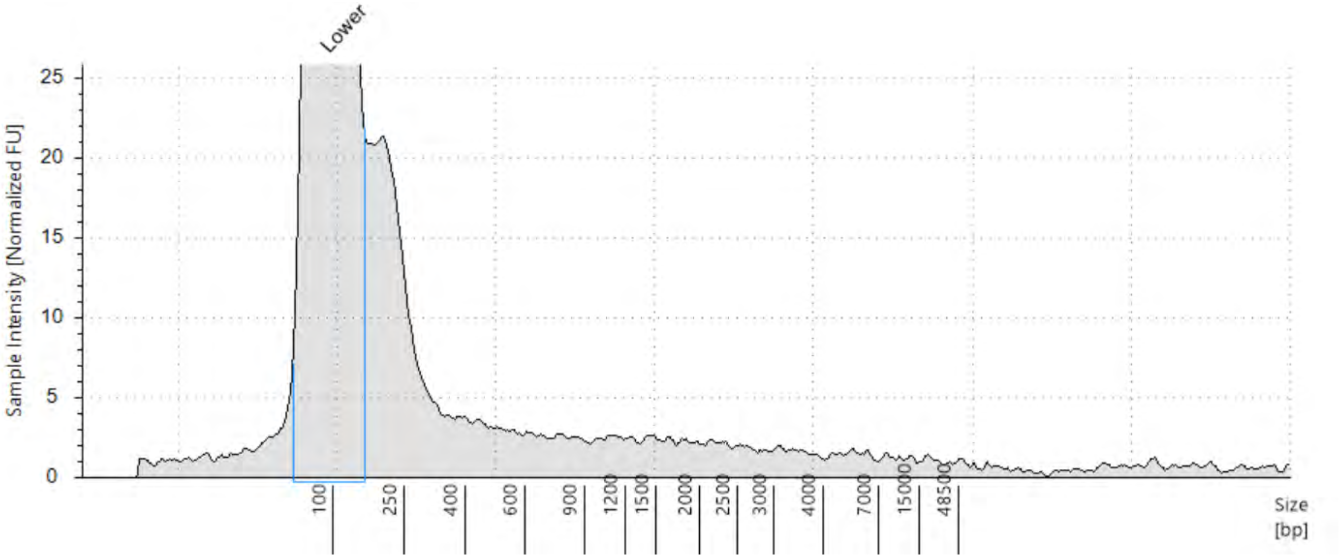

Sample Table

Well	DIN	Conc. [ng/µl]	Sample Description	Alert	Observations
G6	-	1.43	S-834-T4-D	▲	Sample concentration outside functional range for DIN

Peak Table

Size [bp]	Calibrated Conc. [ng/µl]	Assigned Conc. [ng/µl]	% Integrated Area	From [bp]	To [bp]	Peak Comment	Observations
100	8.50	8.50	-	59	150		Lower Marker

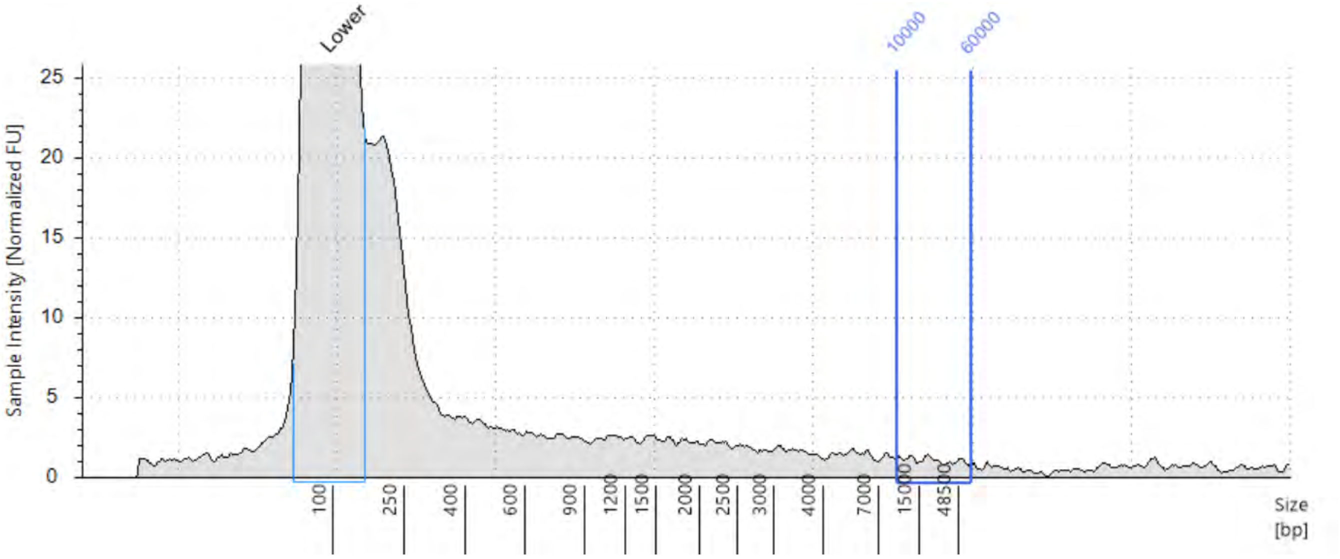

Region Table

From [bp]	To [bp]	Average Size [bp]	Conc. [ng/µl]	Region Molarity [nmol/l]	% of Total	Region Comment	Color
10000	60000	25327	0.0329	0.00416	2.30		■

H6: S-835-T4-D

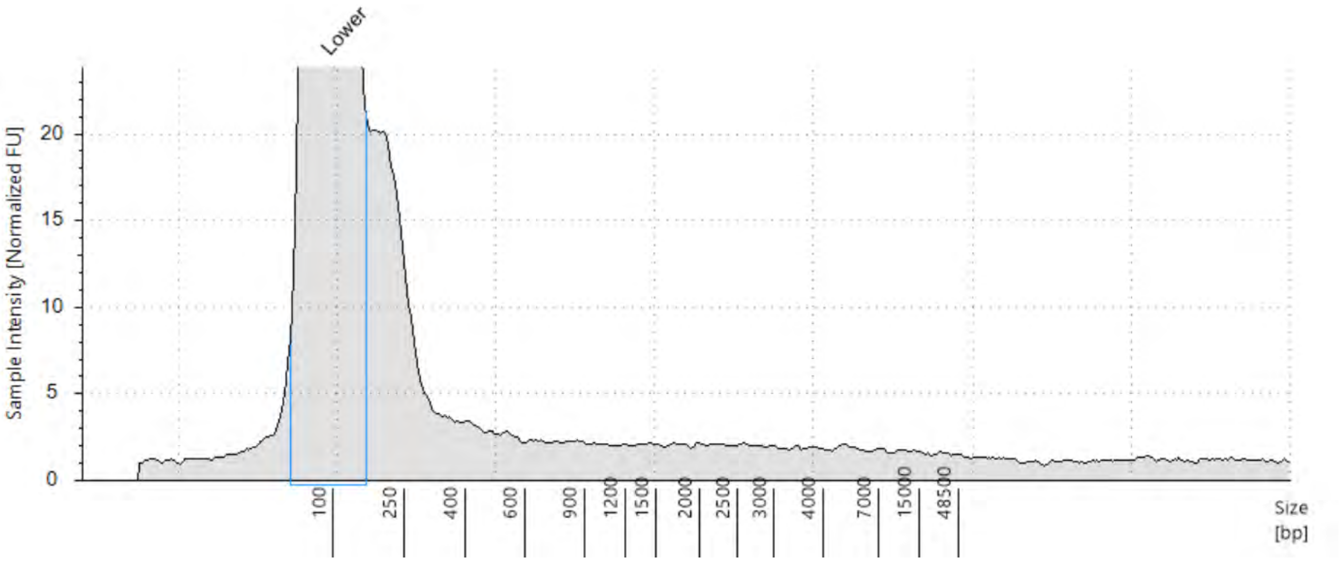

Sample Table

Well	DIN	Conc. [ng/µl]	Sample Description	Alert	Observations
H6	-	1.25	S-835-T4-D	▲	Sample concentration outside functional range for DIN

Peak Table

Size [bp]	Calibrated Conc. [ng/µl]	Assigned Conc. [ng/µl]	% Integrated Area	From [bp]	To [bp]	Peak Comment	Observations
100	8.50	8.50	-	57	153		Lower Marker

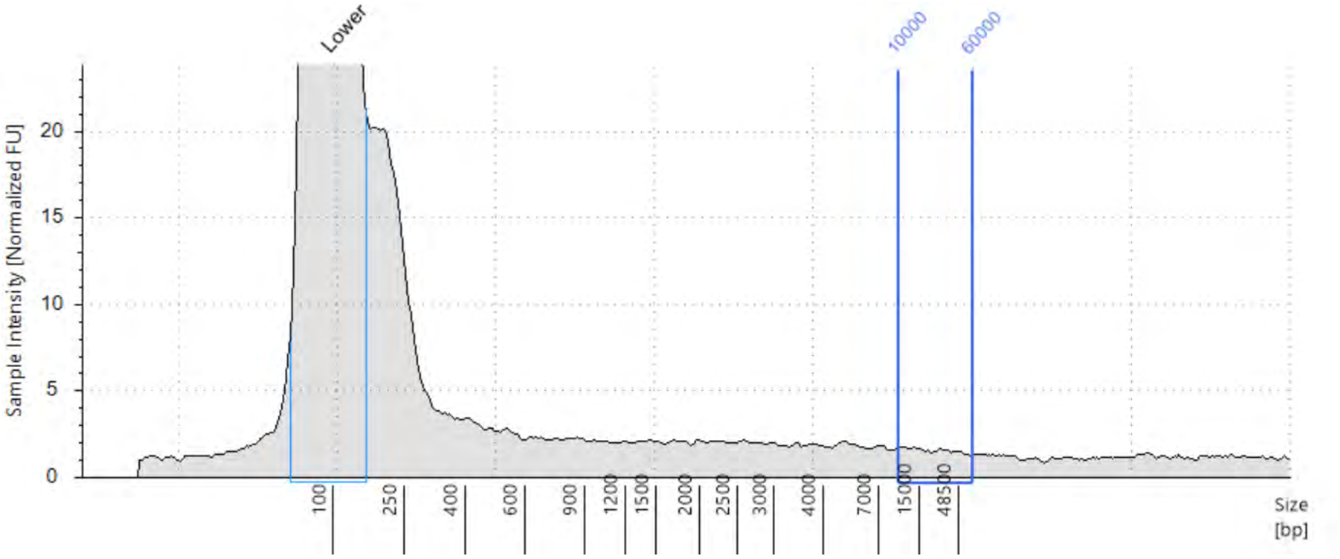

Region Table

From [bp]	To [bp]	Average Size [bp]	Conc. [ng/µl]	Region Molarity [nmol/l]	% of Total	Region Comment	Color
10000	60000	25442	0.0446	0.00538	3.57		■

 © Agilent Technologies, Inc. 2017

A7: S-1056-T4-D

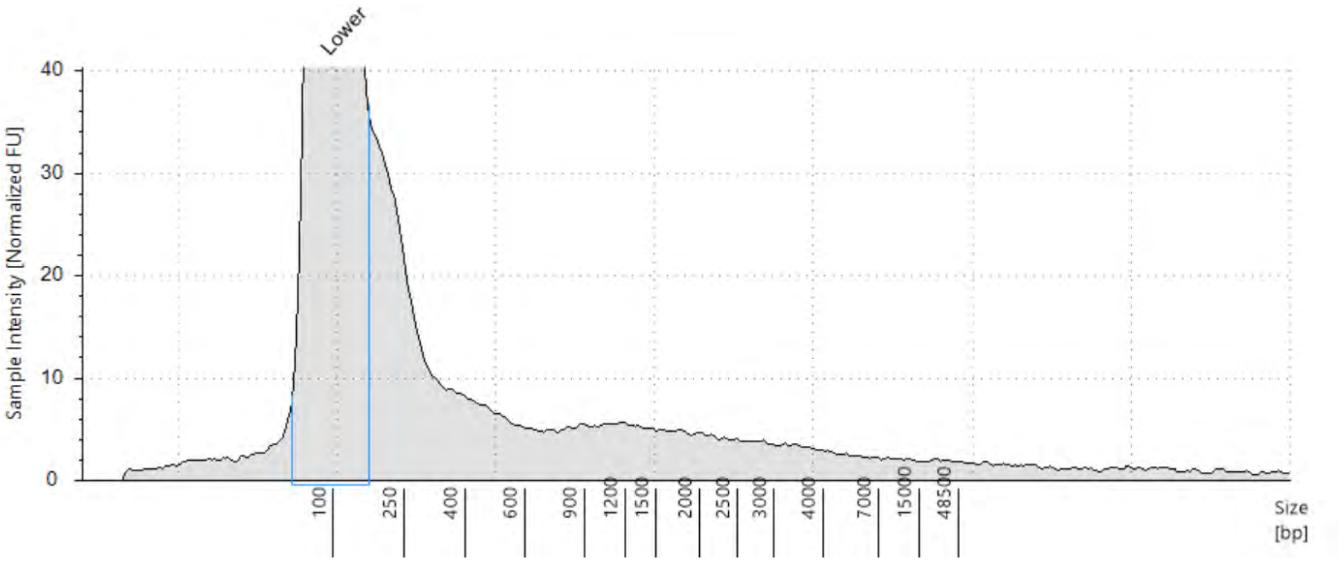

Sample Table

Well	DIN	Conc. [ng/µl]	Sample Description	Alert	Observations
A7	-	2.22	S-1056-T4-D	▲	Sample concentration outside functional range for DIN

Peak Table

Size [bp]	Calibrated Conc. [ng/µl]	Assigned Conc. [ng/µl]	% Integrated Area	From [bp]	To [bp]	Peak Comment	Observations
100	8.50	8.50	-	59	158		Lower Marker

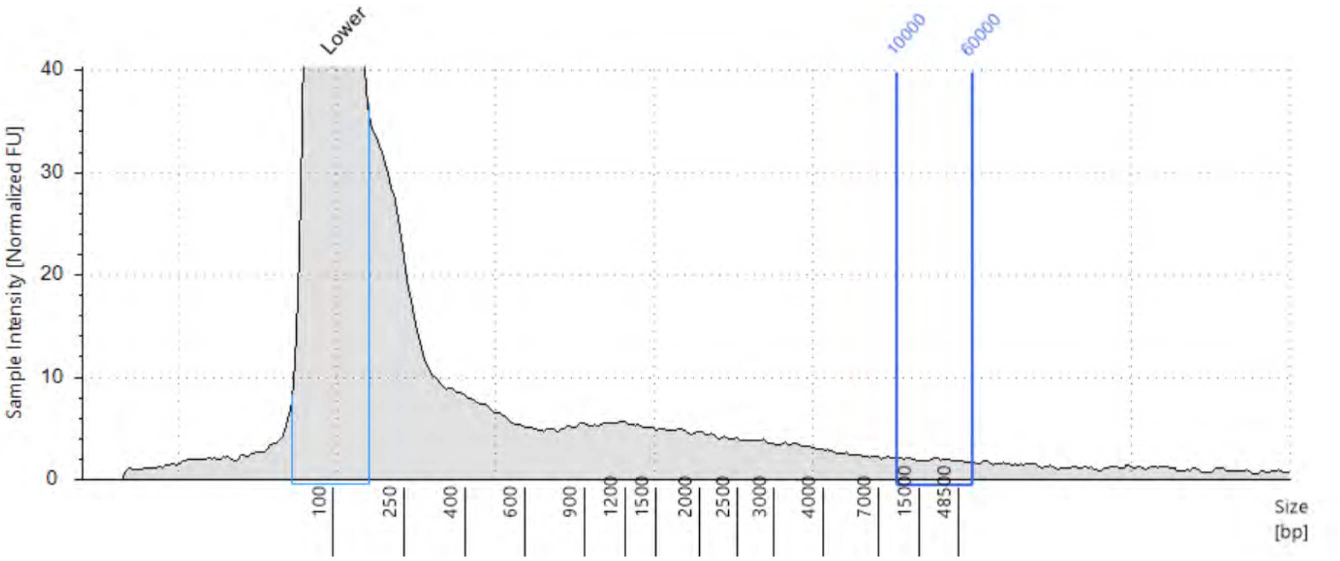

Region Table

From [bp]	To [bp]	Average Size [bp]	Conc. [ng/µl]	Region Molarity [nmol/l]	% of Total	Region Comment	Color
10000	60000	25715	0.0550	0.00637	2.48		■

B7: S-1057-T4-D

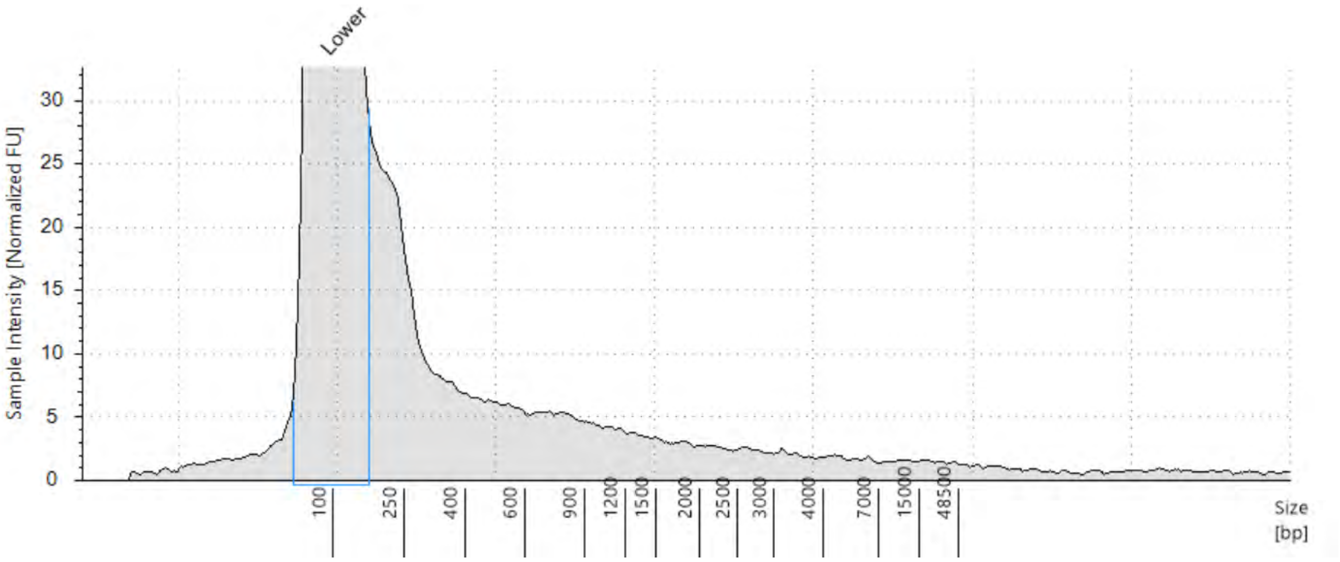

Sample Table

Well	DIN	Conc. [ng/µl]	Sample Description	Alert	Observations
B7	-	1.84	S-1057-T4-D	▲	Sample concentration outside functional range for DIN

Peak Table

Size [bp]	Calibrated Conc. [ng/µl]	Assigned Conc. [ng/µl]	% Integrated Area	From [bp]	To [bp]	Peak Comment	Observations
100	8.50	8.50	-	59	158		Lower Marker

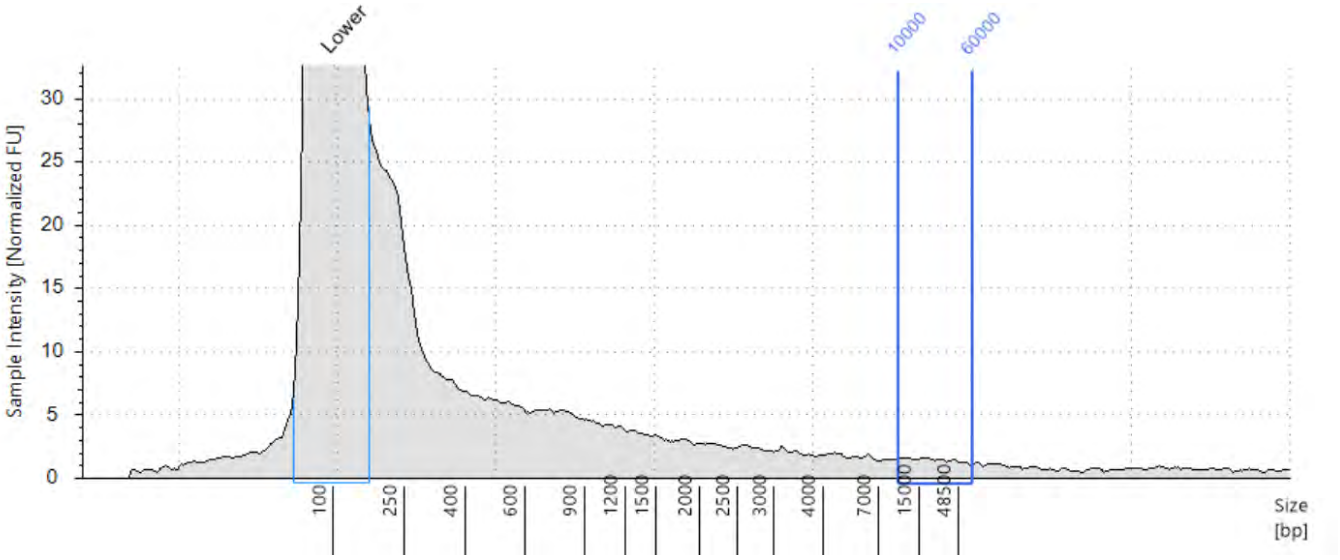

Region Table

From [bp]	To [bp]	Average Size [bp]	Conc. [ng/µl]	Region Molarity [nmol/l]	% of Total	Region Comment	Color
10000	60000	25150	0.0427	0.00536	2.32		■

C7: S-1058-T4-D

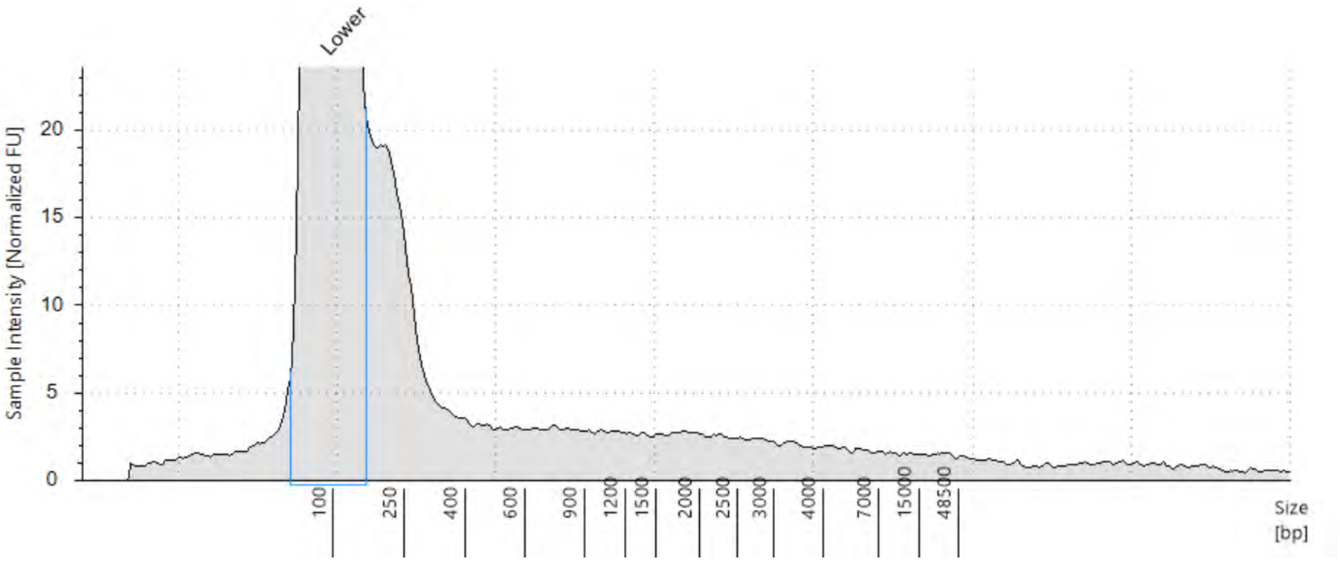

Sample Table

Well	DIN	Conc. [ng/µl]	Sample Description	Alert	Observations
C7	-	1.32	S-1058-T4-D	▲	Sample concentration outside functional range for DIN

Peak Table

Size [bp]	Calibrated Conc. [ng/µl]	Assigned Conc. [ng/µl]	% Integrated Area	From [bp]	To [bp]	Peak Comment	Observations
100	8.50	8.50	-	58	153		Lower Marker

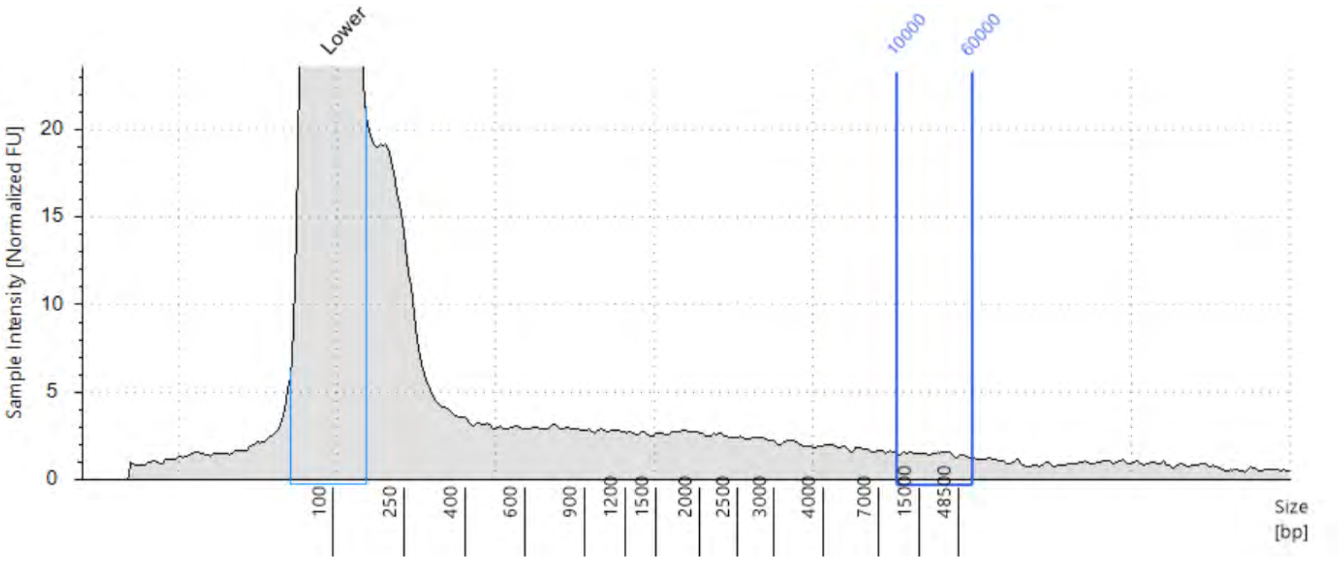

Region Table

From [bp]	To [bp]	Average Size [bp]	Conc. [ng/µl]	Region Molarity [nmol/l]	% of Total	Region Comment	Color
10000	60000	25792	0.0404	0.00508	3.06		■

D7: S-1059-T4-D

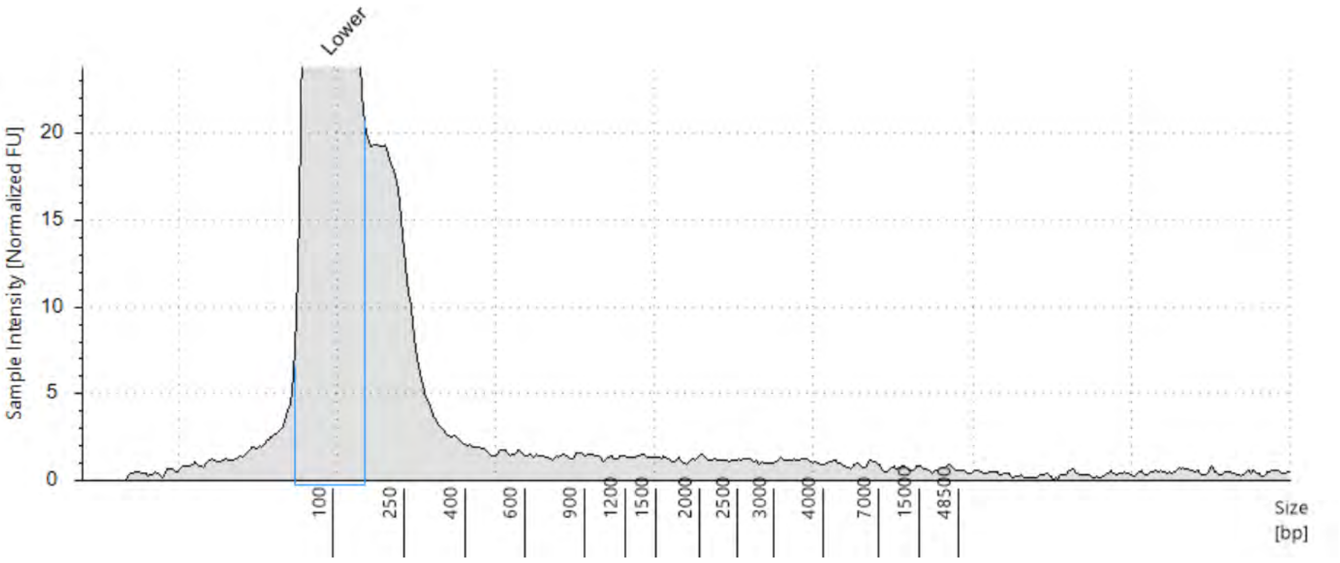

Sample Table

Well	DIN	Conc. [ng/µl]	Sample Description	Alert	Observations
D7	-	0.987	S-1059-T4-D	▲	Sample concentration outside functional range for DIN

Peak Table

Size [bp]	Calibrated Conc. [ng/µl]	Assigned Conc. [ng/µl]	% Integrated Area	From [bp]	To [bp]	Peak Comment	Observations
100	8.50	8.50	-	60	150		Lower Marker

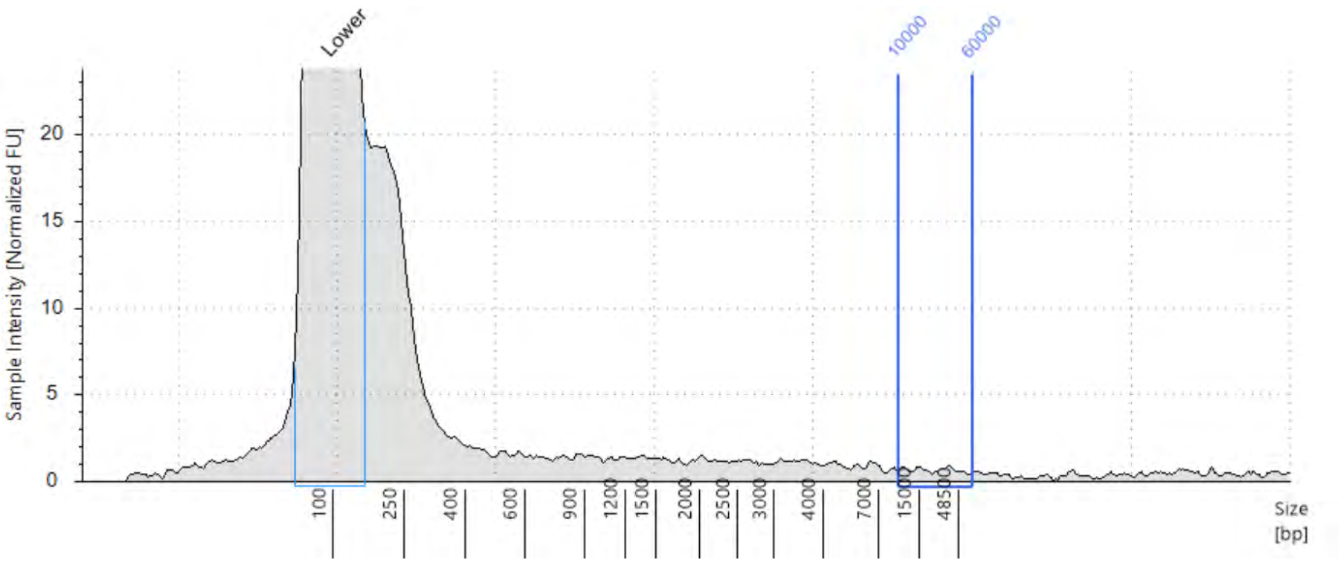

Region Table

From [bp]	To [bp]	Average Size [bp]	Conc. [ng/µl]	Region Molarity [nmol/l]	% of Total	Region Comment	Color
10000	60000	24714	0.00942	0.00258	0.95		■

E7: S-1060-T4-D

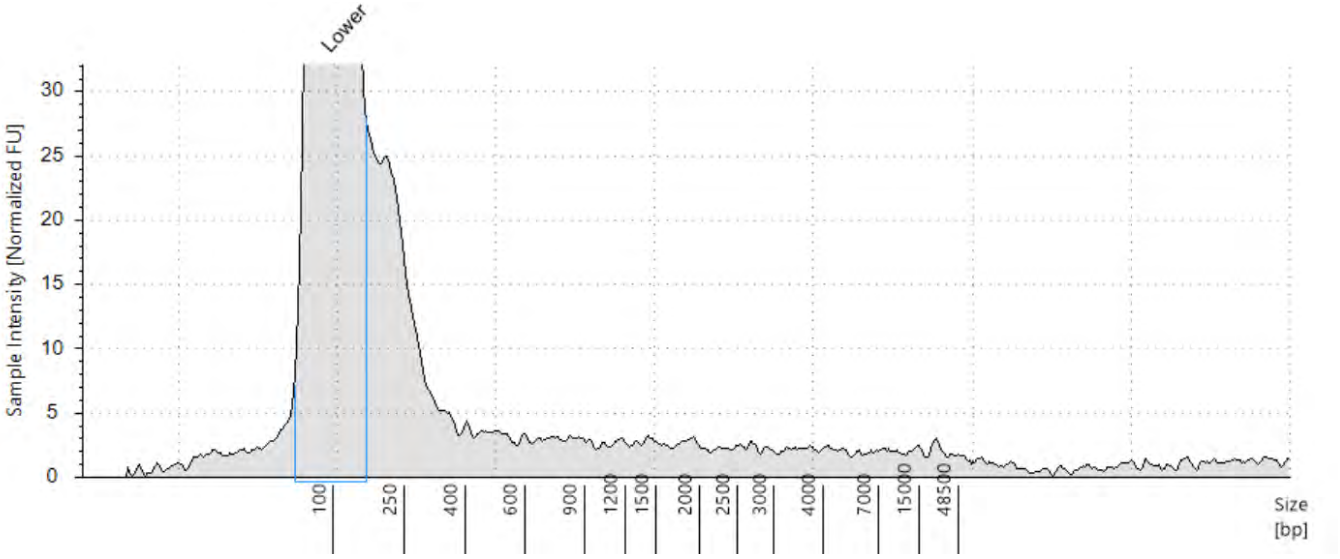

Sample Table

Well	DIN	Conc. [ng/µl]	Sample Description	Alert	Observations
E7	-	1.62	S-1060-T4-D	▲	Sample concentration outside functional range for DIN

Peak Table

Size [bp]	Calibrated Conc. [ng/µl]	Assigned Conc. [ng/µl]	% Integrated Area	From [bp]	To [bp]	Peak Comment	Observations
100	8.50	8.50	-	60	153		Lower Marker

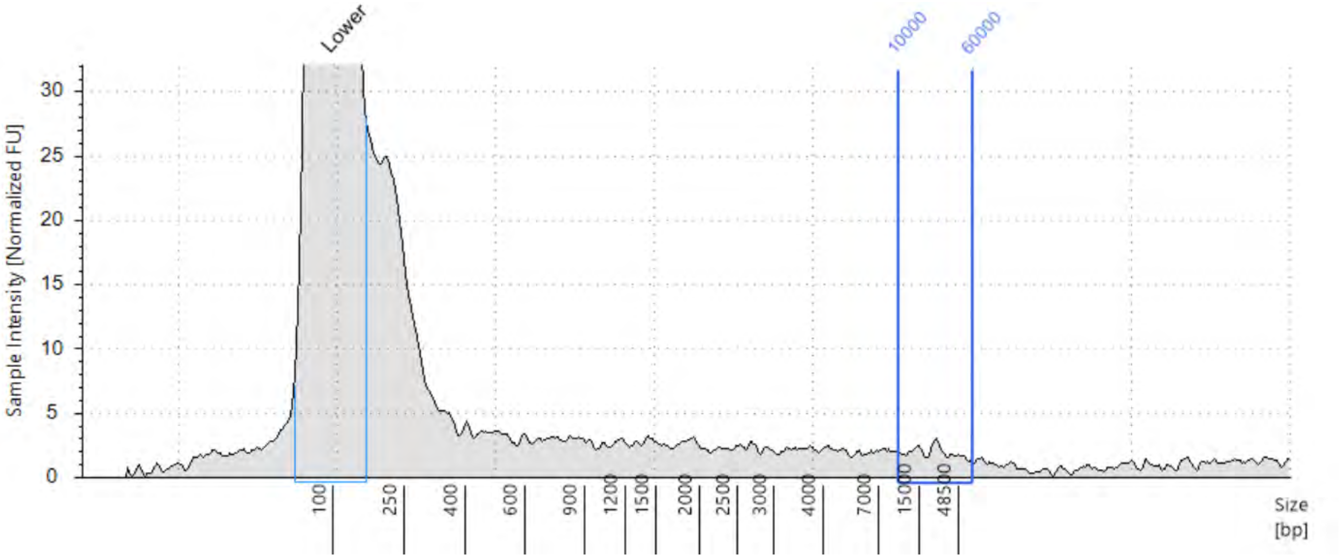

Region Table

From [bp]	To [bp]	Average Size [bp]	Conc. [ng/µl]	Region Molarity [nmol/l]	% of Total	Region Comment	Color
10000	60000	24657	0.0594	0.00727	3.66		■

F7: S-821-T4-DSS

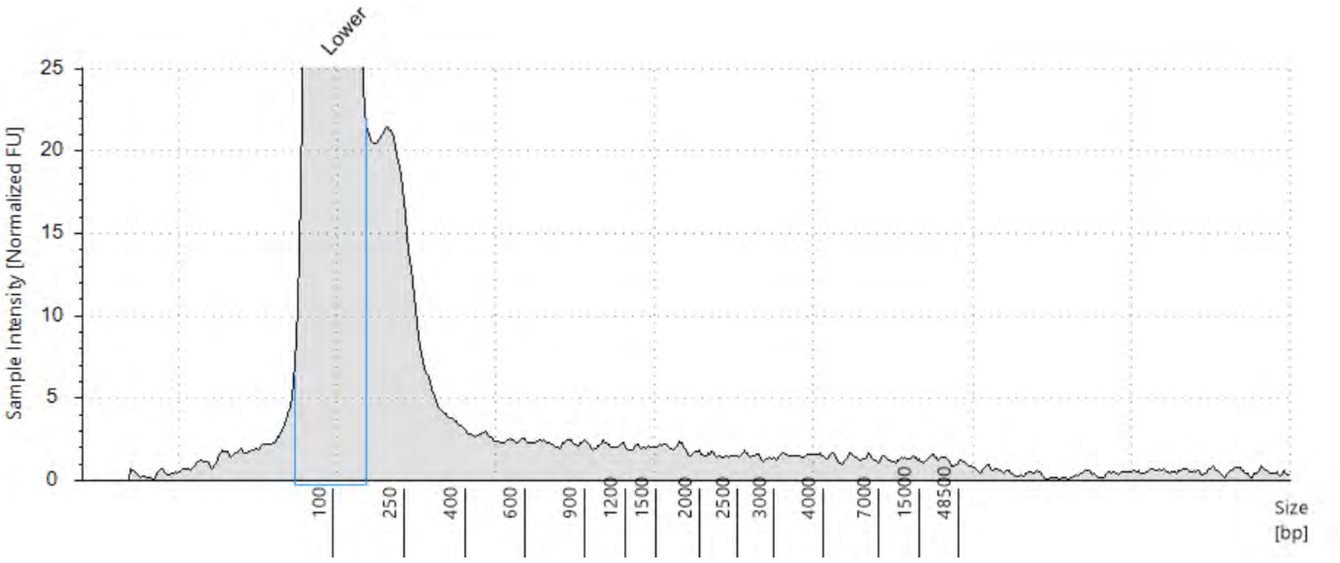

Sample Table

Well	DIN	Conc. [ng/µl]	Sample Description	Alert	Observations
F7	-	1.23	S-821-T4-DSS	▲	Sample concentration outside functional range for DIN

Peak Table

Size [bp]	Calibrated Conc. [ng/µl]	Assigned Conc. [ng/µl]	% Integrated Area	From [bp]	To [bp]	Peak Comment	Observations
100	8.50	8.50	-	60	153		Lower Marker

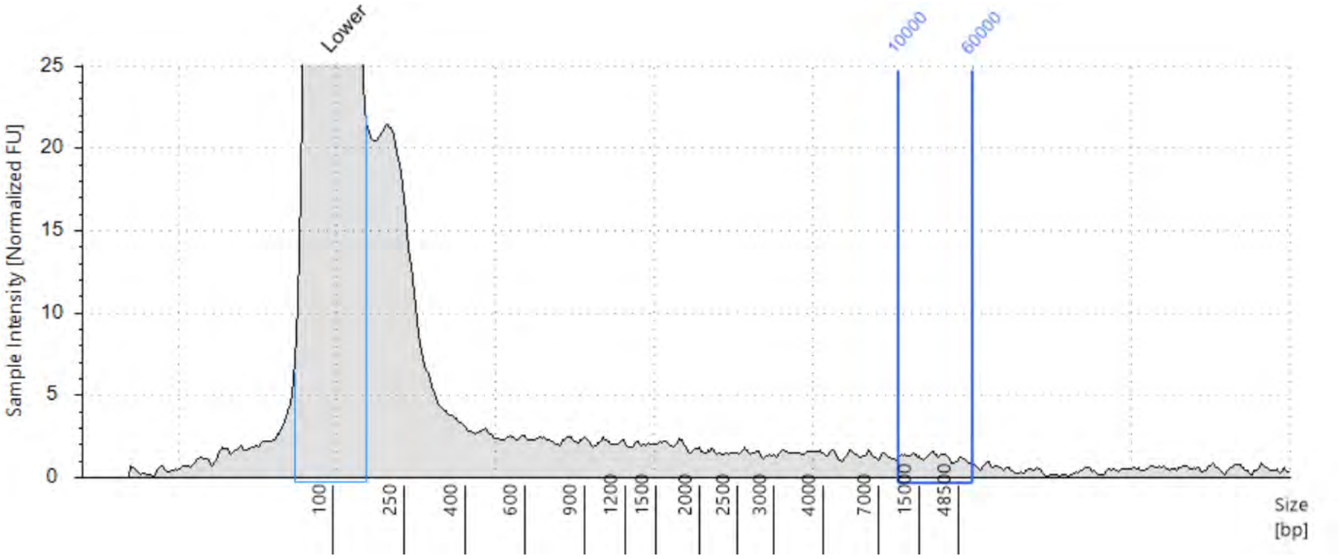

Region Table

From [bp]	To [bp]	Average Size [bp]	Conc. [ng/µl]	Region Molarity [nmol/l]	% of Total	Region Comment	Color
10000	60000	24716	0.0317	0.00477	2.58		■

G7: S-822-T4-DSS

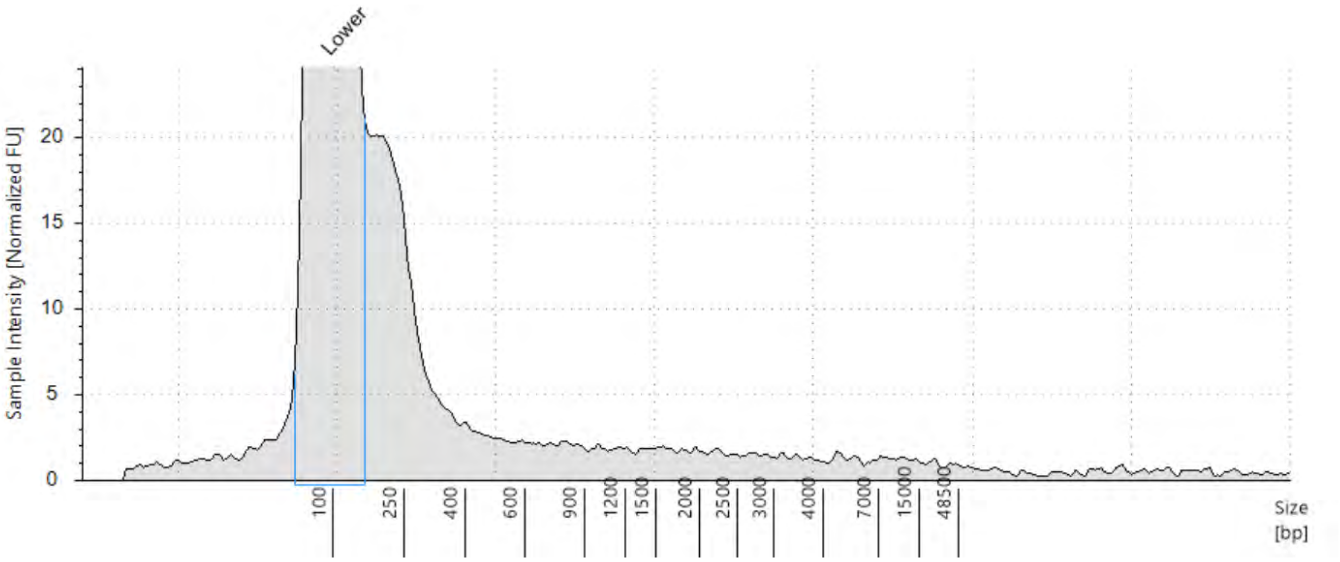

Sample Table

Well	DIN	Conc. [ng/µl]	Sample Description	Alert	Observations
G7	-	1.25	S-822-T4-DSS	▲	Sample concentration outside functional range for DIN

Peak Table

Size [bp]	Calibrated Conc. [ng/µl]	Assigned Conc. [ng/µl]	% Integrated Area	From [bp]	To [bp]	Peak Comment	Observations
100	8.50	8.50	-	60	150		Lower Marker

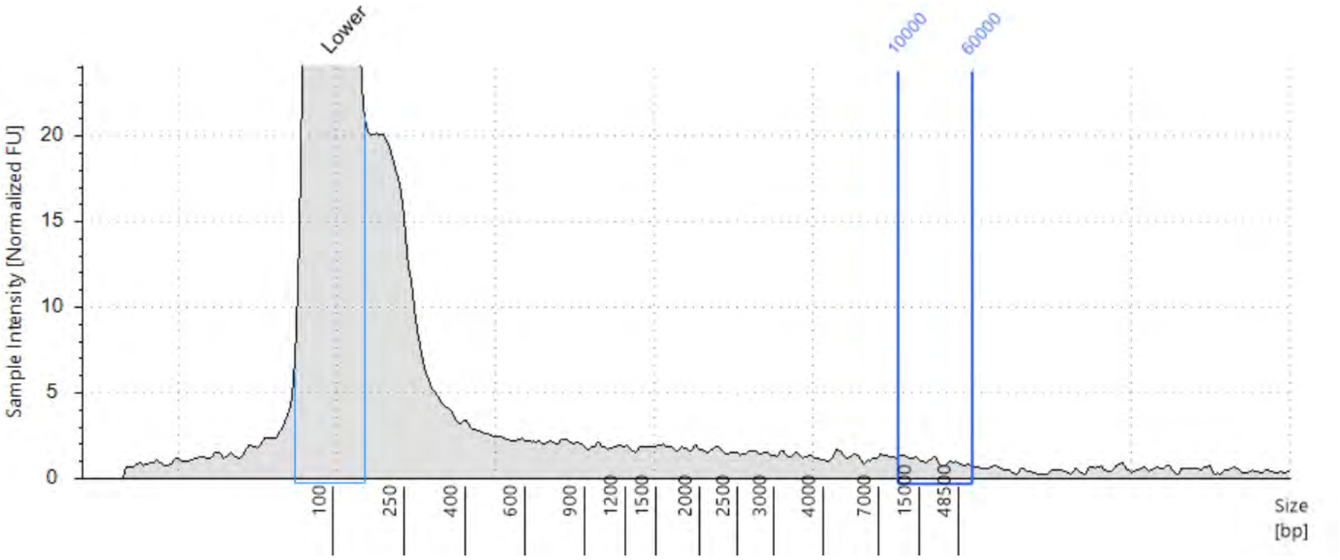

Region Table

From [bp]	To [bp]	Average Size [bp]	Conc. [ng/µl]	Region Molarity [nmol/l]	% of Total	Region Comment	Color
10000	60000	24281	0.0260	0.00424	2.09		■

H7: S-823-T4-DSS

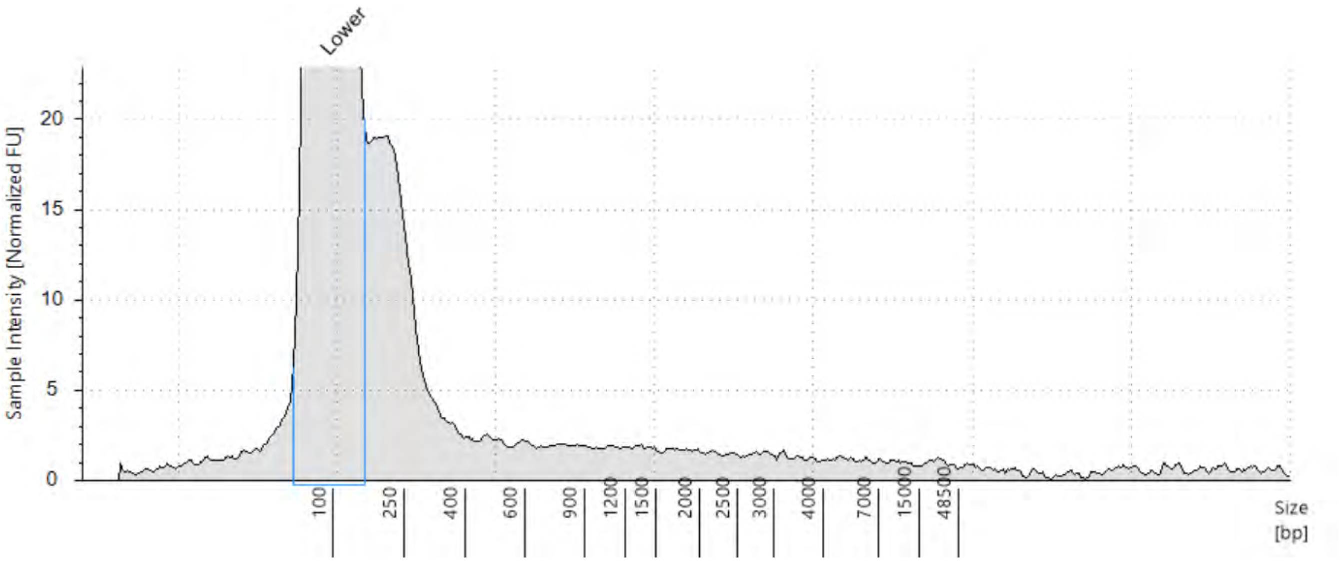

Sample Table

Well	DIN	Conc. [ng/µl]	Sample Description	Alert	Observations
H7	-	1.15	S-823-T4-DSS	▲	Sample concentration outside functional range for DIN

Peak Table

Size [bp]	Calibrated Conc. [ng/µl]	Assigned Conc. [ng/µl]	% Integrated Area	From [bp]	To [bp]	Peak Comment	Observations
100	8.50	8.50	-	60	150		Lower Marker

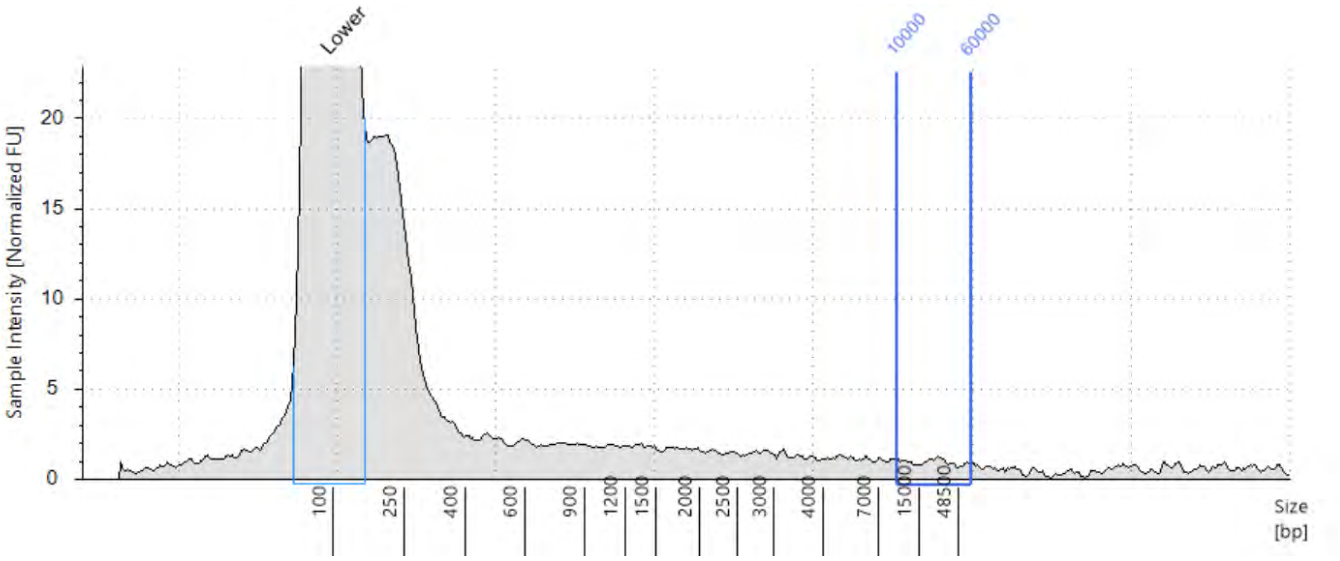

Region Table

From [bp]	To [bp]	Average Size [bp]	Conc. [ng/µl]	Region Molarity [nmol/l]	% of Total	Region Comment	Color
10000	60000	25374	0.0221	0.00366	1.93		■

 © Agilent Technologies, Inc. 2017

A8: S-824-T4-DSS

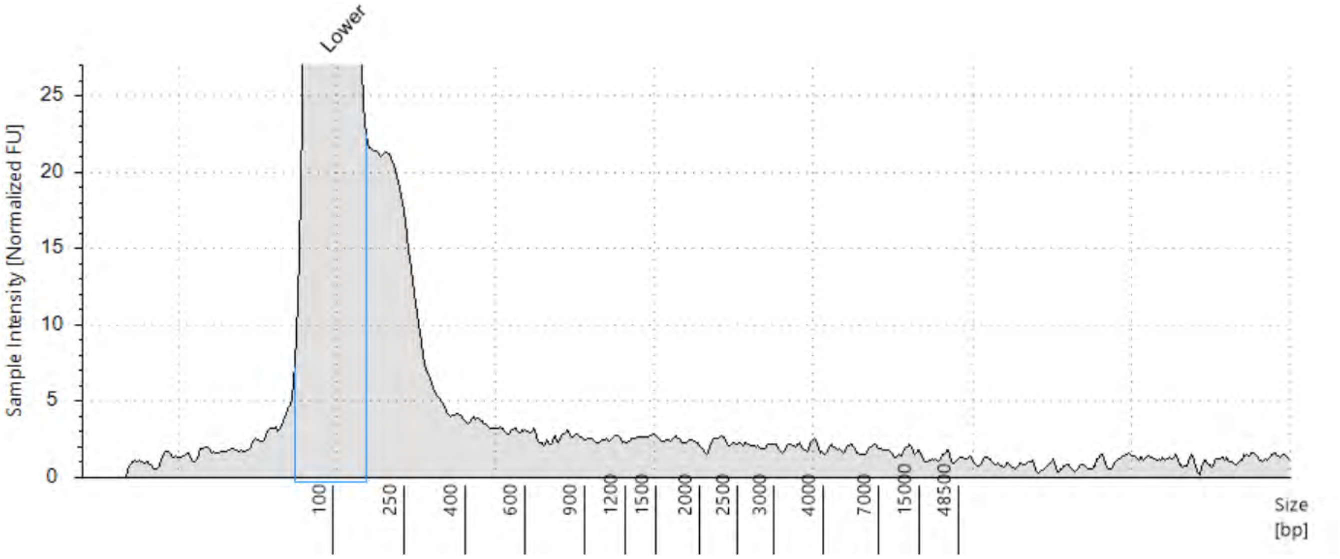

Sample Table

Well	DIN	Conc. [ng/µl]	Sample Description	Alert	Observations
A8	-	1.53	S-824-T4-DSS	▲	Sample concentration outside functional range for DIN

Peak Table

Size [bp]	Calibrated Conc. [ng/µl]	Assigned Conc. [ng/µl]	% Integrated Area	From [bp]	To [bp]	Peak Comment	Observations
100	8.50	8.50	-	61	155		Lower Marker

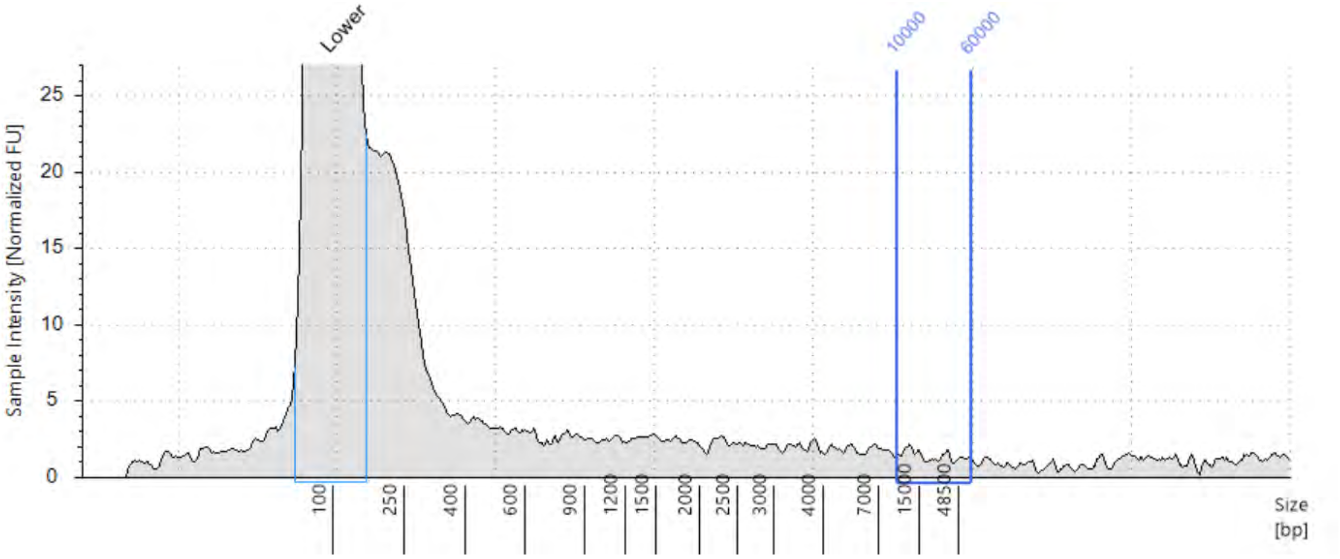

Region Table

From [bp]	To [bp]	Average Size [bp]	Conc. [ng/µl]	Region Molarity [nmol/l]	% of Total	Region Comment	Color
10000	60000	25208	0.0346	0.00560	2.25		■

B8: S-825-T4-DSS

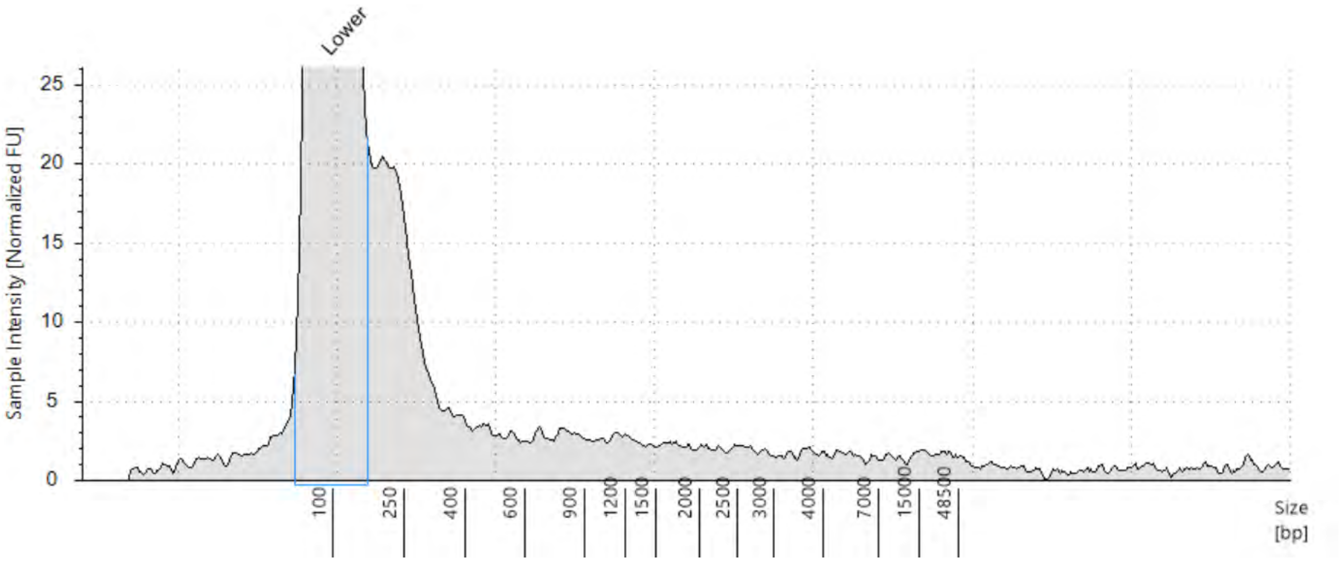

Sample Table

Well	DIN	Conc. [ng/µl]	Sample Description	Alert	Observations
B8	-	1.44	S-825-T4-DSS	▲	Sample concentration outside functional range for DIN

Peak Table

Size [bp]	Calibrated Conc. [ng/µl]	Assigned Conc. [ng/µl]	% Integrated Area	From [bp]	To [bp]	Peak Comment	Observations
100	8.50	8.50	-	60	155		Lower Marker

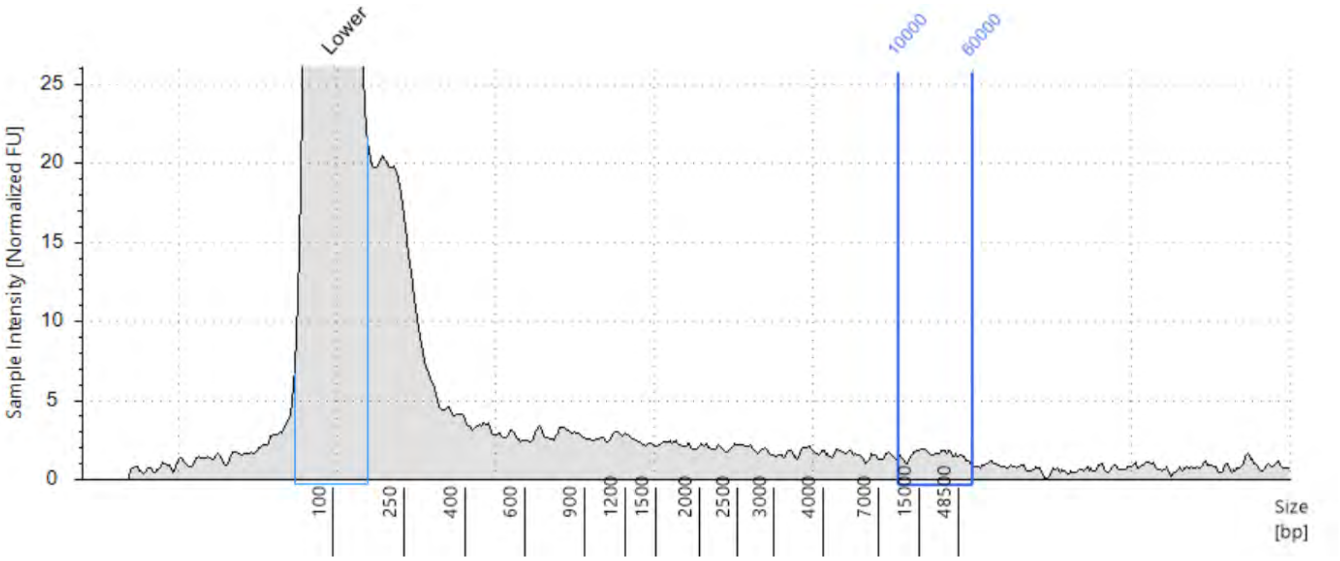

Region Table

From [bp]	To [bp]	Average Size [bp]	Conc. [ng/µl]	Region Molarity [nmol/l]	% of Total	Region Comment	Color
10000	60000	25127	0.0499	0.00596	3.45		■

 © Agilent Technologies, Inc. 2017

C8: S-1046-T4-DSS

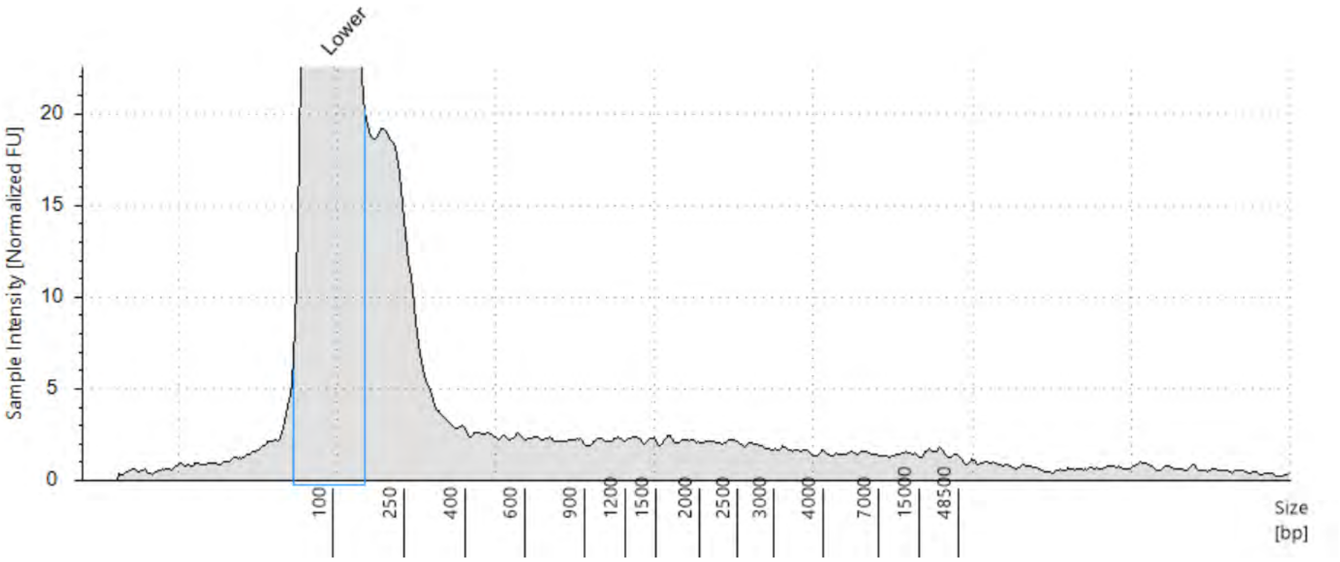

Sample Table

Well	DIN	Conc. [ng/µl]	Sample Description	Alert	Observations
C8	-	1.05	S-1046-T4-DSS	▲	Sample concentration outside functional range for DIN

Peak Table

Size [bp]	Calibrated Conc. [ng/µl]	Assigned Conc. [ng/µl]	% Integrated Area	From [bp]	To [bp]	Peak Comment	Observations
100	8.50	8.50	-	59	150		Lower Marker

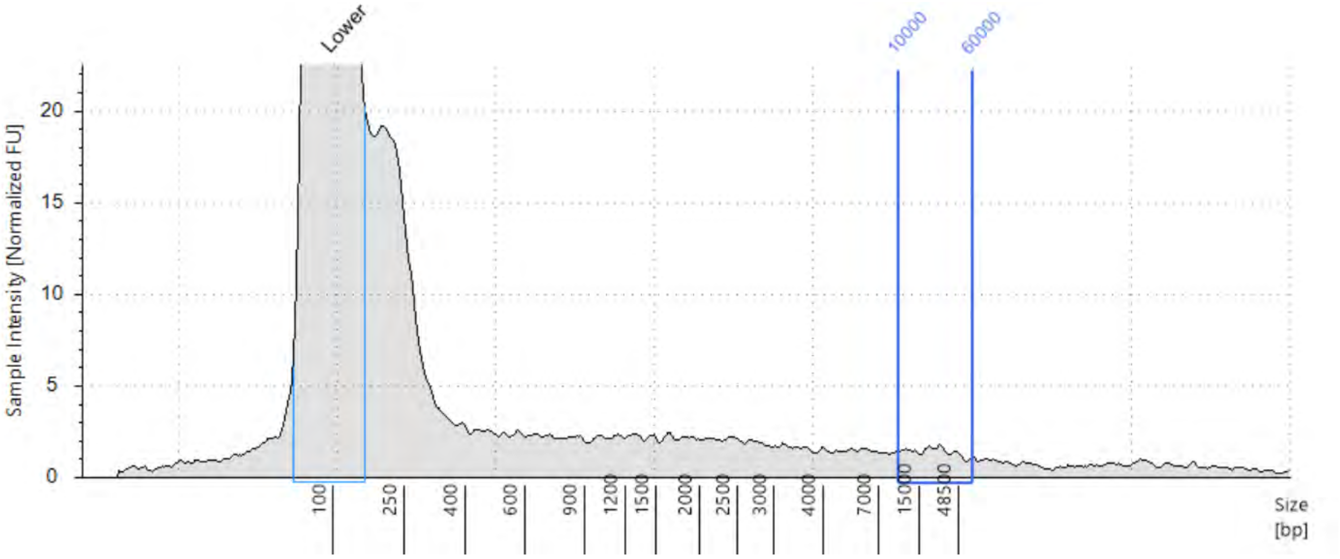

Region Table

From [bp]	To [bp]	Average Size [bp]	Conc. [ng/µl]	Region Molarity [nmol/l]	% of Total	Region Comment	Color
10000	60000	24763	0.0279	0.00543	2.66		■

D8: S-1047-T4-DSS

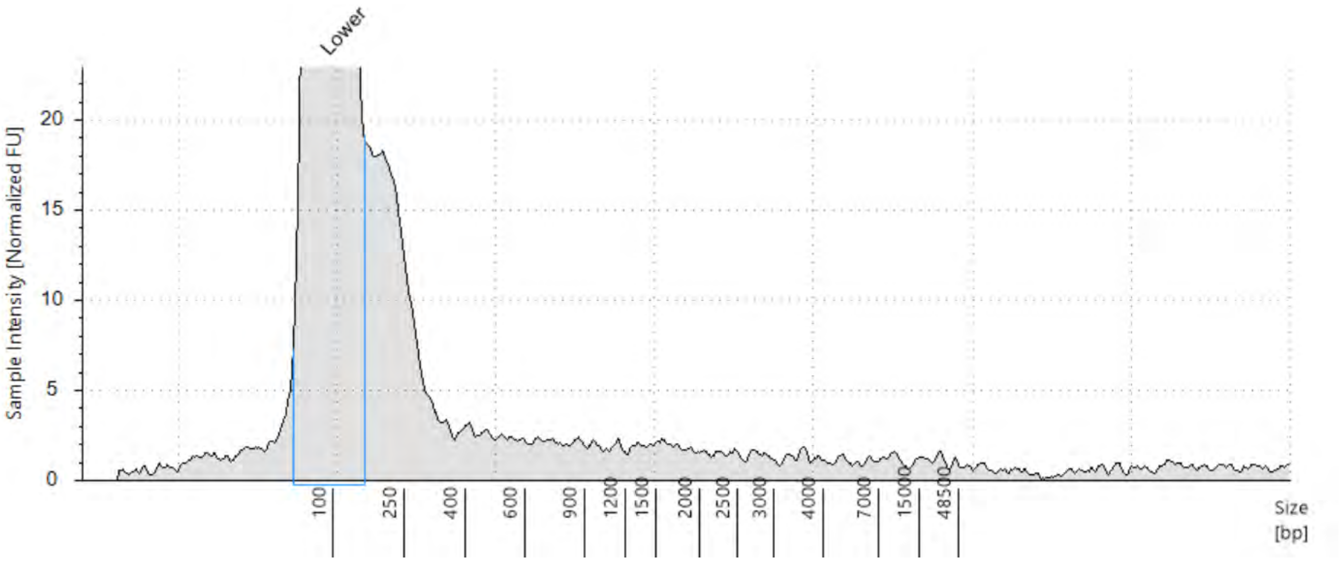

Sample Table

Well	DIN	Conc. [ng/µl]	Sample Description	Alert	Observations
D8	-	1.21	S-1047-T4-DSS	▲	Sample concentration outside functional range for DIN

Peak Table

Size [bp]	Calibrated Conc. [ng/µl]	Assigned Conc. [ng/µl]	% Integrated Area	From [bp]	To [bp]	Peak Comment	Observations
100	8.50	8.50	-	60	150		Lower Marker

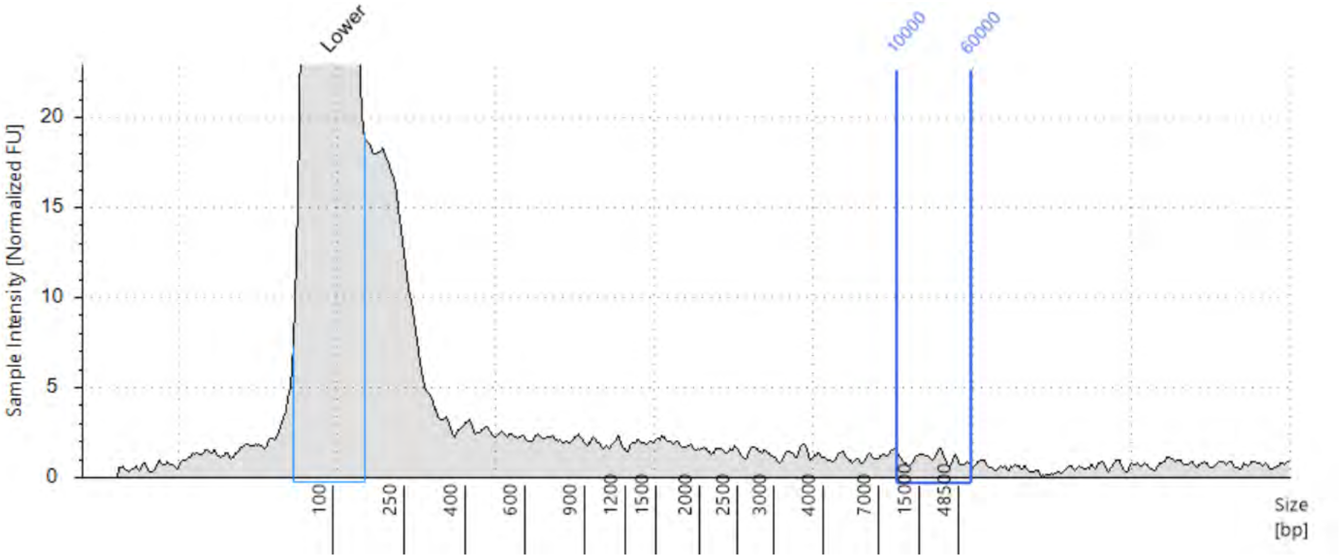

Region Table

From [bp]	To [bp]	Average Size [bp]	Conc. [ng/µl]	Region Molarity [nmol/l]	% of Total	Region Comment	Color
10000	60000	25228	0.0279	0.00390	2.31		■

 © Agilent Technologies, Inc. 2017

E8: S-1048-T4-DSS

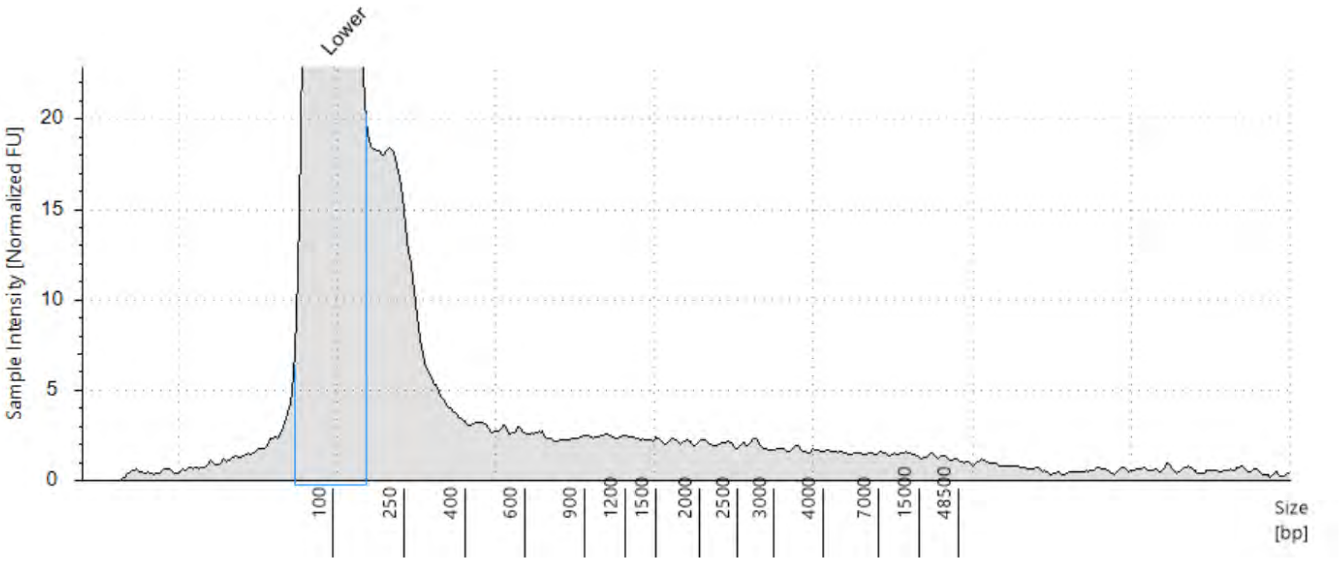

Sample Table

Well	DIN	Conc. [ng/µl]	Sample Description	Alert	Observations
E8	-	1.37	S-1048-T4-DSS	▲	Sample concentration outside functional range for DIN

Peak Table

Size [bp]	Calibrated Conc. [ng/µl]	Assigned Conc. [ng/µl]	% Integrated Area	From [bp]	To [bp]	Peak Comment	Observations
100	8.50	8.50	-	60	153		Lower Marker

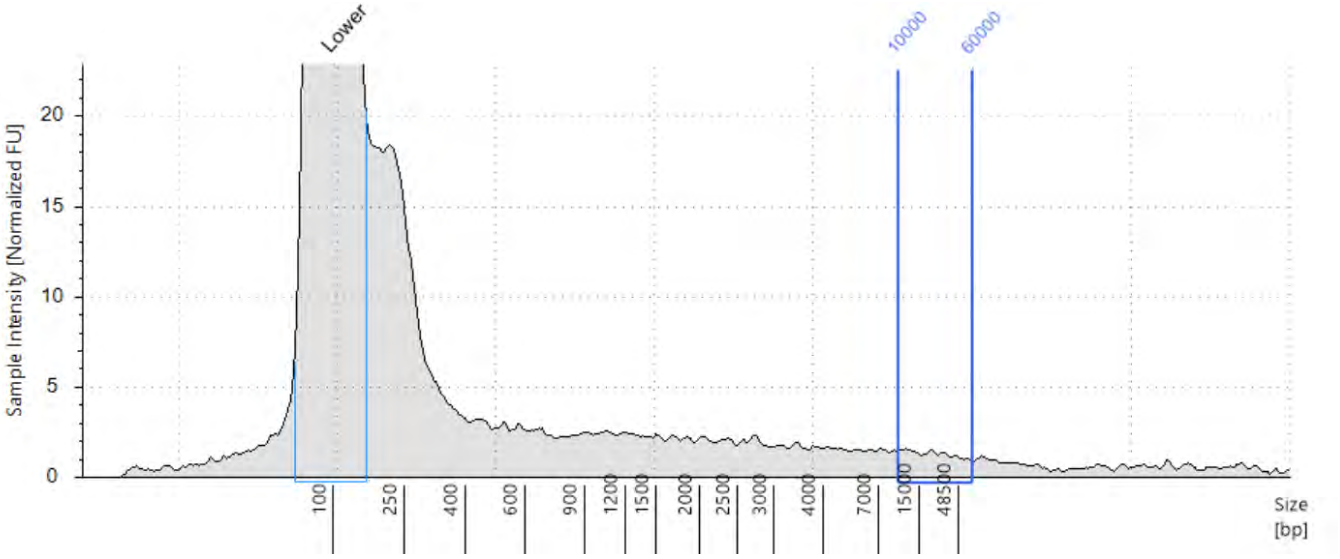

Region Table

From [bp]	To [bp]	Average Size [bp]	Conc. [ng/µl]	Region Molarity [nmol/l]	% of Total	Region Comment	Color
10000	60000	24594	0.0447	0.00521	3.26		■

 © Agilent Technologies, Inc. 2017

F8: S-1049-T4-DSS

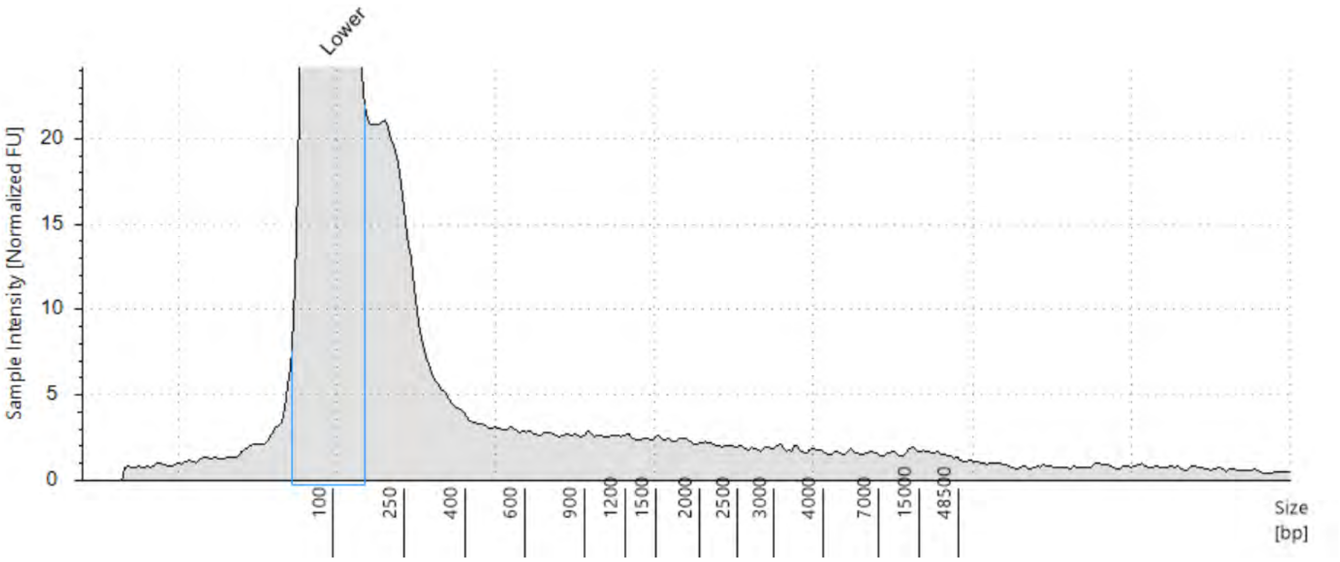

Sample Table

Well	DIN	Conc. [ng/µl]	Sample Description	Alert	Observations
F8	-	1.51	S-1049-T4-DSS	▲	Sample concentration outside functional range for DIN

Peak Table

Size [bp]	Calibrated Conc. [ng/µl]	Assigned Conc. [ng/µl]	% Integrated Area	From [bp]	To [bp]	Peak Comment	Observations
100	8.50	8.50	-	59	153		Lower Marker

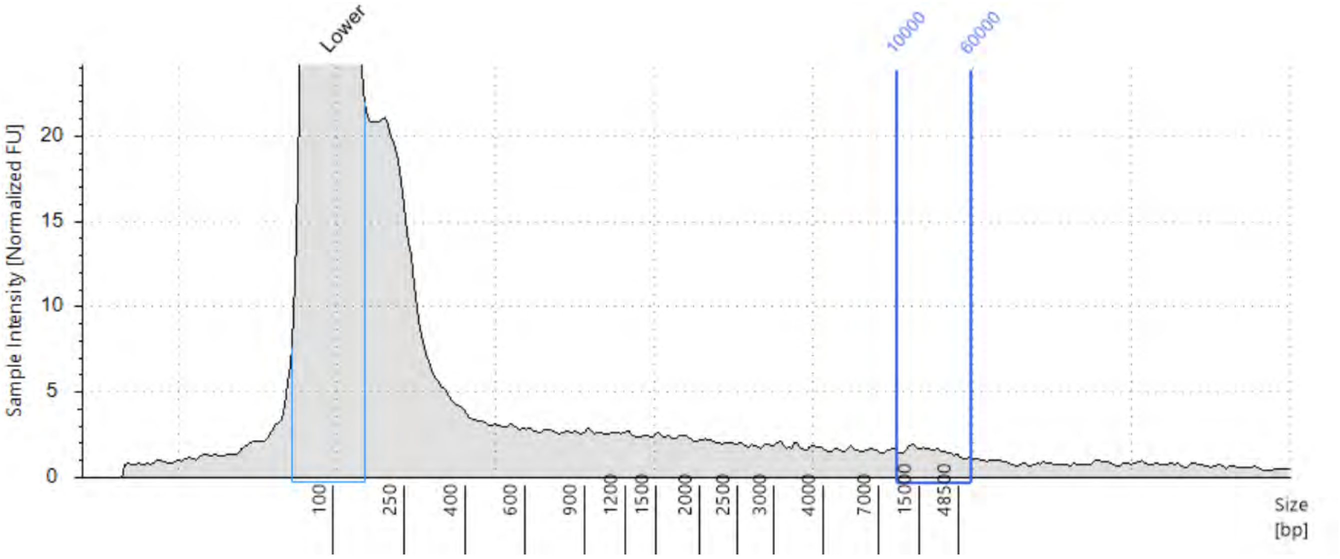

Region Table

From [bp]	To [bp]	Average Size [bp]	Conc. [ng/µl]	Region Molarity [nmol/l]	% of Total	Region Comment	Color
10000	60000	24637	0.0531	0.00593	3.53		■

 © Agilent Technologies, Inc. 2017

G8: S-1050-T4-DSS

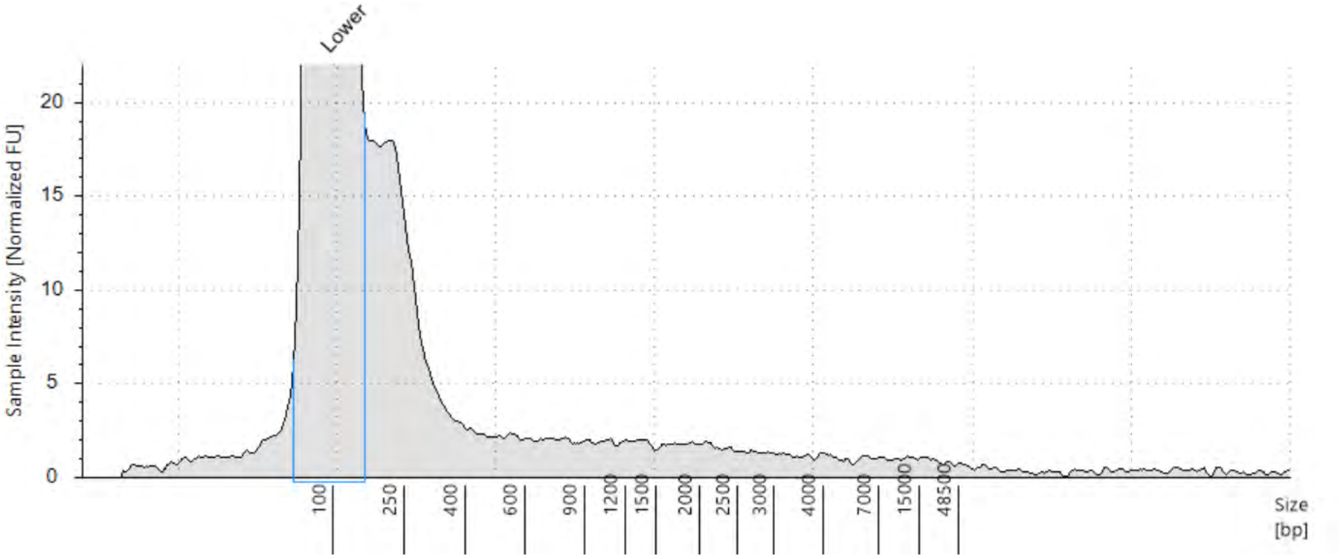

Sample Table

Well	DIN	Conc. [ng/µl]	Sample Description	Alert	Observations
G8	-	1.18	S-1050-T4-DSS	▲	Sample concentration outside functional range for DIN

Peak Table

Size [bp]	Calibrated Conc. [ng/µl]	Assigned Conc. [ng/µl]	% Integrated Area	From [bp]	To [bp]	Peak Comment	Observations
100	8.50	8.50	-	60	150		Lower Marker

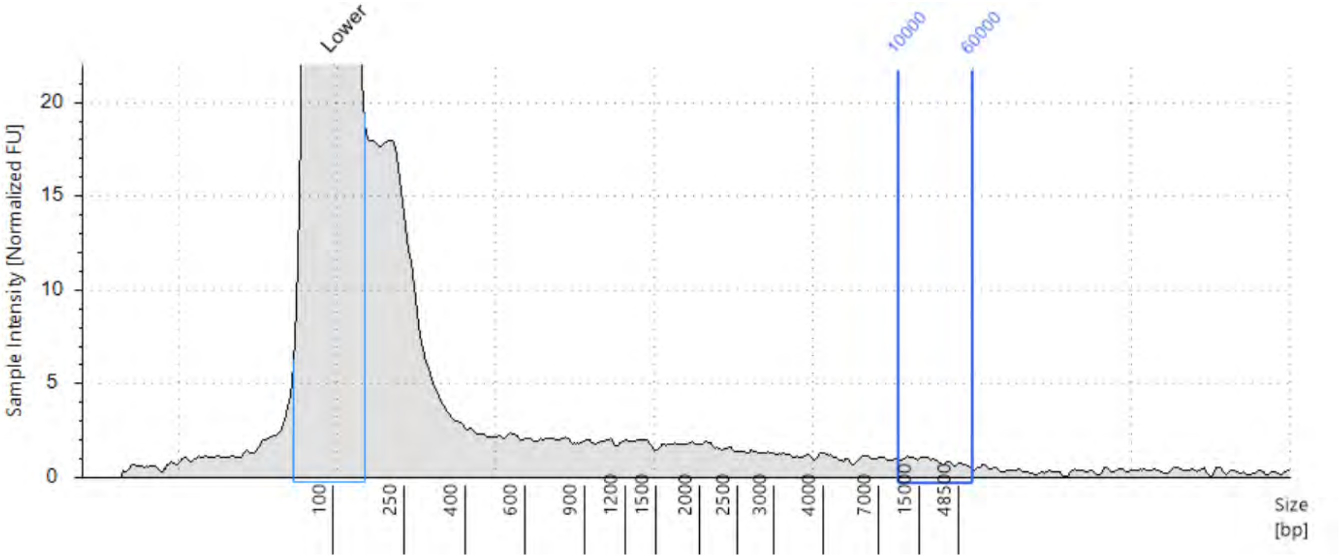

Region Table

From [bp]	To [bp]	Average Size [bp]	Conc. [ng/µl]	Region Molarity [nmol/l]	% of Total	Region Comment	Color
10000	60000	23949	0.0265	0.00362	2.25		■

H8: S-876-T5-D

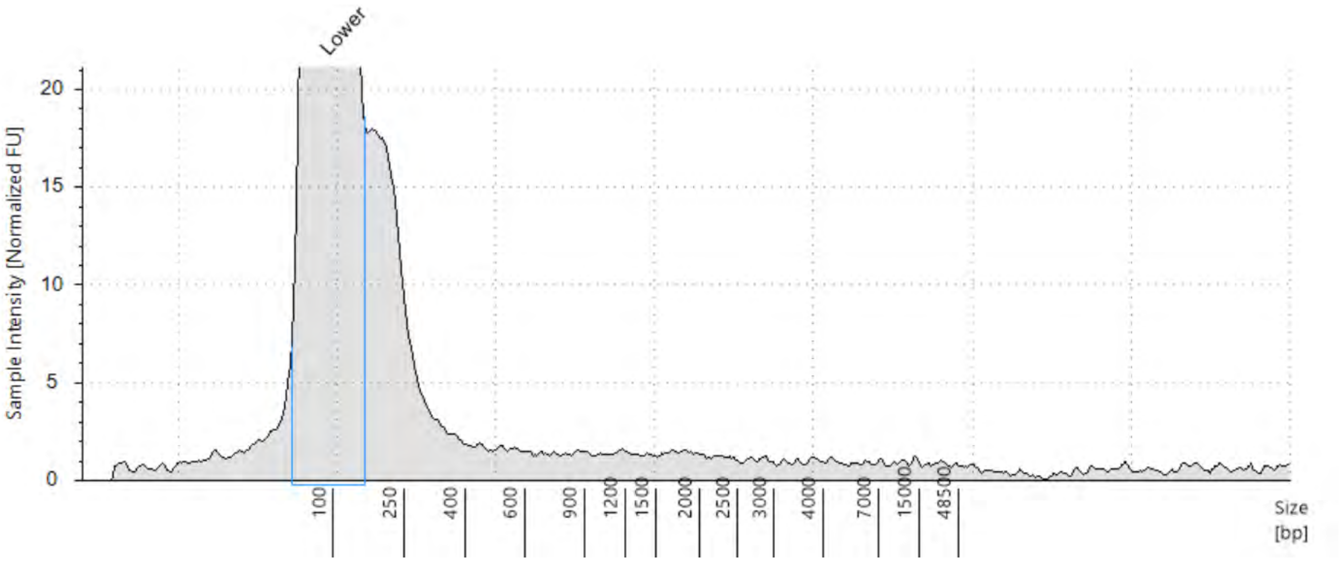

Sample Table

Well	DIN	Conc. [ng/µl]	Sample Description	Alert	Observations
H8	-	0.846	S-876-T5-D	▲	Sample concentration outside functional range for DIN

Peak Table

Size [bp]	Calibrated Conc. [ng/µl]	Assigned Conc. [ng/µl]	% Integrated Area	From [bp]	To [bp]	Peak Comment	Observations
100	8.50	8.50	-	59	151		Lower Marker

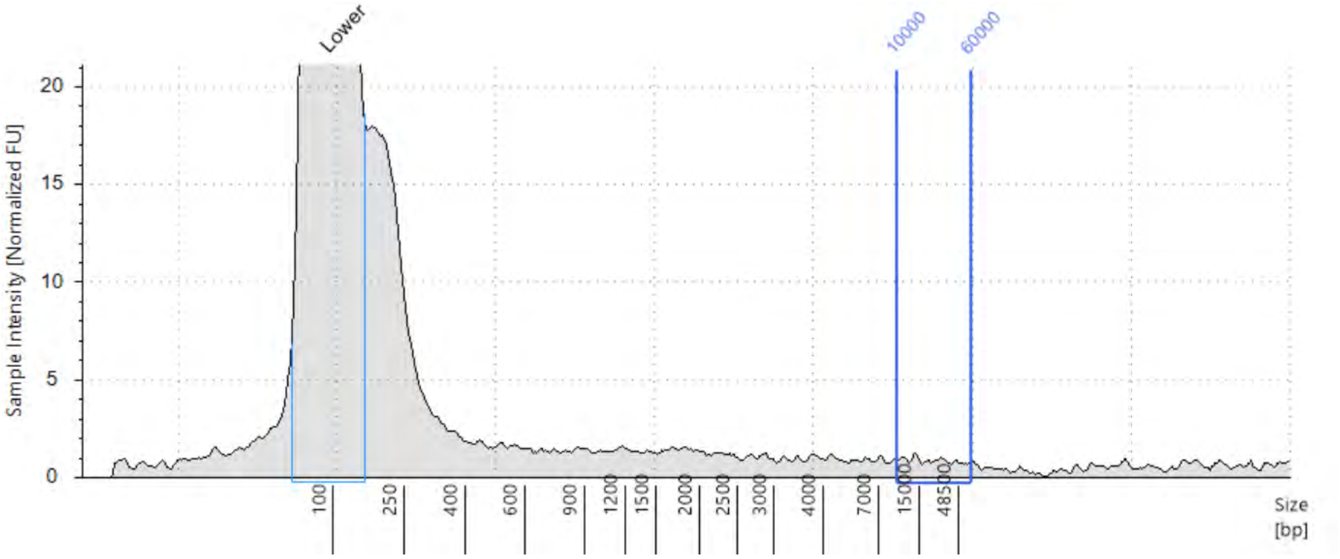

Region Table

From [bp]	To [bp]	Average Size [bp]	Conc. [ng/µl]	Region Molarity [nmol/l]	% of Total	Region Comment	Color
10000	60000	24983	0.0159	0.00330	1.88		■

A9: S-877-T5-D

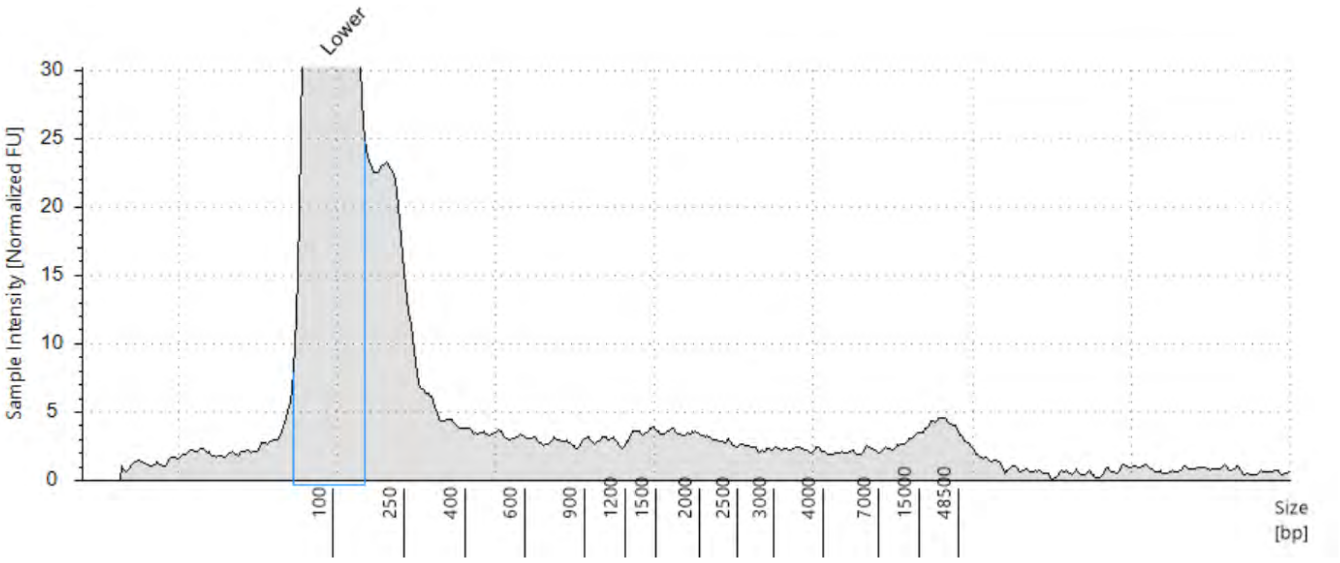

Sample Table

Well	DIN	Conc. [ng/µl]	Sample Description	Alert	Observations
A9	-	1.19	S-877-T5-D	▲	Sample concentration outside functional range for DIN

Peak Table

Size [bp]	Calibrated Conc. [ng/µl]	Assigned Conc. [ng/µl]	% Integrated Area	From [bp]	To [bp]	Peak Comment	Observations
100	8.50	8.50	-	60	153		Lower Marker

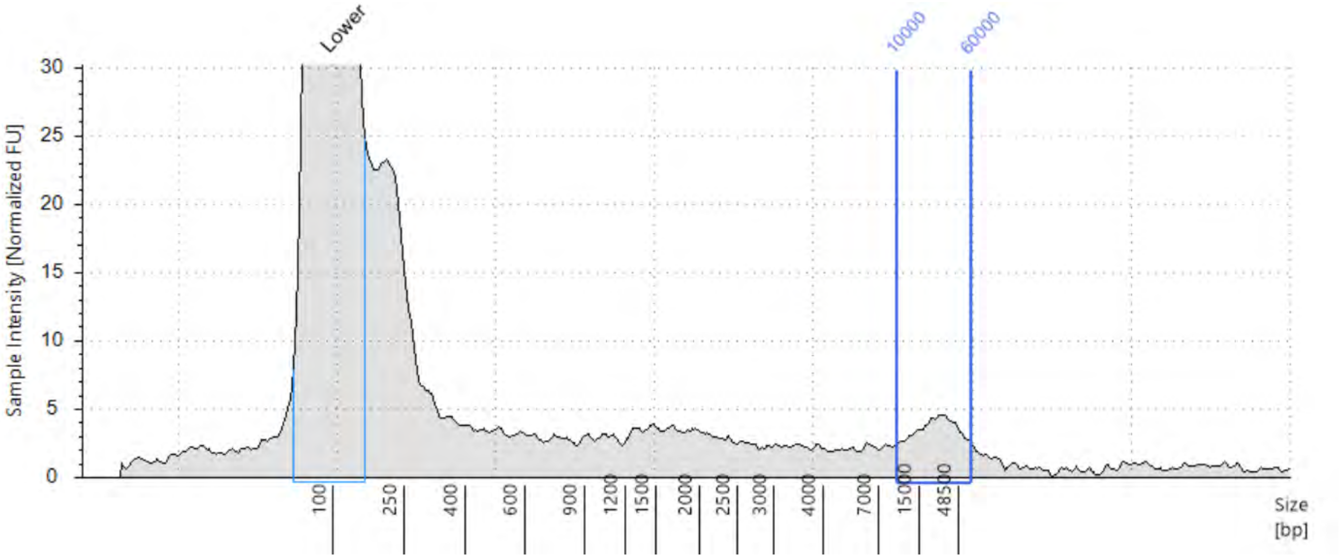

Region Table

From [bp]	To [bp]	Average Size [bp]	Conc. [ng/µl]	Region Molarity [nmol/l]	% of Total	Region Comment	Color
10000	60000	26303	0.0960	0.0129	8.10		■

B9: S-878-T5-D

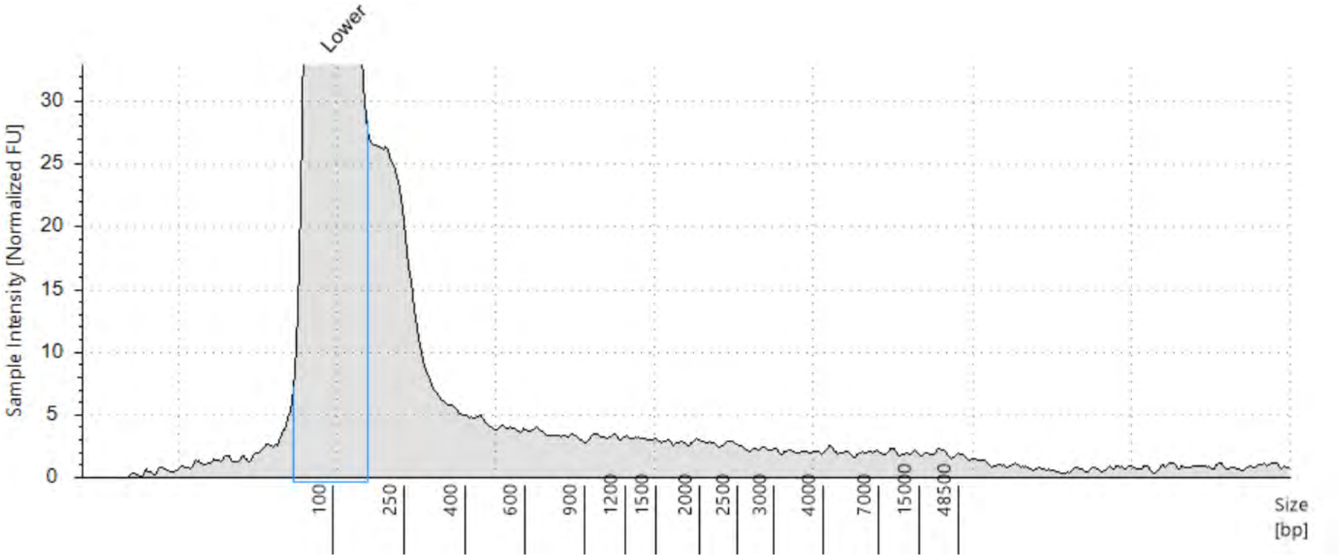

Sample Table

Well	DIN	Conc. [ng/µl]	Sample Description	Alert	Observations
B9	-	1.50	S-878-T5-D	▲	Sample concentration outside functional range for DIN

Peak Table

Size [bp]	Calibrated Conc. [ng/µl]	Assigned Conc. [ng/µl]	% Integrated Area	From [bp]	To [bp]	Peak Comment	Observations
100	8.50	8.50	-	60	155		Lower Marker

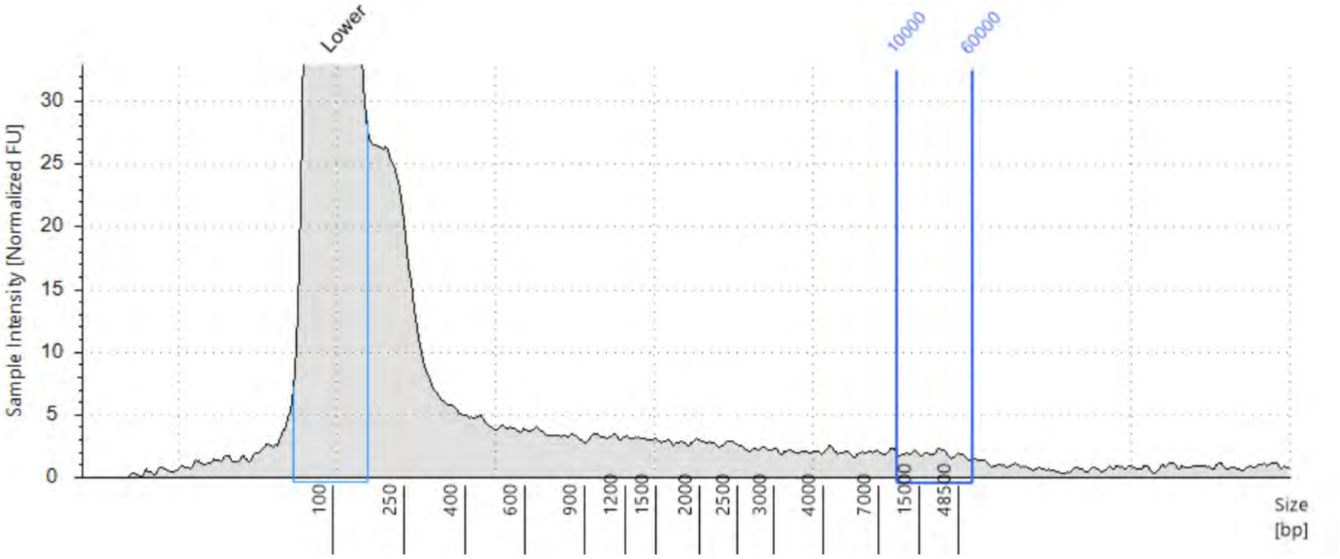

Region Table

From [bp]	To [bp]	Average Size [bp]	Conc. [ng/µl]	Region Molarity [nmol/l]	% of Total	Region Comment	Color
10000	60000	25486	0.0401	0.00676	2.68		■

C9: S-879-T5-D

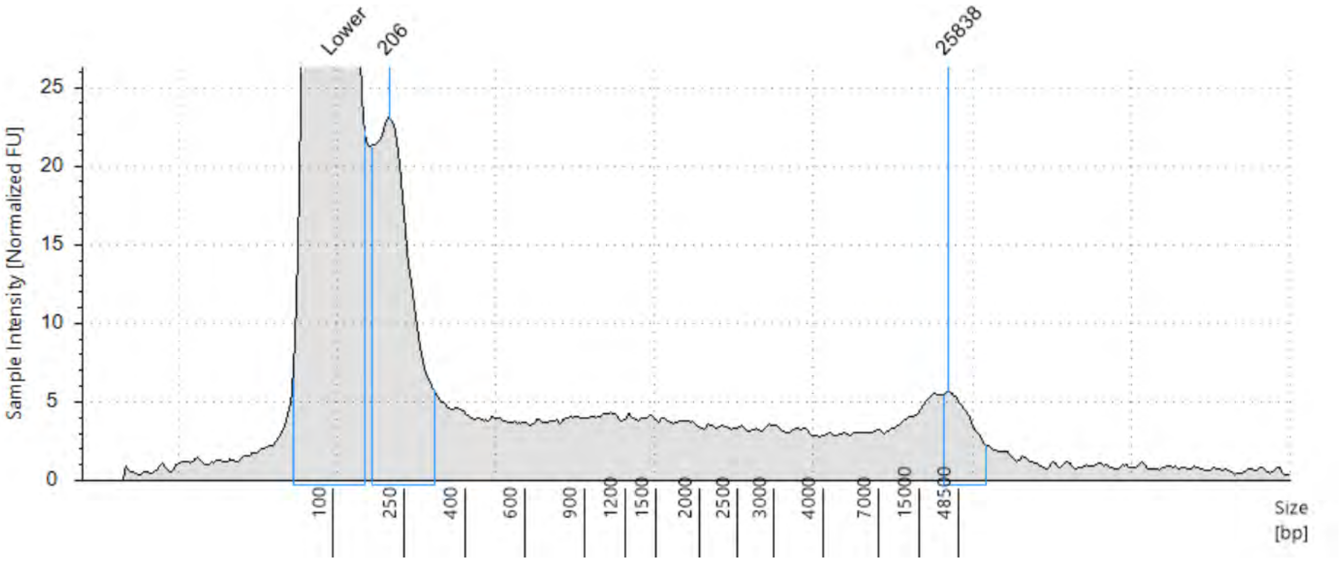

Sample Table

Well	DIN	Conc. [ng/µl]	Sample Description	Alert	Observations
C9	-	1.87	S-879-T5-D	▲	Sample concentration outside functional range for DIN

Peak Table

Size [bp]	Calibrated Conc. [ng/µl]	Assigned Conc. [ng/µl]	% Integrated Area	From [bp]	To [bp]	Peak Comment	Observations
100	8.50	8.50	-	59	150		Lower Marker
206	0.618	-	86.08	164	315		
25838	0.0998	-	13.92	23717	>60000		

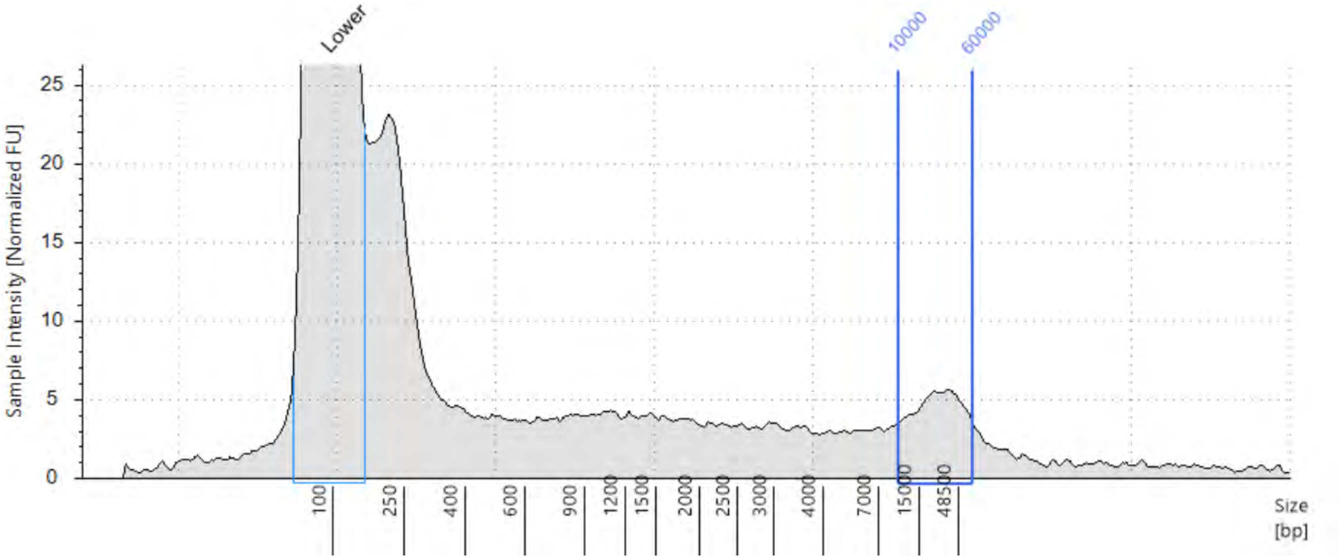

Region Table

From [bp]	To [bp]	Average Size [bp]	Conc. [ng/µl]	Region Molarity [nmol/l]	% of Total	Region Comment	Color
10000	60000	26713	0.191	0.0169	10.21		■

 © Agilent Technologies, Inc. 2017

D9: S-880-T5-D

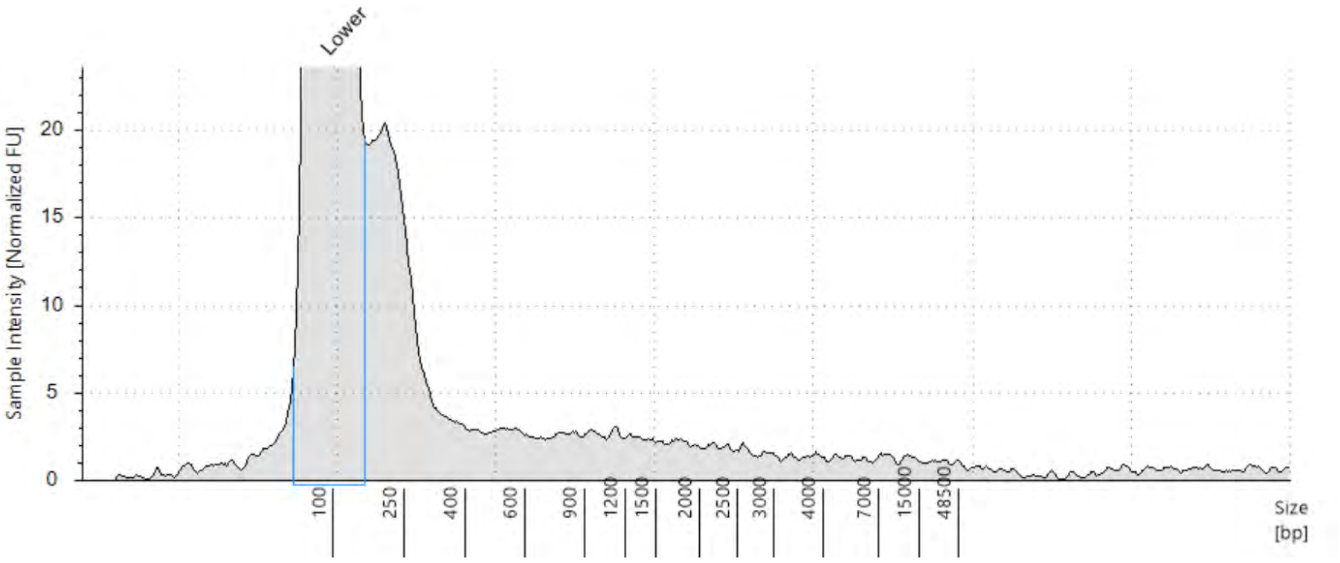

Sample Table

Well	DIN	Conc. [ng/µl]	Sample Description	Alert	Observations
D9	-	1.17	S-880-T5-D	▲	Sample concentration outside functional range for DIN

Peak Table

Size [bp]	Calibrated Conc. [ng/µl]	Assigned Conc. [ng/µl]	% Integrated Area	From [bp]	To [bp]	Peak Comment	Observations
100	8.50	8.50	-	60	150		Lower Marker

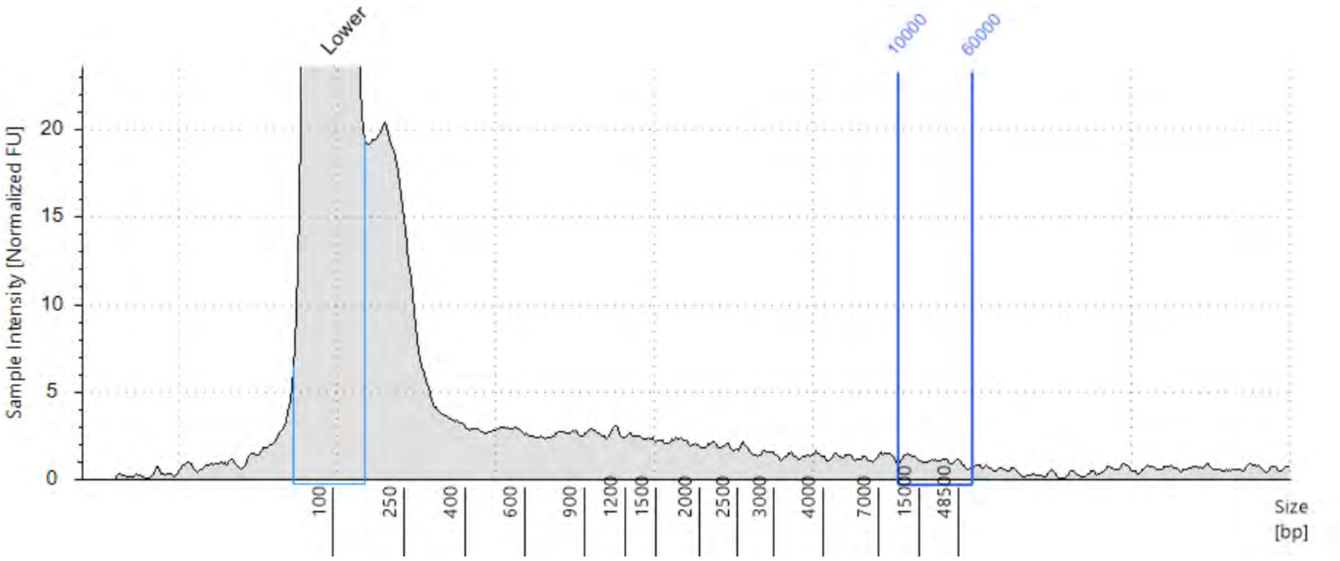

Region Table

From [bp]	To [bp]	Average Size [bp]	Conc. [ng/µl]	Region Molarity [nmol/l]	% of Total	Region Comment	Color
10000	60000	24181	0.0194	0.00432	1.66		■

 © Agilent Technologies, Inc. 2017

E9: S-1101-T5-D

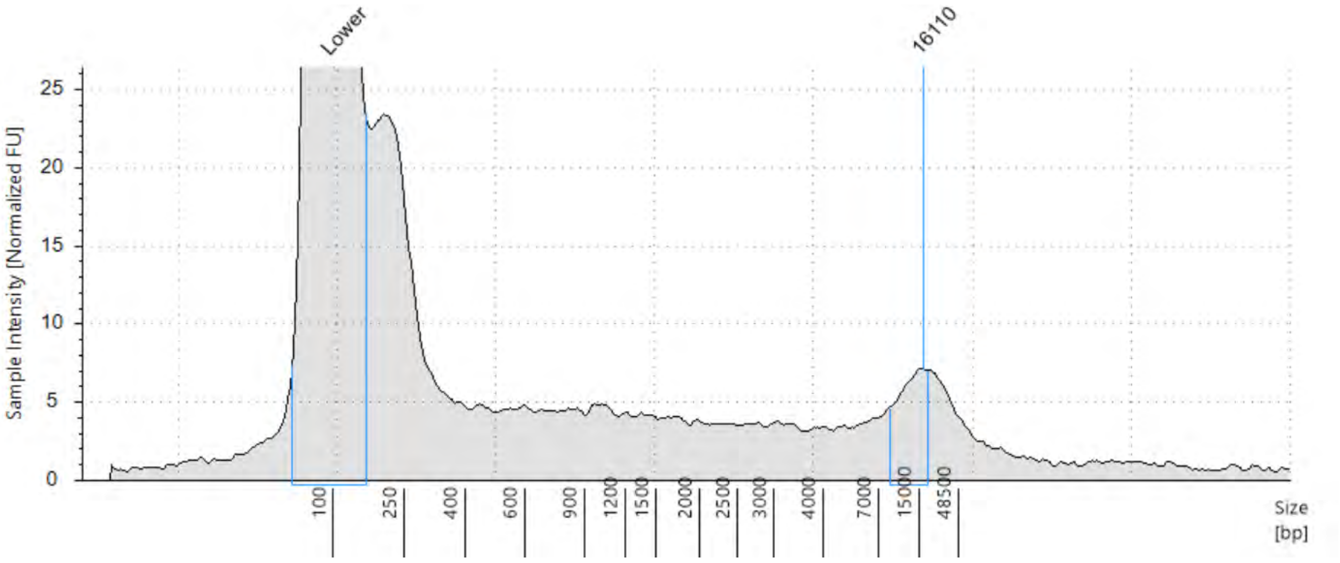

Sample Table

Well	DIN	Conc. [ng/µl]	Sample Description	Alert	Observations
E9	-	2.17	S-1101-T5-D	▲	Sample concentration outside functional range for DIN

Peak Table

Size [bp]	Calibrated Conc. [ng/µl]	Assigned Conc. [ng/µl]	% Integrated Area	From [bp]	To [bp]	Peak Comment	Observations
100	8.50	8.50	-	59	153		Lower Marker
16110	0.132	-	100.00	8721	17395		

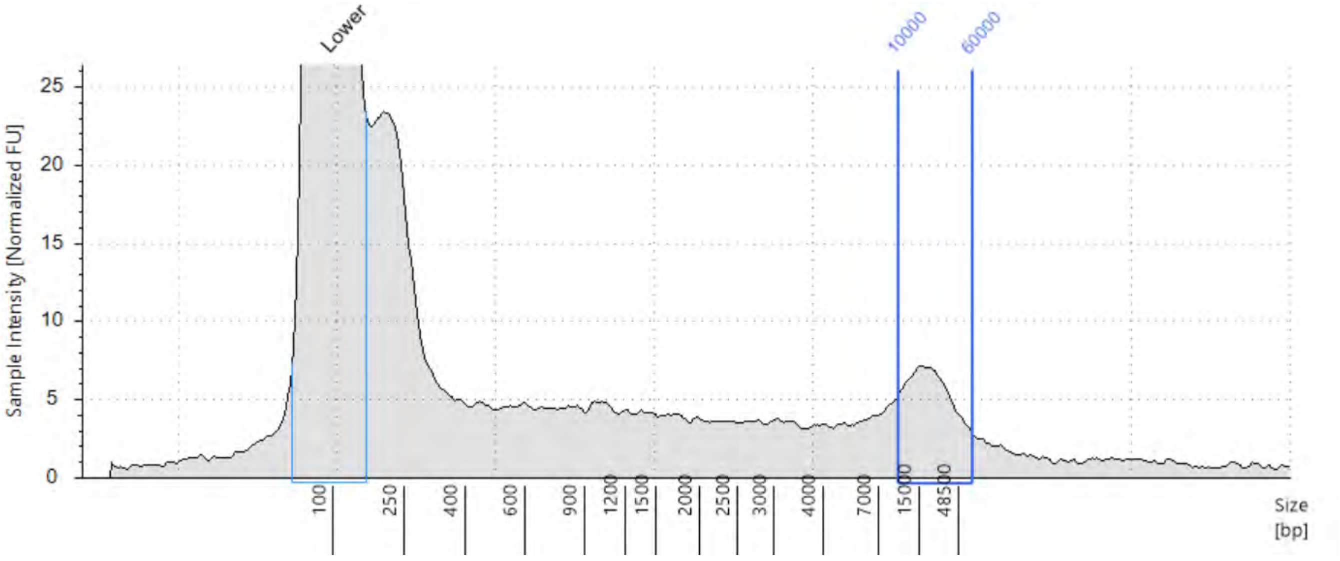

Region Table

From [bp]	To [bp]	Average Size [bp]	Conc. [ng/µl]	Region Molarity [nmol/l]	% of Total	Region Comment	Color
10000	60000	23231	0.239	0.0224	11.00		■

F9: S-1102-T5-D

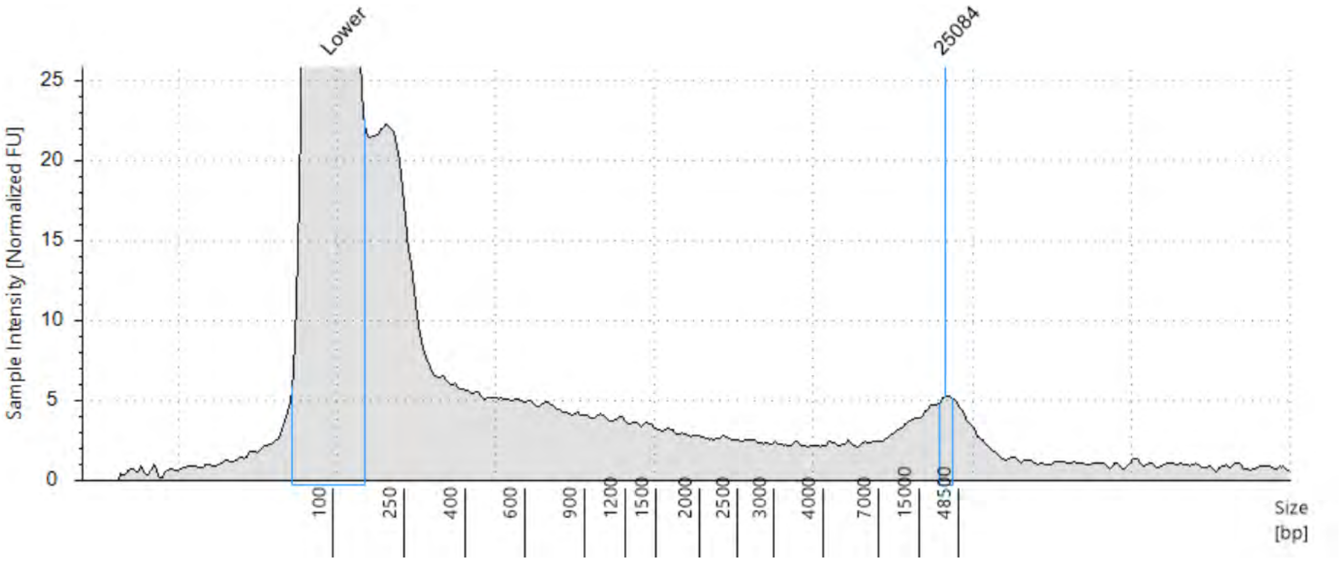

Sample Table

Well	DIN	Conc. [ng/µl]	Sample Description	Alert	Observations
F9	-	2.02	S-1102-T5-D	▲	Sample concentration outside functional range for DIN

Peak Table

Size [bp]	Calibrated Conc. [ng/µl]	Assigned Conc. [ng/µl]	% Integrated Area	From [bp]	To [bp]	Peak Comment	Observations
100	8.50	8.50	-	59	150		Lower Marker
25084	0.0447	-	100.00	21962	30932		

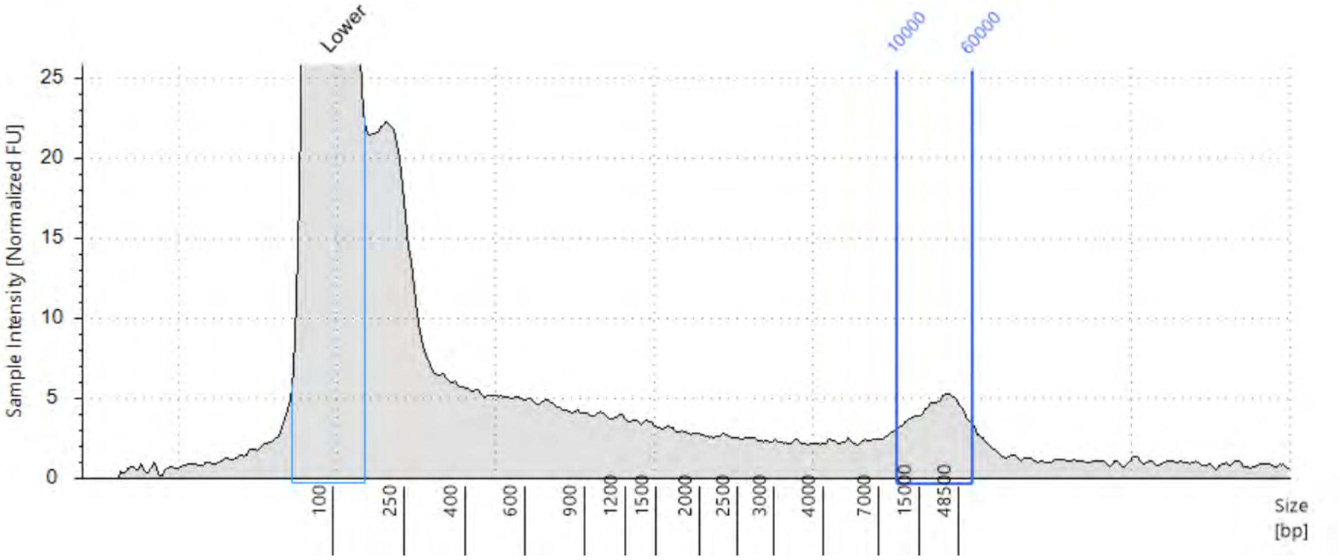

Region Table

From [bp]	To [bp]	Average Size [bp]	Conc. [ng/µl]	Region Molarity [nmol/l]	% of Total	Region Comment	Color
10000	60000	26947	0.182	0.0155	9.00		■

G9: S-1103-T5-D

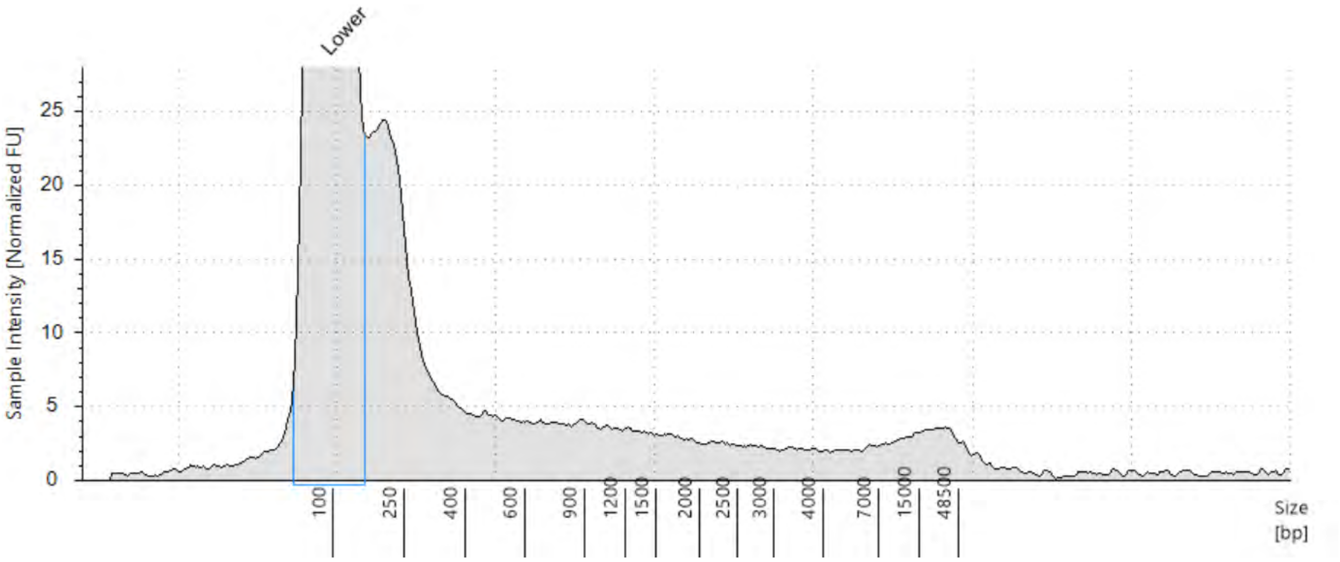

Sample Table

Well	DIN	Conc. [ng/µl]	Sample Description	Alert	Observations
G9	-	1.75	S-1103-T5-D	▲	Sample concentration outside functional range for DIN

Peak Table

Size [bp]	Calibrated Conc. [ng/µl]	Assigned Conc. [ng/µl]	% Integrated Area	From [bp]	To [bp]	Peak Comment	Observations
100	8.50	8.50	-	59	150		Lower Marker

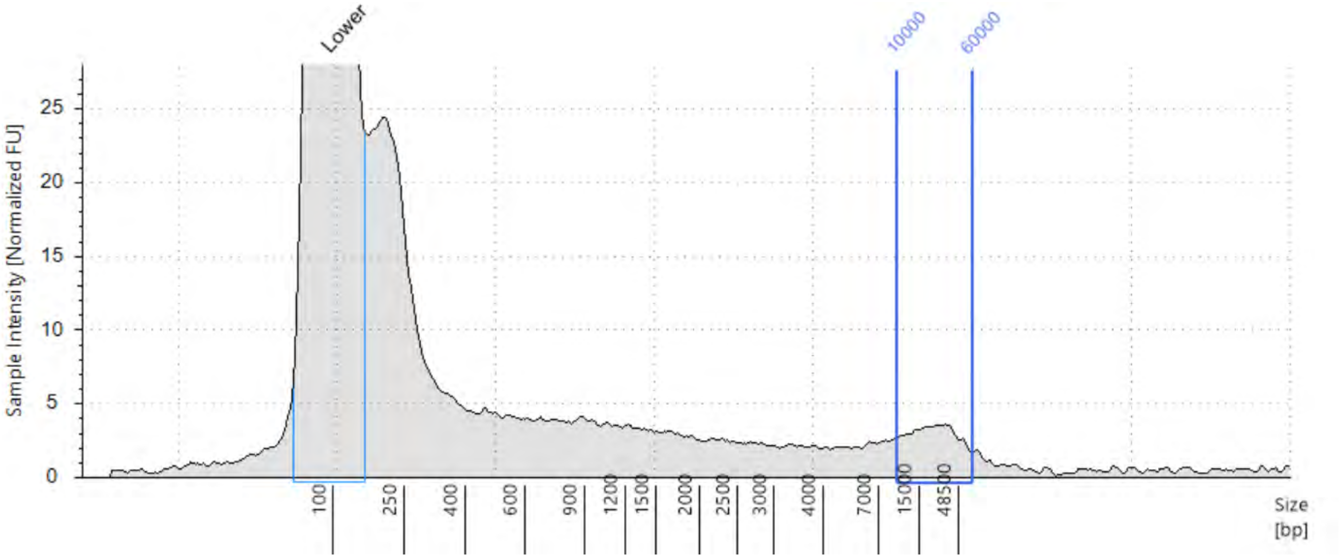

Region Table

From [bp]	To [bp]	Average Size [bp]	Conc. [ng/µl]	Region Molarity [nmol/l]	% of Total	Region Comment	Color
10000	60000	24951	0.122	0.0121	6.97		■

H9: S-1104-T5-D

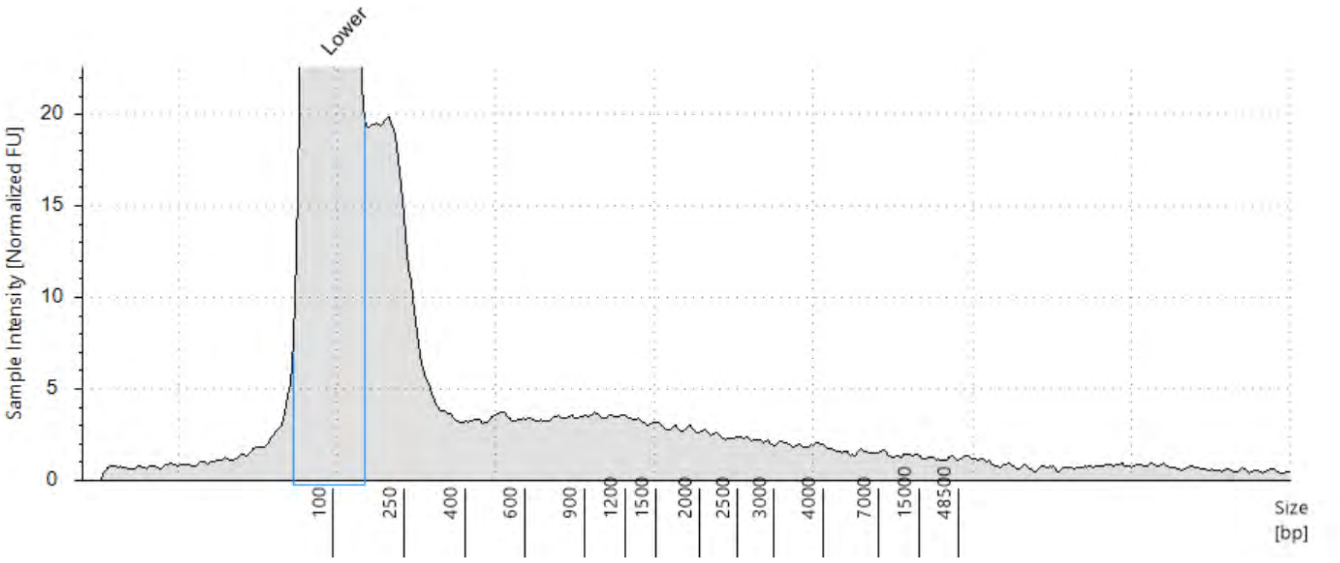

Sample Table

Well	DIN	Conc. [ng/µl]	Sample Description	Alert	Observations
H9	-	1.32	S-1104-T5-D	▲	Sample concentration outside functional range for DIN

Peak Table

Size [bp]	Calibrated Conc. [ng/µl]	Assigned Conc. [ng/µl]	% Integrated Area	From [bp]	To [bp]	Peak Comment	Observations
100	8.50	8.50	-	59	151		Lower Marker

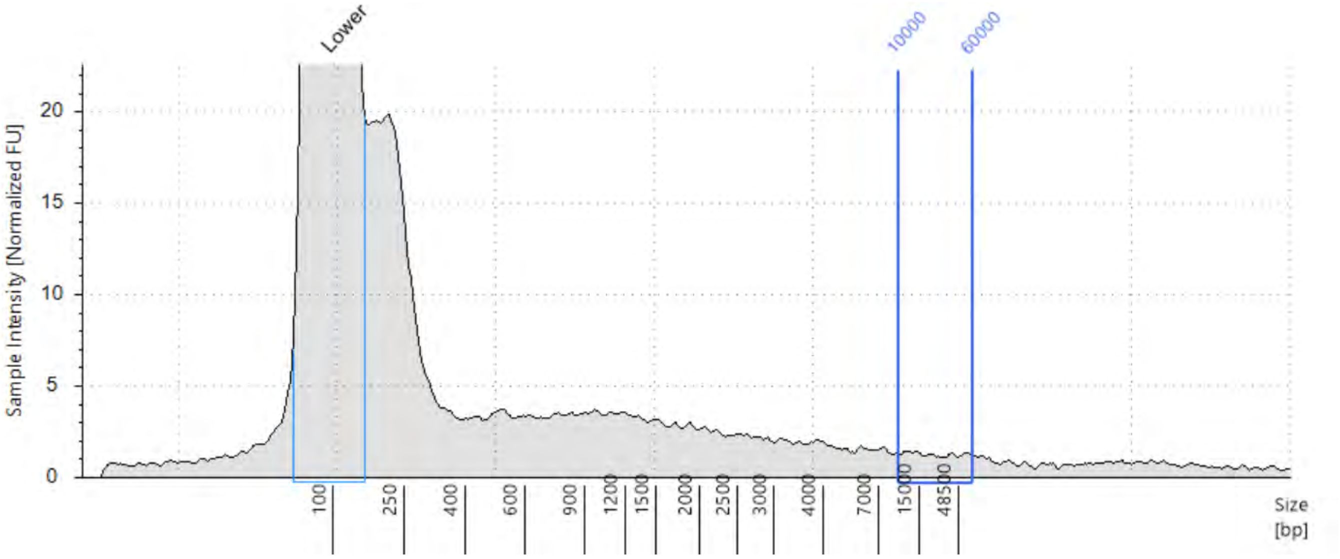

Region Table

From [bp]	To [bp]	Average Size [bp]	Conc. [ng/µl]	Region Molarity [nmol/l]	% of Total	Region Comment	Color
10000	60000	26145	0.0295	0.00471	2.23		■

 © Agilent Technologies, Inc. 2017

A10: S-1105-T5-D

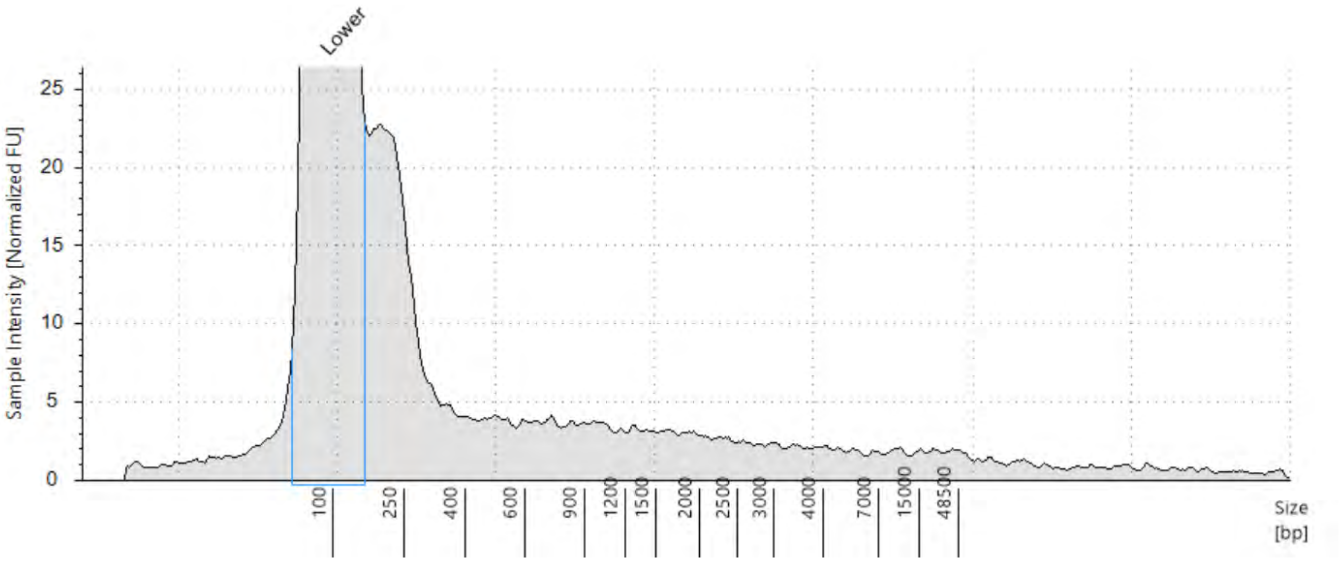

Sample Table

Well	DIN	Conc. [ng/µl]	Sample Description	Alert	Observations
A10	-	1.45	S-1105-T5-D	▲	Sample concentration outside functional range for DIN

Peak Table

Size [bp]	Calibrated Conc. [ng/µl]	Assigned Conc. [ng/µl]	% Integrated Area	From [bp]	To [bp]	Peak Comment	Observations
100	8.50	8.50	-	59	152		Lower Marker

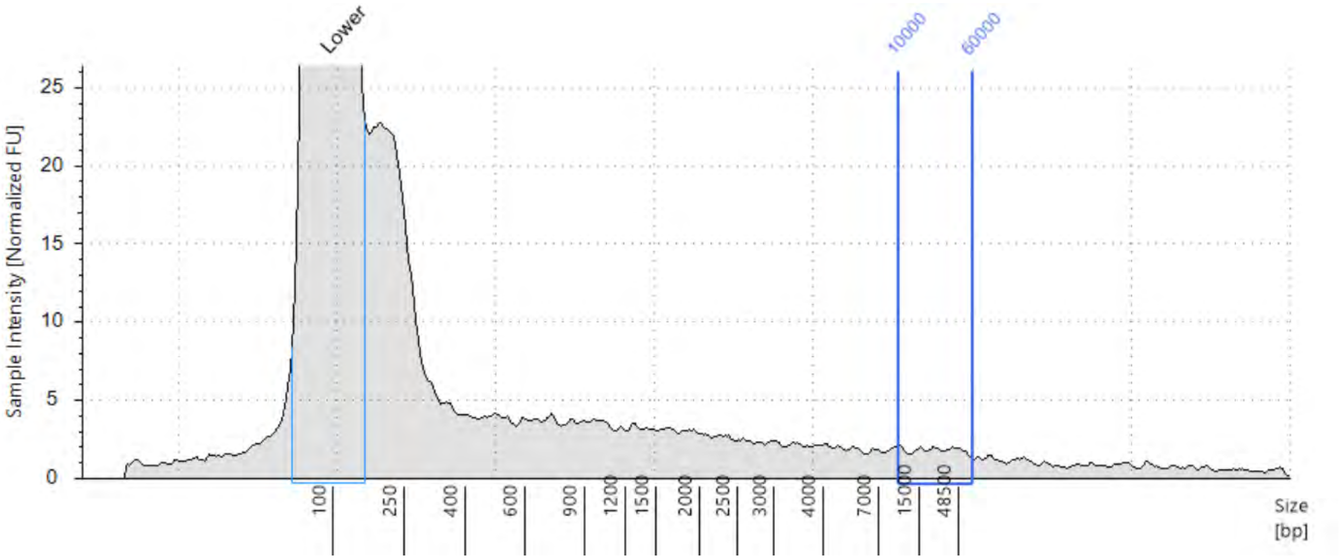

Region Table

From [bp]	To [bp]	Average Size [bp]	Conc. [ng/µl]	Region Molarity [nmol/l]	% of Total	Region Comment	Color
10000	60000	25788	0.0482	0.00670	3.32		■

 © Agilent Technologies, Inc. 2017

B10: S-862-T5-DE

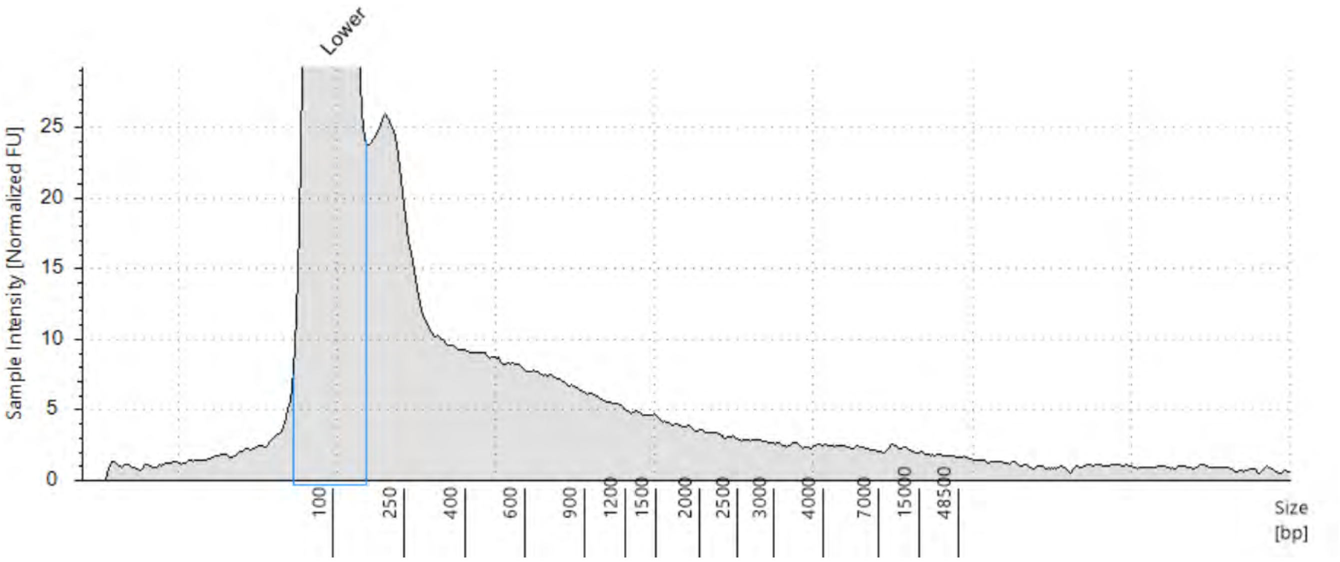

Sample Table

Well	DIN	Conc. [ng/µl]	Sample Description	Alert	Observations
B10	-	2.45	S-862-T5-DE	▲	Sample concentration outside functional range for DIN

Peak Table

Size [bp]	Calibrated Conc. [ng/µl]	Assigned Conc. [ng/µl]	% Integrated Area	From [bp]	To [bp]	Peak Comment	Observations
100	8.50	8.50	-	60	153		Lower Marker

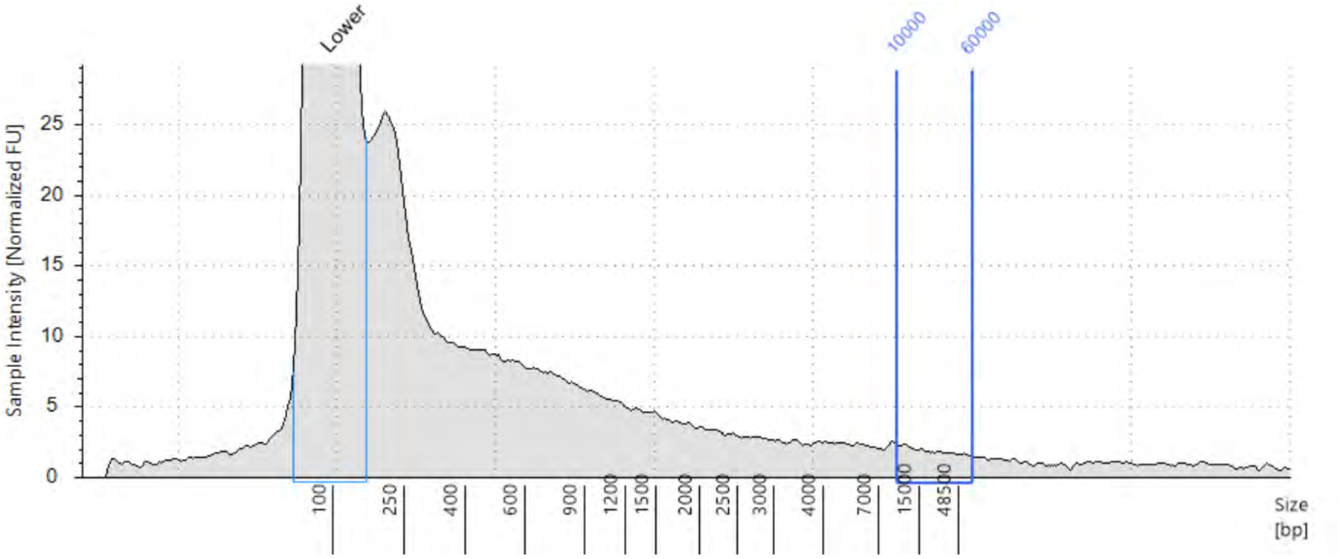

Region Table

From [bp]	To [bp]	Average Size [bp]	Conc. [ng/µl]	Region Molarity [nmol/l]	% of Total	Region Comment	Color
10000	60000	25413	0.0643	0.00730	2.63		■

C10: S-863-T5-DE

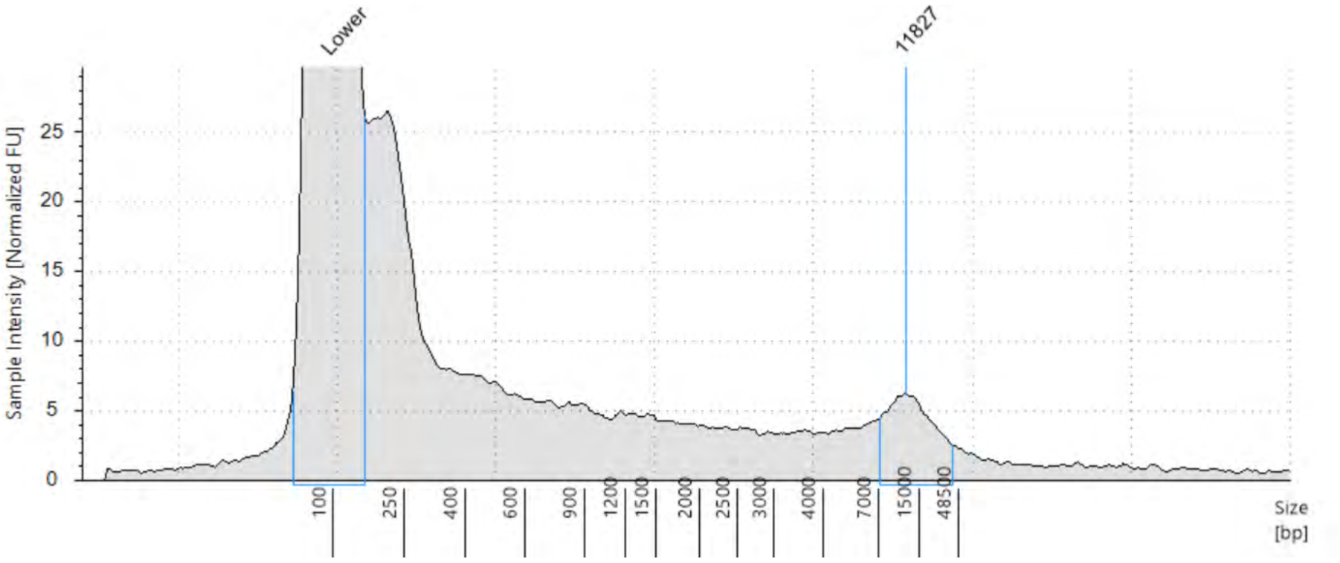

Sample Table

Well	DIN	Conc. [ng/µl]	Sample Description	Alert	Observations
C10	-	2.45	S-863-T5-DE	▲	Sample concentration outside functional range for DIN

Peak Table

Size [bp]	Calibrated Conc. [ng/µl]	Assigned Conc. [ng/µl]	% Integrated Area	From [bp]	To [bp]	Peak Comment	Observations
100	8.50	8.50	-	59	151		Lower Marker
11827	0.201	-	100.00	7268	30808		

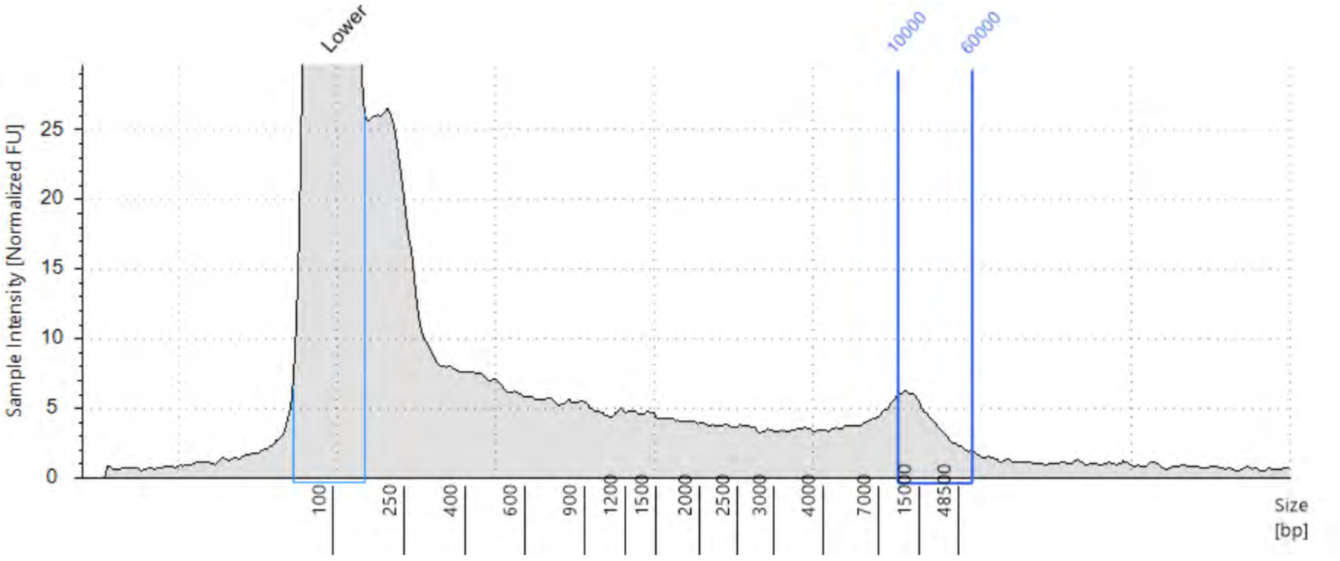

Region Table

From [bp]	To [bp]	Average Size [bp]	Conc. [ng/µl]	Region Molarity [nmol/l]	% of Total	Region Comment	Color
10000	60000	21212	0.169	0.0180	6.90		■

© Agilent Technologies, Inc. 2017

D10: S-864-T5-DE

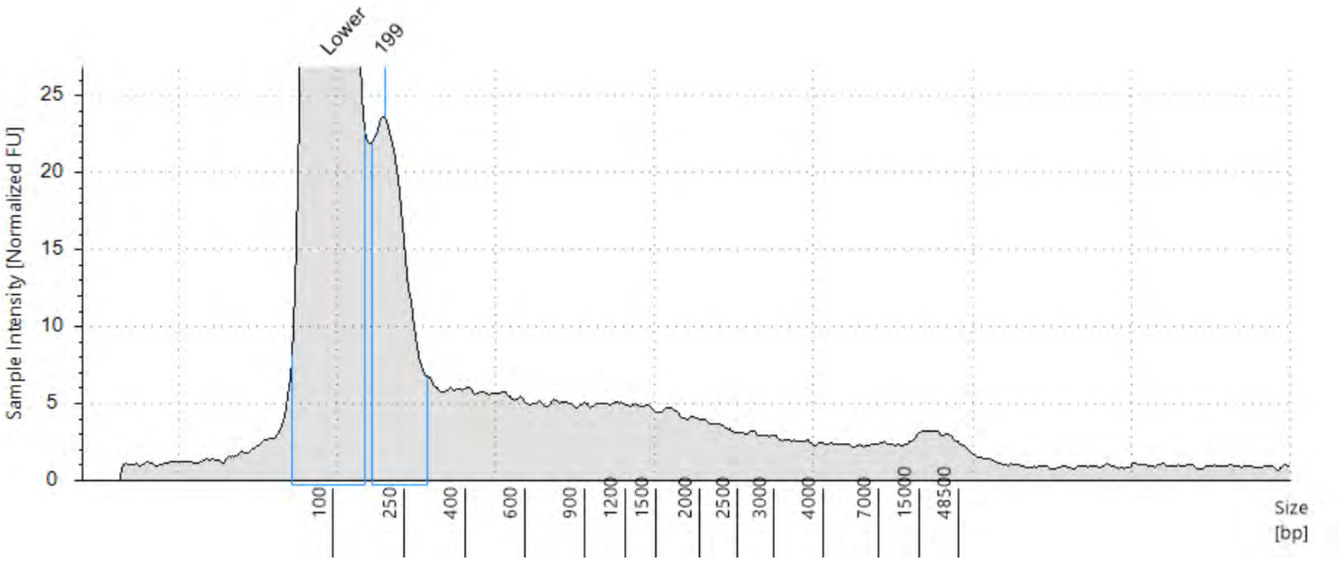

Sample Table

Well	DIN	Conc. [ng/µl]	Sample Description	Alert	Observations
D10	-	1.74	S-864-T5-DE	▲	Sample concentration outside functional range for DIN

Peak Table

Size [bp]	Calibrated Conc. [ng/µl]	Assigned Conc. [ng/µl]	% Integrated Area	From [bp]	To [bp]	Peak Comment	Observations
100	8.50	8.50	-	59	153		Lower Marker
199	0.541	-	100.00	167	301		

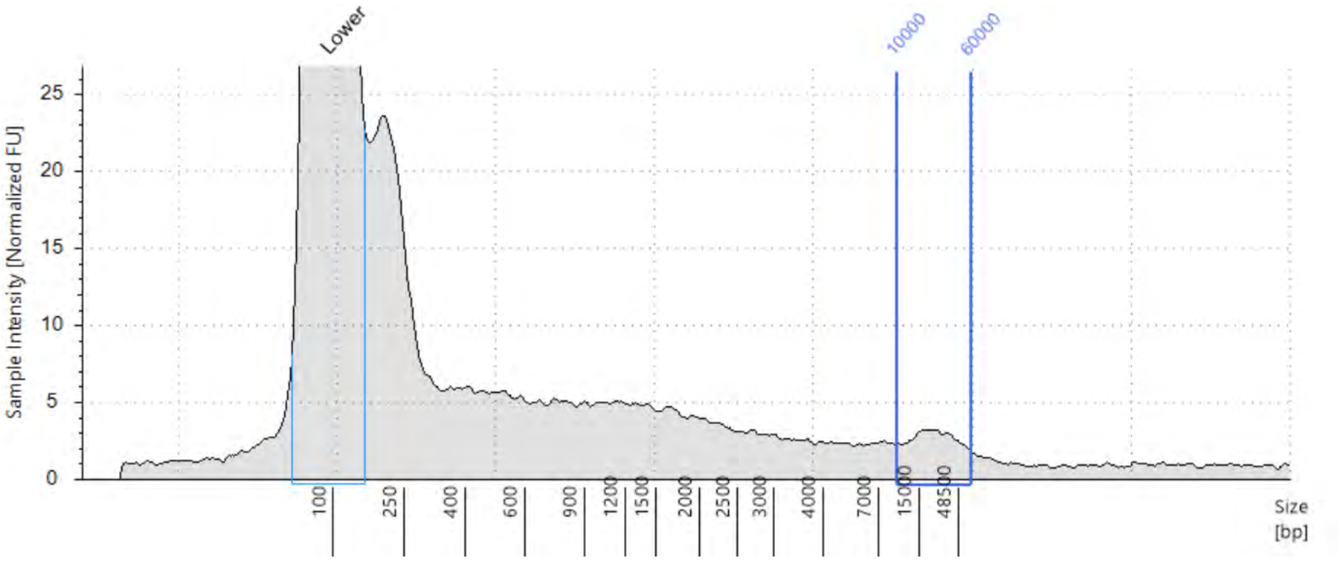

Region Table

From [bp]	To [bp]	Average Size [bp]	Conc. [ng/µl]	Region Molarity [nmol/l]	% of Total	Region Comment	Color
10000	60000	25439	0.0864	0.00958	4.96		■

 © Agilent Technologies, Inc. 2017

E10: S-865-T5-DE

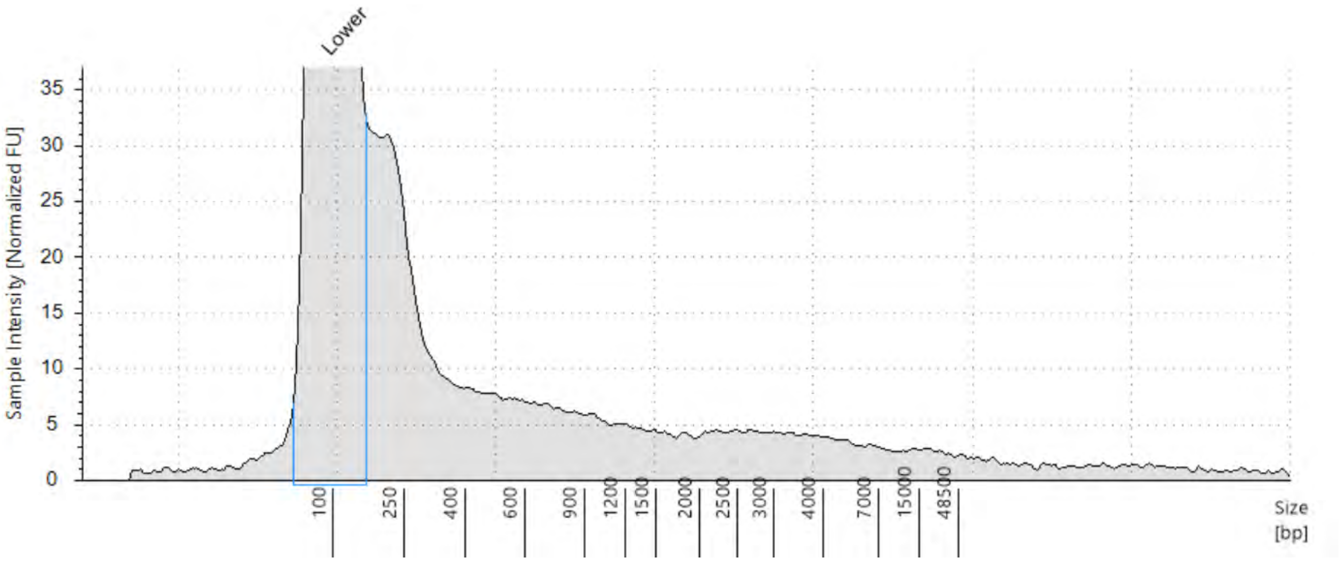

Sample Table

Well	DIN	Conc. [ng/µl]	Sample Description	Alert	Observations
E10	-	1.86	S-865-T5-DE	▲	Sample concentration outside functional range for DIN

Peak Table

Size [bp]	Calibrated Conc. [ng/µl]	Assigned Conc. [ng/µl]	% Integrated Area	From [bp]	To [bp]	Peak Comment	Observations
100	8.50	8.50	-	59	152		Lower Marker

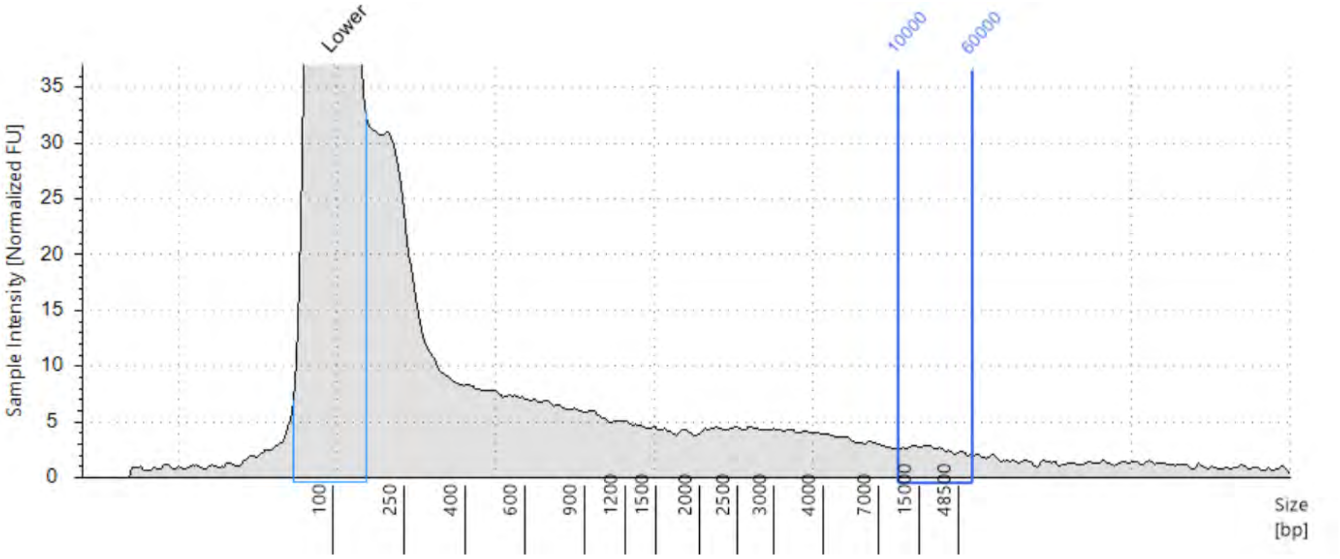

Region Table

From [bp]	To [bp]	Average Size [bp]	Conc. [ng/µl]	Region Molarity [nmol/l]	% of Total	Region Comment	Color
10000	60000	25240	0.0223	0.00936	1.20		■

F10: S-1086-T5-DE

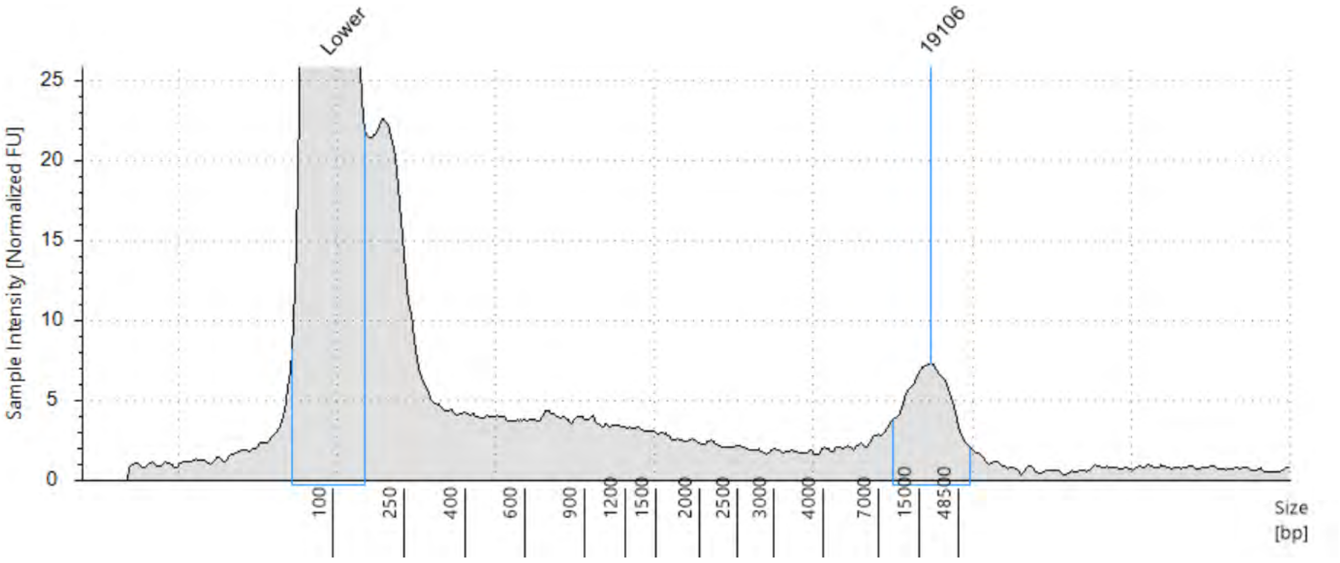

Sample Table

Well	DIN	Conc. [ng/µl]	Sample Description	Alert	Observations
F10	-	1.71	S-1086-T5-DE	▲	Sample concentration outside functional range for DIN

Peak Table

Size [bp]	Calibrated Conc. [ng/µl]	Assigned Conc. [ng/µl]	% Integrated Area	From [bp]	To [bp]	Peak Comment	Observations
100	8.50	8.50	-	59	153		Lower Marker
19106	0.234	-	100.00	9388	59466		

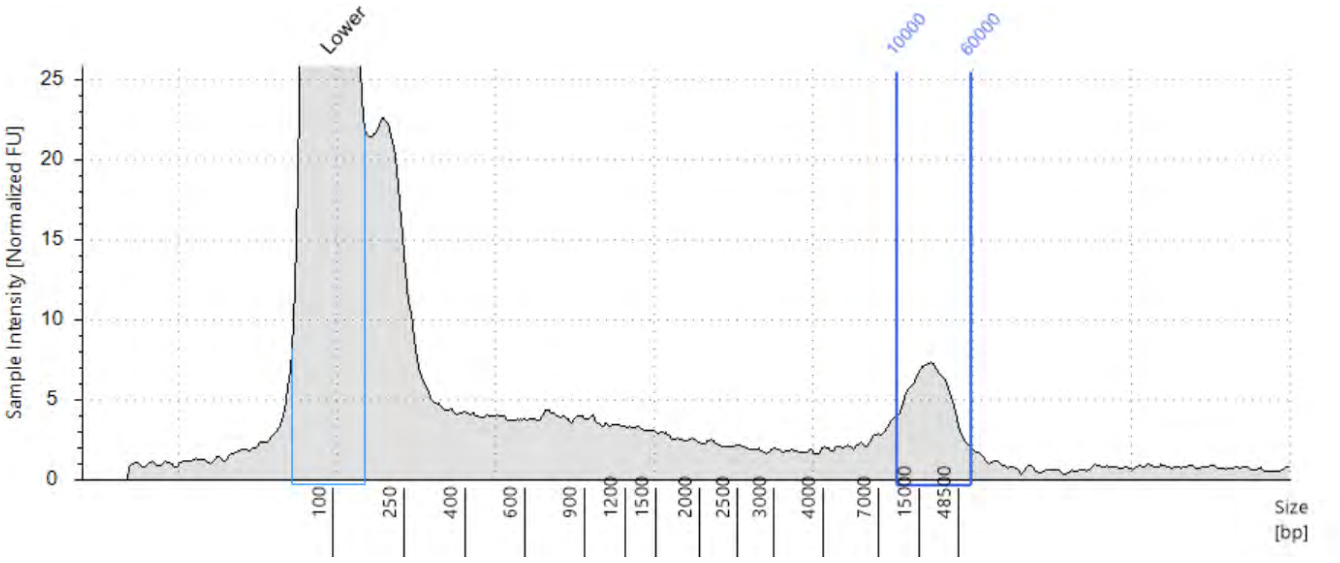

Region Table

From [bp]	To [bp]	Average Size [bp]	Conc. [ng/µl]	Region Molarity [nmol/l]	% of Total	Region Comment	Color
10000	60000	22823	0.227	0.0210	13.27		■

G10: S-1087-T5-DE

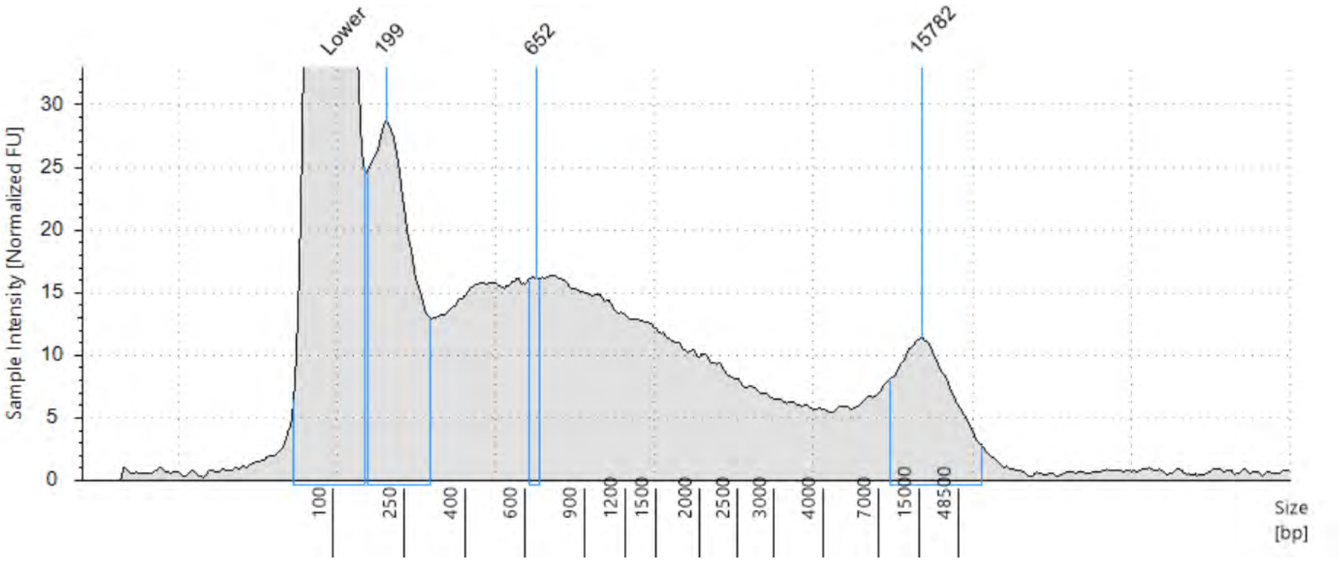

Sample Table

Well	DIN	Conc. [ng/µl]	Sample Description	Alert	Observations
G10	1.3	4.59	S-1087-T5-DE	⚠	Sample concentration outside functional range for DIN

Peak Table

Size [bp]	Calibrated Conc. [ng/µl]	Assigned Conc. [ng/µl]	% Integrated Area	From [bp]	To [bp]	Peak Comment	Observations
100	8.50	8.50	-	60	150		Lower Marker
199	0.914	-	62.07	155	308		
652	0.110	-	7.48	623	664		
15782	0.448	-	30.45	8798	>60000		

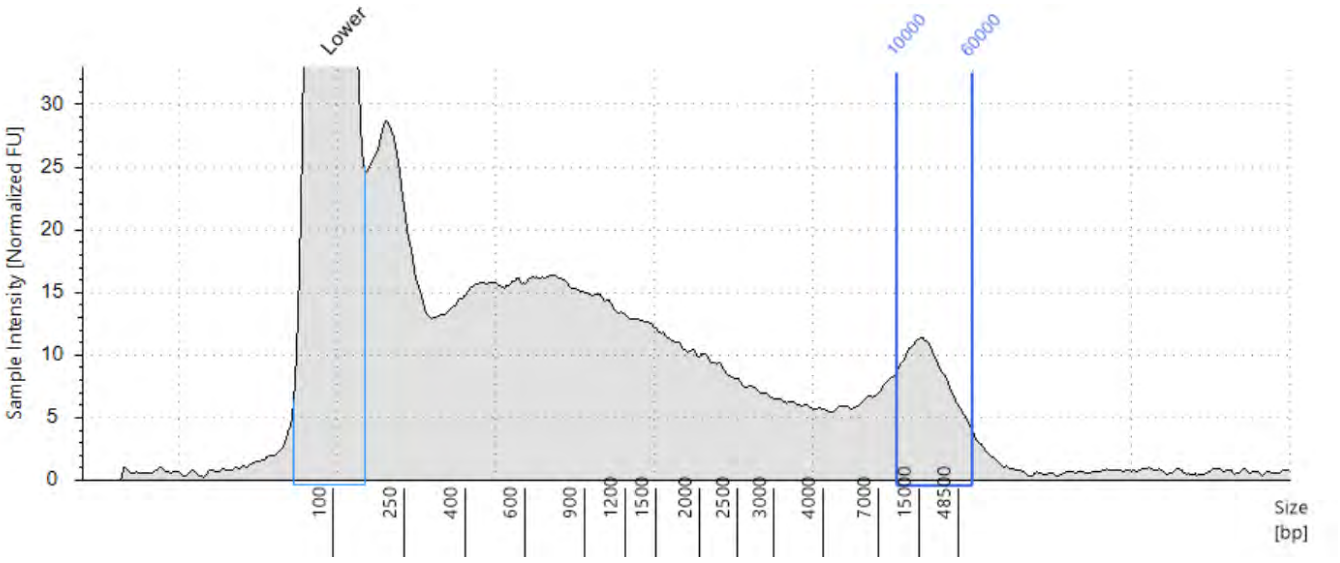

Region Table

From [bp]	To [bp]	Average Size [bp]	Conc. [ng/µl]	Region Molarity [nmol/l]	% of Total	Region Comment	Color
10000	60000	22893	0.391	0.0361	8.51		■

H10: S-1088-T5-DE

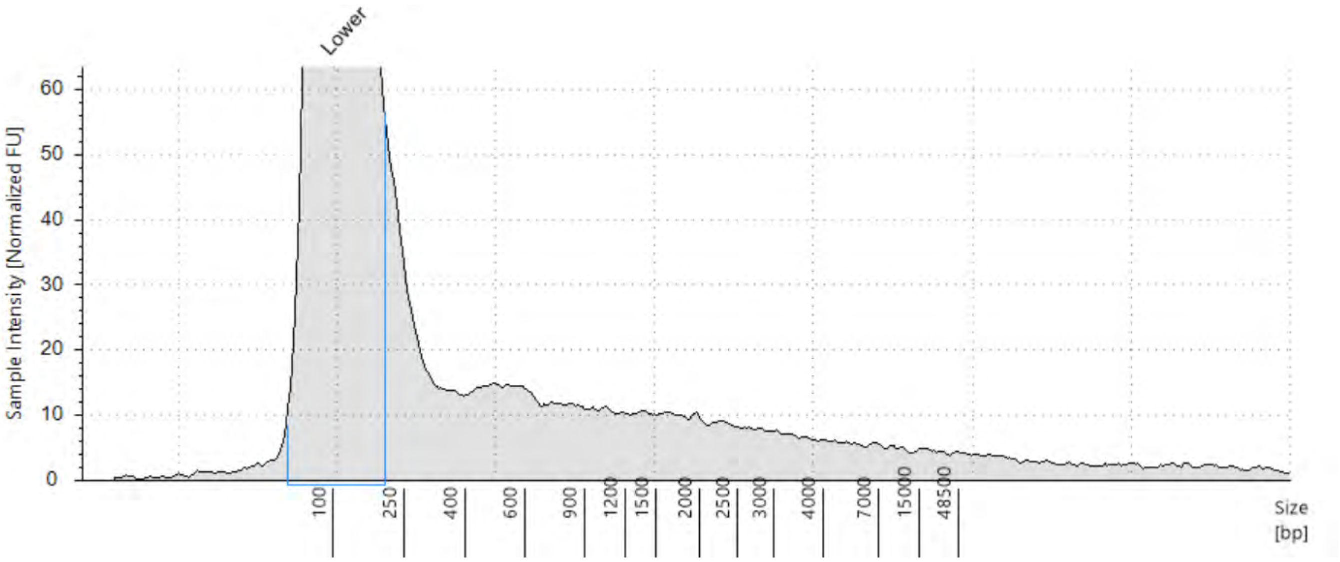

Sample Table

Well	DIN	Conc. [ng/µl]	Sample Description	Alert	Observations
H10	-	2.81	S-1088-T5-DE	▲	Sample concentration outside functional range for DIN

Peak Table

Size [bp]	Calibrated Conc. [ng/µl]	Assigned Conc. [ng/µl]	% Integrated Area	From [bp]	To [bp]	Peak Comment	Observations
100	8.50	8.50	-	55	196		Lower Marker

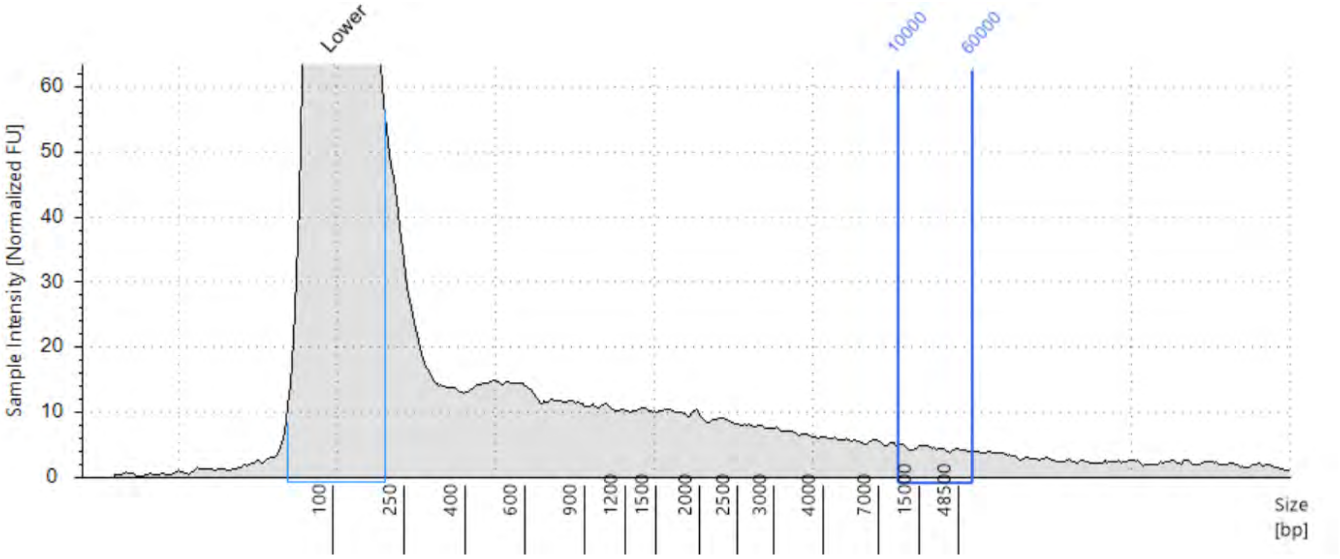

Region Table

From [bp]	To [bp]	Average Size [bp]	Conc. [ng/µl]	Region Molarity [nmol/l]	% of Total	Region Comment	Color
10000	60000	25761	0.117	0.0112	4.16		■

A11: S-1089-T5-DE

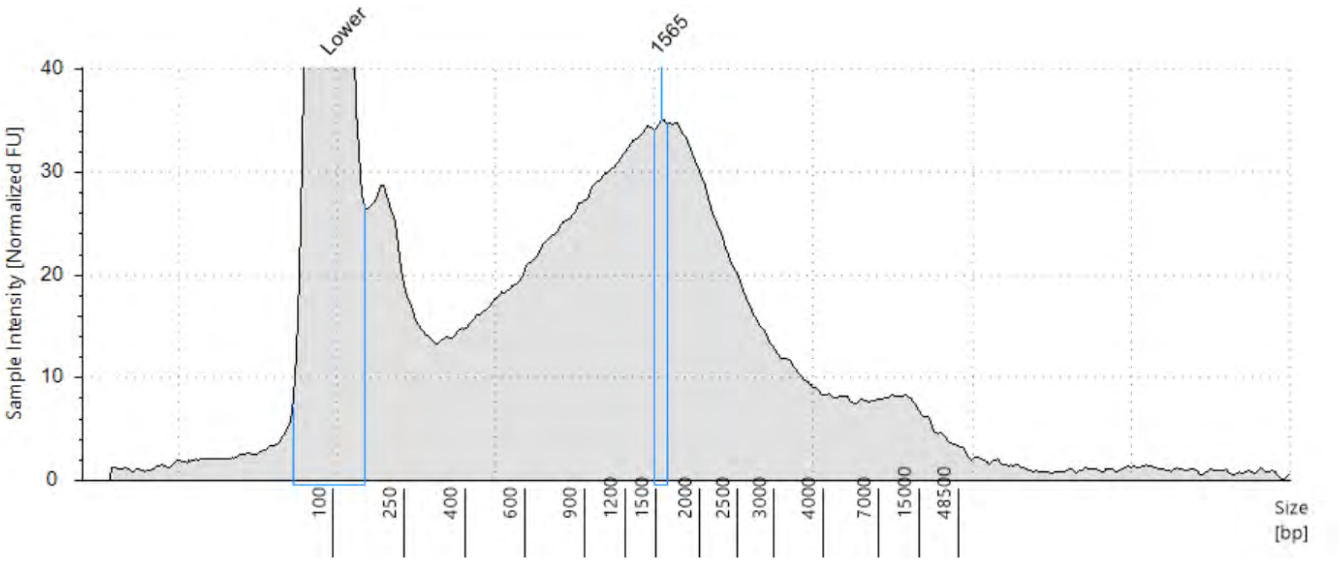

Sample Table

Well	DIN	Conc. [ng/µl]	Sample Description	Alert	Observations
A11	2.7	7.39	S-1089-T5-DE	⚠	Sample concentration outside recommended range

Peak Table

Size [bp]	Calibrated Conc. [ng/µl]	Assigned Conc. [ng/µl]	% Integrated Area	From [bp]	To [bp]	Peak Comment	Observations
100	8.50	8.50	-	59	150		Lower Marker
1565	0.333	-	100.00	1482	1622		

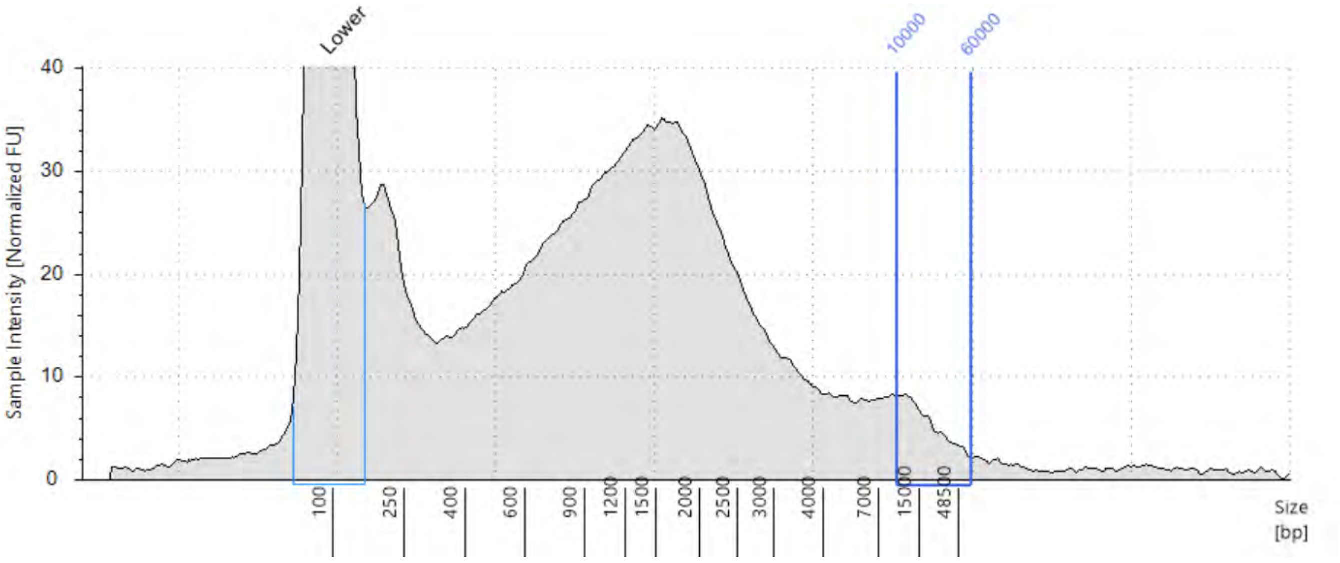

Region Table

From [bp]	To [bp]	Average Size [bp]	Conc. [ng/µl]	Region Molarity [nmol/l]	% of Total	Region Comment	Color
10000	60000	21512	0.237	0.0242	3.20		■

B11: S-1090-T5-DE

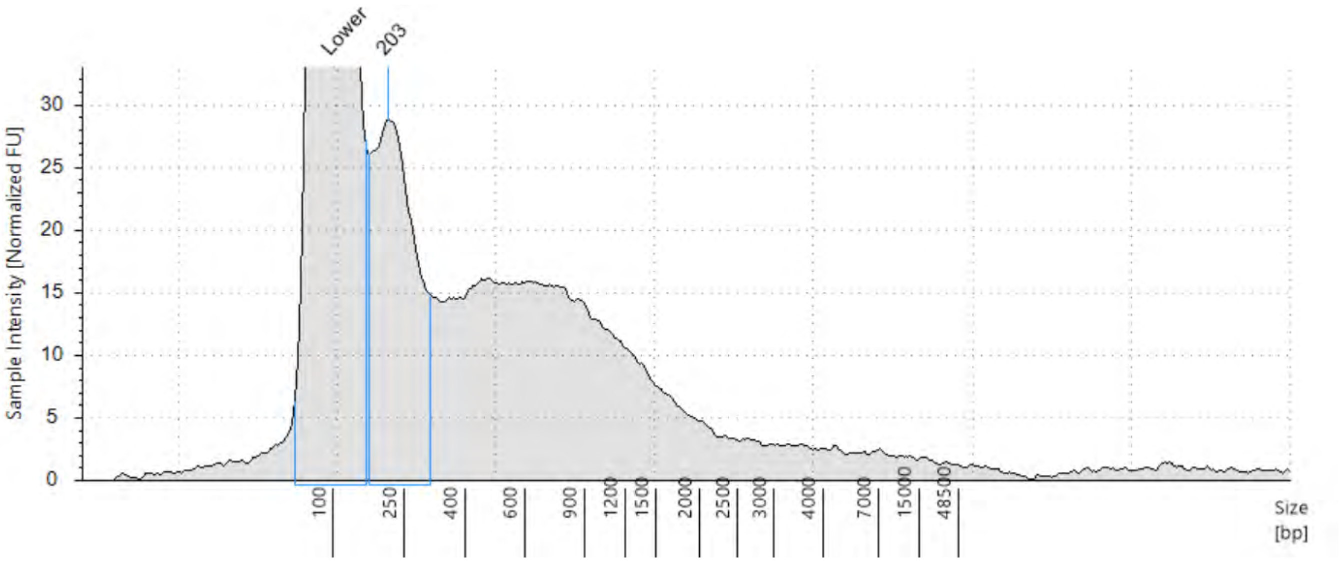

Sample Table

Well	DIN	Conc. [ng/µl]	Sample Description	Alert	Observations
B11	1.3	3.75	S-1090-T5-DE	⚠	Sample concentration outside functional range for DIN

Peak Table

Size [bp]	Calibrated Conc. [ng/µl]	Assigned Conc. [ng/µl]	% Integrated Area	From [bp]	To [bp]	Peak Comment	Observations
100	8.50	8.50	-	61	151		Lower Marker
203	0.967	-	100.00	160	307		

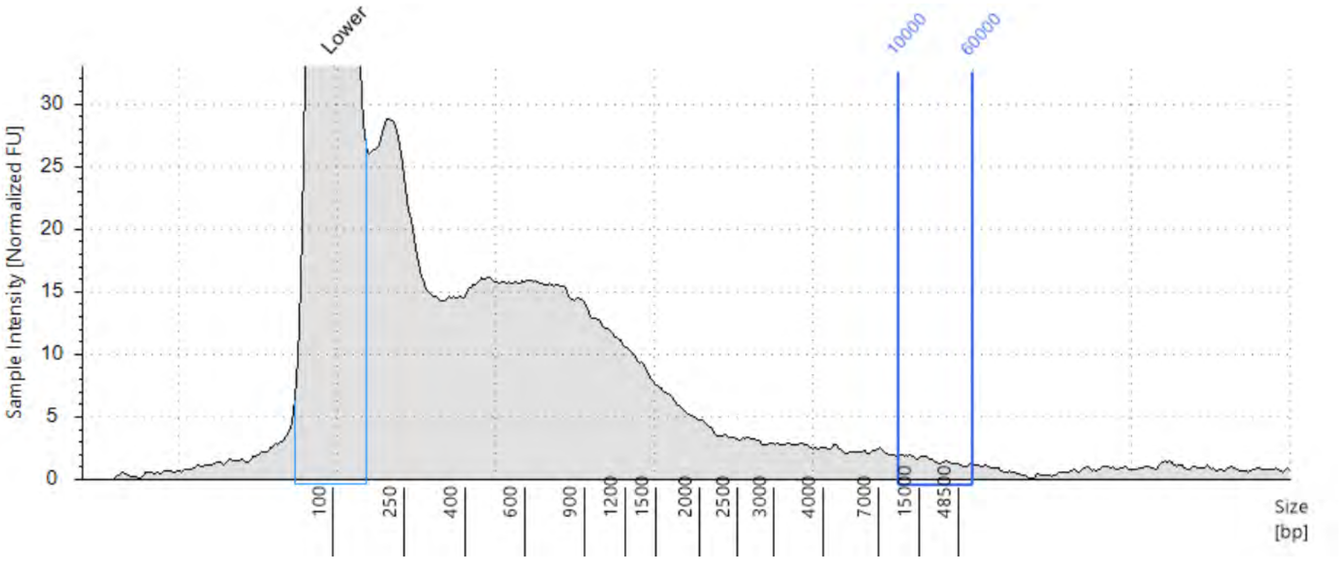

Region Table

From [bp]	To [bp]	Average Size [bp]	Conc. [ng/µl]	Region Molarity [nmol/l]	% of Total	Region Comment	Color
10000	60000	23882	0.0529	0.00654	1.41		■

 © Agilent Technologies, Inc. 2017

C11: S-866-T5-DSS

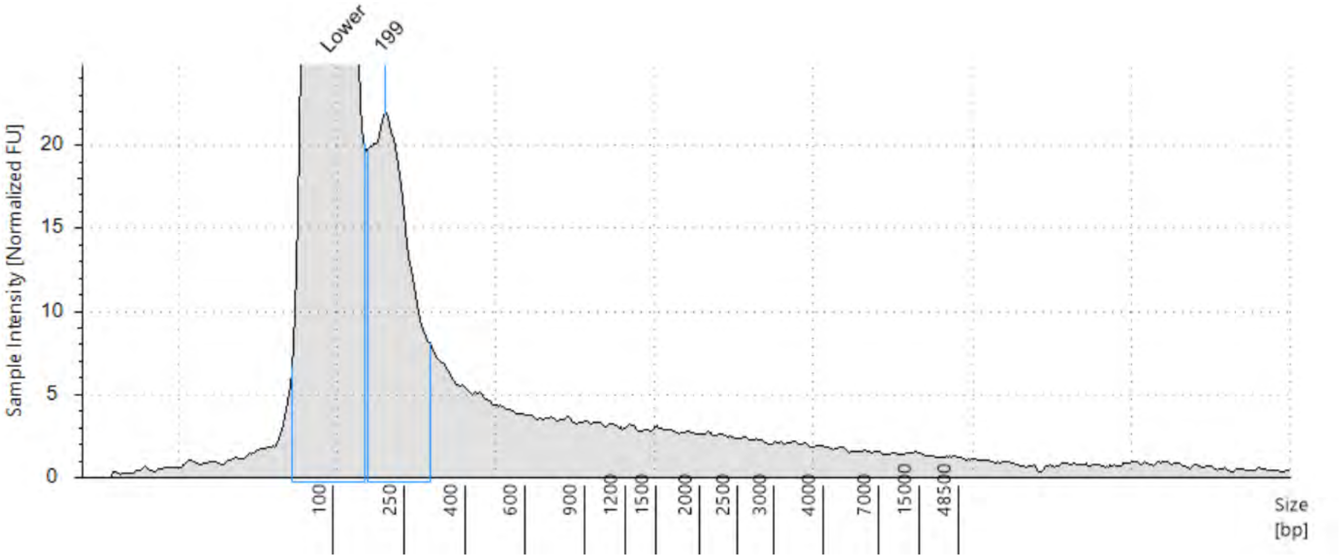

Sample Table

Well	DIN	Conc. [ng/µl]	Sample Description	Alert	Observations
C11	-	1.64	S-866-T5-DSS	▲	Sample concentration outside functional range for DIN

Peak Table

Size [bp]	Calibrated Conc. [ng/µl]	Assigned Conc. [ng/µl]	% Integrated Area	From [bp]	To [bp]	Peak Comment	Observations
100	8.50	8.50	-	59	150		Lower Marker
199	0.645	-	100.00	158	308		

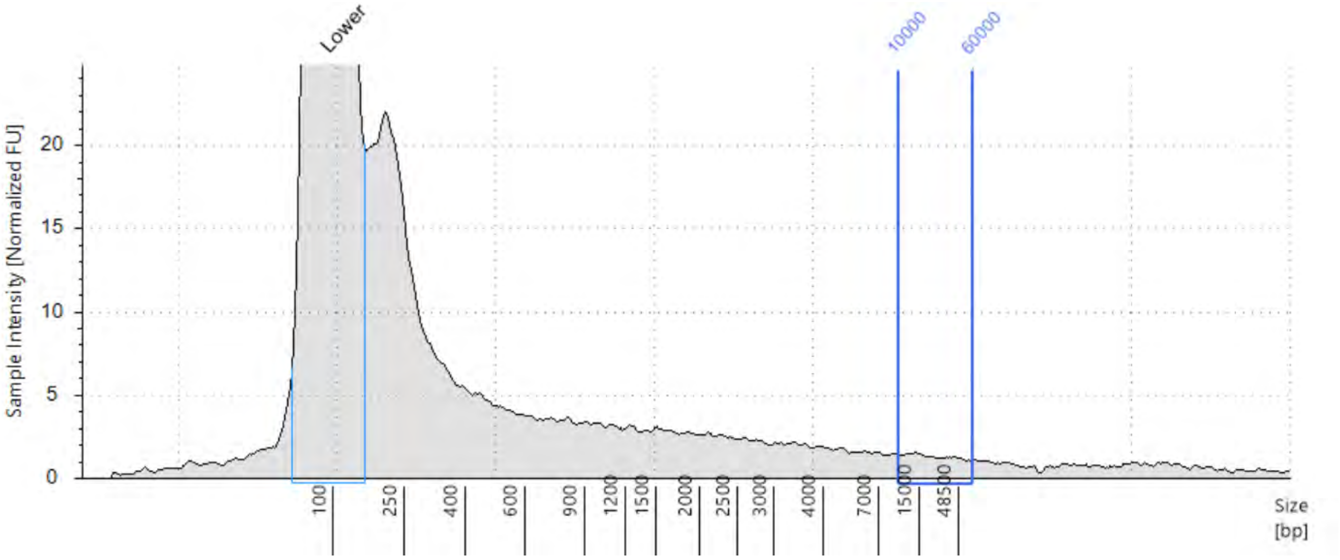

Region Table

From [bp]	To [bp]	Average Size [bp]	Conc. [ng/µl]	Region Molarity [nmol/l]	% of Total	Region Comment	Color
10000	60000	24957	0.0421	0.00510	2.57		■

D11: S-867-T5-DSS

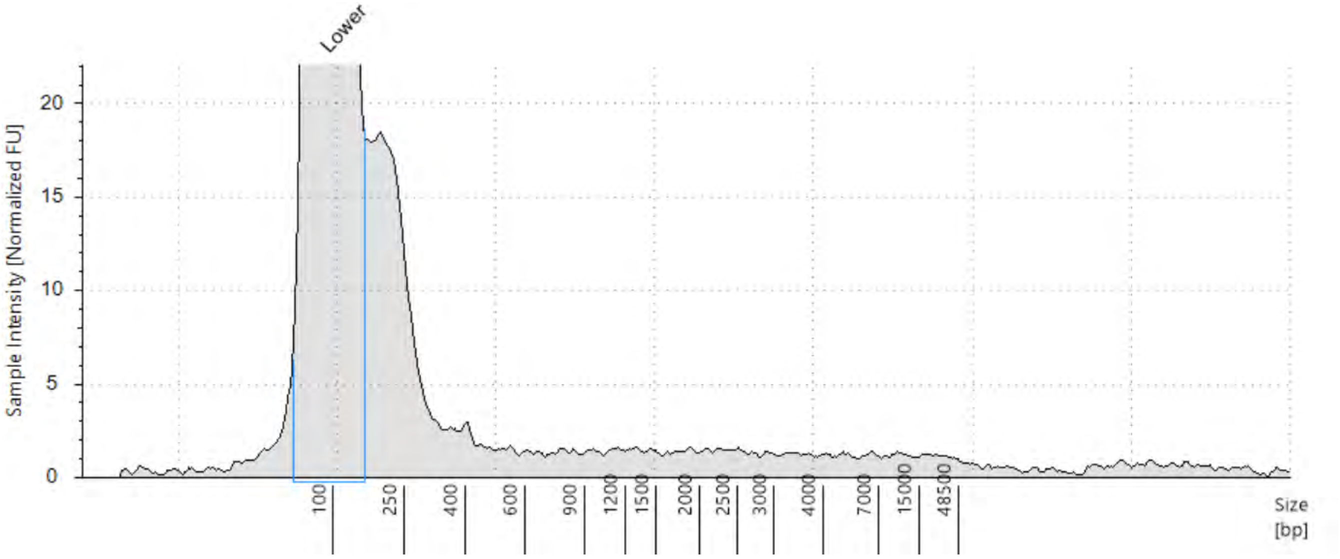

Sample Table

Well	DIN	Conc. [ng/µl]	Sample Description	Alert	Observations
D11	-	0.888	S-867-T5-DSS	▲	Sample concentration outside functional range for DIN

Peak Table

Size [bp]	Calibrated Conc. [ng/µl]	Assigned Conc. [ng/µl]	% Integrated Area	From [bp]	To [bp]	Peak Comment	Observations
100	8.50	8.50	-	59	150		Lower Marker

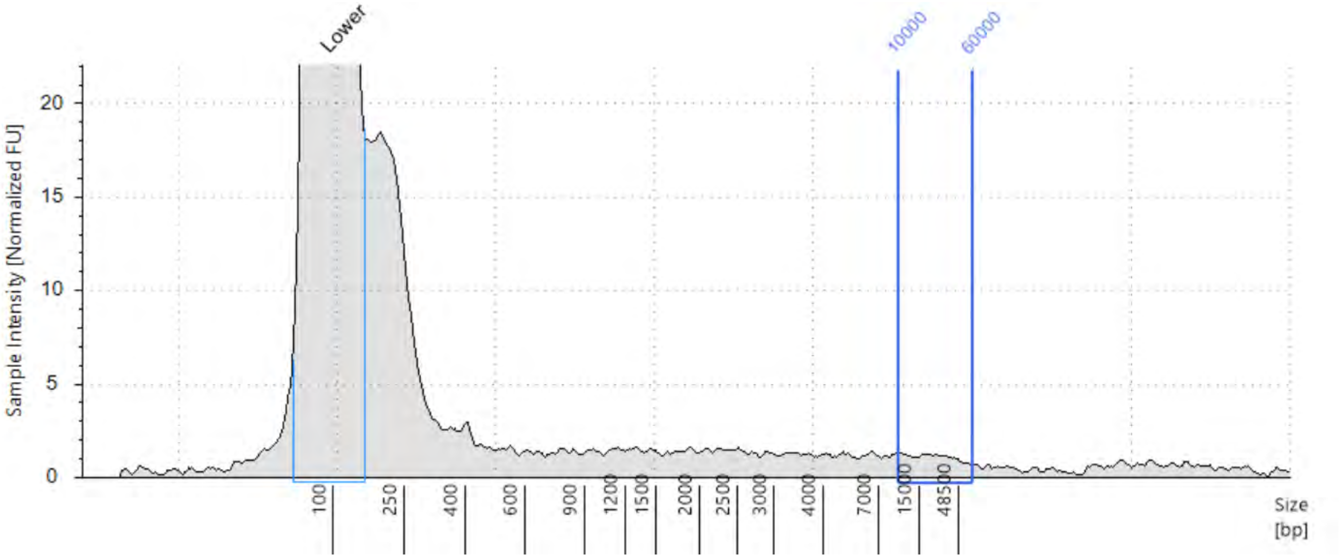

Region Table

From [bp]	To [bp]	Average Size [bp]	Conc. [ng/µl]	Region Molarity [nmol/l]	% of Total	Region Comment	Color
10000	60000	24173	0.0266	0.00431	3.00		■

 © Agilent Technologies, Inc. 2017

E11: S-868-T5-DSS

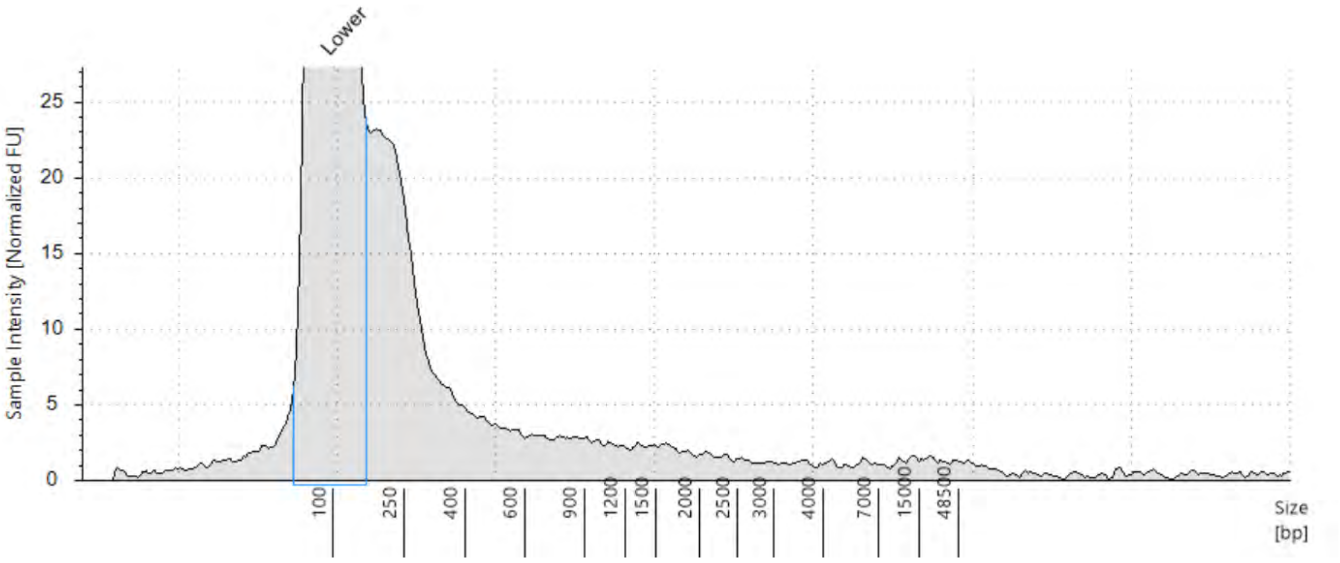

Sample Table

Well	DIN	Conc. [ng/µl]	Sample Description	Alert	Observations
E11	-	1.56	S-868-T5-DSS	▲	Sample concentration outside functional range for DIN

Peak Table

Size [bp]	Calibrated Conc. [ng/µl]	Assigned Conc. [ng/µl]	% Integrated Area	From [bp]	To [bp]	Peak Comment	Observations
100	8.50	8.50	-	60	153		Lower Marker

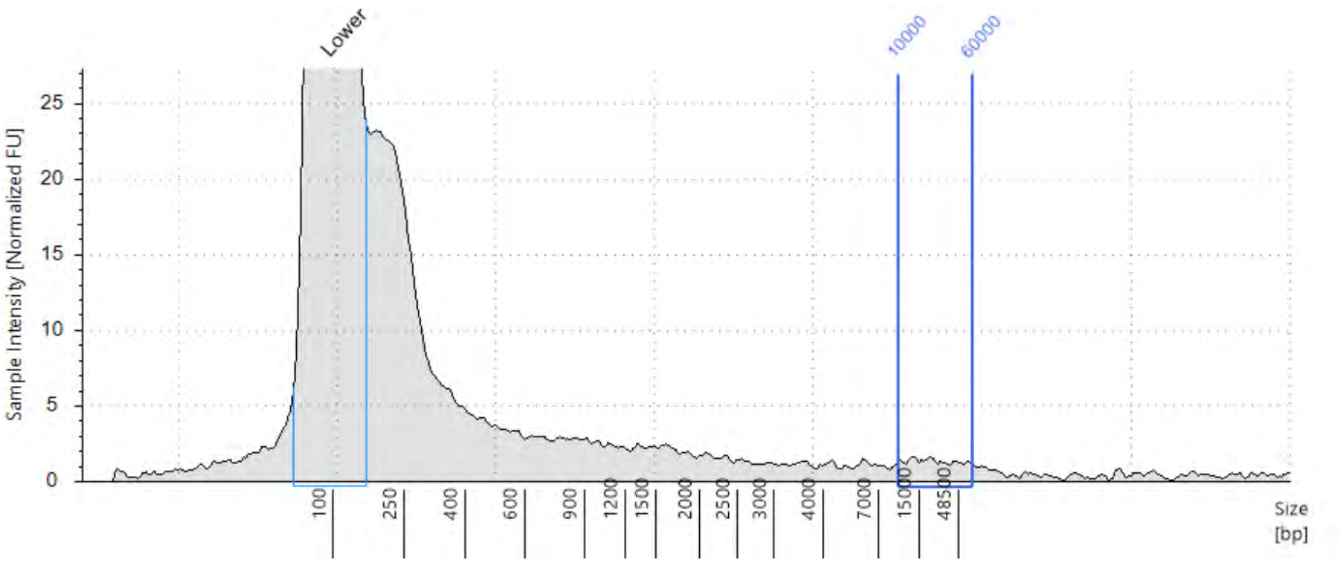

Region Table

From [bp]	To [bp]	Average Size [bp]	Conc. [ng/µl]	Region Molarity [nmol/l]	% of Total	Region Comment	Color
10000	60000	25900	0.0481	0.00522	3.10		■

F11: S-869-T5-DSS

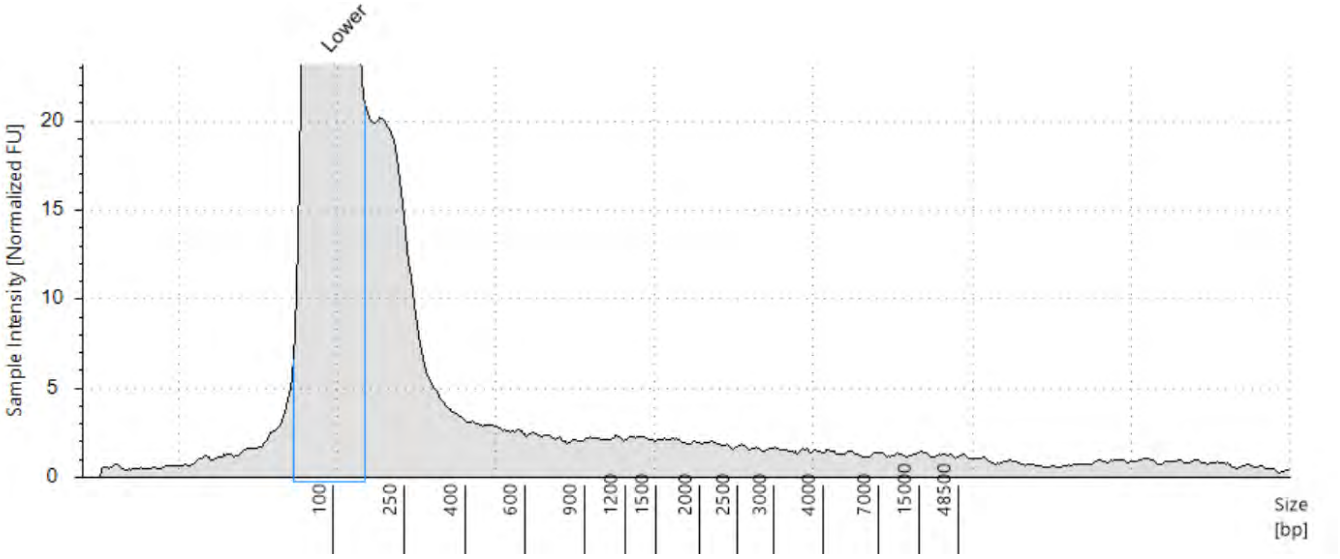

Sample Table

Well	DIN	Conc. [ng/µl]	Sample Description	Alert	Observations
F11	-	1.45	S-869-T5-DSS	▲	Sample concentration outside functional range for DIN

Peak Table

Size [bp]	Calibrated Conc. [ng/µl]	Assigned Conc. [ng/µl]	% Integrated Area	From [bp]	To [bp]	Peak Comment	Observations
100	8.50	8.50	-	60	151		Lower Marker

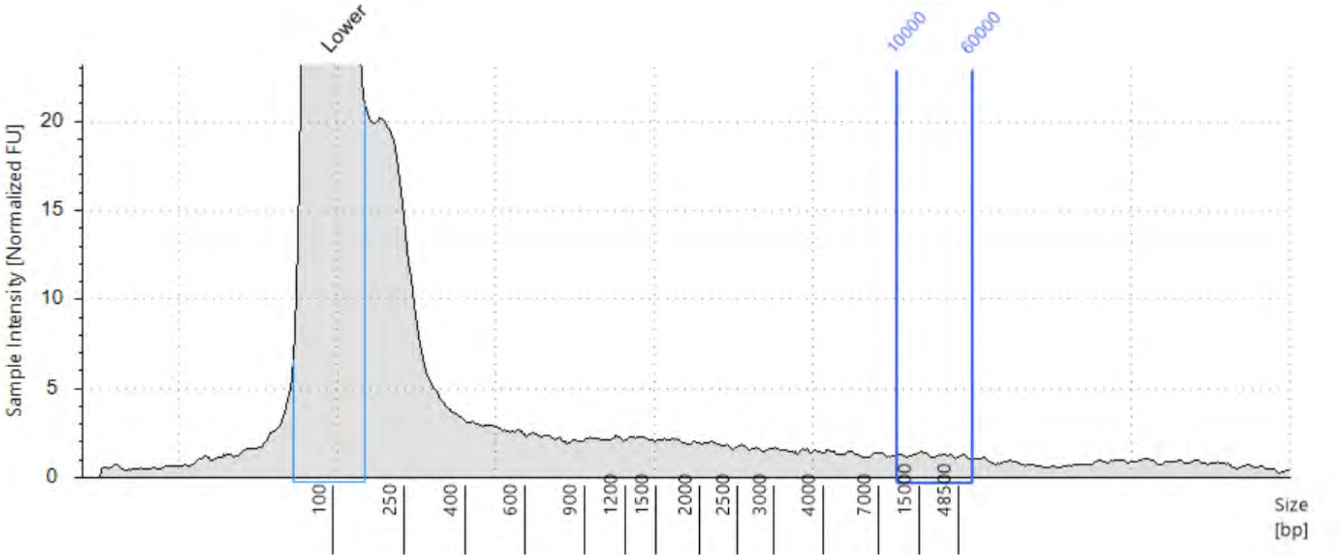

Region Table

From [bp]	To [bp]	Average Size [bp]	Conc. [ng/µl]	Region Molarity [nmol/l]	% of Total	Region Comment	Color
10000	60000	25811	0.0451	0.00486	3.12		■

G11: S-870-T5-DSS

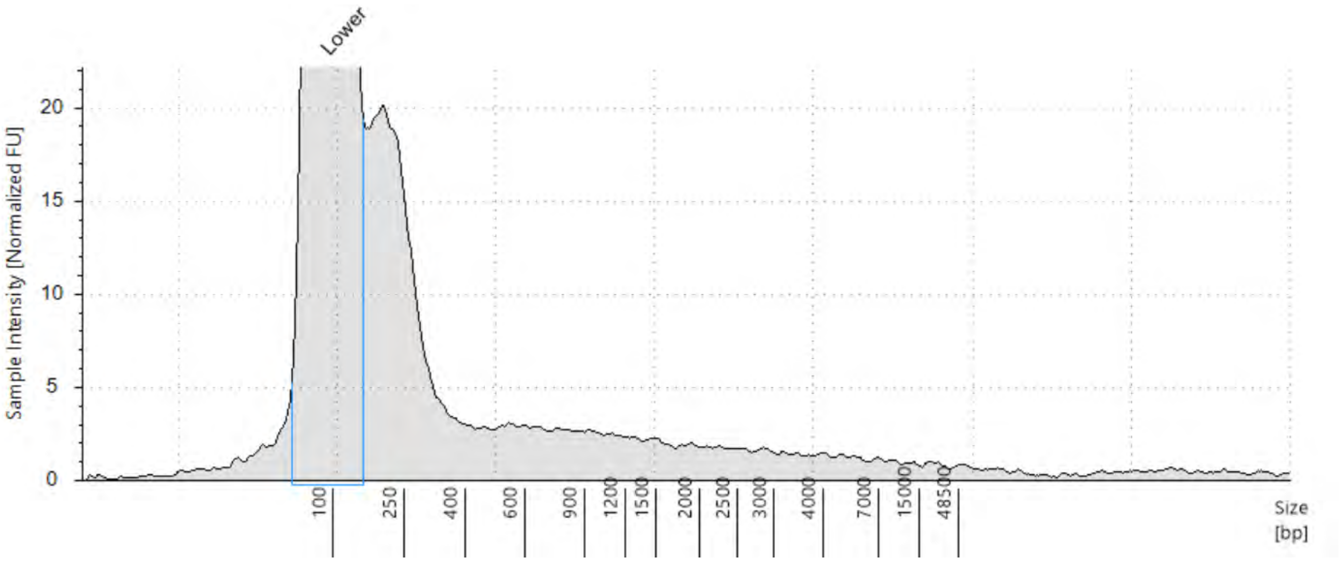

Sample Table

Well	DIN	Conc. [ng/µl]	Sample Description	Alert	Observations
G11	-	1.32	S-870-T5-DSS	▲	Sample concentration outside functional range for DIN

Peak Table

Size [bp]	Calibrated Conc. [ng/µl]	Assigned Conc. [ng/µl]	% Integrated Area	From [bp]	To [bp]	Peak Comment	Observations
100	8.50	8.50	-	58	148		Lower Marker

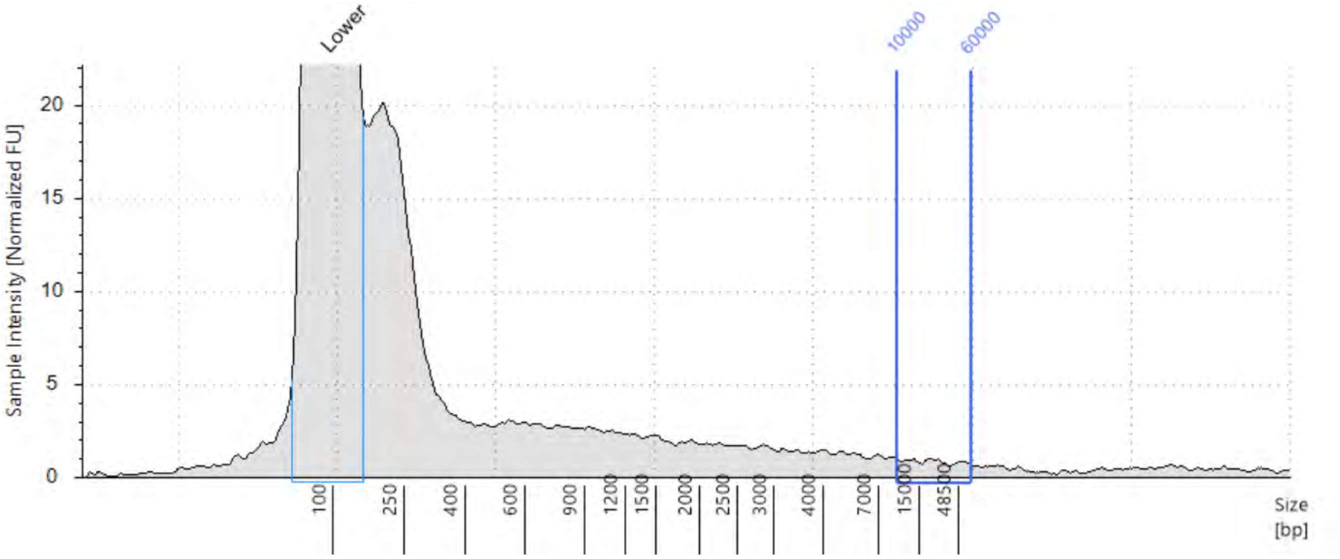

Region Table

From [bp]	To [bp]	Average Size [bp]	Conc. [ng/µl]	Region Molarity [nmol/l]	% of Total	Region Comment	Color
10000	60000	25511	0.0254	0.00328	1.93		■

 © Agilent Technologies, Inc. 2017

H11: S-1091-T5-DSS

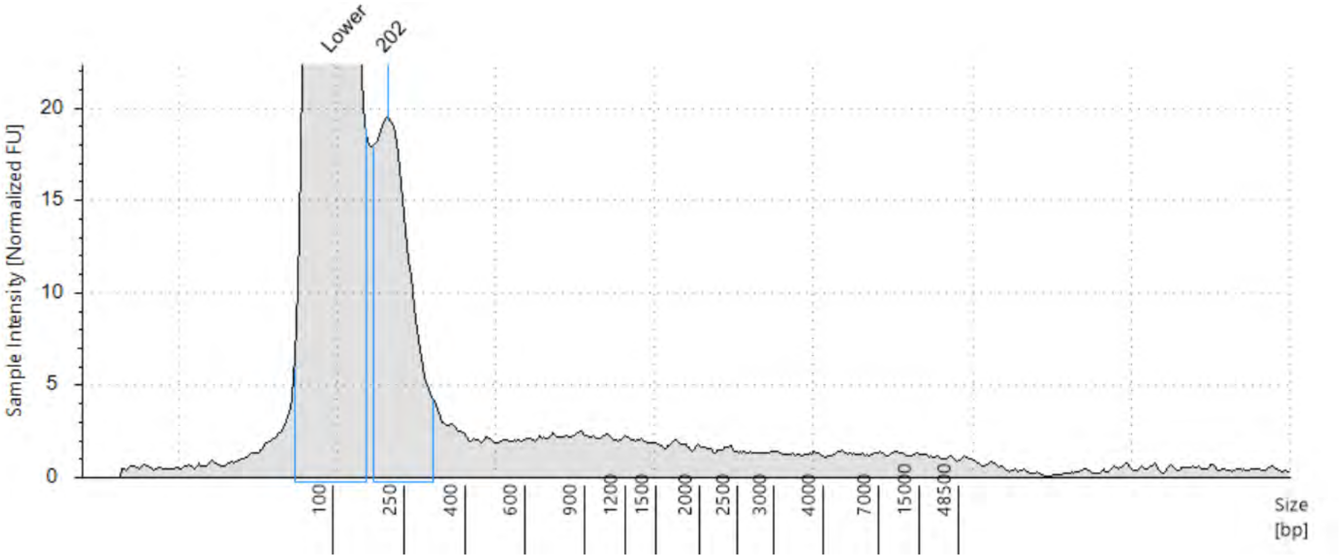

Sample Table

Well	DIN	Conc. [ng/µl]	Sample Description	Alert	Observations
H11	-	1.15	S-1091-T5-DSS	▲	Sample concentration outside functional range for DIN

Peak Table

Size [bp]	Calibrated Conc. [ng/µl]	Assigned Conc. [ng/µl]	% Integrated Area	From [bp]	To [bp]	Peak Comment	Observations
100	8.50	8.50	-	60	152		Lower Marker
202	0.533	-	100.00	166	314		

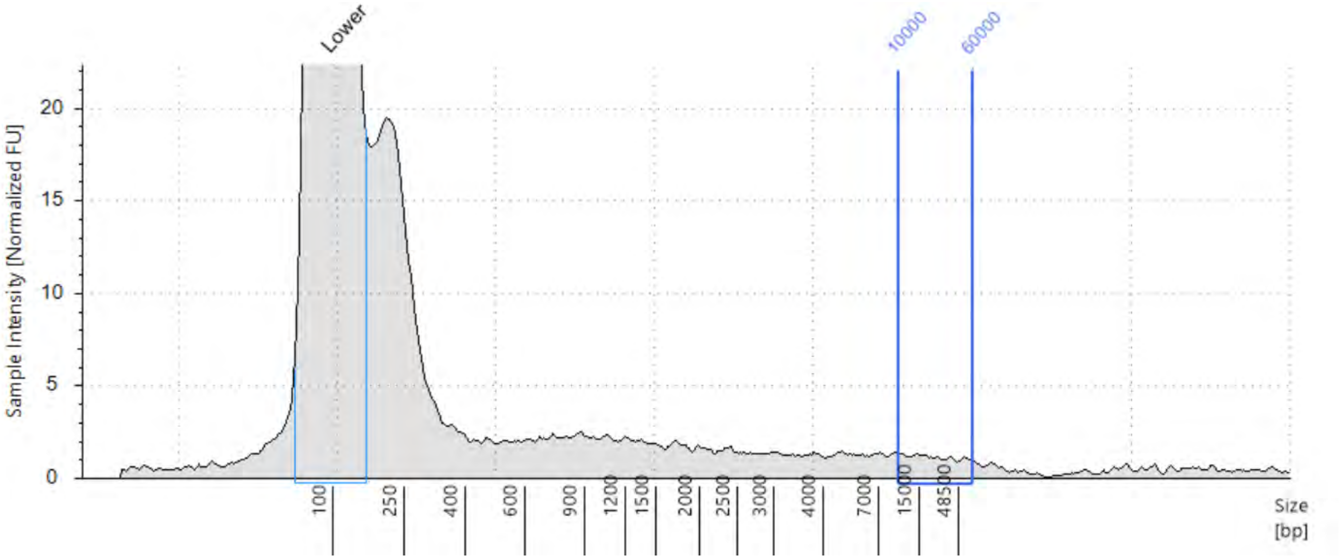

Region Table

From [bp]	To [bp]	Average Size [bp]	Conc. [ng/µl]	Region Molarity [nmol/l]	% of Total	Region Comment	Color
10000	60000	25484	0.0363	0.00454	3.17		■

 © Agilent Technologies, Inc. 2017

A12: S-1092-T5-DSS

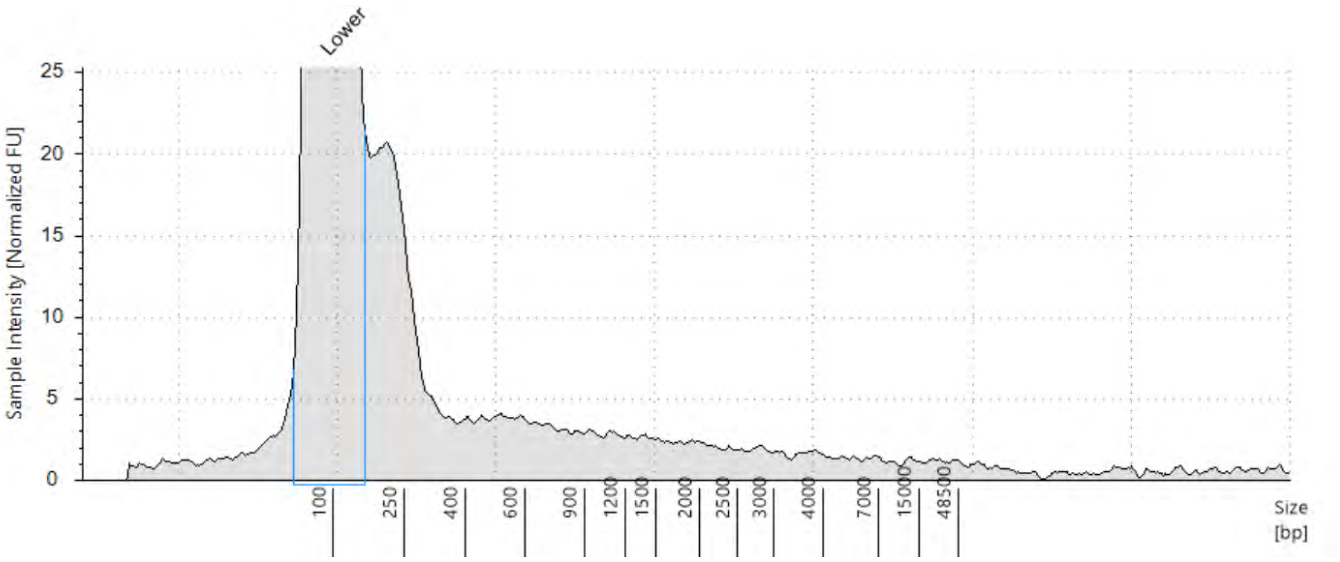

Sample Table

Well	DIN	Conc. [ng/µl]	Sample Description	Alert	Observations
A12	-	1.49	S-1092-T5-DSS	▲	Sample concentration outside functional range for DIN

Peak Table

Size [bp]	Calibrated Conc. [ng/µl]	Assigned Conc. [ng/µl]	% Integrated Area	From [bp]	To [bp]	Peak Comment	Observations
100	8.50	8.50	-	60	152		Lower Marker

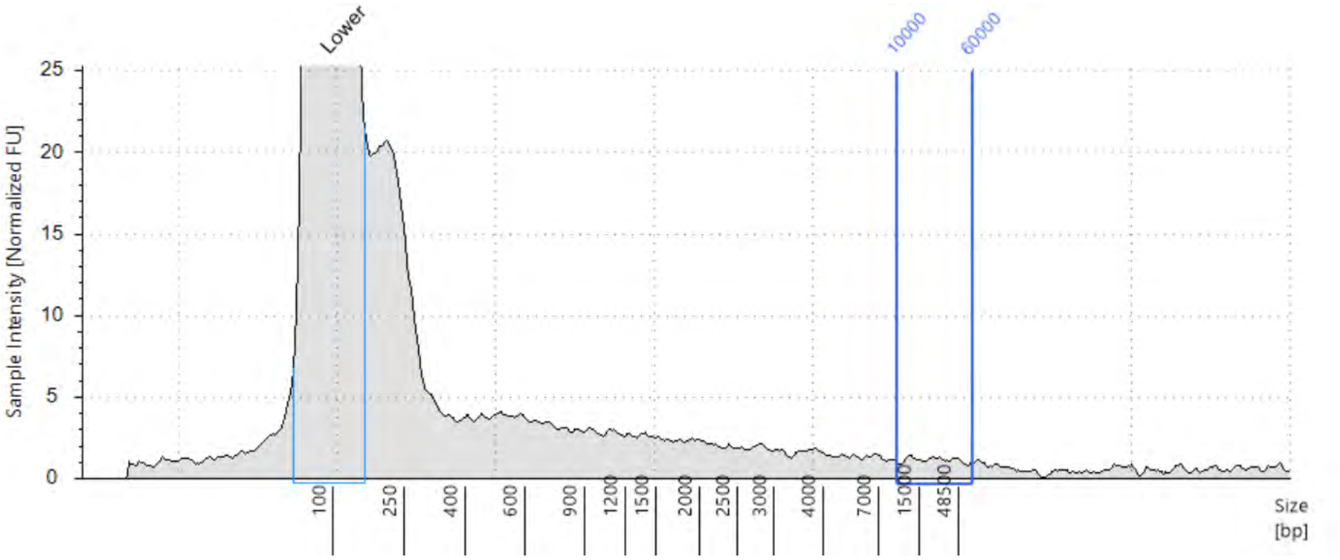

Region Table

From [bp]	To [bp]	Average Size [bp]	Conc. [ng/µl]	Region Molarity [nmol/l]	% of Total	Region Comment	Color
10000	60000	25841	0.0358	0.00424	2.40		■

 © Agilent Technologies, Inc. 2017

B12: S-1093-T5-DSS

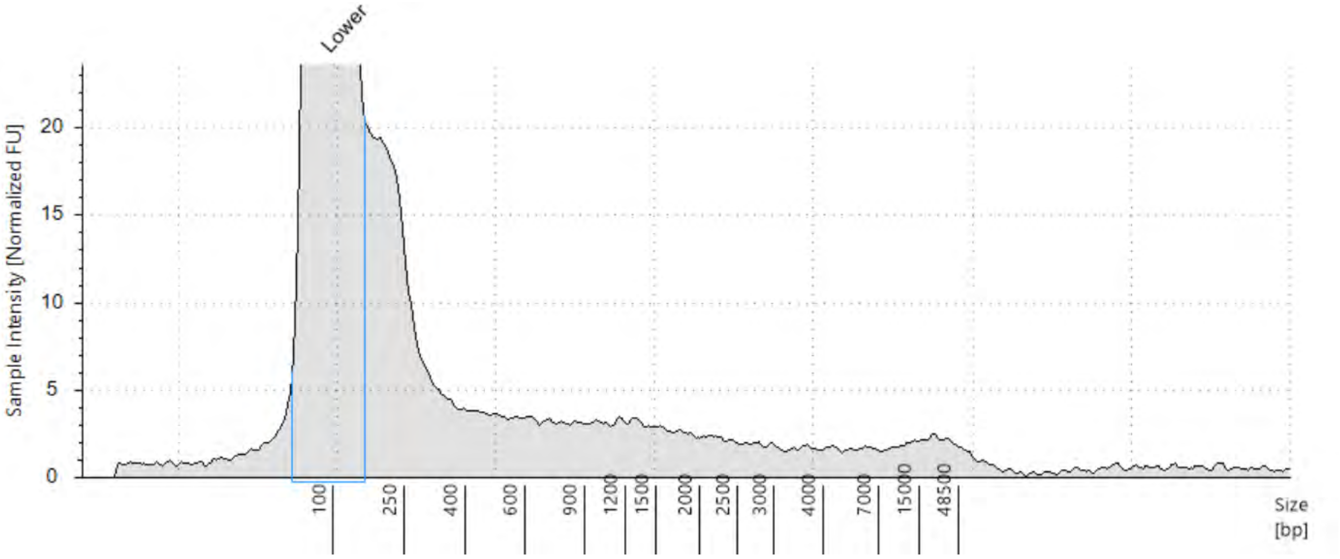

Sample Table

Well	DIN	Conc. [ng/µl]	Sample Description	Alert	Observations
B12	-	1.51	S-1093-T5-DSS	▲	Sample concentration outside functional range for DIN

Peak Table

Size [bp]	Calibrated Conc. [ng/µl]	Assigned Conc. [ng/µl]	% Integrated Area	From [bp]	To [bp]	Peak Comment	Observations
100	8.50	8.50	-	58	149		Lower Marker

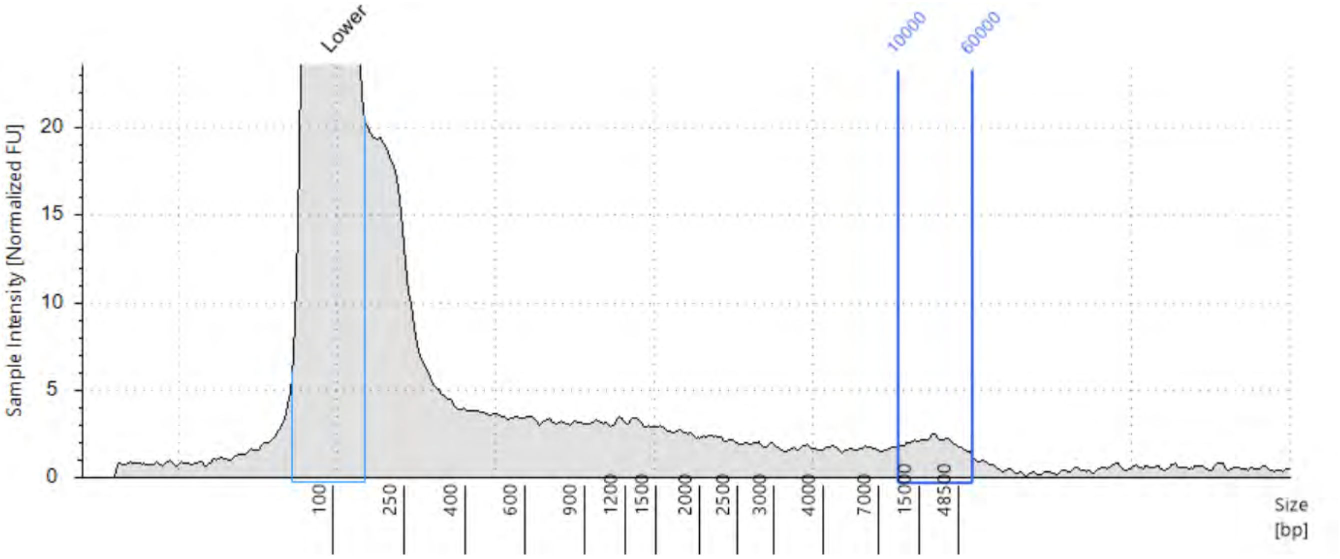

Region Table

From [bp]	To [bp]	Average Size [bp]	Conc. [ng/µl]	Region Molarity [nmol/l]	% of Total	Region Comment	Color
10000	60000	25100	0.0785	0.00784	5.21		■

C12: S-1094-T5-DSS

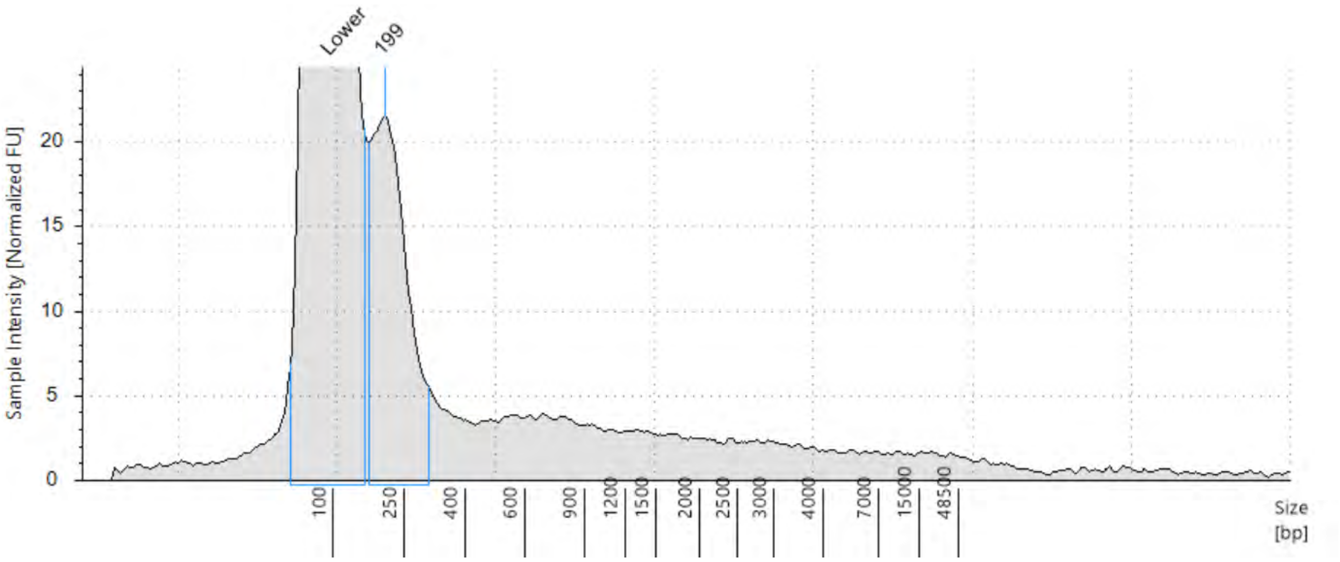

Sample Table

Well	DIN	Conc. [ng/µl]	Sample Description	Alert	Observations
C12	-	1.57	S-1094-T5-DSS	▲	Sample concentration outside functional range for DIN

Peak Table

Size [bp]	Calibrated Conc. [ng/µl]	Assigned Conc. [ng/µl]	% Integrated Area	From [bp]	To [bp]	Peak Comment	Observations
100	8.50	8.50	-	58	150		Lower Marker
199	0.568	-	100.00	161	305		

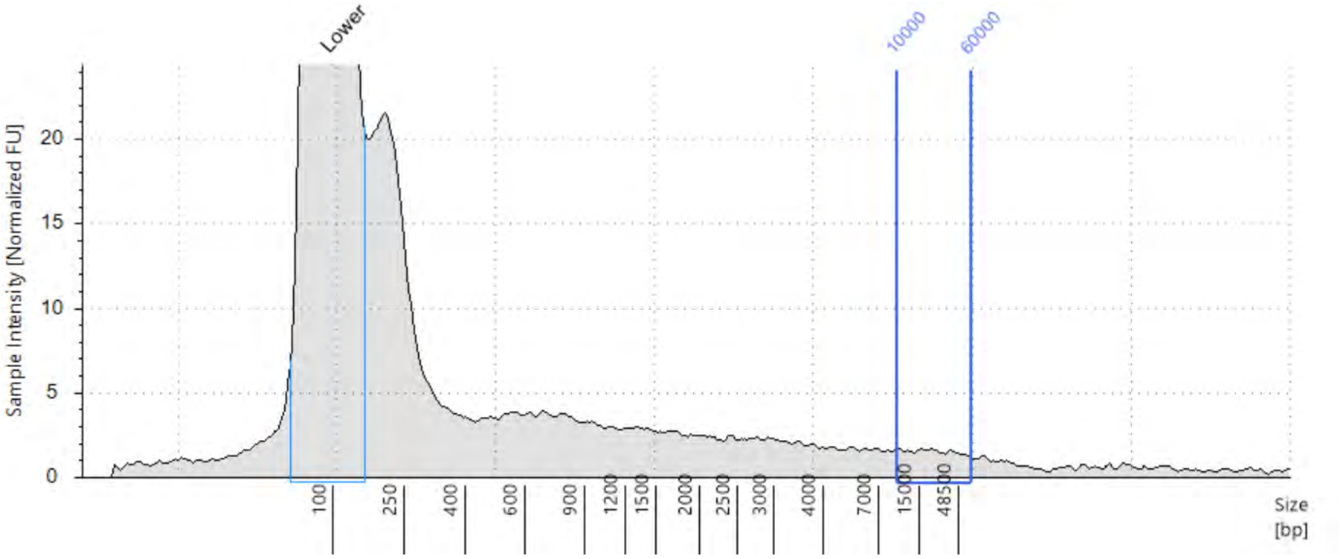

Region Table

From [bp]	To [bp]	Average Size [bp]	Conc. [ng/µl]	Region Molarity [nmol/l]	% of Total	Region Comment	Color
10000	60000	25614	0.0566	0.00575	3.62		■

 © Agilent Technologies, Inc. 2017

D12: S-1095-T5-DSS

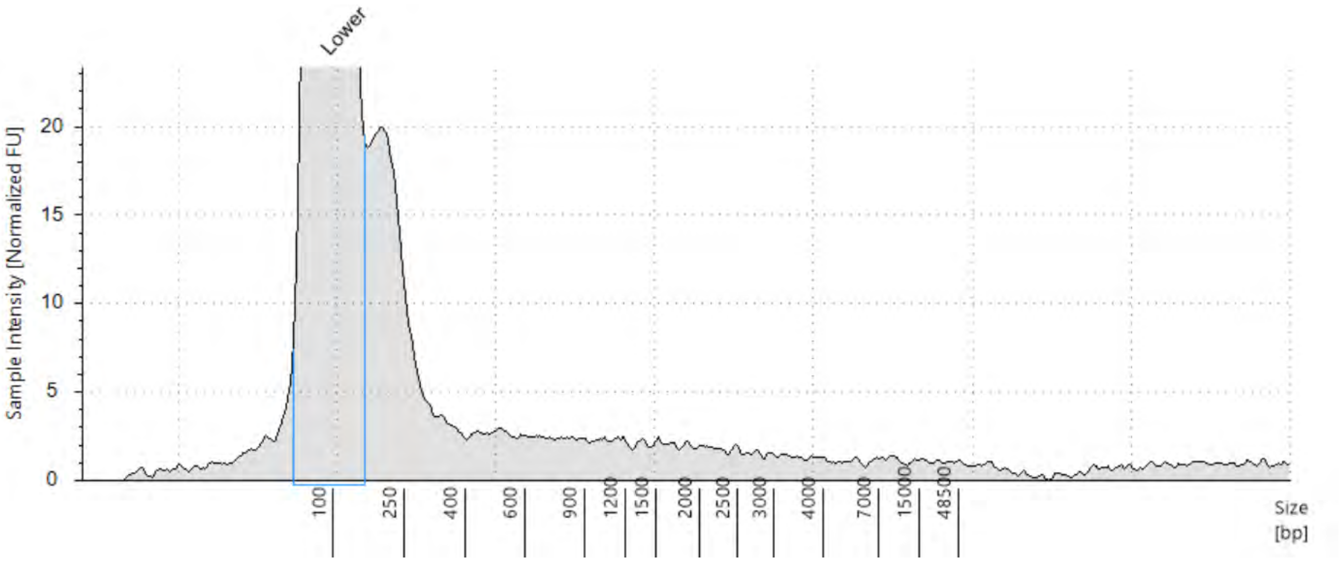

Sample Table

Well	DIN	Conc. [ng/µl]	Sample Description	Alert	Observations
D12	-	1.28	S-1095-T5-DSS	▲	Sample concentration outside functional range for DIN

Peak Table

Size [bp]	Calibrated Conc. [ng/µl]	Assigned Conc. [ng/µl]	% Integrated Area	From [bp]	To [bp]	Peak Comment	Observations
100	8.50	8.50	-	59	149		Lower Marker

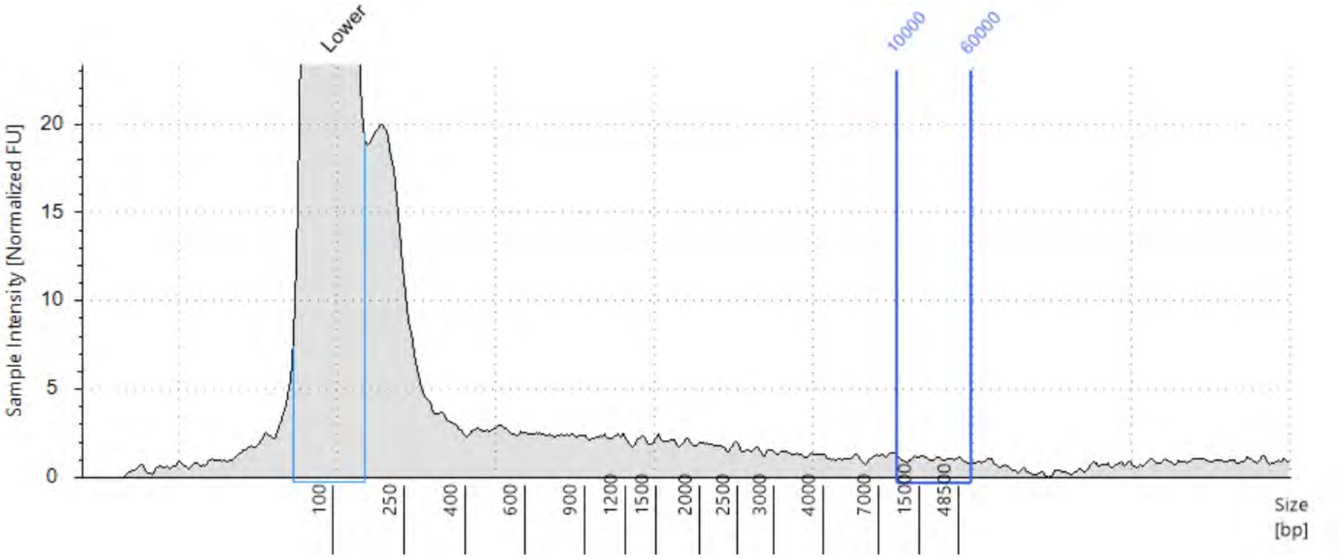

Region Table

From [bp]	To [bp]	Average Size [bp]	Conc. [ng/µl]	Region Molarity [nmol/l]	% of Total	Region Comment	Color
10000	60000	25913	0.0300	0.00390	2.35		■

E12: S-881-T5-E

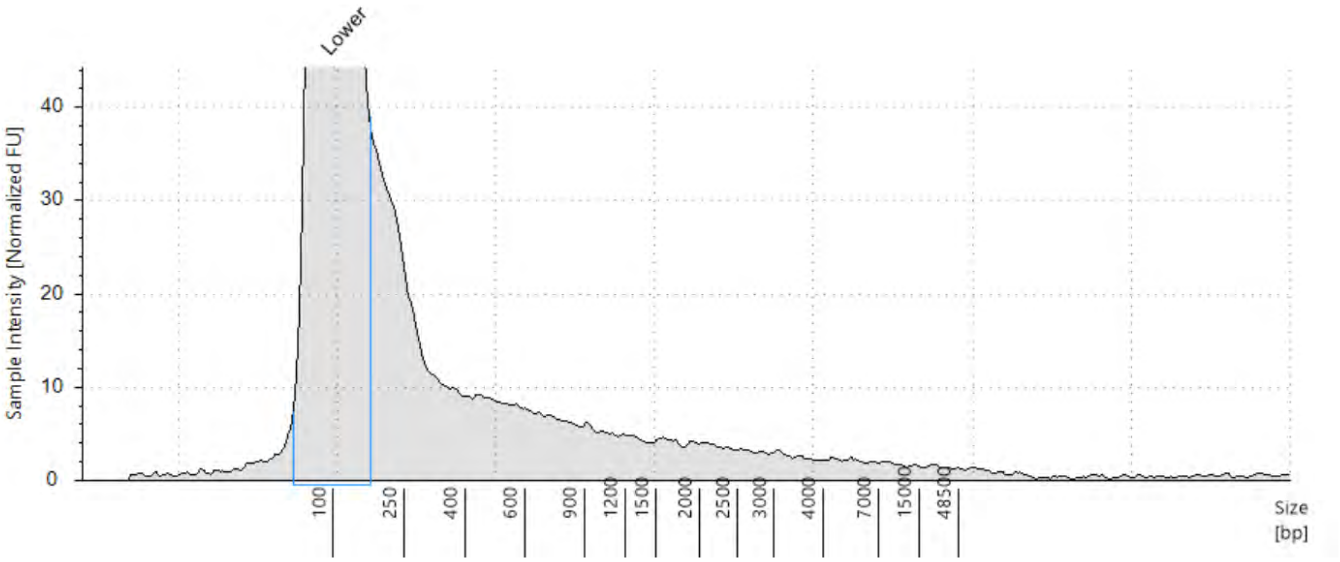

Sample Table

Well	DIN	Conc. [ng/µl]	Sample Description	Alert	Observations
E12	-	2.13	S-881-T5-E	▲	Sample concentration outside functional range for DIN

Peak Table

Size [bp]	Calibrated Conc. [ng/µl]	Assigned Conc. [ng/µl]	% Integrated Area	From [bp]	To [bp]	Peak Comment	Observations
100	8.50	8.50	-	59	160		Lower Marker

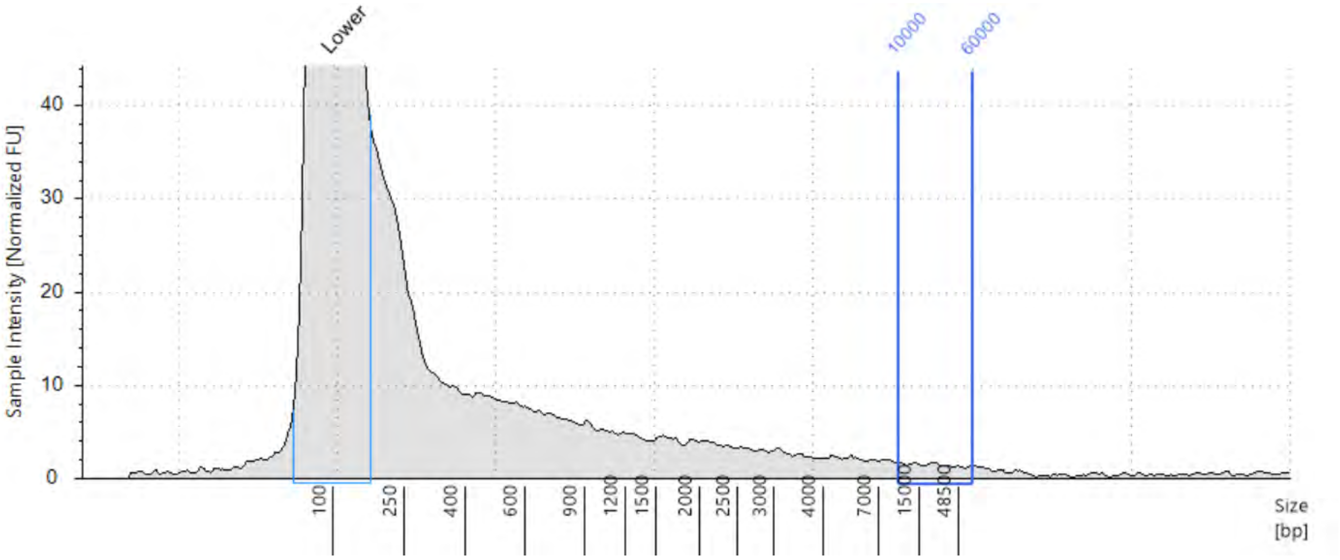

Region Table

From [bp]	To [bp]	Average Size [bp]	Conc. [ng/µl]	Region Molarity [nmol/l]	% of Total	Region Comment	Color
10000	60000	25691	0.0382	0.00499	1.79		■

F12: S-882-T5-E

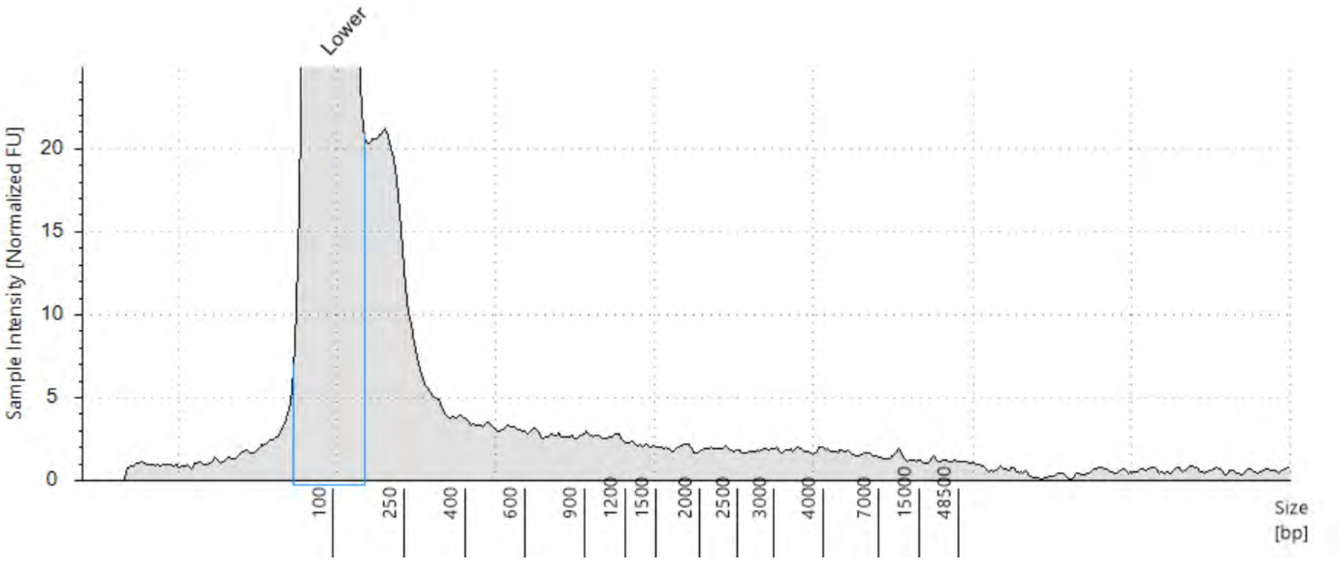

Sample Table

Well	DIN	Conc. [ng/µl]	Sample Description	Alert	Observations
F12	-	1.47	S-882-T5-E	▲	Sample concentration outside functional range for DIN

Peak Table

Size [bp]	Calibrated Conc. [ng/µl]	Assigned Conc. [ng/µl]	% Integrated Area	From [bp]	To [bp]	Peak Comment	Observations
100	8.50	8.50	-	60	150		Lower Marker

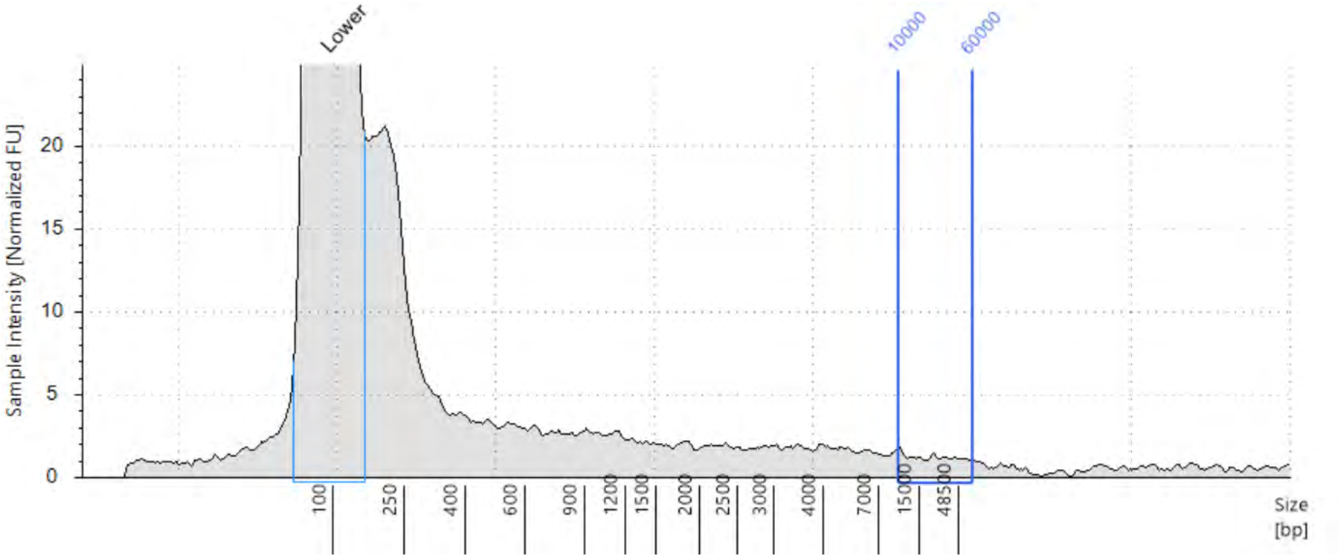

Region Table

From [bp]	To [bp]	Average Size [bp]	Conc. [ng/µl]	Region Molarity [nmol/l]	% of Total	Region Comment	Color
10000	60000	25556	0.0425	0.00499	2.89		■

G12: S-883-T5-E

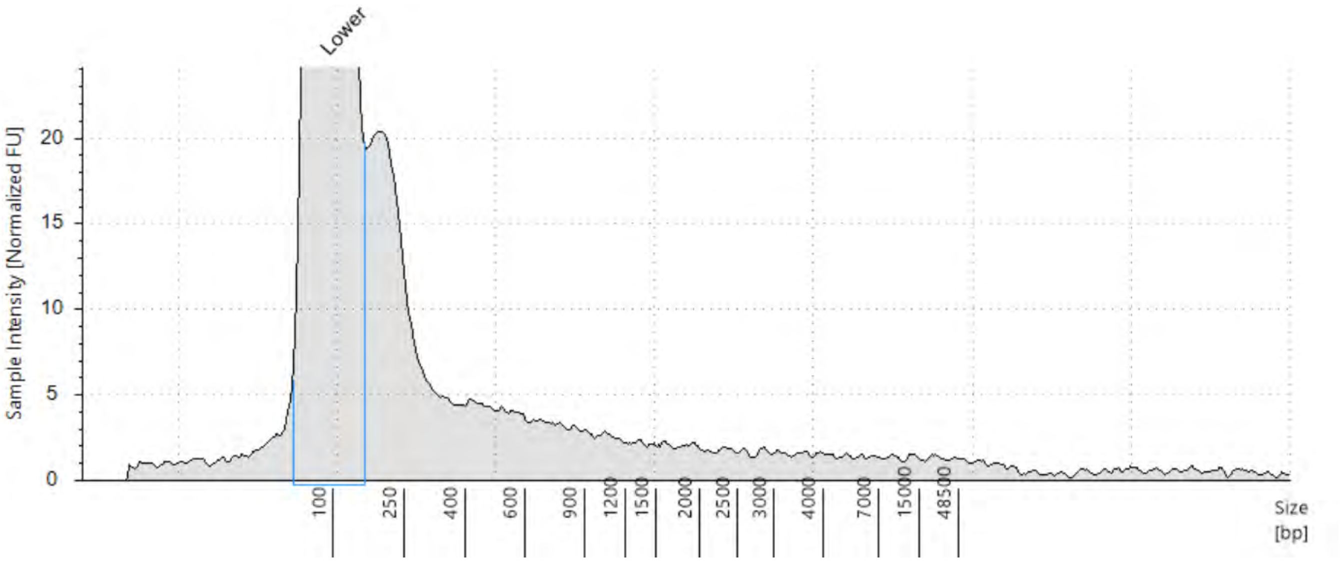

Sample Table

Well	DIN	Conc. [ng/µl]	Sample Description	Alert	Observations
G12	-	1.45	S-883-T5-E	▲	Sample concentration outside functional range for DIN

Peak Table

Size [bp]	Calibrated Conc. [ng/µl]	Assigned Conc. [ng/µl]	% Integrated Area	From [bp]	To [bp]	Peak Comment	Observations
100	8.50	8.50	-	59	150		Lower Marker

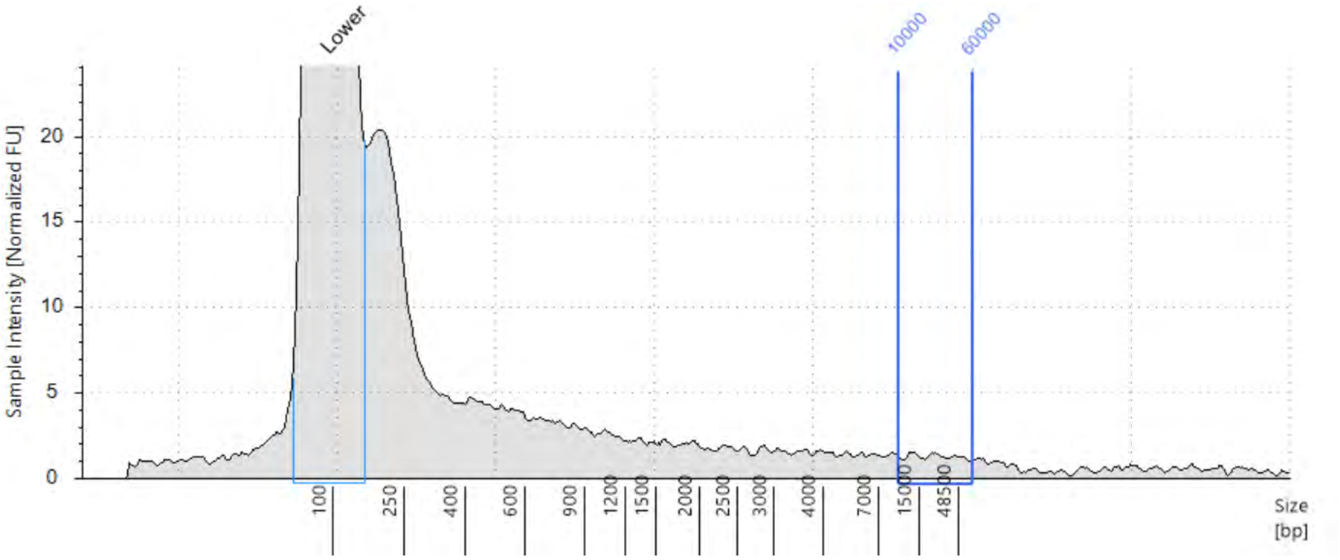

Region Table

From [bp]	To [bp]	Average Size [bp]	Conc. [ng/µl]	Region Molarity [nmol/l]	% of Total	Region Comment	Color
10000	60000	25627	0.0426	0.00503	2.94		

 © Agilent Technologies, Inc. 2017

H12: S-884-T5-E

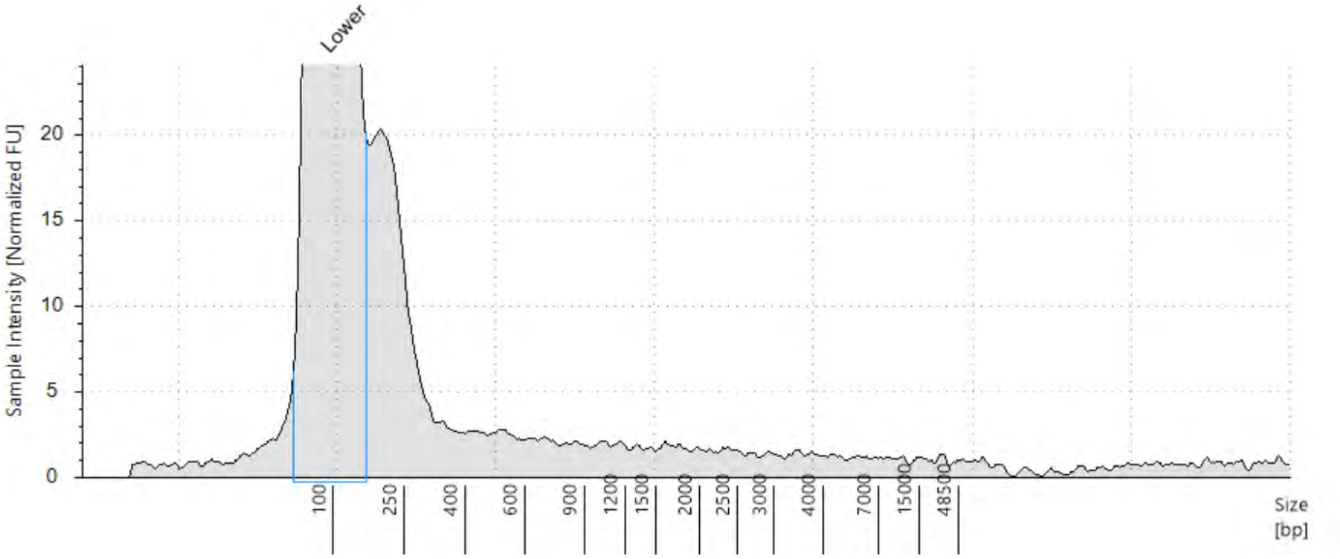

Sample Table

Well	DIN	Conc. [ng/µl]	Sample Description	Alert	Observations
H12	-	1.23	S-884-T5-E	▲	Sample concentration outside functional range for DIN

Peak Table

Size [bp]	Calibrated Conc. [ng/µl]	Assigned Conc. [ng/µl]	% Integrated Area	From [bp]	To [bp]	Peak Comment	Observations
100	8.50	8.50	-	60	153		Lower Marker

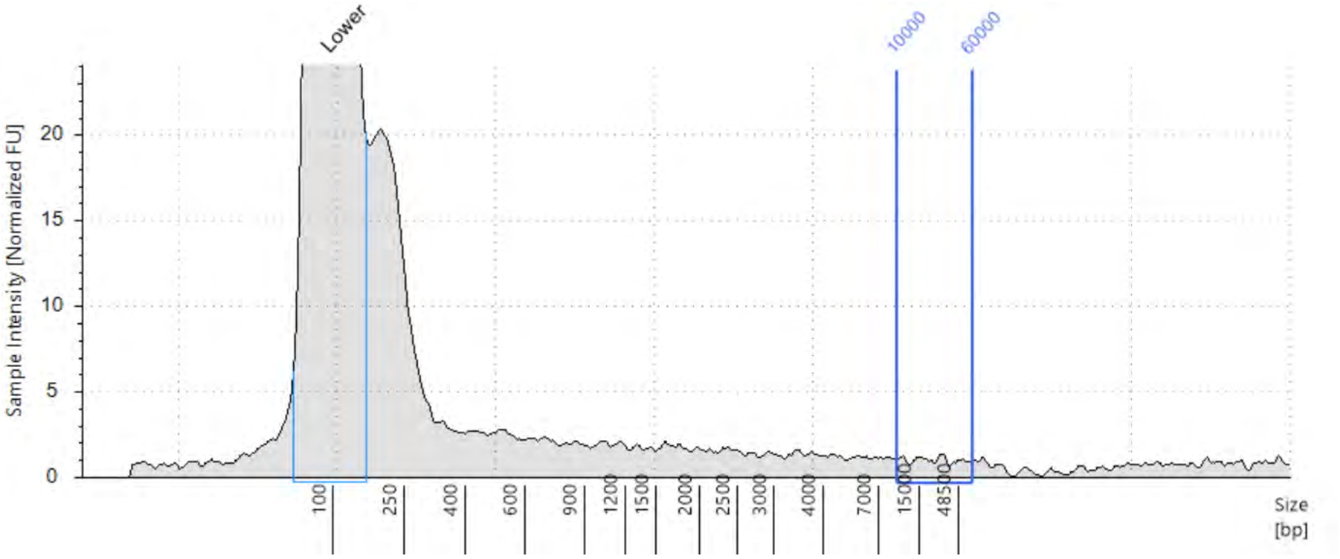

Region Table

From [bp]	To [bp]	Average Size [bp]	Conc. [ng/µl]	Region Molarity [nmol/l]	% of Total	Region Comment	Color
10000	60000	25917	0.0301	0.00386	2.45		■

© Agilent Technologies, Inc. 2017

Filename: 2020-06-10-02.gDNA

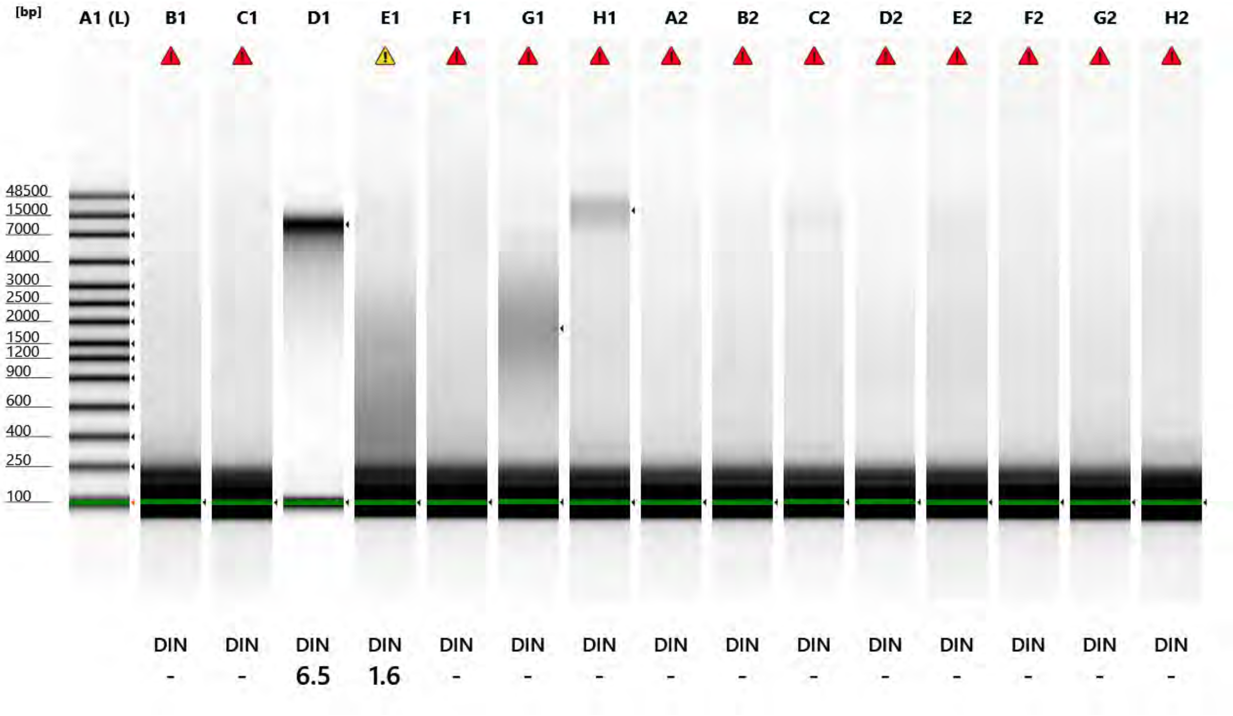

Default image (Contrast 50%), Image is Scaled to Sample

Sample Info

Well	DIN	Conc. [ng/µl]	Sample Description	Alert	Observations
A1	-	76.1	Ladder		Ladder
B1	-	1.81	S-1106-T5-E	▲	Sample concentration outside functional range for DIN
C1	-	1.54	S-1107-T5-E	▲	Sample concentration outside functional range for DIN
D1	6.5	26.3	S-1108-T5-E		
E1	1.6	4.38	S-1109-T5-E	⚠	Sample concentration outside functional range for DIN
F1	-	2.45	S-1110-T5-E	▲	Sample concentration outside functional range for DIN
G1	-	2.39	S-872-T5-ESS	▲	Sample concentration outside functional range for DIN
H1	-	1.77	S-886-T5-SS	▲	Sample concentration outside functional range for DIN
A2	-	1.46	S-887-T5-SS	▲	Sample concentration outside functional range for DIN
B2	-	1.56	S-888-T5-SS	▲	Sample concentration outside functional range for DIN
C2	-	1.55	S-889-T5-SS	▲	Sample concentration outside functional range for DIN
D2	-	0.969	S-890-T5-SS	▲	Sample concentration outside functional range for DIN
E2	-	1.72	S-1111-T5-SS	▲	Sample concentration outside functional range for DIN
F2	-	1.54	S-1112-T5-SS	▲	Sample concentration outside functional range for DIN
G2	-	1.38	S-1113-T5-SS	▲	Sample concentration outside functional range for DIN
H2	-	1.19	S-1114-T5-SS	▲	Sample concentration outside functional range for DIN

 © Agilent Technologies, Inc. 2017

A1: Ladder

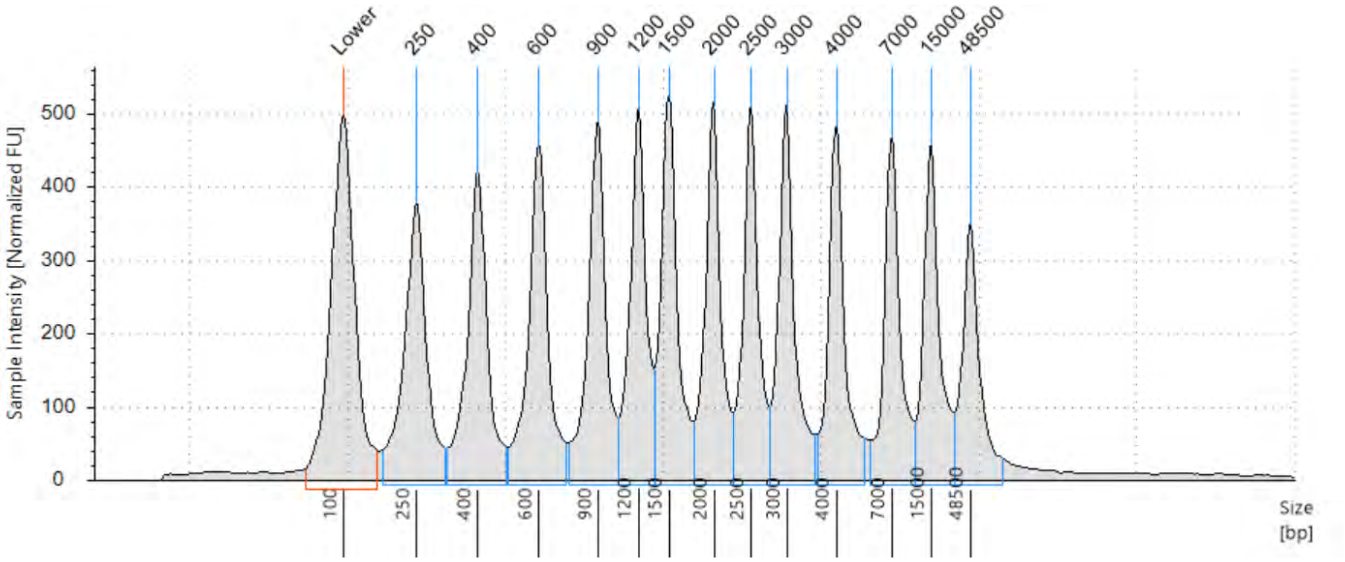

Sample Table

Well	DIN	Conc. [ng/µl]	Sample Description	Alert	Observations
A1	-	76.1	Ladder		Ladder

Peak Table

Size [bp]	Calibrated Conc. [ng/µl]	Assigned Conc. [ng/µl]	% Integrated Area	From [bp]	To [bp]	Peak Comment	Observations
100	8.50	8.50	-	62	152		Lower Marker
250	6.04	-	8.10	162	313		
400	6.07	-	8.14	316	486		
600	6.28	-	8.42	490	725		
900	6.21	-	8.32	738	1039		
1200	5.97	-	8.00	1039	1348		
1500	6.26	-	8.38	1348	1762		
2000	5.89	-	7.90	1762	2254		
2500	5.63	-	7.55	2254	2748		
3000	5.94	-	7.96	2748	3532		
4000	5.45	-	7.30	3587	5292		
7000	5.29	-	7.09	5656	11058		
15000	5.22	-	7.00	11058	23121		
48500	4.37	-	5.86	23121	>60000		

 © Agilent Technologies, Inc. 2017

B1: S-1106-T5-E

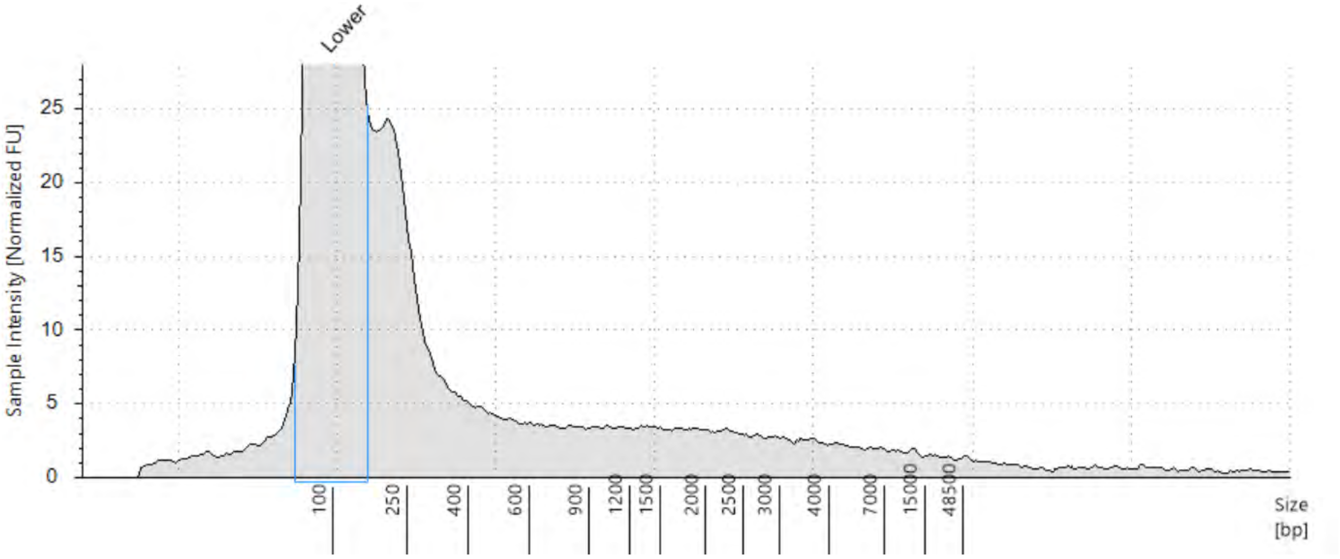

Sample Table

Well	DIN	Conc. [ng/µl]	Sample Description	Alert	Observations
B1	-	1.81	S-1106-T5-E	▲	Sample concentration outside functional range for DIN

Peak Table

Size [bp]	Calibrated Conc. [ng/µl]	Assigned Conc. [ng/µl]	% Integrated Area	From [bp]	To [bp]	Peak Comment	Observations
100	8.50	8.50	-	61	152		Lower Marker

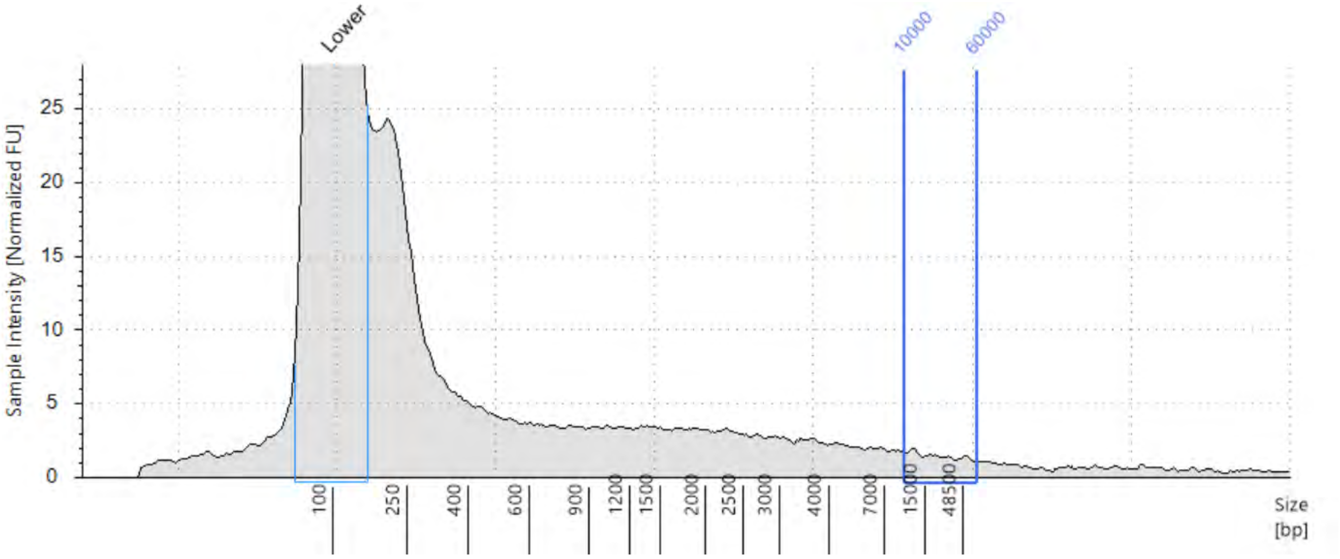

Region Table

From [bp]	To [bp]	Average Size [bp]	Conc. [ng/µl]	Region Molarity [nmol/l]	% of Total	Region Comment	Color
10000	60000	24875	0.0478	0.00551	2.64		

 © Agilent Technologies, Inc. 2017

C1: S-1107-T5-E

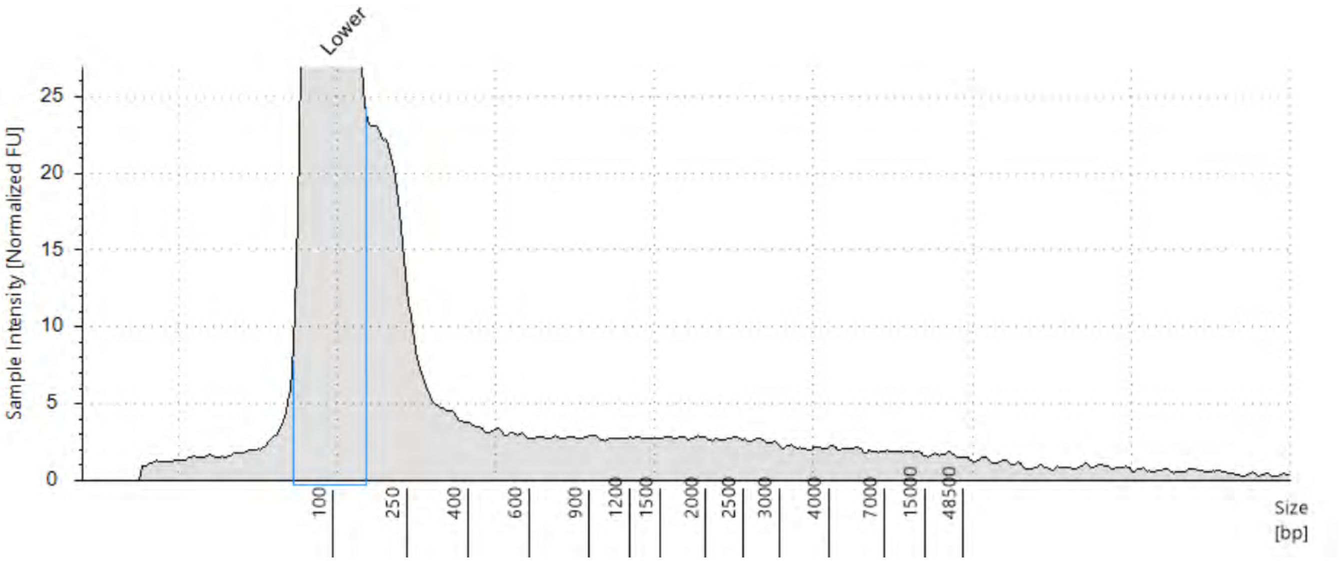

Sample Table

Well	DIN	Conc. [ng/µl]	Sample Description	Alert	Observations
C1	-	1.54	S-1107-T5-E	▲	Sample concentration outside functional range for DIN

Peak Table

Size [bp]	Calibrated Conc. [ng/µl]	Assigned Conc. [ng/µl]	% Integrated Area	From [bp]	To [bp]	Peak Comment	Observations
100	8.50	8.50	-	60	150		Lower Marker

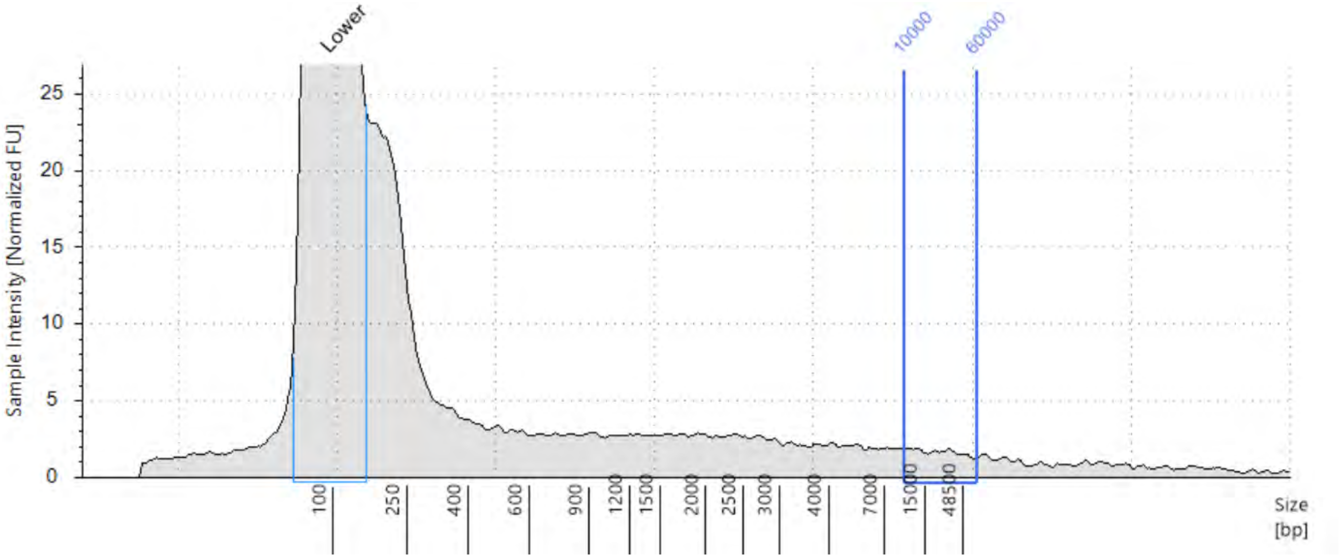

Region Table

From [bp]	To [bp]	Average Size [bp]	Conc. [ng/µl]	Region Molarity [nmol/l]	% of Total	Region Comment	Color
10000	60000	25024	0.0545	0.00633	3.55		■

D1: S-1108-T5-E

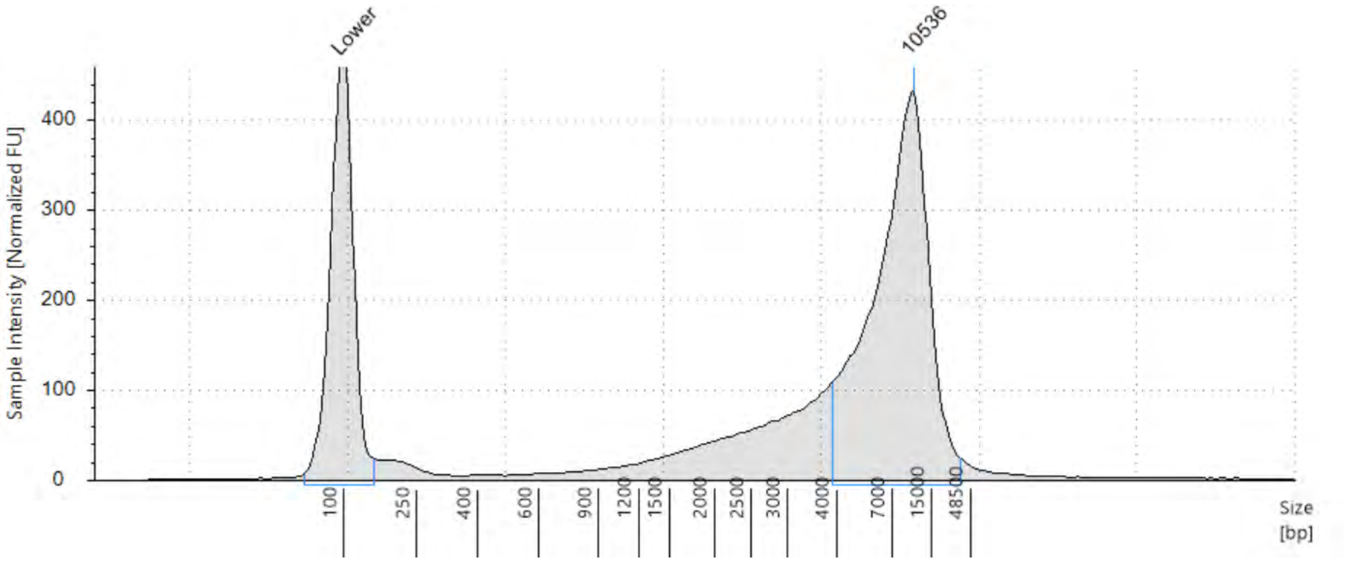

Sample Table

Well	DIN	Conc. [ng/µl]	Sample Description	Alert	Observations
D1	6.5	26.3	S-1108-T5-E		

Peak Table

Size [bp]	Calibrated Conc. [ng/µl]	Assigned Conc. [ng/µl]	% Integrated Area	From [bp]	To [bp]	Peak Comment	Observations
100	8.50	8.50	-	61	147		Lower Marker
10536	17.7	-	100.00	3924	26225		

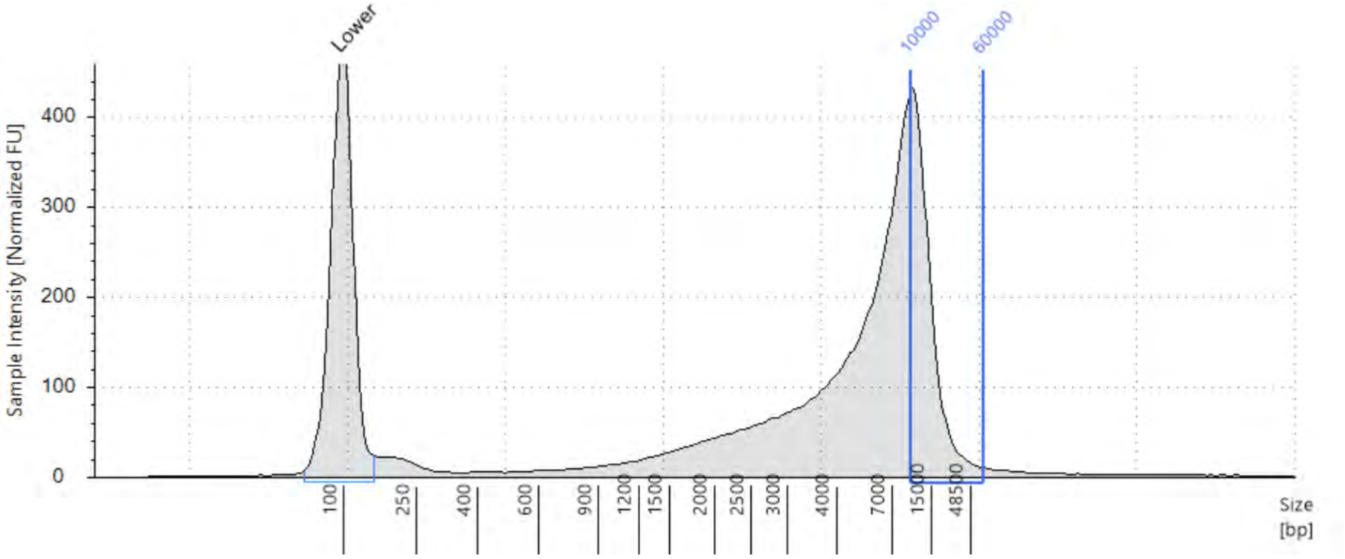

Region Table

From [bp]	To [bp]	Average Size [bp]	Conc. [ng/µl]	Region Molarity [nmol/l]	% of Total	Region Comment	Color
10000	60000	14727	6.83	0.810	25.98		

E1: S-1109-T5-E

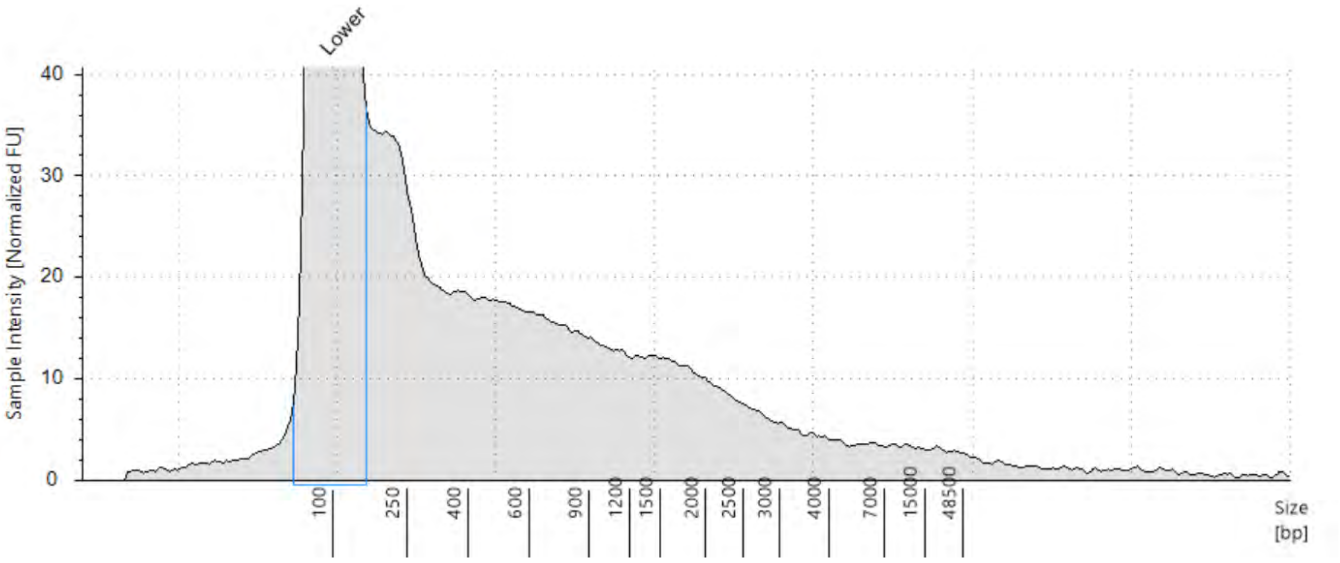

Sample Table

Well	DIN	Conc. [ng/µl]	Sample Description	Alert	Observations
E1	1.6	4.38	S-1109-T5-E	⚠	Sample concentration outside functional range for DIN

Peak Table

Size [bp]	Calibrated Conc. [ng/µl]	Assigned Conc. [ng/µl]	% Integrated Area	From [bp]	To [bp]	Peak Comment	Observations
100	8.50	8.50	-	61	153		Lower Marker

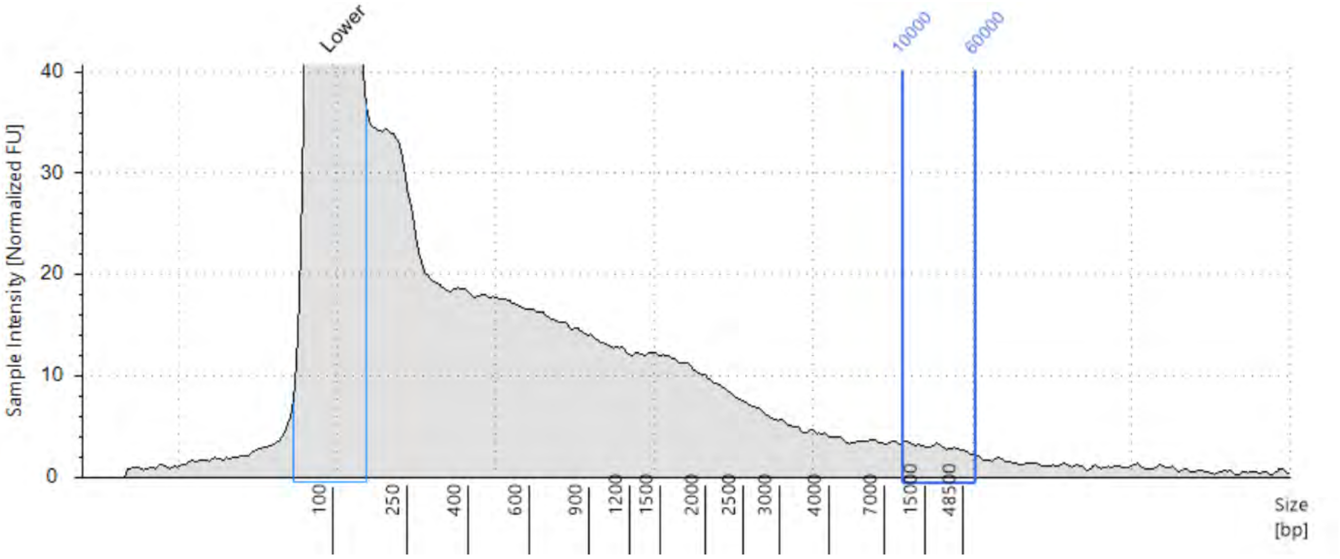

Region Table

From [bp]	To [bp]	Average Size [bp]	Conc. [ng/µl]	Region Molarity [nmol/l]	% of Total	Region Comment	Color
10000	60000	24798	0.106	0.0110	2.43		■

F1: S-1110-T5-E

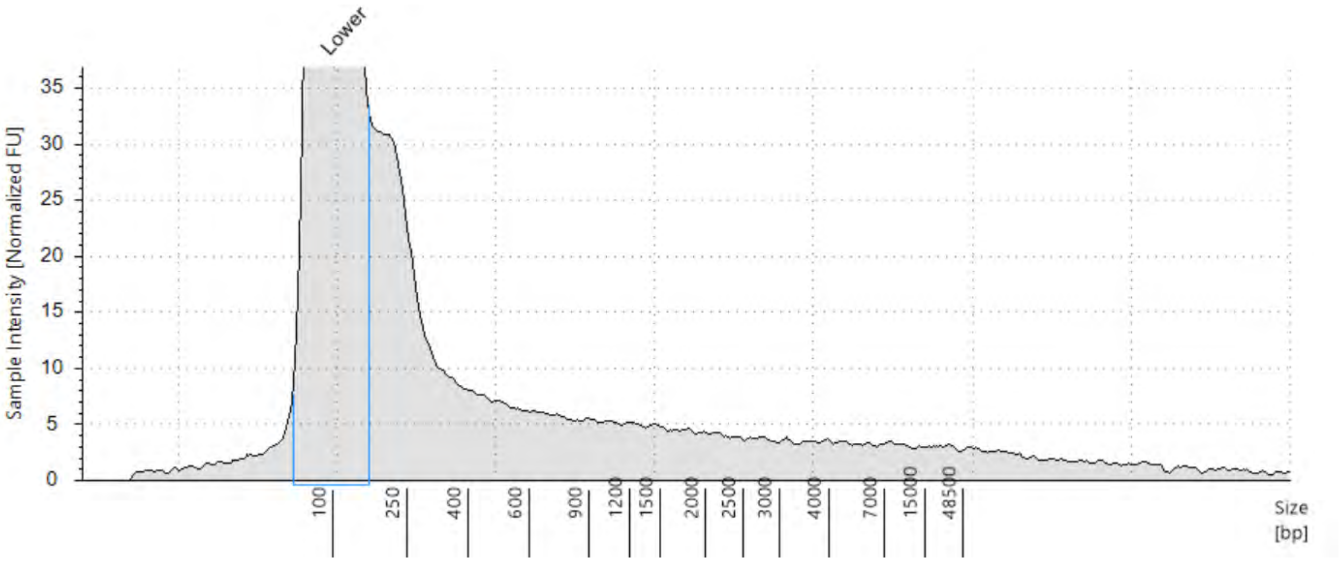

Sample Table

Well	DIN	Conc. [ng/µl]	Sample Description	Alert	Observations
F1	-	2.45	S-1110-T5-E	▲	Sample concentration outside functional range for DIN

Peak Table

Size [bp]	Calibrated Conc. [ng/µl]	Assigned Conc. [ng/µl]	% Integrated Area	From [bp]	To [bp]	Peak Comment	Observations
100	8.50	8.50	-	60	155		Lower Marker

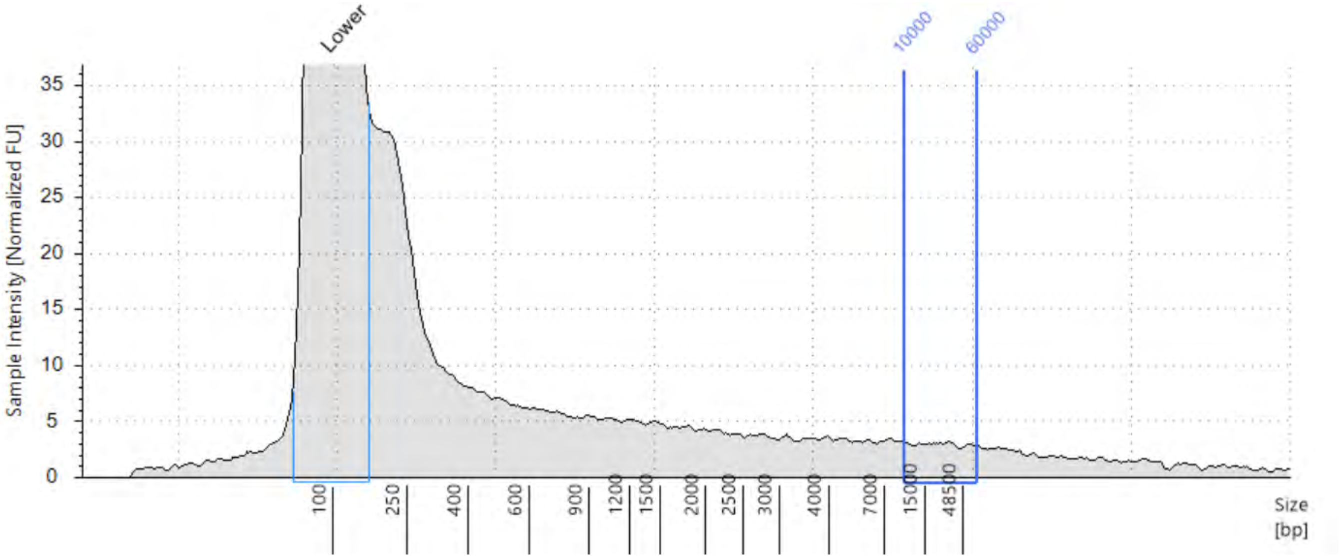

Region Table

From [bp]	To [bp]	Average Size [bp]	Conc. [ng/µl]	Region Molarity [nmol/l]	% of Total	Region Comment	Color
10000	60000	26120	0.102	0.0100	4.18		■

G1: S-872-T5-ESS

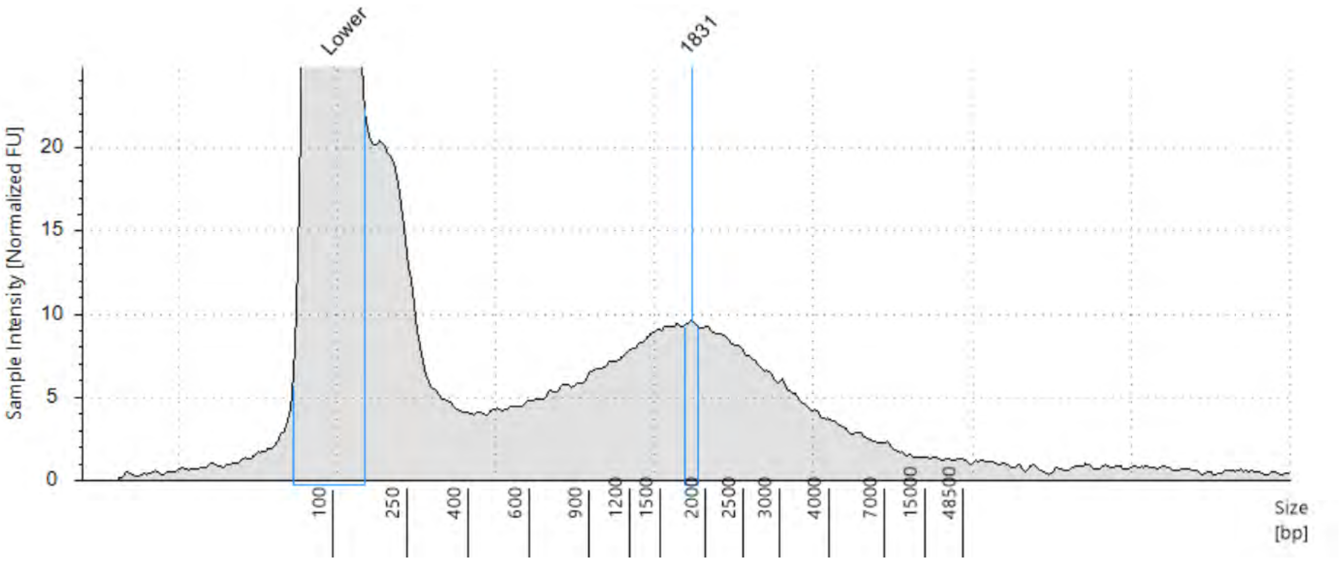

Sample Table

Well	DIN	Conc. [ng/µl]	Sample Description	Alert	Observations
G1	-	2.39	S-872-T5-ESS	▲	Sample concentration outside functional range for DIN

Peak Table

Size [bp]	Calibrated Conc. [ng/µl]	Assigned Conc. [ng/µl]	% Integrated Area	From [bp]	To [bp]	Peak Comment	Observations
100	8.50	8.50	-	60	148		Lower Marker
1831	0.0868	-	100.00	1753	1912		

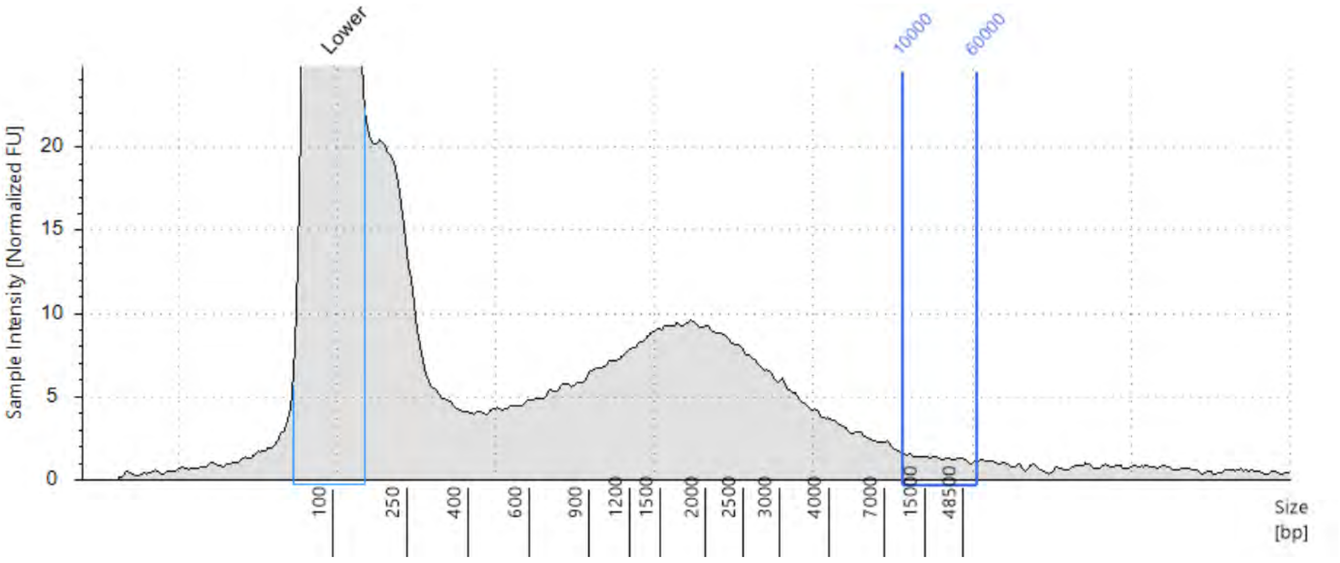

Region Table

From [bp]	To [bp]	Average Size [bp]	Conc. [ng/µl]	Region Molarity [nmol/l]	% of Total	Region Comment	Color
10000	60000	25474	0.0400	0.00502	1.68		■

 © Agilent Technologies, Inc. 2017

H1: S-886-T5-SS

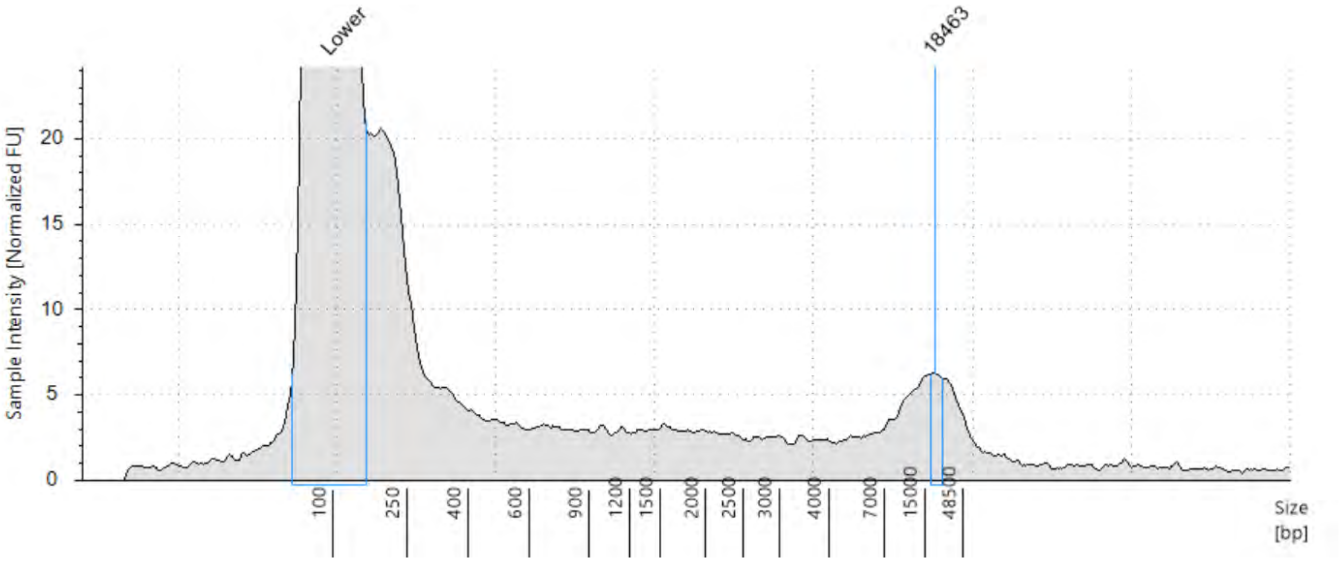

Sample Table

Well	DIN	Conc. [ng/µl]	Sample Description	Alert	Observations
H1	-	1.77	S-886-T5-SS	▲	Sample concentration outside functional range for DIN

Peak Table

Size [bp]	Calibrated Conc. [ng/µl]	Assigned Conc. [ng/µl]	% Integrated Area	From [bp]	To [bp]	Peak Comment	Observations
100	8.50	8.50	-	60	150		Lower Marker
18463	0.0453	-	100.00	17087	21008		

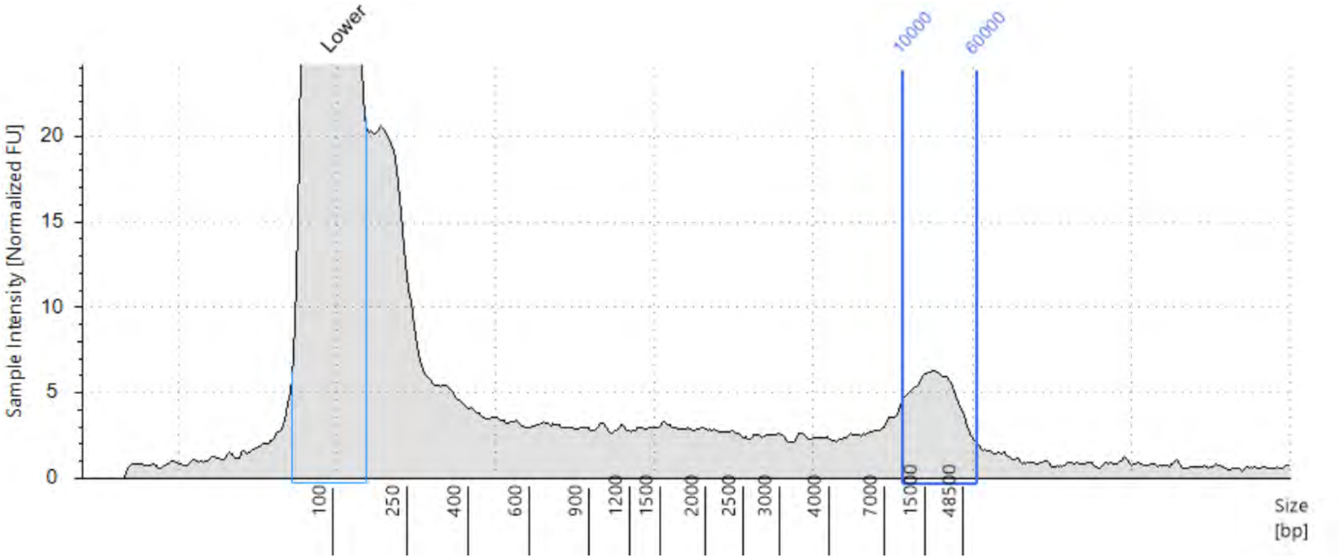

Region Table

From [bp]	To [bp]	Average Size [bp]	Conc. [ng/µl]	Region Molarity [nmol/l]	% of Total	Region Comment	Color
10000	60000	23389	0.217	0.0198	12.23		■

 © Agilent Technologies, Inc. 2017

A2: S-887-T5-SS

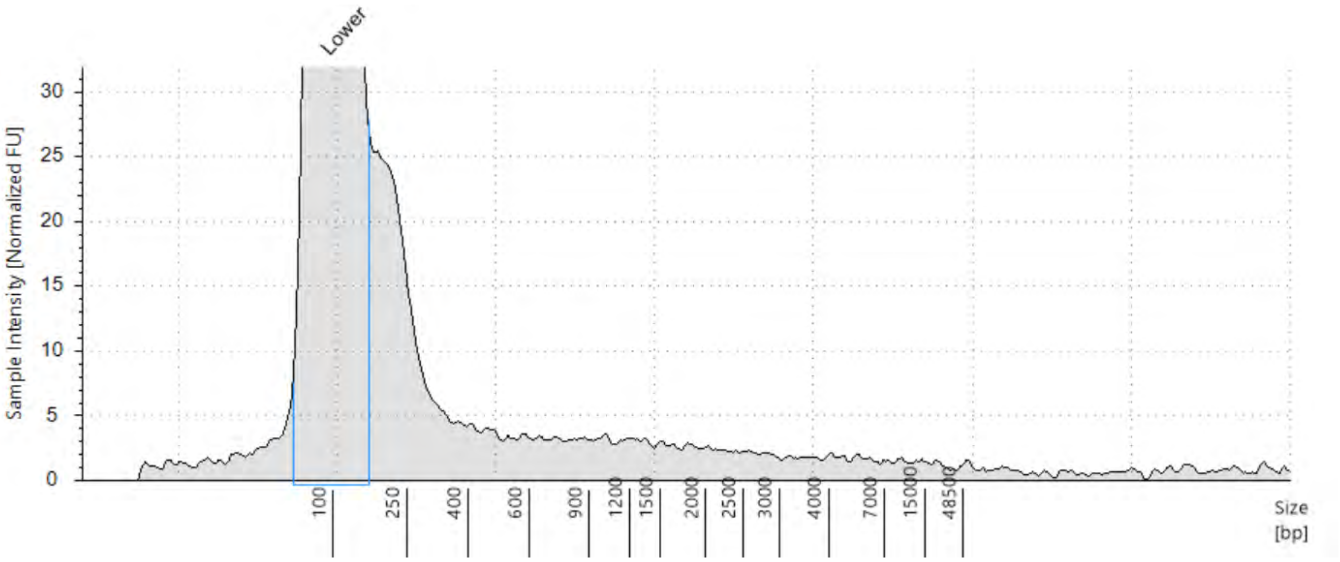

Sample Table

Well	DIN	Conc. [ng/µl]	Sample Description	Alert	Observations
A2	-	1.46	S-887-T5-SS	▲	Sample concentration outside functional range for DIN

Peak Table

Size [bp]	Calibrated Conc. [ng/µl]	Assigned Conc. [ng/µl]	% Integrated Area	From [bp]	To [bp]	Peak Comment	Observations
100	8.50	8.50	-	60	155		Lower Marker

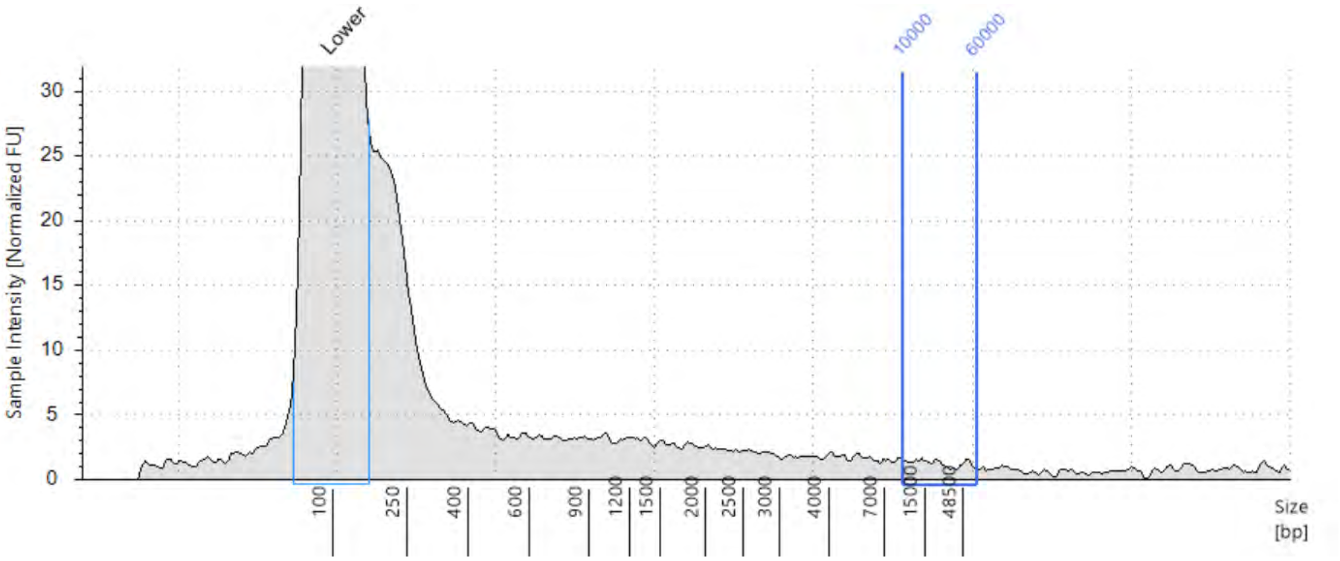

Region Table

From [bp]	To [bp]	Average Size [bp]	Conc. [ng/µl]	Region Molarity [nmol/l]	% of Total	Region Comment	Color
10000	60000	25895	0.0294	0.00443	2.02		■

B2: S-888-T5-SS

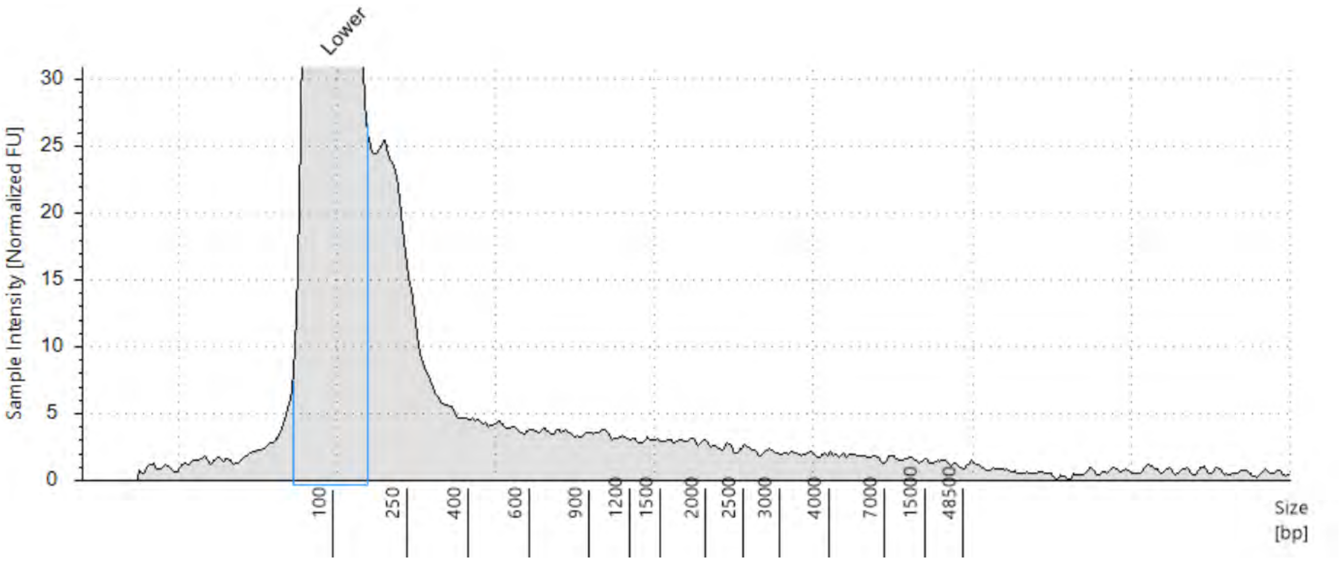

Sample Table

Well	DIN	Conc. [ng/µl]	Sample Description	Alert	Observations
B2	-	1.56	S-888-T5-SS	▲	Sample concentration outside functional range for DIN

Peak Table

Size [bp]	Calibrated Conc. [ng/µl]	Assigned Conc. [ng/µl]	% Integrated Area	From [bp]	To [bp]	Peak Comment	Observations
100	8.50	8.50	-	60	152		Lower Marker

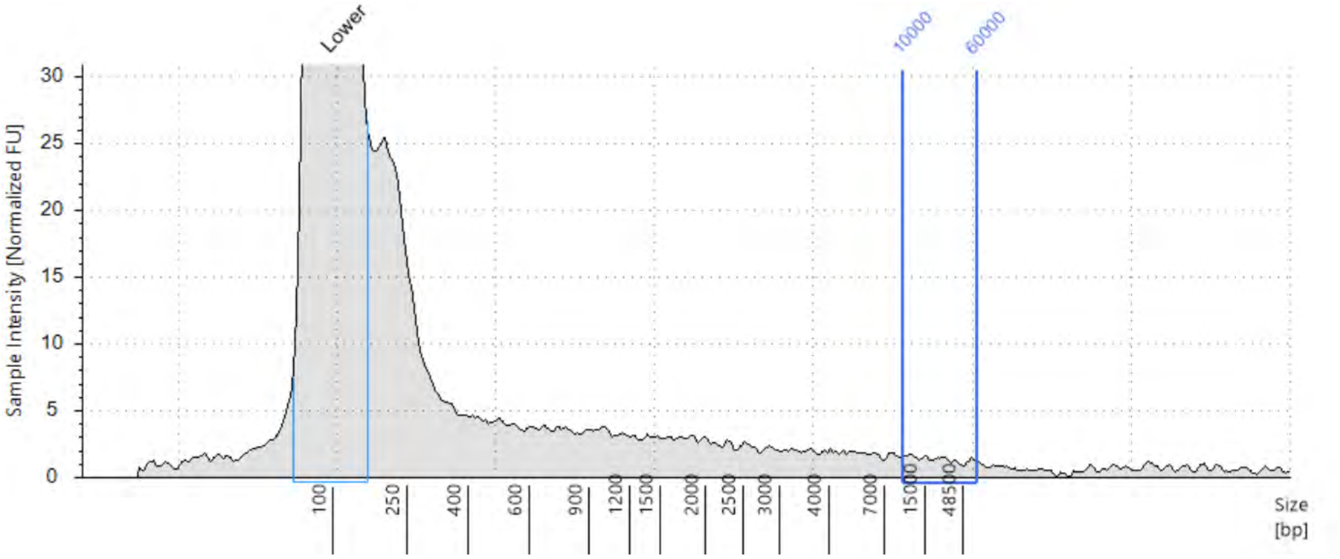

Region Table

From [bp]	To [bp]	Average Size [bp]	Conc. [ng/µl]	Region Molarity [nmol/l]	% of Total	Region Comment	Color
10000	60000	25661	0.0344	0.00481	2.20		■

C2: S-889-T5-SS

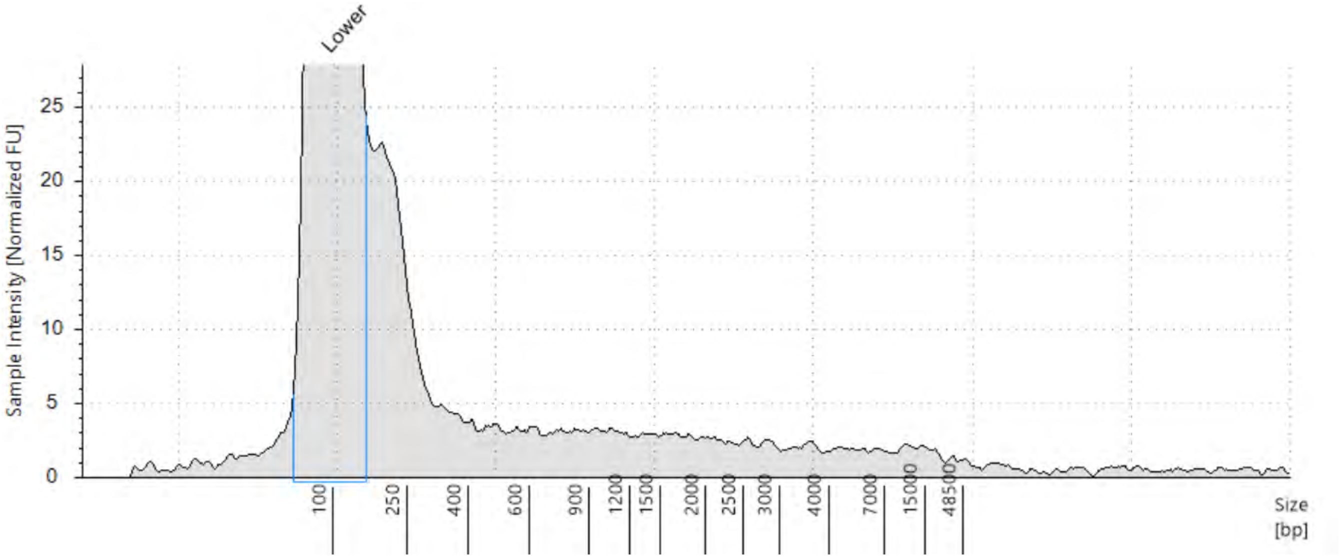

Sample Table

Well	DIN	Conc. [ng/µl]	Sample Description	Alert	Observations
C2	-	1.55	S-889-T5-SS	▲	Sample concentration outside functional range for DIN

Peak Table

Size [bp]	Calibrated Conc. [ng/µl]	Assigned Conc. [ng/µl]	% Integrated Area	From [bp]	To [bp]	Peak Comment	Observations
100	8.50	8.50	-	60	150		Lower Marker

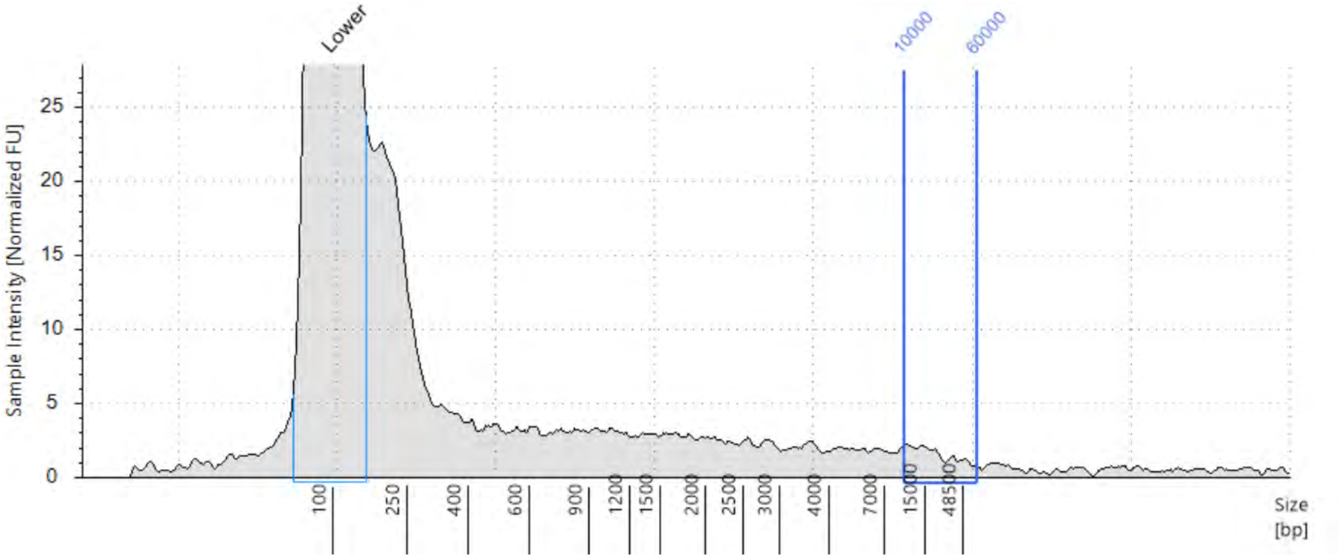

Region Table

From [bp]	To [bp]	Average Size [bp]	Conc. [ng/µl]	Region Molarity [nmol/l]	% of Total	Region Comment	Color
10000	60000	22681	0.0540	0.00655	3.48		■

 © Agilent Technologies, Inc. 2017

D2: S-890-T5-SS

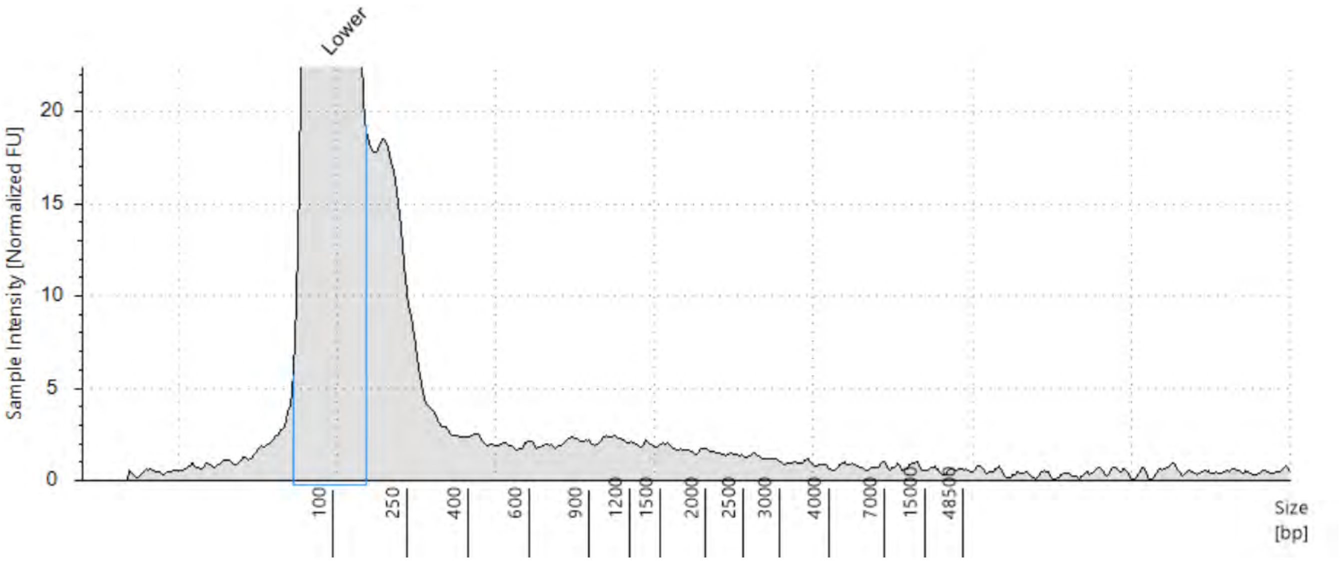

Sample Table

Well	DIN	Conc. [ng/µl]	Sample Description	Alert	Observations
D2	-	0.969	S-890-T5-SS	▲	Sample concentration outside functional range for DIN

Peak Table

Size [bp]	Calibrated Conc. [ng/µl]	Assigned Conc. [ng/µl]	% Integrated Area	From [bp]	To [bp]	Peak Comment	Observations
100	8.50	8.50	-	60	150		Lower Marker

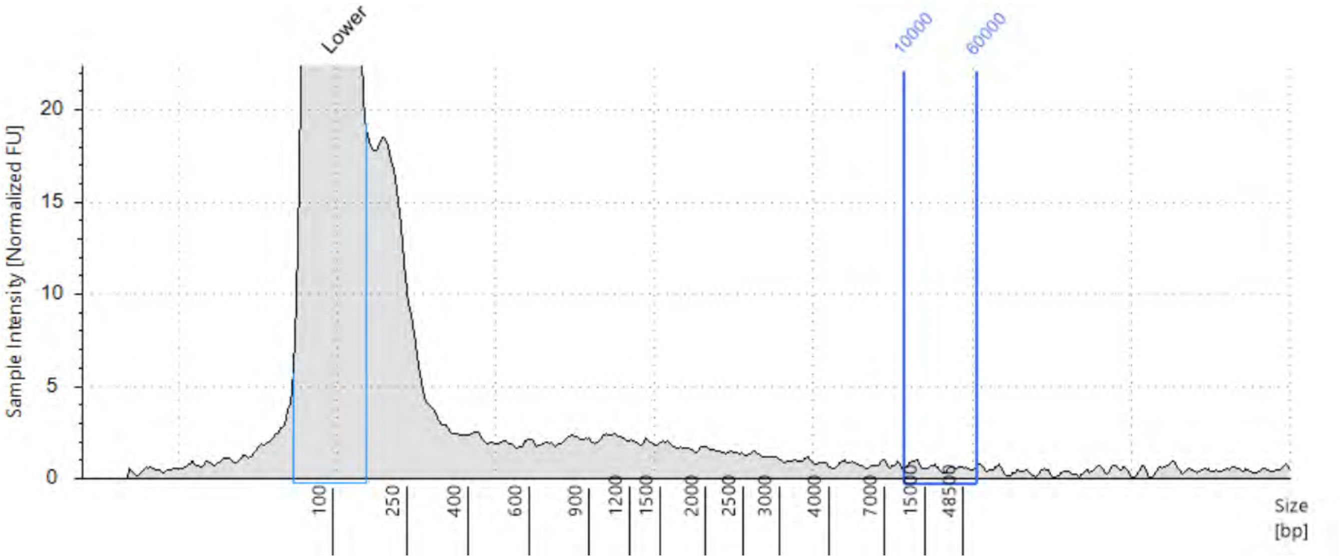

Region Table

From [bp]	To [bp]	Average Size [bp]	Conc. [ng/µl]	Region Molarity [nmol/l]	% of Total	Region Comment	Color
10000	60000	25343	0.00698	0.00242	0.72		■

 © Agilent Technologies, Inc. 2017

E2: S-1111-T5-SS

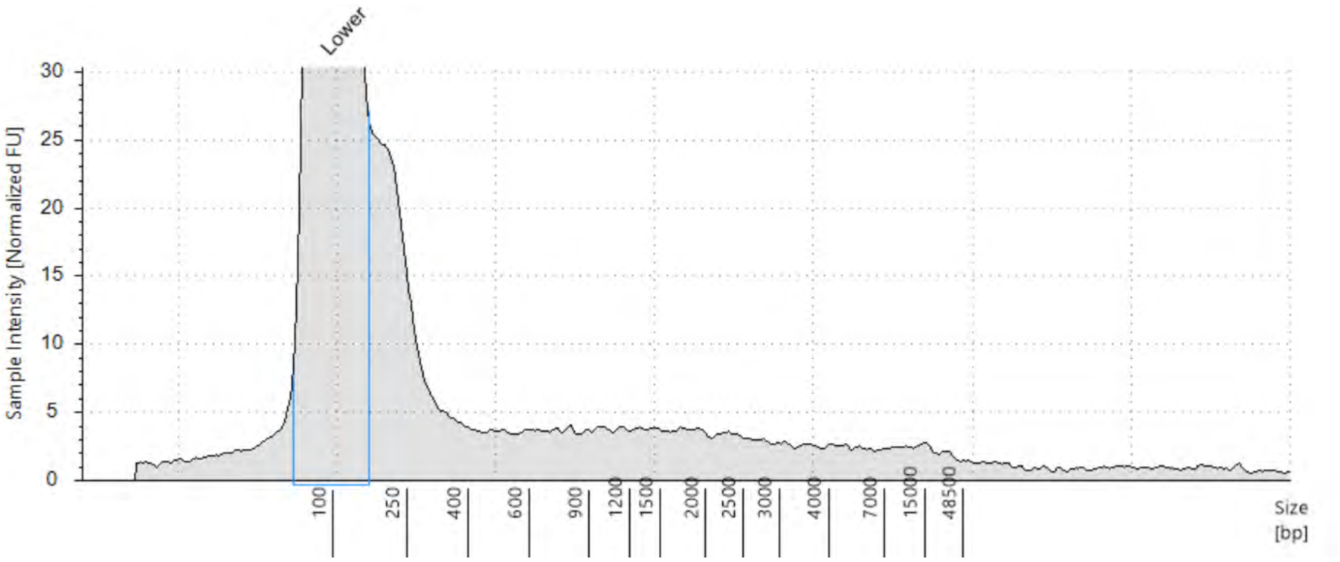

Sample Table

Well	DIN	Conc. [ng/µl]	Sample Description	Alert	Observations
E2	-	1.72	S-1111-T5-SS	▲	Sample concentration outside functional range for DIN

Peak Table

Size [bp]	Calibrated Conc. [ng/µl]	Assigned Conc. [ng/µl]	% Integrated Area	From [bp]	To [bp]	Peak Comment	Observations
100	8.50	8.50	-	60	155		Lower Marker

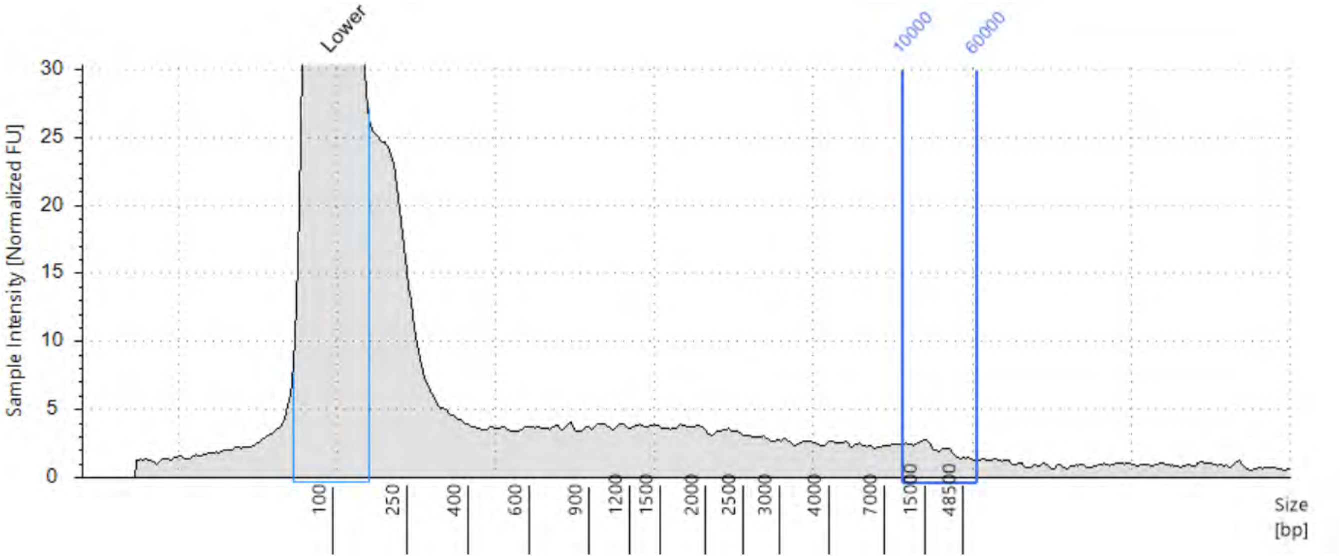

Region Table

From [bp]	To [bp]	Average Size [bp]	Conc. [ng/µl]	Region Molarity [nmol/l]	% of Total	Region Comment	Color
10000	60000	23515	0.0651	0.00759	3.80		■

 © Agilent Technologies, Inc. 2017

F2: S-1112-T5-SS

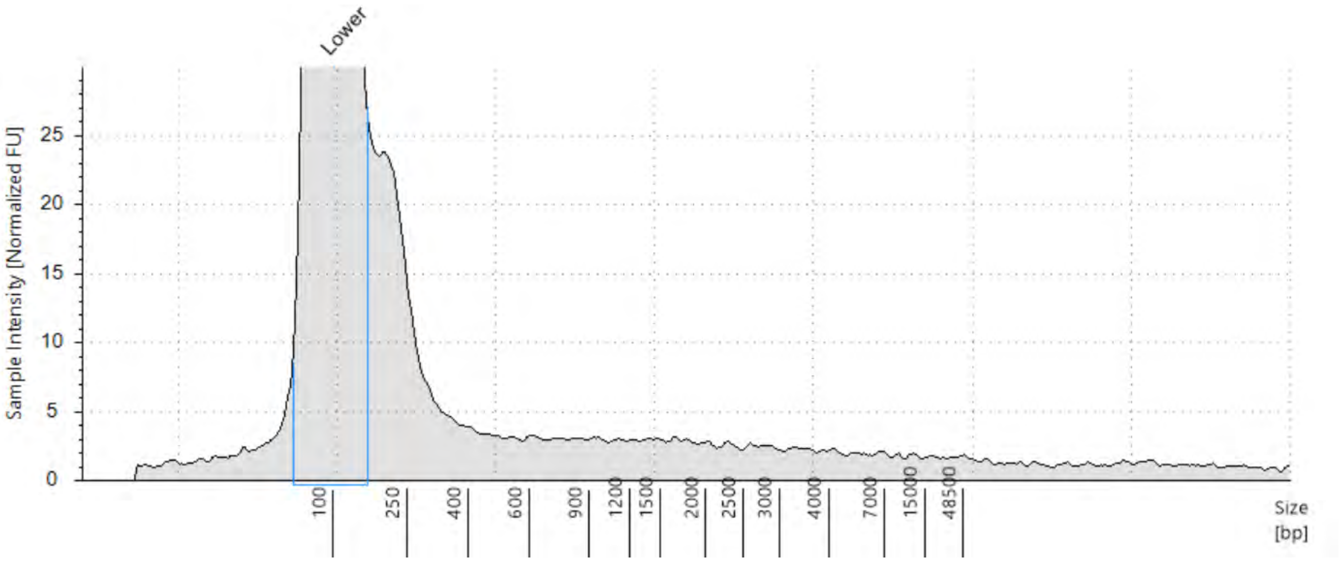

Sample Table

Well	DIN	Conc. [ng/µl]	Sample Description	Alert	Observations
F2	-	1.54	S-1112-T5-SS	▲	Sample concentration outside functional range for DIN

Peak Table

Size [bp]	Calibrated Conc. [ng/µl]	Assigned Conc. [ng/µl]	% Integrated Area	From [bp]	To [bp]	Peak Comment	Observations
100	8.50	8.50	-	60	153		Lower Marker

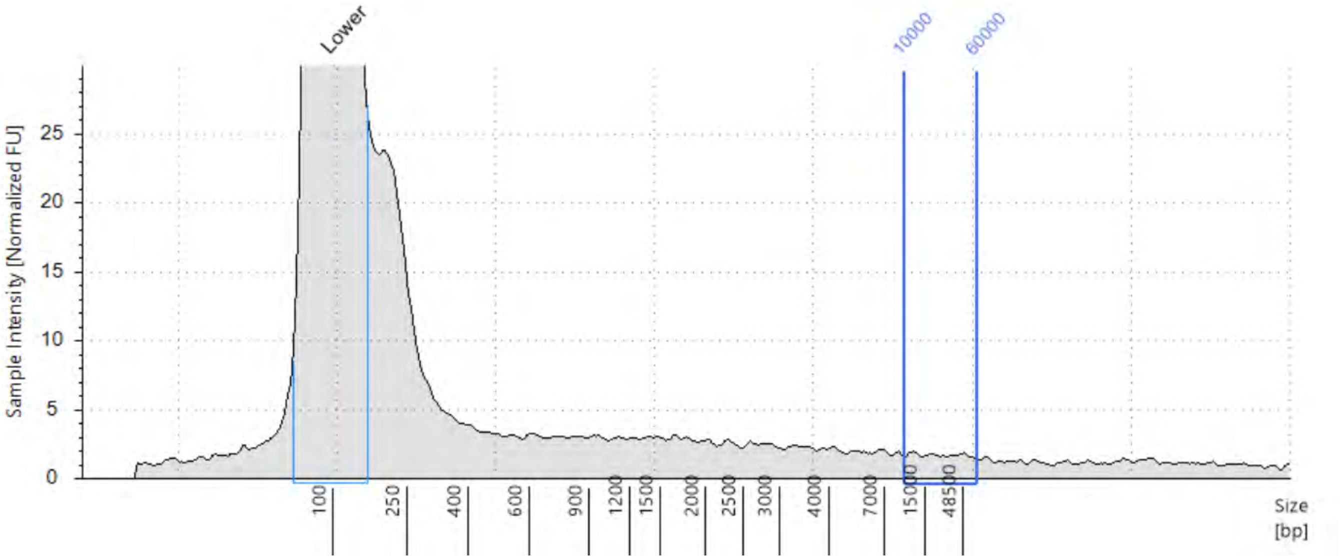

Region Table

From [bp]	To [bp]	Average Size [bp]	Conc. [ng/µl]	Region Molarity [nmol/l]	% of Total	Region Comment	Color
10000	60000	26120	0.0487	0.00576	3.16		■

G2: S-1113-T5-SS

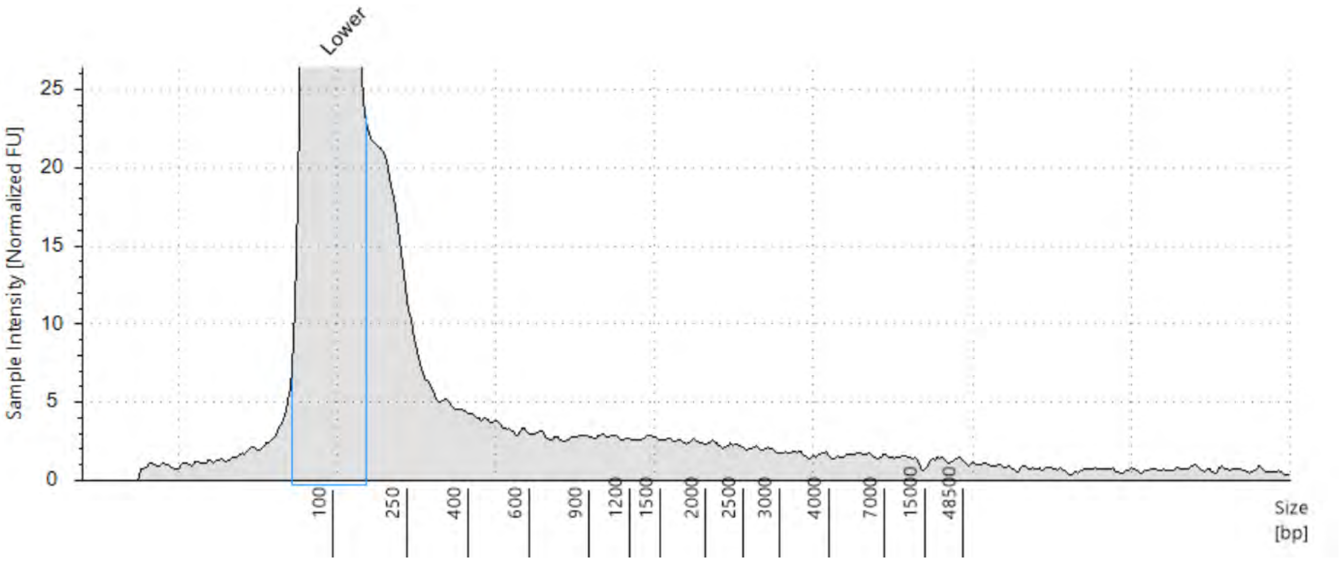

Sample Table

Well	DIN	Conc. [ng/µl]	Sample Description	Alert	Observations
G2	-	1.38	S-1113-T5-SS	▲	Sample concentration outside functional range for DIN

Peak Table

Size [bp]	Calibrated Conc. [ng/µl]	Assigned Conc. [ng/µl]	% Integrated Area	From [bp]	To [bp]	Peak Comment	Observations
100	8.50	8.50	-	59	150		Lower Marker

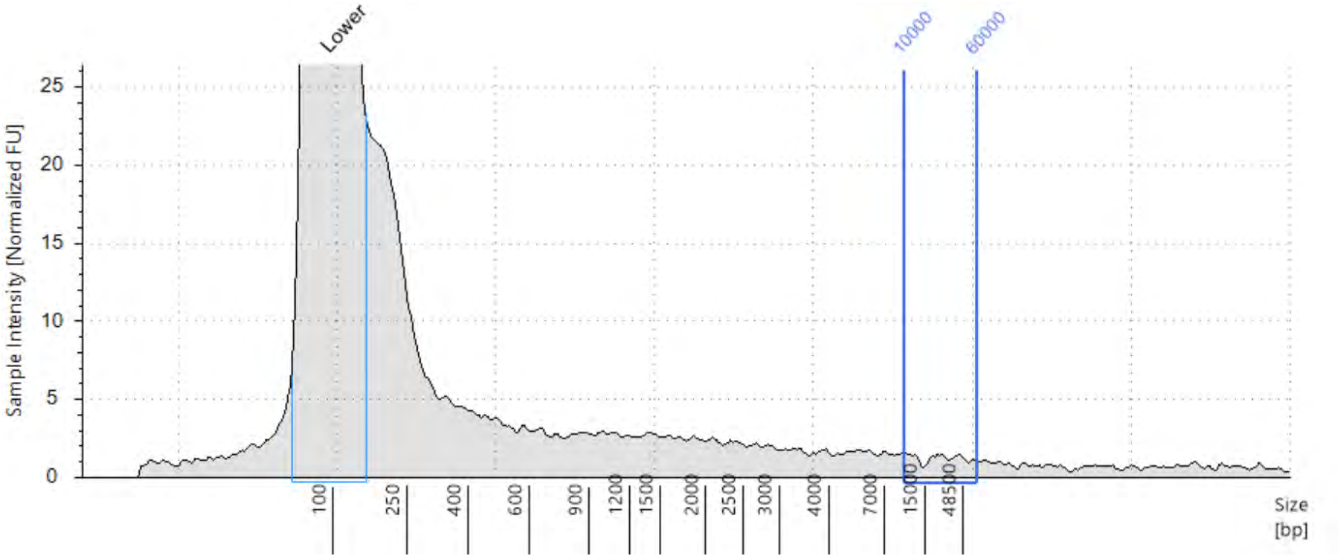

Region Table

From [bp]	To [bp]	Average Size [bp]	Conc. [ng/µl]	Region Molarity [nmol/l]	% of Total	Region Comment	Color
10000	60000	25839	0.0325	0.00470	2.35		■

H2: S-1114-T5-SS

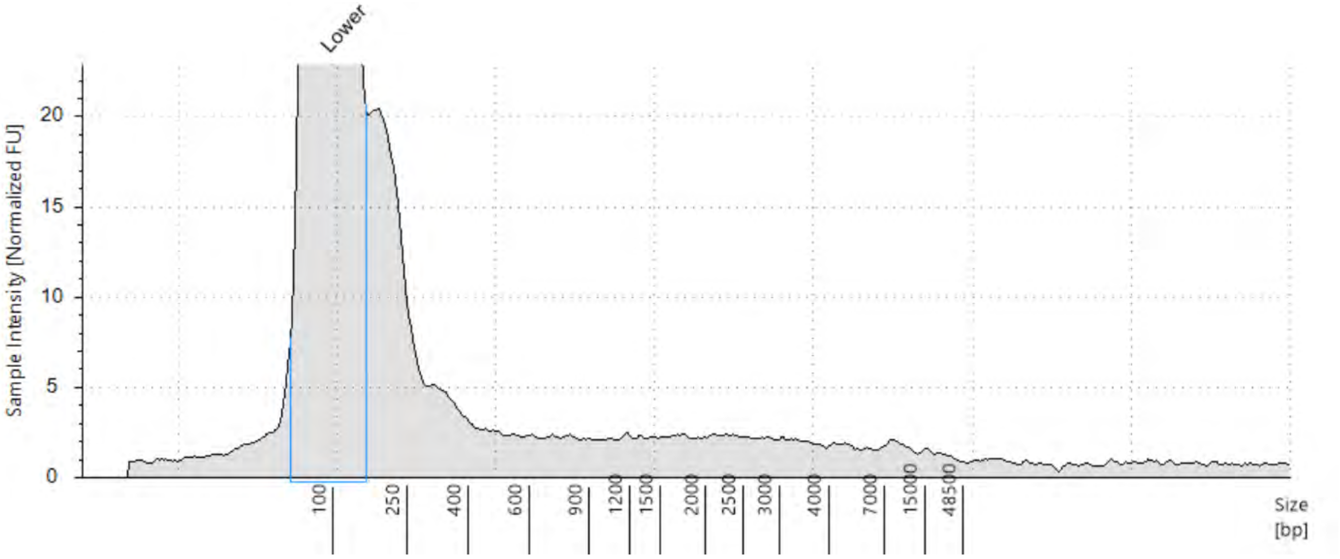

Sample Table

Well	DIN	Conc. [ng/µl]	Sample Description	Alert	Observations
H2	-	1.19	S-1114-T5-SS	▲	Sample concentration outside functional range for DIN

Peak Table

Size [bp]	Calibrated Conc. [ng/µl]	Assigned Conc. [ng/µl]	% Integrated Area	From [bp]	To [bp]	Peak Comment	Observations
100	8.50	8.50	-	59	150		Lower Marker

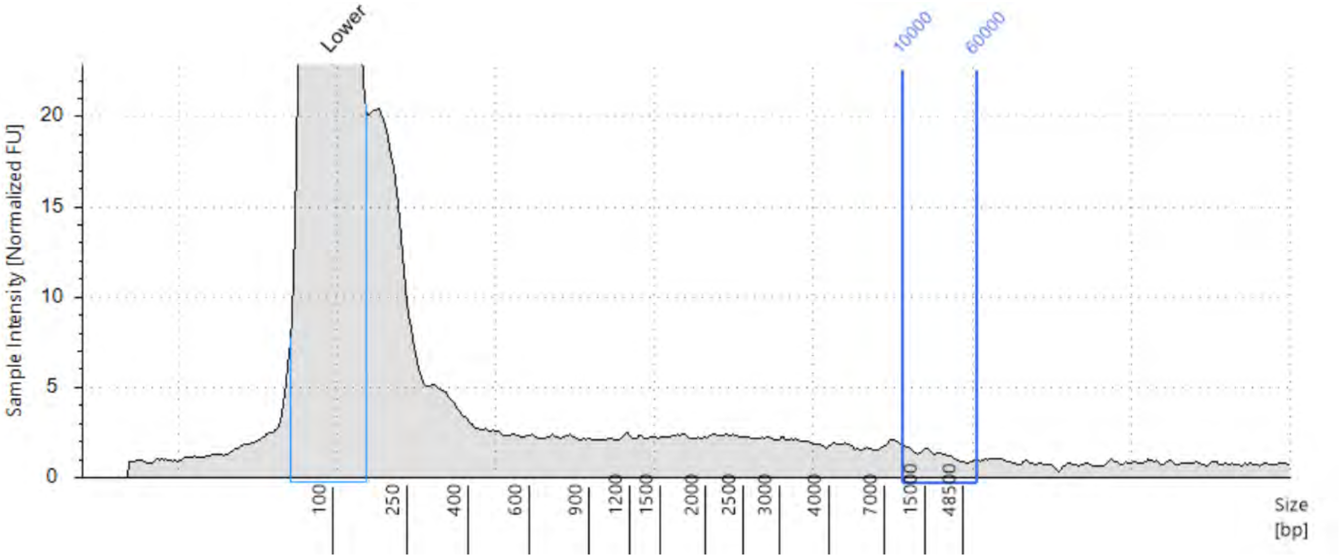

Region Table

From [bp]	To [bp]	Average Size [bp]	Conc. [ng/µl]	Region Molarity [nmol/l]	% of Total	Region Comment	Color
10000	60000	23221	0.0326	0.00478	2.74		■

Filename: 2020-06-10-03.gDNA

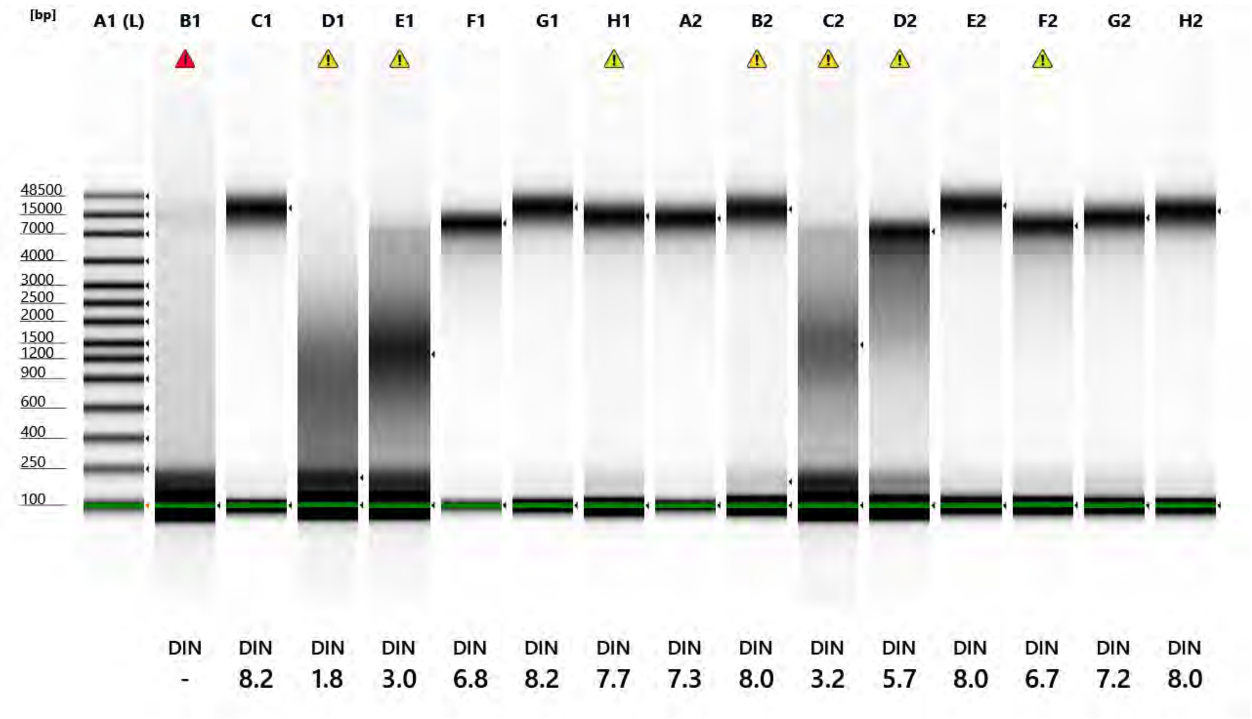

Default image (Contrast 50%), Image is Scaled to Sample

Sample Info

Well	DIN	Conc. [ng/µl]	Sample Description	Alert	Observations
A1	-	79.8	Ladder		Ladder
B1	-	2.22	S-1115-T5-SS	▲	Sample concentration outside functional range for DIN
C1	8.2	14.3	LSL51		
D1	1.8	5.01	LSL52	⚠	Sample concentration outside recommended range
E1	3.0	5.97	LSL53	⚠	Sample concentration outside recommended range
F1	6.8	29.3	LSL54		
G1	8.2	13.8	LSL55		
H1	7.7	6.84	LSL56	⚠	Sample concentration outside recommended range
A2	7.3	20.3	LSL57		
B2	8.0	6.62	LSL58	⚠	Sample concentration outside recommended range
C2	3.2	3.94	LSL59	⚠	Sample concentration outside functional range for DIN
D2	5.7	5.84	LSL60	⚠	Sample concentration outside recommended range
E2	8.0	10.4	LSL61		
F2	6.7	8.25	LSL62	⚠	Sample concentration outside recommended range
G2	7.2	10.4	LSL63		
H2	8.0	11.2	LSL64		

 © Agilent Technologies, Inc. 2017

A1: Ladder

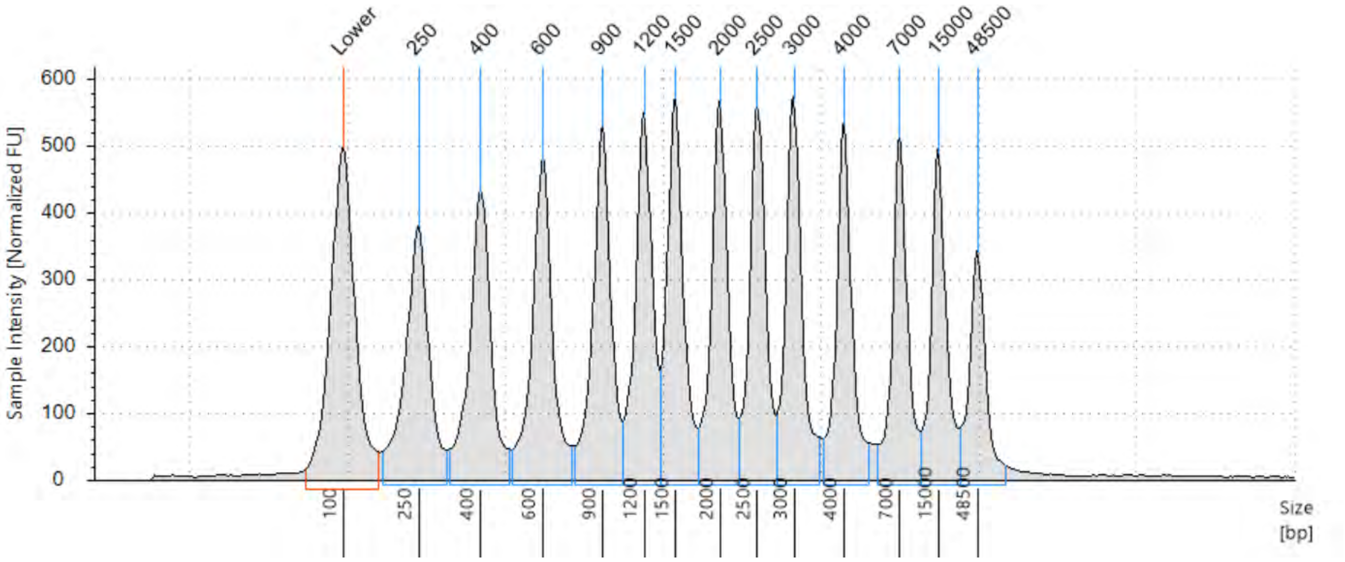

Sample Table

Well	DIN	Conc. [ng/µl]	Sample Description	Alert	Observations
A1	-	79.8	Ladder		Ladder

Peak Table

Size [bp]	Calibrated Conc. [ng/µl]	Assigned Conc. [ng/µl]	% Integrated Area	From [bp]	To [bp]	Peak Comment	Observations
100	8.50	8.50	-	63	152		Lower Marker
250	6.19	-	7.82	162	308		
400	6.41	-	8.10	315	484		
600	6.72	-	8.49	492	728		
900	6.75	-	8.53	741	1034		
1200	6.75	-	8.53	1034	1354		
1500	6.62	-	8.37	1354	1732		
2000	6.56	-	8.30	1732	2236		
2500	6.40	-	8.10	2236	2757		
3000	6.37	-	8.05	2757	3464		
4000	5.81	-	7.34	3544	5152		
7000	5.44	-	6.88	5581	10511		
15000	5.29	-	6.68	10511	22531		
48500	3.81	-	4.82	22531	>60000		

© Agilent Technologies, Inc. 2017

B1: S-1115-T5-SS

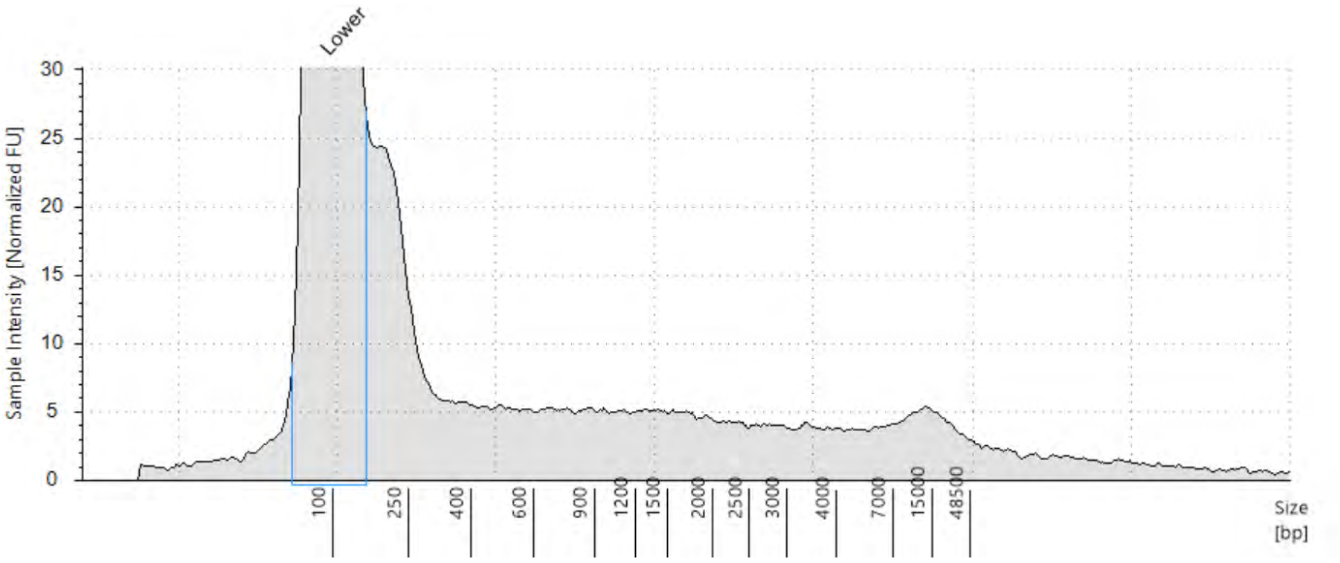

Sample Table

Well	DIN	Conc. [ng/µl]	Sample Description	Alert	Observations
B1	-	2.22	S-1115-T5-SS	▲	Sample concentration outside functional range for DIN

Peak Table

Size [bp]	Calibrated Conc. [ng/µl]	Assigned Conc. [ng/µl]	% Integrated Area	From [bp]	To [bp]	Peak Comment	Observations
100	8.50	8.50	-	61	150		Lower Marker

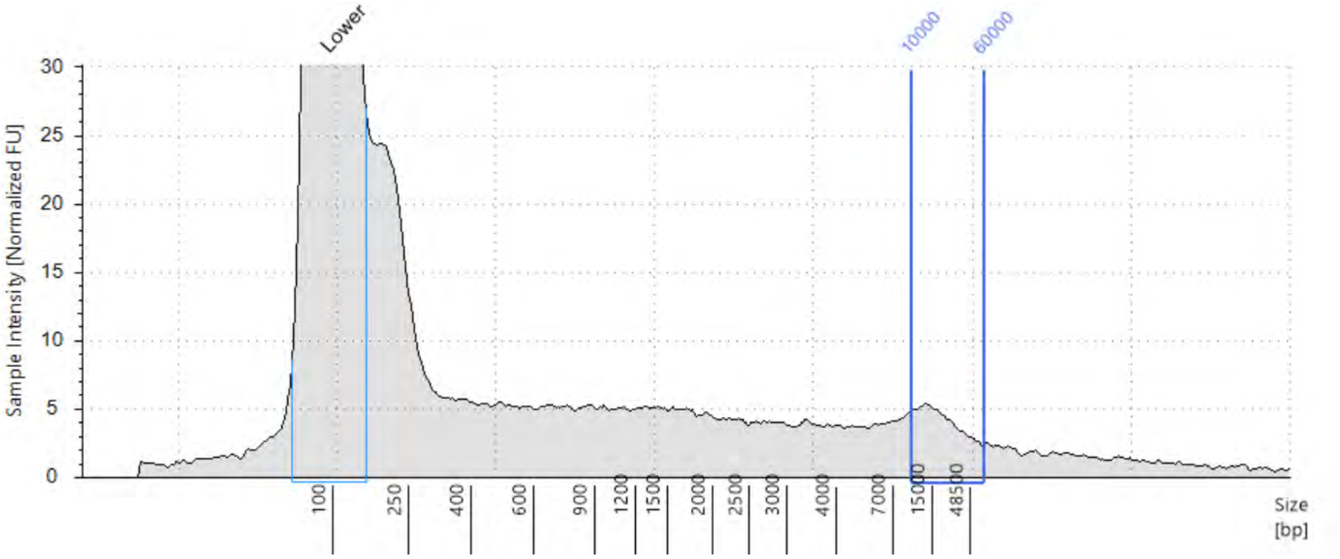

Region Table

From [bp]	To [bp]	Average Size [bp]	Conc. [ng/µl]	Region Molarity [nmol/l]	% of Total	Region Comment	Color
10000	60000	22978	0.154	0.0157	6.96		■

 © Agilent Technologies, Inc. 2017

Filename: Lounsberry_T5_Samples_AllSpecies_AllReplicates_3.19.19.gDNA

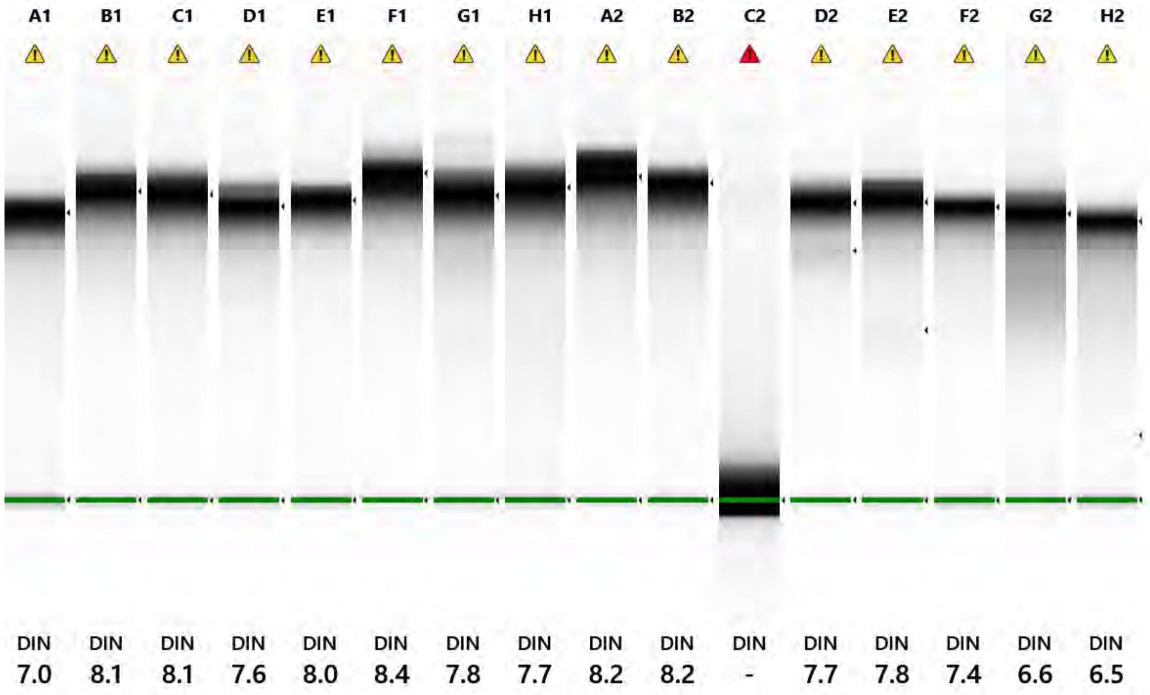

Default image (Contrast 50%), Image is Scaled to Sample

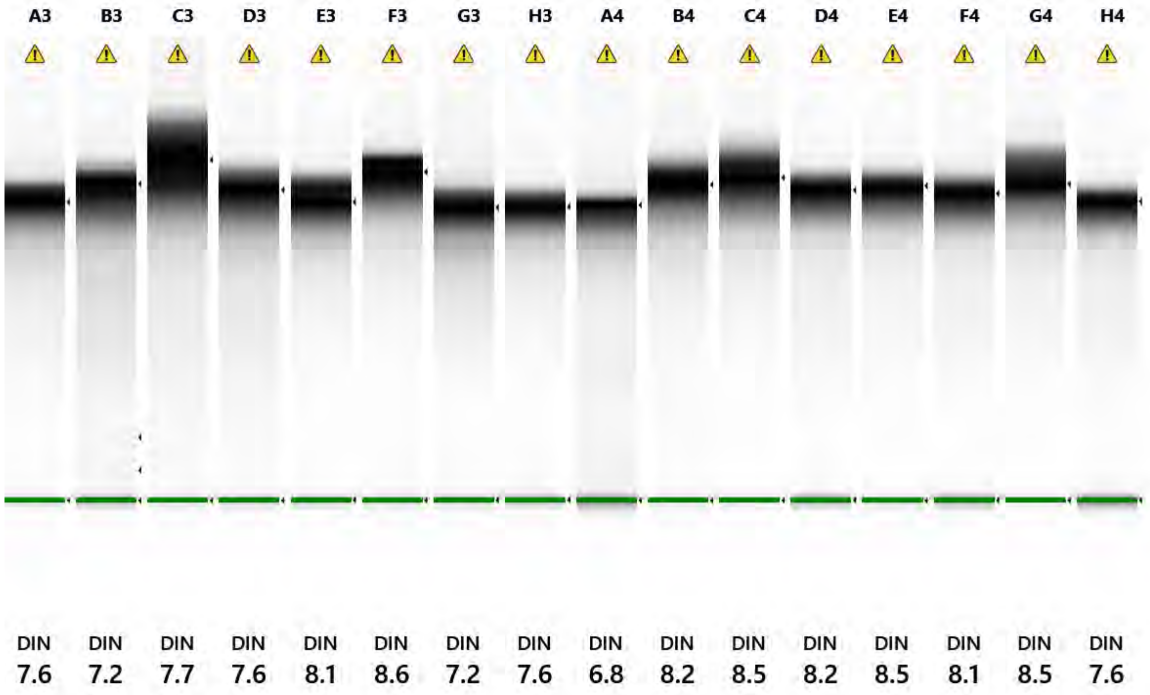

Default image (Contrast 50%), Image is Scaled to Sample

 © Agilent Technologies, Inc. 2017

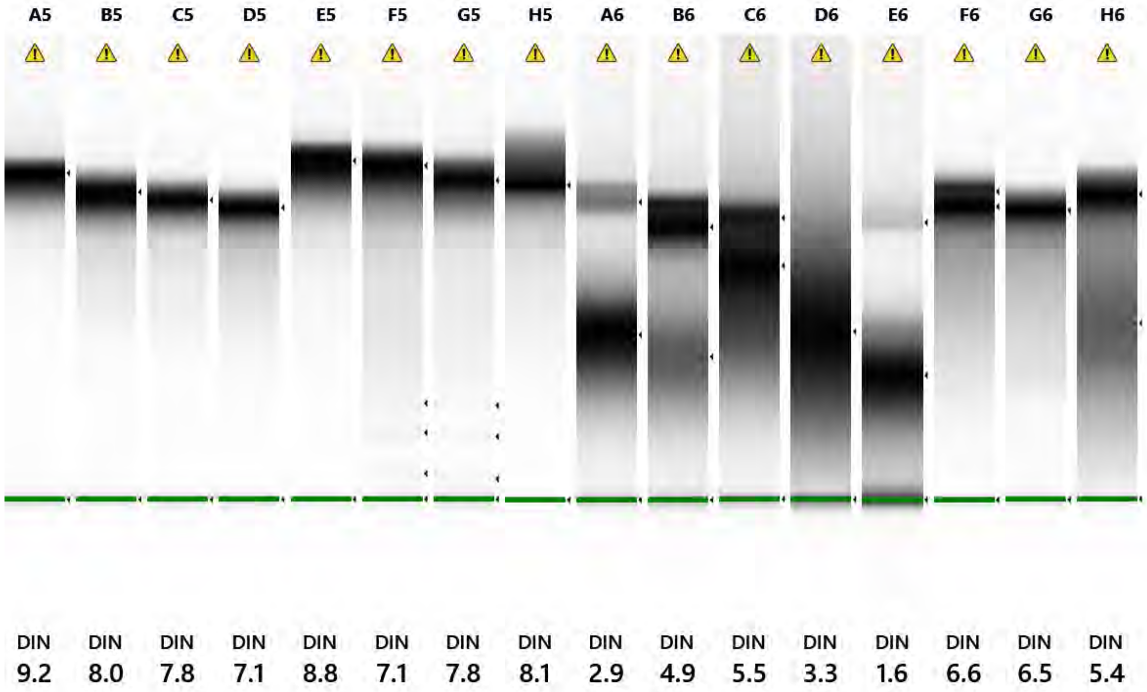

Default image (Contrast 50%), Image is Scaled to Sample

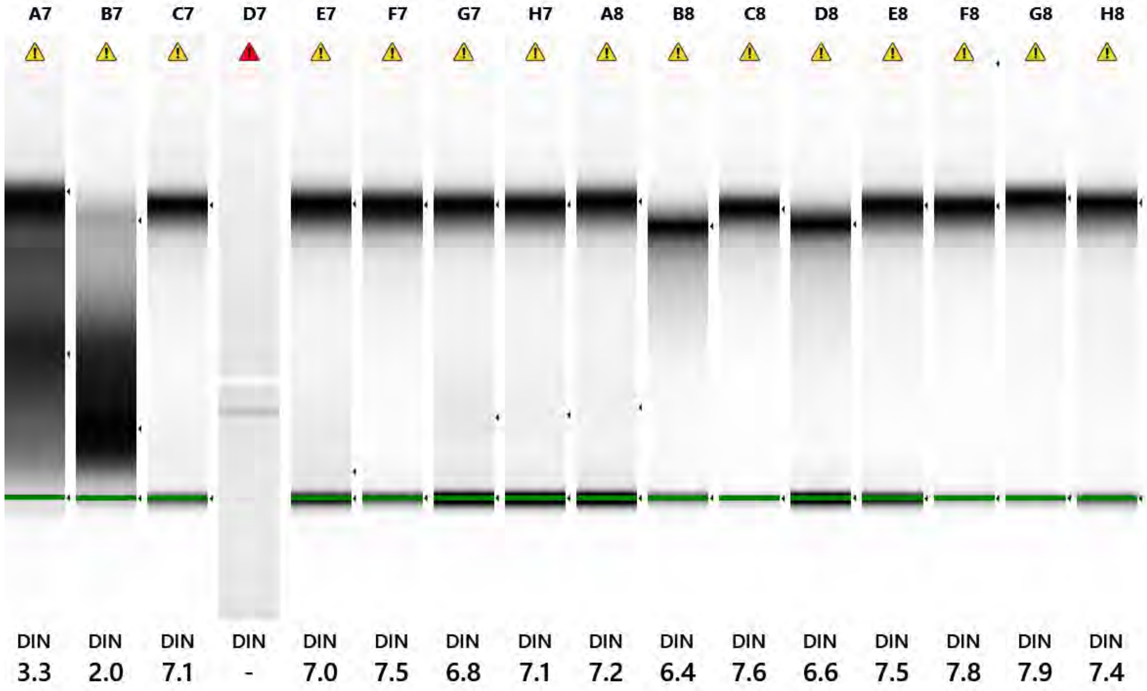

Default image (Contrast 50%), Image is Scaled to Sample

© Agilent Technologies, Inc. 2017

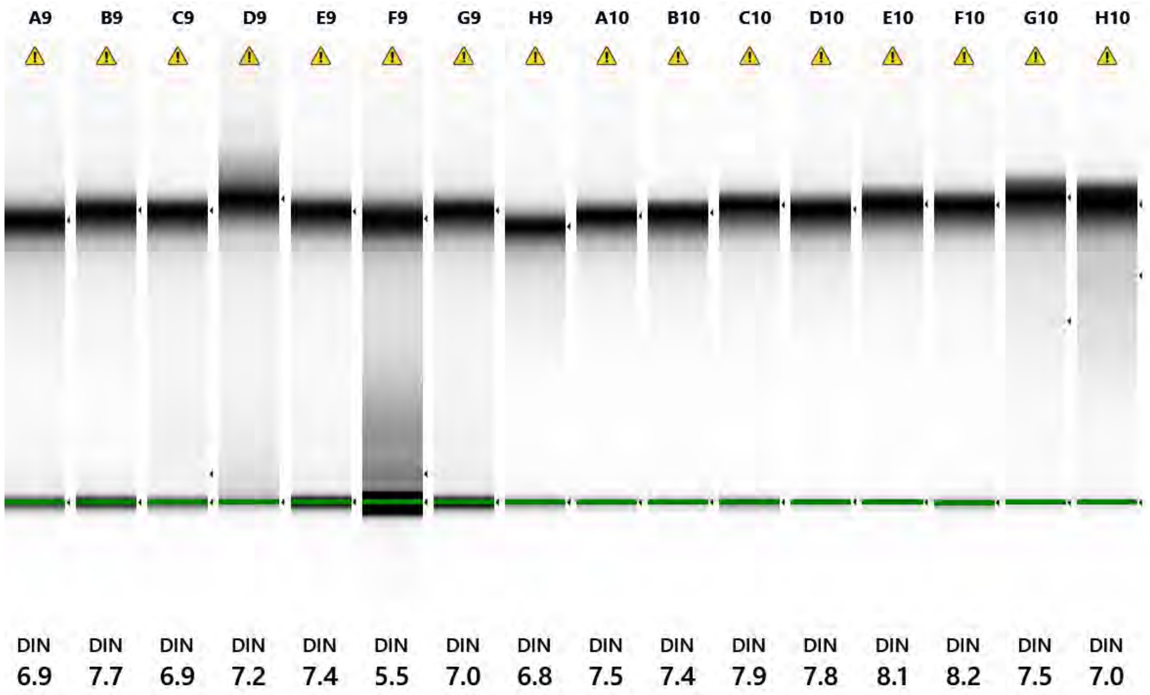

Default image (Contrast 50%), Image is Scaled to Sample

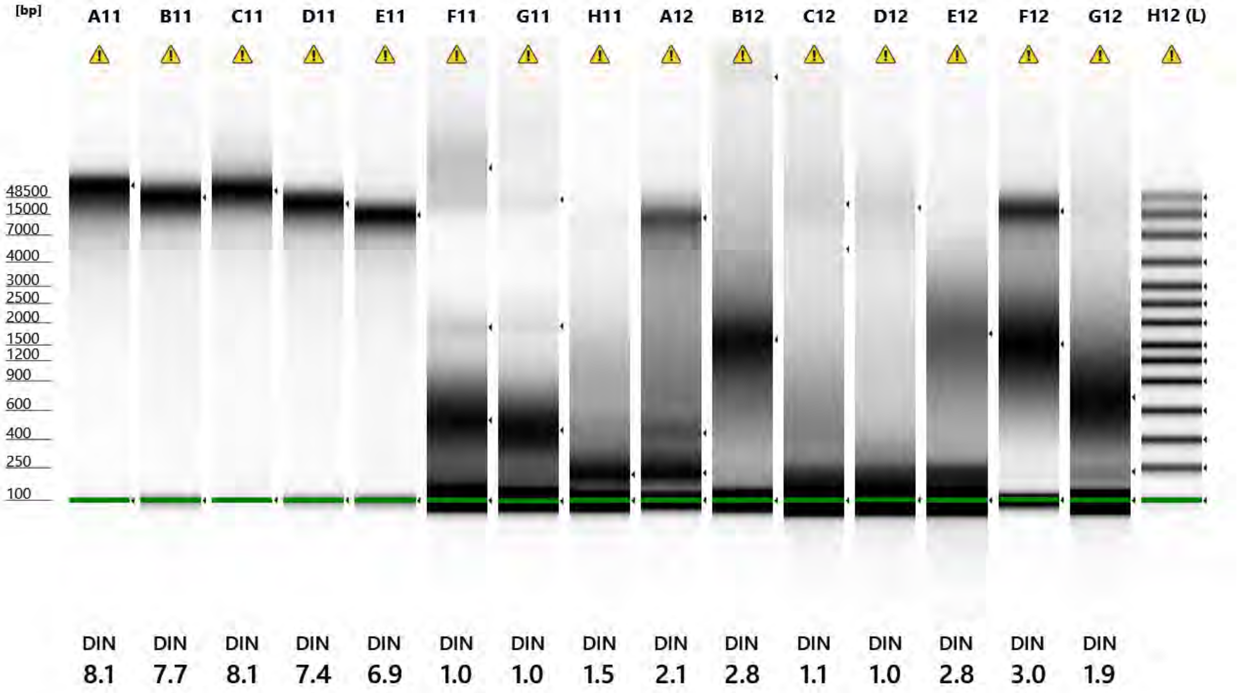

Default image (Contrast 50%), Image is Scaled to Sample

© Agilent Technologies, Inc. 2017

Sample Info

Well	DIN	Conc. [ng/µl]	Sample Description	Alert	Observations
A1	7.0	114	M181	⚠	Caution! Expired ScreenTape device; Sample concentration outside recommended range
B1	8.1	292	M182	⚠	Caution! Expired ScreenTape device; Sample concentration outside recommended range
C1	8.1	240	M183	⚠	Caution! Expired ScreenTape device; Sample concentration outside recommended range
D1	7.6	176	M184	⚠	Caution! Expired ScreenTape device; Sample concentration outside recommended range
E1	8.0	146	M185	⚠	Caution! Expired ScreenTape device; Sample concentration outside recommended range
F1	8.4	409	M298	⚠	Caution! Expired ScreenTape device; Sample concentration outside functional range for DIN
G1	7.8	256	M299	⚠	Caution! Expired ScreenTape device; Sample concentration outside recommended range
H1	7.7	217	M484	⚠	Caution! Expired ScreenTape device; Sample concentration outside recommended range
A2	8.2	446	M485	⚠	Caution! Expired ScreenTape device; Sample concentration outside functional range for DIN
B2	8.2	252	M486	⚠	Caution! Expired ScreenTape device; Sample concentration outside recommended range
C2	-	0.937	M186	▲	Caution! Expired ScreenTape device; Sample concentration outside functional range for DIN
D2	7.7	274	M187	⚠	Caution! Expired ScreenTape device; Sample concentration outside recommended range
E2	7.8	262	M188	⚠	Caution! Expired ScreenTape device; Sample concentration outside recommended range
F2	7.4	116	M189	⚠	Caution! Expired ScreenTape device; Sample concentration outside recommended range
G2	6.6	318	M190	⚠	Caution! Expired ScreenTape device; Sample concentration outside functional range for DIN
H2	6.5	127	M300	⚠	Caution! Expired ScreenTape device; Sample concentration outside recommended range
A3	7.6	283	M301	⚠	Caution! Expired ScreenTape device; Sample concentration outside recommended range
B3	7.2	193	M487	⚠	Caution! Expired ScreenTape device; Sample concentration outside recommended range
C3	7.7	675	M488	⚠	Caution! Expired ScreenTape device; Sample concentration outside functional range for DIN
D3	7.6	264	M489	⚠	Caution! Expired ScreenTape device; Sample concentration outside recommended range
E3	8.1	209	M196	⚠	Caution! Expired ScreenTape device; Sample concentration outside recommended range
F3	8.6	286	M197	⚠	Caution! Expired ScreenTape device; Sample concentration outside recommended range
G3	7.2	253	M198	⚠	Caution! Expired ScreenTape device; Sample concentration outside recommended range
H3	7.6	130	M199	⚠	Caution! Expired ScreenTape device; Sample concentration outside recommended range
A4	6.8	80.3	M200	⚠	Caution! Expired ScreenTape device
B4	8.2	436	M304	⚠	Caution! Expired ScreenTape device; Sample concentration outside functional range for DIN
C4	8.5	526	M305	⚠	Caution! Expired ScreenTape device; Sample concentration outside functional range for DIN
D4	8.2	110	M493	⚠	Caution! Expired ScreenTape device; Sample concentration outside recommended range
E4	8.5	248	M494	⚠	Caution! Expired ScreenTape device; Sample concentration outside recommended range
F4	8.1	108	M495	⚠	Caution! Expired ScreenTape device; Sample concentration outside recommended range
G4	8.5	553	M206	⚠	Caution! Expired ScreenTape device; Sample concentration outside functional range for DIN
H4	7.6	70.5	M207	⚠	Caution! Expired ScreenTape device
A5	9.2	243	M208	⚠	Caution! Expired ScreenTape device; Sample concentration outside recommended range
B5	8.0	337	M209	⚠	Caution! Expired ScreenTape device; Sample concentration outside functional range for DIN
C5	7.8	240	M210	⚠	Caution! Expired ScreenTape device; Sample concentration outside recommended range
D5	7.1	280	M308	⚠	Caution! Expired ScreenTape device; Sample concentration outside recommended range
E5	8.8	639	M309	⚠	Caution! Expired ScreenTape device; Sample concentration outside functional range for DIN
F5	7.1	591	M499	⚠	Caution! Expired ScreenTape device; Sample concentration outside functional range for DIN
G5	7.8	263	M500	⚠	Caution! Expired ScreenTape device; Sample concentration outside recommended range
H5	8.1	2780	M501	⚠	Caution! Expired ScreenTape device; Sample concentration outside functional range for DIN
A6	2.9	379	M216	⚠	Caution! Expired ScreenTape device; Sample concentration outside functional range for DIN
B6	4.9	307	M217	⚠	Caution! Expired ScreenTape device; Sample concentration outside functional range for DIN
C6	5.5	458	M218	⚠	Caution! Expired ScreenTape device; Sample concentration outside functional range for DIN
D6	3.3	236	M219	⚠	Caution! Expired ScreenTape device; Sample concentration outside recommended range
E6	1.6	68.8	M220	⚠	Caution! Expired ScreenTape device
F6	6.6	785	M312	⚠	Caution! Expired ScreenTape device; Sample concentration outside functional range for DIN
G6	6.5	407	M313	⚠	Caution! Expired ScreenTape device; Sample concentration outside functional range for DIN
H6	5.4	998	M481	⚠	Caution! Expired ScreenTape device; Sample concentration outside functional range for DIN
A7	3.3	777	M482	⚠	Caution! Expired ScreenTape device; Sample concentration outside functional range for DIN; DIN Edited (Marker position changed)
B7	2.0	377	M483	⚠	Caution! Expired ScreenTape device; Sample concentration outside functional range for DIN
C7	7.1	48.3	C388	⚠	Caution! Expired ScreenTape device
D7	-		C389	▲	Marker(s) not detected; Caution! Expired ScreenTape device
E7	7.0	33.7	C631	⚠	Caution! Expired ScreenTape device
F7	7.5	47.2	C632	⚠	Caution! Expired ScreenTape device
G7	6.8	21.6	C633	⚠	Caution! Expired ScreenTape device
H7	7.1	20.1	C634	⚠	Caution! Expired ScreenTape device
A8	7.2	22.1	C635	⚠	Caution! Expired ScreenTape device
B8	6.4	68.7	C390	⚠	Caution! Expired ScreenTape device
C8	7.6	123	C391	⚠	Caution! Expired ScreenTape device; Sample concentration outside recommended range
D8	6.6	24.6	C636	⚠	Caution! Expired ScreenTape device
E8	7.5	30.8	C637	⚠	Caution! Expired ScreenTape device
F8	7.8	85.3	C638	⚠	Caution! Expired ScreenTape device
G8	7.9	80.9	C639	⚠	Caution! Expired ScreenTape device
H8	7.4	50.0	C640	⚠	Caution! Expired ScreenTape device
A9	6.9	44.7	C394	⚠	Caution! Expired ScreenTape device
B9	7.7	33.8	C395	⚠	Caution! Expired ScreenTape device
C9	6.9	49.1	C646	⚠	Caution! Expired ScreenTape device
D9	7.2	77.4	C647	⚠	Caution! Expired ScreenTape device

 © Agilent Technologies, Inc. 2017

E9	7.4	28.3	C648	⚠	Caution! Expired ScreenTape device
F9	5.5	8.65	C649	⚠	Caution! Expired ScreenTape device; Sample concentration outside recommended range
G9	7.0	29.8	C650	⚠	Caution! Expired ScreenTape device
H9	6.8	69.2	C398	⚠	Caution! Expired ScreenTape device
A10	7.5	97.3	C399	⚠	Caution! Expired ScreenTape device
B10	7.4	127	C656	⚠	Caution! Expired ScreenTape device; Sample concentration outside recommended range
C10	7.9	65.5	C657	⚠	Caution! Expired ScreenTape device
D10	7.8	141	C658	⚠	Caution! Expired ScreenTape device; Sample concentration outside recommended range
E10	8.1	193	C659	⚠	Caution! Expired ScreenTape device; Sample concentration outside recommended range
F10	8.2	87.2	C660	⚠	Caution! Expired ScreenTape device
G10	7.5	264	C402	⚠	Caution! Expired ScreenTape device; Sample concentration outside recommended range
H10	7.0	297	C403	⚠	Caution! Expired ScreenTape device; Sample concentration outside recommended range
A11	8.1	502	C666	⚠	Caution! Expired ScreenTape device; Sample concentration outside functional range for DIN
B11	7.7	86.3	C667	⚠	Caution! Expired ScreenTape device
C11	8.1	252	C668	⚠	Caution! Expired ScreenTape device; Sample concentration outside recommended range
D11	7.4	83.3	C669	⚠	Caution! Expired ScreenTape device
E11	6.9	59.7	C670	⚠	Caution! Expired ScreenTape device
F11	1.0	13.4	M191	⚠	Caution! Expired ScreenTape device
G11	1.0	12.4	M195	⚠	Caution! Expired ScreenTape device
H11	1.5	9.98	M201	⚠	Caution! Expired ScreenTape device; Sample concentration outside recommended range
A12	2.1	36.4	M202	⚠	Caution! Expired ScreenTape device
B12	2.8	18.8	C392	⚠	Caution! Expired ScreenTape device
C12	1.1	3.17	C393	⚠	Caution! Expired ScreenTape device; Sample concentration outside functional range for DIN
D12	1.0	3.16	C396	⚠	Caution! Expired ScreenTape device; Sample concentration outside functional range for DIN
E12	2.8	3.74	W859	⚠	Caution! Expired ScreenTape device; Sample concentration outside functional range for DIN
F12	3.0	61.1	W874	⚠	Caution! Expired ScreenTape device
G12	1.9	7.99	W885	⚠	Caution! Expired ScreenTape device; Sample concentration outside recommended range
H12	-	352	LADDER	⚠	Caution! Expired ScreenTape device; Ladder

© Agilent Technologies, Inc. 2017

A1: M181

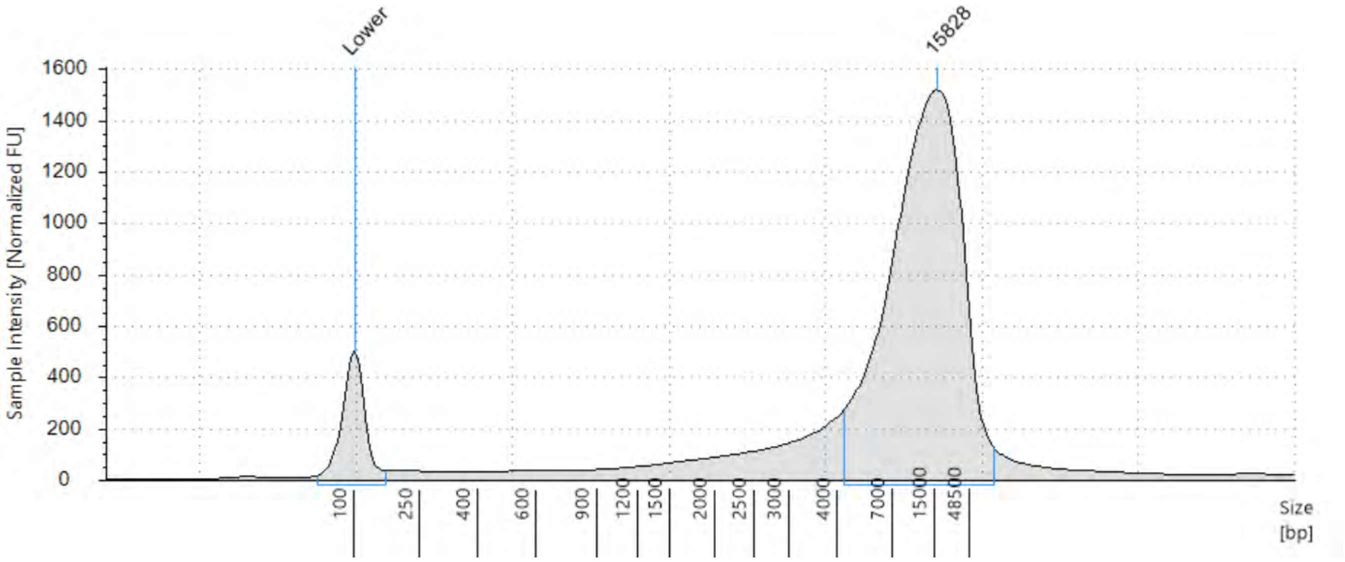

Sample Table

Well	DIN	Conc. [ng/µl]	Sample Description	Alert	Observations
A1	7.0	114	M181	⚠	Caution! Expired ScreenTape device; Sample concentration outside recommended range

Peak Table

Size [bp]	Calibrated Conc. [ng/µl]	Assigned Conc. [ng/µl]	% Integrated Area	From [bp]	To [bp]	Peak Comment	Observations
100	8.50	8.50	-	59	152		Lower Marker
15828	83.3	-	96.43	4291	>60000		
-	-	-	-	-	-		Sample Well

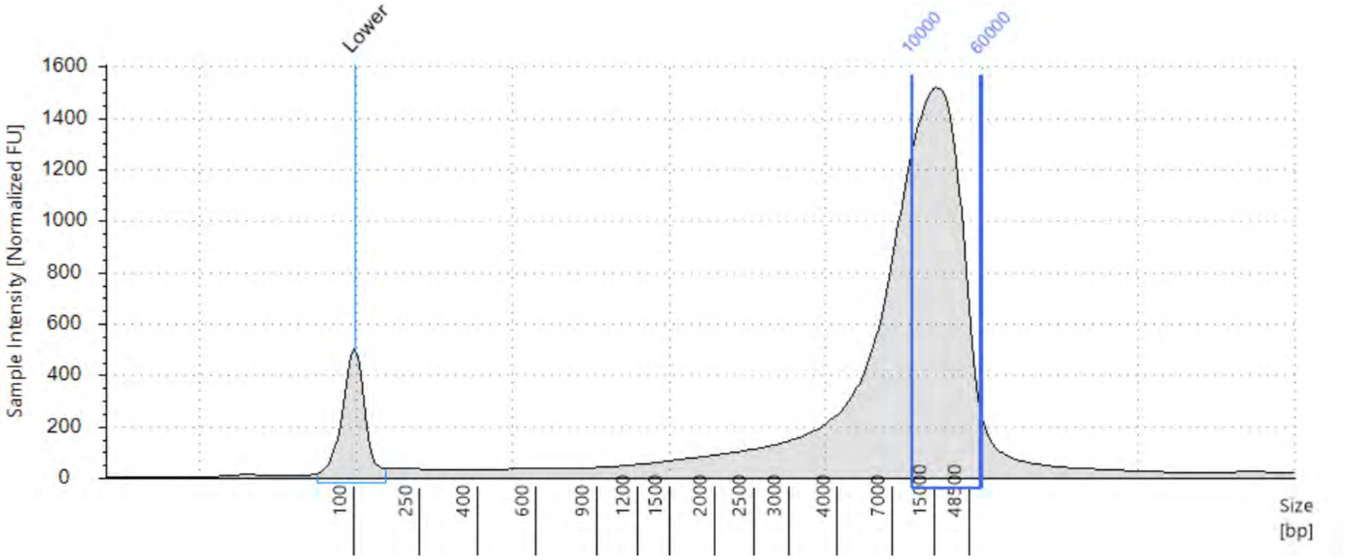

Region Table

From [bp]	To [bp]	Average Size [bp]	Conc. [ng/µl]	Region Molarity [nmol/l]	% of Total	Region Comment	Color
10000	60000	20353	53.7	4.96	47.28		■

 © Agilent Technologies, Inc. 2017

B1: M182

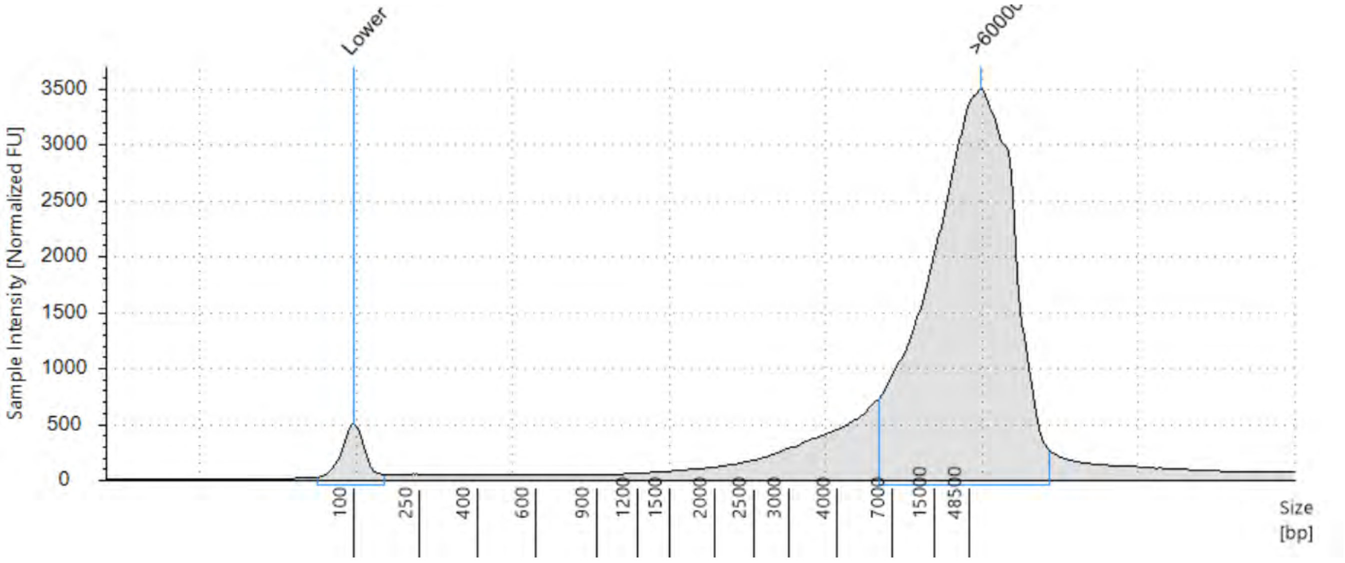

Sample Table

Well	DIN	Conc. [ng/µl]	Sample Description	Alert	Observations
B1	8.1	292	M182	⚠	Caution! Expired ScreenTape device; Sample concentration outside recommended range

Peak Table

Size [bp]	Calibrated Conc. [ng/µl]	Assigned Conc. [ng/µl]	% Integrated Area	From [bp]	To [bp]	Peak Comment	Observations
100	8.50	8.50	-	60	152		Lower Marker
>60000	219	-	91.58	6184	>60000		
-	-	-	-	-	-		Sample Well

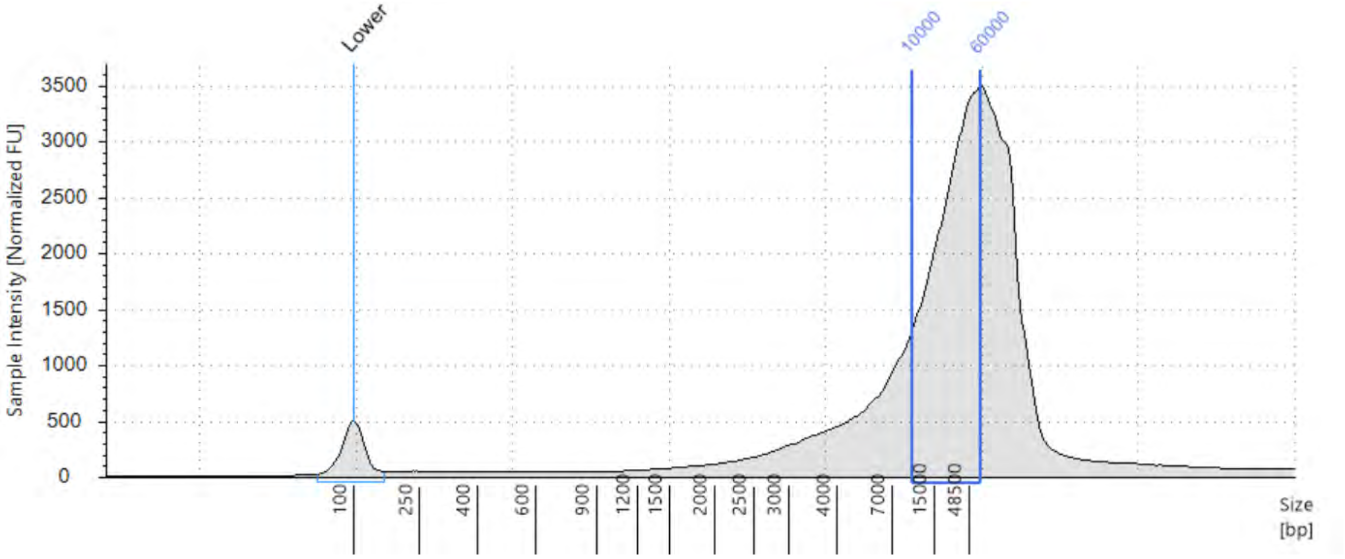

Region Table

From [bp]	To [bp]	Average Size [bp]	Conc. [ng/µl]	Region Molarity [nmol/l]	% of Total	Region Comment	Color
10000	60000	29565	111	7.85	37.98		■

 © Agilent Technologies, Inc. 2017

C1: M183

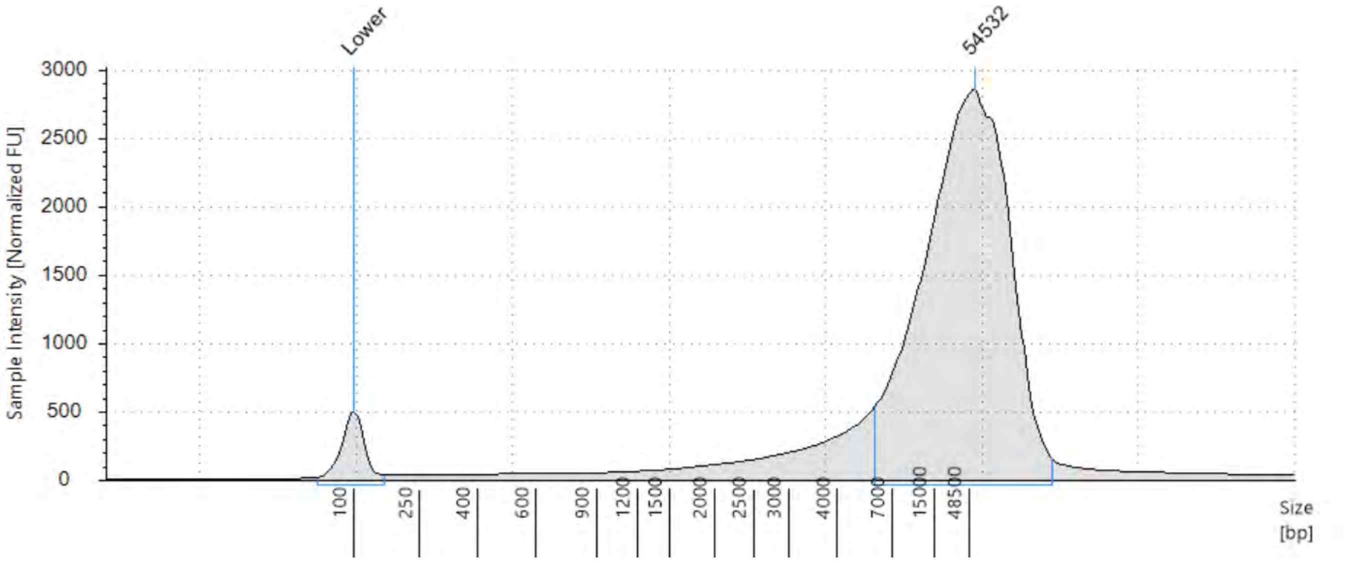

Sample Table

Well	DIN	Conc. [ng/µl]	Sample Description	Alert	Observations
C1	8.1	240	M183	⚠	Caution! Expired ScreenTape device; Sample concentration outside recommended range

Peak Table

Size [bp]	Calibrated Conc. [ng/µl]	Assigned Conc. [ng/µl]	% Integrated Area	From [bp]	To [bp]	Peak Comment	Observations
100	8.50	8.50	-	60	154		Lower Marker
54532	191	-	93.13	5928	>60000		
-	-	-	-	-	-		Sample Well

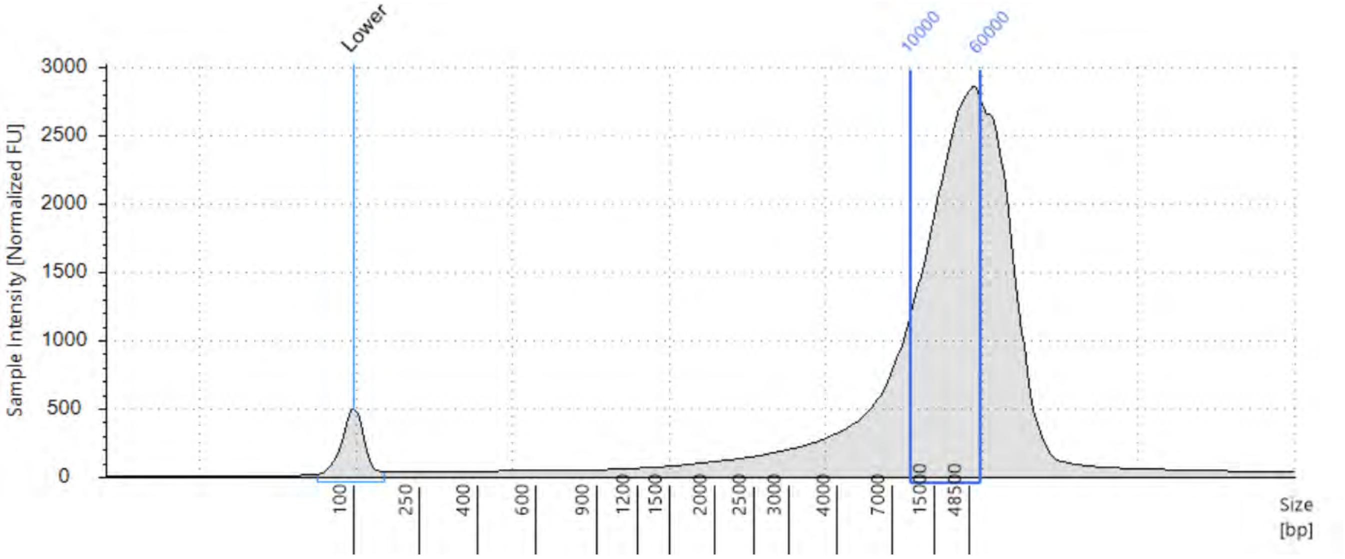

Region Table

From [bp]	To [bp]	Average Size [bp]	Conc. [ng/µl]	Region Molarity [nmol/l]	% of Total	Region Comment	Color
10000	60000	29119	105	7.57	43.67		■

 © Agilent Technologies, Inc. 2017

D1: M184

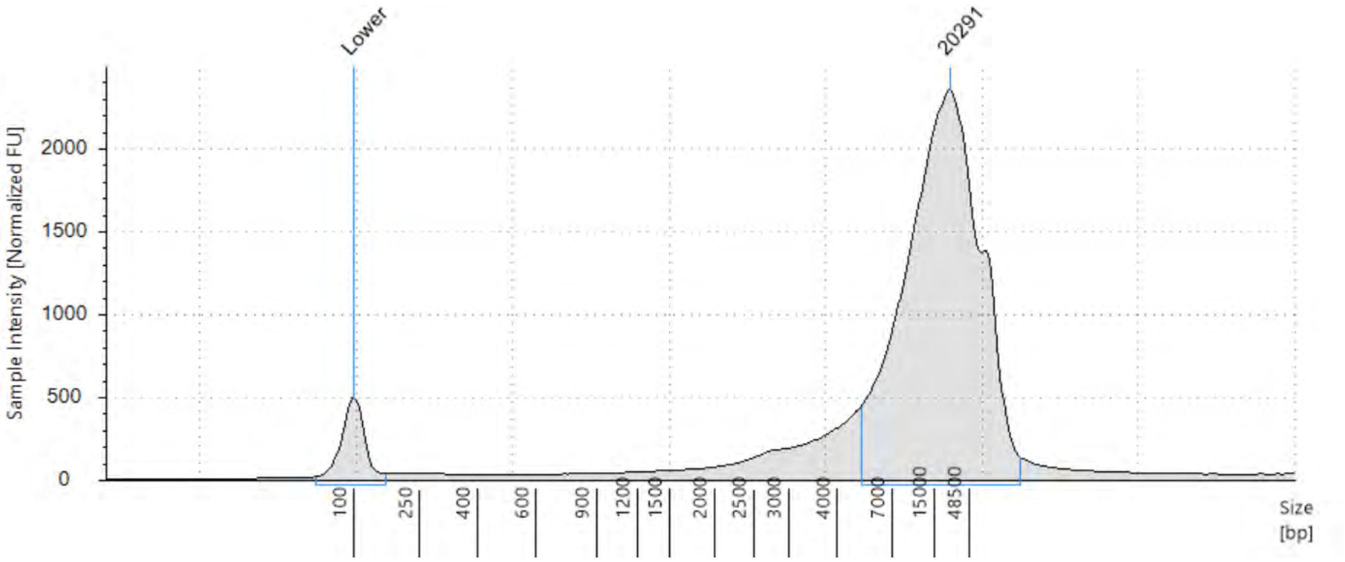

Sample Table

Well	DIN	Conc. [ng/µl]	Sample Description	Alert	Observations
D1	7.6	176	M184	⚠	Caution! Expired ScreenTape device; Sample concentration outside recommended range

Peak Table

Size [bp]	Calibrated Conc. [ng/µl]	Assigned Conc. [ng/µl]	% Integrated Area	From [bp]	To [bp]	Peak Comment	Observations
100	8.50	8.50	-	59	154		Lower Marker
20291	134	-	92.14	5139	>60000		
-	-	-	-	-	-		Sample Well

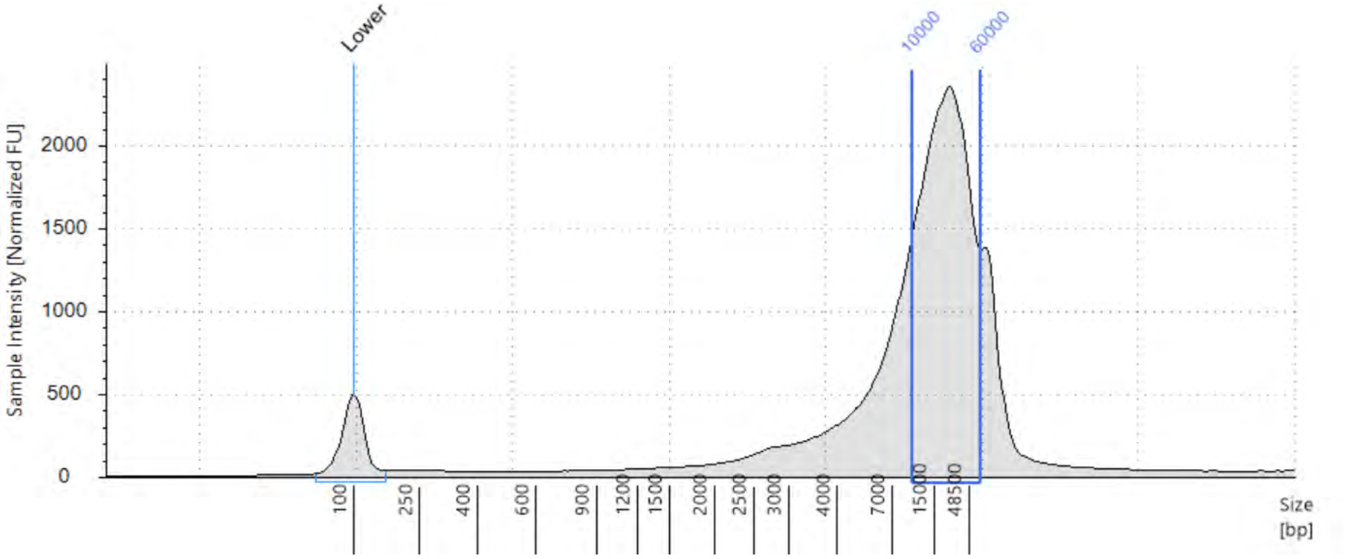

Region Table

From [bp]	To [bp]	Average Size [bp]	Conc. [ng/µl]	Region Molarity [nmol/l]	% of Total	Region Comment	Color
10000	60000	24554	89.3	7.30	50.74		■

E1: M185

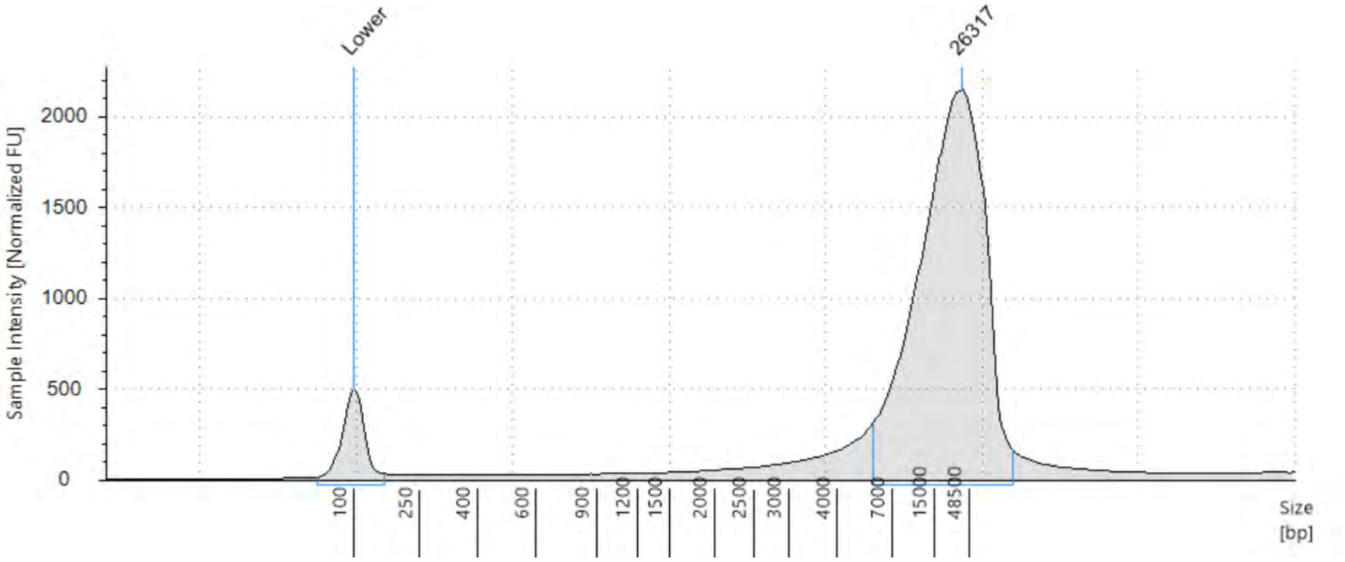

Sample Table

Well	DIN	Conc. [ng/µl]	Sample Description	Alert	Observations
E1	8.0	146	M185	⚠	Caution! Expired ScreenTape device; Sample concentration outside recommended range

Peak Table

Size [bp]	Calibrated Conc. [ng/µl]	Assigned Conc. [ng/µl]	% Integrated Area	From [bp]	To [bp]	Peak Comment	Observations
100	8.50	8.50	-	60	152		Lower Marker
26317	112	-	92.49	5764	>60000		
-	-	-	-	-	-		Sample Well

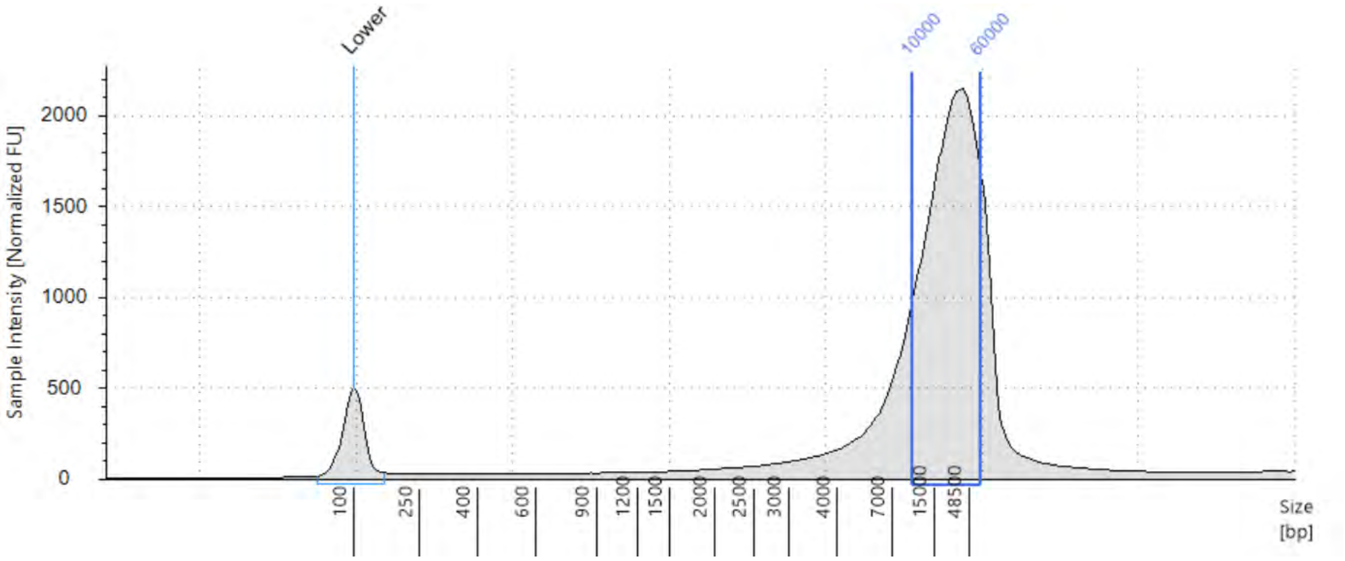

Region Table

From [bp]	To [bp]	Average Size [bp]	Conc. [ng/µl]	Region Molarity [nmol/l]	% of Total	Region Comment	Color
10000	60000	27271	81.8	6.14	55.98		■

© Agilent Technologies, Inc. 2017

F1: M298

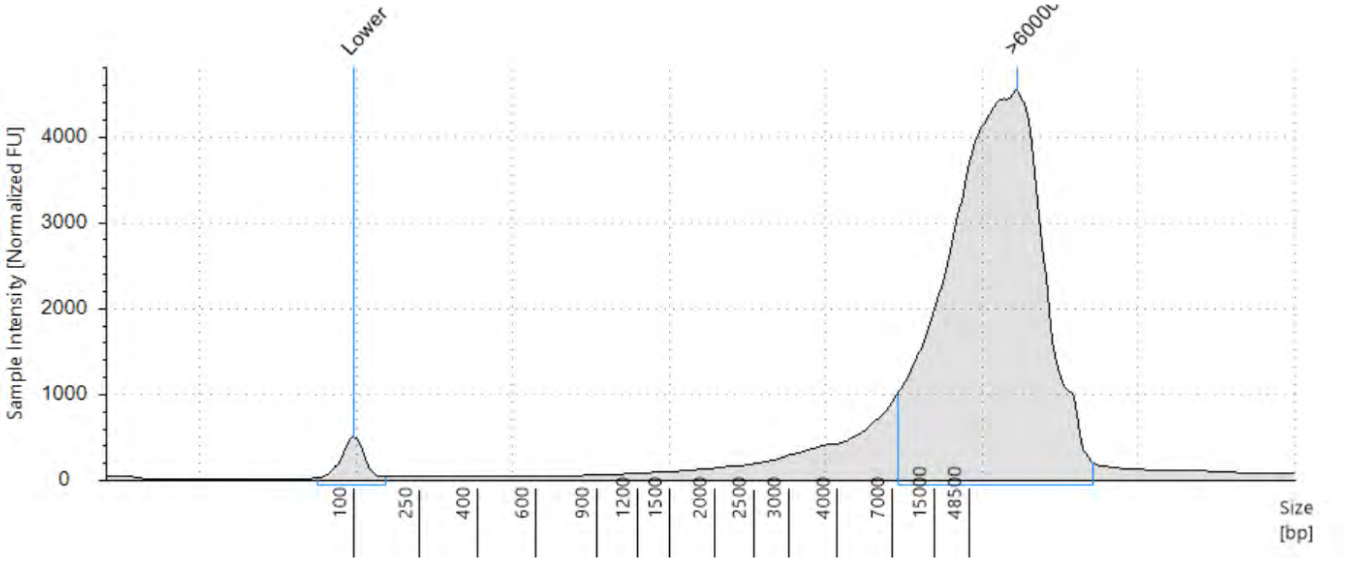

Sample Table

Well	DIN	Conc. [ng/µl]	Sample Description	Alert	Observations
F1	8.4	409	M298	⚠	Caution! Expired ScreenTape device; Sample concentration outside functional range for DIN

Peak Table

Size [bp]	Calibrated Conc. [ng/µl]	Assigned Conc. [ng/µl]	% Integrated Area	From [bp]	To [bp]	Peak Comment	Observations
100	8.50	8.50	-	60	155		Lower Marker
>60000	332	-	94.57	7877	>60000		
-	-	-	-	-	-		Sample Well

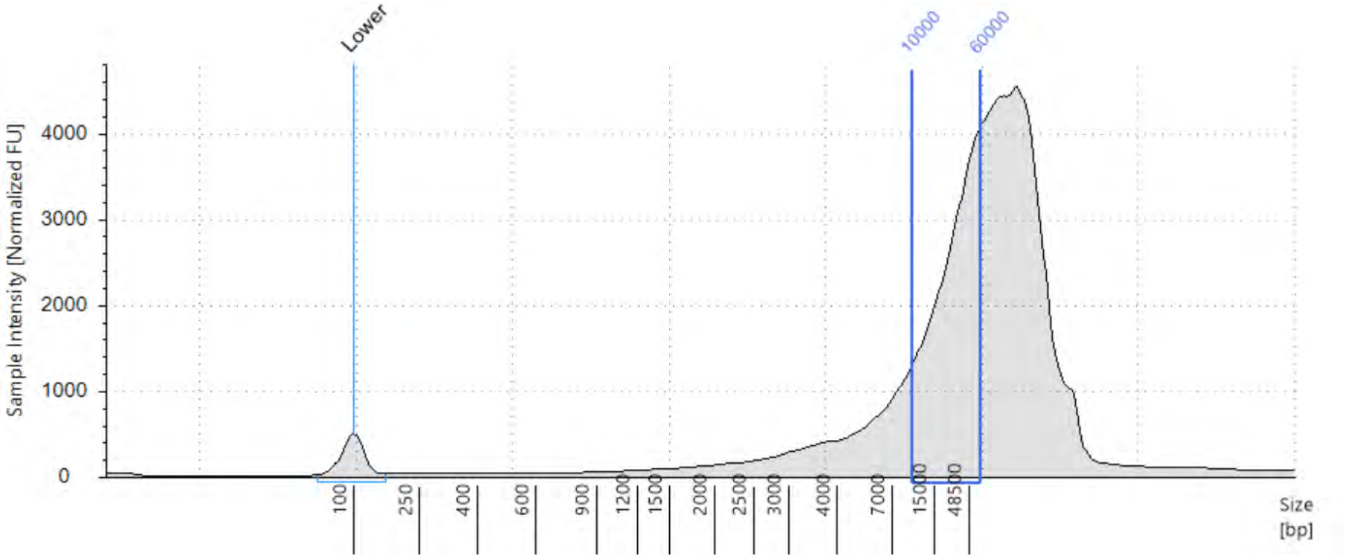

Region Table

From [bp]	To [bp]	Average Size [bp]	Conc. [ng/µl]	Region Molarity [nmol/l]	% of Total	Region Comment	Color
10000	60000	30885	119	8.22	29.18		■

 © Agilent Technologies, Inc. 2017

G1: M299

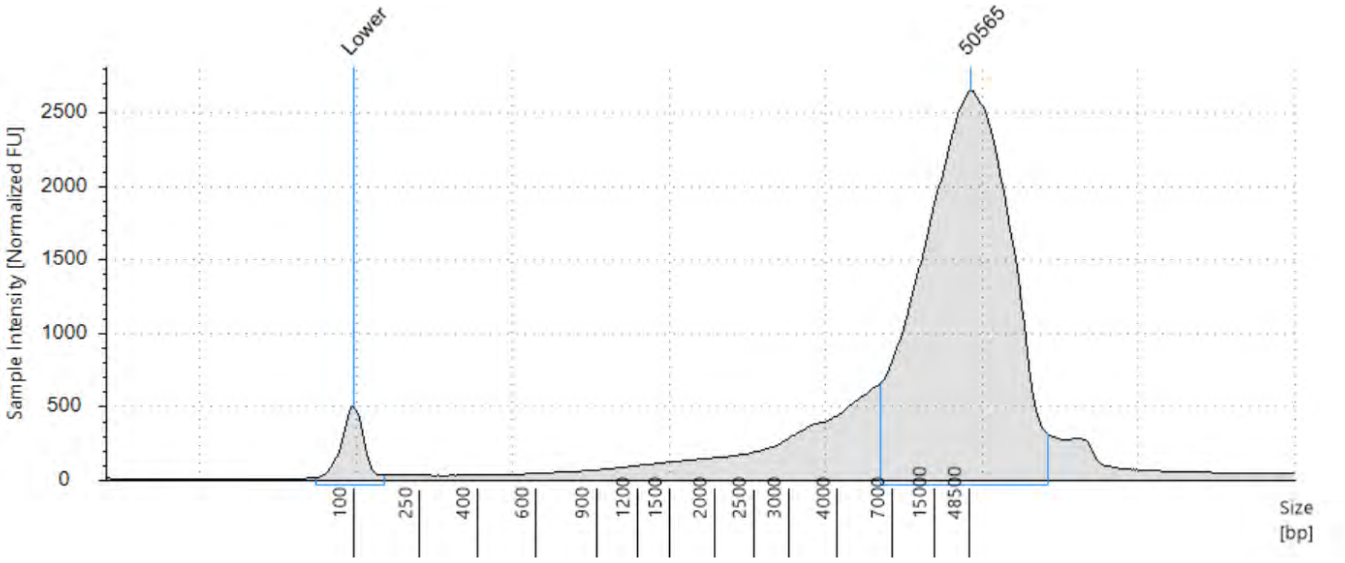

Sample Table

Well	DIN	Conc. [ng/µl]	Sample Description	Alert	Observations
G1	7.8	256	M299	⚠	Caution! Expired ScreenTape device; Sample concentration outside recommended range

Peak Table

Size [bp]	Calibrated Conc. [ng/µl]	Assigned Conc. [ng/µl]	% Integrated Area	From [bp]	To [bp]	Peak Comment	Observations
100	8.50	8.50	-	59	152		Lower Marker
50565	186	-	96.09	6273	>60000		
-	-	-	-	-	-		Sample Well

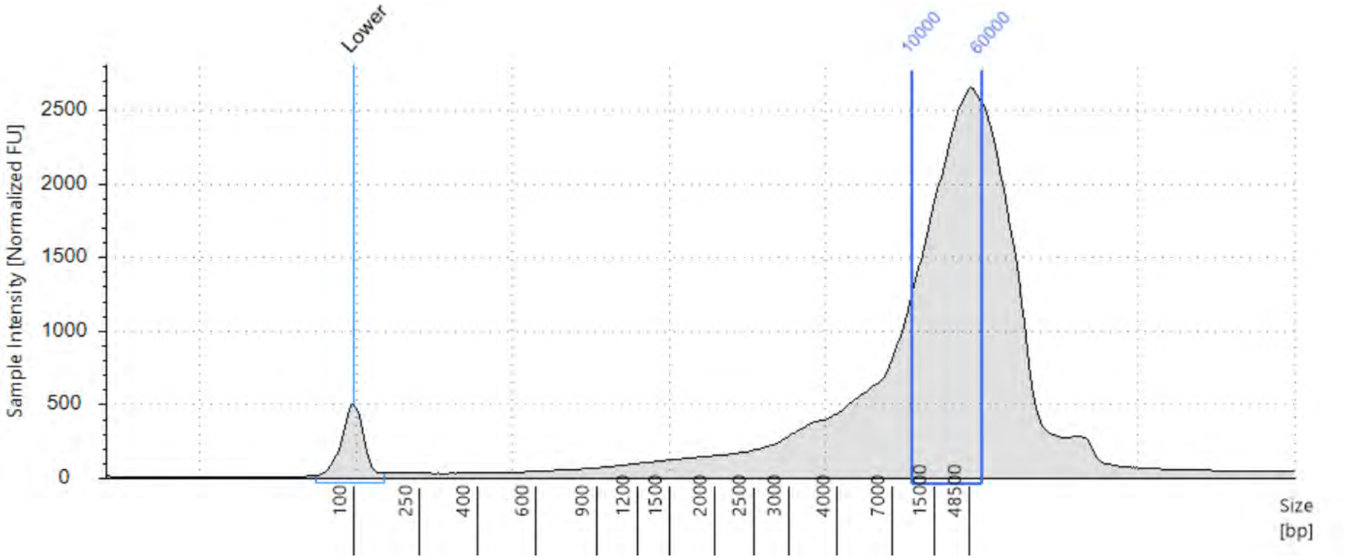

Region Table

From [bp]	To [bp]	Average Size [bp]	Conc. [ng/µl]	Region Molarity [nmol/l]	% of Total	Region Comment	Color
10000	60000	28561	102	7.53	40.03		■

 © Agilent Technologies, Inc. 2017

D2: M187

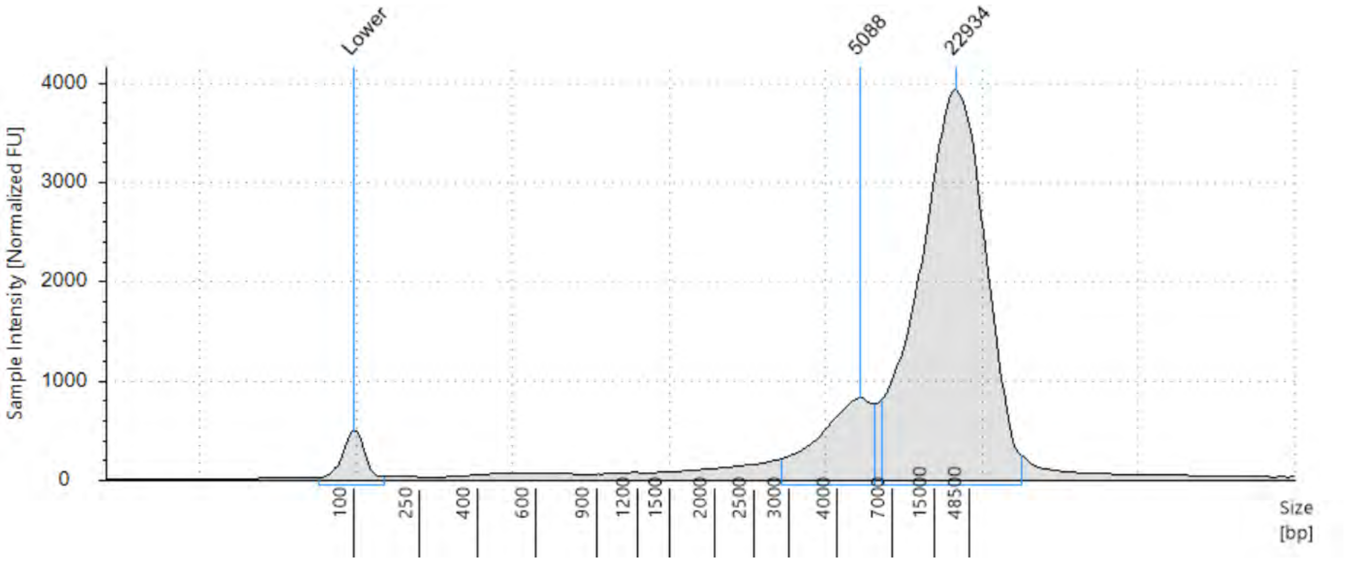

Sample Table

Well	DIN	Conc. [ng/µl]	Sample Description	Alert	Observations
D2	7.7	274	M187	⚠	Caution! Expired ScreenTape device; Sample concentration outside recommended range

Peak Table

Size [bp]	Calibrated Conc. [ng/µl]	Assigned Conc. [ng/µl]	% Integrated Area	From [bp]	To [bp]	Peak Comment	Observations
100	8.50	8.50	-	60	153		Lower Marker
5088	33.2	-	13.41	2913	5871		
22934	206	-	83.23	6306	>60000		
-	-	-	-	-	-		Sample Well

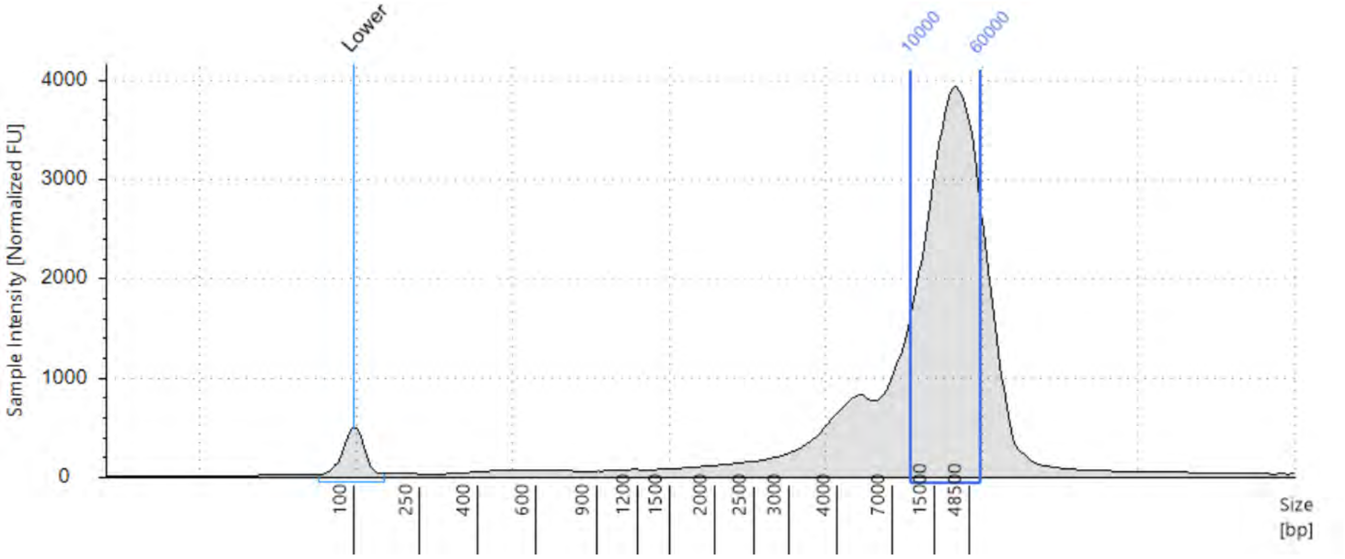

Region Table

From [bp]	To [bp]	Average Size [bp]	Conc. [ng/µl]	Region Molarity [nmol/l]	% of Total	Region Comment	Color
10000	60000	27138	151	11.4	55.20		■

 © Agilent Technologies, Inc. 2017

E2: M188

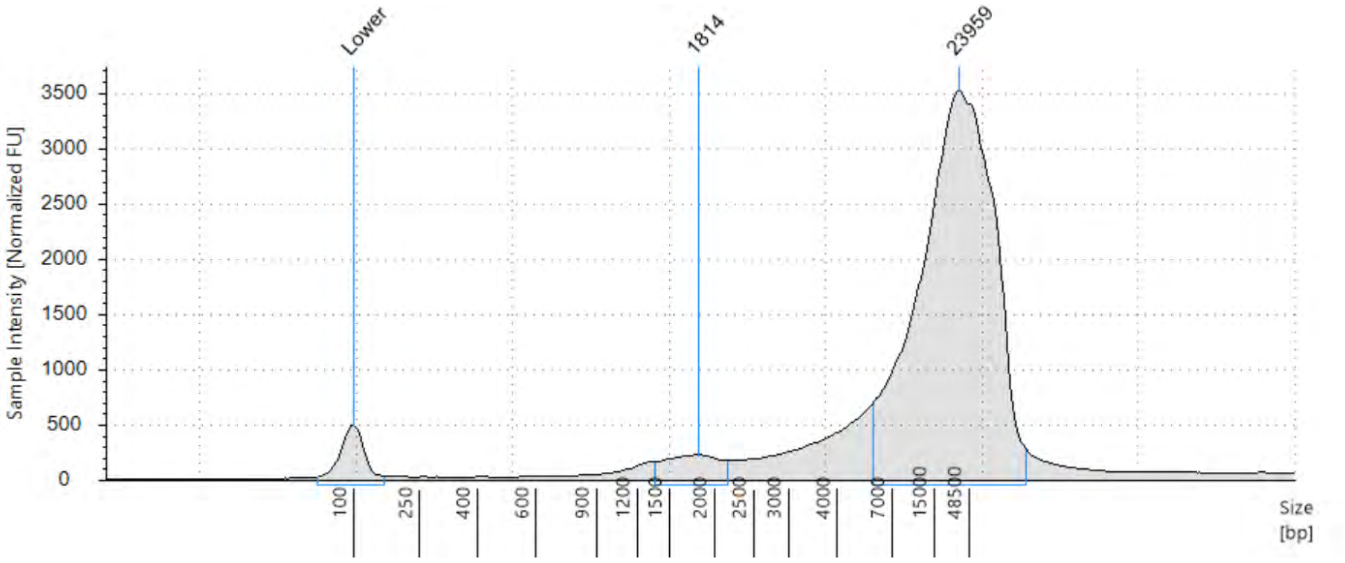

Sample Table

Well	DIN	Conc. [ng/µl]	Sample Description	Alert	Observations
E2	7.8	262	M188	⚠	Caution! Expired ScreenTape device; Sample concentration outside recommended range

Peak Table

Size [bp]	Calibrated Conc. [ng/µl]	Assigned Conc. [ng/µl]	% Integrated Area	From [bp]	To [bp]	Peak Comment	Observations
100	8.50	8.50	-	60	154		Lower Marker
1814	9.13	-	4.29	1361	2170		
23959	197	-	92.62	5782	>60000		
-	-	-	-	-	-		Sample Well

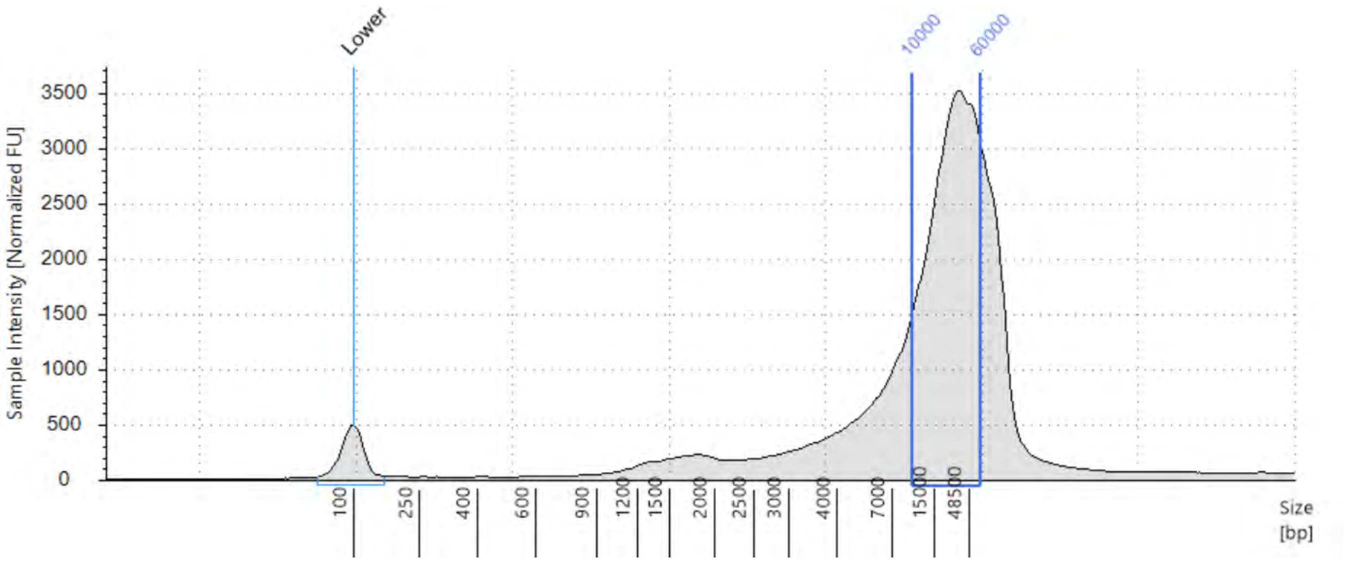

Region Table

From [bp]	To [bp]	Average Size [bp]	Conc. [ng/µl]	Region Molarity [nmol/l]	% of Total	Region Comment	Color
10000	60000	27980	127	9.32	48.40		■

 © Agilent Technologies, Inc. 2017

F2: M189

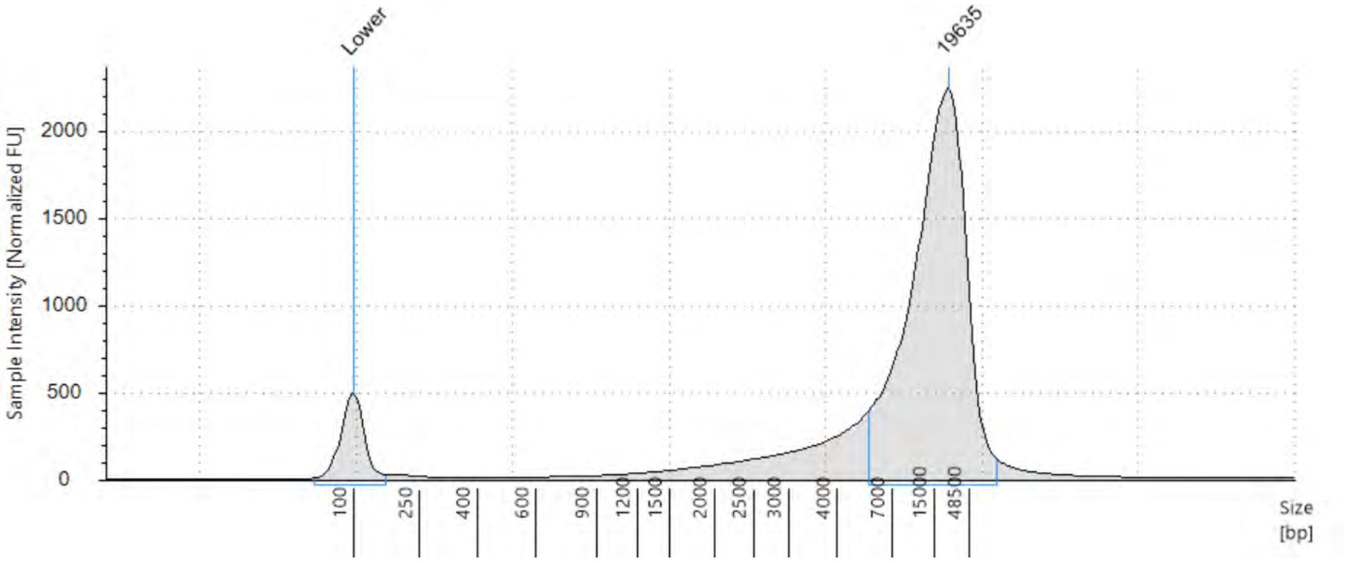

Sample Table

Well	DIN	Conc. [ng/µl]	Sample Description	Alert	Observations
F2	7.4	116	M189	⚠	Caution! Expired ScreenTape device; Sample concentration outside recommended range

Peak Table

Size [bp]	Calibrated Conc. [ng/µl]	Assigned Conc. [ng/µl]	% Integrated Area	From [bp]	To [bp]	Peak Comment	Observations
100	8.50	8.50	-	58	156		Lower Marker
19635	89.4	-	98.20	5558	>60000		
-	-	-	-	-	-		Sample Well

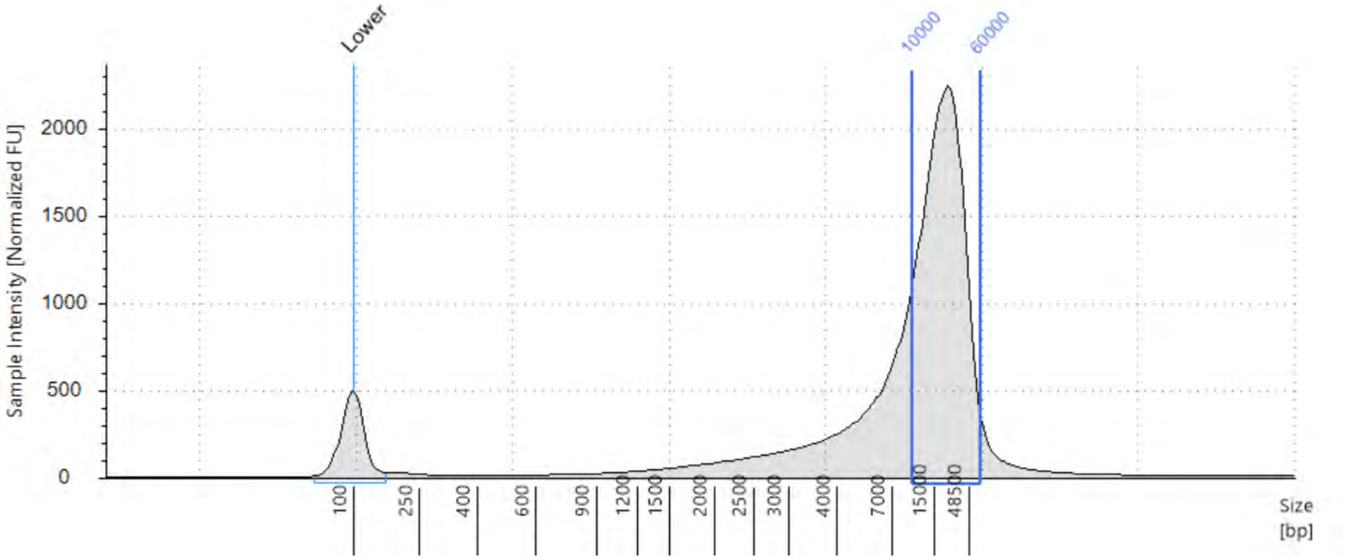

Region Table

From [bp]	To [bp]	Average Size [bp]	Conc. [ng/µl]	Region Molarity [nmol/l]	% of Total	Region Comment	Color
10000	60000	21859	70.2	6.02	60.52		■

© Agilent Technologies, Inc. 2017

G2: M190

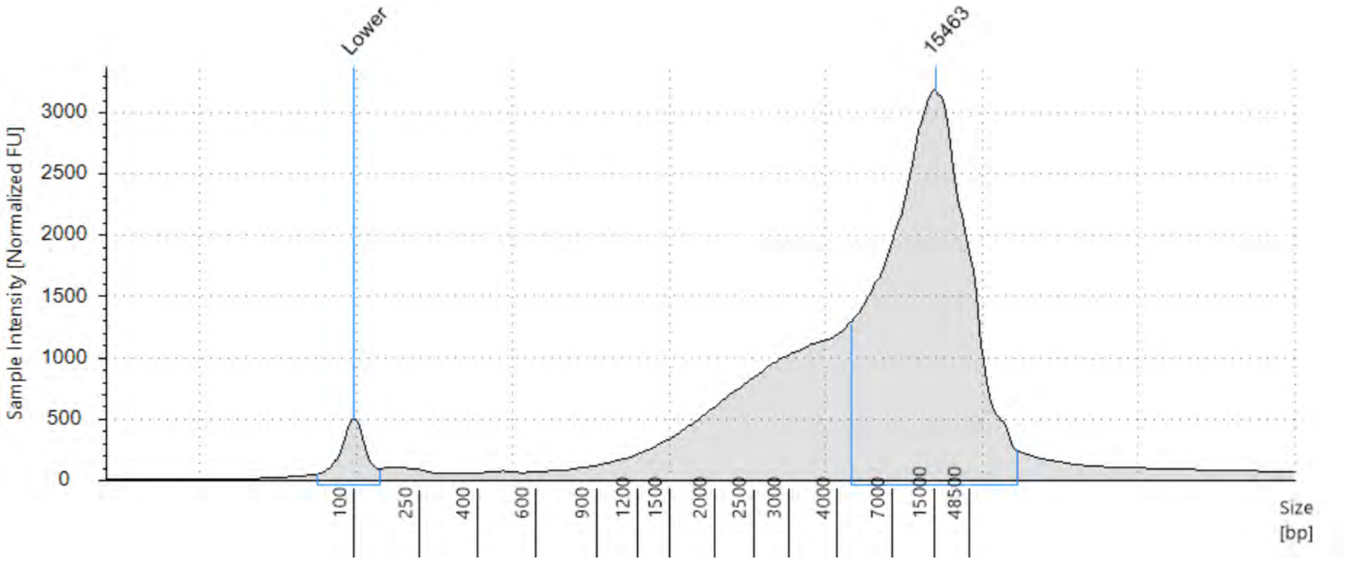

Sample Table

Well	DIN	Conc. [ng/µl]	Sample Description	Alert	Observations
G2	6.6	318	M190	⚠	Caution! Expired ScreenTape device; Sample concentration outside functional range for DIN

Peak Table

Size [bp]	Calibrated Conc. [ng/µl]	Assigned Conc. [ng/µl]	% Integrated Area	From [bp]	To [bp]	Peak Comment	Observations
100	8.50	8.50	-	60	143		Lower Marker
15463	188	-	98.32	4602	>60000		
-	-	-	-	-	-		Sample Well

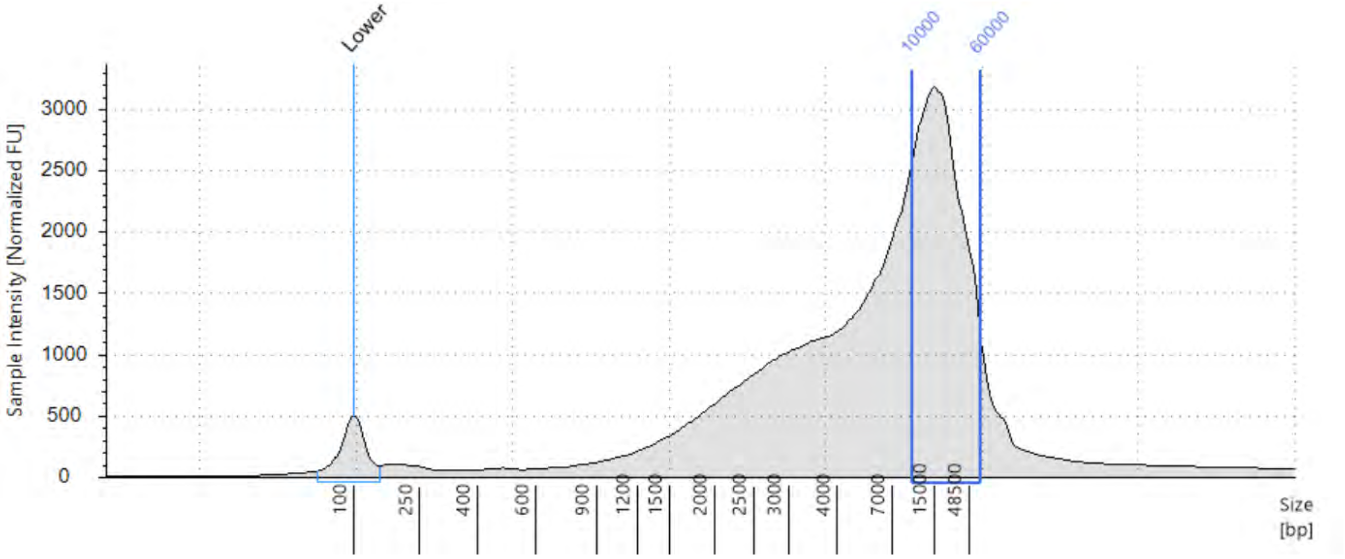

Region Table

From [bp]	To [bp]	Average Size [bp]	Conc. [ng/µl]	Region Molarity [nmol/l]	% of Total	Region Comment	Color
10000	60000	22478	110	9.77	34.70		■

H2: M300

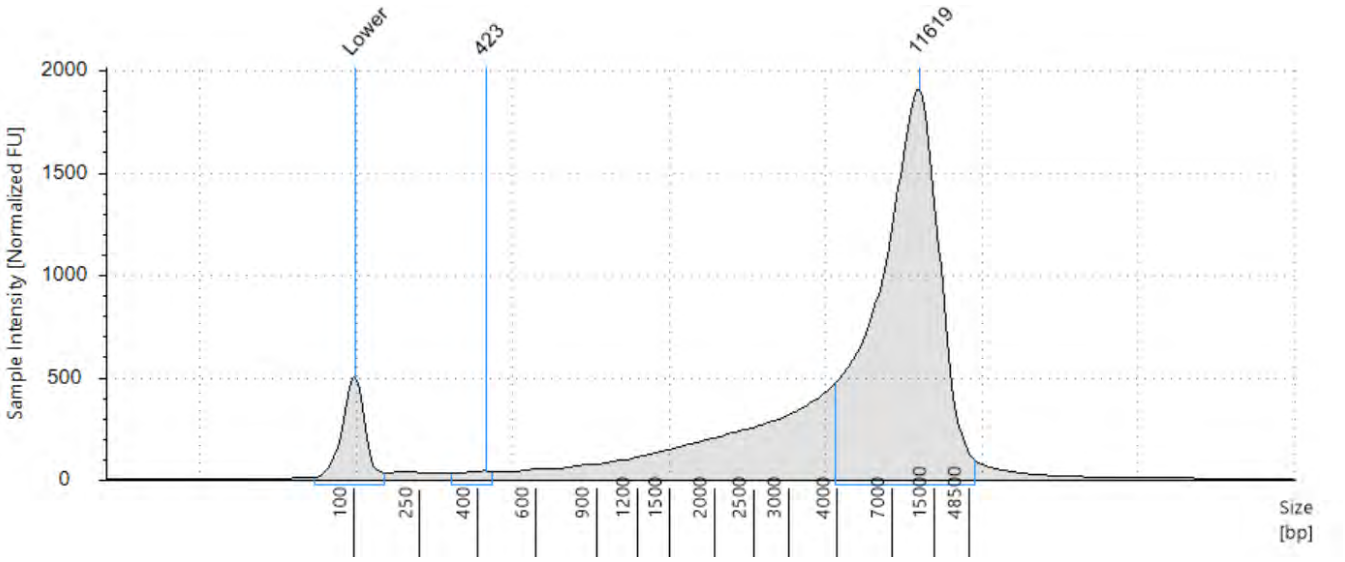

Sample Table

Well	DIN	Conc. [ng/µl]	Sample Description	Alert	Observations
H2	6.5	127	M300	⚠	Caution! Expired ScreenTape device; Sample concentration outside recommended range

Peak Table

Size [bp]	Calibrated Conc. [ng/µl]	Assigned Conc. [ng/µl]	% Integrated Area	From [bp]	To [bp]	Peak Comment	Observations
100	8.50	8.50	-	58	152		Lower Marker
423	0.912	-	1.04	323	443		
11619	86.2	-	97.96	3949	53827		
-	-	-	-	-	-		Sample Well

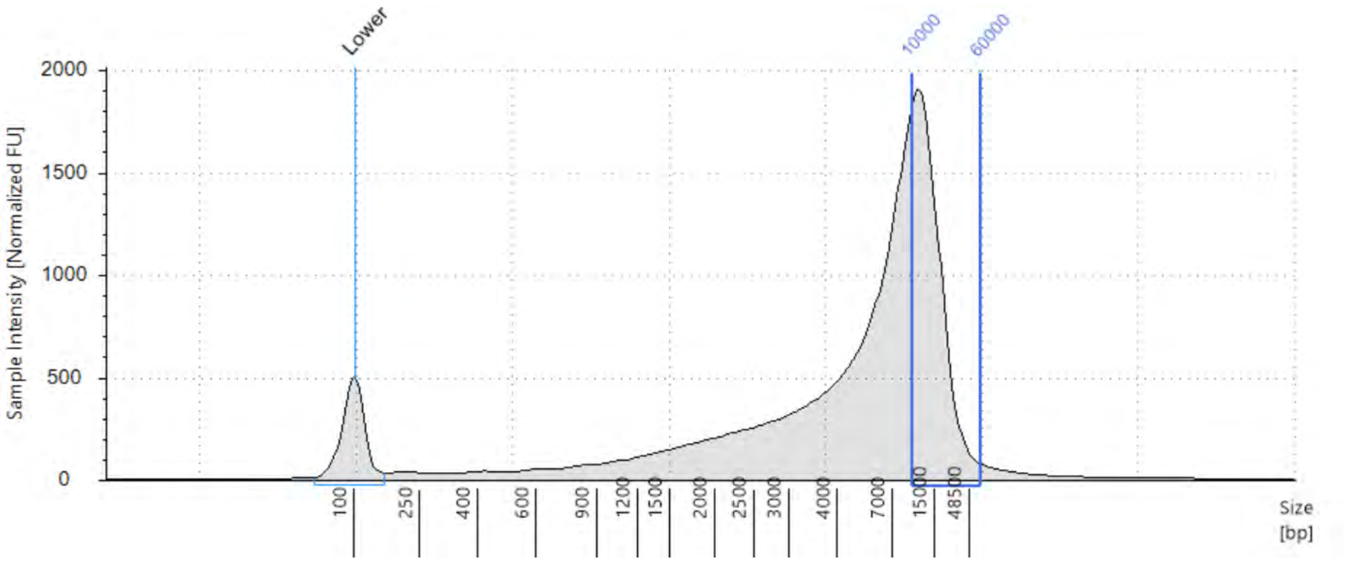

Region Table

From [bp]	To [bp]	Average Size [bp]	Conc. [ng/µl]	Region Molarity [nmol/l]	% of Total	Region Comment	Color
10000	60000	15602	41.3	4.60	32.40		■

 © Agilent Technologies, Inc. 2017

A3: M301

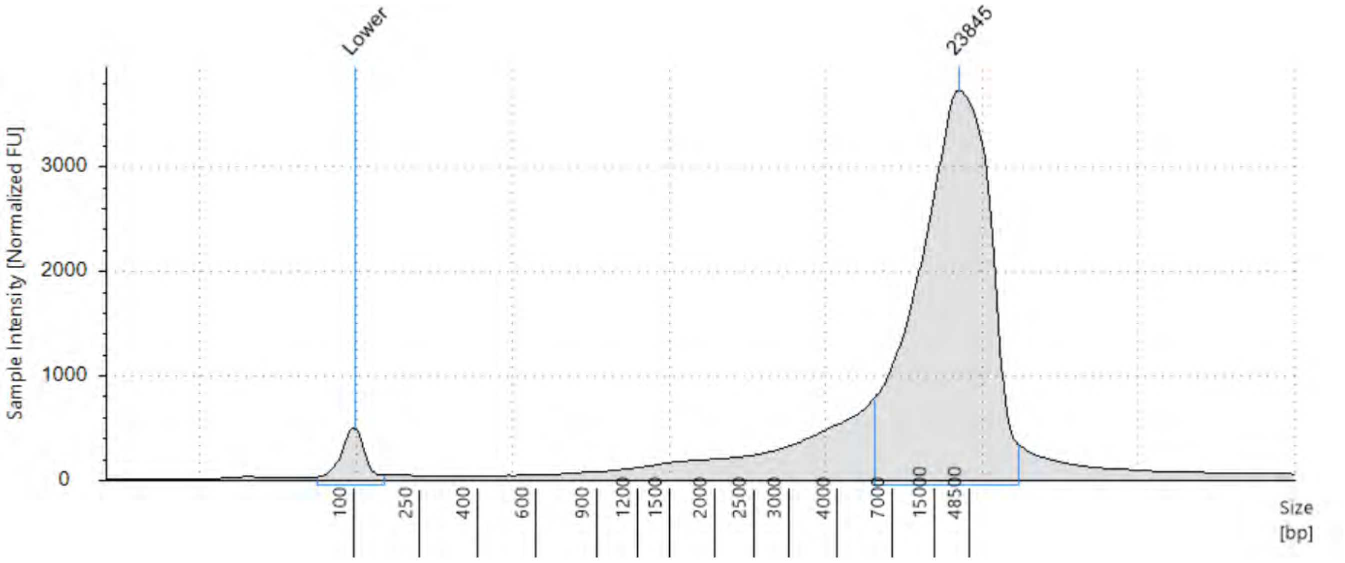

Sample Table

Well	DIN	Conc. [ng/µl]	Sample Description	Alert	Observations
A3	7.6	283	M301	⚠	Caution! Expired ScreenTape device; Sample concentration outside recommended range

Peak Table

Size [bp]	Calibrated Conc. [ng/µl]	Assigned Conc. [ng/µl]	% Integrated Area	From [bp]	To [bp]	Peak Comment	Observations
100	8.50	8.50	-	60	152		Lower Marker
23845	205	-	95.31	5863	>60000		
-	-	-	-	-	-		Sample Well

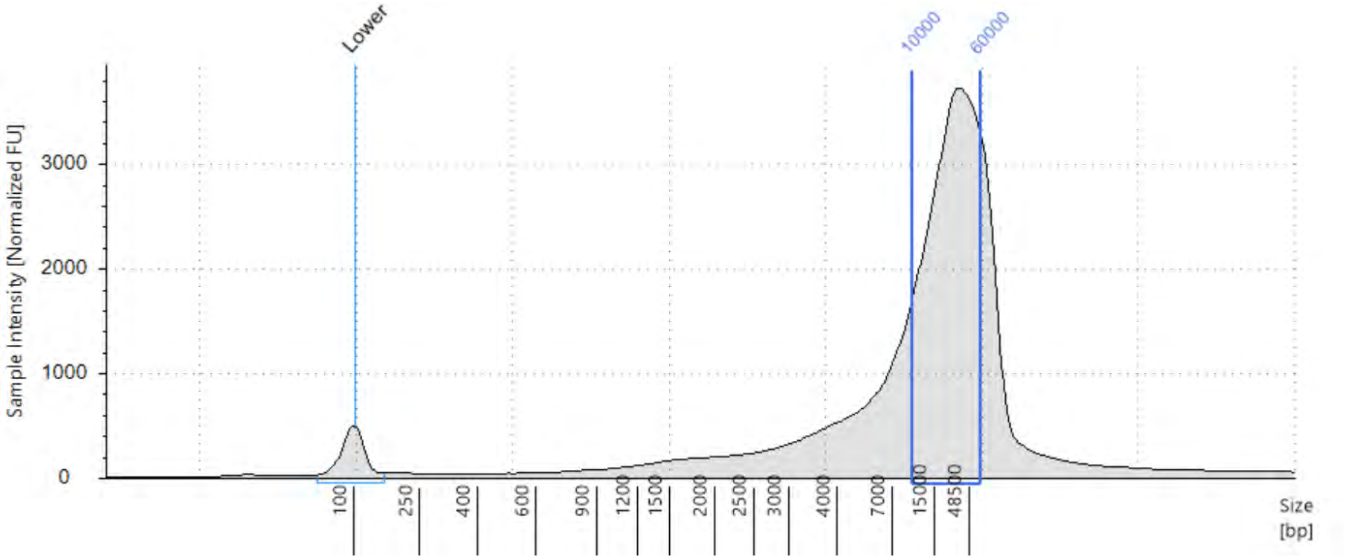

Region Table

From [bp]	To [bp]	Average Size [bp]	Conc. [ng/µl]	Region Molarity [nmol/l]	% of Total	Region Comment	Color
10000	60000	27977	140	10.4	49.55		■

© Agilent Technologies, Inc. 2017

E3: M196

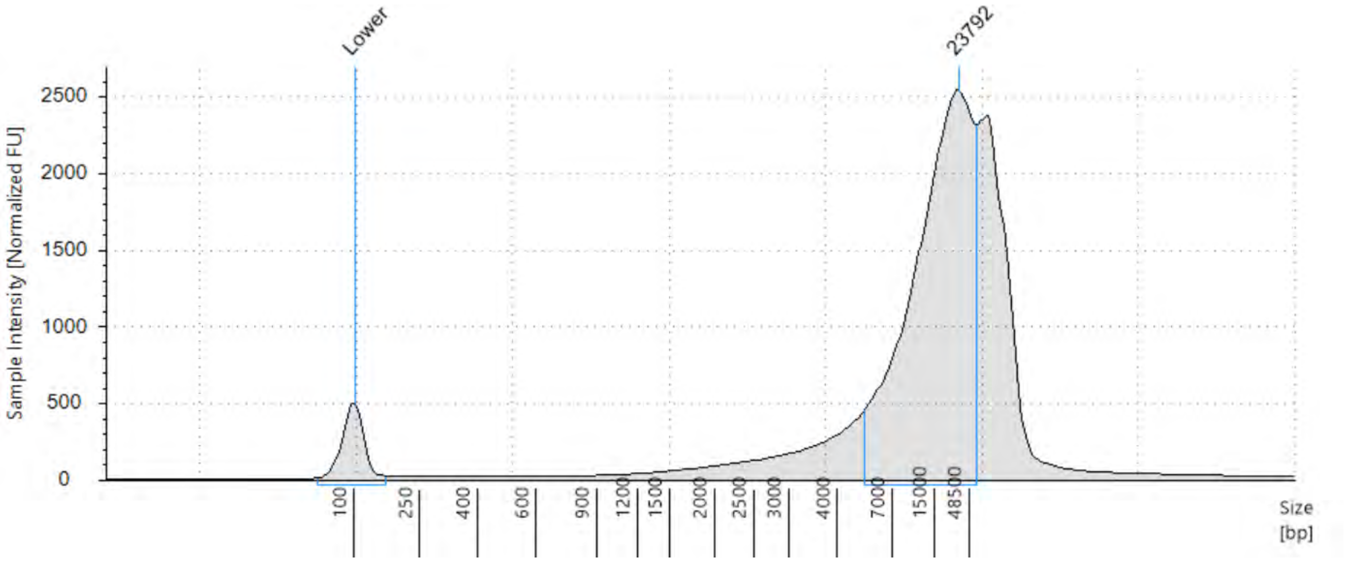

Sample Table

Well	DIN	Conc. [ng/µl]	Sample Description	Alert	Observations
E3	8.1	209	M196	⚠	Caution! Expired ScreenTape device; Sample concentration outside recommended range

Peak Table

Size [bp]	Calibrated Conc. [ng/µl]	Assigned Conc. [ng/µl]	% Integrated Area	From [bp]	To [bp]	Peak Comment	Observations
100	8.50	8.50	-	60	152		Lower Marker
23792	118	-	94.97	5280	55792		
-	-	-	-	-	-		Sample Well

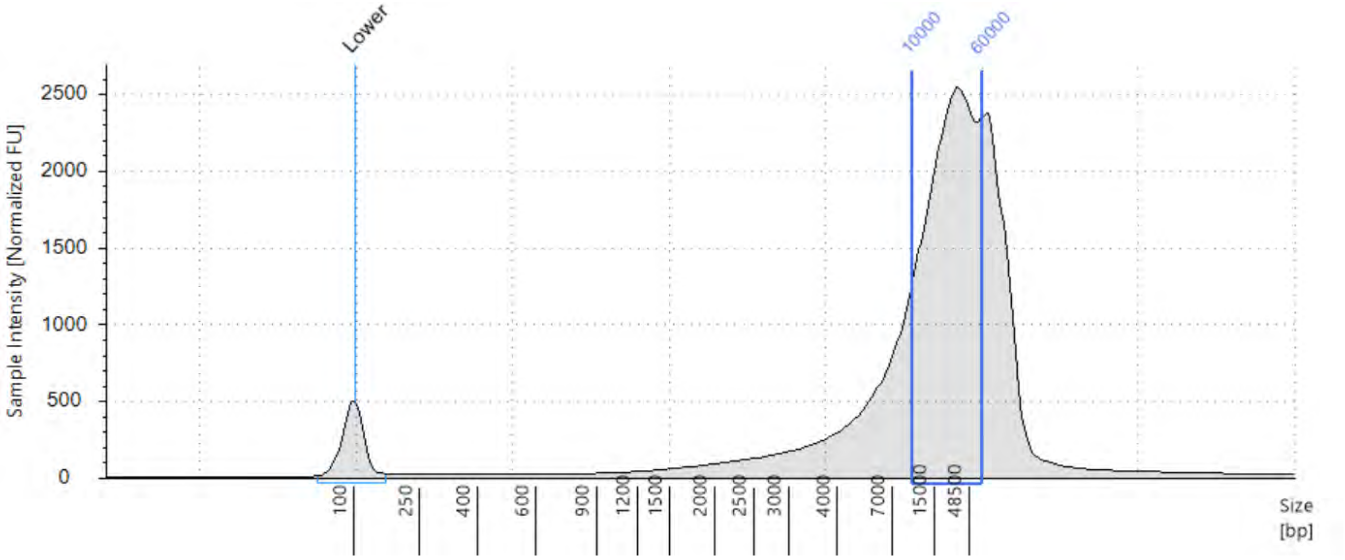

Region Table

From [bp]	To [bp]	Average Size [bp]	Conc. [ng/µl]	Region Molarity [nmol/l]	% of Total	Region Comment	Color
10000	60000	27420	102	7.72	48.82		■

© Agilent Technologies, Inc. 2017

F3: M197

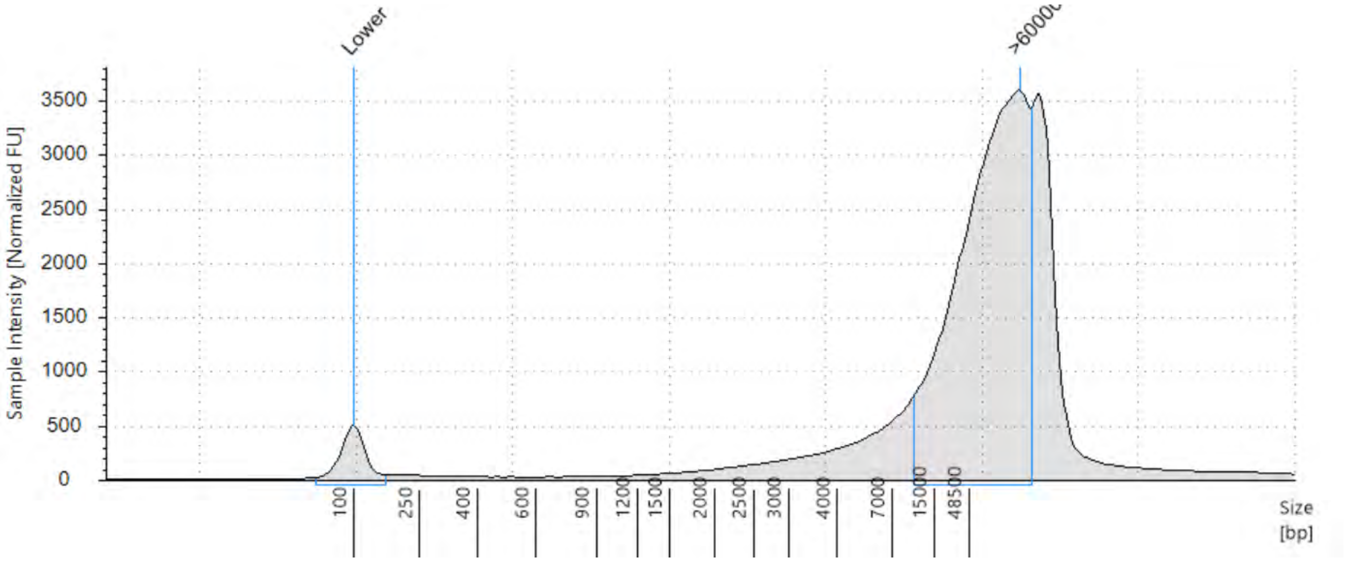

Sample Table

Well	DIN	Conc. [ng/µl]	Sample Description	Alert	Observations
F3	8.6	286	M197	⚠	Caution! Expired ScreenTape device; Sample concentration outside recommended range

Peak Table

Size [bp]	Calibrated Conc. [ng/µl]	Assigned Conc. [ng/µl]	% Integrated Area	From [bp]	To [bp]	Peak Comment	Observations
100	8.50	8.50	-	58	155		Lower Marker
>60000	174	-	93.68	10375	>60000		
-	-	-	-	-	-		Sample Well

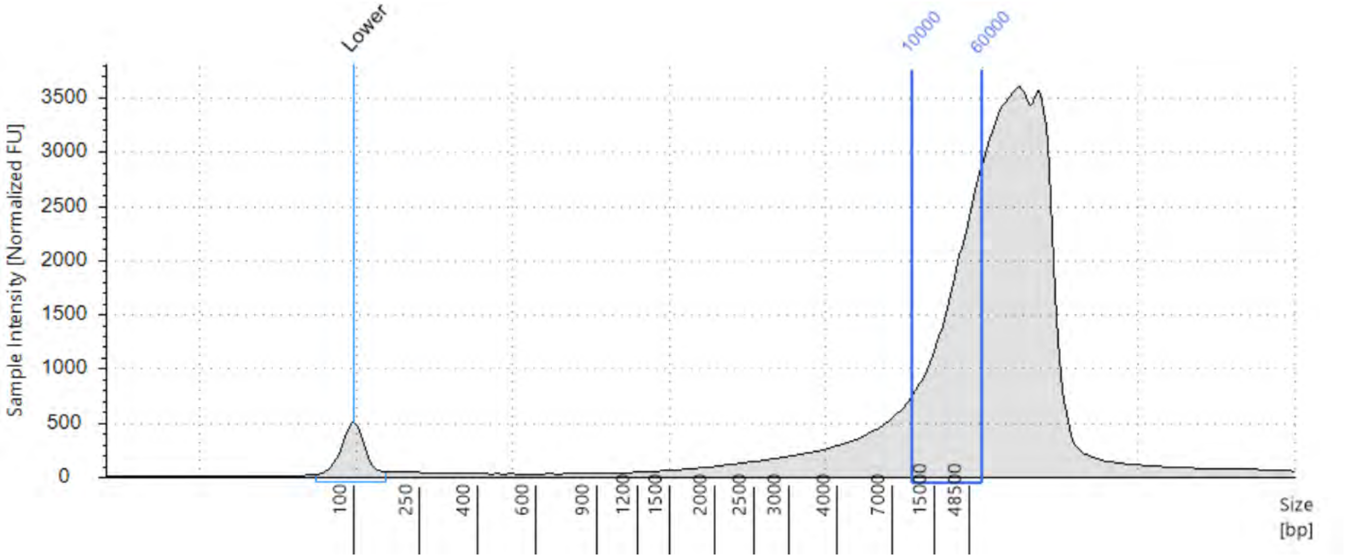

Region Table

From [bp]	To [bp]	Average Size [bp]	Conc. [ng/µl]	Region Molarity [nmol/l]	% of Total	Region Comment	Color
10000	60000	31017	70.1	4.76	24.52		■

G3: M198

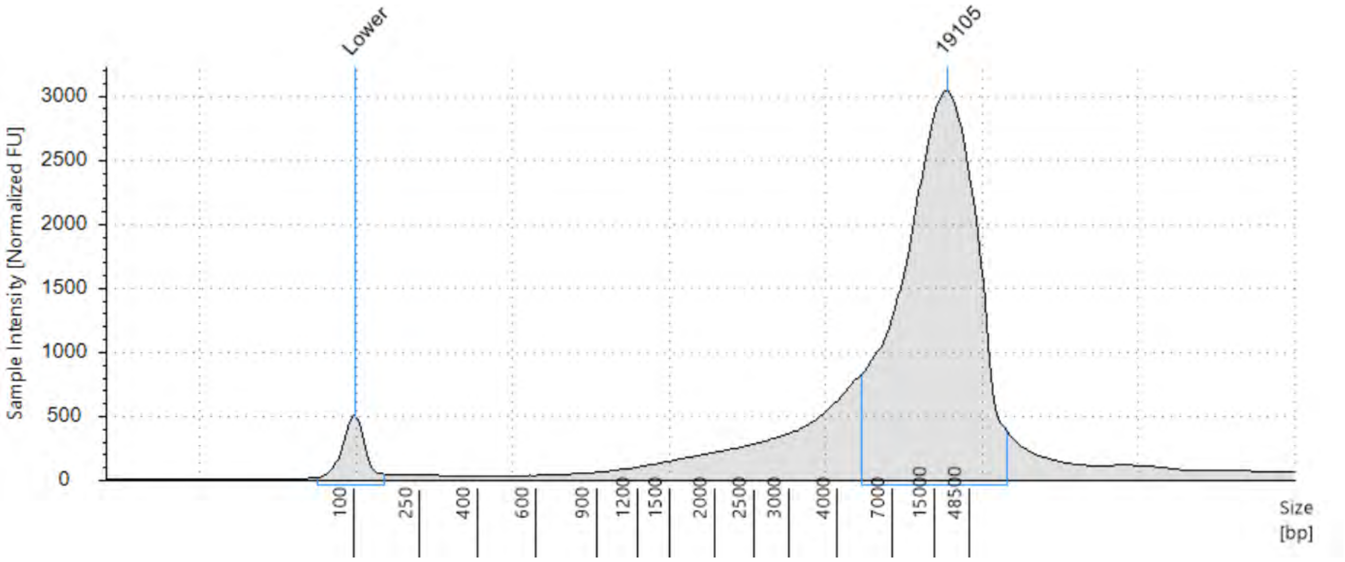

Sample Table

Well	DIN	Conc. [ng/µl]	Sample Description	Alert	Observations
G3	7.2	253	M198	⚠	Caution! Expired ScreenTape device; Sample concentration outside recommended range

Peak Table

Size [bp]	Calibrated Conc. [ng/µl]	Assigned Conc. [ng/µl]	% Integrated Area	From [bp]	To [bp]	Peak Comment	Observations
100	8.50	8.50	-	59	151		Lower Marker
19105	174	-	96.46	5148	>60000		
-	-	-	-	-	-		Sample Well

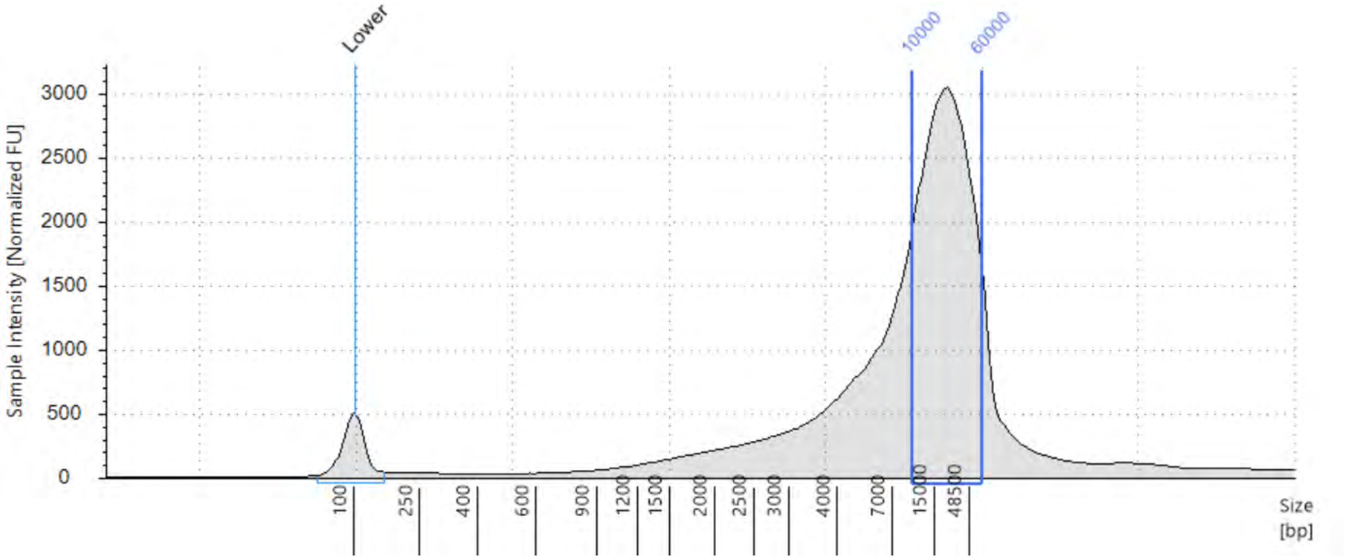

Region Table

From [bp]	To [bp]	Average Size [bp]	Conc. [ng/µl]	Region Molarity [nmol/l]	% of Total	Region Comment	Color
10000	60000	24718	121	9.94	47.97		■

H3: M199

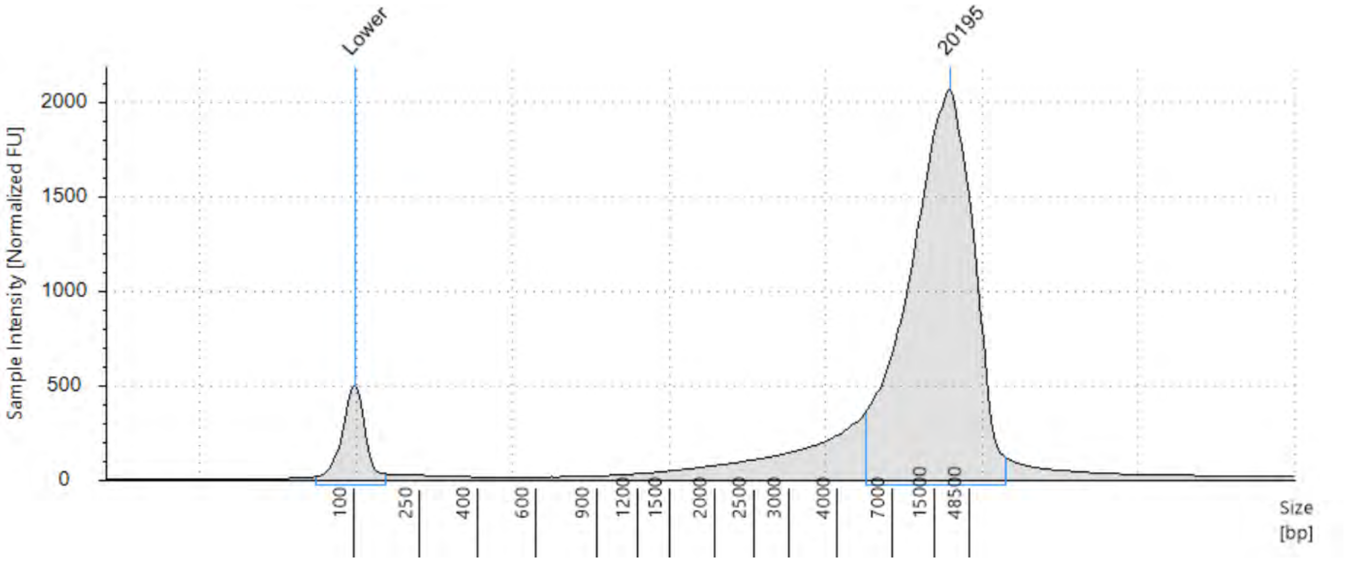

Sample Table

Well	DIN	Conc. [ng/µl]	Sample Description	Alert	Observations
H3	7.6	130	M199	⚠	Caution! Expired ScreenTape device; Sample concentration outside recommended range

Peak Table

Size [bp]	Calibrated Conc. [ng/µl]	Assigned Conc. [ng/µl]	% Integrated Area	From [bp]	To [bp]	Peak Comment	Observations
100	8.50	8.50	-	58	153		Lower Marker
20195	100	-	95.36	5364	>60000		
-	-	-	-	-	-		Sample Well

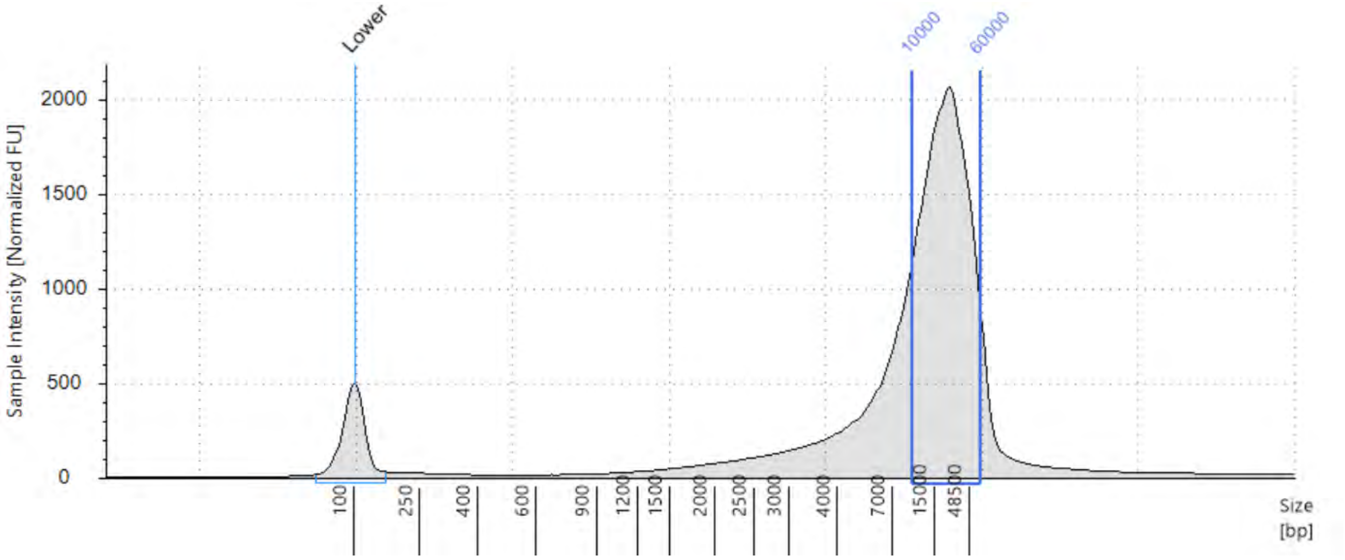

Region Table

From [bp]	To [bp]	Average Size [bp]	Conc. [ng/µl]	Region Molarity [nmol/l]	% of Total	Region Comment	Color
10000	60000	24253	76.7	6.31	58.85		■

 © Agilent Technologies, Inc. 2017

A4: M200

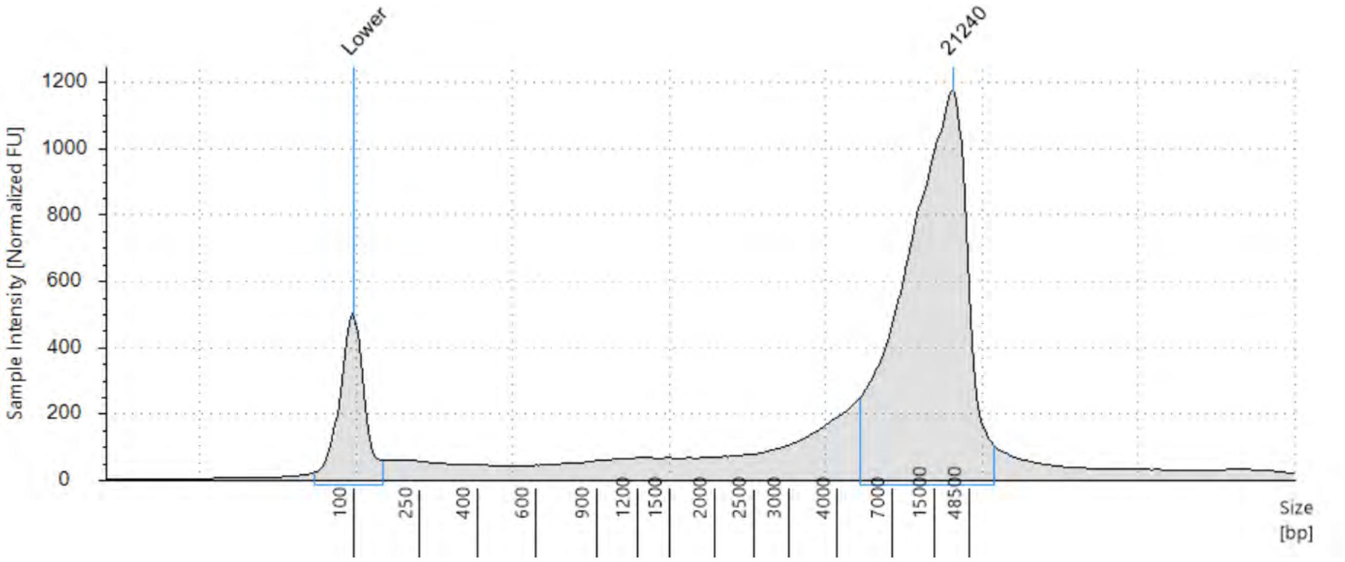

Sample Table

Well	DIN	Conc. [ng/µl]	Sample Description	Alert	Observations
A4	6.8	80.3	M200	⚠	Caution! Expired ScreenTape device

Peak Table

Size [bp]	Calibrated Conc. [ng/µl]	Assigned Conc. [ng/µl]	% Integrated Area	From [bp]	To [bp]	Peak Comment	Observations
100	8.50	8.50	-	57	149		Lower Marker
21240	50.5	-	96.65	5045	>60000		
-	-	-	-	-	-		Sample Well

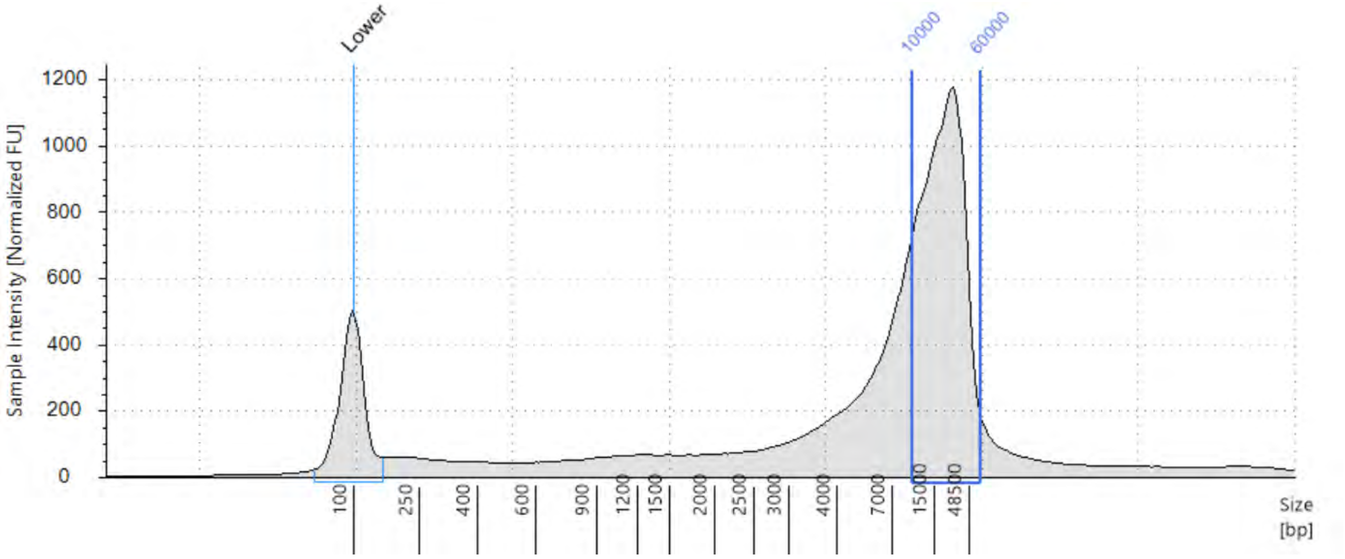

Region Table

From [bp]	To [bp]	Average Size [bp]	Conc. [ng/µl]	Region Molarity [nmol/l]	% of Total	Region Comment	Color
10000	60000	21686	35.9	3.11	44.72		■

 © Agilent Technologies, Inc. 2017

B4: M304

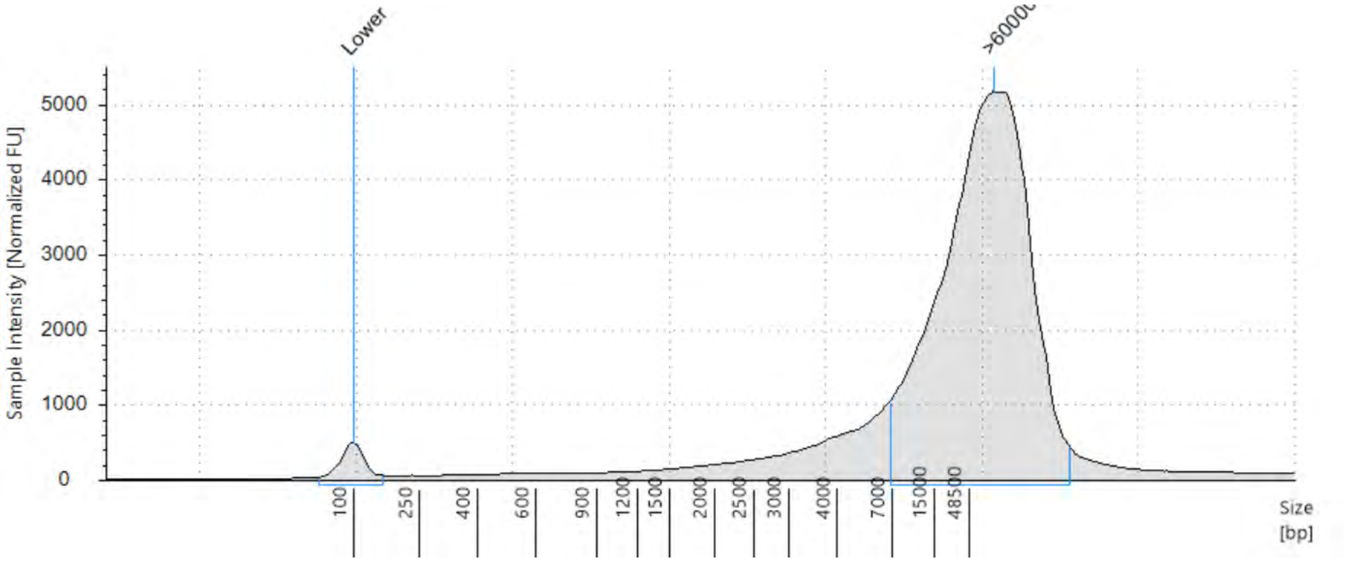

Sample Table

Well	DIN	Conc. [ng/µl]	Sample Description	Alert	Observations
B4	8.2	436	M304	⚠	Caution! Expired ScreenTape device; Sample concentration outside functional range for DIN

Peak Table

Size [bp]	Calibrated Conc. [ng/µl]	Assigned Conc. [ng/µl]	% Integrated Area	From [bp]	To [bp]	Peak Comment	Observations
100	8.50	8.50	-	61	149		Lower Marker
>60000	343	-	95.32	6891	>60000		
-	-	-	-	-	-		Sample Well

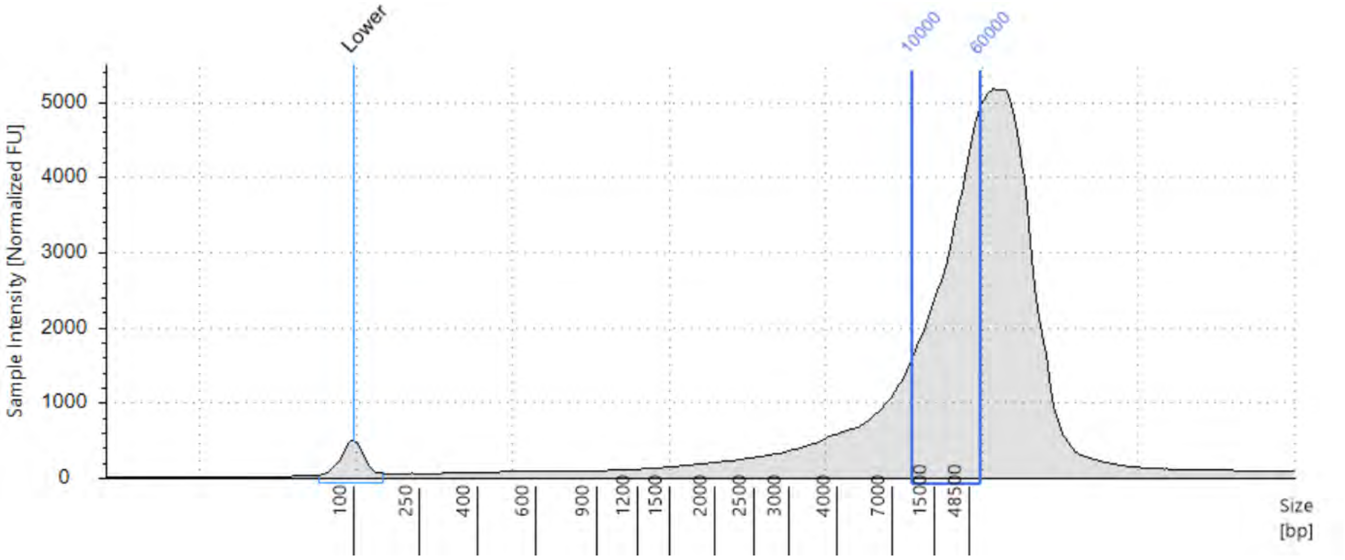

Region Table

From [bp]	To [bp]	Average Size [bp]	Conc. [ng/µl]	Region Molarity [nmol/l]	% of Total	Region Comment	Color
10000	60000	30921	140	9.68	32.11		■

 © Agilent Technologies, Inc. 2017

C4: M305

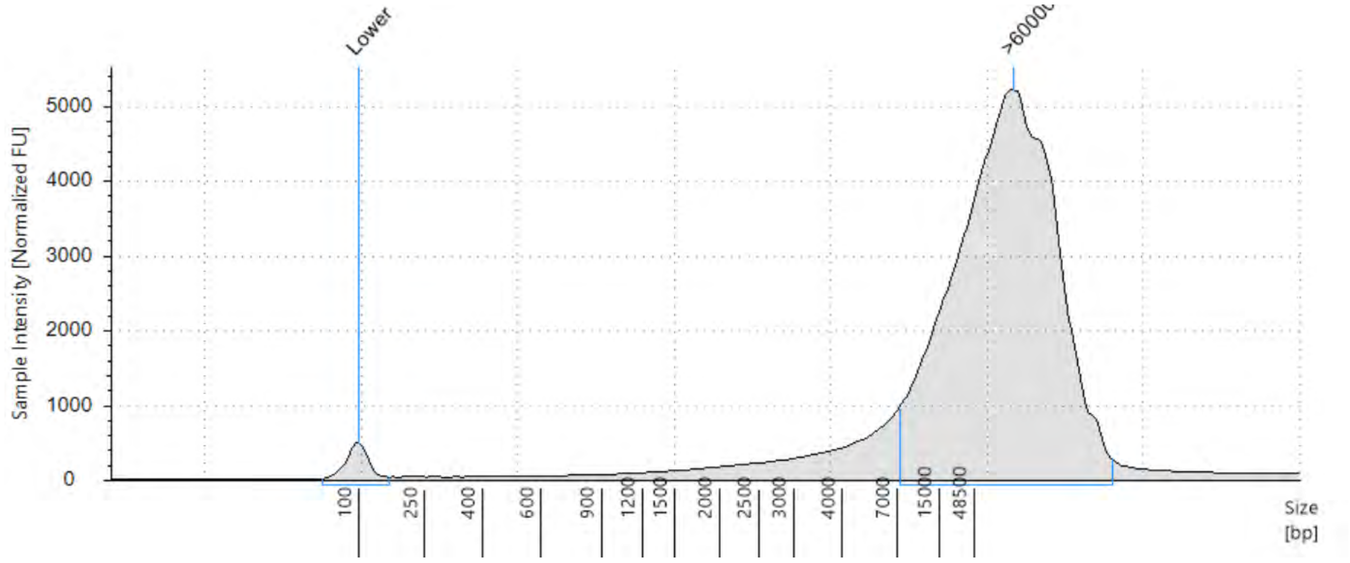

Sample Table

Well	DIN	Conc. [ng/µl]	Sample Description	Alert	Observations
C4	8.5	526	M305	⚠	Caution! Expired ScreenTape device; Sample concentration outside functional range for DIN

Peak Table

Size [bp]	Calibrated Conc. [ng/µl]	Assigned Conc. [ng/µl]	% Integrated Area	From [bp]	To [bp]	Peak Comment	Observations
100	8.50	8.50	-	60	151		Lower Marker
>60000	442	-	96.17	7347	>60000		
-	-	-	-	-	-		Sample Well

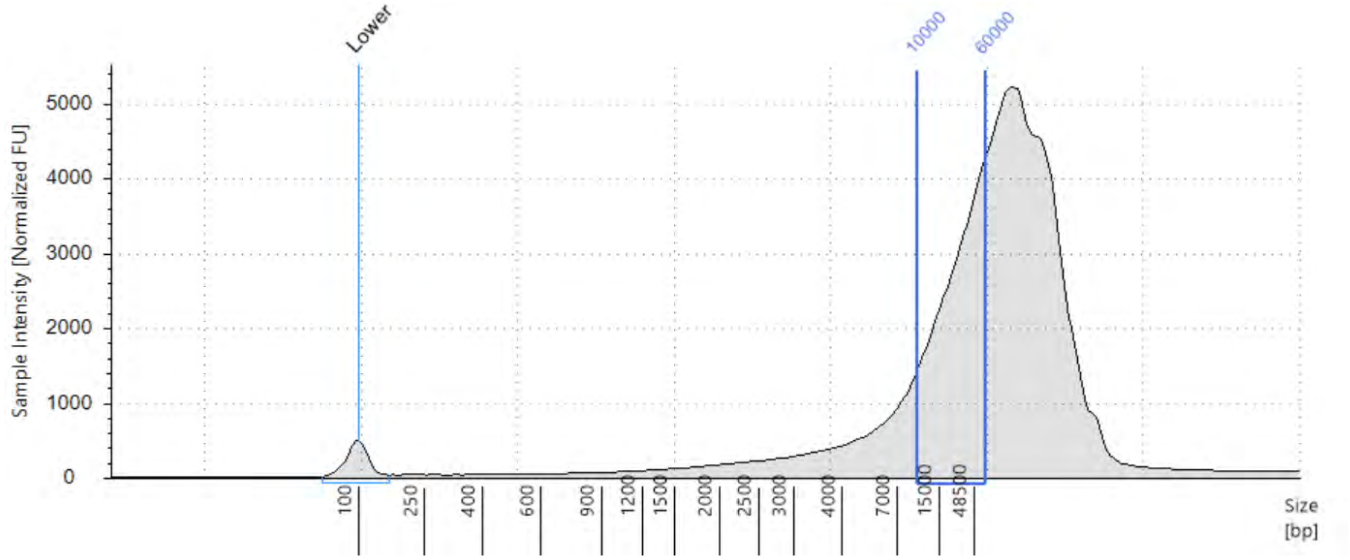

Region Table

From [bp]	To [bp]	Average Size [bp]	Conc. [ng/µl]	Region Molarity [nmol/l]	% of Total	Region Comment	Color
10000	60000	29810	138	9.78	26.13		■

G4: M206

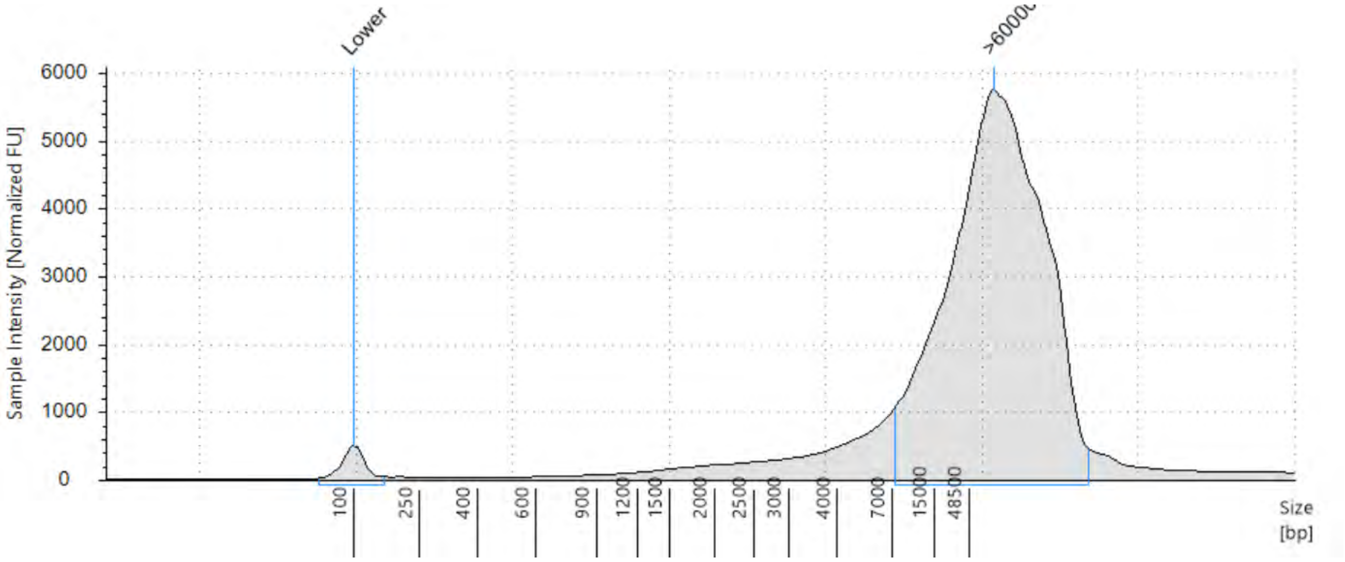

Sample Table

Well	DIN	Conc. [ng/µl]	Sample Description	Alert	Observations
G4	8.5	553	M206	⚠	Caution! Expired ScreenTape device; Sample concentration outside functional range for DIN

Peak Table

Size [bp]	Calibrated Conc. [ng/µl]	Assigned Conc. [ng/µl]	% Integrated Area	From [bp]	To [bp]	Peak Comment	Observations
100	8.50	8.50	-	61	151		Lower Marker
>60000	455	-	96.74	7393	>60000		
-	-	-	-	-	-		Sample Well

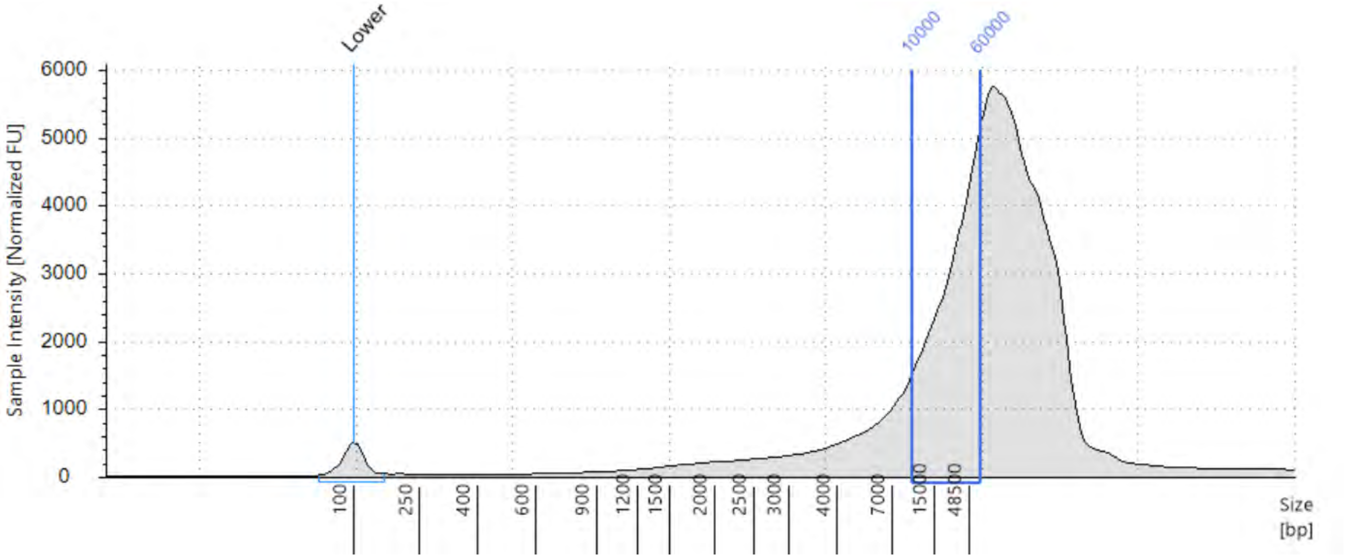

Region Table

From [bp]	To [bp]	Average Size [bp]	Conc. [ng/µl]	Region Molarity [nmol/l]	% of Total	Region Comment	Color
10000	60000	30611	150	10.4	27.08		■

H4: M207

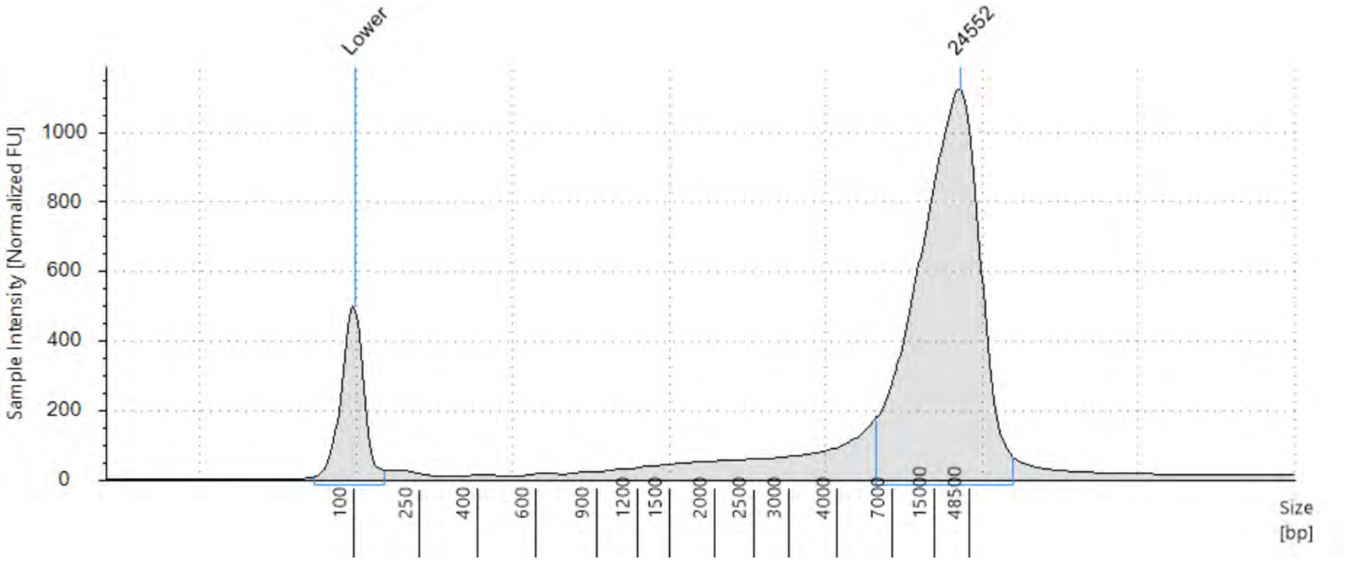

Sample Table

Well	DIN	Conc. [ng/µl]	Sample Description	Alert	Observations
H4	7.6	70.5	M207	⚠	Caution! Expired ScreenTape device

Peak Table

Size [bp]	Calibrated Conc. [ng/µl]	Assigned Conc. [ng/µl]	% Integrated Area	From [bp]	To [bp]	Peak Comment	Observations
100	8.50	8.50	-	57	153		Lower Marker
24552	52.6	-	95.41	5937	>60000		
-	-	-	-	-	-		Sample Well

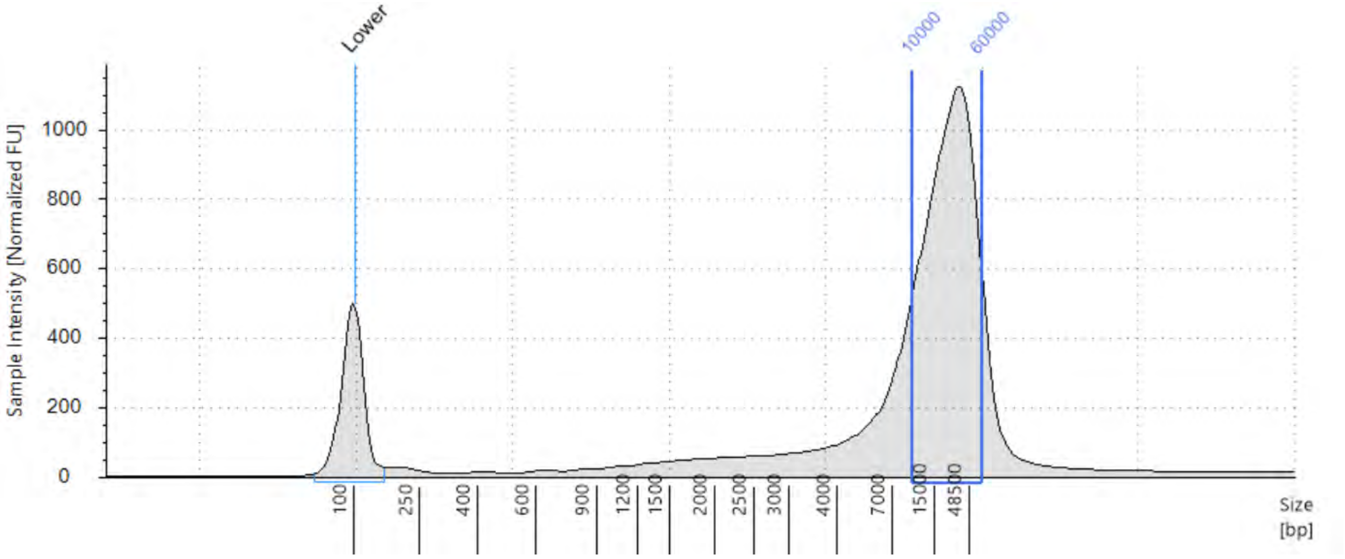

Region Table

From [bp]	To [bp]	Average Size [bp]	Conc. [ng/µl]	Region Molarity [nmol/l]	% of Total	Region Comment	Color
10000	60000	26251	41.1	3.18	58.29		■

 © Agilent Technologies, Inc. 2017

A5: M208

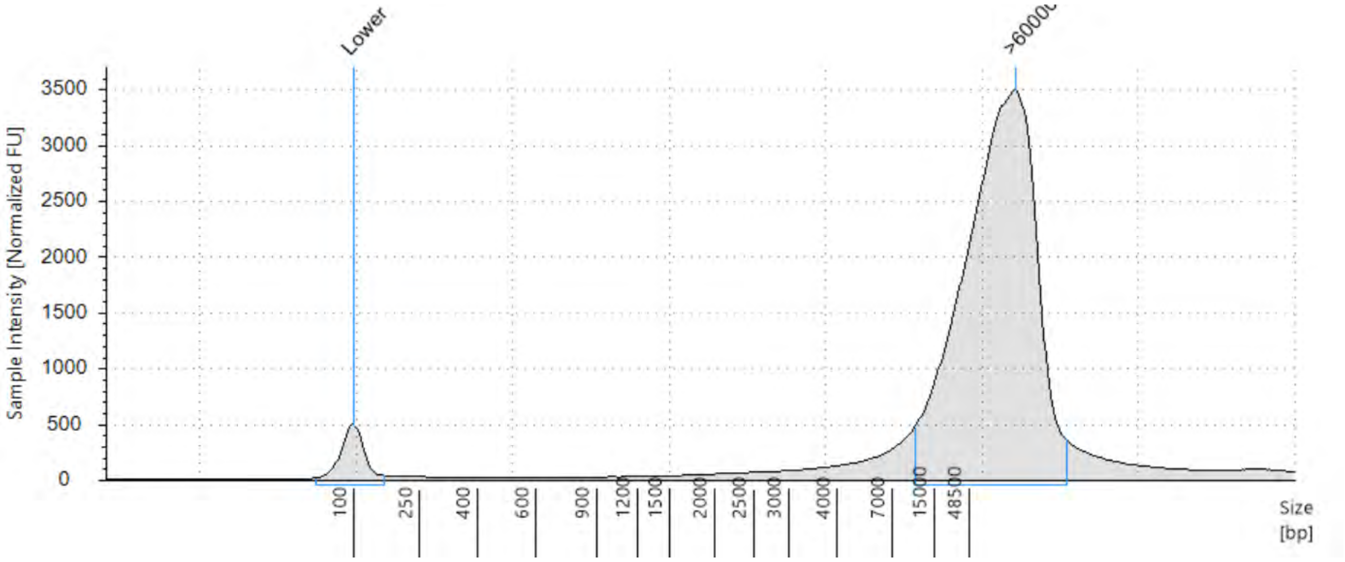

Sample Table

Well	DIN	Conc. [ng/µl]	Sample Description	Alert	Observations
A5	9.2	243	M208	⚠	Caution! Expired ScreenTape device; Sample concentration outside recommended range

Peak Table

Size [bp]	Calibrated Conc. [ng/µl]	Assigned Conc. [ng/µl]	% Integrated Area	From [bp]	To [bp]	Peak Comment	Observations
100	8.50	8.50	-	59	153		Lower Marker
>60000	195	-	92.50	10708	>60000		
-	-	-	-	-	-		Sample Well

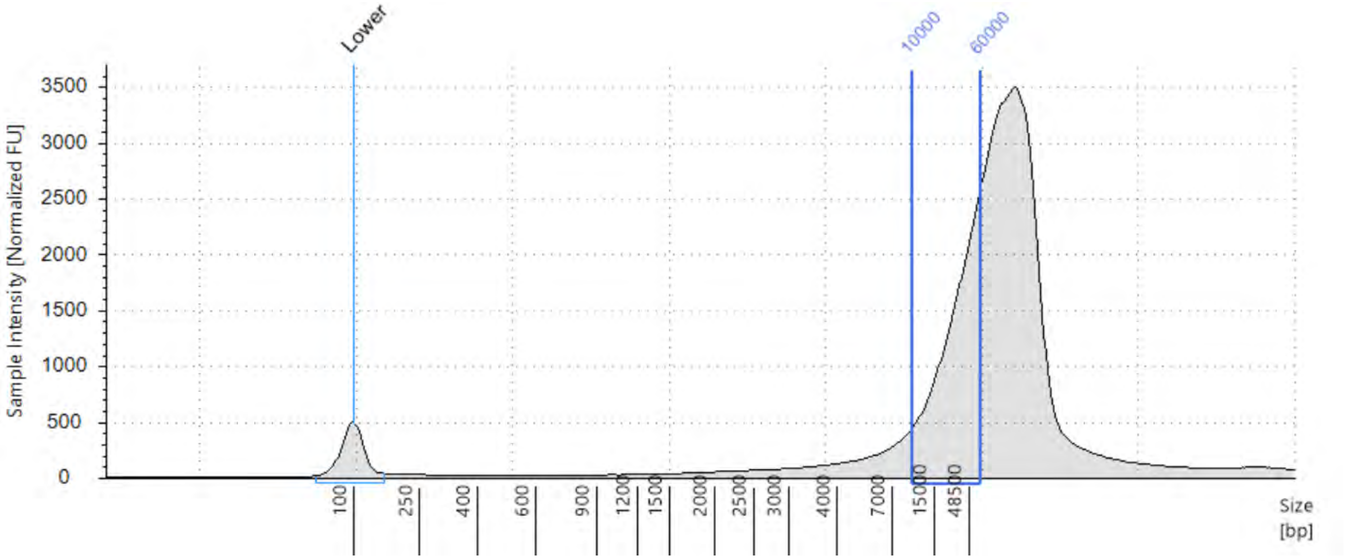

Region Table

From [bp]	To [bp]	Average Size [bp]	Conc. [ng/µl]	Region Molarity [nmol/l]	% of Total	Region Comment	Color
10000	60000	33346	62.8	3.96	25.90		■

B5: M209

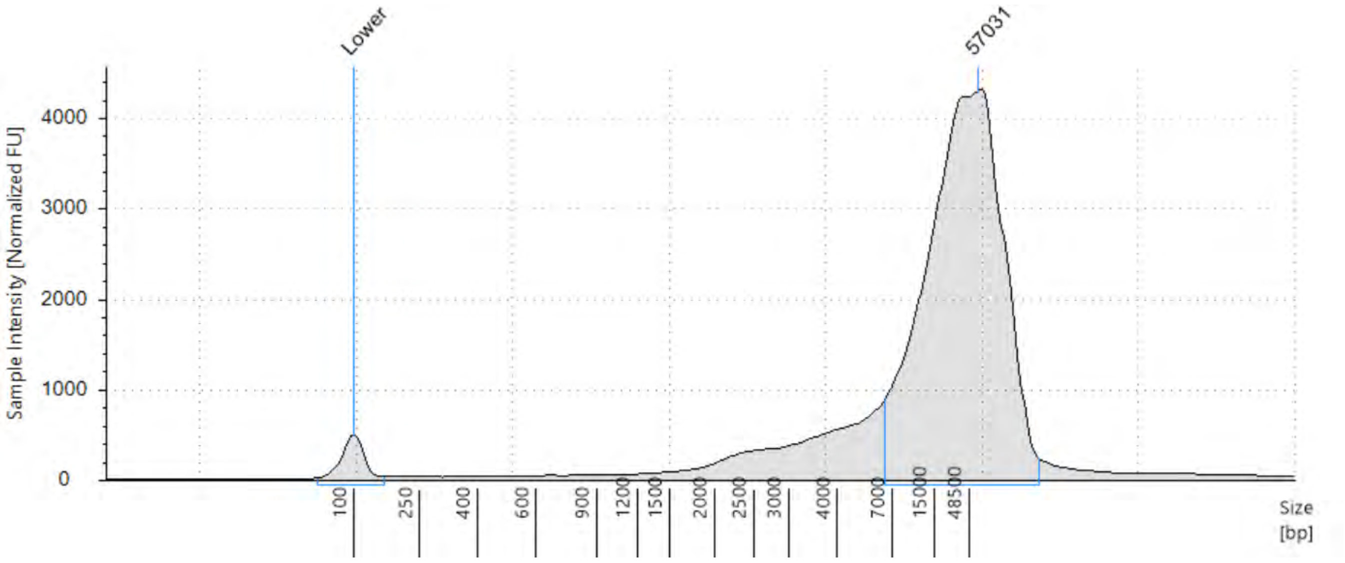

Sample Table

Well	DIN	Conc. [ng/µl]	Sample Description	Alert	Observations
B5	8.0	337	M209	⚠	Caution! Expired ScreenTape device; Sample concentration outside functional range for DIN

Peak Table

Size [bp]	Calibrated Conc. [ng/µl]	Assigned Conc. [ng/µl]	% Integrated Area	From [bp]	To [bp]	Peak Comment	Observations
100	8.50	8.50	-	60	151		Lower Marker
57031	259	-	96.25	6515	>60000		
-	-	-	-	-	-		Sample Well

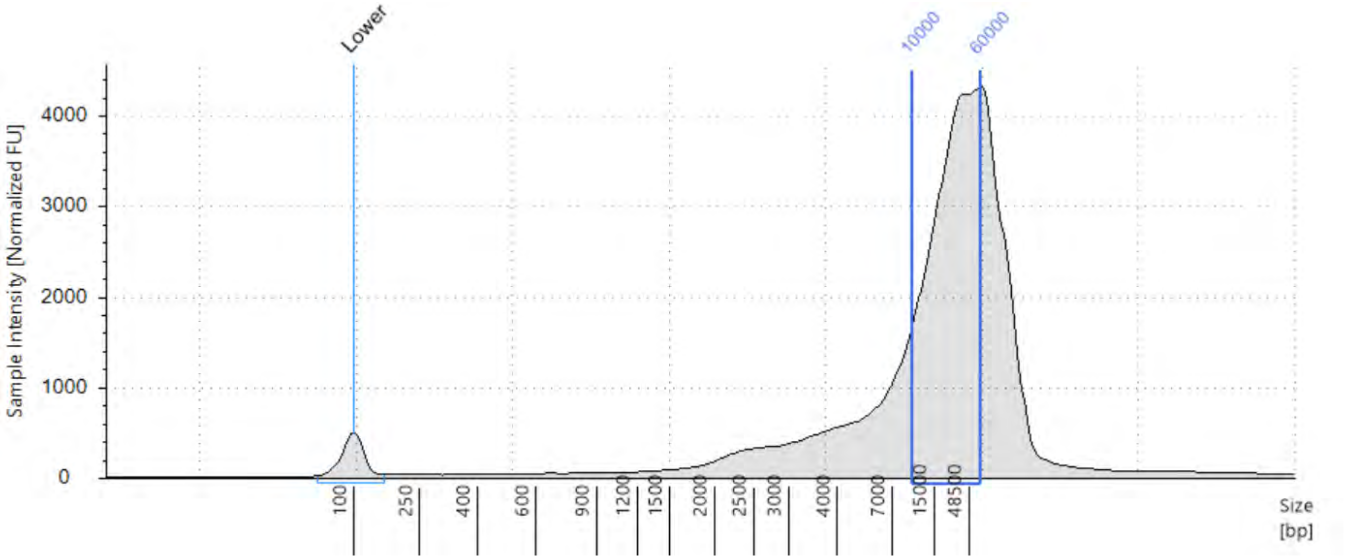

Region Table

From [bp]	To [bp]	Average Size [bp]	Conc. [ng/µl]	Region Molarity [nmol/l]	% of Total	Region Comment	Color
10000	60000	29009	157	11.3	46.53		■

C5: M210

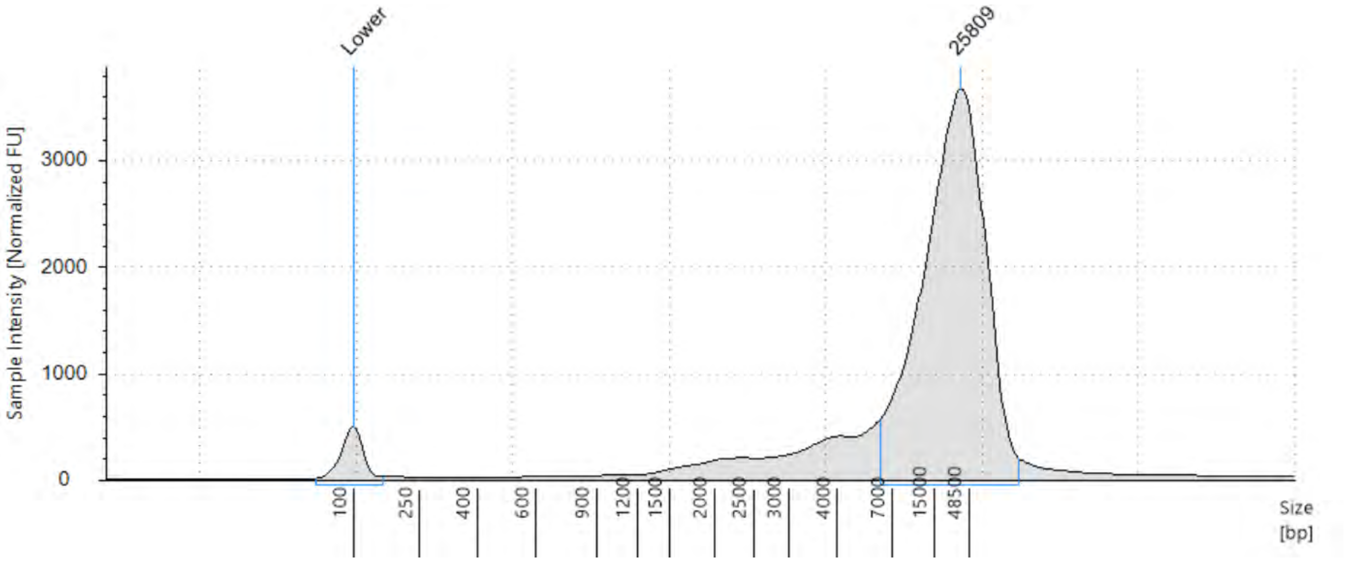

Sample Table

Well	DIN	Conc. [ng/µl]	Sample Description	Alert	Observations
C5	7.8	240	M210	⚠	Caution! Expired ScreenTape device; Sample concentration outside recommended range

Peak Table

Size [bp]	Calibrated Conc. [ng/µl]	Assigned Conc. [ng/µl]	% Integrated Area	From [bp]	To [bp]	Peak Comment	Observations
100	8.50	8.50	-	59	151		Lower Marker
25809	182	-	94.25	6225	>60000		
-	-	-	-	-	-		Sample Well

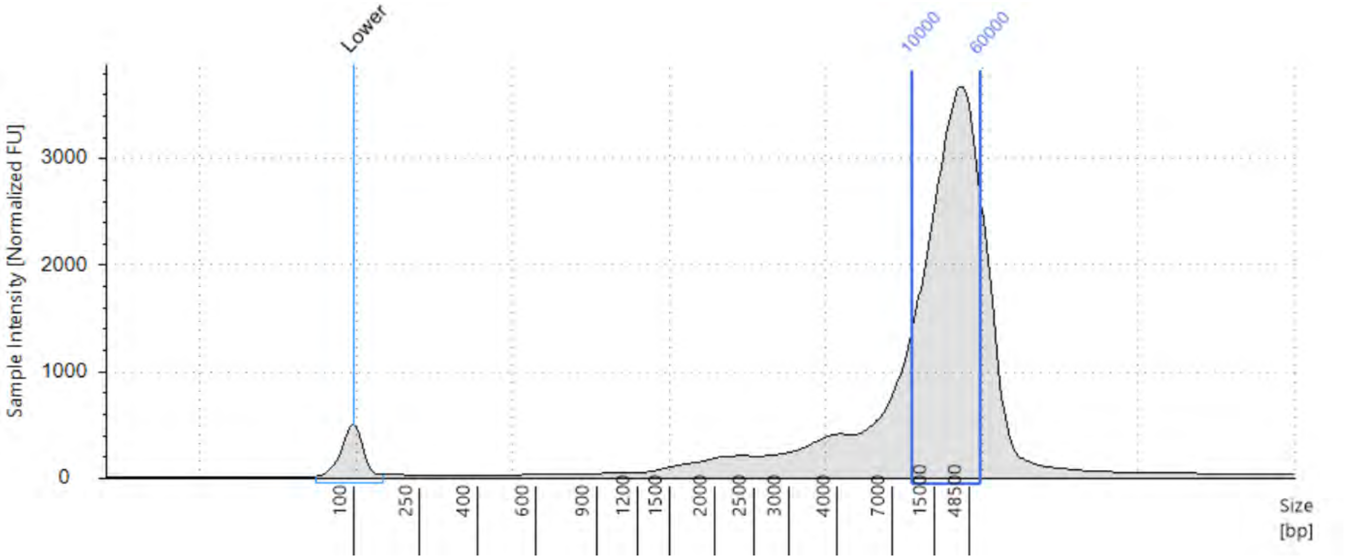

Region Table

From [bp]	To [bp]	Average Size [bp]	Conc. [ng/µl]	Region Molarity [nmol/l]	% of Total	Region Comment	Color
10000	60000	27874	135	9.85	56.02		■

 © Agilent Technologies, Inc. 2017

D5: M308

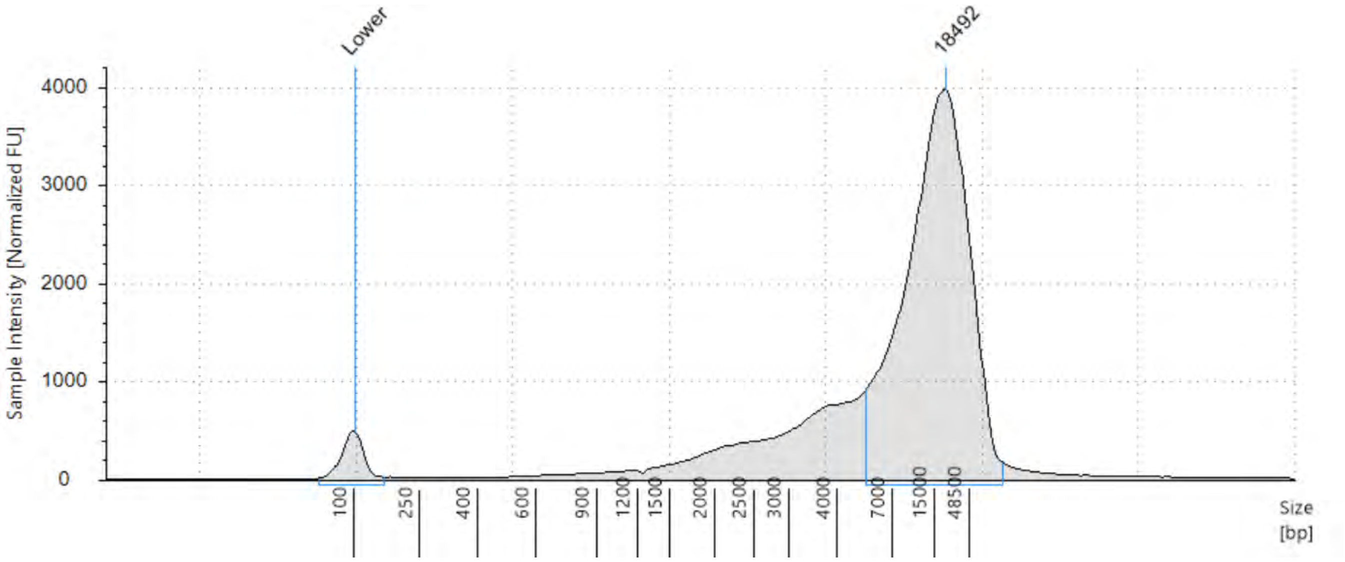

Sample Table

Well	DIN	Conc. [ng/µl]	Sample Description	Alert	Observations
D5	7.1	280	M308	⚠	Caution! Expired ScreenTape device; Sample concentration outside recommended range

Peak Table

Size [bp]	Calibrated Conc. [ng/µl]	Assigned Conc. [ng/µl]	% Integrated Area	From [bp]	To [bp]	Peak Comment	Observations
100	8.50	8.50	-	60	151		Lower Marker
18492	202	-	97.78	5357	>60000		
-	-	-	-	-	-		Sample Well

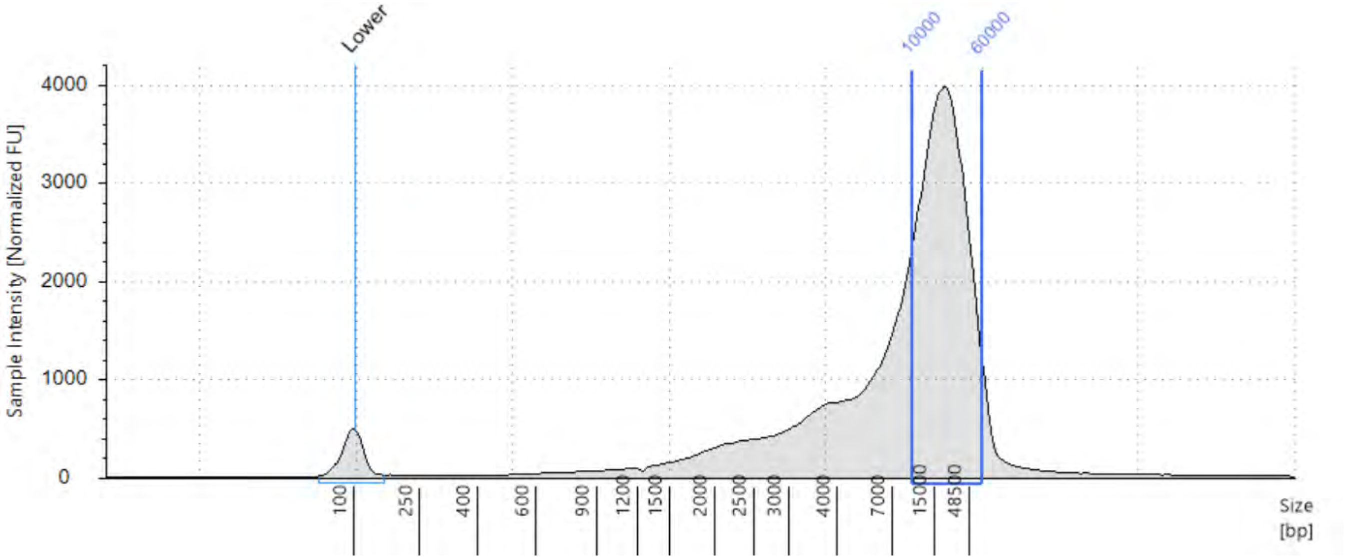

Region Table

From [bp]	To [bp]	Average Size [bp]	Conc. [ng/µl]	Region Molarity [nmol/l]	% of Total	Region Comment	Color
10000	60000	22837	151	12.9	53.69		■

E5: M309

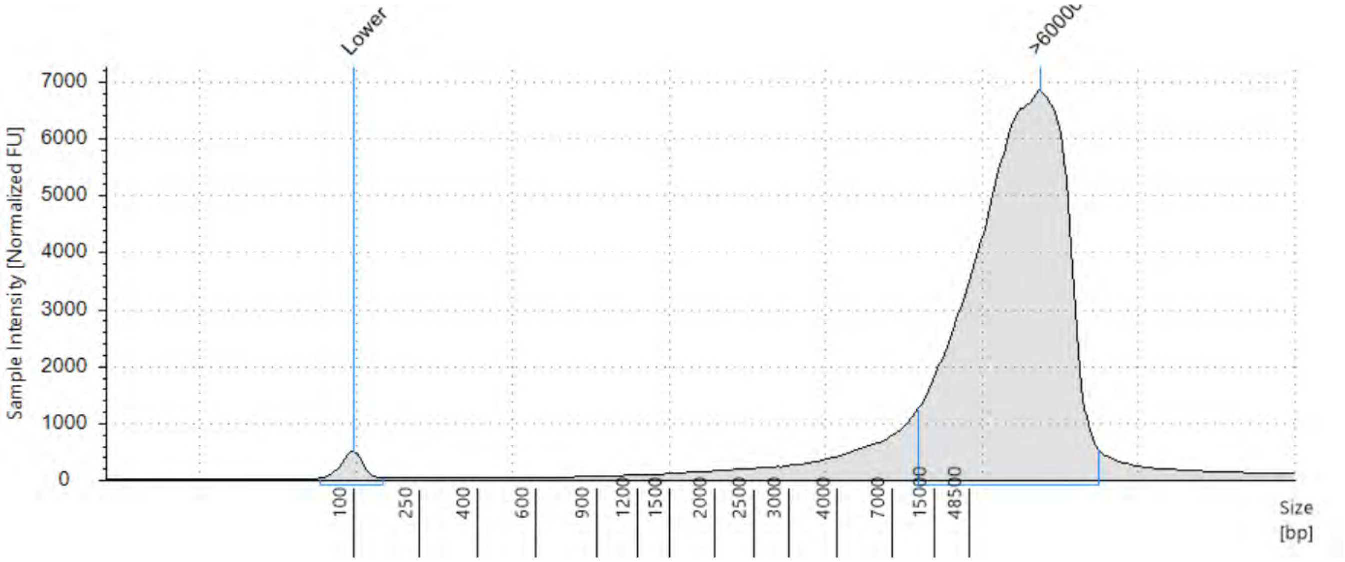

Sample Table

Well	DIN	Conc. [ng/µl]	Sample Description	Alert	Observations
E5	8.8	639	M309	⚠	Caution! Expired ScreenTape device; Sample concentration outside functional range for DIN

Peak Table

Size [bp]	Calibrated Conc. [ng/µl]	Assigned Conc. [ng/µl]	% Integrated Area	From [bp]	To [bp]	Peak Comment	Observations
100	8.50	8.50	-	62	149		Lower Marker
>60000	538	-	97.15	11439	>60000		
-	-	-	-	-	-		Sample Well

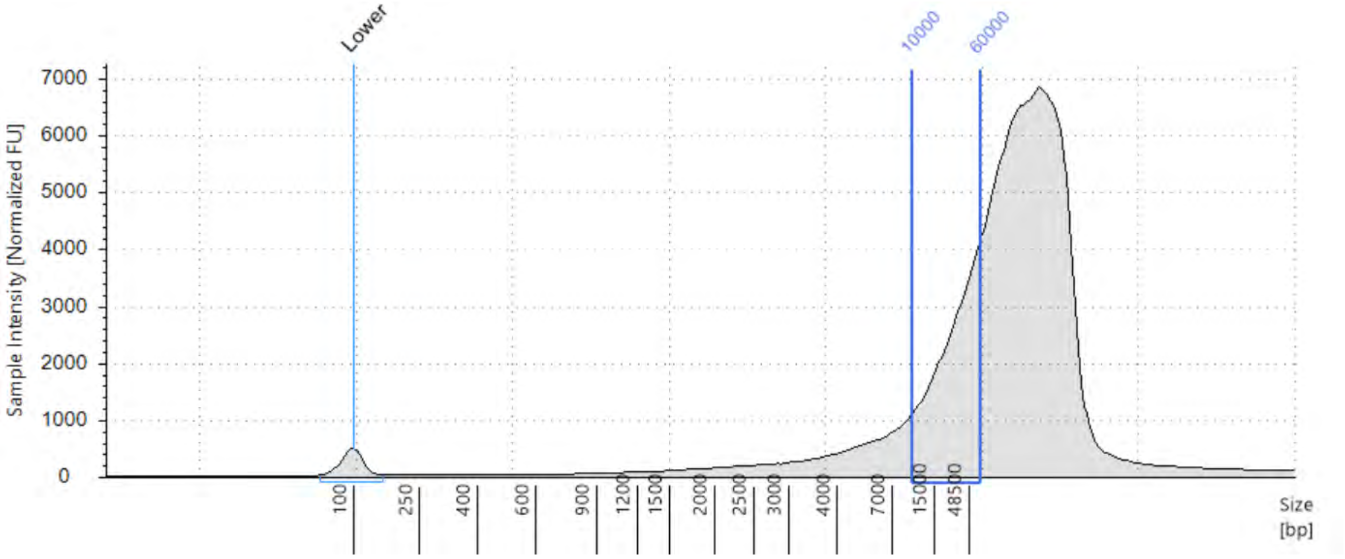

Region Table

From [bp]	To [bp]	Average Size [bp]	Conc. [ng/µl]	Region Molarity [nmol/l]	% of Total	Region Comment	Color
10000	60000	31489	123	8.37	19.29		■

A6: M216

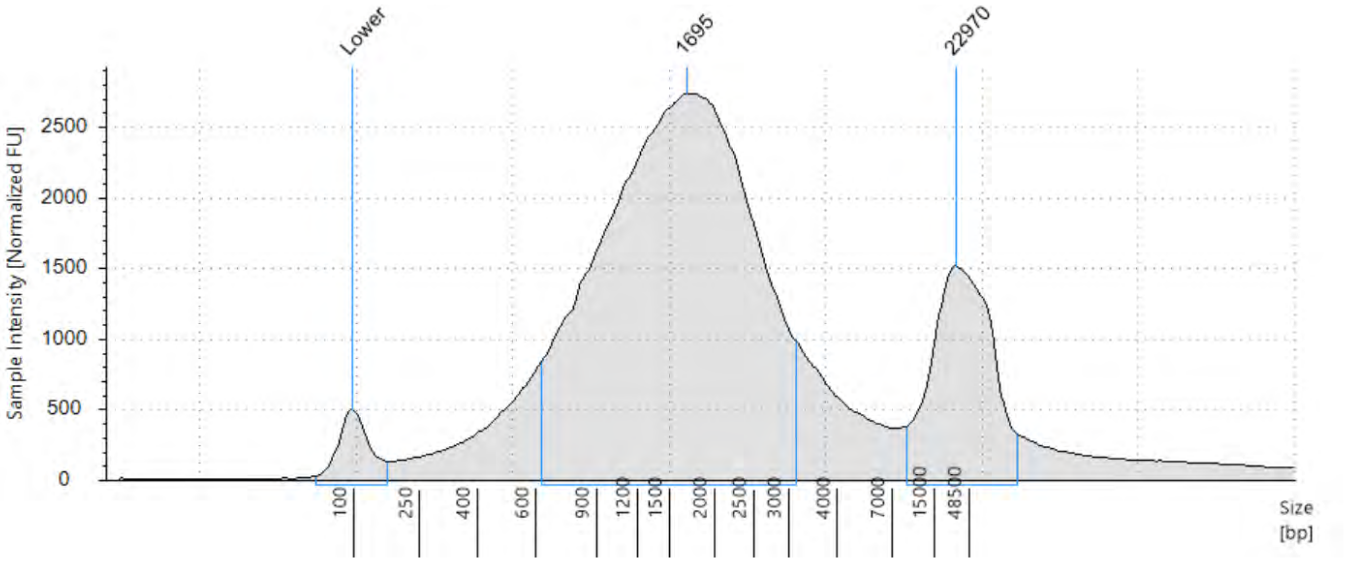

Sample Table

Well	DIN	Conc. [ng/µl]	Sample Description	Alert	Observations
A6	2.9	379	M216	⚠	Caution! Expired ScreenTape device; Sample concentration outside functional range for DIN

Peak Table

Size [bp]	Calibrated Conc. [ng/µl]	Assigned Conc. [ng/µl]	% Integrated Area	From [bp]	To [bp]	Peak Comment	Observations
100	8.50	8.50	-	60	162		Lower Marker
1695	246	-	81.98	629	3165		
22970	53.8	-	17.91	9357	>60000		
-	-	-	-	-	-		Sample Well

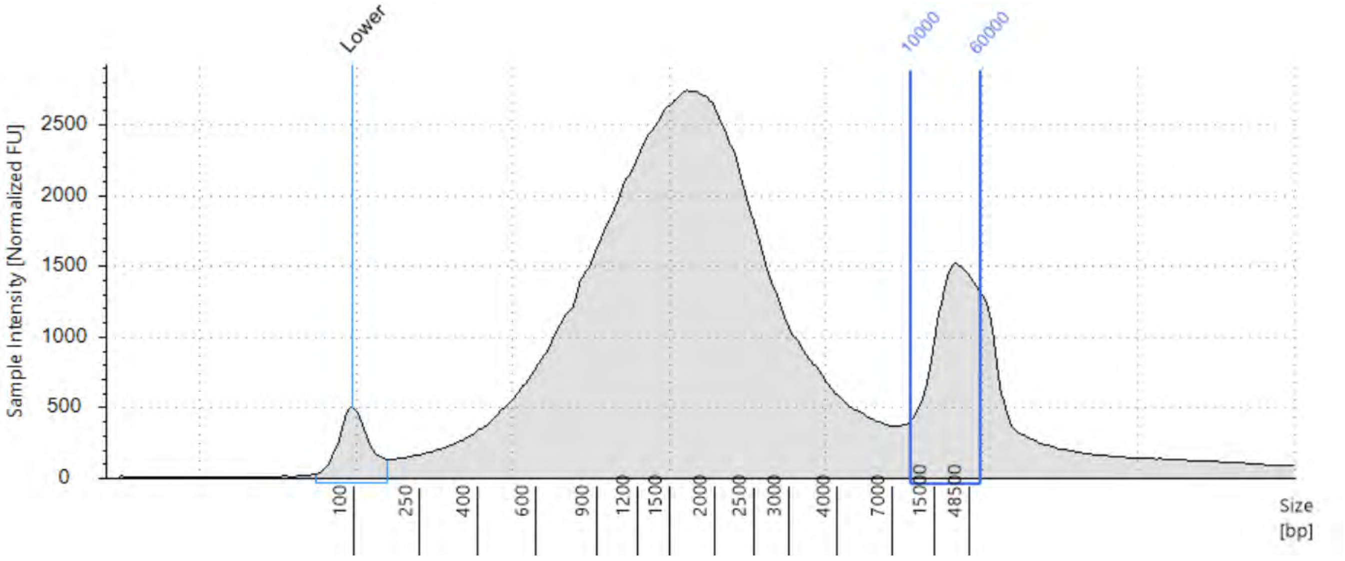

Region Table

From [bp]	To [bp]	Average Size [bp]	Conc. [ng/µl]	Region Molarity [nmol/l]	% of Total	Region Comment	Color
10000	60000	28522	38.9	2.78	10.27		■

B6: M217

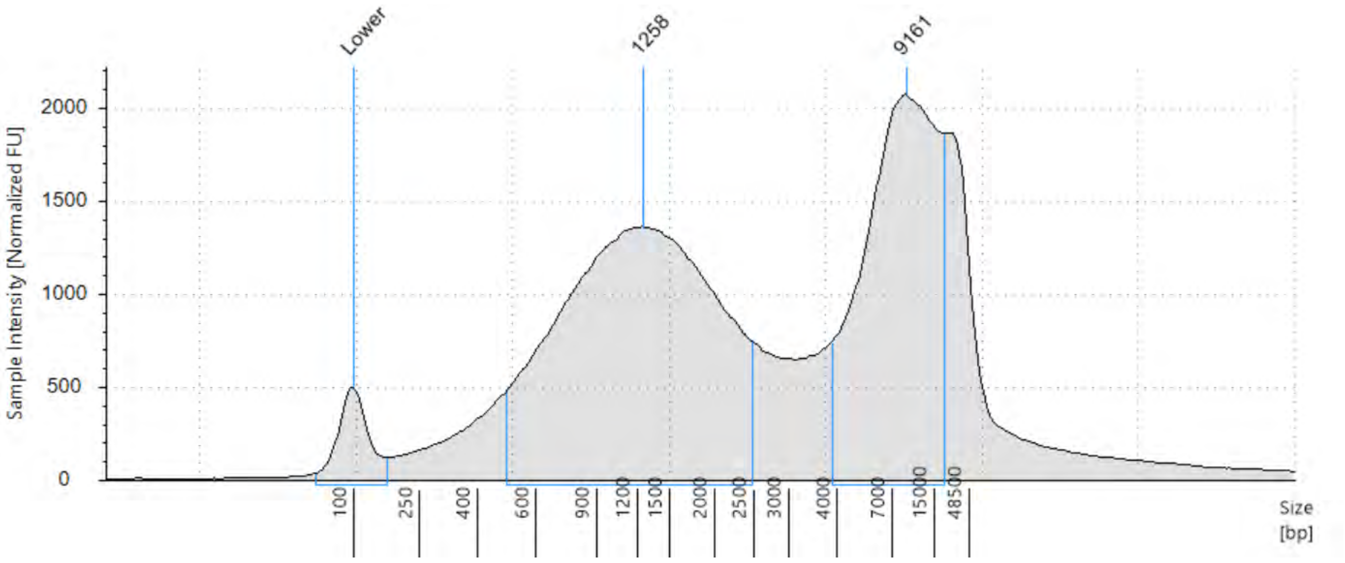

Sample Table

Well	DIN	Conc. [ng/µl]	Sample Description	Alert	Observations
B6	4.9	307	M217	⚠	Caution! Expired ScreenTape device; Sample concentration outside functional range for DIN

Peak Table

Size [bp]	Calibrated Conc. [ng/µl]	Assigned Conc. [ng/µl]	% Integrated Area	From [bp]	To [bp]	Peak Comment	Observations
100	8.50	8.50	-	59	159		Lower Marker
1258	129	-	58.56	491	2507		
9161	90.7	-	41.19	3900	18552		
-	-	-	-	-	-		Sample Well

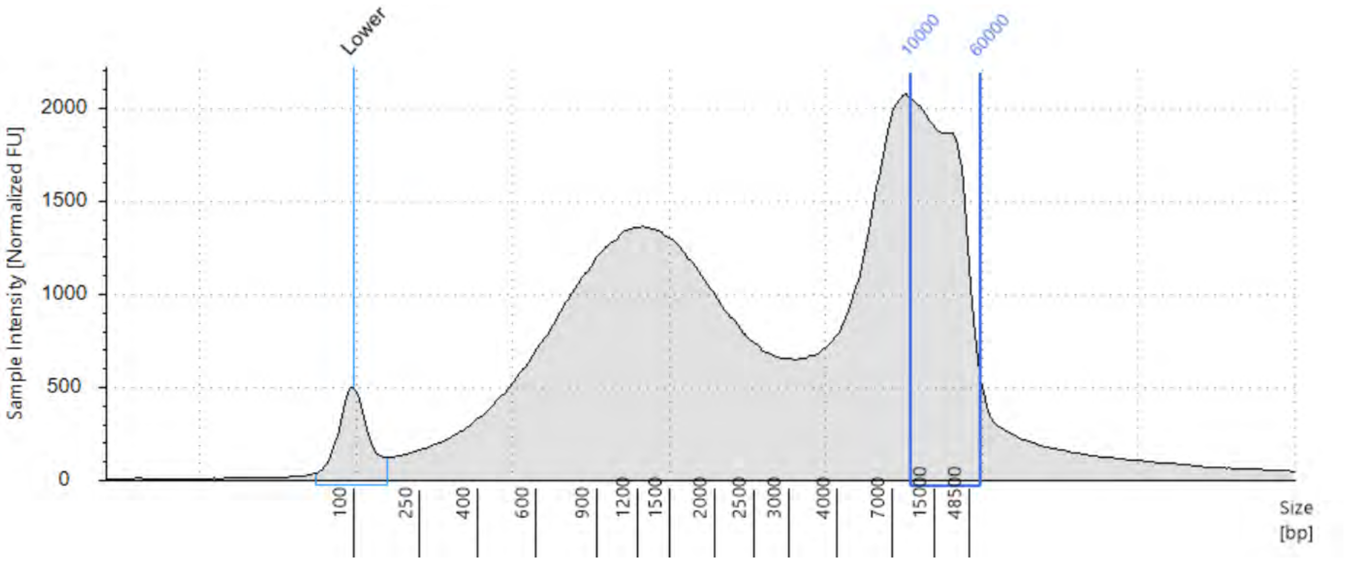

Region Table

From [bp]	To [bp]	Average Size [bp]	Conc. [ng/µl]	Region Molarity [nmol/l]	% of Total	Region Comment	Color
10000	60000	21666	59.9	5.42	19.53		■

 © Agilent Technologies, Inc. 2017

C6: M218

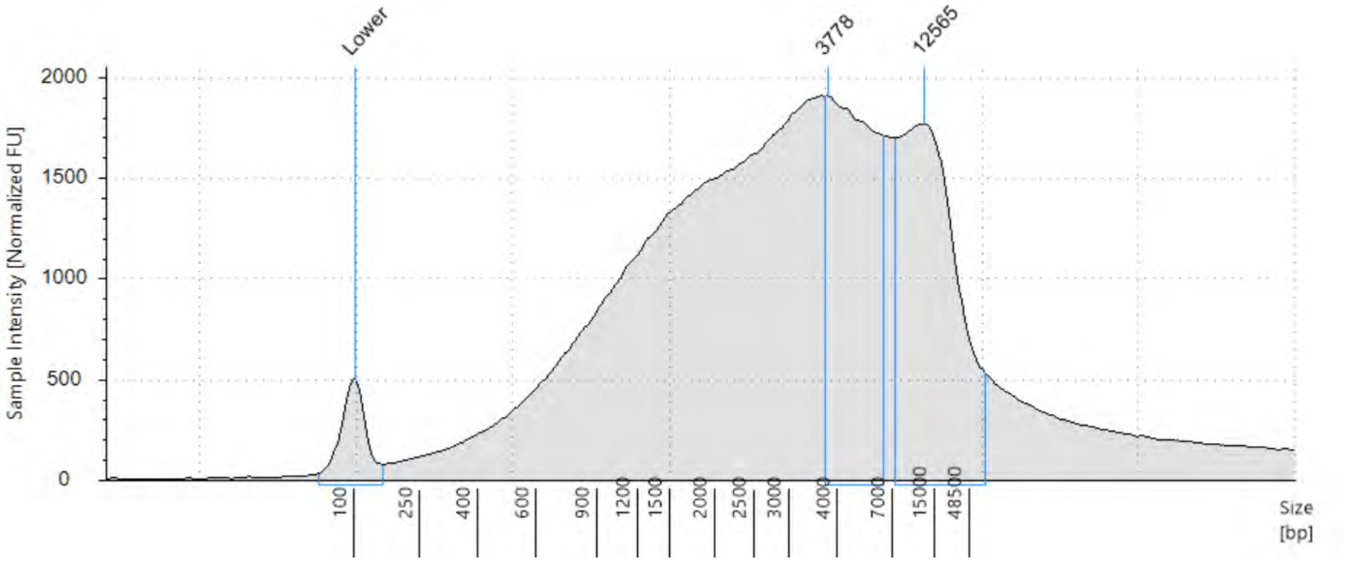

Sample Table

Well	DIN	Conc. [ng/µl]	Sample Description	Alert	Observations
C6	5.5	458	M218	⚠	Caution! Expired ScreenTape device; Sample concentration outside functional range for DIN

Peak Table

Size [bp]	Calibrated Conc. [ng/µl]	Assigned Conc. [ng/µl]	% Integrated Area	From [bp]	To [bp]	Peak Comment	Observations
100	8.50	8.50	-	60	149		Lower Marker
3778	67.1	-	46.17	3718	6412		
12565	75.1	-	51.67	7483	>60000		
-	-	-	-	-	-		Sample Well

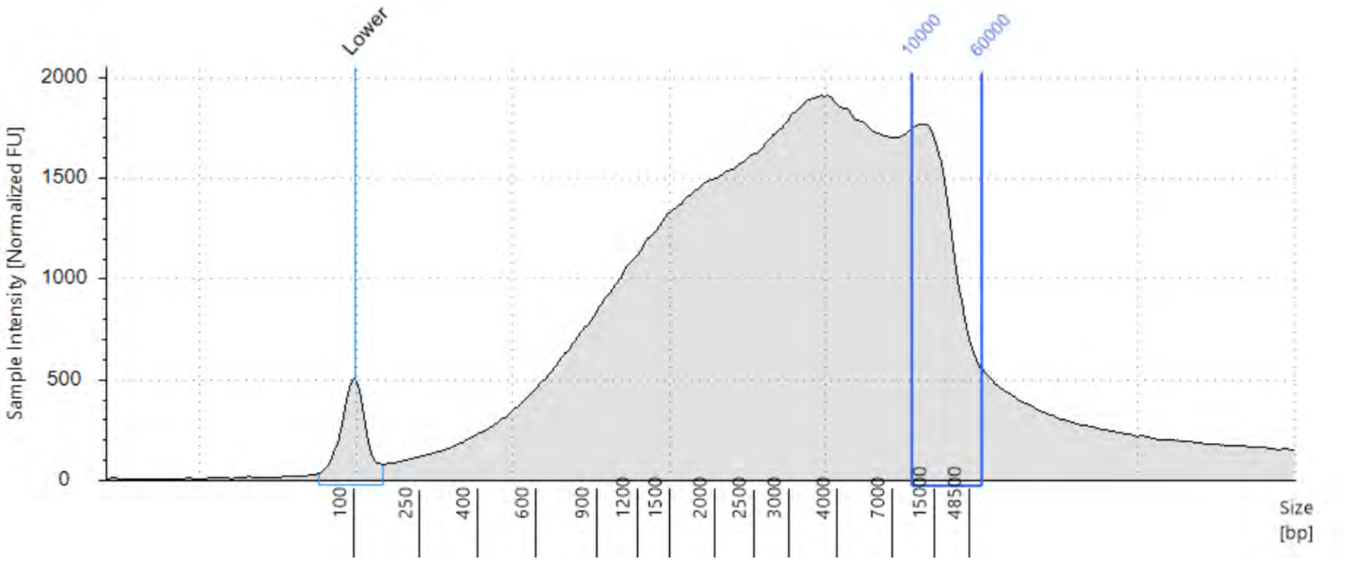

Region Table

From [bp]	To [bp]	Average Size [bp]	Conc. [ng/µl]	Region Molarity [nmol/l]	% of Total	Region Comment	Color
10000	60000	20410	56.5	5.43	12.33		■

 © Agilent Technologies, Inc. 2017

D6: M219

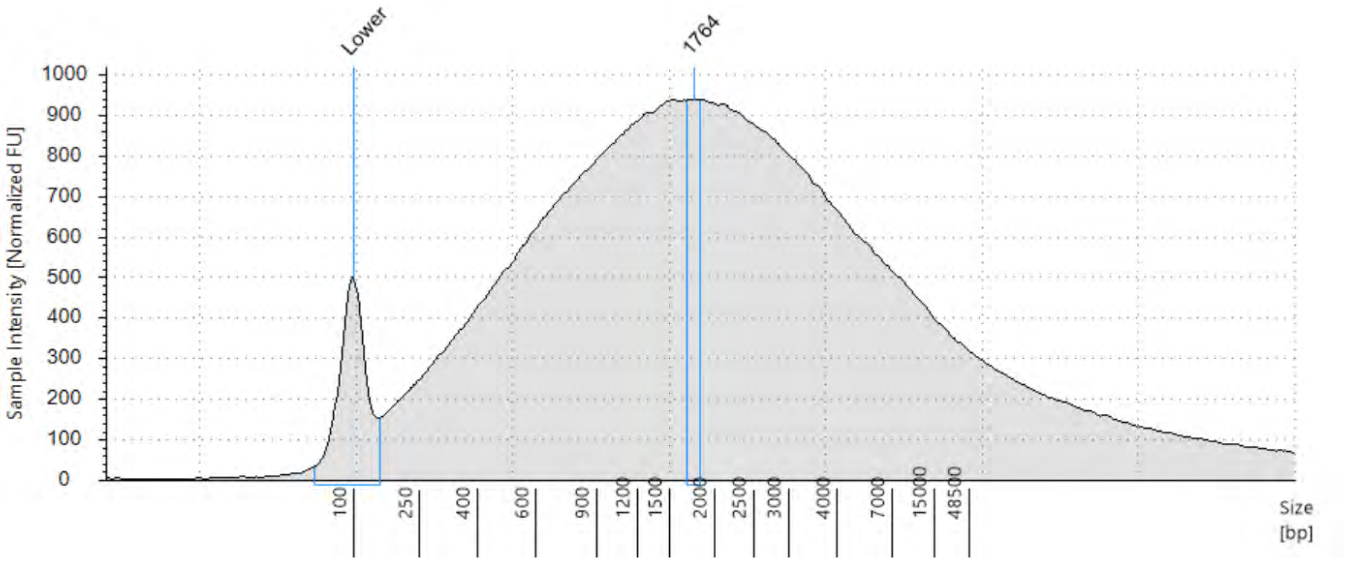

Sample Table

Well	DIN	Conc. [ng/µl]	Sample Description	Alert	Observations
D6	3.3	236	M219	⚠	Caution! Expired ScreenTape device; Sample concentration outside recommended range

Peak Table

Size [bp]	Calibrated Conc. [ng/µl]	Assigned Conc. [ng/µl]	% Integrated Area	From [bp]	To [bp]	Peak Comment	Observations
100	8.50	8.50	-	58	143		Lower Marker
1764	7.74	-	92.97	1689	1841		
-	-	-	-	-	-		Sample Well

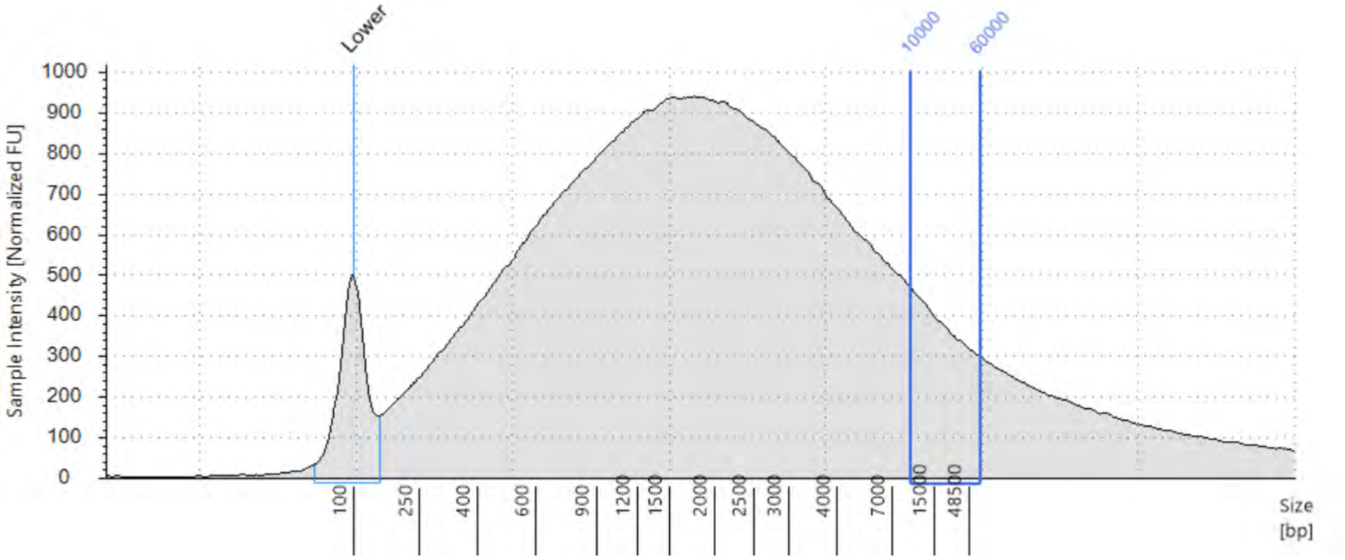

Region Table

From [bp]	To [bp]	Average Size [bp]	Conc. [ng/µl]	Region Molarity [nmol/l]	% of Total	Region Comment	Color
10000	60000	23807	14.3	1.28	6.07		■

E6: M220

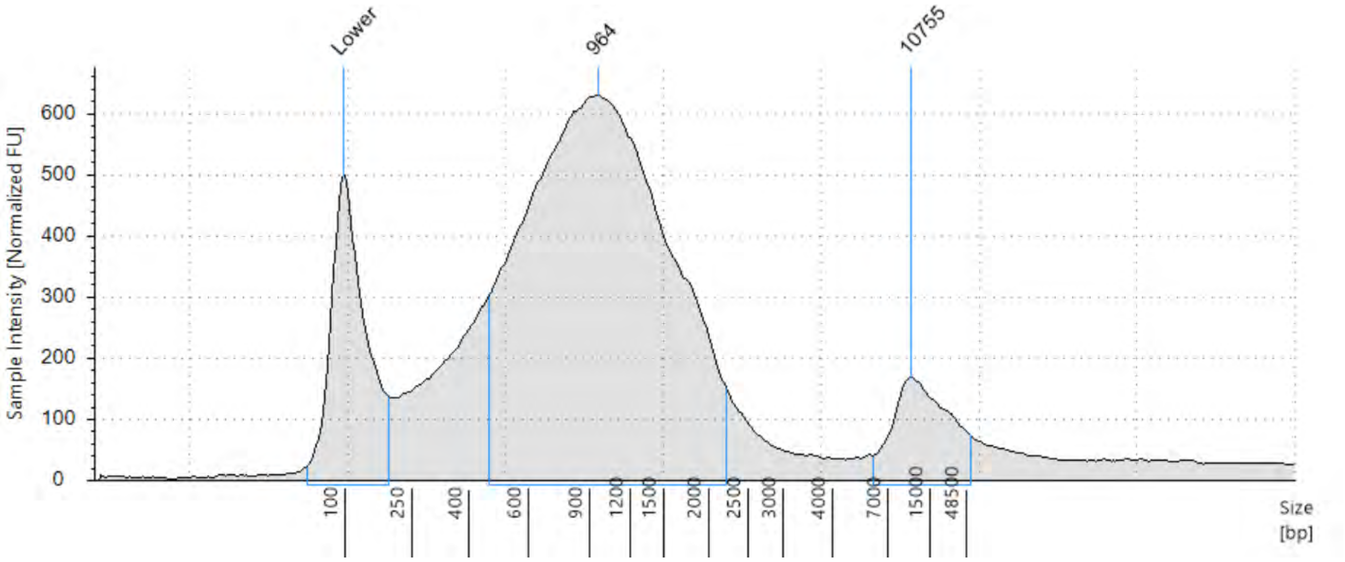

Sample Table

Well	DIN	Conc. [ng/µl]	Sample Description	Alert	Observations
E6	1.6	68.8	M220	⚠	Caution! Expired ScreenTape device

Peak Table

Size [bp]	Calibrated Conc. [ng/µl]	Assigned Conc. [ng/µl]	% Integrated Area	From [bp]	To [bp]	Peak Comment	Observations
100	8.50	8.50	-	61	186		Lower Marker
964	46.0	-	90.52	459	2224		
10755	4.64	-	9.13	6101	53744		
-	-	-	-	-	-		Sample Well

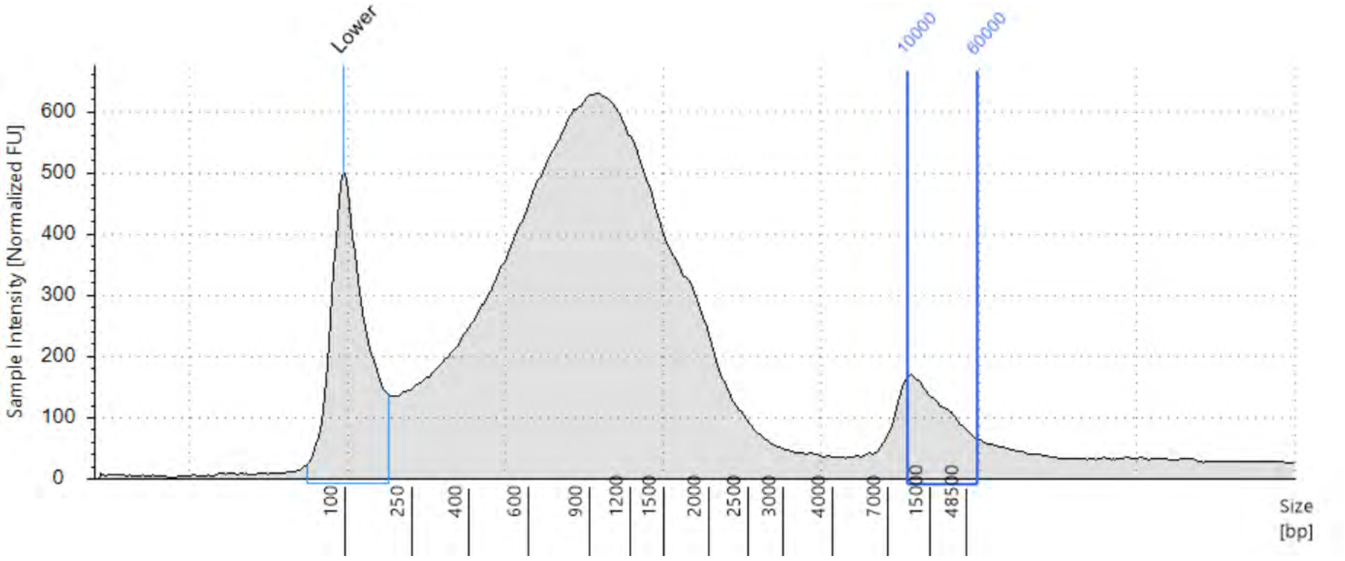

Region Table

From [bp]	To [bp]	Average Size [bp]	Conc. [ng/µl]	Region Molarity [nmol/l]	% of Total	Region Comment	Color
10000	60000	21771	3.63	0.339	5.27		■

 © Agilent Technologies, Inc. 2017

F6: M312

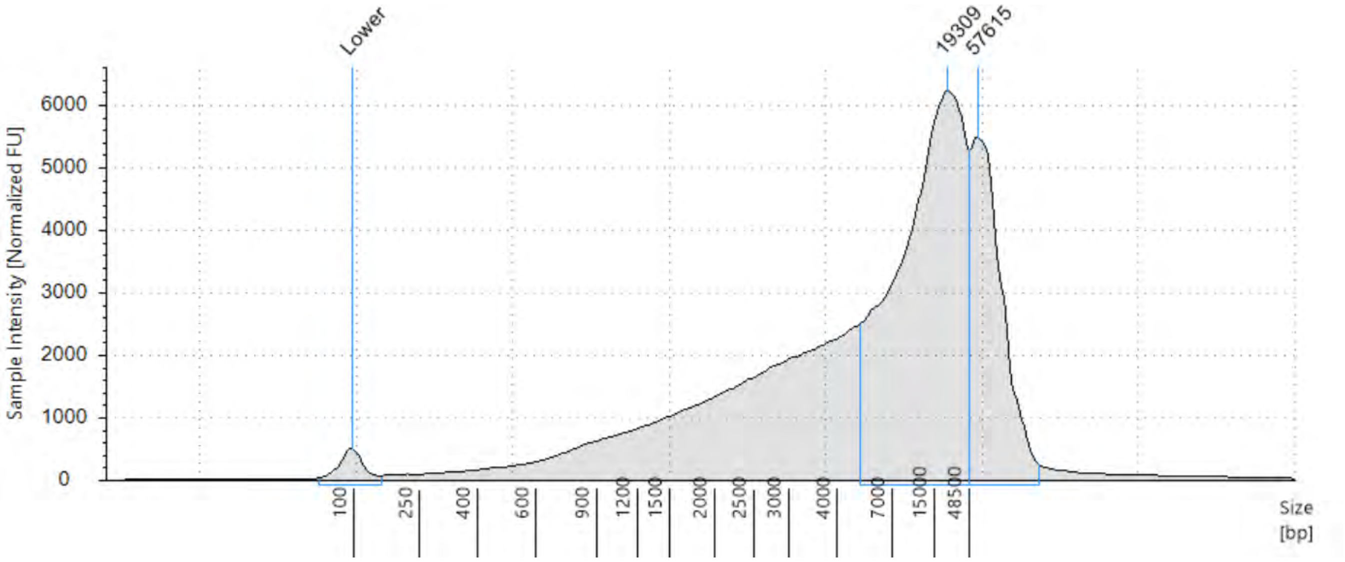

Sample Table

Well	DIN	Conc. [ng/µl]	Sample Description	Alert	Observations
F6	6.6	785	M312	⚠	Caution! Expired ScreenTape device; Sample concentration outside functional range for DIN

Peak Table

Size [bp]	Calibrated Conc. [ng/µl]	Assigned Conc. [ng/µl]	% Integrated Area	From [bp]	To [bp]	Peak Comment	Observations
100	8.50	8.50	-	62	148		Lower Marker
19309	338	-	69.88	5116	49973		
57615	145	-	29.89	49973	>60000		
-	-	-	-	-	-		Sample Well

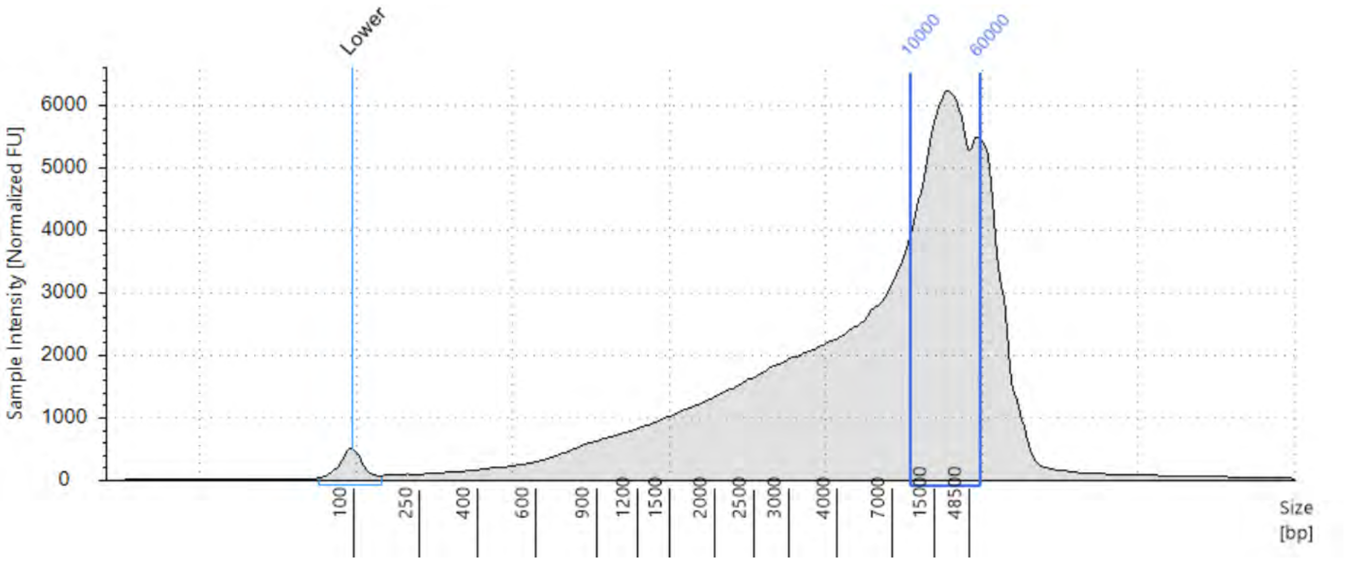

Region Table

From [bp]	To [bp]	Average Size [bp]	Conc. [ng/µl]	Region Molarity [nmol/l]	% of Total	Region Comment	Color
10000	60000	25604	271	21.8	34.57		■

 © Agilent Technologies, Inc. 2017

G6: M313

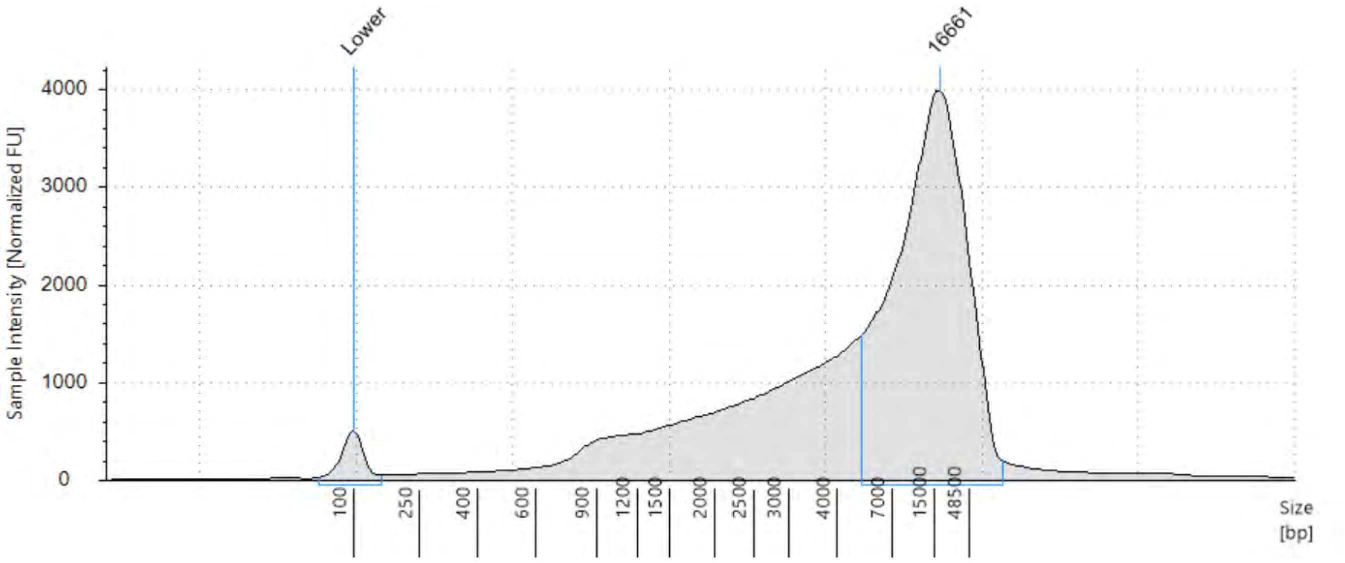

Sample Table

Well	DIN	Conc. [ng/µl]	Sample Description	Alert	Observations
G6	6.5	407	M313	⚠	Caution! Expired ScreenTape device; Sample concentration outside functional range for DIN

Peak Table

Size [bp]	Calibrated Conc. [ng/µl]	Assigned Conc. [ng/µl]	% Integrated Area	From [bp]	To [bp]	Peak Comment	Observations
100	8.50	8.50	-	62	148		Lower Marker
16661	235	-	99.42	5161	>60000		
-	-	-	-	-	-		Sample Well

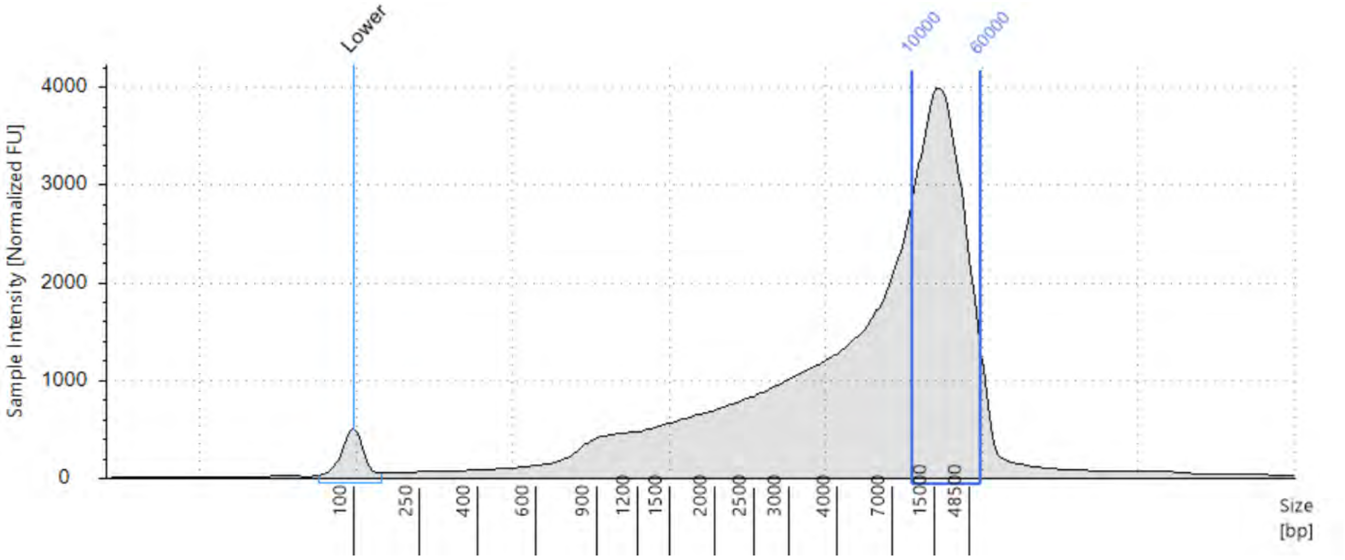

Region Table

From [bp]	To [bp]	Average Size [bp]	Conc. [ng/µl]	Region Molarity [nmol/l]	% of Total	Region Comment	Color
10000	60000	22100	158	14.0	38.86		■

C7: C388

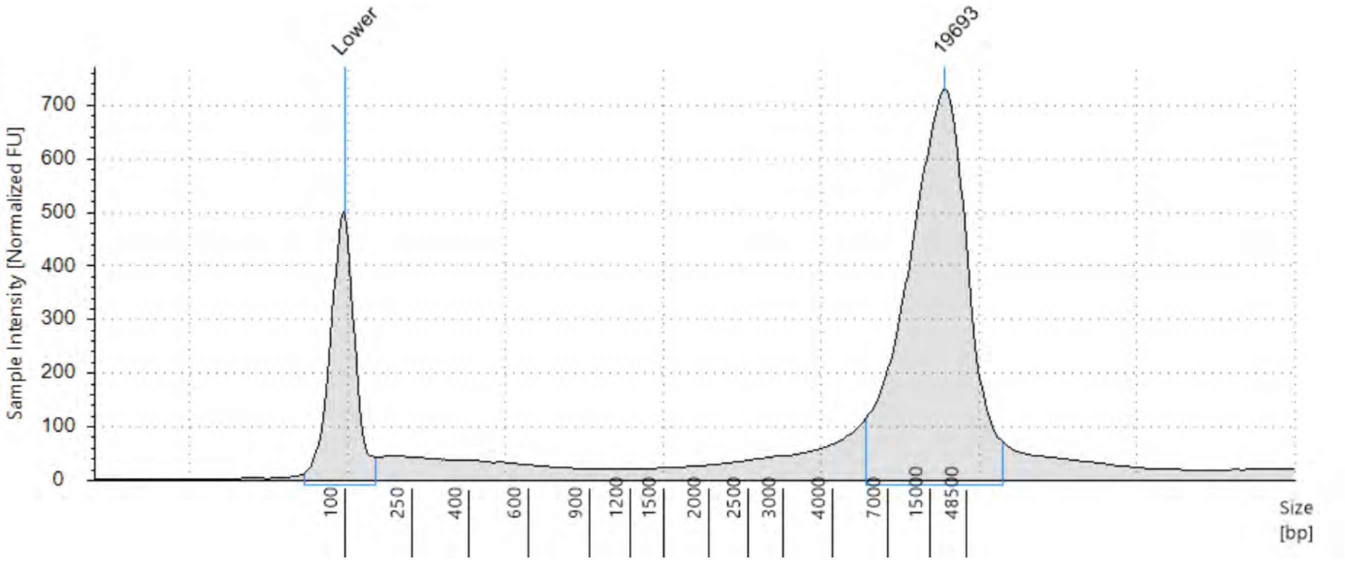

Sample Table

Well	DIN	Conc. [ng/µl]	Sample Description	Alert	Observations
C7	7.1	48.3	C388	⚠	Caution! Expired ScreenTape device

Peak Table

Size [bp]	Calibrated Conc. [ng/µl]	Assigned Conc. [ng/µl]	% Integrated Area	From [bp]	To [bp]	Peak Comment	Observations
100	8.50	8.50	-	57	153		Lower Marker
19693	31.1	-	92.12	5677	>60000		
-	-	-	-	-	-		Sample Well

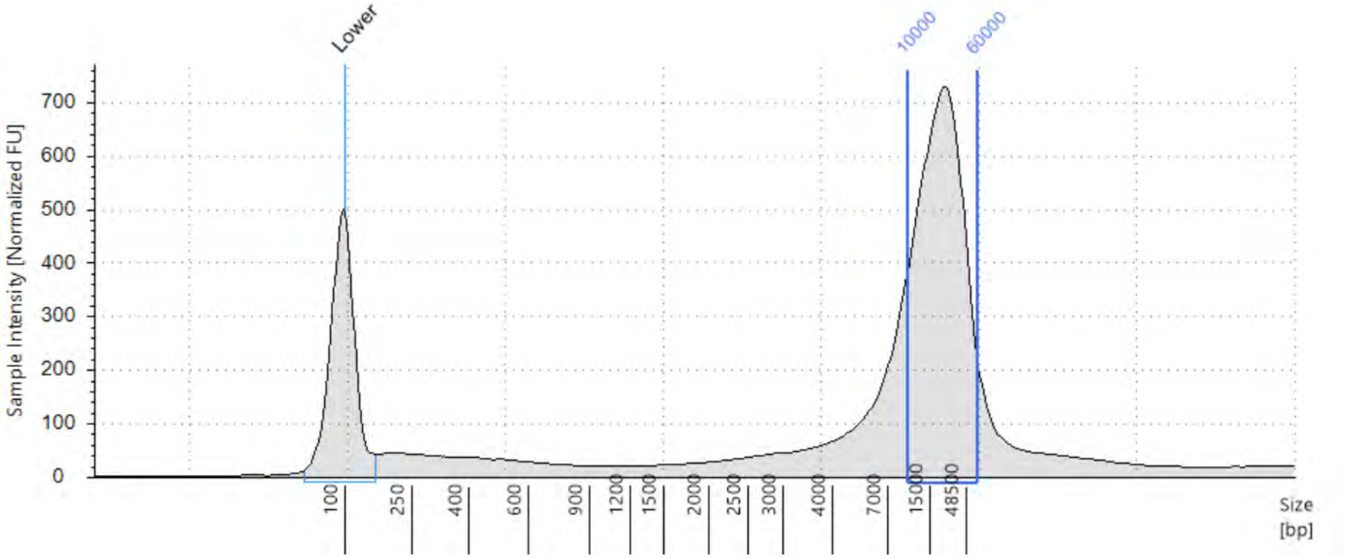

Region Table

From [bp]	To [bp]	Average Size [bp]	Conc. [ng/µl]	Region Molarity [nmol/l]	% of Total	Region Comment	Color
10000	60000	23145	24.2	2.04	50.16		■

 © Agilent Technologies, Inc. 2017

E7: C631

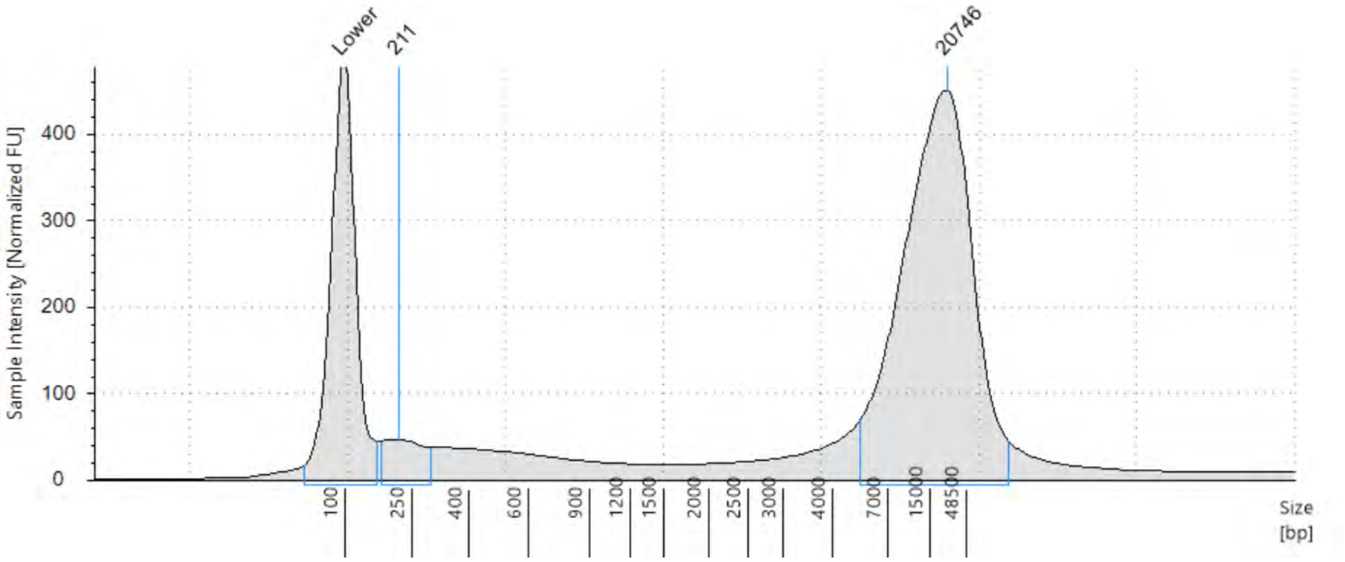

Sample Table

Well	DIN	Conc. [ng/µl]	Sample Description	Alert	Observations
E7	7.0	33.7	C631	⚠	Caution! Expired ScreenTape device

Peak Table

Size [bp]	Calibrated Conc. [ng/µl]	Assigned Conc. [ng/µl]	% Integrated Area	From [bp]	To [bp]	Peak Comment	Observations
100	8.50	8.50	-	57	155		Lower Marker
211	1.31	-	5.28	165	293		
20746	22.1	-	89.53	5315	>60000		
-	-	-	-	-	-		Sample Well

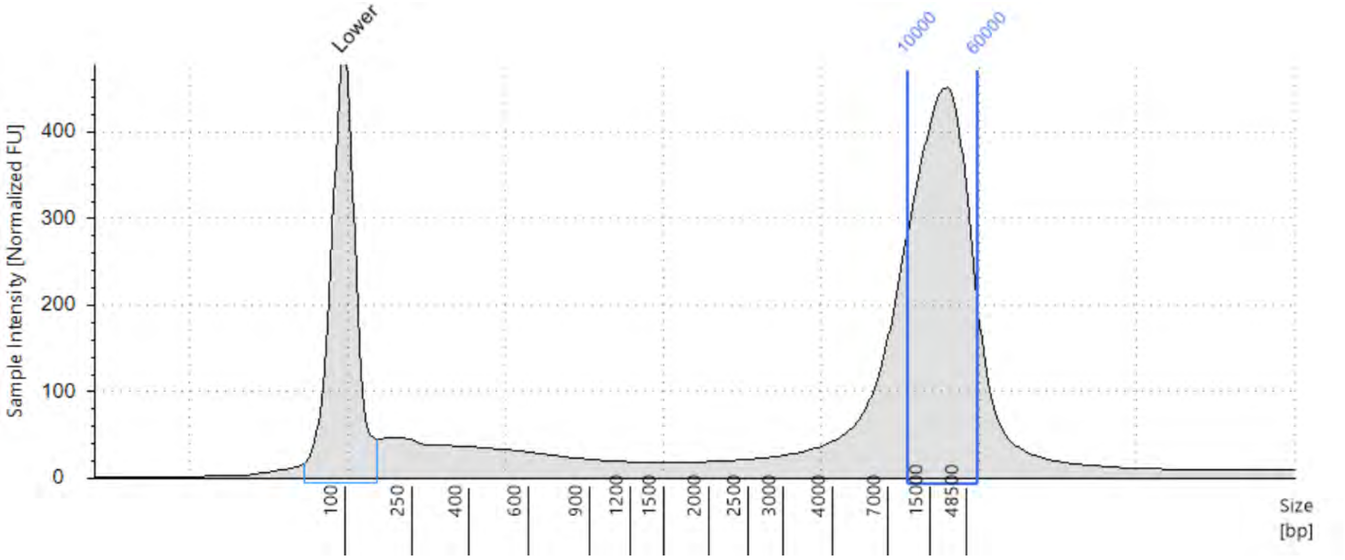

Region Table

From [bp]	To [bp]	Average Size [bp]	Conc. [ng/µl]	Region Molarity [nmol/l]	% of Total	Region Comment	Color
10000	60000	24122	16.2	1.35	48.18		■

 © Agilent Technologies, Inc. 2017

F7: C632

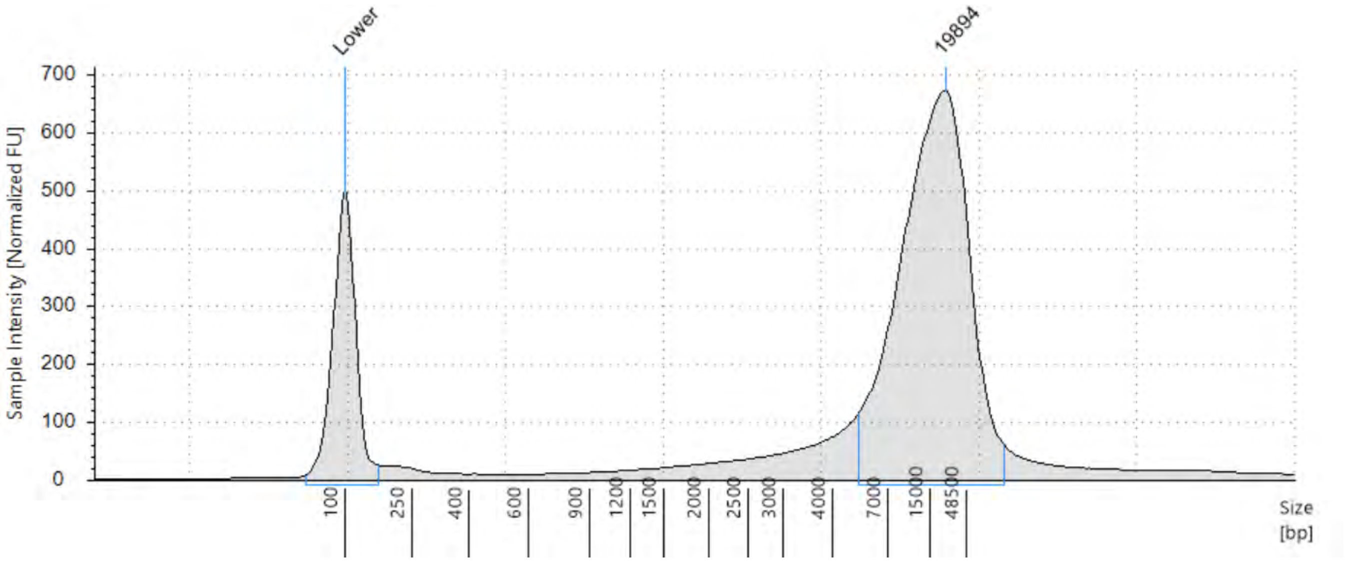

Sample Table

Well	DIN	Conc. [ng/µl]	Sample Description	Alert	Observations
F7	7.5	47.2	C632	⚠	Caution! Expired ScreenTape device

Peak Table

Size [bp]	Calibrated Conc. [ng/µl]	Assigned Conc. [ng/µl]	% Integrated Area	From [bp]	To [bp]	Peak Comment	Observations
100	8.50	8.50	-	58	156		Lower Marker
19894	35.0	-	94.45	5219	>60000		
-	-	-	-	-	-		Sample Well

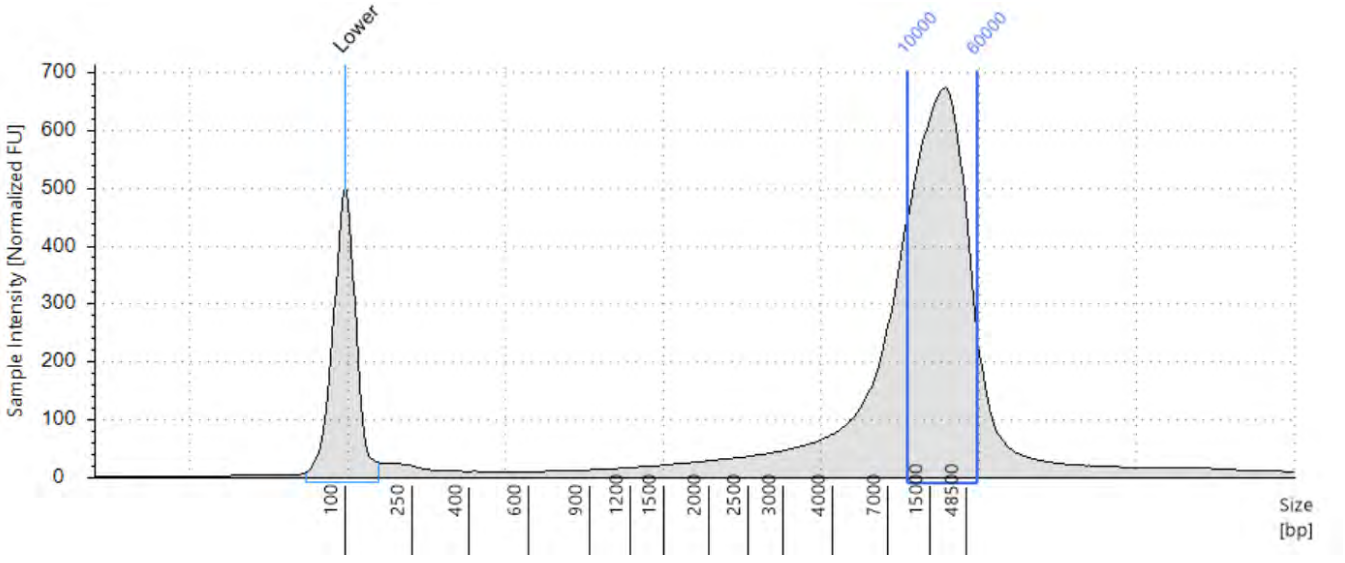

Region Table

From [bp]	To [bp]	Average Size [bp]	Conc. [ng/µl]	Region Molarity [nmol/l]	% of Total	Region Comment	Color
10000	60000	23081	25.4	2.17	53.85		■

G7: C633

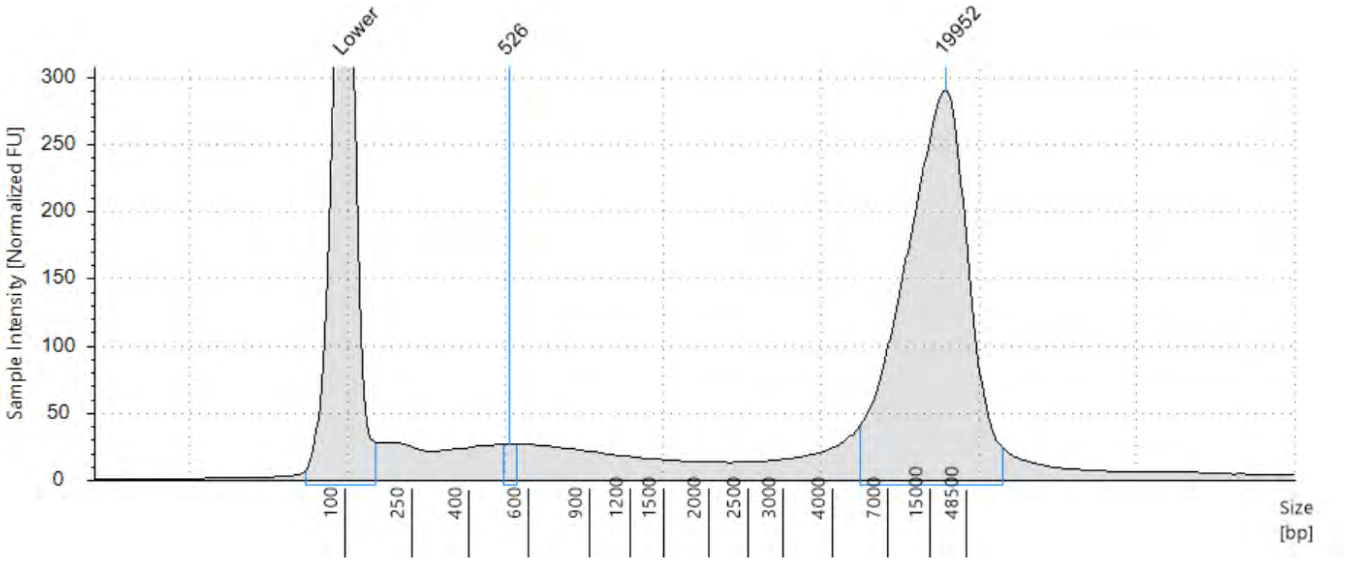

Sample Table

Well	DIN	Conc. [ng/µl]	Sample Description	Alert	Observations
G7	6.8	21.6	C633	⚠	Caution! Expired ScreenTape device

Peak Table

Size [bp]	Calibrated Conc. [ng/µl]	Assigned Conc. [ng/µl]	% Integrated Area	From [bp]	To [bp]	Peak Comment	Observations
100	8.50	8.50	-	57	153		Lower Marker
526	0.252	-	1.67	502	552		
19952	13.9	-	92.31	5258	>60000		
-	-	-	-	-	-		Sample Well

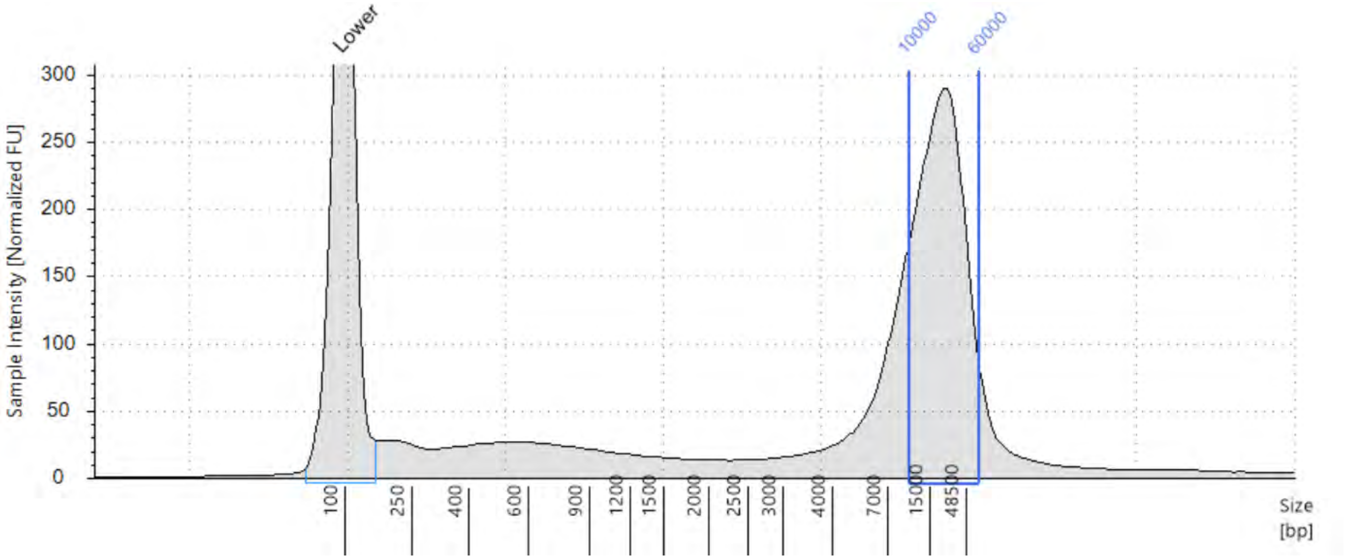

Region Table

From [bp]	To [bp]	Average Size [bp]	Conc. [ng/µl]	Region Molarity [nmol/l]	% of Total	Region Comment	Color
10000	60000	22830	10.3	0.881	47.97		■

H7: C634

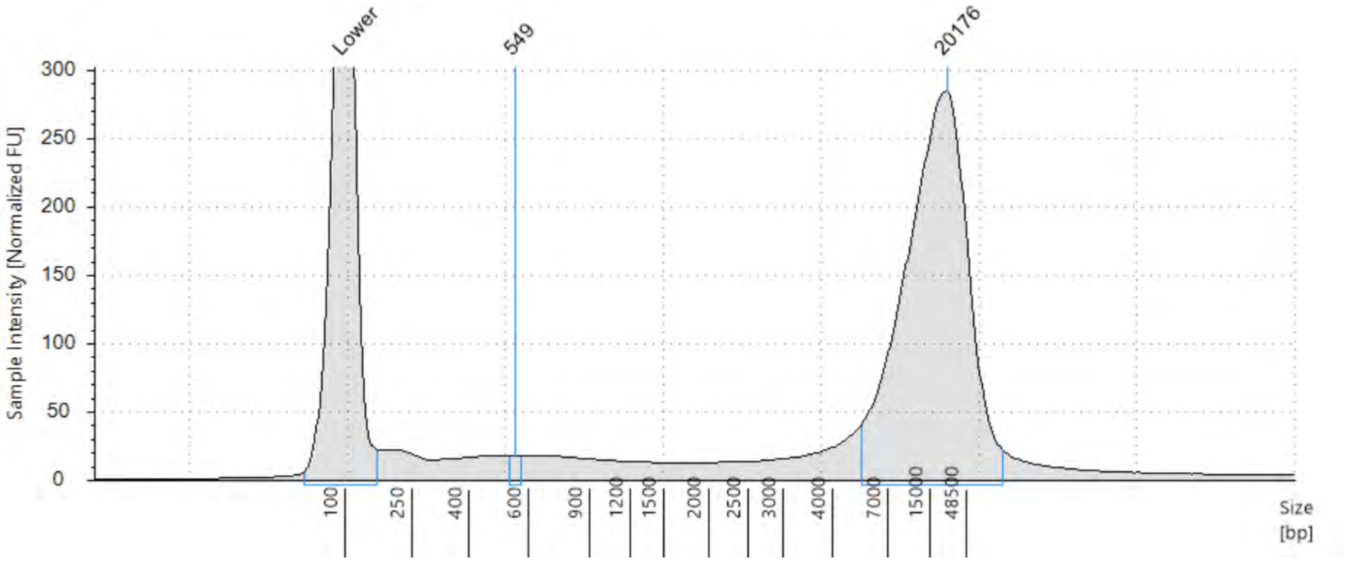

Sample Table

Well	DIN	Conc. [ng/µl]	Sample Description	Alert	Observations
H7	7.1	20.1	C634	⚠	Caution! Expired ScreenTape device

Peak Table

Size [bp]	Calibrated Conc. [ng/µl]	Assigned Conc. [ng/µl]	% Integrated Area	From [bp]	To [bp]	Peak Comment	Observations
100	8.50	8.50	-	57	156		Lower Marker
549	0.156	-	1.09	523	570		
20176	13.6	-	94.83	5351	>60000		
-	-	-	-	-	-		Sample Well

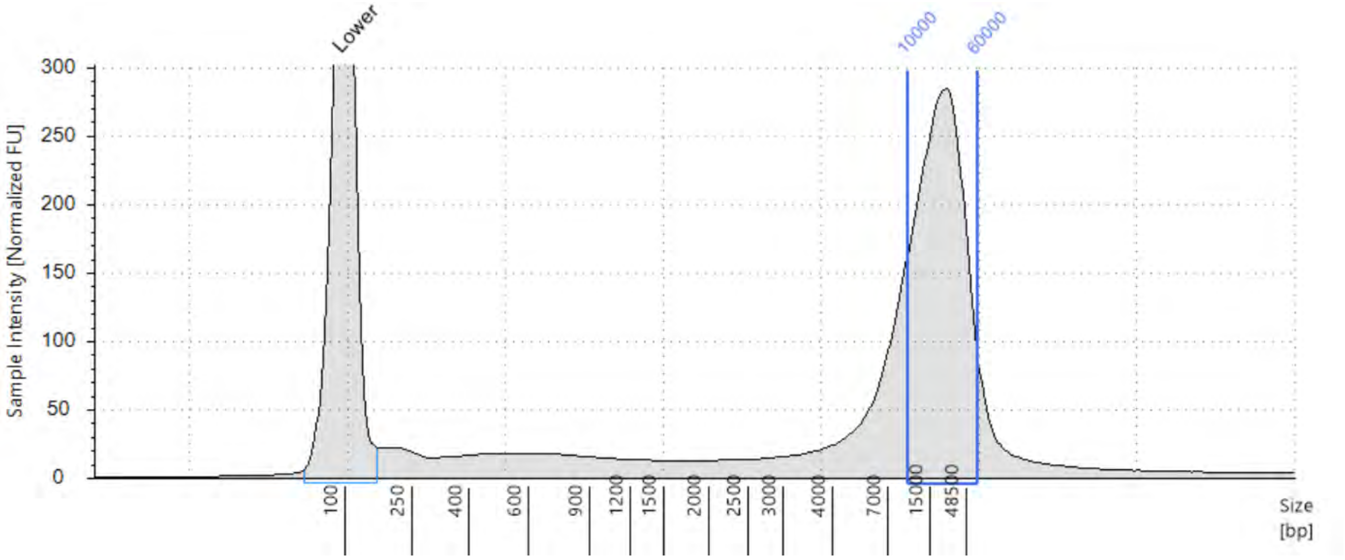

Region Table

From [bp]	To [bp]	Average Size [bp]	Conc. [ng/µl]	Region Molarity [nmol/l]	% of Total	Region Comment	Color
10000	60000	23254	10.2	0.858	51.03		■

 © Agilent Technologies, Inc. 2017

A8: C635

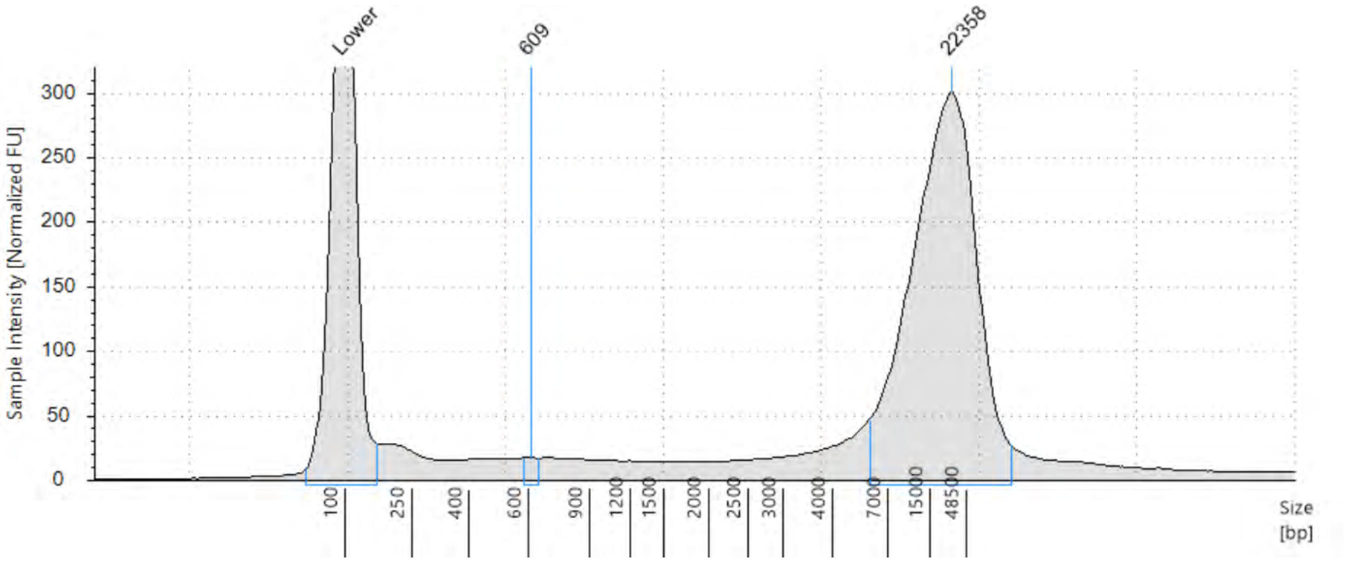

Sample Table

Well	DIN	Conc. [ng/µl]	Sample Description	Alert	Observations
A8	7.2	22.1	C635	⚠	Caution! Expired ScreenTape device

Peak Table

Size [bp]	Calibrated Conc. [ng/µl]	Assigned Conc. [ng/µl]	% Integrated Area	From [bp]	To [bp]	Peak Comment	Observations
100	8.50	8.50	-	58	156		Lower Marker
609	0.155	-	1.03	581	639		
22358	14.5	-	96.40	5827	>60000		
-	-	-	-	-	-		Sample Well

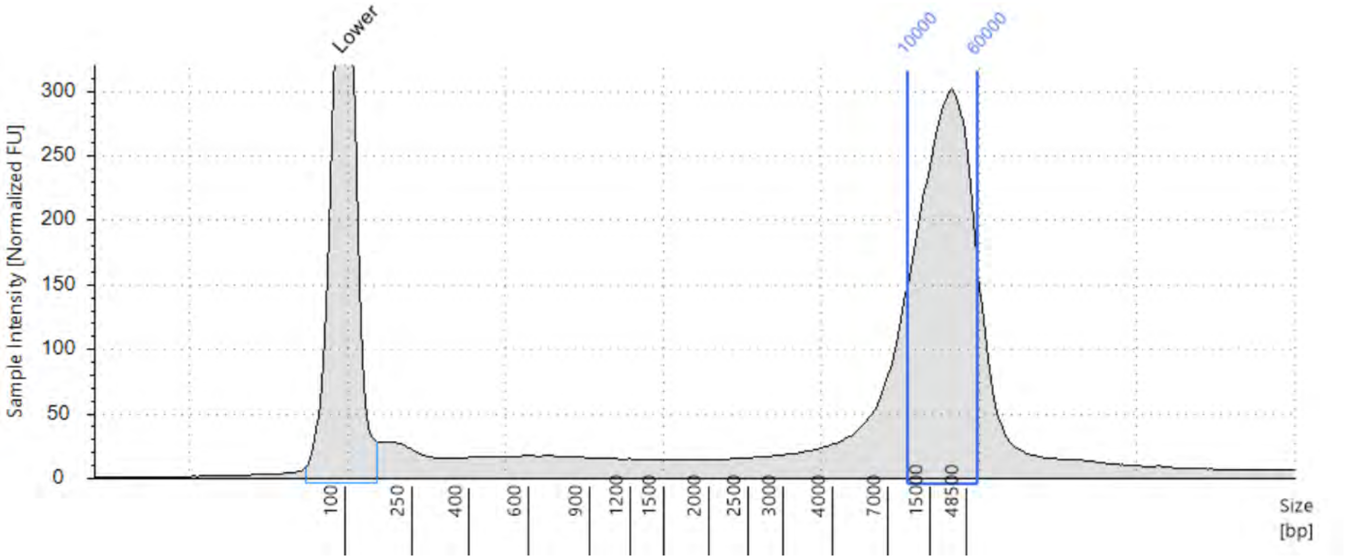

Region Table

From [bp]	To [bp]	Average Size [bp]	Conc. [ng/µl]	Region Molarity [nmol/l]	% of Total	Region Comment	Color
10000	60000	25789	11.0	0.864	49.87		■

 © Agilent Technologies, Inc. 2017

B8: C390

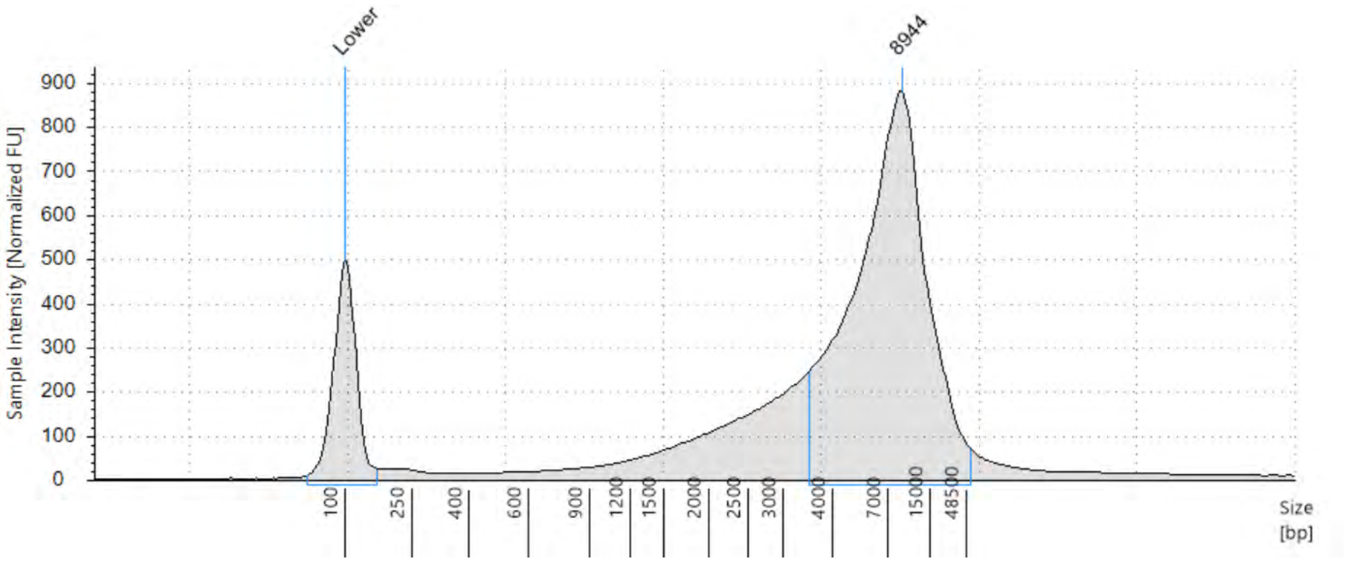

Sample Table

Well	DIN	Conc. [ng/µl]	Sample Description	Alert	Observations
B8	6.4	68.7	C390	⚠	Caution! Expired ScreenTape device

Peak Table

Size [bp]	Calibrated Conc. [ng/µl]	Assigned Conc. [ng/µl]	% Integrated Area	From [bp]	To [bp]	Peak Comment	Observations
100	8.50	8.50	-	59	155		Lower Marker
8944	47.1	-	97.77	3484	53174		
-	-	-	-	-	-		Sample Well

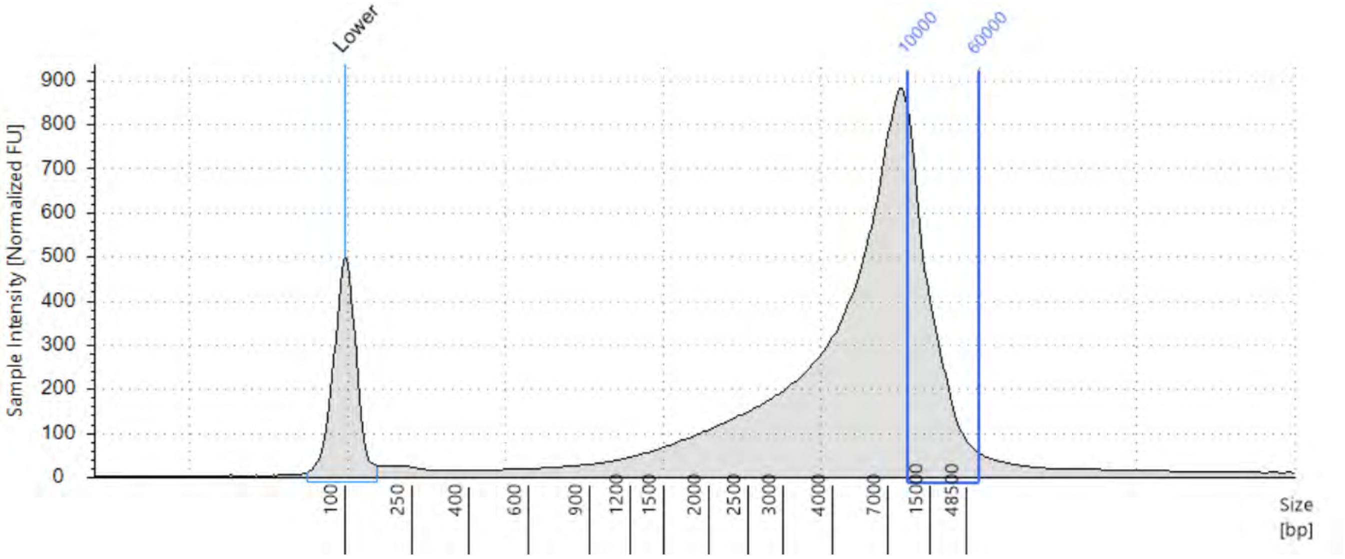

Region Table

From [bp]	To [bp]	Average Size [bp]	Conc. [ng/µl]	Region Molarity [nmol/l]	% of Total	Region Comment	Color
10000	60000	16880	14.5	1.59	21.11		■

C8: C391

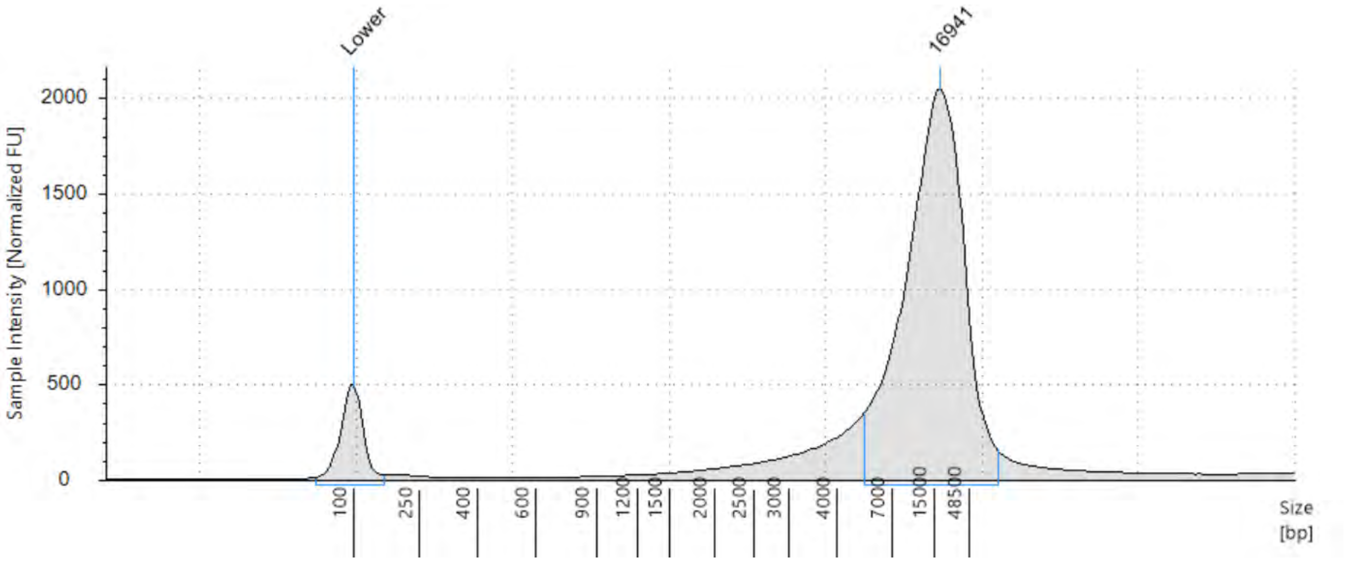

Sample Table

Well	DIN	Conc. [ng/µl]	Sample Description	Alert	Observations
C8	7.6	123	C391	⚠	Caution! Expired ScreenTape device; Sample concentration outside recommended range

Peak Table

Size [bp]	Calibrated Conc. [ng/µl]	Assigned Conc. [ng/µl]	% Integrated Area	From [bp]	To [bp]	Peak Comment	Observations
100	8.50	8.50	-	59	153		Lower Marker
16941	93.2	-	91.65	5289	>60000		
-	-	-	-	-	-		Sample Well

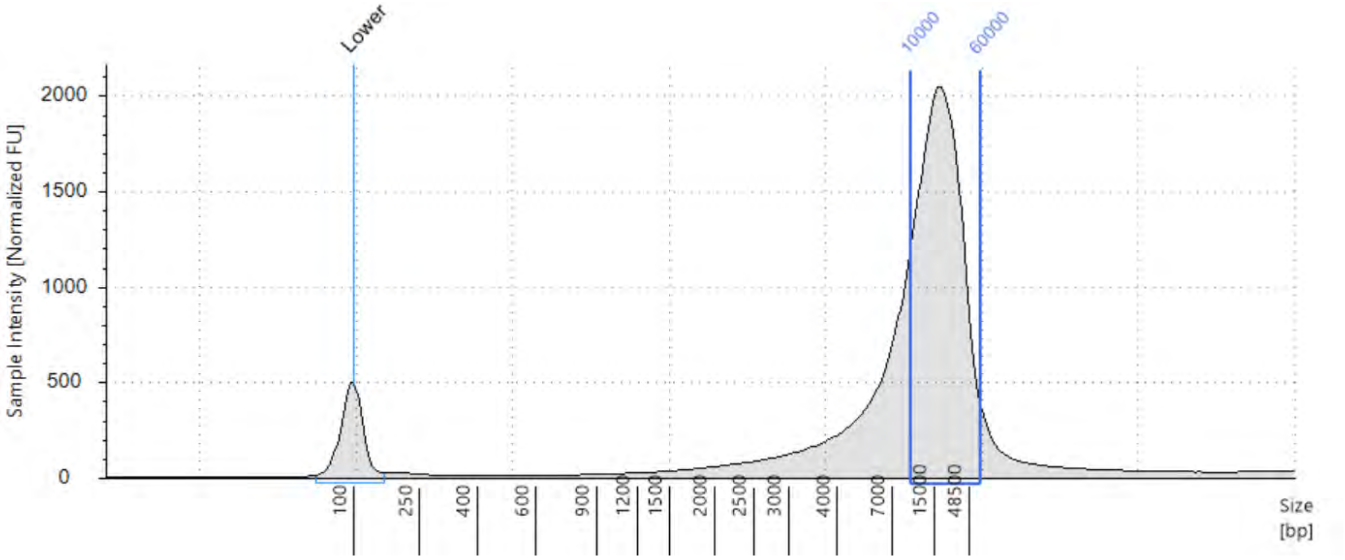

Region Table

From [bp]	To [bp]	Average Size [bp]	Conc. [ng/µl]	Region Molarity [nmol/l]	% of Total	Region Comment	Color
10000	60000	21069	70.7	6.33	57.25		■

© Agilent Technologies, Inc. 2017

D8: C636

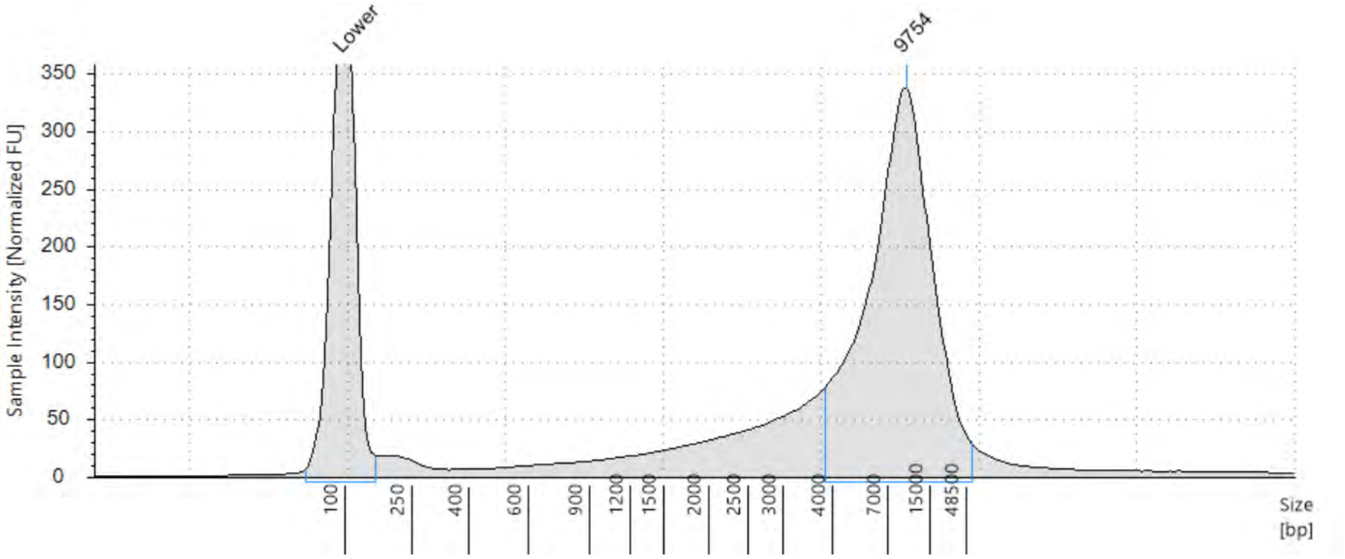

Sample Table

Well	DIN	Conc. [ng/µl]	Sample Description	Alert	Observations
D8	6.6	24.6	C636	⚠	Caution! Expired ScreenTape device

Peak Table

Size [bp]	Calibrated Conc. [ng/µl]	Assigned Conc. [ng/µl]	% Integrated Area	From [bp]	To [bp]	Peak Comment	Observations
100	8.50	8.50	-	57	153		Lower Marker
9754	16.4	-	94.67	3832	54505		
-	-	-	-	-	-		Sample Well

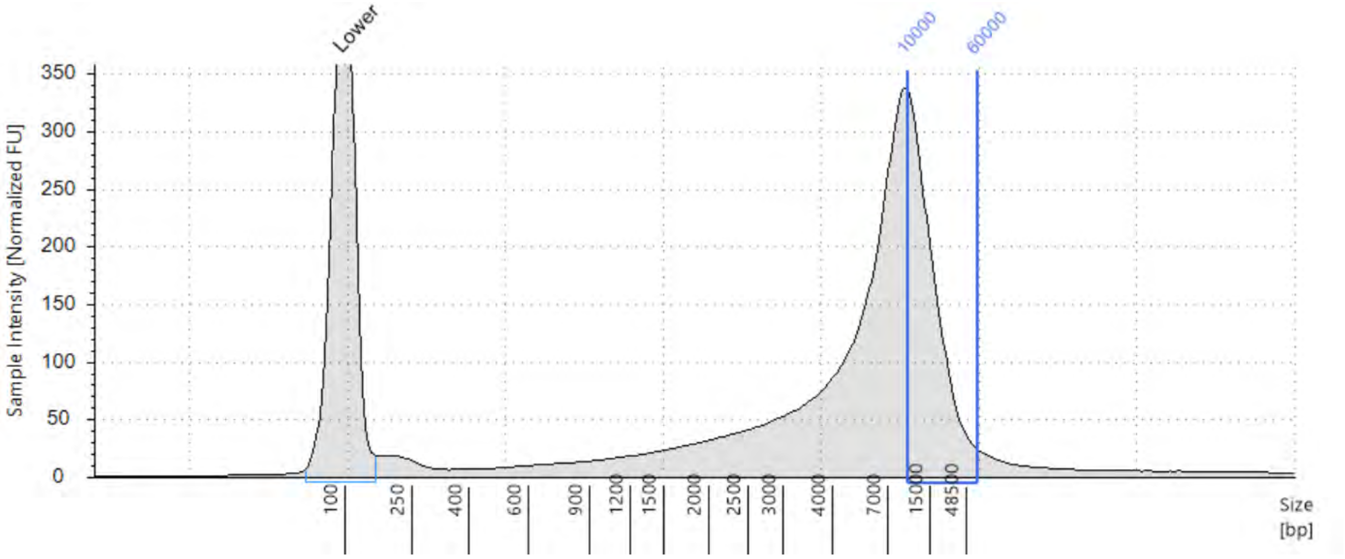

Region Table

From [bp]	To [bp]	Average Size [bp]	Conc. [ng/µl]	Region Molarity [nmol/l]	% of Total	Region Comment	Color
10000	60000	16714	7.03	0.770	28.54		■

E8: C637

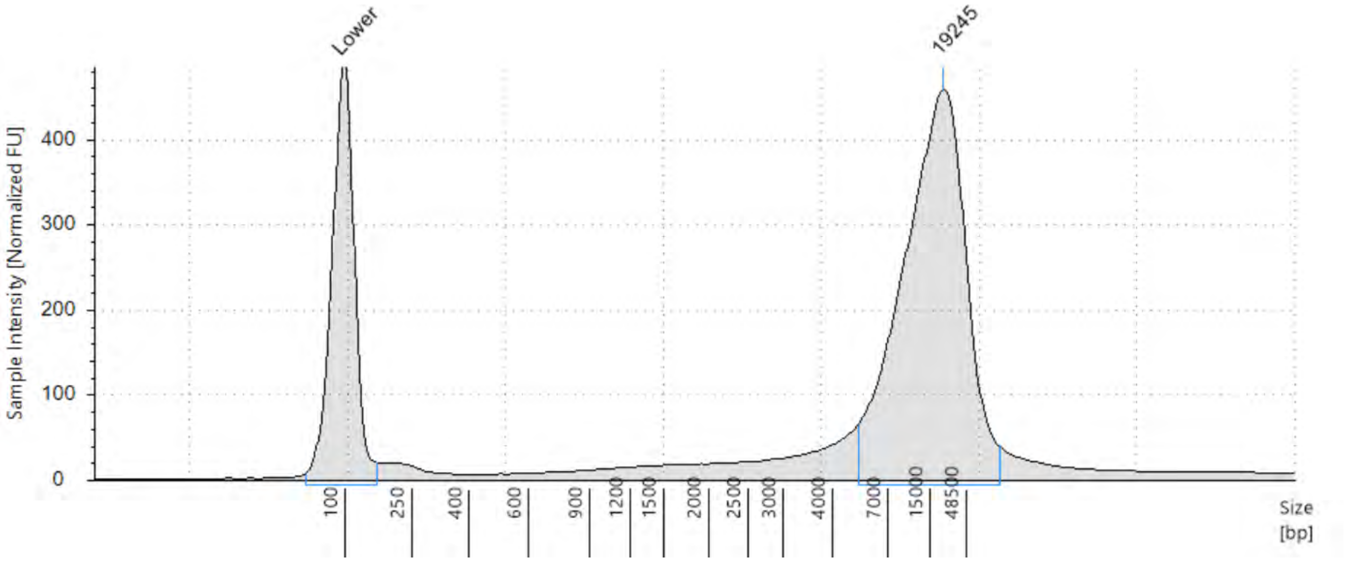

Sample Table

Well	DIN	Conc. [ng/µl]	Sample Description	Alert	Observations
E8	7.5	30.8	C637	⚠	Caution! Expired ScreenTape device

Peak Table

Size [bp]	Calibrated Conc. [ng/µl]	Assigned Conc. [ng/µl]	% Integrated Area	From [bp]	To [bp]	Peak Comment	Observations
100	8.50	8.50	-	58	155		Lower Marker
19245	22.1	-	93.27	5241	>60000		
-	-	-	-	-	-		Sample Well

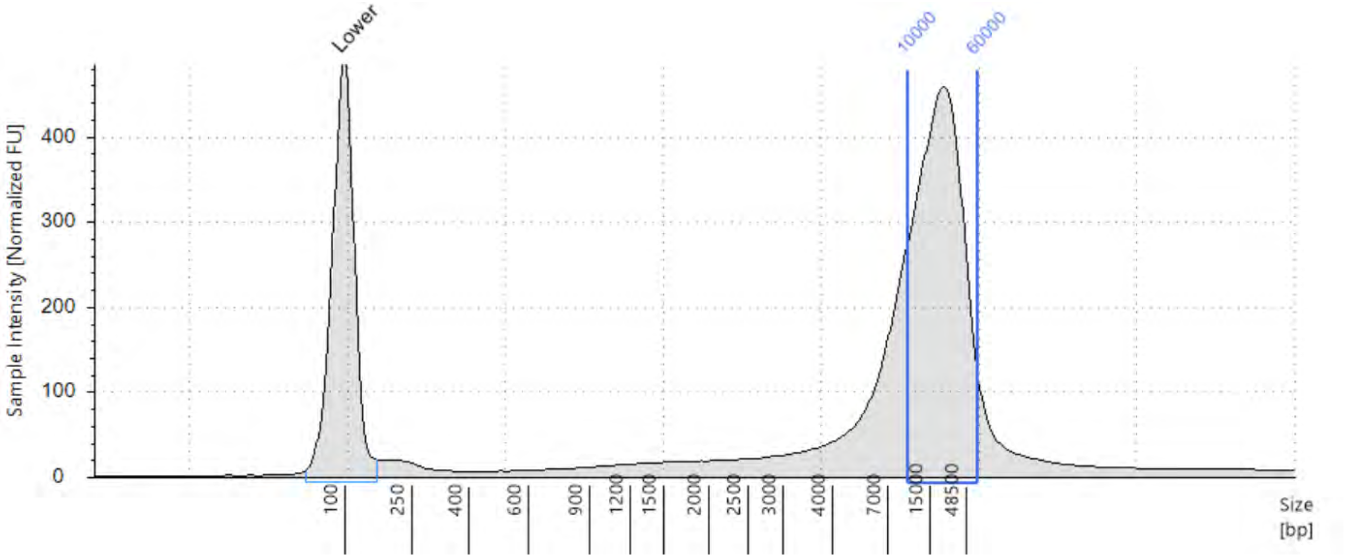

Region Table

From [bp]	To [bp]	Average Size [bp]	Conc. [ng/µl]	Region Molarity [nmol/l]	% of Total	Region Comment	Color
10000	60000	22601	16.6	1.42	53.80		■

F8: C638

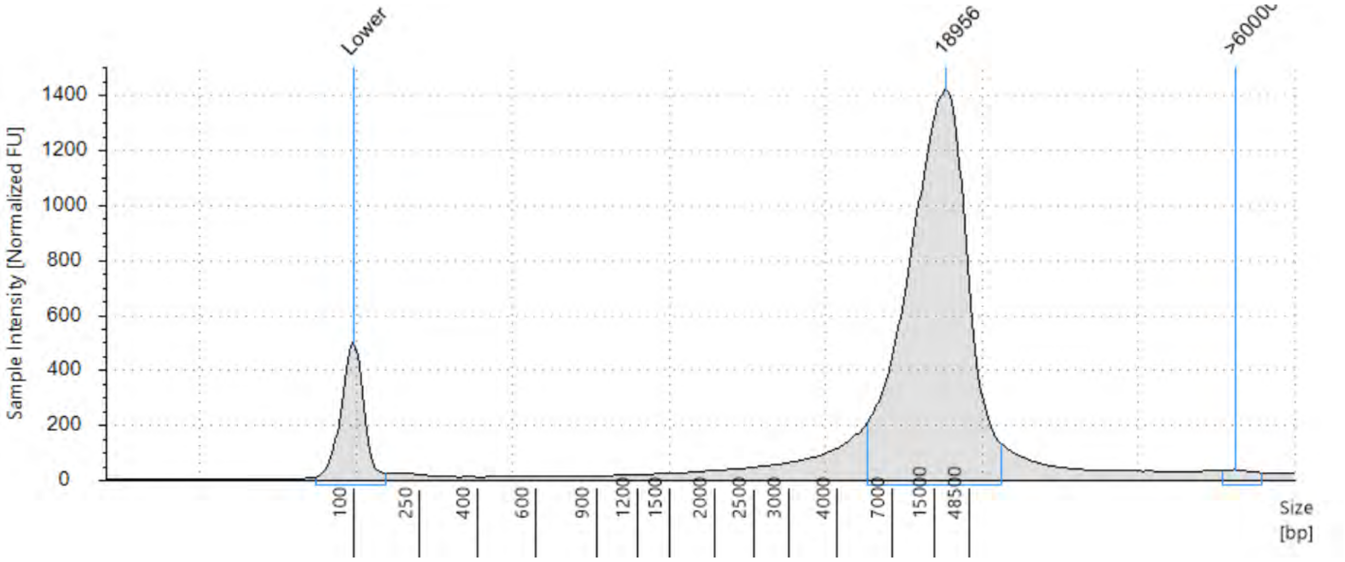

Sample Table

Well	DIN	Conc. [ng/µl]	Sample Description	Alert	Observations
F8	7.8	85.3	C638	⚠	Caution! Expired ScreenTape device

Peak Table

Size [bp]	Calibrated Conc. [ng/µl]	Assigned Conc. [ng/µl]	% Integrated Area	From [bp]	To [bp]	Peak Comment	Observations
100	8.50	8.50	-	58	156		Lower Marker
18956	63.9	-	89.03	5473	>60000		
>60000	0.777	-	1.08	>60000	>60000		
-	-	-	-	-	-		Sample Well

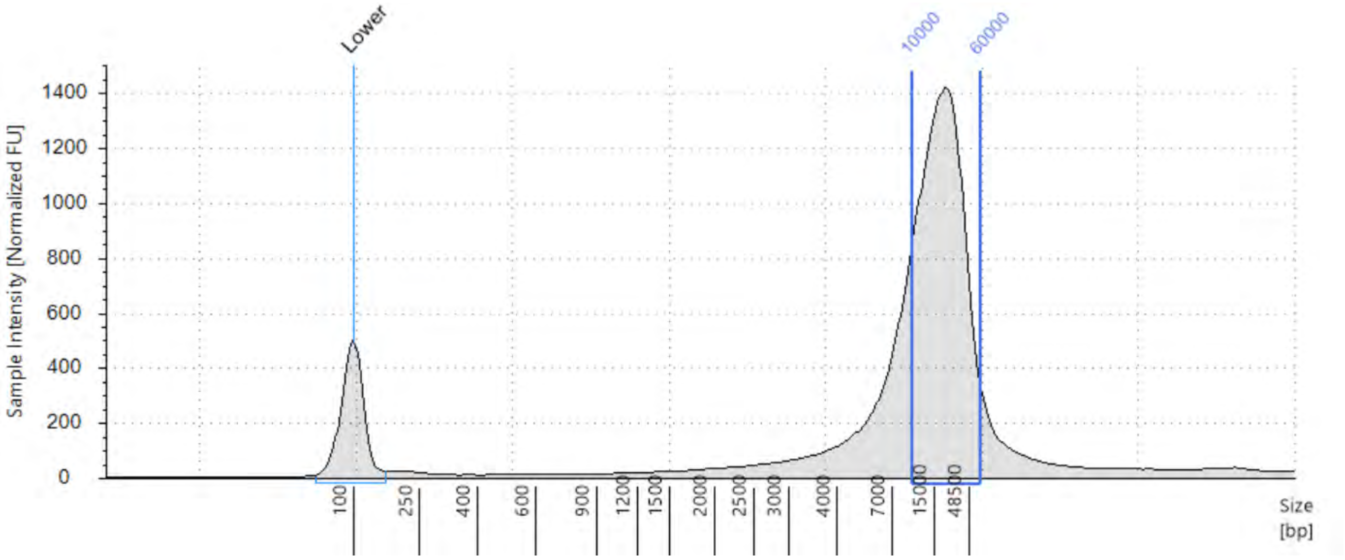

Region Table

From [bp]	To [bp]	Average Size [bp]	Conc. [ng/µl]	Region Molarity [nmol/l]	% of Total	Region Comment	Color
10000	60000	21814	48.8	4.26	57.22		■

G8: C639

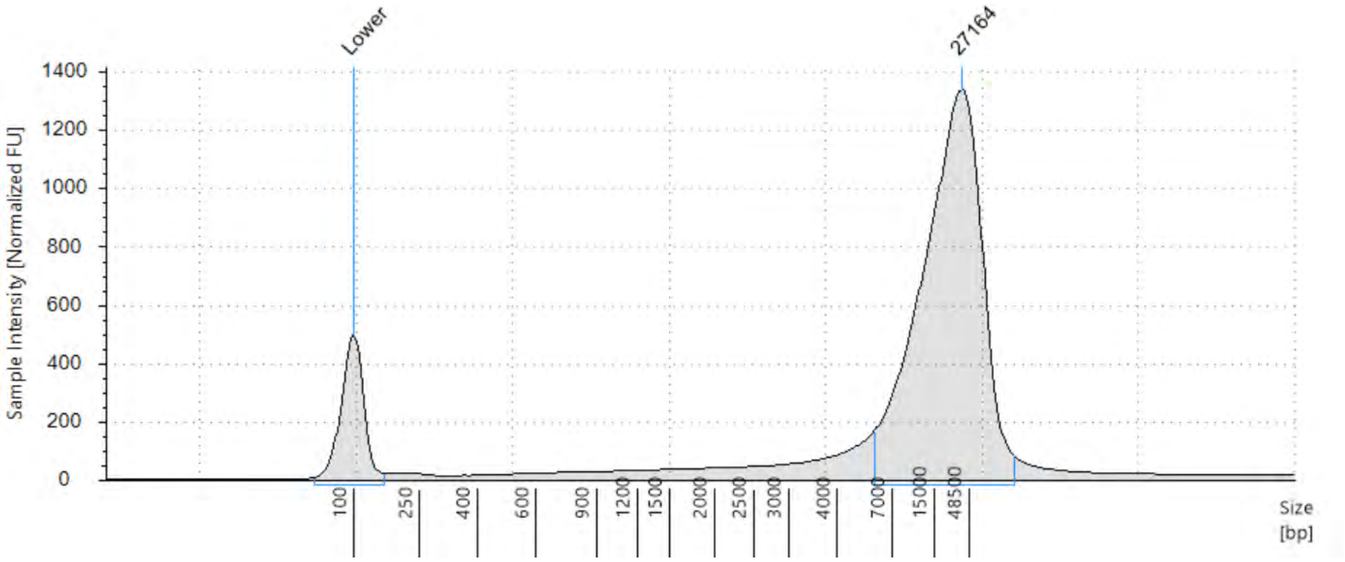

Sample Table

Well	DIN	Conc. [ng/µl]	Sample Description	Alert	Observations
G8	7.9	80.9	C639	⚠	Caution! Expired ScreenTape device

Peak Table

Size [bp]	Calibrated Conc. [ng/µl]	Assigned Conc. [ng/µl]	% Integrated Area	From [bp]	To [bp]	Peak Comment	Observations
100	8.50	8.50	-	58	152		Lower Marker
27164	61.6	-	93.01	5919	>60000		
-	-	-	-	-	-		Sample Well

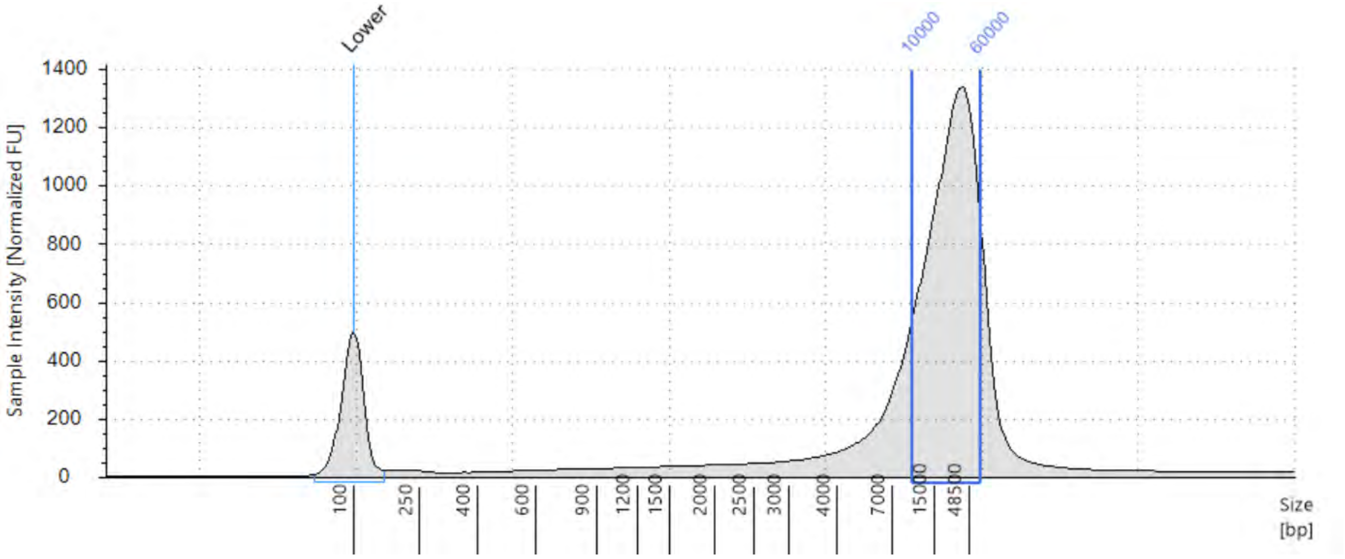

Region Table

From [bp]	To [bp]	Average Size [bp]	Conc. [ng/µl]	Region Molarity [nmol/l]	% of Total	Region Comment	Color
10000	60000	27636	47.5	3.52	58.68		■

 © Agilent Technologies, Inc. 2017

H8: C640

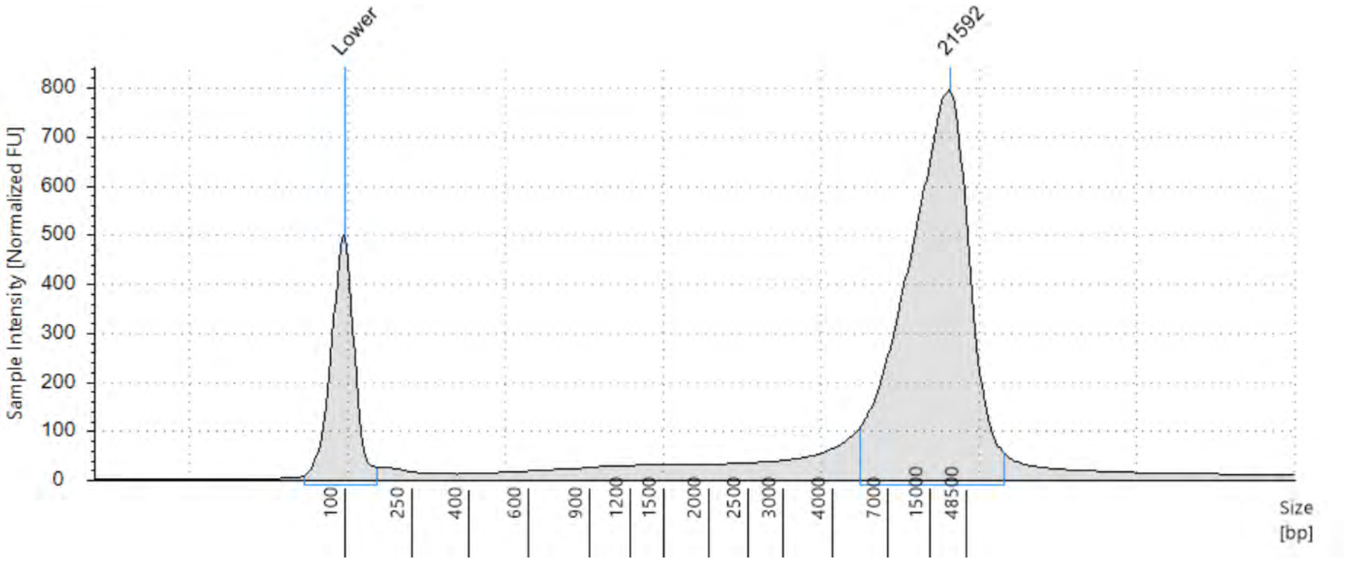

Sample Table

Well	DIN	Conc. [ng/µl]	Sample Description	Alert	Observations
H8	7.4	50.0	C640	⚠	Caution! Expired ScreenTape device

Peak Table

Size [bp]	Calibrated Conc. [ng/µl]	Assigned Conc. [ng/µl]	% Integrated Area	From [bp]	To [bp]	Peak Comment	Observations
100	8.50	8.50	-	58	155		Lower Marker
21592	36.6	-	92.52	5310	>60000		
-	-	-	-	-	-		Sample Well

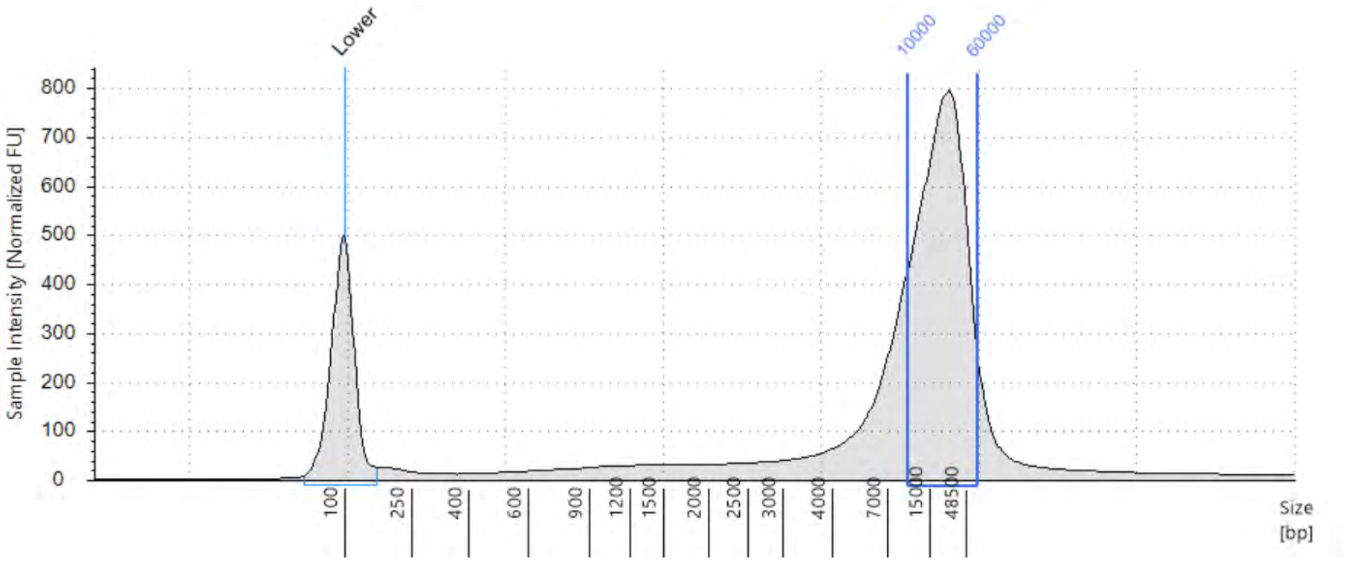

Region Table

From [bp]	To [bp]	Average Size [bp]	Conc. [ng/µl]	Region Molarity [nmol/l]	% of Total	Region Comment	Color
10000	60000	23877	27.4	2.24	54.83		■

© Agilent Technologies, Inc. 2017

A9: C394

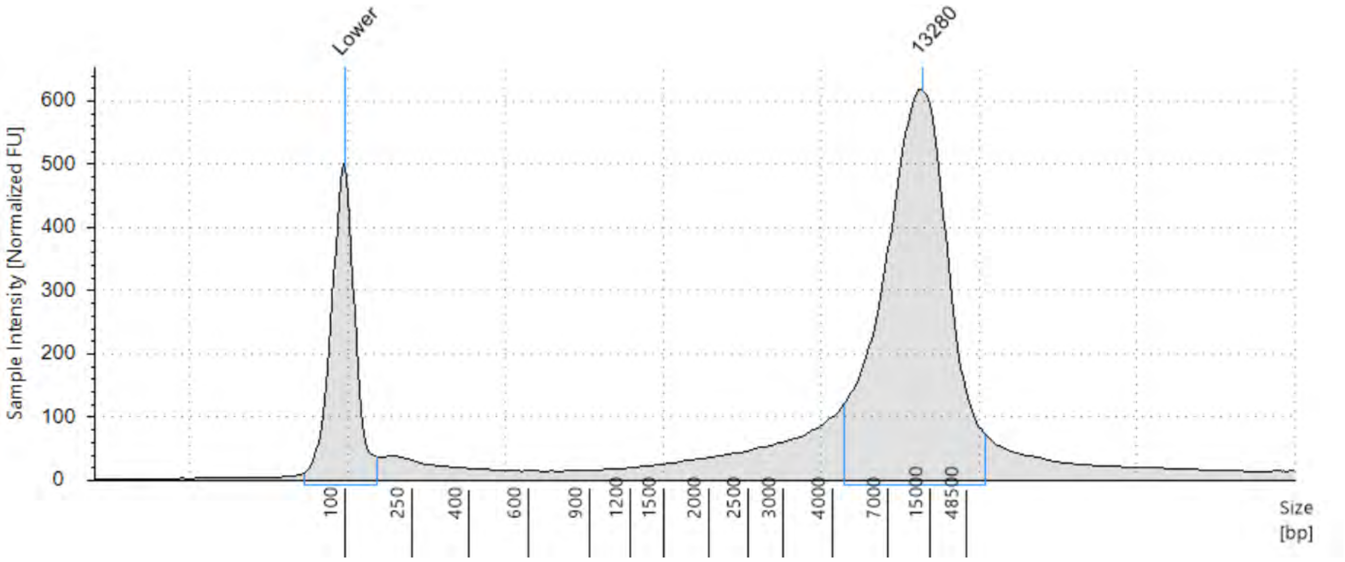

Sample Table

Well	DIN	Conc. [ng/µl]	Sample Description	Alert	Observations
A9	6.9	44.7	C394	⚠	Caution! Expired ScreenTape device

Peak Table

Size [bp]	Calibrated Conc. [ng/µl]	Assigned Conc. [ng/µl]	% Integrated Area	From [bp]	To [bp]	Peak Comment	Observations
100	8.50	8.50	-	57	155		Lower Marker
13280	29.3	-	89.30	4513	>60000		
-	-	-	-	-	-		Sample Well

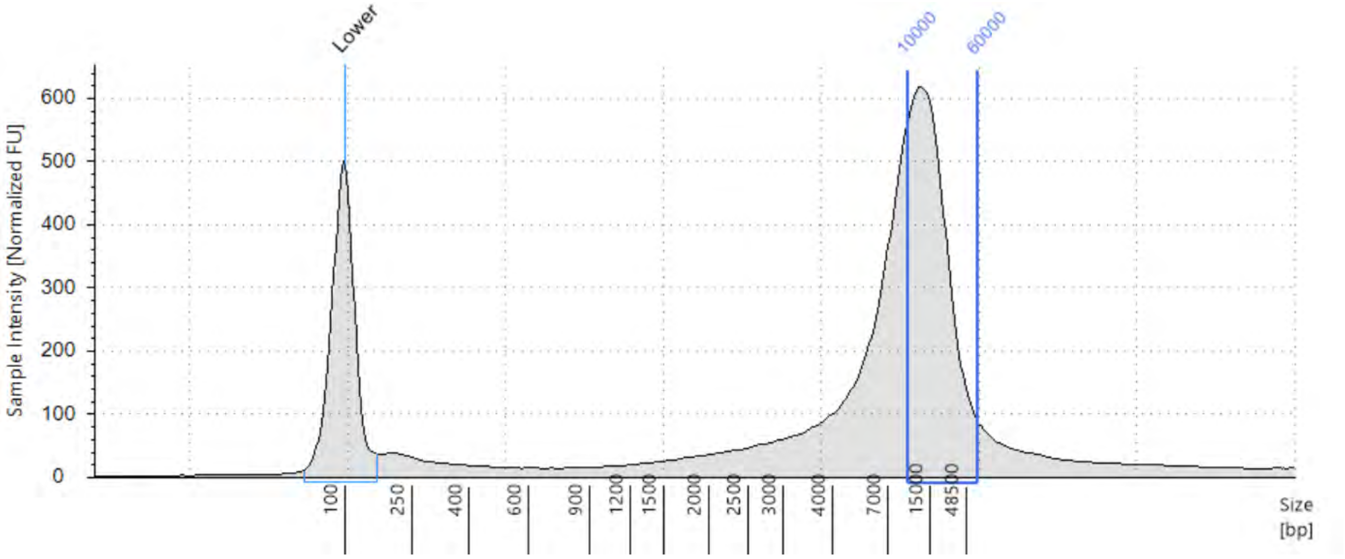

Region Table

From [bp]	To [bp]	Average Size [bp]	Conc. [ng/µl]	Region Molarity [nmol/l]	% of Total	Region Comment	Color
10000	60000	18541	17.8	1.78	39.72		■

B9: C395

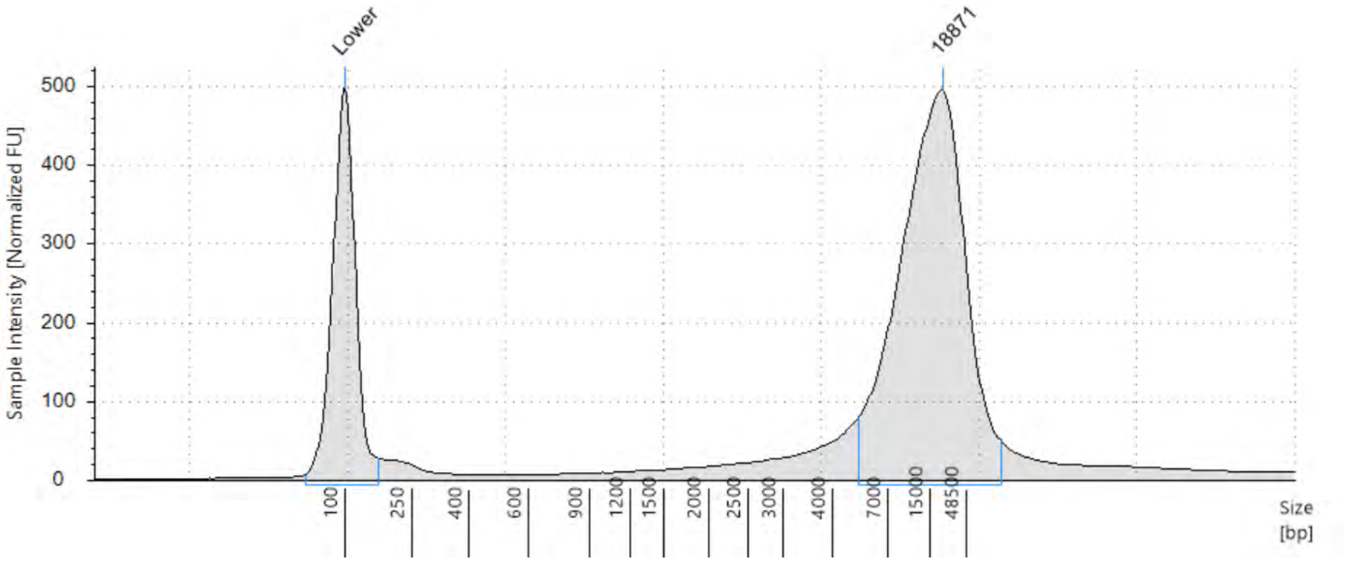

Sample Table

Well	DIN	Conc. [ng/µl]	Sample Description	Alert	Observations
B9	7.7	33.8	C395	⚠	Caution! Expired ScreenTape device

Peak Table

Size [bp]	Calibrated Conc. [ng/µl]	Assigned Conc. [ng/µl]	% Integrated Area	From [bp]	To [bp]	Peak Comment	Observations
100	8.50	8.50	-	59	158		Lower Marker
18871	24.3	-	92.22	5232	>60000		
-	-	-	-	-	-		Sample Well

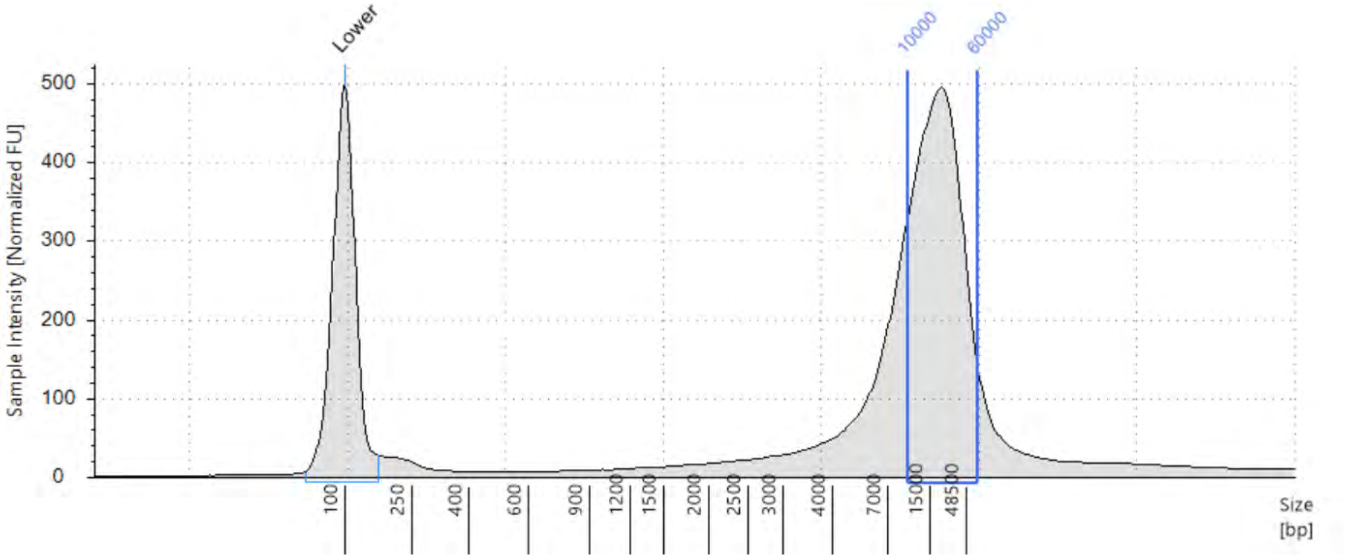

Region Table

From [bp]	To [bp]	Average Size [bp]	Conc. [ng/µl]	Region Molarity [nmol/l]	% of Total	Region Comment	Color
10000	60000	22139	17.7	1.54	52.24		■

C9: C646

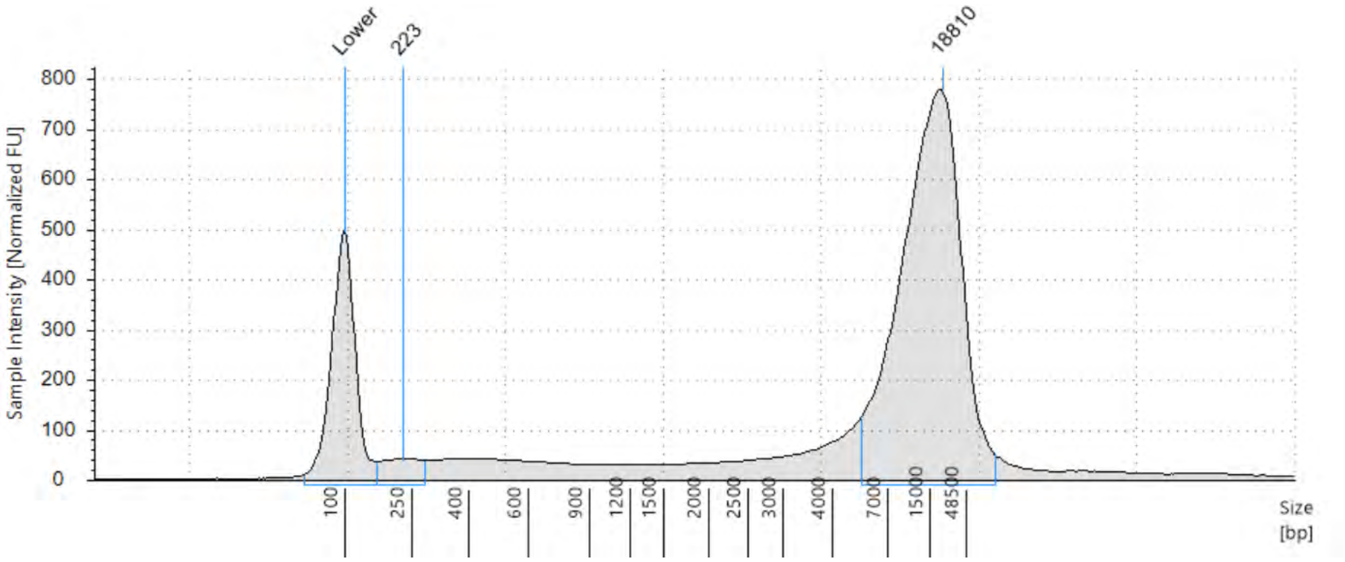

Sample Table

Well	DIN	Conc. [ng/µl]	Sample Description	Alert	Observations
C9	6.9	49.1	C646	⚠	Caution! Expired ScreenTape device

Peak Table

Size [bp]	Calibrated Conc. [ng/µl]	Assigned Conc. [ng/µl]	% Integrated Area	From [bp]	To [bp]	Peak Comment	Observations
100	8.50	8.50	-	57	155		Lower Marker
223	1.17	-	3.20	155	280		
18810	34.1	-	93.47	5341	>60000		
-	-	-	-	-	-		Sample Well

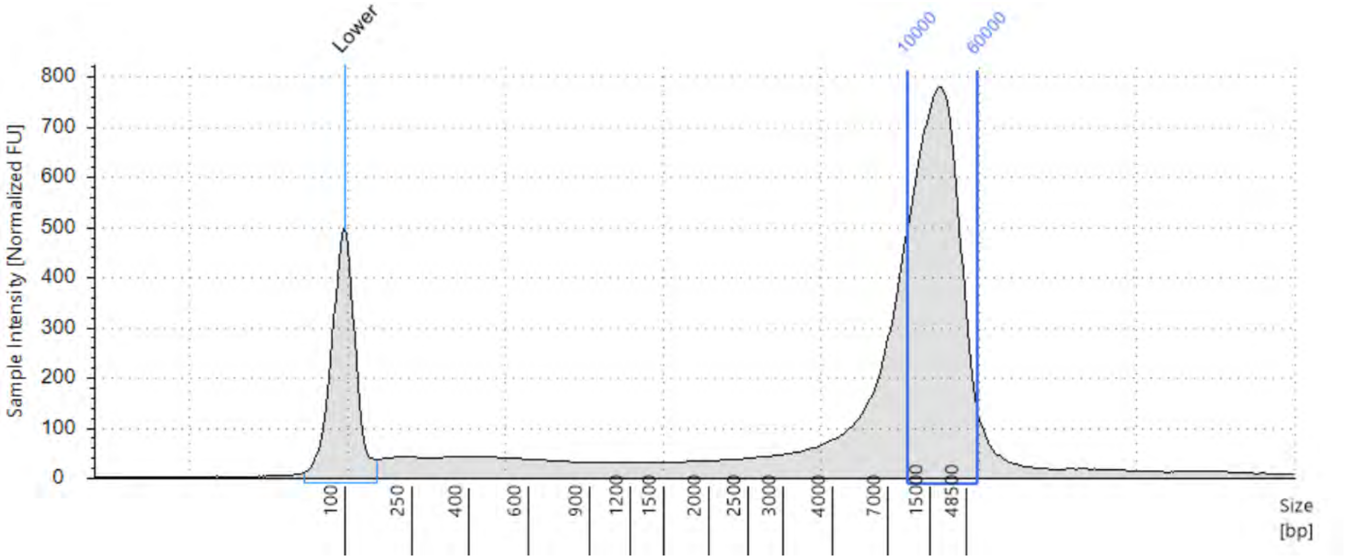

Region Table

From [bp]	To [bp]	Average Size [bp]	Conc. [ng/µl]	Region Molarity [nmol/l]	% of Total	Region Comment	Color
10000	60000	20985	25.7	2.31	52.34		■

 © Agilent Technologies, Inc. 2017

D9: C647

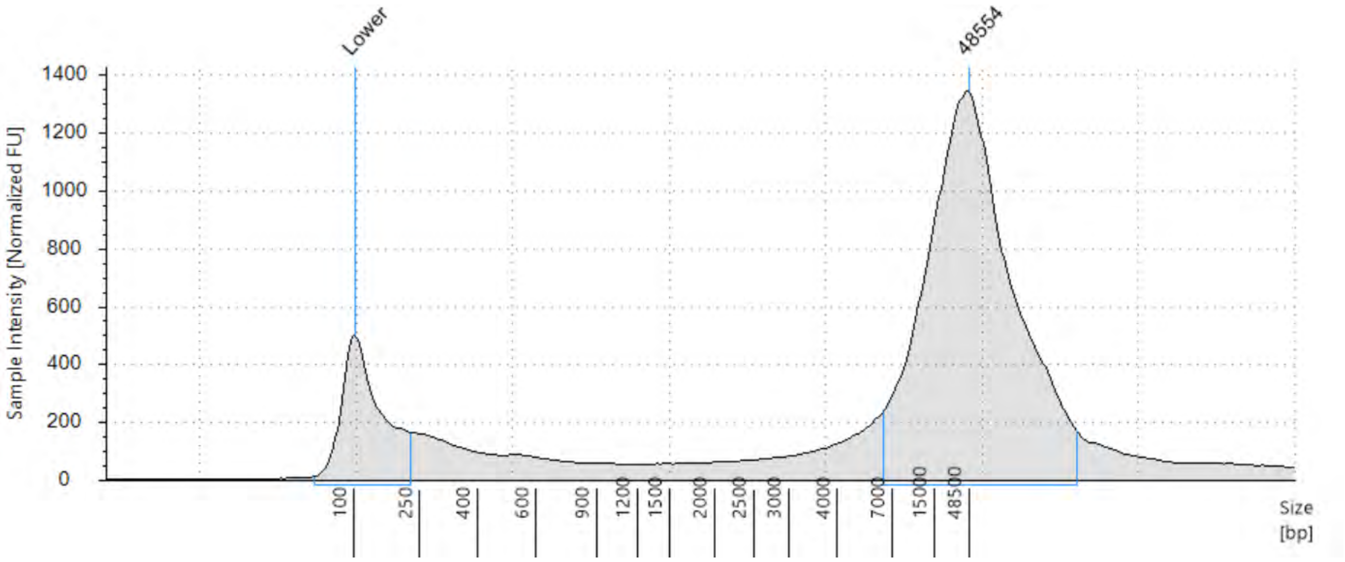

Sample Table

Well	DIN	Conc. [ng/µl]	Sample Description	Alert	Observations
D9	7.2	77.4	C647	⚠	Caution! Expired ScreenTape device

Peak Table

Size [bp]	Calibrated Conc. [ng/µl]	Assigned Conc. [ng/µl]	% Integrated Area	From [bp]	To [bp]	Peak Comment	Observations
100	8.50	8.50	-	57	216		Lower Marker
48554	51.2	-	88.96	6411	>60000		
-	-	-	-	-	-		Sample Well

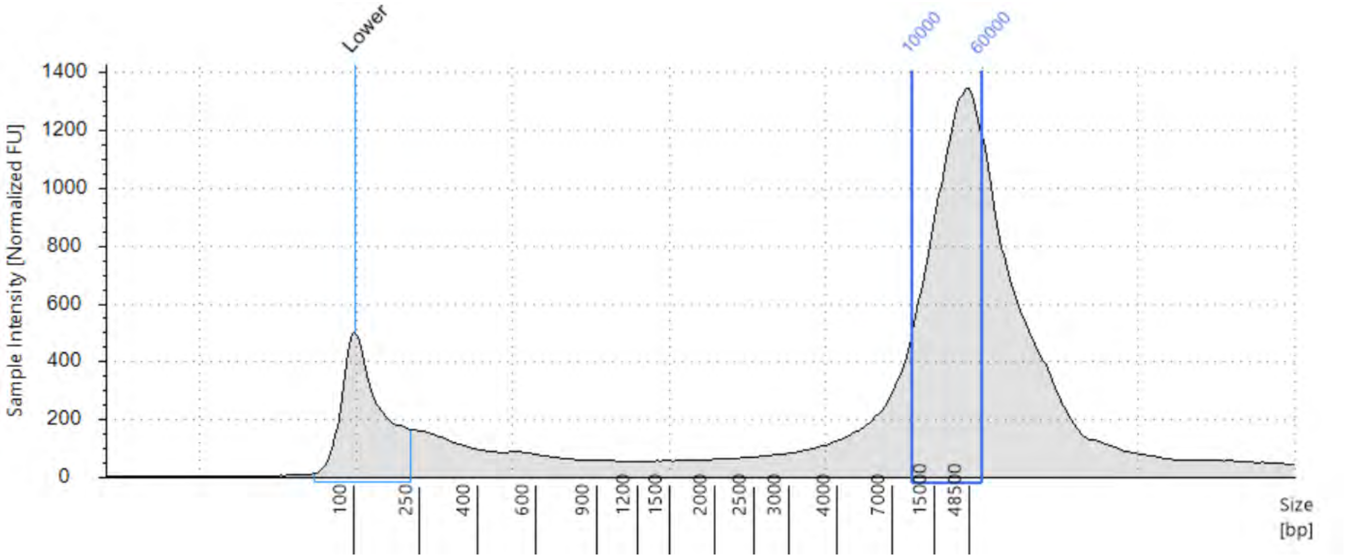

Region Table

From [bp]	To [bp]	Average Size [bp]	Conc. [ng/µl]	Region Molarity [nmol/l]	% of Total	Region Comment	Color
10000	60000	28457	27.5	1.99	35.58		■

E9: C648

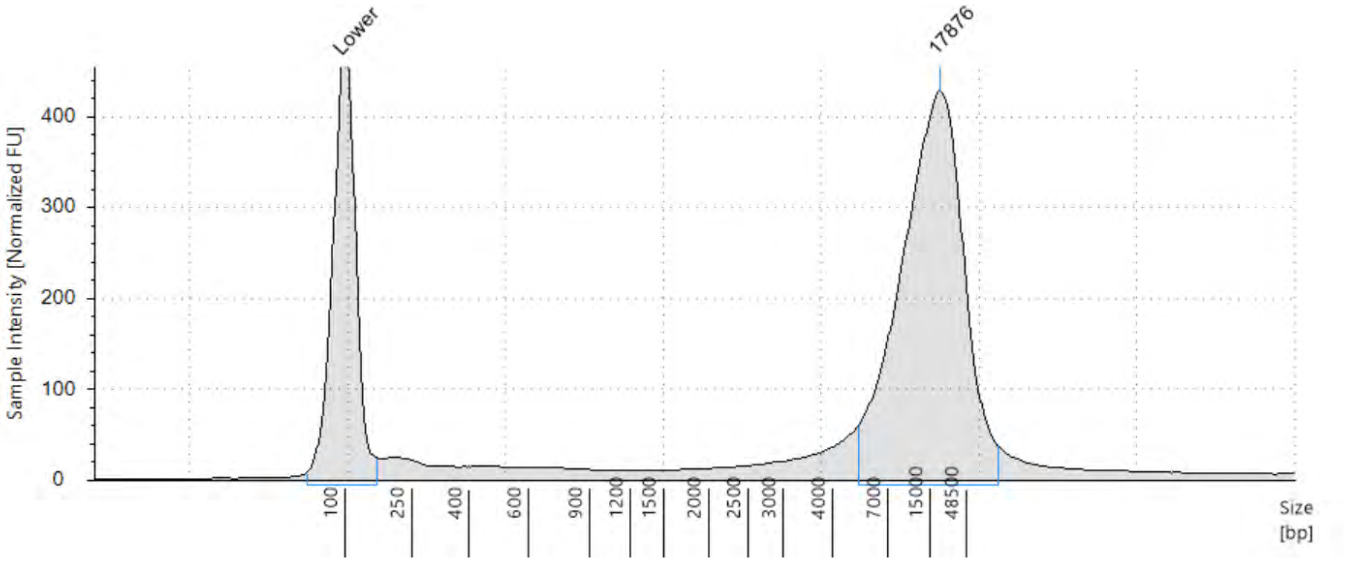

Sample Table

Well	DIN	Conc. [ng/µl]	Sample Description	Alert	Observations
E9	7.4	28.3	C648	⚠	Caution! Expired ScreenTape device

Peak Table

Size [bp]	Calibrated Conc. [ng/µl]	Assigned Conc. [ng/µl]	% Integrated Area	From [bp]	To [bp]	Peak Comment	Observations
100	8.50	8.50	-	59	155		Lower Marker
17876	20.4	-	92.85	5180	>60000		
-	-	-	-	-	-		Sample Well

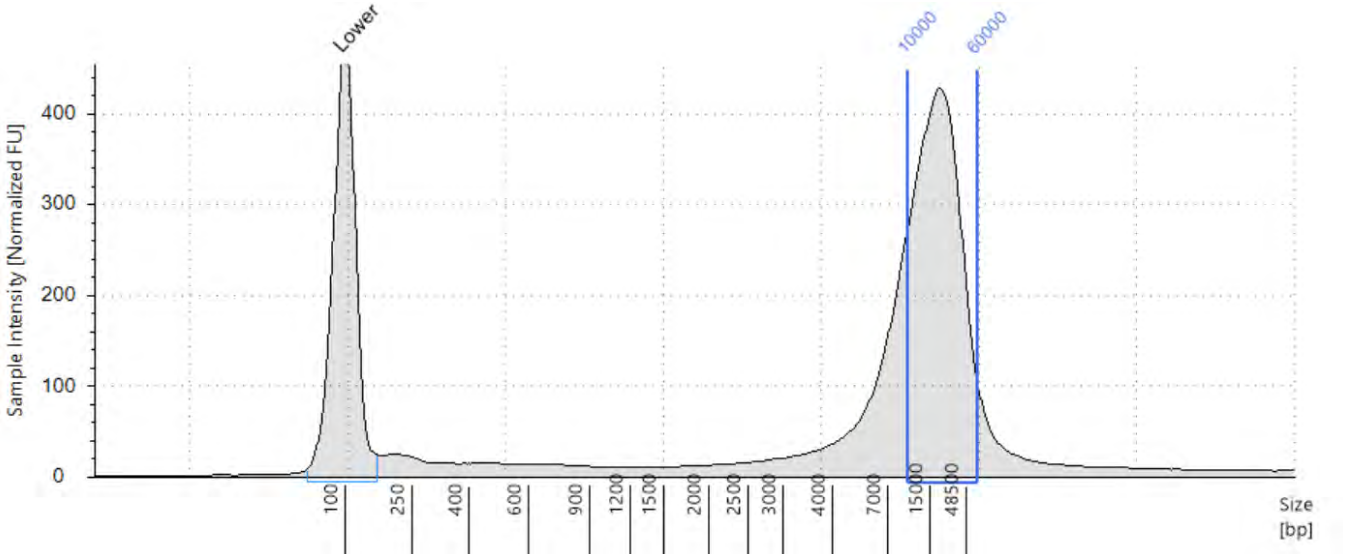

Region Table

From [bp]	To [bp]	Average Size [bp]	Conc. [ng/µl]	Region Molarity [nmol/l]	% of Total	Region Comment	Color
10000	60000	21594	15.1	1.34	53.44		■

F9: C649

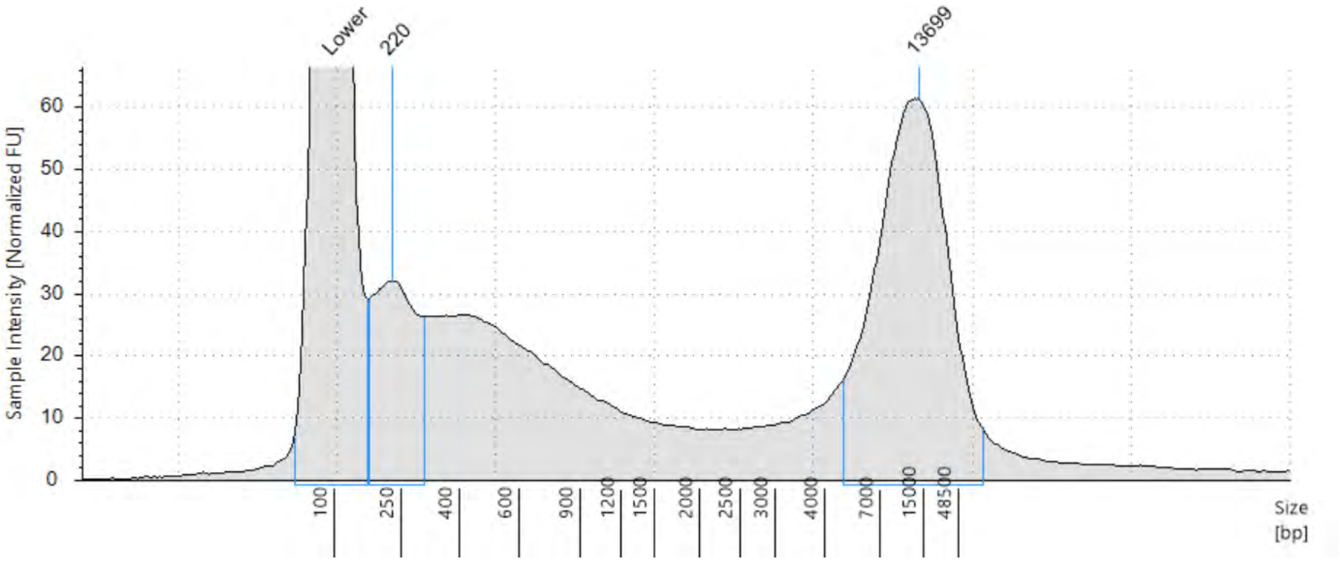

Sample Table

Well	DIN	Conc. [ng/µl]	Sample Description	Alert	Observations
F9	5.5	8.65	C649	⚠	Caution! Expired ScreenTape device; Sample concentration outside recommended range

Peak Table

Size [bp]	Calibrated Conc. [ng/µl]	Assigned Conc. [ng/µl]	% Integrated Area	From [bp]	To [bp]	Peak Comment	Observations
100	8.50	8.50	-	57	156		Lower Marker
220	1.07	-	22.81	159	301		
13699	3.29	-	69.98	4830	>60000		
-	-	-	-	-	-		Sample Well

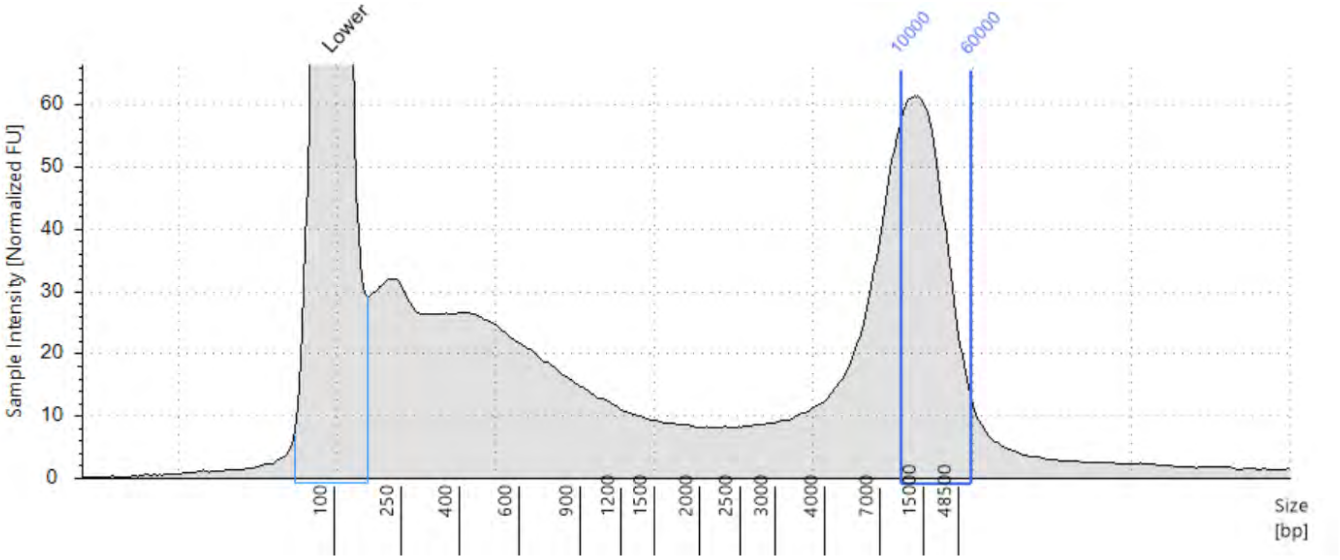

Region Table

From [bp]	To [bp]	Average Size [bp]	Conc. [ng/µl]	Region Molarity [nmol/l]	% of Total	Region Comment	Color
10000	60000	19893	2.02	0.195	23.31		

G9: C650

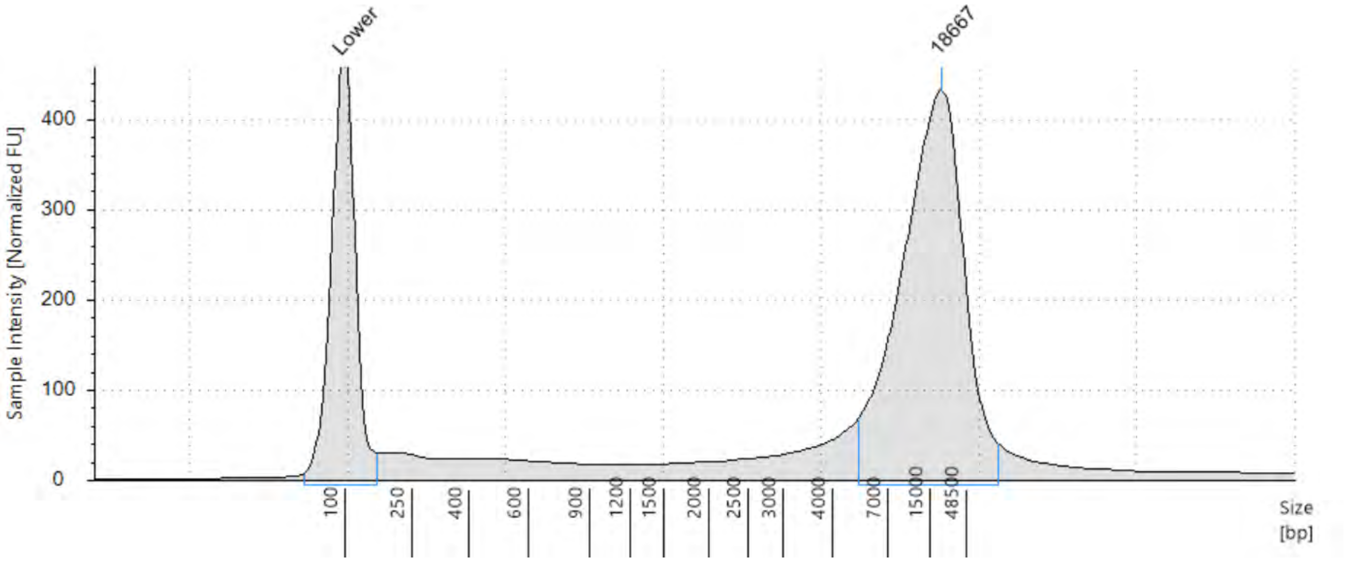

Sample Table

Well	DIN	Conc. [ng/µl]	Sample Description	Alert	Observations
G9	7.0	29.8	C650	⚠	Caution! Expired ScreenTape device

Peak Table

Size [bp]	Calibrated Conc. [ng/µl]	Assigned Conc. [ng/µl]	% Integrated Area	From [bp]	To [bp]	Peak Comment	Observations
100	8.50	8.50	-	57	156		Lower Marker
18667	20.0	-	96.48	5175	>60000		
-	-	-	-	-	-		Sample Well

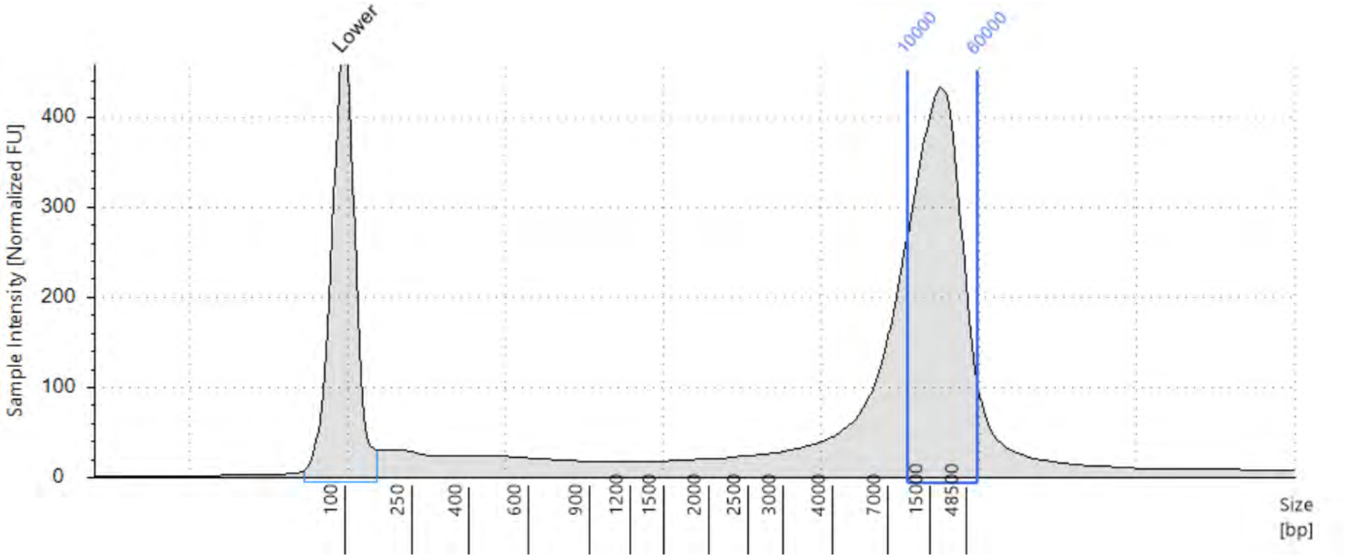

Region Table

From [bp]	To [bp]	Average Size [bp]	Conc. [ng/µl]	Region Molarity [nmol/l]	% of Total	Region Comment	Color
10000	60000	21809	14.7	1.29	49.50		■

H9: C398

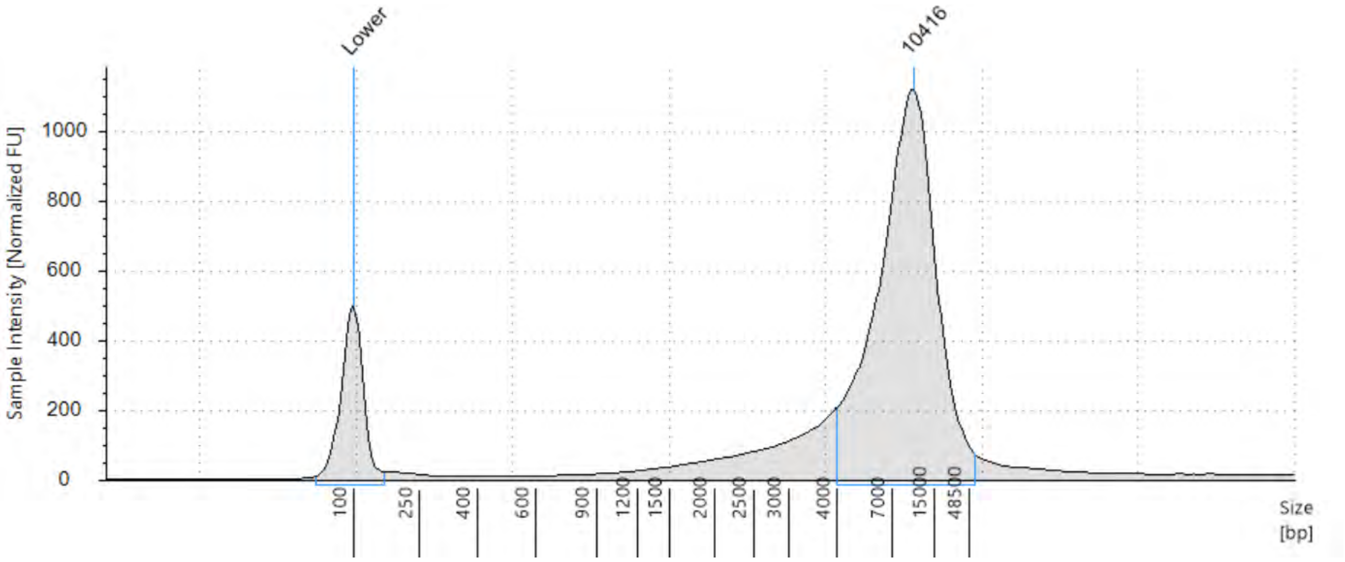

Sample Table

Well	DIN	Conc. [ng/µl]	Sample Description	Alert	Observations
H9	6.8	69.2	C398	⚠	Caution! Expired ScreenTape device

Peak Table

Size [bp]	Calibrated Conc. [ng/µl]	Assigned Conc. [ng/µl]	% Integrated Area	From [bp]	To [bp]	Peak Comment	Observations
100	8.50	8.50	-	58	153		Lower Marker
10416	50.3	-	92.11	3994	54523		
-	-	-	-	-	-		Sample Well

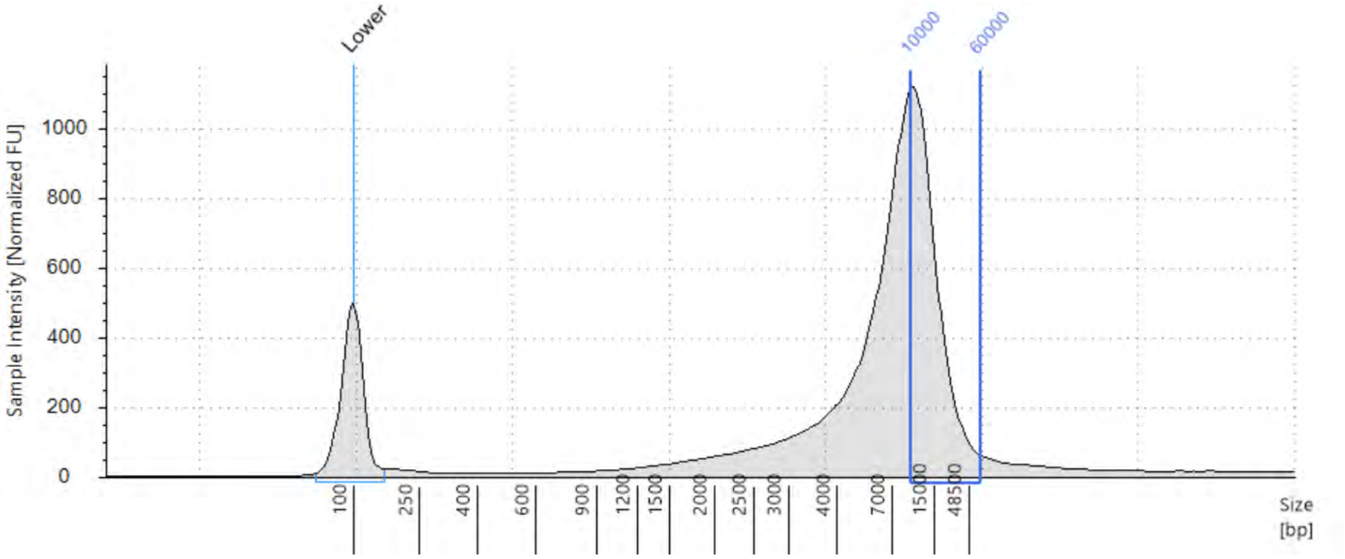

Region Table

From [bp]	To [bp]	Average Size [bp]	Conc. [ng/µl]	Region Molarity [nmol/l]	% of Total	Region Comment	Color
10000	60000	16202	22.9	2.54	33.14		■

 © Agilent Technologies, Inc. 2017

A10: C399

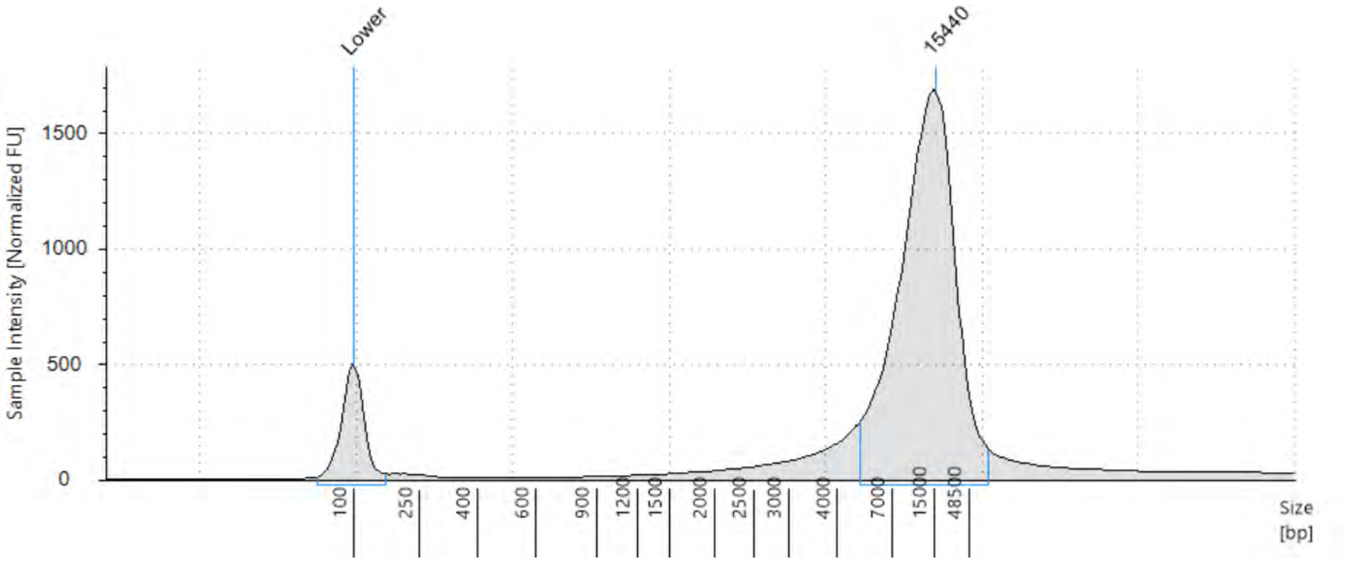

Sample Table

Well	DIN	Conc. [ng/µl]	Sample Description	Alert	Observations
A10	7.5	97.3	C399	⚠	Caution! Expired ScreenTape device

Peak Table

Size [bp]	Calibrated Conc. [ng/µl]	Assigned Conc. [ng/µl]	% Integrated Area	From [bp]	To [bp]	Peak Comment	Observations
100	8.50	8.50	-	60	155		Lower Marker
15440	72.0	-	88.46	5062	>60000		
-	-	-	-	-	-		Sample Well

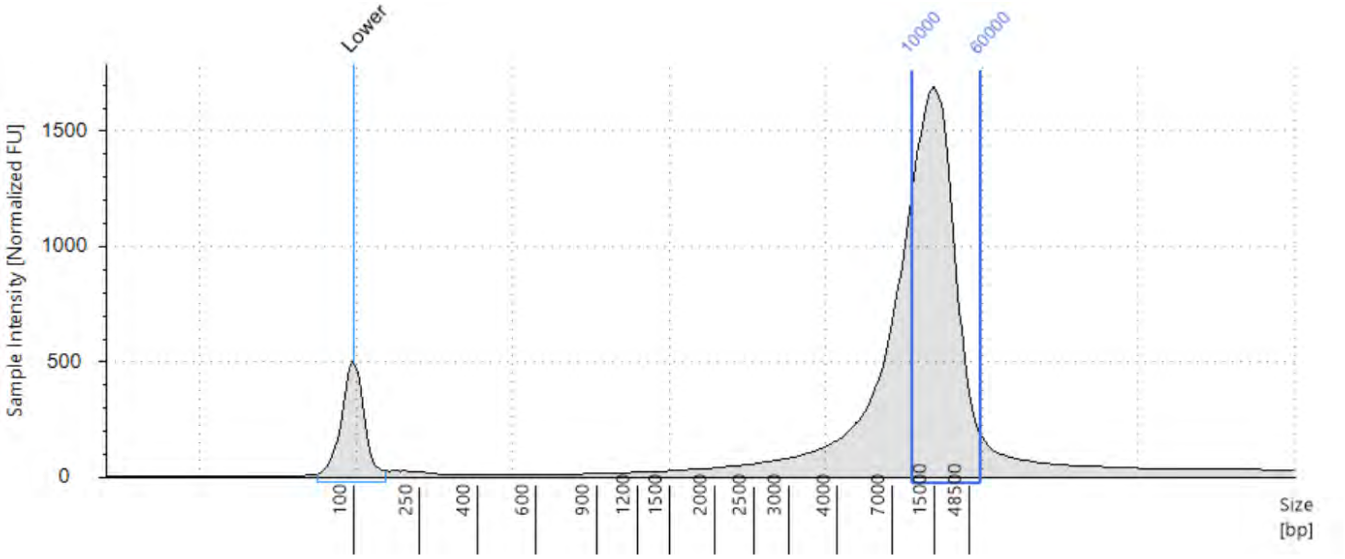

Region Table

From [bp]	To [bp]	Average Size [bp]	Conc. [ng/µl]	Region Molarity [nmol/l]	% of Total	Region Comment	Color
10000	60000	18586	51.1	4.99	52.57		■

B10: C656

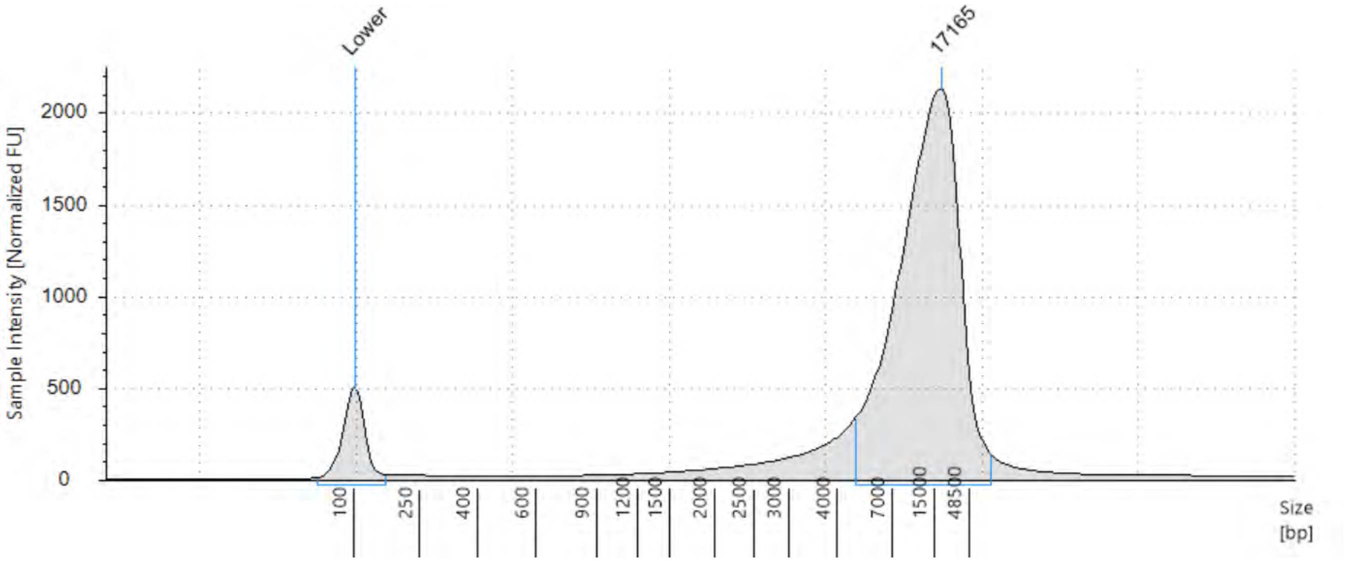

Sample Table

Well	DIN	Conc. [ng/µl]	Sample Description	Alert	Observations
B10	7.4	127	C656	⚠	Caution! Expired ScreenTape device; Sample concentration outside recommended range

Peak Table

Size [bp]	Calibrated Conc. [ng/µl]	Assigned Conc. [ng/µl]	% Integrated Area	From [bp]	To [bp]	Peak Comment	Observations
100	8.50	8.50	-	59	153		Lower Marker
17165	100	-	93.98	4830	>60000		
-	-	-	-	-	-		Sample Well

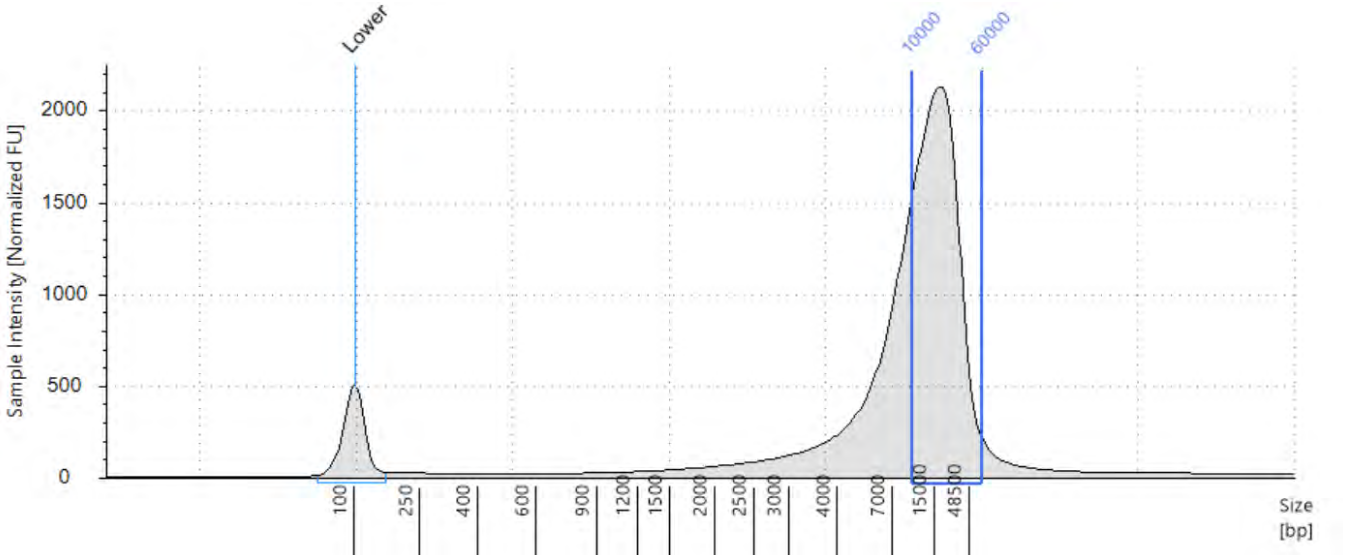

Region Table

From [bp]	To [bp]	Average Size [bp]	Conc. [ng/µl]	Region Molarity [nmol/l]	% of Total	Region Comment	Color
10000	60000	19163	69.9	6.62	55.30		■

C10: C657

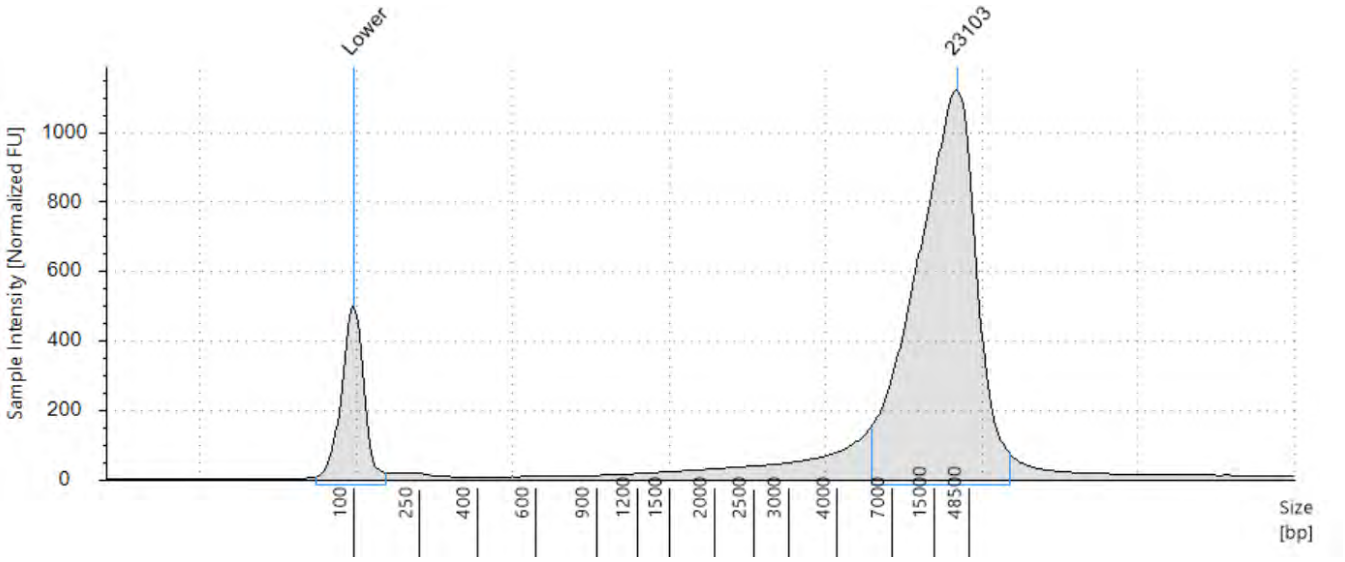

Sample Table

Well	DIN	Conc. [ng/µl]	Sample Description	Alert	Observations
C10	7.9	65.5	C657	⚠	Caution! Expired ScreenTape device

Peak Table

Size [bp]	Calibrated Conc. [ng/µl]	Assigned Conc. [ng/µl]	% Integrated Area	From [bp]	To [bp]	Peak Comment	Observations
100	8.50	8.50	-	59	155		Lower Marker
23103	52.0	-	95.54	5676	>60000		
-	-	-	-	-	-		Sample Well

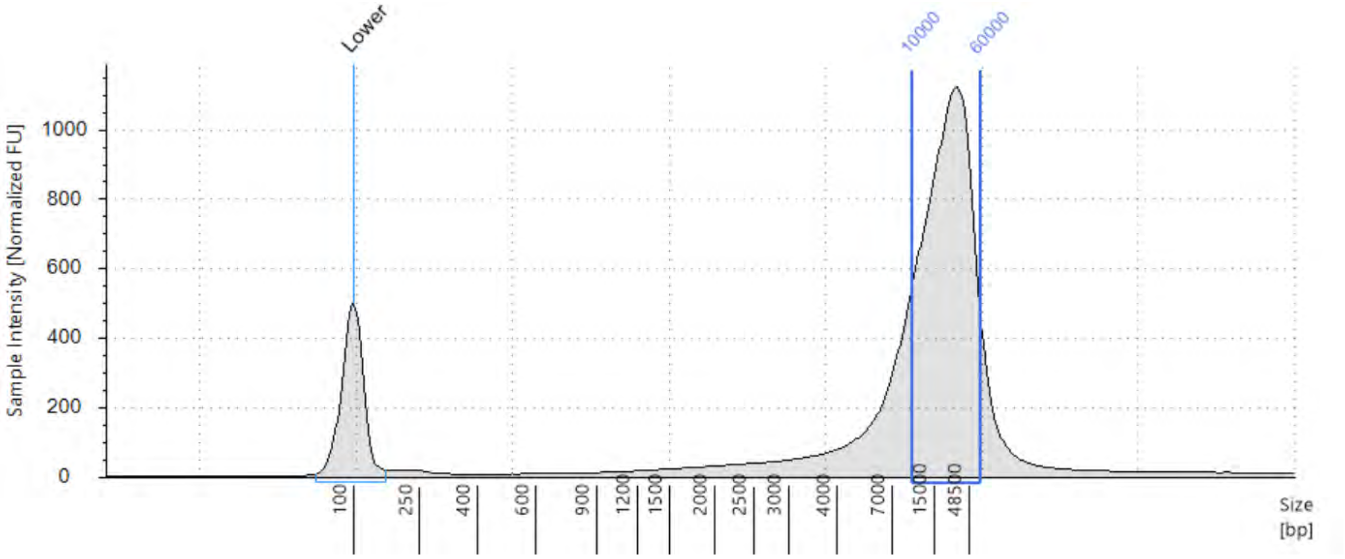

Region Table

From [bp]	To [bp]	Average Size [bp]	Conc. [ng/µl]	Region Molarity [nmol/l]	% of Total	Region Comment	Color
10000	60000	25261	40.5	3.20	61.80		■

 © Agilent Technologies, Inc. 2017

D10: C658

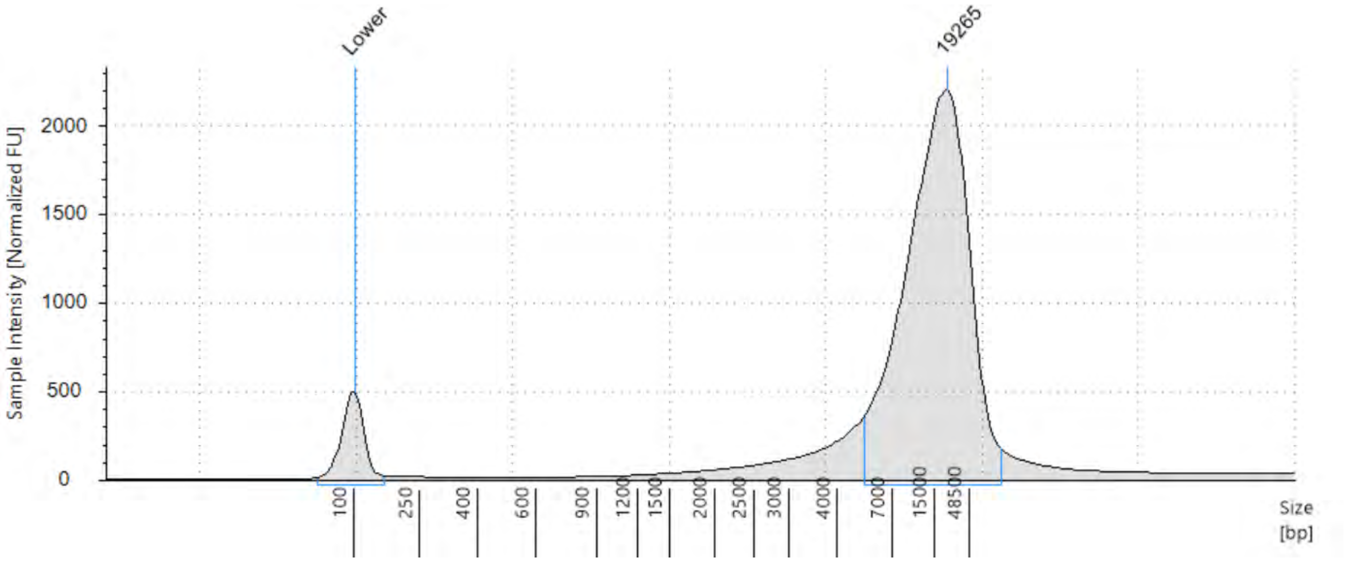

Sample Table

Well	DIN	Conc. [ng/µl]	Sample Description	Alert	Observations
D10	7.8	141	C658	⚠	Caution! Expired ScreenTape device; Sample concentration outside recommended range

Peak Table

Size [bp]	Calibrated Conc. [ng/µl]	Assigned Conc. [ng/µl]	% Integrated Area	From [bp]	To [bp]	Peak Comment	Observations
100	8.50	8.50	-	60	152		Lower Marker
19265	111	-	91.81	5254	>60000		
-	-	-	-	-	-		Sample Well

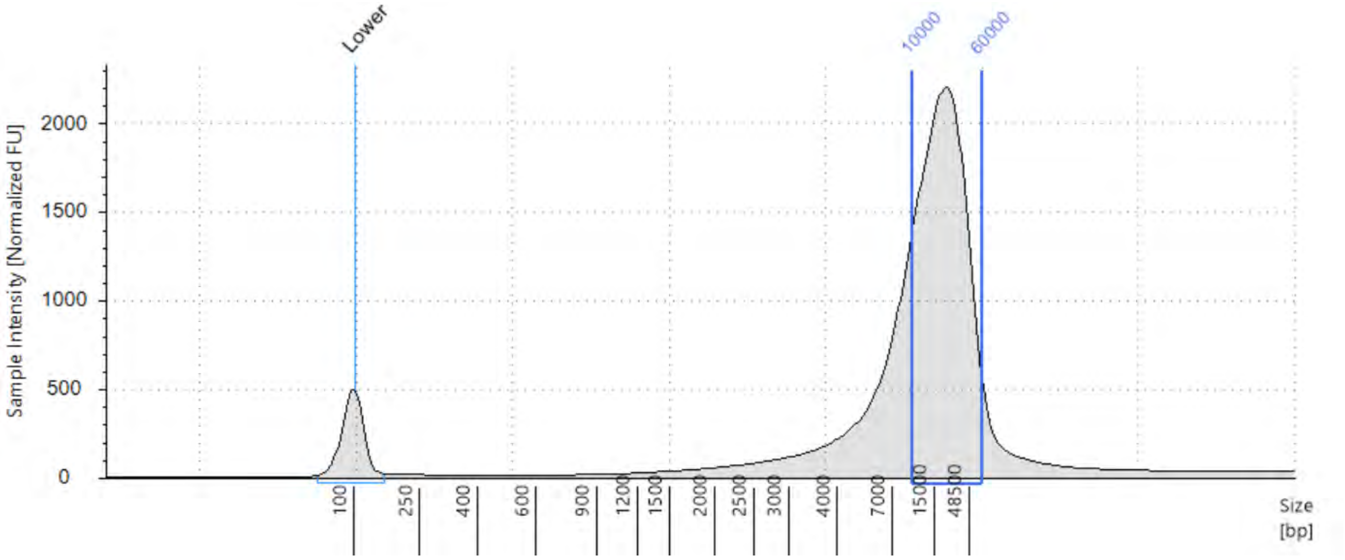

Region Table

From [bp]	To [bp]	Average Size [bp]	Conc. [ng/µl]	Region Molarity [nmol/l]	% of Total	Region Comment	Color
10000	60000	22561	83.5	7.20	59.19		■

E10: C659

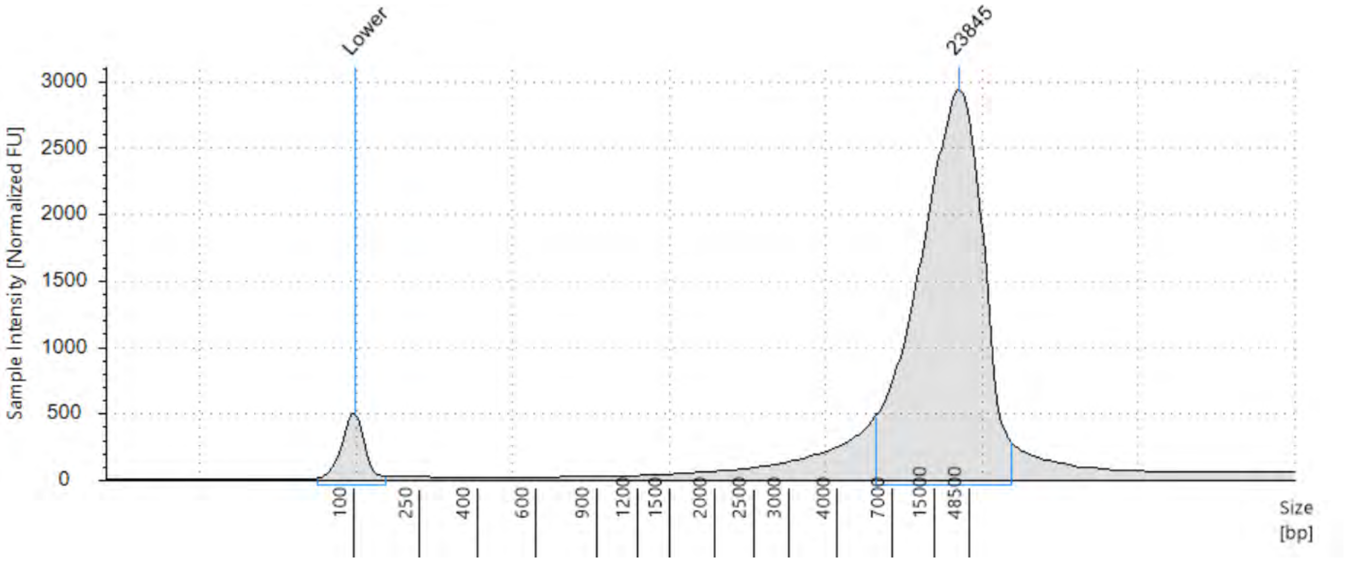

Sample Table

Well	DIN	Conc. [ng/µl]	Sample Description	Alert	Observations
E10	8.1	193	C659	⚠	Caution! Expired ScreenTape device; Sample concentration outside recommended range

Peak Table

Size [bp]	Calibrated Conc. [ng/µl]	Assigned Conc. [ng/µl]	% Integrated Area	From [bp]	To [bp]	Peak Comment	Observations
100	8.50	8.50	-	60	155		Lower Marker
23845	146	-	89.09	5945	>60000		
-	-	-	-	-	-		Sample Well

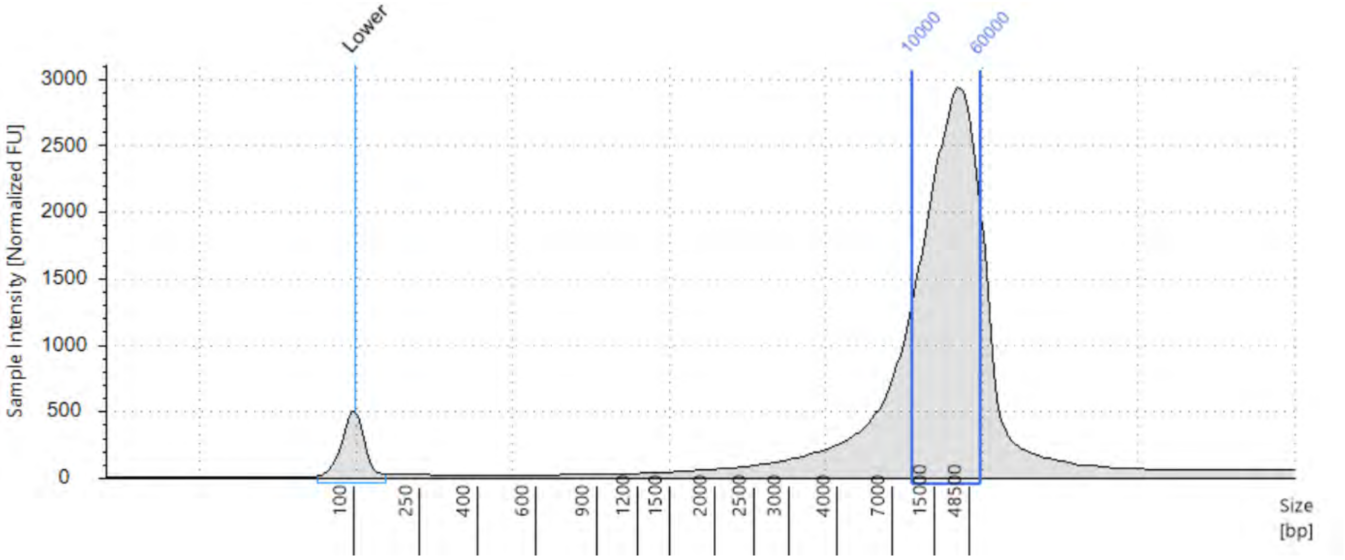

Region Table

From [bp]	To [bp]	Average Size [bp]	Conc. [ng/µl]	Region Molarity [nmol/l]	% of Total	Region Comment	Color
10000	60000	26987	110	8.31	57.04		■

F10: C660

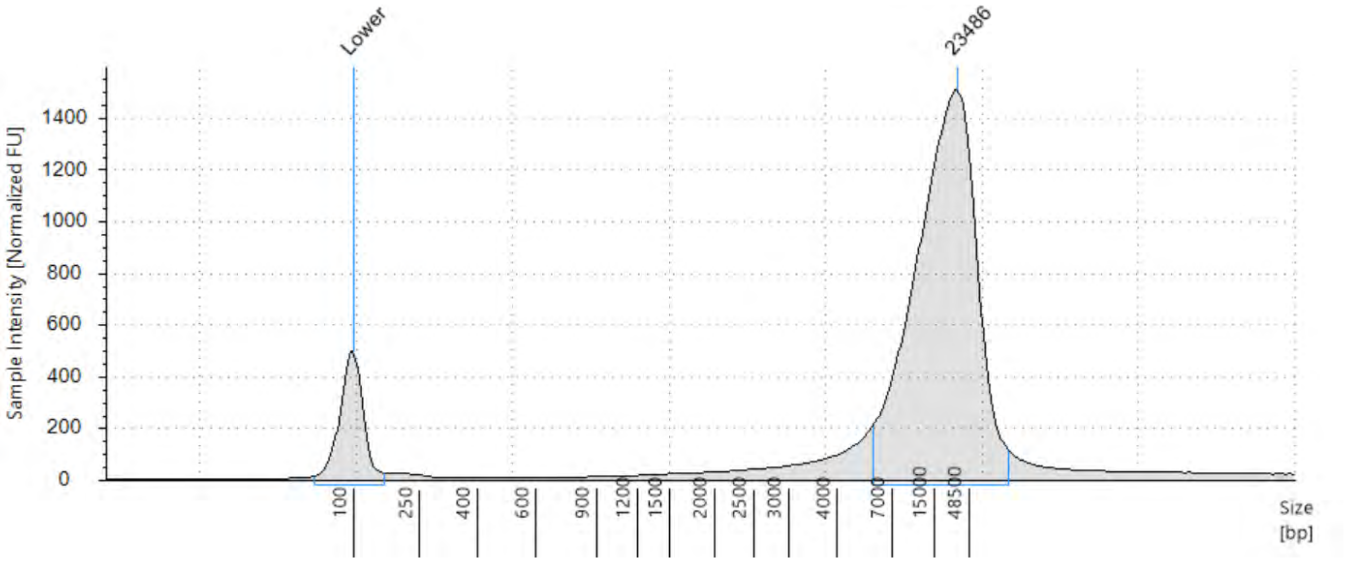

Sample Table

Well	DIN	Conc. [ng/µl]	Sample Description	Alert	Observations
F10	8.2	87.2	C660	⚠	Caution! Expired ScreenTape device

Peak Table

Size [bp]	Calibrated Conc. [ng/µl]	Assigned Conc. [ng/µl]	% Integrated Area	From [bp]	To [bp]	Peak Comment	Observations
100	8.50	8.50	-	58	154		Lower Marker
23486	68.5	-	92.27	5829	>60000		
-	-	-	-	-	-		Sample Well

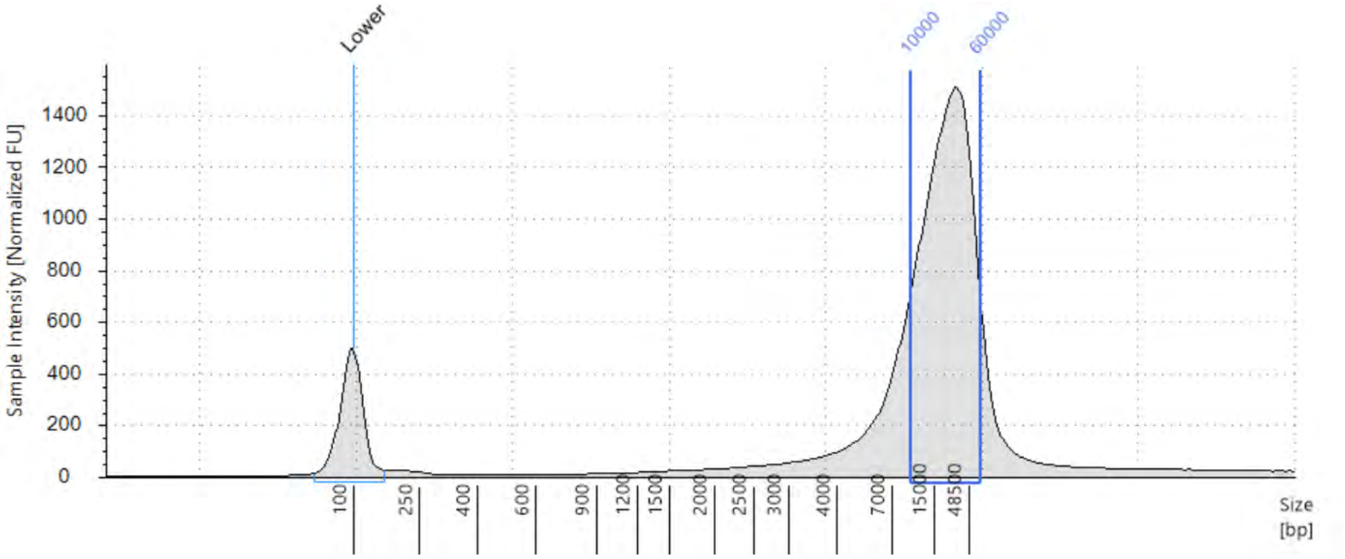

Region Table

From [bp]	To [bp]	Average Size [bp]	Conc. [ng/µl]	Region Molarity [nmol/l]	% of Total	Region Comment	Color
10000	60000	25349	53.6	4.23	61.40		■

 © Agilent Technologies, Inc. 2017

G10: C402

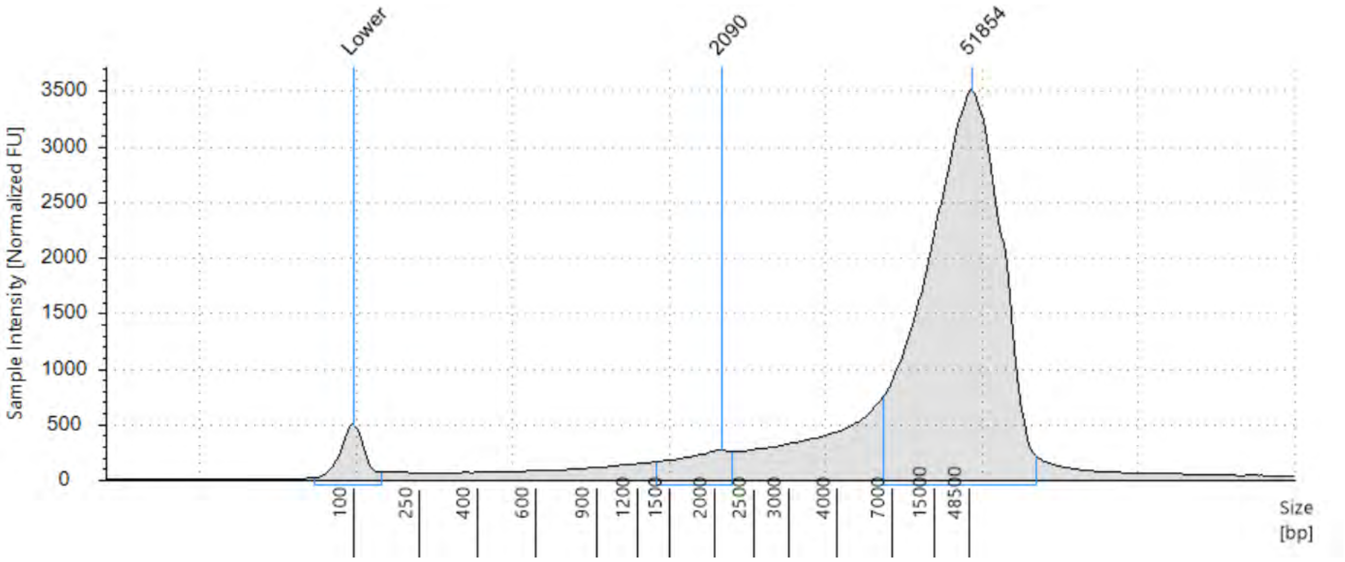

Sample Table

Well	DIN	Conc. [ng/µl]	Sample Description	Alert	Observations
G10	7.5	264	C402	⚠	Caution! Expired ScreenTape device; Sample concentration outside recommended range

Peak Table

Size [bp]	Calibrated Conc. [ng/µl]	Assigned Conc. [ng/µl]	% Integrated Area	From [bp]	To [bp]	Peak Comment	Observations
100	8.50	8.50	-	58	147		Lower Marker
2090	10.0	-	4.86	1372	2227		
51854	190	-	92.19	6410	>60000		
-	-	-	-	-	-		Sample Well

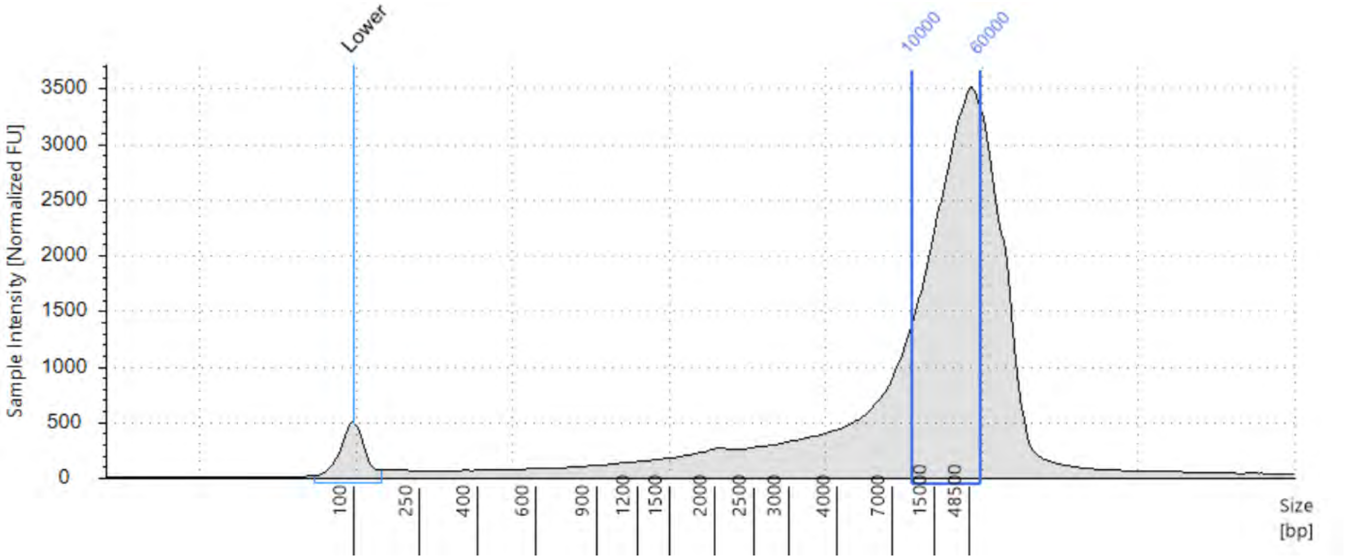

Region Table

From [bp]	To [bp]	Average Size [bp]	Conc. [ng/µl]	Region Molarity [nmol/l]	% of Total	Region Comment	Color
10000	60000	29445	117	8.32	44.30		■

H10: C403

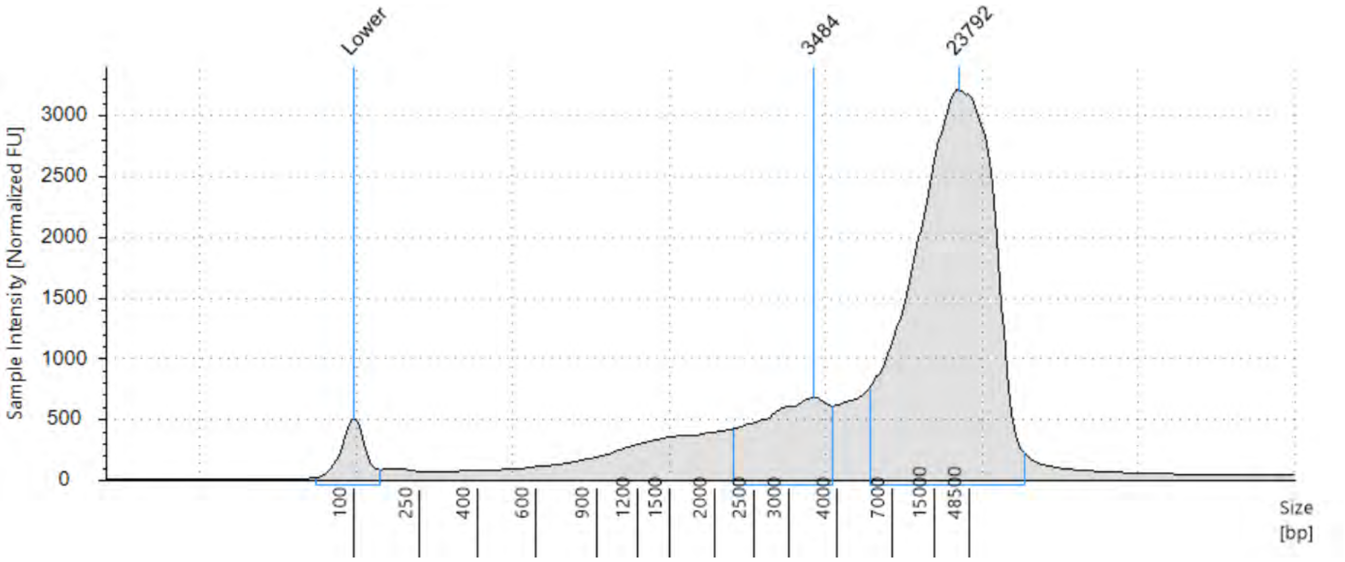

Sample Table

Well	DIN	Conc. [ng/µl]	Sample Description	Alert	Observations
H10	7.0	297	C403	⚠	Caution! Expired ScreenTape device; Sample concentration outside recommended range

Peak Table

Size [bp]	Calibrated Conc. [ng/µl]	Assigned Conc. [ng/µl]	% Integrated Area	From [bp]	To [bp]	Peak Comment	Observations
100	8.50	8.50	-	59	144		Lower Marker
3484	35.1	-	14.47	2246	3899		
23792	194	-	79.66	5584	>60000		
-	-	-	-	-	-		Sample Well

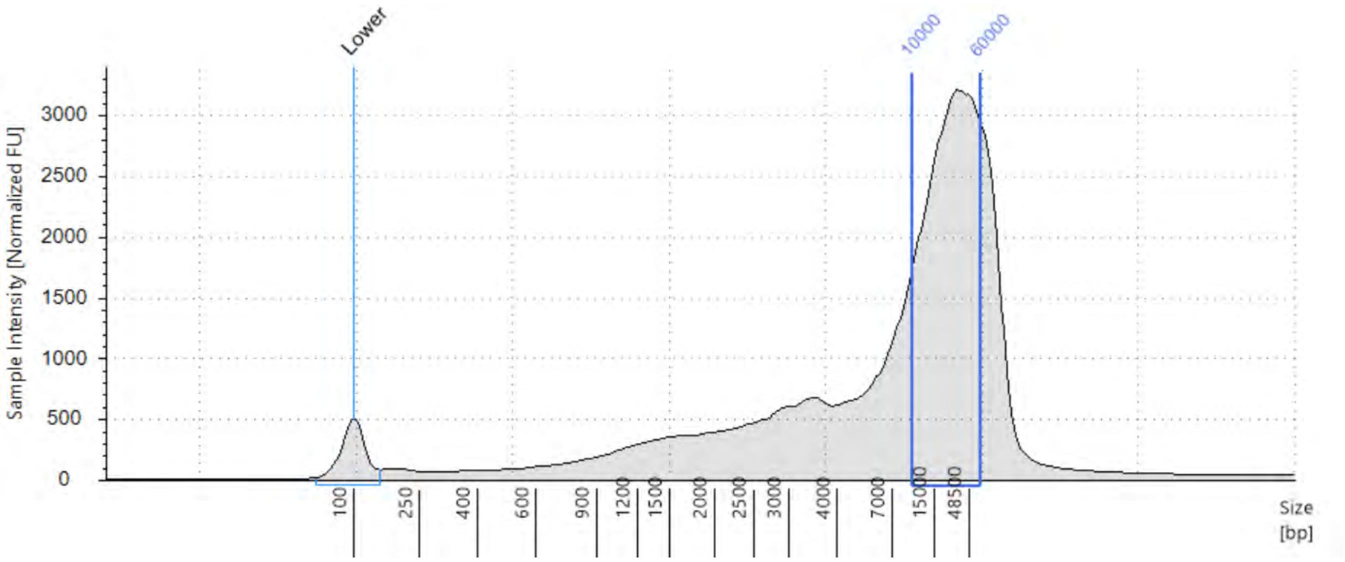

Region Table

From [bp]	To [bp]	Average Size [bp]	Conc. [ng/µl]	Region Molarity [nmol/l]	% of Total	Region Comment	Color
10000	60000	27430	125	9.49	41.99		■

© Agilent Technologies, Inc. 2017

A11: C666

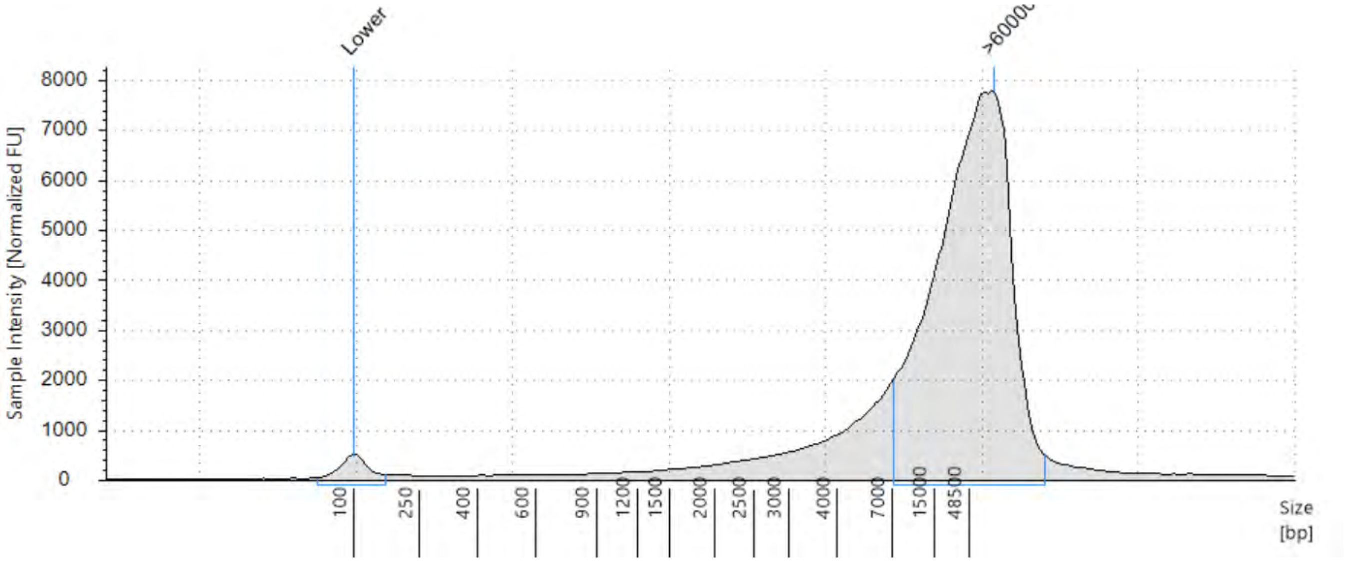

Sample Table

Well	DIN	Conc. [ng/µl]	Sample Description	Alert	Observations
A11	8.1	502	C666	⚠	Caution! Expired ScreenTape device; Sample concentration outside functional range for DIN

Peak Table

Size [bp]	Calibrated Conc. [ng/µl]	Assigned Conc. [ng/µl]	% Integrated Area	From [bp]	To [bp]	Peak Comment	Observations
100	8.50	8.50	-	61	156		Lower Marker
>60000	380	-	96.52	7297	>60000		
-	-	-	-	-	-		Sample Well

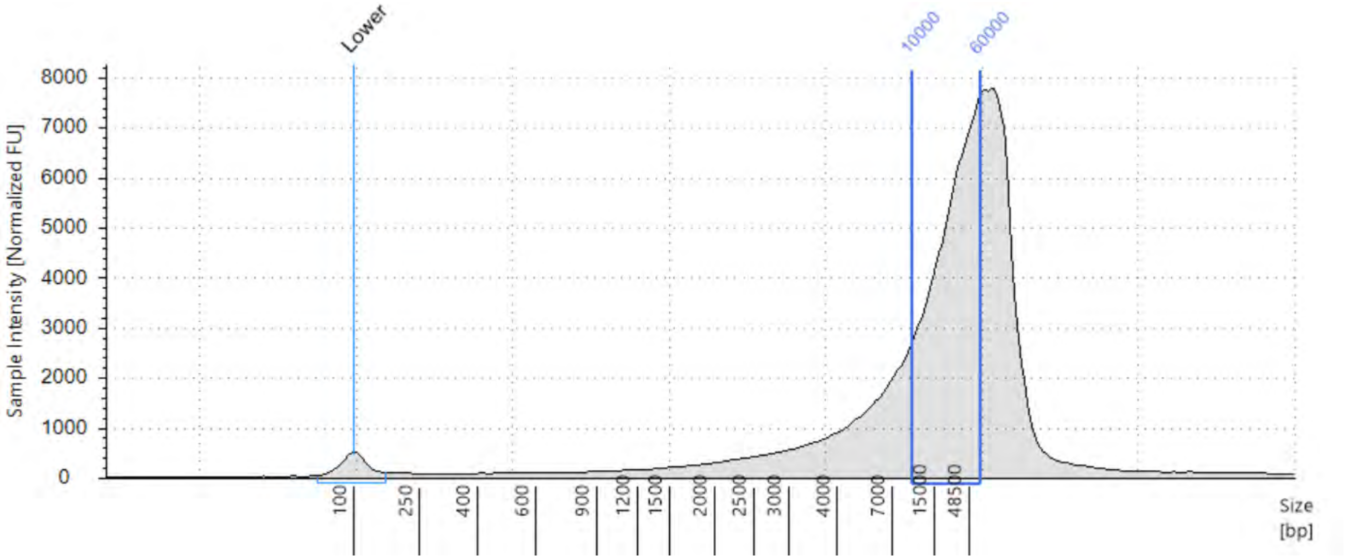

Region Table

From [bp]	To [bp]	Average Size [bp]	Conc. [ng/µl]	Region Molarity [nmol/l]	% of Total	Region Comment	Color
10000	60000	29790	201	14.2	40.14		■

B11: C667

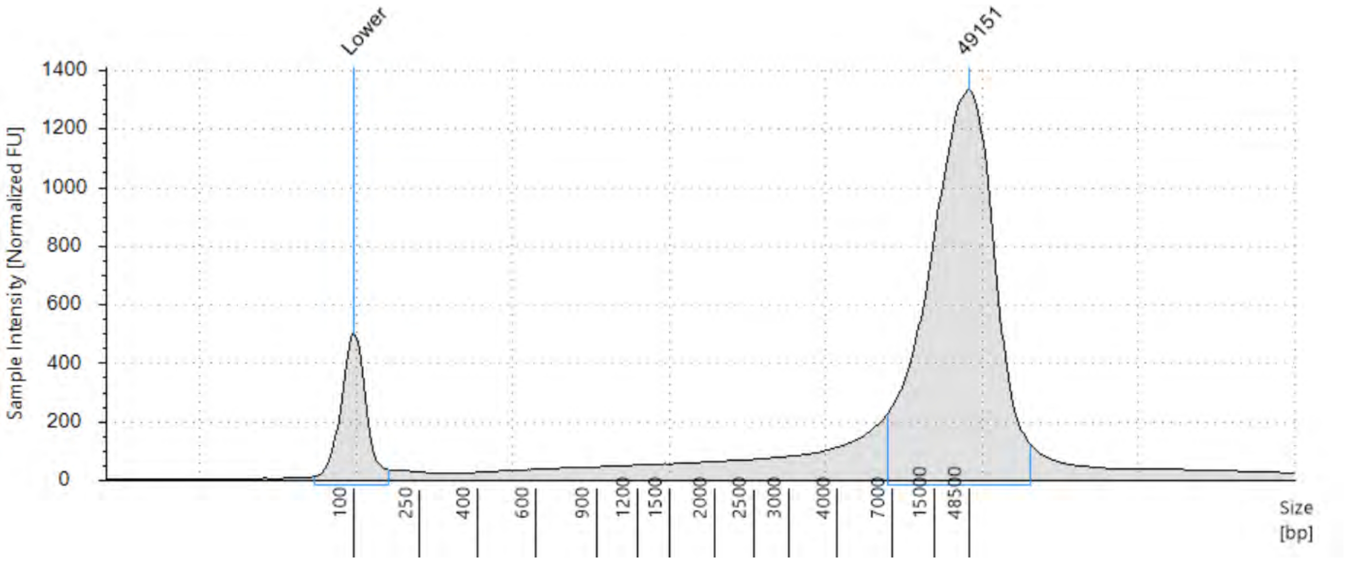

Sample Table

Well	DIN	Conc. [ng/µl]	Sample Description	Alert	Observations
B11	7.7	86.3	C667	⚠	Caution! Expired ScreenTape device

Peak Table

Size [bp]	Calibrated Conc. [ng/µl]	Assigned Conc. [ng/µl]	% Integrated Area	From [bp]	To [bp]	Peak Comment	Observations
100	8.50	8.50	-	58	163		Lower Marker
49151	60.7	-	94.49	6720	>60000		
-	-	-	-	-	-		Sample Well

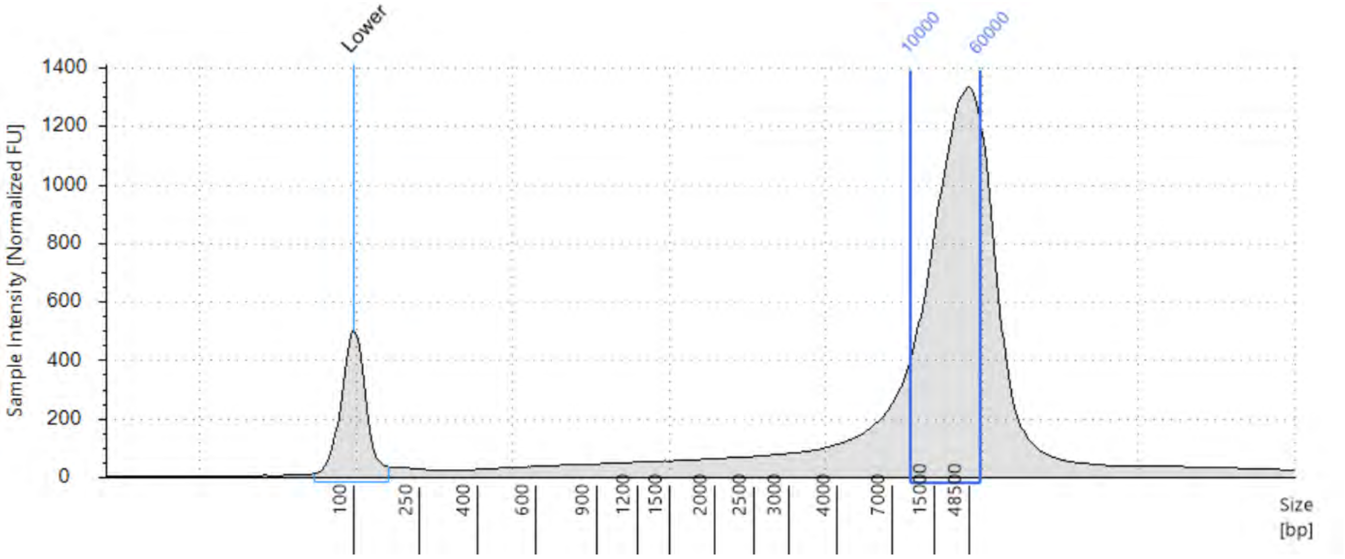

Region Table

From [bp]	To [bp]	Average Size [bp]	Conc. [ng/µl]	Region Molarity [nmol/l]	% of Total	Region Comment	Color
10000	60000	29679	41.7	2.92	48.33		■

C11: C668

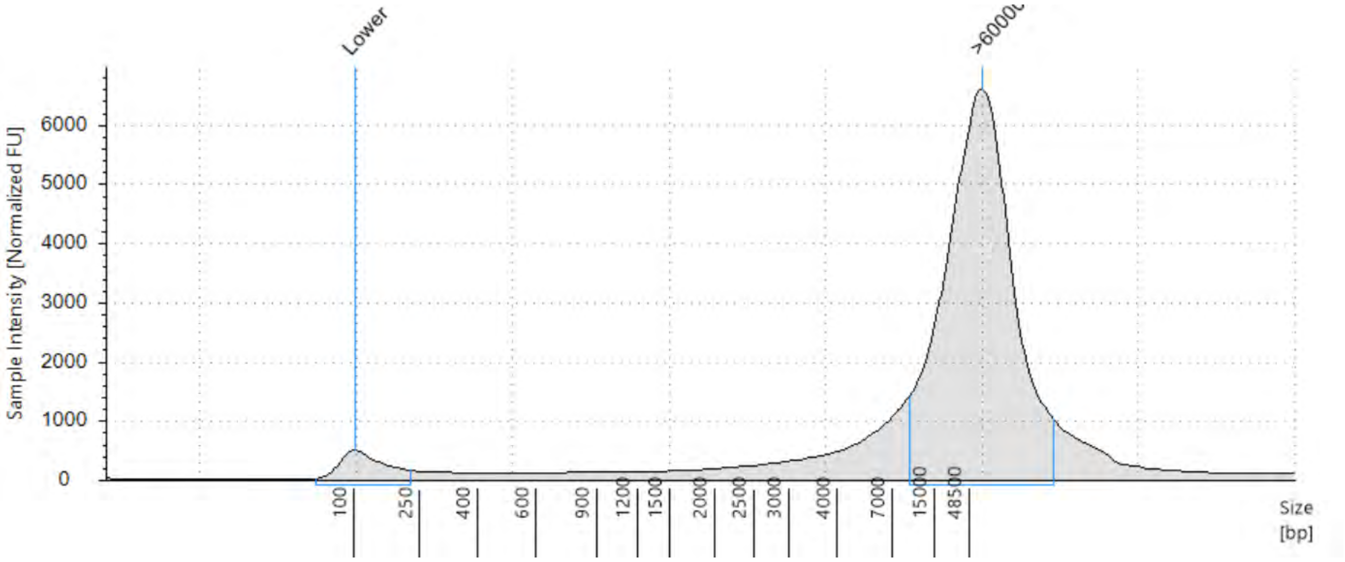

Sample Table

Well	DIN	Conc. [ng/µl]	Sample Description	Alert	Observations
C11	8.1	252	C668	⚠	Caution! Expired ScreenTape device; Sample concentration outside recommended range

Peak Table

Size [bp]	Calibrated Conc. [ng/µl]	Assigned Conc. [ng/µl]	% Integrated Area	From [bp]	To [bp]	Peak Comment	Observations
100	8.50	8.50	-	58	217		Lower Marker
>60000	176	-	91.70	9570	>60000		
-	-	-	-	-	-		Sample Well

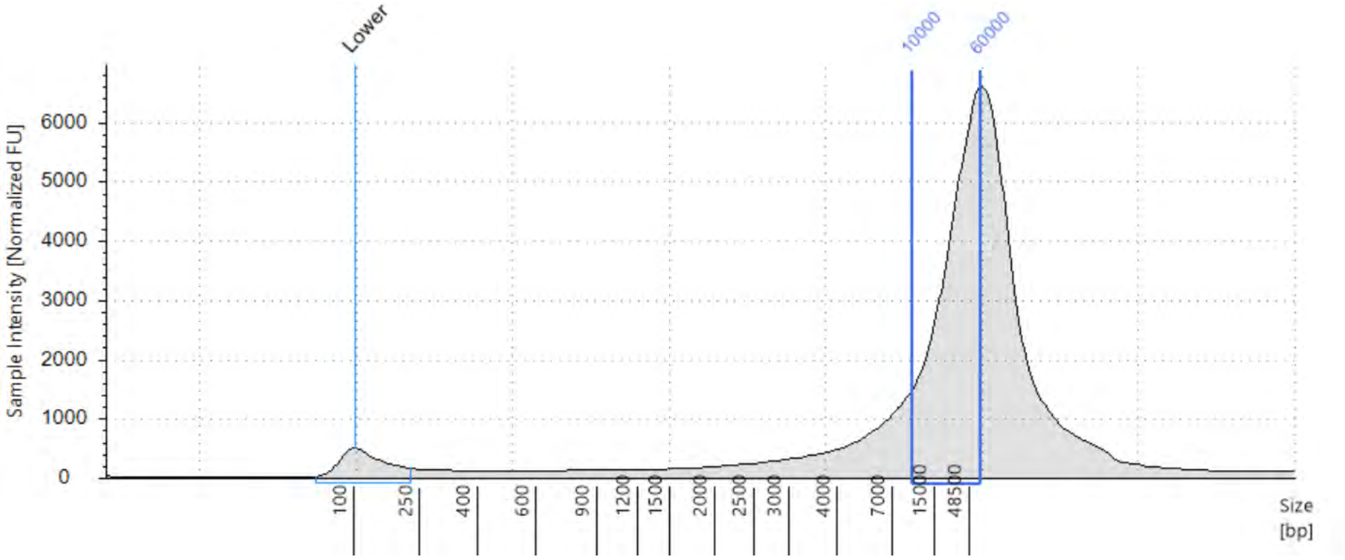

Region Table

From [bp]	To [bp]	Average Size [bp]	Conc. [ng/µl]	Region Molarity [nmol/l]	% of Total	Region Comment	Color
10000	60000	31905	91.8	6.00	36.37		■

 © Agilent Technologies, Inc. 2017

D11: C669

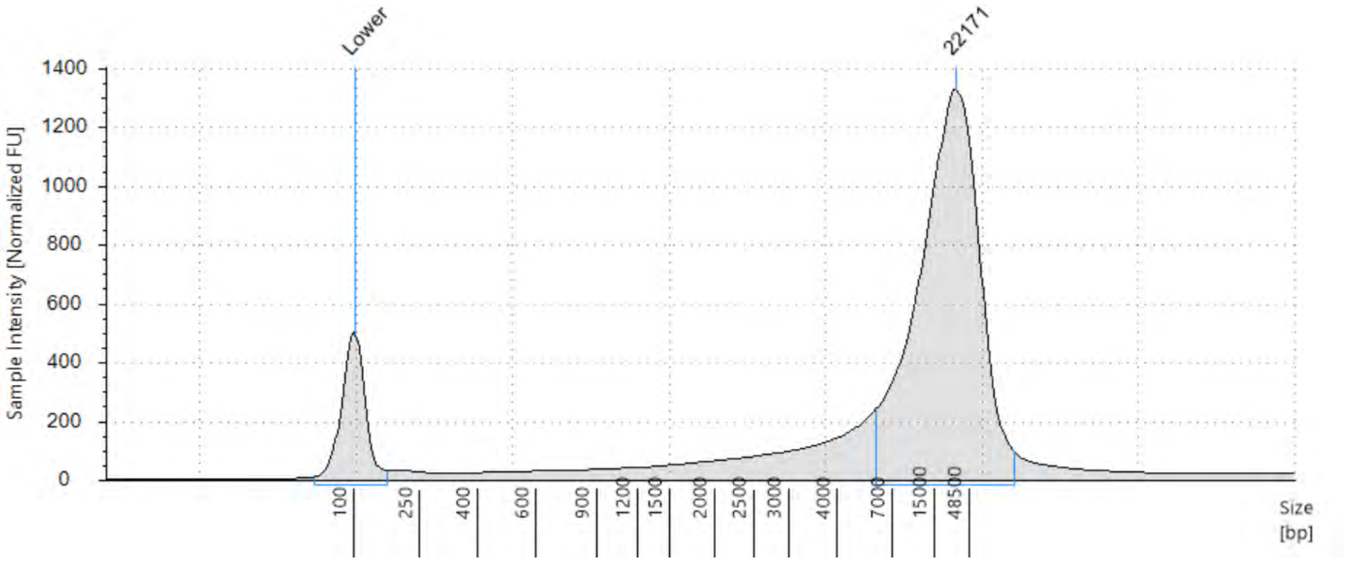

Sample Table

Well	DIN	Conc. [ng/µl]	Sample Description	Alert	Observations
D11	7.4	83.3	C669	⚠	Caution! Expired ScreenTape device

Peak Table

Size [bp]	Calibrated Conc. [ng/µl]	Assigned Conc. [ng/µl]	% Integrated Area	From [bp]	To [bp]	Peak Comment	Observations
100	8.50	8.50	-	57	158		Lower Marker
22171	58.8	-	94.15	5933	>60000		
>60000	0.508	-	0.81	>60000	>60000		
-	-	-	-	-	-		Sample Well

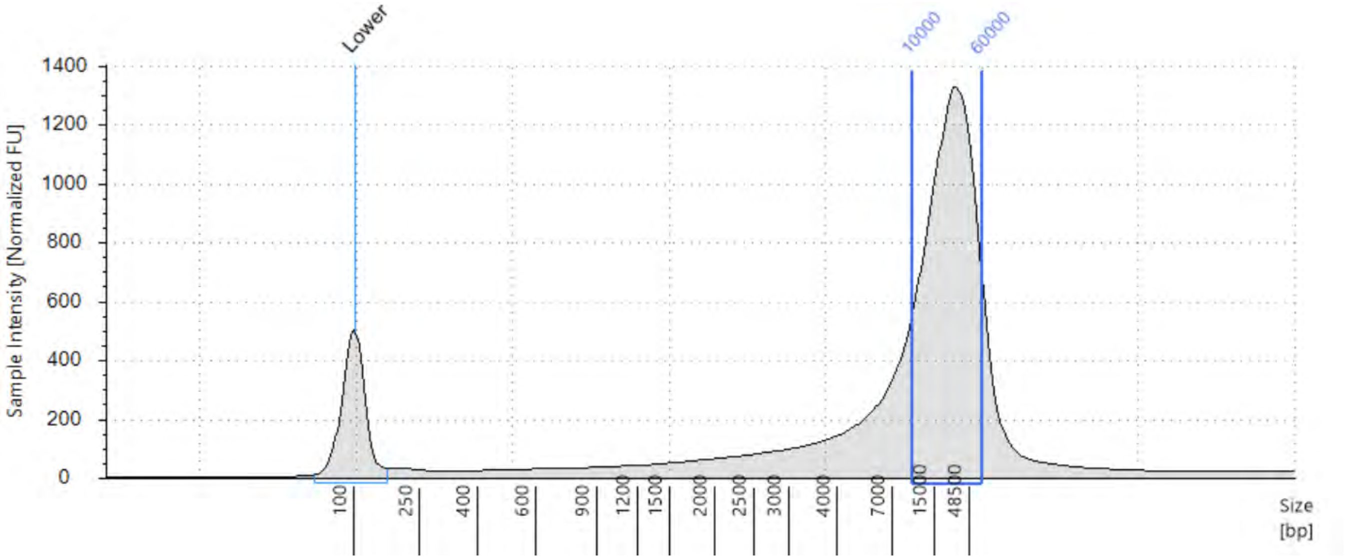

Region Table

From [bp]	To [bp]	Average Size [bp]	Conc. [ng/µl]	Region Molarity [nmol/l]	% of Total	Region Comment	Color
10000	60000	25869	44.7	3.45	53.70		■

E11: C670

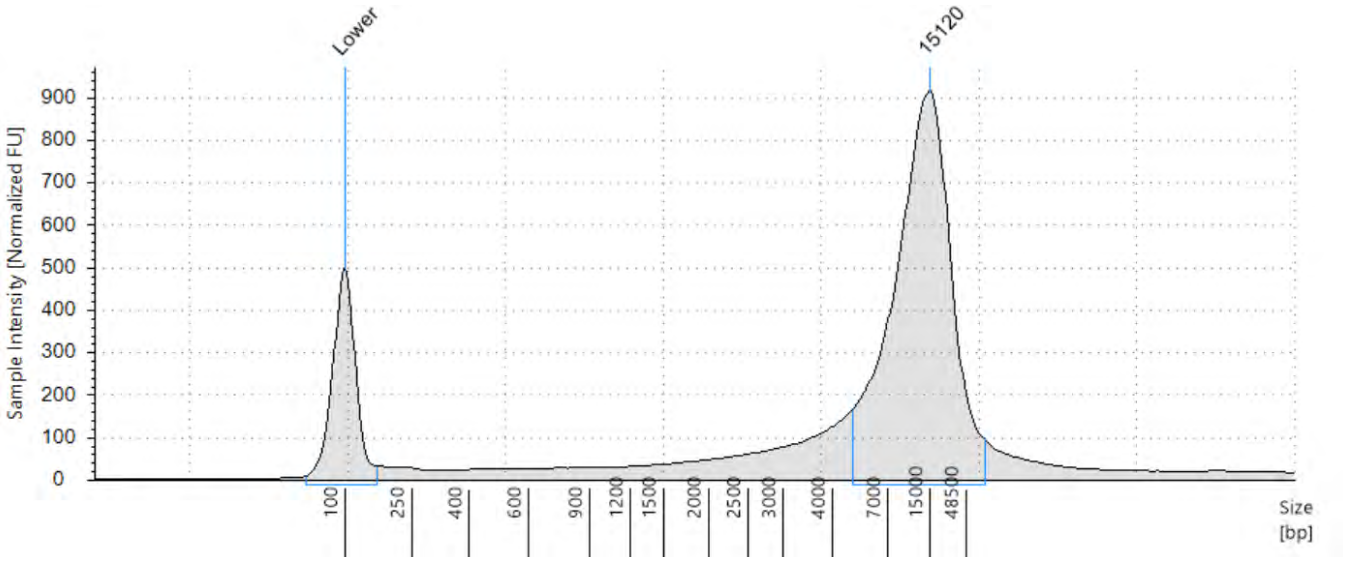

Sample Table

Well	DIN	Conc. [ng/µl]	Sample Description	Alert	Observations
E11	6.9	59.7	C670	⚠	Caution! Expired ScreenTape device

Peak Table

Size [bp]	Calibrated Conc. [ng/µl]	Assigned Conc. [ng/µl]	% Integrated Area	From [bp]	To [bp]	Peak Comment	Observations
100	8.50	8.50	-	58	156		Lower Marker
15120	38.9	-	88.86	4956	>60000		
-	-	-	-	-	-		Sample Well

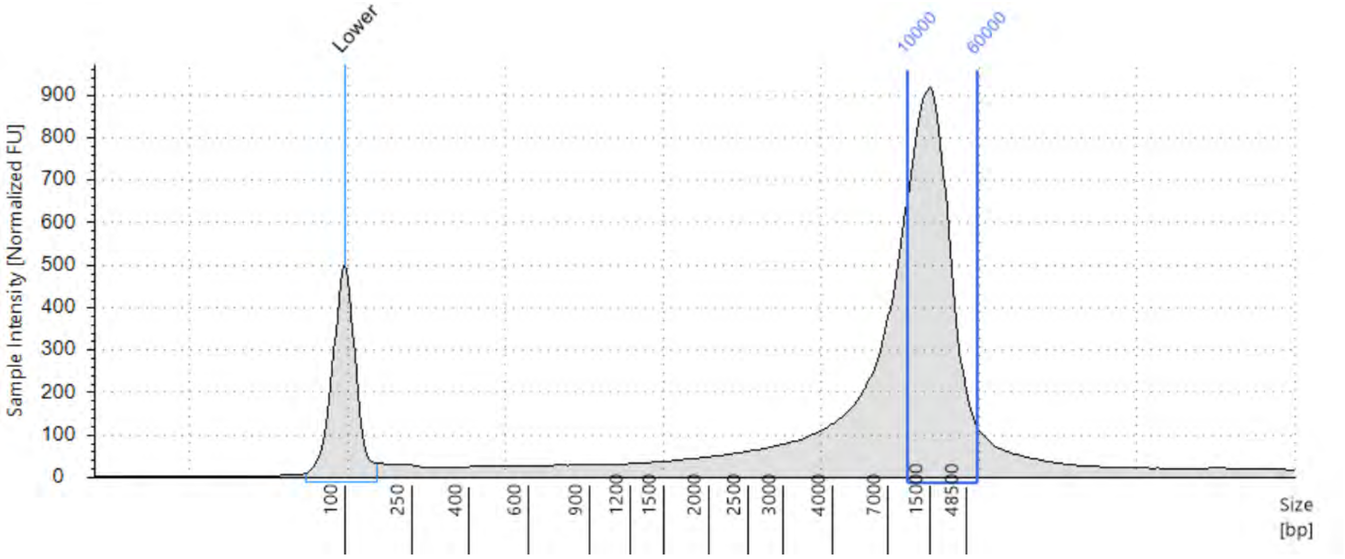

Region Table

From [bp]	To [bp]	Average Size [bp]	Conc. [ng/µl]	Region Molarity [nmol/l]	% of Total	Region Comment	Color
10000	60000	18675	26.9	2.63	45.06		■

 © Agilent Technologies, Inc. 2017

F11: M191

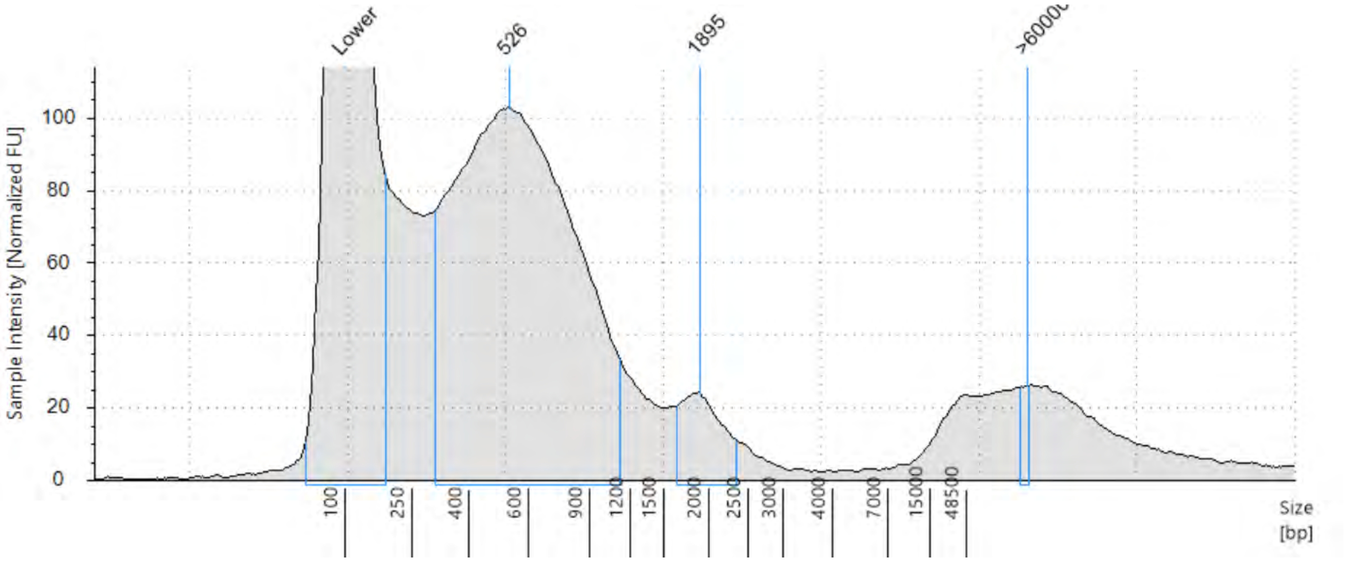

Sample Table

Well	DIN	Conc. [ng/µl]	Sample Description	Alert	Observations
F11	1.0	13.4	M191	⚠	Caution! Expired ScreenTape device

Peak Table

Size [bp]	Calibrated Conc. [ng/µl]	Assigned Conc. [ng/µl]	% Integrated Area	From [bp]	To [bp]	Peak Comment	Observations
100	8.50	8.50	-	59	177		Lower Marker
526	7.31	-	89.90	304	1121		
1895	0.575	-	7.08	1634	2356		
>60000	0.114	-	1.40	>60000	>60000		
-	-	-	-	-	-		Sample Well

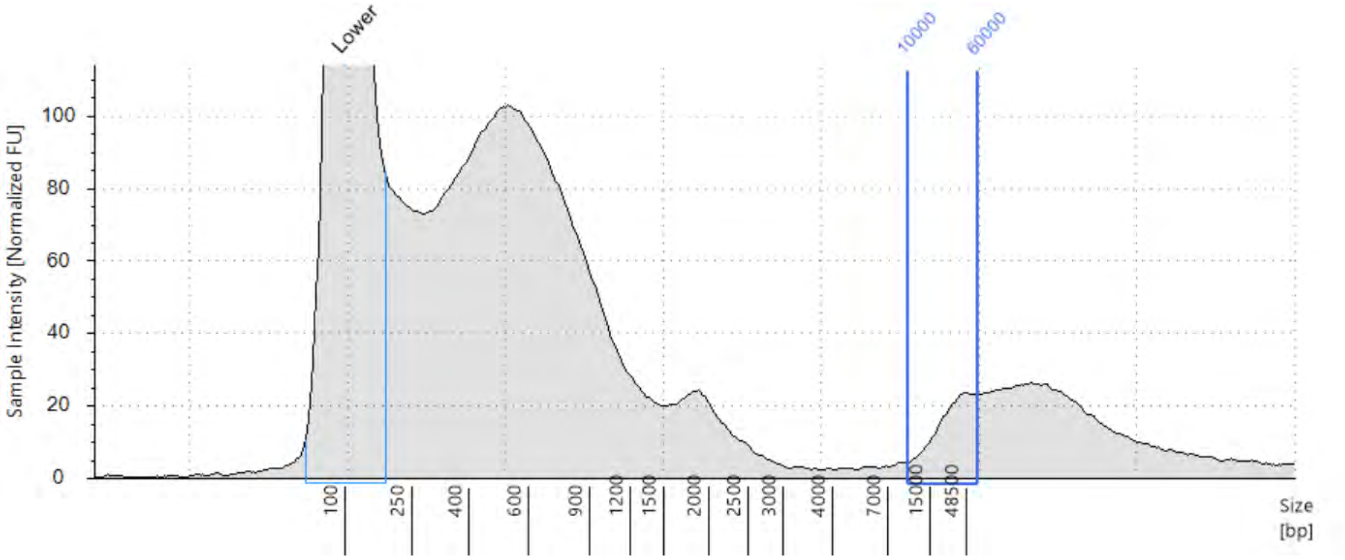

Region Table

From [bp]	To [bp]	Average Size [bp]	Conc. [ng/µl]	Region Molarity [nmol/l]	% of Total	Region Comment	Color
10000	60000	31295	0.516	0.0344	3.84		■

G11: M195

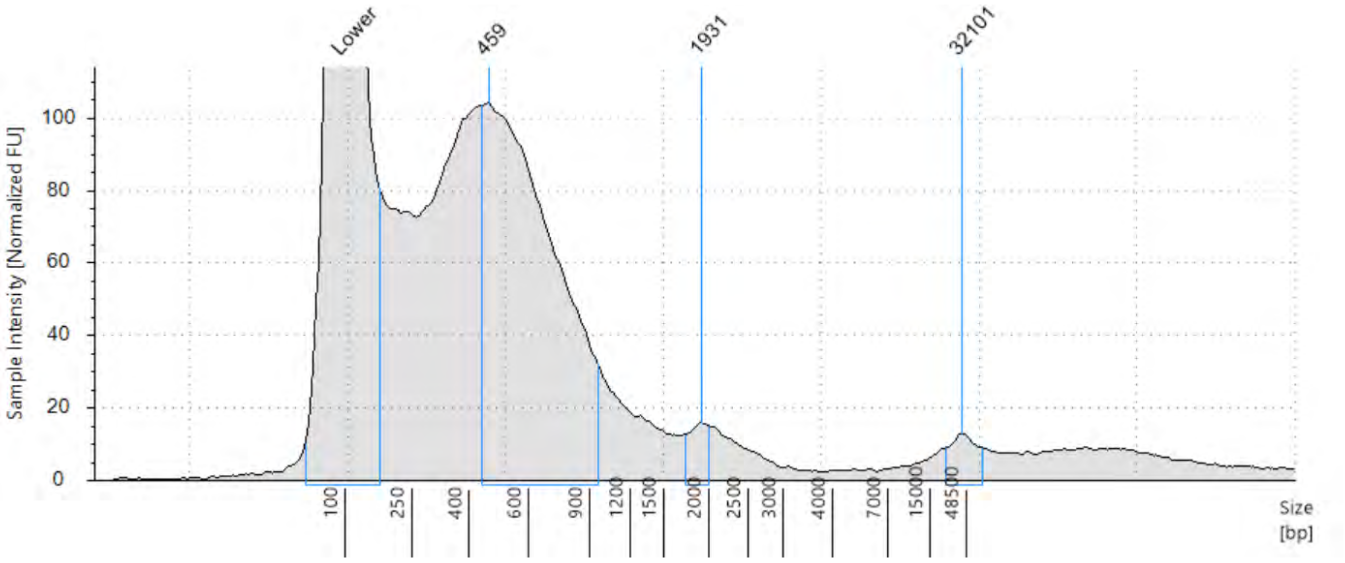

Sample Table

Well	DIN	Conc. [ng/µl]	Sample Description	Alert	Observations
G11	1.0	12.4	M195	⚠	Caution! Expired ScreenTape device

Peak Table

Size [bp]	Calibrated Conc. [ng/µl]	Assigned Conc. [ng/µl]	% Integrated Area	From [bp]	To [bp]	Peak Comment	Observations
100	8.50	8.50	-	59	164		Lower Marker
459	4.69	-	89.46	439	964		
1931	0.185	-	3.52	1737	2010		
32101	0.204	-	3.89	20209	>60000		
-	-	-	-	-	-		Sample Well

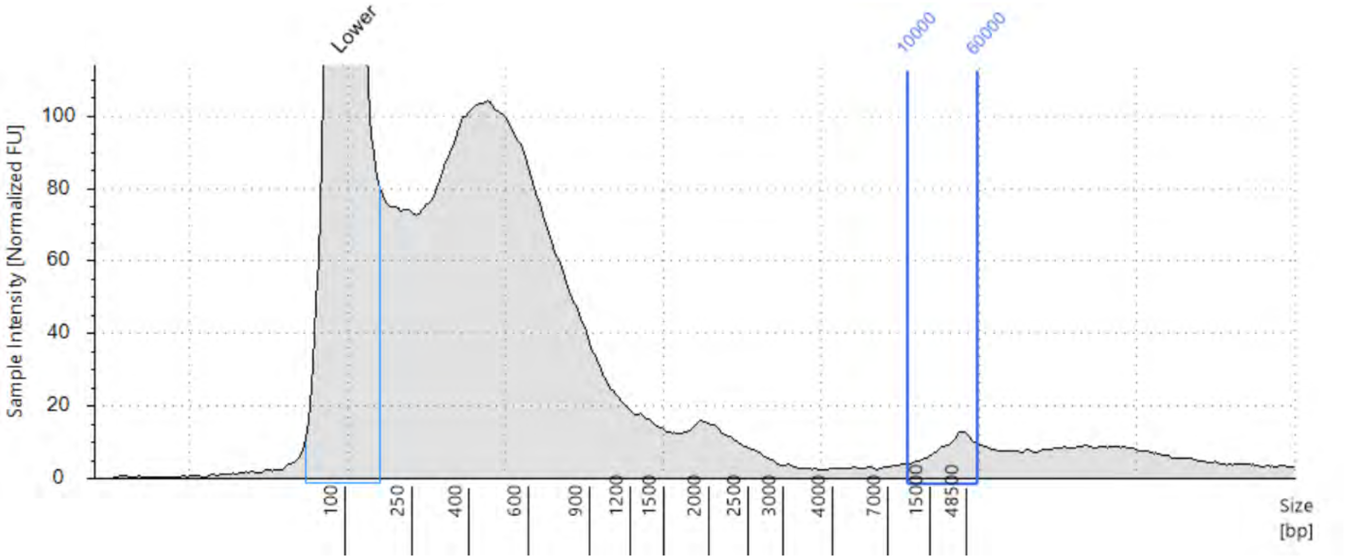

Region Table

From [bp]	To [bp]	Average Size [bp]	Conc. [ng/µl]	Region Molarity [nmol/l]	% of Total	Region Comment	Color
10000	60000	29892	0.297	0.0218	2.39		■

H11: M201

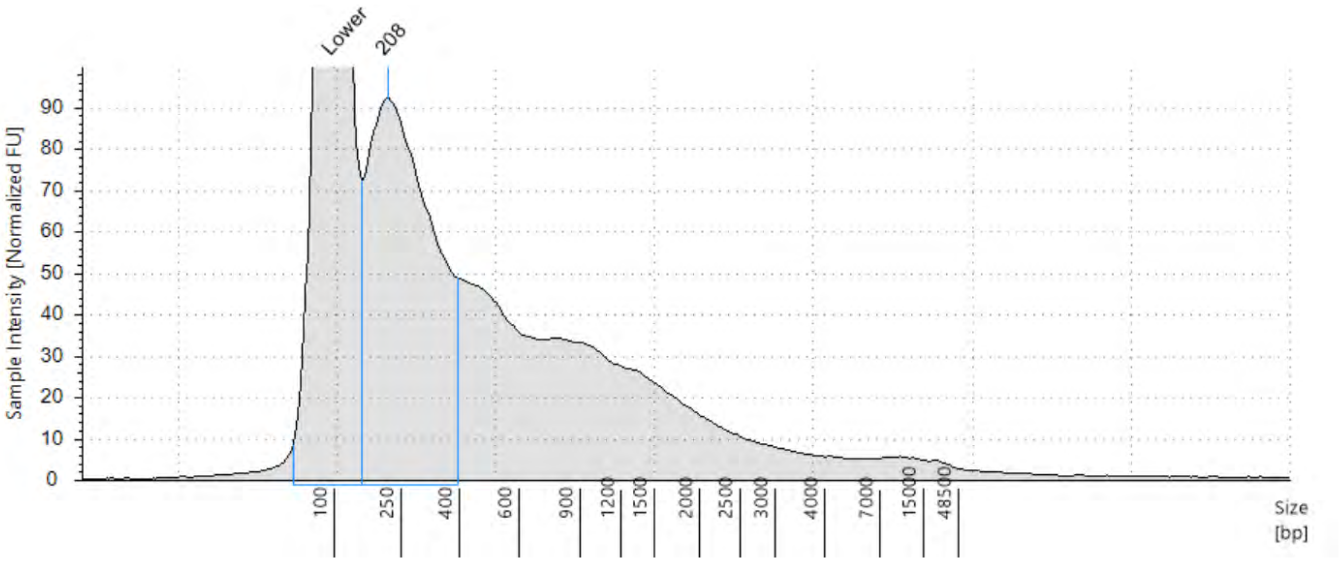

Sample Table

Well	DIN	Conc. [ng/µl]	Sample Description	Alert	Observations
H11	1.5	9.98	M201	⚠	Caution! Expired ScreenTape device; Sample concentration outside recommended range

Peak Table

Size [bp]	Calibrated Conc. [ng/µl]	Assigned Conc. [ng/µl]	% Integrated Area	From [bp]	To [bp]	Peak Comment	Observations
100	8.50	8.50	-	57	147		Lower Marker
208	4.28	-	100.00	147	396		

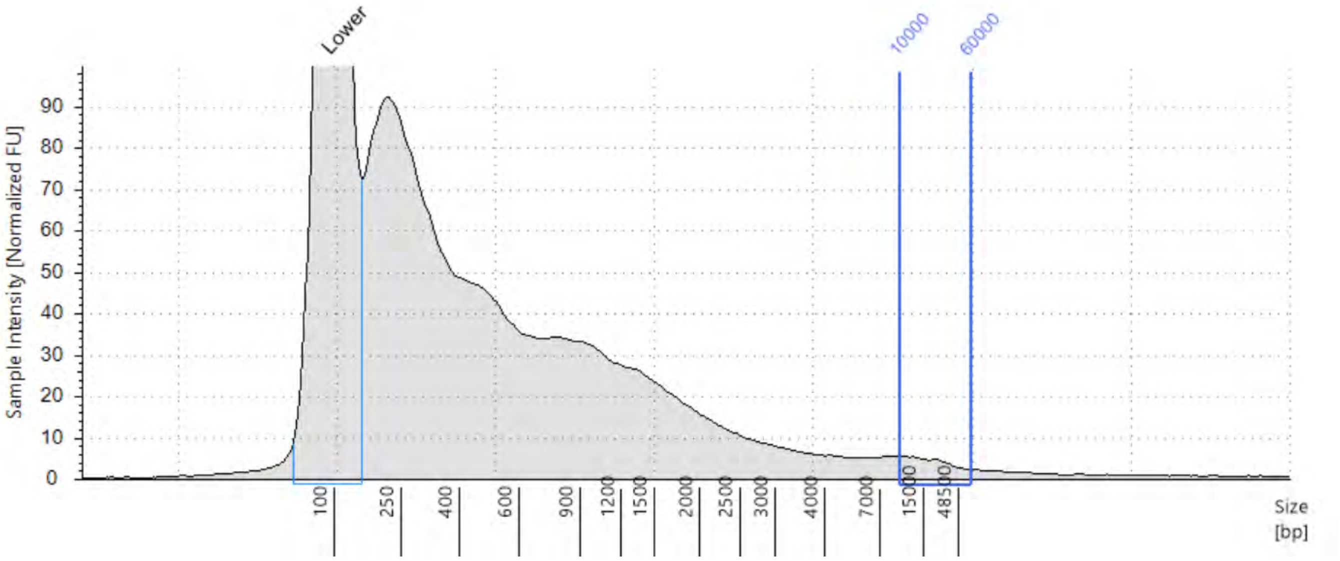

Region Table

From [bp]	To [bp]	Average Size [bp]	Conc. [ng/µl]	Region Molarity [nmol/l]	% of Total	Region Comment	Color
10000	60000	21691	0.150	0.0166	1.51		■

 © Agilent Technologies, Inc. 2017

A12: M202

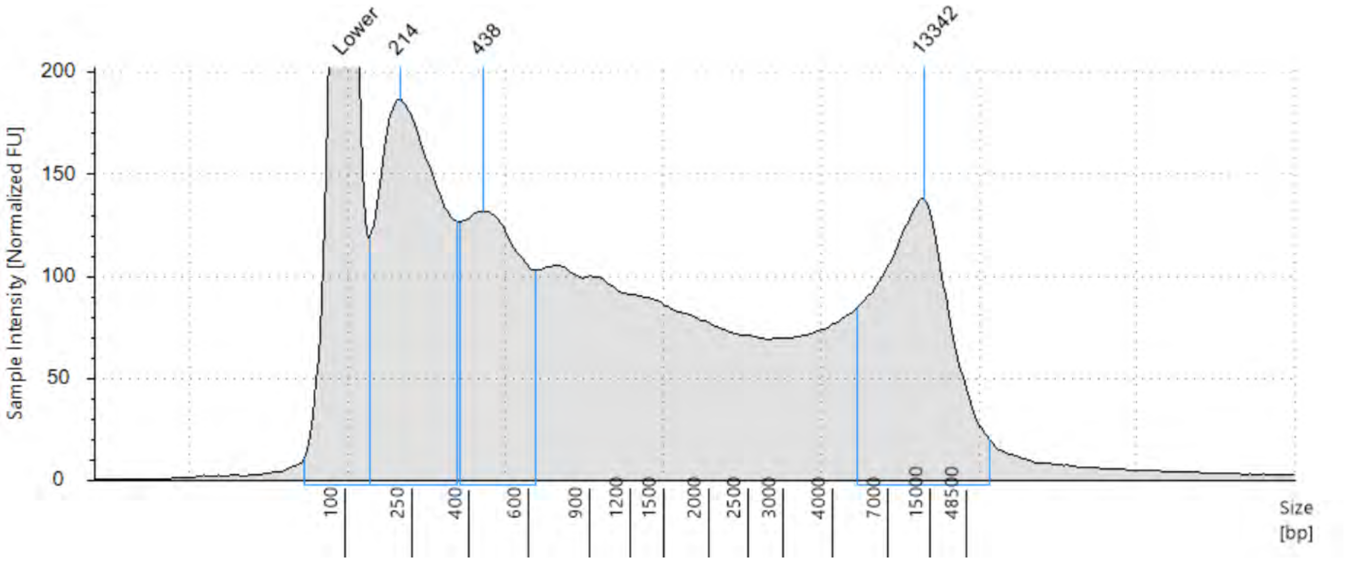

Sample Table

Well	DIN	Conc. [ng/µl]	Sample Description	Alert	Observations
A12	2.1	36.4	M202	⚠	Caution! Expired ScreenTape device

Peak Table

Size [bp]	Calibrated Conc. [ng/µl]	Assigned Conc. [ng/µl]	% Integrated Area	From [bp]	To [bp]	Peak Comment	Observations
100	8.50	8.50	-	57	139		Lower Marker
214	8.05	-	39.63	139	362		
438	5.37	-	26.42	370	626		
13342	6.90	-	33.94	5122	>60000		

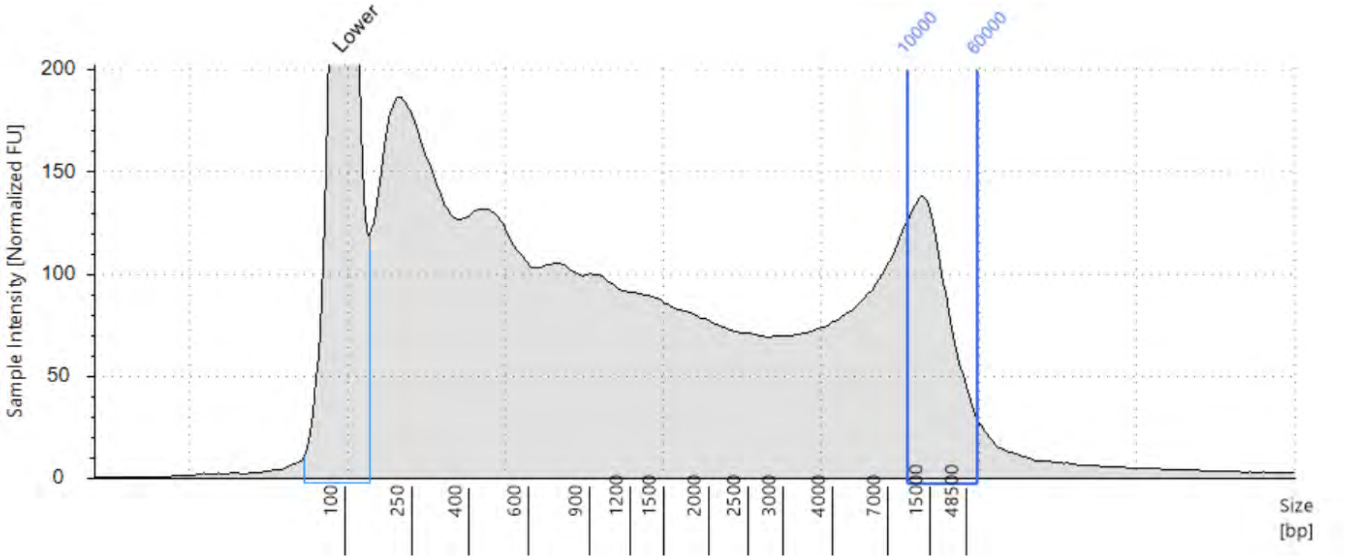

Region Table

From [bp]	To [bp]	Average Size [bp]	Conc. [ng/µl]	Region Molarity [nmol/l]	% of Total	Region Comment	Color
10000	60000	19725	3.81	0.376	10.45		■

B12: C392

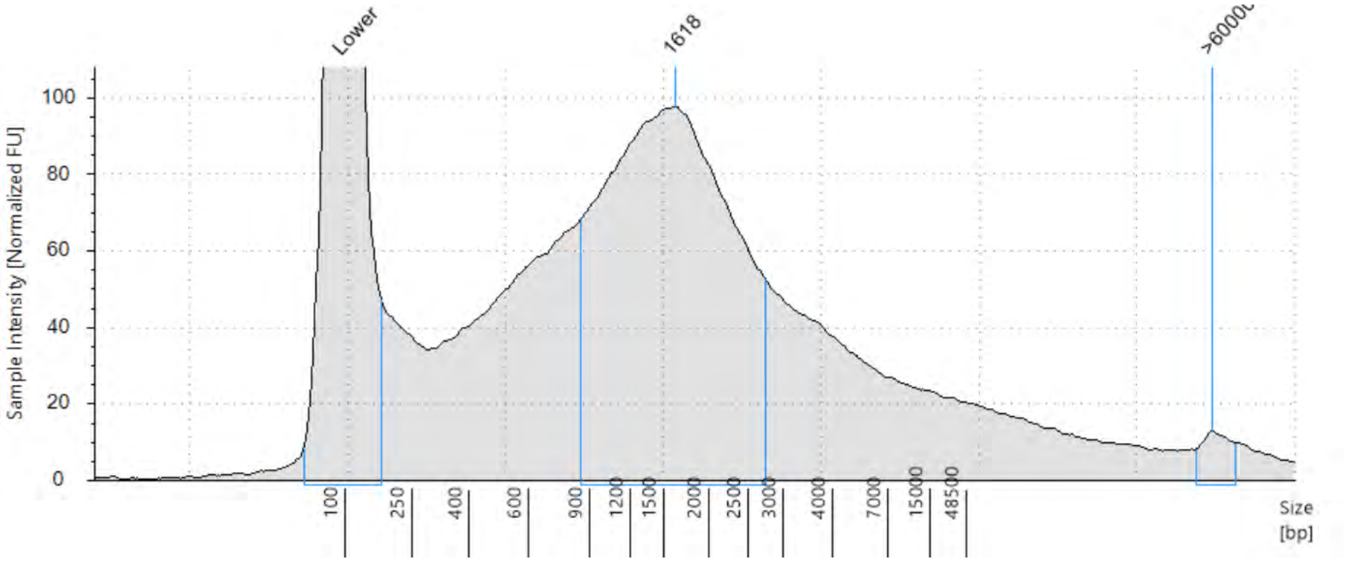

Sample Table

Well	DIN	Conc. [ng/µl]	Sample Description	Alert	Observations
B12	2.8	18.8	C392	⚠	Caution! Expired ScreenTape device

Peak Table

Size [bp]	Calibrated Conc. [ng/µl]	Assigned Conc. [ng/µl]	% Integrated Area	From [bp]	To [bp]	Peak Comment	Observations
100	8.50	8.50	-	57	167		Lower Marker
1618	8.23	-	89.82	853	2740		
>60000	0.221	-	2.41	>60000	>60000		
-	-	-	-	-	-		Sample Well

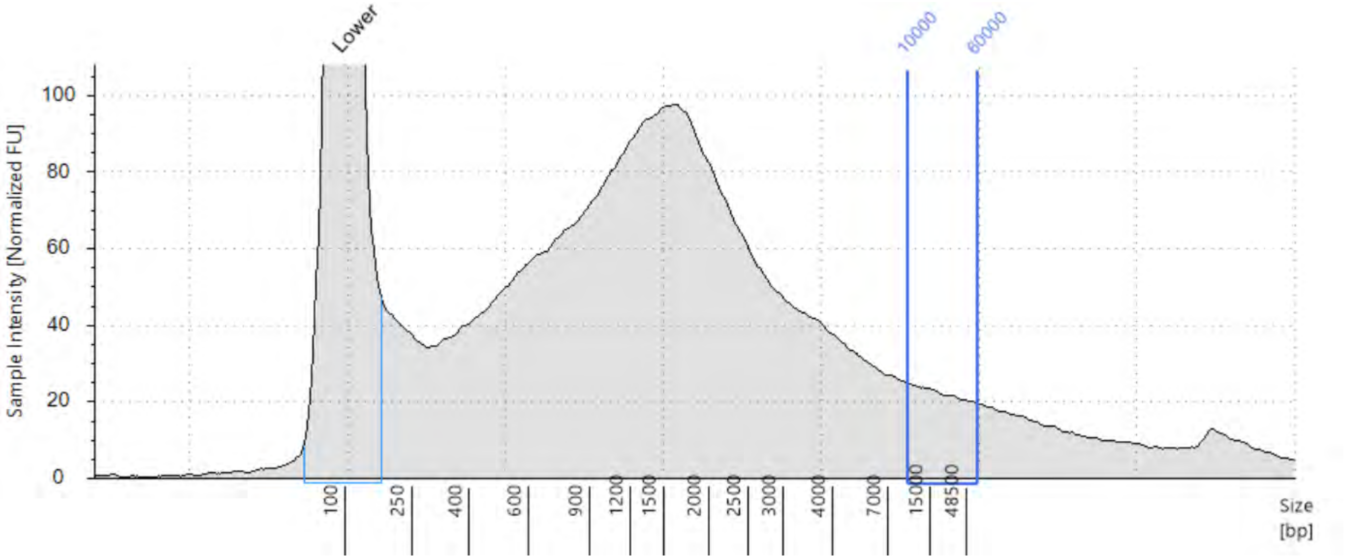

Region Table

From [bp]	To [bp]	Average Size [bp]	Conc. [ng/µl]	Region Molarity [nmol/l]	% of Total	Region Comment	Color
10000	60000	24922	0.828	0.0737	4.40		■

 © Agilent Technologies, Inc. 2017

C12: C393

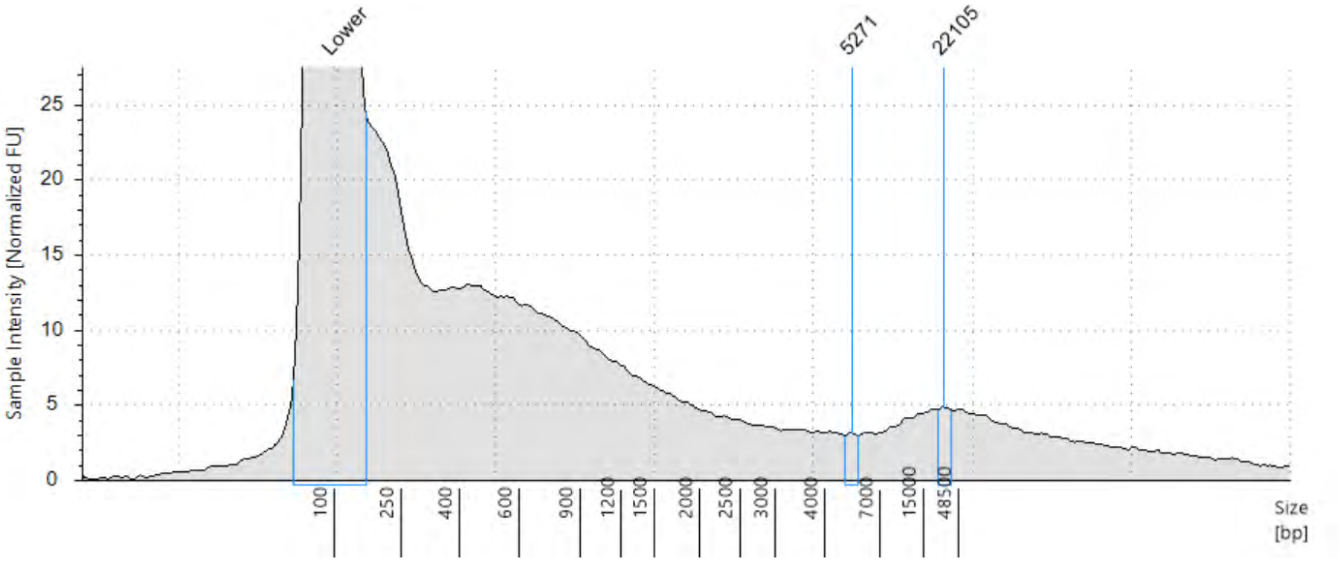

Sample Table

Well	DIN	Conc. [ng/µl]	Sample Description	Alert	Observations
C12	1.1	3.17	C393	⚠	Caution! Expired ScreenTape device; Sample concentration outside functional range for DIN

Peak Table

Size [bp]	Calibrated Conc. [ng/µl]	Assigned Conc. [ng/µl]	% Integrated Area	From [bp]	To [bp]	Peak Comment	Observations
100	8.50	8.50	-	57	155		Lower Marker
5271	0.0218	-	35.01	4912	5578		
22105	0.0405	-	64.99	19477	26266		

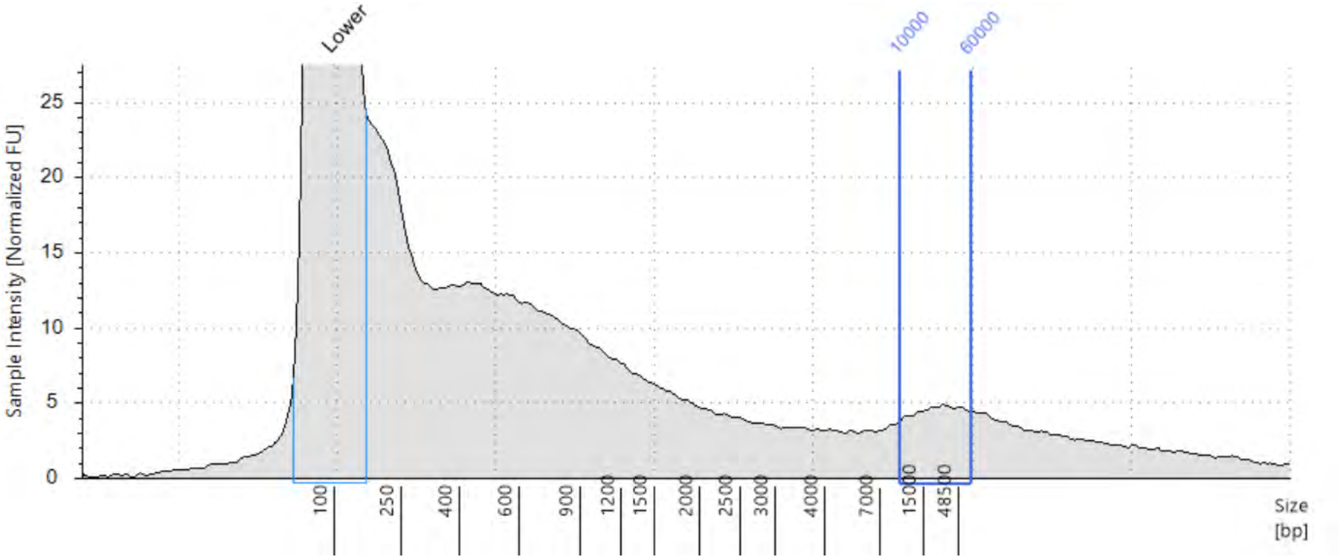

Region Table

From [bp]	To [bp]	Average Size [bp]	Conc. [ng/µl]	Region Molarity [nmol/l]	% of Total	Region Comment	Color
10000	60000	25919	0.173	0.0164	5.48		■

D12: C396

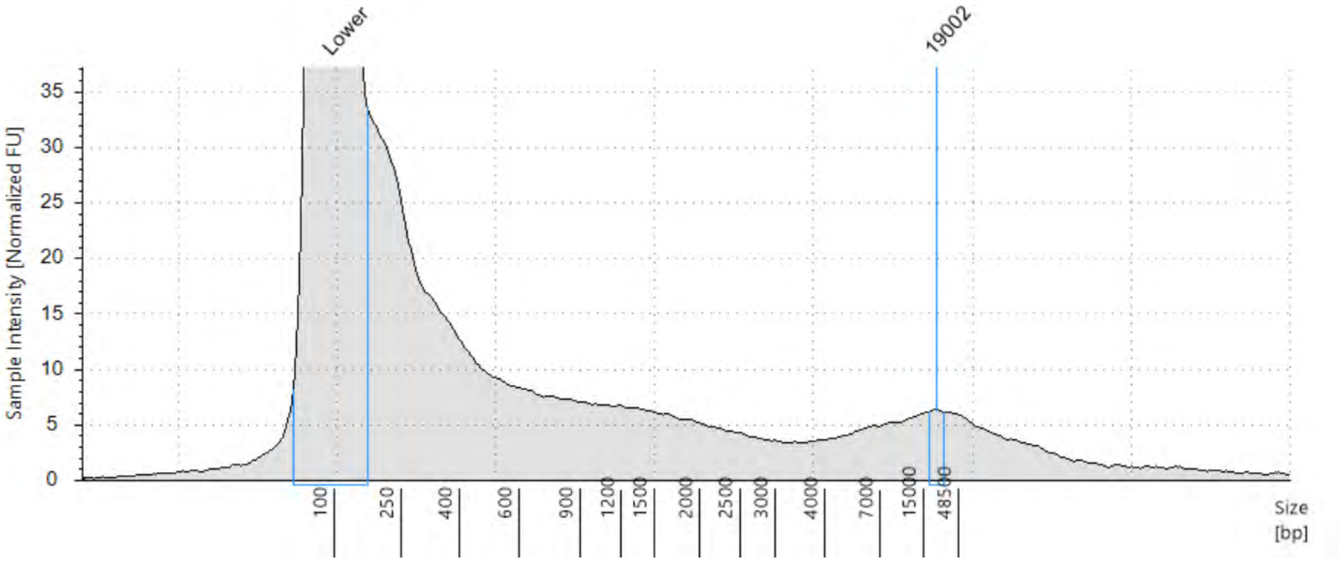

Sample Table

Well	DIN	Conc. [ng/µl]	Sample Description	Alert	Observations
D12	1.0	3.16	C396	⚠	Caution! Expired ScreenTape device; Sample concentration outside functional range for DIN

Peak Table

Size [bp]	Calibrated Conc. [ng/µl]	Assigned Conc. [ng/µl]	% Integrated Area	From [bp]	To [bp]	Peak Comment	Observations
100	8.50	8.50	-	56	157		Lower Marker
19002	0.0550	-	100.00	16714	21621		

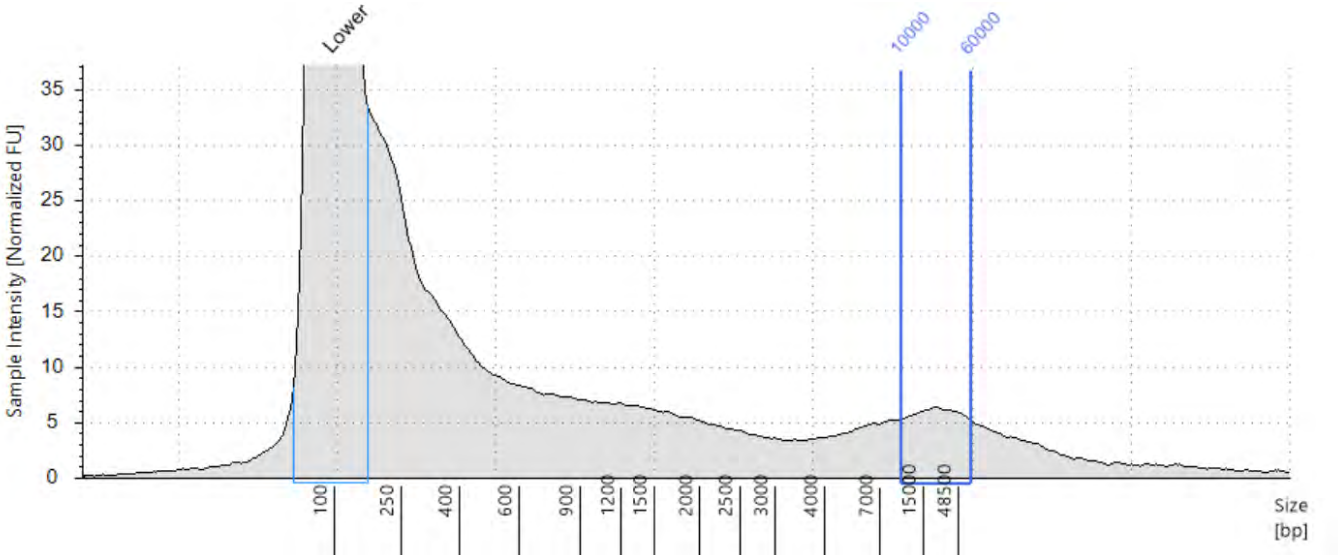

Region Table

From [bp]	To [bp]	Average Size [bp]	Conc. [ng/µl]	Region Molarity [nmol/l]	% of Total	Region Comment	Color
10000	60000	25568	0.235	0.0211	7.42		■

G12: W885

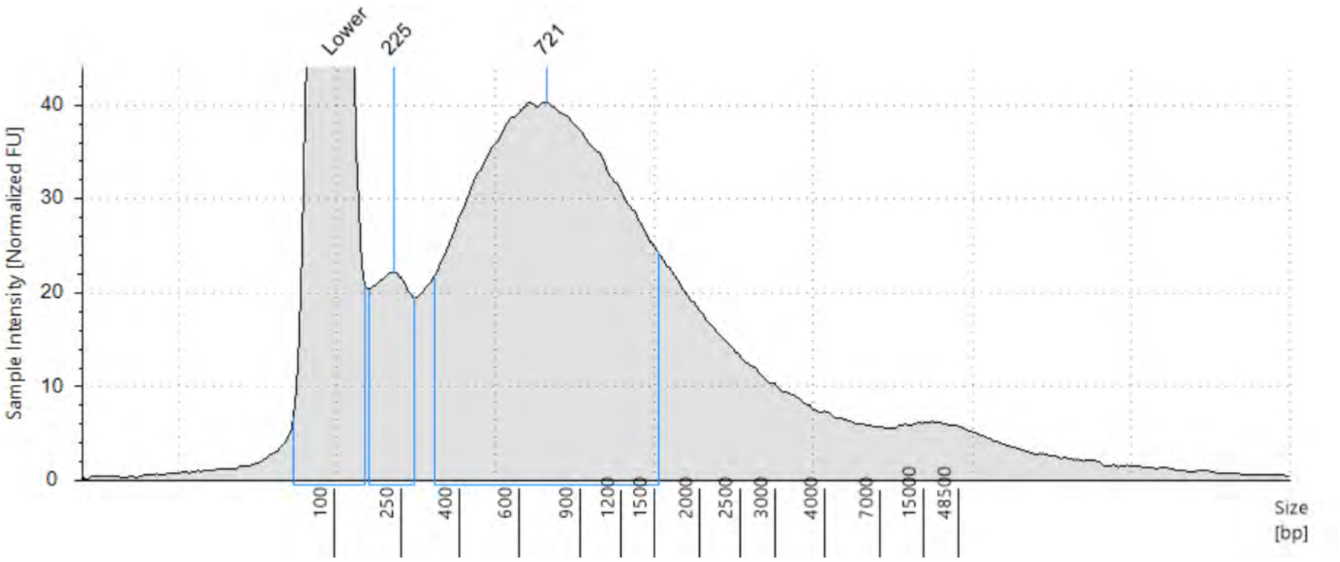

Sample Table

Well	DIN	Conc. [ng/µl]	Sample Description	Alert	Observations
G12	1.9	7.99	W885	⚠	Caution! Expired ScreenTape device; Sample concentration outside recommended range

Peak Table

Size [bp]	Calibrated Conc. [ng/µl]	Assigned Conc. [ng/µl]	% Integrated Area	From [bp]	To [bp]	Peak Comment	Observations
100	8.50	8.50	-	57	153		Lower Marker
225	0.619	-	11.42	162	279		
721	4.81	-	88.58	326	1537		

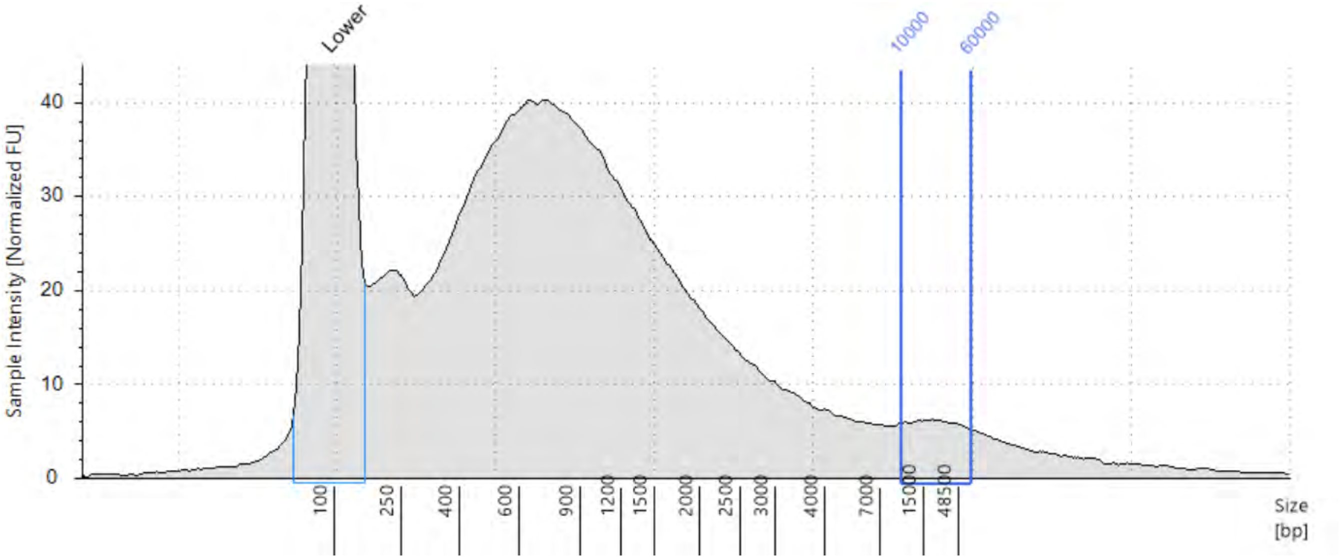

Region Table

From [bp]	To [bp]	Average Size [bp]	Conc. [ng/µl]	Region Molarity [nmol/l]	% of Total	Region Comment	Color
10000	60000	25435	0.246	0.0224	3.07		■

H12: LADDER

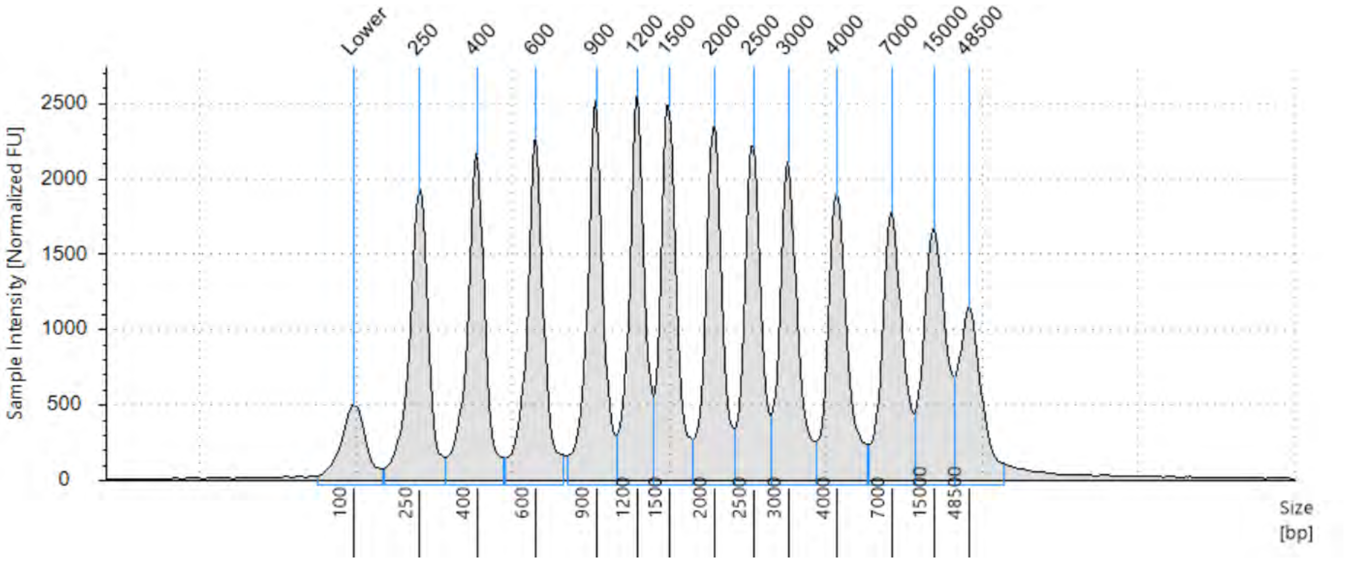

Sample Table

Well	DIN	Conc. [ng/µl]	Sample Description	Alert	Observations
H12	-	352	LADDER	⚠	Caution! Expired ScreenTape device; Ladder

Peak Table

Size [bp]	Calibrated Conc. [ng/µl]	Assigned Conc. [ng/µl]	% Integrated Area	From [bp]	To [bp]	Peak Comment	Observations
100	8.50	8.50	-	60	148		Lower Marker
250	26.3	-	7.48	154	307		
400	27.7	-	7.87	307	481		
600	27.9	-	7.92	485	718		
900	29.6	-	8.41	738	1044		
1200	29.1	-	8.27	1044	1348		
1500	28.9	-	8.22	1348	1748		
2000	28.1	-	7.99	1748	2254		
2500	26.6	-	7.57	2254	2749		
3000	27.7	-	7.88	2749	3536		
4000	26.8	-	7.63	3536	5485		
7000	27.0	-	7.67	5564	10781		
15000	27.1	-	7.71	10781	21986		
48500	19.0	-	5.39	21986	>60000		

© Agilent Technologies, Inc. 2017

Filename: Mussel and Seaworm T1 Data.gDNA

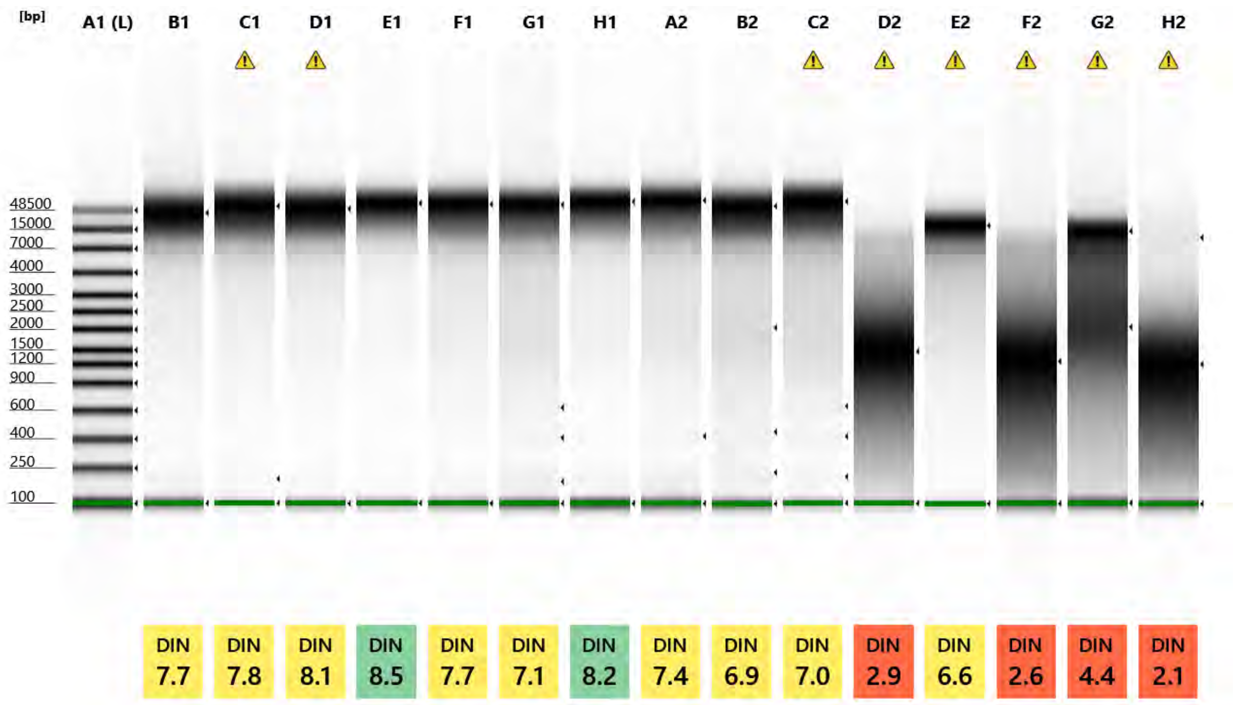

Default image (Contrast 50%), Image is Scaled to Sample

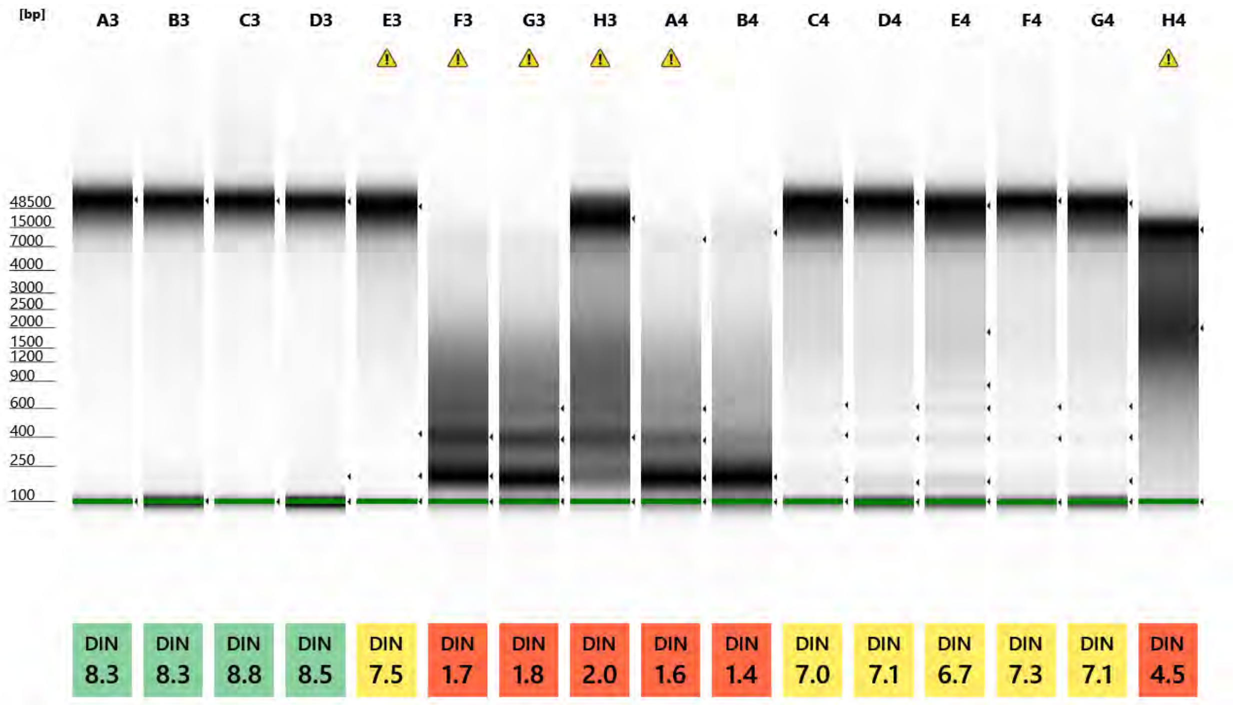

Default image (Contrast 50%), Image is Scaled to Sample

© Agilent Technologies, Inc. 2017

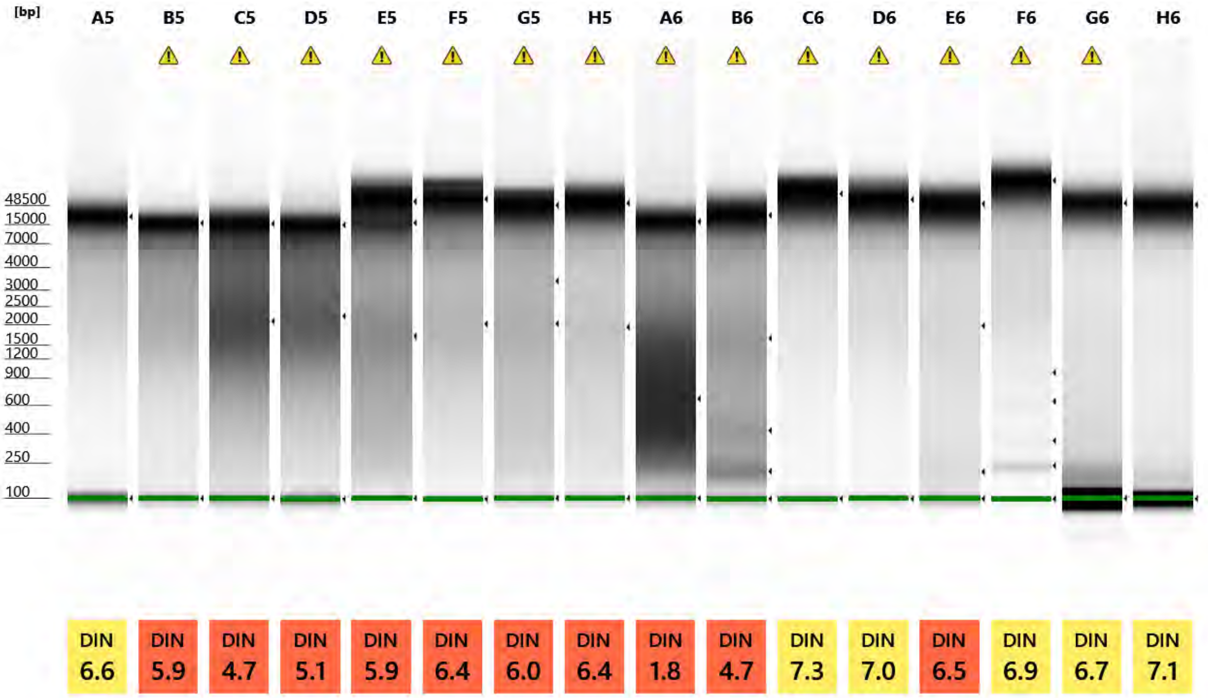

Default image (Contrast 50%), Image is Scaled to Sample

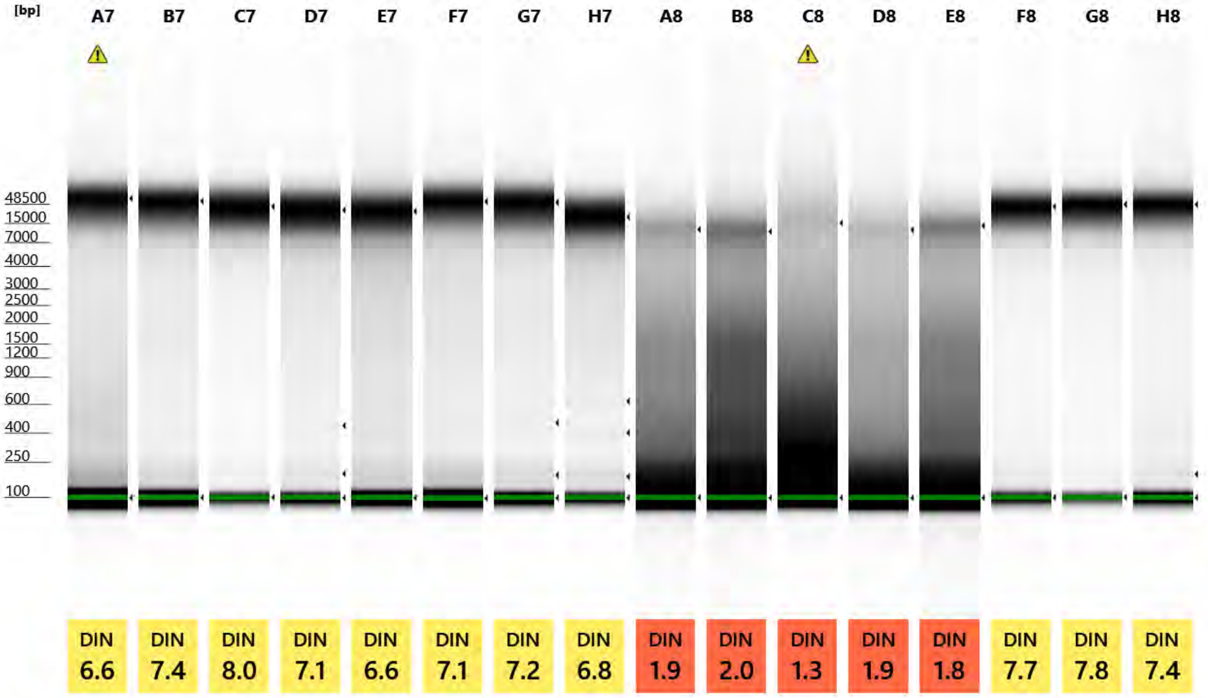

Default image (Contrast 50%), Image is Scaled to Sample

© Agilent Technologies, Inc. 2017

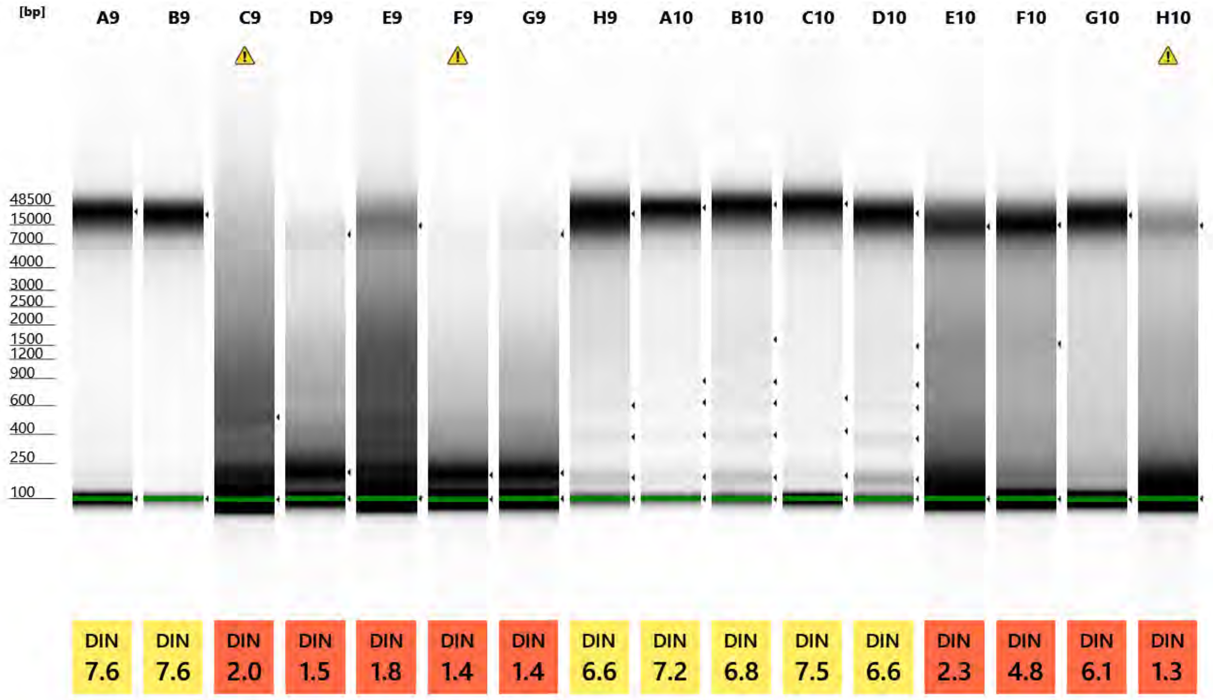

Default image (Contrast 50%), Image is Scaled to Sample

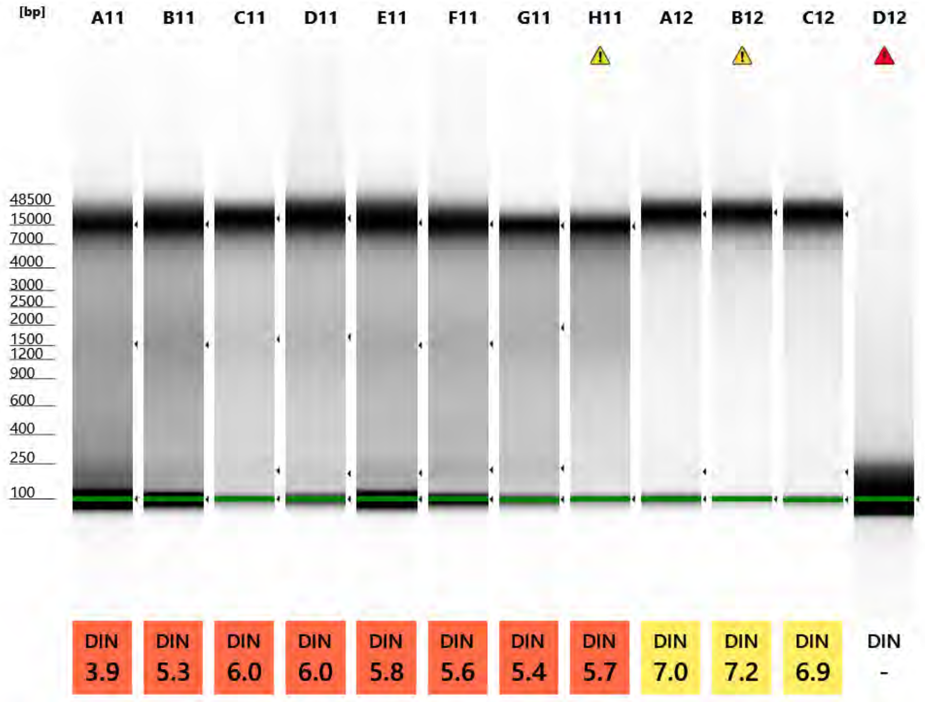

Default image (Contrast 50%), Image is Scaled to Sample

Sample Info

Well	DIN	Conc. [ng/µl]	Sample Description	Alert	Observations
A1	-	71.8	Ladder		Ladder
B1	7.7	67.9	M1		
C1	7.8	201	M2	⚠	Sample concentration outside recommended range
D1	8.1	101	M3	⚠	Sample concentration outside recommended range
E1	8.5	97.2	M4		
F1	7.7	91.1	M5		
G1	7.1	76.7	M6		
H1	8.2	46.0	M7		
A2	7.4	60.3	M8		
B2	6.9	84.1	M9		
C2	7.0	159	M10	⚠	Sample concentration outside recommended range
D2	2.9	302	M11	⚠	Sample concentration outside functional range for DIN
E2	6.6	164	M12	⚠	Sample concentration outside recommended range
F2	2.6	161	M13	⚠	Sample concentration outside recommended range
G2	4.4	144	M14	⚠	Sample concentration outside recommended range
H2	2.1	142	M15	⚠	Sample concentration outside recommended range
A3	8.3	84.2	M16		
B3	8.3	32.4	M17		
C3	8.8	77.1	M18		
D3	8.5	24.5	M19		
E3	7.5	179	M20	⚠	Sample concentration outside recommended range
F3	1.7	203	M21	⚠	Sample concentration outside recommended range
G3	1.8	128	M22	⚠	Sample concentration outside recommended range
H3	2.0	346	M23	⚠	Sample concentration outside functional range for DIN
A4	1.6	118	M24	⚠	Sample concentration outside recommended range
B4	1.4	83.8	M25		
C4	7.0	95.6	M26		
D4	7.1	50.7	M27		
E4	6.7	69.5	M28		
F4	7.3	68.2	M29		
G4	7.1	53.3	M30		
H4	4.5	257	M31	⚠	Sample concentration outside recommended range
A5	6.6	65.9	M32		
B5	5.9	170	M33	⚠	Sample concentration outside recommended range
C5	4.7	251	M34	⚠	Sample concentration outside recommended range
D5	5.1	162	M35	⚠	Sample concentration outside recommended range
E5	5.9	571	M36	⚠	Sample concentration outside functional range for DIN
F5	6.4	537	M37	⚠	Sample concentration outside functional range for DIN
G5	6.0	285	M38	⚠	Sample concentration outside recommended range
H5	6.4	254	M39	⚠	Sample concentration outside recommended range
A6	1.8	264	M40	⚠	Sample concentration outside recommended range
B6	4.7	276	M41	⚠	Sample concentration outside recommended range
C6	7.3	260	M42	⚠	Sample concentration outside recommended range
D6	7.0	266	M43	⚠	Sample concentration outside recommended range
E6	6.5	152	M44	⚠	Sample concentration outside recommended range
F6	6.9	723	M45	⚠	Sample concentration outside functional range for DIN

G6	6.7	6.91	W676	⚠	Sample concentration outside recommended range
H6	7.1	18.0	W677		
A7	6.6	9.01	W678	⚠	Sample concentration outside recommended range
B7	7.4	13.1	W679		
C7	8.0	31.5	W680		
D7	7.1	30.5	W681		
E7	6.6	20.6	W682		
F7	7.1	11.8	W683		
G7	7.2	22.6	W684		
H7	6.8	34.2	W685		
A8	1.9	11.4	W686		
B8	2.0	18.5	W687		
C8	1.3	8.59	W688	⚠	Sample concentration outside recommended range
D8	1.9	10.7	W689		
E8	1.8	11.0	W690		
F8	7.7	25.7	W691		
G8	7.8	36.8	W692		
H8	7.4	20.7	W693		
A9	7.6	20.4	W694		
B9	7.6	54.4	W695		
C9	2.0	8.12	W696	⚠	Sample concentration outside recommended range
D9	1.5	25.4	W697		
E9	1.8	11.6	W698		
F9	1.4	9.70	W699	⚠	Sample concentration outside recommended range
G9	1.4	13.5	W700		
H9	6.6	61.6	W701		
A10	7.2	53.8	W702		
B10	6.8	50.0	W703		
C10	7.5	25.6	W704		
D10	6.6	52.2	W705		
E10	2.3	19.2	W706		
F10	4.8	21.2	W707		
G10	6.1	18.7	W708		
H10	1.3	9.36	W709	⚠	Sample concentration outside recommended range
A11	3.9	19.6	W710		
B11	5.3	37.7	W711		
C11	6.0	92.1	W712		
D11	6.0	73.3	W713		
E11	5.8	23.6	W714		
F11	5.6	51.2	W715		
G11	5.4	76.8	W716		
H11	5.7	116	W717	⚠	Sample concentration outside recommended range
A12	7.0	57.0	W718		
B12	7.2	106	W719	⚠	Sample concentration outside recommended range
C12	6.9	81.6	W720		
D12	-	1.04	buffer	⚠	Sample concentration outside functional range for DIN

© Agilent Technologies, Inc. 2017

A1: Ladder

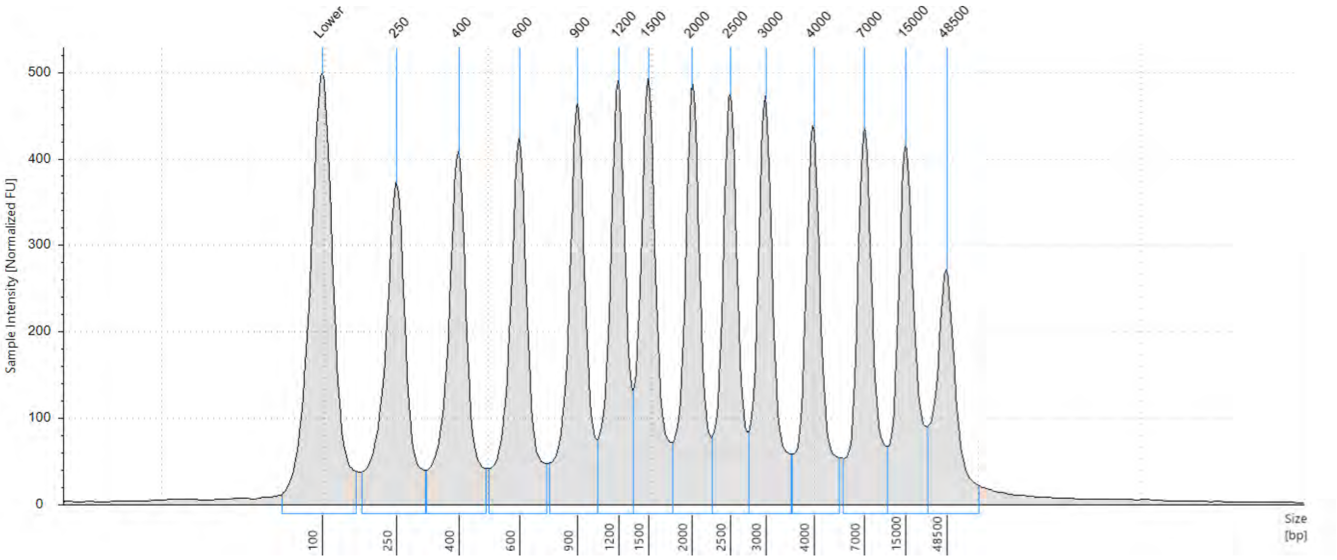

Sample Table

Well	DIN	Conc. [ng/µl]	Sample Description	Alert	Observations
A1	-	71.8	Ladder		Ladder

Peak Table

Size [bp]	Calibrated Conc. [ng/µl]	Assigned Conc. [ng/µl]	% Integrated Area	From [bp]	To [bp]	Peak Comment	Observations
100	8.50	8.50	-	62	151		Lower Marker
250	5.70	-	8.12	162	310		
400	5.71	-	8.14	313	478		
600	5.72	-	8.15	488	724		
900	5.81	-	8.27	738	1034		
1200	5.66	-	8.07	1034	1335		
1500	5.88	-	8.37	1335	1756		
2000	5.58	-	7.96	1756	2245		
2500	5.39	-	7.68	2245	2749		
3000	5.45	-	7.76	2749	3494		
4000	5.18	-	7.38	3523	5292		
7000	5.11	-	7.28	5544	10659		
15000	5.18	-	7.38	10659	22849		
48500	3.81	-	5.43	22849	>60000		

© Agilent Technologies, Inc. 2017

B1: M1

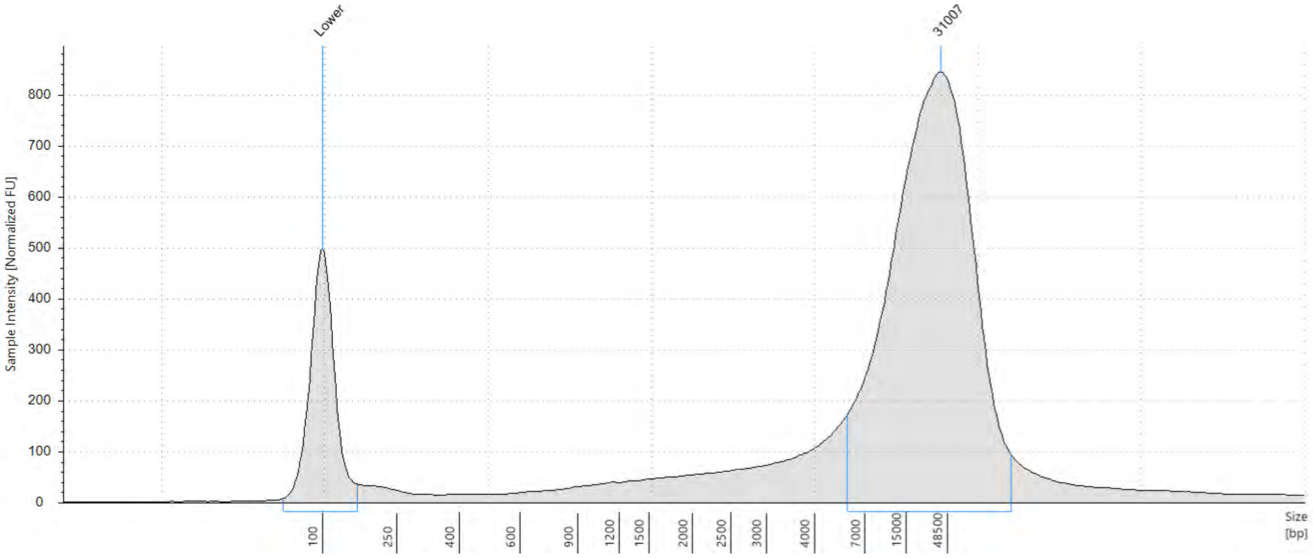

Sample Table

Well	DIN	Conc. [ng/µl]	Sample Description	Alert	Observations
B1	7.7	67.9	M1		

Peak Table

Size [bp]	Calibrated Conc. [ng/µl]	Assigned Conc. [ng/µl]	% Integrated Area	From [bp]	To [bp]	Peak Comment	Observations
100	8.50	8.50	-	62	153		Lower Marker
31007	49.2	-	97.83	5783	>60000		
-	-	-	-	-	-		Sample Well

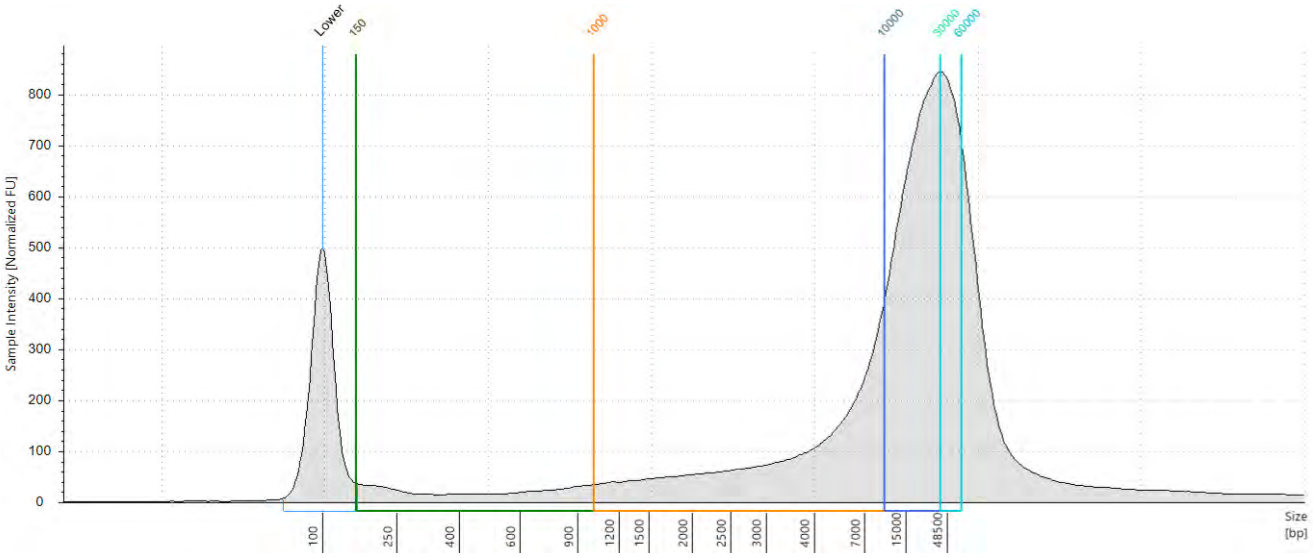

Region Table

From [bp]	To [bp]	Average Size [bp]	Conc. [ng/µl]	Region Molarity [nmol/l]	% of Total	Region Comment	Color
150	10000	4169	19.0	21.9	28.01		
150	1000	504	2.63	14.0	3.88		
1000	10000	4860	16.4	7.93	24.18		
10000	60000	28787	33.6	2.39	49.53		
10000	30000	19322	23.5	2.07	34.57		

© Agilent Technologies, Inc. 2017

30000	60000	50371	10.9	0.357	16.02		

 © Agilent Technologies, Inc. 2017

C1: M2

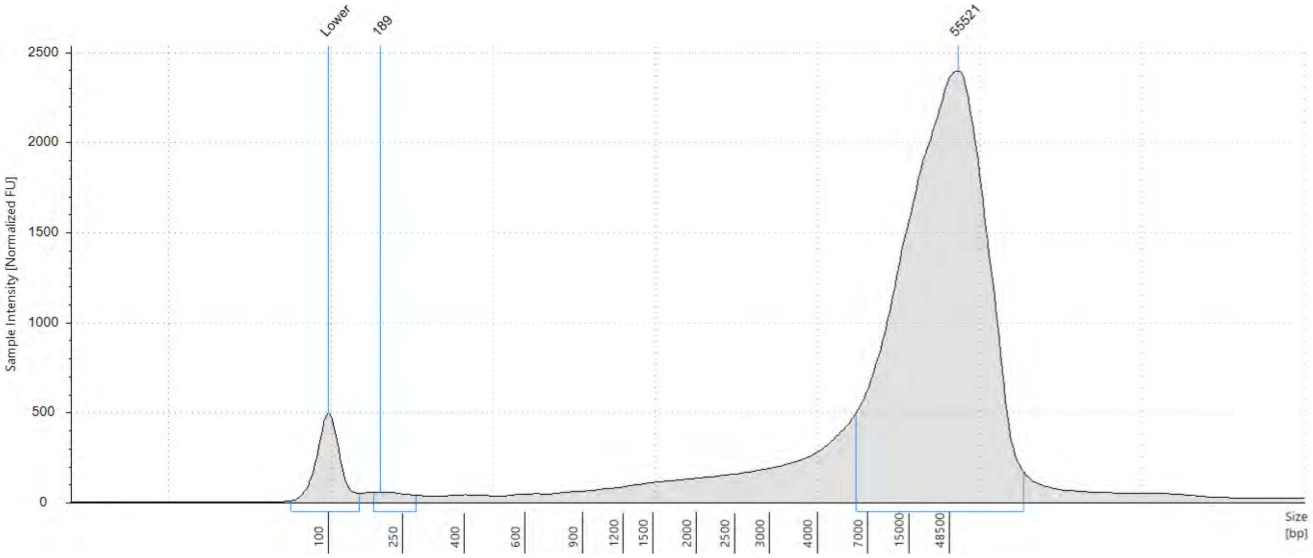

Sample Table

Well	DIN	Conc. [ng/µl]	Sample Description	Alert	Observations
C1	7.8	201	M2	⚠	Sample concentration outside recommended range

Peak Table

Size [bp]	Calibrated Conc. [ng/µl]	Assigned Conc. [ng/µl]	% Integrated Area	From [bp]	To [bp]	Peak Comment	Observations
100	8.50	8.50	-	63	146		Lower Marker
189	1.32	-	0.83	173	277		
55521	154	-	97.04	6154	>60000		
-	-	-	-	-	-		Sample Well

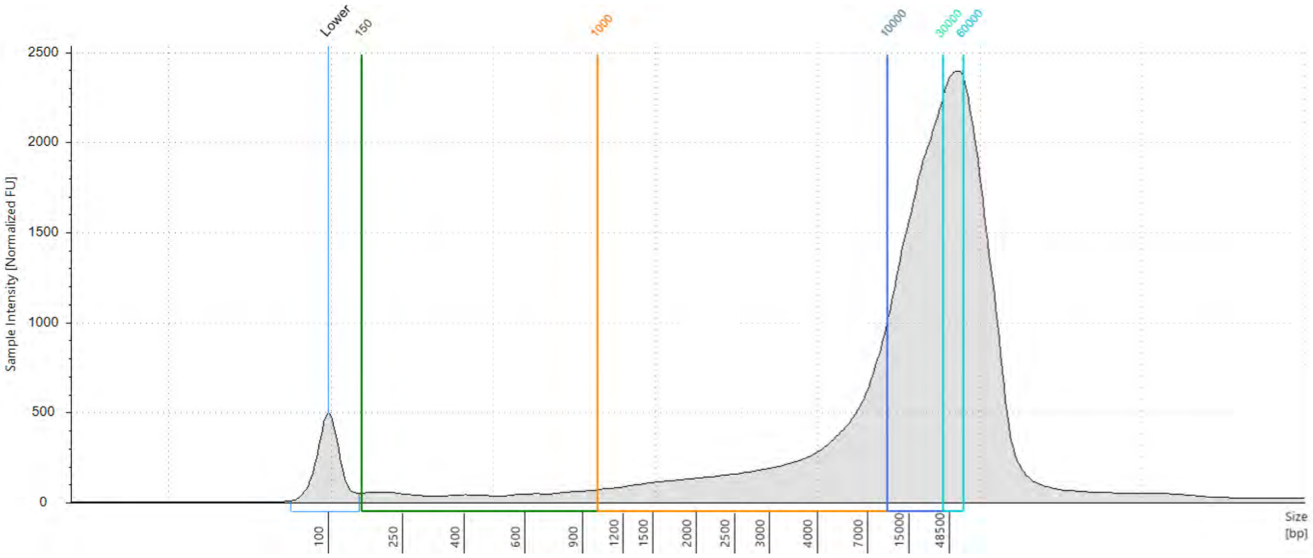

Region Table

From [bp]	To [bp]	Average Size [bp]	Conc. [ng/µl]	Region Molarity [nmol/l]	% of Total	Region Comment	Color
150	10000	4329	51.5	50.8	25.63		
150	1000	512	6.36	30.0	3.16		
1000	10000	4930	45.2	20.9	22.49		
10000	60000	29859	93.3	6.44	46.44		

 © Agilent Technologies, Inc. 2017

10000	30000	19608	63.1	5.50	31.42		
30000	60000	51001	32.2	1.04	16.05		

© Agilent Technologies, Inc. 2017

D1: M3

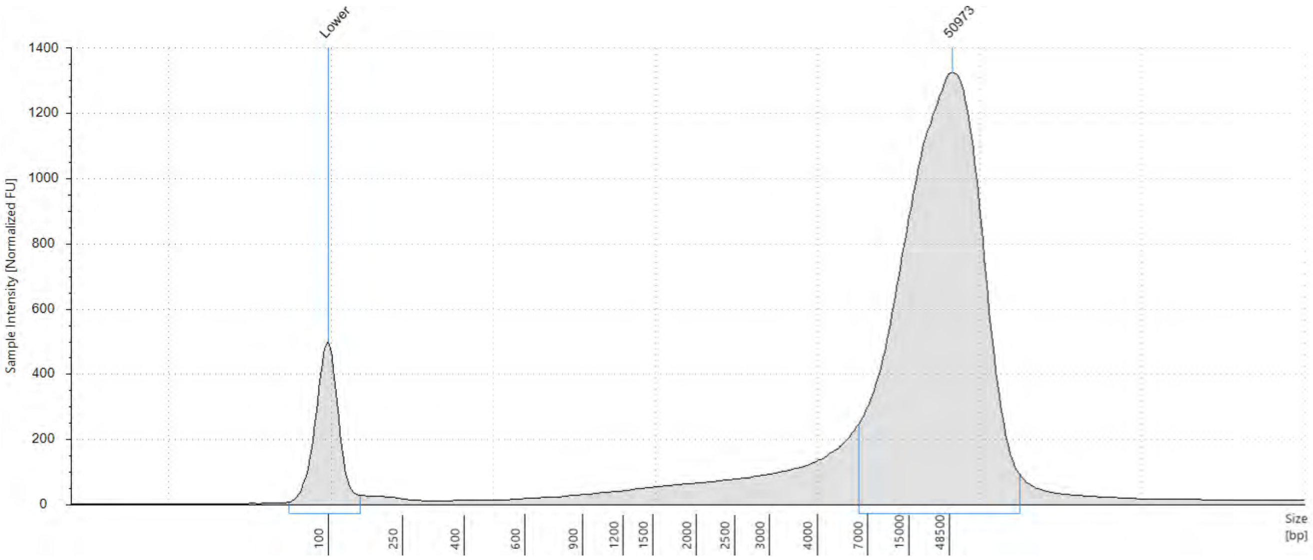

Sample Table

Well	DIN	Conc. [ng/µl]	Sample Description	Alert	Observations
D1	8.1	101	M3	⚠	Sample concentration outside recommended range

Peak Table

Size [bp]	Calibrated Conc. [ng/µl]	Assigned Conc. [ng/µl]	% Integrated Area	From [bp]	To [bp]	Peak Comment	Observations
100	8.50	8.50	-	62	148		Lower Marker
50973	79.1	-	98.10	6363	>60000		
-	-	-	-	-	-		Sample Well

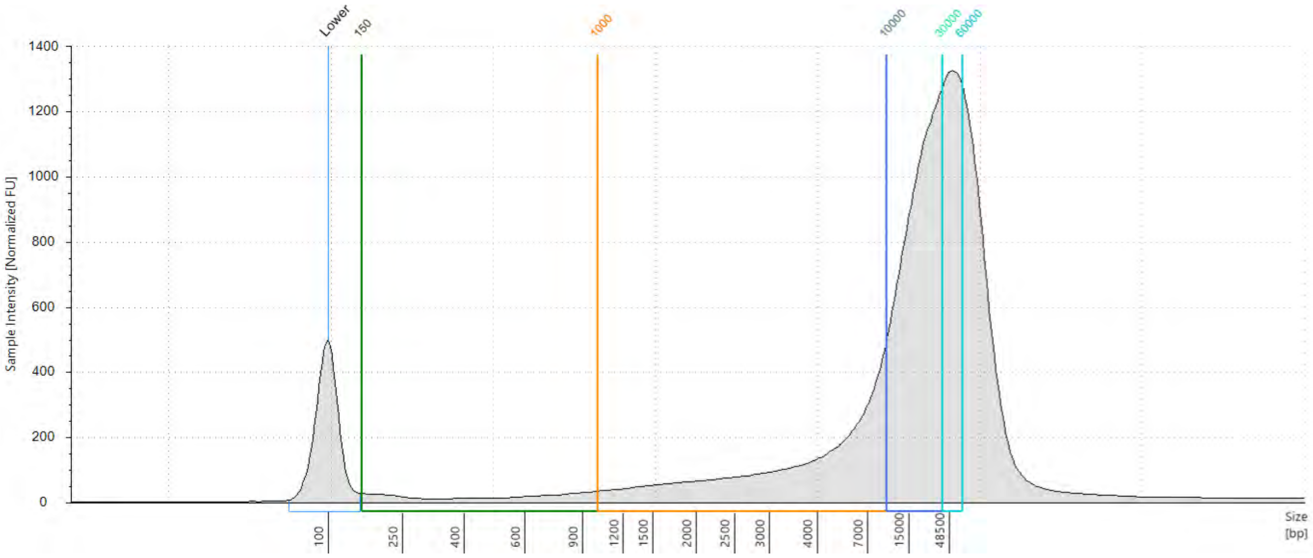

Region Table

From [bp]	To [bp]	Average Size [bp]	Conc. [ng/µl]	Region Molarity [nmol/l]	% of Total	Region Comment	Color
150	10000	4317	23.1	21.4	22.90		
150	1000	530	2.38	11.6	2.36		
1000	10000	4819	20.8	9.80	20.58		
10000	60000	29723	51.4	3.55	50.90		
10000	30000	19266	33.9	2.99	33.56		

 © Agilent Technologies, Inc. 2017

30000	60000	49723	18.6	0.621	18.46		

© Agilent Technologies, Inc. 2017

E1: M4

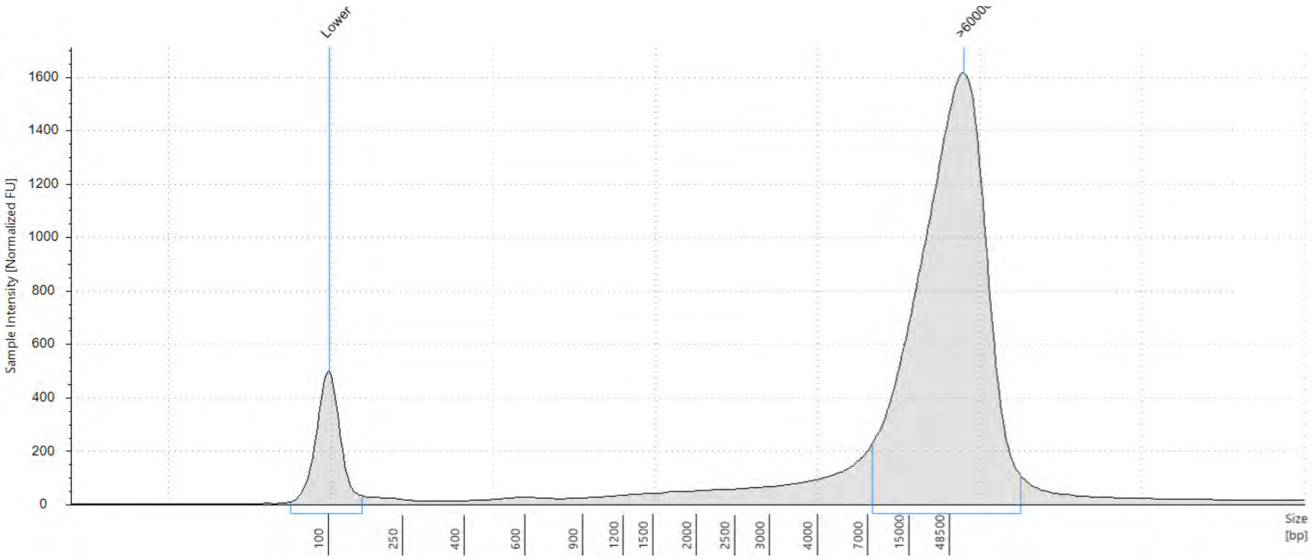

Sample Table

Well	DIN	Conc. [ng/µl]	Sample Description	Alert	Observations
E1	8.5	97.2	M4		

Peak Table

Size [bp]	Calibrated Conc. [ng/µl]	Assigned Conc. [ng/µl]	% Integrated Area	From [bp]	To [bp]	Peak Comment	Observations
100	8.50	8.50	-	62	150		Lower Marker
>60000	77.6	-	98.33	7618	>60000		
-	-	-	-	-	-		Sample Well

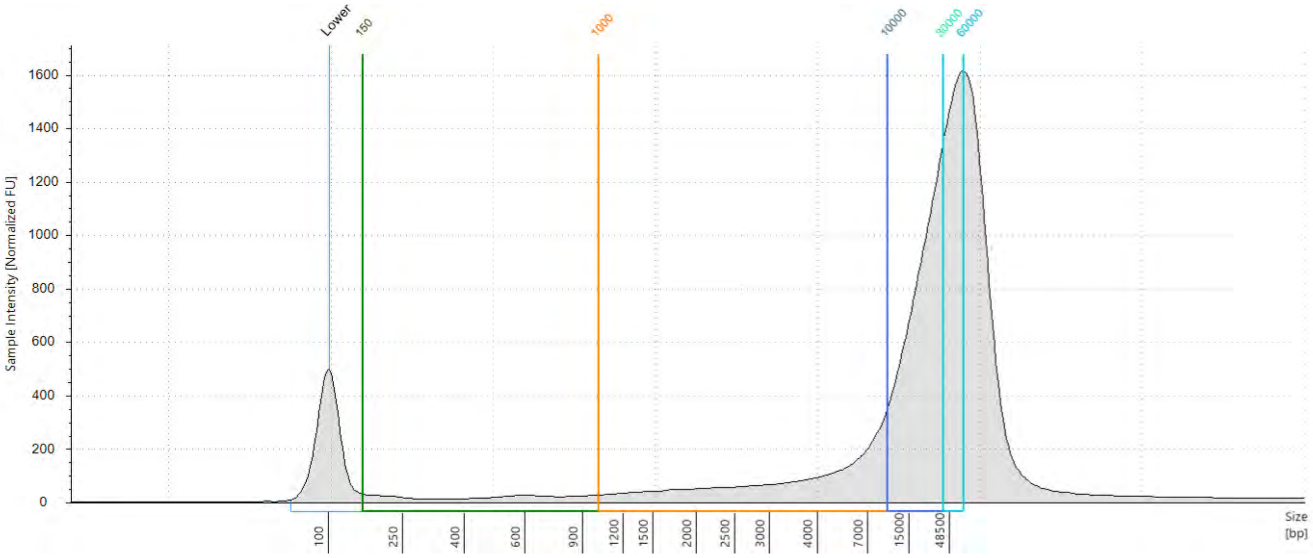

Region Table

From [bp]	To [bp]	Average Size [bp]	Conc. [ng/µl]	Region Molarity [nmol/l]	% of Total	Region Comment	Color
150	10000	4050	16.6	19.3	17.08		
150	1000	512	2.37	12.2	2.43		
1000	10000	4748	14.3	7.09	14.67		
10000	60000	32457	47.7	3.01	49.09		
10000	30000	20015	28.2	2.39	29.03		

 © Agilent Technologies, Inc. 2017

30000	60000	50313	20.6	0.677	21.24		

© Agilent Technologies, Inc. 2017

F1: M5

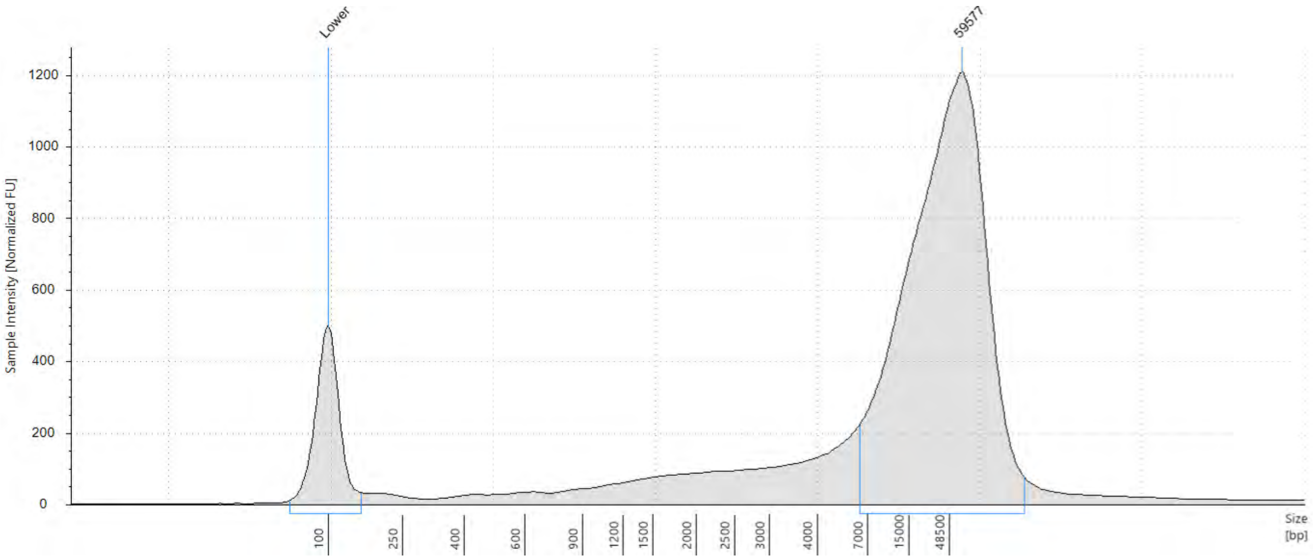

Sample Table

Well	DIN	Conc. [ng/µl]	Sample Description	Alert	Observations
F1	7.7	91.1	M5		

Peak Table

Size [bp]	Calibrated Conc. [ng/µl]	Assigned Conc. [ng/µl]	% Integrated Area	From [bp]	To [bp]	Peak Comment	Observations
100	8.50	8.50	-	62	150		Lower Marker
59577	66.7	-	98.40	6455	>60000		
-	-	-	-	-	-		Sample Well

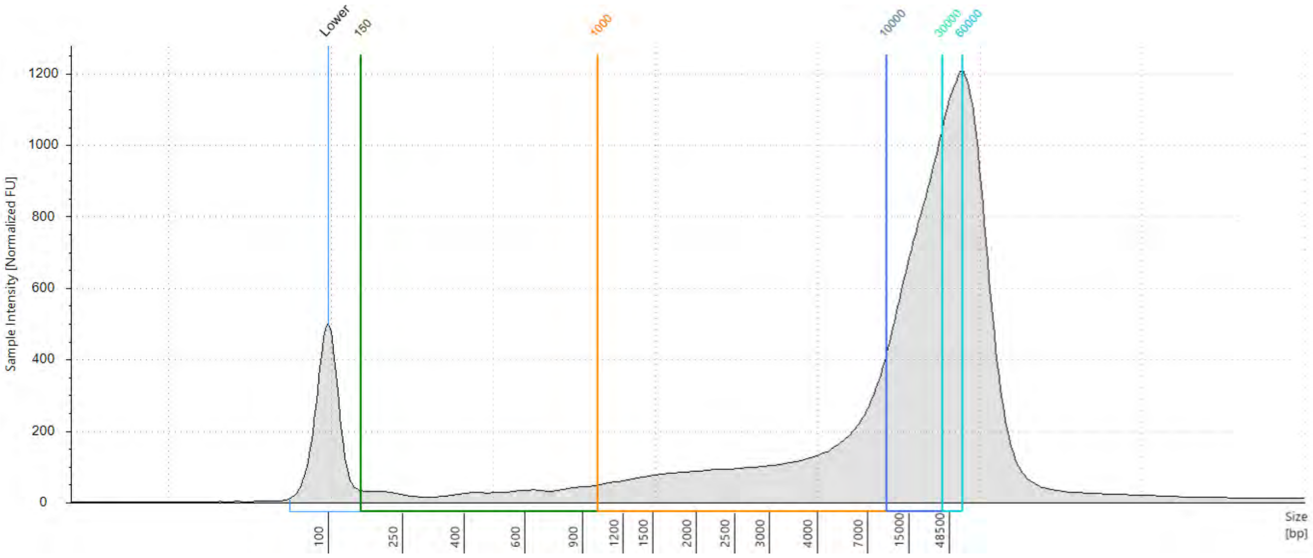

Region Table

From [bp]	To [bp]	Average Size [bp]	Conc. [ng/µl]	Region Molarity [nmol/l]	% of Total	Region Comment	Color
150	10000	3827	25.1	27.3	27.55		
150	1000	542	3.80	16.2	4.17		
1000	10000	4461	21.4	11.2	23.43		
10000	60000	31190	40.5	2.70	44.44		
10000	30000	19626	25.8	2.24	28.25		

 © Agilent Technologies, Inc. 2017

30000	60000	51129	15.6	0.503	17.16		

 © Agilent Technologies, Inc. 2017

G1: M6

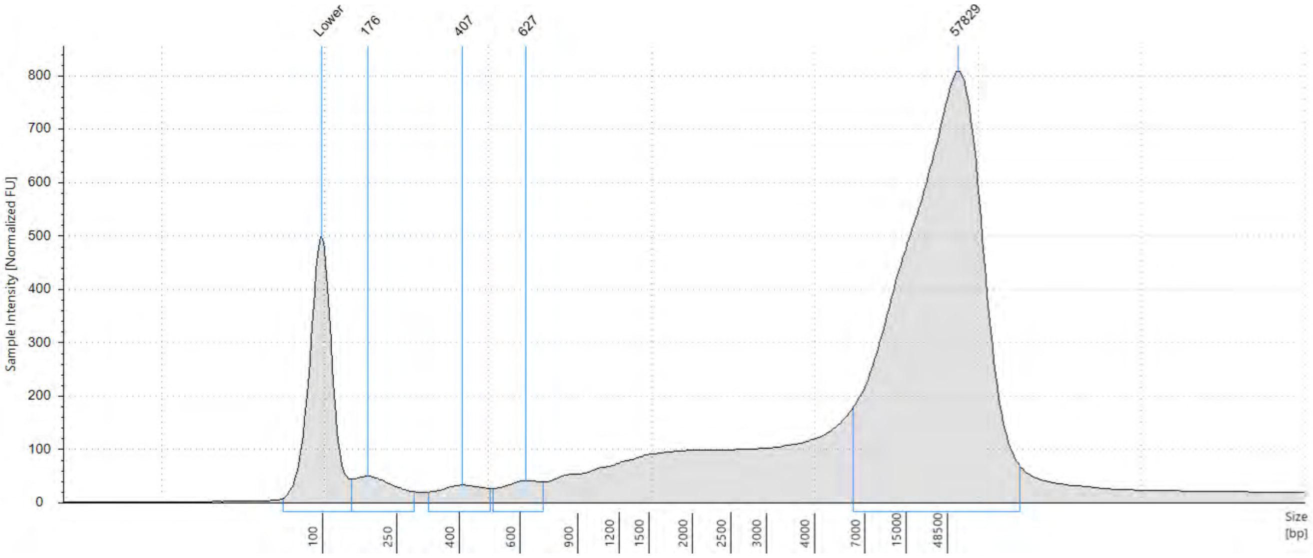

Sample Table

Well	DIN	Conc. [ng/µl]	Sample Description	Alert	Observations
Gl	7.1	76.7	M6		

Peak Table

Size [bp]	Calibrated Conc. [ng/µl]	Assigned Conc. [ng/µl]	% Integrated Area	From [bp]	To [bp]	Peak Comment	Observations
100	8.50	8.50	-	62	143		Lower Marker
176	1.45	-	2.72	143	285		
407	1.02	-	1.91	317	491		
627	1.11	-	2.07	500	705		
57829	48.5	-	90.71	6171	>60000		
-	-	-	-	-	-		Sample Well

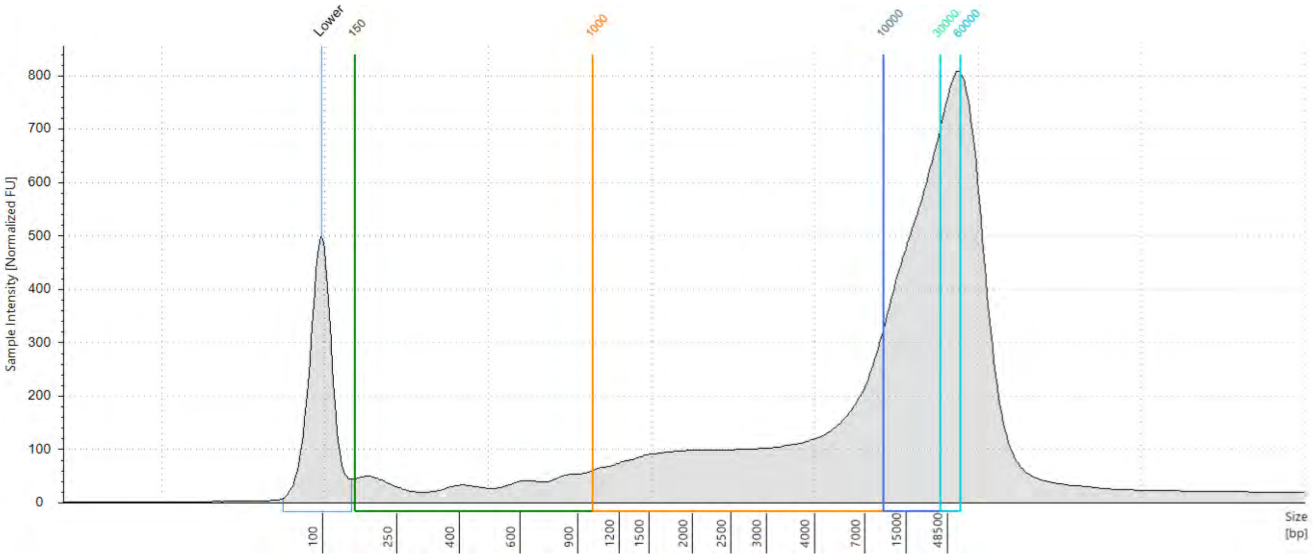

Region Table

From [bp]	To [bp]	Average Size [bp]	Conc. [ng/µl]	Region Molarity [nmol/l]	% of Total	Region Comment	Color
150	10000	3348	26.6	34.6	34.67		
150	1000	529	5.16	22.4	6.73		

1000	10000	4057	21.5	12.3	28.02		
10000	60000	30221	29.3	2.03	38.16		
10000	30000	19032	18.8	1.69	24.53		
30000	60000	50068	11.1	0.367	14.45		

© Agilent Technologies, Inc. 2017

H1: M7

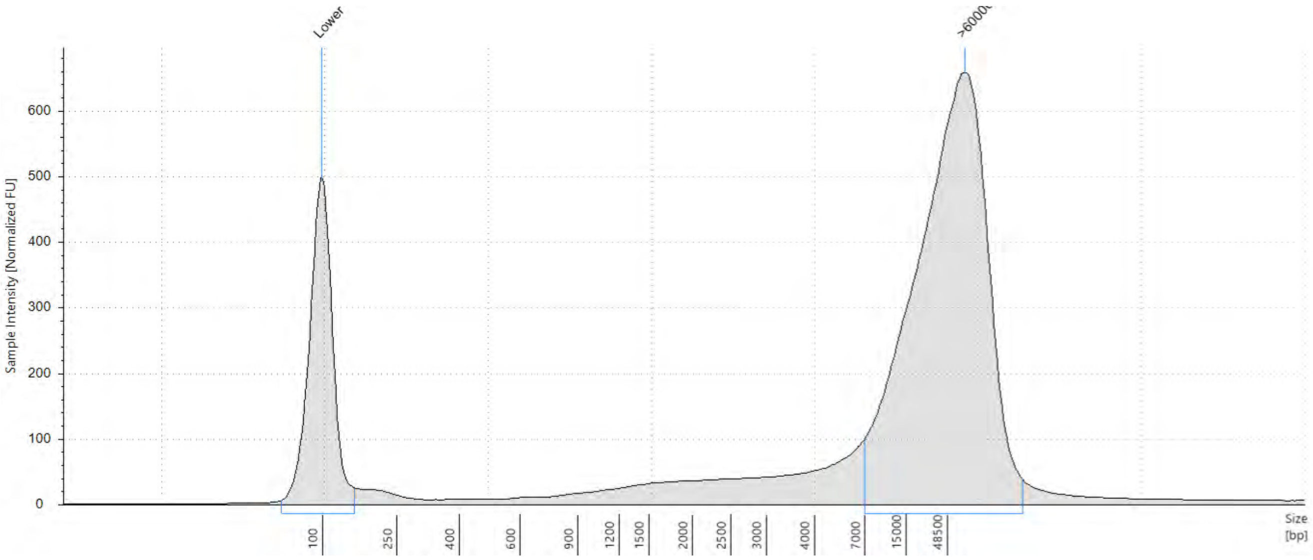

Sample Table

Well	DIN	Conc. [ng/µl]	Sample Description	Alert	Observations
H1	8.2	46.0	M7		

Peak Table

Size [bp]	Calibrated Conc. [ng/µl]	Assigned Conc. [ng/µl]	% Integrated Area	From [bp]	To [bp]	Peak Comment	Observations
100	8.50	8.50	-	61	148		Lower Marker
>60000	35.2	-	97.07	6978	>60000		
-	-	-	-	-	-		Sample Well

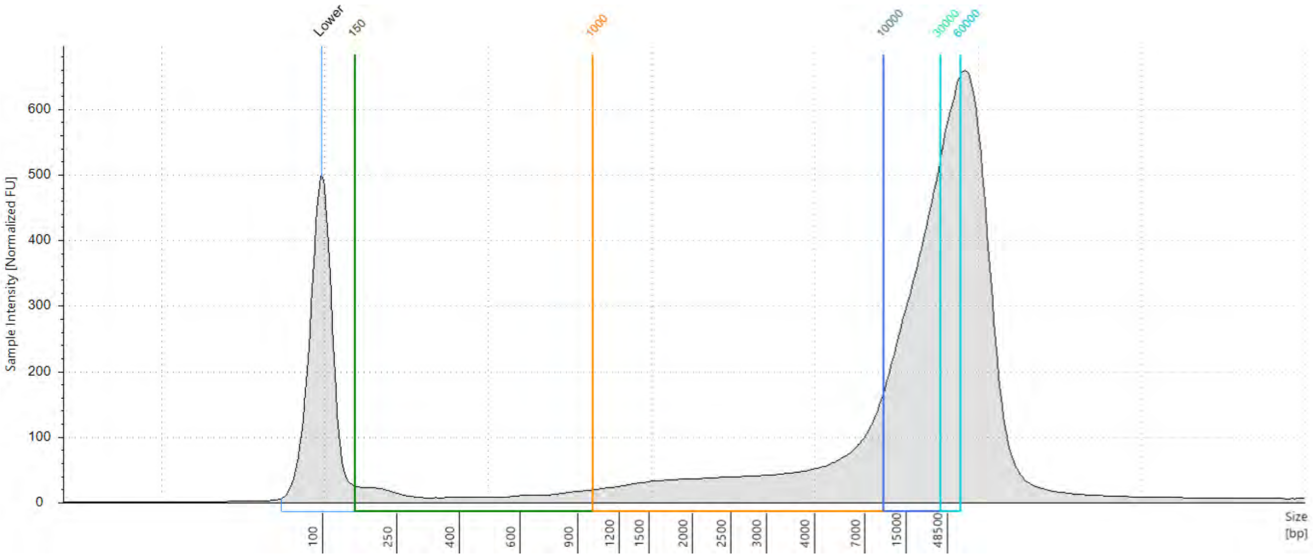

Region Table

From [bp]	To [bp]	Average Size [bp]	Conc. [ng/µl]	Region Molarity [nmol/l]	% of Total	Region Comment	Color
150	10000	3674	10.2	13.4	22.07		
150	1000	485	1.56	8.69	3.39		
1000	10000	4339	8.62	4.71	18.72		
10000	60000	31826	20.2	1.32	43.93		
10000	30000	19564	12.2	1.06	26.47		

 © Agilent Technologies, Inc. 2017

30000	60000	50240	8.50	0.280	18.47		

© Agilent Technologies, Inc. 2017

A2: M8

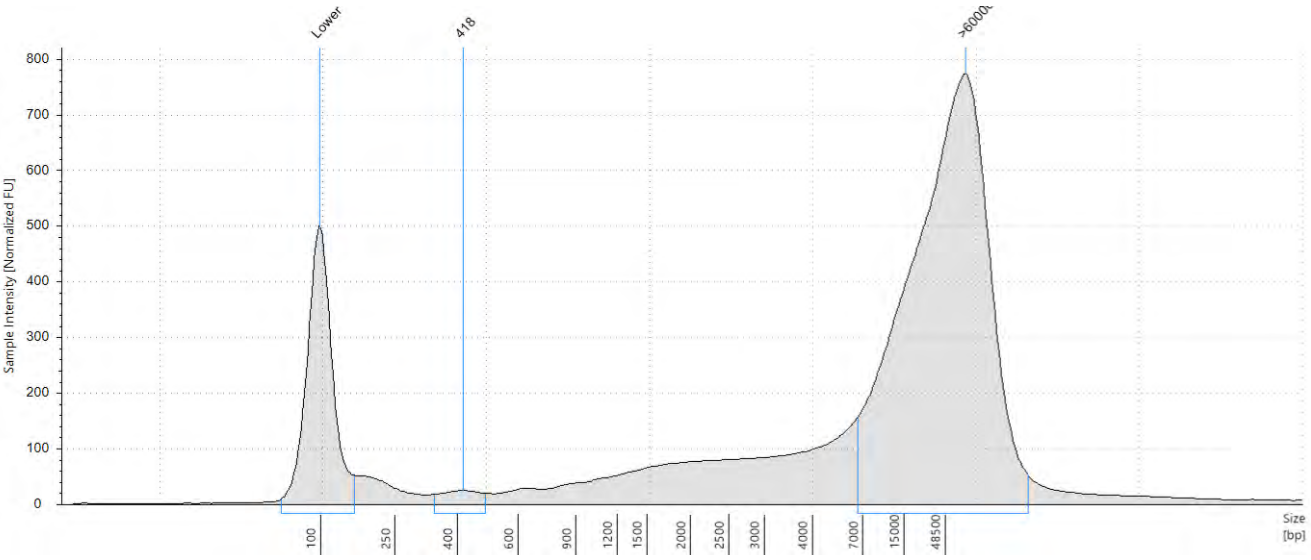

Sample Table

Well	DIN	Conc. [ng/µl]	Sample Description	Alert	Observations
A2	7.4	60.3	M8		

Peak Table

Size [bp]	Calibrated Conc. [ng/µl]	Assigned Conc. [ng/µl]	% Integrated Area	From [bp]	To [bp]	Peak Comment	Observations
100	8.50	8.50	-	62	153		Lower Marker
418	0.595	-	1.39	337	484		
>60000	41.5	-	97.14	6664	>60000		
-	-	-	-	-	-		Sample Well

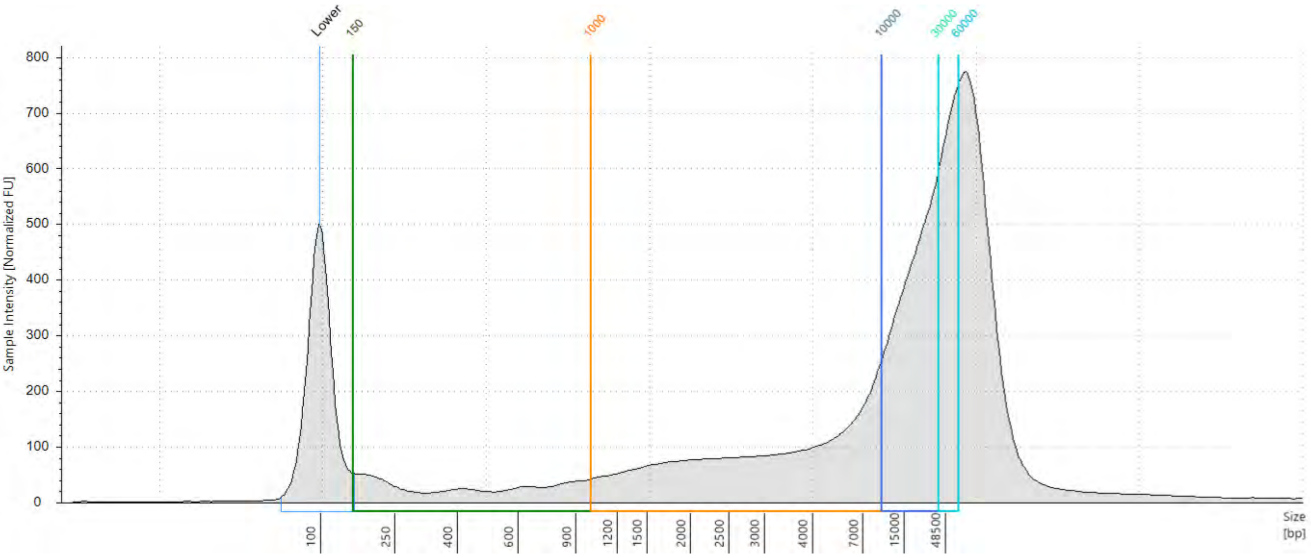

Region Table

From [bp]	To [bp]	Average Size [bp]	Conc. [ng/µl]	Region Molarity [nmol/l]	% of Total	Region Comment	Color
150	10000	3421	19.0	26.3	31.56		
150	1000	495	3.62	17.8	6.01		
1000	10000	4158	15.4	8.64	25.61		
10000	60000	31109	22.3	1.49	36.96		

 © Agilent Technologies, Inc. 2017

10000	30000	19367	14.0	1.23	23.17		
30000	60000	50588	8.79	0.287	14.59		

© Agilent Technologies, Inc. 2017

B2: M9

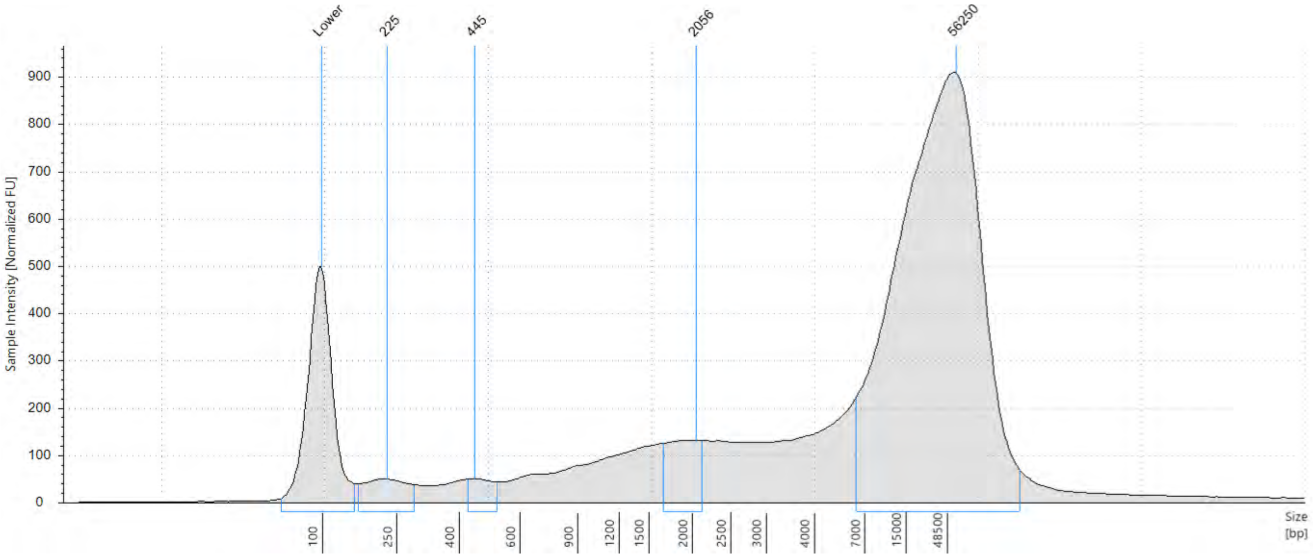

Sample Table

Well	DIN	Conc. [ng/µl]	Sample Description	Alert	Observations
B2	6.9	84.1	M9		

Peak Table

Size [bp]	Calibrated Conc. [ng/µl]	Assigned Conc. [ng/µl]	% Integrated Area	From [bp]	To [bp]	Peak Comment	Observations
100	8.50	8.50	-	61	150		Lower Marker
225	1.42	-	2.39	158	285		
445	0.826	-	1.39	425	515		
2056	3.09	-	5.18	1660	2123		
56250	53.0	-	89.01	6424	>60000		
-	-	-	-	-	-		Sample Well

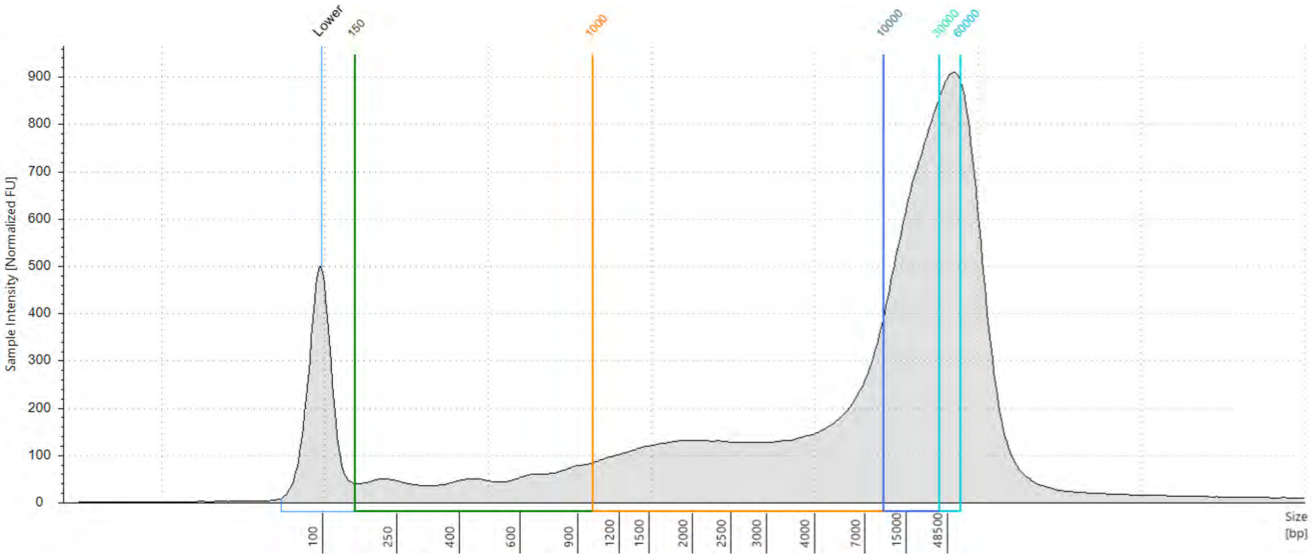

Region Table

From [bp]	To [bp]	Average Size [bp]	Conc. [ng/µl]	Region Molarity [nmol/l]	% of Total	Region Comment	Color
150	10000	3211	32.4	42.7	38.56		
150	1000	542	6.93	27.8	8.24		

1000	10000	3965	25.6	15.1	30.40		
10000	60000	29969	34.1	2.37	40.58		
10000	30000	19401	22.9	2.02	27.18		
30000	60000	51139	12.0	0.385	14.25		

© Agilent Technologies, Inc. 2017

C2: M10

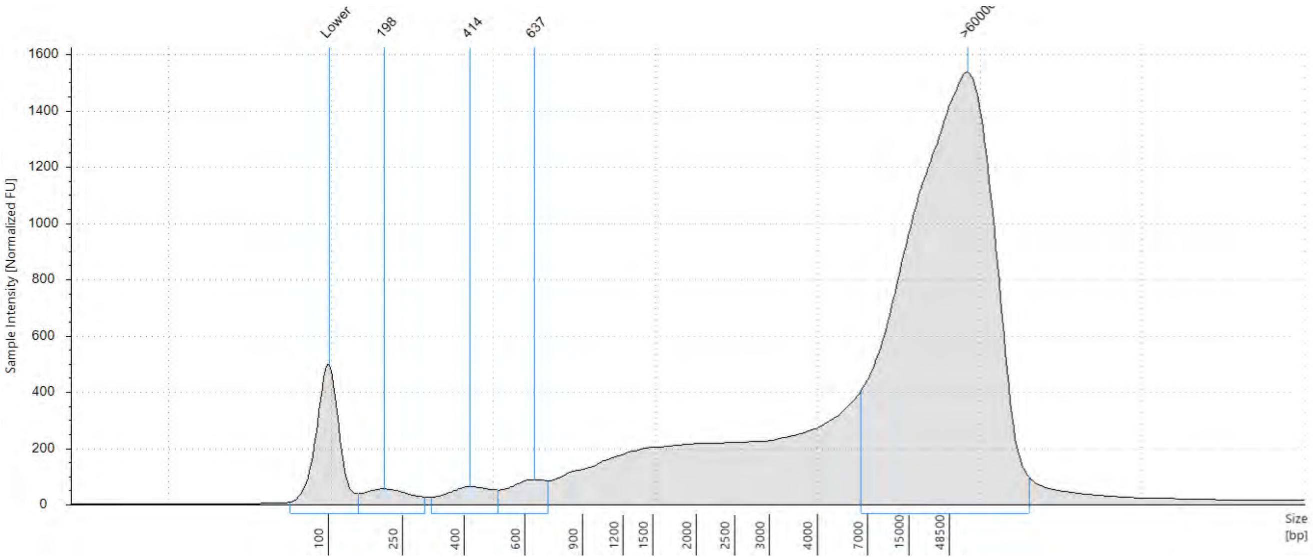

Sample Table

Well	DIN	Conc. [ng/µl]	Sample Description	Alert	Observations
C2	7.0	159	M10	⚠	Sample concentration outside recommended range

Peak Table

Size [bp]	Calibrated Conc. [ng/µl]	Assigned Conc. [ng/µl]	% Integrated Area	From [bp]	To [bp]	Peak Comment	Observations
100	8.50	8.50	-	62	143		Lower Marker
198	1.74	-	1.56	143	294		
414	2.03	-	1.82	310	499		
637	2.37	-	2.12	499	703		
>60000	103	-	92.07	6479	>60000		
-	-	-	-	-	-		Sample Well

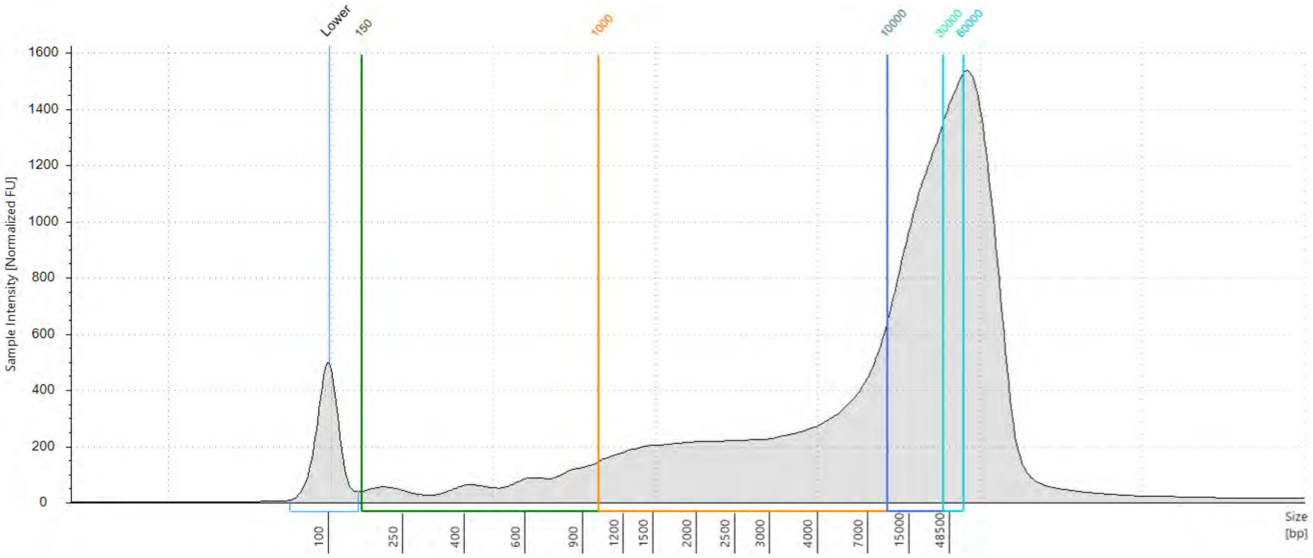

Region Table

From [bp]	To [bp]	Average Size [bp]	Conc. [ng/µl]	Region Molarity [nmol/l]	% of Total	Region Comment	Color
150	10000	3369	58.1	63.8	36.64		green
150	1000	590	9.82	35.9	6.19		red

1000	10000	3963	48.4	28.1	30.53		
10000	60000	29756	58.0	4.05	36.56		
10000	30000	19045	38.1	3.41	24.05		
30000	60000	50049	21.1	0.697	13.29		

© Agilent Technologies, Inc. 2017

D2: M11

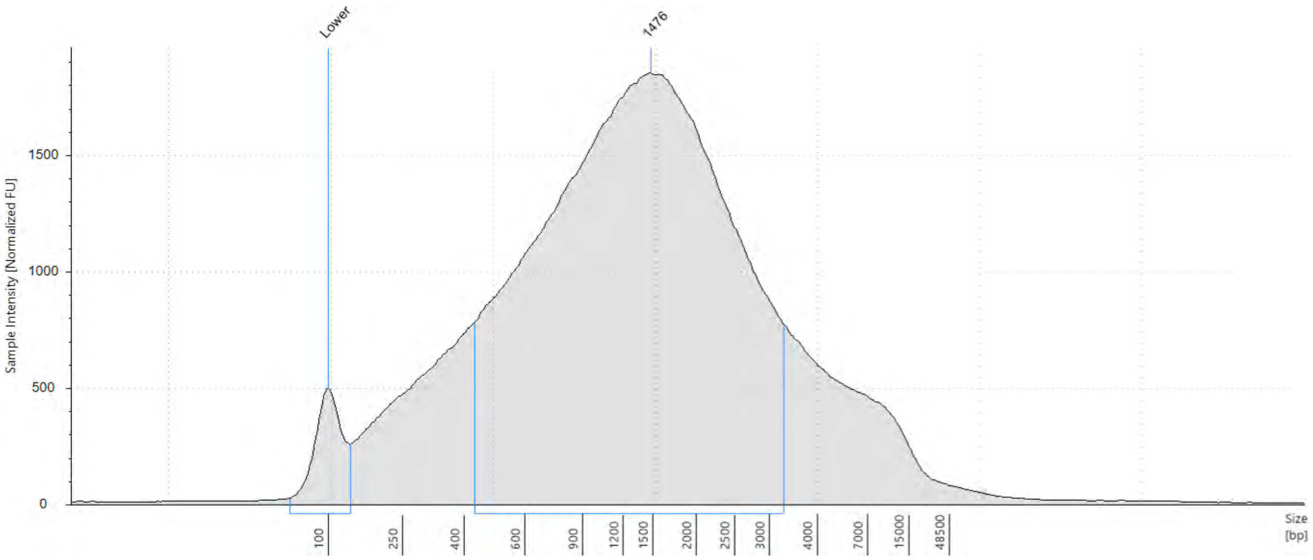

Sample Table

Well	DIN	Conc. [ng/µl]	Sample Description	Alert	Observations
D2	2.9	302	M11	⚠	Sample concentration outside functional range for DIN

Peak Table

Size [bp]	Calibrated Conc. [ng/µl]	Assigned Conc. [ng/µl]	% Integrated Area	From [bp]	To [bp]	Peak Comment	Observations
100	8.50	8.50	-	62	131		Lower Marker
1476	227	-	100.00	429	3266		

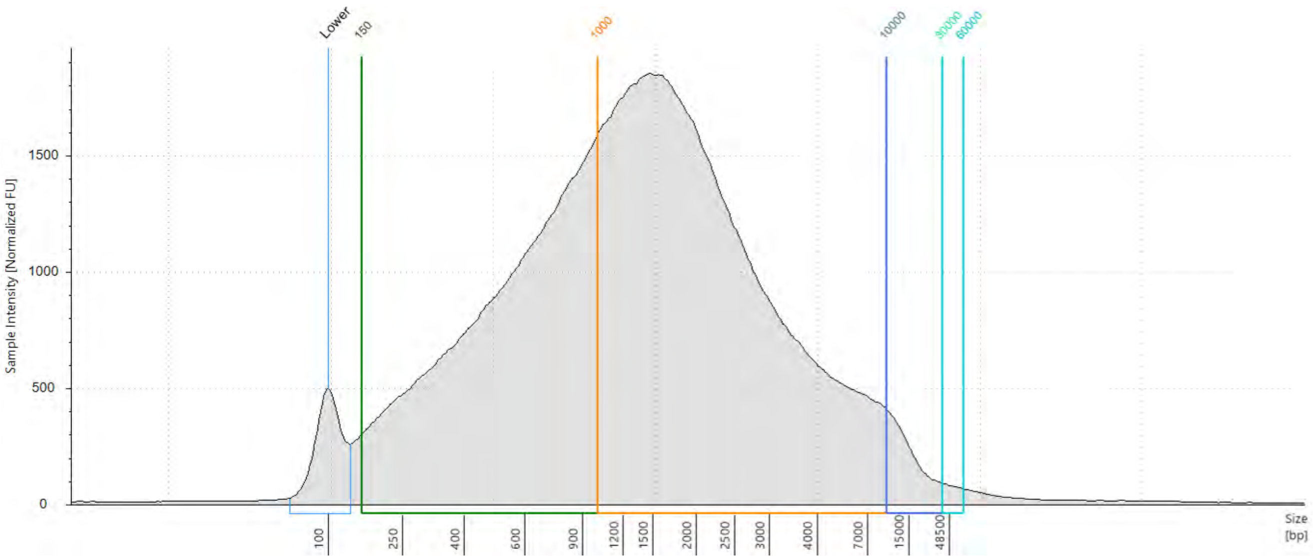

Region Table

From [bp]	To [bp]	Average Size [bp]	Conc. [ng/µl]	Region Molarity [nmol/l]	% of Total	Region Comment	Color
150	10000	1725	291	515	96.54		
150	1000	588	111	366	36.80		
1000	10000	2426	181	150	60.13		
10000	60000	19527	7.43	0.791	2.47		
10000	30000	15555	6.66	0.762	2.21		
30000	60000	49130	0.842	0.0328	0.28		

 © Agilent Technologies, Inc. 2017

© Agilent Technologies, Inc. 2017

E2: M12

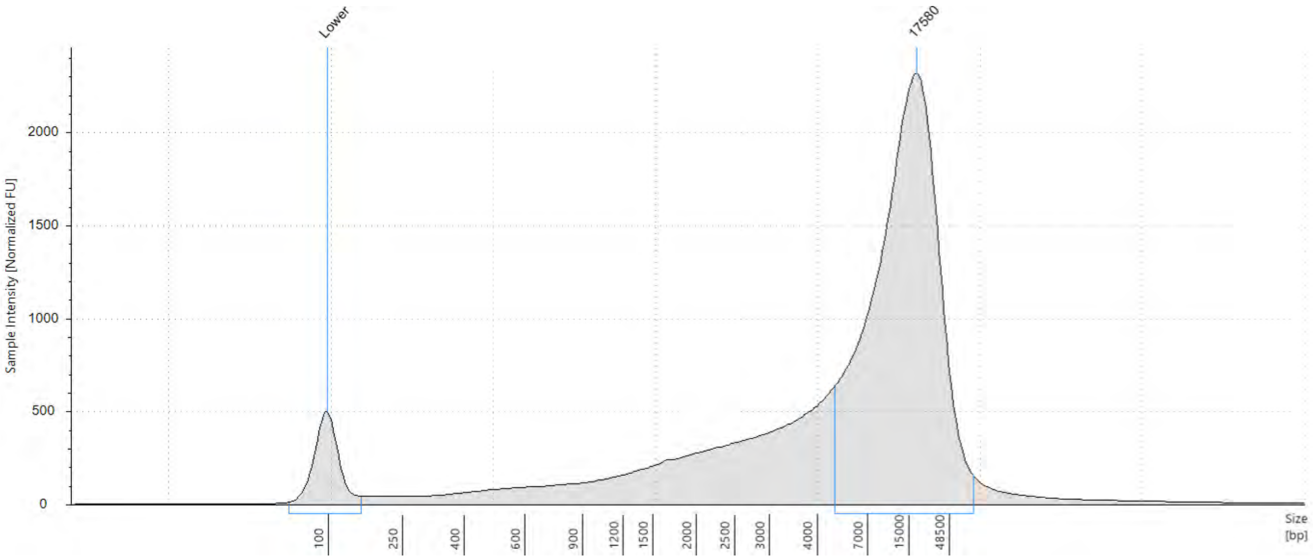

Sample Table

Well	DIN	Conc. [ng/µl]	Sample Description	Alert	Observations
E2	6.6	164	M12	⚠	Sample concentration outside recommended range

Peak Table

Size [bp]	Calibrated Conc. [ng/µl]	Assigned Conc. [ng/µl]	% Integrated Area	From [bp]	To [bp]	Peak Comment	Observations
100	8.50	8.50	-	62	151		Lower Marker
17580	105	-	99.51	4886	>60000		
-	-	-	-	-	-		Sample Well

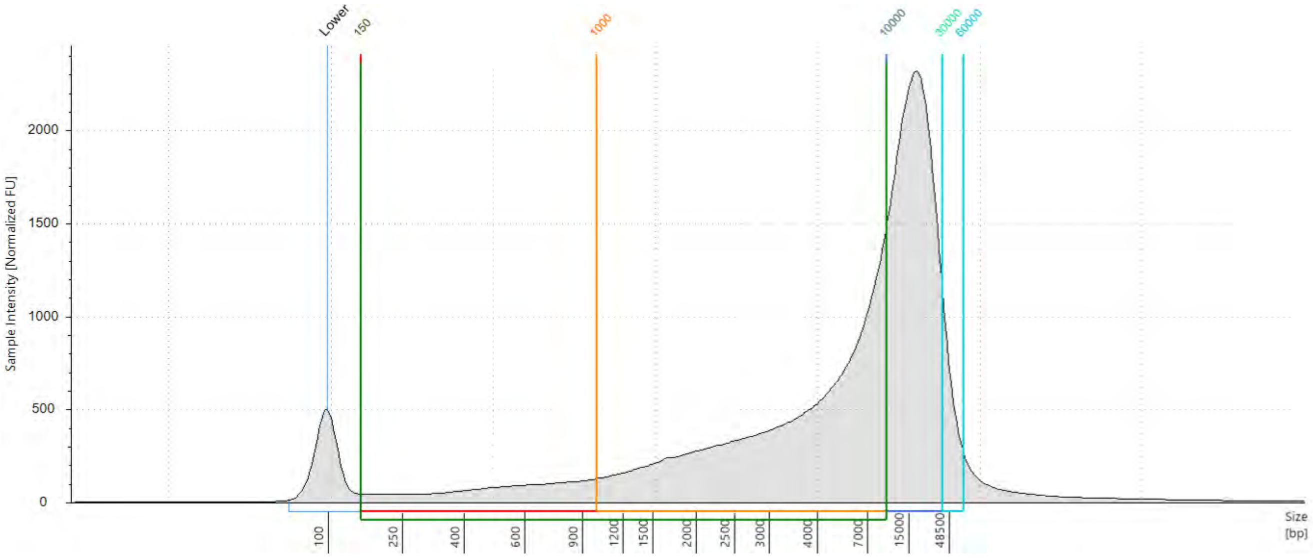

Region Table

From [bp]	To [bp]	Average Size [bp]	Conc. [ng/µl]	Region Molarity [nmol/l]	% of Total	Region Comment	Color
150	10000	4149	85.8	72.5	52.46		(green)
150	1000	573	9.96	36.5	6.09		(red)
1000	10000	4645	76.0	36.2	46.44		(orange)
10000	60000	20910	73.3	6.48	44.83		(blue)
10000	30000	17897	66.0	6.23	40.36		(light green)

30000	60000	47465	8.26	0.295	5.05		

F2: M13

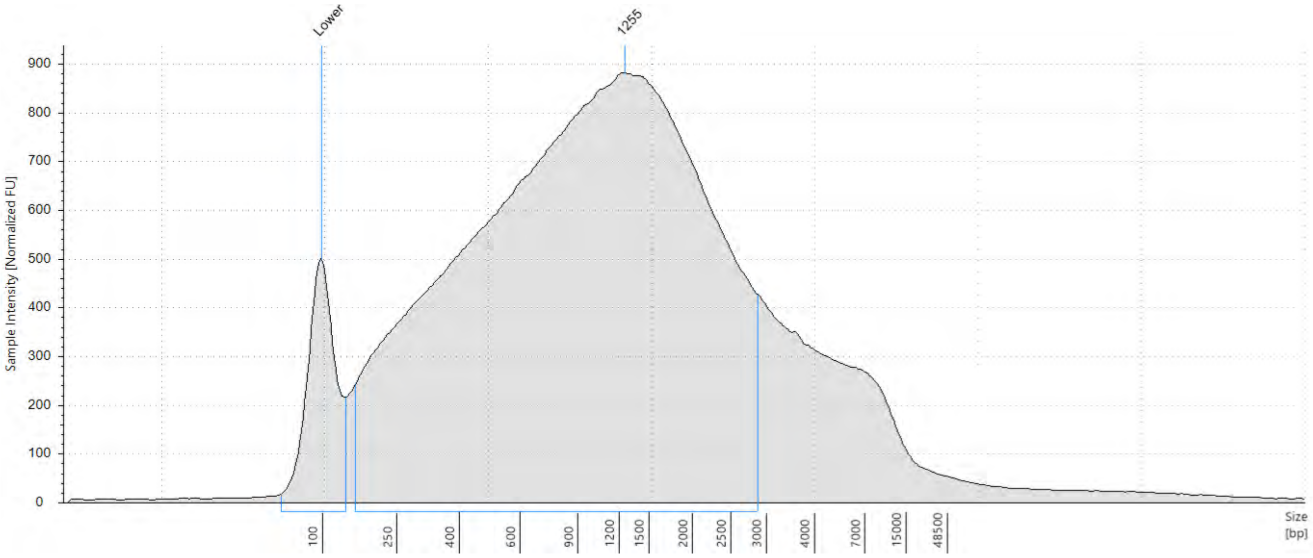

Sample Table

Well	DIN	Conc. [ng/µl]	Sample Description	Alert	Observations
F2	2.6	161	M13	⚠	Sample concentration outside recommended range

Peak Table

Size [bp]	Calibrated Conc. [ng/µl]	Assigned Conc. [ng/µl]	% Integrated Area	From [bp]	To [bp]	Peak Comment	Observations
100	8.50	8.50	-	61	134		Lower Marker
1255	132	-	100.00	151	2871		

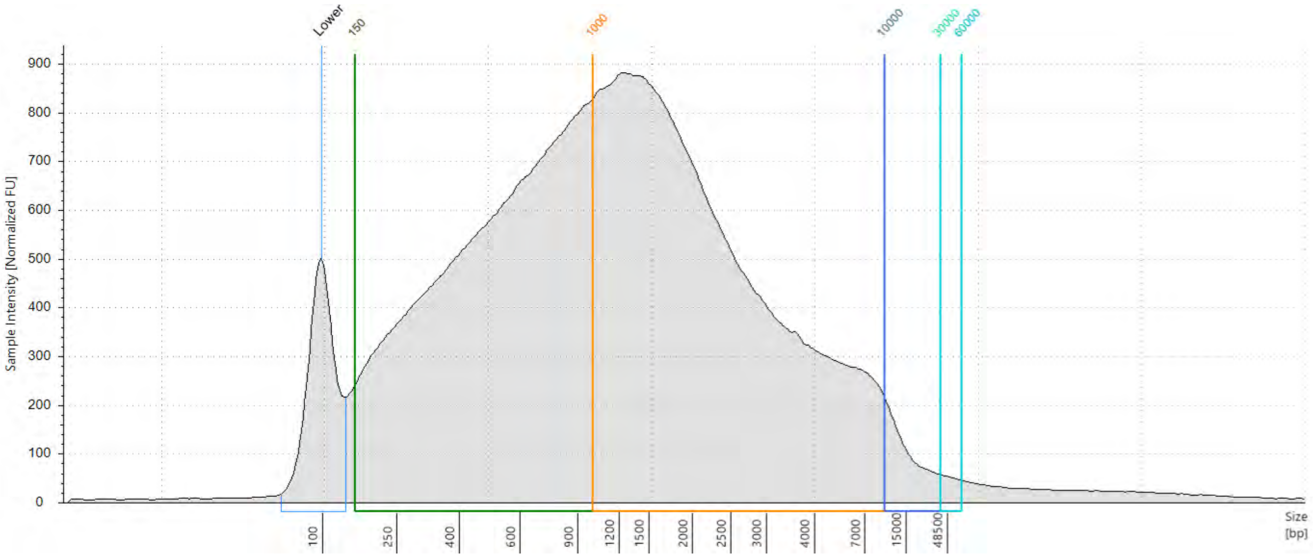

Region Table

From [bp]	To [bp]	Average Size [bp]	Conc. [ng/µl]	Region Molarity [nmol/l]	% of Total	Region Comment	Color
150	10000	1618	153	311	95.13		
150	1000	560	69.4	243	43.25		
1000	10000	2497	83.9	69.2	52.26		
10000	60000	20907	3.72	0.382	2.32		
10000	30000	15829	3.21	0.364	2.00		
30000	60000	49224	0.559	0.0207	0.35		

© Agilent Technologies, Inc. 2017

G2: M14

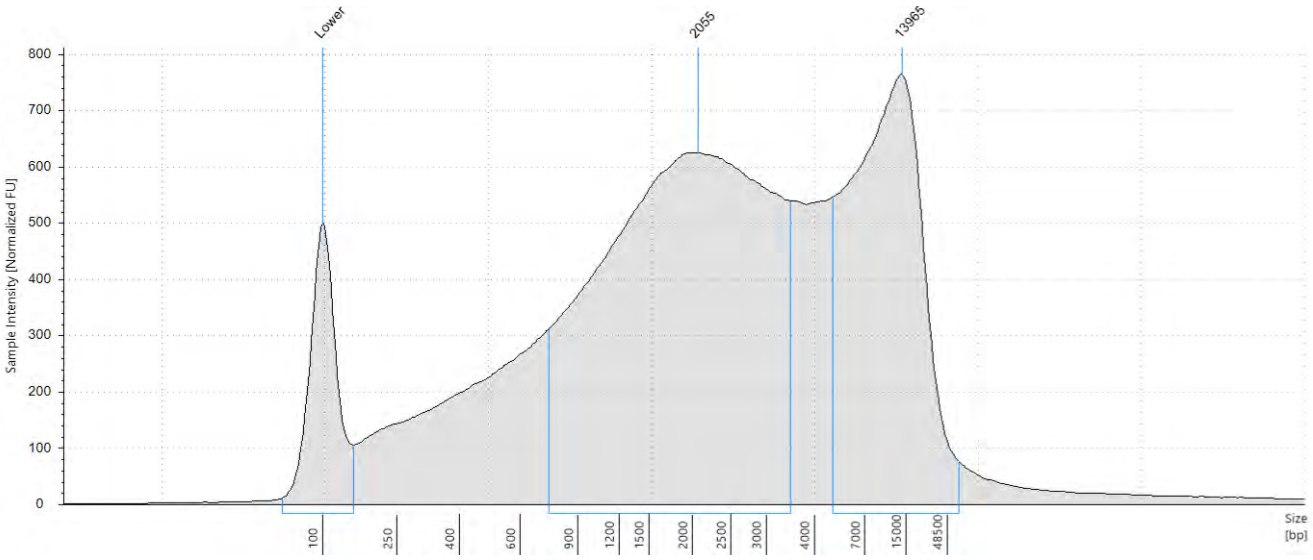

Sample Table

Well	DIN	Conc. [ng/µl]	Sample Description	Alert	Observations
G2	4.4	144	M14	⚠	Sample concentration outside recommended range

Peak Table

Size [bp]	Calibrated Conc. [ng/µl]	Assigned Conc. [ng/µl]	% Integrated Area	From [bp]	To [bp]	Peak Comment	Observations
100	8.50	8.50	-	61	145		Lower Marker
2055	70.5	-	65.77	731	3458		
13965	36.6	-	34.15	4886	57763		
-	-	-	-	-	-		Sample Well

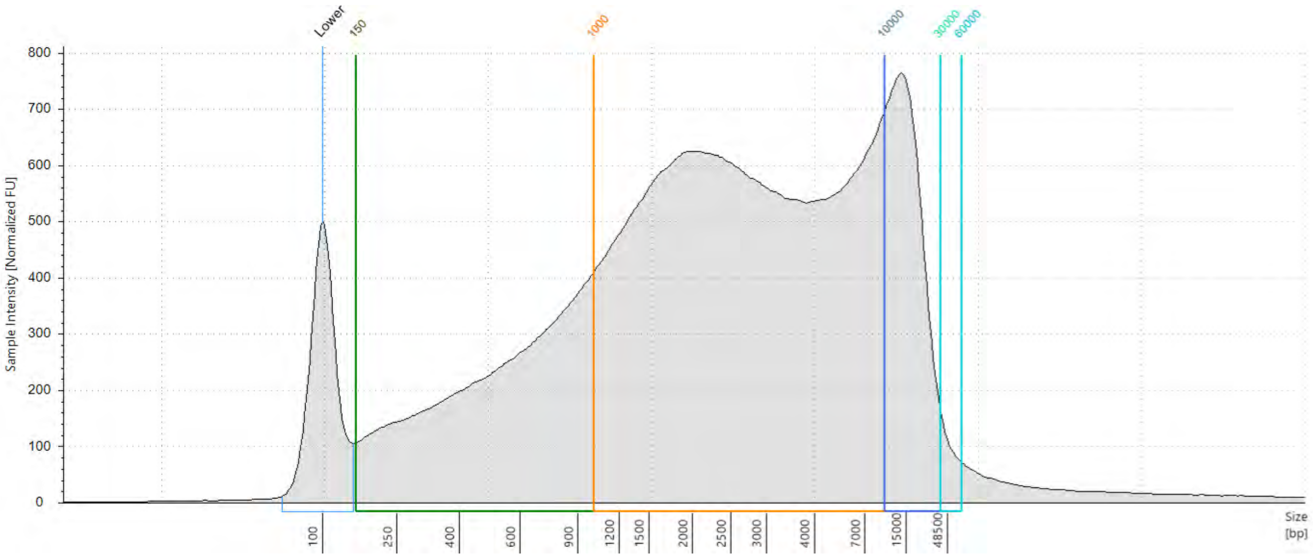

Region Table

From [bp]	To [bp]	Average Size [bp]	Conc. [ng/µl]	Region Molarity [nmol/l]	% of Total	Region Comment	Color
150	10000	2657	122	163	84.61		green
150	1000	575	30.1	104	20.90		red
1000	10000	3343	92.1	60.0	63.95		orange
10000	60000	18271	18.9	1.88	13.14		blue

 © Agilent Technologies, Inc. 2017

10000	30000	16342	17.9	1.85	12.42		
30000	60000	49526	1.17	0.0406	0.81		

© Agilent Technologies, Inc. 2017

H2: M15

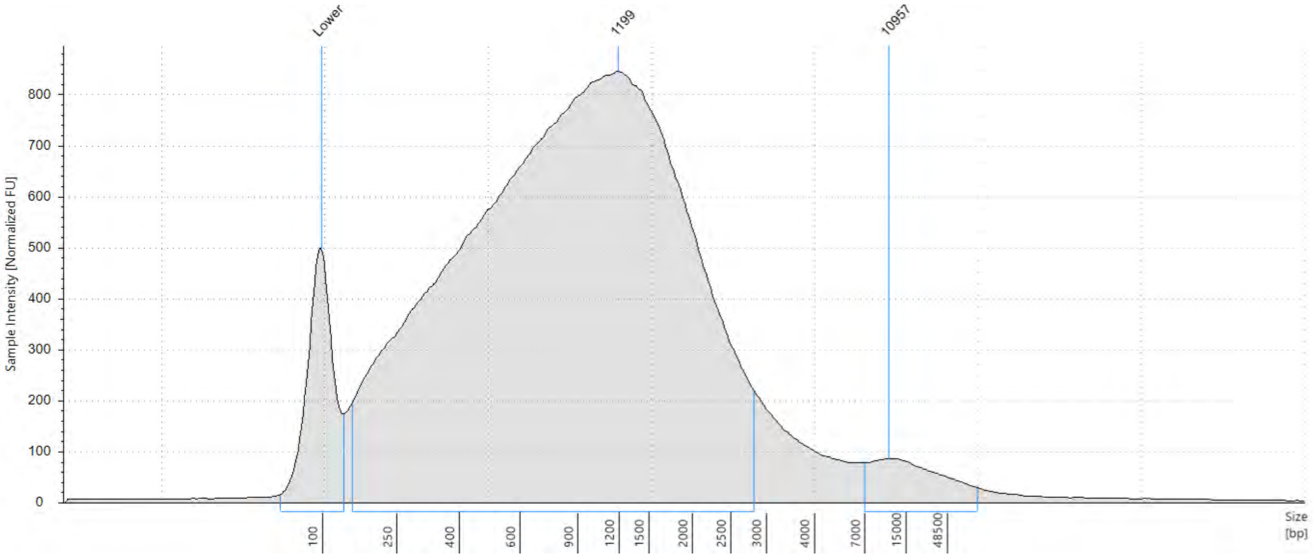

Sample Table

Well	DIN	Conc. [ng/µl]	Sample Description	Alert	Observations
H2	2.1	142	M15	⚠	Sample concentration outside recommended range

Peak Table

Size [bp]	Calibrated Conc. [ng/µl]	Assigned Conc. [ng/µl]	% Integrated Area	From [bp]	To [bp]	Peak Comment	Observations
100	8.50	8.50	-	61	132		Lower Marker
1199	129	-	97.06	146	2830		
10957	3.90	-	2.94	7040	>60000		

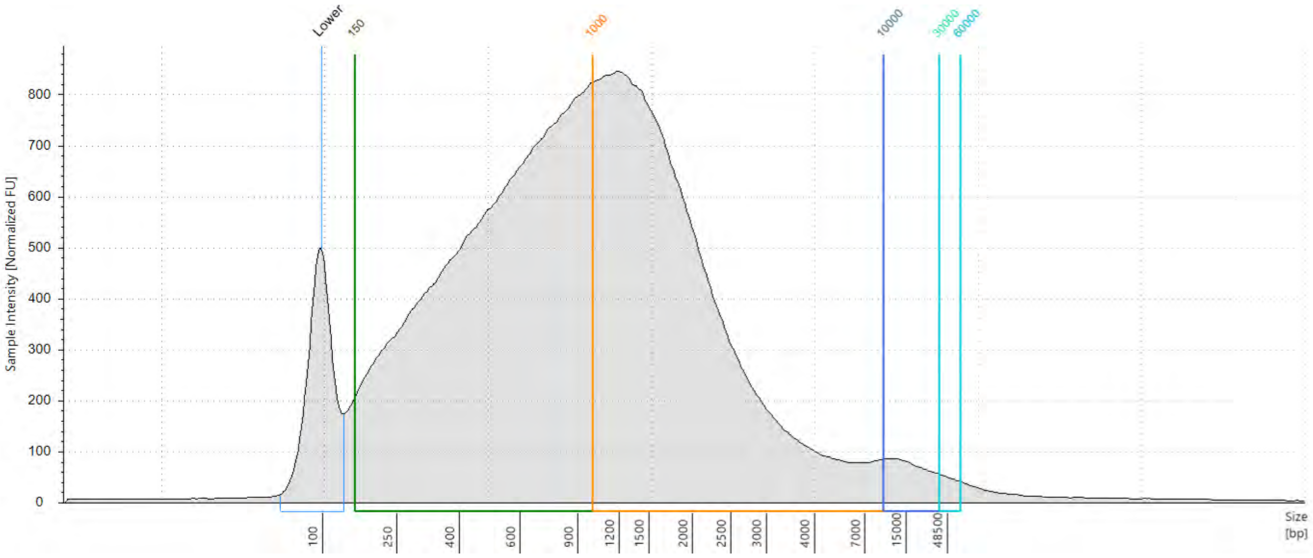

Region Table

From [bp]	To [bp]	Average Size [bp]	Conc. [ng/µl]	Region Molarity [nmol/l]	% of Total	Region Comment	Color
150	10000	1245	137	310	96.39		green
150	1000	566	72.9	250	51.43		red
1000	10000	2016	64.4	61.0	45.43		orange
10000	60000	23676	2.78	0.257	1.97		blue
10000	30000	17390	2.28	0.239	1.61		light green

© Agilent Technologies, Inc. 2017

30000	60000	49934	0.552	0.0202	0.39		

 © Agilent Technologies, Inc. 2017

A3: M16

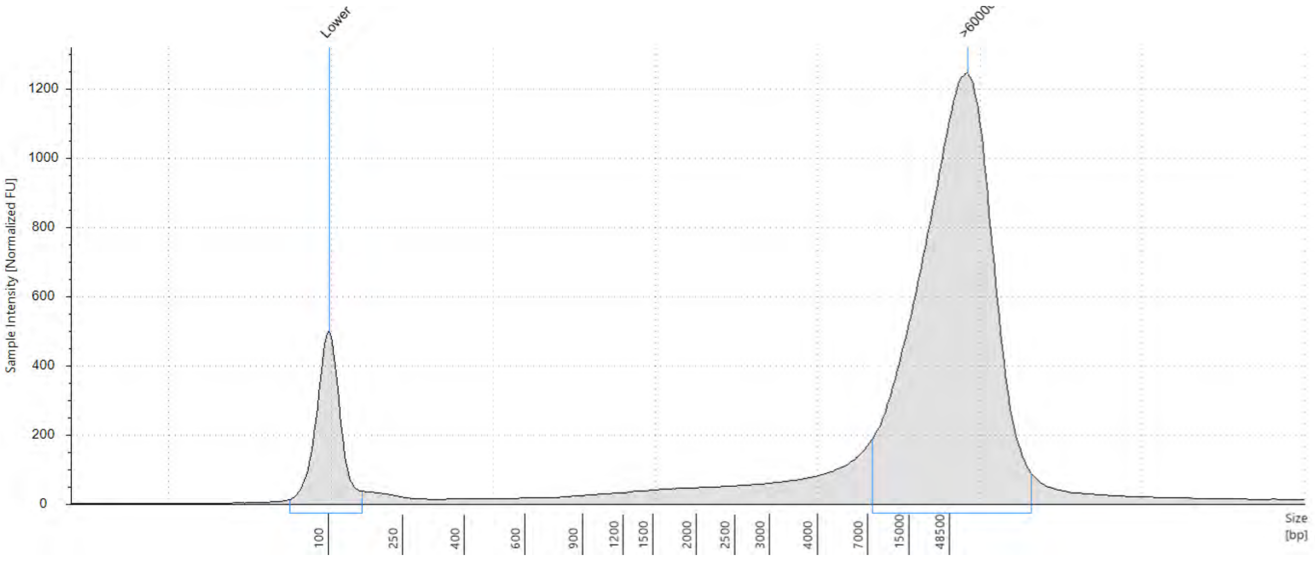

Sample Table

Well	DIN	Conc. [ng/µl]	Sample Description	Alert	Observations
A3	8.3	84.2	M16		

Peak Table

Size [bp]	Calibrated Conc. [ng/µl]	Assigned Conc. [ng/µl]	% Integrated Area	From [bp]	To [bp]	Peak Comment	Observations
100	8.50	8.50	-	62	151		Lower Marker
>60000	64.7	-	97.61	7581	>60000		
-	-	-	-	-	-		Sample Well

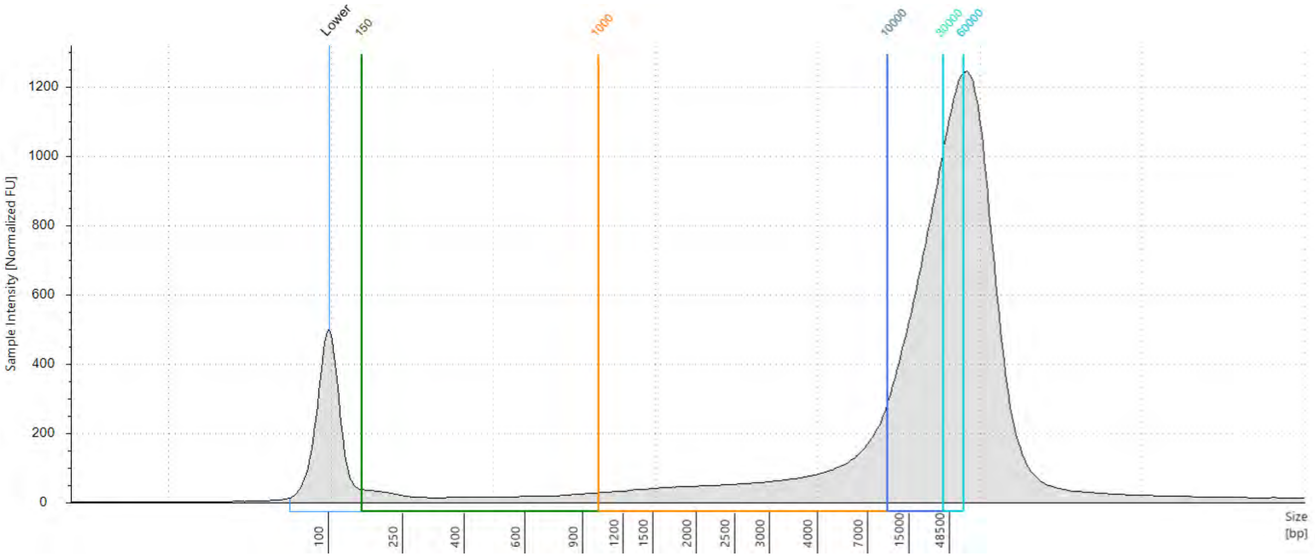

Region Table

From [bp]	To [bp]	Average Size [bp]	Conc. [ng/µl]	Region Molarity [nmol/l]	% of Total	Region Comment	Color
150	10000	3882	15.6	19.0	18.58		
150	1000	490	2.76	12.7	3.27		
1000	10000	4621	12.9	6.34	15.34		
10000	60000	32715	36.7	2.31	43.61		
10000	30000	20052	21.8	1.84	25.90		

30000	60000	51029	15.8	0.508	18.75		

© Agilent Technologies, Inc. 2017

B3: M17

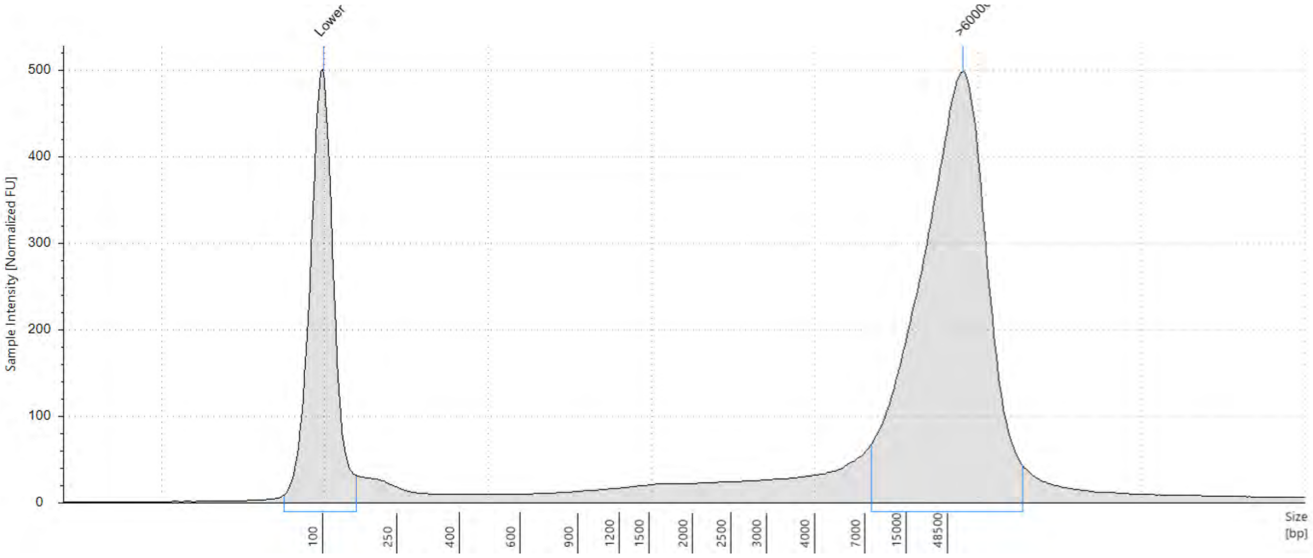

Sample Table

Well	DIN	Conc. [ng/µl]	Sample Description	Alert	Observations
B3	8.3	32.4	M17		

Peak Table

Size [bp]	Calibrated Conc. [ng/µl]	Assigned Conc. [ng/µl]	% Integrated Area	From [bp]	To [bp]	Peak Comment	Observations
100	8.50	8.50	-	62	150		Lower Marker
>60000	23.9	-	98.01	7818	>60000		
-	-	-	-	-	-		Sample Well

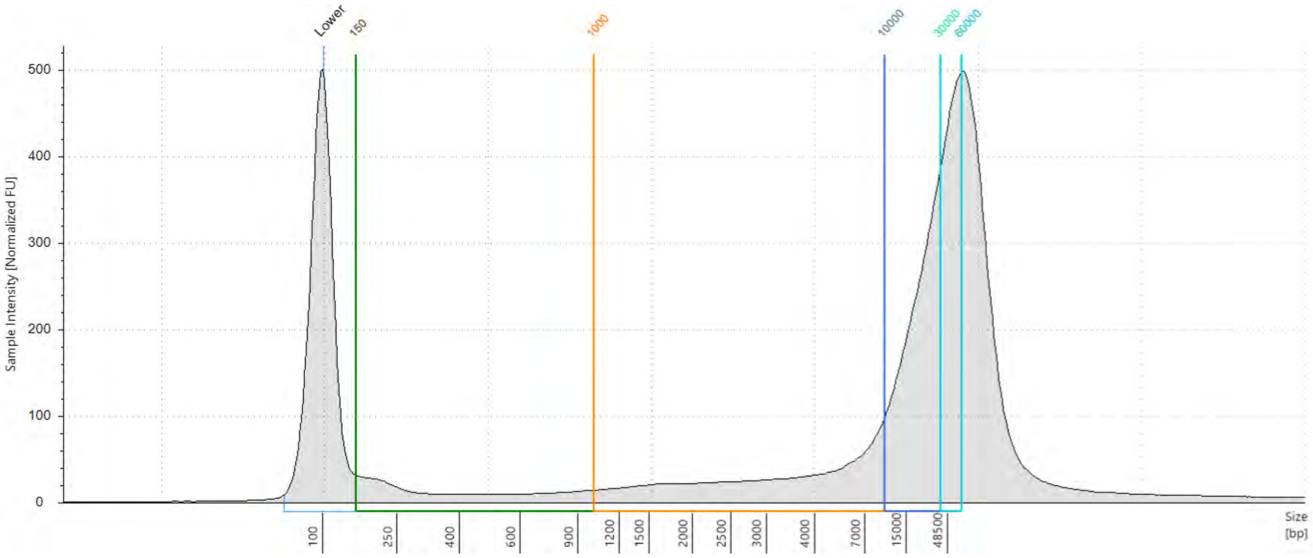

Region Table

From [bp]	To [bp]	Average Size [bp]	Conc. [ng/µl]	Region Molarity [nmol/l]	% of Total	Region Comment	Color
150	10000	3259	6.84	12.7	21.14		
150	1000	437	1.73	9.86	5.36		
1000	10000	4286	5.12	2.87	15.82		
10000	60000	32980	13.9	0.867	43.06		
10000	30000	20067	8.04	0.680	24.85		

 © Agilent Technologies, Inc. 2017

30000	60000	50474	6.23	0.203	19.24		

© Agilent Technologies, Inc. 2017

C3: M18

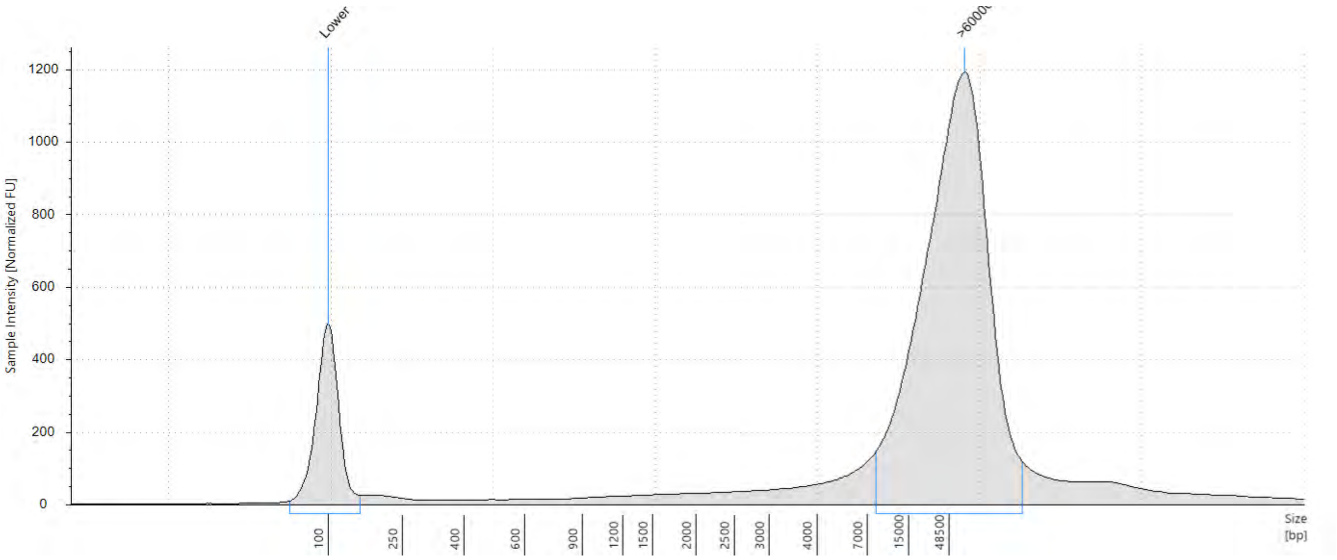

Sample Table

Well	DIN	Conc. [ng/µl]	Sample Description	Alert	Observations
C3	8.8	77.1	M18		

Peak Table

Size [bp]	Calibrated Conc. [ng/µl]	Assigned Conc. [ng/µl]	% Integrated Area	From [bp]	To [bp]	Peak Comment	Observations
100	8.50	8.50	-	62	148		Lower Marker
>60000	59.0	-	98.02	8324	>60000		
-	-	-	-	-	-		Sample Well

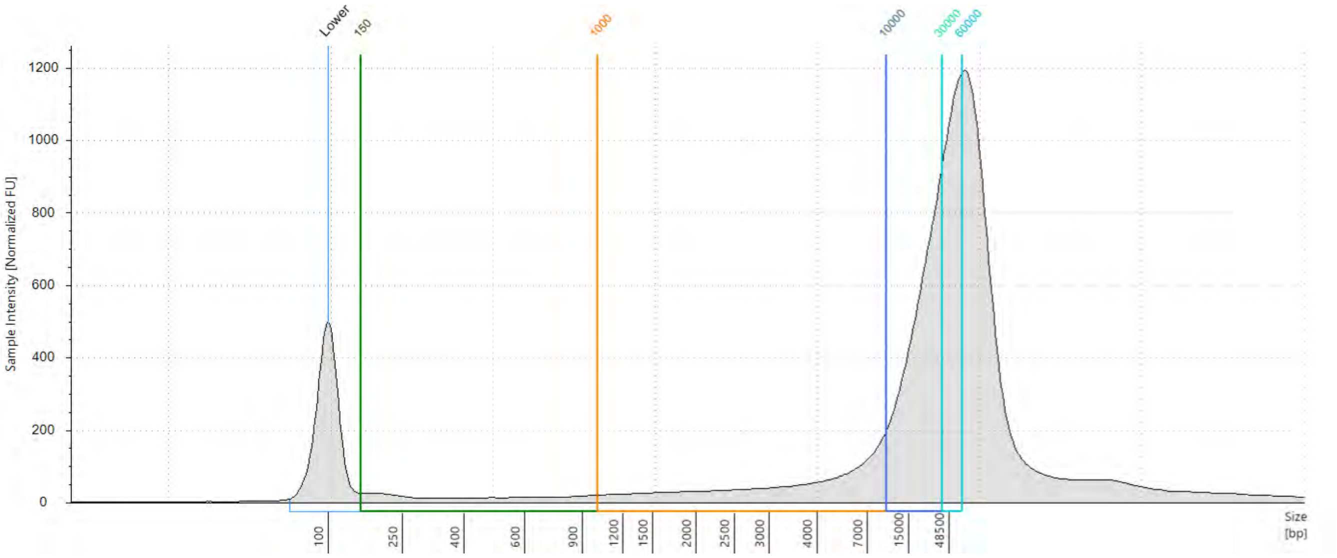

Region Table

From [bp]	To [bp]	Average Size [bp]	Conc. [ng/µl]	Region Molarity [nmol/l]	% of Total	Region Comment	Color
150	10000	3852	10.6	15.3	13.72		
150	1000	480	1.81	10.8	2.35		
1000	10000	4701	8.78	4.61	11.39		
10000	60000	34050	34.2	2.05	44.31		
10000	30000	20621	19.3	1.59	25.02		

 © Agilent Technologies, Inc. 2017

30000	60000	51370	15.7	0.502	20.39		

© Agilent Technologies, Inc. 2017

D3: M19

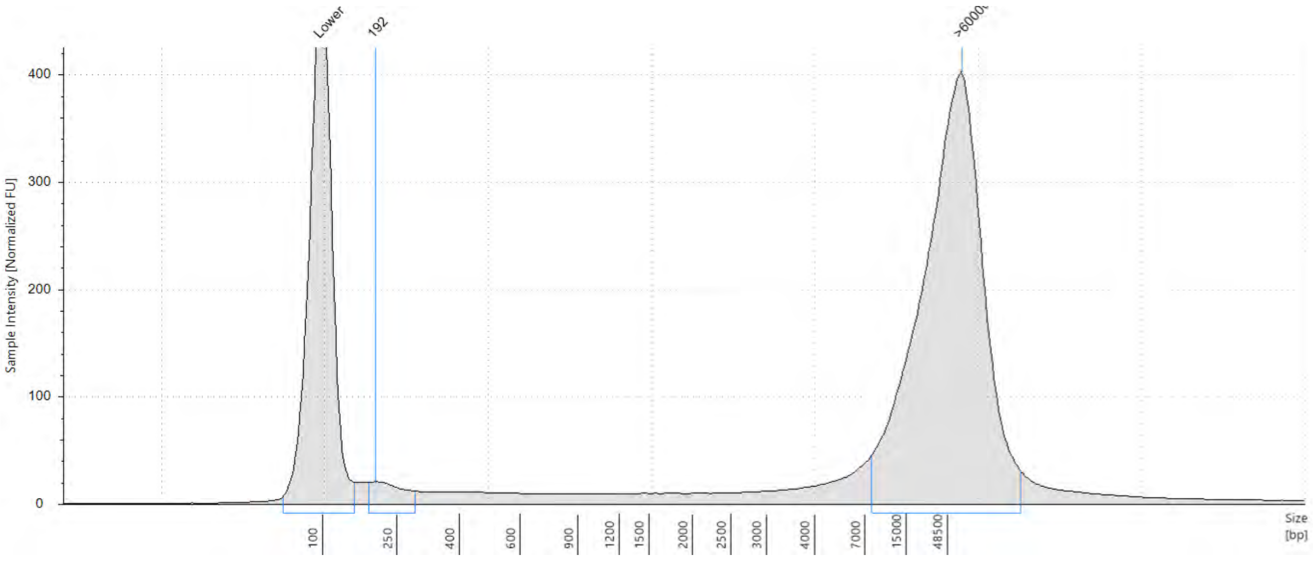

Sample Table

Well	DIN	Conc. [ng/µl]	Sample Description	Alert	Observations
D3	8.5	24.5	M19		

Peak Table

Size [bp]	Calibrated Conc. [ng/µl]	Assigned Conc. [ng/µl]	% Integrated Area	From [bp]	To [bp]	Peak Comment	Observations
100	8.50	8.50	-	62	149		Lower Marker
192	0.506	-	2.62	176	286		
>60000	18.7	-	96.59	7956	>60000		
-	-	-	-	-	-		Sample Well

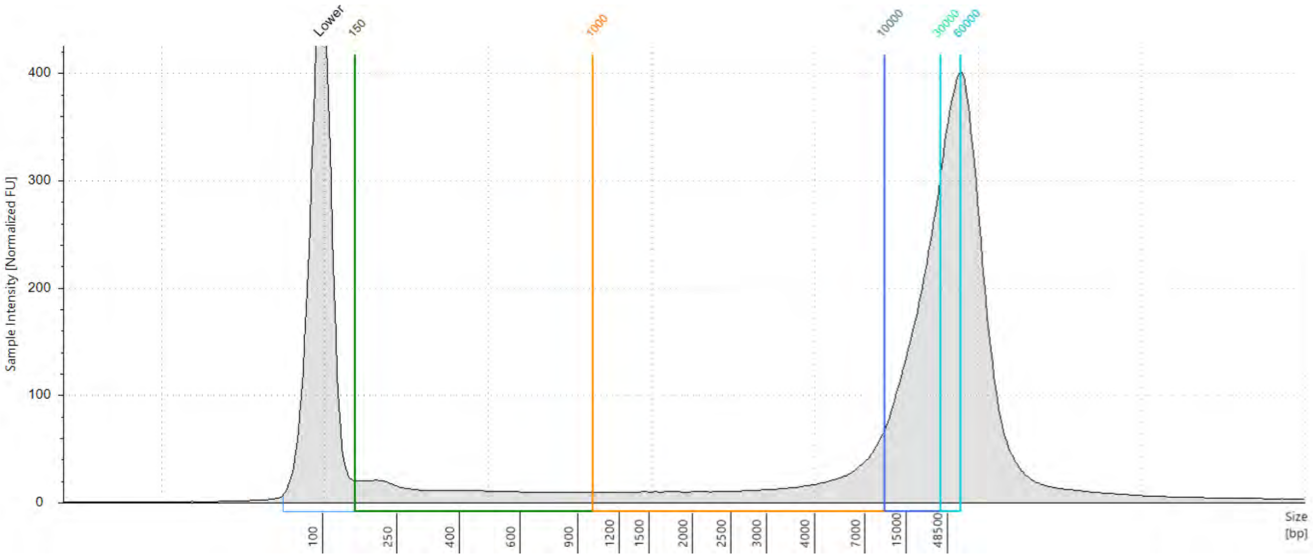

Region Table

From [bp]	To [bp]	Average Size [bp]	Conc. [ng/µl]	Region Molarity [nmol/l]	% of Total	Region Comment	Color
150	10000	3014	4.70	10.6	19.18		
150	1000	438	1.78	9.03	7.26		
1000	10000	4595	2.93	1.58	11.95		
10000	60000	34217	11.4	0.685	46.59		

 © Agilent Technologies, Inc. 2017

10000	30000	20373	6.31	0.525	25.71		
30000	60000	51150	5.40	0.173	22.02		

© Agilent Technologies, Inc. 2017

E3: M20

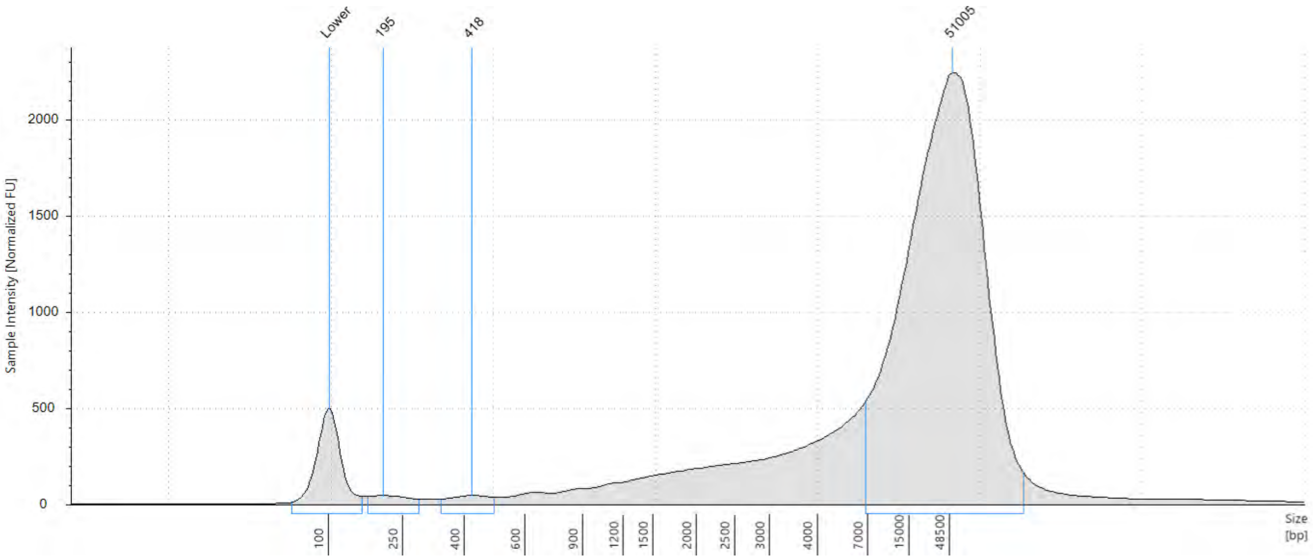

Sample Table

Well	DIN	Conc. [ng/µl]	Sample Description	Alert	Observations
E3	7.5	179	M20	⚠	Sample concentration outside recommended range

Peak Table

Size [bp]	Calibrated Conc. [ng/µl]	Assigned Conc. [ng/µl]	% Integrated Area	From [bp]	To [bp]	Peak Comment	Observations
100	8.50	8.50	-	63	151		Lower Marker
195	1.18	-	0.90	161	282		
418	1.21	-	0.93	333	485		
51005	127	-	97.04	6784	>60000		
-	-	-	-	-	-		Sample Well

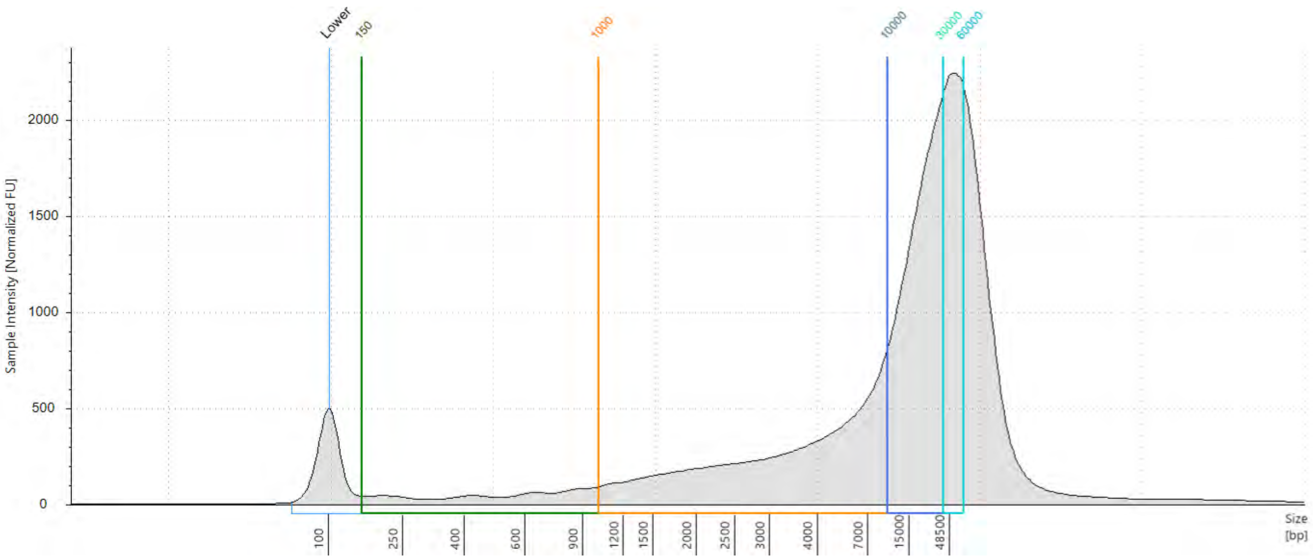

Region Table

From [bp]	To [bp]	Average Size [bp]	Conc. [ng/µl]	Region Molarity [nmol/l]	% of Total	Region Comment	Color
150	10000	3877	54.1	50.4	30.31		
150	1000	564	6.62	26.3	3.71		
1000	10000	4369	47.6	24.2	26.65		

© Agilent Technologies, Inc. 2017

10000	60000	30290	80.9	5.48	45.28		
10000	30000	19435	52.2	4.56	29.23		
30000	60000	49813	30.5	1.01	17.08		

 © Agilent Technologies, Inc. 2017

F3: M21

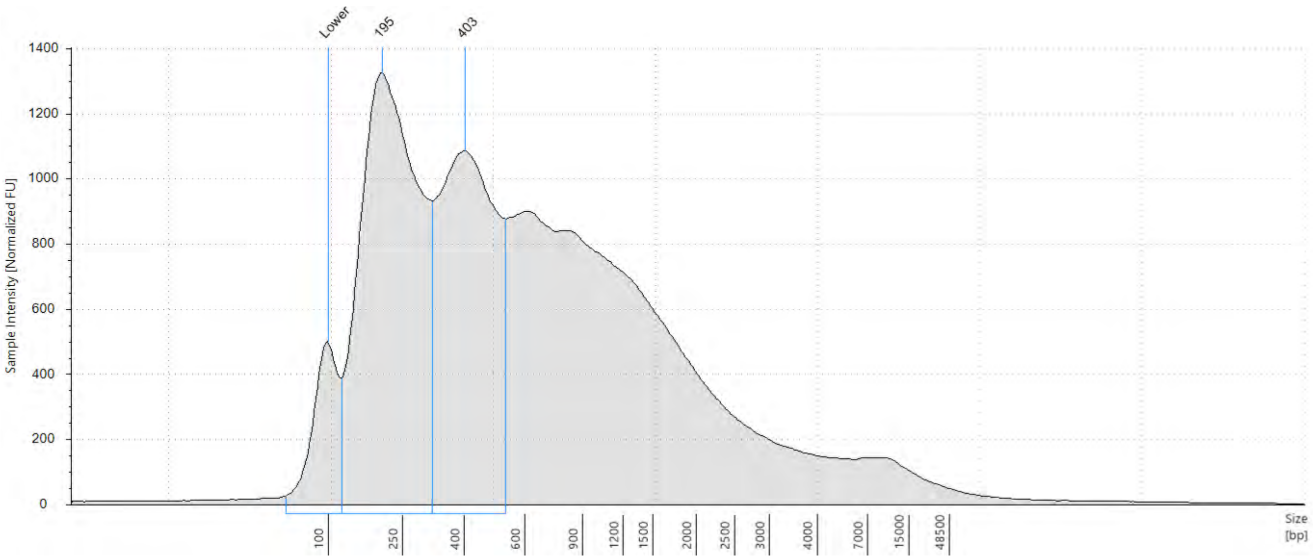

Sample Table

Well	DIN	Conc. [ng/µl]	Sample Description	Alert	Observations
F3	1.7	203	M21	⚠	Sample concentration outside recommended range

Peak Table

Size [bp]	Calibrated Conc. [ng/µl]	Assigned Conc. [ng/µl]	% Integrated Area	From [bp]	To [bp]	Peak Comment	Observations
100	8.50	8.50	-	60	119		Lower Marker
195	52.6	-	55.17	119	313		
403	42.7	-	44.83	313	528		

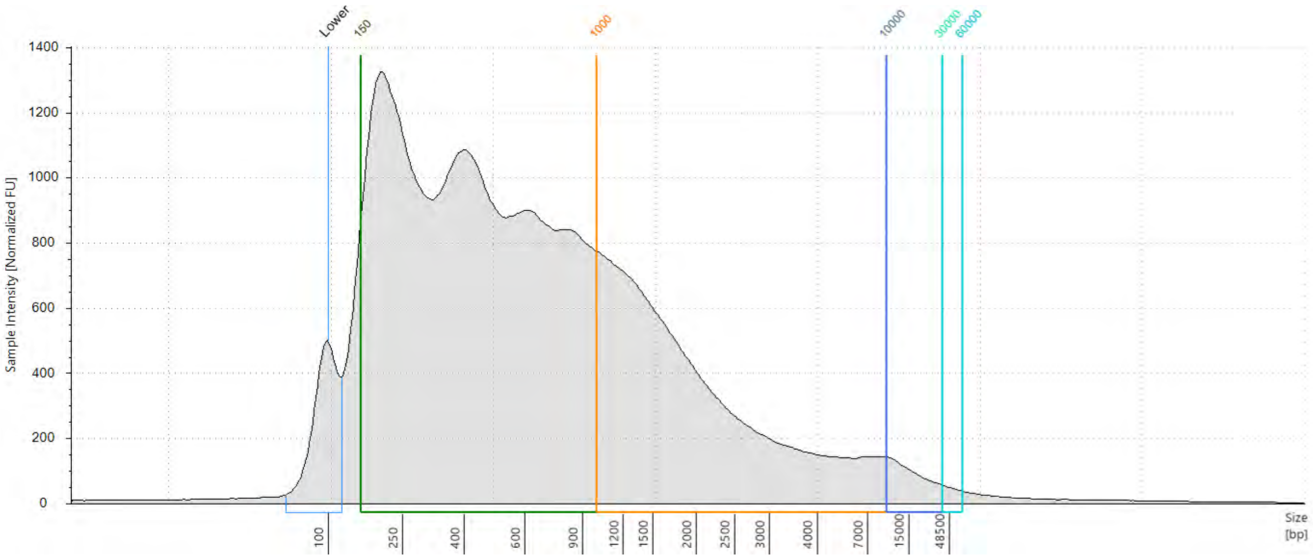

Region Table

From [bp]	To [bp]	Average Size [bp]	Conc. [ng/µl]	Region Molarity [nmol/l]	% of Total	Region Comment	Color
150	10000	1010	190	635	93.88		
150	1000	459	133	584	65.73		
1000	10000	2284	57.6	51.9	28.46		
10000	60000	21695	3.62	0.341	1.79		
10000	30000	16700	3.10	0.324	1.53		

30000	60000	49803	0.563	0.0195	0.28		

G3: M22

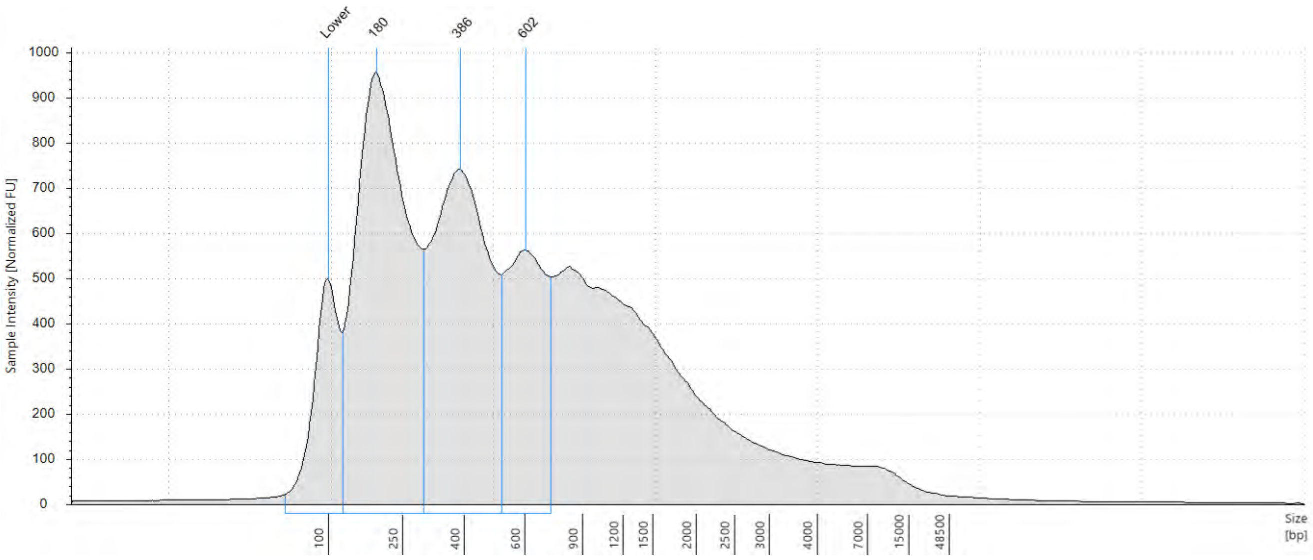

Sample Table

Well	DIN	Conc. [ng/µl]	Sample Description	Alert	Observations
G3	1.8	128	M22	⚠	Sample concentration outside recommended range

Peak Table

Size [bp]	Calibrated Conc. [ng/µl]	Assigned Conc. [ng/µl]	% Integrated Area	From [bp]	To [bp]	Peak Comment	Observations
100	8.50	8.50	-	59	119		Lower Marker
180	33.7	-	43.12	119	293		
386	29.0	-	37.12	293	513		
602	15.4	-	19.76	513	719		

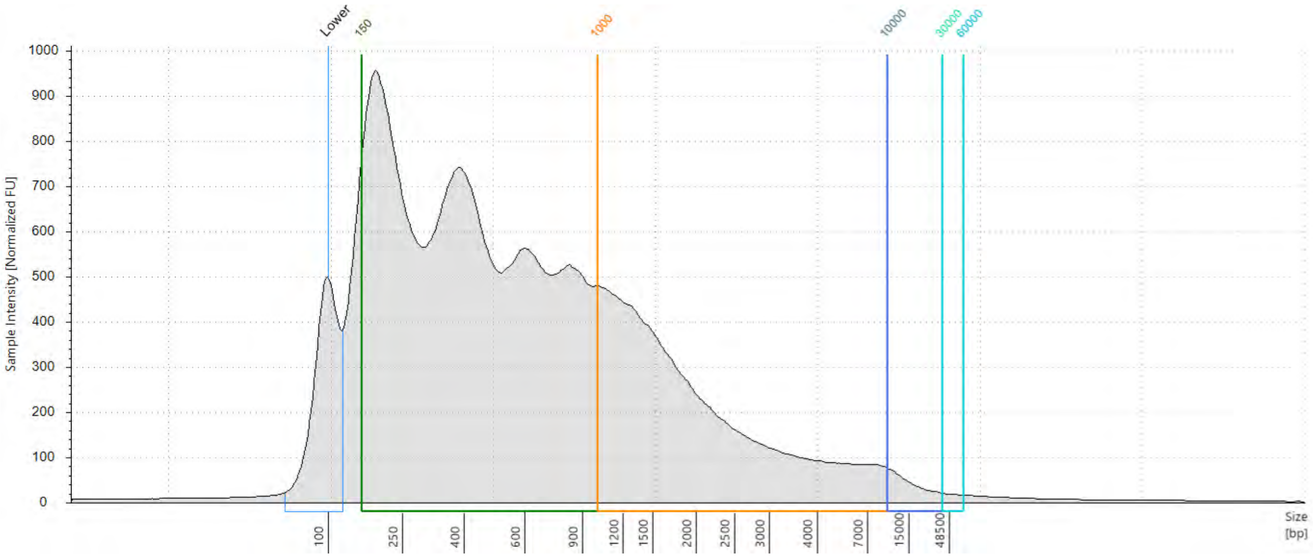

Region Table

From [bp]	To [bp]	Average Size [bp]	Conc. [ng/µl]	Region Molarity [nmol/l]	% of Total	Region Comment	Color
150	10000	981	120	417	93.41		
150	1000	451	85.2	386	66.52		
1000	10000	2270	34.8	31.7	27.19		
10000	60000	20670	1.44	0.153	1.13		

 © Agilent Technologies, Inc. 2017

10000	30000	16160	1.28	0.146	1.00		
30000	60000	50361	0.180	0.00708	0.14		

© Agilent Technologies, Inc. 2017

H3: M23

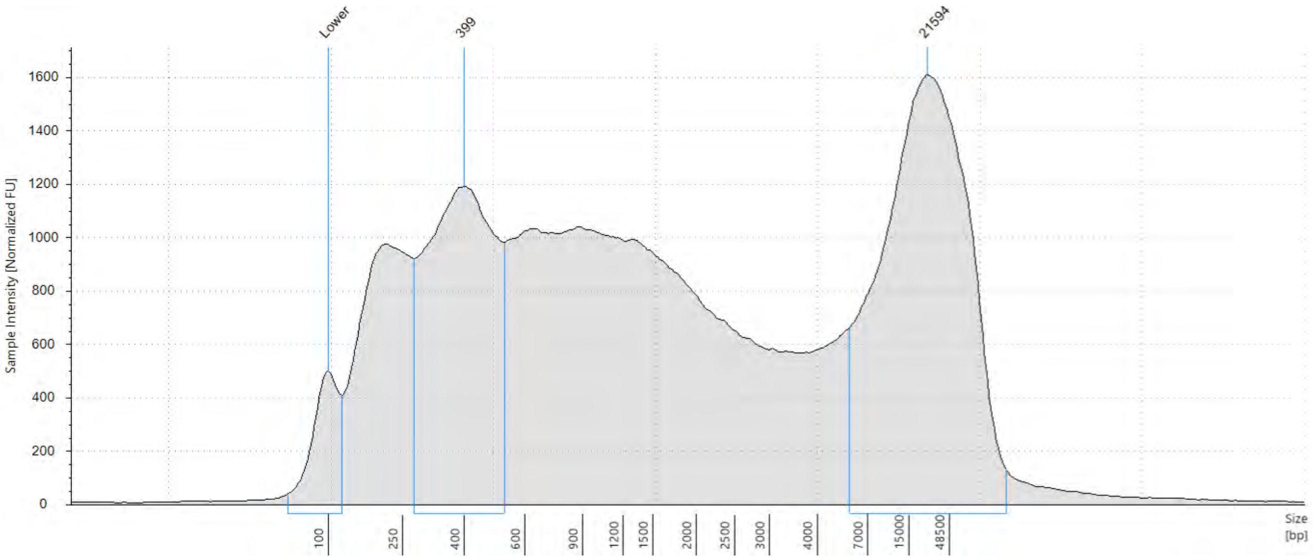

Sample Table

Well	DIN	Conc. [ng/µl]	Sample Description	Alert	Observations
H3	2.0	346	M23	⚠	Sample concentration outside functional range for DIN

Peak Table

Size [bp]	Calibrated Conc. [ng/µl]	Assigned Conc. [ng/µl]	% Integrated Area	From [bp]	To [bp]	Peak Comment	Observations
100	8.50	8.50	-	61	119		Lower Marker
399	55.5	-	37.04	273	523		
21594	94.1	-	62.79	5766	>60000		
-	-	-	-	-	-		Sample Well

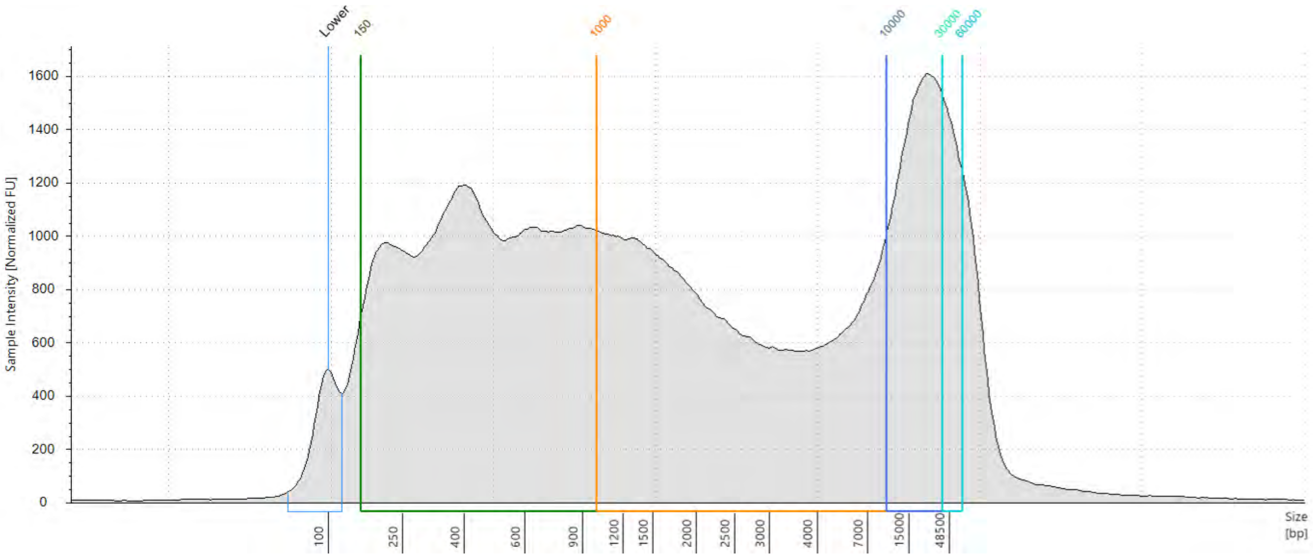

Region Table

From [bp]	To [bp]	Average Size [bp]	Conc. [ng/µl]	Region Molarity [nmol/l]	% of Total	Region Comment	Color
150	10000	1744	261	646	75.28		
150	1000	493	137	555	39.50		
1000	10000	3114	125	92.2	36.02		
10000	60000	27201	62.5	4.71	18.05		

 © Agilent Technologies, Inc. 2017

10000	30000	18835	46.1	4.19	13.30		
30000	60000	50206	17.7	0.586	5.10		

© Agilent Technologies, Inc. 2017

A4: M24

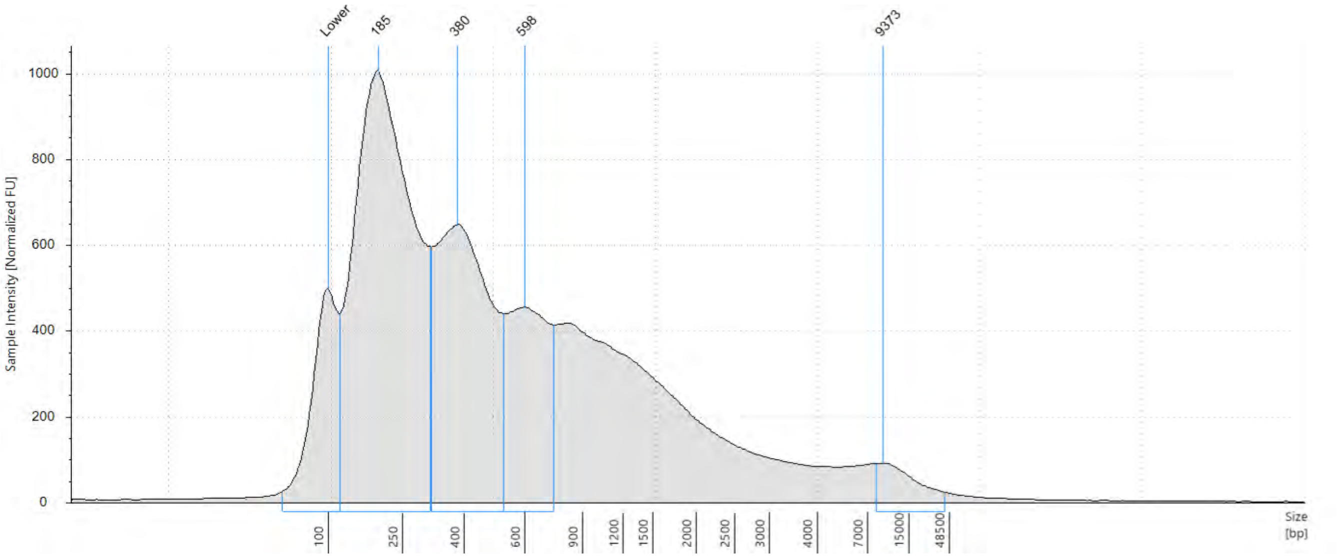

Sample Table

Well	DIN	Conc. [ng/µl]	Sample Description	Alert	Observations
A4	1.6	118	M24	⚠	Sample concentration outside recommended range

Peak Table

Size [bp]	Calibrated Conc. [ng/µl]	Assigned Conc. [ng/µl]	% Integrated Area	From [bp]	To [bp]	Peak Comment	Observations
100	8.50	8.50	-	57	115		Lower Marker
185	40.0	-	50.33	115	307		
380	24.1	-	30.39	310	520		
598	13.0	-	16.40	520	734		
9373	2.29	-	2.88	8230	32993		

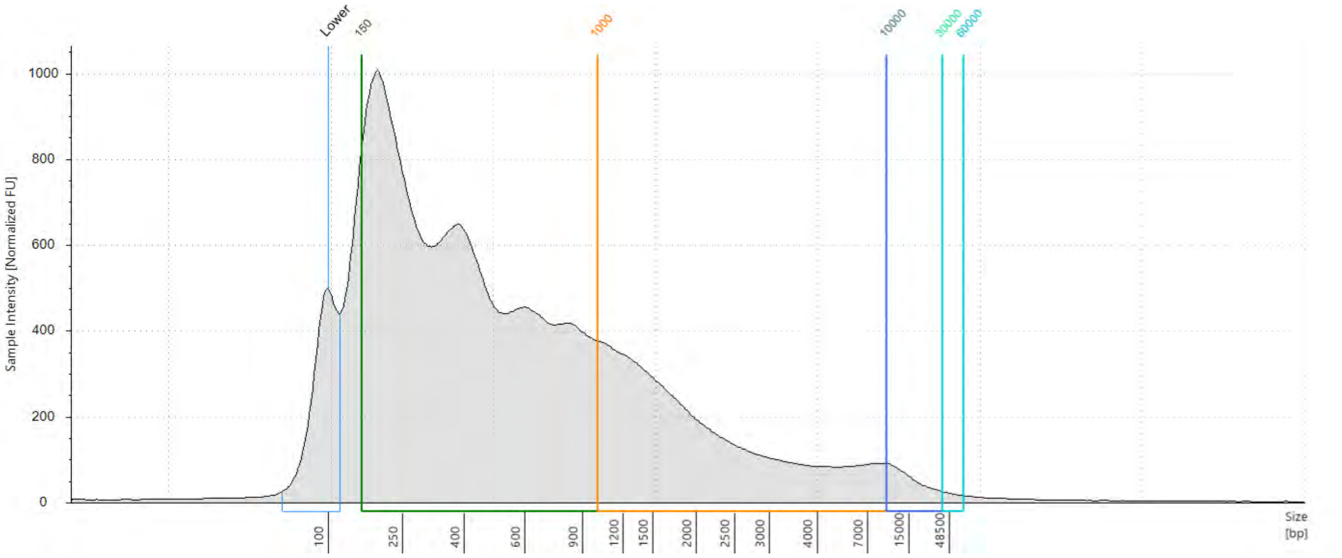

Region Table

From [bp]	To [bp]	Average Size [bp]	Conc. [ng/µl]	Region Molarity [nmol/l]	% of Total	Region Comment	Color
150	10000	952	108	408	91.52		
150	1000	425	79.7	383	67.57		
1000	10000	2407	28.6	25.3	24.20		

 © Agilent Technologies, Inc. 2017

10000	60000	20096	1.90	0.198	1.61		
10000	30000	16085	1.70	0.190	1.44		
30000	60000	49508	0.219	0.00856	0.19		

© Agilent Technologies, Inc. 2017

B4: M25

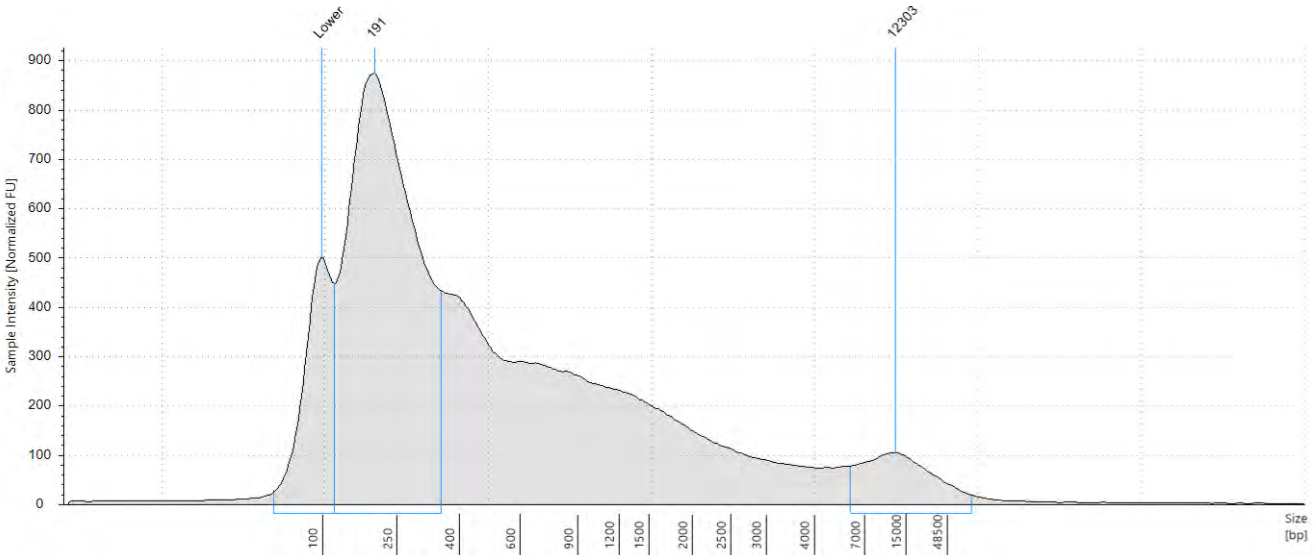

Sample Table

Well	DIN	Conc. [ng/µl]	Sample Description	Alert	Observations
B4	1.4	83.8	M25		

Peak Table

Size [bp]	Calibrated Conc. [ng/µl]	Assigned Conc. [ng/µl]	% Integrated Area	From [bp]	To [bp]	Peak Comment	Observations
100	8.50	8.50	-	55	117		Lower Marker
191	36.3	-	89.22	117	350		
12303	4.39	-	10.78	5996	>60000		

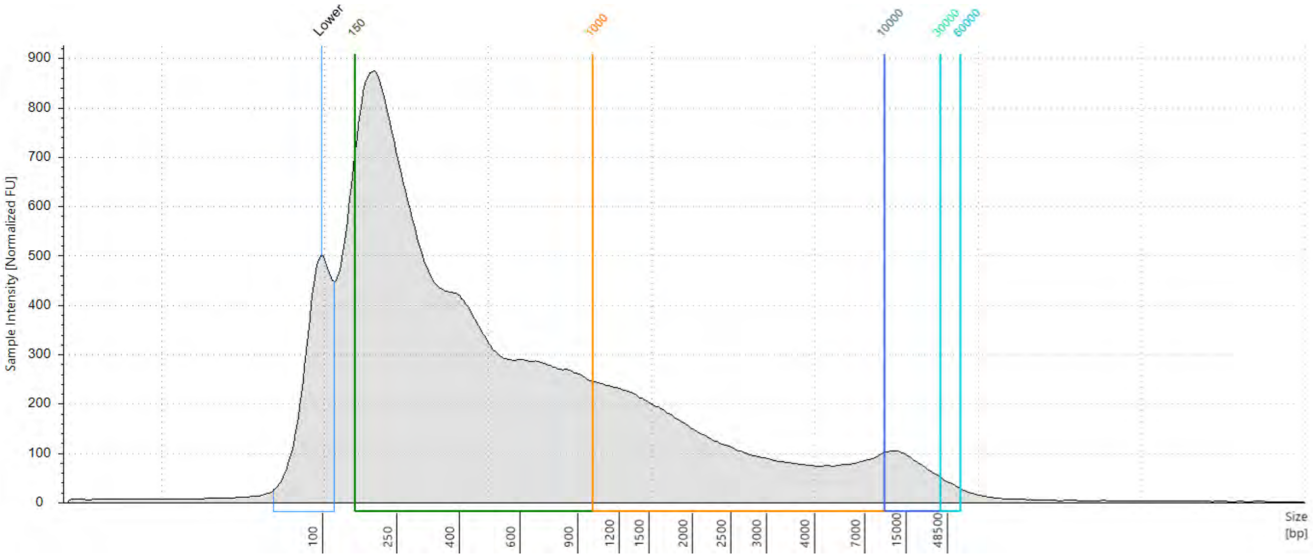

Region Table

From [bp]	To [bp]	Average Size [bp]	Conc. [ng/µl]	Region Molarity [nmol/l]	% of Total	Region Comment	Color
150	10000	982	74.8	300	89.20		
150	1000	399	55.4	283	66.06		
1000	10000	2602	19.6	16.5	23.35		
10000	60000	21503	2.84	0.272	3.39		
10000	30000	16958	2.47	0.259	2.95		

 © Agilent Technologies, Inc. 2017

30000	60000	49484	0.408	0.0149	0.49		

© Agilent Technologies, Inc. 2017

C4: M26

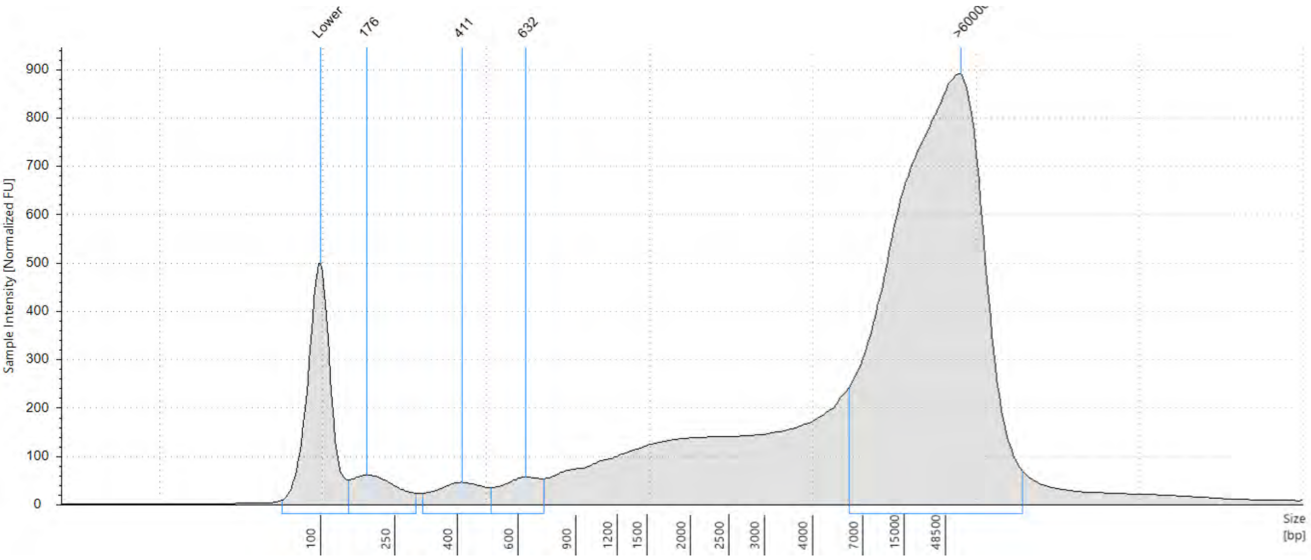

Sample Table

Well	DIN	Conc. [ng/µl]	Sample Description	Alert	Observations
C4	7.0	95.6	M26		

Peak Table

Size [bp]	Calibrated Conc. [ng/µl]	Assigned Conc. [ng/µl]	% Integrated Area	From [bp]	To [bp]	Peak Comment	Observations
100	8.50	8.50	-	62	141		Lower Marker
176	1.93	-	2.88	141	291		
411	1.55	-	2.31	307	499		
632	1.63	-	2.42	499	718		
>60000	61.0	-	90.85	6038	>60000		
-	-	-	-	-	-		Sample Well

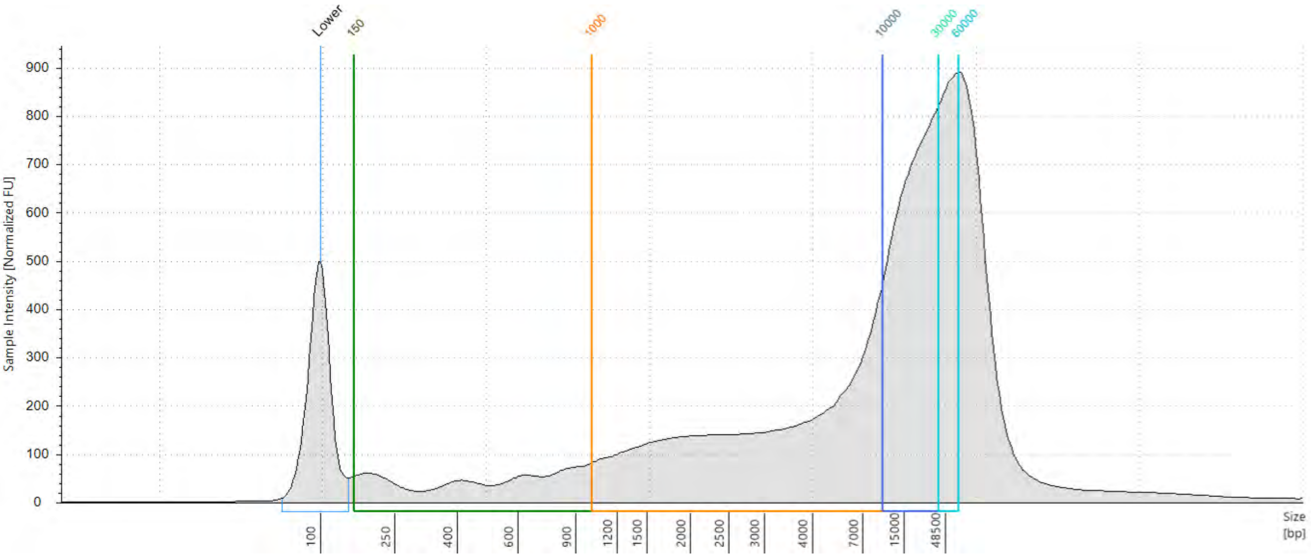

Region Table

From [bp]	To [bp]	Average Size [bp]	Conc. [ng/µl]	Region Molarity [nmol/l]	% of Total	Region Comment	Color
150	10000	3421	37.6	46.2	39.34		
150	1000	535	6.99	29.1	7.32		

 © Agilent Technologies, Inc. 2017

1000	10000	4101	30.7	17.3	32.10		
10000	60000	29362	36.5	2.59	38.19		
10000	30000	19031	24.8	2.23	25.94		
30000	60000	50884	12.5	0.403	13.03		

© Agilent Technologies, Inc. 2017

D4: M27

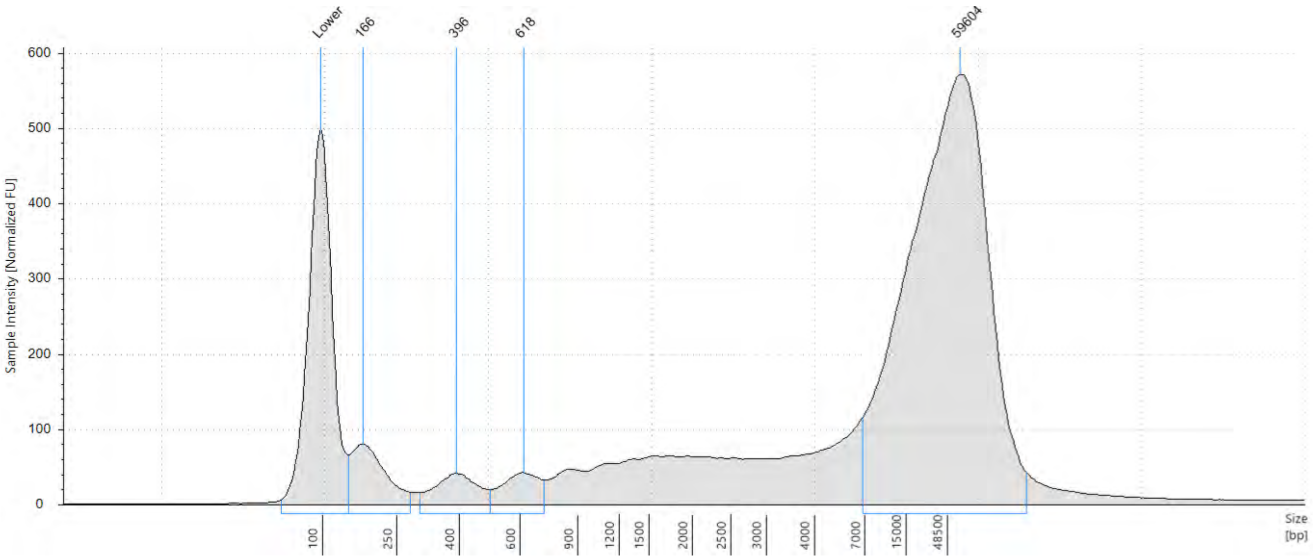

Sample Table

Well	DIN	Conc. [ng/µl]	Sample Description	Alert	Observations
D4	7.1	50.7	M27		

Peak Table

Size [bp]	Calibrated Conc. [ng/µl]	Assigned Conc. [ng/µl]	% Integrated Area	From [bp]	To [bp]	Peak Comment	Observations
100	8.50	8.50	-	61	140		Lower Marker
166	1.90	-	5.09	140	277		
396	1.17	-	3.12	298	495		
618	1.06	-	2.85	495	715		
59604	32.7	-	87.35	6896	>60000		
-	-	-	-	-	-		Sample Well

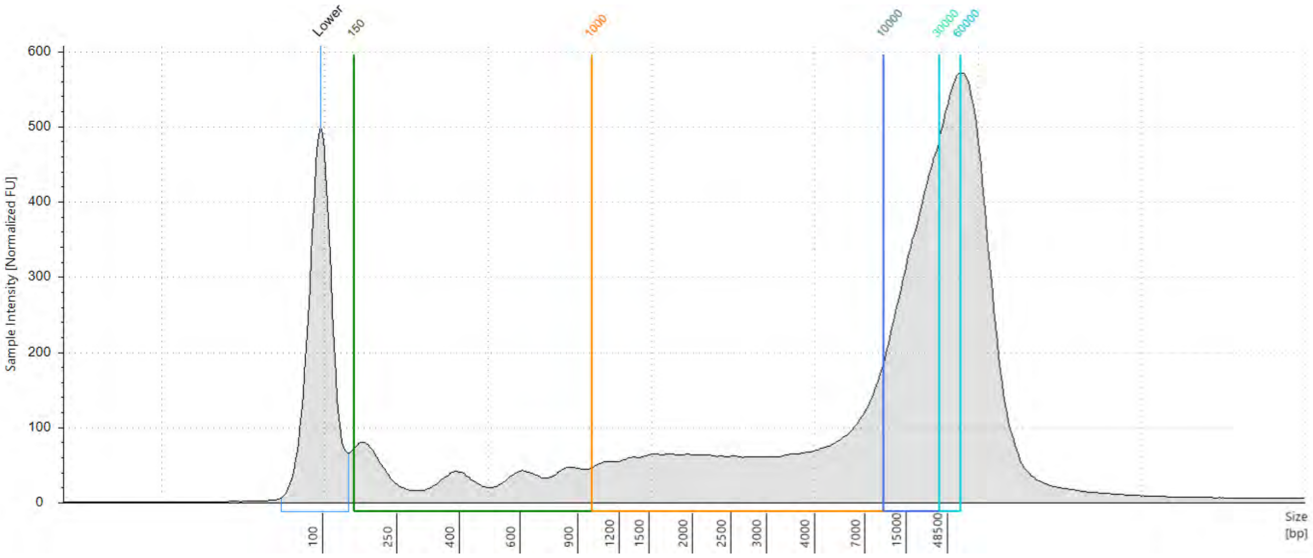

Region Table

From [bp]	To [bp]	Average Size [bp]	Conc. [ng/µl]	Region Molarity [nmol/l]	% of Total	Region Comment	Color
150	10000	2858	17.7	32.7	34.94		
150	1000	485	5.17	25.0	10.18		

1000	10000	3854	12.6	7.77	24.83		
10000	60000	31214	19.3	1.29	37.95		
10000	30000	19626	12.3	1.07	24.16		
30000	60000	51298	7.42	0.238	14.62		

© Agilent Technologies, Inc. 2017

E4: M28

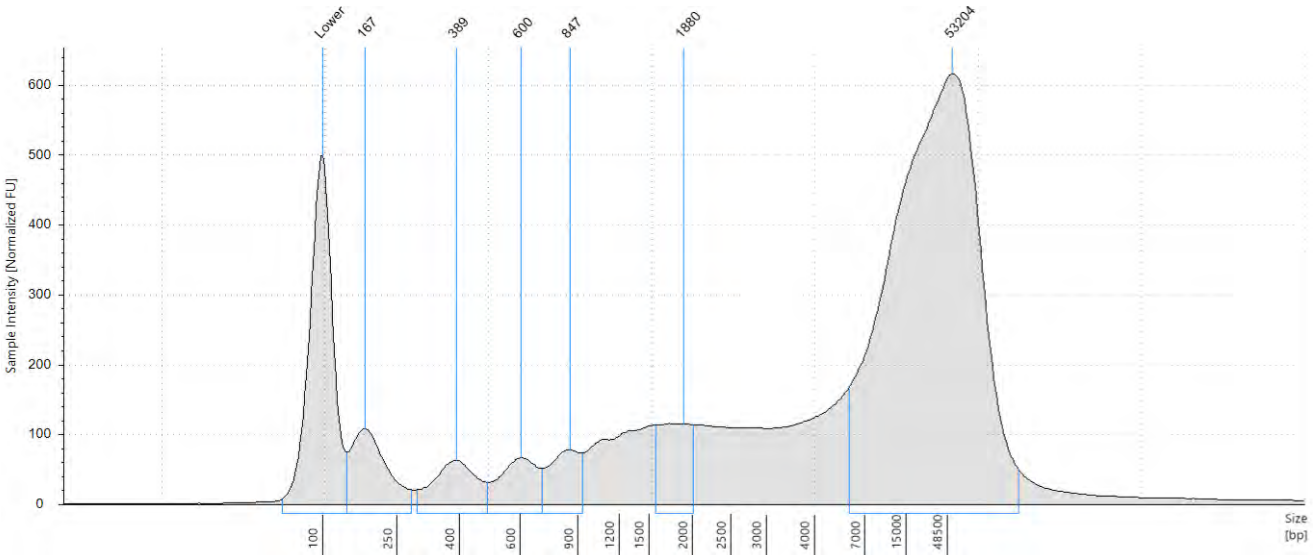

Sample Table

Well	DIN	Conc. [ng/µl]	Sample Description	Alert	Observations
E4	6.7	69.5	M28		

Peak Table

Size [bp]	Calibrated Conc. [ng/µl]	Assigned Conc. [ng/µl]	% Integrated Area	From [bp]	To [bp]	Peak Comment	Observations
100	8.50	8.50	-	61	134		Lower Marker
167	2.75	-	5.32	134	277		
389	1.87	-	3.61	289	480		
600	1.79	-	3.45	480	696		
847	1.77	-	3.43	696	925		
1880	2.84	-	5.48	1563	2005		
53204	40.4	-	78.02	5859	>60000		
-	-	-	-	-	-		Sample Well

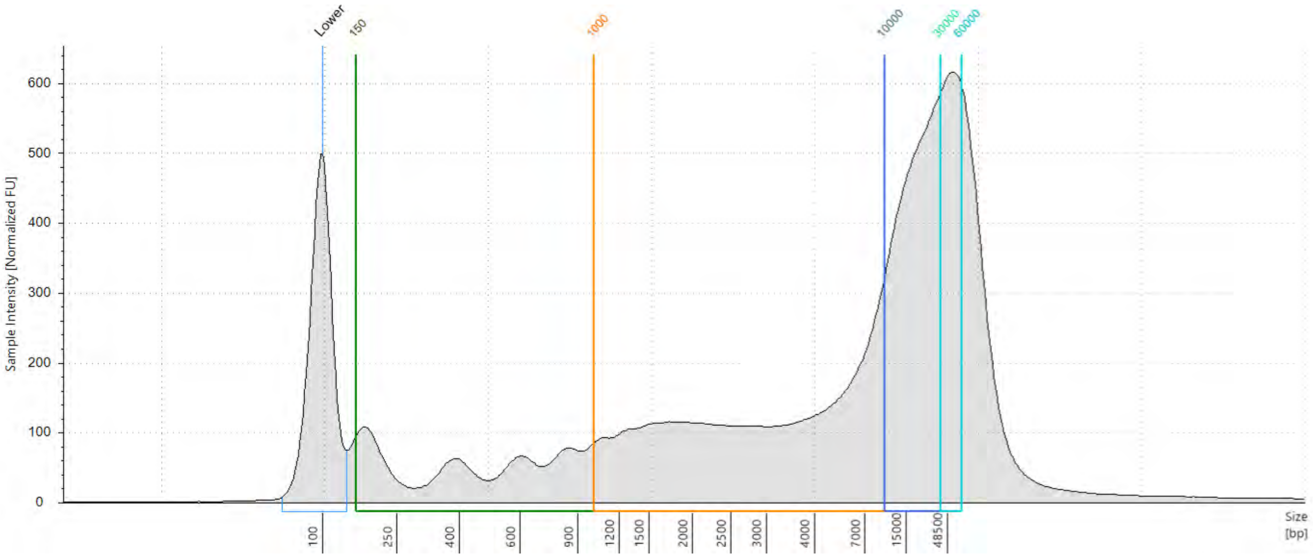

Region Table

From [bp]	To [bp]	Average Size [bp]	Conc. [ng/µl]	Region Molarity [nmol/l]	% of Total	Region Comment	Color

150	10000	3027	31.9	50.2	45.90		
150	1000	509	8.22	36.1	11.83		
1000	10000	3907	23.8	14.2	34.18		
10000	60000	28934	25.2	1.80	36.25		
10000	30000	19224	17.6	1.56	25.27		
30000	60000	50919	8.16	0.263	11.74		

© Agilent Technologies, Inc. 2017

F4: M29

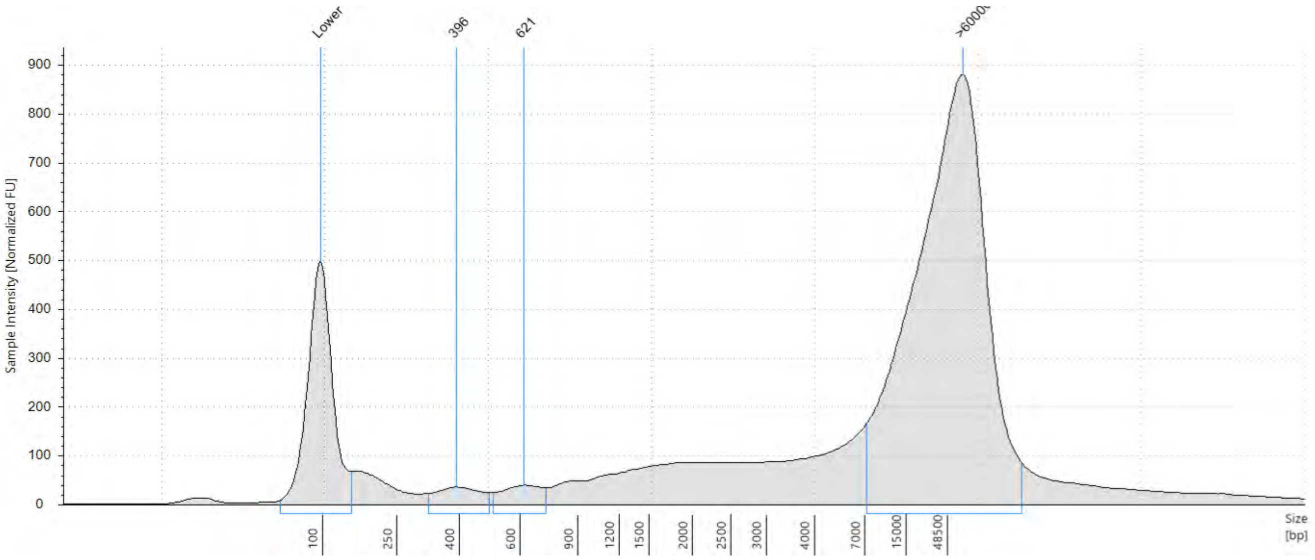

Sample Table

Well	DIN	Conc. [ng/µl]	Sample Description	Alert	Observations
F4	7.3	68.2	M29		

Peak Table

Size [bp]	Calibrated Conc. [ng/µl]	Assigned Conc. [ng/µl]	% Integrated Area	From [bp]	To [bp]	Peak Comment	Observations
100	8.50	8.50	-	61	146		Lower Marker
396	1.01	-	2.21	320	491		
621	1.03	-	2.27	505	726		
>60000	43.1	-	94.50	7335	>60000		
-	-	-	-	-	-		Sample Well

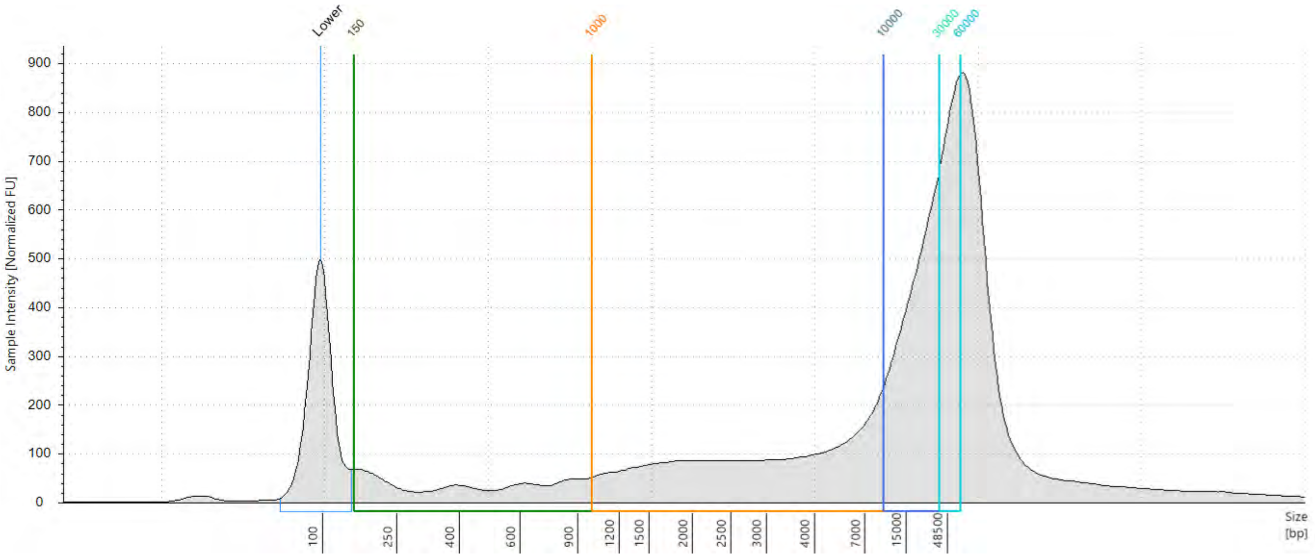

Region Table

From [bp]	To [bp]	Average Size [bp]	Conc. [ng/µl]	Region Molarity [nmol/l]	% of Total	Region Comment	Color
150	10000	3075	21.3	32.9	31.22		
150	1000	495	4.97	23.3	7.29		
1000	10000	3885	16.4	9.73	24.00		

 © Agilent Technologies, Inc. 2017

10000	60000	31891	25.2	1.64	36.94		
10000	30000	19530	15.1	1.32	22.21		
30000	60000	50324	10.6	0.348	15.57		

© Agilent Technologies, Inc. 2017

G4: M30

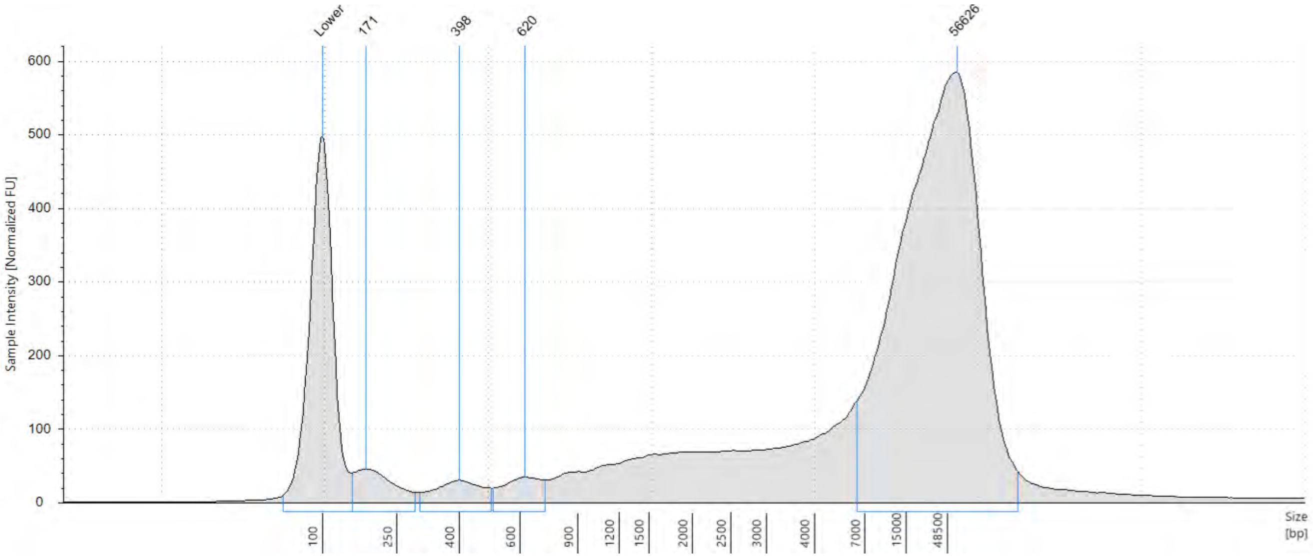

Sample Table

Well	DIN	Conc. [ng/µl]	Sample Description	Alert	Observations
G4	7.1	53.3	M30		

Peak Table

Size [bp]	Calibrated Conc. [ng/µl]	Assigned Conc. [ng/µl]	% Integrated Area	From [bp]	To [bp]	Peak Comment	Observations
100	8.50	8.50	-	62	144		Lower Marker
171	1.26	-	3.32	144	286		
398	0.976	-	2.56	296	494		
620	0.931	-	2.45	499	712		
56626	34.3	-	90.06	6411	>60000		
-	-	-	-	-	-		Sample Well

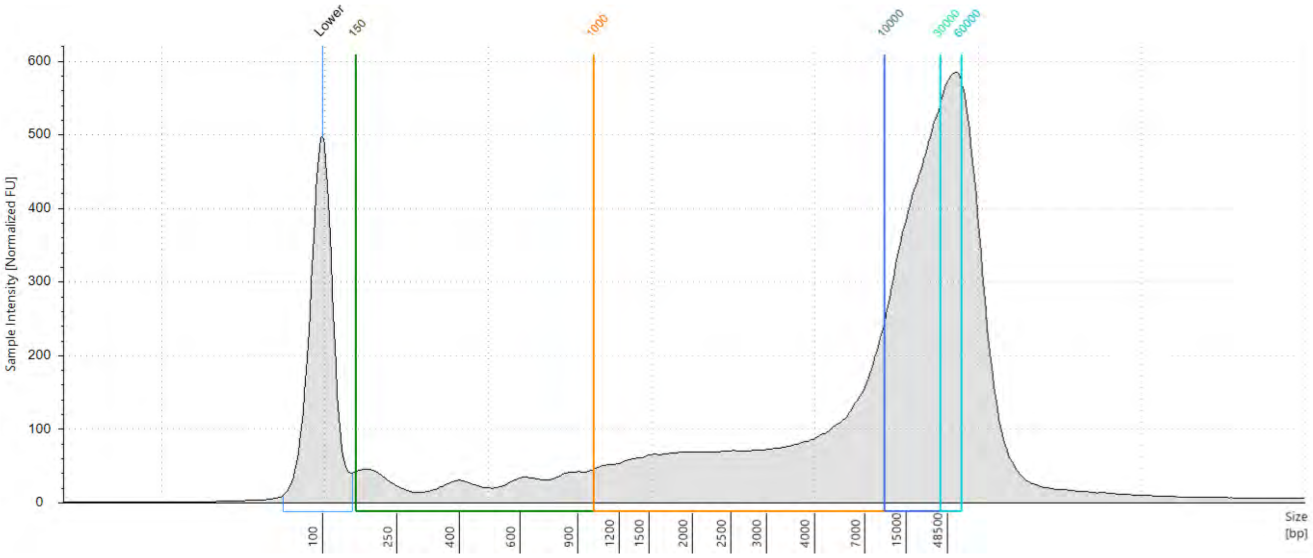

Region Table

From [bp]	To [bp]	Average Size [bp]	Conc. [ng/µl]	Region Molarity [nmol/l]	% of Total	Region Comment	Color
150	10000	3349	19.6	26.9	36.82		
150	1000	519	4.25	18.3	7.98		

1000	10000	4148	15.4	8.66	28.92		
10000	60000	29765	22.1	1.53	41.44		
10000	30000	19490	14.9	1.31	28.02		
30000	60000	50916	7.64	0.246	14.33		

© Agilent Technologies, Inc. 2017

H4: M31

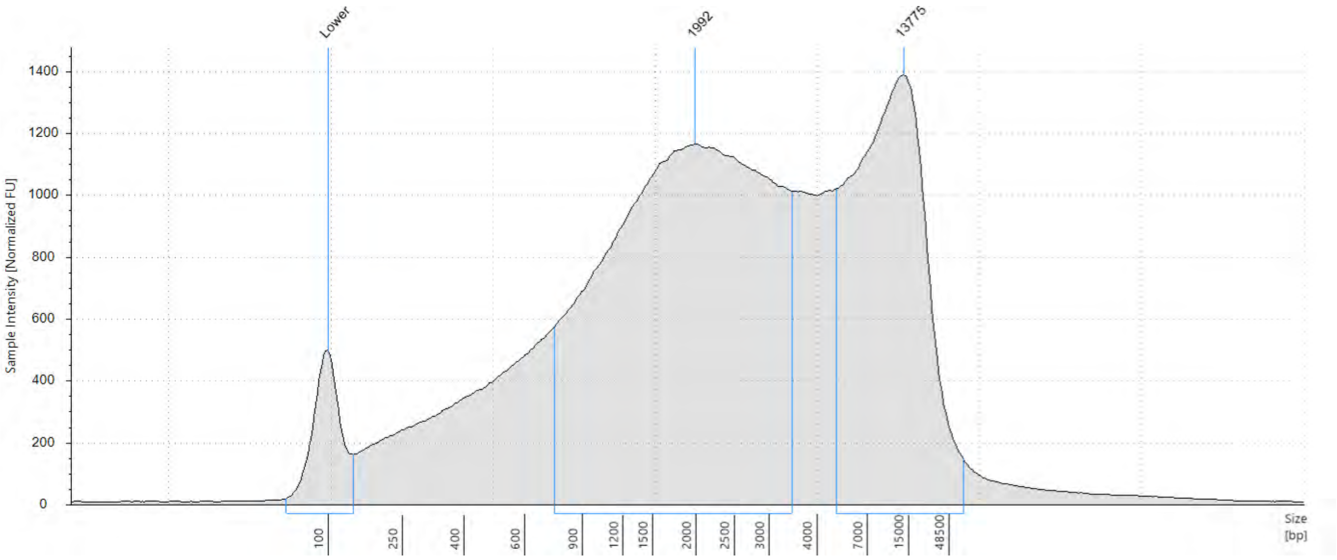

Sample Table

Well	DIN	Conc. [ng/µl]	Sample Description	Alert	Observations
H4	4.5	257	M31	⚠	Sample concentration outside recommended range

Peak Table

Size [bp]	Calibrated Conc. [ng/µl]	Assigned Conc. [ng/µl]	% Integrated Area	From [bp]	To [bp]	Peak Comment	Observations
100	8.50	8.50	-	60	136		Lower Marker
1992	127	-	65.71	739	3441		
13775	66.1	-	34.29	4974	>60000		

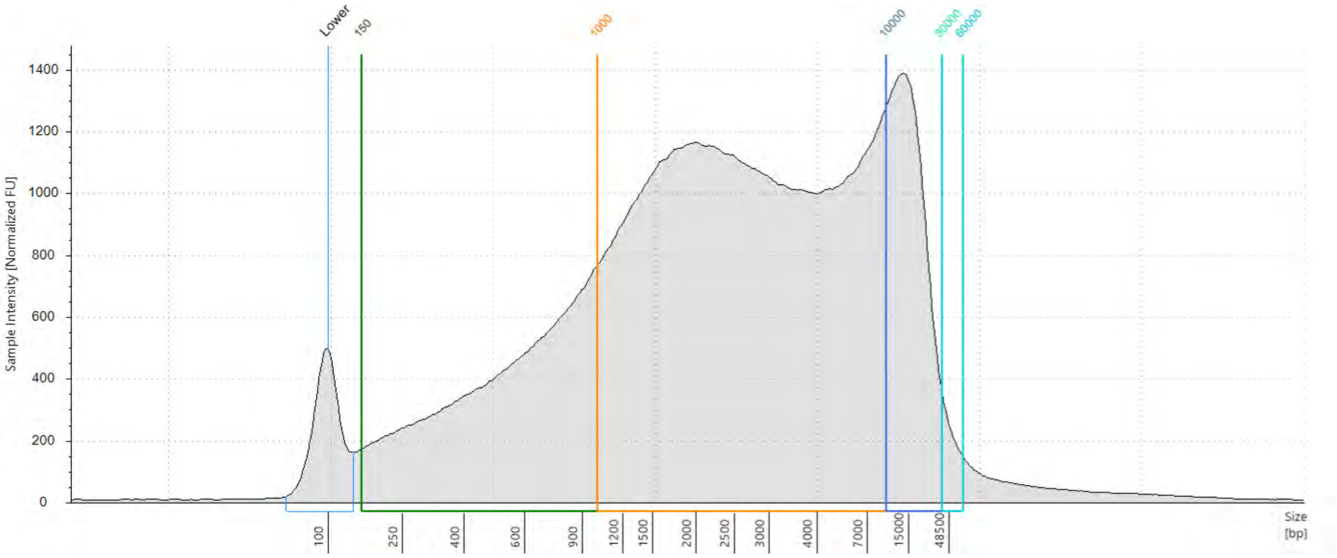

Region Table

From [bp]	To [bp]	Average Size [bp]	Conc. [ng/µl]	Region Molarity [nmol/l]	% of Total	Region Comment	Color
150	10000	2654	217	282	84.25		
150	1000	583	51.2	174	19.88		
1000	10000	3300	166	109	64.60		
10000	60000	18688	35.7	3.52	13.87		
10000	30000	16360	33.3	3.44	12.92		

30000	60000	48474	2.72	0.0974	1.06		

© Agilent Technologies, Inc. 2017

A5: M32

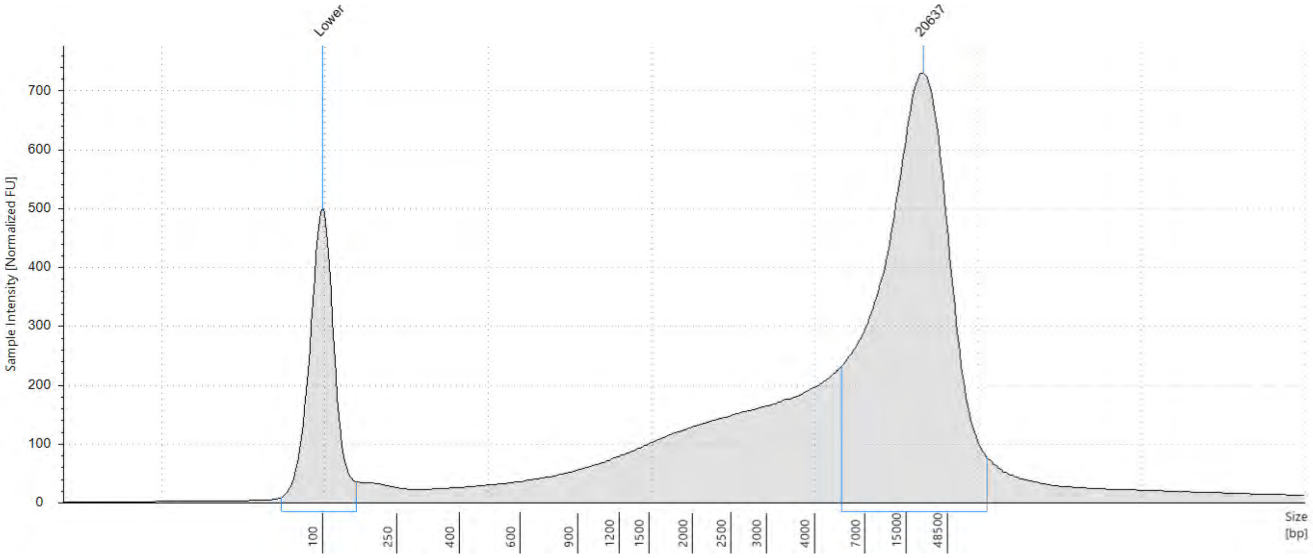

Sample Table

Well	DIN	Conc. [ng/µl]	Sample Description	Alert	Observations
A5	6.6	65.9	M32		

Peak Table

Size [bp]	Calibrated Conc. [ng/µl]	Assigned Conc. [ng/µl]	% Integrated Area	From [bp]	To [bp]	Peak Comment	Observations
100	8.50	8.50	-	61	151		Lower Marker
20637	35.2	-	93.32	5407	>60000		
-	-	-	-	-	-		Sample Well

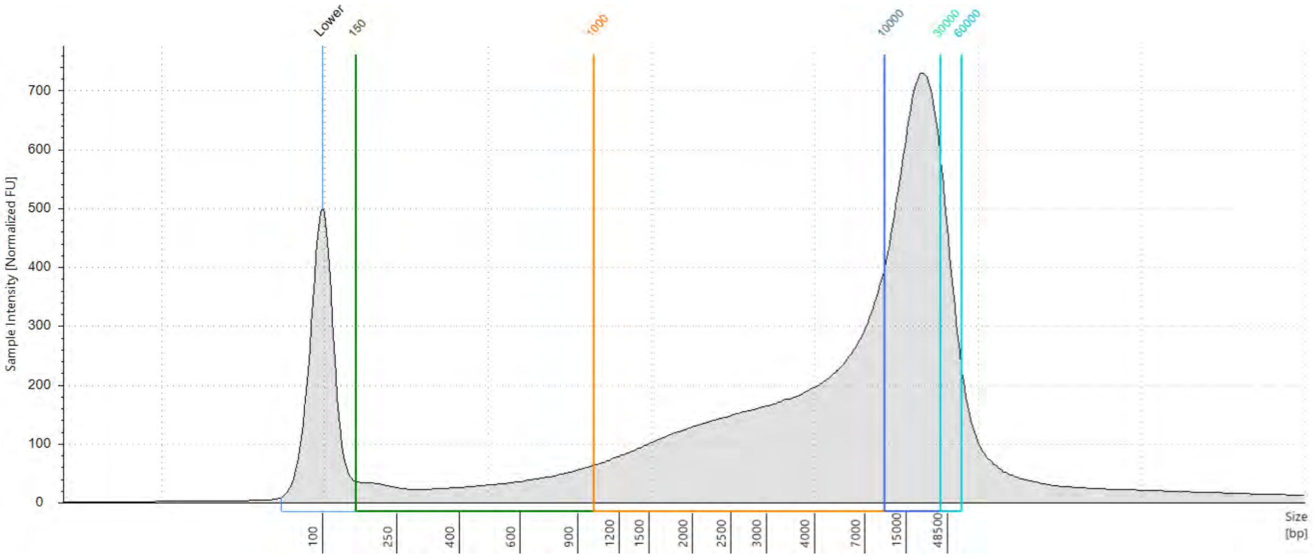

Region Table

From [bp]	To [bp]	Average Size [bp]	Conc. [ng/µl]	Region Molarity [nmol/l]	% of Total	Region Comment	Color
150	10000	3623	32.7	33.1	49.63		
150	1000	555	4.55	18.1	6.90		
1000	10000	4143	28.2	15.1	42.81		
10000	60000	24345	25.6	2.04	38.89		
10000	30000	18930	21.1	1.89	31.98		

 © Agilent Technologies, Inc. 2017

30000	60000	48966	5.04	0.172	7.64		

© Agilent Technologies, Inc. 2017

B5: M33

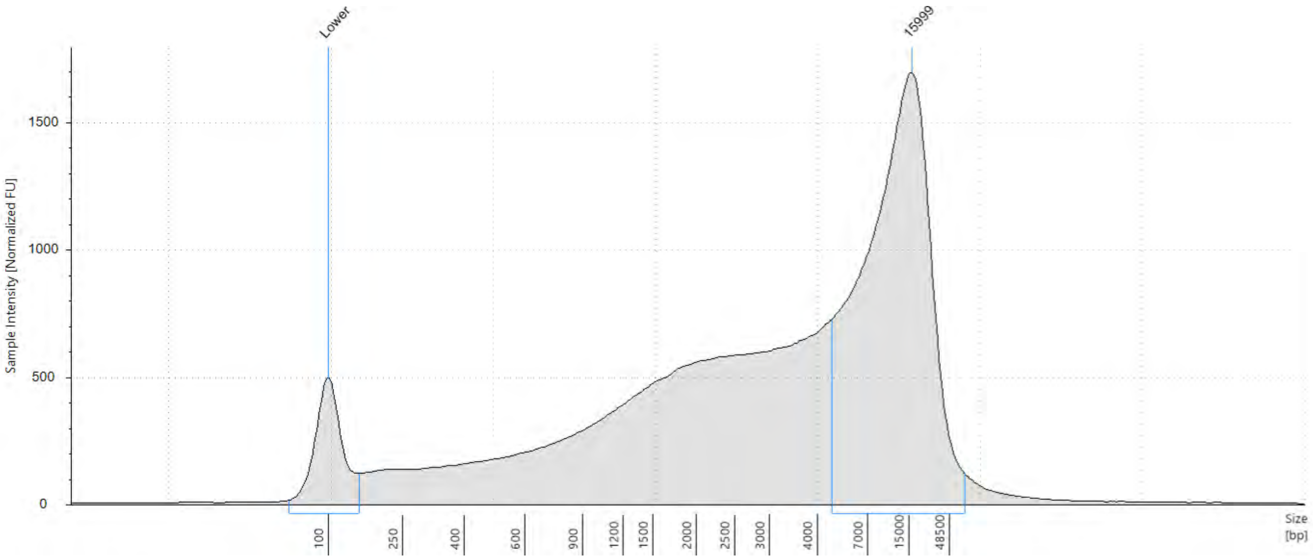

Sample Table

Well	DIN	Conc. [ng/µl]	Sample Description	Alert	Observations
B5	5.9	170	M33	⚠	Sample concentration outside recommended range

Peak Table

Size [bp]	Calibrated Conc. [ng/µl]	Assigned Conc. [ng/µl]	% Integrated Area	From [bp]	To [bp]	Peak Comment	Observations
100	8.50	8.50	-	62	146		Lower Marker
15999	72.5	-	100.00	4751	>60000		

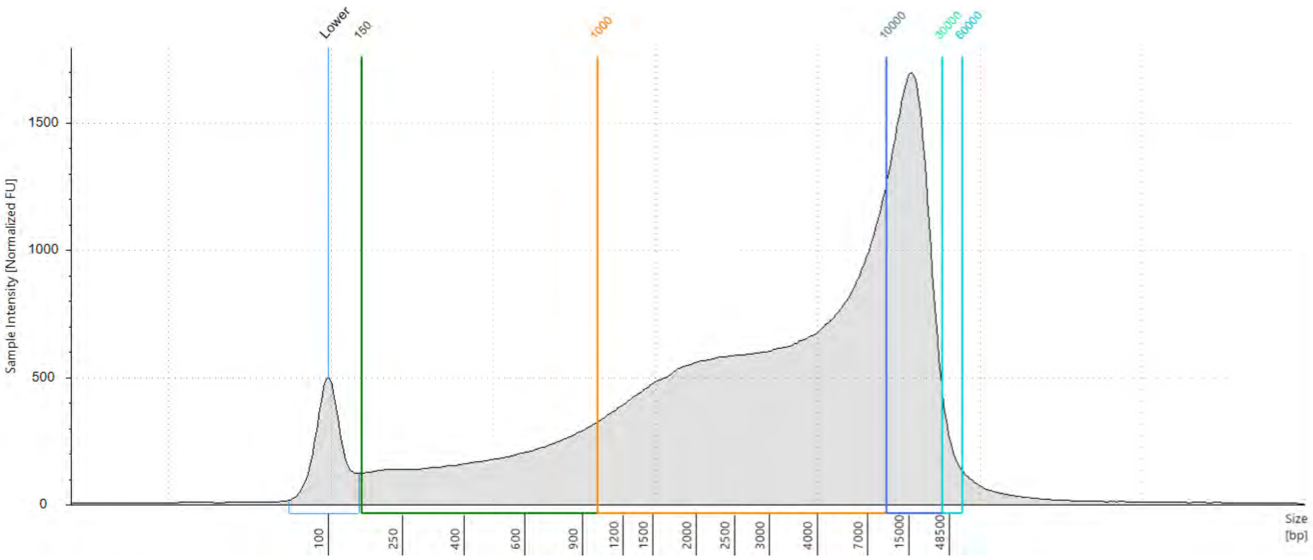

Region Table

From [bp]	To [bp]	Average Size [bp]	Conc. [ng/µl]	Region Molarity [nmol/l]	% of Total	Region Comment	Color
150	10000	3175	123	146	72.49		
150	1000	555	24.0	89.0	14.14		
1000	10000	3820	99.4	57.8	58.49		
10000	60000	18642	44.7	4.30	26.29		
10000	30000	16784	42.0	4.20	24.71		
30000	60000	46527	3.05	0.113	1.79		

 © Agilent Technologies, Inc. 2017

C5: M34

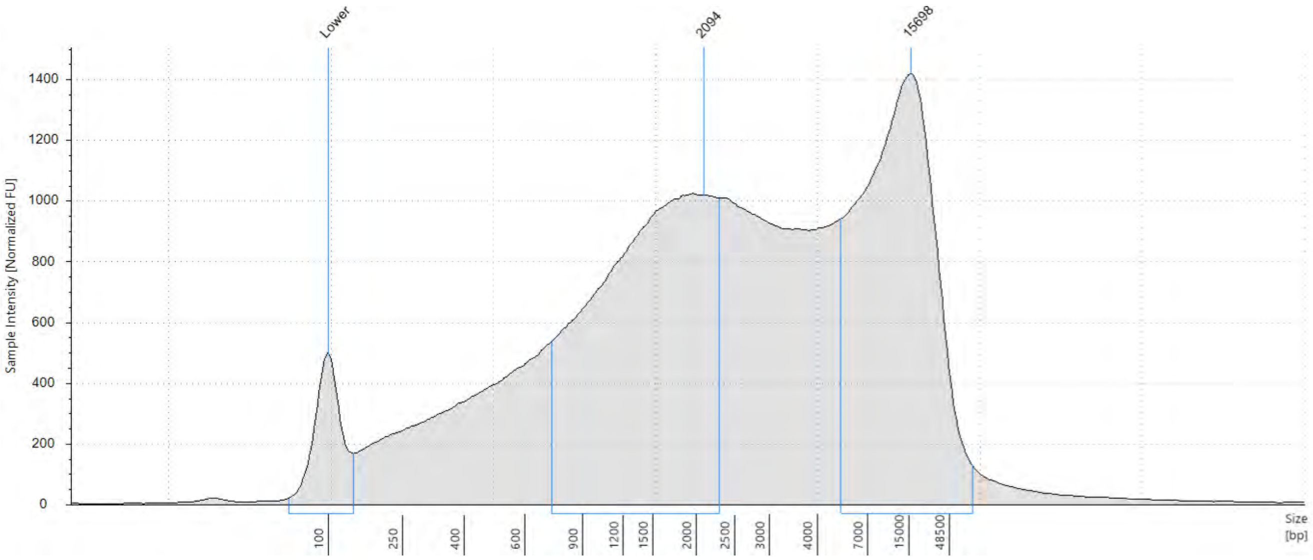

Sample Table

Well	DIN	Conc. [ng/µl]	Sample Description	Alert	Observations
C5	4.7	251	M34	⚠	Sample concentration outside recommended range

Peak Table

Size [bp]	Calibrated Conc. [ng/µl]	Assigned Conc. [ng/µl]	% Integrated Area	From [bp]	To [bp]	Peak Comment	Observations
100	8.50	8.50	-	62	136		Lower Marker
2094	79.0	-	52.94	725	2292		
15698	70.2	-	47.06	5210	>60000		

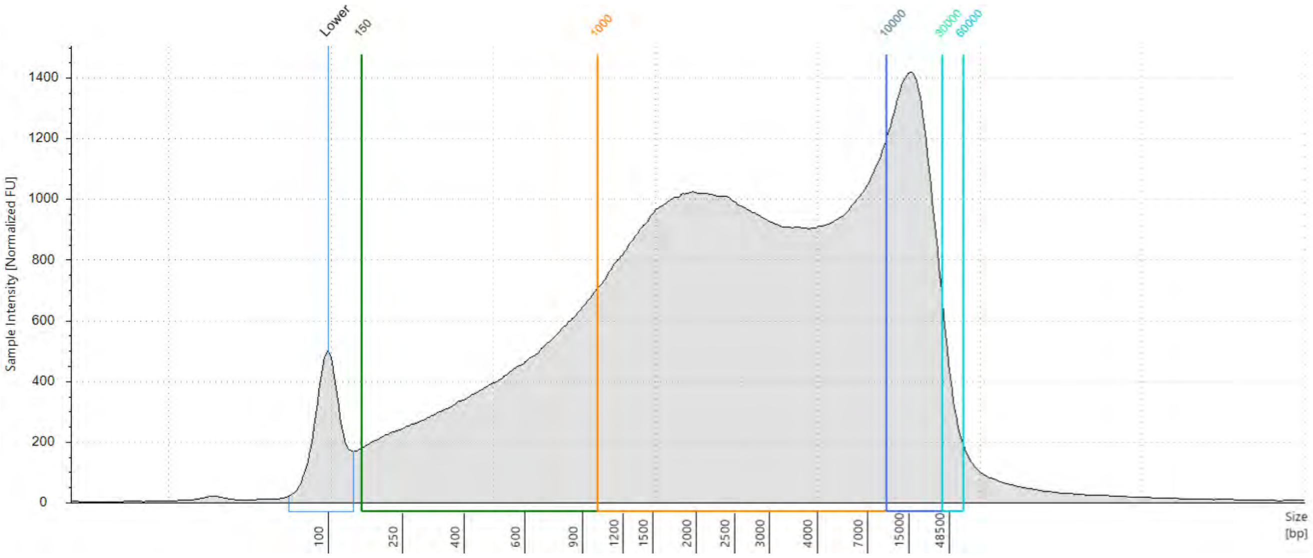

Region Table

From [bp]	To [bp]	Average Size [bp]	Conc. [ng/µl]	Region Molarity [nmol/l]	% of Total	Region Comment	Color
150	10000	2630	204	277	81.25		
150	1000	575	51.3	177	20.40		
1000	10000	3325	154	101	61.08		
10000	60000	20316	42.9	3.96	17.06		
10000	30000	17284	38.7	3.82	15.40		

 © Agilent Technologies, Inc. 2017

30000	60000	47488	4.68	0.169	1.86		

© Agilent Technologies, Inc. 2017

D5: M35

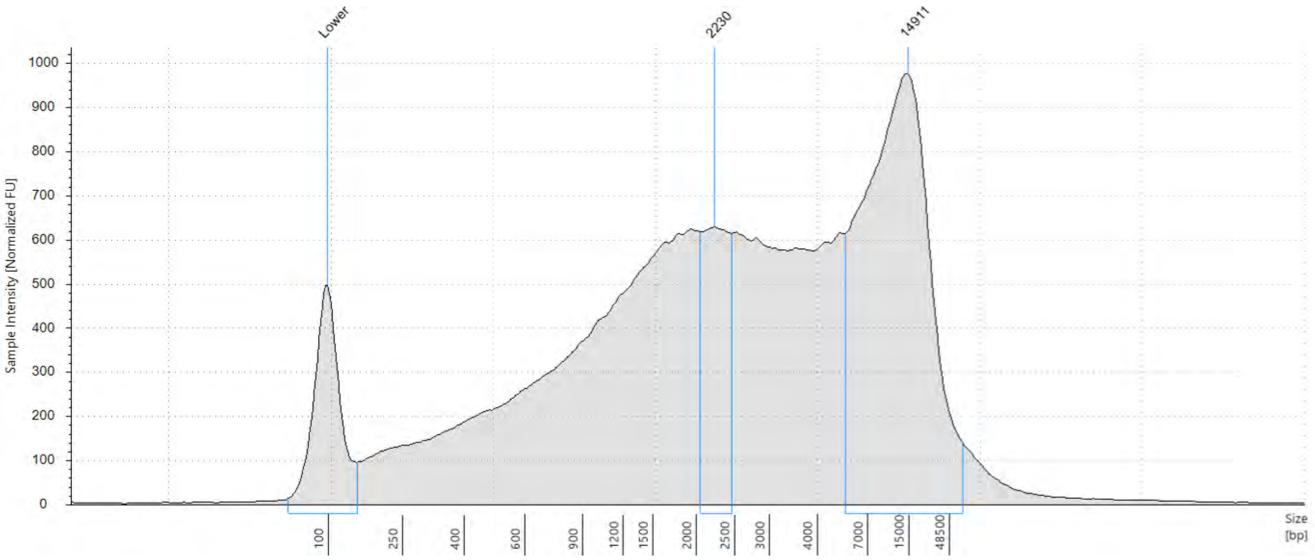

Sample Table

Well	DIN	Conc. [ng/µl]	Sample Description	Alert	Observations
D5	5.1	162	M35	⚠	Sample concentration outside recommended range

Peak Table

Size [bp]	Calibrated Conc. [ng/µl]	Assigned Conc. [ng/µl]	% Integrated Area	From [bp]	To [bp]	Peak Comment	Observations
100	8.50	8.50	-	62	145		Lower Marker
2230	12.2	-	21.10	2056	2479		
14911	45.4	-	78.78	5505	>60000		
-	-	-	-	-	-		Sample Well

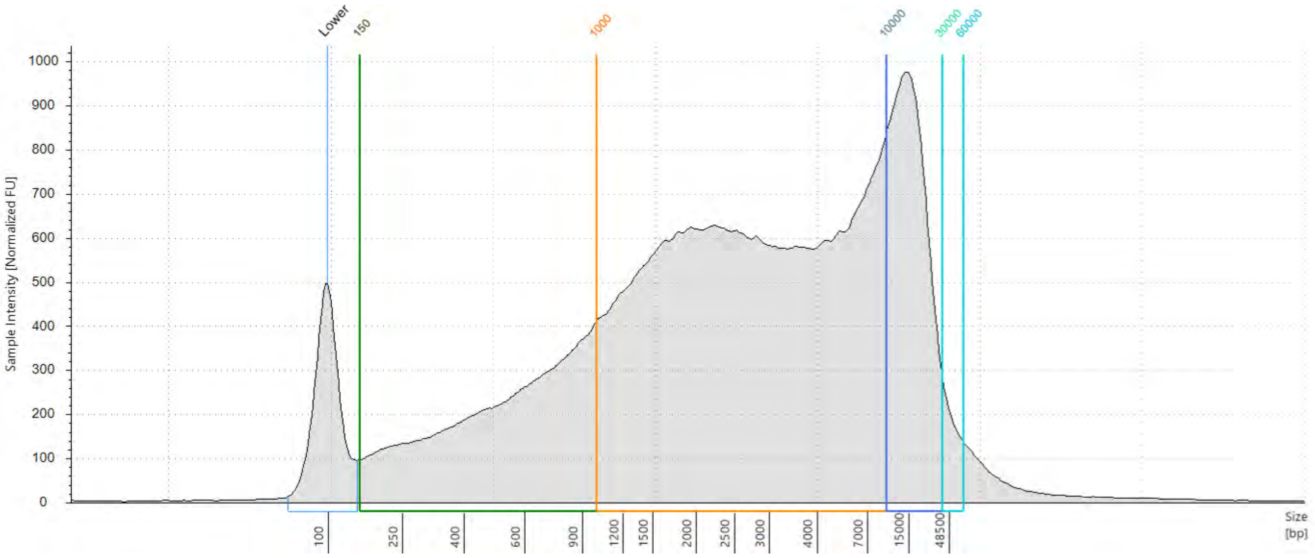

Region Table

From [bp]	To [bp]	Average Size [bp]	Conc. [ng/µl]	Region Molarity [nmol/l]	% of Total	Region Comment	Color
150	10000	2776	131	168	81.20		
150	1000	578	29.8	104	18.42		
1000	10000	3432	102	65.4	62.99		
10000	60000	19329	28.1	2.71	17.38		

 © Agilent Technologies, Inc. 2017

10000	30000	16640	25.8	2.63	15.97		
30000	60000	48099	2.53	0.0911	1.56		

© Agilent Technologies, Inc. 2017

E5: M36

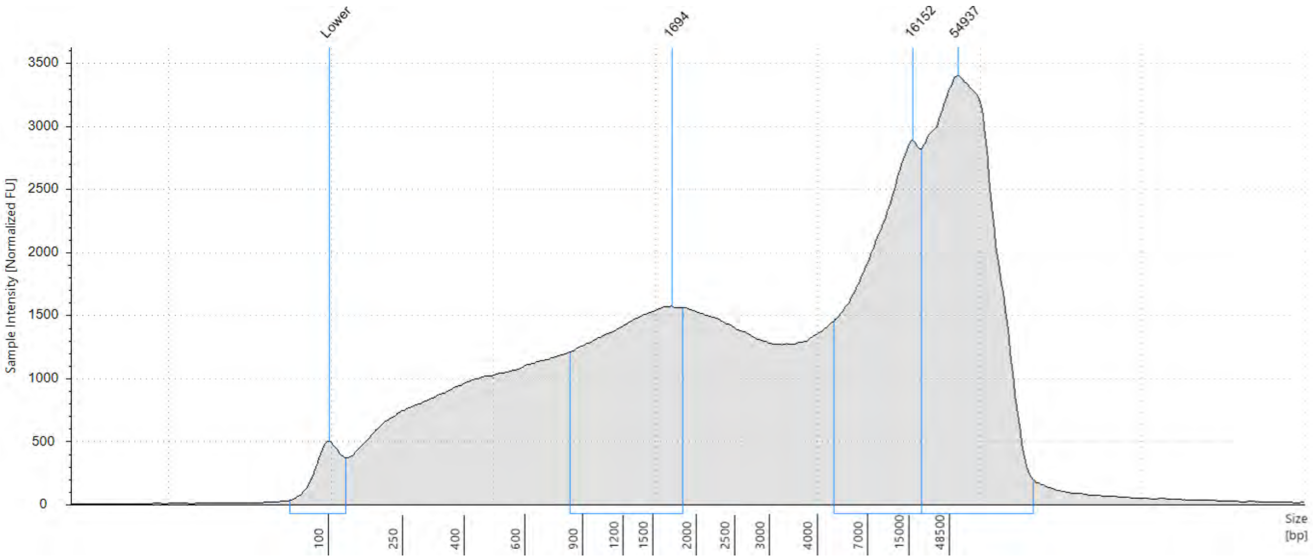

Sample Table

Well	DIN	Conc. [ng/µl]	Sample Description	Alert	Observations
E5	5.9	571	M36	⚠	Sample concentration outside functional range for DIN

Peak Table

Size [bp]	Calibrated Conc. [ng/µl]	Assigned Conc. [ng/µl]	% Integrated Area	From [bp]	To [bp]	Peak Comment	Observations
100	8.50	8.50	-	62	123		Lower Marker
1694	89.7	-	25.88	821	1824		
16152	106	-	30.69	4764	18888		
54937	149	-	43.05	18888	>60000		
-	-	-	-	-	-		Sample Well

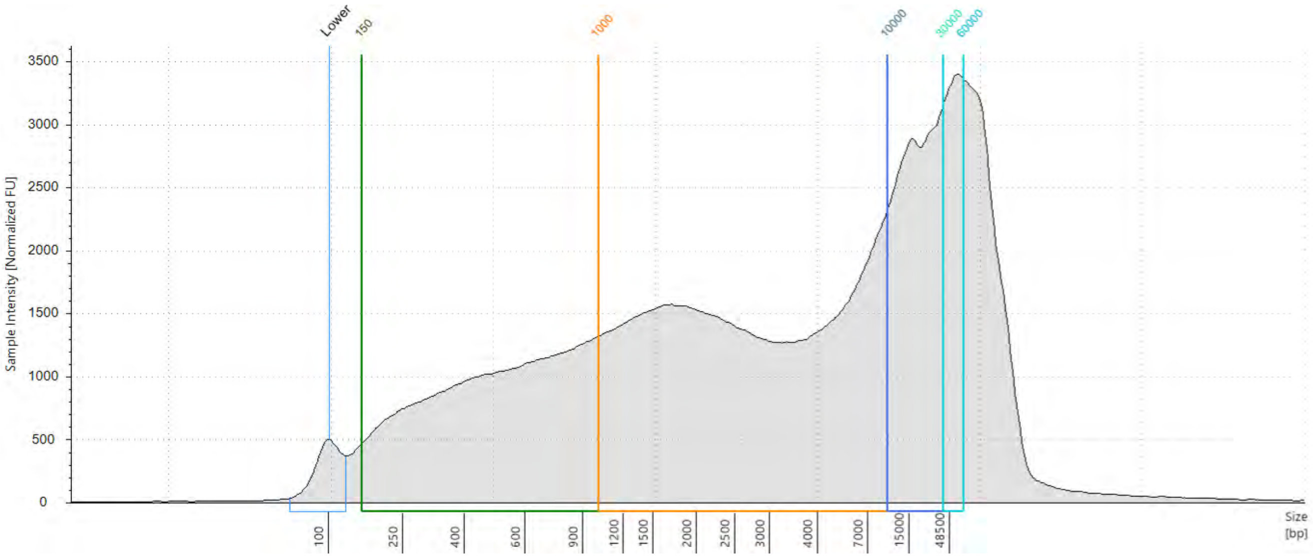

Region Table

From [bp]	To [bp]	Average Size [bp]	Conc. [ng/µl]	Region Molarity [nmol/l]	% of Total	Region Comment	Color
150	10000	2433	364	621	63.83		
150	1000	540	126	465	22.15		
1000	10000	3442	239	157	41.86		

 © Agilent Technologies, Inc. 2017

10000	60000	28426	127	9.46	22.32		
10000	30000	18508	88.7	8.25	15.54		
30000	60000	50723	41.2	1.34	7.21		

© Agilent Technologies, Inc. 2017

F5: M37

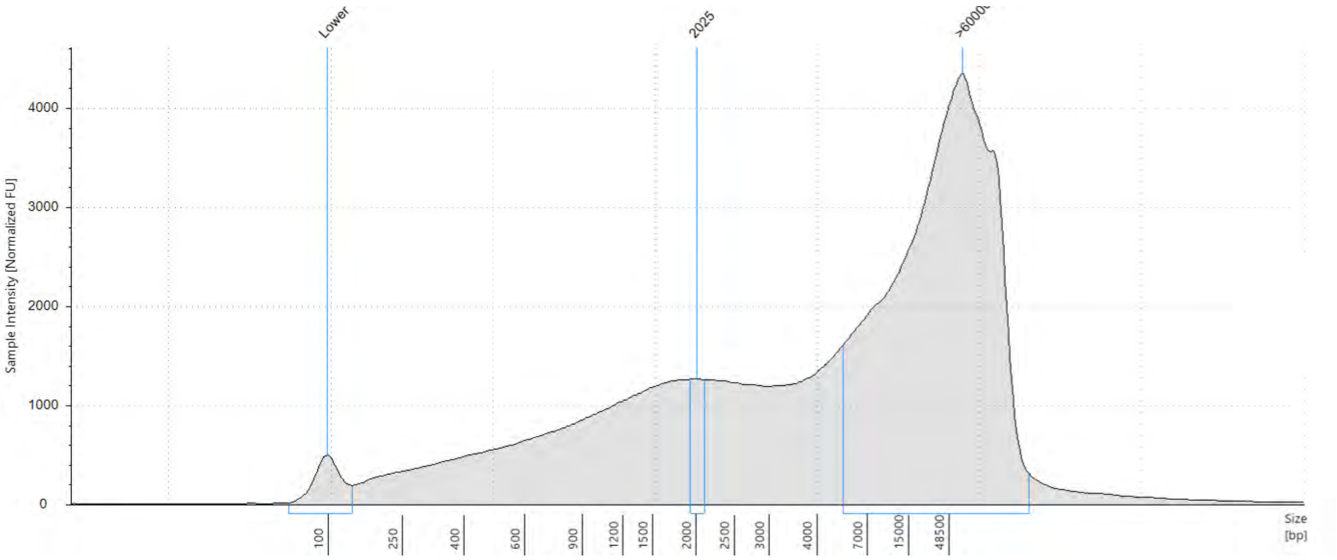

Sample Table

Well	DIN	Conc. [ng/µl]	Sample Description	Alert	Observations
F5	6.4	537	M37	⚠	Sample concentration outside functional range for DIN

Peak Table

Size [bp]	Calibrated Conc. [ng/µl]	Assigned Conc. [ng/µl]	% Integrated Area	From [bp]	To [bp]	Peak Comment	Observations
100	8.50	8.50	-	62	136		Lower Marker
2025	10.9	-	3.60	1938	2109		
>60000	287	-	94.92	5405	>60000		
-	-	-	-	-	-		Sample Well

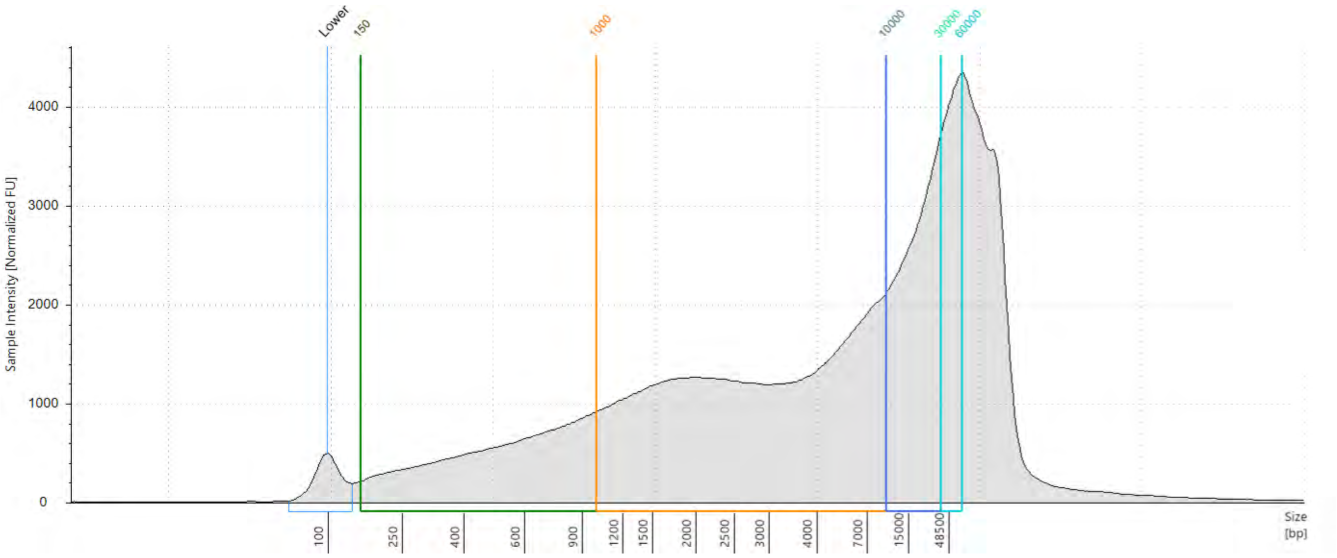

Region Table

From [bp]	To [bp]	Average Size [bp]	Conc. [ng/µl]	Region Molarity [nmol/l]	% of Total	Region Comment	Color
150	10000	2851	287	383	53.36		green
150	1000	571	70.7	248	13.16		red
1000	10000	3611	217	135	40.33		orange
10000	60000	30414	141	9.82	26.25		blue

 © Agilent Technologies, Inc. 2017

10000	30000	19189	92.2	8.31	17.15		
30000	60000	51253	51.9	1.66	9.65		

 © Agilent Technologies, Inc. 2017

G5: M38

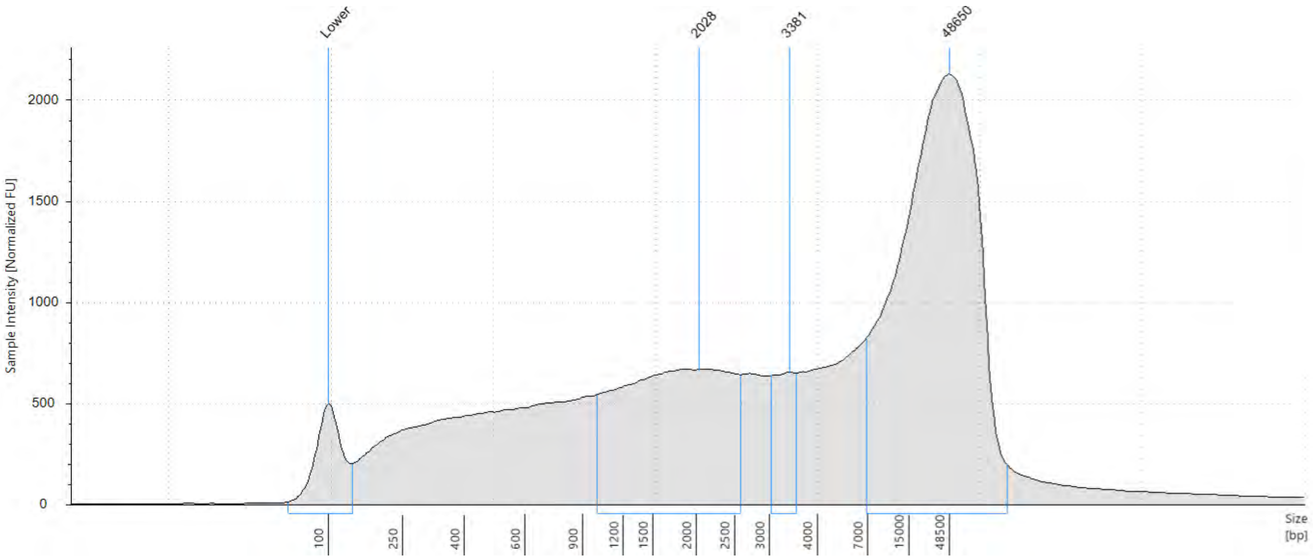

Sample Table

Well	DIN	Conc. [ng/µl]	Sample Description	Alert	Observations
G5	6.0	285	M38	⚠	Sample concentration outside recommended range

Peak Table

Size [bp]	Calibrated Conc. [ng/µl]	Assigned Conc. [ng/µl]	% Integrated Area	From [bp]	To [bp]	Peak Comment	Observations
100	8.50	8.50	-	61	134		Lower Marker
2028	51.9	-	28.73	997	2582		
3381	9.72	-	5.38	3032	3526		
48650	113	-	62.38	6855	>60000		
-	-	-	-	-	-		Sample Well

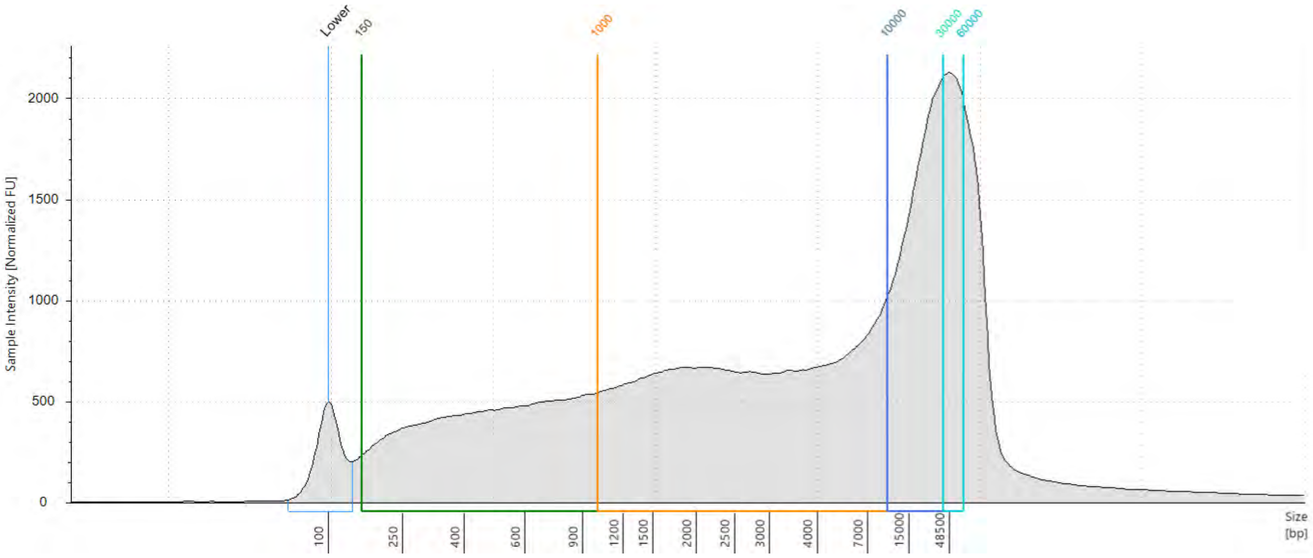

Region Table

From [bp]	To [bp]	Average Size [bp]	Conc. [ng/µl]	Region Molarity [nmol/l]	% of Total	Region Comment	Color
150	10000	2468	170	295	59.46		
150	1000	526	58.5	224	20.52		
1000	10000	3496	112	71.8	39.10		

10000	60000	29416	75.7	5.29	26.53		
10000	30000	19668	52.2	4.56	18.31		
30000	60000	50813	25.2	0.815	8.82		

H5: M39

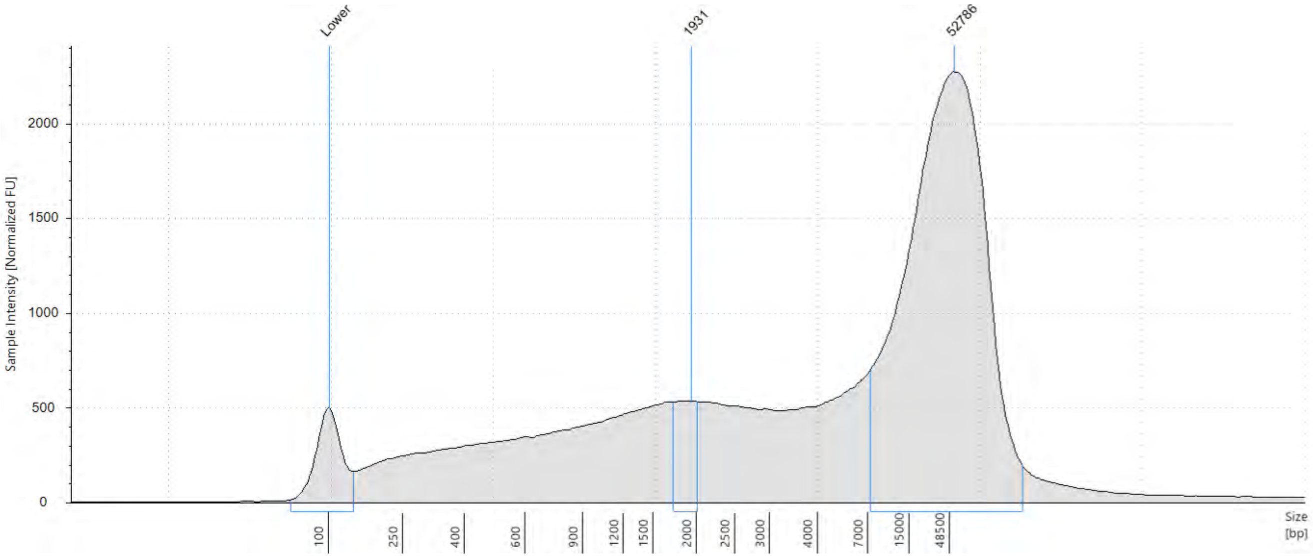

Sample Table

Well	DIN	Conc. [ng/µl]	Sample Description	Alert	Observations
H5	6.4	254	M39	⚠	Sample concentration outside recommended range

Peak Table

Size [bp]	Calibrated Conc. [ng/µl]	Assigned Conc. [ng/µl]	% Integrated Area	From [bp]	To [bp]	Peak Comment	Observations
100	8.50	8.50	-	62	136		Lower Marker
1931	7.61	-	5.64	1715	2002		
52786	123	-	91.21	7344	>60000		
-	-	-	-	-	-		Sample Well

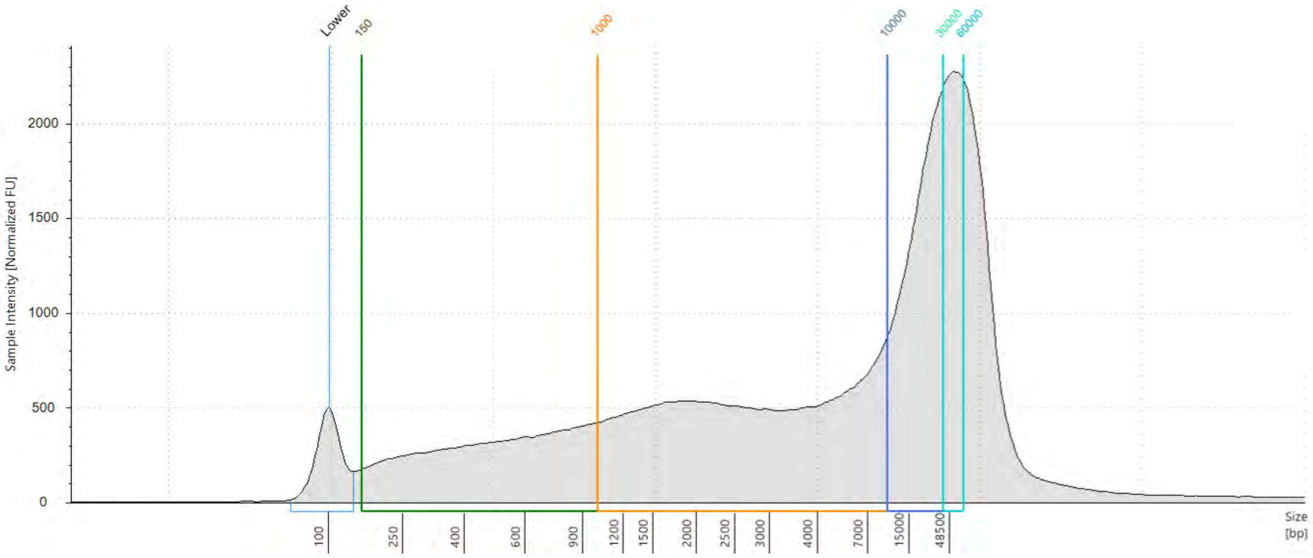

Region Table

From [bp]	To [bp]	Average Size [bp]	Conc. [ng/µl]	Region Molarity [nmol/l]	% of Total	Region Comment	Color
150	10000	2530	129	216	51.00		Green
150	1000	536	41.4	159	16.32		Red
1000	10000	3482	88.4	57.7	34.82		Orange
10000	60000	30625	78.5	5.32	30.93		Blue

 © Agilent Technologies, Inc. 2017

10000	30000	19702	51.2	4.47	20.19		
30000	60000	50911	29.1	0.942	11.45		

A6: M40

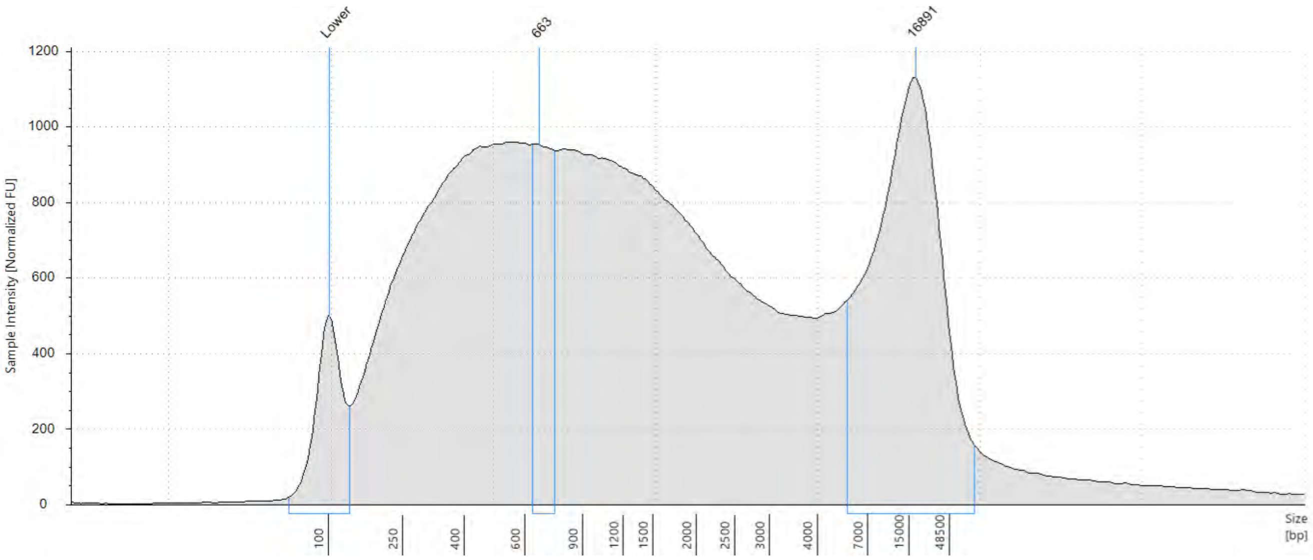

Sample Table

Well	DIN	Conc. [ng/µl]	Sample Description	Alert	Observations
A6	1.8	264	M40	⚠	Sample concentration outside recommended range

Peak Table

Size [bp]	Calibrated Conc. [ng/µl]	Assigned Conc. [ng/µl]	% Integrated Area	From [bp]	To [bp]	Peak Comment	Observations
100	8.50	8.50	-	61	129		Lower Marker
663	12.5	-	19.67	631	738		
16891	50.4	-	79.64	5557	>60000		
-	-	-	-	-	-		Sample Well

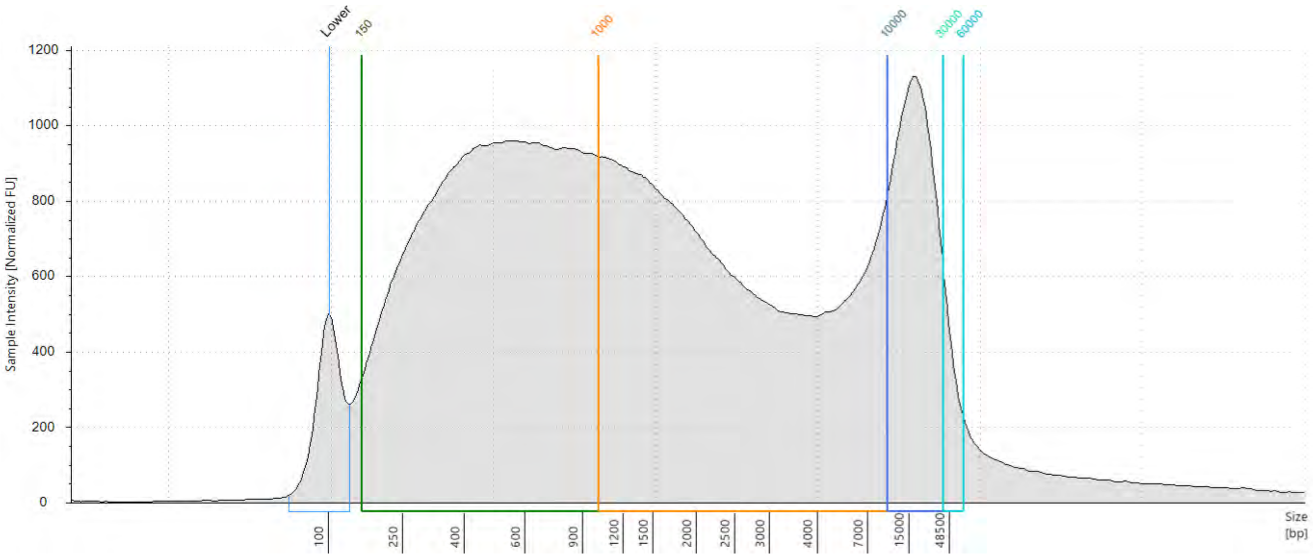

Region Table

From [bp]	To [bp]	Average Size [bp]	Conc. [ng/µl]	Region Molarity [nmol/l]	% of Total	Region Comment	Color
150	10000	1757	217	487	82.01		green
150	1000	524	110	409	41.76		red
1000	10000	3030	107	79.7	40.51		orange
10000	60000	21454	35.1	3.12	13.30		blue

10000	30000	17634	30.7	2.97	11.63		
30000	60000	47150	4.92	0.179	1.86		

© Agilent Technologies, Inc. 2017

B6: M41

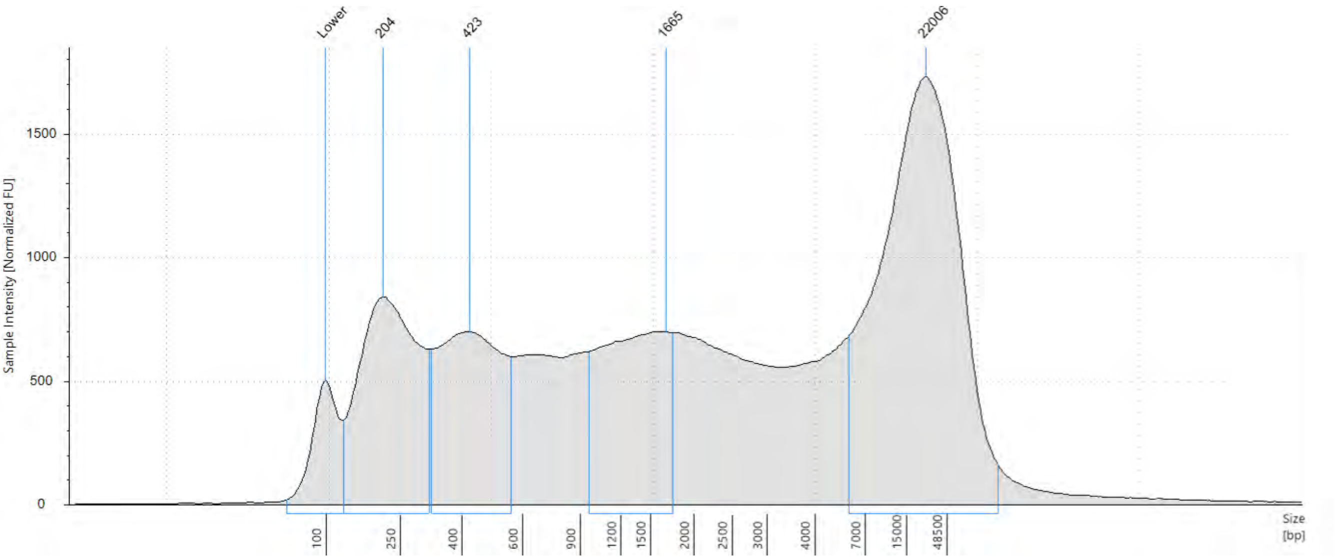

Sample Table

Well	DIN	Conc. [ng/µl]	Sample Description	Alert	Observations
B6	4.7	276	M41	⚠	Sample concentration outside recommended range

Peak Table

Size [bp]	Calibrated Conc. [ng/µl]	Assigned Conc. [ng/µl]	% Integrated Area	From [bp]	To [bp]	Peak Comment	Observations
100	8.50	8.50	-	62	125		Lower Marker
204	32.8	-	17.47	125	311		
423	30.2	-	16.05	318	558		
1665	32.3	-	17.20	958	1742		
22006	90.7	-	48.27	5869	>60000		
-	-	-	-	-	-		Sample Well

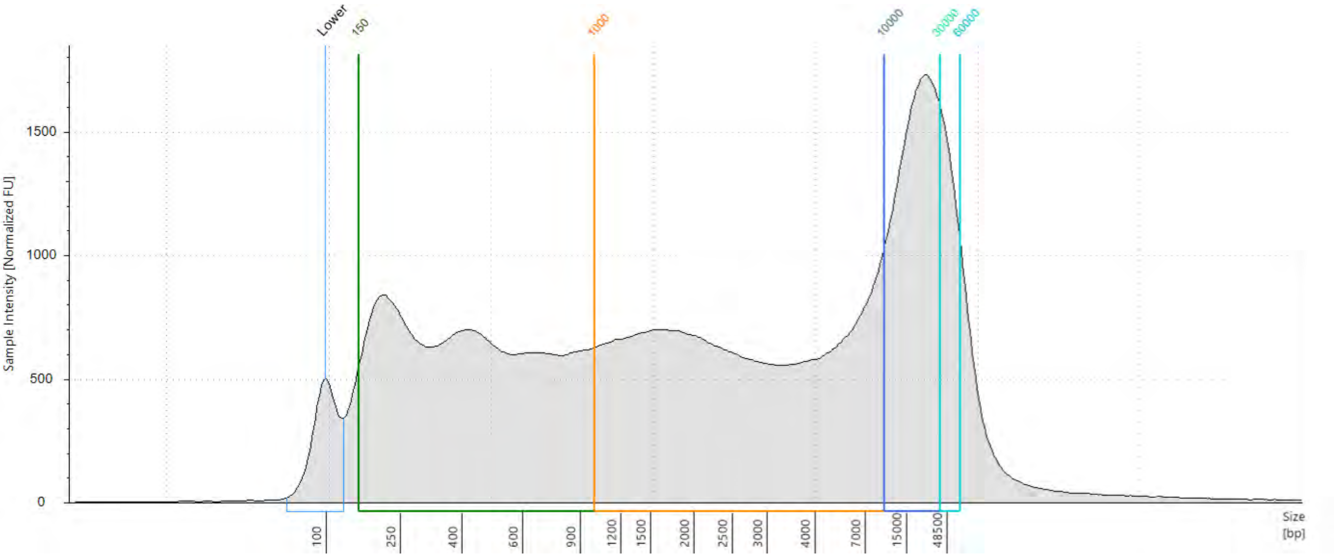

Region Table

From [bp]	To [bp]	Average Size [bp]	Conc. [ng/µl]	Region Molarity [nmol/l]	% of Total	Region Comment	Color
150	10000	2066	196	456	70.76		■
150	1000	472	88.8	384	32.11		■

 © Agilent Technologies, Inc. 2017

1000	10000	3384	107	72.5	38.83		
10000	60000	26405	64.2	4.90	23.22		
10000	30000	18765	48.2	4.38	17.42		
30000	60000	48968	17.3	0.591	6.26		

© Agilent Technologies, Inc. 2017

C6: M42

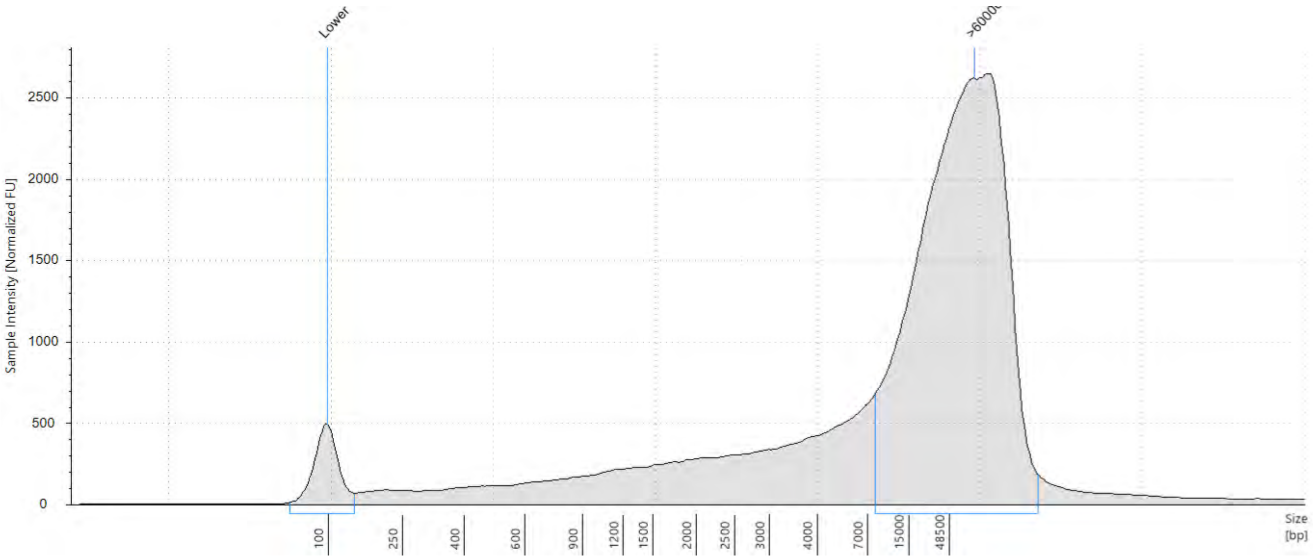

Sample Table

Well	DIN	Conc. [ng/µl]	Sample Description	Alert	Observations
C6	7.3	260	M42	⚠	Sample concentration outside recommended range

Peak Table

Size [bp]	Calibrated Conc. [ng/µl]	Assigned Conc. [ng/µl]	% Integrated Area	From [bp]	To [bp]	Peak Comment	Observations
100	8.50	8.50	-	63	140		Lower Marker
>60000	173	-	93.11	8173	>60000		
-	-	-	-	-	-		Sample Well

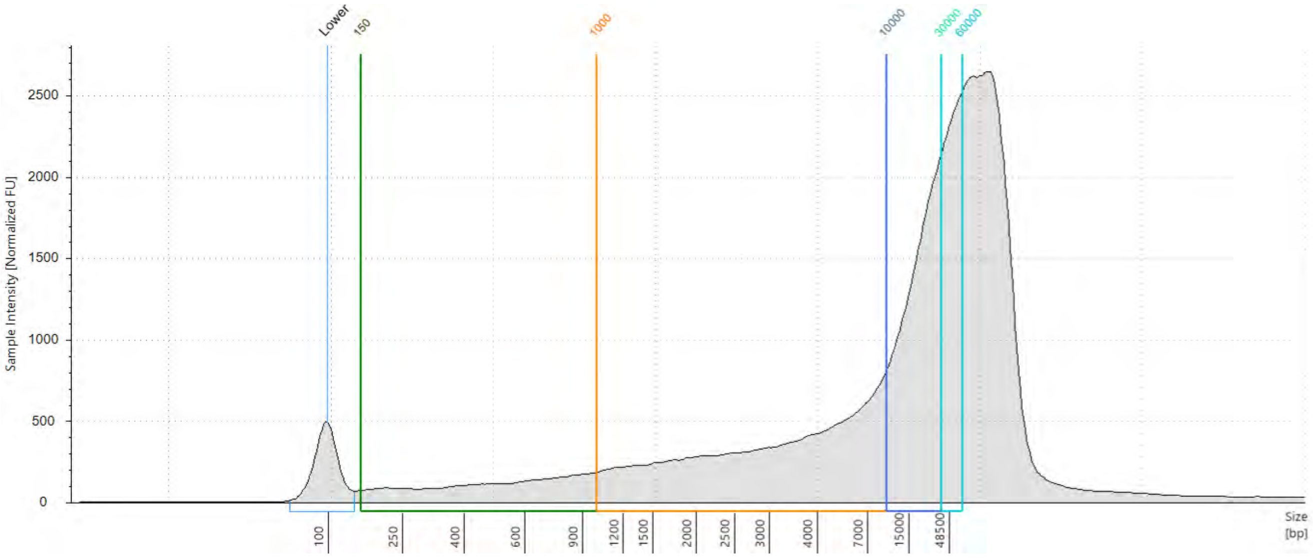

Region Table

From [bp]	To [bp]	Average Size [bp]	Conc. [ng/µl]	Region Molarity [nmol/l]	% of Total	Region Comment	Color
150	10000	3272	79.5	99.0	30.54		
150	1000	551	16.1	63.2	6.20		
1000	10000	4001	63.5	36.0	24.40		
10000	60000	31537	83.9	5.53	32.25		
10000	30000	19816	52.9	4.57	20.32		

 © Agilent Technologies, Inc. 2017

30000	60000	51276	32.9	1.06	12.66		

© Agilent Technologies, Inc. 2017

D6: M43

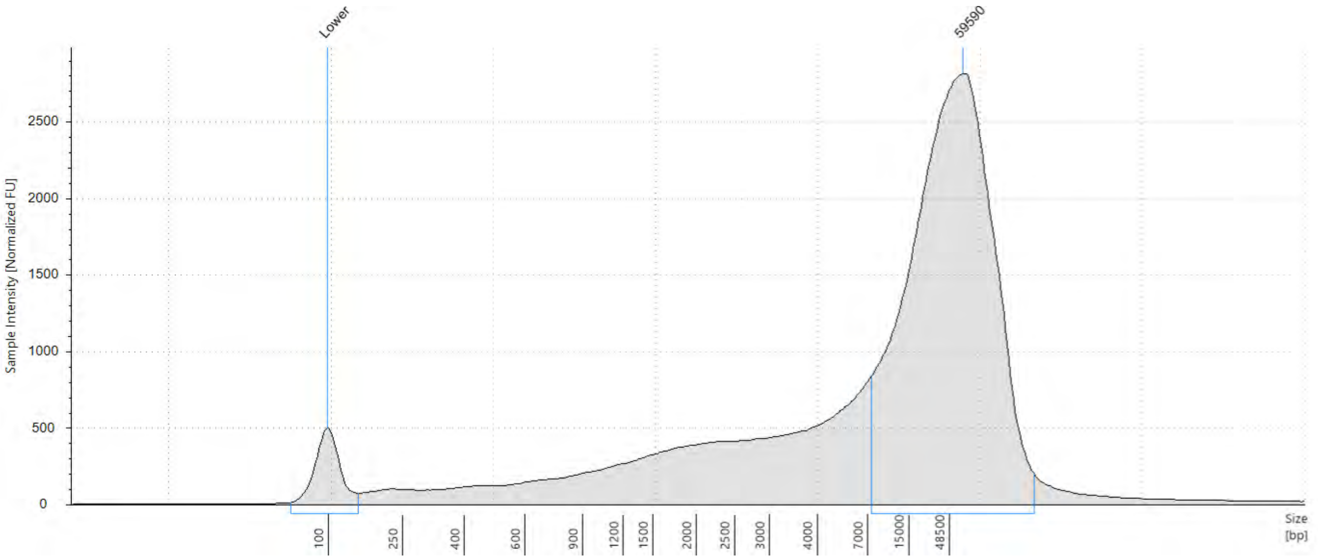

Sample Table

Well	DIN	Conc. [ng/µl]	Sample Description	Alert	Observations
D6	7.0	266	M43	⚠	Sample concentration outside recommended range

Peak Table

Size [bp]	Calibrated Conc. [ng/µl]	Assigned Conc. [ng/µl]	% Integrated Area	From [bp]	To [bp]	Peak Comment	Observations
100	8.50	8.50	-	63	145		Lower Marker
59590	168	-	96.29	7536	>60000		
-	-	-	-	-	-		Sample Well

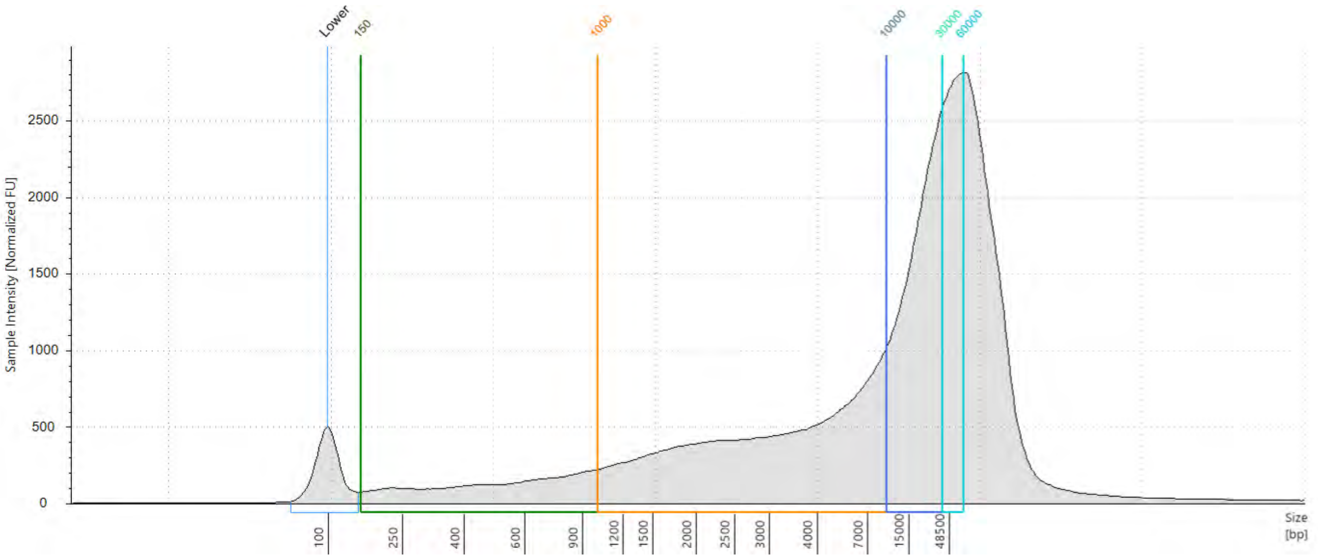

Region Table

From [bp]	To [bp]	Average Size [bp]	Conc. [ng/µl]	Region Molarity [nmol/l]	% of Total	Region Comment	Color
150	10000	3323	97.7	113	36.74		
150	1000	558	17.7	67.7	6.67		
1000	10000	3965	80.1	45.4	30.14		
10000	60000	31074	96.8	6.47	36.43		
10000	30000	19739	62.1	5.39	23.35		

 © Agilent Technologies, Inc. 2017

30000	60000	51050	37.0	1.19	13.92		

© Agilent Technologies, Inc. 2017

E6: M44

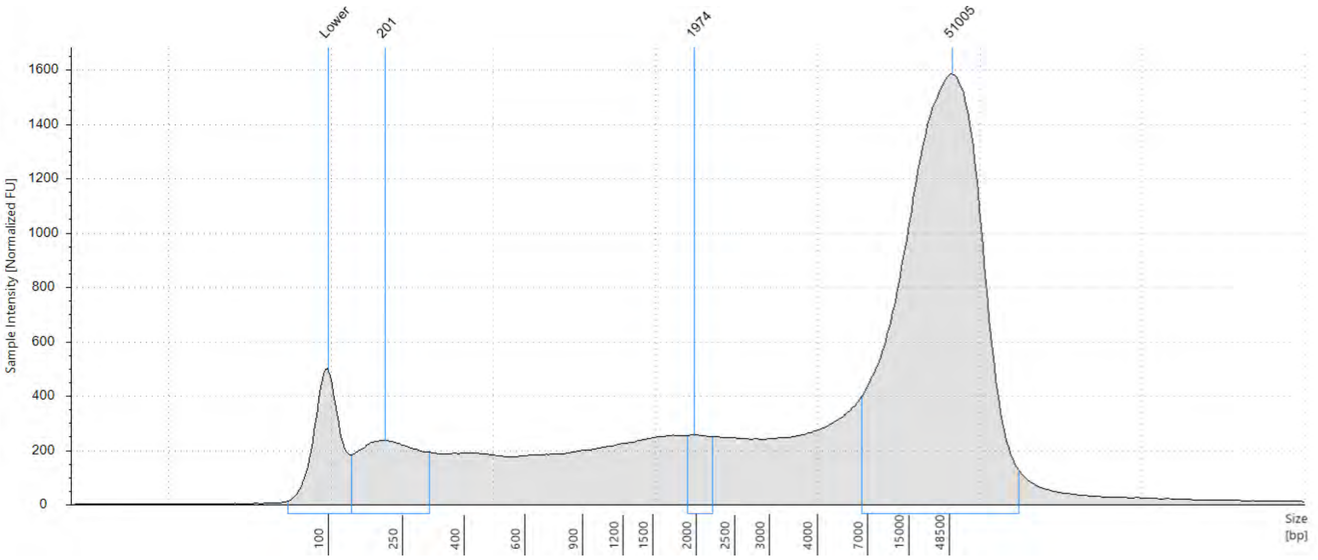

Sample Table

Well	DIN	Conc. [ng/µl]	Sample Description	Alert	Observations
E6	6.5	152	M44	⚠	Sample concentration outside recommended range

Peak Table

Size [bp]	Calibrated Conc. [ng/µl]	Assigned Conc. [ng/µl]	% Integrated Area	From [bp]	To [bp]	Peak Comment	Observations
100	8.50	8.50	-	61	134		Lower Marker
201	9.34	-	9.40	134	306		
1974	3.70	-	3.73	1885	2198		
51005	83.2	-	83.80	6579	>60000		
-	-	-	-	-	-		Sample Well

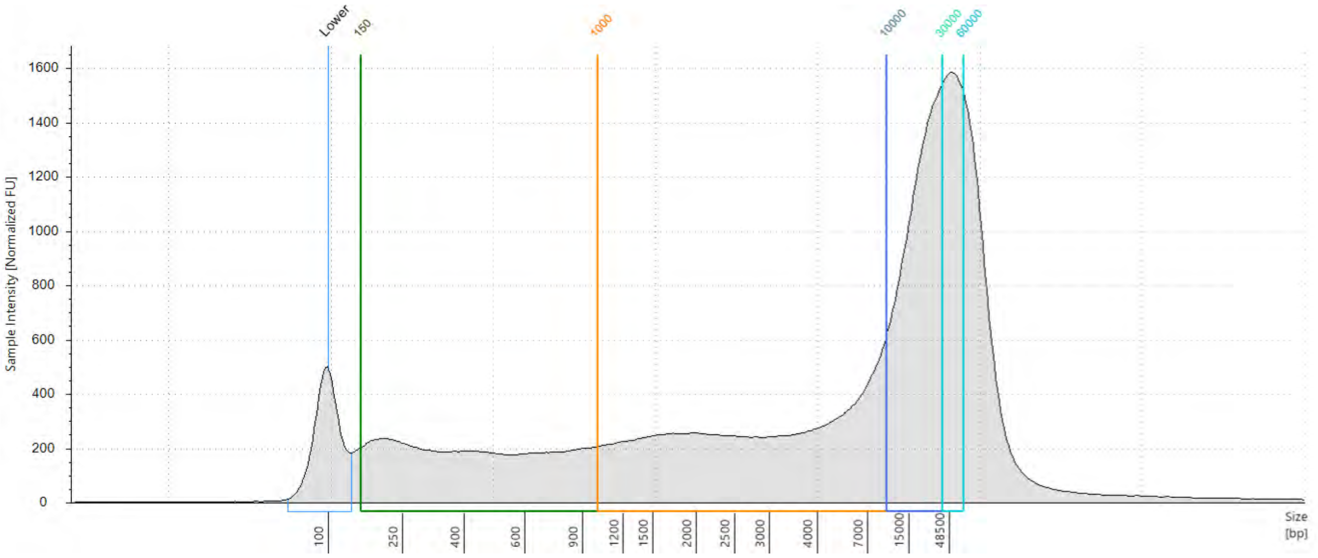

Region Table

From [bp]	To [bp]	Average Size [bp]	Conc. [ng/µl]	Region Molarity [nmol/l]	% of Total	Region Comment	Color
150	10000	2557	70.2	140	46.22		
150	1000	480	25.7	113	16.91		
1000	10000	3758	44.7	27.6	29.41		

10000	60000	29724	54.3	3.75	35.73		
10000	30000	19366	35.9	3.15	23.63		
30000	60000	49726	19.6	0.654	12.89		

© Agilent Technologies, Inc. 2017

F6: M45

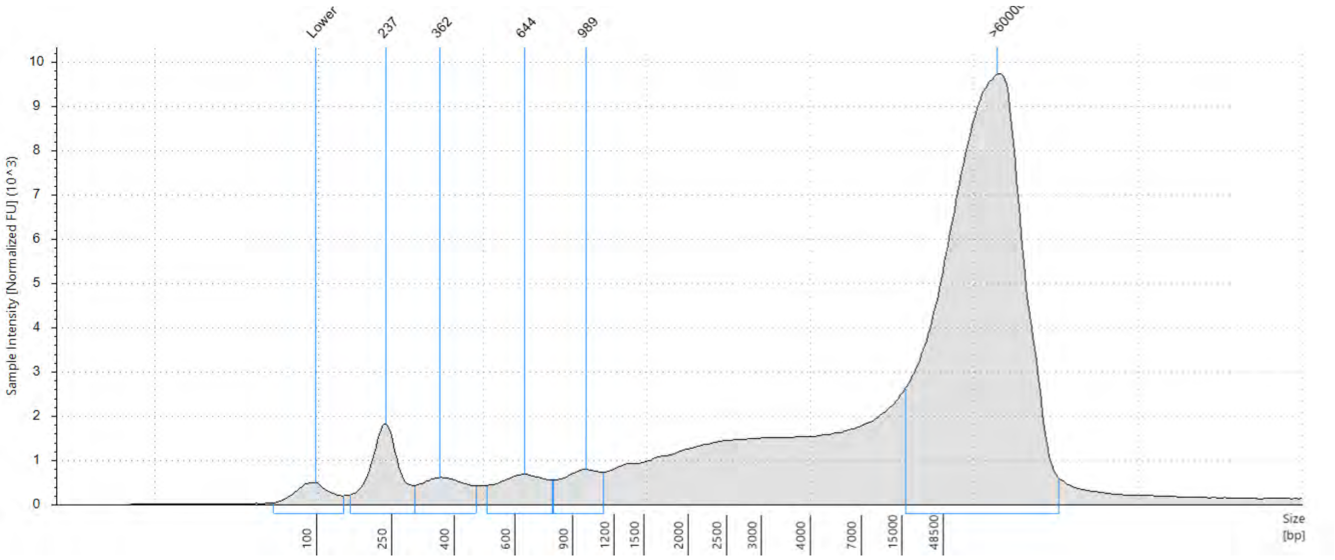

Sample Table

Well	DIN	Conc. [ng/µl]	Sample Description	Alert	Observations
F6	6.9	723	M45	⚠	Sample concentration outside functional range for DIN

Peak Table

Size [bp]	Calibrated Conc. [ng/µl]	Assigned Conc. [ng/µl]	% Integrated Area	From [bp]	To [bp]	Peak Comment	Observations
100	8.50	8.50	-	60	141		Lower Marker
237	26.1	-	5.05	153	299		
362	14.5	-	2.79	299	466		
644	17.1	-	3.30	500	783		
989	15.8	-	3.06	791	1117		
>60000	409	-	79.13	16217	>60000		
-	-	-	-	-	-		Sample Well

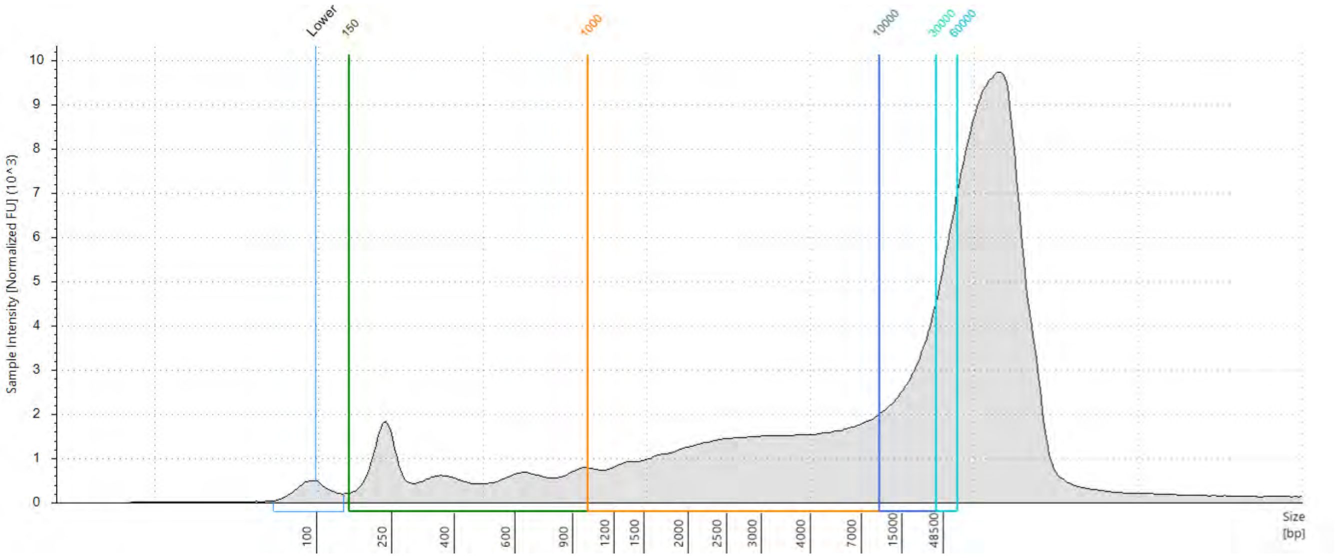

Region Table

From [bp]	To [bp]	Average Size [bp]	Conc. [ng/µl]	Region Molarity [nmol/l]	% of Total	Region Comment	Color
150	10000	2744	248	419	34.35		■

150	1000	472	70.0	313	9.69		
1000	10000	3654	179	106	24.72		
10000	60000	33419	135	8.56	18.61		
10000	30000	19548	76.9	6.79	10.63		
30000	60000	51688	60.5	1.92	8.37		

 © Agilent Technologies, Inc. 2017

G6: W676

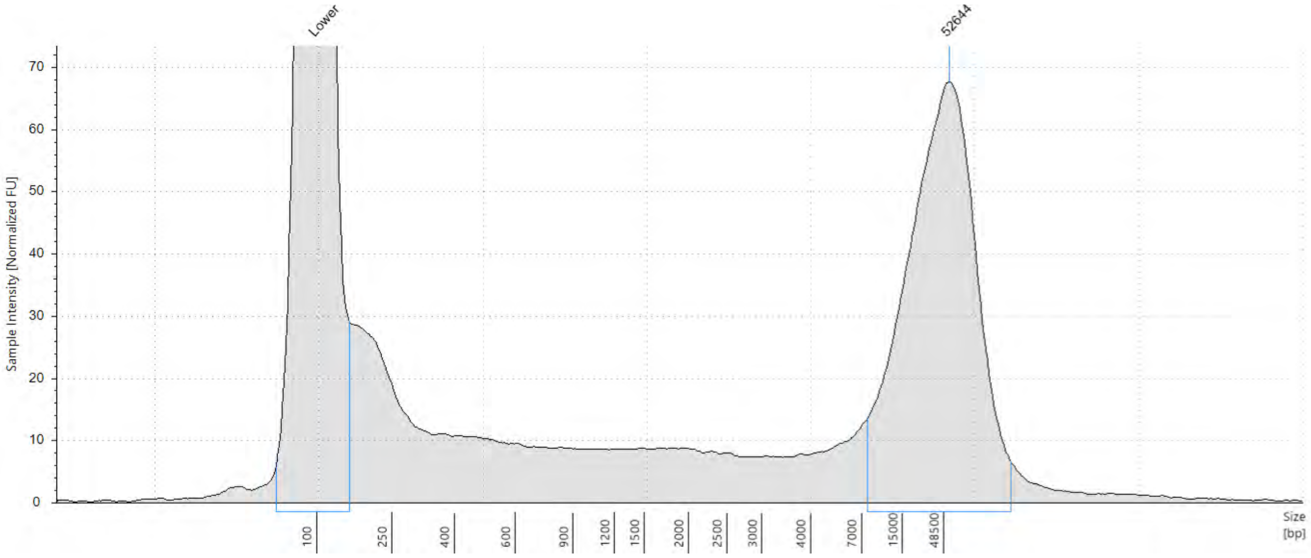

Sample Table

Well	DIN	Conc. [ng/µl]	Sample Description	Alert	Observations
G6	6.7	6.91	W676	⚠	Sample concentration outside recommended range

Peak Table

Size [bp]	Calibrated Conc. [ng/µl]	Assigned Conc. [ng/µl]	% Integrated Area	From [bp]	To [bp]	Peak Comment	Observations
100	8.50	8.50	-	61	148		Lower Marker
52644	3.40	-	100.00	7782	>60000		

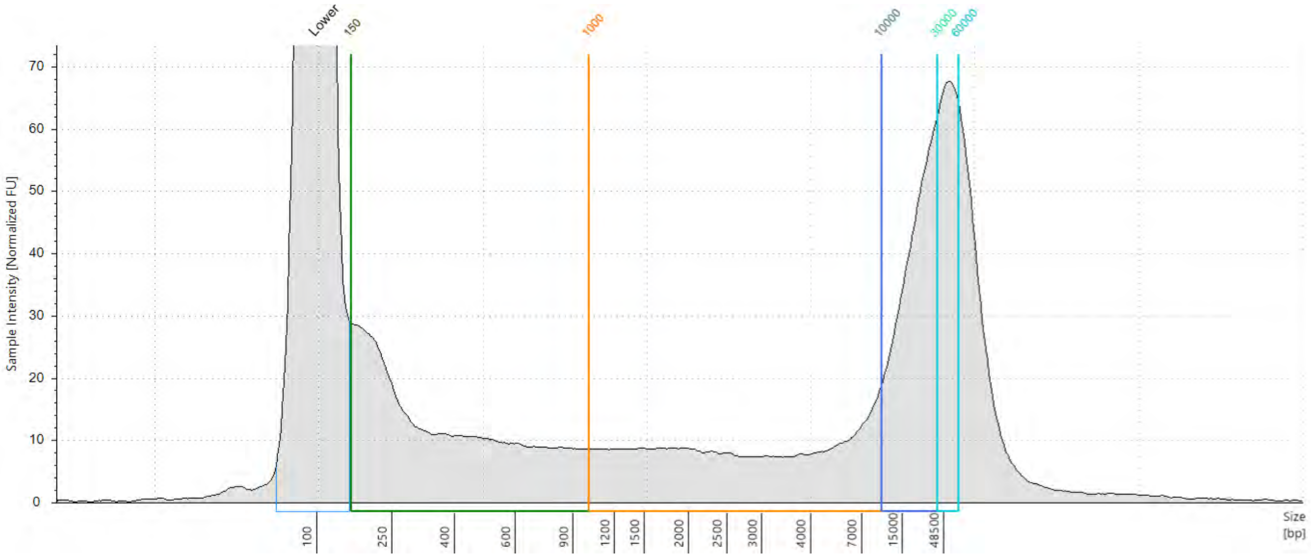

Region Table

From [bp]	To [bp]	Average Size [bp]	Conc. [ng/µl]	Region Molarity [nmol/l]	% of Total	Region Comment	Color
150	10000	1843	3.43	11.1	49.58		
150	1000	410	1.91	10.1	27.57		
1000	10000	3581	1.53	1.04	22.12		
10000	60000	31534	2.32	0.152	33.56		
10000	30000	19953	1.45	0.124	20.95		
30000	60000	50648	0.927	0.0303	13.41		

© Agilent Technologies, Inc. 2017

H6: W677

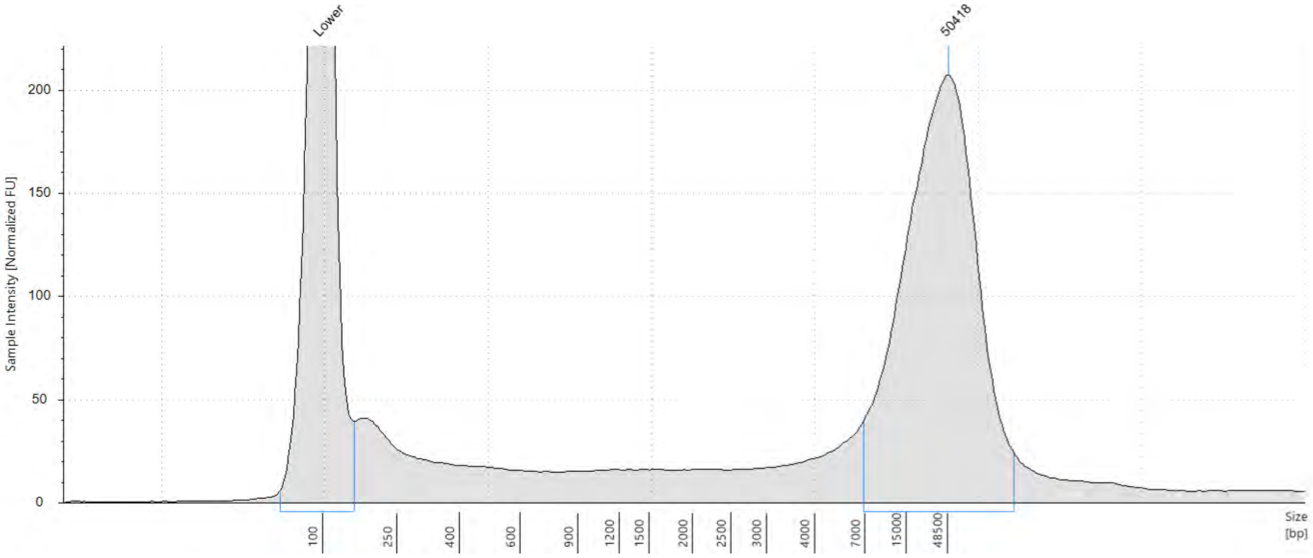

Sample Table

Well	DIN	Conc. [ng/µl]	Sample Description	Alert	Observations
H6	7.1	18.0	W677		

Peak Table

Size [bp]	Calibrated Conc. [ng/µl]	Assigned Conc. [ng/µl]	% Integrated Area	From [bp]	To [bp]	Peak Comment	Observations
100	8.50	8.50	-	60	148		Lower Marker
50418	10.7	-	98.73	6934	>60000		
-	-	-	-	-	-		Sample Well

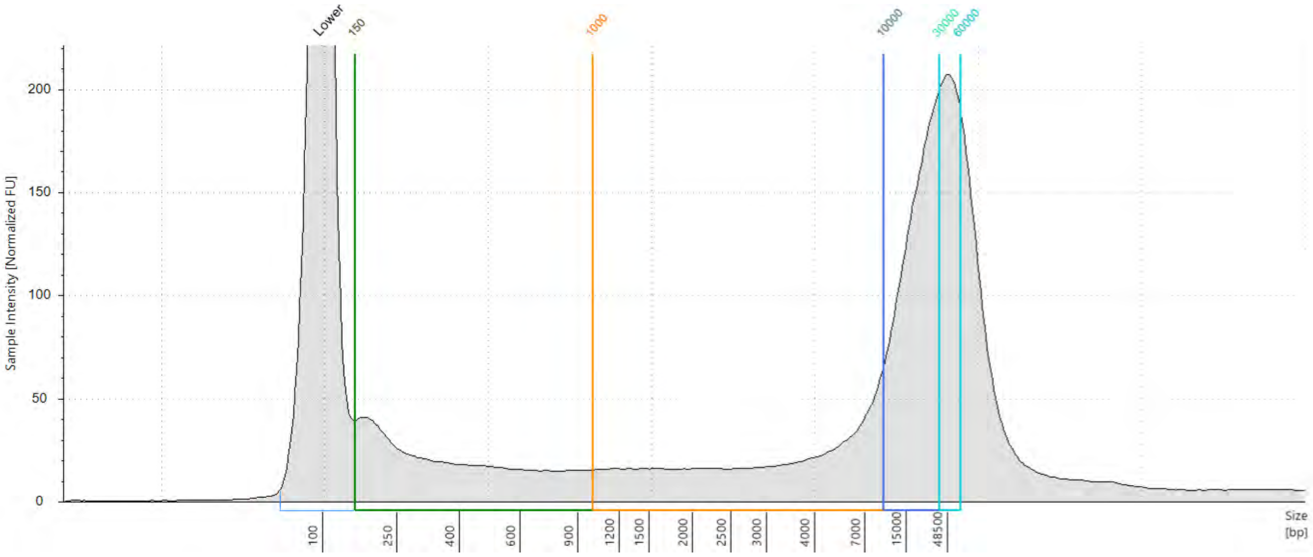

Region Table

From [bp]	To [bp]	Average Size [bp]	Conc. [ng/µl]	Region Molarity [nmol/l]	% of Total	Region Comment	Color
150	10000	2501	6.39	17.0	35.43		
150	1000	422	2.85	14.9	15.82		
1000	10000	4160	3.55	2.13	19.68		
10000	60000	30667	7.36	0.492	40.77		
10000	30000	19835	4.78	0.411	26.50		

 © Agilent Technologies, Inc. 2017

30000	60000	50582	2.75	0.0898	15.23		

© Agilent Technologies, Inc. 2017

A7: W678

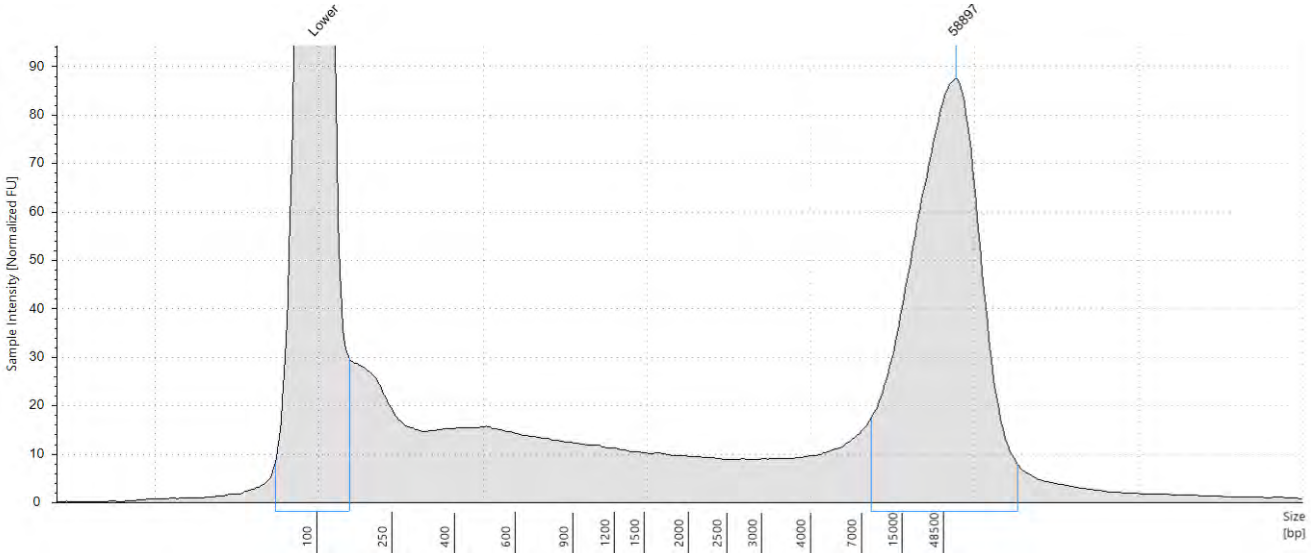

Sample Table

Well	DIN	Conc. [ng/µl]	Sample Description	Alert	Observations
A7	6.6	9.01	W678	⚠	Sample concentration outside recommended range

Peak Table

Size [bp]	Calibrated Conc. [ng/µl]	Assigned Conc. [ng/µl]	% Integrated Area	From [bp]	To [bp]	Peak Comment	Observations
100	8.50	8.50	-	61	149		Lower Marker
58897	4.37	-	98.48	8414	>60000		
-	-	-	-	-	-		Sample Well

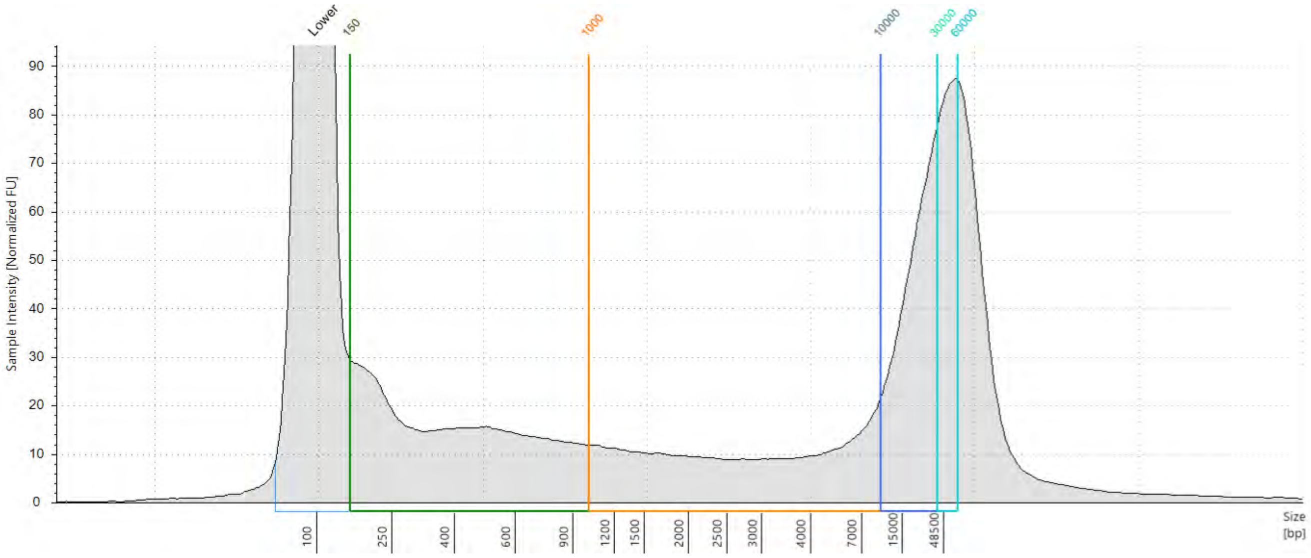

Region Table

From [bp]	To [bp]	Average Size [bp]	Conc. [ng/µl]	Region Molarity [nmol/l]	% of Total	Region Comment	Color
150	10000	1816	4.25	12.5	47.16		
150	1000	441	2.39	11.3	26.50		
1000	10000	3557	1.87	1.23	20.78		
10000	60000	31801	2.74	0.175	30.41		
10000	30000	20272	1.72	0.144	19.05		

 © Agilent Technologies, Inc. 2017

30000	60000	50937	1.09	0.0351	12.11		

© Agilent Technologies, Inc. 2017

B7: W679

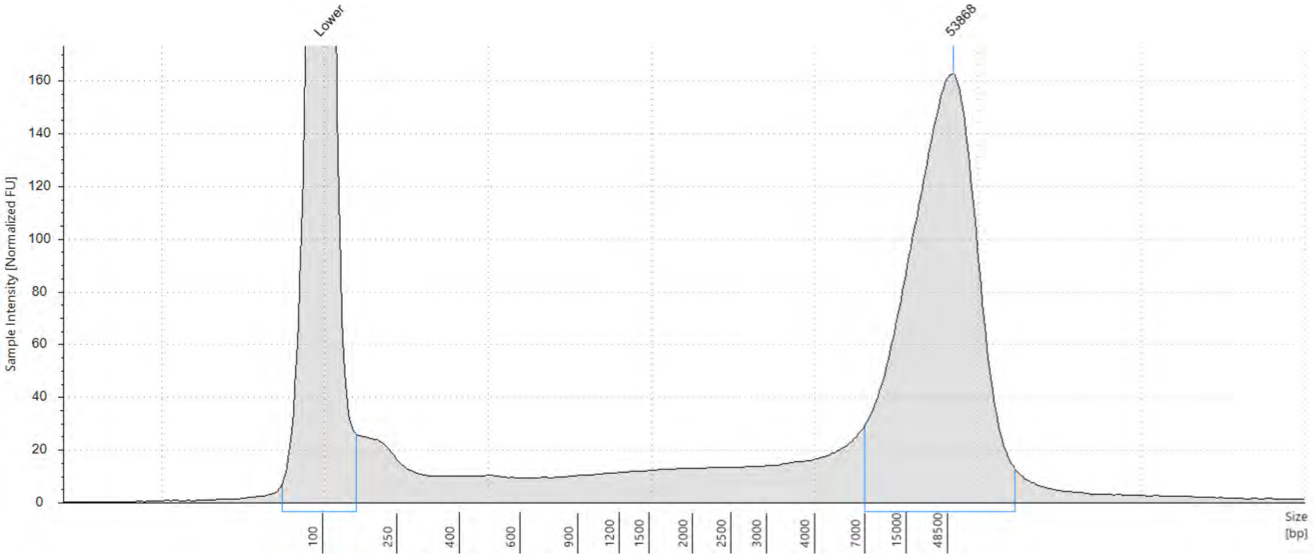

Sample Table

Well	DIN	Conc. [ng/µl]	Sample Description	Alert	Observations
B7	7.4	13.1	W679		

Peak Table

Size [bp]	Calibrated Conc. [ng/µl]	Assigned Conc. [ng/µl]	% Integrated Area	From [bp]	To [bp]	Peak Comment	Observations
100	8.50	8.50	-	61	151		Lower Marker
53868	8.36	-	97.94	6951	>60000		
-	-	-	-	-	-		Sample Well

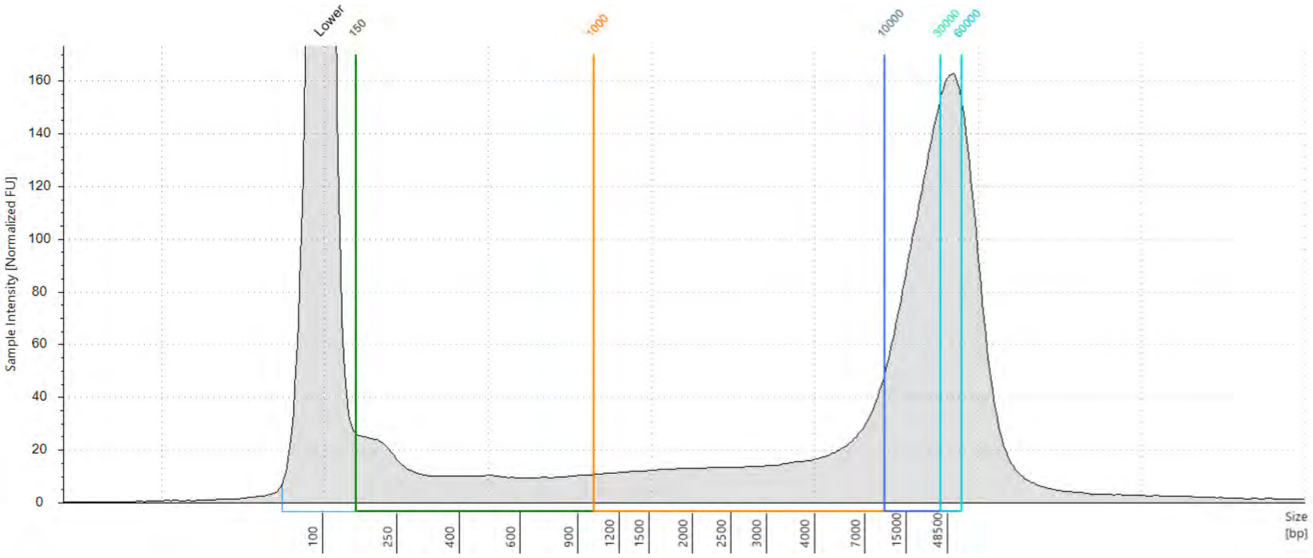

Region Table

From [bp]	To [bp]	Average Size [bp]	Conc. [ng/µl]	Region Molarity [nmol/l]	% of Total	Region Comment	Color
150	10000	2700	4.64	10.9	35.54		
150	1000	431	1.77	9.26	13.56		
1000	10000	4117	2.88	1.68	22.05		
10000	60000	31334	5.71	0.374	43.75		
10000	30000	19964	3.62	0.308	27.69		

© Agilent Technologies, Inc. 2017

30000	60000	50757	2.23	0.0725	17.09		

© Agilent Technologies, Inc. 2017

C7: W680

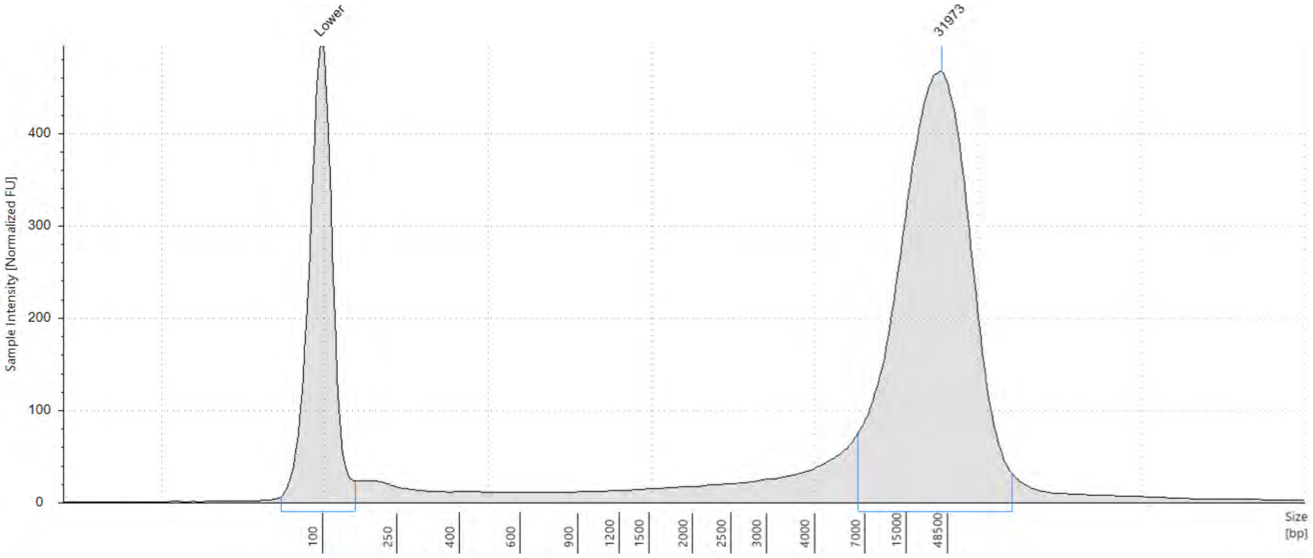

Sample Table

Well	DIN	Conc. [ng/µl]	Sample Description	Alert	Observations
C7	8.0	31.5	W680		

Peak Table

Size [bp]	Calibrated Conc. [ng/µl]	Assigned Conc. [ng/µl]	% Integrated Area	From [bp]	To [bp]	Peak Comment	Observations
100	8.50	8.50	-	60	148		Lower Marker
31973	24.6	-	98.49	6479	>60000		
-	-	-	-	-	-		Sample Well

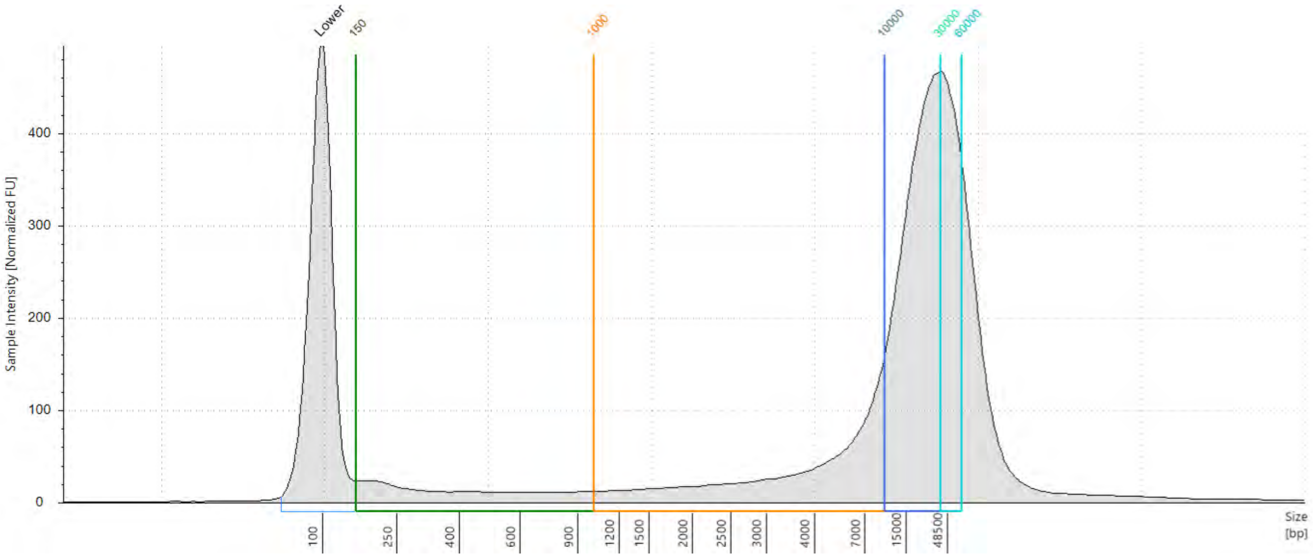

Region Table

From [bp]	To [bp]	Average Size [bp]	Conc. [ng/µl]	Region Molarity [nmol/l]	% of Total	Region Comment	Color
150	10000	3831	7.60	12.3	24.09		
150	1000	444	1.83	9.63	5.79		
1000	10000	4964	5.78	2.73	18.34		
10000	60000	28935	17.9	1.25	56.91		
10000	30000	19471	12.3	1.07	38.94		

 © Agilent Technologies, Inc. 2017

30000	60000	49241	6.07	0.205	19.26		

© Agilent Technologies, Inc. 2017

D7: W681

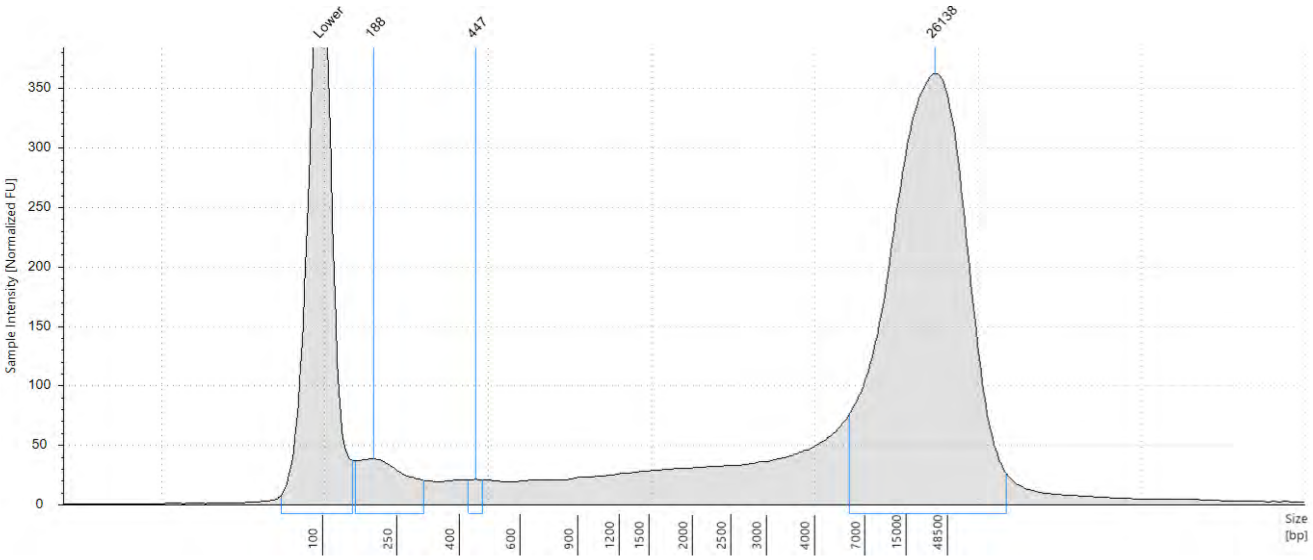

Sample Table

Well	DIN	Conc. [ng/µl]	Sample Description	Alert	Observations
D7	7.1	30.5	W681		

Peak Table

Size [bp]	Calibrated Conc. [ng/µl]	Assigned Conc. [ng/µl]	% Integrated Area	From [bp]	To [bp]	Peak Comment	Observations
100	8.50	8.50	-	61	146		Lower Marker
188	1.31	-	5.86	151	307		
447	0.191	-	0.86	426	468		
26138	20.5	-	91.88	5946	>60000		
-	-	-	-	-	-		Sample Well

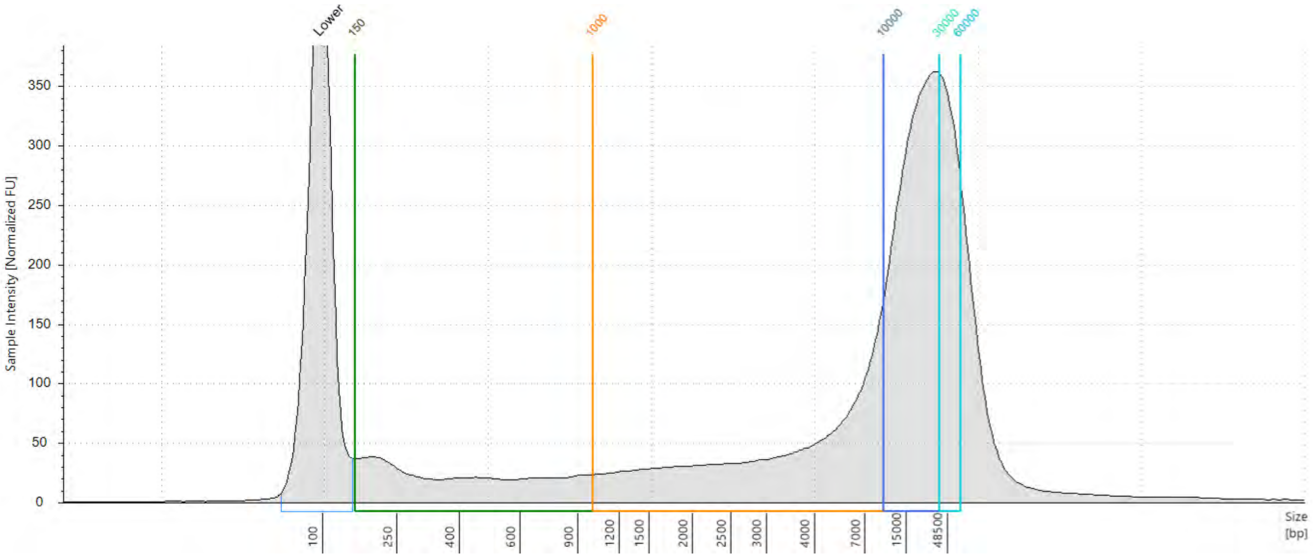

Region Table

From [bp]	To [bp]	Average Size [bp]	Conc. [ng/µl]	Region Molarity [nmol/l]	% of Total	Region Comment	Color
150	10000	3318	11.5	20.5	37.64		
150	1000	456	3.35	16.2	11.00		
1000	10000	4519	8.15	4.29	26.71		

10000	60000	28036	14.9	1.08	48.98		
10000	30000	19195	10.7	0.949	35.09		
30000	60000	50069	4.55	0.151	14.92		

© Agilent Technologies, Inc. 2017

E7: W682

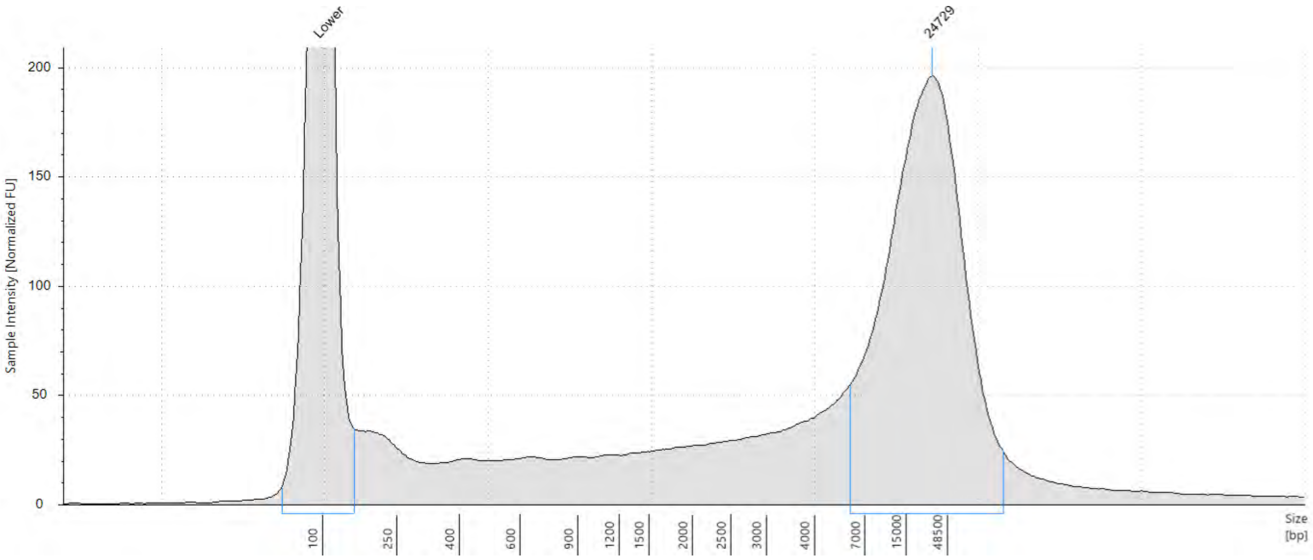

Sample Table

Well	DIN	Conc. [ng/µl]	Sample Description	Alert	Observations
E7	6.6	20.6	W682		

Peak Table

Size [bp]	Calibrated Conc. [ng/µl]	Assigned Conc. [ng/µl]	% Integrated Area	From [bp]	To [bp]	Peak Comment	Observations
100	8.50	8.50	-	61	148		Lower Marker
24729	10.9	-	96.95	5980	>60000		
-	-	-	-	-	-		Sample Well

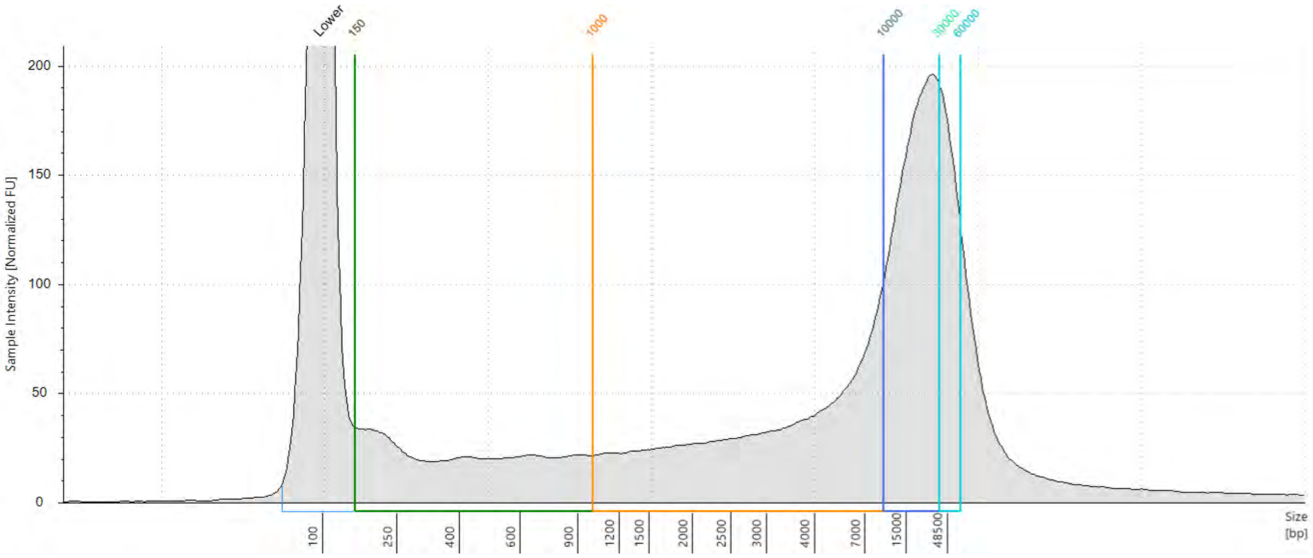

Region Table

From [bp]	To [bp]	Average Size [bp]	Conc. [ng/µl]	Region Molarity [nmol/l]	% of Total	Region Comment	Color
150	10000	2940	9.65	18.5	46.79		
150	1000	465	3.25	15.0	15.76		
1000	10000	4200	6.42	3.56	31.11		
10000	60000	27367	7.84	0.579	38.01		
10000	30000	19181	5.77	0.513	27.99		

30000	60000	49891	2.23	0.0742	10.81		

© Agilent Technologies, Inc. 2017

F7: W683

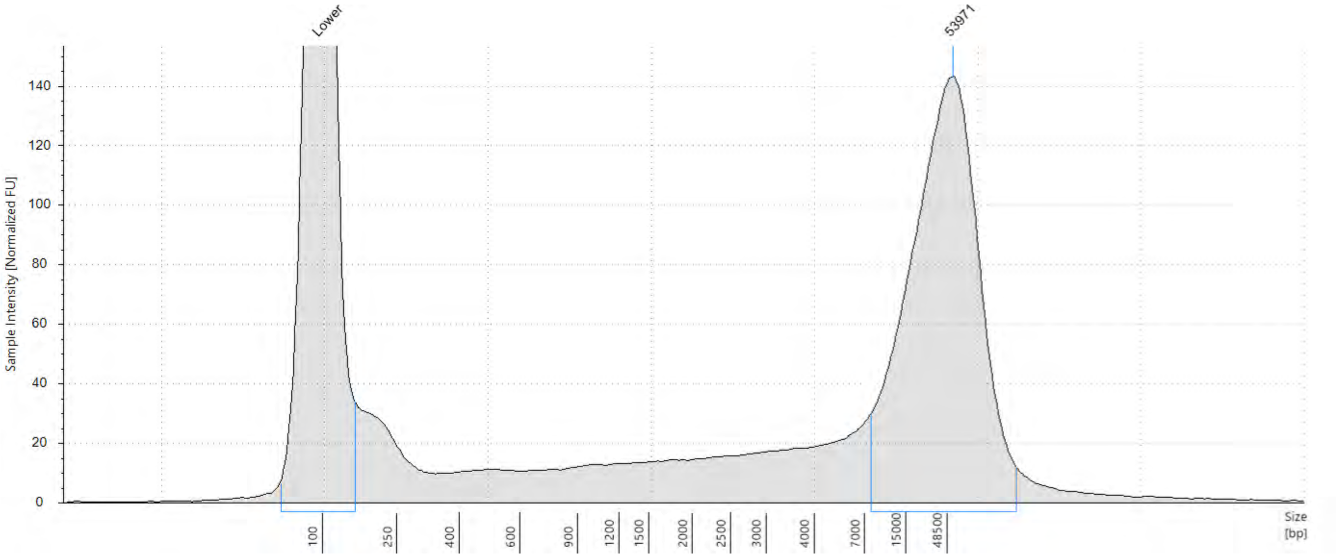

Sample Table

Well	DIN	Conc. [ng/µl]	Sample Description	Alert	Observations
F7	7.1	11.8	W683		

Peak Table

Size [bp]	Calibrated Conc. [ng/µl]	Assigned Conc. [ng/µl]	% Integrated Area	From [bp]	To [bp]	Peak Comment	Observations
100	8.50	8.50	-	61	153		Lower Marker
53971	6.85	-	99.24	7996	>60000		
-	-	-	-	-	-		Sample Well

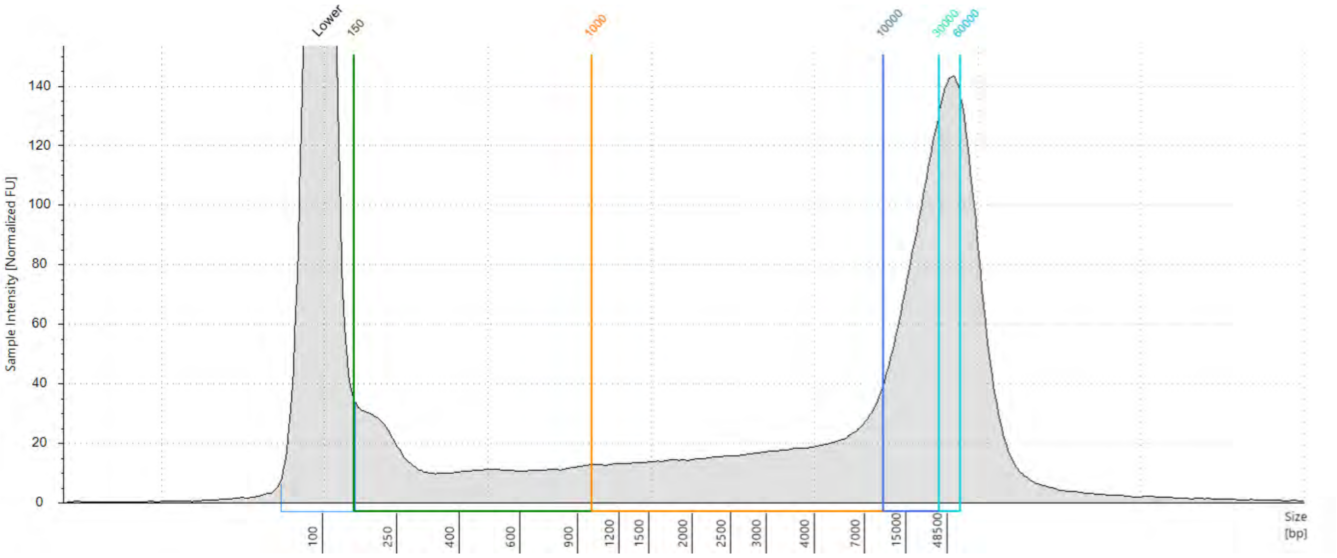

Region Table

From [bp]	To [bp]	Average Size [bp]	Conc. [ng/µl]	Region Molarity [nmol/l]	% of Total	Region Comment	Color
150	10000	2457	4.85	12.2	41.08		
150	1000	428	1.92	10.4	16.26		
1000	10000	3817	2.94	1.79	24.91		
10000	60000	31787	4.62	0.300	39.19		
10000	30000	20095	2.89	0.246	24.48		

 © Agilent Technologies, Inc. 2017

30000	60000	51077	1.85	0.0594	15.64		

© Agilent Technologies, Inc. 2017

G7: W684

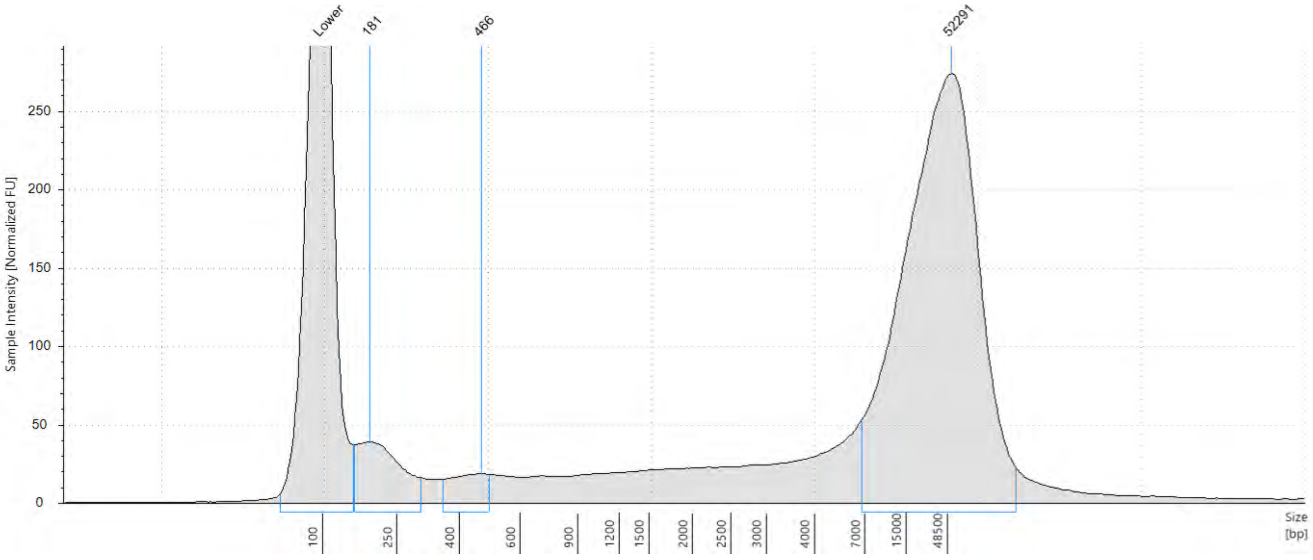

Sample Table

Well	DIN	Conc. [ng/µl]	Sample Description	Alert	Observations
G7	7.2	22.6	W684		

Peak Table

Size [bp]	Calibrated Conc. [ng/µl]	Assigned Conc. [ng/µl]	% Integrated Area	From [bp]	To [bp]	Peak Comment	Observations
100	8.50	8.50	-	60	147		Lower Marker
181	1.21	-	7.24	150	301		
466	0.472	-	2.82	355	488		
52291	14.6	-	87.35	6766	>60000		
-	-	-	-	-	-		Sample Well

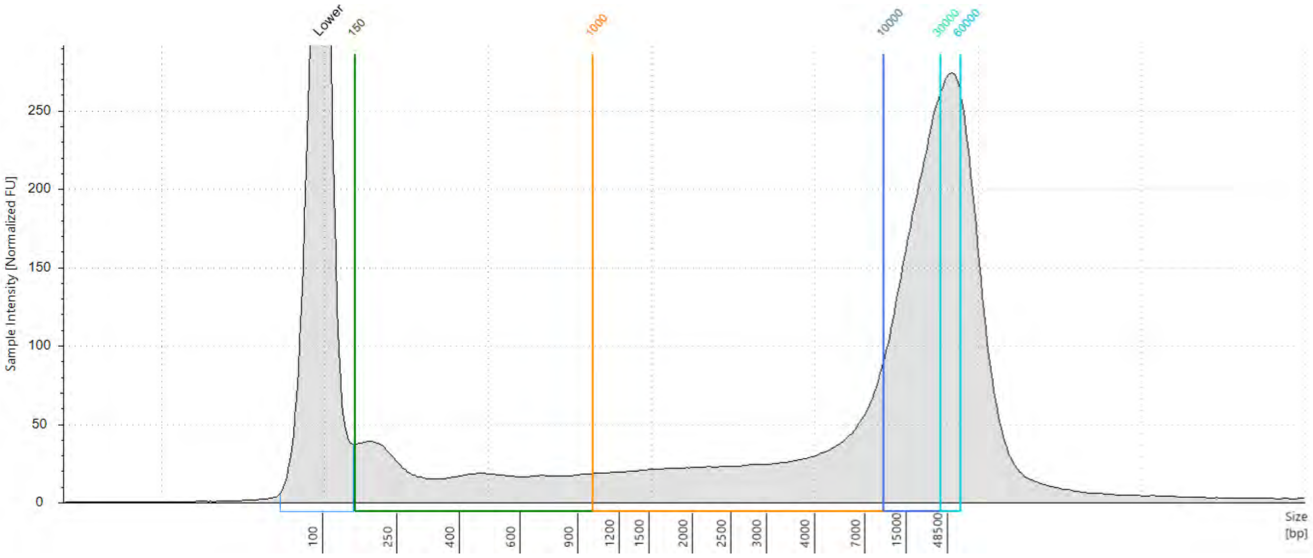

Region Table

From [bp]	To [bp]	Average Size [bp]	Conc. [ng/µl]	Region Molarity [nmol/l]	% of Total	Region Comment	Color
150	10000	2849	7.94	17.3	35.12		
150	1000	445	2.88	14.4	12.73		
1000	10000	4225	5.08	2.91	22.46		

 © Agilent Technologies, Inc. 2017

10000	60000	30553	9.74	0.653	43.08		
10000	30000	19678	6.28	0.542	27.77		
30000	60000	50073	3.68	0.122	16.30		

© Agilent Technologies, Inc. 2017

H7: W685

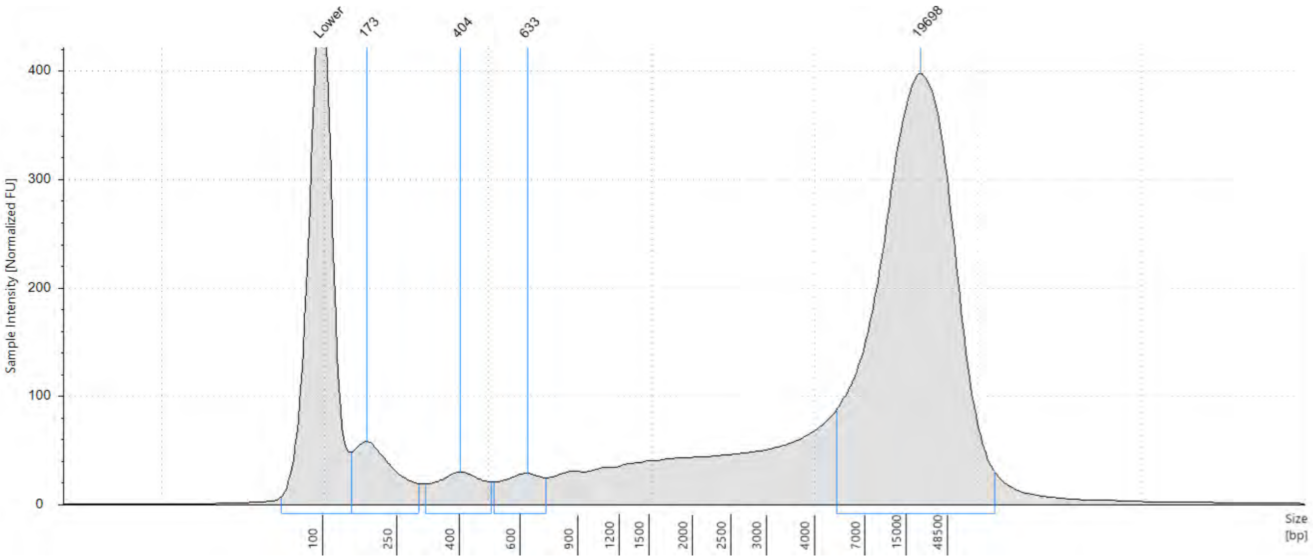

Sample Table

Well	DIN	Conc. [ng/µl]	Sample Description	Alert	Observations
H7	6.8	34.2	W685		

Peak Table

Size [bp]	Calibrated Conc. [ng/µl]	Assigned Conc. [ng/µl]	% Integrated Area	From [bp]	To [bp]	Peak Comment	Observations
100	8.50	8.50	-	61	143		Lower Marker
173	1.67	-	6.54	143	294		
404	0.980	-	3.83	310	495		
633	0.805	-	3.15	505	719		
19698	22.0	-	86.09	5131	>60000		
-	-	-	-	-	-		Sample Well

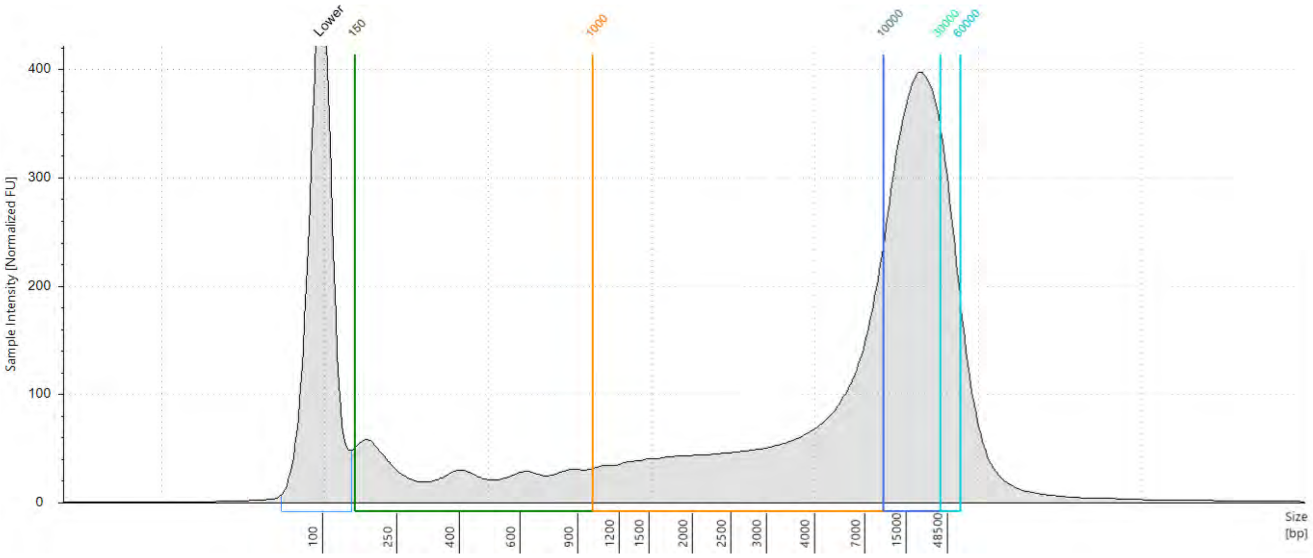

Region Table

From [bp]	To [bp]	Average Size [bp]	Conc. [ng/µl]	Region Molarity [nmol/l]	% of Total	Region Comment	Color
150	10000	3384	15.7	26.4	45.91		
150	1000	462	4.24	20.4	12.41		

1000	10000	4479	11.5	6.00	33.58		
10000	60000	25012	16.2	1.28	47.29		
10000	30000	18398	12.6	1.17	36.94		
30000	60000	48214	3.84	0.134	11.25		

© Agilent Technologies, Inc. 2017

A8: W686

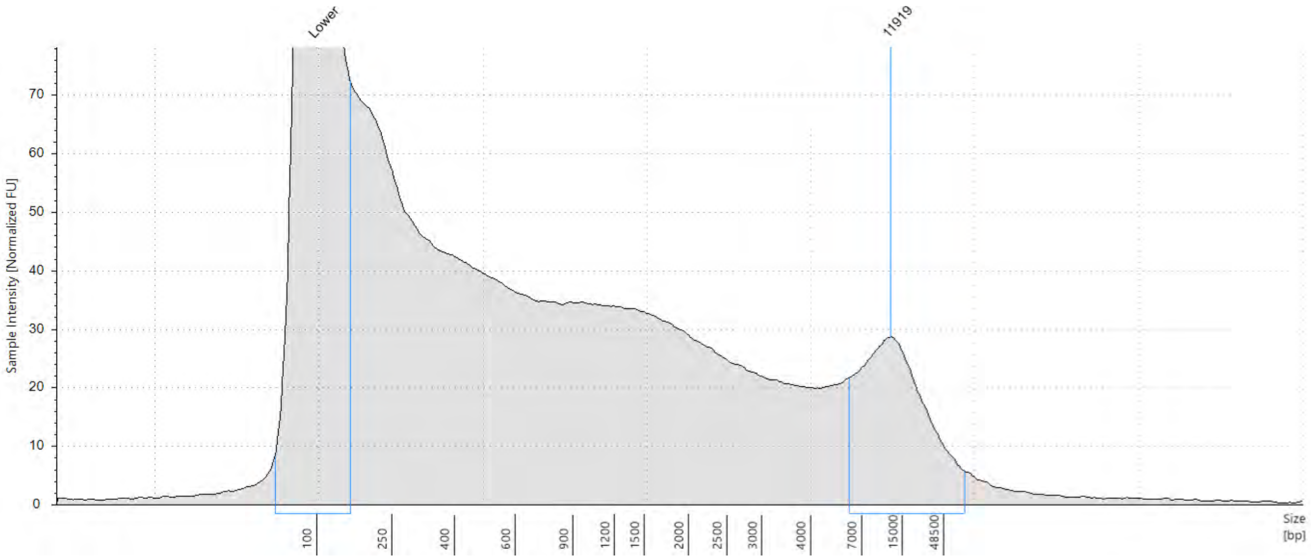

Sample Table

Well	DIN	Conc. [ng/µl]	Sample Description	Alert	Observations
A8	1.9	11.4	W686		

Peak Table

Size [bp]	Calibrated Conc. [ng/µl]	Assigned Conc. [ng/µl]	% Integrated Area	From [bp]	To [bp]	Peak Comment	Observations
100	8.50	8.50	-	60	151		Lower Marker
11919	1.26	-	98.86	6149	>60000		
-	-	-	-	-	-		Sample Well

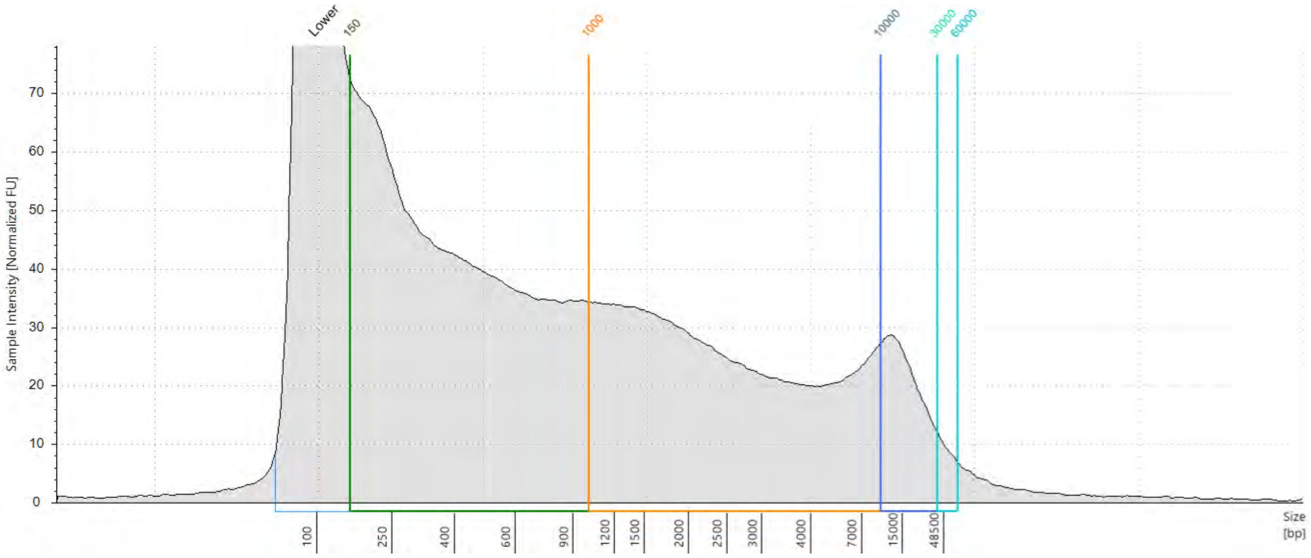

Region Table

From [bp]	To [bp]	Average Size [bp]	Conc. [ng/µl]	Region Molarity [nmol/l]	% of Total	Region Comment	Color
150	10000	1512	10.3	31.9	90.18		
150	1000	440	6.02	28.7	52.67		
1000	10000	3006	4.31	3.22	37.75		
10000	60000	21183	0.813	0.0768	7.12		
10000	30000	16869	0.712	0.0734	6.23		

30000	60000	49481	0.111	0.00395	0.97		

© Agilent Technologies, Inc. 2017

B8: W687

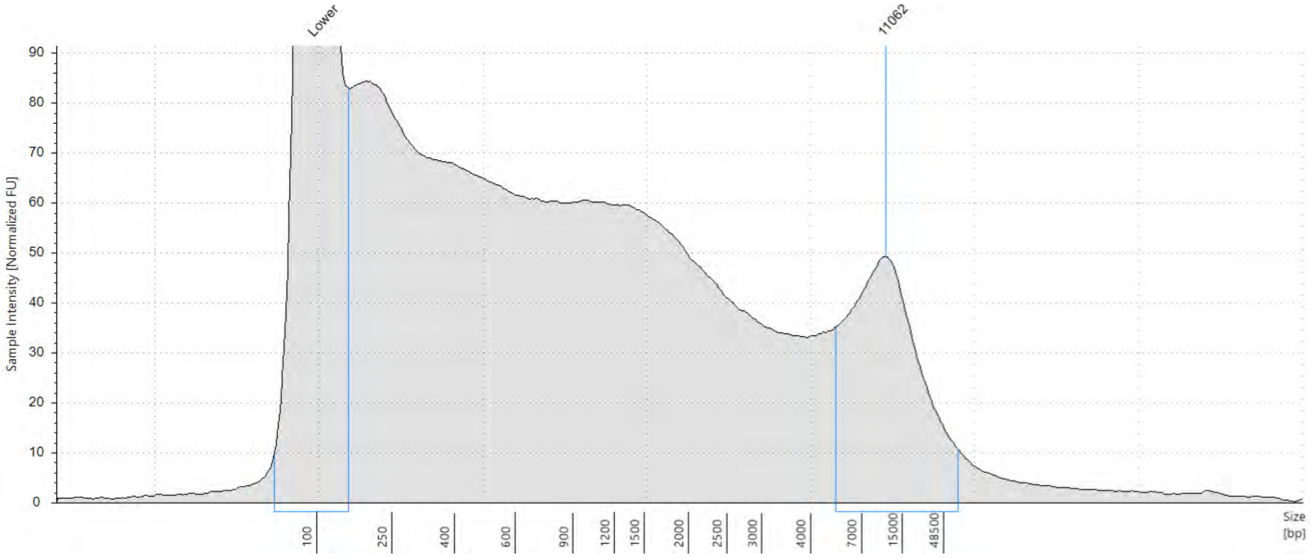

Sample Table

Well	DIN	Conc. [ng/µl]	Sample Description	Alert	Observations
B8	2.0	18.5	W687		

Peak Table

Size [bp]	Calibrated Conc. [ng/µl]	Assigned Conc. [ng/µl]	% Integrated Area	From [bp]	To [bp]	Peak Comment	Observations
100	8.50	8.50	-	60	148		Lower Marker
11062	2.34	-	100.00	5291	>60000		

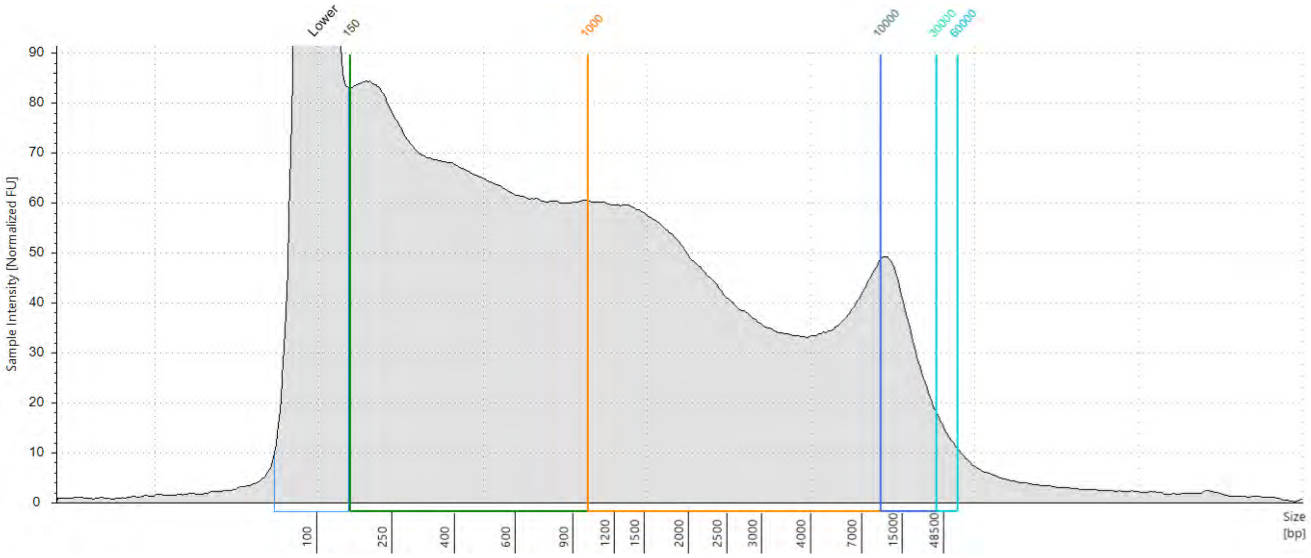

Region Table

From [bp]	To [bp]	Average Size [bp]	Conc. [ng/µl]	Region Molarity [nmol/l]	% of Total	Region Comment	Color
150	10000	1602	16.7	46.5	90.14		
150	1000	464	9.26	41.0	50.06		
1000	10000	3005	7.47	5.60	40.35		
10000	60000	20856	1.29	0.123	6.97		
10000	30000	16617	1.13	0.118	6.12		
30000	60000	49654	0.170	0.00597	0.92		

C8: W688

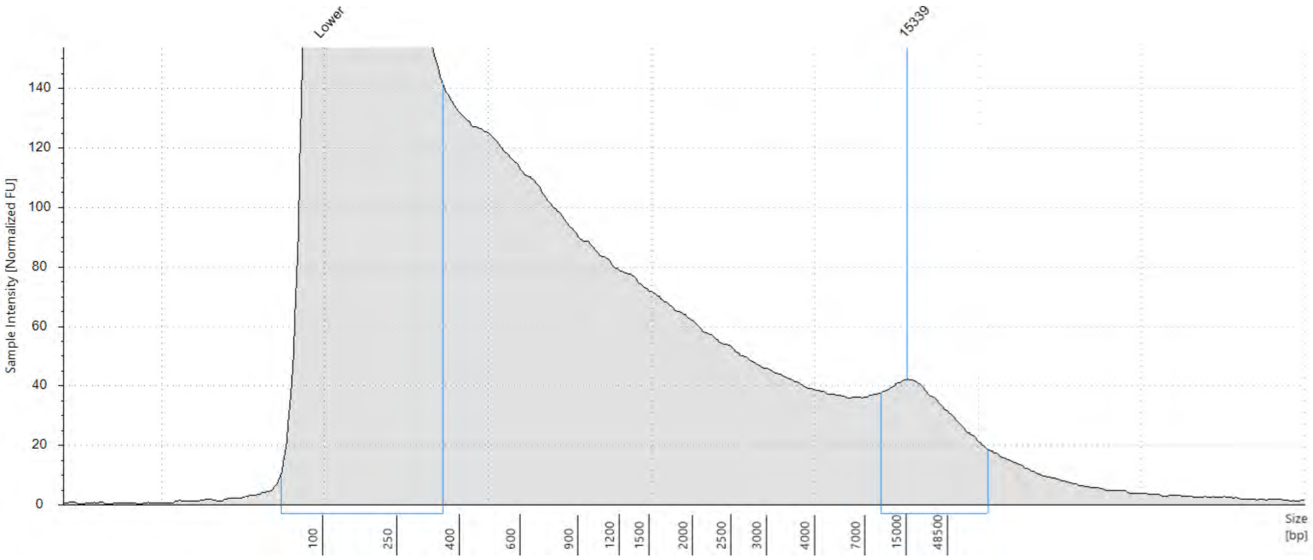

Sample Table

Well	DIN	Conc. [ng/µl]	Sample Description	Alert	Observations
C8	1.3	8.59	W688	⚠	Sample concentration outside recommended range

Peak Table

Size [bp]	Calibrated Conc. [ng/µl]	Assigned Conc. [ng/µl]	% Integrated Area	From [bp]	To [bp]	Peak Comment	Observations
100	8.50	8.50	-	60	353		Lower Marker
15339	0.806	-	100.00	9400	>60000		

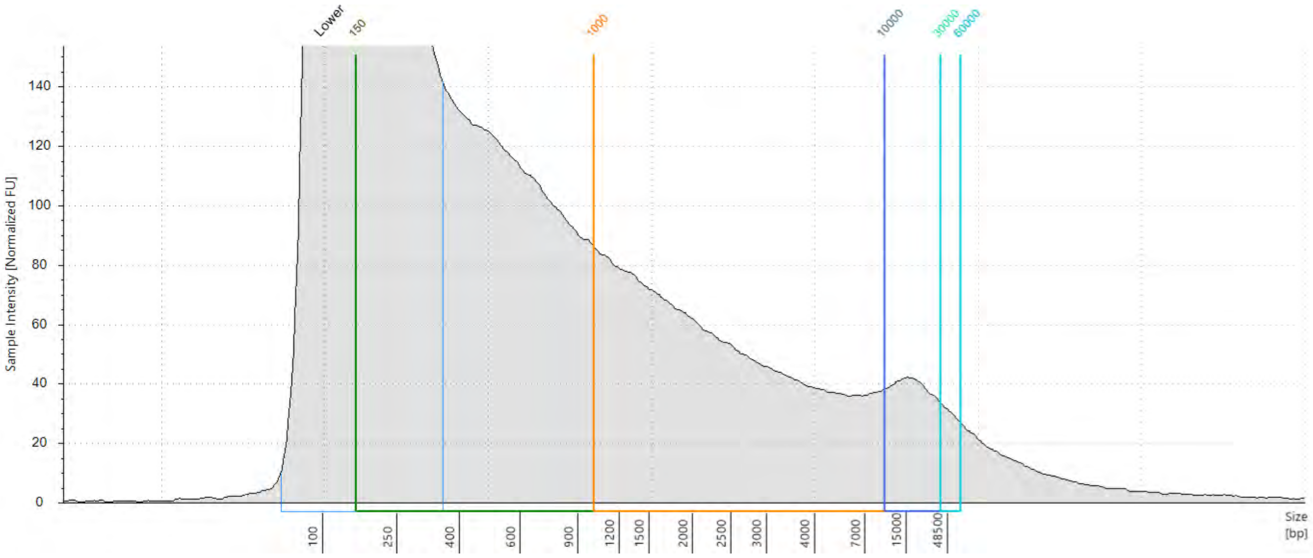

Region Table

From [bp]	To [bp]	Average Size [bp]	Conc. [ng/µl]	Region Molarity [nmol/l]	% of Total	Region Comment	Color
150	10000	1630	7.45	41.0	86.75		
150	1000	598	3.89	38.2	45.24		
1000	10000	2758	3.59	2.83	41.83		
10000	60000	24895	0.641	0.0536	7.46		
10000	30000	17861	0.501	0.0490	5.84		
30000	60000	49398	0.150	0.00520	1.75		

 © Agilent Technologies, Inc. 2017

D8: W689

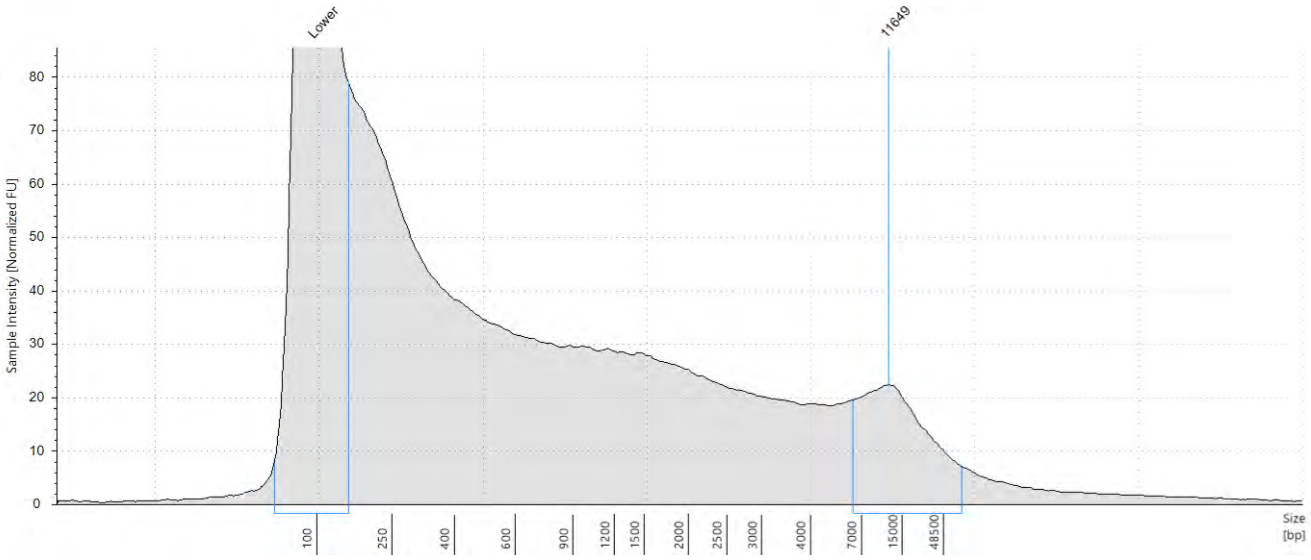

Sample Table

Well	DIN	Conc. [ng/µl]	Sample Description	Alert	Observations
D8	1.9	10.7	W689		

Peak Table

Size [bp]	Calibrated Conc. [ng/µl]	Assigned Conc. [ng/µl]	% Integrated Area	From [bp]	To [bp]	Peak Comment	Observations
100	8.50	8.50	-	60	148		Lower Marker
11649	1.01	-	97.03	6455	>60000		
-	-	-	-	-	-		Sample Well

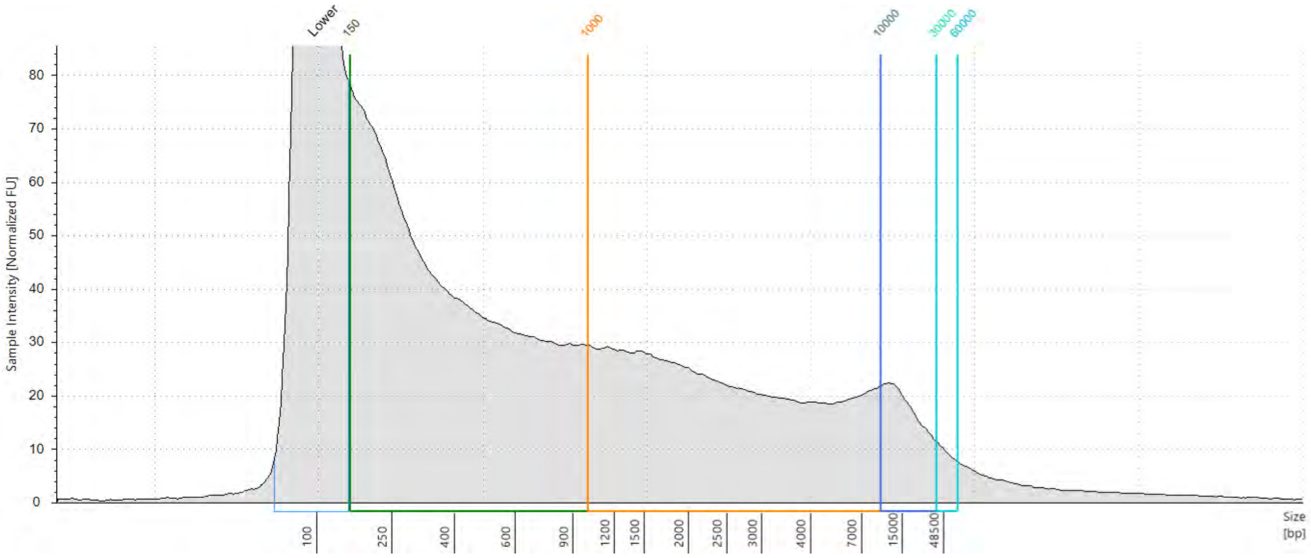

Region Table

From [bp]	To [bp]	Average Size [bp]	Conc. [ng/µl]	Region Molarity [nmol/l]	% of Total	Region Comment	Color
150	10000	1452	9.63	31.8	90.15		
150	1000	420	5.85	29.0	54.78		
1000	10000	3014	3.80	2.83	35.59		
10000	60000	22504	0.673	0.0620	6.31		
10000	30000	17139	0.569	0.0585	5.33		

© Agilent Technologies, Inc. 2017

30000	60000	49898	0.114	0.00401	1.06		

E8: W690

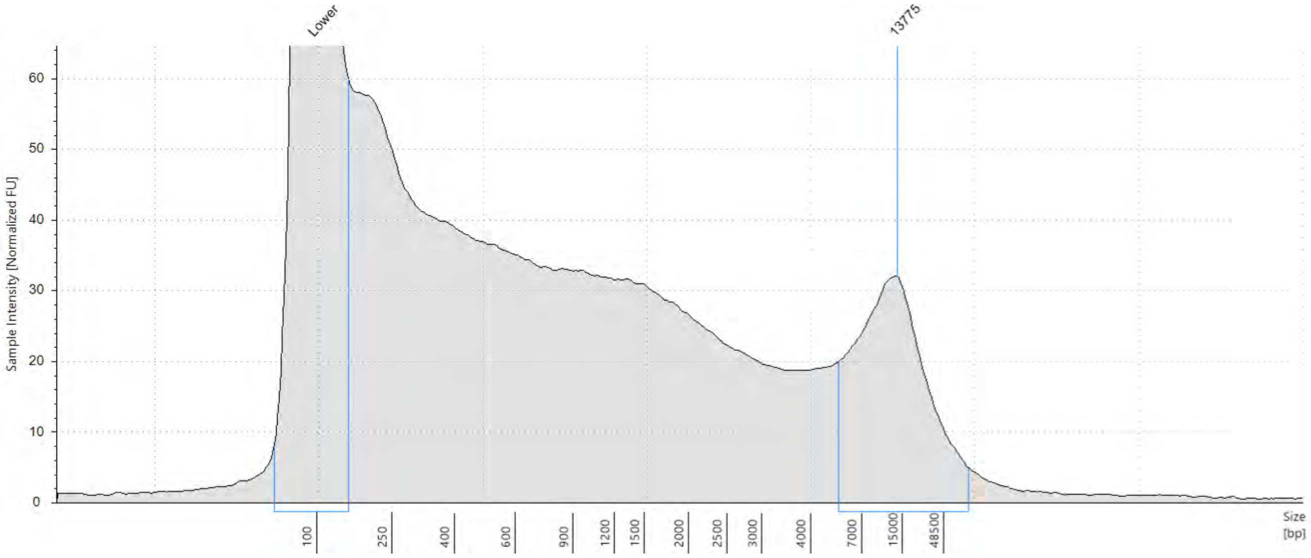

Sample Table

Well	DIN	Conc. [ng/µl]	Sample Description	Alert	Observations
E8	1.8	11.0	W690		

Peak Table

Size [bp]	Calibrated Conc. [ng/µl]	Assigned Conc. [ng/µl]	% Integrated Area	From [bp]	To [bp]	Peak Comment	Observations
100	8.50	8.50	-	60	149		Lower Marker
13775	1.54	-	100.00	5457	>60000		

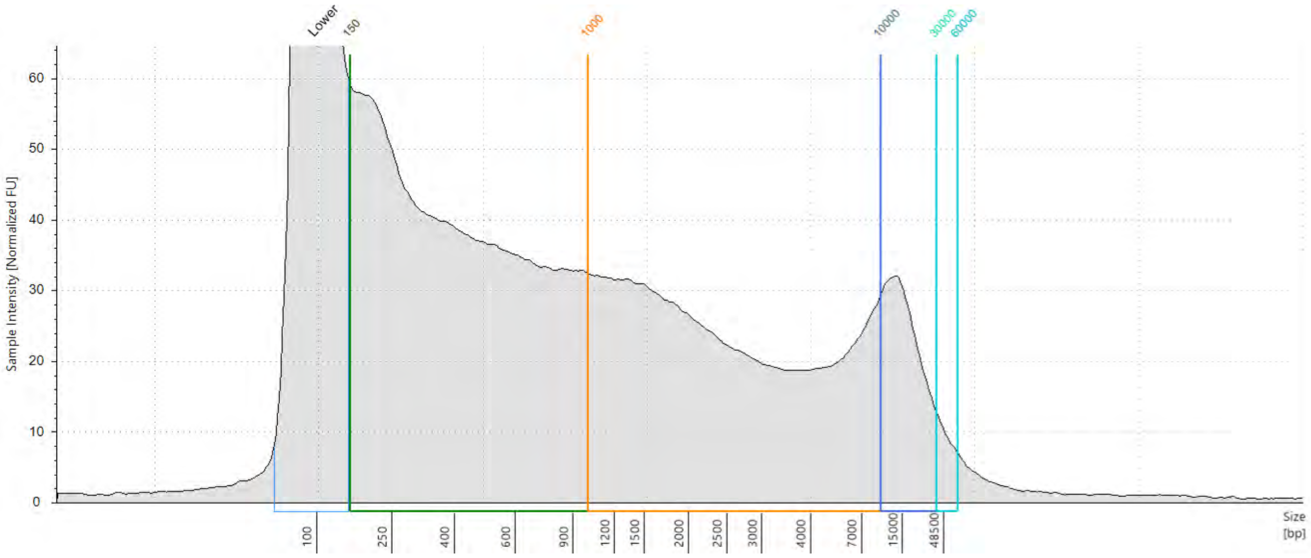

Region Table

From [bp]	To [bp]	Average Size [bp]	Conc. [ng/µl]	Region Molarity [nmol/l]	% of Total	Region Comment	Color
150	10000	1556	9.70	28.9	88.52		
150	1000	449	5.61	25.9	51.21		
1000	10000	3049	4.12	3.08	37.55		
10000	60000	20655	0.934	0.0896	8.52		
10000	30000	16665	0.826	0.0859	7.54		
30000	60000	49130	0.118	0.00426	1.08		

 © Agilent Technologies, Inc. 2017

F8: W691

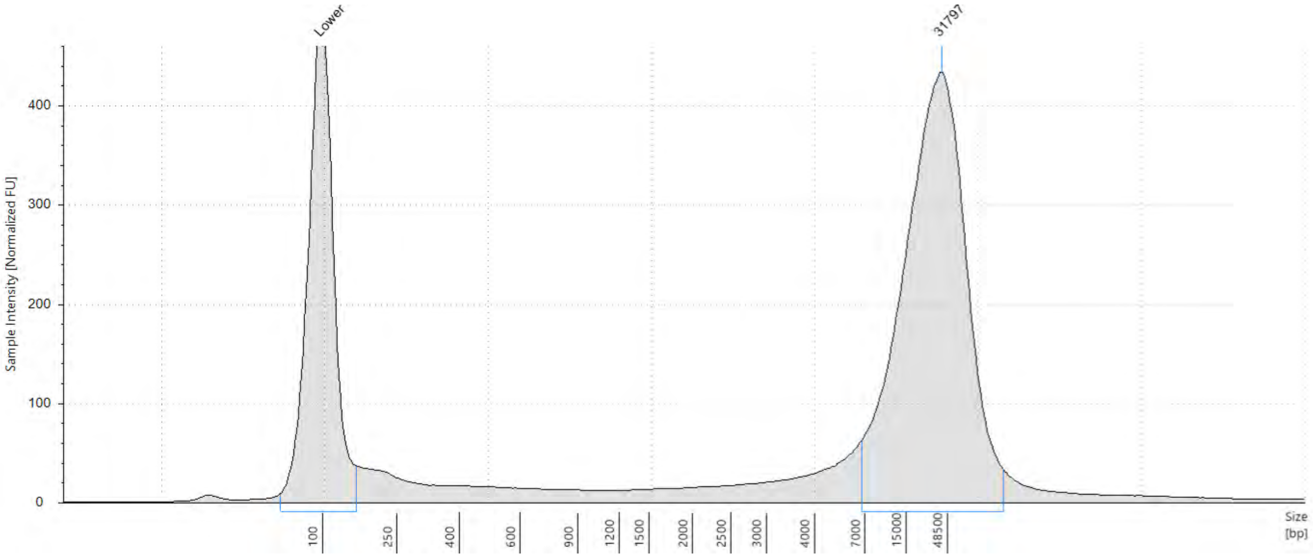

Sample Table

Well	DIN	Conc. [ng/µl]	Sample Description	Alert	Observations
F8	7.7	25.7	W691		

Peak Table

Size [bp]	Calibrated Conc. [ng/µl]	Assigned Conc. [ng/µl]	% Integrated Area	From [bp]	To [bp]	Peak Comment	Observations
100	8.50	8.50	-	60	151		Lower Marker
31797	18.5	-	98.37	6750	>60000		
-	-	-	-	-	-		Sample Well

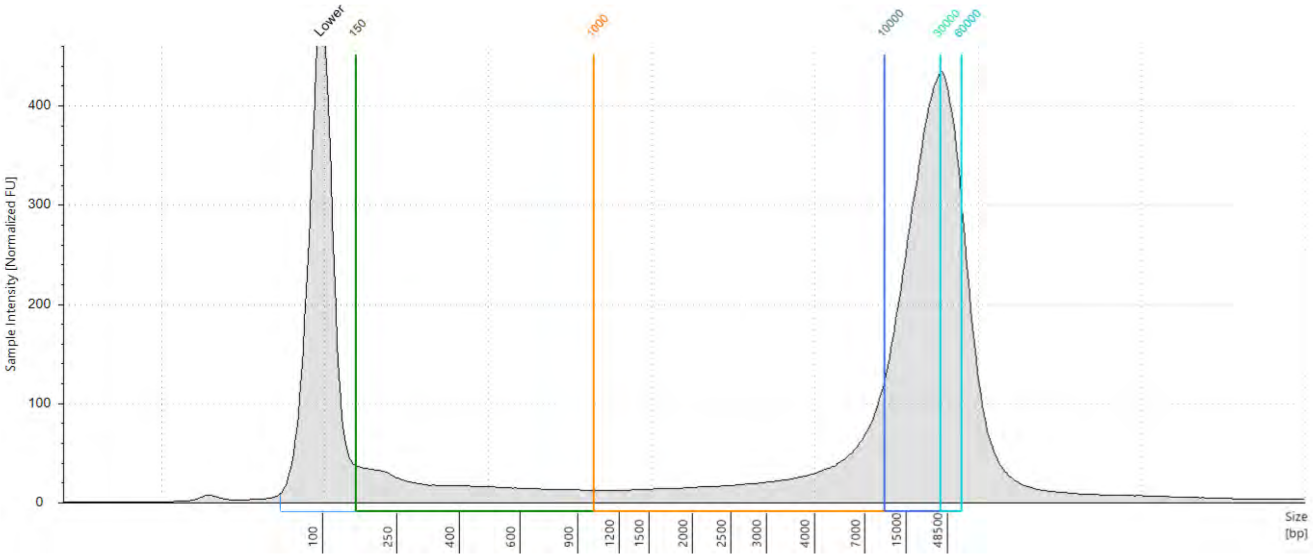

Region Table

From [bp]	To [bp]	Average Size [bp]	Conc. [ng/µl]	Region Molarity [nmol/l]	% of Total	Region Comment	Color
150	10000	3303	6.90	14.9	26.84		
150	1000	422	2.44	12.8	9.48		
1000	10000	4906	4.47	2.18	17.40		
10000	60000	29553	14.4	0.971	55.88		
10000	30000	20261	9.94	0.831	38.68		

© Agilent Technologies, Inc. 2017

30000	60000	50245	4.78	0.157	18.59		

© Agilent Technologies, Inc. 2017

G8: W692

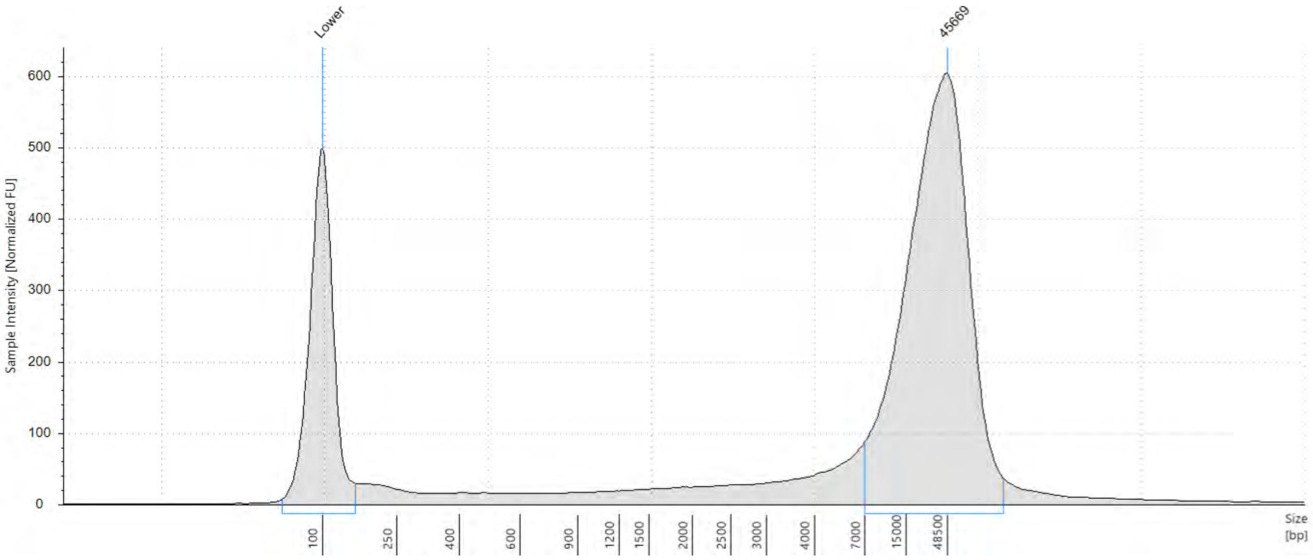

Sample Table

Well	DIN	Conc. [ng/µl]	Sample Description	Alert	Observations
G8	7.8	36.8	W692		

Peak Table

Size [bp]	Calibrated Conc. [ng/µl]	Assigned Conc. [ng/µl]	% Integrated Area	From [bp]	To [bp]	Peak Comment	Observations
100	8.50	8.50	-	61	148		Lower Marker
45669	27.3	-	98.38	6899	>60000		
-	-	-	-	-	-		Sample Well

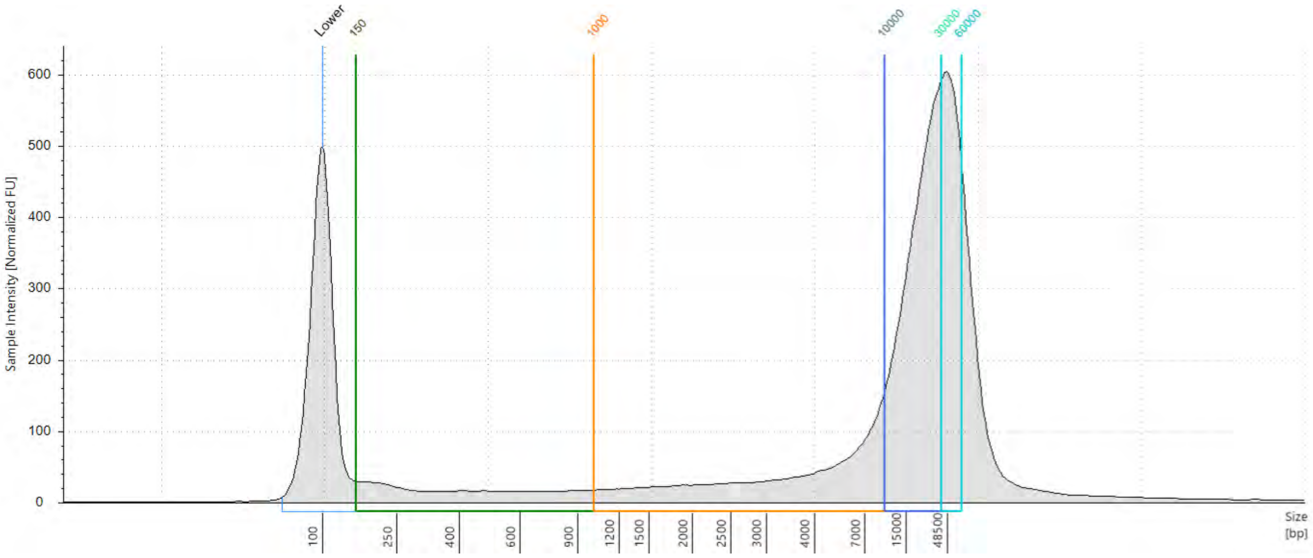

Region Table

From [bp]	To [bp]	Average Size [bp]	Conc. [ng/µl]	Region Molarity [nmol/l]	% of Total	Region Comment	Color
150	10000	3470	9.27	15.9	25.21		
150	1000	460	2.52	12.4	6.85		
1000	10000	4633	6.76	3.47	18.39		
10000	60000	30726	21.3	1.40	57.97		
10000	30000	20093	13.8	1.16	37.55		

 © Agilent Technologies, Inc. 2017

30000	60000	50102	8.03	0.265	21.85		

© Agilent Technologies, Inc. 2017

H8: W693

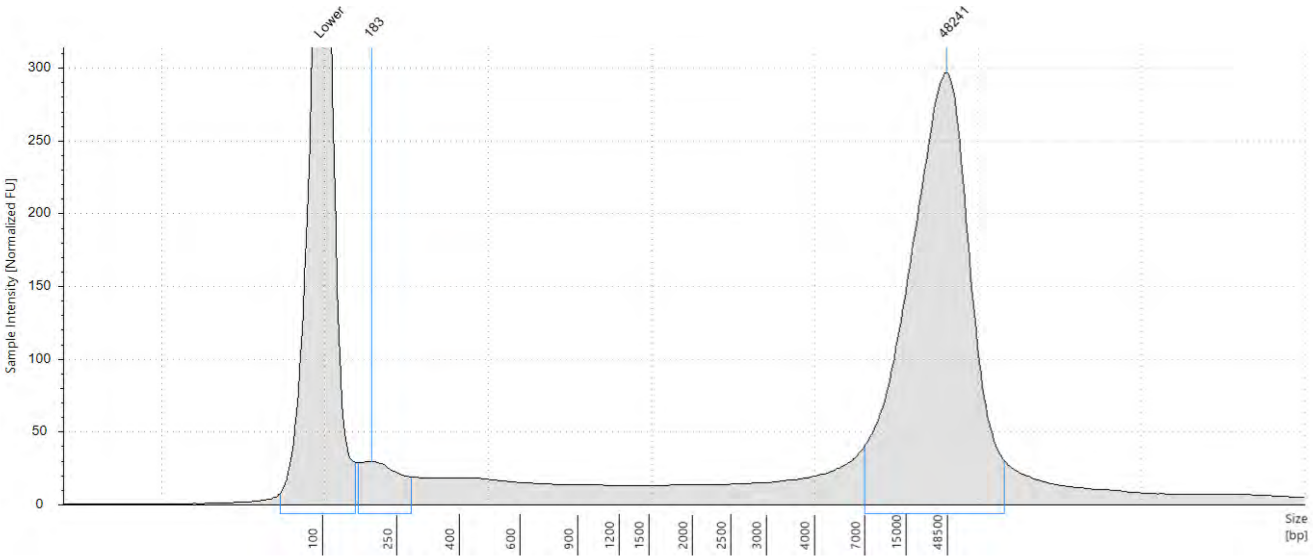

Sample Table

Well	DIN	Conc. [ng/µl]	Sample Description	Alert	Observations
H8	7.4	20.7	W693		

Peak Table

Size [bp]	Calibrated Conc. [ng/µl]	Assigned Conc. [ng/µl]	% Integrated Area	From [bp]	To [bp]	Peak Comment	Observations
100	8.50	8.50	-	59	149		Lower Marker
183	0.845	-	5.83	154	279		
48241	13.2	-	91.08	6983	>60000		
-	-	-	-	-	-		Sample Well

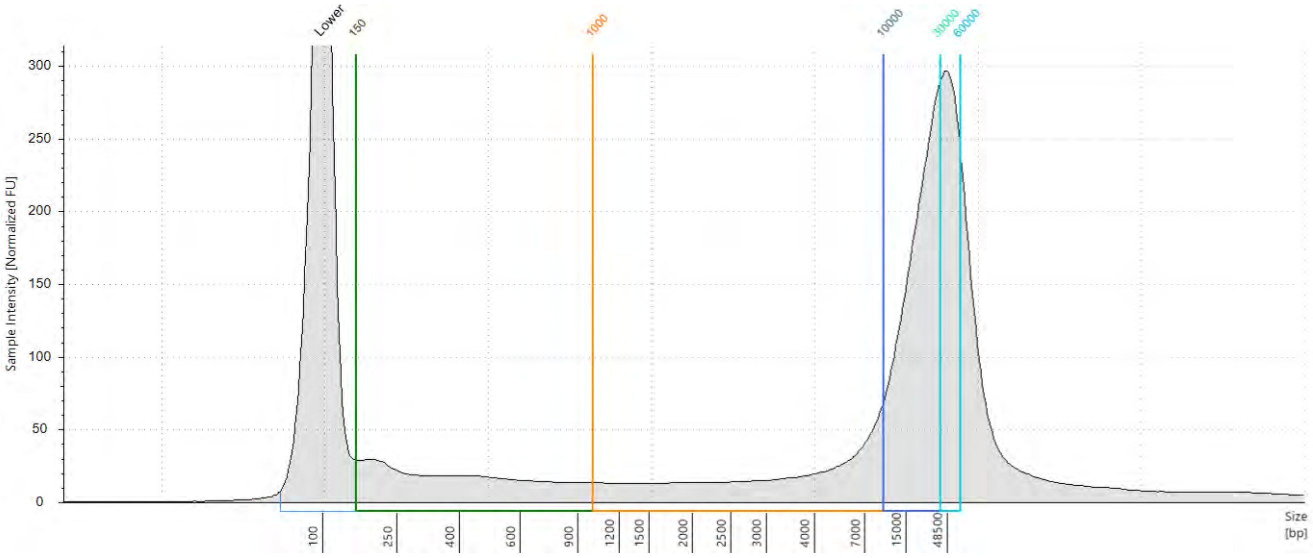

Region Table

From [bp]	To [bp]	Average Size [bp]	Conc. [ng/µl]	Region Molarity [nmol/l]	% of Total	Region Comment	Color
150	10000	2629	5.92	14.6	28.61		
150	1000	440	2.60	12.7	12.60		
1000	10000	4338	3.32	1.89	16.07		
10000	60000	31009	9.87	0.642	47.72		

 © Agilent Technologies, Inc. 2017

10000	30000	20386	6.38	0.532	30.87		
30000	60000	50308	3.74	0.123	18.10		

© Agilent Technologies, Inc. 2017

A9: W694

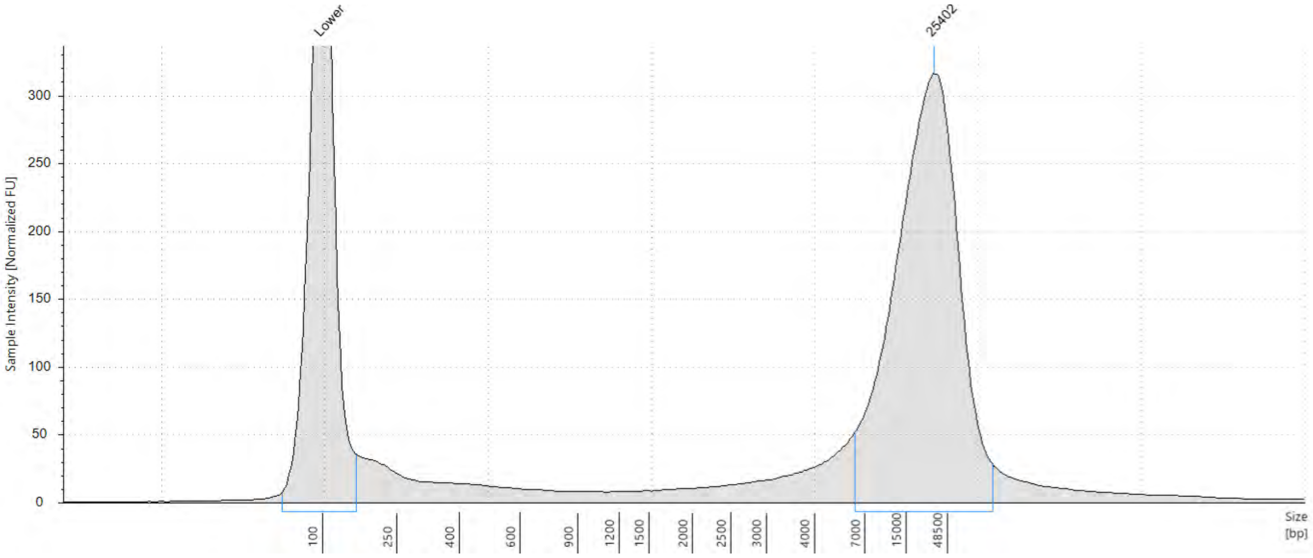

Sample Table

Well	DIN	Conc. [ng/µl]	Sample Description	Alert	Observations
A9	7.6	20.4	W694		

Peak Table

Size [bp]	Calibrated Conc. [ng/µl]	Assigned Conc. [ng/µl]	% Integrated Area	From [bp]	To [bp]	Peak Comment	Observations
100	8.50	8.50	-	61	151		Lower Marker
25402	14.1	-	97.27	6244	>60000		
-	-	-	-	-	-		Sample Well

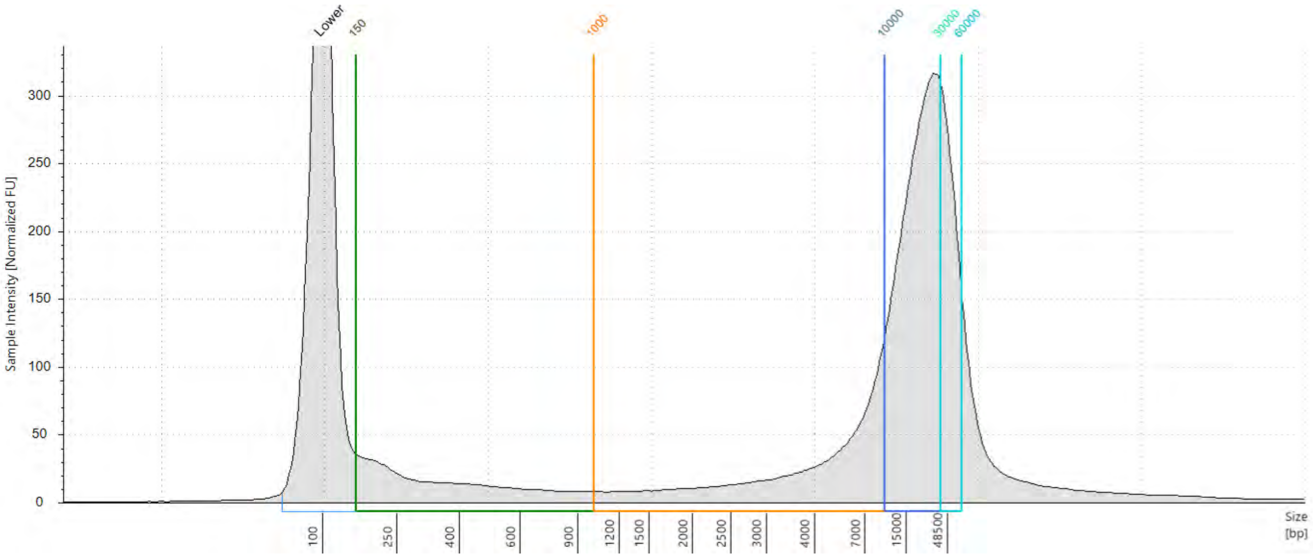

Region Table

From [bp]	To [bp]	Average Size [bp]	Conc. [ng/µl]	Region Molarity [nmol/l]	% of Total	Region Comment	Color
150	10000	3567	6.15	13.1	30.06		
150	1000	395	2.10	11.4	10.26		
1000	10000	5229	4.06	1.76	19.84		
10000	60000	27531	11.4	0.824	55.90		
10000	30000	19588	8.40	0.726	41.09		

30000	60000	49290	3.29	0.111	16.10		

© Agilent Technologies, Inc. 2017

B9: W695

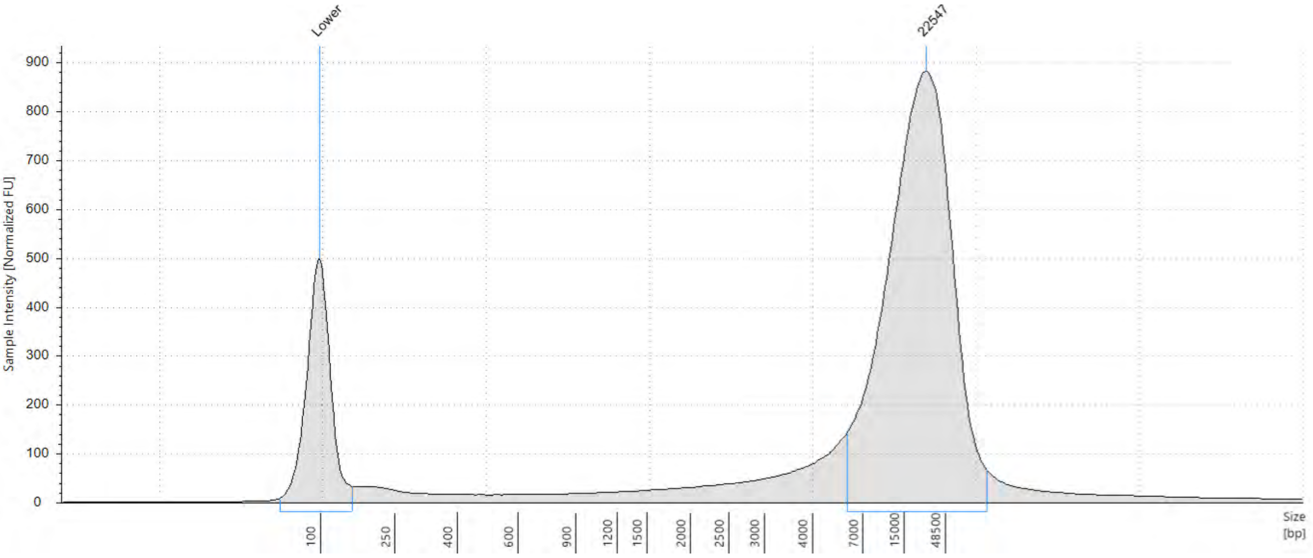

Sample Table

Well	DIN	Conc. [ng/µl]	Sample Description	Alert	Observations
B9	7.6	54.4	W695		

Peak Table

Size [bp]	Calibrated Conc. [ng/µl]	Assigned Conc. [ng/µl]	% Integrated Area	From [bp]	To [bp]	Peak Comment	Observations
100	8.50	8.50	-	61	148		Lower Marker
22547	41.2	-	98.80	5852	>60000		
-	-	-	-	-	-		Sample Well

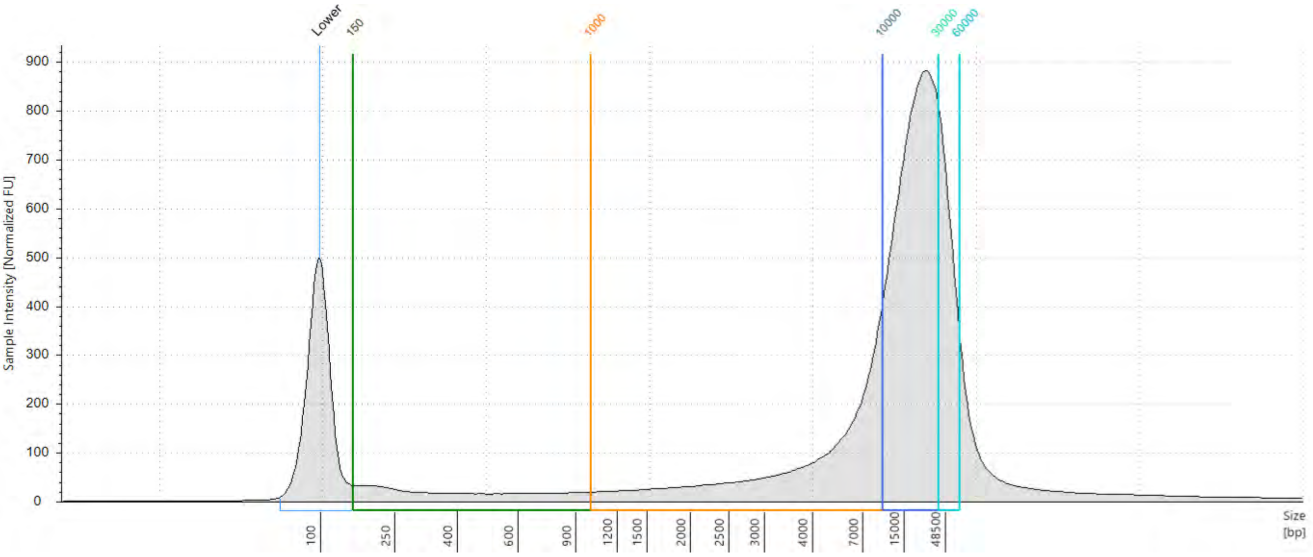

Region Table

From [bp]	To [bp]	Average Size [bp]	Conc. [ng/µl]	Region Molarity [nmol/l]	% of Total	Region Comment	Color
150	10000	4440	15.3	19.0	28.15		
150	1000	449	2.68	13.7	4.93		
1000	10000	5334	12.6	5.34	23.25		
10000	60000	25738	33.3	2.54	61.18		
10000	30000	19007	25.6	2.28	47.12		

© Agilent Technologies, Inc. 2017

30000	60000	47981	8.35	0.291	15.36		

© Agilent Technologies, Inc. 2017

C9: W696

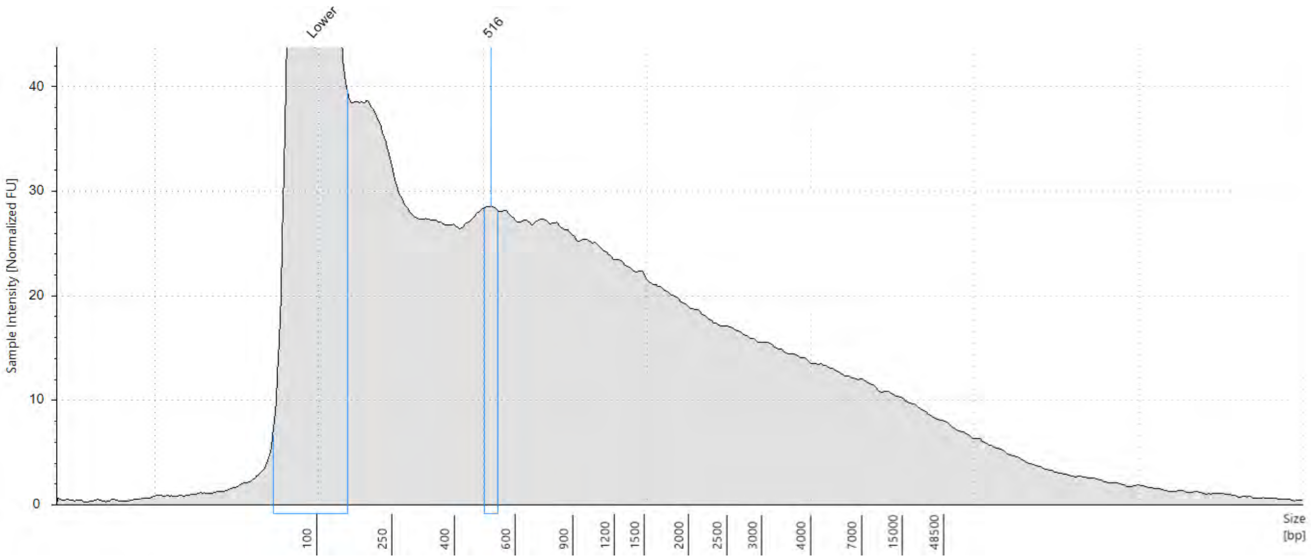

Sample Table

Well	DIN	Conc. [ng/µl]	Sample Description	Alert	Observations
C9	2.0	8.12	W696	⚠	Sample concentration outside recommended range

Peak Table

Size [bp]	Calibrated Conc. [ng/µl]	Assigned Conc. [ng/µl]	% Integrated Area	From [bp]	To [bp]	Peak Comment	Observations
100	8.50	8.50	-	60	148		Lower Marker
516	0.263	-	95.48	492	540		
-	-	-	-	-	-		Sample Well

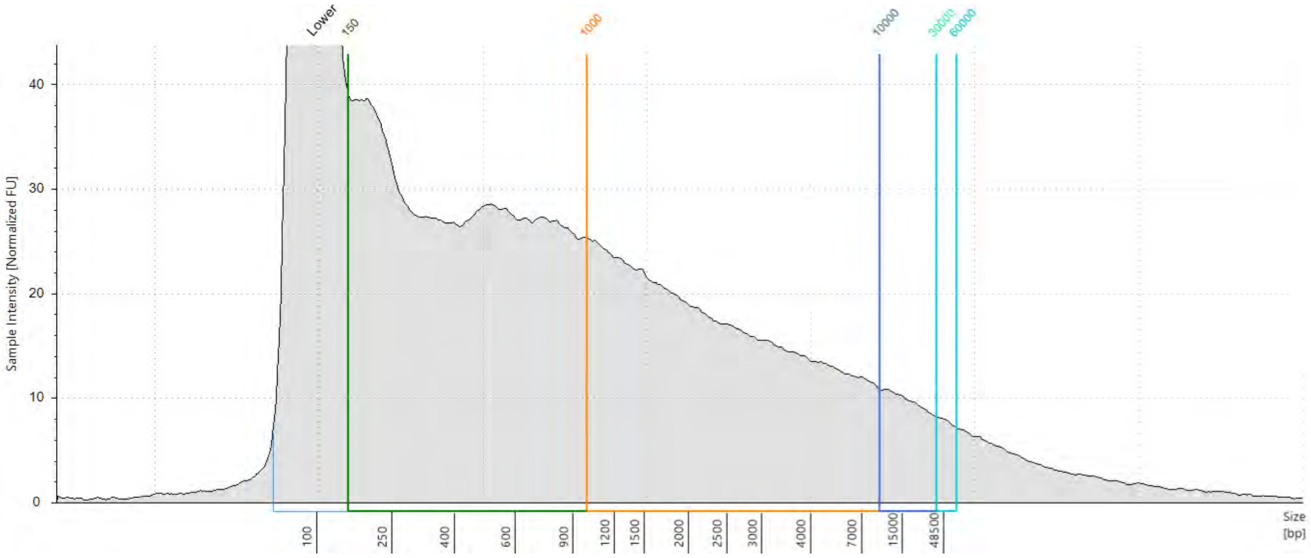

Region Table

From [bp]	To [bp]	Average Size [bp]	Conc. [ng/µl]	Region Molarity [nmol/l]	% of Total	Region Comment	Color
150	10000	1459	7.22	21.2	89.01		
150	1000	466	4.21	18.9	51.89		
1000	10000	2820	3.03	2.37	37.38		
10000	60000	25340	0.415	0.0358	5.12		
10000	30000	17833	0.323	0.0327	3.98		

30000	60000	50493	0.0994	0.00346	1.22		

© Agilent Technologies, Inc. 2017

D9: W697

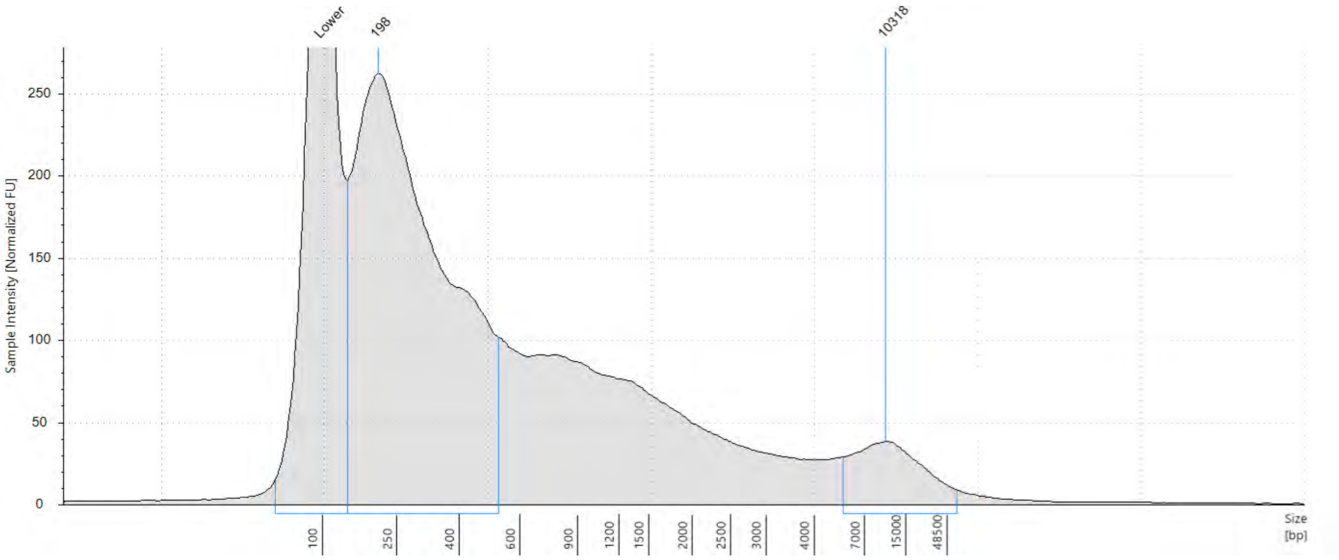

Sample Table

Well	DIN	Conc. [ng/µl]	Sample Description	Alert	Observations
D9	1.5	25.4	W697		

Peak Table

Size [bp]	Calibrated Conc. [ng/µl]	Assigned Conc. [ng/µl]	% Integrated Area	From [bp]	To [bp]	Peak Comment	Observations
100	8.50	8.50	-	56	136		Lower Marker
198	13.6	-	89.84	136	518		
10318	1.54	-	10.16	5473	56706		

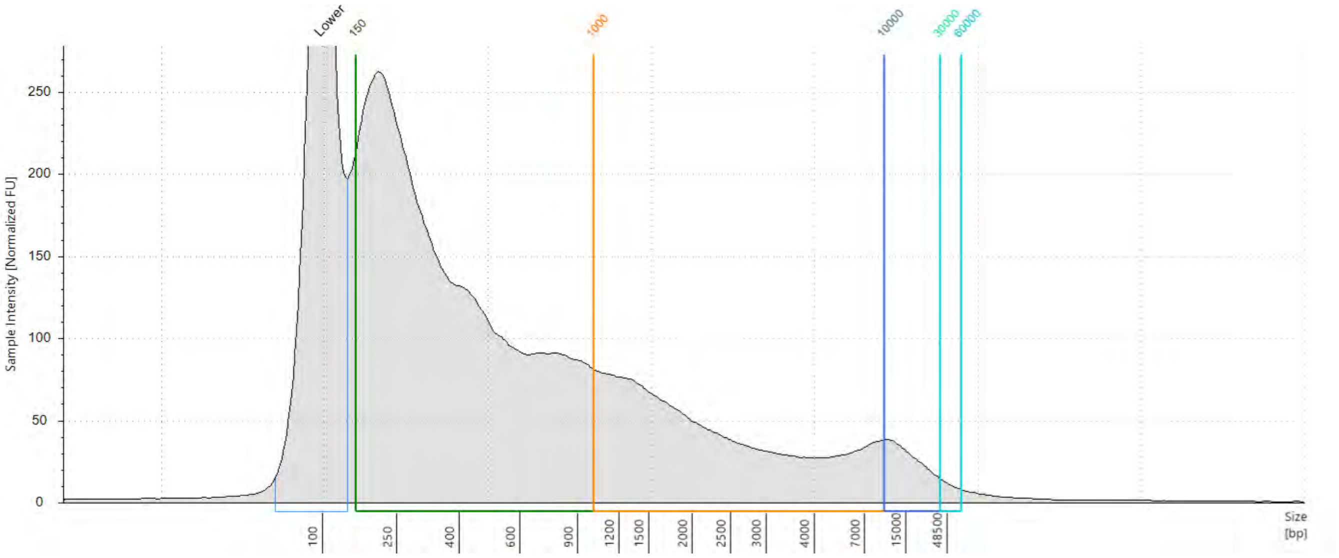

Region Table

From [bp]	To [bp]	Average Size [bp]	Conc. [ng/µl]	Region Molarity [nmol/l]	% of Total	Region Comment	Color
150	10000	1047	23.5	91.0	92.42		
150	1000	405	17.1	85.8	67.16		
1000	10000	2711	6.47	5.34	25.49		
10000	60000	20472	0.875	0.0865	3.44		
10000	30000	16499	0.775	0.0828	3.05		

 © Agilent Technologies, Inc. 2017

30000	60000	48267	0.110	0.00420	0.43		

© Agilent Technologies, Inc. 2017

E9: W698

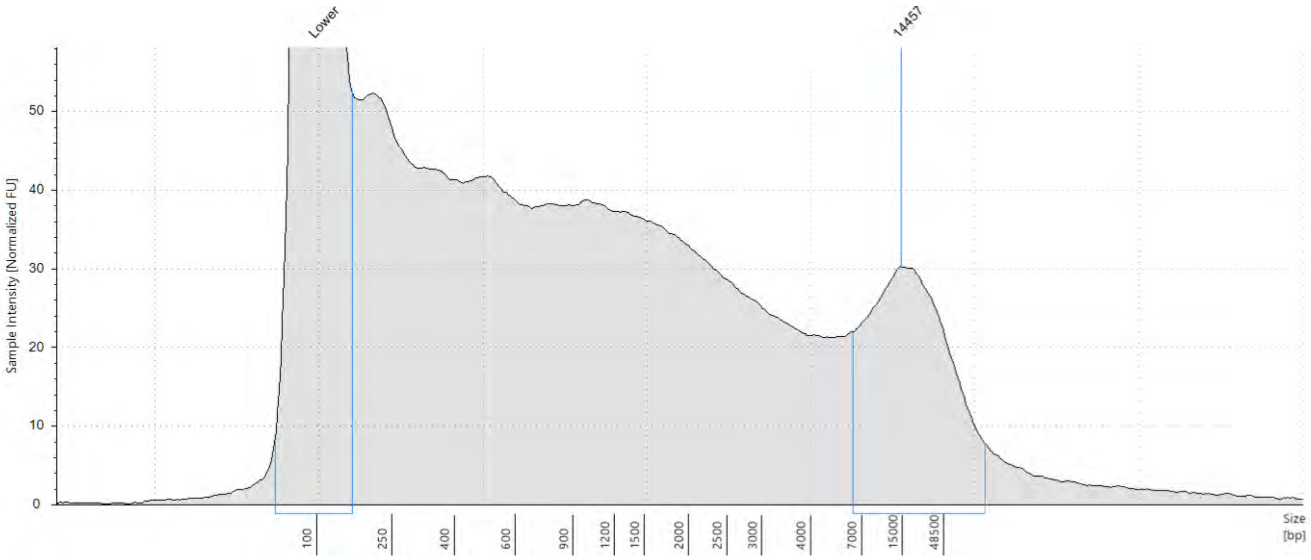

Sample Table

Well	DIN	Conc. [ng/µl]	Sample Description	Alert	Observations
E9	1.8	11.6	W698		

Peak Table

Size [bp]	Calibrated Conc. [ng/µl]	Assigned Conc. [ng/µl]	% Integrated Area	From [bp]	To [bp]	Peak Comment	Observations
100	8.50	8.50	-	60	153		Lower Marker
14457	1.61	-	97.40	6380	>60000		
-	-	-	-	-	-		Sample Well

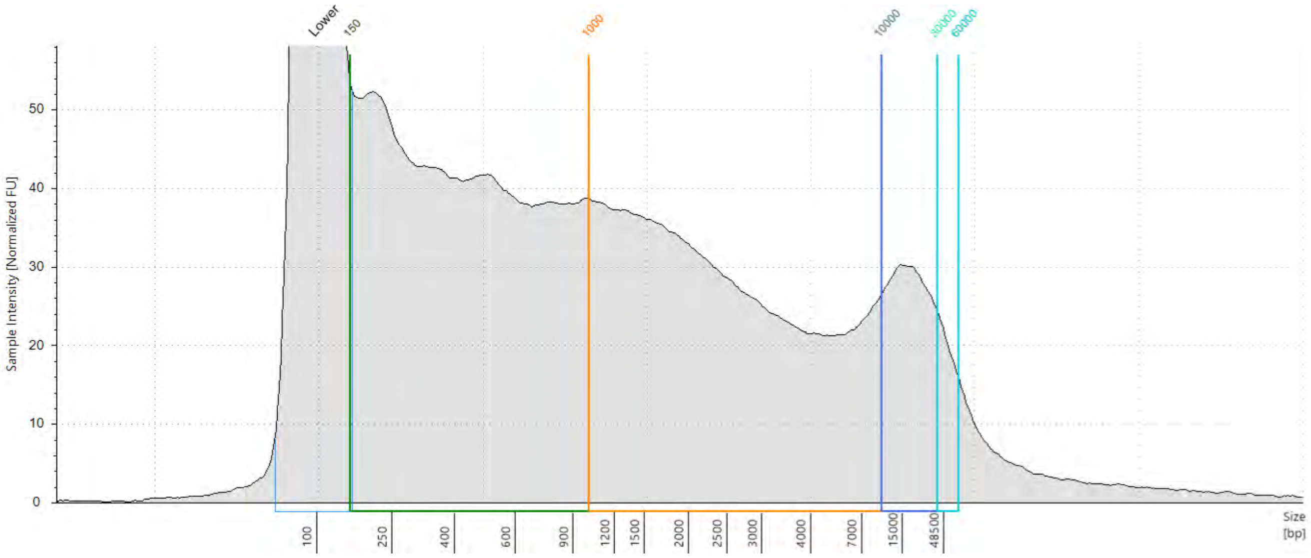

Region Table

From [bp]	To [bp]	Average Size [bp]	Conc. [ng/µl]	Region Molarity [nmol/l]	% of Total	Region Comment	Color
150	10000	1572	9.99	27.9	86.12		
150	1000	468	5.47	24.5	47.16		
1000	10000	2909	4.55	3.44	39.21		
10000	60000	24290	1.10	0.0933	9.51		
10000	30000	17814	0.877	0.0857	7.57		

 © Agilent Technologies, Inc. 2017

30000	60000	48740	0.244	0.00858	2.10		

© Agilent Technologies, Inc. 2017

F9: W699

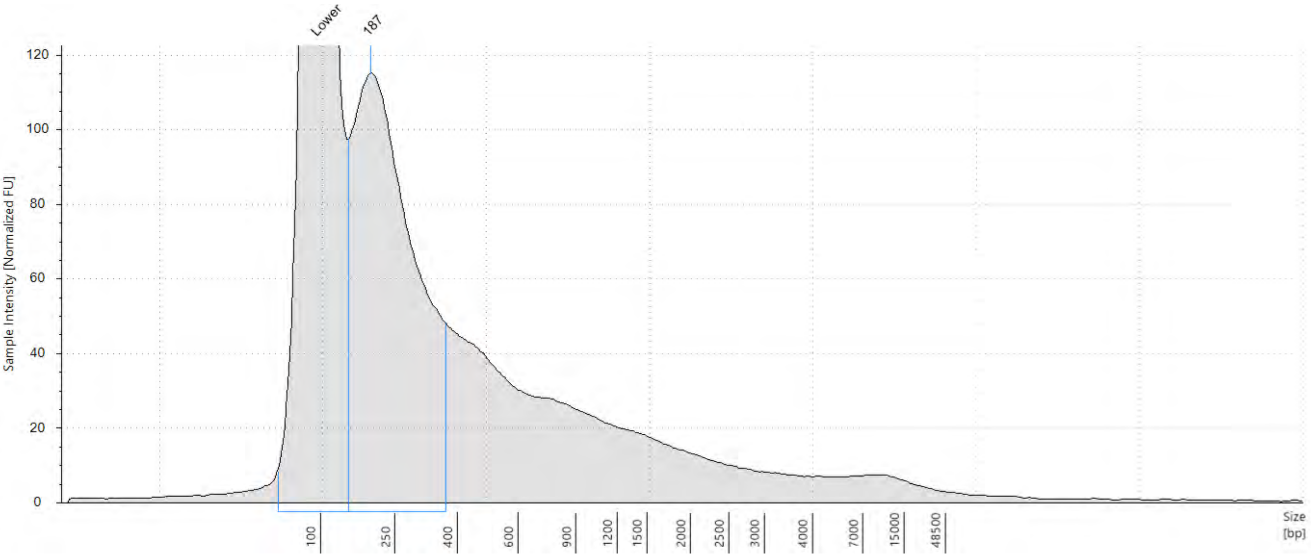

Sample Table

Well	DIN	Conc. [ng/µl]	Sample Description	Alert	Observations
F9	1.4	9.70	W699	⚠	Sample concentration outside recommended range

Peak Table

Size [bp]	Calibrated Conc. [ng/µl]	Assigned Conc. [ng/µl]	% Integrated Area	From [bp]	To [bp]	Peak Comment	Observations
100	8.50	8.50	-	60	143		Lower Marker
187	4.75	-	100.00	143	368		

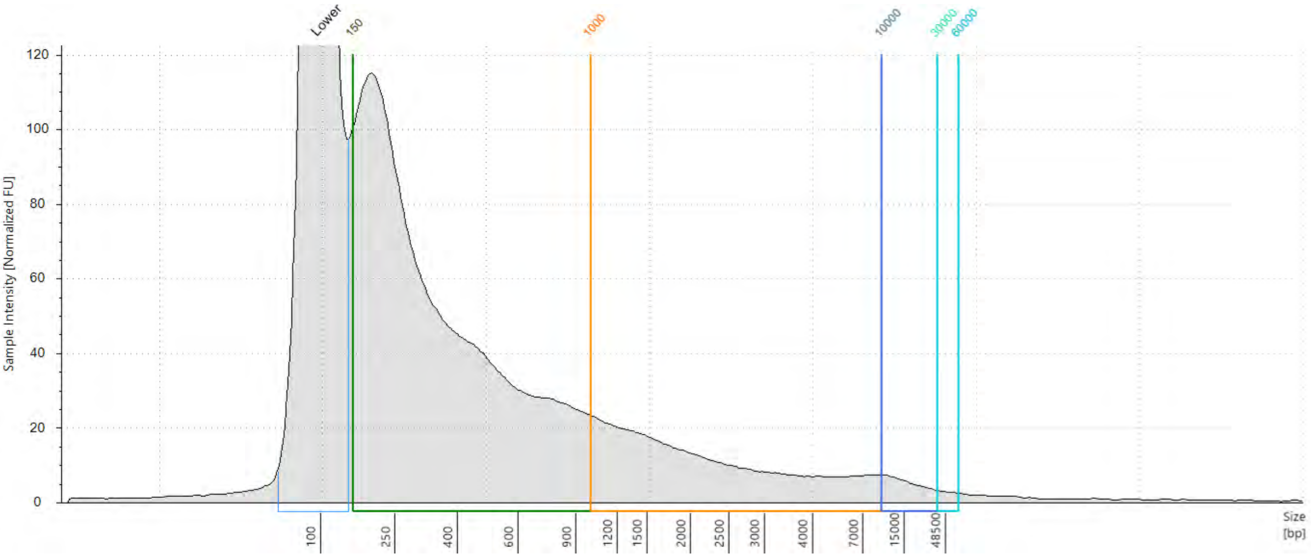

Region Table

From [bp]	To [bp]	Average Size [bp]	Conc. [ng/µl]	Region Molarity [nmol/l]	% of Total	Region Comment	Color
150	10000	850	9.11	40.5	93.96		
150	1000	378	7.24	38.9	74.69		
1000	10000	2591	1.89	1.63	19.47		
10000	60000	22095	0.184	0.0188	1.90		
10000	30000	16748	0.157	0.0178	1.62		
30000	60000	49407	0.0291	0.00119	0.30		

 © Agilent Technologies, Inc. 2017

G9: W700

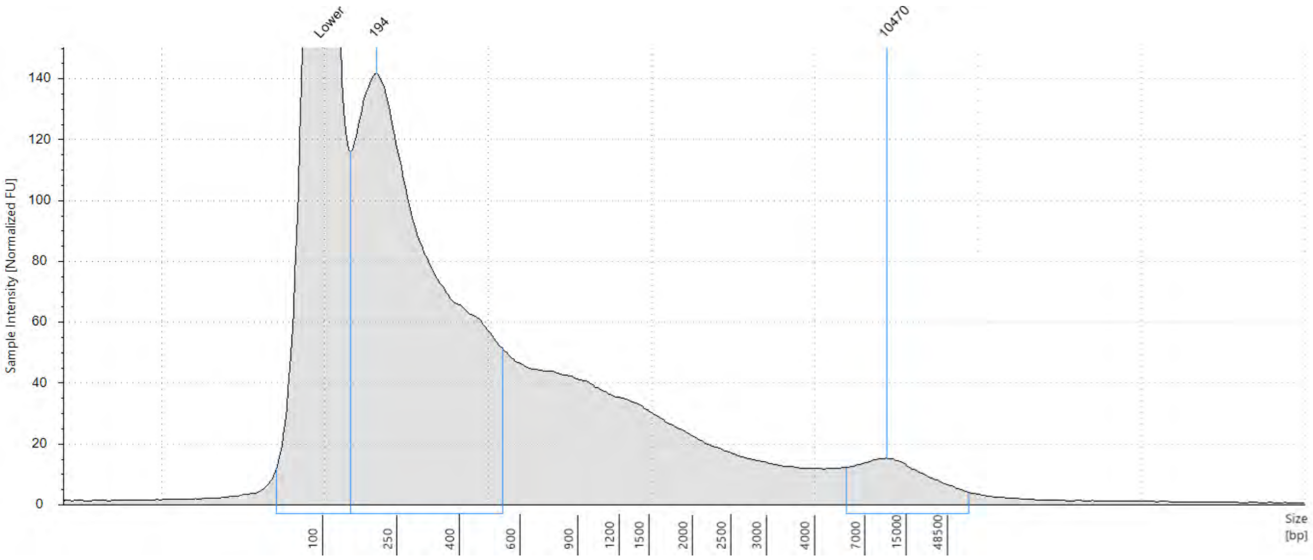

Sample Table

Well	DIN	Conc. [ng/µl]	Sample Description	Alert	Observations
G9	1.4	13.5	W700		

Peak Table

Size [bp]	Calibrated Conc. [ng/µl]	Assigned Conc. [ng/µl]	% Integrated Area	From [bp]	To [bp]	Peak Comment	Observations
100	8.50	8.50	-	57	141		Lower Marker
194	7.58	-	91.57	141	532		
10470	0.697	-	8.43	5695	>60000		

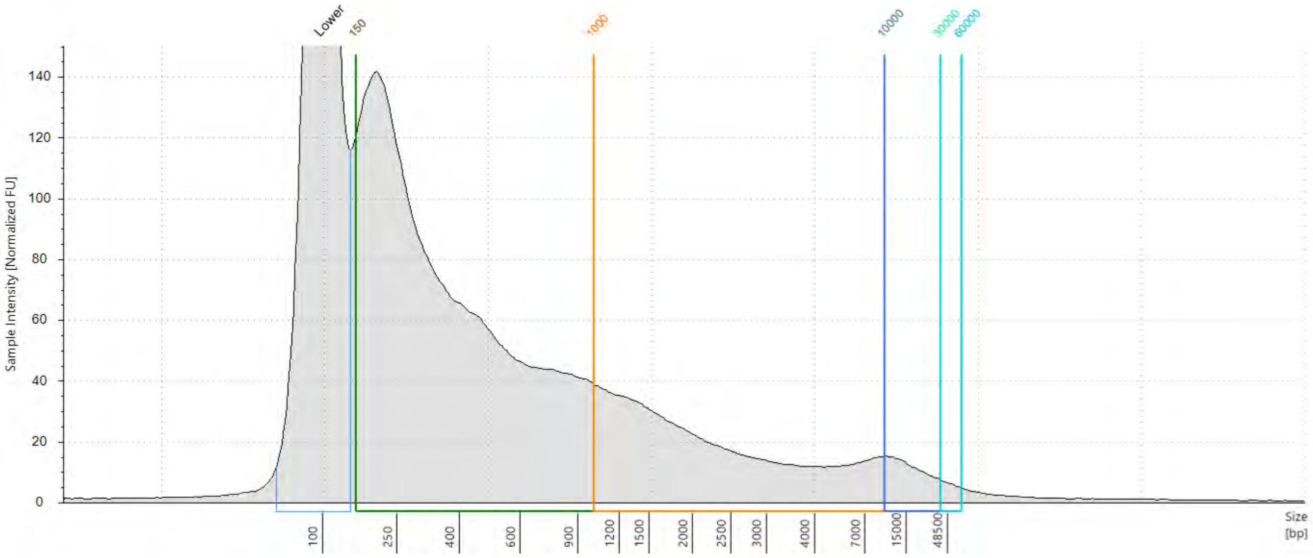

Region Table

From [bp]	To [bp]	Average Size [bp]	Conc. [ng/µl]	Region Molarity [nmol/l]	% of Total	Region Comment	Color
150	10000	968	12.5	50.8	92.77		
150	1000	398	9.39	48.2	69.80		
1000	10000	2649	3.12	2.64	23.19		
10000	60000	22364	0.405	0.0385	3.01		
10000	30000	17050	0.344	0.0363	2.56		

© Agilent Technologies, Inc. 2017

30000	60000	49833	0.0666	0.00244	0.49		

© Agilent Technologies, Inc. 2017

H9: W701

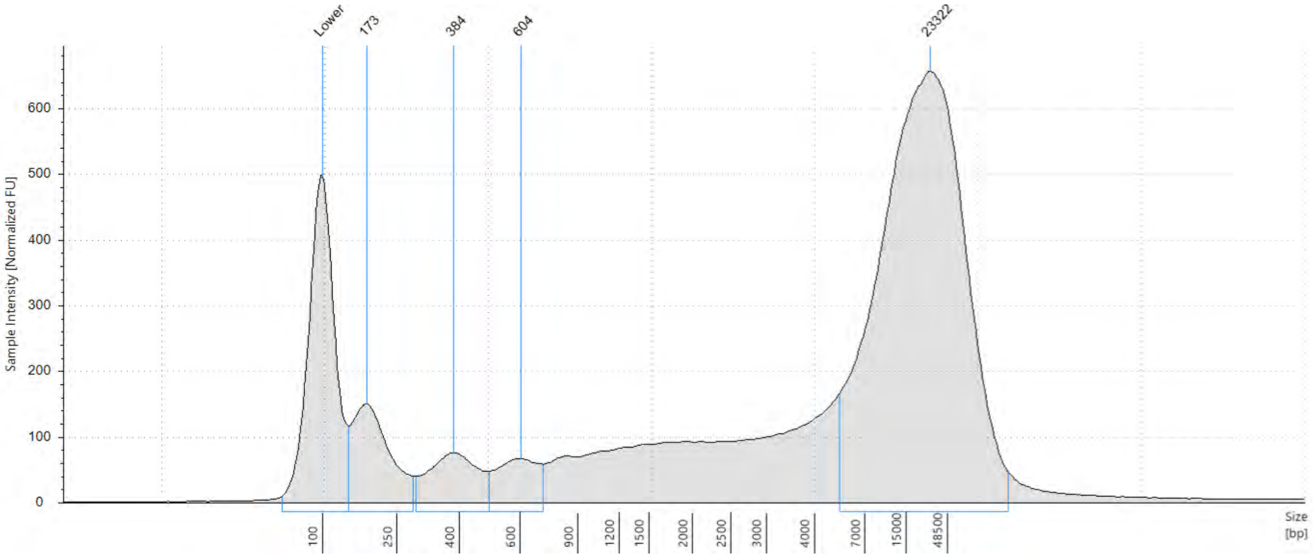

Sample Table

Well	DIN	Conc. [ng/µl]	Sample Description	Alert	Observations
H9	6.6	61.6	W701		

Peak Table

Size [bp]	Calibrated Conc. [ng/µl]	Assigned Conc. [ng/µl]	% Integrated Area	From [bp]	To [bp]	Peak Comment	Observations
100	8.50	8.50	-	61	138		Lower Marker
173	3.61	-	7.98	138	282		
384	2.37	-	5.23	288	487		
604	1.83	-	4.04	487	706		
23322	36.9	-	81.66	5324	>60000		
-	-	-	-	-	-		Sample Well

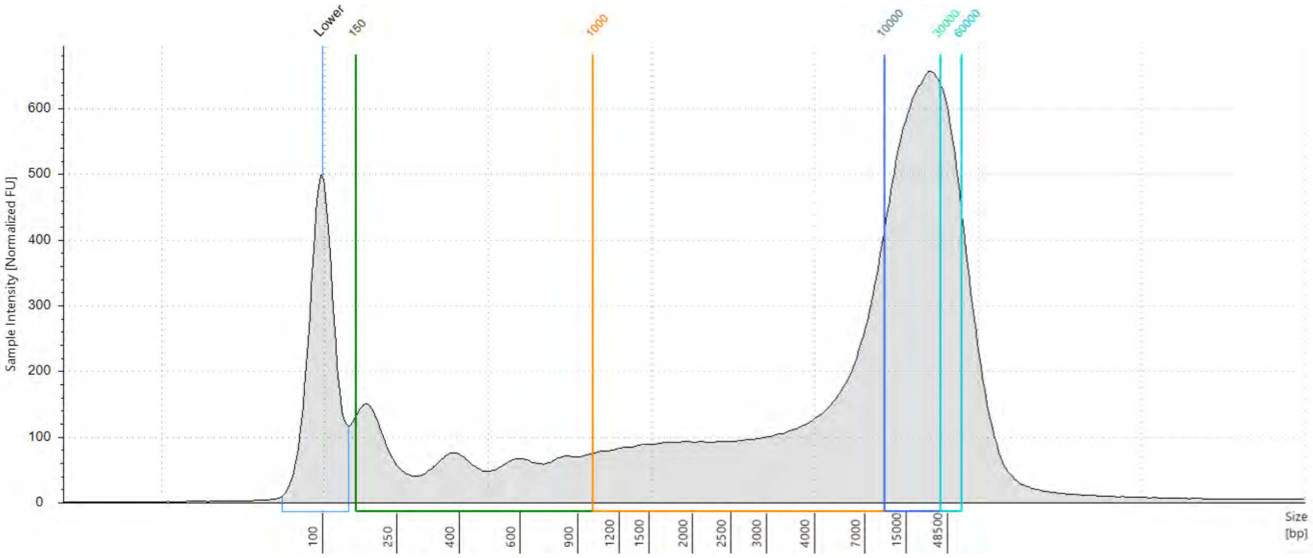

Region Table

From [bp]	To [bp]	Average Size [bp]	Conc. [ng/µl]	Region Molarity [nmol/l]	% of Total	Region Comment	Color
150	10000	3107	29.5	55.2	47.85		
150	1000	463	9.22	44.1	14.96		

 © Agilent Technologies, Inc. 2017

1000	10000	4311	20.3	11.2	32.98		
10000	60000	26876	25.5	1.93	41.31		
10000	30000	18751	18.9	1.72	30.66		
30000	60000	49847	7.07	0.235	11.47		

A10: W702

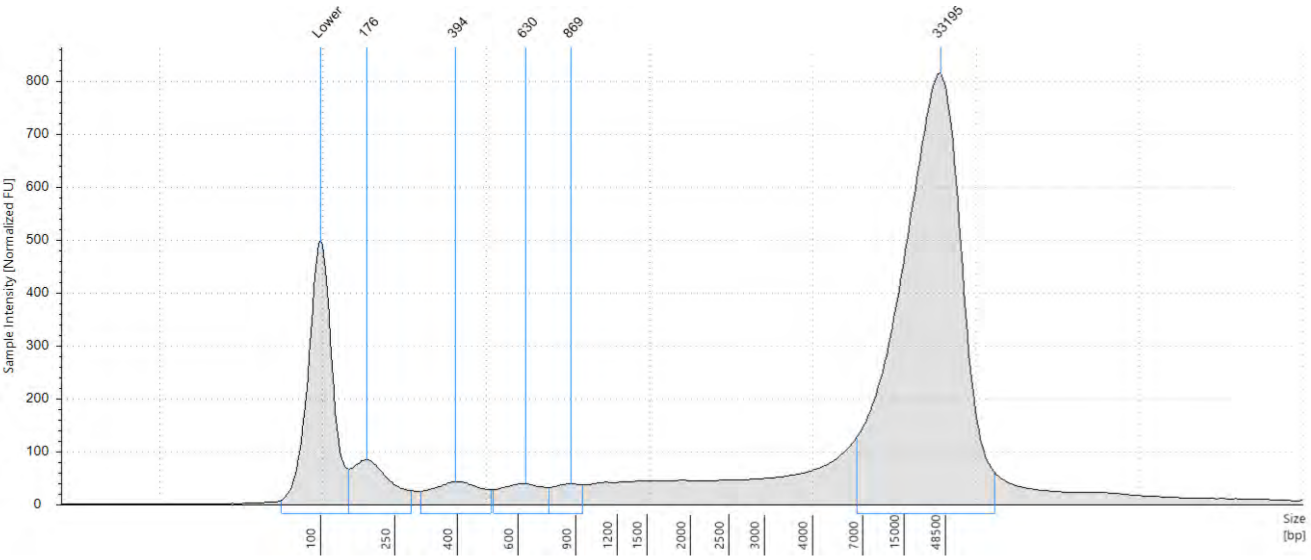

Sample Table

Well	DIN	Conc. [ng/µl]	Sample Description	Alert	Observations
A10	7.2	53.8	W702		

Peak Table

Size [bp]	Calibrated Conc. [ng/µl]	Assigned Conc. [ng/µl]	% Integrated Area	From [bp]	To [bp]	Peak Comment	Observations
100	8.50	8.50	-	62	141		Lower Marker
176	2.23	-	5.29	141	281		
394	1.43	-	3.39	303	498		
630	1.14	-	2.71	508	744		
869	0.743	-	1.76	744	940		
33195	35.4	-	83.96	6537	>60000		
-	-	-	-	-	-		Sample Well

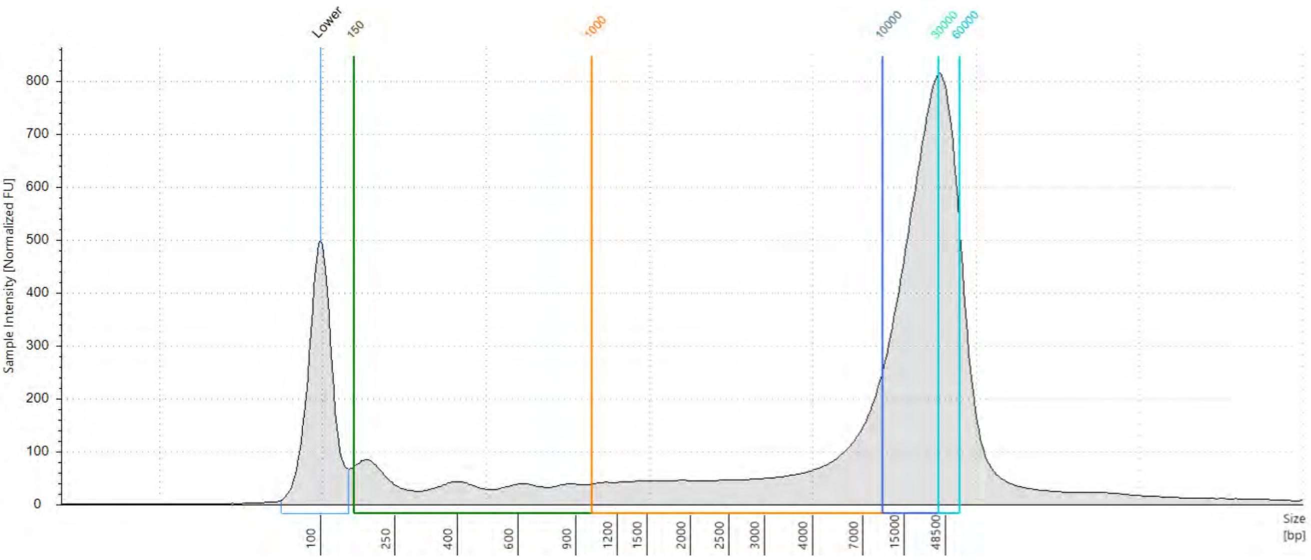

Region Table

From [bp]	To [bp]	Average Size [bp]	Conc. [ng/µl]	Region Molarity [nmol/l]	% of Total	Region Comment	Color
150	10000	3151	17.1	34.3	31.76		

© Agilent Technologies, Inc. 2017

150	1000	454	5.65	28.1	10.49		
1000	10000	4497	11.5	6.25	21.33		
10000	60000	29760	28.3	1.92	52.67		
10000	30000	20040	19.2	1.62	35.61		
30000	60000	49843	9.88	0.328	18.36		

© Agilent Technologies, Inc. 2017

B10: W703

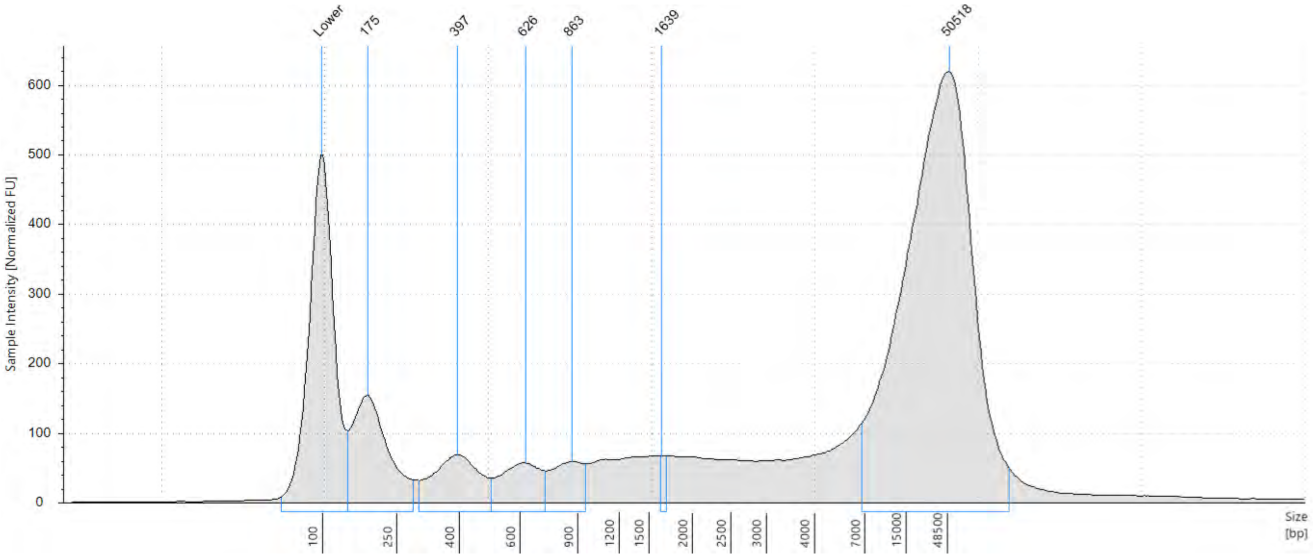

Sample Table

Well	DIN	Conc. [ng/µl]	Sample Description	Alert	Observations
B10	6.8	50.0	W703		

Peak Table

Size [bp]	Calibrated Conc. [ng/µl]	Assigned Conc. [ng/µl]	% Integrated Area	From [bp]	To [bp]	Peak Comment	Observations
100	8.50	8.50	-	61	138		Lower Marker
175	3.69	-	9.66	138	284		
397	2.09	-	5.45	296	496		
626	1.52	-	3.99	496	717		
863	1.28	-	3.36	717	951		
1639	0.270	-	0.71	1624	1684		
50518	28.8	-	75.43	6775	>60000		
-	-	-	-	-	-		Sample Well

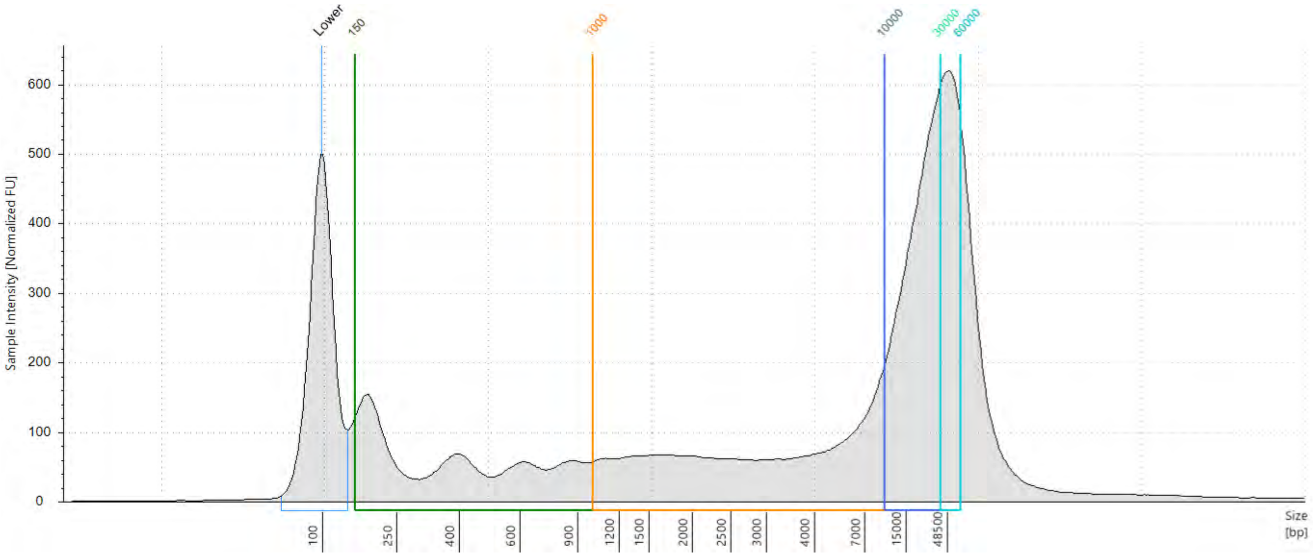

Region Table

From [bp]	To [bp]	Average Size [bp]	Conc. [ng/µl]	Region Molarity [nmol/l]	% of Total	Region Comment	Color

150	10000	2454	20.7	50.2	41.50		
150	1000	444	8.42	42.6	16.85		
1000	10000	3819	12.4	7.73	24.75		
10000	60000	30829	21.0	1.40	42.07		
10000	30000	19907	13.6	1.17	27.23		
30000	60000	50675	7.91	0.257	15.83		

© Agilent Technologies, Inc. 2017

C10: W704

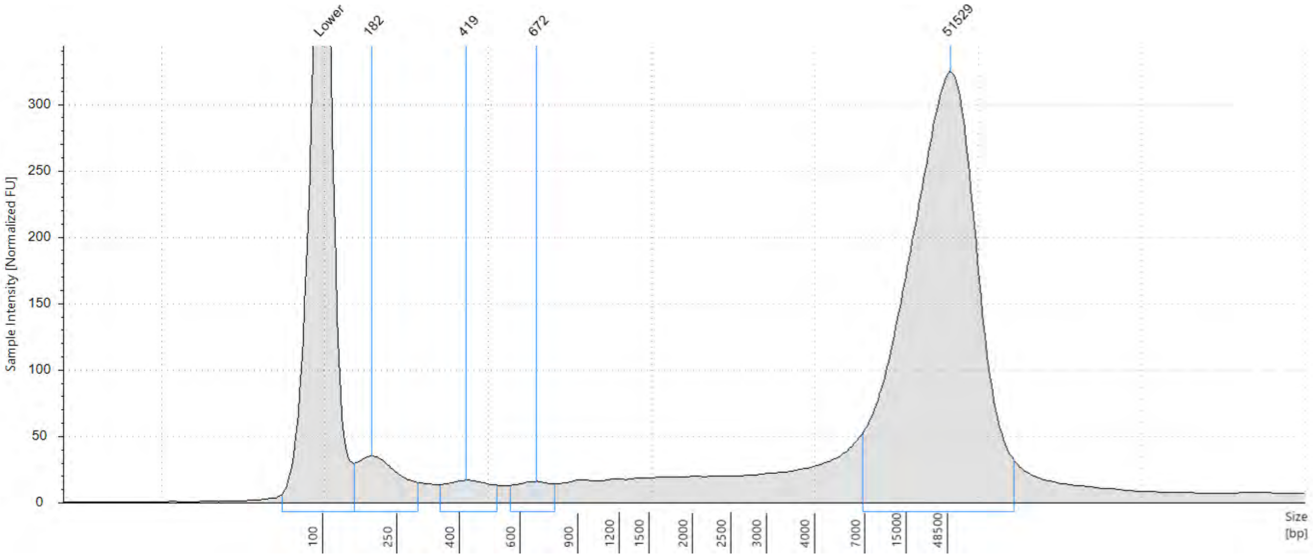

Sample Table

Well	DIN	Conc. [ng/µl]	Sample Description	Alert	Observations
C10	7.5	25.6	W704		

Peak Table

Size [bp]	Calibrated Conc. [ng/µl]	Assigned Conc. [ng/µl]	% Integrated Area	From [bp]	To [bp]	Peak Comment	Observations
100	8.50	8.50	-	61	148		Lower Marker
182	1.06	-	5.47	148	291		
419	0.530	-	2.72	345	515		
672	0.406	-	2.09	560	764		
51529	16.8	-	86.10	6811	>60000		
-	-	-	-	-	-		Sample Well

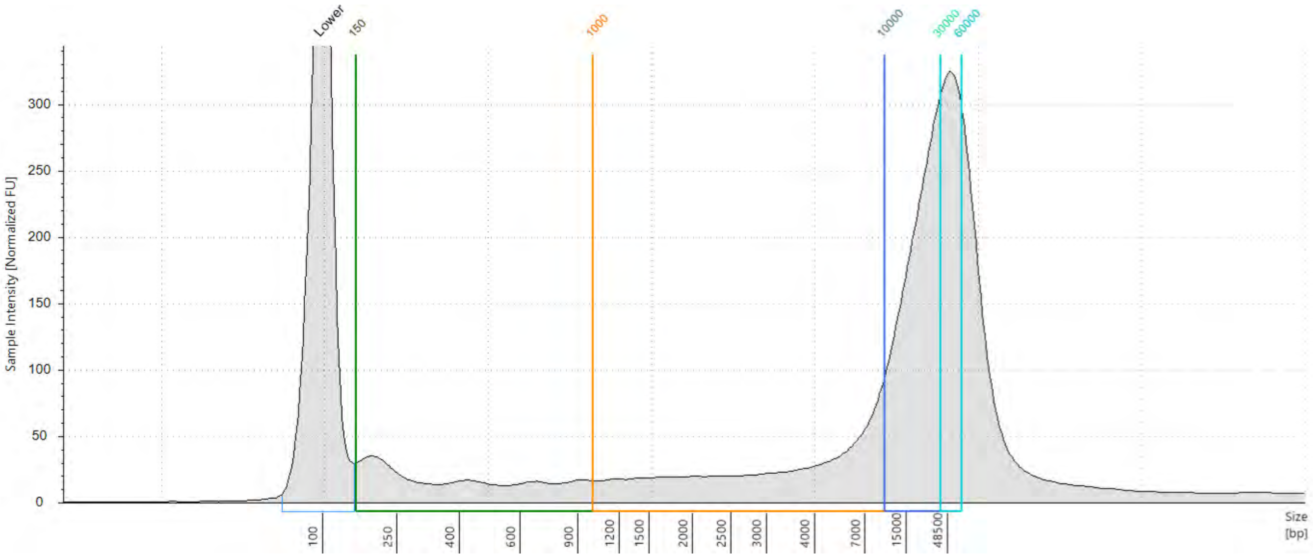

Region Table

From [bp]	To [bp]	Average Size [bp]	Conc. [ng/µl]	Region Molarity [nmol/l]	% of Total	Region Comment	Color
150	10000	2951	7.41	15.8	28.93		
150	1000	444	2.61	13.1	10.18		

1000	10000	4323	4.82	2.70	18.81		
10000	60000	31331	11.5	0.752	44.90		
10000	30000	19954	7.26	0.618	28.33		
30000	60000	50599	4.52	0.147	17.64		

© Agilent Technologies, Inc. 2017

D10: W705

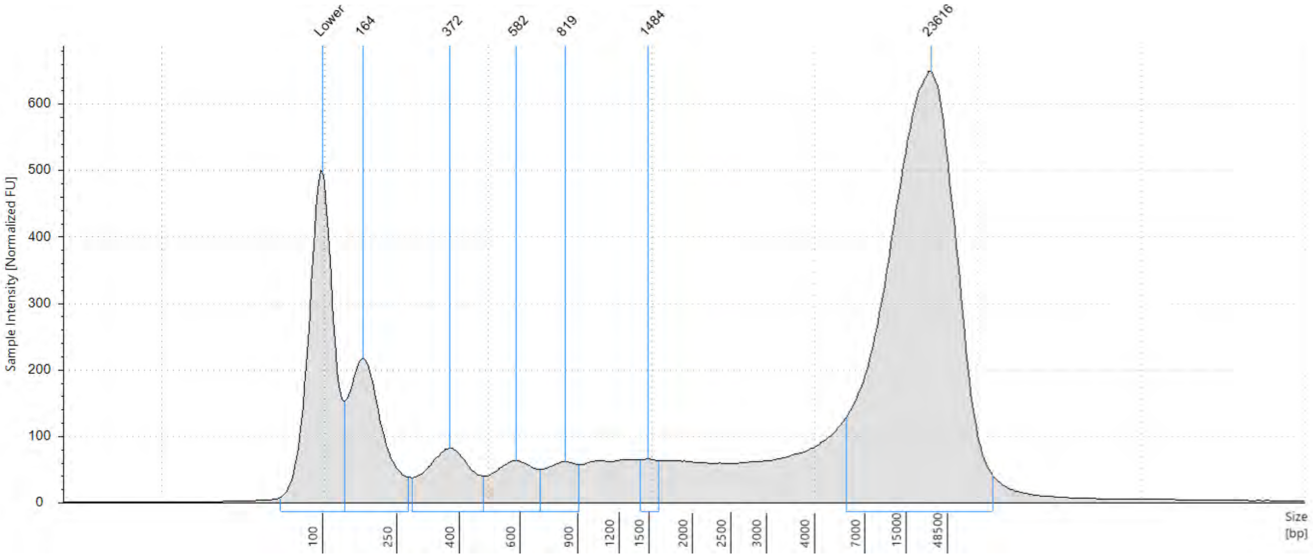

Sample Table

Well	DIN	Conc. [ng/µl]	Sample Description	Alert	Observations
D10	6.6	52.2	W705		

Peak Table

Size [bp]	Calibrated Conc. [ng/µl]	Assigned Conc. [ng/µl]	% Integrated Area	From [bp]	To [bp]	Peak Comment	Observations
100	8.50	8.50	-	60	132		Lower Marker
164	4.91	-	11.66	132	271		
372	2.44	-	5.78	280	470		
582	1.73	-	4.12	470	687		
819	1.31	-	3.11	687	904		
1484	0.725	-	1.72	1408	1599		
23616	30.4	-	72.26	5697	>60000		
-	-	-	-	-	-		Sample Well

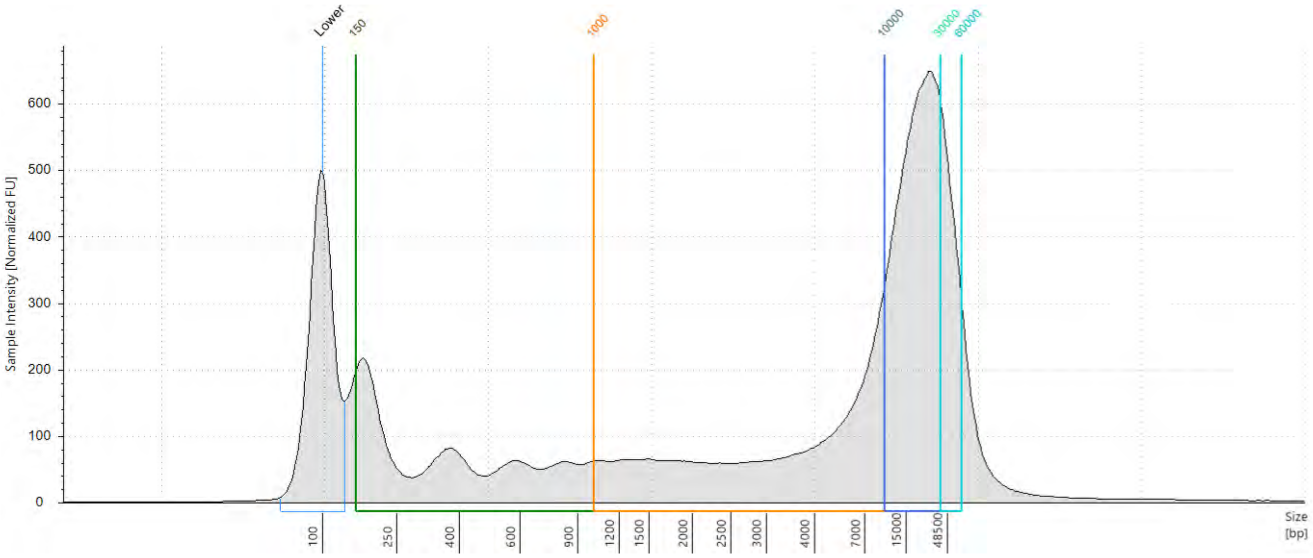

Region Table

From [bp]	To [bp]	Average Size [bp]	Conc. [ng/µl]	Region Molarity [nmol/l]	% of Total	Region Comment	Color

150	10000	2853	24.6	59.4	47.06		
150	1000	428	9.81	51.3	18.79		
1000	10000	4446	14.8	8.16	28.37		
10000	60000	26026	23.5	1.79	45.04		
10000	30000	19262	18.3	1.62	35.13		
30000	60000	49620	5.66	0.189	10.85		

© Agilent Technologies, Inc. 2017

E10: W706

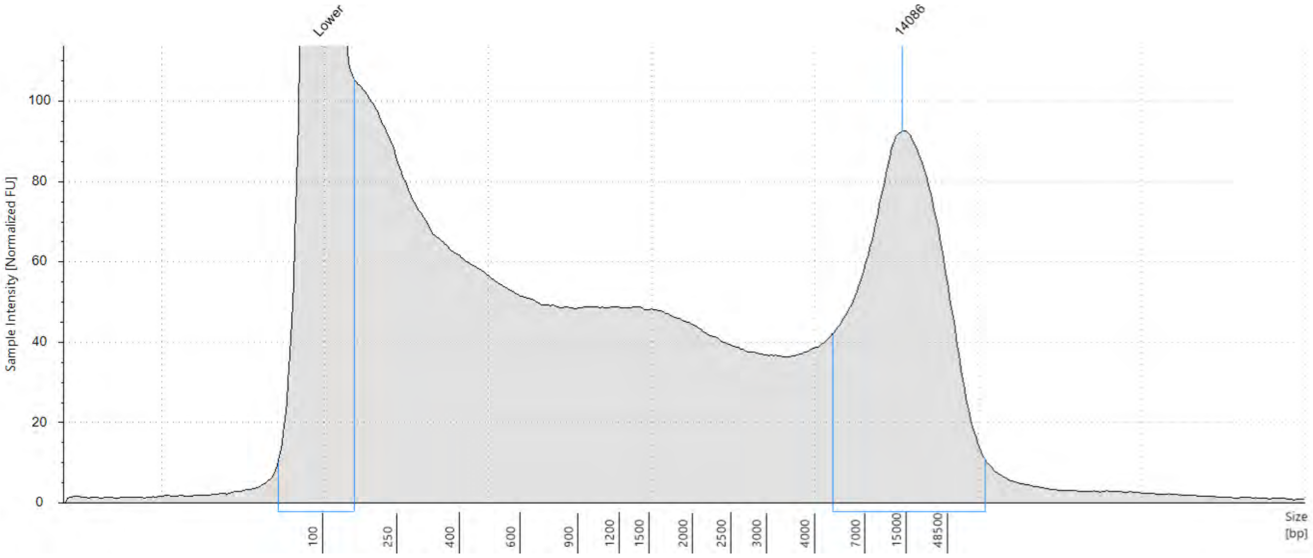

Sample Table

Well	DIN	Conc. [ng/µl]	Sample Description	Alert	Observations
E10	2.3	19.2	W706		

Peak Table

Size [bp]	Calibrated Conc. [ng/µl]	Assigned Conc. [ng/µl]	% Integrated Area	From [bp]	To [bp]	Peak Comment	Observations
100	8.50	8.50	-	59	148		Lower Marker
14086	4.93	-	99.05	4915	>60000		
-	-	-	-	-	-		Sample Well

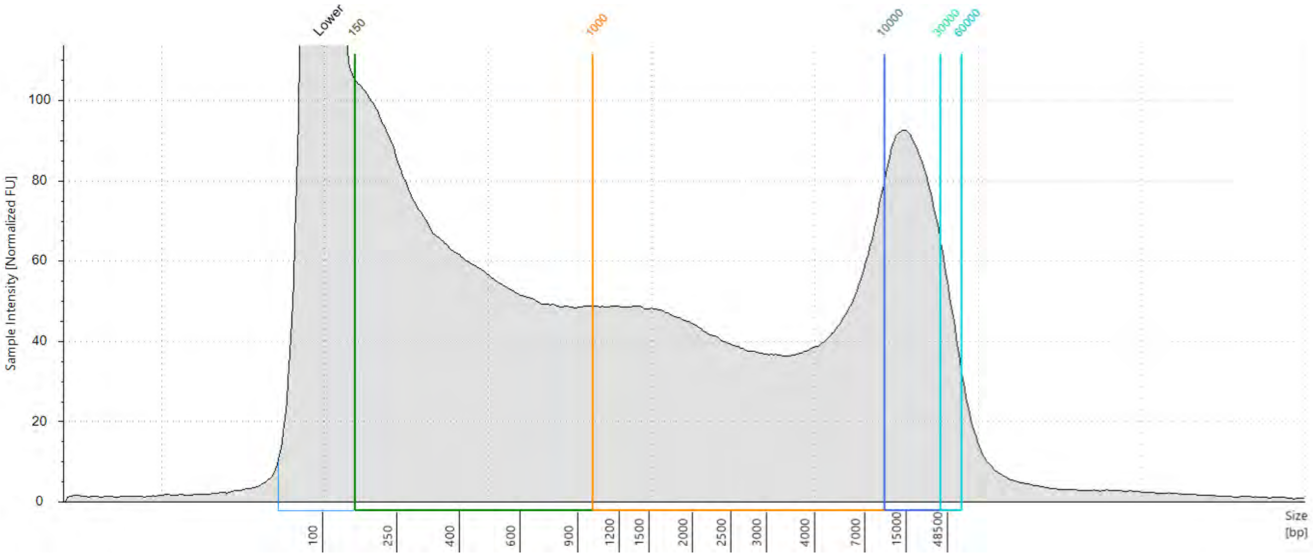

Region Table

From [bp]	To [bp]	Average Size [bp]	Conc. [ng/µl]	Region Molarity [nmol/l]	% of Total	Region Comment	Color
150	10000	1802	15.3	44.6	80.05		
150	1000	436	8.38	39.9	43.73		
1000	10000	3424	7.00	4.76	36.52		
10000	60000	23051	3.16	0.273	16.46		
10000	30000	17592	2.61	0.254	13.60		

 © Agilent Technologies, Inc. 2017

30000	60000	48193	0.599	0.0211	3.13		

© Agilent Technologies, Inc. 2017

F10: W707

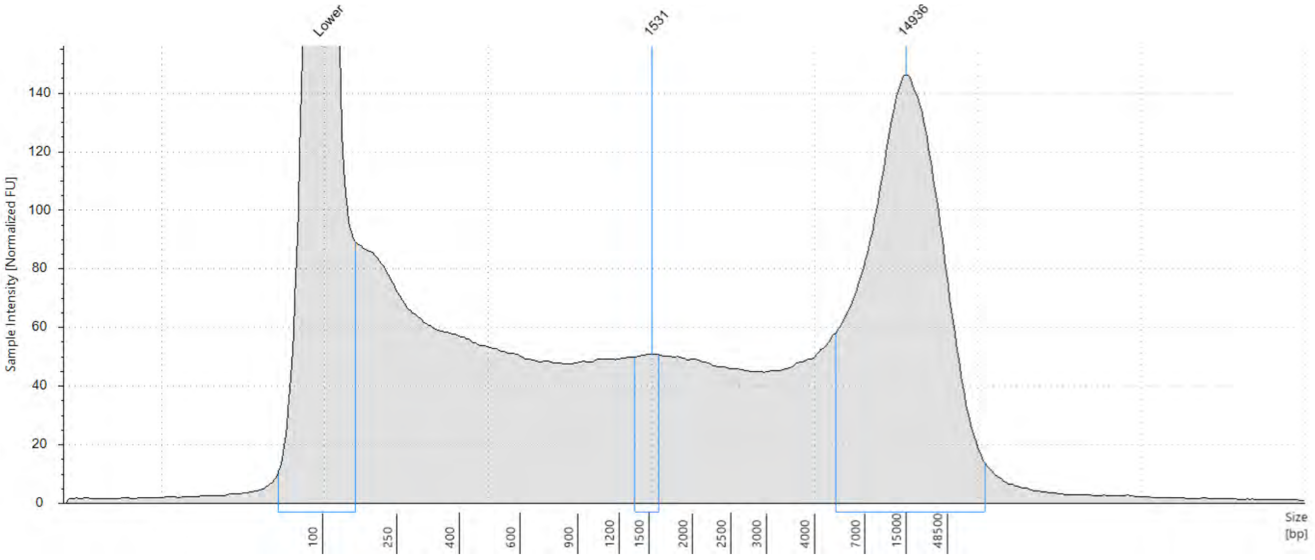

Sample Table

Well	DIN	Conc. [ng/µl]	Sample Description	Alert	Observations
F10	4.8	21.2	W707		

Peak Table

Size [bp]	Calibrated Conc. [ng/µl]	Assigned Conc. [ng/µl]	% Integrated Area	From [bp]	To [bp]	Peak Comment	Observations
100	8.50	8.50	-	59	151		Lower Marker
1531	0.675	-	8.73	1353	1603		
14936	7.06	-	91.27	5068	>60000		

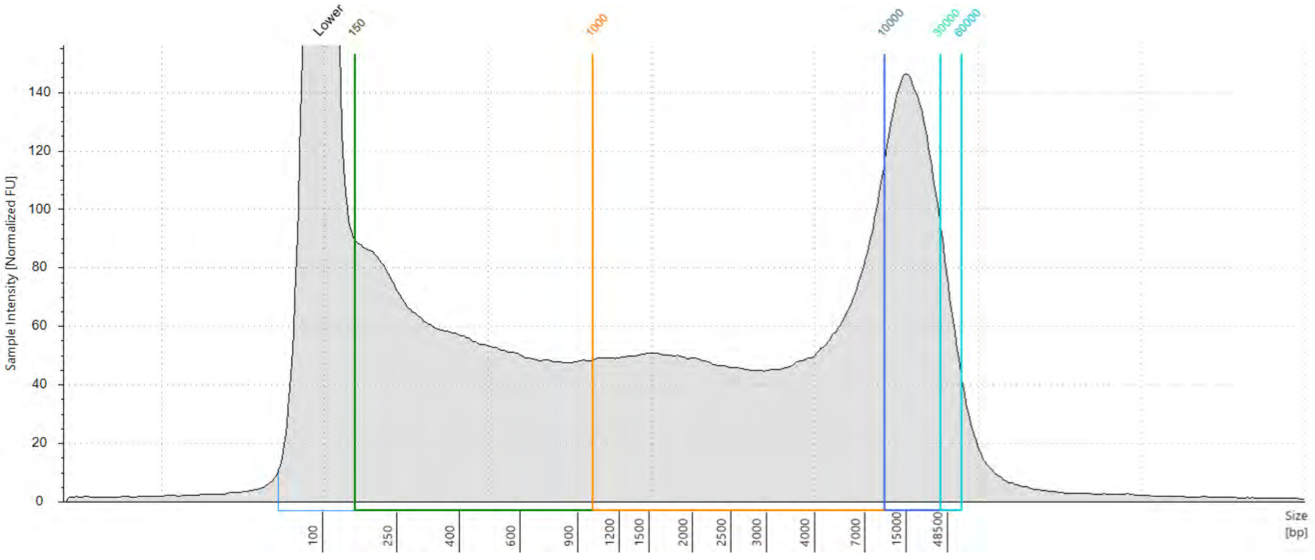

Region Table

From [bp]	To [bp]	Average Size [bp]	Conc. [ng/µl]	Region Molarity [nmol/l]	% of Total	Region Comment	Color
150	10000	2158	15.8	40.1	74.23		
150	1000	449	7.47	34.9	35.18		
1000	10000	3695	8.32	5.28	39.22		
10000	60000	22655	4.71	0.408	22.21		
10000	30000	17704	3.98	0.384	18.75		

 © Agilent Technologies, Inc. 2017

30000	60000	48765	0.805	0.0278	3.79		

© Agilent Technologies, Inc. 2017

G10: W708

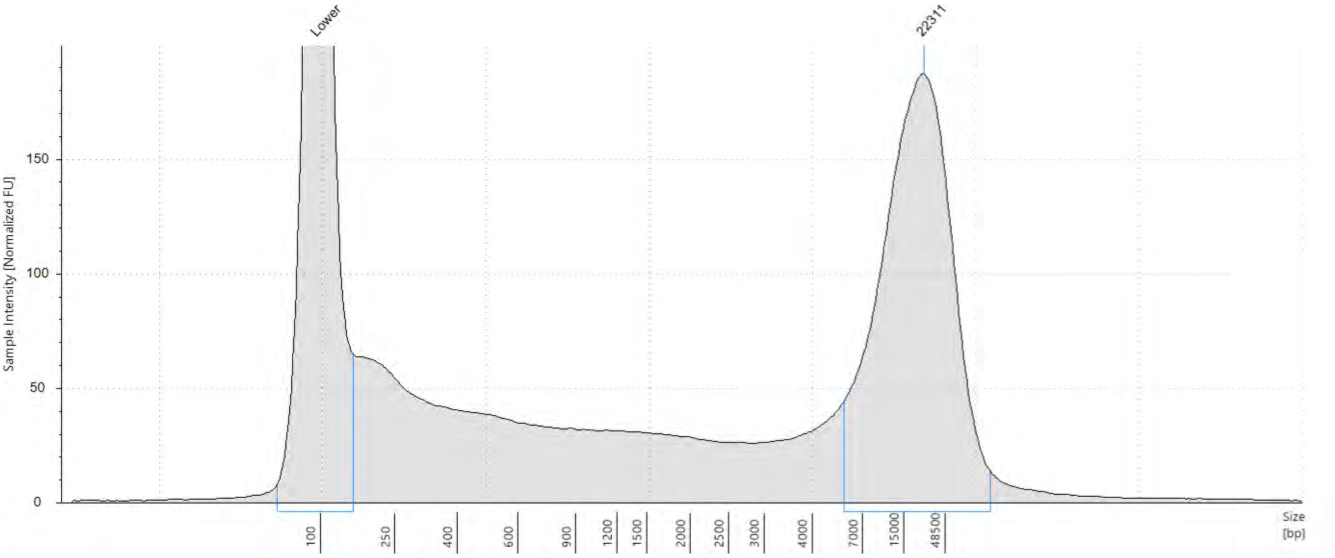

Sample Table

Well	DIN	Conc. [ng/µl]	Sample Description	Alert	Observations
G10	6.1	18.7	W708		

Peak Table

Size [bp]	Calibrated Conc. [ng/µl]	Assigned Conc. [ng/µl]	% Integrated Area	From [bp]	To [bp]	Peak Comment	Observations
100	8.50	8.50	-	60	153		Lower Marker
22311	8.71	-	99.15	5744	>60000		
-	-	-	-	-	-		Sample Well

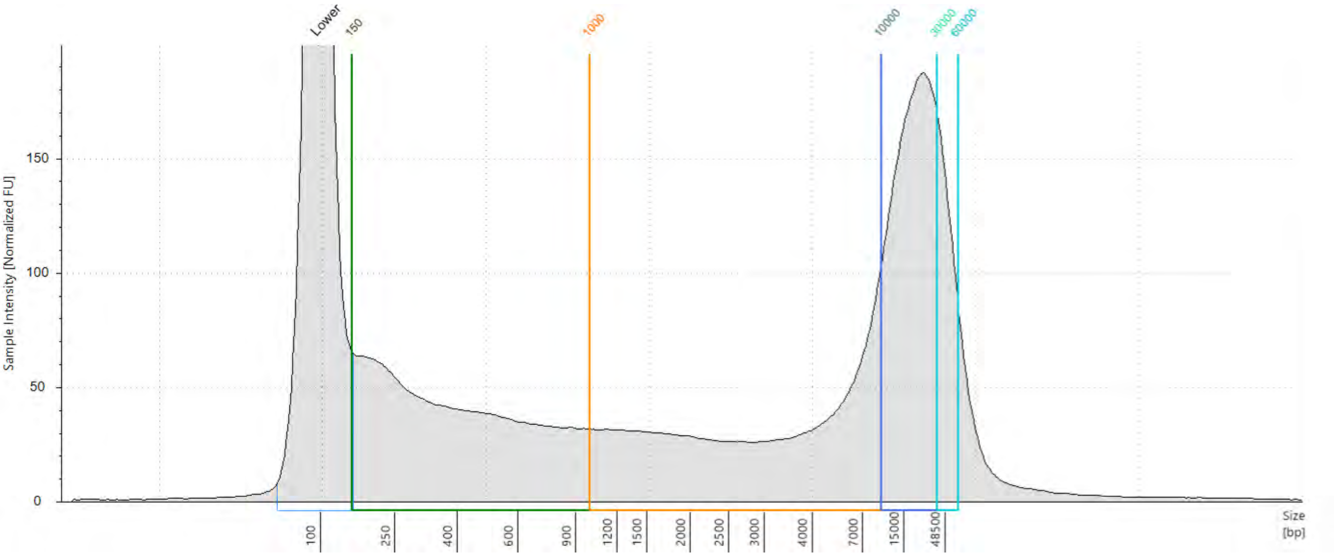

Region Table

From [bp]	To [bp]	Average Size [bp]	Conc. [ng/µl]	Region Molarity [nmol/l]	% of Total	Region Comment	Color
150	10000	2184	11.0	30.0	58.74		
150	1000	439	5.51	26.6	29.42		
1000	10000	3942	5.52	3.42	29.45		
10000	60000	25695	6.70	0.521	35.79		
10000	30000	18864	5.23	0.473	27.90		

30000	60000	49475	1.61	0.0542	8.58		

© Agilent Technologies, Inc. 2017

H10: W709

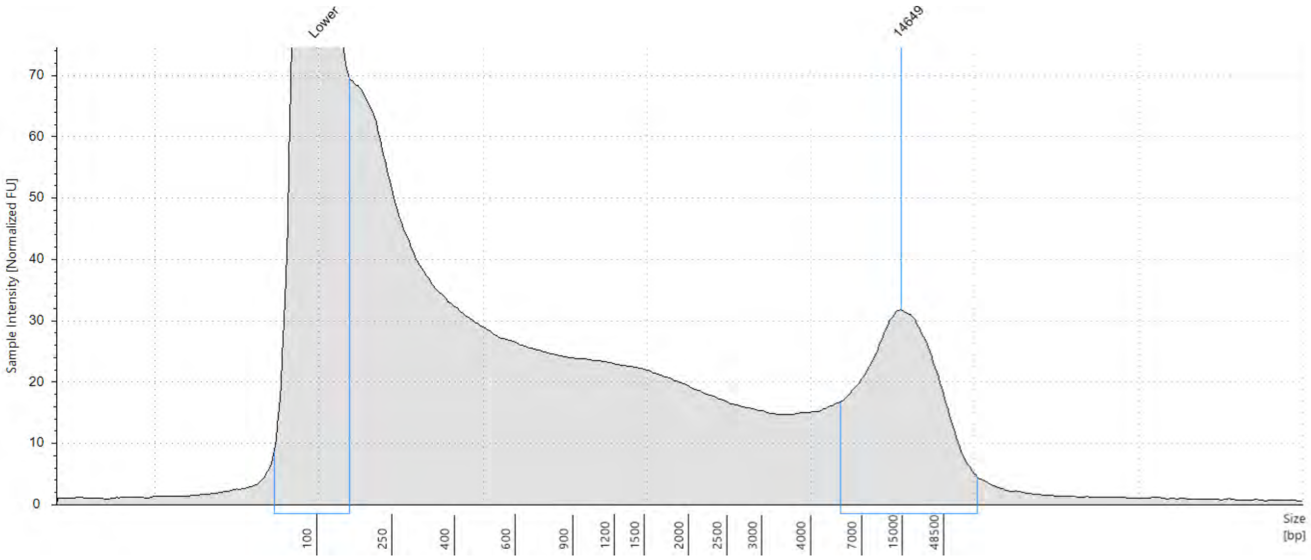

Sample Table

Well	DIN	Conc. [ng/µl]	Sample Description	Alert	Observations
H10	1.3	9.36	W709	⚠	Sample concentration outside recommended range

Peak Table

Size [bp]	Calibrated Conc. [ng/µl]	Assigned Conc. [ng/µl]	% Integrated Area	From [bp]	To [bp]	Peak Comment	Observations
100	8.50	8.50	-	60	149		Lower Marker
14649	1.63	-	100.00	5561	>60000		

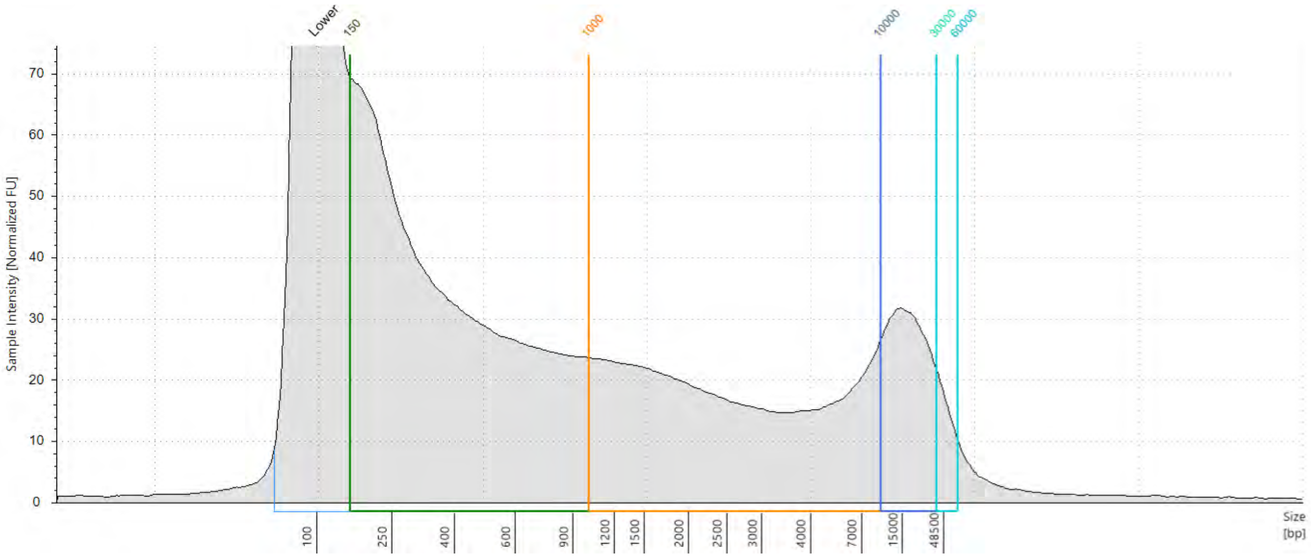

Region Table

From [bp]	To [bp]	Average Size [bp]	Conc. [ng/µl]	Region Molarity [nmol/l]	% of Total	Region Comment	Color
150	10000	1496	7.89	26.5	84.29		
150	1000	416	4.88	24.4	52.12		
1000	10000	3207	3.03	2.19	32.37		
10000	60000	22864	1.09	0.0952	11.69		
10000	30000	17863	0.925	0.0896	9.88		
30000	60000	49290	0.186	0.00641	1.98		

 © Agilent Technologies, Inc. 2017

© Agilent Technologies, Inc. 2017

A11: W710

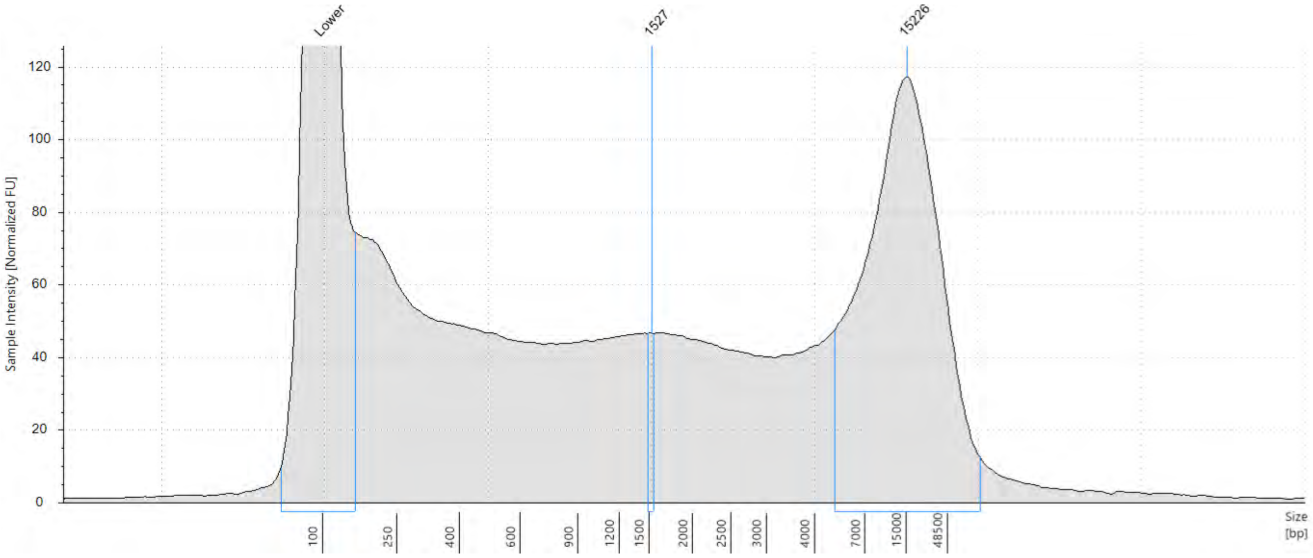

Sample Table

Well	DIN	Conc. [ng/µl]	Sample Description	Alert	Observations
A11	3.9	19.6	W710		

Peak Table

Size [bp]	Calibrated Conc. [ng/µl]	Assigned Conc. [ng/µl]	% Integrated Area	From [bp]	To [bp]	Peak Comment	Observations
100	8.50	8.50	-	60	148		Lower Marker
1527	0.185	-	3.05	1484	1541		
15226	5.89	-	96.95	4991	>60000		

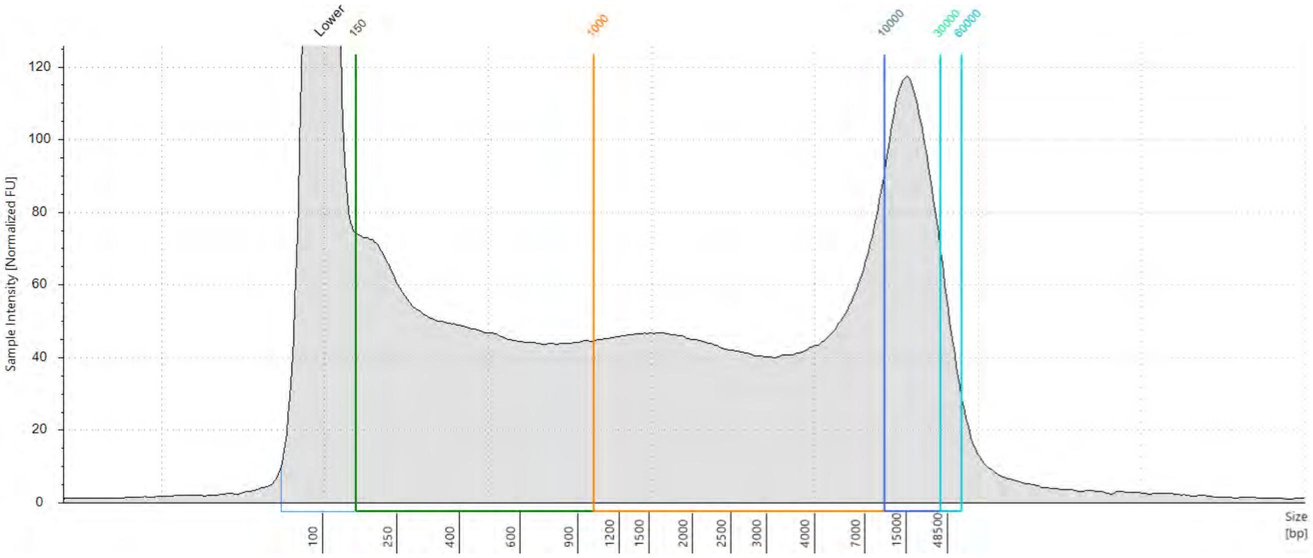

Region Table

From [bp]	To [bp]	Average Size [bp]	Conc. [ng/µl]	Region Molarity [nmol/l]	% of Total	Region Comment	Color
150	10000	2095	14.8	36.8	75.47		
150	1000	457	7.01	31.7	35.68		
1000	10000	3551	7.85	5.08	39.97		
10000	60000	21951	3.89	0.342	19.79		
10000	30000	17455	3.32	0.323	16.91		

 © Agilent Technologies, Inc. 2017

30000	60000	47710	0.622	0.0220	3.16		

© Agilent Technologies, Inc. 2017

B11: W711

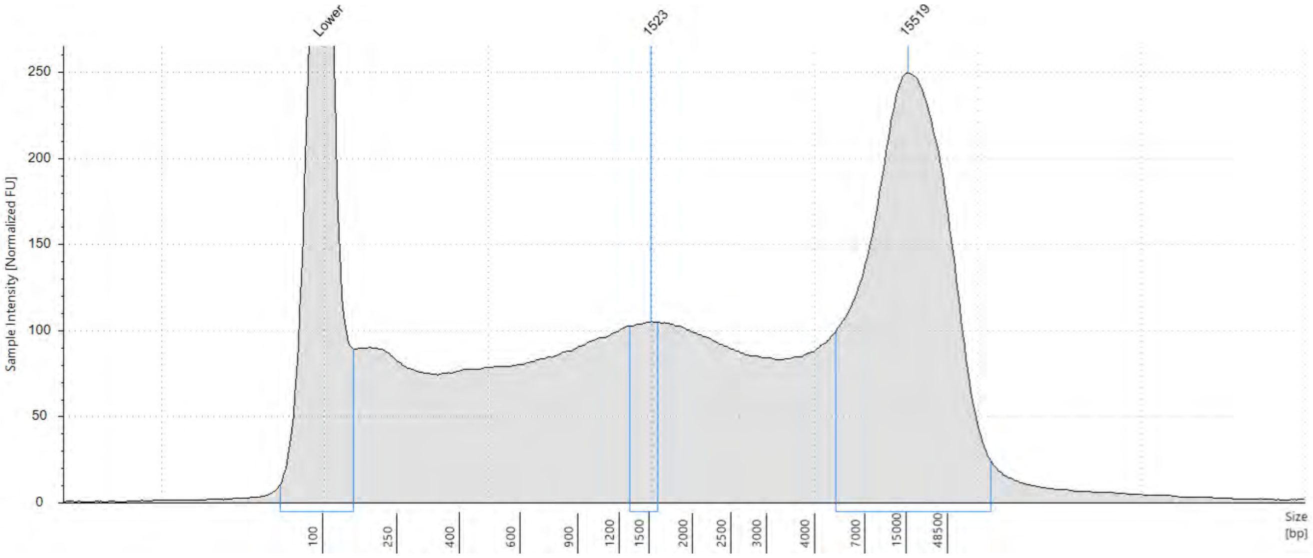

Sample Table

Well	DIN	Conc. [ng/µl]	Sample Description	Alert	Observations
B11	5.3	37.7	W711		

Peak Table

Size [bp]	Calibrated Conc. [ng/µl]	Assigned Conc. [ng/µl]	% Integrated Area	From [bp]	To [bp]	Peak Comment	Observations
100	8.50	8.50	-	60	148		Lower Marker
1523	1.67	-	10.85	1306	1594		
15519	13.6	-	88.06	5070	>60000		
-	-	-	-	-	-		Sample Well

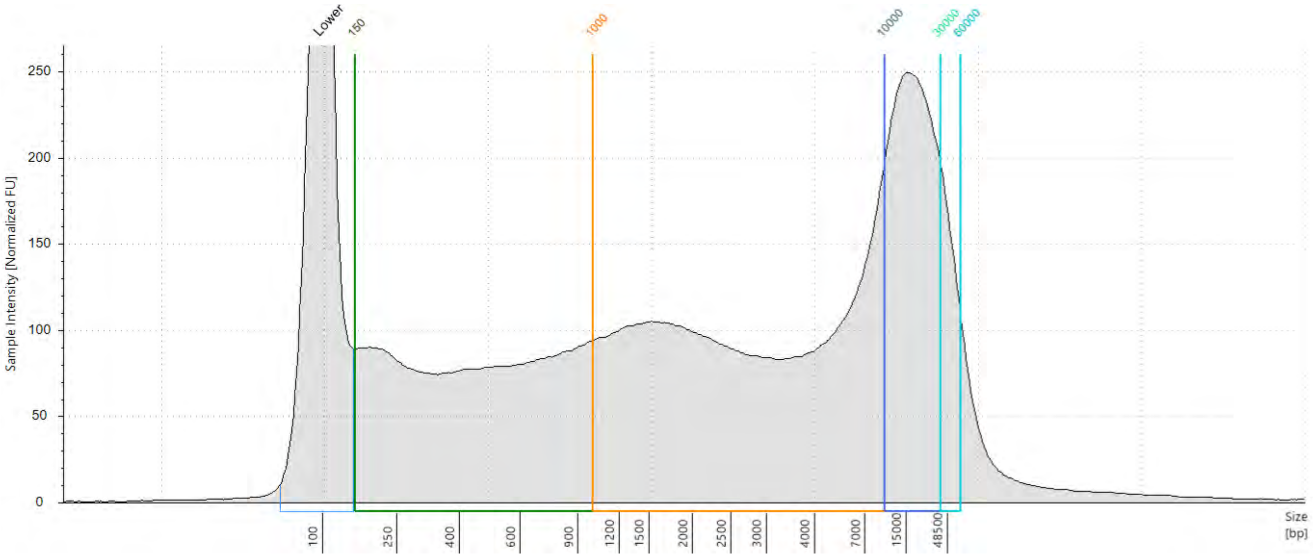

Region Table

From [bp]	To [bp]	Average Size [bp]	Conc. [ng/µl]	Region Molarity [nmol/l]	% of Total	Region Comment	Color
150	10000	2292	26.9	56.0	71.50		
150	1000	495	10.8	45.4	28.75		
1000	10000	3496	16.2	10.7	42.94		
10000	60000	24092	9.19	0.765	24.38		

 © Agilent Technologies, Inc. 2017

10000	30000	18025	7.45	0.709	19.77		
30000	60000	49521	1.89	0.0637	5.01		

 © Agilent Technologies, Inc. 2017

C11: W712

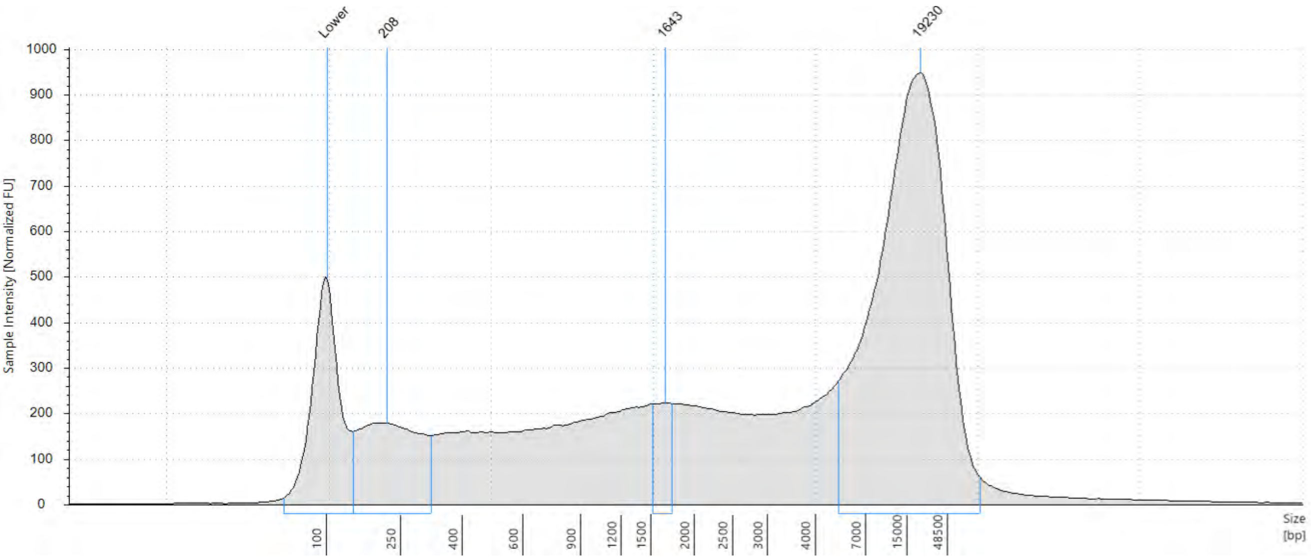

Sample Table

Well	DIN	Conc. [ng/µl]	Sample Description	Alert	Observations
C11	6.0	92.1	W712		

Peak Table

Size [bp]	Calibrated Conc. [ng/µl]	Assigned Conc. [ng/µl]	% Integrated Area	From [bp]	To [bp]	Peak Comment	Observations
100	8.50	8.50	-	59	138		Lower Marker
208	7.08	-	13.82	138	313		
1643	2.48	-	4.84	1513	1720		
19230	41.4	-	80.94	5147	>60000		
-	-	-	-	-	-		Sample Well

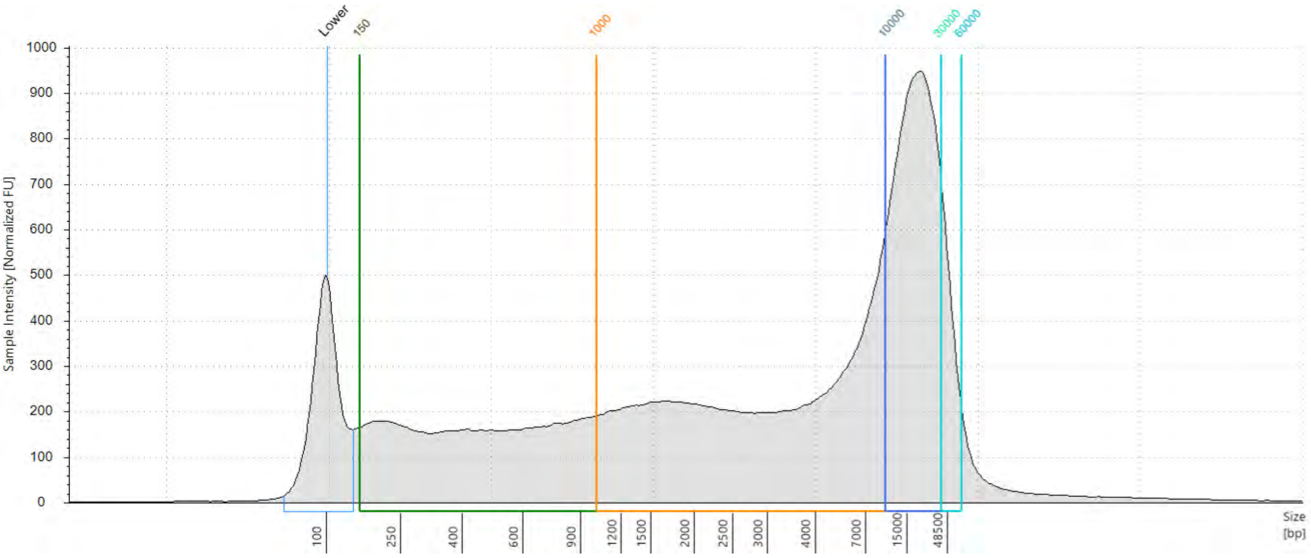

Region Table

From [bp]	To [bp]	Average Size [bp]	Conc. [ng/µl]	Region Molarity [nmol/l]	% of Total	Region Comment	Color
150	10000	2613	58.6	111	63.65		
150	1000	496	21.2	88.6	23.01		
1000	10000	3810	37.6	23.1	40.80		

10000	60000	22966	30.6	2.56	33.25		
10000	30000	18236	25.7	2.39	27.86		
30000	60000	46910	5.51	0.199	5.99		

© Agilent Technologies, Inc. 2017

D11: W713

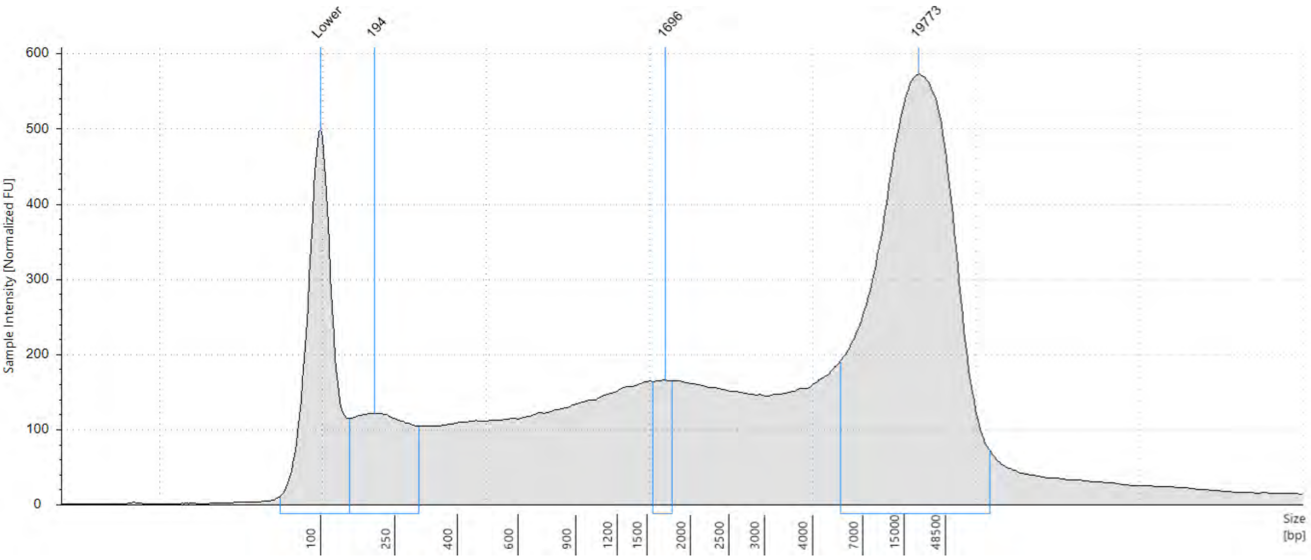

Sample Table

Well	DIN	Conc. [ng/µl]	Sample Description	Alert	Observations
D11	6.0	73.3	W713		

Peak Table

Size [bp]	Calibrated Conc. [ng/µl]	Assigned Conc. [ng/µl]	% Integrated Area	From [bp]	To [bp]	Peak Comment	Observations
100	8.50	8.50	-	61	143		Lower Marker
194	4.58	-	12.21	143	300		
1696	1.94	-	5.18	1562	1776		
19773	30.4	-	81.23	5489	>60000		
-	-	-	-	-	-		Sample Well

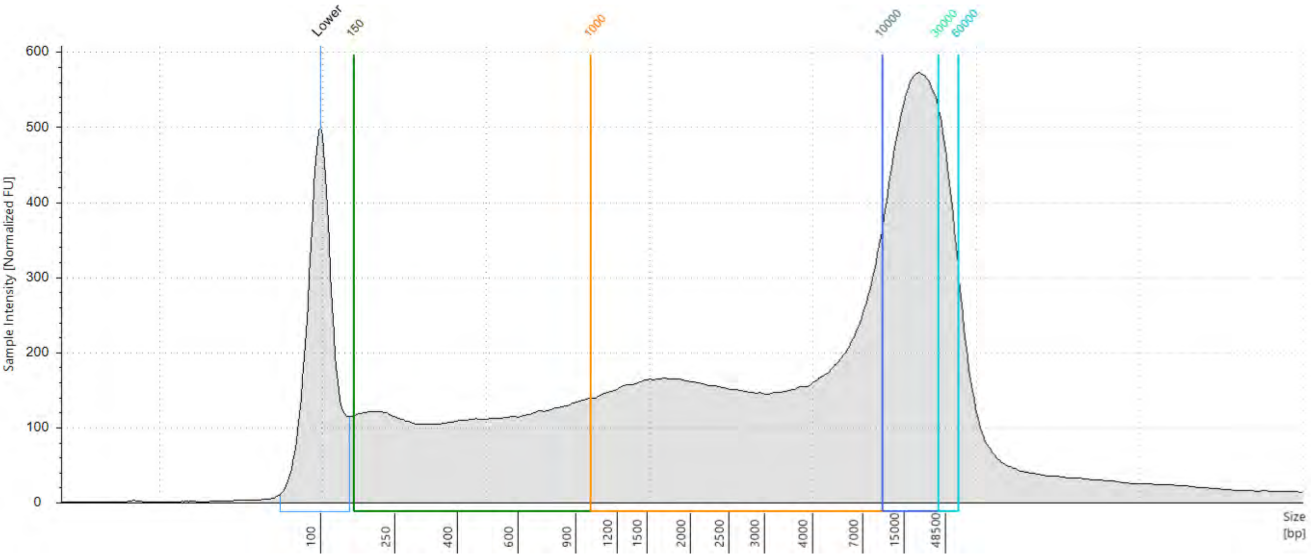

Region Table

From [bp]	To [bp]	Average Size [bp]	Conc. [ng/µl]	Region Molarity [nmol/l]	% of Total	Region Comment	Color
150	10000	2528	43.6	82.6	59.55		
150	1000	501	15.6	64.8	21.26		
1000	10000	3655	28.2	17.9	38.44		

10000	60000	25537	21.9	1.72	29.86		
10000	30000	18465	16.8	1.55	22.94		
30000	60000	48552	5.49	0.190	7.50		

© Agilent Technologies, Inc. 2017

E11: W714

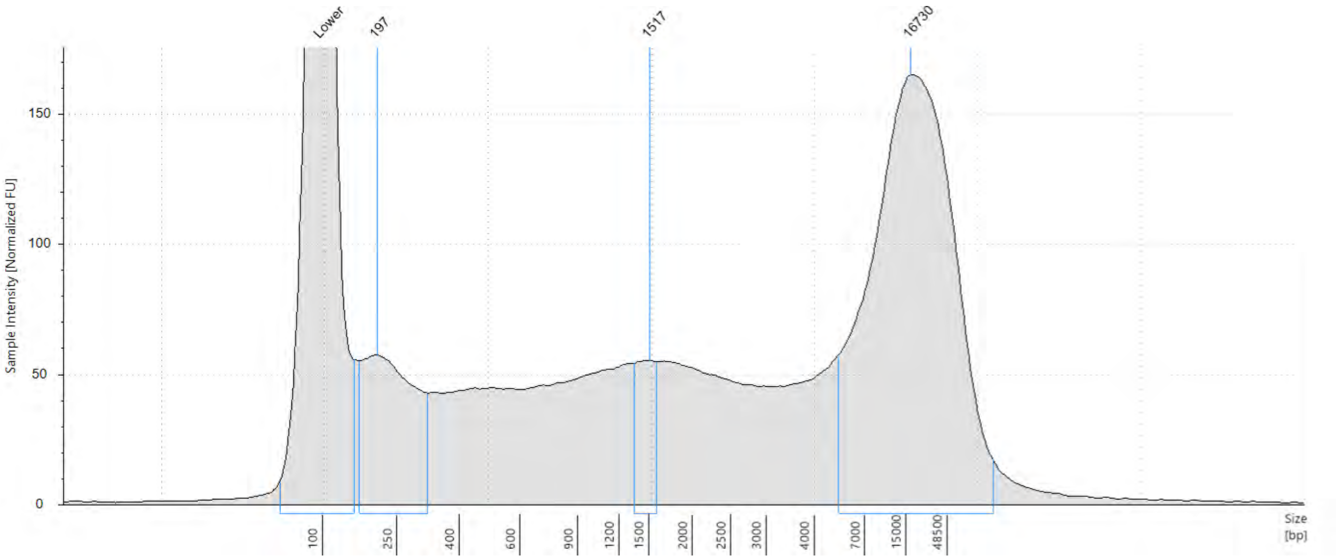

Sample Table

Well	DIN	Conc. [ng/µl]	Sample Description	Alert	Observations
E11	5.8	23.6	W714		

Peak Table

Size [bp]	Calibrated Conc. [ng/µl]	Assigned Conc. [ng/µl]	% Integrated Area	From [bp]	To [bp]	Peak Comment	Observations
100	8.50	8.50	-	60	148		Lower Marker
197	2.07	-	16.96	158	315		
1517	0.755	-	6.17	1354	1587		
16730	9.40	-	76.87	5291	>60000		

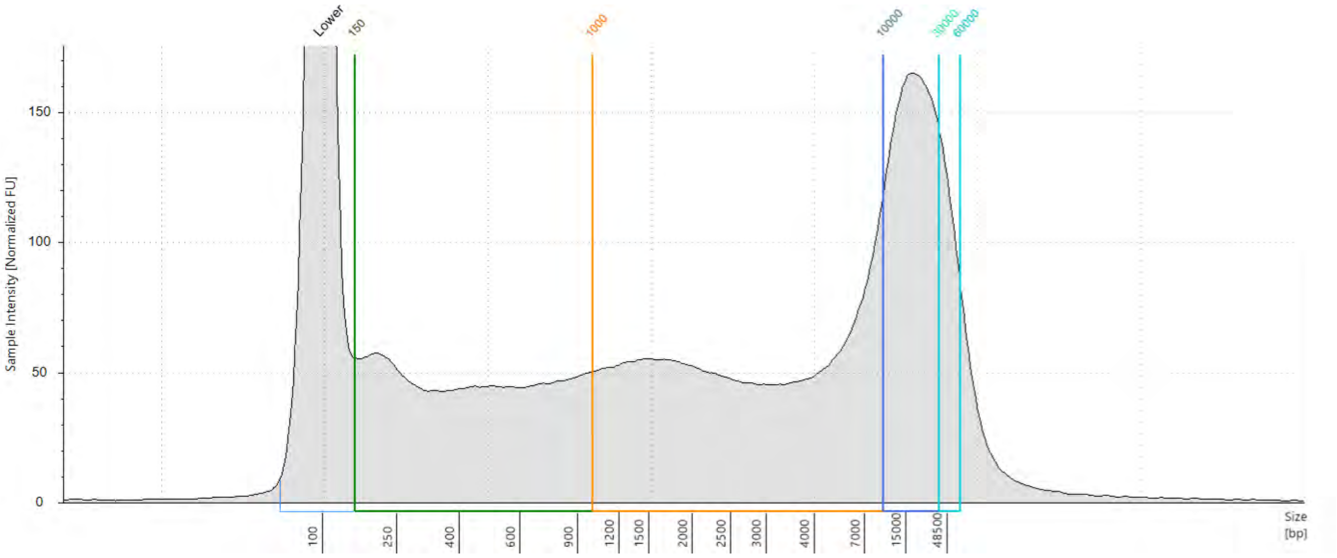

Region Table

From [bp]	To [bp]	Average Size [bp]	Conc. [ng/µl]	Region Molarity [nmol/l]	% of Total	Region Comment	Color
150	10000	2354	15.9	34.3	67.37		
150	1000	481	6.53	28.3	27.66		
1000	10000	3652	9.42	6.09	39.89		
10000	60000	25171	6.50	0.519	27.52		

 © Agilent Technologies, Inc. 2017

10000	30000	18420	5.12	0.475	21.67		
30000	60000	49643	1.50	0.0503	6.35		

F11: W715

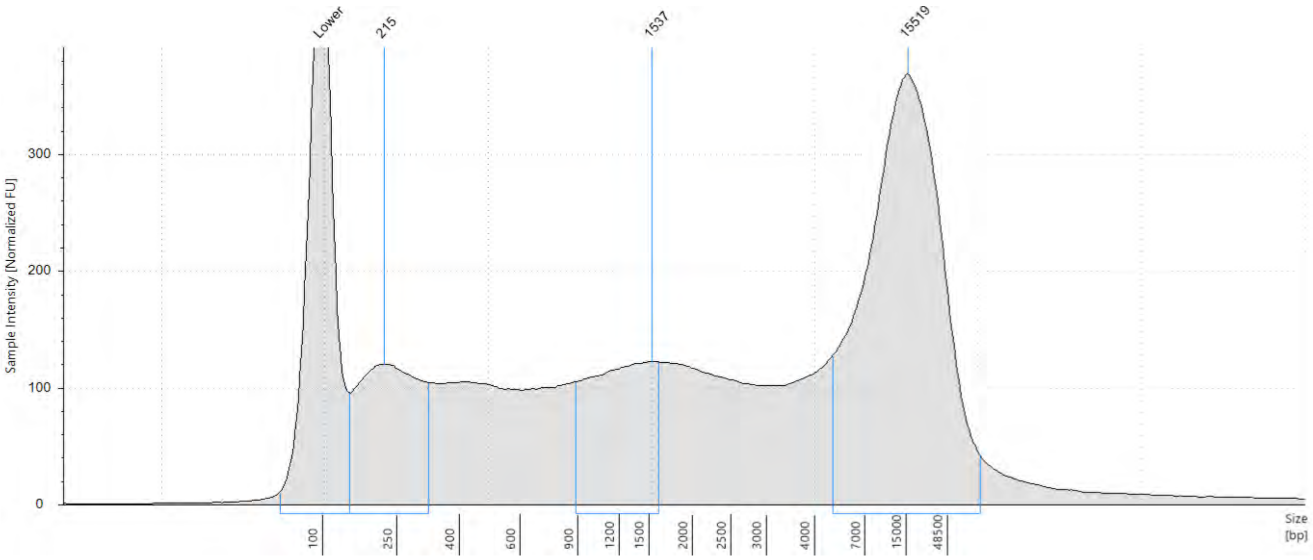

Sample Table

Well	DIN	Conc. [ng/µl]	Sample Description	Alert	Observations
F11	5.6	51.2	W715		

Peak Table

Size [bp]	Calibrated Conc. [ng/µl]	Assigned Conc. [ng/µl]	% Integrated Area	From [bp]	To [bp]	Peak Comment	Observations
100	8.50	8.50	-	60	140		Lower Marker
215	5.14	-	17.25	140	318		
1537	5.62	-	18.83	888	1609		
15519	19.0	-	63.65	4917	>60000		
-	-	-	-	-	-		Sample Well

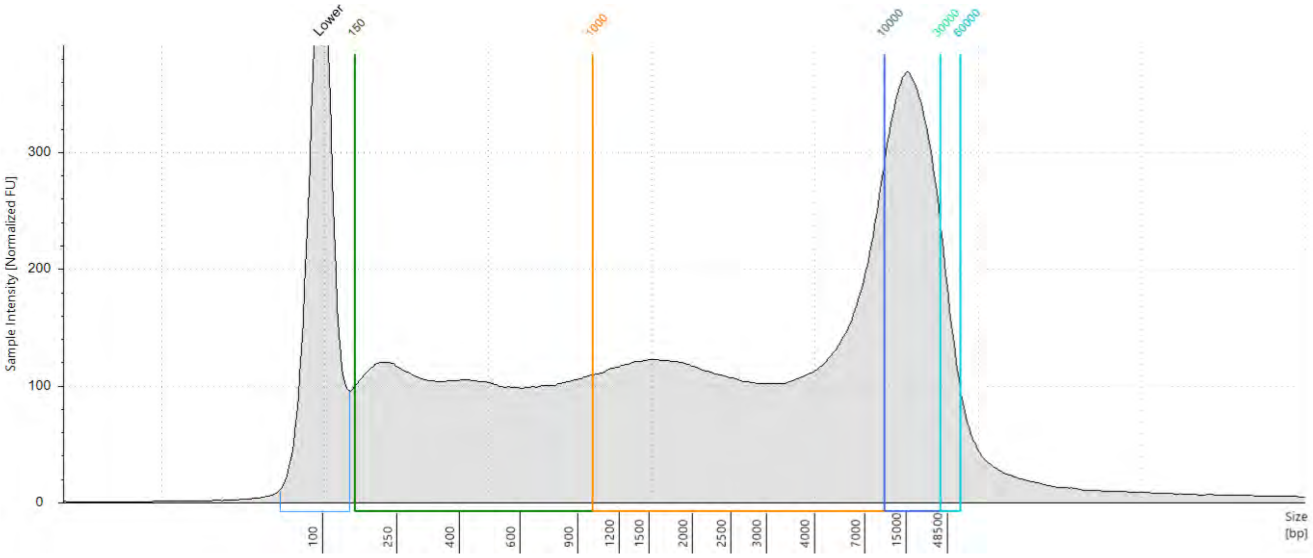

Region Table

From [bp]	To [bp]	Average Size [bp]	Conc. [ng/µl]	Region Molarity [nmol/l]	% of Total	Region Comment	Color
150	10000	2372	35.5	75.3	69.43		
150	1000	482	14.6	62.0	28.45		
1000	10000	3680	21.1	13.4	41.16		

 © Agilent Technologies, Inc. 2017

10000	60000	22331	12.9	1.13	25.31		
10000	30000	17768	11.1	1.07	21.67		
30000	60000	48764	2.06	0.0710	4.02		

© Agilent Technologies, Inc. 2017

G11: W716

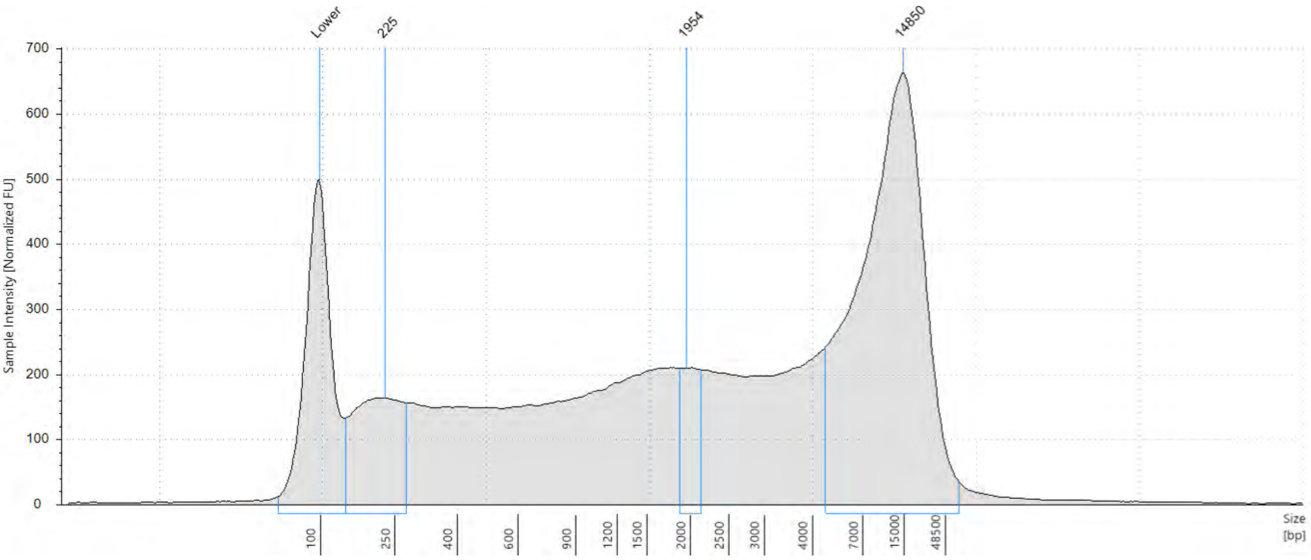

Sample Table

Well	DIN	Conc. [ng/µl]	Sample Description	Alert	Observations
G11	5.4	76.8	W716		

Peak Table

Size [bp]	Calibrated Conc. [ng/µl]	Assigned Conc. [ng/µl]	% Integrated Area	From [bp]	To [bp]	Peak Comment	Observations
100	8.50	8.50	-	60	138		Lower Marker
225	5.45	-	15.01	138	274		
1954	2.79	-	7.68	1867	2140		
14850	28.1	-	77.30	4672	>60000		

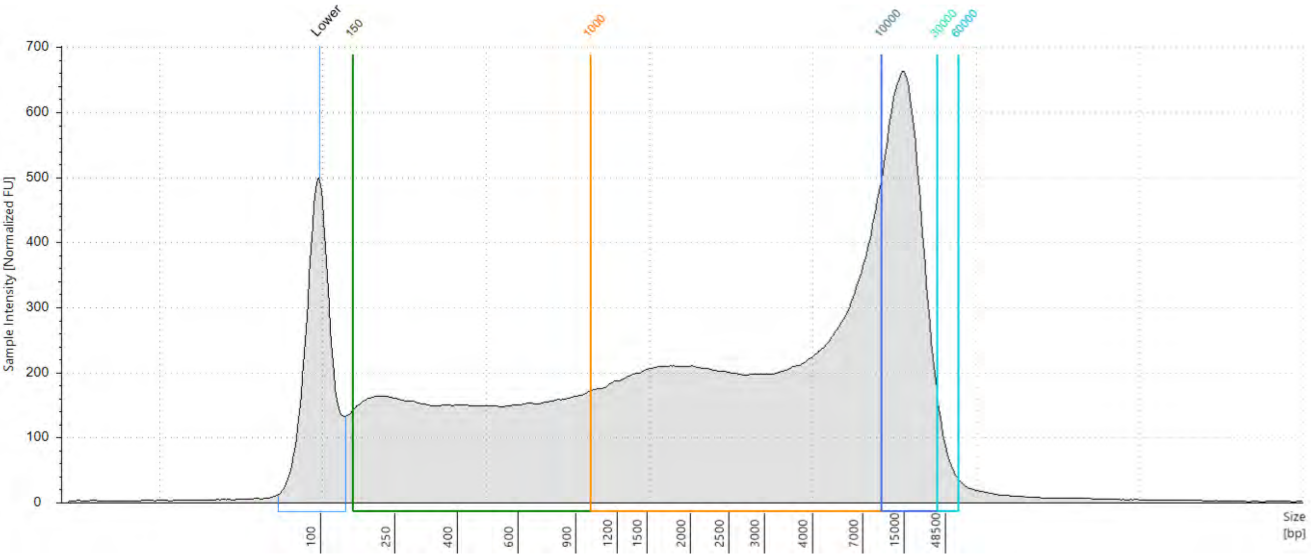

Region Table

From [bp]	To [bp]	Average Size [bp]	Conc. [ng/µl]	Region Molarity [nmol/l]	% of Total	Region Comment	Color
150	10000	2602	58.7	110	76.41		
150	1000	493	20.8	87.2	27.06		
1000	10000	3763	38.0	23.5	49.52		
10000	60000	18246	17.3	1.70	22.57		

© Agilent Technologies, Inc. 2017

10000	30000	16642	16.5	1.67	21.48		
30000	60000	47720	0.960	0.0355	1.25		

© Agilent Technologies, Inc. 2017

H11: W717

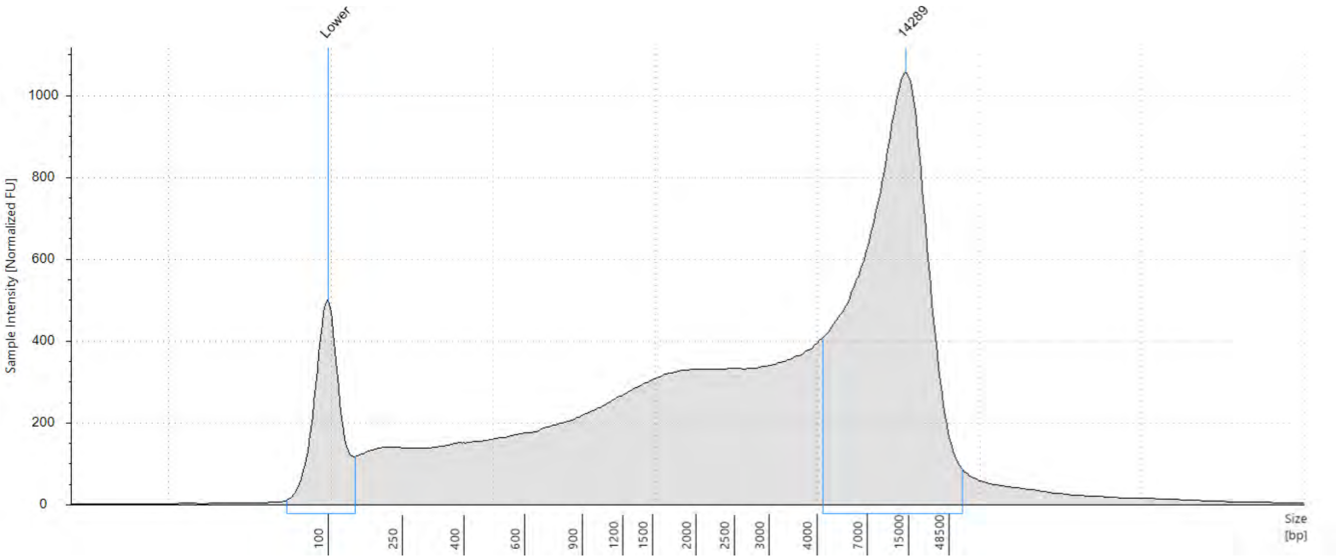

Sample Table

Well	DIN	Conc. [ng/µl]	Sample Description	Alert	Observations
H11	5.7	116	W717	⚠	Sample concentration outside recommended range

Peak Table

Size [bp]	Calibrated Conc. [ng/µl]	Assigned Conc. [ng/µl]	% Integrated Area	From [bp]	To [bp]	Peak Comment	Observations
100	8.50	8.50	-	60	138		Lower Marker
14289	48.6	-	100.00	4256	59563		

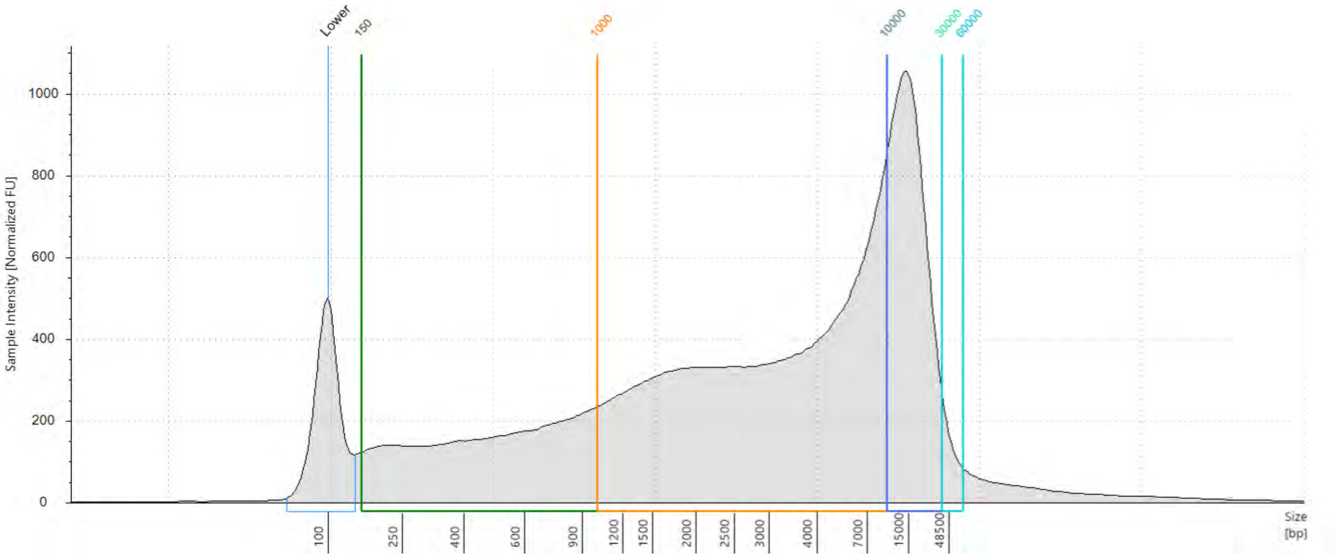

Region Table

From [bp]	To [bp]	Average Size [bp]	Conc. [ng/µl]	Region Molarity [nmol/l]	% of Total	Region Comment	Color
150	10000	3025	85.3	123	73.49		
150	1000	530	22.2	86.1	19.10		
1000	10000	3909	63.4	37.1	54.55		
10000	60000	18526	27.6	2.69	23.75		
10000	30000	16564	25.9	2.63	22.33		
30000	60000	48077	1.85	0.0662	1.59		

 © Agilent Technologies, Inc. 2017

© Agilent Technologies, Inc. 2017

A12: W718

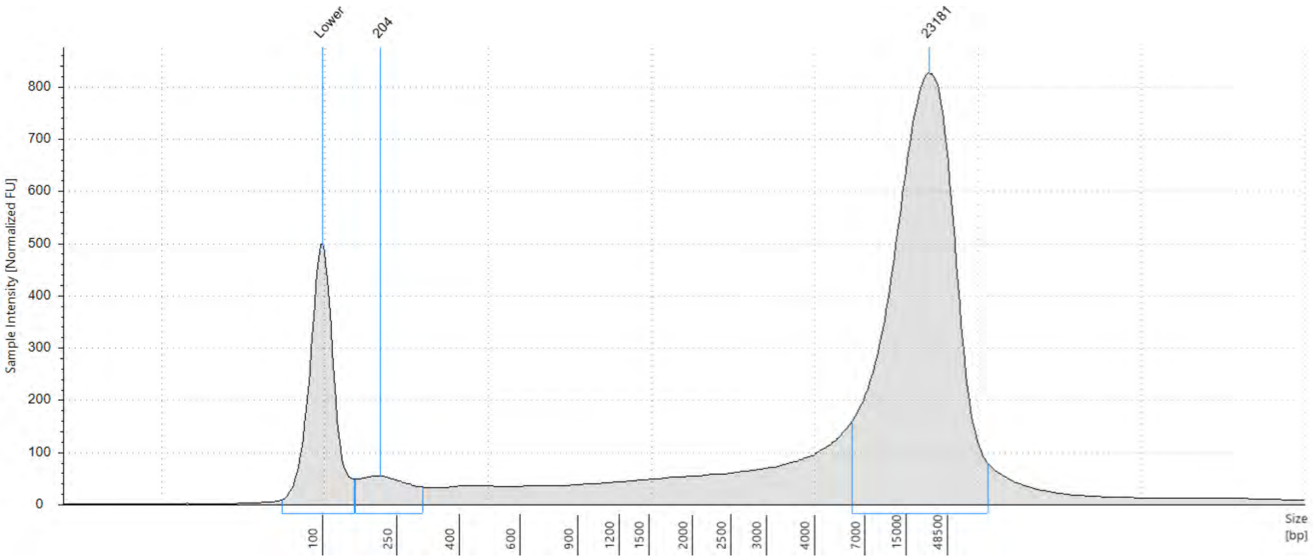

Sample Table

Well	DIN	Conc. [ng/µl]	Sample Description	Alert	Observations
A12	7.0	57.0	W718		

Peak Table

Size [bp]	Calibrated Conc. [ng/µl]	Assigned Conc. [ng/µl]	% Integrated Area	From [bp]	To [bp]	Peak Comment	Observations
100	8.50	8.50	-	61	148		Lower Marker
204	1.86	-	4.66	150	302		
23181	37.7	-	94.52	6050	>60000		
-	-	-	-	-	-		Sample Well

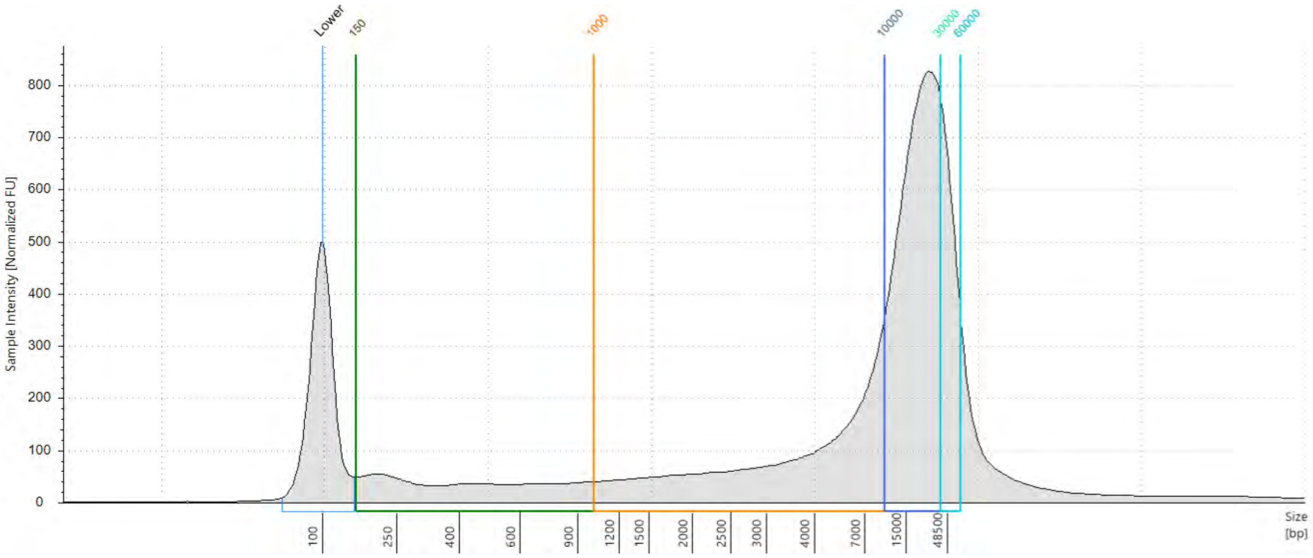

Region Table

From [bp]	To [bp]	Average Size [bp]	Conc. [ng/µl]	Region Molarity [nmol/l]	% of Total	Region Comment	Color
150	10000	3557	20.6	32.5	36.18		
150	1000	472	5.38	24.9	9.42		
1000	10000	4668	15.3	7.66	26.81		
10000	60000	26289	30.4	2.28	53.37		

10000	30000	19195	23.1	2.03	40.44		
30000	60000	48166	8.04	0.279	14.10		

© Agilent Technologies, Inc. 2017

B12: W719

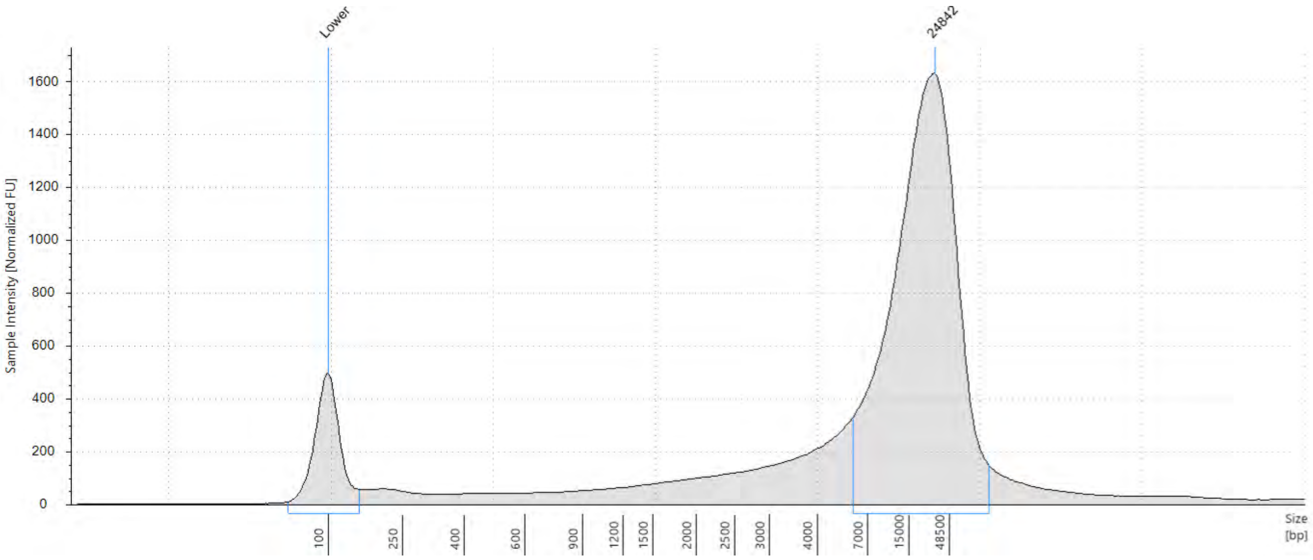

Sample Table

Well	DIN	Conc. [ng/µl]	Sample Description	Alert	Observations
B12	7.2	106	W719	⚠	Sample concentration outside recommended range

Peak Table

Size [bp]	Calibrated Conc. [ng/µl]	Assigned Conc. [ng/µl]	% Integrated Area	From [bp]	To [bp]	Peak Comment	Observations
100	8.50	8.50	-	61	148		Lower Marker
24842	71.4	-	97.25	5938	>60000		
-	-	-	-	-	-		Sample Well

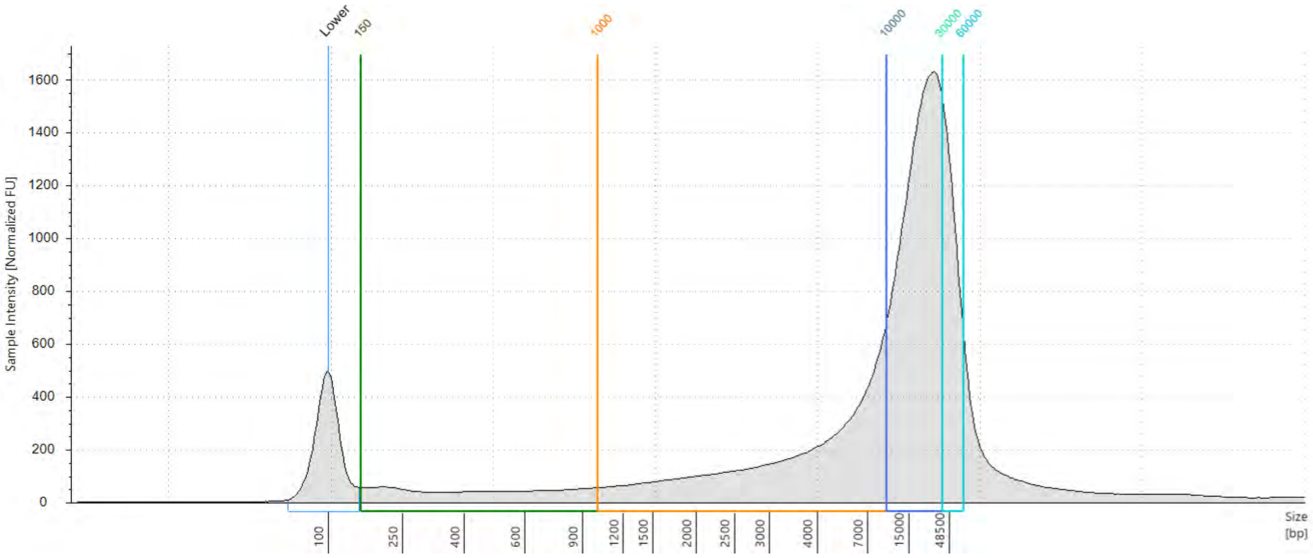

Region Table

From [bp]	To [bp]	Average Size [bp]	Conc. [ng/µl]	Region Molarity [nmol/l]	% of Total	Region Comment	Color
150	10000	4004	35.7	41.8	33.80		
150	1000	491	6.16	28.0	5.83		
1000	10000	4769	29.6	13.9	28.01		
10000	60000	26408	57.6	4.30	54.56		
10000	30000	19430	44.3	3.87	41.90		

30000	60000	49189	14.6	0.494	13.85		

© Agilent Technologies, Inc. 2017

C12: W720

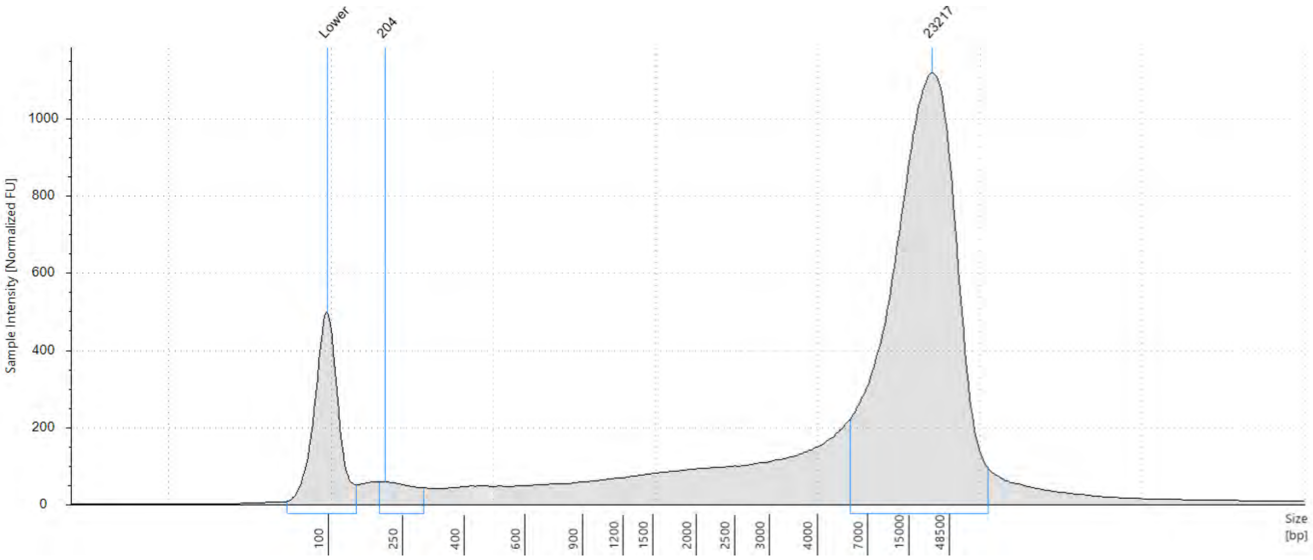

Sample Table

Well	DIN	Conc. [ng/µl]	Sample Description	Alert	Observations
C12	6.9	81.6	W720		

Peak Table

Size [bp]	Calibrated Conc. [ng/µl]	Assigned Conc. [ng/µl]	% Integrated Area	From [bp]	To [bp]	Peak Comment	Observations
100	8.50	8.50	-	61	143		Lower Marker
204	1.40	-	2.55	190	295		
23217	53.2	-	96.60	5796	>60000		
-	-	-	-	-	-		Sample Well

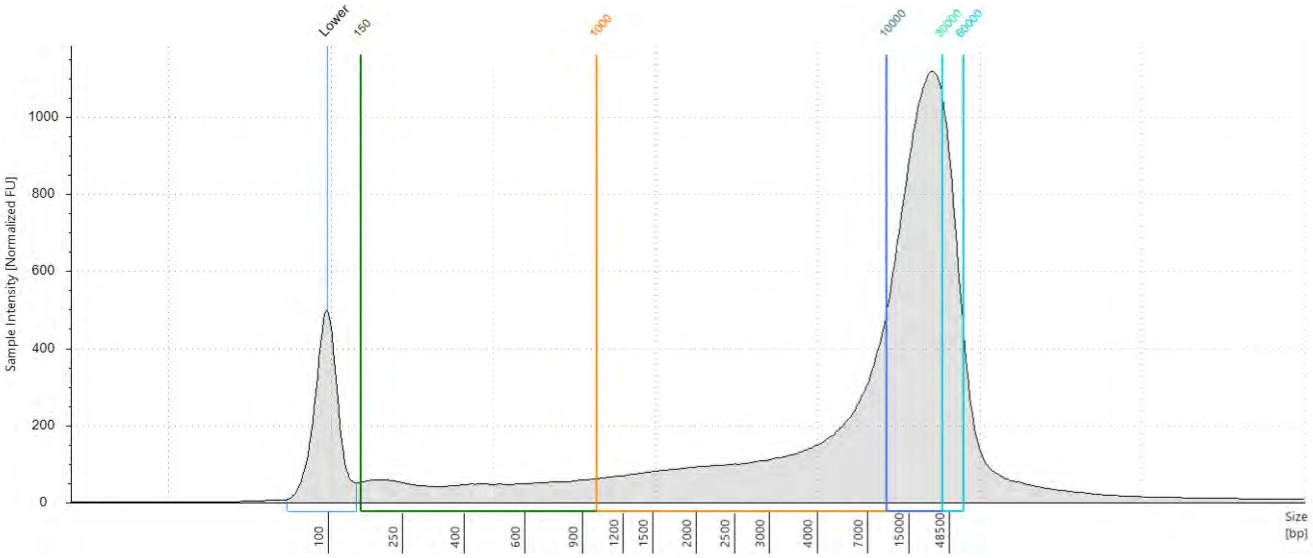

Region Table

From [bp]	To [bp]	Average Size [bp]	Conc. [ng/µl]	Region Molarity [nmol/l]	% of Total	Region Comment	Color
150	10000	3607	31.7	44.1	38.82		
150	1000	496	7.15	31.5	8.76		
1000	10000	4543	24.6	12.7	30.13		
10000	60000	26168	42.4	3.19	51.95		

 © Agilent Technologies, Inc. 2017

10000	30000	19183	32.3	2.85	39.56		
30000	60000	48159	11.0	0.384	13.52		

© Agilent Technologies, Inc. 2017

D12: buffer

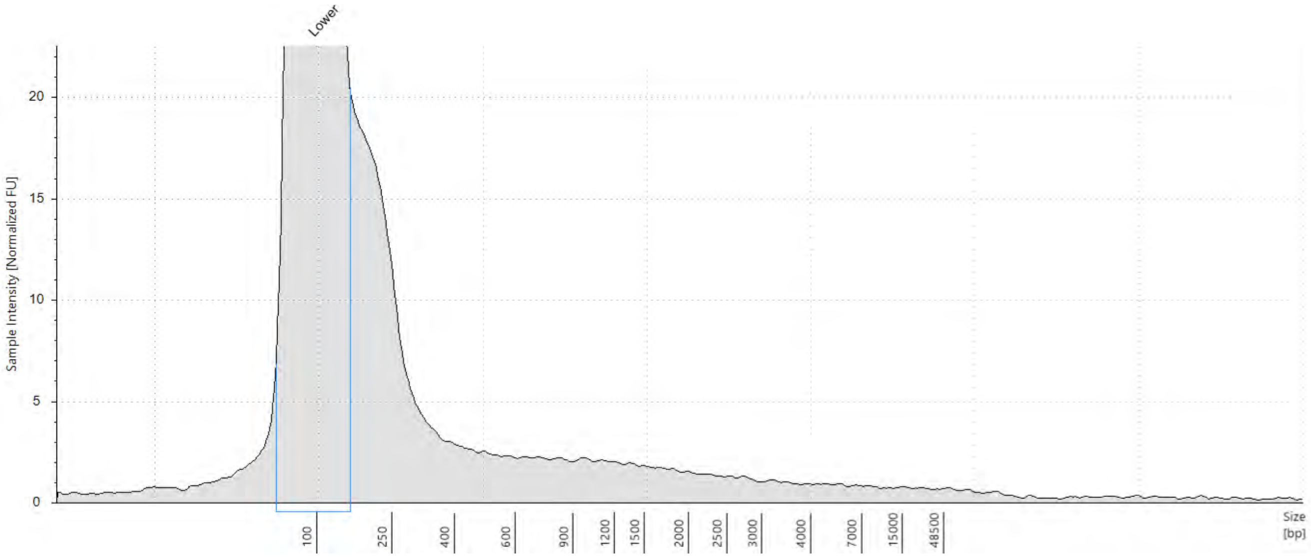

Sample Table

Well	DIN	Conc. [ng/µl]	Sample Description	Alert	Observations
D12	-	1.04	buffer	⚠	Sample concentration outside functional range for DIN

Peak Table

Size [bp]	Calibrated Conc. [ng/µl]	Assigned Conc. [ng/µl]	% Integrated Area	From [bp]	To [bp]	Peak Comment	Observations
100	8.50	8.50	-	61	151		Lower Marker

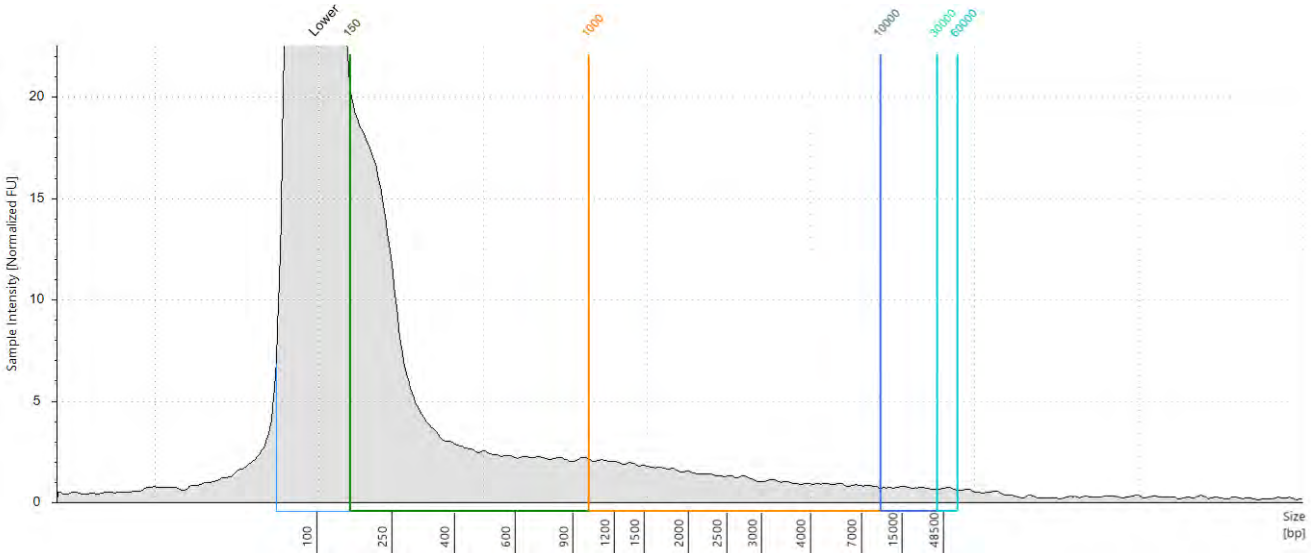

Region Table

From [bp]	To [bp]	Average Size [bp]	Conc. [ng/µl]	Region Molarity [nmol/l]	% of Total	Region Comment	Color
150	10000	861	0.933	5.33	89.54		
150	1000	326	0.761	5.15	73.09		
1000	10000	2701	0.173	0.187	16.62		
10000	60000	26581	0.0166	0.00266	1.59		
10000	30000	18071	0.0126	0.00239	1.21		
30000	60000	50635	0.00457	0.000302	0.44		

Filename: T5_Initials&Errors_4-3-2019.gDNA

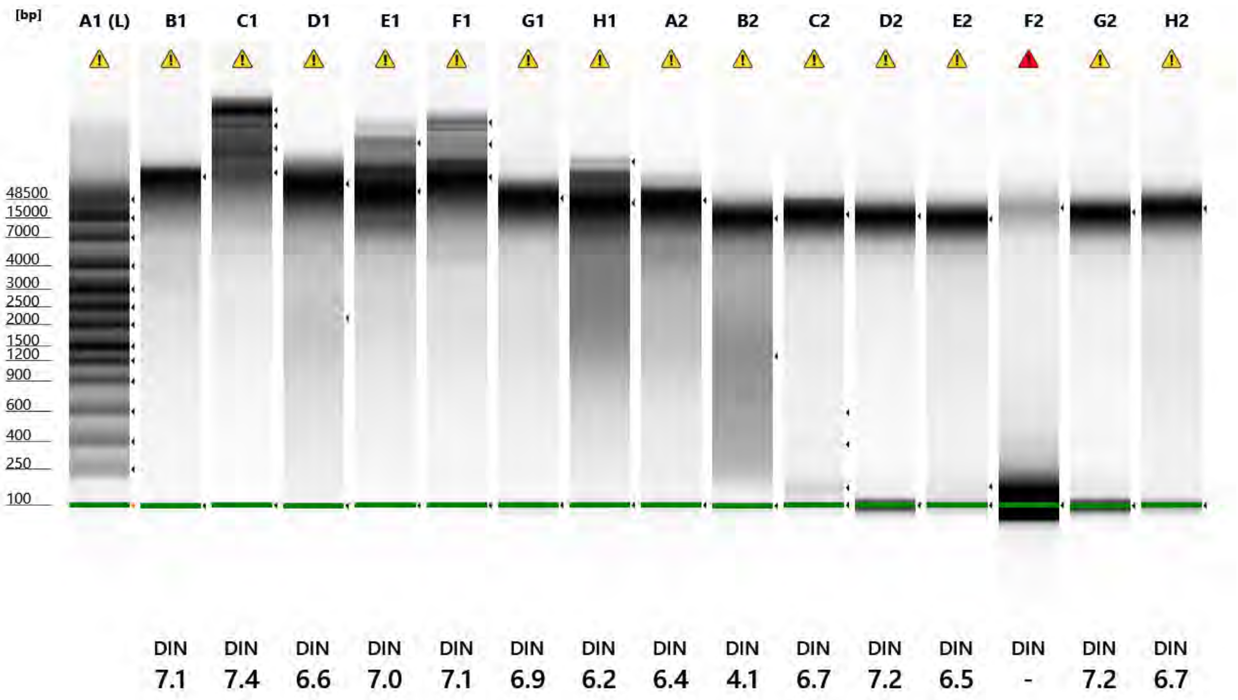

Default image (Contrast 50%), Image is Scaled to Sample

Sample Info

Well	DIN	Conc. [ng/µl]	Sample Description	Alert	Observations
A1	-	1820	Ladder	⚠	Caution! Expired ScreenTape device; Ladder; Sizing Changed
B1	7.1	811	M221	⚠	Caution! Expired ScreenTape device; Sample concentration outside functional range for DIN; DIN Edited (Ladder sizing changed)
C1	7.4	4120	M222	⚠	Caution! Expired ScreenTape device; Sample concentration outside functional range for DIN; DIN Edited (Marker position changed); DIN Edited (Ladder sizing changed)
D1	6.6	1490	M224	⚠	Caution! Expired ScreenTape device; Sample concentration outside functional range for DIN; DIN Edited (Marker position changed); DIN Edited (Ladder sizing changed)
E1	7.0	2300	M225	⚠	Caution! Expired ScreenTape device; Sample concentration outside functional range for DIN; DIN Edited (Marker position changed); DIN Edited (Ladder sizing changed)
F1	7.1	1330	M314	⚠	Caution! Expired ScreenTape device; Sample concentration outside functional range for DIN; DIN Edited (Ladder sizing changed)
G1	6.9	278	M315	⚠	Caution! Expired ScreenTape device; Sample concentration outside recommended range; DIN Edited (Ladder sizing changed)
H1	6.2	814	M1	⚠	Caution! Expired ScreenTape device; Sample concentration outside functional range for DIN; DIN Edited (Ladder sizing changed)
A2	6.4	432	M2	⚠	Caution! Expired ScreenTape device; Sample concentration outside functional range for DIN; DIN Edited (Ladder sizing changed)
B2	4.1	492	M3	⚠	Caution! Expired ScreenTape device; Sample concentration outside functional range for DIN; DIN Edited (Ladder sizing changed)
C2	6.7	264	C671	⚠	Caution! Expired ScreenTape device; Sample concentration outside recommended range; DIN Edited (Ladder sizing changed)
D2	7.2	37.1	C672	⚠	Caution! Expired ScreenTape device; DIN Edited (Ladder sizing changed)
E2	6.5	110	C673	⚠	Caution! Expired ScreenTape device; Sample concentration outside recommended range; DIN Edited (Ladder sizing changed)
F2	-	2.24	C674	▲	Caution! Expired ScreenTape device; Sample concentration outside functional range for DIN; DIN Edited (Ladder sizing changed)
G2	7.2	33.3	C675	⚠	Caution! Expired ScreenTape device; DIN Edited (Ladder sizing changed)
H2	6.7	98.8	C404	⚠	Caution! Expired ScreenTape device; DIN Edited (Ladder sizing changed)

A1: Ladder

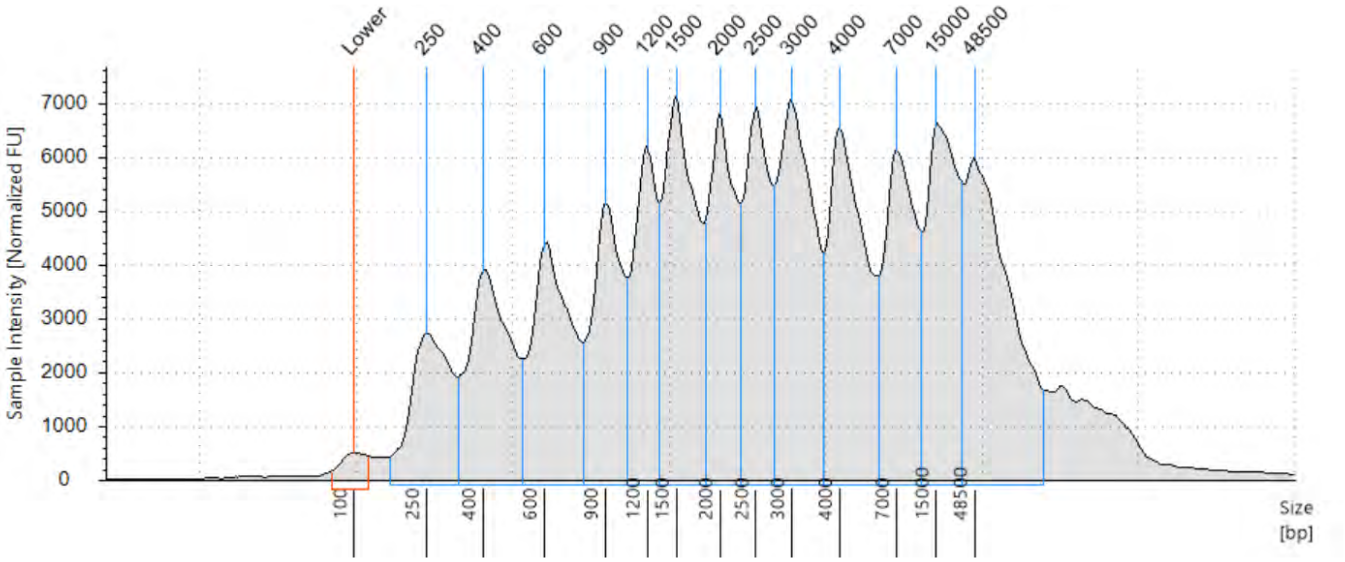

Sample Table

Well	DIN	Conc. [ng/µl]	Sample Description	Alert	Observations
A1	-	1820	Ladder	⚠	Caution! Expired ScreenTape device; Ladder; Sizing Changed

Peak Table

Size [bp]	Calibrated Conc. [ng/µl]	Assigned Conc. [ng/µl]	% Integrated Area	From [bp]	To [bp]	Peak Comment	Observations
100	8.50	8.50	-	75	120		edited Lower Marker
250	75.1	-	4.27	158	327		
400	112	-	6.34	327	521		
600	121	-	6.87	521	783		
900	106	-	6.03	783	1049		
1200	103	-	5.84	1049	1320		
1500	164	-	9.30	1320	1820		
2000	125	-	7.09	1820	2273		
2500	125	-	7.10	2273	2739		
3000	179	-	10.18	2739	3645		
4000	168	-	9.55	3645	5862		
7000	137	-	7.78	5862	11188		
15000	147	-	8.33	11188	24553		
48500	199	-	11.33	24552	>60000		

© Agilent Technologies, Inc. 2017

B1: M221

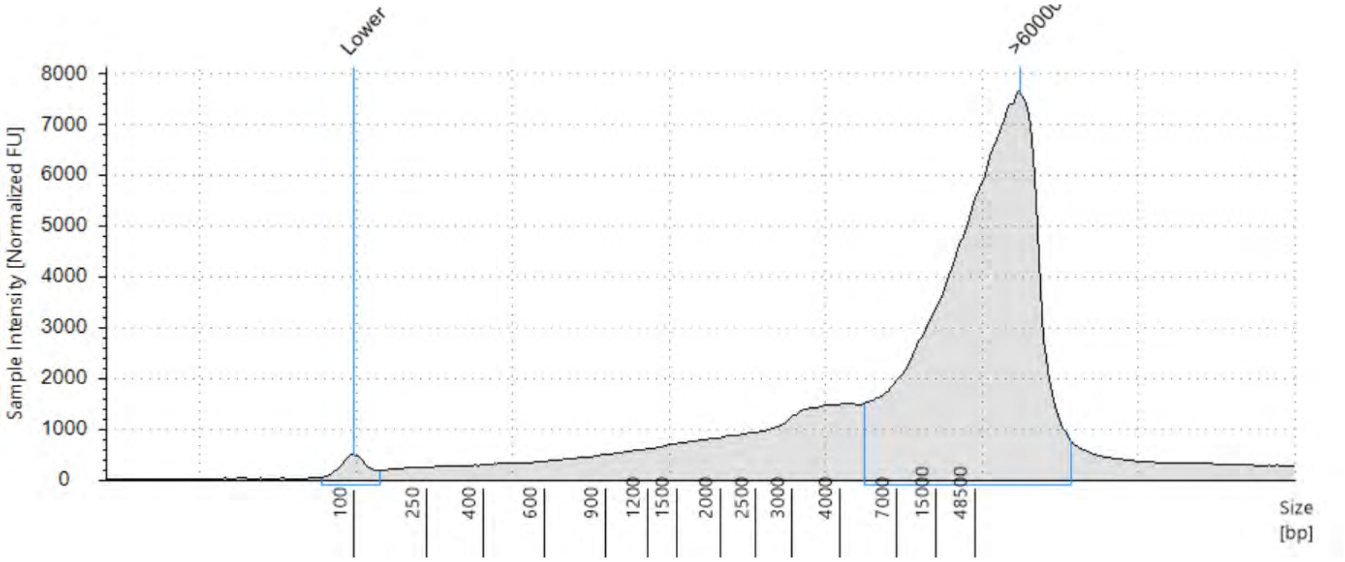

Sample Table

Well	DIN	Conc. [ng/µl]	Sample Description	Alert	Observations
B1	7.1	811	M221	⚠	Caution! Expired ScreenTape device; Sample concentration outside functional range for DIN; DIN Edited (Ladder sizing changed)

Peak Table

Size [bp]	Calibrated Conc. [ng/µl]	Assigned Conc. [ng/µl]	% Integrated Area	From [bp]	To [bp]	Peak Comment	Observations
100	8.50	8.50	-	68	140		Lower Marker
>60000	529	-	87.49	5151	>60000		
-	-	-	-	-	-		Sample Well

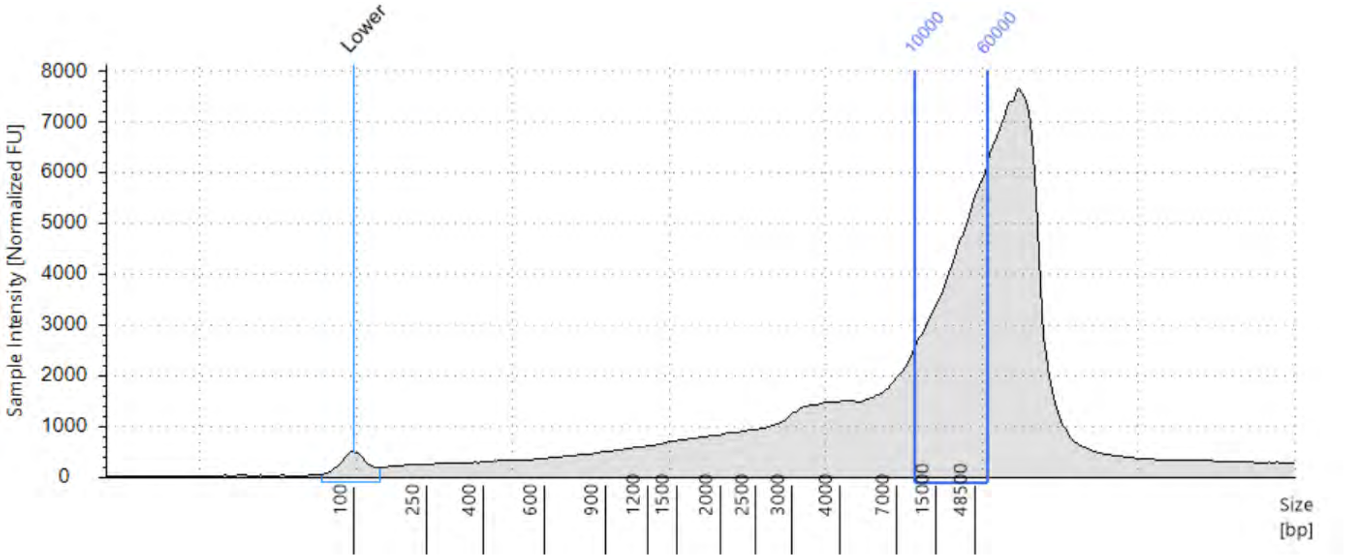

Region Table

From [bp]	To [bp]	Average Size [bp]	Conc. [ng/µl]	Region Molarity [nmol/l]	% of Total	Region Comment	Color
10000	60000	30214	202	14.2	24.95		■

 © Agilent Technologies, Inc. 2017

C1: M222

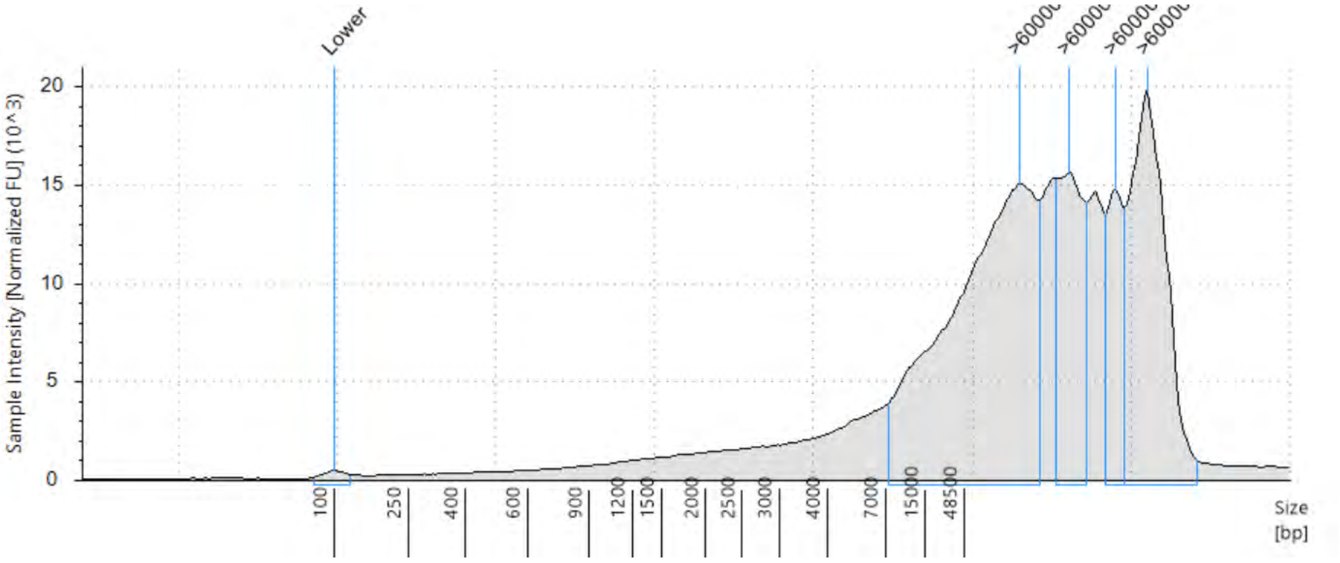

Sample Table

Well	DIN	Conc. [ng/µl]	Sample Description	Alert	Observations
C1	7.4	4120	M222	⚠	Caution! Expired ScreenTape device; Sample concentration outside functional range for DIN; DIN Edited (Marker position changed); DIN Edited (Ladder sizing changed)

Peak Table

Size [bp]	Calibrated Conc. [ng/µl]	Assigned Conc. [ng/µl]	% Integrated Area	From [bp]	To [bp]	Peak Comment	Observations
100	8.50	8.50	-	78	121		edited Lower Marker
>60000	1430	-	43.38	7377	>60000		
>60000	466	-	14.08	>60000	>60000		
>60000	273	-	8.27	>60000	>60000		
>60000	807	-	24.41	>60000	>60000		
-	-	-	-	-	-		Sample Well

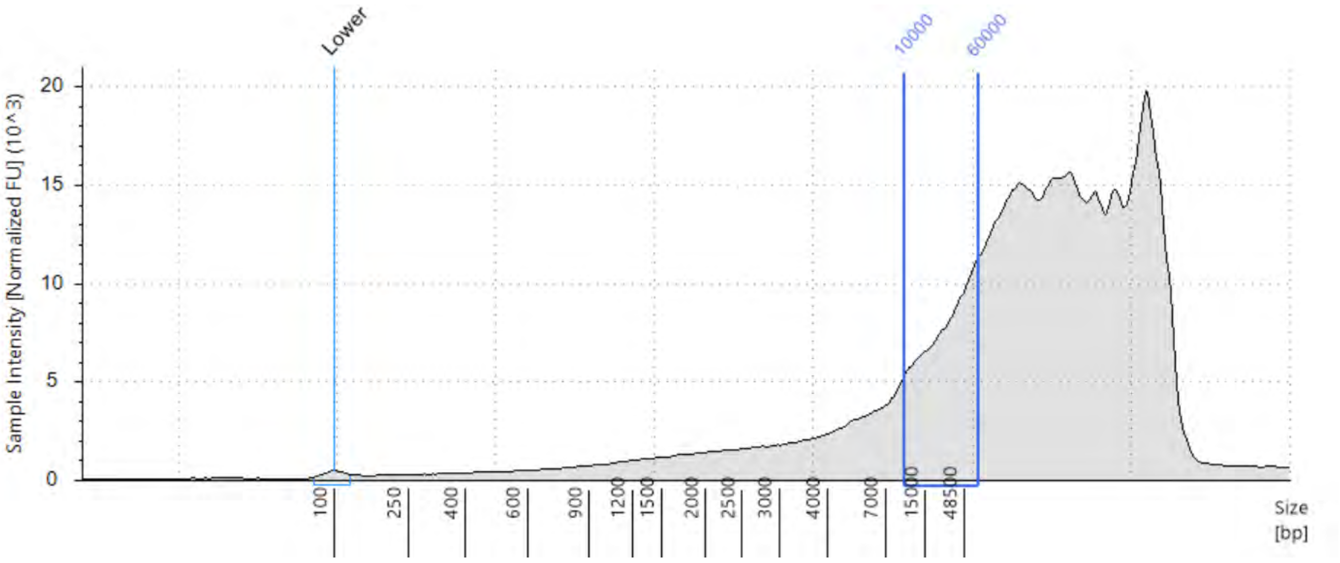

Region Table

From [bp]	To [bp]	Average Size [bp]	Conc. [ng/µl]	Region Molarity [nmol/l]	% of Total	Region Comment	Color
10000	60000	29733	548	39.2	13.28		

D1: M224

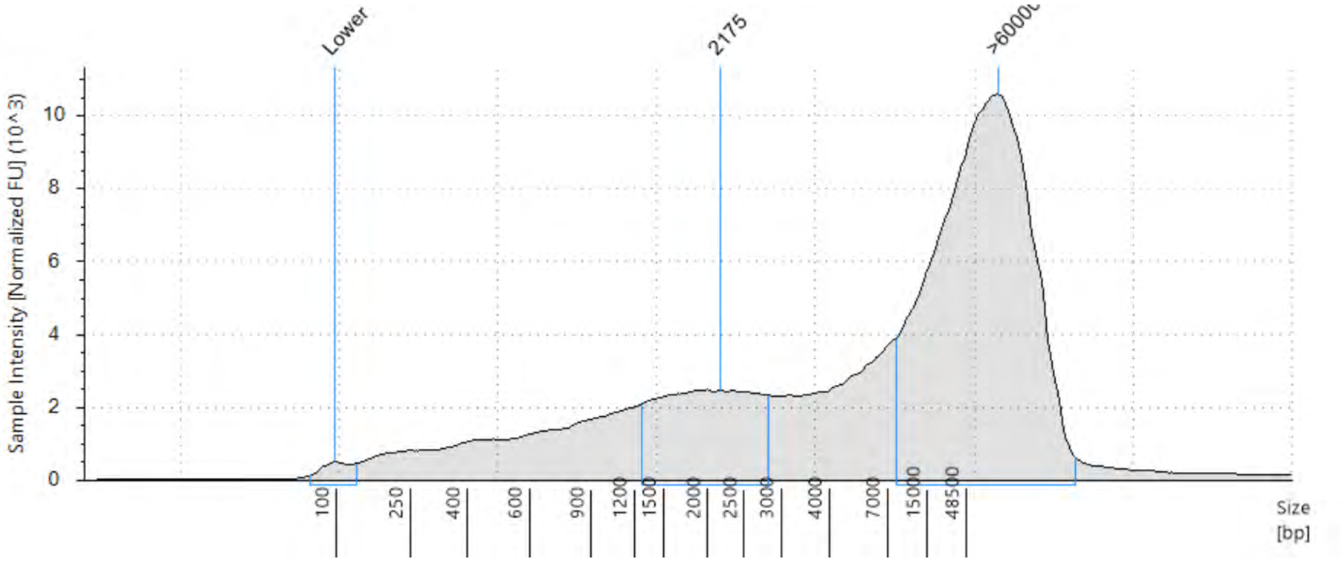

Sample Table

Well	DIN	Conc. [ng/µl]	Sample Description	Alert	Observations
D1	6.6	1490	M224	⚠	Caution! Expired ScreenTape device; Sample concentration outside functional range for DIN; DIN Edited (Marker position changed); DIN Edited (Ladder sizing changed)

Peak Table

Size [bp]	Calibrated Conc. [ng/µl]	Assigned Conc. [ng/µl]	% Integrated Area	From [bp]	To [bp]	Peak Comment	Observations
100	8.50	8.50	-	73	130		edited Lower Marker
2175	199	-	18.73	1285	2822		
>60000	829	-	77.93	8328	>60000		
-	-	-	-	-	-		Sample Well

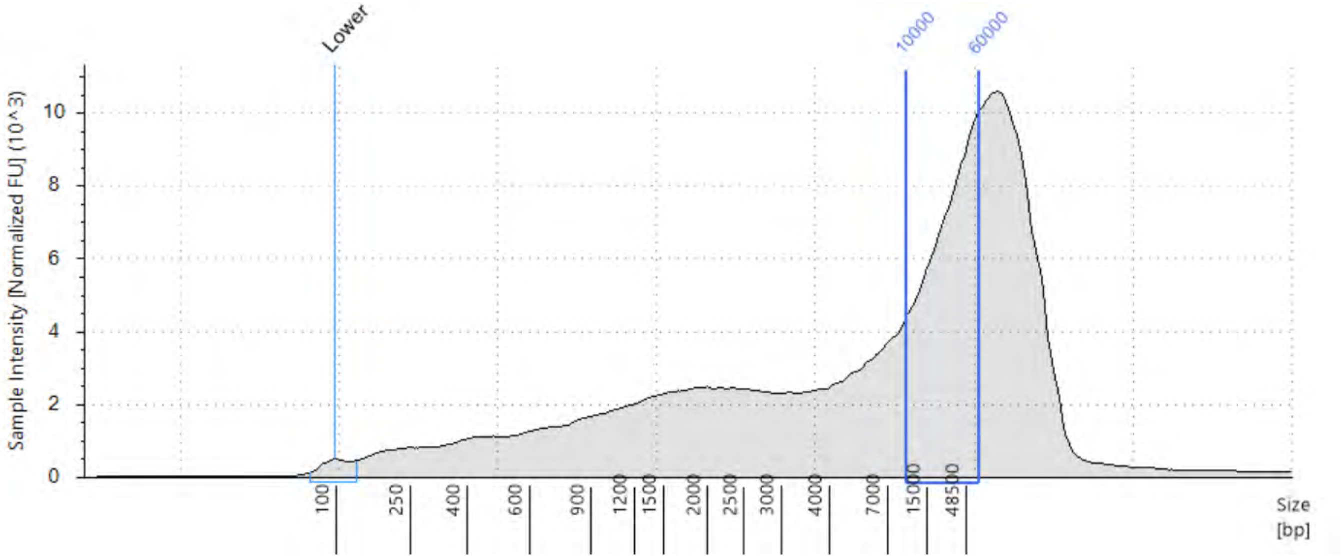

Region Table

From [bp]	To [bp]	Average Size [bp]	Conc. [ng/µl]	Region Molarity [nmol/l]	% of Total	Region Comment	Color
10000	60000	30111	356	25.1	23.91		■

 © Agilent Technologies, Inc. 2017

E1: M225

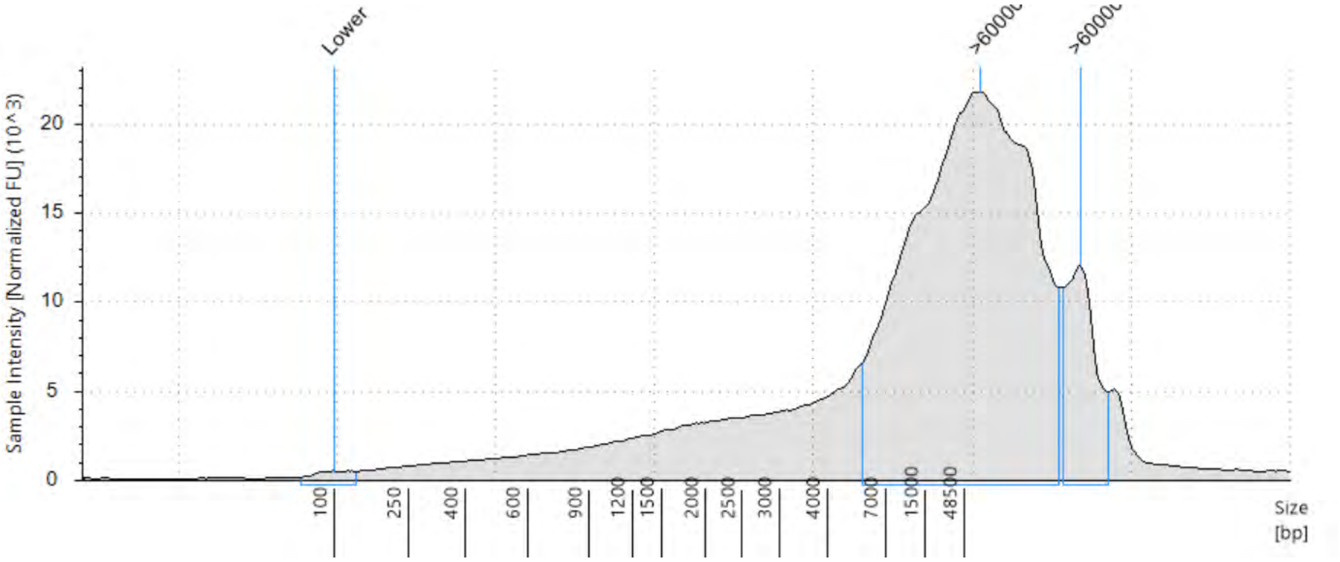

Sample Table

Well	DIN	Conc. [ng/µl]	Sample Description	Alert	Observations
E1	7.0	2300	M225	⚠	Caution! Expired ScreenTape device; Sample concentration outside functional range for DIN; DIN Edited (Marker position changed); DIN Edited (Ladder sizing changed)

Peak Table

Size [bp]	Calibrated Conc. [ng/µl]	Assigned Conc. [ng/µl]	% Integrated Area	From [bp]	To [bp]	Peak Comment	Observations
100	8.50	8.50	-	66	132		edited Lower Marker
>60000	1420	-	84.02	5574	>60000		
>60000	199	-	11.78	>60000	>60000		
-	-	-	-	-	-		Sample Well

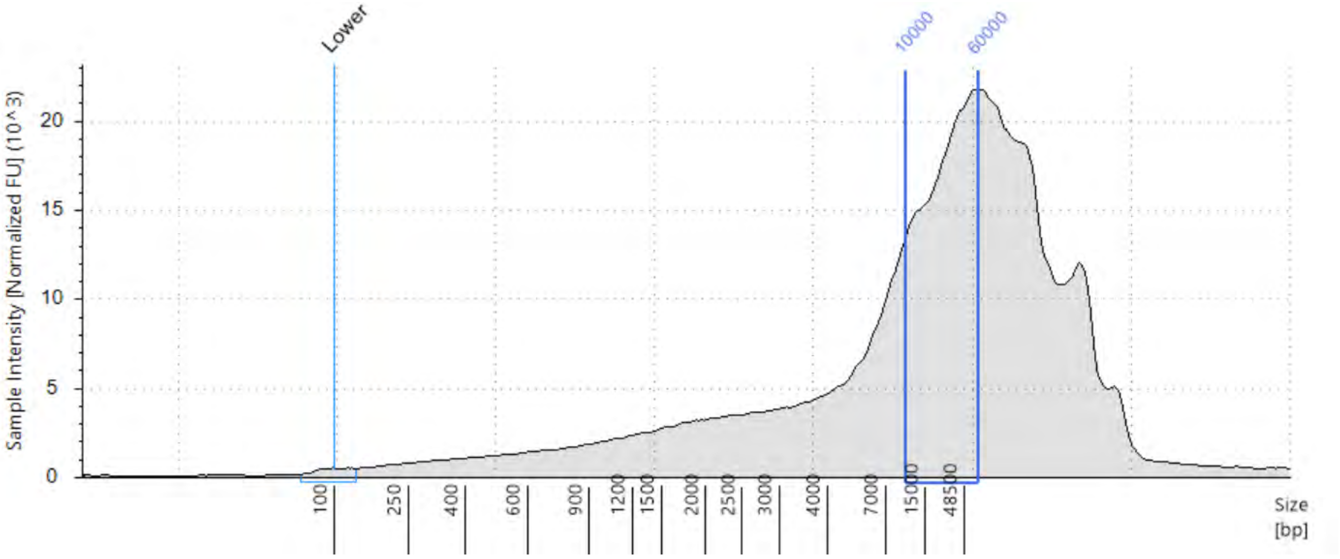

Region Table

From [bp]	To [bp]	Average Size [bp]	Conc. [ng/µl]	Region Molarity [nmol/l]	% of Total	Region Comment	Color
10000	60000	29153	603	43.6	26.23		■

 © Agilent Technologies, Inc. 2017

F1: M314

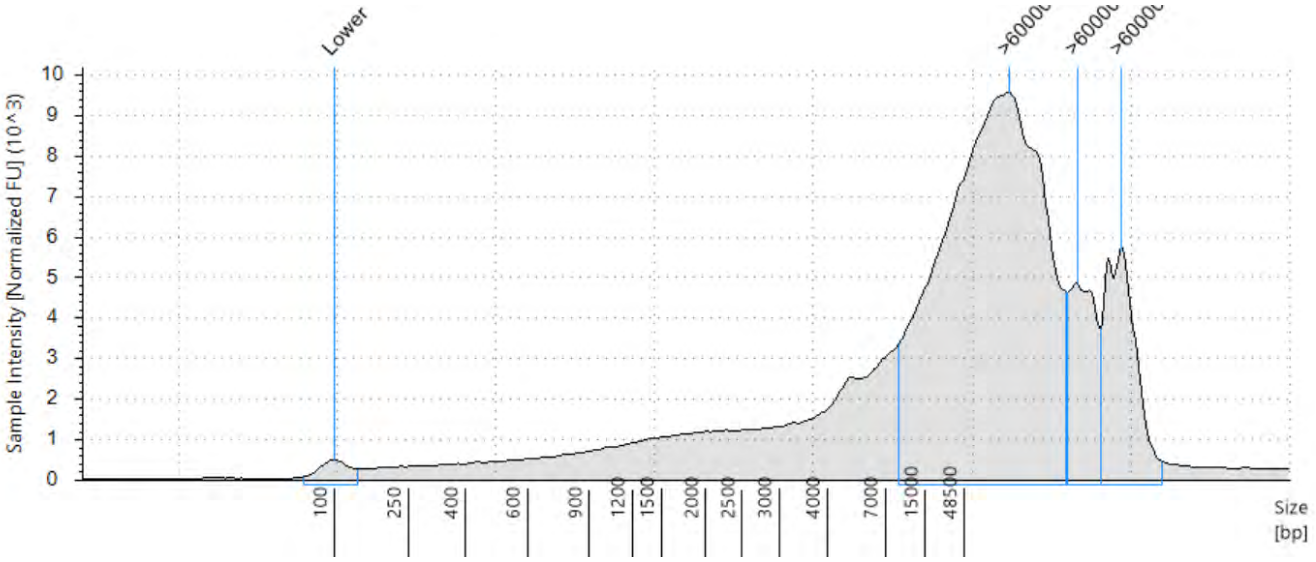

Sample Table

Well	DIN	Conc. [ng/µl]	Sample Description	Alert	Observations
F1	7.1	1330	M314	⚠	Caution! Expired ScreenTape device; Sample concentration outside functional range for DIN; DIN Edited (Ladder sizing changed)

Peak Table

Size [bp]	Calibrated Conc. [ng/µl]	Assigned Conc. [ng/µl]	% Integrated Area	From [bp]	To [bp]	Peak Comment	Observations
100	8.50	8.50	-	68	133		Lower Marker
>60000	723	-	72.65	8918	>60000		
>60000	95.5	-	9.60	>60000	>60000		
>60000	132	-	13.30	>60000	>60000		
-	-	-	-	-	-		Sample Well

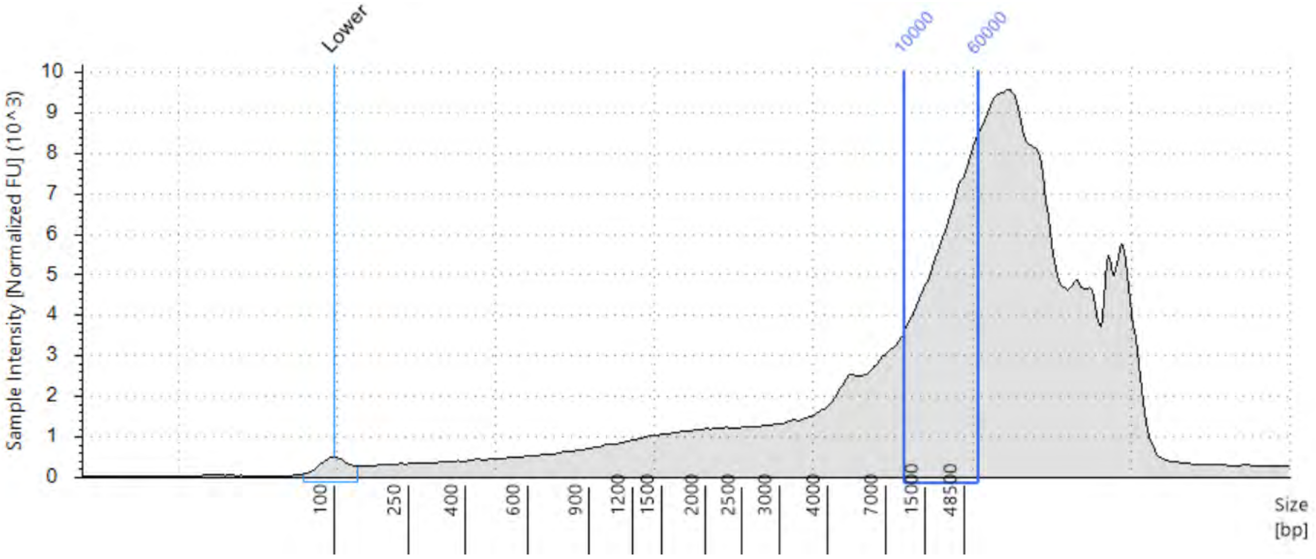

Region Table

From [bp]	To [bp]	Average Size [bp]	Conc. [ng/µl]	Region Molarity [nmol/l]	% of Total	Region Comment	Color
10000	60000	30055	272	19.1	20.49		■

 © Agilent Technologies, Inc. 2017

G1: M315

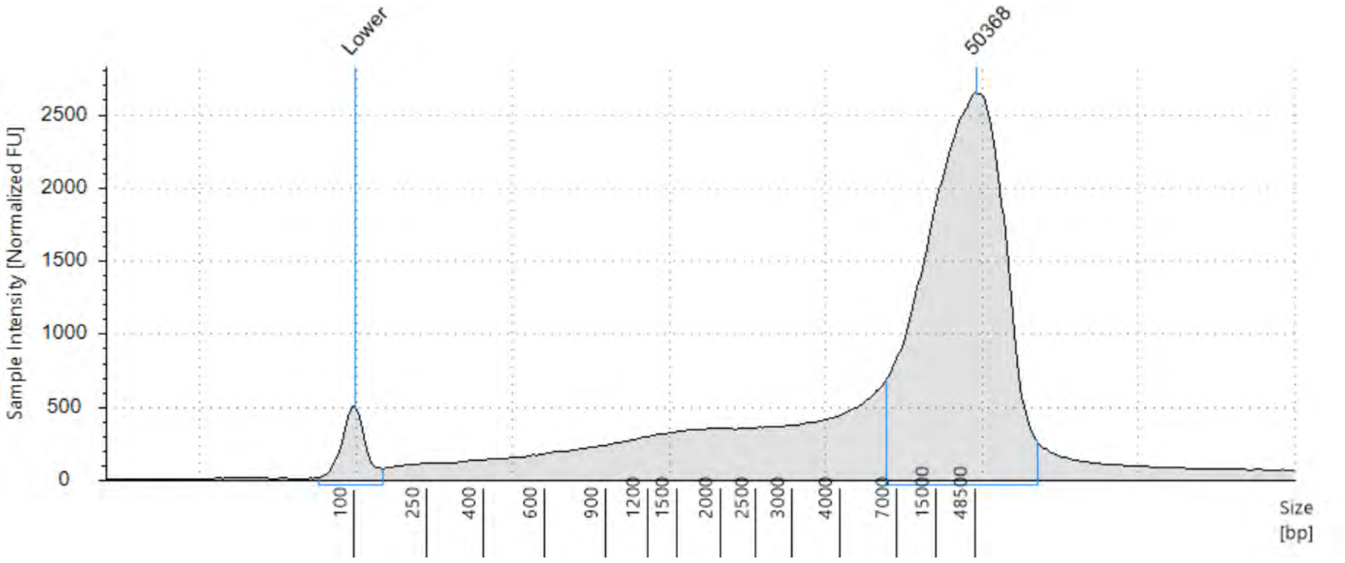

Sample Table

Well	DIN	Conc. [ng/µl]	Sample Description	Alert	Observations
G1	6.9	278	M315	⚠	Caution! Expired ScreenTape device; Sample concentration outside recommended range; DIN Edited (Ladder sizing changed)

Peak Table

Size [bp]	Calibrated Conc. [ng/µl]	Assigned Conc. [ng/µl]	% Integrated Area	From [bp]	To [bp]	Peak Comment	Observations
100	8.50	8.50	-	64	143		Lower Marker
50368	167	-	91.35	6321	>60000		
-	-	-	-	-	-		Sample Well

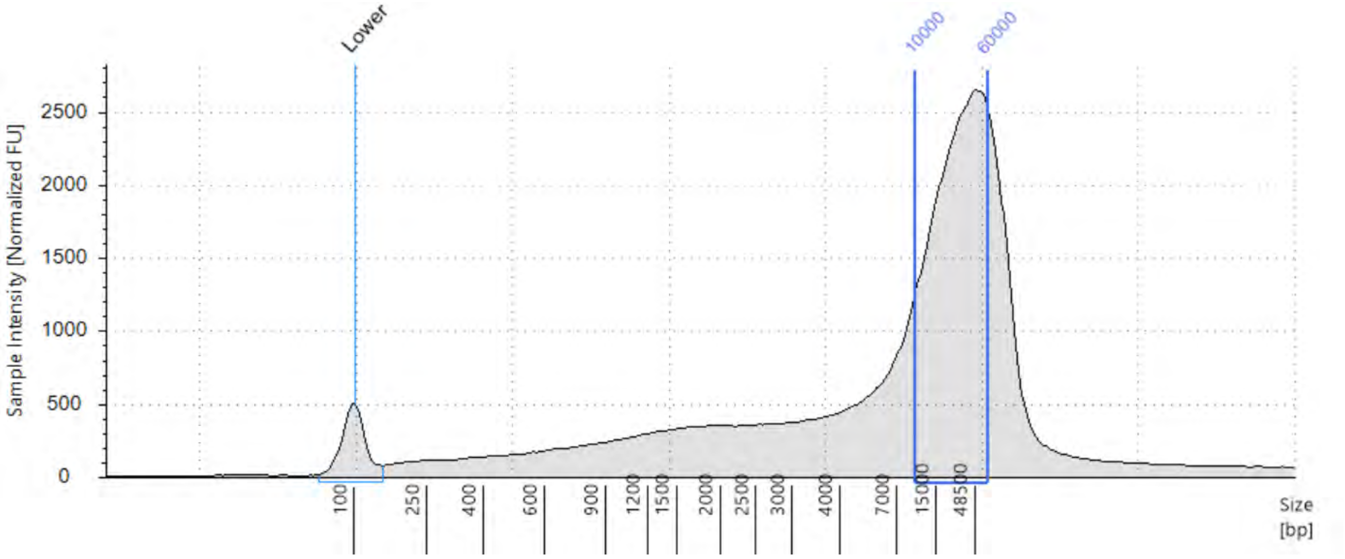

Region Table

From [bp]	To [bp]	Average Size [bp]	Conc. [ng/µl]	Region Molarity [nmol/l]	% of Total	Region Comment	Color
10000	60000	29397	109	7.76	39.18		■

C2: C671

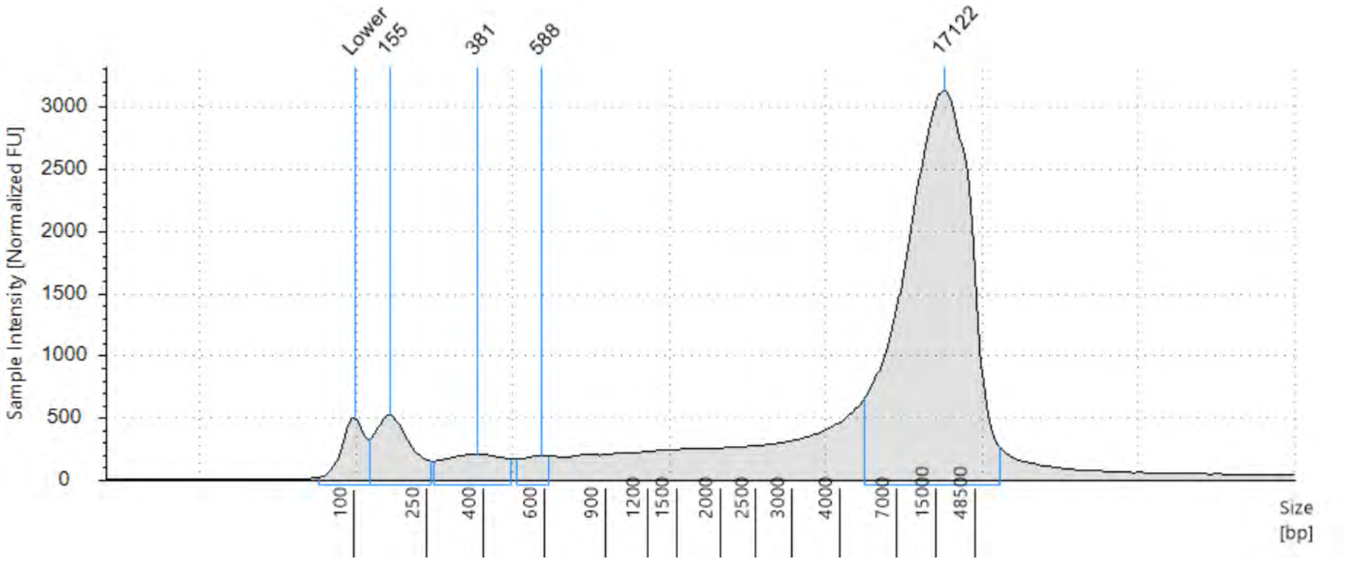

Sample Table

Well	DIN	Conc. [ng/µl]	Sample Description	Alert	Observations
C2	6.7	264	C671	⚠	Caution! Expired ScreenTape device; Sample concentration outside recommended range; DIN Edited (Ladder sizing changed)

Peak Table

Size [bp]	Calibrated Conc. [ng/µl]	Assigned Conc. [ng/µl]	% Integrated Area	From [bp]	To [bp]	Peak Comment	Observations
100	8.50	8.50	-	63	120		Lower Marker
155	14.1	-	6.91	120	258		
381	8.85	-	4.33	263	480		
588	3.74	-	1.83	497	615		
17122	164	-	80.14	5108	>60000		
-	-	-	-	-	-		Sample Well

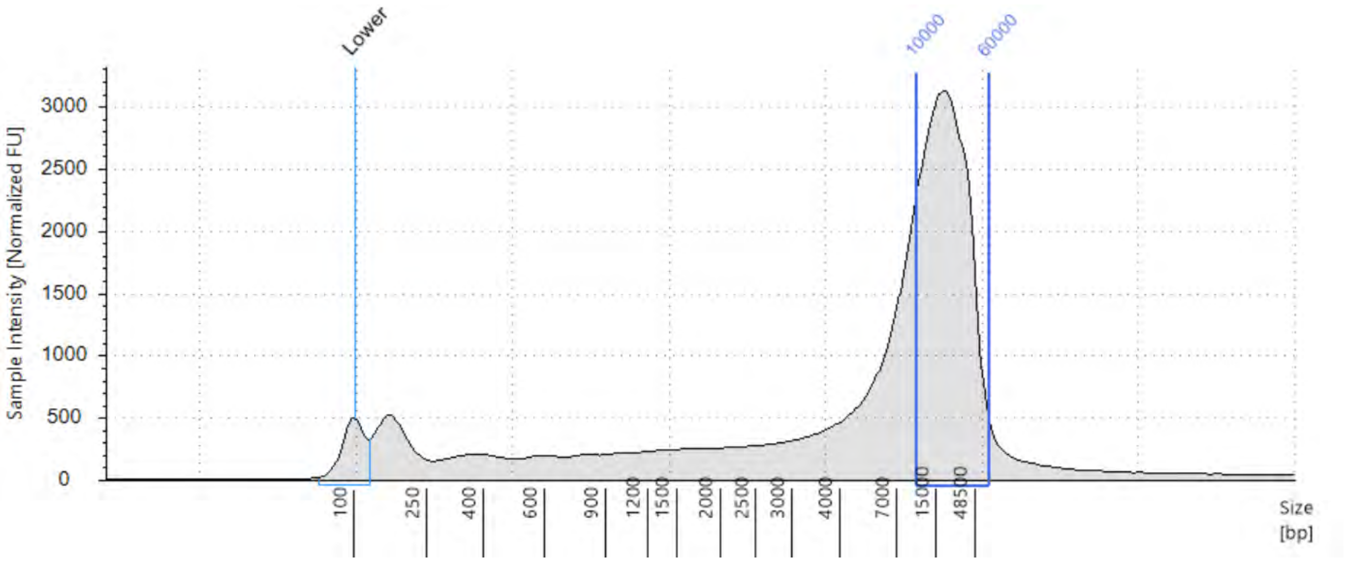

Region Table

From [bp]	To [bp]	Average Size [bp]	Conc. [ng/µl]	Region Molarity [nmol/l]	% of Total	Region Comment	Color
10000	60000	22143	118	10.2	44.70		■

D2: C672

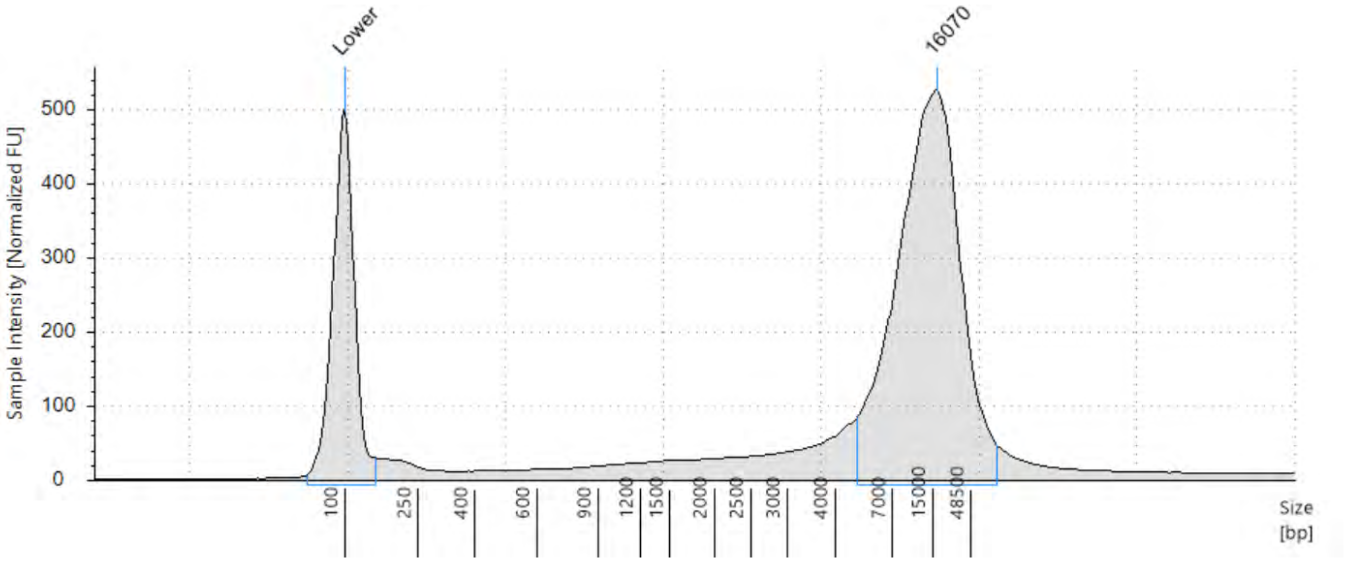

Sample Table

Well	DIN	Conc. [ng/µl]	Sample Description	Alert	Observations
D2	7.2	37.1	C672	⚠	Caution! Expired ScreenTape device; DIN Edited (Ladder sizing changed)

Peak Table

Size [bp]	Calibrated Conc. [ng/µl]	Assigned Conc. [ng/µl]	% Integrated Area	From [bp]	To [bp]	Peak Comment	Observations
100	8.50	8.50	-	62	145		Lower Marker
16070	26.6	-	92.14	4949	>60000		
-	-	-	-	-	-		Sample Well

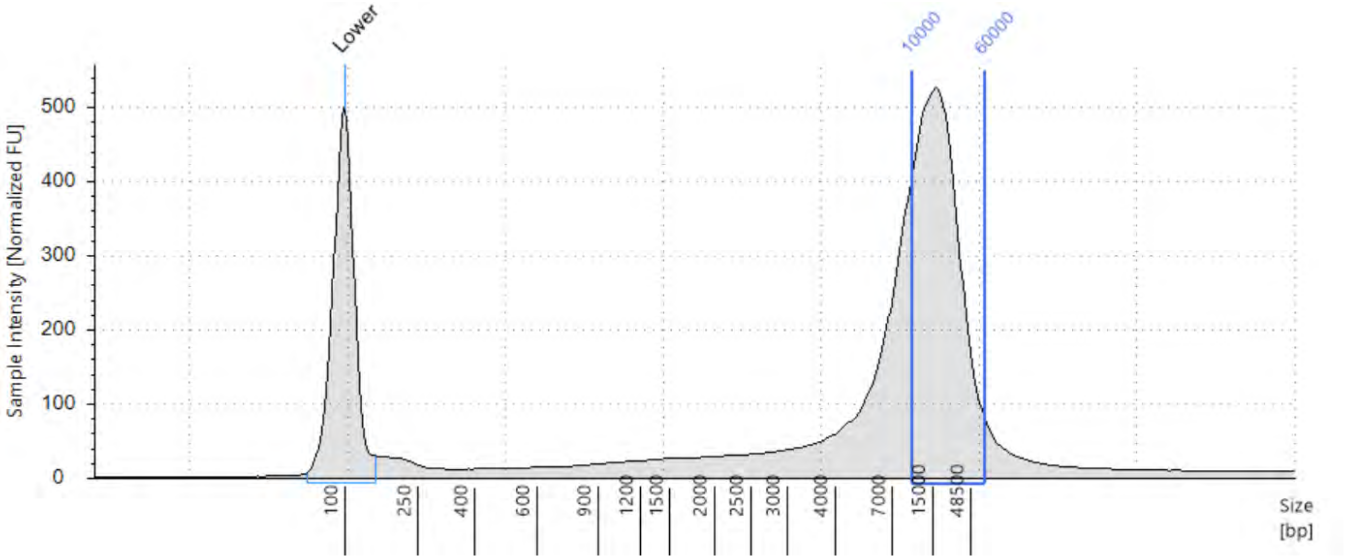

Region Table

From [bp]	To [bp]	Average Size [bp]	Conc. [ng/µl]	Region Molarity [nmol/l]	% of Total	Region Comment	Color
10000	60000	20169	18.4	1.71	49.51		■

© Agilent Technologies, Inc. 2017

E2: C673

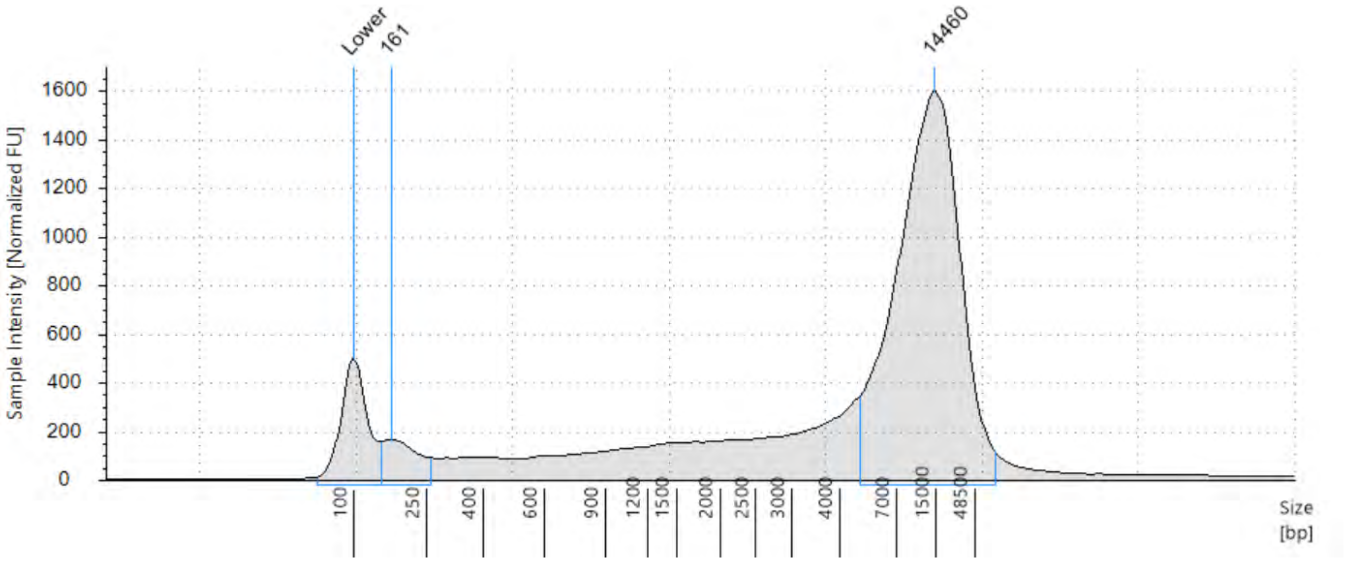

Sample Table

Well	DIN	Conc. [ng/µl]	Sample Description	Alert	Observations
E2	6.5	110	C673	⚠	Caution! Expired ScreenTape device; Sample concentration outside recommended range; DIN Edited (Ladder sizing changed)

Peak Table

Size [bp]	Calibrated Conc. [ng/µl]	Assigned Conc. [ng/µl]	% Integrated Area	From [bp]	To [bp]	Peak Comment	Observations
100	8.50	8.50	-	62	140		Lower Marker
161	3.79	-	5.10	140	258		
14460	67.1	-	90.16	4862	>60000		
-	-	-	-	-	-		Sample Well

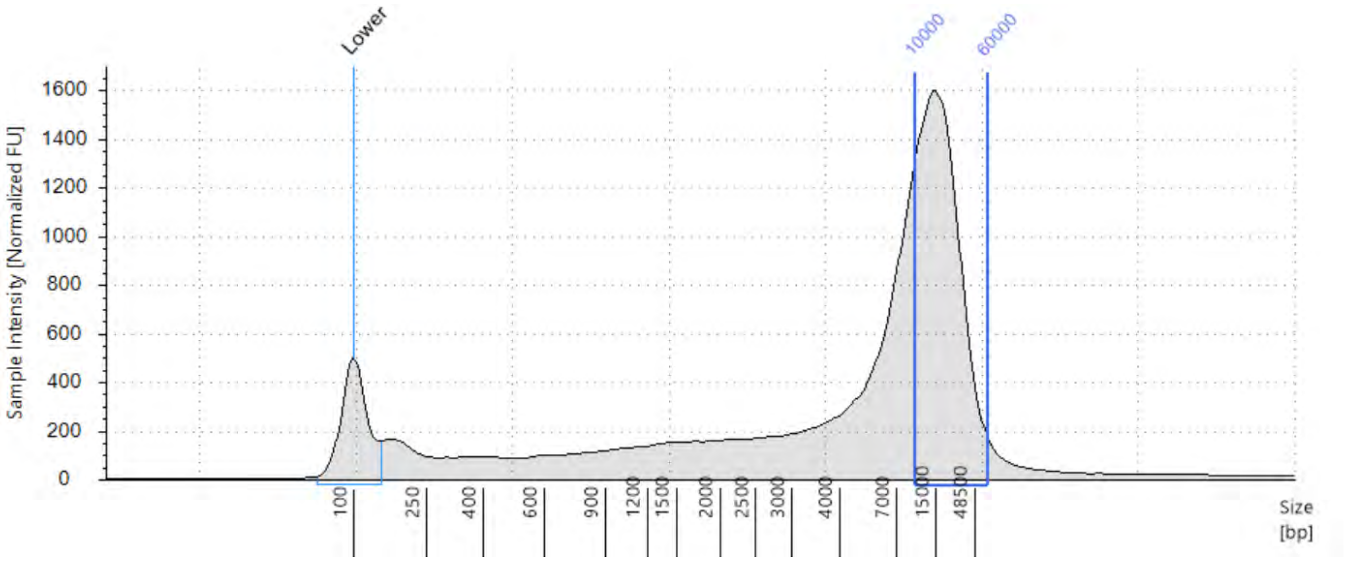

Region Table

From [bp]	To [bp]	Average Size [bp]	Conc. [ng/µl]	Region Molarity [nmol/l]	% of Total	Region Comment	Color
10000	60000	19322	44.2	4.20	40.01		■

 © Agilent Technologies, Inc. 2017

F2: C674

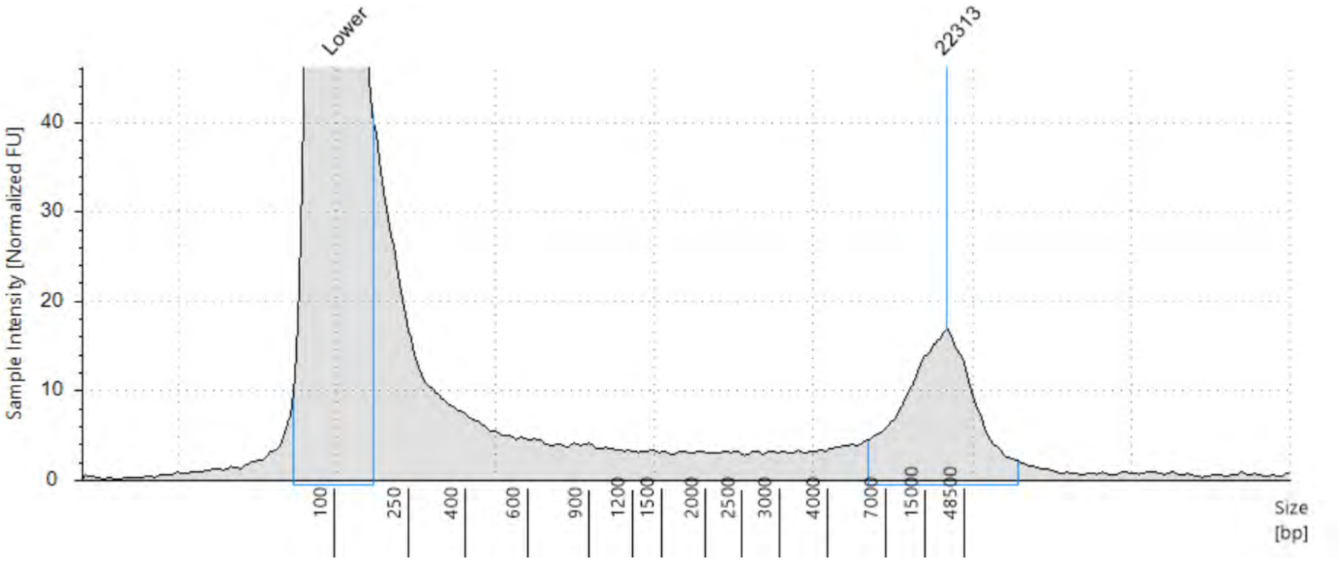

Sample Table

Well	DIN	Conc. [ng/µl]	Sample Description	Alert	Observations
F2	-	2.24	C674	▲	Caution! Expired ScreenTape device; Sample concentration outside functional range for DIN; DIN Edited (Ladder sizing changed)

Peak Table

Size [bp]	Calibrated Conc. [ng/µl]	Assigned Conc. [ng/µl]	%Integrated Area	From [bp]	To [bp]	Peak Comment	Observations
100	8.50	8.50	-	59	162		Lower Marker
22313	0.671	-	100.00	5940	>60000		

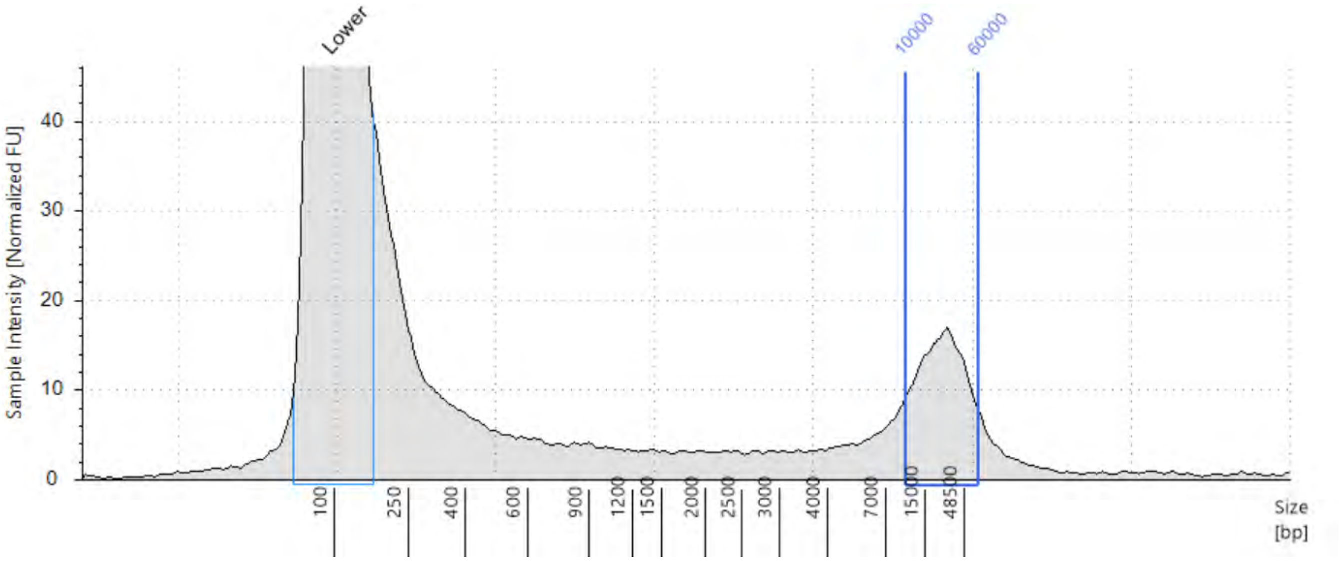

Region Table

From [bp]	To [bp]	Average Size [bp]	Conc. [ng/µl]	Region Molarity [nmol/l]	% of Total	Region Comment	Color
10000	60000	25399	0.499	0.0409	22.27		■

 © Agilent Technologies, Inc. 2017

G2: C675

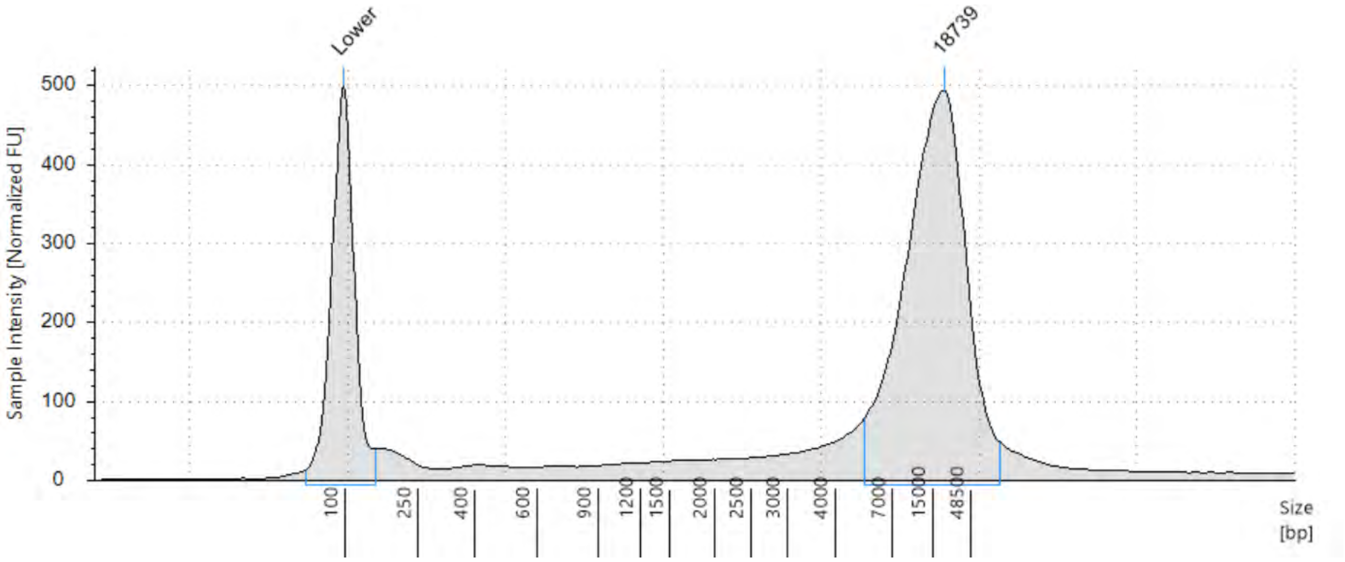

Sample Table

Well	DIN	Conc. [ng/µl]	Sample Description	Alert	Observations
G2	7.2	33.3	C675	⚠	Caution! Expired ScreenTape device; DIN Edited (Ladder sizing changed)

Peak Table

Size [bp]	Calibrated Conc. [ng/µl]	Assigned Conc. [ng/µl]	% Integrated Area	From [bp]	To [bp]	Peak Comment	Observations
100	8.50	8.50	-	62	147		Lower Marker
18739	22.8	-	94.61	5353	>60000		
-	-	-	-	-	-		Sample Well

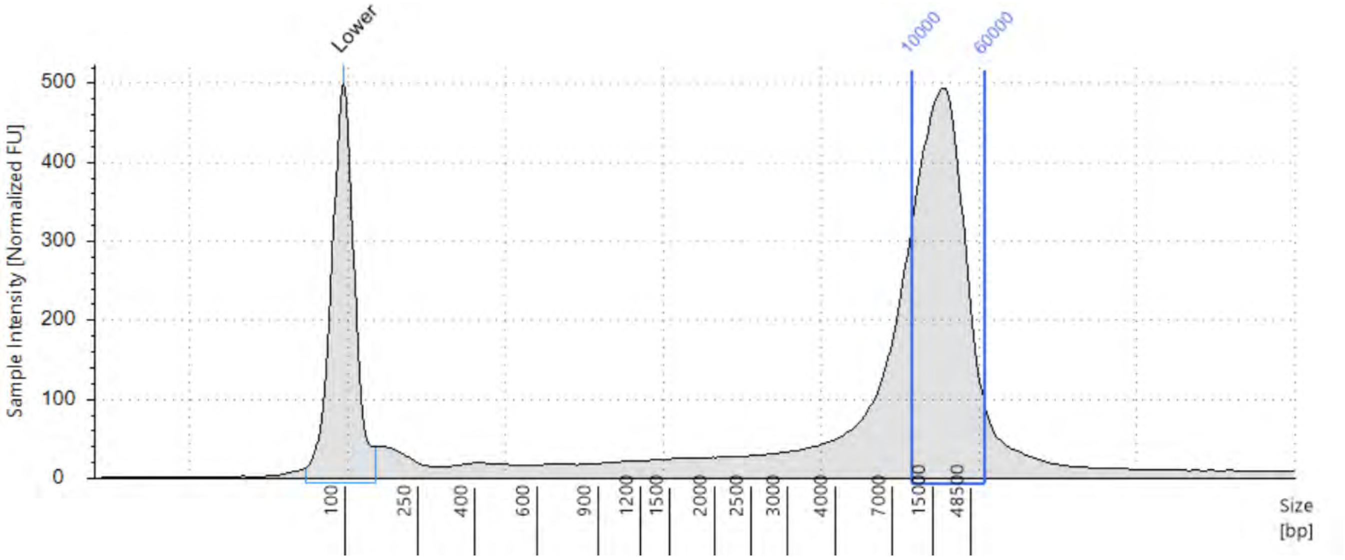

Region Table

From [bp]	To [bp]	Average Size [bp]	Conc. [ng/µl]	Region Molarity [nmol/l]	% of Total	Region Comment	Color
10000	60000	21653	17.2	1.50	51.65		■

© Agilent Technologies, Inc. 2017

H2: C404

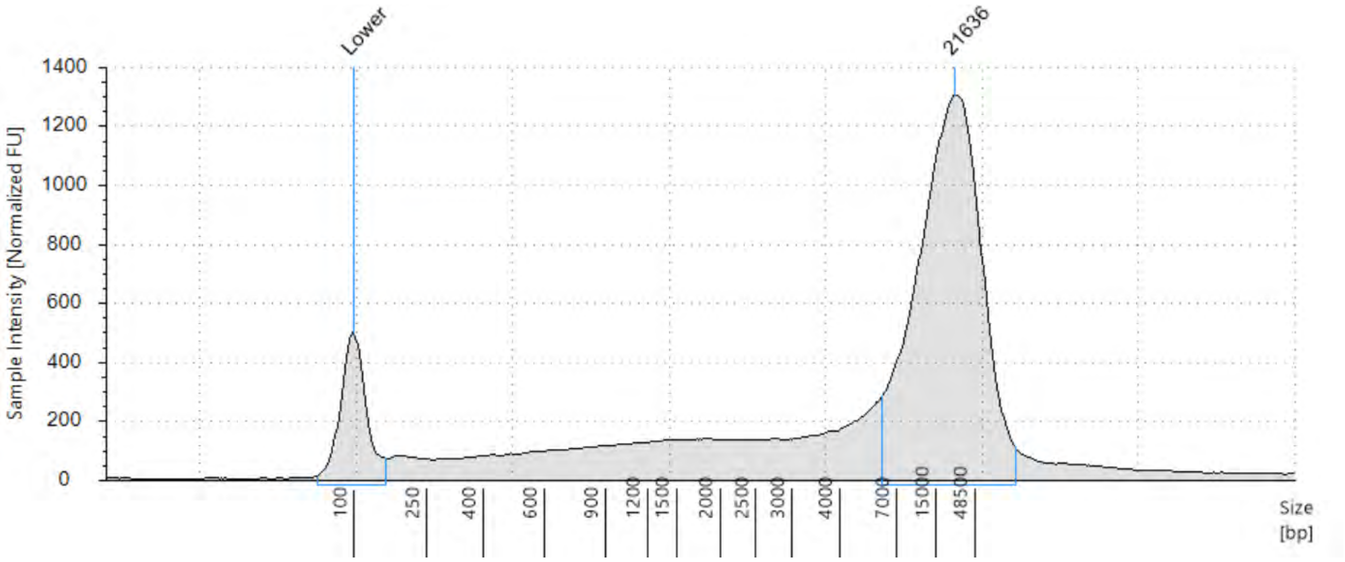

Sample Table

Well	DIN	Conc. [ng/µl]	Sample Description	Alert	Observations
H2	6.7	98.8	C404	⚠	Caution! Expired ScreenTape device; DIN Edited (Ladder sizing changed)

Peak Table

Size [bp]	Calibrated Conc. [ng/µl]	Assigned Conc. [ng/µl]	% Integrated Area	From [bp]	To [bp]	Peak Comment	Observations
100	8.50	8.50	-	63	149		Lower Marker
21636	59.0	-	93.03	6096	>60000		
-	-	-	-	-	-		Sample Well

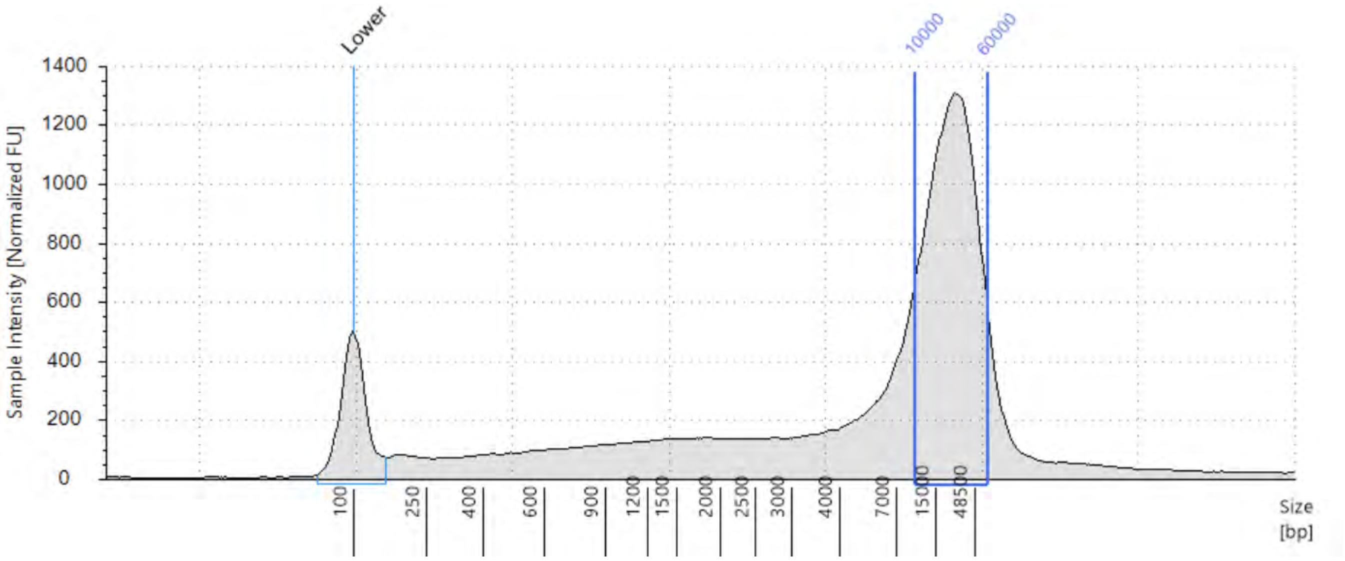

Region Table

From [bp]	To [bp]	Average Size [bp]	Conc. [ng/µl]	Region Molarity [nmol/l]	% of Total	Region Comment	Color
10000	60000	25537	46.4	3.64	46.95		

© Agilent Technologies, Inc. 2017

Filename: T5_Initials&Errors_4-3-2019_p2.gDNA

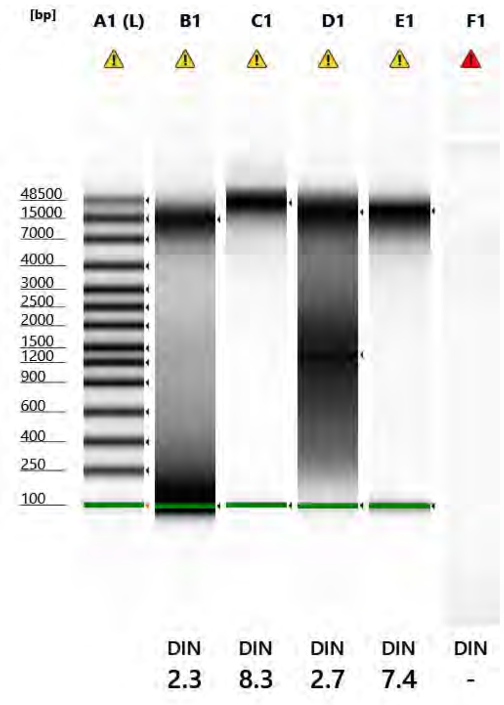

Default image (Contrast 50%), Image is Scaled to Sample

Sample Info

Well	DIN	Conc. [ng/µl]	Sample Description	Alert	Observations
A1	-	559	Ladder	⚠	Caution! Expired ScreenTape device; Ladder
B1	2.3	45.9	C405	⚠	Caution! Expired ScreenTape device
C1	8.3	362	M186	⚠	Caution! Expired ScreenTape device; Sample concentration outside functional range for DIN
D1	2.7	808	M482	⚠	Caution! Expired ScreenTape device; Sample concentration outside functional range for DIN
E1	7.4	86.4	C389	⚠	Caution! Expired ScreenTape device
F1	-			▲	Marker(s) not detected; Caution! Expired ScreenTape device

© Agilent Technologies, Inc. 2017

A1: Ladder

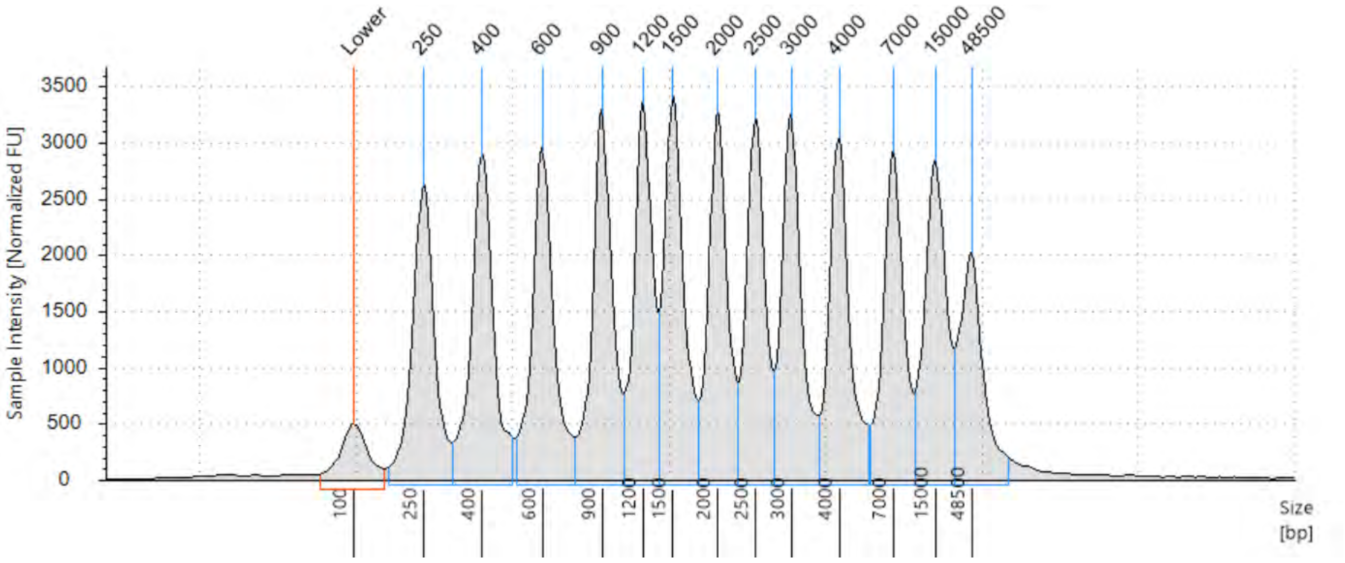

Sample Table

Well	DIN	Conc. [ng/µl]	Sample Description	Alert	Observations
A1	-	559	Ladder	⚠	Caution! Expired ScreenTape device; Ladder

Peak Table

Size [bp]	Calibrated Conc. [ng/µl]	Assigned Conc. [ng/µl]	% Integrated Area	From [bp]	To [bp]	Peak Comment	Observations
100	8.50	8.50	-	64	150		Lower Marker
250	40.2	-	7.16	158	315		
400	44.2	-	7.87	315	488		
600	44.1	-	7.85	501	742		
900	45.5	-	8.10	749	1049		
1200	44.3	-	7.89	1049	1355		
1500	47.8	-	8.51	1355	1770		
2000	43.8	-	7.81	1770	2254		
2500	43.1	-	7.68	2254	2748		
3000	45.8	-	8.15	2748	3536		
4000	43.3	-	7.72	3536	5442		
7000	42.2	-	7.51	5518	10374		
15000	43.6	-	7.76	10374	21167		
48500	33.8	-	6.02	21167	>60000		

© Agilent Technologies, Inc. 2017

B1: C405

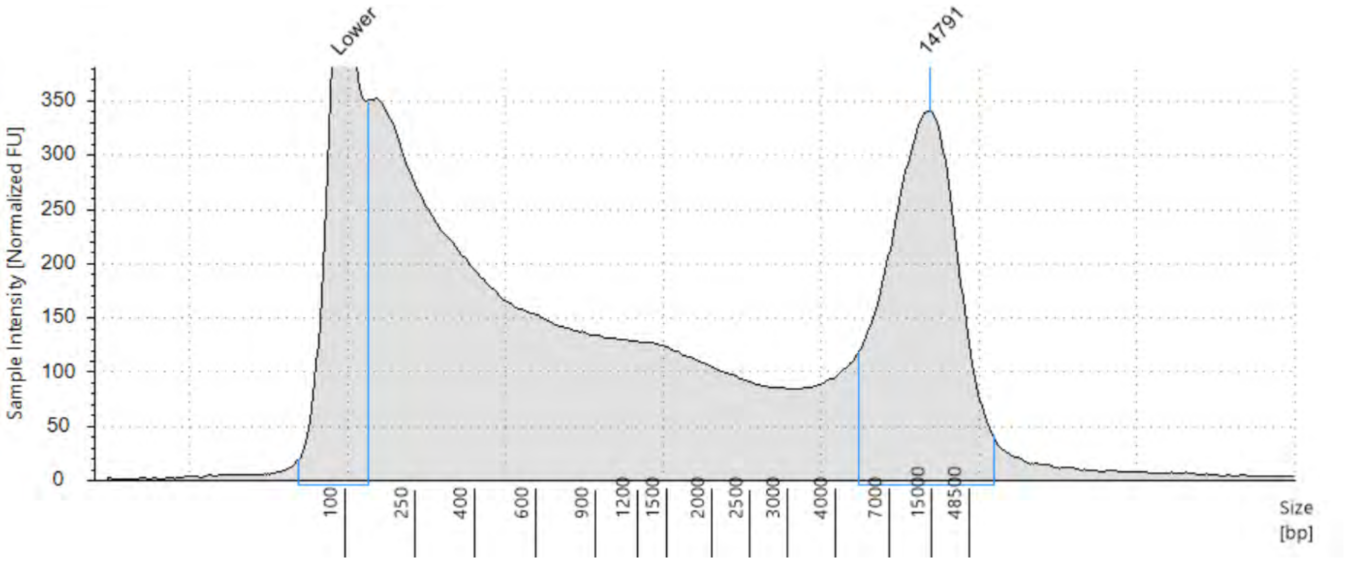

Sample Table

Well	DIN	Conc. [ng/µl]	Sample Description	Alert	Observations
B1	2.3	45.9	C405	⚠	Caution! Expired ScreenTape device

Peak Table

Size [bp]	Calibrated Conc. [ng/µl]	Assigned Conc. [ng/µl]	% Integrated Area	From [bp]	To [bp]	Peak Comment	Observations
100	8.50	8.50	-	56	137		Lower Marker
14791	12.4	-	96.22	5162	>60000		
-	-	-	-	-	-		Sample Well

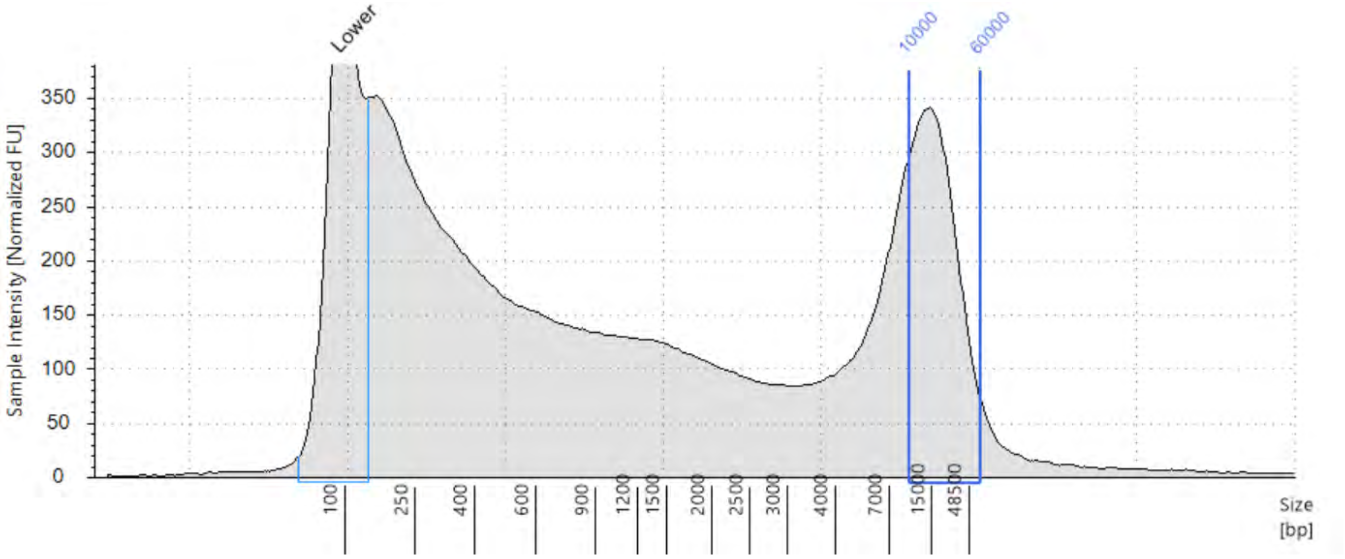

Region Table

From [bp]	To [bp]	Average Size [bp]	Conc. [ng/µl]	Region Molarity [nmol/l]	% of Total	Region Comment	Color
10000	60000	20309	8.00	0.755	17.44		■

C1: M186

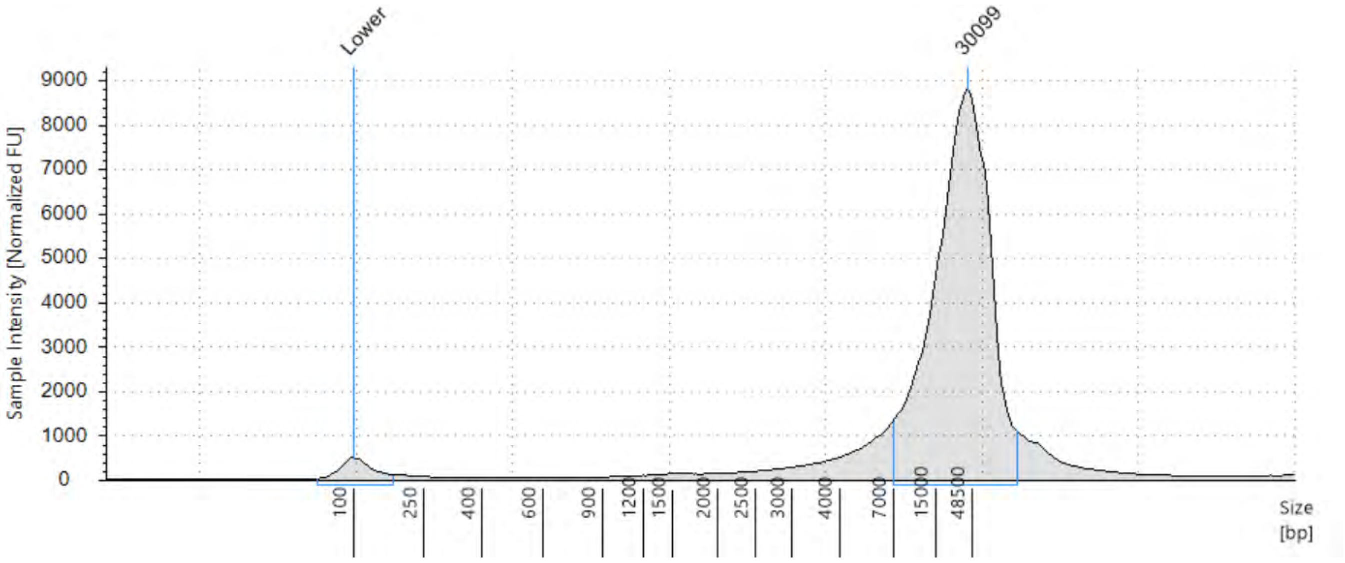

Sample Table

Well	DIN	Conc. [ng/µl]	Sample Description	Alert	Observations
C1	8.3	362	M186	⚠	Caution! Expired ScreenTape device; Sample concentration outside functional range for DIN

Peak Table

Size [bp]	Calibrated Conc. [ng/µl]	Assigned Conc. [ng/µl]	% Integrated Area	From [bp]	To [bp]	Peak Comment	Observations
100	8.50	8.50	-	62	166		Lower Marker
30099	277	-	94.84	6995	>60000		
-	-	-	-	-	-		Sample Well

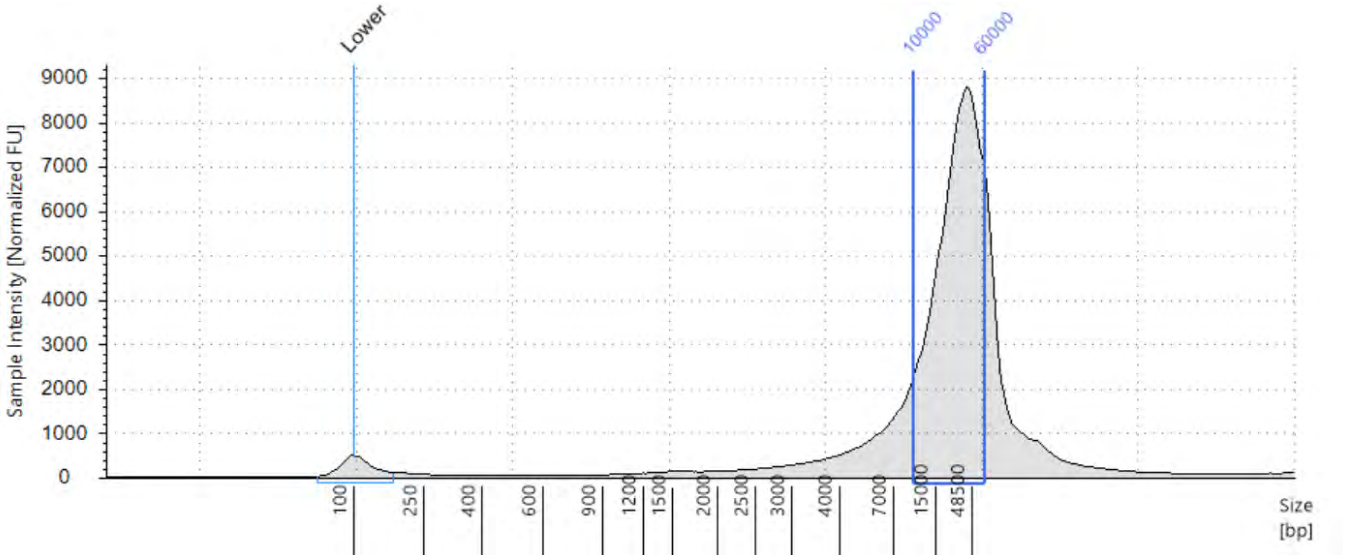

Region Table

From [bp]	To [bp]	Average Size [bp]	Conc. [ng/µl]	Region Molarity [nmol/l]	% of Total	Region Comment	Color
10000	60000	29483	213	14.6	58.75		■

E1: C389

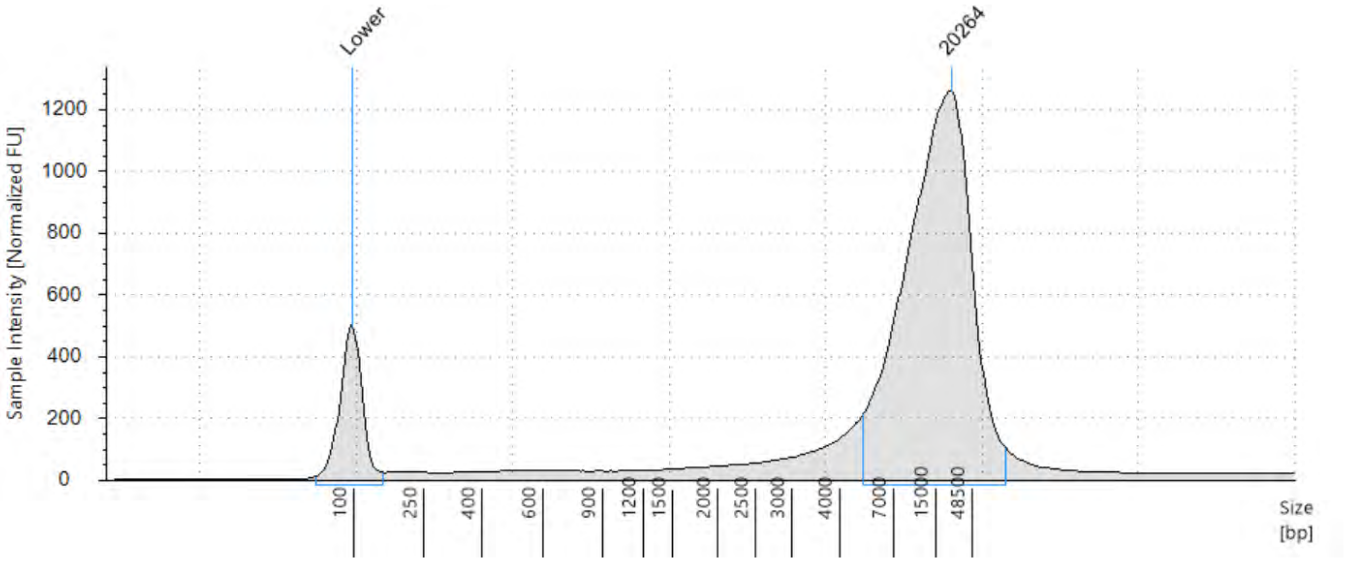

Sample Table

Well	DIN	Conc. [ng/µl]	Sample Description	Alert	Observations
E1	7.4	86.4	C389	⚠	Caution! Expired ScreenTape device

Peak Table

Size [bp]	Calibrated Conc. [ng/µl]	Assigned Conc. [ng/µl]	% Integrated Area	From [bp]	To [bp]	Peak Comment	Observations
100	8.50	8.50	-	62	149		Lower Marker
20264	64.4	-	89.94	5162	>60000		
-	-	-	-	-	-		Sample Well

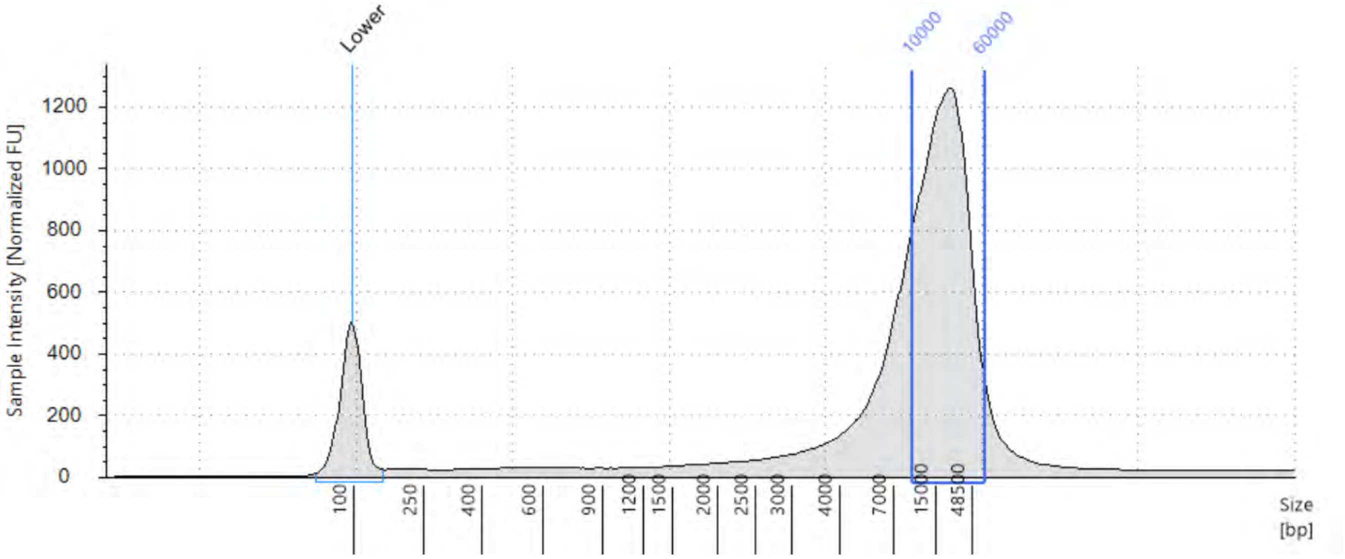

Region Table

From [bp]	To [bp]	Average Size [bp]	Conc. [ng/µl]	Region Molarity [nmol/l]	% of Total	Region Comment	Color
10000	60000	22520	47.4	4.07	54.81		■

© Agilent Technologies, Inc. 2017

Filename: T5_Worm_EtOH&Initials_4-26-2019.gDNA

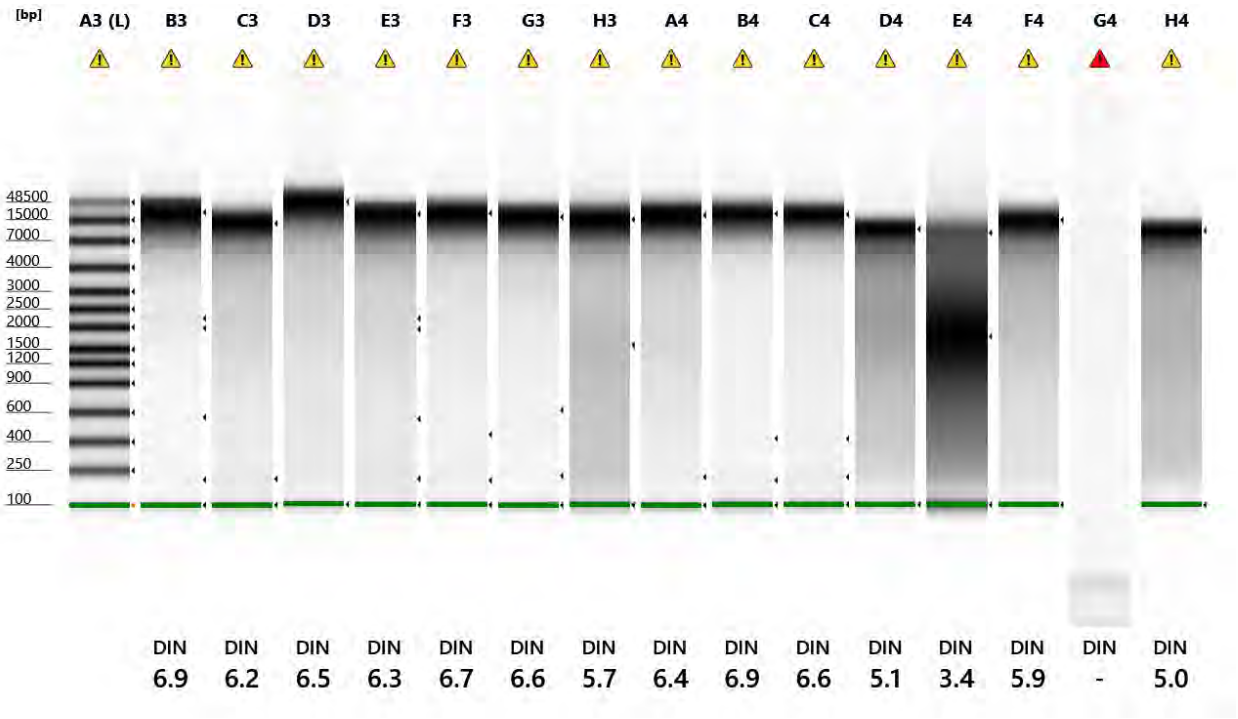

Default image (Contrast 50%), Image is Scaled to Sample

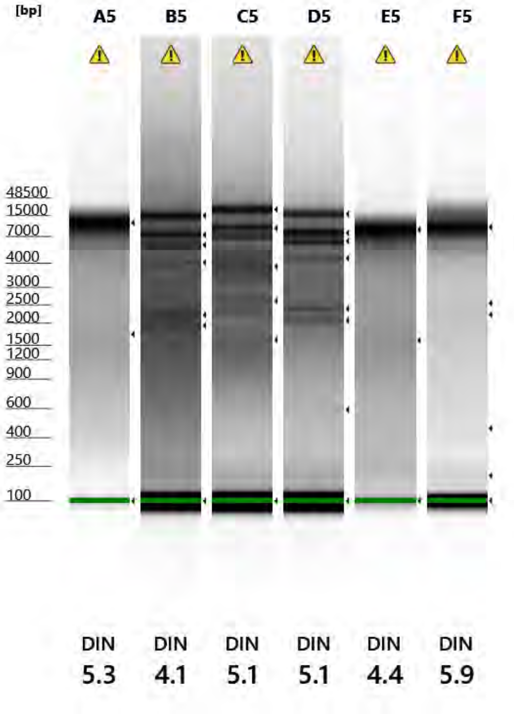

Default image (Contrast 50%), Image is Scaled to Sample

Sample Info

Well	DIN	Conc. [ng/µl]	Sample Description	Alert	Observations
A3	-	459	Ladder	⚠	Caution! Expired ScreenTape device; Ladder
B3	6.9	204	W896	⚠	Caution! Expired ScreenTape device; Sample concentration outside recommended range
C3	6.2	191	W897	⚠	Caution! Expired ScreenTape device; Sample concentration outside recommended range
D3	6.5	217	W898	⚠	Caution! Expired ScreenTape device; Sample concentration outside recommended range
E3	6.3	261	W899	⚠	Caution! Expired ScreenTape device; Sample concentration outside recommended range
F3	6.7	231	W900	⚠	Caution! Expired ScreenTape device; Sample concentration outside recommended range
G3	6.6	173	W1121	⚠	Caution! Expired ScreenTape device; Sample concentration outside recommended range

H3	5.7	175	W1122	⚠	Caution! Expired ScreenTape device; Sample concentration outside recommended range
A4	6.4	179	W1123	⚠	Caution! Expired ScreenTape device; Sample concentration outside recommended range
B4	6.9	108	W1124	⚠	Caution! Expired ScreenTape device; Sample concentration outside recommended range
C4	6.6	135	W1125	⚠	Caution! Expired ScreenTape device; Sample concentration outside recommended range
D4	5.1	266	W891	⚠	Caution! Expired ScreenTape device; Sample concentration outside recommended range
E4	3.4	179	W892	⚠	Caution! Expired ScreenTape device; Sample concentration outside recommended range
F4	5.9	301	W893	⚠	Caution! Expired ScreenTape device; Sample concentration outside functional range for DIN
G4	-		W894	▲	Marker(s) not detected; Caution! Expired ScreenTape device
H4	5.0	346	W895	⚠	Caution! Expired ScreenTape device; Sample concentration outside functional range for DIN
A5	5.3	306	W1116	⚠	Caution! Expired ScreenTape device; Sample concentration outside functional range for DIN
B5	4.1	38.7	W1117	⚠	Caution! Expired ScreenTape device
C5	5.1	25.9	W1118	⚠	Caution! Expired ScreenTape device
D5	5.1	20.8	W1119	⚠	Caution! Expired ScreenTape device
E5	4.4	143	W1120	⚠	Caution! Expired ScreenTape device; Sample concentration outside recommended range
F5	5.9	25.8	F1	⚠	Caution! Expired ScreenTape device

 © Agilent Technologies, Inc. 2017

A3: Ladder

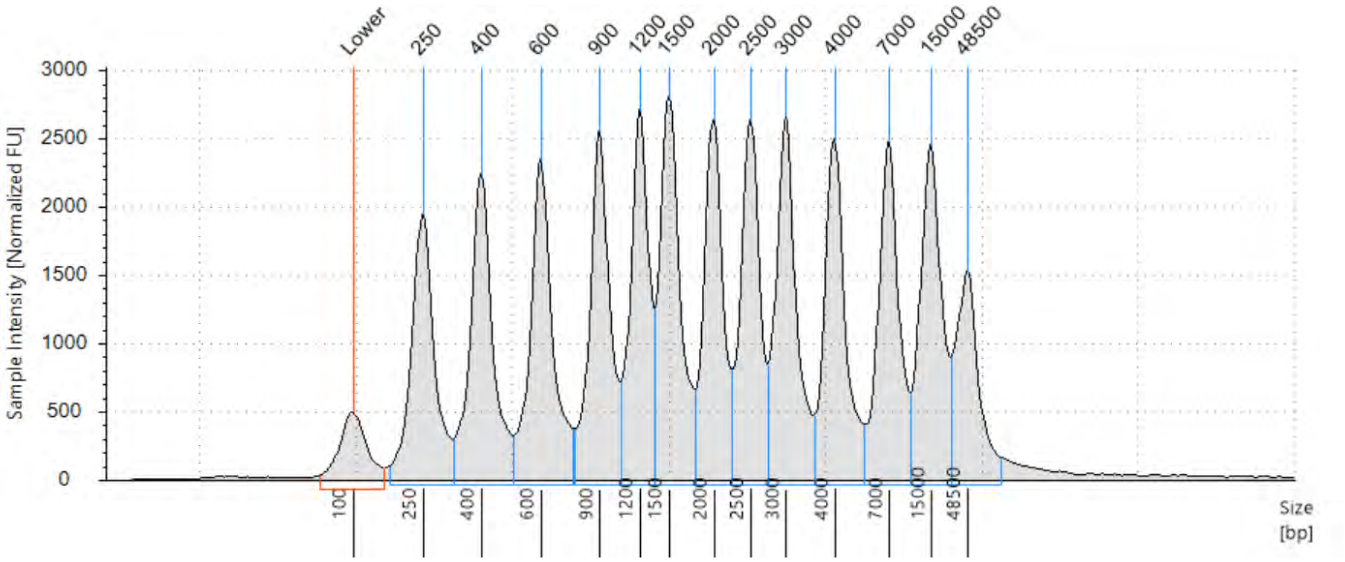

Sample Table

Well	DIN	Conc. [ng/µl]	Sample Description	Alert	Observations
A3	-	459	Ladder	⚠	Caution! Expired ScreenTape device; Ladder

Peak Table

Size [bp]	Calibrated Conc. [ng/µl]	Assigned Conc. [ng/µl]	% Integrated Area	From [bp]	To [bp]	Peak Comment	Observations
100	8.50	8.50	-	65	149		Lower Marker
250	30.8	-	6.69	162	320		
400	34.8	-	7.56	320	497		
600	36.3	-	7.89	501	752		
900	36.0	-	7.82	758	1049		
1200	35.4	-	7.69	1049	1342		
1500	41.3	-	8.97	1342	1777		
2000	35.7	-	7.75	1777	2236		
2500	35.8	-	7.78	2236	2729		
3000	39.1	-	8.49	2729	3532		
4000	36.7	-	7.96	3532	5401		
7000	36.4	-	7.90	5401	10247		
15000	37.8	-	8.20	10247	21451		
48500	24.4	-	5.30	21451	>60000		

B3: W896

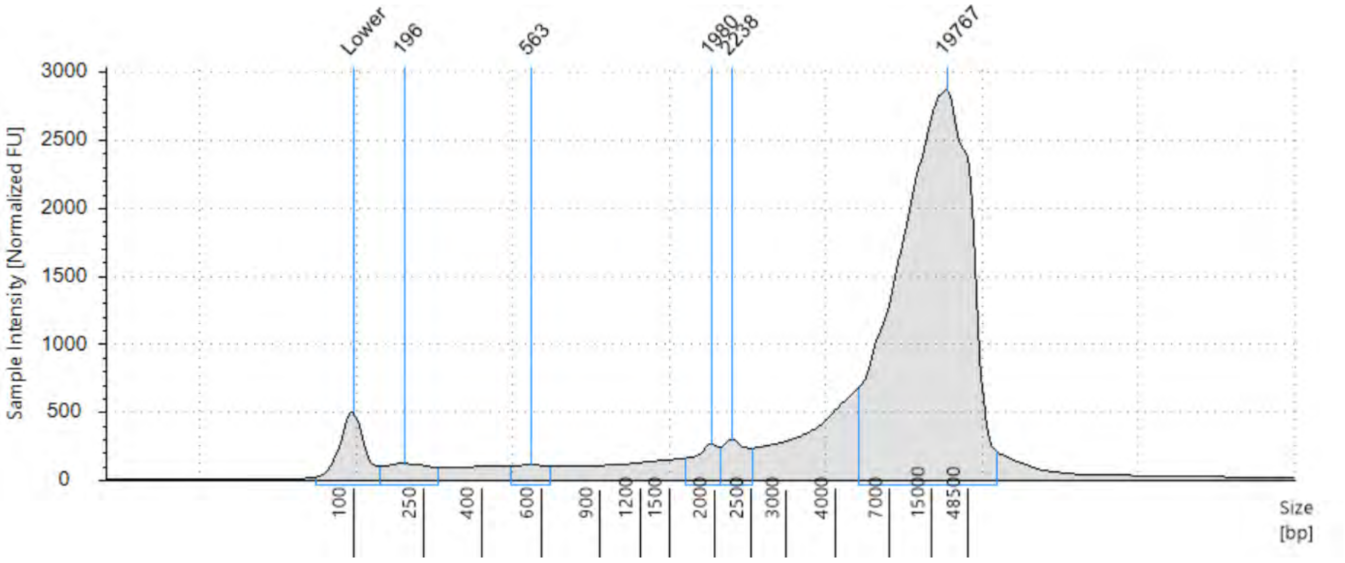

Sample Table

Well	DIN	Conc. [ng/µl]	Sample Description	Alert	Observations
B3	6.9	204	W896	⚠	Caution! Expired ScreenTape device; Sample concentration outside recommended range

Peak Table

Size [bp]	Calibrated Conc. [ng/µl]	Assigned Conc. [ng/µl]	% Integrated Area	From [bp]	To [bp]	Peak Comment	Observations
100	8.50	8.50	-	61	142		Lower Marker
196	3.59	-	2.22	142	282		
563	2.42	-	1.49	490	640		
1980	4.41	-	2.72	1665	2080		
2238	5.13	-	3.16	2080	2525		
19767	144	-	88.75	5126	>60000		
-	-	-	-	-	-		Sample Well

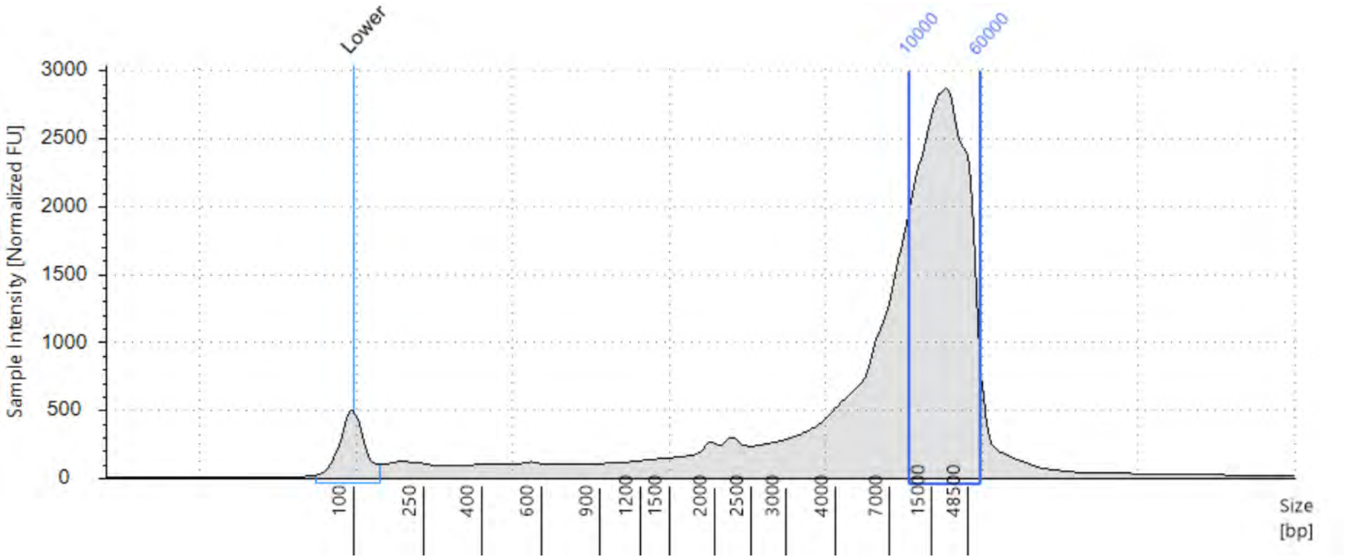

Region Table

From [bp]	To [bp]	Average Size [bp]	Conc. [ng/µl]	Region Molarity [nmol/l]	% of Total	Region Comment	Color
10000	60000	23923	105	8.76	51.26		■

 © Agilent Technologies, Inc. 2017

C3: W897

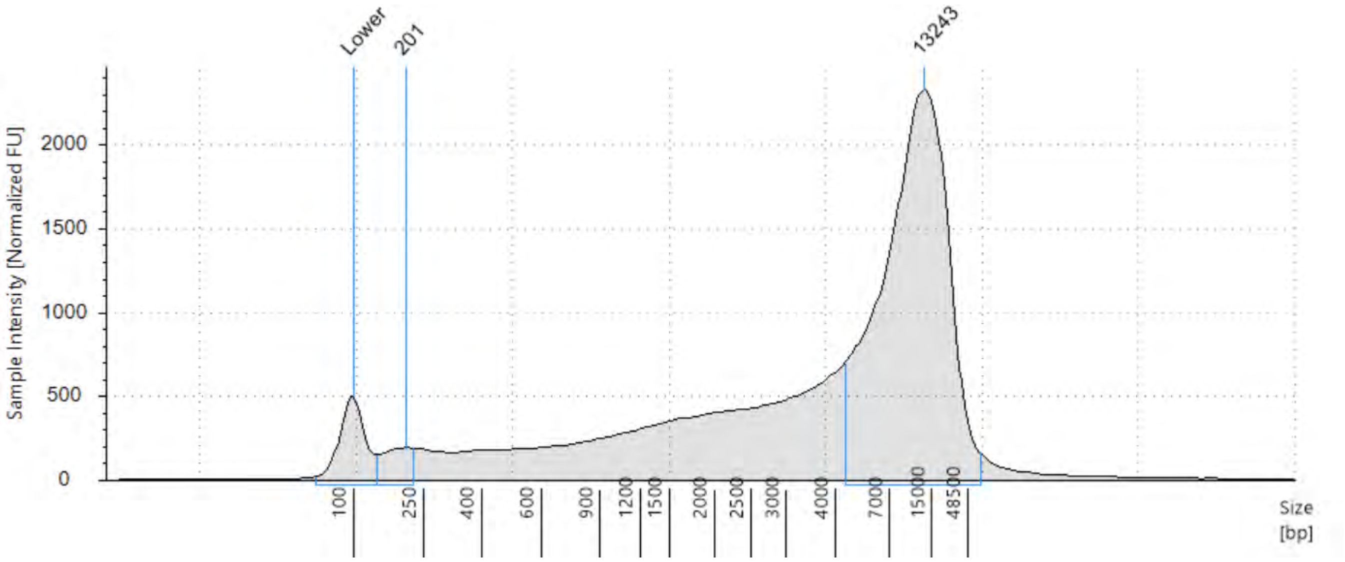

Sample Table

Well	DIN	Conc. [ng/µl]	Sample Description	Alert	Observations
C3	6.2	191	W897	⚠	Caution! Expired ScreenTape device; Sample concentration outside recommended range

Peak Table

Size [bp]	Calibrated Conc. [ng/µl]	Assigned Conc. [ng/µl]	% Integrated Area	From [bp]	To [bp]	Peak Comment	Observations
100	8.50	8.50	-	61	137		Lower Marker
201	3.75	-	3.44	137	219		
13243	105	-	96.56	4424	>60000		

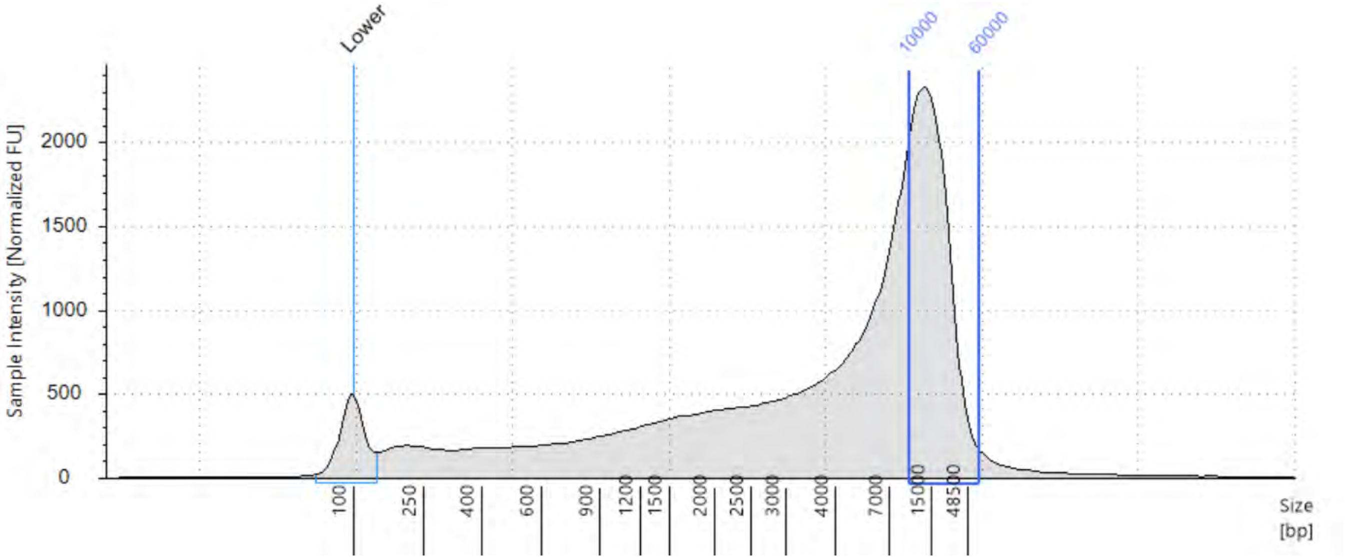

Region Table

From [bp]	To [bp]	Average Size [bp]	Conc. [ng/µl]	Region Molarity [nmol/l]	% of Total	Region Comment	Color
10000	60000	17573	61.0	6.17	31.88		■

 © Agilent Technologies, Inc. 2017

D3: W898

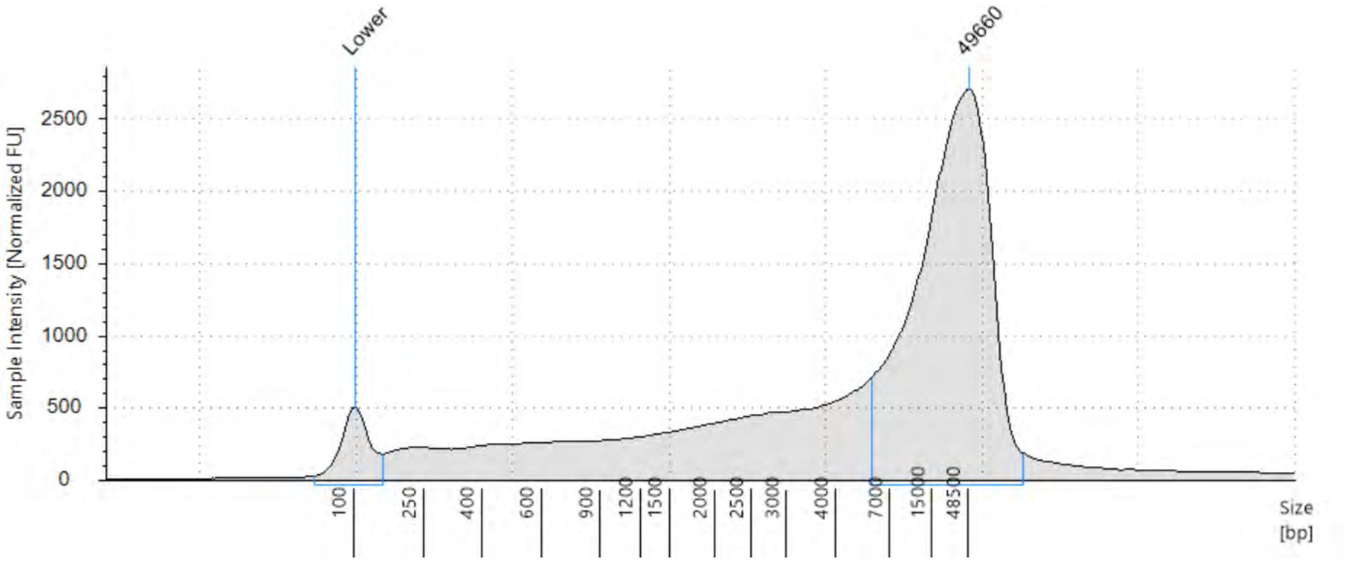

Sample Table

Well	DIN	Conc. [ng/µl]	Sample Description	Alert	Observations
D3	6.5	217	W898	⚠	Caution! Expired ScreenTape device; Sample concentration outside recommended range

Peak Table

Size [bp]	Calibrated Conc. [ng/µl]	Assigned Conc. [ng/µl]	% Integrated Area	From [bp]	To [bp]	Peak Comment	Observations
100	8.50	8.50	-	59	143		Lower Marker
49660	120	-	96.54	5811	>60000		
-	-	-	-	-	-		Sample Well

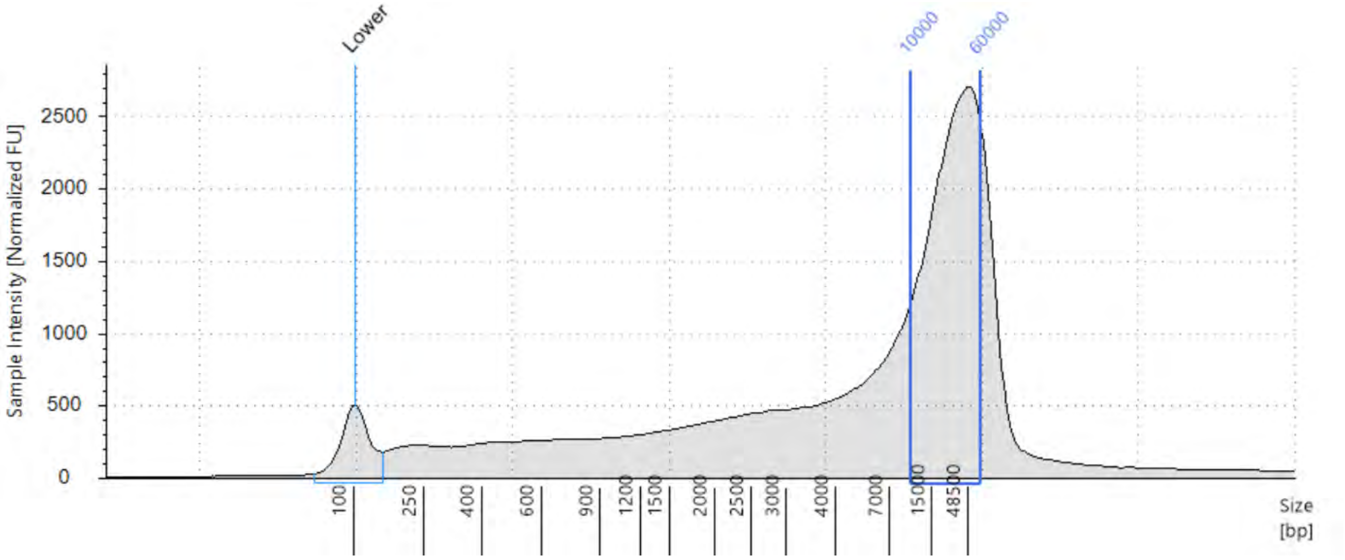

Region Table

From [bp]	To [bp]	Average Size [bp]	Conc. [ng/µl]	Region Molarity [nmol/l]	% of Total	Region Comment	Color
10000	60000	28467	78.8	5.75	36.29		■

 © Agilent Technologies, Inc. 2017

F3: W900

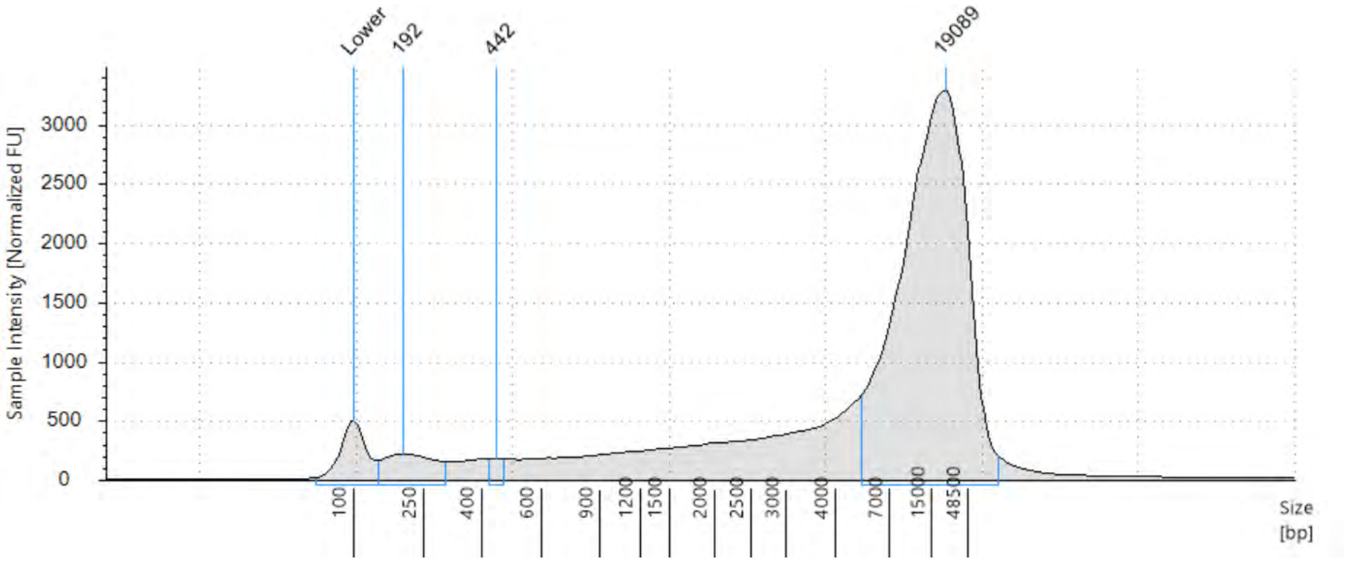

Sample Table

Well	DIN	Conc. [ng/µl]	Sample Description	Alert	Observations
F3	6.7	231	W900	⚠	Caution! Expired ScreenTape device; Sample concentration outside recommended range

Peak Table

Size [bp]	Calibrated Conc. [ng/µl]	Assigned Conc. [ng/µl]	% Integrated Area	From [bp]	To [bp]	Peak Comment	Observations
100	8.50	8.50	-	61	137		Lower Marker
192	7.38	-	4.60	137	297		
442	1.49	-	0.93	422	462		
19089	149	-	92.95	5254	>60000		
-	-	-	-	-	-		Sample Well

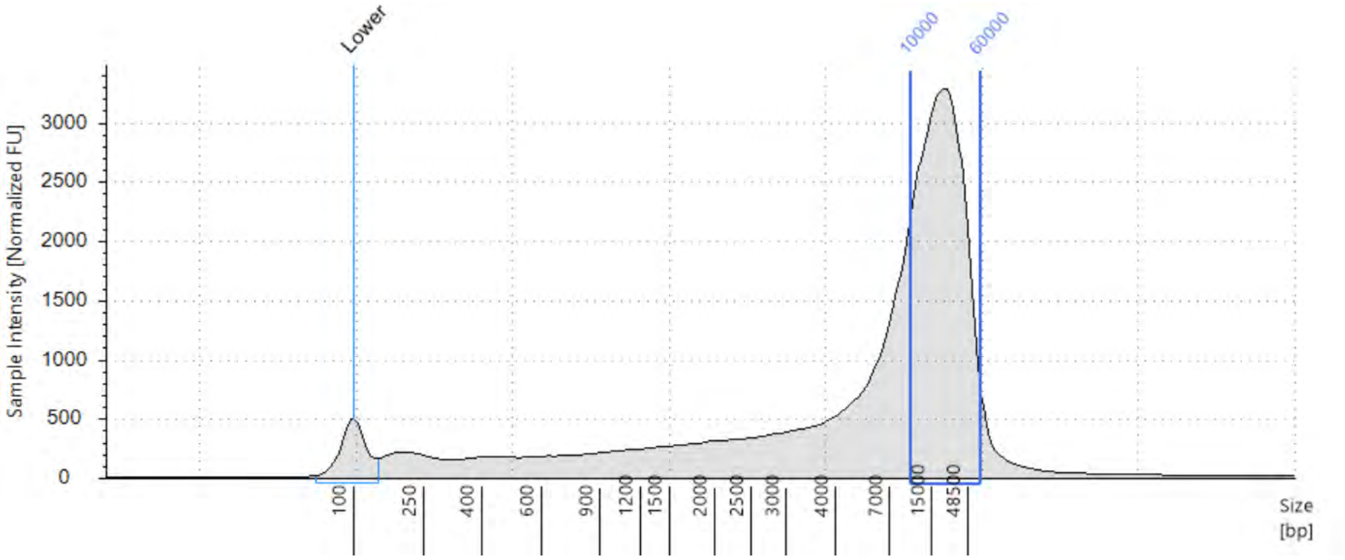

Region Table

From [bp]	To [bp]	Average Size [bp]	Conc. [ng/µl]	Region Molarity [nmol/l]	% of Total	Region Comment	Color
10000	60000	22402	109	9.43	47.39		■

G3: W1121

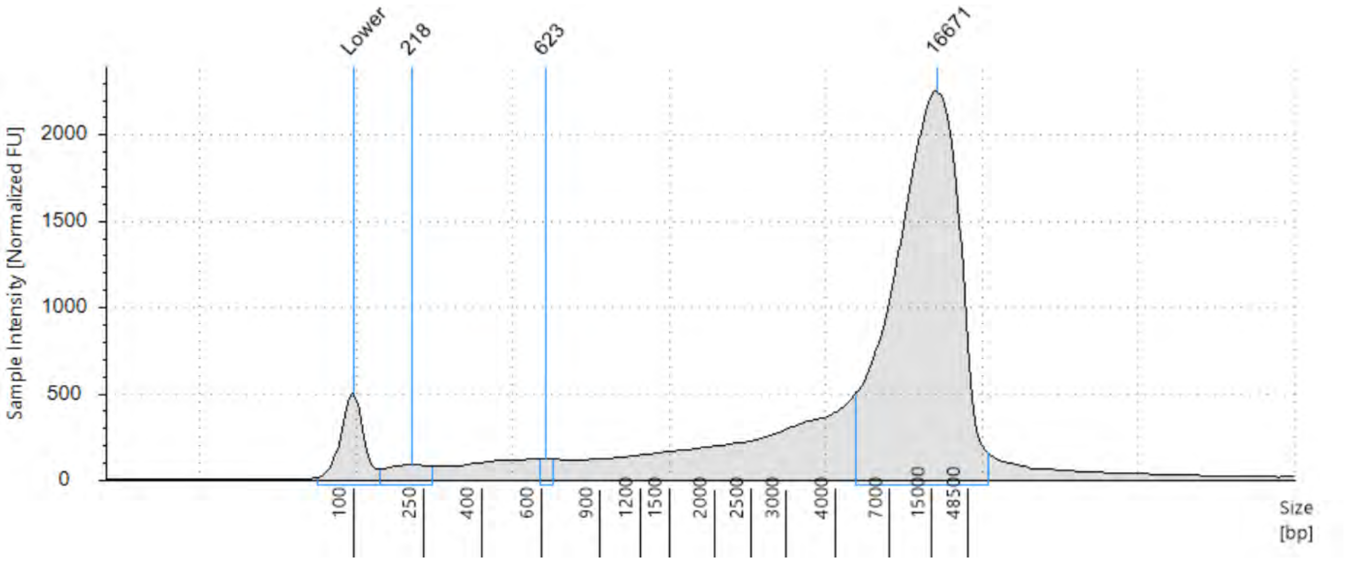

Sample Table

Well	DIN	Conc. [ng/µl]	Sample Description	Alert	Observations
G3	6.6	173	W1121	⚠	Caution! Expired ScreenTape device; Sample concentration outside recommended range

Peak Table

Size [bp]	Calibrated Conc. [ng/µl]	Assigned Conc. [ng/µl]	% Integrated Area	From [bp]	To [bp]	Peak Comment	Observations
100	8.50	8.50	-	62	142		Lower Marker
218	2.60	-	2.20	142	270		
623	1.14	-	0.96	595	652		
16671	112	-	94.18	4916	>60000		
-	-	-	-	-	-		Sample Well

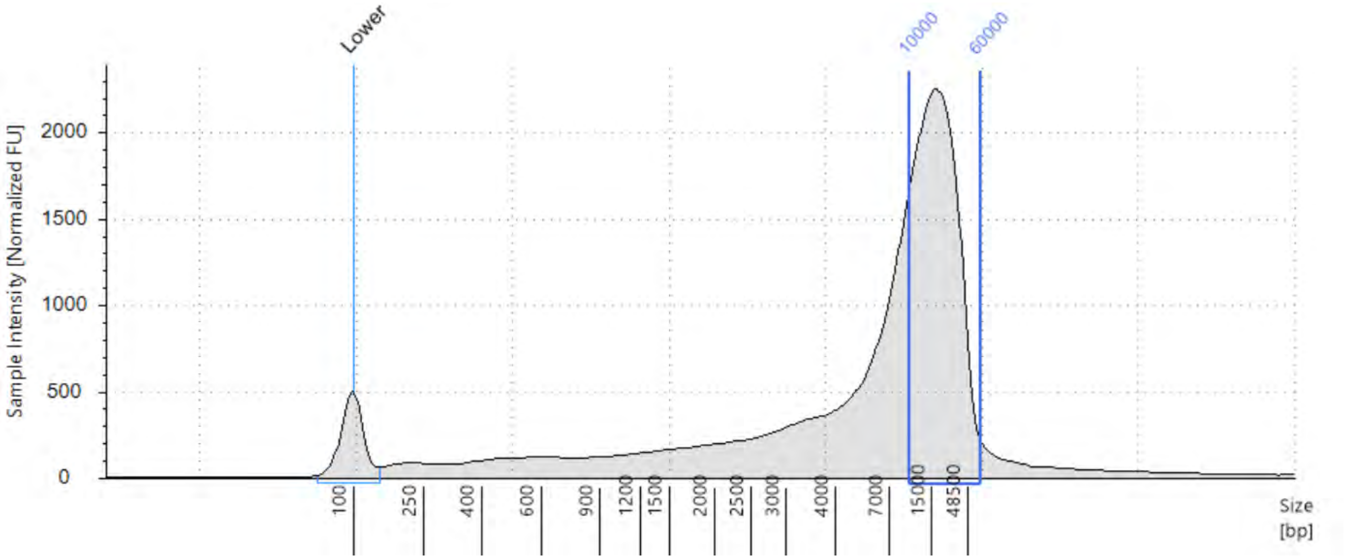

Region Table

From [bp]	To [bp]	Average Size [bp]	Conc. [ng/µl]	Region Molarity [nmol/l]	% of Total	Region Comment	Color
10000	60000	19691	78.4	7.32	45.37		■

H3: W1122

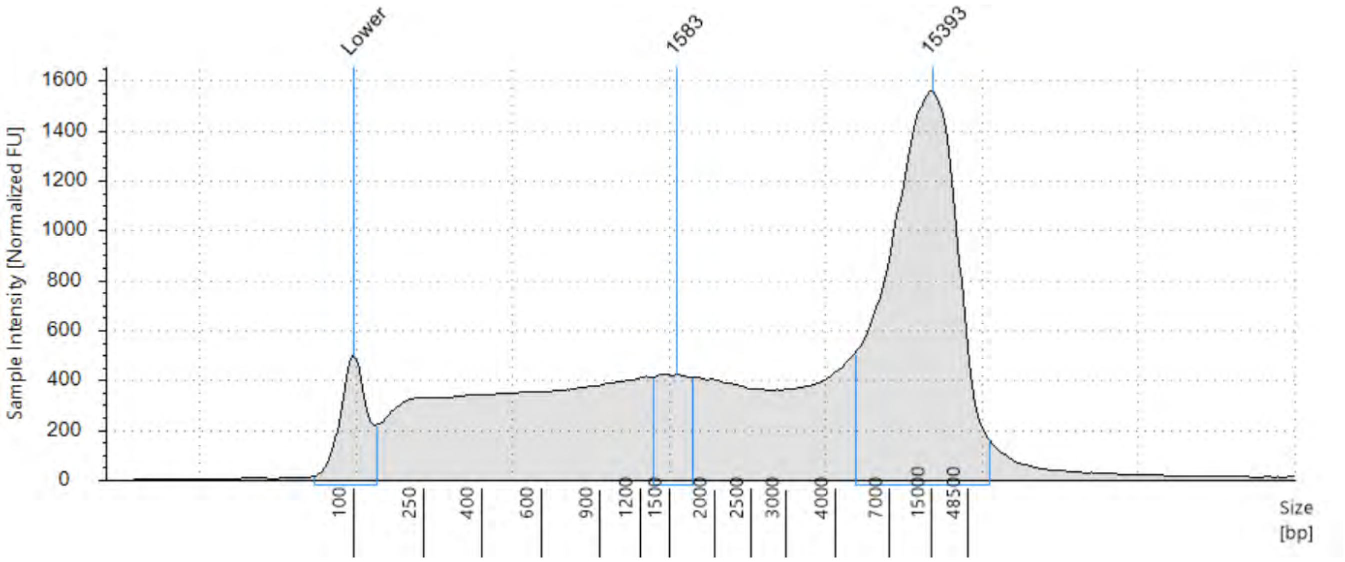

Sample Table

Well	DIN	Conc. [ng/µl]	Sample Description	Alert	Observations
H3	5.7	175	W1122	⚠	Caution! Expired ScreenTape device; Sample concentration outside recommended range

Peak Table

Size [bp]	Calibrated Conc. [ng/µl]	Assigned Conc. [ng/µl]	% Integrated Area	From [bp]	To [bp]	Peak Comment	Observations
100	8.50	8.50	-	60	135		Lower Marker
1583	9.68	-	11.63	1330	1755		
15393	71.9	-	86.34	4927	>60000		
-	-	-	-	-	-		Sample Well

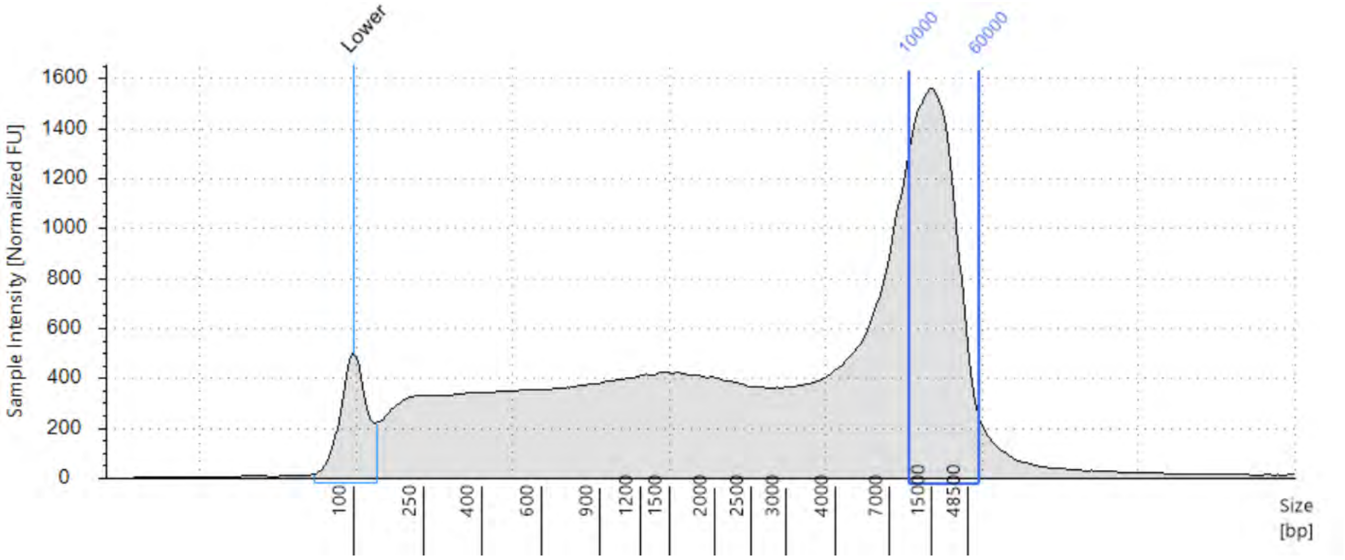

Region Table

From [bp]	To [bp]	Average Size [bp]	Conc. [ng/µl]	Region Molarity [nmol/l]	% of Total	Region Comment	Color
10000	60000	19804	45.9	4.32	26.28		■

© Agilent Technologies, Inc. 2017

A4: W1123

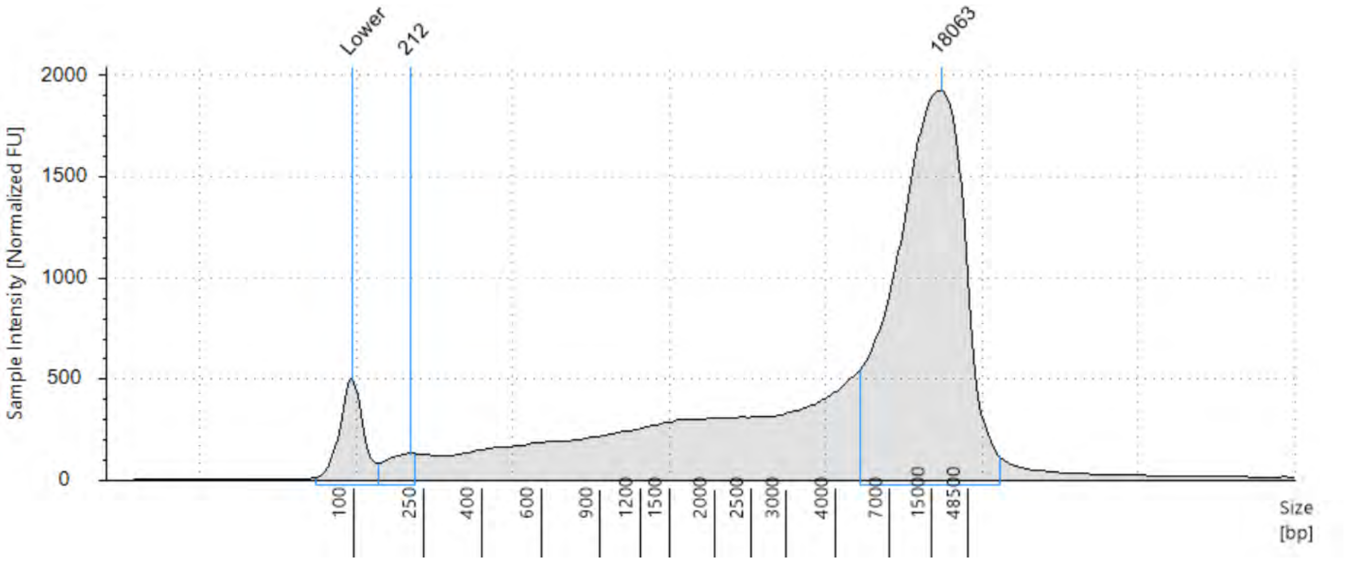

Sample Table

Well	DIN	Conc. [ng/µl]	Sample Description	Alert	Observations
A4	6.4	179	W1123	⚠	Caution! Expired ScreenTape device; Sample concentration outside recommended range

Peak Table

Size [bp]	Calibrated Conc. [ng/µl]	Assigned Conc. [ng/µl]	% Integrated Area	From [bp]	To [bp]	Peak Comment	Observations
100	8.50	8.50	-	61	139		Lower Marker
212	2.64	-	2.51	139	227		
18063	99.7	-	94.59	5204	>60000		
-	-	-	-	-	-		Sample Well

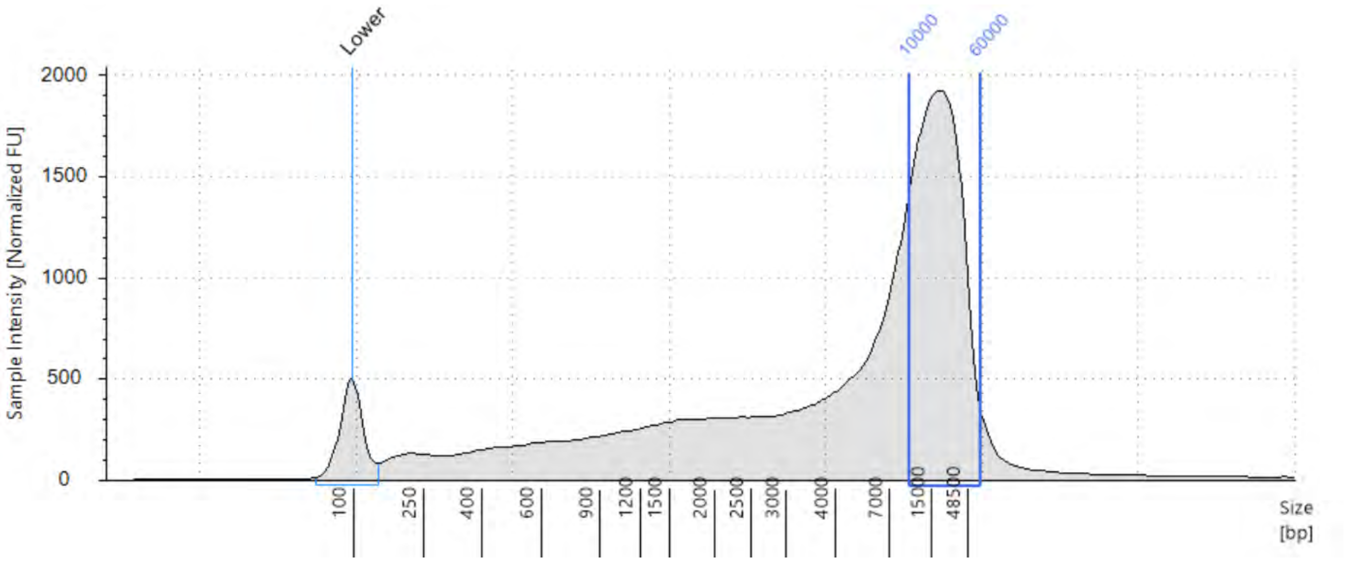

Region Table

From [bp]	To [bp]	Average Size [bp]	Conc. [ng/µl]	Region Molarity [nmol/l]	% of Total	Region Comment	Color
10000	60000	21326	69.9	6.22	38.99		■

 © Agilent Technologies, Inc. 2017

B4: W1124

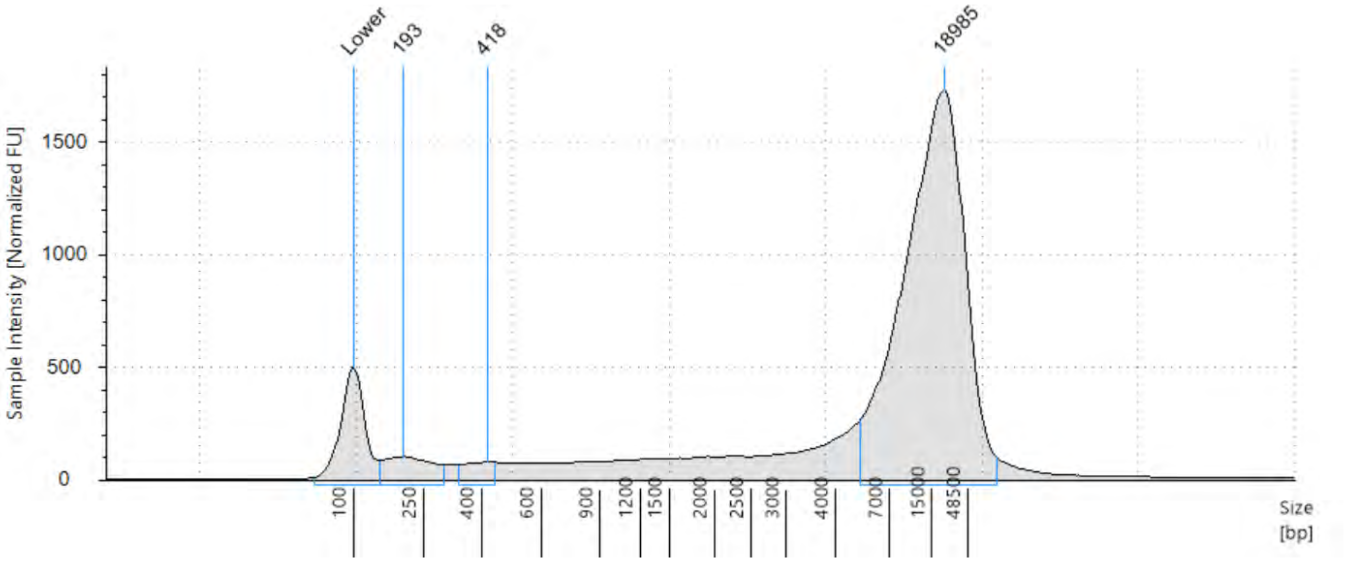

Sample Table

Well	DIN	Conc. [ng/µl]	Sample Description	Alert	Observations
B4	6.9	108	W1124	⚠	Caution! Expired ScreenTape device; Sample concentration outside recommended range

Peak Table

Size [bp]	Calibrated Conc. [ng/µl]	Assigned Conc. [ng/µl]	% Integrated Area	From [bp]	To [bp]	Peak Comment	Observations
100	8.50	8.50	-	60	140		Lower Marker
193	3.51	-	4.27	140	297		
418	1.68	-	2.04	331	438		
18985	75.0	-	91.09	5188	>60000		
-	-	-	-	-	-		Sample Well

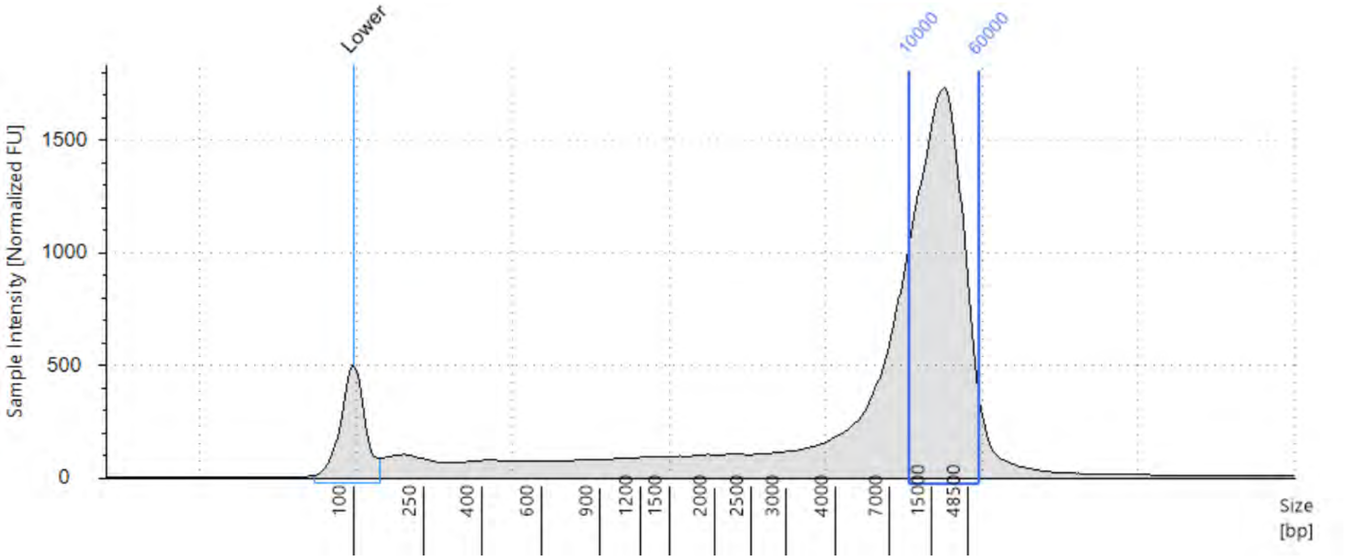

Region Table

From [bp]	To [bp]	Average Size [bp]	Conc. [ng/µl]	Region Molarity [nmol/l]	% of Total	Region Comment	Color
10000	60000	21765	56.6	4.95	52.58		■

C4: W1125

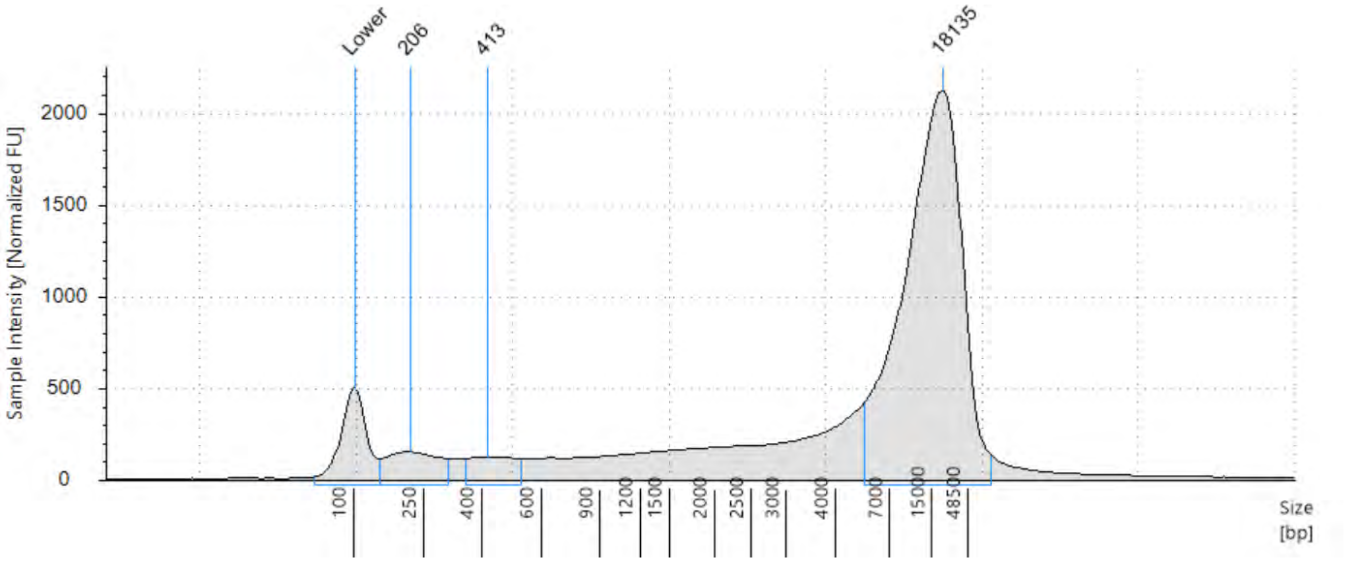

Sample Table

Well	DIN	Conc. [ng/µl]	Sample Description	Alert	Observations
C4	6.6	135	W1125	⚠	Caution! Expired ScreenTape device; Sample concentration outside recommended range

Peak Table

Size [bp]	Calibrated Conc. [ng/µl]	Assigned Conc. [ng/µl]	% Integrated Area	From [bp]	To [bp]	Peak Comment	Observations
100	8.50	8.50	-	59	139		Lower Marker
206	5.46	-	5.80	139	304		
413	3.92	-	4.16	347	518		
18135	84.0	-	89.15	5349	>60000		
-	-	-	-	-	-		Sample Well

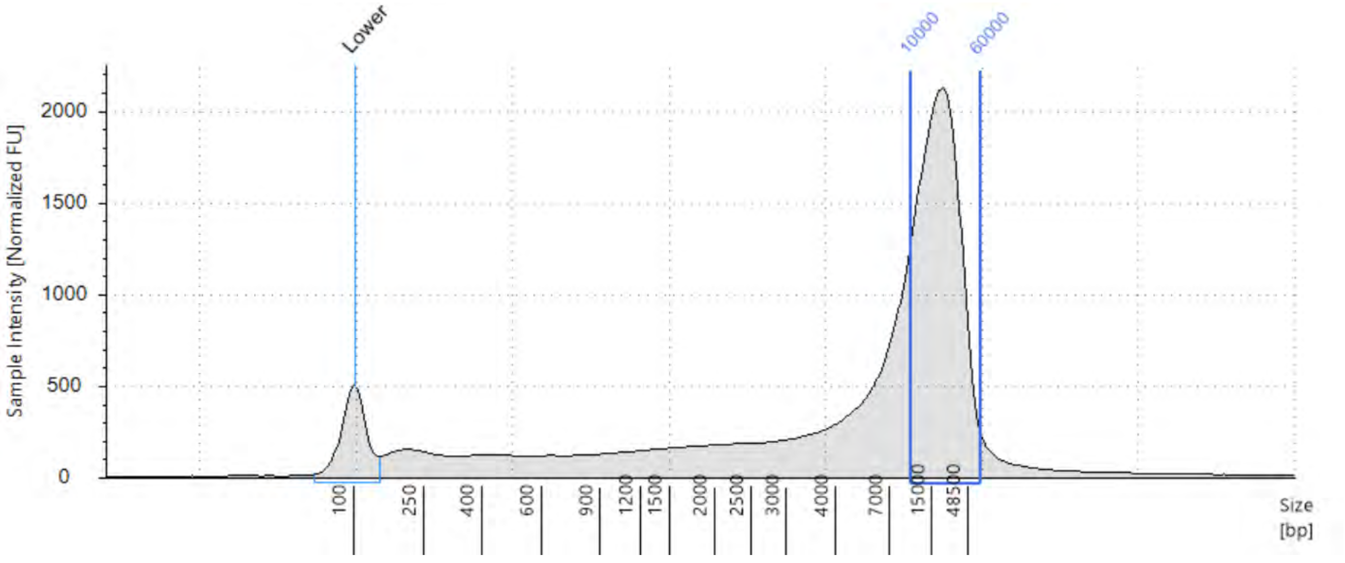

Region Table

From [bp]	To [bp]	Average Size [bp]	Conc. [ng/µl]	Region Molarity [nmol/l]	% of Total	Region Comment	Color
10000	60000	20483	63.0	5.66	46.61		■

© Agilent Technologies, Inc. 2017

D4: W891

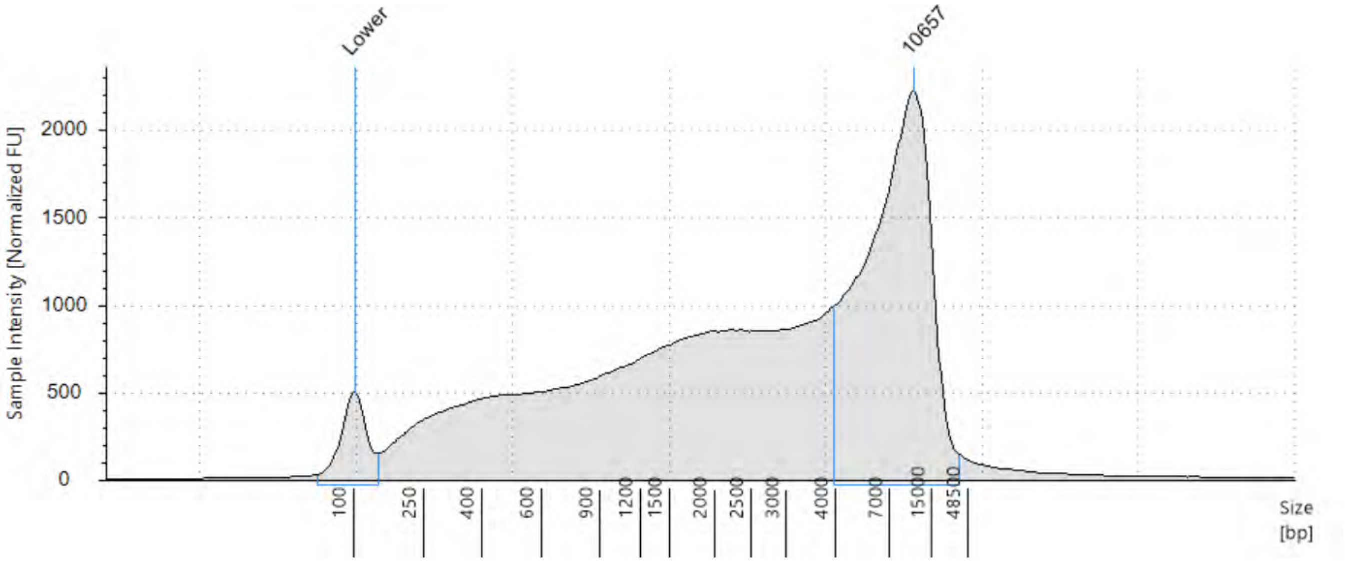

Sample Table

Well	DIN	Conc. [ng/µl]	Sample Description	Alert	Observations
D4	5.1	266	W891	⚠	Caution! Expired ScreenTape device; Sample concentration outside recommended range

Peak Table

Size [bp]	Calibrated Conc. [ng/µl]	Assigned Conc. [ng/µl]	% Integrated Area	From [bp]	To [bp]	Peak Comment	Observations
100	8.50	8.50	-	61	135		Lower Marker
10657	97.7	-	100.00	3933	25216		

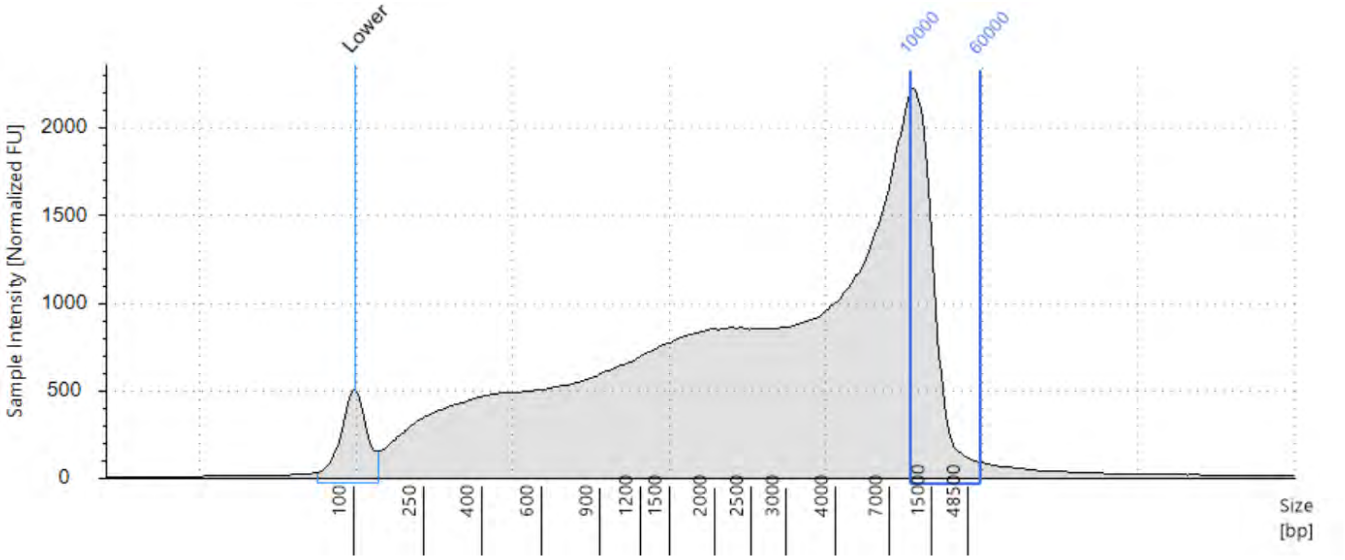

Region Table

From [bp]	To [bp]	Average Size [bp]	Conc. [ng/µl]	Region Molarity [nmol/l]	% of Total	Region Comment	Color
10000	60000	14973	35.4	4.11	13.32		■

 © Agilent Technologies, Inc. 2017

E4: W892

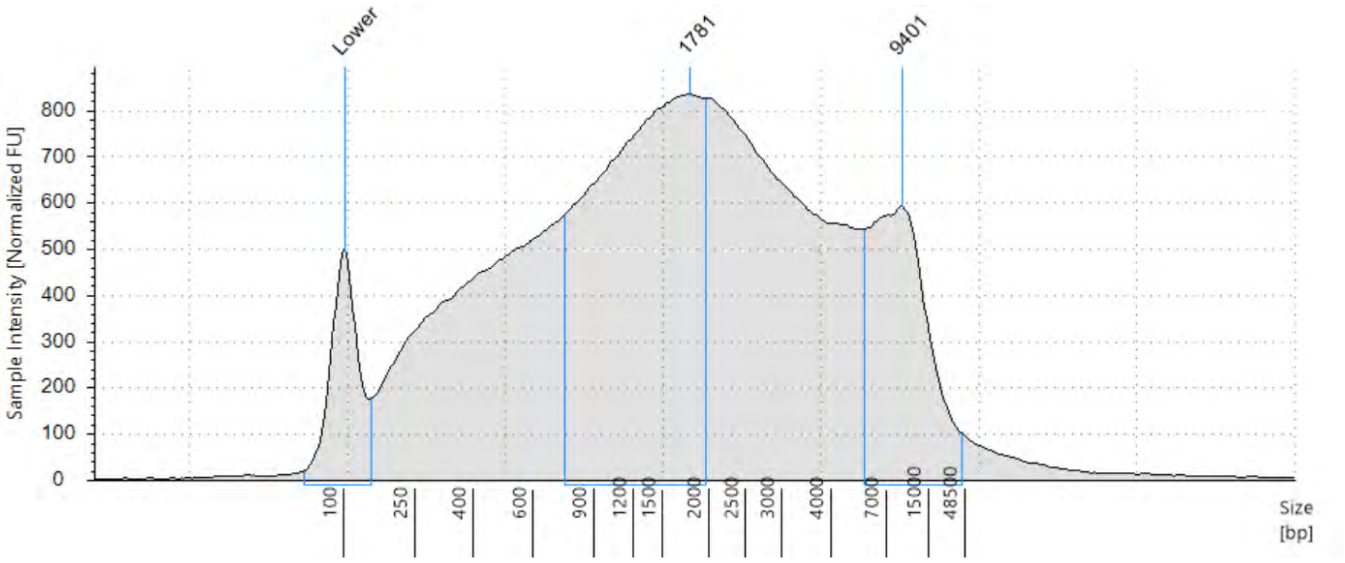

Sample Table

Well	DIN	Conc. [ng/µl]	Sample Description	Alert	Observations
E4	3.4	179	W892	⚠	Caution! Expired ScreenTape device; Sample concentration outside recommended range

Peak Table

Size [bp]	Calibrated Conc. [ng/µl]	Assigned Conc. [ng/µl]	% Integrated Area	From [bp]	To [bp]	Peak Comment	Observations
100	8.50	8.50	-	60	140		Lower Marker
1781	56.5	-	72.00	740	1978		
9401	22.0	-	28.00	5664	39121		

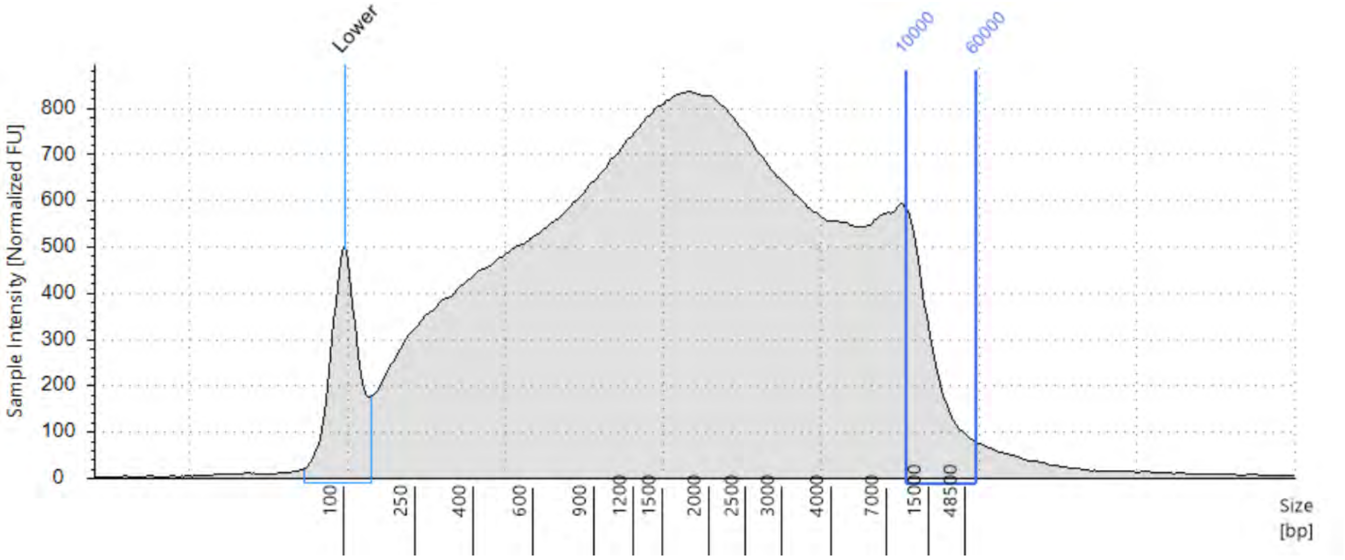

Region Table

From [bp]	To [bp]	Average Size [bp]	Conc. [ng/µl]	Region Molarity [nmol/l]	% of Total	Region Comment	Color
10000	60000	18121	10.0	1.09	5.61		■

 © Agilent Technologies, Inc. 2017

F4: W893

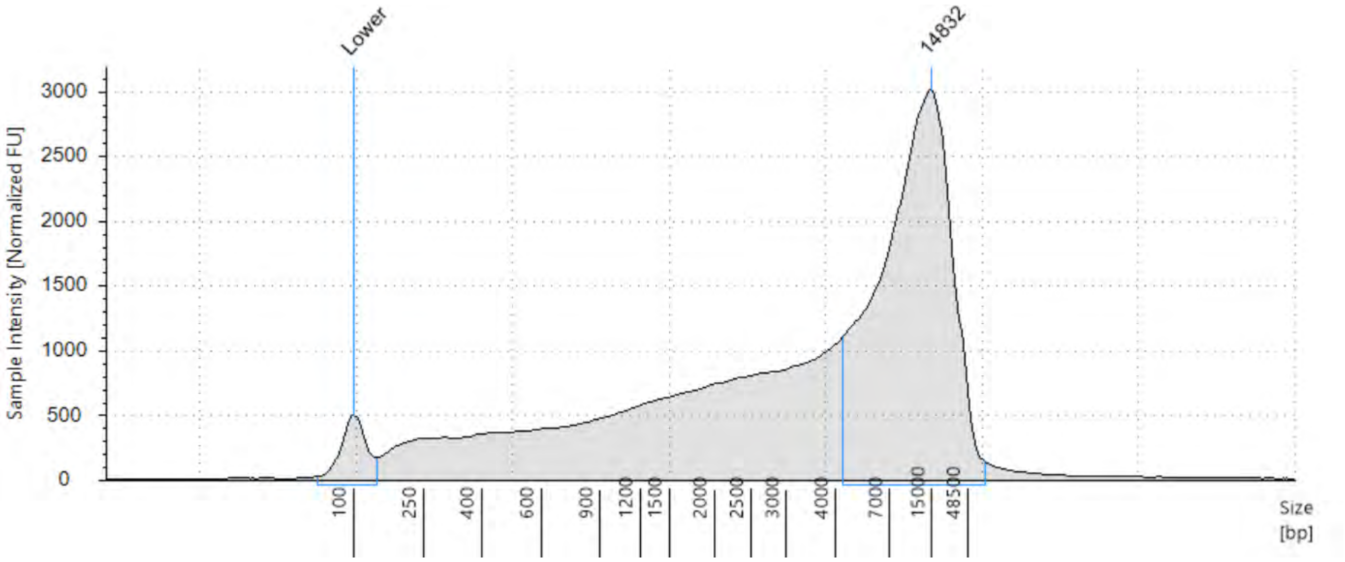

Sample Table

Well	DIN	Conc. [ng/µl]	Sample Description	Alert	Observations
F4	5.9	301	W893	⚠	Caution! Expired ScreenTape device; Sample concentration outside functional range for DIN

Peak Table

Size [bp]	Calibrated Conc. [ng/µl]	Assigned Conc. [ng/µl]	% Integrated Area	From [bp]	To [bp]	Peak Comment	Observations
100	8.50	8.50	-	62	135		Lower Marker
14832	146	-	99.50	4309	>60000		
-	-	-	-	-	-		Sample Well

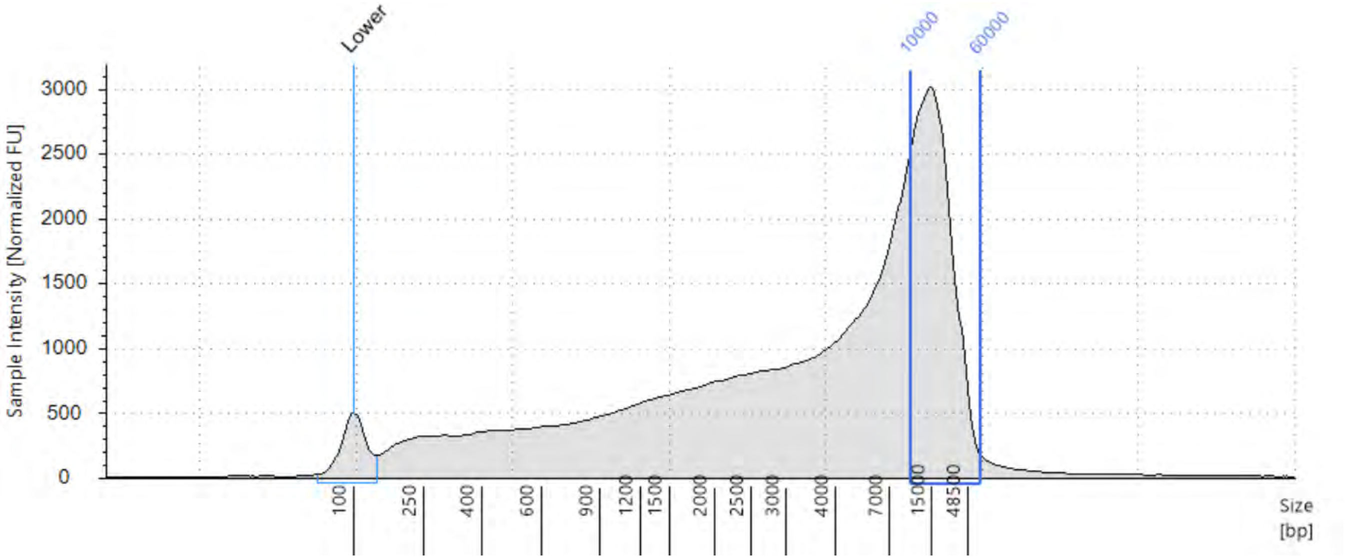

Region Table

From [bp]	To [bp]	Average Size [bp]	Conc. [ng/µl]	Region Molarity [nmol/l]	% of Total	Region Comment	Color
10000	60000	18017	83.8	8.36	27.83		■

 © Agilent Technologies, Inc. 2017

G4: W894

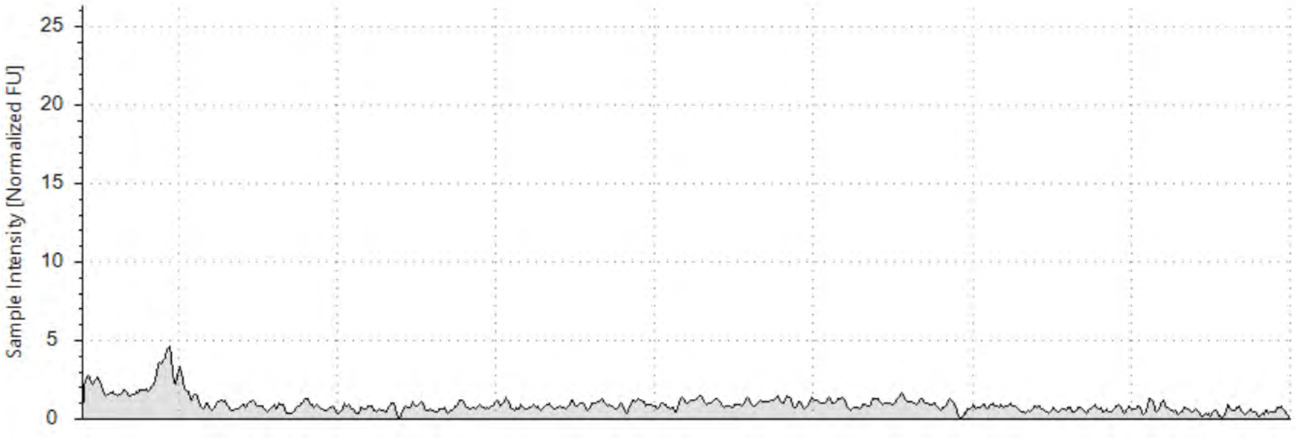

Sample Table

Well	DIN	Conc. [ng/µl]	Sample Description	Alert	Observations
G4	-		W894	▲	Marker(s) not detected; Caution! Expired ScreenTape device

© Agilent Technologies, Inc. 2017

H4: W895

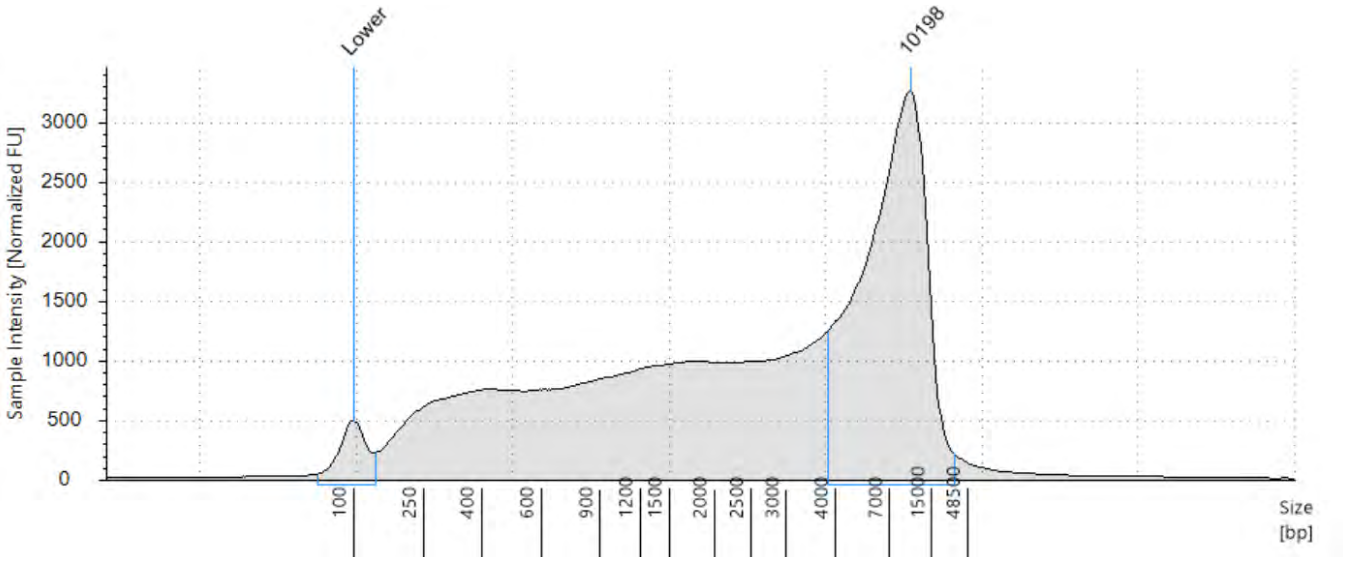

Sample Table

Well	DIN	Conc. [ng/µl]	Sample Description	Alert	Observations
H4	5.0	346	W895	⚠	Caution! Expired ScreenTape device; Sample concentration outside functional range for DIN

Peak Table

Size [bp]	Calibrated Conc. [ng/µl]	Assigned Conc. [ng/µl]	% Integrated Area	From [bp]	To [bp]	Peak Comment	Observations
100	8.50	8.50	-	61	132		Lower Marker
10198	132	-	100.00	3834	22505		

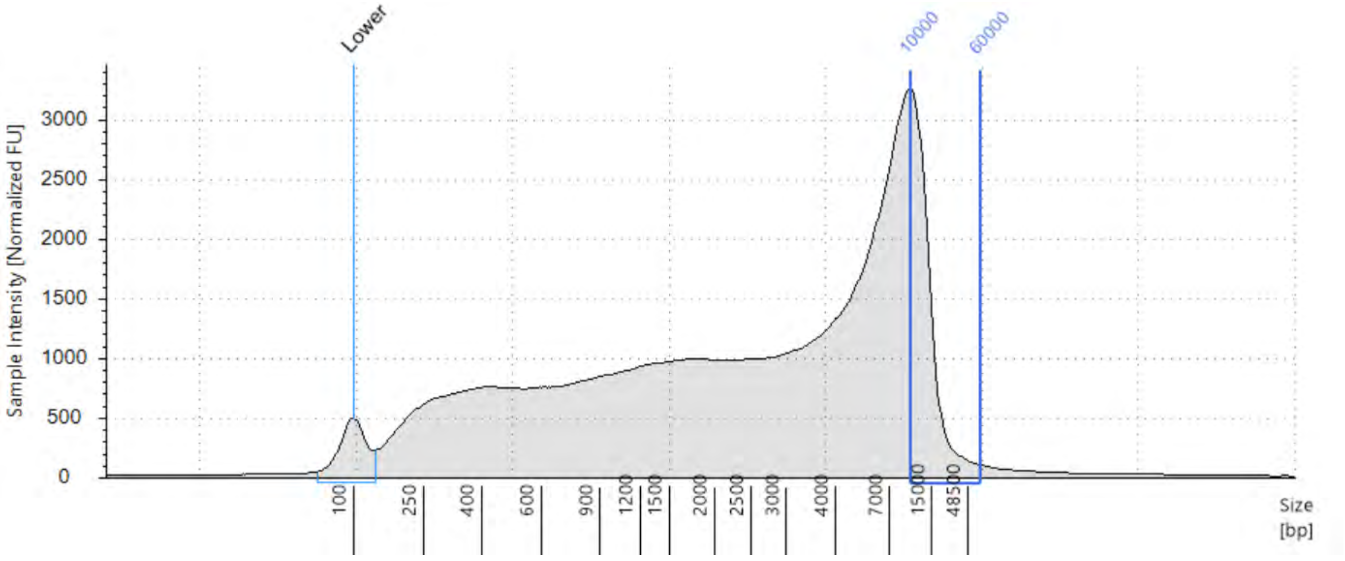

Region Table

From [bp]	To [bp]	Average Size [bp]	Conc. [ng/µl]	Region Molarity [nmol/l]	% of Total	Region Comment	Color
10000	60000	14688	41.6	4.94	12.01		■

 © Agilent Technologies, Inc. 2017

A5: W1116

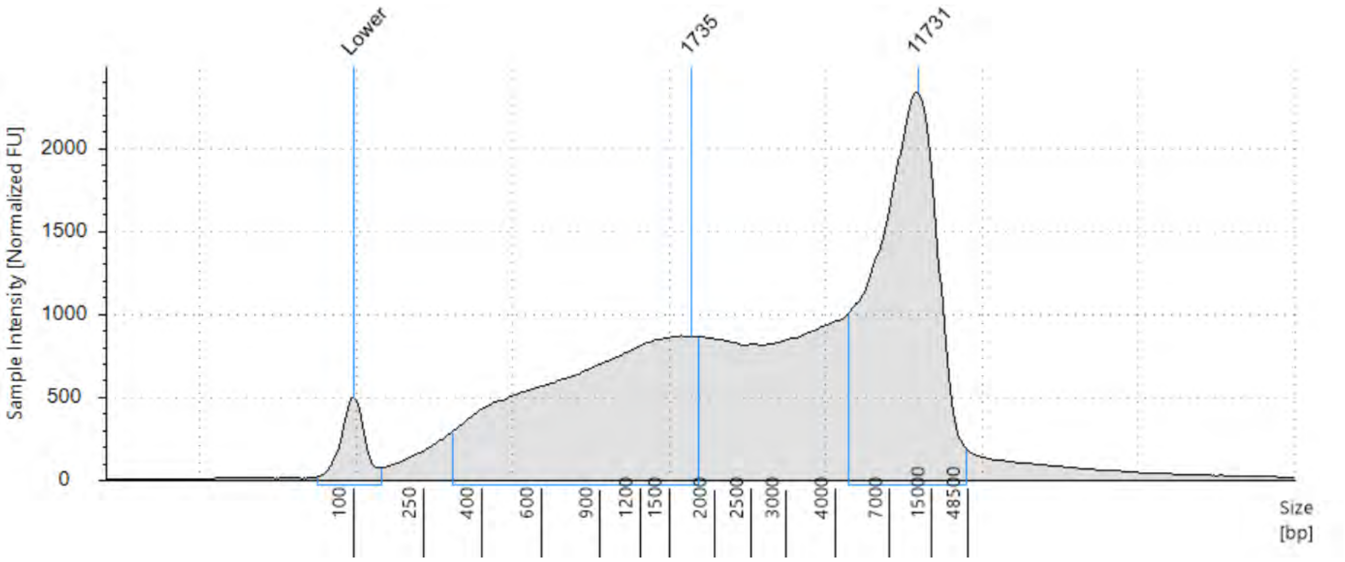

Sample Table

Well	DIN	Conc. [ng/µl]	Sample Description	Alert	Observations
A5	5.3	306	W1116	⚠	Caution! Expired ScreenTape device; Sample concentration outside functional range for DIN

Peak Table

Size [bp]	Calibrated Conc. [ng/µl]	Assigned Conc. [ng/µl]	% Integrated Area	From [bp]	To [bp]	Peak Comment	Observations
100	8.50	8.50	-	62	143		Lower Marker
1735	99.9	-	48.27	314	1812		
11731	107	-	51.73	4585	47199		

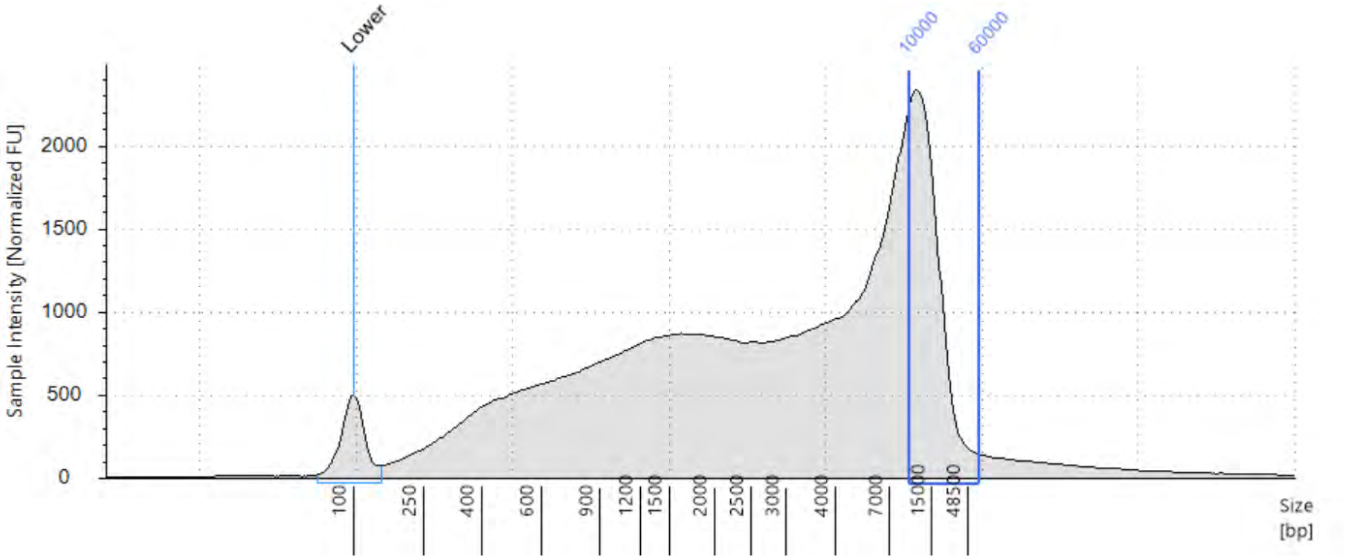

Region Table

From [bp]	To [bp]	Average Size [bp]	Conc. [ng/µl]	Region Molarity [nmol/l]	% of Total	Region Comment	Color
10000	60000	15995	51.5	5.69	16.85		■

B5: W1117

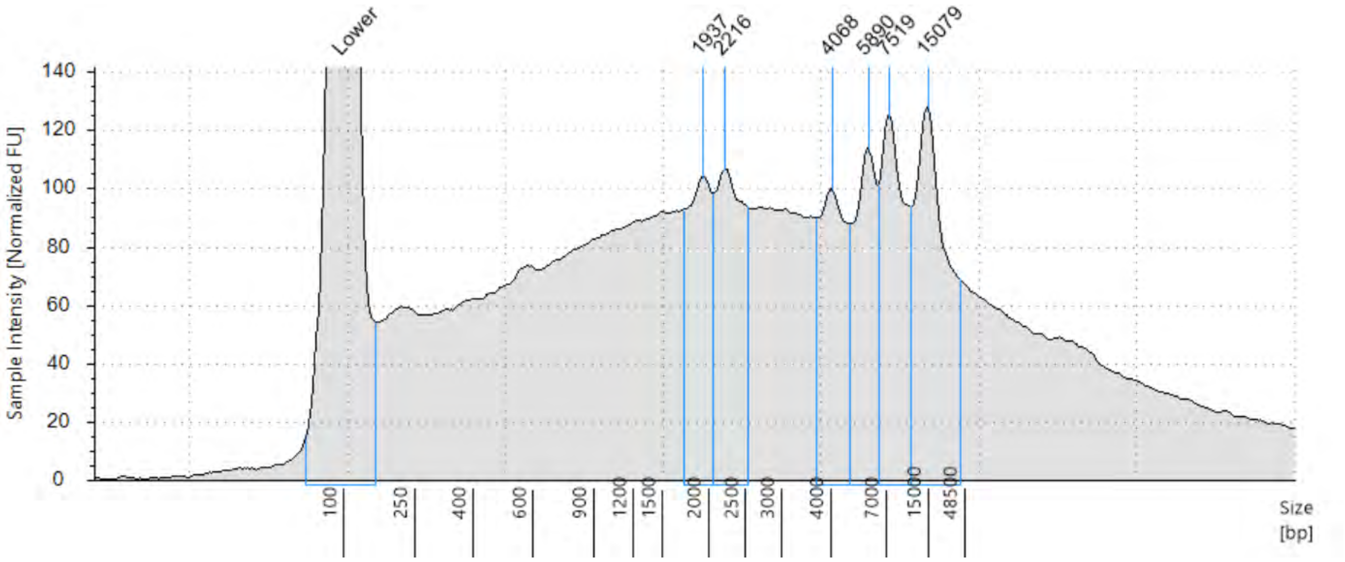

Sample Table

Well	DIN	Conc. [ng/µl]	Sample Description	Alert	Observations
B5	4.1	38.7	W1117	⚠	Caution! Expired ScreenTape device

Peak Table

Size [bp]	Calibrated Conc. [ng/µl]	Assigned Conc. [ng/µl]	% Integrated Area	From [bp]	To [bp]	Peak Comment	Observations
100	8.50	8.50	-	60	149		Lower Marker
1937	1.82	-	13.55	1711	2057		
2216	2.18	-	16.26	2057	2527		
4068	2.03	-	15.11	3664	4895		
5890	1.78	-	13.28	4895	6508		
7519	2.25	-	16.77	6508	11191		
15079	2.89	-	21.52	11191	31680		
-	-	-	-	-	-		Sample Well

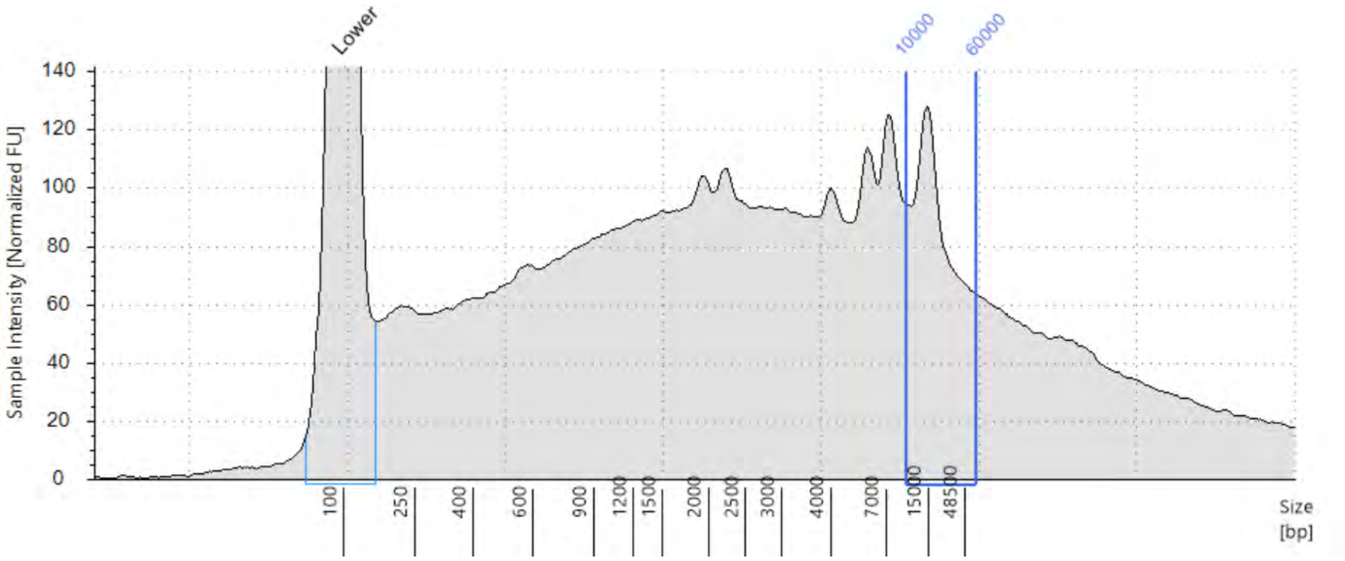

Region Table

From [bp]	To [bp]	Average Size [bp]	Conc. [ng/µl]	Region Molarity [nmol/l]	% of Total	Region Comment	Color
10000	60000	23014	3.86	0.347	9.96		■

C5: W1118

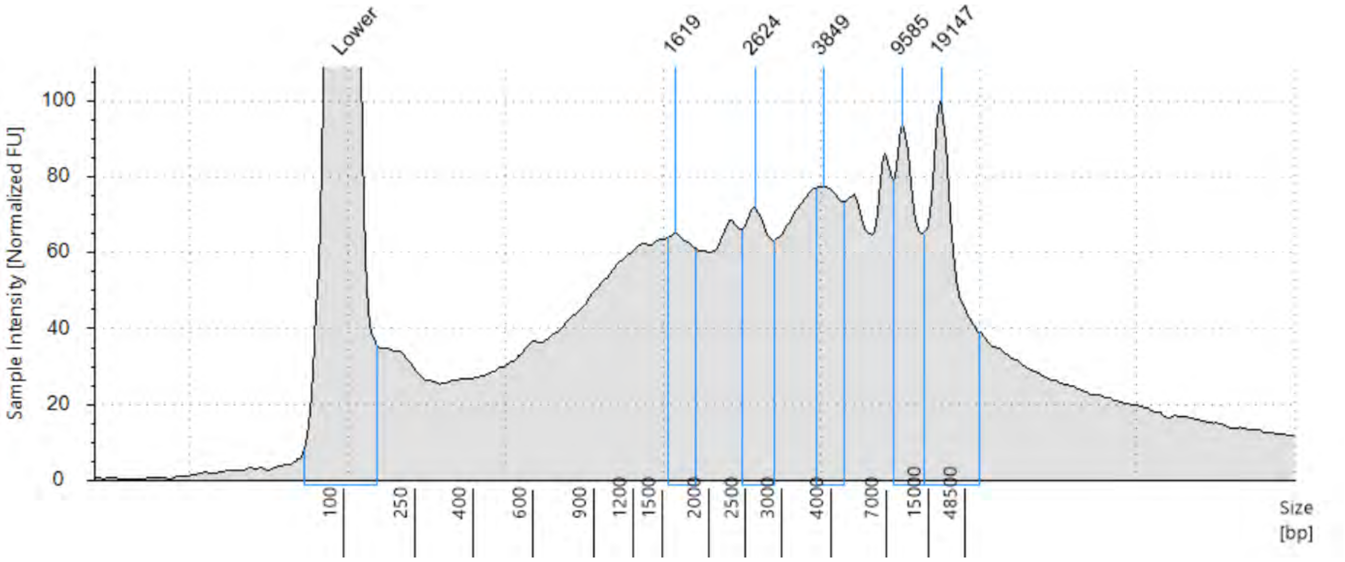

Sample Table

Well	DIN	Conc. [ng/µl]	Sample Description	Alert	Observations
C5	5.1	25.9	W1118	⚠	Caution! Expired ScreenTape device

Peak Table

Size [bp]	Calibrated Conc. [ng/µl]	Assigned Conc. [ng/µl]	% Integrated Area	From [bp]	To [bp]	Peak Comment	Observations
100	8.50	8.50	-	59	151		Lower Marker
1619	1.14	-	13.28	1549	1846		
2624	1.40	-	16.22	2457	2894		
3849	1.31	-	15.19	3697	4559		
9585	1.57	-	18.18	8062	13886		
19147	2.27	-	26.37	13886	>60000		
-	-	-	-	-	-		Sample Well

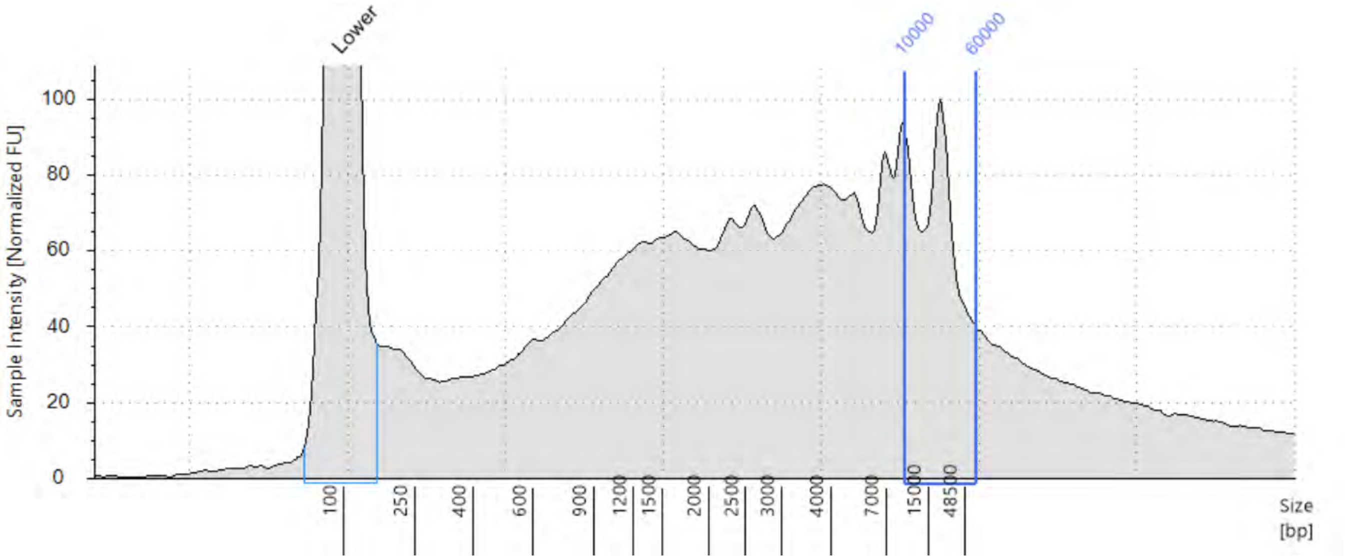

Region Table

From [bp]	To [bp]	Average Size [bp]	Conc. [ng/µl]	Region Molarity [nmol/l]	% of Total	Region Comment	Color
10000	60000	22683	3.08	0.274	11.86		■

© Agilent Technologies, Inc. 2017

D5: W1119

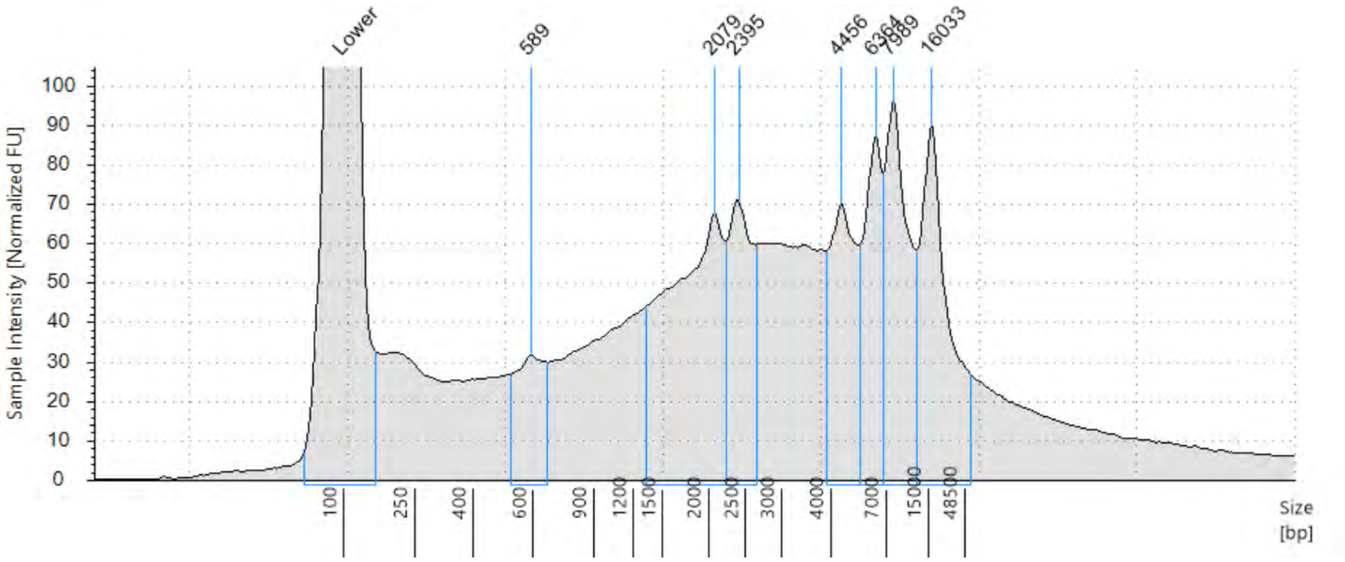

Sample Table

Well	DIN	Conc. [ng/µl]	Sample Description	Alert	Observations
D5	5.1	20.8	W1119	⚠	Caution! Expired ScreenTape device

Peak Table

Size [bp]	Calibrated Conc. [ng/µl]	Assigned Conc. [ng/µl]	% Integrated Area	From [bp]	To [bp]	Peak Comment	Observations
100	8.50	8.50	-	59	149		Lower Marker
589	0.691	-	6.10	512	653		
2079	2.73	-	24.06	1324	2222		
2395	1.31	-	11.55	2222	2642		
4456	1.39	-	12.28	3890	5363		
6364	1.19	-	10.47	5363	6834		
7989	1.69	-	14.87	6834	12195		
16033	1.87	-	16.48	12195	54759		
-	-	-	-	-	-		Sample Well

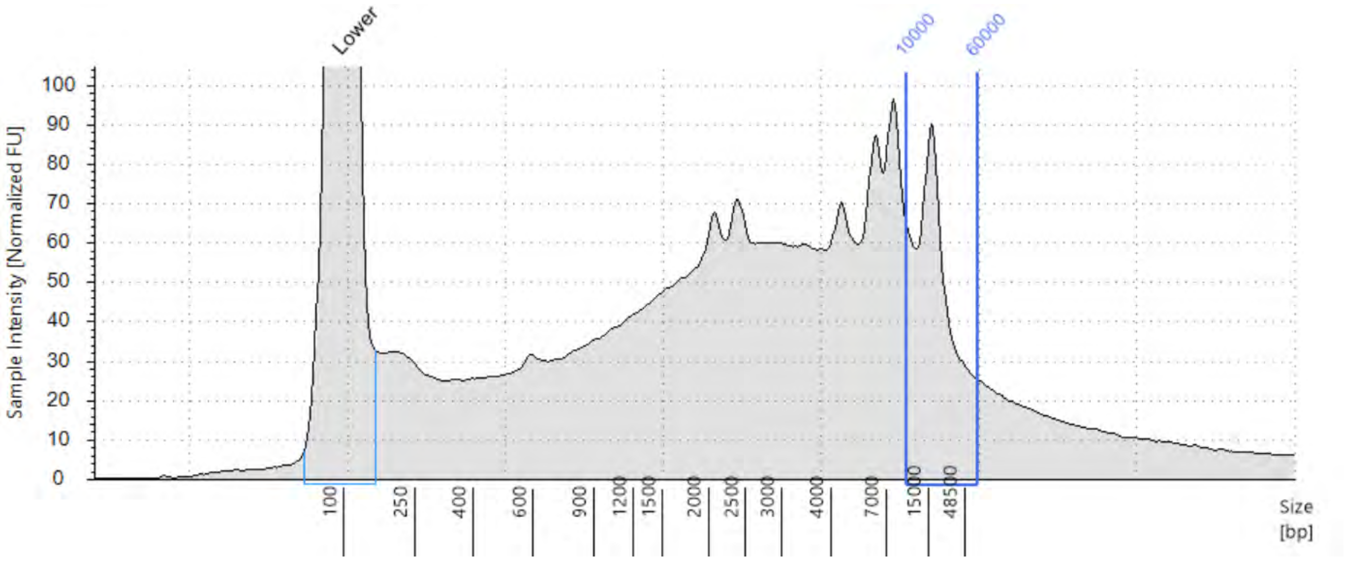

Region Table

From [bp]	To [bp]	Average Size [bp]	Conc. [ng/µl]	Region Molarity [nmol/l]	% of Total	Region Comment	Color
10000	60000	21255	2.36	0.219	11.34		■

 © Agilent Technologies, Inc. 2017

E5: W1120

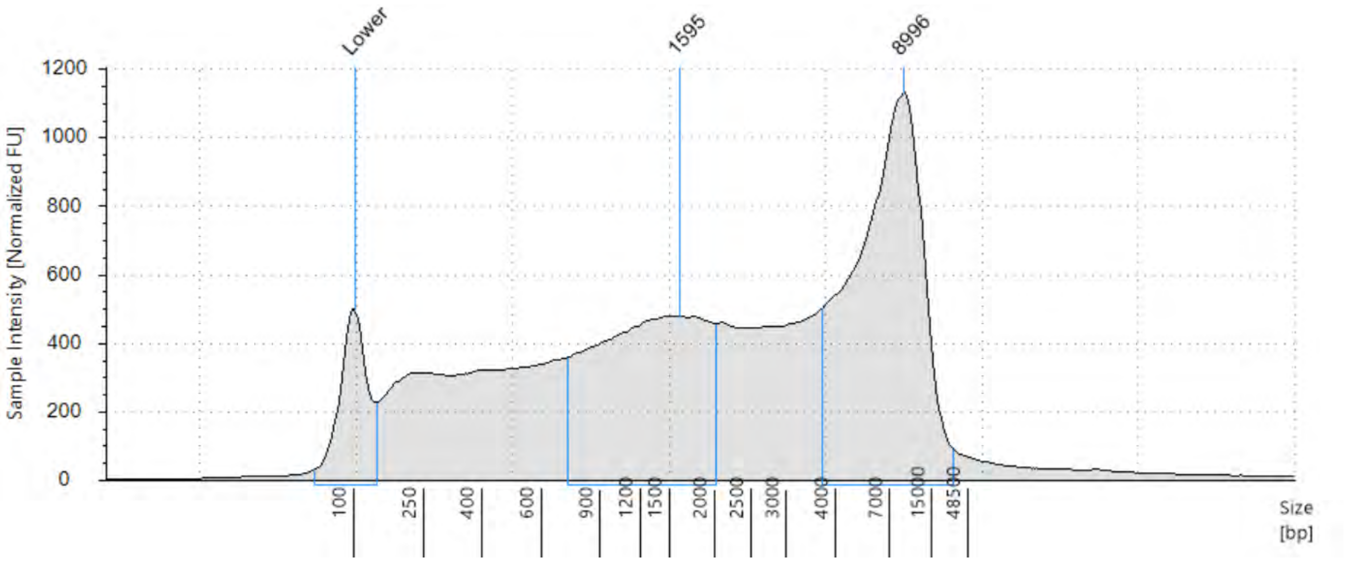

Sample Table

Well	DIN	Conc. [ng/µl]	Sample Description	Alert	Observations
E5	4.4	143	W1120	⚠	Caution! Expired ScreenTape device; Sample concentration outside recommended range

Peak Table

Size [bp]	Calibrated Conc. [ng/µl]	Assigned Conc. [ng/µl]	% Integrated Area	From [bp]	To [bp]	Peak Comment	Observations
100	8.50	8.50	-	58	134		Lower Marker
1595	34.7	-	42.24	716	2018		
8996	47.2	-	57.58	3684	21987		
-	-	-	-	-	-		Sample Well

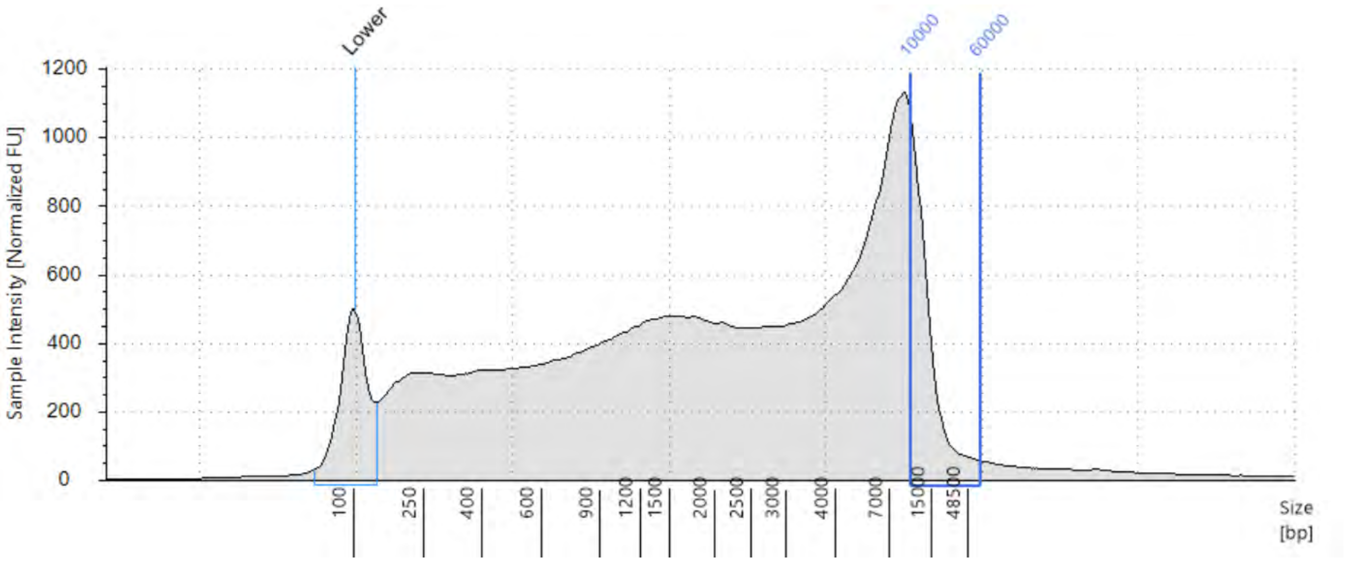

Region Table

From [bp]	To [bp]	Average Size [bp]	Conc. [ng/µl]	Region Molarity [nmol/l]	% of Total	Region Comment	Color
10000	60000	15580	12.0	1.41	8.42		■

 © Agilent Technologies, Inc. 2017
